# Long-term loss in extent and current protection of terrestrial ecosystem diversity in the temperate and tropical Americas

**DOI:** 10.1371/journal.pone.0234960

**Published:** 2020-06-30

**Authors:** Patrick J. Comer, Jon C. Hak, Carmen Josse, Regan Smyth

**Affiliations:** 1 NatureServe, Boulder, CO, United States of America; 2 EcoCiencia, Quito, Ecuador; 3 NatureServe, Arlington, VA, United States of America; Indiana State University, UNITED STATES

## Abstract

Documenting changes in ecosystem extent and protection is essential to understanding status of biodiversity and related ecosystem services and have direct applications to measuring Essential Biodiversity Variables, Targets under the Convention on Biological Diversity (CBD), and IUCN Red List of Ecosystems. We developed both potential and current distribution maps of terrestrial ecosystem types for the temperate and tropical Americas; with “potential” estimating where a type would likely occur today had there not been prior land conversion for modern land uses. We utilized a hierarchical classification to describe and map natural ecosystem types at six levels of thematic detail, with lower thematic levels defining more units each with narrower floristic range than upper levels. Current land use/land cover was derived using available global data on human land use intensity and combined with the potential distribution maps to estimate long-term change in extent for each ecosystem type. We also assessed representation of ecosystem types within protected areas as defined by IUCN I-VI land status categories. Of the 749 ecosystem types assessed, represented at 5^th^ (n = 315) vs. 6^th^ (n = 433) levels of the classification hierarchy, 5 types (1.6%) and 31 types (7.1%), respectively, have lost >90% of their potential extent. Some 66 types (20.9%) and 141 types (32.5%), respectively, have lost >50% of their potential extent; thus, crossing thresholds of Vulnerable status under IUCN Red List criterion A3. For ecosystem type representation within IUCN protected area classes, with reference to potential extent of each type, 111 (45.3%) and 125 (28.8%) of types, respectively, have higher representation (>17%) than CBD 2020 targets. Twelve types (3.8%) and 23 (5.3%) of types, respectively, are represented with <1% within protected areas. We illustrate an option for visualizing and reporting on CBD targets (2020 and proposed post-2020) for ecosystem representativeness using both potential extent as a baseline.

## Introduction

Accelerating landscape change threatens biodiversity worldwide [1]. Therefore, knowledge of trends in the extent of ecosystems, as well as their proportional representation in protected areas, each provide a foundation for conservation action. Because natural pattern and process influence the composition of communities in the short run, and define selective pressures on organisms over evolutionary timeframes, loss of areal extent should correlate with a decrease in niche diversity, the pool of characteristic species, and variability in key ecological processes [2, 3]. Protected area designation is a key strategy for conserving biodiversity [4] and so tracking progress on ecological representativeness of protected areas remains an important indication of progress in conservation.

There are also several global initiatives that should benefit from this knowledge. Under the United Nations Convention on Biological Diversity Strategic Plan 2011–2020 [5], a series of targets were established to support conservation action and monitor progress. Among the Plan’s stated targets, Target 11 states that… “By 2020, at least 17 per cent of terrestrial and inland water, …especially areas of particular importance for biodiversity and ecosystem services, are conserved through effectively and equitably managed, ecologically representative and well-connected systems of protected areas and other effective area-based conservation measures and integrated into the wider landscapes and seascapes.” The meaning of “ecologically representative” has been interpreted variously, with one common interpretation being the surface area of ecoregions [6].

Ecoregions, such as those developed by the World Wildlife Fund (WWF) [7] are well established worldwide. Regional biogeography, climate, and physiography underlie the definition of each ecoregion. Within a given ecoregion, one could expect to find recurring patterns of upland and wetland ecosystems. The WWF ecoregion schema is commonly utilized for measuring progress against Target 11 [8]. However, some have raised concerns that ecoregions on their own fail to truly represent diversity in ecosystems, ecological and evolutionary processes, and genetics, as intended by the Convention [6]. One response to this concern would be to describe and map ecosystem types that have been defined in an established ecological classification and depict the natural patterns within each ecoregion.

In parallel to the UN Convention targets, the International Union for Conservation of Nature (IUCN) supports risk assessment of ecosystem types [9] as a complement to the Red List of Threatened Species [10, 11]. Bland et al. [12] described the IUCN framework for risk assessment of ecosystem types. Under that framework, four major criteria and subset measurements address threatening processes that lead to rangewide ecosystem collapse. Criterion A measures trends in declining distribution of a given ecosystem type, estimating the proportional change over recent centuries, recent decades, and into upcoming decades. Criterion B aims to assess risk to types with restricted current distributions. Criteria C and D aim to gauge trends in environmental degradation and disruption of biotic processes, respectively. Again, the definition of ecosystem type to be assessed is critical to the successful application of this red listing process. Units must describe recurrent pattern in biotic composition in response to geophysical settings and dynamic ecological processes all with sufficient level of detail to support practical conservation status measurement [13].

In 2013, a set of “Essential Biodiversity Variables” (EBVs) was proposed under the Convention on Biological Diversity (CBD) as key measurements required for study, reporting, and management of biodiversity change [14]. These variables are intended to serve as a bridge between biological observations and summary indicators for use by policymakers, and must fulfill criteria on scalability, temporal sensitivity, feasibility, and relevance. While still very much in development, there is a logical role for mapped ecosystem classifications of high thematic detail to serve as the basis for EBVs related to community composition and ecosystem structure. For example, under community composition, trends in loss of ecosystems–if defined at a thematic detail resulting in 10s -100s of types per country–could serve as an indicator under taxonomic diversity. Under ecosystem structure, trends in ecosystem extent and fragmentation for these similarly-defined ecosystem types would be quite informative to decision makers.

However, we have lacked a comprehensive ecosystem classification of sufficient thematic detail, along with type descriptions and associated map products, to support these types of analysis across the Americas. While a number of maps exist at regional [15], continental [16, 17], and global [18] extents, nearly all utilize thematic classifications with a limited number of land unit descriptors that don’t differentiate floristic composition among types. For example, [17] differentiate 10 forest map classes for South America, and distinguish humid, dry, flooded, temperate, and montane forests, as well as variants within these using leaf phenology classes, such as deciduous, semi-deciduous, and evergreen.

Some maps developed at national scales, such as the land use maps of Mexico’s National Institute of Geography and Statistics (INEGI) [19], have used aerial photo interpretation to map vegetation and land use classes, and some have shown their utility for tracking change in key ecosystem characteristics [20], but the classification used is not formally tied to regional or global classification standards. Still others working in Latin America have made substantial contributions toward using internationally standardized classifications of high thematic detail. Josse et al. [21] generated a map of the Amazon drainages of Peru and Bolivia using the NatureServe terrestrial ecological systems classification [22]. Sayre et al. [23] generated a map of current locations for over 600 terrestrial ecosystem types across South America. Josse et al. [24] followed these using the same classification for a similar map product focused on the northern and central Andean countries. In the United States, considerable advances have been made in both ecological classification [25, 26] and mapping terrestrial ecosystems [27, 28].

Building from these prior efforts, our objective was to document patterns of loss and current protection for terrestrial ecosystem types using newly developed ecosystem classifications and maps applicable across much of the Americas. By providing estimates of rangewide potential and current extent, these maps form a practical foundation for trend assessment of terrestrial ecosystems under UN Convention Target 11, and for establishing measurable targets for terrestrial ecosystem conservation post 2020 [29, 6]. These map products should also contribute directly to continental-scaled applications of the IUCN Red List of Ecosystems, and to developing measures of Essential Biodiversity Variables for ecosystem composition. The units assessed are also of sufficient thematic detail, and sufficiently described to be the focal units of on the ground conservation action.

## Materials and methods

This effort focused on temperate North America, all of Latin America, and the Caribbean. The project area includes approximately 32.6 million km^2^ or nearly 22% of the global land surface. The aim was to produce both “potential” and “current” distribution for major terrestrial ecosystem types that would be suitable for continental-scale assessment and planning, but also include units suitable for on-the-ground conservation action. The “potential distribution” includes biophysical conditions where each type might occur today had there not been any prior intensive human intervention. “Current distribution” then accounts for those areas of intensive intervention and conversion, as of approximately 2010. For this effort an effective minimum map unit size, or mapped pixel resolution, ranged from 270m to 450m.

### Target map legend

For this effort, multiple forms of ecological classification were used. The International Vegetation Classification (IVC) under development by NatureServe and partners was used to define the target map legend at multiple levels of detail [30]. The hierarchical structure of this classification follows that established as a federal standard for vegetation description in the United States with broad units at upper levels defined by vegetation physiognomy, followed by progressively narrow units at lower levels defined by vegetation floristic composition [31]. It was recently revised and updated for use across the Americas [30]. The full spectrum, from “natural” to “cultural” vegetation types are encompassed by the IVC, but for purposes of this effort, only “natural” vegetation types were treated. As noted above, since natural pattern and process influences the composition of natural communities in the short run, and selective pressures on species over evolutionary timeframes, changing extent in novel ecosystems (those derived from agricultural, forestry, or urban/industrial land uses), while significant and worthwhile, is in our view, less urgent and informative for biodiversity conservation than is tracking trends in natural ecosystems.

Table 1 provides an example of the IVC hierarchy from Temperate North America, with defining characteristics and numbers of described units at each level (as of 2019). Here, tallgrass prairie types have been well described at all levels of the hierarchy down to the association level, where multiple dominant and diagnostic species are used to define a given type. With a longer history of systematic classification and description, the nearly 6,000 associations describe natural vegetation types within the conterminous United States. While this level of thematic detail is not currently feasible to map on a continental scale, IVC group and alliance levels are increasingly feasible to target in regional and national map legends. Across Latin America and the Caribbean, the IVC macrogroup is the finest level that is fully developed.

**Table 1:** International vegetation classification hierarchy, including example classification units from temperate North America. The number of natural types documented (as of 2019) within each hierarchical level from temperate Canada south throughout South America.

Level No.	Level Name	Defining Characteristics	No. Types	Example
1	Class	Life Form Physiognomy	6	Grassland & Shrubland
2	Subclass	Global Physiognomy	13	Temperate & Boreal Grassland & Shrubland
3	Formation	Global Physiognomy	36	Temperate Grassland & Shrubland
4	Division	Continental Floristics	150	Great Plains Grassland & Shrubland
5	Macrogroup	Subcontinental Floristics	370	Great Plains Tallgrass Prairie
6	Group	Regional Floristics	564*	Northern Great Plains Tallgrass Prairie
7	Alliance	Subregional Floristics	1,452*	*Schizachyrium scoparium - Bouteloua curtipendula* Northern Grassland
8	Association	Local Floristics	7,015*	*Schizachyrium scoparium - Bouteloua curtipendula - Hesperostipa spartea - (Pascopyrum smithii)* Grassland

*numbers apply to USA and adjacent Canada; classification incompletely developed at lower levels for Latin America and Caribbean.

For this effort, within the larger mapping and assessment area extending from temperate Canada throughout Latin America, there were 315 IVC macrogroups mapped.

### Terrestrial ecological systems classification

The NatureServe terrestrial ecological systems classification [25, 22] is an integrated abiotic/biotic classification that built upon numerous national and local classifications across the Americas. Each classification unit is defined as a recurring set of plant community types that share similar geophysical settings and natural disturbance regimes. It was developed in the early 2000s, prior to the current version of the IVC hierarchy, and describes over 1,500 upland and wetland units, that have been in wide usage for mapping and assessment at regional, national, and multi-national scales [27, 21, 23, 28, 24]. Relative to the IVC hierarchy, terrestrial ecological systems concepts roughly correspond to the group level (Level 6), with some equating with the alliance level (Level 7). Therefore, using the example in Table 1, a given terrestrial ecological system would correspond to the Northern Great Tallgrass Prairie dominated by tallgrass species such as *Andropogon gerardii*, *Sorghastrum nutans*, *Panicum virgatum*, *Schizachyrium scoparium*, and *Bouteloua curtipendula*. Each type has been described and given a generalized distribution, including state, province, country and ecoregion. The terrestrial ecological system classification provides the most detailed set of concepts for this effort, and were completed for assessment throughout temperate and tropical North America, extending south through Panama. Each ecological system type can be readily aggregated to Levels 1–5 of the IVC hierarchy, so we may readily use that hierarchy for display and analysis at the broader classification levels.

For this effort, within the mapping and assessment area extending from temperate Canada south through Central America and the Caribbean, there were 623 ecological system types mapped. Type descriptions for all types addressed in this project are provided in Supplementary Information (S1 Appendix for macrogroups and S2 Appendix for ecological systems).

### Mapping methods

Mapping methods are detailed in Supplementary Information (S3 Appendix–Map Methods Detail). Briefly, spatial modeling used georeferenced samples that had been labeled to each type (IVC macrogroup or NatureServe ecological system) and combined these with map surfaces reflecting climate, landform, and soils to depict a “potential” distribution of types on the target legend. The IVC macrogroup was the target legend for the broader hemisphere-wide mapping extent (from temperate Canada south throughout Latin America and the Caribbean). The thematically finer ecological system types were the target legend for temperate and tropical North America only (from temperate Canada south through Central America and Caribbean). The two “potential” distribution outputs were then combined with other mapped information (see below) to measure ecosystem type loss and protected area status across their respective map extents. See Supplementary Information (S3 Appendix) for a detailed discussion of map validation.

### Measuring type loss from land conversion

A composite map for current land use (*ca*. 2010) was developed for the entire study area by combining products from LANDFIRE (30m pixel resolution, circa 2003 [in the USA], GlobCover (270m pixel resolution, circa 2009), and GlobeLand30 (30m pixel resolution circa 2000–2010) [32]. Investigation of GlobeLand30 indicated inaccuracies in predicting deforestation, especially in tropical forest regions. See S4 Appendix for detail showing how classes from GlobeCover and GlobeLand30 were combined to depict developed, agriculture, and surface water classes. Likewise, substantial areas of ruderal vegetation (vegetation with no natural analog resulting from prior land clearing and abandonment) common in portions of the USA were not reflected in either GlobeCover or GlobeLand30 data sets. Therefore, a combined map product was developed and summarized at 270m pixel resolution to best approximate urban, industrial, agricultural, and ruderal land cover. We had no means to evaluate the performance of this combined map, but validation statistics of the component map layers could be consulted by users to provide insights into overall map accuracy.

This layer was then combined with the potential distribution map of vegetation macrogroups to indicate current extent of macrogroups and land use classes *ca*. 2010 for the entire study area. That is, where current land use classes overlap with natural ecosystem types from the potential distribution map, that overlapping area is presumed to have been converted from natural ecosystem type to current land use class. Likewise, this layer was also combined with the potential distribution map of ecological systems to indicate current extent of ecological systems and land use classes *ca*. 2010 for the temperate and tropical North America portion of the study area. These map combinations resulted in two estimates of extent for each target map legend class of each natural classification unit (macrogroup or ecological system): the “potential” extent and the “current” (ca. 2010) extent. These calculations provide an estimate of loss in area for each ecosystem over recent centuries.

**Fig 1:**
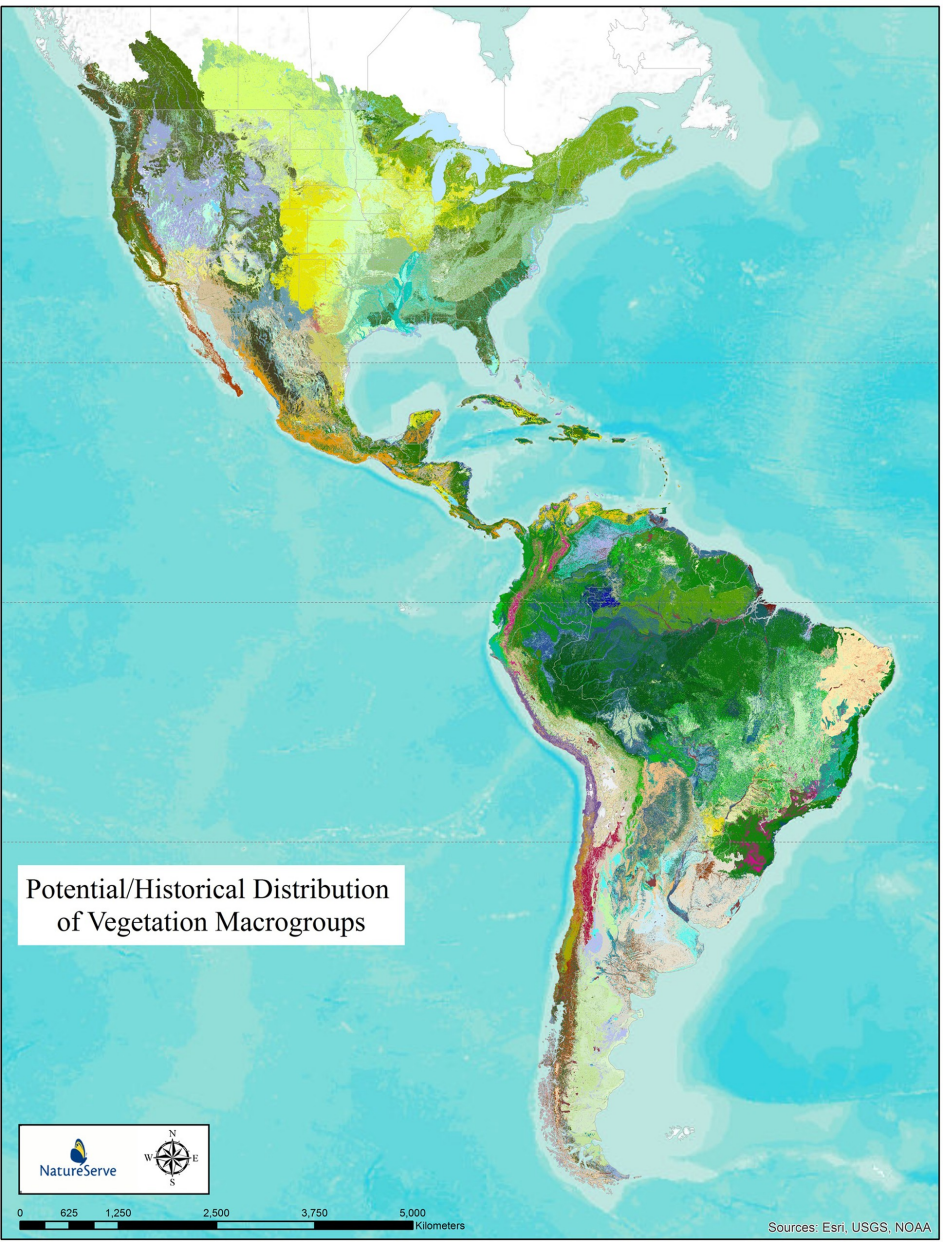
Potential/historical distribution of vegetation macrogroups. Potential distribution of 315 vegetation macrogroups across Temperate and Tropical North and South America. Individual types are too numerous to list in a legend, but this provides a depiction of patterns that will be discernable in part in subsequent figures.

**Fig 2:**
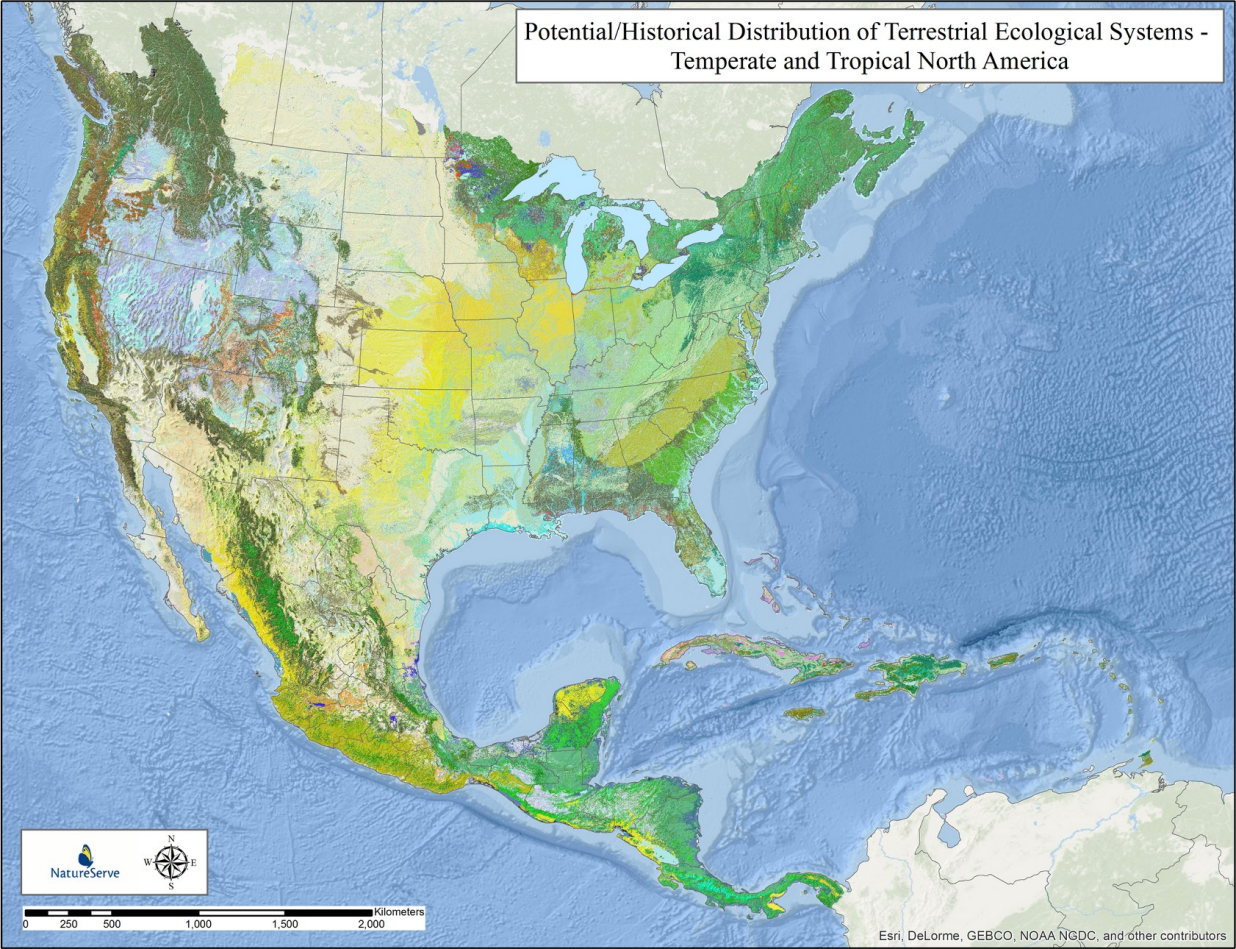
Potential/historical distribution of terrestrial ecological systems—temperate and tropical North America. Potential distribution of 623 terrestrial ecological system units in Temperate and Tropical North America. Individual types are too numerous to list in a legend, but this provides a depiction of patterns that will be discernable in part in subsequent figures.

We then visualize these loss estimates per-type by applying that number to the potential distribution maps (Figs 1–2 below) to depict distributions in terms of loss classes (>95% loss down to <10% loss).

### Ecosystem type representation in protected areas

We then conducted an ecosystem “gap analysis” [33] as one initial step toward prioritizing conservation actions. The IUCN has established a globally-applicable measure of conservation land status that includes 6 protected areas categories [34]. These six categories range from Category I representing “Strict Nature Reserve” to Category VI representing “Protected area with sustainable use of natural resources.” Given the numbers of ecosystems being assessed, our intent was not to analyze differential levels of protection among IUCN I-VI classes, but more simply to document representativeness within any of the classes.

Next, we overlaid protected area information [35] on current extent of all ecological system types in North America and macrogroups across the hemisphere. That enabled us to then compare these area estimates with rangewide estimates of both historical extent and current extent, resulting in proportional estimates of current protection by type (i.e., proportion of “potential” extent currently protected, and proportion of “current” extent currently protected).

We then visualized these per-type protection estimates by applying the calculated proportion of potential extent protected to the potential distribution maps (Figs 1 and 2 below) to depict distributions in terms of relative protection classes (>70% protected down to 0–1% protected).

## Results

Fig 1 depicts the resulting potential distribution map for North and South America, displayed at the level of IVC macrogroup (5^th^ Level of the classification hierarchy). Summary statistics for each of the 315 mapped macrogroups is found in Supplementary Information (S5 Appendix). Fig 2 depicts the resulting map for Temperate and Tropical North America and Caribbean, displayed at the level of terrestrial ecological system types (~6^th^ Level of the classification hierarchy). Summary statistics for each of the 623 mapped terrestrial ecological system types is found in Supplementary Information (S6 Appendix).

### Potential extent–macrogroups across North and South America

Forest and Woodland vegetation classes encompassed 55.3% of the area, with Tropical forests encompassing 30.3% and Temperate forests 25% (Fig 1). Shrubland and Grassland vegetation classes extended over 28.6%, with most (17%) as Cool Semi-Desert Scrub and Grassland. Desert & Semi-Desert extended over 14.1% of the study area, split almost evenly between warm and cool desert types. High Montane vegetation and Open Rock vegetation types encompassed nearly 2% of the area, while Aquatic vegetation encompassed < 1%. The overall potential extent of macrogroup types across the study area varied from a maximum of 2,211,332 km^2^ for Great Plains Mixedgrass & Fescue Prairie down to 13 types that each had <1,000 km^2^.

### Potential extent—terrestrial ecological systems in temperate and tropical North America and Caribbean

Across this area (Fig 2), the potential Forest and Woodland vegetation classes encompassed 54.6% of the area, with Tropical forests encompassing 11.2% and Temperate forests 43.4%. Shrubland and Grassland vegetation classes extended over 25.3%, with most (20.9%) as Cool Semi-Desert Scrub and Grassland. Desert & Semi-Desert extended over 19.1% of the study area, split almost evenly between warm (10.2%) and cool (8.9%) desert types. High Montane vegetation and Open Rock vegetation types, and Aquatic vegetation encompassed < 1%. The overall potential extent of types in North America and Caribbean varied from a maximum of 620,875 km^2^ for Northwestern Great Plains Mixedgrass Prairie down to 211 types, each with <1,000 km^2^.

### Long-term loss in extent–macrogroups across North and South America

**Fig 3:**
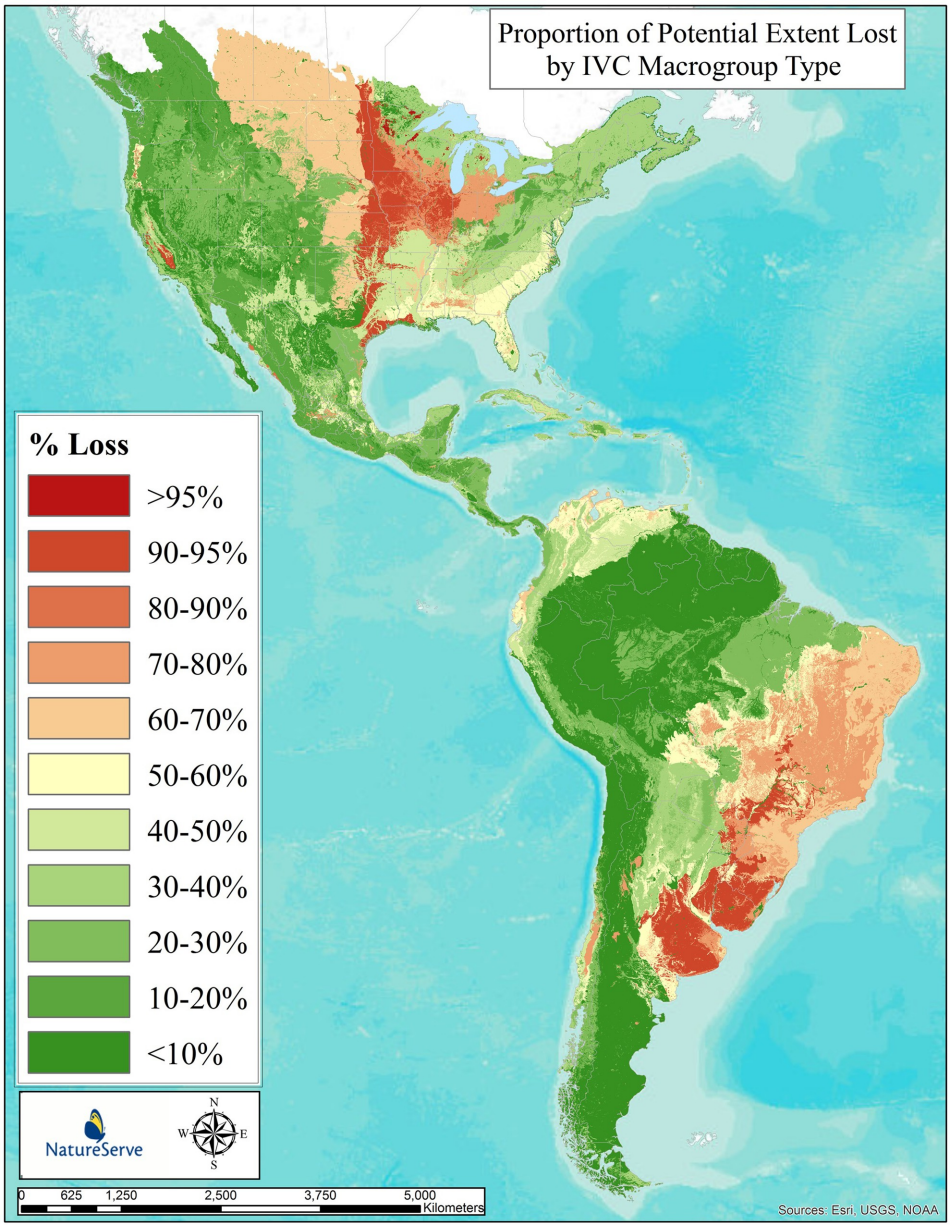
Proportion of potential extent lost by IVC macrogroup. Proportional loss to land conversion by type of 315 vegetation macrogroups across North and South America. Individual types are displayed in terms of their individual loss estimate mapped according to their potential extent, providing a generalize pattern for all types combined.

Fig 3 depicts our estimated loss in potential extent for the 315 mapped terrestrial macrogroups. This form of visualizing loss takes the per-type loss estimate and applies that number to the potential distribution map (Fig 1) and then depicts distributions in terms of loss classes (>95% loss down to <10% loss). The long-term loss in extent of macrogroup types in North and South America varied by major vegetation class and across the continental geography. Importantly, this measure captures complete conversion of type (e.g., to intensive agriculture or urban/industrial land uses) and does *not* consider vegetation alteration or degradation.

**Fig 4:**
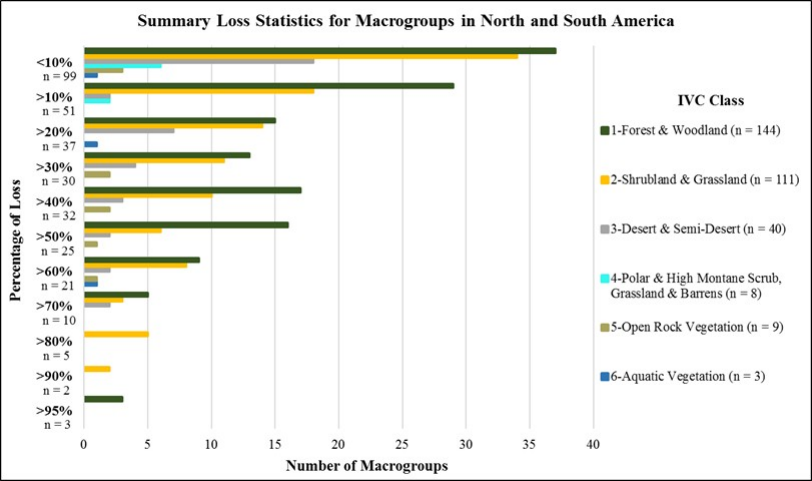
Summary loss statistics for macrogroups in North and South America. (number of types per IVC Class by category of long-term loss in area).

Just 5 macrogroup types (1.6%) scored in the >90% loss category (Fig 4). These included temperate grasslands from North America, such as the Central Lowlands Tallgrass Prairie and the Californian Annual & Perennial Grassland (S1 Appendix). Fifteen types (4.8%) scored in the 70–90% loss category. These included additional temperate grasslands in South America, such as the Humid Pampas Grassland, Parana Upland Savanna. They also include forest and savanna types in North America, such as the Central Midwest Oak Forest, Woodland & Savanna, and the Southeastern Coastal Plain Evergreen Oak—Mixed Hardwood Forest. Forty-six types (14.6%) scored in the 50–70% loss category. Among many other types, these included the Cerrado Savanna, Atlantic, Cerrado, and Caatinga Seasonal Dry Forests of Brazil, and the Brazilian Atlantic Humid Forest. In North America, this category includes Longleaf Pine Woodland in the southeastern United States, the Central Midwest Mesic Forest, and Great Plains Mixedgrass & Fescue Prairie.

The IUCN Red List of Ecosystem criteria for long-term reduction in distribution (criterion A3 [12]) include three thresholds for scoring types as Vulnerable (>50%), Endangered (>70%), or Critically Endangered (>90%). Applying these thresholds, fully 66 macrogroups (20.9%) score within Red List categories of CR, EN, or VU.

Sixty-two types (19.7%) scored in the 30–50% loss category. These include South America’s Northern Andean Paramo, Cerrado Humid Forest, Guajiran Seasonal Dry Forest, and Llanos Humid Forest. In North America, this category includes the Laurentian-Acadian Mesic Hardwood—Conifer Forest, Californian Forest & Woodland, and Caribbean Lowland Humid Forest. Overall, 88 types (27.9%) scored in the 10–20% loss category, and 99 types, or nearly one third (31.4%), fall into the <10% loss category.

### Long-term loss in extent—terrestrial ecological systems in temperate and tropical North America and Caribbean

**Fig 5:**
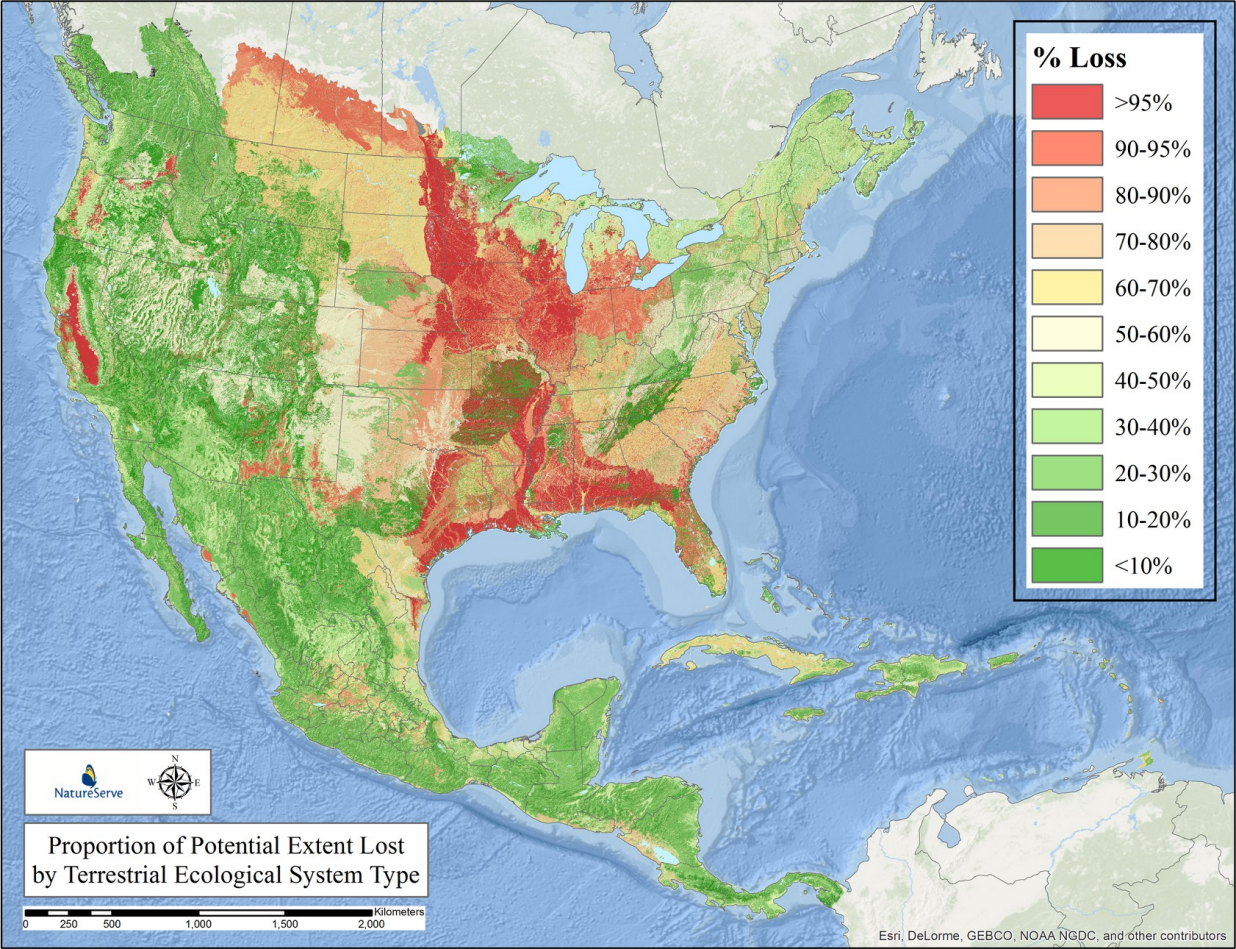
Proportion of potential extent lost by terrestrial ecological system. Proportional loss to land conversion by type of 623 terrestrial ecological system units in Temperate and Tropical North America. Individual types are displayed in terms of their individual loss estimate mapped according to their potential extent, providing a generalize pattern for all types combined.

Fig 5 depicts our estimated loss in potential extent for the 623 mapped terrestrial ecological system types. By comparing Fig 5 to Fig 3, one can see the effect of analysis at a finer-level of ecological classification, and much finer patterns begin to emerge in the North American study area.

**Fig 6:**
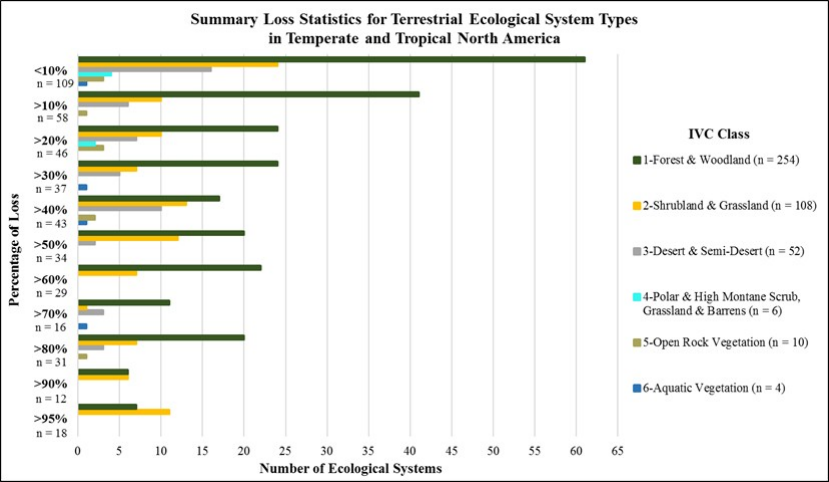
Summary loss statistics for terrestrial ecological systems in North America. (number of types per IVC Class by category of long-term loss in area).

Thirty-one types (7.1%) scored in the >90% loss category (Fig 6). These included temperate grasslands from North America, such as the Northern Tallgrass Prairie, North-Central Interior Oak Savanna, Texas Blackland Tallgrass Prairie, East Gulf Coastal Plain Interior Upland Longleaf Pine Woodland, Mississippi River Low Floodplain (Bottomland) Forest, Columbia Basin Palouse Prairie, and California Central Valley Mixed Oak Savanna (S2 Appendix). Forty-seven types (10.8%) scored in the 70–90% loss category. These included additional temperate grasslands, forest and woodland types, such as the Northern Great Plains Fescue-Mixed Grass Prairie, Great Lakes Wet-Mesic Lakeplain Prairie, North-Central Interior Beech-Maple Forest, and Southeastern Interior Longleaf Pine Woodland. Sixty-three types (14.5%) scored in the 50–70% loss category. Among many other types, these included the Northwestern Great Plains Mixedgrass Prairie, Central Mixedgrass Prairie, Southern Piedmont Dry Oak-(Pine) Forest and Woodland, Allegheny-Cumberland Dry Oak Forest and Woodland, North-Central Interior Floodplain, and Guerreran Savanna in south-central Mexico.

Again, given IUCN Red List of Ecosystem criteria for long-term reduction in distribution (criterion A3 [12]), fully 141 ecological systems (32.5%) score within Red List categories of CR, EN, or VU.

Eighty types (18.4%) scored in the 30–50% loss category. These include the Inter-Mountain Basins Big Sagebrush Shrubland, Laurentian-Acadian Northern Hardwood Forest, Crosstimbers Oak Forest and Woodland, Central Mexican Mixed Desert Scrub, and Central American Caribbean Seasonal Evergreen Lowland Forest. One hundred four types (24%) scored in the 10–20% loss category, and 109 types (25%) fall into the <10% loss category. Among the least converted types are Inter-Mountain Basins Mixed Salt Desert Scrub, Rocky Mountain Subalpine Dry-Mesic Spruce-Fir Forest and Woodland, Colorado Plateau Pinyon-Juniper Woodland, and many other types occurring in arid, high elevation, and other environments where land conversion for other land uses is most challenging.

### Ecosystem type representation in protected areas

#### Vegetation macrogroups

**Fig 7:**
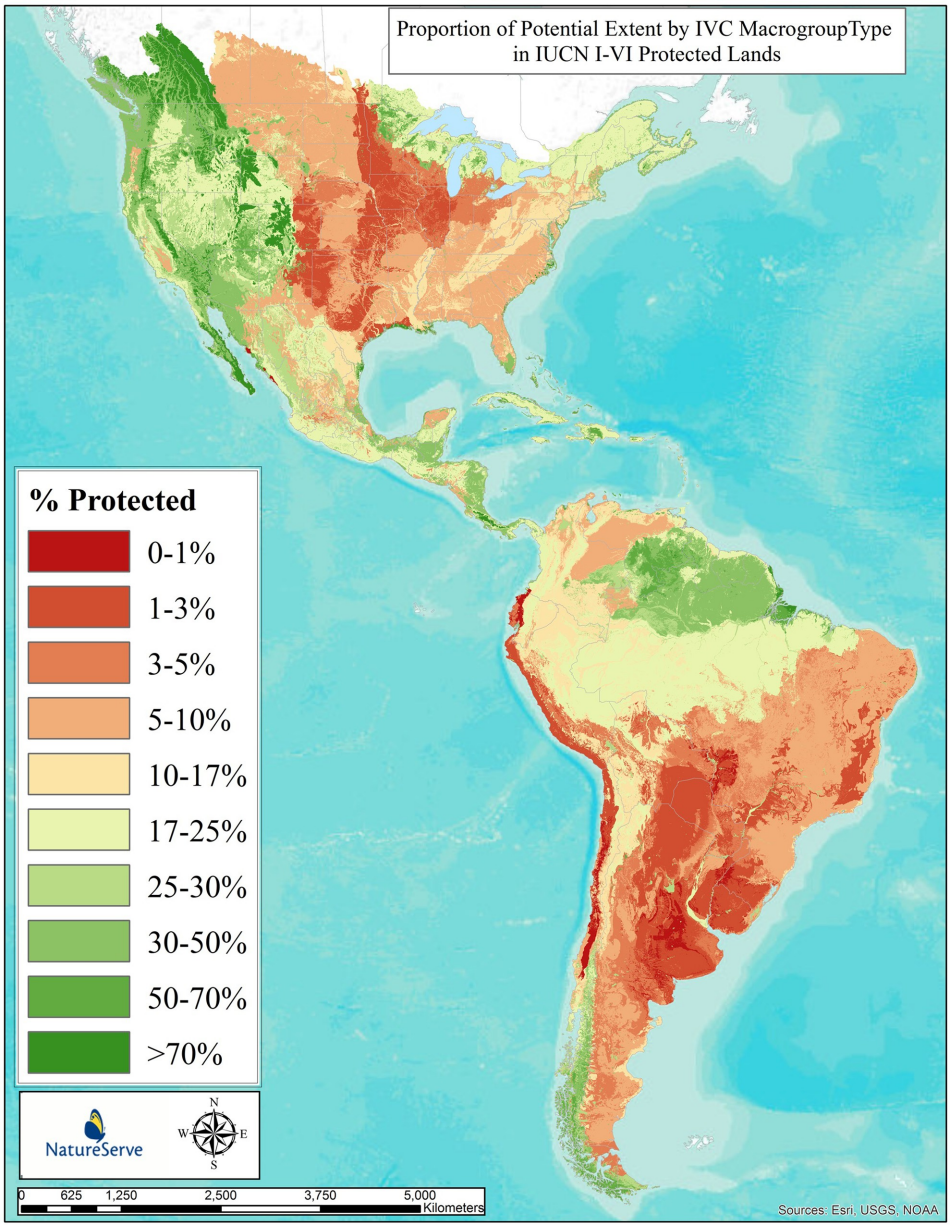
Proportion of potential extent by IVC macrogroup in IUCN I-VI protected lands. Proportion of potential extent within IUCN I-VI protected lands by type of 315 vegetation macrogroups across North and South America. Individual types are displayed in terms of their individual percentage protected estimate mapped according to their potential extent, providing a generalize pattern for all types combined.

Fig 7 depicts results for representation of 315 macrogroups in protected areas of North and South America, as defined by IUCN classes I-VI. Similar to Figs 3 and 5, this form of visualizing loss takes the per-type protected representation estimate and applies that number to the potential distribution map (Fig 1) and then depicts distributions in terms of percent protected classes (>70% protected down to <1% protected).

**Fig 8:**
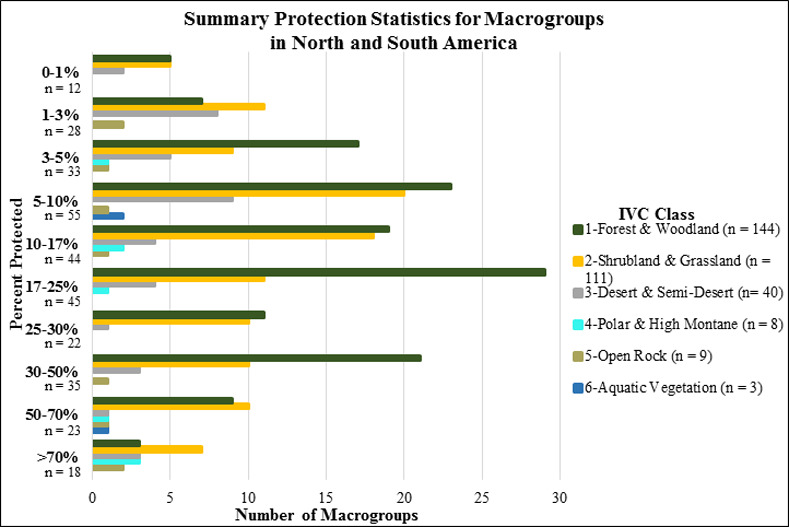
Summary protection statistics for macrogroups in North and South America. (number of types by IVC Class by proportion of potential extent occurring within IUCN I-VI lands).

The proportional extent protected of macrogroup types in North and South America varied by major vegetation class and across the continental geography. Some 41 macrogroup types (13%) scored in the >50% protected category (Fig 8). These included Rocky Mountain Subalpine-High Montane Forest, Intermountain Singleleaf Pinyon—Juniper Woodland, Viscaino-Baja California Desert Scrub, Central Guianan Montane Humid Forest, Amazon Delta Swamp Forest, and Magellanian Montane Tundra (S1 Appendix). Eleven types (11%) scored in the 30–50% protected category. These included Northern Amazon Humid Forest, Eastern Guianan Humid Forest, Mesoamerican Lowland Humid Forest, Central Rocky Mountain Dry Lower Montane-Foothill Forest, and Mojave-Sonoran Semi-Desert Scrub. Sixty-seven types (21.3%) scored in the 17–30% protected category. Among many other types, some of the most extensive types include Central Amazon Humid Forest, Great Basin-Intermountain Tall Sagebrush Steppe & Shrubland, Laurentian-Acadian Mesic Hardwood—Conifer Forest, and Xeric Puna Succulent Scrub. Forty-four types (14%) scored in the 10–17% protected category. These include Appalachian-Northeastern Oak—Hardwood–Pine Forest & Woodland, Western Amazon Lowland Humid Forest, and Guajiran Seasonal Dry Forest. Fifty-five types (17.4%) scored in the 5–10% protected category. These include Great Plains Mixedgrass & Fescue Prairie, Southern & South-Central Oak–Pine Forest & Woodland, Cerrado Savanna, Caatinga Seasonal Dry Forest, and Patagonian Dry Grassland & Shrubland. Thirty-three types (10.5%) were in the 3–5% protected category. Twenty-eight (8.8%) were in the 1–3% protected category, and 12 types (3.8%) scored in the 0–1% protected category. Among the most extensive types in this unprotected category are concentrated in the Southern Cone of South America, including Pampean Freshwater Marsh, Wet Meadow & Shrubland, Espinal Deciduous Forest & Woodland, Pantanal Floodplain Forest, Chilean Mediterranean Sclerophyllous Forest, Western Ecuadorian Humid Forest, and Southern Chaco Xeromorphic Scrub & Savanna.

Overall, 111 types (45.3%) scored at or above the 17% protection category, as described by Aichi Target 11 of the Convention on Biodiversity. On the other hand, fully 128 types (40.6%) have less than 10% of their potential extent represented in protected areas. Therefore, as measured through vegetation macrogroups, there appears to be substantial work yet to be done to achieve the intended 2020 target in the Americas.

#### Terrestrial ecosystem types

**Fig 9:**
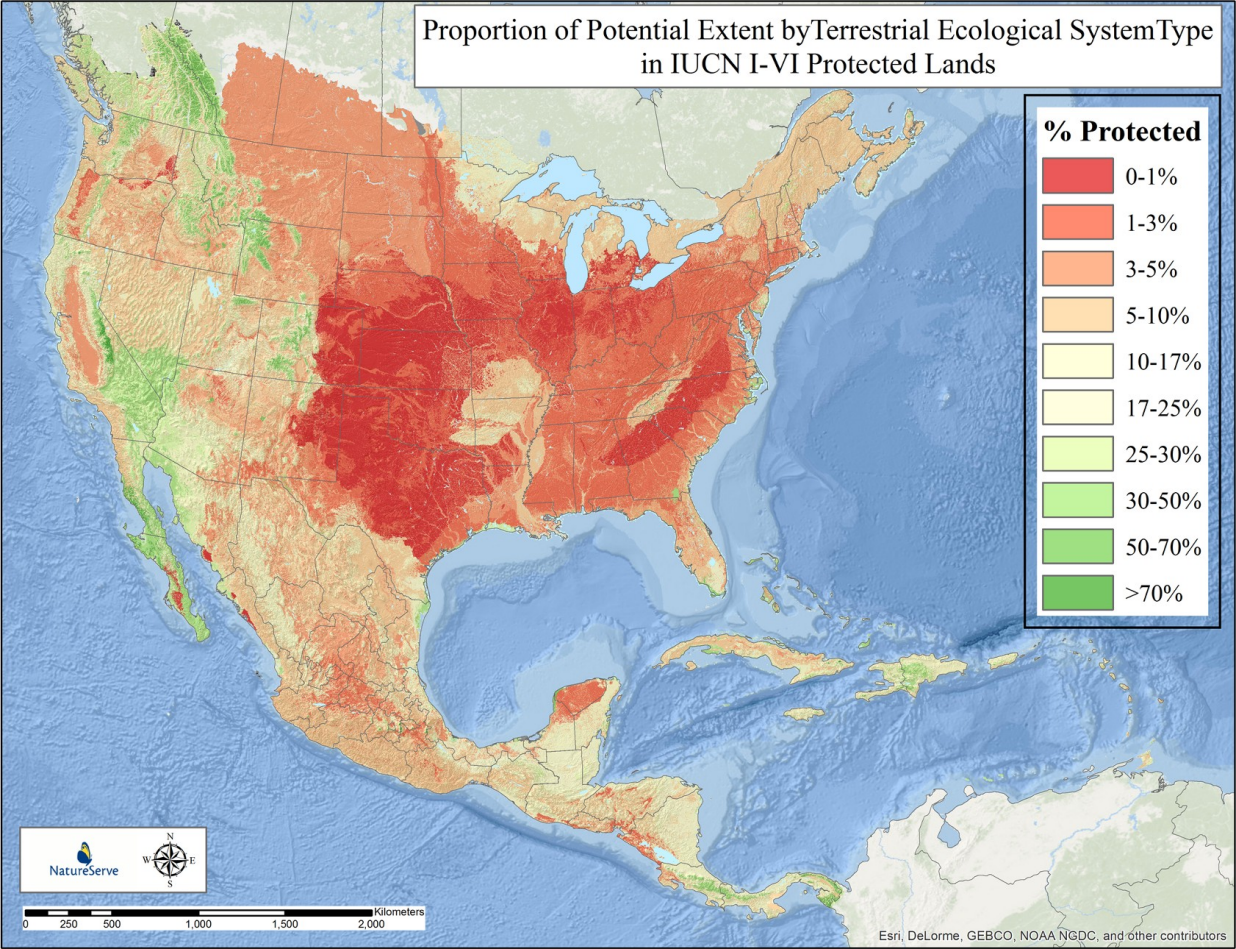
Proportion of potential extent by terrestrial ecological system in IUCN I-VI protected lands. Proportion of potential extent within IUCN I-VI protected lands by type of 623 terrestrial ecological system units in Temperate and Tropical North America. Individual types are displayed in terms of their individual percentage protected estimate mapped according to their potential extent, providing a generalize pattern for all types combined.

**Fig 10:**
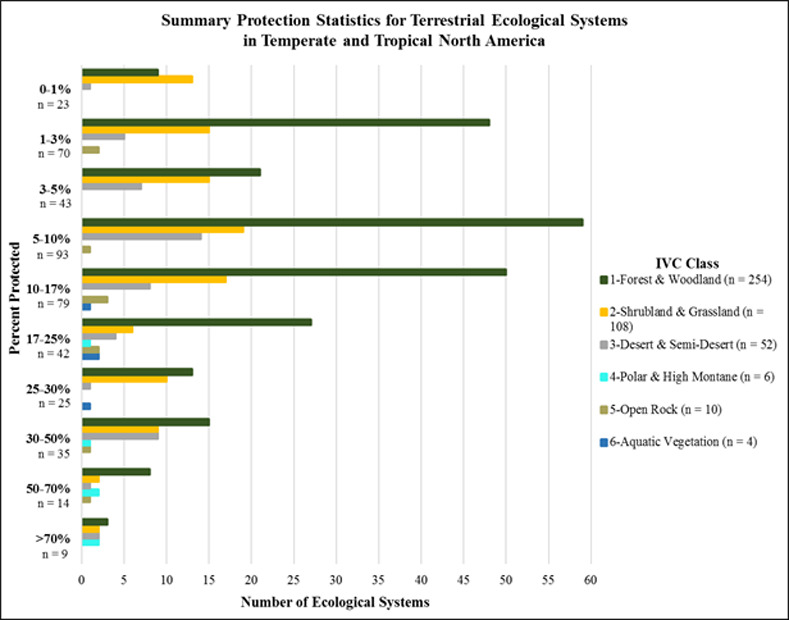
Summary protection statistics for terrestrial ecological systems in temperate and tropical North America. (number of types by IVC Class by proportion of potential extent occurring within IUCN I-VI lands).

The proportional extent protected of ecological system types in North America and the Caribbean also varied by major vegetation class and across the continental geography (Fig 9). Some 13 system types (5.3%) scored in the >50% protected category (Fig 10). These included Northern Rocky Mountain Subalpine Woodland and Parkland, Mediterranean California Subalpine Woodland, Northern Viscaino Coastal Plain Maguey-Boojum Desert Scrub, and Premontane Cloud Forest (*Bosque Pluvial Premontano)* in the Chocó-Darién (S2 Appendix). Thirty-five types (8.9%) scored in the 30–50% protected category. These included Rocky Mountain Subalpine Dry-Mesic Spruce-Fir Forest and Woodland, Mojave Mid-Elevation Mixed Desert Scrub, Caribbean Coastal Mangrove, Hispaniola Montane and Upper Montane Pine Forest, and Talamancan Lower Montane Wet Oak Forest. Sixty-seven types (15.4%) scored in the 17–30% protected category. Among many other types, these included Sonora-Mojave Creosotebush-White Bursage Desert Scrub, Colorado Plateau Mixed Bedrock Canyon and Tableland, Central American Caribbean Evergreen Lowland Forest, and Petén Lowland Alluvial Seasonal Forest on Calcareous Soil. Eighty types (18.4%) scored in the 10–17% protected category. These include Meso-American Premontane Semi-deciduous Forest, Sonoran Paloverde-Mixed Cacti Desert Scrub, Madrean Lower Montane Pine-Oak Forest and Woodland, and Caribbean Wet Submontane/Lowland Forest. Ninety-three types (21.4%) scored in the 5–10% protected category. These include Inter-Mountain Basins Big Sagebrush Shrubland, Laurentian-Acadian Northern Hardwood Forest, Guerreran Dry Deciduous Forest, Sinaloan Dry Deciduous Forest, and Mexican Upper Montane Pine-Oak Forest and Woodland. Forty-three types (9.9%) were in the 3–5% protected category. Seventy types (16.1%) were in the 1–3% protected category, and 23 types (5.3%) scored in the 0–1% protected category. These include Southeastern Great Plains Tallgrass Prairie, Southern Piedmont Dry Oak-(Pine) Forest and Woodland, North-Central Interior Beech-Maple Forest, Texas Blackland Tallgrass Prairie, and East Gulf Coastal Plain Jackson Plain Prairie and Barrens.

Overall, 125 types (28.8%) scored at or above the 17% protection category, as described by Aichi Target 11 of the Convention on Biodiversity. On the other hand, fully 229 types (52.7%) have less than 10% of their potential extent represented in protected areas. Similar to results for macrogroups on a hemispheric scale, if measured through ecological systems, there appears to be substantial work yet to be done to achieve the intended 2020 target in North America.

## Discussion

### Ecosystem loss

This study provides one benchmark for approximating potential or pre-industrial historical extent of most terrestrial ecosystems across much of the Americas; excluding boreal and arctic North America. The “potential” distribution maps cannot fully account for the vagaries of human population densities and land uses in concentrated areas throughout the Holocene [36, 37], but can provide one input to these estimates, and these estimates could be especially meaningful for ecosystem types that have been severely converted or degraded in recent centuries.

As compared with analogous global efforts [38] we used data of substantially finer thematic and spatial resolution. We therefore provide much greater detail and reliability in our estimates. This study clearly shows that, over the past several centuries, ecosystem conversion has been concentrated in most productive and accessible lands throughout the hemisphere. These results largely conform with prior estimates of severe and concentrated loss of temperate grasslands in both North America [39–41], and the South American Pampas [42]. The results also roughly coincide with prior estimates for forests and woodlands across the eastern USA [43–44] and Brazil’s Atlantic Forests [45] and Cerrado [46], as well as in tropical dry forests [47], and the coastal woodlands and shrublands of Chile [48]. Nearly 21% of vegetation macrogroups and 33% of ecological system types, respectively, have lost >50% of their potential extent; thus, crossing thresholds of at least Vulnerable status under the IUCN Red List of Ecosystems.

However, our primary contribution is a standardized classification of terrestrial ecosystems on a near-hemispheric scale, and a set of maps that match those standards. Providing a complement to prior efforts that may have been more anecdotal, qualitative, and/or limited to political jurisdiction, we now have a mapped basis and quantitative measures for a large proportion of recognized ecosystem types across the hemisphere. In effect, for the first time, we have a “common currency” for describing status and trend in terrestrial ecosystems that harmonized concepts across political jurisdictions and is readily linked to emerging global standards [13]. Our approach–utilizing hierarchically structured ecological classification—brings flexibility for application to a range of conservation-related decision-making processes. For example, the IVC macrogroup level (along with higher levels of the hierarchy) may be completely appropriate for defining and global tracking of Essential Biodiversity Variables for ecosystem composition [14]. Meanwhile, finer levels of classification, as illustrated here with ecological systems, are more likely suitable for IUCN ecosystem red listing [12], as well as for carrying forward into ecosystem restoration on the ground [49].

### Ecosystem protection

Given trends in ecosystem loss, one primary response is to ensure that some proportion of their remaining extent is secured or restored within some form of protected land with a management emphasis on conservation. Investments in many country’s conservation lands are known to be concentrated among ecosystems with limited potential for agriculture [50]. We identified patterns in conservation land protection that are roughly similar to those of long-term loss, where opportunities for protection roughly correspond with the amount of ecosystem remaining. These results also largely conform with related analyses at national scales [51–52, 29]. Overall, large proportions of ecosystem types are severely under-represented in protected lands, at least as defined using the combined IUCN I-VI categories. These maps should provide increasingly precise direction as to where conservation actions could be concentrated to ensure adequate representation of ecological diversity within protected areas. With some 41% of macrogroups and 53% of ecological systems occurring with <10% under protective status, we are substantially underachieving the 17% goal set out under Aichi target 11.

The results reported here also do not differentiate among the IUCN I-VI categories that represent a wide spectrum of protective actions on the ground, from strict nature reserves (I) to protected areas with sustainable natural resource use (VI). Therefore, some ecosystems could be found with relatively high overall proportions protected, but those areas could be skewed toward one end of the spectrum or another.

Importantly, this measure captures proportion of the *potential* extent currently protected, as opposed to simply considering the proportion of *current* extent currently protected, as is common in many “gap analysis” efforts [33]. This is of particular interest where high rates of ecosystem conversion have already occurred and there is a desire to report on relative protection status ecosystem diversity under Aichi target 11 of the Convention on Biodiversity [5, 4]. There, recommendations to protect “at least 17%” of representative and connected ecosystems might be rigorously addressed using both potential and current distribution maps for terrestrial ecosystem types. One recent example of the application of these data applied to prioritizing places for conservation action in temperate grasslands of the North American Great Plains [53].

These same data may be combined with others depicting human footprint information [54] to better indicate where current extent of ecosystems is more or less likely to be in poor condition and to indicate trends in ecosystem degradation.

### Limitations of data and methods

This study represents the first approximation of long-term trends in extent and protection for terrestrial ecosystem types across the Americas where ecosystem types have been defined at relatively fine thematic resolution. Given constraints of available resources and knowledge, this effort should be augmented and refined by subsequent analysis with advancing global/continental input data and/or targeted mapping at more local scales. The location and extent of ecosystem types depicted by our potential distribution maps should approximate patterns in terrestrial ecosystems where they would occur today had there not been intensive agriculture and urban land uses that have characterized the modern technologies. That being said, users of this map should acknowledge inherent limitations in data used in modeling. Error in those data could propagate to the application of subsequent assessments. For example, as discussed in supplementary materials (S3 Appendix–Map Methods Detail), there were practical limitations on modeling of types that occur in relatively small fragments. These could include threatened ecosystems with naturally small distributions or linear patterns (such as in riparian and coastal ecosystems) with critically diminished distributions.

In addition, these maps depict current knowledge of terrestrial ecosystem types defined using the structure and approach of current classification systems. Ongoing investment is required for ecological classification, description, and characterization of reference conditions for key ecological attributes of species composition, geophysical settings, and dynamic ecological processes, in order to advance our understanding of these ecosystem types and determine if any prior assumptions were in error. One example where there is an ongoing collaboration in vegetation classification development is with the United States National Vegetation Classification [55]. There, methods, tools, and expert roles are in place to facilitate advancement of the classification.

Users of this analysis should take care to compare classification concepts from other local studies to the concepts used here. For example, in one prior treatment of Brazilian Atlantic Forests [56], all forest area within coastal ecoregions were treated together, with approximate historic extent of 1.5M km^2^. In contrast, our analysis includes forty distinct types in this same area; some with affinities for adjacent Cerrado, Caatinga, and Parana regions. Several forest macrogroups classified with floristic affinities for the Brazilian Atlantic Forest (dry, humid, montane forest, and open savanna) have a total estimated historical extent around 700,000 km^2^. This example illustrates where both conceptual and classification issues, as well as technical issues of map production and area estimation, all likely factor into explaining differing estimates.

Another source of complexity in comparing these findings with other studies comes from the reality that much of the current extent of major vegetation types are altered and degraded at varying levels of severity. This introduces considerable uncertainty and inconsistency. For example, while most prior treatments of longleaf pine woodlands in the southeast USA estimate loss >90%, our estimates for the several distinct classified ecological system types varied from 60% to >95. Much of this difference could be explained by the large proportion of severely degraded and altered forest that stands where longleaf pine woodlands likely occurred until the 20^th^ century [57]. We anticipate many similar cases to this, where prior studies of a particular ecosystem type discounted current areas where it in fact remains, but in severely degraded form.

Our sequential approach using inductive modeling, utilizing multiple hierarchical levels of ecological classification, followed by expert review and refinement, appears to provide an adequate map product for the intended purpose of continental-or regional-scale assessment. The strength of this approach is that it takes full advantage of local vegetation maps, available field reference samples, and global map surfaces–mostly satellite derived–to simultaneously generate plausible distributions of all map classes. This facilitates iterative steps of review and refinement as errors are detected and/or new input data become available. Depending on the degree of local knowledge and mapped information, this method could be applied anywhere in the world. As noted in S3 Appendix, the first attempt to apply this methodology was for the African continent [58]. By comparison, there are a greater number of studies involving ecological classification and mapping in the Americas. However, initial efforts here and in Africa indicate much about the feasibility of applying this method anywhere in the world.

A primary limitation of this method is that the sequential modeling still required expert review and refinement. While this is difficult to complete in a fully repeatable manner, the need for regional expertise in each ecosystem type remains and introduces the subjective judgment that may not be consistent across individual experts. Therefore, the approach remains vulnerable to some degree based upon the individuals involved in the mapping effort. The primary remedy to this situation is to provide more opportunities for engagement and review of the mapping process [59].

A second limitation is the potential error introduced by spatially skewed reference sample data. Since reference samples are the foundation of inductive models, particularly uncommon types may be poorly represented among available samples. Spatial skew in these can result in distorted model output that may not be apparent, even to those most expert in a given ecosystem type. Errors of commission are most likely for the dominant types, while errors of omission are most likely for rare types [60]. In order to address these deficiencies, considerable new effort is needed to acquire reliable georeferenced data for all vegetation types we desire on our map legends. These data could come through increased sharing among researchers, coordinated and targeted field campaigns, and tapping the potential for acquisition through technology and citizen science.

Finally, given the results of map validation, applied at the three levels of spatial resolution (point location vs. 1km^2^ vs. 5km^2^), the intended use of the map at 270m to 450m pixel resolutions for trend assessment appears to be supported. One could augment our analysis by deploying a fuzzy-set approach to map evaluation [61]. These approaches can account for relative similarity among map classes so that error is viewed in light of that relative similarity. This is increasingly relevant in vegetation mapping where the level of thematic detail matches those that were treated in this study.

## Conclusions

There is an increasingly urgent need to document trends in extent, condition, and protection status for ecosystem types to support public policy and conservation action. A robust map representation of ecosystem diversity enables measurement of the primary evaluation criteria under the IUCN Red List of Ecosystems, and results provide a key indication of ecosystem health and sustainability. Gauging conservation actions by ecosystem type, such as is depicted here using IUCN protected area categories, provides appropriately precise indication of conservation investment by governments and civil society that should point the way toward new investments that are efficient and effective.

This effort in the Americas—encompassing 22% of the global land surface—demonstrates methods and outputs suitable for worldwide application at continental scales; albeit more challenging in parts of the globe with a more limited history of ecosystem classification and mapping. We hope that the rich text, tabular, and map data set accompanying this study provide a foundation for deepened analysis and conservation action across the Americas.

## Supporting information

S1 Appendix (DOCX)

S2 Appendix (DOCX)

S3 Appendix (DOCX)

S4 Appendix (DOCX)

S5 Appendix (XLSX)

S6 Appendix (XLSX)
