# Supplementary material for: Long-term loss in extent and current protection of terrestrial ecosystem diversity in the temperate and tropical Americas
Source: PLoS One. 2020 Jun 30;15(6):e0234960. doi: 10.1371/journal.pone.0234960 (PMC7326196; doi:10.1371/journal.pone.0234960)
Supplement: S1 Appendix — (DOCX) [file pone.0234960.s001.docx]

S1 Table of Contents

[1. Forest & Woodland 11](#_Toc532988054)

[1.A. Tropical Forest & Woodland 11](#_Toc532988055)

[1.A.1. Tropical Dry Forest & Woodland 11](#_Toc532988056)

[D099. Caribbean-Mesoamerican Dry Forest & Woodland 11](#_Toc532988057)

[M296. Caribbean-Mesoamerican Pine Dry Forest 17](#_Toc532988058)

[M134. Caribbean Coastal Lowland Dry Forest 21](#_Toc532988059)

[M294. Caribbean Dry Limestone Forest 25](#_Toc532988060)

[M561. Caribbean-Mesoamerican Seasonal Dry Forest 26](#_Toc532988061)

[M562. Pacific Mesoamerican Seasonal Dry Forest 28](#_Toc532988062)

[D219. Colombian-Venezuelan Dry Forest 30](#_Toc532988063)

[M563. Guajiran Seasonal Dry Forest 32](#_Toc532988064)

[M565. Llanos Seasonal Dry Forest 34](#_Toc532988065)

[M566. Tumbes Guayaquil Seasonal Dry Forest 36](#_Toc532988066)

[M573. Northern Andean Seasonal Dry Forest 38](#_Toc532988067)

[D220. Guianan Dry Forest 40](#_Toc532988068)

[M567. Central Guianan Seasonal Dry Forest 42](#_Toc532988069)

[D221. Brazilian-Parana Dry Forest 43](#_Toc532988070)

[M572. Caatinga Seasonal Dry Forest 45](#_Toc532988071)

[M872. Cerradâo Sclerophyllous Woodland 47](#_Toc532988072)

[M570. Cerrado Seasonal Dry Forest 49](#_Toc532988073)

[M568. Brazilian Atlantic Seasonal Dry Forest 51](#_Toc532988074)

[M571. Parana Seasonal Dry Forest 53](#_Toc532988075)

[D222. Tropical Andean Montane Dry Forest 55](#_Toc532988076)

[M575. Bolivian-Tucuman Seasonal Dry Forest 56](#_Toc532988077)

[M574. Central Andean Seasonal Dry Forest 58](#_Toc532988078)

[1.A.2. Tropical Lowland Humid Forest 60](#_Toc532988079)

[D091. Caribbean-Mesoamerican Lowland Humid Forest [Low - Poorly Documented] 60](#_Toc532988080)

[M281. Caribbean Lowland Humid Forest [Low - Poorly Documented] 62](#_Toc532988081)

[M578. Mesoamerican Lowland Humid Forest 64](#_Toc532988082)

[M873. Mesoamerican Submontane Humid Forest 68](#_Toc532988083)

[D224. Colombian-Venezuelan Lowland Humid Forest 70](#_Toc532988084)

[M581. Choco-Darien Humid Forest 71](#_Toc532988085)

[M582. Western Ecuadorian Humid Forest 73](#_Toc532988086)

[M580. Catatumbo Magdalena Humid Forest 75](#_Toc532988087)

[M579. Guajiran Humid Forest 77](#_Toc532988088)

[M583. Llanos Humid Forest 79](#_Toc532988089)

[D225. Guianan Lowland Humid Forest 81](#_Toc532988090)

[M586. Eastern Guianan Humid Forest 83](#_Toc532988091)

[M585. Central Guianan Humid Forest 84](#_Toc532988092)

[M584. Western Guianan Humid Forest 86](#_Toc532988093)

[M587. Orinoquian Humid Forest 88](#_Toc532988094)

[D226. Amazonian Lowland Humid Forest 90](#_Toc532988095)

[M593. Central Amazon Humid Forest 92](#_Toc532988096)

[M592. Northern Amazon Humid Forest 93](#_Toc532988097)

[M590. Southwestern Amazon Lowland Humid Forest 95](#_Toc532988098)

[M591. Southwestern Amazon Subandean Humid Forest 97](#_Toc532988099)

[M588. Western Amazon Lowland Humid Forest 99](#_Toc532988100)

[M589. Western Amazon Subandean Humid Forest 101](#_Toc532988101)

[D227. Brazilian-Parana Lowland Humid Forest 102](#_Toc532988102)

[M597. Cerrado Humid Forest 104](#_Toc532988103)

[M595. Brazilian Atlantic Humid Forest 106](#_Toc532988104)

[M596. Parana Humid Forest 108](#_Toc532988105)

[1.A.3. Tropical Montane Humid Forest 110](#_Toc532988106)

[D228. Caribbean-Mesoamerican Montane Humid Forest [Low - Poorly Documented] 110](#_Toc532988107)

[M598. Caribbean Montane Humid Forest [Low - Poorly Documented] 112](#_Toc532988108)

[M601. Mesoamerican Montane Pine-Oak Forest 114](#_Toc532988109)

[M600. Mesoamerican Montane Humid Forest 116](#_Toc532988110)

[M602. Southern Mesoamerican Montane Humid Forest 118](#_Toc532988111)

[D229. Guianan Montane Humid Forest 121](#_Toc532988112)

[M604. Eastern Guianan Montane Humid Forest 122](#_Toc532988113)

[M603. Central Guianan Montane Humid Forest 124](#_Toc532988114)

[D231. Tropical Andean Montane Humid Forest 126](#_Toc532988115)

[M613. Bolivian-Tucuman Lower Montane Humid Forest 128](#_Toc532988116)

[M612. Bolivian-Tucuman Montane & Upper Montane Humid Forest 130](#_Toc532988117)

[M611. Central Andean (Yungas) Lower Montane Humid Forest 132](#_Toc532988118)

[M610. Central Andean (Yungas) Montane & Upper Montane Humid Forest 134](#_Toc532988119)

[M615. Eastern Subandean Ridge Montane Humid Forest 135](#_Toc532988120)

[M614. Moist Puna Humid Forest 137](#_Toc532988121)

[M607. Northern Andean Lower Montane Humid Forest 139](#_Toc532988122)

[M606. Northern Andean Montane & Upper Montane Humid Forest 141](#_Toc532988123)

[M609. Northern Andean Venezuelan Coastal Ridge Forest 143](#_Toc532988124)

[D232. Brazilian-Parana Montane Humid Forest 144](#_Toc532988125)

[M616. Brazilian Atlantic Montane Humid Forest 146](#_Toc532988126)

[1.A.4. Tropical Flooded & Swamp Forest 148](#_Toc532988127)

[D093. Caribbean-Central American Flooded & Swamp Forest 148](#_Toc532988128)

[M618. Caribbean Floodplain Forest [Low - Poorly Documented] 150](#_Toc532988129)

[M617. Caribbean Swamp Forest 152](#_Toc532988130)

[M620. Mesoamerican Floodplain Forest 155](#_Toc532988131)

[M619. Mesoamerican Coastal Plain Swamp Forest 158](#_Toc532988132)

[D233. Colombian-Venezuelan Flooded & Swamp Forest 161](#_Toc532988133)

[M622. Choco-Darien Floodplain Forest 162](#_Toc532988134)

[M621. Guajiran Flooded Forest 165](#_Toc532988135)

[M625. Guayaquil Flooded & Swamp Forest 167](#_Toc532988136)

[M624. Llanos Flooded & Swamp Forest 168](#_Toc532988137)

[D234. Guianan Flooded & Swamp Forest 170](#_Toc532988138)

[M626. Guianan Riparian Forest 172](#_Toc532988139)

[M627. Guianan Swamp Forest 174](#_Toc532988140)

[M628. Orinoco Delta Swamp Forest 176](#_Toc532988141)

[D235. Tropical Andean Riparian & Flooded Forest 178](#_Toc532988142)

[M631. Bolivian-Tucuman Dry Valley Riparian Forest 179](#_Toc532988143)

[M632. Eastern Subandean Ridge Flooded Forest 181](#_Toc532988144)

[M630. Central Andean Riparian Forest 183](#_Toc532988145)

[M629. Northern Andean Riparian Forest 185](#_Toc532988146)

[D236. Amazonian Flooded & Swamp Forest 186](#_Toc532988147)

[M640. Amazon Delta Swamp Forest 188](#_Toc532988148)

[M638. Central Amazon Floodplain Forest 190](#_Toc532988149)

[M637. Northern Amazon Floodplain Forest 192](#_Toc532988150)

[M639. South-Central Amazon Floodplain Forest 194](#_Toc532988151)

[M636. Southern Amazon Swamp Forest 196](#_Toc532988152)

[M635. Southwestern Amazon Floodplain Forest 197](#_Toc532988153)

[M633. Western Amazon Floodplain Forest 199](#_Toc532988154)

[M634. Western Amazon Swamp Forest 201](#_Toc532988155)

[D237. Brazilian-Parana Flooded & Swamp Forest 203](#_Toc532988156)

[M641. Brazilian Atlantic Coastal Plain Swamp Forest 205](#_Toc532988157)

[M642. Parana Floodplain Forest 207](#_Toc532988158)

[M646. Pantanal Floodplain Forest 209](#_Toc532988159)

[M643. Cerrado Floodplain Forest 211](#_Toc532988160)

[M644. Beni Chiquitano Swamp Forest 213](#_Toc532988161)

[M645. Beni Floodplain Forest 214](#_Toc532988162)

[D238. Chaco Flooded & Swamp Forest & Woodland 216](#_Toc532988163)

[M650. Southern Chaco Floodplain Forest & Woodland 218](#_Toc532988164)

[M647. Northern Chaco Floodplain Forest & Woodland 220](#_Toc532988165)

[M649. Northern Chaco Palm Swamp 222](#_Toc532988166)

[M648. Northern Chaco Riparian Scrub & Woodland 224](#_Toc532988167)

[1.B. Temperate & Boreal Forest & Woodland 225](#_Toc532988168)

[1.B.1. Warm Temperate Forest & Woodland 225](#_Toc532988169)

[D239. Chilean Warm Temperate Forest & Woodland 226](#_Toc532988170)

[M652. Chilean Mediterranean Sclerophyllous Forest 227](#_Toc532988171)

[M653. Chilean Mediterranean Deciduous Forest 229](#_Toc532988172)

[D240. Southeastern South American Warm Temperate Forest & Woodland 231](#_Toc532988173)

[M654. Espinal Deciduous Forest & Woodland 233](#_Toc532988174)

[D006. Southeastern North American Forest & Woodland 235](#_Toc532988175)

[M885. Southeastern Coastal Plain Evergreen Oak - Mixed Hardwood Forest 239](#_Toc532988176)

[1.B.2. Cool Temperate Forest & Woodland 241](#_Toc532988177)

[D241. Valdivian Cool Temperate Forest 241](#_Toc532988178)

[M656. Valdivian Lower Montane Deciduous Forest 243](#_Toc532988179)

[M655. Valdivian Lower Montane Evergreen Forest 245](#_Toc532988180)

[M657. Valdivian Montane & Upper Montane Deciduous Forest 247](#_Toc532988181)

[M658. Valdivian Montane & Upper Montane Evergreen Forest 249](#_Toc532988182)

[D242. Magellanian Cool Temperate Forest 251](#_Toc532988183)

[M659. Magellanian Temperate Evergreen Forest 252](#_Toc532988184)

[D008. Eastern North American Forest & Woodland 254](#_Toc532988185)

[M502. Appalachian-Northeastern Oak - Hardwood - Pine Forest & Woodland 257](#_Toc532988186)

[M883. Appalachian-Interior-Northeastern Mesic Forest 261](#_Toc532988187)

[M882. Central Midwest Mesic Forest 263](#_Toc532988188)

[M159. Laurentian-Acadian Pine - Hardwood Forest & Woodland 266](#_Toc532988189)

[D194. Rocky Mountain Forest & Woodland 269](#_Toc532988190)

[M501. Central Rocky Mountain Dry Lower Montane-Foothill Forest 274](#_Toc532988191)

[M500. Central Rocky Mountain Mesic Lower Montane Forest 283](#_Toc532988192)

[D192. Vancouverian Forest & Woodland 288](#_Toc532988193)

[M886. Southern Vancouverian Dry Foothill Forest & Woodland 293](#_Toc532988194)

[D326. North American Great Plains Forest & Woodland 295](#_Toc532988195)

[M151. Great Plains Forest & Woodland 298](#_Toc532988196)

[1.B.3. Temperate Flooded & Swamp Forest 301](#_Toc532988197)

[D243. Pampean Temperate Flooded & Swamp Forest 301](#_Toc532988198)

[M661. Espinal Floodplain Forest 303](#_Toc532988199)

[D244. Chilean Mediterranean Flooded & Swamp Forest 305](#_Toc532988200)

[M662. Chilean Mediterranean & Desert Riparian & Flooded Forest 307](#_Toc532988201)

[D245. Valdivian Temperate Flooded & Swamp Forest 308](#_Toc532988202)

[M663. Valdivian Temperate Flooded & Swamp Forest 310](#_Toc532988203)

[D246. Northern Patagonian Flooded Forest 312](#_Toc532988204)

[M664. Monte Floodplain Forest 314](#_Toc532988205)

[D011. Eastern North American-Great Plains Flooded & Swamp Forest 316](#_Toc532988206)

[M503. Central Hardwood Swamp Forest 319](#_Toc532988207)

[M504. Laurentian-Acadian-North Atlantic Coastal Flooded & Swamp Forest 322](#_Toc532988208)

[D062. Southeastern North American Flooded & Swamp Forest 327](#_Toc532988209)

[M161. Pond-cypress Basin Swamp 330](#_Toc532988210)

[M033. Southern Coastal Plain Basin Swamp & Flatwoods 333](#_Toc532988211)

[M154. Southern Great Plains Floodplain Forest & Woodland 337](#_Toc532988212)

[D195. Rocky Mountain-Great Basin Montane Flooded & Swamp Forest 340](#_Toc532988213)

[M034. Rocky Mountain-Great Basin Montane Riparian & Swamp Forest 343](#_Toc532988214)

[D013. Western North American Interior Flooded Forest 348](#_Toc532988215)

[M660. Mexican Interior Riparian Forest 351](#_Toc532988216)

[M036. Interior Warm & Cool Desert Riparian Forest 353](#_Toc532988217)

[D193. Vancouverian Flooded & Swamp Forest 356](#_Toc532988218)

[M035. Vancouverian Flooded & Swamp Forest 360](#_Toc532988219)

[1.B.4. Boreal Forest & Woodland 364](#_Toc532988220)

[D014. North American Boreal Forest & Woodland 364](#_Toc532988221)

[M495. Eastern North American Boreal Forest 369](#_Toc532988222)

[M496. West-Central North American Boreal Forest & Woodland 374](#_Toc532988223)

[1.B.5. Boreal Flooded & Swamp Forest 384](#_Toc532988224)

[D016. North American Boreal Flooded & Swamp Forest 384](#_Toc532988225)

[M299. North American Boreal Conifer Poor Swamp 388](#_Toc532988226)

[2. Shrub & Herb Vegetation 391](#_Toc532988227)

[2.A. Tropical Grassland, Savanna & Shrubland 391](#_Toc532988228)

[2.A.1. Tropical Lowland Grassland, Savanna & Shrubland 391](#_Toc532988229)

[D094. Caribbean-Mesoamerican Lowland Grassland, Savanna & Shrubland 391](#_Toc532988230)

[M671. Caribbean Dry Scrub 393](#_Toc532988231)

[M672. Northern Mesoamerican Pine Savanna 396](#_Toc532988232)

[M673. Northern Mesoamerican Savanna & Shrubland 400](#_Toc532988233)

[D124. Amazonian Savanna & Shrubland 403](#_Toc532988234)

[M346. Central Amazon Savanna 405](#_Toc532988235)

[M345. Western Amazon Savanna 407](#_Toc532988236)

[D126. Brazilian-Parana Lowland Grassland, Savanna & Shrubland 408](#_Toc532988237)

[M684. Brazilian Atlantic Coastal Plain Savanna & Woodland 410](#_Toc532988238)

[M688. Parana Upland Savanna & Shrubland 412](#_Toc532988239)

[M685. Cerrado Savanna 414](#_Toc532988240)

[D249. Colombian-Venezuelan Lowland Grassland, Savanna & Shrubland 416](#_Toc532988241)

[M676. Llanos Upland Savanna 418](#_Toc532988242)

[D250. Guianan Lowland & Upland Grassland, Savanna & Shrubland 420](#_Toc532988243)

[M681. Eastern Guianan Savanna & Shrubland 421](#_Toc532988244)

[M679. Central Guianan Savanna & Shrubland 423](#_Toc532988245)

[M680. Western Guianan Savanna& Shrubland 425](#_Toc532988246)

[2.A.2. Tropical Montane Grassland & Shrubland 427](#_Toc532988247)

[D134. Tropical Andean Grassland & Shrubland 427](#_Toc532988248)

[M377. Bolivian-Tucuman Montane Grassland & Shrubland 429](#_Toc532988249)

[M696. Central Andean (Yungas) Upper Montane Grassland & Shrubland 431](#_Toc532988250)

[M375. Northern Andean Montane & Upper Montane Grassland & Shrubland 433](#_Toc532988251)

[M378. Moist Puna Grassland & Scrub 434](#_Toc532988252)

[M694. Northern Andean Paramo 436](#_Toc532988253)

[D135. Caribbean-Mesoamerican Montane & High Montane Grassland & Shrubland [Low - Poorly Documented] 438](#_Toc532988254)

[M691. Mesoamerican Montane Grassland & Shrubland 440](#_Toc532988255)

[D252. Guianan Montane Grassland & Shrubland 441](#_Toc532988256)

[M693. Tepuyan Mesic Grass & Forb Meadow 443](#_Toc532988257)

[M692. Tepuyan Sclerophyllous Shrubland 445](#_Toc532988258)

[D253. Brazilian-Parana Montane Grassland & Shrubland 447](#_Toc532988259)

[M699. Brazilian-Parana Montane Grassland, Savanna & Forb Meadow 449](#_Toc532988260)

[2.A.3. Tropical Scrub & Herb Coastal Vegetation 450](#_Toc532988261)

[D254. Caribbean-Mesoamerican Dune & Coastal Grassland & Shrubland 451](#_Toc532988262)

[M700. Caribbean-Mesoamerican Coastal Dune & Beach 452](#_Toc532988263)

[D255. Tropical Western Atlantic Dune & Coastal Grassland & Shrubland 455](#_Toc532988264)

[M702. Brazilian Atlantic Coastal Beach & Dune 457](#_Toc532988265)

[M701. Eastern Guianan Coastal Rocky Shore & Beach 459](#_Toc532988266)

[D256. Tropical Eastern Pacific Dune & Coastal Grassland & Shrubland 461](#_Toc532988267)

[M703. Tropical Eastern Pacific Coastal Beach & Dune 463](#_Toc532988268)

[2.B. Temperate & Boreal Grassland & Shrubland 464](#_Toc532988269)

[2.B.1. Mediterranean Scrub & Grassland 465](#_Toc532988270)

[D273. Chilean Mediterranean Scrub, Grassland & Forb Meadow 465](#_Toc532988271)

[M742. Central Chilean Interior Scrub 466](#_Toc532988272)

[M741. Central Chilean Coastal Scrub 468](#_Toc532988273)

[M743. Southern Andean Mediterranean Montane Scrub & Forb Meadow 470](#_Toc532988274)

[D274. Chaco-Espinal Scrub & Grassland 472](#_Toc532988275)

[M744. Chaco Serrano Scrub & Grassland 474](#_Toc532988276)

[M745. Monte Scrub & Grassland 476](#_Toc532988277)

[D327. Californian Scrub & Grassland 478](#_Toc532988278)

[M043. Californian Chaparral 485](#_Toc532988279)

[M044. Californian Coastal Scrub 488](#_Toc532988280)

[M045. Californian Annual & Perennial Grassland 492](#_Toc532988281)

[2.B.2. Temperate Grassland & Shrubland 495](#_Toc532988282)

[D141. Pampean Grassland & Shrubland 495](#_Toc532988283)

[M392. Semi-Arid Pampa Grassland & Shrubland 497](#_Toc532988284)

[M748. Humid Pampa Grassland & Shrubland 499](#_Toc532988285)

[D144. Patagonian Grassland & Shrubland 501](#_Toc532988286)

[M749. Patagonian Dry Grassland & Shrubland 503](#_Toc532988287)

[M750. Patagonian Mesic Grassland & Shrubland 505](#_Toc532988288)

[D023. Central North American Grassland & Shrubland 507](#_Toc532988289)

[M054. Central Lowlands Tallgrass Prairie 511](#_Toc532988290)

[M051. Great Plains Mixedgrass & Fescue Prairie 515](#_Toc532988291)

[M053. Western Great Plains Shortgrass Prairie 519](#_Toc532988292)

[M052. Great Plains Sand Grassland & Shrubland 525](#_Toc532988293)

[M158. Great Plains Comanchian Scrub & Open Vegetation 529](#_Toc532988294)

[D024. Eastern North American Grassland & Shrubland 531](#_Toc532988295)

[M506. Appalachian Rocky Felsic & Mafic Scrub & Grassland 536](#_Toc532988296)

[M509. Central Interior Acidic Scrub & Grassland 539](#_Toc532988297)

[M508. Central Interior Calcareous Scrub & Grassland 542](#_Toc532988298)

[M505. Laurentian-Acadian Acidic Rocky Scrub & Grassland 546](#_Toc532988299)

[M507. Laurentian-Acadian Calcareous Scrub & Grassland 550](#_Toc532988300)

[D022. Western North American Grassland & Shrubland 553](#_Toc532988301)

[M049. Southern Rocky Mountain Montane Shrubland 557](#_Toc532988302)

[M048. Central Rocky Mountain Montane-Foothill Grassland & Shrubland 560](#_Toc532988303)

[M168. Rocky Mountain-Vancouverian Subalpine-High Montane Mesic Meadow 566](#_Toc532988304)

[M172. Northern Vancouverian Lowland-Montane Grassland & Shrubland 571](#_Toc532988305)

[D061. Western North American Interior Chaparral 574](#_Toc532988306)

[M094. Cool Interior Chaparral 578](#_Toc532988307)

[M091. Warm Interior Chaparral 581](#_Toc532988308)

[D102. Southeastern North American Grassland & Shrubland 586](#_Toc532988309)

[M162. Florida Peninsula Scrub & Herb 589](#_Toc532988310)

[M309. Southeastern Coastal Plain Patch Prairie 593](#_Toc532988311)

[M308. Southern Barrens & Glade 597](#_Toc532988312)

[2.B.4. Temperate to Polar Scrub & Herb Coastal Vegetation 602](#_Toc532988313)

[D279. Pampean Dune & Coastal Grassland & Shrubland 602](#_Toc532988314)

[M755. Atlantic Coast & La Plata Delta Beach & Dune 604](#_Toc532988315)

[D281. Patagonian Dune & Coastal Grassland & Shrubland 606](#_Toc532988316)

[M757. Patagonian Coastal Grassland & Shrubland 607](#_Toc532988317)

[D026. Eastern North American Coastal Scrub & Herb Vegetation 609](#_Toc532988318)

[M060. Eastern North American Coastal Beach & Rocky Shore 615](#_Toc532988319)

[M057. Eastern North American Coastal Dune & Grassland 618](#_Toc532988320)

[D027. Pacific North American Coastal Scrub & Herb Vegetation 622](#_Toc532988321)

[M059. Pacific Coastal Beach & Dune 626](#_Toc532988322)

[M058. Pacific Coastal Cliff & Bluff 630](#_Toc532988323)

[2.C. Shrub & Herb Wetland 633](#_Toc532988324)

[2.C.1. Tropical Bog & Fen 633](#_Toc532988325)

[D259. Guianan Bog 633](#_Toc532988326)

[M706. Tepuyan Bog 635](#_Toc532988327)

[D260. Andean Montane Bog 636](#_Toc532988328)

[M708. Tropical Andes Upper Montane Bog 638](#_Toc532988329)

[2.C.2. Temperate to Polar Bog & Fen 640](#_Toc532988330)

[D282. Southern Andean Montane Bog 640](#_Toc532988331)

[M758. Southern Andean Montane Bog 642](#_Toc532988332)

[D283. Magellanian Bog & Fen 644](#_Toc532988333)

[M759. Magellanian Anti-Boreal Bog & Fen 645](#_Toc532988334)

[D029. North American Bog & Fen 647](#_Toc532988335)

[M876. North American Boreal & Subboreal Bog & Acidic Fen 650](#_Toc532988336)

[M877. North American Boreal & Subboreal Alkaline Fen 656](#_Toc532988337)

[M063. North Pacific Bog & Fen 660](#_Toc532988338)

[D324. Atlantic & Gulf Coastal Plain Pocosin 666](#_Toc532988339)

[M065. Southeastern Coastal Bog & Fen 669](#_Toc532988340)

[2.C.3. Tropical Freshwater Marsh, Wet Meadow & Shrubland 672](#_Toc532988341)

[D262. Caribbean-Mesoamerican Freshwater Marsh, Wet Meadow & Shrubland 673](#_Toc532988342)

[M710. Caribbean Freshwater Marsh, Wet Meadow & Shrubland 674](#_Toc532988343)

[M711. Mesoamerican Freshwater Marsh, Wet Meadow & Shrubland 677](#_Toc532988344)

[D263. Colombian-Venezuelan Freshwater Marsh, Flooded Savanna & Shrubland 679](#_Toc532988345)

[M715. Llanos Flooded Savanna 681](#_Toc532988346)

[D264. Guianan Freshwater Marsh, Wet Meadow & Shrubland 683](#_Toc532988347)

[M717. Central Guianan Flooded Savanna 685](#_Toc532988348)

[M718. Western Guianan Flooded Savanna & Shrubland 686](#_Toc532988349)

[M707. Orinoquian Floodplain Peat Meadow & Marsh 688](#_Toc532988350)

[M720. Orinoquian Floodplain Marsh & Flooded Savanna 690](#_Toc532988351)

[D265. Tropical Andean Freshwater Marsh, Wet Meadow & Shrubland 692](#_Toc532988352)

[M863. Tropical Andean Pondshore & Wet Meadow 693](#_Toc532988353)

[M722. Andean Puna Wet Meadow 695](#_Toc532988354)

[M721. Northern Andean Wet Meadow 697](#_Toc532988355)

[D266. Amazonian Freshwater Marsh, Wet Meadow & Shrubland 699](#_Toc532988356)

[M709. Amazon Delta Peat Marsh 701](#_Toc532988357)

[M724. Amazonian-Guianan White Sand Flooded Savanna & Shrubland 703](#_Toc532988358)

[M726. Lower Amazon Wet Meadow & Shrubland 704](#_Toc532988359)

[M725. Upper Amazon Wet Meadow & Shrubland 706](#_Toc532988360)

[D267. Parana-Brazilian Freshwater Marsh, Wet Meadow & Shrubland 708](#_Toc532988361)

[M729. Pantanal Floodplain Wet Meadow & Shrubland 710](#_Toc532988362)

[M730. Parana Floodplain Wet Meadow & Shrubland 712](#_Toc532988363)

[M727. Cerrado Flooded Savanna 714](#_Toc532988364)

[M728. Beni Flooded Savanna 715](#_Toc532988365)

[D268. Chaco Freshwater Marsh, Flooded Savanna & Shrubland 717](#_Toc532988366)

[M734. Eastern Chaco Marsh & Flooded Savanna 719](#_Toc532988367)

[M732. Chaco Riparian Marsh & Shrubland 721](#_Toc532988368)

[2.C.4. Temperate to Polar Freshwater Marsh, Wet Meadow & Shrubland 723](#_Toc532988369)

[D284. South American Temperate Freshwater Marsh, Wet Meadow & Shrubland 723](#_Toc532988370)

[M760. Pampean Freshwater Marsh, Wet Meadow & Shrubland 725](#_Toc532988371)

[D031. Western North American Temperate & Boreal Freshwater Marsh, Wet Meadow & Shrubland 726](#_Toc532988372)

[M888. Arid West Interior Freshwater Marsh 730](#_Toc532988373)

[M074. Western North American Vernal Pool 734](#_Toc532988374)

[M073. Vancouverian Lowland Marsh, Wet Meadow & Shrubland 738](#_Toc532988375)

[M075. Western North American Montane-Subalpine-Boreal Marsh, Wet Meadow & Shrubland 744](#_Toc532988376)

[D032. Southwestern North American Warm Desert Freshwater Marsh & Bosque 749](#_Toc532988377)

[M076. Warm Desert Lowland Freshwater Marsh, Wet Meadow & Shrubland 752](#_Toc532988378)

[D323. Eastern North American Temperate & Boreal Freshwater Marsh, Wet Meadow & Shrubland 754](#_Toc532988379)

[M061. Eastern North American Cool Temperate Seep 762](#_Toc532988380)

[M069. Eastern North American Marsh, Wet Meadow & Shrubland 767](#_Toc532988381)

[M881. Eastern North American Riverscour Vegetation 770](#_Toc532988382)

[M071. Great Plains Marsh, Wet Meadow, Shrubland & Playa 773](#_Toc532988383)

[D322. Atlantic & Gulf Coastal Marsh, Wet Meadow & Shrubland 776](#_Toc532988384)

[M066. Atlantic & Gulf Coastal Fresh-Oligohaline Tidal Marsh 782](#_Toc532988385)

[M067. Atlantic & Gulf Coastal Plain Wet Prairie & Marsh 787](#_Toc532988386)

[2.C.5. Salt Marsh 791](#_Toc532988387)

[D269. Eastern Pacific Coastal Salt Marsh 791](#_Toc532988388)

[M737. Mesoamerican-South American Pacific Coastal Salt Marsh 793](#_Toc532988389)

[M736. Mexican Pacific Coastal Salt Marsh 795](#_Toc532988390)

[D270. South American Lowlands Interior Brackish Marsh 796](#_Toc532988391)

[M738. Chaco-Espinal Brackish Marsh 798](#_Toc532988392)

[D271. Andean Salt Marsh 800](#_Toc532988393)

[M739. Central Andean Altiplano Salt Flats 801](#_Toc532988394)

[D272. South American Pacific Desert Salt Flats 803](#_Toc532988395)

[M740. South American Pacific Desert Salt Flats 805](#_Toc532988396)

[D285. South American Temperate Salt Marsh 807](#_Toc532988397)

[M762. South American Temperate Interior Brackish Marsh 808](#_Toc532988398)

[M763. Temperate & Austral Atlantic Coastal Salt Marsh 810](#_Toc532988399)

[M761. Southern Andean Montane Salt Marsh 812](#_Toc532988400)

[D033. North American Great Plains Saline Marsh 814](#_Toc532988401)

[M077. Great Plains Saline Wet Meadow & Marsh 816](#_Toc532988402)

[D034. North American Atlantic & Gulf Coastal Salt Marsh 818](#_Toc532988403)

[M079. North American Atlantic & Gulf Coastal Salt Marsh 822](#_Toc532988404)

[D035. Temperate & Boreal Pacific Coastal Salt Marsh 825](#_Toc532988405)

[M081. North American Pacific Coastal Salt Marsh 829](#_Toc532988406)

[D036. North American Western Interior Brackish Marsh, Playa & Shrubland 832](#_Toc532988407)

[M082. Warm & Cool Desert Alkali-Saline Marsh, Playa & Shrubland 837](#_Toc532988408)

[D037. Tropical Atlantic Coastal Salt Marsh 839](#_Toc532988409)

[M735. Tropical Western Atlantic-Caribbean Salt Marsh 841](#_Toc532988410)

[3. Desert & Semi-Desert 844](#_Toc532988411)

[3.A. Warm Desert & Semi-Desert Woodland, Scrub & Grassland 844](#_Toc532988412)

[3.A.1. Tropical Thorn Woodland 844](#_Toc532988413)

[D287. Caribbean-Northern Mesoamerican Xeromorphic Scrub & Woodland 844](#_Toc532988414)

[M765. Caribbean-Northern Mesoamerican Xeromorphic Scrub & Woodland 846](#_Toc532988415)

[D288. Colombian-Venezuelan Xeromorphic Scrub & Woodland 847](#_Toc532988416)

[M766. Guajiran Xeromorphic Scrub & Woodland 849](#_Toc532988417)

[M767. Tumbesian Xeromorphic Scrub & Woodland 851](#_Toc532988418)

[D289. Interandean Valley Xeromorphic Scrub & Woodland 853](#_Toc532988419)

[M770. Bolivian-Tucuman Xeromorphic Scrub & Woodland 854](#_Toc532988420)

[M769. Central Andean Xeromorphic Scrub & Woodland 856](#_Toc532988421)

[M768. Northern Andean Xeromorphic Scrub & Woodland 858](#_Toc532988422)

[D290. Chaco Xeromorphic Scrub & Woodland 860](#_Toc532988423)

[M773. Southern Chaco Xeromorphic Scrub & Woodland 862](#_Toc532988424)

[M772. Northeastern Chaco Xeromorphic Scrub & Woodland 864](#_Toc532988425)

[M771. Northwestern Chaco Xeromorphic Scrub & Woodland 866](#_Toc532988426)

[3.A.2. Warm Desert & Semi-Desert Scrub & Grassland 868](#_Toc532988427)

[D291. Tropical Andean Xeromorphic Scrub & Grassland 868](#_Toc532988428)

[M777. Bolivian-Tucuman Interandean Xeromorphic Scrub & Grassland 869](#_Toc532988429)

[M776. Central Interandean Xeromorphic Scrub & Grassland 871](#_Toc532988430)

[M775. Northern Interandean Xeromorphic Scrub & Grassland 873](#_Toc532988431)

[D292. Brazilian-Parana Xeromorphic Scrub & Grassland 875](#_Toc532988432)

[M779. Caatinga Dense Scrub & Forb Meadow 877](#_Toc532988433)

[M778. Caatinga Xeromorphic Scrub 878](#_Toc532988434)

[D293. Chaco Xeromorphic Scrub, Grassland & Savanna 880](#_Toc532988435)

[M141. Chaco Xeromorphic Cliff & Other Rock Vegetation 882](#_Toc532988436)

[M781. Southern Chaco Xeromorphic Scrub & Savanna 884](#_Toc532988437)

[M780. Northern Chaco Xeromorphic Scrub & Savanna 886](#_Toc532988438)

[D294. South American Pacific Semi-Desert Scrub & Grassland 887](#_Toc532988439)

[M784. Chilean Mediterranean Coastal Semi-Desert Scrub & Grassland 889](#_Toc532988440)

[M785. Chilean Mediterranean Interior Semi-Desert Scrub & Grassland 891](#_Toc532988441)

[M861. Sechura Atacama Semi-Desert Cliff & Pavement 893](#_Toc532988442)

[M782. Sechura Atacama Semi-Desert Riparian Scrub 895](#_Toc532988443)

[M783. Sechura Atacama Semi-Desert Scrub 897](#_Toc532988444)

[D039. North American Warm Desert Scrub & Grassland 899](#_Toc532988445)

[M130. Tamaulipan Scrub & Grassland 902](#_Toc532988446)

[M086. Chihuahuan Desert Scrub 905](#_Toc532988447)

[M087. Chihuahuan Semi-Desert Grassland 912](#_Toc532988448)

[M088. Mojave-Sonoran Semi-Desert Scrub 919](#_Toc532988449)

[M089. Viscaino-Baja California Desert Scrub 926](#_Toc532988450)

[M117. North American Warm Semi-Desert Cliff, Scree & Rock Vegetation 929](#_Toc532988451)

[M092. North American Warm-Desert Xeric-Riparian Scrub 933](#_Toc532988452)

[M512. North American Warm Desert Ruderal Scrub & Grassland 936](#_Toc532988453)

[3.B. Cool Semi-Desert Scrub & Grassland 940](#_Toc532988454)

[3.B.1. Cool Semi-Desert Scrub & Grassland 940](#_Toc532988455)

[D318. Andean Cool Semi-Desert Cliff, Scree & Other Rock Vegetation 940](#_Toc532988456)

[M862. Andean Cool Semi-Desert Rock Vegetation 942](#_Toc532988457)

[D117. Patagonian Cool Semi-Desert Scrub & Grassland 943](#_Toc532988458)

[M790. Patagonian Semi-Desert Scrub 945](#_Toc532988459)

[D295. Tropical Andean Cool Semi-Desert Scrub & Grassland 947](#_Toc532988460)

[M787. Xeric Puna Succulent Scrub 949](#_Toc532988461)

[D296. Mediterranean-Southern Andean Cool Semi-Desert Scrub & Grassland 951](#_Toc532988462)

[M788. Mediterranean Andean Cool Semi-Desert Scrub & Grassland 952](#_Toc532988463)

[M789. Monte Cool Semi-Desert Scrub & Grassland 954](#_Toc532988464)

[D040. Western North American Cool Semi-Desert Scrub & Grassland 956](#_Toc532988465)

[M171. Great Basin-Intermountain Dry Shrubland & Grassland 961](#_Toc532988466)

[M170. Great Basin-Intermountain Dwarf Sagebrush Steppe & Shrubland 966](#_Toc532988467)

[M169. Great Basin-Intermountain Tall Sagebrush Steppe & Shrubland 971](#_Toc532988468)

[M095. Great Basin-Intermountain Xeric-Riparian Scrub 977](#_Toc532988469)

[M093. Great Basin Saltbush Scrub 979](#_Toc532988470)

[M118. Intermountain Basins Cliff, Scree & Badland Sparse Vegetation 985](#_Toc532988471)

[4. Polar & High Montane Scrub, Grassland & Barrens 988](#_Toc532988472)

[4.A. Tropical High Montane Scrub & Grassland 988](#_Toc532988473)

[4.A.1. Tropical High Montane Scrub & Grassland 989](#_Toc532988474)

[D298. Tropical & Mediterranean Andean High Montane Scrub & Grassland 989](#_Toc532988475)

[M794. High Andean Xeric Puna Bunch Grassland 990](#_Toc532988476)

[M793. High Andean Moist Puna Bunch Grassland 992](#_Toc532988477)

[M792. High Northern Andean Super-Paramo 994](#_Toc532988478)

[4.B. Temperate to Polar Alpine & Tundra Vegetation 996](#_Toc532988479)

[4.B.1. Temperate & Boreal Alpine Tundra 996](#_Toc532988480)

[D299. Southern Andean High Montane Tundra 996](#_Toc532988481)

[M795. Southern Andean Alpine Tundra 998](#_Toc532988482)

[D300. Magellanian High Montane Tundra 1000](#_Toc532988483)

[M796. Magellanian Montane Tundra 1002](#_Toc532988484)

[D042. Eastern North American Alpine Tundra 1003](#_Toc532988485)

[M131. Eastern North American Alpine Tundra 1008](#_Toc532988486)

[D043. Western North American Alpine Tundra 1010](#_Toc532988487)

[M099. Rocky Mountain-Sierran Alpine Tundra 1015](#_Toc532988488)

[M101. Vancouverian Alpine Tundra 1020](#_Toc532988489)

[5. Aquatic Vegetation 1024](#_Toc532988490)

[5.A. Saltwater Aquatic Vegetation 1024](#_Toc532988491)

[5.A.2. Benthic Macroalgae Saltwater Vegetation 1024](#_Toc532988492)

[D047. Temperate Intertidal Shore 1024](#_Toc532988493)

[M106. Temperate Pacific Seaweed Intertidal Vegetation 1027](#_Toc532988494)

[5.A.3. Benthic Vascular Saltwater Vegetation 1029](#_Toc532988495)

[D064. Temperate Seagrass Aquatic Vegetation 1029](#_Toc532988496)

[M184. Temperate Pacific Seagrass Intertidal Vegetation 1034](#_Toc532988497)

[5.B. Freshwater Aquatic Vegetation 1036](#_Toc532988498)

[5.B.1. Tropical Freshwater Aquatic Vegetation 1036](#_Toc532988499)

[D097. Neotropical Freshwater Aquatic Vegetation 1036](#_Toc532988500)

[M291. Neotropical Floating & Submerged Freshwater Marsh [Low - Poorly Documented] 1037](#_Toc532988501)

[5.B.2. Temperate to Polar Freshwater Aquatic Vegetation 1039](#_Toc532988502)

[D319. Temperate South American Freshwater Aquatic Vegetation 1039](#_Toc532988503)

[M865. Temperate South American Freshwater Aquatic Vegetation 1041](#_Toc532988504)

[D049. North American Freshwater Aquatic Vegetation 1043](#_Toc532988505)

[M109. Western North American Freshwater Aquatic Vegetation 1045](#_Toc532988506)

[6. Open Rock Vegetation 1049](#_Toc532988507)

[6.A. Tropical Open Rock Vegetation 1049](#_Toc532988508)

[6.A.1. Tropical Cliff, Scree & Other Rock Vegetation 1049](#_Toc532988509)

[D311. Brazilian-Parana Cliff, Scree & Rock Vegetation 1049](#_Toc532988510)

[M867. Brazilian-Parana Cliff, Scree & Rock Vegetation 1051](#_Toc532988511)

[D310. Guianan Montane Cliff, Scree & Rock Vegetation 1053](#_Toc532988512)

[M851. Tepuyan Cliff, Scree & Rock Vegetation 1054](#_Toc532988513)

[D312. Tropical Andean Cliff, Scree & Rock Vegetation 1056](#_Toc532988514)

[M855. Bolivian-Tucuman Cliff, Scree & Rock Vegetation 1058](#_Toc532988515)

[M853. Northern Andean Cliff, Scree & Rock Vegetation 1060](#_Toc532988516)

[M856. Moist Puna Cliff, Scree & Rock Vegetation 1061](#_Toc532988517)

[6.B. Temperate & Boreal Open Rock Vegetation 1063](#_Toc532988518)

[6.B.1. Temperate & Boreal Cliff, Scree & Other Rock Vegetation 1063](#_Toc532988519)

[D051. Eastern North American Temperate Cliff, Scree & Rock Vegetation 1063](#_Toc532988520)

[M111. Eastern North American Cliff & Rock Vegetation 1066](#_Toc532988521)

[M116. Great Plains Cliff, Scree & Rock Vegetation 1069](#_Toc532988522)

[M115. Great Plains Badlands Vegetation 1072](#_Toc532988523)

[D052. Western North American Temperate Cliff, Scree & Rock Vegetation 1074](#_Toc532988524)

[M887. Western North American Cliff, Scree & Rock Vegetation 1077](#_Toc532988525)

1. Forest & Woodland

Tropical, temperate and boreal forests, woodlands and tree savannas characterized by broadly mesomorphic (including scleromorphic) tree growth forms (including *broad-leaved, needle-leaved, sclerophyllous, palm, bamboo trees*, and *tree ferns*), typically with at least 10% cover (but tropical tree savannas up to 40% cover, when trees <8 m tall), irregular horizontal spacing of vegetation structure, and spanning humid to seasonally dry tropical to boreal and subalpine climates and wet to dry substrate conditions. Includes native forests, as well as managed, and some plantation forests where human management is infrequent.

1.A. Tropical Forest & Woodland

Tropical forests found at lowland and montane elevations including tropical dry forests, and lowland to montane humid forests (tropical rainforests) and tropical forested wetlands, where frost is essentially absent at sea level.

1.A.1. Tropical Dry Forest & Woodland

Tropical Dry Forest & Woodland is dominated by broad-leaved drought-deciduous, semi-deciduous, and small-leaved or sclerophyllous evergreen trees where rainfall is lower, often associated with more strongly seasonal, tropical climates, rainshadows, or drying winds. At continental scales, the largest areas occur between 10° and 23°N and S latitude.

1. Forest & Woodland

1.A.1.Ea. Caribbean-Mesoamerican Dry Forest & Woodland

D099. Caribbean-Mesoamerican Dry Forest & Woodland

Type Concept Sentence: This division includes seasonally drought-deciduous to semi-deciduous tropical forests distributed from sea level up to 1400 m elevation throughout the Caribbean Basin, southern Gulf of Mexico, and in the Pacific Basin from the Gulf of California through Panama.

OVERVIEW

*Hierarchy Level: Division

*Placement in Hierarchy: 1.A.1.Ea. Tropical Dry Forest & Woodland (F003)

Elcode: D099

*Scientific Name: *Bursera simaruba - Enterolobium cyclocarpum - Ceiba aesculifolia* Dry Forest & Woodland Division

*Common (Translated Scientific) Name: Gumbo Limbo - Elephant Ear - Pochote Dry Forest & Woodland Division

*Colloquial Name: Caribbean-Mesoamerican Dry Forest & Woodland

*Type Concept: This division includes tropical forests characterized by a dry season of several months, and found close to the coast or in interior valleys throughout Mexico, Central America, and the Caribbean Basin. Forests tend to have low stature (5-15 m height), high density of small and medium-sized trees, and typically have a single layer, with somewhat open canopies and no emergent trees. The herb layer may be poorly developed or completely lacking. Floristic diversity is low compared to more humid tropical forests, and a few species may dominate the stands. The following list of species are among those that are diagnostic for this division: *Astronium graveolens, Bombacopsis quinata, Bursera simaruba, Bursera excelsa, Bursera fagaroides var. elongata, Bursera instabilis, Bursera morelensis, Calycophyllum candidissimum, Ceiba aesculifolia, Ceiba acuminata, Celtis ehrenbergiana, Coccoloba diversifolia, Conzattia sericea, Enterolobium cyclocarpum, Erythroxylum areolatum, Eugenia axillaris, Exostema caribaeum, Exothea paniculata, Guazuma ulmifolia, Guettarda krugii, Guaiacum sanctum, Guapira obtusata, Gymnanthes lucida, Hypelate trifoliata, Jarilla heterophylla, Krugiodendron ferreum, Maclura tinctoria, Metopium toxiferum, Plumeria obtusa, Sideroxylon foetidissimum, Sideroxylon salicifolium, Martinella obovata*, and *Tabebuia ochracea*. Variation in the dry season period, topography, and substrate determine the specific characteristics of the vegetation types in this division since all of these have great importance in dry forests as determinants of variation in water availability. Seasonal climates with 600-1500 mm rainfall per year and the dry season of two to several months are characteristic. These forests most typically occur in sandy lowlands, littoral or sub-littoral flatlands with rock outcrops and higher terraces facing the sea, karstic flats, towerlike karstic hills, humic carbonate soils, and shallow red ferrallitic soils. These forests tend to occur adjacent and/transitional to more humid submontane tropical forests, or towards more xeric tropical thornscrub vegetation types.

*Diagnostic Characteristics: Seasonally deciduous and semi-deciduous forest occurring in tropical latitudes of Mexico, the Gulf of Mexico, Central America, and Caribbean Basin. Species diagnostic for this division include *Astronium graveolens, Bombacopsis quinata, Bursera simaruba, Bursera excelsa, Bursera fagaroides var. elongata, Bursera instabilis, Bursera morelensis, Calycophyllum candidissimum, Ceiba aesculifolia, Ceiba acuminata, Celtis ehrenbergiana, Coccoloba diversifolia, Conzattia sericea, Enterolobium cyclocarpum, Erythroxylum areolatum, Eugenia axillaris, Exostema caribaeum, Exothea paniculata, Guazuma ulmifolia, Guettarda krugii, Guaiacum sanctum, Guapira obtusata, Gymnanthes lucida, Hypelate trifoliata, Jarilla heterophylla, Krugiodendron ferreum, Maclura tinctoria, Martinella obovata, Metopium toxiferum, Plumeria obtusa, Sideroxylon foetidissimum, Sideroxylon salicifolium*, and *Tabebuia ochracea*.

*Classification Comments: Owing to extensive land conversion and degradation (e.g., from livestock grazing), there remains much ambiguity and unevenness in documentation of this division. More work is needed to fully differentiate this type from slightly more humid and evergreen-dominant, more xeric thornscrub, and tropical *Quercus*-dominated woodland vegetation types.

*Similar NVC Types [if applicable]:

| **Elcode** | **Scientific or Colloquial Name** | **Note** |
| --- | --- | --- |
|  |  |  |

Similar NVC Types General Comments [optional]:

VEGETATION

Physiognomy and Structure Summary: These forests have low stature (5-15 m; extremes up to 25 m height), high density of small and medium-sized trees, and typically have single-layer, somewhat open canopies with no emergent trees. Forests in the humid end of the range are semi-deciduous and taller (up to 20-25 m high), while the forests that grow in areas of lower rainfall are nearly 100% deciduous and generally have a lower height and more open canopy below 15 m. The herb layer may be poorly developed or completely lacking. The dry forests of south Florida and the Caribbean tend to have a greater density of individual stems and shorter canopy heights than tropical dry forests in the mainland Neotropics. Density varies from 14,000 stems >2.5 cm/ha in Puerto Rico dry forest to 4600 stems/ha in Florida (Gillespie 2006, Lugo et al. 2006). Drought-deciduousness is the principal adaptive mode of tropical dry deciduous forests, and at the dry extremes, small evergreen trees are important (Lugo et al. 2006). In the Caribbean, the canopy is seasonally open; there are some truly deciduous species in these forests, with early-successional forests dominated by broadleaf semi-deciduous species and late-successional forests dominated by broadleaf evergreen species. Also in the Caribbean, there tends to be a notable lack of lianas compared with dry forests in the mainland Neotropics (Gillespie 2006). Despite relatively low species diversity, high lifeform diversity is common and is accompanied by diversity in plant habit, leaf size and structure, drought tolerance and growth seasonality; this diversity is attributed to habitat heterogeneity coupled with strong rainfall seasonality (Lugo et al. 1978, Medina 1995, as cited in Lugo et al. 2006). Epiphytic orchid and bromeliad species are often found in areas where frosts or anthropogenic disturbances have not occurred in a long time. During the dry season there is an accumulation of litter because much vegetation is deciduous (Holbrook et al. 1995) and sunlight penetrates to the forest floor which reduces the rate of decomposition by decreasing the relative soil moisture (Pennington et al. 2006). The floral and fruiting phenology is highly seasonal and many species flower synchronously during the transition between the dry season and the rainy season when the trees are still leafless (Bullock et al. 1995). Where these forests occur on karst, they are characterized by trees of small diameter, high tree density, and leaf scleromorphy. These stands have a tendency to show signs of being exposed to frequent drought conditions. This is probably due to the rapid rate of runoff and infiltration of rainwater, low water storage in shallow soils, and high sunlight. But this varies depending on the landscape position and the substrate. For example, at the base of limestone "mogotes" or towerlike "haystack mountains" the vegetation may quickly transition to mesic, closed canopy of evergreen species 25-30 m tall, while on sideslopes and tops the vegetation is a deciduous woodland with trees of 16-18 m height and sclerophyllous leaves. In Cuban mogotes, the slope forest has a 10- to 16-m high open canopy of deciduous trees with barrel-like trunks and abundant columnar cacti, but can quickly grade to a shrubland dominated by terrestrial bromeliads and diverse sclerophyllous shrubs and trees.

Physiognomy and Structure Table [optional]:

| **Physiognomy-Structure Category** | **Prevailing Height (m)** | **Height Range (opt.)** | **Mean % Cover** | **Cover Range (opt.)** |
| --- | --- | --- | --- | --- |
|  |  |  |  | - |

Floristics Summary: The following list of species are among those that are diagnostic for this division: *Astronium graveolens, Bombacopsis quinata, Bursera simaruba, Bursera fagaroides var. elongata (= Bursera odorata), Bursera instabilis, Bursera morelensis, Calycophyllum candidissimum, Ceiba aesculifolia, Ceiba acuminata, Celtis ehrenbergiana (= Celtis pallida), Coccoloba diversifolia, Conzattia sericea, Enterolobium cyclocarpum, Erythroxylum areolatum, Eugenia axillaris, Exostema caribaeum, Exothea paniculata, Guazuma ulmifolia, Guettarda krugii, Guaiacum sanctum, Guapira obtusata, Gymnanthes lucida, Hypolate trifoliate, Jarilla heterophylla (= Jarilla chocola), Krugiodendron ferreum, Maclura tinctoria, Martinella obovata (= Tabebuia cordata), Metopium toxiferum, Pinus elliottii var. densa, Plumeria obtusa, Sideroxylon foetidissimum, Sideroxylon salicifolium*, and *Tabebuia ochracea*. Characteristic pine species in the Caribbean include *Pinus caribaea* (*var. bahamensis, var. caribaea*, and *var. hondurensis*), and *Pinus tropicalis*. Diagnostic shrubs include *Chrysobalanus icaco, Coccothrinax argentata, Ilex cassine, Sideroxylon salicifolium, Tetrazygia bicolor*, and *Thrinax morrisii*. Similar open pine forests occur in the northern Bahama Archipelago and along the coast in western and eastern Cuba where they are known as pine barrens or pine woodlands and are dominated by *Pinus caribaea, Pinus tropicalis*, and *Pinus cubensis*, respectively, accompanied by a similar set of species such as the palms *Coccothrinax argentata, Coccothrinax miraguana, Coccothrinax orientalis*, among others. Given the harsh conditions of the Antillean dry forests, those relatively few species that thrive under the stress are able to dominate sites (Lugo et al. 2006). Dominant species in Puerto Rico coastal dry forest are *Coccoloba krugii, Erithalis fruticosa, Exostema caribaeum, Guettarda krugii, Gymnanthes lucida, Pisonia albida, Savia sessiliflora*, and *Thouinia striata var. portoricensis*. Endemism in these Caribbean forests is very high and represents about 50% of the characteristic plant species.

The division also includes pine-oak dry forests in western Cuba, where the oak species *Quercus oleoides* also occurs in southern Mexico and Central America. Mesoamerican dry forests occur on soils of variable depth, texture, and alkalinity, and typically have a closed canopy 10-25 m high depending on the type and nutrient contents of the soil. A moderate to high diversity of tropical deciduous tree species dominate the multi-tiered tree canopy. The structure and composition vary along the large distributional range, which includes mountainous topographies that influence moisture availability and soil characteristics. Characteristic genera include *Astronium, Bursera, Ceiba, Cassia, Calycophyllum, Cochlospermum, Cordia, Enterolobium, Ficus, Gyrocarpus, Lysiloma, Plumeria, Platymiscium, Pterocarpus, Thouinidium, Zanthoxylum*, among others. At the northwestern extreme in Sinaloa, Mexico, these forests include *Conzattia sericea, Jarilla heterophylla, Bursera inopinnata, Ceiba acuminata, Martinella obovata, Ipomoea arborescens, Lysiloma watsonii, Choclosperma vitifolium, Pachycereus pecten-aboriginum, Stenocereus thurberi, Mardensia edulis, Senna bicapsularis (= Cassia emarginata)*, and *Tillandsia inflata*. Further south from Jalisco through Oaxaca, common species include *Achatocarpus oaxacanus, Aphipterygium glaucum, Bombax ellipicum, Bombax palmeri, Bursera fagaroides, Bursera instabilis, Bursera longipes, Bursera morelensis, Bursera fagaroides var. elongata, Ceiba aesculifolia, Coccoloba* spp., *Pseudosmodingium perniciosum*, and *Tabebuia palmeri*. Dry inter-montane valleys of south-central Mexico (e.g., Balsas dry forest) include *Agave pedunculifera, Bursera ariensis, Bursera diversifolia, Bursera hintonii, Ceiba aesculifolia, Cochlospermum vitifolium, Conzattia multiflora, Cordia elaeagnoides, Cyrtocarpa procera, Ficus cotinifolia, Ficus goldmanii, Ficus kellermanni, Ficus petiolaris, Haematoxylon brasiletto, Heliocarpus reticulatus, Lysiloma divaricatum, Pterocarpus orbiculatus, Ruprechtia fusca, Tabebuia impetiginosa*, and *Vitex pyramidata*. Throughout the northern Yucatán Peninsula, most of its distribution is related to limestone outcrops of coral origin, up to hilly terrain and supports a seasonal climate with low annual rainfall. Characteristic species include *Beaucarnea pliabilis, Caesalpinia gaumeri, Caesalpinia vesicaria, Diospyros cuneata, Guaiacum sanctum, Hampea trilobata, Lemaireocereus griseus, Lemairocereus aragonii, Lysiloma latisiliquum, Manilkara sapota, Parmentiera aculeata, Plumeria obtusa, Pseudophoenix* sp., and *Pterocereus gaumeri*. Further south along the Pacific Central American coast, extending to the Azuero Peninsula of Panama, characteristic species include *Acacia collinsii, Adenocalymma inundatum, Albizia caribea, Allophyllus occidentalis, Andira inermis, Apeiba* spp., *Ardisia revoluta, Arrabidaea mollissima, Astronium graveolens, Bauhinia glabra, Bombacopsis quinata, Bursera simaruba, Calycophyllum candidissimum, Casearia arguta, Cedrela odorata, Ceiba aesculifolia, Chomelia spinosa, Cochlospermum vitifolium, Combretum farinosum, Cydista diversifolia, Enterolobium cyclocarpum, Exostema mexicanum, Ficus* spp., *Genipa americana, Guaiacum sanctum, Guarea excelsa, Guazuma ulmifolia, Gyrocarpus americanus, Hemiangium excelsum, Jacquinia pungens, Lonchocarpus phaseolifolius, Lonchocarpus phlebophyllus, Luehea candida, Maclura tinctoria, Platymiscium pleiostachyum, Sabal allenii, Sideroxylon capiri (= Mastichodendron capiri), Simarouba amara, Simarouba glauca, Spondias mombin, Stemmadenia obovata, Sterculia apetala, Swietenia humilis, Swietenia macrophylla, Tabebuia ochracea, Thouinidium decandrum, Trichilia colimana*, and *Zanthoxylum setulosum*.

Naturalized species that may be common or may dominate ruderal dry forests include *Vachellia farnesiana (= Acacia farnesiana), Leucaena leucocephala, Melicoccus bijugatus, Parkinsonia aculeata, Prosopis juliflora*, and *Tamarindus indica*. Some of the most widespread exotic trees and shrubs in Puerto Rico and the Virgin Islands are *Albizia lebbeck, Leucaena leucocephala, Pinus caribaea, Schinus molle, Spathodea campanulata, Syzygium jambos, Tabebuia rosea*, and *Terminalia catappa* (Brandeis et al. 2009b, Chinea and Helmer 2009).

*Floristics Table [Med - High Confidence]:

*Number of Plots: *Cover Scale Used:

| **Physiognomy-Structure Category** | **Taxon Name** | **Specific Growth Form (opt.)** | **Const- ancy** | **Mean % Cover** | **Cover Range (opt.)** | **Differ-ential** | **Diagnostic Combin- ation** |
| --- | --- | --- | --- | --- | --- | --- | --- |
|  |  |  |  |  | - |  |  |

Dynamics: Caribbean and Mesoamerican dry forests tend to be exposed to harsh environmental conditions that, depending on their intensity, can cause damage or diebacks, such as seasonal water deficit, nutrient stress, hurricane-force winds and salt spray, and saltwater storm surge. This has influenced the development of structural and physiological mechanisms, making them resilient to disturbance. Among the more outstanding ones are a high resistance to wind (short stature), a high proportion of root biomass, high soil carbon and nutrient accumulation below ground, the ability of most tree species to resprout, and high nutrient use efficiency (Lugo et al. 2006). Fire is not thought to be part of the natural dynamics of these dry forests, but hurricanes often are, which naturally results in considerable heterogeneity in habitat structure and food availability on small spatial scales. This structuring of coastal dry forest by frequent natural disturbance may favor their resilience to anthropogenic disturbance and fragmentation.

ENVIRONMENT

Environmental Description: This division includes tropical forests characterized by a dry season of several months occurring close to the coast or in interior valleys throughout Mexico, Central America, the Caribbean Basin, and south Florida. There, mean annual temperatures in the area of distribution range from 23°C (74°F) in the north to 26°C (77°F) in the Lower Keys. Caribbean dry forests occurring on the mainland in Florida and the Upper Keys are periodically exposed to short-term frost and their flora is composed of a subset of native tropical trees that can withstand rare frost events. Mean annual temperature in the West Indies distribution is around 25°C. Precipitation primarily occurs from May or June to October and ranges from 1650 mm along the Atlantic coast decreasing southward to less than 1000 mm in the Lower Keys (Gillespie 2006). Annual precipitation in the distributional range of this forest in Cuba is less than 1500 mm in the west part of the range and increases towards the east. Precipitation in the distribution range of this forest in Puerto Rico and over most of the islands of Culebra and Vieques ranges from 600 to 1100 mm per year (Brandeis et al. 2006), with two dry seasons, the longer one from December to April and a shorter one from June to August. Throughout Mexico and Central America, these seasonally dry deciduous to semi-deciduous forests are distributed from sea level up to 1400 m elevation in the Pacific basin from Mexico to Panama. Precipitation generally varies from 600 to 1600 mm/year and annual mean temperatures are over 24°C. These forests experience at least one distinct dry season of 4-6 months/year.

Dry forests may often be exposed to highly stressful conditions given the combination of environmental features such as low moisture availability, long dry seasons, decadal cycles of pronounced drought, wind exposure and salt spray in littoral locations. These forests are also periodically exposed to hurricane conditions, with effects that span from flooding with seawater to defoliation, treefall and other structural changes due to strong winds.

Limestone is the dominant substrate in Caribbean dry forests, and in the Yucatán Peninsula of Mexico, with skeletal organic soils with minor mineral components, rarely exceeding 20 cm in depth (Snyder et al. 1990, cited in Gillespie 2006). In the Greater Antilles the distribution of dry forests is indicative of limestone substrates occurring in narrow strips on the northern and southern coastal areas. These also include karst formations with steep slopes and plateaus of towerlike karstic hills up to 300-600 m elevation, with bare karstic rock or more-or-less eroded skeletal soils, or limestone cliffs, and the narrow valleys and gorges in between. Rocky limestone soils have low water-holding capacity and nutritional limitations imposed by their calcareous composition. In flat low-lying limestone archipelagos, such as the Bahamas, the Cayman Islands, Mona and Anegada, dry forests and shrublands dominate. In volcanic, low mountainous islands of the Lesser Antilles, dry forests dominate except for protected sites and ravines where moist forest can grow (Lugo et al. 2006). In Cuba, the dry pine forests are found primarily on acidic soils that have little water-retention capacity and are poor in essential elements. The principal soil types on which they occur are quartziferous sands, pseudo-spodosols in the west and lateritic soils in the east. Only pine trees, which have an ectomycorrhizal symbiosis with fungi, are capable of obtaining in this way a sufficient amount of nutrients to achieve the size of trees. In the Bahamas, pine rockland occurs on relatively flat, moderately to well-drained terrain, from 2-7 m above sea level (Snyder et al. 1990). The oolitic limestone is at or very near the surface, and there is very little soil development. Soils are generally composed of small accumulations of nutrient-poor sand, marl, clayey loam, and organic debris in depressions and crevices in the rock surface. Drainage varies according to the porosity of the limestone substrate, but is generally rapid. Consequently, most sites are wet for only short periods following heavy rains. During the rainy season, however, some sites may be shallowly inundated by slow-flowing surface water for up to 60 days each year (FNAI 2010a).

Dry forests throughout Mexico and Central America occur across a wider diversity of local climate conditions, landforms, and substrates than in the Caribbean. In Tamaulipas, Mexico, these forests occur across lower elevation slopes of the eastern Sierra Madre Oriental, Sierra de San Carlos, Sierra de Tamaulipas, and plateaus of northeastern Mexico. In Sinaloa they are found in canyons and steep slopes with thin, sandy soils. These areas typically experience two wet seasons (winter and mid-summer) and two dry seasons each year. This favors deciduous plant species with well-developed root storage systems, able to rapidly respond to wet seasons. In Nayarit these forests occur at elevations from sea level up through 1,300m, always on slopes. These forests occur on soils of variable depth, texture, and alkalinity. Throughout Guerrero they are found in canyons and steep slopes with soils that are shallow, sandy, nutrient poor, and have low water holding capacity. These areas (extending south through Central America) typically experience one distinct wet season (July-November) and one dry season (December-June) each year. In Guerrero, this vegetation occurs between 0 - 900 m elevation with mean annual precipitation varying between 400-1200 mm, and mean annual temperature around 25<sup>o</sup>C. These communities transition into thorn scrub at the drier end of its gradient along lower elevation borders, and to semi-evergreen forest further upslope or at the valley bottoms. In the Motagua Valley of Guatemala, and nearby valleys in Honduras, this vegetation usually occurs on deep, rich soils with annual rainfall between 1000 and 1500 mm, but can reach up to 2000 mm and an average temperature of 24<sup>o</sup>C. With increasing elevation surrounding these valleys, there is an abrupt transition to premontane wet forests. Throughout the remaining Pacific coast of Central America, these forests grow on a variety of soils and topography, with substrates often derived from volcanic activity or from limestones or sandstones. In general, these tend to be more fertile soils than in other parts of the range of this division. Weather conditions that characterize this southerly range are more constant, with an average annual precipitation ranging up to 2000 mm, always with a dry season of 4-6 months, but the exact period varies according to locality.

DISTRIBUTION

*Geographic Range: This division is found throughout the Caribbean, south Florida, Caribbean coastal South America, Gulf Coast of Mexico and Pacific Coast of Mexico and Central America.

Nations: BS, BZ, CO, CR, CU, DO, GT, HN, HT, JM, KN, MQ, MX, NI, PA, PR, SV, TC, TT, US, VE, VG?, VI, XC, XD

States/Provinces: FL

USFS Ecoregions (2007) [optional]:

Omernik Ecoregions L3, L4 [optional]:

MLRAs [optional]:

PLOT SAMPLING AND ANALYSIS

*Plot Analysis Summary [Med - High Confidence]:

*Plots Used to Define the Type [Med - High Confidence]:

CONFIDENCE LEVEL

USNVC Confidence Level: High

USNVC Confidence Comments [optional]:

HIERARCHY

*Lower Level NVC Types:

| **Elcode** | **Scientific or Colloquial Name** |
| --- | --- |
| M296 | Caribbean-Mesoamerican Pine Dry Forest |
| M134 | Caribbean Coastal Lowland Dry Forest |
| M294 | Caribbean Dry Limestone Forest |
| M561 | Caribbean-Mesoamerican Seasonal Dry Forest |
| M562 | Pacific Mesoamerican Seasonal Dry Forest |
| M514 | Caribbean Ruderal Dry Forest |

DISCUSSION

Discussion [optional]:

CONCEPT HISTORY

*Recent Concept Lineage [if applicable]:

| **Date** | **Predecessor** | **Note** |
| --- | --- | --- |
|  |  |  |

RELATED CONCEPTS

Supporting Concepts [optional]:

| **Relationship to NVC** | **Supporting Concept Name** | **Short Citation** | **Note** |
| --- | --- | --- | --- |
|  |  |  |  |

AUTHORSHIP

*Primary Concept Source [if applicable]: C. Josse and P. Comer, in Faber-Langendoen et al. (2015)

| **Relationship to NVC** | **Name Used in Source** | **Short Citation** | **Note** |
| --- | --- | --- | --- |
|  |  |  |  |

*Author of Description: P. Comer and C. Josse

Acknowledgments [optional]: J. Franklin

Version Date: 07 Jan 2016

REFERENCES

*References [Required if used in text]:

Borhidi, A. 1991. Phytogeography and vegetation ecology of Cuba. Akademiai Kiado. Budapest, Hungary. 858 pp. plus color plates and map by A. Borhidi and O. Muniz (1970) inside of back cover.

Brandeis, T. J., E. H. Helmer, H. Marcano-Vega, and A. E. Lugo. 2009b. Climate shapes the novel plant communities that form after deforestation in Puerto Rico and the US Virgin Islands. Forest Ecology and Management 258(7):1704-1718.

Brandeis, T., M. Delaney, L. Royer, and B. Parresol. 2009a. Allometric equations for predicting Puerto Rican dry forest biomass and volume. Pages 197-202 in: R. E. McRoberts, G. A. Reams, P. C. Van Deusen, and W. H. McWilliams, editors. Proceedings of the eighth annual forest inventory and analysis symposium; 2006 October 16-19; Monterey, CA. General Technical Report WO-79. USDA Forest Service, Washington, DC. [http://www.srs.fs.usda.gov/pubs/17281]

Brown, D. E., editor. 1982a. Biotic communities of the American Southwest-United States and Mexico. Desert Plants Special Issue 4(1-4):1-342.

Bullock, S. H., H.A. Mooney, and E. Medina, editors. 1995. Seasonally dry tropical forests. Cambridge University Press, Cambridge, UK. 521 pp.

Chinea, J. D., and E. H. Helmer. 2009. Diversity and composition of tropical secondary forests recovering from large-scale clearing: Results from the 1990 inventory in Puerto Rico. Forest Ecology and Management 180(1):227-240.

Dansereau, P. 1966. Studies on the vegetation of Puerto Rico. Part I. Description and integration of the plant-communities. University of Puerto Rico, Institute of Caribbean Sciences. Special Publication No. 1. Mayagüez, Puerto Rico. 287 pp.

FNAI [Florida Natural Areas Inventory]. 2010a. Guide to the natural communities of Florida: 2010 edition. Florida Natural Areas Inventory, Tallahassee, FL. 228 pp. [https://fnai.org/naturalcommguide.cfm]

Faber-Langendoen, D., J. Drake, S. Gawler, M. Hall, C. Josse, G. Kittel, S. Menard, C. Nordman, M. Pyne, M. Reid, L. Sneddon, K. Schulz, J. Teague, M. Russo, K. Snow, and P. Comer, editors. 2010-2018. Divisions, Macrogroups and Groups for the Revised U.S. National Vegetation Classification. NatureServe, Arlington, VA. plus appendices. [in preparation]

Franklin, J., J. Ripplinger, E. H. Freid, H. Marcano-Vega, and D. W. Steadman. 2015. Regional variation in Caribbean dry forest tree species composition. Plant Ecology DOI 10.1007/s11258-015-0474-8.

Gillespie, T. W. 2006. Diversity, biogeography and conservation of woody plants in tropical dry forest of south Florida. Pages 383-394 in: R. T. Pennington, G. P. Lewis, and J. A. Ratter, editors. Neotropical Savannas and Seasonally Dry Forest. Systematics Association special volume (69). CRC Press.

Gillespie, T., A. Grijalva, and C. Farris. 2000. Diversity, composition, and structure of tropical dry forests in Central America. Plant Ecology 147:37-47.

Helmer, E. H., T. J. Brandeis, A. E. Lugo, and T. Kennaway. 2008. Factors influencing spatial pattern in tropical forest clearance and stand age: Implications for carbon storage and species diversity. Journal of Geophysical Research 113:G02S04. [http://dx.doi.org/10.1029/2007JG000568]

Holbrook, N. M., J. L. Whitbeck, and H. A. Mooney. 1995. Drought responses of neotropical dry forest trees. Pages 234-276 in: H. A. Mooney, S. H. Bullock, and E. Medina, editors. Seasonally Dry Tropical Forests. Cambridge University Press, Cambridge.

Janzen, D. H. 1988. Tropical dry forests: The most endangered major tropical ecosystem. Pages 130-137 in: E. O. Wilson, editor. Biodiversity. National Academic Press, Washington DC.

Leiva, J. A., R. Mata, O. J. Rocha, and M. V. Gutiérrez Soto. 2009. Cronología de la regeneración del bosque tropical seco en Santa Rosa, Guanacaste, Costa Rica. I. Características edáficas. Rev. Biol. Trop. (Int. J. Trop. Biol.) 57(3):801-815.

Lugo, A. E., E. Medina, J. C. Trejo-Torres, and E. Helmer. 2006. Botanical and ecological basis for the resilience of Antillean Dry Forests. Pages 359-381 in: R. T. Pennington, G. P. Lewis, and J. A. Ratter, editors. Neotropical savannas and seasonally dry forests: Plant diversity, biogeography and conservation. CRC Press, Boca Raton, FL.

Lugo, A. E., L. M. Castro, A. Vale, T. del Mar López, E. H. Prieto, A. G. Martinó, A. R. Puente Rolón, A. G. Tossas, D. A. McFarlane, T. Miller, A. Rodríguez, J. Lundberg, J. Thomlinson, J. Colón, J. H. Schellekens, O. Ramos, and E. Helmer. 2001. Puerto Rican karst: A vital resource. General Technical Report WO- 65. USDA Forest Service, Washington, DC. [http://www.fs.fed.us/global/iitf/karst.pdf]

Martinuzzi, S., A. E. Lugo, T. H. Brandeis, and E. H. Helmer. 2013. Case study: Geographic distribution and level of novelty of Puerto Rican Forests. Pages 81-87 in: R. J. Hobbs, E. S. Higgs and C. M. Hall, editors. Novel Ecosystems: Intervening in the New Ecological World Order. John Wiley & Sons. Ltd.

Miller, A. C., 2007. Fire history of Caribbean Pine (Pinus caribaea var. bahamensis (Griseb.) W.H. Barrett & Golfari) forests on Abaco Island, The Bahamas. M.S. Research Paper, Department of Geography, University of Tennessee, Knoxville. 92 pp.

Murphy, P. G., and A. E. Lugo. 1995. Dry forests of Central America and the Caribbean. Pages 9-34 in: S. H. Bullock, H. A. Mooney, and E. Medina, editors. Seasonally Dry Tropical Forest. Cambridge University Press, Cambridge.

O'Brien, J. J., J. K. Hiers, M. A. Callaham, R. J. Mitchell, and S. B. Jack. 2008. Interactions among overstory structure, seedling life-history traits, and fire in frequently burned neotropical pine forests. Ambio 37:542-547.

Pennington, R. T., G. P. Lewis, and J. A. Ratter. 2006b. An overview of the plant diversity, biogeography and conservation of neotropical savannas and seasonally dry forests. Pages 1-29 in: R. T. Pennington, G. P. Lewis, and J. A. Ratter, editors. Neotropical savannas and seasonally dry forests: Plant diversity, biogeography and conservation. CRC Press, Boca Raton, FL.

Rojas-Sandoval, J. and P. Acevedo-Rodriguez. 2015. Naturalization and invasion of alien plants in Puerto Rico and the Virgin Islands. Biological Invasions 17:149-163.

Ross, M. S., J. J. O'Brien, and L. J. Flynn. 1992. Ecological site classification of Florida Keys terrestrial habitats. Biotropica 24:488-502.

Rzedowski, J. 1986. Vegetacion de Mexico. Editorial Limusa, Mexico. 432 pp.

Sabogal, C. 1992. Regeneration of tropical dry forest in Central America, with examples from Nicaragua. Journal of Vegetation Science 3:407-416.

Smith, I. K., and J. L. Vankat. 1992. Dry evergreen forest (coppice) communities of North Andros Island, Bahamas. Bulletin of the Torrey Botanical Club 119:181-191.

Snyder, J. R., A. Herndon, and W. B. Robertson, Jr. 1990. South Florida rockland. Pages 230-277 in: R. L. Myers and J. J. Ewel, editors. Ecosystems of Florida. University of Central Florida Press, Orlando.

Stout, I. J., and W. R. Marion. 1993. Pine flatwoods and xeric pine forests of the southern (lower) coastal plain. Pages 373-446 in: W. H. Martin, S. G. Boyce, and A. C. Echternacht, editors. 1993. Biodiversity of the southeastern United States: Lowland terrestrial communities. John Wiley and Sons, New York.

Tolentino, L., and M. Peña. 1998. Inventario de la vegetacion y uso de la tierra en la Republica Dominicana. Moscosoa 10:179-202.

1. Forest & Woodland

1.A.1.Ea. Caribbean-Mesoamerican Dry Forest & Woodland

M296. Caribbean-Mesoamerican Pine Dry Forest

Type Concept Sentence: This macrogroup is characterized by forests that include in their composition either pine or oak species growing alongside other broadleaf species in tropical lowland, seasonally dry settings, with additional soil moisture due to the substrate composition. Natural communities within this macrogroup are distributed in the southern Florida Peninsula and the Florida Keys, the Bahamian Archipelago, Cuba, the Gulf and Caribbean coast of Mexico, reaching south into Costa Rica. The south Florida slash pine forest or pine rockland is the community represented in Florida and is characterized by an open canopy of *Pinus elliottii var. densa* with a patchy understory of tropical and temperate shrubs and palms and a rich herbaceous layer of mostly perennial species, including numerous species endemic to southern Florida.

OVERVIEW

*Hierarchy Level: Macrogroup

*Placement in Hierarchy: 1.A.1.Ea. Caribbean-Mesoamerican Dry Forest & Woodland (D099)

Elcode: M296

*Scientific Name: *Coccothrinax argentata - Pinus elliottii var. densa - Pinus caribaea* Dry Forest Macrogroup

*Common (Translated Scientific) Name: Florida Silver Palm - Florida Slash Pine - Caribbean Pine Dry Forest Macrogroup

*Colloquial Name: Caribbean-Mesoamerican Pine Dry Forest

*Type Concept: This macrogroup encompasses tropical and subtropical forests of open to semi-open canopy, 8-12 m tall, characterized by a diverse shrub/subcanopy layer composed of species of palm and hardwood mostly of Caribbean floristic affinity. The uniqueness of the flora associated with the South Florida Slash Pine forest has long been recognized; it has been estimated that nearly one-third of the taxa found in this type are restricted to it, including half of southern Florida's endemic plants. Shrubs include *Chrysobalanus icaco, Coccothrinax argentata, Ilex cassine, Sideroxylon salicifolium, Tetrazygia bicolor*, and *Leucothrinax morrisii*. More common and representative (if not diagnostic) species on the mainland and Florida Keys include *Byrsonima lucida, Dodonaea viscosa, Guettarda elliptica, Guettarda scabra*, and *Serenoa repens*. Similar open pine forests occur in the northern Bahama Archipelago and along the coast in western and eastern Cuba where they are known as pine barrens or pine woodlands and are dominated by *Pinus caribaea, Pinus tropicalis*, and *Pinus cubensis*, respectively, accompanied by a similar set of species such as the palms *Coccothrinax argentata, Coccothrinax miraguana, Coccothrinax orientalis, Coccothrinax yuraguana*, and *Leucothrinax morrisii*. Other species include *Rhus copallinum, Tabebuia bahamensis, Tabebuia dubia, Tabebuia lepidophylla, Tabebuia shaferi, Zamia pumila*, and species of *Byrsonima, Guettarda*, and *Tetrazygia*. The macrogroup also includes pine-oak dry forests in western Cuba, where the oak species *Quercus oleoides* also occurs in the Dry Evergreen Oak forest type distributed in Mesoamerica and included in the macrogroup.

~South Florida Slash Pine Rockland Group (G005)$$ is a pyrogenic community, a defining characteristic shared by all the other community types within the macrogroup, especially those dominated by pine species. In general, forests in this macrogroup experience a dry season of several months. Specific communities can be dry-mesic or xeric depending on landscape position and substrate. The macrogroup occurs in lowlands and low hills, littoral or sub-littoral flatlands on limestone or on thin sandy soils over limestone, or on light gray quartz sand or soils derived from sandstone or serpentine bedrock in the case of communities in Cuba. All these different substrates are nutrient-poor and drain very rapidly. Consequently, most sites are wet for only short periods following heavy rains.

*Diagnostic Characteristics: *Pinus elliottii var. densa* is the dominant and characteristic canopy tree and is nearly always present in stands of the Florida pine rockland type; other pine species are characteristic in other parts of the distribution of the type. Diagnostic tropical shrubs include *Coccothrinax argentata, Leucothrinax morrisii, Sideroxylon salicifolium, Chrysobalanus icaco, Ilex cassine*, and *Tetrazygia bicolor*. Different shrubs are found on the Miami Ridge than in the Florida Keys. *Leucothrinax morrisii* is a typical shrub only in the Florida Keys and together with *Coccothrinax argentata* are shared understory species with the Cuban and Bahamian pine woodlands. More common and representative (if not diagnostic) species on the mainland and Florida Keys include *Byrsonima lucida, Dodonaea viscosa, Guettarda elliptica, Guettarda scabra*, and *Serenoa repens*.

*Classification Comments: This description is best developed for the Florida part of the range and further work is needed to make it a more comprehensive rangewide description. The Florida communities bear a strong resemblance to the rocky pinelands in the Caribbean, particularly in the eastern Bahamian Archipelago on Grand Bahama, Abaco, New Providence, and Andros islands. These Bahamian pinelands have a canopy of *Pinus caribaea var. bahamensis*, but are similar to the South Florida types in substrate, physiognomy, and tropical understory species (Snyder et al. 1990). The southwestern Florida *Pinus elliottii var. densa*-dominated flatwoods on sands not influenced by limestone are not included in this macrogroup. The flatwoods tend to have *Serenoa repens*, an evergreen palm, as a common low shrub, and *Aristida beyrichiana* as a common grass. The flatwoods lack the tropical broadleaf evergreen shrubs characteristic of the pine rockland.

*Similar NVC Types [if applicable]:

| **Elcode** | **Scientific or Colloquial Name** | **Note** |
| --- | --- | --- |
|  |  |  |

Similar NVC Types General Comments [optional]:

VEGETATION

Physiognomy and Structure Summary: The slash pine forest in this macrogroup is characterized by an open canopy, generally with multiple age classes, of the needle-leaved evergreen conifer *Pinus elliottii var. densa*, associated with a high diversity of palms, hardwoods and herbaceous plants in the understory, most derived from the tropical flora of the West Indies (Snyder et al. 1990). Many of these species vary in height depending on fire frequency, getting taller with time since fire (FNAI 2010a).

Physiognomy and Structure Table [optional]:

| **Physiognomy-Structure Category** | **Prevailing Height (m)** | **Height Range (opt.)** | **Mean % Cover** | **Cover Range (opt.)** |
| --- | --- | --- | --- | --- |
|  |  |  |  | - |

Floristics Summary: *Pinus elliottii var. densa* is the characteristic and often dominant pine species of the macrogroup distribution in the continental United States. Characteristic pine species in the communities further south and in the Caribbean include *Pinus caribaea* (*var. bahamensis, var. caribaea*, and *var. hondurensis*), and *Pinus tropicalis*. It has been estimated that nearly one-third of the taxa found in the south Florida pine rockland are restricted to it, including half of southern Florida's endemic plants (Stout and Marion 1993). Shrubs include *Chrysobalanus icaco, Coccothrinax argentata, Ilex cassine, Sideroxylon salicifolium, Tetrazygia bicolor*, and *Leucothrinax morrisii (= Thrinax morrisii)*. More common and representative (if not diagnostic) species on the mainland and Florida Keys include *Byrsonima lucida, Dodonaea viscosa, Guettarda elliptica, Guettarda scabra*, and *Serenoa repens*. Similar open pine forests occur in the northern Bahama Archipelago and along the coast in western and eastern Cuba where they are known as pine barrens or pine woodlands and are dominated by *Pinus caribaea, Pinus tropicalis*, and *Pinus cubensis*, respectively, accompanied by a similar set of species such as the palms *Coccothrinax argentata, Coccothrinax miraguana, Coccothrinax orientalis, Coccothrinax yuraguana*, and *Leucothrinax morrisii*. Other species include *Byrsonima lucida, Dodonaea viscosa, Guettarda elliptica, Guettarda scabra, Rhus copallinum, Serenoa repens, Tabebuia bahamensis, Tabebuia dubia, Tabebuia lepidophylla, Tabebuia shaferi*, and *Zamia pumila* (T. Armentano pers. comm. 2003). The macrogroup also includes pine-oak dry forests in western Cuba, where the oak species *Quercus oleoides* also occurs in the Dry Evergreen Oak forest type distributed in Mesoamerica and included in the macrogroup.

*Floristics Table [Med - High Confidence]:

*Number of Plots:

*Cover Scale Used:

| **Physiognomy-Structure Category** | **Taxon Name** | **Specific Growth Form (opt.)** | **Const- ancy** | **Mean % Cover** | **Cover Range (opt.)** | **Differ-ential** | **Diagnostic Combin- ation** |
| --- | --- | --- | --- | --- | --- | --- | --- |
|  |  |  |  |  | - |  |  |

Dynamics: Pine rockland is maintained by regular fire (O'Brien et al. 2008), and susceptible to other natural disturbances such as hurricanes, frost events, and sea-level rise (Ross et al. 1994, as cited in FNAI 2010a). Fires historically burned on an interval of around 3 to 7 years (Snyder et al. 1990) and were typically started by lightning strikes during the frequent summer thunderstorms. Presently, prescribed fire must be periodically introduced into pine rocklands to sustain community structure, prevent invasion by woody species, maintain high herbaceous diversity (Loope and Dunevitz 1981, as cited in FNAI 2010a), and prevent succession to rockland hammock. The ecotone between pine rockland and rockland hammock in Florida is abrupt when regular fire is present in the system. In the absence of fire, the pine rockland may be replaced by hardwood species within several decades (Stout and Marion 1993). Fire is also reported as part of the natural dynamic of dry, pre-montane, mixed *Pinus caribaea - Quercus oleoides* forests of Belize and Honduras, another community represented within this macrogroup. See also Kennedy and Horn (2008).

Hurricanes and storms can have a major impact on pine rocklands. High winds can significantly affect plant structure or composition by causing canopy and subcanopy mortality (Platt et al. 2000, as cited in FNAI 2010a), resulting in subsequent stimulation of shrub or herbaceous growth. Pine rocklands near the coast may be temporarily inundated by saltwater during severe storms, which can kill or damage vegetation (Snyder et al. 1990). Rare frost events bringing below-freezing temperatures can reduce tropical hardwoods. Because tropical and subtropical plants in pine rocklands are more exposed to below-freezing temperatures in the relatively open understory, they are more likely to succumb to freeze damage than their counterparts in sheltered rockland hammocks (FNAI 2010a). The area of pine rockland in the Florida Keys has been reduced since the 1930s. This is at least partially due to increased ground and soil salinity resulting from a 15-cm local rise in sea level that has occurred since that time (Ross et al. 1994, as cited in FNAI 2010a).

The fire cycle is central to succession in these pine forest communities in the Caribbean, and palms, shrubs and hardwood trees are dynamic structural components. That is, they are relatively sensitive to fires not intense enough to cause the complete replacement of the stand, but recover quickly thereafter. This is surely true for shrubs and hardwoods, which resprout prolifically after fire, but palms grow very slowly and do not resprout. Besides intensity, season of fire is also an important component in the fire regime of pine forests in the Caribbean, as it may influence fuel consumption, fire intensity, and forest development during the post-burn period (Liu and Menges 2005 and references therein). In addition to its effects on existing vegetation, a very intense fire may hinder recovery of the plant community (Spier and Snyder 1998), thereby constraining the development of fuel loads in the post-burn period. Thus the effect of different fire cycles, natural or human-caused, has the capacity to result in different structure and composition of the pine forest ecosystems. In addition to fires, hurricanes are another major natural disturbance affecting the distribution, composition and structure of the pine forests.

ENVIRONMENT

Environmental Description: *Climate*: Mean annual temperatures in the area of distribution of the macrogroup range from 23°C (74°F) in the north to 26°C (77°F) in the Lower Keys. Mean annual temperature in the West Indies distribution of the macrogroup is around 25°C. Precipitation primarily occurs from May or June to October and ranges from 1650 mm along the Atlantic coast decreasing southward to less than 1000 mm in the Lower Keys (Gillespie 2006). Annual precipitation in the distributional range of this forest in Cuba is less than 1500 mm in the west part of the range and increases towards the east.

*Soil/Substrate*: Limestone is the dominant substrate in the macrogroup distribution in Florida and the Bahamas, with skeletal organic soils with minor mineral components, rarely exceeding 20 cm in depth (Snyder et al. 1990, as cited in Gillespie 2006). In Cuba, the pine forests included in this macrogroup are found primarily on acidic soils that have little water-retention capacity and are poor in essential elements. The principal soil types on which they occur are quartziferous sands, pseudo-spodosols in the west and lateritic soils in the east. Only pine trees, which have an ectomycorrhizal symbiosis with fungi, are capable of obtaining in this way a sufficient amount of nutrients to achieve the size of trees. In Florida and the Bahamas, pine rockland occurs on relatively flat, moderately to well-drained terrain, from 2-7 m above sea level (Snyder et al. 1990). The oolitic limestone is at or very near the surface, and there is very little soil development. Soils are generally composed of small accumulations of nutrient-poor sand, marl, clayey loam, and organic debris in depressions and crevices in the rock surface. Organic acids occasionally dissolve the surface limestone causing collapsed depressions in the surface rock called solution holes (Outcalt 1997b). Drainage varies according to the porosity of the limestone substrate, but is generally rapid. Consequently, most sites are wet for only short periods following heavy rains. During the rainy season, however, some sites may be shallowly inundated by slow-flowing surface water for up to 60 days each year (FNAI 2010a).

The macrogroup occurs in lowlands and low hills, littoral or sublittoral flatlands on limestone or on thin sandy soils over limestone, or on light gray quartz sand or soils derived from sandstone or serpentine bedrock in the case of communities in Cuba. All these different substrates are nutrient-poor and drain very rapidly. Consequently, most sites are wet for only short periods following heavy rains.

DISTRIBUTION

*Geographic Range: Natural communities within this macrogroup are distributed in the southern Florida peninsula and the Florida Keys. In the Caribbean, pine forests are found in the Bahamas, Turks and Caicos Islands, Cuba, and Hispaniola. In Cuba, conifer forests occur in the eastern and western ends of the island.

Nations: BS, BZ, CR, CU, DO, GT, HN, HT, MX, NI, SV?, TC, US, XC, XD

States/Provinces: FL

USFS Ecoregions (2007) [optional]: 411A:CC

Omernik Ecoregions L3, L4 [optional]:

MLRAs [optional]:

PLOT SAMPLING AND ANALYSIS

*Plot Analysis Summary [Med - High Confidence]:

*Plots Used to Define the Type [Med - High Confidence]:

CONFIDENCE LEVEL

USNVC Confidence Level: Moderate

USNVC Confidence Comments [optional]:

HIERARCHY

*Lower Level NVC Types:

| **Elcode** | **Scientific or Colloquial Name** |
| --- | --- |
| G484 | East Cuban Pine Woodland |
| G482 | Caribbean Pine-Oak Woodland |
| G483 | Mesoamerican Pine-Oak Dry Forest |

DISCUSSION

Discussion [optional]:

CONCEPT HISTORY

*Recent Concept Lineage [if applicable]:

| **Date** | **Predecessor** | **Note** |
| --- | --- | --- |
| 2013-08-28 | M006 Caribbean & Central American Pine - Oak Forest Macrogroup | M006 reworked as M296, M561, M562 |

RELATED CONCEPTS

Supporting Concepts [optional]:

| **Relationship to NVC** | **Supporting Concept Name** | **Short Citation** | **Note** |
| --- | --- | --- | --- |
| > | Pine Forest | Duever et al. 1986 |  |
| = | Pine Rockland | FNAI 2010a |  |
| = | Rockland Pine Forest | Davis 1943 |  |

AUTHORSHIP

*Primary Concept Source [if applicable]: Faber-Langendoen et al. (2014)

| **Relationship to NVC** | **Name Used in Source** | **Short Citation** | **Note** |
| --- | --- | --- | --- |
|  |  |  |  |

*Author of Description: C. Josse and C.W. Nordman

Acknowledgments [optional]:

Version Date: 08 Jan 2015

REFERENCES

*References [Required if used in text]:

Armentano, Tom. Personal communication. National Park Service, Everglades National Park, Homestead, FL.

Comer, P., D. Faber-Langendoen, R. Evans, S. Gawler, C. Josse, G. Kittel, S. Menard, C. Nordman, M. Pyne, M. Reid, M. Russo, K. Schulz, K. Snow, J. Teague, and R. White. 2003-present. Ecological systems of the United States: A working classification of U.S. terrestrial systems. NatureServe, Arlington, VA.

Davis, J. H., Jr. 1943. The natural features of southern Florida, especially the vegetation, and the Everglades. Florida Department of Conservation, Geologic Survey. Geologic Bulletin No. 25. Tallahassee, FL.

De Las Heras, J., M. Bonilla, and L. W. Martinez. 2006. Germination after heat treatments of Pinus tropicalis Morelet and Pinus caribaea Morelet var. caribaea seeds of west Cuban forests. Annals of Forest Science 63:469-475.

Duever, M. J., J. E. Carlson, J. F. Meeder, L. C. Duever, L. H. Gunderson, L. A. Riopelle, T. R. Alexander, R. L. Myers, and D. P. Spangler. 1986. The Big Cypress National Preserve. National Audubon Society Research Report No. 8. National Audubon Society, New York. 444 pp.

FNAI [Florida Natural Areas Inventory]. 2010a. Guide to the natural communities of Florida: 2010 edition. Florida Natural Areas Inventory, Tallahassee, FL. 228 pp. [https://fnai.org/naturalcommguide.cfm]

Faber-Langendoen, D., J. Drake, S. Gawler, M. Hall, C. Josse, G. Kittel, S. Menard, C. Nordman, M. Pyne, M. Reid, L. Sneddon, K. Schulz, J. Teague, M. Russo, K. Snow, and P. Comer, editors. 2010-2018. Divisions, Macrogroups and Groups for the Revised U.S. National Vegetation Classification. NatureServe, Arlington, VA. plus appendices. [in preparation]

Franklin, J., and D. W. Steadman. 2013. The winter bird communities in pine woodland vs. broadleaf forest on Abaco, The Bahamas. Caribbean Naturalist 3:1-18.

Gillespie, T. W. 2006. Diversity, biogeography and conservation of woody plants in tropical dry forest of south Florida. Pages 383-394 in: R. T. Pennington, G. P. Lewis, and J. A. Ratter, editors. Neotropical Savannas and Seasonally Dry Forest. Systematics Association special volume (69). CRC Press.

Kennedy, L. M., and S. P. Horn. 2008. Postfire vegetation recovery in highland pine forests of the Dominican Republic. Biotropica 40:412-421.

Liu, H., and E. S. Menges. 2005. Winter fires promote greater vital rates in the Florida Keys than summer fires. Ecology 86(6):1483-1495.

McPherson, B. F. 1973. Vegetation map of the Big Cypress National Preserve. Figure 5.1 [back cover sleeve] in: M. J. Duever, J. E. Carlson, J. F. Meeder, L. C. Duever, L. H. Gunderson, L. A. Riopelle, T. R. Alexander, R. L. Myers, and D. P. Spangler. 1986. The Big Cypress National Preserve. National Audubon Society Research Report No. 8. National Audubon Society, New York.

Menges, E. S., and M. A. Deyrup. 2001. Postfire survival in south Florida slash pine: Interacting effects of fire intensity, fire season, vegetation, burn size, and bark beetles. International Journal of Wildland Fire 10:53-63.

Miller, A. C., 2007. Fire history of Caribbean Pine (Pinus caribaea var. bahamensis (Griseb.) W.H. Barrett & Golfari) forests on Abaco Island, The Bahamas. M.S. Research Paper, Department of Geography, University of Tennessee, Knoxville. 92 pp.

Negron-Ortiz, V., and D. L. Gorchov. 2000. Effects of fire season and post-fire herbivory on the cycad Zamia pumila (Zamiaceae) in slash pine savanna, Everglades National Park, Florida. International Journal of Plant Sciences 161:659-669.

O'Brien, J. J., J. K. Hiers, M. A. Callaham, R. J. Mitchell, and S. B. Jack. 2008. Interactions among overstory structure, seedling life-history traits, and fire in frequently burned neotropical pine forests. Ambio 37:542-547.

Outcalt, K. W. 1997b. An old-growth definition for tropical and subtropical forests in Florida. General Technical Report SRS-013. USDA Forest Service, Southern Research Station, Asheville, NC. 8 pp.

Robbins, L. E., and R. L. Myers. 1992. Seasonal effects of prescribed burning in Florida: A review. Tall Timbers Research, Miscellaneous Publication Number 8, Tallahassee, FL.

Snyder, J. R., A. Herndon, and W. B. Robertson, Jr. 1990. South Florida rockland. Pages 230-277 in: R. L. Myers and J. J. Ewel, editors. Ecosystems of Florida. University of Central Florida Press, Orlando.

Spier, L. P., and J. R. Snyder. 1998. Effects of wet- and dryseason fires on Jacquemontia curtisii, a south Florida pine forest endemic. Natural Areas Journal 18:350-357.

Stout, I. J., and W. R. Marion. 1993. Pine flatwoods and xeric pine forests of the southern (lower) coastal plain. Pages 373-446 in: W. H. Martin, S. G. Boyce, and A. C. Echternacht, editors. 1993. Biodiversity of the southeastern United States: Lowland terrestrial communities. John Wiley and Sons, New York.

USFWS [U.S. Fish and Wildlife Service]. 1998b. South Florida multi-species recovery plan. U.S. Fish and Wildlife Service, South Florida Ecological Services Office. [http://www.fws.gov/verobeach/Programs/Recovery/vbms5.html]

1. Forest & Woodland

1.A.1.Ea. Caribbean-Mesoamerican Dry Forest & Woodland

M134. Caribbean Coastal Lowland Dry Forest

Type Concept Sentence: This vegetation is found in flat lowlands and low hills (ca. 300 m elevation) with a pronounced dry season along south Florida and the Antilles. Forests have low stature, high density of small and medium-sized trees, and have single-layer canopies with no emergent trees. The herb layer is poorly developed or completely lacking. Floristic diversity is low compared to more humid forests, and high species dominance is common. Variation in the dry season period, topography, and substrate determine the specific characteristics of the vegetation communities in this macrogroup since all of these have great importance in dry forests as determinants of variation in water availability.

OVERVIEW

*Hierarchy Level: Macrogroup

*Placement in Hierarchy: 1.A.1.Ea. Caribbean-Mesoamerican Dry Forest & Woodland (D099)

Elcode: M134

*Scientific Name: *Bursera simaruba - Coccoloba diversifolia - Eugenia* sp. Caribbean Coastal Lowland Dry Forest Macrogroup

*Common (Translated Scientific) Name: Gumbo Limbo - Tie-tongue - Stopper species Caribbean Coastal Lowland Dry Forest Macrogroup

*Colloquial Name: Caribbean Coastal Lowland Dry Forest

*Type Concept: This macrogroup encompass tropical and subtropical forests characterized by a dry season of several months that occur in coastal lowlands and low hills, littoral or sublittoral flatlands with rock outcrops and higher terraces facing the sea, on limestone coral shelves, humic carbonate soils, shallow red ferralitic soils, or sandy soils of stabilized, old coastal dunes in south Florida, the Florida Keys, the Greater Antilles, and other Caribbean islands such as those of the Bahamas and Virgin Islands archipelagos. The species composition and structure of these forests vary depending upon the substrate and climate across their distribution. They are broadleaf semi-deciduous to evergreen forests with a canopy between 6-10 m of height. The density of stems tends to be very high. The woody understory is mostly evergreen. The herb layer is poorly developed or completely lacking. In the continental U.S., the Caribbean dry forest occurs in two settings: the hardwood hammock forest in southern Florida, on elevated outcrops of limestone, and the strand forest - a narrow band of hardwood forest and tall shrublands lying just inland of the coastal dune system in south Florida. The latter occur on stabilized, old coastal dunes, often with substantial shell components. In both cases the vegetation is characterized by hardwood species with tropical affinities, with *Eugenia axillaris* as the most commonly shared species. The Florida hammock forest occurs in three discrete regions, including the Florida Keys, southeastern Big Cypress, and the Miami Rock Ridge. Tropical hardwood species likely to be encountered include *Ardisia escallonoides, Bursera simaruba, Coccoloba diversifolia, Eugenia axillaris*, and *Guapira discolor*. The northward ranges of these species are limited by the incidence of frost. These forests tend to have a dense canopy that produces deeper shade, less evaporation, and lower air temperature than surrounding vegetation in these locations. This microclimate, in combination with high water tables, tends to keep humidity levels high. A number of orchid and bromeliad species thrive in such conditions. Unlike much U.S. coastal plain vegetation, fire is a major threat to these hardwood hammock forests.

Dry forests of the Caribbean islands share some features with the Florida dry forests, such as the high density of stems, low stature and relative low floristic diversity, with several shared species among the dominant ones (*Bursera simaruba, Coccoloba diversifolia, Eugenia foetida, Gymnanthes lucida*). Diagnostic species of this type include *Amyris elemifera, Bursera simaruba, Bucida buceras, Coccoloba diversifolia, Coccoloba uvifera, Coccoloba krugii, Eugenia axillaris, Eugenia foetida, Erithalis fruticosa, Exostema caribaeum, Haematoxylum campechianum, Gymnanthes lucida, Guettarda krugii, Guaiacum officinale, Guaiacum sanctum, Jacquinia armillaris, Krugiodendron ferreum, Nectandra coriacea, Pisonia albida, Sabal palmetto, Simarouba glauca, Savia sessiliflora*, and *Thouinia striata var. portoricensis*. These forests also share the limestone substrate which is widespread among the coastal Antilles. Caribbean coastal dry forests in Cuba are slightly taller and have two canopy layers; with the upper layer reaching 12-15 m. This macrogroup also includes lowland semi-deciduous forests on richer substrates in Cuba, Hispaniola, and several smaller Caribbean islands. However, centuries of human occupation and agricultural land use have largely replaced these natural forests described in the literature as more diverse, semi-deciduous, and with the canopy up to 15-20 m height.

*Diagnostic Characteristics: Diagnostic characteristics for this forest macrogroup are low stature with few or no emergent trees, poorly distinguished canopy layers, a high density of stems resulting from the majority of tree species having a tendency to develop several main stems (to coppice), and a dry season severe enough to select for drought-deciduous or evergreen, drought-tolerant trees. Among the species likely to be encountered throughout are *Bursera simaruba, Coccoloba diversifolia*, and *Eugenia axillaris*. The tree species of the tropical hammocks of Florida are a subset of the native Caribbean dry forests species that can withstand extremely rare frost events (Gillespie 2006).

*Classification Comments: The distinction between this macrogroup and ~Caribbean Dry Limestone Forest Macrogroup (M294)$$ should be clarified, since the latter refers to plant communities that grow on limestone substrates in dry climates, but also in other dry geophysical settings within humid climates. Calcium carbonate-rich soils create physiological and nutrient stress on the plant communities growing on them which results in distinct structure and composition.

*Similar NVC Types [if applicable]:

| **Elcode** | **Scientific or Colloquial Name** | **Note** |
| --- | --- | --- |
|  |  |  |

Similar NVC Types General Comments [optional]:

VEGETATION

Physiognomy and Structure Summary: The dry forests of south Florida and the Caribbean have a greater density of individual stems and shorter canopy heights than tropical dry forests in the mainland Neotropics (Gillespie 2006, Lugo et al. 2006). Density varies from 14,000 stems >2.5 cm/ha in Puerto Rico dry forest to 4600 stems/ha in Florida. Canopy height ranges between 5 and 10 m, with a higher mean height in Florida hammock forest stands than in Puerto Rico dry forest, and few trees reaching 18-20 m height. The canopy is seasonally open; however, there are few truly deciduous species in these forests, with early-successional forests dominated by broadleaf semi-deciduous species and late-successional forests dominated by broadleaf evergreen species. Drought-deciduousness is the principal adaptive mode of tropical dry deciduous forests, and at the dry extremes, small evergreen trees are important (Lugo et al. 2006). There is a notable lack of lianas compared with dry forests in the mainland Neotropics (Gillespie 2006). Despite relatively low species diversity, lifeform diversity is common and is accompanied by diversity in plant habit, leaf size and structure, drought tolerance and growth seasonality; this diversity is attributed to habitat heterogeneity coupled with strong rainfall seasonality (Lugo et al. 1978, Medina 1995, as cited in Lugo et al. 2006). In south Florida, dry tropical forests occurring in sites with higher mean temperature and lower precipitation have greater stand density, while higher precipitation and lower temperatures result in greater canopy heights (Gillespie 2006). Epiphytic orchid and bromeliad species are often found in areas where frosts or anthropogenic disturbances have not occurred in a long time.

Physiognomy and Structure Table [optional]:

| **Physiognomy-Structure Category** | **Prevailing Height (m)** | **Height Range (opt.)** | **Mean % Cover** | **Cover Range (opt.)** |
| --- | --- | --- | --- | --- |
|  |  |  |  | - |

Floristics Summary: The vegetation is characterized by hardwood species with tropical affinities. Common species in south Florida, the Bahamas, Cuba and Puerto Rico are *Amyris elemifera, Bursera simaruba, Coccoloba diversifolia, Coccoloba uvifera, Eugenia axillaris, Eugenia foetida, Gymnanthes lucida, Krugiodendron ferreum, Nectandra coriacea, Sabal palmetto*, and *Simarouba glauca*. Dominant species in Puerto Rico coastal dry forest are *Coccoloba krugii, Erithalis fruticosa, Exostema caribaeum, Guettarda krugii, Gymnanthes lucida, Pisonia albida, Savia sessiliflora*, and *Thouinia striata var. portoricensis*. Given the harsh conditions of the Antillean dry forests, those relatively few species that thrive under the stress are able to dominate sites (Lugo et al. 2006). Endemism is very high and represents about 50% of the species. The northern extent of this macrogroup is limited by periodic freezes and cold-tolerance of tropical constituent species, such as *Ardisia escallonoides, Eugenia axillaris, Exothea paniculata, Guapira discolor, Metopium toxiferum, Nectandra coriacea*, and *Piscidia piscipula* (Johnson and Muller 1993a).

*Floristics Table [Med - High Confidence]:

*Number of Plots:

*Cover Scale Used:

| **Physiognomy-Structure Category** | **Taxon Name** | **Specific Growth Form (opt.)** | **Const- ancy** | **Mean % Cover** | **Cover Range (opt.)** | **Differ-ential** | **Diagnostic Combin- ation** |
| --- | --- | --- | --- | --- | --- | --- | --- |
|  |  |  |  |  | - |  |  |

Dynamics: Along the Florida distribution of this macrogroup, the coastal maritime hammocks are prone to disturbance from hurricanes, which can include extremely high winds, and in coastal areas salt spray, and saltwater storm surge. The rockland habitats on the Florida mainland are mostly inland and not subject to the salt spray and storm surge associated with hurricanes. However, winter freezes sometimes occur and result in damage of the tropical woody plants. In the Florida Keys, freezing temperatures are very unusual.

Drought-deciduousness is the principal adaptive mode of tropical dry deciduous forests, and at the dry extremes, small evergreen trees are important (Lugo et al. 2006). There is a notable lack of lianas compared with dry forests in the mainland Neotropics (Gillespie 2006). Despite relatively low species diversity, lifeform diversity is common and is accompanied by diversity in plant habit, leaf size and structure, drought tolerance and growth seasonality; this diversity is attributed to habitat heterogeneity coupled with strong rainfall seasonality (Lugo et al. 1978, Medina 1995, as cited in Lugo et al. 2006). Caribbean dry forests have to cope with highly stressful conditions given the combination of environmental features such as low moisture availability, long dry seasons, decadal cycles of pronounced drought, wind exposure and salt spray in littoral locations. These forests are also periodically exposed to hurricane conditions with effects that span from flooding with seawater to defoliation, treefall and other structural changes due to strong winds.

Overall, Caribbean coastal dry forests are exposed to harsh environmental conditions that, depending on their intensity, can cause damage or diebacks, such as seasonal water deficit, nutrient stress, strong winds and salt spray, and saltwater storm surge. This has influenced the development of structural and physiological mechanisms to cope, making them very resilient to disturbance. Among the more outstanding ones are a high resistance to wind (short stature), a high proportion of root biomass, high soil carbon and nutrient accumulation below ground, the ability of most tree species to resprout, and high nutrient use efficiency (Lugo et al. 2006).

Fire is not part of the natural dynamics of Caribbean coastal dry forests (though many dry forests are now subject to anthropogenic fires). This is why many examples occur alongside natural firebreaks, such as the leeward side of exposed limestone (Robertson 1955), moats created by limestone solution (Duever et al. 1986), and elevated outcrops above marshes, scrub cypress, or sometimes mangrove swamps (Snyder et al. 1990), or isolated on ridges in pine woodlands.

ENVIRONMENT

Environmental Description: *Climate*: Caribbean dry forests occurring on the mainland in Florida and the Upper Keys are periodically exposed to short-term frost and their flora is composed of a subset of native tropical trees that can withstand rare frost events. Mean annual temperatures in the Caribbean dry forest region range from 23°C (74°F) in the north to 26°C (77°F) in the Lower Keys. Precipitation primarily occurs from June to October and ranges from 1650 mm along the Atlantic coast decreasing southward to less than 1000 mm in the Lower Keys (Gillespie 2006). Precipitation in the distribution range of this forest in Puerto Rico and over most of the islands of Culebra and Vieques ranges from 600 to 1100 mm per year (Brandeis et al. 2009), with two dry seasons. U.S. Virgin Islands examples may be even drier. Some of the U.S. Virgin Islands examples reach 1200 mm per year.

*Soil/Substrate*: Limestone is the dominant substrate in Caribbean dry forests of Florida and the Bahamas, with skeletal organic soils with minor mineral components, rarely exceeding 20 cm in depth (Snyder et al. 1990, as cited in Gillespie 2006). In Florida, the coastal maritime hammocks are found on stabilized coastal dunes, often with substantial shell components. In the Greater Antilles the distribution of dry forests is indicative of limestone substrates occurring in narrow strips on the northern and southern coastal areas. Isolated inland, ultramafic soils associated with limestone also support dry forests. In flat low-lying limestone archipelagos, such as the Bahamas, the Cayman Islands, Mona and Anegada, dry forests and shrublands dominate. In volcanic, low mountainous islands of the Lesser Antilles, dry forests dominate except for protected sites and ravines where moist forest can grow (Lugo et al. 2006).

Caribbean dry forests have to cope with highly stressful conditions given the combination of environmental features such as low moisture availability, long dry seasons, decadal cycles of pronounced drought, wind exposure and salt spray in littoral locations. These forests are also periodically exposed to hurricane conditions with effects that span from flooding with seawater to defoliation, treefall and other structural changes due to strong winds.

DISTRIBUTION

*Geographic Range: This macrogroup is found in south Florida, the Florida Keys, the Bahamas, Cayman Islands, Cuba, Hispaniola, Jamaica, Leeward Islands, Puerto Rico, Trinidad and Tobago, and Windward Islands.

Nations: BS, CU, DO, HT, JM, MQ, PR, TC, TT, US, VE, VG?, VI, XD

States/Provinces: FL

USFS Ecoregions (2007) [optional]: 232D:CC, 232G:CC, 411A:CC

Omernik Ecoregions L3, L4 [optional]:

MLRAs [optional]:

PLOT SAMPLING AND ANALYSIS

*Plot Analysis Summary [Med - High Confidence]:

*Plots Used to Define the Type [Med - High Confidence]:

CONFIDENCE LEVEL

USNVC Confidence Level: High

USNVC Confidence Comments [optional]:

HIERARCHY

*Lower Level NVC Types:

| **Elcode** | **Scientific or Colloquial Name** |
| --- | --- |
| G476 | Caribbean Coastal Dry Evergreen Forest |
| G765 | Caribbean Hardwood Hammock & Coastal Strand Forest |

DISCUSSION

Discussion [optional]:

CONCEPT HISTORY

*Recent Concept Lineage [if applicable]:

| **Date** | **Predecessor** | **Note** |
| --- | --- | --- |
|  |  |  |

RELATED CONCEPTS

Supporting Concepts [optional]:

| **Relationship to NVC** | **Supporting Concept Name** | **Short Citation** | **Note** |
| --- | --- | --- | --- |
| = | Rockland Forest | Ross et al. 1992 |  |
| = | Tropical Hammock | Snyder et al. 1990 |  |

AUTHORSHIP

*Primary Concept Source [if applicable]: C. Josse, in Faber-Langendoen et al. (2014)

| **Relationship to NVC** | **Name Used in Source** | **Short Citation** | **Note** |
| --- | --- | --- | --- |
|  |  |  |  |

*Author of Description: C. Josse, C.W. Nordman, and D. Faber-Langendoen

Acknowledgments [optional]:

Version Date: 30 Oct 2015

REFERENCES

*References [Required if used in text]:

Acevedo-Rodriguez, P., and collaborators. 1996. Flora of St. John, U.S. Virgin Islands. Memoirs of the New York Botanical Garden 78:1-581.

Armentano, Tom. Personal communication. National Park Service, Everglades National Park, Homestead, FL.

Brandeis, T., M. Delaney, L. Royer, and B. Parresol. 2009a. Allometric equations for predicting Puerto Rican dry forest biomass and volume. Pages 197-202 in: R. E. McRoberts, G. A. Reams, P. C. Van Deusen, and W. H. McWilliams, editors. Proceedings of the eighth annual forest inventory and analysis symposium; 2006 October 16-19; Monterey, CA. General Technical Report WO-79. USDA Forest Service, Washington, DC. [http://www.srs.fs.usda.gov/pubs/17281]

Drew, R. D., and N. S. Schomer. 1984. An ecological characterization of the Caloosahatchee River/Big Cypress watershed. USDI Fish and Wildlife Service. FWS/OBS-82/58.2. 225 pp.

Duever, M. J., J. E. Carlson, J. F. Meeder, L. C. Duever, L. H. Gunderson, L. A. Riopelle, T. R. Alexander, R. L. Myers, and D. P. Spangler. 1986. The Big Cypress National Preserve. National Audubon Society Research Report No. 8. National Audubon Society, New York. 444 pp.

FNAI [Florida Natural Areas Inventory]. 1990. Guide to the natural communities of Florida. Florida Natural Areas Inventory and Florida Department of Natural Resources, Tallahassee. 111 pp.

Faber-Langendoen, D., J. Drake, S. Gawler, M. Hall, C. Josse, G. Kittel, S. Menard, C. Nordman, M. Pyne, M. Reid, L. Sneddon, K. Schulz, J. Teague, M. Russo, K. Snow, and P. Comer, editors. 2010-2018. Divisions, Macrogroups and Groups for the Revised U.S. National Vegetation Classification. NatureServe, Arlington, VA. plus appendices. [in preparation]

Franklin, J., and D. W. Steadman. 2013. The winter bird communities in pine woodland vs. broadleaf forest on Abaco, The Bahamas. Caribbean Naturalist 3:1-18.

Gillespie, T. W. 2006. Diversity, biogeography and conservation of woody plants in tropical dry forest of south Florida. Pages 383-394 in: R. T. Pennington, G. P. Lewis, and J. A. Ratter, editors. Neotropical Savannas and Seasonally Dry Forest. Systematics Association special volume (69). CRC Press.

Johnson, A. F., and J. W. Muller. 1993a. An assessment of Florida's remaining coastal upland natural communities: Final summary report. The Nature Conservancy, Florida Natural Areas Inventory, Tallahassee. 37 pp.

Lugo, A. E., E. Medina, J. C. Trejo-Torres, and E. Helmer. 2006. Botanical and ecological basis for the resilience of Antillean Dry Forests. Pages 359-381 in: R. T. Pennington, G. P. Lewis, and J. A. Ratter, editors. Neotropical savannas and seasonally dry forests: Plant diversity, biogeography and conservation. CRC Press, Boca Raton, FL.

Robertson, W. B., Jr. 1955. An analysis of the breeding-bird populations of tropical Florida in relation to the vegetation. Ph.D. thesis, University of Illinois, Urbana.

Ross, M. S., J. J. O'Brien, and L. J. Flynn. 1992. Ecological site classification of Florida Keys terrestrial habitats. Biotropica 24:488-502.

Smith, I. K., and J. L. Vankat. 1992. Dry evergreen forest (coppice) communities of North Andros Island, Bahamas. Bulletin of the Torrey Botanical Club 119:181-191.

Snyder, J. R., A. Herndon, and W. B. Robertson, Jr. 1990. South Florida rockland. Pages 230-277 in: R. L. Myers and J. J. Ewel, editors. Ecosystems of Florida. University of Central Florida Press, Orlando.

1. Forest & Woodland

1.A.1.Ea. Caribbean-Mesoamerican Dry Forest & Woodland

M294. Caribbean Dry Limestone Forest

Type Concept Sentence: These are relatively species-poor dry forests but with high plant endemism. They occur in seasonal climates with 800-1500 mm rainfall per year and the dry season can last two to several months. The canopy is somewhat open, mostly deciduous and 6-15 m height, with emergent trees only in locations with higher precipitation. A lower layer may or may not be present and typically is mostly evergreen, ground vegetation is sparse. Lower annual precipitation on a limestone substrate and shallow soils results in a dense shrub layer about 2-4 m high.

OVERVIEW

*Hierarchy Level: Macrogroup

*Placement in Hierarchy: 1.A.1.Ea. Caribbean-Mesoamerican Dry Forest & Woodland (D099)

Elcode: M294

*Scientific Name: Caribbean Dry Limestone Forest Macrogroup

*Common (Translated Scientific) Name: Caribbean Dry Limestone Forest Macrogroup

*Colloquial Name: Caribbean Dry Limestone Forest

*Type Concept:

*Diagnostic Characteristics:

*Classification Comments:

*Similar NVC Types [if applicable]:

| **Elcode** | **Scientific or Colloquial Name** | **Note** |
| --- | --- | --- |
|  |  |  |

Similar NVC Types General Comments [optional]:

VEGETATION

Physiognomy and Structure Summary:

Physiognomy and Structure Table [optional]:

| **Physiognomy-Structure Category** | **Prevailing Height (m)** | **Height Range (opt.)** | **Mean % Cover** | **Cover Range (opt.)** |
| --- | --- | --- | --- | --- |
|  |  |  |  | - |

Floristics Summary:

*Floristics Table [Med - High Confidence]:

*Number of Plots:

*Cover Scale Used:

| **Physiognomy-Structure Category** | **Taxon Name** | **Specific Growth Form (opt.)** | **Const- ancy** | **Mean % Cover** | **Cover Range (opt.)** | **Differ-ential** | **Diagnostic Combin- ation** |
| --- | --- | --- | --- | --- | --- | --- | --- |
|  |  |  |  |  | - |  |  |

Dynamics:

ENVIRONMENT

Environmental Description:

DISTRIBUTION

*Geographic Range: This macrogroup is found in Cuba, Jamaica, and Puerto Rico, and likely in other Caribbean islands.

Nations: CU, DO, JM, PR

States/Provinces:

USFS Ecoregions (2007) [optional]:

Omernik Ecoregions L3, L4 [optional]:

MLRAs [optional]:

PLOT SAMPLING AND ANALYSIS

*Plot Analysis Summary [Med - High Confidence]:

*Plots Used to Define the Type [Med - High Confidence]:

CONFIDENCE LEVEL

USNVC Confidence Level: Low - Poorly Documented

USNVC Confidence Comments [optional]:

HIERARCHY

*Lower Level NVC Types:

| **Elcode** | **Scientific or Colloquial Name** |
| --- | --- |
| G478 | East Caribbean Karstic Forest |
| G477 | West Caribbean Karstic Forest |

DISCUSSION

Discussion [optional]:

CONCEPT HISTORY

*Recent Concept Lineage [if applicable]:

| **Date** | **Predecessor** | **Note** |
| --- | --- | --- |
| 2013-04-24 | M293 Caribbean Dry Broadleaf Forest Macrogroup | M293 merged into M294 |

RELATED CONCEPTS

Supporting Concepts [optional]:

| **Relationship to NVC** | **Supporting Concept Name** | **Short Citation** | **Note** |
| --- | --- | --- | --- |
|  |  |  |  |

AUTHORSHIP

*Primary Concept Source [if applicable]: Faber-Langendoen et al.

| **Relationship to NVC** | **Name Used in Source** | **Short Citation** | **Note** |
| --- | --- | --- | --- |
|  |  |  |  |

*Author of Description:

Acknowledgments [optional]:

Version Date: 08 Jan 2015

REFERENCES

*References [Required if used in text]:

Faber-Langendoen, D., J. Drake, S. Gawler, M. Hall, C. Josse, G. Kittel, S. Menard, C. Nordman, M. Pyne, M. Reid, L. Sneddon, K. Schulz, J. Teague, M. Russo, K. Snow, and P. Comer, editors. 2010-2018. Divisions, Macrogroups and Groups for the Revised U.S. National Vegetation Classification. NatureServe, Arlington, VA. plus appendices. [in preparation]

1. Forest & Woodland

1.A.1.Ea. Caribbean-Mesoamerican Dry Forest & Woodland

M561. Caribbean-Mesoamerican Seasonal Dry Forest

Type Concept Sentence: Este macrogrupo incluye desde bosque seco caducifolio estacional hasta los bosques semi-deciduos distribuidos desde el nivel del mar hasta los 1.400 m de altitud en la cuenca del Caribe desde México hasta Panamá.

This macrogroup includes seasonally dry deciduous to semi-deciduous forests distributed from sea level up to 1400 m elevation in the Caribbean basin from Mexico to Panama.

OVERVIEW

*Hierarchy Level: Macrogroup

*Placement in Hierarchy: 1.A.1.Ea. Caribbean-Mesoamerican Dry Forest & Woodland (D099)

Elcode: M561

*Scientific Name: Caribbean-Mesoamerican Seasonal Dry Forest Macrogroup

*Common (Translated Scientific) Name: Caribbean-Mesoamerican Seasonal Dry Forest Macrogroup

*Colloquial Name: Caribbean-Mesoamerican Seasonal Dry Forest

*Type Concept:

*Diagnostic Characteristics:

*Classification Comments:

*Similar NVC Types [if applicable]:

| **Elcode** | **Scientific or Colloquial Name** | **Note** |
| --- | --- | --- |
|  |  |  |

Similar NVC Types General Comments [optional]:

VEGETATION

Physiognomy and Structure Summary:

Physiognomy and Structure Table [optional]:

| **Physiognomy-Structure Category** | **Prevailing Height (m)** | **Height Range (opt.)** | **Mean % Cover** | **Cover Range (opt.)** |
| --- | --- | --- | --- | --- |
|  |  |  |  | - |

Floristics Summary:

*Floristics Table [Med - High Confidence]:

*Number of Plots:

*Cover Scale Used:

| **Physiognomy-Structure Category** | **Taxon Name** | **Specific Growth Form (opt.)** | **Const- ancy** | **Mean % Cover** | **Cover Range (opt.)** | **Differ-ential** | **Diagnostic Combin- ation** |
| --- | --- | --- | --- | --- | --- | --- | --- |
|  |  |  |  |  | - |  |  |

Dynamics:

ENVIRONMENT

Environmental Description:

DISTRIBUTION

*Geographic Range: This macrogroup occurs in the Caribbean basin from Mexico to Panama.

Nations: BZ, CR, GT, HN, MX, NI, PA

States/Provinces:

USFS Ecoregions (2007) [optional]:

Omernik Ecoregions L3, L4 [optional]:

MLRAs [optional]:

PLOT SAMPLING AND ANALYSIS

*Plot Analysis Summary [Med - High Confidence]:

*Plots Used to Define the Type [Med - High Confidence]:

CONFIDENCE LEVEL

USNVC Confidence Level: Unassigned

USNVC Confidence Comments [optional]:

HIERARCHY

*Lower Level NVC Types:

| **Elcode** | **Scientific or Colloquial Name** |
| --- | --- |
|  |  |

DISCUSSION

Discussion [optional]:

CONCEPT HISTORY

*Recent Concept Lineage [if applicable]:

| **Date** | **Predecessor** | **Note** |
| --- | --- | --- |
| 2013-08-28 | M006 Caribbean & Central American Pine - Oak Forest Macrogroup | M006 reworked as M296, M561, M562 |

RELATED CONCEPTS

Supporting Concepts [optional]:

| **Relationship to NVC** | **Supporting Concept Name** | **Short Citation** | **Note** |
| --- | --- | --- | --- |
|  |  |  |  |

AUTHORSHIP

*Primary Concept Source [if applicable]:

| **Relationship to NVC** | **Name Used in Source** | **Short Citation** | **Note** |
| --- | --- | --- | --- |
|  |  |  |  |

*Author of Description:

Acknowledgments [optional]:

Version Date: 08 Jan 2015

REFERENCES

*References [Required if used in text]:

Bullock, S. H., H.A. Mooney, and E. Medina, editors. 1995. Seasonally dry tropical forests. Cambridge University Press, Cambridge, UK. 521 pp.

Faber-Langendoen, D., J. Drake, S. Gawler, M. Hall, C. Josse, G. Kittel, S. Menard, C. Nordman, M. Pyne, M. Reid, L. Sneddon, K. Schulz, J. Teague, M. Russo, K. Snow, and P. Comer, editors. 2010-2018. Divisions, Macrogroups and Groups for the Revised U.S. National Vegetation Classification. NatureServe, Arlington, VA. plus appendices. [in preparation]

1. Forest & Woodland

1.A.1.Ea. Caribbean-Mesoamerican Dry Forest & Woodland

M562. Pacific Mesoamerican Seasonal Dry Forest

Type Concept Sentence: Este macrogrupo incluye bosque seco caducifolio estacional y los bosques semi-deciduos distribuidos desde el nivel del mar hasta los 1.400 m de altitud en la cuenca del Pacífico desde México hasta Panamá.

This macrogroup includes seasonally dry deciduous to semi-deciduous forests distributed from sea level up to 1400 m elevation in the Pacific basin from Mexico to Panama.

OVERVIEW

*Hierarchy Level: Macrogroup

*Placement in Hierarchy: 1.A.1.Ea. Caribbean-Mesoamerican Dry Forest & Woodland (D099)

Elcode: M562

*Scientific Name: Pacific Mesoamerican Seasonal Dry Forest Macrogroup

*Common (Translated Scientific) Name: Pacific Mesoamerican Seasonal Dry Forest Macrogroup

*Colloquial Name: Pacific Mesoamerican Seasonal Dry Forest

*Type Concept: Este macrogrupo incluye bosque seco caducifolio estacional y bosques semi-deciduos distribuidos desde el nivel del mar hasta los 1.400 m de altitud en la cuenca del Pacífico desde México hasta Panamá. Precipitacion generalmente oscila entre 1.000 y 1.600 mm / año y temperaturas medias anuales son de más de 24 grados C. Los bosques de este macrogrupo experimentan al menos una estación seca de 4-6 meses / año. Estos bosques ocurren en suelos de profundidad variable, textura, y la alcalinidad, y típicamente tienen un dosel de 10-25 m de altura, cerrado en función del contenido y tipo de nutrientes de los suelos. Una moderada a alta diversidad de especies de árboles caducifolios tropicales dominan el dosel de los árboles de varios niveles. La estructura y composición varían a lo largo del amplio rango de distribución del macrogrupo, que incluye las topografías montañosas que influyen en la disponibilidad de humedad y características del suelo. Géneros característicos con varias especies o especies diagnósticas incluyen *Astronium, Bursera, Ceiba, Cassia, Calycophyllum, Cochlospermum, Cordia, Enterolobium, Ficus, Gyrocarpus, Lysiloma, Plumeria, Platymiscium, Pterocarpus, Thouinidium, Zanthoxylum*, y otros.

This macrogroup includes seasonally dry deciduous to semi-deciduous forests distributed from sea level up to 1400 m elevation in the Pacific basin from Mexico to Panama. Precipitation generally varies from 1000 to 1600 mm/year and annual mean temperatures are over 24°C. Forests in this macrogroup experience at least one distinct dry season of 4-6 months/year. These forests occur on soils of variable depth, texture, and alkalinity, and typically have a closed canopy 10-25 m high depending on the type and nutrient contents of the soil. A moderate to high diversity of tropical deciduous tree species dominate the multi-tiered tree canopy. The structure and composition vary along the large distributional range of the macrogroup, which includes mountainous topographies that influence moisture availability and soil characteristics. Characteristic genera with several species or diagnostic species include *Astronium, Bursera, Ceiba, Cassia, Calycophyllum, Cochlospermum, Cordia, Enterolobium, Ficus, Gyrocarpus, Lysiloma, Plumeria, Platymiscium, Pterocarpus, Thouinidium, Zanthoxylum*, among others.

*Diagnostic Characteristics:

*Classification Comments:

*Similar NVC Types [if applicable]:

| **Elcode** | **Scientific or Colloquial Name** | **Note** |
| --- | --- | --- |
|  |  |  |

Similar NVC Types General Comments [optional]:

VEGETATION

Physiognomy and Structure Summary:

Physiognomy and Structure Table [optional]:

| **Physiognomy-Structure Category** | **Prevailing Height (m)** | **Height Range (opt.)** | **Mean % Cover** | **Cover Range (opt.)** |
| --- | --- | --- | --- | --- |
|  |  |  |  | - |

Floristics Summary:

*Floristics Table [Med - High Confidence]:

*Number of Plots:

*Cover Scale Used:

| **Physiognomy-Structure Category** | **Taxon Name** | **Specific Growth Form (opt.)** | **Const- ancy** | **Mean % Cover** | **Cover Range (opt.)** | **Differ-ential** | **Diagnostic Combin- ation** |
| --- | --- | --- | --- | --- | --- | --- | --- |
|  |  |  |  |  | - |  |  |

Dynamics:

ENVIRONMENT

Environmental Description:

DISTRIBUTION

*Geographic Range: This macrogroup is distributed from sea level up to 1400 m elevation in the Pacific basin from Mexico to Panama.

Nations: CR, GT, HN, MX, NI, PA, SV

States/Provinces:

USFS Ecoregions (2007) [optional]:

Omernik Ecoregions L3, L4 [optional]:

MLRAs [optional]:

PLOT SAMPLING AND ANALYSIS

*Plot Analysis Summary [Med - High Confidence]:

*Plots Used to Define the Type [Med - High Confidence]:

CONFIDENCE LEVEL

USNVC Confidence Level: Unassigned

USNVC Confidence Comments [optional]:

HIERARCHY

*Lower Level NVC Types:

| **Elcode** | **Scientific or Colloquial Name** |
| --- | --- |
|  |  |

DISCUSSION

Discussion [optional]:

CONCEPT HISTORY

*Recent Concept Lineage [if applicable]:

| **Date** | **Predecessor** | **Note** |
| --- | --- | --- |
| 2013-08-28 | M006 Caribbean & Central American Pine - Oak Forest Macrogroup | M006 reworked as M296, M561, M562 |

RELATED CONCEPTS

Supporting Concepts [optional]:

| **Relationship to NVC** | **Supporting Concept Name** | **Short Citation** | **Note** |
| --- | --- | --- | --- |
|  |  |  |  |

AUTHORSHIP

*Primary Concept Source [if applicable]: C. Josse, in Faber-Langendoen et al. (2014)

| **Relationship to NVC** | **Name Used in Source** | **Short Citation** | **Note** |
| --- | --- | --- | --- |
|  |  |  |  |

*Author of Description: C. Josse

Acknowledgments [optional]:

Version Date: 08 Jan 2015

REFERENCES

*References [Required if used in text]:

Faber-Langendoen, D., J. Drake, S. Gawler, M. Hall, C. Josse, G. Kittel, S. Menard, C. Nordman, M. Pyne, M. Reid, L. Sneddon, K. Schulz, J. Teague, M. Russo, K. Snow, and P. Comer, editors. 2010-2018. Divisions, Macrogroups and Groups for the Revised U.S. National Vegetation Classification. NatureServe, Arlington, VA. plus appendices. [in preparation]

Frankie, G. W., H. G. Baker, and P. A. Opler. 1974. Comparative phenological studies of trees in tropical wet and dry forests in the lowlands of Costa Rica. Journal of Ecology 62:881-919.

Frankie, G. W., S. B. Vinson, M. A. Rizzardi, T. L. Griswold, S. O'Keefe, and R. R. Snelling. 1997. Diversity and abundance of bee visiting a mass flowering tree species in disturbed seasonal dry forest, Costa Rica. Journal of the Lansas Entomological Society 70(4):281-296.

Frankie, G. W., W. A. Haber, P. A. Opler, and K. S. Bawa. 1983. Characteristics and organization of the large bee pollination system in the Costa Rican dry forest. Pages 411-447 in: C. E. Jones and R. J. Little, editors. Handbook of experimental pollination biology. Van Nostrand & Reinhold Co., New York.

Frankie, G. W., W. A. Haber, S. B. Vinson, K. S. Bawa, P. S. Ronchi, and N. Zamora. 2004. Flowering phenology and pollination systems diversity in the seasonal dry forest. Pages 17-29 in: G. W. Frankie, A. Mata and S. B. Vinson, editors. Biodiversity conservation in Costa Rica: Learning the lessons in a seasonal dry forest. University of California, Berkeley.

Gillespie, T., A. Grijalva, and C. Farris. 2000. Diversity, composition, and structure of tropical dry forests in Central America. Plant Ecology 147:37-47.

1. Forest & Woodland

1.A.1.Ei. Colombian-Venezuelan Dry Forest

D219. Colombian-Venezuelan Dry Forest

Type Concept Sentence:

OVERVIEW

*Hierarchy Level: Division

*Placement in Hierarchy: 1.A.1.Ei. Tropical Dry Forest & Woodland (F003)

Elcode: D219

*Scientific Name: Colombian-Venezuelan Dry Forest Division

*Common (Translated Scientific) Name: Colombian-Venezuelan Dry Forest Division

*Colloquial Name: Colombian-Venezuelan Dry Forest

*Type Concept:

*Diagnostic Characteristics:

*Classification Comments:

*Similar NVC Types [if applicable]:

| **Elcode** | **Scientific or Colloquial Name** | **Note** |
| --- | --- | --- |
|  |  |  |

Similar NVC Types General Comments [optional]:

VEGETATION

Physiognomy and Structure Summary:

Physiognomy and Structure Table [optional]:

| **Physiognomy-Structure Category** | **Prevailing Height (m)** | **Height Range (opt.)** | **Mean % Cover** | **Cover Range (opt.)** |
| --- | --- | --- | --- | --- |
|  |  |  |  | - |

Floristics Summary:

*Floristics Table [Med - High Confidence]:

*Number of Plots: *Cover Scale Used:

| **Physiognomy-Structure Category** | **Taxon Name** | **Specific Growth Form (opt.)** | **Const- ancy** | **Mean % Cover** | **Cover Range (opt.)** | **Differ-ential** | **Diagnostic Combin- ation** |
| --- | --- | --- | --- | --- | --- | --- | --- |
|  |  |  |  |  | - |  |  |

Dynamics:

ENVIRONMENT

Environmental Description:

DISTRIBUTION

*Geographic Range:

Nations: CO, EC, PE, VE

States/Provinces:

USFS Ecoregions (2007) [optional]:

Omernik Ecoregions L3, L4 [optional]:

MLRAs [optional]:

PLOT SAMPLING AND ANALYSIS

*Plot Analysis Summary [Med - High Confidence]:

*Plots Used to Define the Type [Med - High Confidence]:

CONFIDENCE LEVEL

USNVC Confidence Level: Unassigned

USNVC Confidence Comments [optional]:

HIERARCHY

*Lower Level NVC Types:

| **Elcode** | **Scientific or Colloquial Name** |
| --- | --- |
| M563 | Guajiran Seasonal Dry Forest |
| M565 | Llanos Seasonal Dry Forest |
| M566 | Tumbes Guayaquil Seasonal Dry Forest |
| M573 | Northern Andean Seasonal Dry Forest |

DISCUSSION

Discussion [optional]:

CONCEPT HISTORY

*Recent Concept Lineage [if applicable]:

| **Date** | **Predecessor** | **Note** |
| --- | --- | --- |
|  |  |  |

RELATED CONCEPTS

Supporting Concepts [optional]:

| **Relationship to NVC** | **Supporting Concept Name** | **Short Citation** | **Note** |
| --- | --- | --- | --- |
|  |  |  |  |

AUTHORSHIP

*Primary Concept Source [if applicable]:

| **Relationship to NVC** | **Name Used in Source** | **Short Citation** | **Note** |
| --- | --- | --- | --- |
|  |  |  |  |

*Author of Description:

Acknowledgments [optional]:

Version Date:

REFERENCES

*References [Required if used in text]:

Faber-Langendoen, D., J. Drake, S. Gawler, M. Hall, C. Josse, G. Kittel, S. Menard, C. Nordman, M. Pyne, M. Reid, L. Sneddon, K. Schulz, J. Teague, M. Russo, K. Snow, and P. Comer, editors. 2010-2018. Divisions, Macrogroups and Groups for the Revised U.S. National Vegetation Classification. NatureServe, Arlington, VA. plus appendices. [in preparation]

1. Forest & Woodland

1.A.1.Ei. Colombian-Venezuelan Dry Forest

M563. Guajiran Seasonal Dry Forest

Type Concept Sentence: Seasonally dry deciduous to semi-deciduous forests distributed along the Caribbean coast of Colombia and Venezuela and inland at Lake Maracaibo in Venezuela and also in the foothills of the Sierra Nevada de Santa Marta and the Perija range in Colombia. Usually occur on alluvial-colluvial lowlands between sea level and 500 m elevation. They can attain a relatively complex, multi-layered structure with a dense canopy. In the dry end of their range these forests have a more open canopy and fewer strata. In general those growing at higher elevations have more moisture available from precipitation and fog.

OVERVIEW

*Hierarchy Level: Macrogroup

*Placement in Hierarchy: 1.A.1.Ei. Colombian-Venezuelan Dry Forest (D219)

Elcode: M563

*Scientific Name: Guajiran Seasonal Dry Forest Macrogroup

*Common (Translated Scientific) Name: Guajiran Seasonal Dry Forest Macrogroup

*Colloquial Name: Guajiran Seasonal Dry Forest

*Type Concept: This macrogroup includes seasonally dry deciduous to semi-deciduous forests distributed along the Caribbean coast of Colombia and Venezuela and inland, surrounding Lake Maracaibo in Venezuela and also on the foothills of Sierra Nevada de Santa Marta and the Perija range in Colombia, usually growing on alluvial-colluvial lowlands between sea level and 500 m elevation, depending on the location. They can attain a relatively complex, multi-layered structure with a dense canopy or, in the dry end of their gradient, have a more open canopy and less strata. In general those growing in the upper elevations have more moisture available (from precipitation and fog). Characteristic species from sub-humid to dry forests are *Cordia panamensis, Swartzia pinnata, Ocotea glandulosa, Hura crepitans, Cedrela odorata, Carapa guianensis, Roystonea olerace, Anacardium excelsum, Calycophyllum candidissimum, Brosimum alicastrum, Trophis racemosa, Simira klugii, Belencita nitida, Centrolobium paraense, Maytenus karstenii, Melicoccus bijugatus, Gustavia tejerae, Eugenia oblongifolia, Myroxylon balsamum, Triplaris lindeniana, Machaerium* spp., *Tabebuia chrysantha, Tabebuia serratifolia, Tabebuia heterophylla (= Tabebuia pentaphylla), Trichilia pleeana, Allophylus racemosus (= Allophylus occidentalis), Trophis racemosa, Eugenia mcvaughii, Acacia glomerosa, Lochocarpus punctatus, Coccoloba fallax, Guazuma ulmifolia, Enterolobium cf. cyclocarpum, Morisonia americana, Bursera graveolens, Ficus prinoides, Sabal mauritiiformis, Bactris minor, Bourreria cumanensis, Erythroxylum cumanense, Brownea penduliflora, Bauhinia multinervia (= Bauhinia megalandra), Senna bicapsularis (= Cassia emarginata), Calliandra caracasana, Inga punctata, Erythrina poeppigiana, Platymiscium diadelphum, Guaiacum officinale, Cochlospermum vitifolium, Jacquemontia cumanensis, Stenocereus griseus*.

*Diagnostic Characteristics:

*Classification Comments:

*Similar NVC Types [if applicable]:

| **Elcode** | **Scientific or Colloquial Name** | **Note** |
| --- | --- | --- |
|  |  |  |

Similar NVC Types General Comments [optional]:

VEGETATION

Physiognomy and Structure Summary:

Physiognomy and Structure Table [optional]:

| **Physiognomy-Structure Category** | **Prevailing Height (m)** | **Height Range (opt.)** | **Mean % Cover** | **Cover Range (opt.)** |
| --- | --- | --- | --- | --- |
|  |  |  |  | - |

Floristics Summary:

*Floristics Table [Med - High Confidence]:

*Number of Plots:

*Cover Scale Used:

| **Physiognomy-Structure Category** | **Taxon Name** | **Specific Growth Form (opt.)** | **Const- ancy** | **Mean % Cover** | **Cover Range (opt.)** | **Differ-ential** | **Diagnostic Combin- ation** |
| --- | --- | --- | --- | --- | --- | --- | --- |
|  |  |  |  |  | - |  |  |

Dynamics:

ENVIRONMENT

Environmental Description:

DISTRIBUTION

*Geographic Range: Caribbean coast of Colombia, Venezuela and Trinidad and Tobago.

Nations: CO, TT, VE

States/Provinces:

USFS Ecoregions (2007) [optional]:

Omernik Ecoregions L3, L4 [optional]:

MLRAs [optional]:

PLOT SAMPLING AND ANALYSIS

*Plot Analysis Summary [Med - High Confidence]:

*Plots Used to Define the Type [Med - High Confidence]:

CONFIDENCE LEVEL

USNVC Confidence Level: Unassigned

USNVC Confidence Comments [optional]:

HIERARCHY

*Lower Level NVC Types:

| **Elcode** | **Scientific or Colloquial Name** |
| --- | --- |
|  |  |

DISCUSSION

Discussion [optional]:

CONCEPT HISTORY

*Recent Concept Lineage [if applicable]:

| **Date** | **Predecessor** | **Note** |
| --- | --- | --- |
| 2013-05-10 | M564 Cauca Magdalena Seasonal Dry Forest Macrogroup | M564 concept covered by M563 |

RELATED CONCEPTS

Supporting Concepts [optional]:

| **Relationship to NVC** | **Supporting Concept Name** | **Short Citation** | **Note** |
| --- | --- | --- | --- |
|  |  |  |  |

AUTHORSHIP

*Primary Concept Source [if applicable]: C. Josse, in Faber-Langendoen et al. (2014)

| **Relationship to NVC** | **Name Used in Source** | **Short Citation** | **Note** |
| --- | --- | --- | --- |
|  |  |  |  |

*Author of Description: C. Josse

Acknowledgments [optional]:

Version Date: 08 Jan 2015

REFERENCES

*References [Required if used in text]:

Faber-Langendoen, D., J. Drake, S. Gawler, M. Hall, C. Josse, G. Kittel, S. Menard, C. Nordman, M. Pyne, M. Reid, L. Sneddon, K. Schulz, J. Teague, M. Russo, K. Snow, and P. Comer, editors. 2010-2018. Divisions, Macrogroups and Groups for the Revised U.S. National Vegetation Classification. NatureServe, Arlington, VA. plus appendices. [in preparation]

1. Forest & Woodland

1.A.1.Ei. Colombian-Venezuelan Dry Forest

M565. Llanos Seasonal Dry Forest

Type Concept Sentence: Seasonally dry deciduous forests occurring across the Llanos in Colombia and Venezuela, and at the eastern limit of the Llanos where it borders Orinoquian moist forests. These forests are between 6 and 14 m in stature, not very dense (about 50-60% canopy coverage), and dominated by deciduous species. They form a mosaic with the savannas. In many cases what is now left is a small patch of forest, although originally they covered large areas. These forests grow on topographically higher ground than the surrounding savannas, such as in high plains or mesas, and on low hills with moderately drained, high nutrient content soils.

OVERVIEW

*Hierarchy Level: Macrogroup

*Placement in Hierarchy: 1.A.1.Ei. Colombian-Venezuelan Dry Forest (D219)

Elcode: M565

*Scientific Name: Llanos Seasonal Dry Forest Macrogroup

*Common (Translated Scientific) Name: Llanos Seasonal Dry Forest Macrogroup

*Colloquial Name: Llanos Seasonal Dry Forest

*Type Concept: This macrogroup includes seasonally dry, deciduous forests occurring across the Llanos in Colombia and Venezuela, and those of the eastern limit of the Llanos with the Orinoquian moist forests. Overall, they are forests between 6 and 14 m tall, not very dense (about 50-60% coverage) and with dominance of deciduous species. They form a mosaic with the savannas; in many cases what is now left is a small patch, although originally they covered large areas. This forest grows on topographically higher ground than the surrounding savannas, such as in high plains or mesas, and low hills on moderately drained soils and with high nutrient content. Diagnostic species are *Acrocomia sclerocarpa, Annona jahnii, Apeiba tibourbou, Bursera simaruba, Cassia moschata, Enterolobium cyclocarpum, Godmania aesculifolia, Melicoccus bijugatus, Platymiscium pinnatum, Ruprechtia hamanii, Spondias mombin, Xilopia aromatica, Randia aculeata, Cereus* sp., *Bourreria cumanensis, Gyrocarpus americanus, Maclura tinctoria (= Chlorophora tinctoria), Hymenaea courbaril, Bocageopsis multiflora, Parkia pendula, Qualea rosea, Licania subrachnophylla, Sclerolobium bracteosum, Tabebuia serratifolia, Tabebuia billbergii, Capparis odoratissima, Pithecellobium tortum, Guapira pacurero, Erythroxylum havanense*, and *Pithecellobium oblongum*.

*Diagnostic Characteristics:

*Classification Comments:

*Similar NVC Types [if applicable]:

| **Elcode** | **Scientific or Colloquial Name** | **Note** |
| --- | --- | --- |
|  |  |  |

Similar NVC Types General Comments [optional]:

VEGETATION

Physiognomy and Structure Summary:

Physiognomy and Structure Table [optional]:

| **Physiognomy-Structure Category** | **Prevailing Height (m)** | **Height Range (opt.)** | **Mean % Cover** | **Cover Range (opt.)** |
| --- | --- | --- | --- | --- |
|  |  |  |  | - |

Floristics Summary:

*Floristics Table [Med - High Confidence]:

*Number of Plots:

*Cover Scale Used:

| **Physiognomy-Structure Category** | **Taxon Name** | **Specific Growth Form (opt.)** | **Const- ancy** | **Mean % Cover** | **Cover Range (opt.)** | **Differ-ential** | **Diagnostic Combin- ation** |
| --- | --- | --- | --- | --- | --- | --- | --- |
|  |  |  |  |  | - |  |  |

Dynamics:

ENVIRONMENT

Environmental Description:

DISTRIBUTION

*Geographic Range:

Nations: CO, VE

States/Provinces:

USFS Ecoregions (2007) [optional]:

Omernik Ecoregions L3, L4 [optional]:

MLRAs [optional]:

PLOT SAMPLING AND ANALYSIS

*Plot Analysis Summary [Med - High Confidence]:

*Plots Used to Define the Type [Med - High Confidence]:

CONFIDENCE LEVEL

USNVC Confidence Level: Unassigned

USNVC Confidence Comments [optional]:

HIERARCHY

*Lower Level NVC Types:

| **Elcode** | **Scientific or Colloquial Name** |
| --- | --- |
|  |  |

DISCUSSION

Discussion [optional]:

CONCEPT HISTORY

*Recent Concept Lineage [if applicable]:

| **Date** | **Predecessor** | **Note** |
| --- | --- | --- |
| 2013-01-17 | M344 Orinoquian Savanna Macrogroup | M344 reconfigured into M676 & part of M565 |

RELATED CONCEPTS

Supporting Concepts [optional]:

| **Relationship to NVC** | **Supporting Concept Name** | **Short Citation** | **Note** |
| --- | --- | --- | --- |
|  |  |  |  |

AUTHORSHIP

*Primary Concept Source [if applicable]: C. Josse, in Faber-Langendoen et al. (2014)

| **Relationship to NVC** | **Name Used in Source** | **Short Citation** | **Note** |
| --- | --- | --- | --- |
|  |  |  |  |

*Author of Description: C. Josse

Acknowledgments [optional]:

Version Date: 17 Apr 2014

REFERENCES

*References [Required if used in text]:

Faber-Langendoen, D., J. Drake, S. Gawler, M. Hall, C. Josse, G. Kittel, S. Menard, C. Nordman, M. Pyne, M. Reid, L. Sneddon, K. Schulz, J. Teague, M. Russo, K. Snow, and P. Comer, editors. 2010-2018. Divisions, Macrogroups and Groups for the Revised U.S. National Vegetation Classification. NatureServe, Arlington, VA. plus appendices. [in preparation]

1. Forest & Woodland

1.A.1.Ei. Colombian-Venezuelan Dry Forest

M566. Tumbes Guayaquil Seasonal Dry Forest

Type Concept Sentence: Dry deciduous to semi-deciduous forests growing in areas with a dry season of more than four months on the coastal plain of Ecuador. Occurs up to about 500 m elevation in the hills of the coastal range and to about 900 m elevation in the foothills of the Andes in the south. In Peru these forests are distributed at higher elevation in the Amotape hills and further south along the western foothills of the Andes to northern Lambayeque. The uneven canopies of these forests are typically 15-20 m tall, with a large proportion of deciduous species and feature a dense and semi-deciduous shrub layer.

OVERVIEW

*Hierarchy Level: Macrogroup

*Placement in Hierarchy: 1.A.1.Ei. Colombian-Venezuelan Dry Forest (D219)

Elcode: M566

*Scientific Name: Tumbes Guayaquil Seasonal Dry Forest Macrogroup

*Common (Translated Scientific) Name: Tumbes Guayaquil Seasonal Dry Forest Macrogroup

*Colloquial Name: Tumbes Guayaquil Seasonal Dry Forest

*Type Concept: The macrogroup represents the dry deciduous to semi-deciduous forests growing on the coastal plain of Ecuador with a dry season of more than 4 months. They reach up to about 500 m altitude in the hills of the coastal range located in this region and to about 900 m in the foothills of the Andes Mountains to the south, close to the border with Peru. In Peru it is distributed higher up, to 1500-2000 m elevation in the Amotape hills and further south in the western foothills of the Andes up to northern Lambayeque. In general these forest are 15 20 m tall, have an uneven canopy with a large proportion of deciduous species, and a dense and semi-deciduous shrub layer. Diagnostic species are *Ceiba trischistandra, Cavanillesia platanifolia, Cochlospermum vitifolium, Eriotheca ruizii, Erythrina velutina, Erythrina smithiana, Tabebuia chrysantha, Ziziphus thyrsiflora, Trichilia hirta, Senna mollissima, Guazuma ulmifolia, Phyllanthus anisobolus, Rauvolfia tetraphylla, Carica parviflora, Triplaris cumingiana, Pithecellobium excelsum, Cordia lutea, Coccoloba ruiziana, Capparis heterophylla, Capparis* spp., *Achatocarpus nigricans, Simira ecuadoriensis, Geoffroea spinosa, Machaerium millei, Piscidia carthagenensis, Caesalpinia glabrata, Bauhinia aculeata, Terminalia oblonga, Terminalia valverdae, Gallesia integrifolia, Loxopterygium huasango, Bursera graveolens, Centrolobium ochroxylum, Miroxylon balsamum, Hura cf. crepitans, Ficus jacobii, Delostoma integrifolium*. On higher grounds, with additional seasonal moisture from fog enshrouding these forests, typical species are *Centrolobium ochroxylum, Brosimum alicastrum, Alseis eggersii, Lonchocarpus* sp., *Ficus trigonata, Clarisia racemosa, Ficus* spp., *Pochota trinitensis (= Bombacopsis trinitensis), Pseudobombax millei, Clavija eggersii, Erythroxylum patens, Trichilia elegans, Gustavia pubescens, Ampelocera* sp., *Acnistus arborescens, Zanthoxylum fagara, Myrcia splendens*, among others.

*Diagnostic Characteristics:

*Classification Comments:

*Similar NVC Types [if applicable]:

| **Elcode** | **Scientific or Colloquial Name** | **Note** |
| --- | --- | --- |
|  |  |  |

Similar NVC Types General Comments [optional]:

VEGETATION

Physiognomy and Structure Summary:

Physiognomy and Structure Table [optional]:

| **Physiognomy-Structure Category** | **Prevailing Height (m)** | **Height Range (opt.)** | **Mean % Cover** | **Cover Range (opt.)** |
| --- | --- | --- | --- | --- |
|  |  |  |  | - |

Floristics Summary:

*Floristics Table [Med - High Confidence]:

*Number of Plots:

*Cover Scale Used:

| **Physiognomy-Structure Category** | **Taxon Name** | **Specific Growth Form (opt.)** | **Const- ancy** | **Mean % Cover** | **Cover Range (opt.)** | **Differ-ential** | **Diagnostic Combin- ation** |
| --- | --- | --- | --- | --- | --- | --- | --- |
|  |  |  |  |  | - |  |  |

Dynamics:

ENVIRONMENT

Environmental Description:

DISTRIBUTION

*Geographic Range:

Nations: EC, PE

States/Provinces:

USFS Ecoregions (2007) [optional]:

Omernik Ecoregions L3, L4 [optional]:

MLRAs [optional]:

PLOT SAMPLING AND ANALYSIS

*Plot Analysis Summary [Med - High Confidence]:

*Plots Used to Define the Type [Med - High Confidence]:

CONFIDENCE LEVEL

USNVC Confidence Level: Unassigned

USNVC Confidence Comments [optional]:

HIERARCHY

*Lower Level NVC Types:

| **Elcode** | **Scientific or Colloquial Name** |
| --- | --- |
|  |  |

DISCUSSION

Discussion [optional]:

CONCEPT HISTORY

*Recent Concept Lineage [if applicable]:

| **Date** | **Predecessor** | **Note** |
| --- | --- | --- |
|  |  |  |

RELATED CONCEPTS

Supporting Concepts [optional]:

| **Relationship to NVC** | **Supporting Concept Name** | **Short Citation** | **Note** |
| --- | --- | --- | --- |
|  |  |  |  |

AUTHORSHIP

*Primary Concept Source [if applicable]: C. Josse, in Faber-Langendoen et al. (2014)

| **Relationship to NVC** | **Name Used in Source** | **Short Citation** | **Note** |
| --- | --- | --- | --- |
|  |  |  |  |

*Author of Description: C. Josse

Acknowledgments [optional]:

Version Date: 17 Apr 2014

REFERENCES

*References [Required if used in text]:

Faber-Langendoen, D., J. Drake, S. Gawler, M. Hall, C. Josse, G. Kittel, S. Menard, C. Nordman, M. Pyne, M. Reid, L. Sneddon, K. Schulz, J. Teague, M. Russo, K. Snow, and P. Comer, editors. 2010-2018. Divisions, Macrogroups and Groups for the Revised U.S. National Vegetation Classification. NatureServe, Arlington, VA. plus appendices. [in preparation]

1. Forest & Woodland

1.A.1.Ei. Colombian-Venezuelan Dry Forest

M573. Northern Andean Seasonal Dry Forest

Type Concept Sentence: Subhumid forests of the northern Andes. This forest type occurs on some of the lower slopes of the Andes in Venezuela, Colombia and Ecuador, generally at the foothills of intermontane valleys or in transitions to seasonally dry areas. Composition varies depending on the floristic region occurring at lower elevations. Nevertheless, these forests share the characteristic physiognomy of seasonally dry tropical forest, with an uneven canopy, less dense understory, fewer epiphytes and shorter stature than more moist forests. The floristic composition includes members of the Fabaceae, Bombacacae, Meliaceae, Bignoniaceae, and Burseraceae as dominant in the canopy layer, and Euphorbiaceae, Asteraceae and Cactaceae in the understory.

OVERVIEW

*Hierarchy Level: Macrogroup

*Placement in Hierarchy: 1.A.1.Ei. Colombian-Venezuelan Dry Forest (D219)

Elcode: M573

*Scientific Name: Northern Andean Seasonal Dry Forest Macrogroup

*Common (Translated Scientific) Name: Northern Andean Seasonal Dry Forest Macrogroup

*Colloquial Name: Northern Andean Seasonal Dry Forest

*Type Concept: This macrogroup represents sub-humid forests of the northern Andes. This type of forest occurs in some of the lower slopes of the Andes in Venezuela, Colombia and Ecuador, generally at the foothills of intermontane valleys or in the transition to seasonally dry areas. The specific composition may vary depending on the floristic region of contact further down in the lowlands, but they share the characteristic physiognomy of seasonally dry tropical forest, with an uneven canopy, less dense understory, fewer epiphytes and shorter stature. The floristic composition includes members of the Fabaceae, Bombacacae, Meliaceae, Bignoniaceae, and Burseraceae as dominant in the canopy layer, and Euphorbiaceae, Asteraceae and Cactaceae in the understory.

*Diagnostic Characteristics:

*Classification Comments: A recent paper by DRYFLOR (2016) indicates that the northern inter-Andean dry forests have more floristic similarity with northern coastal dry forests in Colombia and Venezuela than with montane Andean forests to the south. In addition, the northern Andes are placed in the same biogeographic area (NeoGranadian Region) as western Colombia and northern Venezuela by Rivas-Martinez et al. (2011).

*Similar NVC Types [if applicable]:

| **Elcode** | **Scientific or Colloquial Name** | **Note** |
| --- | --- | --- |
|  |  |  |

Similar NVC Types General Comments [optional]:

VEGETATION

Physiognomy and Structure Summary:

Physiognomy and Structure Table [optional]:

| **Physiognomy-Structure Category** | **Prevailing Height (m)** | **Height Range (opt.)** | **Mean % Cover** | **Cover Range (opt.)** |
| --- | --- | --- | --- | --- |
|  |  |  |  | - |

Floristics Summary:

*Floristics Table [Med - High Confidence]:

*Number of Plots:

*Cover Scale Used:

| **Physiognomy-Structure Category** | **Taxon Name** | **Specific Growth Form (opt.)** | **Const- ancy** | **Mean % Cover** | **Cover Range (opt.)** | **Differ-ential** | **Diagnostic Combin- ation** |
| --- | --- | --- | --- | --- | --- | --- | --- |
|  |  |  |  |  | - |  |  |

Dynamics:

ENVIRONMENT

Environmental Description:

DISTRIBUTION

*Geographic Range:

Nations: CO, EC, PE, VE

States/Provinces:

USFS Ecoregions (2007) [optional]:

Omernik Ecoregions L3, L4 [optional]:

MLRAs [optional]:

PLOT SAMPLING AND ANALYSIS

*Plot Analysis Summary [Med - High Confidence]:

*Plots Used to Define the Type [Med - High Confidence]:

CONFIDENCE LEVEL

USNVC Confidence Level: Unassigned

USNVC Confidence Comments [optional]:

HIERARCHY

*Lower Level NVC Types:

| **Elcode** | **Scientific or Colloquial Name** |
| --- | --- |
|  |  |

DISCUSSION

Discussion [optional]:

CONCEPT HISTORY

*Recent Concept Lineage [if applicable]:

| **Date** | **Predecessor** | **Note** |
| --- | --- | --- |
|  |  |  |

RELATED CONCEPTS

Supporting Concepts [optional]:

| **Relationship to NVC** | **Supporting Concept Name** | **Short Citation** | **Note** |
| --- | --- | --- | --- |
|  |  |  |  |

AUTHORSHIP

*Primary Concept Source [if applicable]: C. Josse, in Faber-Langendoen et al. (2014)

| **Relationship to NVC** | **Name Used in Source** | **Short Citation** | **Note** |
| --- | --- | --- | --- |
|  |  |  |  |

*Author of Description: C. Josse

Acknowledgments [optional]:

Version Date: 17 Apr 2014

REFERENCES

*References [Required if used in text]:

DRYFLOR. 2016. Plant diversity patterns in neotropical dry forests and their conservation implications. Science 353:1383-1387 (with supplementary material).

Faber-Langendoen, D., J. Drake, S. Gawler, M. Hall, C. Josse, G. Kittel, S. Menard, C. Nordman, M. Pyne, M. Reid, L. Sneddon, K. Schulz, J. Teague, M. Russo, K. Snow, and P. Comer, editors. 2010-2018. Divisions, Macrogroups and Groups for the Revised U.S. National Vegetation Classification. NatureServe, Arlington, VA. plus appendices. [in preparation]

Rivas-Martínez, S., G. Navarro, A. Penas, and M. Costa. 2011. Biogeographic map of South America. A preliminary survey. International Journal of Geobotanical Research 1:21-40.

1. Forest & Woodland

1.A.1.Ej. Guianan Dry Forest

D220. Guianan Dry Forest

Type Concept Sentence:

OVERVIEW

*Hierarchy Level: Division

*Placement in Hierarchy: 1.A.1.Ej. Tropical Dry Forest & Woodland (F003)

Elcode: D220

*Scientific Name: Guianan Dry Forest Division

*Common (Translated Scientific) Name: Guianan Dry Forest Division

*Colloquial Name: Guianan Dry Forest

*Type Concept:

*Diagnostic Characteristics:

*Classification Comments:

*Similar NVC Types [if applicable]:

| **Elcode** | **Scientific or Colloquial Name** | **Note** |
| --- | --- | --- |
|  |  |  |

Similar NVC Types General Comments [optional]:

VEGETATION

Physiognomy and Structure Summary:

Physiognomy and Structure Table [optional]:

| **Physiognomy-Structure Category** | **Prevailing Height (m)** | **Height Range (opt.)** | **Mean % Cover** | **Cover Range (opt.)** |
| --- | --- | --- | --- | --- |
|  |  |  |  | - |

Floristics Summary:

*Floristics Table [Med - High Confidence]:

*Number of Plots: *Cover Scale Used:

| **Physiognomy-Structure Category** | **Taxon Name** | **Specific Growth Form (opt.)** | **Const- ancy** | **Mean % Cover** | **Cover Range (opt.)** | **Differ-ential** | **Diagnostic Combin- ation** |
| --- | --- | --- | --- | --- | --- | --- | --- |
|  |  |  |  |  | - |  |  |

Dynamics:

ENVIRONMENT

Environmental Description:

DISTRIBUTION

*Geographic Range:

Nations: BR?, VE

States/Provinces:

USFS Ecoregions (2007) [optional]:

Omernik Ecoregions L3, L4 [optional]:

MLRAs [optional]:

PLOT SAMPLING AND ANALYSIS

*Plot Analysis Summary [Med - High Confidence]:

*Plots Used to Define the Type [Med - High Confidence]:

CONFIDENCE LEVEL

USNVC Confidence Level: Unassigned

USNVC Confidence Comments [optional]:

HIERARCHY

*Lower Level NVC Types:

| **Elcode** | **Scientific or Colloquial Name** |
| --- | --- |
| M567 | Central Guianan Seasonal Dry Forest |

DISCUSSION

Discussion [optional]:

CONCEPT HISTORY

*Recent Concept Lineage [if applicable]:

| **Date** | **Predecessor** | **Note** |
| --- | --- | --- |
|  |  |  |

RELATED CONCEPTS

Supporting Concepts [optional]:

| **Relationship to NVC** | **Supporting Concept Name** | **Short Citation** | **Note** |
| --- | --- | --- | --- |
|  |  |  |  |

AUTHORSHIP

*Primary Concept Source [if applicable]:

| **Relationship to NVC** | **Name Used in Source** | **Short Citation** | **Note** |
| --- | --- | --- | --- |
|  |  |  |  |

*Author of Description:

Acknowledgments [optional]:

Version Date:

REFERENCES

*References [Required if used in text]:

Faber-Langendoen, D., J. Drake, S. Gawler, M. Hall, C. Josse, G. Kittel, S. Menard, C. Nordman, M. Pyne, M. Reid, L. Sneddon, K. Schulz, J. Teague, M. Russo, K. Snow, and P. Comer, editors. 2010-2018. Divisions, Macrogroups and Groups for the Revised U.S. National Vegetation Classification. NatureServe, Arlington, VA. plus appendices. [in preparation]

1. Forest & Woodland

1.A.1.Ej. Guianan Dry Forest

M567. Central Guianan Seasonal Dry Forest

Type Concept Sentence: Seasonal semi-deciduous forests of the northwestern and northern piedmont of the Guiana Shield in Venezuela, located between the Orinoco Llanos and semi-evergreen and evergreen forests of the Guiana Shield. Canopy height is 15-20 m. Diagnostic species are *Cochlospermum orinocense, Elaeoluma glabrescens, Erisma uncinatum, Eschweilera subglandulosa, Galipea davisii, Licania canescens, Licania cruegeriana, Licania densiflora, Macrolobium bifolium, Parinari excelsa, Terminalia amazonia*, and *Vochysia glaberrima*.

OVERVIEW

*Hierarchy Level: Macrogroup

*Placement in Hierarchy: 1.A.1.Ej. Guianan Dry Forest (D220)

Elcode: M567

*Scientific Name: Central Guianan Seasonal Dry Forest Macrogroup

*Common (Translated Scientific) Name: Central Guianan Seasonal Dry Forest Macrogroup

*Colloquial Name: Central Guianan Seasonal Dry Forest

*Type Concept: Seasonal semi-deciduous forests of the northwestern and northern piedmont of the Guiana Shield in Venezuela, located between the Orinoco Llanos and semi-evergreen and evergreen forests of the Guiana Shield. These forests are 15-20 m tall. Diagnostic species are *Parinari excelsa, Licania canescens, Licania cruegeriana, Licania densiflora, Vochysia glaberrima, Elaeoluma glabrescens, Cochlospermum orinocense, Terminalia amazonia, Galipea davisii, Macrolobium bifolium, Eschweilera subglandulosa, Erisma uncinatum*.

*Diagnostic Characteristics:

*Classification Comments:

*Similar NVC Types [if applicable]:

| **Elcode** | **Scientific or Colloquial Name** | **Note** |
| --- | --- | --- |
|  |  |  |

Similar NVC Types General Comments [optional]:

VEGETATION

Physiognomy and Structure Summary:

Physiognomy and Structure Table [optional]:

| **Physiognomy-Structure Category** | **Prevailing Height (m)** | **Height Range (opt.)** | **Mean % Cover** | **Cover Range (opt.)** |
| --- | --- | --- | --- | --- |
|  |  |  |  | - |

Floristics Summary:

*Floristics Table [Med - High Confidence]:

*Number of Plots:

*Cover Scale Used:

| **Physiognomy-Structure Category** | **Taxon Name** | **Specific Growth Form (opt.)** | **Const- ancy** | **Mean % Cover** | **Cover Range (opt.)** | **Differ-ential** | **Diagnostic Combin- ation** |
| --- | --- | --- | --- | --- | --- | --- | --- |
|  |  |  |  |  | - |  |  |

Dynamics:

ENVIRONMENT

Environmental Description:

DISTRIBUTION

*Geographic Range:

Nations: BR?, VE

States/Provinces:

USFS Ecoregions (2007) [optional]:

Omernik Ecoregions L3, L4 [optional]:

MLRAs [optional]:

PLOT SAMPLING AND ANALYSIS

*Plot Analysis Summary [Med - High Confidence]:

*Plots Used to Define the Type [Med - High Confidence]:

CONFIDENCE LEVEL

USNVC Confidence Level: Unassigned

USNVC Confidence Comments [optional]:

HIERARCHY

*Lower Level NVC Types:

| **Elcode** | **Scientific or Colloquial Name** |
| --- | --- |
|  |  |

DISCUSSION

Discussion [optional]:

CONCEPT HISTORY

*Recent Concept Lineage [if applicable]:

| **Date** | **Predecessor** | **Note** |
| --- | --- | --- |
|  |  |  |

RELATED CONCEPTS

Supporting Concepts [optional]:

| **Relationship to NVC** | **Supporting Concept Name** | **Short Citation** | **Note** |
| --- | --- | --- | --- |
|  |  |  |  |

AUTHORSHIP

*Primary Concept Source [if applicable]: C. Josse, in Faber-Langendoen et al. (2014)

| **Relationship to NVC** | **Name Used in Source** | **Short Citation** | **Note** |
| --- | --- | --- | --- |
|  |  |  |  |

*Author of Description: C. Josse

Acknowledgments [optional]:

Version Date: 17 Apr 2014

REFERENCES

*References [Required if used in text]:

Faber-Langendoen, D., J. Drake, S. Gawler, M. Hall, C. Josse, G. Kittel, S. Menard, C. Nordman, M. Pyne, M. Reid, L. Sneddon, K. Schulz, J. Teague, M. Russo, K. Snow, and P. Comer, editors. 2010-2018. Divisions, Macrogroups and Groups for the Revised U.S. National Vegetation Classification. NatureServe, Arlington, VA. plus appendices. [in preparation]

1. Forest & Woodland

1.A.1.Ek. Brazilian-Parana Dry Forest

D221. Brazilian-Parana Dry Forest

Type Concept Sentence:

OVERVIEW

*Hierarchy Level: Division

*Placement in Hierarchy: 1.A.1.Ek. Tropical Dry Forest & Woodland (F003)

Elcode: D221

*Scientific Name: Brazilian-Parana Dry Forest Division

*Common (Translated Scientific) Name: Brazilian-Parana Dry Forest Division

*Colloquial Name: Brazilian-Parana Dry Forest

*Type Concept:

*Diagnostic Characteristics:

*Classification Comments:

*Similar NVC Types [if applicable]:

| **Elcode** | **Scientific or Colloquial Name** | **Note** |
| --- | --- | --- |
|  |  |  |

Similar NVC Types General Comments [optional]:

VEGETATION

Physiognomy and Structure Summary:

Physiognomy and Structure Table [optional]:

| **Physiognomy-Structure Category** | **Prevailing Height (m)** | **Height Range (opt.)** | **Mean % Cover** | **Cover Range (opt.)** |
| --- | --- | --- | --- | --- |
|  |  |  |  | - |

Floristics Summary:

*Floristics Table [Med - High Confidence]:

*Number of Plots: *Cover Scale Used:

| **Physiognomy-Structure Category** | **Taxon Name** | **Specific Growth Form (opt.)** | **Const- ancy** | **Mean % Cover** | **Cover Range (opt.)** | **Differ-ential** | **Diagnostic Combin- ation** |
| --- | --- | --- | --- | --- | --- | --- | --- |
|  |  |  |  |  | - |  |  |

Dynamics:

ENVIRONMENT

Environmental Description:

DISTRIBUTION

*Geographic Range:

Nations: BO, BR, PY

States/Provinces:

USFS Ecoregions (2007) [optional]:

Omernik Ecoregions L3, L4 [optional]:

MLRAs [optional]:

PLOT SAMPLING AND ANALYSIS

*Plot Analysis Summary [Med - High Confidence]:

*Plots Used to Define the Type [Med - High Confidence]:

CONFIDENCE LEVEL

USNVC Confidence Level: Unassigned

USNVC Confidence Comments [optional]:

HIERARCHY

*Lower Level NVC Types:

| **Elcode** | **Scientific or Colloquial Name** |
| --- | --- |
| M572 | Caatinga Seasonal Dry Forest |
| M872 | Cerradâo Sclerophyllous Woodland |
| M570 | Cerrado Seasonal Dry Forest |
| M568 | Brazilian Atlantic Seasonal Dry Forest |
| M571 | Parana Seasonal Dry Forest |

DISCUSSION

Discussion [optional]:

CONCEPT HISTORY

*Recent Concept Lineage [if applicable]:

| **Date** | **Predecessor** | **Note** |
| --- | --- | --- |
|  |  |  |

RELATED CONCEPTS

Supporting Concepts [optional]:

| **Relationship to NVC** | **Supporting Concept Name** | **Short Citation** | **Note** |
| --- | --- | --- | --- |
|  |  |  |  |

AUTHORSHIP

*Primary Concept Source [if applicable]:

| **Relationship to NVC** | **Name Used in Source** | **Short Citation** | **Note** |
| --- | --- | --- | --- |
|  |  |  |  |

*Author of Description:

Acknowledgments [optional]:

Version Date:

REFERENCES

*References [Required if used in text]:

Faber-Langendoen, D., J. Drake, S. Gawler, M. Hall, C. Josse, G. Kittel, S. Menard, C. Nordman, M. Pyne, M. Reid, L. Sneddon, K. Schulz, J. Teague, M. Russo, K. Snow, and P. Comer, editors. 2010-2018. Divisions, Macrogroups and Groups for the Revised U.S. National Vegetation Classification. NatureServe, Arlington, VA. plus appendices. [in preparation]

1. Forest & Woodland

1.A.1.Ek. Brazilian-Parana Dry Forest

M572. Caatinga Seasonal Dry Forest

Type Concept Sentence: Seasonal deciduous forests that grow in the Caatinga (Brazil), in places with more moisture, nutrient availability, or richer soils than other Caatinga habitats. Occurs on residual reliefs occupying slightly peripheral positions along the western and southwestern boundaries of the Caatinga (i.e., karstic or limestone substrates in Bahia and northern Minas Gerais). Canopy height is 15-20 m, with lianas and some epiphytes. Besides typical Caatinga tree species such as *Myracrodrun urundeuva, Schinopsis brasiliensis*, and *Tabebuia impetiginosa*, diagnostic species are *Bauhinia trichosepala, Caesalpinia bracteosa, Pseudopiptadenia brenanii, Pseudopiptadenia contorta, Piptadenia viridifolia*, and *Plathymenia reticulata*.

OVERVIEW

*Hierarchy Level: Macrogroup

*Placement in Hierarchy: 1.A.1.Ek. Brazilian-Parana Dry Forest (D221)

Elcode: M572

*Scientific Name: Caatinga Seasonal Dry Forest Macrogroup

*Common (Translated Scientific) Name: Caatinga Seasonal Dry Forest Macrogroup

*Colloquial Name: Caatinga Seasonal Dry Forest

*Type Concept: The macrogroup represents the seasonally dry deciduous forests that grow in the Brazilian phytogeographic region known as Caatinga, in places with improved moisture or nutrient availability due to topographic location (i.e., slopes of small ridges receiving more precipitation or humidity), or location in areas with richer soils on residual reliefs occupying slightly peripheral positions along the western and southwestern boundaries of the Caatinga (i.e., karstic or limestone substrates in Bahia sites and north of Minas Gerais). They are forest 15-20 m high, with lianas vines and some epiphytes. Besides some typical Caatinga tree species, such as *Schinopsis brasiliensis, Tabeabuia impetiginosa, Myracrodrun urundeuva, Pterogyne nitens*, diagnostic species of this group are *Bauhinia trichosepala, Caesalpinia bracteosa, Pseudopiptadenia brenanii, Pseudopiptadenia contorta, Piptadenia viridifolia, Acacia monacantha, Plathymenia reticulata, Hymenaea martiana*, and genera *Blanchetiodendron, Goniorrhachis*, and *Mysanthus*.

*Diagnostic Characteristics:

*Classification Comments:

*Similar NVC Types [if applicable]:

| **Elcode** | **Scientific or Colloquial Name** | **Note** |
| --- | --- | --- |
|  |  |  |

Similar NVC Types General Comments [optional]:

VEGETATION

Physiognomy and Structure Summary:

Physiognomy and Structure Table [optional]:

| **Physiognomy-Structure Category** | **Prevailing Height (m)** | **Height Range (opt.)** | **Mean % Cover** | **Cover Range (opt.)** |
| --- | --- | --- | --- | --- |
|  |  |  |  | - |

Floristics Summary:

*Floristics Table [Med - High Confidence]:

*Number of Plots:

*Cover Scale Used:

| **Physiognomy-Structure Category** | **Taxon Name** | **Specific Growth Form (opt.)** | **Const- ancy** | **Mean % Cover** | **Cover Range (opt.)** | **Differ-ential** | **Diagnostic Combin- ation** |
| --- | --- | --- | --- | --- | --- | --- | --- |
|  |  |  |  |  | - |  |  |

Dynamics:

ENVIRONMENT

Environmental Description:

DISTRIBUTION

*Geographic Range:

Nations: BR

States/Provinces:

USFS Ecoregions (2007) [optional]:

Omernik Ecoregions L3, L4 [optional]:

MLRAs [optional]:

PLOT SAMPLING AND ANALYSIS

*Plot Analysis Summary [Med - High Confidence]:

*Plots Used to Define the Type [Med - High Confidence]:

CONFIDENCE LEVEL

USNVC Confidence Level: Unassigned

USNVC Confidence Comments [optional]:

HIERARCHY

*Lower Level NVC Types:

| **Elcode** | **Scientific or Colloquial Name** |
| --- | --- |
|  |  |

DISCUSSION

Discussion [optional]:

CONCEPT HISTORY

*Recent Concept Lineage [if applicable]:

| **Date** | **Predecessor** | **Note** |
| --- | --- | --- |
|  |  |  |

RELATED CONCEPTS

Supporting Concepts [optional]:

| **Relationship to NVC** | **Supporting Concept Name** | **Short Citation** | **Note** |
| --- | --- | --- | --- |
|  |  |  |  |

AUTHORSHIP

*Primary Concept Source [if applicable]: C. Josse, in Faber-Langendoen et al. (2014)

| **Relationship to NVC** | **Name Used in Source** | **Short Citation** | **Note** |
| --- | --- | --- | --- |
|  |  |  |  |

*Author of Description: C. Josse

Acknowledgments [optional]:

Version Date: 17 Apr 2014

REFERENCES

*References [Required if used in text]:

Faber-Langendoen, D., J. Drake, S. Gawler, M. Hall, C. Josse, G. Kittel, S. Menard, C. Nordman, M. Pyne, M. Reid, L. Sneddon, K. Schulz, J. Teague, M. Russo, K. Snow, and P. Comer, editors. 2010-2018. Divisions, Macrogroups and Groups for the Revised U.S. National Vegetation Classification. NatureServe, Arlington, VA. plus appendices. [in preparation]

1. Forest & Woodland

1.A.1.Ek. Brazilian-Parana Dry Forest

M872. Cerradâo Sclerophyllous Woodland

Type Concept Sentence: Sclerophyllous woodland of the Cerrado biogeographic region, distributed in Brazil, Bolivia and Paraguay. A transitional type between seasonal dry forests and the Cerrado savannas, with a woody cover >50% and trees 7-15 m high and a rich and varied shrub and grassland component. Characteristic species vary according to the location, soils, and climatic conditions, but are generally seasonal and show xeromorphic features. Soils vary from mesotrophic with high calcium content to lateritic and acidic or very shallow. Species composition is diverse with many endemics.

OVERVIEW

*Hierarchy Level: Macrogroup

*Placement in Hierarchy: 1.A.1.Ek. Brazilian-Parana Dry Forest (D221)

Elcode: M872

*Scientific Name: Cerradâo Sclerophyllous Woodland Macrogroup

*Common (Translated Scientific) Name: Cerradâo Sclerophyllous Woodland Macrogroup

*Colloquial Name: Cerradâo Sclerophyllous Woodland

*Type Concept: Sclerophyllous woodland of the Cerrado biogeographic region, distributed in Brazil, Bolivia and Paraguay. It is a transitional type between the seasonal dry forest and the Cerrado savannas, with woody cover >50% and trees 7-15 m high, with a rich and varied shrub and grassland component. Characteristic species vary according to the location, soils and climatic conditions. In general they are seasonal and show xeromorphic features. The soils where they develop vary widely, from mesotrophic with high calcium content to lateritic and acidic, or very shallow. Characteristic species are *Hirtella glandulosa, Emmotum nitens, Sclerolobium paniculatum, Vochysia haenkeana, Virola sebifera, Blaepharocalyx salicifolius, Siphoneugena densiflora, Ocotea spixiana, Phoebe erythropoda, Callisthene major, Sclerolobium paniculatum, Siparuna guianensis*, and *Cardiopetalum calophyllum*, and for Bolivia cerradoes: *Magonia pubescens, Qualea grandiflora, Qualea multiflora, Qualea parviflora, Agonandra brasiliensis, Salvertia convallariodora, Caryocar brasiliense, Eriotheca gracilipes, Hancornia speciosa, Ouratea hexasperma, Callisthene fasciculata, Callisthene hassleri, Callisthene microphylla, Copaifera langsdorfii, Antonia ovata, Bonyunia antoniifolia, Cariniana multiflora, Pterodon emarginatus, Kielmeyera coriacea, Dipteryx alata, Plathymenia reticulata, Terminalia argentea, Bowdichia virgilioides, Xylopia aromatica, Allagoptera leucocalyx, Anacardium humile, Pseudobombax longiflorum, Himatanthus obovatus, Machaerium acutifolium, Guettarda viburnoides, Zamia boliviana, Lafoensia pacari, Priogymnanthus hasslerianus, Ananas ananassoides, Bromelia villosa*, among others.

*Diagnostic Characteristics:

*Classification Comments:

*Similar NVC Types [if applicable]:

| **Elcode** | **Scientific or Colloquial Name** | **Note** |
| --- | --- | --- |
|  |  |  |

Similar NVC Types General Comments [optional]:

VEGETATION

Physiognomy and Structure Summary:

Physiognomy and Structure Table [optional]:

| **Physiognomy-Structure Category** | **Prevailing Height (m)** | **Height Range (opt.)** | **Mean % Cover** | **Cover Range (opt.)** |
| --- | --- | --- | --- | --- |
|  |  |  |  | - |

Floristics Summary:

*Floristics Table [Med - High Confidence]:

*Number of Plots:

*Cover Scale Used:

| **Physiognomy-Structure Category** | **Taxon Name** | **Specific Growth Form (opt.)** | **Const- ancy** | **Mean % Cover** | **Cover Range (opt.)** | **Differ-ential** | **Diagnostic Combin- ation** |
| --- | --- | --- | --- | --- | --- | --- | --- |
|  |  |  |  |  | - |  |  |

Dynamics:

ENVIRONMENT

Environmental Description:

DISTRIBUTION

*Geographic Range:

Nations: BO, BR, PY

States/Provinces:

USFS Ecoregions (2007) [optional]:

Omernik Ecoregions L3, L4 [optional]:

MLRAs [optional]:

PLOT SAMPLING AND ANALYSIS

*Plot Analysis Summary [Med - High Confidence]:

*Plots Used to Define the Type [Med - High Confidence]:

CONFIDENCE LEVEL

USNVC Confidence Level: Unassigned

USNVC Confidence Comments [optional]:

HIERARCHY

*Lower Level NVC Types:

| **Elcode** | **Scientific or Colloquial Name** |
| --- | --- |
|  |  |

DISCUSSION

Discussion [optional]:

CONCEPT HISTORY

*Recent Concept Lineage [if applicable]:

| **Date** | **Predecessor** | **Note** |
| --- | --- | --- |
| 2013-09-10 | M569 Chiquitano Seasonal Dry Forest Macrogroup | M569 split between M570 and M872 |

RELATED CONCEPTS

Supporting Concepts [optional]:

| **Relationship to NVC** | **Supporting Concept Name** | **Short Citation** | **Note** |
| --- | --- | --- | --- |
|  |  |  |  |

AUTHORSHIP

*Primary Concept Source [if applicable]: C. Josse, in Faber-Langendoen et al. (2014)

| **Relationship to NVC** | **Name Used in Source** | **Short Citation** | **Note** |
| --- | --- | --- | --- |
|  |  |  |  |

*Author of Description: C. Josse

Acknowledgments [optional]:

Version Date: 17 Apr 2014

REFERENCES

*References [Required if used in text]:

Faber-Langendoen, D., J. Drake, S. Gawler, M. Hall, C. Josse, G. Kittel, S. Menard, C. Nordman, M. Pyne, M. Reid, L. Sneddon, K. Schulz, J. Teague, M. Russo, K. Snow, and P. Comer, editors. 2010-2018. Divisions, Macrogroups and Groups for the Revised U.S. National Vegetation Classification. NatureServe, Arlington, VA. plus appendices. [in preparation]

1. Forest & Woodland

1.A.1.Ek. Brazilian-Parana Dry Forest

M570. Cerrado Seasonal Dry Forest

Type Concept Sentence: Seasonal deciduous forest distributed across the Cerrado in Brazil, on soils derived from limestone and basalt and with improved moisture and nutrient availability which allow the growth of forests instead of treed savannas. Includes seasonal forests with the same floristic affinity but with more extensive, continuous distribution in the Bolivian Chiquitano region adjacent to the southwest of the Cerrado. Forests range from partially open, mid-sized forests to 20-25 m high multi-strata forests with abundant lianas. Diagnostic species include *Acosmium cardenasii, Amburana cearensis, Anadenanthera colubrina, Anadenanthera peregrina, Cariniana estrellensis, Holocalyx balansae, Machaerium scleroxylon*, and *Pterogyne nitens*.

OVERVIEW

*Hierarchy Level: Macrogroup

*Placement in Hierarchy: 1.A.1.Ek. Brazilian-Parana Dry Forest (D221)

Elcode: M570

*Scientific Name: Cerrado Seasonal Dry Forest Macrogroup

*Common (Translated Scientific) Name: Cerrado Seasonal Dry Forest Macrogroup

*Colloquial Name: Cerrado Seasonal Dry Forest

*Type Concept: This macrogroup represents the seasonal deciduous forest distributed across the Cerrado in Brazil, on topographies or soils (limestone- and basalt-derived) with improved moisture and nutrient availability which allow the growth of forests instead of woody savannas. It includes also seasonal forests with the same floristic affinity but with more extensive, continuous distribution in the Bolivian lowlands adjacent to the southwest of the Cerrado, known as the Chiquitano region. Forests in this macrogroup range from partially open mid-sized forests to multi-strata forest, 20-25 m high, with abundant lianas. Diagnostic species include *Amburana cearensis, Anadenanthera colubrina, Cariniana estrellensis, Cassia ferruginea, Cedrela fissilis, Centrolobium tomentosum, Chloroleucon tenuiflorum, Dilodendron bipinnatum, Guazuma ulmifolia, Jacaranda caroba, Lonchocarpus domingensis (= Lonchocarpus sericeus), Physocalymma scaberrimum, Platycyamus regnellii, Trichilia elegans, Zanthoxylum rhoifolium, Anadenanthera peregrina var. falcata, Tabebuia impetiginosa, Aspidosperma cf. subincanum, Astronium urundeuva, Lacistema aggregatum, Dilodendron bipinnatum, Astronium fraxinifolium, Guarea guidonia (= Guarea trichilioides), Acrocomia sclerocarpa, Callisthene fasciculata*. The Chiquitano forests share mostly the same species with a few others such as *Eriotheca roseorum, Guibourtia chodatiana, Holocalyx balansae, Machaerium scleroxylon, Piptadenia viridiflora, Peltogyne heterophylla, Platypodium elegans, Pseudobombax marginatum, Pterogyne nitens, Acosmium cardenasii, Talisia esculenta, Ocotea cernua, Nectandra megapotamica, Lonchocarpus nudiflorens, Cedrela fissilis*.

*Diagnostic Characteristics:

*Classification Comments:

*Similar NVC Types [if applicable]:

| **Elcode** | **Scientific or Colloquial Name** | **Note** |
| --- | --- | --- |
|  |  |  |

Similar NVC Types General Comments [optional]:

VEGETATION

Physiognomy and Structure Summary:

Physiognomy and Structure Table [optional]:

| **Physiognomy-Structure Category** | **Prevailing Height (m)** | **Height Range (opt.)** | **Mean % Cover** | **Cover Range (opt.)** |
| --- | --- | --- | --- | --- |
|  |  |  |  | - |

Floristics Summary:

*Floristics Table [Med - High Confidence]:

*Number of Plots:

*Cover Scale Used:

| **Physiognomy-Structure Category** | **Taxon Name** | **Specific Growth Form (opt.)** | **Const- ancy** | **Mean % Cover** | **Cover Range (opt.)** | **Differ-ential** | **Diagnostic Combin- ation** |
| --- | --- | --- | --- | --- | --- | --- | --- |
|  |  |  |  |  | - |  |  |

Dynamics:

ENVIRONMENT

Environmental Description:

DISTRIBUTION

*Geographic Range:

Nations: BO, BR

States/Provinces:

USFS Ecoregions (2007) [optional]:

Omernik Ecoregions L3, L4 [optional]:

MLRAs [optional]:

PLOT SAMPLING AND ANALYSIS

*Plot Analysis Summary [Med - High Confidence]:

*Plots Used to Define the Type [Med - High Confidence]:

CONFIDENCE LEVEL

USNVC Confidence Level: Unassigned

USNVC Confidence Comments [optional]:

HIERARCHY

*Lower Level NVC Types:

| **Elcode** | **Scientific or Colloquial Name** |
| --- | --- |
|  |  |

DISCUSSION

Discussion [optional]:

CONCEPT HISTORY

*Recent Concept Lineage [if applicable]:

| **Date** | **Predecessor** | **Note** |
| --- | --- | --- |
| 2013-09-10 | M569 Chiquitano Seasonal Dry Forest Macrogroup | M569 split between M570 and M872 |

RELATED CONCEPTS

Supporting Concepts [optional]:

| **Relationship to NVC** | **Supporting Concept Name** | **Short Citation** | **Note** |
| --- | --- | --- | --- |
|  |  |  |  |

AUTHORSHIP

*Primary Concept Source [if applicable]: C. Josse, in Faber-Langendoen et al. (2014)

| **Relationship to NVC** | **Name Used in Source** | **Short Citation** | **Note** |
| --- | --- | --- | --- |
|  |  |  |  |

*Author of Description: C. Josse

Acknowledgments [optional]:

Version Date: 17 Apr 2014

REFERENCES

*References [Required if used in text]:

Faber-Langendoen, D., J. Drake, S. Gawler, M. Hall, C. Josse, G. Kittel, S. Menard, C. Nordman, M. Pyne, M. Reid, L. Sneddon, K. Schulz, J. Teague, M. Russo, K. Snow, and P. Comer, editors. 2010-2018. Divisions, Macrogroups and Groups for the Revised U.S. National Vegetation Classification. NatureServe, Arlington, VA. plus appendices. [in preparation]

1. Forest & Woodland

1.A.1.Ek. Brazilian-Parana Dry Forest

M568. Brazilian Atlantic Seasonal Dry Forest

Type Concept Sentence: Seasonal semi-deciduous forests of the eastern Brazilian Shield, fringing the Atlantic evergreen and semi-evergreen forests in transition to the Cerrado and the Caatinga to the west. Also occupy favorable sites within the Agreste and the Caatinga. Among the common species are *Allophylus sericeus, Aloysia virgata, Apeiba tibourbou, Aspidosperma macrocarpon, Astronium urundeuva, Basiloxylon brasiliense, Cavanillesia arborea, Cedrela fissilis, Chorisia speciosa, Luehea divaricata, Phyllanthus acuminatus*, and *Schinopsis brasiliensis*. The forests transitional to the Cerrado in Parana and Sao Paulo states are associated with poor, sandy soils and their composition has more affinities with the Cerrado flora (i.e., *Acosmium dasycarpum, Acrocomia totai, Agonandra brasiliensis, Alibertia concolor, Alibertia macrophylla, Anadenanthera peregrina, Astronium fraxinifolium, Callisthene major*).

OVERVIEW

*Hierarchy Level: Macrogroup

*Placement in Hierarchy: 1.A.1.Ek. Brazilian-Parana Dry Forest (D221)

Elcode: M568

*Scientific Name: Brazilian Atlantic Seasonal Dry Forest Macrogroup

*Common (Translated Scientific) Name: Brazilian Atlantic Seasonal Dry Forest Macrogroup

*Colloquial Name: Brazilian Atlantic Seasonal Dry Forest

*Type Concept: Seasonal semi-deciduous forests of the eastern Brazilian shield, fringing the Atlantic evergreen and semi-evergreen forests in transition to the Cerrado and the Caatinga to the west, and also occupying favorable sites within the Agreste and the Caatinga. Those to the west of the Caatinga usually are on nutrient-rich soils derived from limestones. Among the common species are *Apeiba tibourbou, Basiloxylon brasiliense, Luehea divaricata, Molopanthera paniculata, Phyllanthus acuminatus, Cnidoscolus urens, Hymenaea courbaril, Chorisia speciosa, Cereus calcirupicola, Allophylus sericeus, Aloysia virgata, Cavanillesia arborea, Cedrela fissilis, Schinopsis brasiliensis, Astronium urundeuva, Aspidosperma macrocarpon*. The forests transitional to the Cerrado in the states of Parana and Sao Paulo tend to be associated with poor sandy soils, and their composition is different, with more affinities with the Cerrado flora, i.e., *Acosmium dasycarpum, Acrocomia totai, Agonandra brasiliensis, Alibertia concolor, Alibertia macrophylla, Anadenanthera peregrina, Astronium fraxinifolium, Callisthene major, Dalbergia miscolobium, Diospyros hispida, Gomidesia lindeniana, Guatteria sellowiana, Luehea paniculata, Machaerium acutifolium, Margaritaria nobilis, Miconia* spp.

*Diagnostic Characteristics:

*Classification Comments:

*Similar NVC Types [if applicable]:

| **Elcode** | **Scientific or Colloquial Name** | **Note** |
| --- | --- | --- |
|  |  |  |

Similar NVC Types General Comments [optional]:

VEGETATION

Physiognomy and Structure Summary:

Physiognomy and Structure Table [optional]:

| **Physiognomy-Structure Category** | **Prevailing Height (m)** | **Height Range (opt.)** | **Mean % Cover** | **Cover Range (opt.)** |
| --- | --- | --- | --- | --- |
|  |  |  |  | - |

Floristics Summary:

*Floristics Table [Med - High Confidence]:

*Number of Plots:

*Cover Scale Used:

| **Physiognomy-Structure Category** | **Taxon Name** | **Specific Growth Form (opt.)** | **Const- ancy** | **Mean % Cover** | **Cover Range (opt.)** | **Differ-ential** | **Diagnostic Combin- ation** |
| --- | --- | --- | --- | --- | --- | --- | --- |
|  |  |  |  |  | - |  |  |

Dynamics:

ENVIRONMENT

Environmental Description:

DISTRIBUTION

*Geographic Range:

Nations: BR, PY

States/Provinces:

USFS Ecoregions (2007) [optional]:

Omernik Ecoregions L3, L4 [optional]:

MLRAs [optional]:

PLOT SAMPLING AND ANALYSIS

*Plot Analysis Summary [Med - High Confidence]:

*Plots Used to Define the Type [Med - High Confidence]:

CONFIDENCE LEVEL

USNVC Confidence Level: Unassigned

USNVC Confidence Comments [optional]:

HIERARCHY

*Lower Level NVC Types:

| **Elcode** | **Scientific or Colloquial Name** |
| --- | --- |
|  |  |

DISCUSSION

Discussion [optional]:

CONCEPT HISTORY

*Recent Concept Lineage [if applicable]:

| **Date** | **Predecessor** | **Note** |
| --- | --- | --- |
|  |  |  |

RELATED CONCEPTS

Supporting Concepts [optional]:

| **Relationship to NVC** | **Supporting Concept Name** | **Short Citation** | **Note** |
| --- | --- | --- | --- |
|  |  |  |  |

AUTHORSHIP

*Primary Concept Source [if applicable]: C. Josse, in Faber-Langendoen et al. (2014)

| **Relationship to NVC** | **Name Used in Source** | **Short Citation** | **Note** |
| --- | --- | --- | --- |
|  |  |  |  |

*Author of Description: C. Josse

Acknowledgments [optional]:

Version Date: 17 Apr 2014

REFERENCES

*References [Required if used in text]:

Faber-Langendoen, D., J. Drake, S. Gawler, M. Hall, C. Josse, G. Kittel, S. Menard, C. Nordman, M. Pyne, M. Reid, L. Sneddon, K. Schulz, J. Teague, M. Russo, K. Snow, and P. Comer, editors. 2010-2018. Divisions, Macrogroups and Groups for the Revised U.S. National Vegetation Classification. NatureServe, Arlington, VA. plus appendices. [in preparation]

1. Forest & Woodland

1.A.1.Ek. Brazilian-Parana Dry Forest

M571. Parana Seasonal Dry Forest

Type Concept Sentence: Semi-deciduous, subhumid and well-drained forests of the Parana floristic region in Paraguay transitional to the Chaco, but with a drier climate and more sandy substrates. Forest stature is lower with a more open canopy and with lower plant diversity than Chaco. Species diagnostic to this system include *Alchornea triplinervia, Apuleia leiocarpa, Copaifera langsdorfii, Cupania vernalis, Dendropanax cuneatus, Diatenopteryx sorbifolia, Hellieta apiculata, Holocalyx balansae, Luehea divaricata, Matayba alaeganoides, Nectandra megapotamica, Ocotea puberula, Plinia rivularis, Styrax leprosum*, and *Syagrus romanzoffiana*.

OVERVIEW

*Hierarchy Level: Macrogroup

*Placement in Hierarchy: 1.A.1.Ek. Brazilian-Parana Dry Forest (D221)

Elcode: M571

*Scientific Name: Parana Seasonal Dry Forest Macrogroup

*Common (Translated Scientific) Name: Parana Seasonal Dry Forest Macrogroup

*Colloquial Name: Parana Seasonal Dry Forest

*Type Concept: Semi-deciduous, sub-humid and well-drained forests of the Parana floristic region in Paraguay transitional to the Chaco, in a climate drier than that of the rest of the Parana floristic region and on more sandy substrates. The changes are manifested in the structure, which is shorter, with a more open canopy and lower plant diversity. The following list of species is diagnostic for this macrogroup: *Hellieta apiculata, Syagrus romanzoffiana, Ocotea puberula, Nectandra megapotamica, Apuleia leiocarpa, Copaifera langsdorfii, Alchornea triplinervia, Plinia rivularis (= Myrciaria rivularis), Holocalyx balansae, Dendropanax cuneatus, Luehea divaricata, Cupania vernalis, Matayba alaeganoides, Diatenopteryx sorbifolia, Styrax leprosum*.

*Diagnostic Characteristics:

*Classification Comments:

*Similar NVC Types [if applicable]:

| **Elcode** | **Scientific or Colloquial Name** | **Note** |
| --- | --- | --- |
|  |  |  |

Similar NVC Types General Comments [optional]:

VEGETATION

Physiognomy and Structure Summary:

Physiognomy and Structure Table [optional]:

| **Physiognomy-Structure Category** | **Prevailing Height (m)** | **Height Range (opt.)** | **Mean % Cover** | **Cover Range (opt.)** |
| --- | --- | --- | --- | --- |
|  |  |  |  | - |

Floristics Summary:

*Floristics Table [Med - High Confidence]:

*Number of Plots:

*Cover Scale Used:

| **Physiognomy-Structure Category** | **Taxon Name** | **Specific Growth Form (opt.)** | **Const- ancy** | **Mean % Cover** | **Cover Range (opt.)** | **Differ-ential** | **Diagnostic Combin- ation** |
| --- | --- | --- | --- | --- | --- | --- | --- |
|  |  |  |  |  | - |  |  |

Dynamics:

ENVIRONMENT

Environmental Description:

DISTRIBUTION

*Geographic Range:

Nations: PY

States/Provinces:

USFS Ecoregions (2007) [optional]:

Omernik Ecoregions L3, L4 [optional]:

MLRAs [optional]:

PLOT SAMPLING AND ANALYSIS

*Plot Analysis Summary [Med - High Confidence]:

*Plots Used to Define the Type [Med - High Confidence]:

CONFIDENCE LEVEL

USNVC Confidence Level: Unassigned

USNVC Confidence Comments [optional]:

HIERARCHY

*Lower Level NVC Types:

| **Elcode** | **Scientific or Colloquial Name** |
| --- | --- |
|  |  |

DISCUSSION

Discussion [optional]:

CONCEPT HISTORY

*Recent Concept Lineage [if applicable]:

| **Date** | **Predecessor** | **Note** |
| --- | --- | --- |
|  |  |  |

RELATED CONCEPTS

Supporting Concepts [optional]:

| **Relationship to NVC** | **Supporting Concept Name** | **Short Citation** | **Note** |
| --- | --- | --- | --- |
|  |  |  |  |

AUTHORSHIP

*Primary Concept Source [if applicable]: C. Josse, in Faber-Langendoen et al. (2014)

| **Relationship to NVC** | **Name Used in Source** | **Short Citation** | **Note** |
| --- | --- | --- | --- |
|  |  |  |  |

*Author of Description: C. Josse

Acknowledgments [optional]:

Version Date: 17 Apr 2014

REFERENCES

*References [Required if used in text]:

Faber-Langendoen, D., J. Drake, S. Gawler, M. Hall, C. Josse, G. Kittel, S. Menard, C. Nordman, M. Pyne, M. Reid, L. Sneddon, K. Schulz, J. Teague, M. Russo, K. Snow, and P. Comer, editors. 2010-2018. Divisions, Macrogroups and Groups for the Revised U.S. National Vegetation Classification. NatureServe, Arlington, VA. plus appendices. [in preparation]

1. Forest & Woodland

1.A.1.El. Tropical Andean Montane Dry Forest

D222. Tropical Andean Montane Dry Forest

Type Concept Sentence:

OVERVIEW

*Hierarchy Level: Division

*Placement in Hierarchy: 1.A.1.El. Tropical Dry Forest & Woodland (F003)

Elcode: D222

*Scientific Name: Tropical Andean Montane Dry Forest Division

*Common (Translated Scientific) Name: Tropical Andean Montane Dry Forest Division

*Colloquial Name: Tropical Andean Montane Dry Forest

*Type Concept:

*Diagnostic Characteristics:

*Classification Comments:

*Similar NVC Types [if applicable]:

| **Elcode** | **Scientific or Colloquial Name** | **Note** |
| --- | --- | --- |
|  |  |  |

Similar NVC Types General Comments [optional]:

VEGETATION

Physiognomy and Structure Summary:

Physiognomy and Structure Table [optional]:

| **Physiognomy-Structure Category** | **Prevailing Height (m)** | **Height Range (opt.)** | **Mean % Cover** | **Cover Range (opt.)** |
| --- | --- | --- | --- | --- |
|  |  |  |  | - |

Floristics Summary:

*Floristics Table [Med - High Confidence]:

*Number of Plots: *Cover Scale Used:

| **Physiognomy-Structure Category** | **Taxon Name** | **Specific Growth Form (opt.)** | **Const- ancy** | **Mean % Cover** | **Cover Range (opt.)** | **Differ-ential** | **Diagnostic Combin- ation** |
| --- | --- | --- | --- | --- | --- | --- | --- |
|  |  |  |  |  | - |  |  |

Dynamics:

ENVIRONMENT

Environmental Description:

DISTRIBUTION

*Geographic Range:

Nations: AR, CO, EC, PE, VE

States/Provinces:

USFS Ecoregions (2007) [optional]:

Omernik Ecoregions L3, L4 [optional]:

MLRAs [optional]:

PLOT SAMPLING AND ANALYSIS

*Plot Analysis Summary [Med - High Confidence]:

*Plots Used to Define the Type [Med - High Confidence]:

CONFIDENCE LEVEL

USNVC Confidence Level: Unassigned

USNVC Confidence Comments [optional]:

HIERARCHY

*Lower Level NVC Types:

| **Elcode** | **Scientific or Colloquial Name** |
| --- | --- |
| M575 | Bolivian-Tucuman Seasonal Dry Forest |
| M574 | Central Andean Seasonal Dry Forest |

DISCUSSION

Discussion [optional]:

CONCEPT HISTORY

*Recent Concept Lineage [if applicable]:

| **Date** | **Predecessor** | **Note** |
| --- | --- | --- |
|  |  |  |

RELATED CONCEPTS

Supporting Concepts [optional]:

| **Relationship to NVC** | **Supporting Concept Name** | **Short Citation** | **Note** |
| --- | --- | --- | --- |
|  |  |  |  |

AUTHORSHIP

*Primary Concept Source [if applicable]:

| **Relationship to NVC** | **Name Used in Source** | **Short Citation** | **Note** |
| --- | --- | --- | --- |
|  |  |  |  |

*Author of Description:

Acknowledgments [optional]:

Version Date:

REFERENCES

*References [Required if used in text]:

Faber-Langendoen, D., J. Drake, S. Gawler, M. Hall, C. Josse, G. Kittel, S. Menard, C. Nordman, M. Pyne, M. Reid, L. Sneddon, K. Schulz, J. Teague, M. Russo, K. Snow, and P. Comer, editors. 2010-2018. Divisions, Macrogroups and Groups for the Revised U.S. National Vegetation Classification. NatureServe, Arlington, VA. plus appendices. [in preparation]

1. Forest & Woodland

1.A.1.El. Tropical Andean Montane Dry Forest

M575. Bolivian-Tucuman Seasonal Dry Forest

Type Concept Sentence: Semi-deciduous forests of the subhumid intermontane valleys of central south Bolivia (Santa Cruz, Chuquisaca and Tarija) and northwestern Argentina (south to Salta) in the floristic region known as Boliviano-Tucumano. Forests are dense to semi-dense with canopies 20-25 m high, frequent presence of woody lianas, and several subcanopy strata. Occurs at 800-1900 m elevation. Diagnostic species of the upper belt include *Anadenanthera macrocarpa, Cupania vernalis, Diatenopteryx sorbifolia, Erythrina falcata, Parapiptadenia excelsa, Terminalia triflora*, and *Tipuana tipu*. Characteristic species of the lower belt are *Anadenanthera macrocarpa, Astronium urundeuva, Caesalpinia pluviosa, Calycophyllum multiflorum, Cedrela fissilis, Eriotheca roseorum, Myroxylon peruiferum, Phyllostylon rhamnoides*, and *Xylosma pubescens*.

OVERVIEW

*Hierarchy Level: Macrogroup

*Placement in Hierarchy: 1.A.1.El. Tropical Andean Montane Dry Forest (D222)

Elcode: M575

*Scientific Name: Bolivian-Tucuman Seasonal Dry Forest Macrogroup

*Common (Translated Scientific) Name: Bolivian-Tucuman Seasonal Dry Forest Macrogroup

*Colloquial Name: Bolivian-Tucuman Seasonal Dry Forest

*Type Concept: Semi-deciduous forests of the subhumid intermontane valleys of central southern Bolivia (Santa Cruz, Chuquisaca and Tarija) and northwestern Argentina, south down to Salta, in the floristic region known as Boliviano-Tucumano, growing from 800-1900 m elevation. They are dense to semi-dense forests with canopy 20-25 m high, frequent presence of woody lianas, and several subcanopy strata. The following species are diagnostic in the upper belt: *Parapiptadenia excelsa, Tipuana tipu, Cupania vernalis, Diatenopteryx sorbifolia, Acacia polyphylla, Anadenanthera macrocarpa, Erythrina falcata, Terminalia triflora*, while the lower is characterized by *Calycophyllum multiflorum, Phyllostylon rhamnoides, Myroxylon peruiferum, Eriotheca roseorum, Caesalpinia pluviosa, Anadenanthera macrocarpa, Gleditsia amorphoides, Cedrela fissilis, Xylosma pubescens, Astronium urundeuva*.

*Diagnostic Characteristics:

*Classification Comments:

*Similar NVC Types [if applicable]:

| **Elcode** | **Scientific or Colloquial Name** | **Note** |
| --- | --- | --- |
|  |  |  |

Similar NVC Types General Comments [optional]:

VEGETATION

Physiognomy and Structure Summary:

Physiognomy and Structure Table [optional]:

| **Physiognomy-Structure Category** | **Prevailing Height (m)** | **Height Range (opt.)** | **Mean % Cover** | **Cover Range (opt.)** |
| --- | --- | --- | --- | --- |
|  |  |  |  | - |

Floristics Summary:

*Floristics Table [Med - High Confidence]:

*Number of Plots:

*Cover Scale Used:

| **Physiognomy-Structure Category** | **Taxon Name** | **Specific Growth Form (opt.)** | **Const- ancy** | **Mean % Cover** | **Cover Range (opt.)** | **Differ-ential** | **Diagnostic Combin- ation** |
| --- | --- | --- | --- | --- | --- | --- | --- |
|  |  |  |  |  | - |  |  |

Dynamics:

ENVIRONMENT

Environmental Description:

DISTRIBUTION

*Geographic Range:

Nations: AR, BO

States/Provinces:

USFS Ecoregions (2007) [optional]:

Omernik Ecoregions L3, L4 [optional]:

MLRAs [optional]:

PLOT SAMPLING AND ANALYSIS

*Plot Analysis Summary [Med - High Confidence]:

*Plots Used to Define the Type [Med - High Confidence]:

CONFIDENCE LEVEL

USNVC Confidence Level: Unassigned

USNVC Confidence Comments [optional]:

HIERARCHY

*Lower Level NVC Types:

| **Elcode** | **Scientific or Colloquial Name** |
| --- | --- |
|  |  |

DISCUSSION

Discussion [optional]:

CONCEPT HISTORY

*Recent Concept Lineage [if applicable]:

| **Date** | **Predecessor** | **Note** |
| --- | --- | --- |
|  |  |  |

RELATED CONCEPTS

Supporting Concepts [optional]:

| **Relationship to NVC** | **Supporting Concept Name** | **Short Citation** | **Note** |
| --- | --- | --- | --- |
|  |  |  |  |

AUTHORSHIP

*Primary Concept Source [if applicable]: C. Josse, in Faber-Langendoen et al. (2014)

| **Relationship to NVC** | **Name Used in Source** | **Short Citation** | **Note** |
| --- | --- | --- | --- |
|  |  |  |  |

*Author of Description: C. Josse

Acknowledgments [optional]:

Version Date: 17 Apr 2014

REFERENCES

*References [Required if used in text]:

Faber-Langendoen, D., J. Drake, S. Gawler, M. Hall, C. Josse, G. Kittel, S. Menard, C. Nordman, M. Pyne, M. Reid, L. Sneddon, K. Schulz, J. Teague, M. Russo, K. Snow, and P. Comer, editors. 2010-2018. Divisions, Macrogroups and Groups for the Revised U.S. National Vegetation Classification. NatureServe, Arlington, VA. plus appendices. [in preparation]

1. Forest & Woodland

1.A.1.El. Tropical Andean Montane Dry Forest

M574. Central Andean Seasonal Dry Forest

Type Concept Sentence: Semi-deciduous forests of the lower montane belt of the eastern slopes of the Andes from northern Peru to south-central Bolivia. The canopy is 20-25 m high, and the forests have several understory strata and abundant woody lianas. Two floristic compositions occur: a northern type restricted to the eastern portion of the Huancabamba - Maranon valley area of northern Peru and southern Ecuador, and a southern type in inter-Andean valleys of the Yungas from central Peru to Cochabamba department in Bolivia. Forest are characterized by numerous species shared with the Chiquitano deciduous forests of Santa Cruz, representing disjunct 'islands' of Brazilian-Paranense flora in the Yungas.

OVERVIEW

*Hierarchy Level: Macrogroup

*Placement in Hierarchy: 1.A.1.El. Tropical Andean Montane Dry Forest (D222)

Elcode: M574

*Scientific Name: Central Andean Seasonal Dry Forest Macrogroup

*Common (Translated Scientific) Name: Central Andean Seasonal Dry Forest Macrogroup

*Colloquial Name: Central Andean Seasonal Dry Forest

*Type Concept: Semi-deciduous, sub-humid forests distributed in the lower montane belt of the eastern slopes of the Andes from northern Peru to south-central Bolivia. They occur in areas with medium or moderate effect of orographic rainshadow. Structurally, they are partially deciduous forests with a dense canopy 20-25 m high and several levels of understory characteristically presenting abundant woody lianas. Relative to their floristic composition there are two groups: a northern and a southern one. The former is restricted to the eastern portion of the Huancabamba - Maranon valley area of northern Peru and southern Ecuador. The southern type occurs in inter-Andean valleys of the Yungas from central Peru to the Cochabamba department in Bolivia and are characterized by numerous species shared with the Chiquitano deciduous forests of Santa Cruz, representing disjunct "islands" of Brazilian-Paranense flora in the Yungas, of great biogeographical interest. Characteristic species are *Schinopsis brasiliensis, Cariniana estrellensis, Astronium urundeuva, Aspidosperma cylindrocarpon, Aspidosperma macrocarpon, Zeyheria tuberculosa, Ceiba boliviana, Ceiba pubiflora, Cereus tacuaralensis, Brasiliopuntia brasiliensis (= Opuntia brasiliensis), Maytenus ilicifolia, Hedyosmum angustifolium, Clusia ducuoides, Juglans boliviana, Hymenaea courbaril, Piptadenia buchtienii, Piptadenia viridiflora, Anadenanthera colubrina, Maclura tinctoria, Stylogine ambigua, Aiphanes aculeata, Cinchona calisaya, Cavanillesia umbellata, Apuleia leiocarpa, Caesalpinia floribunda, Cedrela fissilis, Cedrela lilloi, Centrolobium tomentosum, Chrysophyllum gonocarpum, Combretum leprosum, Lonchocarpus macrocarpus, Machaerium guanaiense, Kielmeyera paniculata, Physocalymma scaberrimum, Attalea phalerata, Gallesia integrifolia, Machaerium scleroxylon, Spondias mombin, Tabebuia ochracea, Tabebuia roseoalba, Amburana cearensis, Myrocarpus frondosus, Sweetia fruticosa, Pachystroma longifolium*, and *Copaifera langsdorfii*.

*Diagnostic Characteristics:

*Classification Comments:

*Similar NVC Types [if applicable]:

| **Elcode** | **Scientific or Colloquial Name** | **Note** |
| --- | --- | --- |
|  |  |  |

Similar NVC Types General Comments [optional]:

VEGETATION

Physiognomy and Structure Summary:

Physiognomy and Structure Table [optional]:

| **Physiognomy-Structure Category** | **Prevailing Height (m)** | **Height Range (opt.)** | **Mean % Cover** | **Cover Range (opt.)** |
| --- | --- | --- | --- | --- |
|  |  |  |  | - |

Floristics Summary:

*Floristics Table [Med - High Confidence]:

*Number of Plots:

*Cover Scale Used:

| **Physiognomy-Structure Category** | **Taxon Name** | **Specific Growth Form (opt.)** | **Const- ancy** | **Mean % Cover** | **Cover Range (opt.)** | **Differ-ential** | **Diagnostic Combin- ation** |
| --- | --- | --- | --- | --- | --- | --- | --- |
|  |  |  |  |  | - |  |  |

Dynamics:

ENVIRONMENT

Environmental Description:

DISTRIBUTION

*Geographic Range:

Nations: BO, EC, PE

States/Provinces:

USFS Ecoregions (2007) [optional]:

Omernik Ecoregions L3, L4 [optional]:

MLRAs [optional]:

PLOT SAMPLING AND ANALYSIS

*Plot Analysis Summary [Med - High Confidence]:

*Plots Used to Define the Type [Med - High Confidence]:

CONFIDENCE LEVEL

USNVC Confidence Level: Unassigned

USNVC Confidence Comments [optional]:

HIERARCHY

*Lower Level NVC Types:

| **Elcode** | **Scientific or Colloquial Name** |
| --- | --- |
|  |  |

DISCUSSION

Discussion [optional]:

CONCEPT HISTORY

*Recent Concept Lineage [if applicable]:

| **Date** | **Predecessor** | **Note** |
| --- | --- | --- |
|  |  |  |

RELATED CONCEPTS

Supporting Concepts [optional]:

| **Relationship to NVC** | **Supporting Concept Name** | **Short Citation** | **Note** |
| --- | --- | --- | --- |
|  |  |  |  |

AUTHORSHIP

*Primary Concept Source [if applicable]: C. Josse, in Faber-Langendoen et al. (2014)

| **Relationship to NVC** | **Name Used in Source** | **Short Citation** | **Note** |
| --- | --- | --- | --- |
|  |  |  |  |

*Author of Description: C. Josse

Acknowledgments [optional]:

Version Date: 17 Apr 2014

REFERENCES

*References [Required if used in text]:

Faber-Langendoen, D., J. Drake, S. Gawler, M. Hall, C. Josse, G. Kittel, S. Menard, C. Nordman, M. Pyne, M. Reid, L. Sneddon, K. Schulz, J. Teague, M. Russo, K. Snow, and P. Comer, editors. 2010-2018. Divisions, Macrogroups and Groups for the Revised U.S. National Vegetation Classification. NatureServe, Arlington, VA. plus appendices. [in preparation]

1.A.2. Tropical Lowland Humid Forest

Tropical Lowland Humid Forest is dominated by broad-leaved evergreen trees, often with multiple complex strata and growth forms in lowland to submontane or premontane elevations with aseasonal to moderately seasonal rainfall and warm temperatures.

1. Forest & Woodland

1.A.2.Eg. Caribbean-Mesoamerican Lowland Humid Forest

D091. Caribbean-Mesoamerican Lowland Humid Forest

Type Concept Sentence:

OVERVIEW

*Hierarchy Level: Division

*Placement in Hierarchy: 1.A.2.Eg. Tropical Lowland Humid Forest (F020)

Elcode: D091

*Scientific Name: Caribbean-Mesoamerican Lowland Humid Forest Division

*Common (Translated Scientific) Name: Caribbean-Mesoamerican Lowland Humid Forest Division

*Colloquial Name: Caribbean-Mesoamerican Lowland Humid Forest

*Type Concept:

*Diagnostic Characteristics:

*Classification Comments:

*Similar NVC Types [if applicable]:

| **Elcode** | **Scientific or Colloquial Name** | **Note** |
| --- | --- | --- |
|  |  |  |

Similar NVC Types General Comments [optional]:

VEGETATION

Physiognomy and Structure Summary:

Physiognomy and Structure Table [optional]:

| **Physiognomy-Structure Category** | **Prevailing Height (m)** | **Height Range (opt.)** | **Mean % Cover** | **Cover Range (opt.)** |
| --- | --- | --- | --- | --- |
|  |  |  |  | - |

Floristics Summary:

*Floristics Table [Med - High Confidence]:

*Number of Plots: *Cover Scale Used:

| **Physiognomy-Structure Category** | **Taxon Name** | **Specific Growth Form (opt.)** | **Const- ancy** | **Mean % Cover** | **Cover Range (opt.)** | **Differ-ential** | **Diagnostic Combin- ation** |
| --- | --- | --- | --- | --- | --- | --- | --- |
|  |  |  |  |  | - |  |  |

Dynamics:

ENVIRONMENT

Environmental Description:

DISTRIBUTION

*Geographic Range:

Nations: BS, BZ, CR, CU, DO, GT, HN, JM, MQ, MX, NI, PA, PR, SV, TT, VE, VI, XD

States/Provinces:

USFS Ecoregions (2007) [optional]:

Omernik Ecoregions L3, L4 [optional]:

MLRAs [optional]:

PLOT SAMPLING AND ANALYSIS

*Plot Analysis Summary [Med - High Confidence]:

*Plots Used to Define the Type [Med - High Confidence]:

CONFIDENCE LEVEL

USNVC Confidence Level: Low - Poorly Documented

USNVC Confidence Comments [optional]:

HIERARCHY

*Lower Level NVC Types:

| **Elcode** | **Scientific or Colloquial Name** |
| --- | --- |
| M281 | Caribbean Lowland Humid Forest |
| M578 | Mesoamerican Lowland Humid Forest |
| M873 | Mesoamerican Submontane Humid Forest |

DISCUSSION

Discussion [optional]:

CONCEPT HISTORY

*Recent Concept Lineage [if applicable]:

| **Date** | **Predecessor** | **Note** |
| --- | --- | --- |
|  |  |  |

RELATED CONCEPTS

Supporting Concepts [optional]:

| **Relationship to NVC** | **Supporting Concept Name** | **Short Citation** | **Note** |
| --- | --- | --- | --- |
|  |  |  |  |

AUTHORSHIP

*Primary Concept Source [if applicable]: Faber-Langendoen et al.

| **Relationship to NVC** | **Name Used in Source** | **Short Citation** | **Note** |
| --- | --- | --- | --- |
|  |  |  |  |

*Author of Description:

Acknowledgments [optional]:

Version Date:

REFERENCES

*References [Required if used in text]:

Faber-Langendoen, D., J. Drake, S. Gawler, M. Hall, C. Josse, G. Kittel, S. Menard, C. Nordman, M. Pyne, M. Reid, L. Sneddon, K. Schulz, J. Teague, M. Russo, K. Snow, and P. Comer, editors. 2010-2018. Divisions, Macrogroups and Groups for the Revised U.S. National Vegetation Classification. NatureServe, Arlington, VA. plus appendices. [in preparation]

1. Forest & Woodland

1.A.2.Eg. Caribbean-Mesoamerican Lowland Humid Forest

M281. Caribbean Lowland Humid Forest

Type Concept Sentence: These are moist forests with high canopy closure and usually without emergent trees. They are located in the lowlands of the Caribbean islands, in areas that do not have a regular dry season and usually with an average monthly rainfall of 100 mm or more, or where water stress is intermittent but very short.

Bosques húmedos con un dosel alto, cerrado y generalmente sin árboles emergentes. Se encuentra en las tierras bajas de las islas del Caribe, en áreas que no presentan una estación seca regular y generalmente con una precipitación promedio mensual de 100 mm o más, o donde el estrés hídrico es intermitente pero muy corto.

OVERVIEW

*Hierarchy Level: Macrogroup

*Placement in Hierarchy: 1.A.2.Eg. Caribbean-Mesoamerican Lowland Humid Forest (D091)

Elcode: M281

*Scientific Name: Caribbean Lowland Humid Forest Macrogroup

*Common (Translated Scientific) Name: Caribbean Lowland Humid Forest Macrogroup

*Colloquial Name: Caribbean Lowland Humid Forest

*Type Concept:

*Diagnostic Characteristics:

*Classification Comments:

*Similar NVC Types [if applicable]:

| **Elcode** | **Scientific or Colloquial Name** | **Note** |
| --- | --- | --- |
|  |  |  |

Similar NVC Types General Comments [optional]:

VEGETATION

Physiognomy and Structure Summary:

Physiognomy and Structure Table [optional]:

| **Physiognomy-Structure Category** | **Prevailing Height (m)** | **Height Range (opt.)** | **Mean % Cover** | **Cover Range (opt.)** |
| --- | --- | --- | --- | --- |
|  |  |  |  | - |

Floristics Summary:

*Floristics Table [Med - High Confidence]:

*Number of Plots:

*Cover Scale Used:

| **Physiognomy-Structure Category** | **Taxon Name** | **Specific Growth Form (opt.)** | **Const- ancy** | **Mean % Cover** | **Cover Range (opt.)** | **Differ-ential** | **Diagnostic Combin- ation** |
| --- | --- | --- | --- | --- | --- | --- | --- |
|  |  |  |  |  | - |  |  |

Dynamics: Diversity of above-ground plant functional groups (species that share morphological, chemical, structural or life history characteristics) determines the role of biodiversity in ecosystem functioning such as nutrient cycling, forest regeneration and successional patterns. Diversity of animal functional groups determines a number of key ecological processes such as trophic structure, nutrient cycling, and the system's resilience to disturbance. Community composition/diversity /structure affects species diversity and several ecosystem-level processes. Gap dynamics provide light, the major environmental limiting factor to plant growth in the closed-canopy humid tropical forest, and maintains the forest in shifting mosaic steady state.

Biotic interactions: pollination (bees, butterflies, beetles, moths, bats, and hummingbirds) is important for reproductive success and pollinators influence the frequency and distribution pattern of plant species; seed dispersal is executed by fruit-eating birds, mammals and ants, is important for reproductive success, and seed dispersal agents affect food webs in tropical forests by making available reproductive resources to other consumers and influencing the frequency and distribution pattern of plant species, especially woody species; seed predation is important for reproductive success and seed predation affects population recruitment and establishment of diverse plant species (e.g., palms and legumes). Seed predators occasionally act as dispersers. Seed predation is a specialized form of herbivory. Vertebrates involved are often objects of hunting by humans. Herbivores, including insects, parasitic fungi, and vertebrates, affect vigor and mortality of plants of all sizes, especially understory seedlings, and influences food chain and species composition of understory. The presence of top predators controls the populations of small mammals and herbivores. Species diversity and composition of soil biota, e.g., mycorrhizae, fungi, microbes, soil mesofauna such as leaf-cutter ants, termites, nematodes, collembola, dung beetles, etc., are fundamental for nutrient cycling and soil structure.

Disturbance regimes from catastrophic natural causes, e.g., hurricanes, rare catastrophic floods, or multiple landslides, or volcanism, or earthquakes, rare extreme cold fronts, rare extreme droughts, are rare events that can be very important for ecological dynamics. Create canopy gaps of great size allowing pioneer species to colonize and initiate successional processes, e.g., hurricanes play a major role in landscape-scale dynamics of forests on Caribbean islands. Fire due to dry spell or prolonged dry seasons or human activities: Certain species might be maintained because of this big, very rare catastrophic event. For example, mahogany thrives on fire outbreaks. Background disturbances, such as small gaps, small landslides, downbursts, normal cold fronts, and normal seasonal precipitation variability. Important for creating and maintaining habitat heterogeneity and species and structural diversity, preventing competitive exclusion. Drives regeneration.

Spatial integration and coverage (e.g., connectivity by riparian habitats) allowing migration of animals and plants outside of lowland forest: Allow to define at landscape level integrity of ecosystem. Allow to assess the extent of potential for species extinction. Spatial integration important for species to maintain contact with all habitats required for life cycles.

Biogeochemical dynamics (referring to regional and global processes such as global warming, ozone depletion, CO2 concentration, atmospheric and soil pollution, etc.): Affects basic ecosystem functioning at both global and local levels. Soil type or fertility: Affects forest primary productivity and species richness. Soil type is also relevant to tree mortality rate, treefall frequency, forest regeneration mode, and stand turnover time (Hartshorn 1990).

ENVIRONMENT

Environmental Description: Major factors that determine variation in community types within lowland tropical moist forest include precipitation, temperature, topography, edaphic conditions, and natural disturbance. The amount of rainfall and length of dry season determine the occurrences of evergreen forest or seasonally dry forest. Yearly extreme temperature fluctuations result in cold-front stressed forests in southwestern Amazonia and the southern Atlantic region and non-cold-front stressed forests in Mexico and Central America.: Zonation may occur depending on whether the forest is on a plain, or rolling hills, or foothills of a mountain range. Edaphic conditions (soil quality or fertility) can create special community types. Forests on white sand soil, on clay soil, or over limestone/ultrabasic rock differ considerably in species composition. Natural disturbance includes hurricanes and landslides. Hurricanes are the most frequent causes of landslides.

DISTRIBUTION

*Geographic Range: Northern part of eastern Cuba, northern Jamaica, eastern Dominican Republic, northern Puerto Rico, Trinidad and Tobago and the Lesser Antilles in small areas.

Parte norte de Cuba oriental, norte de Jamaica, el este de República Dominicana, el norte de Puerto Rico, Trinidad y Tobago y áreas pequeñas en las Antillas Menores.

Nations: BS, CU, DO, JM, MQ, PR, TT, VE, VI, XD

States/Provinces:

USFS Ecoregions (2007) [optional]:

Omernik Ecoregions L3, L4 [optional]:

MLRAs [optional]:

PLOT SAMPLING AND ANALYSIS

*Plot Analysis Summary [Med - High Confidence]:

*Plots Used to Define the Type [Med - High Confidence]:

CONFIDENCE LEVEL

USNVC Confidence Level: Low - Poorly Documented

USNVC Confidence Comments [optional]:

HIERARCHY

*Lower Level NVC Types:

| **Elcode** | **Scientific or Colloquial Name** |
| --- | --- |
| G455 | Caribbean Seasonal Evergreen Lowland Rainforest |
| G456 | Caribbean Evergreen Lowland Rainforest |
| G845 | Caribbean Lowland Ruderal Rainforest |

DISCUSSION

Discussion [optional]:

CONCEPT HISTORY

*Recent Concept Lineage [if applicable]:

| **Date** | **Predecessor** | **Note** |
| --- | --- | --- |
| 2013-01-04 | M282 Caribbean Lowland Seasonal Evergreen Rainforest Macrogroup | M282 merged into M281 |

RELATED CONCEPTS

Supporting Concepts [optional]:

| **Relationship to NVC** | **Supporting Concept Name** | **Short Citation** | **Note** |
| --- | --- | --- | --- |
|  |  |  |  |

AUTHORSHIP

*Primary Concept Source [if applicable]: Faber-Langendoen et al. (2014)

| **Relationship to NVC** | **Name Used in Source** | **Short Citation** | **Note** |
| --- | --- | --- | --- |
|  |  |  |  |

*Author of Description:

Acknowledgments [optional]:

Version Date: 08 Jan 2015

REFERENCES

*References [Required if used in text]:

Faber-Langendoen, D., J. Drake, S. Gawler, M. Hall, C. Josse, G. Kittel, S. Menard, C. Nordman, M. Pyne, M. Reid, L. Sneddon, K. Schulz, J. Teague, M. Russo, K. Snow, and P. Comer, editors. 2010-2018. Divisions, Macrogroups and Groups for the Revised U.S. National Vegetation Classification. NatureServe, Arlington, VA. plus appendices. [in preparation]

Hartshorn, G. S. 1990. An overview of Neotropical forest dynamics. Pages 585-599 in: A. H. Gentry, editor. Four Neotropical rainforests. Yale University Press, New Haven.

1. Forest & Woodland

1.A.2.Eg. Caribbean-Mesoamerican Lowland Humid Forest

M578. Mesoamerican Lowland Humid Forest

Type Concept Sentence: Evergreen to semi-evergreen humid forests of the coastal plain (both Pacific and Caribbean), coastal ridges and inland lowlands and low hills of Central America, and the humid, tropical coasts of southern Mexico.

OVERVIEW

*Hierarchy Level: Macrogroup

*Placement in Hierarchy: 1.A.2.Eg. Caribbean-Mesoamerican Lowland Humid Forest (D091)

Elcode: M578

*Scientific Name: Mesoamerican Lowland Humid Forest Macrogroup

*Common (Translated Scientific) Name: Mesoamerican Lowland Humid Forest Macrogroup

*Colloquial Name: Mesoamerican Lowland Humid Forest

*Type Concept: The macrogroup includes forests developed under conditions of high year-round precipitation, as well as those with a short dry season. They grow on soils of sedimentary (limestone) or igneous origin (ash or basalt), and metamorphic in some cases. These are very diverse, dense, 30-m high forests with emergents up to 40 m tall, with several strata, rich in palms, epiphytes and lianas. The composition varies based on their geographic location. Communities of the Caribbean basin side include *Anaxagorea costaricensis, Aspidosperma megalocarpon, Capparis pittieri, Carpotroche platyptera, Cassipourea elliptica, Dialium guianense, Magnolia yocoronte, Manilkara zapota, Pentaclethra macroloba, Virola guatemalensis, Virola koschnyi, Vochysia guatemalensis*, among many others. On the Pacific basin side, a sample of characteristic species includes *Alchornea costaricensis, Anacardium excelsum, Andira inermis, Apeiba aspera, Apeiba tibourbou, Ardisia cutteri, Aspidosperma megalocarpon, Brosimum utile, Brosimum utile, Carapa guianensis, Caryocar costaricense, Heisteria longipes, Huberodendron patinoi, Iriartea deltoidea, Iriartea gigantea, Manilkara zapota (= Achras zapota), Minquartia guianensis, Parkia pendula, Peltogyne purpurea, Poulsenia armata, Protium copal, Qualea paraensis, Scheelea rostrata, Schizolobium parahyba, Socratea exorrhiza, Sorocea pubivena, Symphonia globulifera, Talisia nervosa, Terminalia lucida, Tetragastris panamensis, Vantanea barbourii, Vatairea lundellii*, and *Welfia georgii*. The semi-evergreen forests of the Peten on limestone substrates include *Alseis yucatanensis, Ampelocera hottlei, Aspidosperma cruenta, Astronium graveolens, Orbignya cohune (= Attalea cohune), Bernoullia flammea, Brosimum alicastrum, Bursera simaruba, Calophyllum antillanum (= Calophyllum brasiliense), Clusia salvinii, Cordia dodecandra, Cupania belicensis, Cupania prisca, Crysophila stauracantha, Chione chiapasensis, Dendropanax arboreus, Drypetes laterifolia, Drypetes brownei, Eugenia capuli, Hirtella triandra (= Hirtella americana), Laetia thamnia, Lonchocarpus castilloi, Manilkara chicle, Matayba apetala (= Matayba oppositifolia), Metopium brownei, Omphalea oleifera, Pouteria amygdalina, Pouteria campechiana, Pouteria reticulata, Protium copal, Pseudobombax ellipticum, Pseudolmedia spuria, Sabal mauritiiformis, Schizolobium parahyba, Sebastiana longicuspis*, and *Simira salvadorensis*.

*Diagnostic Characteristics:

*Classification Comments:

*Similar NVC Types [if applicable]:

| **Elcode** | **Scientific or Colloquial Name** | **Note** |
| --- | --- | --- |
|  |  |  |

Similar NVC Types General Comments [optional]:

VEGETATION

Physiognomy and Structure Summary:

Physiognomy and Structure Table [optional]:

| **Physiognomy-Structure Category** | **Prevailing Height (m)** | **Height Range (opt.)** | **Mean % Cover** | **Cover Range (opt.)** |
| --- | --- | --- | --- | --- |
|  |  |  |  | - |

Floristics Summary:

*Floristics Table [Med - High Confidence]:

*Number of Plots:

*Cover Scale Used:

| **Physiognomy-Structure Category** | **Taxon Name** | **Specific Growth Form (opt.)** | **Const- ancy** | **Mean % Cover** | **Cover Range (opt.)** | **Differ-ential** | **Diagnostic Combin- ation** |
| --- | --- | --- | --- | --- | --- | --- | --- |
|  |  |  |  |  | - |  |  |

Dynamics: Diversity of above-ground plant functional groups (species that share morphological, chemical, structural or life history characteristics): Determines the role of biodiversity in ecosystem functioning such as nutrient cycling, forest regeneration and successional patterns. Diversity of animal functional groups: Such diversity determines a number of key ecological processes such as trophic structure, nutrient cycling, system's resilience to disturbance. Community composition/diversity /structure: Affects species diversity and several ecosystem-level processes. Gap dynamics: Provides light, the major environmental limiting factor to plant growth in the closed-canopy tropical forest, and maintains the forest in shifting mosaic steady state.

Biotic interactions: Pollination (bees, butterflies, beetles, moths, bats, and hummingbirds): Important for reproductive success: pollinators influence the frequency and distribution pattern of plant species. Biotic interactions: seed dispersal executed by fruit-eating birds (e.g., toucans and cotingas), mammals (e.g., tapirs, peccaries, monkeys, bats, etc.) and ants: Important for reproductive success: seed dispersal agents affect food webs in tropical forests by making available reproductive resources to other consumers and Influence the frequency and distribution pattern of plant species, especially woody species. The composite seed shadow generated by the entire forest is the base of the food chain for a large number of animals (Janzen 1983). Biotic interactions: seed predation (parrots, cracids, beetles, agoutis, etc.): Important for reproductive success: seed predation affects population recruitment and establishment of diverse plant species (e.g., palms and legumes). Seed predators occasionally act as dispersers. Seed predation is a specialized form of herbivory. Vertebrates involved are often objects of hunting by humans. Biotic interactions: herbivores including insects (e.g., caterpillars, leaf-cutter ants), parasitic fungi, and vertebrates (e.g., peccaries, tapirs, deer, some monkeys): Herbivory affects vigor and mortality of plants of all sizes, especially understory seedlings, and Influences food chain and species composition of understory. Biotic interactions: presence of top predators: Controls the populations of small mammals and herbivores. Army ants form an important ecological system as predators of insects and species complex that follow them (e.g., parasitic flies, birds etc.). Army ants are also critical for maintenance of vegetation structure. Species diversity and composition of soil biota, e.g., mycorrhizae, fungi, microbes, soil mesofauna such as leaf-cutter ants, termites, nematodes, collembola, dung beetles, etc.: Fundamental for nutrient cycling and soil structure. Species diversity and composition of understory vegetation: Determine the mechanism of forest regeneration and indicate the health of successional change.

Disturbance regimes from catastrophic natural causes, e.g., hurricanes (hurricanes occur between 10-20 degree north and south of Equator), rare catastrophic floods, or multiple landslides, or volcanism, or earthquakes, rare extreme cold fronts, rare extreme droughts: These are extremely rare events that can be very important for ecological dynamics. Create canopy gaps of great size allowing pioneer species, e.g., *Cercropia* sp. to colonize and initiate successional processes, e.g., hurricanes play a major role in landscape-scale dynamics of forests on Caribbean islands. Fire due to dry spell or prolonged dry seasons or human activities: Certain species might be maintained because of this big, very rare catastrophic event. For example, mahogany thrives on fire outbreaks. Background disturbances: Small gaps, small landslides, downbursts, normal cold fronts, and normal seasonal precipitation variability. Important for creating and maintaining habitat heterogeneity and species and structural diversity, preventing competitive exclusion. Drives regeneration. Spatial integration and coverage (e.g., connectivity by riparian habitats) allowing migration of animals and plants outside of lowland forest: Allow to define at landscape level integrity of ecosystem. Allow to assess the extent of potential for species extinction. Spatial integration important for species to maintain contact with all habitats required for life cycles. Biogeochemical dynamics (referring to regional and global processes such as global warming, ozone depletion, CO2 concentration, atmospheric and soil pollution, etc.): Affects basic ecosystem functioning at both global and local levels. Soil type or fertility: Affects forest primary productivity and species richness. Soil type is also relevant to tree mortality rate, treefall frequency, forest regeneration mode, and stand turnover time (Hartshorn 1990).

ENVIRONMENT

Environmental Description: These major factors determine variation in community types within lowland tropical moist forest. Precipitation: The amount of rainfall and length of dry season determine the occurrences of evergreen forest or seasonally dry forest. Temperature: Yearly extreme temperature fluctuations result in cold-front stressed forests in southwestern Amazonia and the southern Atlantic region and non-cold-front stressed forests in Mexico and Central America. Topography: Zonation may occur depending on whether the forest is on a plain, or rolling hills, or foothills of a mountain range. Edaphic conditions (soil quality or fertility) can create special community types. Forests on white sand soil, on clay soil, or over limestone/ultrabasic rock differ considerably in species composition. Natural disturbance includes earthquakes, hurricanes and landslides. Earthquakes or hurricanes are the most frequent causes of landslides. Earthquake prone areas cover Central America, while the hurricane belt stretches from Mexico to Central America, and from the Caribbean islands to Yucatan.

DISTRIBUTION

*Geographic Range: Mexico and Central America.

Nations: BZ, CR, GT, HN, MX, NI, PA, SV

States/Provinces:

USFS Ecoregions (2007) [optional]:

Omernik Ecoregions L3, L4 [optional]:

MLRAs [optional]:

PLOT SAMPLING AND ANALYSIS

*Plot Analysis Summary [Med - High Confidence]:

*Plots Used to Define the Type [Med - High Confidence]:

CONFIDENCE LEVEL

USNVC Confidence Level: Unassigned

USNVC Confidence Comments [optional]:

HIERARCHY

*Lower Level NVC Types:

| **Elcode** | **Scientific or Colloquial Name** |
| --- | --- |
|  |  |

DISCUSSION

Discussion [optional]:

CONCEPT HISTORY

*Recent Concept Lineage [if applicable]:

| **Date** | **Predecessor** | **Note** |
| --- | --- | --- |
|  |  |  |

RELATED CONCEPTS

Supporting Concepts [optional]:

| **Relationship to NVC** | **Supporting Concept Name** | **Short Citation** | **Note** |
| --- | --- | --- | --- |
|  |  |  |  |

AUTHORSHIP

*Primary Concept Source [if applicable]: C. Josse, in Faber-Langendoen et al. (2014)

| **Relationship to NVC** | **Name Used in Source** | **Short Citation** | **Note** |
| --- | --- | --- | --- |
|  |  |  |  |

*Author of Description: C. Josse

Acknowledgments [optional]:

Version Date: 08 Jan 2015

REFERENCES

*References [Required if used in text]:

Attiwill, P. M. 1994. The disturbance of forest ecosystems: The ecological basis for conservative management. Forest Ecology and Management 63(2):247-300.

Challenger, A. 1998. Utilización y conservación: de los ecosistemas terrestres de México, pasado, presente y futuro. CONABIO.

Chambers, J. Q., G. P. Asner, D. C. Morton, L. O. Anderson, S. S. Saatchi, F. D. Espírito-Santo, M. Palace, and C. Souza, Jr. 2007b. Regional ecosystem structure and function: Ecological insights from remote sensing of tropical forests. Trends in Ecology and Evolution 22(8):414-423.

Chazdon, R. L. 2003. Tropical forest recovery: Legacies of human impact and natural disturbances. Perspectives in Plant Ecology, Evolution and Systematics 6(1):51-71.

Coley, P. D. 1982. Rates of herbivory on different tropical trees. Pages 123-132 in: E. G. Leigh, Jr., A. S. Rand, and D. M. Windsor, editors. The ecology of a tropical forest: Seasonal rhythms and long-term changes. Second edition. Smithsonian Institution, Washington, DC.

Condit, R. 1997. Forest turnover, diversity, and CO2. Trends in Ecology and Evolution 12:249-250.

De la Cruz, M., and R. Dirzo. 1987. A survey of the standing levels of herbivory in seedlings from a Mexican rain forest. Biotropica 19:98-106.

Denslow, J. S. 1996. Functional group diversity and responses to disturbance. Pages 127-151 in: G. H. Orians, R. Dirzo, and J. H. Cushman, editors. Biodiversity and ecosystem processes in tropical forests. Springer-Verlag, New York.

Dirso, R. 2001. Tropical forests: Biodiversity, ecological processes, and global environmental change. In: F. S. Chapin and O. E. Sala, editors. Future biodiversity scenarios. [in press].

Dirso, R., and A. Miranda. 1990. Contemporary Neotropical defaunation and forest structure, function, and diversity - a sequel to John Terborgh. Conservation Biology 4(4):444-447.

Emmons, L. H. 1991. Jaguars. In: J. Seidensticker and S. Lumpkin, editors. Great cats. Fog City Press, San Francisco.

Ewel, J. 1983. Succession. Pages 217-223 in: F. B. Golley, editor. Ecosystems of the World 14A. Tropical rain forest ecosystems: Structure and function. Elsevier Scientific Publication Company, New York.

Ewel, J. J., and S. W. Bigelow. 1996. Plant life-forms and tropical ecosystem functioning. Pages 101-126 in: G. H. Orians, R. Dirzo, and J. H. Cushman, editors. Biodiversity and ecosystem processes in tropical forests. Springer-Verlag, New York.

Faber-Langendoen, D., J. Drake, S. Gawler, M. Hall, C. Josse, G. Kittel, S. Menard, C. Nordman, M. Pyne, M. Reid, L. Sneddon, K. Schulz, J. Teague, M. Russo, K. Snow, and P. Comer, editors. 2010-2018. Divisions, Macrogroups and Groups for the Revised U.S. National Vegetation Classification. NatureServe, Arlington, VA. plus appendices. [in preparation]

Gardner, T. A., J. Barlow, R. Chazdon, R. M. Ewers, C. A. Harvey, C. A. Peres, and N. S. Sodhi. 2009. Prospects for tropical forest biodiversity in a human-modified world. Ecology Letters 12(6):561-582.

Gentry, A. H., editor. 1990. Four Neotropical rainforests. Yale University Press, New Haven.

Guariguata, M. R., R. L. Chazdon, J. S. Denslow, J. M. Dupuy, and L. Anderson. 1997. Structure and floristics of secondary and old-growth forest stands in lowland Costa Rica. Plant ecology 132(1):107-120.

Hartshorn, G. S. 1990. An overview of Neotropical forest dynamics. Pages 585-599 in: A. H. Gentry, editor. Four Neotropical rainforests. Yale University Press, New Haven.

Henderson, A., G. Galeano, and R. Bernal. 1995. Field guide to the palms of the Americas. Princeton University Press, Princeton, NJ. 352 pp.

Hunter, M. L., editor. 1999. Maintaining biodiversity in forest ecosystems. Cambridge University Press, Cambridge.

Janzen, D. H. 1983b. Food webs: Who eats what, why, how, and with what effects in a tropical forest? Pages 167-182 in: F. B. Golley, editor. Ecosystems of the World 14A. Tropical rain forest ecosystems: Structure and function. Elsevier Scientific Publication Company, New York.

Laurance, W. F., and R. O. Bierregaard, editors. 1997. Tropical forest remnants: Ecology, management, and conservation of fragmented communities. University of Chicago Press, Chicago.

Malhi, Y., and J. Grace. 2000. Tropical forests and atmospheric carbon dioxide. Trends in Ecology and Evolution 15:332-337.

McDade, L. A., K. S. Bawa, H. A. Hespenheide, and G. S. Hartshorn, editors. 1994. La Selva. University of Chicago Press, Chicago. 486 pp.

Nepstad, D. C., P. R. Moutinho, C. Uhl, I. C. Vieira, and J. M. C. da Silva. 1996. The ecological importance of forest remnants in an eastern Amazonian frontier landscape. Pages 133-150 in: J. Schelhas and R. Greenberg, editors. Forest patches in tropical landscape. Island Press, Washington, DC.

Orians, G. H., R. Dirzo, and J. H. Cushman, editors. 1996. Biodiversity and ecosystem processes in tropical forests. Springer-Verlag, New York.

Phillips, O. 1997. The changing ecology of a tropical forest. Biodiversity and Conservation 6:291-311.

Prance, G. T. 1989. American tropical forests Pages 99-132 in: H. Lieth and M. J. A. Werger, editors. Ecosystems of the World 14B. Tropical rain forest ecosystems: Biogeographical and ecological studies. Elsevier Scientific Publication Co., New York.

Rejmánek, M. 1996. Species richness and resistance to invasions. Pages 153-172 in: G. H. Orians, R. Dirzo, and J. H. Cushman, editors. Biodiversity and ecosystem processes in tropical forests. Springer-Verlag, New York.

Ricklefs, R. E. 1987. Community diversity: Relative roles of local and regional processes. Science 235(4785):167-171.

Voss, R. S., and L. H. Emmons. 1996. Mammal diversity in Neotropical lowland rainforest: A preliminary assessment. Bulletin of the American Museum of Natural History 230:1-115.

Whitmore, T. C. 1990. An introduction to tropical rain forests. Oxford York University Press, New York.

Whitmore, T. C., and J. A. Sayer, editors. 1992. Tropical deforestation and species extinction. Chapman and Hall, London.

1. Forest & Woodland

1.A.2.Eg. Caribbean-Mesoamerican Lowland Humid Forest

M873. Mesoamerican Submontane Humid Forest

Type Concept Sentence:

OVERVIEW

*Hierarchy Level: Macrogroup

*Placement in Hierarchy: 1.A.2.Eg. Caribbean-Mesoamerican Lowland Humid Forest (D091)

Elcode: M873

*Scientific Name: Mesoamerican Submontane Humid Forest Macrogroup

*Common (Translated Scientific) Name: Mesoamerican Submontane Humid Forest Macrogroup

*Colloquial Name: Mesoamerican Submontane Humid Forest

*Type Concept:

*Diagnostic Characteristics:

*Classification Comments:

*Similar NVC Types [if applicable]:

| **Elcode** | **Scientific or Colloquial Name** | **Note** |
| --- | --- | --- |
|  |  |  |

Similar NVC Types General Comments [optional]:

VEGETATION

Physiognomy and Structure Summary:

Physiognomy and Structure Table [optional]:

| **Physiognomy-Structure Category** | **Prevailing Height (m)** | **Height Range (opt.)** | **Mean % Cover** | **Cover Range (opt.)** |
| --- | --- | --- | --- | --- |
|  |  |  |  | - |

Floristics Summary:

*Floristics Table [Med - High Confidence]:

*Number of Plots:

*Cover Scale Used:

| **Physiognomy-Structure Category** | **Taxon Name** | **Specific Growth Form (opt.)** | **Const- ancy** | **Mean % Cover** | **Cover Range (opt.)** | **Differ-ential** | **Diagnostic Combin- ation** |
| --- | --- | --- | --- | --- | --- | --- | --- |
|  |  |  |  |  | - |  |  |

Dynamics:

ENVIRONMENT

Environmental Description:

DISTRIBUTION

*Geographic Range:

Nations: BZ, CR, GT, HN, NI, PA

States/Provinces:

USFS Ecoregions (2007) [optional]:

Omernik Ecoregions L3, L4 [optional]:

MLRAs [optional]:

PLOT SAMPLING AND ANALYSIS

*Plot Analysis Summary [Med - High Confidence]:

*Plots Used to Define the Type [Med - High Confidence]:

CONFIDENCE LEVEL

USNVC Confidence Level: Unassigned

USNVC Confidence Comments [optional]:

HIERARCHY

*Lower Level NVC Types:

| **Elcode** | **Scientific or Colloquial Name** |
| --- | --- |
|  |  |

DISCUSSION

Discussion [optional]:

CONCEPT HISTORY

*Recent Concept Lineage [if applicable]:

| **Date** | **Predecessor** | **Note** |
| --- | --- | --- |
|  |  |  |

RELATED CONCEPTS

Supporting Concepts [optional]:

| **Relationship to NVC** | **Supporting Concept Name** | **Short Citation** | **Note** |
| --- | --- | --- | --- |
|  |  |  |  |

AUTHORSHIP

*Primary Concept Source [if applicable]:

| **Relationship to NVC** | **Name Used in Source** | **Short Citation** | **Note** |
| --- | --- | --- | --- |
|  |  |  |  |

*Author of Description:

Acknowledgments [optional]:

REFERENCES

*References [Required if used in text]:

Faber-Langendoen, D., J. Drake, S. Gawler, M. Hall, C. Josse, G. Kittel, S. Menard, C. Nordman, M. Pyne, M. Reid, L. Sneddon, K. Schulz, J. Teague, M. Russo, K. Snow, and P. Comer, editors. 2010-2018. Divisions, Macrogroups and Groups for the Revised U.S. National Vegetation Classification. NatureServe, Arlington, VA. plus appendices. [in preparation]

1. Forest & Woodland

1.A.2.Eh. Colombian-Venezuelan Lowland Humid Forest

D224. Colombian-Venezuelan Lowland Humid Forest

Type Concept Sentence:

OVERVIEW

*Hierarchy Level: Division

*Placement in Hierarchy: 1.A.2.Eh. Tropical Lowland Humid Forest (F020)

Elcode: D224

*Scientific Name: Colombian-Venezuelan Lowland Humid Forest Division

*Common (Translated Scientific) Name: Colombian-Venezuelan Lowland Humid Forest Division

*Colloquial Name: Colombian-Venezuelan Lowland Humid Forest

*Type Concept:

*Diagnostic Characteristics:

*Classification Comments:

*Similar NVC Types [if applicable]:

| **Elcode** | **Scientific or Colloquial Name** | **Note** |
| --- | --- | --- |
|  |  |  |

Similar NVC Types General Comments [optional]:

VEGETATION

Physiognomy and Structure Summary:

Physiognomy and Structure Table [optional]:

| **Physiognomy-Structure Category** | **Prevailing Height (m)** | **Height Range (opt.)** | **Mean % Cover** | **Cover Range (opt.)** |
| --- | --- | --- | --- | --- |
|  |  |  |  | - |

Floristics Summary:

*Floristics Table [Med - High Confidence]:

*Number of Plots: *Cover Scale Used:

| **Physiognomy-Structure Category** | **Taxon Name** | **Specific Growth Form (opt.)** | **Const- ancy** | **Mean % Cover** | **Cover Range (opt.)** | **Differ-ential** | **Diagnostic Combin- ation** |
| --- | --- | --- | --- | --- | --- | --- | --- |
|  |  |  |  |  | - |  |  |

Dynamics:

ENVIRONMENT

Environmental Description:

DISTRIBUTION

*Geographic Range:

Nations: CO, CR, EC, PA, VE

States/Provinces:

USFS Ecoregions (2007) [optional]:

Omernik Ecoregions L3, L4 [optional]:

MLRAs [optional]:

PLOT SAMPLING AND ANALYSIS

*Plot Analysis Summary [Med - High Confidence]:

*Plots Used to Define the Type [Med - High Confidence]:

CONFIDENCE LEVEL

USNVC Confidence Level: Unassigned

USNVC Confidence Comments [optional]:

HIERARCHY

*Lower Level NVC Types:

| **Elcode** | **Scientific or Colloquial Name** |
| --- | --- |
| M581 | Choco-Darien Humid Forest |
| M582 | Western Ecuadorian Humid Forest |
| M580 | Catatumbo Magdalena Humid Forest |
| M579 | Guajiran Humid Forest |
| M583 | Llanos Humid Forest |

DISCUSSION

Discussion [optional]:

CONCEPT HISTORY

*Recent Concept Lineage [if applicable]:

| **Date** | **Predecessor** | **Note** |
| --- | --- | --- |
|  |  |  |

RELATED CONCEPTS

Supporting Concepts [optional]:

| **Relationship to NVC** | **Supporting Concept Name** | **Short Citation** | **Note** |
| --- | --- | --- | --- |
|  |  |  |  |

AUTHORSHIP

*Primary Concept Source [if applicable]:

| **Relationship to NVC** | **Name Used in Source** | **Short Citation** | **Note** |
| --- | --- | --- | --- |
|  |  |  |  |

*Author of Description:

Acknowledgments [optional]:

Version Date:

REFERENCES

*References [Required if used in text]:

Faber-Langendoen, D., J. Drake, S. Gawler, M. Hall, C. Josse, G. Kittel, S. Menard, C. Nordman, M. Pyne, M. Reid, L. Sneddon, K. Schulz, J. Teague, M. Russo, K. Snow, and P. Comer, editors. 2010-2018. Divisions, Macrogroups and Groups for the Revised U.S. National Vegetation Classification. NatureServe, Arlington, VA. plus appendices. [in preparation]

1. Forest & Woodland

1.A.2.Eh. Colombian-Venezuelan Lowland Humid Forest

M581. Choco-Darien Humid Forest

Type Concept Sentence: Rainforests that occur along the Pacific coast from southern Panama to northern Ecuador. Includes the forests of the non-flooded plains and those of the foothills of the Darien Mountains in Panama, and of the western Andes in Colombia and Ecuador, up to 600 m elevation. Also includes forests along the coast of the Gulf of Uraba in northwestern Colombia. Forests are evergreen, tall, hyper-humid, multi-strata, and have abundant palm trees. They have high biodiversity derived from the Darien and the Choco centers of endemism.

OVERVIEW

*Hierarchy Level: Macrogroup

*Placement in Hierarchy: 1.A.2.Eh. Colombian-Venezuelan Lowland Humid Forest (D224)

Elcode: M581

*Scientific Name: Choco-Darien Humid Forest Macrogroup

*Common (Translated Scientific) Name: Choco-Darien Humid Forest Macrogroup

*Colloquial Name: Choco-Darien Humid Forest

*Type Concept: The macrogroup represents the rainforests that occur on the Pacific Coast from southern Panama to northern Ecuador and Colombia. It includes the forests of the lowland, non-flooded plains and those of the foothills of the Darien Mountains in Panama, and of the western Andes in Colombia and northwestern Ecuador, up to approximately 600-800 m elevation; it also includes the forests of the coast of the Gulf of Uraba, in northwest Colombia. These are evergreen, very tall, hyper-humid forests with several strata, and outstanding biodiversity richness from strongly characterized centers of endemism of the Darien and the Choco. Characteristic tree species are *Alchornea polyantha, Anacardium excelsum, Apeiba aspera, Billia columbiana, Brosimum utile, Castilla elastica ssp. costaricana (= Castilla panamensis), Cephaelis elata, Cephaelis elata, Cordia alliodora, Coussapoa eggersii, Dipteryx panamensis, Elaeagia utilis, Eschweilera awaensis, Eschweilera pachyderma, Eschweilera verruculosa, Exarata chocoensis, Grias* spp., *Guettarda chiriquense, Huberodendron patinoi, Humiriastrum procerum, Hyeronima oblonga, Malpighia glabra, Oenocarpus panamanus, Pentaclethra* spp., *Perebea guianensis, Pourouma aspera, Pourouma chocoana, Pseudolmedia laevigata, Sorocea* sp., *Symphonia globulifera, Tababuia* spp., *Terminalia amazonica, Virola dixonii, Virola* spp., *Weinmannia putumayensis, Wettinia radiata*. Palm species are abundant and dominate the understory, among them are *Attalea colenda, Jessenia polycarpa, Phytelephas seemanni, Phytelephas* spp., *Welfia regia*.

*Diagnostic Characteristics:

*Classification Comments:

*Similar NVC Types [if applicable]:

| **Elcode** | **Scientific or Colloquial Name** | **Note** |
| --- | --- | --- |
|  |  |  |

Similar NVC Types General Comments [optional]:

VEGETATION

Physiognomy and Structure Summary:

Physiognomy and Structure Table [optional]:

| **Physiognomy-Structure Category** | **Prevailing Height (m)** | **Height Range (opt.)** | **Mean % Cover** | **Cover Range (opt.)** |
| --- | --- | --- | --- | --- |
|  |  |  |  | - |

Floristics Summary:

*Floristics Table [Med - High Confidence]:

*Number of Plots:

*Cover Scale Used:

| **Physiognomy-Structure Category** | **Taxon Name** | **Specific Growth Form (opt.)** | **Const- ancy** | **Mean % Cover** | **Cover Range (opt.)** | **Differ-ential** | **Diagnostic Combin- ation** |
| --- | --- | --- | --- | --- | --- | --- | --- |
|  |  |  |  |  | - |  |  |

Dynamics:

ENVIRONMENT

Environmental Description: Chocó rainforest is the wettest region in the world with annual precipitation >=9000 mm, and is an important center of rainforest endemism.

DISTRIBUTION

*Geographic Range: The macrogroup occurs on the Pacific Coast from southern Panama to northern Ecuador and Colombia.

Nations: CO, CR, EC, PA

States/Provinces:

USFS Ecoregions (2007) [optional]:

Omernik Ecoregions L3, L4 [optional]:

MLRAs [optional]:

PLOT SAMPLING AND ANALYSIS

*Plot Analysis Summary [Med - High Confidence]:

*Plots Used to Define the Type [Med - High Confidence]:

CONFIDENCE LEVEL

USNVC Confidence Level: Unassigned

USNVC Confidence Comments [optional]:

HIERARCHY

*Lower Level NVC Types:

| **Elcode** | **Scientific or Colloquial Name** |
| --- | --- |
|  |  |

DISCUSSION

Discussion [optional]:

CONCEPT HISTORY

*Recent Concept Lineage [if applicable]:

| **Date** | **Predecessor** | **Note** |
| --- | --- | --- |
|  |  |  |

RELATED CONCEPTS

Supporting Concepts [optional]:

| **Relationship to NVC** | **Supporting Concept Name** | **Short Citation** | **Note** |
| --- | --- | --- | --- |
|  |  |  |  |

AUTHORSHIP

*Primary Concept Source [if applicable]: C. Josse, in Faber-Langendoen et al. (2014)

| **Relationship to NVC** | **Name Used in Source** | **Short Citation** | **Note** |
| --- | --- | --- | --- |
|  |  |  |  |

*Author of Description: C. Josse

Acknowledgments [optional]:

Version Date: 08 Jan 2015

REFERENCES

*References [Required if used in text]:

Faber-Langendoen, D., J. Drake, S. Gawler, M. Hall, C. Josse, G. Kittel, S. Menard, C. Nordman, M. Pyne, M. Reid, L. Sneddon, K. Schulz, J. Teague, M. Russo, K. Snow, and P. Comer, editors. 2010-2018. Divisions, Macrogroups and Groups for the Revised U.S. National Vegetation Classification. NatureServe, Arlington, VA. plus appendices. [in preparation]

1. Forest & Woodland

1.A.2.Eh. Colombian-Venezuelan Lowland Humid Forest

M582. Western Ecuadorian Humid Forest

Type Concept Sentence: Evergreen to semi-evergreen humid forests of the coastal plain, coastal ridges and low Andean slopes of western Ecuador, from sea level to 300-400 m elevation. Although year-round precipitation is high, it is not as high as in the Choco. Some of these forests experience a dry season. They grow on soils of sedimentary or igneous origin (ash or basalt), mainly of the Latosols group with clayish texture and good organic matter content. Prominent tree species are *Brosimum utile, Carapa megistocarpa, Coussapoa eggersii, Dialyanthera otoba, Otoba cf. novogranatensis, Quararibea coloradorum, Virola dixonii*, and several palm species.

OVERVIEW

*Hierarchy Level: Macrogroup

*Placement in Hierarchy: 1.A.2.Eh. Colombian-Venezuelan Lowland Humid Forest (D224)

Elcode: M582

*Scientific Name: Western Ecuadorian Humid Forest Macrogroup

*Common (Translated Scientific) Name: Western Ecuadorian Humid Forest Macrogroup

*Colloquial Name: Western Ecuadorian Humid Forest

*Type Concept: Evergreen to semi-evergreen humid forests of the coastal plain, coastal ridges and low Andean slopes of western Ecuador, up to 300-400 m elevation. Although still developed under conditions of high year-round precipitation, the annual rainfall is not as high as in the Choco, and in part of their distribution, these forests experience a dry season. They grow on soils of sedimentary or igneous origin (ash or basalt), mainly of the Latosols group with clayish texture and good organic matter content. Prominent tree species are *Virola dixonii, Quararibea coloradorum, Otoba cf. novogranatensis, Dialyanthera otoba, Cespedesia spathulata, Brosimum utile, Aniba perutilis, Pourouma bicolor, Coussapoa eggersii, Coussapoa herthae, Perebea cf. angustifolia, Protium* sp., *Caryodaphnopsis theobromifolia, Daphnopsis oculta, Cedrela odorata, Carapa megistocarpa, Carapa nicaraguensis, Chrysophyllum argenteum, Chrysophyllum venezuelanense, Clarisia racemosa, Clarisia biflora, Guarea kunthiana, Matisia grandifolia, Matisia palenquiana*. Palms include *Aiphanes tricuspidata, Attalea colenda, Bactris gasipaes, Bactris setulosa, Jessenia bataua, Oenocarpus bataua, Oenocarpus mapora*. Semi-deciduous species include *Pseudobombax millei, Centrolobium ochroxylum, Sapindus saponaria, Zanthoxylum acuminatum*.

*Diagnostic Characteristics:

*Classification Comments:

*Similar NVC Types [if applicable]:

| **Elcode** | **Scientific or Colloquial Name** | **Note** |
| --- | --- | --- |
|  |  |  |

Similar NVC Types General Comments [optional]:

VEGETATION

Physiognomy and Structure Summary:

Physiognomy and Structure Table [optional]:

| **Physiognomy-Structure Category** | **Prevailing Height (m)** | **Height Range (opt.)** | **Mean % Cover** | **Cover Range (opt.)** |
| --- | --- | --- | --- | --- |
|  |  |  |  | - |

Floristics Summary:

*Floristics Table [Med - High Confidence]:

*Number of Plots:

*Cover Scale Used:

| **Physiognomy-Structure Category** | **Taxon Name** | **Specific Growth Form (opt.)** | **Const- ancy** | **Mean % Cover** | **Cover Range (opt.)** | **Differ-ential** | **Diagnostic Combin- ation** |
| --- | --- | --- | --- | --- | --- | --- | --- |
|  |  |  |  |  | - |  |  |

Dynamics:

ENVIRONMENT

Environmental Description:

DISTRIBUTION

*Geographic Range:

Nations: EC

States/Provinces:

USFS Ecoregions (2007) [optional]:

Omernik Ecoregions L3, L4 [optional]:

MLRAs [optional]:

PLOT SAMPLING AND ANALYSIS

*Plot Analysis Summary [Med - High Confidence]:

*Plots Used to Define the Type [Med - High Confidence]:

CONFIDENCE LEVEL

USNVC Confidence Level: Unassigned

USNVC Confidence Comments [optional]:

HIERARCHY

*Lower Level NVC Types:

| **Elcode** | **Scientific or Colloquial Name** |
| --- | --- |
|  |  |

DISCUSSION

Discussion [optional]:

CONCEPT HISTORY

*Recent Concept Lineage [if applicable]:

| **Date** | **Predecessor** | **Note** |
| --- | --- | --- |
|  |  |  |

RELATED CONCEPTS

Supporting Concepts [optional]:

| **Relationship to NVC** | **Supporting Concept Name** | **Short Citation** | **Note** |
| --- | --- | --- | --- |
|  |  |  |  |

AUTHORSHIP

*Primary Concept Source [if applicable]: C. Josse, in Faber-Langendoen et al. (2014)

| **Relationship to NVC** | **Name Used in Source** | **Short Citation** | **Note** |
| --- | --- | --- | --- |
|  |  |  |  |

*Author of Description: C. Josse

Acknowledgments [optional]:

Version Date: 17 Apr 2014

REFERENCES

*References [Required if used in text]:

Faber-Langendoen, D., J. Drake, S. Gawler, M. Hall, C. Josse, G. Kittel, S. Menard, C. Nordman, M. Pyne, M. Reid, L. Sneddon, K. Schulz, J. Teague, M. Russo, K. Snow, and P. Comer, editors. 2010-2018. Divisions, Macrogroups and Groups for the Revised U.S. National Vegetation Classification. NatureServe, Arlington, VA. plus appendices. [in preparation]

1. Forest & Woodland

1.A.2.Eh. Colombian-Venezuelan Lowland Humid Forest

M580. Catatumbo Magdalena Humid Forest

Type Concept Sentence: Dense, tall evergreen forests with a stature of 30-40 m, emergent trees as high as 60 m, and various strata. Partially and periodically flooded. Occurs along the southern shore of Lake Maracaibo and in the foothills of the Cordillera de Perija in Colombia. Forms part of the Catatumbo Refugia with important connections to the Amazonian flora. Found also in the lower Magdalena and Cauca valleys, and very localized in the lower slopes of the Sierra Nevada de Santa Marta in Colombia. Diagnostic species include *Acosmium panamensis, Anacardium excelsum, Cariniana pyriformis, Faramea capillipes, Gustavia hexapetala, Miconia barbinervis, Miconia mocquerysii, Pterygota colombiana, Sterculia apetala, Trichilia maynasiana, Trichilia pleeana*, and *Vochysia lehmannii*.

OVERVIEW

*Hierarchy Level: Macrogroup

*Placement in Hierarchy: 1.A.2.Eh. Colombian-Venezuelan Lowland Humid Forest (D224)

Elcode: M580

*Scientific Name: Catatumbo Magdalena Humid Forest Macrogroup

*Common (Translated Scientific) Name: Catatumbo Magdalena Humid Forest Macrogroup

*Colloquial Name: Catatumbo Magdalena Humid Forest

*Type Concept: Evergreen dense, tall forests 30-40 m high with emergents up to 60 m, and various strata. It is partially and periodically flooded and occurs at the southern shore of Lake Maracaibo and in adjacent parts of Colombia. It forms part of the Catatumbo Refugia with important connections to the Amazonian flora. It is found also in the lower Magdalena and Cauca valleys, and very localized in the lower slopes of the Sierra Nevada de Santa Marta. The following list of species is diagnostic for this macrogroup: *Cariniana pyriformis, Anacardium excelsum, Acosmium panamense, Gustavia hexapetala, Ceiba pentandra, Sterculia apetala, Trichilia pleeana, Trichilia maynasiana, Faramea capillipes, Miconia mocquerysii, Miconia barbinervis, Miconia nervosa, Pterygota colombiana, Vochysia lehmannii, Rhodospatha perezii, Spathiphyllum perezii, Pentaclethra macroloba, Calophyllum antillanum (= Calophyllum brasiliense), Parkia pendula, Tapirira guianensis, Carapa guianensis, Eschweilera microcalyx, Clathrotropis brachypetala, Humiriastrum colombianum, Protium pedicelatum, Pseudolmedia rigida, Couma macrocarpa, Pterocarpus officinalis, Swartzia cuspidata, Sterculia steyermarkii, Terminalia amazonia, Grias foetida, Iranthera ulei, Caryocar amygdaliferum, Swietenia macrophylla, Aspidosperma dugandii, Aspidosperma curranii, Bombacopsis quinata, Cedrela fissilis, Cedrela mexicana, Brosimum* sp.

*Diagnostic Characteristics:

*Classification Comments:

*Similar NVC Types [if applicable]:

| **Elcode** | **Scientific or Colloquial Name** | **Note** |
| --- | --- | --- |
|  |  |  |

Similar NVC Types General Comments [optional]:

VEGETATION

Physiognomy and Structure Summary:

Physiognomy and Structure Table [optional]:

| **Physiognomy-Structure Category** | **Prevailing Height (m)** | **Height Range (opt.)** | **Mean % Cover** | **Cover Range (opt.)** |
| --- | --- | --- | --- | --- |
|  |  |  |  | - |

Floristics Summary:

*Floristics Table [Med - High Confidence]:

*Number of Plots:

*Cover Scale Used:

| **Physiognomy-Structure Category** | **Taxon Name** | **Specific Growth Form (opt.)** | **Const- ancy** | **Mean % Cover** | **Cover Range (opt.)** | **Differ-ential** | **Diagnostic Combin- ation** |
| --- | --- | --- | --- | --- | --- | --- | --- |
|  |  |  |  |  | - |  |  |

Dynamics:

ENVIRONMENT

Environmental Description:

DISTRIBUTION

*Geographic Range:

Nations: CO, VE

States/Provinces:

USFS Ecoregions (2007) [optional]:

Omernik Ecoregions L3, L4 [optional]:

MLRAs [optional]:

PLOT SAMPLING AND ANALYSIS

*Plot Analysis Summary [Med - High Confidence]:

*Plots Used to Define the Type [Med - High Confidence]:

CONFIDENCE LEVEL

USNVC Confidence Level: Unassigned

USNVC Confidence Comments [optional]:

HIERARCHY

*Lower Level NVC Types:

| **Elcode** | **Scientific or Colloquial Name** |
| --- | --- |
|  |  |

DISCUSSION

Discussion [optional]:

CONCEPT HISTORY

*Recent Concept Lineage [if applicable]:

| **Date** | **Predecessor** | **Note** |
| --- | --- | --- |
|  |  |  |

RELATED CONCEPTS

Supporting Concepts [optional]:

| **Relationship to NVC** | **Supporting Concept Name** | **Short Citation** | **Note** |
| --- | --- | --- | --- |
|  |  |  |  |

AUTHORSHIP

*Primary Concept Source [if applicable]: C. Josse, in Faber-Langendoen et al. (2014)

| **Relationship to NVC** | **Name Used in Source** | **Short Citation** | **Note** |
| --- | --- | --- | --- |
|  |  |  |  |

*Author of Description: C. Josse

Acknowledgments [optional]:

Version Date: 17 Apr 2014

REFERENCES

*References [Required if used in text]:

Faber-Langendoen, D., J. Drake, S. Gawler, M. Hall, C. Josse, G. Kittel, S. Menard, C. Nordman, M. Pyne, M. Reid, L. Sneddon, K. Schulz, J. Teague, M. Russo, K. Snow, and P. Comer, editors. 2010-2018. Divisions, Macrogroups and Groups for the Revised U.S. National Vegetation Classification. NatureServe, Arlington, VA. plus appendices. [in preparation]

1. Forest & Woodland

1.A.2.Eh. Colombian-Venezuelan Lowland Humid Forest

M579. Guajiran Humid Forest

Type Concept Sentence: Evergreen humid to hyper-humid forests with a stature of 25-30 m, many strata, and a dense understory consisting of palms and tree ferns. Occurs on coastal ridges of northern Venezuela. Its elevational range varies depending on the mountain range and slope aspect, but generally occurs between 500 and 1300-1500 m elevation on north-facing slopes and from 800-1600 m on south-facing slopes. Diagnostic species include *Bactris setulosa, Eschweilera trinitensis, Euterpe* spp., *Ficus apollinaris, Guapira ferruginea, Guarea kunthiana, Gustavia hexapetala, Gustavia parviflora, Heliocarpus popayanensis, Myrcianthes karsteniana, Nectandra pichurim, Pachira insignis, Poulsenia armata, Ruellia chrysantha, Turpinia heterophylla*, and *Zinowiewia australis*.

OVERVIEW

*Hierarchy Level: Macrogroup

*Placement in Hierarchy: 1.A.2.Eh. Colombian-Venezuelan Lowland Humid Forest (D224)

Elcode: M579

*Scientific Name: Guajiran Humid Forest Macrogroup

*Common (Translated Scientific) Name: Guajiran Humid Forest Macrogroup

*Colloquial Name: Guajiran Humid Forest

*Type Concept: Evergreen humid to hyper-humid forest 25-30 m high with many strata and a dense understory with palms and tree ferns. It occurs on coastal ridges of northern Venezuela. Its altitudinal location varies depending on the mountain and the slope aspect, but generally between 500 and 1300-1500 m elevation on northern slopes and from 800-1600 m on southern slopes. Diagnostic species include *Bactris setulosa, Eschweilera trinitensis, Euterpe* spp., *Ficus apollinaris, Guapira ferruginea, Guarea kunthiana, Gustavia hexapetala, Gustavia parviflora, Heliocarpus popayanensis, Myrcianthes karsteniana, Nectandra pichurim, Pachira insignis, Poulsenia armata, Ruellia chrysantha, Turpinia heterophylla*, and *Zinowiewia australis*.

*Diagnostic Characteristics:

*Classification Comments:

*Similar NVC Types [if applicable]:

| **Elcode** | **Scientific or Colloquial Name** | **Note** |
| --- | --- | --- |
|  |  |  |

Similar NVC Types General Comments [optional]:

VEGETATION

Physiognomy and Structure Summary:

Physiognomy and Structure Table [optional]:

| **Physiognomy-Structure Category** | **Prevailing Height (m)** | **Height Range (opt.)** | **Mean % Cover** | **Cover Range (opt.)** |
| --- | --- | --- | --- | --- |
|  |  |  |  | - |

Floristics Summary:

*Floristics Table [Med - High Confidence]:

*Number of Plots:

*Cover Scale Used:

| **Physiognomy-Structure Category** | **Taxon Name** | **Specific Growth Form (opt.)** | **Const- ancy** | **Mean % Cover** | **Cover Range (opt.)** | **Differ-ential** | **Diagnostic Combin- ation** |
| --- | --- | --- | --- | --- | --- | --- | --- |
|  |  |  |  |  | - |  |  |

Dynamics:

ENVIRONMENT

Environmental Description:

DISTRIBUTION

*Geographic Range:

Nations: VE

States/Provinces:

USFS Ecoregions (2007) [optional]:

Omernik Ecoregions L3, L4 [optional]:

MLRAs [optional]:

PLOT SAMPLING AND ANALYSIS

*Plot Analysis Summary [Med - High Confidence]:

*Plots Used to Define the Type [Med - High Confidence]:

CONFIDENCE LEVEL

USNVC Confidence Level: Unassigned

USNVC Confidence Comments [optional]:

HIERARCHY

*Lower Level NVC Types:

| **Elcode** | **Scientific or Colloquial Name** |
| --- | --- |
|  |  |

DISCUSSION

Discussion [optional]:

CONCEPT HISTORY

*Recent Concept Lineage [if applicable]:

| **Date** | **Predecessor** | **Note** |
| --- | --- | --- |
|  |  |  |

RELATED CONCEPTS

Supporting Concepts [optional]:

| **Relationship to NVC** | **Supporting Concept Name** | **Short Citation** | **Note** |
| --- | --- | --- | --- |
|  |  |  |  |

AUTHORSHIP

*Primary Concept Source [if applicable]: C. Josse, in Faber-Langendoen et al. (2014)

| **Relationship to NVC** | **Name Used in Source** | **Short Citation** | **Note** |
| --- | --- | --- | --- |
|  |  |  |  |

*Author of Description: C. Josse

Acknowledgments [optional]:

Version Date: 17 Apr 2014

REFERENCES

*References [Required if used in text]:

Faber-Langendoen, D., J. Drake, S. Gawler, M. Hall, C. Josse, G. Kittel, S. Menard, C. Nordman, M. Pyne, M. Reid, L. Sneddon, K. Schulz, J. Teague, M. Russo, K. Snow, and P. Comer, editors. 2010-2018. Divisions, Macrogroups and Groups for the Revised U.S. National Vegetation Classification. NatureServe, Arlington, VA. plus appendices. [in preparation]

1. Forest & Woodland

1.A.2.Eh. Colombian-Venezuelan Lowland Humid Forest

M583. Llanos Humid Forest

Type Concept Sentence: Forests of the altitudinal gradient ranging from the Llanos lowlands to the lower eastern Andean slopes, up to 600 m elevation. These diverse forests are distributed from northern Venezuela to central Colombia where climates range from very humid to seasonal. In general, they grow on rich Quaternary deposits, with well-drained soils from medium and low alluvial fans that form terraces, tables and hills. Characteristic species are *Attalea maracaibensis, Bombacopsis quinata, Calycophyllum candidissimum, Cochlospermum vitifolium, Couroupita guinanensis, Luehea cymulosa, Luehea ferruginea, Platymiscium pinnatum, Pradosia caracasana, Sloanea terniflora*, and *Symmeria paniculata*.

OVERVIEW

*Hierarchy Level: Macrogroup

*Placement in Hierarchy: 1.A.2.Eh. Colombian-Venezuelan Lowland Humid Forest (D224)

Elcode: M583

*Scientific Name: Llanos Humid Forest Macrogroup

*Common (Translated Scientific) Name: Llanos Humid Forest Macrogroup

*Colloquial Name: Llanos Humid Forest

*Type Concept: This macrogroup includes the forest of the altitudinal gradient from the Llanos lowlands to the lower eastern Andean slopes, up to 600 m elevation. In the long belt they are distributed from northern Venezuela to central Colombia. They cover a wide elevational range and climates from very humid to seasonal, therefore the forests included are diverse. In general, they grow on rich Quaternary deposits, with well-drained soils from medium and low alluvial fans, which currently form terraces, tables and hills. Characteristic species are *Attalea maracaibensis, Bombacopsis quinata, Calycophyllum candidissimum, Couroupita guinanensis, Guazuma ulmifolia, Luehea ferruginea, Luehea cymulosa, Pradosia caracasana, Cochlospermum vitifolium, Genipa americana, Platymiscium pinnatum, Symmeria paniculata, Sloanea terniflora, Swietenia macrophylla, Tabebuia chrysantha, Sapium biglandulosum, Pterocarpus acapulcensis, Rupechtria ramiflora, Sorocea sprucei, Guatteria pilosula, Syagrus sancona, Trichanthea gigantea, Clarisia biflora, Spondias mombin, Celtis iguanaea, Astronium graveolens, Guazuma ulmifolia, Trichilia* spp. In the upper part of their distribution are *Protium heptaphyllum, Vochysia lehmannii, Inga spuria, Ardisia foetida, Clusia minor, Ficus* spp.

*Diagnostic Characteristics:

*Classification Comments:

*Similar NVC Types [if applicable]:

| **Elcode** | **Scientific or Colloquial Name** | **Note** |
| --- | --- | --- |
|  |  |  |

Similar NVC Types General Comments [optional]:

VEGETATION

Physiognomy and Structure Summary:

Physiognomy and Structure Table [optional]:

| **Physiognomy-Structure Category** | **Prevailing Height (m)** | **Height Range (opt.)** | **Mean % Cover** | **Cover Range (opt.)** |
| --- | --- | --- | --- | --- |
|  |  |  |  | - |

Floristics Summary:

*Floristics Table [Med - High Confidence]:

*Number of Plots:

*Cover Scale Used:

| **Physiognomy-Structure Category** | **Taxon Name** | **Specific Growth Form (opt.)** | **Const- ancy** | **Mean % Cover** | **Cover Range (opt.)** | **Differ-ential** | **Diagnostic Combin- ation** |
| --- | --- | --- | --- | --- | --- | --- | --- |
|  |  |  |  |  | - |  |  |

Dynamics:

ENVIRONMENT

Environmental Description:

DISTRIBUTION

*Geographic Range:

Nations: CO, VE

States/Provinces:

USFS Ecoregions (2007) [optional]:

Omernik Ecoregions L3, L4 [optional]:

MLRAs [optional]:

PLOT SAMPLING AND ANALYSIS

*Plot Analysis Summary [Med - High Confidence]:

*Plots Used to Define the Type [Med - High Confidence]:

CONFIDENCE LEVEL

USNVC Confidence Level: Unassigned

USNVC Confidence Comments [optional]:

HIERARCHY

*Lower Level NVC Types:

| **Elcode** | **Scientific or Colloquial Name** |
| --- | --- |
|  |  |

DISCUSSION

Discussion [optional]:

CONCEPT HISTORY

*Recent Concept Lineage [if applicable]:

| **Date** | **Predecessor** | **Note** |
| --- | --- | --- |
|  |  |  |

RELATED CONCEPTS

Supporting Concepts [optional]:

| **Relationship to NVC** | **Supporting Concept Name** | **Short Citation** | **Note** |
| --- | --- | --- | --- |
|  |  |  |  |

AUTHORSHIP

*Primary Concept Source [if applicable]: C. Josse, in Faber-Langendoen et al. (2014)

| **Relationship to NVC** | **Name Used in Source** | **Short Citation** | **Note** |
| --- | --- | --- | --- |
|  |  |  |  |

*Author of Description: C. Josse

Acknowledgments [optional]:

Version Date: 17 Apr 2014

REFERENCES

*References [Required if used in text]:

Faber-Langendoen, D., J. Drake, S. Gawler, M. Hall, C. Josse, G. Kittel, S. Menard, C. Nordman, M. Pyne, M. Reid, L. Sneddon, K. Schulz, J. Teague, M. Russo, K. Snow, and P. Comer, editors. 2010-2018. Divisions, Macrogroups and Groups for the Revised U.S. National Vegetation Classification. NatureServe, Arlington, VA. plus appendices. [in preparation]

1. Forest & Woodland

1.A.2.Ei. Guianan Lowland Humid Forest

D225. Guianan Lowland Humid Forest

Type Concept Sentence:

OVERVIEW

*Hierarchy Level: Division

*Placement in Hierarchy: 1.A.2.Ei. Tropical Lowland Humid Forest (F020)

Elcode: D225

*Scientific Name: Guianan Lowland Humid Forest Division

*Common (Translated Scientific) Name: Guianan Lowland Humid Forest Division

*Colloquial Name: Guianan Lowland Humid Forest

*Type Concept:

*Diagnostic Characteristics:

*Classification Comments:

*Similar NVC Types [if applicable]:

| **Elcode** | **Scientific or Colloquial Name** | **Note** |
| --- | --- | --- |
|  |  |  |

Similar NVC Types General Comments [optional]:

VEGETATION

Physiognomy and Structure Summary:

Physiognomy and Structure Table [optional]:

| **Physiognomy-Structure Category** | **Prevailing Height (m)** | **Height Range (opt.)** | **Mean % Cover** | **Cover Range (opt.)** |
| --- | --- | --- | --- | --- |
|  |  |  |  | - |

Floristics Summary:

*Floristics Table [Med - High Confidence]:

*Number of Plots: *Cover Scale Used:

| **Physiognomy-Structure Category** | **Taxon Name** | **Specific Growth Form (opt.)** | **Const- ancy** | **Mean % Cover** | **Cover Range (opt.)** | **Differ-ential** | **Diagnostic Combin- ation** |
| --- | --- | --- | --- | --- | --- | --- | --- |
|  |  |  |  |  | - |  |  |

Dynamics:

ENVIRONMENT

Environmental Description:

DISTRIBUTION

*Geographic Range:

Nations: BR, CO?, GF, GY, SR, VE

States/Provinces:

USFS Ecoregions (2007) [optional]:

Omernik Ecoregions L3, L4 [optional]:

MLRAs [optional]:

PLOT SAMPLING AND ANALYSIS

*Plot Analysis Summary [Med - High Confidence]:

*Plots Used to Define the Type [Med - High Confidence]:

CONFIDENCE LEVEL

USNVC Confidence Level: Unassigned

USNVC Confidence Comments [optional]:

HIERARCHY

*Lower Level NVC Types:

| **Elcode** | **Scientific or Colloquial Name** |
| --- | --- |
| M586 | Eastern Guianan Humid Forest |
| M585 | Central Guianan Humid Forest |
| M584 | Western Guianan Humid Forest |
| M587 | Orinoquian Humid Forest |

DISCUSSION

Discussion [optional]:

CONCEPT HISTORY

*Recent Concept Lineage [if applicable]:

| **Date** | **Predecessor** | **Note** |
| --- | --- | --- |
|  |  |  |

RELATED CONCEPTS

Supporting Concepts [optional]:

| **Relationship to NVC** | **Supporting Concept Name** | **Short Citation** | **Note** |
| --- | --- | --- | --- |
|  |  |  |  |

AUTHORSHIP

*Primary Concept Source [if applicable]:

| **Relationship to NVC** | **Name Used in Source** | **Short Citation** | **Note** |
| --- | --- | --- | --- |
|  |  |  |  |

*Author of Description:

Acknowledgments [optional]:

Version Date:

REFERENCES

*References [Required if used in text]:

Faber-Langendoen, D., J. Drake, S. Gawler, M. Hall, C. Josse, G. Kittel, S. Menard, C. Nordman, M. Pyne, M. Reid, L. Sneddon, K. Schulz, J. Teague, M. Russo, K. Snow, and P. Comer, editors. 2010-2018. Divisions, Macrogroups and Groups for the Revised U.S. National Vegetation Classification. NatureServe, Arlington, VA. plus appendices. [in preparation]

1. Forest & Woodland

1.A.2.Ei. Guianan Lowland Humid Forest

M586. Eastern Guianan Humid Forest

Type Concept Sentence: Evergreen forests of Guyana and Surinam with canopies averaging 25-40 m high with emergents reaching up to 60 m. These forests are in part seasonal and include both mixed forests with no clear dominant species and monodominant forests that probably result from infertile soils. Typical species include *Alexa imperatricis, Couratari coriacea, Dicymbe corimbosa, Dinizia excelsa, Eperua falcata, Eschweilera sagotiana, Goupia glabra, Licania heteromorpha, Licania laxiflora, Licania venosa, Mora gonggrijpii, Parinari excelsa, Pentaclethra macroloba, Protium decandrum, Quiina indigofera*, and numerous palm species. Also included are semi-deciduous forests of lower statured that grow on poor, coarse sand soils.

OVERVIEW

*Hierarchy Level: Macrogroup

*Placement in Hierarchy: 1.A.2.Ei. Guianan Lowland Humid Forest (D225)

Elcode: M586

*Scientific Name: Eastern Guianan Humid Forest Macrogroup

*Common (Translated Scientific) Name: Eastern Guianan Humid Forest Macrogroup

*Colloquial Name: Eastern Guianan Humid Forest

*Type Concept: Evergreen forests of Guyana and Surinam, 25-40 m tall, some of them with emergents up to 60 m. These forests are in part seasonal and include, in addition to mixed forests with no clear dominant species, a number of communities with a single dominant, probably linked to infertile soils or a combination of soils and topography. Some of the typical species are *Licania venosa, Licania laxiflora, Licania heteromorpha, Mora gonggrijpii, Eschweilera sagotiana, Protium decandrum, Pentaclethra macroloba, Quiina indigofera, Alexa imperatricis, Goupia glabra, Eperua falcata, Parinari excelsa, Couratari coriacea, Dinizia excelsa, Dicymbe corimbosa*, and numerous palm species such as *Jessenia bataua, Geonoma paniculigera, Oenocarpus bacaba, Attalea sagotti, Iriartea hexorrhiza, Astrocaryum sciophilum*. The macrogroup includes also edafoxerophyllous, semi-deciduous smaller forests which grow on poor white, coarse sand soils.

*Diagnostic Characteristics:

*Classification Comments:

*Similar NVC Types [if applicable]:

| **Elcode** | **Scientific or Colloquial Name** | **Note** |
| --- | --- | --- |
|  |  |  |

Similar NVC Types General Comments [optional]:

VEGETATION

Physiognomy and Structure Summary:

Physiognomy and Structure Table [optional]:

| **Physiognomy-Structure Category** | **Prevailing Height (m)** | **Height Range (opt.)** | **Mean % Cover** | **Cover Range (opt.)** |
| --- | --- | --- | --- | --- |
|  |  |  |  | - |

Floristics Summary:

*Floristics Table [Med - High Confidence]:

*Number of Plots:

*Cover Scale Used:

| **Physiognomy-Structure Category** | **Taxon Name** | **Specific Growth Form (opt.)** | **Const- ancy** | **Mean % Cover** | **Cover Range (opt.)** | **Differ-ential** | **Diagnostic Combin- ation** |
| --- | --- | --- | --- | --- | --- | --- | --- |
|  |  |  |  |  | - |  |  |

Dynamics:

ENVIRONMENT

Environmental Description:

DISTRIBUTION

*Geographic Range:

Nations: GF, GY, SR

States/Provinces:

USFS Ecoregions (2007) [optional]:

Omernik Ecoregions L3, L4 [optional]:

MLRAs [optional]:

PLOT SAMPLING AND ANALYSIS

*Plot Analysis Summary [Med - High Confidence]:

*Plots Used to Define the Type [Med - High Confidence]:

CONFIDENCE LEVEL

USNVC Confidence Level: Unassigned

USNVC Confidence Comments [optional]:

HIERARCHY

*Lower Level NVC Types:

| **Elcode** | **Scientific or Colloquial Name** |
| --- | --- |
|  |  |

DISCUSSION

Discussion [optional]:

CONCEPT HISTORY

*Recent Concept Lineage [if applicable]:

| **Date** | **Predecessor** | **Note** |
| --- | --- | --- |
|  |  |  |

RELATED CONCEPTS

Supporting Concepts [optional]:

| **Relationship to NVC** | **Supporting Concept Name** | **Short Citation** | **Note** |
| --- | --- | --- | --- |
|  |  |  |  |

AUTHORSHIP

*Primary Concept Source [if applicable]: C. Josse, in Faber-Langendoen et al. (2014)

| **Relationship to NVC** | **Name Used in Source** | **Short Citation** | **Note** |
| --- | --- | --- | --- |
|  |  |  |  |

*Author of Description: C. Josse

Acknowledgments [optional]:

Version Date: 17 Apr 2014

REFERENCES

*References [Required if used in text]:

Faber-Langendoen, D., J. Drake, S. Gawler, M. Hall, C. Josse, G. Kittel, S. Menard, C. Nordman, M. Pyne, M. Reid, L. Sneddon, K. Schulz, J. Teague, M. Russo, K. Snow, and P. Comer, editors. 2010-2018. Divisions, Macrogroups and Groups for the Revised U.S. National Vegetation Classification. NatureServe, Arlington, VA. plus appendices. [in preparation]

1. Forest & Woodland

1.A.2.Ei. Guianan Lowland Humid Forest

M585. Central Guianan Humid Forest

Type Concept Sentence: Humid, evergreen forests of the peneplain of the Caura-Paragua river basins occurring on well-drained or occasionally flooded Ultisols. The canopy averages 15-18 m high, with emergent trees reaching 30 m. Diagnostic species are *Catostemma commune, Dialium guianense, Eschweilera subglandulosa, Gustavia coriacea, Lecythis corrugata, Micrandra minor, Oenocarpus bacaba, Protium sagotianum*, and *Simaba cedron*. To the east, this vegetation type also includes the foothill forests of the Gran Sabana plateau in Venezuela and Brazil and the Pakaraima and Roraima mountains of Guyana. Characteristic species are *Brownea coccinea, Crudia oblonga, Eschweilera sagotiana, Licania heteromorpha, Licania venosa*, and *Mora gonggrijpii*.

OVERVIEW

*Hierarchy Level: Macrogroup

*Placement in Hierarchy: 1.A.2.Ei. Guianan Lowland Humid Forest (D225)

Elcode: M585

*Scientific Name: Central Guianan Humid Forest Macrogroup

*Common (Translated Scientific) Name: Central Guianan Humid Forest Macrogroup

*Colloquial Name: Central Guianan Humid Forest

*Type Concept: The macrogroup includes the humid forests of the peneplain of the Caura-Paragua river basins on well-drained or occasionally flooded Ultisols. Evergreen and dense forest, of medium height (15-18 m average), but with emerging trees up to 30 m and relatively few small trees. Diagnostic composition includes *Gustavia coriacea, Simaba cedron, Dialium guianense, Protium sagotianum, Alexa confusa, Oenocarpus bacaba, Bocageopsis multiflora, Lecythis corrugata, Micrandra minor, Eschweilera subglandulosa, Catostemma commune*. To the east in Venezuela and Brazil, it also includes the piedmontane forests of the Gran Sabana plateau and in Guyana those of the Pakaraima and Roraima mountains, with red soils derived from volcanic intrusive rocks; some of these soils can be derived from the underlying laterite. Among the characteristic species of this type are *Crudia oblonga, Brownea coccinea, Icania venosa, Licania laxiflora, Licania heteromorpha, Mora gonggrijpii, Eschweilera sagotiana, Protium decandrum, Pentaclethra macroloba, Quiina indigofera*, and *Alexa imperatricis*.

*Diagnostic Characteristics:

*Classification Comments:

*Similar NVC Types [if applicable]:

| **Elcode** | **Scientific or Colloquial Name** | **Note** |
| --- | --- | --- |
|  |  |  |

Similar NVC Types General Comments [optional]:

VEGETATION

Physiognomy and Structure Summary:

Physiognomy and Structure Table [optional]:

| **Physiognomy-Structure Category** | **Prevailing Height (m)** | **Height Range (opt.)** | **Mean % Cover** | **Cover Range (opt.)** |
| --- | --- | --- | --- | --- |
|  |  |  |  | - |

Floristics Summary:

*Floristics Table [Med - High Confidence]:

*Number of Plots:

*Cover Scale Used:

| **Physiognomy-Structure Category** | **Taxon Name** | **Specific Growth Form (opt.)** | **Const- ancy** | **Mean % Cover** | **Cover Range (opt.)** | **Differ-ential** | **Diagnostic Combin- ation** |
| --- | --- | --- | --- | --- | --- | --- | --- |
|  |  |  |  |  | - |  |  |

Dynamics:

ENVIRONMENT

Environmental Description:

DISTRIBUTION

*Geographic Range:

Nations: BR, GY, VE

States/Provinces:

USFS Ecoregions (2007) [optional]:

Omernik Ecoregions L3, L4 [optional]:

MLRAs [optional]:

PLOT SAMPLING AND ANALYSIS

*Plot Analysis Summary [Med - High Confidence]:

*Plots Used to Define the Type [Med - High Confidence]:

CONFIDENCE LEVEL

USNVC Confidence Level: Unassigned

USNVC Confidence Comments [optional]:

HIERARCHY

*Lower Level NVC Types:

| **Elcode** | **Scientific or Colloquial Name** |
| --- | --- |
|  |  |

DISCUSSION

Discussion [optional]:

CONCEPT HISTORY

*Recent Concept Lineage [if applicable]:

| **Date** | **Predecessor** | **Note** |
| --- | --- | --- |
|  |  |  |

RELATED CONCEPTS

Supporting Concepts [optional]:

| **Relationship to NVC** | **Supporting Concept Name** | **Short Citation** | **Note** |
| --- | --- | --- | --- |
|  |  |  |  |

AUTHORSHIP

*Primary Concept Source [if applicable]: C. Josse, in Faber-Langendoen et al. (2014)

| **Relationship to NVC** | **Name Used in Source** | **Short Citation** | **Note** |
| --- | --- | --- | --- |
|  |  |  |  |

*Author of Description: C. Josse

Acknowledgments [optional]:

Version Date: 17 Apr 2014

REFERENCES

*References [Required if used in text]:

Faber-Langendoen, D., J. Drake, S. Gawler, M. Hall, C. Josse, G. Kittel, S. Menard, C. Nordman, M. Pyne, M. Reid, L. Sneddon, K. Schulz, J. Teague, M. Russo, K. Snow, and P. Comer, editors. 2010-2018. Divisions, Macrogroups and Groups for the Revised U.S. National Vegetation Classification. NatureServe, Arlington, VA. plus appendices. [in preparation]

1. Forest & Woodland

1.A.2.Ei. Guianan Lowland Humid Forest

M584. Western Guianan Humid Forest

Type Concept Sentence: Forests of the westernmost extension of the Guianan rainforest in Brazil, Colombia, and Venezuela. Forests of the Colombian Guainía region are of lower stature and more interdigitated with white sand savannas and shrublands than those further east, whereas those of the upper Orinoco basin, including the Caura basin in Venezuela, are dense, tall forests with a rather open understory. The following species and genera are typical: *Aldina macrophylla, Eperua leucantha, Micrandra spruceana, Calliandra, Cladonia, Clusia, Eperua, Gustavia, Hevea, Humiria, Ilex, Micrandra, Monopteryx, Ormosia, Pradosia*, and *Qualea*.

OVERVIEW

*Hierarchy Level: Macrogroup

*Placement in Hierarchy: 1.A.2.Ei. Guianan Lowland Humid Forest (D225)

Elcode: M584

*Scientific Name: Western Guianan Humid Forest Macrogroup

*Common (Translated Scientific) Name: Western Guianan Humid Forest Macrogroup

*Colloquial Name: Western Guianan Humid Forest

*Type Concept: Forests of the westernmost extension of the Guianan rainforest in Brazil, Colombia, and Venezuela. The rainforests of the upper Orinoco basin, including the Caura basin in Venezuela, are dense tall forests with a rather open understory. Representative species are *Lecointea amazonica, Clathrotropis glaucophylla, Peltogyne venosa, Erisma uncinatum, Oenocarpus, Socratea, Leopoldinia, Ocotea* spp., *Nectandra* spp. The forests in the Colombian Guainia region are of lower stature and more interdigitated with white sand savannas and shrublands. In their composition the following species and genera are typical: *Eperua leucantha, Aldina macrophylla, Micrandra spruceana, Hevea, Qualea, Clusia, Eperua, Micrandra, Humiria, Ilex, Ormosia, Pradosia, Calliandra, Cladonia, Gustavia, Monopteryx*.

*Diagnostic Characteristics:

*Classification Comments:

*Similar NVC Types [if applicable]:

| **Elcode** | **Scientific or Colloquial Name** | **Note** |
| --- | --- | --- |
|  |  |  |

Similar NVC Types General Comments [optional]:

VEGETATION

Physiognomy and Structure Summary:

Physiognomy and Structure Table [optional]:

| **Physiognomy-Structure Category** | **Prevailing Height (m)** | **Height Range (opt.)** | **Mean % Cover** | **Cover Range (opt.)** |
| --- | --- | --- | --- | --- |
|  |  |  |  | - |

Floristics Summary:

*Floristics Table [Med - High Confidence]:

*Number of Plots:

*Cover Scale Used:

| **Physiognomy-Structure Category** | **Taxon Name** | **Specific Growth Form (opt.)** | **Const- ancy** | **Mean % Cover** | **Cover Range (opt.)** | **Differ-ential** | **Diagnostic Combin- ation** |
| --- | --- | --- | --- | --- | --- | --- | --- |
|  |  |  |  |  | - |  |  |

Dynamics:

ENVIRONMENT

Environmental Description:

DISTRIBUTION

*Geographic Range:

Nations: CO?, VE

States/Provinces:

USFS Ecoregions (2007) [optional]:

Omernik Ecoregions L3, L4 [optional]:

MLRAs [optional]:

PLOT SAMPLING AND ANALYSIS

*Plot Analysis Summary [Med - High Confidence]:

*Plots Used to Define the Type [Med - High Confidence]:

CONFIDENCE LEVEL

USNVC Confidence Level: Unassigned

USNVC Confidence Comments [optional]:

HIERARCHY

*Lower Level NVC Types:

| **Elcode** | **Scientific or Colloquial Name** |
| --- | --- |
|  |  |

DISCUSSION

Discussion [optional]:

CONCEPT HISTORY

*Recent Concept Lineage [if applicable]:

| **Date** | **Predecessor** | **Note** |
| --- | --- | --- |
|  |  |  |

RELATED CONCEPTS

Supporting Concepts [optional]:

| **Relationship to NVC** | **Supporting Concept Name** | **Short Citation** | **Note** |
| --- | --- | --- | --- |
|  |  |  |  |

AUTHORSHIP

*Primary Concept Source [if applicable]: C. Josse, in Faber-Langendoen et al. (2014)

| **Relationship to NVC** | **Name Used in Source** | **Short Citation** | **Note** |
| --- | --- | --- | --- |
|  |  |  |  |

*Author of Description: C. Josse

Acknowledgments [optional]:

Version Date: 17 Apr 2014

REFERENCES

*References [Required if used in text]:

Faber-Langendoen, D., J. Drake, S. Gawler, M. Hall, C. Josse, G. Kittel, S. Menard, C. Nordman, M. Pyne, M. Reid, L. Sneddon, K. Schulz, J. Teague, M. Russo, K. Snow, and P. Comer, editors. 2010-2018. Divisions, Macrogroups and Groups for the Revised U.S. National Vegetation Classification. NatureServe, Arlington, VA. plus appendices. [in preparation]

1. Forest & Woodland

1.A.2.Ei. Guianan Lowland Humid Forest

M587. Orinoquian Humid Forest

Type Concept Sentence: Upland forests of the Orinoco Delta and those of the valleys of the lower reaches of tributary streams. In general, these forests occupy non-flooded plains and low hills, although lower-elevation tracts are flooded temporarily or sporadically. Canopies are up to 25-30 m tall, generally evergreen and with three arboreal strata. Palms are abundant and in lower or less well-drained sites, and the legume *Mora excelsa* is often dominant.

OVERVIEW

*Hierarchy Level: Macrogroup

*Placement in Hierarchy: 1.A.2.Ei. Guianan Lowland Humid Forest (D225)

Elcode: M587

*Scientific Name: Orinoquian Humid Forest Macrogroup

*Common (Translated Scientific) Name: Orinoquian Humid Forest Macrogroup

*Colloquial Name: Orinoquian Humid Forest

*Type Concept: The macrogroup includes upland forests of the Orinoco Delta and those of the valleys of the lower reaches of tributary streams of the Orinoco. In general it occupy non-flooded plains and low hills, but the lower parts are flooded temporarily or sporadically. Forests are up to 25-30 m tall, generally evergreen and with three arboreal strata. Palms are abundant and in lower or less drainage sites *Mora excelsa* is clearly dominant.

*Diagnostic Characteristics:

*Classification Comments:

*Similar NVC Types [if applicable]:

| **Elcode** | **Scientific or Colloquial Name** | **Note** |
| --- | --- | --- |
|  |  |  |

Similar NVC Types General Comments [optional]:

VEGETATION

Physiognomy and Structure Summary:

Physiognomy and Structure Table [optional]:

| **Physiognomy-Structure Category** | **Prevailing Height (m)** | **Height Range (opt.)** | **Mean % Cover** | **Cover Range (opt.)** |
| --- | --- | --- | --- | --- |
|  |  |  |  | - |

Floristics Summary:

*Floristics Table [Med - High Confidence]:

*Number of Plots:

*Cover Scale Used:

| **Physiognomy-Structure Category** | **Taxon Name** | **Specific Growth Form (opt.)** | **Const- ancy** | **Mean % Cover** | **Cover Range (opt.)** | **Differ-ential** | **Diagnostic Combin- ation** |
| --- | --- | --- | --- | --- | --- | --- | --- |
|  |  |  |  |  | - |  |  |

Dynamics:

ENVIRONMENT

Environmental Description:

DISTRIBUTION

*Geographic Range:

Nations: GY, VE

States/Provinces:

USFS Ecoregions (2007) [optional]:

Omernik Ecoregions L3, L4 [optional]:

MLRAs [optional]:

PLOT SAMPLING AND ANALYSIS

*Plot Analysis Summary [Med - High Confidence]:

*Plots Used to Define the Type [Med - High Confidence]:

CONFIDENCE LEVEL

USNVC Confidence Level: Unassigned

USNVC Confidence Comments [optional]:

HIERARCHY

*Lower Level NVC Types:

| **Elcode** | **Scientific or Colloquial Name** |
| --- | --- |
|  |  |

DISCUSSION

Discussion [optional]:

CONCEPT HISTORY

*Recent Concept Lineage [if applicable]:

| **Date** | **Predecessor** | **Note** |
| --- | --- | --- |
|  |  |  |

RELATED CONCEPTS

Supporting Concepts [optional]:

| **Relationship to NVC** | **Supporting Concept Name** | **Short Citation** | **Note** |
| --- | --- | --- | --- |
|  |  |  |  |

AUTHORSHIP

*Primary Concept Source [if applicable]: C. Josse, in Faber-Langendoen et al. (2014)

| **Relationship to NVC** | **Name Used in Source** | **Short Citation** | **Note** |
| --- | --- | --- | --- |
|  |  |  |  |

*Author of Description: C. Josse

Acknowledgments [optional]:

Version Date: 17 Apr 2014

REFERENCES

*References [Required if used in text]:

Faber-Langendoen, D., J. Drake, S. Gawler, M. Hall, C. Josse, G. Kittel, S. Menard, C. Nordman, M. Pyne, M. Reid, L. Sneddon, K. Schulz, J. Teague, M. Russo, K. Snow, and P. Comer, editors. 2010-2018. Divisions, Macrogroups and Groups for the Revised U.S. National Vegetation Classification. NatureServe, Arlington, VA. plus appendices. [in preparation]

1. Forest & Woodland

1.A.2.Ej. Amazonian Lowland Humid Forest

D226. Amazonian Lowland Humid Forest

Type Concept Sentence:

OVERVIEW

*Hierarchy Level: Division

*Placement in Hierarchy: 1.A.2.Ej. Tropical Lowland Humid Forest (F020)

Elcode: D226

*Scientific Name: Amazonian Lowland Humid Forest Division

*Common (Translated Scientific) Name: Amazonian Lowland Humid Forest Division

*Colloquial Name: Amazonian Lowland Humid Forest

*Type Concept:

*Diagnostic Characteristics:

*Classification Comments:

*Similar NVC Types [if applicable]:

| **Elcode** | **Scientific or Colloquial Name** | **Note** |
| --- | --- | --- |
|  |  |  |

Similar NVC Types General Comments [optional]:

VEGETATION

Physiognomy and Structure Summary:

Physiognomy and Structure Table [optional]:

| **Physiognomy-Structure Category** | **Prevailing Height (m)** | **Height Range (opt.)** | **Mean % Cover** | **Cover Range (opt.)** |
| --- | --- | --- | --- | --- |
|  |  |  |  | - |

Floristics Summary:

*Floristics Table [Med - High Confidence]:

*Number of Plots: *Cover Scale Used:

| **Physiognomy-Structure Category** | **Taxon Name** | **Specific Growth Form (opt.)** | **Const- ancy** | **Mean % Cover** | **Cover Range (opt.)** | **Differ-ential** | **Diagnostic Combin- ation** |
| --- | --- | --- | --- | --- | --- | --- | --- |
|  |  |  |  |  | - |  |  |

Dynamics:

ENVIRONMENT

Environmental Description:

DISTRIBUTION

*Geographic Range:

Nations: BO, BR, CO, EC, PE

States/Provinces:

USFS Ecoregions (2007) [optional]:

Omernik Ecoregions L3, L4 [optional]:

MLRAs [optional]:

PLOT SAMPLING AND ANALYSIS

*Plot Analysis Summary [Med - High Confidence]:

*Plots Used to Define the Type [Med - High Confidence]:

CONFIDENCE LEVEL

USNVC Confidence Level: Unassigned

USNVC Confidence Comments [optional]:

HIERARCHY

*Lower Level NVC Types:

| **Elcode** | **Scientific or Colloquial Name** |
| --- | --- |
| M593 | Central Amazon Humid Forest |
| M592 | Northern Amazon Humid Forest |
| M594 | Southern Amazon Humid Forest |
| M590 | Southwestern Amazon Lowland Humid Forest |
| M591 | Southwestern Amazon Subandean Humid Forest |
| M588 | Western Amazon Lowland Humid Forest |
| M589 | Western Amazon Subandean Humid Forest |

DISCUSSION

Discussion [optional]:

CONCEPT HISTORY

*Recent Concept Lineage [if applicable]:

| **Date** | **Predecessor** | **Note** |
| --- | --- | --- |
|  |  |  |

RELATED CONCEPTS

Supporting Concepts [optional]:

| **Relationship to NVC** | **Supporting Concept Name** | **Short Citation** | **Note** |
| --- | --- | --- | --- |
|  |  |  |  |

AUTHORSHIP

*Primary Concept Source [if applicable]:

| **Relationship to NVC** | **Name Used in Source** | **Short Citation** | **Note** |
| --- | --- | --- | --- |
|  |  |  |  |

*Author of Description:

Acknowledgments [optional]:

Version Date:

REFERENCES

*References [Required if used in text]:

Faber-Langendoen, D., J. Drake, S. Gawler, M. Hall, C. Josse, G. Kittel, S. Menard, C. Nordman, M. Pyne, M. Reid, L. Sneddon, K. Schulz, J. Teague, M. Russo, K. Snow, and P. Comer, editors. 2010-2018. Divisions, Macrogroups and Groups for the Revised U.S. National Vegetation Classification. NatureServe, Arlington, VA. plus appendices. [in preparation]

1. Forest & Woodland

1.A.2.Ej. Amazonian Lowland Humid Forest

M593. Central Amazon Humid Forest

Type Concept Sentence: Rainforests distributed in the central and lower Amazon Basin east of Manaus in the interfluves along the main stem and the northern Uatuma Trombetas region. Forests are very tall, diverse and multi-layered, with emergents up to 40 m tall and many epiphytes and lianas.

OVERVIEW

*Hierarchy Level: Macrogroup

*Placement in Hierarchy: 1.A.2.Ej. Amazonian Lowland Humid Forest (D226)

Elcode: M593

*Scientific Name: Central Amazon Humid Forest Macrogroup

*Common (Translated Scientific) Name: Central Amazon Humid Forest Macrogroup

*Colloquial Name: Central Amazon Humid Forest

*Type Concept: Evergreen Amazon forests distributed in the central and lower Amazon Basin east of Manaus, in the interfluves along the main stem and towards the northern Uatuma Trombetas region. Very tall, diverse and multi-layered forest, with emergents up to 40 m tall and rich in epiphytes and lianas.

*Diagnostic Characteristics:

*Classification Comments:

*Similar NVC Types [if applicable]:

| **Elcode** | **Scientific or Colloquial Name** | **Note** |
| --- | --- | --- |
|  |  |  |

Similar NVC Types General Comments [optional]:

VEGETATION

Physiognomy and Structure Summary:

Physiognomy and Structure Table [optional]:

| **Physiognomy-Structure Category** | **Prevailing Height (m)** | **Height Range (opt.)** | **Mean % Cover** | **Cover Range (opt.)** |
| --- | --- | --- | --- | --- |
|  |  |  |  | - |

Floristics Summary:

*Floristics Table [Med - High Confidence]:

*Number of Plots:

*Cover Scale Used:

| **Physiognomy-Structure Category** | **Taxon Name** | **Specific Growth Form (opt.)** | **Const- ancy** | **Mean % Cover** | **Cover Range (opt.)** | **Differ-ential** | **Diagnostic Combin- ation** |
| --- | --- | --- | --- | --- | --- | --- | --- |
|  |  |  |  |  | - |  |  |

Dynamics:

ENVIRONMENT

Environmental Description:

DISTRIBUTION

*Geographic Range:

Nations: BR

States/Provinces:

USFS Ecoregions (2007) [optional]:

Omernik Ecoregions L3, L4 [optional]:

MLRAs [optional]:

PLOT SAMPLING AND ANALYSIS

*Plot Analysis Summary [Med - High Confidence]:

*Plots Used to Define the Type [Med - High Confidence]:

CONFIDENCE LEVEL

USNVC Confidence Level: Unassigned

USNVC Confidence Comments [optional]:

HIERARCHY

*Lower Level NVC Types:

| **Elcode** | **Scientific or Colloquial Name** |
| --- | --- |
|  |  |

DISCUSSION

Discussion [optional]:

CONCEPT HISTORY

*Recent Concept Lineage [if applicable]:

| **Date** | **Predecessor** | **Note** |
| --- | --- | --- |
|  |  |  |

RELATED CONCEPTS

Supporting Concepts [optional]:

| **Relationship to NVC** | **Supporting Concept Name** | **Short Citation** | **Note** |
| --- | --- | --- | --- |
|  |  |  |  |

AUTHORSHIP

*Primary Concept Source [if applicable]: C. Josse, in Faber-Langendoen et al. (2014)

| **Relationship to NVC** | **Name Used in Source** | **Short Citation** | **Note** |
| --- | --- | --- | --- |
|  |  |  |  |

*Author of Description: C. Josse

Acknowledgments [optional]:

Version Date: 17 Apr 2014

REFERENCES

*References [Required if used in text]:

Faber-Langendoen, D., J. Drake, S. Gawler, M. Hall, C. Josse, G. Kittel, S. Menard, C. Nordman, M. Pyne, M. Reid, L. Sneddon, K. Schulz, J. Teague, M. Russo, K. Snow, and P. Comer, editors. 2010-2018. Divisions, Macrogroups and Groups for the Revised U.S. National Vegetation Classification. NatureServe, Arlington, VA. plus appendices. [in preparation]

1. Forest & Woodland

1.A.2.Ej. Amazonian Lowland Humid Forest

M592. Northern Amazon Humid Forest

Type Concept Sentence: Rainforests of the sedimentary plains of the northwestern Amazon region. In Colombia they are represented by the forests in the Caqueta, Guaviare, Apaporis, and Vaupes river basins. These are tall, evergreen, dense forests of interfluves and high, well-drained riverbanks. Common species include *Astrocaryum aculeatum, Caryocar glabrum, Clathrotropis macrocarpa, Clusia spathulifolia, Couma catingae, Dacryodes cf. roraimensis, Goupia glabra, Leopoldinia piassaba, Mauritia carana, Mezilaurus itauba, Pouteria ucuqui, Protium grandifolium, Rhodognaphalopsis brevipes, Scleronema micranthum, Sloanea aff. macroana, Socratea exorrhiza, Swartzia schomburgkii, Tachigali aff. paniculata, Virola calophylloidea*, and many other palms.

OVERVIEW

*Hierarchy Level: Macrogroup

*Placement in Hierarchy: 1.A.2.Ej. Amazonian Lowland Humid Forest (D226)

Elcode: M592

*Scientific Name: Northern Amazon Humid Forest Macrogroup

*Common (Translated Scientific) Name: Northern Amazon Humid Forest Macrogroup

*Colloquial Name: Northern Amazon Humid Forest

*Type Concept: Rainforests of the sedimentary plains of the northwestern Amazon region; in Brazil they are distributed in the basin of the Japura and Negro rivers, up to the limit of the white sands Guianan forests in Venezuela, including in this area semi-evergreen forests of the northern fringe of the Amazonian region in transition to more seasonal forests in the north. In Colombia they are represented by the forests north of the Caqueta River up to the Guaviare and east in the basins of the Apaporis and Vaupes. These are tall, evergreen, dense forests of the interfluves and high, well-drained riverbanks. Among the common species are *Goupia glabra, Clathrotropis macrocarpa, Dacryodes cf. roraimensis, Sloanea aff. macroana, Pouteria ucuqui, Scleronema micranthum, Virola calophylloidea, Swartzia schomburgkii, Protium grandifolium, Mezilaurus itauba, Tachigali aff. paniculata, Mauritia carana, Rhodognaphalopsis brevipes, Couma catingae, Clusia spathulifolia, Dialium* sp., *Caryocar glabrum, Socratea exorrhiza, Astrocaryum aculeatum, Leopoldinia piassaba*, and many other palms.

*Diagnostic Characteristics:

*Classification Comments:

*Similar NVC Types [if applicable]:

| **Elcode** | **Scientific or Colloquial Name** | **Note** |
| --- | --- | --- |
|  |  |  |

Similar NVC Types General Comments [optional]:

VEGETATION

Physiognomy and Structure Summary:

Physiognomy and Structure Table [optional]:

| **Physiognomy-Structure Category** | **Prevailing Height (m)** | **Height Range (opt.)** | **Mean % Cover** | **Cover Range (opt.)** |
| --- | --- | --- | --- | --- |
|  |  |  |  | - |

Floristics Summary:

*Floristics Table [Med - High Confidence]:

*Number of Plots:

*Cover Scale Used:

| **Physiognomy-Structure Category** | **Taxon Name** | **Specific Growth Form (opt.)** | **Const- ancy** | **Mean % Cover** | **Cover Range (opt.)** | **Differ-ential** | **Diagnostic Combin- ation** |
| --- | --- | --- | --- | --- | --- | --- | --- |
|  |  |  |  |  | - |  |  |

Dynamics:

ENVIRONMENT

Environmental Description:

DISTRIBUTION

*Geographic Range:

Nations: BR, CO

States/Provinces:

USFS Ecoregions (2007) [optional]:

Omernik Ecoregions L3, L4 [optional]:

MLRAs [optional]:

PLOT SAMPLING AND ANALYSIS

*Plot Analysis Summary [Med - High Confidence]:

*Plots Used to Define the Type [Med - High Confidence]:

CONFIDENCE LEVEL

USNVC Confidence Level: Unassigned

USNVC Confidence Comments [optional]:

HIERARCHY

*Lower Level NVC Types:

| **Elcode** | **Scientific or Colloquial Name** |
| --- | --- |
|  |  |

DISCUSSION

Discussion [optional]:

CONCEPT HISTORY

*Recent Concept Lineage [if applicable]:

| **Date** | **Predecessor** | **Note** |
| --- | --- | --- |
|  |  |  |

RELATED CONCEPTS

Supporting Concepts [optional]:

| **Relationship to NVC** | **Supporting Concept Name** | **Short Citation** | **Note** |
| --- | --- | --- | --- |
|  |  |  |  |

AUTHORSHIP

*Primary Concept Source [if applicable]: C. Josse, in Faber-Langendoen et al. (2014)

| **Relationship to NVC** | **Name Used in Source** | **Short Citation** | **Note** |
| --- | --- | --- | --- |
|  |  |  |  |

*Author of Description: C. Josse

Acknowledgments [optional]:

Version Date: 17 Apr 2014

REFERENCES

*References [Required if used in text]:

Faber-Langendoen, D., J. Drake, S. Gawler, M. Hall, C. Josse, G. Kittel, S. Menard, C. Nordman, M. Pyne, M. Reid, L. Sneddon, K. Schulz, J. Teague, M. Russo, K. Snow, and P. Comer, editors. 2010-2018. Divisions, Macrogroups and Groups for the Revised U.S. National Vegetation Classification. NatureServe, Arlington, VA. plus appendices. [in preparation]

1. Forest & Woodland

1.A.2.Ej. Amazonian Lowland Humid Forest

M590. Southwestern Amazon Lowland Humid Forest

Type Concept Sentence: Amazonian forests east and south of the Ucayali River in Peru, including the mid and lower basins of the Madre de Dios River in Peru and Bolivia and the area between the Jurua and Yavari rivers in Brazil. Although annual precipitation is high, these forests experience seasonality in rainfall. *Bertholletia excelsa* and *Hevea brasiliensis* inhabit these forests, which also include large tracts of mostly monodominant *Guadua* forests.

OVERVIEW

*Hierarchy Level: Macrogroup

*Placement in Hierarchy: 1.A.2.Ej. Amazonian Lowland Humid Forest (D226)

Elcode: M590

*Scientific Name: Southwestern Amazon Lowland Humid Forest Macrogroup

*Common (Translated Scientific) Name: Southwestern Amazon Lowland Humid Forest Macrogroup

*Colloquial Name: Southwestern Amazon Lowland Humid Forest

*Type Concept: Amazonian forests east and south of the Ucayali River in Peru, Madre de Dios mid and lower basin in Peru and Bolivia, and the areas between Jurua and Yavari in Brazil. Though very humid due to total annual precipitation, this region is characterized by its seasonality and because the topography is more irregular due to the presence of rolling plains and high hills like Serra do Divisor in the Purus area. It is the area of distribution of the Brazil nut or *Bertholletia excelsa*; other common species are *Hevea brasiliensis, Couratari guianensis, Couratari macrosperma, Jacaranda copaia ssp. spectabilis, Apuleia leiocarpa, Spondias mombin (= Spondias lutea), Caryocar villosum, Capirona decorticans, Heisteria nitida, Iryanthera juruensis, Dypteris odorata, Pseudolmedia macrophylla, Pseudolmedia laevis, Diploon cuspidatum, Parkia pendula, Copaifera reticulata, Castilla ulei, Swietenia macrophylla*, among many others. Included in this macrogroup are the very large extensions of mostly monodominant *Guadua* (bamboo) forests.

*Diagnostic Characteristics:

*Classification Comments:

*Similar NVC Types [if applicable]:

| **Elcode** | **Scientific or Colloquial Name** | **Note** |
| --- | --- | --- |
|  |  |  |

Similar NVC Types General Comments [optional]:

VEGETATION

Physiognomy and Structure Summary:

Physiognomy and Structure Table [optional]:

| **Physiognomy-Structure Category** | **Prevailing Height (m)** | **Height Range (opt.)** | **Mean % Cover** | **Cover Range (opt.)** |
| --- | --- | --- | --- | --- |
|  |  |  |  | - |

Floristics Summary:

*Floristics Table [Med - High Confidence]:

*Number of Plots:

*Cover Scale Used:

| **Physiognomy-Structure Category** | **Taxon Name** | **Specific Growth Form (opt.)** | **Const- ancy** | **Mean % Cover** | **Cover Range (opt.)** | **Differ-ential** | **Diagnostic Combin- ation** |
| --- | --- | --- | --- | --- | --- | --- | --- |
|  |  |  |  |  | - |  |  |

Dynamics:

ENVIRONMENT

Environmental Description:

DISTRIBUTION

*Geographic Range:

Nations: BO, BR, PE

States/Provinces:

USFS Ecoregions (2007) [optional]:

Omernik Ecoregions L3, L4 [optional]:

MLRAs [optional]:

PLOT SAMPLING AND ANALYSIS

*Plot Analysis Summary [Med - High Confidence]:

*Plots Used to Define the Type [Med - High Confidence]:

CONFIDENCE LEVEL

USNVC Confidence Level: Unassigned

USNVC Confidence Comments [optional]:

HIERARCHY

*Lower Level NVC Types:

| **Elcode** | **Scientific or Colloquial Name** |
| --- | --- |
|  |  |

DISCUSSION

Discussion [optional]:

CONCEPT HISTORY

*Recent Concept Lineage [if applicable]:

| **Date** | **Predecessor** | **Note** |
| --- | --- | --- |
|  |  |  |

RELATED CONCEPTS

Supporting Concepts [optional]:

| **Relationship to NVC** | **Supporting Concept Name** | **Short Citation** | **Note** |
| --- | --- | --- | --- |
|  |  |  |  |

AUTHORSHIP

*Primary Concept Source [if applicable]: C. Josse, in Faber-Langendoen et al. (2014)

| **Relationship to NVC** | **Name Used in Source** | **Short Citation** | **Note** |
| --- | --- | --- | --- |
|  |  |  |  |

*Author of Description: C. Josse

Acknowledgments [optional]:

Version Date: 17 Apr 2014

REFERENCES

*References [Required if used in text]:

Faber-Langendoen, D., J. Drake, S. Gawler, M. Hall, C. Josse, G. Kittel, S. Menard, C. Nordman, M. Pyne, M. Reid, L. Sneddon, K. Schulz, J. Teague, M. Russo, K. Snow, and P. Comer, editors. 2010-2018. Divisions, Macrogroups and Groups for the Revised U.S. National Vegetation Classification. NatureServe, Arlington, VA. plus appendices. [in preparation]

1. Forest & Woodland

1.A.2.Ej. Amazonian Lowland Humid Forest

M591. Southwestern Amazon Subandean Humid Forest

Type Concept Sentence: Humid to hyper-humid forests growing between 500 and 1000-1300 m elevation on the sub-Andean hills of the southwestern Amazon Basin in Peru and Bolivia. These forests occur on relatively poor, sandy loam soils that are well-drained. Characteristic species are *Apeiba membranacea, Cavanillesia hylogeiton, Clarisia biflora, Clarisia racemosa, Dipteryx odorata, Elaeagia mariae, Elaeagia obovata, Eschweilera andina, Eschweilera coriacea, Euterpe precatoria, Geonoma macrostachys, Iriartea deltoidea, Manilkara excelsa, Oenocarpus bataua, Sloanea fragrans, Sterculia apeibophylla*, and *Talauma boliviana*. Among the predominantly Amazonian floristic composition, some Andean elements such as *Elaeagia obovata, Ladenbergia magnifolia, Ladenbergia oblongifolia, Ladenbergia sericea, Podocarpus celatus, Podocarpus magnifolius, Prumnopitys harmsiana*, and *Weinmannia pinnata* are present.

OVERVIEW

*Hierarchy Level: Macrogroup

*Placement in Hierarchy: 1.A.2.Ej. Amazonian Lowland Humid Forest (D226)

Elcode: M591

*Scientific Name: Southwestern Amazon Subandean Humid Forest Macrogroup

*Common (Translated Scientific) Name: Southwestern Amazon Subandean Humid Forest Macrogroup

*Colloquial Name: Southwestern Amazon Subandean Humid Forest

*Type Concept: Humid to hyper-humid forests growing above 500 and up to 1000-1300 m elevation on the sub-Andean hills of the southwestern Amazon Basin in Peru and Bolivia. They have relatively poor, well-drained sandy loam soils. Characteristic species are *Apeiba membranacea, Cavanillesia hylogeiton, Clarisia biflora, Clarisia racemosa, Dipteryx odorata, Elaeagia mariae, Elaeagia obovata, Eschweilera andina, Eschweilera coriacea, Euterpe precatoria, Geonoma macrostachys, Iriartea deltoidea, Manilkara excelsa, Oenocarpus bataua, Sloanea fragrans, Sterculia apeibophylla*, and *Talauma boliviana*. Among the predominantly Amazonian floristic composition, some Andean elements such as *Elaeagia obovata, Ladenbergia magnifolia, Ladenbergia oblongifolia, Ladenbergia sericea, Podocarpus celatus, Podocarpus magnifolius, Prumnopitys harmsiana*, and *Weinmannia pinnata* are present.

*Diagnostic Characteristics:

*Classification Comments:

*Similar NVC Types [if applicable]:

| **Elcode** | **Scientific or Colloquial Name** | **Note** |
| --- | --- | --- |
|  |  |  |

Similar NVC Types General Comments [optional]:

VEGETATION

Physiognomy and Structure Summary:

Physiognomy and Structure Table [optional]:

| **Physiognomy-Structure Category** | **Prevailing Height (m)** | **Height Range (opt.)** | **Mean % Cover** | **Cover Range (opt.)** |
| --- | --- | --- | --- | --- |
|  |  |  |  | - |

Floristics Summary:

*Floristics Table [Med - High Confidence]:

*Number of Plots:

*Cover Scale Used:

| **Physiognomy-Structure Category** | **Taxon Name** | **Specific Growth Form (opt.)** | **Const- ancy** | **Mean % Cover** | **Cover Range (opt.)** | **Differ-ential** | **Diagnostic Combin- ation** |
| --- | --- | --- | --- | --- | --- | --- | --- |
|  |  |  |  |  | - |  |  |

Dynamics:

ENVIRONMENT

Environmental Description:

DISTRIBUTION

*Geographic Range:

Nations: BO, PE

States/Provinces:

USFS Ecoregions (2007) [optional]:

Omernik Ecoregions L3, L4 [optional]:

MLRAs [optional]:

PLOT SAMPLING AND ANALYSIS

*Plot Analysis Summary [Med - High Confidence]:

*Plots Used to Define the Type [Med - High Confidence]:

CONFIDENCE LEVEL

USNVC Confidence Level: Unassigned

USNVC Confidence Comments [optional]:

HIERARCHY

*Lower Level NVC Types:

| **Elcode** | **Scientific or Colloquial Name** |
| --- | --- |
|  |  |

DISCUSSION

Discussion [optional]:

CONCEPT HISTORY

*Recent Concept Lineage [if applicable]:

| **Date** | **Predecessor** | **Note** |
| --- | --- | --- |
|  |  |  |

RELATED CONCEPTS

Supporting Concepts [optional]:

| **Relationship to NVC** | **Supporting Concept Name** | **Short Citation** | **Note** |
| --- | --- | --- | --- |
|  |  |  |  |

AUTHORSHIP

*Primary Concept Source [if applicable]: C. Josse, in Faber-Langendoen et al. (2014)

| **Relationship to NVC** | **Name Used in Source** | **Short Citation** | **Note** |
| --- | --- | --- | --- |
|  |  |  |  |

*Author of Description: C. Josse

Acknowledgments [optional]:

Version Date: 17 Apr 2014

REFERENCES

*References [Required if used in text]:

Faber-Langendoen, D., J. Drake, S. Gawler, M. Hall, C. Josse, G. Kittel, S. Menard, C. Nordman, M. Pyne, M. Reid, L. Sneddon, K. Schulz, J. Teague, M. Russo, K. Snow, and P. Comer, editors. 2010-2018. Divisions, Macrogroups and Groups for the Revised U.S. National Vegetation Classification. NatureServe, Arlington, VA. plus appendices. [in preparation]

1. Forest & Woodland

1.A.2.Ej. Amazonian Lowland Humid Forest

M588. Western Amazon Lowland Humid Forest

Type Concept Sentence: Rainforests of the Amazon Basin in southern Colombia, Ecuador, northwest of the Ucayali River in Peru, and west of the Japura River in Brazil. Very tall, multi-stratified evergreen forests, with abundant lianas and epiphytes, growing on different landforms, including riverbanks and higher terraces, peneplains, low hills and depressed, but well-drained terrain. These are among the forests with the greatest diversity of tree species. Most diversified or prominent among their floristic composition are members of the families Fabaceae, Myristicaceae, Bombacaceae, Meliaceae, Vochysiaceae, Lauraceae, Rubiaceae, and Arecaceae.

OVERVIEW

*Hierarchy Level: Macrogroup

*Placement in Hierarchy: 1.A.2.Ej. Amazonian Lowland Humid Forest (D226)

Elcode: M588

*Scientific Name: Western Amazon Lowland Humid Forest Macrogroup

*Common (Translated Scientific) Name: Western Amazon Lowland Humid Forest Macrogroup

*Colloquial Name: Western Amazon Lowland Humid Forest

*Type Concept: Rainforests of the Amazon Basin in southern Colombia, all of Ecuador, northwest of the Ucayali River in Peru, and west of the Japura River in Brazil. Very tall, multi-stratified evergreen forests, with abundant lianas and epiphytes, growing on different landforms, from riverbanks and higher terraces, to peneplains, low hills and also more depressed, but well-drained terrain. It is considered one of the most diverse forests in tree species. Most diversified or prominent among their floristic composition are members of the following families: Fabaceae, Myristicaceae, Bombacaceae, Meliaceae, Vochysiaceae, Lauraceae, Rubiaceae, and Arecaceae.

*Diagnostic Characteristics:

*Classification Comments:

*Similar NVC Types [if applicable]:

| **Elcode** | **Scientific or Colloquial Name** | **Note** |
| --- | --- | --- |
|  |  |  |

Similar NVC Types General Comments [optional]:

VEGETATION

Physiognomy and Structure Summary:

Physiognomy and Structure Table [optional]:

| **Physiognomy-Structure Category** | **Prevailing Height (m)** | **Height Range (opt.)** | **Mean % Cover** | **Cover Range (opt.)** |
| --- | --- | --- | --- | --- |
|  |  |  |  | - |

Floristics Summary:

*Floristics Table [Med - High Confidence]:

*Number of Plots:

*Cover Scale Used:

| **Physiognomy-Structure Category** | **Taxon Name** | **Specific Growth Form (opt.)** | **Const- ancy** | **Mean % Cover** | **Cover Range (opt.)** | **Differ-ential** | **Diagnostic Combin- ation** |
| --- | --- | --- | --- | --- | --- | --- | --- |
|  |  |  |  |  | - |  |  |

Dynamics:

ENVIRONMENT

Environmental Description:

DISTRIBUTION

*Geographic Range:

Nations: BR, CO, EC, PE

States/Provinces:

USFS Ecoregions (2007) [optional]:

Omernik Ecoregions L3, L4 [optional]:

MLRAs [optional]:

PLOT SAMPLING AND ANALYSIS

*Plot Analysis Summary [Med - High Confidence]:

*Plots Used to Define the Type [Med - High Confidence]:

CONFIDENCE LEVEL

USNVC Confidence Level: Unassigned

USNVC Confidence Comments [optional]:

HIERARCHY

*Lower Level NVC Types:

| **Elcode** | **Scientific or Colloquial Name** |
| --- | --- |
|  |  |

DISCUSSION

Discussion [optional]:

CONCEPT HISTORY

*Recent Concept Lineage [if applicable]:

| **Date** | **Predecessor** | **Note** |
| --- | --- | --- |
|  |  |  |

RELATED CONCEPTS

Supporting Concepts [optional]:

| **Relationship to NVC** | **Supporting Concept Name** | **Short Citation** | **Note** |
| --- | --- | --- | --- |
|  |  |  |  |

AUTHORSHIP

*Primary Concept Source [if applicable]: C. Josse, in Faber-Langendoen et al. (2014)

| **Relationship to NVC** | **Name Used in Source** | **Short Citation** | **Note** |
| --- | --- | --- | --- |
|  |  |  |  |

*Author of Description: C. Josse

Acknowledgments [optional]:

Version Date: 17 Apr 2014

REFERENCES

*References [Required if used in text]:

Faber-Langendoen, D., J. Drake, S. Gawler, M. Hall, C. Josse, G. Kittel, S. Menard, C. Nordman, M. Pyne, M. Reid, L. Sneddon, K. Schulz, J. Teague, M. Russo, K. Snow, and P. Comer, editors. 2010-2018. Divisions, Macrogroups and Groups for the Revised U.S. National Vegetation Classification. NatureServe, Arlington, VA. plus appendices. [in preparation]

1. Forest & Woodland

1.A.2.Ej. Amazonian Lowland Humid Forest

M589. Western Amazon Subandean Humid Forest

Type Concept Sentence: Forests growing from 500 to 1000-1300 m elevation on the sub-Andean hills of the western Amazon Basin from Sierra de la Macarena in Colombia to central Peru. These are multi stratified forests 20-30 m tall,, growing in hyper-humid conditions on well-drained, somewhat acidic soils. The composition is typical of western Amazonian forests but with a few Andean elements from the genera *Miconia, Myrcia, Ocotea, Schefflera*, and *Weinmannia*.

OVERVIEW

*Hierarchy Level: Macrogroup

*Placement in Hierarchy: 1.A.2.Ej. Amazonian Lowland Humid Forest (D226)

Elcode: M589

*Scientific Name: Western Amazon Subandean Humid Forest Macrogroup

*Common (Translated Scientific) Name: Western Amazon Subandean Humid Forest Macrogroup

*Colloquial Name: Western Amazon Subandean Humid Forest

*Type Concept: This macrogroup includes the forests growing above 500 and up to 1000-1300 m elevation on the sub-Andean hills of the western Amazon Basin from Sierra de la Macarena in Colombia to central Peru. These are 20-30 m tall, multi-stratified forests, growing in hyper-humid conditions, on well-drained, somewhat acidic soils. The composition is typical of western Amazonian forests but with a few Andean elements from the genera *Miconia, Myrcia, Ocotea, Schefflera*, and *Weinmannia*.

*Diagnostic Characteristics:

*Classification Comments:

*Similar NVC Types [if applicable]:

| **Elcode** | **Scientific or Colloquial Name** | **Note** |
| --- | --- | --- |
|  |  |  |

Similar NVC Types General Comments [optional]:

VEGETATION

Physiognomy and Structure Summary:

Physiognomy and Structure Table [optional]:

| **Physiognomy-Structure Category** | **Prevailing Height (m)** | **Height Range (opt.)** | **Mean % Cover** | **Cover Range (opt.)** |
| --- | --- | --- | --- | --- |
|  |  |  |  | - |

Floristics Summary:

*Floristics Table [Med - High Confidence]:

*Number of Plots:

*Cover Scale Used:

| **Physiognomy-Structure Category** | **Taxon Name** | **Specific Growth Form (opt.)** | **Const- ancy** | **Mean % Cover** | **Cover Range (opt.)** | **Differ-ential** | **Diagnostic Combin- ation** |
| --- | --- | --- | --- | --- | --- | --- | --- |
|  |  |  |  |  | - |  |  |

Dynamics:

ENVIRONMENT

Environmental Description:

DISTRIBUTION

*Geographic Range:

Nations: CO, EC, PE

States/Provinces:

USFS Ecoregions (2007) [optional]:

Omernik Ecoregions L3, L4 [optional]:

MLRAs [optional]:

PLOT SAMPLING AND ANALYSIS

*Plot Analysis Summary [Med - High Confidence]:

*Plots Used to Define the Type [Med - High Confidence]:

CONFIDENCE LEVEL

USNVC Confidence Level: Unassigned

USNVC Confidence Comments [optional]:

HIERARCHY

*Lower Level NVC Types:

| **Elcode** | **Scientific or Colloquial Name** |
| --- | --- |
|  |  |

DISCUSSION

Discussion [optional]:

CONCEPT HISTORY

*Recent Concept Lineage [if applicable]:

| **Date** | **Predecessor** | **Note** |
| --- | --- | --- |
|  |  |  |

RELATED CONCEPTS

Supporting Concepts [optional]:

| **Relationship to NVC** | **Supporting Concept Name** | **Short Citation** | **Note** |
| --- | --- | --- | --- |
|  |  |  |  |

AUTHORSHIP

*Primary Concept Source [if applicable]: C. Josse, in Faber-Langendoen et al. (2014)

| **Relationship to NVC** | **Name Used in Source** | **Short Citation** | **Note** |
| --- | --- | --- | --- |
|  |  |  |  |

*Author of Description: C. Josse

Acknowledgments [optional]:

Version Date: 17 Apr 2014

REFERENCES

*References [Required if used in text]:

Faber-Langendoen, D., J. Drake, S. Gawler, M. Hall, C. Josse, G. Kittel, S. Menard, C. Nordman, M. Pyne, M. Reid, L. Sneddon, K. Schulz, J. Teague, M. Russo, K. Snow, and P. Comer, editors. 2010-2018. Divisions, Macrogroups and Groups for the Revised U.S. National Vegetation Classification. NatureServe, Arlington, VA. plus appendices. [in preparation]

1. Forest & Woodland

1.A.2.Ek. Brazilian-Parana Lowland Humid Forest

D227. Brazilian-Parana Lowland Humid Forest

Type Concept Sentence:

OVERVIEW

*Hierarchy Level: Division

*Placement in Hierarchy: 1.A.2.Ek. Tropical Lowland Humid Forest (F020)

Elcode: D227

*Scientific Name: Brazilian-Parana Lowland Humid Forest Division

*Common (Translated Scientific) Name: Brazilian-Parana Lowland Humid Forest Division

*Colloquial Name: Brazilian-Parana Lowland Humid Forest

*Type Concept:

*Diagnostic Characteristics:

*Classification Comments:

*Similar NVC Types [if applicable]:

| **Elcode** | **Scientific or Colloquial Name** | **Note** |
| --- | --- | --- |
|  |  |  |

Similar NVC Types General Comments [optional]:

VEGETATION

Physiognomy and Structure Summary:

Physiognomy and Structure Table [optional]:

| **Physiognomy-Structure Category** | **Prevailing Height (m)** | **Height Range (opt.)** | **Mean % Cover** | **Cover Range (opt.)** |
| --- | --- | --- | --- | --- |
|  |  |  |  | - |

Floristics Summary:

*Floristics Table [Med - High Confidence]:

*Number of Plots: *Cover Scale Used:

| **Physiognomy-Structure Category** | **Taxon Name** | **Specific Growth Form (opt.)** | **Const- ancy** | **Mean % Cover** | **Cover Range (opt.)** | **Differ-ential** | **Diagnostic Combin- ation** |
| --- | --- | --- | --- | --- | --- | --- | --- |
|  |  |  |  |  | - |  |  |

Dynamics:

ENVIRONMENT

Environmental Description:

DISTRIBUTION

*Geographic Range:

Nations: AR, BO, BR, PY

States/Provinces:

USFS Ecoregions (2007) [optional]:

Omernik Ecoregions L3, L4 [optional]:

MLRAs [optional]:

PLOT SAMPLING AND ANALYSIS

*Plot Analysis Summary [Med - High Confidence]:

*Plots Used to Define the Type [Med - High Confidence]:

CONFIDENCE LEVEL

USNVC Confidence Level: Unassigned

USNVC Confidence Comments [optional]:

HIERARCHY

*Lower Level NVC Types:

| **Elcode** | **Scientific or Colloquial Name** |
| --- | --- |
| M597 | Cerrado Humid Forest |
| M595 | Brazilian Atlantic Humid Forest |
| M596 | Parana Humid Forest |

DISCUSSION

Discussion [optional]:

CONCEPT HISTORY

*Recent Concept Lineage [if applicable]:

| **Date** | **Predecessor** | **Note** |
| --- | --- | --- |
|  |  |  |

RELATED CONCEPTS

Supporting Concepts [optional]:

| **Relationship to NVC** | **Supporting Concept Name** | **Short Citation** | **Note** |
| --- | --- | --- | --- |
|  |  |  |  |

AUTHORSHIP

*Primary Concept Source [if applicable]:

| **Relationship to NVC** | **Name Used in Source** | **Short Citation** | **Note** |
| --- | --- | --- | --- |
|  |  |  |  |

*Author of Description:

Acknowledgments [optional]:

Version Date:

REFERENCES

*References [Required if used in text]:

Faber-Langendoen, D., J. Drake, S. Gawler, M. Hall, C. Josse, G. Kittel, S. Menard, C. Nordman, M. Pyne, M. Reid, L. Sneddon, K. Schulz, J. Teague, M. Russo, K. Snow, and P. Comer, editors. 2010-2018. Divisions, Macrogroups and Groups for the Revised U.S. National Vegetation Classification. NatureServe, Arlington, VA. plus appendices. [in preparation]

1. Forest & Woodland

1.A.2.Ek. Brazilian-Parana Lowland Humid Forest

M597. Cerrado Humid Forest

Type Concept Sentence: Moist to subhumid forests of the Cerrado and Chiquitania that transition to more humid Amazon forests. Also includes Cerrado forests with additional moisture availability such as riparian and gallery forests. The multi-strata forests can reach 25 m high, and grow on well-drained soil in seasonal climates. Species composition is predominantly from the Cerrado floristic region with Amazonian elements. Characteristic species are *Albizia niopoides, Cariniana estrellensis, Cedrela fissilis, Erytrochiton fallax, Hymenaea courbaril, Ocotea guianensis, Poeppigia procera, Protium heptaphyllum, Sapium marmieri, Sterculia apetala*, and in riparian situations *Apuleia leiocarpa, Callisthene major, Cariniana rubra, Cheiloclinum cognatum, Copaifera langsdorfii, Guarea kunthiana*, and *Guettarda viburnoides*.

OVERVIEW

*Hierarchy Level: Macrogroup

*Placement in Hierarchy: 1.A.2.Ek. Brazilian-Parana Lowland Humid Forest (D227)

Elcode: M597

*Scientific Name: Cerrado Humid Forest Macrogroup

*Common (Translated Scientific) Name: Cerrado Humid Forest Macrogroup

*Colloquial Name: Cerrado Humid Forest

*Type Concept: The macrogroup includes moist to sub-humid forests of the Cerrado and Chiquitania in transitional locations to the Amazon Basin or with additional moisture availability in the case of riparian, gallery forests. Multi-strata forests that can reach 25 m high, growing on well-drained ground and in a seasonal but humid climate. The predominant composition is from the Cerrado floristic region but with Amazonian elements. Some of the characteristic species are *Ocotea guianensis, Spondias mombin, Physocalymma scaberrimum, Hymenaea courbaril, Poeppigia procera, Casearia gossypiosperma, Cordia alliodora, Cedrela fissilis, Chusquea ramosissima, Pourouma cecropiifolia, Erytrochiton fallax, Phenakospermum guianensis, Albizia niopoides, Attalea phalerata, Cariniana estrellensis, Gallesia integrifolia, Protium heptaphyllum, Sapium marmieri, Sterculia apetala, Inga laurina (= Inga fagifolia), Inga ingoides*, and in riparian situations *Apuleia leiocarpa, Copaifera langsdorfii, Hymenaea courbaril, Guettarda viburnoides, Callisthene major, Cariniana rubra, Cheiloclinum cognatum, Guarea guidonia, Guarea kunthiana*.

*Diagnostic Characteristics:

*Classification Comments:

*Similar NVC Types [if applicable]:

| **Elcode** | **Scientific or Colloquial Name** | **Note** |
| --- | --- | --- |
|  |  |  |

Similar NVC Types General Comments [optional]:

VEGETATION

Physiognomy and Structure Summary:

Physiognomy and Structure Table [optional]:

| **Physiognomy-Structure Category** | **Prevailing Height (m)** | **Height Range (opt.)** | **Mean % Cover** | **Cover Range (opt.)** |
| --- | --- | --- | --- | --- |
|  |  |  |  | - |

Floristics Summary:

*Floristics Table [Med - High Confidence]:

*Number of Plots:

*Cover Scale Used:

| **Physiognomy-Structure Category** | **Taxon Name** | **Specific Growth Form (opt.)** | **Const- ancy** | **Mean % Cover** | **Cover Range (opt.)** | **Differ-ential** | **Diagnostic Combin- ation** |
| --- | --- | --- | --- | --- | --- | --- | --- |
|  |  |  |  |  | - |  |  |

Dynamics:

ENVIRONMENT

Environmental Description:

DISTRIBUTION

*Geographic Range:

Nations: BO, BR

States/Provinces:

USFS Ecoregions (2007) [optional]:

Omernik Ecoregions L3, L4 [optional]:

MLRAs [optional]:

PLOT SAMPLING AND ANALYSIS

*Plot Analysis Summary [Med - High Confidence]:

*Plots Used to Define the Type [Med - High Confidence]:

CONFIDENCE LEVEL

USNVC Confidence Level: Unassigned

USNVC Confidence Comments [optional]:

HIERARCHY

*Lower Level NVC Types:

| **Elcode** | **Scientific or Colloquial Name** |
| --- | --- |
|  |  |

DISCUSSION

Discussion [optional]:

CONCEPT HISTORY

*Recent Concept Lineage [if applicable]:

| **Date** | **Predecessor** | **Note** |
| --- | --- | --- |
|  |  |  |

RELATED CONCEPTS

Supporting Concepts [optional]:

| **Relationship to NVC** | **Supporting Concept Name** | **Short Citation** | **Note** |
| --- | --- | --- | --- |
|  |  |  |  |

AUTHORSHIP

*Primary Concept Source [if applicable]: C. Josse, in Faber-Langendoen et al. (2014)

| **Relationship to NVC** | **Name Used in Source** | **Short Citation** | **Note** |
| --- | --- | --- | --- |
|  |  |  |  |

*Author of Description: C. Josse

Acknowledgments [optional]:

Version Date: 17 Apr 2014

REFERENCES

*References [Required if used in text]:

Faber-Langendoen, D., J. Drake, S. Gawler, M. Hall, C. Josse, G. Kittel, S. Menard, C. Nordman, M. Pyne, M. Reid, L. Sneddon, K. Schulz, J. Teague, M. Russo, K. Snow, and P. Comer, editors. 2010-2018. Divisions, Macrogroups and Groups for the Revised U.S. National Vegetation Classification. NatureServe, Arlington, VA. plus appendices. [in preparation]

1. Forest & Woodland

1.A.2.Ek. Brazilian-Parana Lowland Humid Forest

M595. Brazilian Atlantic Humid Forest

Type Concept Sentence: Lowland evergreen rainforests of the Brazilian Atlantic coastal plain and low slopes of the coastal ridge up to 200 m elevation. They are distributed from 6°S to almost 30°S latitude. Tall, multi-strata forests with dense shrubby undergrowth. Lianas and vascular epiphytes are abundant and diverse, as well as the herbaceous layer with numerous representatives of the Marantaceae and Strelitziaceae families. Several centers of endemism, with restricted-range plant and animal species, occur. Dominant families of the canopy layer are Fabaceae, Bignoniaceae, Meliaceae and Sapotaceae.

OVERVIEW

*Hierarchy Level: Macrogroup

*Placement in Hierarchy: 1.A.2.Ek. Brazilian-Parana Lowland Humid Forest (D227)

Elcode: M595

*Scientific Name: Brazilian Atlantic Humid Forest Macrogroup

*Common (Translated Scientific) Name: Brazilian Atlantic Humid Forest Macrogroup

*Colloquial Name: Brazilian Atlantic Humid Forest

*Type Concept: Lowland evergreen rainforests of the Brazilian Atlantic coastal plain and low slopes of the coastal ridge up to 200 m elevation. They are distributed in a belt from 6°S to almost 30°S latitude, and are currently very fragmented. They are tall, multi-layered forests, with a dense shrubby undergrowth. Lianas and vascular epiphytes are highly abundant and diverse, as well as the herbaceous layer, with numerous members of the Marantaceae and Strelitziaceae. Along this large latitudinal gradient, several centers of endemism developed with both plant and animal species with very restricted distributions. Overall, dominant families of the canopy layer are Fabaceae, Bignoniaceae, Meliaceae and Sapotaceae.

*Diagnostic Characteristics:

*Classification Comments:

*Similar NVC Types [if applicable]:

| **Elcode** | **Scientific or Colloquial Name** | **Note** |
| --- | --- | --- |
|  |  |  |

Similar NVC Types General Comments [optional]:

VEGETATION

Physiognomy and Structure Summary:

Physiognomy and Structure Table [optional]:

| **Physiognomy-Structure Category** | **Prevailing Height (m)** | **Height Range (opt.)** | **Mean % Cover** | **Cover Range (opt.)** |
| --- | --- | --- | --- | --- |
|  |  |  |  | - |

Floristics Summary:

*Floristics Table [Med - High Confidence]:

*Number of Plots:

*Cover Scale Used:

| **Physiognomy-Structure Category** | **Taxon Name** | **Specific Growth Form (opt.)** | **Const- ancy** | **Mean % Cover** | **Cover Range (opt.)** | **Differ-ential** | **Diagnostic Combin- ation** |
| --- | --- | --- | --- | --- | --- | --- | --- |
|  |  |  |  |  | - |  |  |

Dynamics:

ENVIRONMENT

Environmental Description:

DISTRIBUTION

*Geographic Range:

Nations: BR

States/Provinces:

USFS Ecoregions (2007) [optional]:

Omernik Ecoregions L3, L4 [optional]:

MLRAs [optional]:

PLOT SAMPLING AND ANALYSIS

*Plot Analysis Summary [Med - High Confidence]:

*Plots Used to Define the Type [Med - High Confidence]:

CONFIDENCE LEVEL

USNVC Confidence Level: Unassigned

USNVC Confidence Comments [optional]:

HIERARCHY

*Lower Level NVC Types:

| **Elcode** | **Scientific or Colloquial Name** |
| --- | --- |
|  |  |

DISCUSSION

Discussion [optional]:

CONCEPT HISTORY

*Recent Concept Lineage [if applicable]:

| **Date** | **Predecessor** | **Note** |
| --- | --- | --- |
|  |  |  |

RELATED CONCEPTS

Supporting Concepts [optional]:

| **Relationship to NVC** | **Supporting Concept Name** | **Short Citation** | **Note** |
| --- | --- | --- | --- |
|  |  |  |  |

AUTHORSHIP

*Primary Concept Source [if applicable]: C. Josse, in Faber-Langendoen et al. (2014)

| **Relationship to NVC** | **Name Used in Source** | **Short Citation** | **Note** |
| --- | --- | --- | --- |
|  |  |  |  |

*Author of Description: C. Josse

Acknowledgments [optional]:

Version Date: 17 Apr 2014

REFERENCES

*References [Required if used in text]:

Faber-Langendoen, D., J. Drake, S. Gawler, M. Hall, C. Josse, G. Kittel, S. Menard, C. Nordman, M. Pyne, M. Reid, L. Sneddon, K. Schulz, J. Teague, M. Russo, K. Snow, and P. Comer, editors. 2010-2018. Divisions, Macrogroups and Groups for the Revised U.S. National Vegetation Classification. NatureServe, Arlington, VA. plus appendices. [in preparation]

1. Forest & Woodland

1.A.2.Ek. Brazilian-Parana Lowland Humid Forest

M596. Parana Humid Forest

Type Concept Sentence: Subtropical humid to subhumid forests of southern Brazil, eastern Paraguay and northeastern Argentina. Forests have dense cover and are multi-strata and mostly evergreen, with a limited number of deciduous trees. Most of the distribution occurs on a basaltic plateau that abuts to the lower floodplains of the Parana and Uruguay rivers. A few hills are present, which are part of the Serra Geral ridge in Brazil. Although there are floristic affinities with the Brazilian Atlantic forests, characteristic tree species are *Apuleia leiocarpa, Astronium fraxinifolium, Cabralea canjerana, Cariniana estrellensis, Peltophorum dubium, Syagrus romanzoffiana*, and *Tabebuia heptaphylla*.

OVERVIEW

*Hierarchy Level: Macrogroup

*Placement in Hierarchy: 1.A.2.Ek. Brazilian-Parana Lowland Humid Forest (D227)

Elcode: M596

*Scientific Name: Parana Humid Forest Macrogroup

*Common (Translated Scientific) Name: Parana Humid Forest Macrogroup

*Colloquial Name: Parana Humid Forest

*Type Concept: Subtropical humid to sub-humid forests of southern Brazil, eastern Paraguay and northeastern Argentina. Dense cover, multi-strata, mostly evergreen with a limited admixture of deciduous trees among the canopy and emergent species. Most of the distribution occurs on a basaltic plateau which abuts to the lower floodplains of the Parana and Uruguay rivers; a few hills or sierras are present which are part of the Serra Geral ridge in Brazil. Their strongest floristic affinity is with the Brazilian Atlantic forests, but it is a distinct assemblage of which some characteristic tree species are *Apuleia leiocarpa, Cabralea canjerana, Cedrela fissilis, Aspidosperma polyneuron, Tabebuia heptaphylla, Tabebuia impetiginosa, Anadenanthera colubrina, Astronium fraxinifolium, Syagrus romanzoffiana, Euterpe edulis, Annona amambayensis, Cariniana estrellensis, Machaerium stipitatum, Balfourodendron riedelianum, Parapiptadenia rigida, Peltophorum dubium, Patagonula americana, Pterogyne nitens, Cordia trichotoma, Enterolobium contortisiliquum, Myrocarpus frondosus, Matayba elaeagnoides, Allophylus edulis, Garcinia brasiliensis, Myrciaria rivularis, Alsophila cuspidata, Sorocea bonplandii*, among many others.

*Diagnostic Characteristics:

*Classification Comments:

*Similar NVC Types [if applicable]:

| **Elcode** | **Scientific or Colloquial Name** | **Note** |
| --- | --- | --- |
|  |  |  |

Similar NVC Types General Comments [optional]:

VEGETATION

Physiognomy and Structure Summary:

Physiognomy and Structure Table [optional]:

| **Physiognomy-Structure Category** | **Prevailing Height (m)** | **Height Range (opt.)** | **Mean % Cover** | **Cover Range (opt.)** |
| --- | --- | --- | --- | --- |
|  |  |  |  | - |

Floristics Summary:

*Floristics Table [Med - High Confidence]:

*Number of Plots:

*Cover Scale Used:

| **Physiognomy-Structure Category** | **Taxon Name** | **Specific Growth Form (opt.)** | **Const- ancy** | **Mean % Cover** | **Cover Range (opt.)** | **Differ-ential** | **Diagnostic Combin- ation** |
| --- | --- | --- | --- | --- | --- | --- | --- |
|  |  |  |  |  | - |  |  |

Dynamics:

ENVIRONMENT

Environmental Description:

DISTRIBUTION

*Geographic Range:

Nations: AR, BR, PY

States/Provinces:

USFS Ecoregions (2007) [optional]:

Omernik Ecoregions L3, L4 [optional]:

MLRAs [optional]:

PLOT SAMPLING AND ANALYSIS

*Plot Analysis Summary [Med - High Confidence]:

*Plots Used to Define the Type [Med - High Confidence]:

CONFIDENCE LEVEL

USNVC Confidence Level: Unassigned

USNVC Confidence Comments [optional]:

HIERARCHY

*Lower Level NVC Types:

| **Elcode** | **Scientific or Colloquial Name** |
| --- | --- |
|  |  |

DISCUSSION

Discussion [optional]:

CONCEPT HISTORY

*Recent Concept Lineage [if applicable]:

| **Date** | **Predecessor** | **Note** |
| --- | --- | --- |
|  |  |  |

RELATED CONCEPTS

Supporting Concepts [optional]:

| **Relationship to NVC** | **Supporting Concept Name** | **Short Citation** | **Note** |
| --- | --- | --- | --- |
|  |  |  |  |

AUTHORSHIP

*Primary Concept Source [if applicable]: C. Josse, in Faber-Langendoen et al. (2014)

| **Relationship to NVC** | **Name Used in Source** | **Short Citation** | **Note** |
| --- | --- | --- | --- |
|  |  |  |  |

*Author of Description: C. Josse

Acknowledgments [optional]:

Version Date: 17 Apr 2014

REFERENCES

*References [Required if used in text]:

Faber-Langendoen, D., J. Drake, S. Gawler, M. Hall, C. Josse, G. Kittel, S. Menard, C. Nordman, M. Pyne, M. Reid, L. Sneddon, K. Schulz, J. Teague, M. Russo, K. Snow, and P. Comer, editors. 2010-2018. Divisions, Macrogroups and Groups for the Revised U.S. National Vegetation Classification. NatureServe, Arlington, VA. plus appendices. [in preparation]

1.A.3. Tropical Montane Humid Forest

Tropical Montane Humid Forest is dominated by broad-leaved evergreen trees, with increasingly small leaves and stems, often gnarly, with dense crowns as elevations increase. These forests are generally found within 23°N and S latitude of the equator between 1000 and 3500 m in elevation.

1. Forest & Woodland

1.A.3.Eg. Caribbean-Mesoamerican Montane Humid Forest

D228. Caribbean-Mesoamerican Montane Humid Forest

Type Concept Sentence:

OVERVIEW

*Hierarchy Level: Division

*Placement in Hierarchy: 1.A.3.Eg. Tropical Montane Humid Forest (F004)

Elcode: D228

*Scientific Name: Caribbean-Mesoamerican Montane Humid Forest Division

*Common (Translated Scientific) Name: Caribbean-Mesoamerican Montane Humid Forest Division

*Colloquial Name: Caribbean-Mesoamerican Montane Humid Forest

*Type Concept:

*Diagnostic Characteristics:

*Classification Comments:

*Similar NVC Types [if applicable]:

| **Elcode** | **Scientific or Colloquial Name** | **Note** |
| --- | --- | --- |
|  |  |  |

Similar NVC Types General Comments [optional]:

VEGETATION

Physiognomy and Structure Summary:

Physiognomy and Structure Table [optional]:

| **Physiognomy-Structure Category** | **Prevailing Height (m)** | **Height Range (opt.)** | **Mean % Cover** | **Cover Range (opt.)** |
| --- | --- | --- | --- | --- |
|  |  |  |  | - |

Floristics Summary:

*Floristics Table [Med - High Confidence]:

*Number of Plots: *Cover Scale Used:

| **Physiognomy-Structure Category** | **Taxon Name** | **Specific Growth Form (opt.)** | **Const- ancy** | **Mean % Cover** | **Cover Range (opt.)** | **Differ-ential** | **Diagnostic Combin- ation** |
| --- | --- | --- | --- | --- | --- | --- | --- |
|  |  |  |  |  | - |  |  |

Dynamics:

ENVIRONMENT

Environmental Description:

DISTRIBUTION

*Geographic Range:

Nations: BR?, CR, CU, DO, GT, HN, HT, JM, KN, MQ, MX, NI, PA, PR, SV, XC, XD

States/Provinces:

USFS Ecoregions (2007) [optional]:

Omernik Ecoregions L3, L4 [optional]:

MLRAs [optional]:

PLOT SAMPLING AND ANALYSIS

*Plot Analysis Summary [Med - High Confidence]:

*Plots Used to Define the Type [Med - High Confidence]:

CONFIDENCE LEVEL

USNVC Confidence Level: Low - Poorly Documented

USNVC Confidence Comments [optional]:

HIERARCHY

*Lower Level NVC Types:

| **Elcode** | **Scientific or Colloquial Name** |
| --- | --- |
| M598 | Caribbean Montane Humid Forest |
| M601 | Mesoamerican Montane Pine-Oak Forest |
| M600 | Mesoamerican Montane Humid Forest |
| M602 | Southern Mesoamerican Montane Humid Forest |

DISCUSSION

Discussion [optional]:

CONCEPT HISTORY

*Recent Concept Lineage [if applicable]:

| **Date** | **Predecessor** | **Note** |
| --- | --- | --- |
| 2013-01-04 | D090 Caribbean & Central American Montane & Cloud Forest Division | replaced |

RELATED CONCEPTS

Supporting Concepts [optional]:

| **Relationship to NVC** | **Supporting Concept Name** | **Short Citation** | **Note** |
| --- | --- | --- | --- |
|  |  |  |  |

AUTHORSHIP

*Primary Concept Source [if applicable]:

| **Relationship to NVC** | **Name Used in Source** | **Short Citation** | **Note** |
| --- | --- | --- | --- |
|  |  |  |  |

*Author of Description:

Acknowledgments [optional]:

Version Date:

REFERENCES

*References [Required if used in text]:

Faber-Langendoen, D., J. Drake, S. Gawler, M. Hall, C. Josse, G. Kittel, S. Menard, C. Nordman, M. Pyne, M. Reid, L. Sneddon, K. Schulz, J. Teague, M. Russo, K. Snow, and P. Comer, editors. 2010-2018. Divisions, Macrogroups and Groups for the Revised U.S. National Vegetation Classification. NatureServe, Arlington, VA. plus appendices. [in preparation]

1. Forest & Woodland

1.A.3.Eg. Caribbean-Mesoamerican Montane Humid Forest

M598. Caribbean Montane Humid Forest

Type Concept Sentence: In the Caribbean, forests between 700 and 1600 m altitude, grow on mountain summits that penetrate the base of trade wind clouds. Therefore, most of these forests are cloud forests. The cloud forest, variously called elfin woodland, mossy forest, montane thicket, or dwarf forest, is characterized by gnarled, open-crowned trees less than 7 m tall, high stem density, high basal area, small diameters, and slow growth rates, with greater abundance of epiphytes, palms, and tree ferns than lowland forests. Leaves tend to be coriaceous and grouped toward the ends of the branches. Dwarf stature of trees may be attributed to strong winds and water-saturated soils. Tree roots form a tight mat on the surface.

OVERVIEW

*Hierarchy Level: Macrogroup

*Placement in Hierarchy: 1.A.3.Eg. Caribbean-Mesoamerican Montane Humid Forest (D228)

Elcode: M598

*Scientific Name: Caribbean Montane Humid Forest Macrogroup

*Common (Translated Scientific) Name: Caribbean Montane Humid Forest Macrogroup

*Colloquial Name: Caribbean Montane Humid Forest

*Type Concept:

*Diagnostic Characteristics:

*Classification Comments:

*Similar NVC Types [if applicable]:

| **Elcode** | **Scientific or Colloquial Name** | **Note** |
| --- | --- | --- |
|  |  |  |

Similar NVC Types General Comments [optional]:

VEGETATION

Physiognomy and Structure Summary:

Physiognomy and Structure Table [optional]:

| **Physiognomy-Structure Category** | **Prevailing Height (m)** | **Height Range (opt.)** | **Mean % Cover** | **Cover Range (opt.)** |
| --- | --- | --- | --- | --- |
|  |  |  |  | - |

Floristics Summary:

*Floristics Table [Med - High Confidence]:

*Number of Plots:

*Cover Scale Used:

| **Physiognomy-Structure Category** | **Taxon Name** | **Specific Growth Form (opt.)** | **Const- ancy** | **Mean % Cover** | **Cover Range (opt.)** | **Differ-ential** | **Diagnostic Combin- ation** |
| --- | --- | --- | --- | --- | --- | --- | --- |
|  |  |  |  |  | - |  |  |

Dynamics: Landslides and hurricanes are the key triggers of dynamic processes of these forests. Substrate and topography and their interaction with the vegetation are the most important factors for the survival of these forests during hurricanes - probably the single most important natural trigger of the successional dynamic. Surviving trees have their roots securely anchored in the substrate. These factors are also critical for regulating surface runoff and maintaining the water balance under very humid conditions on exposed ridges and steep slopes. Forest recovery after disturbance is slow. Monitoring of dwarf forest in Puerto Rico's Luquillo Mountains showed that it can take up to 20 years for woody species to establish and after that their growth rate is very slow. It took almost 35 years until the canopy closing decreased the grass and fern cover (Weaver 2008). Moreover, the succession process is often subjected to setbacks due to periodic hurricane disturbance. This study also showed that hurricanes cause delayed mortality, with declines in biomass and stem numbers exceeding ingrowth during 15 years after Hurricane Hugo hit. Another important finding of this study is that more than half of the arborescent species growing in dwarf forest, where they play a prominent role in post disturbance recovery, are endemic to Puerto Rico (Weaver 2008). Cloud forests are known as places of high endemism but not necessarily as areas with rich biotas (Weaver 2000, 2008).

ENVIRONMENT

Environmental Description: Ecosystems of this macrogroup occur above 700 m elevation in areas with mean annual precipitation >1600 mm, frequently or seasonally surrounded by clouds, and on different topographies but mostly slopes, exposed ridges, and ravines. Forests growing on exposed areas are of smaller stature and very dense. Taller forests grow on protected areas on lower slopes to the leeward of ridges or spurs. With montane forests, one of the most critical climatic factors is the frequency and duration of the cloud cover; condensation can contribute 10% or more of the precipitation amount that these forests receive. In the Caribbean, the trade winds forming clouds have saline components which have an effect on the chemistry of the ecophysiology of these forests. Cloud cover causes less solar radiation, lower temperatures, decreased transpiration and lower photosynthetic rates, resulting in lower growth rates and lower nutrient-cycling rates. The efficiency shown by these forests in the use of nutrients is high though, which is important to avoid nutrient loss due to leaching (Silver et al. 2001).

DISTRIBUTION

*Geographic Range: This type of forest is distributed in the Caribbean islands with mountains above 600-700 m elevation and on different geologies and substrates. This system is found in Cuba, Dominican Republic, Jamaica, Puerto Rico and mountainous islands of the Lesser Antilles.

Nations: CU, DO, HT, JM, KN, MQ, PR, XC, XD, XE

States/Provinces:

USFS Ecoregions (2007) [optional]:

Omernik Ecoregions L3, L4 [optional]:

MLRAs [optional]:

PLOT SAMPLING AND ANALYSIS

*Plot Analysis Summary [Med - High Confidence]:

*Plots Used to Define the Type [Med - High Confidence]:

CONFIDENCE LEVEL

USNVC Confidence Level: Low - Poorly Documented

USNVC Confidence Comments [optional]:

HIERARCHY

*Lower Level NVC Types:

| **Elcode** | **Scientific or Colloquial Name** |
| --- | --- |
| G451 | Caribbean Montane Cloud Forest & Scrub |
| G449 | Caribbean Montane Rocky Riverine Scrub |
| G446 | Caribbean Moist Montane Mixed Pine - Broad-leaved Forest |
| G448 | Caribbean Wet Montane Forest |
| G447 | Caribbean Wet Montane Serpentine Forest & Scrub |
| G846 | Caribbean Montane Ruderal Forest |

DISCUSSION

Discussion [optional]:

CONCEPT HISTORY

*Recent Concept Lineage [if applicable]:

| **Date** | **Predecessor** | **Note** |
| --- | --- | --- |
| 2013-05-10 | M599 Caribbean Montane Pine Forest Macrogroup | M599 concept covered by M598 |

RELATED CONCEPTS

Supporting Concepts [optional]:

| **Relationship to NVC** | **Supporting Concept Name** | **Short Citation** | **Note** |
| --- | --- | --- | --- |
|  |  |  |  |

AUTHORSHIP

*Primary Concept Source [if applicable]:

| **Relationship to NVC** | **Name Used in Source** | **Short Citation** | **Note** |
| --- | --- | --- | --- |
|  |  |  |  |

*Author of Description: C. Josse

Acknowledgments [optional]:

Version Date: 08 Jan 2015

REFERENCES

*References [Required if used in text]:

Faber-Langendoen, D., J. Drake, S. Gawler, M. Hall, C. Josse, G. Kittel, S. Menard, C. Nordman, M. Pyne, M. Reid, L. Sneddon, K. Schulz, J. Teague, M. Russo, K. Snow, and P. Comer, editors. 2010-2018. Divisions, Macrogroups and Groups for the Revised U.S. National Vegetation Classification. NatureServe, Arlington, VA. plus appendices. [in preparation]

Silver, W. L., E. Marin-Spiotta, and A. E. Lugo. 2001. El Caribe. En: M. Kappelle and A. D. Brown, editors. Bosques nublados del Neotrópico. Instituto Nacional de Biodiversidad, INBio, Santo Domingo de Heredia, Costa Rica. 704 pp.

TNC [The Nature Conservancy]. 2004a. Greater Caribbean Ecoregional Plan. An ecoregional plan for Puerto Rico: Portfolio design. Unpublished report. The Nature Conservancy, Arlington, VA.

Weaver, P. L. 2000. Elfin woodland recovery 30 years after a plane wreck in Puerto Rico's Luquillo Mountains. Caribbean Journal of Science 36(1-2):1-9.

Weaver, P. L. 2008. Dwarf forest recovery after disturbance in the Luquillo Mountains of Puerto Rico. Caribbean Journal of Science 44(2):150-163.

1. Forest & Woodland

1.A.3.Eg. Caribbean-Mesoamerican Montane Humid Forest

M601. Mesoamerican Montane Pine-Oak Forest

Type Concept Sentence: En Mesoamérica, los bosques de pino-encino se encuentran en México, Guatemala, Honduras y el norte de Nicaragua. Los árboles de pino nunca cruzaron la depresión nicaragüense durante la migración desde las regiones Holárticas hacia el Neotrópico.

In Meso-America, pine-oak forests are found in Mexico, Guatemala, Honduras and northern Nicaragua. Pine trees never crossed the Nicaraguan depression during migration from Holarctic regions into the Neotropics.

OVERVIEW

*Hierarchy Level: Macrogroup

*Placement in Hierarchy: 1.A.3.Eg. Caribbean-Mesoamerican Montane Humid Forest (D228)

Elcode: M601

*Scientific Name: Mesoamerican Montane Pine-Oak Forest Macrogroup

*Common (Translated Scientific) Name: Mesoamerican Montane Pine-Oak Forest Macrogroup

*Colloquial Name: Mesoamerican Montane Pine-Oak Forest

*Type Concept:

*Diagnostic Characteristics:

*Classification Comments:

*Similar NVC Types [if applicable]:

| **Elcode** | **Scientific or Colloquial Name** | **Note** |
| --- | --- | --- |
|  |  |  |

Similar NVC Types General Comments [optional]:

VEGETATION

Physiognomy and Structure Summary:

Physiognomy and Structure Table [optional]:

| **Physiognomy-Structure Category** | **Prevailing Height (m)** | **Height Range (opt.)** | **Mean % Cover** | **Cover Range (opt.)** |
| --- | --- | --- | --- | --- |
|  |  |  |  | - |

Floristics Summary:

*Floristics Table [Med - High Confidence]:

*Number of Plots:

*Cover Scale Used:

| **Physiognomy-Structure Category** | **Taxon Name** | **Specific Growth Form (opt.)** | **Const- ancy** | **Mean % Cover** | **Cover Range (opt.)** | **Differ-ential** | **Diagnostic Combin- ation** |
| --- | --- | --- | --- | --- | --- | --- | --- |
|  |  |  |  |  | - |  |  |

Dynamics: Climate Regime (vertical and horizontal precipitation, temperature): Determines the occurrence and distribution of dominant flora and sets the boundaries for adjacent vegetation type. Slope stability and landslide regime: Slope stability determines the frequency of landslides triggered by earthquakes and high rainfalls. Landslide regime in turn determines landslide disturbance patterns and creates landscape heterogeneity. Fire regime: Affects vegetation structure. Affects vegetation or faunal composition. With altered fire regime, Increase pine's susceptibility to diseases and pests. Succession after disturbance (storms, hurricanes, landslides): Increases heterogeneity of vegetation structure and between-habitat (beta) diversity, important for patch dynamics. Hydrological regime and fluvial dynamics: Along with the cover and structure of the vegetation, hydrological regime and fluvial dynamics determine the water yield and runoff rates. Fluvial dynamics associated with large rainfall events provides mineral seedbeds for pine regeneration. Viable populations of frugivorous and granivorous species: Regulate seed dispersal of pioneer, secondary and primary forest tree and shrub species. Acorn specific and pine seed specific in pine/oak forest. Viable populations of mycorrhiza and fungal decomposers: Maintain decomposition and symbiotic relations with key tree species such as oak. Maintain the nutrient availability to species at ground level in closed forest. Vegetation structure (size, age class, strata): Provide diversity in micro-habitats and niches. Mean and Maximum Fire Disturbance Area: Fire is the principal disturbance regime and occurs with regularity.

ENVIRONMENT

Environmental Description:

DISTRIBUTION

*Geographic Range: Mexico, Guatemala, Honduras and northern Nicaragua.

Nations: BR?, GT, HN, MX, NI, SV

States/Provinces:

USFS Ecoregions (2007) [optional]:

Omernik Ecoregions L3, L4 [optional]:

MLRAs [optional]:

PLOT SAMPLING AND ANALYSIS

*Plot Analysis Summary [Med - High Confidence]:

*Plots Used to Define the Type [Med - High Confidence]:

CONFIDENCE LEVEL

USNVC Confidence Level: Unassigned

USNVC Confidence Comments [optional]:

HIERARCHY

*Lower Level NVC Types:

| **Elcode** | **Scientific or Colloquial Name** |
| --- | --- |
|  |  |

DISCUSSION

Discussion [optional]:

CONCEPT HISTORY

*Recent Concept Lineage [if applicable]:

| **Date** | **Predecessor** | **Note** |
| --- | --- | --- |
|  |  |  |

RELATED CONCEPTS

Supporting Concepts [optional]:

| **Relationship to NVC** | **Supporting Concept Name** | **Short Citation** | **Note** |
| --- | --- | --- | --- |
|  |  |  |  |

AUTHORSHIP

*Primary Concept Source [if applicable]:

| **Relationship to NVC** | **Name Used in Source** | **Short Citation** | **Note** |
| --- | --- | --- | --- |
|  |  |  |  |

*Author of Description:

Acknowledgments [optional]:

Version Date: 08 Jan 2015

REFERENCES

*References [Required if used in text]:

Darrow, W. K., and T. A. Zanoni. 1993. El pino de La Española (Pinus occidentalis Swartz): Un pino subtropical poco conocido de potencial económico. Moscosoa 7:15-37.

Faber-Langendoen, D., J. Drake, S. Gawler, M. Hall, C. Josse, G. Kittel, S. Menard, C. Nordman, M. Pyne, M. Reid, L. Sneddon, K. Schulz, J. Teague, M. Russo, K. Snow, and P. Comer, editors. 2010-2018. Divisions, Macrogroups and Groups for the Revised U.S. National Vegetation Classification. NatureServe, Arlington, VA. plus appendices. [in preparation]

Horn, S. P., K. Orvis, L. M. Kennedy, and M. Clark. 2000. Prehistoric fires in the highlands of the Dominican Republic: Evidence from charcoal in soils and sediments. Caribbean Journal of Science 36:10-18.

1. Forest & Woodland

1.A.3.Eg. Caribbean-Mesoamerican Montane Humid Forest

M600. Mesoamerican Montane Humid Forest

Type Concept Sentence: Broadly defined as tropical forests occurring in lower montane (1000-2500 m elevation), upper montane (2500-3500 m), and subalpine (3500-3800 m) with mean annual precipitation between 1000 and 5000 mm and high relative humidity, with high proportions contributed by cloud or mist.

OVERVIEW

*Hierarchy Level: Macrogroup

*Placement in Hierarchy: 1.A.3.Eg. Caribbean-Mesoamerican Montane Humid Forest (D228)

Elcode: M600

*Scientific Name: Mesoamerican Montane Humid Forest Macrogroup

*Common (Translated Scientific) Name: Mesoamerican Montane Humid Forest Macrogroup

*Colloquial Name: Mesoamerican Montane Humid Forest

*Type Concept:

*Diagnostic Characteristics:

*Classification Comments:

*Similar NVC Types [if applicable]:

| **Elcode** | **Scientific or Colloquial Name** | **Note** |
| --- | --- | --- |
|  |  |  |

Similar NVC Types General Comments [optional]:

VEGETATION

Physiognomy and Structure Summary:

Physiognomy and Structure Table [optional]:

| **Physiognomy-Structure Category** | **Prevailing Height (m)** | **Height Range (opt.)** | **Mean % Cover** | **Cover Range (opt.)** |
| --- | --- | --- | --- | --- |
|  |  |  |  | - |

Floristics Summary:

*Floristics Table [Med - High Confidence]:

*Number of Plots:

*Cover Scale Used:

| **Physiognomy-Structure Category** | **Taxon Name** | **Specific Growth Form (opt.)** | **Const- ancy** | **Mean % Cover** | **Cover Range (opt.)** | **Differ-ential** | **Diagnostic Combin- ation** |
| --- | --- | --- | --- | --- | --- | --- | --- |
|  |  |  |  |  | - |  |  |

Dynamics: Régimen deslizamiento de tierra: múltiple por año de extensión areal variable crea patrón de vegetación. Sucesión tras deslizamiento de tierra: (de Ecuador Andes) géneros intolerantes a la sombra que se mantienen en derrumbes recientes, incluyendo *Blechnum, Equisetum, Piper, Baccharis, Senecio, Miconia*, y *Chusquea*. Régimen hidrológico: flujo / descarga o régimen de la erosión: la producción de agua y la tasa de escorrentía varía según la ubicación. Componentes faunísticos clave importantes para las redes alimentarias incluyen: roedores, colibríes, ranas, arañas y moluscos terrestres. Interacciones bióticas incluyen: tanques de bromelias de apoyo rica fauna incluyendo renacuajos e insectos. Agentes de polinización y dispersión: la diversidad de abejas y lepidópteros y taxones de plantas adaptadas a dípteros y la polinización colibrí; por ejemplo, los colibríes de Trochilinae abundantes en 1000-2000m elevación son polinizadores importantes para *Centropogon, Fuchsia, Vaccinieae*, y bromelias, los dípteros importantes para las orquídeas pleurothallid. Top depredadores regulan pequeñas poblaciones de mamíferos que red alimentaria impacto.

Landslide regime: multiple per year of variable areal extent, creates vegetation pattern. Succession following landslide: (from Ecuadoran Andes) shade-intolerant genera maintained in recent landslides, including *Blechnum, Equisetum, Piper, Baccharis, Senecio, Miconia*, and *Chusquea*. Hydrologic regime: flow/discharge or erosion regime: water yield and runoff rate varies by location. Key faunal components important for food webs include rodents, hummingbirds, frogs, spiders and terrestrial molluscs. Biotic interactions include bromeliad tanks supporting rich fauna including tadpoles and insects. Pollination and dispersal agents: diversity of bees and lepidopterans and plant taxa adapted to dipteran and hummingbird pollination; e.g., hummingbirds from Trochilinae abundant at 1000-2000m elevation are important pollinators for *Centropogon, Fuchsia, Vaccinieae*, and bromeliads, the dipterans important for pleurothallid orchids. Top predators regulate small mammal populations that impact food web.

ENVIRONMENT

Environmental Description: Régimen de humedad atmosférica: la precipitación media anual: 2000-4000mm; la niebla / nubes contribuyen 5-20% de la precipitación anual, alta humedad relativa anual >90%, con una media de temperatura anual: 6-22C. El relieve topográfico (posición de inclinación y estabilidad): Forma de relieve variable (acantilado, cresta, cóncavo / convexo pendiente más altos bosques nublados a menudo se encuentran en la posición media ladera con pendiente moderada y suelos más profundos.

Atmospheric moisture regime: mean annual rainfall: 2000-4000 mm; mist/clouds contributing 5-20% of annual precipitation. High annual relative humidity >90%. Mean annual temperature is 6-22°C. Topographic relief (slope position and stability): Variable landform (cliff, ridge, concave/convex slope). Tallest cloud forests often found on midslope position with moderate steepness and deeper soils.

DISTRIBUTION

*Geographic Range: Sierra Madre Oriental of Mexico south to Honduras and Nicaragua

Nations: CR, GT, HN, MX, NI, PA

States/Provinces:

USFS Ecoregions (2007) [optional]:

Omernik Ecoregions L3, L4 [optional]:

MLRAs [optional]:

PLOT SAMPLING AND ANALYSIS

*Plot Analysis Summary [Med - High Confidence]:

*Plots Used to Define the Type [Med - High Confidence]:

CONFIDENCE LEVEL

USNVC Confidence Level: Unassigned

USNVC Confidence Comments [optional]:

HIERARCHY

*Lower Level NVC Types:

| **Elcode** | **Scientific or Colloquial Name** |
| --- | --- |
|  |  |

DISCUSSION

Discussion [optional]:

CONCEPT HISTORY

*Recent Concept Lineage [if applicable]:

| **Date** | **Predecessor** | **Note** |
| --- | --- | --- |
|  |  |  |

RELATED CONCEPTS

Supporting Concepts [optional]:

| **Relationship to NVC** | **Supporting Concept Name** | **Short Citation** | **Note** |
| --- | --- | --- | --- |
|  |  |  |  |

AUTHORSHIP

*Primary Concept Source [if applicable]:

| **Relationship to NVC** | **Name Used in Source** | **Short Citation** | **Note** |
| --- | --- | --- | --- |
|  |  |  |  |

*Author of Description:

Acknowledgments [optional]:

Version Date: 08 Jan 2015

REFERENCES

*References [Required if used in text]:

Bruijnzeel, L. A., and J. Proctor. 1995. Hydrology and biogeochemistry of tropical montane forests: What do we really know? Page 38-78 in: L. S. Hamilton, J. O. Juvik, and F. N. Scatena, editors. Tropical montane cloud forests. Springer-Verlag, New York.

Davis, S. D., V. H. Heywood, O. Herrera-MacBryde, J. Villalobos, and A. C. Hamilton, editors. 1997. Centers of plant diversity: A guide and strategy for their conservation. Volume 3, The Americas. World Wide Fund for Nature and World Conservation Union, Cambridge, UK.

Eisenberg, J. F. 1989. Mammals of the Neotropics. The Northern Neotropics. Volume 1: Panama, Colombia, Venezuela, Guyana, Suriname, French Guiana. The University of Chicago Press, Chicago.

Faber-Langendoen, D., J. Drake, S. Gawler, M. Hall, C. Josse, G. Kittel, S. Menard, C. Nordman, M. Pyne, M. Reid, L. Sneddon, K. Schulz, J. Teague, M. Russo, K. Snow, and P. Comer, editors. 2010-2018. Divisions, Macrogroups and Groups for the Revised U.S. National Vegetation Classification. NatureServe, Arlington, VA. plus appendices. [in preparation]

Gentry, A. H. 1995b. Patterns of diversity and floristic composition in Neotropical montane forests. Pages 103-126 in: S. P. Churchill, H. Balslev, E. Forero, and J. L. Luteyn editors. Biodiversity and conservation of Neotropical montane forests. The New York Botanical Garden, Bronx.

Hamilton, L. S. 1995. Montane cloud forest conservation and research: A synopsis. Mountain Research and Development 15:259-266.

Hamilton, L. S., J. O. Jurik and F. N. Scatena, editors. 1995. Tropical montane cloud forests. Ecological Studies 110, Springer Verlag, New York.

INEGI. 2005 Guía para la interpretacion de la información cartografic: La vegetación y uso del suelo.

Long, A. J. 1995. The importance of tropical montane cloud forests for endemic and threatened birds. Pages 79-106 in: L. S. Hamilton, J. O. Juvik, and F. N. Scatena, editors. Tropical montane cloud forests. Springer-Verlag, New York.

Luteyn, J. L., and S. P. Churchill. 2000. Vegetation of the tropical Andes. Pages 281-310 in: D. L. Lentz, editor. Imperfect balance: Landscape transformations in the Precolumbian Americas. Columbia University Press, New York.

Nadkarni, N. M., and N. T. Wheelwright. 2000. Monteverde: Ecology and conservation of a tropical cloud forest. Oxford University Press, New York.

Stadtmuller, T. 1987. Los bosques nublados en el trópico húmedo. University of the United Nations (Tokyo) and Centro Agronómico Tropical de Investigación y Enseñanza CATIE. Turrialba. 85 pp.

Webster, G. L. 1995. The panorama of Neotropical cloud forests. Pages 53-77 in: S. P. Churchill, H. Balslev, E. Forero, and J. L. Luteyn, editors. Biodiversity and Conservation of Neotropical Montane Forests. The New York Botanical Garden, Bronx.

Young, K. R., and B. León. 1999. Peru's humid eastern montane forests: An overview of their physical settings, biological diversity, human use and settlement, and conservation needs. DIVA, Technical Report no 5. 97 pp.

1. Forest & Woodland

1.A.3.Eg. Caribbean-Mesoamerican Montane Humid Forest

M602. Southern Mesoamerican Montane Humid Forest

Type Concept Sentence: Los bosques de robles costarricenses son generalmente mono- o bi-específicos a nivel del dosel y albergan bambúes del género Chusquea en el sotobosque. Estos bosques son extremadamente mixtos en el sentido de que se componen tanto de elementos tropicales como templados, donde robles, alisos holárticos y conejos conviven con vegetación y fauna tropical. El volumen de madera y la biomasa son muy altos en estos bosques de roble de Costa Rica y se encuentran entre los valores más altos conocidos.

Costa Rican oak forests are generally mono- or bi-specific at the canopy level and harbor bamboos of the genus *Chusquea* in the understorey. Costa Rican oak forests are extremely mixed in the sense that they are made up of both temperate and tropical elements: holarctic oaks, alders, and rabbits occur together with tropical vegetation and fauna. Wood volume and biomass in these Costa Rican oak forests and are among the highest values known.

OVERVIEW

*Hierarchy Level: Macrogroup

*Placement in Hierarchy: 1.A.3.Eg. Caribbean-Mesoamerican Montane Humid Forest (D228)

Elcode: M602

*Scientific Name: Southern Mesoamerican Montane Humid Forest Macrogroup

*Common (Translated Scientific) Name: Southern Mesoamerican Montane Humid Forest Macrogroup

*Colloquial Name: Southern Mesoamerican Montane Humid Forest

*Type Concept:

*Diagnostic Characteristics:

*Classification Comments:

*Similar NVC Types [if applicable]:

| **Elcode** | **Scientific or Colloquial Name** | **Note** |
| --- | --- | --- |
|  |  |  |

Similar NVC Types General Comments [optional]:

VEGETATION

Physiognomy and Structure Summary:

Physiognomy and Structure Table [optional]:

| **Physiognomy-Structure Category** | **Prevailing Height (m)** | **Height Range (opt.)** | **Mean % Cover** | **Cover Range (opt.)** |
| --- | --- | --- | --- | --- |
|  |  |  |  | - |

Floristics Summary:

*Floristics Table [Med - High Confidence]:

*Number of Plots:

*Cover Scale Used:

| **Physiognomy-Structure Category** | **Taxon Name** | **Specific Growth Form (opt.)** | **Const- ancy** | **Mean % Cover** | **Cover Range (opt.)** | **Differ-ential** | **Diagnostic Combin- ation** |
| --- | --- | --- | --- | --- | --- | --- | --- |
|  |  |  |  |  | - |  |  |

Dynamics:

ENVIRONMENT

Environmental Description: Las comunidades de los bosques de roble forman una zonificación distinguible a lo largo de gradientes de elevación. En Costa Rica, desde zonas más bajas hacia arriba, el roble comienza a dominar a unos 2000 m de altitud. Por encima de 2.000 m, en las partes altas del bosque montano bajo, el roble se encuentra como un género codominante, junto con especies de los géneros Ocotea y Nectandra. Por encima de c. 2300 m, hasta c. 3000 m, en las partes más bajas de la comunidad montano alta, el roble domina la capa de dosel (35 a 45 m) y se asocia con géneros montano bajos (por ejemplo, *Tovomitopsis, Hyeronima, Guarea, Sapium, Billia, Alfaroa* y *Phoebe*) en el la capa del subdosel (hasta 20 m) y con las palmas enanas (por ejemplo, *Chamaedorea* y *Geonoma*) y gesneriáceas en el sotobosque. Los bosques de roble son a menudo dominados por especies de *Quercus, Podocarpus* (una conífera) y *Magnolia* en el dosel, y *Weinmannia, Vaccinium, Viburnum, Ocotea, Prunus, Styrax, Symplocos, Cornus, Ilex, Miconia*, y Clusia en el subdosel. Aquí, el bambú *Chusquea* caracteriza el sotobosque. Por encima de c. 3000 m, hasta un máximo de 3.400 m, el bosque montano alto y las comunidades subalpinas de roble (altura máx. de 15 a 20 m) se acompaña del arbusto ericoide Comarostaphylis y géneros leñosos como *Schefflera, Gaiadendron, Drymis, Weinmannia, Vaccinium, Brunellia, Buddleja, Escallonia* y *Miconia*. Las epífitas abundan en todas las comunidades de los bosques de roble de montaña. Sin embargo, las orquídeas y bromelias epifitas dominan entre c. 2000 y c. 3000 m, mientras que los briófitos y líquenes se encuentran principalmente entre c. 2500 y 3400 m.

Oak forest communities form distinguishable zonation along elevation gradients. In Costa Rica, from lower elevations upward, oak starts to dominate at about 2000 m. Above 2000 m, in the upper parts of lower montane communities, oak is found as a codominant genus, together with lauraceaous species of genera *Ocotea* and *Nectandra*. Above about 2300 m and up to about 3000 m, in the lower parts of upper montane communities, oak dominates the canopy layer (35-45 m) and is associated with lower montane genera (e.g., *Alfaroa, Billia, Guarea, Hyeronima, Phoebe, Sapium, Tovomitopsis*) in the subcanopy layer (up to 20 m) and with dwarf palms (e.g., *Chamaedorea* and *Geonoma*) and gesneriads in the understorey. The oak forests are often dominated by species of *Quercus, Podocarpus* (a conifer), and *Magnolia* in the canopy, and *Cornus, Ilex, Miconia, Ocotea, Prunus, Styrax, Symplocos, Vaccinium, Viburnum, Weinmannia*, and the strangler *Clusia* in the subcanopy. Here, the *Chusquea* bamboo characterizes the understory. Above about 3000 m and up to a maximum of 3400 m, in the upper montane and subalpine communities, oak (maximum height 15-20 m) is accompanied by the ericad *Comarostaphylis* and genera such as *Brunellia, Buddleja, Drymis, Escallonia, Gaiadendron, Miconia, Schefflera, Vaccinium*, and *Weinmannia*. Epiphytes abound in all montane oak forest communities. However, epiphytic orchids and bromeliads dominate between about 2000 and 3000 m, while bryophytes and lichens are mainly found between about 2500 and 3400 m.

DISTRIBUTION

*Geographic Range:

Nations: CR, PA

States/Provinces:

USFS Ecoregions (2007) [optional]:

Omernik Ecoregions L3, L4 [optional]:

MLRAs [optional]:

PLOT SAMPLING AND ANALYSIS

*Plot Analysis Summary [Med - High Confidence]:

*Plots Used to Define the Type [Med - High Confidence]:

CONFIDENCE LEVEL

USNVC Confidence Level: Unassigned

USNVC Confidence Comments [optional]:

HIERARCHY

*Lower Level NVC Types:

| **Elcode** | **Scientific or Colloquial Name** |
| --- | --- |
|  |  |

DISCUSSION

Discussion [optional]:

CONCEPT HISTORY

*Recent Concept Lineage [if applicable]:

| **Date** | **Predecessor** | **Note** |
| --- | --- | --- |
|  |  |  |

RELATED CONCEPTS

Supporting Concepts [optional]:

| **Relationship to NVC** | **Supporting Concept Name** | **Short Citation** | **Note** |
| --- | --- | --- | --- |
|  |  |  |  |

AUTHORSHIP

*Primary Concept Source [if applicable]:

| **Relationship to NVC** | **Name Used in Source** | **Short Citation** | **Note** |
| --- | --- | --- | --- |
|  |  |  |  |

*Author of Description:

Acknowledgments [optional]:

Version Date: 08 Jan 2015

REFERENCES

*References [Required if used in text]:

Faber-Langendoen, D., J. Drake, S. Gawler, M. Hall, C. Josse, G. Kittel, S. Menard, C. Nordman, M. Pyne, M. Reid, L. Sneddon, K. Schulz, J. Teague, M. Russo, K. Snow, and P. Comer, editors. 2010-2018. Divisions, Macrogroups and Groups for the Revised U.S. National Vegetation Classification. NatureServe, Arlington, VA. plus appendices. [in preparation]

Horn, S. P., K. Orvis, L. M. Kennedy, and M. Clark. 2000. Prehistoric fires in the highlands of the Dominican Republic: Evidence from charcoal in soils and sediments. Caribbean Journal of Science 36:10-18.

Kappelle, M. 1996. Los Bosques de Roble (Quercus) de la Cordillera de Talamanca, Costa Rica: Biodiversidad, Ecología, Conservación y Desarrollo. Universidad de Amsterdam y Instituto Nacional de Biodiversidad (INBio). Amsterdam - Santo Domingo de Heredia, Costa Rica. 336 pp.

Kappelle, M., and N. Zamora. 1995. Changes in woody species richness along an altitudinal gradient in Talamancan montane Quercus forest, Costa Rica. Pages 135-148 in: S. P. Churchill, H. Balslev, E. Forero and J. L. Luteyn, editors. Biodiversity and conservation of Neotropical montane forests. The New York Botanical Garden, Bronx.

1. Forest & Woodland

1.A.3.Eh. Guianan Montane Humid Forest

D229. Guianan Montane Humid Forest

Type Concept Sentence:

OVERVIEW

*Hierarchy Level: Division

*Placement in Hierarchy: 1.A.3.Eh. Tropical Montane Humid Forest (F004)

Elcode: D229

*Scientific Name: Guianan Montane Humid Forest Division

*Common (Translated Scientific) Name: Guianan Montane Humid Forest Division

*Colloquial Name: Guianan Montane Humid Forest

*Type Concept:

*Diagnostic Characteristics:

*Classification Comments:

*Similar NVC Types [if applicable]:

| **Elcode** | **Scientific or Colloquial Name** | **Note** |
| --- | --- | --- |
|  |  |  |

Similar NVC Types General Comments [optional]:

VEGETATION

Physiognomy and Structure Summary:

Physiognomy and Structure Table [optional]:

| **Physiognomy-Structure Category** | **Prevailing Height (m)** | **Height Range (opt.)** | **Mean % Cover** | **Cover Range (opt.)** |
| --- | --- | --- | --- | --- |
|  |  |  |  | - |

Floristics Summary:

*Floristics Table [Med - High Confidence]:

*Number of Plots: *Cover Scale Used:

| **Physiognomy-Structure Category** | **Taxon Name** | **Specific Growth Form (opt.)** | **Const- ancy** | **Mean % Cover** | **Cover Range (opt.)** | **Differ-ential** | **Diagnostic Combin- ation** |
| --- | --- | --- | --- | --- | --- | --- | --- |
|  |  |  |  |  | - |  |  |

Dynamics:

ENVIRONMENT

Environmental Description:

DISTRIBUTION

*Geographic Range:

Nations: BR, GF, GY, SR, VE

States/Provinces:

USFS Ecoregions (2007) [optional]:

Omernik Ecoregions L3, L4 [optional]:

MLRAs [optional]:

PLOT SAMPLING AND ANALYSIS

*Plot Analysis Summary [Med - High Confidence]:

*Plots Used to Define the Type [Med - High Confidence]:

CONFIDENCE LEVEL

USNVC Confidence Level: Unassigned

USNVC Confidence Comments [optional]:

HIERARCHY

*Lower Level NVC Types:

| **Elcode** | **Scientific or Colloquial Name** |
| --- | --- |
| M604 | Eastern Guianan Montane Humid Forest |
| M603 | Central Guianan Montane Humid Forest |

DISCUSSION

Discussion [optional]:

CONCEPT HISTORY

*Recent Concept Lineage [if applicable]:

| **Date** | **Predecessor** | **Note** |
| --- | --- | --- |
|  |  |  |

RELATED CONCEPTS

Supporting Concepts [optional]:

| **Relationship to NVC** | **Supporting Concept Name** | **Short Citation** | **Note** |
| --- | --- | --- | --- |
|  |  |  |  |

AUTHORSHIP

*Primary Concept Source [if applicable]:

| **Relationship to NVC** | **Name Used in Source** | **Short Citation** | **Note** |
| --- | --- | --- | --- |
|  |  |  |  |

*Author of Description:

Acknowledgments [optional]:

Version Date:

REFERENCES

*References [Required if used in text]:

Faber-Langendoen, D., J. Drake, S. Gawler, M. Hall, C. Josse, G. Kittel, S. Menard, C. Nordman, M. Pyne, M. Reid, L. Sneddon, K. Schulz, J. Teague, M. Russo, K. Snow, and P. Comer, editors. 2010-2018. Divisions, Macrogroups and Groups for the Revised U.S. National Vegetation Classification. NatureServe, Arlington, VA. plus appendices. [in preparation]

1. Forest & Woodland

1.A.3.Eh. Guianan Montane Humid Forest

M604. Eastern Guianan Montane Humid Forest

Type Concept Sentence: Complex, multi-strata rainforests occurring on metamorphic hills in the central and southern parts of the Eastern Guiana Shield in Guyana, Suriname and Brazil at 500 to 1200 m elevation. These forests can be found on narrow ridges with steep slopes or, more often, on broad and flat plateaus with shallow soils with lateritic gravel. Slope forests are on deep clay soils and are taller and denser. Plateau forests have a more open canopy with a lower stature. Characteristic species are *Amphirrhox longifolia, Aspidosperma* spp., *Spermacoce alata, Chimarrhis* sp., *Faramea lourteigiana, Leandra agrestis, Leandra divaricata, Parinari rodoplphii, Petrea macrostachya, Rheedia macrophylla, Rinorea* spp., *Stylosanthes guianensis*, and *Trattinnickia* spp.

OVERVIEW

*Hierarchy Level: Macrogroup

*Placement in Hierarchy: 1.A.3.Eh. Guianan Montane Humid Forest (D229)

Elcode: M604

*Scientific Name: Eastern Guianan Montane Humid Forest Macrogroup

*Common (Translated Scientific) Name: Eastern Guianan Montane Humid Forest Macrogroup

*Colloquial Name: Eastern Guianan Montane Humid Forest

*Type Concept: Complex, multi-strata rainforest distributed on metamorphic hills scattered in the central and southern parts of eastern Guiana, in Guyana, Suriname and Brazil between 500 and 1200 m elevation. The ridges can be narrow and very steep, but more often are broad and flat plateaus with shallow soils with lateritic gravel. The slopes are deep clay soils and support the taller, more dense forest, while the tops have a more open-canopy, lower forest. Characteristic species and genera of this forest are *Chimarrhis* sp., *Parinari rodoplphii, Pouteria* spp., *Trattinnickia* spp., *Aspidosperma* spp., *Rheedia macrophylla, Amphirrhox longifolia, Rinorea* spp., *Petrea macrostachya, Stylosanthes guianensis, Faramea lourteigiana, Psychotria pungens, Psychotria uliginosa, Leandra agrestis, Leandra divaricata, Spermacoce alata (= Borreria alata)*.

*Diagnostic Characteristics:

*Classification Comments:

*Similar NVC Types [if applicable]:

| **Elcode** | **Scientific or Colloquial Name** | **Note** |
| --- | --- | --- |
|  |  |  |

Similar NVC Types General Comments [optional]:

VEGETATION

Physiognomy and Structure Summary:

Physiognomy and Structure Table [optional]:

| **Physiognomy-Structure Category** | **Prevailing Height (m)** | **Height Range (opt.)** | **Mean % Cover** | **Cover Range (opt.)** |
| --- | --- | --- | --- | --- |
|  |  |  |  | - |

Floristics Summary:

*Floristics Table [Med - High Confidence]:

*Number of Plots:

*Cover Scale Used:

| **Physiognomy-Structure Category** | **Taxon Name** | **Specific Growth Form (opt.)** | **Const- ancy** | **Mean % Cover** | **Cover Range (opt.)** | **Differ-ential** | **Diagnostic Combin- ation** |
| --- | --- | --- | --- | --- | --- | --- | --- |
|  |  |  |  |  | - |  |  |

Dynamics:

ENVIRONMENT

Environmental Description:

DISTRIBUTION

*Geographic Range:

Nations: BR?, GF, GY, SR, VE

States/Provinces:

USFS Ecoregions (2007) [optional]:

Omernik Ecoregions L3, L4 [optional]:

MLRAs [optional]:

PLOT SAMPLING AND ANALYSIS

*Plot Analysis Summary [Med - High Confidence]:

*Plots Used to Define the Type [Med - High Confidence]:

CONFIDENCE LEVEL

USNVC Confidence Level: Unassigned

USNVC Confidence Comments [optional]:

HIERARCHY

*Lower Level NVC Types:

| **Elcode** | **Scientific or Colloquial Name** |
| --- | --- |
|  |  |

DISCUSSION

Discussion [optional]:

CONCEPT HISTORY

*Recent Concept Lineage [if applicable]:

| **Date** | **Predecessor** | **Note** |
| --- | --- | --- |
|  |  |  |

RELATED CONCEPTS

Supporting Concepts [optional]:

| **Relationship to NVC** | **Supporting Concept Name** | **Short Citation** | **Note** |
| --- | --- | --- | --- |
|  |  |  |  |

AUTHORSHIP

*Primary Concept Source [if applicable]: C. Josse, in Faber-Langendoen et al. (2014)

| **Relationship to NVC** | **Name Used in Source** | **Short Citation** | **Note** |
| --- | --- | --- | --- |
|  |  |  |  |

*Author of Description: C. Josse

Acknowledgments [optional]:

Version Date: 17 Apr 2014

REFERENCES

*References [Required if used in text]:

Faber-Langendoen, D., J. Drake, S. Gawler, M. Hall, C. Josse, G. Kittel, S. Menard, C. Nordman, M. Pyne, M. Reid, L. Sneddon, K. Schulz, J. Teague, M. Russo, K. Snow, and P. Comer, editors. 2010-2018. Divisions, Macrogroups and Groups for the Revised U.S. National Vegetation Classification. NatureServe, Arlington, VA. plus appendices. [in preparation]

1. Forest & Woodland

1.A.3.Eh. Guianan Montane Humid Forest

M603. Central Guianan Montane Humid Forest

Type Concept Sentence: Lower and upper montane humid forests of the central Guianan Highlands in Venezuela, Guyana and Brazil, including the slopes of the Tepuis, the Gran Sabana, and some interior plateaus of the massifs, from 400-2500 m elevation. Also included are relict Guianan forest on Colombian sierras. Forests are evergreen, with canopies that are 15-30 m high. At higher elevations, the forests are lower in stature and more open. Characteristic species for the lower montane type include *Matayba macrolepis, Rinorea guianensis*, and *Virola surinamensis*. Upper montane forests are dominated by the genera *Bonnetia, Clusia, Cyrilla, Matayba, Podocarpus, Sloanea*, and *Vochysia*.

OVERVIEW

*Hierarchy Level: Macrogroup

*Placement in Hierarchy: 1.A.3.Eh. Guianan Montane Humid Forest (D229)

Elcode: M603

*Scientific Name: Central Guianan Montane Humid Forest Macrogroup

*Common (Translated Scientific) Name: Central Guianan Montane Humid Forest Macrogroup

*Colloquial Name: Central Guianan Montane Humid Forest

*Type Concept: Lower and upper montane forests of the central Guiana Highlands in Venezuela, Guyana and Brazil, on the slopes of the Tepuis and the Gran Sabana, and some interior plateaus of the massifs, from 400-2500 m elevation. Also included are those of the Guianan relict sierras in Colombia. These are evergreen, humid forests with sclerophyllous leaves and 15-30 m high; those of higher elevations are shorter and more open. Depending on the location, diagnostic species assemblages for the lower montane type are *Protium heptaphyllum, Parahancornia surrogata, Pleonostoma clematis, Roupala montana, Matayba macrolepis, Rinorea guianensis, Oenocarpus bataua, Macairea schultesii, Molongum nitidum, Schefflera aff. roraimae, Ocotea sp. Remijia* sp., or *Virola surinamensis, Licania alba, Licania densiflora, Parinari excelsa, Inga punctata, Mora gonggrijpii, Hymenaea courbaril, Clathrotropis brachypetala, Lecointea amazonica, Cedrela odorata, Erisma uncinatum, Qualea dinizii, Tabebuia stenocalyx*, while upper montane forests are dominated by genera *Bonnetia, Sloanea, Matayba, Vochysia, Podocarpus, Cyrilla racemiflora, Perissocarpa, Hedyosmum, Prunus, Schefflera, Vismia*, and *Clusia*, with some very restricted species endemics.

*Diagnostic Characteristics:

*Classification Comments:

*Similar NVC Types [if applicable]:

| **Elcode** | **Scientific or Colloquial Name** | **Note** |
| --- | --- | --- |
|  |  |  |

Similar NVC Types General Comments [optional]:

VEGETATION

Physiognomy and Structure Summary:

Physiognomy and Structure Table [optional]:

| **Physiognomy-Structure Category** | **Prevailing Height (m)** | **Height Range (opt.)** | **Mean % Cover** | **Cover Range (opt.)** |
| --- | --- | --- | --- | --- |
|  |  |  |  | - |

Floristics Summary:

*Floristics Table [Med - High Confidence]:

*Number of Plots:

*Cover Scale Used:

| **Physiognomy-Structure Category** | **Taxon Name** | **Specific Growth Form (opt.)** | **Const- ancy** | **Mean % Cover** | **Cover Range (opt.)** | **Differ-ential** | **Diagnostic Combin- ation** |
| --- | --- | --- | --- | --- | --- | --- | --- |
|  |  |  |  |  | - |  |  |

Dynamics:

ENVIRONMENT

Environmental Description:

DISTRIBUTION

*Geographic Range:

Nations: BR, GY, VE

States/Provinces:

USFS Ecoregions (2007) [optional]:

Omernik Ecoregions L3, L4 [optional]:

MLRAs [optional]:

PLOT SAMPLING AND ANALYSIS

*Plot Analysis Summary [Med - High Confidence]:

*Plots Used to Define the Type [Med - High Confidence]:

CONFIDENCE LEVEL

USNVC Confidence Level: Unassigned

USNVC Confidence Comments [optional]:

HIERARCHY

*Lower Level NVC Types:

| **Elcode** | **Scientific or Colloquial Name** |
| --- | --- |
|  |  |

DISCUSSION

Discussion [optional]:

CONCEPT HISTORY

*Recent Concept Lineage [if applicable]:

| **Date** | **Predecessor** | **Note** |
| --- | --- | --- |
|  |  |  |

RELATED CONCEPTS

Supporting Concepts [optional]:

| **Relationship to NVC** | **Supporting Concept Name** | **Short Citation** | **Note** |
| --- | --- | --- | --- |
|  |  |  |  |

AUTHORSHIP

*Primary Concept Source [if applicable]: C. Josse, in Faber-Langendoen et al. (2014)

| **Relationship to NVC** | **Name Used in Source** | **Short Citation** | **Note** |
| --- | --- | --- | --- |
|  |  |  |  |

*Author of Description: C. Josse

Acknowledgments [optional]:

Version Date: 17 Apr 2014

REFERENCES

*References [Required if used in text]:

Faber-Langendoen, D., J. Drake, S. Gawler, M. Hall, C. Josse, G. Kittel, S. Menard, C. Nordman, M. Pyne, M. Reid, L. Sneddon, K. Schulz, J. Teague, M. Russo, K. Snow, and P. Comer, editors. 2010-2018. Divisions, Macrogroups and Groups for the Revised U.S. National Vegetation Classification. NatureServe, Arlington, VA. plus appendices. [in preparation]

1. Forest & Woodland

1.A.3.Ej. Tropical Andean Montane Humid Forest

D231. Tropical Andean Montane Humid Forest

Type Concept Sentence:

OVERVIEW

*Hierarchy Level: Division

*Placement in Hierarchy: 1.A.3.Ej. Tropical Montane Humid Forest (F004)

Elcode: D231

*Scientific Name: Tropical Andean Montane Humid Forest Division

*Common (Translated Scientific) Name: Tropical Andean Montane Humid Forest Division

*Colloquial Name: Tropical Andean Montane Humid Forest

*Type Concept:

*Diagnostic Characteristics:

*Classification Comments:

*Similar NVC Types [if applicable]:

| **Elcode** | **Scientific or Colloquial Name** | **Note** |
| --- | --- | --- |
|  |  |  |

Similar NVC Types General Comments [optional]:

VEGETATION

Physiognomy and Structure Summary:

Physiognomy and Structure Table [optional]:

| **Physiognomy-Structure Category** | **Prevailing Height (m)** | **Height Range (opt.)** | **Mean % Cover** | **Cover Range (opt.)** |
| --- | --- | --- | --- | --- |
|  |  |  |  | - |

Floristics Summary:

*Floristics Table [Med - High Confidence]:

*Number of Plots: *Cover Scale Used:

| **Physiognomy-Structure Category** | **Taxon Name** | **Specific Growth Form (opt.)** | **Const- ancy** | **Mean % Cover** | **Cover Range (opt.)** | **Differ-ential** | **Diagnostic Combin- ation** |
| --- | --- | --- | --- | --- | --- | --- | --- |
|  |  |  |  |  | - |  |  |

Dynamics:

ENVIRONMENT

Environmental Description:

DISTRIBUTION

*Geographic Range:

Nations: AR, BO, CO, EC, PE, VE

States/Provinces:

USFS Ecoregions (2007) [optional]:

Omernik Ecoregions L3, L4 [optional]:

MLRAs [optional]:

PLOT SAMPLING AND ANALYSIS

*Plot Analysis Summary [Med - High Confidence]:

*Plots Used to Define the Type [Med - High Confidence]:

CONFIDENCE LEVEL

USNVC Confidence Level: Unassigned

USNVC Confidence Comments [optional]:

HIERARCHY

*Lower Level NVC Types:

| **Elcode** | **Scientific or Colloquial Name** |
| --- | --- |
| M613 | Bolivian-Tucuman Lower Montane Humid Forest |
| M612 | Bolivian-Tucuman Montane & Upper Montane Humid Forest |
| M611 | Central Andean (Yungas) Lower Montane Humid Forest |
| M610 | Central Andean (Yungas) Montane & Upper Montane Humid Forest |
| M615 | Eastern Subandean Ridge Montane Humid Forest |
| M614 | Moist Puna Humid Forest |
| M607 | Northern Andean Lower Montane Humid Forest |
| M606 | Northern Andean Montane & Upper Montane Humid Forest |
| M609 | Northern Andean Venezuelan Coastal Ridge Forest |
| M608 | Northern Andean Santa Marta Montane Humid Forest |

DISCUSSION

Discussion [optional]:

CONCEPT HISTORY

*Recent Concept Lineage [if applicable]:

| **Date** | **Predecessor** | **Note** |
| --- | --- | --- |
|  |  |  |

RELATED CONCEPTS

Supporting Concepts [optional]:

| **Relationship to NVC** | **Supporting Concept Name** | **Short Citation** | **Note** |
| --- | --- | --- | --- |
|  |  |  |  |

AUTHORSHIP

*Primary Concept Source [if applicable]:

| **Relationship to NVC** | **Name Used in Source** | **Short Citation** | **Note** |
| --- | --- | --- | --- |
|  |  |  |  |

*Author of Description:

Acknowledgments [optional]:

Version Date:

REFERENCES

*References [Required if used in text]:

Faber-Langendoen, D., J. Drake, S. Gawler, M. Hall, C. Josse, G. Kittel, S. Menard, C. Nordman, M. Pyne, M. Reid, L. Sneddon, K. Schulz, J. Teague, M. Russo, K. Snow, and P. Comer, editors. 2010-2018. Divisions, Macrogroups and Groups for the Revised U.S. National Vegetation Classification. NatureServe, Arlington, VA. plus appendices. [in preparation]

1. Forest & Woodland

1.A.3.Ej. Tropical Andean Montane Humid Forest

M613. Bolivian-Tucuman Lower Montane Humid Forest

Type Concept Sentence: Includes several types of Bolivian-Tucuman forests distributed in the sub-Andean belt of the eastern Andes from central Bolivia to northwestern Argentina. They occupy an elevational range from 600 to 1500-1900 m in seasonal, humid to subhumid areas. These are tall forests, with canopy heights of 25-30 m and several layers of understory and abundant epiphytes. They are dominated by evergreen and seasonally deciduous tree species, including *Blepharocalyx salicifolius, Cedrela lilloi, Citronella apogon, Cordyline dracaenoides, Juglans australis, Lonchocarpus lilloi, Nectandra angusta, Ocotea monzonensis, Phoebe porphyria, Roupala meissneri*, and *Tabebuia lapacho*.

OVERVIEW

*Hierarchy Level: Macrogroup

*Placement in Hierarchy: 1.A.3.Ej. Tropical Andean Montane Humid Forest (D231)

Elcode: M613

*Scientific Name: Bolivian-Tucuman Lower Montane Humid Forest Macrogroup

*Common (Translated Scientific) Name: Bolivian-Tucuman Lower Montane Humid Forest Macrogroup

*Colloquial Name: Bolivian-Tucuman Lower Montane Humid Forest

*Type Concept: This macrogroup brings together several types of Bolivian-Tucuman forests, distributed in the sub-Andean belt of the eastern Andes, from central Bolivia to the northwest of Argentina. They occupy an altitudinal range from 600 m to 1500-1900 m elevation, in seasonal, humid to subhumid areas. These are tall forests, with a canopy 25-30 m high, several layers of understory and abundant epiphytes. They are dominated by evergreen and seasonally deciduous tree species. *Phoebe porphyria, Juglans australis, Nectandra angusta, Ocotea monzonensis, Cedrela lilloi, Tabebuia lapacho, Lonchocarpus lilloi, Cordyline dracaenoides, Blepharocalyx salicifolius, Roupala meissneri, Citronella apogon* are some of the common species.

*Diagnostic Characteristics:

*Classification Comments:

*Similar NVC Types [if applicable]:

| **Elcode** | **Scientific or Colloquial Name** | **Note** |
| --- | --- | --- |
|  |  |  |

Similar NVC Types General Comments [optional]:

VEGETATION

Physiognomy and Structure Summary:

Physiognomy and Structure Table [optional]:

| **Physiognomy-Structure Category** | **Prevailing Height (m)** | **Height Range (opt.)** | **Mean % Cover** | **Cover Range (opt.)** |
| --- | --- | --- | --- | --- |
|  |  |  |  | - |

Floristics Summary:

*Floristics Table [Med - High Confidence]:

*Number of Plots:

*Cover Scale Used:

| **Physiognomy-Structure Category** | **Taxon Name** | **Specific Growth Form (opt.)** | **Const- ancy** | **Mean % Cover** | **Cover Range (opt.)** | **Differ-ential** | **Diagnostic Combin- ation** |
| --- | --- | --- | --- | --- | --- | --- | --- |
|  |  |  |  |  | - |  |  |

Dynamics:

ENVIRONMENT

Environmental Description:

DISTRIBUTION

*Geographic Range:

Nations: AR, BO

States/Provinces:

USFS Ecoregions (2007) [optional]:

Omernik Ecoregions L3, L4 [optional]:

MLRAs [optional]:

PLOT SAMPLING AND ANALYSIS

*Plot Analysis Summary [Med - High Confidence]:

*Plots Used to Define the Type [Med - High Confidence]:

CONFIDENCE LEVEL

USNVC Confidence Level: Unassigned

USNVC Confidence Comments [optional]:

HIERARCHY

*Lower Level NVC Types:

| **Elcode** | **Scientific or Colloquial Name** |
| --- | --- |
|  |  |

DISCUSSION

Discussion [optional]:

CONCEPT HISTORY

*Recent Concept Lineage [if applicable]:

| **Date** | **Predecessor** | **Note** |
| --- | --- | --- |
|  |  |  |

RELATED CONCEPTS

Supporting Concepts [optional]:

| **Relationship to NVC** | **Supporting Concept Name** | **Short Citation** | **Note** |
| --- | --- | --- | --- |
|  |  |  |  |

AUTHORSHIP

*Primary Concept Source [if applicable]: C. Josse, in Faber-Langendoen et al. (2014)

| **Relationship to NVC** | **Name Used in Source** | **Short Citation** | **Note** |
| --- | --- | --- | --- |
|  |  |  |  |

*Author of Description: C. Josse

Acknowledgments [optional]:

Version Date: 17 Apr 2014

REFERENCES

*References [Required if used in text]:

Faber-Langendoen, D., J. Drake, S. Gawler, M. Hall, C. Josse, G. Kittel, S. Menard, C. Nordman, M. Pyne, M. Reid, L. Sneddon, K. Schulz, J. Teague, M. Russo, K. Snow, and P. Comer, editors. 2010-2018. Divisions, Macrogroups and Groups for the Revised U.S. National Vegetation Classification. NatureServe, Arlington, VA. plus appendices. [in preparation]

1. Forest & Woodland

1.A.3.Ej. Tropical Andean Montane Humid Forest

M612. Bolivian-Tucuman Montane & Upper Montane Humid Forest

Type Concept Sentence: Evergreen to seasonally deciduous forests growing between 2000 and 4000 m elevation on the eastern slopes of the Andes from central Bolivia south to Catamarca Province in western Argentina. Upper montane forests have an open canopy and are dominated by *Polylepis* species (*Polylepis neglecta* and *Polylepis crista-galli* in Bolivia; *Polylepis australis* in Argentina). Forests occurring just above 2000 m have a more complex, multi-strata structure, a continuous canopy at 15-20 m height, and are more diverse, with the families Podocarpaceae and Myrtaceae dominant.

OVERVIEW

*Hierarchy Level: Macrogroup

*Placement in Hierarchy: 1.A.3.Ej. Tropical Andean Montane Humid Forest (D231)

Elcode: M612

*Scientific Name: Bolivian-Tucuman Montane & Upper Montane Humid Forest Macrogroup

*Common (Translated Scientific) Name: Bolivian-Tucuman Montane & Upper Montane Humid Forest Macrogroup

*Colloquial Name: Bolivian-Tucuman Montane & Upper Montane Humid Forest

*Type Concept: Evergreen to seasonally deciduous forests growing between 2000-4000 m elevation on the eastern slopes of the Andes from central Bolivia south to Catamarca province in western Argentina. Upper montane forests have an open canopy and are dominated by *Polylepis* species (*Polylepis neglecta, Polylepis crista-gallii*, in Bolivia; *Polylepis australis* in Argentina). Forests occurring just above 2000 m have a more complex, multi-strata structure, a continuous canopy at 15-20 m height, and are more diverse, with Podocarpaceae and Myrtaceae as dominant families.

*Diagnostic Characteristics:

*Classification Comments:

*Similar NVC Types [if applicable]:

| **Elcode** | **Scientific or Colloquial Name** | **Note** |
| --- | --- | --- |
|  |  |  |

Similar NVC Types General Comments [optional]:

VEGETATION

Physiognomy and Structure Summary:

Physiognomy and Structure Table [optional]:

| **Physiognomy-Structure Category** | **Prevailing Height (m)** | **Height Range (opt.)** | **Mean % Cover** | **Cover Range (opt.)** |
| --- | --- | --- | --- | --- |
|  |  |  |  | - |

Floristics Summary:

*Floristics Table [Med - High Confidence]:

*Number of Plots:

*Cover Scale Used:

| **Physiognomy-Structure Category** | **Taxon Name** | **Specific Growth Form (opt.)** | **Const- ancy** | **Mean % Cover** | **Cover Range (opt.)** | **Differ-ential** | **Diagnostic Combin- ation** |
| --- | --- | --- | --- | --- | --- | --- | --- |
|  |  |  |  |  | - |  |  |

Dynamics:

ENVIRONMENT

Environmental Description:

DISTRIBUTION

*Geographic Range:

Nations: AR, BO

States/Provinces:

USFS Ecoregions (2007) [optional]:

Omernik Ecoregions L3, L4 [optional]:

MLRAs [optional]:

PLOT SAMPLING AND ANALYSIS

*Plot Analysis Summary [Med - High Confidence]:

*Plots Used to Define the Type [Med - High Confidence]:

CONFIDENCE LEVEL

USNVC Confidence Level: Unassigned

USNVC Confidence Comments [optional]:

HIERARCHY

*Lower Level NVC Types:

| **Elcode** | **Scientific or Colloquial Name** |
| --- | --- |
|  |  |

DISCUSSION

Discussion [optional]:

CONCEPT HISTORY

*Recent Concept Lineage [if applicable]:

| **Date** | **Predecessor** | **Note** |
| --- | --- | --- |
|  |  |  |

RELATED CONCEPTS

Supporting Concepts [optional]:

| **Relationship to NVC** | **Supporting Concept Name** | **Short Citation** | **Note** |
| --- | --- | --- | --- |
|  |  |  |  |

AUTHORSHIP

*Primary Concept Source [if applicable]: C. Josse, in Faber-Langendoen et al. (2014)

| **Relationship to NVC** | **Name Used in Source** | **Short Citation** | **Note** |
| --- | --- | --- | --- |
|  |  |  |  |

*Author of Description: C. Josse

Acknowledgments [optional]:

Version Date: 17 Apr 2014

REFERENCES

*References [Required if used in text]:

Faber-Langendoen, D., J. Drake, S. Gawler, M. Hall, C. Josse, G. Kittel, S. Menard, C. Nordman, M. Pyne, M. Reid, L. Sneddon, K. Schulz, J. Teague, M. Russo, K. Snow, and P. Comer, editors. 2010-2018. Divisions, Macrogroups and Groups for the Revised U.S. National Vegetation Classification. NatureServe, Arlington, VA. plus appendices. [in preparation]

1. Forest & Woodland

1.A.3.Ej. Tropical Andean Montane Humid Forest

M611. Central Andean (Yungas) Lower Montane Humid Forest

Type Concept Sentence: Forests growing between 1200-1400 m and 1700-2000 m elevation on the upper slopes and ridges of sub-Andean hills that are either exposed to the rains and mists (palm grove physiognomy) or have less steep slopes and deeper soils (forest physiognomy). Always in areas with humid to hyper-humid climates. Usually distributed on deep and well-drained humic soils. The variant dominated by the palm *Dictyocaryum lamarckianum* is less diverse, whereas the forest variant is floristically diverse. Characteristic species are *Ceiba boliviana, Cinchona* spp., *Cyathea* spp., *Escallonia pendula, Guatteria boliviana, Juglans boliviana, Ladenbergia* spp., *Mauria heterophylla, Nectandra cissiflora, Nectandra cuneato-cordata, Podocarpus oleifolius, Saurauia peruviana*, and *Toxicodendron striatum*.

OVERVIEW

*Hierarchy Level: Macrogroup

*Placement in Hierarchy: 1.A.3.Ej. Tropical Andean Montane Humid Forest (D231)

Elcode: M611

*Scientific Name: Central Andean (Yungas) Lower Montane Humid Forest Macrogroup

*Common (Translated Scientific) Name: Central Andean (Yungas) Lower Montane Humid Forest Macrogroup

*Colloquial Name: Central Andean (Yungas) Lower Montane Humid Forest

*Type Concept: This macrogroup includes forests growing at 1200-1400 m and 1700-2000 m elevation. They occupy upper slopes and ridges of the sub-Andean hills, well exposed to the rains and mists (palm grove physiognomy) or less steep slopes and deeper soils (forest physiognomy), always in areas with humid to hyper-humid climate. It is usually distributed on deep and well-drained humic soils. The variant dominated by the palm *Dictyocaryum lamarckianum* is less diverse, while the diverse forest variant is extremely floristically diverse. Characteristic species are *Juglans neotropica, Juglans boliviana, Saurauia peruviana, Saurauia spectabilis, Podocarpus oleifolius, Mauria heterophylla, Toxicodendron striatum, Myroxylon balsamum, Nectandra cissiflora, Nectandra cuneato-cordata, Myrcia splendens, Myrcia fallax, Myrcia mollis, Ceiba boliviana, Escallonia pendula, Guatteria boliviana, Dendropanax arboreus, Protium heptaphyllum, Cinchona* spp., *Ladenbergia* spp., *Cyathea* spp., among others.

*Diagnostic Characteristics:

*Classification Comments:

*Similar NVC Types [if applicable]:

| **Elcode** | **Scientific or Colloquial Name** | **Note** |
| --- | --- | --- |
|  |  |  |

Similar NVC Types General Comments [optional]:

VEGETATION

Physiognomy and Structure Summary:

Physiognomy and Structure Table [optional]:

| **Physiognomy-Structure Category** | **Prevailing Height (m)** | **Height Range (opt.)** | **Mean % Cover** | **Cover Range (opt.)** |
| --- | --- | --- | --- | --- |
|  |  |  |  | - |

Floristics Summary:

*Floristics Table [Med - High Confidence]:

*Number of Plots:

*Cover Scale Used:

| **Physiognomy-Structure Category** | **Taxon Name** | **Specific Growth Form (opt.)** | **Const- ancy** | **Mean % Cover** | **Cover Range (opt.)** | **Differ-ential** | **Diagnostic Combin- ation** |
| --- | --- | --- | --- | --- | --- | --- | --- |
|  |  |  |  |  | - |  |  |

Dynamics:

ENVIRONMENT

Environmental Description:

DISTRIBUTION

*Geographic Range:

Nations: BO, CO, EC, PE

States/Provinces:

USFS Ecoregions (2007) [optional]:

Omernik Ecoregions L3, L4 [optional]:

MLRAs [optional]:

PLOT SAMPLING AND ANALYSIS

*Plot Analysis Summary [Med - High Confidence]:

*Plots Used to Define the Type [Med - High Confidence]:

CONFIDENCE LEVEL

USNVC Confidence Level: Unassigned

USNVC Confidence Comments [optional]:

HIERARCHY

*Lower Level NVC Types:

| **Elcode** | **Scientific or Colloquial Name** |
| --- | --- |
|  |  |

DISCUSSION

Discussion [optional]:

CONCEPT HISTORY

*Recent Concept Lineage [if applicable]:

| **Date** | **Predecessor** | **Note** |
| --- | --- | --- |
|  |  |  |

RELATED CONCEPTS

Supporting Concepts [optional]:

| **Relationship to NVC** | **Supporting Concept Name** | **Short Citation** | **Note** |
| --- | --- | --- | --- |
|  |  |  |  |

AUTHORSHIP

*Primary Concept Source [if applicable]: C. Josse, in Faber-Langendoen et al. (2014)

| **Relationship to NVC** | **Name Used in Source** | **Short Citation** | **Note** |
| --- | --- | --- | --- |
|  |  |  |  |

*Author of Description: C. Josse

Acknowledgments [optional]:

Version Date: 17 Apr 2014

REFERENCES

*References [Required if used in text]:

Faber-Langendoen, D., J. Drake, S. Gawler, M. Hall, C. Josse, G. Kittel, S. Menard, C. Nordman, M. Pyne, M. Reid, L. Sneddon, K. Schulz, J. Teague, M. Russo, K. Snow, and P. Comer, editors. 2010-2018. Divisions, Macrogroups and Groups for the Revised U.S. National Vegetation Classification. NatureServe, Arlington, VA. plus appendices. [in preparation]

1. Forest & Woodland

1.A.3.Ej. Tropical Andean Montane Humid Forest

M610. Central Andean (Yungas) Montane & Upper Montane Humid Forest

Type Concept Sentence: Dense evergreen, multi-strata forests, 15-20 m tall, growing between about 2200 and 3500 m elevation on the eastern slopes of the Andes of Peru and Bolivia in humid to hyper-humid climates. Biomass of epiphytes and woody lianas is high. Floristic composition includes a base of tropical Andean tree genera (e.g., *Brunellia, Clusia, Hesperomeles, Ilex, Miconia, Myrsine, Oreopanax, Podocarpus*, and *Weinmannia*) with species composition and dominance varying by latitude. Includes also low, semi-sclerophyllous evergreen forest dominated by *Polylepis* species and growing between 3000 and 4500 m elevation on rugged Andean highlands, especially glacial cirques and slopes with boulders.

OVERVIEW

*Hierarchy Level: Macrogroup

*Placement in Hierarchy: 1.A.3.Ej. Tropical Andean Montane Humid Forest (D231)

Elcode: M610

*Scientific Name: Central Andean (Yungas) Montane & Upper Montane Humid Forest Macrogroup

*Common (Translated Scientific) Name: Central Andean (Yungas) Montane & Upper Montane Humid Forest Macrogroup

*Colloquial Name: Central Andean (Yungas) Montane & Upper Montane Humid Forest

*Type Concept: Dense, evergreen, 15-20 m tall, multi-strata forests growing between about 2200 and 3500 m elevation on the eastern slopes of the Andes of Peru and Bolivia under humid to hyper-humid climate. They hold a large biomass of epiphytes and woody lianas. In addition to a common floristic base of tropical Andean tree genera (i.e., *Podocarpus, Weinmannia, Oreopanax, Hesperomeles, Clusia, Miconia, Myrsine, Brunellia, Ilex*) species level composition and dominance vary along their latitudinal distribution. This macrogroup also includes low, semi-sclerophyllous, evergreen forest dominated by *Polylepis* species, growing between 3000 and 4500 m elevation on rugged Andean highlands, especially glacial cirques and slopes with boulders.

*Diagnostic Characteristics:

*Classification Comments:

*Similar NVC Types [if applicable]:

| **Elcode** | **Scientific or Colloquial Name** | **Note** |
| --- | --- | --- |
|  |  |  |

Similar NVC Types General Comments [optional]:

VEGETATION

Physiognomy and Structure Summary:

Physiognomy and Structure Table [optional]:

| **Physiognomy-Structure Category** | **Prevailing Height (m)** | **Height Range (opt.)** | **Mean % Cover** | **Cover Range (opt.)** |
| --- | --- | --- | --- | --- |
|  |  |  |  | - |

Floristics Summary:

*Floristics Table [Med - High Confidence]:

*Number of Plots:

*Cover Scale Used:

| **Physiognomy-Structure Category** | **Taxon Name** | **Specific Growth Form (opt.)** | **Const- ancy** | **Mean % Cover** | **Cover Range (opt.)** | **Differ-ential** | **Diagnostic Combin- ation** |
| --- | --- | --- | --- | --- | --- | --- | --- |
|  |  |  |  |  | - |  |  |

Dynamics:

ENVIRONMENT

Environmental Description:

DISTRIBUTION

*Geographic Range:

Nations: BO, PE

States/Provinces:

USFS Ecoregions (2007) [optional]:

Omernik Ecoregions L3, L4 [optional]:

MLRAs [optional]:

PLOT SAMPLING AND ANALYSIS

*Plot Analysis Summary [Med - High Confidence]:

*Plots Used to Define the Type [Med - High Confidence]:

CONFIDENCE LEVEL

USNVC Confidence Level: Unassigned

USNVC Confidence Comments [optional]:

HIERARCHY

*Lower Level NVC Types:

| **Elcode** | **Scientific or Colloquial Name** |
| --- | --- |
|  |  |

DISCUSSION

Discussion [optional]:

CONCEPT HISTORY

*Recent Concept Lineage [if applicable]:

| **Date** | **Predecessor** | **Note** |
| --- | --- | --- |
|  |  |  |

RELATED CONCEPTS

Supporting Concepts [optional]:

| **Relationship to NVC** | **Supporting Concept Name** | **Short Citation** | **Note** |
| --- | --- | --- | --- |
|  |  |  |  |

AUTHORSHIP

*Primary Concept Source [if applicable]: C. Josse, in Faber-Langendoen et al. (2014)

| **Relationship to NVC** | **Name Used in Source** | **Short Citation** | **Note** |
| --- | --- | --- | --- |
|  |  |  |  |

*Author of Description: C. Josse

Acknowledgments [optional]:

Version Date: 17 Apr 2014

REFERENCES

*References [Required if used in text]:

Faber-Langendoen, D., J. Drake, S. Gawler, M. Hall, C. Josse, G. Kittel, S. Menard, C. Nordman, M. Pyne, M. Reid, L. Sneddon, K. Schulz, J. Teague, M. Russo, K. Snow, and P. Comer, editors. 2010-2018. Divisions, Macrogroups and Groups for the Revised U.S. National Vegetation Classification. NatureServe, Arlington, VA. plus appendices. [in preparation]

1. Forest & Woodland

1.A.3.Ej. Tropical Andean Montane Humid Forest

M615. Eastern Subandean Ridge Montane Humid Forest

Type Concept Sentence: Forests growing on steep and rugged topography of the lower eastern branches of the Cordillera Oriental in southeastern Ecuador and northeastern Peru. These forests usually occur from 1500 to 2200 m elevation in humid to hyper-humid conditions due to precipitation and fog, and situated on a variety of substrates including igneous and metamorphic rocks. Canopy cover is >70% and canopy height is 20-30 m. Forests can be interspersed with patches of several hectares dominated by one or a few species of tall trees (e.g., *Podocarpus oleifolius*) or shrubs (*Alzatea verticillata, Dictyocaryum lamarckianum, Graffenrieda emarginata*). Floristic composition predominantly consists of Andean genera.

OVERVIEW

*Hierarchy Level: Macrogroup

*Placement in Hierarchy: 1.A.3.Ej. Tropical Andean Montane Humid Forest (D231)

Elcode: M615

*Scientific Name: Eastern Subandean Ridge Montane Humid Forest Macrogroup

*Common (Translated Scientific) Name: Eastern Subandean Ridge Montane Humid Forest Macrogroup

*Colloquial Name: Eastern Subandean Ridge Montane Humid Forest

*Type Concept: This macrogroup covers forests growing on very steep and rugged topography of the lower eastern branches of the Cordillera Oriental in southeastern Ecuador and northeastern Peru, on igneous and metamorphic rocks and a wide range of substrates, usually between 1500 m and 2200 m elevation in humid to hyper-humid conditions due to precipitation and fog. Canopy cover is >70% and trees are 20-30 m tall, interspersed with patches of several hectares dominated by one or a few species of tall trees (i.e., *Podocarpus oleifolius*) or else shrublands (*Alzatea verticillata, Graffenrieda emarginata*, and the palm *Dictyocaryum lamarckianum*). Their floristic composition is predominantly of Andean genera, as compared with other forests in this belt which show a mixed Amazonian and Andean flora. The species assemblages include several diverse genera such as *Cinchona, Clusia, Beilschmiedia, Hieronyma, Mauria, Nectandra, Ocotea, Oreopanax, Siparuna, Weinmannia, Ceroxylon*, and many other palms. *Axinaea, Vismia*, and *Chusquea*, take over disturbed areas after landslides or mass movements.

*Diagnostic Characteristics:

*Classification Comments:

*Similar NVC Types [if applicable]:

| **Elcode** | **Scientific or Colloquial Name** | **Note** |
| --- | --- | --- |
|  |  |  |

Similar NVC Types General Comments [optional]:

VEGETATION

Physiognomy and Structure Summary:

Physiognomy and Structure Table [optional]:

| **Physiognomy-Structure Category** | **Prevailing Height (m)** | **Height Range (opt.)** | **Mean % Cover** | **Cover Range (opt.)** |
| --- | --- | --- | --- | --- |
|  |  |  |  | - |

Floristics Summary:

*Floristics Table [Med - High Confidence]:

*Number of Plots:

*Cover Scale Used:

| **Physiognomy-Structure Category** | **Taxon Name** | **Specific Growth Form (opt.)** | **Const- ancy** | **Mean % Cover** | **Cover Range (opt.)** | **Differ-ential** | **Diagnostic Combin- ation** |
| --- | --- | --- | --- | --- | --- | --- | --- |
|  |  |  |  |  | - |  |  |

Dynamics:

ENVIRONMENT

Environmental Description:

DISTRIBUTION

*Geographic Range:

Nations: EC, PE

States/Provinces:

USFS Ecoregions (2007) [optional]:

Omernik Ecoregions L3, L4 [optional]:

MLRAs [optional]:

PLOT SAMPLING AND ANALYSIS

*Plot Analysis Summary [Med - High Confidence]:

*Plots Used to Define the Type [Med - High Confidence]:

CONFIDENCE LEVEL

USNVC Confidence Level: Unassigned

USNVC Confidence Comments [optional]:

HIERARCHY

*Lower Level NVC Types:

| **Elcode** | **Scientific or Colloquial Name** |
| --- | --- |
|  |  |

DISCUSSION

Discussion [optional]:

CONCEPT HISTORY

*Recent Concept Lineage [if applicable]:

| **Date** | **Predecessor** | **Note** |
| --- | --- | --- |
|  |  |  |

RELATED CONCEPTS

Supporting Concepts [optional]:

| **Relationship to NVC** | **Supporting Concept Name** | **Short Citation** | **Note** |
| --- | --- | --- | --- |
|  |  |  |  |

AUTHORSHIP

*Primary Concept Source [if applicable]: C. Josse, in Faber-Langendoen et al. (2014)

| **Relationship to NVC** | **Name Used in Source** | **Short Citation** | **Note** |
| --- | --- | --- | --- |
|  |  |  |  |

*Author of Description: C. Josse

Acknowledgments [optional]:

Version Date: 17 Apr 2014

REFERENCES

*References [Required if used in text]:

Faber-Langendoen, D., J. Drake, S. Gawler, M. Hall, C. Josse, G. Kittel, S. Menard, C. Nordman, M. Pyne, M. Reid, L. Sneddon, K. Schulz, J. Teague, M. Russo, K. Snow, and P. Comer, editors. 2010-2018. Divisions, Macrogroups and Groups for the Revised U.S. National Vegetation Classification. NatureServe, Arlington, VA. plus appendices. [in preparation]

1. Forest & Woodland

1.A.3.Ej. Tropical Andean Montane Humid Forest

M614. Moist Puna Humid Forest

Type Concept Sentence: Forests occurring at high elevations near the treeline in the ecotone with high Andean grasslands. Canopies are 3-10 m high and discontinuous, formed by *Polylepis* and other tree genera as well as shrubs. The open canopy allows the development of a dense herbaceous lower stratum that includes ferns. Characteristic woody species comprise several members of the genera *Polylepis, Buddleia, Barnadesia*, and *Oreopanax*, and the species *Berberis commutata, Berberis rariflora, Escallonia corymbosa, Gynoxys psilophylla, Hesperomeles pernettyioides, Mutisia weberbauerii*, and *Schinus microphyllus*.

OVERVIEW

*Hierarchy Level: Macrogroup

*Placement in Hierarchy: 1.A.3.Ej. Tropical Andean Montane Humid Forest (D231)

Elcode: M614

*Scientific Name: Moist Puna Humid Forest Macrogroup

*Common (Translated Scientific) Name: Moist Puna Humid Forest Macrogroup

*Colloquial Name: Moist Puna Humid Forest

*Type Concept: Forests occurring in high elevations at the treeline, in the ecotone with high Andean grasslands. They are 3-10 m tall and have a discontinuous canopy formed by *Polylepis* and other tree genera, as well as shrubs. The open canopy allows the development of a dense herbaceous lower stratum, including ferns. Characteristic woody species include several species of *Polylepis* and *Buddleia, Hesperomeles pernettyioides, Escallonia corymbosa, Mutisia weberbauerii, Oreopanax* spp., *Berberis commutata, Berberis rariflora, Gynoxys psilophylla, Schinus microphyllus, Barnadesia* sp.

*Diagnostic Characteristics:

*Classification Comments:

*Similar NVC Types [if applicable]:

| **Elcode** | **Scientific or Colloquial Name** | **Note** |
| --- | --- | --- |
|  |  |  |

Similar NVC Types General Comments [optional]:

VEGETATION

Physiognomy and Structure Summary:

Physiognomy and Structure Table [optional]:

| **Physiognomy-Structure Category** | **Prevailing Height (m)** | **Height Range (opt.)** | **Mean % Cover** | **Cover Range (opt.)** |
| --- | --- | --- | --- | --- |
|  |  |  |  | - |

Floristics Summary:

*Floristics Table [Med - High Confidence]:

*Number of Plots:

*Cover Scale Used:

| **Physiognomy-Structure Category** | **Taxon Name** | **Specific Growth Form (opt.)** | **Const- ancy** | **Mean % Cover** | **Cover Range (opt.)** | **Differ-ential** | **Diagnostic Combin- ation** |
| --- | --- | --- | --- | --- | --- | --- | --- |
|  |  |  |  |  | - |  |  |

Dynamics:

ENVIRONMENT

Environmental Description:

DISTRIBUTION

*Geographic Range:

Nations: BO, EC, PE

States/Provinces:

USFS Ecoregions (2007) [optional]:

Omernik Ecoregions L3, L4 [optional]:

MLRAs [optional]:

PLOT SAMPLING AND ANALYSIS

*Plot Analysis Summary [Med - High Confidence]:

*Plots Used to Define the Type [Med - High Confidence]:

CONFIDENCE LEVEL

USNVC Confidence Level: Unassigned

USNVC Confidence Comments [optional]:

HIERARCHY

*Lower Level NVC Types:

| **Elcode** | **Scientific or Colloquial Name** |
| --- | --- |
|  |  |

DISCUSSION

Discussion [optional]:

CONCEPT HISTORY

*Recent Concept Lineage [if applicable]:

| **Date** | **Predecessor** | **Note** |
[truncated: 3,458,911 more chars]
