# Supplementary material for: Long-term loss in extent and current protection of terrestrial ecosystem diversity in the temperate and tropical Americas
Source: PLoS One. 2020 Jun 30;15(6):e0234960. doi: 10.1371/journal.pone.0234960 (PMC7326196; doi:10.1371/journal.pone.0234960)
Supplement: S2 Appendix — (DOCX) [file pone.0234960.s002.docx]

S2 Table of Contents

[1. Forest & Woodland 16](#_Toc532991579)

[1.A. Tropical Forest & Woodland 16](#_Toc532991580)

[1.A.1. Tropical Dry Forest & Woodland 16](#_Toc532991581)

[1.A.1.Ea. Caribbean‑Mesoamerican Dry Forest & Woodland 16](#_Toc532991582)

[M134. Caribbean Coastal Lowland Dry Forest 16](#_Toc532991583)

[CES411.421 Caribbean Coastal Dry Evergreen Forest 16](#_Toc532991584)

[CES411.419 Caribbean Semi‑deciduous Lowland Forest 17](#_Toc532991585)

[CES411.287 South Florida Hardwood Hammock 18](#_Toc532991586)

[CES411.369 Southeast Florida Coastal Strand and Maritime Hammock 19](#_Toc532991587)

[CES411.368 Southwest Florida Coastal Strand and Maritime Hammock 19](#_Toc532991588)

[M294. Caribbean Dry Limestone Forest 20](#_Toc532991589)

[CES411.457 Caribbean Edapho‑Xerophilous "Mogote" Complex 20](#_Toc532991590)

[CES411.465 Caribbean Submontane/Montane Karstic Forest 21](#_Toc532991591)

[M296. Caribbean‑Mesoamerican Pine Dry Forest 22](#_Toc532991592)

[CES411.463 Bahamas Pine Barrens 22](#_Toc532991593)

[CES401.294 Bosque Seco Siempreverde de Encino (*Quercus*) 22](#_Toc532991594)

[CES411.468 Cuban Lowland Pine Forest on Ferritic Soils 23](#_Toc532991595)

[CES411.469 Cuban Lowland Pine Woodland on Sand 23](#_Toc532991596)

[CES411.432 Cuban Sandstone Mixed Pine‑Broad‑leaved Forest 24](#_Toc532991597)

[CES411.435 Cuban Serpentine Mixed Pine‑Broad‑leaved Forest 25](#_Toc532991598)

[CES401.300 San Lucan Evergreen Forest and Woodland 25](#_Toc532991599)

[M561. Caribbean‑Mesoamerican Seasonal Dry Forest 26](#_Toc532991600)

[CES401.293 Bosque Seco de Motagua y Valles Secos de Honduras 26](#_Toc532991601)

[CES401.309 Bosque Seco Deciduo de Yucatán 26](#_Toc532991602)

[CES301.982 Bosque Semideciduo de Tamaulipas 27](#_Toc532991603)

[M562. Pacific Mesoamerican Seasonal Dry Forest 28](#_Toc532991604)

[CES401.312 Bosque Seco Deciduo de Darién 28](#_Toc532991605)

[CES401.289 Bosque Seco Deciduo de Guerrero 29](#_Toc532991606)

[CES401.302 Bosque Seco Deciduo de Sinaloa 30](#_Toc532991607)

[CES403.606 Bosque Semideciduo Premontano Mesoamericano 31](#_Toc532991608)

[CES401.298 Bosque Semi‑perennifolio de Nayarit Guerrero 32](#_Toc532991609)

[CES401.299 San Lucan Dry Deciduous Forest 33](#_Toc532991610)

[1.A.1.Ei. Colombian‑Venezuelan Dry Forest 33](#_Toc532991611)

[M563. Guajiran Seasonal Dry Forest 33](#_Toc532991612)

[CES411.439 Venezuelan Coastal Piedmontane Semi‑deciduous Forest 33](#_Toc532991613)

[1.A.2. Tropical Lowland Humid Forest 34](#_Toc532991614)

[1.A.2.Eg. Caribbean‑Mesoamerican Lowland Humid Forest 34](#_Toc532991615)

[M281. Caribbean Lowland Humid Forest 34](#_Toc532991616)

[CES411.500 Caribbean Lowland Moist Serpentine Woodland 34](#_Toc532991617)

[CES411.426 Caribbean Seasonal Evergreen Lowland Forest 35](#_Toc532991618)

[CES411.427 Caribbean Seasonal Evergreen Submontane/Lowland Forest 36](#_Toc532991619)

[CES411.424 Caribbean Wet Submontane/Lowland Forest 38](#_Toc532991620)

[M578. Mesoamerican Lowland Humid Forest 39](#_Toc532991621)

[CES402.604 Bosque Estacional Siempreverde de Colinas de Carso del Petén 39](#_Toc532991622)

[CES402.580 Bosque Siempreverde de Tierras Bajas Centroamericano Caribeño 39](#_Toc532991623)

[CES402.597 Bosque Siempreverde de Tierras Bajas del Pacífico Mesoamericano 40](#_Toc532991624)

[CES402.581 Bosque Siempreverde Estacional de Tierras Bajas del Caribe Centroamericano 41](#_Toc532991625)

[CES402.600 Bosque Siempreverde Estacional de Tierras Bajas del Pacífico Centroamericano 41](#_Toc532991626)

[M873. Mesoamerican Submontane Humid Forest 42](#_Toc532991627)

[CES403.323 Bosque Húmedo Submontano del Norte de Mesoamérica 42](#_Toc532991628)

[CES402.607 Bosque Muy Húmedo Submontano de Talamanca 43](#_Toc532991629)

[1.A.2.Eh. Colombian‑Venezuelan Lowland Humid Forest 43](#_Toc532991630)

[M581. Choco‑Darien Humid Forest 43](#_Toc532991631)

[CES402.616 Bosque Pluvial de Tierra Firme del Chocó‑Darién 43](#_Toc532991632)

[CES402.614 Bosque Pluvial Premontano del Chocó‑Darién 44](#_Toc532991633)

[1.A.2.Ei. Guianan Lowland Humid Forest 44](#_Toc532991634)

[M587. Orinoquian Humid Forest 44](#_Toc532991635)

[CES404.351 Bosque Aluvial de la Guayana Oriental 44](#_Toc532991636)

[1.A.3. Tropical Montane Humid Forest 45](#_Toc532991637)

[1.A.3.Eg. Caribbean‑Mesoamerican Montane Humid Forest 45](#_Toc532991638)

[M598. Caribbean Montane Humid Forest 45](#_Toc532991639)

[CES411.450 Caribbean Moist Montane Mixed Pine‑Broad‑leaved Forest 45](#_Toc532991640)

[CES411.471 Caribbean Montane Serpentine Shrubland 46](#_Toc532991641)

[CES411.455 Caribbean Montane Wet Elfin Forest 46](#_Toc532991642)

[CES411.429 Caribbean Montane Wet Serpentine Woodland 47](#_Toc532991643)

[CES411.451 Caribbean Montane Wet Short Shrubland 48](#_Toc532991644)

[CES411.430 Caribbean Wet Montane Forest 49](#_Toc532991645)

[CES411.470 Hispaniola Montane and Upper Montane Pine Forest 49](#_Toc532991646)

[M600. Mesoamerican Montane Humid Forest 50](#_Toc532991647)

[CES403.315 Bosque Mesófilo de Montaña Mexicano 50](#_Toc532991648)

[CES403.324 Bosque Montano Alto de Coníferas y Mixto del Norte de Mesoamérica 51](#_Toc532991649)

[CES403.319 Bosque Siempreverde Nublado del Norte de Mesoamérica 52](#_Toc532991650)

[M601. Mesoamerican Montane Pine‑Oak Forest 53](#_Toc532991651)

[CES403.318 Bosque de Encino‑Pino de [Guerrero] México 53](#_Toc532991652)

[CES403.321 Bosque Montano Alto de Pino Encino del Norte de Mesoamérica 53](#_Toc532991653)

[CES403.320 Bosque Montano Bajo de Pino Encino del Norte de Mesoamérica 55](#_Toc532991654)

[M602. Southern Mesoamerican Montane Humid Forest 55](#_Toc532991655)

[CES402.609 Bosque de Roble Muy Húmedo Montano Alto de Talamanca 55](#_Toc532991656)

[CES402.608 Bosque de Roble Muy Húmedo Montano Bajo de Talamanca 56](#_Toc532991657)

[1.A.4. Tropical Flooded & Swamp Forest 56](#_Toc532991658)

[1.A.4.Ed. Caribbean‑Central American Flooded & Swamp Forest 56](#_Toc532991659)

[M618. Caribbean Floodplain Forest 56](#_Toc532991660)

[CES411.420 Caribbean Floodplain Forest 56](#_Toc532991661)

[CES402.579 Caribbean Seasonal Evergreen Gallery Forest 57](#_Toc532991662)

[M617. Caribbean Swamp Forest 58](#_Toc532991663)

[CES411.453 Caribbean Coastal Palm Swamp 58](#_Toc532991664)

[CES411.366 South Florida Bayhead Swamp 58](#_Toc532991665)

[M619. Mesoamerican Coastal Plain Swamp Forest 59](#_Toc532991666)

[CES402.586 Bosque Pantanoso Costero Mesoamérico 59](#_Toc532991667)

[CES402.585 Yolillal Costero Mesoamérico 60](#_Toc532991668)

[M620. Mesoamerican Floodplain Forest 60](#_Toc532991669)

[CES402.602 Bosque Aluvial de Tierras Bajas del Petén 60](#_Toc532991670)

[CES402.603 Bosque Aluvial Estacional de Tierras Bajas de Suelos Calcáreos del Petén 61](#_Toc532991671)

[CES402.584 Bosque Siempreverde Aluvial de Mesoamérica 61](#_Toc532991672)

[CES401.295 Bosque y Arbustal Semideciduo de Galería de Mesoamérica 62](#_Toc532991673)

[1.A.4.Ei. Colombian‑Venezuelan Flooded & Swamp Forest 62](#_Toc532991674)

[M622. Choco‑Darien Floodplain Forest 62](#_Toc532991675)

[CES402.582 Palmar Pantanoso de Tierras Bajas del Chocó‑Darién 62](#_Toc532991676)

[1.A.4.Ej. Guianan Flooded & Swamp Forest 63](#_Toc532991677)

[M628. Orinoco Delta Swamp Forest 63](#_Toc532991678)

[CES404.380 Pantano Mixto con Palmas del Delta del Orinoco 63](#_Toc532991679)

[1.A.5. Mangrove 63](#_Toc532991680)

[1.A.5.Ua. Atlantic‑Caribbean & East Pacific Mangrove 63](#_Toc532991681)

[M004. Eastern Pacific Mangrove 63](#_Toc532991682)

[CES402.599 Manglar Estuarino y de la Costa del Pacifico 63](#_Toc532991683)

[CES402.596 Manglar Mixto con Mora Estuarino del Pacifico 64](#_Toc532991684)

[M005. Western Atlantic & Caribbean Mangrove 65](#_Toc532991685)

[CES411.444 Caribbean Coastal Mangrove 65](#_Toc532991686)

[CES402.578 Manglar Costero y de Estuario del Caribe 66](#_Toc532991687)

[1.B. Temperate & Boreal Forest & Woodland 67](#_Toc532991688)

[1.B.1. Warm Temperate Forest & Woodland 67](#_Toc532991689)

[1.B.1.Na. Southeastern North American Forest & Woodland 67](#_Toc532991690)

[M007. Longleaf Pine Woodland 67](#_Toc532991691)

[CES203.254 Atlantic Coastal Plain Fall‑line Sandhills Longleaf Pine Woodland 67](#_Toc532991692)

[CES203.281 Atlantic Coastal Plain Upland Longleaf Pine Woodland 69](#_Toc532991693)

[CES203.265 Central Atlantic Coastal Plain Wet Longleaf Pine Savanna and Flatwoods 70](#_Toc532991694)

[CES203.382 Central Florida Pine Flatwoods 71](#_Toc532991695)

[CES203.496 East Gulf Coastal Plain Interior Upland Longleaf Pine Woodland 72](#_Toc532991696)

[CES203.375 East Gulf Coastal Plain Near‑Coast Pine Flatwoods 74](#_Toc532991697)

[CES203.284 Florida Longleaf Pine Sandhill 74](#_Toc532991698)

[CES411.381 South Florida Pine Flatwoods 75](#_Toc532991699)

[CES411.367 South Florida Pine Rockland 76](#_Toc532991700)

[CES203.536 Southern Atlantic Coastal Plain Wet Pine Savanna and Flatwoods 77](#_Toc532991701)

[CES203.497 Southern Atlantic Coastal Plain Xeric River Dune 78](#_Toc532991702)

[CES203.891 West Gulf Coastal Plain Stream Terrace Sandyland Longleaf Pine Woodland 79](#_Toc532991703)

[CES203.293 West Gulf Coastal Plain Upland Longleaf Pine Forest and Woodland 81](#_Toc532991704)

[CES203.191 West Gulf Coastal Plain Wet Longleaf Pine Savanna and Flatwoods 83](#_Toc532991705)

[M885. Southeastern Coastal Plain Evergreen Oak ‑ Mixed Hardwood Forest 84](#_Toc532991706)

[CES203.464 Central and South Texas Coastal Fringe Forest and Woodland 84](#_Toc532991707)

[CES203.261 Central Atlantic Coastal Plain Maritime Forest 85](#_Toc532991708)

[CES203.503 East Gulf Coastal Plain Maritime Forest 87](#_Toc532991709)

[CES203.513 Mississippi Delta Maritime Forest 88](#_Toc532991710)

[CES203.537 Southern Atlantic Coastal Plain Maritime Forest 89](#_Toc532991711)

[CES203.560 Southern Coastal Plain Dry Upland Hardwood Forest 90](#_Toc532991712)

[CES203.494 Southern Coastal Plain Oak Dome and Hammock 92](#_Toc532991713)

[CES203.466 West Gulf Coastal Plain Chenier and Upper Texas Coastal Fringe Forest and Woodland 93](#_Toc532991714)

[M008. Southern Mesic Mixed Broadleaf Forest 94](#_Toc532991715)

[CES203.079 Crowley's Ridge Mesic Loess Slope Forest 94](#_Toc532991716)

[CES203.481 East Gulf Coastal Plain Northern Loess Bluff Forest 95](#_Toc532991717)

[CES203.477 East Gulf Coastal Plain Northern Mesic Hardwood Slope Forest 96](#_Toc532991718)

[CES203.556 East Gulf Coastal Plain Southern Loess Bluff Forest 98](#_Toc532991719)

[CES203.242 Southern Atlantic Coastal Plain Mesic Hardwood Forest 99](#_Toc532991720)

[CES203.502 Southern Coastal Plain Limestone Forest 100](#_Toc532991721)

[CES203.476 Southern Coastal Plain Mesic Slope Forest 101](#_Toc532991722)

[CES203.280 West Gulf Coastal Plain Mesic Hardwood Forest 103](#_Toc532991723)

[1.B.1.Nc. Californian Forest & Woodland 104](#_Toc532991724)

[M009. Californian Forest & Woodland 104](#_Toc532991725)

[CES206.935 California Central Valley Mixed Oak Savanna 104](#_Toc532991726)

[CES206.922 California Coastal Closed‑Cone Conifer Forest and Woodland 105](#_Toc532991727)

[CES206.937 California Coastal Live Oak Woodland and Savanna 106](#_Toc532991728)

[CES206.936 California Lower Montane Blue Oak‑Foothill Pine Woodland and Savanna 107](#_Toc532991729)

[CES206.920 Central and Southern California Mixed Evergreen Woodland 108](#_Toc532991730)

[CES206.923 Mediterranean California Lower Montane Black Oak‑Conifer Forest and Woodland 108](#_Toc532991731)

[CES206.919 Mediterranean California Mixed Evergreen Forest 109](#_Toc532991732)

[CES206.909 Mediterranean California Mixed Oak Woodland 110](#_Toc532991733)

[CES206.938 Southern California Oak Woodland and Savanna 111](#_Toc532991734)

[1.B.1.Nd. Madrean‑Balconian Forest & Woodland 112](#_Toc532991735)

[M015. Balconian Forest & Woodland 112](#_Toc532991736)

[CES303.656 Edwards Plateau Dry‑Mesic Slope Forest and Woodland 112](#_Toc532991737)

[CES303.660 Edwards Plateau Limestone Savanna and Woodland 113](#_Toc532991738)

[CES303.038 Edwards Plateau Mesic Canyon 114](#_Toc532991739)

[CES303.657 Llano Uplift Acidic Forest, Woodland and Glade 115](#_Toc532991740)

[M010. Madrean Lowland Evergreen Woodland 117](#_Toc532991741)

[CES301.712 Bosque de Tascate de Guerrero 117](#_Toc532991742)

[CES305.795 Madrean Encinal 119](#_Toc532991743)

[CES301.730 Madrean Juniper Savanna 122](#_Toc532991744)

[CES305.797 Madrean Pinyon‑Juniper Woodland 124](#_Toc532991745)

[M011. Madrean Montane Forest & Woodland 126](#_Toc532991746)

[CES305.282 Bosque de Pino Ponderosa de la Sierra Madre 126](#_Toc532991747)

[CES305.281 Bosque de Pino y Aliso de la Sierra Madre 127](#_Toc532991748)

[CES305.796 Madrean Lower Montane Pine‑Oak Forest and Woodland 127](#_Toc532991749)

[CES305.798 Madrean Upper Montane Conifer‑Oak Forest and Woodland 129](#_Toc532991750)

[1.B.2. Cool Temperate Forest & Woodland 131](#_Toc532991751)

[1.B.2.Na. Eastern North American Forest & Woodland 131](#_Toc532991752)

[M883. Appalachian‑Interior‑Northeastern Mesic Forest 131](#_Toc532991753)

[CES202.593 Appalachian (Hemlock)‑Northern Hardwood Forest 131](#_Toc532991754)

[CES202.887 South‑Central Interior Mesophytic Forest 132](#_Toc532991755)

[CES202.373 Southern and Central Appalachian Cove Forest 133](#_Toc532991756)

[CES202.029 Southern Appalachian Northern Hardwood Forest 134](#_Toc532991757)

[CES202.342 Southern Piedmont Mesic Forest 136](#_Toc532991758)

[M502. Appalachian‑Northeastern Oak ‑ Hardwood ‑ Pine Forest & Woodland 137](#_Toc532991759)

[CES202.359 Allegheny‑Cumberland Dry Oak Forest and Woodland 137](#_Toc532991760)

[CES202.598 Appalachian Shale Barrens 138](#_Toc532991761)

[CES202.596 Central and Southern Appalachian Montane Oak Forest 139](#_Toc532991762)

[CES202.591 Central Appalachian Dry Oak‑Pine Forest 140](#_Toc532991763)

[CES202.600 Central Appalachian Pine‑Oak Rocky Woodland 141](#_Toc532991764)

[CES202.592 Northeastern Interior Dry‑Mesic Oak Forest 142](#_Toc532991765)

[CES202.590 Northeastern Interior Pine Barrens 143](#_Toc532991766)

[CES203.069 Northern Atlantic Coastal Plain Calcareous Ravine 144](#_Toc532991767)

[CES203.475 Northern Atlantic Coastal Plain Hardwood Forest 145](#_Toc532991768)

[CES203.302 Northern Atlantic Coastal Plain Maritime Forest 146](#_Toc532991769)

[CES203.269 Northern Atlantic Coastal Plain Pitch Pine Barrens 147](#_Toc532991770)

[CES202.331 Southern Appalachian Montane Pine Forest and Woodland 148](#_Toc532991771)

[CES202.886 Southern Appalachian Oak Forest 149](#_Toc532991772)

[CES202.457 Southern Ridge and Valley / Cumberland Dry Calcareous Forest 150](#_Toc532991773)

[M882. Central Midwest Mesic Forest 152](#_Toc532991774)

[CES202.693 North‑Central Interior Beech‑Maple Forest 152](#_Toc532991775)

[CES202.696 North‑Central Interior Maple‑Basswood Forest 153](#_Toc532991776)

[CES202.043 Ozark‑Ouachita Mesic Hardwood Forest 154](#_Toc532991777)

[M012. Central Midwest Oak Forest, Woodland & Savanna 154](#_Toc532991778)

[CES202.047 North‑Central Interior Dry Oak Forest and Woodland 154](#_Toc532991779)

[CES202.046 North‑Central Interior Dry‑Mesic Oak Forest and Woodland 156](#_Toc532991780)

[CES202.698 North‑Central Interior Oak Savanna 157](#_Toc532991781)

[CES202.727 North‑Central Oak Barrens 158](#_Toc532991782)

[M014. Laurentian‑Acadian Mesic Hardwood ‑ Conifer Forest 159](#_Toc532991783)

[CES201.565 Acadian Low‑Elevation Spruce‑Fir‑Hardwood Forest 159](#_Toc532991784)

[CES201.566 Acadian‑Appalachian Montane Spruce‑Fir Forest 159](#_Toc532991785)

[CES202.028 Central and Southern Appalachian Spruce‑Fir Forest 160](#_Toc532991786)

[CES201.564 Laurentian‑Acadian Northern Hardwood Forest 161](#_Toc532991787)

[CES201.563 Laurentian‑Acadian Pine‑Hemlock‑Hardwood Forest 162](#_Toc532991788)

[CES103.020 Laurentian‑Acadian Sub‑boreal Aspen‑Birch Forest 163](#_Toc532991789)

[CES103.426 Laurentian‑Acadian Sub‑boreal Mesic Balsam Fir‑Spruce Forest 164](#_Toc532991790)

[CES202.704 Paleozoic Plateau Bluff and Talus 165](#_Toc532991791)

[M159. Laurentian‑Acadian Pine ‑ Hardwood Forest & Woodland 166](#_Toc532991792)

[CES103.075 Laurentian Jack Pine‑Red Pine Forest 166](#_Toc532991793)

[CES201.718 Laurentian Pine‑Oak Barrens 166](#_Toc532991794)

[CES201.719 Laurentian‑Acadian Northern Pine‑(Oak) Forest 167](#_Toc532991795)

[CES103.425 Laurentian‑Acadian Sub‑boreal Dry‑Mesic Pine‑Black Spruce‑Hardwood Forest 167](#_Toc532991796)

[CES103.424 Northern Dry Jack Pine‑Red Pine‑Hardwood Woodland 168](#_Toc532991797)

[M016. Southern & South‑Central Oak ‑ Pine Forest & Woodland 169](#_Toc532991798)

[CES205.896 Bastrop Lost Pines Forest and Woodland 169](#_Toc532991799)

[CES205.682 Crosstimbers Oak Forest and Woodland 170](#_Toc532991800)

[CES203.072 Crowley's Ridge Sand Forest 171](#_Toc532991801)

[CES203.506 East Gulf Coastal Plain Interior Shortleaf Pine‑Oak Forest 172](#_Toc532991802)

[CES203.483 East Gulf Coastal Plain Northern Dry Upland Hardwood Forest 174](#_Toc532991803)

[CES203.482 East Gulf Coastal Plain Northern Loess Plain Oak‑Hickory Upland 175](#_Toc532991804)

[CES205.679 East‑Central Texas Plains Post Oak Savanna and Woodland 176](#_Toc532991805)

[CES203.531 Lower Mississippi River Dune Woodland and Forest 177](#_Toc532991806)

[CES203.071 Mississippi River Alluvial Plain Dry‑Mesic Loess Slope Forest 178](#_Toc532991807)

[CES202.306 Ouachita Montane Oak Forest 179](#_Toc532991808)

[CES202.707 Ozark‑Ouachita Dry Oak Woodland 180](#_Toc532991809)

[CES202.708 Ozark‑Ouachita Dry‑Mesic Oak Forest 180](#_Toc532991810)

[CES202.325 Ozark‑Ouachita Shortleaf Pine‑Bluestem Woodland 181](#_Toc532991811)

[CES202.313 Ozark‑Ouachita Shortleaf Pine‑Oak Forest and Woodland 182](#_Toc532991812)

[CES202.268 Piedmont Hardpan Woodland and Forest 183](#_Toc532991813)

[CES202.319 Southeastern Interior Longleaf Pine Woodland 185](#_Toc532991814)

[CES202.332 Southern Appalachian Low‑Elevation Pine Forest 186](#_Toc532991815)

[CES203.241 Southern Atlantic Coastal Plain Dry and Dry‑Mesic Oak Forest 188](#_Toc532991816)

[CES202.898 Southern Interior Low Plateau Dry‑Mesic Oak Forest 189](#_Toc532991817)

[CES202.339 Southern Piedmont Dry Oak‑(Pine) Forest and Woodland 190](#_Toc532991818)

[CES203.378 West Gulf Coastal Plain Pine‑Hardwood Forest 192](#_Toc532991819)

[CES203.056 West Gulf Coastal Plain Sandhill Oak and Shortleaf Pine Forest and Woodland 193](#_Toc532991820)

[1.B.2.Nb. Rocky Mountain Forest & Woodland 194](#_Toc532991821)

[M501. Central Rocky Mountain Dry Lower Montane‑Foothill Forest 194](#_Toc532991822)

[CES306.959 Middle Rocky Mountain Montane Douglas‑fir Forest and Woodland 194](#_Toc532991823)

[CES306.805 Northern Rocky Mountain Dry‑Mesic Montane Mixed Conifer Forest 196](#_Toc532991824)

[CES306.958 Northern Rocky Mountain Foothill Conifer Wooded Steppe 198](#_Toc532991825)

[CES306.030 Northern Rocky Mountain Ponderosa Pine Woodland and Savanna 200](#_Toc532991826)

[CES303.650 Northwestern Great Plains‑Black Hills Ponderosa Pine Woodland and Savanna 203](#_Toc532991827)

[CES306.955 Rocky Mountain Foothill Limber Pine‑Juniper Woodland 205](#_Toc532991828)

[M500. Central Rocky Mountain Mesic Lower Montane Forest 207](#_Toc532991829)

[CES204.086 East Cascades Mesic Montane Mixed‑Conifer Forest and Woodland 207](#_Toc532991830)

[CES306.802 Northern Rocky Mountain Mesic Montane Mixed Conifer Forest 208](#_Toc532991831)

[CES306.837 Northern Rocky Mountain Western Larch Savanna 209](#_Toc532991832)

[M020. Rocky Mountain Subalpine‑High Montane Forest 210](#_Toc532991833)

[CES304.776 Inter‑Mountain Basins Aspen‑Mixed Conifer Forest and Woodland 210](#_Toc532991834)

[CES304.790 Inter‑Mountain Basins Subalpine Limber‑Bristlecone Pine Woodland 213](#_Toc532991835)

[CES306.807 Northern Rocky Mountain Subalpine Woodland and Parkland 215](#_Toc532991836)

[CES303.957 Northwestern Great Plains Highland White Spruce Woodland 217](#_Toc532991837)

[CES306.813 Rocky Mountain Aspen Forest and Woodland 218](#_Toc532991838)

[CES306.814 Rocky Mountain Bigtooth Maple Ravine Woodland 220](#_Toc532991839)

[CES306.820 Rocky Mountain Lodgepole Pine Forest 220](#_Toc532991840)

[CES306.828 Rocky Mountain Subalpine Dry‑Mesic Spruce‑Fir Forest and Woodland 222](#_Toc532991841)

[CES306.830 Rocky Mountain Subalpine Mesic‑Wet Spruce‑Fir Forest and Woodland 224](#_Toc532991842)

[CES306.819 Rocky Mountain Subalpine‑Montane Limber‑Bristlecone Pine Woodland 225](#_Toc532991843)

[M021. Sierra Madre High Montane Forest 226](#_Toc532991844)

[CES305.283 Bosque Subalpino de Oyamel de la Sierra Madre 226](#_Toc532991845)

[CES403.327 Bosque Subalpino de Pino de la Sierra Madre 227](#_Toc532991846)

[M022. Southern Rocky Mountain Lower Montane Forest 227](#_Toc532991847)

[CES306.823 Southern Rocky Mountain Dry‑Mesic Montane Mixed Conifer Forest and Woodland 227](#_Toc532991848)

[CES306.825 Southern Rocky Mountain Mesic Montane Mixed Conifer Forest and Woodland 230](#_Toc532991849)

[CES306.649 Southern Rocky Mountain Ponderosa Pine Savanna 231](#_Toc532991850)

[CES306.648 Southern Rocky Mountain Ponderosa Pine Woodland 233](#_Toc532991851)

[1.B.2.Nc. Western North American Pinyon ‑ Juniper Woodland & Scrub 235](#_Toc532991852)

[M026. Intermountain Singleleaf Pinyon ‑ Juniper Woodland 235](#_Toc532991853)

[CES304.082 Columbia Plateau Western Juniper Woodland and Savanna 235](#_Toc532991854)

[CES304.773 Great Basin Pinyon‑Juniper Woodland 238](#_Toc532991855)

[CES304.772 Inter‑Mountain Basins Curl‑leaf Mountain‑mahogany Woodland and Shrubland 240](#_Toc532991856)

[CES304.782 Inter‑Mountain Basins Juniper Savanna 243](#_Toc532991857)

[M027. Southern Rocky Mountain‑Colorado Plateau Two‑needle Pinyon ‑ Juniper Woodland 245](#_Toc532991858)

[CES304.766 Colorado Plateau Pinyon‑Juniper Shrubland 245](#_Toc532991859)

[CES304.767 Colorado Plateau Pinyon‑Juniper Woodland 247](#_Toc532991860)

[CES306.834 Southern Rocky Mountain Juniper Woodland and Savanna 250](#_Toc532991861)

[CES306.835 Southern Rocky Mountain Pinyon‑Juniper Woodland 252](#_Toc532991862)

[1.B.2.Nd. Vancouverian Forest & Woodland 255](#_Toc532991863)

[M886. Southern Vancouverian Dry Foothill Forest & Woodland 255](#_Toc532991864)

[CES204.085 East Cascades Oak‑Ponderosa Pine Forest and Woodland 255](#_Toc532991865)

[CES204.845 North Pacific Dry Douglas‑fir‑(Madrone) Forest and Woodland 257](#_Toc532991866)

[CES204.852 North Pacific Oak Woodland 258](#_Toc532991867)

[M023. Southern Vancouverian Montane‑Foothill Forest 259](#_Toc532991868)

[CES206.918 California Montane Jeffrey Pine‑(Ponderosa Pine) Woodland 259](#_Toc532991869)

[CES206.917 Klamath‑Siskiyou Lower Montane Serpentine Mixed Conifer Woodland 261](#_Toc532991870)

[CES206.914 Klamath‑Siskiyou Upper Montane Serpentine Mixed Conifer Woodland 262](#_Toc532991871)

[CES206.916 Mediterranean California Dry‑Mesic Mixed Conifer Forest and Woodland 263](#_Toc532991872)

[CES206.915 Mediterranean California Mesic Mixed Conifer Forest and Woodland 264](#_Toc532991873)

[CES206.928 Mediterranean California Mesic Serpentine Woodland and Chaparral 265](#_Toc532991874)

[CES204.101 Sierran‑Intermontane Desert Western White Pine‑White Fir Woodland 266](#_Toc532991875)

[M024. Vancouverian Coastal Rainforest 267](#_Toc532991876)

[CES206.921 California Coastal Redwood Forest 267](#_Toc532991877)

[CES204.846 North Pacific Broadleaf Landslide Forest and Shrubland 269](#_Toc532991878)

[CES204.098 North Pacific Dry‑Mesic Silver Fir‑Western Hemlock‑Douglas‑fir Forest 269](#_Toc532991879)

[CES204.842 North Pacific Hypermaritime Western Red‑cedar‑Western Hemlock Forest 270](#_Toc532991880)

[CES204.073 North Pacific Lowland Mixed Hardwood‑Conifer Forest 271](#_Toc532991881)

[CES204.001 North Pacific Maritime Dry‑Mesic Douglas‑fir‑Western Hemlock Forest 272](#_Toc532991882)

[CES204.002 North Pacific Maritime Mesic‑Wet Douglas‑fir‑Western Hemlock Forest 274](#_Toc532991883)

[CES204.097 North Pacific Mesic Western Hemlock‑Silver Fir Forest 275](#_Toc532991884)

[CES204.841 North Pacific Seasonal Sitka Spruce Forest 276](#_Toc532991885)

[CES204.883 North Pacific Wooded Volcanic Flowage 277](#_Toc532991886)

[M025. Vancouverian Subalpine‑High Montane Forest 278](#_Toc532991887)

[CES206.913 Mediterranean California Red Fir Forest 278](#_Toc532991888)

[CES206.910 Mediterranean California Subalpine Woodland 279](#_Toc532991889)

[CES204.837 North Pacific Maritime Mesic Subalpine Parkland 280](#_Toc532991890)

[CES204.838 North Pacific Mountain Hemlock Forest 281](#_Toc532991891)

[CES206.911 Northern California Mesic Subalpine Woodland 282](#_Toc532991892)

[CES206.912 Sierra Nevada Subalpine Lodgepole Pine Forest and Woodland 282](#_Toc532991893)

[1.B.2.Ne. North American Great Plains Forest & Woodland 284](#_Toc532991894)

[M151. Great Plains Forest & Woodland 284](#_Toc532991895)

[CES205.688 Eastern Great Plains Tallgrass Aspen Parkland 284](#_Toc532991896)

[CES303.680 Great Plains Wooded Draw and Ravine 284](#_Toc532991897)

[CES303.681 Northwestern Great Plains Aspen Forest and Parkland 285](#_Toc532991898)

[CES303.667 Western Great Plains Dry Bur Oak Forest and Woodland 286](#_Toc532991899)

[1.B.3. Temperate Flooded & Swamp Forest 287](#_Toc532991900)

[1.B.3.Na. Eastern North American‑Great Plains Flooded & Swamp Forest 287](#_Toc532991901)

[M029. Central Hardwood Floodplain Forest 287](#_Toc532991902)

[CES202.608 Central Appalachian River Floodplain 287](#_Toc532991903)

[CES202.609 Central Appalachian Stream and Riparian 288](#_Toc532991904)

[CES202.694 North‑Central Interior Floodplain 289](#_Toc532991905)

[CES202.705 South‑Central Interior Large Floodplain 290](#_Toc532991906)

[CES202.706 South‑Central Interior Small Stream and Riparian 291](#_Toc532991907)

[M503. Central Hardwood Swamp Forest 292](#_Toc532991908)

[CES202.018 Central Interior Highlands and Appalachian Sinkhole and Depression Pond 292](#_Toc532991909)

[CES202.605 North‑Central Interior and Appalachian Rich Swamp 293](#_Toc532991910)

[CES202.700 North‑Central Interior Wet Flatwoods 294](#_Toc532991911)

[CES202.336 Piedmont Upland Depression Swamp 295](#_Toc532991912)

[CES203.479 South‑Central Interior / Upper Coastal Plain Flatwoods 296](#_Toc532991913)

[CES203.480 South‑Central Interior / Upper Coastal Plain Wet Flatwoods 297](#_Toc532991914)

[M028. Great Plains Flooded & Swamp Forest 299](#_Toc532991915)

[CES303.676 Northwestern Great Plains Floodplain 299](#_Toc532991916)

[CES303.677 Northwestern Great Plains Riparian 300](#_Toc532991917)

[CES303.678 Western Great Plains Floodplain 301](#_Toc532991918)

[CES303.956 Western Great Plains Riparian 302](#_Toc532991919)

[M504. Laurentian‑Acadian‑North Atlantic Coastal Flooded & Swamp Forest 304](#_Toc532991920)

[CES201.562 Acadian Sub‑boreal Spruce Flat 304](#_Toc532991921)

[CES201.726 Great Lakes Wooded Dune and Swale 304](#_Toc532991922)

[CES202.069 High Allegheny Wetland 306](#_Toc532991923)

[CES201.575 Laurentian‑Acadian Alkaline Conifer‑Hardwood Swamp 308](#_Toc532991924)

[CES201.587 Laurentian‑Acadian Floodplain Forest 309](#_Toc532991925)

[CES202.604 North‑Central Appalachian Acidic Swamp 309](#_Toc532991926)

[CES201.574 Northern Appalachian‑Acadian Conifer‑Hardwood Acidic Swamp 310](#_Toc532991927)

[CES203.522 Northern Atlantic Coastal Plain Basin Peat Swamp 311](#_Toc532991928)

[CES203.520 Northern Atlantic Coastal Plain Basin Swamp and Wet Hardwood Forest 311](#_Toc532991929)

[CES203.374 Northern Atlantic Coastal Plain Pitch Pine Lowland 312](#_Toc532991930)

[CES203.070 Northern Atlantic Coastal Plain Riparian and Floodplain 313](#_Toc532991931)

[CES203.282 Northern Atlantic Coastal Plain Tidal Swamp 313](#_Toc532991932)

[1.B.3.Nb. Southeastern North American Flooded & Swamp Forest 314](#_Toc532991933)

[M161. Pond‑cypress Basin Swamp 314](#_Toc532991934)

[CES203.245 Atlantic Coastal Plain Clay‑Based Carolina Bay Wetland 314](#_Toc532991935)

[CES411.365 South Florida Cypress Dome 316](#_Toc532991936)

[CES411.290 South Florida Dwarf Cypress Savanna 316](#_Toc532991937)

[CES203.251 Southern Coastal Plain Nonriverine Cypress Dome 317](#_Toc532991938)

[M033. Southern Coastal Plain Basin Swamp & Flatwoods 318](#_Toc532991939)

[CES203.557 East Gulf Coastal Plain Southern Loblolly‑Hardwood Flatwoods 318](#_Toc532991940)

[CES203.193 Lower Mississippi River Flatwoods 319](#_Toc532991941)

[CES203.304 Southern Atlantic Coastal Plain Nonriverine Swamp and Wet Hardwood Forest 320](#_Toc532991942)

[CES203.384 Southern Coastal Plain Nonriverine Basin Swamp 322](#_Toc532991943)

[CES203.548 West Gulf Coastal Plain Nonriverine Wet Hardwood‑Pine Flatwoods 323](#_Toc532991944)

[CES203.278 West Gulf Coastal Plain Pine‑Hardwood Flatwoods 324](#_Toc532991945)

[M032. Southern Coastal Plain Evergreen Hardwood ‑ Conifer Swamp 325](#_Toc532991946)

[CES203.252 Atlantic Coastal Plain Streamhead Seepage Swamp, Pocosin and Baygall 325](#_Toc532991947)

[CES203.501 Southern Coastal Plain Hydric Hammock 326](#_Toc532991948)

[CES203.505 Southern Coastal Plain Seepage Swamp and Baygall 327](#_Toc532991949)

[CES203.372 West Gulf Coastal Plain Seepage Swamp and Baygall 328](#_Toc532991950)

[M031. Southern Coastal Plain Floodplain Forest 329](#_Toc532991951)

[CES203.247 Atlantic Coastal Plain Blackwater Stream Floodplain Forest 329](#_Toc532991952)

[CES203.249 Atlantic Coastal Plain Small Blackwater River Floodplain Forest 331](#_Toc532991953)

[CES203.250 Atlantic Coastal Plain Small Brownwater River Floodplain Forest 333](#_Toc532991954)

[CES203.299 East Gulf Coastal Plain Freshwater Tidal Wooded Swamp 334](#_Toc532991955)

[CES203.489 East Gulf Coastal Plain Large River Floodplain Forest 335](#_Toc532991956)

[CES203.559 East Gulf Coastal Plain Small Stream and River Floodplain Forest 336](#_Toc532991957)

[CES203.490 Mississippi River Bottomland Depression 338](#_Toc532991958)

[CES203.195 Mississippi River Low Floodplain (Bottomland) Forest 339](#_Toc532991959)

[CES203.190 Mississippi River Riparian Forest 340](#_Toc532991960)

[CES203.065 Red River Large Floodplain Forest 341](#_Toc532991961)

[CES203.240 Southern Atlantic Coastal Plain Tidal Wooded Swamp 342](#_Toc532991962)

[CES203.493 Southern Coastal Plain Blackwater River Floodplain Forest 343](#_Toc532991963)

[CES202.324 Southern Piedmont Large Floodplain Forest 344](#_Toc532991964)

[CES202.323 Southern Piedmont Small Floodplain and Riparian Forest 346](#_Toc532991965)

[CES203.488 West Gulf Coastal Plain Large River Floodplain Forest 348](#_Toc532991966)

[CES203.459 West Gulf Coastal Plain Near‑Coast Large River Swamp 349](#_Toc532991967)

[CES203.487 West Gulf Coastal Plain Small Stream and River Forest 350](#_Toc532991968)

[M154. Southern Great Plains Floodplain Forest & Woodland 351](#_Toc532991969)

[CES203.714 Central Texas Coastal Prairie Riparian 351](#_Toc532991970)

[CES203.713 Central Texas Coastal Prairie River Floodplain 351](#_Toc532991971)

[CES203.715 Columbia Bottomlands Forest and Woodland 352](#_Toc532991972)

[CES303.651 Edwards Plateau Floodplain Terrace 353](#_Toc532991973)

[CES303.652 Edwards Plateau Riparian 354](#_Toc532991974)

[CES205.710 Southeastern Great Plains Floodplain Forest 355](#_Toc532991975)

[1.B.3.Nc. Rocky Mountain‑Great Basin Montane Flooded & Swamp Forest 357](#_Toc532991976)

[M034. Rocky Mountain‑Great Basin Montane Riparian & Swamp Forest 357](#_Toc532991977)

[CES304.768 Columbia Basin Foothill Riparian Woodland and Shrubland 357](#_Toc532991978)

[CES304.045 Great Basin Foothill and Lower Montane Riparian Woodland and Shrubland 358](#_Toc532991979)

[CES306.803 Northern Rocky Mountain Conifer Swamp 359](#_Toc532991980)

[CES306.804 Northern Rocky Mountain Lower Montane Riparian Woodland and Shrubland 360](#_Toc532991981)

[CES304.060 Northern Rocky Mountain Wooded Vernal Pool 361](#_Toc532991982)

[CES306.821 Rocky Mountain Lower Montane‑Foothill Riparian Woodland and Shrubland 362](#_Toc532991983)

[CES306.833 Rocky Mountain Subalpine‑Montane Riparian Woodland 363](#_Toc532991984)

[1.B.3.Nd. Western North American Interior Flooded Forest 364](#_Toc532991985)

[M036. Interior Warm & Cool Desert Riparian Forest 364](#_Toc532991986)

[CES206.946 California Central Valley Riparian Woodland and Shrubland 364](#_Toc532991987)

[CES206.944 Mediterranean California Foothill and Lower Montane Riparian Woodland and Shrubland 365](#_Toc532991988)

[CES206.945 Mediterranean California Serpentine Foothill and Lower Montane Riparian Woodland and Seep 366](#_Toc532991989)

[CES302.748 North American Warm Desert Lower Montane Riparian Woodland and Shrubland 367](#_Toc532991990)

[CES302.753 North American Warm Desert Riparian Woodland and Shrubland 368](#_Toc532991991)

[CES301.716 Rio Grande Delta Thorn Woodland 369](#_Toc532991992)

[CES302.759 Sonoran Fan Palm Oasis 370](#_Toc532991993)

[CES301.990 Tamaulipan Floodplain 371](#_Toc532991994)

[CES301.991 Tamaulipan Palm Grove Riparian Forest 373](#_Toc532991995)

[M660. Mexican Interior Riparian Forest 374](#_Toc532991996)

[CES305.279 Arbustal Ripario Montano Mexicano 374](#_Toc532991997)

[CES403.316 Bosque y Arbustal Ripario Montano Bajo Mexicano 374](#_Toc532991998)

[1.B.3.Ng. Vancouverian Flooded & Swamp Forest 375](#_Toc532991999)

[M035. Vancouverian Flooded & Swamp Forest 375](#_Toc532992000)

[CES204.090 North Pacific Hardwood‑Conifer Swamp 375](#_Toc532992001)

[CES204.875 North Pacific Intertidal Freshwater Wetland 376](#_Toc532992002)

[CES204.869 North Pacific Lowland Riparian Forest and Shrubland 376](#_Toc532992003)

[CES204.866 North Pacific Montane Riparian Woodland and Shrubland 377](#_Toc532992004)

[1.B.4. Boreal Forest & Woodland 378](#_Toc532992005)

[1.B.4.Na. North American Boreal Forest & Woodland 378](#_Toc532992006)

[M495. Eastern North American Boreal Forest 378](#_Toc532992007)

[CES103.022 Boreal Jack Pine-Black Spruce Forest 378](#_Toc532992008)

[1.B.5. Boreal Flooded & Swamp Forest 379](#_Toc532992009)

[1.B.5.Na. North American Boreal Flooded & Swamp Forest 379](#_Toc532992010)

[M299. North American Boreal Conifer Poor Swamp 379](#_Toc532992011)

[CES103.724 Eastern Boreal‑Sub‑boreal Conifer Acidic Swamp and Treed Poor Fen 379](#_Toc532992012)

[2. Shrub & Herb Vegetation 380](#_Toc532992013)

[2.A. Tropical Grassland, Savanna & Shrubland 380](#_Toc532992014)

[2.A.1. Tropical Lowland Grassland, Savanna & Shrubland 380](#_Toc532992015)

[2.A.1.Ea. Caribbean‑Mesoamerican Lowland Grassland, Savanna & Shrubland 380](#_Toc532992016)

[M671. Caribbean Dry Scrub 380](#_Toc532992017)

[CES411.422 Caribbean Coastal Thornscrub 380](#_Toc532992018)

[CES411.423 Caribbean Dry Karst Shrubland 381](#_Toc532992019)

[CES411.464 Caribbean Serpentine Dry Scrub 381](#_Toc532992020)

[M672. Northern Mesoamerican Pine Savanna 382](#_Toc532992021)

[CES402.590 Sabana de Pino de Mesoamérica 382](#_Toc532992022)

[CES402.621 Sabana Inundada de Pino de Mesoamérica 383](#_Toc532992023)

[M673. Northern Mesoamerican Savanna & Shrubland 384](#_Toc532992024)

[CES401.310 Sabana de Campeche Veracruz 384](#_Toc532992025)

[CES401.290 Sabana de Guerrero 384](#_Toc532992026)

[2.A.2. Tropical Montane Grassland & Shrubland 385](#_Toc532992027)

[2.A.2.Eb. Caribbean‑Mesoamerican Montane & High Montane Grassland & Shrubland 385](#_Toc532992028)

[M691. Mesoamerican Montane Grassland & Shrubland 385](#_Toc532992029)

[CES402.610 Herbazal Montano Alto de Talamanca 385](#_Toc532992030)

[CES305.284 Zacatonal Transvolcánico y de la Sierra Madre 386](#_Toc532992031)

[2.A.3. Tropical Scrub & Herb Coastal Vegetation 386](#_Toc532992032)

[2.A.3.Ee. Caribbean‑Mesoamerican Dune & Coastal Grassland & Shrubland 386](#_Toc532992033)

[M700. Caribbean‑Mesoamerican Coastal Dune & Beach 386](#_Toc532992034)

[CES402.601 Colinas de Carso del Litoral de Belize 386](#_Toc532992035)

[CES411.271 South Florida Shell Hash Beach 387](#_Toc532992036)

[CES411.272 Southeast Florida Beach 387](#_Toc532992037)

[CES411.276 Southwest Florida Beach 388](#_Toc532992038)

[2.A.3.Eg. Tropical Eastern Pacific Dune & Coastal Grassland & Shrubland 388](#_Toc532992039)

[M703. Tropical Eastern Pacific Coastal Beach & Dune 388](#_Toc532992040)

[CES402.598 Vegetacion de Playas Marinas del Pacifico 388](#_Toc532992041)

[2.B. Temperate & Boreal Grassland & Shrubland 389](#_Toc532992042)

[2.B.1. Mediterranean Scrub & Grassland 389](#_Toc532992043)

[2.B.1.Na. Californian Scrub & Grassland 389](#_Toc532992044)

[M045. Californian Annual & Perennial Grassland 389](#_Toc532992045)

[CES206.942 California Central Valley and Southern Coastal Grassland 389](#_Toc532992046)

[CES206.943 California Mesic Serpentine Grassland 390](#_Toc532992047)

[M043. Californian Chaparral 391](#_Toc532992048)

[CES206.929 California Maritime Chaparral 391](#_Toc532992049)

[CES206.926 California Mesic Chaparral 392](#_Toc532992050)

[CES206.927 California Xeric Serpentine Chaparral 393](#_Toc532992051)

[CES206.150 Klamath‑Siskiyou Xeromorphic Serpentine Savanna and Chaparral 394](#_Toc532992052)

[CES206.931 Northern and Central California Dry‑Mesic Chaparral 395](#_Toc532992053)

[CES206.930 Southern California Dry‑Mesic Chaparral 396](#_Toc532992054)

[M044. Californian Coastal Scrub 397](#_Toc532992055)

[CES206.906 Mediterranean California Coastal Bluff 397](#_Toc532992056)

[CES206.932 Northern California Coastal Scrub 397](#_Toc532992057)

[CES206.933 Southern California Coastal Scrub 398](#_Toc532992058)

[2.B.2. Temperate Grassland & Shrubland 399](#_Toc532992059)

[2.B.2.Nb. Central North American Grassland & Shrubland 399](#_Toc532992060)

[M054. Central Lowlands Tallgrass Prairie 399](#_Toc532992061)

[CES205.683 Central Tallgrass Prairie 399](#_Toc532992062)

[CES202.695 North‑Central Interior Sand and Gravel Tallgrass Prairie 400](#_Toc532992063)

[CES205.686 Northern Tallgrass Prairie 401](#_Toc532992064)

[CES205.685 Southern Tallgrass Prairie 402](#_Toc532992065)

[CES205.684 Texas Blackland Tallgrass Prairie 404](#_Toc532992066)

[CES203.550 Texas‑Louisiana Coastal Prairie 405](#_Toc532992067)

[CES202.312 Arkansas Valley Prairie and Woodland 406](#_Toc532992068)

[M158. Great Plains Comanchian Scrub & Open Vegetation 407](#_Toc532992069)

[CES303.041 Edwards Plateau Limestone Shrubland 407](#_Toc532992070)

[CES303.725 Llano Estacado Caprock Escarpment and Breaks Shrubland and Steppe 408](#_Toc532992071)

[M051. Great Plains Mixedgrass & Fescue Prairie 409](#_Toc532992072)

[CES303.659 Central Mixedgrass Prairie 409](#_Toc532992073)

[CES303.451 Northern Great Plains Fescue‑Mixed Grass Prairie 410](#_Toc532992074)

[CES303.674 Northwestern Great Plains Mixedgrass Prairie 411](#_Toc532992075)

[CES303.662 Northwestern Great Plains Shrubland 414](#_Toc532992076)

[CES303.817 Western Great Plains Foothill and Piedmont Grassland 416](#_Toc532992077)

[CES303.673 Western Great Plains Tallgrass Prairie 417](#_Toc532992078)

[M052. Great Plains Sand Grassland & Shrubland 418](#_Toc532992079)

[CES303.670 Western Great Plains Sand Prairie 418](#_Toc532992080)

[CES303.671 Western Great Plains Sandhill Steppe 420](#_Toc532992081)

[M053. Western Great Plains Shortgrass Prairie 422](#_Toc532992082)

[CES303.668 Western Great Plains Mesquite Scrub Woodland and Shrubland 422](#_Toc532992083)

[CES303.672 Western Great Plains Shortgrass Prairie 422](#_Toc532992084)

[2.B.2.Nc. Eastern North American Grassland & Shrubland 426](#_Toc532992085)

[M506. Appalachian Rocky Felsic & Mafic Scrub & Grassland 426](#_Toc532992086)

[CES202.347 Eastern Serpentine Woodland 426](#_Toc532992087)

[CES202.348 Southern and Central Appalachian Mafic Glade and Barrens 427](#_Toc532992088)

[CES202.297 Southern Appalachian Granitic Dome 428](#_Toc532992089)

[CES202.294 Southern Appalachian Grass and Shrub Bald 429](#_Toc532992090)

[CES202.327 Southern Appalachian Rocky Summit 431](#_Toc532992091)

[CES202.328 Southern Piedmont Glade and Barrens 432](#_Toc532992092)

[CES202.329 Southern Piedmont Granite Flatrock and Outcrop 433](#_Toc532992093)

[M509. Central Interior Acidic Scrub & Grassland 435](#_Toc532992094)

[CES202.692 Central Interior Highlands Dry Acidic Glade and Barrens 435](#_Toc532992095)

[CES202.337 Cumberland Sandstone Glade and Barrens 436](#_Toc532992096)

[M508. Central Interior Calcareous Scrub & Grassland 437](#_Toc532992097)

[CES202.602 Central Appalachian Alkaline Glade and Woodland 437](#_Toc532992098)

[CES202.691 Central Interior Highlands Calcareous Glade and Barrens 438](#_Toc532992099)

[CES202.354 Eastern Highland Rim Prairie and Barrens 439](#_Toc532992100)

[CES202.334 Nashville Basin Limestone Glade and Woodland 440](#_Toc532992101)

[CES202.024 Southern Ridge and Valley Calcareous Glade and Woodland 441](#_Toc532992102)

[M505. Laurentian‑Acadian Acidic Rocky Scrub & Grassland 442](#_Toc532992103)

[CES201.019 Laurentian Acidic Rocky Outcrop 442](#_Toc532992104)

[CES201.571 Northern Appalachian‑Acadian Rocky Heath Outcrop 443](#_Toc532992105)

[M507. Laurentian‑Acadian Calcareous Scrub & Grassland 443](#_Toc532992106)

[CES201.721 Great Lakes Alvar 443](#_Toc532992107)

[CES201.572 Laurentian‑Acadian Calcareous Rocky Outcrop 444](#_Toc532992108)

[2.B.2.Nf. Western North American Grassland & Shrubland 445](#_Toc532992109)

[M048. Central Rocky Mountain Montane‑Foothill Grassland & Shrubland 445](#_Toc532992110)

[CES304.792 Columbia Basin Palouse Prairie 445](#_Toc532992111)

[CES204.087 North Pacific Montane Shrubland 445](#_Toc532992112)

[CES306.801 Northern Rocky Mountain Avalanche Chute Shrubland 446](#_Toc532992113)

[CES306.040 Northern Rocky Mountain Lower Montane, Foothill and Valley Grassland 447](#_Toc532992114)

[CES306.994 Northern Rocky Mountain Montane‑Foothill Deciduous Shrubland 449](#_Toc532992115)

[CES306.961 Northern Rocky Mountain Subalpine Deciduous Shrubland 450](#_Toc532992116)

[CES306.806 Northern Rocky Mountain Subalpine‑Upper Montane Grassland 451](#_Toc532992117)

[M168. Rocky Mountain‑Vancouverian Subalpine‑High Montane Mesic Meadow 452](#_Toc532992118)

[CES206.940 Mediterranean California Subalpine Meadow 452](#_Toc532992119)

[CES204.099 North Pacific Alpine and Subalpine Dry Grassland 453](#_Toc532992120)

[CES204.100 North Pacific Montane Grassland 453](#_Toc532992121)

[CES306.829 Rocky Mountain Subalpine‑Montane Mesic Meadow 454](#_Toc532992122)

[CES306.824 Southern Rocky Mountain Montane‑Subalpine Grassland 455](#_Toc532992123)

[M049. Southern Rocky Mountain Montane Shrubland 457](#_Toc532992124)

[CES306.818 Rocky Mountain Gambel Oak‑Mixed Montane Shrubland 457](#_Toc532992125)

[CES306.822 Rocky Mountain Lower Montane‑Foothill Shrubland 459](#_Toc532992126)

[M050. Southern Vancouverian Lowland Grassland & Shrubland 460](#_Toc532992127)

[CES206.941 California Northern Coastal Grassland 460](#_Toc532992128)

[CES204.089 North Pacific Herbaceous Bald and Bluff 461](#_Toc532992129)

[CES204.088 North Pacific Hypermaritime Shrub and Herbaceous Headland 462](#_Toc532992130)

[CES204.858 Willamette Valley Upland Prairie and Savanna 463](#_Toc532992131)

[2.B.2.Ng. Western North American Interior Chaparral 464](#_Toc532992132)

[M094. Cool Interior Chaparral 464](#_Toc532992133)

[CES206.925 California Montane Woodland and Chaparral 464](#_Toc532992134)

[CES304.001 Great Basin Semi‑Desert Chaparral 465](#_Toc532992135)

[M091. Warm Interior Chaparral 466](#_Toc532992136)

[CES302.905 Chaparral Transvolcanico 466](#_Toc532992137)

[CES302.031 Madrean Oriental Chaparral 467](#_Toc532992138)

[CES302.741 Mogollon Chaparral 469](#_Toc532992139)

[CES302.757 Sonora‑Mojave Semi‑Desert Chaparral 470](#_Toc532992140)

[2.B.2.Nh. Southeastern North American Grassland & Shrubland 471](#_Toc532992141)

[M162. Florida Peninsula Scrub & Herb 471](#_Toc532992142)

[CES203.380 Florida Dry Prairie 471](#_Toc532992143)

[CES203.057 Florida Peninsula Inland Scrub 473](#_Toc532992144)

[M309. Southeastern Coastal Plain Patch Prairie 474](#_Toc532992145)

[CES203.478 Southern Coastal Plain Blackland Prairie and Woodland 474](#_Toc532992146)

[CES203.377 West Gulf Coastal Plain Northern Calcareous Prairie 476](#_Toc532992147)

[CES203.379 West Gulf Coastal Plain Southern Calcareous Prairie 477](#_Toc532992148)

[M308. Southern Barrens & Glade 478](#_Toc532992149)

[CES203.364 West Gulf Coastal Plain Catahoula Barrens 478](#_Toc532992150)

[CES203.371 West Gulf Coastal Plain Nepheline Syenite Glade 479](#_Toc532992151)

[2.B.4. Temperate to Polar Scrub & Herb Coastal Vegetation 480](#_Toc532992152)

[2.B.4.Na. Eastern North American Coastal Scrub & Herb Vegetation 480](#_Toc532992153)

[M060. Eastern North American Coastal Beach & Rocky Shore 480](#_Toc532992154)

[CES203.266 Florida Panhandle Beach Vegetation 480](#_Toc532992155)

[CES203.544 Gulf Coast Chenier Plain Beach 481](#_Toc532992156)

[CES201.586 Laurentian‑Acadian Lakeshore Beach 482](#_Toc532992157)

[CES203.469 Louisiana Beach 482](#_Toc532992158)

[CES203.301 Northern Atlantic Coastal Plain Sandy Beach 483](#_Toc532992159)

[CES203.535 Southern Atlantic Coastal Plain Florida Beach 484](#_Toc532992160)

[CES203.383 Southern Atlantic Coastal Plain Sea Island Beach 485](#_Toc532992161)

[CES203.463 Texas Coast Beach 485](#_Toc532992162)

[M057. Eastern North American Coastal Dune & Grassland 486](#_Toc532992163)

[CES201.573 Acadian‑North Atlantic Rocky Coast 486](#_Toc532992164)

[CES203.500 East Gulf Coastal Plain Dune and Coastal Grassland 486](#_Toc532992165)

[CES201.026 Great Lakes Dune 487](#_Toc532992166)

[CES203.264 Northern Atlantic Coastal Plain Dune and Swale 488](#_Toc532992167)

[CES203.895 Northern Atlantic Coastal Plain Heathland and Grassland 489](#_Toc532992168)

[CES203.273 Southern Atlantic Coastal Plain Dune and Maritime Grassland 490](#_Toc532992169)

[CES203.539 Southwest Florida Dune and Coastal Grassland 491](#_Toc532992170)

[CES203.465 Texas Coast Dune and Coastal Grassland 492](#_Toc532992171)

[CES203.543 Texas Saline Coastal Prairie 493](#_Toc532992172)

[2.B.4.Nb. Pacific North American Coastal Scrub & Herb Vegetation 494](#_Toc532992173)

[M059. Pacific Coastal Beach & Dune 494](#_Toc532992174)

[CES302.003 Duna Costera de Sonora y la Península de Baja California 494](#_Toc532992175)

[CES206.907 Mediterranean California Northern Coastal Dune 495](#_Toc532992176)

[CES206.908 Mediterranean California Southern Coastal Dune 496](#_Toc532992177)

[CES200.881 North Pacific Maritime Coastal Sand Dune and Strand 497](#_Toc532992178)

[M058. Pacific Coastal Cliff & Bluff 498](#_Toc532992179)

[CES204.094 North Pacific Coastal Cliff and Bluff 498](#_Toc532992180)

[2.C. Shrub & Herb Wetland 499](#_Toc532992181)

[2.C.2. Temperate to Polar Bog & Fen 499](#_Toc532992182)

[2.C.2.Na. North American Bog & Fen 499](#_Toc532992183)

[M877. North American Boreal & Subboreal Alkaline Fen 499](#_Toc532992184)

[CES201.585 Laurentian‑Acadian Alkaline Fen 499](#_Toc532992185)

[CES306.831 Rocky Mountain Subalpine‑Montane Fen 500](#_Toc532992186)

[M876. North American Boreal & Subboreal Bog & Acidic Fen 501](#_Toc532992187)

[CES201.580 Acadian Maritime Bog 501](#_Toc532992188)

[CES201.583 Eastern Boreal‑Sub‑boreal Acidic Basin Fen 502](#_Toc532992189)

[CES202.606 North‑Central Interior and Appalachian Acidic Peatland 503](#_Toc532992190)

[M063. North Pacific Bog & Fen 504](#_Toc532992191)

[CES206.953 Mediterranean California Serpentine Fen 504](#_Toc532992192)

[CES206.952 Mediterranean California Subalpine‑Montane Fen 504](#_Toc532992193)

[CES204.063 North Pacific Bog and Fen 505](#_Toc532992194)

[2.C.2.Nb. Atlantic & Gulf Coastal Plain Pocosin 506](#_Toc532992195)

[M065. Southeastern Coastal Bog & Fen 506](#_Toc532992196)

[CES203.893 Atlantic Coastal Plain Northern Bog 506](#_Toc532992197)

[CES203.267 Atlantic Coastal Plain Peatland Pocosin and Canebrake 507](#_Toc532992198)

[2.C.3. Tropical Freshwater Marsh, Wet Meadow & Shrubland 509](#_Toc532992199)

[2.C.3.Ef. Caribbean‑Mesoamerican Freshwater Marsh, Wet Meadow & Shrubland 509](#_Toc532992200)

[M710. Caribbean Freshwater Marsh, Wet Meadow & Shrubland 509](#_Toc532992201)

[CES411.467 Caribbean Emergent Herbaceous Estuary 509](#_Toc532992202)

[CES411.286 South Florida Everglades Sawgrass Marsh 509](#_Toc532992203)

[CES411.485 South Florida Slough, Gator Hole and Willow Head 510](#_Toc532992204)

[CES411.370 South Florida Wet Marl Prairie 511](#_Toc532992205)

[M711. Mesoamerican Freshwater Marsh, Wet Meadow & Shrubland 512](#_Toc532992206)

[CES402.589 Vegetacion Palustre Mesoamérica 512](#_Toc532992207)

[2.C.4. Temperate to Polar Freshwater Marsh, Wet Meadow & Shrubland 513](#_Toc532992208)

[2.C.4.Nb. Western North American Temperate & Boreal Freshwater Marsh, Wet Meadow & Shrubland 513](#_Toc532992209)

[M888. Arid West Interior Freshwater Marsh 513](#_Toc532992210)

[CES304.059 Inter‑Mountain Basins Interdunal Swale Wetland 513](#_Toc532992211)

[CES300.729 North American Arid West Emergent Marsh 513](#_Toc532992212)

[CES302.747 North American Warm Desert Cienega 515](#_Toc532992213)

[M073. Vancouverian Lowland Marsh, Wet Meadow & Shrubland 516](#_Toc532992214)

[CES204.854 North Pacific Avalanche Chute Shrubland 516](#_Toc532992215)

[CES204.865 North Pacific Shrub Swamp 517](#_Toc532992216)

[CES200.877 Temperate Pacific Freshwater Emergent Marsh 517](#_Toc532992217)

[CES200.878 Temperate Pacific Freshwater Mudflat 518](#_Toc532992218)

[CES204.874 Willamette Valley Wet Prairie 519](#_Toc532992219)

[M893. Western North American Montane Marsh, Wet Meadow & Shrubland 520](#_Toc532992220)

[CES304.084 Columbia Plateau Silver Sagebrush Seasonally Flooded Shrub‑Steppe 520](#_Toc532992221)

[CES306.812 Rocky Mountain Alpine‑Montane Wet Meadow 520](#_Toc532992222)

[CES306.832 Rocky Mountain Subalpine‑Montane Riparian Shrubland 521](#_Toc532992223)

[CES200.998 Temperate Pacific Subalpine‑Montane Wet Meadow 522](#_Toc532992224)

[M074. Western North American Vernal Pool 523](#_Toc532992225)

[CES304.057 Columbia Plateau Vernal Pool 523](#_Toc532992226)

[CES206.948 Northern California Claypan Vernal Pool 525](#_Toc532992227)

[2.C.4.Nc. Southwestern North American Warm Desert Freshwater Marsh & Bosque 526](#_Toc532992228)

[M076. Warm Desert Lowland Freshwater Marsh, Wet Meadow & Shrubland 526](#_Toc532992229)

[CES302.752 North American Warm Desert Riparian Mesquite Bosque 526](#_Toc532992230)

[2.C.4.Nd. Eastern North American Temperate & Boreal Freshwater Marsh, Wet Meadow & Shrubland 526](#_Toc532992231)

[M061. Eastern North American Cool Temperate Seep 526](#_Toc532992232)

[CES202.300 Southern and Central Appalachian Bog and Fen 526](#_Toc532992233)

[M069. Eastern North American Marsh, Wet Meadow & Shrubland 528](#_Toc532992234)

[CES205.687 Eastern Great Plains Wet Meadow, Prairie and Marsh 528](#_Toc532992235)

[CES202.033 Great Lakes Freshwater Estuary and Delta 529](#_Toc532992236)

[CES202.027 Great Lakes Wet‑Mesic Lakeplain Prairie 530](#_Toc532992237)

[CES201.594 Laurentian‑Acadian Freshwater Marsh 531](#_Toc532992238)

[CES201.582 Laurentian‑Acadian Wet Meadow‑Shrub Swamp 531](#_Toc532992239)

[CES202.899 North‑Central Interior Freshwater Marsh 532](#_Toc532992240)

[CES202.701 North‑Central Interior Wet Meadow‑Shrub Swamp 533](#_Toc532992241)

[CES201.722 Northern Great Lakes Coastal Marsh 533](#_Toc532992242)

[M881. Eastern North American Riverscour Vegetation 535](#_Toc532992243)

[CES202.036 Cumberland Riverscour 535](#_Toc532992244)

[CES202.703 Ozark‑Ouachita Riparian 536](#_Toc532992245)

[M071. Great Plains Marsh, Wet Meadow, Shrubland & Playa 537](#_Toc532992246)

[CES303.654 Edwards Plateau Playa 537](#_Toc532992247)

[CES303.661 Great Plains Prairie Pothole 538](#_Toc532992248)

[CES303.666 Western Great Plains Closed Depression Wetland & Playa 539](#_Toc532992249)

[CES303.675 Western Great Plains Open Freshwater Depression Wetland 541](#_Toc532992250)

[2.C.4.Ne. Atlantic & Gulf Coastal Marsh, Wet Meadow & Shrubland 542](#_Toc532992251)

[M066. Atlantic & Gulf Coastal Fresh‑Oligohaline Tidal Marsh 542](#_Toc532992252)

[CES203.259 Atlantic Coastal Plain Embayed Region Tidal Freshwater Marsh 542](#_Toc532992253)

[CES203.507 Florida Big Bend Fresh and Oligohaline Tidal Marsh 543](#_Toc532992254)

[CES203.467 Gulf Coast Chenier Plain Fresh and Oligohaline Tidal Marsh 543](#_Toc532992255)

[CES203.470 Mississippi Delta Fresh and Oligohaline Tidal Marsh 544](#_Toc532992256)

[CES203.516 Northern Atlantic Coastal Plain Fresh and Oligohaline Tidal Marsh 545](#_Toc532992257)

[CES203.376 Southern Atlantic Coastal Plain Fresh and Oligohaline Tidal Marsh 546](#_Toc532992258)

[CES203.472 Texas Coast Fresh and Oligohaline Tidal Marsh 547](#_Toc532992259)

[M067. Atlantic & Gulf Coastal Plain Wet Prairie & Marsh 547](#_Toc532992260)

[CES203.890 Central Florida Herbaceous Pondshore 547](#_Toc532992261)

[CES203.491 Central Florida Wet Prairie and Herbaceous Seep 548](#_Toc532992262)

[CES203.558 East Gulf Coastal Plain Depression Pond 548](#_Toc532992263)

[CES203.192 East Gulf Coastal Plain Savanna and Wet Prairie 550](#_Toc532992264)

[CES203.077 Floridian Highlands Freshwater Marsh 550](#_Toc532992265)

[CES203.258 Southeastern Coastal Plain Interdunal Wetland 551](#_Toc532992266)

[CES203.044 Southeastern Coastal Plain Natural Lakeshore 552](#_Toc532992267)

[CES203.262 Southern Atlantic Coastal Plain Depression Pond 553](#_Toc532992268)

[CES203.078 Southern Coastal Plain Herbaceous Seep and Bog 554](#_Toc532992269)

[CES203.541 Texas‑Louisiana Coastal Prairie Pondshore 555](#_Toc532992270)

[2.C.5. Salt Marsh 556](#_Toc532992271)

[2.C.5.El. Eastern Pacific Coastal Salt Marsh 556](#_Toc532992272)

[M737. Mesoamerican‑South American Pacific Coastal Salt Marsh 556](#_Toc532992273)

[CES402.591 Herbazal Pantanoso Halófilo Costero de Mesoamérica 556](#_Toc532992274)

[CES402.592 Salina Mesoamérica 556](#_Toc532992275)

[M736. Mexican Pacific Coastal Salt Marsh 557](#_Toc532992276)

[CES302.005 Herbazal Pantanoso Costero de Sonora y la Península de Baja California 557](#_Toc532992277)

[2.C.5.Na. North American Great Plains Saline Marsh 557](#_Toc532992278)

[M077. Great Plains Saline Wet Meadow & Marsh 557](#_Toc532992279)

[CES303.669 Western Great Plains Saline Depression Wetland 557](#_Toc532992280)

[2.C.5.Nb. North American Atlantic & Gulf Coastal Salt Marsh 559](#_Toc532992281)

[M079. North American Atlantic & Gulf Coastal Salt Marsh 559](#_Toc532992282)

[CES201.578 Acadian Coastal Salt Marsh 559](#_Toc532992283)

[CES201.579 Acadian Estuary Marsh 559](#_Toc532992284)

[CES203.260 Atlantic Coastal Plain Embayed Region Tidal Salt and Brackish Marsh 560](#_Toc532992285)

[CES203.257 Atlantic Coastal Plain Indian River Lagoon Tidal Marsh 561](#_Toc532992286)

[CES203.508 Florida Big Bend Salt and Brackish Tidal Marsh 562](#_Toc532992287)

[CES203.468 Gulf Coast Chenier Plain Salt and Brackish Tidal Marsh 562](#_Toc532992288)

[CES301.461 Laguna Madre Salt and Brackish Tidal Flat 564](#_Toc532992289)

[CES203.471 Mississippi Delta Salt and Brackish Tidal Marsh 564](#_Toc532992290)

[CES203.303 North‑Central Gulf of Mexico Salt and Brackish Tidal Marsh 565](#_Toc532992291)

[CES203.519 Northern Atlantic Coastal Plain Tidal Salt Marsh 566](#_Toc532992292)

[CES203.270 Southern Atlantic Coastal Plain Salt and Brackish Tidal Marsh 567](#_Toc532992293)

[CES203.473 Texas Coast Salt and Brackish Tidal Marsh 568](#_Toc532992294)

[2.C.5.Nc. Temperate & Boreal Pacific Coastal Salt Marsh 569](#_Toc532992295)

[M081. North American Pacific Coastal Salt Marsh 569](#_Toc532992296)

[CES200.091 Temperate Pacific Tidal Salt and Brackish Marsh 569](#_Toc532992297)

[2.C.5.Nd. North American Western Interior Brackish Marsh, Playa & Shrubland 570](#_Toc532992298)

[M082. Warm & Cool Desert Alkali‑Saline Marsh, Playa & Shrubland 570](#_Toc532992299)

[CES304.998 Inter‑Mountain Basins Alkaline Closed Depression 570](#_Toc532992300)

[CES304.780 Inter‑Mountain Basins Greasewood Flat 571](#_Toc532992301)

[CES304.786 Inter‑Mountain Basins Playa 574](#_Toc532992302)

[CES302.751 North American Warm Desert Playa 574](#_Toc532992303)

[CES301.717 Tamaulipan Saline Lake 575](#_Toc532992304)

[2.C.5.Ue. Tropical Atlantic Coastal Salt Marsh 575](#_Toc532992305)

[M735. Tropical Western Atlantic‑Caribbean Salt Marsh 575](#_Toc532992306)

[CES411.460 Caribbean Salt Flat and Pond 575](#_Toc532992307)

[3. Desert & Semi‑Desert 576](#_Toc532992308)

[3.A. Warm Desert & Semi‑Desert Woodland, Scrub & Grassland 576](#_Toc532992309)

[3.A.1. Tropical Thorn Woodland 576](#_Toc532992310)

[3.A.1.Ea. Caribbean‑Northern Mesoamerican Xeromorphic Scrub & Woodland 576](#_Toc532992311)

[M765. Caribbean‑Northern Mesoamerican Xeromorphic Scrub & Woodland 576](#_Toc532992312)

[CES401.291 Matorral Espinoso de Guerrero 576](#_Toc532992313)

[CES401.308 Matorral Xerofítico del Valle de Motagua 577](#_Toc532992314)

[3.A.2. Warm Desert & Semi‑Desert Scrub & Grassland 578](#_Toc532992315)

[3.A.2.Na. North American Warm Desert Scrub & Grassland 578](#_Toc532992316)

[M086. Chihuahuan Desert Scrub 578](#_Toc532992317)

[CES302.731 Chihuahuan Creosotebush Desert Scrub 578](#_Toc532992318)

[CES302.734 Chihuahuan Mixed Desert and Thornscrub 580](#_Toc532992319)

[CES302.017 Chihuahuan Mixed Salt Desert Scrub 583](#_Toc532992320)

[CES302.737 Chihuahuan Stabilized Coppice Dune and Sand Flat Scrub 584](#_Toc532992321)

[CES302.738 Chihuahuan Succulent Desert Scrub 585](#_Toc532992322)

[M087. Chihuahuan Semi‑Desert Grassland 586](#_Toc532992323)

[CES302.735 Apacherian‑Chihuahuan Semi‑Desert Grassland and Steppe 586](#_Toc532992324)

[CES302.732 Chihuahuan Gypsophilous Grassland and Steppe 589](#_Toc532992325)

[CES302.061 Chihuahuan Loamy Plains Desert Grassland 590](#_Toc532992326)

[CES302.736 Chihuahuan Sandy Plains Semi‑Desert Grassland 592](#_Toc532992327)

[CES302.746 Chihuahuan‑Sonoran Desert Bottomland and Swale Grassland 593](#_Toc532992328)

[M088. Mojave‑Sonoran Semi‑Desert Scrub 595](#_Toc532992329)

[CES302.744 North American Warm Desert Active and Stabilized Dune 595](#_Toc532992330)

[CES302.756 Sonora‑Mojave Creosotebush‑White Bursage Desert Scrub 596](#_Toc532992331)

[CES302.760 Sonoran Granite Outcrop Desert Scrub 598](#_Toc532992332)

[CES302.035 Sonoran Mid‑Elevation Desert Scrub 599](#_Toc532992333)

[CES302.761 Sonoran Paloverde‑Mixed Cacti Desert Scrub 600](#_Toc532992334)

[M512. North American Warm Desert Ruderal Scrub & Grassland 602](#_Toc532992335)

[CES302.733 Apacherian‑Chihuahuan Mesquite Upland Scrub 602](#_Toc532992336)

[M117. North American Warm Semi‑Desert Cliff, Scree & Rock Vegetation 603](#_Toc532992337)

[CES302.743 North American Warm Desert Badland 603](#_Toc532992338)

[CES302.745 North American Warm Desert Bedrock Cliff and Outcrop 604](#_Toc532992339)

[CES302.750 North American Warm Desert Pavement 605](#_Toc532992340)

[CES302.754 North American Warm Desert Volcanic Rockland 606](#_Toc532992341)

[M092. North American Warm‑Desert Xeric‑Riparian Scrub 606](#_Toc532992342)

[CES302.755 North American Warm Desert Wash 606](#_Toc532992343)

[M130. Tamaulipan Scrub & Grassland 607](#_Toc532992344)

[CES301.713 Matorral Crasicaule 607](#_Toc532992345)

[CES301.714 Matorral Submontano 608](#_Toc532992346)

[CES301.986 Tamaulipan Calcareous Thornscrub 609](#_Toc532992347)

[CES301.989 Tamaulipan Caliche Grassland 610](#_Toc532992348)

[CES301.987 Tamaulipan Clay Grassland 611](#_Toc532992349)

[CES301.462 Tamaulipan Loma Shrubland and Grassland 611](#_Toc532992350)

[CES301.984 Tamaulipan Mesquite Upland Scrub 612](#_Toc532992351)

[CES301.983 Tamaulipan Mixed Deciduous Thornscrub 613](#_Toc532992352)

[CES301.992 Tamaulipan Ramadero 614](#_Toc532992353)

[CES301.711 Tamaulipan Saline Thornscrub 615](#_Toc532992354)

[CES301.985 Tamaulipan Savanna Grassland 616](#_Toc532992355)

[CES301.460 South Texas Sand Sheet Grassland 617](#_Toc532992356)

[M089. Viscaino‑Baja California Desert Scrub 618](#_Toc532992357)

[CES302.013 Gulf of California Coast Torchwood‑Cardon Desert Scrub 618](#_Toc532992358)

[CES302.740 Magdalena Plain Desert Scrub 618](#_Toc532992359)

[CES302.739 Matorral Desértico de Magdalena Barrancas 619](#_Toc532992360)

[CES302.006 Matorral Desértico de Maguey y Cirio de la Planicie Costera del Norte de Viscaino 620](#_Toc532992361)

[CES302.007 Northern Viscaino White Bursage‑Agave Inland Low Desert Scrub 621](#_Toc532992362)

[CES401.301 San Lucan Thornscrub 621](#_Toc532992363)

[3.B. Cool Semi‑Desert Scrub & Grassland 622](#_Toc532992364)

[3.B.1. Cool Semi‑Desert Scrub & Grassland 622](#_Toc532992365)

[3.B.1.Ne. Western North American Cool Semi‑Desert Scrub & Grassland 622](#_Toc532992366)

[M093. Great Basin Saltbush Scrub 622](#_Toc532992367)

[CES304.783 Inter‑Mountain Basins Mat Saltbush Shrubland 622](#_Toc532992368)

[CES304.784 Inter‑Mountain Basins Mixed Salt Desert Scrub 624](#_Toc532992369)

[CES302.749 Sonora‑Mojave Mixed Salt Desert Scrub 626](#_Toc532992370)

[M171. Great Basin‑Intermountain Dry Shrubland & Grassland 627](#_Toc532992371)

[CES304.763 Colorado Plateau Blackbrush‑Mormon‑tea Shrubland 627](#_Toc532992372)

[CES304.993 Columbia Basin Foothill and Canyon Dry Grassland 629](#_Toc532992373)

[CES304.775 Inter‑Mountain Basins Active and Stabilized Dune 630](#_Toc532992374)

[CES304.787 Inter‑Mountain Basins Semi‑Desert Grassland 632](#_Toc532992375)

[CES304.788 Inter‑Mountain Basins Semi‑Desert Shrub‑Steppe 634](#_Toc532992376)

[CES302.742 Mojave Mid‑Elevation Mixed Desert Scrub 636](#_Toc532992377)

[CES304.793 Southern Colorado Plateau Sand Shrubland 638](#_Toc532992378)

[M170. Great Basin‑Intermountain Dwarf Sagebrush Steppe & Shrubland 639](#_Toc532992379)

[CES304.762 Colorado Plateau Mixed Low Sagebrush Shrubland 639](#_Toc532992380)

[CES304.080 Columbia Plateau Low Sagebrush Steppe 641](#_Toc532992381)

[CES304.770 Columbia Plateau Scabland Shrubland 642](#_Toc532992382)

[CES304.794 Wyoming Basins Dwarf Sagebrush Shrubland and Steppe 644](#_Toc532992383)

[M169. Great Basin‑Intermountain Tall Sagebrush Steppe & Shrubland 646](#_Toc532992384)

[CES304.083 Columbia Plateau Steppe and Grassland 646](#_Toc532992385)

[CES304.774 Great Basin Xeric Mixed Sagebrush Shrubland 647](#_Toc532992386)

[CES304.777 Inter‑Mountain Basins Big Sagebrush Shrubland 649](#_Toc532992387)

[CES304.778 Inter‑Mountain Basins Big Sagebrush Steppe 652](#_Toc532992388)

[CES304.785 Inter‑Mountain Basins Montane Sagebrush Steppe 655](#_Toc532992389)

[M095. Great Basin‑Intermountain Xeric‑Riparian Scrub 657](#_Toc532992390)

[CES304.781 Inter‑Mountain Basins Wash 657](#_Toc532992391)

[M118. Intermountain Basins Cliff, Scree & Badland Sparse Vegetation 658](#_Toc532992392)

[CES304.765 Colorado Plateau Mixed Bedrock Canyon and Tableland 658](#_Toc532992393)

[CES304.081 Columbia Plateau Ash and Tuff Badland 659](#_Toc532992394)

[CES304.779 Inter‑Mountain Basins Cliff and Canyon 660](#_Toc532992395)

[CES304.789 Inter‑Mountain Basins Shale Badland 661](#_Toc532992396)

[CES304.791 Inter‑Mountain Basins Volcanic Rock and Cinder Land 661](#_Toc532992397)

[4. Polar & High Montane Scrub, Grassland & Barrens 662](#_Toc532992398)

[4.B. Temperate to Polar Alpine & Tundra Vegetation 662](#_Toc532992399)

[4.B.1. Temperate & Boreal Alpine Tundra 662](#_Toc532992400)

[4.B.1.Na. Eastern North American Alpine Tundra 662](#_Toc532992401)

[M131. Eastern North American Alpine Tundra 662](#_Toc532992402)

[CES201.567 Acadian‑Appalachian Alpine Tundra 662](#_Toc532992403)

[4.B.1.Nb. Western North American Alpine Tundra 663](#_Toc532992404)

[M099. Rocky Mountain‑Sierran Alpine Tundra 663](#_Toc532992405)

[CES206.899 Mediterranean California Alpine Bedrock and Scree 663](#_Toc532992406)

[CES206.939 Mediterranean California Alpine Dry Tundra 663](#_Toc532992407)

[CES206.900 Mediterranean California Alpine Fell‑Field 664](#_Toc532992408)

[CES306.809 Rocky Mountain Alpine Bedrock and Scree 665](#_Toc532992409)

[CES306.810 Rocky Mountain Alpine Dwarf‑Shrubland 665](#_Toc532992410)

[CES306.811 Rocky Mountain Alpine Fell‑Field 666](#_Toc532992411)

[CES306.816 Rocky Mountain Alpine Turf 667](#_Toc532992412)

[M101. Vancouverian Alpine Tundra 669](#_Toc532992413)

[CES204.853 North Pacific Alpine and Subalpine Bedrock and Scree 669](#_Toc532992414)

[CES204.862 North Pacific Dry and Mesic Alpine Dwarf‑Shrubland, Fell‑Field and Meadow 670](#_Toc532992415)

[5. Aquatic Vegetation 670](#_Toc532992416)

[5.A. Saltwater Aquatic Vegetation 670](#_Toc532992417)

[5.A.2. Benthic Macroalgae Saltwater Vegetation 670](#_Toc532992418)

[5.A.2.Wb. Temperate Intertidal Shore 670](#_Toc532992419)

[M106. Temperate Pacific Seaweed Intertidal Vegetation 670](#_Toc532992420)

[CES204.879 Temperate Pacific Intertidal Flat 670](#_Toc532992421)

[5.A.3. Benthic Vascular Saltwater Vegetation 671](#_Toc532992422)

[5.A.3.We. Temperate Seagrass Aquatic Vegetation 671](#_Toc532992423)

[M184. Temperate Pacific Seagrass Intertidal Vegetation 671](#_Toc532992424)

[CES200.882 North Pacific Maritime Eelgrass Bed 671](#_Toc532992425)

[5.B. Freshwater Aquatic Vegetation 672](#_Toc532992426)

[5.B.2. Temperate to Polar Freshwater Aquatic Vegetation 672](#_Toc532992427)

[5.B.2.Na. North American Freshwater Aquatic Vegetation 672](#_Toc532992428)

[M109. Western North American Freshwater Aquatic Vegetation 672](#_Toc532992429)

[CES200.876 Temperate Pacific Freshwater Aquatic Bed 672](#_Toc532992430)

[6. Open Rock Vegetation 673](#_Toc532992431)

[6.B. Temperate & Boreal Open Rock Vegetation 673](#_Toc532992432)

[6.B.1. Temperate & Boreal Cliff, Scree & Other Rock Vegetation 673](#_Toc532992433)

[6.B.1.Na. Eastern North American Temperate Cliff, Scree & Rock Vegetation 673](#_Toc532992434)

[M111. Eastern North American Cliff & Rock Vegetation 673](#_Toc532992435)

[CES202.689 Central Interior Acidic Cliff and Talus 673](#_Toc532992436)

[CES202.690 Central Interior Calcareous Cliff and Talus 673](#_Toc532992437)

[CES202.309 Cumberland Acidic Cliff and Rockhouse 674](#_Toc532992438)

[CES203.492 East Gulf Coastal Plain Dry Chalk Bluff 675](#_Toc532992439)

[CES201.025 Great Lakes Acidic Rocky Shore and Cliff 676](#_Toc532992440)

[CES201.569 Laurentian‑Acadian Acidic Cliff and Talus 676](#_Toc532992441)

[CES201.570 Laurentian‑Acadian Calcareous Cliff and Talus 677](#_Toc532992442)

[CES202.601 North‑Central Appalachian Acidic Cliff and Talus 677](#_Toc532992443)

[CES202.603 North‑Central Appalachian Circumneutral Cliff and Talus 678](#_Toc532992444)

[CES202.330 Southern Appalachian Montane Cliff and Talus 679](#_Toc532992445)

[CES202.356 Southern Interior Calcareous Cliff 680](#_Toc532992446)

[CES202.386 Southern Piedmont Cliff 681](#_Toc532992447)

[M115. Great Plains Badlands Vegetation 682](#_Toc532992448)

[CES303.663 Western Great Plains Badlands 682](#_Toc532992449)

[M116. Great Plains Cliff, Scree & Rock Vegetation 683](#_Toc532992450)

[CES303.664 Southwestern Great Plains Canyon 683](#_Toc532992451)

[CES303.665 Western Great Plains Cliff and Outcrop 684](#_Toc532992452)

[6.B.1.Nb. Western North American Temperate Cliff, Scree & Rock Vegetation 685](#_Toc532992453)

[M887. Western North American Cliff, Scree & Rock Vegetation 685](#_Toc532992454)

[CES206.903 Central California Coast Ranges Cliff and Canyon 685](#_Toc532992455)

[CES206.902 Klamath‑Siskiyou Cliff and Outcrop 685](#_Toc532992456)

[CES206.905 Mediterranean California Serpentine Barrens 686](#_Toc532992457)

[CES204.092 North Pacific Active Volcanic Rock and Cinder Land 686](#_Toc532992458)

[CES204.093 North Pacific Montane Massive Bedrock, Cliff and Talus 687](#_Toc532992459)

[CES204.095 North Pacific Serpentine Barren 688](#_Toc532992460)

[CES306.815 Rocky Mountain Cliff, Canyon and Massive Bedrock 688](#_Toc532992461)

[CES206.901 Sierra Nevada Cliff and Canyon 689](#_Toc532992462)

[CES206.904 Southern California Coast Ranges Cliff and Canyon 690](#_Toc532992463)

[X. Not linked to hierarchy 691](#_Toc532992464)

[. 691](#_Toc532992465)

[. 691](#_Toc532992466)

[. 691](#_Toc532992467)

[. 691](#_Toc532992468)

[CES100.728 North American Glacier and Ice Field 691](#_Toc532992469)

1. Forest & Woodland

1.A. Tropical Forest & Woodland

1.A.1. Tropical Dry Forest & Woodland

1.A.1.Ea. Caribbean‑Mesoamerican Dry Forest & Woodland

M134. Caribbean Coastal Lowland Dry Forest

CES411.421 Caribbean Coastal Dry Evergreen Forest

**Primary Division:** Caribbean (411)

**Land Cover Class:** Forest and Woodland

**Spatial Scale & Pattern:** Large patch

**Required Classifiers:** Natural/Semi‑natural; Vegetated (>10% vasc.); Upland

**Diagnostic Classifiers:** Humus carbonate soils; Limestone

**Concept Summary:** This system represents tropical forests characterized by a dry season of several months, that occur in coastal lowlands, littoral or sub‑littoral flatlands with rock outcrops and higher terraces facing the sea, on limestone coral shelves, humic carbonate soils, shallow red ferrallitic soils, or sandy soils close to the coast in the Greater Antilles and other Caribbean islands such as those of the Bahamas and Virgin Islands archipelagos. The species composition and structure of these forests vary depending upon the substrate and climate across their distribution. They are evergreen forests, or at least most of the dominant tree species are evergreen, with thick, sclerophyllous, small leaves and only a third of the trees deciduous or semi‑deciduous (Wadsworth 1964, cited in Murphy and Lugo 1995). They have relative low floristic diversity and a tendency to have high species dominance. The canopy is somewhat open, between 6‑10 m in height or taller in the case of occurrences in Cuba and sites in St. John where they have two canopy layers, with the upper layer reaching 12‑15 m and occasional emergents up to 20 m tall. The density of stems tends to be very high. The woody understory is mostly evergreen. The herb layer is poorly developed or completely lacking. Species composition varies depending on past uses, substrate, and local climate. The following list of species is diagnostic for this system: *Bursera simaruba, Coccoloba diversifolia, Erythroxylum areolatum, Eugenia axillaris, Exostema caribaeum, Exothea paniculata, Guettarda krugii, Guaiacum sanctum, Guapira obtusata, Gymnanthes lucida, Metopium toxiferum, Sideroxylon foetidissimum*, and *Sideroxylon salicifolium*. Common accompanying species are *Pisonia albida, Pictetia aculeata, Thouinia striata var. portoricensis, Coccoloba krugii, Erithalis fruticosa, Guettarda elliptica, Lysiloma latisiliquum (= Lysiloma bahamense), Thrinax radiata, Ficus aurea, Capparis cynophallophora, Capparis flexuosa, Chrysophyllum oliviforme, Tabernaemontana amblyocarpa, Caesalpinia* spp., *Ateleia gummifera, Eugenia foetida, Eugenia confusa, Erythroxylum rotundifolium, Bourreria succulenta, Amyris elemifera, Krugiodendron ferreum, Bucida buceras, Terminalia neglecta, Chionanthus ligustrinus (= Linociera ligustrina), Chrysobalanus icaco, Colubrina* spp., *Randia aculeata, Coccothrinax littoralis*, and *Sabal parviflora*. The species composition reported for St. John includes as dominants *Guapira fragrans (= Pisonia fragrans), Nectandra coriacea (= Ocotea coriacea), Coccoloba microstachya, Maytenus laevigata, Bourreria succulenta*, and *Tabebuia heterophylla*.

DISTRIBUTION

**Range:** This system is found in Cuba, the Dominican Republic, Jamaica, Puerto Rico, Trinidad, the Bahamas, Cayman Islands, and the Virgin Islands.

**Divisions:** 411:C

**Nations:** BS, CU, DO, JM, PR, TT, VI

CONCEPT

**Environment:** Precipitation in the distribution range of this forest in Puerto Rico and over most of the islands of Culebra and Vieques ranges from 600 to 1100 mm per year (Brandeis et al. 2006), with two dry seasons, the longer one from December to April and a shorter one from June to August. The annual precipitation range is somewhat higher across much of the distribution of this forest type (800‑1300 mm) (Murphy and Lugo 1995).

Limestone is the dominant substrate in Caribbean coastal dry forests, with skeletal organic soils with minor mineral components, rarely exceeding 20 cm in depth (Snyder et al. 1990, cited in Gillespie 2006). In the Greater Antilles the distribution of dry forests is indicative of limestone substrates occurring in narrow strips on the northern and southern coastal areas. Rocky limestone soils have low water‑holding capacity and nutritional limitations imposed by their calcareous composition. Isolated inland, ultramafic soils associated with limestone also support dry forests. In flat low‑lying limestone archipelagos, such as the Bahamas, the Cayman Islands, Mona and Anegada, dry forests and shrublands dominate. In volcanic, low mountainous islands of the Lesser Antilles, dry forests dominate except for protected sites and ravines where moist forest can grow (Lugo et al. 2006).

Caribbean dry forests have to cope with highly stressful conditions given the combination of environmental features such as low moisture availability, long dry seasons, decadal cycles of pronounced drought, wind exposure and salt spray in littoral locations. These forests are also periodically exposed to hurricane conditions with effects that span from flooding with seawater to treefall and other structural changes due to strong winds.

**Dynamics:** Caribbean coastal dry forests are exposed to harsh environmental conditions that, depending on their intensity, can cause damage or diebacks, such as seasonal water deficit, nutrient stress, strong winds and salt spray, and saltwater storm surge. This has influenced in the development of structural and physiological mechanisms to cope, making them very resilient to disturbance. Among the more outstanding ones are a high resistance to wind (short stature), a high proportion of root biomass, high soil carbon and nutrient accumulation below ground, the ability of most tree species to resprout, and high nutrient use efficiency (Lugo et al. 2006). Fire is not part of the natural dynamics of Caribbean coastal dry forests, but hurricanes are, which naturally results in considerable heterogeneity in habitat structure and food availability on small spatial scales. This structuring of coastal dry forest by frequent natural disturbance may favor their resilience to anthropogenic disturbance and fragmentation.

SOURCES

**References:** Acevedo‑Rodriguez et al. 1996, Areces‑Mallea et al. 1999, Borhidi 1991, Brandeis et al. 2006, Franklin and Steadman 2013, Franklin et al. 2015, Gillespie 2006, Josse et al. 2003*, Lugo et al. 2006, Martinuzzi et al. 2013, Murphy and Lugo 1995, Snyder et al. 1990, Tolentino and Peña 1998

**Version:** 30 Oct 2015 **Stakeholders:** Caribbean, Latin America, U.S. Territories

**Concept Author:** C. Josse **LeadResp:** Latin America

CES411.419 Caribbean Semi‑deciduous Lowland Forest

**Primary Division:** Caribbean (411)

**Land Cover Class:** Forest and Woodland

**Spatial Scale & Pattern:** Large patch

**Required Classifiers:** Natural/Semi‑natural; Vegetated (>10% vasc.); Upland

**Diagnostic Classifiers:** Tropical brown soils

**Concept Summary:** This system is found in lowlands and low hills (ca. 300 m elevation) that are characterized by a dry season. It is composed of two canopy layers with the upper canopy 18‑25 m tall and about 75% deciduous species. The woody understory, 6‑12 m, is mostly evergreen. The herb layer is poorly developed or completely lacking. The prevailing conditions determine if this forest type is deciduous or semi‑deciduous. In sandy or rocky areas with nutrient‑poor soils, forests are lower in height and include a spiny sclerophyllous shrub layer. The following list of species is diagnostic for this system: *Acacia muricata, Allophylus cominia, Amyris balsamifera, Andira inermis, Ateleia cubensis, Brya ebenus, Byrsonima spicata, Capparis* spp., *Catalpa macrocarpa (= Catalpa punctata), Cedrela odorata (= Cedrela mexicana), Coccoloba* spp., *Copernicia baileyana, Copernicia sueroana, Copernicia textilis, Cordia laevigata, Diospyros crassinervis, Diospyros halesioides, Eugenia confusa, Ficus citrifolia, Hymenaea courbaril, Manilkara jaimiqui, Manilkara bidentata, Maytenus buxifolia, Myrcia citrifolia, Myrciaria floribunda, Phyllostylon brasiliensis, Picramnia pentandra, Guapira fragrans (= Pisonia fragrans), Pisonia subcordata, Savia sessiliflora, Swietenia mahagoni, Tabebuia heterophylla (= Tabebuia pallida), Tabebuia shaferi, Trichilia hirta, Trichilia pallida*, and *Zanthoxylum martinicense*. In Puerto Rico, the following species are typical: *Bucida buceras, Citharexylum spinosum (= Citharexylum fruticosum), Coccoloba diversifolia, Cordia laevigata, Guaiacum officinale, Guazuma ulmifolia, Lonchocarpus domingensis*, and *Rauvolfia nitida*. The species composition reported for St. John includes as dominants *Inga laurina, Byrsonima spicata, Acacia muricata, Nectandra coriacea (= Ocotea coriacea), Tabebuia heterophylla, Faramea occidentalis, Chionanthus compactus*, and *Guazuma ulmifolia*.

**Comments:** Various references show that composition across sites representative of CES411.421 and CES411.419 is not totally differential because some species among the top dominant ones are present in both types, for example *Guapira fragrans* or *Tabebuia heterophylla*. Thus, a higher stature and density of the stand, as well as the predominance of mesophyllous and deciduous instead of sclerophyllous, evergreen foliage, are key features to distinguish this type.

DISTRIBUTION

**Range:** This system is found in Cuba, the Dominican Republic, the Lesser Antilles, Puerto Rico, the coast of Venezuela, and the Virgin Islands.

**Divisions:** 411:C

**Nations:** CU, DO, PR, VE, VI, XD

CONCEPT

**Environment:** In the Greater Antilles the distribution of dry forests is indicative of limestone substrates occurring in narrow strips on the northern and southern coastal areas. Isolated inland, ultramafic soils associated with limestone also support dry forests. Annual precipitation ranges from 1500 mm to less than 1000 mm with one or two long and pronounced dry seasons. Mean temperatures between 24‑27°C are typical throughout the area of distribution. This type of forest with local variations occurs throughout moister areas, in protected uplands with more elevational range, drainage areas, and coastal protected valleys.

**Dynamics:** Overall, Caribbean coastal dry forests are exposed to harsh environmental conditions that, depending on their intensity, can cause damage or diebacks, such as seasonal water deficit, nutrient stress, strong winds and salt spray, and saltwater storm surge. This has influenced the development of structural and physiological mechanisms to cope, making them very resilient to disturbance. Among the more outstanding ones are a high resistance to wind (short stature), a high proportion of root biomass, high soil carbon and nutrient accumulation below ground, the ability of most tree species to resprout, and high nutrient use efficiency (Lugo et al. 2006).<br />

Fire is not part of the natural dynamics of Caribbean coastal dry forests (though many dry forests are now subject to anthropogenic fires).

SOURCES

**References:** Areces‑Mallea et al. 1999, Borhidi 1991, Dansereau 1966, Dominica Ministry of Agriculture and Environment n.d., Figueroa Colon 1996, Helmer et al. 2002, International Institute of Tropical Forestry n.d., Josse et al. 2003*, Lugo et al. 2006, Martinuzzi et al. 2013, Murphy and Lugo 1995, TNC 2000, TNC 2004a, Tolentino and Peña 1998

**Version:** 30 Oct 2015 **Stakeholders:** Caribbean, Latin America, U.S. Territories

**Concept Author:** C. Josse **LeadResp:** Latin America

CES411.287 South Florida Hardwood Hammock

**Primary Division:** Caribbean (411)

**Land Cover Class:** Forest and Woodland

**Spatial Scale & Pattern:** Large patch

**Required Classifiers:** Natural/Semi‑natural; Vegetated (>10% vasc.); Upland

**Diagnostic Classifiers:** Forest and Woodland (Treed); Alkaline Soil; Broad‑Leaved Evergreen Tree

**National Mapping Codes:** EVT 2333; ESLF 4139; ESP 1333

**Concept Summary:** This rockland tropical hammock system, as currently defined, occurs only in extreme southern Florida. It consists of upland hardwood forest on elevated ridges of limestone in three discrete major regions; the Keys, southeastern Big Cypress, and the Miami Rock Ridge. Tropical hardwood species are diagnostic of the system. Among the species likely to be encountered throughout are *Bursera simaruba, Coccoloba diversifolia*, and *Eugenia axillaris*. *Quercus laurifolia* is one of the few temperate species which attains prominence in this system. These forests tend to have a dense canopy that produces deeper shade, less evaporation, and lower air temperature than surrounding vegetation. This microclimate, in combination with high water tables, tends to keep humidity levels high. A number of orchid and bromeliad species thrive in such conditions. Unlike most coastal plain systems, fire is a major threat to South Florida Hardwood Hammock (CES411.287). For this reason, many examples occur alongside natural firebreaks.

DISTRIBUTION

**Range:** This system is endemic to south Florida.

**Divisions:** 411:C

**TNC Ecoregions:** 54:C

**Nations:** US

**Subnations:** FL

**Map Zones:** 56:C

**USFS Ecomap Regions:** 411A:CC

CONCEPT

**Environment:** This system occurs in three discrete regions of south Florida. Underlying geology and soils are somewhat different among these regions, and the juxtaposition of the system may be somewhat unique. Generally, soils are highly organic with uneven and widely ranging thickness (Snyder et al. 1990). These forests tend to have a dense canopy that produces deeper shade, less evaporation, and lower air temperature than surrounding vegetation. This microclimate, in combination with high water tables, tends to keep humidity levels high and the community quite mesic (FNAI 1990). Unlike most coastal plain ecological systems, fire is a major threat to South Florida Hardwood Hammock (CES411.287). For this reason, many examples occur alongside natural firebreaks, such as the leeward side of exposed limestone (Robertson 1955), moats created by limestone solution (Duever et al. 1986), and elevated outcrops above marshes, scrub cypress, or sometimes mangrove swamps (Snyder et al. 1990).

**Vegetation:** There tends not to be strong dominance in these forests, so the principal species list can be long. Tropical hardwood species are diagnostic of the system, although few are common or dominant in all regions where these hammocks occur (Snyder et al. 1990). Among the species likely to be encountered throughout are *Bursera simaruba, Coccoloba diversifolia*, and *Eugenia axillaris*. The northward ranges of these species are limited by the incidence of frosts (Drew and Schomer 1984). Other typical dominant tree species, in no real order, are *Metopium toxiferum, Swietenia mahagoni, Zanthoxylum fagara, Gymnanthes lucida (= Ateramnus lucidus), Piscidia piscipula*, and *Pithecellobium keyense* (T. Armentano pers. comm.). Other species can include *Lysiloma latisiliquum, Nectandra coriacea, Ficus aurea, Sideroxylon foetidissimum, Eugenia foetida, Guapira discolor, Coccoloba uvifera, Leucothrinax morrisii (= Thrinax morrisii), Thrinax radiata, Erithalis fruticosa, Krugiodendron ferreum, Casasia clusiifolia, Erithalis fruticosa, Byrsonima lucida*, and *Capparis flexuosa*.

**Dynamics:** Groundwater and seasonal pooling and drying of the soil are important dynamics. There is organic soil accumulation, thick in some areas and thin in others. Solution‑eroded limestone provides wet pockets and dry patches in the environment. Thick organic soil helps maintain high levels of moisture in the system. Hurricanes are a part of the natural dynamics of this ecological system. Fire is very infrequent, due to the protection of this ecological system, many examples occur alongside natural firebreaks.

SOURCES

**References:** Armentano pers. comm., Comer et al. 2003*, Davis 1943, Drew and Schomer 1984, Duever et al. 1986, Enge et al. 2002, Eyre 1980, FNAI 2010a, Harshberger 1914a, LANDFIRE 2007a, Robertson 1955, Ross et al. 1992, Snyder et al. 1990

**Version:** 14 Jan 2014 **Stakeholders:** Southeast

**Concept Author:** R. Evans **LeadResp:** Southeast

CES411.369 Southeast Florida Coastal Strand and Maritime Hammock

**Primary Division:** Caribbean (411)

**Land Cover Class:** Forest and Woodland

**Spatial Scale & Pattern:** Linear

**Required Classifiers:** Natural/Semi‑natural; Vegetated (>10% vasc.); Upland

**Diagnostic Classifiers:** Forest and Woodland (Treed)

**National Mapping Codes:** EVT 2337; ESLF 4143; ESP 1337

**Concept Summary:** This ecological system occurs as a narrow band of hardwood forest and shrublands along the Atlantic Coast of southeastern Florida (approximately Volusia County southward). It is found on stabilized, old, coastal dunes, often with substantial shell components. The vegetation is characterized by hardwood species with tropical affinities, such as *Guapira discolor* and *Exothea paniculata*. As such, the northern extent of this type is limited by periodic freezes. This system is closely related to both inland tropical hammocks and southwest Florida maritime hammocks, and may share some species overlap with each.

**Comments:** This system may be distinguished from southwest Florida maritime hammocks by geographic location, presence of certain indicator species lacking from southwest type (*Guapira discolor* and *Exothea paniculata*), and relatively harsher coastal exposure. It is distinguished from maritime hammocks further north which contain temperate species including *Persea borbonia, Quercus virginiana, Magnolia grandiflora*, and *Juniperus virginiana var. silicicola* (Johnson and Muller 1993a). Thatch palms (*Leucothrinax morrisii, Thrinax radiata*) are found in rockland hammocks, but absent from maritime hammocks.

DISTRIBUTION

**Range:** Endemic to south Florida.

**Divisions:** 411:C

**TNC Ecoregions:** 54:C

**Nations:** US

**Subnations:** FL

**Map Zones:** 56:C

**USFS Ecomap Regions:** 232G:CC, 411A:CC

CONCEPT

**Environment:** This system occurs along the coast on stabilized, old coastal dunes, often with substantial shell components. The northern extent of this type is limited by periodic freezes.

**Dynamics:** The northern extent of this type is limited by periodic freezes and lack of cold tolerance of tropical plants, such as *Guapira discolor* and *Exothea paniculata* (Johnson and Muller 1993a). Maritime hammocks are relatively stable forest communities, as long as the canopy remains intact and the underlying landform is stable (FNAI 1990). Surface fires may help to maintain the open understory (Landfire 2007a). The shrub‑dominated, coastal strand communities are considered ecotonal, and historically burned more frequently than maritime hammocks, possibly every 4‑5 years (Austin and Coleman‑Marois 1977). However, there is some disagreement on this point. There is little information on natural fire frequency in coastal strand (FNAI 2010a). The low stature of strand is due to the influence of storms and the ongoing salt spray pruning (FNAI 2010a). Fire is not needed to explain the shrub‑dominated vegetation of coastal strands (Landfire 2007a).

SOURCES

**References:** Austin and Coleman‑Marois 1977, Comer et al. 2003*, Eyre 1980, FNAI 2010a, Johnson and Muller 1993a, LANDFIRE 2007a

**Version:** 14 Jan 2014 **Stakeholders:** Southeast

**Concept Author:** R. Evans, after Johnson and Muller (1993a) **LeadResp:** Southeast

CES411.368 Southwest Florida Coastal Strand and Maritime Hammock

**Primary Division:** Caribbean (411)

**Land Cover Class:** Forest and Woodland

**Spatial Scale & Pattern:** Linear

**Required Classifiers:** Natural/Semi‑natural; Vegetated (>10% vasc.); Upland

**Diagnostic Classifiers:** Forest and Woodland (Treed); Coast

**National Mapping Codes:** EVT 2336; ESLF 4142; ESP 1336

**Concept Summary:** This ecological system occurs as a narrow band of hardwood forest and strand lying just inland of the coastal dune system in southwestern Florida. It is found on stabilized, old, coastal dunes, often with substantial shell components. The vegetation is characterized by hardwood species with tropical affinities. As such, the northern extent of this type is limited by periodic freezes and cold tolerance of tropical constituent species, such as *Piscidia piscipula* and *Eugenia axillaris*. This system is closely related to both inland tropical hammocks and southeast Florida maritime hammocks, and may share some species overlap with each.

**Comments:** This system may be distinguished from southeast Florida maritime hammocks by geographic location, presence/absence of certain indicator species, and relatively less harsh coastal exposure. It is distinguished from maritime hammocks further north which contain temperate species including *Persea borbonia, Quercus virginiana, Magnolia grandiflora*, and *Juniperus virginiana var. silicicola* (Johnson and Muller 1993a).

DISTRIBUTION

**Range:** Endemic to south Florida.

**Divisions:** 411:C

**TNC Ecoregions:** 54:C

**Nations:** US

**Subnations:** FL

**Map Zones:** 56:C

**USFS Ecomap Regions:** 232D:CC, 411A:CC

CONCEPT

**Environment:** This system occurs along the coast on stabilized, old coastal dunes, often with substantial shell components. The northern extent of this type is limited by periodic freezes.

**Dynamics:** The northern extent of this type is limited by periodic freezes and lack of cold tolerance of tropical plants, such as *Piscidia piscipula* and *Eugenia axillaris* (Johnson and Muller 1993a). Maritime hammocks are relatively stable forest communities, as long as the canopy remains intact and the underlying landform is stable (FNAI 1990). Surface fires may help to maintain the open understory (Landfire 2007a). The shrub‑dominated, coastal strand communities are considered ecotonal, and historically burned more frequently than maritime hammocks, possibly every 4‑5 years (Austin and Coleman‑Marois 1977). However, there is some disagreement on this point. There is little information on natural fire frequency in coastal strand (FNAI 2010a). The low stature of strand is due to the influence of storms and the ongoing salt spray pruning (FNAI 2010a). Fire is not needed to explain the shrub‑dominated vegetation of coastal strands (Landfire 2007a).

SOURCES

**References:** Comer et al. 2003*, Eyre 1980, FNAI 2010a, Johnson 1994b, Johnson and Muller 1993a, LANDFIRE 2007a

**Version:** 14 Jan 2014 **Stakeholders:** Southeast

**Concept Author:** R. Evans, after Johnson and Muller (1993a) **LeadResp:** Southeast

M294. Caribbean Dry Limestone Forest

CES411.457 Caribbean Edapho‑Xerophilous "Mogote" Complex

**Primary Division:** Caribbean (411)

**Land Cover Class:** Forest and Woodland

**Spatial Scale & Pattern:** Small patch

**Required Classifiers:** Natural/Semi‑natural; Vegetated (>10% vasc.); Upland

**Diagnostic Classifiers:** Limestone

**Concept Summary:** This system includes the steep slopes and plateaus of towerlike karstic hills up to 300‑600 m elevation, with bare karstic rock or more‑or‑less eroded skeletal soils, or limestone cliffs, and the narrow valleys and gorges in between. Puerto Rican karst forests, regardless of rainfall conditions, share common characteristics, including physiognomy and leaf characteristics. Karst forests are characterized by trees of small diameter, high tree density, and leaf scleromorphy. Stands have a tendency to show signs of being exposed to frequent drought conditions. Even in the moist and wet karst belt, forests have a high proportion of deciduous tree species and show a high degree of scleromorphism (Chinea 1980). This is probably due to the rapid rate of runoff and infiltration of rainwater, low water storage in shallow soils, and high sunlight. Depending on the position and the substrate. At the base of mogotes the forest can be mesic with a closed canopy of evergreen species 25‑30 m tall. On slopes and tops the vegetation is a deciduous forest/woodland with trees of 16‑18 m and sclerophyllous leaves. In Cuban mogotes, the slope forest has a 10‑ to 16‑m high open canopy of deciduous trees with barrel‑like trunks and abundant columnar cacti, but can grade to a shrubland dominated by terrestrial bromeliads and diverse sclerophyllous shrubs and trees. The following list of species is diagnostic for this system: *Bombacopsis cubensis, Gaussia princeps, Spathelia brittonii, Thrinax punctulata, Omphalea hypoleuca, Microcycas calocoma, Plumeria emarginata, Trichilia havanensis, Hohenbergia penduliflora, Vriesea dissitiflora, Tillandsia* spp., *Ceratopyxis verbenacea, Eugenia galleata, Psidium vicentinum, Malpighia roigiana, Guettarda calcicola, Agave tubulata, Leptocereus assurgens, Siemensia pendula, Pilosocereus brooksianus, Agave* spp., *Coccothrinax elegans, Tabebuia albicans, Alvaradoa arborescens, Plumeria* spp., *Swietenia mahagoni, Colubrina elliptica, Catalpa brevipes, Zanthoxylum spinosum, Cordia alliodora, Dendropanax arboreus, Bernardia dichotoma, Eugenia monticola (= Eugenia maleolens), Forsteronia corymbosa*. In Puerto Rico, the following species are common: *Dendropanax arboreus* and *Quararibea turbinata* in the mesic forest, *Coccoloba diversifolia* and *Bursera simaruba* in the deciduous forest, and *Clusia rosea* on the cliffs.

DISTRIBUTION

**Range:** This system is found in Cuba, Dominican Republic, Jamaica, and Puerto Rico.

**Divisions:** 411:C

**Nations:** CU, DO, JM, PR

CONCEPT

**Environment:** In northern Puerto Rico karst, mogotes are isolated, steep‑sided hills or towers that rise out of the blanket sand deposits. Mogotes may be aligned in ridges along which they form a series of sawteeth. Solution caves are visible on the sides of the mogotes, but they don't usually pass through the hill. Mogotes have a rounded or pointed hard cap, generally 5 to 10 m thick. Reprecipitated limestone on slopes tends to form nearly vertical slopes. Since the rate of this process is dependent on climatic factors which are not uniform around the hill, the mogote tends to become asymmetric, with a steep slope on one side and a gentler slope on the other. The ecological system is called a complex because of the diversity of vegetation types resulting from ecological gradients due to different exposures to precipitation, wind and substrates, with deep fertile soils in valleys and shallow, rocky, and infertile soils on tops of mogotes, and slopes exhibiting intermediate edaphic conditions.

**Dynamics:** Droughts and hurricanes are the main drivers of the natural dynamics of this system. Low rainfall intensities of 76 mm/d have a recurrence interval of 1 year while high rainfall intensities of >305 mm/d are possible during hurricane conditions or when low‑pressure systems become stationary. These events have a recurrence interval of 100 years (Gómez Gómez 1984). Forests and other natural ecosystems of the limestone region recover quickly from hurricanes and storms (Wadsworth and Englerth 1959, cited in Lugo et al. 2001). Moreover, these events transport vast amounts of freshwater to the island and trigger many ecologically beneficial functions such as the reproduction of karst forest plants and animals, and the maintenance of the hydrological cycle of the karst area.

SOURCES

**References:** Borhidi 1991, Chinea 1980, Dansereau 1966, Figueroa Colon 1996, Gómez Gómez 1984, Josse et al. 2003*, Lugo et al. 2001, Pool and Morris 1979

**Version:** 08 Jan 2015 **Stakeholders:** Caribbean, Latin America, U.S. Territories

**Concept Author:** C. Josse **LeadResp:** Latin America

CES411.465 Caribbean Submontane/Montane Karstic Forest

**Primary Division:** Caribbean (411)

**Land Cover Class:** Forest and Woodland

**Spatial Scale & Pattern:** Small patch

**Required Classifiers:** Natural/Semi‑natural; Vegetated (>10% vasc.); Upland

**Diagnostic Classifiers:** Humus carbonate soils

**Concept Summary:** This system occurs as small patches in submontane or montane rainforest zones, below 600 m elevation in Puerto Rico and up to 1100 m in higher mountains with karst outcrops. It is composed of drought‑tolerant deciduous trees with open canopy layers, 6‑8 m tall. The shrub layer is 2‑3 m high and very dense. Rocks and trunks are covered by mosses and epiphytes. The following list of species is diagnostic for this system: *Thouinia clarensis, Fadyenia hookeri (= Garrya fadyenii), Mahonia tenuifolia (= Berberis tenuifolia), Coccothrinax trinitensis, Terminalia neglecta, Ocotea floribunda, Tabebuia sauvallei, Tabebuia bibracteolata, Bernardia dichotoma, Citharexylum matheanum, Savia sessiliflora, Erythroxylum clarense, Karwinskia potrerilloana, Psychotria martii, Zanthoxylum cubense, Agave* and Cactaceae. In Puerto Rico, the following species are typical: *Coccoloba diversifolia, Bursera simaruba, Bucida buceras*, and *Zanthoxylum martinicense*. Other characteristic species include *Thouinia striata, Nectandra coriacea (= Ocotea coriacea), Tetrazygia elaeagnoides, Gaussia attenuata, Rondeletia inermis, Guettarda scabra, Eugenia confusa, Eugenia* spp., *Coccothrinax barbadensis (= Coccothrinax alta), Leucothrinax morrisii (= Thrinax morrisii)*, and *Aiphanes minima (= Aiphanes acanthophylla)*. In Jamaica common species are *Sideroxylon portoricense (= Bumelia nigra), Cedrela odorata, Cinnamomum montanum, Coccoloba swartzii, Guapira fragrans, Nectandra patens*, and *Pisonia subcordata*.

DISTRIBUTION

**Range:** This system is found in Cuba, Jamaica, and Puerto Rico.

**Divisions:** 411:C

**Nations:** CU, JM, PR

CONCEPT

**Dynamics:** Droughts and hurricanes are the main drivers of the natural dynamics of this system. Low rainfall intensities of 76 mm/d have a recurrence interval of 1 year while high rainfall intensities of >305 mm/d are possible during hurricane conditions or when low‑pressure systems become stationary. These events have a recurrence interval of 100 years (Gómez Gómez 1984). Forests and other natural ecosystems of the limestone region recover quickly from hurricanes and storms (Wadsworth and Englerth 1959, cited in Lugo et al. 2001). Moreover, these events transport vast amounts of freshwater to the island and trigger many ecologically beneficial functions such as the reproduction of karst forest plants and animals, and the maintenance of the hydrological cycle of the karst area.

SOURCES

**References:** Areces‑Mallea et al. 1999, Borhidi 1991, Chinea 1980, Dansereau 1966, Figueroa Colon 1996, Gómez Gómez 1984, Josse et al. 2003*, Little and Wadsworth 1964, Lugo et al. 2001, TNC 2000, TNC 2004a

**Version:** 08 Jan 2015 **Stakeholders:** Caribbean, Latin America, U.S. Territories

**Concept Author:** C. Josse **LeadResp:** Latin America

M296. Caribbean‑Mesoamerican Pine Dry Forest

CES411.463 Bahamas Pine Barrens

**Primary Division:** Caribbean (411)

**Land Cover Class:** Forest and Woodland

**Spatial Scale & Pattern:** Small patch

**Required Classifiers:** Natural/Semi‑natural; Vegetated (>10% vasc.); Upland

**Diagnostic Classifiers:** Limestone

**Concept Summary:** These are open pine woodlands on limestones. The canopy is formed by pines and silver‑thatch palm and reaches between 4‑10 m high. There is substantial grass coverage. These woodlands occur on the boundary between wetland and upland situations. The following list of species is diagnostic for this system: *Coccothrinax argentea, Ernodea littoralis, Pinus caribaea var. bahamensis, Sabal palmetto, Setaria pumila (= Setaria glauca), Tabebuia bahamensis, Tetrazygia bicolor, Vernonia bahamense*, and *Zanthoxylum fagara*.

DISTRIBUTION

**Range:** Grand Bahama and Abaco on the Little Bahama Bank, and Andros and New Providence on the Great Bahama Bank.

**Divisions:** 411:C

**Nations:** BS

CONCEPT

**Environment:** These woodlands occur on the boundary between wetland and upland situations.

**Dynamics:** In addition to fires, hurricanes are the major natural disturbance affecting the distribution, composition and structure of the pine forests.

SOURCES

**References:** Borhidi 1991, Josse et al. 2003*, WWF and IUCN 1997

**Version:** 08 Jan 2015 **Stakeholders:** Caribbean, Latin America

**Concept Author:** C. Josse **LeadResp:** Latin America

CES401.294 Bosque Seco Siempreverde de Encino (*Quercus*)

**Primary Division:** Dry Meso‑America (401)

**Land Cover Class:** Forest and Woodland

**Spatial Scale & Pattern:** Small patch

**Required Classifiers:** Natural/Semi‑natural; Vegetated (>10% vasc.); Upland

**Diagnostic Classifiers:** Lowland [Foothill]; Forest and Woodland (Treed); Ustic

**Concept Summary:** Este sistema abarca una gran variedad de comunidades caracterizadas por la presencia de *Quercus* en tierras bajas tropicales. Las comunidades son azonales, es decir que ocurren por causa de determinados sustratos, más que por el clima. Este sistema representa a los bosques puros de *Quercus* o comunidades mixtas con especies de los bosques deciduos o semideciduos circundantes. Son más comunes en la vertiente del Caribe de México, pero hay ejemplos de ellos hasta en Costa Rica. En su mayoría ha sido muy alterado y actualmente la mayor parte de su extensión se ha convertido en pastos de jaragua, una graminea introducida, sabanas pastizal o plantaciones. La siguiente lista de especies es diagnóstica para este sistema: *Acrocomia aculeata, Annona reticulata, Apeiba tibourbou, Byrsonima crassifolia, Cochlospermum vitifolium, Cordia alliodora, Curatella americana, Guazuma ulmifolia, Luehea candida, Luehea speciosa, Quercus affinis, Quercus glaucescens, Quercus oleoides, Quercus peduncularis, Quercus sororia, Spondias mombin, Tabebuia rosea, Zinowiewia integerrima, Zuelania guidonia*.

This system encompasses a variety of communities characterized by the presence of evergreen *Quercus* in tropical lowland communities. Communities are azonal, i.e., they occur because of certain substrates, rather than the climate. This system represents a pure oak or mixed forest with species from surrounding deciduous forests. They are more common on the Caribbean side of Mexico, but there are examples of them south to Costa Rica. Mostly it has been altered and now most of its length has been converted to jaragua pastures, an introduced graminea, savanna grassland or plantations. The following list of species is diagnostic for this system: *Acrocomia aculeata, Annona reticulata, Apeiba tibourbou, Byrsonima crassifolia, Cochlospermum vitifolium, Cordia alliodora, Curatella americana, Guazuma ulmifolia, Luehea candida, Luehea speciosa, Quercus affinis, Quercus glaucescens, Quercus oleoides, Quercus peduncularis, Quercus sororia, Spondias mombin, Tabebuia rosea, Zinowiewia integerrima*, and *Zuelania guidonia*.

DISTRIBUTION

**Divisions:** 401:C

**Nations:** CR, GT?, HN?, MX, NI?, SV?

CONCEPT

**Environment:** Generalmente en terrenos colinados, en las partes altas. Suelos de origen volcánico, afloramientos de rocas ígneas, suelos derivados de roca basáltica y suelos latosólicos ácidos arcillosos y con presencia de cantos de grava cuarzosa. Bien drenados, sobre los 200 m de altitud y con clima estacional.

Usually on upper slopes. Volcanic soils (pumice / ash), outcrops of igneous rocks, well‑drained soils derived from basaltic rock and clay latosols, other acidic soils and the presence of quartz gravel ridges, about 200 m above sea level and seasonal climate.

**Vegetation:** Bosque bajo de hasta 15 m y abierto o con el dosel más continuo y cerrado en las asociaciones mixtas con especies de los bosques estacionales.

**Dynamics:** Quedan pocos remanentes, principalmente convertido en pastos.

SOURCES

**References:** Janzen 1983a, Josse et al. 2003*, Pennington and Sarukhán 1998

**Version:** 08 Jan 2015 **Stakeholders:** Latin America

**Concept Author:** C. Josse **LeadResp:** Latin America

CES411.468 Cuban Lowland Pine Forest on Ferritic Soils

**Primary Division:** Caribbean (411)

**Land Cover Class:** Forest and Woodland

**Spatial Scale & Pattern:** Small patch

**Required Classifiers:** Natural/Semi‑natural; Vegetated (>10% vasc.); Upland

**Diagnostic Classifiers:** Latosols

**Concept Summary:** Found covering all the ridges and slopes on the northern part of the Cajálbana hills of western Cuba, on ferritic soils, with a closed canopy strongly dominated by *Pinus caribaea var. caribaea*. Typical accompanying species of this low‑altitude pine forest include *Neomazaea phialanthoides, Coccothrinax yuraguana*, and *Phania cajalbanica*. A well‑developed herb layer is dominated by grasses (e.g., *Andropogon gracilis, Aristida refracta*) is present. Also on the ferritic soils of the foothills of subcoastal plains of eastern Cuba, it develops a lowland pine forest very rich in endemic species. The rather closed canopy of this forest is strongly dominated by *Pinus cubensis* with *Dracaena cubensis, Coccothrinax orientalis*, and *Guatteria moralesii* also present. Both the shrub and herbaceous layers are well‑developed in this community. Characteristic species are *Sideroxylon cubense (= Bumelia cubensis), Callicarpa oblanceolata, Casearia bissei, Casearia moaensis, Chaetocarpus oblongatus, Cyrilla cubensis, Eugenia pinetorum, Guettarda crassipes, Guettarda ferruginea, Jacquinia roigii, Myrtus ophiticola, Ossaea pauciflora, Phyllanthus myrtilloides ssp. erythrinus, Psidium parviflorum, Rhynchospora lindeniana, Schmidtottia sessiliflora*, and *Schmidtottia shaferi*.

DISTRIBUTION

**Range:** Lowlands of western and eastern Cuba.

**Divisions:** 411:C

**Nations:** CU

CONCEPT

**Environment:** On ferritic soils of the ridges and slopes on the northern part of the Cajálbana hills of western Cuba, and of the subcoastal plain between Moa and Baracoa in eastern Cuba.

**Dynamics:** In addition to fires, hurricanes are the major natural disturbance affecting the distribution, composition and structure of the pine forests.

SOURCES

**References:** Areces‑Mallea et al. 1999, Borhidi 1991, Josse et al. 2003*

**Version:** 08 Jan 2015 **Stakeholders:** Caribbean, Latin America

**Concept Author:** C. Josse **LeadResp:** Latin America

CES411.469 Cuban Lowland Pine Woodland on Sand

**Primary Division:** Caribbean (411)

**Land Cover Class:** Forest and Woodland

**Spatial Scale & Pattern:** Small patch

**Required Classifiers:** Natural/Semi‑natural; Vegetated (>10% vasc.); Upland

**Diagnostic Classifiers:** Quartz allitic soils

**Concept Summary:** Originally a pine forest with loose canopy and a shrub layer rich in species, growing on nutrient‑poor, light gray quartz sand. Logged forests have been replaced by scrub and savanna. Occurs on the hillsides of Isla de Pinos, Cuba. The following list of species is diagnostic for this system: *Acoelorraphe wrightii, Byrsonima crassifolia, Byrsonima wrightiana, Chaetolepis cubensis, Cladium mariscus ssp. jamaicense (= Cladium jamaicense), Coccothrinax miraguama, Colpothrinax wrightii, Kalmiella aggregata, Dichanthelium longiligulatum (= Panicum longiligulatum), Pinus caribaea var. caribaea, Pinus tropicalis, Syngonanthus insularis, Tabebuia lepidophylla*, and *Xyris longibracteata*.

DISTRIBUTION

**Divisions:** 411:C

**Nations:** CU

CONCEPT

**Environment:** [from M296] *Climate*: Mean annual temperatures in the area of distribution of the type range from 23°C (74°F) in the north to 26°C (77°F) in the Lower Keys. Mean annual temperature in the West Indies distribution of the macrogroup is around 25°C. Precipitation primarily occurs from May or June to October and ranges from 1650 mm along the Atlantic coast decreasing southward to less than 1000 mm in the Lower Keys (Gillespie 2006). Annual precipitation in the distributional range of this forest in Cuba is less than 1500 mm in the west part of the range and increases towards the east.

*Soil/Substrate*: Limestone is the dominant substrate in the macrogroup distribution in Florida and the Bahamas, with skeletal organic soils with minor mineral components, rarely exceeding 20 cm in depth (Snyder et al. 1990, as cited in Gillespie 2006). In Cuba, the pine forests included in this macrogroup are found primarily on acidic soils that have little water‑retention capacity and are poor in essential elements. The principal soil types on which they occur are quartziferous sands, pseudo‑spodosols in the west and lateritic soils in the east. Only pine trees, which have an ectomycorrhizal symbiosis with fungi, are capable of obtaining in this way a sufficient amount of nutrients to achieve the size of trees. In Florida and the Bahamas, pine rockland occurs on relatively flat, moderately to well‑drained terrain, from 2‑7 m above sea level (Snyder et al. 1990). The oolitic limestone is at or very near the surface, and there is very little soil development. Soils are generally composed of small accumulations of nutrient‑poor sand, marl, clayey loam, and organic debris in depressions and crevices in the rock surface. Organic acids occasionally dissolve the surface limestone causing collapsed depressions in the surface rock called solution holes (Outcalt 1997b). Drainage varies according to the porosity of the limestone substrate, but is generally rapid. Consequently, most sites are wet for only short periods following heavy rains. During the rainy season, however, some sites may be shallowly inundated by slow‑flowing surface water for up to 60 days each year (FNAI 2010a).

The macrogroup occurs in lowlands and low hills, littoral or sublittoral flatlands on limestone or on thin sandy soils over limestone, or on light gray quartz sand or soils derived from sandstone or serpentine bedrock in the case of communities in Cuba. All these different substrates are nutrient‑poor and drain very rapidly. Consequently, most sites are wet for only short periods following heavy rains.

**Dynamics:** In addition to fires, hurricanes and landslides are the major natural disturbances affecting the distribution, composition and structure of the pine forests.

SOURCES

**References:** Areces‑Mallea et al. 1999, Borhidi 1991, Josse et al. 2003*

**Version:** 08 Jan 2015 **Stakeholders:** Caribbean, Latin America

**Concept Author:** C. Josse **LeadResp:** Latin America

CES411.432 Cuban Sandstone Mixed Pine‑Broad‑leaved Forest

**Primary Division:** Caribbean (411)

**Land Cover Class:** Forest and Woodland

**Spatial Scale & Pattern:** Large patch

**Required Classifiers:** Natural/Semi‑natural; Vegetated (>10% vasc.); Upland

**Diagnostic Classifiers:** White sand

**Concept Summary:** This ecological system is found in the sandstone belt of western Cuba, from lowlands to submontane zones, on yellow soils derived from slatey sandstone rocks. The canopy is rather closed with pines, palms and evergreen trees. In Pinar del Rio the mixed pine‑oak type occurs. The understory is rich in species of Melastomataceae. The following list of species is diagnostic for this system in the canopy layer: *Calophyllum calaba ssp. pinetorum, Clusia rosea, Matayba apetala (= Matayba oppositifolia), Pinus caribaea var. caribaea, Pinus tropicalis, Quercus oleoides ssp. sagraeana*, and *Xylopia aromatica*; in the understory: *Acoelorraphe wrightii, Befaria cubensis, Byrsonima crassifolia, Coccothrinax miraguana, Curatella americana, Leptocoryphium lanatum, Miconia ibaguensis, Phyllanthus junceus, Rhus copallinum, Tabebuia lepidophylla, Tetrazygia delicatula, Trachypogon filifolius, Vaccinium cubense, Xylopia aromatica*, and *Zamia silicea*. In early‑seral stages or degraded conditions the canopy is fairly open, forming a woodland with *Byrsonima crassifolia, Curatella americana*, and grasses *Eragrostis cubensis, Paepalanthus seslerioides*, and *Syngonanthus insularis*.

DISTRIBUTION

**Range:** Submontane sandstone belt of Sierra de los Organos and Rosario ranges, in Pinar del Rio province in West Cuba.

**Divisions:** 411:C

**Nations:** CU

CONCEPT

**Environment:** Submontane belt on the slatey sandstones of western Cuba.

**Dynamics:** In addition to fires, hurricanes are the major natural disturbance affecting the distribution, composition and structure of the pine forests.

SOURCES

**References:** Areces‑Mallea et al. 1999, Borhidi 1991, Josse et al. 2003*

**Version:** 08 Jan 2015 **Stakeholders:** Caribbean, Latin America

**Concept Author:** C. Josse **LeadResp:** Latin America

CES411.435 Cuban Serpentine Mixed Pine‑Broad‑leaved Forest

**Primary Division:** Caribbean (411)

**Land Cover Class:** Forest and Woodland

**Spatial Scale & Pattern:** Large patch

**Required Classifiers:** Natural/Semi‑natural; Vegetated (>10% vasc.); Upland

**Diagnostic Classifiers:** Serpentine

**Concept Summary:** On ferritic soils of lowlands and hilly serpentine areas of the Sagua‑Baracoa range in eastern Cuba. The canopy of forests growing on deep soils is high and relatively open with a well‑developed shrub layer. On cliffs or submontane rocky substrate, the canopy cover is only 30‑50%. The following list of species is diagnostic for this system: *Agave shaferi, Anemia coriacea, Anemia nipensis, Sideroxylon cubense (= Bumelia cubensis), Casearia* spp., *Coccothrinax orientalis, Coccothrinax yuraguana, Cyrilla cubensis, Cyrilla nipensis, Dracaena cubensis, Eugenia pinetorum, Malpighia cnide, Neobracea valenzuelana, Ossaea acunae, Paspalum breve, Pinus cubensis, Psidium parviflorum, Rondeletia myrtacea, Tabebuia dubia, Tabebuia pinetorum, Tabebuia shaferi*, and *Vaccinium alainii*.

DISTRIBUTION

**Range:** Eastern Cuba

**Divisions:** 411:C

**Nations:** CU

CONCEPT

**Environment:** In the foothill of serpentine ranges of eastern Cuba, on ferritic soils.

**Dynamics:** In addition to fires, hurricanes are the major natural disturbance affecting the distribution, composition and structure of the pine forests.

SOURCES

**References:** Areces‑Mallea et al. 1999, Borhidi 1991, Josse et al. 2003*

**Version:** 08 Jan 2015 **Stakeholders:** Caribbean, Latin America

**Concept Author:** C. Josse **LeadResp:** Latin America

CES401.300 San Lucan Evergreen Forest and Woodland

**Primary Division:** Dry Meso‑America (401)

**Land Cover Class:** Forest and Woodland

**Spatial Scale & Pattern:** Large patch

**Required Classifiers:** Natural/Semi‑natural; Vegetated (>10% vasc.); Upland

**Diagnostic Classifiers:** Lowland [Foothill]; Forest and Woodland (Treed); Aridic

**Concept Summary:** This pine‑oak forest system is limited in distribution to the Cape region of southern Baja California. It is found along high‑elevation granitic sideslopes and plateaus of Sierra de la Laguna. Several endemic pine and oak species dominate. The following list of species is diagnostic for this system: *Pinus lagunae, Quercus devia*.

DISTRIBUTION

**Divisions:** 401:C

**Nations:** MX

**Subnations:** MXBS

CONCEPT

**Environment:** It is found along high elevation granitic side slopes and plateaus of Sierra de la Laguna.

**Vegetation:** There is little extant documentation of vegetative structure and composition for this system.

**Dynamics:** natural fire regime not documented

SOURCES

**References:** Brown et al. 1998, Ffolliott and Ortega‑Rubio 1999, Josse et al. 2003*, Rzedowski 1986

**Version:** 17 Jul 2003 **Stakeholders:** Latin America

**Concept Author:** C. Josse **LeadResp:** Latin America

M561. Caribbean‑Mesoamerican Seasonal Dry Forest

CES401.293 Bosque Seco de Motagua y Valles Secos de Honduras

**Primary Division:** Dry Meso‑America (401)

**Land Cover Class:** Forest and Woodland

**Spatial Scale & Pattern:** Large patch

**Required Classifiers:** Natural/Semi‑natural; Vegetated (>10% vasc.); Upland

**Diagnostic Classifiers:** Lowland [Lowland]; Forest and Woodland (Treed); Udic; Ustic

**Concept Summary:** Este sistema ocurre generalmente sobre suelos profundos y ricos de tierras bajas con un clima estacional tropical/subtropical (4‑6 meses secos), con precipitaciones anuales entre 1000 y 1500 mm, aunque puede llegar a los 2000 mm y una temperatura media sobre 24°C. Occurre solo en valles secos de Gutemala y Honduras, y a mayor altitud, este sistema colinda con bosques premontanos húmedos. La siguiente lista de las especies es de diagnóstica para este sistema: *Andira inermis, Apeiba* spp., *Ardisia revoluta, Astronium graveolens, Bombacopsis quinata, Bursera simaruba, Calycophyllum candidissimum, Casearia arguta, Cavanillesia platanifolia, Ceiba aesculifolia, Chomelia spinosa, Cochlospermum vitifolium, Enterolobium cyclocarpum, Ficus* spp., *Genipa americana, Guarea excelsa, Guazuma ulmifolia, Hymenaea courbaril, Jacquinia pungens, Luehea candida, Maclura tinctoria, Manilkara zapota, Mastichodendron capiri, Samanea saman (= Pithecellobium saman), Simarouba glauca, Spondias mombin, Sterculia apetala, Swietenia macrophylla, Tabebuia ochracea, Tabebuia* spp., *Thouinidium decandrum, Trichilia colimana, Zanthoxylum setulosum*.

This system usually occurs on deep, rich soils of lowland seasonal tropical / subtropical climate (4‑6 months dry), with annual rainfall between 1000 and 1500 mm, but can reach up to 2000 mm and an average temperature of 24oC. It occurs in dry valleys of Guatemala and Honduras, and as altitude increases, this system borders premontane wet forest. The following list of species is diagnostic for this system: *Andira inermis, Apeiba* spp., *Ardisia revoluta, Astronium graveolens, Bombacopsis quinata, Bursera simaruba, Calycophyllum candidissimum, Casearia arguta, Cavanillesia platanifolia, Ceiba aesculifolia, Chomelia spinosa, Cochlospermum vitifolium, Enterolobium cyclocarpum, Ficus* spp., *Genipa americana, Guarea excelsa, Guazuma ulmifolia, Hymenaea courbaril, Jacquinia pungens, Luehea candida, Maclura tinctoria, Manilkara zapota, Mastichodendron capiri, Samanea saman, Simarouba glauca, Spondias mombin, Sterculia apetala, Swietenia macrophylla, Tabebuia ochracea, Tabebuia* spp., *Thouinidium decandrum, Trichilia colimana*, and *Zanthoxylum setulosum*.

DISTRIBUTION

**Divisions:** 401:C

**Nations:** GT, HN

CONCEPT

**Environment:** Estos sistemas ocurren en mesetas, terrazas coluviales, laderas con afloramientos rocosos y calas protegidas. Basáltica derivado de los suelos, o de cenizas volcánicas, o arcilloso y generalmente bien drenados.

These system occur on plateaus, colluvial terraces, slopes with rock outcrops and sheltered coves. Basaltic derived soils, or from volcanic ashes, or clayish and usually well drained.

**Vegetation:** Canopy 20 m tall, emergents up to 30 m, with most species deciduous and large, spreading crowns. The woody understory 10‑20 m tall with more evergreen species, slender or leaning trunks with small open crowns. Shrub layer is 2‑5 m tall and ground layer is sparse, except in openings.

**Dynamics:** Los procesos clave y las interacciones son similares a los de otros tipos de bosque seco.

SOURCES

**References:** Janzen 1983a, Josse et al. 2003*, WWF and IUCN 1997

**Version:** 08 Jan 2015 **Stakeholders:** Caribbean, Latin America

**Concept Author:** C. Josse **LeadResp:** Latin America

CES401.309 Bosque Seco Deciduo de Yucatán

**Primary Division:** Dry Meso‑America (401)

**Land Cover Class:** Forest and Woodland

**Spatial Scale & Pattern:** Large patch

**Required Classifiers:** Natural/Semi‑natural; Vegetated (>10% vasc.); Upland

**Diagnostic Classifiers:** Lowland [Lowland]; Forest and Woodland (Treed); Ustic

**Concept Summary:** Es un sistema que en la mayoría de su distribución se relaciona con afloramientos de calizas de origen coralino, relieve plano a colinado y soporta un clima muy estacional con baja precipitación anual. En general el bosque es de estatura baja a media, con la mayoría de las especies deciduas, algunas comunidades más cercanas a la costa se caracterizan por poseer numerosas cactáceas arborescentes. Los suelos son variables y generalmente muy bien drenados. La siguiente lista de las especies es diagnóstica para este sistema: *Acacia* sp., *Bauhinia jennigsii, Beaucarnea pliabilis, Bursera simaruba, Caesalpinia gaumeri, Caesalpinia vesicaria, Ceiba aesculifolia, Diospyros cuneata, Guaiacum sanctum, Gymnopodium floribundum, Hampea trilobata, Jatropha gaumeri, Lemaireocereus griseus, Lemairocereus aragonii, Lonchocarpus rugosus, Lysiloma latisiliquum, Manilkara sapota, Metopium brownei, Parmentiera aculeata, Piscidia piscipula, Plumeria obtusa, Pseudophoenix* sp., *Pterocereus gaumeri, Thrinax radiata, Vitex gaumeri*.

In most of its distribution, this system is related to limestone outcrops of coral origin, up to hilly terrain and supports a seasonal climate with low annual rainfall. Overall the forest is short to medium, with mostly deciduous species. Some communities closest to the coast are characterized by numerous arborescent cacti. Soils are variable and generally well‑drained. The following list of species is diagnostic for this system: *Acacia* sp., *Bauhinia jennigsii, Beaucarnea pliabilis, Bursera simaruba, Caesalpinia gaumeri, Caesalpinia vesicaria, Ceiba aesculifolia, Diospyros cuneata, Guaiacum sanctum, Gymnopodium floribundum, Hampea trilobata, Jatropha gaumeri, Lemaireocereus griseus, Lemairocereus aragonii, Lonchocarpus rugosus, Lysiloma latisiliquum, Manilkara sapota, Metopium brownei, Parmentiera aculeata, Piscidia piscipula, Plumeria obtusa, Pseudophoenix* sp., *Pterocereus gaumeri, Thrinax radiata*, and *Vitex gaumeri*.

DISTRIBUTION

**Range:** Yucatan Peninsula and immediate surroundings.

**Divisions:** 401:C

**Nations:** BZ, MX

CONCEPT

**Environment:** Ocurre en las planicies costeras y colinas sobre calizas porosas o suelos superficiales sobre calizas, con clima estacionalmente seco y precipitación anual entre 1000‑2000 mm. Hay diferencias de la profundidad del suelo que inciden en la estructura del bosque, con suelos mas delgados generando un bosque mas bajo, pero de troncos gruesos.

Occurs on coastal plain and low hills on porous limestone or shallow soil on limestone, with seasonal dry climate and annual precipitation somewhat less than 1500 mm.

**Vegetation:** Dense mid stature, 8‑15 m forest, deciduous forest.

**Dynamics:** En estos bosques la principal fuente de disturbio natural son los huracanes (Morales 1993, Boose et al. 2003). La frecuencia de estos eventos es altamente variable si se calcula por décadas, pero un promedio general es 0.7 huracanes por año. La intensidad también varía y son más comunes los de menor intensidad. El frente de impacto y su posterior dirección siguen diferentes patrones pero los más comunes implican que el área más afectada es el norte y noreste de la Península. Estos patrones de impacto posiblemente generan una diversidad de habitats que influyen en la distribución de los tipos de bosque seco. Gracias a la estructura de los árboles y a la alta proporción de biomasa radicular, el daño causado por vientos o huracanes derriba pocos árboles, produciendo claros pequeños y/o pocos claros grandes (Dickinson et al. 2001), lo clave es que no causan remoción o disturbios importantes de suelo. La regeneración se da fundamentalmente por retoños, adaptación que se facilita por la cantidad de biomasa radicular que provee mayor circulación del agua disponible, nutrientes del suelo y reservas de materia orgánica. Esta estrategia de recuperación causa una composición con alta dominancia y poca diversidad. A este factor se añade la presencia humana en la Península que data de siglos de activa utilización de los recursos por una población importante y que introdujo el fuego como un mecanismo de manejo de la vegetación. El efecto de los huracanes también tiene incidencia por el nivel de inundación debido a la precipitación y la duración de la presencia de zonas inundadas, especialmente con aguas salobres, pues esto causa mortalidad incluso en mayor grado que la caída de árboles por los vientos.

In these forests the main source of natural disturbance are hurricanes (Morales 1993, Boose et al. 2003). The frequency of these events is highly variable when calculated for decades, but an overall average is 0.7 hurricanes per year. The intensity also varies but lower intensity is more common. Different patterns form based on distance to the costal impact area. The most affected area is north and northeast of the peninsula. These patterns of impact may generate a variety of habitats that influence the distribution of dry forest types. Thanks to the structure of the trees and the high proportion of root biomass, wind damage from hurricanes fells few trees, causing small gaps and/or a few large clearings (Dickinson et al. 2001); the key is to not cause removal or significant disturbance to soil. Regeneration occurs primarily by suckers, and adaptation is facilitated by the amount of root biomass which provides greater flow of available water, soil nutrients and organic matter reserves. This recovery strategy causes a composition with high dominance and low diversity. Human presence in the peninsula is centuries‑long and active use of resources includes introduced fire as a mechanism for vegetation management. The effect of hurricanes also has implications for the level of flooding due to rainfall and the duration of the presence of water in flooded areas, especially saltwater, as this causes mortality to an even greater degree than treefall from wind.

SOURCES

**References:** Boose et al. 2003, Dickinson et al. 2001, Diekmann et al. 2007, Josse et al. 2003*, Meyrat et al. 2001, Morales 1993, Pennington and Sarukhán 1998

**Version:** 08 Jan 2015 **Stakeholders:** Latin America

**Concept Author:** C. Josse **LeadResp:** Latin America

CES301.982 Bosque Semideciduo de Tamaulipas

**Primary Division:** Madrean Semidesert (301)

**Land Cover Class:** Forest and Woodland

**Spatial Scale & Pattern:** Matrix

**Required Classifiers:** Natural/Semi‑natural; Vegetated (>10% vasc.); Upland

**Diagnostic Classifiers:** Lowland [Foothill]; Forest and Woodland (Treed); Tropical/Subtropical [Tropical Xeric]; Broad‑Leaved Deciduous Tree

**Concept Summary:** Este sistema ecológico se encuentra a partir de bajas pendientes de elevación de la Sierra Madre Oriental, Sierra de San Carlos, Sierra de Tamaulipas, y mesetas del noreste de México. Las especies dominantes o indicadores incluyen *Acacia farnesiana, Celtis ehrenbergiana (= Celtis pallida), Celtis laevigata var. reticulata, Dasylirion longissimum, Ebenopsis ebano, Prosopis glandulosa*, y *Ulmus crassifolia*. La epífita conocida como musgo español, *Tillandsia usneoides*, a menudo crece en las ramas de los árboles.

This ecological system occurs from lower elevation slopes of the eastern Sierra Madre Oriental, Sierra de San Carlos, Sierra de Tamaulipas, and plateaus of northeastern Mexico. Dominant or indicator species include *Acacia farnesiana, Celtis ehrenbergiana, Celtis laevigata var. reticulata, Dasylirion longissimum, Ebenopsis ebano, Prosopis glandulosa*, and *Ulmus crassifolia*. This epiphyte *Tillandsia usneoides* often grows on tree branches.

DISTRIBUTION

**Range:** Se encuentra a partir de bajas pendientes de elevación de la oriental Sierra Madre Oriental, Sierra de San Carlos, Sierra de Tamaulipas, y mesetas del noreste de México.Occurs from lower elevation slopes of the eastern Sierra Madre Oriental, Sierra de San Carlos, Sierra de Tamaulipas, and plateaus of northeastern Mexico.

**Divisions:** 301:C

**TNC Ecoregions:** 30:C

**Nations:** MX

**Subnations:** MXCO, MXNU, MXTM

**Map Zones:** 36:?

CONCEPT

SOURCES

**References:** Brown 1982a, Brown et al. 1998, Comer et al. 2003*

**Version:** 08 Jan 2015 **Stakeholders:** Latin America, Southeast

**Concept Author:** NatureServe Western Ecology Team **LeadResp:** Southeast

M562. Pacific Mesoamerican Seasonal Dry Forest

CES401.312 Bosque Seco Deciduo de Darién

**Primary Division:** Dry Meso‑America (401)

**Land Cover Class:** Forest and Woodland

**Spatial Scale & Pattern:** Small patch

**Required Classifiers:** Natural/Semi‑natural; Vegetated (>10% vasc.); Upland

**Diagnostic Classifiers:** Lowland [Lowland]; Forest and Woodland (Treed); Ustic

**Concept Summary:** Bosques secos caducifolios se encontraban en diferentes partes de la costa pacífica de Panamá, como en la Península de Azuero y en la ensenada de Garachiné, sin embargo hoy quedan pequeños y escasos remanentes. Estos bosques estacionales no son muy diversos y en su mayor parte han sido remplazados por sabanas y pastizales. La siguiente lista de las especies es de diagnóstica para este sistema: *Albizia caribaea, Bombacopsis quinata, Prosopis juliflora, Sabal allenii*.

Dry deciduous forests were found in different parts of the Pacific Coast of Panama, as in the Azuero Peninsula and Garachiné Cove; however, today they are small and few remain. These seasonal forests are not very diverse and for the most part have been replaced by savannas and grasslands. The following list of species is diagnostic for this system: *Albizia caribaea, Bombacopsis quinata, Prosopis juliflora*, and *Sabal allenii*.

DISTRIBUTION

**Divisions:** 401:C

**TNC Ecoregions:** NT0224:C

**Nations:** PA

CONCEPT

**Environment:** Planicie costera con clima estacional en la costa Pacífica de Panamá.

**Vegetation:** Bosque muy abierto y de mediana estatura.

**Dynamics:** En bosques como éstos, con precipitación limitada y estacional, los procesos de ciclaje de nutrientes son característicamente muy especializados y eficientes y por lo tanto las características edáficas juegan también un papel clave en los procesos de regeneración del bosque. Estudios edáficos realizados en bosques de distinta edad en Santa Rosa, Costa Rica han encontrado una alta heterogeneidad de suelos a muy pequeña escala relacionada con la alta heterogeneidad espacial del ambiente físico y sobre todo de los usos, que aparte del efecto directo sobre el suelo pueden originar erosión tanto eólica como hídrica. Los datos indican que los cambios observados en el suelo son resultado de la presencia anual de fuego, la adición de materia orgánica y minerales al suelo conforme la regeneración avanza, las condiciones microclimáticas más benignas gracias al desarrollo progresivo del bosque, el creciente ciclaje de nutrientes, y la predominancia de texturas franco‑arenosas en los suelos examinados. Estos cambios en las propiedades del suelo con la sucesión pueden tener importantes consecuencias sobre la fisiología y la fenología de las diversas formas de vida vegetal observadas durante la regeneración de los bosques tropicales estacionalmente secos (Leiva et al. 2009). El estudio también observó que bosques más maduros tienen mayor desarrollo de la biomasa radical (Raich 1980) y suelos con mejor estructura y aireación y mayor disponibilidad de cationes (Ca, Mg, K, Na y CIC), pero el contenido de agua disponible para las plantas disminuye. Esto podría modificar la severidad de la sequía experimentada por diferentes formas de vida en diferentes estados de sucesión, así como las respuestas fenológicas de las plantas, que podrían experimentar déficit hídricos más severos y mayor competencia por los recursos del suelo en estados más avanzados de sucesión (Leiva et al. 2009).

Debido a los extremos característicos de la estacionalidad, las especies de plantas muestran estrechas relaciones entre polinizadores (abejas) y la flor dentro de períodos limitados durante las estaciones secas o de lluvia. La posterior dispersión de semillas es realizada por las aves, pequeños mamíferos y hormigas. Se piensa que la herbivoría natural es mucho más reducida en la mayoría de los bosques secos ahora con respecto a los niveles históricos, lo que resulta en la estructura de la vegetación alterada (cuando no se trata de alteraciones producidas por el sobrepastoreo o por el fuego). La dinámica en el sistema de agua de la superficie y del subsuelo pueden proporcionar fuentes críticas de humedad durante los periodos de sequía más largos, afectando el establecimiento de las plantas y su reclutamiento.

In these forests, with limited seasonal rainfall, nutrient‑cycling processes are typically very specialized and efficient and therefore soil characteristics also play a key role in the processes of forest regeneration. Soil tests conducted in forests of different ages in Gunacaste National Park, Costa Rica, found a high heterogeneity of soils at very small scale related to the high spatial heterogeneity of the physical environment and above all uses, apart from the direct effect on the soil, can cause both wind and water erosion. The data indicate that the observed changes in soil resulting from the annual presence of fire, the addition of organic matter and minerals down as regeneration progresses, more benign microclimate thanks to the progressive development of forests, increasing nutrient cycling and the predominance of French‑gritty textures in soils examined. These changes in soil properties with the succession may have important consequences on the physiology and phenology of the various forms of plant life observed during regeneration of the seasonally dry tropical forests (Leiva et al. 2009). The study also found that mature forests have greater development of root biomass (Raich 1980) and better soil structure and aeration and increased availability of cations (Ca, Mg, K, Na and CIC), but the content of water available for plant decreases. This could modify the severity of drought experienced by different life forms in different stages of succession, and phenological responses of plants, they may experience more severe water deficit and increased competition for land resources in more advanced stages of succession (Lewis et al. 2009).

Because of characteristic extremes in seasonality, plant species show close relationships between certain pollinators (bees) and flowers within limited periods during rainy or dry seasons. Subsequent seed dispersal is mediated by birds, small mammals, and ants. Natural herbivory is thought to be much reduced in most dry forests from historic levels, resulting in altered vegetation structure (where not then overgrazed or otherwise altered by human‑induced fire). The dynamics in the surface and subsurface water system may provide critical sources of moisture during the longest dry periods, affecting plant establishment and recruitment.

SOURCES

**References:** Gillespie et al. 2000, Janzen 1988, Josse et al. 2003*, Leiva et al. 2009, Lewis et al. 1995, Maass 1995, Raich 1980, WWF and IUCN 1997

**Version:** 08 Jan 2015 **Stakeholders:** Latin America

**Concept Author:** C. Josse **LeadResp:** Latin America

CES401.289 Bosque Seco Deciduo de Guerrero

**Primary Division:** Dry Meso‑America (401)

**Land Cover Class:** Forest and Woodland

**Spatial Scale & Pattern:** Large patch

**Required Classifiers:** Natural/Semi‑natural; Vegetated (>10% vasc.); Upland

**Diagnostic Classifiers:** Lowland [Foothill]; Forest and Woodland (Treed); Aridic

**Concept Summary:** Se encuentra este sistema de bosque seco tropical caducifolio de Jalisco sur a través de Oaxaca. Encontrado en cañones y empinadas laderas con suelos arenosos finos, estas áreas típicamente experimentan una temporada húmeda distinta (Mayo a Noviembre) cada año. Una moderada a alta diversidad de especies de árboles de hojas caducas tropicales dominan las copas de árboles en niveles un complejo multi alrededor de 9‑12 m de altura. Este bosque tiene una alta densidad de pequeños tallos y lianas. La estructura y composición varían a lo largo de la gran distribución de este sistema forestal, que incluye las topografías montañosas que influyen en la disponibilidad de humedad y el carácter del suelo. La siguiente lista de las especies es de diagnóstica para este sistema: *Achatocarpus oaxacanus, Aphipterygium glaucum, Bombax ellipicum, Bombax palmeri, Bursera fagaroides, Bursera instabilis, Bursera longipes, Bursera morelensis, Bursera fagaroides var. elongata (= Bursera odorata), Caesalpinia coccinea, Ceiba aesculifolia, Cephalocereus* spp., *Coccoloba* spp., *Cyrtocarpa procera, Gyrocarpus mocinnoi, Hibiscus kochii, Jatropha alamani, Jatropha cordata, Leucaena esculenta, Lonchocarpus* spp., *Lysiloma* spp., *Pachycereus* spp., *Plumeria rubra, Pseudosmodingium perniciosum, Tabebuia palmeri*. Balsas dry forest: *Agave pedunculifera, Bursera ariensis, Bursera diversifolia, Bursera hintonii, Ceiba aesculifolia, Cochlospermum vitifolium, Conzattia multiflora, Cordia elaeagnoides, Cyrtocarpa procera, Ficus cotinifolia, Ficus goldmanii, Ficus kellermanni, Ficus petiolaris, Haematoxylon brasiletto, Heliocarpus reticulatus, Lysiloma divaricatum, Pterocarpus orbiculatus, Ruprechtia fusca, Tabebuia impetiginosa* y *Vitex pyramidata*.

This dry tropical deciduous forest system is found from Jalisco south through Oaxaca, Mexico. It is found in canyons and steep slopes with thin sandy soils. These areas typically experience one distinct wet season (May‑November) each year. A moderate to high diversity of tropical deciduous tree species dominate a complex multi‑tiered tree canopy around 9‑12 m tall. This forest has a high density of small stems and lianas. The structure and composition vary along the large distributional range of this forest system, which includes mountainous topographies that influence moisture availability and soil characteristics. The following list of species is diagnostic for this system: *Achatocarpus oaxacanus, Aphipterygium glaucum, Bombax ellipicum, Bombax palmeri, Bursera fagaroides, Bursera instabilis, Bursera longipes, Bursera morelensis, Bursera fagaroides var. elongata (= Bursera odorata), Caesalpinia coccinea, Ceiba aesculifolia, Cephalocereus* spp., *Coccoloba* spp., *Cyrtocarpa procera, Gyrocarpus mocinnoi, Hibiscus kochii, Jatropha alamani, Jatropha cordata, Leucaena esculenta, Lonchocarpus* spp., *Lysiloma* spp., *Pachycereus* spp., *Plumeria rubra, Pseudosmodingium perniciosum*, and *Tabebuia palmeri*. Balsas dry forest: *Agave pedunculifera, Bursera ariensis, Bursera diversifolia, Bursera hintonii, Ceiba aesculifolia, Cochlospermum vitifolium, Conzattia multiflora, Cordia elaeagnoides, Cyrtocarpa procera, Ficus cotinifolia, Ficus goldmanii, Ficus kellermanni, Ficus petiolaris, Haematoxylon brasiletto, Heliocarpus reticulatus, Lysiloma divaricatum, Pterocarpus orbiculatus, Ruprechtia fusca, Tabebuia impetiginosa*, and *Vitex pyramidata*.

DISTRIBUTION

**Range:** Mexico from Jalisco south through Oaxaca.

**Divisions:** 401:C

**TNC Ecoregions:** NT0205:C

**Nations:** MX

**Subnations:** MXGJ, MXGU, MXJA, MXMI, MXMO, MXNA, MXOA, MXPU

CONCEPT

**Environment:** Encontrado en cañones y laderas escarpadas con suelos arenosos de capacidad de retención de agua poco profundos, pobres en nutrientes y bajos, estas áreas suelen experimentar una temporada distinta húmeda (julio de noviembre) y una estación seca (diciembre a junio) cada año. Este sistema se encuentra con entre 0 a 900 msnm con precipitación media anual varía entre 400 a 1200 mm, y la temperatura media anual de alrededor de 25°C. Este sistema de transiciones en matorral espinoso en el extremo más seco de su gradiente a lo largo de las fronteras de elevación menores, y para los bosques de hoja perenne semi pendiente ascendente más o en los fondos de los valles. Régimen natural de incendios no está documentada para este sistema, sin embargo el fuego se utiliza para gestionar los pastos introducidos.

Found in canyons and steep slopes with shallow, nutrient‑poor sandy soils with low water‑holding capacity. These areas typically experience one distinct wet season (July‑November) and one dry season (December‑June) each year. This system occurs between 0‑900 m asl with mean annual precipitation varying between 400‑1200 mm, and mean annual temperature around 25°C. This system transitions into thornscrub at the drier end of its gradient along lower elevation borders, and to semi‑evergreen forest further upslope or at the valley bottoms. Natural fire regime is not documented for this system; however, fire is used to manage the introduced pastures.

**Vegetation:** These are structurally complex, often with three layers of foliage, and tallest trees reaching over 15m high, but lower canopy trees commonly do not exceed 8 m. Lianas are well developed. Deciduous trees dominate steep slopes while a mix with evergreen species is common in canyon bottoms. Deciduous phases expose extensive thorn‑bearing trees and cacti, as well as bright and robust flowering by tree species of Tabebuia and Ipomoea.

**Dynamics:** Este bosque ha desarrollado mecanismos de reciclaje muy ajustados para evitar la pérdida de nutrientes del sistema. Algunos de estos mecanismos incluyen una capa de hojarasca densa de hasta 8,2 Mg ha, la inmovilización microbiana de los nutrientes durante la estación seca, la reabsorción de nutrientes antes de la abscisión de la hoja, la resistencia de los bosques a los incendios, y la estabilidad de los agregados del suelo elevado (Maass et al. 2005 y sus referencias). La dinámica natural está impulsada principalmente por el estrés y la muerte regresiva relacionados con la sequía.

This forest has evolved tight recycling mechanisms to avoid nutrient loss from the system. Some of these mechanisms include a dense leaf litter layer of up to 8.2 Mgha, microbial immobilization of nutrients during the dry season, nutrient reabsorption prior to leaf abscission, forest resistance to fires, and high soil aggregate stability (Maass et al. 2005 and references therein). Natural dynamics is mainly driven by drought‑related stress and dieback.

SOURCES

**References:** Fernández et al. 1998, Josse et al. 2003*, Maass et al. 2005, Nava Cruz 2006, Rzedowski 1986

**Version:** 08 Jan 2015 **Stakeholders:** Latin America

**Concept Author:** C. Josse **LeadResp:** Latin America

CES401.302 Bosque Seco Deciduo de Sinaloa

**Primary Division:** Dry Meso‑America (401)

**Land Cover Class:** Forest and Woodland

**Spatial Scale & Pattern:** Large patch

**Required Classifiers:** Natural/Semi‑natural; Vegetated (>10% vasc.); Upland

**Diagnostic Classifiers:** Lowland [Foothill]; Forest and Woodland (Treed); Aridic

**Concept Summary:** Este sistema de bosque subtropical seco se encuentra en todo el sur de Sonora y el oeste de Sinaloa. Encontrado en cañones y laderas escarpadas con suelos delgados de arena, estas áreas tienen dos estaciones húmedas (invierno y mediados de verano) y dos estaciones secas cada año. Esto favorece a las especies de plantas de hoja caduca con sistemas de raíces de almacenamiento bien desarrollados capaces de responder rápidamente a las estaciones húmedas. Una moderada a alta diversidad de especies de árboles caducifolios tropicales dominan un complejo dosel de varios niveles. Se considera que este sistema soporta una rica fauna de aves y reptiles. La siguiente lista de especies es diagnóstica para este sistema: *Conzattia sericea, Jarilla heterophylla (= Jarilla chocola), Bursera inopinnata, Ceiba acuminata, Tabebuia cordata, Ipomoea arborescens, Lysiloma watsonii, Choclosperma vitifolium, Senna bicapsularis (= Cassia emarginata), Pachycereus pecten‑aboriginum, Stenocereus thurberi, Mardensia edulis, Tillandsia inflata*.

This dry subtropical forest system is found throughout southern Sonora and western Sinaloa, Mexico. Found in canyons and steep slopes with thin sandy soils, these areas typically experience two wet seasons (winter and mid‑summer) and two dry seasons each year. This favors deciduous plant species with well‑developed root‑storage systems able to rapidly respond to wet seasons. A moderate to high diversity of tropical deciduous tree species dominate a complex multi‑tiered tree canopy. This system is commonly viewed as supporting a very rich fauna of songbirds and reptiles. The following list of species is diagnostic for this system: *Conzattia sericea, Jarilla heterophylla, Bursera inopinnata, Ceiba acuminata, Tabebuia cordata, Ipomoea arborescens, Lysiloma watsonii, Choclosperma vitifolium, Senna bicapsularis, Pachycereus pecten‑aboriginum, Stenocereus thurberi, Mardensia edulis, Tillandsia inflata*.

DISTRIBUTION

**Divisions:** 401:C

**Nations:** MX

**Subnations:** MXSI, MXSO

CONCEPT

**Environment:** Encontrado en cañones y laderas escarpadas con suelos delgados y arenosos. Estas zonas suelen experimentar dos estaciones húmedas (invierno y mediados de verano) y dos estaciones secas cada año. Esto favorece a las especies de plantas de hoja caduca con sistemas de almacenamiento de raíces bien desarrolladas, capaces de responder rápidamente a las estaciones húmedas.<br />

Found in canyons and steep slopes with thin, sandy soils. These areas typically experience two wet seasons (winter and mid‑summer) and two dry seasons each year. This favors deciduous plant species with well‑developed root storage systems, able to rapidly respond to wet seasons.

**Vegetation:** These are structurally complex, often with three layers of foliage, and tallest trees reaching over 18 m high, but lower canopy trees commonly do not exceed 8 m. Lianas are well‑developed. Deciduous trees dominate steep slopes while a mix with evergreen species is common in canyon bottoms. Deciduous phases expose extensive thorn‑bearing trees and cacti, as well as bright and robust flowering by tree species of *Tabebuia* and *Ipomoea*.

**Dynamics:** Este sistema es un sistema de de transición hacia el matorral espinoso Sinaloense a lo largo de las fronteras de menor elevación y hacia los bosques abiertos Madreanos a mayor altitud. Hacia el sur, es probable una transición hacia sistemas forestales semi‑perennes. Régimen natural de incendios no está documentado para este sistema.

This system transitions into Sinaloan thornscrub along lower elevation borders and Madrean woodlands further upslope. To the south, there is likely a transition into more southerly semi‑evergreen forest systems. Natural fire regime is not documented for this system.

SOURCES

**References:** Brown 1982a, Josse et al. 2003*, Rzedowski 1986

**Version:** 08 Jan 2015 **Stakeholders:** Latin America

**Concept Author:** C. Josse **LeadResp:** Latin America

CES403.606 Bosque Semideciduo Premontano Mesoamericano

**Primary Division:** Meso‑American Seasonal Highlands (403)

**Land Cover Class:** Forest and Woodland

**Spatial Scale & Pattern:** Large patch

**Required Classifiers:** Natural/Semi‑natural; Vegetated (>10% vasc.); Upland

**Diagnostic Classifiers:** Montane [Lower Montane]; Forest and Woodland (Treed); Tropical/Subtropical [Tropical Pluviseasonal]

**Concept Summary:** El sistema representa las comunidades boscosas premontanas semideciduas que crecen entre los 500 m de altitud y 1000‑1200 m a partir de donde empieza el bosque mixto con pinos y robles. Se encuentra en ambas vertientes y generalmente a continuación de los bosques semideciduos de tierras bajas. Posiblemente se incluyen comunidades sucesionales producto de la alteración de bosques húmedos de las vertientes más estacionales del Pacífico. La siguiente lista de las especies es de diagnóstica para este sistema: *Quercus sapotifolia, Quercus oleoides, Pinus caribaea, Guazuma ulmifolia, Acacia pennatula, Bursera bipinnata, Bursera simaruba, Cordia alliodora, Trema micrantha (= Cordia dentata), Cedrela odorata, Ceiba aesculifolia, Castilla elastica, Gliricidia sepium, Serjania, Calliandra* sp., *Crescentia alata, Tecoma stans, Coccoloba caracasana, Cochlospermum vitifolium, Trophis racemosa, Rauvolfia tetraphylla, Gyrocarpus americanus, Pseudobombax ellipticum, Vismia ferruginea, Enterolobium cyclocarpum*.

The system represents the semi‑deciduous premontane forest communities growing between 500 and 1000‑1200 m from where the mixed pine and oak forest begins. It lies on both Pacific and Caribbean sides and generally occurs just above semi‑deciduous lowland forest. The following list of species is diagnostic for this system: *Quercus sapotifolia, Quercus oleoides, Pinus caribaea, Guazuma ulmifolia, Acacia pennatula, Bursera bipinnata, Bursera simaruba, Cordia alliodora, Trema micrantha, Cedrela odorata, Ceiba aesculifolia, Castilla elastica, Gliricidia sepium, Serjania, Calliandra* sp., *Crescentia alata, Tecoma stans, Coccoloba caracasana, Cochlospermum vitifolium, Trophis racemosa, Rauvolfia tetraphylla, Gyrocarpus americanus, Pseudobombax ellipticum, Vismia ferruginea, Enterolobium cyclocarpum*.

DISTRIBUTION

**Divisions:** 403:C

**TNC Ecoregions:** NT0209:C

**Nations:** CR, GT, HN, NI

CONCEPT

**Environment:** Base de las cordilleras y contrafuertes montañosos, sobre sustrato sedimentario aluvial y volcánico, en ocasiones sobre suelos pedregosos, o limoso arenosos, siempre bien drenados.

Found on low mountain ridges and alluvial sedimentary and volcanic substrate, sometimes on stony soils, or sandy loam, always well‑drained.

**Vegetation:** Bosque abierto de 10 a 15 m de alto, con individuos de hasta 20 m. Principalmente latifoliado, semideciduo y por lo general con pocos bejucos y sin palmas.

**Dynamics:** Parcialmente de origen secundario, experimenta quemas periódicas.

SOURCES

**References:** Josse et al. 2003*, Meyrat et al. 2001

**Version:** 08 Jan 2015 **Stakeholders:** Latin America

**Concept Author:** C. Josse **LeadResp:** Latin America

CES401.298 Bosque Semi‑perennifolio de Nayarit Guerrero

**Primary Division:** Dry Meso‑America (401)

**Land Cover Class:** Forest and Woodland

**Spatial Scale & Pattern:** Large patch

**Required Classifiers:** Natural/Semi‑natural; Vegetated (>10% vasc.); Upland

**Diagnostic Classifiers:** Lowland [Foothill]; Forest and Woodland (Treed); Ustic

**Concept Summary:** Estos sistema se desarrolla en altitudes desde el nivel del mar hasta 1300 m de altitud. La precipitación media varía de 1000‑1600 mm / año, y las temperaturas medias anuales están entre 20° a 28°C. Estos bosques crecen en suelos de profundidad, textura, y alcalinidad variables. Por lo general son bosques densos que alcanzan alturas de 40 m. Especies de hoja perenne son más frecuentes en las capas subdosel. Las epífitas y pteridofitas son comunes pero menos que en los bosques siempreverdes. La siguiente lista de especies es diagnóstica para este sistema: *Brosimum alicastrum, Bursera excelsa, Celtis monoica, Astronium graveolens, Bursera arborea, Enterolobium cyclocarpum, Ficus* spp., *Hura polyandra, Licania cervantesii, Tabebuia donnell‑smithii (= Roseodendron donnell‑smithii), Swietenia humilis, Tabebuia donnell‑smithii, Tabebuia impetiginosa, Cordia elaeagnoides*.

<br />This system occurs at elevations ranging from sea level up through 1300 m. Precipitation varies from 1000‑1600 mm/year, and annual mean temperatures are between 20‑28°C. These forests occur on soils of variable depth, texture, and alkalinity. These are typically dense forests reaching heights of 40 m. Evergreen species are most prevalent in subcanopy layers. Epiphytes and pteridophytes are common but less so than in evergreen forests. The following list of species is diagnostic for this system: *Brosimum alicastrum, Bursera excelsa, Celtis monoica, Astronium graveolens, Bursera arborea, Enterolobium cyclocarpum, Ficus* spp., *Hura polyandra, Licania cervantesii, Tabebuia donnell‑smithii (= Roseodendron donnell‑smithii), Swietenia humilis, Tabebuia donnell‑smithii, Tabebuia impetiginosa, Cordia elaeagnoides*.

DISTRIBUTION

**Divisions:** 401:C

**Nations:** MX

**Subnations:** MXCL, MXGU, MXNA

CONCEPT

**Environment:** Estos sistemas se encuentran en altitudes desde el nivel del mar hasta 1300 metros, siempre en pendientes. Las precipitaciones varían desde 1000‑1600mm / año y temperaturas medias anuales entre 20° a 28°C. Estos bosques se desarrollan en suelos de profundidad, textura, y alcalinidad variables.

These systems occur at elevations from sea level up through 1300 m, always on slopes. Precipitation varies from 1000‑1600 mm/year and annual mean temperatures between 20‑28°C. These forests occur on soils of variable depth, texture, and alkalinity.

**Vegetation:** These are typically dense forests reaching heights of 40 m. Evergreen species are most prevalent in subcanopy layers. Epiphytes and pteridophytes are common, but less so than in evergreen forests.

SOURCES

**References:** Josse et al. 2003*, Rzedowski 1986

**Version:** 08 Jan 2015 **Stakeholders:** Latin America

**Concept Author:** C. Josse **LeadResp:** Latin America

CES401.299 San Lucan Dry Deciduous Forest

**Primary Division:** Dry Meso‑America (401)

**Land Cover Class:** Forest and Woodland

**Spatial Scale & Pattern:** Large patch

**Required Classifiers:** Natural/Semi‑natural; Vegetated (>10% vasc.); Upland

**Diagnostic Classifiers:** Lowland [Foothill]; Forest and Woodland (Treed); Aridic

**Concept Summary:** This dry tropical forest system is limited in distribution to the Cape region of southern Baja California. It is found in canyons and lowlands with thin rocky or sandy soils. A moderate to low diversity of tropical deciduous tree species dominate. These are structurally simple and of low species diversity relative to dry forests of mainland Mexico and Central America. The following list of species is diagnostic for this system: *Jatropha cinerea, Bursera microphylla, Fouquieria diguetii, Albizia occidentalis, Lysiloma candida, Lysiloma divaricata, Indigofera fruticosa, Senna bicapsularis (= Cassia emarginata), Plumeria acutifolia, Cercidium peninsulare, Ebenopsis confinis (= Pithecellobium confine)*, and *Karwinskia humboldtiana*.

DISTRIBUTION

**Divisions:** 401:C

**Nations:** MX

**Subnations:** MXBS

CONCEPT

**Environment:** Found in canyons and lowlands with thin, sandy soils, though with a high organic content. These areas receive 316‑482 mm of precipitation per year, with a dry season from late October through July. Mean monthly temperatures range from 21.5‑23.6°C. Pacific slopes receive greater rainfall and experience generally lower temperatures than the Gulf side of the Cape region.

**Vegetation:** These are structurally simple and of low species diversity, relative to dry forests of mainland Mexico and Central America. Trees reach heights of 14 m, and canopy density varies widely.

**Dynamics:** Natural fire regime is not documented.

SOURCES

**References:** Brown et al. 1998, Ffolliott and Ortega‑Rubio 1999, Josse et al. 2003*, Rzedowski 1986

**Version:** 17 Jul 2003 **Stakeholders:** Latin America

**Concept Author:** C. Josse **LeadResp:** Latin America

1.A.1.Ei. Colombian‑Venezuelan Dry Forest

M563. Guajiran Seasonal Dry Forest

CES411.439 Venezuelan Coastal Piedmontane Semi‑deciduous Forest

**Primary Division:** Caribbean (411)

**Land Cover Class:** Forest and Woodland

**Spatial Scale & Pattern:** Large patch

**Required Classifiers:** Natural/Semi‑natural; Vegetated (>10% vasc.); Upland

**Concept Summary:** Semi‑deciduous, dense forests with 2‑3 strata and up to 25 m high. Its altitudinal location varies depending on the hill and the aspect, but occurs on most of the coastal ridges and highlands (500‑800/1200 m or 200‑600 m elevation). In the northeastern hills this forest reaches the littoral. The following list of species is diagnostic for this system: *Tabebuia chrysantha, Tabebuia serratifolia, Tabebuia heterophylla (= Tabebuia pentaphylla), Trichilia pleeana, Allophylus racemosus (= Allophylus occidentalis), Trophis racemosa, Eugenia mcvaughii, Acacia glomerosa, Lochocarpus punctatus, Coccoloba fallax, Talisia hexaphylla, Cordia panamensis, Swartzia pinnata, Ocotea glandulosa, Hura crepitans, Cedrela odorata, Carapa guianensis, Roystonea oleracea*.

DISTRIBUTION

**Divisions:** 411:C

**Nations:** CO, TT, VE

CONCEPT

**Environment:** Found on plains and terraces in well‑drained lowlands, hills and foothills up to 500 m elevation. Average annual rainfall is 700‑1500 mm with one or two dry seasons during the year. In the distribution there is a moisture gradient that goes from the coast with less precipitation and drying conditions on the sea winds, plains and hills up to more sheltered inner areas with greater precipitation.

En planicies y terrazas bien drenadas de las tierras bajas, colinas y piedemontes hasta los 500 m de altitud. Precipitación promedio anual 700‑1500 mm con una o dos estaciones secas durante el año. En su distribución existe un gradiente de humedad que va desde la costa con menor precipitación y con condiciones desecantes por los vientos marinos, hasta planicies y colinas del interior mas resguardadas y con una precipitación mayor.

**Dynamics:** In these forests, with limited seasonal rainfall, nutrient‑cycling processes are typically very specialized and efficient and therefore soil characteristics also play a key role in the processes of forest regeneration. Because of characteristic extremes in seasonality, plant species show close relationships between certain pollinator (bees) and flowers within limited periods during rain or dry seasons. Subsequent seed dispersal is mediated by birds, small mammals, and ants. Natural herbivory is thought to be much reduced in most dry forests from historic levels, resulting in altered vegetation structure (where not then overgrazed or otherwise altered by human‑induced fire). The dynamics in the surface and subsurface water system may provide critical sources of moisture during the longest dry periods, affecting plant establishment and recruitment.

SOURCES

**References:** Devillers and Devillers‑Terschuren 1996, Gillespie et al. 2000, Huber and Alarcón 1988, Josse et al. 2003*, Maass 1995, Rangel 2001

**Version:** 08 Jan 2015 **Stakeholders:** Caribbean, Latin America

**Concept Author:** C. Josse **LeadResp:** Latin America

1.A.2. Tropical Lowland Humid Forest

1.A.2.Eg. Caribbean‑Mesoamerican Lowland Humid Forest

M281. Caribbean Lowland Humid Forest

CES411.500 Caribbean Lowland Moist Serpentine Woodland

**Primary Division:** Caribbean (411)

**Land Cover Class:** Forest and Woodland

**Spatial Scale & Pattern:** Large patch

**Required Classifiers:** Natural/Semi‑natural; Vegetated (>10% vasc.); Upland

**Diagnostic Classifiers:** Serpentine

**Concept Summary:** This system occurs below 400 m elevation on poor, acidic ferralitic soils in the serpentine areas of eastern Cuba and southwestern Puerto Rico. There are two canopy layers, mostly sclerophyllous and lauraceous trees and shrubs. The upper vegetative canopy tends to be open, with a dense lower stratum. Succulents are common. The following list of species is diagnostic for this system in Puerto Rico: *Pilosocereus royenii, Thouinia striata var. portoricensis, Plumeria alba, Croton lucidus, Pictetia aculeata*, and *Comocladia dodonaea*.

DISTRIBUTION

**Range:** This system is found in Cuba and Puerto Rico.

**Divisions:** 411:C

**Nations:** CU, PR

CONCEPT

**Environment:** Occurs on ferralitic soils derived from serpentine bedrock, with annual precipitation of 1800‑3200 mm and mean annual temperature of 18‑24°C.

**Dynamics:** Diversity of above‑ground plant functional groups (species that share morphological, chemical, structural or life history characteristics) determines the role of biodiversity in ecosystem functioning such as nutrient cycling, forest regeneration and successional patterns. Diversity of animal functional groups determines a number of key ecological processes such as trophic structure, nutrient cycling, and the system's resilience to disturbance. Community composition/diversity /structure affects species diversity and several ecosystem‑level processes. Gap dynamics provide light, the major environmental limiting factor to plant growth in the closed‑canopy humid tropical forest, and maintains the forest in shifting mosaic steady state.

Biotic interactions: pollination (bees, butterflies, beetles, moths, bats, and hummingbirds) is important for reproductive success and pollinators influence the frequency and distribution pattern of plant species; seed dispersal is executed by fruit‑eating birds, mammals and ants, is important for reproductive success, and seed dispersal agents affect food webs in tropical forests by making available reproductive resources to other consumers and influencing the frequency and distribution pattern of plant species, especially woody species; seed predation is important for reproductive success and seed predation affects population recruitment and establishment of diverse plant species (e.g., palms and legumes). Seed predators occasionally act as dispersers. Seed predation is a specialized form of herbivory. Vertebrates involved are often objects of hunting by humans. Herbivores, including insects, parasitic fungi, and vertebrates, affect vigor and mortality of plants of all sizes, especially understory seedlings, and influences food chain and species composition of understory. The presence of top predators controls the populations of small mammals and herbivores. Species diversity and composition of soil biota, e.g., mycorrhizae, fungi, microbes, soil mesofauna such as leaf‑cutter ants, termites, nematodes, collembola, dung beetles, etc., are fundamental for nutrient cycling and soil structure.

Disturbance regimes from catastrophic natural causes, e.g., hurricanes, rare catastrophic floods, or multiple landslides, or volcanism, or earthquakes, rare extreme cold fronts, rare extreme droughts, are rare events that can be very important for ecological dynamics. Create canopy gaps of great size allowing pioneer species to colonize and initiate successional processes, e.g., hurricanes play a major role in landscape‑scale dynamics of forests on Caribbean islands. Fire due to dry spell or prolonged dry seasons or human activities: Certain species might be maintained because of this big, very rare catastrophic event. For example, mahogany thrives on fire outbreaks. Background disturbances, such as small gaps, small landslides, downbursts, normal cold fronts, and normal seasonal precipitation variability. Important for creating and maintaining habitat heterogeneity and species and structural diversity, preventing competitive exclusion. Drives regeneration.

Spatial integration and coverage (e.g., connectivity by riparian habitats) allowing migration of animals and plants outside of lowland forest: Allow to define at landscape level integrity of ecosystem. Allow to assess the extent of potential for species extinction. Spatial integration important for species to maintain contact with all habitats required for life cycles.

Biogeochemical dynamics (referring to regional and global processes such as global warming, ozone depletion, CO2 concentration, atmospheric and soil pollution, etc.): Affects basic ecosystem functioning at both global and local levels. Soil type or fertility: Affects forest primary productivity and species richness. Soil type is also relevant to tree mortality rate, treefall frequency, forest regeneration mode, and stand turnover time (Hartshorn 1990).

SOURCES

**References:** Areces‑Mallea et al. 1999, Dansereau 1966, Figueroa Colon 1996, Garcia 1991, Hartshorn 1990, Helmer et al. 2002, Josse et al. 2003*

**Version:** 08 Jan 2015 **Stakeholders:** Caribbean, Latin America, U.S. Territories

**Concept Author:** C. Josse **LeadResp:** Latin America

CES411.426 Caribbean Seasonal Evergreen Lowland Forest

**Primary Division:** Caribbean (411)

**Land Cover Class:** Forest and Woodland

**Spatial Scale & Pattern:** Large patch

**Required Classifiers:** Natural/Semi‑natural; Vegetated (>10% vasc.); Upland

**Diagnostic Classifiers:** Ferralitic red soils

**Concept Summary:** This system occurs on calcareous and alluvial soils below 400 m elevation in moist climates. In Puerto Rico this system refers to the forests of the wide flatlands or valleys of the karst belt, where very little of the original extent is left. It has an open canopy, 20‑25 m high, with emergents and a second denser layer, 8‑15 m high. About 70% of canopy species are evergreen. Lianas are abundant. Few drought‑tolerant epiphytes are present. Much of this forest has disappeared. Now open pastures and agricultural crops replace it. The following list of species is diagnostic for this system: *Andira inermis, Guettarda scabra, Guettarda odorata, Dendropanax arboreus, Guazuma ulmifolia, Hymenaea courbaril, Quararibea turbinata, Ceiba pentandra, Roystonea regia, Bucida buceras, Luehea speciosa, Lonchocarpus heptaphyllus (= Lonchocarpus latifolius), Lonchocarpus* sp., *Chamaecrista glandulosa var. mirabilis (= Cassia mirabilis), Cordia collococca, Cordia gerascanthus, Ficus stahlii, Pithecellobium cubense, Cojoba arborea (= Pithecellobium arboreum), Oxandra lanceolata, Crescentia cujete, Melicoccus bijugatus, Spondias mombin, Manilkara bidentata*, and *Margaritaria nobilis (= Phyllanthus nobilis)*.

**Comments:** Besides moisture availability and lowland distribution, the composition of this ecological system is influenced by the substrate and past land use. Extensive areas in the islands can represent secondary forest of different ages and grown after such distinct land uses as shade coffee plantations, pastures or sugar cane. All these factors play a role in the current composition. *Andira inermis* is a widespread species after pasture land use, while *Guettarda scabra* is characteristic of karst substrate.

DISTRIBUTION

**Range:** This system occurs in the Bahamas, Cuba, Jamaica, Martinique, Puerto Rico (includes forest on white sands in the alluvial valleys within the karst belt), and the Virgin Islands.

**Divisions:** 411:C

**Nations:** BS, CU, JM, MQ, PR, VI

CONCEPT

**Environment:** Major factors that determine variation in community types within lowland tropical moist forest include precipitation, temperature, topography, edaphic conditions, and natural disturbance. The amount of rainfall and length of dry season determine the occurrences of evergreen forest or seasonally dry forest. Yearly extreme temperature fluctuations result in cold‑front stressed forests in southwestern Amazonia and the southern Atlantic region and non‑cold‑front stressed forests in Mexico and Central America.: Zonation may occur depending on whether the forest is on a plain, or rolling hills, or foothills of a mountain range. Edaphic conditions (soil quality or fertility) can create special community types. Forests on white sand soil, on clay soil, or over limestone/ultrabasic rock differ considerably in species composition. Natural disturbance includes hurricanes and landslides. Hurricanes are the most frequent causes of landslides.

**Dynamics:** Diversity of above‑ground plant functional groups (species that share morphological, chemical, structural or life history characteristics) determines the role of biodiversity in ecosystem functioning such as nutrient cycling, forest regeneration and successional patterns. Diversity of animal functional groups determines a number of key ecological processes such as trophic structure, nutrient cycling, and the system's resilience to disturbance. Community composition/diversity /structure affects species diversity and several ecosystem‑level processes. Gap dynamics provide light, the major environmental limiting factor to plant growth in the closed‑canopy humid tropical forest, and maintains the forest in shifting mosaic steady state.

Biotic interactions: pollination (bees, butterflies, beetles, moths, bats, and hummingbirds) is important for reproductive success and pollinators influence the frequency and distribution pattern of plant species; seed dispersal is executed by fruit‑eating birds, mammals and ants, is important for reproductive success, and seed dispersal agents affect food webs in tropical forests by making available reproductive resources to other consumers and influencing the frequency and distribution pattern of plant species, especially woody species; seed predation is important for reproductive success and seed predation affects population recruitment and establishment of diverse plant species (e.g., palms and legumes). Seed predators occasionally act as dispersers. Seed predation is a specialized form of herbivory. Vertebrates involved are often objects of hunting by humans. Herbivores, including insects, parasitic fungi, and vertebrates, affect vigor and mortality of plants of all sizes, especially understory seedlings, and influences food chain and species composition of understory. The presence of top predators controls the populations of small mammals and herbivores. Species diversity and composition of soil biota, e.g., mycorrhizae, fungi, microbes, soil mesofauna such as leaf‑cutter ants, termites, nematodes, collembola, dung beetles, etc., are fundamental for nutrient cycling and soil structure.

Disturbance regimes from catastrophic natural causes, e.g., hurricanes, rare catastrophic floods, or multiple landslides, or volcanism, or earthquakes, rare extreme cold fronts, rare extreme droughts, are rare events that can be very important for ecological dynamics. Create canopy gaps of great size allowing pioneer species to colonize and initiate successional processes, e.g., hurricanes play a major role in landscape‑scale dynamics of forests on Caribbean islands. Fire due to dry spell or prolonged dry seasons or human activities: Certain species might be maintained because of this big, very rare catastrophic event. For example, mahogany thrives on fire outbreaks. Background disturbances, such as small gaps, small landslides, downbursts, normal cold fronts, and normal seasonal precipitation variability. Important for creating and maintaining habitat heterogeneity and species and structural diversity, preventing competitive exclusion. Drives regeneration.

Spatial integration and coverage (e.g., connectivity by riparian habitats) allowing migration of animals and plants outside of lowland forest: Allow to define at landscape level integrity of ecosystem. Allow to assess the extent of potential for species extinction. Spatial integration important for species to maintain contact with all habitats required for life cycles.

Biogeochemical dynamics (referring to regional and global processes such as global warming, ozone depletion, CO2 concentration, atmospheric and soil pollution, etc.): Affects basic ecosystem functioning at both global and local levels. Soil type or fertility: Affects forest primary productivity and species richness. Soil type is also relevant to tree mortality rate, treefall frequency, forest regeneration mode, and stand turnover time (Hartshorn 1990).

SOURCES

**References:** Areces‑Mallea et al. 1999, Borhidi 1991, Dansereau 1966, Hartshorn 1990, Helmer et al. 2002, International Institute of Tropical Forestry n.d., Josse et al. 2003*, Lugo et al. 2001

**Version:** 30 Oct 2015 **Stakeholders:** Caribbean, Latin America, U.S. Territories

**Concept Author:** C. Josse **LeadResp:** Latin America

CES411.427 Caribbean Seasonal Evergreen Submontane/Lowland Forest

**Primary Division:** Caribbean (411)

**Land Cover Class:** Forest and Woodland

**Spatial Scale & Pattern:** Large patch

**Required Classifiers:** Natural/Semi‑natural; Vegetated (>10% vasc.); Upland

**Diagnostic Classifiers:** Ferralitic red soils

**Concept Summary:** This system occurs between (200) 400 and 800 m elevation, under moist climate conditions on soils derived from volcanic and sedimentary geologies. The canopy is 20‑25 m high, is not densely closed, and emergents are common. The second stratum is closed, and terrestrial ferns dominate the herb layer. Lichens and bryophytes grow on trunks. Different mountains (and islands) have different composition. This type of forest has been replaced by coffee plantations or other crops in a significant part of its original extent. The following list of species is diagnostic for this system: *Dipholis jubilla, Sideroxylon salicifolium (= Dipholis salicifolia), Cedrela odorata (= Cedrela mexicana), Calophyllum antillanum (= Calophyllum calaba), Ziziphus rhodoxylon, Calyptronoma occidentalis, Zanthoxylum martinicense, Zanthoxylum cubense, Sapium laurifolium (= Sapium jamaicense), Matayba apetala (= Matayba oppositifolia), Pseudolmedia spuria, Cupania glabra, Roystonea regia, Chrysophyllum argenteum, Oxandra lanceolata, Dendropanax arboreus, Laplacea haematoxylon*, and *Lonchocarpus heptaphyllus (= Lonchocarpus latifolius)*. The tree fern *Alsophila bryophila (= Cyathea pubescens)* can be common in the understory. In St. John in the Virgin Islands, the species assemblage for this type of forest includes *Andira inermis, Amyris elemifera, Swietenia mahagoni, Melicoccus bijugatus, Casearia guianensis, Eugenia monticola, Eugenia rhombea, Zanthoxylum monophyllum, Adenanthera pavonina*, and *Acacia muricata*.

DISTRIBUTION

**Range:** This system is found in Cuba, the Dominican Republic, Jamaica, Puerto Rico, Venezuela, and the Virgin Islands.

**Divisions:** 411:C

**Nations:** CU, DO, JM, PR, VE, VI

CONCEPT

**Environment:** Major factors that determine variation in community types within lowland tropical moist forest include precipitation, temperature, topography, edaphic conditions, and natural disturbance. The amount of rainfall and length of dry season determine the occurrences of evergreen forest or seasonally dry forest. Yearly extreme temperature fluctuations result in cold‑front stressed forests in southwestern Amazonia and the southern Atlantic region and non‑cold‑front stressed forests in Mexico and Central America.: Zonation may occur depending on whether the forest is on a plain, or rolling hills, or foothills of a mountain range. Edaphic conditions (soil quality or fertility) can create special community types. Forests on white sand soil, on clay soil, or over limestone/ultrabasic rock differ considerably in species composition. Natural disturbance includes hurricanes and landslides. Hurricanes are the most frequent causes of landslides.

**Dynamics:** Diversity of above‑ground plant functional groups (species that share morphological, chemical, structural or life history characteristics) determines the role of biodiversity in ecosystem functioning such as nutrient cycling, forest regeneration and successional patterns. Diversity of animal functional groups determines a number of key ecological processes such as trophic structure, nutrient cycling, and the system's resilience to disturbance. Community composition/diversity /structure affects species diversity and several ecosystem‑level processes. Gap dynamics provide light, the major environmental limiting factor to plant growth in the closed‑canopy humid tropical forest, and maintains the forest in shifting mosaic steady state.

Biotic interactions: pollination (bees, butterflies, beetles, moths, bats, and hummingbirds) is important for reproductive success and pollinators influence the frequency and distribution pattern of plant species; seed dispersal is executed by fruit‑eating birds, mammals and ants, is important for reproductive success, and seed dispersal agents affect food webs in tropical forests by making available reproductive resources to other consumers and influencing the frequency and distribution pattern of plant species, especially woody species; seed predation is important for reproductive success and seed predation affects population recruitment and establishment of diverse plant species (e.g., palms and legumes). Seed predators occasionally act as dispersers. Seed predation is a specialized form of herbivory. Vertebrates involved are often objects of hunting by humans. Herbivores, including insects, parasitic fungi, and vertebrates, affect vigor and mortality of plants of all sizes, especially understory seedlings, and influences food chain and species composition of understory. The presence of top predators controls the populations of small mammals and herbivores. Species diversity and composition of soil biota, e.g., mycorrhizae, fungi, microbes, soil mesofauna such as leaf‑cutter ants, termites, nematodes, collembola, dung beetles, etc., are fundamental for nutrient cycling and soil structure.

Disturbance regimes from catastrophic natural causes, e.g., hurricanes, rare catastrophic floods, or multiple landslides, or volcanism, or earthquakes, rare extreme cold fronts, rare extreme droughts, are rare events that can be very important for ecological dynamics. Create canopy gaps of great size allowing pioneer species to colonize and initiate successional processes, e.g., hurricanes play a major role in landscape‑scale dynamics of forests on Caribbean islands. Fire due to dry spell or prolonged dry seasons or human activities: Certain species might be maintained because of this big, very rare catastrophic event. For example, mahogany thrives on fire outbreaks. Background disturbances, such as small gaps, small landslides, downbursts, normal cold fronts, and normal seasonal precipitation variability. Important for creating and maintaining habitat heterogeneity and species and structural diversity, preventing competitive exclusion. Drives regeneration.

Spatial integration and coverage (e.g., connectivity by riparian habitats) allowing migration of animals and plants outside of lowland forest: Allow to define at landscape level integrity of ecosystem. Allow to assess the extent of potential for species extinction. Spatial integration important for species to maintain contact with all habitats required for life cycles.

Biogeochemical dynamics (referring to regional and global processes such as global warming, ozone depletion, CO2 concentration, atmospheric and soil pollution, etc.): Affects basic ecosystem functioning at both global and local levels. Soil type or fertility: Affects forest primary productivity and species richness. Soil type is also relevant to tree mortality rate, treefall frequency, forest regeneration mode, and stand turnover time (Hartshorn 1990).

SOURCES

**References:** Areces‑Mallea et al. 1999, Borhidi 1991, Dansereau 1966, Hartshorn 1990, Helmer et al. 2002, International Institute of Tropical Forestry n.d., Josse et al. 2003*

**Version:** 30 Oct 2015 **Stakeholders:** Caribbean, Latin America, U.S. Territories

**Concept Author:** C. Josse **LeadResp:** Latin America

CES411.424 Caribbean Wet Submontane/Lowland Forest

**Primary Division:** Caribbean (411)

**Land Cover Class:** Forest and Woodland

**Spatial Scale & Pattern:** Large patch

**Required Classifiers:** Natural/Semi‑natural; Vegetated (>10% vasc.); Upland

**Diagnostic Classifiers:** Yellowish red soils

**Concept Summary:** This system is found below 800 m elevation on yellowish red soils. The canopy is closed, 30‑35 m high, with three tree layers. The canopy's dominant species vary from island to island. Along creeks, palms are frequent in the understory. The following list of species is diagnostic for this system: *Carapa guianensis, Clusia rosea, Calophyllum utile, Calophyllum jacquinii, Calophyllum antillanum (= Calophyllum calaba), Sloanea curatellifolia, Sloanea berteriana, Ormosia krugii, Guarea guidonia, Cupania americana, Ficus* spp., *Roystonea regia, Psidium montanum, Dacryodes excelsa, Manilkara bidentata, Meliosma herbertii, Tetragastris balsamifera, Buchenavia tetraphylla (= Buchenavia capitata), Ocotea leucoxylon, Cinnamomum montanum (= Phoebe montana), Bactris cubensis, Prestoea acuminata var. montana (= Prestoea montana), Calyptronoma plumeriana (= Calyptronoma clementis)*, and *Calyptronoma occidentalis*. In addition, *Cecropia* spp., *Schefflera morototonii (= Didymopanax morototonii)*, and *Ochroma pyramidale* are common in cleared sites.

DISTRIBUTION

**Range:** This system is found in Cuba, the Dominican Republic, Jamaica, the Lesser Antilles, and Puerto Rico.

**Divisions:** 411:C

**Nations:** CU, DO, JM, PR, XD

CONCEPT

**Environment:** [from M281] Major factors that determine variation in community types within lowland tropical moist forest include precipitation, temperature, topography, edaphic conditions, and natural disturbance. The amount of rainfall and length of dry season determine the occurrences of evergreen forest or seasonally dry forest. Yearly extreme temperature fluctuations result in cold‑front stressed forests in southwestern Amazonia and the southern Atlantic region and non‑cold‑front stressed forests in Mexico and Central America.: Zonation may occur depending on whether the forest is on a plain, or rolling hills, or foothills of a mountain range. Edaphic conditions (soil quality or fertility) can create special community types. Forests on white sand soil, on clay soil, or over limestone/ultrabasic rock differ considerably in species composition. Natural disturbance includes hurricanes and landslides. Hurricanes are the most frequent causes of landslides.

**Dynamics:** Diversity of above‑ground plant functional groups (species that share morphological, chemical, structural or life history characteristics) determines the role of biodiversity in ecosystem functioning such as nutrient cycling, forest regeneration and successional patterns. Diversity of animal functional groups determines a number of key ecological processes such as trophic structure, nutrient cycling, and the system's resilience to disturbance. Community composition/diversity /structure affects species diversity and several ecosystem‑level processes. Gap dynamics provide light, the major environmental limiting factor to plant growth in the closed‑canopy humid tropical forest, and maintains the forest in shifting mosaic steady state.

Biotic interactions: pollination (bees, butterflies, beetles, moths, bats, and hummingbirds) is important for reproductive success and pollinators influence the frequency and distribution pattern of plant species; seed dispersal is executed by fruit‑eating birds, mammals and ants, is important for reproductive success, and seed dispersal agents affect food webs in tropical forests by making available reproductive resources to other consumers and influencing the frequency and distribution pattern of plant species, especially woody species; seed predation is important for reproductive success and seed predation affects population recruitment and establishment of diverse plant species (e.g., palms and legumes). Seed predators occasionally act as dispersers. Seed predation is a specialized form of herbivory. Vertebrates involved are often objects of hunting by humans. Herbivores, including insects, parasitic fungi, and vertebrates, affect vigor and mortality of plants of all sizes, especially understory seedlings, and influences food chain and species composition of understory. The presence of top predators controls the populations of small mammals and herbivores. Species diversity and composition of soil biota, e.g., mycorrhizae, fungi, microbes, soil mesofauna such as leaf‑cutter ants, termites, nematodes, collembola, dung beetles, etc., are fundamental for nutrient cycling and soil structure.

Disturbance regimes from catastrophic natural causes, e.g., hurricanes, rare catastrophic floods, or multiple landslides, or volcanism, or earthquakes, rare extreme cold fronts, rare extreme droughts, are rare events that can be very important for ecological dynamics. Create canopy gaps of great size allowing pioneer species to colonize and initiate successional processes, e.g., hurricanes play a major role in landscape‑scale dynamics of forests on Caribbean islands. Fire due to dry spell or prolonged dry seasons or human activities: Certain species might be maintained because of this big, very rare catastrophic event. For example, mahogany thrives on fire outbreaks. Background disturbances, such as small gaps, small landslides, downbursts, normal cold fronts, and normal seasonal precipitation variability. Important for creating and maintaining habitat heterogeneity and species and structural diversity, preventing competitive exclusion. Drives regeneration.

Spatial integration and coverage (e.g., connectivity by riparian habitats) allowing migration of animals and plants outside of lowland forest: Allow to define at landscape level integrity of ecosystem. Allow to assess the extent of potential for species extinction. Spatial integration important for species to maintain contact with all habitats required for life cycles.

Biogeochemical dynamics (referring to regional and global processes such as global warming, ozone depletion, CO2 concentration, atmospheric and soil pollution, etc.): Affects basic ecosystem functioning at both global and local levels. Soil type or fertility: Affects forest primary productivity and species richness. Soil type is also relevant to tree mortality rate, treefall frequency, forest regeneration mode, and stand turnover time (Hartshorn 1990).

SOURCES

**References:** Areces‑Mallea et al. 1999, Borhidi 1991, Dansereau 1966, Dominica Ministry of Agriculture and Environment n.d., Figueroa Colon 1996, Hartshorn 1990, Helmer et al. 2002, International Institute of Tropical Forestry n.d., Josse et al. 2003*, TNC 2000, Tolentino and Peña 1998, Weaver 1990

**Version:** 08 Jan 2015 **Stakeholders:** Caribbean, Latin America, U.S. Territories

**Concept Author:** C. Josse **LeadResp:** Latin America

M578. Mesoamerican Lowland Humid Forest

CES402.604 Bosque Estacional Siempreverde de Colinas de Carso del Petén

**Primary Division:** Moist Meso‑America (402)

**Land Cover Class:** Forest and Woodland

**Spatial Scale & Pattern:** Large patch

**Required Classifiers:** Natural/Semi‑natural; Vegetated (>10% vasc.); Upland

**Diagnostic Classifiers:** Lowland [Foothill, Lowland]; Forest and Woodland (Treed); Ustic

**Concept Summary:** Este sistema representa las comunidades boscosas siempreverdes estacionales que crecen sobre suelos calcáreos o derivados de calizas y sobre colinas cársticas, generalmente hacia el interior, no costeros. Este tipo de sistema ocupa grandes extensiones en Honduras, Belice, Guatemala y sur de México. La topografía comprende terrenos colinados o accidentados. Por tratarse de suelos fértiles, gran parte de su distribución está dedicada a la agricultura por lo que actualmente quedan pocos remanentes en buen estado. La siguiente lista de especies es diagnóstica para este sistema: *Alseis yucatanensis, Ampelocera hottlei, Aspidosperma cruenta, Astronium graveolens, Orbignya cohune (= Attalea cohune), Bernoullia flammea, Brosimum alicastrum, Bursera simaruba, Calophyllum antillanum (= Calophyllum brasiliense), Cedrela odorata, Ceiba pentandra, Clusia salvinii, Cordia dodecandra, Cupania belicensis, Cupania prisca, Crysophila stauracantha, Chione chiapasensis, Dendropanax arboreus, Drypetes laterifolia, Drypetes brownei, Eugenia capuli, Ficus* spp., *Hirtella triandra (= Hirtella americana), Laetia thamnia, Lonchocarpus castilloi, Manilkara chicle, Matayba oppositifolia, Metopium brownei, Omphalea oleifera, Passiflora mayarum, Pimenta dioica, Pouteria amygdalina, Pouteria campechiana, Pouteria reticulata, Protium copal, Pseudobombax ellipticum, Pseudolmedia spuria, Sabal mauritiiformis, Schizolobium parahyba, Sebastiana longicuspis, Simira salvadorensis, Spondias mombin, Stemmadenia donnell‑smithii, Swietenia macrophylla, Talisia olivaeformis, Terminalia amazonia, Trichilia minutiflora, Trophis racemosa, Vatairea lundellii, Vitex gaumeri, Zuelania guidonia*. Understory: *Adiantum pulverulatum, Malvaviscus arboreus, Piper jacquemontianum, Psychotria pubescens, Pteris longifolia, Tectaria heracleifolia, Ichnanthus lanceolatus*.

This system represents the seasonal evergreen forest communities growing on calcareous soils derived from limestone or karst hills and generally inland instead of coastal. This type occupies large areas in Honduras, Belize, Guatemala and southern Mexico. The topography comprises hilly or difficult terrain. Being fertile soils, much of its distribution is devoted to agriculture and there are now few remaining unaltered examples. The above list of species is diagnostic for this system.

DISTRIBUTION

**Range:** This type occupies large areas in Honduras, Belize, Guatemala and southern Mexico.

**Divisions:** 402:C

**Nations:** BZ, GT, HN, MX

CONCEPT

**Environment:** Ocurren en colinas cársticas sobre terreno ondulado o accidentado. Suelos fértiles, superficiales en las pendientes y por lo tanto propensos a la erosión. Clima con una estación seca de 3 a 4 meses.

Occurs in karst hills on corrugated or rough terrain. Fertile surface on slopes and therefore prone to erosion. Climate with a dry season of 3‑4 months.

**Vegetation:** Bosques de 25‑30 m de alto, con dosel irregular debido a que los claros son comunes por la caída de árboles. Muchas de las especies del dosel, pierden sus hojas en la estación seca.

**Dynamics:** Bosque maduro, muy afectado por las quemas y clareos para agricultura.

SOURCES

**References:** Josse et al. 2003*, Meyrat et al. 2001, Pennington and Sarukhán 1998, WWF and IUCN 1997

**Version:** 08 Jan 2015 **Stakeholders:** Latin America

**Concept Author:** C. Josse **LeadResp:** Latin America

CES402.580 Bosque Siempreverde de Tierras Bajas Centroamericano Caribeño

**Primary Division:** Moist Meso‑America (402)

**Land Cover Class:** Forest and Woodland

**Spatial Scale & Pattern:** Large patch

**Required Classifiers:** Natural/Semi‑natural; Vegetated (>10% vasc.); Upland

**Diagnostic Classifiers:** Lowland [Foothill]; Forest and Woodland (Treed); Udic

**Concept Summary:** Sistema que representa los bosques altos, húmedos siempreverdes y bien drenados de la vertiente atlántica de Centroamérica y México. Crecen sobre suelos de origen sedimentario (calizo) o ígneo (cenizas o basalto), principalmente del grupo de los latosoles con textura arcillosa y buenos contenidos de materia orgánica. El relieve es generalmente colinado o accidentado en las colinas bajas de serranías costeras y estribaciones de montañas del interior. La siguiente lista de las especies es de diagnóstica para este sistema: *Anaxagorea costaricensis, Aspidosperma megalocarpon, Bursera simaruba, Calophyllum antillanum (= Calophyllum brasiliense), Capparis pittieri, Carpotroche platyptera, Cassipourea elliptica, Cordia gerascanthus, Cynometra retusa, Dalbergia tucurensis, Dendropanax arboreus, Dialium guianense, Dussia macroprophyllata, Dypterix panamensis, Eschweilera mexicana, Faramea suerrencis, Guarea rhopalocarpa, Guatteria anomala, Hernandia didymantha, Hyeronima alchorneoides, Laetia procera, Lecythis costaricensis, Licania hypoleuca, Licania platypus, Magnolia yocoronte, Manilkara zapota, Mauria sessiflora, Ocotea* sp., *Ormosia* sp., *Pentaclethra macroloba, Perebea angustifolia, Pouteria neglecta, Quararibea bracteolosa, Sloanea tuerckheimii, Socratea durissima, Socratea exorrhiza, Spondias radlkoferi, Swietenia macrophylla, Symphonia globulifera, Terminalia amazonia, Virola guatemalensis, Virola koschnyi, Vochysia guatemalensis, Welfia georgii*.

System representing the tall, moist, well‑drained evergreen forests of the Atlantic slope of Central America and Mexico. It occurs on soils of sedimentary origin (limestone) or igneous (ash or basalt), mainly latosols with loamy texture and high organic matter content. The topography is generally hilly or in low coastal hills and foothills of inland mountains. The above list of species is diagnostic for this system.

DISTRIBUTION

**Divisions:** 402:C

**Nations:** CR, HN, MX, NI, PA

CONCEPT

**Environment:** Se encuentran sobre terrenos colinados a accidentados, de suelos bien drenados y de origen sedimentario. Alfisoles, ultisoles, latosoles e inceptisoles, pueden presentar alta concentración de materia orgánica en el horizonte superficial. Tierras bajas y piedemontes con clima húmedo, si es estacional, la estación seca es corta. La precipitación anual generalmente es >3000 mm.<br />

They are on well‑drained soils of sedimentary origin: Alfisols, Ultisols, and Inceptisols; Latosols can present high concentration of organic matter in the surface horizon. Lowlands and foothills in wet weather; if seasonal, the dry season is short. Annual rainfall is usually >3000 mm.

**Vegetation:** Bosques multiestratificados, con abundantes palmas en el sotobosque, de dosel cerrado y en promedio de 30 m de alto, con emergentes hasta 40‑50 m.

**Dynamics:** Bosque maduro sujeto a intervención.

SOURCES

**References:** Aide and Cavelier 1994, Josse et al. 2003*, Meyrat et al. 2001, Parrotta et al. 1997, Pennington and Sarukhán 1998, WWF and IUCN 1997

**Version:** 08 Jan 2015 **Stakeholders:** Latin America

**Concept Author:** C. Josse **LeadResp:** Latin America

CES402.597 Bosque Siempreverde de Tierras Bajas del Pacífico Mesoamericano

**Primary Division:** Moist Meso‑America (402)

**Land Cover Class:** Forest and Woodland

**Spatial Scale & Pattern:** Large patch

**Required Classifiers:** Natural/Semi‑natural; Vegetated (>10% vasc.); Upland

**Diagnostic Classifiers:** Lowland [Foothill, Lowland]; Forest and Woodland (Treed); Udic

**Concept Summary:** Este sistema que representa los bosques altos, húmedos siempreverdes y bien drenados de la vertiente pacífica de Centroamérica, México, el Chocó colombiano y la costa norte del Ecuador. Crecen sobre suelos de origen sedimentario o ígneo (cenizas o basalto), principalmente del grupo de los latosoles con textura arcillosa y buenos contenidos de materia orgánica. Generalmente en relieve colinado o accidentado, en las colinas bajas de serranías costeras y estribaciones de montañas del interior. La siguiente lista de especies es diagnóstica para este sistema: *Manilkara zapota (= Achras zapota), Alchornea costaricensis, Anacardium excelsum, Andira inermis, Apeiba aspera, Apeiba tibourbou, Ardisia cutteri, Aspidosperma megalocarpon, Brosimum utile, Brosimum utile, Carapa guianensis, Caryocar costaricense, Heisteria longipes, Huberodendron patinoi, Iriartea deltoidea, Iriartea gigantea, Minquartia guianensis, Parkia pendula, Peltogyne purpurea, Poulsenia armata, Protium copal, Qualea paraensis, Scheelea rostrata, Schizolobium parahyba, Socratea exorrhiza, Sorocea pubivena, Symphonia globulifera, Symphonia globulifera, Talisia nervosa, Terminalia lucida, Tetragastris panamensis, Vantanea barbourii, Vatairea lundellii, Welfia georgii*. Hills: *Astronium* sp., *Browneopsis* sp., *Caryodaphnopsis theobromifolia, Catoblastus* sp., *Ceiba pentandra, Coussapoa eggersii (= Coussapoa villosa ssp. eggersii), Coussapoa herthae, Daphnopsis oculta, Endlicheria* sp., *Eschweilera* sp., *Guarea pterorhachis, Guettarda* sp., *Jessenia bataua, Metteniusa nucifera, Otoba cf. novogranatensis, Perebea cf. angustifolia, Poulsenia armata, Pourouma bicolor, Protium* sp., Quararibea coloradorum, Sloanea sp., *Virola dixonii*. Palms: *Iriartea deltoidea, Oenocarpus bataua, Welfia regia, Wettinia quinaria*.

This system represents the high, moist, well‑drained evergreen forests of the Pacific slope of Central America, Mexico, the Colombian Chocó and the northern coast of Ecuador. Grows on soils of sedimentary or igneous origin (ash or basalt), mainly group latosols with clay texture and good organic matter content. Usually in hilly or mountainous terrain, low hills in coastal mountains and foothills of mountains inland. The above list of species is diagnostic for this system.

DISTRIBUTION

**Divisions:** 402:C

**Nations:** CR, MX, PA

CONCEPT

**Environment:** Relieve colinado hasta accidentado sobre las colinas de serranías costeras bajas, planicies sedimentarias marinas, latosoles e inceptisoles. Clima tropical muy húmedo con precipitación annual usualmente >4000 mm.

Hilly relief on the hills to rugged coastal mountains, low, marine sedimentary plains and Inceptisols and Latosols. Humid tropical climate, with annual rainfall ususally >4000 mm.

**Vegetation:** Bosques multiestratificados, con abundantes palmas en el sotobosque, de dosel cerrado y de 40‑50 m de alto. Los troncos y ramas están cargados de epífitas y lianas.

**Dynamics:** Bosque maduro sujeto a intervención.

SOURCES

**References:** Conservation International 1992a, Janzen 1983a, Josse et al. 2003*, Meyrat et al. 2001, Pennington and Sarukhán 1998

**Version:** 08 Jan 2015 **Stakeholders:** Latin America

**Concept Author:** C. Josse **LeadResp:** Latin America

CES402.581 Bosque Siempreverde Estacional de Tierras Bajas del Caribe Centroamericano

**Primary Division:** Moist Meso‑America (402)

**Land Cover Class:** Forest and Woodland

**Spatial Scale & Pattern:** Large patch

**Required Classifiers:** Natural/Semi‑natural; Vegetated (>10% vasc.); Upland

**Diagnostic Classifiers:** Lowland [Lowland]; Forest and Woodland (Treed); Udic

**Concept Summary:** Bosques estacionales siempreverdes de tierras bajas y suelos bien drenados de la vertiente atlántica de Centro América. Generalmente sobre terrenos colinados o accidentados, en sustratos sedimentarios en las partes bajas y metamórficos en las partes altas, por ejemplo cuarcíticos. La siguiente lista de las especies es de diagnóstica para este sistema: *Alseis yucatanensis, Apeiba membranacea, Aphananthe monoica, Attalea butyracea, Brosimum alicastrum, Bursera simaruba, Carapa guianensis, Castilla tunu, Cedrela odorata, Chamaedorea* spp., *Coccoloba barbadensis, Cyathea* sp., *Euterpe precatoria, Ficus insipida, Guettarda combsii, Liquidambar styraciflua, Manilkara zapota, Miconia* sp., *Mouriri myrtilloides, Pimenta dioica, Podocarpus guatemalensis, Pourouma aspera, Pseudolmedia oxyphyllaria, Schippia concolor, Sloanea terniflora, Spondias mombin, Swietenia macrophylla, Symphonia globulifera, Tabebuia rosea, Terminalia amazonia, Virola brachycarpa, Vismia ferruginea, Vochysia hondurensis, Xylopia frutescens, Zuelania guidonia*.

Evergreen seasonal lowland forests and well‑drained soils of the Atlantic Coast of Central America. Generally found on rugged terrain, in sedimentary or metapmorphic substrates in the lower vs. higher locations, respectively.

DISTRIBUTION

**Divisions:** 402:C

**Nations:** CR, HN, MX, NI

CONCEPT

**Environment:** Ocurre sobre suelos arcillosos o arenoso‑arcillosos con relieve colinado. Clima estacional.

Occurs on clay or sandy clay soils with hilly relief. Seasonal climate.

**Vegetation:** Bosque denso de 30 m de alto y multiestratificado.

**Dynamics:** Bosque maduro sujeto a intervención.

SOURCES

**References:** Josse et al. 2003*, Meyrat et al. 2001, Pennington and Sarukhán 1998, WWF and IUCN 1997

**Version:** 08 Jan 2015 **Stakeholders:** Latin America

**Concept Author:** C. Josse **LeadResp:** Latin America

CES402.600 Bosque Siempreverde Estacional de Tierras Bajas del Pacífico Centroamericano

**Primary Division:** Moist Meso‑America (402)

**Land Cover Class:** Forest and Woodland

**Spatial Scale & Pattern:** Large patch

**Required Classifiers:** Natural/Semi‑natural; Vegetated (>10% vasc.); Upland

**Diagnostic Classifiers:** Lowland [Foothill, Lowland]; Forest and Woodland (Treed); Udic

**Concept Summary:** Bosques estacionales siempreverdes de tierras bajas y suelos bien drenados de la vertiente pacífica de Centro América, ej. Darién panameño. La siguiente lista de especies es diagnóstica para este sistema: *Alchornea costaricensis, Alchornea latifolia, Ampelocera* sp., *Anacardium excelsum, Aspidosperma megalocarpon, Bombacopsis quinata, Bombacopsis sessilis, Brosimum alicastrum, Brosimum guianense, Brosimum utile, Caryocar costaricense, Senna spectabilis (= Cassia spectabilis), Castilla elastica, Cavanillesia platanifolia, Ceiba pentandra, Cochlospermum williamsii, Cordia alliodora, Schefflera morototonii (= Didymopanax morototonii), Dipteryx panamensis, Enterolobium cyclocarpum, Enterolobium guatemalense, Inga oerstediana, Jacaranda copaia, Jacaranda lasiogyne, Licania hypoleuca, Luehea seemannii, Myroxylon balsamum, Oenocarpus panamanus, Peltogyne purpurea, Pourouma aspera, Pouteria reticulata, Pseudolmedia rigida, Sloanea laurifolia, Sorocea sarcocarpa, Sterculia recordiana, Swartzia haughtii, Tabebuia* spp., *Trichilia pallida, Trichilia pleeana, Virola riedii, Virola sebifera*.

Seasonal evergreen lowland forests on well‑drained soils of the Pacific slope of Central America, e.g., Panama's Darien.

DISTRIBUTION

**Divisions:** 402:C

**Nations:** CR?, GT, PA, SV

CONCEPT

**Environment:** Generalmente sobre ultisoles profundos, arcillosos y bien drenados. Relieve colinado y clima húmedo con una estacionalidad ligera.

Generally on deep, loamy, well‑drained Ultisols. Hilly terrain and humid with a slight seasonality.

**Vegetation:** Tall (up to 35 m), dense forest with some deciduous species, with an understorey rich in palms, *Bactris, Geonoma, Oenocarpus*.

**Dynamics:** Bosque maduro.

SOURCES

**References:** Josse et al. 2003*, Meyrat et al. 2001, WWF and IUCN 1997

**Version:** 08 Jan 2015 **Stakeholders:** Latin America

**Concept Author:** C. Josse **LeadResp:** Latin America

M873. Mesoamerican Submontane Humid Forest

CES403.323 Bosque Húmedo Submontano del Norte de Mesoamérica

**Primary Division:** Meso‑American Seasonal Highlands (403)

**Land Cover Class:** Forest and Woodland

**Spatial Scale & Pattern:** Large patch

**Required Classifiers:** Natural/Semi‑natural; Vegetated (>10% vasc.); Upland

**Diagnostic Classifiers:** Lowland [Foothill]; Forest and Woodland (Treed); Udic

**Concept Summary:** Este sistema corresponde a los bosques humedos y muy húmedos que crecen en las estribaciones bajas y cerros del norte de Centroamérica desde los 600‑800 m hasta los 1300‑1500 m de altitud y con precipitación superior a 2000 mm anuales. Se tata de bosques de especies latifoliadas y siempreverdes, en algunas comunidades hay palmas y en general la estructura de los bosques es compleja, con numerosas epífitas, lianas y varios estratos leñosos, además de un dosel cerrado que alcanza los 20‑40 m de alto. El tipo más húmedo ocurre sobre las vertientes del Caribe. La siguiente lista de especies es diagnóstica para este sistema: *Aspidosperma cruenta, Astrocaryum mexicanum, Calatola costaricensis, Calophyllum antillanum (= Calophyllum brasiliense), Chamaedorea tepejilote, Chamaedorea* sp., *Colpothrinax cookii, Dendropanax arboreus, Euterpe precatoria, Ficus* spp., *Hedyosmum mexicanum, Ilex guianensis, Magnolia* sp., *Myrcia splendens, Nectandra* sp., *Persea schiedeana, Pouteria* spp., *Schizolobium parahyba, Symphonia globulifera, Terminalia amazonia, Virola koschnyi, Vismia* spp., *Vochysia hondurensis*.

<br />This system corresponds to the moist and wet forests growing on the lower slopes and hills of northern Central America to 1300‑1500 m altitude and with more than 2000 mm annual precipitation. It is tata forest and evergreen broadleaf species, in some communities there are palms and overall forest structure is complex, with numerous epiphytes, woody vines and various strata, and a closed canopy reaching 20‑40 m tall. The wet type occurs on the slopes of the Caribbean. The above list of species is diagnostic for this system.

DISTRIBUTION

**Divisions:** 403:C

**TNC Ecoregions:** NT0112:C, NT0162:C

**Nations:** BZ, GT, HN, NI

CONCEPT

**Environment:** Región montañosa de origen terciario con suelos molisoles, alfisoles, ultisoles, bien drenados y de alto contenido orgánico. En algunas partes puede haber afloramientos de roca, aunque no es común.

Highlands of tertiary origin with soils including Mollisols, Alfisols, Ultisols, that are well‑drained and with high organic content. In some parts it may be rock outcrops, although not common.

**Vegetation:** Bosques de especies latifoliadas y siempreverdes, en algunas comunidades hay palmas y en general la estructura de los bosques es compleja, con numerosas epífitas, lianas y varios estratos leñosos, además de un dosel cerrado que alcanza los 20‑40 m de alto.

SOURCES

**References:** Josse et al. 2003*

**Version:** 08 Jan 2015 **Stakeholders:** Latin America

**Concept Author:** C. Josse **LeadResp:** Latin America

CES402.607 Bosque Muy Húmedo Submontano de Talamanca

**Primary Division:** Moist Meso‑America (402)

**Land Cover Class:** Forest and Woodland

**Spatial Scale & Pattern:** Large patch

**Required Classifiers:** Natural/Semi‑natural; Vegetated (>10% vasc.); Upland

**Diagnostic Classifiers:** Montane [Lower Montane]; Forest and Woodland (Treed); Tropical/Subtropical [Tropical Pluvial]

**Concept Summary:** Este sistema agrupa los bosques muy húmedos premontanos de la vertiente Atlántica en Costa Rica y de ambas vertientes en las montañas de la región occidental y las del área del Canal de Panamá. En Panamá constituyen una gran parte de los sistemas montanos ya que hay relativamente poco territorio sobre los 1500‑1600 m de altitud, sin embargo no se cuenta con información suficiente para discriminar tipos o unidades diferentes dentro del sistema. La siguiente lista de especies es diagnóstica para este sistema: *Brosimum utile, Calophyllum longifolium, Calophyllum brasiliense var. rekoi, Micropholis crotonoides, Vochysia ferruginea, Billia columbiana, Alchornea latifolia, Hieronyma guatemalensis, Hirtella racemosa, Meliosma vernicosa, Pouteria* sp., *Podocarpus cf. oleifolius, Terminalia amazonia, Sacoglottis amazonica*, Lauraceae spp., *Euterpe macrospadix, Welfia georgii, Socratea durissima, Euterpe precatoria, Wettinia augusta*, tree ferns.

This system groups premontane wet forests of the Atlantic slope in Costa Rica and both slopes in the mountains of the western region and the area of the Panama Canal. In Panama it constitutes a large part of montane systems and there is relatively little territory about 1500‑1600 m above sea level; however, there is insufficient information to discriminate different types or units within the system. The above list of species is diagnostic for this system.

DISTRIBUTION

**Divisions:** 402:C

**TNC Ecoregions:** NT0115:C, NT0122:C, NT0167:C

**Nations:** CR, PA

CONCEPT

**Environment:** Estribaciones bajas y muy húmedas de las montañas de Costa Rica y occidente de Panamá, sobre suelos ácidos, arcillosos y generalmente bien drenados.

Low and very humid mountain slopes of Costa Rica and western Panama, on acidic, clayey and generally well‑drained soils.

**Vegetation:** Bosque denso de 30 a 40 m de alto, de estructura compleja y cargado de epífitas. En el sotobosque son abundantes las palmas y los helechos arborescentes.

**Dynamics:** Bosque maduro

SOURCES

**References:** Josse et al. 2003*, Meyrat et al. 2001, WWF and IUCN 1997

**Version:** 08 Jan 2015 **Stakeholders:** Latin America

**Concept Author:** C. Josse **LeadResp:** Latin America

1.A.2.Eh. Colombian‑Venezuelan Lowland Humid Forest

M581. Choco‑Darien Humid Forest

CES402.616 Bosque Pluvial de Tierra Firme del Chocó‑Darién

**Primary Division:** Moist Meso‑America (402)

**Required Classifiers:** Natural/Semi‑natural; Vegetated (>10% vasc.)

DISTRIBUTION

**Divisions:** 402:C

**Nations:** CO, EC, PA?

CONCEPT

SOURCES

**References:** Latin American Ecology Working Group n.d.*

**Stakeholders:** Latin America

**Concept Author:** C. Josse **LeadResp:** Latin America

CES402.614 Bosque Pluvial Premontano del Chocó‑Darién

**Primary Division:** Moist Meso‑America (402)

**Land Cover Class:** Forest and Woodland

**Spatial Scale & Pattern:** Large patch

**Required Classifiers:** Natural/Semi‑natural; Vegetated (>10% vasc.); Upland

**Diagnostic Classifiers:** Lowland [Foothill]; Forest and Woodland (Treed); Sideslope; Tropical/Subtropical [Tropical Pluvial]

**Concept Summary:** Este sistema agrupa los bosques húmedos y muy húmedos de la Cordillera de Darién en Panamá y las colinas de los contrafuertes andinos occidentales en la región del Chocó colombiano, por sobre los 600 m de altitud aproximadamente. Se trata de bosques de gran estatura y alta diversidad. La siguiente lista de especies es diagnóstica para este sistema: *Alchornea polyantha, Anacardium excelsum, Billia columbiana, Brosimum guianense, Brosimum utile, Cephaelis elata, Cephaelis elata, Dipteryx panamensis, Elaeagia utilis, Eschweilera verruculosa, Guettarda chiriquense, Oenocarpus panamanus, Perebea guianensis, Pourouma aspera, Pourouma chocoana, Sorocea* sp., *Weinmannia putumayensis, Welfia regia, Wettinia radiata*.

This system groups moist and wet forests of the Cordillera de Panama and Darien in the hills of the western Andean foothills in the Chocó region of Colombia, above about 600 m altitude. These are very tall forests with high diversity. The above list of species is diagnostic for this system.

DISTRIBUTION

**Divisions:** 402:C

**TNC Ecoregions:** NT0115:C, NT0122:C

**Nations:** CO, PA

CONCEPT

**Environment:** Estribaciones bajas y medias, muy húmedas y frecuentemente nubladas de las serranías de San Blas y Darién al oriente de Panamá, sobre suelos ácidos, arcillosos y generalmente bien drenados. Principalmente de origen volcánico.

Low and medium, very moist and often cloudy in the mountains of San Blas and Darien in eastern Panama, on acidic, well‑drained loamy soils and generally foothills. Mainly of volcanic origin.

**Vegetation:** Bosque de 30‑40 m de alto, varios estratos leñosos, abundancia de palmas en el sotobosque y numerosas epífitas. Siempreverde.

**Dynamics:** El sistema describe al bosque maduro, aunque se ha especulado sobre el la posibilidad de que sean bosques sucesionales debido a la larga historia de ocupación humana en la zona. Se encuentran regularmente especies secundarias dominantes.

<br />The system describes the mature forest, although it has been speculated that they may be successional forests due to the long history of human occupation in the area. They are regularly key secondary species.

SOURCES

**References:** Josse et al. 2003*, Kappelle and Brown 2001, Meyrat et al. 2001, WWF and IUCN 1997, Zuluaga 1987

**Version:** 08 Jan 2015 **Stakeholders:** Latin America

**Concept Author:** C. Josse **LeadResp:** Latin America

1.A.2.Ei. Guianan Lowland Humid Forest

M587. Orinoquian Humid Forest

CES404.351 Bosque Aluvial de la Guayana Oriental

**Primary Division:** Guiana Uplands and Highlands (404)

**Land Cover Class:** Forest and Woodland

**Spatial Scale & Pattern:** Large patch

**Required Classifiers:** Natural/Semi‑natural; Vegetated (>10% vasc.); Upland

**Diagnostic Classifiers:** Lowland [Lowland]; Forest and Woodland (Treed)

**Concept Summary:** Ocupan partes del Delta Alto del Orinoco y se extienden por los valles de la cuenca baja de rios tributarios del Orinoco. En general se trata de planos no inundables y terrenos colinados, aunque las partes bajas pueden inundarse temporal o esporádicamente. Ombroclima húmedo. Son bosques de hasta 25‑30 m de alto, siempreverdes y generalmente con tres estratos arboreos. Las palmas son abundantes y en sitios mas bajos o con menor drenaje, la especie Mora excelsa es claramente dominante. Pueden haber algunas especies deciduas en el dosel. The following list of species is diagnostic for this system: *Licania densiflora, Licania alba, Eschweilera decolorans, Gustavia poeppigiana, Gustavia augusta, Tabebuia capitata, Trichilia pleeana, Tetragastris altissima, Catostemma commune, Virola surinamensis, Alexa imperatricis, Mora excelsa, Sterculia pruriens, Peltogyne venosa, Clathrotropis brachypetala, Manilkara bidentata, Terminalia amazonia, Simarouba amara, Ceiba pentandra, Erythrina* sp., *Triplaris surinamensis*.

DISTRIBUTION

**Divisions:** 404:C

**Nations:** GY, VE

CONCEPT

**Environment:** Ocupan partes del Delta Alto del Orinoco y se extienden por los valles de la cuenca baja de rios tributarios del Orinoco. En general se trata de planos no inundables y terrenos colinados, aunque las partes bajas pueden inundarse temporal o esporádicamente. Ombroclima húmedo.

**Vegetation:** Son bosques de hasta 25‑30 m de alto, siempreverdes y generalmente con tres estratos arboreos. Las palmas son abundantes y en sitios mas bajos o con menor drenaje, la especie Mora excelsa es claramente dominante. Pueden haber algunas especies deciduas en el dosel.

SOURCES

**References:** Berry et al. 1995, Huber 1995, Josse et al. 2003*

**Version:** 11 Apr 2003 **Stakeholders:** Latin America

**Concept Author:** C. Josse **LeadResp:** Latin America

1.A.3. Tropical Montane Humid Forest

1.A.3.Eg. Caribbean‑Mesoamerican Montane Humid Forest

M598. Caribbean Montane Humid Forest

CES411.450 Caribbean Moist Montane Mixed Pine‑Broad‑leaved Forest

**Primary Division:** Caribbean (411)

**Land Cover Class:** Forest and Woodland

**Spatial Scale & Pattern:** Matrix

**Required Classifiers:** Natural/Semi‑natural; Vegetated (>10% vasc.); Upland

**Diagnostic Classifiers:** Tropical yellow soils

**Concept Summary:** Found between 800 and 2100 m elevation on acidic yellow soils derived from sandstone and andesitic tuffs in southeastern Cuba, and on red acidic clay soils in the Cordillera Central of Hispaniola. Two canopy layers. The top canopy is formed by *Pinus maestrensis* in Cuba, and by *Pinus occidentalis* in Hispaniola. The following list of species is diagnostic for this system: *Pinus maestrensis, Myrsine coriacea, Weinmannia pinnata, Ilex macfadyenii, Clethra cubensis, Myrica punctata, Cyathea arborea, Alsophila aspera, Pinus occidentalis, Ilex microwrightioides, Ilex tuerckheimii, Eupatorium illitium, Gnaphalium eggersii, Vernonia stenophylla, Psychotria dolichocalyx, Sideroxylon repens, Buddleia domingensis, Senecio buchii, Tournefortia selleana, Magnolia pallescens, Didymopanax tremulus, Tabebuia vinosa*.

DISTRIBUTION

**Divisions:** 411:C

**Nations:** CU, DO, XE

CONCEPT

**Environment:** Distribution is highly modified by disturbance regime. Growing under a wide range of physical parameters, soils can be volcanic, sedimentary and alluvial substrates. Most pine forests are found above 2200 m in the Cordillera Central of Hispaniola.

**Dynamics:** Landslides and hurricanes are the key triggers of dynamic processes of these forests. Substrate and topography and their interaction with the vegetation are the most important factors for the survival of these forests during hurricanes ‑ probably the single most important natural trigger of the successional dynamic. Surviving trees have their roots securely anchored in the substrate. These factors are also critical for regulating surface runoff and maintaining the water balance under very humid conditions on exposed ridges and steep slopes. Forest recovery after disturbance is slow. Monitoring of dwarf forest in Puerto Rico's Luquillo Mountains showed that it can take up to 20 years for woody species to establish and after that their growth rate is very slow. It took almost 35 years until the canopy closing decreased the grass and fern cover (Weaver 2008). Moreover, the succession process is often subjected to setbacks due to periodic hurricane disturbance. This study also showed that hurricanes cause delayed mortality, with declines in biomass and stem numbers exceeding ingrowth during 15 years after Hurricane Hugo hit. Another important finding of this study is that more than half of the arborescent species growing in dwarf forest, where they play a prominent role in post disturbance recovery, are endemic to Puerto Rico (Weaver 2008). Cloud forests are known as places of high endemism but not necessarily as areas with rich biotas (Weaver 2000, 2008).

SOURCES

**References:** Areces‑Mallea et al. 1999, Borhidi 1991, Josse et al. 2003*, Silver et al. 2001, Weaver 2000, Weaver 2008

**Version:** 08 Jan 2015 **Stakeholders:** Caribbean, Latin America

**Concept Author:** C. Josse **LeadResp:** Latin America

CES411.471 Caribbean Montane Serpentine Shrubland

**Primary Division:** Caribbean (411)

**Land Cover Class:** Shrubland

**Spatial Scale & Pattern:** Small patch

**Required Classifiers:** Natural/Semi‑natural; Vegetated (>10% vasc.); Upland

**Diagnostic Classifiers:** Serpentine

**Concept Summary:** This system occurs on ferrallitic soils derived from serpentine bedrock, between 600 and 1000 m elevation and higher, in humid conditions, as a result of fog condensation. Dense scrub, 4‑6 m high, with some emergents up to 10 m. Very rich in endemics. The following list of species is diagnostic for this system: *Ilex berteroi, Ilex alainii (= Ilex victorini), Ilex hypaneura, Ilex shaferi, Laplacea moaensis, Laplacea benitoensis, Clusia moaensis, Clusia callosa, Clusia monocarpa, Rauvolfia salicifolia, Byrsonima biflora, Myrica shaferi, Cyrilla cubensis, Myrcia retivenia, Coccoloba reflexa, Bourreria pauciflora, Callicarpa lancifolia, Clusia nipens, Jacaranda arborea, Eugenia mensuraensis*.

DISTRIBUTION

**Divisions:** 411:C

**Nations:** CU

CONCEPT

**Environment:** This system occurs on ferrallitic soils derived from serpentine bedrock, between 600 and 1000 m elevation and higher, in humid conditions, as a result of fog condensation.

**Dynamics:** Damage from passing hurricanes that cause breakage and subsequent forking of larger specimen trees results in uneven forest canopy that allows additional light to penetrate and encourages growth in adventitious or second growth species that may not be part of the climax forest type. Hurricanes play a major role in controlling composition and complexity of forest vegetation and periodic disruption is variable due to storm direction and intensity.

SOURCES

**References:** Borhidi 1991, Josse et al. 2003*

**Version:** 08 Jan 2015 **Stakeholders:** Caribbean, Latin America

**Concept Author:** C. Josse **LeadResp:** Latin America

CES411.455 Caribbean Montane Wet Elfin Forest

**Primary Division:** Caribbean (411)

**Land Cover Class:** Forest and Woodland

**Spatial Scale & Pattern:** Small patch

**Required Classifiers:** Natural/Semi‑natural; Vegetated (>10% vasc.); Upland

**Diagnostic Classifiers:** Tropical yellow soils

**Concept Summary:** This system tends to occur above 1600 m elevation; in Puerto Rico, it is usually above 700 m, but lower elevations are possible, especially under conditions of high precipitation. Stands have a closed but irregular canopy which is typically 6‑12 m high. Trees have gnarled trunks, compact crowns and small leaves. The shrub layer is almost impenetrable. Tree ferns and epiphytes are abundant. Forest floor, tree trunks and branches are covered by bryophytes. The following list of species is diagnostic for this system: *Myrsine microphylla, Nectandra reticularis, Sapium maestrense, Persea anomala, Symplocos leonis, Cyrilla racemiflora, Weinmannia pinnata, Torralbasia cuneifolia, Alsophila aspera, Didymopanax tremulus, Podocarpus aristulatus, Cyathea arborea, Cyathea balanocarpa, Vaccinium leonis, Miconia turquinensis, Tabebuia turquinensis, Tabebuia rigida, Tabebuia vinosa, Hedyosmum cubense, Henriettea ekmanii*, and *Duranta fletcheriana*. In windswept mountain ridges and summits from 500‑1350 m a.s.l. in Puerto Rico and islands of the Lesser Antilles, the following species are typical: *Cyrilla racemiflora, Prestoea acuminata var. montana (= Prestoea montana), Magnolia splendens, Podocarpus coriaceus, Dacryodes excelsa, Croton poecilanthus, Ternstroemia luquillensis, Ternstroemia subsessilis, Miconia laevigata, Micropholis garciniifolia, Micropholis guyanensis, Ocotea leucoxylon, Ocotea spathulata* and stunted trees of *Sloanea* spp.

DISTRIBUTION

**Range:** This system is found in Cuba, the Dominican Republic, Jamaica, the Lesser Antilles, and Puerto Rico.

**Divisions:** 411:C

**Nations:** CU, DO, JM, PR, XD

CONCEPT

**Environment:** Ecosystems of this macrogroup occur above 700 m elevation in areas with mean annual precipitation >1600 mm, frequently or seasonally surrounded by clouds, and on different topographies but mostly slopes, exposed ridges, and ravines. Forests growing on exposed areas are of smaller stature and very dense. Taller forests grow on protected areas on lower slopes to the leeward of ridges or spurs. With montane forests, one of the most critical climatic factors is the frequency and duration of the cloud cover; condensation can contribute 10% or more of the precipitation amount that these forests receive. In the Caribbean, the trade winds forming clouds have saline components which have an effect on the chemistry of the ecophysiology of these forests. Cloud cover causes less solar radiation, lower temperatures, decreased transpiration and lower photosynthetic rates, resulting in lower growth rates and lower nutrient‑cycling rates. The efficiency shown by these forests in the use of nutrients is high though, which is important to avoid nutrient loss due to leaching (Silver et al. 2001).

**Dynamics:** Landslides and hurricanes are the key triggers of dynamic processes of these forests. Substrate and topography and their interaction with the vegetation are the most important factors for the survival of these forests during hurricanes ‑ probably the single most important natural trigger of the successional dynamic. Surviving trees have their roots securely anchored in the substrate. These factors are also critical for regulating surface runoff and maintaining the water balance under very humid conditions on exposed ridges and steep slopes. Forest recovery after disturbance is slow. Monitoring of dwarf forest in Puerto Rico's Luquillo Mountains showed that it can take up to 20 years for woody species to establish and after that their growth rate is very slow. It took almost 35 years until the canopy closing decreased the grass and fern cover (Weaver 2008). Moreover, the succession process is often subjected to setbacks due to periodic hurricane disturbance. This study also showed that hurricanes cause delayed mortality, with declines in biomass and stem numbers exceeding ingrowth during 15 years after Hurricane Hugo hit. Another important finding of this study is that more than half of the arborescent species growing in dwarf forest, where they play a prominent role in post disturbance recovery, are endemic to Puerto Rico (Weaver 2008). Cloud forests are known as places of high endemism but not necessarily as areas with rich biotas (Weaver 2000, 2008).

SOURCES

**References:** Areces‑Mallea et al. 1999, Beard 1949, Borhidi 1991, Byer and Weaver 1977, Dansereau 1966, Dominica Ministry of Agriculture and Environment n.d., Figueroa Colon 1996, Helmer et al. 2002, International Institute of Tropical Forestry n.d., Josse et al. 2003*, Silver et al. 2001, TNC 2000, Tolentino and Peña 1998, Weaver 1990, Weaver 1991, Weaver 2000, Weaver 2008, Weaver et al. 1986

**Version:** 08 Jan 2015 **Stakeholders:** Caribbean, Latin America, U.S. Territories

**Concept Author:** C. Josse **LeadResp:** Latin America

CES411.429 Caribbean Montane Wet Serpentine Woodland

**Primary Division:** Caribbean (411)

**Land Cover Class:** Forest and Woodland

**Spatial Scale & Pattern:** Large patch

**Required Classifiers:** Natural/Semi‑natural; Vegetated (>10% vasc.); Upland

**Diagnostic Classifiers:** Serpentine

**Concept Summary:** This system occurs between 400 and 900 m elevation, on poor acidic ferrallitic soils in the serpentine areas of the Crystal and Moa mountains of eastern Cuba and western Puerto Rico. It has an open canopy, 15‑22 m high. The lower stratum, 5‑12 m, is dense. Most of the trees and shrubs are sclerophyllous. Lianas are common, but the density and diversity of epiphytes decrease. The following list of species is diagnostic for this system: *Calophyllum utile, Podocarpus ekmanii, Dipholis jubilla, Ocotea leucoxylon, Ocotea* spp., *Hyeronima nipensis, Tabebuia dubia, Byrsonima spicata (= Byrsonima coriacea), Byrsonima orientensis, Matayba domingensis, Bonnetia cubensis, Magnolia cubensis, Pinus cubensis, Chionanthus domingensis, Tetrazygia cristalensis, Byrsonima biflora, Ilex berteroi*. In addition, species of *Psychotria, Myrica, Eugenia, Baccharis, Ossaea, Eupatorium* and *Vernonia* are typical in the shrub layer. In Puerto Rico, the following species are typical: *Alsophila brooksii, Calyptranthes peduncularis, Calyptranthes triflora, Cordia bellonis, Crescentia portoricensis, Croton impressus, Diospyros revoluta, Eugenia glabrata, Gesneria pauciflora, Lunania ekmanii, Mikania stevensiana, Myrcia maricaensis, Phialanthus grandifolius, Phialanthus myrtilloides, Thelypteris hastata var. heterodoxa, Xylosma pachyphyllum, Xylosma* sp., *Cyathea arborea, Cnemidaria horrida, Gleichenia nervosa (= Dicanopteris nervosa), Sticherus bifidus, Magnolia splendens, Magnolia portoricensis, Schefflera gleasonii (= Didymopanax gleasonii), Micropholis guyanensis (= Micropholis chrysophylloides)*, and *Croton poecilanthus*.

DISTRIBUTION

**Range:** This system is found in the Crystal and Moa mountains of eastern Cuba and western Puerto Rico.

**Divisions:** 411:C

**Nations:** CU, PR

CONCEPT

**Environment:** [from M598] Ecosystems of this type occur above 700 m elevation in areas with mean annual precipitation >1600 mm, frequently or seasonally surrounded by clouds, and on different topographies but mostly slopes, exposed ridges, and ravines. Forests growing on exposed areas are of smaller stature and very dense. Taller forests grow on protected areas on lower slopes to the leeward of ridges or spurs. With montane forests, one of the most critical climatic factors is the frequency and duration of the cloud cover; condensation can contribute 10% or more of the precipitation amount that these forests receive. In the Caribbean, the trade winds forming clouds have saline components which have an effect on the chemistry of the ecophysiology of these forests. Cloud cover causes less solar radiation, lower temperatures, decreased transpiration and lower photosynthetic rates, resulting in lower growth rates and lower nutrient‑cycling rates. The efficiency shown by these forests in the use of nutrients is high though, which is important to avoid nutrient loss due to leaching (Silver et al. 2001).

**Dynamics:** Landslides and hurricanes are the key triggers of dynamic processes of these forests. Substrate and topography and their interaction with the vegetation are the most important factors for the survival of these forests during hurricanes ‑ probably the single most important natural trigger of the successional dynamic. Surviving trees have their roots securely anchored in the substrate. These factors are also critical for regulating surface runoff and maintaining the water balance under very humid conditions on exposed ridges and steep slopes. Forest recovery after disturbance is slow. Monitoring of dwarf forest in Puerto Rico's Luquillo Mountains showed that it can take up to 20 years for woody species to establish and after that their growth rate is very slow. It took almost 35 years until the canopy closing decreased the grass and fern cover (Weaver 2008). Moreover, the succession process is often subjected to setbacks due to periodic hurricane disturbance. This study also showed that hurricanes cause delayed mortality, with declines in biomass and stem numbers exceeding ingrowth during 15 years after Hurricane Hugo hit. Another important finding of this study is that more than half of the arborescent species growing in dwarf forest, where they play a prominent role in post disturbance recovery, are endemic to Puerto Rico (Weaver 2008). Cloud forests are known as places of high endemism but not necessarily as areas with rich biotas (Weaver 2000, 2008).

SOURCES

**References:** Areces‑Mallea et al. 1999, Borhidi 1991, Figueroa Colon 1996, Helmer et al. 2002, International Institute of Tropical Forestry n.d., Josse et al. 2003*, Silver et al. 2001, TNC 2004a, Weaver 2000, Weaver 2008

**Version:** 08 Jan 2015 **Stakeholders:** Caribbean, Latin America, U.S. Territories

**Concept Author:** C. Josse **LeadResp:** Latin America

CES411.451 Caribbean Montane Wet Short Shrubland

**Primary Division:** Caribbean (411)

**Land Cover Class:** Shrubland

**Spatial Scale & Pattern:** Small patch

**Required Classifiers:** Natural/Semi‑natural; Vegetated (>10% vasc.); Upland

**Diagnostic Classifiers:** Reddish yellow soils

**Concept Summary:** This system occurs on mountain peaks or summits. In Puerto Rico, it is found on the highest peaks of Luquillo Mountains (900‑1050 m elevation) and Cordillera Central; in Cuba, on steep rocky ridges of the highest peaks of Sierra Maestra, between 1800 and 1970 m. It is dominated by short scrub, 1.5‑2 m high, with many thorny shrubs and herbaceous‑leaved succulents. The following list of species is diagnostic for this system in Cuba: *Ilex nunezii, Ilex turquinensis, Myrica cacuminis, Lobelia cacuminis, Eupatorium* sp., *Vernonia* sp., *Weinmannia pinnata, Persea similis, Viburnum villosum, Agave pendentata, Pleurothalis* spp., *Lepanthes* spp., *Mitracarpus acunae, Cassia turquinae, Juniperus saxicola, Schoepfia stenophylla*, and *Eugenia maestrensis*. In Puerto Rico and Martinique, the following species are typical: *Eugenia borinquensis, Alsophila bryophila (= Cyathea bryophila), Tabebuia rigida, Marcgravia sintenisii, Ocotea spathulata, Henriettea squamulosa, Micropholis garciniifolia, Daphnopsis philippiana, Ardisia luquillensis, Clidemia cymosa (= Heterotrichum cymosum)*, and *Gonocalyx portoricensis*. On mountain summits of St. Kitts and Nevis *Hedyosmum arborescens, Podocarpus coriaceus, Clusia rosea, Myrsine coriacea, Cyathea arborea*, are common.

DISTRIBUTION

**Range:** This system is found in Cuba, Puerto Rico, Martinique, and islands of the Lesser Antilles with mountain ridges.

**Divisions:** 411:C

**Nations:** CU, KN, MQ, PR, XD

CONCEPT

**Environment:** Growing above 600 m elevation, associated with high rainfall, extremely high moisture levels, frequent overcast conditions, and high winds. The soil is often waterlogged, but due to the gradient of the slope, runoff is high.

**Dynamics:** Damage from passing hurricanes that cause breakage and subsequent forking of larger specimen trees results in uneven forest canopy that allows additional light to penetrate and encourages growth in adventitious or second growth species that may not be part of the climax forest type. Hurricanes play a major role in controlling composition and complexity of forest vegetation and periodic disruption is variable due to storm direction and intensity.

SOURCES

**References:** Areces‑Mallea et al. 1999, Beard 1949, Borhidi 1991, Josse et al. 2003*, TNC 2004a, Weaver et al. 1986

**Version:** 08 Jan 2015 **Stakeholders:** Caribbean, Latin America, U.S. Territories

**Concept Author:** C. Josse **LeadResp:** Latin America

CES411.430 Caribbean Wet Montane Forest

**Primary Division:** Caribbean (411)

**Land Cover Class:** Forest and Woodland

**Spatial Scale & Pattern:** Large patch

**Required Classifiers:** Natural/Semi‑natural; Vegetated (>10% vasc.); Upland

**Diagnostic Classifiers:** Tropical yellow soils

**Concept Summary:** This system is found over 800 m and up to 1600 m elevation on yellowish or red ferrallitic soils or clay‑loam derived from limestones. In mountains exposed to higher precipitation, it is found as low as 450 m. Remnants of these evergreen tall forests can be found in the mountains of Jamaica, Cuba, and Puerto Rico. Examples have a closed or open canopy, 15‑25 m high, consisting of microphylls and notophylls. When in good condition, the upper layer is closed and has a second layer with abundant palms, tree ferns and epiphytes, all of them rich in species. *Prestoea acuminata var. montana (= Prestoea montana)* and ferns dominate areas after deforestation or hurricanes. The following list of species is diagnostic for this system: *Magnolia* spp., *Cyrilla racemiflora, Solanum acropterum, Ocotea ekmanii, Nectandra krugii (= Ocotea krugii), Ocotea cernua, Nectandra coriacea (= Ocotea coriacea), Myrsine coriacea, Clusia tetrastigma, Gomidesia lindeniana, Alchornea latifolia, Calophyllum jacquinii, Matayba apetala, Miconia punctata, Cyathea arborea, Cyathea balanocarpa, Cyathea cubensis, Torralbasia cuneifolia, Brunellia comocladiifolia, Weinmannia pinnata, Lasianthus lanceolatus, Ilex macfadyenii, Cleyera nimanimae, Clethra occidentalis, Prunus occidentalis* and *Podocarpus* spp. In Puerto Rico, the following species are typical: *Banara portoricensis, Brachionidium ciliolatum, Myrcia margarettiae (= Eugenia margarettiae), Gonocalyx concolor, Habenaria amalfitana (= Habenaria dussii), Ternstroemia luquillensis*, and *Ternstroemia subsessilis*. In Cuba: *Carapa guianensis, Calophyllum utile, Sloanea curatellifolia, Dipholis jubilla, Bactris cubensis*, and *Calyptronoma plumeriana (= Calyptronoma clementis)*.

DISTRIBUTION

**Range:** This system occurs in Cuba, the Dominican Republic, Jamaica, Puerto Rico, and in some of the Lesser Antilles islands.

**Divisions:** 411:C

**Nations:** CU, DO, JM, PR, XD

CONCEPT

**Environment:** Ecosystems of this macrogroup occur above 700 m elevation in areas with mean annual precipitation >1600 mm, frequently or seasonally surrounded by clouds, and on different topographies but mostly slopes, exposed ridges, and ravines. Forests growing on exposed areas are of smaller stature and very dense. Taller forests grow on protected areas on lower slopes to the leeward of ridges or spurs. With montane forests, one of the most critical climatic factors is the frequency and duration of the cloud cover; condensation can contribute 10% or more of the precipitation amount that these forests receive. In the Caribbean, the trade winds forming clouds have saline components which have an effect on the chemistry of the ecophysiology of these forests. Cloud cover causes less solar radiation, lower temperatures, decreased transpiration and lower photosynthetic rates, resulting in lower growth rates and lower nutrient‑cycling rates. The efficiency shown by these forests in the use of nutrients is high though, which is important to avoid nutrient loss due to leaching (Silver et al. 2001).

**Dynamics:** Landslides and hurricanes are the key triggers of dynamic processes of these forests. Substrate and topography and their interaction with the vegetation are the most important factors for the survival of these forests during hurricanes ‑ probably the single most important natural trigger of the successional dynamic. Surviving trees have their roots securely anchored in the substrate. These factors are also critical for regulating surface runoff and maintaining the water balance under very humid conditions on exposed ridges and steep slopes. Forest recovery after disturbance is slow. Monitoring of dwarf forest in Puerto Rico's Luquillo Mountains showed that it can take up to 20 years for woody species to establish and after that their growth rate is very slow. It took almost 35 years until the canopy closing decreased the grass and fern cover (Weaver 2008). Moreover, the succession process is often subjected to setbacks due to periodic hurricane disturbance. This study also showed that hurricanes cause delayed mortality, with declines in biomass and stem numbers exceeding ingrowth during 15 years after Hurricane Hugo hit. Another important finding of this study is that more than half of the arborescent species growing in dwarf forest, where they play a prominent role in post disturbance recovery, are endemic to Puerto Rico (Weaver 2008). Cloud forests are known as places of high endemism but not necessarily as areas with rich biotas (Weaver 2000, 2008).

SOURCES

**References:** Areces‑Mallea et al. 1999, Borhidi 1991, Dansereau 1966, Devillers and Devillers‑Terschuren 1996, Dominica Ministry of Agriculture and Environment n.d., Figueroa Colon 1996, Helmer et al. 2002, International Institute of Tropical Forestry n.d., Josse et al. 2003*, Silver et al. 2001, TNC 2000, TNC 2004a, Walter 1971, Weaver 1990, Weaver 2000, Weaver 2008

**Version:** 08 Jan 2015 **Stakeholders:** Caribbean, Latin America, U.S. Territories

**Concept Author:** C. Josse **LeadResp:** Latin America

CES411.470 Hispaniola Montane and Upper Montane Pine Forest

**Primary Division:** Caribbean (411)

**Land Cover Class:** Forest and Woodland

**Spatial Scale & Pattern:** Small patch

**Required Classifiers:** Natural/Semi‑natural; Vegetated (>10% vasc.); Upland

**Concept Summary:** This system is found above 2200 m elevation in the Cordillera Central of Hispaniola and in Massif du Nord in Haiti. Forests are characterized by a fairly open and monospecific canopy of *Pinus occidentalis*, with many endemic shrubs and ferns in the understory or an herbaceous layer dominated by the tussock grass *Danthonia domingensis*. The following list of species is diagnostic for this system: *Pinus occidentalis, Lyonia urbaniana, Lyonia tuerkheimii, Gaultheria domingensis, Fadyenia hookeri (= Garrya fadyenii), Senecio* spp., *Oxandra lanceolata, Hypericum pycnophyllum, Weinmannia pinnata, Vaccinium cubense, Cojoba arborea (= Pithecellobium arboreum), Juniperus gracilior, Juniperus eckmanii, Podocarpus buchii, Pteridium aquilinum, Calamagrostis leonardii, Agrostis hyemalis, Danthonia domingensis, Verbena domingensis*.

DISTRIBUTION

**Range:** This system is found above 2200 m elevation in the Cordillera Central of Hispaniola and in Massif du Nord in Haiti.

**Divisions:** 411:C

**Nations:** DO, HT, XE

CONCEPT

**Environment:** Occurs on elevations above 1900 m and up to 3000 m, with a dry season of 3‑5 months.

**Dynamics:** In these seasonal, open forests fire is a natural disturbance factor triggering dynamic processes, originally caused by lightning and then intensified by human intervention (Horn et al. 2000). Given their distribution on mountain slopes, landslides and hurricanes also play a role in the dynamic processes of these forests.

SOURCES

**References:** Areces‑Mallea et al. 1999, Horn et al. 2000, Josse et al. 2003*, Silver et al. 2001, Tolentino and Peña 1998

**Version:** 08 Jan 2015 **Stakeholders:** Caribbean, Latin America

**Concept Author:** C. Josse **LeadResp:** Latin America

M600. Mesoamerican Montane Humid Forest

CES403.315 Bosque Mesófilo de Montaña Mexicano

**Primary Division:** Meso‑American Seasonal Highlands (403)

**Land Cover Class:** Forest and Woodland

**Spatial Scale & Pattern:** Large patch

**Required Classifiers:** Natural/Semi‑natural; Vegetated (>10% vasc.); Upland

**Diagnostic Classifiers:** Montane [Montane, Lower Montane]; Forest and Woodland (Treed); Udic

**Concept Summary:** Cloud forests in Mexico are transitional forest communities occurring in tropical and subtropical humid mountains located south of the 25°N parallel, at elevations mostly between 1250 and 2500 m. They are located in areas of high relative humidity, on steep or irregular topography, often in protected ravines. These areas are more humid than pine, pine‑oak and oak forests, warmer than high‑elevation conifer forests, and cooler than those that support the development of tropical plant formations (González‑Espinosa et al. 2011). Floristically, this forest type is one of the ecosystems that better expresses transitional conditions between tropical and temperate biogeographic realms. It shows close floristic affinities to deciduous forests of North America, equivalent forests of eastern Asia, and montane forests in the Andean region of South America (Alcántara et al. 2002). They are easily distinguishable from other forest systems by the abundance of epiphytes and reduction in woody climbers. The distribution of cloud forests in Mexico is archipelago‑like; this, the great variety of habitats and the wide contact between Holartic and Neotropical floras, make this forest floristically very rich. It is considered that 10% of the vascular plant species of Mexico are found in the country's cloud forests, which only cover between 0.5‑1.0% of the national territory. Plant species endemism is also extremely high in these forests. the following list of species is diagnostic for this system: *Carpinus caroliniana, Chiranthodendron pentadactylon, Liquidambar styraciflua, Oreomunnea mexicana, Oreopanax echinops*, and *Podocarpus matudae*, although none of these species occurs throughout the distribution of this ecological system (González‑Espinosa et al. 2011). Genera with most of their species better distributed in Mexican cloud forest than in any other type of Mexican vegetation are *Clethra, Magnolia, Meliosma, Styrax, Symplocos*, and *Ternstroemia* (Alcántara et al. 2002). Also *Cyathea* (tree fern) and many moss species are characteristic.

DISTRIBUTION

**Range:** Tropical and subtropical humid mountains located south of the 25°N parallel, in Mexico and Guatemala at elevations mostly between 1250 and 2500 m.

**Divisions:** 403:C

**TNC Ecoregions:** NT0113:C, NT0114:C, NT0146:C, NT0177:C

**Nations:** GT, MX

**Subnations:** MXCL, MXCP, MXJA, MXMI, MXNA, MXOA, MXTM, MXVE

CONCEPT

**Environment:** En México, los bosques nubosos en general, aparecen como parches aislados dentro de elevación oscila entre 600 y 2900 m (en su mayoría por encima de 1.500 m) y están rodeadas de vegetación xerofítica, bosques de *Quercus* o pino, o bosques mixtos. La topografía es abrupta, con pendientes bastante pronunciadas. La temperatura media anual es de 13‑14°C (hasta 18°C en las partes inferiores del cinturón altitudinal), y la media de precipitación anual es de 1200 a 1500 mm, aunque también se encuentran en lugares donde la precipitación media anual supera los 5.000 mm. Por lo general ocurren en suelos profundos, bien drenados, se originó a partir de piedra caliza o rocas metamórficas, a menudo afloran. Las extensiones de bosque nuboso más grandes de México se encuentran en la Sierra Madre Oriental, la Sierra Norte de Oaxaca (Northern Range Oaxaca), la Sierra Madre del Sur, las montañas del norte de Chiapas y la Sierra Madre de Chiapas (González‑Espinosa et al. 2011). Al igual que en otras regiones del mundo donde se producen estos bosques, su hábitat se considera único entre los ecosistemas terrestres: está fuertemente ligada a los procesos de formación de nubes y un resultantes cerca de la saturación atmosférica constante.

In Mexico, cloud forests generally appear as isolated patches within elevation ranges from 600‑2900 m (mostly above 1500 m) and are surrounded by xeric vegetation, *Quercus* or *Pinus* forests, or mixed forests. The topography is abrupt with fairly steep slopes. Mean annual temperature is 13‑14°C (up to 18°C in the lower portions of the altitudinal belt), and mean annual rainfall is 1200‑1500 mm, though they are also found in places where the average annual precipitation exceeds 5000 mm. They usually occur on deep, well‑drained soils, originated from limestone or metamorphic rocks, often outcropping. The largest cloud forest tracts in Mexico are located in the Sierra Madre Oriental, the Sierra Norte de Oaxaca (Northern Oaxaca Range), the Sierra Madre del Sur, the Northern Mountains of Chiapas and the Sierra Madre de Chiapas (González‑Espinosa et al. 2011). As in other regions of the world where these forests occur, their habitat is considered unique among terrestrial ecosystems; it is strongly linked to processes of cloud formation and a resulting near constant atmospheric saturation.

**Vegetation:** This system includes typically dense forests, 25‑30 m high, with a cold‑deciduous overstory and evergreen understory (e.g., tree ferns, etc.). Epiphytes such as bryophytes (liverworts and mosses), ferns and fern allies, and flowering plants (mainly Bromeliaceae, Orchidaceae and Araceae) are significant in both species diversity and biomass.

**Dynamics:** La perturbación natural es causada principalmente por los deslizamientos de tierra, por lo que la sucesión secundaria es un proceso clave que define los aspectos estructurales y de composición de los bosques de niebla. Perturbación de bosque nublado y húmedo tiende a conducir a las comunidades de roble dominado.

Natural disturbance is primarily caused by landslides, so secondary succession is a key process defining structural and compositional aspects of cloud forests. Disturbance of humid cloud forest tends to lead to oak‑dominated communities.

SOURCES

**References:** Alcántara et al. 2002, Cayuela et al. 2006, González‑Espinosa et al. 2011, INEGI 2005, Josse et al. 2003*, Ochoa‑Gaona and González‑Espinosa 2000, Pattanavibool and Dearden 2002, Pennington and Sarukhán 1998, Ponce‑Reyes et al. 2012, Rojas‑Soto et al. 2012, Rzedowski 1978, Rzedowski 1986, Toledo‑Aceves et al. 2014

**Version:** 08 Jan 2015 **Stakeholders:** Latin America

**Concept Author:** C. Josse **LeadResp:** Latin America

CES403.324 Bosque Montano Alto de Coníferas y Mixto del Norte de Mesoamérica

**Primary Division:** Meso‑American Seasonal Highlands (403)

**Land Cover Class:** Forest and Woodland

**Spatial Scale & Pattern:** Large patch

**Required Classifiers:** Natural/Semi‑natural; Vegetated (>10% vasc.); Upland

**Diagnostic Classifiers:** Montane [Upper Montane]; Forest and Woodland (Treed); Udic

**Concept Summary:** En las partes más altas de montañas de Guatemala y Honduras, crecen comunidades dominadas por coníferas. Un ejemplo típico de este sistema se encuentra sobre los 1500 m en Sierra de las Minas, en Guatemala. En Honduras también se conoce de bosques mixtos y rodales puros de pinos entre los 1800 y 2800 m. Generalmente los rodales puros se encuentran en pendientes que reciben poca precipitación y además experimentan quemas cada cierto tiempo. En estos casos el sotobosque es muy pobre y está dominado por especies de gramíneas. La siguiente lista de especies es diagnóstica para este sistema: *Abies guatemalensis, Alnus jorulensis, Alsophila salvinii, Culcita coniifolia, Cyathea divergens, Dicksonia sellowiana, Ilex* spp., *Juglans guatemalensis, Juniperus standleyi, Lophosoria quadripinnata, Pinus strobiformis (= Pinus ayacahuite), Podocarpus oleifolius, Quercus sapotifolia, Quercus* spp., *Taxus globosa, Weinmannia pinnata, Weinmannia tuerckheimii*.

In the higher parts of the mountains of Guatemala and Honduras are located communities dominated by conifers. A typical example of this system is found at about 1500 m in the Sierra de las Minas in Guatemala. In Honduras mixed and pure stands of pine forests are known between 1800 and 2800 m. Generally pure stands are found on slopes that receive little precipitation and also experience burning every so often. In these cases the understory is very poor and is dominated by grasses. The above list of species is diagnostic for this system.

DISTRIBUTION

**Divisions:** 403:C

**TNC Ecoregions:** NT0303:C

**Nations:** GT, HN

CONCEPT

**Environment:** Pendientes altas generalmente muy inclinadas, a veces rocosas. Suelos bien drenados y de textura variable.

<br />Generally high steep slopes, sometimes rocky. Well‑drained soils of variable texture.

**Vegetation:** Bosque siempreverde de cobertura variable, densa o abierta. De 10‑25 m de alto aunque hay emergentes de hasta 40 m. Puede haber helechos arborescentes y epífitas terrestres. En algunas localidades pueden ocurrir poblaciones puras de Abies o Pinus. En las crestas de cerros la cobertura del dosel es muy baja.

**Dynamics:** Mature forest

SOURCES

**References:** Josse et al. 2003*, Meyrat et al. 2001, WWF and IUCN 1997

**Version:** 08 Jan 2015 **Stakeholders:** Latin America

**Concept Author:** C. Josse **LeadResp:** Latin America

CES403.319 Bosque Siempreverde Nublado del Norte de Mesoamérica

**Primary Division:** Meso‑American Seasonal Highlands (403)

**Land Cover Class:** Forest and Woodland

**Spatial Scale & Pattern:** Large patch

**Required Classifiers:** Natural/Semi‑natural; Vegetated (>10% vasc.); Upland

**Diagnostic Classifiers:** Montane [Upper Montane]; Forest and Woodland (Treed); Udic

**Concept Summary:** Los bosques nubosos se encuentran en zonas de alta humedad relativa, ambientes montanos, topografía irregular, una capa de humus profunda, y el clima relativamente templado. En México y en otros lugares que se distribuyen entre los 600 y 3000 m de altitud, pero se encuentran de manera óptima entre 1250 y 2450 m. Son fácilmente distinguibles de otros sistemas forestales por la abundancia de epífitas y la reducción en la densidad de trepadoras leñosas. Con el aumento de la elevación, la altura del dosel de los bosques nubosos es inferior a la de los bosques de tierras bajas; los árboles exhiben coronas compactas y troncos retorcidos; contrafuertes, lianas, palmas y hojas tienden a ser más pequeños, más gruesos y más duros, al parecer una adaptación a la transpiración suprimida debido a la alta humedad atmosférica. Generos característicos de los bosques de niebla mexicanos son: *Clethra, Magnolia, Meliosma, Styrax, Symplocos* y *Ternstroemia* con varias especies cada uno. Otras especies comunes en México, Guatemala y Honduras son: *Quercus crispifolia, Quercus bumelioides, Quercus insignis, Quercus cortesii, Quercus lancifolia, Quercus laurina, Quercus xalapensis, Oreopanax xalapensis, Oreopanax* spp., *Phoebe helicterifolia, Alsophila salvinii, Persea donnell‑smithii, Persea sessilis, Persea schiedeana, Podocarpus oleifolius, Podocarpus guatemalensis, Weinmannia pinnata, Magnolia hondurensis, Alfaroa costaricensis, Alfaroa hondurensis, Billia hippocastanum, Brunellia mexicana, Prunus brachybotrya, Olmediella betschleriana, Amphitecna montana, Pithecellobium vulcanorum*.

Cloud forests are located in areas of high relative humidity, montane environments, irregular topography, a deep litter layer, and relatively temperate climate. In Mexico and elsewhere they are distributed between 600 and 3000 m elevation but are found optimally between 1250 and 2450 m. They are easily distinguishable from other forest systems by the abundance of epiphytes and reduction in woody climbers. With increasing elevation, the canopy height of cloud forests is lower than that of lowland forests; trees exhibit compact crowns and gnarled trunks; buttresses, lianas, palms, and leaves tend to be smaller, thicker, and harder, apparently an adaptation to suppressed transpiration due to high atmospheric moisture. Genera characteristic of Mexican cloud forests are *Clethra, Magnolia, Meliosma, Styrax, Symplocos*, and *Ternstroemia* with several species each. Other common species in Mexico, Guatemala and Honduras are listed above.

DISTRIBUTION

**Range:** Neotropical cloud forests extend from 23°N to 25°S latitude, roughly from mid‑Mexico to northeastern Argentina.

**Divisions:** 403:C

**TNC Ecoregions:** NT0112:C, NT0146:C, NT0177:C

**Nations:** GT, HN, MX

**Subnations:** MXCP, MXSL, MXVE

CONCEPT

**Environment:** Bosques nublados neotropicales se extienden desde 23°N a 25°S, aproximadamente desde el centro de México hasta el noreste de Argentina. El típico bosque nublado, húmedo y denso, generalmente se encuentra en las cordilleras, de 1.000 a 3.000 m, con nubes relativamente continuas, cubriendo el bosque. El parche más septentrional del bosque nuboso parece ser el Rancho del Cielo, a 23°N, en la Sierra Madre Oriental de México, entre 1000 y 1500 m. En Mesoamérica, los bosques nubosos en general, aparecen como parches aislados rodeados de diferentes tipos de vegetación. Por lo general, la precipitación anual es de más de 1500 mm y la temperatura media inferior a 18°C. Los suelos son poco profundos, pero con alto contenido de materia orgánica.

Neotropical cloud forests extend from 23°N to 25°S latitude, roughly from mid‑Mexico to northeastern Argentina. The typical cloud forest, humid and dense, is generally found on mountain ranges, from 1000 to 3000 m, with relatively continuous cloud cover at the vegetation level, blanketing the forest. The northernmost stand of cloud forest appears to be the Rancho del Cielo, at 23°N latitude in the Sierra Madre Oriental of Mexico, between 1000 and 1500 m. In Meso‑America, cloud forests generally appear as isolated patches surrounded by different types of vegetation. Usually anual precipitation is more than 1500 mm and mean temperature lower than 18°C. Soils are shallow but with high organic matter content.

**Vegetation:** Dense, mostly evergreen forest, 15 ‑25 m canopy with emergents up to 30 m. Epiphytes such as bryophytes (liverworts and mosses), ferns and fern allies, and flowering plants (mainly Bromeliaceae, Orchidaceae and Araceae) are significant in both species diversity and biomass. Some locales of low elevation in Guatemala contain palm species (*Colpothrinax cookii*).

**Dynamics:** La perturbación natural es causada principalmente por los deslizamientos de tierra, por lo que la sucesión secundaria es un proceso clave que define los aspectos estructurales y de composición de los bosques de niebla.

Disturbance is primarily caused by landslides, so secondary succession is a key process defining structural and compositional aspects of cloud forests.

SOURCES

**References:** Alcántara et al. 2002, Josse et al. 2003*, Kappelle and Brown 2001, Meyrat et al. 2001, Pennington and Sarukhán 1998, Ponce‑Reyes et al. 2012, Rojas‑Soto et al. 2012

**Version:** 08 Jan 2015 **Stakeholders:** Latin America

**Concept Author:** C. Josse **LeadResp:** Latin America

M601. Mesoamerican Montane Pine‑Oak Forest

CES403.318 Bosque de Encino‑Pino de [Guerrero] México

**Primary Division:** Meso‑American Seasonal Highlands (403)

**Land Cover Class:** Forest and Woodland

**Spatial Scale & Pattern:** Large patch

**Required Classifiers:** Natural/Semi‑natural; Vegetated (>10% vasc.); Upland

**Diagnostic Classifiers:** Montane [Upper Montane]; Forest and Woodland (Treed); Ustic

**Concept Summary:** This system occurs on mountain slopes in the southern Sierra Madre Occidental, Transvolcanic ranges, and mountain slopes of Mexico, extending south into Central America. These forests and woodlands are composed of Madrean pines and evergreen oaks intermingled with patchy shrublands on most mid‑elevation slopes (2300‑2400 m elevation). The following list of species is diagnositc for this system: *Cleyera theaoides, Solanum nigricans, Litsea glaucescens, Pinus oaxacana, Pinus oocarpa, Prunus serotina, Quercus crassifolia, Quercus laurina, Quercus rugosa, Rapanea juergensenii*.

DISTRIBUTION

**Divisions:** 403:C

**TNC Ecoregions:** NT0308:C, NT0309:C, NT0310:C

**Nations:** MX

**Subnations:** MXCP, MXDU, MXGJ, MXGU, MXHI, MXJA, MXMI, MXNU, MXOA, MXPU, MXSL, MXVE, MXZA

CONCEPT

**Environment:** Encontrado en suelos moderadamente profundos que a menudo son calcáreos y de textura franco arcillosa. Ocurren en elevaciones entre 2.300 y 2.400 m, donde las temperaturas de congelación casi nunca, o nunca, se producen.

Found on moderately deep soils that are often calcareous and of clay loam texture. They occur at elevations between 2300 and 2400 m where freezing temperatures seldom, if ever, occur.

**Vegetation:** Pines and oaks define a complex overstory driven by patchy landslide, fire and wind.

**Dynamics:** La frecuencia de incendios de superficie: 20 años. Lafrecuencia de incendios del dosel: 150‑250 años. Estabilidad de taludes determina la frecuencia de deslizamientos de tierra provocados por terremotos y altas precipitaciones. La frecuencia de deslizamientos a su vez determina los patrones de perturbación y crea heterogeneidad en el paisaje.

Surface fire frequency is 20 years. Crown fire frequency is 150‑250 years. Slope stability determines the frequency of landslides triggered by earthquakes and high rainfalls. Landslide regime in turn determines landslide disturbance patterns and creates landscape heterogeneity.

SOURCES

**References:** Josse et al. 2003*, Rzedowski 1986, Velazquez et al. 2000

**Version:** 08 Jan 2015 **Stakeholders:** Latin America

**Concept Author:** C. Josse **LeadResp:** Latin America

CES403.321 Bosque Montano Alto de Pino Encino del Norte de Mesoamérica

**Primary Division:** Meso‑American Seasonal Highlands (403)

**Land Cover Class:** Forest and Woodland

**Spatial Scale & Pattern:** Large patch

**Required Classifiers:** Natural/Semi‑natural; Vegetated (>10% vasc.); Upland

**Diagnostic Classifiers:** Montane [Montane]; Forest and Woodland (Treed); Udic

**Concept Summary:** Este sistema agrupa los bosques nublados mixtos de montañas del norte de Centro América caracterizados por la asociación de especies de pino y encino (roble), y representa el límite de la influencia de la flora boreal en el Neotrópico caracterizada por una alta diversidad de coníferas. Se trata de un sistema transicional entre el templado de pino encino o mesófilo de montaña y los robledales tropicales montanos de Costa Rica hasta Colombia y se distribuye desde el sur de Chiapas, México hasta el noroeste de Nicaragua. La diversidad de coníferas y de robles es una de las mas altas del mundo porque se trata del centro de especiación del genero *Pinus*. Es un sistema influenciado por la neblina y por lo tanto con humedad ambiental casi constante a pesar de la estacionalidad que pudiera tener. Esta es una de las principales diferencias con el sistema de Bosque de pino‑encino montano de la zona templada de México, que se desarrolla en un ambiente más seco. El bosque montano nublado de pino encino se encuentra generalmente entre los 1500 y 2300 m y ocurre en ambas vertientes: Pacífica y Caribe. Se han registrado hasta 36 especies de Encino (roble) y 11 especies de Pino en comunidades de bosque maduro. La siguiente lista de especies es diagnóstica para este sistema: *Abies guatemalensis, Acer skutchii, Alnus jorulensis, Arbutus xalapensis, Bocconia glaucifolia, Cornus disciflora, Culcita coniifolia, Hesperocyparis lusitanica (= Cupressus lusitanica), Drimys tuerckheimii, Juglans guatemalensis, Juniperus comitana, Liquidambar styraciflua, Miconia theaezans, Morella cerifera (= Myrica cerifera), Nectandra* spp., *Persea* spp., *Phoebe acuminatissima, Pinus strobiformis (= Pinus ayacahuite), Pinus chiapensis, Pinus hartwegii, Pinus maximinoi, Pinus patula ssp. tecunumanii, Pinus pseudostrobus, Podocarpus maturai, Podocarpus montana, Podocarpus oleifolius, Quercus benthamii, Quercus corrugata, Quercus cortesii, Quercus lancifolia, Quercus laurina, Quercus ovandensis, Quercus rugosa, Quercus sapotaefolia, Saurauia kegeliana, Saurauia scabrida, Weinmannia tuerckheimii*.

This system groups the mixed mountain cloud forests of northern Central America characterized by the association of pine and encinal (oak), and represents the limit of the influence of boreal flora in the Neotropics characterized by a high diversity of conifers. This is a transitional system between the temperate pine‑oak or cloud forests and the tropical montane oak forests of Costa Rica to Colombia, and is distributed from southern Chiapas, Mexico, to northwestern Nicaragua. The diversity of conifers and oaks is one of the highest in the world because it is the center of speciation of the genus *Pinus*. It is a system influenced by the mist and therefore almost constant, although it may have seasonal humidity. This is one of the main differences with the system of forest montane pine‑oak forests of the temperate zone of Mexico, which develops in a dry environment. Cloudy montane pine‑oak forest is generally between 1500 and 2300 m and occurs in both strands: Pacific and Caribbean. Up to 36 species of encinal (oak) and 11 species of pine have been recorded in mature forest communities. The above list of species is diagnostic for this system.

DISTRIBUTION

**Range:** Se distribuye sobre los 1500 m y hasta los 2300 m de altitud desde el sur de Chiapas, Mexico hasta el noroeste de Nicaragua, sobre la Sierra Madre de Chiapas, las montañas del sur de Guatemala, una importante porción en el centro de Honduras, ocurrencias menores en el norte del Salvador y el noroccidente de Nicaragua.Found between about 1500‑2300 m elevation from southern Chiapas, Mexico, to northwestern Nicaragua, on the Sierra Madre de Chiapas, the southern mountains of Guatemala, a large portion in central Honduras, and minor occurrences in northern El Salvador and northwestern Nicaragua.

**Divisions:** 403:C

**TNC Ecoregions:** NT0303:C

**Nations:** GT, HN, MX, NI, SV

CONCEPT

**Environment:** Pendientes de media y alta montaña a menudo de origen volcánico, con precipitación de 1500‑3000 mm anuales, aunque generalmente más de 2000 mm y además con influencia de la neblina. Las lluvias ocurren principalemnte en el verano y normalmente los periodos secos van de 0‑4 meses al año. Se ubica a mayor altitud en el lado Pacífico que en el Atlántico. La temperatura media de estos bosques varía entre 12°C y 20°C dependiendo de la altitud. Estos bosques se presentan en condiciones de temperaturas moderadas y de alta humedad atmosférica. La nubosidad es un factor importante para mantener la humedad atmosférica ya que reduce la incidencia de la radiación solar y la intensidad lumínica, provocando un descenso en la temperatura. Al ubicarse en las partes medias y altas de las cuencas hidrográficas, con capacidad para recibir y conservar gran cantidad de humedad gracias a la vegetación y los suelos, cumplen un papel importante en la regulación del sistema hídrico aguas abajo (Luna et al. 2001).

Moderate slopes and high elevation often volcanic, with annual rainfall of 1500‑3000 mm, but generally more than 2000 mm and also influenced by fog. Principally rainfall occurs in the summer and dry periods are usually 0‑4 months a year. It is located at the highest altitude in the Pacific than in the Atlantic side. The average temperature of these forests varies from 12°C and 20°C depending on the altitude. These forests occur under moderate temperatures and high humidity. The cloudiness is an important factor to maintain the atmospheric humidity and to reduce the incidence of solar radiation and the light intensity, causing a drop in temperature. Being located in the middle and upper parts of watersheds, with capacity to receive and store a considerable amount of moisture through vegetation and soils plays an important role in regulating the water systems downstream (Luna et al. 2001) .

**Vegetation:** Bosque subtropical siempreverde a semideciduo, con el dosel a 30 m de alto y especies latifoliadas y aciculifoliadas. En las pendientes de sotavento el aire cargado de humedad, posibilita la mayor cobertura de herbáceas y epífitas terrestres y arboreas.

**Dynamics:** La cantidad de materia orgánica que se acumula en el horizonte superior generalmente es mucha y forma una capa gruesa que detiene el drenaje con lo que el proceso de mineralización está limitado por condiciones de saturación y anaerobismo. Estos suelos en general son ácidos, poco fértiles y sujetos a fuertes problemas de erosión si se transforma la cobertura vegetal. Las condiciones de humedad atmosférica también se alteran significativamente al remover la cubierta boscosa.

The amount of organic matter that accumulates in the upper horizon is generally very thick and forms a layer that stops thereby draining the mineralization process is limited by saturation conditions and anaerobismo. These soils are generally acidic, infertile and subject to severe erosion problems if the vegetation becomes. Humidity conditions were also significantly altered by removing forest cover.

SOURCES

**References:** Josse et al. 2003*, Kappelle and Brown 2001, Luna et al. 2001, Pérez et al. 2007

**Version:** 08 Jan 2015 **Stakeholders:** Latin America

**Concept Author:** C. Josse **LeadResp:** Latin America

CES403.320 Bosque Montano Bajo de Pino Encino del Norte de Mesoamérica

**Primary Division:** Meso‑American Seasonal Highlands (403)

**Land Cover Class:** Forest and Woodland

**Spatial Scale & Pattern:** Small patch

**Required Classifiers:** Natural/Semi‑natural; Vegetated (>10% vasc.); Upland

**Diagnostic Classifiers:** Montane [Lower Montane]; Forest and Woodland (Treed); Udic

**Concept Summary:** Este sistema representa los bosques mixtos de las estribaciones bajas de las montañas del norte de Centro América, entre 900 y 1500 m de altitud aproximadamente. Agrupa las comunidades que están en el límite superior de los bosques húmedos latifoliados premontanos y bajo los bosques nublados. Posiblemente más característico de las estribaciones del Caribe que reciben los vientos alisios húmedos. La siguiente lista de especies es diagnóstica para este sistema: *Acer negundo var. mexicana, Arbutus xalapensis, Calyptranthes hondurensis, Carpinus caroliniana, Cedrela oaxacensis, Clethra macrophylla, Cleyera theanoides, Ficus* spp., *Inga* spp., *Liquidambar styraciflua, Morella cerifera (= Myrica cerifera), Persea* spp., *Pinus maximinoi, Pinus oocarpa, Pinus patula ssp. tecunumanii, Pinus pseudostrobus, Prunus* spp., *Quercus elliptica (= Quercus hondurensis), Quercus oleoides, Quercus segoviensis (= Quercus peduncularis)*.

This system represents the mixed forests of the lower foothills of the northern mountains of Central America, between 900 and 1500 m altitude. It brings together communities that are at the upper limit of premontane moist broadleaf forests and below cloud forest. Perhaps most characteristic of the Caribbean slopes due to higher moisture brough by the moist trade winds. The above list of species is diagnostic for this system.

DISTRIBUTION

**Divisions:** 403:C

**Nations:** BZ?, GT?, HN, MX?

CONCEPT

**Environment:** Pendientes medias y bajas. Suelos volcánicos antiguos.

Middle and lower slopes on ancient volcanic soils.

**Vegetation:** Bosques siempreverdes de 25‑30 m de alto, con epifitismo medio.

**Dynamics:** Bosque maduros, muy afectados por las plantaciones de café entre 1400 y 1800 m.

Mature forest between 1400 and 1800 m elevation, greatly affected by coffee plantations.

SOURCES

**References:** Josse et al. 2003*, Meyrat et al. 2001

**Version:** 08 Jan 2015 **Stakeholders:** Latin America

**Concept Author:** C. Josse **LeadResp:** Latin America

M602. Southern Mesoamerican Montane Humid Forest

CES402.609 Bosque de Roble Muy Húmedo Montano Alto de Talamanca

**Primary Division:** Moist Meso‑America (402)

**Land Cover Class:** Forest and Woodland

**Spatial Scale & Pattern:** Large patch

**Required Classifiers:** Natural/Semi‑natural; Vegetated (>10% vasc.); Upland

**Diagnostic Classifiers:** Montane [Upper Montane]; Forest and Woodland (Treed); Tropical/Subtropical [Tropical Pluvial]

**Concept Summary:** Las montañas de Costa Rica hacia el sur son diferentes de las del norte de Centroamérica gracias a la barrera que representa la depresión nicaraguense con sus llanuras y lagos y también debido a la presencia de un clima más lluvioso y menos estacional hacia el sur. La Cordillera de Talamanca representa en el sur de Centroamérica la distribución típica de los bosques de robles, que alcanzan su límite sur de distribución en los andes colombianos. Este sistema agrupa los bosques de roble sobre los 2500 m aproximadamente y hasta los 3100‑3200 m snm. Estos bosques son muy húmedos y también tienen una estructura compleja y gran estatura, hacia su límite superior la estatura disminuye y los robles alternan principalmente con especies de Ericaceae. La siguiente lista de especies es diagnóstica para este sistema: *Quercus costaricensis, Ilex lamprophylla, Quercus copeyensis, Myrsine pittieri, Brunellia costaricensis, Drimys granadensis, Clethra gelida, Magnolia* spp., *Ilex vulcanicola, Weinmannia pinnata, Weinmannia* spp., *Schefflera rodriguesiana, Alnus, Buddleia, Escallonia, Miconia, Oreopanax, Prumnopitys standleyi, Podocarpus macrostachys, Cinnamomum* spp., Lauraceae, *Chusquea* spp.

DISTRIBUTION

**Divisions:** 402:C

**TNC Ecoregions:** NT0167:C

**Nations:** CR, PA

CONCEPT

**Environment:** En su mayoría ocurre sobre suelos de origen volcánico, ricos en materia orgánica y generalmente con textura media y drenaje excesivo. La topografía es de pendientes convexas fuertes y muy disectada por la red de drenaje.

**Vegetation:** Bosques densos, de especies latifoliadas y siempreverdes, generalmente dominados por *Quercus* a nivel del dosel pero muy diversos en los estratos leñosos inferiores. Especies de Chusquea son abundantes y comunes en el sotobosque, y la abundancia y diversidad de briofitas es muy alta. En general la estructura de estos bosques es compleja, con numerosas epífitas y varios estratos leñosos, además de un dosel cerrado que alcanza los 30 m de alto y disminuye de estatura conforme aumenta la elevacion.

**Dynamics:** Bosque maduro.

SOURCES

**References:** Josse et al. 2003*, Kappelle and Brown 2001, Meyrat et al. 2001, WWF and IUCN 1997

**Version:** 08 Jan 2015 **Stakeholders:** Latin America

**Concept Author:** C. Josse **LeadResp:** Latin America

CES402.608 Bosque de Roble Muy Húmedo Montano Bajo de Talamanca

**Primary Division:** Moist Meso‑America (402)

**Land Cover Class:** Forest and Woodland

**Spatial Scale & Pattern:** Large patch

**Required Classifiers:** Natural/Semi‑natural; Vegetated (>10% vasc.); Upland

**Diagnostic Classifiers:** Montane [Lower Montane]; Forest and Woodland (Treed); Tropical/Subtropical [Tropical Pluvial]

**Concept Summary:** Las montañas de Costa Rica hacia el sur son diferentes de las del norte de Centroamérica gracias a la barrera que representa la depresión nicaraguense con sus llanuras y lagos y también debido a la presencia de un clima más lluvioso y menos estacional hacia el sur. La Cordillera de Talamanca representa en el sur de Centroamérica la distribución típica de los bosques de robles, que alcanzan su límite sur de distribución en Colombia. Este sistema agrupa los bosques de roble montano bajos y húmedos, con palmas y helechos arborescentes en el sotobosque. Hay algunas diferencias de estructura y composición entre las vertientes Pacífica y Atlántica, ya que la última es más húmeda. La siguiente lista de especies es diagnóstica para este sistema: *Quercus seemannii, Quercus rapurahuensis, Quercus corrugata, Quercus tonduzii, Quercus humboldtii, Billia hippocastanum, Turpinia occidentalis*, Lauraceae spp., *Ardisia* spp., *Cornus disciflora, Magnolia poasana, Podocarpus macrostachys, Roupala complicata, Sapium* spp., *Didymopanax pittieri, Geonoma hoffmaniana, Mollinedia* sp., *Weinamannia pinnata, Geonoma interrupta, Chusquea longifolia*.

DISTRIBUTION

**Divisions:** 402:C

**TNC Ecoregions:** NT0167:C

**Nations:** CR, PA

CONCEPT

**Environment:** En su mayoría ocurre sobre suelos de origen volcánico, ricos en materia orgánica y generalmente con textura media y drenaje excesivo. La topografía es de pendientes convexas fuertes y muy disectada por la red de drenaje.

**Vegetation:** Bosques densos, de especies latifoliadas y siempreverdes, dominados por *Quercus* a nivel del dosel pero muy diversos en los estratos leñosos inferiores. Las palmas son comunes en el sotobosque y en general la estructura de los bosques es compleja, con numerosas epífitas y varios estratos leñosos, además de un dosel cerrado que alcanza los 35‑40 m de alto o más.

**Dynamics:** Bosques maduros, afectados en muchos lugares por la ampliación de la frontera agrícola.

SOURCES

**References:** Josse et al. 2003*, Kappelle and Brown 2001, Meyrat et al. 2001, WWF and IUCN 1997

**Version:** 08 Jan 2015 **Stakeholders:** Latin America

**Concept Author:** C. Josse **LeadResp:** Latin America

1.A.4. Tropical Flooded & Swamp Forest

1.A.4.Ed. Caribbean‑Central American Flooded & Swamp Forest

M618. Caribbean Floodplain Forest

CES411.420 Caribbean Floodplain Forest

**Primary Division:** Caribbean (411)

**Land Cover Class:** Woody Wetland

**Spatial Scale & Pattern:** Large patch

**Required Classifiers:** Natural/Semi‑natural; Vegetated (>10% vasc.); Wetland

**Diagnostic Classifiers:** Aluvial; Riverine / Alluvial

**Concept Summary:** This system occurs in basins and plains along the coast, in the wide valleys of lowland rivers, or on rich, black alluvial soils. It can also occur right behind the mangrove communities in high rainfall and/or abundant river runoff locations. Depending on the duration of the flooding period, forests can have one or more tree layers. The canopy can be 10‑15 m, 15‑18 m, or 20‑25 m high. The following list of species is diagnostic for this system: *Pterocarpus officinalis, Roystonea regia, Roystonea borinquena, Tabebuia angustata, Bucida buceras, Sideroxylon portoricense (= Bucida subinermis), Calophyllum antillanum (= Calophyllum brasiliense), Swietenia mahagoni, Tabernaemontana amblyocarpa, Sabal parviflora, Sabal yapa, Acoelorraphe wrightii, Ficus* spp., *Myrsine cubana, Prestoea acuminata var. montana (= Prestoea montana), Symphonia globulifera, Melicoccus bijugatus, Cladium mariscus ssp. jamaicense (= Cladium jamaicense)*, and *Nephrolepis biserrata*.

DISTRIBUTION

**Range:** This system is found in Cuba, the Dominican Republic, Puerto Rico, and Trinidad and Tobago.

**Divisions:** 411:C

**Nations:** CU, DO, PR, TT

CONCEPT

**Environment:** [from M618] Located on alluvial plains in climates that vary from very humid to seasonal.

**Dynamics:** In the Caribbean, hurricanes constitute a trigger of periodic disturbance that provides long‑term opportunities for species invasions and long‑term ecosystem response in floodplain forests. A study about the effects of a hurricane in a Puerto Rican floodplain palm forest (Frangi and Lugo 1998), showed that the dominant species became more dominant and created low instantaneous tree mortality (1% of stems) and reductions in tree biomass (‑16 Mg/ha/yr) and density, although not in basal area. Five years after the hurricane, the palm floodplain forest had exceeded its pre‑hurricane above‑ground tree biomass, tree density, and basal area. Delayed tree mortality was twice as high as instantaneous tree mortality after the storm and affected dicotyledonous trees more than it did palms. Regeneration of dicotyledonous trees, palms, and tree ferns was influenced by a combination of factors including hydroperiod, light, and space (Frangi and Lugo 1998).

SOURCES

**References:** Areces‑Mallea et al. 1999, Borhidi 1991, Dansereau 1966, Dominica Ministry of Agriculture and Environment n.d., Frangi and Lugo 1998, Josse et al. 2003*, TNC 2004a, Tolentino and Peña 1998

**Version:** 08 Jan 2015 **Stakeholders:** Caribbean, Latin America, U.S. Territories

**Concept Author:** C. Josse **LeadResp:** Latin America

CES402.579 Caribbean Seasonal Evergreen Gallery Forest

**Primary Division:** Moist Meso‑America (402)

**Land Cover Class:** Woody Wetland

**Spatial Scale & Pattern:** Linear

**Required Classifiers:** Natural/Semi‑natural; Vegetated (>10% vasc.); Wetland

**Diagnostic Classifiers:** Lowland [Lowland]; Forest and Woodland (Treed); Riverine / Alluvial; Udic

**Concept Summary:** El sistema integra las comunidades boscosas de las planicies aluviales de cauces fluviales largos y caudalosos que tienen crecidas esporádicas según la cantidad de lluvia en las cuencas altas (arroyo washes). De igual forma, en la estación seca, estos cauces pueden permanecer totalmente secos. En este tipo de sistema los suelos pedregosos o rocosos son comunes y generalmente son ultisoles arcillosos. Los bosques son relativamente abiertos y de estatura media. En algunas partes de su distribución se encuentran rodeados por sabanas de pinos, por lo que los márgenes pueden verse afectados por las quemas. The following list of species is diagnostic for this system: *Aristolochia grandiflora, Bactris major, Bactris mexicana, Belotia campbellii, Bucida buceras, Cassia grandis, Cordia gerascanthus, Balizia leucocalyx, Lonchocarpus guatemalensis, Muntingia calabura, Pachira aquatica, Pterocarpus officinalis, Roystonea regia, Samanea saman, Schizolobium parahyba, Tabebuia rosea, Guadua longifolia, Calophyllum brasiliense var. rekoi, Vochysia hondurensis, Xilopia frutescens, Xilopia aromatica, Alchornea latifolia, Apeiba membranacea, Bactris gassipaeas, Bellucia costaricensis, Guadua macclurei, Quassia amara, Vismia macrophylla, Pera arborea, Zygia longifolia, Chrysobalanus icaco, Eugenia acapulcensis, Eugenia monticola, Tibouchina aspera, Amanoa guianensis, Myrsine coriacea, Croton trinitatis, Alibertia edulis*.

DISTRIBUTION

**Divisions:** 402:C

**Nations:** BZ, HN, NI

CONCEPT

**Environment:** Planicies aluviales con topografía ondulada, mayormente suelos tipo ultisoles arcillosos, drenaje variable. Se encuentran formando galerías cuando están rodeados de sabanas de pino o si no están adyacentes a los bosques pantanosos costeros.

**Vegetation:** Bosques de un 60% de cobertura del dosel y entre 10‑16 m de alto.

**Dynamics:** Bosque maduro, en partes de su distribución afectados por las quemas.

SOURCES

**References:** Josse et al. 2003*, Meyrat et al. 2001

**Version:** 01 Aug 2003 **Stakeholders:** Caribbean, Latin America

**Concept Author:** C. Josse **LeadResp:** Latin America

M617. Caribbean Swamp Forest

CES411.453 Caribbean Coastal Palm Swamp

**Primary Division:** Caribbean (411)

**Land Cover Class:** Woody Wetland

**Spatial Scale & Pattern:** Small patch

**Required Classifiers:** Natural/Semi‑natural; Vegetated (>10% vasc.); Wetland

**Diagnostic Classifiers:** Solonetz; Depressional; Tidal / Estuarine [Freshwater, Oligohaline]

**Concept Summary:** Coastal plain semipermanently or tidally flooded. Fen woods are 8‑15 m high, on peat or limestone soil. Freshwater and transitional halophilic communities. The following list of species is diagnostic for this system: *Acoelorraphe wrightii, Leucothrinax morrisii (= Thrinax morrisii), Coccothrinax argentata, Chrysobalanus icaco, Annona glabra, Sabal parviflora, Bucida palustris, Tabebuia angustata, Fraxinus caroliniana, Guettarda combsii, Ilex cassine, Salix caroliniana (= Salix longipes), Copernicia* spp., and mangrove species. The herbaceous stratum is well‑developed, and consists of *Eleocharis* spp. and *Cladium mariscus ssp. jamaicense*. Communities dominated by the clumping palm species *Acoelorraphe wrightii* occur also in the humid sites of white‑sand areas, usually along or near the drainage network surrounding shallow oligotrophic lakes. The fern *Blechnum serrulatum* often gives substantial coverage to the ground.

DISTRIBUTION

**Divisions:** 411:C

**Nations:** BS, CU, MQ, PR, TT

CONCEPT

**Environment:** Coastal plain in seasonally flooded and semipermanently saturated situations, on peat or limestone soil. Freshwater and transitional halophilic communities. Some of these communities occur associated with *Cladium* marsh.

**Dynamics:** Based on the length of the hydroperiod, flooded forests can be grouped into permanently inundated swamp forest and periodically inundated swamp forest. Swamp forest is usually found on soils that a have high water table, e.g., *Mauritia flexuosa* (palm) swamp in Trinidad grows on land perpetually inundated with 30 to 100 cm of water, while periodically‑inundated swamp occurs in areas subjected to inundation during rainy season. Species richness generally decreases with increasing hydroperiod. Based on the type of dominant species, swamp forests can be conveniently divided into two types: forests dominated by hardwood species and those dominated by palms. Dominance by palms becomes stronger with increasing hydroperiod or soil moisture conditions (Bacon 1990, Lugo et al. 1990).

SOURCES

**References:** Areces‑Mallea et al. 1999, Bacon 1990, Borhidi 1991, Josse et al. 2003*, Lugo et al. 1990

**Version:** 08 Jan 2015 **Stakeholders:** Caribbean, Latin America, U.S. Territories

**Concept Author:** C. Josse **LeadResp:** Latin America

CES411.366 South Florida Bayhead Swamp

**Primary Division:** Caribbean (411)

**Land Cover Class:** Woody Wetland

**Spatial Scale & Pattern:** Large patch

**Required Classifiers:** Natural/Semi‑natural; Vegetated (>10% vasc.); Wetland

**Concept Summary:** This ecological system consists of stands of predominately broad‑leaved hardwoods which are emergent amidst marshes of the south Florida Everglades region. These areas are often called "tree islands" as they occur on slightly elevated sites above the low‑relief marshes. Loveless, writing in 1959, considered them to be "perhaps the most striking botanical feature in the Everglades." Individual islands often have a characteristic shape depending upon the size; large islands are often teardrop‑shaped, smaller islands are circular. Patches range in size from one‑quarter acre to 300 acres or more. These islands often form an abrupt ecotone with adjacent fire‑prone marshes. Fires enter bayhead swamps only under extreme drought conditions and may kill much of the bayhead vegetation and heavily reduce peat accumulation. If left long unburned, bayheads may succeed to hardwood hammocks.

DISTRIBUTION

**Range:** Endemic to south Florida.

**Divisions:** 411:C

**TNC Ecoregions:** 54:C

**Nations:** US

**Subnations:** FL

**Map Zones:** 56:C

CONCEPT

**Environment:** This system occurs on sites elevated above surrounding marshes; they are inundated 2‑6 months during the year, and often found on Gandy Peat soils (Gunderson and Loftus 1993). Tree islands in the northern Everglades occur on acidic, deep peat sites, while southern examples are higher in pH, and shallower peat. Individual islands often have a characteristic shape depending upon the size; large islands are often teardrop‑shaped, smaller islands are circular (Loveless 1959, Gunderson and Loftus 1993). Patches range in size from one‑quarter acre to 300 acres or more.

**Vegetation:** Although plant communities in this system have quite similar floristic composition across the Everglades region, there are suggestions that pH and peat depth vary between northern and southern examples, factors which may influence species composition (Loveless 1959). Stands often support a luxuriant ground layer of ferns.

**Dynamics:** These islands often form an abrupt ecotone with adjacent marshes. Although fires often burn through the marshes, they enter bayhead swamps only under extreme drought conditions. Under these conditions, fires may kill much of the bayhead vegetation and heavily reduce peat accumulation. If left long unburned, bayheads may succeed to hardwood hammocks. Bayheads in some areas are inundated 2‑6 months during the year (Gunderson and Loftus 1993), but hydroperiods may vary from 1‑4 months in the northern to middle part of Taylor Slough; small, higher areas within a bayhead may never be under water (Olmstead et al. 1980b).

SOURCES

**References:** Brandt et al. 2003a, Comer et al. 2003*, Eyre 1980, FNAI 2010a, Gunderson and Loftus 1993, LANDFIRE 2007a, Loveless 1959, Olmsted et al. 1980b, Ugarte et al. 2006, Wade et al. 1980

**Version:** 14 Jan 2014 **Stakeholders:** Southeast

**Concept Author:** R. Evans **LeadResp:** Southeast

M619. Mesoamerican Coastal Plain Swamp Forest

CES402.586 Bosque Pantanoso Costero Mesoamérico

**Primary Division:** Moist Meso‑America (402)

**Land Cover Class:** Woody Wetland

**Spatial Scale & Pattern:** Large patch

**Required Classifiers:** Natural/Semi‑natural; Vegetated (>10% vasc.); Wetland

**Diagnostic Classifiers:** Lowland [Lowland]; Forest and Woodland (Treed); Riverine / Alluvial; Tidal / Estuarine [Oligohaline]; Aquic

**Concept Summary:** Este sistema representa las comunidades costeras sobre suelos saturados debido a lo alto del nivel freático, o que soportan inundación durante buena parte del año. Pueden encontrarse en los márgenes de un yolillal y se caracterizan por ser más diversas y presentar una mezcla de palmas y especies de hoja ancha, aunque algunas de las asociaciones son dominadas por palmas (*Manicaria, Acoelorrhaphe*). El agua es dulce o salobre de baja salinidad. Generalmente están hacia la costa pero en algunas partes llegan hasta más de 300 m de altitud. La siguiente lista de especies es diagnóstica para este sistema: Isthmian Atlantic and Choco‑Darien: *Camnosperma panamensis* (orey, sajales), *Raphia taedigera* (yolillo, matomba), *Euterpe precatoria, Carapa guianensis, Dialyanthera gordoniifolia* (guandales), *Prioria copaifera, Symphonia globulifera, Grias fendleri, Sacoglottis trichogyna, Conocarpus erectus, Cassipourea* sp., *Calophyllum antillanum (= Calophyllum brasiliense)*. Peten and CA Atlantic: *Manicaria saccifera* (manacal), *Roystonea dunlapiana, Roystonea regia, Acoelorraphe wrightii* (tique), *Astrocaryum mexicanum, Astrocaryum alatum, Dialium guianense, Symphonia globulifera, Orbignya cohune (= Attalea cohune), Pentaclethra macroloba, Sabal mauritiiformis, Bactris* spp., *Euterpe aff. oleracea, Crysophila stauracantha*.

DISTRIBUTION

**Divisions:** 402:C

**Nations:** BZ, CO, CR, EC, GT, HN, NI, PA

CONCEPT

**Environment:** Ocurre a lo largo de canales de estuarios y de ríos de la planicie costera, puede crecer adyacente a los manglares e incluso avanzar hasta la playa, así como avanzar tierra adentro a lo largo del recorrido de ríos. Los suelos son hidromórficos de textura arcillosa ‑aunque es común una capa arenosa superficial, el drenaje es defectuoso y puede haber acumulación de turba.

It occurs along channels and river estuaries of the coastal plain. It can grow adjacent to mangroves and even advance to the beach and move inland along the course of rivers. The soil is clayey hydromorphic though it is commonly a shallow sandy layer; drainage is poor and there may be an accumulation of peat.

**Vegetation:** Dense to open forest, 10‑25 m canopy but emergents up to 30 m. Some of the associations are dominated by palms.

**Dynamics:** La dinámica de las mareas y fluviales moderado.

Moderate tidal and fluvial dynamics.

SOURCES

**References:** Ellison 2001, Gómez 1986, Josse et al. 2003*, Meyrat et al. 2001, Rangel et al. 1987

**Version:** 08 Jan 2015 **Stakeholders:** Latin America

**Concept Author:** C. Josse **LeadResp:** Latin America

CES402.585 Yolillal Costero Mesoamérico

**Primary Division:** Moist Meso‑America (402)

**Land Cover Class:** Woody Wetland

**Spatial Scale & Pattern:** Large patch

**Required Classifiers:** Natural/Semi‑natural; Vegetated (>10% vasc.); Wetland

**Diagnostic Classifiers:** Lowland [Lowland]; Forest and Woodland (Treed); Riverine / Alluvial [Whitewater]; Tidal / Estuarine [Oligohaline]; Aquic

**Concept Summary:** Este sistema corresponde a las asociaciones costeras sobre suelos muy recientes que soportan inundación durante buena parte del año. Es muy común la dominancia de la palma yolillo (*Raphia taedigera*), que puede formar rodales monoespecíficos bastante grandes. El agua es dulce o salobre de baja salinidad. La siguiente lista de las especies es de diagnóstica para este sistema: *Symphonia globulifera, Calophyllum antillanum (= Calophyllum brasiliense), Raphia taedigera, Scheelea rostrata, Pterocarpus officinalis, Carapa nicaraguensis, Erythrina* sp., *Acoelorraphe wrightii, Manicaria saccifera, Xilopia* spp., *Isertia hankeana, Alibertia edulis, Psychotria aubletiana*.

DISTRIBUTION

**Divisions:** 402:C

**Nations:** CO, CR, NI, PA

CONCEPT

**Environment:** El sistema está asociado a terrenos planos cercanos a la costa marítima, estuarios y lagunas costeras que se inundan periódicamente o permanecen inundados la mayor parte del año. Los suelos son entisoles e inceptisoles sedimentarios hidromórficos y con mal drenaje.

**Vegetation:** Bosque de 15‑20 m de alto con dosel cerrado, la dominancia por una o dos especies de palmas es común.

**Dynamics:** Active tidal and fluvial dynamics.

SOURCES

**References:** Ellison 2001, Gómez 1986, Josse et al. 2003*, Meyrat et al. 2001, Rangel et al. 1987

**Version:** 08 Jan 2015 **Stakeholders:** Latin America

**Concept Author:** C. Josse **LeadResp:** Latin America

M620. Mesoamerican Floodplain Forest

CES402.602 Bosque Aluvial de Tierras Bajas del Petén

**Primary Division:** Moist Meso‑America (402)

**Land Cover Class:** Woody Wetland

**Spatial Scale & Pattern:** Linear

**Required Classifiers:** Natural/Semi‑natural; Vegetated (>10% vasc.); Wetland

**Diagnostic Classifiers:** Lowland [Lowland]; Shrubland (Shrub‑dominated); Riverine / Alluvial [Whitewater]; Aquic

**Concept Summary:** En Belice las comunidades de suelos aluviales se encuentran en las depresiones formadas por las quebradas y en bancos riparios. Posiblemente por su carácter secundario, tienen una estatura baja y fisonomía predominante arbustiva. La siguiente lista de especies es diagnóstica para este sistema: *Acacia* sp., *Coccoloba* spp., *Guazuma ulmifolia, Guettarda combsii, Hirtella racemosa, Miconia racemosa, Mouriri excelsa, Sabal mauritiiformis, Simarouba glauca, Vochysia hondurensis, Xilopia frutescens, Astrocaryum mexicanum, Calyptrogyne ghiesbreghtiana, Desmoncus orthocanthus*.

DISTRIBUTION

**Divisions:** 402:C

**Nations:** BZ

CONCEPT

**Environment:** Ocurre sobre depósitos aluviales sedimentarios arcillosos con suelos profundos y pobres en calcio. Se inunda ocasionalmente.

**Vegetation:** Vegetación predominantemente arbustiva y abierta.

**Dynamics:** Bosques secundarios por causa de quemas producidas.

SOURCES

**References:** Josse et al. 2003*, Meyrat et al. 2001

**Version:** 08 Jan 2015 **Stakeholders:** Latin America

**Concept Author:** C. Josse **LeadResp:** Latin America

CES402.603 Bosque Aluvial Estacional de Tierras Bajas de Suelos Calcáreos del Petén

**Primary Division:** Moist Meso‑America (402)

**Land Cover Class:** Woody Wetland

**Spatial Scale & Pattern:** Large patch

**Required Classifiers:** Natural/Semi‑natural; Vegetated (>10% vasc.); Wetland

**Diagnostic Classifiers:** Lowland [Lowland]; Forest and Woodland (Treed); Extensive Wet Flat; Udic

**Concept Summary:** Sistema que agrupa las comunidades boscosas siempreverdes que crecen en depresiones del terreno u hondonadas, en suelos ricos en calcio de textura arcillosa y que se inundan estacionalmente alternando con periodos de extrema sequía de varios meses debido al clima estacional. La inundabilidad está asociada al balance entre el escurrimiento, la infiltración y las precipitaciones. Son bosques de unos 10 m de alto, con alta densidad de árboles de diámetros pequeños y con numerosos árboles de troncos retorcidos y/o espinosos. Se encuentran en situaciones heterogéneas inmersos en la matriz de bosque siempreverde estacional de suelos calcáreos y colinas cársticas (Tun Dzul 2007). Principalmente en el sur de la península de Yucatán en México, el Petén en Guatemala y Belice, en Honduras se encuentra muy alterado. La siguiente lista de especies es diagnóstica para este sistema: *Croton nitens, Cameraria latifolia, Haematoxylum campechianum, Bucida buceras, Diospyros anisandra, Metopium brownei, Manilkara zapota, Coccoloba cozumelensis, Coccoloba spicata, Coccoloba diversifolia, Myrcianthes fragrans, Eugenia winzerlingii, Syderoxylon celastrinum, Calophyllum antillanum (= Calophyllum brasiliense), Acacia gaumeri, Lonchocarpus yucatanensis, Vitex gaumeri, Byrsonima bucidaefolia, Hippocratea excelsa, Krugiodendron ferreum, Manilkara zapota* y *Swietenia macrophylla*. *Cladium mariscus ssp. jamaicense (= Cladium jamaicense)* es abundante en el estrato herbáceo.

DISTRIBUTION

**Range:** En los bosques del Petén y sur de la Península de Yucatán.

**Divisions:** 402:C

**Nations:** BZ, GT, MX

CONCEPT

**Environment:** Ocurre en suelos compuestos por residuales de las fracciones insolubles de las rocas carbonatadas y con un alto contenido de arcilla (58%) por lo que tienen poco drenaje tanto interno como superficial, llegando a anegarse hasta 50 cm o mas en la época de lluvias, la alternabilidad entre el anegamiento y el secado del suelo arcilloso hace que se formen pequeños montículos conocidos como relieve gilgai. En los bosques del Petén y sur de la Península de Yucatán este sistema se encuentra en zonas inundables que forman parte de la variada geomorfología de la altiplanicie cárstica que forma la espina dorsal de la península, con áreas de planicie con lomeríos de cimas redondeadas, separados por zonas bajas inundables y mesetas niveladas. La altitud varía de 250 a 340 msnm. Bajo estas depresiones u hondonadas limitadas por las elevaciones calcáreas, pueden haber cavidades con flujo subterráneo vertical u horizontal o disponerse una capa impermeable de terrenos muy planos, que a causa de la poca permeabilidad del suelo, pueden anegarse durante algunos meses con aguas salobres o no, originadas en el nivel freático e inundaciones temporales en época lluviosa, luego de lo cual los suelos se secan. La precipitación anual fluctúa en un rango de 900‑1400 mm y se concentra entre mayo y octubre. La temperatura promedio mensual es >21°C.

**Vegetation:** Bosque medio de 10‑15 m de alto y dosel cerrado o abierto.

**Dynamics:** Bosques sujetos a intervención y fuegos.

SOURCES

**References:** Josse et al. 2003*, Meyrat et al. 2001, Tun Dzul 2007, Turner et al. 2001

**Version:** 08 Jan 2015 **Stakeholders:** Latin America

**Concept Author:** C. Josse **LeadResp:** Latin America

CES402.584 Bosque Siempreverde Aluvial de Mesoamérica

**Primary Division:** Moist Meso‑America (402)

**Land Cover Class:** Woody Wetland

**Spatial Scale & Pattern:** Large patch

**Required Classifiers:** Natural/Semi‑natural; Vegetated (>10% vasc.); Wetland

**Diagnostic Classifiers:** Lowland [Lowland]; Forest and Woodland (Treed); Riverine / Alluvial [Whitewater]; Udic

**Concept Summary:** Este sistema corresponde a los bosques de las planicies aluviales anegados o inundados estacionalmente por corto tiempo y moderadamente drenados. Son bosques altos siempreverdes de varios estratos y dosel cerrado. Puede haber diferencias en la composición entre la vertiente Atlántica y la Pacífica. La siguiente lista de especies es diagnóstica para este sistema: *Adelia triloba, Astrocaryum alatum, Bactris longiseta, Dialyanthera otoba, Piper cenocladum, Pterocarpus officinalis, Clusia* spp., *Allophylus psilospermus, Anaxagorea costaricensis, Astrocaryum alatum, Brosimum panamense, Capparis pittieri, Carpotroche platyptera, Casearia* spp., *Cespedezia macrophylla, Cynometra retusa, Dendropanax arboreus, Gloeospermum diversipetalum, Hedyosmum calloso‑serratum, Hernandia didymantha, Jacaratia* spp., *Laetia procera, Lecythis costaricensis, Mortoniodendron membranaceum, Pentaclethra macroloba, Protium* spp., *Sloanea medusula, Sterculia apetala, Stryphnodendron excelsum, Tomovita nicaraguensis, Veconcibea pleiostemona, Bactris hondurensis, Prestoea decurrens*.

DISTRIBUTION

**Divisions:** 402:C

**Nations:** CR, NI, PA

CONCEPT

**Environment:** Planicies aluviales, suelos tipo ultisoles arcillosos, moderadamente drenados.

**Vegetation:** Bosque de 30‑40 m de alto, con un 80% de cobertura del dosel y estructura compleja con varios estratos y abundancia de epífitas y bejucos.

**Dynamics:** Bosque maduro

SOURCES

**References:** Janzen 1983a, Josse et al. 2003*, Meyrat et al. 2001

**Version:** 08 Jan 2015 **Stakeholders:** Latin America

**Concept Author:** C. Josse **LeadResp:** Latin America

CES401.295 Bosque y Arbustal Semideciduo de Galería de Mesoamérica

**Primary Division:** Dry Meso‑America (401)

**Land Cover Class:** Woody Wetland

**Spatial Scale & Pattern:** Linear

**Required Classifiers:** Natural/Semi‑natural; Vegetated (>10% vasc.); Wetland

**Diagnostic Classifiers:** Lowland [Foothill, Lowland]; Forest and Woodland (Treed); Udic

**Concept Summary:** Este sistema reune las comunidades que ocurren a lo largo de ríos que atraviesan zonas de bosque deciduo o semideciduo y sabanas (naturales o antrópicas). Se trata de complejos de vegetación herbácea, arbustiva y boscosa en diferentes posiciones a lo largo de los bancos del río y terrazas, con diferentes niveles de disturbio natural propios de la dinámica de inundación del río, o debidos a la alteración antrópica. Generalmente la composición de las especies leñosas asemeja la de un bosque más húmedo que el del entorno, debido a la mayor disponibilidad de humedad. El regimen de humedad y anegamiento del sustrato también juegan un papel fundamental. Si se encuentra en terrenos con pendiente, la inundación puede ser muy corta porque los ríos tienen un curso rápido y hay posibilidad de drenaje, en planicies la inundación puede durar varios días o semanas. La siguiente lista de especies es diagnóstica para este sistema: *Cecropia obtusifolia, Salix humboldtiana, Cordia alliodora, Cedrela odorata, Schizolobium parahyba, Castilla elastica, Castilla tunu, Calliandra emarginata, Inga vera, Inga affinis, Vismia* sp., *Ficus insipida, Anacardium excelsum, Annona glabra, Annona reticulata, Astronium graveolens, Brosimum alicastrum, Spondias mombin, Trichilia pittieri, Hernandia didymantha, Caryocar costaricense, Couroupita nicaraguarensis, Chrysophila guaguara, Albizia caribaea, Calophyllum antillanum (= Calophyllum brasiliense), Ochroma, Miconia, Heliconia, Canna, Calathea, Isertia*.

DISTRIBUTION

**Divisions:** 401:C

**Nations:** CR, GT, NI, SV

CONCEPT

**Environment:** Bancos de río y planicies contiguas con sustratos variados, siempre con aportes de limo. Textura arenosa y generalmente suelos bien drenados, aunque con tabla de agua superficial y sujetos a inundaciones esporádicas o estacionales.

**Vegetation:** Complejo de vegetación herbácea graminoide, de forbias, bejucos trepadores y especies arbustivas con bosques de galería sobre los sustratos más estables.

**Dynamics:** Fuentes de alteración natural por la dinámica fluvial y también sujetos a alteración antrópica.

SOURCES

**References:** Josse et al. 2003*, Meyrat et al. 2001, WWF and IUCN 1997

**Version:** 08 Jan 2015 **Stakeholders:** Latin America

**Concept Author:** C. Josse **LeadResp:** Latin America

1.A.4.Ei. Colombian‑Venezuelan Flooded & Swamp Forest

M622. Choco‑Darien Floodplain Forest

CES402.582 Palmar Pantanoso de Tierras Bajas del Chocó‑Darién

**Primary Division:** Moist Meso‑America (402)

**Land Cover Class:** Woody Wetland

**Spatial Scale & Pattern:** Linear

**Required Classifiers:** Natural/Semi‑natural; Vegetated (>10% vasc.); Wetland

**Diagnostic Classifiers:** Lowland [Lowland]; Forest and Woodland (Treed); Riverine / Alluvial; Udic

**Concept Summary:** Bosques de las planicies de inundación dominados por palmas, muy comunes en el Chocó‑Darién, pero se extienden hasta Costa Rica. Los suelos se inundan o saturan por periodos estacionales. La siguiente lista de las especies es de diagnóstica para este sistema: *Wettinia quinaria, Oenocarpus bataua, Cedrela angustifolia, Euterpe oleracea, Manicaria, Jessenia*.

DISTRIBUTION

**Divisions:** 402:C

**Nations:** CO, CR, PA

CONCEPT

**Environment:** Planicies aluviales y bancos de río. Inundados temporalmente.

**Vegetation:** Bosque mixto de palmas y especies de hoja ancha, de 15‑25 m de alto.

**Dynamics:** Dinámica fluvial activa.

SOURCES

**References:** Instituto Geográfico Agustín Codazzi 2000, Josse et al. 2003*

**Version:** 08 Jan 2015 **Stakeholders:** Latin America

**Concept Author:** C. Josse **LeadResp:** Latin America

1.A.4.Ej. Guianan Flooded & Swamp Forest

M628. Orinoco Delta Swamp Forest

CES404.380 Pantano Mixto con Palmas del Delta del Orinoco

**Primary Division:** Guiana Uplands and Highlands (404)

**Land Cover Class:** Woody Wetland

**Spatial Scale & Pattern:** Large patch

**Required Classifiers:** Natural/Semi‑natural; Vegetated (>10% vasc.); Wetland

**Diagnostic Classifiers:** Lowland [Lowland]; Forest and Woodland (Treed); Histosoles; Tidal / Estuarine [Freshwater, Oligohaline]

**Concept Summary:** Delta bajo y medio del Orinoco, aguas dulces a salobres. Ombroclima húmedo. Complejo de pantanos y bosques riparios inundables con abundancia de palmas, de entre 10 y 25 m de alto. Generalmente con una franja de herbaceas/forbias a lo largo de las orillas sin sombra. maduros y en general con poca intervencion. The following list of species is diagnostic for this system: *Symphonia globulifera, Virola surinamensis, Carapa guianensis, Pterocarpus officinalis, Tabebuia fluviatilis, Mora excelsa, Pachira aquatica, Mauritia flexuosa, Manicaria saccifera, Euterpe oleracea, Bactris* sp., *Phenakospermum guianensis*.

DISTRIBUTION

**Divisions:** 404:C

**Nations:** GY, VE

CONCEPT

**Environment:** Delta bajo y medio del Orinoco, aguas dulces a salobres. Ombroclima húmedo.

**Vegetation:** Complejo de pantanos y bosques riparios inundables con abundancia de palmas, de entre 10 y 25 m de alto. Generalmente con una franja de herbaceas/ forbias a lo largo de las orillas sin sombra.

**Dynamics:** Maduros y en general con poca intervencion.

SOURCES

**References:** Berry et al. 1995, Huber 1995, Josse et al. 2003*

**Version:** 11 Apr 2003 **Stakeholders:** Latin America

**Concept Author:** C. Josse **LeadResp:** Latin America

1.A.5. Mangrove

1.A.5.Ua. Atlantic‑Caribbean & East Pacific Mangrove

M004. Eastern Pacific Mangrove

CES402.599 Manglar Estuarino y de la Costa del Pacifico

**Primary Division:** Moist Meso‑America (402)

**Land Cover Class:** Woody Wetland

**Spatial Scale & Pattern:** Linear

**Required Classifiers:** Natural/Semi‑natural; Vegetated (>10% vasc.); Wetland

**Diagnostic Classifiers:** Lowland [Lowland]; Forest and Woodland (Treed); Tidal / Estuarine [Haline, Oligohaline]; Aquic

**Concept Summary:** Es un sistema de zonas mareales con fluctuaciones de hasta más de 5 m. Según su ubicación puede estar permanentemente inundados o soportar dos inundaciones diarias, este gradiente del nivel de inundación del suelo y de salinidad influye en las características estructurales y de composición de la vegetación. En el Pacífico la amplitud de la marea es mayor que en el Caribe, y por tanto los manglares se extienden muy adentro por los deltas de los ríos. Esta dinámica intensa produce un proceso de sucesión con el resultado de que se forman comunidades casi monoespecíficas de Rizophora en las zonas de influencia mareal más directa. En sustratos más estables se encuentran las poblaciones de Avicennia, Laguncularia y Pelliciera y finalmente, Mora megistosperma y Euterpe que están en la transición con los terrenos aluviales. La siguiente lista de las especies es de diagnóstica para este sistema: *Rhizophora mangle, Rhizophora racemosa, Rhizophora x harrisonii, Laguncularia racemosa, Avicennia germinans, Avicennia bicolor, Conocarpus erectus, Pelliciera rhizophorae, Acrostichum aureum*.

DISTRIBUTION

**Divisions:** 401:C, 402:C

**Nations:** CO, CR, EC, GT, HN, MX, NI, PA, SV

CONCEPT

**Environment:** Los manglares ocurren en una planicie fluvial marina con sedimentos aluviales. Los suelos son inceptisoles higromórficos arcillosos. Se trata de un sistema en el ecotono entre los sistemas continentales y marinos y por tanto las especies se distribuyen de acuerdo a sus adpataciones, en un gradiente del nivel de inundación del suelo y de salinidad.

**Vegetation:** Bosques altos o medios y de dosel relativamente abierto. La presencia de raíces zancudas y neumatófores es una de las características más sobresalientes. Los árboles son el componente principal en la mayoría de manglares, aunque también los hay arbustivos; otras formas de vida como hierbas, epífitas, bejucos y lianas, son escasas pero están presentes.

**Dynamics:** Active tidal and fluvial dynamics.

SOURCES

**References:** Ellison 2001, Josse et al. 2003*, Meyrat et al. 2001

**Version:** 08 Jan 2015 **Stakeholders:** Latin America

**Concept Author:** C. Josse **LeadResp:** Latin America

CES402.596 Manglar Mixto con Mora Estuarino del Pacifico

**Primary Division:** Moist Meso‑America (402)

**Land Cover Class:** Woody Wetland

**Spatial Scale & Pattern:** Linear

**Required Classifiers:** Natural/Semi‑natural; Vegetated (>10% vasc.); Wetland

**Diagnostic Classifiers:** Lowland [Lowland]; Forest and Woodland (Treed); Riverine / Alluvial; Tidal / Estuarine [Oligohaline]; Aquic

**Concept Summary:** Es un sistema marginal salobre entre el manglar y los pantanos de agua dulce o bosques saturados de los terrenos aluviales y costeros. Las especies leguminosas *Mora oleifera* y *Mora megistosperma* son características. La siguiente lista de especies es diagnóstica para este sistema: *Mora oleifera, Pterocarpus officinalis, Prioria copaifera, Pachira aquatica, Astrocaryum standleyanum, Montrichardia arborescens, Crinum erubescens*, mangrove species.

DISTRIBUTION

**Divisions:** 402:C

**Nations:** CO, CR, PA

CONCEPT

**Environment:** [de M004] Hogarth (1999) reconoce los siguientes tipos de sistemas de manglares basado en el entorno ambiental. Manglares de franja (dominado por mareas): se caracteriza por un alto rango de mareas en una zona intermareal de poca profundidad a menudo colonizada por manglares. La marea tiene típicamente toda la fuerza oceánica, pero acción la de las olas se difunde rápidamente por el paso a través de una zona intermareal escalonada. Sedimentos y suelos de manglares es probable que sean más dinámicos ya que las mareas depositan y remueven sedimentos de los estuarios y de los ríos interiores. Reciben menos escurrimiento de nutrientes terrestres en comparación con los bosques ribereños. Manglares de cuenca: adyacentes a los manglares de franja hacia el lado interior (hacia tierra). Protegidos de la acción del oleaje, e inundados con poca frecuencia. Con salinidad altamente variable en función de la precipitación, el flujo de las aguas subterráneas, y el aumento de marea local. A menudo exhiben altas tasas de evaporación, lo cual puede resultar en suelos hipersalinos. Debido a las corrientes bajas y poca turbulencia, los manglares de cuenca pueden ser sumideros de nutrientes y sedimentos. Los manglares ribereños: grandes extensiones de manglares se encuentran en los deltas de los ríos, donde los suelos y la salinidad son adecuadas para el desarrollo de manglares (por ejemplo, el delta del Amazonas). Tienen una baj amplitud de mareas y un fuerte flujo de agua dulce que transporta cargas sustanciales de sedimentos, gran parte del cual se deposita en las comunidades de manglar. Se caracterizan por desplazamientos de los canales del río, y por lo una dinámica de expansión hacia el interior, así como hacia el exterior gracias a la sedimentación cambiante en el delta. Manglares arbustivos: se encuentran en ambientes extremos donde los nutrientes y el agua dulce pueden ser limitantes. Los manglares elevados (hammock): el aislamiento relativo de los ríos o el mar lleva a una acumulación en forma de cúpula de turba orgánica sobre depresiones, donde se arraigan los manglares. Manglares en sustratos carbonatados: En las costas de baja energía, donde el carbonato ha acumulado de la descomposición de arrecifes de coral, lo que resulta en los sedimentos de cal y la acumulación de sedimentos. Manglares del interior: Las zonas donde los manglares se encuentran totalmente separadas del mar, a menudo en agujeros de geología cársica u otras depresiones.

[from M004] Hogarth (1999) recognizes the following types of mangrove systems based on environmental setting. Fringe Mangroves (tide‑dominated): characterized by a high tidal range over a shallow intertidal zone that is often colonized by mangrove trees. Tidal water is typically full strength seawater, but wave action is diffused quickly by passage over a stepped intertidal zone. Sediment and mangrove soils are likely to be more dynamic as tides deposit and remove sediments from the sea and from inland river estuaries. Receive less runoff of terrestrial nutrients compared to riverine forests. Basin Mangroves: On the landward side of fringing mangroves in estuaries. Sheltered from wave action, and inundated infrequently. Highly variable salinity depending on rainfall, groundwater flow, and local tidal surges. Often exhibit high evaporation rates, which can result in hypersaline soils. Due to low currents and little turbulence, basin mangroves can be sinks for nutrients and sediment. Riverine Mangroves: Many large expanses of mangroves are located at river deltas where soils and salinity are amenable to mangrove community development (e.g., Amazon delta). Have low tidal ranges, and strong freshwater flow carrying substantial sediment loads, much of which is deposited within the mangrove communities. Characterized by shifting river channels, and typically mangal expanding inland as well as outward in the shifting, sediment‑driven river deltas. Scrub Mangroves: Found in extreme environments where nutrients and freshwater may be limiting. Hammock Mangroves: Relative isolation from rivers or the sea leads to a domed accumulation of organic peat over depressions, where mangroves take root. Carbonate Setting Mangroves: On low‑energy coasts where carbonate has accumulated from coral reef breakdown, resulting in lime sediment and silt accumulation. Inland Mangroves: Areas where the mangroves are completely cut‑off from the sea, often in sink holes or other depressions.

**Dynamics:** [de M004] Condicion: Los manglares presentan vivipary, o el crecimiento precoz de plántulas mientras permanecen pegadas al árbol madre. Cuando se desperenden, los propágulos son fuertes, flotantes, y fácilmente dispersados por el agua. La competencia entre los manglares y especies de árboles que nos son de manglar no es un factor clave en el manglar porque las condiciones hidrológicas y edáficas únicas de los ecosistemas de manglar hacen que sea difícil invadir para otras especies. Los lodos de turba típicos de los manglares tienen un alto contenido de limo y tienden a ser bastante inhóspitos para los invertebrados filtradores. Sin embargo, cangrejos especializados de los grupos sesarmid, portunuid, and ocupodid son extremadamente comunes.

<br />Conectividad y Paisaje Contexto: Los manglares son naturalmente hábitats disyuntos que ocurren a lo largo de las costas y ríos. Ellos tienden a tener distrbutions lineales o de parche pequeño, y por lo tanto no suelen ocurrir como grandes hábitats de matriz. También se dispersan bien con propágulos transmitidas por el agua (a menudo vivíparos). Tienden, por tanto, a no ser tan sensibles a la fragmentación del hábitat como muchos otros hábitats forestales costeros, siempre y cuando los principales procesos ecológicos continúen intactos. Sin embargo, los manglares de franja en particular, pueden verse afectados por el aumento del nivel del mar, ya que su contexto paisajístico es extremadamente limitado y lineal. Estos tipos de sistemas de manglares estarán limitados por la geología y por la fragmentación humana, con la pérdida in‑situ de los sistemas de franja restantes debido a los efectos fisiológicos de la subida del nivel del mar.

[from M004] Condition: Mangroves exhibit vivipary, or the precocious growth of seedlings while still attached to the parent tree. When abscised, the propagules are tough, buoyant, and readily water‑dispersed. Competition between mangrove and non‑mangrove tree species is rarely a key factor in mangal because the unique hydrologic and edaphic conditions of mangrove ecosystems make it difficult for non‑mangrove species to invade. The peaty muds typical of mangroves have a very high silt content and tend to be fairly inhospitable to most suspension and filter‑feeding invertebrates. However, mud‑dwelling sesarmid, portunuid, and ocupodid crabs are extremely common.

Connectivity and Landscape Context: Mangroves are naturally disjunct habitats occurring along coastlines and rivers. They tend to have linear or small‑patch distributions, and therefore do not generally occur as large matrix habitats. They also disperse well with waterborne propagules (often viviparous). They tend therefore not to be as sensitive to habitat fragmentation as many other coastal forest habitats, as long as major ecological processes are intact. However, fringing mangroves in particular may be affected by rising sea level, as their landscape context is extremely limited and linear. These types of mangrove systems will be limited by geology, and by human fragmentation, with *in situ* loss of the remaining fringing systems from physiological effects of rising sea levels.

SOURCES

**References:** Ellison 2001, Gómez 1986, Josse et al. 2003*, Rangel et al. 1987

**Version:** 08 Jan 2015 **Stakeholders:** Latin America

**Concept Author:** C. Josse **LeadResp:** Latin America

M005. Western Atlantic & Caribbean Mangrove

CES411.444 Caribbean Coastal Mangrove

**Primary Division:** Caribbean (411)

**Land Cover Class:** Woody Wetland

**Spatial Scale & Pattern:** Linear

**Required Classifiers:** Natural/Semi‑natural; Vegetated (>10% vasc.); Wetland

**Diagnostic Classifiers:** Peat and mud; Tidal / Estuarine [Haline]

**Concept Summary:** This ecological system represents the oceanward mangrove forest with a tidal flooding regime, distributed along the coasts of the Greater and Lesser Antilles and the Caribbean coast of Colombia and Venezuela. The mangrove forest structure and composition depend on the geomorphic and hydrological processes that characterize its specific location along the coast, with important differences between locations depending on total precipitation amounts, freshwater runoff, and wave action (Cintrón et al. 1978). Mangroves in drier climates and with less freshwater runoff exhibit simpler structure, less leaf fall and a lower rate of tree growth; the salinity in these mangroves approaches that of the seawater and has little change throughout the year. Mangroves on St. John, Virgin Islands, although containing the typical assemblage of species of Caribbean mangroves and associated halophytes, are poorly represented by a narrow strip of vegetation occurring in protected, shallow waters. These forests and, in general, fringe mangroves are dominated by *Rhizophora mangle*. The standard zonation of mangroves consists of *Rhizophora mangle* in the lower and middle intertidal zone, *Avicennia germinans* in the upper intertidal areas that are occasionally flooded, and *Laguncularia racemosa* in patches on higher elevations that are less frequently flooded. Dense mangrove forests do not typically have understory plant associations, except for mangrove seedlings (FNAI 1990).

DISTRIBUTION

**Range:** This system is found in Colombia, the Greater Antilles, Puerto Rico, Virgin Islands, and Venezuela.

**Divisions:** 411:C

**Nations:** CO, CU, DO, JM, PR, VE, VI, XC

CONCEPT

**Environment:** Fringe mangroves occur in close proximity to the ocean, are dominated by *Rhizophora mangle*, and may have leeward zones dominated by *Avicennia germinans* or *Laguncularia racemosa*. These tidal forests can reach 20 m (66 feet) high. Stands occur in frost‑free zones, on soils that are permanently saturated with brackish water and which become inundated during high tides. The brackish environment tends to limit competition from other species. Mangroves are found on fine inorganic muds, muds with high organic content, peat, sand, rock, coral, shells, and some man‑made surfaces if there are sufficient crevices for root attachment. *Avicennia germinans* grows best in soils of high salinity, *Rhizophora mangle* grows best in areas of estuarine salinity with regular flushing, and *Laguncularia racemosa* grows best in areas with freshwater input on sandy soils (FNAI 1990). Mangroves attain larger biomass in areas of low wave‑energy shorelines, river deltas, and floodplains with depositional environments (Odum et al. 1982). Fluctuating tidal waters are important for transporting nutrients, controlling soil salinities, and dispersing propagules, but high wave energy prevents establishment and may destroy their shallow root systems (Odum and McIvor 1990).

**Vegetation:** Stands are dominated by *Rhizophora mangle*.

**Dynamics:** Disturbance in mangrove forests may be caused by large‑scale events such as hurricanes, or clearcutting, but also by small‑scale events such as lightning, causing mangrove trees to die in small areas around lightning strikes, or attack by wood‑boring beetles. The relative importance of these different types of disturbance vary with geography, with some localities more often subjected to the impact of hurricanes or lightning. Recovery from large‑scale disturbance may be slow and may vary depending on species composition and intensity of stress factors subsequent to the disturbance event, with increases in solar exposure, soil temperature and/or salinity capable of inhibiting regeneration (McKee and Feller 1994, cited in Barbour and Billings 2000, Smith et al. 2009), or of influencing the establishment of the pioneer mangrove species, along with other factors such as presence of a seedling source, herbivory, and seed consumption.

Mangroves are considered pioneer species because of their ability to establish on otherwise unvegetated substrates. Once individuals begin to colonize a disturbed area, even‑aged stands are established with little variance in the structure because new development of successive colonizers is arrested by the closed canopy. On shorter time scales, the pulses of the tides and freshwater runoff are very important factors in the dynamics of mangroves because these control the rates of sedimentation and vertical accretion and thus determine their intertidal position. Tidal flooding is also key for the distribution of soil nutrient resources in the coastal mangrove forest. The distribution of the different mangrove species and the mangrove community can experience fluctuations in structure and species composition as a result of changes affecting the hydrologic patterns.

SOURCES

**References:** Areces‑Mallea et al. 1999, Barbour and Billings 2000, Borhidi 1991, Cardona and Botero 1998, Cintrón et al. 1978, Dansereau 1966, Di Nitto et al. n.d., Duke et al. 1998, Ellison 1993, Ellison 2000, Ellison 2006, Field 1995, Gilman et al. 2008, Huber and Alarcón 1988, Josse et al. 2003*, Lewis 2005b, Lovelock and Ellison 2007, Naidoo 1983, Odum and McIvor 1990, Odum et al. 1982, Semeniuk 1994, Smith et al. 2009, Valiela et al. 2001

**Version:** 30 Oct 2015 **Stakeholders:** Caribbean, Latin America, U.S. Territories

**Concept Author:** C. Josse **LeadResp:** Latin America

CES402.578 Manglar Costero y de Estuario del Caribe

**Primary Division:** Moist Meso‑America (402)

**Land Cover Class:** Woody Wetland

**Spatial Scale & Pattern:** Linear

**Required Classifiers:** Natural/Semi‑natural; Vegetated (>10% vasc.); Wetland

**Diagnostic Classifiers:** Lowland [Lowland]; Forest and Woodland (Treed); Shrubland (Shrub‑dominated); Tidal / Estuarine [Haline]; Aquic

**Concept Summary:** Es un sistema de zonas mareales que en la costa atlántica sufre fluctuaciones muy bajas ya que la amplitud mareal es menor a 1 m. Según su ubicación puede estar permanentemente inundado o soportar dos inundaciones diarias, este gradiente del nivel de inundación del suelo y de salinidad influye en las características estructurales y de composición de la vegetación. Forman franjas estrechas a lo largo de la costa. La siguiente lista de las especies es de diagnóstica para este sistema: *Rhizophora mangle, Avicennia germinans, Laguncularia racemosa, Conocarpus erectus, Morella cerifera (= Myrica cerifera), Raphia taedigera, Acoelorraphe wrightii*.

DISTRIBUTION

**Divisions:** 401:C, 402:C

**Nations:** BZ, CR, CU, GT, HN, MX, NI, PA

CONCEPT

**Environment:** Los manglares ocurren en una planicie fluvial marina con sedimentos aluviales. Los suelos son inceptisoles higromórficos arcillosos. Se trata de un sistema en el ecotono entre los sistemas continentales y marinos y por tanto las especies se distribuyen de acuerdo a sus adpataciones, en un gradiente del nivel de inundación del suelo y de salinidad.

**Vegetation:** En la costa atlántica muchos de los manglares son arbustivos de 1‑5 m de alto, aunque también pueden haberlos dominados por árboles, generalmente los más altos están en zonas riparias y lagunares, seguramente debido al mayor aporte de agua dulce y sedimentos.

**Dynamics:** Disturbance in mangrove forests may be caused by large‑scale events such as hurricanes, frost damage or clearcutting, but also by small‑scale events such as lightning, causing mangrove trees to die in small areas around lightning strikes, or attack by wood‑boring beetles. The relative importance of these different types of disturbance varies with geography, with some localities more often subjected to the impact of hurricanes or lightning. Mangroves are considered pioneer species because of their ability to establish on otherwise unvegetated substrates. Once individuals begin to colonize a disturbed area, even‑aged stands are established with little variation in the structure because new development of successive colonizers is arrested by the closed canopy. On shorter time scales, the pulses of the tides and freshwater runoff are very important factors in the dynamics of mangroves because these control the rates of sedimentation and vertical accretion and thus determine their intertidal position.

SOURCES

**References:** Ellison 2001, Josse et al. 2003*, Meyrat et al. 2001

**Version:** 08 Jan 2015 **Stakeholders:** Caribbean, Latin America

**Concept Author:** C. Josse **LeadResp:** Latin America

1.B. Temperate & Boreal Forest & Woodland

1.B.1. Warm Temperate Forest & Woodland

1.B.1.Na. Southeastern North American Forest & Woodland

M007. Longleaf Pine Woodland

CES203.254 Atlantic Coastal Plain Fall‑line Sandhills Longleaf Pine Woodland

**Primary Division:** Gulf and Atlantic Coastal Plain (203)

**Land Cover Class:** Forest and Woodland

**Spatial Scale & Pattern:** Matrix

**Required Classifiers:** Natural/Semi‑natural; Vegetated (>10% vasc.); Upland

**Diagnostic Classifiers:** Forest and Woodland (Treed); Very Short Disturbance Interval; Needle‑Leaved Tree

**National Mapping Codes:** EVT 2346; ESLF 4249; ESP 1346

**Concept Summary:** This system occurs in the Fall‑line Sandhills region of central North Carolina south and west into central Georgia. It is the predominant system in its range, covering most of the natural landscape of the region. It occurs on upland sites ranging from gently rolling, broad ridgetops to steeper sideslopes, as well as locally in mesic swales and terraces. Most soils are well‑drained to excessively‑drained. The vegetation is naturally dominated by *Pinus palustris*. Most associations have an understory of scrub oaks (*Quercus laevis, Quercus marilandica, Quercus incana*, and *Quercus margarettae*). The herb layer is generally well‑developed and dominated by grasses. Wiregrasses (*Aristida stricta* in the north, *Aristida beyrichiana* in the south) dominate in most of the range, but other grasses dominate where these are absent. Forbs, including many legumes and composites, are also abundant. Frequent, low‑intensity fire is the dominant natural ecological force.

**Comments:** This system is distinguished from Atlantic Coastal Plain Upland Longleaf Pine Woodland (CES203.281) based on differences in landscape patterns, prevailing associations, and some floristic differences. Dissected topography with much higher relief, predominance of interbedded sands and clays, and interspersion with seepage wetlands all characterize the Fall‑line Sandhills, in contrast to the low relief, pure sands or loams, and mosaics containing other wetland types in the rest of the Coastal Plain. Some matrix associations in the Fall‑line Sandhills, such as *Pinus palustris / Quercus marilandica / Gaylussacia dumosa / Aristida stricta* Woodland (CEGL003595) are nearly absent in the rest of the Coastal Plain. The abundance of legumes in most Sandhills region associations and their scarcity in most Outer Coastal Plain associations is striking, and is probably related to the differences in prevailing soil texture. This system does not have a biogeographic break in southern South Carolina, as the Outer Coastal Plain systems do. It includes areas with both *Aristida stricta* and *Aristida beyrichiana*. Gopher tortoises (*Gopherus polyphemus*), used as a break in the Outer Coastal Plain systems because of their keystone species role, are not present in the Fall‑line Sandhills. This system is distinguished from Central Atlantic Coastal Plain Wet Longleaf Pine Savanna and Flatwoods (CES203.265) because of the ecological role of saturated wetland conditions in the latter.

DISTRIBUTION

**Range:** This system ranges from central North Carolina to central Georgia, in the Fall‑line Sandhills region (Ecoregion 65c of EPA (2004); 232Bq of Keys et al. (1995)).

**Divisions:** 203:C

**TNC Ecoregions:** 56:C, 57:C

**Nations:** US

**Subnations:** GA, NC, SC

**Map Zones:** 55:C, 58:C

**USFS Ecomap Regions:** 232J:CC

CONCEPT

**Environment:** This system occurs on upland sites in the Fall‑line Sandhills region (Ecoregion 65c of EPA (2004); 232Bq of Keys et al. (1995)). It covers the gently rolling, ancient eolian sands and the steeper side slopes in older formations that make up most of the dissected landscape in this region. Shallow swales, drier stream terraces, and rock outcrops also may support this system. Substrates include interbedded sands and clays, deep sands, and occasional loamy sediments. Soils are generally well‑ to excessively drained and infertile, though local richer, mesic sites occur. All soil types are underlain by a thick clay layer that impedes drainage and creates innumerable headwater creeks; the depth from the surface to this clay layer is very variable. Non‑wetland conditions and frequent fire unify this system within the Fall‑line Sandhills region. Soil texture appears to be the most important driver of differences among associations within the system, with biogeography also important.

**Vegetation:** Vegetation is a set of associations naturally dominated by longleaf pine (*Pinus palustris*). Scrub oaks (*Quercus laevis, Quercus marilandica, Quercus incana*, and *Quercus margarettae*) form an understory in most associations, all but the mesic ones. Low shrubs, most ericaceous, may be abundant. In most of the range, wiregrass (*Aristida stricta* or in the south *Aristida beyrichiana*) is the dominant herb. In central South Carolina both species are absent and various other grass species dominate. Most associations have abundant legumes, as well as composites and other forbs. The abundance of legumes distinguishes this system from Atlantic Coastal Plain Upland Longleaf Pine Woodland (CES203.281), where most associations have few legumes. Many associations have moderate to high species richness, with most of the species in the herb layer. Some mesic associations have among the highest species richness values measured at the 1/10‑hectare scale. Associations on deep, coarse sands may have low species richness but have a distinct set of xerophytic herbs and dwarf‑shrubs.

**Dynamics:** Frequent fire is the predominant natural disturbance in this system. Component communities naturally burned every few years, many averaging as often as every 3 years. Fires are naturally low to moderate in intensity. They burn above‑ground parts of herbs and shrubs, but have little effect on the fire‑tolerant *Pinus palustris* trees. Vegetation recovers very quickly from fires, with live herbaceous biomass often restored in just a few weeks during the growing season. Many plants have their flowering triggered by burning. Fire is important in creating the structure of the vegetation. In the absence of fire, less fire‑tolerant species increase and others invade the system. The scrub oaks and shrubs, kept to low density and mostly reduced to shrub size, become tall and dense and can suppress *Pinus palustris* tree regeneration. Herb layer density and diversity decline. Only on the most excessively drained coarse sands does the vegetation not undergo substantial structural alteration and reduction in species richness after just a few years without burning. The often patchy nature of natural fires (and controlled burns) results in part from the abundance of streamheads that lace the Sandhills region and which tend to restrict fires from sweeping across large acreages.

Canopies are believed to naturally be multi‑aged, consisting of a fine mosaic of small even‑aged groves driven by gap‑phase regeneration. *Pinus palustris* is shade‑intolerant and slow to reach reproductive age, but is very long‑lived. Most plants in these systems appear to be conservative, living a long time and only rarely sexually reproducing or colonizing new sites. Similar conservatism is shown by some of the vertebrates, such as red‑cockaded woodpecker (*Picoides borealis)*. Different dynamics occur in many insect populations, whose individuals are not resilient to fire and must recolonize burned areas from nearby unburned patches.

SOURCES

**References:** Brewer 2008, Comer et al. 2003*, EPA 2004, Eyre 1980, Keys et al. 1995, NatureServe 2011a, Nelson 1986, Nordman 2012, Oswalt et al. 2012, Schafale 2012, Schafale and Weakley 1990, Wahlenberg 1946

**Version:** 21 May 2014 **Stakeholders:** Southeast

**Concept Author:** M. Schafale and R. Evans **LeadResp:** Southeast

CES203.281 Atlantic Coastal Plain Upland Longleaf Pine Woodland

**Primary Division:** Gulf and Atlantic Coastal Plain (203)

**Land Cover Class:** Forest and Woodland

**Spatial Scale & Pattern:** Matrix

**Required Classifiers:** Natural/Semi‑natural; Vegetated (>10% vasc.); Upland

**Diagnostic Classifiers:** Forest and Woodland (Treed); Very Short Disturbance Interval; Needle‑Leaved Tree

**National Mapping Codes:** EVT 2347; ESLF 4250; ESP 1347

**Concept Summary:** This system of upland *Pinus palustris*‑dominated vegetation is found in the Atlantic Coastal Plain of the United States, where it ranges from southern Virginia (where it is nearly extirpated and of very limited extent) to northeastern Florida. This system does not include *Pinus palustris* stands found in the Fall‑line Sandhills, which are accommodated by another ecological system. Examples and associations share the common feature of upland (non‑wetland) moisture regimes and natural exposure to frequent fire. They occur on a variety of well‑ to excessively drained soils, and on the higher parts of upland‑wetland mosaics. The vegetation is naturally dominated by *Pinus palustris*. Most associations have an understory of scrub oaks. The herb layer is generally well‑developed and dominated by grasses, with legumes and composites. *Aristida stricta* primarily dominates in the northern part of its range, and *Aristida beyrichiana* in the southern part. Frequent, low‑intensity fire is the dominant natural ecological force.

**Comments:** This system is distinguished from Central Atlantic Coastal Plain Wet Longleaf Pine Savanna and Flatwoods (CES203.265) because of the ecological role of saturated wetland conditions in the latter. The two systems have much in common, including frequent fire and the same primary dominant tree and herb species. They often occur in the same landscapes. However, floristic differences are well marked, and no associations are shared. This system is distinguished from Atlantic Coastal Plain Fall‑line Sandhills Longleaf Pine Woodland (CES203.254) based on the differences in landscape patterns and prevailing associations in the two regions. Dissected topography with much higher relief, predominance of interbedded sands and clays, and interspersion with seepage wetlands all characterize the Fall‑line Sandhills, in contrast to the low relief, pure sands or loams, and mosaics containing other wetland types in the rest of the Coastal Plain. Some matrix associations in the Fall‑line Sandhills, such as *Pinus palustris / Quercus marilandica / Gaylussacia dumosa / Aristida stricta* Woodland (CEGL003595) are nearly absent in the rest of the Coastal Plain, and there are systematic floristic differences. If this were to be split into a northern and southern component, the distinction would be justified based on differences in climate, flora, and some differences in ecological dynamics. Gopher tortoises (*Gopherus polyphemus*) are an important keystone species in the southern portion of the range. The dominant grass also changes at this approximate point, with *Aristida beyrichiana* dominating herb layers to the south.

DISTRIBUTION

**Range:** This system is found in the Atlantic Coastal Plain (exclusive of the Fall‑line Sandhills) from southern Virginia to northeastern Florida.

**Divisions:** 203:C

**TNC Ecoregions:** 56:C, 57:C

**Nations:** US

**Subnations:** FL, GA, NC, SC, VA

**Map Zones:** 55:C, 58:C, 60:C

**USFS Ecomap Regions:** 232C:CC, 232H:CC, 232I:CC, 232J:CC

CONCEPT

**Environment:** This system occurs on upland sites of the Middle to Outer Atlantic Coastal Plain, on landforms that include loamy to sandy flats, relict beach system deposits, eolian sand deposits, Carolina bay rims (Bennett and Nelson 1991), and occasional low rolling hills. Soils range from mesic to xeric and from sandy to loamy or occasionally clayey. Most natural remnants are on coarse sands, but most examples probably once occurred on loamy soils but have subsequently been converted to agricultural uses since the time of European settlement. Soils are largely acidic and infertile, and the coarsest sands are excessively drained and sterile. The unifying feature of this system is non‑wetland sites that naturally supported frequent fire. As such, it once covered much of the landscape of the Coastal Plain. Variations in soil texture and drainage appear to be a primary driver of differences between associations within the system, with biogeography also important as there is considerable floristic turnover along a northeast‑to‑southwest gradient paralleling the coast. In addition, soil texture varies dramatically along this gradient with finer‑textured soils predominating north of the Neuse River (in North Carolina), and again south of the Great Pee Dee River and north of the Savanna River (in South Carolina).

**Vegetation:** Vegetation is a set of associations that are most naturally woodlands or savannas dominated by *Pinus palustris* and having a well‑developed grassy herb layer. A few associations have sparse herb layers due to excessively drained soils, and a few are dominated by scrub oaks. Other pine species may sometimes be present. Scrub oaks (*Quercus laevis, Quercus incana, Quercus margarettae, Quercus hemisphaerica*, and others) form an understory in most associations, all but the mesic ones. Low shrubs, most ericaceous, are often an important component. In most of the range, *Aristida stricta* is the dominant herb. In the southern and northern parts of the range, it is absent, and various other grass species dominate. Forbs, especially composites, are usually also an important herb component, and lichens are abundant in some associations. Many associations have moderate species richness, with most of the species in the herb layer. Some mesic associations have very high species richness, among the highest values ever measured at the 1/10‑hectare scale. Associations on deep, coarse sands may have low species richness but have a distinct set of xerophytic herbs and dwarf‑shrubs.

**Dynamics:** Frequent fire is the predominant natural disturbance in this ecological system, except on the most excessively drained coarse sands, where the sparse ground cover vegetation limits low intensity fire. Component communities naturally burned every few years, many averaging as often as every 3 years. Fires are naturally low to moderate in intensity. They burn above‑ground parts of herbs and shrubs but have little effect on the fire‑tolerant trees. Vegetation recovers very quickly from fire, with live herbaceous biomass often restored in just a few weeks. Many plants have their flowering triggered by burning. In the absence of fire, less fire‑tolerant species increase and others invade the system. The scrub oaks and shrubs, kept to low density and mostly reduced to shrub size by fire, become tall and dense and can suppress *Pinus palustris* regeneration as well as dramatically reducing the herbaceous layer. Only on the most excessively drained coarse sands does the vegetation not undergo substantial structural alteration and reduction in species richness after just a few years without burning.

Canopies are believed to naturally be multi‑aged, consisting of a fine mosaic of small even‑aged patches driven by gap‑phase regeneration. *Pinus palustris* is shade‑intolerant and slow to reach reproductive age but is very long‑lived.

SOURCES

**References:** Bennett and Nelson 1991, Brewer 2008, Capinera et al. 2004, Comer et al. 2003*, Dakin and Hays 1970, Engeman et al. 2007, Eyre 1980, FNAI 2010a, NatureServe 2011a, Nelson 1986, Oswalt et al. 2012, Rehn and Hebard 1916, Schafale 2012, Schafale and Weakley 1990, Schafale pers. comm., Schuster 1974, Squitier and Capinera 2002, Van Lear et al. 2005, Wahlenberg 1946

**Version:** 23 Apr 2015 **Stakeholders:** East, Southeast

**Concept Author:** R. Evans **LeadResp:** Southeast

CES203.265 Central Atlantic Coastal Plain Wet Longleaf Pine Savanna and Flatwoods

**Primary Division:** Gulf and Atlantic Coastal Plain (203)

**Land Cover Class:** Woody Wetland

**Spatial Scale & Pattern:** Matrix

**Required Classifiers:** Natural/Semi‑natural; Vegetated (>10% vasc.); Wetland

**Diagnostic Classifiers:** Forest and Woodland (Treed); Short Disturbance Interval; Needle‑Leaved Tree

**National Mapping Codes:** EVT 2449; ESLF 9118; ESP 1449

**Concept Summary:** This ecological system of wet *Pinus palustris*‑dominated savannas and flatwoods ranges from southern Virginia to central South Carolina. It was once one of the most extensive systems in the coastward part of its range. Examples and associations share the common features of wet, seasonally saturated, mineral soils and exposure to frequent fire. They occur on a wide range of soil textures, which is an important factor in distinguishing different associations. The vegetation is naturally dominated by *Pinus palustris* or, less frequently, *Pinus serotina*. There is a dense ground cover of herbs and low shrubs; grasses dominate but there is often a large diversity of other herbs. Frequent, low‑intensity fire is the dominant natural ecological force.

**Comments:** This system is distinguished from Southern Atlantic Coastal Plain Wet Pine Savanna and Flatwoods (CES203.536) because of substantial biogeographic differences. The break is placed at the Santee River, which approximates the transition between the ranges of *Aristida stricta* and *Aristida beyrichiana*, which are keystone species in the communities where they occur. This corresponds roughly with the geographic break in the upland longleaf pine systems as well. This system is distinguished from Atlantic Coastal Plain Upland Longleaf Pine Woodland (CES203.281) because of that system's more upland character. However, the two systems have much in common, including frequent fire, the same primary dominant canopy tree, and many herbaceous species. They can also occur in the same landscapes. However, floristic differences are well marked, and no associations are shared. This system occurs primarily in the Outer Coastal Plain, but small patches may occur in atypical landforms in the Fall‑line Sandhills. Sandhills examples are not treated as a separate system, as the upland longleaf pine systems are, because they are confined to sites that more resemble the Outer Coastal Plain. They are distinguished in the Sandhills from Atlantic Coastal Plain Sandhill Seep (CES203.253) by landform and apparent hydrology that is driven by seasonal high water table rather than seepage.

DISTRIBUTION

**Range:** This system ranges from southern Virginia to central South Carolina. To the south, the equivalent system is Southern Atlantic Coastal Plain Wet Pine Savanna and Flatwoods (CES203.536), the range of which includes Georgia and northern Florida.

**Divisions:** 203:C

**TNC Ecoregions:** 57:C

**Nations:** US

**Subnations:** NC, SC, VA

**Map Zones:** 58:C, 60:C

**USFS Ecomap Regions:** 232C:CC, 232H:CC, 232I:CC, 232J:CC

CONCEPT

**Environment:** This system occurs on wet mineral soil sites, primarily in the Middle and Outer Coastal Plain but occasionally in the Fall‑line Sandhills. Landforms include low areas in relict beach ridge systems and eolian sand deposits, and poorly drained clayey, loamy, or sandy flats. They occasionally occur on river terraces above current flood levels. Soils range from clayey to sandy, with no accumulated organic surface layer. Soils are seasonally saturated, due to high water table or poor soil drainage. The unifying feature of this system is wet mineral soils associated with a high frequency of fire. Variation in soil texture appears to be a primary driver of differences between associations within the system, with biogeography also important.

**Vegetation:** Vegetation is a set of associations that are naturally woodlands or savannas dominated by *Pinus palustris* or, less frequently, by *Pinus serotina, Pinus elliottii*, or some combination. Hardwoods are present in any abundance only in examples altered by fire suppression. The ground cover is a dense combination of herbs and low shrubs. A variety of ericaceous shrubs and hollies is common, with density determined by fire history. Grasses naturally dominate the ground cover. *Aristida stricta* often dominates within its range, but *Ctenium aromaticum, Sporobolus pinetorum, Sporobolus teretifolius*, or other grasses may dominate. A great diversity of other herbs is often present, including composites, sedges, insectivorous plants, and variety of showy forbs. Communities in this system are often very high in species richness, with some of the highest values measured anywhere at the 1/10‑hectare, 1/100‑hectare, and 1‑m2 levels. However, some associations are naturally low to moderate in species richness.

**Dynamics:** Frequent fire is the predominant natural disturbance in this system. Communities naturally burned every few years, many averaging as often as every 3 years. Fires are naturally low to moderate in intensity. They burn above‑ground parts of herbs and shrubs but have little effect on the fire‑tolerant trees. Vegetation recovers very quickly from fire, with live herbaceous biomass often restored in just a few weeks during the growing season. Many plants have their flowering triggered by burning, the effects on subsequent establishment are not well‑documented. In the absence of fire, the shrubs increase and hardwoods may invade the system. Herb layer density and diversity decline after a number of years without fire. In time, unburned examples may become nearly indistinguishable from the drier associations of Atlantic Coastal Plain Peatland Pocosin and Canebrake (CES203.267).

<br />Canopies are believed to naturally be multi‑aged, consisting of a mosaic of even‑aged patches driven by gap‑phase regeneration. *Pinus palustris* is shade‑intolerant and slow to reach reproductive age but is very long‑lived, and healthy trees continue to produce more cones as they age beyond 100 years.

SOURCES

**References:** Brewer 2008, Comer et al. 2003*, Eyre 1980, NatureServe 2011a, Nelson 1986, Oswalt et al. 2012, Rehn and Hebard 1916, Schafale 2012, Wahlenberg 1946

**Version:** 21 May 2014 **Stakeholders:** East, Southeast

**Concept Author:** M. Schafale and R. Evans **LeadResp:** Southeast

CES203.382 Central Florida Pine Flatwoods

**Primary Division:** Gulf and Atlantic Coastal Plain (203)

**Land Cover Class:** Mixed Upland and Wetland

**Spatial Scale & Pattern:** Matrix

**Required Classifiers:** Natural/Semi‑natural; Vegetated (>10% vasc.); Upland; Wetland

**Diagnostic Classifiers:** Forest and Woodland (Treed); Woody‑Herbaceous; Short Disturbance Interval; Needle‑Leaved Tree

**National Mapping Codes:** EVT 2453; ESLF 9122; ESP 1453

**Concept Summary:** This system is endemic to Florida, ranging from Levy and St. Johns counties in the north (ca. 30°N latitude) southward to Hillsborough, Osceola and Polk counties. It was once an extensive system within its historic range. As currently conceived, this system includes both "scrubby flatwoods" that occur on well‑drained soils and typical flatwoods that occur on more poorly drained soils. The vegetation is naturally dominated by either *Pinus palustris* or *Pinus elliottii var. elliottii*, and less frequently includes *Pinus serotina*. Examples vary in aspect from well‑developed understory layers or scrub species to more herbaceous, savanna‑like conditions. There is a dense ground cover of low shrubs, grasses, and herbs. Frequent, low‑intensity fire is the dominant natural ecological force.

**Comments:** This system includes at least two predominant expressions which could individually constitute distinct systems. Scrubby flatwoods are much more well‑drained, uplands with characteristically shrubby understories, while flatwoods are much more poorly drained and savanna‑like in aspect (Abrahamson et al. 1984).

DISTRIBUTION

**Range:** Endemic to Florida, ranging in the north from Levy and St. Johns counties southward to Hillsborough and Polk counties. It was once an extensive ecological system within its historic range (Stout and Marion 1993).

**Divisions:** 203:C

**TNC Ecoregions:** 55:C

**Nations:** US

**Subnations:** FL

**Map Zones:** 55:C, 56:C

**USFS Ecomap Regions:** 232D:CC, 232G:CC, 232K:CC

CONCEPT

**Environment:** As currently conceived, this system includes both "scrubby flatwoods" that occur on well‑drained soils and typical mesic and wet flatwoods that occur on more poorly drained soils. Wetter pine flatwoods sites with an herbaceous ground cover are included, these are sometimes called wet pine savannas.

**Vegetation:** The southern limit of this system marks the approximate natural distribution limit for both *Pinus serotina* and *Pinus elliottii var. elliottii* (Abrahamson and Hartnett 1990). The associations comprising this system are not well documented; more information is needed to describe additional communities that are believed to be present. The vegetation varies between examples of this system based on fire history, geographic location, and the soils on which it occurs. The most well‑drained examples may be considered "scrubby flatwoods" that support a characteristic understory layer of xeromorphic adapted species, such as *Quercus geminata, Lyonia fruticosa, Lyonia ferruginea, Sideroxylon tenax (= Bumelia tenax)*, and *Persea humilis*; *Quercus inopina* is especially diagnostic (Abrahamson et al. 1984). These conditions range to examples on more poorly drained soils that include scattered *Pinus elliottii var. elliottii* or *Pinus palustris* over *Serenoa repens* and other species such as *Panicum abscissum* and *Aristida beyrichiana*.

**Dynamics:** Fire is naturally frequent, with a fire‑return time of from one to four years. Disturbances are an important part of the natural functions of this system. In order for these habitats to burn frequently there needs to be enough fine fuel, such as needles from *Pinus palustris* trees, healthy populations of native warm‑season grasses, and evergreen shrubs with volatile oils in their leaves, such as *Ilex glabra, Lyonia* spp., *Morella cerifera, Quercus geminata, Quercus minima, Serenoa repens*, and *Vaccinium* spp. The frequent fires promote flowering, seed production, and seed germination of many plants and provide open areas in patches (Van Lear et al. 2005).

SOURCES

**References:** Abrahamson and Hartnett 1990, Abrahamson et al. 1984, Brewer 2008, Carr et al. 2010, Comer et al. 2003*, Eyre 1980, NatureServe 2011a, Oswalt et al. 2012, Stout and Marion 1993, Van Lear et al. 2005, Wahlenberg 1946

**Version:** 14 Jan 2014 **Stakeholders:** Southeast

**Concept Author:** R. Evans **LeadResp:** Southeast

CES203.496 East Gulf Coastal Plain Interior Upland Longleaf Pine Woodland

**Primary Division:** Gulf and Atlantic Coastal Plain (203)

**Land Cover Class:** Forest and Woodland

**Spatial Scale & Pattern:** Matrix

**Required Classifiers:** Natural/Semi‑natural; Vegetated (>10% vasc.); Upland

**Diagnostic Classifiers:** Forest and Woodland (Treed); Very Short Disturbance Interval; East Gulf Coastal Plain

**National Mapping Codes:** EVT 2349; ESLF 4252; ESP 1349

**Concept Summary:** This ecological system represents *Pinus palustris* forests of rolling, dissected to relatively flat uplands of the East Gulf Coastal Plain. These stands occur primarily in the Southeastern Plains (EPA Ecoregion 65). It is found inland of the Gulf Coast Flatwoods (EPA Ecoregion 75a) and extends landward into the Upper East Gulf Coastal Plain Ecoregion by about 80 km (50 miles). It potentially occupies a much larger geographic area than the related *Pinus palustris* woodlands of the outer coastal area. The characteristic species is *Pinus palustris*, although many stands may support only relictual individuals following a long history of exploitation, harvest, and stand conversion, primarily to agriculture or to planted stands of *Pinus elliottii var. elliottii* or *Pinus taeda*. This system includes stands with a range of soil and moisture conditions. Mesic stands on medium‑ to fine‑textured soils are more typical of the system, although limited xeric areas on deep sands are also present. In natural condition, fire is believed to have been frequent enough to limit development of fire‑intolerant hardwood species as well as *Pinus taeda* and *Pinus echinata*. Although such species may be present or even common in the most mesic stands, they generally do not share dominance in the overstory unless fire has been absent from the stand.

**Comments:** The dominance of *Pinus palustris* in examples of this ecological system may be lost through fire suppression, bark beetle infestations, forestry and agricultural land conversion, and mechanical disturbance. Loss of *Pinus palustris* dominance will fundamentally change the ecological function of the landscape occupied by the system, primarily by altering the fire regime. Without the appropriate fire regime, canopy closure will increase along with shrub dominance, and grasses, forbs and other finer‑fuel components will decline, further altering the fire regime dynamics.

Systems dominated by *Pinus palustris* are subdivided by biogeography, from northeast to southwest across the coastal plains from Virginia to Texas. Longleaf pine‑dominated stands in the rocky submontane areas of the Piedmont as well as the Ridge and Valley (from North Carolina to Alabama) are classified as a separate system, Southeastern Interior Longleaf Pine Woodland (CES202.319).

DISTRIBUTION

**Range:** This system formerly occupied an extensive range across the southern parts of Alabama, northern Panhandle of Florida (north of the Cody Scarp), southern Mississippi, and southwestern Georgia and was also present in limited areas of Louisiana. It has been greatly reduced in its extent, with much of its range now occupied by agriculture or by planted stands of *Pinus taeda*. In southwestern Mississippi, this system is apparently absent (or very rare and limited) west of 91°W longitude to the limits of the alluvial plain and northwest of a line running approximately from the intersection of 31°N latitude and 91°W longitude, northeastward to the city of Jackson, Mississippi. This is consistent with the ranges of "Oak‑Pine" vegetation versus "Longleaf‑Loblolly‑Slash Pines" (generally equivalent to this system) in Shantz and Zon (1924). In southwest Georgia, this ecological system occurs in Coastal Plain areas which drain to the Gulf of Mexico.

**Divisions:** 203:C

**TNC Ecoregions:** 43:C, 53:C

**Nations:** US

**Subnations:** AL, FL, GA, LA, MS

**Map Zones:** 46:C, 55:C, 99:C

**USFS Ecomap Regions:** 231B:CC, 232B:CC, 232C:CC, 232J:CC, 232K:CC

CONCEPT

**Environment:** This system once occupied extensive areas of the East Gulf Coastal Plain from the northern range limits of *Pinus palustris* southward to the inland terminus of the Coastal Flatlands (sensu Peet and Allard (1993); Ecoregion 75a (EPA 2004)). In its natural condition, this system occupied a range of upland soils from clays and loams to deep sands, including weathered and older Ultisols. Due to locally distinctive understory, shrub and herbaceous vegetation associated with differing soil textures, "sandhills" and "loamhills" are generally recognizable as distinctive components of this system. However, they are generally interspersed to such an extent that differentiating them as separate systems is not practical. The topography of this system is generally more rolling than East Gulf Coastal Plain Near‑Coast Pine Flatwoods (CES203.375) to the south. The largest and best examples occupy landscapes where prescribed fire is an active management practice. Localized soil characteristics will determine the specific composition of the lower strata. Ultisols are the dominant soil order and cover most of the range of the system. Ultisols most commonly associated with *Pinus palustris* are the Typic Paleudults and Plinthic Paleudults. More limited areas are occupied by Psamments and other coarser‑textured materials. *Pinus palustris* grows in warm, wet temperate climates characterized by hot summers and mild winters. The annual mean temperatures range from 16‑23°C (60‑74°F), and the annual precipitation ranges from 1090 to 1750 mm (43‑69 inches) (Boyer 1990). Fall is the driest season of the year, although periods of drought during the growing season are not unusual (Boyer 1990).

**Vegetation:** Occurrences of this system are typically more‑or‑less open‑canopy stands (woodlands) dominated by the evergreen needle‑leaved tree *Pinus palustris*. In parts of the range, and on more rolling topography, other pines may be present, including *Pinus echinata* and *Pinus taeda*. These may increase or become codominant with extended fire‑return times. Unless fire suppression is extreme, deciduous trees generally do not share dominance in the canopy. More mesic stands (e.g., those on finer‑textured soils) may contain oaks, such as *Quercus falcata, Quercus nigra*, or *Quercus pumila*, and occasionally species favoring more xeric conditions, such as *Quercus marilandica* or *Quercus stellata*, in combination with the more mesic oaks. Even more xeric stands (uncommon in this system) may contain "scrub oaks" such as *Quercus incana, Quercus laevis, Quercus margarettae*, or *Quercus arkansana*. In fire‑suppressed areas, *Quercus falcata, Liquidambar styraciflua, Acer rubrum, Quercus nigra, Nyssa sylvatica, Cornus florida, Callicarpa americana*, and/or *Rhus copallinum* may invade or increase. Some typical mesic to dry‑mesic herbaceous species include *Andropogon ternarius, Andropogon gyrans var. gyrans, Schizachyrium scoparium, Sorghastrum nutans*, and *Panicum virgatum*. *Aristida stricta* or *Aristida beyrichiana* are also dominant or at least present in the herbaceous layer of many more southern and coastward examples. Variation in floristic composition of this wide‑ranging system is related to site conditions, fire‑return interval, and local or regional floristics. The herbaceous layer typically becomes much less diverse with increased fire‑return interval. The wiregrass *Aristida beyrichiana* is not present throughout the range of this system, and even within the range of this species, it tends to be dominant or more abundant in moister sites, particularly in the western part of the system's range (and also in examples of East Gulf Coastal Plain Near‑Coast Pine Flatwoods (CES203.375)).

**Dynamics:** Frequent fire was the predominant natural disturbance in this system, which is now dependent on management with prescribed fire. Component communities naturally burned every few years, many averaging as often as every 3 years. Fires are naturally low to moderate in intensity. They burn above‑ground parts of herbs and shrubs but have little effect on the fire‑tolerant trees. Vegetation recovers very quickly from fire; the perennial species resprout quickly. Many herbaceous plants have their flowering triggered by burning. Frequent fires help maintain more species richness at small sample scales, compared to pinelands of the other regions (Carr et al. 2010). In the absence of fire, hardwoods increase. *Quercus* spp. and shrubs, kept to low density and mostly reduced to shrub size by fire, become tall and dense and can suppress *Pinus palustris* regeneration. Herb layer density and diversity decline without occasional fire. Frequent fire requires a mix of fine fuels composed both of herbaceous (primarily grasses) fine fuels and *Pinus palustris* leaf litter. Consequently, thinning the *Pinus palustris* canopy to low basal area or opening too large gaps, particularly in absence of *Aristida beyrichiana*, can lead to rapid hardwood encroachment due to lack of abundant and continuous fuels necessary for frequent fire (K. Kirkman pers. comm.). Only on the most excessively drained coarse sands does the vegetation not undergo substantial structural alteration and reduction in species richness after a number of years without burning. This is due to the infertile soils. This structural alteration occurs more slowly on these infertile soils, but due to the slow accumulation of fuels, lack of fire can become more pronounced.

Canopies are believed to naturally be multi‑aged, consisting of a fine mosaic of small even‑aged groves driven by gap‑phase regeneration. *Pinus palustris* is shade‑intolerant and slow to reach reproductive age but is very long‑lived. *Pinus palustris* seedlings can survive under a gap opening in canopy >35%. However, they will not move out of grass stage unless the gap fraction is >60%. Because these canopy gaps have less needle fall, the frequent fires which burn there are less intense, which permits *Pinus palustris* seedlings to survive. *Pinus palustris* can also stay in the sapling stage for decades and still take advantage of a gap opening to move into the canopy (Kirkman and Mitchell 2006).

SOURCES

**References:** Boyer 1990, Brewer 2008, Carr et al. 2010, Comer et al. 2003*, EPA 2004, Eyre 1980, FNAI 2010a, Kirkman and Mitchell 2006, NatureServe 2011a, Oswalt et al. 2012, Peet and Allard 1993, Shantz and Zon 1924, Wahlenberg 1946

**Version:** 21 May 2014 **Stakeholders:** Southeast

**Concept Author:** R. Evans, A. Schotz, M. Pyne **LeadResp:** Southeast

CES203.375 East Gulf Coastal Plain Near‑Coast Pine Flatwoods

**Primary Division:** Gulf and Atlantic Coastal Plain (203)

**Land Cover Class:** Mixed Upland and Wetland

**Spatial Scale & Pattern:** Matrix

**Required Classifiers:** Natural/Semi‑natural; Vegetated (>10% vasc.); Upland; Wetland

**Diagnostic Classifiers:** Forest and Woodland (Treed); Extensive Wet Flat; Short Disturbance Interval; Needle‑Leaved Tree

**National Mapping Codes:** EVT 2454; ESLF 9123; ESP 1454

**Concept Summary:** This ecological system of open forests or woodlands occupies broad, sandy flatlands in a relatively narrow band along the northern Gulf of Mexico coast east of the Mississippi River. This range corresponds roughly to the Gulf Coast Flatwoods (EPA Ecoregion 75a). These areas predominantly occur on poorly drained acidic Spodosol soils, which are subject to seasonal inundation as well as droughty conditions. Often called "flatwoods" or "flatlands," they are subject to short fire‑return intervals and seasonally high water tables. Overstory vegetation is characterized by *Pinus palustris* and, to a lesser degree, by *Pinus elliottii var. elliottii*. Understory structure ranges from densely shrubby to open and herbaceous‑dominated, with variation in soils and drainage. The variation includes Scrubby Flatwoods, Mesic Flatwoods, Wet Flatwoods, and Maritime Flatwoods. Fire is naturally frequent; many sites have a fire‑return time of from one to four years.

**Comments:** There was some consideration of splitting out the slash pine flatwoods from this system due to presumed differences in both moisture status and fire history when compared with typical longleaf. There is considerable variation between wet and "non‑wet" flatwoods implied in this system.

DISTRIBUTION

**Range:** This system is conceived of as including wet and dry pine flatwoods of the near‑coastal zone of the East Gulf Coastal Plain, mainly south of the Cody Scarp (Peet and Allard 1993). It corresponds roughly to the Gulf Coast Flatwoods, Ecoregion 75a (EPA 2004).

**Divisions:** 203:C

**TNC Ecoregions:** 53:C

**Nations:** US

**Subnations:** AL, FL, GA, LA, MS

**Map Zones:** 55:C, 99:C

**USFS Ecomap Regions:** 232D:CC, 232L:CC

CONCEPT

**Environment:** This system occupies broad, sandy flatlands which are subject to short fire‑return intervals even though they are subject to seasonally high water tables. Spodosols encourage seasonal saturation, acidity, and high soil iron and aluminum concentrations. These areas are often called "flatwoods" or "flatlands."

**Vegetation:** Overstory vegetation is characterized by *Pinus palustris* and to a lesser degree by *Pinus elliottii*. Some stands include *Pinus serotina*. Shrubs include *Quercus geminata, Quercus minima, Quercus pumila, Serenoa repens, Cyrilla racemiflora, Ilex coriacea, Ilex glabra, Ilex vomitoria*, and *Lyonia lucida*. Herbaceous species may include *Aristida beyrichiana, Ctenium aromaticum, Muhlenbergia expansa, Schizachyrium scoparium, Sporobolus floridanus, Carphephorus pseudoliatris, Sarracenia alata, Agalinis filicaulis, Polygala cymosa, Rhynchospora* spp., and *Helianthus radula*.

**Dynamics:** Fire is naturally frequent, with a fire‑return time of from one to four years. Disturbances are an important part of the natural functions of wet pine savanna and flatwoods. In order for these habitats to burn frequently (every 2‑3 years), there needs to be enough fine fuel, such as needles from *Pinus palustris* trees, healthy populations of native warm‑season grasses, and evergreen shrubs with volatile oils in their leaves, such as *Gaylussacia frondosa, Ilex coriacea, Ilex glabra, Lyonia* spp., *Serenoa repens*, and *Vaccinium* spp. The frequent fires promote flowering, seed production, and seed germination of many plants and provide open areas in patches (Van Lear et al. 2005).

SOURCES

**References:** Brewer 2008, Carr et al. 2010, Comer et al. 2003*, EPA 2004, Eyre 1980, FNAI 2010a, Griffith et al. 2001, NatureServe 2011a, Oswalt et al. 2012, Peet 2006, Peet and Allard 1993, Van Lear et al. 2005, Wahlenberg 1946

**Version:** 21 May 2014 **Stakeholders:** Southeast

**Concept Author:** R. Evans **LeadResp:** Southeast

CES203.284 Florida Longleaf Pine Sandhill

**Primary Division:** Gulf and Atlantic Coastal Plain (203)

**Land Cover Class:** Forest and Woodland

**Spatial Scale & Pattern:** Large patch

**Required Classifiers:** Natural/Semi‑natural; Vegetated (>10% vasc.); Upland

**Diagnostic Classifiers:** Forest and Woodland (Treed); Xeric; Very Short Disturbance Interval; Needle‑Leaved Tree

**National Mapping Codes:** EVT 2356; ESLF 4259; ESP 1356

**Concept Summary:** This system represents stands of *Pinus palustris* on excessively well‑drained, sandy soils in the Outer Coastal Plain and adjacent Inner Coastal Plain of Florida. This includes the "high pine islands" of central Florida, as well as vegetation of extensive areas of sand in the Florida Panhandle, north of the Cody Scarp, including at Eglin Air Force Base (with greater than 100,000 hectares of this ecological system). In central Florida on the Ocala National Forest, these stands are found in relation with sand pine scrub vegetation. This system is represented by larger patches of *Pinus palustris* sandhills, generally ranging from 60 to 4000 hectares in size and larger. In addition to the largest extent at Eglin Air Force Base, examples also occur on the Ocala National Forest, the southern end of the Lake Wales Ridge, the Brooksville Ridge, and in other parts of the Florida Peninsula. Fire is absolutely essential to maintain this system, without which it may be almost completely replaced by scrub vegetation, hardwood trees, *Pinus taeda*, or other non‑*Pinus palustris*‑dominated vegetation.

DISTRIBUTION

**Range:** This ecological system is found in the Outer Coastal Plain and adjacent Inner Coastal Plain of Florida, including the central Florida Peninsula (Ocala National Forest, Brooksville Ridge, southern end of the Lake Wales Ridge) (Abrahamson et al. 1984) and the Florida Panhandle, mainly north of the Cody Scarp (e.g., Eglin Air Force Base).

**Divisions:** 203:C

**TNC Ecoregions:** 53:C, 55:C

**Nations:** US

**Subnations:** FL

**Map Zones:** 55:C, 56:C, 99:C

**USFS Ecomap Regions:** 232D:CC, 232G:CC, 232K:CC

CONCEPT

**Environment:** Surface soils tend to be coarse, with <5% composition of finer‑textured particles (silt and clay), and very low organic content and low moisture‑holding capacity. Soils are typically Entisols (Psamments), with very limited profile development. In the Florida Panhandle soils can be Ultisols. Some soil series associated with this system include the Astatula series (Kalisz 1982), as well as the Lakeland, Tavares, and Orsino series (Abrahamson et al. 1984). Candler is the most extensive soil on sandhills on the ridges of Central Florida (S. Carr pers. comm.) In some cases on the Ocala National Forest the soils may be unusually dark in color at the surface, which has been attributed, in part, to the presence of charcoal. Soils are strongly acidic (pH 4.7‑5.0). Some Central Florida sites have silt or clay in the subsoil contributing to significantly higher extractable bases at the surface when compared to nearby scrub sites (Kalisz 1982). Excluded are areas with a "shallow sand cap" (K. Outcault pers. comm.). On Eglin Air Force Base in the western Florida Panhandle, this ecological system occurs on deep sands on the Citronelle Formation. Psamments are the dominant soil suborder in the areas of Florida where this system is found (NRCS n.d.).

**Vegetation:** Stands of this system typically lack a well‑developed subcanopy, especially in contrast to surrounding *Pinus clausa* scrub vegetation. However, the shrub layer may be well‑developed, even under frequent fire conditions, and appears to be dominated by sprouts of *Quercus laevis* and *Quercus myrtifolia*. A rich herbaceous layer is present. Characteristic species in this stratum are *Aristida beyrichiana* and *Licania michauxii*. In addition, a number of species found primarily in central Florida may also be present, among the most frequent of which is *Chapmannia floridana*. Other geographically limited species may include *Sabal etonia, Polygonella ciliata*, and *Arnoglossum floridanum*.

**Dynamics:** Fire is absolutely essential to maintain this system, without which it may be almost completely replaced by scrub vegetation (in the Florida Peninsula), hardwood trees, *Pinus clausa, Pinus taeda*, or other non‑*Pinus palustris*‑dominated vegetation.

SOURCES

**References:** Abrahamson et al. 1984, Brewer 2008, Carr et al. 2010, Comer et al. 2003*, Eyre 1980, FNAI 2010a, Kalisz 1982, NRCS n.d., NatureServe 2011a, Oswalt et al. 2012, Outcalt pers. comm., Wahlenberg 1946

**Version:** 21 May 2014 **Stakeholders:** Southeast

**Concept Author:** R. Evans and C. Nordman **LeadResp:** Southeast

CES411.381 South Florida Pine Flatwoods

**Primary Division:** Caribbean (411)

**Land Cover Class:** Mixed Upland and Wetland

**Spatial Scale & Pattern:** Matrix

**Required Classifiers:** Natural/Semi‑natural; Vegetated (>10% vasc.); Upland; Wetland

**Diagnostic Classifiers:** Needle‑Leaved Tree

**National Mapping Codes:** EVT 2446; ESLF 9115; ESP 1446

**Concept Summary:** This system is endemic to Florida, ranging from Lee, Desoto, Highlands, and Okeechobee counties southward. It was once an extensive system within its historic range. The vegetation is naturally dominated by *Pinus elliottii var. densa*, being largely outside the natural range of *Pinus serotina, Pinus elliottii var. elliottii*, and *Pinus palustris*. In natural condition, examples are generally open with a variety of low shrub and grass species forming a dense ground cover. Frequent, low‑intensity fire was the dominant natural ecological force, but most areas have undergone long periods without fire, resulting in greater dominance of shrubs and saw palmetto, as well as denser canopies of slash pine.

**Comments:** No associations have currently been described in the USNVC for this system. More information is needed. The floristic composition of this system overlaps Florida Dry Prairie (CES203.380); the primary difference lies in taller and denser shrub cover (especially of *Serenoa repens*) (Huffman and Judd 1998). There is considerable variation between wet and "non‑wet" flatwoods implied in this system.

DISTRIBUTION

**Range:** This system is found in southern Florida, extending north to mid‑peninsula (e.g., Lee, Desoto, Highlands, and Okeechobee counties).

**Divisions:** 203:C, 411:C

**TNC Ecoregions:** 54:C, 55:C

**Nations:** US

**Subnations:** FL

**Map Zones:** 56:C

**USFS Ecomap Regions:** 232D:CC, 232G:CC, 411A:CC

CONCEPT

**Environment:** This system occurs on sandy soils, including Spodosols, which are prone to some saturation or short periods of flooding after summer rains. These flatwoods occur in areas which have some creeks, which provide some natural firebreaks. Similar areas which are very extensive without creeks tend to be Florida Dry Prairie (CES203.380), which naturally burns more frequently.

**Vegetation:** According to Huffman and Judd (1998), examples of this system have generally open canopies composed of *Pinus elliottii var. densa* and, more rarely, *Pinus palustris*. *Serenoa repens, Lyonia lucida, Lyonia fruticosa, Ilex glabra, Vaccinium darrowii, Vaccinium myrsinites*, and *Quercus minima* are common shrubs. Grasses are typically abundant, including *Aristida beyrichiana* and *Schizachyrium scoparium var. stoloniferum*; most other grass and herbaceous species found are in common with Florida Dry Prairie (CES203.380).

**Dynamics:** Frequent, low‑intensity fire was the dominant natural ecological force, but most areas have undergone long periods without fire, resulting in greater dominance of shrubs and saw palmetto, as well as denser canopies of slash pine (Huffman and Judd 1998, Noel et al. 1998). Disturbances are an important part of the natural functions of pine flatwoods. In order for these habitats to burn frequently (every 2‑3 years), there needs to be enough fine fuel, such as needles from *Pinus elliottii var. densa* or *Pinus palustris* trees, healthy populations of native warm‑season grasses, and evergreen shrubs with volatile oils in their leaves, such as *Gaylussacia frondosa, Hypericum tenuifolium, Ilex glabra, Lyonia ferruginea, Lyonia fruticosa, Serenoa repens*, and *Vaccinium myrsinites*. The frequent fires promote flowering, seed production, and seed germination of many plants and provide open areas in patches (Van Lear et al. 2005).

SOURCES

**References:** Brewer 2008, Comer et al. 2003*, Duever et al. 1986, FNAI 2010a, Huffman and Judd 1998, McPherson 1986, NatureServe 2011a, Noel et al. 1998, Oswalt et al. 2012, Stout and Marion 1993, Van Lear et al. 2005, Wahlenberg 1946

**Version:** 14 Jan 2014 **Stakeholders:** Southeast

**Concept Author:** R. Evans and C. Nordman **LeadResp:** Southeast

CES411.367 South Florida Pine Rockland

**Primary Division:** Caribbean (411)

**Land Cover Class:** Forest and Woodland

**Spatial Scale & Pattern:** Large patch

**Required Classifiers:** Natural/Semi‑natural; Vegetated (>10% vasc.); Upland

**Diagnostic Classifiers:** Forest and Woodland (Treed); Circumneutral Soil; Needle‑Leaved Tree

**National Mapping Codes:** EVT 2360; ESLF 4263; ESP 1360

**Concept Summary:** This system includes pinelands of extreme south Florida growing on limestone. The uniqueness of the flora associated with this type has long been recognized, including the number of endemic and West Indian species. Many plant and animal taxa found in this system are restricted to it, including many of south Florida's endemic plants. Unlike pinelands elsewhere in the southeastern coastal plain, *Pinus elliottii var. densa* is the only native pine species in this system. Understory vegetation consists of many hardwood species, including a number with tropical origins, and the herbaceous flora is species‑rich and fire‑adapted.

DISTRIBUTION

**Range:** Davis (1943) mapped this system, which occurred primarily on the Miami ridge bordering the Everglades, with disjunct examples found in the Big Cypress Swamp. Davis estimated there once was 180,000 acres of "Miami region pine" (Davis 1943). McPherson's (1986) map of Big Cypress shows "pine forest," which includes both pine rocklands and pine flatwoods, scattered across the unit. It may be possible to differentiate based on soil type or geology, the pine rockland being in the southeast part of Big Cypress. In the Florida Keys it is found on Big Pine Key, No Name Key, Little Pine Key, Cudjoe Key, and Upper Sugarloaf Key. The Miami Rockridge extends from around downtown Miami southwest to Long Pine Key in Everglades National Park (Miami‑Dade County). Big Pine Key is in Monroe County, and the Big Cypress National Preserve is in Monroe and Collier counties. In addition, pine rockland historically occurred in the upper Florida Keys; pine stumps and remnant species characteristic of pine rockland have been found in one area of Key Largo (Alexander 1953). There has been an estimated 98% decline in the amount of pine rockland habitat on the Miami Rock Ridge in southern Florida, outside of the Everglades National Park where Long Pine Key is protected (Noss et al. 1995, Enge et al. 2002). About 6200 ha (15,000 acres) of pine rockland remain (Enge et al. 2002).

**Divisions:** 411:C

**TNC Ecoregions:** 54:C

**Nations:** US

**Subnations:** FL

**Map Zones:** 56:C

**USFS Ecomap Regions:** 411A:CC

CONCEPT

**Environment:** Pine rockland occurs on relatively flat, moderately to well‑drained terrain from 2‑7 m above sea level (Snyder et al. 1990). Along the southeastern coast of Florida this system occurs on Miami Oolitic Limestone, while in the Big Cypress region (southwest Florida) it is found on outcrops of Tamiami Limestone. Outcrops of weathered oolitic limestone, known locally as pinnacle rock, are common, and solution holes may be present (FNAI 2010a). The oolitic limestone is at or very near the surface, and there is very little soil development. Soils are generally composed of small accumulations of nutrient‑poor sand, marl, clayey loam, and organic debris in depressions and crevices in the rock surface. Organic acids occasionally dissolve the surface limestone causing collapsed depressions in the surface rock called solution holes (Outcalt 1997b). Drainage varies according to the porosity of the limestone substrate, but is generally rapid. Consequently, most sites are wet for only short periods following heavy rains. During the rainy season, however, some sites may be shallowly inundated by slow‑flowing surface water for up to 60 days each year.

**Vegetation:** *Pinus elliottii var. densa* is the only native pine species in this system. Stands have an open canopy, generally with multiple age classes. It has been estimated that nearly one‑third of the taxa found in this system are restricted to it, including half of south Florida's endemic plants (Stout and Marion 1993). The diverse, open shrub/subcanopy layer is composed of more than 100 species of palms and hardwoods (Gann et al. 2009), most derived from the tropical flora of the West Indies (Snyder et al. 1990). Many of these species vary in height depending on fire frequency, getting taller with time since fire. These include *Ardisia escallonoides, Byrsonima lucida, Coccothrinax argentata, Dodonaea viscosa, Guettarda scabra, Metopium toxiferum, Morella cerifera (= Myrica cerifera), Myrsine cubana (= Rapanea punctata), Psidium longipes, Rhus copallinum, Sabal palmetto, Serenoa repens, Sideroxylon salicifolium, Tetrazygia bicolor*, and *Leucothrinax morrisii (= Thrinax morrisii)*. Short‑statured shrubs include *Chiococca alba, Crossopetalum ilicifolium, Morinda royoc*, and *Randia aculeata*. Grasses, forbs, and ferns make up a diverse herbaceous layer ranging from mostly continuous in areas with more soil development and little exposed rock to sparse where more extensive outcroppings of rock occur. Typical herbaceous species include *Andropogon gracilis (= Schizachyrium gracile), Andropogon* spp., *Anemia adiantifolia, Aristida purpurascens, Chamaecrista fasciculata, Chamaesyce* spp., *Croton cascarilla (= Croton linearis), Echites umbellatus, Muhlenbergia capillaris, Pteridium caudatum (= Pteridium aquilinum var. caudatum), Pteris bahamensis, Rhynchospora floridensis, Schizachyrium rhizomatum, Schizachyrium sanguineum, Sorghastrum secundum, Tragia saxicola*, and *Zamia pumila*. The range of this system is largely outside the natural range of *Pinus serotina, Pinus elliottii var. elliottii*, and *Pinus palustris*.

**Dynamics:** Historical accounts show that fire has been frequent over the past several hundred years, perhaps as often as every 1‑4 years (Wade et al. 1980, Bergh and Wisby 1996, Slocum et al. 2003). Without fire, after 15‑20 years, hardwoods will be numerous and quite large (Wade et al. 1980). In the absence of fire, this system may be replaced by hardwoods species within several decades (Stout and Marion 1993). High winds from hurricanes are an infrequent, natural disturbance. Pine rockland in the Florida Keys can be subjected to storm surge associated with hurricanes (Saha et al. 2011).

SOURCES

**References:** Alexander 1953, Bergh and Wisby 1996, Bradley and Gann 1999, Comer et al. 2003*, Davis 1943, Duever et al. 1986, Enge et al. 2002, Eyre 1980, FNAI 2010a, Gann et al. 2014, LANDFIRE 2007a, Loope et al. 1979, McPherson 1986, Noss et al. 1995, Outcalt 1997b, Saha et al. 2011, Slocum et al. 2003, Snyder et al. 1990, Stout and Marion 1993, USFWS 1998b, Wade et al. 1980

**Version:** 07 Jul 2014 **Stakeholders:** Southeast

**Concept Author:** R. Evans **LeadResp:** Southeast

CES203.536 Southern Atlantic Coastal Plain Wet Pine Savanna and Flatwoods

**Primary Division:** Gulf and Atlantic Coastal Plain (203)

**Land Cover Class:** Woody Wetland

**Spatial Scale & Pattern:** Matrix

**Required Classifiers:** Natural/Semi‑natural; Vegetated (>10% vasc.); Wetland

**National Mapping Codes:** EVT 2450; ESLF 9119; ESP 1450

**Concept Summary:** This ecological system of pine‑dominated savannas and/or flatwoods ranges from central South Carolina to northeastern Florida, centered near the coast in southeastern Georgia. It was the former matrix system in this region. This general area has been referred to as the Longleaf Pine Wiregrass Savannas region and the Sea Island Flatwoods Ecoregion (75f). Examples of this system and component community associations share the common features of wet, seasonally saturated, mineral soils and historic exposure to frequent low‑intensity fire. They occur on a wide range of soil textures, which is an important factor in distinguishing different associations. The vegetation is naturally dominated by *Pinus palustris* or, on wetter sites, *Pinus elliottii* or less commonly *Pinus serotina*. Understory conditions may be dramatically altered by fire frequency and seasonality. In natural condition (with frequent fires, including some growing‑season fire), there tends to be a dense ground cover of herbs and low shrubs; grasses can dominate, but there is often a large diversity of other herbs and shrubs.

**Comments:** This system is distinguished from Central Atlantic Coastal Plain Wet Longleaf Pine Savanna and Flatwoods (CES203.265) because of substantial biogeographic differences. The break is placed at the Santee River, which approximates the transition between the ranges of *Aristida stricta* and *Aristida beyrichiana*, which are keystone species in the communities where they occur. This corresponds roughly with the geographic break in the upland longleaf pine systems as well. This system is distinguished from Atlantic Coastal Plain Upland Longleaf Pine Woodland (CES203.281) because of that system's more upland character. However, the two systems have much in common, including frequent fire, the same primary dominant canopy tree, and many herbaceous species. They can also occur in the same landscapes. However, floristic differences are well marked, and no associations are shared.

DISTRIBUTION

**Range:** This system is restricted to the Atlantic Coastal Plain from central South Carolina to northeastern Florida. This general area has been referred to as the Longleaf Pine Wiregrass Savannas region (Platt 1999) and the Sea Island Flatwoods (EPA Ecoregion 75f) (Griffith et al. 2001, 2002).

**Divisions:** 203:C

**TNC Ecoregions:** 56:C

**Nations:** US

**Subnations:** FL, GA, SC

**Map Zones:** 55:C, 58:C

**USFS Ecomap Regions:** 232C:CC, 232J:CC

CONCEPT

**Environment:** This system occurs on wet mineral soil sites, in the middle and outer Coastal Plain. Landforms include low areas in relict beach ridge systems and eolian sand deposits, and poorly drained clayey, loamy, or sandy flats.

**Vegetation:** The best examples are typically open woodlands naturally dominated by *Pinus palustris* or *Pinus elliottii* and/or *Pinus serotina* on wetter sites. In many areas past logging and subsequent lack of frequent growing‑season fire have led to much greater dominance by *Pinus elliottii*. In natural condition, there is typically a dense ground cover of herbs and low shrubs; grasses can dominate, but there is often a large diversity of other herbs and shrubs. The shrubs are mainly *Serenoa repens, Ilex glabra*, and *Ilex coriacea* along with various ericaceous species. These shrub species become especially prominent on sites not frequently burned.

**Dynamics:** Frequent low‑intensity fire is important. Lightning has been an important source of ignition for these fires, especially historically. Disturbances are an important part of the natural functions of wet pine savanna and flatwoods. In order for these habitats to burn frequently (every 2‑3 years), there needs to be enough fine fuel, such as needles from *Pinus palustris* trees, healthy populations of native warm‑season grasses, and evergreen shrubs with volatile oils in their leaves, such as *Gaylussacia frondosa, Ilex coriacea, Ilex glabra, Lyonia* spp., *Serenoa repens*, and *Vaccinium* spp. The frequent fires promote flowering, seed production, and seed germination of many plants and provide open areas in patches (Van Lear et al. 2005).

In the past, wildland fires were started by lightning strikes and deliberately by people, including Native Americans prior to the 1700s. The wet pine savanna may have burned as frequently as every 2‑3 years. Hurricane‑force winds can knock down and break trees, including *Pinus palustris*, but in frequently burned savannas, weakened hardwood midstory trees could be especially prone to blowdown.

SOURCES

**References:** Brewer 2008, Christensen 2000, Comer et al. 2003*, Eyre 1980, FNAI 2010a, Griffith et al. 2001, Griffith et al. 2002, Jensen and Gatrell 2004, LANDFIRE 2007a, NatureServe 2006, NatureServe 2011a, Nelson 1986, Oswalt et al. 2012, Platt 1999, Rheinhardt et al. 2002, Van Lear et al. 2005, Wahlenberg 1946

**Version:** 23 Apr 2015 **Stakeholders:** Southeast

**Concept Author:** R. Evans and C. Nordman **LeadResp:** Southeast

CES203.497 Southern Atlantic Coastal Plain Xeric River Dune

**Primary Division:** Gulf and Atlantic Coastal Plain (203)

**Land Cover Class:** Barren

**Spatial Scale & Pattern:** Large patch

**Required Classifiers:** Natural/Semi‑natural; Unvegetated (<10% vasc.); Upland

**Diagnostic Classifiers:** Dune (Substrate)

**National Mapping Codes:** EVT 2388; ESLF 5319; ESP 1388

**Concept Summary:** This system encompasses a range of vegetation present on inland sand dunes of the Atlantic Coastal Plain of Georgia. These dunes are associated with certain rivers such as the Ohoopee and Canoochee and are apparently eolian in origin, formed of riverine alluvial sands. The sandy soils are deep, coarse, and xeric in nature. The vegetation consists of an assemblage of xeric communities that also occur in other xeric habitats in the Coastal Plain. These include *Pinus palustris ‑ Quercus laevis* communities and a scrub community akin to Inland Florida Scrub, but lacking *Pinus clausa*. This system is distinguished from more typical xeric sandhills of the Coastal Plain by its occurrence on the deep sands of river dunes. Xeric river dunes have a similar fire‑return interval to other upland systems of which *Pinus palustris* is a component, but the fuels are fires tend to be patchy, leaving some unburned areas.

DISTRIBUTION

**Range:** This system is endemic to river‑associated dunes in the South Atlantic Coastal Plain of Georgia, such as along the Ohoopee and Canoochee rivers (Wharton 1978), as well as other watersheds. Reports of similar or related vegetation from North and South Carolina are being investigated.

**Divisions:** 203:C

**TNC Ecoregions:** 56:C

**Nations:** US

**Subnations:** GA

**Map Zones:** 55:C

**USFS Ecomap Regions:** 232J:CC

CONCEPT

**Environment:** These dunes are apparently eolian in origin, formed of reworked riverine alluvial sands. The sandy soils are deep, coarse, and xeric in nature. These deep coarse sand dunes have formed from winds blowing exposed sand from the riverbars in the Pleistocene (Edwards et al. 2013). They occur on the east and northeast sides of rivers which flow southeast (Bozeman 1971) in south Georgia, such as the Altamaha, Ohoopee, Flint, Satilla and Canoochee (Edwards et al. 2013). The dune system is most developed along the east side of the Ohoopee River, which is 35 miles long and about 40,000 acres (Edwards et al. 2013).

**Vegetation:** Upland plant communities include longleaf pine ‑ turkey oak, dwarf oak, oak hammock, and rosemary scrub (Wharton 1978). On the Altamaha River, there is *Pinus palustris, Pinus elliottii, Quercus laevis, Aristida beyrichiana, Quercus hemisphaerica*, and *Quercus virginiana* (these last two may be from examples from which fire has been excluded) (J. Thompson, GDNR, pers. comm.)

**Dynamics:** About half of the woody species are evergreen, but there is greater cover of deciduous shrubs, and there are more shrubs than herbs (Harper 1906). This contrasts with other *Pinus palustris* habitats, which tend to be grass‑dominated with a high diversity of herbs. Both *Ceratiola ericoides* and *Chrysoma pauciflosculosa* are evergreen shrubs and are fire‑sensitive. *Chrysoma pauciflosculosa* seeds are able to spread to newly available open sandy and unburned habitat in local areas where they occur, but *Ceratiola ericoides* seeds are heavy, landing mostly near the mother plant, are inhibited by allelopathy, and generally start germinating after death of mother shrub, in same vicinity (M. Hodges pers. comm.). These shrubs may persist with the sparse *Pinus palustris* due to their metapopulation dynamics; certain subpopulations may be lost to occasional wildland fires, but new subpopulations also form where seeds germinate. While *Ceratiola ericoides* will generally not persist if burned more frequently than every 20 years (Johnson 1982), in some Florida habitats, population models of *Ceratiola ericoides* on Georgia xeric river dune sandhills suggest that burns at least as frequently as every 10 years may be important for maintaining open habitat and promoting recruitment of new shrubs (Schmidt 2006). These river dune habitats are naturally topographically isolated by a river on one (west or southwest) side and typically are adjacent to pine flatwoods on the other (east or northeast) side. Consequently, they have been partially protected from large wildland fires and may have a similar or lower fire‑return interval than typical dry *Pinus palustris* habitats. The accumulation of fuel in these xeric river dune habitats is slow and does not support the frequent continuous fires that can occur in *Pinus palustris* habitats which have higher nutrient availability. The natural fire‑return interval may have varied from 5 to 10 years depending on the fertility of the site and accumulation of fuels (Edwards et al. 2013). Where xeric river dunes are connected to pine flatwoods, fires would have been more frequent, and fires were patchy, leaving many unburned patches (M. Hodges pers. comm.). Fuels include *Pinus palustris* needles and dead leaves of *Aristida purpurascens, Aristida beyrichiana, Quercus laevis, Triplasis americana*, and other plants. Small areas along the Altamaha River sand ridge have broadleaf evergreen tall‑shrub and small‑tree vegetation, called the Georgia River Dune Myrtle Oak Scrub NVC Association (Bozeman 1971). These areas apparently burn only rarely (perhaps at high intensity), if at all, and are somewhat similar to oak scrub found in Florida.

SOURCES

**References:** Ambrose pers. comm., Bozeman 1971, Comer et al. 2003*, Covell et al. 1984, Edwards et al. 2013, Eyre 1980, Harper 1906, Johnson 1982, NatureServe 2011a, Oswalt et al. 2012, Schafale pers. comm., Schmidt 2006, Thompson pers. comm., Wahlenberg 1946, Wharton 1978

**Version:** 21 May 2014 **Stakeholders:** Southeast

**Concept Author:** R. Evans **LeadResp:** Southeast

CES203.891 West Gulf Coastal Plain Stream Terrace Sandyland Longleaf Pine Woodland

**Primary Division:** Gulf and Atlantic Coastal Plain (203)

**Land Cover Class:** Forest and Woodland

**Spatial Scale & Pattern:** Large patch

**Required Classifiers:** Natural/Semi‑natural; Vegetated (>10% vasc.); Upland

**Diagnostic Classifiers:** Forest and Woodland (Treed); Very Short Disturbance Interval; Needle‑Leaved Tree; West Gulf Coastal Plain

**National Mapping Codes:** EVT 2521; ESLF 4283; ESP 1521

**Concept Summary:** These sandhills are dry longleaf pine‑dominated woodlands or savannas found on excessively drained, xeric soils of alluvial origin in the West Gulf Coastal Plain (South Central Plains of EPA) of Texas and formerly Louisiana. They occur on areas of deep sand (ranging in texture from coarse to fine) which are present in quaternary alluvial deposits. The general habitat is on low terraces adjacent to stream floodplains, and adjacent communities may include baygalls and ponds. Precipitation rapidly dissipates via percolation due to the character of the soil. Soils include fine sands, such as fluvial terraces of Bienville‑Alaga soils developed in the Deweyville Formation, and the Tonkawa fine sand, as well as other coarse sands. *Pinus palustris* historically dominated the vegetation of this region across nearly all uplands regardless of soil type or moisture. The importance of frequent fire has been well‑documented for the perpetuation of this and related systems throughout the coastal plains. Stands are dominated by *Pinus palustris*, which often occurs in mixed stands with *Quercus incana, Pinus echinata*, and *Carya texana*. Some small isolated terraces (inclusions) may be dominated by oaks and hickories, with little or no *Pinus palustris*. The oaks generally become denser with fire exclusion, particularly on the small isolated areas. Mesophytic oak species are absent or extremely rare. This type, and other longleaf communities and systems of the West Gulf Coastal Plain, lie outside the range of *Aristida beyrichiana* (wiregrass). Other grasses (*Andropogon* spp., other *Aristida* spp., and *Schizachyrium* spp.) dominate understories which are rich in species diversity.

**Comments:** This system is relatively xeric vegetation, even though it occurs on terraces adjacent to, or within, floodplains. This is the case because the soils are deep and well‑drained sands (often alluvial deposits), with low moisture retention and high permeability. This system is floristically similar to other sandhill longleaf pine systems, but the landform position of the occurrences makes this system unique. This system was formerly part of West Gulf Coastal Plain Upland Longleaf Pine Forest and Woodland (CES203.293) but has been separated out due to its distinctive environment. The herbaceous cover of subtypes 2 and 3 of Bridges and Orzell (1989a) is usually sparse, with considerable exposed sand and foliose lichen cover, and is characterized by numerous West Gulf Coastal Plain endemics (Bridges and Orzell 1989a). Two taxa (*Phlox nivalis ssp. texensis* and *Gaillardia aestivalis var. winkleri*) are nearly endemic to subtype 3 and occur primarily along Village Creek in Hardin County, Texas. *Carex tenax* and *Galium hispidulum* are nearly restricted to subtype 3 and are long‑distance disjuncts from the East Gulf Coastal Plain (Bridges and Orzell 1989a).

DISTRIBUTION

**Range:** This upland ecological system occurs mainly in the Southern Loam Hills Subsection (232Fa) of Texas and formerly Louisiana, apparently ranging south into the Southwest Flatwoods Subsection (232Fb) (Hardin County, Texas). West Gulf Coastal Plain longleaf sandhills are distinctive from those in the East Gulf Coastal Plain because they occur beyond the limits of where wiregrass and sand post oak are dominant.

**Divisions:** 203:C

**TNC Ecoregions:** 41:C

**Nations:** US

**Subnations:** LA?, TX

**Map Zones:** 37:C

**USFS Ecomap Regions:** 232F:CC

CONCEPT

**Environment:** This system is relatively xeric vegetation, even though it occurs on terraces adjacent to, or within, floodplains. This is because the soils are deep and well‑drained sands (often alluvial deposits), with low moisture retention and high permeability. This system usually occurs in deep, well‑drained sandy soils on stream terraces, occurring above medium‑sized perennial creeks that are typically clear and have sandy bottoms. These sites have very fine sands on ridgetops or slightly higher rises in the sandhill terraces. The flat areas with broad sandhills are slightly coarser and hold a little more water. A site for this system can have both fine and coarse sands. The landscape profile starts out with some bottomland hardwoods type with braided bald‑cypress ‑ tupelo, then a slight slope with a wide baygall edge against the sandhill. Sometimes there is a small ribbon of American beech slope forest just above the baygall, then going into the upland sandhill; sometimes it is just a baygall to sandhill transition (J. Singhurst pers. comm.). It represents a distinctive subset of longleaf pine‑dominated vegetation in the inner (landward) portions of the West Gulf Coastal Plain in eastern Texas (and Louisiana).

**Vegetation:** Stands are dominated by *Pinus palustris*, which may form a discontinuous and sparse overstory, and often occurs in mixed stands with *Quercus incana, Quercus margarettae, Quercus marilandica, Pinus echinata*, and *Carya texana*. Some small isolated terraces (inclusions) may be dominated by oaks and hickories, with little or no *Pinus palustris*. The oaks generally become denser with fire exclusion, particularly on the small isolated areas. *Pinus elliottii* and *Pinus taeda* may be present to common in the current landscape. Other mesophytic oak species are absent or extremely rare. This type, and other longleaf communities and systems of the West Gulf Coastal Plain, lies outside the range of *Aristida beyrichiana*. Other grasses (*Andropogon* spp., other non‑wiregrass *Aristida* spp., and *Schizachyrium* spp.) dominate understories rich in species diversity. Some additional small trees may include *Quercus marilandica, Quercus hemisphaerica*, and *Liquidambar styraciflua*. A sparse understory shrub component includes *Vaccinium arboreum* and *Ilex vomitoria*; however, fire suppression allows the shrubs to become more dominant in the midstory. Some other common shrubs may include *Asimina parviflora, Callicarpa americana, Crataegus* spp., *Prunus angustifolia, Prunus gracilis, Rhus aromatica, Ptelea trifoliata var. mollis, Sassafras albidum*, and *Sideroxylon lanuginosum*. Ground cover plant species are tolerant of periodic drought during the growing season, and they also exhibit adaptations to a frequent fire regime. Some characteristic herbs (which vary across environments) include *Schizachyrium scoparium, Aristida desmantha, Berlandiera pumila, Bulbostylis ciliatifolia, Cnidoscolus texanus, Croton argyranthemus, Dichanthelium acuminatum, Eriogonum longifolium, Lespedeza hirta, Liatris elegans var. elegans, Liatris pycnostachya, Opuntia humifusa (= var. humifusa), Paronychia drummondii, Rudbeckia grandiflora var. alismifolia, Ruellia humilis, Silphium laciniatum, Stillingia sylvatica, Stylisma pickeringii var. pattersonii, Tradescantia reverchonii, Tragia urens*, and *Yucca louisianensis*. Some other taxa that may be present include *Ambrosia trifida, Baptisia* sp., *Bulbostylis capillaris, Bulbostylis ciliatifolia, Carex tenax, Cenchrus* sp., *Chamaecrista* sp., *Commelina erecta, Croptilon divaricatum, Cyperus grayoides, Dalea* sp., *Delphinium carolinianum, Echinacea* sp., *Eragrostis secundiflora, Eriogonum multiflorum, Euphorbia corollata, Gaillardia aestivalis var. winkleri, Heterotheca subaxillaris, Hypericum drummondii, Hypericum hypericoides, Hymenopappus artemisiifolius , Lechea tenuifolia, Lechea mucronata, Nuttallanthus canadensis (= Linaria canadensis), Loeflingia squarrosa, Matelea cynanchoides, Mirabilis albida, Monarda punctata, Oenothera heterophylla, Paronychia drummondii, Polanisia erosa ssp. erosa, Polypremum procumbens, Pteridium aquilinum, Rhynchosia* sp., *Scutellaria* sp., *Solidago* spp., *Streptanthus hyacinthoides, Tephrosia* sp., *Tetragonotheca ludoviciana, Thelesperma filifolium, Toxicodendron radicans, Trichostema dichotomum*, and *Vernonia* sp. (J. Singhurst pers. comm.).

In Bridges and Orzell (1989a; table 3 ‑ herbs), the following herbs are "differentials" (present in subtype 3 [or at much greater abundance] than in subtype 2): *Aristida desmantha, Carex tenax, Eriogonum longifolium* [which is in subtypes 1 and 3, but not 2], *Eriogonum multiflorum*, and *Polanisia erosa*. Many "indicators" are present at roughly the same frequency in subtypes 2 and 3.

**Dynamics:** The importance of frequent fire has been widely‑accepted for the perpetuation of *Pinus palustris* systems (Stambaugh et al. 2011a), but fires may actually be less frequent, more patchy and discontinuous than in other related longleaf pine‑dominated systems. The oaks generally become denser with fire exclusion, particularly in small, isolated examples. Lichens (e.g., *Cladonia* spp.) and *Selaginella arenicola ssp. riddellii* also occur along with patches of bare sand. Canopy trees are patchy in distribution, with regeneration in canopy gaps of a quarter acre or less in size, mid‑successional clumps in similar sized patches, and the oldest trees occurring as isolated individuals. The reference condition classes are aggregates of numerous patches well‑dispersed over the landscape. Canopy gaps are created by fire mortality, pest outbreaks, lightning, and windthrow at the scale of individual trees or several trees. Because of the irregular seed production of longleaf pine, canopy gaps may lack regeneration for several years. Uncharacteristic vegetation types include even‑aged canopy stands in which age structure has been homogenized by logging or clearing. Examples include where *Pinus taeda* or *Pinus elliottii* have replaced some or all of the longleaf pine, where midstory oaks and/or low shrubs have become dense due to fire suppression, and where the grass‑dominated ground cover has been lost due to soil disturbance or canopy closure.

SOURCES

**References:** Ajilvsgi 1979, Bray 1906, Bridges and Orzell 1989a, Christensen 1981, Collier 1964, Comer et al. 2003*, Cruikshank and Eldredge 1939, Eyre 1980, Foster et al. 1917, Frost 1993, Glitzenstein et al. 1986, Harcombe et al. 1993, Marks and Harcombe 1981, McWilliams and Lord 1988, Outcalt 1997a, Singhurst 1996, Singhurst pers. comm., Snead and McCulloh 1984, Stambaugh et al. 2011a, TNC 2001, TNC 2003b, Turner 1979, Turner et al. 1999, Van Lear et al. 2005, Williams 1989

**Version:** 14 Jan 2014	**Stakeholders:** Southeast

**Concept Author:** M. Pyne, after I. McWhorter, W. Ledbetter et al.	**LeadResp:** Southeast

CES203.293 West Gulf Coastal Plain Upland Longleaf Pine Forest and Woodland

**Primary Division:** Gulf and Atlantic Coastal Plain (203)

**Land Cover Class:** Forest and Woodland

**Spatial Scale & Pattern:** Matrix

**Required Classifiers:** Natural/Semi‑natural; Vegetated (>10% vasc.); Upland

**Diagnostic Classifiers:** Forest and Woodland (Treed); Very Short Disturbance Interval; Needle‑Leaved Tree; West Gulf Coastal Plain

**National Mapping Codes:** EVT 2348; ESLF 4251; ESP 1348

**Concept Summary:** The common and unifying feature of this system is vegetation naturally dominated by *Pinus palustris*. This was formerly the most extensive system within its natural range in western Louisiana and eastern Texas. In most of the region, longleaf pine is (presently) a distinctive, but rarely dominant, element of existing vegetation. However, this tree historically dominated the vegetation across nearly all uplands regardless of soil type or moisture (excluding wetlands), and longleaf pine forests were among the most valuable economic resources in the region at the turn of the century. Typical sites include sandhills on well‑drained to excessively drained soils, but the type is also found on loamy and clayey upland soils. The importance of frequent fire has been well documented for the perpetuation of this system. This type lies outside the ranges of *Aristida stricta* and *Aristida beyrichiana*, unlike comparable systems east of the Mississippi River, but most stands at least formerly supported open grass‑dominated understories rich in species diversity.

**Comments:** This system was part of what was once considered "the lumber region par excellence of Texas" (Bray 1906). Intensive logging began around 1880 as forests in the northern states were cut out and railroads and logging technologies were moved into the region (Collier 1964, Williams 1989). By 1917, the majority of Texas longleaf had been cut (Foster et al. 1917), and by 1934‑35, loblolly had become the single most prevalent species in 17 southeastern Texas counties (Cruikshank and Eldredge 1939). Overall losses of longleaf pine in Texas have exceeded those of all other southern states (Outcalt 1997); less than 16,200 hectares of mostly second‑growth stands remain (McWilliams and Lord 1988). Land‑use practices continue to degrade remaining examples of longleaf pine communities (Bridges and Orzell 1989a).

DISTRIBUTION

**Range:** The natural range of this system is in the coastal plains of western Louisiana and eastern Texas. Its boundary follows TNC Ecoregion 41 (West Gulf Coastal Plain) closely in western Louisiana, but extends slightly into Ecoregion 40 (Upper West Gulf Coastal Plain) in eastern Texas.

**Divisions:** 203:C

**TNC Ecoregions:** 40:C, 41:C

**Nations:** US

**Subnations:** LA, TX

**Map Zones:** 37:C

**USFS Ecomap Regions:** 231Ef:CCC, 231Eg:CCC, 232Fa:CCC, 232Fb:CCC, 232Fe:CCC, 232Ff:CCC

CONCEPT

**Environment:** This system represents the presumed matrix vegetation type of the inner (landward) portions of the West Gulf Coastal Plain in Louisiana and eastern Texas within the range of *Pinus palustris*. In Louisiana, these are mapped as the Upper Terrace and some smaller landward units (Snead and McCulloh 1984). The system is bounded on the outer (seaward) side by West Gulf Coastal Plain Wet Longleaf Pine Savanna and Flatwoods (CES203.191) and on the inner (landward) side primarily by West Gulf Coastal Plain Pine‑Hardwood Forest (CES203.378) and other hardwood or hardwood‑pine systems. Stands are found on sedimentary Pleistocene formations (particularly the Bentley Formation), to formations of the Tertiary period (particularly the Catahoula and Wilcox formations). Historically, this system was more widely distributed on older, more inland formations of the Eocene and Paleocene epochs. They occupy topography ranging from rolling uplands, to hills and ridges such as those associated with the Kisatchie Wold (or Kisatchie Cuesta) and the Sabine Uplift, and are usually associated with coarse‑textured, well‑drained Ultisols and Alfisols, including loams, sandy loams, loamy sands, and sands, though occurrences may also be found to a lesser extent on tighter soils such as clay loams (Elliott 2011). It is characteristically dissected by small to large streams.

**Vegetation:** Examples are characterized by relatively open‑canopied woodlands dominated by *Pinus palustris* with an herbaceous layer often dominated by graminoids. It often occupied gently rolling uplands with coarse‑textured, well‑drained soils. *Pinus echinata* may be a significant component of some of the stands. *Quercus stellata, Quercus marilandica, Quercus incana, Pinus taeda, Liquidambar styraciflua*, and *Nyssa sylvatica* may also be common components of the canopy or subcanopy. Occurrences that are less frequently burned may develop a significant shrub layer with species including *Callicarpa americana, Vaccinium arboreum, Vaccinium stamineum, Morella cerifera, Ilex vomitoria, Rhus copallinum*, and *Toxicodendron radicans*. Instances with a more optimal fire‑return interval will retain a more open understory with a grassy aspect. Unlike comparable systems east of the Mississippi River, this type lies outside the ranges of *Aristida stricta* and *Aristida beyrichiana*, but most stands historically supported open grass‑dominated understories rich in species diversity. The herbaceous layer is often dominated by grass species such as *Schizachyrium scoparium, Schizachyrium tenerum, Sporobolus junceus, Panicum virgatum, Nassella leucotricha, Andropogon ternarius, Dichanthelium* spp., and *Andropogon virginicus*. *Pteridium aquilinum* may be locally abundant, forming a continuous ground cover. Forbs may be diverse in the herbaceous layer, including species such as *Pityopsis graminifolia, Solidago odora, Tephrosia* spp., *Tragia urens, Euphorbia corollata, Croton argyranthemus, Vernonia texana, Alophia drummondii, Lespedeza virginica, Aristolochia reticulata, Rhynchosia reniformis, Stylosanthes biflora, Opuntia humifusa (= var. humifusa), Cnidoscolus texanus, Stylisma pickeringii var. pattersonii, Rudbeckia grandiflora var. alismifolia, Silphium laciniatum, Ruellia humilis, Liatris pycnostachya*, and *Liatris elegans*. With prolonged absence of fire, hardwoods and *Pinus taeda* may come to dominate the system (Elliott 2011).

In most of the region, *Pinus palustris* is (presently) a distinctive, but rarely dominant, element of existing vegetation (Harcombe et al. 1993). However, this tree historically dominated the vegetation across nearly all uplands regardless of soil type or moisture (excluding wetlands), and longleaf pine forests were among the most valuable economic resources in the region at the turn of the century (Bray 1906).

**Dynamics:** Frequent fire was the predominant natural disturbance in this system, which is now dependent on management with prescribed fire. The importance of frequent surface fire (every 1‑5 years) has been widely accepted for the perpetuation of this system (Stambaugh et al. 2011a and others). Fires are usually low in intensity overall, consuming only shrubs and herbs, but will occasionally kill patches of young pine regeneration and rarely kill individual older trees. Historically, individual fires covered extensive areas. This high fire frequency is dependent on the presence of fine fuels in the form of grasses and pine leaf litter. This ecological system is also affected by hurricane and tornado occurrences every 200 +/‑ years. In mature stands, competition between pine and hardwood trees is also a factor in maintaining species composition.

SOURCES

**References:** Ajilvsgi 1979, Bray 1906, Bridges and Orzell 1989a, Collier 1964, Comer et al. 2003*, Cruikshank and Eldredge 1939, Elliott 2011, Eyre 1980, Foster et al. 1917, Harcombe et al. 1993, LDWF 2005, Marks and Harcombe 1981, McWilliams and Lord 1988, Outcalt 1997a, Smith 1993, Snead and McCulloh 1984, Stambaugh et al. 2011a, Turner et al. 1999, Van Lear et al. 2005, Williams 1989

**Version:** 14 Jan 2014	**Stakeholders:** Southeast

**Concept Author:** R. Evans	**LeadResp:** Southeast

CES203.191 West Gulf Coastal Plain Wet Longleaf Pine Savanna and Flatwoods

**Primary Division:** Gulf and Atlantic Coastal Plain (203)

**Land Cover Class:** Woody Wetland

**Spatial Scale & Pattern:** Matrix

**Required Classifiers:** Natural/Semi‑natural; Vegetated (>10% vasc.); Wetland

**Diagnostic Classifiers:** Forest and Woodland (Treed); Extensive Wet Flat; Very Short Disturbance Interval; Needle‑Leaved Tree; West Gulf Coastal Plain

**National Mapping Codes:** EVT 2451; ESLF 9120; ESP 1451

**Concept Summary:** This system was the historical matrix vegetation of the outer (seaward) portions of the West Gulf Coastal Plain between the coastal prairies and the inner coastal plain in Louisiana and eastern Texas within the range of longleaf pine. These areas are characterized by poorly drained upland soils with high and highly fluctuating water tables. In natural condition, monospecific stands of *Pinus palustris* and species‑rich herbaceous layers characterize this system. Other species in the canopy include *Quercus stellata, Quercus marilandica, Nyssa sylvatica, Quercus laurifolia, Quercus falcata*, and *Liquidambar styraciflua*. Shrubs are typically limited in distribution within the system to local topographic highs and include species such as *Morella cerifera, Ilex vomitoria, Symplocos tinctoria, Cyrilla racemiflora*, and others. Widespread alterations following European settlement, including changes to natural fire regimes, have produced drastic changes to this system, and few large examples are extant. Examples appear to be somewhat more common in western Louisiana than in eastern Texas.

**Comments:** In Louisiana, two Natural Heritage communities (variants) of this system are recognized (Smith 1996b). These two variants are the longleaf pine flatwoods (which are mesic to dry‑mesic [non‑wetland] stands) and the true pine savannas which occupy poorly drained and seasonally saturated/flooded depressional areas and low flats. These two types form an interdigitated mosaic (Smith 1996b), which constitutes this system as here described and defined.

DISTRIBUTION

**Range:** This system is endemic to western Louisiana and eastern Texas, and examples appear to be somewhat more common in western Louisiana.

**Divisions:** 203:C

**TNC Ecoregions:** 31:C, 41:C

**Nations:** US

**Subnations:** LA, TX

**Map Zones:** 37:C, 98:C

**USFS Ecomap Regions:** 232Ea:CCC, 232Fa:CCC, 232Fb:CCC, 232Fe:CCC, 232Ff:CCC

CONCEPT

**Environment:** This system represents the presumed matrix vegetation on relatively recent (Pleistocene) geologic formations within the range of longleaf pine in the outer (seaward) portions of the West Gulf Coastal Plain between the coastal prairies and the inner coastal plain in Louisiana and eastern Texas. In Louisiana, these are mapped as the Intermediate Terrace and the upper Prairie Terrace (Snead and McCulloh 1984), and in Texas as the Lissie Formation and the upper Beaumont Formation (Sellards et al. 1932). The Intermediate Terrace of Snead and McCulloh (1984) includes terraces formerly designated as the Montgomery, Irene, and most of the Bentley. These areas are characterized by poorly drained upland soils with high water tables (Bridges and Orzell 1989a). Landforms include mesic to seasonally saturated low areas and flats, on level to gently rolling uplands. Microtopographic variation is provided by the presence of swales and pimple mounds. Soils are sandy to silty loams that are strongly acidic, nutrient poor, and low in organic constituents. Typically these soils are hydric, with seasonal fluctuations between saturation and droughtiness (Elliott 2011). Within the range of longleaf pine, this system is bounded on the landward side by West Gulf Coastal Plain Upland Longleaf Pine Forest and Woodland (CES203.293).

**Vegetation:** This system may be characterized as having a sparse canopy (under natural fire cycles) dominated by *Pinus palustris*. Other species in the canopy include *Quercus stellata, Quercus marilandica, Nyssa sylvatica, Quercus laurifolia, Quercus falcata*, and *Liquidambar styraciflua*. Shrubs are typically limited in distribution within the system to local topographic highs and include species such as *Morella cerifera, Ilex vomitoria, Symplocos tinctoria, Cyrilla racemiflora*, and others. The herbaceous layer may be highly diverse. Drier sites may be dominated by *Schizachyrium scoparium, Schizachyrium tenerum, Eupatorium rotundifolium*, and others. Wetter sites may not have species showing a clear dominance. Species such as *Liatris* spp., *Xyris* spp., *Rhexia* spp., *Rhynchospora* spp., *Fuirena* spp., *Marshallia graminifolia, Aletris aurea*, and many other species may share dominance in this system. Suppression of fire has lead to increased woody dominance. *Pinus taeda, Pinus elliottii, Liquidambar styraciflua, Nyssa sylvatica*, and *Acer rubrum* may now dominate the canopy of these sites, with a thick understory dominated by *Ilex vomitoria* and *Morella cerifera* (Elliott 2011).

**Dynamics:** Frequent fires (every 1‑4 years), seasonal wetness and low nutrient availability of this ecological system inhibit the establishment of woody understory species and maintain a sparse canopy of longleaf pine (Stambaugh et al. 2011a and others). This frequent fire regime is necessary to maintain the open savanna condition and provides bare ground for *Pinus palustris* regeneration. Current examples must be managed with prescribed fire. Fires are usually low in intensity overall, consuming only shrubs and herbs, but will occasionally kill patches of young pine regeneration and rarely kill individual older trees. Historically, individual fires covered extensive areas. This high fire frequency is dependent on the presence of fine fuels in the form of grasses and other graminoids. Prescribed fire has been used as an attempt to reverse the effects of decades of fire suppression. However, the results of these attempts have been mixed. Uncertainty remains over the frequency of burning necessary to restore fire‑dependent ecosystems; however, a return frequency of every 2‑5 years appears best. Application of burns is often too infrequent, allowing woody understory species to crowd out longleaf or, in hardwood forests, oaks, beeches and other dominant trees. Similarly, burns are ineffective if applied at the wrong life stage of plants or at the wrong point in the growing season. An example: late‑spring to early‑summer burns favor longleaf and associated herbaceous plants, whereas late‑season or winter burns favor woody shrubs. However, prescribed burns, properly applied, are a crucial restoration and management tool in the pyrogenic longleaf pine ecosystems. Canopy gaps are created by fire mortality, lightning, and windthrow from hurricanes and tornados.

SOURCES

**References:** Ajilvsgi 1979, Bridges and Orzell 1989a, Comer et al. 2003*, Elliott 2011, Eyre 1980, LDWF 2005, Marks and Harcombe 1981, McWilliams and Lord 1988, Sellards et al. 1932, Smith 1993, Smith 1996b, Snead and McCulloh 1984, Stambaugh et al. 2011a, Van Lear et al. 2005

**Version:** 02 Oct 2014	**Stakeholders:** Southeast

**Concept Author:** R. Evans	**LeadResp:** Southeast

M885. Southeastern Coastal Plain Evergreen Oak ‑ Mixed Hardwood Forest

CES203.464 Central and South Texas Coastal Fringe Forest and Woodland

**Primary Division:** Gulf and Atlantic Coastal Plain (203)

**Land Cover Class:** Forest and Woodland

**Spatial Scale & Pattern:** Large patch

**Required Classifiers:** Natural/Semi‑natural; Vegetated (>10% vasc.); Upland

**Diagnostic Classifiers:** Forest and Woodland (Treed)

**National Mapping Codes:** EVT 2338; ESLF 4144; ESP 1338

**Concept Summary:** This ecological system includes oak‑dominated forests woodlands, shrublands and savannas occurring on deep sands of the Pleistocene‑aged Ingleside barrier‑strandplain of the central Texas coast and the Holocene‑aged eolian sand deposits of the South Texas Sand Sheet. Topography varies from larger dunes to smaller ridges and swales. Vegetation of this physiognomically variable and dynamic system primarily includes patches (mottes) of forests, woodlands and shrublands dominated by *Quercus fusiformis*. Associated species vary in a north/south manner across the range of this system. Some examples contain dense shrublands dominated (almost to the exclusion of other species) by running clones of *Quercus fusiformis*. Other canopy species in the vicinity of Aransas National Wildlife Refuge, at the northern end of the range, include *Quercus marilandica, Quercus hemisphaerica, Persea borbonia*, and *Celtis laevigata*. In this area, understory species include *Ilex vomitoria, Smilax bona‑nox, Vitis mustangensis*, and/or *Morella cerifera*. Other canopy species on the South Texas Sand Sheet, at the southern end of the range, include *Prosopis glandulosa var. glandulosa, Zanthoxylum hirsutum, Condalia hookeri, Lantana urticoides, Ziziphus obtusifolia var. obtusifolia*, and a very few other species. Many of the species found in the northern parts of the range of this system are absent in the southern occurrences. *Quercus fusiformis ‑ Prosopis glandulosa var. glandulosa / Malvaviscus arboreus var. drummondii* Forest (CEGL007785) can be referred to the southern expression, while *Quercus fusiformis ‑ Persea borbonia* Forest (CEGL002117) represents the northern expression. A characteristic component of the sparse ground cover within the mottes and forests across the entire range is *Malvaviscus arboreus var. drummondii*. Canopy openings are similar in composition to surrounding grasslands. In addition to *Schizachyrium littorale*, other herbaceous species common in canopy openings across the range of this system include *Paspalum plicatulum, Paspalum monostachyum, Andropogon gerardii, Sorghastrum nutans, Muhlenbergia capillaris, Helianthemum georgianum, Croton argyranthemus*, and *Froelichia floridana*. Minor changes in drainage can cause major differences in species composition. On the Ingleside barrier‑strandplain, while *Paspalum monostachyum* may dominate slightly lower areas, deeper swales are typically dominated by *Panicum virgatum, Spartina patens, Fimbristylis* spp., *Hydrocotyle bonariensis, Rhynchospora* spp., *Fuirena* spp., *Eleocharis* spp., and *Cyperus* spp.

**Comments:** More data are needed to better define the boundary and distinction between this system and the surrounding grassland systems. The wooded component of this landscape is considered separately here due to its apparent long‑term stability (>100 years) on the landscape, but some of the factors controlling its occurrence are not known. Live oak taxonomy follows that suggested by Nixon and Muller (1997), where all live oaks of coastal Texas southwest of the Brazos are considered *Quercus fusiformis*, likely introgressed with *Quercus virginiana* and/or the Mexican species *Quercus oleoides*. Though *Quercus fusiformis* is the dominant species across the range of this system, associated species vary in a north/south manner. There are probably more associations to be developed for this system.

DISTRIBUTION

**Range:** This system is endemic to Texas. It is found within 10 km of the coast on deep sands of ancient Pleistocene strandplains (the Ingleside barrier‑strandplain) at its northern extent and within a much greater distance from the coast (100 km) on the Holocene‑aged eolian sand deposits of the South Texas Sand Sheet (primarily Kenedy and Brooks counties but extending into adjacent Jim Hogg, Hidalgo, and Willacy counties) at its southern extent.

**Divisions:** 203:C, 301:C

**TNC Ecoregions:** 30:C, 31:C

**Nations:** US

**Subnations:** TX

**Map Zones:** 36:C

**USFS Ecomap Regions:** 255D:CC

CONCEPT

**Environment:** This system occurs on deep sands of the Pleistocene‑aged Ingleside barrier‑strandplain and the Holocene‑ and Pleistocene‑aged eolian sand deposits of the South Texas Sand Sheet. Ridge and swale topography characterizes these sites, with some large (up to 15 m tall) vegetated dunes present. Topography varies from larger dunes to smaller ridges and swales.

**Vegetation:** Vegetation of this physiognomically variable and dynamic system is dominated by *Quercus fusiformis*. Stands primarily consist of patches (mottes) of forest, woodland and shrubland in a matrix of more open savannas and grasslands. Closed canopy mottes typically occur within a grassland matrix but may become more extensive forests. In the northern range of this system, other canopy components may include *Quercus marilandica, Quercus hemisphaerica, Persea borbonia*, and *Celtis laevigata*. In this area, understory species include *Callicarpa americana, Ilex vomitoria, Smilax bona‑nox, Vitis mustangensis*, and *Morella cerifera*. Characteristic components of the sparse ground cover within the mottes and forests include *Malvaviscus arboreus var. drummondii, Scleria triglomerata*, and *Erythrina herbacea*. A shrubland component of this system is also present in some areas and is sometimes extensive, consisting of a rhizomatous expression of sprouting live oaks referred to locally as "running live oak." This shrubland often appears to be a monoculture of shrubby *Quercus fusiformis* (1.5‑6 m tall), but other species of the oak motte are also found here, including larger *Quercus fusiformis* trees, *Quercus hemisphaerica, Persea borbonia, Morella cerifera* (usually in swales), *Toxicodendron pubescens, Callicarpa americana, Vitis mustangensis, Ilex vomitoria, Erythrina herbacea*, and scattered *Quercus marilandica*. Small openings with *Sorghastrum nutans* hint at what is thought to have been the historical condition of these areas. These "running‑live oak" thickets are thought to be a modified community that is the result of years of fire suppression and severe grazing pressures. Once this shrubland is established, it is difficult to restore the grassland community to these areas. Canopy openings are similar in composition to surrounding grasslands. In addition to *Schizachyrium littorale* and *Paspalum monostachyum*, common components include *Heteropogon contortus, Paspalum plicatulum, Trichoneura elegans, Andropogon gerardii, Sorghastrum nutans, Bothriochloa saccharoides, Muhlenbergia capillaris, Dichanthelium* spp., *Elionurus tripsacoides, Eriogonum multiflorum, Stylosanthes viscosa, Helianthemum georgianum, Croton glandulosus, Paspalum setaceum, Tradescantia humilis, Physalis cinerascens var. spathulifolia, Palafoxia hookeriana, Scleria triglomerata, Thelesperma nuecense, Lechea mucronata, Liatris elegans var. carizzana*, and *Froelichia floridana*. The oak mottes may have expanded at the expense of the oak savanna phase and become more dense in the absence of fire. A maritime component occurs on stabilized dunes composed of deep sand that stretch along San Antonio Bay. This component is characterized by a relatively tall forest (8‑12 m) dominated by *Quercus fusiformis*. Other trees that reach the canopy include *Persea borbonia* and *Quercus hemisphaerica*. The midcanopy is dominated by *Persea borbonia* and *Quercus hemisphaerica* with *Celtis laevigata* and *Quercus marilandica* occurring as occasional associates. The understory includes *Ilex vomitoria* and *Callicarpa americana*. A characteristic member of the sparse ground layer is *Scleria triglomerata*. [continued in Other Comments]

**Dynamics:** Fire, climate, and edaphic factors all likely played a role historically in maintaining a more open structure in this vegetation. Historically, fire likely limited the development of woody cover. Likewise, edaphic conditions limited this system to deep sandy soils. Loss of these natural processes often results in a shift toward a more closed canopy and decrease in native grass cover. Threats to this system include fire suppression, coastal development, invasive exotics, and damage by vehicles.

SOURCES

**References:** Comer et al. 2003*, Elliott 2011, Eyre 1980, Nixon and Muller 1997

**Version:** 14 Feb 2011	**Stakeholders:** Southeast

**Concept Author:** J. Teague, L. Elliott, M. Pyne	**LeadResp:** Southeast

CES203.261 Central Atlantic Coastal Plain Maritime Forest

**Primary Division:** Gulf and Atlantic Coastal Plain (203)

**Land Cover Class:** Forest and Woodland

**Spatial Scale & Pattern:** Large patch

**Required Classifiers:** Natural/Semi‑natural; Vegetated (>10% vasc.); Upland; Wetland

**Diagnostic Classifiers:** Forest and Woodland (Treed); Coast

**National Mapping Codes:** EVT 2361; ESLF 4264; ESP 1361

**Concept Summary:** This system encompasses most woody vegetation of Atlantic Coast barrier islands and similar coastal strands, from Virginia Beach to central South Carolina (south approximately to the Cooper River where the true Sea Islands begin). It includes forests and shrublands whose structure and composition are influenced by salt spray, extreme disturbance events, and the distinctive climate of the immediate coast. Many examples of this system will include a component of *Quercus virginiana* or *Morella cerifera*. Also included are embedded freshwater depressional wetlands dominated by shrubs or small trees, such as *Cornus foemina, Persea palustris*, or *Salix caroliniana*. This system may experience less effects from fire than the equivalent Southern Atlantic Coastal Plain Maritime Forest (CES203.537).

**Comments:** Southern Atlantic Coastal Plain Maritime Forest (CES203.537) occurs south of this system where barrier islands give way to sea islands (central South Carolina, approximately the Cooper River). Sea islands are wider and more extensive and their size may contribute to a greater ecological influence of fire resulting in a greater component of *Pinus elliottii* and *Pinus palustris* in maritime forests occurring there.

Northern Atlantic Coastal Plain Maritime Forest (CES203.302) occurs north of this system where deciduous trees come to prevail in the maritime forests [see Bellis (1992)] at approximately 37°N latitude. There is a zone where both evergreen and deciduous forests occur (from approximately Nags Head, North Carolina, to Virginia Beach, Virginia), making the geographic boundary between the two systems somewhat unclear. The boundary of cold and warm offshore waters near Cape Hatteras may be an important climatic influence. This system is separated from Southern Atlantic Coastal Plain Dune and Maritime Grassland (CES203.273) by the dominance of woody vegetation, which corresponds to increased shelter from salt spray and increased stability of landforms.

DISTRIBUTION

**Range:** This system is found from southernmost Virginia to central South Carolina (south approximately to the Cooper River where the true Sea Islands begin).

**Divisions:** 203:C

**TNC Ecoregions:** 56:C, 57:C

**Nations:** US

**Subnations:** NC, SC, VA

**Map Zones:** 58:C, 60:C

**USFS Ecomap Regions:** 232C:CC, 232Ib:CPP

CONCEPT

**Environment:** This system occurs on barrier islands and on coastal strands where barrier islands are lacking, and is seldom or never found more than 2 or 3 miles from the ocean. Chronic salt spray is an important influence on vegetation structure and composition; however, the extent to which plant communities found in this system are shaped by salt spray varies. Examples closest to the coast are most likely to exhibit classic streamlined canopy shape due to spray sculpting and are less likely to support salt‑intolerant plant species. Heavier salt spray often determines the boundary of this system with Southern Atlantic Coastal Plain Dune and Maritime Grassland (CES203.273). Maritime forest requires some shelter from the ocean, in the form of high dunes or extensive sand flats, in order to develop. This system may occur from the top of interior dunes to wet swales. Soils are sandy, except for mucks in the wettest swamps. Soils range from excessively drained to permanently saturated. They are presumably low in nutrient‑holding capacity, but input of nutrients in salt spray probably makes this system fairly fertile. Topography and apparent moisture may vary widely with little change in vegetation. The ocean's moderation of climate may be a significant factor in the character of this system. A number of plant species extend much farther north in the maritime forests than they do even a few miles inland.

**Vegetation:** Vegetation includes shrublands and forests. Shrubland dominated by salt‑tolerant shrubs such as *Morella cerifera* and *Ilex vomitoria* or by stunted trees often occurs on the seaward edge where salt spray is heavier. Forests are typically dominated by a small set of salt‑tolerant evergreen trees, mainly *Quercus virginiana, Quercus hemisphaerica, Pinus taeda*, and in the southern portions, *Sabal palmetto*. Rare forested wetlands are dominated by a variety of wetland tree species, including *Acer rubrum, Nyssa biflora*, and *Taxodium distichum*. A few of the most sheltered areas near the northern end of the range have forests with deciduous species such as *Fagus grandifolia* and *Quercus falcata*. Also included are embedded freshwater depressional wetlands dominated by shrubs or small trees, such as *Cornus foemina, Persea palustris*, or *Salix caroliniana*. Communities tend to be low in species richness, with all strata limited to a set of salt‑tolerant species.

**Dynamics:** Maritime forests occur in the most stable portions of barrier islands, but the maritime environment is still extremely dynamic. Wind events and hurricanes will have significant impacts on this system. The environment for these forests may be severely altered or destroyed by geologic processes, such as the slow movement of dunes or their catastrophic destruction by storms. Sand movement may also create new sites for this system to occupy. Chronic salt spray and intense salt spray during storms are important influences on vegetation structure and composition; however, the extent to which plant communities found in this system are shaped by salt spray varies. Extreme salt spray or saltwater flooding in storms can severely disturb vegetation, though it recovers if the landforms have not been altered. Mature *Quercus virginiana* trees are fire‑resistant when mature, and their litter also does not easily burn (Stalter and Odum 1993). Fire may have naturally occurred infrequently in this system, but probably was not an important factor. Extreme salt spray or saltwater flooding in storms can severely disturb vegetation, although the vegetation recovers if the landforms have not been altered.

SOURCES

**References:** Bellis 1992, Bellis 1995, Comer et al. 2003*, Drehle 1973, Eaton 1979, Eyre 1980, Gaddy and Kohlsaat 1987, Johnson and Barbour 1990, Jolls pers. comm., NCDENR 2010, Nelson 1986, Saltonstall 2002, Schafale 2012, Schafale and Weakley 1990, Schafale pers. comm., Seneca and Broome 1981, Senter 2003, Stalter and Odum 1993, Ward 1975, Winner 1975, Winner 1979

**Version:** 23 Apr 2015	**Stakeholders:** East, Southeast

**Concept Author:** R. Evans	**LeadResp:** Southeast

CES203.503 East Gulf Coastal Plain Maritime Forest

**Primary Division:** Gulf and Atlantic Coastal Plain (203)

**Land Cover Class:** Forest and Woodland

**Spatial Scale & Pattern:** Large patch

**Required Classifiers:** Natural/Semi‑natural; Vegetated (>10% vasc.); Upland

**Diagnostic Classifiers:** Forest and Woodland (Treed); Coast; East Gulf Coastal Plain

**National Mapping Codes:** EVT 2380; ESLF 4323; ESP 1380

**Concept Summary:** This system encompasses a mosaic of woody vegetation present on barrier islands and near‑coastal strands along the northern Gulf of Mexico, from the Florida panhandle to southern Mississippi. Examples may include forests and/or shrublands that are found in somewhat more protected environments than East Gulf Coastal Plain Dune and Coastal Grassland (CES203.500). Such areas include relatively stabilized coastal dunes, sometimes with a substantial shell component. Vegetation structure and composition are influenced by salt spray, extreme disturbance events, and the distinctive climate of the immediate coast. Stands may be dominated by a variety of needle‑leaved and broad‑leaved evergreen trees, including *Pinus clausa, Pinus elliottii var. elliottii, Pinus palustris, Quercus virginiana, Sabal palmetto, Carya glabra*, and *Carya pallida*. Wetland inclusions may be dominated by *Taxodium ascendens* and *Magnolia virginiana*. The most heavily salt‑influenced examples may appear pruned or sculpted.

DISTRIBUTION

**Range:** This system is found along the northern Gulf of Mexico, from the Florida panhandle to southern Mississippi, restricted to the most coastward part of the "Gulf Coast Flatwoods" (Ecoregion 75a of EPA (2004)).

**Divisions:** 203:C

**TNC Ecoregions:** 53:C

**Nations:** US

**Subnations:** AL, FL, MS

**Map Zones:** 55:C, 99:C

**USFS Ecomap Regions:** 232L:CC

CONCEPT

**Environment:** This system is found on barrier islands and near‑coastal strands, on stable dune‑and‑swale topography in somewhat more protected environments along the northern Gulf of Mexico. More specifically, these areas are generally landward of the foredune and transitional backdune zones. Examples may include forests and/or shrublands that are found in somewhat more protected environments than adjacent dune and coastal grassland vegetation. The system typically includes a series of stabilized dunes and interdune swales oriented parallel to the coastline. Soils are primarily wind‑ and wave‑deposited, well‑drained quartz sands of Appalachian origin (Drehle 1973, Johnson and Barbour 1990), sometimes with a substantial shell component, that have been stabilized long enough to support trees and shrubs. As the forest establishes, soil temperature fluctuations moderate and humus begins to build up over the well‑drained sands, contributing to moisture retention and leading to more mesic conditions, especially in swales where soil moisture is typically higher (FNAI 1990).

**Vegetation:** Stands may be dominated by a variety of needle‑leaved and broad‑leaved evergreen trees, including *Pinus clausa, Pinus elliottii var. elliottii, Pinus palustris, Quercus virginiana, Sabal palmetto, Carya glabra*, and *Carya pallida*. Wetland inclusions may be dominated by *Taxodium ascendens* and *Magnolia virginiana*. Understory trees and shrubs may include *Quercus geminata, Quercus myrtifolia, Ilex vomitoria, Serenoa repens, Morella cerifera, Ilex glabra, Vaccinium arboreum, Juniperus virginiana, Zanthoxylum clava‑herculis, Sideroxylon lanuginosum, Persea borbonia, Conradina canescens*, and *Callicarpa americana*. Herbs may include *Spartina patens, Juncus roemerianus*, and *Panicum virgatum*. Wetland inclusions may contain *Cladium mariscus ssp. jamaicense*.

**Dynamics:** The maritime environment for these forests is extremely dynamic, even though they occur on the most stable portions of barrier islands. Maritime forest systems remain subject to periodic severe physical stresses. The environment for these forests may be severely altered or destroyed by geologic processes such as the slow movement of dunes or their destruction by storms and hurricanes. Sand movement may also create new sites for this system to occupy or degrade them through erosion or sand burial. Chronic salt spray (sea salt aerosol), as well as intense salt spray during storms are important influences on vegetation structure and composition; however, the extent to which plant communities found in this system are shaped by salt spray varies. The most heavily salt‑influenced examples of these systems may appear pruned or sculpted. Extreme salt spray or saltwater flooding in storms can severely disturb vegetation, though it recovers if the landforms have not been altered. Fire may have naturally occurred infrequently in this system, but probably was not an important factor. Mature *Quercus virginiana* trees are fire‑resistant when mature, and their litter also does not easily burn (Stalter and Odum 1993).

Hurricanes frequently make landfall in the northern Gulf of Mexico and have a significant impact on coastal systems. Even when they do not make landfall in the region, the storm surge and wave action generated by an off‑shore storm can have a significant impact. For example, a total of 112 hurricanes made landfall from Wakulla County, Florida, to Hancock County, Mississippi, during the period 1926 to 2005 (Jarrell et al. 1992 with updates); 36 major hurricanes (Category 3 or higher) made landfall along the Gulf Coast from Louisiana to the Florida Panhandle between 1851 and 2004 (Blake et al. 2005). Hurricane‑associated storm surges can overwash the dune system and cause significant erosion and/or sand burial of maritime forests (Landfire 2007a).

The role of fire in this system is poorly documented. The majority of this system occurs on narrow barrier islands along the northern Gulf of Mexico. FNAI (1990) indicates that the mesic conditions and insular locations of well‑developed maritime hammock communities inhibit natural fires, which occur no more frequently than once every 26 to 100 years. Mature *Quercus virginiana* trees are fire‑resistant when mature, and their litter also does not easily burn (Stalter and Odum 1993). Liu et al. (2003), in their study of sediment cores from Little Lake, Alabama, suggested that wildfires have been common in the coastal ecosystems in Alabama; however, they offered no frequency estimates. They did suggest a correlation between hurricanes and fire. This correlation was also supported by Meyers and van Lear (1998) who suggest that interactions between hurricanes and fires once played a major role in the development of ecosystems in the southern U.S., influencing their composition, structure, and pattern on the landscape (Landfire 2007a).

The following fire‑return interval estimates were based on Huffman and Platt (2004) and the return interval in similar ecological systems on the mainland. Fire interval and intensity depend on the patch vegetation type. In *Quercus/Ceratiola ericoides*‑dominated ridges, there is little fuel to sustain surface fires; in this vegetation type, fires are typically replacement fires that burn through the shrub crowns. This return interval was estimated to be 25 to 100 years. These fires were more likely to have occurred following a hurricane or other intense storm‑related event when more fuel became available and fire intensity presumably would have been higher. Pine‑dominated swales and flats most likely burned more frequently than the *Quercus/Ceratiola ericoides*‑dominated ridges. Fires in these swales were primarily light surface fires occurring every four years during the growing season. More intense replacement fires may have occurred following hurricanes, when more fuel was available as a result of storm damage (Landfire 2007a).

SOURCES

**References:** Bellis 1995, Blake et al. 2005, Comer et al. 2003*, Drehle 1973, EPA 2004, Eaton 1979, Eyre 1980, FNAI 2010a, Gaddy and Kohlsaat 1987, Huffman and Platt 2004, Jarrell et al. 1992, Johnson and Barbour 1990, LANDFIRE 2007a, Liu et al. 2003, Meyers and van Lear 1998, NCDENR 2010, Seneca and Broome 1981, Stalter and Odum 1993, Ward 1975, Winner 1975, Winner 1979

**Version:** 14 Jan 2014	**Stakeholders:** Southeast

**Concept Author:** R. Evans	**LeadResp:** Southeast

CES203.513 Mississippi Delta Maritime Forest

**Primary Division:** Gulf and Atlantic Coastal Plain (203)

**Land Cover Class:** Forest and Woodland

**Spatial Scale & Pattern:** Small patch

**Required Classifiers:** Natural/Semi‑natural; Vegetated (>10% vasc.); Upland

**Diagnostic Classifiers:** Forest and Woodland (Treed); Coast

**National Mapping Codes:** EVT 2384; ESLF 4327; ESP 1384

**Concept Summary:** This system includes forests on barrier islands and spits formed during the deltaic shifts of the Mississippi River. It also includes the woody vegetation of salt domes in the Mississippi River deltaic plain. Since natural deltaic processes have been altered, barrier islands are no longer being formed in the Mississippi Delta region and existing barrier islands are undergoing subsidence and beach erosion. Some documented stands that apparently pertain to this system are found on Native American middens (shell mounds) located in the salt marshes of Hancock County, Mississippi. This system currently includes one forested beach ridge located at Grande Isle in Louisiana.

DISTRIBUTION

**Range:** This system is apparently restricted to Louisiana. It is found on barrier islands and spits formed during the deltaic shifts of the Mississippi River.

**Divisions:** 203:C

**TNC Ecoregions:** 31:C, 42:?

**Nations:** US

**Subnations:** LA

**Map Zones:** 98:C

**USFS Ecomap Regions:** 232E:CC

CONCEPT

**Environment:** This system includes forests on barrier islands and spits formed during the deltaic shifts of the Mississippi River. It also includes the woody vegetation of salt domes in the Mississippi River deltaic plain. Some documented stands that apparently pertain to this system are found on Native American middens (shell mounds) located in the salt marshes of Hancock County, Mississippi (Eleuterius and Otvos 1979). This system also includes one forested beach ridge located at Grande Isle in Louisiana.

**Dynamics:** The maritime environment for these forests is extremely dynamic and may be severely altered or destroyed by geologic processes, including catastrophic destruction by storms. Fire may have naturally occurred infrequently in this system, but probably was not an important factor. Mature *Quercus virginiana* trees are fire‑resistant when mature, and their litter also does not easily burn (Stalter and Odum 1993). Maritime forest systems remain subject to periodic severe physical stresses, although less than coastal dune and grassland systems. Vegetation structure and composition are influenced by salt spray (sea salt aerosol) and extreme disturbance events such as hurricanes, erosion, accretion and sand burial. Chronic salt spray, as well as intense salt spray during storms are important influences on vegetation structure and composition; however, the extent to which plant communities found in this system are shaped by salt spray varies. The most heavily salt‑influenced examples of these systems may appear pruned or sculpted. Extreme salt spray or saltwater flooding in storms can severely disturb vegetation, though it recovers if the landforms have not been altered.

Hurricanes frequently make landfall in the northern Gulf of Mexico region, and have a significant impact on coastal systems. Even when they do not make landfall, the storm surge and wave action generated by an off‑shore storm can have a significant impact. A total of 112 hurricanes made landfall from Wakulla County, Florida, to Hancock County, Mississippi, during the period 1926 to 2005 (Jarrell et al. 1992 with updates). From the period 1851 to 2004, 36 major hurricanes (Category 3 or higher) made landfall along the Gulf Coast from Louisiana to the Florida Panhandle (Blake et al. 2005). Hurricane‑associated storm surges can overwash the dune system and cause significant erosion and/or sand burial of maritime forests. Personal observations along coastal areas of the Florida panhandle region in 2005 revealed large areas of vegetation extending several hundred yards inland that were killed or significantly impacted by saltwater inundation (Landfire 2007a).

The role of fire in this system is poorly documented. The majority of this system occurs on narrow barrier islands along the northern Gulf of Mexico. FNAI (1990) indicates that the mesic conditions and insular locations of well‑developed maritime hammock communities inhibit natural fires, which occur no more frequently than once every 26 to 100 years. Liu et. al. (2003), in their study of sediment cores from Little Lake, Alabama, suggested that wildfires have been common in the coastal ecosystems in Alabama; however, they offered no frequency estimates. They did suggest a correlation between hurricanes and fire. This correlation was also supported by Meyers and van Lear (1998) who suggest that hurricane‑fire interactions once played a major role in the development of ecosystems in the southern U.S., influencing their composition, structure, and pattern on the landscape (Landfire 2007a).

The following fire‑return interval estimates were based on the Huffman and Platt (2004) study of fire scars on slash pines on Little St. George Island (Florida) and the return interval in similar ecological systems on the mainland. Fires were primarily light surface fires occurring every four years during the growing season. More intense replacement fires may have occurred following hurricanes, when more fuel was available as a result of storm damage. There is little fuel to sustain surface fires in the *Quercus/Ceratiola ericoides*‑dominated ridges. Fires in this vegetation type are typically replacement fires that burn through the shrub crowns. The return interval here was estimated at 25 to 100 years, and may have occurred following a hurricane or other intense storm‑related event when more fuel was available and fire intensity was higher (Landfire 2007a).

SOURCES

**References:** Barbour et al. 1985, Bellis 1995, Blake et al. 2005, Comer et al. 2003*, Eaton 1979, Eleuterius and Otvos 1979, Eyre 1980, Gaddy and Kohlsaat 1987, Huffman and Platt 2004, Jarrell et al. 1992, Johnson and Barbour 1990, LANDFIRE 2007a, Liu et al. 2003, Meyers and van Lear 1998, Morton 2008, NCDENR 2010, Seneca and Broome 1981, Stalter and Odum 1993, Ward 1975, Winner 1975, Winner 1979

**Version:** 14 Jan 2014	**Stakeholders:** Southeast

**Concept Author:** J. Teague	**LeadResp:** Southeast

CES203.537 Southern Atlantic Coastal Plain Maritime Forest

**Primary Division:** Gulf and Atlantic Coastal Plain (203)

**Land Cover Class:** Forest and Woodland

**Spatial Scale & Pattern:** Large patch

**Required Classifiers:** Natural/Semi‑natural; Vegetated (>10% vasc.); Upland

**Diagnostic Classifiers:** Forest and Woodland (Treed); Coast

**National Mapping Codes:** EVT 2382; ESLF 4325; ESP 1382

**Concept Summary:** This system encompasses a range of woody vegetation present on stabilized upland dunes of barrier islands and near‑coastal strands, from central South Carolina (from approximately the Cooper River) southward to Volusia County, Florida. It includes vegetation whose structure and composition are influenced by salt spray, extreme disturbance events, and the distinctive climate of the immediate coast. Examples are known from the barrier islands of Georgia and Florida, such as Big Talbot Island, Florida, and probably Sapelo Island, Georgia. Most typical stands are dominated by oaks, primarily *Quercus virginiana* and/or *Quercus geminata*. Vegetation may also include different woodland communities often dominated by southern pine species. *Pinus palustris, Pinus serotina*, and *Pinus elliottii var. elliottii* are all important in documented examples. These examples tend to have densely shrubby subcanopies and understories with species such as *Quercus virginiana, Quercus geminata, Quercus hemisphaerica, Quercus chapmanii, Quercus myrtifolia*, and *Magnolia grandiflora*. Unlike maritime vegetation to the north, this system may be more heavily influenced by natural fire regimes that may help to explain the predominance of the fire‑tolerant pine species. It has been postulated that the natural fire‑return interval is from 20 to 30 years.

DISTRIBUTION

**Range:** This system occurs from central South Carolina (Cooper River) southward to approximately Volusia County, Florida (ca. 28°30'N latitude).

**Divisions:** 203:C

**TNC Ecoregions:** 56:C

**Nations:** US

**Subnations:** FL, GA, SC

**Map Zones:** 55:C, 58:C

**USFS Ecomap Regions:** 232C:CC

CONCEPT

**Environment:** The primary range of this system coincides with the Sea Islands, a chain of more than 100 low islands off the Atlantic coast of South Carolina, Georgia, and northern Florida, extending from the Cooper River to the St. Johns River. Many of these islands have a long history of human use and occupation, including Spanish missions and garrisons in the 16th century. In addition, the Sea Islands were the first important cotton‑growing area in North America. The degree to which this system has been altered by these events is unknown.

This system is found on these islands and associated near‑coastal strands, on stable dune and swale topography in somewhat more protected environments. These areas are generally landward of the foredune and transitional backdune zones. Examples typically include forests and/or shrublands that are found in somewhat more protected environments than adjacent dune and coastal grassland vegetation. The system typically includes a series of stabilized dunes and interdune swales oriented parallel to the coastline. Soils are primarily wind‑ and wave‑deposited, well‑drained quartz sands of Appalachian origin (Drehle 1973, Johnson and Barbour 1990), sometimes with a substantial shell component, that have been stabilized long enough to support trees and shrubs. As the forest establishes, soil temperature fluctuations moderate and humus begins to build up over the well‑drained sands, contributing to moisture retention and leading to more mesic conditions, especially in swales where soil moisture is typically higher (FNAI 1990).

**Vegetation:** Most typical stands are dominated by oaks, primarily *Quercus virginiana* and/or *Quercus geminata*. Vegetation may also include different woodland communities often dominated by southern pine species. *Pinus palustris, Pinus serotina*, and *Pinus elliottii var. elliottii* are all important in documented examples. These examples tend to have densely shrubby subcanopies and understories with species such as *Quercus virginiana, Quercus geminata, Quercus hemisphaerica, Quercus chapmanii, Quercus myrtifolia*, and *Magnolia grandiflora*.

**Dynamics:** Maritime forests occur in the most stable portions of barrier islands, but the maritime environment is still extremely dynamic. Wind events and hurricanes will have significant impacts on this system. The environment for these forests may be severely altered or destroyed by geologic processes, such as the slow movement of dunes or their catastrophic destruction by storms. Sand movement may also create new sites for this system to occupy. Extreme salt spray or saltwater flooding in storms can severely disturb vegetation, though it recovers if the landforms have not been altered. Mature *Quercus virginiana* trees are fire‑resistant when mature, and their litter also does not easily burn (Stalter and Odum 1993). Fire may have occurred naturally yet infrequently in this system, but probably was not an important factor.

The vegetation of this system has a structure and composition that is influenced by salt spray (sea salt aerosol), extreme disturbance events, and the distinctive climate of the immediate coast. Extreme salt spray or saltwater flooding in storms can severely disturb vegetation, although the vegetation recovers if the landforms have not been altered. Unlike maritime vegetation to the north, this system may be more heavily influenced by natural fire regimes that may help to explain the predominance of the fire‑tolerant pine species. It has been postulated that the natural fire frequency is from 20 to 30 years.

SOURCES

**References:** Comer et al. 2003*, Eyre 1980, FNAI 2010a, Nelson 1986

**Version:** 14 Jan 2014	**Stakeholders:** Southeast

**Concept Author:** R. Evans	**LeadResp:** Southeast

CES203.560 Southern Coastal Plain Dry Upland Hardwood Forest

**Primary Division:** Gulf and Atlantic Coastal Plain (203)

**Land Cover Class:** Forest and Woodland

**Spatial Scale & Pattern:** Large patch

**Required Classifiers:** Natural/Semi‑natural; Vegetated (>10% vasc.); Upland

**Diagnostic Classifiers:** Forest and Woodland (Treed); Broad‑Leaved Deciduous Tree

**National Mapping Codes:** EVT 2330; ESLF 4136; ESP 1330

**Concept Summary:** This is one of three hardwood‑dominated systems found in the East Gulf Coastal Plain and adjacent areas of central Florida. This type is found in the Southern Coastal Plain and Southeastern Plains (EPA Level III Ecoregion 75 and parts of 65). Examples attributable to this type are typically deciduous or mixed evergreen oak‑dominated forests, often with a pine component present. Although the southern portion of the range of this system overlaps Southern Coastal Plain Oak Dome and Hammock (CES203.494), the latter is dominated by evergreen oak species, and the two should not be confused. The core range of this type extends northward to the approximate historical range of *Pinus palustris*; although most deciduous species do not mimic this range, this boundary does appear to be a reasonable demarcation boundary north of which *Quercus alba* becomes more abundant and south of which *Quercus hemisphaerica* is more diagnostic. Like all hardwood systems of this region, examples occur within a landscape matrix historically occupied by pine‑dominated uplands and consequently this system only occurred in fire‑sheltered locations in naturally small to large patches. Examples of this system tend to occur on sites intermediate in moisture status (mostly dry to dry‑mesic), although occasionally very dry (xeric) stands may also be included. Toward the northern range limits of this system, it may have been less restricted to small patches in fire‑protected locations, and may have been formerly more prevalent on the landscape even in areas heavily influenced by fire.

Important tree species vary geographically and according to previous disturbance. *Quercus hemisphaerica* is a typical species in many examples, with *Quercus stellata, Quercus falcata*, and *Quercus alba* less frequently encountered, but dominant in some stands. The overstory of some examples may be quite diverse, with hickories and other hardwood species often present. Typically mesic sites, as indicated by species indicative of these conditions (e.g., *Fagus grandifolia*), are covered under other systems. *Pinus taeda* is sometimes present, but it is unclear if it is a natural component or has entered only as a result of past cutting. *Pinus glabra* or *Pinus echinata* may also be present in some examples. Stands may be found on slopes above rivers and adjacent to sinkholes, as well as other fire‑infrequent habitats including narrow bands between mesic slopes below and pine‑dominated flats above.

**Comments:** As currently conceived, the Alabama range of this type extends throughout the Southern Hilly Gulf Coastal Plain (Ecoregion 65d), as mapped by the U.S. Environmental Protection Agency (EPA 2004) northward across the Black Belt and into the Fall Line Hills (Ecoregion 65i) to approximately Tuscaloosa (A. Schotz pers. comm.). To the north it is eventually replaced by East Gulf Coastal Plain Northern Dry Upland Hardwood Forest (CES203.483), but along this northern range it occurs in a mosaic with CES203.483 as well as East Gulf Coastal Plain Interior Shortleaf Pine‑Oak Forest (CES203.506). In Mississippi the range extends almost to the same latitude, but this range is confined to Ecoregion 65d.

DISTRIBUTION

**Range:** This system is found in the East Gulf Coastal Plain and adjacent areas of central Florida ranging northward into central Mississippi and Alabama.

**Divisions:** 203:C

**TNC Ecoregions:** 43:C, 53:C, 55:C

**Nations:** US

**Subnations:** AL, FL, GA, MS

**Map Zones:** 46:C, 55:C, 56:C, 99:C

**USFS Ecomap Regions:** 231B:CC, 232B:CC, 232C:CC, 232D:CC, 232J:CC, 232K:CC, 232L:CC

CONCEPT

**Environment:** Topographically, these sites tend to occur on upper to mid slopes, but occasionally on broader uplands with reduced fire frequencies. A range of soils may be present from loamy and clayey to coarse sands, but are generally well‑drained but not excessively drained. Soils are generally acidic, though calcareous soils occur occasionally. Sites are somewhat protected from most natural fires by steep topography and by limited flammability of the vegetation.

**Vegetation:** Vegetation consists of forests dominated by combinations of upland oaks, particularly *Quercus alba, Quercus falcata, Quercus stellata, Quercus margarettae*, and other species. There is some variation between the composition of northern versus southern examples in which evergreen species such as *Quercus nigra* and *Quercus hemisphaerica* become more prominent. *Carya tomentosa (= Carya alba)* and *Carya glabra* may be present. There is some variation in composition with aspect and degree of exposure to fire. More mesophytic species such as *Fagus grandifolia* and *Magnolia grandiflora* are absent or are confined to the understory. *Pinus echinata* may be present in some stands, particularly on drier south‑ and west‑facing slopes, but is typically not dominant. *Pinus taeda* is sometimes present, but it is unclear if it is a natural component or has entered only as a result of past cutting. Some examples of this system will have pine (*Pinus echinata, Pinus glabra, Pinus taeda*) as a natural component, where occasional fires may allow them to regenerate. In most examples, the understory is well‑developed. A well‑developed shrub layer may be present, with *Vaccinium* spp. and *Gaylussacia* spp. most typical. The herb layer is generally sparse; species richness tends to be low but may be richer if fire has played a role in shaping the structure and composition of the stand. The most likely grass taxa (found in open‑understory examples) are *Schizachyrium scoparium, Andropogon* spp., *Chasmanthium* spp., *Dichanthelium* spp., and *Danthonia sericea*.

**Dynamics:** Sites where this system occurs almost invariably grade upslope into pine‑dominated systems, especially stands containing *Pinus palustris* and, to a lesser extent, *Pinus echinata*. If these sites were burned more frequently, the vegetation would likely be replaced by more fire‑tolerant southern pines. Fires that penetrate stands of this type are generally low in intensity and have fairly limited ecological effect. In general, more frequent or intense fire would move the vegetation on the site toward more fire‑tolerant components. Conversely, with the prolonged complete absence of fire, less fire‑tolerant species could invade, causing the vegetation to resemble the more mesic slope forests below.

Frequent surface fires occurred on a 4‑ to 8‑year return interval from both lightning and Native American ignitions. These frequent light surface fires maintained the grassy understory and kept more fire‑tolerant hardwoods and shrubs from capturing the understory and forming a midstory layer. Lightning fires occurred primarily during the spring dry season (April and May) with a secondary peak of Native American and settler burning during the fall (October and November) (Landfire 2007a). Occasionally, during extensive droughts, mixed‑severity or stand‑replacement fires did occur, especially in drier *Pinus echinata*‑dominated stands. Local thunderstorms created gaps on a small but continual basis. More extensive regional disturbances included tropical storms during the growing season and ice storms during winter (in the northern part of the range). Dense stands of middle to older aged *Pinus* species (where present) were susceptible to periodic mortality from bark beetle epidemics (Landfire 2007a).

SOURCES

**References:** Comer et al. 2003*, EPA 2004, Edwards et al. 2013, Engeman et al. 2007, Eyre 1980, FNAI 2010a, LANDFIRE 2007a, Schotz pers. comm., Ware et al. 1993

**Version:** 14 Jan 2014	**Stakeholders:** Southeast

**Concept Author:** M. Pyne and R. Evans	**LeadResp:** Southeast

CES203.494 Southern Coastal Plain Oak Dome and Hammock

**Primary Division:** Gulf and Atlantic Coastal Plain (203)

**Land Cover Class:** Forest and Woodland

**Spatial Scale & Pattern:** Small patch

**Required Classifiers:** Natural/Semi‑natural; Vegetated (>10% vasc.); Upland

**Diagnostic Classifiers:** Forest and Woodland (Treed); Long Disturbance Interval; Broad‑Leaved Evergreen Tree

**Concept Summary:** This small‑patch system occurs in the Southern Coastal Plain (EPA ecoregion 75). Examples are known from some more inland portions of this region as well as the Southeastern Plain (EPA ecoregion 65) in Georgia and Alabama. Relatively dense stands of *Quercus virginiana* and/or *Quercus geminata* are diagnostic of this system. Examples often occupy locally distinct microhabitats that differ from the surrounding landscape, such as shallow depressions or slight topographic highs in a predominantly *Pinus palustris* ‑dominated landscape. Although embedded in a matrix of vegetation with extremely frequent fire regimes, patches of this system are subject to only infrequent or rare fire events. Under more frequent fire regimes, these sites would likely be occupied by *Pinus palustris*. It has been postulated that winter burning regimes have allowed this type to expand. A range of soil and moisture conditions may be present. More mesic examples have relatively thin soils (to 50 cm) above clay, while xeric examples occupy deep (>130 cm) well‑drained sands. Dominant plants of mesic examples include *Quercus virginiana* and *Quercus hemisphaerica*, along with *Diospyros virginiana*. Vines including *Campsis radicans* and *Smilax* spp. dominate the sparse ground cover. In xeric examples, dominants include *Quercus geminata, Pinus palustris, Quercus virginiana, Aristida beyrichiana*, and *Stylisma humistrata*. This system is low in plant species diversity compared to most other habitats in the region.

**Comments:** More diverse stands of upland hardwoods occurring in the same ecoregions should generally be treated under Southern Coastal Plain Dry Upland Hardwood Forest (CES203.560). The core range of this system lies farther south than CES203.560. Closely related stands of vegetation may also occur in near‑coastal environments where they are more obviously influenced by maritime disturbances; these are treated under different ecological systems. In Alabama examples of this system are of very limited extent, but occur inland as far as 60 miles or so from the coast; it is also known from bluffs along the Mobile‑Tensas (A. Schotz pers. comm.).

DISTRIBUTION

**Range:** This system occurs in Florida, adjacent Georgia and in very limited areas of Alabama (A. Schotz pers. comm.).

**Divisions:** 203:C

**TNC Ecoregions:** 53:C, 55:C, 56:C

**Nations:** US

**Subnations:** AL, FL, GA, MS

**Map Zones:** 55:C, 56:C, 99:C

CONCEPT

**Environment:** Examples are thickets or groves of *Quercus* species in a *Pinus* spp.‑dominated landscape (Myers 1990). These typically occupy locally distinct microhabitats that differ from the surrounding landscape, such as shallow depressions or slight topographic highs in a predominantly *Pinus palustris*‑dominated landscape. A range of soil and moisture conditions may be present. As currently defined, this system includes examples across a moisture gradient from mesic to xeric, ranging across parts of the southeastern coastal plains from Georgia to Mississippi. In Georgia, more mesic examples of this system have relatively thin soils (to 50 cm) above clay, while xeric examples occupy deep (>130 cm) well‑drained sands (Drew et al. 1998). In Florida, the xeric hammock typically develops on excessively drained sands where fire exclusion has allowed for the establishment of an oak canopy (FNAI 2010a). This may occur naturally, when the area has isolation from, or significant barriers to, fire. This can also occur as the result of human intervention, as at old homesites where fire was excluded for many years. In these areas, xeric hammock is found as small patches within or near sandhill or scrub. Xeric hammock can also occur on high islands within flatwoods or even on a high, well‑drained ridge within a floodplain. Xeric hammock can occur on barrier islands and in other coastal situations, as an advanced successional stage of coastal scrub.

Along and near the east coast of Florida, from Cape Canaveral and northward, there is more shell or humus in the sand, and a tendency to have hammocks containing *Quercus virginiana* with coastal strand rather than scrub; on the other hand, where there is more dry acidic sand, scrub occurs nearer the coast and *Quercus geminata* hammocks are found further back from the coast (A. Johnson pers. comm.).

**Vegetation:** This concept covers both xeric and more mesic types of oak domes and hammocks. In the more xeric examples (Xeric Hammock of FNAI 2010a), the canopy is more‑or‑less closed and dominated by *Quercus geminata*, although *Quercus chapmanii, Quercus hemisphaerica, Quercus incana, Quercus laevis*, and *Quercus margarettae* may also be common. An emergent canopy of pine, either *Pinus clausa, Pinus elliottii*, or *Pinus palustris* may be present (FNAI 2010a). *Quercus myrtifolia* may form a clonal shrub layer. *Aristida beyrichiana* and *Stylisma humistrata* may also be present. Hammocks that are intermediate in moisture status may have some live oak (*Quercus virginiana*) in the canopy. According to Drew et al. (1998), the dominant taxa of mesic examples are *Quercus hemisphaerica, Quercus nigra*, and *Quercus virginiana*, along with *Diospyros virginiana*. *Campsis radicans* and *Smilax* spp. dominate the sparse ground cover. Examples of this system are low in plant species diversity compared to other habitats in the region. Cabbage palms are a diagnostic component of examples of this system in central Florida (A. Johnson pers. comm.).

**Dynamics:** Although embedded in a matrix of vegetation with extremely frequent fire regimes, patches of this system are subject to only infrequent or rare fire events. Under more frequent fire regimes, these sites would likely be occupied by *Pinus palustris*. Myers (1990) postulated that winter ‑burning regimes have allowed for the expansion of this type. *Quercus geminata* and *Quercus myrtifolia* are both clonal species which establish large rhizome systems capable of quickly resprouting following injury. Xeric hammocks, whether natural or anthropogenic, result from years of fire exclusion, maintained and further enhanced by incombustible oak litter and a sparsity of herbs. The thick bark of *Quercus geminata* makes these trees somewhat resistant to fire. Once they form a canopy that shades the understory, the trees generate a layer of leaf litter that covers open patches of sand and leads to more shaded, mesic ground conditions. The resulting shaded habitat can allow more fire‑intolerant species such as *Magnolia grandiflora* to establish (Daubenmire 1990). Once the canopy is greater than 2 m high, even hot summer burns may not be sufficient to kill the dome, which can become established after only 7 to 16 years of fire exclusion (Guerin 1993). At that stage, oaks would only be killed through a catastrophic burn during dry conditions. Otherwise, the spread of oaks could be halted through mechanical removal or the use of herbicides if the management intent is the re‑establishment of the fire‑maintained community that was replaced by the xeric hammock. Xeric hammocks also form from long unburned oak scrub (Laessle 1958). There is a dynamic tension between the *Quercus*‑dominated patches and the *Pinus*‑dominated matrix. Oak domes are a natural part of the landscape, but can also result from human‑caused fire exclusion. Near the coast, these communities are affected by salt spray (sea salt aerosol). At and near the coast, salt spray maintains the *Quercus geminata* at shrub height as much as does fire; one may observe a gradient of increasingly taller *Quercus geminata* as you move inland and the effect of salt spray becomes diminished (A. Johnson pers. comm.).

SOURCES

**References:** Brewer 2008, Comer et al. 2003*, Daubenmire 1990, Drew et al. 1998, Edwards et al. 2013, Engeman et al. 2007, Eyre 1980, FNAI 2010a, Guerin 1993, Johnson, A. pers. comm., Laessle 1958, Myers 1990a, Schotz pers. comm.

**Version:** 14 Jan 2014	**Stakeholders:** Southeast

**Concept Author:** R. Evans	**LeadResp:** Southeast

CES203.466 West Gulf Coastal Plain Chenier and Upper Texas Coastal Fringe Forest and Woodland

**Primary Division:** Gulf and Atlantic Coastal Plain (203)

**Land Cover Class:** Forest and Woodland

**Spatial Scale & Pattern:** Large patch

**Required Classifiers:** Natural/Semi‑natural; Vegetated (>10% vasc.); Upland

**Diagnostic Classifiers:** Forest and Woodland (Treed)

**National Mapping Codes:** EVT 2339; ESLF 4145; ESP 1339

**Concept Summary:** This system includes a range of woody vegetation typically dominated by *Quercus virginiana* present along the northern Gulf of Mexico, from Vermillion Bay in Louisiana to the upper Texas coast. Landscape position includes shell ridges along the coast and bay margins, coastal salt domes, stranded ancient barrier ridges (Ingleside barrier strandplain), and chenier ridges of the Chenier Plain. In addition to *Quercus virginiana*, other species such as *Celtis laevigata* and *Quercus nigra* may be present to codominant in the canopy which may also include *Carya illinoinensis, Diospyros virginiana, Fraxinus pennsylvanica, Liquidambar styraciflua*, and *Magnolia grandiflora*.

DISTRIBUTION

**Range:** This ecological system is found in small patches along the northern Gulf of Mexico, from Vermillion Bay in Louisiana to the upper Texas coast.

**Divisions:** 203:C

**TNC Ecoregions:** 31:C

**Nations:** US

**Subnations:** LA, TX

**Map Zones:** 37:C, 98:C

**USFS Ecomap Regions:** 232E:CC, 255D:CC

CONCEPT

**Environment:** This system occupies sand and shell ridges (Quaternary deposits) which resulted from ancient abandoned beach ridges associated with migrating shorelines, shell ridges, as well as salt domes near the coast. The Ingleside barrier strandplain, an ancient barrier ridge composed of deep sands and occurring well inland of the current Gulf shoreline, may support occurrences of this system. Most occurrences occupy ridges formed from sediments deposited along ancient shorelines. These ridges (cheniers), which often parallel the coast and are composed of coarse material such as sand or shell, may be up to 3 m above mean sea level. Some occurrences occupy coastal salt domes, which may rise 30 m above the surrounding landscape. The soils are typically Entisols of coarse‑textured material, either sand or shell. The Ecological Site Description, which may be related to this system, is the Coastal Sand ecoclass (Elliott 2011).

**Vegetation:** Typically these forests and woodlands are dominated by *Quercus virginiana*; however, other species such as *Celtis laevigata* and *Quercus nigra* may be present to codominant in the canopy. Other species such as *Liquidambar styraciflua, Carya illinoinensis, Diospyros virginiana, Fraxinus pennsylvanica*, and *Magnolia grandiflora* may also be present in the canopy. The understory is often patchy but may include species such as *Ilex vomitoria, Callicarpa americana, Zanthoxylum clava‑herculis, Crataegus viridis, Sabal minor, Morella cerifera*, and/or *Sideroxylon lanuginosum*. Woody vines present in this system include *Vitis mustangensis, Parthenocissus quinquefolia, Campsis radicans*, and *Toxicodendron radicans*. The two epiphytes *Tillandsia usneoides* and *Pleopeltis polypodioides* may be commonly encountered. The herbaceous layer is usually sparse, but may include species such as *Schizachyrium scoparium, Sanicula canadensis, Malvaviscus arboreus var. drummondii, Elephantopus carolinianus*, and *Oplismenus hirtellus*. *Triadica sebifera* and *Ligustrum sinense* may be important non‑native invaders (Elliott 2011).

**Dynamics:** This ecological system is heterogeneous in physiognomy, including forests, woodlands and shrublands. The Chenier Plain was historically characterized by a prograding coastline replenished by sediments carried to the Gulf of Mexico initially by the Mississippi and subsequently the Atchafalaya and other rivers. It is void of barrier islands and sediments are reworked by waves into beach ridges, sometimes with a substantial shell component. This process has been continuing since the last glacial retreat, and as the coastline prograded, older beach ridges were left as interior ridges surrounded by marsh. These interior beach ridges are referred to as cheniers (from the French word for oak) because they were historically dominated by *Quercus virginiana*. Ridges parallel the coast and are usually 3‑5 m above mean sea level. Though not confined to coastal areas, salt domes are a distinctive feature along the Gulf Coast of upper Texas and Louisiana where they often form a drastic contrast to the low‑lying Coastal Plain sediments surrounding them. Formed by the rise of salt masses which push up overlying strata, salt domes may rise 30 m above the surrounding landscape. The natural vegetation of cheniers and coastal salt domes are quite similar. The Ingleside barrier strandplain is a Pleistocene barrier ridge that is exposed discontinuously along the Texas coast. One of these areas is located northeast of Galveston Bay and supports *Quercus virginiana*‑dominated woodlands included within this ecological system. Shell ridges located along coast and bay margins are typically dominated by halophytic shrubs. Similar vegetation may also be found on coastal dredge spoil. Vegetation structure and composition of occurrences of this system may be influenced by salt spray (on those shell ridges, salt domes and cheniers closest to the gulf), tropical storms and hurricanes, and the distinctive climate of the immediate coast. Studies have shown that chenier forests and woodlands are very important stop‑over sites for neotropical migrants during both spring and fall migration.

SOURCES

**References:** Comer et al. 2003*, Elliott 2011, Eyre 1980, LDWF 2005, Neyland and Meyer 1997

**Version:** 14 Jan 2014	**Stakeholders:** Southeast

**Concept Author:** J. Teague and R. Evans	**LeadResp:** Southeast

M008. Southern Mesic Mixed Broadleaf Forest

CES203.079 Crowley's Ridge Mesic Loess Slope Forest

**Primary Division:** Gulf and Atlantic Coastal Plain (203)

**Land Cover Class:** Forest and Woodland

**Spatial Scale & Pattern:** Large patch

**Required Classifiers:** Natural/Semi‑natural; Vegetated (>10% vasc.); Upland

**Diagnostic Classifiers:** Unglaciated

**National Mapping Codes:** EVT 2322; ESLF 4128; ESP 1322

**Concept Summary:** This ecological system of mesic upland forests is confined to Crowley's Ridge, which extends from Missouri south into Arkansas along the western side of the lower Mississippi River. This vegetation and the ridge itself are very distinctive from that of the adjacent alluvial plain. The ridge is a remnant loess‑capped feature rising from 30 m to over 60 m (100‑200 feet) above the alluvial plain surface, to about 150 m (450 feet) above sea level. The base of the ridge is composed of Tertiary substrates overlain by Quaternary alluvial deposits and capped with up to 15 m (50 feet) of Pleistocene loess. The system is generally composed of mesic forests that occupy ravines between narrow, "finger" ridges and slopes in a highly dissected landscape. The sites tend to be more mesic than sites elsewhere in the southeastern United States. In many cases, these slopes and ravines provide habitat for plant species that are rare or absent from other parts of the alluvial plain (e.g., *Liriodendron tulipifera, Tilia americana*). Canopies are dominated by *Fagus grandifolia, Quercus alba*, and *Liriodendron tulipifera*, with many associates.

**Comments:** This type does not include all forests across the entire extent of southern Crowley's Ridge; excluded are dry and dry‑mesic forests, typically on west‑facing slopes and ridgetops. This system is best developed on southern Crowley's Ridge where loess is most pronounced, and becomes much more isolated and rare on the ridge north of approximately Jonesboro, Arkansas. Conversely, dry‑mesic oak and shortleaf pine communities are rare within this system, becoming dominant on western slopes and in the northern ridge, respectively. The vegetation may share some superficial similarities with types referred to as western mesophytic forests, but it is well‑separated geographically from these. A similar ecological system is East Gulf Coastal Plain Northern Loess Bluff Forest (CES203.481) which occurs farther eastward and is restricted to the loess bluffs east of the Mississippi River. The vegetation of these areas is believed to share a great detail of overlap. They are recognized as distinct for now due to geographic separation; further work may suggest that these two systems should be merged. There are a number of state parks and small natural areas on Crowley's Ridge, including Village Creek State Park, Crowley's Ridge State Park, Wittsburg Natural Area and Chalk Bluff Natural Area (which is toward the northern end of the ridge). All of these have moderate to high‑quality examples of this system.

DISTRIBUTION

**Range:** This system is endemic to Crowley's Ridge (Arkansas, Missouri), which is a distinctive landscape feature in the Mississippi River Alluvial Plain.

**Divisions:** 203:C

**TNC Ecoregions:** 42:C

**Nations:** US

**Subnations:** AR, MO

**Map Zones:** 45:C

**USFS Ecomap Regions:** 234D:CC

CONCEPT

**Environment:** These diverse‑canopy forests occur in ravines in a highly dissected environment. The system is best expressed on southern Crowley's Ridge, Arkansas (Cross County south through Phillips County), with additional limited occurrences to the north, in undisturbed valleys and coves. Deep loessal soil is the most characteristic and diagnostic component of the environment of this system.

**Vegetation:** This system consists of forests that are typically dominated by beech, oaks and other hardwoods. Canopies are dominated by *Fagus grandifolia, Quercus alba*, and *Liriodendron tulipifera* (Clark 1977d), with many associates, including *Magnolia acuminata* and *Tilia americana* (T. Witsell pers. comm. 2013). Other oaks which may be present include *Quercus falcata, Quercus pagoda, Quercus rubra*, and *Quercus velutina*. Due to the apparent richness of the loessal soils, *Ostrya virginiana* is a particularly common species across many of the component community types. Species that may be present in the shrub layer include *Arundinaria gigantea, Asimina triloba, Bignonia capreolata, Lindera benzoin, Parthenocissus quinquefolia, Toxicodendron radicans*, and *Vitis rotundifolia*. Some possible herbs include *Cynoglossum virginianum, Dioscorea quaternata*, and *Sanicula canadensis*.

**Dynamics:** These are stable, generally fire‑sheltered forests, with relatively low fire frequency and intensity. There is some natural disturbance from the effects of windstorms and collapse of the fragile loess. This mesic loess forest type typically experiences surface fires with return intervals of from 30 to greater than 100 years. Mixed‑severity fires will occur approximately every 100 years, opening the canopy with increased mortality. This effect may also be achieved by recurrent, severe insect defoliations or droughts. Straight‑line winds or microbursts may cause blowdowns on a scale of 1 to 100 acres. Stand‑replacement fires happen very infrequently (Landfire 2007a).

SOURCES

**References:** Arkansas Forestry Commission 2010, Clark 1974, Clark 1977d, Comer et al. 2003*, Engeman et al. 2007, Eyre 1980, LANDFIRE 2007a, NatureServe Ecology ‑ Southeastern U.S. unpubl. data, Nelson 2010, Witsell pers. comm.

**Version:** 14 Jan 2014	**Stakeholders:** Midwest, Southeast

**Concept Author:** T. Foti, D. Zollner, and M. Pyne	**LeadResp:** Southeast

CES203.481 East Gulf Coastal Plain Northern Loess Bluff Forest

**Primary Division:** Gulf and Atlantic Coastal Plain (203)

**Land Cover Class:** Forest and Woodland

**Spatial Scale & Pattern:** Large patch

**Required Classifiers:** Natural/Semi‑natural; Vegetated (>10% vasc.); Upland

**Diagnostic Classifiers:** Forest and Woodland (Treed); Loess deposit (undifferentiated); Broad‑Leaved Deciduous Tree

**National Mapping Codes:** EVT 2327; ESLF 4133; ESP 1327

**Concept Summary:** This system is largely confined to steep bluffs bordering the northern portion of the eastern edge of the Mississippi River Alluvial Plain. The geology is typically mapped as the Jackson Formation. These bluffs extend up to 150 m (500 feet) in elevation and from 30 to 60 m (100‑200 feet) above the adjacent plain. They consist of a belt of Pleistocene and Tertiary eolian deposits that are often deeply eroded and very steep, with fertile topsoil and abundant moisture. The vegetation is often richer than surrounding non‑loessal areas, or those with only thin loess deposits. The forests found on these bluffs are intermediate in soil moisture for the region and may best be thought of as mesic. The vegetation may sometimes be referred to as western mesophytic forest and may share some superficial similarities with cove forests of the Interior Highlands. In many cases, these bluffs provide habitat for plant species that are rare or absent from other parts of the Coastal Plain. The composition of these forests changes from north to south along the bluffs; more southerly examples are represented by the East Gulf Coastal Plain Southern Loess Bluff Forest (CES203.556), and these would contain *Magnolia grandiflora* as an important component. As currently defined this system ranges northward from about 32°N latitude (where the Big Black River cuts through the bluffs), and occurs only in the westernmost portions of the Upper East Gulf Coastal Plain, including northern and central Mississippi, western Tennessee, and western Kentucky, being restricted to the northern part of the Loess Bluff Hills (EPA Ecoregion 74a).

**Comments:** Similar ecological systems include East Gulf Coastal Plain Southern Loess Bluff Forest (CES203.556) which occurs further southward in the East Gulf Coastal Plain and has greater dominance by broad‑leaved and needle‑leaved evergreen trees, Southern Coastal Plain Mesic Slope Forest (CES203.476), and East Gulf Coastal Plain Northern Mesic Hardwood Slope Forest (CES203.477). There are other mixed deciduous mesic systems in the West Gulf Coastal Plain as well as other mesic forest systems to the east of this one, in areas other than the loess bluffs.

DISTRIBUTION

**Range:** This system is endemic to the loess bluffs ("Bluff Hills" [Ecoregion 74a] of EPA (2004)) along the eastern edge of the Mississippi River Alluvial Plain in Mississippi, Tennessee, and Kentucky.

**Divisions:** 203:C

**TNC Ecoregions:** 43:C

**Nations:** US

**Subnations:** KY, MS, TN

**Map Zones:** 46:C, 47:C

**USFS Ecomap Regions:** 231H:CC

CONCEPT

**Environment:** This system is largely confined to the lower portions of steep bluffs east of the Mississippi River. These bluffs consist of a belt of Pleistocene and Tertiary eolian deposits (Braun 1950) that are often deeply eroded and very steep, with fertile topsoil and abundant moisture (Miller and Neiswender 1987). The core of this is mapped as the Jackson Formation (Hardeman 1966) and corresponds more broadly with Ecoregion 74a (Bluff Hills) (EPA 2004). These bluffs border the eastern edge of the Mississippi River Alluvial Plain from about 32°N latitude (where the Big Black River cuts through the bluffs) northward to western Tennessee and Kentucky. Examples may extend up to 150 m (500 feet) in elevation and from 30 to 60 m (100‑200 feet) above the adjacent Mississippi Alluvial Plain. In Tennessee the loess soils may be 9‑27.5 m (30‑90 feet deep) (Springer and Elder 1980).

**Vegetation:** Examples of this system have deciduous canopies dominated by *Fagus grandifolia* or this species in combination with *Quercus alba*. The most mesic stands may lack codominance by *Quercus* spp. In addition, a variety of other hardwood species may also be found in the overstory, including *Liriodendron tulipifera, Liquidambar styraciflua, Acer rubrum, Nyssa sylvatica, Fraxinus americana, Magnolia acuminata* (of local distribution), and *Pinus taeda* (in more southern stands). This system is defined as being north of the range of *Magnolia grandiflora*, which excludes the "Beech‑Magnolia" forests of the southern loess bluffs. Some subcanopy components (in addition to canopy species) include *Carpinus caroliniana, Diospyros virginiana, Oxydendrum arboreum, Cornus florida, Acer floridanum (= Acer barbatum), Magnolia macrophylla, Ostrya virginiana, Ulmus alata*, and *Ilex opaca*. Other shrubs and woody vines include *Decumaria barbara, Rhododendron canescens, Toxicodendron radicans, Vitis rotundifolia*, and *Smilax glauca*. Important herbs include *Polystichum acrostichoides, Woodwardia areolata, Osmunda cinnamomea, Mitchella repens*, and *Hexastylis arifolia*. In many cases, these bluffs provide habitat for plant species that are rare or absent from other parts of the Coastal Plain, such as *Magnolia acuminata, Aralia racemosa*, and *Hydrophyllum canadense* (Chester et al. 1997).

**Dynamics:** These are stable, generally fire‑sheltered forests. These forests probably generally exist naturally as old‑growth forests, with canopy dynamics dominated by gap‑phase regeneration. As modeled here, replacement disturbance is over 60% and more likely due to weather‑related events than fire. Included among these are windthrow, lightning, and ice damage, as well as the inclusion of the erosion and mass wastage (Bryant et al. 1993) that give the bluffs their characteristic steepness. Widespread insect or disease mortality has not been reported. Wind/weather/stress replacement frequency is modeled near 240 years, replacement fire return at approximately 385 years, and all fire return frequency at about 85 years. "Open" structure is uncommon, even when defined as canopy closure less than 81%, and may be created by mixed‑severity fire. Surface fire may maintain open conditions, but it does not transition closed classes. Disturbance is presumed to mirror mixed mesophytic forest, occurring primarily in small gaps (less than one‑quarter acre), although the occurrence of aggregates of intolerant species suggests that larger scale disturbances occasionally play a role (Landfire 2007a). In addition, periodic droughts may cause death of or stress to moisture‑requiring canopy trees. There is presumably some natural disturbance from the effects of windstorms, which are relatively frequent in the range of this system.

SOURCES

**References:** Batista and Platt 1997, Braun 1950, Bryant et al. 1993, Chester et al. 1997, Comer et al. 2003*, Delcourt and Delcourt 2000, EPA 2004, Edwards et al. 2013, Engeman et al. 2007, Evans et al. 2009, Eyre 1980, Greenberg et al. 1997, Hardeman 1966, LANDFIRE 2007a, Miller and Neiswender 1987, Springer and Elder 1980

**Version:** 14 Jan 2014	**Stakeholders:** Southeast

**Concept Author:** R. Evans and M. Pyne	**LeadResp:** Southeast

CES203.477 East Gulf Coastal Plain Northern Mesic Hardwood Slope Forest

**Primary Division:** Gulf and Atlantic Coastal Plain (203)

**Land Cover Class:** Forest and Woodland

**Spatial Scale & Pattern:** Large patch

**Required Classifiers:** Natural/Semi‑natural; Vegetated (>10% vasc.); Upland

**Diagnostic Classifiers:** Forest and Woodland (Treed); Slope; Broad‑Leaved Deciduous Tree

**National Mapping Codes:** EVT 2325; ESLF 4131; ESP 1325

**Concept Summary:** This system includes mesic deciduous hardwood forests of inland portions of the East Gulf Coastal Plain, including Alabama, Mississippi, western Kentucky, and western Tennessee. This system covers parts of the more mesic forests in the coastal plain portion of the Western Mesophytic Forest Region referred to as mesophytic mixed hardwoods, as well as mesic forests in the adjacent "Oak‑Pine‑Hickory" region to the south. Examples of this system occur on slopes and ravines between dry uplands and stream bottoms. Mesic forests of the loess bluffs are treated in separate ecological systems, being confined to that landform of steep bluffs and ravines on deep loess. The most characteristic feature of the vegetation in some examples may be *Fagus grandifolia*, but a variety of other hardwood species may also be found in the overstory, and *Fagus grandifolia* may not always be present. Some stands may be dominated by *Fagus grandifolia* and *Quercus alba*, others by *Quercus alba* or *Quercus pagoda* with other mesic hardwoods. In addition, *Pinus taeda* may be common in some examples in the southern portion of the range and, depending on previous disturbance and site conditions, may be locally dominant [see CEGL004763]. To the south this system is replaced by Southern Coastal Plain Mesic Slope Forest (CES203.476), which is within the range of *Pinus glabra* and *Magnolia grandiflora*.

**Comments:** Southern Coastal Plain Mesic Slope Forest (CES203.476) is a similar mesic forest system to the south of this one in the East Gulf Coastal Plain with greater dominance by broad‑leaved evergreen trees. The systems of the loess bluffs to the west of this one, bordering the Mississippi River Alluvial Plain, are treated as distinct and are more extensive and continuous in their extent both vertically and latitudinally [see East Gulf Coastal Plain Northern Loess Bluff Forest (CES203.481) and East Gulf Coastal Plain Southern Loess Bluff Forest (CES203.556)]. One association now (2005) included here (*Quercus alba ‑ Fagus grandifolia / Hydrangea quercifolia ‑ Viburnum acerifolium / Carex picta* Forest (CEGL007213)) has the majority of its occurrences in the interior regions (southern Cumberland Plateau, Ridge and Valley), but its flora contains some Coastal Plain elements as well as more interior ones. It is from a "transition region" where *Quercus rubra* may be present in parts of the upper Coastal Plain and conversely some more southerly affiliated species (e.g., *Decumaria barbara*) range farther north. This association is now affiliated with two different ecological systems.

DISTRIBUTION

**Range:** This system is found in northern and inland portions of the East Gulf Coastal Plain, including Alabama, Mississippi, western Kentucky, and western Tennessee. It does not occur in Arkansas. This area is equivalent to the coastal plain portion of the Western Mesophytic Forest Region of Braun (1950) and the "Oak‑Pine‑Hickory" region of Greller (1988).

**Divisions:** 203:C

**TNC Ecoregions:** 43:C

**Nations:** US

**Subnations:** AL, GA, KY, MS, TN

**Map Zones:** 46:C, 47:C

**USFS Ecomap Regions:** 231B:CC, 231H:CC

CONCEPT

**Environment:** This system occurs along the eastern margin of the Upper Coastal Plain where elevation is greatest and influence of loess is minimal where stands occur as predominantly slope forests in relatively deep, dissected stream valleys. The vegetation in this region has been broadly considered distinct from other coastal plain forests (Bryant et al. 1993, Fralish and Franklin 2002) but has received almost no specific study (Franklin and Kupfer 2004). Although vastly forested when compared to the loess plains to the west (USGS 1992), most of the vegetation is recovering from one or more forms of severe disturbance (Franklin and Kupfer 2004). *Quercus alba* dominates the upland forests, examples of which have been studied in a limited portion of this area by Franklin and Kupfer (2004), but these communities have not been described to the same detail as other ecological systems.

**Vegetation:** The most characteristic feature of the vegetation is a high cover value for *Fagus grandifolia*, but a variety of other hardwood species may also be found in the overstory. Stands are mesic, and some may be dominated by *Fagus grandifolia* and *Quercus alba*, others by *Quercus alba* or *Quercus pagoda* with other mesic hardwoods. This system is defined as being north of the range of *Magnolia grandiflora*, which excludes the "Beech‑Magnolia" forests of the deeper south. From north to south, there is some floristic variability in the component floristics of this system. *Quercus rubra* will be of greater importance north of 35 degrees N latitude, and *Pinus taeda* conversely of greater importance to the south of this boundary. The core concept of this system consists of association types in which *Quercus* spp. can be present in the canopy, but are not dominant; but some may exhibit codominance by *Fagus grandifolia* and *Quercus alba* or other mesic *Quercus* spp. Other important canopy components include *Liriodendron tulipifera, Liquidambar styraciflua, Acer rubrum, Nyssa sylvatica, Fraxinus americana, Magnolia acuminata* (of local distribution), *Magnolia virginiana*, and *Pinus taeda*. Some subcanopy components (in addition to canopy species) include *Carpinus caroliniana, Diospyros virginiana, Oxydendrum arboreum, Cornus florida, Acer floridanum (= Acer barbatum), Magnolia macrophylla* (to the south), *Ostrya virginiana, Ulmus alata*, and *Ilex opaca*. Other shrubs and woody vines include *Decumaria barbara, Rhododendron canescens, Toxicodendron radicans, Vitis rotundifolia*, and *Smilax glauca*. Important herbs include *Polystichum acrostichoides, Woodwardia areolata, Osmunda cinnamomea, Mitchella repens*, and *Hexastylis arifolia*. This system is found north of the distribution of *Pinus glabra* and *Magnolia grandiflora*, which will be absent.

**Dynamics:** These are stable, generally fire‑sheltered forests. There is presumably some natural disturbance from the effects of hurricanes (to the south), or from other windstorms, which are relatively frequent in the range of this system. Most of the vegetation is recovering from one or more forms of severe anthropogenic disturbance (Franklin and Kupfer 2004). Infrequent, low‑intensity surface fires and rare mosaic or replacement fires are typical in this system (Fire Regime Group III) (Landfire 2007a). The mean fire‑return interval (MFRI) is about 35 years with wide year‑to‑year and within‑type variation related to moisture cycles, degree of sheltering, and proximity to more fire‑prone vegetation types. Anthropogenic fire is also part of this variation. Exposure to occasional fires and severe storms may create some canopy disturbances, which can be followed by waves of tree recruitment, growth, and death resulting in changes in the density and structure of tree populations and in consequent fluctuations in forest species composition. Periodic droughts will cause death of or stress to moisture‑requiring canopy trees.

SOURCES

**References:** Batista and Platt 1997, Braun 1950, Bryant et al. 1993, Comer et al. 2003*, Delcourt and Delcourt 2000, Edwards et al. 2013, Engeman et al. 2007, Evans et al. 2009, Eyre 1980, Fralish and Franklin 2002, Franklin and Kupfer 2004, Greenberg et al. 1997, Greller 1988, LANDFIRE 2007a, USGS 1992

**Version:** 14 Jan 2014	**Stakeholders:** Southeast

**Concept Author:** R. Evans, M. Pyne, A. Schotz	**LeadResp:** Southeast

CES203.556 East Gulf Coastal Plain Southern Loess Bluff Forest

**Primary Division:** Gulf and Atlantic Coastal Plain (203)

**Land Cover Class:** Forest and Woodland

**Spatial Scale & Pattern:** Large patch

**Required Classifiers:** Natural/Semi‑natural; Vegetated (>10% vasc.); Upland

**Diagnostic Classifiers:** Forest and Woodland (Treed); Loess deposit (undifferentiated); Broad‑Leaved Deciduous Tree

**National Mapping Codes:** EVT 2329; ESLF 4135; ESP 1329

**Concept Summary:** This system of upland hardwood‑dominated forests is defined as including both the steep loess bluffs bordering the eastern edge of the Mississippi River Alluvial Plain, ranging from south‑central Mississippi to southeastern Louisiana, as well as hardwood vegetation of the "Loess Plains" immediately to the east of these bluffs and ravines. The vegetation is often richer than surrounding non‑loessal areas, or those with only thin loess deposits. At least in some examples of this system, tree species normally associated with bottomland habitats are found to be abundant or even dominant in non‑flooded uplands. In many cases, the bluffs provide habitat "refugia" for plant species that are more common to the north. The general composition of these forests along the bluffs changes from north to south; the more northerly examples are represented in this classification by East Gulf Coastal Plain Northern Loess Bluff Forest (CES203.481), north of the range of *Magnolia grandiflora* and *Pinus glabra*. As currently defined this system ranges from about 32°N latitude (where the Big Black River dissects the bluffs) southward and is restricted to the southern part of the Loess Bluff Hills (EPA Ecoregion 74a).

**Comments:** The vegetation of this system has been poorly studied and documented, and few associations have currently been described in the USNVC for this system. More information is needed. This system meets East Gulf Coastal Plain Interior Shortleaf Pine‑Oak Forest (CES203.506) farther to the east in Louisiana and Mississippi.

DISTRIBUTION

**Range:** This system is endemic to the loess bluffs ("Bluff Hills" [Ecoregion 74a] of EPA (2004)) and the immediately adjacent Southern Rolling Plains (western portion of EPA Ecoregion 74c) along the eastern edge of the Mississippi River Alluvial Plain in southwestern Mississippi and adjacent Louisiana.

**Divisions:** 203:C

**TNC Ecoregions:** 43:C

**Nations:** US

**Subnations:** LA, MS

**Map Zones:** 46:C, 99:C

**USFS Ecomap Regions:** 231H:CC

CONCEPT

**Environment:** This system occupies upland loess bluffs, ravines, and adjacent plains that are considerably higher in elevation than the adjacent Mississippi River Alluvial Plain. These bluffs consist of a belt of Pleistocene and Tertiary eolian deposits (Braun 1950) that are often deeply eroded and very steep, with fertile topsoil and abundant moisture. In many cases, the bluffs provide habitat "refugia" for plant species that are more common to the north (Delcourt and Delcourt 1975).

**Vegetation:** Forest stands of the southern loess bluffs are characteristically dominated by *Fagus grandifolia* and *Magnolia grandiflora*, with *Quercus pagoda, Liquidambar styraciflua*, and other hardwood species, along with *Pinus glabra* and *Pinus taeda*. Vegetation of the loess plains would more likely be dominated by *Quercus pagoda, Liquidambar styraciflua*, and other hardwood species, along with *Pinus taeda*.

**Dynamics:** Considering the southern bluffs in conjunction with a portion of the adjacent plains, along with proximity to the Gulf of Mexico, stands of this system tend to be somewhat less stable and more fire‑prone than the bluffs alone to the north (Landfire 2007a). As modeled here, replacement disturbance has roughly equal probability of occurring by either fire or weather‑related events. The latter include windthrow, lightning and ice damage, as well as the inclusion of the erosion and mass wastage that give the bluffs their characteristic steepness. Widespread insect or disease mortality has not been reported. Wind/weather/stress replacement frequency is modeled near 220 years, replacement fire return at approximately 215 years, and all fire return frequency at about 40 years. "Open" structure is uncommon, even when defined as canopy closure <81%, and may be created by mixed‑severity fire. Surface fire may maintain open conditions, but it does not transition closed classes. Disturbance is presumed to occur primarily in small gaps (less than one‑half acre). The presence of aggregates of intolerant species suggests that larger scale disturbances occasionally play a role, likely more so on the plains (Landfire 2007a). Periodic droughts will cause death of or stress to moisture‑requiring canopy trees.

SOURCES

**References:** Batista and Platt 1997, Braun 1950, Bryant et al. 1993, Comer et al. 2003*, Delcourt and Delcourt 1975, Delcourt and Delcourt 2000, EPA 2004, Edwards et al. 2013, Engeman et al. 2007, Eyre 1980, LANDFIRE 2007a

**Version:** 14 Jan 2014	**Stakeholders:** Southeast

**Concept Author:** R. Wieland and R. Evans	**LeadResp:** Southeast

CES203.242 Southern Atlantic Coastal Plain Mesic Hardwood Forest

**Primary Division:** Gulf and Atlantic Coastal Plain (203)

**Land Cover Class:** Forest and Woodland

**Spatial Scale & Pattern:** Large patch

**Required Classifiers:** Natural/Semi‑natural; Vegetated (>10% vasc.); Upland

**Diagnostic Classifiers:** Forest and Woodland (Treed); Long Disturbance Interval

**National Mapping Codes:** EVT 2343; ESLF 4150; ESP 1343

**Concept Summary:** This upland system of the Atlantic Coastal Plain ranges from Delaware south to interior Georgia in a variety of moist but non‑wetland sites that are naturally sheltered from frequent fire. Such sites include lower slopes and bluffs along streams and rivers in dissected terrain, mesic flats between drier pine‑dominated uplands and floodplains, and local topographic high areas within bottomland terraces or nonriverine wet flats. Soil textures are variable in both texture and pH. The vegetation consists of forests dominated by combinations of trees that include a significant component of mesophytic deciduous hardwood species, such as *Fagus grandifolia* or *Acer floridanum*. Its southern limit is generally exclusive of the natural range of *Pinus glabra* and *Magnolia grandiflora*. Upland and bottomland oaks at the mid range of moisture tolerance are usually also present, particularly *Quercus alba*, but sometimes also *Quercus pagoda, Quercus falcata, Quercus michauxii, Quercus shumardii*, or *Quercus nigra*. *Pinus taeda* is sometimes present, but it is unclear if it is a natural component or has entered only as a result of past cutting. Analogous systems on the Gulf Coastal Plain have pine as a natural component, and this may be true for some examples of this system. Understories are usually well‑developed. Shrub and herb layers may be sparse or moderately dense. Within its range, *Sabal minor* may be a prominent shrub. Species richness may be fairly high in basic sites but is fairly low otherwise.

**Comments:** There remains some uncertainty how this system and other mesic hardwood systems should be divided. There is a broad gradient in climate and species composition from north to south and west. The boundaries at the northern edge of its range (the Chesapeake Bay Lowlands TNC ecoregion) and at the break between the South Atlantic Coastal Plain and East Gulf Coastal Plain ecoregions are boundaries of convenience to create breaks in this broad gradient. At the southern end, the boundary has been better determined (April 2006) to exclude areas within the combined ranges of *Pinus glabra* and *Magnolia grandiflora*, making this system deciduous rather than mixed evergreen‑deciduous. Differences from mesic forests of the Piedmont are sometimes fairly subtle, and species that differentiate them in one part of the range many not work in other parts. In particular, some species that are excluded from the Coastal Plain farther south are common components farther north. In MD and DC, this system can extend into the Piedmont (this area is part of TNC ECO52, but USFS 221C [2005 version]), straddling the fall zone where the Coastal Plain and Piedmont meet. Besides the variation across the range of this system, there are two sets of distinctions within it that may be worthy of consideration for defining separate systems. Acidic and basic substrates have substantial floristic differences. Variants on upland slopes, nonriverine swamp islands, and high ridges in bottomlands could be recognized as separate systems, or the latter two could be treated as part of the systems that surround them. However, the difference between ecological processes in uplands and wetlands separates those surrounded by wetland systems from the surrounding systems. This is especially true in the case of floodplains, which have flood‑carried nutrient input as well as wetness as a difference. Floristic differences may exist between these variants, but they are subtle and do not appear to be definitive.

DISTRIBUTION

**Range:** This system ranges from Delaware south to central Georgia in the Atlantic Coastal Plain. Its southern limit is generally exclusive of the natural range of *Pinus glabra* as mapped by Kossuth and Michael (1990) and *Magnolia grandiflora* as mapped by Outcalt (1990).

**Divisions:** 203:C

**TNC Ecoregions:** 52:C, 56:C, 57:C, 58:C, 61:C

**Nations:** US

**Subnations:** GA, NC, SC, VA

**Map Zones:** 55:C, 58:C, 60:C

**USFS Ecomap Regions:** 221D:CC, 232Ac:CCC, 232B:CC, 232C:CC, 232H:CC, 232I:CC, 232J:CC

CONCEPT

**Environment:** This system occurs in a variety of moist non‑wetland sites that are naturally sheltered from frequent fire. The distribution of these forests is determined by the interaction of local topography and soil texture. Most common are lower slope and bluff examples along streams and rivers in dissected terrain, but some examples occur on mesic flats between drier pine‑dominated uplands and floodplains or on local high areas within bottomland terraces or nonriverine wet flats. Soils cover the full range of mineral soil textures, except the coarsest sands. Richer and more mesic stands occur in more strongly concave and finer‑textured areas. Soils are not saturated for any significant time during the growing season and seldom, if ever, are extremely dry. Soils developed from calcareous materials or rich alluvium may be basic; others are strongly acidic. Sites are protected from most natural fires by steep topography or by surrounding extensive areas of non‑flammable vegetation (Batista and Platt 1997).

**Vegetation:** Stands of this system include a significant component of mesophytic species such as *Fagus grandifolia* or *Acer floridanum (= Acer barbatum)*. Upland and bottomland oaks at the mid range of moisture tolerance are usually also present, particularly *Quercus alba*, but sometimes also *Quercus falcata, Quercus michauxii, Quercus shumardii*, or *Quercus nigra*. Other hardwood components include *Liriodendron tulipifera, Liquidambar styraciflua, Carya cordiformis, Nyssa sylvatica*, and *Magnolia tripetala*. *Pinus taeda* is sometimes present, but it is unclear if it is a natural component or has entered only as a result of past removal of the hardwood canopy and subsequent invasion. Analogous systems on the Gulf Coastal Plain have pine as a natural component, and this may be true for some examples of this system. Understories are usually well‑developed. Shrub and herb layers may be sparse or moderately dense, with the herb layer being forb‑dominated. Some typical smaller trees and shrubs include *Cornus florida, Symplocos tinctoria, Oxydendrum arboreum, Hamamelis virginiana, Morus rubra*, and *Stewartia malacodendron*. Within its range, *Sabal minor* may be a prominent shrub. Some stands may contain *Arundinaria gigantea*. Some typical herbs include *Mitchella repens* and *Hexastylis arifolia*. Species richness may be fairly high in basic sites but is fairly low otherwise.

**Dynamics:** Fire is naturally infrequent to absent in this system. Sites are protected from most natural fires by steep topography or by surrounding extensive areas of non‑flammable vegetation (Landfire 2007a). If fire does penetrate, it is likely to be low in intensity but may have significant ecological effects. These forests probably generally exist naturally as old‑growth forests, with canopy dynamics dominated by gap‑phase regeneration. However, exposure to occasional fires and severe storms may create more frequent and larger canopy disturbances than analogous systems inland. Storm‑related disturbance can be followed by waves of tree recruitment, growth, and death resulting in changes in the density and structure of tree populations and in consequent fluctuations in forest species composition. Disturbances in these forests appear to be critical for both regeneration and change in older stands (Batista and Platt 1997). Periodic droughts will cause death of or stress to moisture‑requiring canopy trees.

SOURCES

**References:** Batista and Platt 1997, Comer et al. 2003*, Edwards et al. 2013, Engeman et al. 2007, Eyre 1980, Flynn 1994, Kossuth and Michael 1990, LANDFIRE 2007a, Nelson 1986, Outcalt 1990, Quarterman and Keever 1962, Schafale 2012, Ware et al. 1993

**Version:** 14 Jan 2014	**Stakeholders:** East, Southeast

**Concept Author:** R. Evans	**LeadResp:** Southeast

CES203.502 Southern Coastal Plain Limestone Forest

**Primary Division:** Gulf and Atlantic Coastal Plain (203)

**Land Cover Class:** Forest and Woodland

**Spatial Scale & Pattern:** Large patch

**Required Classifiers:** Natural/Semi‑natural; Vegetated (>10% vasc.); Upland

**Diagnostic Classifiers:** Forest and Woodland (Treed); Circumneutral Soil; Broad‑Leaved Deciduous Tree

**National Mapping Codes:** EVT 2328; ESLF 4134; ESP 1328

**Concept Summary:** This system represents dry to dry‑mesic deciduous forests of the East Gulf Coastal Plain where limestone, marl, or other calcareous substrates occur near enough to the surface to influence vegetation composition. Examples are most common in the Black Belt region of Alabama and Mississippi, but are also present in more isolated patches in other portions of the region, including western Alabama, eastern Georgia, and southwestern middle Tennessee. Generally, the vegetation consists of forests and woodlands on well‑developed, deep soils. Related, but physiognomically distinct, vegetation surrounding rock outcrops and calcareous prairies is accommodated within other ecological systems.

**Comments:** Examples have been found in the Atlantic Coastal Plain which has led to a range expansion. South Carolina Heritage (B. Pittman/K. Boyle pers. comm.) has an ongoing study related to these communities in that state.

DISTRIBUTION

**Range:** This system occurs in the East Gulf (and rarely the Atlantic) Coastal Plain, most commonly in the Black Belt region of Alabama and Mississippi. It is also present in more isolated patches in other portions of the region, including western Alabama, eastern Georgia, and marginally in southwestern middle Tennessee. It is also apparently found in the Tallahassee Hills/Valdosta Limesink Region EPA 65o (Florida, Georgia).

**Divisions:** 203:C

**TNC Ecoregions:** 43:C, 53:C

**Nations:** US

**Subnations:** AL, GA?, MS, TN

**Map Zones:** 46:C, 55:C, 99:C

**USFS Ecomap Regions:** 231B:CC, 232B:CC, 232J:CC, 232K:CC

CONCEPT

**Environment:** Stands typically occur on ridges and upper to middle slopes of the southern coastal plains where limestone, marl, or other calcareous substrates occur near enough to the surface to influence vegetation composition.

**Vegetation:** Typical stands are dominated by oaks and hickories, particularly species which are indicative of finer‑textured soils and/or a higher base status in the soil (e.g., *Carya carolinae‑septentrionalis, Quercus muehlenbergii, Quercus pagoda, Quercus shumardii, Quercus stellata*). Other hardwood trees include *Fraxinus americana, Liquidambar styraciflua, Acer floridanum (= Acer barbatum)*, and *Aesculus glabra*. The rare *Carya myristiciformis* may also be found in some stands. Understory trees may include *Fraxinus americana* and *Juniperus virginiana var. virginiana*. Early‑successional or fire‑suppressed stands may exhibit greater dominance by *Juniperus virginiana*. More nutrient‑rich or fire‑sheltered stands may exhibit dominance or codominance by *Fraxinus americana, Tilia americana* (most commonly *var. caroliniana*, but *var. heterophylla* along the Chattahoochee River), and/or *Acer floridanum*. Understory trees may include smaller examples of canopy species in addition to *Aesculus pavia var. pavia, Cercis canadensis, Cornus florida, Ostrya virginiana*, and *Ulmus alata*. Shrubs and woody vines may include *Arundinaria gigantea, Berchemia scandens, Bignonia capreolata, Cocculus carolinus, Cornus drummondii, Crataegus* spp., *Euonymus americanus, Euonymus atropurpureus, Frangula caroliniana, Hydrangea quercifolia, Ilex decidua, Menispermum canadense, Parthenocissus quinquefolia, Ptelea trifoliata, Sideroxylon lycioides, Staphylea trifolia, Symphoricarpos orbiculatus, Toxicodendron radicans, Viburnum* spp., and *Vitis* spp. Some typical herbs include *Chasmanthium laxum, Chasmanthium sessiliflorum, Dichanthelium boscii, Lithospermum tuberosum, Polystichum acrostichoides, Sanicula* spp., *Solidago auriculata, Spigelia marilandica, Trillium* spp., and *Verbesina virginica*. The ground layers of some stands may exhibit dominance by native warm‑season grasses and other graminoids, including *Schizachyrium scoparium, Andropogon* spp., *Danthonia* spp., and *Carex cherokeensis*. In addition, *Tillandsia usneoides* may be present as an epiphyte.

**Dynamics:** Fire frequency and intensity are factors determining the relative mixture of deciduous hardwood versus evergreen trees in this system. Frequent surface fires occurred on a 5‑ to 10‑year return interval from both lightning and Native American ignitions. These frequent light surface fires maintained the grassy understory and kept hardwoods and shrubs from dominating the understory and forming a midstory layer. Lightning fires occurred primarily during the spring dry season (April and May) with a secondary peak of Native American and settler burning during the fall (October and November) (Landfire 2007a). Occasionally, during extensive droughts, mixed‑severity or stand‑replacement fires did occur, especially in drier stands, or those containing *Juniperus virginiana* or rarely with *Pinus* species (e.g., *Pinus taeda* and/or *Pinus echinata*). In addition, local thunderstorm‑caused blowdowns created gaps on a small but continual basis. More extensive regional disturbances included tropical storms during the growing season and ice storms during winter (in the northern part of the range). Dense stands of middle to older aged pines (where present) were susceptible to periodic mortality from bark beetle epidemics, and younger *Juniperus virginiana* trees were killed by periodic droughts.

SOURCES

**References:** Comer et al. 2003*, Edwards et al. 2013, Engeman et al. 2007, Eyre 1980, LANDFIRE 2007a, Spetich 2004, TNC 1996c

**Version:** 14 Jan 2014	**Stakeholders:** Southeast

**Concept Author:** A. Schotz and R. Evans	**LeadResp:** Southeast

CES203.476 Southern Coastal Plain Mesic Slope Forest

**Primary Division:** Gulf and Atlantic Coastal Plain (203)

**Land Cover Class:** Forest and Woodland

**Spatial Scale & Pattern:** Large patch

**Required Classifiers:** Natural/Semi‑natural; Vegetated (>10% vasc.); Upland

**Diagnostic Classifiers:** Forest and Woodland (Treed); Slope; Long Disturbance Interval; Broad‑Leaved Evergreen Tree

**National Mapping Codes:** EVT 2357; ESLF 4260; ESP 1357

**Concept Summary:** This forested system of the southern East Gulf and Atlantic coastal plains occurs on steep slopes, bluffs, or sheltered ravines where fire is naturally rare, generally within the natural range of *Pinus glabra* and *Magnolia grandiflora*. Stands are mesic, and vegetation typically includes species such as *Fagus grandifolia, Magnolia grandiflora, Illicium floridanum*, and other species rarely encountered outside this system in the region. Related forests which occur on deep loess soils along the western margin of the region are classified as East Gulf Coastal Plain Southern Loess Bluff Forest (CES203.556). Some component associations are also found in temporarily flooded floodplains adjacent to these slopes, but this is primarily an upland system. The system also includes essentially upland vegetation of Pleistocene terraces, although these are conceptually transitional to creek floodplain systems.

**Comments:** East Gulf Coastal Plain Northern Mesic Hardwood Slope Forest (CES203.477) is a similar mesic forest system to the north of this one in the Upper East Gulf Coastal Plain that has greater dominance by deciduous trees. The systems of the loess bluffs to the west of this one, bordering the Mississippi River Alluvial Plain, are treated as distinct and are more extensive and continuous in their extent both vertically and latitudinally [see East Gulf Coastal Plain Northern Loess Bluff Forest (CES203.481) and East Gulf Coastal Plain Southern Loess Bluff Forest (CES203.556)]. To the north of the combined ranges of *Pinus glabra* and *Magnolia grandiflora* in the Atlantic Coastal Plain, this system is replaced by Southern Atlantic Coastal Plain Mesic Hardwood Forest (CES203.242).

DISTRIBUTION

**Range:** This mesic upland system of the southern (Atlantic and Gulf) coastal plains is found in suitable conditions from southern South Carolina south to northern Florida and west to (and including) the loessal plains of Mississippi and Louisiana. Its range is generally congruent with the natural range of *Pinus glabra* as mapped by Kossuth and Michael (1990) and *Magnolia grandiflora* as mapped by Outcalt (1990).

**Divisions:** 203:C

**TNC Ecoregions:** 43:C, 53:C, 55:P, 56:C

**Nations:** US

**Subnations:** AL, FL, GA, LA, MS, SC

**Map Zones:** 46:C, 55:C, 56:?, 58:C, 99:C

**USFS Ecomap Regions:** 231B:CC, 231H:CC, 232B:CC, 232C:CC, 232D:CC, 232J:CC, 232K:CC, 232L:CC, 234A:CC

CONCEPT

**Environment:** This system is restricted to steep slopes, bluffs, or sheltered ravines where fire is naturally rare. This mesic habitat is confined to very limited, fire‑sheltered areas within the natural ranges of *Pinus glabra* (Kossuth and Michael 1990) and *Magnolia grandiflora* (Outcalt 1990). This system occurs in a variety of moist, non‑wetland sites that are naturally sheltered from frequent fire. These are typically narrow bands of vegetation between floodplain forests and upland communities dominated by *Pinus palustris* (Batista and Platt 1997). Most common are lower slope, bluff, and ravine examples along streams and rivers in dissected terrain, but some examples occur on mesic flats between drier pine‑dominated uplands and floodplains or on local high areas within bottomland terraces or nonriverine wet flats. There may be larger patches where side ‑drains join larger streams. Under closed‑canopy conditions, fire may only partially penetrate this system from adjacent uplands. Soils are typically deep, fine‑textured, and moderately well‑drained. Soils cover the full range of mineral soil textures, except for the coarsest sands. Soils are not saturated for any significant time during the growing season and seldom, if ever, are extremely dry. Soils developed from calcareous materials or rich alluvium may be basic; others are strongly acidic. Richer and more mesic stands occur in more strongly concave and finer‑textured areas. Sites are normally protected from most natural fires by steep topography or by surrounding extensive areas of non‑flammable vegetation. This system occurs in a region of mild winters, high annual rainfall and high evapotranspiration, as well as a high likelihood of hurricane landfall (Ware et al. 1993). These forests may represent relicts derived from the early Tertiary flora (Batista and Platt 1997).

**Vegetation:** Stands are mesic, and vegetation typically includes species such as *Fagus grandifolia, Magnolia grandiflora, Pinus glabra*, and other species rarely encountered outside this system in the region. All woody strata contain a mixture of evergreen and deciduous species. Canopies are diverse; in addition to the aforementioned taxa, other canopy taxa may include *Quercus alba, Quercus pagoda, Quercus michauxii, Quercus falcata, Quercus shumardii, Quercus velutina, Quercus laurifolia, Quercus nigra, Quercus hemisphaerica, Pinus echinata, Pinus taeda, Nyssa sylvatica, Fraxinus americana, Carya tomentosa (= Carya alba)* (in the north), *Carya glabra, Ulmus alata, Ulmus americana, Ulmus rubra, Liriodendron tulipifera*, and *Liquidambar styraciflua* (NatureServe Ecology unpubl. data 2003). The presence of *Pinus taeda* is normal at lower frequencies, but higher ones may indicate past disturbance or removal of the hardwood canopy and subsequent invasion. Additional subcanopy taxa may include *Acer floridanum (= Acer barbatum), Acer rubrum, Oxydendrum arboreum, Carpinus caroliniana ssp. caroliniana, Ostrya virginiana, Prunus caroliniana, Prunus serotina, Symplocos tinctoria, Magnolia macrophylla* (rare to the west), *Halesia diptera, Styrax grandifolius, Sassafras albidum, Ilex opaca, Hamamelis virginiana, Magnolia pyramidata, Tilia americana var. caroliniana, Zanthoxylum clava‑herculis, Crataegus marshallii, Morus rubra*, and *Cornus florida*. The shrub layer can be very diverse. Trees support lianas and epiphytes. Shrubs and woody vines include *Illicium floridanum, Hydrangea quercifolia, Arundinaria gigantea, Halesia diptera, Aesculus pavia, Calycanthus floridus var. floridus, Toxicodendron radicans, Parthenocissus quinquefolia, Viburnum rufidulum, Ilex vomitoria, Berchemia scandens, Vitis rotundifolia, Decumaria barbara, Callicarpa americana, Symplocos tinctoria, Nekemias arborea (= Ampelopsis arborea), Frangula caroliniana, Smilax tamnoides (= Smilax hispida), Gelsemium sempervirens, Sabal minor, Schisandra glabra, Lindera benzoin, Asimina parviflora, Cornus drummondii, Bignonia capreolata*, and *Euonymus americanus*. Except in gaps, herbs are scarce (Batista and Platt 1997). Herbs and herbaceous vines include *Thelypteris kunthii, Cystopteris protrusa, Viola walteri, Polystichum acrostichoides, Galium obtusum, Chasmanthium sessiliflorum, Aristolochia serpentaria, Trillium foetidissimum, Desmodium nudiflorum, Lithospermum tuberosum, Boehmeria cylindrica, Ageratina altissima var. altissima, Sanicula canadensis, Sanicula marilandica, Arisaema dracontium, Tillandsia usneoides, Cryptotaenia canadensis, Adiantum pedatum, Passiflora lutea, Cynoglossum virginianum, Botrychium virginianum, Ranunculus recurvatus, Mikania scandens*, and *Clematis crispa* (NatureServe Ecology unpubl. data 2003).

**Dynamics:** These are stable, fire‑sheltered forests. Fire is naturally infrequent to absent in this system. Sites are protected from most natural fires by steep topography or by surrounding extensive areas of non‑flammable vegetation (Landfire 2007a). If fire does penetrate, it is likely to be low in intensity but may have significant ecological effects. These forests probably generally exist naturally as old‑growth forests, with canopy dynamics dominated by gap‑phase regeneration. There is presumably some natural disturbance from the effects of hurricanes, which are relatively frequent in the range of this system, creating more frequent and larger canopy disturbances than analogous systems inland. Hurricanes can be followed by waves of tree recruitment, growth, and death resulting in changes in the density and structure of tree populations and in consequent fluctuations in forest species composition. Disturbances in these forests appear to be critical for both regeneration and change in older stands (Batista and Platt 1997). Periodic droughts will cause death of or stress to moisture‑requiring canopy trees.

SOURCES

**References:** Batista and Platt 1997, Christensen 2000, Comer et al. 2003*, Edwards et al. 2013, Engeman et al. 2007, Eyre 1980, FNAI 2010a, Flynn 1994, Kossuth and Michael 1990, LANDFIRE 2007a, NatureServe Ecology ‑ Southeastern U.S. unpubl. data, Nelson 1986, Nordman 2013, Outcalt 1990, Quarterman and Keever 1962, Ware et al. 1993

**Version:** 23 Apr 2015	**Stakeholders:** Southeast

**Concept Author:** A. Schotz and R. Evans	**LeadResp:** Southeast

CES203.280 West Gulf Coastal Plain Mesic Hardwood Forest

**Primary Division:** Gulf and Atlantic Coastal Plain (203)

**Land Cover Class:** Forest and Woodland

**Spatial Scale & Pattern:** Large patch

**Required Classifiers:** Natural/Semi‑natural; Vegetated (>10% vasc.); Upland

**Diagnostic Classifiers:** Forest and Woodland (Treed); Very Long Disturbance Interval; Broad‑Leaved Tree

**National Mapping Codes:** EVT 2323; ESLF 4129; ESP 1323

**Concept Summary:** This ecological system is found in limited upland areas, including ravines and sideslopes, of the Gulf Coastal Plain west of the Mississippi River. These areas are topographically isolated from historically fire‑prone, pine‑dominated uplands in eastern Texas, western Louisiana, and southern Arkansas. Sites are often found along slopes above perennial streams in the region. These sites have moderate to high fertility and moisture retention. Soils can be quite variable, ranging from coarse to loamy in surface texture. Most are acidic in surface reactions and less commonly circumneutral. Vegetation indicators are mesic hardwoods such as *Fagus grandifolia, Quercus alba*, and *Ilex opaca*, although scattered, large‑diameter pines (most often *Pinus taeda*) are also often present. Spring‑blooming herbaceous species are typical in the understory of most examples.

**Comments:** Some stands from Macon Ridge in Louisiana (a terrace ecoregion in the Mississippi River Alluvial Plain) are also included here. Some hardwood stands could occur on narrow ridgetops which are isolated from fire, but which would not be "mesic" in their composition or environment. These may be treated as a hardwood‑dominated example of the widespread and variable West Gulf Coastal Plain Pine‑Hardwood Forest (CES203.378). More information is needed.

DISTRIBUTION

**Range:** This system is limited to particular upland areas (especially ravines and sideslopes) of the Gulf Coastal Plain west of the Mississippi River, with some occurrences on Macon Ridge (a terrace ecoregion in the Mississippi River Alluvial Plain) in Louisiana.

**Divisions:** 203:C

**TNC Ecoregions:** 40:C, 41:C, 42:C

**Nations:** US

**Subnations:** AR, LA, TX

**Map Zones:** 37:C, 44:P, 99:C

**USFS Ecomap Regions:** 231E:CC, 232F:CC

CONCEPT

**Environment:** Sites are often found along slopes above perennial streams in the region. These sites have moderate to high fertility and moisture retention. Soils can be quite variable, ranging from coarse to loamy in surface texture. Most are acidic in surface reactions and less commonly circumneutral. It is found on Tertiary formations, from the Willis Formation in the south, northward through Eocene formations; it is primarily restricted to fairly rugged landscapes on ravines, steep slopes and low landscape positions, often near streams. It often occupies lower slope positions and adjacent steep slopes, where topographic position results in moisture accumulation and lower solar insolation. These sites may occur adjacent to bottomlands, but on more well‑drained soils and/or slightly higher topographic positions (Elliott 2011).

**Vegetation:** Examples of this forested system can be diverse. Canopy trees can include *Fagus grandifolia, Magnolia grandiflora, Liquidambar styraciflua, Quercus alba, Quercus shumardii, Quercus pagoda, Quercus falcata, Quercus michauxii* (in wetter examples), *Quercus hemisphaerica* (in drier examples), *Quercus stellata* (in drier examples), *Quercus nigra, Fraxinus americana, Carya tomentosa (= Carya alba), Celtis laevigata, Nyssa sylvatica, Ulmus americana*, and *Pinus taeda*. *Pinus taeda*, and to a lesser extent, *Pinus echinata* may be present to codominant in the overstory. *Quercus rubra* is rare and of limited extent in the range of this ecological system but is attributed to an association which occurs in Hempstead, Howard, Little River, and Sevier counties, Arkansas. Understory trees can include *Carpinus caroliniana, Prunus caroliniana, Ostrya virginiana, Ilex opaca var. opaca, Cornus florida, Acer floridanum (= Acer barbatum)*, and *Acer leucoderme*. *Arundinaria gigantea* may be present in some examples. Other shrubs may include *Persea borbonia, Viburnum acerifolium*, and *Sabal minor*. *Vitis rotundifolia, Smilax* spp., and *Parthenocissus quinquefolia* are commonly encountered woody vines. Herbs can include *Solidago auriculata, Athyrium filix‑femina ssp. Asplenioides, Chasmanthium sessiliflorum, Cynoglossum virginianum*, and *Trillium ludovicianum*. Some occurrences on more calcareous substrates lack *Magnolia grandiflora* and may contain species such as *Tilia americana* and *Styrax* spp., and may have a rich, more calciphilic, vernal forb flora. Such species as *Podophyllum peltatum, Arisaema dracontium, Arisaema triphyllum, Sanguinaria canadensis, Erythronium* spp., *Trillium* spp., and *Polygonatum biflorum* may dominate the ground layer of the forest in the early spring. Later in the year, these species become inconspicuous and are replaced by species such as *Chasmanthium sessiliflorum, Mitchella repens, Sanicula canadensis, Carex* spp., and *Dichanthelium* spp. Ferns, such as *Woodwardia* spp., *Osmunda cinnamomea, Athyrium filix‑femina ssp. asplenioides*, and *Polystichum acrostichoides*, may be conspicuous.

**Dynamics:** The mesic nature of sites occupied by this system, along with the topography of the sites and the limited fine fuel production in the system, results in reduced fire frequency.

SOURCES

**References:** Ajilvsgi 1979, Comer et al. 2003*, Elliott 2011, Eyre 1980, Marks and Harcombe 1981

**Version:** 14 Feb 2011	**Stakeholders:** Southeast

**Concept Author:** R. Evans	**LeadResp:** Southeast

1.B.1.Nc. Californian Forest & Woodland

M009. Californian Forest & Woodland

CES206.935 California Central Valley Mixed Oak Savanna

**Primary Division:** Mediterranean California (206)

**Land Cover Class:** Steppe/Savanna

**Spatial Scale & Pattern:** Matrix

**Required Classifiers:** Natural/Semi‑natural; Vegetated (>10% vasc.); Upland

**Diagnostic Classifiers:** Lowland [Lowland]; Woody‑Herbaceous; Mediterranean [Mediterranean Xeric‑Oceanic]; Deep Soil; Xeric; F‑Landscape/Low Intensity; Quercus lobata, Quercus douglasii

**National Mapping Codes:** EVT 2112; ESLF 5401; ESP 1112

**Concept Summary:** Historically, these savannas occurred on alluvial terraces and flat plains, often with deep, fertile soils, throughout the California Central Valley from Lake Shasta south to Los Angeles County. This system is found from 10‑1200 m (30‑3600 feet) elevation; receiving on average 50 cm (range 25‑100 cm) of precipitation per year, mainly as winter rain. Variable canopy densities in existing occurrences are likely due to variation in soil moisture regime, natural patch dynamics of fire, and land use (fire suppression, livestock grazing, herbivory, etc.). *Quercus lobata* was the characteristic oak species of these savannas, though other species were present, including *Quercus wislizeni, Quercus agrifolia, Quercus douglasii, Aesculus californica, Cercis canadensis var. texensis, Juniperus californica*, and *Nassella pulchra*. There is some evidence that much of the understory prior to the invasion by non‑native annual grasses and forbs was composed of native annual herbs such as *Hemizonia, Eriogonum, Trifolium, Gilia, Navarretia, Lupinus, Calycadenia, Lessingia, Lotus, Daucus*, and *Holocarpha* spp. There is considerable seasonal and annual variation in cover of understory species due to phenology and intra‑annual precipitation and temperature variation.

DISTRIBUTION

**Range:** Historically, this system was found throughout the California Central Valley from Lake Shasta south to Los Angeles County.

**Divisions:** 206:C

**TNC Ecoregions:** 13:C, 15:P, 16:P

**Nations:** US

**Subnations:** CA

**Map Zones:** 3:C, 4:C, 5:C, 6:C

**USFS Ecomap Regions:** 262A:CC, 263A:??, 322A:??, M261A:CP, M261B:CC, M261C:CC, M261E:CC, M261F:CC

CONCEPT

**Environment:** These savannas historically occurred on alluvial terraces and flat plains, often with deep, well‑drained fertile soils, throughout the California Central Valley from Lake Shasta south to Los Angeles County. This system is found from 10‑1200 m (30‑3600 feet) elevation; receiving on average 50 cm (range 25‑100 cm) of precipitation per year, mainly as winter rain. Summers are generally hot and dry. Variable canopy densities in existing occurrences are likely due to variation in soil moisture regime and natural patch dynamics of fire, also intra‑annual precipitation and temperature variability result in variability in cover of plants.

**Vegetation:** *Quercus lobata* was the characteristic oak species of these savannas, though other species were present, including *Quercus wislizeni, Quercus agrifolia, Quercus douglasii, Aesculus californica, Cercis canadensis var. texensis (= Cercis occidentalis), Juniperus californica*, and *Nassella pulchra*. There is some evidence that much of the understory prior to the invasion by non‑native annual grasses and forbs was composed of native annual herbs such as *Hemizonia, Eriogonum, Trifolium, Gilia, Navarretia, Lupinus, Calycadenia, Lessingia, Lotus, Daucus*, and *Holocarpha* spp. There is considerable seasonal and annual variation in cover of understory species due to phenology and intra‑annual precipitation and temperature variation.

**Dynamics:** Fire regime: frequent surface fires since good fuels of grasses, and carried from adjacent grasslands. Summer to early fall; FRI 5‑100+ (Sawyer et al. 2009). Very productive and fire‑prone landscape. From Sawyer et al. (2009): Literature describing post‑fire natural regeneration and long‑term fire recovery of *Quercus lobata* woodlands is minimal. Plants have the ability to survive fire, and stands probably burned frequently and hot with dry grasses and oak litter carrying surface fires. Larger mature trees are usually resistant to moderate‑severity fire because of their thick bark. While seedlings and saplings are top‑killed by such fire, juveniles sprout from root crowns. However, older mature trees that are top‑killed do not have this same ability. Animals such as scrub jays also facilitate regeneration of *Quercus lobata*, because they prefer burned areas as acorn‑caching sites, and buried acorns usually survive fire (Howard 1992, Wills 2006). Hot surface fires may kill large trees that have extensive internal rot, and usually kill small trees. Crown fires will kill a large number of valley oak of all size classes (Howard 1992). Herbivory from ungulates winter range; ground burrowers; oak regeneration is dependent upon bare soil and dispersal from birds/small mammals burial of seeds. Valley oak regeneration to replace mature trees is lower than in other deciduous oak species (Landfire 2007a). Some studies indicate that this is due to a rare occurrence of necessary climate conditions, such as a warm summer followed by several wet years.

SOURCES

**References:** Allen‑Diaz et al. 2007, Barbour and Billings 2000, Barbour and Major 1988, Barbour et al. 2007a, Bolsinger 1988, Brooks and Minnich 2006, Comer et al. 2003*, Griffin 1971, Holland and Keil 1995, Howard 1992a, LANDFIRE 2007a, Mahall et al. 2005, Mayer and Laudenslayer 1988, PRBO Conservation Science 2011, Sawyer and Keeler‑Wolf 1995, Sawyer et al. 2009, Shiflet 1994, WNHP 2011, Wills 2006

**Version:** 14 Jan 2014	**Stakeholders:** West

**Concept Author:** P. Comer and T. Keeler‑Wolf	**LeadResp:** West

CES206.922 California Coastal Closed‑Cone Conifer Forest and Woodland

**Primary Division:** Mediterranean California (206)

**Land Cover Class:** Forest and Woodland

**Spatial Scale & Pattern:** Small patch

**Required Classifiers:** Natural/Semi‑natural; Vegetated (>10% vasc.); Upland

**Diagnostic Classifiers:** Forest and Woodland (Treed); Marine Sedimentary; Cupressus macrocarpa, C. goveniana, C. abramsiana

**National Mapping Codes:** EVT 2177; ESLF 4268; ESP 1177

**Concept Summary:** Small occurrences of this system may be found in scattered locations along California's entire coastline and onto the Channel Islands. They are found on marine sedimentary, non‑metamorphosed features, often with podsols on sterile sandstone. These forests and woodlands are limited to coastal areas with moderate maritime climate and likely receive more annual precipitation than nearby coastal chaparral. Highly localized endemic tree species include *Hesperocyparis macrocarpa, Hesperocyparis goveniana*, and *Hesperocyparis abramsiana* in scattered groves along coastal Mendocino, San Mateo, Santa Cruz, and Monterey counties. *Pinus contorta var. contorta, Pinus contorta var. bolanderi, Pinus muricata, Pinus torreyana*, and *Pinus radiata* are dominant or codominant in these and other occurrences. These occurrences can also include pygmy woodland expressions where nearly lateritic subsoil underlies acidic sands (ancient marine terraces). Stunted and twisted *Pinus contorta var. contorta* stands along the Oregon coast (often called pygmy forests) are also part of this system. Other associated plant species include *Arctostaphylos nummularia, Ledum groenlandicum, Vaccinium ovatum, Gaultheria shallon, Rhododendron macrophyllum*, and *Morella californica*. The lichen and moss component of this system is very diverse, includes *Cladonia* spp., and can be abundant in these communities.

DISTRIBUTION

**Range:** This system is found in scattered locations along California's entire coastline and onto the Channel Islands and possibly just into southern Oregon in southern Coos and Curry counties.

**Divisions:** 206:C

**TNC Ecoregions:** 14:C, 15:C, 16:C

**Nations:** MX, US

**Subnations:** CA, MXBC, OR?

**Map Zones:** 2:C, 3:C, 4:C

**USFS Ecomap Regions:** 261B:CC, 263A:CC

CONCEPT

**Environment:** These woodlands occur in fire‑prone, seasonally dry and nutritionally poor locations, in areas with a Mediterranean climate (Barbour 2007). Found in scattered locations along California's entire coastline and onto the Channel Islands, as well as along the southern Oregon coast and on two small Islands off the coast of Baja California, Mexico. These forests and woodlands are limited to coastal areas with moderate maritime climate and likely receive more annual precipitation than nearby coastal chaparral; fog drip can be an important source of moisture in some stands. They are found on marine sedimentary, non‑metamorphosed features, often with Podsols on sterile sandstone. These occurrences can also include pygmy woodland expressions where nearly lateritic subsoil underlies acidic sands (ancient marine terraces). The soils are excessively well‑drained in most cases, but stands of *Pinus contorta var. bolanderi* occur on poorly drained Spodosols.

**Vegetation:** Highly localized endemic tree species include *Hesperocyparis macrocarpa (= Cupressus macrocarpa), Hesperocyparis goveniana (= Cupressus goveniana)*, and *Hesperocyparis abramsiana (= Cupressus abramsiana)* in scattered groves along coastal Mendocino, San Mateo, Santa Cruz, and Monterey counties. *Pinus contorta var. contorta, Pinus contorta var. bolanderi, Pinus muricata, Pinus torreyana*, and *Pinus radiata* are dominant or codominant in these and other occurrences. These occurrences can also include pygmy woodland expressions where nearly lateritic subsoil underlies acidic sands (ancient marine terraces). Stunted and twisted *Pinus contorta var. contorta* stands along the Oregon coast (often called pygmy forests) are also part of this system. Other associated plant species include *Arctostaphylos nummularia, Ledum groenlandicum, Vaccinium ovatum, Gaultheria shallon, Rhododendron macrophyllum*, and *Morella californica (= Myrica californica)*. The lichen and moss component of this system is very diverse, includes *Cladonia* spp., and can be abundant in these communities.

**Dynamics:** These woodlands typically are found in sharply demarcated localized groves with a single‑aged and monospecific overstory (Barbour 2007). The dominant trees are mostly serotinous in fire response (Davis and Borchert 2006), requiring heat to open the closed cones. Degree of serotiny varies widely across these species, along a continuum of conditions, but all are serotinous to some degree (Keeley and Zedler 1998, Barbour 2007). *Pinus torreyana* is reported to shed seeds from third‑year cones and continuously from those cones for several years (Lanner 1999). The seeds are wingless and large, suggesting they are animal dispersed and cached in the ground which protects them from fires. Most of the closed‑cone conifers are killed in crown fires because they grow in or near highly flammable chaparral (Barbour 2007). Moreover, they self‑prune poorly, typically retaining lower limbs to within a meter of the ground surface (Barbour 2007) so fire easily carries into the canopy. Because they often grow in dense thickets of small‑stemmed individuals, they may burn intensely even in the absence of chaparral. Basically, the fire regime of many closed‑cone conifers is the same as that of the surrounding shrublands and particularly characterizes *Hesperocyparis sargentii, Hesperocyparis forbesii, Hesperocyparis stephensonii, Pinus coulteri*, and *Pinus attenuata* (Landfire 2007a). The typical fire regime for most adjacent communities is known to have a return interval of less than 50 years (Barbour 2007).

Postfire regeneration of these species is closely linked to the frequency of fire relative to cone bank accumulation. For example, *Hesperocyparis sargentii* needs at least 20 years between fires to accumulate a cone bank sufficient to regenerate the stand. *Pinus coulteri* likely needs at least 25 years and preferably 30 years to develop an adequate cone bank. Fires that kill a stand before an adequate cone bank is in place will disappear (immaturity risk) as has been observed in *Hesperocyparis forbesii* and *Hesperocyparis sargentii*. Fire opens closed cones but not all stands necessarily burn in crown fires. Some may burn in ground and surface fires (Landfire 2007a). Severe drought can cause mortality of the trees without triggering seed dispersal; some *Hesperocyparis* species are susceptible to cypress canker, a fungus (*Coryneum cardinale*) (Barbour 2007).

SOURCES

**References:** Barbour 2007, Barbour and Major 1988, Barbour et al. 2007a, Borchert 1985, Comer et al. 2003*, Davis and Borchert 2006, Eyre 1980, Holland and Keil 1995, Keeley and Zedler 1998a, LANDFIRE 2007a, Lanner 2007, Ne'eman et al. 1999, PRBO Conservation Science 2011, Sawyer and Keeler‑Wolf 1995, Sawyer et al. 2009

**Version:** 14 Jan 2014	**Stakeholders:** Latin America, West

**Concept Author:** P. Comer and T. Keeler‑Wolf	**LeadResp:** West

CES206.937 California Coastal Live Oak Woodland and Savanna

**Primary Division:** Mediterranean California (206)

**Land Cover Class:** Steppe/Savanna

**Spatial Scale & Pattern:** Large patch

**Required Classifiers:** Natural/Semi‑natural; Vegetated (>10% vasc.); Upland

**Diagnostic Classifiers:** Woody‑Herbaceous; Sideslope; Mediterranean [Mediterranean Xeric‑Oceanic]; Xeric; F‑Patch/Medium Intensity; Broad‑Leaved Evergreen Tree; Quercus agrifolia

**National Mapping Codes:** EVT 2113; ESLF 5402; ESP 1113

**Concept Summary:** These *Quercus agrifolia*‑dominated woodlands occur throughout the Pacific coastal areas from Sonoma County, California, south to Baja California. Occurrences vary in canopy cover from dense conditions that support sparse understory vegetation of *Rubus ursinus, Symphoricarpos mollis, Heteromeles arbutifolia*, and *Toxicodendron diversilobum*, to more open conditions with perennial bunchgrass understory. The latter typically occur on south‑facing slopes with soils of variable depth. Variable canopy densities in existing occurrences are likely due to variation in soil moisture regime, natural patch dynamics of fire, and land use (fire suppression, livestock grazing, herbivory, etc.).

DISTRIBUTION

**Range:** Pacific coastal areas from Sonoma County, California, south to Baja California.

**Divisions:** 206:C

**TNC Ecoregions:** 15:C, 16:C

**Nations:** MX, US

**Subnations:** CA, MXBC

**Map Zones:** 3:C, 4:C, 5:?

**USFS Ecomap Regions:** 261B:CC, 262A:CC, 263A:CC, M261A:CC, M261B:CC

CONCEPT

**Environment:** This system is found mainly below 500 m elevation in foothill environments (but up to 1200 m) on alluvial terraces, canyon bottoms, streambanks, slopes, and flats. It is typically found within 100 km of the coast, largely within the coastal fog belt (Allen‑Diaz et al. 2007). Soils are moderately to well‑drained, deep, sandy or loamy with high organic matter. More open occurrences with perennial bunchgrass undergrowth are typically on south‑facing slopes with soils of variable depth. Annual precipitation is 40‑80 cm, with January mean minimum daily temperatures of 5‑10°C and July mean maximum daily temperatures of 18‑23°C.

**Dynamics:** From Sawyer et al. (2009): Dominant tree root system contains both roots that tap groundwater and extensive surface‑feeding ones (Callaway 1990, as cited in Sawyer et al. 2009). It is the most susceptible of the California oaks to soil drought.<br />

Fire is the dominant disturbance mechanism. Fire severity can range from high in oak woodlands with a high shrub component to moderate or low in open woodlands and savannas with a grass understory. Historically, fire occurred frequently, and the dominant oaks are resistant to low‑intensity surface fires (Allen‑Diaz et al. 2007). Lightning‑ignited fires are uncommon but human‑ignited fires may have occurred frequently given the propensity of aboriginal cultures to burn foothill environments (Keeley 2002, Landfire 2007a). Fire history does exert some effect on fire mosaic turnover, although the effect appears to be short‑lived. Also, productivity (e.g., high cover of flammable shrubs and grasses) does not seem to be as strong a control on fire occurrence as meteorology (i.e., hot, dry wind events) in these systems (Landfire 2007a).

From Sawyer et al. (2009): Large trees are exceptionally fire‑resistant with the thickest bark of any California oak. They generally recover well from a fire, although severely burned crowns, trunks, and root crowns may require several years to sprout. Smaller trees are less resistant, but even low to moderately severe fires often kill seedlings and saplings. Stands may attain 80 to 100% of their pre‑fire densities within 10 years after fire, though fire‑return intervals in natural conditions vary widely (Steinberg 2002b, Sugihara et al. 2006).

SOURCES

**References:** Allen‑Diaz et al. 2007, Barbour and Billings 2000, Barbour and Major 1988, Callaway and Davis 1993, Callaway and Davis 1998, Comer et al. 2003*, Davis and Borchert 2006, Eyre 1980, Holland and Keil 1995, Keeley 2002, LANDFIRE 2007a, Mensing 1998, PRBO Conservation Science 2011, Sawyer and Keeler‑Wolf 1995, Sawyer et al. 2009, Shiflet 1994, Steinberg 2002b, Sugihara et al. 2006, Van Dyke et al. 2001, WNHP 2011, Wills 2006

**Version:** 14 Jan 2014	**Stakeholders:** Latin America, West

**Concept Author:** P. Comer and T. Keeler‑Wolf	**LeadResp:** West

CES206.936 California Lower Montane Blue Oak‑Foothill Pine Woodland and Savanna

**Primary Division:** Mediterranean California (206)

**Land Cover Class:** Steppe/Savanna

**Spatial Scale & Pattern:** Matrix

**Required Classifiers:** Natural/Semi‑natural; Vegetated (>10% vasc.); Upland

**Diagnostic Classifiers:** Woody‑Herbaceous; Mediterranean [Mediterranean Xeric‑Oceanic]; Ustic; F‑Patch/Low Intensity; Needle‑Leaved Tree; Graminoid; Pinus sabiniana, Quercus douglasii; Savanna‑Woodland Mosaic

**National Mapping Codes:** EVT 2114; ESLF 5403; ESP 1114

**Concept Summary:** This ecological system is primarily found in the valley margins and foothills of the Sierra Nevada and Coast Ranges of California from approximately 120‑1200 m (360‑3600 feet) in elevation on rolling plains or dry slopes. Over a century of anthropogenic changes (especially cutting of oak) have altered the density and distribution of woody vegetation. A high‑quality occurrence often consists of open park‑like stands of *Pinus sabiniana*, with oaks and other various broadleaf tree and shrub species, including *Quercus douglasii, Quercus wislizeni, Quercus agrifolia* (primarily central and southern Coast Ranges), *Quercus lobata, Aesculus californica, Arctostaphylos* spp., *Cercis canadensis var. texensis, Ceanothus cuneatus, Frangula californica, Ribes quercetorum, Juniperus californica*, and *Pinus coulteri* (central and southern Coast Ranges). *Pinus sabiniana* tends to drop out all together in the driest and more southerly sites, which are often dominated by *Quercus douglasii*. The California central coast region may have open stands of just *Juniperus californica*, with a grassy understory. These stands belong here due to proximity to other blue oak and gray pine stands or chaparral, and due to the heavy native or non‑native grass cover. This is distinguished from Great Basin pinyon‑juniper stands, which have little herbaceous understory, and *Pinus monophylla* rather than *Pinus sabiniana*. These stands of only juniper are caused by repeated removal of the oaks by humans and feral pig populations. Northern extensions of this system include *Quercus garryana* as the dominant oak, where it becomes successional to Mediterranean California Lower Montane Black Oak‑Conifer Forest and Woodland (CES206.923). *Pinus sabiniana* density also varies based on intensity or frequency of fire, being less abundant in areas of higher intensity or frequency, hence it is often more abundant on steep, rocky or more mesic north‑facing slope exposures. Historically, understory vegetation included mixed chaparral to perennial bunchgrass. Currently, most occurrences have understories dominated by dense cover of annual species, both native and non‑native. Variable canopy densities in existing occurrences are likely due to variation in soil moisture regime, natural patch dynamics of fire, and land use (fire suppression, livestock grazing, herbivory, etc.).

DISTRIBUTION

**Range:** This system occurs primarily in the valley margins and foothills of the Sierra Nevada and Coast Ranges from approximately 120‑1200 m (360‑3600 feet) elevation, from Shasta County to Kern and northern Los Angeles counties, California. It is unlikely to occur in the southern portion of zone 7 (Modoc Plateau), but this needs to be confirmed with California ecologists.

**Divisions:** 206:C

**TNC Ecoregions:** 5:C, 12:C, 13:C, 14:C, 15:C

**Nations:** US

**Subnations:** CA

**Map Zones:** 2:P, 3:C, 4:C, 5:C, 6:C, 7:P

**USFS Ecomap Regions:** 261B:CC, 262A:CC, 263A:CC, 322A:PP, M242A:??, M242B:??, M261A:CC, M261B:CC, M261C:CC, M261D:CC, M261E:CC, M261F:CC

CONCEPT

**Environment:** Soils are shallow, low in fertility, and moderately to excessively drained with extensive rock fragments. It occurs on valley margins and foothills, rolling plains or dry slopes, and generally steeper and drier slopes than pure blue oak woodlands without foothill pine. Mediterranean climate with mild winter rain (not snow) and very hot summers. This system is extremely drought‑tolerant. The upper elevational limit is 150 m in the north and 900 m in the south.

**Vegetation:** A high‑quality occurrence often consists of open park‑like stands of *Pinus sabiniana*, with oaks and other various broadleaf tree and shrub species, including *Quercus douglasii, Quercus wislizeni, Quercus agrifolia* (primarily central and southern Coast Ranges), *Quercus lobata, Aesculus californica, Arctostaphylos* spp., *Cercis canadensis var. texensis (= Cercis occidentalis), Ceanothus cuneatus, Frangula californica (= Rhamnus californica), Ribes quercetorum, Juniperus californica*, and *Pinus coulteri* (central and southern Coast Ranges). *Pinus sabiniana* tends to drop out all together in the driest and more southerly sites, which are often dominated by *Quercus douglasii*.

SOURCES

**References:** Barbour and Billings 2000, Barbour and Major 1988, Comer et al. 2003*, Eyre 1980, Holland and Keil 1995, Sawyer and Keeler‑Wolf 1995, Sawyer et al. 2009, Shiflet 1994

**Version:** 13 Jan 2012	**Stakeholders:** West

**Concept Author:** P. Comer and T. Keeler‑Wolf	**LeadResp:** West

CES206.920 Central and Southern California Mixed Evergreen Woodland

**Primary Division:** Mediterranean California (206)

**Land Cover Class:** Forest and Woodland

**Spatial Scale & Pattern:** Large patch

**Required Classifiers:** Natural/Semi‑natural; Vegetated (>10% vasc.); Upland

**Diagnostic Classifiers:** Forest and Woodland (Treed); Mediterranean [Mediterranean Xeric‑Oceanic]; Xeric; Broad‑Leaved Evergreen Tree

**National Mapping Codes:** EVT 2014; ESLF 4201; ESP 1014

**Concept Summary:** This system occurs from Monterey, California, south across the outer Central Coast Ranges to crests of Peninsular Ranges. It can occur on metasediments and granitics. In much of this area, conifers are relatively infrequent, *Pinus coulteri* occurs in scattered stands and *Pseudotsuga macrocarpa* picks up in Transverse Ranges south to Mexico. Characteristic tree species include *Quercus chrysolepis, Quercus agrifolia, Quercus kelloggii, Umbellularia californica, Acer macrophyllum*, and *Arbutus menziesii*. Historic fire frequency was likely higher in this system than in similar systems to the north.

DISTRIBUTION

**Range:** Occurs from Monterey, California, south across the outer Central Coast Ranges to crests of Peninsular Ranges, and in Transverse Ranges south to Mexico.

**Divisions:** 206:C

**TNC Ecoregions:** 15:C, 16:C

**Nations:** MX, US

**Subnations:** CA, MXBC

**Map Zones:** 4:C, 5:?

**USFS Ecomap Regions:** 261B:CC, 262A:PP, 322A:PP

CONCEPT

SOURCES

**References:** Barbour and Billings 2000, Barbour and Major 1988, Comer et al. 2003*, Eyre 1980, Holland and Keil 1995, Sawyer and Keeler‑Wolf 1995

**Version:** 17 Mar 2003	**Stakeholders:** Latin America, West

**Concept Author:** P. Comer and T. Keeler‑Wolf	**LeadResp:** West

CES206.923 Mediterranean California Lower Montane Black Oak‑Conifer Forest and Woodland

**Primary Division:** Mediterranean California (206)

**Land Cover Class:** Forest and Woodland

**Spatial Scale & Pattern:** Large patch

**Required Classifiers:** Natural/Semi‑natural; Vegetated (>10% vasc.); Upland

**Diagnostic Classifiers:** Forest and Woodland (Treed); Toeslope/Valley Bottom; Franciscan Formation Soils; Deep Soil; Mineral: W/ A‑Horizon >10 cm

**National Mapping Codes:** EVT 2030; ESLF 4217; ESP 1030

**Concept Summary:** This ecological system is found throughout California's middle and inner North Coast Ranges, as well as the southern and eastern Klamath Mountains from 600‑1600 m (1800‑4850 feet) elevation, and the lower slopes of the western Sierra Nevada. It occurs in valleys and lower slopes on a variety of parent materials, including granitics, metamorphic and Franciscan metasedimentary parent material and deep, well‑developed soils. It is characterized by woodlands or forests of *Pinus ponderosa* with one or more oaks, including *Quercus kelloggii, Quercus garryana, Quercus wislizeni*, or *Quercus chrysolepis*. *Pseudotsuga menziesii* may co‑occur with *Pinus ponderosa*, particularly in the North Coast Ranges and Klamath Mountains. On most sites, the oaks are dominant, forming a dense subcanopy under a more open canopy of the conifers. On many sites, *Quercus kelloggii* is the dominant; in late‑seral stands on more mesic sites, conifers such as *Pinus ponderosa* or *Pseudotsuga menziesii* will form a persistent emergent canopy over the oak. Stands may have shrubby understories (in the Klamath Mountains and Sierra Nevada) and, more rarely, grassy understories (in North Coast Ranges). Common shrubs include *Arctostaphylos viscida, Arctostaphylos manzanita, Ceanothus integerrimus*, and *Toxicodendron diversilobum*. Grasses can include *Festuca californica, Festuca idahoensis*, and *Melica* spp. Historical fire in this system was likely high frequency but of low intensity. Conifer species, such as *Pseudotsuga menziesii*, become more abundant with wildfire suppression.

**Comments:** The floristic and geographic transition from this system to North Pacific Oak Woodland (CES204.852) needs to be further detailed. This system generally has lower tree species richness in the canopy and a lower canopy density than Mediterranean California Dry‑Mesic Mixed Conifer Forest and Woodland (CES206.916), although the oaks can form a dense subcanopy in the mixed conifer system.

DISTRIBUTION

**Range:** This system is found throughout California's middle and inner North Coast Ranges, as well as the Klamath Mountains from 600‑1600 m (1800‑4850 feet) elevation, and the lower slopes of the western Sierra Nevada.

**Divisions:** 206:C

**TNC Ecoregions:** 5:C, 14:C, 15:C

**Nations:** US

**Subnations:** CA, OR

**Map Zones:** 2:C, 3:C, 4:C, 5:P, 6:C, 7:C, 13:?

**USFS Ecomap Regions:** 261B:CC, 262A:CC, 263A:CC, 322A:CC, 341D:PP, 342B:PP, M242A:P?, M242B:PP, M261A:CC, M261B:CC, M261C:CC, M261D:CC, M261E:CC, M261F:CC, M261G:CC

CONCEPT

**Environment:** Mediterranean climate where winter temperatures can be from near freezing to 10°C. Snow occurs in winter at higher elevations, but does not last all season. Annual precipitation is 100 cm (Barbour et al. 2007). Low‑intensity fires are frequent (every 7‑10 year). Elevation ranges between 520 and 1525 m (1700‑5000 feet) in the Coast Ranges, Klamath Mountains and Sierra Nevada on deep often productive soils. North‑facing aspects tend to have more conifers, with more oak dominating on south, east and west exposures.

**Vegetation:** Vegetation is characterized by woodlands or forests of *Pinus ponderosa* with one or more oaks, including *Quercus kelloggii, Quercus garryana, Quercus wislizeni*, or *Quercus chrysolepis*. *Pseudotsuga menziesii* may co‑occur with *Pinus ponderosa*, particularly in the North Coast Ranges and Klamath Mountains. On most sites, the oaks are dominant, forming a dense subcanopy under a more open canopy of the conifers. On many sites, *Quercus kelloggii* is the dominant; in late‑seral stands on more mesic sites, conifers such as *Pinus ponderosa* or *Pseudotsuga menziesii* will form a persistent emergent canopy over the oak. Stands may have shrubby understories (in the Klamath Mountains and Sierra Nevada) and, more rarely, grassy understories (in North Coast Ranges). Common shrubs include *Arctostaphylos viscida, Arctostaphylos manzanita, Ceanothus integerrimus*, and *Toxicodendron diversilobum*. Grasses can include *Festuca californica, Festuca idahoensis*, and *Melica* spp. Historical fire in this system was likely high frequency but of low intensity. Conifer species, such as *Pseudotsuga menziesii*, become more abundant with wildfire suppression.

**Dynamics:** LANDFIRE model information: Historical fire frequency was 5 to 30 years in this type. Fire intensities were probably low in open stands but increased in severity as woodland vegetation transitioned to a denser, closed‑canopy type along watercourses. Vegetation is fire‑tolerant and therefore fire severity is low. The natural fire regime was a type I regime in the upland. With the more dense vegetation and the occurrence of fuel ladders, fire severity would become mixed. The fire regime may reflect a type III in this more mesic habitat.

Insects and disease may impact individual trees (ponderosa pine) locally. Armillaria root rot, western pine beetle, western oak looper, western tent caterpillar, and the pine engraver have the greatest potential for damage.

SOURCES

**References:** Barbour and Billings 2000, Barbour and Major 1988, Barbour et al. 2007a, Comer et al. 2003*, Eyre 1980, Holland and Keil 1995, Sawyer and Keeler‑Wolf 1995

**Version:** 12 Jan 2012	**Stakeholders:** West

**Concept Author:** P. Comer and T. Keeler‑Wolf	**LeadResp:** West

CES206.919 Mediterranean California Mixed Evergreen Forest

**Primary Division:** Mediterranean California (206)

**Land Cover Class:** Forest and Woodland

**Spatial Scale & Pattern:** Large patch

**Required Classifiers:** Natural/Semi‑natural; Vegetated (>10% vasc.); Upland

**Diagnostic Classifiers:** Forest and Woodland (Treed); Franciscan Formation Soils; Broad‑Leaved Evergreen Tree

**National Mapping Codes:** EVT 2043; ESLF 4230; ESP 1043

**Concept Summary:** This ecological system occurs from the Santa Cruz Mountains (and locally in the Santa Lucia Mountains), California, north into southwestern Oregon throughout the outer and middle Coast Ranges on Franciscan Formation soils (metasedimentary sandstones, schists, and shales) with moderate to high rainfall. This system occurs just inland from the redwood belt of this region. It also occurs in southern California in more mesic, protected, cooler sites of the Transverse and Peninsular ranges. Historic fire frequency in this system was higher than for redwood‑dominated systems (every 50‑100 years). It is characterized by mixes of coniferous and broad‑leaved evergreen trees. Characteristic trees include *Pseudotsuga menziesii, Quercus chrysolepis, Notholithocarpus densiflorus (= Lithocarpus densiflorus), Arbutus menziesii, Umbellularia californica*, and *Chrysolepis chrysophylla*. On the eastern fringe of this system, in the western Siskiyous, other conifers occur such as *Pinus ponderosa* and *Chamaecyparis lawsoniana*. In southern California (Transverse and Peninsular ranges), *Pseudotsuga macrocarpa* replaces *Pseudotsuga menziesii* but co‑occurs with *Quercus chrysolepis* and sometimes *Quercus agrifolia*. *Calocedrus decurrens* is occasional. In the southern portion of the range, *Notholithocarpus densiflorus, Arbutus menziesii, Umbellularia californica*, and *Chrysolepis chrysophylla* become less important or are absent. In the Santa Lucia Mountains, stands of *Abies bracteata* are included in this system and are an unusual and unique component. These stands are a mixture of *Abies bracteata* and *Quercus chrysolepis*. The more northerly stands tend to have dense or diverse shrub understories, with *Corylus cornuta, Vaccinium ovatum, Rhododendron macrophyllum, Gaultheria shallon, Quercus sadleriana, Mahonia nervosa*, and *Toxicodendron diversilobum* being common. Southern stands are less diverse and more sparse; *Toxicodendron diversilobum* is the most constant shrub, with *Ribes* spp. occasionally present, along with much *Polystichum munitum*. Especially in the south, stands are restricted to fire‑protected sites (extremely steep, northerly, mesic slopes and coves) where fires from adjacent chaparral systems do not carry.

**Comments:** In northern California, especially around Point Reyes, there are stands dominated by *Umbellularia californica*. These nearly pure stands are a part of this system, as it is a disturbance‑driven species and grows rapidly with full sunlight. With time and succession, other trees will succeed into the canopy. This is in contrast to small patches or individuals of *Umbellularia californica* in some of the various chaparral systems. Here there are no chaparral shrubs in the understory. The presence of *Notholithocarpus densiflorus* in mixed stands of pine and oak is the indicator species for this system in many places in the Coast Ranges throughout northern and central California.

DISTRIBUTION

**Range:** This system occurs from the Santa Lucia and Santa Cruz mountains of California north into southwestern Oregon throughout the outer and middle Coast Ranges and in southern California (Transverse and Peninsular ranges). It occurs in localized areas of the central to northern Sierra Nevada and southern and eastern Klamath Mountains.

**Divisions:** 206:C

**TNC Ecoregions:** 5:C, 14:C

**Nations:** US

**Subnations:** CA, OR

**Map Zones:** 2:C, 3:C, 4:C, 5:?, 6:C, 7:C

**USFS Ecomap Regions:** 263A:CC, M242A:CC, M242B:C?, M261A:CC, M261B:CC, M261C:CC, M261D:CC, M261E:CC, M261F:CC

CONCEPT

SOURCES

**References:** Barbour and Billings 2000, Barbour and Major 1988, Comer et al. 2003*, Eyre 1980, Holland and Keil 1995, Sawyer and Keeler‑Wolf 1995

**Version:** 23 Jan 2006	**Stakeholders:** West

**Concept Author:** P. Comer and T. Keeler‑Wolf	**LeadResp:** West

CES206.909 Mediterranean California Mixed Oak Woodland

**Primary Division:** Mediterranean California (206)

**Land Cover Class:** Forest and Woodland

**Spatial Scale & Pattern:** Large patch

**Required Classifiers:** Natural/Semi‑natural; Vegetated (>10% vasc.); Upland

**Diagnostic Classifiers:** Montane [Lower Montane]; Sideslope; Mediterranean [Mediterranean Xeric‑Oceanic]; Shallow Soil; F‑Patch/Medium Intensity; Quercus kelloggii; Quercus garryana var. breweri

**National Mapping Codes:** EVT 2029; ESLF 4216; ESP 1029

**Concept Summary:** This ecological system is found throughout the Sierra Nevada and Coast Range foothills and lower montane elevations from 600‑1600 m (1800‑4850 feet) on steep, rocky slopes where snow and cold temperatures occur. Fire frequency and intensity drive composition of this system, with *Quercus chrysolepis* dominant with less frequent fires. With frequent annual burning (at lower elevations and on warmer sites), this system is an open to dense woodland of large oaks with well‑developed grassy understories of native perennial bunchgrass. The predominant oaks with the higher frequency fires include *Quercus kelloggii* and *Quercus garryana*, with *Quercus garryana var. garryana* codominant in the central and northern Coast Ranges and *Quercus garryana var. fruticosa* often codominant in the northwestern Coast Ranges as well as portions of the Sierra Nevada. *Quercus chrysolepis* becomes dominant with less frequent fires (but in Oregon this species is not important and occurs in a different system, either Mediterranean California Mixed Evergreen Forest (CES206.919) or Mediterranean California Dry‑Mesic Mixed Conifer Forest and Woodland (CES206.916)). The perennial bunchgrass component includes *Festuca idahoensis, Festuca californica, Elymus glaucus*, and *Danthonia californica* (close to the coast). A variety of native forbs also occur. Other characteristic species include *Toxicodendron diversilobum, Juniperus occidentalis*, and *Ceanothus cuneatus*. This system is similar to North Pacific Oak Woodland (CES204.852) but does not include a conifer component, and *Quercus garryana* is not the only oak.

DISTRIBUTION

**Range:** This system is found throughout the Sierra Nevada and Coast Range foothills and lower montane of California and Oregon at elevations from 600‑1600 m (1800‑4850 feet).

**Divisions:** 206:C

**TNC Ecoregions:** 12:C, 13:C, 14:C, 16:P

**Nations:** US

**Subnations:** CA, OR

**Map Zones:** 2:C, 3:C, 4:C, 5:C, 6:C, 7:C, 13:?

**USFS Ecomap Regions:** 261B:CC, 262A:CC, 263A:CC, 322A:CC, 341D:PP, 342B:CC, M242A:??, M261A:CC, M261B:CC, M261C:CC, M261D:CC, M261E:CC, M261F:CC, M261G:CC

CONCEPT

**Environment:** Climate is Mediterranean, where winter temperatures can be from near freezing to 10°C. Snow occurs in winter at higher elevations, but does not last all season. Annual precipitation is 100 cm (Barbour et al. 2007). Low‑intensity fires are frequent (every 7‑10 years). This system occurs in the foothills of the Coast Ranges and Sierra Nevada between 600‑1600 m (1970‑5250 feet) in elevation on steep rocky slopes.

**Vegetation:** Fire frequency and intensity drive composition of this system, with *Quercus chrysolepis* dominant with less frequent fires. With frequent annual burning (at lower elevations and on warmer sites), this system is an open to dense woodland of large oaks with well‑developed grassy understories of native perennial bunchgrass. The predominant oaks with the higher frequency fires include *Quercus kelloggii* and *Quercus garryana*, with *Quercus garryana var. garryana* codominant in the central and northern Coast Ranges and *Quercus garryana var. fruticosa (= var. breweri)* often codominant in the northwestern Coast Ranges as well as portions of the Sierra Nevada. *Quercus chrysolepis* becomes dominant with less frequent fires (but in Oregon this species is not important). The perennial bunchgrass component includes *Festuca idahoensis, Festuca californica, Elymus glaucus*, and *Danthonia californica* (close to the coast). A variety of native forbs also occur. Other characteristic species include *Toxicodendron diversilobum, Juniperus occidentalis*, and *Ceanothus cuneatus*.

**Dynamics:** LANDFIRE model information: Fire Regime I, primarily short‑interval (e.g., <10 years) surface fires. Surface fires every 3‑10 years maintained an open savanna‑like structure. Fires can be mixed‑severity, especially when closed‑canopy conditions or additional species such as conifers and shrubs are present. Native burning was a significant factor in fire frequency of this type, but return intervals may increase significantly with a little distance from native settlements and valley bottoms.

SOURCES

**References:** Barbour and Billings 2000, Barbour and Major 1988, Barbour et al. 2007a, Comer et al. 2003*, Eyre 1980, Holland and Keil 1995, Sawyer and Keeler‑Wolf 1995

**Version:** 12 Jan 2012	**Stakeholders:** West

**Concept Author:** P. Comer and T. Keeler‑Wolf	**LeadResp:** West

CES206.938 Southern California Oak Woodland and Savanna

**Primary Division:** Mediterranean California (206)

**Land Cover Class:** Steppe/Savanna

**Spatial Scale & Pattern:** Large patch

**Required Classifiers:** Natural/Semi‑natural; Vegetated (>10% vasc.); Upland

**Diagnostic Classifiers:** Woody‑Herbaceous; Mediterranean [Mediterranean Desertic‑Oceanic]; Broad‑Leaved Evergreen Tree; Evergreen Sclerophyllous Shrub; Quercus agrifolia, Q. wislizeni, Q. engelmannii

**National Mapping Codes:** EVT 2118; ESLF 5407; ESP 1118

**Concept Summary:** These oak woodlands and savannas occur in coastal plains, intermountain valleys, and low mountains (such as the San Jacinto Mountains) from Ventura County, California, south into Baja California, Mexico. *Quercus agrifolia, Quercus wislizeni, Quercus engelmannii, Quercus kelloggii*, and/or *Juglans californica* dominate a mixed closed or open canopy. Southern chaparral species such as *Adenostoma fasciculatum, Artemisia californica, Rhus integrifolia, Rhus ovata, Rhus trilobata, Ceanothus* spp., *Ribes* spp., and *Arctostaphylos* spp. are also characteristic. These woodlands may occur as remnant patches on offshore islands, where they include endemic species such as *Quercus tomentella* and *Lyonothamnus floribundus*. The California central coast region may have open stands of just *Juniperus californica*, with a grassy understory. These stands belong here due to proximity to other oak stands or chaparral, and due to the heavy native or non‑native grass cover. This is distinguished from Great Basin pinyon‑juniper stands, which have little herbaceous understory, and *Pinus monophylla* mixed with *Juniperus californica*. These stands of only juniper are caused by repeated removal of the oaks by humans and feral pig populations. Variable canopy densities in existing occurrences are likely due to variation in soil moisture regime, natural patch dynamics of fire, and land use (fire suppression, livestock grazing, herbivory, etc.). Most of these woodlands and savannas have been heavily altered through urban and agricultural development throughout southern California.

DISTRIBUTION

**Range:** This system occurs in coastal plains and intermountain valleys from Ventura County, California, south into Baja California, Mexico.

**Divisions:** 206:C

**TNC Ecoregions:** 15:C, 16:C

**Nations:** MX, US

**Subnations:** CA, MXBC

**Map Zones:** 4:C, 5:C

**USFS Ecomap Regions:** 261B:CC, 262A:CC, 322A:PP, 322C:P?

CONCEPT

**Environment:** This system occurs in coastal plains, intermountain valleys, and low mountains (such as the San Jacinto Mountains). Soils are moderately to well‑drained, deep, sandy or loamy with high organic matter. Elevation ranges from sea level to 2200 m, but generally at less than 1500 m elevation. It is found on variable aspects and topography with rainfall between 13‑102 cm (5‑40 inches).

**Vegetation:** *Quercus agrifolia, Quercus wislizeni, Quercus engelmannii, Quercus kelloggii*, and/or *Juglans californica* dominate a mixed closed or open canopy. Southern chaparral species such as *Adenostoma fasciculatum, Artemisia californica, Rhus integrifolia, Rhus ovata, Rhus trilobata, Ceanothus* spp., *Ribes* spp., and *Arctostaphylos* spp. are also characteristic. These woodlands may occur as remnant patches on offshore islands, where they include endemic species such as *Quercus tomentella* and *Lyonothamnus floribundus*.

**Dynamics:** Variable canopy densities in existing occurrences are likely due to variation in soil moisture regime and natural patch dynamics of fire. Fire reduces the survivorship and growth of juvenile *Quercus engelmannii*, with seedlings especially sensitive. Stands with grassy understories tend to suffer minimal damage, but those with shrubby understories tend to top‑kill the trees, which may sprout and survive (Sawyer et al. 2009). Natural fire‑return intervals are 30‑100 years, and occur primarily in summer to early fall. From Landfire (2007a): Typical regime is frequent, low‑severity fire that likely exert positive influences on overstory productivity and canopy resilience to fire damage. Infrequent isolated areas of stand‑replacement fire create gaps of grasslands that require patch‑gap recruitment and edge recolonization over time. Grass fuels allow very frequent fire, up to annually. A high proportion of seedlings and saplings are top‑killed in low‑ to moderate‑severity fires. Mortality rates of different size trees decrease with increasing height and dbh. Mortality may be as much as 50‑60% for trees less than 40 cm (15.7 inches) dbh. In plants that survive fires, there is a significant amount of resprouting (Lathrop and Osborne 1991, Lawson 1993, Steinberg 2002b).

SOURCES

**References:** Allen‑Diaz et al. 2007, Barbour and Billings 2000, Barbour and Major 1988, Brooks and Minnich 2006, Comer et al. 2003*, Eyre 1980, Holland and Keil 1995, Howard 1992a, LANDFIRE 2007a, Lathrop and Osborne 1991, Lawson 1993, Osborne 1989, PRBO Conservation Science 2011, Principe 2002, Sawyer and Keeler‑Wolf 1995, Sawyer et al. 2009, Shiflet 1994, Steinberg 2002b, WNHP 2011

**Version:** 14 Jan 2014	**Stakeholders:** Latin America, West

**Concept Author:** P. Comer and T. Keeler‑Wolf	**LeadResp:** West

1.B.1.Nd. Madrean‑Balconian Forest & Woodland

M015. Balconian Forest & Woodland

CES303.656 Edwards Plateau Dry‑Mesic Slope Forest and Woodland

**Primary Division:** Western Great Plains (303)

**Land Cover Class:** Forest and Woodland

**Spatial Scale & Pattern:** Large patch

**Required Classifiers:** Natural/Semi‑natural; Vegetated (>10% vasc.); Upland; Wetland

**Diagnostic Classifiers:** Forest and Woodland (Treed); Midslope; Broad‑Leaved Deciduous Tree; Broad‑Leaved Evergreen Tree

**National Mapping Codes:** EVT 2523; ESLF 4331; ESP 1523

**Concept Summary:** This system occurs on dry to mesic, middle slopes of the rolling uplands and escarpments of the Edwards Plateau and similar sites in the adjacent Blackland Prairie region. The canopy is typically dominated or codominated by deciduous trees, including *Quercus buckleyi, Quercus sinuata var. breviloba, Ulmus crassifolia*, and/or *Celtis laevigata var. reticulata*. *Quercus fusiformis* and *Juniperus ashei* are often present and are sometimes codominant with deciduous species of this system. Canopy closure is variable, and this system can be expressed as forests or woodlands. The shrub layer may be well‑represented, especially where the overstory canopy is discontinuous. Species such as *Aesculus pavia var. flavescens, Cercis canadensis var. texensis, Forestiera pubescens, Ungnadia speciosa, Ceanothus herbaceus, Sophora secundiflora, Rhus* spp., *Vitis* spp., and *Garrya ovata* may be present in the shrub layer. With the large amount of exposed rock, frequent accumulation of leaf litter, and significant canopy closure, herbaceous cover is generally sparse, with *Carex planostachys* often present. Woodland forbs such as *Tinantia anomala, Chaptalia texana, Nemophila phacelioides, Salvia roemeriana, Lespedeza texana*, and various ferns may also be present, these often being patchy in distribution.

**Comments:** Further field investigation is needed to better develop the association‑level information for this system.

DISTRIBUTION

**Range:** This system is expected to occur on dry‑mesic slopes in the Edwards Plateau and Lampasas Cutplain.

**Divisions:** 303:C

**TNC Ecoregions:** 29:C

**Nations:** US

**Subnations:** TX

**Map Zones:** 35:C

**USFS Ecomap Regions:** 255E:CC, 315C:C?, 315D:CC, 315G:C?

CONCEPT

**Environment:** This system occurs on dry‑mesic, primarily north‑ and east‑facing limestone slopes in the Edwards Plateau of Texas. In the adjacent Blackland Prairie region, it is found on limestone chalk cuestas (Elliott 2011). Stones and boulders are conspicuous on the soil surface. Soils are generally dark clay to clay loam and shallow. Steep Rocky and Steep Adobe Ecological Sites may be associated with this system (Elliot 2011).

**Vegetation:** The canopy is typically dominated or codominated by deciduous trees, including *Quercus buckleyi, Quercus laceyi, Quercus sinuata var. breviloba, Fraxinus albicans (= Fraxinus texensis), Ulmus crassifolia, Prunus serotina var. eximia, Juglans major*, and/or *Celtis laevigata var. reticulata*. *Quercus fusiformis* and *Juniperus ashei* are often present and are sometimes codominant with deciduous species of this system. Canopy closure is variable, and this system can be expressed as forests or woodlands. The shrub layer may be well‑represented, especially where the overstory canopy is discontinuous. Species such as *Aesculus pavia var. flavescens, Cercis canadensis var. texensis, Forestiera pubescens, Ungnadia speciosa, Ceanothus herbaceus, Frangula caroliniana, Sophora secundiflora, Viburnum rufidulum, Rhus* spp., *Vitis* spp., and *Garrya ovata* may be present in the shrub layer. With the large amount of exposed rock, frequent accumulation of leaf litter, and significant canopy closure, herbaceous cover is generally sparse, with *Carex planostachys* often present. Woodland forbs such as *Tinantia anomala, Chaptalia texana, Nemophila phacelioides, Salvia roemeriana, Lespedeza texana*, and various ferns may also be present, if patchy (Elliott 2011).

SOURCES

**References:** Comer et al. 2003*, Elliott 2011, Eyre 1980

**Version:** 24 Feb 2011	**Stakeholders:** Midwest, Southeast, West

**Concept Author:** L. Elliott and J. Teague	**LeadResp:** Southeast

CES303.660 Edwards Plateau Limestone Savanna and Woodland

**Primary Division:** Western Great Plains (303)

**Land Cover Class:** Forest and Woodland

**Spatial Scale & Pattern:** Large patch

**Required Classifiers:** Natural/Semi‑natural; Vegetated (>10% vasc.); Upland

**National Mapping Codes:** EVT 2383; ESLF 4326; ESP 1383

**Concept Summary:** This upland system occurs primarily on soils derived from chalk or limestone of Cretaceous or Pennsylvanian origin in the Edwards Plateau; it forms the matrix within this ecoregion. It can also occur on limestone in the shortgrass regions of Texas and north into Oklahoma in areas such as the Arbuckle Mountains. This system is typified by a mosaic of evergreen oak forests, woodlands and savannas over shallow soils of rolling uplands and upper slopes within the Edwards Plateau and Lampasas Cutplain. *Quercus fusiformis* or *Juniperus ashei* typically dominate the canopy of this system. Other species may include *Quercus buckleyi, Quercus laceyi, Quercus stellata, Ulmus crassifolia, Fraxinus albicans, Quercus sinuata, Quercus vaseyana, Sophora secundiflora, Mahonia trifoliolata*, and *Diospyros texana*. Physiographic expression of this system varies from dense mottes (patches of forest where canopy cover approaches 100%) interspersed with grasslands to open savannalike woodlands with scattered individual or small groups of trees. Understories can contain various shrubs and graminoids, including *Cercis canadensis var. texensis, Forestiera pubescens, Sideroxylon lanuginosum, Diospyros texana, Rhus trilobata, Bouteloua* spp., *Schizachyrium scoparium, Nassella leucotricha, Carex planostachys, Aristida purpurea, Aristida oligantha, Liatris punctata var. mucronata, Stillingia texana, Symphyotrichum ericoides, Stenaria nigricans, Monarda citriodora*, and *Salvia texana*. Grasslands dominated by *Schizachyrium scoparium* occur in small patches within more closed woodlands and in larger patches between mottes or in open savannalike woodlands with scattered trees. Grasslands in this system tend to grade from shortgrass communities in the west to mixedgrass communities to the east. Substrate (limestone) determines the range of this system within given examples. Some disturbed areas of the western plateau are now dominated by mesquite woodland. Natural mesquite woodlands are believed to have occurred on the deeper soils of adjacent riparian systems.

**Comments:** Distribution in Oklahoma needs to be reviewed. This system is described as a mosaic of grassland and woodland or forest communities. Southern mixedgrass grassland associations of M051 are found in the open savannas of this system.

DISTRIBUTION

**Range:** This system is found primarily within the Edwards Plateau ecoregion but can extend north into Oklahoma and into portions of the Southern Shortgrass region of Texas.

**Divisions:** 303:C

**TNC Ecoregions:** 28:P, 29:C, 33:?

**Nations:** US

**Subnations:** OK, TX

**Map Zones:** 26:C, 34:?, 35:C

**USFS Ecomap Regions:** 255E:CC, 315C:CC, 315D:CC, 315G:CC

CONCEPT

**Environment:** This system is primarily found on Cretaceous limestones of the Edwards Plateau and Limestone (also referred to as Lampasas) Cutplain, but also associated with Pennsylvanian limestones of the Palo Pinto Formation and Winchell, Ranger, Home Creek limestone in the vicinity of Palo Pinto County, as well as on Cretaceous chalk formations in the northern Blackland Prairie and Cretaceous limestones of the western Crosstimbers and Rolling Plains. It ranges north into Oklahoma and is found on rolling to level upland topography, often on plateau tops, but also on gentle slopes. Soils are generally loams, clay loams, or clays, often with limestone parent material apparent. Low Stony Hill, Adobe, Clay Loam, and Shallow Ecological Sites are commonly associated with this system (Elliott 2011). Soil moisture and topography influence this system.

**Vegetation:** This forest and woodland system is dominated by species such as *Quercus fusiformis, Quercus laceyi, Quercus vaseyana, Juniperus ashei*, or *Pinus remota*. Other species may include *Quercus buckleyi, Ulmus crassifolia, Fraxinus albicans (= Fraxinus texensis), Quercus sinuata*, and *Diospyros texana*. Certain uplands may have mottes of *Quercus fusiformis* dominating a savannalike woodland. Physiographic expression varies from dense mottes (patches of forest where canopy cover approaches 100%) interspersed with large or small grassland patches to open savannalike woodlands with scattered individual or small groups of trees. Understories can contain various shrubs and graminoids, including *Cercis canadensis var. texensis, Forestiera pubescens, Sideroxylon lanuginosum, Diospyros texana, Rhus trilobata, Mahonia trifoliolata, Sophora secundiflora, Opuntia engelmannii var. lindheimeri*, and *Cylindropuntia leptocaulis*. Grasses and forbs include *Bouteloua* spp., *Schizachyrium scoparium, Sorghastrum nutans, Bouteloua curtipendula, Bothriochloa barbinodis, Bothriochloa laguroides ssp. torreyana, Nassella leucotricha, Hilaria belangeri, Bouteloua dactyloides, Andropogon gerardii, Bouteloua hirsuta, Bouteloua rigidiseta, Muhlenbergia reverchonii, Muhlenbergia lindheimeri, Carex planostachys, Aristida purpurea, Aristida oligantha, Liatris punctata var. mucronata (= Liatris mucronata), Stillingia texana, Symphyotrichum ericoides, Stenaria nigricans (= Hedyotis nigricans), Monarda citriodora*, and *Salvia texana*. Grasslands dominated by *Schizachyrium scoparium* occur in small patches within more closed woodlands and in larger patches between mottes or in open savannalike woodlands with scattered trees. Grasslands in this system tend to grade from shortgrass communities in the west to mixedgrass communities to the east. Substrate (limestone) determines the range of this system within given examples. The herbaceous stratum is often dominated by non‑native grass species, especially *Bothriochloa ischaemum var. songarica*.

**Dynamics:** Substrate (limestone) and topographic position primarily influence this system. Fire, grazing and browsing may also influence this system.

SOURCES

**References:** Barbour and Billings 1988, Comer et al. 2003*, Elliott 2011, Eyre 1980, Ricketts et al. 1999, TNC 2004b

**Version:** 24 Feb 2011	**Stakeholders:** Midwest, Southeast, West

**Concept Author:** S. Menard and K. Kindscher	**LeadResp:** Southeast

CES303.038 Edwards Plateau Mesic Canyon

**Primary Division:** Western Great Plains (303)

**Land Cover Class:** Forest and Woodland

**Spatial Scale & Pattern:** Linear

**Required Classifiers:** Natural/Semi‑natural; Vegetated (>10% vasc.); Upland

**Diagnostic Classifiers:** Forest and Woodland (Treed); Canyon; Toeslope/Valley Bottom; Ustic; Flood Scouring; Canyon Mosaic

**National Mapping Codes:** EVT 2524; ESLF 4153; ESP 1524

**Concept Summary:** This ecological system is largely endemic to the Edwards Plateau ecoregion and occurs on canyon bottoms, mesic lower slopes and steep canyons, primarily in the Southern Balcones Escarpment, but also in the Eastern Balcones Escarpment. This system also includes cliff faces and lower slopes of boxed canyons occurring as narrow, sometimes long bands in areas often with seeps where moisture is consistently more available than on adjacent slopes. The tree canopy is generally closed. Common components include *Ulmus crassifolia, Juglans major, Quercus buckleyi, Quercus laceyi, Prunus serotina var. eximia* (becoming less common to the north), *Fraxinus albicans* (dominant in the northeastern plateau), *Quercus muehlenbergii*, and *Acer grandidentatum*. Canyon bottoms may have scattered *Quercus macrocarpa*. Substrate (limestone) and topographic position (north and east aspects and lower slopes) are the dominant characteristics of this system. Small seepage areas are often dominated by *Adiantum capillus‑veneris*, with *Thelypteris ovata var. lindheimeri* on nearby moist habitats. Other prominent species include *Buddleja racemosa, Ungnadia speciosa*, and *Toxicodendron radicans ssp. eximium*. Fire probably plays little role in the system, while grazing and browsing (by native as well as exotic ungulates) may play an important role in recruitment and understory composition. Adjacent, drier slopes are usually dominated by various *Quercus* species and *Juniperus ashei*.

DISTRIBUTION

**Range:** Largely endemic to the Edwards Plateau ecoregion and occurs on canyon bottoms, mesic lower slopes and steep canyons, primarily in the Southern Balcones Escarpment, but also in the Eastern Balcones Escarpment.

**Divisions:** 303:C

**TNC Ecoregions:** 29:C

**Nations:** US

**Subnations:** TX

**Map Zones:** 35:C

**USFS Ecomap Regions:** 255E:CC, 315D:CC

CONCEPT

**Environment:** This system is largely endemic to the Edwards Plateau ecoregion. Examples are associated with lower Cretaceous limestones of the Edwards Plateau, often on the Glen Rose or related formations. This system occurs on mesic lower slopes (toeslopes), canyon bottoms, and onto the margins of adjacent valleys of small drainages, primarily in the Southern Balcones Escarpment, but also in the Eastern Balcones Escarpment (also on the Limestone Cutplain). Occurrences are generally found in steep canyons where insolation is minimal, or on lower positions on northern‑ or eastern‑facing slopes. This system also includes areas of cliff faces and lower slopes of boxed canyons occurring as narrow, sometimes long bands in areas often with seeps where moisture is consistently more available than on adjacent slopes. Soils are rich loams, often very rocky, with little soil development. It includes Steep Rocky Ecological Site, in part (Elliott 2011).

**Vegetation:** The tree canopy is generally closed. Composition is variable among examples, depending on moisture status. Common components include *Ulmus crassifolia, Juglans major, Quercus buckleyi, Quercus laceyi, Prunus serotina var. eximia* (becoming less common to the north), *Fraxinus albicans (= Fraxinus texensis)* (dominant in the northeastern plateau), *Quercus muehlenbergii*, and *Acer grandidentatum*. Canyon bottoms may have scattered *Quercus macrocarpa*. Small seepage areas are often dominated by *Adiantum capillus‑veneris*, with *Thelypteris ovata var. lindheimeri* on nearby moist habitats. Other prominent species include *Buddleja racemosa, Ungnadia speciosa*, and *Toxicodendron radicans ssp. eximium*. Mesic examples are characterized by the presence of *Acer grandidentatum*, with *Quercus muehlenbergii* as a common associate, along with *Ulmus crassifolia, Juglans major, Quercus buckleyi, Quercus laceyi, Prunus serotina var. eximia* (becoming less common to the north), and *Fraxinus albicans* (dominant in the northeastern plateau). Mesic herbaceous indicators such as *Aquilegia canadensis* and *Clematis texensis* may be present in these examples (Elliott 2011).

**Dynamics:** Substrate (limestone) and topographic position (northern and eastern aspects and lower slopes) are the dominant characteristics of this system. Fire probably plays little role in the system, while grazing and browsing (by native as well as exotic ungulates) may play an important role in recruitment and understory composition.

SOURCES

**References:** Comer et al. 2003*, Elliott 2011, Eyre 1980, TNC 2004b

**Version:** 24 Feb 2011	**Stakeholders:** Southeast, West

**Concept Author:** L. Elliott and K.A. Schulz	**LeadResp:** Southeast

CES303.657 Llano Uplift Acidic Forest, Woodland and Glade

**Primary Division:** Western Great Plains (303)

**Land Cover Class:** Forest and Woodland

**Spatial Scale & Pattern:** Matrix

**Required Classifiers:** Natural/Semi‑natural; Vegetated (>10% vasc.); Upland; Wetland

**Diagnostic Classifiers:** Isolated Wetland [Strictly Isolated]

**National Mapping Codes:** EVT 2410; ESLF 7159; ESP 1410

**Concept Summary:** This upland matrix system occurs primarily on coarse soils derived from the weathering of underlying Precambrian granites in the Llano Uplift region of Texas. The underlying intrusive granitic bedrock substrate determines the range of this system. It is composed of a mosaic of vegetation types, including closed‑canopy forests, open woodlands, savannas and sparsely vegetated rock outcrops. Common trees include *Quercus marilandica, Quercus fusiformis, Quercus stellata, Carya texana, Ulmus crassifolia*, and *Prosopis glandulosa*. Subcanopy species may include *Diospyros texana, Aloysia gratissima, Ungnadia speciosa, Ziziphus obtusifolia var. obtusifolia, Eysenhardtia texana, Aesculus glabra var. arguta, Opuntia engelmannii var. lindheimeri, Yucca elata, Nolina texana*, and *Cylindropuntia leptocaulis*. Grasslands may be dominated by *Schizachyrium scoparium, Sorghastrum nutans, Panicum virgatum, Bouteloua hirsuta, Bouteloua curtipendula, Nassella leucotricha, Bothriochloa laguroides*, and *Plantago wrightiana*. Granitic glades and barrens are sparsely vegetated by crustose and foliose lichens, several ferns and fern allies, and cacti. This system also includes small (up to 16 m in diameter) shallow depressions that hold rainwater and support wetland flora including the Texas endemic, *Isoetes lithophila*.

**Comments:** This ecological system is defined to include a diversity of vegetation occurring on granitic outcrops and on soils that have developed over these outcrops in central Texas. In comparison to other areas of the U.S. where sparsely vegetated glades and barrens may be defined separately from the woodland surrounding them and/or the woodland separately from the forest (e.g., Southern Piedmont Granite Flatrock and Outcrop (CES202.329) just includes the sparsely vegetated barrens), these different vegetation types are included together here because they occur as an ecological complex or mosaic and they share floristic and geologic affinities that set them apart from the surrounding landscape. In the central mineral region of central Texas, granite glades and barrens are surrounded by areas of deeper soils derived from granite that support denser herbaceous or woody vegetation that includes many species found sparsely on the glades. In the eastern U.S. xeric granite outcrops are generally separated from one another by large areas of mesic to dry‑mesic forests, whereas the granitic outcrops in central Texas are separated from one another by areas of coarse soils derived from the underlying granite. In addition, the xeric nature of the granite outcrops in the eastern U.S. is a stark contrast to the other vegetation in this humid temperate environment. Whereas, west of the dry line, the moisture availability of the granite outcrops in central Texas is not as starkly contrasted with the surrounding landscape. This has been suggested as a reason why the granite glades of central Texas do not support the degree of endemism that is found on the granite outcrops of the eastern U.S. (Walters and Wyatt 1982). The relationship of this ecological system to the granite glades and woodlands occurring in Oklahoma (currently included in Crosstimbers Oak Forest and Woodland (CES205.682)) needs to be further explored.

Currently this system includes dry woodlands on shallow soil and mesic woodlands on deeper soil. The more mesic woodlands tend to support *Carya texana* which is generally absent in other areas on the plateau. Further investigation is needed to determine if the mesic and dry components should be classified as two separate systems.

DISTRIBUTION

**Range:** This system is restricted to the Llano Uplift region of Texas.

**Divisions:** 303:C

**TNC Ecoregions:** 29:C

**Nations:** US

**Subnations:** TX

**Map Zones:** 35:C

**USFS Ecomap Regions:** 315D:CC

CONCEPT

**Environment:** This system is restricted to the Llano Uplift, also known as the central mineral region of Texas. Though named as an uplift because it is an intrusion of Precambrian metamorphic rocks and large granitic massifs, this area is generally lower in elevation than the surrounding Edwards Plateau (Walters and Wyatt 1982, Riskind and Diamond 1988). At a regional scale, it is a topographic bowl, though rock outcrops such as Enchanted Rock often produce dramatic increases in elevation at a local scale. Aside from these massif intrusions, topography is generally level to rolling. The substrate of granites, gneisses and schists determines the range of this system in central Texas. Elevation ranges from 251 to 686 m above sea level (825‑2250 feet). Rainfall averages about 76 cm (30 inches), peaking in May or June and September. The central mineral region occupies approximately 1.5 million hectares in central Texas (Riskind and Diamond 1988). Mineralogy of the granitic material varies, with hornblende schist, graphite schist, quartz‑feldspar gneiss and quartz‑plagioclase‑microcline rock common (Riskind and Diamond 1988). Soils are generally sandy loams, with gravelly soils common. They are generally acidic and coarse, resulting from weathering of the underlying granite. Many areas of exposed bedrock are present. Most frequently encountered Ecological Sites include Shallow Granite, Sandy Loam, Red Savannah, Gravelly Sandy Loam, Shallow Ridge, Granite Gravel, Sandstone Hill, and Granite Hill (Elliott 2011).

**Vegetation:** This system is typified by a mosaic of vegetation types, including mixed oak forests and savannas over coarse soils and sparsely vegetated areas on rock outcrops. Species such as *Quercus marilandica, Quercus fusiformis, Quercus stellata, Carya texana, Ulmus crassifolia*, and *Prosopis glandulosa* may dominate the canopy of this system. Some areas are characterized by dense forest patches (mottes) of *Quercus fusiformis*, with various mixtures of other oaks and shrubs surrounded by open grasslands. Subcanopy species may include *Diospyros texana, Aloysia gratissima, Ungnadia speciosa, Ziziphus obtusifolia var. obtusifolia, Eysenhardtia texana, Aesculus glabra var. arguta, Opuntia engelmannii var. lindheimeri (= Opuntia lindheimeri), Yucca elata, Nolina texana*, and *Cylindropuntia leptocaulis (= Opuntia leptocaulis)*. The ground flora may contain *Schizachyrium scoparium, Sorghastrum nutans, Panicum virgatum, Bouteloua hirsuta, Bouteloua curtipendula, Nassella leucotricha, Eragrostis intermedia, Croton monanthogynus*, and *Plantago wrightiana*. In addition to oak woodlands and grasslands, this system also includes granitic glades and barrens. These are sparsely vegetated areas characterized by crustose and foliose lichens, several ferns and fern allies, and cacti, including *Cheilanthes lindheimeri, Pellaea ternifolia, Selaginella arenicola ssp. riddellii, Selaginella peruviana, Selaginella wrightii, Echinocereus reichenbachii*, and *Echinocereus coccineus*. Other species that may occur in cracks and crevices or slight depressions with shallow, gravelly soil include *Eriogonum tenellum, Lechea san‑sabeana, Sedum nuttallianum, Tripogon spicatus, Plantago wrightiana, Phemeranthus parviflorus (= Talinum parviflorum), Helenium amarum, Campanula reverchonii, Aphanostephus skirrhobasis*, and *Hypericum gentianoides*. Small‑scale shallow vernal pools formed within barrens by weathering of the granitic surface support *Crassula aquatica, Sedum nuttallianum, Phemeranthus parviflorus, Eleocharis montevidensis, Elatine brachysperma, Juncus diffusissimus, Allium canadense, Nothoscordum bivalve, Cooperia drummondii, Lepuropetalon spathulatum, Isoetes melanopoda*, and the Texas endemic *Isoetes lithophila*. Larger pools often exhibit a pattern of zonation of the vegetation as soil accumulates in the center. Crevices in the rock outcrops tend to support scattered, stunted individuals of trees and shrubs found in the adjacent woodland. Endemics or near‑endemics occurring within this ecological system include *Isoetes lithophila, Campanula reverchonii, Eriogonum tenellum var. ramosissimum, Elatine brachysperma, Valerianella texana, Packera texensis, Tradescantia pedicellata, Brazoria enquistii, Indigofera miniata (= var. texana)*, and *Tripogon spicatus*.

**Dynamics:** This ecological system is a complex of vegetation types. The different physiognomies are maintained by an interaction between site conditions and disturbance dynamics. The forest patches, woodlands, savannas and grasslands are thought to have been maintained historically by various fire frequencies and intensities. In the absence of natural or prescribed fire, increased cover of woody vegetation has increased in some occurrences. Native grazing may have also played a role in preventing woody encroachment though the rough terrain of much of this system would have limited the extent of native grazers.

SOURCES

**References:** Comer et al. 2003*, Elliott 2011, Eyre 1980, Riskind and Diamond 1988, Walters and Wyatt 1982, Whitehouse 1933

**Version:** 24 Feb 2011	**Stakeholders:** Southeast

**Concept Author:** J. Teague and L. Elliott	**LeadResp:** Southeast

M010. Madrean Lowland Evergreen Woodland

CES301.712 Bosque de Tascate de Guerrero

**Primary Division:** Madrean Semidesert (301)

**Required Classifiers:** Natural/Semi‑natural; Vegetated (>10% vasc.)

**Concept Summary:** Son bosques formados por árboles escuamifolios (hojas en forma de escama) del género *Juniperus* a los que se les conoce como táscate, enebro o cedro, con una altura promedio de 8 a 15 m de regiones subcálidas templadas y semifrías, siempre en contacto con los bosques de encino, pino‑encino, selva baja caducifolia y matorrales de zonas áridas. Las especies más comunes y de mayor distribución son *Juniperus flaccida, Juniperus deppeana, Juniperus monosperma* y algunas especies del género *Quercus* y *Pinus*. Estas comunidades por lo regular, se encuentran abiertas como consecuencia de las actividades forestales, agrícolas y pecuarias principalmente en el norte del país. Este sistema es una variante sur de la Sabana de Táscate Madreana (CES301.730). Ambas son templadas debido a la presencia de *Juniperus*, pero este sistema ocurre en áreas más xéricas del suroeste de México.

This system is dominated by escuamifolios trees forests (leaf‑shaped flake) of the genus *Juniperus* those who are known as juniper, juniper or cedar, with an average height of 8‑15 m regions warm‑temperate, always in contact with the oak forests, pine‑oak forest, deciduous forest and arid scrub. The most common and most widely distributed species are *Juniperus flaccida, Juniperus deppeana, Juniperus monosperma*, and some species of the genus Quercus and Pinus. In these communities, usually, are open as a result of forest, agricultural and livestock activities mainly in the north. This system is a southern variant of Madrean Juniper Savanna (CES301.730). Both are temperate due to the presence of *Juniperus*, but this is more xeric south western Mexico.

DISTRIBUTION

**Divisions:** 301:C

**Nations:** MX

CONCEPT

**Environment:** [from M010] This type is common in foothills, mountains and plateaus in the Sierra Madre Occidentale and Sierra Madre Orientale in Mexico, Trans‑Pecos Texas, southern New Mexico and Arizona, generally south of the Mogollon Rim. Stands occur on cool aspects of steep scarp slopes, in canyons (including alluvial terraces), on gently sloping alluvial fan piedmonts (bajadas), steeper colluvial slopes and ridges, as well as mesatops. Elevation generally ranges from 1300‑2225 m with high‑elevation stands restricted to warmer southern aspects. Pinyon and juniper woodlands extend down to 760 m elevation in Trans‑Pecos ranges. Oak‑dominated encinal stands may extend down along drainages to 900 m elevation in southern Sonora, but generally range from around 1350 m as woodland savannas on rolling hills intermixed with semi‑desert grasslands. Encinal woodlands extend up to 1650‑2200 m elevation in a mosaic within Madrean montane forests and woodlands (Brown 1982a). At the lowest elevation, encinal generally occupies the rockier substrates or is restricted to drainages within grasslands (Brown 1982a). Ground cover often has high cover of rock or bare ground. *Soil/substrate/hydrology:* Soils are variable, but are generally thin and rocky, but may include deeper clay loamy to gravelly loamy soils, especially on sites dominated by pinyon and junipers.

**Dynamics:** [from M010] Dynamics are complicated by the diverse plant communities present in this macrogroup. The pinyon‑juniper woodlands and savannas included in this macrogroup are represented by what Moir and Carleton (1987) classified as the High Sun Mild climate zone (summer precipitation and warm climate). Romme et al. (2003) developed a pinyon‑juniper classification with three types based on canopy structure, understory composition, and historic fire regime. All three types, pinyon‑juniper grass savanna, pinyon‑juniper shrub woodland, and pinyon‑juniper forest, are included in this macrogroup. However, the pinyon‑juniper grass savanna and a new, ecologically similar type with tree canopy >10% cover (pinyon‑juniper grass open woodland) have the greater aerial extent in the macrogroup (Landis and Bailey 2005, Gori and Bate 2007). Other types are the pinyon‑juniper shrub woodland, represented by pinyon‑juniper trees with an understory of shrubs such as *Quercus turbinella*, and the pinyon‑juniper forest type that has a typically sparse understory and is restricted to dry, rocky areas or following fires (Romme et al. 2003).

Fire dynamics for these types under historic natural conditions (also called natural range of variability (NRV); for pre‑1900 timeframe) are summarized as follows based on (Romme et al. 2003). The fire regime for the pinyon‑juniper grass savanna/pinyon‑juniper grass open woodland includes frequent, low‑severity surface fires that are carried by the herbaceous layer. The low density of trees (5‑20% cover) and high perennial grass cover is maintained by this fire regime. Mean fire interval is estimated to be 12‑43 years (Gori and Bate 2007). The fire regime for the pinyon‑juniper shrub woodland has moderately frequent, high‑severity crown fires that are carried by the shrub and tree layers. After a stand replacing fire the site begins at early seral stage and returns to a moderately dense tree layer with a moderate to dense shrub layer. Succession happens relatively quickly if the shrub layer includes chaparral species that recover rapidly from fire by re‑sprouting or from fire scarified seeds in a seed bank. Mixed‑severity fires may alter this pattern by creating a mosaic of pinyon‑juniper states (early‑, mid‑, and late‑seral). Mean fire interval is estimated to be 23‑81 years (Gori and Bate 2007). The fire regime for the pinyon‑juniper forest type has very infrequent, very high‑severity fires that are carried by tree crowns. The stand dynamics are stable with multi‑age tree canopy and with little change in shrub or herbaceous layers.

Pinyon and juniper stands in this macrogroup have been impacted by human activities over the last century. Historical fire regimes were disrupted following the introduction of livestock (and the 1890s drought). Grazing passively suppresses fire by removing fine fuels needed to carry surface and mixed‑severity fires that likely maintained the structure and composition of pinyon‑juniper savannas and pinyon‑juniper shrub woodlands historically. Active fire suppression was also practiced by the Federal government during the last 100 years (Swetnam and Baisan 1996b). As fire became less frequent, pinyon and juniper trees became denser and subsequent fires became more severe (Gori and Bate 2007). These impacts altered stand dynamics differently depending on stand structure. Fire dynamics under current conditions are summarized for the three major pinyon‑juniper types (pinyon‑juniper grass savanna/open woodland, pinyon‑juniper shrub woodland, and pinyon‑juniper forest) developed by Romme et al. (2003) using canopy structure, understory composition, and historic fire regime and adapted for our use as follows.

The fire regime for the pinyon‑juniper grass savanna/open woodland has a fire frequency that is significantly reduced and fire severity has greatly increased from pre‑1900, from low‑severity surface fires towards high‑severity and stand‑replacing crown fires. Tree density has increased and herbaceous biomass has decreased from historic conditions with active fire suppression and livestock grazing. Currently stands have some very old trees (>300 years) present but not numerous, and are typically dominated by many young trees (<150 years). This type may also occur on sites with more rock soil and less grasses. This type is outside Historic Range of Variation (HRV) for disturbance regime, structure and composition (Gori and Bate 2007).

The fire regime for the pinyon‑juniper shrub woodland has a fire frequency that is reduced and fire severity is somewhat increased from pre‑1900, from low to moderately frequent, high‑severity stand‑replacing fires and moderately frequent mixed‑severity fires that likely maintain this type, toward less frequent, higher severity fires (Gori and Bate 2007). Tree density has increased and herbaceous biomass has decreased from historic conditions with active fire suppression and livestock grazing. Currently most stands have a variable mix of tree and shrubs with few or no very old trees (>300 years) present. With fire suppression, this type maybe outside HRV for disturbance regime, and possibly for structure and composition as recent fires are likely more severe than historic fire in the late 1800s (Romme et al. 2003).

The fire regime for the pinyon‑juniper forest type still has infrequent, high‑severity fires that are carried by tree crowns. The stand dynamics remain relatively stable with little change in density of tree or shrub and herbaceous layers. Currently stands have numerous very old trees (>300 years) present with a multi‑aged structure. Active fire suppression and livestock grazing are thought to have had little impact on fire frequency and severity and the overstory structure and composition with this type remaining within HRV for disturbance regime (Gori and Bate 2007).

Most pinyon‑juniper woodlands in the southwest have high soil erosion potential. Several studies have measured present‑day erosion rates in pinyon‑juniper woodlands, highlighting the importance of herbaceous cover and biological soil crusts (Belnap et al. 2001) in minimizing precipitation runoff and soil loss in pinyon‑juniper woodlands.

Madrean encinal stands included in this macrogroup also vary considerably under historic natural conditions in tree density ranging from very open woodlands and treed savannas (5‑15% cover) with a perennial grass‑dominated understory in uplands, to moderately dense oak woodlands (20‑40% tree cover) in drainages and on north‑facing slopes. The understory of good‑condition stands generally has high cover of perennial grasses and low cover of shrubs such as *Mimosa*, and this good condition of the stand is maintained with frequent fires. Turner et al. (2003) documented a trend from more open woodlands and savannas to denser woodlands with higher cover of species of *Juniperus* and *Prosopis* over the last 150 years. Regeneration of oaks following disturbance is from resprouting rather than acorns because of the dry conditions (Germaine and McPherson 1998).

Although there is not much encinal‑specific information on fire‑return intervals (FRI) available, it is thought to be similar to adjacent ecosystems, primarily the semi‑desert grassland (FRI of 2.5‑10 years) (Wright 1980, Bahre 1985, McPherson 1995, Kaib et al. 1996) and the pine‑oak woodlands (FRI of 3‑7 years) (Wright 1980, Bahre 1985, Swetnam et al. 1992, McPherson 1995, Kaib et al. 1996, Swetnam and Baisan 1996b). Fire season in encinal was probably similar to that of other Madrean woodlands and grasslands, occurring predominantly before the summer monsoon between April and June when vegetation is dry and ignition sources from dry lightning strikes are common (Swetnam and Betancourt 1990). Post disturbance regeneration (such as after stand‑replacing fire) mostly occurs from resprouting from trees roots. Successful regeneration from acorns is related to annual precipitation (Germaine and McPherson 1998). The understory of poor‑condition stands with less frequent fires or experiencing extended drought may have significant shrub invasion by species of *Arctostaphylos* and *Juniperus* and reduction of perennial grass cover (Schussman 2006a).

Over the last century, the woody component in encinal has increased in density over time in the absence of disturbance such as fire (Burgess 1995, Gori and Enquist 2003, Turner et al. 2003, Schussman 2006a). This is correlated to a decrease in fire frequency that is related to a reduction of fine fuels that carry fire because of extensive livestock grazing. Frequent, stand‑replacing fire was likely a key ecological attribute prior to 1890 (Wright 1980, Bahre 1985, McPherson 1995, Kaib et al. 1996). The oak woodlands and savannas included in this macrogroup are characterized by a strong perennial grass layer and are driven by many of the same ecological processes as semi‑desert mixed grassland, primarily frequent fire and drought (USFS 2009). It is generally agreed that fire regime has been altered for encinal by passive fire suppression via removal of fine fuels through livestock grazing, as well as active suppression over the last 100 years. This has reduced the number of surface fires, permitting a buildup in woody fuels, resulting in increased fire severity when fires occur in encinal and adjacent vegetation types such as semi‑desert grasslands and pine‑oak woodlands across much or the southwestern U.S. and adjacent Mexico (Kaib et al. 1996, Swetnam and Baisan 1996). Reduced fire frequency is a disturbance of the natural fire regime and results in increased cover of woody plants (Barton 1999, Muldavin et al. 2002b, Gori and Enquist 2003, Turner et al. 2003). The increase in woody species in the Madrean encinal has changed species composition, in some areas, from oak‑dominated woodlands or savanna to mesquite‑ and/or juniper‑dominated woodlands (Turner et al. 2003).

Livestock grazing in encinal is currently a common practice in both the United States and Mexico, with grazing occurring in virtually all of Mexico's and in roughly 75% of the United States' oak woodlands (McPherson 1995). Livestock grazing can affect the structure and composition of Madrean oak woodlands, as well as soil structure and water infiltration (USFS 2009).

The introduction of the invasive non‑native, perennial grasses *Eragrostis lehmanniana* and *Eragrostis curvula* has greatly impacted many semi‑desert grasslands and encinal in this ecoregion (Cable 1971, Anable et al. 1992, Gori and Enquist 2003). Anable et al. (1992) and Cable (1971) found Lehmann lovegrass is a particularly aggressive invader and alters ecosystem processes, vegetation composition, and species diversity.

Historic fuelwood cutting for mining and domestic use and fencepost cutting was common in stands of this macrogroup in southeastern Arizona until the late 1800s, and is still common in Arizona and northern Mexico today (Bahre 1991, Bennett 1992). Although fuelwood harvesting had dramatic effects historically, its consequences were generally local and short‑lived (Turner et al. 2003). More recently, chemical and mechanical treatments such as chaining and rotochopping have impacted age structure, tree density and cover of many pinyon‑juniper woodlands with current demand for these products continuing to increase (Ffolliott et al. 1979, Gottfried 1987, Dick‑Peddie 1993, Gottfried and Severson 1993).

SOURCES

**References:** Barbour and Billings 2000, Brown et al. 1979, Brown et al. 1998, Dick‑Peddie 1993, INEGI 2005, Latin American Ecology Working Group n.d.*, Shiflet 1994

**Version:** 08 Jan 2015	**Stakeholders:** Latin America

**Concept Author:** C. Josse	**LeadResp:** Latin America

CES305.795 Madrean Encinal

**Primary Division:** Sierra Madre (305)

**Land Cover Class:** Forest and Woodland

**Spatial Scale & Pattern:** Large patch

**Required Classifiers:** Natural/Semi‑natural; Vegetated (>10% vasc.); Upland

**Diagnostic Classifiers:** Montane [Lower Montane]; Lowland [Foothill]; Tropical/Subtropical [Tropical Xeric]; Xeric; F‑Patch/Medium Intensity; Broad‑Leaved Evergreen Tree; Graminoid; Quercus arizonica, Q. emoryi, Q. grisea, Q. oblongifolia Q. toumeyi

**National Mapping Codes:** EVT 2023; ESLF 4210; ESP 1023

**Concept Summary:** Madrean Encinal occurs on foothills, canyons, bajadas and plateaus in the Sierra Madre Occidentale and Sierra Madre Orientale in Mexico, extending north into Trans‑Pecos Texas, southern New Mexico and sub‑Mogollon Arizona. These woodlands are dominated by Madrean evergreen oaks along a low‑slope transition below Madrean Lower Montane Pine‑Oak Forest and Woodland (CES305.796) and Madrean Pinyon‑Juniper Woodland (CES305.797). Lower elevation stands are typically open woodlands or savannas where they transition into desert grasslands, chaparral or in some cases desertscrub. Common evergreen oak species include *Quercus arizonica, Quercus emoryi, Quercus intricata, Quercus grisea, Quercus oblongifolia, Quercus toumeyi*, and in Mexico *Quercus chihuahuensis* and *Quercus albocincta*. Madrean pine, Arizona cypress, pinyon and juniper trees may be present but do not codominate. Chaparral species such as *Arctostaphylos pungens, Cercocarpus montanus, Purshia* spp., *Garrya wrightii, Quercus turbinella, Frangula betulifolia*, or *Rhus* spp. may be present but do not dominate. The graminoid layer is usually prominent between trees in grassland or steppe that is dominated by warm‑season grasses such as *Aristida* spp., *Bouteloua gracilis, Bouteloua curtipendula, Bouteloua rothrockii, Digitaria californica, Eragrostis intermedia, Hilaria belangeri, Leptochloa dubia, Muhlenbergia* spp., *Pleuraphis jamesii*, or *Schizachyrium cirratum*, species typical of Apacherian‑Chihuahuan Semi‑Desert Grassland and Steppe (CES302.735). This system includes seral stands dominated by shrubby Madrean oaks typically with a strong graminoid layer. In transition areas with drier chaparral systems, stands of chaparral are not dominated by Madrean oaks; however, Madrean Encinal may extend down along drainages.

Este encinal se produce en colinas, cañones, bajadas y mesetas de la Sierra Madre Occidental y Sierra Madre Oriental de México, que se extiende hacia el norte en Trans‑Pecos Texas, el sur de Nuevo México y sub‑Mogollon Arizona. Estos bosques están dominados por encinares Madrenses a lo largo de una transición de baja pendiente por debajo del Bosque Madreano Montano Bajo de Pino‑Roble (CES305.796) y Madrean Pinyon‑Juniper Woodland (CES305.797). Rodales a baja elevación son típicamente bosques abiertos o sabanas que transicionan a los pastizales del desierto, chaparral o en algunos casos matorral desértico. Especies de roble de hoja perenne comunes incluyen *Quercus arizonica, Quercus emoryi, Quercus intricata, Quercus grisea, Quercus oblongifolia, Quercus toumeyi*, y en México *Quercus chihuahuensis* y *Quercus albocincta*. Pino madreano, ciprés de Arizona, piñoneros y enebros pueden estar presentes pero no son codominates. Especies de chaparral como *Arctostaphylos pungens, Cercocarpus montanus, Purshia* spp., *Garrya wrightii, Quercus turbinella, Frangula betulifolia*, o *Rhus* spp. pueden estar presentes pero no dominan. La capa de gramíneas suele ser prominente entre los árboles en praderas o estepas que está dominada por pastos de estación cálida tales como *Aristida* spp., *Bouteloua gracilis, Bouteloua curtipendula, Bouteloua rothrockii, Digitaria californica, Eragrostis intermedia, Hilaria belangeri, Leptochloa dubia, Muhlenbergia* spp., *Pleuraphis jamesii* o *Schizachyrium cirratum*, especies típicas del sistema de Pastizales Semi‑desérticos y de la Estepa Apacherian‑Chihuahua (CES302.735). Este sistema incluye rodales suciesionales dominados por robles Madrenses arbustivos típicamente con una capa densa de gramíneas. En las zonas de transición con sistemas de chaparral más seco, se los chaparrales no están dominados por robles Madrenses, sin embargo, este encinal puede extenderse hacia abajo a lo largo de los drenajes.

**Comments:** Although some stands may be shrubby especially in the north, E. Muldavin (pers. comm.) says encinal is considered woodland in Mexico.

DISTRIBUTION

**Range:** This system is found in the Sierra Madre Occidentale and Sierra Madre Orientale of Mexico, Trans‑Pecos Texas, southern New Mexico and southeastern Arizona.

**Divisions:** 305:C

**TNC Ecoregions:** 22:C, 23:C, 24:C, 30:P

**Nations:** MX, US

**Subnations:** AZ, MXCH, MXSO, NM, TX

**Map Zones:** 14:P, 15:C, 24:C, 25:C, 26:C, 27:P

**USFS Ecomap Regions:** 313C:CC, 315A:CC, 321A:CC, 322A:CP, 322B:CC, M313A:CC, M313B:CC

CONCEPT

**Environment:** Madrean Encinal occurs on foothills, canyons, bajadas and plateaus in the Sierra Madre Occidentale and Sierra Madre Orientale in Mexico, extending north into Trans‑Pecos Texas, southern New Mexico and sub‑Mogollon Arizona. In Texas, it is found on often rocky or gravelly soils over various substrates, including Permian limestones of the Guadalupe Mountains, Tertiary igneous formations, sandstone formations, and even colluvial/alluvial substrates at middle elevations in mountainous areas of the Trans‑Pecos. It may also occur on loams and alluvial surfaces.

**Vegetation:** Stands of this system may be present as a shrubland, closed woodland, or open woodland dominated by evergreen oak species including *Quercus arizonica, Quercus emoryi, Quercus intricata, Quercus grisea, Quercus oblongifolia, Quercus toumeyi*, and in Mexico *Quercus chihuahuensis* and *Quercus albocincta*. Madrean pine, Arizona cypress, pinyon and juniper trees may be present but do not codominate. Chaparral species such as *Arctostaphylos pungens, Cercocarpus montanus, Purshia* spp., *Garrya wrightii, Quercus turbinella, Frangula betulifolia (= Rhamnus betulifolia)*, or *Rhus* spp. may be present but do not dominate. The graminoid layer is usually prominent between trees in grassland or steppe that is dominated by warm‑season grasses such as *Aristida* spp., *Bouteloua gracilis, Bouteloua curtipendula, Bouteloua rothrockii, Digitaria californica, Eragrostis intermedia, Hilaria belangeri, Leptochloa dubia, Muhlenbergia* spp., *Pleuraphis jamesii*, or *Schizachyrium cirratum*. These species are also typical of Apacherian‑Chihuahuan Semi‑Desert Grassland and Steppe (CES302.735).

In Texas, this system is typically dominated by oak species such as *Quercus grisea, Quercus emoryi, Quercus hypoleucoides, Quercus arizonica*, and/or *Quercus rugosa*. On limestone, *Quercus mohriana* may be common. Various pine and juniper species, such as *Juniperus deppeana, Pinus cembroides, Pinus edulis* (in the Guadalupe Mountains region), may be conspicuous elements of the canopy. In addition to the oak, pine, and juniper species, other shrubs that may be encountered include *Mimosa aculeaticarpa var. biuncifera, Mimosa dysocarpa, Rhus trilobata*, and *Cercocarpus montanus*. *Viguiera stenoloba, Parthenium incanum*, and other species common to the deserts of lower elevations may be present to common. *Nolina texana, Dasylirion leiophyllum, Cylindropuntia imbricata (= Opuntia imbricata)*, and *Agave* spp. are commonly encountered. The herbaceous layer is typically dominated by graminoids such as *Muhlenbergia emersleyi, Bouteloua curtipendula, Bouteloua gracilis, Bouteloua hirsuta, Bouteloua eriopoda, Piptochaetium fimbriatum*, and *Heteropogon contortus*, but this layer may be sparse. Lower elevation occurrences tend to be more open woodlands and savannas.

**Dynamics:** [from M010] Dynamics are complicated by the diverse plant communities present in this macrogroup. The pinyon‑juniper woodlands and savannas included in this macrogroup are represented by what Moir and Carleton (1987) classified as the High Sun Mild climate zone (summer precipitation and warm climate). Romme et al. (2003) developed a pinyon‑juniper classification with three types based on canopy structure, understory composition, and historic fire regime. All three types, pinyon‑juniper grass savanna, pinyon‑juniper shrub woodland, and pinyon‑juniper forest, are included in this macrogroup. However, the pinyon‑juniper grass savanna and a new, ecologically similar type with tree canopy >10% cover (pinyon‑juniper grass open woodland) have the greater aerial extent in the macrogroup (Landis and Bailey 2005, Gori and Bate 2007). Other types are the pinyon‑juniper shrub woodland, represented by pinyon‑juniper trees with an understory of shrubs such as *Quercus turbinella*, and the pinyon‑juniper forest type that has a typically sparse understory and is restricted to dry, rocky areas or following fires (Romme et al. 2003).

Fire dynamics for these types under historic natural conditions (also called natural range of variability (NRV); for pre‑1900 timeframe) are summarized as follows based on (Romme et al. 2003). The fire regime for the pinyon‑juniper grass savanna/pinyon‑juniper grass open woodland includes frequent, low‑severity surface fires that are carried by the herbaceous layer. The low density of trees (5‑20% cover) and high perennial grass cover is maintained by this fire regime. Mean fire interval is estimated to be 12‑43 years (Gori and Bate 2007). The fire regime for the pinyon‑juniper shrub woodland has moderately frequent, high‑severity crown fires that are carried by the shrub and tree layers. After a stand replacing fire the site begins at early seral stage and returns to a moderately dense tree layer with a moderate to dense shrub layer. Succession happens relatively quickly if the shrub layer includes chaparral species that recover rapidly from fire by re‑sprouting or from fire scarified seeds in a seed bank. Mixed‑severity fires may alter this pattern by creating a mosaic of pinyon‑juniper states (early‑, mid‑, and late‑seral). Mean fire interval is estimated to be 23‑81 years (Gori and Bate 2007). The fire regime for the pinyon‑juniper forest type has very infrequent, very high‑severity fires that are carried by tree crowns. The stand dynamics are stable with multi‑age tree canopy and with little change in shrub or herbaceous layers.

Pinyon and juniper stands in this macrogroup have been impacted by human activities over the last century. Historical fire regimes were disrupted following the introduction of livestock (and the 1890s drought). Grazing passively suppresses fire by removing fine fuels needed to carry surface and mixed‑severity fires that likely maintained the structure and composition of pinyon‑juniper savannas and pinyon‑juniper shrub woodlands historically. Active fire suppression was also practiced by the Federal government during the last 100 years (Swetnam and Baisan 1996b). As fire became less frequent, pinyon and juniper trees became denser and subsequent fires became more severe (Gori and Bate 2007). These impacts altered stand dynamics differently depending on stand structure. Fire dynamics under current conditions are summarized for the three major pinyon‑juniper types (pinyon‑juniper grass savanna/open woodland, pinyon‑juniper shrub woodland, and pinyon‑juniper forest) developed by Romme et al. (2003) using canopy structure, understory composition, and historic fire regime and adapted for our use as follows.

The fire regime for the pinyon‑juniper grass savanna/open woodland has a fire frequency that is significantly reduced and fire severity has greatly increased from pre‑1900, from low‑severity surface fires towards high‑severity and stand‑replacing crown fires. Tree density has increased and herbaceous biomass has decreased from historic conditions with active fire suppression and livestock grazing. Currently stands have some very old trees (>300 years) present but not numerous, and are typically dominated by many young trees (<150 years). This type may also occur on sites with more rock soil and less grasses. This type is outside Historic Range of Variation (HRV) for disturbance regime, structure and composition (Gori and Bate 2007).

The fire regime for the pinyon‑juniper shrub woodland has a fire frequency that is reduced and fire severity is somewhat increased from pre‑1900, from low to moderately frequent, high‑severity stand‑replacing fires and moderately frequent mixed‑severity fires that likely maintain this type, toward less frequent, higher severity fires (Gori and Bate 2007). Tree density has increased and herbaceous biomass has decreased from historic conditions with active fire suppression and livestock grazing. Currently most stands have a variable mix of tree and shrubs with few or no very old trees (>300 years) present. With fire suppression, this type maybe outside HRV for disturbance regime, and possibly for structure and composition as recent fires are likely more severe than historic fire in the late 1800s (Romme et al. 2003).

The fire regime for the pinyon‑juniper forest type still has infrequent, high‑severity fires that are carried by tree crowns. The stand dynamics remain relatively stable with little change in density of tree or shrub and herbaceous layers. Currently stands have numerous very old trees (>300 years) present with a multi‑aged structure. Active fire suppression and livestock grazing are thought to have had little impact on fire frequency and severity and the overstory structure and composition with this type remaining within HRV for disturbance regime (Gori and Bate 2007).

Most pinyon‑juniper woodlands in the southwest have high soil erosion potential. Several studies have measured present‑day erosion rates in pinyon‑juniper woodlands, highlighting the importance of herbaceous cover and biological soil crusts (Belnap et al. 2001) in minimizing precipitation runoff and soil loss in pinyon‑juniper woodlands.

Madrean encinal stands included in this macrogroup also vary considerably under historic natural conditions in tree density ranging from very open woodlands and treed savannas (5‑15% cover) with a perennial grass‑dominated understory in uplands, to moderately dense oak woodlands (20‑40% tree cover) in drainages and on north‑facing slopes. The understory of good‑condition stands generally has high cover of perennial grasses and low cover of shrubs such as *Mimosa*, and this good condition of the stand is maintained with frequent fires. Turner et al. (2003) documented a trend from more open woodlands and savannas to denser woodlands with higher cover of species of *Juniperus* and *Prosopis* over the last 150 years. Regeneration of oaks following disturbance is from resprouting rather than acorns because of the dry conditions (Germaine and McPherson 1998).

Although there is not much encinal‑specific information on fire‑return intervals (FRI) available, it is thought to be similar to adjacent ecosystems, primarily the semi‑desert grassland (FRI of 2.5‑10 years) (Wright 1980, Bahre 1985, McPherson 1995, Kaib et al. 1996) and the pine‑oak woodlands (FRI of 3‑7 years) (Wright 1980, Bahre 1985, Swetnam et al. 1992, McPherson 1995, Kaib et al. 1996, Swetnam and Baisan 1996b). Fire season in encinal was probably similar to that of other Madrean woodlands and grasslands, occurring predominantly before the summer monsoon between April and June when vegetation is dry and ignition sources from dry lightning strikes are common (Swetnam and Betancourt 1990). Post disturbance regeneration (such as after stand‑replacing fire) mostly occurs from resprouting from trees roots. Successful regeneration from acorns is related to annual precipitation (Germaine and McPherson 1998). The understory of poor‑condition stands with less frequent fires or experiencing extended drought may have significant shrub invasion by species of *Arctostaphylos* and *Juniperus* and reduction of perennial grass cover (Schussman 2006a).

Over the last century, the woody component in encinal has increased in density over time in the absence of disturbance such as fire (Burgess 1995, Gori and Enquist 2003, Turner et al. 2003, Schussman 2006a). This is correlated to a decrease in fire frequency that is related to a reduction of fine fuels that carry fire because of extensive livestock grazing. Frequent, stand‑replacing fire was likely a key ecological attribute prior to 1890 (Wright 1980, Bahre 1985, McPherson 1995, Kaib et al. 1996). The oak woodlands and savannas included in this macrogroup are characterized by a strong perennial grass layer and are driven by many of the same ecological processes as semi‑desert mixed grassland, primarily frequent fire and drought (USFS 2009). It is generally agreed that fire regime has been altered for encinal by passive fire suppression via removal of fine fuels through livestock grazing, as well as active suppression over the last 100 years. This has reduced the number of surface fires, permitting a buildup in woody fuels, resulting in increased fire severity when fires occur in encinal and adjacent vegetation types such as semi‑desert grasslands and pine‑oak woodlands across much or the southwestern U.S. and adjacent Mexico (Kaib et al. 1996, Swetnam and Baisan 1996). Reduced fire frequency is a disturbance of the natural fire regime and results in increased cover of woody plants (Barton 1999, Muldavin et al. 2002b, Gori and Enquist 2003, Turner et al. 2003). The increase in woody species in the Madrean encinal has changed species composition, in some areas, from oak‑dominated woodlands or savanna to mesquite‑ and/or juniper‑dominated woodlands (Turner et al. 2003).

Livestock grazing in encinal is currently a common practice in both the United States and Mexico, with grazing occurring in virtually all of Mexico's and in roughly 75% of the United States' oak woodlands (McPherson 1995). Livestock grazing can affect the structure and composition of Madrean oak woodlands, as well as soil structure and water infiltration (USFS 2009).

The introduction of the invasive non‑native, perennial grasses *Eragrostis lehmanniana* and *Eragrostis curvula* has greatly impacted many semi‑desert grasslands and encinal in this ecoregion (Cable 1971, Anable et al. 1992, Gori and Enquist 2003). Anable et al. (1992) and Cable (1971) found Lehmann lovegrass is a particularly aggressive invader and alters ecosystem processes, vegetation composition, and species diversity.

Historic fuelwood cutting for mining and domestic use and fencepost cutting was common in stands of this macrogroup in southeastern Arizona until the late 1800s, and is still common in Arizona and northern Mexico today (Bahre 1991, Bennett 1992). Although fuelwood harvesting had dramatic effects historically, its consequences were generally local and short‑lived (Turner et al. 2003). More recently, chemical and mechanical treatments such as chaining and rotochopping have impacted age structure, tree density and cover of many pinyon‑juniper woodlands with current demand for these products continuing to increase (Ffolliott et al. 1979, Gottfried 1987, Dick‑Peddie 1993, Gottfried and Severson 1993).

SOURCES

**References:** Barbour and Billings 2000, Brown 1982a, Brown et al. 1980, Brown et al. 1998, Comer et al. 2003*, Elliott 2012, Eyre 1980, Muldavin pers. comm., Shiflet 1994

**Version:** 08 Jan 2015	**Stakeholders:** Latin America, Southeast, West

**Concept Author:** K.A. Schulz	**LeadResp:** West

CES301.730 Madrean Juniper Savanna

**Primary Division:** Madrean Semidesert (301)

**Land Cover Class:** Steppe/Savanna

**Spatial Scale & Pattern:** Large patch

**Required Classifiers:** Natural/Semi‑natural; Vegetated (>10% vasc.); Upland

**Diagnostic Classifiers:** Montane [Lower Montane]; Woody‑Herbaceous; Tropical/Subtropical [Tropical Xeric]; Evergreen Sclerophyllous Tree; Succulent Shrub; Juniperus coahuilensis, J. deppeana, J. pinchotii

**National Mapping Codes:** EVT 2116; ESLF 5405; ESP 1116

**Concept Summary:** This Madrean ecological system occurs in lower foothills and plains of southeastern Arizona, southern New Mexico extending into west Texas and Mexico. These savannas have widely spaced mature juniper trees and moderate to high cover of graminoids (>25% cover). The presence of Madrean *Juniperus* spp. such as *Juniperus coahuilensis, Juniperus pinchotii*, and/or *Juniperus deppeana* is diagnostic. *Juniperus monosperma* may be present in some stands; *Juniperus deppeana* has a broader range than this Madrean system and extends north into southern stands of Southern Rocky Mountain Juniper Woodland and Savanna (CES306.834). Stands of *Juniperus pinchotii* may be short and resemble a shrubland. Graminoid species are a mix of those found in Western Great Plains Shortgrass Prairie (CES303.672) and Apacherian‑Chihuahuan Semi‑Desert Grassland and Steppe (CES302.735), with *Bouteloua gracilis* and *Pleuraphis jamesii* being most common. In addition, these areas include succulents such as species of *Yucca, Opuntia*, and *Agave*. Juniper savanna expansion into grasslands has been documented in the last century.

DISTRIBUTION

**Range:** This system is found in southeastern Arizona, southern New Mexico, and extending into west Texas and Mexico. It likely occurs on the west side of the Sacramento and Guadalupe mountains.

**Divisions:** 301:C

**TNC Ecoregions:** 22:C, 24:C, 30:P

**Nations:** MX, US

**Subnations:** AZ, NM, TX

**Map Zones:** 14:P, 15:C, 24:C, 25:C, 26:C, 27:C, 28:?

**USFS Ecomap Regions:** 313B:CC, 313C:CC, 313D:CC, 315A:CC, 315B:CC, 315H:CC, 321A:CC, 322A:CC, 322B:CC, M313A:CC, M313B:CC, M331F:??

CONCEPT

**Environment:** This ecological system occurs on a variety of substrates in rolling landscapes on gentle to level terrain. In Texas, soils are gravelly to shallow to loamy.

**Vegetation:** One of several juniper species may be the dominant overstory, including *Juniperus pinchotii, Juniperus coahuilensis, Juniperus deppeana*, or *Juniperus monosperma*. The system may occur with junipers forming a shrubland, or as a closed woodland, or, more commonly, as an open woodland. Common grasses in the understory include *Bouteloua gracilis* and *Pleuraphis jamesii*. In addition, these areas include succulents such as species of *Yucca, Opuntia*, and *Agave*. In addition, in Texas, *Nolina texana* and *Dasylirion leiophyllum* are commonly encountered. This system typically gives way at lower elevations to grassland, with species such as *Bouteloua gracilis, Bouteloua curtipendula, Bouteloua eriopoda, Muhlenbergia emersleyi*, and *Muhlenbergia setifolia* commonly encountered in the herbaceous layer within their respective ranges.

**Dynamics:** [from M010] Dynamics are complicated by the diverse plant communities present in this macrogroup. The pinyon‑juniper woodlands and savannas included in this macrogroup are represented by what Moir and Carleton (1987) classified as the High Sun Mild climate zone (summer precipitation and warm climate). Romme et al. (2003) developed a pinyon‑juniper classification with three types based on canopy structure, understory composition, and historic fire regime. All three types, pinyon‑juniper grass savanna, pinyon‑juniper shrub woodland, and pinyon‑juniper forest, are included in this macrogroup. However, the pinyon‑juniper grass savanna and a new, ecologically similar type with tree canopy >10% cover (pinyon‑juniper grass open woodland) have the greater aerial extent in the macrogroup (Landis and Bailey 2005, Gori and Bate 2007). Other types are the pinyon‑juniper shrub woodland, represented by pinyon‑juniper trees with an understory of shrubs such as *Quercus turbinella*, and the pinyon‑juniper forest type that has a typically sparse understory and is restricted to dry, rocky areas or following fires (Romme et al. 2003).

Fire dynamics for these types under historic natural conditions (also called natural range of variability (NRV); for pre‑1900 timeframe) are summarized as follows based on (Romme et al. 2003). The fire regime for the pinyon‑juniper grass savanna/pinyon‑juniper grass open woodland includes frequent, low‑severity surface fires that are carried by the herbaceous layer. The low density of trees (5‑20% cover) and high perennial grass cover is maintained by this fire regime. Mean fire interval is estimated to be 12‑43 years (Gori and Bate 2007). The fire regime for the pinyon‑juniper shrub woodland has moderately frequent, high‑severity crown fires that are carried by the shrub and tree layers. After a stand replacing fire the site begins at early seral stage and returns to a moderately dense tree layer with a moderate to dense shrub layer. Succession happens relatively quickly if the shrub layer includes chaparral species that recover rapidly from fire by re‑sprouting or from fire scarified seeds in a seed bank. Mixed‑severity fires may alter this pattern by creating a mosaic of pinyon‑juniper states (early‑, mid‑, and late‑seral). Mean fire interval is estimated to be 23‑81 years (Gori and Bate 2007). The fire regime for the pinyon‑juniper forest type has very infrequent, very high‑severity fires that are carried by tree crowns. The stand dynamics are stable with multi‑age tree canopy and with little change in shrub or herbaceous layers.

Pinyon and juniper stands in this macrogroup have been impacted by human activities over the last century. Historical fire regimes were disrupted following the introduction of livestock (and the 1890s drought). Grazing passively suppresses fire by removing fine fuels needed to carry surface and mixed‑severity fires that likely maintained the structure and composition of pinyon‑juniper savannas and pinyon‑juniper shrub woodlands historically. Active fire suppression was also practiced by the Federal government during the last 100 years (Swetnam and Baisan 1996b). As fire became less frequent, pinyon and juniper trees became denser and subsequent fires became more severe (Gori and Bate 2007). These impacts altered stand dynamics differently depending on stand structure. Fire dynamics under current conditions are summarized for the three major pinyon‑juniper types (pinyon‑juniper grass savanna/open woodland, pinyon‑juniper shrub woodland, and pinyon‑juniper forest) developed by Romme et al. (2003) using canopy structure, understory composition, and historic fire regime and adapted for our use as follows.

The fire regime for the pinyon‑juniper grass savanna/open woodland has a fire frequency that is significantly reduced and fire severity has greatly increased from pre‑1900, from low‑severity surface fires towards high‑severity and stand‑replacing crown fires. Tree density has increased and herbaceous biomass has decreased from historic conditions with active fire suppression and livestock grazing. Currently stands have some very old trees (>300 years) present but not numerous, and are typically dominated by many young trees (<150 years). This type may also occur on sites with more rock soil and less grasses. This type is outside Historic Range of Variation (HRV) for disturbance regime, structure and composition (Gori and Bate 2007).

The fire regime for the pinyon‑juniper shrub woodland has a fire frequency that is reduced and fire severity is somewhat increased from pre‑1900, from low to moderately frequent, high‑severity stand‑replacing fires and moderately frequent mixed‑severity fires that likely maintain this type, toward less frequent, higher severity fires (Gori and Bate 2007). Tree density has increased and herbaceous biomass has decreased from historic conditions with active fire suppression and livestock grazing. Currently most stands have a variable mix of tree and shrubs with few or no very old trees (>300 years) present. With fire suppression, this type maybe outside HRV for disturbance regime, and possibly for structure and composition as recent fires are likely more severe than historic fire in the late 1800s (Romme et al. 2003).

The fire regime for the pinyon‑juniper forest type still has infrequent, high‑severity fires that are carried by tree crowns. The stand dynamics remain relatively stable with little change in density of tree or shrub and herbaceous layers. Currently stands have numerous very old trees (>300 years) present with a multi‑aged structure. Active fire suppression and livestock grazing are thought to have had little impact on fire frequency and severity and the overstory structure and composition with this type remaining within HRV for disturbance regime (Gori and Bate 2007).

Most pinyon‑juniper woodlands in the southwest have high soil erosion potential. Several studies have measured present‑day erosion rates in pinyon‑juniper woodlands, highlighting the importance of herbaceous cover and biological soil crusts (Belnap et al. 2001) in minimizing precipitation runoff and soil loss in pinyon‑juniper woodlands.

Madrean encinal stands included in this macrogroup also vary considerably under historic natural conditions in tree density ranging from very open woodlands and treed savannas (5‑15% cover) with a perennial grass‑dominated understory in uplands, to moderately dense oak woodlands (20‑40% tree cover) in drainages and on north‑facing slopes. The understory of good‑condition stands generally has high cover of perennial grasses and low cover of shrubs such as *Mimosa*, and this good condition of the stand is maintained with frequent fires. Turner et al. (2003) documented a trend from more open woodlands and savannas to denser woodlands with higher cover of species of *Juniperus* and *Prosopis* over the last 150 years. Regeneration of oaks following disturbance is from resprouting rather than acorns because of the dry conditions (Germaine and McPherson 1998).

Although there is not much encinal‑specific information on fire‑return intervals (FRI) available, it is thought to be similar to adjacent ecosystems, primarily the semi‑desert grassland (FRI of 2.5‑10 years) (Wright 1980, Bahre 1985, McPherson 1995, Kaib et al. 1996) and the pine‑oak woodlands (FRI of 3‑7 years) (Wright 1980, Bahre 1985, Swetnam et al. 1992, McPherson 1995, Kaib et al. 1996, Swetnam and Baisan 1996b). Fire season in encinal was probably similar to that of other Madrean woodlands and grasslands, occurring predominantly before the summer monsoon between April and June when vegetation is dry and ignition sources from dry lightning strikes are common (Swetnam and Betancourt 1990). Post disturbance regeneration (such as after stand‑replacing fire) mostly occurs from resprouting from trees roots. Successful regeneration from acorns is related to annual precipitation (Germaine and McPherson 1998). The understory of poor‑condition stands with less frequent fires or experiencing extended drought may have significant shrub invasion by species of *Arctostaphylos* and *Juniperus* and reduction of perennial grass cover (Schussman 2006a).

Over the last century, the woody component in encinal has increased in density over time in the absence of disturbance such as fire (Burgess 1995, Gori and Enquist 2003, Turner et al. 2003, Schussman 2006a). This is correlated to a decrease in fire frequency that is related to a reduction of fine fuels that carry fire because of extensive livestock grazing. Frequent, stand‑replacing fire was likely a key ecological attribute prior to 1890 (Wright 1980, Bahre 1985, McPherson 1995, Kaib et al. 1996). The oak woodlands and savannas included in this macrogroup are characterized by a strong perennial grass layer and are driven by many of the same ecological processes as semi‑desert mixed grassland, primarily frequent fire and drought (USFS 2009). It is generally agreed that fire regime has been altered for encinal by passive fire suppression via removal of fine fuels through livestock grazing, as well as active suppression over the last 100 years. This has reduced the number of surface fires, permitting a buildup in woody fuels, resulting in increased fire severity when fires occur in encinal and adjacent vegetation types such as semi‑desert grasslands and pine‑oak woodlands across much or the southwestern U.S. and adjacent Mexico (Kaib et al. 1996, Swetnam and Baisan 1996). Reduced fire frequency is a disturbance of the natural fire regime and results in increased cover of woody plants (Barton 1999, Muldavin et al. 2002b, Gori and Enquist 2003, Turner et al. 2003). The increase in woody species in the Madrean encinal has changed species composition, in some areas, from oak‑dominated woodlands or savanna to mesquite‑ and/or juniper‑dominated woodlands (Turner et al. 2003).

Livestock grazing in encinal is currently a common practice in both the United States and Mexico, with grazing occurring in virtually all of Mexico's and in roughly 75% of the United States' oak woodlands (McPherson 1995). Livestock grazing can affect the structure and composition of Madrean oak woodlands, as well as soil structure and water infiltration (USFS 2009).

The introduction of the invasive non‑native, perennial grasses *Eragrostis lehmanniana* and *Eragrostis curvula* has greatly impacted many semi‑desert grasslands and encinal in this ecoregion (Cable 1971, Anable et al. 1992, Gori and Enquist 2003). Anable et al. (1992) and Cable (1971) found Lehmann lovegrass is a particularly aggressive invader and alters ecosystem processes, vegetation composition, and species diversity.

Historic fuelwood cutting for mining and domestic use and fencepost cutting was common in stands of this macrogroup in southeastern Arizona until the late 1800s, and is still common in Arizona and northern Mexico today (Bahre 1991, Bennett 1992). Although fuelwood harvesting had dramatic effects historically, its consequences were generally local and short‑lived (Turner et al. 2003). More recently, chemical and mechanical treatments such as chaining and rotochopping have impacted age structure, tree density and cover of many pinyon‑juniper woodlands with current demand for these products continuing to increase (Ffolliott et al. 1979, Gottfried 1987, Dick‑Peddie 1993, Gottfried and Severson 1993).

SOURCES

**References:** Barbour and Billings 2000, Brown et al. 1979, Brown et al. 1998, Comer et al. 2003*, Dick‑Peddie 1993, Elliott 2012, INEGI 2005, Shiflet 1994

**Version:** 02 Oct 2014	**Stakeholders:** Latin America, Southeast, West

**Concept Author:** P. Comer	**LeadResp:** West

CES305.797 Madrean Pinyon‑Juniper Woodland

**Primary Division:** Sierra Madre (305)

**Land Cover Class:** Forest and Woodland

**Spatial Scale & Pattern:** Matrix

**Required Classifiers:** Natural/Semi‑natural; Vegetated (>10% vasc.); Upland

**Diagnostic Classifiers:** Montane [Lower Montane]; Tropical/Subtropical [Tropical Xeric]; Shallow Soil; Xeric; F‑Patch/Medium Intensity; Needle‑Leaved Tree; Evergreen Sclerophyllous Shrub; Pinus cembroides, Juniperus deppeana

**National Mapping Codes:** EVT 2025; ESLF 4212; ESP 1025

**Concept Summary:** This system occurs on foothills, mountains and plateaus in the Sierra Madre Occidentale and Sierra Madre Orientale in Mexico, Trans‑Pecos Texas, southern New Mexico and Arizona, generally south of the Mogollon Rim. Substrates are variable, but soils are generally dry and rocky. The presence of *Pinus cembroides, Pinus discolor*, or other Madrean trees and shrubs is diagnostic of this woodland system. *Juniperus coahuilensis, Juniperus deppeana, Juniperus pinchotii, Juniperus monosperma*, and/or *Pinus edulis* may be present to dominant. Madrean oaks such as *Quercus arizonica, Quercus emoryi, Quercus grisea*, or *Quercus mohriana* may be codominant. *Pinus ponderosa* is absent or sparse. If present, understory layers are variable and may be dominated by shrubs or graminoids.

**Comments:** According to USFS TES mapping (USDA 2001), *Quercus grisea* woodlands (Madrean Encinal) occur on both sides of the Guadalupe Mountains and in the southeastern portion of the Sacramento Mountains. This suggests that the associated pinyon and juniper woodlands are Madrean Pinyon‑Juniper Woodland (CES305.797). In Culberson County, Texas, *Pinus edulis* and *Juniperus monosperma* indicate the relationship of this system to other pinyon‑juniper systems to the north, but other components of these occurrences recommend the relationship to this Madrean system.

DISTRIBUTION

**Range:** This system occurs in the Sierra Madre Occidentale and Sierra Madre Orientale of Mexico, Trans‑Pecos Texas, southern New Mexico and Arizona, generally south of the Mogollon Rim. It occurs on the west side of the Sacramento Mountains but may transition into Southern Rocky Mountain Pinyon‑Juniper Woodland (CES306.835) or Southern Rocky Mountain Juniper Woodland and Savanna (CES306.834) on the eastern side.

**Divisions:** 305:C

**TNC Ecoregions:** 22:C, 24:C, 30:C

**Nations:** MX, US

**Subnations:** AZ, NM, TX

**Map Zones:** 14:C, 15:C, 24:C, 25:C, 26:C, 27:C, 28:?

**USFS Ecomap Regions:** 313B:CC, 313C:CC, 313D:CP, 315A:CC, 315B:CC, 315H:CC, 321A:CC, 322A:CC, 322B:CC, 331I:??, M313A:CC, M313B:CC, M331F:??

CONCEPT

**Environment:** This woodland system is common in foothills, mountains and plateaus in the Sierra Madre Occidentale and Sierra Madre Orientale in Mexico, Trans‑Pecos Texas, southern New Mexico and Arizona, generally south of the Mogollon Rim. Elevation generally ranges from 1300‑2225 m with high‑elevation stands restricted to warmer southern aspects.

*Climate:* Climate is semi‑arid with drought not uncommon. Summers are hot and winters are mild with cold periods and occasional snows. The mean annual precipitation ranges from 40‑50 cm with approximately two‑thirds occurring during the Arizona monsoon season from July to September, often as high‑intensity convective storms. May and June are typically dry. Stands typically occur on nearly level to steep, rocky slopes.

*Physiography/landform:* Stands occur on cool aspects of steep scarp slopes, in canyons (including alluvial terraces), on gently sloping alluvial fan piedmonts (bajadas), steeper colluvial slopes and ridges, as well as mesatops. Pinyon and juniper woodlands extend down to 760 m elevation in Trans‑Pecos ranges. At the lowest elevation, encinal generally occupies the rockier substrates or is restricted to drainages within grasslands (Brown 1982a).

*Soil/substrate/hydrology:* Soils are variable, but are generally shallow, rocky, calcareous, but may include deeper clay loamy to gravelly loamy soils. Parent materials include andesite, rhyolite, limestone, basalt, colluvium and alluvium (Sullivan 1993c, Pavek 1994b, Tirmenstein 1999i, Hauser 2007b).

**Vegetation:** Vegetation is characterized by an open to moderately dense tree canopy dominated by pinyon and juniper trees 2‑5 m tall. The presence of pinyons *Pinus cembroides, Pinus discolor, Pinus remota*, or *Pinus edulis* with Madrean elements in the understory is diagnostic of this ecosystem. *Juniperus coahuilensis, Juniperus deppeana*, and *Juniperus pinchotii* are character species that are often present to dominant. *Pinus edulis* and *Juniperus monosperma* may be the dominants in the northern distribution in combination with Madrean shrub and/or graminoid elements. *Pinus ponderosa* is absent or scattered. Understory layers are variable, ranging from sparse to dense grass or shrub layers. If Madrean oak trees such as *Quercus arizonica, Quercus emoryi*, or *Quercus grisea* are present, then they do not dominate the tree canopy. Common shrub species may include chaparral, desert scrub or lower montane shrubs such as *Arctostaphylos pungens, Canotia holacantha, Ceanothus greggii, Cercocarpus montanus, Mimosa dysocarpa, Quercus turbinella*, or *Rhus trilobata*. Perennial grasses such as *Bouteloua curtipendula, Bouteloua eriopoda, Bouteloua gracilis, Muhlenbergia emersleyi, Muhlenbergia pauciflora, Piptochaetium fimbriatum*, or *Piptochaetium pringlei* are present in many stands and may form an herbaceous layer. The vegetation description is based on several references, including Brown (1982a), Gottfried (1992), Dick‑Peddie (1993), Muldavin et al. (2000b), and Gori and Bate (2007).

**Dynamics:** Dynamics are complicated by the variation in physiognomy and diverse plant communities present in this system. The pinyon‑juniper woodlands and savannas included in this system are represented by what Moir and Carleton (1987) classified as the High Sun Mild climate zone (summer precipitation and warm climate). Romme et al. (2003) developed a pinyon‑juniper classification with three types based on canopy structure, understory composition, and historic fire regime. All three types, pinyon‑juniper grass savanna, pinyon‑juniper shrub woodland, and pinyon‑juniper forest, are included in this system. For this model an ecologically similar type, pinyon‑juniper grass open woodland (with tree canopy >10% cover), was added to the pinyon‑juniper grass savanna making this the more widespread type (Landis and Bailey 2005, Gori and Bate 2007). The other types are the pinyon‑juniper shrub woodland, represented by pinyon‑juniper trees with an understory of shrubs such as *Quercus turbinella*, and the pinyon‑juniper forest type that has a typically sparse understory and is restricted to dry, rocky areas where it is protected from fires (Romme et al. 2003).<br />

Fire dynamics for these types under historical natural conditions (also called natural range of variability (NRV) for pre‑1900 timeframe) are summarized below based on (Romme et al. 2003).

The fire regime for the pinyon‑juniper grass savanna/pinyon‑juniper grass open woodland includes frequent, low‑severity surface fires that are carried by the herbaceous layer. The low density of trees (5‑20% cover) and high perennial grass cover is maintained by this fire regime. Mean fire interval is estimated to be 12‑43 years (Gori and Bate 2007).

The fire regime for the pinyon‑juniper shrub woodland is described as moderately frequent, high‑severity crown fires that are carried by the shrub and tree layers. After a stand‑replacing fire the site begins at early‑seral stage and returns to a moderately dense tree layer with a moderate to dense shrub layer. Succession happens relatively quickly if the shrub layer includes chaparral species that recover rapidly from fire by re‑sprouting or from fire‑scarified seeds in a seed bank. Mixed‑severity fires may alter this pattern by creating a mosaic of pinyon‑juniper states (early‑, mid‑, and late‑seral). Mean fire interval is estimated to be 23‑81 years (Gori and Bate 2007).

The fire regime for the pinyon‑juniper forest type is characterized by very infrequent, very high‑severity fires that are carried by tree crowns. The stand dynamics are stable with a multi‑age tree canopy and with little change in shrub or herbaceous layers.

The historical fire season was probably similar to that of other Madrean woodlands and grasslands, occurring predominantly before the summer monsoon between April and June when vegetation is dry and ignition sources from dry lightning strikes are common (Swetnam and Betancourt 1990).

Other important ecological processes include climate, drought, insect infestations, pathogens, herbivory and seed dispersal by birds and small mammals.

<br />Juniper berries and pinyon nut crops are primarily utilized by birds and small mammals (Johnsen 1962, McCulloch 1969, Short et al. 1977, Salomonson 1978, Balda 1987, Gottfried et al. 1995, Tirmenstein 1999i). Large mammals, such as mule deer (*Odocoileus hemionus*), white‑tailed deer (*Odocoileus virginianus*) and elk (*Cervus elaphus*), eat leaves and seeds of both species and browse woodland grasses, forbs and shrubs, including *Artemisia tridentata, Cercocarpus montanus, Quercus gambelii*, and *Purshia stansburiana* (Short and McCulloch 1977). The most important dispersers of juniper and pinyon seeds are birds. Juniper seeds that pass through the digestive tract of birds and other herbivores germinate faster than uneaten seeds (Johnsen 1962, Tirmenstein 1999i). The primary dispersers of pinyon seeds, i.e., scrub jays (*Aphelocoma californica*), pinyon jays (*Gymnorhinus cyanocephalus*), Steller's jays (*Cyanocitta stelleri*) and Clark's nutcrackers (*Nucifraga columbiana*), cache hundreds of thousands of pinyon seeds during mast crop years, many of which are never recovered (Balda and Bateman 1971, Vander Wall and Balda 1977, Ligon 1978, Pavek 1994b). This seed dispersal mechanism is a good example of a co‑evolved, mutualistic, plant‑vertebrate relationship (Vander Wall et al. 1981, Evans 1988, Lanner 1996) and would be at risk with loss of trees or dispersers. In addition, small mammals, such as cliff chipmunk (*Neotamias dorsalis*) and rock squirrel (*Otospermophilus variegatus*), compete with birds (Christensen and Whitham 1993).

There are many insects, pathogens, and plant parasites that attack pinyon and juniper trees (Gottfried et al. 1995, Rogers 1995, Weber et al. 1999). For pinyon, there are at least seven insects, plus a fungus (black stain root disease (*Leptographium wageneri*), and pinyon dwarf mistletoe (*Arceuthobium divaricatum*). These insects are normally present in these woodland stands, and during drought‑induced water stress outbreaks may cause local to regional mortality (Wilson and Tkacz 1992, Gottfried et al. 1995, Rogers 1995). Most insect‑related pinyon mortality in the West is caused by pinyon ips beetle (*Ips confusus)* (Rogers 1993).

Most pinyon‑juniper woodlands in the Southwest have high soil erosion potential. Several studies have measured present‑day erosion rates in pinyon‑juniper woodlands, highlighting the importance of herbaceous cover and biological soil crusts (Belnap et al. 2001) in minimizing precipitation runoff and soil loss in pinyon‑juniper woodlands.

SOURCES

**References:** Bahre 1991, Balda 1987, Balda and Bateman 1971, Barton 1999, Belnap 2001, Belnap et al. 2001, Bennett 1992, Betancourt et al. 1993, Breshears et al. 2005, Christensen and Whitham 1993, Comer et al. 2003*, Cully and Knight 1987, Davis and Turner 1986, Dick‑Peddie 1993, Elliott 2012, Evans 1988, Eyre 1980, Ffolliott et al. 1979, Floyd et al. 2006, Gori and Bate 2007, Gottfried 1987, Gottfried 1992, Gottfried and Severson 1993, Gottfried et al. 1995, Hauser 2007b, Heil and Herring 1999, Hollander and Vander Wall 2004, Johnsen 1962, Landis and Bailey 2005, Lanner 1996, Ligon 1978, McAuliffe and Van Devender 1998, McCulloch 1969, Mehringer and Wigand 1990, Miller and Tausch 2001, Moir and Carleton 1987, Muldavin et al. 2002b, Pavek 1994b, Rogers 1993, Rogers 1995, Romme et al. 2003, Rosentreter and Belnap 2003, Salomonson 1978, Shiflet 1994, Short and McCulloch 1977, Short et al. 1977, Sivinski and Knight 1996, Sivinski and Lightfoot 1994, Sullivan 1993c, Swetnam and Baisan 1996a, Swetnam and Betancourt 1990, Tirmenstein 1999i, Turner et al. 2003, USFWS 1986, Van Devender 1977, Van Devender 1990, Vander Wall and Balda 1977, Vander Wall et al. 1981, Weber et al. 1999, Wilson and Tkacz 1992

**Version:** 03 Nov 2015	**Stakeholders:** Latin America, Southeast, West

**Concept Author:** P. Comer	**LeadResp:** West

M011. Madrean Montane Forest & Woodland

CES305.282 Bosque de Pino Ponderosa de la Sierra Madre

**Primary Division:** Sierra Madre (305)

**Land Cover Class:** Forest and Woodland

**Spatial Scale & Pattern:** Large patch

**Required Classifiers:** Natural/Semi‑natural; Vegetated (>10% vasc.); Upland

**Diagnostic Classifiers:** Montane [Lower Montane]; Forest and Woodland (Treed); Ustic

**Concept Summary:** This is a Madrean variant of the widespread and well‑studied Southern Rocky Mountain Ponderosa Pine Woodland (CES306.648) common throughout the cordillera of the Rocky Mountains. It is also found primarily in the Sierra Madre Occidental. These woodlands occur at the lower treeline/ecotone between dry deciduous forests and pine‑oak forests typically in warm, dry, exposed sites. Elevations range from less than 400 to 1500 m. Occurrences are found on all slopes and aspects; however, moderately steep to very steep slopes or ridgetops are most common. Much like the Rocky Mountain system, this system likely occurs on igneous, metamorphic, and sedimentary material derived soils, with characteristic features of good aeration and drainage, coarse textures, circumneutral to slightly acidic pH, an abundance of mineral material, rockiness, and periods of drought during the growing season. Mixed fire regimes and surface fires of variable return interval likely maintain these woodlands, depending on climate, degree of soil development, and understory density. The following species are diagnostic for this system: *Pinus ponderosa, Abies concolor*.

DISTRIBUTION

**Range:** This system is found in the Sierra Madre Occidental and Sierra Madre Oriental of Mexico, Trans‑Pecos Texas, southern New Mexico and Arizona, generally south of the Mogollon Rim.

**Divisions:** 305:C

**Nations:** MX

**Subnations:** MXCH, MXDU, MXSO

CONCEPT

**Environment:** These woodlands occur at the lower treeline/ecotone between dry deciduous forests and pine‑oak forests, typically in warm, dry, exposed sites. Elevations range from less than 400 to 1500 m. Occurrences are found on all slopes and aspects; however, moderately steep to very steep slopes or ridgetops are most common. Much like the Rocky Mountain system, this system likely occurs on igneous, metamorphic, and sedimentary material derived soils, with characteristic features of good aeration and drainage, coarse textures, circumneutral to slightly acidic pH, an abundance of mineral material, rockiness, and periods of drought during the growing season.

**Vegetation:** These are not well‑documented systems, but likely share many structural attributes with Rocky Mountain systems, but likely include many evergreen oaks, other shrubs and herbaceous species of Madrean affinity.

**Dynamics:** Mixed fire regimes and surface fires of variable return interval likely maintain these woodlands, depending on climate, degree of soil development, and understory density.

SOURCES

**References:** Brown et al. 1998, Josse et al. 2003*, Rzedowski 1986, Velazquez et al. 2000

**Version:** 08 Jan 2015	**Stakeholders:** Latin America

**Concept Author:** C. Josse	**LeadResp:** Latin America

CES305.281 Bosque de Pino y Aliso de la Sierra Madre

**Primary Division:** Sierra Madre (305)

**Land Cover Class:** Forest and Woodland

**Spatial Scale & Pattern:** Large patch

**Required Classifiers:** Natural/Semi‑natural; Vegetated (>10% vasc.); Upland

**Diagnostic Classifiers:** Montane [Upper Montane]; Forest and Woodland (Treed); Ustic

**Concept Summary:** This system occurs on high mountains and plateaus throughout Mexico. These forests and woodlands are composed of Madrean pines, alder, intermingled with patchy shrublands and bunchgrasses on many mid‑ to high‑elevation slopes (2700‑3500 m elevation). Some stands have moderate cover of perennial graminoids in the form of bunchgrasses and forbs. Fires are frequent, with perhaps more crown fires than ponderosa pine woodlands, which tend to have more frequent surface fires on gentle slopes. Alder likely plays a similar ecological role to species of aspen in Rocky Mountain forests, with fire and wind providing frequent canopy openings, and this alder species exploiting disturbance, especially on soils with high moisture‑holding capacity. The following list of species is diagnostic for this system: *Pinus montezumae, Pinus michoacanus, Pinus oocarpa, Alnus firmifolia, Arbutus glandulosa, Buddleia paryflora, Penstemon gentianoides, Senecio cineraroides, Symphoricarpos microphyllus, Muhlenbergia macroura, Festuca tolucensis, Alchemilla procumbens*, and *Arenaria lycopodioides*.

DISTRIBUTION

**Divisions:** 305:C

**Nations:** MX

**Subnations:** MXCH, MXDU, MXSO

CONCEPT

**Environment:** These woodlands occur above pine‑oak forests on dissected landscapes with rolling‑to‑steep slopes, and on lava flows, between 2700‑3500 m elevation. Soils tend to be shallow with gravelly sandy loam textures.

**Vegetation:** These are not well documented systems, but likely share many structural attributes with Rocky Mountain systems.

**Dynamics:** Mixed fire regimes and surface fires of variable return interval likely maintain these woodlands, depending on climate, degree of soil development, and understory density.

SOURCES

**References:** Josse et al. 2003*, Rzedowski 1986, Velazquez et al. 2000

**Version:** 08 Jan 2015	**Stakeholders:** Latin America

**Concept Author:** C. Josse	**LeadResp:** Latin America

CES305.796 Madrean Lower Montane Pine‑Oak Forest and Woodland

**Primary Division:** Sierra Madre (305)

**Land Cover Class:** Forest and Woodland

**Spatial Scale & Pattern:** Large patch

**Required Classifiers:** Natural/Semi‑natural; Vegetated (>10% vasc.); Upland

**Diagnostic Classifiers:** Montane [Montane]; Tropical/Subtropical [Tropical Xeric]; Shallow Soil; Xeric; F‑Patch/High Intensity; Needle‑Leaved Tree; Evergreen Sclerophyllous Shrub; Quercus arizonica, Q. emoryi, Q. grisea, Q. oblongifolia Q. toumeyi; Pinus discolor, P. leiophylla, P. engelmannii

**National Mapping Codes:** EVT 2024; ESLF 4211; ESP 1024

**Concept Summary:** This ecological system occurs on mountains and plateaus in the Sierra Madre Occidental and Sierra Madre Oriental in Mexico, Trans‑Pecos Texas, southern New Mexico and Arizona, generally south of the Mogollon Rim. These forests and woodlands are composed of Madrean pines (*Pinus arizonica, Pinus engelmannii, Pinus leiophylla*, or *Pinus strobiformis*) and evergreen oaks (*Quercus arizonica, Quercus emoryi*, or *Quercus grisea*) intermingled with patchy shrublands on most mid‑elevation slopes (1500‑2300 m elevation). Other tree species include *Hesperocyparis arizonica, Juniperus deppeana, Pinus cembroides, Pinus discolor, Pinus ponderosa* (with Madrean pines or oaks), and *Pseudotsuga menziesii*. Subcanopy and shrub layers may include typical encinal and chaparral species such as *Agave* spp., *Arbutus arizonica, Arctostaphylos pringlei, Arctostaphylos pungens, Garrya wrightii, Nolina* spp., *Quercus hypoleucoides, Quercus rugosa*, and *Quercus turbinella*. Some stands have moderate cover of perennial graminoids such as *Muhlenbergia emersleyi, Muhlenbergia longiligula, Muhlenbergia straminea*, and *Schizachyrium cirratum*. Fires are frequent with perhaps more crown fires than ponderosa pine woodlands, which tend to have more frequent surface fires on gentle slopes.

DISTRIBUTION

**Range:** This system is found in the Sierra Madre Occidental and Sierra Madre Oriental of Mexico, Trans‑Pecos Texas, southern New Mexico and Arizona, generally south of the Mogollon Rim.

**Divisions:** 305:C

**TNC Ecoregions:** 22:C, 24:C

**Nations:** MX, US

**Subnations:** AZ, NM, TX

**Map Zones:** 14:C, 15:C, 24:C, 25:C, 26:C, 27:P, 28:?

**USFS Ecomap Regions:** 313B:CC, 313C:CC, 313D:C?, 315A:CC, 315H:CC, 321A:CC, 322A:CC, 322B:CC, M313A:CC, M313B:CC, M331F:??, M331G:??

CONCEPT

**Environment:** This system is found on mountains and plateaus, on gently rolling landscapes or rugged slopes. In the Davis Mountains of Texas, it occurs on Tertiary igneous substrates, but may also occur on sandstone and limestone substrates, such as in the Guadalupe Mountains region. Soils are often rocky but also include mountain loams.

**Vegetation:** These forests and woodlands are composed of Madrean pines (*Pinus arizonica, Pinus engelmannii, Pinus leiophylla*, or *Pinus strobiformis*) and evergreen oaks (*Quercus arizonica, Quercus emoryi*, or *Quercus grisea*) intermingled with patchy shrublands on most mid‑elevation slopes (1500‑2300 m elevation). Other tree species include *Hesperocyparis arizonica (= Cupressus arizonica), Juniperus deppeana, Pinus cembroides, Pinus discolor, Pinus ponderosa* (with Madrean pines or oaks), and *Pseudotsuga menziesii*. Subcanopy and shrub layers may include typical encinal and chaparral species such as *Agave* spp., *Arbutus arizonica, Arctostaphylos pringlei, Arctostaphylos pungens, Garrya wrightii, Nolina* spp., *Quercus hypoleucoides, Quercus rugosa*, and *Quercus turbinella*. Some stands have moderate cover of perennial graminoids such as *Muhlenbergia emersleyi, Muhlenbergia longiligula, Muhlenbergia straminea (= Muhlenbergia virescens)*, and *Schizachyrium cirratum*.

Texas occurrences are typically dominated by *Pinus ponderosa* (or *Pinus arizonica* in the Chisos), but oak species such as *Quercus emoryi, Quercus grisea, Quercus x pauciloba*, and *Quercus gambelii* may be present to codominant. The subcanopy and shrub layer are typically not dense and may include species of the canopy as well as *Quercus hypoleucoides, Juniperus deppeana, Cercocarpus montanus, Holodiscus dumosus, Symphoricarpos* spp., *Nolina* spp., *Cylindropuntia imbricata*, and *Mimosa aculeaticarpa var. biuncifera*. *Pinus cembroides* and, in the Guadalupe Mountains, *Pinus edulis* becomes a common component, particularly at the lower elevational limits of this type and in more xeric situations. The herbaceous layer is typically dominated by graminoids, including *Andropogon gerardii, Blepharoneuron tricholepis, Bothriochloa barbinodis, Bothriochloa laguroides ssp. torreyana, Bouteloua curtipendula, Bouteloua gracilis, Bouteloua hirsuta, Eragrostis intermedia, Hesperostipa neomexicana, Heteropogon contortus, Koeleria macrantha, Muhlenbergia dubia, Muhlenbergia emersleyi, Muhlenbergia montana, Muhlenbergia pauciflora, Muhlenbergia rigida, Panicum bulbosum, Piptochaetium fimbriatum, Schizachyrium cirratum*, and *Schizachyrium scoparium*.

**Dynamics:** [from M011] Under historic natural conditions (also called natural range of variability, NRV), lower to mid‑elevation stands in this macrogroups varied from open woodlands (10‑20% cover) with pines dominating the overstory and perennial bunch grass dominating the understory to moderately dense woodlands (20‑40% tree cover) with less dense herbaceous layer and more tree and shrub cover. Lower elevation tree line of pines is primarily controlled by dry season water stress (Barton 1993). Fire and drought are the primary disturbances of this ecosystem (USFS 2009).

Information on fire return intervals is varied depending on elevation zone with fires frequently starting at lower elevations and burning upslope into the montane zone. Lower montane elevation pine‑oak stands had frequent, low intensity surface fires (mean fire return every 6‑14 years) as a result of lightning ignitions primarily between early spring and summer (Bahre 1985, Swetnam et al. 1992, 2001, Kaib et al. 1996, Schussman and Gori 2006, Swetnam and Baisan 1996b). However, minimum fire‑free periods of 20‑30 years are necessary for pines to establish and become resistant (thick bark) to surface fires (Barton et al. 2001). More frequent fire favors oaks and other sprouting species over pines and other conifers, which can alter stand composition. Less frequent fire (FRI >50 years) results in more conifer recruitment and denser vegetation that can lead to higher intensity, mixed‑severity and patches of stand‑replacing fires that also favors oaks and other sprouting species (Danzer et al. 1996, Barton 1999, Barton et al. 2001, Schussman and Gori 2006). For stands with inclusions of Ponderosa Pine Woodland in the Madrean Conifer‑Oak Forest and Woodland, the historic mean fire‑return interval is similar (Smith 2006). In Arizona and New Mexico, Swetnam and Baisan (1996b) found the historic mean fire‑return interval ranges from 2 to 17 years for fires scarring one or more trees, and 4 to 36 years for fires scarring between 10% and 25% of trees between the years of 1700 and 1900. However, in the more mesic subalpine fir communities a fire return interval of up to 400 years is not uncommon.

Herbivory by native herbivores in the Madrean montane conifer‑oak forests and woodlands is variable in this type. For more open stands with grass‑dominated understory herbivores are similar to semi‑desert grasslands. Large herbivores include browsers like Coues' white‑tailed deer (*Odocoileus virginianus couesi*), mule deer (*Odocoileus hemionus*), elk (*Cervus elaphus*), and rodents such as yellow nosed cotton rat (*Sigmodon ochrognathus*), white‑throated wood rat (*Neotoma albigula*), southern pocket gopher (*Thomomys umbrinus*), Apache squirrel (*Sciurus nayaritensis*), Arizona gray squirrel (*Sciurus arizonensis*), porcupine (*Erethizon dorsatum*), Bailey's pocket mouse (*Chaetodipus baileyi*), and eastern cotton tail (*Sylvilagus floridanus*) are common in the Madrean pine‑oak woodlands (Schussman and Gori 2006, Majka et al. 2007). Southwestern forest trees have been host to several species of insects, pathogenic fungi, and parasitic plants, however there are no accounts of historic insect outbreak, fungi or parasitic plant periodicity (Dahms and Geils 1997).

A good condition/proper functioning occurrence of Madrean Montane Conifer‑Oak Forest and Woodland ecosystem is large and uninterrupted; the surrounding landscape is also in good condition with soils that have not been excessively eroded. Weeds are few. There is a diversity of stand age and size classes in response to a functioning natural fire regime. For the majority of the type (lower montane pine‑oak woodlands) that is frequent (mean fire return every 6‑14 years), low‑intensity surface fires with occasional fire free periods of 20‑30 years minimum to allow for conifers to establish and become resistant (thick bark) to surface fires. For upper montane conifer oak and mixed conifer forests, the historical fire regime would have less frequent fires, mixed‑severity and occasional stand‑replacing fires.

A poor condition/non‑functioning occurrence is highly fragmented, or much reduced in size from its historic extent; the surrounding landscape is in poor condition either with highly eroding soils, many non‑native species or a large percentage of the surrounding landscape has been converted to exurban development. Over time passive (livestock grazing) and active fire suppression would result high density of trees and heavy fuel loading that would lead to large, high‑severity, stand‑replacing fires in stands of the montane conifer‑oak forests.

SOURCES

**References:** Barbour and Billings 2000, Brown 1982a, Brown et al. 1998, Comer et al. 2003*, Elliott 2012, Eyre 1980

**Version:** 02 Oct 2014	**Stakeholders:** Latin America, Southeast, West

**Concept Author:** P. Comer	**LeadResp:** West

CES305.798 Madrean Upper Montane Conifer‑Oak Forest and Woodland

**Primary Division:** Sierra Madre (305)

**Land Cover Class:** Forest and Woodland

**Spatial Scale & Pattern:** Large patch

**Required Classifiers:** Natural/Semi‑natural; Vegetated (>10% vasc.); Upland

**Diagnostic Classifiers:** Montane [Montane]; Forest and Woodland (Treed); Tropical/Subtropical [Tropical Xeric]; Xeric; F‑Patch/Medium Intensity; Abies coahuilensis, Quercus hypoleucoides, Q. rugosa

**National Mapping Codes:** EVT 2026; ESLF 4213; ESP 1026

**Concept Summary:** This ecological system occurs at the upper elevations in the Sierra Madre Occidental and Sierra Madre Oriental of Mexico with disjunct and limited occurrences at the highest elevations of the Chisos and Guadalupe mountains in Texas. In the U.S., it is restricted to north and east aspects at high elevations (1980‑2440 m) in the Sky Islands (Chiricahua, Huachuca, Pinaleno, Santa Catalina, and Santa Rita mountains) and along the Nantanes Rim. It is more common in Mexico and does not occur north of the Mogollon Rim. The vegetation is characterized by large‑ and small‑patch forests and woodlands dominated by *Pseudotsuga menziesii, Abies coahuilensis*, or *Abies lowiana* and Madrean oaks such as *Quercus arizonica, Quercus emoryi, Quercus grisea, Quercus hypoleucoides, Quercus rugosa*, and *Quercus toumeyi*. If *Quercus gambelii* is prominent in the shrub layer, then other Madrean elements are present. This system may include stands of *Quercus gravesii* woodlands. It is similar to Southern Rocky Mountain Dry‑Mesic Montane Mixed Conifer Forest and Woodland (CES306.823) which typically lacks Madrean elements.

**Comments:** Texas experts for mapzone 26 feel that this system does not occur in that zone. This system was identified at the highest elevations of the Chisos and Guadalupe mountains in Texas during the 2007‑2014 mapping of ecological systems for that state (Elliott 2012).

DISTRIBUTION

**Range:** This system is found in the Sierra Madre Occidental and Sierra Madre Oriental of Mexico. In the U.S., it is restricted to north and east aspects at high elevations (1980‑2440 m) in the Sky Islands (Chiricahua, Huachuca, Pinaleno, Santa Catalina, and Santa Rita mountains) and along the Nantanes Rim. It also has limited distribution in Texas on the highest mountain areas of the Guadalupe and Chisos mountains, but is lacking from high elevations of the Davis Mountains.

**Divisions:** 302:C, 305:C, 306:C

**TNC Ecoregions:** 21:C, 22:C, 24:C

**Nations:** MX, US

**Subnations:** AZ, MXCH, MXSO, NM

**Map Zones:** 15:C, 24:?, 25:C, 26:C, 27:?, 28:?

**USFS Ecomap Regions:** 313D:??, 321A:CC, 322B:??, M313A:CC, M313B:CC

CONCEPT

**Environment:** In Texas, this system occurs on Permian limestone in the Guadalupe Mountains, and in the Chisos Mountains, it primarily occurs on Tertiary igneous formations and associated colluvial and alluvial deposits from these formations. In the Chisos Mountains, it occurs on Igneous Hill and Mountain soils; in the Guadalupe Mountains in occurs on Victorio‑Lorenz‑Rock outcrop complex.

**Vegetation:** The vegetation is characterized by large‑ and small‑patch forests and woodlands dominated by *Pseudotsuga menziesii, Abies coahuilensis*, or *Abies lowiana (= Abies concolor var. lowiana)*, and Madrean oaks such as *Quercus arizonica, Quercus emoryi, Quercus grisea, Quercus hypoleucoides, Quercus rugosa*, and *Quercus toumeyi*. In Texas, the characteristic dominants of the system are *Pseudotsuga menziesii* and *Pinus strobiformis*, though *Pinus ponderosa, Juniperus* spp., and *Pinus cembroides* or *Pinus edulis* may also be present to common. The shrub and subcanopy are typically sparse and generally dominated by species from the canopy. In some areas, *Quercus gambelii* may form dense shrub patches. The herbaceous layer is typically dominated by graminoids, including species such as *Blepharoneuron tricholepis, Festuca arizonica, Koeleria macrantha, Muhlenbergia pauciflora, Piptochaetium fimbriatum*, and *Poa fendleriana*.

**Dynamics:** [from M011] Under historic natural conditions (also called natural range of variability, NRV), lower to mid‑elevation stands in this macrogroups varied from open woodlands (10‑20% cover) with pines dominating the overstory and perennial bunch grass dominating the understory to moderately dense woodlands (20‑40% tree cover) with less dense herbaceous layer and more tree and shrub cover. Lower elevation tree line of pines is primarily controlled by dry season water stress (Barton 1993). Fire and drought are the primary disturbances of this ecosystem (USFS 2009).

Information on fire return intervals is varied depending on elevation zone with fires frequently starting at lower elevations and burning upslope into the montane zone. Lower montane elevation pine‑oak stands had frequent, low intensity surface fires (mean fire return every 6‑14 years) as a result of lightning ignitions primarily between early spring and summer (Bahre 1985, Swetnam et al. 1992, 2001, Kaib et al. 1996, Schussman and Gori 2006, Swetnam and Baisan 1996b). However, minimum fire‑free periods of 20‑30 years are necessary for pines to establish and become resistant (thick bark) to surface fires (Barton et al. 2001). More frequent fire favors oaks and other sprouting species over pines and other conifers, which can alter stand composition. Less frequent fire (FRI >50 years) results in more conifer recruitment and denser vegetation that can lead to higher intensity, mixed‑severity and patches of stand‑replacing fires that also favors oaks and other sprouting species (Danzer et al. 1996, Barton 1999, Barton et al. 2001, Schussman and Gori 2006). For stands with inclusions of Ponderosa Pine Woodland in the Madrean Conifer‑Oak Forest and Woodland, the historic mean fire‑return interval is similar (Smith 2006). In Arizona and New Mexico, Swetnam and Baisan (1996b) found the historic mean fire‑return interval ranges from 2 to 17 years for fires scarring one or more trees, and 4 to 36 years for fires scarring between 10% and 25% of trees between the years of 1700 and 1900. However, in the more mesic subalpine fir communities a fire return interval of up to 400 years is not uncommon.

Herbivory by native herbivores in the Madrean montane conifer‑oak forests and woodlands is variable in this type. For more open stands with grass‑dominated understory herbivores are similar to semi‑desert grasslands. Large herbivores include browsers like Coues' white‑tailed deer (*Odocoileus virginianus couesi*), mule deer (*Odocoileus hemionus*), elk (*Cervus elaphus*), and rodents such as yellow nosed cotton rat (*Sigmodon ochrognathus*), white‑throated wood rat (*Neotoma albigula*), southern pocket gopher (*Thomomys umbrinus*), Apache squirrel (*Sciurus nayaritensis*), Arizona gray squirrel (*Sciurus arizonensis*), porcupine (*Erethizon dorsatum*), Bailey's pocket mouse (*Chaetodipus baileyi*), and eastern cotton tail (*Sylvilagus floridanus*) are common in the Madrean pine‑oak woodlands (Schussman and Gori 2006, Majka et al. 2007). Southwestern forest trees have been host to several species of insects, pathogenic fungi, and parasitic plants, however there are no accounts of historic insect outbreak, fungi or parasitic plant periodicity (Dahms and Geils 1997).

A good condition/proper functioning occurrence of Madrean Montane Conifer‑Oak Forest and Woodland ecosystem is large and uninterrupted; the surrounding landscape is also in good condition with soils that have not been excessively eroded. Weeds are few. There is a diversity of stand age and size classes in response to a functioning natural fire regime. For the majority of the type (lower montane pine‑oak woodlands) that is frequent (mean fire return every 6‑14 years), low‑intensity surface fires with occasional fire free periods of 20‑30 years minimum to allow for conifers to establish and become resistant (thick bark) to surface fires. For upper montane conifer oak and mixed conifer forests, the historical fire regime would have less frequent fires, mixed‑severity and occasional stand‑replacing fires.

A poor condition/non‑functioning occurrence is highly fragmented, or much reduced in size from its historic extent; the surrounding landscape is in poor condition either with highly eroding soils, many non‑native species or a large percentage of the surrounding landscape has been converted to exurban development. Over time passive (livestock grazing) and active fire suppression would result high density of trees and heavy fuel loading that would lead to large, high‑severity, stand‑replacing fires in stands of the montane conifer‑oak forests.

SOURCES

**References:** Comer et al. 2003*, Elliott 2012

**Version:** 02 Oct 2014	**Stakeholders:** Latin America, Southeast, West

**Concept Author:** P. Comer	**LeadResp:** West

1.B.2. Cool Temperate Forest & Woodland

1.B.2.Na. Eastern North American Forest & Woodland

M883. Appalachian‑Interior‑Northeastern Mesic Forest

CES202.593 Appalachian (Hemlock)‑Northern Hardwood Forest

**Primary Division:** Central Interior and Appalachian (202)

**Land Cover Class:** Forest and Woodland

**Spatial Scale & Pattern:** Matrix

**Required Classifiers:** Natural/Semi‑natural; Vegetated (>10% vasc.); Upland

**Diagnostic Classifiers:** Mesotrophic Soil; Needle‑Leaved Tree; Broad‑Leaved Deciduous Tree; Pinus spp. ‑ Tsuga canadensis

**National Mapping Codes:** EVT 2370; ESLF 4313; ESP 1370

**Concept Summary:** This forested system of the eastern U.S. ranges from central New England west to Lake Erie and south to the higher elevations of Virginia and West Virginia. It is one of the matrix forest types in the northern part of the Central Interior and Appalachian Division. Northern hardwoods such as *Acer saccharum, Betula alleghaniensis*, and *Fagus grandifolia* are characteristic, either forming a deciduous canopy or mixed with *Tsuga canadensis* (or in some cases *Pinus strobus*). Other common and sometimes dominant trees include *Quercus* spp. (most commonly *Quercus rubra*), *Liriodendron tulipifera, Prunus serotina, Acer rubrum*, and *Betula lenta*. It is of more limited extent and more ecologically constrained in the southern part of its range in northern parts of Virginia and West Virginia.

**Comments:** Northward this system is replaced by Laurentian‑Acadian Pine‑Hemlock‑Hardwood Forest (CES201.563) and Laurentian‑Acadian Northern Hardwood Forest (CES201.564), but the systems overlap on the Allegheny Plateau and in central New England. USFS ecological province lines provide a general delimiter, with areas in Provinces 211 and M211 mostly falling into the Laurentian‑Acadian systems, and areas in Provinces 221 and M221 falling into this Appalachian system. The range of *Liriodendron tulipifera* is a good approximator for the northern limit of this system's range.

DISTRIBUTION

**Range:** This system is found from southern New Hampshire south to Virginia and West Virginia, and possibly in adjacent Kentucky.

**Divisions:** 202:C

**TNC Ecoregions:** 48:C, 49:C, 52:C, 59:C, 60:C, 61:C

**Nations:** US

**Subnations:** CT, KY?, MA, MD, NH, NJ, NY, OH?, PA, VA, WV

**Map Zones:** 53:C, 60:C, 61:C, 62:C, 63:C, 64:C, 65:C, 66:C

**USFS Ecomap Regions:** 211E:CC, 211Fc:CCC, 211Fd:CCC, 211G:CC, 221Aa:CCC, 221Ae:CCC, 221Af:CCC, 221Ag:CCC, 221Ah:CCC, 221Ai:CCC, 221Am:CCC, 221B:CC, 221D:CC, 221E:CC, 221F:CC, 222I:CC, M221A:CC, M221B:CC, M221C:CP

CONCEPT

**Environment:** This system occurs predominantly on mesic sites over a broad range of topographic conditions, such as protected low and midslopes and valley bottoms, at elevations from 305 to 1360 m. Soils are usually acidic and retain some moisture except during severe droughts. They are moderately well‑drained to well‑drained loamy or silty soils, and are rocky and usually deep in depressions among boulders. Forests in this system are also associated with high‑elevation periglacial boulderfields. In the Central Appalachian center of its range, its ecological amplitude is somewhat broader, and it becomes the matrix forest in some areas of Pennsylvania, Maryland, West Virginia. At Shenandoah National Park, this system spans a broad range of environmental settings from steep west‑facing slopes to south‑facing gentle slopes.

**Vegetation:** The canopy is characterized and often usually dominated by northern hardwoods (e.g., *Fagus grandifolia* and *Acer saccharum*), often with *Tsuga canadensis*, but may also contain large amounts of *Pinus strobus* and *Quercus* spp. *Tsuga canadensis* can dominate the canopy on cool/moist sites at higher elevations and in shaded coves, valley bottoms and riparian areas. Bottomlands and toeslopes may also contain *Fraxinus americana*, as well as *Platanus occidentalis* (Whitney 1990). Other common associates may include *Acer rubrum, Prunus serotina, Betula lenta, Tilia americana, Pinus strobus, Liriodendron tulipifera, Quercus* spp., and *Magnolia acuminata*. The subcanopy and shrub layers are usually well‑developed and may include *Viburnum lantanoides (= Viburnum alnifolium), Viburnum acerifolium, Hamamelis virginiana*, and *Cornus alternifolia*. A dense, low to high shrub layer of *Rhododendron maximum* and sometimes *Kalmia latifolia* is sometimes present. Common herbaceous species include *Maianthemum canadense, Onoclea sensibilis, Huperzia lucidula (= Lycopodium lucidulum), Dryopteris carthusiana (= Dryopteris spinulosa), Oxalis montana*, and *Mitchella repens* (Lutz 1930, Braun 1950).

**Dynamics:** In general, this system is dominated by long‑lived, mesophytic species that form multi‑layered uneven‑aged forests. Canopy dynamics are dominated by single and multiple disturbances encouraging gap phase regeneration (Abrams and Orwig 1996). Larger disturbances include windthrow, insect attack and icestorms. Although stand‑replacing wind events are rare, small to medium blowdown events are more common and occur at greater frequency on the plateau and exposed sideslopes (Ruffner and Abrams 2003).This system is currently being devastated in large parts of its range by the hemlock woolly adelgid (*Adelges tsugae*). This sucking insect is continuing to cause close to 100% mortality in some areas as it spreads from the north into the southern United States. The insect will most likely cause canopy hemlocks to be replaced by other canopy trees. Historically, this system was probably only subject to occasional fires. Fires that did occur may have been catastrophic and may have led to even‑aged stands of pine and hemlock. Fire suppression appears to have increased the extent of this system at the expense of oak‑pine systems.

Fire Regime Description (from Landfire 2007a): Historically, this system was probably only subject to occasional fires. Fires that did occur may have been catastrophic and may have led to even‑aged stands of pine and hemlock. Due to the predominance of cool, moist site conditions, surface and replacement fires are extremely rare, occurring at 700‑ to 1000‑year intervals. Most protected sites are essentially fire‑free. The principal cause of fuel formation leading to fire in northern hardwood ecosystems is broad‑scale, storm‑driven windthrow of catastrophic proportions (Hough 1936, Runkle 1982). The importance of red maple, sweet birch, northern red oak, and especially black cherry in contemporary Central Appalachian examples of this community group reflects secondary succession following catastrophic logging and fire disturbances in the early part of the twentieth century. Sugar maple and beech, both abundant in understory layers and locally codominant in the overstory, appear positioned to assume dominance as current secondary stands mature. However, beech bark disease and excessive deer browsing are serious threats to the future viability of the largest stands on Allegheny Mountain (VDNH 2007).

SOURCES

**References:** Abrams and Orwig 1996, Braun 1950, Braun 2001, Comer et al. 2003*, Connolly et al. 2007, Delcourt and Delcourt 1988, Edinger et al. 2014a, Ellison et al. 2005, Evans et al. 2009, Eyre 1980, Fike 1999, Gawler and Cutko 2010, Hough 1936, Hough 1963, Ison 2000, LANDFIRE 2007a, Litvaitis 2003, Lorimer and Frelich 1994, Lutz 1930, NYNHP 2013b, ONHD unpubl. data, Orwig and Foster 1998, Orwig et al. 2012, PNHP 2002, Paradis et al. 2007, Ruffner and Abrams 2003, Runkle 1981, Runkle 1982, Runkle 1985, Schafale 2012, Sperduto and Nichols 2004, Spies 2004, Swain and Kearsley 2011, VDNH 2007, Whitney 1990a, Whitney 1990b

**Version:** 14 Jan 2014	**Stakeholders:** East, Midwest, Southeast

**Concept Author:** S.C. Gawler, R. White, R. Evans, M. Pyne	**LeadResp:** East

CES202.887 South‑Central Interior Mesophytic Forest

**Primary Division:** Central Interior and Appalachian (202)

**Land Cover Class:** Forest and Woodland

**Spatial Scale & Pattern:** Large patch

**Required Classifiers:** Natural/Semi‑natural; Vegetated (>10% vasc.); Upland

**Diagnostic Classifiers:** Forest and Woodland (Treed); Sideslope; Unglaciated; Eutrophic Soil; Broad‑Leaved Deciduous Tree

**National Mapping Codes:** EVT 2321; ESLF 4127; ESP 1321

**Concept Summary:** These high‑diversity, predominately deciduous forests occur on deep and enriched soils (in some cases due to, or enhanced by, the presence of limestone or related base‑rich geology), in non‑montane settings and usually in somewhat protected landscape positions such as coves or lower slopes. The core distribution of this system lies in the Cumberland and Allegheny plateaus, extending into the adjacent southern Ridge and Valley and portions of the Interior Low Plateau where it is located entirely south of the glacial boundary. Dominant species include *Acer saccharum, Fagus grandifolia, Liriodendron tulipifera, Tilia americana, Quercus rubra, Magnolia acuminata*, and *Juglans nigra*. The abundance of *Tsuga canadensis*, which may be a component of some stands, is being rapidly reduced by the hemlock woolly adelgid (*Adelges tsugae*). The canopy trees may grow very large in undisturbed areas. The herb layer is very rich, often with abundant spring ephemerals. Many examples may be bisected by small streams.

**Comments:** Southern and Central Appalachian Cove Forest (CES202.373) (Ecoregions 51 and 59) is being treated as a separate system. The concept of this type (CES202.887) is more‑or‑less consistent with the "Mixed Mesophytic Communities" of both the Mixed Mesophytic Forest Region and the non‑coastal plain portion of the Western Mesophytic Forest Region, extending north into unglaciated portions of the Beech‑Maple Forest Region, of Braun (1950) and Greller (1988). There is much variability in different examples of this system across its range, with the composition of some occurrences in the escarpment of the Cumberland Plateau approaching that of examples of Southern and Central Appalachian Cove Forest (CES202.373). The Allegheny Front is adopted as the divide between these two similar systems: material to the west goes to this system, and material to the east goes to Southern and Central Appalachian Cove Forest (CES202.373). In limited areas of the region, some stands may contain hemlock (*Tsuga canadensis*). These are noteworthy on a local basis, as the tree is less well distributed in the range of this system than it is in corresponding environments at higher elevation in the Appalachians or to the north.

DISTRIBUTION

**Range:** This system occurs in southeastern Ohio east to Virginia, West Virginia, Kentucky, Tennessee, Georgia, and Alabama, with disjunct occurrences in unglaciated southwestern Pennsylvania and southwestern New York. This range is more‑or‑less consistent with the "Mixed Mesophytic" and "Western Mesophytic" (non‑coastal plain portion only) forest regions of Braun (1950) and Greller (1988), although it does extend into unglaciated portions of the "Beech‑Maple" region to the north. Thus, this system is most extensive in the Cumberland and Allegheny plateaus, as well as the unglaciated Interior Low Plateau, and becomes relatively limited in extent towards its western limit in the Ozark Hills of Illinois, and towards its northern limit in southwestern New York.. It is replaced in the Upper East Gulf Coastal Plain by other systems. Its range also includes the southern Ridge and Valley from Tennessee (and adjacent southwestern Virginia) to Alabama. Parts of the Cumberland Mountains (EPA 69 in Kentucky and Tennessee) are instead occupied by Southern and Central Appalachian Cove Forest (CES202.373). North‑Central Interior Beech‑Maple Forest (CES202.693) replaces this one in EPA 72b of Indiana.

**Divisions:** 202:C

**TNC Ecoregions:** 44:C, 49:C, 50:C, 60:C

**Nations:** US

**Subnations:** AL, GA, IL, IN, KY, NY, OH, PA, TN, VA, WV

**Map Zones:** 47:C, 48:C, 49:C, 53:C, 57:C, 61:C, 62:C, 63:C

**USFS Ecomap Regions:** 211G:CC, 221E:CC, 221F:C?, 221H:CC, 221J:CC, 223B:CC, 223D:CC, 223E:CC, 223F:CC, 231C:CC, 231D:CC, M221Ca:CCC

CONCEPT

**Environment:** These high‑diversity deciduous forests occur on deep and enriched soils, usually in somewhat protected landscape positions such as coves or lower slopes.

**Vegetation:** Dominant tree species include *Acer saccharum, Fagus grandifolia, Liriodendron tulipifera, Tilia americana, Quercus rubra, Magnolia acuminata*, and *Juglans nigra*. *Tsuga canadensis* may be a component of some stands. Shrubs include *Calycanthus floridus, Hydrangea quercifolia, Lindera benzoin, Staphylea trifolia*, and others. The herb layer is very rich, often with abundant spring ephemerals. Some typical species include *Actaea racemosa, Athyrium filix‑femina ssp. asplenioides, Caulophyllum thalictroides, Collinsonia verticillata, Galium circaezans, Hydrastis canadensis, Panax quinquefolius, Podophyllum peltatum, Polystichum acrostichoides, Sanguinaria canadensis, Stylophorum diphyllum*, and many others.

SOURCES

**References:** Braun 1950, Comer et al. 2003*, Edinger et al. 2014a, Evans et al. 2009, Eyre 1980, Greller 1988, ONHD unpubl. data

**Version:** 06 Jun 2017	**Stakeholders:** East, Midwest, Southeast

**Concept Author:** M. Pyne and R. Evans	**LeadResp:** Southeast

CES202.373 Southern and Central Appalachian Cove Forest

**Primary Division:** Central Interior and Appalachian (202)

**Land Cover Class:** Forest and Woodland

**Spatial Scale & Pattern:** Large patch

**Required Classifiers:** Natural/Semi‑natural; Vegetated (>10% vasc.); Upland

**Diagnostic Classifiers:** Forest and Woodland (Treed); Broad‑Leaved Tree

**National Mapping Codes:** EVT 2318; ESLF 4124; ESP 1318

**Concept Summary:** This system consists of mesophytic hardwood or hemlock‑hardwood forests of sheltered topographic positions in the Southern Blue Ridge and central Appalachian Mountains. Examples are generally found on concave slopes that promote moist conditions. The system includes acidic and "rich" coves that may be distinguished by individual plant communities based on perceived differences in soil fertility and species richness (rich examples have higher diversity and density in the herbaceous layer). Both acidic and rich coves may occur in the same site, with the acidic coves potentially creeping out of the draw‑up to at least midslope on well‑protected north‑facing slopes. Characteristic species in the canopy include *Aesculus flava, Acer saccharum, Fraxinus americana, Tilia americana, Carya cordiformis, Liriodendron tulipifera, Halesia tetraptera, Tsuga canadensis, Fagus grandifolia, Magnolia acuminata*, and *Magnolia fraseri*.

**Comments:** This system is best distinguished from others in its range by the combination of sheltered topography, low elevation, and mesophytic flora with high species richness. Canopies can sometimes become depauperate after repeated logging. It does not include rich, mesophytic "cove" forests further west in the Cumberland Plateau and Interior Low Plateau, even though some of these approach or exceed Appalachian examples in their species composition and occur on similar sites. They are interpreted as variability within South‑Central Interior Mesophytic Forest (CES202.887). In West Virginia and north, the Allegheny front is adopted as the divide between these two similar systems: examples to the west go to South‑Central Interior Mesophytic Forest (CES202.887), and examples to the east go to this system.

DISTRIBUTION

**Range:** This system occurs in the southern and central Appalachian Mountains, ranging into the Cumberland Mountains of Kentucky and Tennessee. This range is more‑or‑less consistent with the "Oak‑Chestnut" forest region of Braun (1950) and Greller (1988), versus the "Mixed Mesophytic" and "Western Mesophytic" forest regions to the west. In West Virginia and north, the Allegheny front is the boundary between this and South‑Central Interior Mesophytic Forest (CES202.887).

**Divisions:** 202:C

**TNC Ecoregions:** 50:C, 51:C, 52:C, 59:C, 61:P

**Nations:** US

**Subnations:** GA, KY, MD, NC, SC, TN, VA, WV

**Map Zones:** 53:C, 57:C, 61:C, 62:C

**USFS Ecomap Regions:** M221A:CC, M221B:CC, M221Cb:CCC, M221Cc:CCC, M221Cd:CCC, M221D:CC

CONCEPT

**Environment:** This mixed mesophytic forest system occurs on moist, topographically protected areas such as coves, V‑shaped valley bottoms and ravines, and north‑ and east‑facing toeslopes in a dissected landscape. It generally occurs below 1525 m (5000 feet) elevation. The dissected topography creates strong gradients in microclimate and soil moisture and fertility at the local (watershed) scale (Hutchins et al. 1976, Iverson et al. 1997, Morris and Boerner 1998). This forest type developed primarily on mesic, sheltered landscape positions (e.g., lower concave slopes, coves, ravines) but also occurred on some dry‑mesic slopes, where presumably fire was infrequent (Wade et al. 2000). This system has two primary components, an acidic cove of lower soil fertility that ranges from the lowest slope positions up the slope on north‑facing protected slopes, and a rich, high‑fertility cove forest that tends to occur only at the lowest slope positions. Both are sheltered from wind and may be shaded by concave topography, which promotes moist conditions. Local slopes are usually concave. Bedrock may be of numerous types. Acidic rocks, such as felsic igneous and metamorphic rocks, support rich cove forests in a more limited range of sites than do basic rocks, such as mafic metamorphic rocks or marble. Soils may be rocky or fine‑textured, and may be residual, alluvial, or colluvial. In the southern Appalachians, the hemlock "phase" of this ("acidic cove forest") often occurs between "richer" examples of Southern and Central Appalachian Cove Forest (CES202.373) in the lowest areas and Southern Appalachian Oak Forest (CES202.886) on the midslopes.

**Vegetation:** This system is among the most diverse in the United States, containing more than 30 canopy tree species. Vegetation consists of forests dominated by various combinations of mesophytic species, usually with many different species of primarily deciduous trees present. *Acer saccharum, Liriodendron tulipifera, Tilia americana, Tilia americana var. heterophylla, Fraxinus americana, Aesculus flava, Fagus grandifolia, Betula lenta, Magnolia acuminata, Magnolia fraseri, Quercus rubra, Halesia tetraptera, Prunus serotina*, and *Tsuga canadensis* are the most frequent dominant canopy species (Braun 1950, Muller 1982). Witness tree data (from early land surveys) and studies of old‑growth forests suggest that mixed‑oak forests were generally more abundant on the landscape than mixed‑mesophytic forests prior to European settlement (Beatley 1959, McCarthy et al. 1987, Abrams et al. 1995, Dyer 2001, McCarthy et al. 2001, Rentch et al. 2003). Canopies are generally very diverse, with all species potentially occurring in one 20x50‑meter plot in rich cove areas. A well‑developed herb layer, often very dense and usually high in species richness, is present in all but the acid coves. Well‑developed and fairly diverse subcanopy and shrub layers are often also present in all but the acid coves. Ulrey (1999) listed *Caulophyllum thalictroides, Actaea racemosa (= Cimicifuga racemosa), Laportea canadensis, Osmorhiza claytonii, Sanguinaria canadensis, Viola canadensis, Acer saccharum, Aesculus flava, Carya cordiformis*, and *Tilia americana var. heterophylla* as characteristic species.

**Dynamics:** This system is naturally dominated by stable, uneven‑aged forests, with canopy dynamics dominated by gap‑phase regeneration on a fine scale. Occasional extreme wind or ice events may disturb larger patches. In the absence of frequent or catastrophic disturbance, environmental gradients formed by the dissected topography determine forest composition (Hutchins et al. 1976, Muller 1982, Iverson et al. 1997, Dyer 2001). Most of the component species are among the less fire‑tolerant in the region. The mixed‑mesophytic forest type is fire regime class III, surface fires with return intervals 30‑100+ years (Wade et al. 2000). Mixed‑severity fires may occur approximately every 500 years opening the canopy with increased mortality. Straight‑line winds or microbursts may cause blowdowns on a scale of one to 100 acres. Stand‑replacement fires happen very infrequently. Current composition and structure of this system is influenced by the absence of fire, deer herbivory, and non‑native invasive species (plants, animals, insects and disease). The absence of fire is causing an expansion of some of the characteristic mesic taxa out of coves, potentially replacing previous oak‑dominated vegetation on drier and more exposed sites than those typically associated with "mesic" vegetation.

SOURCES

**References:** Abrams et al. 1995, Beatley 1959, Braun 1950, Brown and Smith 2000, Comer et al. 2003*, Delcourt et al. 1998, DuMond 1970, Dyer 2001, Edwards et al. 2013, Evans et al. 2009, Eyre 1980, Frost 1998, Gettman 1974, Guyette et al. 2003, Hutchins et al. 1976, Iverson et al. 1997, Küchler 1964, LANDFIRE 2007a, McCarthy et al. 1987, McCarthy et al. 2001, McNab and Avers 1994, Morris and Boerner 1998, Muller 1982, Nelson 1986, Patterson 1994, Rentch et al. 2003, Schafale 2012, Schafale and Weakley 1990, Schmidt et al. 2002, Simon 2011, Simon 2015, Tobe et al. 1992, Ulrey 1999, Wade et al. 2000

**Version:** 28 Apr 2016	**Stakeholders:** East, Southeast

**Concept Author:** M. Schafale, M. Pyne, R. White, R. Evans	**LeadResp:** Southeast

CES202.029 Southern Appalachian Northern Hardwood Forest

**Primary Division:** Central Interior and Appalachian (202)

**Land Cover Class:** Forest and Woodland

**Spatial Scale & Pattern:** Large patch

**Required Classifiers:** Natural/Semi‑natural; Vegetated (>10% vasc.); Upland

**Diagnostic Classifiers:** Montane; Broad‑Leaved Tree

**National Mapping Codes:** EVT 2309; ESLF 4115; ESP 1309

**Concept Summary:** This ecological system consists of hardwood forests of the higher elevation zones of the Southern Appalachians, generally above 1372 m (4500 feet) elevation within its primary range. Included are classic northern hardwood forests, dominated by various combinations of mesophytic hardwoods, which interfinger with high‑elevation oak forests downslope or on more exposed aspects. The combination of elevation and aspect provides habitat for this system. Included in this system are limited areas locally known as "beech gaps" and "boulderfields." Stands are dominated by various combinations of Appalachian mesophytic trees, including *Acer saccharum, Aesculus flava, Betula alleghaniensis, Fagus grandifolia*, and *Tsuga canadensis*. In addition, *Prunus serotina* and *Tilia americana var. heterophylla* are occasionally abundant. *Quercus rubra* may be present but is not dominant. In Kentucky, this system is of extremely limited extent, being restricted to areas on Black Mountain (the highest mountain in the state) above about 915‑1100 m (3000‑3600 feet) elevation. Black Mountain is apparently higher in elevation than adjacent areas in Tennessee and Virginia.

**Comments:** This system does not include high‑elevation *Quercus rubra* associations, which are placed in related Central and Southern Appalachian Montane Oak Forest (CES202.596). Even though they may occur in the same elevational zone as the mesophytic northern hardwood forests, they occupy a different habitat (drier and more exposed aspects), and comprise a different set of plant associations. They differ from the mesophytic northern hardwood forests in the dominance of oaks and the probable importance of fire as a process. The border of this system with adjacent systems is usually gradational. The transition to Central and Southern Appalachian Spruce‑Fir Forest (CES202.028) that often adjoins at higher elevation is marked by a gradual shift in canopy dominance from hardwoods to conifers. The transition to lower elevation hardwood forest systems is similarly marked by a gradual turnover of dominant trees but may be more subtle because more species are shared. The transition to Southern and Central Appalachian Cove Forest (CES202.373) is particularly gradual, being marked mainly by the addition of species without loss of species. The non‑forested systems that occur in the same elevational zone may have transition zones of open woody vegetation, though some have sharp borders. In relatively undisturbed stands, the canopy composition and structure are the best way to determine the boundary of this system.

This system is similar to the northern hardwood forests of the northeastern U.S., i.e., Laurentian‑Acadian Northern Hardwood Forest (CES201.564), but differs in having a southern mountain climate (shorter winters, less extreme cold temperatures, shorter summer days), lacking a history of glaciation, and in having a flora and fauna with many Southern Appalachian endemics. A few characteristic dominants of the northern hardwoods are lacking, including *Betula papyrifera* and *Populus tremuloides*. It differs from Appalachian (Hemlock)‑Northern Hardwood Forest (CES202.593) in its more montane setting and its flora and fauna having many Southern Appalachian endemics. The northern hardwoods in the Ridge and Valley are primarily included in CES202.593. The northern boundary of this system follows a gradual northward transition through central and northern Virginia.

DISTRIBUTION

**Range:** This system is primarily found in the Southern Blue Ridge, where it ranges from northwestern Georgia, western North Carolina and eastern Tennessee northward to southern Virginia. In Kentucky, this system is restricted to the Cumberland Mountains in the extreme southeastern corner of that state.

**Divisions:** 202:C

**TNC Ecoregions:** 50:C, 51:C, 59:P

**Nations:** US

**Subnations:** GA, KY, NC, TN, VA

**Map Zones:** 53:C, 57:C, 61:C

**USFS Ecomap Regions:** 221A:CC, 221B:CC, 221E:CC, 231A:CC, M221A:CC, M221Bc:CCC, M221C:CC, M221D:CC

CONCEPT

**Environment:** The habitat for this system in the Southern Blue Ridge includes cooler, moister slopes and more‑or‑less concave landforms, at elevations from 1220‑1680 m (4000‑5500 feet), occasionally extending up to nearly 1830 m (6000 feet). It is most prevalent on north‑ to east‑facing slopes, but can occur on a variety of landforms and aspects within this elevational range, tending to be more predominant towards its upper limits, where it transitions to spruce‑ or spruce‑hardwood‑dominated types. Elevation and orographic effects make the climate cool and wet, with significant moisture input from fog as well as high rainfall. Strong winds, ice glaze, and extreme cold may occur but are less important than in Central and Southern Appalachian Spruce‑Fir Forest (CES202.028). Soils are generally very rocky, with the matrix ranging from well‑weathered parent material to coarse colluvial boulder deposits. Soils are probably moist but not saturated most of the time. Any kind of bedrock may be present. Limited areas support boulderfields. In related areas of Kentucky, this system is of extremely limited extent. It is found as low as about 915 m (3000 feet) on exposed northwest‑facing slopes on Black Mountain, the highest mountain in the state. Its elevational range here is lower than in the Southern Blue Ridge. Black Mountain is higher in elevation than adjacent areas in Tennessee and Virginia, which apparently lack examples of this system.

**Vegetation:** This vegetation consists of forests dominated by various combinations of *Acer saccharum, Aesculus flava, Betula alleghaniensis, Fagus grandifolia*, and *Tsuga canadensis*. *Prunus serotina* and *Tilia americana var. heterophylla* are occasionally abundant. *Quercus rubra* may be present but is not dominant; it dominates the warmer, more exposed aspects in this elevational range, and these stands are part of Central and Southern Appalachian Montane Oak Forest (CES202.596), not Southern Appalachian Northern Hardwood Forest (CES202.029). Lower strata usually include a dense herb layer and often a well‑developed deciduous shrub layer as well. Limited areas may have a dense evergreen shrub layer. Plant species richness ranges from fairly high to very low. Kentucky occurrences will of necessity lack the species that are endemic to the higher elevation Southern Appalachians from Georgia north to Virginia.

**Dynamics:** This system is naturally dominated by stable, uneven‑aged forests, with canopy dynamics dominated by gap‑phase regeneration on a fine to medium scale. Occasional extreme wind or ice events disturb larger patches on exposed slopes. Fire appears to be uncommon under natural conditions, perhaps extremely rare in the more mesic portions. In contrast, fire may be important in regeneration of *Quercus rubra* in stands of Central and Southern Appalachian Montane Oak Forest (CES202.596), and may be crucial in maintaining its dominance in these drier sites. Many *Quercus rubra* forests now appear to be succeeding to mesophytic hardwoods in the absence of fire. Little is known about natural fire behavior. Fires are likely to be low in intensity because of limited flammability of the vegetation and prevailing moist conditions, but most of the component tree species are probably not very tolerant of fire.

SOURCES

**References:** Comer et al. 2003*, Evans et al. 2009, Eyre 1980, Lohman and Watson 1943, Schafale 2012, Schafale and Weakley 1990

**Version:** 28 Mar 2014	**Stakeholders:** East, Southeast

**Concept Author:** M. Schafale and R. Evans	**LeadResp:** Southeast

CES202.342 Southern Piedmont Mesic Forest

**Primary Division:** Central Interior and Appalachian (202)

**Land Cover Class:** Forest and Woodland

**Spatial Scale & Pattern:** Large patch

**Required Classifiers:** Natural/Semi‑natural; Vegetated (>10% vasc.); Upland

**Diagnostic Classifiers:** Forest and Woodland (Treed); Very Long Disturbance Interval; Broad‑Leaved Deciduous Tree

**National Mapping Codes:** EVT 2316; ESLF 4122; ESP 1316

**Concept Summary:** This system encompasses mixed deciduous hardwood or occasionally hardwood‑pine forests of mesic sites in the Piedmont of the southeastern United States. Most examples occur on lower or north‑facing slopes where topography creates mesic moisture conditions. A mix of a small number of mesophytic trees is usually dominant, with *Fagus grandifolia* most prominent. Both acidic and basic substrates are currently included in this concept, as are certain heath bluffs, where dense shrub layers of mesophytic ericaceous shrubs may occur beneath an open tree canopy. Fire is naturally infrequent in this system, due to the slopes and moist conditions. If fire does penetrate, it is likely to be low in intensity and may not have significant ecological effects. Vegetation consists of forests dominated by combinations of trees that include a significant component of mesophytic species. *Fagus grandifolia* is almost always abundant and is often strongly dominant. *Quercus rubra, Liriodendron tulipifera*, and *Acer rubrum* may be abundant. In basic soil examples, *Fraxinus americana* and *Acer floridanum* are also abundant. A well‑developed understory is usually present. Herbs range from fairly dense in basic examples to sparse in acidic examples, and may be nearly absent in a few. The composition of all lower strata varies substantially with soil acidity.

**Comments:** This system is distinguished from Southern Piedmont Dry Oak‑(Pine) Forest and Woodland (CES202.339) by the significant component of mesophytic tree species, particularly *Fagus grandifolia*, as well as by occurrence on mesic topographic sites. Some oaks may also be present. It is distinguished from Southern Piedmont Small Floodplain and Riparian Forest (CES202.323) and Southern Piedmont Large Floodplain Forest (CES202.324) by the absence of characteristic alluvial or bottomland species, along with upland position. This boundary can be somewhat difficult to place, as some alluvial species will occur upslope in basic soils, and some mesic forests will extend onto higher terraces in bottomlands. This system is closely related to Southern Atlantic Coastal Plain Mesic Hardwood Forest (CES203.242) and in the northern part of the range may be very similar except for the geologic substrate. Farther south, there is a greater floristic difference between the two. This system is related to the cove forest systems of the southern Appalachians but lacks a number of species characteristic of those regions. These species are present in increasing numbers as one goes west in the Piedmont. The westernmost Piedmont has some examples of well‑developed Southern and Central Appalachian Cove Forest (CES202.373) in the more mountainous portions. Distinct subsets of this system, which could be recognized as different systems, are the basic/circumneutral and acidic examples, and also the shrubby heath bluffs.

DISTRIBUTION

**Range:** This ecological system ranges throughout the southern Piedmont, from Virginia to Alabama.

**Divisions:** 202:C

**TNC Ecoregions:** 52:C

**Nations:** US

**Subnations:** AL, GA, NC, SC, VA

**Map Zones:** 54:C, 59:C, 60:C, 61:C

**USFS Ecomap Regions:** 231A:CC, 231I:CC

CONCEPT

**Environment:** Examples occur on lower slopes or on north‑facing slopes, where topography creates mesic moisture conditions. This system may occur on any kind of rock type, with rock chemistry being an important determinant of variation. Most soils are acidic, but those formed on mafic rocks often are circumneutral to basic. The moist conditions and slope limit natural fire intensity and frequency.

**Vegetation:** These forests are dominated by mesophytic tree species. *Fagus grandifolia* is almost always abundant and is often strongly dominant. *Quercus rubra, Liriodendron tulipifera*, and *Acer rubrum* may be abundant. In examples on basic soils, *Fraxinus americana* and *Acer floridanum (= Acer barbatum)* are also abundant. A well‑developed understory is usually present. Shrubs are generally sparse to moderate in density, except on heath bluffs. Herbs range from fairly dense in basic examples to sparse in acidic examples. The composition of all lower strata varies substantially with soil acidity. Basic examples have fairly diverse plants, especially herbs, which may include a number of species shared with Southern and Central Appalachian Cove Forest (CES202.373). The more common acidic examples have fewer plant species, though generally they have a higher species richness than other drier ecological systems.

**Dynamics:** Fire is naturally infrequent in this system, due to the slopes and moist conditions. If fire does occur, it is likely to be low in intensity and may not have significant ecological effects. These forests generally exist naturally as old‑growth forests, with canopy dynamics dominated by gap‑phase regeneration. Small to occasional medium‑sized canopy gaps created by wind are likely the primary form of natural disturbance, though infrequent fires might create gaps. Most of the prevailing species are shade‑tolerant. Most are not very fire‑tolerant. The mesophytic forest type is fire regime class III, surface fires with return intervals of 20 to 70 years (Landfire 2007a). Mixed‑severity fires may occur approximately every 100 years depending on climatic conditions. Disturbance may also occur by recurrent, severe insect defoliations or droughts. Ice, straight‑line winds or microbursts may cause blow‑downs on a scale of 1 to 10 acres. Stand‑replacement fires happen very infrequently. Low‑intensity surface fires, whether natural or set by Native Americans, would have maintained the more fire‑resistant *Castanea dentata* and oak species.

SOURCES

**References:** Anderson 1999a, Batista and Platt 1997, Comer et al. 2003*, Edwards et al. 2013, Engeman et al. 2007, Eyre 1980, LANDFIRE 2007a, Nelson 1986, Peet et al. 2013, Schafale 2012, Schafale and Weakley 1990, Simon and Hayden 2014, Simon pers. comm.

**Version:** 02 Oct 2015	**Stakeholders:** East, Southeast

**Concept Author:** M. Schafale and R. Evans	**LeadResp:** Southeast

M502. Appalachian‑Northeastern Oak ‑ Hardwood ‑ Pine Forest & Woodland

CES202.359 Allegheny‑Cumberland Dry Oak Forest and Woodland

**Primary Division:** Central Interior and Appalachian (202)

**Land Cover Class:** Forest and Woodland

**Spatial Scale & Pattern:** Large patch

**Required Classifiers:** Natural/Semi‑natural; Vegetated (>10% vasc.); Upland

**Diagnostic Classifiers:** Acidic Soil; Broad‑Leaved Tree

**National Mapping Codes:** EVT 2317; ESLF 4123; ESP 1317

**Concept Summary:** This system encompasses dry hardwood forests on predominately acidic substrates in the Allegheny and Cumberland plateaus, as well as acidic sandstone ridges in the southern Ridge and Valley. Its range is more‑or‑less consistent with the "Mixed Mesophytic Forest Region" of Braun (1950) and Greller (1988), although it is not a mesic forest type. These forests are typically dominated by *Quercus alba, Quercus falcata, Quercus montana, Quercus coccinea*, with lesser amounts of *Acer rubrum, Carya glabra*, and *Carya tomentosa*. Small inclusions of *Pinus echinata* and/or *Pinus virginiana* may occur, particularly adjacent to escarpments or following fire. In addition, *Pinus strobus* may be prominent in some stands in the absence of fire. It occurs in a variety of situations, including on nutrient‑poor or acidic soils. Sprouts of *Castanea dentata* can often be found where it was formerly a common tree.

**Comments:** Related forests on more base‑rich substrates may be classified as examples of Southern Ridge and Valley / Cumberland Dry Calcareous Forest (CES202.457), where this distinction may be made. Eastward and northward, this system transitions into Central Appalachian Dry Oak‑Pine Forest (CES202.591). The dividing line between them in West Virginia is the Allegheny Front.

DISTRIBUTION

**Range:** This system is centered on the Allegheny and Cumberland plateaus from northern Alabama north to Ohio, West Virginia, and possibly western Pennsylvania.

**Divisions:** 202:C

**TNC Ecoregions:** 49:C, 50:C

**Nations:** US

**Subnations:** AL, GA, KY, OH, PA?, TN, VA, WV

**Map Zones:** 48:C, 53:C, 57:C, 61:C, 62:C

**USFS Ecomap Regions:** 221E:CC, 221Ha:CCC, 221Hb:CCC, 221Hc:CCC, 221He:CCC, 221J:CC, 231Ca:CCC, 231Cb:CCC, 231Cc:CCC, 231Cd:CCC, 231Db:CCC, 231Dc:CCC, M221Ba:CC?, M221Bb:CCC, M221Bc:CCC, M221Be:CCC, M221Ca:CCC

CONCEPT

**Environment:** This system is most likely found on predominantly nutrient‑poor or acidic substrates in the Allegheny and Cumberland plateaus, and acidic, weather‑resistant ridges in the southern Ridge and Valley.

**Vegetation:** These forests are typically dominated by *Quercus alba, Quercus falcata, Quercus montana (= Quercus prinus), Quercus coccinea, Acer rubrum, Carya glabra*, and *Carya tomentosa (= Carya alba)*. These occur in a variety of situations, most likely on nutrient‑poor or acidic soils and, to a much lesser extent, on circumneutral soils. Sprouts of *Castanea dentata* can often be found where it was formerly a common tree. Small inclusions of *Pinus echinata* and/or *Pinus virginiana* may occur, particularly adjacent to escarpments or following fire. In addition, *Pinus strobus* may be prominent in some stands in the absence of fire.

SOURCES

**References:** Braun 1950, Comer et al. 2003*, Evans et al. 2009, Eyre 1980, Greller 1988, ONHD unpubl. data

**Version:** 06 Jun 2017	**Stakeholders:** East, Midwest, Southeast

**Concept Author:** R. Evans, M. Pyne, C. Nordman	**LeadResp:** Southeast

CES202.598 Appalachian Shale Barrens

**Primary Division:** Central Interior and Appalachian (202)

**Land Cover Class:** Forest and Woodland

**Spatial Scale & Pattern:** Small patch

**Required Classifiers:** Natural/Semi‑natural; Vegetated (>10% vasc.); Upland

**Diagnostic Classifiers:** Sideslope; Talus (Substrate); Unglaciated; Unconsolidated

**National Mapping Codes:** EVT 2340; ESLF 4147; ESP 1340

**Concept Summary:** This system encompasses the distinctive shale barrens of the Central and Southern Appalachians at low to mid elevations. The exposure and lack of soil create extreme conditions for plant growth. Vegetation is mostly classified as woodland, overall, but may include large open areas of sparse vegetation. Dominant trees are primarily *Quercus montana* and *Pinus virginiana*, although on higher‑pH substrates the common trees include *Juniperus virginiana* and *Fraxinus americana*. Shale barren endemics are diagnostic in the herb layer. The substrate includes areas of solid rock as well as unstable areas of shale scree, usually steeply sloped. The fully exposed areas are extremely dry. These barrens are high in endemic species.

**Comments:** Examples of related barrens in the "Knobs" region of Kentucky are included in Central Interior Highlands Dry Acidic Glade and Barrens (CES202.692), not here. The southern range limit is not completely clear. "Central Appalachian Shale Barrens" (sensu VDNH) are the "core" concept. The bluestone shale barrens of West Virginia are placed in this system even though many of the endemics are not present there; the same is true at the northern periphery of this system in Pennsylvania.

DISTRIBUTION

**Range:** This system is found from southern Pennsylvania south to Virginia and extreme eastern Tennessee. Application of the concept south of Virginia is uncertain. It is not attributed to Kentucky.

**Divisions:** 202:C

**TNC Ecoregions:** 50:P, 51:P, 59:C

**Nations:** US

**Subnations:** MD, NC?, PA, TN, VA, WV

**Map Zones:** 57:C, 61:C

**USFS Ecomap Regions:** M221Ac:CCC, M221Be:CCC

CONCEPT

**Environment:** This system is found at low to mid elevations in the Central and Southern Appalachians. Most shale barrens occur between 305 and 610 m (1000‑2000 feet) elevation and have a generally southern exposure. Slopes are steep and often undercut by a stream at the base. Soils are thin, with a layer weathered rock fragments covering the surface. The exposure and lack of soil create extreme conditions for plant growth. The chemistry and pH vary somewhat from site to site, and this variability may be reflected in the vegetation. The substrate includes areas of solid rock as well as unstable areas of shale scree, usually steeply sloped.

**Vegetation:** Although stunted trees of several species such as *Quercus montana (= Quercus prinus), Pinus virginiana*, and *Carya glabra* are common, Central Appalachian Shale Barrens are strongly characterized by their open physiognomy and by a suite of uncommon and rare plants found almost exclusively in these habitats (Fleming et al. 2004). Endemic or near‑endemic shale barren species include *Arabis serotina, Clematis albicoma, Clematis viticaulis* (also endemic to Virginia), *Eriogonum allenii, Oenothera argillicola, Packera antennariifolia*, and *Trifolium virginicum*. Other more‑or‑less widespread and characteristic herbaceous species of Virginia shale barrens include *Carex pensylvanica, Schizachyrium scoparium, Danthonia spicata, Deschampsia flexuosa var. flexuosa, Phlox subulata, Paronychia montana, Selaginella rupestris, Antennaria virginica, Potentilla canadensis, Helianthus laevigatus, Brickellia eupatorioides var. eupatorioides, Blephilia ciliata, Erysimum capitatum var. capitatum* (Bath and Alleghany counties).

SOURCES

**References:** Comer et al. 2003*, Eyre 1980, Fleming et al. 2004, Keener 1970, Platt 1951, Schafale 2012, TDNH unpubl. data 2018

**Version:** 05 May 2008	**Stakeholders:** East, Southeast

**Concept Author:** S.C. Gawler	**LeadResp:** East

CES202.596 Central and Southern Appalachian Montane Oak Forest

**Primary Division:** Central Interior and Appalachian (202)

**Land Cover Class:** Forest and Woodland

**Spatial Scale & Pattern:** Large patch

**Required Classifiers:** Natural/Semi‑natural; Vegetated (>10% vasc.); Upland

**Diagnostic Classifiers:** Montane; Forest and Woodland (Treed); Ridge/Summit/Upper Slope; Unglaciated; Broad‑Leaved Deciduous Tree; Quercus ‑ Carya

**National Mapping Codes:** EVT 2320; ESLF 4126; ESP 1320

**Concept Summary:** This generally oak‑dominated system is found in the central and southern Appalachian Mountains. These high‑elevation deciduous forests occur on exposed sites, including ridgecrests and south‑ to west‑facing slopes, mostly between 915 and 1372 m (3000‑4500 feet) elevation, less commonly ranging up to 1680 m (5500 feet). In most associations attributed to this system, the soils are thin, weathered, nutrient‑poor, low in organic matter, and acidic. The forests are dominated by *Quercus* spp. (most commonly *Quercus rubra* and *Quercus alba*), with the individual trees in high‑elevation red oak examples often stunted or wind‑flagged. *Castanea dentata* sprouts are also common, but the importance of chestnut in these forests has been dramatically altered by chestnut blight. *Ilex montana, Hamamelis virginiana*, and *Rhododendron prinophyllum* (in Virginia and West Virginia) are characteristic shrubs.

**Comments:** This system may be interfingered with the non‑oak‑dominated Southern Appalachian Northern Hardwood Forest (CES202.029), particularly between 1220 and 1525 m (4000‑5000 feet) elevation. Above 1372 m (4500 feet) elevation and below spruce‑fir communities, this system may be replaced on certain aspects by Southern Appalachian Northern Hardwood Forest (CES202.029).

DISTRIBUTION

**Range:** This system is found at higher elevations of the central and southern Appalachian Mountains, Virginia and West Virginia to Georgia. In Kentucky, this system is restricted to the Cumberland Mountains in the extreme southeastern corner of that state. In West Virginia, this system is found in the Ridge and Valley.

**Divisions:** 202:C

**TNC Ecoregions:** 50:C, 51:C, 59:C

**Nations:** US

**Subnations:** GA, KY, MD?, NC, SC, TN, VA, WV

**Map Zones:** 53:P, 57:C, 61:C

**USFS Ecomap Regions:** M221A:CC, M221B:CP, M221Cb:CCC, M221Cc:CCC, M221Cd:CCC, M221D:CC

CONCEPT

**Environment:** The habitat for this system includes high ridgelines and exposed upper slopes, primarily on south‑ to west‑facing aspects, mostly between 915 and 1372 m (3000‑4500 feet) elevation, and less commonly ranging up to 1680 m (5500 feet).It generally occurs as a transition between Southern Appalachian Oak Forest (CES202.886) and more mesic Southern Appalachian Northern Hardwood Forest (CES202.029) that occurs on less‑exposed ridgetops and cooler, moister upper slopes (e.g., north‑ and east‑facing aspects). At high elevations (e.g., above 1372 m [4500 feet]), this system is generally less common than Southern Appalachian Northern Hardwood Forest (CES202.029), since the habitat on most slopes at this elevation tends to favor those species adapted to a more mesic environment. Rockslides occur periodically due to the steep slopes, and severe rockslides can cause stand replacement. Ice storms occur frequently and cause extensive damage to older dwarfed trees. Fire occurs at moderate frequency and probably needed in the long run to promote growth of fire tolerant *Quercus* and maintain their dominance. Some rare examples may be too rocky to burn, and even these have mostly closed canopies and produce a substantial leaf litter layer in most places (M. Schafale pers. comm. 2013).

**Vegetation:** Examples of this system may be dominated by *Quercus rubra* in some cases, and by *Quercus alba* and/or *Quercus montana (= Quercus prinus)* in others. The trees may be stunted, or at least not as tall as they would be in other systems farther downslope. Species richness is low to moderate. Tree associates include *Prunus serotina, Acer rubrum, Betula lenta*, and *Betula alleghaniensis*. Typical small trees and shrubs include *Ilex montana, Hamamelis virginiana, Acer pensylvanicum, Menziesia pilosa, Rhododendron prinophyllum, Vaccinium pallidum, Corylus cornuta var. cornuta*, and sprouts of *Castanea dentata*. The understory is usually dominated by ericaceous shrubs, but some communities are dominated by graminoid species or ferns. *Dennstaedtia punctilobula, Carex pensylvanica*, and *Deschampsia flexuosa* are common. Only rarely are the communities dominated by other herbs.

**Dynamics:** The communities of this system occur on exposed high ridges in the Appalachians. They are subject to frequent ice in winter, wind storms in the summer and high winds throughout the year. Natural old‑growth forest examples have trees reproducing in small to medium‑sized canopy gaps created by the death of individual or small groups of trees. Wind and ice storms are the main cause of tree mortality. Breakage of trees and of branches by ice storms can additionally produce partial canopy opening over large areas (M. Schafale pers. comm.). In addition, lightning‑caused fires may create surface fires that change the understory composition and inhibit some ericaceous shrub species in some areas. Fire is naturally at moderate or low frequency, but appears to be important in structuring the vegetation. In many locations, fire exclusion and competing understory vegetation are factors in poor oak regeneration, with replacement by more mesophytic species such as *Acer saccharum* (Fleming et al. 2005). Fire likely was crucial for reducing the competitive advantage of these species. Presettlement forests are likely to have experienced lightning‑caused fires every 40‑60 years (Fleming et al. 2005). Fires likely were more frequent than this farther south. Rockslides cause severe disturbance in occasional locations, initiating a primary succession that may last many years. Despite the high elevation, *Castanea dentata* had been a fairly substantial component of this system and can still be seen as rotting stumps in the forest.

SOURCES

**References:** Comer et al. 2003*, DuMond 1970, Edwards et al. 2013, Evans et al. 2009, Eyre 1980, Gettman 1974, Greenberg et al. 2011, LANDFIRE 2007a, Nelson 1986, Patterson 1994, Schafale 2012, Schafale and Weakley 1990, Schafale pers. comm., Simon 2011, Simon 2015, Southern Group of State Foresters 2013, Whittaker 1956, Woods et al. 2002

**Version:** 28 Apr 2016	**Stakeholders:** East, Southeast

**Concept Author:** R. White, M. Pyne, R. Evans, M. Schafale, S.C. Gawler	**LeadResp:** Southeast

CES202.591 Central Appalachian Dry Oak‑Pine Forest

**Primary Division:** Central Interior and Appalachian (202)

**Land Cover Class:** Forest and Woodland

**Spatial Scale & Pattern:** Matrix

**Required Classifiers:** Natural/Semi‑natural; Vegetated (>10% vasc.); Upland

**Diagnostic Classifiers:** Forest and Woodland (Treed); Ridge/Summit/Upper Slope; Acidic Soil; Pinus (strobus, rigida, echinata, virginiana) ‑ Quercus prinus

**National Mapping Codes:** EVT 2369; ESLF 4312; ESP 1369

**Concept Summary:** These oak and oak‑pine forests cover large areas in the low‑ to mid‑elevation Central Appalachians and middle Piedmont. The topography and landscape position range from rolling hills to steep slopes, with occasional occurrences on more level, ancient alluvial fans. In the highly dissected fall zone of Maryland and the District of Columbia, where the Piedmont and Coastal Plain meet, it is also found on dry knolls capped with Pleistocene‑ and Tertiary‑aged fluvial cobble and gravel terrace deposits. Soils are typically coarse and infertile; they may be deep (on glacial deposits in the northern and terrace deposits in the southern parts of the system's range), or more commonly shallow, on rocky slopes of acidic rock (shale, sandstone, other acidic igneous or metamorphic rock). The well‑drained soils and exposure create dry conditions. The forest is mostly closed‑canopy but can include patches of more open woodlands. It is dominated by a variable mixture of dry‑site oak and pine species, most typically *Quercus montana, Pinus virginiana*, and *Pinus strobus*, but sometimes *Quercus alba* and/or *Quercus coccinea*. The system may include areas of oak forest, pine forest (usually small), and mixed oak‑pine forest. Heath shrubs such as *Vaccinium pallidum, Gaylussacia baccata*, and *Kalmia latifolia* are common in the understory and often form a dense layer. Embedded submesic ravines and concave landforms support slightly more diverse forests characterized by mixtures of oaks, several hickories, *Cornus florida*, and sometimes *Liriodendron tulipifera*. Small hillslope pockets with impeded drainage may support small isolated wetlands with *Acer rubrum* and *Nyssa sylvatica* characteristic. Disturbance agents include fire, windthrow, and ice damage. Increased site disturbance generally leads to secondary forest vegetation with a greater proportion of *Pinus virginiana* and weedy hardwoods such as *Acer rubrum*.

**Comments:** This system occurs in drier settings than the other matrix oak forest system of the division, Northeastern Interior Dry‑Mesic Oak Forest (CES202.592), except in New York and New England, which are mostly out of the range of CES202.592. In that system, *Quercus rubra, Quercus alba, Quercus velutina*, and/or *Quercus coccinea* and *Carya* spp. are the typical dominants rather than *Quercus montana*. It includes the system formerly segregated as Southern Piedmont Dry Oak‑Heath Forest (CES202.023). Its analog from central Virginia south is Southern Piedmont Dry Oak‑(Pine) Forest and Woodland (CES202.339), which has somewhat more southern floristics, for example, the typical presence of *Pinus taeda*.

DISTRIBUTION

**Range:** This system is found from central New England through Pennsylvania and south to the Roanoke River in southern Virginia. It is primarily Appalachian but overlaps slightly into the upper Piedmont and fall zone in Virginia, Maryland and the District of Columbia.

**Divisions:** 202:C

**TNC Ecoregions:** 52:C, 58:C, 59:C, 60:C, 61:C

**Nations:** US

**Subnations:** CT, DC, MA, MD, ME, NH, NJ, NY, OH, PA, RI, VA, VT, WV

**Map Zones:** 57:P, 60:C, 61:C, 63:C, 64:C, 65:C, 66:C

**USFS Ecomap Regions:** 211E:CC, 211F:CC, 211G:CC, 211I:CC, 221Ae:CCC, 221Af:CCC, 221Ag:CCC, 221Ah:CCC, 221Ai:CCC, 221Ak:CCC, 221Am:CCC, 221An:CCP, 221B:CC, 221D:CC, 232A:CC, M221A:CC, M221Ba:CCC, M221Bb:CCC, M221Bd:CCC, M221Bf:CCC, M221Da:CCC

CONCEPT

**Environment:** These oak and oak‑pine forests cover large areas in the low‑ to mid‑elevation central Appalachians and middle Piedmont. The topography and landscape position range from rolling hills to steep slopes, with occasional occurrences on more level, ancient alluvial fans. The soils are coarse and infertile; they may be deep (on glacial deposits in the northern part of the system's range), or more commonly shallow, on rocky slopes of acidic rock (shale, sandstone, other acidic igneous or metamorphic rock). The well‑drained soils and exposure create dry conditions. In the highly dissected fall zone of Maryland and the District of Columbia, where the Piedmont and Coastal Plain meet, it is also found on dry knolls capped with Pleistocene‑ and Tertiary‑aged fluvial cobble and gravel terrace deposits.

**Vegetation:** Stands of this forest system are mostly closed‑canopied but can include more open woodlands. They are dominated by a variable mixture of dry‑site oak and pine species, including *Quercus montana (= Quercus prinus), Pinus virginiana*, and *Pinus strobus*. The system may include areas of pine forest and mixed oak‑pine forest. Heath shrubs such as *Vaccinium pallidum, Gaylussacia baccata*, and *Kalmia latifolia* are common in the understory. Within these forests, hillslope pockets with impeded drainage may support small isolated wetlands with *Acer rubrum* and *Nyssa sylvatica* characteristic.

**Dynamics:** Disturbance agents include fire, windthrow, and ice damage.

SOURCES

**References:** Comer et al. 2003*, Edinger et al. 2014a, Eyre 1980, Gawler and Cutko 2010, LANDFIRE 2007a, ONHD unpubl. data, Sperduto and Nichols 2004

**Version:** 23 Jan 2012	**Stakeholders:** East, Midwest, Southeast

**Concept Author:** S.C. Gawler	**LeadResp:** East

CES202.600 Central Appalachian Pine‑Oak Rocky Woodland

**Primary Division:** Central Interior and Appalachian (202)

**Land Cover Class:** Forest and Woodland

**Spatial Scale & Pattern:** Large patch

**Required Classifiers:** Natural/Semi‑natural; Vegetated (>10% vasc.); Upland

**Diagnostic Classifiers:** Forest and Woodland (Treed); Shrubland (Shrub‑dominated); Woody‑Herbaceous; Ridge/Summit/Upper Slope; Acidic Soil; Pinus (strobus, rigida, echinata, virginiana) ‑ Quercus prinus

**National Mapping Codes:** EVT 2377; ESLF 4320; ESP 1377

**Concept Summary:** This system encompasses open or patchily wooded hilltops and outcrops or rocky slopes in the Central Appalachians, High Allegheny Plateau, and Lower New England / Northern Piedmont. It occurs mostly at lower elevations, but occasionally up to 1220 m (4000 feet) in West Virginia. The substrate rock is generally granitic or of other acidic lithology, although near the northern limit of its range in New England, examples can also occur on intermediate, base‑rich, or mafic bedrock including traprock. The vegetation is patchy, with woodland as well as open portions. *Pinus rigida* (and within its range *Pinus virginiana*) is diagnostic and often mixed with xerophytic *Quercus* spp. and sprouts of *Castanea dentata*. In New England, some examples lack pine and feature *Juniperus virginiana* or *Ostrya virginiana* as important codominants with oak. Some areas have a fairly well‑developed heath shrub layer, others a graminoid layer, the latter particularly common under oaks or other deciduous trees. Conditions are dry and for the most part nutrient‑poor, and at many, if not most, sites, a history of fire is evident. In the Central Appalachians ecoregion, this system is rarely found on sandy soils rather than rock.

**Comments:** The northern extent of this system in central New England may overlap with Northern Appalachian‑Acadian Rocky Heath Outcrop (CES201.571), which has *Picea* spp. prominent. The southern extent overlaps with Southern Appalachian Montane Pine Forest and Woodland (CES202.331), which is characterized by *Pinus pungens*. The present type may have some *Pinus pungens* (from southern Pennsylvania south) but generally has other pines as well. This type is differentiated from the similar Central Appalachian Dry Oak‑Pine Forest (CES202.591) by its mosaic nature of wooded and open patches, as opposed to being merely a "thin forest." New England dry‑rich forest/woodlands (e.g., those on traprock ridges) are also housed here, expanding the concept beyond pitch pine diagnostics. Pike Knob, West Virginia, with its disjunct red pine woodlands, is put into this system.

DISTRIBUTION

**Range:** This system occurs from central New England south to Virginia and West Virginia, with peripheral occurrences in southeastern Ohio and easternmost Kentucky.

**Divisions:** 202:C

**TNC Ecoregions:** 49:C, 50:C, 52:C, 59:C, 60:C, 61:C, 64:C

**Nations:** US

**Subnations:** CT, KY, MA, MD?, ME, NH, NJ, NY, OH, PA, VA, VT, WV

**Map Zones:** 53:C, 57:P, 60:C, 61:C, 62:C, 63:C, 64:C, 65:C, 66:C

**USFS Ecomap Regions:** 211E:CC, 211F:CC, 221A:CC, 221B:CC, M211Bb:CCC, M211Bd:CCC, M211C:CC, M221A:CC, M221B:CP

CONCEPT

**Environment:** This system occurs mostly at lower elevations, but occasionally up to 1220 m (4000 feet) in West Virginia. The substrate rock is generally granitic or of other acidic lithology, although near the northern limit of its range in New England, examples can also occur on intermediate, base‑rich, or mafic bedrock including traprock.

This system contains species‑poor, fire‑influenced, mixed woodlands of xeric, exposed montane habitats. They are typically located on convex, south to west facets of steep spur ridges, narrow rocky crests, and cliff tops. Pine ‑ oak / heath woodlands are widespread throughout both the Ridge and Valley and Blue Ridge provinces in western Virginia. They occur at elevations from below 305 m (1000 feet) to more than 1220m (4000 feet) on various substrates, but most commonly on acidic, sedimentary and metasedimentary substrates, e.g., sandstone, quartzite, and shale. A few stands occur on Piedmont monadnocks and foothills. Soils are very infertile, shallow, and droughty (VDNH 2007).

The type is restricted to poor, dry sites which have been disturbed in the recent past by heavy cutting, fire, or both. It is found on thin, rocky soils in the mountainous areas. Soils are strongly acidic and devoid of nutrients. Precipitation is low in the shale barrens of eastern West Virginia and adjacent states (Eyre 1980).

**Vegetation:** Short‑statured *Pinus pungens* and *Pinus rigida* are usually the dominants forming an open overstory, often with codominant *Quercus montana*. Less important tree associates include *Quercus coccinea, Pinus virginiana*, and *Sassafras albidum*. Except in the Piedmont, *Quercus ilicifolia* is characteristically abundant in the shrub layer, along with various ericaceous species. Colonial shrubs usually preempt available microhabitats for most herbaceous species, but *Pteridium aquilinum var. latiusculum* and *Xerophyllum asphodeloides* are often competitive enough to achieve significant cover (VDNH 2007). The globally rare *Carex polymorpha* is locally associated with these woodlands (VDNH 2007).

**Dynamics:** Periodic fire is an important ecological process that provides opportunities for regeneration of both pines and less competitive herbaceous species, while setting back successional encroachment of potential overstory oaks (especially chestnut oak). On cliffs and other very rocky sites, the vegetation is self‑perpetuating due to extreme edaphic conditions. (VDNH 2007). Fire is the most common disturbance type, but frost pockets and late‑spring frosts have been also documented. If disturbances occur very frequently (every 2‑3 years), *Quercus ilicifolia* may be replaced by low shrubs, grasses, ferns, and other herbs. If disturbances are infrequent, canopy trees can outgrow the shade‑intolerant *Quercus ilicifolia*. Suppression of white pine and increase in Virginia pine were accomplished through low‑intensity fires in shortleaf pine ‑ oak forests in Georgia (Hubbard et al. 2004).

SOURCES

**References:** Comer et al. 2003*, Edinger et al. 2014a, Eyre 1980, Gawler and Cutko 2010, Hubbard et al. 2004, LANDFIRE 2007a, Metzler and Barrett 2006, ONHD unpubl. data, Sperduto 2005, Sperduto and Nichols 2004, VDNH 2007

**Version:** 23 Jan 2012	**Stakeholders:** East, Midwest, Southeast

**Concept Author:** S.C. Gawler	**LeadResp:** East

CES202.592 Northeastern Interior Dry‑Mesic Oak Forest

**Primary Division:** Central Interior and Appalachian (202)

**Land Cover Class:** Forest and Woodland

**Spatial Scale & Pattern:** Matrix

**Required Classifiers:** Natural/Semi‑natural; Vegetated (>10% vasc.); Upland; Wetland

**Diagnostic Classifiers:** Lowland; Forest and Woodland (Treed); Acidic Soil; Quercus ‑ Carya

**National Mapping Codes:** EVT 2303; ESLF 4109; ESP 1303

**Concept Summary:** These oak‑dominated forests are one of the matrix forest systems in the northeastern and north‑central U.S. Occurring in dry‑mesic settings, they are typically closed‑canopy forests, though there may be areas of patchy‑canopy woodlands. They cover large expanses at low to mid elevations, where the topography is flat to gently rolling, occasionally steep. Soils are mostly acidic and relatively infertile but not strongly xeric. Local areas of calcareous bedrock, or colluvial pockets, may support forests typical of richer soils. Oak species characteristic of dry‑mesic conditions (e.g., *Quercus rubra, Quercus alba, Quercus velutina*, and *Quercus coccinea*) and *Carya* spp. (particularly *Carya tomentosa, Carya glabra, Carya ovalis, Carya ovata*, and *Carya pallida*) are dominant in mature stands. *Quercus montana* may be present but is generally less important than the other oak species. *Castanea dentata* was a prominent tree before chestnut blight eradicated it as a canopy constituent. *Acer rubrum, Betula lenta*, and *Betula alleghaniensis* may be common associates; *Acer saccharum* is occasional. With a long history of human habitation, many of the forests are early‑ to mid‑successional, where *Pinus strobus, Pinus virginiana*, or *Liriodendron tulipifera* may be dominant or codominant. Within these forests, hillslope pockets with impeded drainage may support small isolated wetlands, including non‑forested seeps or forested wetlands with *Acer rubrum, Quercus bicolor*, or *Nyssa sylvatica* characteristic.

**Comments:** The oak‑dominated forest matrix in this region spans a range of elevational and moisture regimes, reflected in different ecological systems. Those in drier settings, within the general range of this system, are placed in either Allegheny‑Cumberland Dry Oak Forest and Woodland (CES202.359) or Central Appalachian Dry Oak‑Pine Forest (CES202.591).

DISTRIBUTION

**Range:** This system is found from southern New York west through Ohio and Pennsylvania and south to Virginia. It does not extend to the southernmost part of Virginia, except in the Ridge and Valley.

**Divisions:** 202:C

**TNC Ecoregions:** 49:C, 52:C, 59:C, 60:C, 61:C

**Nations:** US

**Subnations:** MA, MD, NJ, NY, OH, PA, VA, WV

**Map Zones:** 53:C, 57:C, 60:C, 61:C, 62:C, 63:C, 64:C

**USFS Ecomap Regions:** 211F:CC, 211G:CC, 221Ae:CCC, 221Am:CCC, 221B:CC, 221D:CC, 221F:CC, M221A:CC, M221B:CC, M221Da:CCC

CONCEPT

**Environment:** These oak‑dominated forests are one of the matrix forest systems in the northeastern and north‑central U.S. Occurring in dry‑mesic settings, they are typically closed‑canopy forests, though there may be areas of patchy‑canopy woodlands. They cover large expanses at low to mid elevations, where the topography is flat to gently rolling, occasionally steep. The typical landscape position is midslope to toeslope, transitioning to more xeric systems on the upper slopes and ridges. Soils are acidic and relatively infertile but not strongly xeric.

**Vegetation:** Mature stands are dominated by oak species characteristic of dry‑mesic conditions (e.g., *Quercus rubra, Quercus alba, Quercus velutina*, and *Quercus coccinea*), along with various *Carya* spp. (particularly *Carya tomentosa (= Carya alba), Carya glabra, Carya ovalis, Carya ovata*, and *Carya pallida*). In addition, *Quercus montana (= Quercus prinus)* may be present but is generally less important than the other oak species. *Castanea dentata* was a prominent tree before chestnut blight eradicated it as a canopy constituent. *Acer rubrum* and *Betula lenta* are frequently common associates. Local areas of calcareous bedrock may support forests typical of richer soils (e.g., with *Acer saccharum* and/or *Quercus muehlenbergii*). Common shrubs include *Viburnum acerifolium, Hamamelis virginiana, Corylus* spp., and *Smilax* spp., as well as heaths such as *Kalmia latifolia, Vaccinium* spp., and *Gaylussacia* spp. Herbs, forbs, and ferns are usually sparse to moderate in density.

**Dynamics:** This system is naturally dominated by stable, uneven‑aged forests, with canopy dynamics dominated by gap‑phase regeneration. Most oaks are long‑lived, with typical age of mortality ranging from 200 to 400 years. *Quercus coccinea* and *Quercus velutina* are shorter‑lived with typical ages being approximately 50 to 100 years, while *Quercus alba* can live as long as 600 years. Extreme wind or ice storms occasionally create larger canopy openings.

This forest system is characterized by low‑severity surface fires that cause variable structure and composition based on fire frequency and intensity. The great majority of historical fires were generated by Native Americans.

Open woodlands developed within a moderate burning regime, (fire‑return intervals of 5 to 15 years), and canopy closure occurred with greater fire‑return intervals. Shade‑tolerant, fire‑sensitive trees such as *Acer saccharum* regenerated beneath oak‑hickory canopies when fire was excluded over several decades. With continued fire exclusion, *Acer saccharum* and other late‑successional species gradually replaced overstory oaks and hickories as forest gaps closed (Sutherland et al. 2003), generating a mosaic of vegetation types formed with varying fire history (Cutter and Guyette 1994). A recent study on fire history of a *Quercus rubra* stand in West Virginia revealed that fire intervals ranged from 7 to 32 years from 1846 to 2002, in contrast to intervals of 7 to 15 years prior to the fire control era. These results were consistent with previous research in the oak forests of Ohio, Maryland, and Missouri (Schuler and McClain 2003).

SOURCES

**References:** Braun 1950, Comer et al. 2003*, Cutter and Guyette 1994, Edinger et al. 2014a, Eyre 1980, Greller 1988, LANDFIRE 2007a, ONHD unpubl. data, Schuler and McClain 2003, Sutherland et al. 2003, USFS 1995, Vanderhorst and Streets 2006

**Version:** 20 Aug 2015	**Stakeholders:** East, Midwest, Southeast

**Concept Author:** S.C. Gawler	**LeadResp:** East

CES202.590 Northeastern Interior Pine Barrens

**Primary Division:** Central Interior and Appalachian (202)

**Land Cover Class:** Forest and Woodland

**Spatial Scale & Pattern:** Large patch

**Required Classifiers:** Natural/Semi‑natural; Vegetated (>10% vasc.); Upland

**Diagnostic Classifiers:** Sandplains/Glacial Outwash or Flats; Glaciated; Oligotrophic Soil; Acidic Soil; Sand Soil Texture; F‑Landscape/Medium Intensity; Pinus rigida

**National Mapping Codes:** EVT 2354; ESLF 4257; ESP 1354

**Concept Summary:** These pine barrens occur on glacial sandplains of the inland regions of the northeastern U.S., with a disjunction to the distinctive till plain shrublands in the Poconos of eastern Pennsylvania. Substrates include outwash plains, stabilized sand dunes, and glacial till. The soils are consequently coarse‑textured, acidic, mostly well‑drained to xeric, and low in nutrients. *Pinus rigida* is the usual dominant, and cover may range from closed‑canopy forest to (more typically) open woodlands. *Quercus rubra, Pinus strobus*, and *Betula populifolia* are common associates. A tall‑shrub layer of *Quercus ilicifolia* and/or *Quercus prinoides* is commonly present, although portions of some barrens (or occasionally the entire barrens) lack the scrub oak component. A well‑developed low‑shrub layer is typical, with lowbush *Vaccinium* spp., *Gaylussacia baccata*, and *Comptonia peregrina* characteristic, with *Rhododendron canadense* characteristic on the slightly more mesic microsites of the Poconos. The system is often a physiognomic patchwork, ranging from nearly closed‑canopy forest to open pine woodlands, to scrub oak shrublands, to herbaceous/dwarf‑shrub frost pockets. Grassy areas dominated by *Schizachyrium scoparium* with *Lupinus perennis, Lespedeza capitata*, and other forbs provide habitat for several rare invertebrates. Small changes in elevation can create pockets with saturated soil, where shrubs such as *Corylus americana, Cephalanthus occidentalis, Vaccinium corymbosum*, and *Alnus* spp. form dense cover. These barrens always have a history of recurrent fires, and fire is required to maintain them.

**Comments:** This system can include patches, or sometimes larger areas, of mesic pitch pine ‑ shrub vegetation. For instance, Pennsylvania's "mesic till barrens" are somewhat less dry than the typical barrens in this system, but fit otherwise. Elsewhere in these barrens, wet pitch pine pockets can occur in (usually small) topographic depressions.

DISTRIBUTION

**Range:** This system is restricted to interior south‑central New England; Colchester, Vermont; eastern New York; and the Pennsylvania Poconos.

**Divisions:** 202:C

**TNC Ecoregions:** 60:C, 61:C, 63:C, 64:C

**Nations:** US

**Subnations:** CT, MA, ME, NH, NY, PA, RI, VT

**Map Zones:** 64:C, 65:C, 66:C

**USFS Ecomap Regions:** 211E:CC, 211Fd:CCC, 221Af:CCC, 221Ag:CCC, 221Ai:CCC, 221Ak:CCC, 221Al:CCC, 221Bc:CCC

CONCEPT

**Environment:** This system is confined to flat to gently rolling plains with sandy soils that are coarse‑textured, acidic, mostly well‑drained to xeric, and low in nutrients.

**Dynamics:** Fire regime includes frequent stand‑replacing events and lower intensity surface fires. Periodic severe wildfires with 40‑ to 100‑year intervals have produced oak‑pine mixtures over extensive areas of uplands, while more frequent severe fires have created mixtures of pitch pine and shrub oaks. Pitch pine younger than 20‑40 years may produce stump sprouts after top‑killing fire (Andresen 1959). If not top‑killed, pines may recover from fire by sprouting from branches and trunk. Pitch pine has. Additionally, pitch pine is quick to maturity and to produce seeds. Frequent fires of moderate to high intensity/severity eventually eliminate all other tree species except for scrub oak and pitch pine, which has thick, fire‑resistant bark and is a prolific seed producer. Fires, especially large wildfires, have been a major factor in the development of the present differences among forest stands on similar sites in the Pine Barrens. Abandoned upland sites generally progress from a grass or shrubland (MFRI of 2‑3? years) to pitch pine/scrub oak woodland (5‑25 years) to pure pitch pine forest with heath/oak scrublands (30‑60 years) to pitch pine/tree‑sized oak forest (60‑100 years) to oak‑hickory forest (100‑200 years) (Landfire 2007a).

SOURCES

**References:** Andresen 1959, Burns and Honkala 1990a, Comer et al. 2003, Copenheaver et al. 2000, Eberhardt and Latham 2000, Edinger et al. 2014a, Eyre 1980, Fike 1999, Forman 1979, Gawler and Cutko 2010, Gray and Dawson 2004, Kurczewski and Boyle 2000, LANDFIRE 2007a, Latham et al. 1996, Latham et al. n.d., Little 1979c, MNHESP 2007, Maurice et al. 2004, McCormick 1979, Meilleur et al. 1997, Motzkin et al. 1999, Motzkin et al. 2002, NatureServe 2005b, Olsvig 1980, Petraitis and Latham 1999, Schweitzer and Rawinski 1988*, Seischab and Bernard 1996, Sperduto and Nichols 2004, Swain and Kearsley 2011, Thompson 1995, USFS 2002b, Wibiralske et al. 2004

**Version:** 14 Jan 2014	**Stakeholders:** East

**Concept Author:** D.S. Schweitzer and T.J. Rawinski (1988)	**LeadResp:** East

CES203.069 Northern Atlantic Coastal Plain Calcareous Ravine

**Primary Division:** Gulf and Atlantic Coastal Plain (203)

**Land Cover Class:** Mixed Upland and Wetland

**Spatial Scale & Pattern:** Linear

**Required Classifiers:** Natural/Semi‑natural; Vegetated (>10% vasc.); Upland; Wetland

**Diagnostic Classifiers:** Ravine; Slope; Seepage‑Fed Sloping; Calcareous

**Concept Summary:** This system occurs on dry to mesic slopes and saturated bottoms of dissected ravine systems in the northern Atlantic Coastal Plain where erosion has exposed Tertiary‑aged shell deposits, limesands, or aboriginal shell middens. These calcium‑bearing sediments produce soils that range from slightly acidic to circumneutral and moderately to very strongly calcareous. The fertile soils support a rich diversity of plant species that distinguishes this system from the more widespread dry‑mesic, acidic (poor) ravines. This system includes mostly deciduous upland forests and woodlands on slopes and low interfluves and forested seepage wetlands found in saturated stream valley bottoms. Species composition varies with the environmental setting , but all habitats are characterized by species indicative of high base status soils. The communities of this system often contain species that are disjunct from their primary ranges in the mountains or Piedmont, such as Erigeron pulchellus, *Actaea pachypoda, Caltha palustris, Pedicularis lanceolata, Solidago flexicaulis, Quercus muehlenbergii, Verbesina virginica var. virginica, Hexalectris spicata, Corallorhiza wisteriana, Campanulastrum americanum, Celastrus scandens, Muhlenbergia sobolifera, Muhlenbergia tenuiflora, Sanicula marilandica*, and *Thalictrum revolutum*.

DISTRIBUTION

**Range:** This system is known from the northern Atlantic Coastal Plain of Virginia and Maryland, possibly ranging north into Delaware and New Jersey.

**Divisions:** 203:C

**TNC Ecoregions:** 57:C, 58:C

**Nations:** US

**Subnations:** DE?, MD, NJ?, VA

**Map Zones:** 60:C

CONCEPT

**Environment:** This system occurs on dry to mesic slopes and saturated bottoms of dissected ravine systems in the northern Atlantic Coastal Plain where erosion has exposed Tertiary‑aged shell deposits, limesands or aboriginal shell middens that have been exposed by downcutting streams or on river fronting bluffs along the northern Atlantic Coastal Plain of Virginia, Maryland, Delaware and New Jersey. It includes mesic and dry uplands and groundwater‑saturated wetlands associated with these fertile, base‑rich soils. Occurrences are typically linear or small patch and uncommon.

**Vegetation:** Forests of mesic slopes and low interfluves are characterized by *Fagus grandifolia, Liriodendron tulipifera, Quercus alba, Carya cordiformis*, and *Quercus rubra* in the overstory. Other canopy dominants and associates may include *Quercus muehlenbergii, Fraxinus americana, Tilia americana*, and *Acer floridanum (= Acer barbatum)*. The understory is often dense and may include *Asimina triloba, Lindera benzoin, Viburnum prunifolium, Ulmus rubra, Ilex opaca, Magnolia tripetala*, and *Cercis canadensis var. canadensis*. The ground cover is lush though sometimes patchy and may include *Podophyllum peltatum, Arisaema triphyllum, Sanguinaria canadensis, Circaea lutetiana ssp. canadensis, Maianthemum racemosum ssp. racemosum, Cardamine concatenata*, and *Polystichum acrostichoides*. More locally abundant herbs include *Cystopteris protrusa, Deparia acrostichoides, Diplazium pycnocarpon, Actaea racemosa (= Cimicifuga racemosa), Phegopteris hexagonoptera, Nemophila aphylla*, and *Actaea pachypoda*.

Drier, more southerly facing convex slopes are characterized by a more open canopy of *Quercus muehlenbergii*; common associates may include *Acer floridanum, Carya cordiformis, Fagus grandifolia, Fraxinus americana, Quercus alba, Quercus rubra*, and *Quercus montana (= Quercus prinus)*. The understory may include *Juniperus virginiana var. virginiana, Cercis canadensis var. canadensis, Dirca palustris, Ilex opaca var. opaca, Sideroxylon lycioides*, and *Viburnum rufidulum*. The herb layer is usually patchy but contains a diversity of species, including *Aquilegia canadensis, Erigeron pulchellus var. pulchellus, Bromus pubescens, Dichanthelium boscii, Verbesina virginica var. virginica, Campanulastrum americanum, Smallanthus uvedalius, Silphium trifoliatum var. trifoliatum, Desmodium pauciflorum, Hexalectris spicata*, and *Piptochaetium avenaceum*.

Forested seepage wetlands are often found along stream bottoms and at the base of slopes. Braided streams and hummock‑and‑hollow microtopography are characteristic of the environmental setting. The tree canopy is characterized by *Fraxinus pennsylvanica, Acer rubrum, Liquidambar styraciflua, Nyssa biflora*, and others. The shrub layer is composed of *Lindera benzoin, Morella cerifera (= Myrica cerifera)*, and *Cornus foemina*. Vines are abundant, especially *Decumaria barbara*. The herbaceous layer is characterized by *Caltha palustris, Carex bromoides, Packera aurea (= Senecio aureus), Scirpus lineatus, Thelypteris palustris, Pedicularis lanceolata, Carex tetanica, Liparis loeselii*, and *Carex granularis* on drier hummocks, and *Saururus cernuus, Bidens laevis, Pilea fontana, Glyceria striata*, and *Impatiens capensis* in wetter hollows and seepage rivulets.

**Dynamics:** Natural erosion of steep bluffs exposed by downcutting streams.

SOURCES

**References:** Comer et al. 2003*, Eyre 1980, Fleming 2002b, McAvoy and Harrison 2012, Patterson 2008c, Ware and Ware 1992

**Version:** 14 Jan 2014	**Stakeholders:** East, Southeast

**Concept Author:** NCR Review Team	**LeadResp:** East

CES203.475 Northern Atlantic Coastal Plain Hardwood Forest

**Primary Division:** Gulf and Atlantic Coastal Plain (203)

**Land Cover Class:** Forest and Woodland

**Spatial Scale & Pattern:** Large patch

**Required Classifiers:** Natural/Semi‑natural; Vegetated (>10% vasc.); Upland

**Diagnostic Classifiers:** Forest and Woodland (Treed); Long Disturbance Interval; Broad‑Leaved Deciduous Tree

**National Mapping Codes:** EVT 2324; ESLF 4130; ESP 1324

**Concept Summary:** This ecological system comprises dry hardwood forests largely dominated by oaks, ranging from sandy glacial and outwash deposits of Cape Cod, Massachusetts, and Long Island, New York, south to the Coastal Plain portions of Maryland and Virginia south to about the James River. *Quercus alba, Quercus montana (= Quercus prinus), Quercus coccinea*, and *Quercus rubra* are typical, and *Ilex opaca* is sometimes present. *Pinus* species may be codominant in some areas, for example the mixture of oaks with *Pinus virginiana* or *Pinus echinata* on very xeric, relict inland dunes. In the northern half of the range, conditions can grade to dry‑mesic, reflected in the local abundance of *Fagus grandifolia*. These forests occur on acidic, sandy to gravelly soils with a thick duff layer, often with an ericaceous shrub layer. From New Jersey south to Virginia, this system also includes oak‑beech/heath forests on steep slopes.

**Comments:** This system grades into other hardwood types of the northeastern U.S. as one moves inland and northward. North of Cape Cod, similar forests are treated as part of Central Appalachian Dry Oak‑Pine Forest (CES202.591). In Delaware and New York these coastal forests are apparently distinct (fauna, flora and substrate are distinct) from more inland forests. The southern part of this type's range overlaps with Southern Atlantic Coastal Plain Mesic Hardwood Forest (CES203.242); where they overlap, they are separated based on moisture regime, with the drier forests (often with an ericaceous shrub layer) going to this type.

DISTRIBUTION

**Range:** This system ranges from sandy glacial and outwash deposits of Massachusetts and Long Island, New York, south to the Coastal Plain portions of Maryland and Virginia, south to about the James River, with historic occurrences (and possibly some extant remnants) in eastern Pennsylvania.

**Divisions:** 202:C, 203:C

**TNC Ecoregions:** 52:P, 58:C, 61:C, 62:C

**Nations:** US

**Subnations:** CT, DC, DE, MA, MD, NJ, NY, PA, VA

**Map Zones:** 60:C, 65:C, 66:C

**USFS Ecomap Regions:** 221Ab:CCC, 221Ac:CCC, 221Ad:CCC, 221An:CCC, 221Db:CPP, 232Ab:CCC, 232Ac:CCC, 232Ad:CCC, 232Ha:CCC, 232Hb:CCC, 232Hc:CCC, 232Hd:CCC, 232Ib:CCC

CONCEPT

SOURCES

**References:** Comer et al. 2003*, Edinger et al. 2014a, Eyre 1980, NatureServe Explorer 2009a

**Version:** 05 Feb 2009	**Stakeholders:** East, Southeast

**Concept Author:** R. Evans	**LeadResp:** East

CES203.302 Northern Atlantic Coastal Plain Maritime Forest

**Primary Division:** Gulf and Atlantic Coastal Plain (203)

**Land Cover Class:** Forest and Woodland

**Spatial Scale & Pattern:** Large patch

**Required Classifiers:** Natural/Semi‑natural; Vegetated (>10% vasc.); Upland; Wetland

**Diagnostic Classifiers:** Forest and Woodland (Treed); Coast

**National Mapping Codes:** EVT 2379; ESLF 4322; ESP 1379

**Concept Summary:** This system encompasses a range of woody vegetation present on barrier islands, maritime shores and near‑coastal strands, from Fisherman's Island, Virginia (the northern range limit of *Quercus virginiana* and the southernmost tip of the Delmava Peninsula) northward to the extent of the Atlantic Coastal Plain. It includes forests and shrublands whose structure and composition are influenced by proximity to marine environments, including both upland and wetland. Vegetation includes narrow bands of forests with often stunted trees with contorted branches and dense vine layers. A range of trees may be present depending upon actual location and degree of protection from most extreme maritime influences. Common trees include *Prunus serotina, Pinus taeda, Ilex opaca, Quercus stellata, Juniperus virginiana, Pinus rigida, Pinus virginiana, Amelanchier canadensis*, and *Celtis occidentalis*. These trees are also found in less extreme or non‑maritime settings; this system is distinguished as much by the structure of the vegetation as its composition. *Morella pensylvanica* is a characteristic shrub, and *Smilax rotundifolia* and *Vitis rotundifolia* are characteristic vines. *Morella cerifera* is often present south of central New Jersey.

**Comments:** In New York this concept includes Maritime Holly Forest, Maritime Post Oak Forest, Maritime Beech Forest, Maritime Red Cedar Forest (Edinger et al. 2002).

DISTRIBUTION

**Range:** This system ranges from Fisherman's Island, Virginia northward to Massachusetts along the extent of the Atlantic Coastal Plain.

**Divisions:** 203:C

**TNC Ecoregions:** 58:C, 62:C

**Nations:** US

**Subnations:** DE, MA, MD, NH, NJ, NY, VA

**Map Zones:** 60:C, 65:C

**USFS Ecomap Regions:** 221Ab:CCC, 221Ac:CCC, 221Ad:CCC, 221An:CCC, 232Ab:CCC, 232Hb:CCC, 232Hc:CCC, 232Hd:CCP, 232Ib:C??

CONCEPT

**Environment:** This system occurs in marine coastal areas on sandy soils, usually in low interdunal areas behind primary or secondary dunes. In the glaciated portion of the range, it also occurs on till or morainal bluffs fronting the ocean, or on drowned drumlins on coastal islands. Examples also occur on sill or sand deposits in salt marsh islands. Soils range from well‑drained on higher topographic positions to mesic in lower positions.

**Vegetation:** Vegetation includes narrow bands of forest with often stunted trees with contorted branches and wilted leaves and dense vine layers (Edinger et al. 2002). A range of trees may be present depending upon actual location and degree of protection from most extreme maritime influences. Species include many deciduous hardwoods as well as *Pinus rigida* and *Pinus virginiana*. A rare *Pinus rigida* variant is found in Delaware (Cape Henlopen) and New York. Common trees include *Prunus serotina, Pinus taeda, Ilex opaca, Quercus stellata, Juniperus virginiana, Pinus rigida, Pinus virginiana, Amelanchier canadensis*, and *Celtis occidentalis*. *Morella pensylvanica* is a characteristic shrub, and *Smilax rotundifolia* and *Vitis rotundifolia* are characteristic vines. *Morella cerifera* is often present south of central New Jersey.

**Dynamics:** Salt spray, high winds, dune deposition, sand shifting and blasting, and occasional overwash during extreme disturbance events.

SOURCES

**References:** Backman 1984, Busby and Motzkin 2009, Clark 1986b, Comer et al. 2003*, Edinger et al. 2014a, Elliman 2005, Eyre 1980, Foster and Motzkin 1999, NYNHP 2013a, Sperduto and Nichols 2004

**Version:** 02 Jan 2015	**Stakeholders:** East, Southeast

**Concept Author:** R. Evans, G. Fleming, P. Coulling, L. Sneddon	**LeadResp:** East

CES203.269 Northern Atlantic Coastal Plain Pitch Pine Barrens

**Primary Division:** Gulf and Atlantic Coastal Plain (203)

**Land Cover Class:** Forest and Woodland

**Spatial Scale & Pattern:** Large patch

**Required Classifiers:** Natural/Semi‑natural; Vegetated (>10% vasc.); Upland

**Diagnostic Classifiers:** Forest and Woodland (Treed); Xeric; F‑Patch/High Intensity; Needle‑Leaved Tree

**National Mapping Codes:** EVT 2355; ESLF 4258; ESP 1355

**Concept Summary:** This system comprises a group of dry pitch pine woodlands and forests of deep sandy soils ranging from Cape Cod (Massachusetts) south through Long Island (New York) and the famous Pine Barrens of the New Jersey Coastal Plain, with occasional occurrences north to southernmost Maine and south to the Anacostia watershed (Maryland). The vegetation is characterized by a tree canopy of *Pinus rigida* with a tall‑shrub layer dominated by *Quercus ilicifolia* and a low‑shrub layer characterized by *Vaccinium pallidum* and/or *Vaccinium angustifolium*. The system is heavily influenced by fire, the composition and structure of its components varying with fire frequency. In general, tree oaks are more prevalent in those stands having a longer fire‑return interval; fire frequencies of 8‑10 years foster the growth of "pine plains," i.e., dwarf pine stands 1 m in height. Pine barrens with a history of more‑or‑less biennial burns for lowbush blueberry production may have very few trees and be characterized as sandplain grasslands. Dwarf‑shrubs such as *Arctostaphylos uva‑ursi, Vaccinium angustifolium, Vaccinium pallidum*, and *Hudsonia ericoides* typify the field layer of pine plains and sandplain grasslands. *Schizachyrium scoparium* is the most common grass (in close proximity to the coast, it may be represented by its close relative *Schizachyrium littorale*).

Scrub oak stands may occur without pine cover, particularly in low‑lying areas that do not intersect the water table, where cold‑air drainage inhibits pine growth. North of the glacial boundary, heathlands characterized by *Arctostaphylos uva‑ursi, Corema conradii*, and *Morella pensylvanica*, and grasslands characterized by *Schizachyrium littorale, Schizachyrium scoparium*, and *Danthonia spicata* occur as small (or occasionally large) patches. The Pine Barrens of New Jersey are very similar in structure and composition to those north of the glacial boundary but are characterized by additional species, such as *Quercus marilandica, Quercus stellata, Pyxidanthera barbulata, Leiophyllum buxifolium*, and others. Where the water table is close to the surface, pitch pine lowland vegetation (described as a separate system) occurs.

**Comments:** This system includes the New Jersey Pine Barrens, the uniqueness of which has long been recognized, and the system is well‑studied and summarized in a number of recent treatments (Forman 1979, Buckhholz and Good 1982, Gibson et al. 1999).

DISTRIBUTION

**Range:** This system is found in the Atlantic Coastal Plain from Delaware Bay northward through the New Jersey Coastal Plain and Long Island (New York) to Cape Cod, Massachusetts, with peripheral occurrences in Pennsylvania (historic), New Hampshire (historic), and southern Maine (Kennebunk Plains and Wells Barren).

**Divisions:** 203:C

**TNC Ecoregions:** 58:C, 62:C

**Nations:** US

**Subnations:** DE, MA, MD, ME, NH, NJ, NY, PA, RI

**Map Zones:** 60:C, 65:C

**USFS Ecomap Regions:** 221Ab:CCC, 221Ac:CCP, 221An:CCC, 232A:CC, 232Hc:CCC

CONCEPT

**Environment:** This system typically occurs on deep well‑drained sand deposits. In the coastal regions of the glaciated Northeast, it occurs on outwash plains and morainal deposits. In New Jersey, it occurs on Cohansey sand, which is sometimes overlain with hilltop gravel deposits.

**Vegetation:** The uniqueness of the New Jersey Pine Barrens flora has long been recognized (Stone 1911, Harshberger 1916). More recent treatments by Forman (1979) and Buckhholz and Good (1982) have compiled much of the available information. *Pinus rigida* is the dominant and characteristic species of this system. It may be found in well‑developed tree form or as a short‑statured, shrubby ecotype. *Pinus rigida* may occur as the sole dominant or occur with a variety of oak species, especially *Quercus marilandica, Quercus stellata*, and *Quercus ilicifolia*. In some examples *Pinus echinata* may co‑occur.

**Dynamics:** Different fire frequencies and intensities interrupt succession, accounting for variations in forest composition. Periodic severe wildfires with 40‑ to 100‑year intervals have produced oak‑pine mixtures over extensive areas of uplands, while more frequent severe fires have created mixtures of pitch pine and shrub oaks. The most frequent and severe fires have created the pine plains (Landfire 2007a).

SOURCES

**References:** Backman 1984, Buckhholz and Good 1982, Comer et al. 2003*, Edinger et al. 2014a, Eyre 1980, Finton 1998, Forman 1979, Gawler and Cutko 2010, Gibson et al. 1999, Givnish 1981, Harshberger 1916, Jordan et al. 2003, LANDFIRE 2007a, Motzkin and Foster 2002, Stone 1911, Swain and Kearsley 2011, Wacker 1979, Walker and Solecki 1999, Windisch 1990, Windisch 1994, Windisch 1999

**Version:** 14 Jan 2014	**Stakeholders:** East, Southeast

**Concept Author:** L. Sneddon and K. Straskosch Walz	**LeadResp:** East

CES202.331 Southern Appalachian Montane Pine Forest and Woodland

**Primary Division:** Central Interior and Appalachian (202)

**Land Cover Class:** Forest and Woodland

**Spatial Scale & Pattern:** Large patch

**Required Classifiers:** Natural/Semi‑natural; Vegetated (>10% vasc.); Upland

**Diagnostic Classifiers:** Montane; Forest and Woodland (Treed); Needle‑Leaved Tree

**National Mapping Codes:** EVT 2352; ESLF 4255; ESP 1352

**Concept Summary:** This system consists of predominantly evergreen woodlands (or more rarely forests) occupying very exposed, convex, often rocky south‑ and west‑facing slopes, ridge spurs, crests, and clifftops in the Central Appalachians, Southern Ridge and Valley and Southern Blue Ridge. They occur at moderate to upper elevations (450‑1200 m [1500‑4000 feet]), with the more southerly examples at the higher elevations. In the Southern Blue Ridge, this system is best developed above 700 m (2300 feet) in elevation. The underlying rock is acidic and sedimentary or metasedimentary (e.g., quartzites, sandstones and shales). The soils are very infertile, shallow and droughty. A thick, poorly decomposed duff layer, along with dead wood and highly volatile ericaceous shrubs, creates a strongly fire‑prone habitat. Most examples are dominated by *Pinus pungens*, often with *Pinus rigida* and/or *Pinus virginiana*, and occasionally *Tsuga caroliniana*. The canopy is usually patchy to open, but areas of closed canopy may be present, especially where *Tsuga caroliniana* is prominent. Fire is a very important ecological process in this system. Pines may be able to maintain dominance due to edaphic conditions, such as very shallow soil or extreme exposure in some areas which can produce sustained drought conditions, but most sites appear eventually to succeed to oak dominance in the absence of fire. Fire is also presumably a strong influence on vegetation structure, producing a more open woodland canopy structure and more herbaceous ground cover.

**Comments:** This system is related to Central Appalachian Pine‑Oak Rocky Woodland (CES202.600), which is distinguished by a mixed or deciduous canopy and absence of *Pinus pungens*. Stands with *Pinus echinata* present are generally accommodated by Southern Appalachian Low‑Elevation Pine Forest (CES202.332). The relationship between these two systems may need further clarification. This system is distinguished by occurrence as small patches on the most extreme topography, as well as by the species of pines dominating. However, *Pinus echinata* may codominate in Southern Appalachian Low‑Elevation Pine Forest (CES202.332) at times. Sites that would support this system under a natural fire regime, but which have lost the pines by logging, southern pine beetle or senescence in the absence of fire, should probably be regarded as degraded examples of this system. However, they become virtually indistinguishable from Southern Appalachian Oak Forest (CES202.886) and Central Appalachian Pine‑Oak Rocky Woodland (CES202.600) over time.

DISTRIBUTION

**Range:** This system is centered on the Southern Blue Ridge, from northern Georgia and South Carolina north through Virginia, with outlying occurrences north through the Central Appalachians to a small incursion in the northern Blue Ridge of south‑central Pennsylvania.

**Divisions:** 202:C

**TNC Ecoregions:** 49:C, 50:C, 51:C, 52:C, 59:C

**Nations:** US

**Subnations:** GA, KY, MD, NC, OH, PA, SC, TN, VA, WV

**Map Zones:** 53:C, 54:C, 57:C, 59:C, 60:C, 61:C

**USFS Ecomap Regions:** M221D:CC

CONCEPT

**Environment:** This system occurs on ridgetops, usually only on the sharpest and narrowest spur ridges, and adjacent convex upper slopes. These sites are the extreme of convex landforms. Rapid drainage of rainfall and exposure to wind, sun and lightning are probably the important characteristics. Bedrock may be of any acidic type, including felsic igneous and metamorphic rocks, sandstone and quartzite. Soils are shallow and rocky residual soils. Fire appears to be an important factor.

**Vegetation:** Vegetation consists of open forests or woodlands dominated by *Pinus pungens*, often with *Pinus rigida* or less commonly *Tsuga caroliniana*, and sometimes with *Pinus virginiana* or rarely *Pinus echinata* codominant. In examples that have not had fire in a long time, *Quercus montana (= Quercus prinus), Quercus coccinea*, or other oaks are usually present and are sometimes abundant, as are *Nyssa sylvatica* and *Acer rubrum*. *Castanea dentata* may also have once been abundant. A dense heath shrub layer is almost always present. *Kalmia latifolia* is the most typical dominant, but species of *Rhododendron, Vaccinium*, or *Gaylussacia* may be dominant. Herbs are usually sparse but probably were more abundant and shrubs less dense when fires occurred more frequently.

**Dynamics:** Fire is apparently a very important process in this system (Harrod and White 1999). Pines may be able to maintain dominance due to shallow soils and extreme exposure in some areas, but most sites appear eventually to succeed to oak dominance in the absence of fire. Fire is also presumably a strong influence on vegetation structure, producing a more open woodland canopy structure and more herbaceous ground cover. Occurrence in highly exposed sites may make this system more prone to ignition, but most fires probably spread from adjacent oak forests. Fires could be expected to show more extreme behavior in this system than in oaks forests under similar conditions, due to the flammability of the vegetation and the dry, windy and steep location. Both high‑intensity fires and lower‑intensity fires probably occurred naturally. Natural stands probably include both even‑aged and uneven‑aged canopies.

Southern pine beetle outbreaks are an important disturbance in this system, at least under present conditions. Beetle outbreaks can kill all the pines without creating the conditions for the pines to regenerate. If the pines are lost, the distinction between this system and Southern Appalachian Oak Forest (CES202.886) or Central Appalachian Pine‑Oak Rocky Woodland (CES202.600) becomes blurred.

SOURCES

**References:** Comer et al. 2003*, DuMond 1970, Edwards et al. 2013, Evans et al. 2009, Eyre 1980, Gettman 1974, Harrod and White 1999, Nelson 1986, Schafale 2012, Schafale and Weakley 1990, Simon 2011, Simon 2015, Tobe et al. 1992

**Version:** 29 Apr 2016	**Stakeholders:** East, Midwest, Southeast

**Concept Author:** M. Schafale, R. Evans, M. Pyne, R. White	**LeadResp:** Southeast

CES202.886 Southern Appalachian Oak Forest

**Primary Division:** Central Interior and Appalachian (202)

**Land Cover Class:** Forest and Woodland

**Spatial Scale & Pattern:** Large patch

**Required Classifiers:** Natural/Semi‑natural; Vegetated (>10% vasc.); Upland

**Diagnostic Classifiers:** Forest and Woodland (Treed); Ridge/Summit/Upper Slope; Unglaciated; Broad‑Leaved Deciduous Tree; Quercus ‑ Carya

**National Mapping Codes:** EVT 2315; ESLF 4121; ESP 1315

**Concept Summary:** This system consists of predominantly dry‑mesic (to dry) forests occurring on open and exposed topography at lower to mid elevations in the Southern Blue Ridge and Southern Ridge and Valley ecoregions. This is the upland forest that characterizes much of the lower elevations of these areas. The geology and soils can range from acidic to circumneutral or basic, and the vegetation varies accordingly. Soils are usually deep residual soils but are often rocky. Some shallow soils and colluvium may be present locally, but shallow soil environments are more extreme and have more pine. These forests are typically dominated by oaks, especially *Quercus montana, Quercus alba, Quercus rubra, Quercus velutina*, and *Quercus coccinea*, with varying amounts of *Carya* spp., *Nyssa sylvatica, Acer rubrum*, and other species such as *Pinus strobus* and *Fraxinus americana*. Historically, *Castanea dentata* was a dominant or codominant in many of these communities until its virtual elimination by the chestnut blight fungus (*Cryphonectria parasitica*) during the early 1900s. Some areas (usually on drier sites) now have dense evergreen heath shrub layers of *Kalmia latifolia*, with *Rhododendron maximum* on more mesic sites. Some other areas have deciduous heath‑dominated layers, sometimes consisting of *Vaccinium* spp. or *Gaylussacia* spp. This system concept also includes many successional communities that have been impacted by logging or agriculture, such as types dominated by *Liriodendron tulipifera, Pinus* spp., and *Robinia pseudoacacia*. This system is naturally dominated by stable, uneven‑aged forests, with canopy dynamics dominated by gap‑phase regeneration. Most oaks are long‑lived with typical age of mortality ranging from 200 to 400 years. Scarlet and black oaks are shorter lived with typical ages being approximately 50 to 100 years, while white oaks can live as long as 600 years.

**Comments:** This system is distinguished from the oak forests of the Piedmont by substantial floristic differences that probably are determined by biogeography as well as climate and topography. Compositional differences were more pronounced in the past, when *Castanea dentata* was a major species in this system and not in Piedmont oak forests. This system is distinguished from most other systems in its primary range by the canopy dominance of oaks (other than strong dominance by red oak) without a large component of yellow pines (*Pinus echinata, Pinus virginiana, Pinus pungens*) in the canopy. It shares those characteristics with Allegheny‑Cumberland Dry Oak Forest and Woodland (CES202.359), which might be thought of as a subtype of this system on the more exposed and acidic substrates. The environment is intermediate within the region in topography and moisture. Northward this system grades into Northeastern Interior Dry‑Mesic Oak Forest (CES202.592), which occurs in similar environmental conditions. This southern Appalachian system is characterized by the presence, in most occurrences, of plant species of southern Appalachian affinity, such as *Magnolia fraseri, Gaylussacia ursina, Rhododendron calendulaceum*, etc.

DISTRIBUTION

**Range:** This system ranges throughout the southern Appalachians, from northern Georgia and South Carolina north into the Southern Blue Ridge of Virginia to the Roanoke River in the Blue Ridge, and slightly farther south in the Ridge and Valley. It occurs in very limited montane outliers in the Piedmont, and possibly on Pine/Black Mountain in Kentucky.

**Divisions:** 202:C

**TNC Ecoregions:** 50:C, 51:C, 52:C

**Nations:** US

**Subnations:** GA, KY, NC, SC, TN, VA, WV

**Map Zones:** 53:C, 57:C, 59:C, 61:P

**USFS Ecomap Regions:** 231Aa:CCC, M221C:C?, M221D:CC

CONCEPT

**Environment:** This system occurs on open slopes, ridgetops, lower elevation peaks, and higher parts of broad valley bottoms, at low to moderate elevations. Soils are usually deep residual soils, but are often rocky. Some shallow soils, colluvium, and other soils may be present locally within the system, but shallow soil environments are more extreme and have more *Pinus* spp. than this system. Moisture levels are intermediate for the region. Soil chemistry and topography are important determinants of different associations within the system. Topography, elevation, and soil depth are the most important factors separating this system from others.

**Vegetation:** These forests are dominated by *Quercus* species, most typically *Quercus alba, Quercus coccinea, Quercus montana (= Quercus prinus), Quercus rubra*, and *Quercus velutina* with varying amounts of *Acer rubrum, Carya* spp., *Fraxinus americana, Nyssa sylvatica*, and other species. Less typical are stands dominated by other hardwood species, or by *Pinus strobus*. Historically, *Castanea dentata* was a dominant or codominant in many of these communities until its virtual elimination by the chestnut blight fungus (*Cryphonectria parasitica*) during the early 1900s. Subcanopies and shrub layers are usually well‑developed. Some areas (usually on drier sites) now have dense evergreen heath shrub layers of *Kalmia latifolia*, with *Rhododendron maximum* on more mesic sites. Some other areas have deciduous heath‑dominated layers, sometimes consisting of *Vaccinium* spp. or *Gaylussacia* spp. Herbs, forbs and ferns are usually sparse to moderate in density.

There are drier phase stands of this system which are related in terms of their canopy composition to examples of Allegheny‑Cumberland Dry Oak Forest and Woodland (CES202.359), but this type is found to the west of the Southern Blue Ridge ecoregion. These drier stands are primarily dominated by *Quercus montana* and *Quercus coccinea* with associated species such as *Carya pallida, Oxydendrum arboreum, Quercus falcata*, and *Quercus velutina*, and shrub layers dominated by evergreen or deciduous heaths such as *Kalmia latifolia, Vaccinium arboreum*, and *Vaccinium pallidum*, with *Galax urceolata* and *Gaultheria procumbens* in the ground layer. This phase would include associations such as CEGL006271 and CEGL008431. This includes the Dry Oak Deciduous Heath Forest & Woodlands and Dry Oak Evergreen Heath Forest & Woodlands Ecological Zones of Simon and Hayden (2014). Examples of the dry‑mesic phase of this system are typically dominated by *Quercus alba* and *Quercus rubra*, possibly with *Quercus montana* and/or *Carya* spp. in some associations. Understory species may include *Acer saccharum, Cornus florida*, and *Hamamelis virginiana*.

**Dynamics:** This system is naturally dominated by stable, uneven‑aged forests. Extreme wind or ice storms occasionally create larger canopy openings. Natural old‑growth forest examples have trees reproducing in small to medium‑sized canopy gaps created by the death of individual or small groups of trees. Fire occurred fairly frequently in presettlement times, though there is some dispute whether most of the fires were natural or anthropogenic in origin (Abrams 1992, Delcourt and Delcourt 1997). Fires were usually low‑intensity surface fires. The dominant species are fairly fire‑tolerant, making most fires non‑catastrophic. Fire may be important for favoring oak dominance over more mesophytic tree species within some of the topographic range of this system. Fire also can be expected to have a moderate effect on vegetation structure, producing a somewhat more open canopy and less dense understory and shrub layer than currently seen in most examples. Fire frequency or intensity may be important for determining the boundary between this system and both the more mesic and the drier systems. Virtually all examples have been strongly affected by the introduction of the chestnut blight, which killed all of the *Castanea dentata* trees, eliminating it as a canopy dominant. Past logging affected most occurrences. Regenerated forest canopies are even‑aged, or have a more even‑aged structure. Extreme wind or ice storms occasionally create larger canopy openings, which may provide particularly good sites for *Quercus* regeneration. Virtually all examples have been strongly affected by introduction of chestnut blight (*Cryphonectria parasitica*), which killed *Castanea dentata* trees, eliminating it as a canopy dominant. The introduction, and now widespread establishment, of gypsy moth (*Lymantria dispar*) that favors oaks as food has also affected these forests by causing widespread mortality of overstory trees depending on topographic position and precipitation amounts around defoliation events. Past logging, and now lack of fire, has affected most occurrences by changing canopies to an even‑aged, or more even‑aged, structure with an understory of shade‑tolerant but fire‑intolerant species such as *Pinus strobus, Acer rubrum*, and *Acer pensylvanicum*. The removal of *Castanea dentata* from the overstory of these forests is thought to have benefited *Carya* spp., and their persistence and continued recruitment in contemporary oak‑hickory forests may reflect fire exclusion in recent decades.

SOURCES

**References:** Abrams 1992, Comer et al. 2003*, Delcourt and Delcourt 1997, DuMond 1970, Edwards et al. 2013, Evans et al. 2009, Eyre 1980, Gettman 1974, Nelson 1986, Patterson 1994, Schafale 2012, Schafale and Weakley 1990, Simon 2011, Simon 2015, Simon and Hayden 2014, Tobe et al. 1992, Woods et al. 2002

**Version:** 29 Apr 2016	**Stakeholders:** East, Southeast

**Concept Author:** M. Schafale, R. Evans, M. Pyne, R. White	**LeadResp:** Southeast

CES202.457 Southern Ridge and Valley / Cumberland Dry Calcareous Forest

**Primary Division:** Central Interior and Appalachian (202)

**Land Cover Class:** Forest and Woodland

**Spatial Scale & Pattern:** Large patch, Matrix

**Required Classifiers:** Natural/Semi‑natural; Vegetated (>10% vasc.); Upland

**Diagnostic Classifiers:** Forest and Woodland (Treed); Circumneutral Soil; Broad‑Leaved Tree

**National Mapping Codes:** EVT 2376; ESLF 4319; ESP 1376

**Concept Summary:** This system includes dry to dry‑mesic calcareous forests of the Southern Ridge and Valley region of Alabama and Georgia, extending north into Tennessee, Kentucky, Virginia and adjacent West Virginia. It includes calcareous forests on lower escarpments of the Cumberland Plateau and other related areas. Examples occur on a variety of different landscape positions and occur on generally deeper soils than glade systems of the same regions. This system is distinguished from those farther north in the Ridge and Valley by its relatively southern location in the region, in an area which is transitional to the "Oak‑Pine‑Hickory" region. High‑quality and historic examples are typically dominated by combinations of *Quercus* species and *Carya* species, sometimes with *Pinus* species and/or *Juniperus virginiana* as a significant component in certain landscape positions and with particular successional histories. These forests occur in a variety of habitats and are the matrix vegetation type that covers portions of the landscape under natural conditions. Examples can occur on a variety of topographic and landscape positions including valley floors, sideslopes, and lower to midslopes. Fire frequency and intensity are factors determining the relative mixture of deciduous hardwood versus evergreen trees in this system. Much of this system is currently composed of successional forests that have arisen after repeated cutting, clearing, and cultivation of the original forests. The range of this system is primarily composed of circumneutral substrates, which exert an expected influence on the composition of the vegetation.

**Comments:** To the north, this transitions into Northeastern Interior Calcareous Oak Forest (CES202.452). This system (CES202.457) is defined as distinct because of its location in the portion of the Ridge and Valley region which is transitional to the "Oak‑Pine‑Hickory" region (Greller 1988). Southern Ridge and Valley / Cumberland Dry Calcareous Forest (CES202.457) occurs primarily in the "Southern Limestone/Dolomite Valleys and Low Rolling Hills" (67f) and the "Southern Shale Valleys" (67g) of Griffith et al. (2001), as well as calcareous parts of 68b and 68c (where it is more limited in extent). In addition, the system could be found in drier, more exposed portions of 66f, "Limestone Valleys and Coves" (Griffith et al. 2001), but most of this terrain is probably more mesic and concave. This ecoregion and "Southern Sedimentary Ridges" (66e) are part of the "Blue Ridge" but are clearly transitional to the Ridge and Valley region. Ecoregion 66e is more likely too acidic to support this system. It is also possible in the "Carter Hills" (EPA Ecoregion 70h of Woods et al. (2002)) of Kentucky and in limited portions of related parts of EPA Ecoregion 70 (Western Allegheny Plateau) in Kentucky.

DISTRIBUTION

**Range:** This system is endemic to the Southern Ridge and Valley and the Cumberland Plateau escarpment in Alabama, Georgia, Tennessee, Kentucky, and southwestern Virginia.

**Divisions:** 202:C

**TNC Ecoregions:** 50:C, 59:C

**Nations:** US

**Subnations:** AL, GA, KY, TN, VA

**Map Zones:** 48:C, 53:C, 57:C, 61:C

**USFS Ecomap Regions:** 221Jb:CCC, 222J:CC, 231Cc:CCC, 231D:CC, M221A:CC

CONCEPT

**Environment:** Examples of this forest and woodland system occur usually on dry sites, on a variety of topographic and landscape positions, including sideslopes (particularly south‑ and west‑facing ones), ridges, and knobs, as well as valley floors, depending on where the base‑rich rock is present or crops out, and where the soils are influenced by calcareous/circumneutral geology. Elevation is generally between 200 and 500 m. In some landscapes, the ridges and ridgetops will more likely be composed of sandstones and other more weather‑resistant and acidic materials.

**Vegetation:** Natural vegetation consists of forests (or woodlands) dominated most typically by *Quercus alba, Quercus muehlenbergii, Quercus stellata*, and *Quercus shumardii*, with varying amounts of *Carya* spp., *Acer saccharum, Acer floridanum (= Acer barbatum), Acer leucoderme, Acer rubrum*, and other species. This system concept also includes successional communities that have been impacted by logging or agriculture, including upland forest types dominated by *Liriodendron tulipifera, Pinus* spp., *Juniperus virginiana*, and *Robinia pseudoacacia*.

**Dynamics:** Fire frequency and intensity are factors determining the relative mixture of deciduous hardwood versus evergreen trees in this system. Presettlement fire‑return intervals are believed to have ranged from 3 to 14 years from both lightning and Native American ignitions. These frequent surface fires maintained the grassy understory and kept hardwoods and shrubs from dominating the understory and forming a midstory layer. These fires occurred in the dormant season with occasional growing‑season mosaic fires (most likely occurring infrequently once or twice every 20 to 25 years) (Landfire 2007a). Occasionally, during extensive droughts, mixed‑severity or stand‑replacement fires could occur, especially in drier stands or those containing *Juniperus virginiana*. In addition, local thunderstorm‑caused blowdowns and windthrow created gaps on a small but continual basis. More extensive regional disturbances included winter ice storms. Dense stands of middle to older aged pines (where present) were susceptible to periodic mortality from bark beetle epidemics, and younger *Juniperus virginiana* trees were killed by periodic droughts.

SOURCES

**References:** Comer et al. 2003*, Evans et al. 2009, Eyre 1980, Greller 1988, Griffith et al. 2001, LANDFIRE 2007a, TNC 1996c, Woods et al. 2002

**Version:** 07 Oct 2016	**Stakeholders:** East, Southeast

**Concept Author:** R. Evans and M. Pyne	**LeadResp:** Southeast

M882. Central Midwest Mesic Forest

CES202.693 North‑Central Interior Beech‑Maple Forest

**Primary Division:** Central Interior and Appalachian (202)

**Land Cover Class:** Forest and Woodland

**Spatial Scale & Pattern:** Large patch

**Required Classifiers:** Natural/Semi‑natural; Vegetated (>10% vasc.); Upland

**National Mapping Codes:** EVT 2313; ESLF 4119; ESP 1313

**Concept Summary:** This system is found primarily along the southern Great Lakes ranging from central Indiana to southern Ontario. It is typically found on flat to rolling uplands to steep slopes with rich loam soils over glacial till. This system is characterized by a dense tree canopy that forms a thick layer of humus and leaf litter leading to a dense and rich herbaceous layer. *Acer saccharum* and *Fagus grandifolia* comprise up to 80% of the canopy. Canopy associates can include *Quercus rubra, Tilia americana*, and *Liriodendron tulipifera* with *Carpinus caroliniana* and *Ostrya virginiana* common in the understory and subcanopy. The relative dominance of sugar maple compared to other tree species varies across the range of this system based on regional climate and microclimate. The herbaceous layer is very diverse and typically includes spring ephemerals. Some common species include *Arisaema triphyllum, Osmorhiza claytonii, Polygonatum biflorum*, and *Trillium grandiflorum*. The primary natural disturbance influencing this system includes wind‑driven gap dynamics. Conversion to agriculture has significantly decreased the range of this system, and very few large stands remain intact.

**Comments:** North‑Central Interior Wet Flatwoods (CES202.700) may co‑occur in close proximity to this system on clay‑plain landscapes. This is on richer sites than the corresponding Appalachian (Hemlock)‑Northern Hardwood Forest (CES202.593).

DISTRIBUTION

**Range:** This system is located in the southern Great Lakes from central Indiana north into southern Ontario, and east to northwestern Pennsylvania and western New York.

**Divisions:** 202:C

**TNC Ecoregions:** 36:C, 45:C, 47:P, 48:C

**Nations:** CA, US

**Subnations:** IN, MI, NY, OH, ON, PA

**Map Zones:** 47:C, 49:C, 51:C, 52:C, 62:P, 63:C

**USFS Ecomap Regions:** 221F:CC, 222H:CC, 222Ia:CCC, 222Ja:CCC, 222Jb:CCC, 222Jc:CCC, 222Je:CCC, 222Jg:CCC, 222Jh:CCC, 222Ji:CCC, 222K:CC, 222L:CC, 222M:CC, 222Ua:CCC, 222Ud:CCC, 222Ue:CCC, 223G:PP, 251D:CC

CONCEPT

**Environment:** This system is typically found on flat to rolling uplands to steep slopes with rich loam soils over glacial till. It occurs principally on medium‑ or fine‑textured ground moraine, medium‑ or fine‑textured end moraine, and silty/clayey glacial lakeplains. Sand dunes and sandy lakeplains can support these systems where proximity to the Great Lakes modifies local climate (within 10‑20 miles of the shore, evapotranspiration conditions are suitable for mesic forest). Prevalent topographic positions of this community are gentle to moderate slopes and level areas with moderate to good drainage. Where mesic southern forest occurs on steeper slopes, it is often associated with northern to eastern exposures which receive low amounts of direct sunlight and are characterized by a cool, moist microclimate.

It can occur on a variety of soil types, but loam is the predominant texture. The diversity of soils which can support this system include sand, sandy loam, loamy sand, loam, silt loam, silty clay loam, clay loam, and clay. Soils are typically well‑drained with high water‑holding capacity and high nutrient and soil organism content. High soil fertility is maintained by nutrient inputs from the decomposition of deciduous leaves which enrich the top layer of soil (Cohen 2004).

**Dynamics:** Small‑gap development and replacement due to tree death is the prevalent disturbance factor influencing this system. Catastrophic fire and/or wind can impact this system over long return intervals but are rare. Tree canopy tends to be closed so understory plants receive little light after leaf‑out in the spring. This system could form large stands or be part of a large forested landscape in conjunction with other forested types, resulting in a relatively high proportion of forest interior to forest edge.

SOURCES

**References:** Barbour and Billings 1988, Barnes 1991, Beaman 1970, Brewer 1980, Cain 1935, Cohen 2004, Comer and Albert 1997, Comer et al. 1995a, Comer et al. 1998, Comer et al. 2003*, Curtis 1959, Donnelly and Murphy 1987, Edinger et al. 2014a, Eyre 1980, Horsley et al. 2003, Howell et al. 2005, Kost et al. 2007, ONHD unpubl. data, Palik and Murphy 1990, Parker 1989, Parker et al. 1985, Robertson and Robertson 1995, Rooney and Waller 2003, Witter et al. 2005

**Version:** 14 Jan 2014	**Stakeholders:** Canada, East, Midwest, Southeast

**Concept Author:** S. Menard	**LeadResp:** Midwest

CES202.696 North‑Central Interior Maple‑Basswood Forest

**Primary Division:** Central Interior and Appalachian (202)

**Land Cover Class:** Forest and Woodland

**Spatial Scale & Pattern:** Large patch

**Required Classifiers:** Natural/Semi‑natural; Vegetated (>10% vasc.); Upland

**National Mapping Codes:** EVT 2314; ESLF 4120; ESP 1314

**Concept Summary:** This system is primarily found in the prairie forest border region of Minnesota, Wisconsin, and Iowa, but it can range north into northern Minnesota and Wisconsin and south to central Illinois, central Missouri, and eastern Kansas. This forest system is distinguished by underlying mesic soils and the predominance of mesic deciduous species forming a moderately dense to dense canopy. Examples of this system occur on valley slopes and bottoms often with northern or eastern aspects. Soils are moderately well‑drained, fertile, and medium to deep loams that have developed from glacial till or loess parent material. *Acer saccharum* typifies this system, with *Tilia americana, Quercus rubra*, and *Ostrya virginiana* as common associates. The dense canopy allows for a rich mixture of shrub and herbaceous species in the understory. Examples of common herbaceous species include *Anemone quinquefolia, Adiantum pedatum, Arisaema triphyllum*, and *Sanicula* spp. Spring ephemeral herbaceous species are characteristic of this system, including *Aplectrum hyemale, Cardamine* spp., *Claytonia virginica, Dicentra cucullaria, Diplazium pycnocarpon, Erythronium americanum, Hydrastis canadensis, Phlox divaricata*, and *Trillium flexipes*. Dynamic processes such as wind and fire can impact this system over long return cycles; however, the most immediate threats to remaining examples of this system are grazing, unsustainable logging, and conversion to agriculture.

**Comments:** Where *Quercus alba* is prominent in the upper Midwest, this system can be difficult to separate from related oak‑dominated systems [see Effigy Mounds NPS map (Hop et al. 2005)]. *Quercus alba* in combination with *Acer saccharum*, and with a more mesic understory, is classified as the present system.

DISTRIBUTION

**Range:** This system ranges from northern Minnesota and Wisconsin south to eastern Kansas and Nebraska and southeast to central Illinois, Missouri, and possibly western Indiana.

**Divisions:** 201:C, 202:C, 205:C

**TNC Ecoregions:** 36:C, 37:C, 38:?, 45:C, 46:C, 47:C, 48:C

**Nations:** US

**Subnations:** IA, IL, IN, KS, MI, MN, MO, NE, WI

**Map Zones:** 39:C, 40:C, 41:C, 42:C, 43:C, 44:P, 49:C, 50:C, 51:C, 52:C

**USFS Ecomap Regions:** 212J:CP, 212Q:CC, 212S:CP, 212T:CP, 212X:CP, 212Y:C?, 212Z:CP, 222K:CC, 222L:CC, 222M:CC, 222R:CC, 251B:CC, 251H:CC

CONCEPT

**Environment:** This system is found primarily on mesic soils that are moderately well‑drained and fertile. These are mostly moderate to deep loams that have developed from glacial till or loess. This system occurs near the prairie‑forest border, and the closer to this border, the stronger the association this system has with natural firebreaks. These sites are typically on the east and north sides of rivers, lakes, and wetlands and topographically protected areas on valley slopes and bottoms often with northern or eastern aspects (Kucera and McDermott 1955, Grimm 1984, Moran n.d.).

**Vegetation:** Mesic deciduous trees form a moderately dense to dense canopy in examples of this system. *Acer saccharum* is the most common tree species forming the majority of the canopy and sapling layers. Common associates include *Tilia americana, Quercus rubra*, and *Ostrya virginiana*. The understory contains a rich mixture of shrub and herbaceous species such as *Anemone quinquefolia, Adiantum pedatum, Arisaema triphyllum*, and *Sanicula* spp. This system is found west and north of where *Fagus grandifolia* is a reliable and dominant member of the canopy.

**Dynamics:** Wind and fire can impact this system over long return intervals but are rare. Small‑gap development and replacement due to tree death is more frequent than catastrophic fire or wind (Bray 1956, Grimm 1984). Tree canopy tends to be closed so understory plants receive little light after leaf‑out in the spring. Old‑growth stands may not vary greatly in species composition from mature managed forest but have different structural characteristics, including more snags, coarse woody debris, and large trees (McHale et al. 1999). This provides different habitats for wildlife and other non‑plant species. This system could form large stands or be part of a large forested landscape in conjunction with other forested types, resulting in a relatively high proportion of forest interior to forest edge.

SOURCES

**References:** Alverson et al. 1988, Augustine and Frelich 1998, Barbour and Billings 1988, Bohlen et al. 2004, Bray 1956, Comer et al. 2003*, Eyre 1980, Grimm 1984, Groffman et al. 2004, Gundale 2002, Hale et al. 2005, Hop et al. 2005, Howell et al. 2005, Kost et al. 2007, Kourtev et al. 1999, Kucera and McDermott 1955, McHale et al. 1999, Moran n.d., Nelson 2010, ONHD unpubl. data, Rolfsmeier and Steinauer 2010, Rooney and Waller 2003, Suárez et al. 2004, WDNR 2015, Wiegmann and Waller 2006

**Version:** 14 Jan 2014	**Stakeholders:** Midwest, Southeast

**Concept Author:** S. Menard and K. Kindscher	**LeadResp:** Midwest

CES202.043 Ozark‑Ouachita Mesic Hardwood Forest

**Primary Division:** Central Interior and Appalachian (202)

**Land Cover Class:** Forest and Woodland

**Spatial Scale & Pattern:** Small patch

**Required Classifiers:** Natural/Semi‑natural; Vegetated (>10% vasc.); Upland

**Diagnostic Classifiers:** Forest and Woodland (Treed); Ozark/Ouachita

**National Mapping Codes:** EVT 2334; ESLF 4140; ESP 1334

**Concept Summary:** This system is found on lower slopes, toeslopes and valley bottoms within the Ozark and Ouachita regions, as well as on north slopes. In the Ozarks, *Quercus rubra* increases in abundance compared to dry‑mesic habitats, and *Acer saccharum* is sometimes a leading dominant. On more alkaline moist soils, *Quercus muehlenbergii, Tilia americana*, and *Cercis canadensis* may be common. In the Boston Mountains, mesic forests may also be common on protected slopes and terraces next to streams. Here, *Fagus grandifolia* may be the leading dominant, with codominants of *Acer saccharum, Liquidambar styraciflua, Tilia americana, Magnolia acuminata, Magnolia tripetala*, and others. Similar habitats occur in the western Ouachita Mountains.

DISTRIBUTION

**Range:** This system is found within the Ozarks and Ouachita Mountains of Missouri, Arkansas, and Oklahoma.

**Divisions:** 202:C

**TNC Ecoregions:** 38:C, 39:C

**Nations:** US

**Subnations:** AR, MO, OK

**Map Zones:** 44:C, 49:P

**USFS Ecomap Regions:** 223A:CC, 231E:CC, 231G:CC, M223A:CC, M231A:CC

CONCEPT

**Environment:** This system may be found on a wide range of topographic positions. It includes mixed mesophytic forests, seeps/springs and smaller riparian areas. This system is found on primarily north‑ and east‑facing aspects, lower slopes, toeslopes, small valley bottoms and terraces, as well as other protected slopes and ravines along intermittent and/or ephemeral streams. Distribution is influenced by local conditions affecting moisture, aspect, elevation and soil productivity. Closed conditions are multiple canopy usually late‑seral forests. Stands of this system are generally small, isolated, and/or disjunct and are generally "embedded" in a larger landscape matrix. These communities are maintained primarily through naturally occurring circumstances such as aspect, elevation, soil moisture conditions, and soil productivity, except for mortality or other disturbance‑induced openings or gaps.

**Vegetation:** Dominant or characteristic trees in examples of this system may include *Quercus alba, Quercus rubra, Acer floridanum (= Acer barbatum), Acer saccharum, Fagus grandifolia, Liquidambar styraciflua, Quercus muehlenbergii*, and *Tilia americana*. The understory may contain *Cercis canadensis, Magnolia tripetala*, and/or *Magnolia acuminata*. Some common shrubs include *Asimina triloba* and *Lindera benzoin*. Stands will typically have diverse ground layers. Some typical herbs include *Podophyllum peltatum* and *Hybanthus concolor*.

**Dynamics:** This type has a lower fire frequency than drier (uphill) types and experiences primarily low‑intensity surface fire with occasional mosaic (mixed‑severity) or replacement fire. Mean fire‑return interval (MFI) is about 25 years with wide year‑to‑year and within‑type variation related to moisture cycles, degree of sheltering and proximity to more fire‑prone types. Anthropogenic fire is considered and contributes to within‑type MFRI variation. Drought and moisture cycles play a strong role interacting with fire and insect and disease damage. Other natural disturbances may include wind and ice (Landfire 2007a).

SOURCES

**References:** Arkansas Forestry Commission 2010, Barnes 1991, Comer et al. 2003*, Engeman et al. 2007, Eyre 1980, Foti and Glenn 1990, LANDFIRE 2007a, Nelson 2010

**Version:** 14 Jan 2014	**Stakeholders:** Midwest, Southeast

**Concept Author:** R. Evans, D. Faber‑Langendoen	**LeadResp:** Southeast

M012. Central Midwest Oak Forest, Woodland & Savanna

CES202.047 North‑Central Interior Dry Oak Forest and Woodland

**Primary Division:** Central Interior and Appalachian (202)

**Land Cover Class:** Forest and Woodland

**Spatial Scale & Pattern:** Large patch

**Required Classifiers:** Natural/Semi‑natural; Vegetated (>10% vasc.); Upland

**Diagnostic Classifiers:** Forest and Woodland (Treed); Outwash plain; Sand Soil Texture; Intermediate Disturbance Interval; F‑Patch/Medium Intensity

**National Mapping Codes:** EVT 2311; ESLF 4117; ESP 1311

**Concept Summary:** This system is found throughout the glaciated regions of the Midwest, typically in gently rolling to flat landscapes. It can occur on uplands within the prairie matrix or within the context of dry‑mesic oak‑hickory forests and oak savannas. These are common on rolling glacial moraines and outwash plains. Soils are typically well‑drained to excessively drained Mollisols or Alfisols that range from sand to sandy loam in texture. Historically, this type was quite extensive in Michigan, Indiana, Illinois, Missouri, Iowa, Wisconsin, and Minnesota. It is distinguished from other forested systems within the region by a dry edaphic condition that is transitional between dry prairies, oak barrens, or savannas and dry‑mesic oak‑hickory forests and woodlands. Forest cover can range from dense to moderately open canopy. Fire‑resistant oak species, in particular *Quercus velutina, Quercus macrocarpa, Quercus coccinea*, and *Quercus ellipsoidalis*, dominate the overstory. *Carya glabra, Prunus serotina*, and *Sassafras albidum* are also common in portions of the range of this system. Depending on range of distribution and overstory canopy density, the understory may include species such as *Gaylussacia baccata* (in Michigan, Wisconsin, and Minnesota), *Vaccinium angustifolium*, and *Rhus aromatica*, and/or a mixture of woodland and grassland species, including *Schizachyrium scoparium, Deschampsia flexuosa*, and *Carex pensylvanica*. Extreme drought, along with periodic ground and crown fire events, constitute the main natural processes for this type and likely maintained a more open canopy structure that supported oak regeneration. In fact, many current examples of this type have resulted from long‑term fire suppression and conversion of oak barrens to these forests and woodlands. Fire suppression may also account for examples of this system with the more dry‑mesic understory. It likely has allowed for other associates such as *Quercus rubra* and *Fraxinus americana* to become more prevalent. Extensive conversion for agriculture in the surrounding landscape with more productive soils has fragmented and isolated examples of this system. It is found primarily within the "corn belt" of the United States, and remaining large areas of this system are likely under considerable pressure due to conversion to pastureland and urban development.

**Comments:** This system is related to North‑Central Interior Dry‑Mesic Oak Forest and Woodland (CES202.046), which has white oak, red oak, and bur oak, and occurs on somewhat deeper soils; the present system has oak savannas and oak‑hickory and occurs on sandplains. Applying this concept difference to drawing lines on the ground can be tricky.

DISTRIBUTION

**Range:** Found throughout the glaciated regions of the Midwest.

**Divisions:** 202:C, 205:P

**TNC Ecoregions:** 35:P, 36:C, 37:?, 44:?, 45:C, 46:C, 47:?, 48:C

**Nations:** US

**Subnations:** IL, IN, MI, MN, MO, ND, OH, WI

**Map Zones:** 38:P, 39:P, 40:P, 41:C, 42:C, 43:C, 44:P, 47:P, 49:C, 50:C, 51:C, 52:C

**USFS Ecomap Regions:** 212H:CC, 222H:CC, 222Ja:CCC, 222Jc:CCC, 222Je:CCC, 222Jg:CCC, 222Jh:CCC, 222Ji:CCC, 222Ua:CCC, 223A:CP, 223G:CC, 251B:CC, 251C:CC, 251D:CC, 251E:CC, 251H:CC, 255A:CC, 331F:CC, 331M:CC, 332B:PP

CONCEPT

**Environment:** This system can occur on uplands within the prairie matrix or within the context of dry‑mesic oak‑hickory forests and oak savannas. These are common on rolling glacial moraines and outwash plains and, less frequently, old dunes. Soils are typically well‑drained to excessively drained Mollisols or Alfisols that range from sand to sandy loam in texture. Dry soils or landscape position (steep slopes, upper slopes, south‑ or west‑facing aspect) favor the formation of this system. Historically, this type was quite extensive in Michigan, Indiana, Illinois, Missouri, Iowa, Wisconsin, and Minnesota. It is distinguished from other forested systems within the region by a dry edaphic condition that is transitional between dry prairies, oak barrens, or savannas and dry‑mesic oak‑hickory forests and woodlands.

**Vegetation:** Forest cover can range from a dense to moderately open canopy. Fire‑resistant oak species, in particular *Quercus velutina, Quercus macrocarpa, Quercus coccinea*, and *Quercus ellipsoidalis*, dominate the overstory. *Carya glabra, Prunus serotina*, and *Sassafras albidum* are also common in portions of the range of this system. Depending on range of distribution and overstory canopy density, the understory may include species such as *Gaylussacia baccata* (in MI, WI, and MN), *Vaccinium angustifolium*, and *Rhus aromatica*, and/or a mixture of woodland and grassland species, including *Schizachyrium scoparium, Deschampsia flexuosa*, and *Carex pensylvanica*.

**Dynamics:** Extreme drought, along with periodic ground and crown fire events, constitute the main natural processes for this type and likely maintained a more open canopy structure that supported oak regeneration. In fact, many current examples of this type have resulted from long‑term fire suppression and conversion of oak barrens to these forests and woodlands. Frequency of fires necessary to maintain this system varied, largely depending on soil fertility and drainage, with more fertile and mesic sites requiring more frequent fires. Fire‑return intervals of 5‑20 years would typically maintain a woodland or oak grub shrubland, while fire‑return intervals of 20‑50 years would typically maintain a closed canopy oak forest (Landfire 2007a). Fire suppression may also account for examples of this system with the more dry‑mesic understory. It likely has allowed for other associates such as *Quercus rubra* and *Fraxinus americana* to become more prevalent.

SOURCES

**References:** Abrams 1992, Archambault et al. 1989, Archambault et al. 1990, Comer and Albert 1997, Comer et al. 1995a, Comer et al. 1999, Comer et al. 2003*, Davidson et al. 2001, Eyre 1980, Healy 1997, Kost et al. 2007, LANDFIRE 2007a, MNNHP 1993, Nowacki and Abrams 2008, ONHD unpubl. data, Rooney 2001, Stroke and Anderson 1992, WDNR 2015

**Version:** 14 Jan 2014	**Stakeholders:** Midwest, Southeast

**Concept Author:** P. Comer, K. Kindscher, S. Menard, D. Faber‑Langendoen	**LeadResp:** Midwest

CES202.046 North‑Central Interior Dry‑Mesic Oak Forest and Woodland

**Primary Division:** Central Interior and Appalachian (202)

**Land Cover Class:** Forest and Woodland

**Spatial Scale & Pattern:** Large patch

**Required Classifiers:** Natural/Semi‑natural; Vegetated (>10% vasc.); Upland

**Diagnostic Classifiers:** Forest and Woodland (Treed); Udic; F‑Patch/Low Intensity; Quercus ‑ Carya

**National Mapping Codes:** EVT 2310; ESLF 4116; ESP 1310

**Concept Summary:** This system is found throughout the glaciated regions of the Midwest, typically in gently rolling landscapes. It can occur on uplands within the prairie matrix and near floodplains, or on rolling glacial moraines and among kettle‑kame topography. Soils are typically well‑drained Mollisols or Alfisols that range from loamy to sandy loam or even coarse sands in texture. Historically, this type was quite extensive in Michigan, Indiana, Illinois, Missouri, Iowa, Wisconsin, and Minnesota. Well over 700,000 hectares likely occurred in southern Michigan alone (ca. 1800). It is distinguished from other forested systems within the region by a dry‑mesic edaphic condition that is transitional between dry oak forests and woodlands and mesic hardwood forests, such as maple‑basswood forests. Forest cover can range from a dense to moderately open canopy and there is commonly a dense shrub layer. Fire‑resistant oak species, in particular *Quercus macrocarpa, Quercus rubra*, and/or *Quercus alba*, dominate the overstory. *Carya* spp., including *Carya ovata, Carya cordiformis*, and *Carya tomentosa*, are diagnostic in portions of the range of this system. Depending on site location and overstory canopy density, the understory may include species such as *Amelanchier* spp., *Aralia nudicaulis, Corylus americana, Desmodium glutinosum, Maianthemum stellatum, Osmunda claytoniana, Phryma leptostachya, Trillium grandiflorum*, and *Viburnum acerifolium*. Occasionally, prairie grasses such as *Andropogon gerardii* and *Panicum virgatum* may be present. Fire constitutes the main natural process for this type and likely maintained a more open canopy structure to support oak regeneration. Historic fire frequency was likely highest in the prairie‑forest border areas. Fire suppression may account for the more closed oak forest examples of this system with the more mesic understory. It likely has allowed for other associates, such as *Acer saccharum, Acer rubrum, Celtis occidentalis, Liriodendron tulipifera, Ostrya virginiana*, and *Juglans nigra*, to become more prevalent, especially in upland areas along floodplains. Periodic drought, intensified by local conditions, such as slope, southern exposure, or sandy soil, also inhibit growth of mesophytic trees. Extensive conversion for agriculture has fragmented this system. Continued fire suppression has also resulted in succession to mesic hardwoods, such that in many locations, no oak species are regenerating. Remaining large areas of this system are likely under considerable pressure due to conversion to agriculture, pastureland, and urban development.

**Comments:** This is the predominant oak system for the upper Midwest, and is more extensive on the landscape than the related North‑Central Interior Dry Oak Forest and Woodland (CES202.047).

DISTRIBUTION

**Range:** This system is found throughout the glaciated regions of the Midwest south of the tension zone.

**Divisions:** 202:C, 205:C

**TNC Ecoregions:** 35:C, 36:C, 44:?, 45:C, 46:C, 47:?, 48:C

**Nations:** US

**Subnations:** IA, IL, IN, KS, MI, MN, MO, ND, NE, OH, SD, WI

**Map Zones:** 31:C, 38:C, 39:C, 40:C, 41:?, 42:C, 43:C, 44:C, 47:P, 49:C, 50:C, 51:C, 52:C

**USFS Ecomap Regions:** 222H:CC, 222Ja:CCC, 222Jb:CCC, 222Jc:CCC, 222Je:CCC, 222Jg:CCC, 222Jh:CCC, 222Ji:CCC, 222K:CC, 222L:CC, 222M:CC, 222Ua:CCC, 222Ue:CCC, 223G:CC, 251B:CC, 251C:CC, 251D:CC, 251H:CC

CONCEPT

**Environment:** This system can occur on uplands within the prairie matrix and near floodplains, or on rolling glacial moraines and kettle‑kame topography. Soils are typically well‑drained Mollisols or Alfisols that range from loamy to sandy loam or even coarse sands in texture. Historically, this type was quite extensive in Michigan, Indiana, Illinois, Missouri, Iowa, Wisconsin, and Minnesota. Well over 700,000 hectares likely occurred in southern Michigan alone (ca. 1800). It is distinguished from other forested systems within the region by a dry‑mesic edaphic condition that is transitional between dry oak forests and woodlands and mesic hardwood forests, such as maple‑basswood forests.

**Vegetation:** Forest cover can range from a dense to moderately open canopy and there is commonly a dense shrub layer. Fire‑resistant oak species, in particular *Quercus macrocarpa, Quercus rubra*, and/or *Quercus alba*, dominate the overstory. *Carya* spp., including *Carya ovata, Carya cordiformis*, and *Carya tomentosa*, are diagnostic in portions of the range of this system. Depending on site location and overstory canopy density, the understory may include species such as *Corylus americana, Amelanchier* spp., *Maianthemum stellatum, Caulophyllum thalictroides, Laportea canadensis, Trillium grandiflorum, Aralia nudicaulis*, and *Urtica dioica*. Occasionally, prairie grasses such as *Andropogon gerardii* and *Panicum virgatum* may be present. Fire suppression likely has allowed for other associates, such as *Acer saccharum, Celtis occidentalis, Liriodendron tulipifera, Ostrya virginiana*, and *Juglans nigra*, to become more prevalent, especially in upland areas along floodplains.

**Dynamics:** Fire constitutes the main natural process for this type and frequent surface fires combined with uncommon crown fires maintained a more open canopy and subcanopy structure to allow oak regeneration. Historic fire frequency was highest in the prairie‑forest border areas and declined further from prairies and behind natural firebreaks. Frequency of fires necessary to maintain this system varied, largely depending on soil fertility and drainage, with more fertile and mesic sites requiring more frequent fires. Fire‑return intervals of 15‑25 years would typically maintain a woodland, while fire‑return intervals of 25‑50 years would typically maintain a closed‑canopy oak forest (Landfire 2007a). Fire suppression accounts for many of the more closed oak forest examples of this system with the more mesic understory (Abrams 1992, Lorimer 2001). Fire suppression has allowed for other associates, such as *Acer saccharum, Celtis occidentalis, Juglans nigra, Liriodendron tulipifera, Ostrya virginiana*, and invasive shrubs, to become more prevalent, especially in more mesic upland areas or along floodplains (Rogers et al. 2008). Periodic drought, intensified by local conditions like slope, southern exposure, or sandy soil, also inhibit growth of mesophytic trees. Some stands currently in this system were more open savanna stands but fire suppression has allowed them to succeed to the more close‑canopy oak woodland or forest. A continued lack of fire in many of those stands will result in succession to more mesophytic forest types. Gap‑phase dynamics producing multi‑structured, uneven‑aged stands operate most noticeably in North‑Central Interior Beech‑Maple Forest (CES202.693) but also influence succession in this system. Canopy gap formation originates through localized stem breakage resulting from wind (Runkle 1982), glaze or ice storms (Lemon 1961), attack by oak wilt fungus (*Chalara quercina*), and episodic defoliation caused by insects such as gypsy moth (*Lymantria dispar*).

SOURCES

**References:** Abrams 1986, Abrams 1992, Archambault et al. 1989, Archambault et al. 1990, Comer and Albert 1997, Comer et al. 1995a, Comer et al. 2003*, Davidson et al. 2001, Eyre 1980, Healy 1997, Kost et al. 2007, LANDFIRE 2007a, Lemon 1961, Lorimer 2001, MNNHP 1993, Nelson 2010, Nowacki and Abrams 2008, ONHD unpubl. data, Rogers et al. 2008, Rolfsmeier and Steinauer 2010, Rooney 2001, Runkle 1982, Stroke and Anderson 1992, WDNR 2015

**Version:** 14 Jan 2014	**Stakeholders:** Midwest, Southeast

**Concept Author:** P. Comer, K. Kindscher, S. Menard, D. Faber‑Langendoen	**LeadResp:** Midwest

CES202.698 North‑Central Interior Oak Savanna

**Primary Division:** Central Interior and Appalachian (202)

**Land Cover Class:** Steppe/Savanna

**Spatial Scale & Pattern:** Matrix

**Required Classifiers:** Natural/Semi‑natural; Vegetated (>10% vasc.); Upland

**National Mapping Codes:** EVT 2394; ESLF 5410; ESP 1394

**Concept Summary:** This system is found primarily in the northern glaciated regions of the Midwest with the largest concentration in the prairie‑forest border ecoregion. It is typically found on rolling outwash plains, hills and ridges. Soils are typically moderately well‑ to well‑drained deep loams. This system is typified by scattered trees over a continual understory of prairie and woodland grasses and forbs. *Quercus macrocarpa* is the most common tree species and can range from 10‑60% cover. The understory is dominated by tallgrass prairie species such as *Andropogon gerardii* and *Schizachyrium scoparium* associated with several forb species. Historically, frequent fires maintained this savanna system within its range and would have restricted tree canopies to 10‑30%. Fire suppression in the region has allowed trees to establish more dense canopies. Periodic, strong wind disturbances and browsing also impact this system. Much of this system has also been converted to urban use or agriculture, and thus its range has decreased considerably.

DISTRIBUTION

**Range:** This system is found throughout the northern glaciated regions of the Midwest. Its main concentration, where it was likely the matrix type, is within the Prairie Forest Border of Minnesota, Wisconsin, Iowa, and Illinois. Conversion to urban uses and agriculture and fire suppression have significantly impacted the range of this system.

**Divisions:** 201:?, 202:C, 205:C

**TNC Ecoregions:** 35:C, 36:C, 45:P, 46:C, 47:P

**Nations:** US

**Subnations:** IA, IL, IN, MI, MN, MO, OH, WI

**Map Zones:** 39:P, 40:C, 41:C, 42:C, 43:P, 44:P, 49:C, 50:C, 51:P, 52:C

**USFS Ecomap Regions:** 212K:CP, 212Q:CP, 222Jb:CCP, 222Jc:CCC, 222Je:CCC, 222Jf:CCC, 222Jg:CCC, 222Jh:CCC, 222Ji:CCC, 222K:CC, 222L:CC, 222M:CC, 222N:CC, 222Ua:CCC, 222Ud:CCC, 222Ue:CCC, 251A:CC, 251B:CC

CONCEPT

**Environment:** This system is typically found on rolling tillplains, hills, and ridges in the glaciated Midwest. Soils are typically moderately well‑ to well‑drained deep loams and fertile. Because fire is critical to maintaining this system, it is not found in fire‑protected portions of the landscape.

**Vegetation:** *Quercus macrocarpa* is the most common tree species and can range from 10‑60% cover. The understory is dominated by tallgrass prairie species such as *Andropogon gerardii, Calamagrostis canadensis*, and *Schizachyrium scoparium* associated with several forb species.

**Dynamics:** Historically, frequent fires maintained this savanna system within its range and would have restricted tree canopies to 10‑30% cover with some portions having up to 60% tree canopy. On average, surface fires were very frequent (1‑ to 5‑year return intervals) and maintained the open, herbaceous understory. Canopy trees were replaced when periodic longer fire‑return intervals, due to chance, multi‑year wet climatic cycles, or lack of burning by Native Americans, allowed oak seedlings to grow large enough to survive surface fires when they returned. If fire is absent for more than about 20‑40 years, a site will transition to oak woodland/forest (Cottam 1949, Curtis 1959, Grimm 1981). Fire suppression in the region has allowed trees to establish more consistent dense canopies. Periodic, strong wind disturbances and browsing/grazing also impact this system through modification to the herbaceous layer and tree seedlings.

SOURCES

**References:** Abella et al. 2001, Albert 1995b, Anderson and Bowles 1999, Apfelbaum and Haney 1991, Bowles and McBride 1994, Cohen 2004, Comer et al. 1995a, Comer et al. 1998, Comer et al. 2003*, Cottam 1949, Curtis 1959, Edinger et al. 2014a, Grimm 1981, Kost et al. 2007, LANDFIRE 2007a, Nelson 2010, Nuzzo 1986, ONHD unpubl. data, WDNR 2015

**Version:** 14 Jan 2014	**Stakeholders:** Midwest, Southeast

**Concept Author:** S. Menard	**LeadResp:** Midwest

CES202.727 North‑Central Oak Barrens

**Primary Division:** Central Interior and Appalachian (202)

**Land Cover Class:** Steppe/Savanna

**Spatial Scale & Pattern:** Large patch

**Required Classifiers:** Natural/Semi‑natural; Vegetated (>10% vasc.); Upland

**Diagnostic Classifiers:** Woody‑Herbaceous

**National Mapping Codes:** EVT 2395; ESLF 5411; ESP 1395

**Concept Summary:** This system occurs on well‑drained, coarse‑textured sandy soils derived from glacial outwash, end moraine formations, lakeplain dune systems, and broad sandy river terraces in the north‑central U.S. into Ontario, Canada. Soils range from almost pure sand, to loamy sand, to sandy loam. The soils have low fertility, organic matter, and moisture‑retention capacity. Factors which affect seasonal soil moisture are strongly related to variation in this type. This oak barrens system is a scrubby, open‑treed system dominated by graminoids and shrubs. Canopy structure varies from a dominant herbaceous ground layer with sparse, scattered "savanna" canopy (5‑30%), through oak‑dominated scrub, to a more closed woodland canopy (30‑80%). The canopy layer is dominated by *Quercus velutina*, with some *Quercus ellipsoidalis, Quercus macrocarpa*, and *Quercus alba* (the latter more common eastward and in woodland conditions). Occasional *Pinus banksiana* can occur in the northern parts of the range. Species found in the herb layer include *Ambrosia psilostachya, Amphicarpaea bracteata, Artemisia ludoviciana, Andropogon gerardii, Calamovilfa longifolia, Carex pensylvanica, Carex* spp., *Comandra umbellata, Dichanthelium* spp., *Hesperostipa spartea, Koeleria macrantha, Lupinus perennis, Schizachyrium scoparium, Sorghastrum nutans*, and *Tephrosia virginiana*. Fire was an important factor in maintaining this system. Oak wilt, droughts and, in some northern sites, frosts during the growing season also reduce tree cover.

**Comments:** Black oak woodland variants may occur in this system, but because *Quercus velutina* and *Quercus ellipsoidalis* can sprout after stems have been killed by fires, stands generally have a somewhat scrubby structure that can vary from 10‑60% cover over time. Some stands may occur on fairly mesic sands. In New England and (most of) New York, similar settings are occupied by pitch pine ‑ oak barrens (Northeastern Interior Pine Barrens (CES202.590)) which are characterized by *Quercus ilicifolia*, not *Quercus ellipsoidalis*.

DISTRIBUTION

**Range:** This system is found in the north‑central U.S. from North Dakota to western New York and westernmost Pennsylvania (mostly historic there) and into Ontario, Canada.

**Divisions:** 202:C

**TNC Ecoregions:** 35:C, 36:C, 45:C, 46:C, 47:C, 48:C, 49:C

**Nations:** CA, US

**Subnations:** IL, IN, MI, MN, ND, NY, OH, ON, PA, WI

**Map Zones:** 39:C, 40:C, 41:C, 42:C, 43:C, 49:C, 50:C, 51:C, 52:C, 63:C

**USFS Ecomap Regions:** 212Ha:CCP, 212Hb:CCC, 222I:CC, 222Ja:CCC, 222Jb:CCC, 222Jc:CCC, 222Je:CCC, 222Jf:CCC, 222Jg:CCC, 222Jh:CCC, 222Ji:CCP, 222K:CC, 222L:CC, 222M:CP, 222R:CC, 222Ua:CCC, 222Ud:CCC, 222Ue:CCC

CONCEPT

**Environment:** This system occurs on well‑drained, coarse‑textured sandy soils derived from glacial outwash, end moraine formations, lakeplain dune systems, broad sandy river terraces, and sometimes on colluvium below sandstone bluffs. Soils range from almost pure sand, to loamy sand, to sandy loam. The soils have low fertility, organic matter, and moisture‑retention capacity. Factors which affect seasonal soil moisture are strongly related to variation in this type.

**Vegetation:** This oak barrens system is a scrubby, open‑treed system dominated by graminoids and shrubs. Canopy structure varies from a dominant herbaceous ground layer with sparse, scattered "savanna" canopy (5‑30%), through oak‑dominated scrub, to a more closed woodland canopy (30‑80%). The canopy layer is dominated by *Quercus velutina*, with some *Quercus ellipsoidalis, Quercus macrocarpa*, and *Quercus alba* (the latter more common eastward and in woodland conditions). Occasional *Pinus banksiana* can occur in the northern parts of the range. Species found in the herb layer include *Ambrosia psilostachya, Amphicarpaea bracteata, Artemisia ludoviciana, Andropogon gerardii, Calamovilfa longifolia, Carex pensylvanica, Carex* spp., *Comandra umbellata, Sorghastrum nutans, Hesperostipa spartea (= Stipa spartea)*, and *Schizachyrium scoparium*.

**Dynamics:** Fire was an important factor in maintaining this system. Oak wilt and droughts also reduce tree cover. For more fertile sites, surface fires were very frequent (1‑ to 5‑year return intervals) and important for maintaining the open canopy and herbaceous understory. This system was not as fire‑dependent as more mesic savannas and woodlands, due to the relatively infertile and often droughty soils on which it occurred. Some examples retained an open canopy without frequent fires (Whitford and Whitford 1971). Canopy trees were replaced when periodic longer fire‑return intervals, due to chance, multi‑year wet climatic cycles, or lack of burning by Native Americans, allowed oak seedlings to grow large enough to survive surface fires when they returned. If fire is absent for more than about 20‑40 years, a site will transition to oak woodland/forest (Curtis 1959).

SOURCES

**References:** Chapman et al. 1994, Comer and Albert 1997, Comer et al. 1995a, Comer et al. 1998, Comer et al. 2003*, Curtis 1959, Eyre 1980, Kost et al. 2007, LANDFIRE 2007a, Nuzzo 1986, ONHD unpubl. data, WDNR 2015, Whitford and Whitford 1971

**Version:** 14 Jan 2014	**Stakeholders:** Canada, East, Midwest

**Concept Author:** D. Faber‑Langendoen	**LeadResp:** Midwest

M014. Laurentian‑Acadian Mesic Hardwood ‑ Conifer Forest

CES201.565 Acadian Low‑Elevation Spruce‑Fir‑Hardwood Forest

**Primary Division:** Laurentian‑Acadian (201)

**Land Cover Class:** Forest and Woodland

**Spatial Scale & Pattern:** Matrix, Large patch

**Required Classifiers:** Natural/Semi‑natural; Vegetated (>10% vasc.); Upland

**Diagnostic Classifiers:** Lowland; Forest and Woodland (Treed); Mesotrophic Soil; Oligotrophic Soil; Picea (glauca, mariana, rubens) ‑ Abies

**National Mapping Codes:** EVT 2373; ESLF 4316; ESP 1373

**Concept Summary:** This system represents the Acadian and northern Appalachian red spruce‑fir forest that extends to the southern boreal region of southeastern Canada. The low‑ to mid‑elevation forests are dominated by *Picea rubens* and *Abies balsamea*. *Picea mariana* and *Picea glauca* may be present. *Betula alleghaniensis* is the most common codominant, and *Acer rubrum, Acer saccharum*, and *Fagus grandifolia* are sometimes present. The upland soils are acidic and usually rocky, mostly well‑ to moderately well‑drained but with some somewhat poorly drained patches at the slope bottoms. This is the matrix forest type in the lower‑elevation northern portions of this division. This system may include earlier successional patches in which *Populus* spp. and *Betula* spp. are dominant or mixed with *Picea* and *Abies* that will develop into spruce‑fir forests. Blowdowns with subsequent gap regeneration are the most frequent form of natural disturbance, with large‑scale fires important at longer return intervals.

**Comments:** Differences between this and the seasonally flooded Acadian Sub‑boreal Spruce Flat (CES201.562) are not well‑defined; data from Canada may help to resolve this.

DISTRIBUTION

**Range:** This system is found in northern New England, northern New York and adjacent Canada and is occasional southwards.

**Divisions:** 201:C, 202:C

**TNC Ecoregions:** 60:C, 61:C, 63:C

**Nations:** CA, US

**Subnations:** ME, NB, NH, NY, PA, QC?, VT

**Map Zones:** 64:C, 65:C, 66:C

**USFS Ecomap Regions:** 211A:CC, 211B:CC, 211C:CC, 211D:CC, 211F:CC, 211I:CC, 221Ak:CCC, 221Al:CCC, M211B:CC, M211C:CC, M211D:CC

CONCEPT

SOURCES

**References:** Comer et al. 2003*, Edinger et al. 2014a, Eyre 1980, Gawler and Cutko 2010, Lorimer 1977, Sperduto and Nichols 2004

**Version:** 20 Aug 2007	**Stakeholders:** Canada, East, Midwest

**Concept Author:** S.C. Gawler	**LeadResp:** East

CES201.566 Acadian‑Appalachian Montane Spruce‑Fir Forest

**Primary Division:** Laurentian‑Acadian (201)

**Land Cover Class:** Forest and Woodland

**Spatial Scale & Pattern:** Large patch

**Required Classifiers:** Natural/Semi‑natural; Vegetated (>10% vasc.); Upland

**Diagnostic Classifiers:** Montane; Forest and Woodland (Treed); Ridge/Summit/Upper Slope; Picea (glauca, mariana, rubens) ‑ Abies

**National Mapping Codes:** EVT 2374; ESLF 4317; ESP 1374

**Concept Summary:** This is the matrix forest system in the montane spruce‑fir region of the northern Appalachian Mountains, extending east through the Canadian Maritimes. It occurs mostly upwards of 457 m (1500 feet) elevation and is restricted to progressively higher elevations southward. Northward, it is often contiguous with Acadian Low‑Elevation Spruce‑Fir‑Hardwood Forest (CES201.565). This system often forms a mosaic of strongly coniferous patches and mixed patches, with occasional smaller inclusions of northern hardwoods, but is overall more than 50% coniferous. *Picea rubens* and *Abies balsamea* are the dominant conifers. Gaps formed by wind, snow, ice, and harvesting are the major replacement agents; fires may be important but only over a long return interval.

**Comments:** This system can occupy an intermediate elevation position between Acadian Low‑Elevation Spruce‑Fir‑Hardwood Forest (CES201.565) and Acadian‑Appalachian Subalpine Woodland and Heath‑Krummholz (CES201.568), and it could arguably be combined with one of those, probably the former. However, in the southern part of its range, it often occurs without either of these other systems. It is distinguished, in concept, from Acadian Low‑Elevation Spruce‑Fir‑Hardwood Forest (CES201.565) by the presence or greater abundance of montane species such as *Sorbus americana* or *Sorbus decora, Dryopteris campyloptera, Oxalis montana*, etc., and by occurring at higher positions in the toposequence. It is generally above northern hardwood forests, while Acadian Low‑Elevation Spruce‑Fir‑Hardwood Forest (CES201.565) is generally below (or at similar elevations to) northern hardwood forests. More careful review is needed to determine if it should remain a separate system.

DISTRIBUTION

**Range:** This system is found at higher elevations of northern New England and the Adirondacks, extending north along the mountains and higher hills into Canada and occurring southward in the Catskills.

**Divisions:** 201:C, 202:C

**TNC Ecoregions:** 60:C, 61:C, 63:C

**Nations:** US

**Subnations:** MA, ME, NH, NY, VT

**Map Zones:** 64:C, 65:C, 66:C

**USFS Ecomap Regions:** 211Ia:CCC, 221A:CC, M211A:CC, M211B:CC, M211C:CC, M211Df:CCC

CONCEPT

SOURCES

**References:** Comer et al. 2003*, Edinger et al. 2014a, Eyre 1980, Gawler and Cutko 2010, Sperduto and Nichols 2004

**Version:** 20 Aug 2007	**Stakeholders:** East

**Concept Author:** S.C. Gawler	**LeadResp:** East

CES202.028 Central and Southern Appalachian Spruce‑Fir Forest

**Primary Division:** Central Interior and Appalachian (202)

**Land Cover Class:** Forest and Woodland

**Spatial Scale & Pattern:** Large patch

**Required Classifiers:** Natural/Semi‑natural; Vegetated (>10% vasc.); Upland

**Diagnostic Classifiers:** Montane; Forest and Woodland (Treed); Needle‑Leaved Tree

**National Mapping Codes:** EVT 2350; ESLF 4253; ESP 1350

**Concept Summary:** This system consists of forests in the highest elevation zone of the Blue Ridge and parts of the Central Appalachians, generally dominated by *Picea rubens, Abies fraseri*, or by a mixture of spruce and fir. *Abies fraseri* is the constituent fir from Mount Rogers in Virginia southward. Examples occur above 1676 m (5500 feet) in the Southern Blue Ridge, but as low as 975 m (3200 feet) at the northern range in West Virginia, and may range up to the highest peaks. Elevation and orographic effects make the climate cool and wet, with heavy moisture input from fog as well as high rainfall. Strong winds, extreme cold, rime ice, and other extreme weather are periodically important.

**Comments:** The border of this system with adjacent systems is often gradational. The non‑forested systems that occur in the same elevational zone may have transition zones of open woody vegetation, though some have sharp borders. The transition to Southern Appalachian Northern Hardwood Forest (CES202.029) or other systems that adjoin at lower elevations is marked by a gradual shift in canopy dominance from conifers to hardwoods. In relatively undisturbed stands, the canopy composition and structure are the best way to determine the boundary of this system.

This system is similar to the spruce‑fir systems of the northern Appalachians and the boreal forests but differs in having less frequent natural fire, having southern seasonal dynamics (shorter winters, less extreme cold temperatures, lack of long summer days), lacking a history of glaciation, and in a flora and fauna that has southern Appalachian endemics and lacks some characteristic northern species. High‑elevation spruce‑fir in West Virginia is placed in this system because its location well below the glacial boundary and presence of species of more southern affinity (e.g., *Rhododendron maximum* and *Vaccinium erythrocarpum*) differentiate it from the northern Appalachian system.

DISTRIBUTION

**Range:** This system ranges from the Balsam Mountains and Great Smoky Mountains of North Carolina and Tennessee northward to the mountains of western Virginia and eastern West Virginia.

**Divisions:** 202:C

**TNC Ecoregions:** 51:C, 59:C

**Nations:** US

**Subnations:** NC, TN, VA, WV

**Map Zones:** 57:C, 61:C

**USFS Ecomap Regions:** M221A:CC, M221B:CC, M221C:CC, M221D:CC

CONCEPT

**Environment:** This system occurs at elevations typically above about 1300 m (4300 feet), up to the highest peaks. Species distribution follows an elevational gradient, with *Picea rubens*‑dominated stands occurring between 1370 and 1675 m, mixed stands between 1675 and 1890 m, and *Abies fraseri* stands above 1890 m (Whittaker 1956 cited in Nicholas and Zedaker 1989). Examples occur on most of the landforms that are present in this elevational range; most sites are strongly exposed and convex in shape. Elevation and orographic effects make the climate cool and wet, with heavy moisture input from fog as well as high rainfall. Strong winds, extreme cold, rime ice, and other extreme weather are periodically important factors in the structure and dynamics of this vegetation. Concentration of air pollutants has been implicated as an important anthropogenic stress in recent years. In recent decades, the balsam woolly adelgid (*Adelges piceae*), an introduced insect, has killed almost all of the mature *Abies fraseri*. The saplings are not susceptible, resulting in many dense stands of young trees. Soils are generally very rocky, with the matrix ranging from well‑weathered parent material to organic deposits over boulders. Soils may be saturated for long periods from a combination of precipitation and seepage. Any kind of bedrock may be present, but most sites have erosion‑resistant felsic igneous or metamorphic rocks (White et al. 1993).

**Vegetation:** Vegetation consists primarily of forests dominated by *Picea rubens, Abies fraseri*, or occasionally by *Sorbus americana*. *Betula alleghaniensis, Tsuga canadensis*, and *Quercus rubra* are the only other locally common canopy species. *Acer rubrum, Betula lenta, Magnolia acuminata*, and *Magnolia fraseri* may occur. Lower strata are most typically dominated by mosses, ferns or forbs, but a few associations have dense shrub layers of *Rhododendron catawbiense, Rhododendron maximum*, or *Vaccinium erythrocarpum*.

**Dynamics:** This system is naturally dominated by stable, uneven‑aged forests, with canopy dynamics dominated by gap‑phase regeneration on a fine scale, as well as larger disturbances resulting primarily from ice storms (Nicholas and Zedaker 1989). Despite the extreme climate, *Picea rubens* is long‑lived (300‑400 or more years) (White et al. 1993). Both *Picea* and *Abies* seedlings are shade‑tolerant, and advanced regeneration is important in stand dynamics. Natural disturbances are primarily wind and ice storms, but may include debris avalanches or very rarely lightning fires (White 1984b, Nicholas and Zedaker 1989, White et al. 1993). Occasional extreme wind events disturb larger patches on the most exposed slopes. Fire is a very rare event under natural conditions, due to the wetness and limited flammability of the undergrowth (Korstian 1937 cited in White et al. 1993), and return intervals have been estimated between 500 and 1000 years or more. If fires do occur, they are likely to be catastrophic, because few of the species are at all fire‑tolerant. Anthropogenic fires fueled by logging slash were extremely destructive, turning large expanses of this system into grass‑shrub‑hardwood scrub (e.g., Dolly Sods, Graveyard Fields) that has not recovered to conifer dominance after 100 years.

<br />Estimates of the loss in extent of the Southern Appalachian spruce‑fir forest range from 50% (White 1984c) to 90% (Korstian 1937 cited in Nicholas and Zedaker 1989). The primary disturbances are weather‑related, including ice storms and windthrow, occurring at intervals of 100 to 200 years. There have been multiple events of wind and ice damage in single‑ and multiple‑tree patches that have cumulatively damaged a lot of the canopy in spruce forests (M. Schafale pers. comm. 2013). Rare extreme weather events are also important large‑scale disturbances. In contrast to northern stands of *Picea‑Abies* vegetation, insect outbreaks are not important disturbances (M. Schafale pers. comm. 2013). Windthrow produces dense *Abies* seedlings if overstory is mature (Eyre 1980). In general, fire is extremely rare in Southern Appalachian *Picea‑Abies* vegetation, and fire is not a primary factor in its successional dynamics.

SOURCES

**References:** Comer et al. 2003*, Eyre 1980, Korstian 1937, Lohman and Watson 1943, Nicholas and Zedaker 1989, Pyle and Schafale 1985, Schafale 2012, Schafale pers. comm., TDNH unpubl. data 2018, USFS 1973, USFS 1997, White 1984b, White and Cogbill 1992, White et al. 1993, Whittaker 1956

**Version:** 14 Jan 2014	**Stakeholders:** East, Southeast

**Concept Author:** M. Schafale and R. Evans	**LeadResp:** Southeast

CES201.564 Laurentian‑Acadian Northern Hardwood Forest

**Primary Division:** Laurentian‑Acadian (201)

**Land Cover Class:** Forest and Woodland

**Spatial Scale & Pattern:** Matrix

**Required Classifiers:** Natural/Semi‑natural; Vegetated (>10% vasc.); Upland

**Diagnostic Classifiers:** Montane; Forest and Woodland (Treed); Eutrophic Soil; Mesotrophic Soil; Broad‑Leaved Tree; Acer saccharum ‑ Betula spp.

**National Mapping Codes:** EVT 2302; ESLF 4108; ESP 1302

**Concept Summary:** These northern hardwood forests range across New England and adjacent Canada, south to New York and possibly northern Pennsylvania and west to Minnesota. They occur in various dry‑mesic to wet‑mesic settings at low to moderate elevations, generally less than 610 m (2000 feet), throughout the Laurentian‑Acadian Division. *Acer saccharum, Betula alleghaniensis*, and *Fagus grandifolia* are the dominant trees (the latter only east of northern Wisconsin). *Tsuga canadensis* or, in the Northeast, *Picea rubens* are common minor canopy associates. *Ostrya virginiana* is frequent but not dominant. Oak is a minor component and absent from northern regions. Successional stands may be dominated by *Populus tremuloides, Betula papyrifera, Acer rubrum, Fraxinus americana, Prunus serotina*, sometimes with scattered *Pinus strobus*. Soils range from moderately nutrient‑poor to quite enriched, with associated shifts in the herb flora. This system can include large expanses of rich forest in areas of limestone or similar bedrock, as well as forests that are relatively poor floristically in areas of granitic (or similar) bedrock or acidic till. Blowdowns or snow and ice loading, with subsequent gap regeneration, are the most frequent form of natural disturbance.

**Comments:** An east‑west separation between the Laurentian and Acadian regions was considered, but the hardwoods component is essentially similar (though beech drops out in the most western part of this system). It appears to be more of a gradient, with beech and hobblebush dropping out and fire frequency probably a little greater in the western portion. A possible split at Lake Michigan could be considered if one could make a better case than just beech. Hemlock‑hardwood inclusions in the East may be part of this system where the matrix and surroundings are predominantly hardwood, but where hemlock and pine are prevalent, as in ravines or cool slopes, Laurentian‑Acadian Pine‑Hemlock‑Hardwood Forest (CES201.563) is the appropriate system. A clearer set of floristic and ecological criteria is needed to sort out the southern limits of this Laurentian‑Acadian type in relation to related vegetation to the south. For example, currently, we do not report this system in 211F and 211G, the High Allegheny Plateau [see instead Appalachian (Hemlock) ‑Northern Hardwood Forest (CES202.593)], but stands in cooler microclimates of these regions may have a strong resemblance to Laurentian‑Acadian types.

DISTRIBUTION

**Range:** This system occurs in northern New England and northern New York west across the upper Great Lakes to northern Minnesota, and adjacent Canada; occasional southwards.

**Divisions:** 201:C, 202:C

**TNC Ecoregions:** 47:C, 48:C, 60:C, 61:C, 63:C, 64:C

**Nations:** CA, US

**Subnations:** MA, ME, MI, MN, NB, NH, NS, NY, ON, PA?, QC, VT, WI

**Map Zones:** 41:C, 50:C, 51:C, 63:C, 64:C, 65:C, 66:C

**USFS Ecomap Regions:** 211A:CC, 211B:CC, 211C:CC, 211D:CC, 211E:CC, 211I:CC, 211J:CC, 212Ha:CCC, 212Hb:CCC, 212Hc:CCC, 212Hd:CCC, 212He:CCC, 212Hf:CCC, 212Hg:CCC, 212Hh:CCC, 212Hi:CCC, 212Hj:CCC, 212Hk:CCC, 212Hl:CCC, 212Hm:CCC, 212J:CC, 212K:CC, 212L:CC, 212M:CC, 212N:CC, 212Q:CC, 212Ra:CCC, 212Rb:CCC, 212Rc:CCC, 212Rd:CCC, 212Re:CCC, 212S:CC, 212T:CC, 212X:CC, 212Y:CC, 212Z:CC, 222I:CC, 222Ja:CCC, 222Jf:CCC, 222Ud:CCC, 222Ue:CCC, M211A:CC, M211B:CC, M211C:CC, M211D:CC

CONCEPT

SOURCES

**References:** Comer and Albert 1997, Comer et al. 1995a, Comer et al. 1998, Comer et al. 2003*, Edinger et al. 2014a, Eyre 1980, Gawler and Cutko 2010, Kost et al. 2007, Sperduto and Nichols 2004, WDNR 2015

**Version:** 04 Feb 2009	**Stakeholders:** Canada, East, Midwest

**Concept Author:** S.C. Gawler	**LeadResp:** East

CES201.563 Laurentian‑Acadian Pine‑Hemlock‑Hardwood Forest

**Primary Division:** Laurentian‑Acadian (201)

**Land Cover Class:** Forest and Woodland

**Spatial Scale & Pattern:** Matrix

**Required Classifiers:** Natural/Semi‑natural; Vegetated (>10% vasc.); Upland

**Diagnostic Classifiers:** Lowland; Forest and Woodland (Treed); Pinus spp. ‑ Tsuga canadensis

**National Mapping Codes:** EVT 2366; ESLF 4308; ESP 1366

**Concept Summary:** This north‑temperate forest system ranges from the northeastern U.S. and adjacent Canada west to the Great Lakes and upper Midwest. The mesic to dry‑mesic forests usually occur on low‑nutrient soils at low elevations, mostly less than 610 m (2000 feet). Canopy dominants include *Pinus strobus, Tsuga canadensis*, and *Quercus rubra* in varying percentages. *Acer rubrum* is also quite common; *Betula lenta* may be common at the southern periphery of this system's range. *Quercus velutina* and *Quercus alba* are essentially absent from this system, being more representative of systems in the Central Interior‑Appalachian Division to the south. This is a widespread, matrix forest type for the more temperate portions of this division. Gap replacement and infrequent fire are the major natural regeneration modes.

**Comments:** *Tsuga canadensis* is useful to separate this system from Laurentian‑Acadian Northern Pine‑(Oak) Forest (CES201.719), but does not always occur in this system. Hemlock draws in USFS Section 222L (Baraboo) could be considered as remnants of this system rather than an inclusion in the hardwood matrix, as they are very distinctive from the surrounding forest and have the northern representative flora. In the East, northern hardwoods other than beech, e.g., sugar maple, are rarely found in this system. This system and Appalachian (Hemlock)‑Northern Hardwood Forest (CES202.593) grade into one another in southern New York and northern Pennsylvania; the presence of *Liriodendron tulipifera* is diagnostic for the Division 202 Appalachian (Hemlock)‑Northern Hardwood Forest (CES202.593). A clearer set of floristic and ecological criteria is needed to sort out the southern limits of this Laurentian‑Acadian type in relation to related vegetation to the south. For example, currently, we do not report this system in 211F and 211G, the High Allegheny Plateau [see instead Appalachian (Hemlock)‑Northern Hardwood Forest (CES202.593)], but stands in cooler microclimates of these regions may have a strong resemblance to Laurentian‑Acadian types.

DISTRIBUTION

**Range:** New England west to the Great Lakes and northern Minnesota.

**Divisions:** 201:C

**TNC Ecoregions:** 47:C, 48:C, 60:C, 61:C, 63:C, 64:C

**Nations:** CA, US

**Subnations:** MA, ME, MI, MN, NB, NH, NS, NY, ON, PA?, QC, VT, WI

**Map Zones:** 41:C, 50:C, 51:C, 63:C, 64:C, 65:C, 66:C

**USFS Ecomap Regions:** 211A:CC, 211B:CC, 211C:CC, 211D:CC, 211E:CC, 211Ia:CCC, 211Jb:CCC, 211Jc:CCC, 212Ha:CCC, 212Hb:CCC, 212Hc:CCC, 212Hd:CCC, 212He:CCC, 212Hf:CCC, 212Hg:CCC, 212Hh:CCC, 212Hi:CCC, 212Hj:CCC, 212Hk:CCC, 212Hl:CCC, 212Hm:CCC, 212J:CC, 212K:CC, 212L:CC, 212M:CC, 212N:CC, 212Ra:CCC, 212Rb:CCC, 212Rc:CCC, 212Rd:CCC, 212Re:CCC, 212S:CC, 212T:CC, 212X:CC, 212Y:CC, 212Z:CC, 221Ah:CCC, 221Ai:CCC, 221Ak:CCC, 221Al:CCC, 222Ja:CCC, 222Jf:CCC, 222L:CC, 222Ud:CC?, 222Ue:CCC, M211A:CC, M211B:CC, M211C:CC, M211D:CC

CONCEPT

SOURCES

**References:** Comer and Albert 1997, Comer et al. 1995a, Comer et al. 1998, Comer et al. 2003*, Edinger et al. 2014a, Eyre 1980, Gawler and Cutko 2010, Kost et al. 2007, Sperduto and Nichols 2004, WDNR 2015, Whitney 1984

**Version:** 20 Aug 2007	**Stakeholders:** Canada, East, Midwest

**Concept Author:** S.C. Gawler	**LeadResp:** East

CES103.020 Laurentian‑Acadian Sub‑boreal Aspen‑Birch Forest

**Primary Division:** Boreal (103)

**Land Cover Class:** Forest and Woodland

**Spatial Scale & Pattern:** Matrix

**Required Classifiers:** Natural/Semi‑natural; Vegetated (>10% vasc.); Upland

**Diagnostic Classifiers:** Forest and Woodland (Treed); Boreal [Boreal Continental]; Intermediate Disturbance Interval

**National Mapping Codes:** EVT 2301; ESLF 4107; ESP 1301

**Concept Summary:** These early‑successional boreal hardwood forests and woodlands are widespread throughout the eastern subboreal region of Canada, extending into parts of the Laurentian‑Acadian region, but more localized eastward. They originate naturally after fires and blowdowns, but more commonly originate after logging of conifer or mixed conifer‑hardwood systems. *Populus tremuloides* and *Betula papyrifera* are the most important tree species. This system is maintained by repeated disturbance within 50‑year return intervals and would otherwise succeed to conifer systems. Localized stands of mixed conifer‑hardwoods (pines and spruces) can occur in this type, but are more typically part of conifer systems.

**Comments:** As defined here, these are deciduous forest‑dominated systems; mixed conifer‑hardwoods areas will go in the appropriate conifer forest system. In addition, this system is primarily eastern subboreal, because some northern hardwood species do occur; in the Laurentian‑Acadian region, successional aspen‑birch or red maple stands would be placed within the appropriate mature Laurentian‑Acadian forest system (e.g., aspen‑birch stands in Maine are placed within Acadian Low‑Elevation Spruce‑Fir‑Hardwood Forest (CES201.565). The perspective here is that *Picea rubens* (red spruce) is not a boreal species; stands of *Picea rubens* often contain many typical northern hardwood associates, rather than *Populus tremuloides* or *Betula papyrifera*. It is not clear how naturally this system occurs in the upper Midwest given catastrophic fires in the 1800s‑early 1900s; is the extensive aspen‑birch found in northern Minnesota this system or should those be considered part of northern hardwoods or spruce‑fir? A workable approach for now would be to restrict this system to northernmost Minnesota and southern Lake Superior [see USFS Ecomap Regions].

The original "Boreal Aspen‑Birch Forest" system was not well defined in Canada. As described it only covered the subboreal region of eastern Canada. It has been renamed to reflect that concept. This system is closely related to Laurentian‑Acadian Sub‑boreal Mesic Balsam Fir‑Spruce Forest (CES103.426), and within the IVC these are part of Laurentian Sub‑boreal Mesic Balsam Fir ‑ Spruce ‑ Hardwood Forest Group (G048). It may be that a series of aspen‑birch systems separate from the spruce‑fir systems are needed for Eastern Boreal, Central Subboreal, Central Boreal, Western Boreal and Western Subboreal regions of the North American Boreal Forest. Or, conversely, this system (CES103.020) could be lumped within CES103.426, and the distinction treated here and elsewhere at the alliance level, below system.

DISTRIBUTION

**Range:** This system is found in the hemi‑boreal region of the Upper Great Lakes and southeastern Canada from northwestern Ontario and northern Minnesota east to Quebec (and possibly northern portions of the Canadian Maritimes).

**Divisions:** 103:C, 201:C

**TNC Ecoregions:** 47:C, 48:C

**Nations:** CA, US

**Subnations:** LB, MI, MN, NB?, NF, ON, QC, WI

**Map Zones:** 41:C, 50:C, 51:C

**USFS Ecomap Regions:** 212Ha:CPP, 212Hf:CPP, 212Hl:CPP, 212J:CP, 212Lb:CCC, 212M:CC, 212R:CP, 212S:CP, 212T:CP, 212X:CP, 212Y:CP

CONCEPT

SOURCES

**References:** Brandt 2009, Comer et al. 2003*, Eyre 1980, Kost et al. 2007

**Version:** 15 Oct 2012	**Stakeholders:** Canada, East, Midwest

**Concept Author:** D. Faber‑Langendoen and S. Gawler	**LeadResp:** Midwest

CES103.426 Laurentian‑Acadian Sub‑boreal Mesic Balsam Fir‑Spruce Forest

**Primary Division:** Boreal (103)

**Land Cover Class:** Forest and Woodland

**Required Classifiers:** Natural/Semi‑natural; Vegetated (>10% vasc.); Upland

**Concept Summary:** This ecological system represents the mesic southern or subboreal eastern boreal forest, ranging from northwestern Ontario to eastern Canada's Atlantic provinces and extending into the U.S. in northeastern Minnesota, Isle Royale, and near‑coastal areas of Lake Superior shores in northern Wisconsin and Michigan. The low‑elevation forests are dominated by *Picea glauca* and *Abies balsamea*. *Picea mariana* is often present, along with occasional *Pinus banksiana*. Codominant boreal hardwoods include *Populus tremuloides* and *Betula papyrifera*. Northern hardwoods, such as *Acer saccharum* and *Tilia americana* are relatively minor. The shrub and herb layers are variable, decreasing as the percent conifer cover increases. Common shrub species include *Acer spicatum, Alnus viridis, Corylus cornuta, Diervilla lonicera*, and *Lonicera canadensis*. The moss layer ranges from discontinuous to continuous. These upland forests typically occur on loamy soils over bedrock in scoured bedrock uplands and loamy, rocky, or sandy soils on glacial moraines, till plains and outwash plains, and moisture conditions range from well‑drained to somewhat poorly drained. Wetter sites may contain *Alnus incana ssp. rugosa, Calamagrostis canadensis*, and *Equisetum* spp. This is the matrix forest type in many parts of its range. This group may include earlier‑successional patches, in which *Populus* spp. and *Betula* spp. are dominant or mixed with *Picea* and *Abies*, that will develop into spruce‑fir forests. Blowdown with subsequent gap regeneration is the most frequent form of natural disturbance, with large‑scale fires important at longer return intervals. Insect infestations, in particular by *Choristoneura fumiferana* (spruce budworm), also can impact this group.

**Comments:** The transition zone from the Boreal (including subboreal) Forest formation (where *Abies balsamea* is the dominant tree species) to the Cool Temperate Forest (where *Acer saccharum* is the dominant tree species) is difficult to untangle, but depends on the increasing abundance of northern hardwood tree species and more cool‑temperate shrubs and herbs. Where these species occur with the boreal conifers, they are placed in 1.B.2 Cool Temperate Forest Formation (F008). Forest associations typical of this transition zone are mixedwood associations dominated by *Betula alleghaniensis* or *Acer rubrum* and *Abies balsamea* or *Acer rubrum* and *Abies balsamea*. These associations are currently placed in Cool Temperate Forest under Laurentian‑Acadian Sub‑boreal Mesic Balsam Fir‑Spruce Forest (CES103.426). In Quebec (C. Morneau pers. comm. 2009), the most northerly cool temperate transition zone extends between 47° and 49°N latitude, including Bas‑St. Laurent and Gaspesie regions.

A separate aspen‑birch system is recognized in the eastern subboreal region, Laurentian‑Acadian Sub‑boreal Aspen‑Birch Forest (CES103.020), though these are lumped together at the group level (G048).

DISTRIBUTION

**Range:** This system ranges in Canada from northwestern Ontario (possibly eastern Manitoba) to eastern Canada's Atlantic provinces and extending into the U.S. in northeastern Minnesota, Isle Royale, and near‑coastal areas of Lake Superior shores in northern Wisconsin and Michigan. Its range westward is marked by a shift towards greater *Picea glauca* dominance and lower *Abies balsamea* dominance.

**Divisions:** 103:C

**TNC Ecoregions:** 47:C, 48:C, 63:?, 64:?

**Nations:** CA, US

**Subnations:** LB, MB?, MI, MN, NB?, NF, ON, QC, WI

CONCEPT

**Environment:** These upland forests typically occur on loamy soils over bedrock in scoured bedrock uplands and loamy, rocky, or sandy soils on glacial moraines, till plains and outwash plains (Minnesota DNR 2003). Moisture conditions range from well‑drained to somewhat poorly drained. Climate typically is characterized by cool, even temperatures, shorter growing season, and deep and sometimes severe winter snowfall. In the southern part of their range in the Great Lakes states, they occur along northern Great Lakes shorelines and on islands in Lake Superior. Cold temperate to boreal. Soils are typically neutral to acidic, shallow sandy, sandy‑loam, or loamy‑sand. Some examples occur on heavier, mesic silty or clay loams that are more alkaline in nature. Along Great Lakes shorelines, these soils overlay limestone or volcanic bedrock.

**Vegetation:** *Picea glauca* typically dominates on drier sites or is codominant with *Abies balsamea* on more mesic sites. In some mesic to wet‑mesic examples, *Abies balsamea* dominates. This group includes several successional stages, including earlier‑successional patches in which *Populus* spp. and *Betula* spp. are dominant. Mid‑successional stands often contain stands mixed with *Picea* and *Abies*, that will develop into spruce‑fir forests. The shrub and herb layers are variable, decreasing as the percent conifer cover increases. Common shrub species include *Acer spicatum, Corylus cornuta, Diervilla lonicera*, and *Lonicera canadensis*. The composition and density of the herbaceous layer can vary among associations and location. Typically, *Aralia nudicaulis, Eurybia macrophylla, Clintonia borealis*, and *Maianthemum canadense* are common understory species. The moss layer ranges from discontinuous to continuous. Wetter sites may contain *Alnus incana ssp. rugosa, Calamagrostis canadensis*, and *Equisetum* spp. Additional diagnostic shrub and herb species of this subboreal type will be added through further analyses.

**Dynamics:** These forests are affected by windthrow, insect defoliation, and infrequent fires. Forests closer to the Great Lakes shorelines occur on shallower soils and are more likely to experience more serious windthrow and snap‑off of larger trees. Mammalian herbivory also can impact forest stands. Selective herbivory by white‑tailed deer and moose (*Alces americanus*) can alter the composition and structure and favor browse‑tolerant species such as *Picea glauca*. These forests typically regenerate from gap‑phase dynamics.

SOURCES

**References:** Curtis 1959, Eyre 1980, Faber‑Langendoen et al. 2012*, Heinselman 1996, Kost et al. 2007, Minnesota DNR 2003, WDNR 2015, Wisconsin DNR 2009a

**Version:** 05 Sep 2012	**Stakeholders:** Canada, Midwest

**Concept Author:** Faber‑Langendoen, in Faber‑Langendoen et al. (2012)	**LeadResp:** Midwest

CES202.704 Paleozoic Plateau Bluff and Talus

**Primary Division:** (202)

**Concept Summary:** This system is found in the driftless regions of southeastern Minnesota, southwestern Wisconsin, and northern Iowa and Illinois. This region was not glaciated like the surrounding areas and thus is predominated by rolling hills and bluff outcrops. This system is found primarily on blufftops and dry upper slopes along the Upper Mississippi River, although it can range into bordering regions such as the Baraboo Hills in Wisconsin. This system contains a mosaic of woodlands, savannas, prairies and sparsely vegetated limestone, dolomite, and/or sandstone outcrops, with occasional talus, especially algific talus. Soils range from thin to moderately deep and are moderately to excessively well-drained with a high mineral content. Woodlands consist of primarily a mixture of oak species such as *Quercus macrocarpa, Quercus rubra, Quercus muehlenbergii*, and *Quercus alba*. *Acer saccharum, Betula alleghaniensis*, and conifer species such as *Pinus* spp. and *Tsuga canadensis* may occur on more mesic and protected areas within this system. Prairie openings (also called "goat prairies") contain *Schizachyrium scoparium* and *Bouteloua curtipendula* with scattered *Juniperus virginiana*. Historically, fire was the most important dynamic maintaining these systems, however, fire suppression within the region has allowed more canopy cover and thus very few prairie openings remain. Algific talus harbors a number of unusual Pleistocene relict species, including plants and snails.

DISTRIBUTION

**Range:** This system is found within the Paleozoic Plateau (aka Driftless Region) of southeastern Minnesota, southwestern Wisconsin and northern Iowa and Illinois.

**Nations:** US

**Subnations:** IA, IL, MN, WI

CONCEPT

**Environment:** This system is found on an unglaciated landscape that is predominated by rolling hills and bluff outcrops. This system is found primarily on blufftops and dry upper slopes along the Upper Mississippi River, although it can range into bordering regions such as the Baraboo Hills in Wisconsin. This system contains limestone, dolomite, and/or sandstone outcrops, with occasional talus, especially algific talus. Soils are primarily loess and range from thin to moderately deep and are moderately to excessively well-drained with a high mineral content.

**Vegetation:** Woodlands consist of primarily a mixture of oak species such as *Quercus macrocarpa, Quercus rubra, Quercus muehlenbergii*, and *Quercus alba*. *Acer saccharum, Betula alleghaniensis*, and conifer species such as *Pinus* spp. and *Tsuga canadensis* may occur on more mesic and protected areas within this system. Prairie openings (also called "goat prairies") contain *Schizachyrium scoparium* and *Bouteloua curtipendula* with scattered *Juniperus virginiana*. Historically, fire was the most important dynamic maintaining these systems, however, fire suppression within the region has allowed more canopy cover and thus very few prairie openings remain. Algific talus harbors a number of unusual Pleistocene relict species, including plants and snails.

**Dynamics:** This is a diverse system with different ecological processes necessary for different aspects. Fire is important for maintaining the prairie and dry oak aspects of the system, but the steep slope and thin soil reduce the suitability for many other species, so fire frequency does not need to be as high as in more fertile prairies and oak woodlands. The prolonged absence of fire will favor shrub and tree invasion of the prairie and an increase in mesophytic trees and shrubs in the oak forests and woodlands (Nowacki and Abrams 2008). The cooler, more mesic aspects of the system with significant conifers (*Pinus strobus, Pinus resinosa*, and *Tsuga canadensis*) occur in protected ravines or on steep slopes with little soil development, and fire is not important in establishing or maintaining these communities (McIntosh 1950, Kline and Cottam 1979). These communities occur where there are cooler summer soil and air temperatures and on this soiled sites over acidic bedrock (McIntosh 1950, Adams and Loucks 1971).

SOURCES

**References:** Comer et al. 2003*

**Version:** 02 Oct 2014	**Stakeholders:**

**Concept Author:** P. Comer

M159. Laurentian‑Acadian Pine ‑ Hardwood Forest & Woodland

CES103.075 Laurentian Jack Pine‑Red Pine Forest

**Primary Division:** Boreal (103)

**Land Cover Class:** Forest and Woodland

**Required Classifiers:** Natural/Semi‑natural; Vegetated (>10% vasc.); Upland

DISTRIBUTION

**Divisions:** 103:C

**Nations:** CA, US

**Subnations:** MI, ON, WI

CONCEPT

SOURCES

**References:** Comer et al. 2003*

**Stakeholders:** Canada, Midwest

**Concept Author:** P. Comer	**LeadResp:** Midwest

CES201.718 Laurentian Pine‑Oak Barrens

**Primary Division:** Laurentian‑Acadian (201)

**Land Cover Class:** Steppe/Savanna

**Spatial Scale & Pattern:** Matrix

**Required Classifiers:** Natural/Semi‑natural; Vegetated (>10% vasc.); Upland

**Diagnostic Classifiers:** Woody‑Herbaceous

**National Mapping Codes:** EVT 2407; ESLF 5423; ESP 1407

**Concept Summary:** These pine‑oak barrens occur in the northern and western Great Lakes region. They occur on sandplains/outwash habitats, with droughty, infertile sand or loamy sands and frequent fires (every 5‑30 years). *Pinus banksiana, Pinus resinosa, Quercus ellipsoidalis*, and *Pinus strobus* are common overstory dominants. Prairie species are common throughout much of the range of the type. Common shrub and ground cover species include *Andropogon gerardii, Carex pensylvanica, Corylus americana, Schizachyrium scoparium*, and *Vaccinium angustifolium*. Oak grubs may be common under frequent burning. Catastrophic burns may create open bracken grasslands.

**Comments:** his system covers the Great Lakes barrens. The eastern U.S. pine barrens fall into Northeastern Interior Pine Barrens (CES202.590) described under the Central Interior‑Appalachian Division (202). The more southern North‑Central Oak Barrens (CES202.727) overlaps this type along the "tension zone" of Minnesota and Wisconsin. Northward, this system is differentiated from more boreal systems with *Pinus banksiana* by absence of *Picea mariana* and the presence of many prairie species. Within the pine barrens landscape this system overlaps with Laurentian‑Acadian Northern Pine‑(Oak) Forest (CES201.719), which may occupy pine barrens sites that have not burned for more than 50 years.

DISTRIBUTION

**Range:** Occurs in the northern and western Great Lakes region.

**Divisions:** 201:C

**TNC Ecoregions:** 45:P, 47:C, 48:C

**Nations:** CA, US

**Subnations:** MI, MN, ON, WI

**Map Zones:** 41:C, 50:C, 51:C

**USFS Ecomap Regions:** 212Ha:CCC, 212Hb:CCC, 212Hc:CCC, 212He:CCC, 212Hg:CCC, 212Hj:CCC, 212Hk:CCC, 212Hl:CCP, 212J:CP, 212K:CC, 212L:CP, 212N:CC, 212Ra:CCC, 212Rb:CCP, 212Rc:CCP, 212Re:CCP, 212Sc:CCP, 212Sn:CCC, 212Sq:CCP, 212Tb:CCC, 212Tc:CCC, 212Te:CCC, 212X:CP, 222Ja:CCC, 222Jb:CCC, 222R:CC, 222Ud:CCC

CONCEPT

**Environment:** These barrens occur on sandy outwash plains, glacial lakeplains, and broad riverine terraces. Soils are generally infertile, coarse‑textured, and acidic sands and loamy sands. The landscape is flat to gently rolling.

**Dynamics:** Fire and droughty soil conditions maintain the characteristic open tree/shrub canopy and prairie‑like understory of this system. The dry, relatively infertile soil limits the rate of tree growth, while periodic fires remove most tree regeneration and, less commonly, canopy trees. Fires are also necessary for regeneration of *Pinus banksiana*. Sites with finer‑textured and more fertile soils need greater fire frequency, while sites with coarser‑textured, less fertile soils need less frequent fires to maintain this system. The historical fire‑return interval is 5‑20 years (Landfire 2007a). Fire‑return intervals of 20‑30 years result in abundant woody cover. Occasional frost during the growing season, sustained drought, and catastrophic winds can kill canopy trees (*Quercus* spp. would be more affected by frost) (Kost et al. 2007).

SOURCES

**References:** Comer et al. 1995a, Comer et al. 1998, Comer et al. 2003*, Curtis 1959, Eyre 1980, Kost et al. 2007, LANDFIRE 2007a, Vogl 1964, WDNR 2015

**Version:** 14 Jan 2014	**Stakeholders:** Canada, Midwest

**Concept Author:** D. Faber‑Langendoen	**LeadResp:** Midwest

CES201.719 Laurentian‑Acadian Northern Pine‑(Oak) Forest

**Primary Division:** Laurentian‑Acadian (201)

**Land Cover Class:** Forest and Woodland

**Spatial Scale & Pattern:** Matrix

**Required Classifiers:** Natural/Semi‑natural; Vegetated (>10% vasc.); Upland

**Diagnostic Classifiers:** Forest and Woodland (Treed)

**National Mapping Codes:** EVT 2362; ESLF 4265; ESP 1362

**Concept Summary:** This is a pine‑dominated, or occasionally pine‑oak, forest system that is typically found on nutrient‑poor soils, or on moderately rich soils in the upper Midwest, northeastern U.S., and adjacent Canada, in a variety of topographic settings. Soils are loamy to sandy, varying from thin soil over bedrock to deeper soils, sometimes sandy. Sites are xeric to subxeric, but less strongly than barrens and sandplains. The dominant fire regime varies from 100‑200 years for *Pinus strobus* and *Pinus resinosa*. Other boreal conifers, or in the East *Picea rubens*, may occasionally be present. Canopy structure is mostly closed but can be partially open. Conifers typically dominate the canopy, but codominates may include hardwoods, especially *Quercus rubra* or *Acer rubrum*, but also *Populus tremuloides* or *Betula papyrifera*. The shrub and field layers can be somewhat dense to sparse.

**Comments:** This system is dominated by white pine and red pine forests, which are found primarily in the Great Lakes and subboreal region, but extend eastward to Acadia. Where *Pinus strobus* is a codominant with *Tsuga canadensis*, stands most typically are placed within Laurentian‑Acadian Pine‑Hemlock‑Hardwood Forest (CES201.563).

DISTRIBUTION

**Range:** This system is found in the upper midwestern and northeastern United States and adjacent Canada.

**Divisions:** 102:?, 103:?, 201:C

**TNC Ecoregions:** 46:?, 47:C, 48:C, 61:C, 63:C

**Nations:** CA, US

**Subnations:** MB, ME, MI, MN, NB, NH, NS, NY, ON, PA?, PE?, QC, VT, WI

**Map Zones:** 41:C, 50:C, 51:C, 63:C, 64:C, 65:C, 66:C

**USFS Ecomap Regions:** 211A:CP, 211B:CC, 211C:CC, 211D:CC, 211E:CC, 211J:CP, 212Ha:CCC, 212Hb:CCC, 212Hc:CCC, 212Hd:CCC, 212He:CCC, 212Hf:CCC, 212Hg:CCC, 212Hh:CCC, 212Hi:CCC, 212Hj:CCC, 212Hk:CCC, 212Hl:CCC, 212Hm:CCC, 212Jb:CCC, 212Jc:CCC, 212Jo:CCC, 212Ka:CCC, 212Kb:CCC, 212La:CCC, 212Lb:CCC, 212Lc:CCC, 212Ld:CCC, 212Le:CCC, 212Ma:CCC, 212Mb:CCC, 212Na:CCC, 212Nb:CCC, 212Nc:CCC, 212Nd:CCC, 212Qa:CCC, 212Qb:CCC, 212Qc:CCC, 212Qd:CCC, 212Ra:CCC, 212Rb:CCC, 212Rc:CCC, 212Rd:CCC, 212Re:CCC, 212Sb:CCC, 212Sc:CCC, 212Sn:CCC, 212Sq:CCC, 212Ta:CCC, 212Tb:CCC, 212Tc:CCC, 212Te:CCC, 212Tf:CCC, 212Xa:CCC, 212Xb:CCC, 212Xc:CCC, 212Xd:CCC, 212Xe:CCC, 212Xf:CCC, 212Ya:CCC, 212Za:CCC, 212Zb:CCC, 212Zc:CCC, 221Al:CCC, 222I:CP, 222Ja:CCC, 222Ud:CCC, 222Ue:CCC, M211A:CC, M211Bd:CCC, M211C:CP, M211D:CP

CONCEPT

SOURCES

**References:** Comer et al. 1995a, Comer et al. 1998, Comer et al. 2003*, Edinger et al. 2014a, Eyre 1980, Frelich 1992, Gawler and Cutko 2010, Heinselman 1973, Kost et al. 2007, Sperduto and Nichols 2004, WDNR 2015, Whitney 1986, Whitney 1987

**Version:** 04 Mar 2004	**Stakeholders:** Canada, East, Midwest

**Concept Author:** D. Faber‑Langendoen and S.C. Gawler	**LeadResp:** Midwest

CES103.425 Laurentian‑Acadian Sub‑boreal Dry‑Mesic Pine‑Black Spruce‑Hardwood Forest

**Primary Division:** Boreal (103)

**Land Cover Class:** Forest and Woodland

**Required Classifiers:** Natural/Semi‑natural; Vegetated (>10% vasc.); Upland

**Concept Summary:** This subboreal forest ecological system is found on dry‑mesic nutrient‑poor soils in a variety of topographic settings. It ranges from northwestern Ontario to eastern Canada, and southward into Minnesota, the Great Lakes region, and very locally into northwestern Maine. Soils are loamy to sandy, varying from nutrient‑poor, thin soils over bedrock to deeper soils, sometimes sandy. Sites are typically dry‑mesic. The dominant fire regime varies from 50‑100 years. *Pinus banksiana, Pinus resinosa*, and *Picea mariana* are characteristic overstory species, with *Pinus strobus* occasionally common, over much of the range, but east of the Great Lakes, *Picea mariana* becomes increasingly dominant with *Abies balsamea* as an important associate. Canopy structure is mostly closed but can be partially open. Conifers typically dominate the canopy, but boreal hardwoods (*Populus tremuloides, Betula papyrifera*) may codominate. As time since fire increases, *Picea mariana* may dominate. Tree regeneration includes *Abies balsamea, Betula papyrifera, Populus tremuloides*, and *Picea mariana*. The shrub and field layers can be very open to somewhat dense (5‑75% cover). Characteristic low‑shrub and herb species include *Amelanchier* spp., *Vaccinium angustifolium, Diervilla lonicera, Cornus canadensis, Linnaea borealis, Doellingeria umbellata*, and *Eurybia macrophylla*. Older *Picea mariana* stands may be strongly dominated by feathermosses.

**Comments:** In Quebec (C. Morneau pers. comm. 2009), *Picea mariana* is far more common than *Pinus banksiana* in the boreal forest. Secondly, forests composed of a mixture of *Picea mariana* and *Abies balsamea* with a feathermoss carpet on the ground are very common east of 74°W longitude and north of 48°N latitude where climate undergoes a maritime influence and where *Pinus banksiana* gradually becomes absent. *Picea mariana ‑ Picea rubens / Rhododendron canadense / Cladonia* spp. Swamp Woodland (CEGL006421), in the present system, represents spruce‑lichen woodlands at the boreal‑temperate forest interface.

At this time, this system excludes xeric *Pinus banksiana* and *Picea mariana* stands, which are placed in their own system, Northern Dry Jack Pine‑Red Pine‑Hardwood Woodland (CES103.424), found on dry, poor sites, where there is a low density of *Pinus banksiana* trees resulting in a woodland condition. Lichens are dominant. Woodland physiognomy and lichen dominance distinguish that system from this system, which has more of a closed canopy and feathermosses and herbs are more abundant. See also Minnesota DNR (2003), which separates Northern Dry‑Sand Pine Woodland (FDn12) and Northern Dry‑Bedrock Pine‑(Oak) Woodland (FDn22), and belong with CES103.424, from the dry‑mesic *Pinus banksiana* and *Picea mariana* Forests and Woodlands (FDn32 and FDn33), which belong with this system.

DISTRIBUTION

**Range:** This system ranges from northwestern Ontario to eastern Canada, and southward into Minnesota, the Great Lakes region, and very locally into northwestern Maine.

**Divisions:** 103:C

**TNC Ecoregions:** 47:C, 48:C, 63:C

**Nations:** CA, US

**Subnations:** LB, MB?, MI, MN, NB, NF, ON, QC, WI

CONCEPT

**Environment:** Soils are loamy to sandy, varying from nutrient‑poor, thin soil over bedrock to deeper soils, sometimes sandy. Sites are typically on dry‑mesic to dry sites, but not commonly found on xeric sandplains or bedrock sites.

**Vegetation:** *Pinus banksiana* and *Picea mariana* are characteristic overstory species. In the Upper Great Lakes region, *Pinus banksiana* may intermix with *Pinus resinosa*. Canopy structure is mostly closed but can be partially open. Conifers typically dominate the canopy, but boreal hardwoods (*Populus tremuloides, Betula papyrifera*) may codominate. As time since fire increases, *Picea mariana* may dominate. Tree regeneration includes *Abies balsamea, Betula papyrifera, Populus tremuloides*, and *Picea mariana*. Characteristic low‑shrub and herb species include *Amelanchier* spp., *Vaccinium angustifolium, Diervilla lonicera, Cornus canadensis, Linnaea borealis, Doellingeria umbellata (= Aster umbellatus)*, and *Eurybia macrophylla*. Older *Picea mariana* stands may be strongly dominated by feathermosses (Minnesota DNR 2003).

SOURCES

**References:** Eyre 1980, Faber‑Langendoen et al. 2012*, Heinselman 1973, Minnesota DNR 2003

**Version:** 05 Sep 2012	**Stakeholders:** Canada, East, Midwest

**Concept Author:** K.A. Schulz, in Faber‑Langendoen et al. (2012)	**LeadResp:** Midwest

CES103.424 Northern Dry Jack Pine‑Red Pine‑Hardwood Woodland

**Primary Division:** Boreal (103)

**Land Cover Class:** Forest and Woodland

**Required Classifiers:** Natural/Semi‑natural; Vegetated (>10% vasc.); Upland

**Concept Summary:** This conifer woodland is found throughout the eastern southern or subboreal regions of eastern Canada, extending into the Upper Midwest and Northeast parts of the United States. It occurs on dry nutrient‑poor sand plains and along rocky ridges, often adjacent to rivers and lakes, and along talus slopes. The canopy ranges from patchy to continuous and is dominated by a mix of primarily conifer and hardwood species. In some examples, canopy trees may be stunted. *Pinus banksiana* is the most frequent conifer species, although *Pinus resinosa, Pinus strobus, Picea mariana*, or *Picea glauca* can be common and may dominate some sites. Hardwood species vary in cover from 25‑90% of the canopy. *Quercus ellipsoidalis* is a restricted dominant in the Midwest part of the range of this system, along with *Quercus macrocarpa* and *Quercus rubra*. More common are *Betula papyrifera* and *Populus* spp. In areas of open bedrock, species typical of bedrock outcrops and shallow soils can be found and include *Danthonia spicata, Poa alsodes, Elymus trachycaulus, Maianthemum canadense, Schizachne purpurascens*, and *Oryzopsis asperifolia*. The nonvascular layer can be absent or present with up to 30% cover. In the open bedrock areas, this layer consists mainly of the lichens and mosses. Infrequent fire is the primary dynamic, with catastrophic fires occurring approximately every 150‑200 years with surface fires every 50‑200 years.

DISTRIBUTION

**Range:** This system ranges in Canada from northwestern Ontario (possibly eastern Manitoba) to eastern Canada's Atlantic provinces and extending into the U.S. in northeastern Minnesota, Isle Royale, and near‑coastal areas of Lake Superior shores in northern Wisconsin and Michigan.

**Divisions:** 103:C

**TNC Ecoregions:** 47:C, 48:C, 63:C

**Nations:** CA, US

**Subnations:** LB?, MB?, MI, MN, NB, NF, ON, QC, WI

CONCEPT

**Environment:** Examples of this system occur on rocky ridgetops, high slopes, and terraces sometimes along rivers or lakeshores, including Great Lakes shorelines. These areas are dry, well‑drained sites, often with exposed bedrock. Soils range from bare bedrock and talus slopes to rocky, shallow loams and deep sands. Those stands on bedrock may have occasional cracks in the underlying bedrock resulting in pockets of relatively deep (15‑20 cm) soil. Bare rock (with crustose lichens) can cover up to 50% of the area.

**Vegetation:** The canopy ranges from scattered trees to a moderately dense canopy. Stands are a mix of conifer species, occasionally with hardwood species. In some examples, canopy trees may be stunted. The conifers in most examples are dominated by *Pinus banksiana*. *Pinus resinosa, Pinus strobus, Picea mariana*, or *Picea glauca* can be common and may dominate some sites. Hardwood species vary in cover from 25‑90% of the canopy. *Quercus ellipsoidalis* is a restricted dominant in the Midwest part of the range, with *Quercus macrocarpa* or *Quercus rubra, Betula papyrifera*, and *Populus* spp. occurring more commonly. Shrubs may be absent to dense and include *Amelanchier* spp., *Diervilla lonicera, Corylus cornuta, Juniperus communis, Prunus pensylvanica, Salix bebbiana*, and *Vaccinium angustifolium*. Herbaceous species vary across the range of this type. Some typical species include *Danthonia spicata, Poa alsodes, Elymus trachycaulus (= Agropyron trachycaulum), Maianthemum canadense, Schizachne purpurascens*, and *Oryzopsis asperifolia*. The nonvascular layer can be absent or present with up to 30% cover. In the open bedrock areas, this layer consists mainly of the lichens and mosses. Lichen species may include *Cladonia rangiferina* and *Cladonia arbuscula ssp. mitis (= Cladonia mitis)*. Mosses include *Dicranum* spp., *Pleurozium schreberi*, and *Polytrichum* spp.

SOURCES

**References:** Faber‑Langendoen et al. 2012*, Minnesota DNR 2005a

**Version:** 29 Aug 2012	**Stakeholders:** Canada, East, Midwest

**Concept Author:** D. Faber‑Langendoen, in Faber‑Langendoen et al. (2012)	**LeadResp:** Midwest

M016. Southern & South‑Central Oak ‑ Pine Forest & Woodland

CES205.896 Bastrop Lost Pines Forest and Woodland

**Primary Division:** Eastern Great Plains (205)

**Land Cover Class:** Forest and Woodland

**Spatial Scale & Pattern:** Large patch

**Required Classifiers:** Natural/Semi‑natural; Vegetated (>10% vasc.); Upland

**Diagnostic Classifiers:** Forest and Woodland (Treed); Needle‑Leaved Tree

**National Mapping Codes:** EVT 2358; ESLF 4261; ESP 1358

**Concept Summary:** This system, dominated by *Pinus taeda*, is endemic to central Texas. Locally this is known as the "Bastrop Pines." Examples may share similarities, in terms of the vegetation, with Coastal Plain pine‑hardwood systems to the east (in TNC Ecoregions 40 and 41) but differ in the fact that this system contains only loblolly pine which is generally considered successional in the more eastern systems. The vegetation includes a range of communities (that have yet to be defined) that range from very dry to xeric uplands to dry and even mesic areas with different suites of hardwood associates. The *Pinus taeda* of this region is genetically different than strains to the east; it has much greater drought tolerance. It is possible that this area was one of the epicenters of early southern pine colonization of the Coastal Plain based on fossil pollen evidence.

**Comments:** No associations have currently been described in the NVC for this system. More information is needed.

DISTRIBUTION

**Range:** This system is endemic to central Texas.

**Divisions:** 205:C

**TNC Ecoregions:** 32:C

**Nations:** US

**Subnations:** TX

**Map Zones:** 32:?, 35:?, 36:C, 37:P

**USFS Ecomap Regions:** 255C:CC

CONCEPT

**Environment:** Stands of this system occur on dissected uplands. Sandy soils characterize this system with typical Ecological Sites being deep sand, sandy, and sandy loam. It may also occupy gravelly sites associated with more recent geologic strata. Sandy Eocene formations such as Carrizo, Sparta, and Queen City formations are most frequently associated with this system, though it may also occur on the Reklaw (another Eocene) Formation (Elliott 2010).

**Vegetation:** This system is dominated by *Pinus taeda*, often with *Quercus stellata* and *Quercus marilandica* present to codominant. *Quercus incana, Quercus margarettae, Carya texana, Ulmus crassifolia, Celtis* spp., and *Juniperus virginiana* may also be present. *Vaccinium arboreum* is a frequent shrub component. Other shrub and woody vine species that may be present include *Sideroxylon lanuginosum, Callicarpa americana, Ilex vomitoria, Toxicodendron* spp., *Rhus aromatica, Smilax bona‑nox, Parthenocissus quinquefolia*, and *Vitis* spp. A grassy herbaceous layer may be present with *Schizachyrium scoparium* commonly encountered, but other species include *Andropogon gerardii, Nassella leucotricha, Sporobolus junceus, Paspalum plicatulum, Paspalum setaceum, Aristida* spp., *Sporobolus clandestinus, Digitaria cognata, Dichanthelium oligosanthes var. scribnerianum*, and *Dichanthelium oligosanthes*. Forbs are conspicuous and include *Heterotheca subaxillaris, Euphorbia corollata, Monarda citriodora, Galactia volubilis, Liatris aspera, Brazoria truncata, Diodia teres*, and many others (Elliott 2011).

**Dynamics:** Local accumulations of pine needles result in a patchy distribution of herbaceous cover. This system bears some resemblance to pine woodlands and forests farther to the east and may represent a western, more xeric outlier of these similar systems.

SOURCES

**References:** Comer et al. 2003*, Diamond pers. comm., Elliott 2011

**Version:** 17 Feb 2011	**Stakeholders:** Southeast

**Concept Author:** R. Evans and M. Pyne	**LeadResp:** Southeast

CES205.682 Crosstimbers Oak Forest and Woodland

**Primary Division:** Eastern Great Plains (205)

**Land Cover Class:** Forest and Woodland

**Spatial Scale & Pattern:** Matrix

**Required Classifiers:** Natural/Semi‑natural; Vegetated (>10% vasc.); Upland

**National Mapping Codes:** EVT 2308; ESLF 4114; ESP 1308

**Concept Summary:** This system is primarily found within central Texas and Oklahoma, ranging north to southeastern Kansas and east into eastern Oklahoma. It is distinct from the surrounding prairie by the higher density of tree species. The area consists of irregular plains with primarily sandy to loamy Ustalf soils that range from shallow to moderately deep. Rainfall can be moderate, but somewhat erratic, therefore moisture is often limiting during part of the growing season. Short, stunted *Quercus stellata* and *Quercus marilandica* characterize and dominate this system. Other species, such as *Carya texana, Carya cordiformis, Quercus prinoides, Ulmus crassifolia*, and other *Quercus* spp., can also be present within their respective ranges. The understory often contains species typical of the surrounding prairies, in particular *Schizachyrium scoparium*. Shrubs such as *Rhus* spp. may also be present. Drought, grazing, and fire are the primary natural processes that affect this system. Overgrazing and conversion to agriculture, along with fire suppression, have led to the invasion of some areas by problematic brush species such as *Juniperus virginiana* and *Juniperus ashei* and *Prosopis glandulosa* farther south in Texas and Oklahoma. It has also led to decreases in native grass cover allowing for annual grasses and forbs to invade.

**Comments:** This system currently includes woodlands of the Arbuckle Mountains, as well as a disjunct occurrence in the Wichita Mountains of Oklahoma comprised of the following member: *Quercus fusiformis ‑ Quercus stellata / Schizachyrium scoparium* Granite Woodland (CEGL004937) (B. Hoagland pers. comm. 2005). This vegetation could also be considered an outlier of Edwards Plateau Limestone Savanna and Woodland (CES303.660).

DISTRIBUTION

**Range:** This system is primarily found within central Texas and Oklahoma, with the northern extent reaching into southeastern Kansas in the Cross Timbers (EPA level III ecoregion 29). It also includes the "Lower Canadian Hills" and "Osage Cuestas" in eastern Oklahoma and the Edwards Plateau Woodland, Semiarid Edwards Plateau and Broken Red Plains of Texas (37e, 40b, 30a, 30d, 27i of EPA, respectively).

**Divisions:** 205:C, 303:C

**TNC Ecoregions:** 28:C, 29:C, 32:C, 33:C

**Nations:** US

**Subnations:** AR, KS, MO?, OK, TX

**Map Zones:** 32:C, 34:P, 35:C, 38:?, 43:C, 44:C

**USFS Ecomap Regions:** 231G:CC, 251E:CC, 251H:CC, 255A:CC, 255B:C?, 255E:CC, 315C:CC, 315D:CC, 315G:CC, 321B:CC

CONCEPT

**Environment:** This system is located on irregular plains composed of sandy to loamy Ustalf soils. These soils range from shallow to moderately deep. Rainfall can be moderate, but sporadic, leading to periods of limiting moisture. This system also includes smaller patch woodlands dominated by *Quercus stellata* occurring over Mollisols and scattered throughout the limestone uplands of the eastern Edwards Plateau and Lampasas Cutplain of Texas, locally referred to as "Redlands" (B. Carr pers. comm. 2005). The eastern occurrences of this system are associated with sandy members of the Cretaceous Woodbine Formation, while western occurrences occupy soils derived from the sands of the Cretaceous Trinity Group (such as Paluxy, Antler, and Twin Mountain‑Travis Peak sands). Further west, in the fringe of the western Crosstimbers, the system occurs on more rugged, rocky and gravelly sites derived from Pennsylvanian formations. The landforms are gently rolling, moderately dissected uplands, and irregular plains becoming more rugged in the western fringe of the distribution of this system. Soils are sands or sandy loams, some with a claypan. Ecological Sites typical of the eastern expressions include Sandy Loam, Tight Sandy Loam, Claypan Prairie, Sandstone Hill, and Sandy. Those more typical of the western expressions include Sandy Loam, Loamy Sand, Tight Sandy Loam, Sandy, and Clay Loam (Elliott 2011).

**Vegetation:** This system is generally described as a savanna or woodland, distinguished by its dominance by short, stunted *Quercus stellata* and/or *Quercus marilandica*. It occurs in southwest/northeast‑trending bands separated by the Grand Prairie. Other species in the canopy may include *Ulmus crassifolia, Quercus fusiformis, Celtis laevigata*, and *Juniperus virginiana*. The understory may have been historically dominated by *Schizachyrium scoparium*, but current understory composition may be largely determined by land‑use history and grazing pressure. *Carya texana, Carya cordiformis*, and *Quercus prinoides* are lacking from Texas examples and are mainly present in stands of this system in the northern Crosstimbers of Oklahoma (L. Elliott pers. comm. 2011). In the east, where precipitation is greater, tallgrass species such as *Andropogon gerardii* and *Sorghastrum nutans* may be important components of the understory or occupy prairie patches. In the drier west, shortgrass species such as *Bouteloua dactyloides (= Buchloe dactyloides)* become more conspicuous. Other graminoid species that may be present include *Schizachyrium scoparium, Paspalum setaceum, Sporobolus compositus, Bouteloua curtipendula, Bouteloua hirsuta, Bouteloua rigidiseta, Bothriochloa laguroides ssp. torreyana, Nassella leucotricha*, and *Aristida* spp. Non‑native species such as *Cynodon dactylon* and *Bothriochloa ischaemum var. songarica* frequently dominate the herbaceous layer. With the disruption of a natural fire cycle, branching of overstory species may be continuous to near ground level, reducing light penetration and leading to reduced herbaceous cover. The shrub layer may contain species such as *Smilax bona‑nox, Rhus glabra, Rhus trilobata, Crataegus* spp., and *Symphoricarpos orbiculatus*. Sites dominated by *Prosopis glandulosa*, sometimes with *Ziziphus obtusifolia* as a common shrub component, are particularly common to the west. Sites dominated by junipers (including *Juniperus virginiana, Juniperus ashei*, and *Juniperus pinchotii*, depending on the site) are also frequently encountered. Prairie openings and inclusions tend to occur on tighter soils. Shrubs such as *Rhus* spp. may also be present. Other species may include *Celtis laevigata, Cercis canadensis, Cotinus obovatus, Fraxinus albicans (= Fraxinus texensis), Gleditsia triacanthos, Juniperus ashei, Juniperus virginiana var. virginiana, Quercus fusiformis, Quercus buckleyi, Quercus velutina, Ulmus alata*, and *Ulmus americana* (Elliott 2011).

**Dynamics:** Drought, grazing, and fire primarily influence this system. Overgrazing and conversion to agriculture have allowed for the invasion of eastern red‑cedar (*Juniperus virginiana*), Ashe's juniper (*Juniperus ashei*), and honey mesquite (*Prosopis glandulosa*) in some areas. Decreases in native grass cover associated with overgrazing can also lead to an increase in invasive annual grasses and forbs.

SOURCES

**References:** Barbour and Billings 1988, Burns and Honkala 1990b, Comer et al. 2003*, Elliott 2011, Elliott, L. pers. comm., Eyre 1980, Griffith et al. 2004, Hoagland 2000, Hoagland pers. comm., Ricketts et al. 1999

**Version:** 17 Feb 2011	**Stakeholders:** Midwest, Southeast

**Concept Author:** S. Menard and K. Kindscher	**LeadResp:** Midwest

CES203.072 Crowley's Ridge Sand Forest

**Primary Division:** Gulf and Atlantic Coastal Plain (203)

**Land Cover Class:** Forest and Woodland

**Spatial Scale & Pattern:** Large patch

**Required Classifiers:** Natural/Semi‑natural; Vegetated (>10% vasc.); Upland

**Diagnostic Classifiers:** Sand; Unglaciated

**National Mapping Codes:** EVT 2510; ESLF 4332; ESP 1510

**Concept Summary:** This system of upland shortleaf pine ‑ hardwood forests is confined to Crowley's Ridge on the western side of the Mississippi River. This vegetation is very distinctive from that of the adjacent alluvial plain, and the ridge itself also contrasts sharply with the adjacent alluvial plain. Crowley's Ridge is a remnant loess‑capped feature rising from 30 m to over 60 m (100‑200 feet) above the alluvial plain surface, to about 150 m (450 feet) above sea level. The base of the northern ridge is composed of Tertiary substrates overlain by alluvial deposits and capped with generally thin layers of Pleistocene loess. The Pleistocene alluvial deposits are often sandy, and in a very limited area, there are outcrops of sandstone of uncertain origin. Forests on the ridgetops are dominated by *Pinus echinata* with varying amounts of *Quercus alba, Quercus rubra, Quercus falcata, Quercus stellata, Carya texana*, and *Quercus velutina*. Loess slopes and ravines are dominated by mesic or dry‑mesic hardwood forests such as those of the southern ridge, but are of relatively limited extent.

**Comments:** This system has been little studied, with the best description in Clark (1974). The presettlement and then‑current distribution were mapped, and several sites were sampled. Clark classed the predominant community as Oak‑Hickory‑Pine, with shortleaf pine dominance ranging from 12‑56% and combined white oak and post oak, the most abundant oaks, ranging from 24‑60%.

DISTRIBUTION

**Range:** This system is endemic to Crowley's Ridge in the Mississippi River Alluvial Plain of Arkansas and Missouri (Nelson 2010).

**Divisions:** 203:C

**TNC Ecoregions:** 42:C

**Nations:** US

**Subnations:** AR, MO

**Map Zones:** 45:C

**USFS Ecomap Regions:** 234D:CC

CONCEPT

**Environment:** These forests occur on sandy ridges and slopes in a dissected environment. The system is best expressed on northern Crowley's Ridge, but there are limited occurrences on the southern ridge as well, on sandy, exposed sites. They generally lie to the east of hydroxeric Pleistocene terrace flatwoods (now usually converted to cropland) that burned frequently. Those fires would have continued into these dry to dry‑mesic forests, thereby increasing the fire frequency.

**Vegetation:** This system consists of forests that are typically dominated by shortleaf pine with oaks and other hardwoods. Depending upon local soil moisture and other factors, canopy oaks can vary from *Quercus stellata* and *Quercus falcata* on the driest sites to *Quercus alba* and other oaks on more mesic sites. Associated species in the subcanopy and understory vary along this moisture gradient as well (refer to association‑level descriptions for more details).

**Dynamics:** These are fire‑adapted forests. There is presumably some natural disturbance from the effects of windstorms and collapse of the fragile loess. This vegetation is classed as Fire Regime I, with frequent surface fire (mean fire‑return interval is approximately five years) and less frequent mixed fire. In addition, straight‑line winds or microbursts may cause blowdowns on a scale of 1 to 100 acres. Stand‑replacement fires happen very infrequently (Landfire 2007a).

SOURCES

**References:** Arkansas Forestry Commission 2010, Clark 1974, Comer et al. 2003*, Engeman et al. 2007, Eyre 1980, LANDFIRE 2007a, NatureServe Ecology ‑ Southeastern U.S. unpubl. data, Nelson 2010, Zollner pers. comm.

**Version:** 14 Jan 2014	**Stakeholders:** Midwest, Southeast

**Concept Author:** T. Foti, D. Zollner, M. Pyne	**LeadResp:** Southeast

CES203.506 East Gulf Coastal Plain Interior Shortleaf Pine‑Oak Forest

**Primary Division:** Gulf and Atlantic Coastal Plain (203)

**Land Cover Class:** Forest and Woodland

**Spatial Scale & Pattern:** Matrix

**Required Classifiers:** Natural/Semi‑natural; Vegetated (>10% vasc.); Upland

**Diagnostic Classifiers:** Forest and Woodland (Treed); Short Disturbance Interval

**National Mapping Codes:** EVT 2372; ESLF 4315; ESP 1372

**Concept Summary:** This forested ecological system of the East Gulf Coastal Plain occurs most extensively on generally rolling uplands north of the range of *Pinus palustris*. It was the historical matrix in large areas of the region in Alabama and Mississippi, particularly from about 32°30'N latitude (the approximate local northern limit of the historic range of *Pinus palustris*), north to about 35°N latitude (the approximate limit where relatively extensive examples of *Pinus echinata* are replaced by predominantly hardwood‑dominated systems). It is also understood that isolated examples of this system may occur both north and south of these boundaries in limited areas, including in the "Florida Parishes" of Louisiana. Stands tend to occur on generally well‑drained sandy or clayey soils with dry to dry‑mesic moisture regimes. *Pinus echinata* is the dominant pine species of the generalized "dry and dry‑mesic oak‑pine" forest type in the Gulf Coastal Plain and is the most characteristic floristic component of this system. The actual amount of *Pinus echinata* present varies based on a number of factors, but intact examples of this system often include stands that are dominated by *Pinus echinata* grading into stands with a mixture of upland hardwoods. Locally, on mid to lower slopes, *Pinus taeda* may be a component, extending further upslope in the absence of fire. Fire is possibly the most important natural process affecting the floristic composition and vegetation structure of this system, although fire‑return intervals are lower than those associated with East Gulf Coastal Plain Interior Upland Longleaf Pine Woodland (CES203.496). *Pinus echinata* may have difficulty replacing itself in the absence of fire, particularly on sites other than the driest ones. Local topographic conditions affecting natural fire compartment size generally lend themselves to this fire frequency, although some examples may have more frequent fires and some less than this generalized value. Where fire is most frequent the system may develop a relatively pure canopy of *Pinus echinata* typified by a very open woodland structure with scattered overstory trees and an herbaceous‑dominated understory; such examples are rare on the modern landscape. More typical are areas in which *Quercus* spp., *Carya* spp., *Liquidambar styraciflua, Liriodendron tulipifera, Acer* spp., and *Nyssa sylvatica* have become prominent in the midstory and even overstory and in which herbaceous patches are rare. Although the general distributional boundaries described above indicate where this system formed an historical landscape matrix, smaller patches of the system may also be present in limited areas both north and south of these boundaries. Although some sources map the native range of shortleaf pine throughout a relatively large area of western Tennessee, the actual distribution of the species appears to be much more confined and almost absent from the Coastal Plain; when present, it occurs in only small stands on dry southwestern aspects.

**Comments:** The range of this system overlaps with East Gulf Coastal Plain Northern Dry Upland Hardwood Forest (CES203.483) in the Fall Line Hills ecoregion (65i) of Alabama and in the Southern Hilly Gulf Coastal Plain ecoregion (65d) of Mississippi and may overlap to some degree with Southern Coastal Plain Dry Upland Hardwood Forest (CES203.560) as well. In parts of the overlapping range (including the Oakmulgee Ranger District of the Talladega National Forest), these types occur in a mosaic which is difficult to interpret environmentally and ecologically (A. Schotz pers. comm.). East Gulf Coastal Plain Northern Loess Plain Oak‑Hickory Upland (CES203.482) replaces this system along the northern and northwestern boundary in Tennessee.

DISTRIBUTION

**Range:** This system is restricted to the East Gulf Coastal Plain; it was the historical matrix in large areas of the region in Alabama and Mississippi, particularly between about 32°30'N latitude and about 35°N latitude. In southwestern Mississippi, this system is apparently dominant on the landscape west of 91°W longitude to the limits of the alluvial plain and northwest of a line running approximately from the intersection of 31°N latitude and 91°W longitude, northeastward to the city of Jackson, Mississippi, extending at least to about 34°N latitude. This is consistent with the ranges of Oak‑Pine vegetation (generally equivalent to this system) versus Longleaf‑Loblolly‑Slash Pines in Shantz and Zon (1924). There are also limited and sporadic occurrences in the "Florida Parishes" of Louisiana (LNHP 2009).

**Divisions:** 203:C

**TNC Ecoregions:** 43:C

**Nations:** US

**Subnations:** AL, LA, MS, TN?

**Map Zones:** 46:C, 47:?, 99:C

**USFS Ecomap Regions:** 231B:CC, 231H:CC

CONCEPT

**Environment:** The core distribution of this system lies between about 32°30'N latitude and about 35°N latitude; more localized occurrences may be found as small patches both north and south of these boundaries embedded in other systems. The belted character of this region, in the form of inner lowlands and cuestas and other low‑ridge landforms (Bowman 1911, Fenneman 1938), the associated diversity of soil types, and differences in settlement history appear to account for the importance of shortleaf pine in the Gulf Coast region when compared to the Atlantic Coastal Plain (White and Lloyd 1998). Cuestas and other hills create strong environmental gradients which, coupled with soil characteristics, promote a variety of mixed pine and pine‑hardwood vegetation in this region; local differences in topography, parent material, and exposure influence site characteristics, resulting in numerous different plant communities. This system primarily occupies the dry and dry‑mesic portion of regional moisture gradients. Wide variation in vegetation composition across this gradient is also strongly related to fire frequency and intensity (White and Lloyd 1998). Generally to the south and southeast it grades into longleaf pine‑dominated system(s), and to the north into hardwood‑dominated ones.

**Vegetation:** This system is primarily composed of forest or woodland vegetation dominated by trees generally up to about 33 m (100 feet) in height. Individual patches or stands may be predominantly evergreen, predominantly deciduous, or mixed. The canopy will be primarily relatively closed (greater than 60%), but some areas may exhibit lower canopy closures, either as a result of repeated surface fires, timber removal, or other disturbances. This system includes the Shortleaf Pine‑Oak Cover Type (Eyre 1980) as expressed in the Upper East Gulf Coastal Plain. In contrast to most of the Atlantic Coastal Plain, *Pinus echinata* is a much more ecologically and economically important species across much of the Gulf Coastal Plain, both presently and historically (Mohr 1901, Harper 1920, 1943). The actual vegetation composition depends greatly upon local site conditions, ongoing management, and disturbance history of an area. Locally, the species that comprise the system are strongly influenced by soil, slope, and aspect (Eyre 1980). Examples may be composed of various mixtures of pines and hardwoods. Although the actual amount of *Pinus echinata* present varies based on a number of factors, intact examples of this system often include stands that are dominated by *Pinus echinata* grading into stands with a mixture of upland hardwoods. Where fire is most frequent the system may develop a relatively pure canopy of shortleaf typified by a very open woodland structure with scattered overstory trees and an herbaceous‑dominated understory; such examples are rare on the modern landscape. More typical are areas in which *Pinus echinata* trees occur in mixture with *Quercus* spp. and *Carya* spp. Many such areas also support *Liquidambar styraciflua, Liriodendron tulipifera, Acer* spp., and *Nyssa sylvatica*, and even *Pinus taeda*. When these species are prominent in the overstory and midstory it is generally though to be indicative of fire suppression. *Quercus alba* and *Quercus stellata* are common hardwood components, particularly in later‑seral or higher‑quality stands, typically combined with *Carya tomentosa (= Carya alba), Carya pallida, Carya glabra*, and other *Carya* spp. Higher‑quality areas may exhibit somewhat open canopies. Other tree species indicative of recent disturbance and/or fire suppression are *Quercus nigra, Quercus hemisphaerica, Quercus falcata*, and *Quercus velutina*. Subcanopies will typically contain *Cornus florida, Oxydendrum arboreum, Nyssa sylvatica*, and *Liquidambar styraciflua*. The patchy shrub layer includes *Vaccinium arboreum, Vaccinium elliottii, Asimina parviflora, Aesculus pavia, Hamamelis virginiana, Callicarpa americana, Hypericum hypericoides, Gelsemium sempervirens, Vitis rotundifolia*, and *Arundinaria gigantea*. Herbs, which may be few and sparse, include *Cnidoscolus urens var. stimulosus (= Cnidoscolus stimulosus), Indigofera caroliniana, Aristolochia serpentaria, Piptochaetium avenaceum, Chasmanthium sessiliflorum, Elephantopus tomentosus, Hexastylis arifolia, Iris verna, Rudbeckia fulgida, Solidago juncea, Euphorbia pubentissima, Mitchella repens*, and *Desmodium* spp. (NatureServe Ecology unpubl. data 2003). Other associates may include *Smilax* spp., *Symphyotrichum* spp., *Coreopsis* spp., *Lespedeza* spp., *Viola pedata, Mimosa microphylla, Antennaria* spp., *Clitoria mariana, Senna* spp., *Chasmanthium latifolium, Dichanthelium* spp., *Andropogon* spp., *Schizachyrium scoparium*, and *Carex* spp. (Lawson 1990).

**Dynamics:** The frequent presence of surface fire is important in order to support the reproduction of *Pinus echinata*, which is a critical species characteristic to the system. *Pinus echinata* is a shade‑intolerant species and does not survive or grow well when fire‑suppressed. Outbreaks of *Dendroctonus frontalis* (Southern Pine Beetle) also play an important role in shaping the dynamics of this system and the balance of pine versus hardwood dominance over time. Young shortleaf pines are generally slower growing and slower to dominate a site than *Pinus taeda* or many hardwood competitors, but they usually will endure competition longer than the common associate, *Pinus taeda*. *Pinus echinata* can maintain dominance on most sites after it overtops competing vegetation, but in general hardwoods cannot be eliminated from pine sites. On very good sites (i.e., with high site index), however, it may not outgrow competing species such as sweetgum and red maple (Lawson 1990).

SOURCES

**References:** Bowman 1911, Chester et al. 1993, Comer et al. 2003*, Eyre 1980, Fenneman 1938, Harper 1920b, Harper 1943, LNHP 2009, Landers 1989, Lawson 1990, Mohr 1901, NatureServe Ecology ‑ Southeastern U.S. unpubl. data, Nordman pers. comm., Schotz pers. comm., Shantz and Zon 1924, White and Lloyd 1998

**Version:** 20 Aug 2015	**Stakeholders:** Southeast

**Concept Author:** R. Evans and A. Schotz	**LeadResp:** Southeast

CES203.483 East Gulf Coastal Plain Northern Dry Upland Hardwood Forest

**Primary Division:** Gulf and Atlantic Coastal Plain (203)

**Land Cover Class:** Forest and Woodland

**Spatial Scale & Pattern:** Matrix

**Required Classifiers:** Natural/Semi‑natural; Vegetated (>10% vasc.); Upland

**Diagnostic Classifiers:** Forest and Woodland (Treed); Broad‑Leaved Deciduous Tree

**National Mapping Codes:** EVT 2307; ESLF 4113; ESP 1307

**Concept Summary:** This system represents dry, upland, predominantly hardwood forests of limited portions of the East Gulf Coastal Plain of western Kentucky and Tennessee, northern Mississippi and Alabama. The core range of this type lies within the Northern Hilly Coastal Plain (EPA Level IV Ecoregion 65e), which includes the Northern Pontotoc Ridge (222Cf), Upper Loam Hills (222Cg), and Northern Loessal Hills (222Ce) Ecomap subsections. These areas occupy the eastern margin of the Upper East Gulf Coastal Plain where elevation is greatest and influence of loess is less than adjacent areas to the west. The vegetation has been broadly considered distinct from other coastal plain forests but has received almost no specific study. Although vastly forested when compared to the loess plains to the west, most of the vegetation is recovering from one or more forms of severe disturbance. *Quercus alba* dominates the upland forests which have been studied in a limited portion of this area, but communities have not been described to the same detail as in other ecological systems.

**Comments:** The range of this system overlaps with East Gulf Coastal Plain Interior Shortleaf Pine‑Oak Forest (CES203.506) in the Fall Line Hills (Ecoregion 65i) of Alabama and in the Southern Hilly Gulf Coastal Plain (Ecoregion 65d) of Mississippi and may overlap to some degree with Southern Coastal Plain Dry Upland Hardwood Forest (CES203.560) at its southern boundary as well. In parts of the overlapping range (including the Oakmulgee Ranger District of the Talladega National Forest), these types occur in a mosaic which is difficult to interpret environmentally and ecologically (A. Schotz pers. comm.). The vegetation of this system has received almost no specific study and is extremely poorly documented.

DISTRIBUTION

**Range:** This system is found in the Coastal Plain of western Kentucky and Tennessee, ranging south to northern Mississippi and Alabama.

**Divisions:** 203:C

**TNC Ecoregions:** 43:C

**Nations:** US

**Subnations:** AL, KY, MS, TN

**Map Zones:** 46:C, 47:C

**USFS Ecomap Regions:** 231B:CC, 231H:CC

CONCEPT

**Environment:** The most northern examples (e.g., western Tennessee and Kentucky) occur along the eastern margin of the East Gulf Coastal Plain where elevation is greatest and influence of loess is minimal, and where they occur as predominantly slope forests in relatively deep, dissected stream valleys. The vegetation in this region has been broadly considered distinct from other coastal plain forests (Bryant et al. 1993, Fralish and Franklin 2002) but has received almost no specific study (Franklin and Kupfer 2004). Although vastly forested when compared to the loess plains to the west (USGS 1992), most of the vegetation is recovering from one or more forms of severe disturbance (Franklin and Kupfer 2004). *Quercus alba* dominates the upland forests which have been studied in a limited portion of this area (Franklin and Kupfer 2004), but communities have not been described to the same detail as in other ecological systems.

**Vegetation:** Stands may contain *Aesculus pavia, Carya tomentosa (= Carya alba), Carya glabra, Carya pallida, Carya* spp., *Celtis laevigata, Iris verna var. smalliana, Kalmia latifolia, Liquidambar styraciflua, Liriodendron tulipifera, Ostrya virginiana, Oxydendrum arboreum, Quercus alba, Quercus falcata, Quercus marilandica, Quercus muehlenbergii, Quercus pagoda, Quercus stellata, Quercus velutina, Styrax grandifolius, Vaccinium arboreum, Vaccinium* spp., and *Vaccinium stamineum*.

**Dynamics:** Fire suppression and the resulting greater understory density and resulting cooler conditions on the forest floor affect this system.

SOURCES

**References:** Bryant et al. 1993, Comer et al. 2003*, Evans et al. 2009, Eyre 1980, Fralish and Franklin 2002, Franklin and Kupfer 2004, Keys et al. 1995, Smalley et al. 1996, Springer and Elder 1980, USGS 1992

**Version:** 05 Apr 2007	**Stakeholders:** Southeast

**Concept Author:** R. Evans and M. Pyne	**LeadResp:** Southeast

CES203.482 East Gulf Coastal Plain Northern Loess Plain Oak‑Hickory Upland

**Primary Division:** Gulf and Atlantic Coastal Plain (203)

**Land Cover Class:** Forest and Woodland

**Spatial Scale & Pattern:** Matrix

**Required Classifiers:** Natural/Semi‑natural; Vegetated (>10% vasc.); Upland

**Diagnostic Classifiers:** Forest and Woodland (Treed); Loess deposit (undifferentiated); Broad‑Leaved Tree

**National Mapping Codes:** EVT 2306; ESLF 4112; ESP 1306

**Concept Summary:** This is the former matrix hardwood system flanking the loess bluffs of the most northern portions of the Upper East Gulf Coastal Plain of western Tennessee, western Kentucky, possibly southern Illinois, and northern Mississippi. The core distribution of this system is mapped as the Loess Plains (EPA Ecoregion 74b). Extensive forests once covered this broad area of generally flat to rolling uplands. Most have been cleared for agriculture due to the rich, productive soils derived from relatively thick loess deposits. The areal extent of this forested system has been so heavily reduced that the component community types remain undocumented and speculative at best. Typical stands would contain oaks and other hardwoods. Some typical canopy dominants include *Quercus falcata, Quercus alba, Carya tomentosa, Quercus stellata, Quercus marilandica*, and *Quercus velutina*. Scattered successional stands would be dominated by *Juniperus virginiana var. virginiana*. In addition, *Liquidambar styraciflua* and *Liriodendron tulipifera* may be present.

**Comments:** The southern boundary of this system has not been clearly delineated; Omernik (EPA 2004) Ecoregion 74b extends farther south than the presumed boundary of this system. For now, the boundary is assumed to occur in northern Mississippi at the latitude of the junction of Omernik (EPA 2004) Ecoregion 65e and Ecoregion 65d (around 34°N). To the east, this system grades into East Gulf Coastal Plain Northern Dry Upland Hardwood Forest (CES203.483). The two types may be similar and difficult to distinguish where they come together, but the former is believed to be more mesic and richer floristically due to the influence of the loessal soils. However, it is also rare due the fertility of the soils for agriculture. More work is needed to better quantify the differences between these types and their exact boundaries.

DISTRIBUTION

**Range:** This system would have occupied the most northern portions of the Upper East Gulf Coastal Plain of western Tennessee, western Kentucky, possibly southern Illinois, and northern Mississippi. Its core distribution is mapped by EPA (2004) as the Loess Plains (EPA Ecoregion 74b). Today it is reduced to remnant forest patches in a largely agricultural landscape.

**Divisions:** 203:C

**TNC Ecoregions:** 43:C

**Nations:** US

**Subnations:** IL?, KY, MS, TN

**Map Zones:** 46:C, 47:C, 49:?

**USFS Ecomap Regions:** 231H:CC

CONCEPT

**Environment:** The habitat for this system is a broad area of generally flat to rolling uplands. Soils included in this system in western Tennessee are rich, productive, and silty, being derived from relatively thick loess deposits. Most of the soils have fragipans and some are poorly drained (Springer and Elder 1980).

**Vegetation:** Typical stands would contain oaks and other hardwoods. Some typical canopy dominants include *Quercus falcata, Quercus alba, Carya tomentosa (= Carya alba), Quercus stellata, Quercus marilandica*, and *Quercus velutina*. Scattered successional stands would be dominated by *Juniperus virginiana var. virginiana*. In addition, *Liquidambar styraciflua* and *Liriodendron tulipifera* may be present.

**Dynamics:** Most of the landscape in which this was the matrix system was cleared of forests for settlement and agriculture during the nineteenth and early twentieth century and very few sites remain in primary forest condition. Fire frequency and severity are classified as Fire Regime Group I, with frequent, low‑intensity surface fires. The mean fire‑return interval (MFRI) is about 15 years with wide year‑to‑year and within‑type variation related to moisture cycles, degree of sheltering, and proximity to more fire‑prone vegetation types. Anthropogenic fire may have contributed to presettlement fire frequency (Landfire 2007a). When sites are cleared for settlement or agriculture, *Liquidambar styraciflua* is a major component of the replacement successional forest, in addition to other wind‑blown or bird‑dispersed trees such as *Acer rubrum, Celtis* spp., *Fraxinus americana, Juglans nigra, Juniperus virginiana, Liquidambar styraciflua, Liriodendron tulipifera, Prunus serotina, Robinia pseudoacacia, Sassafras albidum, Ulmus americana*, and the exotic *Ailanthus altissima*. In addition, *Baccharis halimifolia* is a native increaser shrub that will colonize disturbed sites.

SOURCES

**References:** Bryant et al. 1993, Comer et al. 2003*, EPA 2004, Edwards et al. 2013, Engeman et al. 2007, Evans et al. 2009, Eyre 1980, Greenberg et al. 1997, LANDFIRE 2007a, Springer and Elder 1980

**Version:** 14 Jan 2014	**Stakeholders:** Midwest, Southeast

**Concept Author:** R. Evans and M. Pyne	**LeadResp:** Southeast

CES205.679 East‑Central Texas Plains Post Oak Savanna and Woodland

**Primary Division:** Eastern Great Plains (205)

**Land Cover Class:** Forest and Woodland

**Spatial Scale & Pattern:** Matrix

**Required Classifiers:** Natural/Semi‑natural; Vegetated (>10% vasc.); Upland

**National Mapping Codes:** EVT 2519; ESLF 4158; ESP 1519

**Concept Summary:** This ecological system is found in east‑central Texas in a broad, northeast/southwest‑trending band located west of the Upper West Gulf Coastal Plain, northwest of the Coastal Prairie, and east and south of the Blackland Prairie ecoregions. It exhibits some floristic and physiognomic variation across this northeast‑southwest gradient, losing some eastern species and picking up some species with more western affinities. It is distinguished from the nearby prairie by the higher density of trees and diversity of woody species. The system differs from the floristically similar Crosstimbers Oak Forest and Woodland (CES205.682) in that it generally occurs on Tertiary (primarily Eocene) geologic formations on the east‑central Texas Plains, while the related Crosstimbers ecological system occupies Cretaceous and older formations of the interior plains. Floristically, Post Oak Savanna (at least north of the Colorado River) contains species of more eastern affinities such as *Callicarpa americana, Sassafras albidum, Cornus florida, Vaccinium arboreum, Ulmus alata*, and particularly *Ilex vomitoria*, the latter species being absent from Crosstimbers Oak Forest and Woodland (CES205.682). Post Oak Savanna generally occurs on sandy or loamy soils, often underlain by a claypan subsoil. Rainfall ranges from about 120 cm in the northeastern part of the range to about 70 cm in the southwest, where it becomes increasingly erratic. Therefore moisture is often limiting during part of the growing season. The system was historically characterized as having significant areas of graminoid cover with species composition resembling that of nearby prairie systems, punctuated by short, stunted woodlands and forests dominated by *Quercus stellata* and *Quercus marilandica*. Drought, grazing, and fire are the primary natural processes that affect this system. Much of this system has been impacted by conversion to improved pasture or crop production. Overgrazing and fire suppression have led to increased woody cover on most extant occurrences and the invasion of some areas by problematic brush species such as *Juniperus virginiana var. virginiana* and *Prosopis glandulosa* in the southern part of the system's range. These factors have also led to decreases in native grass cover allowing for annual grasses and forbs to invade.

**Comments:** Vegetation of East‑Central Texas Plains Xeric Sandyland (CES205.897) can be embedded within the matrix‑forming East‑Central Texas Plains Post Oak Savanna and Woodland (CES205.679). East‑Central Texas Plains Xeric Sandyland (CES205.897) was formerly called Crosstimbers Southern Xeric Sandhill but has been renamed to reflect this relationship.

DISTRIBUTION

**Range:** This ecological system is found in east‑central Texas in a broad, northeast/southwest‑trending band located west of the Upper West Gulf Coastal Plain, northwest of the Coastal Prairie, and east and south of the Blackland Prairie ecoregions. An arm extends along the Red River in north Texas.

**Divisions:** 203:P, 205:C

**TNC Ecoregions:** 31:C, 32:C, 40:C

**Nations:** US

**Subnations:** OK, TX

**Map Zones:** 32:P, 35:P, 36:C, 37:C

**USFS Ecomap Regions:** 231Ef:CCC, 231Eg:CCC, 231Eo:CCC, 255A:CP, 255Ba:CCC, 255C:CC, 315E:CC

CONCEPT

**Environment:** This system is typically located on irregular plains in the East Central Texas Plains (Level III Ecoregion 33) of EPA (Griffith et al. 2004), composed of sedimentary formations of Tertiary age, including Eocene sands such the Queen City, Sparta, and Carrizo sands, as well as the Wilcox and Claiborne groups. The system also occupies other Tertiary formations such as the Goliad and Willis formations, as well as portions of the Quaternary Willis Formation. This system occupies gently rolling to hilly topography. It is moderately dissected by drainages. It usually occurs on sandy to sandy loam soils, often with a marked clay subsurface horizon. Soils of this system are generally Alfisols, are typically acidic to neutral, and range from shallow to moderately deep. Typical Ecological Sites include Claypan Savannah, Claypan Prairie, Sandy Loam, Sandy, and Deep Sand (Elliott 2011). Rainfall ranges from about 120 cm in the northeastern part of the range to about 70 cm in the southwest, where it becomes increasingly erratic.

**Vegetation:** This system represents a transition from the woodlands and forests of East Texas to the prairies to the west, specifically the Blackland Prairie. Savannas and woodlands are typically dominated by *Quercus stellata, Quercus marilandica*, and *Carya texana*. Large areas of woodland, particularly in the south and east, are dominated or codominated by *Quercus fusiformis* or *Quercus virginiana* (east of the Brazos River). Other species, such as *Quercus incana* (on more xeric sites), *Ulmus alata, Ulmus crassifolia, Quercus nigra, Juniperus virginiana*, and *Prosopis glandulosa*, can also be present in the overstory. To the east, *Quercus falcata, Quercus nigra, Liquidambar styraciflua, Pinus echinata, Pinus taeda*, and *Carya tomentosa (= Carya alba)* may be conspicuous in the overstory. Shrubs may attain significant cover in the understory, with species including *Ilex vomitoria* (often dominant), *Callicarpa americana, Sideroxylon lanuginosum, Crataegus* spp., *Ilex decidua, Toxicodendron radicans, Smilax bona‑nox, Juniperus virginiana*, and *Symphoricarpos orbiculatus*. To the south, this system grades into vegetation more characteristic of southern Texas, with *Quercus fusiformis* and *Prosopis glandulosa* becoming the primary overstory components, and shrubs of southern Texas such as *Acacia rigidula, Forestiera angustifolia, Condalia hookeri, Colubrina texensis, Eysenhardtia texana, Opuntia engelmannii var. lindheimeri*, and *Diospyros texana* becoming increasingly conspicuous understory components. To the east, *Vaccinium arboreum, Morella cerifera, Diospyros virginiana*, and *Cornus florida* may be common components of the understory. On some sites, *Ilex vomitoria* can form a nearly continuous, sometimes impenetrable, dense shrub layer. Mid‑ and tallgrass species, including *Schizachyrium scoparium, Sorghastrum nutans*, and *Panicum virgatum*, are frequent in the understory where light penetration supports herbaceous cover, and also form prairie patches within the savanna, particularly on tighter soils. Other grasses present include *Andropogon gerardii, Bothriochloa laguroides ssp. torreyana, Paspalum plicatulum* (to the south), *Nassella leucotricha, Dichanthelium* spp., *Aristida* spp., and *Sporobolus cryptandrus*. Non‑native grass species such as *Bothriochloa ischaemum var. songarica, Paspalum notatum*, and *Cynodon dactylon* may dominate some sites. Forbs are often conspicuous and may include species such as *Croton capitatus, Gaillardia pulchella, Monarda punctata, Rudbeckia hirta, Phlox drummondii, Commelina erecta, Acalypha radians, Verbesina virginica, Aphanostephus skirrhobasis, Froelichia gracilis, Cnidoscolus texanus*, and many others (Elliott 2011).

**Dynamics:** Drought, grazing, and fire are the primary natural processes that affect this system. This system is intricately tied with some occurrences of West Gulf Coastal Plain Herbaceous Seep and Bog (CES203.194). The sandy soils and underlying geologic strata that support this system serve as recharge areas for groundwater that supports seeps and bogs along hillsides and at the heads of drainages supporting West Gulf Coastal Plain Herbaceous Seep and Bog.

SOURCES

**References:** Barbour and Billings 1988, Bartlett 1995, Bezanson 2000, Campbell 1925, Comer et al. 2003*, Elliott 2011, Eyre 1980, Griffith et al. 2004, Loucks 1999, MacRoberts and MacRoberts 2004, MacRoberts et al. 2002a, MacRoberts et al. 2002b, McBride 1933, Midwood et al. 1998, Parmalee 1955, Ricketts et al. 1999, Singhurst et al. 2004, Smeins and Diamond 1986a, Stambaugh et al. 2011b, TPDW 2012a, Tharp 1926, Ward and Nixon 1992

**Version:** 14 Jan 2014	**Stakeholders:** Midwest, Southeast

**Concept Author:** L. Elliott and J. Teague	**LeadResp:** Southeast

CES203.531 Lower Mississippi River Dune Woodland and Forest

**Primary Division:** Gulf and Atlantic Coastal Plain (203)

**Land Cover Class:** Forest and Woodland

**Spatial Scale & Pattern:** Large patch

**Required Classifiers:** Natural/Semi‑natural; Vegetated (>10% vasc.); Upland

**Diagnostic Classifiers:** Forest and Woodland (Treed); Dune (Substrate); Sand Soil Texture

**National Mapping Codes:** EVT 2381; ESLF 4324; ESP 1381

**Concept Summary:** This system represents the vegetation of sand dunes and related eolian features of the lower Mississippi River Alluvial Valley in Missouri and Arkansas. These Pleistocene dunes were overlooked or unrecognized until the late 1970s. This fact coupled with long periods of weathering and human disturbance, as well as proximity to a terrace mapped as "prairie" in General Land Office records, has led to considerable confusion regarding this type. These dunes are west of Crowley's Ridge and near the Black and White rivers, above the normal flood level of the Mississippi. Examples in Missouri occur amidst a series of low‑lying, anastomosing channels that have helped to protect them from extensive alteration more typical in Arkansas where the uplands have been largely cleared. The uppermost portions of the dunes support a xeric community similar to sandhills of the West Gulf Coastal Plain (WGCP), but are outside the natural range of *Quercus incana*, a diagnostic species typical of the WGCP examples. Instead the dunes support very open *Quercus stellata* woodlands with *Schizachyrium scoparium* and abundant lichen cover (presumably *Cladonia* spp.), along with *Opuntia* sp. Less edaphically extreme slopes support more closed‑canopied forests in which *Quercus stellata* is still important, along with *Quercus falcata* and possibly other species. In many instances, distinctive wetlands imbedded within this system are also present (Lower Mississippi River Dune Pond (CES203.189)). Called "sand ponds" in Arkansas, these depressions have silty bottoms and perched water tables. The margins of these ponds are rimmed by *Quercus phellos* and have *Quercus lyrata*.

**Comments:** Heineke (1987) states that this large area of eolian sand dunes occurs "mainly in a long band to the west of Crowley's Ridge," and occupies approximately 1000 square kilometers (400 square miles) in discrete fields of up to 78 square kilometers (30 square miles) each. The dunes consist of a layer of sand or sandy loam over an impervious sublayer (Heineke 1987). Depressions in the dune fields (e.g., Lower Mississippi River Dune Pond (CES203.189)) are one of the principal habitats for the rare shrub *Lindera melissifolia*.

DISTRIBUTION

**Range:** Lower Mississippi River Alluvial Valley in Missouri (Ripley County, Sand Ponds Natural Area) and Arkansas. In Arkansas, examples occur in Clay, Jackson, Lawrence, and Woodruff counties.

**Divisions:** 202:?, 203:C

**TNC Ecoregions:** 42:C

**Nations:** US

**Subnations:** AR, MO

**Map Zones:** 45:C

**USFS Ecomap Regions:** 234D:CC

CONCEPT

**Environment:** This system represents the vegetation of sand dunes and related eolian features of the lower Mississippi River Alluvial Valley in Missouri and Arkansas. These Pleistocene dunes were overlooked or unrecognized until the late 1970s (Saucier 1978). This fact coupled with long periods of weathering and human disturbance, as well as proximity to a terrace mapped as "prairie" in General Land Office records, has led to considerable confusion regarding this type (T. Foti pers. comm.). These dunes are west of Crowley's Ridge and near the Black and White rivers, above the normal flood level of the Mississippi. Examples in Missouri occur amidst a series of low‑lying, anastomosing channels that have helped to protect them from extensive alteration more typical in Arkansas where the uplands have been largely cleared. The uppermost portions of the dunes support a xeric community similar to sandhills of the West Gulf Coastal Plain.

**Vegetation:** The uppermost portions of the dunes support a xeric community of very open *Quercus stellata* woodlands with *Schizachyrium scoparium* and abundant lichen cover (presumably *Cladonia* spp.), along with *Opuntia* sp. Less edaphically extreme slopes support more closed‑canopied forests in which *Quercus stellata* is still important, along with *Quercus falcata* and possibly other species. The margins of these ponds are rimmed by *Quercus phellos* and have *Quercus lyrata* (Heineke 1987).

SOURCES

**References:** Comer et al. 2003*, Eyre 1980, Heineke 1987, Nelson 2010, Saucier 1978

**Version:** 26 Jan 2005	**Stakeholders:** Midwest, Southeast

**Concept Author:** T. Foti and R. Evans	**LeadResp:** Southeast

CES203.071 Mississippi River Alluvial Plain Dry‑Mesic Loess Slope Forest

**Primary Division:** Gulf and Atlantic Coastal Plain (203)

**Land Cover Class:** Forest and Woodland

**Spatial Scale & Pattern:** Large patch

**Required Classifiers:** Natural/Semi‑natural; Vegetated (>10% vasc.); Upland

**Diagnostic Classifiers:** Unglaciated

**National Mapping Codes:** EVT 2509; ESLF 4155; ESP 1509

**Concept Summary:** This system of dry‑mesic upland forests occurs most extensively on west‑facing loess slopes on southern Crowley's Ridge, with more limited occurrences on northern Crowley's Ridge and in the erosional slopes and hills that bound the Grand Prairie terrace of Arkansas and Macon Ridge in Louisiana and Arkansas. The vegetation is very distinctive from that of the adjacent alluvial plain, and the sites themselves, which occur on distinct slopes that rise above the alluvial plain surface, also contrast sharply with it. Occurrences of this system generally comprise dry‑mesic forests that occupy west‑facing slopes and narrow, "finger" ridgetops in a highly dissected landscape. In many cases, these slopes provide habitat for plant species that are uncommon in other parts of the alluvial plain. Forests on the ridgetops are dominated by *Quercus alba, Quercus rubra* (Crowley's Ridge only), *Quercus falcata, Quercus pagoda, Quercus stellata, Carya texana, Quercus shumardii*, and *Quercus velutina*.

**Comments:** This system is best developed on southern Crowley's Ridge where loess is most pronounced and is more isolated and less extensive elsewhere. More associations may need to be developed.

DISTRIBUTION

**Range:** This system is endemic to well‑drained sites on Crowley's Ridge (Arkansas, Missouri) and Macon Ridge (Louisiana/Arkansas), along the eastern slopes of the Grand Prairie terrace in Arkansas, and perhaps other such sites in the Mississippi River Alluvial Plain, including Missouri and extreme western Kentucky and Tennessee.

**Divisions:** 203:C

**TNC Ecoregions:** 42:C

**Nations:** US

**Subnations:** AR, KY, LA, MO, TN

**Map Zones:** 45:C, 47:C

**USFS Ecomap Regions:** 234A:CC, 234D:CC, 234Eb:CCC

CONCEPT

**Environment:** These forests occur on narrow ridgetops and slopes in a highly dissected environment. The system is best documented from southern Crowley's Ridge, Arkansas (Cross County south through Phillips County), with additional occurrences on the northern ridge, on the eastern border of the Grand Prairie terrace in Arkansas, on Macon Ridge (Louisiana/Arkansas) and probably on other upland sites within the alluvial plain, including Missouri and extreme western Kentucky and Tennessee. Loess soil is a characteristic and diagnostic component of the environment of this system.

**Vegetation:** This system consists of forests that are typically dominated by oaks and other hardwoods. Depending upon local soil moisture and other factors, canopy composition can vary from *Quercus stellata*‑ and *Quercus falcata*‑dominated on the driest sites to *Quercus alba* and other oaks on more mesic sites. Associated species in the subcanopy and understory vary along this moisture gradient.

**Dynamics:** These are fire‑maintained forests. In Arkansas, they generally lie to the east of hydroxeric Pleistocene terrace flatwoods or prairies (now usually converted to cropland) that burned frequently. Those fires would have continued into these dry to dry‑mesic forests. There is presumably also some natural disturbance from the effects of windstorms and collapse of the fragile loess.

<br />This loess forest type is Fire Regime Group III, surface fires with return intervals of 30 to 100 or more years. Mixed‑severity fires will occur approximately every 100 years, opening the canopy with increased mortality. This effect may also be achieved by recurrent, severe insect defoliations or droughts. Straight‑line winds or microbursts may cause blowdowns on a scale of 1 to 100 acres. Stand‑replacement fires happen very infrequently (Landfire 2007a).

SOURCES

**References:** Clark 1974, Comer et al. 2003*, Edwards et al. 2013, Engeman et al. 2007, Eyre 1980, LANDFIRE 2007a, NatureServe Ecology ‑ Southeastern U.S. unpubl. data, Nelson 2010, Rentch et al. 2003

**Version:** 14 Jan 2014	**Stakeholders:** Midwest, Southeast

**Concept Author:** T. Foti and M. Pyne	**LeadResp:** Southeast

CES202.306 Ouachita Montane Oak Forest

**Primary Division:** Central Interior and Appalachian (202)

**Land Cover Class:** Forest and Woodland

**Spatial Scale & Pattern:** Large patch

**Required Classifiers:** Natural/Semi‑natural; Vegetated (>10% vasc.); Upland

**Diagnostic Classifiers:** Montane; Forest and Woodland (Treed); Broad‑Leaved Tree; Ozark/Ouachita

**National Mapping Codes:** EVT 2312; ESLF 4118; ESP 1312

**Concept Summary:** This system represents hardwood forests of the highest elevations of the Ouachita, Rich, and Black Fork mountains of Arkansas and Oklahoma (about 790‑850 m [2600‑2800 feet]). Vegetation consists of either forests or open woodlands dominated by *Quercus alba* or *Quercus stellata*. Canopy trees are often stunted due to the effects of ice, wind and cold conditions, in combination with fog, shallow soils over rock, and periodic severe drought. Some stands form almost impenetrable thickets.

**Comments:** *Quercus alba ‑ Carya tomentosa / Ostrya virginiana / Carex pensylvanica ‑ Schizachyrium scoparium* Forest (CEGL007818) is taller and less influenced by wind and ice. It is no longer included in this system.

DISTRIBUTION

**Range:** This system is found at the highest elevations of the Ouachita, Rich, and Black Fork mountains of Arkansas and Oklahoma (about 790‑850 m [2600‑2800 feet]).

**Divisions:** 202:C

**TNC Ecoregions:** 39:C

**Nations:** US

**Subnations:** AR, OK

**Map Zones:** 44:C

**USFS Ecomap Regions:** M231A:CC

CONCEPT

**Environment:** This system is restricted to the highest elevations of the Ouachita, Rich, and Black Fork mountains of Arkansas and Oklahoma (about 790‑850 m [2600‑2800 feet]). Ecological factors include the effects of ice, wind and cold, in combination with fog, shallow soils over rock, and periodic severe drought.

**Vegetation:** The vegetation of this system consists of either forests or open woodlands dominated by *Quercus alba* or *Quercus stellata*. Some examples may have *Quercus marilandica var. ashei*; herb layers may contain *Carex pensylvanica* and/or *Carex ouachitana*. In addition, *Quercus rubra, Quercus stellata, Carya texana*, and *Quercus marilandica* may occur as minor components of the canopy. Associated woody species of minor importance include *Amelanchier arborea, Acer rubrum var. rubrum, Sassafras albidum, Vaccinium pallidum, Vaccinium stamineum, Rubus* spp., *Nyssa sylvatica, Hamamelis virginiana, Rhus copallinum, Rhus glabra, Pinus echinata, Chionanthus virginicus, Ulmus alata, Smilax* spp., and *Rubus* spp. The ground layer may have a mosaic of sedge‑dominated and lichen/moss‑dominated areas. In addition to *Carex pensylvanica* and *Carex ouachitana*, herbaceous species include *Carex albicans var. albicans, Carex nigromarginata, Deschampsia flexuosa, Schizachyrium scoparium, Elymus* spp., *Maianthemum racemosum, Hypericum* sp., *Baptisia* sp., *Cynoglossum virginianum var. virginianum, Agalinis* sp., *Dichanthelium* spp., *Solidago ulmifolia*, and *Solidago* spp.

**Dynamics:** Canopy trees are often stunted due to the effects of ice, wind and cold conditions, in combination with fog, shallow soils over rock, and periodic severe drought.

SOURCES

**References:** Comer et al. 2003*, Eyre 1980

**Version:** 23 Feb 2010	**Stakeholders:** Southeast

**Concept Author:** T. Foti and R. Evans	**LeadResp:** Southeast

CES202.707 Ozark‑Ouachita Dry Oak Woodland

**Primary Division:** Central Interior and Appalachian (202)

**Land Cover Class:** Forest and Woodland

**Spatial Scale & Pattern:** Large patch

**Required Classifiers:** Natural/Semi‑natural; Vegetated (>10% vasc.); Upland

**Diagnostic Classifiers:** Ozark/Ouachita

**National Mapping Codes:** EVT 2364; ESLF 4306; ESP 1364

**Concept Summary:** This system occurs in the Ozark and Ouachita Highlands and far western portions of the Interior Low Plateau regions along gentle to steep slopes and over bluff escarpments with southerly to westerly aspects. Parent material can range from calcareous to acidic with very shallow, well‑ to excessively well‑drained soils, sometimes with a fragipan that causes "xero‑hydric" moisture conditions. Historically, this system primarily exhibited a woodland structure with related composition and processes, but now most stands have a more closed canopy. Oak species such as *Quercus stellata, Quercus marilandica*, and *Quercus coccinea* dominate this system with an understory of grassland species such as *Schizachyrium scoparium* and shrub species such as *Vaccinium arboreum*. Drought stress is the major dynamic influencing and maintaining this system. Some examples are flatwoods with fragipans; in these examples *Quercus stellata* is the major dominant. In addition, *Quercus alba, Quercus falcata*, and/or *Carya texana* may be present in some stands.

**Comments:** Dry‑mesic to mesic oaks were separated from dry oak per the suggestion of Missouri [see Ozark‑Ouachita Dry‑Mesic Oak Forest (CES202.708)]. This separation may need to be further reviewed.

DISTRIBUTION

**Range:** This system occurs in the Western Interior Highlands of the Ozark, Ouachita, and western Interior Low Plateau regions.

**Divisions:** 202:C

**TNC Ecoregions:** 38:C, 39:C, 44:C

**Nations:** US

**Subnations:** AR, IL, MO, OK

**Map Zones:** 43:C, 44:C, 49:P

**USFS Ecomap Regions:** 223A:CC, 231E:CC, 231G:CC, M223A:CC, M231A:CC

CONCEPT

**Environment:** This system occurs along gentle to steep slopes and over bluff escarpments with southerly to westerly aspects in the Ozark and Ouachita Highlands and far western portions of the Interior Low Plateau regions. Parent material can range from calcareous to acidic with very shallow, well‑ to excessively well‑drained soils, sometimes with a fragipan that causes "xero‑hydric" moisture conditions. Conditions are drier than those of the dry oak woodlands.

**Vegetation:** Oak species such as *Quercus stellata, Quercus marilandica*, and *Quercus coccinea* dominate this system with an understory of grassland species such as *Schizachyrium scoparium* and shrub species such as *Vaccinium arboreum*. Drought stress is the major dynamic influencing and maintaining this system. On flatwoods with fragipans, *Quercus stellata* is the major dominant. *Quercus alba, Quercus falcata*, and/or *Carya texana* may be present in some stands. Other species that may be present include *Schizachyrium scoparium, Ulmus alata*, and *Vaccinium arboreum*.

SOURCES

**References:** Comer et al. 2003*, Eyre 1980, Nelson 2010

**Version:** 18 Apr 2012	**Stakeholders:** Midwest, Southeast

**Concept Author:** S. Menard and T. Nigh	**LeadResp:** Midwest

CES202.708 Ozark‑Ouachita Dry‑Mesic Oak Forest

**Primary Division:** Central Interior and Appalachian (202)

**Land Cover Class:** Forest and Woodland

**Spatial Scale & Pattern:** Matrix

**Required Classifiers:** Natural/Semi‑natural; Vegetated (>10% vasc.); Upland

**Diagnostic Classifiers:** Ozark/Ouachita

**National Mapping Codes:** EVT 2304; ESLF 4110; ESP 1304

**Concept Summary:** This system is found throughout the Ozark and Ouachita Highlands ranging to the western edge of the Interior Low Plateau. It is the matrix system of this region and occurs on dry‑mesic to mesic, gentle to moderately steep slopes. Soils are typically moderately to well‑drained and more fertile than those associated with oak woodlands. A closed canopy of oak species (*Quercus rubra* and *Quercus alba*) often associated with hickory species (*Carya* spp.) typifies this system. *Acer saccharum* (or *Acer floridanum* to the south) may occur on more mesic examples of this system. Wind, drought, lightning, and occasional fires can influence this system.

**Comments:** Dry‑mesic to mesic oaks were separated from dry oak (Ozark‑Ouachita Dry Oak Woodland (CES202.707)) per the suggestion of Missouri. This separation may need to be further reviewed. Likewise, the distribution of this system versus the one farther north (North‑Central Interior Dry‑Mesic Oak Forest and Woodland (CES202.046)) needs to be reviewed. Currently the glacial line separates the two systems.

DISTRIBUTION

**Range:** This system is found throughout the Ozark and Ouachita Highlands, reaching to the western Interior Low Plateau of Illinois.

**Divisions:** 202:C

**TNC Ecoregions:** 37:P, 38:C, 39:C, 44:C

**Nations:** US

**Subnations:** AR, IL, KS?, MO, OK

**Map Zones:** 32:P, 43:?, 44:C, 49:C

**USFS Ecomap Regions:** 223A:CC, 231E:CC, 231G:CC, M223A:CC, M231A:CC

CONCEPT

**Environment:** This is the matrix system of this region and occurs on dry‑mesic to mesic, gentle to moderately steep slopes. Soils are typically moderately to well‑drained and more fertile than those associated with oak woodlands.

**Vegetation:** A closed canopy of oak species (*Quercus rubra, Quercus muehlenbergii*, and *Quercus alba*) often associated with hickory species (*Carya* spp.) typifies this system. *Acer saccharum* (or *Acer floridanum (= Acer barbatum)* to the south) may occur in more mesic examples. Some stands in the western edge of the Interior Low Plateau (eastern range limit of the system) may contain *Quercus montana (= Quercus prinus)*. Some other species which may be present include *Carex pensylvanica, Carya tomentosa (= Carya alba), Carya cordiformis, Carya glabra, Carya ovata, Cercis canadensis, Cornus florida, Fagus grandifolia, Fraxinus americana, Gleditsia triacanthos, Gymnocladus dioicus, Hybanthus concolor, Juglans nigra, Juniperus virginiana, Lindera benzoin, Liquidambar styraciflua, Maclura pomifera, Ostrya virginiana, Quercus alba, Quercus falcata, Quercus marilandica, Quercus shumardii, Quercus velutina, Schizachyrium scoparium, Smilax* spp., *Ulmus americana, Ulmus serotina*, and *Vitis aestivalis*.

**Dynamics:** Wind, drought, lightning, and occasional fires can influence this system.

SOURCES

**References:** Comer et al. 2003*, Eyre 1980, Nelson 2010

**Version:** 18 Apr 2012	**Stakeholders:** Midwest, Southeast

**Concept Author:** S. Menard	**LeadResp:** Midwest

CES202.325 Ozark‑Ouachita Shortleaf Pine‑Bluestem Woodland

**Primary Division:** Central Interior and Appalachian (202)

**Land Cover Class:** Forest and Woodland

**Spatial Scale & Pattern:** Matrix

**Required Classifiers:** Natural/Semi‑natural; Vegetated (>10% vasc.); Upland

**Diagnostic Classifiers:** Forest and Woodland (Treed); Very Short Disturbance Interval; Needle‑Leaved Tree; Ozark/Ouachita

**National Mapping Codes:** EVT 2507; ESLF 4281; ESP 1507

**Concept Summary:** This system represents woodlands of the Ouachita and Ozark mountains region of Arkansas, adjacent Oklahoma, and southern Missouri in which *Pinus echinata* is the canopy dominant, and the understory is characterized by *Andropogon gerardii, Schizachyrium scoparium*, and other prairie plants. Although examples of this system occur throughout this region, there is local variation in the extent to which they were present. The center of distribution is the northern and western Ouachita Mountains, and it is best developed in large, dry, and flat to gently undulating portions of the landscape which carry fire well, creating extensive natural fire compartments. In the Ouachitas, the system occurs on the northern Hogback Ridges excluding the Novaculite areas to the south. These are large, gently sloping, east/west‑trending ridges of sandstone and shale, the south‑facing slopes of which constitute large fire compartments. In nearly all examples, *Pinus echinata* occurs with a variable mixture of hardwood species. The exact composition of the hardwoods is much more closely related to aspect and topographic factors than is the pine component. In the Ozark Highlands this system is less extensive but was historically prominent where sandstone‑derived soils are common. In Missouri and Oklahoma, this system occurs on gently dissected upland cherty plains (in addition to sandstone ridges).

**Comments:** This system is primarily confined to gently to moderately sloping, upland plains and is distinguished from Ozark‑Ouachita Shortleaf Pine‑Oak Forest and Woodland (CES202.313), which occurs on more steeply dissected ridges and steep southwest‑facing slopes. The abundance of prairie flora also distinguishes this system from the shortleaf pine‑oak woodland.

DISTRIBUTION

**Range:** This system occurs in the Ouachita and Ozark mountains region of Arkansas, adjacent Oklahoma, and southern Missouri.

**Divisions:** 202:C

**TNC Ecoregions:** 38:C, 39:C

**Nations:** US

**Subnations:** AR, MO, OK

**Map Zones:** 44:C

**USFS Ecomap Regions:** 223A:CC, M223A:CC, M231A:CC

CONCEPT

**Environment:** This system occurs throughout the Ouachita and Ozark mountains region, and there is some local variation in the extent to which it is present. The system is best developed in large portions of the landscape which are flat to gently undulating and which would carry fire well, creating extensive natural fire compartments. In the Ouachitas, the system occurs on the northern Hogback Ridges, which are large, gently sloping, east/west‑trending ridges of sandstone and shale, the south‑facing slopes of which constitute large fire compartments. In nearly all examples, *Pinus echinata* occurs with a variable mixture of hardwood species. The exact composition of the hardwoods is much more closely related to aspect and topographic factors than is the pine component. In the Ozark Highlands this system is less extensive but was historically prominent where sandstone‑derived soils are common. In Missouri and Oklahoma, this system occurs on gently dissected upland cherty plains (in addition to sandstone ridges). This system is primarily confined to gently to moderately sloping, upland plains (larger fire compartments) and is thereby distinguished from shortleaf pine‑oak woodland, which occurs on more steeply dissected ridges and steep southwest‑facing slopes (smaller fire compartments). In the Ouachitas, the primary pine‑bluestem landscape lies to the north of the two tallest ridges, Blackfork Mountain and Rich Mountain, which form a rainshadow by orographic lifting of the moisture‑laden winds from the Gulf of Mexico that strongly influence the climate of this region; precipitation on those ridges can be as high as 147 cm (58 inches) annually, while just to the north, it may fall to 117 cm (46 inches) (T. Foti pers. comm. 2013).

**Vegetation:** In the northern part of this geographic area *Pinus echinata*, xeric oaks and some hickory dominate the overstory with a high percentage of oak on steep north slopes and on *Quercus stellata* flats. Associated species include *Quercus marilandica* and *Carya tomentosa (= Carya alba)* on drier sites and to the west *Carya texana*. In some examples of this system, the aggregate importance of hardwoods may be greater than pine, especially on subxeric and mesic sites (Dale and Ware 1999). Pine is often the canopy emergent on upper slopes. Stand density increases with available moisture. Typical shrubs may include *Vaccinium arboreum, Vaccinium pallidum*, and *Vaccinium stamineum*, but these patches are rare. Various bluestem grasses, legumes and other forbs dominate the understory (herbaceous layer).

**Dynamics:** This system is Fire Regime Group I (Landfire 2007a), with frequent surface fires. Area fire frequency is 3 to 4 years, and the mean fire‑return interval ranges from 1 to 12 years (Masters et al. 1995). Annual fire was common historically, such as in the 1800s. Replacement and mixed‑severity fires are infrequent, every 100 to 1000 years. Stand‑replacement fires occurred mostly under extreme drought conditions during the growing season. The impact of native ungulate grazing (buffalo and elk) was negligible, but fire generally maintained these open woodlands. Drought and moist cycles play a strong role interacting with both fire and native grazing. Other disturbance types include ice storms, wind events, and insect infestations. These disturbances can add significantly to downed woody debris, which can add fuel and increase fire intensity when that downed material is dry and burns. *Pinus echinata* has shorter needles and is not as susceptible to ice as *Pinus taeda*, which is more common further south in Arkansas.

<br />Fire is an important dynamic process, which maintains open woodland conditions and can promote oak and pine regeneration. Today the region consists largely of closed‑canopy forests, though relatively frequent fires prior to Euro‑American settlement created and maintained forests, woodlands, savannas and glades (Stambaugh and Guyette 2006). Prior to 1820, fires were most frequent in areas with low topographic roughness, such as flat or gently sloping lands away from ravines and creeks (Stambaugh and Guyette 2008). For the next hundred years, fires increased as population increased (Stambaugh and Guyette 2006, 2008), until about 1930 when very effective fire‑suppression practices began (Guldin et al. 2005). During the 1800s, these fires helped maintain *Pinus echinata* woodlands with floristically rich understory vegetation of prairie grasses and forbs (Hedrick et al. 2007). There is a very low rate of fire ignitions from lightning strikes in the area, nearly all ignitions are caused by people (Stambaugh and Guyette 2006).

SOURCES

**References:** Comer et al. 2003*, Dale and Ware 1999, Eyre 1980, Foti pers. comm., Guldin et al. 2005, Hedrick et al. 2007, LANDFIRE 2007a, Masters et al. 1995, Stambaugh and Guyette 2006, Stambaugh and Guyette 2008, USFS 1999

**Version:** 14 Jan 2014	**Stakeholders:** Midwest, Southeast

**Concept Author:** T. Foti, R. Masters, D. Zollner	**LeadResp:** Southeast

CES202.313 Ozark‑Ouachita Shortleaf Pine‑Oak Forest and Woodland

**Primary Division:** Central Interior and Appalachian (202)

**Land Cover Class:** Forest and Woodland

**Spatial Scale & Pattern:** Matrix

**Required Classifiers:** Natural/Semi‑natural; Vegetated (>10% vasc.); Upland

**Diagnostic Classifiers:** Forest and Woodland (Treed); Short Disturbance Interval; Needle‑Leaved Tree; Ozark/Ouachita

**National Mapping Codes:** EVT 2367; ESLF 4310; ESP 1367

**Concept Summary:** This system represents forests and woodlands of the Ouachita and Ozark mountains region of Arkansas, adjacent Oklahoma, and southern Missouri in which *Pinus echinata* is an important or dominant component. Although examples of this system occur throughout this region, there is local variation in the extent to which they were present. For example, in the Ozark Highlands, this system was historically prominent only in the southeastern part where sandstone‑derived soils were common, and in the southern part on soils derived from chert, being excluded from or diminished in other areas by non‑conducive soils. In contrast, pine was virtually ubiquitous in the historical forests of the Ouachitas. In nearly all cases (at least in the Ouachitas), *Pinus echinata* occurs with a variable mixture of hardwood species. The exact composition of the hardwoods is much more closely related to aspect and topographic factors than is the pine component. In some examples of this system, the aggregate importance of hardwoods may be greater than pine, especially on subxeric and mesic sites.

**Comments:** This system (CES202.313) is distinguished from the equivalent Appalachian system (CES202.332) at its western extent in central Tennessee by the absence of *Pinus virginiana* and *Quercus montana*, which do not cross the Mississippi River.

DISTRIBUTION

**Range:** This system occurs in the Ouachita and Ozark mountains region of Arkansas, adjacent Oklahoma, and southern Missouri.

**Divisions:** 202:C

**TNC Ecoregions:** 38:C, 39:C

**Nations:** US

**Subnations:** AR, MO, OK

**Map Zones:** 32:C, 44:C, 49:P

**USFS Ecomap Regions:** 223A:CC, 231Ee:CCC, 231Gc:CCC, M223A:CC, M231A:CC

CONCEPT

**Environment:** In the Ozark Highlands, this system was historically prominent only in the southeastern part, where sandstone derived soils were common (USFS 1999) and in the southern part on soils derived from chert; being limited in other areas by non‑conducive soils. In contrast, pine was "virtually ubiquitous in the historical forests of the Ouachitas" (USFS 1999). In nearly all cases (at least in the Ouachitas), Pinus echinata occurs with a variable mixture of hardwood species. The exact composition of the hardwoods is much more closely related to aspect and topographic factors than is the pine component (Dale and Ware 1999).

**Vegetation:** Stands of this system typically contain *Pinus echinata* with various oak species, including *Quercus alba, Quercus rubra, Quercus falcata, Quercus stellata, Quercus velutina*, and *Quercus marilandica*. In some examples of this system, the aggregate importance of hardwoods may be greater than pine, especially on subxeric and mesic sites (Dale and Ware 1999). Typical shrubs include *Vaccinium arboreum, Vaccinium pallidum*, and *Vaccinium stamineum*. Characteristic herbs include *Schizachyrium scoparium, Chasmanthium sessiliflorum, Solidago ulmifolia, Monarda russeliana*, and *Echinacea pallida*.

**Dynamics:** Fire is an important dynamic process, which maintains open woodland conditions and can promote oak and pine regeneration. Fires have historically occurred more frequently than once every 10 years (Hedrick et al. 2007). Today the region consists largely of closed‑canopy forests, though relatively frequent fires prior to Euro‑American settlement created and maintained forests, woodlands, savannas and glades (Stambaugh and Guyette 2006). Prior to 1820, fires were most frequent in areas with relatively low topographic roughness, such as flat or gently sloping lands away from ravines and creeks (Stambaugh and Guyette 2008). For the next hundred years, fires increased as population increased (Stambaugh and Guyette 2006, 2008), until about 1930 when very effective fire‑suppression practices began (Guldin et al. 2005). During the 1800s, these fires helped maintain *Pinus echinata* and hardwood forests with floristically rich understory vegetation of grasses and forbs (Hedrick et al. 2007). There is a very low rate of fire ignitions from lightning strikes in the Ozark Highlands area, nearly all ignitions are caused by people (Stambaugh and Guyette 2006). However, fires started by lightning could become very large, since ignitions may occur associated with drought, high winds, drying fuels, and decreasing humidity. The number of lightning strike‑initiated wildfires is higher in the Ouachita Mountains and Boston Mountains. In these areas, presettlement wildland fires were ignited by Native Americans and by lightning (Foti and Glenn 1990). Other disturbances include wind, tornados, drought, and ice storms. These disturbances can open forest canopies and add significantly to downed woody debris, which can add fuel and lead to increased fire intensity when that downed material is dry and burns. *Pinus echinata* has shorter needles and is not as susceptible to ice as *Pinus taeda*, which is more common further south in Arkansas.

SOURCES

**References:** Comer et al. 2003*, Dale and Ware 1999, Eyre 1980, Foti and Glenn 1990, Guldin et al. 2005, Hedrick et al. 2007, Nelson 2010, Stambaugh and Guyette 2006, Stambaugh and Guyette 2008, USFS 1999

**Version:** 14 Jan 2014	**Stakeholders:** Midwest, Southeast

**Concept Author:** T. Foti and R. Evans	**LeadResp:** Southeast

CES202.268 Piedmont Hardpan Woodland and Forest

**Primary Division:** Central Interior and Appalachian (202)

**Land Cover Class:** Forest and Woodland

**Spatial Scale & Pattern:** Large patch

**Required Classifiers:** Natural/Semi‑natural; Vegetated (>10% vasc.); Upland

**Diagnostic Classifiers:** Forest and Woodland (Treed); Clay Soil Texture; Broad‑Leaved Tree

**National Mapping Codes:** EVT 2342; ESLF 4149; ESP 1342

**Concept Summary:** This system of the southern Piedmont occurs in places where a particularly dense clay hardpan has developed over a range of typically mafic rocks, sometimes with more limited areas of shallow glade‑like vegetation. In the deeper soil portions of this system, the density of the clay, in combination with its shrink‑swell properties, limits water and root penetration into the soil and creates xeric conditions for plants despite the presence of deep soil. Possibly the most typical expression of this system in North and South Carolina is an open forest or woodland of *Quercus stellata*, with *Quercus marilandica* as a characteristic associate. The open canopy leads to a better developed herb layer than in most Piedmont forests, one that is usually grassy. In Virginia, typical canopy trees include *Quercus alba, Carya glabra*, and *Fraxinus americana*. Some of these sites may have once supported open prairies or prairie savannas when they burned more frequently. Fire was probably once the most important natural dynamic process, but the universal elimination of fire in the Piedmont makes this difficult to observe on most of the modern landscape.

**Comments:** This system is distinguished from others in the Piedmont by occurrence on distinctive substrates. These include hardpan soils in the Triassic basins, as well as on soils derived from gabbro and on acidic metasediments in the Carolina Slate Belt. Despite the contrast in vegetation, this system will sometimes grade quite gradually into Piedmont Upland Depression Swamp (CES202.336), with which it often co‑occurs. One of the best sites in South Carolina is the Little Mountain Creek area near Edgefield, SC (Simon and Hayden 2014).

DISTRIBUTION

**Range:** As currently known, this system is found in the Piedmont of Maryland, Virginia, North Carolina, South Carolina and Georgia. Its status in Alabama is not known. Its occurrence may be more frequent in the Triassic basins, but it is not restricted to them.

**Divisions:** 202:C

**TNC Ecoregions:** 52:C, 61:C

**Nations:** US

**Subnations:** GA, MD, NC, SC, VA

**Map Zones:** 54:C, 59:C, 60:C, 61:C

**USFS Ecomap Regions:** 221B:CC, 221D:CC, 221J:CC, 231A:CC, 231D:CC, 231I:CC, M221A:CC, M221B:CC

CONCEPT

**Environment:** This system occurs in places in the southern Piedmont where a particularly dense clay hardpan, typically composed of Montmorillonite, has developed. The substrate is typically mafic igneous or metamorphic rock (gabbro, basalt, diabase, or amphibolite) but occasionally is slate. The density of the clay, or its shrink‑swell properties, limits penetration of water into the soil and limits penetration of roots, creating xeric conditions for plants despite the presence of deep soil. These areas generally occur on unusually flat uplands but may occur on tops of narrower ridges. Only a minority of these substrates form the distinctive soil conditions of this system. Local topography that promotes runoff is important to forming this system. Areas with these soil conditions but with concave topography perch water and support Piedmont depressional wetlands. Soils in most examples are basic or circumneutral, but those formed from slate are somewhat acidic. In Virginia and adjacent Maryland, this system occupies one of the largest Triassic basins in eastern North America. It includes a mix of sedimentary rocks, especially siltstone, mixed with igneous intrusions. The igneous rocks weather to form more mafic soils, while the sedimentary rocks are more acidic. The local landscape may best be thought of as a lowland, in comparison with the surrounding and prevailing topography.

**Vegetation:** Vegetation consists of xerophytic species, most typically consisting of open forests or woodlands of *Quercus stellata*, with *Quercus marilandica* as a characteristic associate in North and South Carolina. In Virginia and adjacent Maryland, *Quercus alba, Fraxinus americana*, and *Carya glabra* are common canopy components. The open canopy leads to a better developed herb layer than in most Piedmont forests, one that is usually grassy. Some of these sites may have once supported open prairies or prairie savannas when they burned more frequently. A significant flora of shade‑intolerant herbs with prairie affinities is present in open areas on these soils. Typical understory species include *Juniperus virginiana, Cercis canadensis, Diospyros virginiana, Vaccinium arboreum, Ulmus alata*, and *Chionanthus virginicus*. Shrubs may be sparse or dense, with *Viburnum rafinesqueanum, Viburnum prunifolium, Vaccinium stamineum*, and *Vaccinium pallidum* being typical. The most common herbs are *Danthonia spicata* and *Schizachyrium scoparium*. Other herbs include *Clematis ochroleuca, Sericocarpus linifolius (= Aster solidagineus), Hieracium venosum, Hieracium gronovii, Hypericum hypericoides, Symphyotrichum dumosum (= Aster dumosus), Lespedeza* spp., *Oenothera fruticosa, Liatris pilosa (= Liatris graminifolia)*, and *Solidago* spp. (Schafale and Weakley 1990). In contrast to upland forests of adjacent portions of the Virginia Piedmont, there seems to be a greater abundance of hickory in examples of this ecological system (Farrell and Ware 1991, Ware 1992).

**Dynamics:** Fire was probably once the most important natural dynamic process, but the universal elimination of fire in the Piedmont makes this difficult to determine. Both the drier character of the sites and the distinctive soil conditions interact with one another to retard woody succession. These factors would presumably have interacted with the fire regime to promote more open vegetation on these sites. This would presumably lead to a greater probability that these open woodland conditions would prevail for a longer period than they would on more typical soils. Fire would have kept canopies open and would have promoted a more diverse, grass‑dominated herb layer. Bison may have once been a significant grazing influence on this system. These sites are now of limited extent and it is harder to determine how these past disturbance factors operated in the larger landscape.

SOURCES

**References:** Comer et al. 2003*, EPA 2004, Edwards et al. 2013, Engeman et al. 2007, Eyre 1980, Farrell and Ware 1991, Nelson 1986, Nordman 2013, Peet et al. 2013, Schafale 2012, Schafale and Weakley 1990, Simon and Hayden 2014, Southeastern Ecology Working Group n.d., Ware 1992, Wharton 1978

**Version:** 01 Oct 2015	**Stakeholders:** East, Southeast

**Concept Author:** M. Schafale, R. Evans, G. Fleming, M. Pyne	**LeadResp:** Southeast

CES202.319 Southeastern Interior Longleaf Pine Woodland

**Primary Division:** Central Interior and Appalachian (202)

**Land Cover Class:** Forest and Woodland

**Spatial Scale & Pattern:** Large patch

**Required Classifiers:** Natural/Semi‑natural; Vegetated (>10% vasc.); Upland

**Diagnostic Classifiers:** Forest and Woodland (Treed); Short Disturbance Interval; Needle‑Leaved Tree

**National Mapping Codes:** EVT 2351; ESLF 4254; ESP 1351

**Concept Summary:** This system encompasses the fire‑maintained non‑Coastal Plain woodlands and forests where *Pinus palustris* is a dominant or codominant canopy species. Its current range includes the outer Piedmont of Georgia and the Carolinas and various parts of Alabama, including the Talladega upland region (quartzite‑slate transition) and the Cumberland Plateau, as well as, at least historically, the intervening Ridge and Valley. Examples occur on rolling to somewhat mountainous upland slopes in North Carolina, South Carolina, Georgia, and Alabama. They are believed to naturally be open woodlands with grassy ground cover, but many are now closed forests with dense shrubs or with little ground cover. *Pinus palustris* is either dominant, codominant, or present in circumstances that indicate former dominance or codominance. *Pinus echinata, Quercus coccinea, Quercus falcata, Quercus marilandica, Quercus montana, Quercus stellata*, and *Quercus velutina* are frequent associates, often codominating. *Carya pallida* and *Sassafras albidum* are also frequent trees. Some of the most frequently encountered grasses include *Andropogon* spp., *Chasmanthium laxum, Danthonia spicata, Dichanthelium commutatum, Panicum virgatum, Piptochaetium avenaceum, Schizachyrium scoparium*, and *Sorghastrum nutans*. Important forbs include *Coreopsis major, Euphorbia corollata, Helianthus microcephalus, Pityopsis graminifolia, Solidago odora, Tephrosia virginiana*, and the fern *Pteridium aquilinum*.

**Comments:** This system is closely related to the upland longleaf pine systems of the Coastal Plain and other southern open pine woodlands, with which it shares the ecological importance of fire, much of its flora and presumably fauna, and probably canopy dynamics. It is distinguished by the distinctive non‑Coastal Plain soils and landscape, with its greater topographic relief, and by some floristic and compositional differences. It probably had less frequent natural fire and a somewhat more mixed canopy, with additional pine species in addition to oaks.

This system is distinguished from all other Piedmont and interior systems in having *Pinus palustris*, an indicator of frequent fire, as a dominant species. However, universal logging and fire suppression have blurred the distinction and have made many former examples indistinguishable from one of these other systems. This system should be recognized where there remains evidence of its past occurrence in the form of remnant flora.

One South Carolina site is on the massive and extensive Liberty Hill granitoid pluton between Lancaster and Camden (Kershaw County), along the Catawba River (B. Pittman pers. comm.).

DISTRIBUTION

**Range:** This system once occurred in parts of the mostly outer Piedmont, from central North Carolina to Alabama, where it extends into the adjacent Ridge and Valley in northeastern Alabama and northwest Georgia. More extensive areas are now largely, if not exclusively, restricted to south‑central North Carolina (outer Piedmont) and eastern Alabama (Talladega upland), as well as the Cumberland Plateau and at least historically, the Ridge and Valley of Alabama. Smaller remnants are found in very limited areas of South Carolina and Georgia (such as Pine Mountain).

**Divisions:** 202:C

**TNC Ecoregions:** 50:C, 52:C

**Nations:** US

**Subnations:** AL, GA, NC, SC

**Map Zones:** 48:C, 54:C, 59:C

**USFS Ecomap Regions:** 231A:CC, 231C:CC, 231D:CC, 231I:CC

CONCEPT

**Environment:** This system occurs in upland settings, which may range from gently rolling to rugged and mountainous. Geologic substrates vary. Most portions are dry, but occasional moist areas and seepage wetlands occur. The primary influence on the system is frequent fire, associated with a location near a fire‑prone portion of the Coastal Plain or with other factors. Apparently once widespread along the Fall‑line, remnants are now largely limited to two clusters, in eastern Alabama and adjacent Georgia and in south‑central North Carolina. There are also examples on the Piedmont portion of the Sumter National Forest, on the Long Cane Ranger District and possibly on the Enoree Ranger District. The former occurs on rugged terrain associated with the extension of geologic belts of the Blue Ridge. The latter is on gently to moderately rolling topography of metasedimentary and volcanic rocks. Most common on the poorest soils in the Piedmont of eastern Alabama, *Pinus palustris* was "a prominent constituent of the upland forests of nearly every county" but, by the first half of the twentieth century, "grew too scattered to be logged economically" (Harper 1943). About 35% of the original forest there was estimated to have been evergreen; the most common pines were *Pinus taeda, Pinus palustris*, and *Pinus echinata* (Harper 1943). However, this estimate is likely to have been low, since much *Pinus palustris* logging, turpentining and regeneration failure had already occurred prior to Harper's time (J.M. Varner pers. comm.). Today, montane *Pinus palustris* occurs mainly on ridgelines and south to southwesterly slopes (USFWS 2005), but was previously found on nearly all upland sites surveyed in Coosa County, Alabama (Reed 1905). In northwest Georgia, *Pinus palustris* occurs above 300 m (1000 feet) elevation, and it occurs up to nearly 600 m (2000 feet) in Talladega County, Alabama (Harper 1905).

**Vegetation:** Vegetation consists of open woodlands or forests. *Pinus palustris* is either dominant, codominant, or present in circumstances that indicate former dominance or codominance. *Pinus echinata, Pinus taeda, Quercus falcata, Quercus stellata, Quercus montana (= Quercus prinus), Quercus coccinea*, and *Quercus velutina* are frequent associates, often codominating. Alteration of fire regimes and universal logging have made the natural condition of the vegetation somewhat uncertain. Almost certainly *Pinus palustris* was more abundant than it usually is at present, but very likely some component of other pines and oaks was present. Under conditions of frequent fire, understories and shrub layers were sparse and the grassy herb layer dense. Some of the most frequently encountered grasses include *Andropogon* spp., *Chasmanthium laxum, Danthonia spicata, Dichanthelium commutatum (= Panicum commutatum), Panicum virgatum, Piptochaetium avenaceum, Schizachyrium scoparium*, and *Sorghastrum nutans*. Some frequent forbs include *Tephrosia virginiana, Solidago odora*, and *Pteridium aquilinum* (Andrews 1917, Peet 2006). Other frequently dominant species, such as *Piptochaetium avenaceum* and *Danthonia spicata*, are not characteristic of Coastal Plain longleaf pine systems. Many other grasses and forbs are shared with the upland longleaf pine systems of the Coastal Plain. There is no evidence that *Aristida stricta* or *Aristida beyrichiana* were present in stands of this system, as these species are confined to the coastal plains. In remnant examples, where fire suppression has affected vegetation structure, the ground cover is often shrubby, with dense ericaceous shrubs leaving little space for herbs. Examples that have been burned recently often have ground cover dominated by shrubs and hardwood sprouts, with somewhat increased herb cover.

**Dynamics:** The dynamics of this system are strongly dominated by fire. The needles of *Pinus palustris* are an important fuel source for low‑intensity fires. Fires probably once occurred at frequencies similar to those in the Coastal Plain but more frequently than in any other Piedmont ecological system. Evidence suggests fire frequencies of once every two to four years, with some annual fires (Bale 2009). Modern fire suppression has allowed *Pinus taeda* and *Quercus* spp. to increase in density, along with shrubs, and has resulted in the decrease in cover and diversity of the herb layer. Reproduction of *Pinus palustris* has been largely eliminated by the lack of fire, and the rooting of feral hogs (*Sus scrofa*). Where the canopy was also logged, *Pinus palustris* has often been completely eliminated, leaving the system indistinguishable from logged examples of Southern Piedmont Dry Oak‑(Pine) Forest and Woodland (CES202.339). Because *Pinus palustris* and some of the canopy species naturally associated with it are fairly resilient to fire, and many have the ability to sprout, reintroduction of fire can return this system to its natural composition and structure, but this must be done gradually. Despite frequent fire, canopy dynamics were probably naturally dominated by gap‑phase regeneration, with trees reproducing in small to medium‑sized gaps created by wind storms and hot spots in fires. *Pinus palustris* is a long‑lived tree, which continues to produce greater numbers of cones after age 100.

SOURCES

**References:** Andrews 1917, Bale 2009, Comer et al. 2003*, Eyre 1980, Harper 1905, Harper 1943, Klaus 2006, LANDFIRE 2007a, Maceina et al. 2000, NatureServe 2011a, Peet 2006, Peet et al. 2013, Pittman pers. comm., Reed 1905, Schafale 2012, Schafale and Weakley 1990, Schafale pers. comm., Simon and Hayden 2014, USFWS 2005, Varner et al. 2003a, Varner et al. 2003b

**Version:** 14 Jan 2014	**Stakeholders:** Southeast

**Concept Author:** M. Schafale and R. Evans	**LeadResp:** Southeast

CES202.332 Southern Appalachian Low‑Elevation Pine Forest

**Primary Division:** Central Interior and Appalachian (202)

**Land Cover Class:** Forest and Woodland

**Spatial Scale & Pattern:** Large patch

**Required Classifiers:** Natural/Semi‑natural; Vegetated (>10% vasc.); Upland

**Diagnostic Classifiers:** Forest and Woodland (Treed); Acidic Soil; Short Disturbance Interval; Needle‑Leaved Tree

**National Mapping Codes:** EVT 2353; ESLF 4256; ESP 1353

**Concept Summary:** This ecological system consists of *Pinus echinata*‑ and *Pinus virginiana*‑dominated forests in the lower elevation Southern Appalachians and adjacent Piedmont and Cumberland Plateau, extending into the Interior Low Plateau of Indiana, Kentucky and Tennessee. Examples can occur on a variety of topographic and landscape positions, including ridgetops, upper and midslopes, as well as lower elevations (generally below 700 m [2300 feet]) in the Southern Appalachians such as mountain valleys. Examples occur on a variety of acidic bedrock types. Frequent, low‑intensity fires coupled with severe fires may have been the sole factor favoring the occurrence of this system instead of hardwood forests in the absence of fire. Under current conditions, stands are dominated by *Pinus echinata* or *Pinus virginiana*. *Pinus rigida* may sometimes be present. Hardwoods are sometimes abundant, especially dry‑site oaks such as *Quercus falcata, Quercus montana*, and *Quercus coccinea*, but also *Carya glabra, Acer rubrum*, and others. The shrub layer may be well‑developed, with *Gaylussacia baccata, Kalmia latifolia, Rhododendron minus, Vaccinium pallidum*, or other acid‑tolerant species most characteristic. Herbs are usually sparse but may include *Pityopsis graminifolia* and *Tephrosia virginiana*.

**Comments:** This system and its component associations are among the least studied in the Southern Appalachians (Harrod and White 1999). Settlement, universal logging, pine beetle outbreaks, and the lack of fire potentially have altered their character and blurred their boundaries more than most systems in the region. The situation is further complicated by the potential for pine‑dominated forests to have been both created and destroyed in different places by these disturbances.

The relationship between this system and Southern Appalachian Montane Pine Forest and Woodland (CES202.331) may need further clarification. Southern Appalachian Low‑Elevation Pine Forest (CES202.332) is distinguished by its occurrence as large patches on lower terrain (generally below 700 m [2300 feet]) and less extreme topography. The vegetation of the two systems may overlap due to the factors outlined above, but *Pinus pungens*, and to some extent *Pinus rigida*, are more typical of the former, while *Pinus echinata* and *Pinus virginiana* are more typical of the latter. Pine‑dominated stands in the Piedmont portions of the Sumter National Forest are best affiliated with a pine‑dominated "phase" of Southern Piedmont Dry Oak‑(Pine) Forest and Woodland (CES202.339), rather than with this system. These stands would be dominated by *Pinus echinata* with hardwoods and possibly *Pinus taeda*. In contrast to more montane low‑elevation stands, *Pinus virginiana* should be absent or present at very low cover.

Presently the shortleaf pine‑dominated vegetation of the Interior Low Plateau (ILP), including examples in southern Indiana and the Tennessee portion of Land Between the Lakes, is included in this system. Frost (1998) treats the ILP region in a different fire‑return‑interval class than the core range of this system, although local variation may overwhelm the broad regional differences. If more detailed information becomes available to document important ecological differences between these areas, a new system may be required.

This system (CES202.332) at its western extent in central Tennessee and Kentucky would be distinguished from equivalent Ozarkian systems (e.g., Ozark‑Ouachita Shortleaf Pine‑Oak Forest and Woodland (CES202.313)) by the presence of *Pinus virginiana* and *Quercus montana*, which do not cross the Mississippi River.

DISTRIBUTION

**Range:** This system is found primarily in the Appalachian regions of Kentucky and the Southern Blue Ridge in northern Georgia, western North Carolina, southeastern Tennessee, the Cumberlands of Alabama, parts of the Interior Low Plateau (e.g., the Knobs Region of Kentucky and southern Indiana and the western Highland Rim of Tennessee), and southwestern Virginia. Any possible stands in the Piedmont would be found in the western foothills portions adjacent to the mountains.

**Divisions:** 202:C

**TNC Ecoregions:** 44:C, 50:C, 51:C, 52:C

**Nations:** US

**Subnations:** AL, GA, IN, KY, NC, SC, TN, VA

**Map Zones:** 47:C, 48:C, 53:C, 54:C, 57:C, 59:C

**USFS Ecomap Regions:** 221H:CC, 221J:CC, 223D:CC, 223E:CC, 231A:CC, 231C:CC, 231D:CC, 231I:CC, M221A:CC, M221C:CC, M221D:CC

CONCEPT

**Environment:** This system occurs on ridgetops, upper and midslopes, in mountain valleys and the lower ranges. It is found on south‑ and southwest‑facing slopes (Whittaker 1956). Bedrock may be a variety of types, but the system may be limited to acidic substrates. Fire is undoubtedly a very important and necessary influence.

**Vegetation:** Vegetation consists of closed to open forests or woodlands dominated by *Pinus echinata* or *Pinus virginiana*. *Pinus rigida* may sometimes be present. Hardwoods are sometimes abundant, especially dry‑site oaks such as *Quercus falcata, Quercus montana (= Quercus prinus)*, and *Quercus coccinea*, but also *Carya glabra, Acer rubrum*, and others. An extensive hardwood component may partly be the result of fire suppression. The shrub layer may be well‑developed, with *Vaccinium pallidum, Gaylussacia baccata*, or other acid‑tolerant species most characteristic. Herbs are usually sparse but may include *Pityopsis graminifolia* and *Tephrosia virginiana*. Herbs probably were more abundant and shrubs less dense when fires occurred more frequently, and the communities of this system may have been grassy under more natural conditions, with *Schizachyrium scoparium* being a typical component, possibly with *Danthonia* sp.

**Dynamics:** Fire is clearly an important influence on the dynamics of this ecological system, and frequent, low‑intensity fires coupled with occasional severe fires (Harrod and White 1999, Fesenmyer and Christenson 2010) are thought to have been the primary factor leading to the occurrence of this system rather than hardwood forests on dry sites in the absence of fire. Fires probably were frequent and of low intensity, or a mix of low and higher intensity. Over many decades, accumulation of dead biomass can predispose these forests to catastrophic fire. However, even in the absence of fire, successional changes are normally restricted (possibly ending with oak domination) because most sites are infertile and dry (Murphy and Nowacki 1997). Fire probably is important for determining the dominance of pine species, the component of hardwoods, and the overall vegetation structure. *Pinus echinata* is fairly resilient to fire once mature, while *Pinus virginiana* individuals are fairly susceptible to fire but well‑adapted to establishing in areas opened by intense fire.

Southern pine beetles (*Dendroctonus frontalis*) are an important disturbance and threat in this system, at least under present conditions and severe outbreaks can kill all the pines without creating the conditions for the pines to regenerate. Effects of logging and past clearing as well as lack of fire make understanding of this system's natural character and dynamics difficult. An extensive hardwood component may partly be the result of lack of fire. Some pine‑dominated areas appear to be successional stands established in former hardwood forests after logging or cultivation, and would not be expected to have the same dynamics or ecosystem characteristics as natural pine forests maintained by fire. In natural pine forests, with adequate seed and seedlings, logging may allow pines to regenerate or, without adequate seedlings and with lack of fire may lead to a change in composition to hardwoods. This might also alter canopy composition as well as structure. In many cases, several prescribed fires or a combination of fire and thinning treatments will be necessary to restore these ecosystems (Elliott and Vose 2005).

SOURCES

**References:** Comer et al. 2003*, DuMond 1970, Edwards et al. 2013, Elliott and Vose 2005, Elliott et al. 2011, Evans et al. 2009, Eyre 1980, Fesenmyer and Christensen 2010, Frost 1998, Gettman 1974, Harper 1943, Harrod and White 1999, Murphy and Nowacki 1997, NatureServe 2002, Nelson 1986, Patterson 1994, Schafale 2012, Schafale and Weakley 1990, Simon 2011, Simon 2015, Tobe et al. 1992, Wendel and Smith 1990, Whittaker 1956

**Version:** 28 Apr 2016	**Stakeholders:** East, Midwest, Southeast

**Concept Author:** M. Schafale, R. Evans, R. White	**LeadResp:** Southeast

CES203.241 Southern Atlantic Coastal Plain Dry and Dry‑Mesic Oak Forest

**Primary Division:** Gulf and Atlantic Coastal Plain (203)

**Land Cover Class:** Forest and Woodland

**Spatial Scale & Pattern:** Large patch

**Required Classifiers:** Natural/Semi‑natural; Vegetated (>10% vasc.); Upland

**Diagnostic Classifiers:** Long Disturbance Interval; Broad‑Leaved Tree

**National Mapping Codes:** EVT 2335; ESLF 4141; ESP 1335

**Concept Summary:** This system encompasses oak‑dominated forests of somewhat fire‑sheltered dry to dry‑mesic sites in the Mid‑Atlantic and South Atlantic coastal plains from southeastern Virginia to Georgia. Sites where this system occurs are somewhat protected from most natural fires by some combination of steeper topography, isolation from the spread of fire, and limited flammability of the vegetation. If fires were more frequent, the vegetation would likely be replaced by more fire‑tolerant southern pines, especially *Pinus palustris*.

**Comments:** There remains some uncertainty how this system and other dry and dry‑mesic hardwood systems should be divided. There is a broad gradient in climate and species composition from north to south and west. The boundaries at the north edge of the Mid‑Atlantic Coastal Plain ecoregion and at the break between the South Atlantic Coastal Plain and East Gulf Coastal Plain ecoregions are boundaries of convenience to create breaks in this broad gradient. Better boundaries may be possible. Differences from comparable systems in the Piedmont are sometimes fairly subtle, and species that differentiate them in one part of the range many not work in other parts. In particular, some species that are excluded from the Coastal Plain farther south are common components farther north. The boundary with Southern Atlantic Coastal Plain Mesic Hardwood Forest (CES203.242) and with adjacent *Pinus palustris*‑dominated systems may be blurred by fire suppression.

Should there be a separate system for calcareous elements, comparable to Southern Coastal Plain Limestone Forest (CES203.502)? An example association for this base‑rich phase (or system) is *Carya glabra ‑ Tilia americana var. caroliniana ‑ Acer floridanum / Trillium maculatum* Forest (CEGL004747).

DISTRIBUTION

**Range:** This system ranges from southeastern Virginia (south of the James River) south to southeastern Georgia in the Atlantic Coastal Plain.

**Divisions:** 203:C

**TNC Ecoregions:** 56:C, 57:C

**Nations:** US

**Subnations:** GA, NC, SC, VA

**Map Zones:** 55:C, 58:C, 60:C

**USFS Ecomap Regions:** 232C:CC, 232H:CC, 232I:CC, 232J:CC

CONCEPT

**Environment:** This system occurs in dry‑mesic to dry but not xeric sites, generally on upper to midslopes in bluff systems, but occasionally it occurs on broader uplands or on the highest parts of non‑flooded river terraces. Soils are generally acidic, though calcareous soils occur occasionally (as in Carya glabra ‑ Tilia americana var. caroliniana ‑ Acer barbatum / Trillium maculatum Forest (CEGL004747)). Soils are loamy to clayey and well‑drained but not excessively drained. Similar sites with coarse sandy soils tend to support other ecological systems, in part due to the influence of more frequent fire. Sites are somewhat protected from most natural fires by steep topography and by limited flammability of the vegetation. Fires that penetrate them are generally low in intensity and have fairly limited ecological effect.

**Vegetation:** Vegetation consists of forests dominated by combinations of upland oaks, particularly *Quercus alba, Quercus falcata*, and *Quercus stellata*. In the northern part of the range, *Quercus rubra* may be a component, while in the southern part, evergreen species such as *Quercus nigra* or *Quercus hemisphaerica* become more prominent. Hickories (*Carya* spp.) are also prominent, including *Carya tomentosa (= Carya alba), Carya glabra*, and *Carya pallida*. Other woody plants may include *Tilia americana var. caroliniana, Acer floridanum (= Acer barbatum), Aesculus pavia, Osmanthus americanus var. americanus, Ilex glabra, Ilex opaca, Vaccinium arboreum, Vaccinium elliottii*, and *Clethra alnifolia*. Some typical herbs are *Trillium maculatum* and *Chasmanthium sessiliflorum*. There is some variation in composition with aspect and degree of exposure to fire. *Pinus echinata* may be present in some stands, particularly on drier south‑ and west‑facing slopes but is typically not dominant. *Pinus taeda* is sometimes present, but it is unclear if it is a natural component or has entered only as a result of past cutting. More mesophytic species such as *Fagus grandifolia* and *Magnolia grandiflora* are absent or are confined to the understory. Analogous systems on the Gulf Coastal Plain have pine as a natural component, and this may be true for some examples of this system as well, where occasional fires may allow them to regenerate. A well‑developed shrub layer may be present, with *Vaccinium* spp. and *Gaylussacia* spp. most typical. The herb layer is generally sparse, and species richness tends to be low. In examples where fires have occurred, the understory is open and savannalike and dominated by grasses and forbs rather than shrubs.

**Dynamics:** Fire is intermediate in frequency in this system, being less frequent than in adjacent *Pinus palustris*‑dominated stands, and more frequent than in mesic hardwood stands below. This fire regime is an important factor separating it from adjacent *Pinus palustris*‑dominated systems. If fire does penetrate, it is likely to be low in intensity and have somewhat limited ecological effects. However, there is some evidence that this system has expanded into areas once occupied by *Pinus palustris* as fire has been suppressed (Ware et al. 1993). There may have been a shifting boundary between these systems, driven by variation in fire frequency. These forests probably generally naturally existed as old‑growth forests, with canopy dynamics dominated by gap‑phase regeneration. However, exposure to occasional fires and hurricanes may create more frequent and larger canopy disturbances than analogous systems inland.

Frequent surface fires occurred on a 5‑ to 10‑year return interval from both lightning and Native American ignitions. These frequent light surface fires would have maintained a grassy understory and kept more fire‑tolerant hardwoods and shrubs from capturing the understory and forming a midstory layer. Lightning fires occurred primarily during the spring dry season (April and May) with a secondary peak of Native American and settler burning during the fall (October and November) (Landfire 2007a). Occasionally, during extensive droughts, mixed‑severity or stand‑replacement fires did occur, especially in drier stand dominated of codominated by *Pinus echinata*.

Local blowdown winds associated with thunderstorms created gaps on a small but continual basis. More extensive regional disturbances included tropical storms during the growing season and ice storms during winter (in the northern part of the range). Dense stands of middle to older aged pines (where present) were susceptible to periodic mortality from bark beetle epidemics (Landfire 2007a).

SOURCES

**References:** Bennett and Nelson 1991, Comer et al. 2003*, Edwards et al. 2013, Engeman et al. 2007, Eyre 1980, LANDFIRE 2007a, Nelson 1986, Nordman 2013, Rehn and Hebard 1916, Schafale 2012, Schafale and Weakley 1990, Ware et al. 1993

**Version:** 14 Jan 2014	**Stakeholders:** East, Southeast

**Concept Author:** R. Evans and M. Schafale	**LeadResp:** Southeast

CES202.898 Southern Interior Low Plateau Dry‑Mesic Oak Forest

**Primary Division:** Central Interior and Appalachian (202)

**Land Cover Class:** Forest and Woodland

**Spatial Scale & Pattern:** Matrix

**Required Classifiers:** Natural/Semi‑natural; Vegetated (>10% vasc.); Upland

**National Mapping Codes:** EVT 2305; ESLF 4111; ESP 1305

**Concept Summary:** This system of upland hardwood‑dominated forests occurs in the Interior Low Plateau region of the southeastern United States along ridgetops and slopes of various aspects. The system includes essentially all upland hardwood stands of the region except for mesic hardwood forests (which are accommodated by South‑Central Interior Mesophytic Forest (CES202.887)). The floristic expression of different stands included in this system varies considerably with aspect and soil type. Included here are a variety of associations ranging along a moisture gradient from submesic to drier ones. The submesic to dry‑mesic expressions tend to be found on midslopes with northerly to easterly aspects, and the drier ones on southerly to westerly aspects and on broad ridges. Parent material can range from calcareous to acidic with very shallow, well‑ to excessively well‑drained soils in the drier expressions and moderately well‑drained soils in the submesic to dry‑mesic ones. The canopy closure of this system ranges from closed to somewhat open in the drier examples. Historically, these examples may have been more open under conditions of more frequent fire.

A number of different *Quercus* species may dominate stands of this system, with *Carya* species also prominent. In some drier examples on more acidic substrates, *Quercus montana* is typical over most of the range, reflecting relations with other Appalachian systems to the east. In addition, *Quercus stellata, Quercus marilandica*, and *Quercus coccinea* will also share dominance or be prominent in many of the drier examples. *Quercus muehlenbergii* and/or *Quercus shumardii* may appear in drier examples with high base status. *Quercus alba* may also be present but not typically dominant. In the submesic to dry‑mesic examples, *Quercus alba* will typically exhibit dominance, possibly with *Quercus velutina* or *Quercus falcata*. The understories are typically shrub‑ and small tree‑dominated, with the typical species varying with aspect, soil, and moisture relations.

**Comments:** The range of this system is consistent with the non‑coastal plain portion of the "Western Mesophytic" Forest region of Braun (1950), Keever (1971), and Greller (1988). To the glaciated north, it is replaced by North‑Central Interior Dry‑Mesic Oak Forest and Woodland (CES202.046) or North‑Central Interior Dry Oak Forest and Woodland (CES202.047).

DISTRIBUTION

**Range:** This system occurs in the southeastern Interior Highlands of the Interior Low Plateau region, including southern Indiana and a small part of southeastern Ohio.

**Divisions:** 202:C

**TNC Ecoregions:** 44:C

**Nations:** US

**Subnations:** AL, IL, IN, KY, OH, TN

**Map Zones:** 47:C, 48:C, 49:C, 53:C

**USFS Ecomap Regions:** 223B:CC, 223D:CC, 223E:CC, 223F:CC

CONCEPT

**Environment:** This system encompasses a variety of associations ranging along a moisture gradient from submesic to drier ones. The submesic to dry‑mesic expressions tend to be found on midslopes with northerly to easterly aspects, the drier ones on southerly to westerly aspects and on broad ridges. Parent material can range from calcareous to acidic with very shallow, well‑ to excessively well‑drained soils in the drier expressions and moderately well‑drained soils in the submesic to dry‑mesic ones.

**Vegetation:** A number of different *Quercus* species may dominate stands of this system, with *Carya* species also prominent. In the drier examples, *Quercus montana (= Quercus prinus)* is typical over most of the range, reflecting relations with other Appalachian systems to the east. In addition, *Quercus stellata, Quercus marilandica*, and *Quercus coccinea* will also share dominance or be prominent in many of the drier examples. *Quercus muehlenbergii* and/or *Quercus shumardii* may appear in drier examples with high base status. *Quercus alba* may also be present but not typically dominant. In the submesic to dry‑mesic examples, *Quercus alba* will typically exhibit dominance, possibly with *Quercus velutina* or *Quercus falcata*. The understories are typically shrub‑ and small tree‑dominated, with the typical species varying with aspect, soil, and moisture relations. Some typical species include *Cornus florida, Cercis canadensis, Oxydendrum arboreum, Vaccinium pallidum, Vaccinium stamineum, Vaccinium arboreum*, other highbush *Vaccinium* species, *Kalmia latifolia, Viburnum acerifolium, Styrax americanus*, and others. Some more open and drier stands may exhibit an understory of grassland species such as *Schizachyrium scoparium, Danthonia spicata*, and others. Forbs of the Fabaceae (e.g., *Desmodium*) and Asteraceae (e.g., *Helianthus*) will be prominent in many examples.

SOURCES

**References:** Braun 1950, Comer et al. 2003*, Evans et al. 2009, Eyre 1980, Greller 1988, Keever 1971, ONHD unpubl. data

**Version:** 22 Jan 2008	**Stakeholders:** Midwest, Southeast

**Concept Author:** M. Pyne	**LeadResp:** Southeast

CES202.339 Southern Piedmont Dry Oak‑(Pine) Forest and Woodland

**Primary Division:** Central Interior and Appalachian (202)

**Land Cover Class:** Forest and Woodland

**Spatial Scale & Pattern:** Matrix

**Required Classifiers:** Natural/Semi‑natural; Vegetated (>10% vasc.); Upland

**Diagnostic Classifiers:** Forest and Woodland (Treed); Needle‑Leaved Tree; Broad‑Leaved Tree

**National Mapping Codes:** EVT 2368; ESLF 4311; ESP 1368

**Concept Summary:** This system encompasses the prevailing upland forests of the southern Piedmont from Alabama north to central and southern Virginia. High‑quality and historic examples are typically dominated by combinations of upland oaks, sometimes with pines as a significant component, especially in the southern portions of the region. These forests occur in a variety of habitats and, under natural conditions, were the matrix vegetation type covering most of the landscape. Much of this system is currently composed of successional forests that have arisen after repeated cutting, clearing, and cultivation of original oak‑hickory and oak‑hickory‑pine forests. Stands of these forests are dominated by combinations of upland oaks, particularly *Quercus alba, Quercus rubra, Quercus velutina, Quercus stellata, Quercus coccinea*, and *Quercus falcata*, along with *Carya glabra, Carya tomentosa*, and other *Carya* spp. Other common tree species include *Pinus taeda, Pinus echinata, Pinus virginiana, Acer rubrum, Liquidambar styraciflua*, and *Liriodendron tulipifera*. There is considerable variation in this widespread matrix system. In particular, there are "dry‑mesic" as well as "dry" components, as well as stands with codominance by *Pinus echinata*, and distinctive stands dominated by *Quercus montana* with other dry‑site species on the summits of hills called monadnocks. There are particular associations that represent this variation.

**Comments:** Although these forests have often been called "oak‑hickory" (Braun 1950) or "oak‑pine‑hickory" (Küchler 1964, Greller 1989, Skeen et al. 1993), Monk et al. (1990) concluded there was insufficient abundance of hickory to justify including this genus in the name of such forests. There are fairly dramatic differences in the amount of pine present across the modern day Piedmont landscape, with it being especially prevalent in South Carolina, Georgia, and Alabama (USGS 1992). To some extent, the prevalence of pine in these southern portions of the region may represent natural or presettlement conditions (Nelson 1957, Cowell 1998). It is possible that the more heavily mixed or pine‑dominated forests of the southern Piedmont should be recognized as a different ecological system, but distinguishing natural examples is difficult given a long history of land‑use impacts and resulting vegetational changes in the region (Brender 1974, Cowell 1998). In addition, Skeen et al. (1993) assert that "the oak‑hickory‑pine designation may be reflective of past land use and disturbance history and that the steady‑state typal forest of the southeastern Piedmont is in reality oak‑hickory‑yellow poplar."

There are fairly clear variations within this system between dry and dry‑mesic forests and also between those on acidic or basic soils. These might warrant separate ecological systems, but the similar canopy composition and similar dynamics tie them together, and those distinctions may best be made at the association level. Some of this variation (e.g., dry‑mesic, dry, pine‑dominated, monadnocks) has been recognized at the association level, and at finer spatial scales may be reliably mapped, as in Simon and Hayden (2014).

Large areas once dominated by oak‑hickory forests now have successional pine forest. This may be regarded as a distinct phase of this system for mapping purposes.

DISTRIBUTION

**Range:** This system ranges throughout the Piedmont from Alabama to Virginia. In Virginia, it is primarily central and southern, but extends into a narrow portion of northern Virginia in the Piedmont ecoregion.

**Divisions:** 202:C

**TNC Ecoregions:** 52:C

**Nations:** US

**Subnations:** AL, GA, NC, SC, VA

**Map Zones:** 54:C, 59:C, 60:C, 61:C

**USFS Ecomap Regions:** 231A:CC, 231Dd:CCC, 231I:CC

CONCEPT

**Environment:** This system occurs on upland ridges and upper to midslopes, occupying most of the uplands. Moisture conditions, determined by topography, are dry to dry‑mesic. This system may occur on soils derived from any kind of rock type, with rock chemistry being an important determinant of variation. Soils include almost the full range of upland soils, with only the shallowest rocky soils and those with extreme clay hardpans excluded.

The Piedmont has mostly gently rolling topography ranging from 90 to 365 m (300‑1200 feet) elevation. Several erosion‑resistant metamorphic and igneous rock types have been left as monadnocks that stand 60 to 305 m (200‑1000 feet) above the surrounding landscape. Average annual precipitation is 110‑122 cm (44‑48 inches). The presettlement vegetation as described by early explorers and the first settlers was a mosaic of forest and open woodland, with interspersed savannas or prairies (Lederer 1672, Logan 1859). The prairie component was located on the flat to convex and gently rolling uplands of the larger fire compartments. The largest of these in the southern part of the range was up to five miles wide without a tree or only a few blackjack oaks (Logan 1859).

This ecological system encompasses the prevailing upland forests of the southern Piedmont. High‑quality and historic examples are typically dominated by combinations of upland oaks, sometimes with pines as a significant component, especially in the southern portions of the region. These forests occur in a variety of habitats and, under natural conditions, were the matrix upland vegetation type covering most of the landscape.

The Piedmont Monadnock Forest (Schafale and Weakley 1990) is included within this broad type. Stands are dominated by *Quercus montana*, and occur mainly on resistant ridges (monadnocks) over felsic rocks of the Piedmont, including quartzite, rhyolite, and pyrophyllite. Soils are well‑drained, acidic and nutrient‑poor. Lightning strikes and high winds are common in these exposed locations (Schafale and Weakley 1990).

**Vegetation:** Vegetation consists of forests dominated by combinations of upland oaks, particularly *Quercus alba, Quercus rubra, Quercus velutina, Quercus stellata, Quercus coccinea*, and *Quercus falcata*, along with *Carya glabra, Carya tomentosa (= Carya alba)*, and other *Carya* spp. Other common tree species include *Pinus taeda, Pinus echinata, Pinus virginiana, Acer rubrum, Liquidambar styraciflua*, and *Liriodendron tulipifera*. A well‑developed understory and shrub layer is generally present, with species varying with soil chemistry. The herb layer is sparse to at most moderate in density. Before natural fires were suppressed, the forests presumably had less understory and shrub component and probably a grassy herb layer. There is considerable variation in this widespread matrix system. In particular, there are "dry‑mesic" as well as "dry" components, as well as stands with codominance by *Pinus echinata*, and distinctive stands dominated by *Quercus montana (= Quercus prinus)* with other dry‑site species on the summits of hills called monadnocks. There are particular associations that represent this variation. There is some uncertainly about the composition and physiognomy of this system under presettlement conditions, in particular with some debate about the relative importance of *Quercus* spp., *Carya* spp., and *Liriodendron tulipifera*, as well as the role of *Pinus echinata* and its increasing abundance to the south.

**Dynamics:** In successional forests recovering from clearcutting or cultivation, *Pinus taeda, Pinus echinata*, and/or *Pinus virginiana* typically dominate for a number of decades, with *Quercus* spp., *Carya* spp., and other hardwoods gradually invading the understory.

Fire was probably an important natural disturbance in this system, affecting vegetation structure and composition of the lower strata. It may have been important in favoring oaks and pines over other trees. Fires were likely almost always low‑intensity surface fires. Native American burning was also important in the Piedmont (Cowell 1998). These forests appear to occur naturally as predominantly old‑growth, with canopy dynamics dominated by gap‑phase regeneration. Small to medium‑sized canopy gaps created by wind are the primary natural disturbance at present, and probably were in the past as well. Fire likely created some small to medium‑sized gaps in the past also, and likely caused all canopy gaps to persist longer. The dominant tree species are capable of living for several centuries.

Fire and grazing are possibly the most important natural processes affecting the floristic composition and vegetation structure of this system (Landfire 2007a). The presence of frequent (2‑5 years) surface fire is important in order to support the reproduction of *Pinus echinata* and the development of diverse herbaceous understories. *Pinus echinata* is a shade‑intolerant species and does not compete and regenerate well when fire is absent. Where fire occurs at an appropriate frequency, the stand may develop a relatively pure canopy of *Pinus echinata*, typified by a very open woodland structure with scattered overstory trees and an herbaceous‑dominated understory (Landfire 2007a).

The frequency of fire is variable across the landscape to create a mosaic of vegetation. However, most agree that the fire‑return interval was relatively short. Fire may have been as frequent as every two to three years. Brewer (2001) compared the current tree species composition to bearing tree records in the upper coastal plain of northern Mississippi and found that *Pinus echinata* and more fire‑tolerant species such as *Quercus velutina* and *Quercus stellata* were prevalent on the landscape, indicating a greater fire frequency. Without a short fire‑return interval, community succession tends to favor upland mixed pine‑xeric hardwood forests or hardwood‑dominated forests. Landers (1989) inferred a fire‑return interval of 10 times per century for pure stands of *Pinus echinata*.

<br />Lightning fires occurred primarily during the spring dry season (April and May) with a second peak of Native American burning during the fall (October and November). Occasionally, during extensive droughts, mixed‑severity or stand‑replacement fires did occur, especially on drier pine‑dominated sites. Local thunderstorms and outbreaks of southern pine beetle (*Dendroctonus frontalis*) created gaps on a small but continual basis. More extensive regional disturbances included tropical storms during the growing season, ice storms during winter, and tornadoes throughout the year (Landfire 2007a).

SOURCES

**References:** Braun 1950, Brender 1974, Brewer 2001, Comer et al. 2003*, Cowell 1998, Eyre 1980, Gibbon 1966, Godfrey 1982b, Greller 1989, Küchler 1964, LANDFIRE 2007a, Landers 1989, Lederer 1672, Logan 1859, McDonald et al. 2002, Mistretta 1984, Monk et al. 1990, Nelson 1957, Nelson 1986, Nowak 2002, Oosting 1942, Peet et al. 2013, Schafale 2012, Schafale and Weakley 1990, Simon and Hayden 2014, Simon pers. comm., Skeen et al. 1993, Taecker 2007, Taverna et al. 2005, Trimble 1974, Trimble 2008, USGS 1992, USGS 2015b, VDNH unpubl. data

**Version:** 02 Oct 2015	**Stakeholders:** East, Southeast

**Concept Author:** M. Schafale, R. Evans, M. Pyne	**LeadResp:** Southeast

CES203.378 West Gulf Coastal Plain Pine‑Hardwood Forest

**Primary Division:** Gulf and Atlantic Coastal Plain (203)

**Land Cover Class:** Forest and Woodland

**Spatial Scale & Pattern:** Matrix

**Required Classifiers:** Natural/Semi‑natural; Vegetated (>10% vasc.); Upland

**Diagnostic Classifiers:** Forest and Woodland (Treed); Short Disturbance Interval; Needle‑Leaved Tree; Broad‑Leaved Deciduous Tree

**National Mapping Codes:** EVT 2371; ESLF 4314; ESP 1371

**Concept Summary:** This West Gulf Coastal Plain ecological system consists of forests and woodlands dominated by *Pinus taeda* and/or *Pinus echinata* in combination with a variety of dry to dry‑mesic site hardwood species. This type was the historical matrix vegetation (dominant vegetation type) for large portions of the West Gulf Coastal Plain landward of the range of *Pinus palustris*, where it replaced *Pinus palustris*‑dominated vegetation. In this region of southern Arkansas, northwestern Louisiana, and parts of eastern Texas, this type was historically present on nearly all uplands in the region except on the most edaphically limited sites (droughty sands, calcareous clays, and shallow soil barrens/rock outcrops). Such sites are underlain by loamy to fine‑textured soils of variable depths. These are upland sites on ridgetops and adjacent sideslopes, with moderate fertility and moisture retention. This type was also present in more limited areas within the range of *Pinus palustris* (in the West Gulf Coastal Plain), where it was confined more typically to sideslopes and other locations not dominated by *Pinus palustris*. There are no known "fidel" herbaceous species or any local endemic or globally rare plant species, and overall this system may have supported relatively low levels of vascular plant species diversity. This system has undergone major transformations since European settlement of the region.

DISTRIBUTION

**Range:** This system is restricted to the West Gulf Coastal Plain of Arkansas, Louisiana and Texas.

**Divisions:** 203:C

**TNC Ecoregions:** 40:C, 41:C

**Nations:** US

**Subnations:** AR, LA, TX

**Map Zones:** 37:C, 44:C

**USFS Ecomap Regions:** 231E:CC, 232F:CC, 234E:PP

CONCEPT

**Environment:** In southern Arkansas, northwestern Louisiana, and parts of eastern Texas, this type was historically present on nearly all uplands in the region except on the most edaphically limited sites (droughty sands, calcareous clays, and shallow soil barrens/rock outcrops). Such sites are underlain by loamy to fine‑textured soils of variable depths and generally are Alfisols or Ultisols. These are upland sites on ridgetops and adjacent sideslopes, with moderate fertility and moisture retention. In Texas, this system occurs over a wide variety of landforms, with drier expressions occurring on hilltops and ridges. It occupies slopes and lower landscape positions, where conditions are more mesic, and composition of the system varies across these gradients. It is found on numerous Cenozoic sedimentary formations and some Cretaceous formations of the Mesozoic era. These formations range from sandstone, shale, alluvium, and conglomerate, to marl, with glauconitic formations (Weches) and tuffaceous formations (Catahoula) present (Elliott 2011).

**Vegetation:** Examples of this system are forests and woodlands dominated by *Pinus taeda* and/or *Pinus echinata* in combination with a host of dry to dry‑mesic site hardwood species, such as *Quercus alba, Quercus falcata*, and *Quercus stellata*. Stands on narrow ridgetops, which can be isolated from the effects of fire, may exhibit greater dominance by hardwoods. Other species that may occur include *Quercus margarettae, Quercus velutina, Carya tomentosa (= Carya alba), Carya texana, Cornus florida, Crataegus* spp., *Ostrya virginiana, Symplocos tinctoria, Morella cerifera, Vaccinium arboreum, Vaccinium elliottii, Viburnum acerifolium*, and *Viburnum dentatum*. Woody vines in this system may be conspicuous and often include *Smilax bona‑nox, Vitis* spp. (often *Vitis rotundifolia* or *Vitis mustangensis* to the south), *Parthenocissus quinquefolia*, and *Toxicodendron radicans*. The herbaceous layer is generally sparse (often less than 20% cover), with *Schizachyrium scoparium, Chasmanthium laxum, Chasmanthium sessiliflorum, Dichanthelium sphaerocarpon*, and *Pteridium aquilinum* often present to dominant. The importance of *Acer floridanum (= Acer barbatum), Acer leucoderme*, and *Liquidambar styraciflua* may increase with the absence of fire. In parts of southeastern Texas, the hardwood component of stands may contain *Quercus nigra, Quercus virginiana, Quercus falcata, Liquidambar styraciflua*, and *Ulmus* spp. Shrubs include *Ilex vomitoria, Cornus florida, Morella cerifera, Callicarpa americana*, and *Vaccinium arboreum* (Elliott 2011).

**Dynamics:** Forests with dense tree cover (especially evergreen cover) have reduced shrub and herbaceous cover. Herbaceous cover may be additionally limited by dense litter accumulation. Few occurrences of this system can be considered old‑growth.

SOURCES

**References:** Comer et al. 2003*, Elliott 2011, Eyre 1980, Griffith et al. 2004, LNHP 2009, Marks and Harcombe 1981

**Version:** 23 May 2013	**Stakeholders:** Southeast

**Concept Author:** R. Evans and T. Foti	**LeadResp:** Southeast

CES203.056 West Gulf Coastal Plain Sandhill Oak and Shortleaf Pine Forest and Woodland

**Primary Division:** Gulf and Atlantic Coastal Plain (203)

**Land Cover Class:** Forest and Woodland

**Spatial Scale & Pattern:** Large patch

**Required Classifiers:** Natural/Semi‑natural; Vegetated (>10% vasc.); Upland

**Diagnostic Classifiers:** Forest and Woodland (Treed); Needle‑Leaved Tree; Broad‑Leaved Deciduous Tree

**National Mapping Codes:** EVT 2378; ESLF 4321; ESP 1378

**Concept Summary:** This ecological system occurs west of the Mississippi River primarily outside the natural range of longleaf pine (*Pinus palustris*) and less commonly within this range. Like other sandhill systems of the Gulf and Atlantic coastal plains, this type is found on uplands underlain with deep, coarse sandy soils. These sites are typified by low fertility and moisture retention, which contribute to open tree canopies with usually less than 60% canopy closure. Sparse understory vegetation and abundant patches of bare soil are indicative of this system. Vegetation indicators are species tolerant of droughty sites, especially *Quercus incana* and *Quercus arkansana*, but also *Quercus marilandica* and *Quercus stellata*. *Pinus echinata* is usually present, and *Pinus palustris* is absent (or perhaps at low frequency within its range). This system supports a large concentration of vascular plant endemics, near endemics, and a number of plant species with high fidelity to sandhills in the region. Elsewhere in the Atlantic and Gulf coastal plains, including most of the adjacent outer West Gulf Coastal Plain ecoregion, these site conditions are closely associated with longleaf pine.

**Comments:** In Arkansas (at least), this system is most closely affiliated with the "Briley‑Alaga‑Bibb" Soil Association (MUID=AR039 in STATSGO).

DISTRIBUTION

**Range:** This system occurs west of the Mississippi River primarily outside the natural range of longleaf pine (*Pinus palustris*).

**Divisions:** 203:C

**TNC Ecoregions:** 39:C, 40:C, 41:C

**Nations:** US

**Subnations:** AR, LA, TX

**Map Zones:** 37:C

**USFS Ecomap Regions:** 231Ee:CCC, 232F:CC

CONCEPT

**Environment:** This system type is found on droughty uplands underlain with deep, coarse sandy soils. It is generally associated with Eocene sand formations such as Carrizo, Sparta, and Queen City sands, including the Betis, Darco, Letney, Tehran, Tonkawa, and other Grossarenic or Psammentic soil series. It is also found on sands derived from the Pliocene Willis formation (Elliott 2011). These sites are typified by low fertility and moisture retention. In particular, these are found on deep sands on generally high, convex landforms, and often display a relatively open overstory canopy.

**Vegetation:** Examples of this system may occur as pine dominated woodlands, with *Pinus palustris* dominating some sites within its range, and *Pinus echinata* dominating areas where *Pinus palustris* is absent. *Pinus taeda* is naturally less common, but in the current landscape, it is common and sometimes dominant. Pines may co‑dominate along with deciduous species, or the canopy may be dominated by oaks and other deciduous species including *Quercus stellata, Quercus marilandica, Quercus incana, Quercus falcata, Quercus margarettae*, and *Carya texana* (Elliott 2011). Other deciduous trees present may include *Sassafras albidum, Liquidambar styraciflua*, and *Quercus nigra*. The shrub stratum can be fairly well‑developed, and includes shorter individuals of canopy species in addition to such species as *Callicarpa americana, Ilex vomitoria, Vaccinium arboreum, Rhus aromatica, Asimina parviflora, Cornus florida*, and *Smilax bona‑nox*. The herbaceous layer may be quite well‑developed or relatively patchy (with areas of bare sandy soil exposed). Commonly encountered species include *Schizachyrium scoparium, Pteridium aquilinum, Aristida desmantha, Ambrosia psilostachya, Cnidoscolus texanus, Rudbeckia hirta, Dichanthelium dichotomum, Pityopsis graminifolia, Croton argyranthemus, Tragia urticifolia, Froelichia floridana, Matelea cynanchoides, Opuntia humifusa, Sporobolus junceus, Triplasis purpurea, Bulbostylis ciliatifolia, Chamaecrista fasciculata, Berlandiera pumila, Commelina erecta var. angustifolia, Stylisma pickeringii, Tetragonotheca ludoviciana, Tradescantia reverchonii, Rhynchosia* spp., *Tephrosia* spp., *Yucca louisianensis*, as well as the fern ally *Selaginella arenicola ssp. riddellii* (Elliott 2011). All described community types in this system tend to support relatively open wooded canopies (<60% closure), and one type is described as essentially treeless. A degraded expression of this type has been described [see CEGL007507], but this is treated under the semi‑natural ecological system. Other types are floristically differentiated, with special importance placed on the occurrence of *Quercus arkansana*.

**Dynamics:** The primary natural processes controlling this system are droughty, deep sandy soils, and a natural fire regime. Fire is believed to have been a critical natural disturbance process which affected the vegetation structure and likely the species composition of communities in this system. There are several indirect pieces of evidence which suggest this: (1) *Pinus echinata* is intolerant of competition, and young stems are generally slower growing and slower to dominate sites than either *Pinus taeda* or many hardwood species (Lawson 1990); (2) *Pinus echinata* regeneration decreases dramatically with time since fire (Ferguson 1958); and (3) *Pinus echinata* has the ability to resprout. Watson (1986) postulates that most seedlings of *Pinus echinata* are killed during the periodic fires, and the mature trees are spared. This prevents the formation of thickets. This paper implies that low fuel levels accompany the sparse vegetation of these sandy areas, leading to a somewhat longer fire‑return interval, which suits *Pinus echinata*. A variety of fire‑return intervals have been estimated for *Pinus echinata* vegetation. Garren (1943) proposed an 8‑ to 10 ‑year return interval, Landers (1989) inferred a regime of 10 per century, and Martin and Smith (1993) estimated a 5‑ to 15 ‑year interval, however, none of these estimates were specific to *Pinus echinata* on sandhills. Many such sites in the region lack well ‑developed and continuous fine fuels necessary to ignite and spread fires, possibly due to site infertility and droughtiness (R. Evans pers. obs., L. Smith pers. comm.).

SOURCES

**References:** Ajilvsgi 1979, Comer et al. 2003*, Elliott 2011, Evans, R. pers. comm., Eyre 1980, Ferguson 1958, Garren 1943, Landers 1989, Lawson 1990, Marks and Harcombe 1981, Martin and Smith 1993, Smith, L. pers. comm., Turner et al. 1999, Watson 1986

**Version:** 14 Jan 2014	**Stakeholders:** Southeast

**Concept Author:** R. Evans	**LeadResp:** Southeast

1.B.2.Nb. Rocky Mountain Forest & Woodland

M501. Central Rocky Mountain Dry Lower Montane‑Foothill Forest

CES306.959 Middle Rocky Mountain Montane Douglas‑fir Forest and Woodland

**Primary Division:** Rocky Mountain (306)

**Land Cover Class:** Forest and Woodland

**Spatial Scale & Pattern:** Matrix

**Required Classifiers:** Natural/Semi‑natural; Vegetated (>10% vasc.); Upland

**Diagnostic Classifiers:** Montane [Montane, Lower Montane]; Forest and Woodland (Treed); Aridic; Intermediate Disturbance Interval; F‑Patch/Medium Intensity; F‑Landscape/Medium Intensity; Needle‑Leaved Tree; RM Montane Mesic Mixed Conifer; Moderate (100‑500 yrs) Persistence

**National Mapping Codes:** EVT 2166; ESLF 4266; ESP 1166

**Concept Summary:** This ecological system occurs throughout the middle Rocky Mountains of central and southern Idaho (Lemhi, Beaverhead and Lost River ranges), south and east into the greater Yellowstone region, and south and east into the Wind River, Gros Ventre and Bighorn ranges of Wyoming. It extends north into Montana on the east side of the Continental Divide, north to about the McDonald Pass area, and into the Rocky Mountain Front region of Montana. This is a *Pseudotsuga menziesii*‑dominated system without the maritime floristic composition; these are forests and woodlands occurring in the Central Rockies where the southern monsoon influence is lessened and maritime climate regime is not important. This system includes extensive *Pseudotsuga menziesii* forests, occasionally with *Pinus flexilis* on calcareous substrates, and *Pinus contorta* at higher elevations. True firs, such as *Abies concolor, Abies grandis*, and *Abies lasiocarpa*, are absent in these occurrences, but *Picea engelmannii* can occur in some stands. Understory components include shrubs such as *Physocarpus malvaceus, Juniperus communis, Symphoricarpos oreophilus*, and *Mahonia repens*, and graminoids such as *Calamagrostis rubescens, Carex rossii*, and *Leucopoa kingii*. The fire regime is of mixed severity with moderate frequency. This system often occurs at the lower treeline immediately above valley grasslands, or sagebrush steppe and shrublands. Sometimes there may be a "bath‑tub ring" of *Pinus ponderosa* at lower elevations or *Pinus flexilis* between the valley non‑forested and the solid *Pseudotsuga menziesii* forest. In the Wyoming Basins, this system occurs as isolated stands of *Pseudotsuga menziesii*, with *Artemisia tridentata, Pseudoroegneria spicata, Leucopoa kingii*, and *Carex rossii*.

DISTRIBUTION

**Range:** This system occurs throughout the middle Rocky Mountains of central and southern Idaho (Lemhi, Beaverhead and Lost River ranges), south and east into the greater Yellowstone region, and south and east into the Wind River, Gros Ventre and Bighorn ranges of Wyoming. It extends north into Montana on the east side of the Continental Divide to the Rocky Mountain Front and includes all of the Beaverhead Mountains Section (M332E) (Bailey et al. 1994). It may also occur in scattered patches in southeastern Oregon.

**Divisions:** 304:C, 306:C

**TNC Ecoregions:** 6:P, 7:?, 8:C, 9:C, 10:C

**Nations:** US

**Subnations:** ID, MT, OR?, WY

**Map Zones:** 9:?, 10:C, 18:?, 19:C, 20:?, 21:C, 22:C, 29:C

**USFS Ecomap Regions:** 342A:CC, 342C:CP, 342D:CP, 342J:CP, M331A:CC, M331B:CC, M331D:CP, M331J:CC, M332A:CC, M332B:CC, M332E:CC, M332F:CC

CONCEPT

**Environment:** These are forests and woodlands occurring in the Central Rockies where the southern monsoon influence is lessened and maritime climate regime is not important. These *Pseudotsuga menziesii* forests occur under a comparatively drier and more continental climate regime, and at higher elevations than in the Pacific Northwest. Elevations range from less than 1000 m in the central Rocky Mountains to over 2400 m in the Wyoming Rockies. Lower‑elevation stands typically occupy protected northern exposures or mesic ravines and canyons, often on steep slopes. At higher elevations, these forests occur primarily on southerly aspects or ridgetops and plateaus.

Annual precipitation ranges from 50‑100 cm with moderate snowfall and a greater proportion falling during the growing season. Monsoonal summer rains can contribute a significant proportion of the annual precipitation in the southern portion of the range.

Soils are highly variable and derived from diverse parent materials. *Pseudotsuga menziesii* forests are reported by most studies (Pfister et al. 1977, Steele et al. 1983, Mauk and Henderson 1984) to show no particular affinities to geologic substrates. Rock types can include extrusive volcanics in the Yellowstone region, and sedimentary rocks elsewhere in the Rockies. The soils are typically slightly acidic (pH 5.0‑6.0), well‑drained, and well‑aerated. They can be derived from moderately deep colluvium or shallow‑jointed bedrock and are usually gravelly or rocky.

**Vegetation:** This is a *Pseudotsuga menziesii*‑dominated system without the maritime floristic composition; it includes extensive *Pseudotsuga menziesii* forests, occasionally with *Pinus flexilis* on calcareous substrates and *Pinus contorta* at higher elevations. *Picea engelmannii* can occur in some stands; however, true firs, such as *Abies concolor, Abies grandis*, and *Abies lasiocarpa*, are absent. Understory components include shrubs such as *Artemisia tridentata, Physocarpus malvaceus, Juniperus communis, Symphoricarpos oreophilus*, and *Mahonia repens*, and graminoids such as *Calamagrostis rubescens, Carex rossii, Leucopoa kingii*, and *Pseudoroegneria spicata*. Sometimes there may be a "bath‑tub ring" of *Pinus ponderosa* at lower elevations or *Pinus flexilis* between the valley non‑forested and the solid *Pseudotsuga menziesii* forest.

**Dynamics:** Successional relationships in this group are complex. *Pseudotsuga menziesii* is less shade‑tolerant than some montane trees such as *Abies concolor* or *Picea engelmannii*, and seedlings compete poorly in deep shade. At drier locales, seedlings may be favored by moderate shading, such as by a canopy of *Pinus flexilis*, which helps to minimize drought stress. In some locations, much of these forests have been logged or burned during European settlement, and present‑day stands are second‑growth forests dating from fire, logging, or other stand‑replacing disturbances (Mauk and Henderson 1984). *Pseudotsuga menziesii* forests were probably subject to a moderate‑severity fire regime in presettlement times, with fire‑return intervals of 30‑100 years. Many of the important tree species in these forests are fire‑adapted (*Populus tremuloides, Pinus contorta*) (Pfister et al. 1977). Some stands may have higher tree‑stem density than historically, due largely to fire suppression (Steele et al. 1983).

<br />LANDFIRE developed a state‑and‑transition vegetation dynamics VDDT model for this system which has five classes in total (LANDFIRE 2007a, BpS 2111660). These are summarized as:

A) (10% of type in this stage) Tree cover is 0‑100%. Dominated by graminoids and seedling/sapling Douglas‑fir and possibly lodgepole pine. Understory may be dominated by *Calamagrostis rubescens* and/or *Carex* spp. Shrub species such as *Symphoricarpos* spp. may be present. Succession occurs in approximately 40 years, and the class moves to a mid‑open state. Replacement fire occurs every 500 years, and mixed fire occurs every 200 years. If this class experiences no fire in 20 years, it will move to class B, a mid‑closed state. Wind/weather events occur infrequently (probability of 0.001), but the class is maintained in this state.

B) Mid Development 1 Closed (tree‑dominated ‑ 10% of type in this stage): Tree cover is 41‑100%. Relatively dense pole and some medium Douglas‑fir and possibly lodgepole pine. The understory is open and relatively depauperate. Understory may be dominated by *Calamagrostis rubescens* and/or *Carex* spp. This class persists for 80 years, then moves to a late‑closed stage. Replacement fire occurs every 200 years, and mixed fire every 50 years, causing a transition to a mid‑open stage. Insect/disease outbreaks occur with a probability of 0.005 and can move the class to a mid‑open state. Also, wind/weather stress causes a change to a mid‑open state with a probability of 0.001. Although reviewers recommended removing insects/disease from this class, it was decided by Region 1 insect experts that some insect damage is likely for the class B forest types. The insects to be concerned about at low levels are Douglas‑fir pole beetle and western spruce budworm.

C) Mid Development 1 Open (tree‑dominated ‑ 10% of type in this stage): Tree cover is 21‑40%. Open pole and medium Douglas‑fir that may have lodgepole pine with patchy graminoid cover and dispersed shrubs such as *Symphoricarpos* spp. Understory may be dominated by *Calamagrostis rubescens* and/or *Carex* spp. Conifer heights range between 5‑20 m but adjusted to eliminate class overlap. This class can persist for 60 years, then moves to a late‑open stage. Replacement fire occurs every 200 years, and mixed fire every 40 years. Without fire for 58 years, this class can move to a mid‑closed state. Insect/disease outbreaks and wind/weather events occur with a probability of .005, and maintain this class in a mid‑open state.

D) Late Development 1 Open (conifer‑dominated ‑ 50% of type in this stage): Tree cover is 21‑40%. Open canopy of medium to large Douglas‑fir with a graminoid and shrub understory with highly variable understory cover. Lodgepole pine may be present. Understory may be dominated by *Symphoricarpos* spp., *Calamagrostis rubescens*, and/or *Carex* spp. Heights can exceed 25 m up to approximately 30 m. Replacement fire occurs every 500 years, and mixed fire every 50 years. Without fire for 45 years, this class can move to a late‑closed state. Insect disturbance occurs every 10 years but does not move this class to another class. Wind/weather stress also occurs, with a probability of 0.008, but does not cause a transition to another class.

<br />E) Late Development 1 Closed (conifer‑dominated ‑ 20% of type in this stage): Tree cover is 41‑100%. Multi‑storied Douglas‑fir, sometimes with lodgepole pine present. Understory with variable cover often dominated by *Calamagrostis rubescens, Carex* spp., *Symphoricarpos* spp., and/or *Physocarpus malvaceus*. Heights can exceed 25 m up to approximately 30 m. Replacement fire occurs every 200 years, and mixed fire every 30 years, causing a transition back to a late‑open state. Insect outbreaks occur frequently, probability of 0.01, and cause a transition to an open state. Wind/weather stress occurs with a probability of 0.005 and causes a transition to a late‑open state.

Fire regime is predominantly mixed‑severity (Fire Regime III) with a MFI of approximately 20‑50 years (Houston 1973, Arno and Gruell 1983, Fischer and Clayton 1983, Littell 2002, Korb et al. in prep.). Mixed‑severity fires are generally characterized as spatially heterogeneous (LANDFIRE 2007a, BpS 2111660). Fire regime in more northern stands is predominantly mixed with a MFI of approximately 35‑50 years (Crane and Fischer 1986, Bradley et al. 1992) (LANDFIRE 2007a, BpS 1911660).

Nutrient cycling, specifically carbon cycling, is an important ecological process within many ecological systems. Biological decomposition in ponderosa pine forests is more limited than biological production, resulting in accumulation of organic materials, especially in the absence of fire (Harvey 1994, Graham and Jain 2005).

SOURCES

**References:** Amman 1977, Anderson 2003b, Arno and Gruell 1983, Bailey et al. 1994, Bell et al. 2009, Bradley et al. 1992b, Burns and Honkala 1990a, Comer et al. 2003*, Crane and Fischer 1986, Dale et al. 2001, Eyre 1980, Fischer and Bradley 1987, Graham and Jain 2005, Harvey 1994, Houston 1973, Korb et al. 2018, LANDFIRE 2007a, Littell 2002, Mauk and Henderson 1984, McKenzie et al. 2004, McKenzie et al. 2008, Mote et al. 2014, Pfister et al. 1977, Shafer et al. 2014, Steele et al. 1983, Steinberg 2002e, Stevens‑Rumann et al. 2017, Westerling et al. 2006

**Version:** 06 Nov 2018	**Stakeholders:** West

**Concept Author:** M.S. Reid	**LeadResp:** West

CES306.805 Northern Rocky Mountain Dry‑Mesic Montane Mixed Conifer Forest

**Primary Division:** Rocky Mountain (306)

**Land Cover Class:** Forest and Woodland

**Spatial Scale & Pattern:** Matrix

**Required Classifiers:** Natural/Semi‑natural; Vegetated (>10% vasc.); Upland

**Diagnostic Classifiers:** Montane [Montane]; Forest and Woodland (Treed); Ustic; Short Disturbance Interval; F‑Patch/Low Intensity; Needle‑Leaved Tree; Abies grandis ‑ Mixed

**National Mapping Codes:** EVT 2045; ESLF 4232; ESP 1045

**Concept Summary:** This ecological system is composed of highly variable montane coniferous forests found in the interior Pacific Northwest, from southernmost interior British Columbia, eastern Washington, eastern Oregon, northern Idaho, western and north‑central Montana, and south along the east slope of the Cascades in Washington and Oregon. In central Montana it occurs on mountain islands (the Snowy Mountains). This system is associated with a submesic climate regime with annual precipitation ranging from 50 to 100 cm, with a maximum in winter or late spring. Winter snowpacks typically melt off in early spring at lower elevations. Elevations range from 460 to 1920 m. Most occurrences of this system are dominated by a mix of *Pseudotsuga menziesii* and *Pinus ponderosa* (but there can be one without the other) and other typically seral species, including *Pinus contorta, Pinus monticola* (not in central Montana), and *Larix occidentalis* (not in central Montana). *Picea engelmannii* (or *Picea glauca* or their hybrid) becomes increasingly common towards the eastern edge of the range. The nature of this forest system is a matrix of large patches dominated or codominated by one or combinations of the above species; *Abies grandis* (a fire‑sensitive, shade‑tolerant species not occurring in central Montana) has increased on many sites once dominated by *Pseudotsuga menziesii* and *Pinus ponderosa*, which were formerly maintained by low‑severity wildfire. Presettlement fire regimes may have been characterized by frequent, low‑intensity surface fires that maintained relatively open stands of a mix of fire‑resistant species. Under present conditions the fire regime is mixed severity and more variable, with stand‑replacing fires more common, and the forests are more homogeneous. With vigorous fire suppression, longer fire‑return intervals are now the rule, and multi‑layered stands of *Pseudotsuga menziesii, Pinus ponderosa*, and/or *Abies grandis* provide fuel "ladders," making these forests more susceptible to high‑intensity, stand‑replacing fires. They are very productive forests which have been priorities for timber production. They rarely form either upper or lower timberline forests. Understories are dominated by graminoids, such as *Pseudoroegneria spicata, Calamagrostis rubescens, Carex geyeri*, and *Carex rossii*, that may be associated with a variety of shrubs, such as *Acer glabrum, Juniperus communis, Physocarpus malvaceus, Symphoricarpos albus, Spiraea betulifolia*, or *Vaccinium membranaceum* on mesic sites. *Abies concolor* and *Abies grandis x concolor* hybrids in central Idaho (the Salmon Mountains) are included here but have very restricted range in this area. *Abies concolor* and *Abies grandis* in the Blue Mountains of Oregon are probably hybrids of the two and mostly *Abies grandis*.

**Comments:** Need to re‑assess the concept of this system in relation to Northern Rocky Mountain Western Larch Savanna (CES306.837) and East Cascades Mesic Montane Mixed‑Conifer Forest and Woodland (CES204.086). In PNV (PAGs) concept, this is mostly *Pseudotsuga menziesii*, moist *Pinus ponderosa* series, dry *Abies grandis* or warm, dry *Abies lasiocarpa* series in the Canadian Rockies, northern Middle Rockies, East Cascades and Okanagan ecoregions. Everett et al. (2000) indicate that in the eastern Cascades of Washington this system forms fire polygons due to abrupt north and south topography with presettlement fire‑return intervals of 11‑12 years typically covering less than 810 ha. Currently, fires have 40‑ to 45‑year return intervals with thousands of hectares in size. Northern Rocky Mountain Western Larch Savanna (CES306.837) is a large‑patch type that occurs typically within this matrix or Northern Rocky Mountain Mesic Montane Mixed Conifer Forest (CES306.802) matrix. We need to define the percent cover of larch over 50% or over 75% relative cover of all trees for an occurrence to be placed in Northern Rocky Mountain Western Larch Savanna (CES306.837). This needs to be relative because these look(ed) like ponderosa savanna in places. East Cascades Mesic Montane Mixed‑Conifer Forest and Woodland (CES204.086) has North Pacific floristic composition, and is mostly east Cascades ecoregion, peripheral in Okanagan ecoregion, and west Cascades. PAGs most of the *Abies grandis*, dry western red‑cedar and western hemlock in the east Cascades. Environmentally, it is equivalent to Northern Rocky Mountain Mesic Montane Mixed Conifer Forest (CES306.802). Contrasting this system (CES306.805) with Rocky Mountain Subalpine Dry‑Mesic Spruce‑Fir Forest and Woodland (CES306.828) and Rocky Mountain Subalpine Mesic‑Wet Spruce‑Fir Forest and Woodland (CES306.830) is important in the Middle Rockies ecoregion and Oregon.

DISTRIBUTION

**Range:** This system is found in the interior Pacific Northwest, from southern interior British Columbia south and east into Oregon, Idaho (including north and central Idaho, down to the Boise Mountains), and western Montana, and south along the east slope of the Cascades in Washington and Oregon.

**Divisions:** 204:C, 304:P, 306:C

**TNC Ecoregions:** 2:P, 4:C, 6:C, 7:C, 8:C, 26:C, 68:C

**Nations:** CA, US

**Subnations:** BC, ID, MT, OR, WA:S3S4

**Map Zones:** 1:C, 7:C, 8:C, 9:C, 10:C, 16:?, 17:?, 18:P, 19:C, 20:C

**USFS Ecomap Regions:** 331A:CC, 331D:C?, 341G:PP, 342C:CC, 342D:CC, 342H:CC, 342I:CC, M242B:CC, M242C:CC, M242D:CC, M331A:CC, M331D:CC, M332A:CC, M332B:CC, M332D:CP, M332E:CC, M332F:CC, M332G:CC, M333A:CC, M333B:CC, M333C:CC, M333D:CC

CONCEPT

**Environment:** This interior Pacific Northwest montane coniferous forest ecological system ranges from southernmost interior British Columbia, eastern Washington, and eastern Oregon across northern Idaho, western and north‑central Montana extending east out on mountain islands (the Snowy Mountains) in the northwestern Great Plains and south along the east slope of the Cascades in Washington and Oregon. It has a submesic climate regime with annual precipitation ranging from 50 to 100 cm, with a maximum in winter or late spring. Winter snowpacks typically melt off in early spring at lower elevations. Stands are often dry in late summer when fire season begins. Elevations range from 460 to 1920 m. Substrates are variable, but it often occurs on shallow rocky soils.

**Vegetation:** This system is generally dominated by a mix of *Pseudotsuga menziesii* and *Pinus ponderosa* (but there can be one without the other) and other typically seral species, including *Pinus contorta, Pinus monticola*, and *Larix occidentalis*. *Picea engelmannii* (or *Picea glauca* or their hybrid) becomes increasingly common towards the eastern edge of the range. *Abies grandis* (a fire‑sensitive, shade‑tolerant species not occurring in central Montana) has increased on many sites once dominated by *Pseudotsuga menziesii* and *Pinus ponderosa*. Understories are often dominated by graminoids, such as *Pseudoroegneria spicata, Calamagrostis rubescens, Carex geyeri*, and *Carex rossii*, which may be associated with a variety of shrubs, such as *Acer glabrum, Juniperus communis, Physocarpus malvaceus, Symphoricarpos albus, Spiraea betulifolia*, or *Vaccinium membranaceum* on mesic sites. *Abies concolor* and *Abies grandis x concolor* hybrids in central Idaho (the Salmon Mountains) are included here but have very restricted range in this area.

**Dynamics:** LANDFIRE developed several state‑and‑transition vegetation dynamics VDDT models for this system. Some mapzone teams created multiple models for different dominant trees. Below is a model with five classes from mountains of eastern Oregon (LANDFIRE 2007a, BpS 0910450). These are summarized as:

A) Early Development 1 All Structures (10% of type in this stage): Tree cover is 0‑20%. Open stand of ponderosa pine and other tree seedlings mixed with grasses and shrubs. Early‑seral dominant species include ceanothus, scouler willow, *Bromus*, some sedges and grasses. We use Comp/Maintenance to hold a portion of this class back in an extended shrub‑dominated stage. Also, we use AltSucc. without TSD to allow a portion of this type to succeed to class B ‑ mid‑closed.

B) Mid Development 1 Closed (tree‑dominated ‑ 5% of type in this stage): Tree cover is 41‑100%. Closed stands of 5‑20 inches dbh early‑seral tree species. Forests in this type rarely if ever exceed 80% canopy closure even in closed, dense conditions.

C) Mid Development 1 Open (tree‑dominated ‑ 30% of type in this stage): Tree cover is 11‑40%. Open stands of 5‑20 inches dbh early‑seral tree species. Dominant understory plants include elk sedge, pinegrass, common snowberry, rose, mountain‑mahogany (wetter), heartleaf arnica and lupines. This class has low probability of replacement fire due to discontinuous fuel in these open stands. A small portion of the class succeeds to class E ‑ late‑closed.

D) Late Development 1 Open (conifer‑dominated ‑ 45% of type in this stage): Tree cover is 11‑40%. Open stands of 20+ inches dbh early‑seral tree species. Dominant understory plants include elk sedge, pinegrass, common snowberry, rose, mountain‑mahogany (wetter), heartleaf arnica and lupines.

E) Late Development 1 Closed (conifer‑dominated ‑ 10% of type in this stage): Tree cover is 41‑100%. Closed stands of 20+ inches dbh early‑seral tree tree species. Forests in this PNVG rarely if ever exceed 80% canopy closure even in closed, dense conditions. This class has relatively high probability of replacement fires, due to the dense understory, though it is less than the probability of replacement fire in the mid‑closed.

Typical disturbance regimes under natural conditions include frequent, low‑intensity underburns that maintain open stands of fire‑resistant trees. Much more infrequent mixed‑severity and stand‑replacement wildfire occurred and tended to generate mosaics of older, larger trees and younger regeneration. Endemic bark beetles produced patch mortality. Rarer epidemic bark beetle outbreaks caused larger‑scale overstory mortality and released understory trees. Defoliator outbreaks also caused fir mortality in some areas. Defoliation by spruce budworm is now more widespread than historically. Root diseases may play a significant role in later‑seral forests in this environment (LANDFIRE 2007a, BpS 0910450).

Nutrient cycling, specifically carbon cycling, is an important ecological process within many ecological systems. Biological decomposition in ponderosa pine forests is more limited than biological production, resulting in accumulation of organic materials, especially in the absence of fire (Harvey 1994, Graham and Jain 2005).

SOURCES

**References:** Amman 1977, Anderson 2003b, Burns and Honkala 1990a, Comer et al. 2003*, Cooper et al. 1987, Crawford and Johnson 1985, Dale et al. 2001, Daubenmire and Daubenmire 1968, Eyre 1980, Graham and Jain 2005, Habeck 1992a, Habeck 1992d, Harvey 1994, Howard 2003b, Howard 2003c, Howard and Aleksoff 2000, LANDFIRE 2007a, Lillybridge et al. 1995, McKenzie et al. 2004, McKenzie et al. 2008, Mote et al. 2014, NCC 2002, Pfister et al. 1977, Schmid 1988, Shafer et al. 2014, Steele and Geier‑Hayes 1995, Steele et al. 1981, Steen and Coupé 1997, Steinberg 2002e, Stevens‑Rumann et al. 2017, Topik 1989, Topik et al. 1988, Uchytil 1991g, WNHP unpubl. data 2018, Westerling et al. 2006, Williams and Lillybridge 1983

**Version:** 06 Nov 2018	**Stakeholders:** Canada, West

**Concept Author:** M.S. Reid	**LeadResp:** West

CES306.958 Northern Rocky Mountain Foothill Conifer Wooded Steppe

**Primary Division:** Rocky Mountain (306)

**Land Cover Class:** Steppe/Savanna

**Spatial Scale & Pattern:** Large patch

**Required Classifiers:** Natural/Semi‑natural; Vegetated (>10% vasc.); Upland

**Diagnostic Classifiers:** Woody‑Herbaceous; Shallow Soil; Aridic; Short Disturbance Interval; F‑Patch/Low Intensity; F‑Landscape/Low Intensity; Needle‑Leaved Tree

**National Mapping Codes:** EVT 2165; ESLF 5426; ESP 1165

**Concept Summary:** This inland Pacific Northwest ecological system occurs in the foothills of the northern Rocky Mountains in the Columbia Plateau region and west along the foothills of the Modoc Plateau and eastern Cascades into southern interior British Columbia. It also occurs east across Idaho into the eastern foothills of the Montana Rockies. The system may also occur on the lower treeline slopes of the Wyoming Rockies. These wooded steppes occur at the lower treeline/ecotone between grasslands or shrublands and forests and woodlands, typically on warm, dry, exposed sites too droughty to support a closed tree canopy. This is not a fire‑maintained system. The "savanna" character results from a climate‑edaphic interaction that results in widely scattered trees over shrubs or grasses, and even in the absence of fire, a "woodland" or "forest" structure will not be obtained. Elevations range from less than 500 m in British Columbia to 1600 m in the central Idaho mountains. Occurrences are found on all slopes and aspects; however, moderately steep to very steep slopes or ridgetops are most common. This system can occur in association with cliff and canyon systems. It generally occurs on glacial till, glacio‑fluvial sand and gravel, dune, basaltic rubble, colluvium, to deep loess or volcanic ash‑derived soils, with characteristic features of good aeration and drainage, coarse textures, circumneutral to slightly acidic pH, an abundance of mineral material, rockiness, and periods of drought during the growing season. These can also occur on areas of sand dunes, scablands, and pumice where the edaphic conditions limit tree abundance. *Pinus ponderosa* (*var. ponderosa* and *var. scopulorum*) and *Pseudotsuga menziesii* are the predominant conifers (not always together); *Pinus flexilis* may be present or common in the tree canopy. In interior British Columbia, *Pseudotsuga menziesii* is the characteristic canopy dominant. In transition areas with big sagebrush steppe systems, *Purshia tridentata, Artemisia tridentata ssp. wyomingensis, Artemisia tridentata ssp. tridentata*, and *Artemisia tripartita* may be common in fire‑protected sites such as rocky areas. Deciduous shrubs, such as *Physocarpus malvaceus, Symphoricarpos albus*, or *Spiraea betulifolia*, can be abundant in more northerly sites or more moist climates. Important grass species include *Pseudoroegneria spicata, Poa secunda, Hesperostipa* spp., *Achnatherum* spp., and *Elymus elymoides*.

**Comments:** This is not a fire‑maintained system; it occurs on sites too droughty to support a closed tree canopy. It does burn with a high‑frequency / low‑intensity regime, but fire is not carried because of the sparse vegetation of the edaphically constrained sites (rock outcrops, dunes, super‑dry, sparse trees over shrubs and sometimes grasses but widely spaced). True "savannas" with grassy understories and high‑frequency / low‑intensity fires are now placed into Northern Rocky Mountain Ponderosa Pine Woodland and Savanna (CES306.030). Ponderosa woodlands and "steppes" in eastern Wyoming, eastern and central Montana, including the Missouri River Breaks, are now included in Northwestern Great Plains‑Black Hills Ponderosa Pine Woodland and Savanna (CES303.650). Southern Rocky Mountain Ponderosa Pine Woodland (CES306.648) and Southern Rocky Mountain Ponderosa Pine Savanna (CES306.649) mostly contain *Pinus ponderosa var. scopulorum, Pinus ponderosa var. brachyptera*, and *Pinus arizonica var. arizonica*. The FRIS site describes different varieties of *Pinus ponderosa* and associated species. This ecological system of the northern Rockies is primarily *Pinus ponderosa var. ponderosa*. Johansen and Latta (2003) have mapped the distribution of two varieties (*Pinus ponderosa var. scopulorum* and *Pinus ponderosa var. ponderosa*) using mitochondrial DNA. They hybridize along the Continental Divide in Montana backing up the FRIS information.

DISTRIBUTION

**Range:** This system is found in the Fraser River drainage of southern British Columbia south along the Cascades into the Modoc Plateau of California, and the northern Rocky Mountains of Washington and Oregon. In the northeastern part of its range, it extends across the northern Rocky Mountains west of the Continental Divide into northwestern Montana and south to the Snake River Plain in Idaho. In Oregon, it is most common in south‑central Oregon, in lands managed by the Lakeview District of the BLM, and by the adjacent Fremont and Deschutes national forests. It also occurs on the marginal lands coming south out of the Blue Mountains, on the edge of the northern Basin and Range.

**Divisions:** 204:C, 304:C, 306:C

**TNC Ecoregions:** 4:C, 6:C, 7:C, 8:C, 9:C, 10:C, 26:C, 68:C

**Nations:** CA, US

**Subnations:** BC, ID, MT, OR, WA:S3S5, WY

**Map Zones:** 1:C, 7:C, 8:C, 9:C, 10:C, 12:?, 18:P, 19:C, 20:?, 21:C

**USFS Ecomap Regions:** 331A:CP, 342B:CC, 342C:CC, 342D:CC, 342H:CP, 342I:CC, 342J:C?, M331A:PP, M332A:PP, M332B:PP, M332D:PP, M332E:PP, M332F:PP, M332G:PP, M333A:PP

CONCEPT

**Environment:** These wooded steppes occur at the lower treeline/ecotone between grasslands or shrublands and forests and woodlands, typically on warm, dry, exposed sites too droughty to support a closed tree canopy. The "savanna" character results from a climate‑edaphic interaction that results in widely scattered trees over shrubs or grasses, and even in the absence of fire, a "woodland" or "forest" structure will not be obtained. Elevations range from less than 500 m in British Columbia to 1600 m in the central Idaho mountains. Occurrences are found on all slopes and aspects; however, moderately steep to very steep slopes or ridgetops are most common. This system can occur in association with cliff and canyon systems. It generally occurs on glacial till, glacio‑fluvial sand and gravel, dune, basaltic rubble, colluvium, to deep loess or volcanic ash‑derived soils, with characteristic features of good aeration and drainage, coarse textures, circumneutral to slightly acidic pH, an abundance of mineral material, rockiness, and periods of drought during the growing season. These can also occur on areas of sand dunes, scablands, and pumice where the edaphic conditions limit tree abundance.

**Vegetation:** *Pinus ponderosa* (vars. *ponderosa* and *scopulorum*) and *Pseudotsuga menziesii* are the predominant conifers (not always together); *Pinus flexilis* may be present or common in the tree canopy. In interior British Columbia, *Pseudotsuga menziesii* is the characteristic canopy dominant. In transition areas with big sagebrush steppe systems, *Purshia tridentata, Artemisia tridentata ssp. wyomingensis, Artemisia tridentata ssp. tridentata*, and *Artemisia tripartita* may be common in fire‑protected sites such as rocky areas. Deciduous shrubs, such as *Physocarpus malvaceus, Symphoricarpos albus*, or *Spiraea betulifolia*, can be abundant in more northerly sites or more moist climates. Important grass species include *Pseudoroegneria spicata, Poa secunda, Hesperostipa* spp., *Achnatherum* spp., and *Elymus elymoides*.

**Dynamics:** This is not a fire‑maintained system. Periodic drought that limits tree establishment is the driving factor in this system. The concept is that of the climate‑edaphic interaction that results in widely scattered trees over "shrub‑steppe" of sage, bitterbrush, or sparsely distributed grasses. Tree growth is likely episodic, with regeneration episodes in years with available moisture. Tree density is limited in some areas by available growing space due to rocky conditions of the site. The tree canopy in this system will never reach woodland density or close due to the interaction of climate and edaphic factors, even in the absence of fire. This system burns occasionally, but the vegetation is sparse enough that fires are typically not carried through the stand. Fire frequency is speculated to be 30‑50 years. This type usually has little surface fuel and replacement fires would be a function of extreme conditions, such as very high winds (LANDFIRE 2007a). Western pine beetle is a significant disturbance and especially affects larger trees, while parasitic mistletoe can cause tree mortality in young and small trees.

LANDFIRE developed a state‑and‑transition vegetation dynamics VDDT model for this system which has four classes in total (LANDFIRE 2007a, BpS 0911650). These are summarized with some modifications below:

A) Early Development 1 All Structures (10% of type in this stage): Dominated by bunchgrasses, mountain sagebrush and seed/sapling‑sized Douglas‑fir. Limber pine and ponderosa pine may be present in varying amounts.

 <br />B) Mid Development 1 Closed (tree‑dominated ‑ 2% of type in this stage): Tree cover is 31‑100%. Relatively dense pole‑ and/or large‑sized Douglas‑fir. Limber pine and ponderosa pine may be present in varying amounts. Sagebrush has largely dropped out of the stand. Mixed‑severity fire may open up the canopy; however, vegetation is generally too sparse to carry fire through stand and is more affected by drought.

C) Mid Development 1 Open (tree‑dominated ‑ 8% of type in this stage): Tree cover is 0‑30%. Open poles of Douglas‑fir with bunchgrass and sagebrush understory. Limber pine and ponderosa pine may be present in varying amounts. Surface fires may help maintain the open condition; however, vegetation is generally too sparse to carry fire through stand and is more affected by drought.

D) Late Development 1 Open (conifer‑dominated ‑ 80% of type in this stage): Tree cover is 0‑30%. Widely spaced, open canopy of medium‑ to large‑diameter Douglas‑fir with bunchgrass and sagebrush understory. Canopy fuels are discontinuous. Limber pine and ponderosa pine may be present in varying amounts. Surface fires may help maintain the open condition; however, vegetation is generally too sparse to carry fire through stand except under extreme conditions.

 <br />LANDFIRE modeled fire regime as predominantly (70%) frequent, low‑severity fires with an MFI of approximately 30 years. Mixed‑severity fires occur with a typical frequency of 30‑50 years primarily in dense stands (classes B and E). Native American burning may have occurred in many of these low‑elevation forests. Limber pine may be affected by blister rust (LANDFIRE 2007a, BpS 0911650). However, this system generally has low surface fuels and is too sparse to carry fire through stand except under extreme conditions so fires are patchy.

Nutrient cycling, specifically carbon cycling, is an important ecological process within many ecological systems. Biological decomposition in ponderosa pine forests is more limited than biological production, resulting in accumulation of organic materials, especially in the absence of fire (Harvey 1994, Graham and Jain 2005).

SOURCES

**References:** Belnap 2001, Belnap et al. 2001, Burns and Honkala 1990a, Camp et al. 1997, Comer et al. 2002, Comer et al. 2003*, Cooper et al. 1987, Dale et al. 2001, Daubenmire and Daubenmire 1968, Everett et al. 2000, Eyre 1980, Franklin and Dyrness 1973, Garfin et al. 2014, Graham and Jain 2005, Habeck 1992a, Habeck 1992d, Harvey 1994, Howard 2003b, Howard 2003c, Johansen and Latta 2003, LANDFIRE 2007a, Littell et al. 2009, Mauk and Henderson 1984, Mehl 1992, Meidinger and Pojar 1991, Mote et al. 2014, Pfister et al. 1977, Reid et al. 1999, Rice et al. 2012a, Schmid 1988, Shafer et al. 2014, Shiflet 1994, Steinberg 2002e, Stevens‑Rumann et al. 2017, USFS 1993a, WNHP 2011, WNHP unpubl. data 2018, Westerling et al. 2006, Youngblood and Mauk 1985

**Version:** 22 May 2018	**Stakeholders:** Canada, West

**Concept Author:** Western Ecology Group	**LeadResp:** West

CES306.030 Northern Rocky Mountain Ponderosa Pine Woodland and Savanna

**Primary Division:** Rocky Mountain (306)

**Land Cover Class:** Forest and Woodland

**Spatial Scale & Pattern:** Matrix

**Required Classifiers:** Natural/Semi‑natural; Vegetated (>10% vasc.); Upland

**Diagnostic Classifiers:** Ridge/Summit/Upper Slope; Very Shallow Soil; Mineral: W/ A‑Horizon <10 cm; Sand Soil Texture; Aridic; Intermediate Disturbance Interval [Periodicity/Polycyclic Disturbance]; F‑Patch/Medium Intensity; Needle‑Leaved Tree; Graminoid; Pinus ponderosa with grassy understory; Pinus ponderosa with shrubby understory

**National Mapping Codes:** EVT 2053; ESLF 4240; ESP 1053

**Concept Summary:** This inland Pacific Northwest ecological system occurs in the foothills of the northern Rocky Mountains in the Columbia Plateau region and west along the foothills of the Modoc Plateau and eastern Cascades into southern interior British Columbia. These woodlands and savannas occur at the lower treeline/ecotone between grasslands or shrublands and more mesic coniferous forests typically in warm, dry, exposed sites. Elevations range from less than 500 m in British Columbia to 1600 m in the central Idaho mountains. Occurrences are found on all slopes and aspects; however, moderately steep to very steep slopes or ridgetops are most common. This ecological system generally occurs on glacial till, glacio‑fluvial sand and gravel, dune, basaltic rubble, colluvium, to deep loess or volcanic ash‑derived soils, with characteristic features of good aeration and drainage, coarse textures, circumneutral to slightly acidic pH, an abundance of mineral material, rockiness, and periods of drought during the growing season. In the Oregon "pumice zone" this system occurs as matrix‑forming, extensive woodlands on rolling pumice plateaus and other volcanic deposits. These woodlands in the eastern Cascades, Okanagan and northern Rockies regions receive winter and spring rains, and thus have a greater spring "green‑up" than the drier woodlands in the central Rockies. *Pinus ponderosa* (primarily *var. ponderosa*) is the predominant conifer; *Pseudotsuga menziesii* may be present in the tree canopy but is usually absent. In southern interior British Columbia, *Pseudotsuga menziesii* or *Pinus flexilis* may form woodlands or fire‑maintained savannas with and without *Pinus ponderosa var. ponderosa* at the lower treeline transition into grassland or shrub‑steppe. The understory can be shrubby, with *Artemisia tridentata, Arctostaphylos patula, Arctostaphylos uva‑ursi, Cercocarpus ledifolius, Physocarpus malvaceus, Purshia tridentata, Symphoricarpos oreophilus* or *Symphoricarpos albus, Prunus virginiana, Amelanchier alnifolia*, and *Rosa* spp. common species. Understory vegetation in the true savanna occurrences is predominantly fire‑resistant grasses and forbs that resprout following surface fires; shrubs, understory trees and downed logs are uncommon. These more open stands support grasses such as *Pseudoroegneria spicata, Hesperostipa* spp., *Achnatherum* spp., dry *Carex* species (*Carex inops*), *Festuca idahoensis*, or *Festuca campestris*. The more mesic portions of this system may include *Calamagrostis rubescens* or *Carex geyeri*, species more typical of Northern Rocky Mountain Dry‑Mesic Montane Mixed Conifer Forest (CES306.805). Mixed fire regimes and surface fires of variable return intervals maintain these woodlands typically with a shrub‑dominated or patchy shrub layer, depending on climate, degree of soil development, and understory density. This includes the northern race of Interior Ponderosa Pine old‑growth (USFS Region 6, USFS Region 1). Historically, many of these woodlands and savannas lacked the shrub component resulting from 3‑ to 7‑year fire‑return intervals.

**Comments:** Hot, dry Douglas‑fir types with grass are included here. Southern Rocky Mountain Ponderosa Pine Woodland (CES306.648) and Southern Rocky Mountain Ponderosa Pine Savanna (CES306.649) contain mostly *Pinus ponderosa var. scopulorum* and *Pinus arizonica var. arizonica*. The FRIS site describes different varieties of *Pinus ponderosa* and associated species. Johansen and Latta (2003) have mapped the distribution of the two varieties using mitochondrial DNA. They hybridize along the Continental Divide in Montana backing up the FRIS information. Another ponderosa pine system remains to be defined and described for the woodlands and savannas occurring in central and eastern Montana and the Black Hills region. These "northwestern Great Plains ponderosa pine woodlands" are likely to have a floristic component that is more northern Great Plains mixedgrass in nature, as well as being open woodlands generally found in a grassland matrix. Further work is need to identify the geographic and conceptual boundaries between Northern Rocky Mountain Ponderosa Pine Woodland and Savanna (CES306.030) and the northwestern Great Plains system.

Meeting of Pacific Northwest ecologists for Landfire concluded that the "true savanna" of high‑frequency / low‑intensity fires and grassy understories is now minimally in existence. Most areas that may have been savanna in the past are now more nearly closed‑canopy woodlands/forests. Conclusion was that these true savannas should be included with this woodland system, rather than with the climatically‑edaphically controlled Northern Rocky Mountain Foothill Conifer Wooded Steppe (CES306.958). Hence, the "true fire‑maintained savanna" is included in this woodland system.

<br />Louisa Evers (pers. comm. 2006) notes that she has not found any evidence that ponderosa pine savanna existed historically in north‑central and central Oregon. In north‑central Oregon, the savanna would have been oak or pine‑oak. In central Oregon, it may well have been western juniper. Condition surveys of the Cascades Forest Reserve and General Land Office survey notes suggest that ponderosa pine formed a woodland with grassy understories, but still was often referred to as open‑parklike. Conversely pine‑oak and Douglas‑fir‑oak savannas appeared to have once been quite common in the Willamette Valley (and are classified in North Pacific Oak Woodland (CES204.852)).

DISTRIBUTION

**Range:** This system is found in the Fraser River drainage of southern British Columbia south along the Cascades and northern Rocky Mountains of Washington, Oregon and California. In the northeastern part of its range, it extends across the northern Rocky Mountains west of the Continental Divide into northwestern Montana, south to the Snake River Plain in Idaho, and east into the foothills of western Montana.

**Divisions:** 204:C, 304:C, 306:C

**TNC Ecoregions:** 4:C, 6:C, 7:C, 8:C, 9:C, 10:C, 26:?, 33:?, 68:C

**Nations:** CA, US

**Subnations:** BC, ID, MT, NV?, OR, WA:S2, WY

**Map Zones:** 1:C, 2:C, 7:C, 8:C, 9:C, 10:C, 18:P, 19:C, 20:?, 30:?

**USFS Ecomap Regions:** 331A:CC, 342B:CC, 342C:CC, 342D:CP, 342H:CC, 342I:CC, M242B:CC, M242C:CC, M242D:CC, M261A:C?, M261D:CC, M261G:CC, M331A:PP, M331J:PP, M332A:CC, M332B:CC, M332D:CP, M332E:CC, M332F:CC, M332G:CC, M333A:CC, M333B:CC, M333C:CC, M333D:CC

CONCEPT

**Environment:** This ecological system within the region occurs at the lower treeline/ecotone between grasslands or shrublands and more mesic coniferous forests typically in warm, dry, exposed sites at elevations ranging from 500‑1600 m (1600‑5248 feet). These woodlands receive winter and spring rains, and thus have a greater spring "green‑up" than the drier ponderosa woodlands in the Colorado and New Mexico Rockies. In eastern Washington, precipitation varies from 36‑76 cm (14‑30 inches) with most occurring as snowfall (WNHP 2011). It can occur on all slopes and aspects; however, it commonly occurs on moderately steep to very steep slopes or ridgetops. This ecological system generally occurs on most geological substrates from weathered rock to glacial deposits to eolian deposits (e.g., glacial till, glacio‑fluvial sand and gravel, dunes, basaltic rubble, colluvium, to deep loess or volcanic ash‑derived soils) (WNHP 2011). Characteristic soil features include good aeration and drainage, coarse textures, circumneutral to slightly acidic pH, an abundance of mineral material, and periods of drought during the growing season. Some occurrences may occur as edaphic climax communities on very skeletal, infertile and/or excessively drained soils, such as pumice, cinder or lava fields, and scree slopes. In the Oregon "pumice zone" this system occurs as matrix‑forming, extensive woodlands on rolling pumice plateaus and other volcanic deposits. Surface textures are highly variable in this ecological system ranging from sand to loam and silt loam. Exposed rock and bare soil consistently occur to some degree in all the associations.

**Vegetation:** *Pinus ponderosa* (primarily *var. ponderosa*) is the predominant conifer; *Pseudotsuga menziesii* may be present in the tree canopy but is usually absent. In southern interior British Columbia, *Pseudotsuga menziesii* or *Pinus flexilis* may form woodlands or fire‑maintained savannas with and without *Pinus ponderosa var. ponderosa* at the lower treeline transition into grassland or shrub‑steppe. The understory can be shrubby, with *Artemisia tridentata, Arctostaphylos patula, Arctostaphylos uva‑ursi, Cercocarpus ledifolius, Physocarpus malvaceus, Purshia tridentata, Symphoricarpos oreophilus* or *Symphoricarpos albus, Prunus virginiana, Amelanchier alnifolia*, and *Rosa* spp. common species. Understory vegetation in the true savanna occurrences is predominantly fire‑resistant grasses and forbs that resprout following surface fires; shrubs, understory trees and downed logs are uncommon. These more open stands support grasses such as *Pseudoroegneria spicata, Hesperostipa* spp., *Achnatherum* spp., dry *Carex* species (*Carex inops*), *Festuca idahoensis*, or *Festuca campestris*. The more mesic portions of this system may include *Calamagrostis rubescens* or *Carex geyeri*.

**Dynamics:** Summer drought and frequent, low‑severity fires create woodlands composed of widely spaced, large trees with small scattered clumps of dense, even‑aged stands which regenerated in forest gaps or were protected from fire due to higher soil moisture or topographic protection. Closed‑canopy or dense stands were also part of the historical range of stand variability but under natural disturbance regimes are a minor component of that landscape. Mixed fire regimes and surface fires of variable return intervals maintain these woodlands typically with a shrub‑dominated or patchy shrub layer, depending on climate, degree of soil development, and understory density. Historically, many of these woodlands and savannas lacked the shrub component resulting from low‑severity but high‑frequency fires (2 ‑ to 10‑year fire‑return intervals). Some sites, because of low productivity, naturally lacked a dense shrub understory. Mixed‑severity fires had a return interval of 25‑75 years while stand‑replacing fire occurred at an interval of >100 years (Arno 1980, Fischer and Bradley 1987). The latter two intervals only occurred on 20‑25% of stands within the landscape while surface fires were the dominant fire regime on over 75% of stands (Landfire 2007a). Presettlement fires were triggered by lightning strikes or deliberately set fires by Native Americans.

*Pinus ponderosa* is a drought‑resistant, shade‑intolerant conifer which usually occurs at lower treeline in the major ranges of the western United States. Establishment of ponderosa pine is erratic and believed to be linked to periods of adequate soil moisture and good seed crops as well as fire frequencies, which allow seedlings to reach sapling size.

Western pine beetle is another significant disturbance and especially affects larger trees. Bark beetle outbreaks are highly related to stand density. Denser stands in relation to site capacity will favor outbreaks, which will decrease as trees are thinned (Landfire 2007a). Mistletoe can cause tree mortality in young and small trees. Fires and insect outbreaks resulted in a landscape consisting of a mosaic of open forests of large trees (most abundant patch), small denser patches of trees, and openings (Franklin et al. 2008). White‑headed woodpecker, pygmy nuthatch, and flammulated owl are indicators of healthy ponderosa pine woodlands. All these birds prefer mature trees in an open woodland setting (Jones 1998, Levad 1998 Winn 1998, as cited in Rondeau 2001).

LANDFIRE developed several state‑and‑transition vegetation dynamics VDDT models for this system across its range and dry or mesic conditions. This model is typical of much of the range and has five classes in total (LANDFIRE 2007a, BpS 1910530). These are summarized as:

A) Early Development 1 Open (5% of type in this stage): Fire‑maintained grass/forb and/or seedlings and saplings. Seedling/sapling size class would be less than 5 inches in diameter. There would be no large patches (10‑100 acres) of large or old‑growth trees due to poor site conditions and abundance of rock outcroppings. However, dispersed large‑diameter fire‑remnant ponderosa pines and snag trees could be present. These large‑diameter trees would have a density of less than one tree per acre. Grass species are the dominant lifeform in this class attaining maximum heights of 3 feet and patchy in distribution (25‑75% cover).

B) Mid Development 1 Closed (tree‑dominated ‑ 10% of type in this stage): Tree cover is 41‑60%. Closed ponderosa pine pole and medium‑diameter stand; may have Douglas‑fir as incidentals. Larger, old‑growth trees may be present in this class, though the pole and medium‑diameter class (5‑21 inches) occurring between these large trees is most abundant and characteristic of this class. May see large‑diameter snags, dead and downed trees present. High‑density stunted pole stands are counted here; may see insect/disease here.

C) Mid Development 1 Open (tree‑dominated ‑ 20% of type in this stage): Tree cover is 0‑40%. Open ponderosa pine pole and medium‑diameter stand that may have Douglas‑fir as incidentals. Larger, old‑growth trees may be present in this class, the pole and medium‑diameter (5‑21 inches) trees are characteristic for this class. These patches have probably had recent fire or are drier so they retain a more open condition.

D) Late Development 1 Open (conifer‑dominated ‑ 55% of type in this stage): Tree cover is 0‑40%. Fire‑maintained open, park‑like ponderosa pine; nearly any fire maintains; Douglas‑fir may be seen as incidentals or in patches, but not a major component of the overstory. The overstory is characterized by large and very large ponderosa pine and isolated Douglas‑fir. Understory is dominated by grasses and is relatively open. Seedlings are very infrequent, with <10% cover and usually occurring in patches.

E) Late Development 1 Close (conifer‑dominated ‑ 5% of type in this stage): Tree cover is 41‑60%. High‑density, multi‑storied ponderosa pine stand; Douglas‑fir regeneration on some sites. Thickets of various size classes distributed within the class and may be interspersed with large snags.

Frequent, non‑lethal surface fires were the dominant disturbance factor, occurring every 3‑30 years (Arno 1980, Arno and Petersen 1983, Fischer and Bradley 1987). Three‑year fire‑return intervals are likely very localized and associated with Native American burning. However, there is some disagreement as to the extent of Native burning. More median fire‑return intervals were likely about 15 years. Mixed‑severity fires likely occurred about every 50 years, again, depending on the vegetative state. Stand‑replacement fires likely occurred in stands and small patches on the order of a few hundred acres every 300‑700 years depending on the vegetative state. Some authors note that little information is available regarding the exact nature of stand‑replacement fire severity in this BpS (LANDFIRE 2007a, BpS 1910530). Western pine beetle can attack large ponderosa pine in any canopy density (LANDFIRE 2007a, BpS 1910530).

Nutrient cycling, specifically carbon cycling, is an important ecological process within many ecological systems. However, biological decomposition in ponderosa pine forests is more limited than biological production, resulting in accumulation of organic materials, especially in the absence of fire (Harvey 1994, Graham and Jain 2005).

SOURCES

**References:** Arno 1980, Arno and Peterson 1983, Burns and Honkala 1990a, Camp et al. 1997, Comer et al. 2003*, Cooper et al. 1987, Dale et al. 2001, Daubenmire and Daubenmire 1968, Everett et al. 2000, Evers pers. comm., Eyre 1980, Fischer and Bradley 1987, Franklin and Dyrness 1973, Franklin et al. 2008, Habeck 1992a, Hessburg et al. 2005, Howard 2003b, Howard 2003c, Johansen and Latta 2003, LANDFIRE 2007a, Littell et al. 2009, Mauk and Henderson 1984, McKenzie et al. 2004, McKenzie et al. 2008, Mehl 1992, Meidinger and Pojar 1991, Mote et al. 2014, NCC 2002, Pfister et al. 1977, Reid et al. 1999, Rice et al. 2012a, Rondeau 2001, Schmid 1988, Shafer et al. 2014, Shiflet 1994, Stevens‑Rumann et al. 2017, USFS 1993a, WNHP 2011, WNHP unpubl. data 2018, Westerling et al. 2006, Western Ecology Working Group n.d., Youngblood and Mauk 1985

**Version:** 22 May 2018	**Stakeholders:** Canada, West

**Concept Author:** NatureServe Western Ecology Team	**LeadResp:** West

CES303.650 Northwestern Great Plains‑Black Hills Ponderosa Pine Woodland and Savanna

**Primary Division:** Western Great Plains (303)

**Land Cover Class:** Forest and Woodland

**Spatial Scale & Pattern:** Large patch

**Required Classifiers:** Natural/Semi‑natural; Vegetated (>10% vasc.); Upland

**Diagnostic Classifiers:** Ridge/Summit/Upper Slope; Very Shallow Soil; Mineral: W/ A‑Horizon <10 cm; Sand Soil Texture; Aridic; Intermediate Disturbance Interval [Periodicity/Polycyclic Disturbance]; F‑Patch/Medium Intensity; Needle‑Leaved Tree; Pinus ponderosa with grassy understory; Pinus ponderosa with shrubby understory

**National Mapping Codes:** EVT 2179; ESLF 4280; ESP 1179

**Concept Summary:** This system occurs throughout the northwestern Great Plains along areas that border the Rocky Mountains. The expansion of this system within the central Great Plains may be due to fire suppression. These can be physiognomically variable, ranging from very sparse patches of trees on drier sites, to nearly closed‑canopy forest stands on north slopes or in draws where available soil moisture is higher. This system occurs primarily on gentle to steep slopes along escarpments, buttes, canyons, rock outcrops or ravines and can grade into one of the surrounding prairie systems or the Great Plains canyon system. Soils typically range from well‑drained loamy sands to sandy loams formed in colluvium, weathered sandstone, limestone, scoria or eolian sand. This system is primarily dominated by *Pinus ponderosa* but may include a sparse to relatively dense understory of *Juniperus scopulorum, Thuja*, or *Cercocarpus* with just a few scattered trees. Deciduous trees are an important component in some areas (western Dakotas, Black Hills) and are sometimes codominant with the pines, including *Fraxinus pennsylvanica, Betula papyrifera, Quercus macrocarpa, Ulmus americana, Acer negundo*, and *Populus tremuloides*. Along the Missouri Breaks in north‑central Montana, woodlands dominated by *Pseudotsuga menziesii* are in similar ecological settings as *Pinus ponderosa* in the Great Plains and are included in this system. In the breaks where it occurs, *Pseudotsuga menziesii* has a very open canopy over grassy undergrowth, predominantly composed of *Pseudoroegneria spicata*, with little to no shrubs present. Important or common shrub species with ponderosa pine can include *Arctostaphylos uva‑ursi, Mahonia repens, Yucca glauca, Symphoricarpos* spp., *Prunus virginiana, Juniperus communis, Juniperus horizontalis, Amelanchier alnifolia, Rhus trilobata*, and *Physocarpus monogynus*. The herbaceous understory can range from sparse to a dense layer with species typifying the surrounding prairie system, with mixedgrass species common, such as *Andropogon gerardii, Bouteloua curtipendula, Carex inops ssp. heliophila, Carex filifolia, Danthonia intermedia, Koeleria macrantha, Nassella viridula, Oryzopsis asperifolia, Pascopyrum smithii, Piptatheropsis micrantha*, and *Schizachyrium scoparium*. Timber cutting and other disturbances have degraded many examples of this system within the Great Plains. However, some good examples may occur along the Pine Ridge escarpment and Pine Ridge district of the Nebraska National Forest in Nebraska.

**Comments:** In this Great Plains region, what were previously called Northern Rocky Mountain Foothill Conifer Wooded Steppe (CES306.958), Southern Rocky Mountain Ponderosa Pine Woodland (CES303.648) and Southern Rocky Mountain Ponderosa Pine Savanna (CES306.826) are now included in this new system. Physiognomically, this is a variable system, with everything from sparse woodlands on breaks and scoria bluffs to dense closed‑canopy stands in the Black Hills included.

Southern Rocky Mountain Ponderosa Pine Woodland (CES306.648) is now defined to occur in the montane zones of the Bighorns (USFS section M331B) and Laramie Range (USFS section M331I) and to the west and south of these mountains. It will also occur in other isolated mountain ranges of central Wyoming, but not in eastern Wyoming. It does not occur farther north than Wyoming; all Montana ponderosa pine woodlands are placed into either this Northwest Great Plains system or into Northern Rocky Mountain Ponderosa Pine Woodland and Savanna (CES306.030), as appropriate. The southern extent is hard to determine, but farther south in Colorado, there is more *Juniperus, Pinus edulis*, and *Quercus gambelii*. This system certainly occurs in New Mexico, but stands at the Black Mesa in western Oklahoma and in southeastern Colorado may also be viewed as having the southwestern affinities.

<br />In the Pine Escarpments of Nebraska, pine communities can range from open canopies with grassy understories to more closed canopies. Included within these areas are also several rocky outcrops, which probably should be included within the system as they are often intermingled with the savanna. The more closed‑canopy examples may be more similar to Southern Rocky Mountain Ponderosa Pine Woodland (CES306.648) but are included in this system for now.

DISTRIBUTION

**Range:** This system is found in central and eastern Montana, the western Dakotas, eastern Wyoming (east of the Bighorns), the Black Hills, and south into the Sand Hills of Nebraska and northeastern Colorado (north of Pawnee National Grasslands to Cedar Point near Limon and south). In Montana, it occurs along the Missouri River breaks, around the Little Belts and Snowy mountains, in south‑central Montana between the Bighorns and the Black Hills (along the Tongue and Powder rivers), and other areas of eastern Montana. In Wyoming, it is found around the Black Hills and Bear Lodge Mountains, and in isolated areas of eastern Wyoming on bluffs and rock outcrops, and along "breaks." Whether this system occurs in Kansas is uncertain.

**Divisions:** 303:C, 306:C

**TNC Ecoregions:** 25:C, 26:C, 27:C, 33:C, 34:?

**Nations:** US

**Subnations:** KS?, MT, ND, NE, SD, WY

**Map Zones:** 20:C, 29:C, 30:C, 31:C, 33:C, 39:?, 40:?

**USFS Ecomap Regions:** 331C:C?, 331D:CC, 331E:CC, 331F:CC, 331G:CC, 331H:CC, 331K:CC, 331L:CC, 331M:CC, 331N:CC, 332A:C?, 332B:C?, 332C:CC, 332D:C?, 332E:C?, M334A:CC

CONCEPT

**Environment:** The ponderosa pine system is found in a matrix of northwestern Great Plains grassland systems along escarpments and in foothills and mountains in the Black Hills. It is often surrounded by mixedgrass or tallgrass prairie, in places where available soil moisture is higher, or soils are more coarse and rocky. Some stands are found adjacent to major creek bottoms and the lower toeslope and footslope positions. In some cases, these woodlands or savannas may occur where fire suppression has allowed trees to become established (in areas where deciduous trees are more abundant) (Girard et al. 1987). These are typically not in the same setting as Rocky Mountain ponderosa pine, where ponderosa pine forms woodlands at lower treeline and grades into mixed montane conifer systems at higher elevations. These are physiognomically variable woodlands, ranging from very sparse patches of trees on drier, often rocky sites, to nearly closed‑canopy forest stands on north slopes or in draws where available soil moisture is higher. This system occurs primarily on gentle to steep slopes along escarpments, buttes, canyons, rock outcrops or ravines and can grade into the Great Plains canyons the surrounding mixedgrass prairie systems (Hoffman and Alexander 1987). Soils typically range from well‑drained loamy sands to loams formed in colluvium, weathered sandstone, limestone, calcareous shales, scoria or eolian sand (Hoffman and Alexander 1987, Hansen and Hoffman 1988).

**Vegetation:** This system is primarily dominated by *Pinus ponderosa* but may include a sparse to relatively dense understory of *Juniperus scopulorum, Thuja*, or *Cercocarpus* with just a few scattered trees. Deciduous trees are an important component in some areas (western Dakotas, Black Hills) and are sometimes codominant with the pines, including *Fraxinus pennsylvanica, Betula papyrifera, Quercus macrocarpa, Ulmus americana, Acer negundo*, and *Populus tremuloides*. Along the Missouri Breaks in north‑central Montana, woodlands dominated by *Pseudotsuga menziesii* are in similar ecological settings as *Pinus ponderosa* in the Great Plains and are included in this system. In the breaks where it occurs, *Pseudotsuga menziesii* has a very open canopy over grassy undergrowth, predominantly composed of *Pseudoroegneria spicata*, with little to no shrubs present. Important or common shrub species with ponderosa pine can include *Arctostaphylos uva‑ursi, Mahonia repens, Yucca glauca, Symphoricarpos* spp., *Prunus virginiana, Juniperus communis, Juniperus horizontalis, Amelanchier alnifolia, Rhus trilobata*, and *Physocarpus monogynus*. The herbaceous understory can range from sparse to a dense layer with species typifying the surrounding prairie system, with mixedgrass species common, such as *Andropogon gerardii, Bouteloua curtipendula, Carex inops ssp. heliophila, Carex filifolia, Danthonia intermedia, Koeleria macrantha, Nassella viridula, Oryzopsis asperifolia, Pascopyrum smithii, Piptatheropsis micrantha (= Piptatherum micranthum)*, and *Schizachyrium scoparium*.

**Dynamics:** Marriot and Faber‑Langendoen (2000) report different fire regimes for ponderosa pine communities in the Black Hills, with their "Dry Group" more typically having frequent surface fires and the "Mesic Group" having infrequent catastrophic fires (every 100‑200 years). The Dry Group of associations includes lower elevation foothill savanna associations, and the mesic group somewhat higher elevation, north‑slope, swale associations. K. Kindscher (pers. comm. 2007) believes that almost all the stands in Nebraska were there at the time of settlement and are not a result of pine expansion due to fire suppression; in addition, at least some have disappeared, such as the one in southern Nebraska (Franklin County). It is possible, however, that some areas of this system have expanded in size due to fire suppression, but this needs substantiation.

LANDFIRE developed several a state‑and‑transition vegetation dynamics VDDT models for this system for different map zones and savanna vs low elevation woodland stands. Shone in the grassland model for Map Zone 29 which has five classes in total (LANDFIRE 2007a, BpS 2911792). These are summarized as:<br />

A) Early Development 1 All Structures (5% of type in this stage): This community is dominated by herbaceous and woody species, including the graminoids needlegrasses, western wheatgrass, bluebunch wheatgrass, sedges, Idaho fescue and little bluestem in moister areas, and various shrubs including skunkbush and snowberry. Ponderosa pine seedlings are scattered and found in small clumps. Little bluestem will also be an indicator species. Number of years in this class is variable depending on climatic patterns and fire disturbances. This class typically ends at 30 years in this model. Without fire for 25 years, this class can move to a mid‑closed stage.

B) Mid Development 1 Closed (2% of type in this stage): Tree cover is 0‑50%. Multi‑story stand of small and medium trees with saplings and seedlings coming in as clumps. Understory is sparse. Some juniper might be present ‑ could be an outlier. Grasses and shrubs are shaded out. This class lasts approximately 70 years, then moves to a late‑closed stage. Low‑severity surface fires occur every 15 years and move this stage to a mid‑open stage. Replacement fires occur infrequently, approximately every 300 years. Insect/disease was modeled at approximately occurring every 50 years, not causing a transition.

C) Mid Development 1 Open (8% of type in this stage): Tree cover is 0‑50%. Predominantly single‑story stands with a few pockets of regeneration. Low shrubs such as snowberry and skunkbush and poison ivy are dominant as well as grasses and forbs. Graminoids could have up to 70‑80% cover. Rocky Mountain juniper present in patches (Rocky Mountain juniper is not common on the Pine Ridge in Nebraska). *Carex* spp. and little bluestem will also be indicator species. This class lasts approximately 50 years then goes to a late‑open stage. Without fire for 40 years, this could transition back to a mid‑closed stage. Low‑severity surface fires occur every 15 years, maintaining this class. Replacement fires occur very infrequently (modeled at 0.0015 probability).

D) Late Development 1 Open (80% of type in this stage): Tree cover is 0‑50%. Predominantly single‑story stands of large ponderosa pine with pockets of smaller size classes (replacement). Snowberry, skunkbush and patches of Rocky Mountain juniper. Understory is dominated by shrub species and grasses and poison ivy. Graminoids could have up to 70‑80% cover. *Carex* spp. and little bluestem will also be indicator species. It is thought that class D, the late‑open stage, should occupy approximately 80% of the historical landscape. Low‑severity fires occur every 15 years and maintain this stage. Replacement fires occur very infrequently (0.0015 probability). If no fire occurs after 40 years, this class could transition to the late‑closed stage. Insect/disease occurs every 50 years and maintains this stage.

E) Late Development 1 Closed (5% of type in this stage): Tree cover is 51‑100%. This is a somewhat uniform late‑development stage, multi‑story stands of large, medium, small and seedling ponderosa pine. Shrubs and grasses are sparse. This type generally exceeds 70% canopy cover. dbh is less in this class than late‑open. Low‑severity surface fires occur every 15 years and cause a transition back to the late‑open stage. Replacement fires occur every 300 years. Insect/disease occurs every 250 years, causing a transition back to the late‑open stage. Drought can also occur ‑ every 500 years, causing a transition to the late‑open stage.

<br />Generally, the fire regime is characterized by frequent fire‑return interval of low‑severity surface fire. The presence of abundant fire‑scarred trees in multi‑aged stands supports a prevailing historical model for ponderosa pine forests in which recurrent surface fires affected heterogeneous forest structure (Brown 2006). Mixed‑severity fire occurs in closed‑canopy conditions and stand‑replacement fire is very infrequent (300+ years) (LANDFIRE 2007a, BpS 2911792). Low‑severity fires are frequent and range from <10 years to more than 20 years (Fischer and Clayton 1983, Brown and Sieg 1999), but probably not more than 40 years at the high end (3‑70 years range). The MFRI is approximately 12‑15 years for low‑severity fires (LANDFIRE 2007a, BpS 2911792).

There is considerable debate over the role of mixed‑severity and surface fires in the historical range of variability in this and other ponderosa pine forests in the northern and central Rockies (Veblen et al. 2000, Baker and Ehle 2001, 2003, Barrett 2004a, b). However, Brown (2006) argues that surface fire was the dominant mode of fire disturbance and that the role of mixed‑severity fires is overstated. For MZs 29 and 30, it was suggested that mixed fire be removed from this savanna model; reviewers agreed, and therefore mixed fire is not in this model (LANDFIRE 2007a, BpS 2911792).

Variation in precipitation and temperature interacting with fire, tip moths and ungulate grazing affects pine regeneration. Windthrow, storm damage and mountain pine beetles were minor disturbances in this type unless stands reach high densities. The interactions among drought, insects and disease are not well understood (LANDFIRE 2007a, BpS 2911792). *Pinus ponderosa ‑ Juniperus scopulorum* savanna in the southern Black Hills has lots of rock exposure or sparsely grassed soils, which probably protected some of the juniper seed trees from being wiped out by fire (LANDFIRE 2007a, BpS 2911792).

Nutrient cycling, specifically carbon cycling, is an important ecological process within many ecological systems. However, biological decomposition in ponderosa pine forests is more limited than biological production, resulting in accumulation of organic materials, especially in the absence of fire (Harvey 1994, Graham and Jain 2005).

SOURCES

**References:** Baker and Ehle 2001, Baker et al. 2003, Barrett 2004a, Barrett 2004b, Belnap 2001, Belnap et al. 2001, Bock and Bock 1984, Brown 2006, Brown and Sieg 1999, Burns and Honkala 1990a, Comer et al. 2003*, Dale et al. 2001, Eyre 1980, Fischer and Clayton 1983, Girard 1985, Girard et al. 1987, Girard et al. 1989, Graham and Jain 2005, Hansen and Hoffman 1988, Harvey 1994, Hoffman and Alexander 1987, Howard 2003b, Howard 2003c, LANDFIRE 2007a, Marriott and Faber‑Langendoen 2000, McKenzie et al. 2004, McKenzie et al. 2008, Reid et al. 1999, Rolfsmeier and Steinauer 2010, Schmid 1988, Shafer et al. 2014, Stevens‑Rumann et al. 2017, Thilenius 1972, Veblen et al. 2000, Westerling et al. 2006

**Version:** 22 May 2018	**Stakeholders:** Midwest, West

**Concept Author:** M.S. Reid	**LeadResp:** West

CES306.955 Rocky Mountain Foothill Limber Pine‑Juniper Woodland

**Primary Division:** Rocky Mountain (306)

**Land Cover Class:** Forest and Woodland

**Spatial Scale & Pattern:** Large patch

**Required Classifiers:** Natural/Semi‑natural; Vegetated (>10% vasc.); Upland

**Diagnostic Classifiers:** Lowland [Foothill]; Forest and Woodland (Treed); Very Shallow Soil; Mineral: W/ A‑Horizon <10 cm; Sand Soil Texture; Aridic; Long Disturbance Interval; F‑Patch/High Intensity; Needle‑Leaved Tree; Pinus flexilis, Juniperus scopulorum, J. osteosperma

**National Mapping Codes:** EVT 2049; ESLF 4236; ESP 1049

**Concept Summary:** This ecological system occurs in foothill and lower montane zones in the Rocky Mountains from northern Montana south to central Colorado and on escarpments across Wyoming extending out into the western Great Plains. Elevation ranges from 1000‑2440 m. It occurs generally below continuous forests of *Pseudotsuga menziesii* or *Pinus ponderosa* and can occur in large stands well within the zone of continuous forests in the northeastern Rocky Mountains. It is restricted to shallow soils and fractured bedrock derived from a variety of parent material, including limestone, sandstone, dolomite, granite and colluvium. Soils have a high rock component (typically over 50% cover) and are coarse‑ to fine‑textured, often gravelly and calcareous. Slopes are typically moderately steep to steep. At lower montane elevations, it is limited to the most xeric aspects on rock outcrops, and at lower elevations to the relatively mesic north aspects. Fire is infrequent and spotty because rocky substrates prevent a continuous vegetation canopy needed to spread. Vegetation is characterized by an open‑tree canopy or patchy woodland that is dominated by *Pinus flexilis, Juniperus osteosperma*, or *Juniperus scopulorum*. *Pinus edulis* is not present. A sparse to moderately dense short‑shrub layer, if present, may include a variety of shrubs, such as *Arctostaphylos uva‑ursi, Artemisia nova, Artemisia tridentata, Cercocarpus ledifolius, Cercocarpus montanus, Dasiphora fruticosa ssp. floribunda, Ericameria nauseosa, Juniperus horizontalis, Purshia tridentata, Rhus trilobata, Rosa woodsii, Shepherdia canadensis* (important in Montana stands), *Symphoricarpos albus*, or *Symphoricarpos oreophilus*. Herbaceous layers are generally sparse, but range to moderately dense, and are typically dominated by perennial graminoids such as *Bouteloua gracilis, Festuca idahoensis, Festuca campestris, Danthonia intermedia, Leucopoa kingii, Hesperostipa comata, Koeleria macrantha, Piptatheropsis micrantha, Poa secunda*, or *Pseudoroegneria spicata*. Within this ecological system, there may be small patches of grassland or shrubland composed of some of the above species.

**Comments:** These limber pine and juniper woodlands are a subset of the scarp woodlands found throughout the northwestern Great Plains (CNHP 2010).

DISTRIBUTION

**Range:** This system occurs in foothill and lower montane zones in the Rocky Mountains from northern Montana south to central Colorado and on escarpments across Wyoming, extending out into the western Great Plains. Elevation ranges from 1000‑2400 m. This system may also occur in southeastern Idaho, though it would not be common there. It is also very likely to occur north into Canada along the Front Range of Alberta, in similar ecological settings.

**Divisions:** 303:C, 306:C

**TNC Ecoregions:** 8:C, 9:C, 10:C, 20:C, 25:P, 26:C, 27:C

**Nations:** CA?, US

**Subnations:** AB?, CO, MT, ND, SD, WY

**Map Zones:** 16:C, 19:C, 20:C, 21:P, 22:C, 28:C, 29:C, 30:C, 31:?, 33:C, 40:?

**USFS Ecomap Regions:** 331D:CC, 331F:CC, 331G:CC, 331H:CC, 331K:CP, 331N:CC, 332C:CC, 342A:CC, 342E:CC, 342F:CC, 342G:CC, M242D:PP, M331A:CC, M331B:CC, M331D:CC, M331E:CC, M331I:CC, M331J:CC, M332D:CC, M334A:??

CONCEPT

**Environment:** This ecological system occurs in foothill and lower montane zones in the Rocky Mountains from northern Montana south to central Colorado and on exposed, windswept escarpments and other geographic breaks across Wyoming extending out into the northwestern Great Plains. Elevation typically ranges from 1000‑2400 m. It occurs generally below continuous forests of *Pseudotsuga menziesii* or *Pinus ponderosa* but can occur in large stands well within the zone of continuous forests in the northeastern Rocky Mountains. In Wyoming, some limber pine stands are found up to 2440 m (8000 feet) elevation and are still included in this system.

*Climate:* This woodland system occurs in a semi‑arid, cool‑temperate climate. Annual precipitation patterns and amounts are variable but are typically below 500 mm annual precipitation with much occurring in winter as snow or spring rain.

*Physiography/landform:* Stands occur on moderately steep to steep slopes on all aspects but are most common on dry south‑ and west‑facing slopes. At higher elevations, it is limited to the most xeric aspects on rock outcrops, and at lower elevations to the relatively mesic north aspects.

*Soil/substrate/hydrology:* It is restricted to shallow soils and fractured bedrock derived from a variety of parent material, including limestone and calcareous sandstone, but also dolomite, granite, gneiss, quartzite, rhyolite, schist, shale and colluvium. Some stands are on eroded substrates and resemble "badlands" while others may occur on lava flows. Soils are typically shallow and have a high rock component (skeletal) with typically over 50% cover of surface rock. They are often coarse‑textured, such as gravelly, sandy loams or loams, but may include alkaline clays. Exposed soil is common, and many stands have over 50% cover of bare soil. Soil pH is typically neutral or slightly alkaline, but ranges from acidic to alkaline.

**Vegetation:** Vegetation is characterized by an open‑tree canopy or patchy woodland that is dominated by either *Pinus flexilis, Juniperus osteosperma*, or *Juniperus scopulorum*. *Pinus edulis* is not present. A sparse to moderately dense short‑shrub layer, if present, may include a variety of shrubs, such as *Arctostaphylos uva‑ursi, Artemisia nova, Artemisia tridentata, Cercocarpus ledifolius, Cercocarpus montanus, Dasiphora fruticosa ssp. floribunda, Ericameria nauseosa, Juniperus horizontalis, Purshia tridentata, Rhus trilobata, Rosa woodsii, Shepherdia canadensis* (important in Montana stands), *Symphoricarpos albus*, or *Symphoricarpos oreophilus*. Herbaceous layers are generally sparse, but range to moderately dense, and are typically dominated by perennial graminoids such as *Bouteloua gracilis, Festuca idahoensis, Festuca campestris, Danthonia intermedia, Leucopoa kingii, Hesperostipa comata, Koeleria macrantha, Piptatheropsis micrantha (= Piptatherum micranthum), Poa secunda*, or *Pseudoroegneria spicata*.

**Dynamics:** The processes shaping the distribution and persistence of scarp woodlands is not well understood (CNHP 2010). The interaction of wind, fire, and topography is thought to have played a major role in the current pattern of occurrences. These woodlands are not physiologically limited to a particular substrate, but are generally found on larger, relatively high escarpments, and not on smaller or more gently sloping breaks. The abrupt topographic changes may act as natural firebreaks. In addition, the typically sparse vegetation of the breaks in comparison with the adjacent deeper soils does not allow grassland fires to carry into the woodland understory (CNHP 2010).

Although some of the conifers that are typically codominant in *Pinus flexilis* stands are late‑successional species, they are not likely to displace *Pinus flexilis*. This is because most of these stands occur on harsh sites where *Pinus flexilis* is more competitive than most other conifer species. These stands are generally considered to be topographic or edaphic "climax" stands (Cooper 1975, Eyre 1980). Even in stands at lower elevations, such as prairie breaks, it is unlikely that other coniferous species will become dominant (Eyre 1980). Because *Pinus flexilis* occurs over a broad range of elevations, it can also be important as a post‑fire seral species on drier sites in the Rocky Mountains (Cooper 1975, Peet 1988). Peet (1978a) reported apparent competitive displacement with *Pinus flexilis* in Colorado. He noted that *Pinus flexilis* may dominate xeric sites from low to high elevations, except where *Pinus aristata* or *Pinus albicaulis* occur. There, *Pinus flexilis* is largely restricted to lower elevation, rocky sites. Peet (1978a) also reported that *Pinus flexilis* occurs in the less xeric *Pinus contorta* and *Pinus ponderosa* habitats. However, the higher elevation *Pinus flexilis* stands would be included in Rocky Mountain Subalpine‑Montane Limber‑Bristlecone Pine Woodland (CES306.819).

Birds and small mammals often eat and cache the large, wingless pine seeds. Most important is the Clark's nutcracker, which can transport the seeds long distances and cache them on exposed windswept sites (Lanner and Vander Wall 1980, Lanner 1985, 1996). This results in the regeneration of pines in clumps from forgotten caches (Woodmansee 1977, Eyre 1980, Steele et al. 1983).

Fire history information is lacking and has a wide range, making modeling difficult. As a whole, fire has occurred in this community in relation to fuel types adjacent to and within the woodland site. On shallow, rocky sites fire may have occurred less frequently. On deeper‑soiled sites, the associated vegetation is more robust and would support a more frequent fire‑return interval.

Given the uncertainty about the fire frequencies of this ecological system, it is predicted to vary from 30 to 80 years for mixed‑severity fire and over 200 years for replacement fires (LANDFIRE 2007a). Fire is likely infrequent and spotty because rocky substrates prevent a continuous vegetation canopy that is needed for fire to spread.

LANDFIRE developed a state‑and‑transition vegetation dynamics VDDT model for this system which has three classes in total (LANDFIRE 2007a, BpS 2010490). These classes are summarized as:

A) Early Development 1 All Structures (30% of type in this stage): Grass/forb/shrub/seedling ‑ usually post‑fire. Cover is 0‑30%. Shrub height 0‑1.0 m. The first 25 years dominated by shrub/herbaceous. Toward end of class increasing pine/juniper. When pine/juniper becomes dominant it has 10‑20% cover. Height of pine/juniper reaching 15 m (48 feet). On shallow, rocky sites, seedlings tend to establish in protected areas, such as sheltered spaces in rocky outcrops. On these sites there is little grass or herb competition. On deeper‑soiled sites, there is a significant herbaceous component and seedlings are established from bird seed caches and seed from limber pine and juniper that were not killed. This class lasts for 50 years or less. Replacement fire occurs every 250 years.

B) Mid Development 1 Open (30% of type in this stage): Tree cover is 21‑40%. Tree height <10 m. Trees are established, but typically short and widely spaced. Grasses and herbs are sparse in shallow, rocky soils. On deeper‑soil sites grasses and shrubs are prevalent. This class lasts until trees are approximately 100 years old, and then succeeds to Class C. Other indicator species might be *Cercocarpus montanus*. Replacement fire occurs every 200 years.

C) Late Development 1 Closed (40% of type in this stage). Tree cover is 41‑60%. Tree height <10 m. Mature trees greater than 100 years old. On shallow, rocky sites trees dominate the site with sparse shrub‑grass understory. On deeper‑soil sites mature trees are codominant with shrub‑grass understory with an increasing component of younger age class limber pine and juniper that will shade out shrubs and eventually leave a woodland site dominated by pine or pine‑juniper overstory and grass understory. It is possible that limber pine might not occur in this stage in some areas. Replacement fire occurs every 200 years. Insect/disease occur with a probability of 0.0016 (every 625 years, or 0.16% of this class each year), returning the class to class A.

SOURCES

**References:** Belnap 2001, Belnap et al. 2001, Breshears et al. 2005, Burns and Honkala 1990a, Comer et al. 2003*, Eyre 1980, Hoff et al. 1980, Johnson 2001, LANDFIRE 2007a, Lanner 1985, Lanner 1996, Lanner and Vander Wall 1980, Peet 1978a, Peet 1988, Rosentreter and Belnap 2003, Scher 2002, Schmidt and McDonald 1990, Steele et al. 1983, Thilenius et al. 1995, Woodmansee 1977, Zlatnik 1999e

**Version:** 04 Nov 2015	**Stakeholders:** Canada, Midwest, West

**Concept Author:** G. Jones and K.A. Schulz	**LeadResp:** West

M500. Central Rocky Mountain Mesic Lower Montane Forest

CES204.086 East Cascades Mesic Montane Mixed‑Conifer Forest and Woodland

**Primary Division:** North American Pacific Maritime (204)

**Land Cover Class:** Forest and Woodland

**Spatial Scale & Pattern:** Large patch

**Required Classifiers:** Natural/Semi‑natural; Vegetated (>10% vasc.); Upland

**Diagnostic Classifiers:** Forest and Woodland (Treed); Udic; Very Long Disturbance Interval; F‑Landscape/Medium Intensity; Needle‑Leaved Tree; Abies grandis ‑ Mixed; Tsuga heterophylla, Thuja plicata; Pseudotsuga menziesii; Long (>500 yrs) Persistence

**National Mapping Codes:** EVT 2018; ESLF 4205; ESP 1018

**Concept Summary:** This ecological system occurs on the upper east slopes of the Cascades in Washington, south of Lake Chelan and south to Mount Hood in Oregon. Elevations range from 610 to 1220 m (2000‑4000 feet) in a very restricted range occupying less than 5% of the forested landscape in the east Cascades. This system is associated with a submesic climate regime with annual precipitation ranging from 100 to 200 cm (40‑80 inches) and maximum winter snowpacks that typically melt off in spring at lower elevations. This ecological system is composed of variable montane coniferous forests typically below Pacific silver fir forests along the crest east of the Cascades. This system also includes montane forests along rivers and slopes, and in mesic "coves" which were historically protected from wildfires. Most occurrences of this system are dominated by a mix of *Pseudotsuga menziesii* with *Abies grandis* and/or *Tsuga heterophylla*. Several other conifers can dominate or codominate, including *Thuja plicata, Pinus contorta, Pinus monticola*, and *Larix occidentalis*. *Abies grandis* and other fire‑sensitive, shade‑tolerant species dominate forests on many sites once dominated by *Pseudotsuga menziesii* and *Pinus ponderosa*, which were formerly maintained by wildfire. They are very productive forests in the eastern Cascades which have been priority stands for timber production. *Mahonia nervosa, Linnaea borealis, Paxistima myrsinites, Acer circinatum, Spiraea betulifolia, Symphoricarpos hesperius, Cornus nuttallii, Rubus parviflorus*, and *Vaccinium membranaceum* are common shrub species. The composition of the herbaceous layer reflects local climate and degree of canopy closure and contains species more restricted to the Cascades, for example, *Achlys triphylla, Anemone deltoidea*, and *Vancouveria hexandra*. Typically, stand‑replacement fire‑return intervals are 150‑500 years with moderate‑severity fire‑return intervals of 50‑100 years.

**Comments:** Includes *Tsuga heterophylla* and *Thuja plicata* associations and moister *Abies grandis* associations in eastern Cascades.

DISTRIBUTION

**Range:** This ecological system occurs on the upper east slopes of the Cascades in Washington, south of Lake Chelan and south to Mount Hood in Oregon.

**Divisions:** 204:C

**TNC Ecoregions:** 4:C

**Nations:** CA, US

**Subnations:** BC, OR, WA:S3S4

**Map Zones:** 1:C, 7:C, 9:P

**USFS Ecomap Regions:** 242A:CC, 342H:CP, 342I:CC, M242B:CC, M242C:CC, M242D:CC, M261G:CC

CONCEPT

**Environment:** This ecological system occurs on the upper east slopes of the Cascades in Washington, south of Lake Chelan and south to Mount Hood in Oregon. Elevations range from 610 to 1220 m (2000‑4000 feet) in a very restricted range occupying less than 5% of the forested landscape in the east Cascades. This system is associated with a submesic climate regime with annual precipitation ranging from 100 to 200 cm (40‑80 inches) and maximum winter snowpacks that typically melt off in spring at lower elevations. This ecological system is composed of variable montane coniferous forests typically below Pacific silver fir forests along the crest east of the Cascades. This system also includes montane forests along rivers and slopes, and in mesic "coves" which were historically protected from wildfires.

**Vegetation:** Most occurrences of this system are dominated by a mix of *Pseudotsuga menziesii* with *Abies grandis* and/or *Tsuga heterophylla*. Several other conifers can dominate or codominate, including *Thuja plicata, Pinus contorta, Pinus monticola*, and *Larix occidentalis*. *Abies grandis* and other fire‑sensitive, shade‑tolerant species dominate forests on many sites once dominated by *Pseudotsuga menziesii* and *Pinus ponderosa*, which were formerly maintained by wildfire. They are very productive forests in the eastern Cascades which have been priority stands for timber production. *Mahonia nervosa, Linnaea borealis, Paxistima myrsinites, Acer circinatum, Spiraea betulifolia, Symphoricarpos hesperius, Cornus nuttallii, Rubus parviflorus*, and *Vaccinium membranaceum* are common shrub species. The composition of the herbaceous layer reflects local climate and degree of canopy closure and contains species more restricted to the Cascades, for example, *Achlys triphylla, Anemone deltoidea*, and *Vancouveria hexandra*.

**Dynamics:** Typically, stand‑replacement fire‑return intervals are 150‑500 years with moderate‑severity fire‑return intervals of 50‑100 years. Landfire VDDT models: R#MCONm Eastside mixed conifer moist (GF/DF) model is applied with stages A‑B‑E.

SOURCES

**References:** Comer et al. 2003*, Eyre 1980, Hessburg et al. 1999, Hessburg et al. 2000, Lillybridge et al. 1995, Topik 1989, Topik et al. 1988, WNHP unpubl. data 2018

**Version:** 31 Mar 2005	**Stakeholders:** Canada, West

**Concept Author:** R. Crawford	**LeadResp:** West

CES306.802 Northern Rocky Mountain Mesic Montane Mixed Conifer Forest

**Primary Division:** Rocky Mountain (306)

**Land Cover Class:** Forest and Woodland

**Spatial Scale & Pattern:** Matrix

**Required Classifiers:** Natural/Semi‑natural; Vegetated (>10% vasc.); Upland

**Diagnostic Classifiers:** Forest and Woodland (Treed); Udic; Very Long Disturbance Interval; F‑Landscape/Medium Intensity; Needle‑Leaved Tree; Tsuga heterophylla and Thuja plicata; Long (>500 yrs) Persistence

**National Mapping Codes:** EVT 2047; ESLF 4234; ESP 1047

**Concept Summary:** This ecological system occurs in the Northern Rockies of western Montana west into northeastern Washington and southern British Columbia. These are vegetation types dominated by *Tsuga heterophylla* and *Thuja plicata* in most cases, found in areas influenced by incursions of mild, wet, Pacific maritime air masses. Much of the annual precipitation occurs as rain, but where snow does occur, it can generally be melted by rain during warm winter storms. Occurrences generally are found on all slopes and aspects but grow best on sites with high soil moisture, such as toeslopes and bottomlands. At the periphery of its distribution, this system is confined to moist canyons and cooler, moister aspects. Generally these are moist, non‑flooded or upland sites that are not saturated yearlong. Along with *Tsuga heterophylla* and *Thuja plicata, Pseudotsuga menziesii* commonly shares the canopy, and *Pinus monticola, Pinus contorta, Abies grandis, Taxus brevifolia*, and *Larix occidentalis* are major associates. Mesic *Abies grandis* associations are included in this system, and *Abies grandis* is often the dominant in these situations; *Tsuga heterophylla* and *Thuja plicata* can both be absent. *Cornus nuttallii* may be present in some situations. *Picea engelmannii, Abies lasiocarpa*, and *Pinus ponderosa* may be present but only on the coldest or warmest and driest sites. *Linnaea borealis, Paxistima myrsinites, Alnus incana, Acer glabrum, Spiraea betulifolia, Symphoricarpos hesperius, Cornus canadensis, Rubus parviflorus, Menziesia ferruginea*, and *Vaccinium membranaceum* are common shrub species. The composition of the herbaceous layer reflects local climate and degree of canopy closure; it is typically highly diverse in all but closed‑canopy conditions. Important forbs and ferns include *Actaea rubra, Anemone piperi, Aralia nudicaulis, Asarum caudatum, Clintonia uniflora, Coptis occidentalis, Thalictrum occidentale, Tiarella trifoliata, Trientalis borealis, Trillium ovatum, Viola glabella, Gymnocarpium dryopteris, Polystichum munitum*, and *Adiantum pedatum*. Typically, stand‑replacement, fire‑return intervals are 150‑500 years, with moderate‑severity fire intervals of 50‑100 years.

DISTRIBUTION

**Range:** This system occurs in the Northern Rockies of western Montana west into northeastern Washington and southern British Columbia.

**Divisions:** 306:C

**TNC Ecoregions:** 7:C, 8:C, 68:C

**Nations:** CA, US

**Subnations:** BC, ID, MT, OR, WA:S3S4, WY?

**Map Zones:** 1:C, 8:P, 9:C, 10:C, 19:C

**USFS Ecomap Regions:** 331A:CC, M331A:PP, M332A:CC, M332B:CP, M332E:C?, M332F:C?, M332G:CC, M333A:CC, M333B:CC, M333C:CC, M333D:CC

CONCEPT

**Environment:** These forests are found in areas influenced by incursions of mild, wet, Pacific maritime air masses. Much of the annual precipitation occurs as rain, but where snow does occur, it can generally be melted by rain during warm winter storms. Occurrences generally are found on all slopes and aspects but grow best on sites with high soil moisture, such as toeslopes and bottomlands. At the periphery of its distribution, this system is confined to moist canyons and cooler, moister aspects. Generally these are moist, non‑flooded or upland sites that are not saturated yearlong.

**Vegetation:** Along with *Tsuga heterophylla* and *Thuja plicata, Pseudotsuga menziesii* commonly shares the canopy, and *Pinus monticola, Pinus contorta, Abies grandis, Taxus brevifolia*, and *Larix occidentalis* are major associates. Mesic *Abies grandis* associations are included in this system, and *Abies grandis* is often the dominant in these situations; *Tsuga heterophylla* and *Thuja plicata* can both be absent. *Cornus nuttallii* may be present in some situations. *Picea engelmannii, Abies lasiocarpa*, and *Pinus ponderosa* may be present but only on the coldest or warmest and driest sites. *Linnaea borealis, Paxistima myrsinites, Alnus incana, Acer glabrum, Spiraea betulifolia, Symphoricarpos hesperius (= Symphoricarpos mollis ssp. hesperius), Cornus canadensis, Rubus parviflorus, Menziesia ferruginea*, and *Vaccinium membranaceum* are common shrub species. The composition of the herbaceous layer reflects local climate and degree of canopy closure; it is typically highly diverse in all but closed‑canopy conditions. Important forbs and ferns include *Actaea rubra, Anemone piperi, Aralia nudicaulis, Asarum caudatum, Clintonia uniflora, Coptis occidentalis, Thalictrum occidentale, Tiarella trifoliata, Trientalis borealis, Trillium ovatum, Viola glabella, Gymnocarpium dryopteris, Polystichum munitum*, and *Adiantum pedatum*.

**Dynamics:** Typically, stand‑replacement, fire‑return intervals are 150‑500 years, with moderate‑severity fire intervals of 50‑100 years.

SOURCES

**References:** Comer et al. 2003*, Cooper et al. 1987, Daubenmire and Daubenmire 1968, Eyre 1980, Meidinger and Pojar 1991, NCC 2002, Pfister et al. 1977, WNHP unpubl. data 2018

**Version:** 23 Jan 2006	**Stakeholders:** Canada, West

**Concept Author:** M.S. Reid	**LeadResp:** West

CES306.837 Northern Rocky Mountain Western Larch Savanna

**Primary Division:** Rocky Mountain (306)

**Land Cover Class:** Forest and Woodland

**Spatial Scale & Pattern:** Large patch

**Required Classifiers:** Natural/Semi‑natural; Vegetated (>10% vasc.); Upland

**Diagnostic Classifiers:** Forest and Woodland (Treed); Udic; Very Long Disturbance Interval; F‑Landscape/Medium Intensity; Other Floristics/Dominants [User‑defined]; Moderate (100‑500 yrs) Persistence

**National Mapping Codes:** EVT 2010; ESLF 4103; ESP 1010

**Concept Summary:** This ecological system is restricted to the interior montane zone of the Pacific Northwest in northern Idaho and adjacent Montana, Washington, Oregon, and in southeastern interior British Columbia. It also appears in the east Cascades of Washington. Winter snowpacks typically melt off in early spring at lower elevations. Elevations range from 680 to 2195 m (2230‑7200 feet), and sites include drier, lower montane settings of toeslopes and ash deposits. This system is composed of open‑canopied "savannas" of the deciduous conifer *Larix occidentalis*, which may have been initiated following stand‑replacing crownfires of other conifer systems, but are maintained by a higher frequency, surface‑fire regime. These savannas are found in settings where low‑intensity, high‑frequency fires create open larch woodlands, often with the undergrowth dominated by low‑growing *Arctostaphylos uva‑ursi, Calamagrostis rubescens, Linnaea borealis, Spiraea betulifolia, Vaccinium cespitosum*, or *Xerophyllum tenax*. Less frequent or absence of fire creates mixed‑dominance stands with often shrubby undergrowth; *Vaccinium cespitosum* is common, and taller shrubs can include *Acer glabrum, Ceanothus velutinus, Shepherdia canadensis, Physocarpus malvaceus, Rubus parviflorus*, or *Vaccinium membranaceum*. Fire suppression has led to invasion of the more shade‑tolerant tree species *Abies grandis, Abies lasiocarpa, Picea engelmannii*, or *Tsuga* spp. and loss of much of the single‑story canopy woodlands.

**Comments:** Stands initiated following crownfires in areas with stand‑replacing fire frequencies greater than 150 years are included in the more mesic adjacent forest systems (Northern Rocky Mountain Mesic Montane Mixed Conifer Forest (CES306.802) and Northern Rocky Mountain Dry‑Mesic Montane Mixed Conifer Forest (CES306.805)). This is a fire‑dependent system and was much more extensive in the past; it is now very patchy in distribution. Most *Larix occidentalis* is a seral component of the dry‑mesic mixed montane forest.

DISTRIBUTION

**Range:** This ecological system is restricted to the interior montane zone of the Pacific Northwest in northern Idaho and adjacent Montana, Washington, Oregon, and in southeastern interior British Columbia. It also appears in the east Cascades of Washington.

**Divisions:** 204:C, 306:C

**TNC Ecoregions:** 3:C, 4:C, 6:P, 7:C, 8:P, 68:C

**Nations:** CA?, US

**Subnations:** BC?, ID, MT, OR, WA:S1

**Map Zones:** 1:C, 7:C, 8:P, 9:P, 10:C, 19:C

**USFS Ecomap Regions:** 331A:CC, 342I:??, M242D:CC, M332A:CC, M332B:CP, M332E:C?, M332F:C?, M332G:CC, M333A:CC, M333B:CC, M333C:CC, M333D:CC

CONCEPT

**Dynamics:** *Larix occidentalis* is a long‑lived species (in excess of 700 years in the northern Rocky Mountains), and thus stands fitting this concept are themselves long‑persisting; the life of *Larix*‑dominated stands probably does not much exceed 250 years due to various mortality sources and the ingrowth of shade‑tolerant species. Occurrences of this ecological system are generated by stand‑replacing fire, the fire‑return interval for which is speculated to be on the order of 80 to 200 years. These sites may be maintained in a seral status for hundreds of years due to the fact that *Larix occidentalis* is a long‑lived species and the understory is often dominated by *Pseudotsuga*, which will grow into the upper canopy. The potential dominants *Abies lasiocarpa, Picea engelmannii*, or *Abies grandis* are slow to establish on these sites and grow slowly presenting the distinct probability, given the fire‑return intervals for this type, that the "climax" (long‑term stable) condition is never realized.

It has been noted in northern Idaho that, following disturbance (particularly logging) in some mesic‑site occurrences, *Larix occidentalis* does not necessarily succeed itself, the first tree‑dominated successional stages being dominated by *Pseudotsuga menziesii, Pinus contorta*, or less frequently by more shade‑tolerant species (Cooper et al. 1987); this response is a consequence of the episodic nature of favorable cone crop years in *Larix occidentalis*.

Landfire VDDT models: #RMCONm and #RMCONdy classes B, C, & D.

SOURCES

**References:** Agee 1993, Comer et al. 2003*, Cooper et al. 1987, Daubenmire and Daubenmire 1968, Eyre 1980, Hessburg et al. 1999, Hessburg et al. 2000, Johnson and Clausnitzer 1992, Johnson and Simon 1987, Leavell 2000, Lillybridge et al. 1995, Pfister et al. 1977, Steele et al. 1981, WNHP unpubl. data 2018, Williams et al. 1995

**Version:** 01 Sep 2005	**Stakeholders:** Canada, West

**Concept Author:** R.C. Crawford and M.S. Reid	**LeadResp:** West

M020. Rocky Mountain Subalpine‑High Montane Forest

CES304.776 Inter‑Mountain Basins Aspen‑Mixed Conifer Forest and Woodland

**Primary Division:** Inter‑Mountain Basins (304)

**Land Cover Class:** Forest and Woodland

**Spatial Scale & Pattern:** Matrix

**Required Classifiers:** Natural/Semi‑natural; Vegetated (>10% vasc.); Upland

**Diagnostic Classifiers:** Forest and Woodland (Treed); Needle‑Leaved Tree; Broad‑Leaved Deciduous Tree; Aspen ‑ Conifer Mix

**National Mapping Codes:** EVT 2061; ESLF 4302; ESP 1061

**Concept Summary:** This ecological system occurs on montane slopes and plateaus in Utah, western Colorado, northern Arizona, eastern Nevada, southern Idaho, western Wyoming, and in north‑central Montana in the Big Snowy Mountains. It also occurs in localized settings in the Klamath Mountains of California, as well as in the Sierra Nevada and adjacent Great Basin mountains (Inyo, White, Warner, and Modoc Plateau). Elevations range from 1700 to 2800 m. Occurrences are typically on gentle to steep slopes on any aspect but are often found on clay‑rich soils in intermontane valleys. Soils are derived from alluvium, colluvium and residuum from a variety of parent materials but most typically occur on sedimentary rocks. The tree canopy is composed of a mix of deciduous and coniferous species, codominated by *Populus tremuloides* and conifers, including *Pseudotsuga menziesii, Abies concolor, Abies lasiocarpa, Abies magnifica, Picea engelmannii, Picea x albertiana, Picea pungens, Pinus contorta, Pinus flexilis, Pinus jeffreyi, Pinus contorta var. murrayana*, and *Pinus ponderosa*. As the stands age, cover of *Populus tremuloides* may be slowly reduced until the conifer species become dominant. Common shrubs include *Amelanchier alnifolia, Prunus virginiana, Acer grandidentatum, Symphoricarpos oreophilus, Juniperus communis, Paxistima myrsinites, Rosa woodsii, Spiraea betulifolia, Symphoricarpos albus*, or *Mahonia repens*. Herbaceous species include *Bromus carinatus, Calamagrostis rubescens, Carex geyeri, Elymus glaucus, Poa* spp., and *Achnatherum, Hesperostipa, Nassella*, and/or *Piptochaetium* spp. *Achillea millefolium, Arnica cordifolia*, Asteraceae spp., *Erigeron* spp., *Galium boreale, Geranium viscosissimum, Lathyrus* spp., *Lupinus argenteus, Mertensia arizonica, Mertensia lanceolata, Maianthemum stellatum, Osmorhiza berteroi*, and *Thalictrum fendleri*. Most occurrences at present represent a late‑seral stage of aspen changing to a pure conifer occurrence. Nearly a hundred years of fire suppression and livestock grazing have converted much of the pure aspen occurrences to the present‑day aspen‑conifer forest and woodland ecological system. This is the typical meadow edge aspen‑conifer setting in the Sierra Nevada where frequently, due to fire suppression, the conifers are replacing aspens.

**Comments:** This system represents the drier, fairly stable upland aspen mixed conifer stands in the intermountain western U.S. where periodic disturbance such as die‑back from drought is thought to maintain the mixed deciduous‑conifer composition. These drier stands often have a more open tree canopy where aspen is not shaded out by conifers. Aspen and conifers each make of over a quarter of tree canopy. This system should not be confused with the relatively short‑lived, mixed conifer‑deciduous, mid‑seral stages of conifer‑dominated forest and woodland systems.

DISTRIBUTION

**Range:** This system occurs on montane slopes and plateaus in Utah, eastern Nevada, southern Idaho, western and central Wyoming (in the Bighorn Mountains), and in north‑central Montana in the Big Snowy Mountains. Elevations range from 1700 to 2800 m.

**Divisions:** 304:C, 306:C

**TNC Ecoregions:** 6:C, 9:C, 11:C, 18:C, 19:P, 26:C

**Nations:** US

**Subnations:** AZ, CO, ID, MT, NV, UT, WY

**Map Zones:** 3:C, 6:C, 8:?, 9:C, 10:C, 12:C, 15:C, 16:C, 17:P, 18:C, 19:C, 20:C, 21:C, 22:P, 23:C, 24:C, 25:?, 27:P, 28:C, 29:C

**USFS Ecomap Regions:** 313A:CC, 313B:CC, 315H:??, 331D:C?, 331J:CC, 341A:CC, 341B:CC, 341F:CC, 341G:CP, 342B:CP, 342C:CC, 342D:CC, 342E:CC, 342G:CP, 342J:CC, M242C:??, M313B:CC, M331A:CC, M331B:CC, M331D:CC, M331E:CC, M331F:CC, M331G:CC, M331H:CC, M331I:CC, M331J:CP, M332A:CP, M332B:C?, M332D:CC, M332E:CC, M332F:CC, M333A:CC, M333B:CC, M333C:C?, M333D:C?, M341A:CC, M341B:CC, M341C:CC, M341D:CC

CONCEPT

**Environment:** This ecological system is found on montane slopes and high plateaus in Utah, western Colorado, northern Arizona, eastern Nevada, southern Idaho, and western Wyoming from 1700 to 2800 m elevation. Climate is temperate with cold winters. Mean annual precipitation is greater than 38 cm and typically greater than 50 cm. Although often drier, sites are similar to Rocky Mountain Aspen Forest and Woodland (CES306.813) with regards to environmental characteristics. Topography is variable, with sites ranging from level to steep slopes. Aspect varies according to the limiting factors. Occurrences at high elevations are restricted by cold temperatures and are found on warmer southern aspects. At lower elevations aspen is restricted by lack of moisture and is found on cooler north aspects and mesic microsites such as seeps and drainages. Soils are derived from alluvium, colluvium and residuum from a variety of parent materials and may include sedimentary, metamorphic or igneous rocks, but it appears to grow best on sedimentary rocks such as limestone and calcareous or neutral shales, or basalt (Mueggler 1988). Soil texture ranges from sandy loam to clay loam. This system represents a stable mixed aspen ‑ conifer woodlands typically found on broad plateaus where periodic disturbance such as die‑back from drought is thought to maintain the mixed deciduous‑conifer composition. It is sometimes confused with the relatively short‑lived, mid‑seral stages of conifer‑dominated forest and woodland systems such as Rocky Mountain Subalpine Dry‑Mesic Spruce‑Fir Forest and Woodland (CES306.828), Rocky Mountain Subalpine Mesic‑Wet Spruce‑Fir Forest and Woodland (CES306.830), or Southern Rocky Mountain Mesic Montane Mixed Conifer Forest and Woodland (CES306.825). Distribution of this ecological system is primarily limited by adequate soil moisture required to meet its high evapotranspiration demand (Mueggler 1988). Secondarily, its range is limited by the length of the growing season or low temperatures (Mueggler 1988). The environmental description is based on several other references, including DeByle and Winokur (1985), Mueggler (1988), Howard (1996), Reid et al. (1999), Bartos (2001), Comer et al. (2002), Tuhy et al. (2002), and Sawyer et al. (2009).

**Vegetation:** The open to moderately closed canopy is composed of a mix of deciduous and coniferous species, codominated by *Populus tremuloides* and conifers, including *Pseudotsuga menziesii, Abies concolor, Abies lasiocarpa, Picea engelmannii, Picea x albertiana (= Picea glauca x engelmannii), Picea pungens, Pinus contorta, Pinus flexilis*, and *Pinus ponderosa*. The sparse to moderately dense understory may be structurally complex and includes tall‑shrub, short‑shrub and herbaceous layers, or it may be simple with just an herbaceous layer or sparse. Because of the open growth form of *Populus tremuloides*, more light can penetrate the canopy than in a pure conifer occurrence. If present, the tall‑shrub layer may be dominated by *Amelanchier alnifolia, Prunus virginiana*, or *Acer grandidentatum*, and short‑shrub layer by *Symphoricarpos oreophilus, Juniperus communis*, or *Mahonia repens*. Other common shrubs include *Paxistima myrsinites, Rosa woodsii, Spiraea betulifolia, Symphoricarpos albus*, and in wet areas *Salix scouleriana*. Where the herbaceous layer is dense, it is often dominated by graminoids such as *Bromus carinatus, Calamagrostis rubescens, Carex geyeri, Elymus glaucus, Poa* spp., and species of *Achnatherum, Hesperostipa, Nassella*, and/or *Piptochaetium*. Sparse herbaceous layers are generally a more even mixture of forbs such as *Achillea millefolium, Arnica cordifolia, Eucephalus engelmannii (= Aster engelmannii), Erigeron speciosus, Fragaria vesca, Galium boreale, Geranium viscosissimum, Lathyrus* spp., *Lupinus argenteus, Mertensia arizonica, Mertensia lanceolata, Maianthemum stellatum, Osmorhiza berteroi (= Osmorhiza chilensis)*, and *Thalictrum fendleri*. Annuals are typically uncommon. The exotic species *Poa pratensis* and *Taraxacum officinale* are more common in livestock‑impacted occurrences (Mueggler 1988). The vegetation description is based on several references, including DeByle and Winokur (1985), Mueggler (1988), Howard (1996), Reid et al. (1999), Bartos (2001), Comer et al. (2002), Tuhy et al. (2002), and Sawyer et al. (2009).

**Dynamics:** *Populus tremuloides* is a fast‑growing deciduous tree that reaches 20 m in height and forms clones that can be ancient, although the stems are relatively short‑lived (up to 150 years in the western U.S.) (Howard 1996, Sawyer et al. 2009). It is thin‑barked and stems are readily killed by fire, although the clone will usually resprout after burning or other disturbance (Howard 1996). It is a fire‑adapted species that generally needs a large disturbance to establish and maintain dominance in a forest stand. Mixed aspen ‑ conifer forests are generally seral and, in the absence of stand‑replacing disturbance such as fire, will slowly convert to a conifer‑dominated forest (Mueggler 1988). Although the young conifer trees in these occurrences are susceptible to fire, older individuals develop self‑pruned lower branches and develop a thick corky bark that makes them resistant to surface fires. The natural fire‑return interval is approximately 20 to 50 years for seral occurrences (Hardy and Arno 1996). Intervals that approach 100 years are typical of late‑seral occurrences (Hardy and Arno 1996).

However, this system represents stable mixed aspen ‑ conifer woodlands typically found on broad plateaus in the interior western U.S. where periodic disturbance such as die‑back from drought or other disturbance is thought to maintain the mixed deciduous‑conifer composition and not allow conifers to dominate and shade out the aspen (Tuhy et al. 2002). Sudden aspen decline (SAD) results in root mortality with subsequent effects on tree canopy and clone persistence. It appears to be triggered by severe drought (Worrall et al. 2010). This may have increasing impact on these forests. More research is needed to clarify the dynamics of this system as it is sometimes confused with the relatively short‑lived, mid‑seral stages of conifer‑dominated forest and woodland systems such as Rocky Mountain Subalpine Dry‑Mesic Spruce‑Fir Forest and Woodland (CES306.828), Rocky Mountain Subalpine Mesic‑Wet Spruce‑Fir Forest and Woodland (CES306.830), or Southern Rocky Mountain Mesic Montane Mixed Conifer Forest and Woodland (CES306.825).

LANDFIRE developed a state‑and‑transition vegetation dynamics VDDT model for this system which has five classes in total (LANDFIRE 2007a, BpS 1810610). The model represents a fire maintained, seral mixed aspen ‑ conifer types that succeeds to a conifer dominated types without mixed‑severity fire (mean FRI of 20 years). The classes are summarized as:

A) Early Development 1 All Structures (14% of type in this stage): Grass/forb and aspen suckers <12 feet tall. Generally, this is expected to occur 1‑3 years post‑disturbance. Fire is absent. Succession to class B after 10 years.

B) Mid Development 1 Closed (tree‑dominated ‑ 40% of type in this stage): Tree cover is 41‑100%. Aspen saplings over 12 feet tall dominate. Canopy cover is highly variable. Replacement fire occurs every 60 years on average. Mixed‑severity fire (average FRI of 40 years) does not change the successional age of these stands, although this fire consumes litter and woody debris and may stimulate suckering. Succession to class C after 30 years.

C) Mid Development 1 Closed (tree‑dominated ‑ 35% of type in this stage): Tree cover is 41‑100%. Aspen trees 5‑16 inches dbh. Canopy cover is highly variable. Conifer seedlings and saplings may be present. Replacement fire occurs every 60 years on average. Mixed‑severity fire (mean FRI of 40 years), while thinning some trees, promotes suckering and maintains vegetation in this class. Insect/diseases outbreaks occur every 200 years on average with 80% of times causing stand thinning (transition to class B) and 20% of times causing stand replacement (transition to class A). Conifer encroachment causes a succession to class D after 40 years.

D) Late Development 1 Open (tree‑dominated ‑ 10% of type in this stage): Tree cover is 0‑40%. Aspen dominate, making up 80% of the overstory. Conifers which escape fire, or are the more fire‑resistant species, are present in the understory and will likely cause the progressive suppression of aspen. Mixed‑severity fire (20‑year MFI) keeps this stand open, kills young conifers and maintains aspen (max FRI from Baker 1925). Replacement fire occurs every 60 years on average. In the absence of any fire for at least 100 years, the stand will become closed and dominated by conifers (transition to class E).

E) Late Development 1 Closed (conifer‑dominated ‑ 1% of type in this stage): Tree cover is 41‑80%. Conifers dominate at 100+ years. Aspen over 16 inches dbh, uneven sizes of mixed conifer and main overstory is conifers. Greater than 50% conifer in the overstory. FRI for replacement fire is every 60 years. Mixed‑severity fire (mean FRI of 20 years) causes a transition to class D. Insect/disease outbreaks will thin older conifers (transition to class D) every 300 years on average.

From (LANDFIRE 2007a, BpS 1810610): "This is a strongly fire‑adapted community, more so than BpS 1011 (Rocky Mountains Aspen Woodland and Forest), with FRIs varying for mixed‑severity fire with the encroachment of conifers. It is important to understand that aspen is considered a fire‑proof vegetation type that does not burn during the normal lightning season, yet evidence of fire scars and historical studies show that native burning was the only source of fire that occurred mostly during the spring and fall. BpS 1061 has elements of Fire Regime Groups II, III and IV. Mean FRI for replacement fire is every 60 years on average in most development classes. Replacement fire is absent during early development (as for stable aspen, BpS 1011) and has a mean FRI of 100 years between 80 and 100 years in the open condition. The FRI of mixed‑severity fire increases from 40 years in stands <100 years to 60 years in stands >100 years with conifer encroachment."

Under presettlement conditions, disease and insect mortality did not appear to have major effects; however, older aspen stands would be susceptible to outbreaks every 200 years on average. We assumed that 20% of outbreaks resulted in heavy insect/disease stand‑replacing events (average return interval 1000 years), whereas 80% of outbreaks would thin older trees >40 years (average return interval 250 years). Older conifers (>100 years) would experience insect/disease outbreaks every 300 years on average (LANDFIRE 2007a, BpS 1810610).

SOURCES

**References:** Adams 2010, Baker 1925, Bartos 2001, Bartos 2008, Bartos and Campbell 1998, Bell et al. 2009, Bethers et al. 2010, Bradley et al. 1992a, Brandt et al. 2003b, Comer et al. 2002, Comer et al. 2003*, Dale et al. 2001, DeByle 1985b, DeByle and Winokur 1985, DeVelice et al. 1986, Elliott and Baker 2004, Eyre 1980, Garfin et al. 2014, Graham et al. 1990, Hardy and Arno 1996, Henderson et al. 1977, Howard 1996a, Jones and DeByle 1985, LANDFIRE 2007a, Morelli and Carr 2011, Mote et al. 2014, Mueggler 1988, Niinemets and Valladares 2006, Reid et al. 1999, Rogers 2002, Romme et al. 2001, Sawyer and Keeler‑Wolf 1995, Sawyer et al. 2009, Schier et al. 1985, Scott et al. 1980, Sexton et al. 2006, Shafer et al. 2014, Shepperd 2008, Shiflet 1994, Spracklen et al. 2009, Tuhy et al. 2002, Westerling et al. 2006, Worrall et al. 2008a, Worrall et al. 2010, Youngblood and Mauk 1985, Youngblood and Mueggler 1981

**Version:** 23 May 2018	**Stakeholders:** West

**Concept Author:** NatureServe Western Ecology Team	**LeadResp:** West

CES304.790 Inter‑Mountain Basins Subalpine Limber‑Bristlecone Pine Woodland

**Primary Division:** Inter‑Mountain Basins (304)

**Land Cover Class:** Forest and Woodland

**Spatial Scale & Pattern:** Large patch

**Required Classifiers:** Natural/Semi‑natural; Vegetated (>10% vasc.); Upland

**Diagnostic Classifiers:** Montane [Upper Montane, Montane]; Forest and Woodland (Treed); Ridge; Ridge/Summit/Upper Slope; Temperate [Temperate Continental]; Xeric; Pinus longaeva, P. flexilis

**National Mapping Codes:** EVT 2020; ESLF 4207; ESP 1020

**Concept Summary:** This ecological system extends from the Mojave Desert and Sierra Nevada across the central Great Basin to the central Wasatch and western Uinta mountains. These open woodlands are typically found on high‑elevation ridges and rocky slopes above subalpine forests and woodlands. Site are harsh, exposed to desiccating winds with rocky substrates and a short growing season that limit plant growth. Parent materials include dolomitic, limestone or granitic rocks. Occurrences can be found on all aspects but are more common on southwestern exposures on steep convex slopes and ridges between 2530 and 3600 m (8300‑12,000 feet). Stands are strongly dominated by *Pinus flexilis* and/or *Pinus longaeva*. *Pinus monophylla* may be present in lower‑elevation stands. If present, shrub and herbaceous layers are generally sparse and composed of xeric shrubs, graminoids and cushion plants. Associated species may include *Antennaria rosea, Arenaria kingii, Artemisia tridentata, Cercocarpus intricatus, Chamaebatiaria millefolium, Cymopterus cinerarius, Elymus elymoides, Erigeron pygmaeus, Eriogonum ovalifolium, Festuca brachyphylla, Koeleria macrantha, Linanthus pungens, Ribes cereum*, or *Ribes montigenum*.

DISTRIBUTION

**Range:** This system extends from the Mojave Desert and Sierra Nevada across the Great Basin to the central Wasatch and extreme western Uinta mountains.

**Divisions:** 304:C, 306:?

**TNC Ecoregions:** 9:?, 11:C, 12:C, 18:C, 19:C

**Nations:** US

**Subnations:** CA, NV, UT

**Map Zones:** 6:P, 7:?, 9:?, 12:C, 13:C, 16:C, 17:C, 18:P

**USFS Ecomap Regions:** 322A:CC, 331J:CC, 341A:CC, 341D:CC, 341E:CC, 341F:CC, 341G:CC, 342B:CC, 342J:C?, M261E:CC, M331D:PP, M341A:CC, M341D:CC

CONCEPT

**Environment:** This ecological system extends from the Mojave Desert and eastern Sierra Nevada across the central Great Basin to the central Wasatch and western Uinta mountains. These open woodlands are typically found on high‑elevation ridges and rocky slopes above subalpine forests and woodlands, sometimes extending down into the montane zone. Sites are harsh, exposed to desiccating winds, with rocky substrates and a short growing season that limit plant growth. Occurrences can be found on all aspects but are more common on southwestern exposures on steep convex slopes and ridges between 2530 and 3600 m (8300‑12,000 feet) elevation. Most sites are droughty, with gravel in the shallow subsurface horizons. Surface textures vary depending upon substrate, which are best represented on colluvium derived from limestone and dolomite or Tertiary and Cretaceous sandstone parent materials. Steep slopes, high‑intensity summer convection storms, and only partial ground cover for interception often result in severe sheet erosion of fine particles. This usually leads to the development of gravel pavements. Additional erosion can be expected from wind action. High insolation and wind during the winter usually result in reduced snowpack accumulations. However, soils can be expected to freeze. The sparsity of shrubs, forbs, grasses, and litter, in addition to the widely spaced trees, usually means that fire does not carry easily. Individual trees may be ignited from lightning, but seldom is an entire occurrence burned. The environmental description is based on several other references, including Graybosch and Buchanan (1983), Lanner (1983), Holland (1986b), Holland and Keil (1995), Nachlinger and Reese (1996), Reid et al. (1999), Fryer (2004), Thorne et al. (2007), and Sawyer et al. (2009).

**Vegetation:** Vegetation is characterized by a typically open tree canopy (<25% cover) with heights ranging from 1‑2 m (krummholz) to over 10 m. *Pinus flexilis* and/or *Pinus longaeva* dominate the tree canopy, alone or in combination. *Pinus longaeva* stands tend to occur at higher elevation with less mixed canopies. Other trees present to codominant include *Picea engelmannii, Pseudotsuga menziesii, Populus tremuloides*, or *Abies concolor*. In the Sierra Nevada stands, *Pinus albicaulis, Pinus balfouriana*, and/or *Pinus contorta var. murrayana* may be present. Understory layers, if present, are sparse to moderately dense and composed of xeric shrubs, graminoids and cushion plants. Characteristic shrubs include *Arctostaphylos patula, Artemisia arbuscula, Artemisia tridentata ssp. vaseyana, Ericameria discoidea, Juniperus communis, Mahonia repens, Ribes cereum*, and *Ribes montigenum*. *Cercocarpus intricatus, Cercocarpus ledifolius*, or *Chrysolepis sempervirens* frequently occur in stands in the Sierra Nevada. The herbaceous layer is typically sparse. Associated herbaceous species are diverse given the wide elevational range, with alpine species occurring near the upper treeline and montane and subalpine species below. Associated species may include *Antennaria rosea, Aquilegia scopulorum, Arabis drummondii, Arenaria congesta, Arenaria kingii, Astragalus kentrophyta, Astragalus platytropis, Calamagrostis rubescens, Carex rossii, Cirsium eatonii, Cymopterus cinerarius, Cymopterus nivalis, Elymus elymoides, Eriogonum gracilipes, Eriogonum holmgrenii, Eriogonum ovalifolium, Erigeron pygmaeus, Erigeron tener, Festuca brachyphylla, Koeleria macrantha, Linanthus pungens (= Leptodactylon pungens), Packera werneriifolia, Penstemon leiophyllus, Poa fendleriana, Phlox pulvinata, Trifolium gymnocarpon*, and *Trisetum spicatum*. *Selaginella watsonii* is common in some high‑elevation stands. The vegetation description is based on several references, including Graybosch and Buchanan (1983), Lanner (1983), Holland (1986b), Holland and Keil (1995), Nachlinger and Reese (1996), Reid et al. (1999), Fryer (2004), Thorne et al. (2007), and Sawyer et al. (2009).

**Dynamics:** Both *Pinus longaeva* and *Pinus flexilis* are slow‑growing, long‑lived trees that are intolerant of shade. *Pinus longaeva* may attain nearly 4900 years in age and 12 m in height, whereas *Pinus flexilis* may live 1000 years and attain 18 m in height. Bristlecone pine branches retain needles for as long as 30 years, whereas limber pine needles are lost after only several years. Bristlecone pine trees produce dense, resinous wood that is resistant to rot and disease. Mature trees have massive, contorted trunks with mostly dead and gnarled wood (Sawyer et al. 2009). Tree‑ring data over the last 4000 years indicate that droughts of 200 years or more have occurred.

Natural regeneration of both species appears to be closely associated with caching of the large wingless seeds, primarily by Clark's nutcracker (*Nucifraga columbiana*) (Lanner and Vander Wall 1980). Germination of cached seeds often results in the multi‑stemmed clumps characteristic of these sites, although the species may produce multiple stems from boles damaged near the ground. Germination and rooting will sometimes be restricted to crevices in rock. *Pinus longaeva* has smaller winged seeds and should be wind‑disseminated. However, caching by nutcrackers does take place, especially when other *Pinus* species are also available (Dr. R. Lanner pers. comm.). The longevity of individuals enables stands to persist for centuries between times of favorable seedling establishment (Keeley and Zedler 1998). Stands are subject to long, intense droughts.

<br />These pines have relatively thin bark adapted to survive only low‑severity surface fires. However, fires seldom destroy stands due to the sparse nature of the canopy cover of trees and abundant bare ground. When fire occurs on high‑elevation sites, they are usually small, low‑severity surface fires (Bradley et al. 1992).

*Pinus longaeva* and *Pinus flexilis* are both experiencing mountain pine beetle (*Dendroctonus ponderosae*) infestations throughout much of their ranges (Lanner 1983). Logan and Powell (2001) provide information on the ecology and management of mountain pine beetles in high‑elevation ecosystems. Gibson et al. (2008) reported recently detected mortality of *Pinus longaeva* in the Great Basin, including 100 acres in 2005, 60 acres in 2006, and 300 acres in 2007, all within the Snake Range in east‑central Nevada (aerial detection surveys). Western dwarf mistletoe (*Arceuthobium campylopodum*) infests Great Basin bristlecone pines in southern Nevada and Utah (Mathiasen and Hawksworth 1990).

SOURCES

**References:** Barbour et al. 2007a, Bradley et al. 1992a, Comer et al. 2003*, Eyre 1980, Fryer 2004, Gibson et al. 2008, Graybosch and Buchanan 1983, Hiebert and Hamrick 1984b, Holland 1986b, Holland and Keil 1995, Keeley and Zedler 1998a, Kilpatrick and Biondi 2013, LANDFIRE 2007a, LaMarche and Mooney 1972, Lanner 1983, Lanner and Vander Wall 1980, Lanner pers. comm., Logan and Powell 2001, Logan and Powell 2005, Logan et al. 2010, Mathiasen and Hawksworth 1990, McKinney et al. 2007, Nachlinger and Reese 1996, Reid et al. 1999, Sawyer and Keeler‑Wolf 1995, Sawyer et al. 2009, Schoettle and Sniezko 2007a, Schoettle et al. 2008, Smith and Hoffman 2000, TNC 2013, Thorne et al. 2007

**Version:** 02 Apr 2014	**Stakeholders:** West

**Concept Author:** NatureServe Western Ecology Team	**LeadResp:** West

CES306.807 Northern Rocky Mountain Subalpine Woodland and Parkland

**Primary Division:** Rocky Mountain (306)

**Land Cover Class:** Forest and Woodland

**Spatial Scale & Pattern:** Large patch

**Required Classifiers:** Natural/Semi‑natural; Vegetated (>10% vasc.); Upland

**Diagnostic Classifiers:** Montane [Upper Montane]; Forest and Woodland (Treed); Ridge/Summit/Upper Slope; Oligotrophic Soil; Very Short Disturbance Interval; W‑Patch/High Intensity; W‑Patch/Medium Intensity; W‑Landscape/Medium Intensity; Larix lyallii; Upper Treeline; Long (>500 yrs) Persistence

**National Mapping Codes:** EVT 2046; ESLF 4233; ESP 1046

**Concept Summary:** This ecological system of the Northern Rockies, Cascade Range, and northeastern Olympic Mountains is typically a high‑elevation mosaic of stunted tree clumps, open woodlands, and herb‑ or dwarf‑shrub‑dominated openings, occurring above closed forest ecosystems and below alpine communities. It includes open areas with clumps of *Pinus albicaulis*, as well as woodlands dominated by *Pinus albicaulis* or *Larix lyallii*. In the Cascade Range and northeastern Olympic Mountains, the tree clump pattern is one manifestation, but these are also woodlands with an open canopy, without a tree clump/opening patchiness to them; in fact, that is quite common with *Pinus albicaulis*. The climate is typically very cold in winter and dry in summer. In the Cascades and Olympic Mountains, the climate is more maritime in nature and wind is not as extreme. The upper and lower elevational limits, due to climatic variability and differing topography, vary considerably; in interior British Columbia, this system occurs between 1000 and 2100 m elevation, and in northwestern Montana, it occurs up to 2380 m. Landforms include ridgetops, mountain slopes, glacial trough walls and moraines, talus slopes, landslides and rockslides, and cirque headwalls and basins. Some sites have little snow accumulation because of high winds and sublimation. *Larix lyallii* stands generally occur at or near upper treeline on north‑facing cirques or slopes where snowfields persist until June or July. In this harsh, often windswept environment, trees are often stunted and flagged from damage associated with wind and blowing snow and ice crystals, especially at the upper elevations of the type. The stands or patches often originate when *Picea engelmannii, Larix lyallii*, or *Pinus albicaulis* colonize a sheltered site such as the lee side of a rock. *Abies lasiocarpa* can then colonize in the shelter of the *Picea engelmannii* and may form a dense canopy by branch‑layering. Major disturbances are windthrow and snow avalanches. Fire is known to occur infrequently in this system, at least where woodlands are present; lightning damage to individual trees is common, but sparse canopies and rocky terrain limit the spread of fire.

These high‑elevation coniferous woodlands are dominated by *Pinus albicaulis, Abies lasiocarpa*, and/or *Larix lyallii*, with occasional *Picea engelmannii*. In the Cascades and Olympics, *Abies lasiocarpa* sometimes dominates the tree layer without *Pinus albicaulis*, though in this dry parkland *Tsuga mertensiana* and *Abies amabilis* are largely absent. The undergrowth is usually somewhat depauperate, but some stands support a near sward of heath plants, such as *Phyllodoce glanduliflora, Phyllodoce empetriformis, Empetrum nigrum, Cassiope mertensiana*, and *Kalmia polifolia*, and can include a slightly taller layer of *Ribes montigenum, Salix brachycarpa, Salix glauca, Salix planifolia, Vaccinium membranaceum, Vaccinium myrtillus*, or *Vaccinium scoparium* that may be present to codominant. The herbaceous layer is sparse under dense shrub canopies or may be dense where the shrub canopy is open or absent. *Vahlodea atropurpurea, Luzula glabrata var. hitchcockii*, and *Juncus parryi* are the most commonly associated graminoids.

In the mountains of northwestern and west‑central Wyoming, where this upper‑treeline system reaches the edge of its geographic range, the vegetation usually has the form of an open woodland, and only rarely as scattered groves of trees. At the highest elevations, *Pinus albicaulis* usually has a wind‑stunted shrub form. On lower, more favorable sites, upright but wind‑shaped *Pinus albicaulis* forms woodlands, sometimes with *Pinus contorta* as a codominant or even the dominant species. With decreased altitude, where this system merges into the subalpine forests, *Picea engelmannii* and *Abies lasiocarpa* become common tree species as well.

**Comments:** There is a proposal to either split the dry, subalpine *Pinus albicaulis* woodlands of the Blue Mountains (Oregon) and northern Nevada into a different system; or else to include them in Rocky Mountain Subalpine‑Montane Limber‑Bristlecone Pine Woodland (CES306.819). For Landfire, these *Pinus albicaulis* woodlands were included in this subalpine parkland system, but ecologically and floristically they are more similar to Rocky Mountain dry subalpine woodlands. In addition, there is a proposal and discussion that tree ribbon spruce‑fir woodlands in scattered ranges of southern Wyoming are more ecologically "parklands"; possibly those areas could be included in this system.

DISTRIBUTION

**Range:** This system occurs in the northern Rocky Mountains, west into the Cascade Range and northeastern Olympic Mountains, and east into the mountain "islands" of central Montana.

**Divisions:** 204:C, 306:C

**TNC Ecoregions:** 3:C, 7:C, 8:C, 9:P, 26:C, 68:C

**Nations:** CA, US

**Subnations:** AB, BC, ID, MT, WA:S4, WY

**Map Zones:** 1:C, 7:?, 9:P, 10:C, 12:C, 16:?, 18:C, 19:C, 20:C, 21:C, 22:?, 29:?

**USFS Ecomap Regions:** 342A:CC, 342F:CP, 342H:CC, 342I:CC, M242A:CC, M242C:CC, M242D:CC, M331A:CC, M331B:CP, M331D:CC, M332A:CC, M332B:CC, M332D:CC, M332E:CC, M332F:CC, M332G:CC, M333A:CC, M333B:CC, M333C:CC, M333D:CC

CONCEPT

**Environment:** This ecological system of the Northern Rockies, Cascade Range, and northeastern Olympic Mountains is typically a high‑elevation mosaic of stunted tree clumps, open woodlands, and herb‑ or dwarf‑shrub‑dominated openings, occurring above closed forest ecosystems and below alpine communities. The upper and lower elevational limits, due to climatic variability and differing topography, vary considerably from 1000‑3200 m depending on latitude. In interior British Columbia, this system occurs between 1000 and 2100 m elevation, and in northwestern Montana, it occurs up to 2380 m. In west‑central Wyoming, this system occurs on various landforms over an elevational range from 2230 to 3200 m (Steele et al. 1983).

*Climate:* The climate is typically very cold in winter and dry in summer. Mean annual precipitation ranges from 60‑180 cm, occurring mostly in the winter. Yearly snow accumulations are often over 3 m in the northern Cascades and 2‑3 m in the Rockies. Some sites have little snow accumulation because of high winds and sublimation. In the Cascades and Olympic Mountains, the climate is more maritime in nature and wind is not as extreme.

*Physiography/Landform:* Landforms include ridgetops, mountain slopes, glacial trough walls and moraines, talus slopes, landslides and rockslides, and cirque headwalls and basins. Sites may be nearly level to steep sloping, on all aspects. Some stands occur at treeline in mesic, protected pockets away from the extremely harsh environmental conditions. It is not tied to particular aspects (Steele et al. 1983).

*Soil/substrate/hydrology:* Soils are generally lithic, well‑to excessively drained, and coarse‑textured such as shallow, gravelly sands or loams, but may include silt and clay loams. Soils are derived from colluvium, glacial till and residuum from a variety of volcanic, igneous, sedimentary and metamorphic geologic formations.

**Vegetation:** These high‑elevation coniferous woodlands are dominated by *Pinus albicaulis, Abies lasiocarpa*, and/or *Larix lyallii*, with occasional *Picea engelmannii*. In the Cascades and Olympics, *Abies lasiocarpa* sometimes dominates the tree layer without *Pinus albicaulis*, though in this dry parkland *Tsuga mertensiana* and *Abies amabilis* are largely absent. The undergrowth is usually somewhat depauperate, but some stands support a near sward of heath plants, such as *Phyllodoce glanduliflora, Phyllodoce empetriformis, Empetrum nigrum, Cassiope mertensiana*, and *Kalmia polifolia*, and can include a slightly taller layer of *Ribes montigenum, Salix brachycarpa, Salix glauca, Salix planifolia, Vaccinium membranaceum, Vaccinium myrtillus*, or *Vaccinium scoparium* that may be present to codominant. The herbaceous layer is sparse under dense shrub canopies or may be dense where the shrub canopy is open or absent. *Vahlodea atropurpurea (= Deschampsia atropurpurea), Luzula glabrata var. hitchcockii*, and *Juncus parryi* are the most commonly associated graminoids.

<br />In the mountains of northwestern and west‑central Wyoming, where this upper‑treeline system reaches the edge of its geographic range, the vegetation usually has the form of an open woodland, and only rarely as scattered groves of trees. At the highest elevations, *Pinus albicaulis* usually has a wind‑stunted shrub form. On lower, more favorable sites, upright but wind‑shaped *Pinus albicaulis* forms woodlands, sometimes with *Pinus contorta* as a codominant or even the dominant species. With decrease in altitude, where this system merges into the subalpine forests, *Picea engelmannii* and *Abies lasiocarpa* become common tree species as well.

**Dynamics:** *Pinus albicaulis* is a slow‑growing, long‑lived conifer that is common at higher elevations in the upper subalpine zone. It typically occurs in a mosaic of tree islands and meadows where it often colonizes sites and creates habitat for less hardy tree species. In lower subalpine forests, it is a seral species, establishing after a large disturbance such as stand‑replacing fire or avalanche, or it is restricted to dry, rocky ridges where it competes well with shade‑tolerant tree species. Without disturbance it will be overtopped in 100‑120 years by faster growing, shade‑tolerant species such as *Abies lasiocarpa, Picea engelmannii, Pseudotsuga menziesii*, and *Tsuga mertensiana*. Although crown fires and hot ground fires kill *Pinus albicaulis*, it tolerates low‑intensity ground fires that will kill the shade‑tolerant understory. Fire intervals range from 30‑300 years.

In this harsh, often windswept environment, trees are often stunted and flagged from damage associated with wind and blowing snow and ice crystals, especially at the upper elevations of the type. The stands or patches often originate when *Picea engelmannii, Larix lyallii*, or *Pinus albicaulis* colonize a sheltered site such as the lee side of a rock. *Abies lasiocarpa* can then colonize in the shelter of the *Picea engelmannii* and may form a dense canopy by branch‑layering. Major disturbances are windthrow and snow avalanches. Fire is known to occur infrequently in this system, at least where woodlands are present; lightning damage to individual trees is common, but sparse canopies and rocky terrain limit the spread of fire. *Larix lyallii* is a very slow‑growing, long‑lived tree, with individuals up to 1000 years in age. It is generally shade‑intolerant; however, extreme environmental conditions limit potentially competing trees. In the Cascades and Olympic Mountains, the climate is more maritime in nature and wind is not as extreme, but summer drought is a more important process than in the related North Pacific Maritime Mesic Subalpine Parkland (CES204.837). In northwestern and west‑central Wyoming, *Pinus albicaulis* is the initial colonizer, and trees of other species become established in the micro‑sites that it creates (Callaway 1998, cited in Greater Yellowstone Coordinating Committee 2011). In the highest‑elevation stands where *Pinus albicaulis* usually is the only tree present, vegetation dynamics are relatively simple: stands start out with rather dense overstories and sparse undergrowth and develop more open overstories and denser undergrowth over time. At lower elevations, *Pinus contorta* dominates some stands soon after fire, and the long‑lived, more shade‑tolerant *Pinus albicaulis* become dominant over time (Steele et al. 1983). As in the Pacific Northwest, fire has, in the past, been a minor process (compared to the subalpine forests at lower elevations): lightning starts many fires, but they rarely spread (Steele et al. 1983).

<br />Birds and small mammals often eat and cache the large, wingless pine seeds and are responsible for the dispersal of this species. Most important is the Clark's nutcracker, which can transport the seeds long distances and cache them on exposed windswept and burned‑over sites. This results in the regeneration of pines in clumps from forgotten caches (Eyre 1980, Burns and Honkala 1990a, Schmidt and McDonald 1990, Steel et al. 1983).

The mountain pine beetle (*Dendroctonus ponderosae*) has killed many mature trees in the past, during epidemics where populations of the beetles build up in lower elevation *Pinus contorta* stands, then move up into the *Pinus albicaulis* (Burns and Honkala 1990a, Schmidt and McDonald 1990, Steel et al. 1983).

SOURCES

**References:** Arno 1970, Arno and Habeck 1972, Burns and Honkala 1990a, Callaway 1998, Comer et al. 2003*, Cooper et al. 1999, Ecosystems Working Group 1998, Eyre 1980, Greater Yellowstone Coordinating Committee 2011, Johnson 2004, Johnson and Swanson 2005, Keane and Parsons 2010, Kendall and Keane 2001, LANDFIRE 2007a, Lillybridge et al. 1995, Littell et al. 2009, Macfarlane et al. 2009, Meidinger and Pojar 1991, NCC 2002, Rice et al. 2012a, Steele et al. 1983, WNDD 2013, WNHP 2011, WNHP unpubl. data 2018, Williams and Lillybridge 1983, Williams and Smith 1990

**Version:** 03 Feb 2017	**Stakeholders:** Canada, West

**Concept Author:** M.S. Reid	**LeadResp:** West

CES303.957 Northwestern Great Plains Highland White Spruce Woodland

**Primary Division:** Western Great Plains (303)

**Land Cover Class:** Forest and Woodland

**Spatial Scale & Pattern:** Large patch

**Required Classifiers:** Natural/Semi‑natural; Vegetated (>10% vasc.); Upland

**Diagnostic Classifiers:** Temperate [Temperate Continental]; Picea glauca

**National Mapping Codes:** EVT 2048; ESLF 4235; ESP 1048

**Concept Summary:** This uncommon system is limited to relatively high‑elevation outliers of montane environments in the northwestern Great Plains. Best known areas of this system are small portions of the Black Hills of Wyoming and South Dakota and the Cypress Upland of southern Alberta and Saskatchewan. These highland areas have a cooler climate than surrounding mixedgrass prairie. In the Black Hills, these woodlands occur as small or large patches within the ponderosa pine matrix, from about 1740 to 2135 m (5700‑7000 feet); at lower elevations, they are restricted to north‑facing slopes. At the higher elevations, they are found on level or gently sloping areas. In other locations, this woodland system is limited to sideslopes and depressions, likely adjoining riparian zones, where snow is well‑retained. Soils vary widely from deep to quite shallow. *Picea glauca* is the characteristic conifer, but other trees can include *Pinus ponderosa, Populus tremuloides*, and *Betula papyrifera*. Undergrowth shrubs typically include *Arctostaphylos uva‑ursi, Juniperus communis, Linnaea borealis, Symphoricarpos albus*, and *Vaccinium scoparium*. Disturbance regimes are not well‑documented for this system, but likely include periodic windthrow as well as fire spreading from adjacent, lower elevation woodlands and grasslands.

DISTRIBUTION

**Range:** This system is limited to relatively high‑elevation outliers of montane environments in the northwestern Great Plains. Best known areas of this system are small portions of the Black Hills of Wyoming and South Dakota and the Cypress Upland of southern Alberta and Saskatchewan. It may also occur in very small stands of the Bighorn Mountains of north‑central Wyoming and south‑central Montana.

**Divisions:** 303:C, 306:C

**TNC Ecoregions:** 25:C, 26:C

**Nations:** CA, US

**Subnations:** AB, MT?, SD, SK, WY

**Map Zones:** 29:C, 30:?, 31:?

**USFS Ecomap Regions:** M331B:CP, M334A:CC

CONCEPT

**Environment:** This system is limited to relatively high‑elevation outliers of montane environments in the northwestern Great Plains of the U.S. and southern Canada. These highland areas have a cooler and more mesic climate than surrounding mixedgrass prairie. In the Black Hills, these woodlands occur as small or large patches within the ponderosa pine matrix, from about 1740 to 2135 m (5700‑7000 feet) elevation; at lower elevations, they are restricted to north‑facing slopes. At the higher elevations, they are found on level or gently sloping areas. In other locations, this woodland system is limited to sideslopes and depressions, likely adjoining riparian zones, where snow is well‑retained. Geology is generally dominated by limestone, granite, slate and schist. Soils vary widely from deep to quite shallow. In the Cypress Hills of Alberta and Saskatchewan, the elevations where this system is found range up to 1466 m; generally these woodlands occur on north‑facing slopes or near small springs and seeps.

**Dynamics:** Disturbance regimes are not well‑documented for this system, but likely include periodic windthrow as well as fire spreading from adjacent, lower elevation or drier woodlands and grasslands. There is some debate about whether mixed‑severity fire would have occurred in this type based on tree‑ring and historical evidence; estimated at a 100‑year return interval (Landfire 2007a). Stand‑replacing disturbances are primarily associated with climatic fluctuations and include fire and insect (in late‑development classes only, mountain pine beetles create larger patch sizes; Ips beetles create smaller patches). Snowbreak and windthrow events may occur. The majority of the insect outbreaks generally occur in late‑development stands but in periods of drought (such as that which the forest is currently experiencing), tree mortality is occurring in ponderosa pine that are less than 18 cm (7 inches) dbh. Surface and stand‑replacing fire events occur in this system. Stand‑replacing fires were likely most common in higher elevation and northern slopes that were primarily dominated by spruce, with surface fires occurring most often in the moist ponderosa pine.

SOURCES

**References:** Baker and Ehle 2001, Barrow 2009, Brown 2003, Brown 2006, Comer et al. 2003*, ESWG 1995, Graves 1899, Henderson et al. 2002, Hoffman and Alexander 1987, LANDFIRE 2007a, Marriott and Faber‑Langendoen 2000, Marriott et al. 1999, Rogers 1982, Shinneman and Baker 1997, WNHP 2011

**Version:** 14 Jan 2014	**Stakeholders:** Canada, Midwest, West

**Concept Author:** P. Comer	**LeadResp:** West

CES306.813 Rocky Mountain Aspen Forest and Woodland

**Primary Division:** Rocky Mountain (306)

**Land Cover Class:** Forest and Woodland

**Spatial Scale & Pattern:** Large patch

**Required Classifiers:** Natural/Semi‑natural; Vegetated (>10% vasc.); Upland

**Diagnostic Classifiers:** Forest and Woodland (Treed); Long Disturbance Interval; F‑Patch/Medium Intensity; F‑Landscape/Medium Intensity; Broad‑Leaved Deciduous Tree; Populus tremuloides

**National Mapping Codes:** EVT 2011; ESLF 4104; ESP 1011

**Concept Summary:** This widespread ecological system is more common in the southern and central Rocky Mountains but occurs in the montane and subalpine zones throughout much of the western U.S. and north into Canada. An eastern extension occurs along the Rocky Mountains foothill front and in mountain "islands" in Montana (Big Snowy and Highwood mountains), and the Black Hills of South Dakota. In California, this system is only found on the east side of the Sierra Nevada adjacent to the Great Basin. Large stands are found in the Inyo and White mountains, while small stands occur on the Modoc Plateau. In western Alberta, it occurs only in the Upper Foothills subregion, and north of there transitions to Western North American Boreal Mesic Birch‑Aspen Forest (CES105.108). Elevations generally range from 1525 to 3050 m (5000‑10,000 feet), but occurrences can be found at lower elevations in some regions, especially in the Canadian Rockies. Distribution of this ecological system is primarily limited by adequate soil moisture required to meet its high evapotranspiration demand. Secondarily, it is limited by the length of the growing season or low temperatures. These are upland forests and woodlands dominated by *Populus tremuloides* without a significant conifer component (<25% relative tree cover). The understory structure may be complex with multiple shrub and herbaceous layers, or simple with just an herbaceous layer. The herbaceous layer may be dense or sparse, dominated by graminoids or forbs. In California, *Symphyotrichum spathulatum* is a common forb. Associated shrub species include *Symphoricarpos* spp., *Rubus parviflorus, Amelanchier alnifolia*, and *Arctostaphylos uva‑ursi*. Occurrences of this system originate and are maintained by stand‑replacing disturbances such as avalanches, crown fire, insect outbreak, disease and windthrow, or clearcutting by man or beaver, within the matrix of conifer forests. It differs from Northwestern Great Plains Aspen Forest and Parkland (CES303.681), which is limited to plains environments. In Texas, this system occurs as small patches within the higher elevation conifer systems of the Guadalupe, Davis, and Chisos mountains. These patches are considered relictual remnants in this southwestern extension of this more commonly encountered type further north.

**Comments:** The scattered occurrences in Trans‑Pecos of Texas are of interest as they represent disjunct outliers of the type occurring under highly limited circumstances.

DISTRIBUTION

**Range:** This system is more common in the central and southern Rocky Mountains extending south to the Sacramento Mountains, however, it occurs in the montane and subalpine zones throughout much of the western U.S. and north into Canada, as well as west into California. Elevations generally range from 1525 to 3050 m (5000‑10,000 feet), but occurrences can be found at lower elevations in some regions. Very small occurrences may be found in a few scattered locations of the Trans‑Pecos of Texas.

**Divisions:** 204:C, 206:P, 304:C, 306:C

**TNC Ecoregions:** 1:P, 3:C, 4:P, 5:P, 7:C, 8:C, 9:C, 11:C, 12:P, 18:C, 19:C, 20:C, 21:P, 25:C, 26:C, 81:P

**Nations:** CA, US

**Subnations:** AB, AZ, BC, CA, CO, ID, MT, NM, NV, OR, SD, TX, UT, WA:S2, WY

**Map Zones:** 1:C, 3:C, 6:C, 7:C, 8:?, 9:C, 10:C, 12:C, 13:?, 15:C, 16:C, 17:C, 18:C, 19:C, 20:C, 21:C, 22:C, 23:C, 24:P, 25:C, 26:C, 27:C, 28:C, 29:C

**USFS Ecomap Regions:** 313A:CC, 313B:CC, 313D:CC, 315H:PP, 321A:CC, 322A:CC, 331A:CC, 331F:CC, 331G:CC, 331I:C?, 331J:CC, 331K:CP, 331N:CP, 332F:??, 341A:CC, 341B:CC, 341C:CC, 341D:CC, 341E:CC, 341F:CC, 341G:CC, 342A:CC, 342B:CC, 342C:CC, 342D:CC, 342E:CC, 342F:CC, 342G:CC, 342H:CC, 342I:CP, 342J:CC, M242B:CP, M242C:CC, M242D:CC, M261D:CC, M261E:CC, M261G:CC, M313A:CC, M313B:CC, M331A:CC, M331B:CC, M331D:CC, M331E:CC, M331F:CC, M331G:CC, M331H:CC, M331I:CC, M331J:CC, M332A:CC, M332B:CP, M332D:CC, M332E:CC, M332F:CC, M332G:CC, M333A:CC, M333B:CC, M333C:CP, M333D:CC, M334A:CC, M341A:CC, M341B:CC, M341C:CC, M341D:CC

CONCEPT

**Environment:** This widespread montane and subalpine ecological system is more common in the central and southern Rocky Mountains extending south to the Sacramento Mountains of New Mexico, west into the high plateaus of the Colorado Plateau and ranges of the Great Basin into the eastern Sierra Nevada, and north into the Canadian Rockies. Eastern extensions occur along the Rocky Mountains foothill front and in mountain "islands" in Montana (Big Snowy and Highwood mountains), and the Black Hills of South Dakota. Very small occurrences may be found in a few scattered locations of the Trans‑Pecos of Texas. Elevations generally range from 1525 to 3050 m (5000‑10,000 feet), but occurrences can be found at lower elevations in some regions. Climate is temperate with a relatively long growing season, typically cold winters and deep snow. Mean annual precipitation is greater than 38 cm (15 inches) and typically greater than 51 cm (20 inches), except in semi‑arid environments where occurrences are restricted to mesic microsites such as seeps or large snow drifts. Distribution of this ecological system is primarily limited by adequate soil moisture required to meet its high evapotranspiration demand (Mueggler 1988). Secondarily, its range is limited by the length of the growing season or low temperatures (Mueggler 1988). Topography is variable; sites range from level to steep slopes. Aspect varies according to the limiting factors. Occurrences at high elevations are restricted by cold temperatures and are found on warmer southern aspects. At lower elevations occurrences are restricted by lack of moisture and are found on cooler north aspects and mesic microsites. The soils are typically deep and well‑developed, with rock often absent from the soil. Soil texture ranges from sandy loam to clay loam. Parent materials are variable and may include sedimentary, metamorphic or igneous rocks, but it appears to grow best on limestone, basalt, and calcareous or neutral shales (Mueggler 1988). In Texas, this system occurs on high mountain slopes, valleys and ridges at higher elevations on Permian limestone (Guadalupe Mountains) and igneous substrates (Davis and Chisos mountains). The environmental description is based on several other references, including Henderson et al. (1977), Bartos (1979), Bartos and Mueggler (1979), Eyre (1980), Hess and Wasser (1982), DeByle and Winokur (1985), Johnston and Hendzel (1985), Youngblood and Mauk (1985), DeVelice et al. (1986), Mueggler (1988), Powell (1988a), Knight (1994), Shiflet (1994), Bartos and Campbell (1998), Reid et al. (1999), Neely et al. (2001), Comer et al. (2002), Tuhy et al. (2002), Minnich (2007), and NatureServe Explorer (2009).

**Vegetation:** These are cold‑deciduous, broad‑leaved upland forests and woodlands dominated by *Populus tremuloides* without a significant conifer component (<25% relative tree cover). The tree canopy ranges from 5‑20 m tall and may be open to closed. Conifers may be present but never codominant and include *Abies concolor, Abies lasiocarpa, Picea engelmannii, Picea pungens, Pinus ponderosa*, and *Pseudotsuga menziesii*. Because of the open growth form of *Populus tremuloides*, enough light can penetrate for lush understory development. Depending on available soil moisture and other factors such as disturbance, the understory structure may be complex with multiple shrub and herbaceous layers, or simple with just an herbaceous layer. The herbaceous layer may be dense or sparse, dominated by graminoids or forbs depending on available soil moisture and other factors such as disturbance. Associated shrub species include *Amelanchier alnifolia, Arctostaphylos uva‑ursi, Artemisia tridentata, Juniperus communis, Prunus virginiana, Ribes montigenum, Robinia neomexicana, Rosa woodsii, Rubus parviflorus, Shepherdia canadensis, Symphoricarpos* spp., and the dwarf‑shrubs *Mahonia repens* and *Vaccinium* spp. Numerous mesic forbs and graminoids may be present to dominant. Common graminoids may include *Bromus carinatus, Calamagrostis rubescens, Carex siccata (= Carex foenea), Carex geyeri, Carex rossii, Elymus glaucus, Elymus trachycaulus, Festuca thurberi, Hesperostipa comata*, and *Muhlenbergia montana*. Associated forbs may include *Achillea millefolium, Eucephalus engelmannii (= Aster engelmannii), Delphinium* spp., *Geranium viscosissimum, Heracleum sphondylium, Ligusticum filicinum, Lupinus argenteus, Osmorhiza berteroi (= Osmorhiza chilensis), Pteridium aquilinum, Rudbeckia occidentalis, Thalictrum fendleri, Valeriana occidentalis, Wyethia amplexicaulis*, and many others. Exotic grasses such as the perennials *Poa pratensis* and *Bromus inermis* and the annual *Bromus tectorum* are often common in occurrences disturbed by grazing. The over 60 associations included in this system document its heterogeneous nature. The vegetation description is based on several references, including Henderson et al. (1977), Eyre (1980), Hess and Wasser (1982), DeByle and Winokur (1985), Youngblood and Mauk (1985), DeVelice et al. (1986), Mueggler (1988), Powell (1988a), Knight (1994), Shiflet (1994), Reid et al. (1999), Neely et al. (2001), NCC (2002), Comer et al. (2002), Tuhy et al. (2002), Minnich (2007), and NatureServe Explorer (2009).

**Dynamics:** Occurrences in this ecological system often originate, and are likely maintained by, stand‑replacing disturbances such as crown fire, disease and windthrow, or clearcutting by man or beaver. The stems of these thin‑barked, clonal trees are easily killed by surface fires, but they can quickly and vigorously resprout in densities of up to 30,000 stems per hectare (Knight 1994). As dbh increases beyond 15 cm, *Populus tremuloides* stems become increasingly resistant to fire mortality, and large stems may survive low‑severity surface fire but usually show fire damage (Brown and DeByle 1987). The stems are relatively short‑lived (100‑150 years), and the stand will succeed to longer‑lived conifer forest if undisturbed. Occurrences are favored by fire in the conifer zone (Mueggler 1988). With adequate disturbance a clone may live many centuries. Although *Populus tremuloides* produces abundant seeds, seedling survival is rare because the long moist conditions required to establish them are rare in the habitats that it occurs in. Superficial soil drying will kill seedlings (Knight 1994).

Although many diseases and insects attack *Populus tremuloides* (DeByle and Winokur 1985), under presettlement conditions, disease and insect mortality did not appear to have major effects; however, older aspen stands would be susceptible to outbreaks every 200 years on average (LANDFIRE 2007a, BpS:1210110). Sudden aspen decline (SAD) results in root mortality with subsequent effects on tree canopy and clone persistence. It appears to be triggered by severe drought (Worrall et al. 2010).

This system is also important habitat and browse for many species of wildlife, including various birds, beaver, snowshoe hare and large ungulates such as deer, elk and moose (DeByle and Winokur 1985). Concentrated use by elk can significantly impact stands (DeByle and Winokur 1985).

LANDFIRE developed a state‑and‑transition vegetation dynamics VDDT model for this system which has three classes in total (LANDFIRE 2007a, BpS 2810110). These are summarized as:

A) Early Development 1 All Structures (5% of type in this stage): Aspen suckers less than 6 feet tall and abundant. Grasses and forbs resprout vigorously with high cover. Often densely vegetated.

B) Mid Development 1 Closed (pole‑sized tree‑dominated ‑ 35% of type in this stage): Tree cover is 21‑100%. Aspen over 6 feet tall dominate. Canopy cover highly variable, but usually dense. Understory also usually dense.

C) Late Development 1 Closed (tree‑dominated ‑ 60% of type in this stage): Tree cover is 21‑100%. Aspen trees 9+ inches dbh. Canopy cover is highly variable, but usually dense. Understory dense. Lots of dead and downed material.

Fire, insects and disease. In absence of disturbance, may stay aspen. Fire will generally come from adjacent systems. Surface fire would generally affect the margins of stands as a result of fire on adjacent vegetation types. Mixed fire may occur, but is undocumented (LANDFIRE 2007a, BpS 2810110).

SOURCES

**References:** Adams 2010, Bartos 1979, Bartos 2008, Bartos and Campbell 1998, Bartos and Mueggler 1979, Bell et al. 2009, Bethers et al. 2010, Brandt et al. 2003b, Brown and DeByle 1987, Comer et al. 2002, Comer et al. 2003*, Dale et al. 2001, DeByle 1985b, DeByle and Winokur 1985, DeVelice et al. 1986, Downing and Pettapiece 2006, Elliott 2012, Elliott and Baker 2004, Eyre 1980, Fairweather et al. 2008, Graham et al. 1990, Henderson et al. 1977, Hess and Wasser 1982, Howard 1996a, Johnston and Hendzel 1985, Jones and DeByle 1985, Keammerer 1974a, Keammerer 1974b, Knight 1994, LANDFIRE 2007a, Minnich 2007b, Morelli and Carr 2011, Mueggler 1988, NCC 2002, Natural Regions Committee 2006, NatureServe Explorer 2011, Neely et al. 2001, Niinemets and Valladares 2006, Powell 1988a, Reid et al. 1999, Rogers 2002, Romme et al. 2001, Schier et al. 1985, Scott et al. 1980, Shepperd et al. 2006, Shiflet 1994, Spracklen et al. 2009, Tuhy et al. 2002, WNHP unpubl. data 2018, Westerling et al. 2006, Willoughby 2007, Worrall et al. 2008a, Worrall et al. 2008b, Worrall et al. 2010, Youngblood and Mauk 1985

**Version:** 23 May 2018	**Stakeholders:** Canada, Midwest, Southeast, West

**Concept Author:** M.S. Reid	**LeadResp:** West

CES306.814 Rocky Mountain Bigtooth Maple Ravine Woodland

**Primary Division:** Rocky Mountain (306)

**Land Cover Class:** Forest and Woodland

**Spatial Scale & Pattern:** Large patch

**Required Classifiers:** Natural/Semi‑natural; Vegetated (>10% vasc.); Upland

**Diagnostic Classifiers:** Colluvial slope; Ravine; Stream terrace (undifferentiated); Toeslope; Mineral: W/ A‑Horizon <10 cm; Unconsolidated; Broad‑Leaved Deciduous Tree; Acer grandidentatum

**National Mapping Codes:** EVT 2012; ESLF 4105; ESP 1012

**Concept Summary:** This ecological system occurs in cool ravines, on toeslopes and slump benches associated with riparian areas in the northern and central Wasatch Range and Tavaputs Plateau extending into southern Idaho, as well as in scattered localities in southwestern Utah, central Arizona and New Mexico. Substrates are typically rocky colluvial or alluvial soils with favorable soil moisture. These woodlands are dominated by *Acer grandidentatum* but may include mixed stands codominated by *Quercus gambelii* or with scattered conifers. Some stands may include *Acer negundo* or *Populus tremuloides* as minor components. It also occurs on steeper, north‑facing slopes at higher elevations, often adjacent to Rocky Mountain Gambel Oak‑Mixed Montane Shrubland (CES306.818) or Rocky Mountain Aspen Forest and Woodland (CES306.813).

**Comments:** What was once included in this system in Texas has been reclassified as Madrean Mesic Canyon Forest and Woodland (CES302.454).

DISTRIBUTION

**Range:** Occurs in the northern and central Wasatch Range and Tavaputs Plateau extending into southern Idaho, as well as in scattered localities in southwestern Utah, central Arizona and New Mexico.

**Divisions:** 302:?, 304:?, 306:C

**TNC Ecoregions:** 6:C, 9:C, 18:P, 21:P

**Nations:** US

**Subnations:** ID, NM, UT, WY?

**Map Zones:** 15:C, 16:C, 17:C, 18:C, 23:P, 24:P, 25:C, 26:C

**USFS Ecomap Regions:** 315A:PP, 321A:PP, 341A:CC, 342C:C?, 342D:CC, 342E:CC, 342J:CC, M313A:CC, M331D:CC, M331E:CC, M341A:CP, M341B:CC, M341C:CP

CONCEPT

SOURCES

**References:** Comer et al. 2003*, Gehlbach 1967, Ream 1964, Shiflet 1994

**Version:** 03 Nov 2014	**Stakeholders:** Southeast, West

**Concept Author:** M.S. Reid	**LeadResp:** West

CES306.820 Rocky Mountain Lodgepole Pine Forest

**Primary Division:** Rocky Mountain (306)

**Land Cover Class:** Forest and Woodland

**Spatial Scale & Pattern:** Matrix

**Required Classifiers:** Natural/Semi‑natural; Vegetated (>10% vasc.); Upland

**Diagnostic Classifiers:** Acidic Soil; Very Shallow Soil; Mineral: W/ A‑Horizon <10 cm; Ustic; Long Disturbance Interval; F‑Patch/High Intensity [Seasonality/Fall Fire]; F‑Landscape/High Intensity; Needle‑Leaved Tree; Pinus contorta; Moderate (100‑500 yrs) Persistence

**National Mapping Codes:** EVT 2050; ESLF 4237; ESP 1050

**Concept Summary:** This ecological system is widespread in upper montane to subalpine elevations of the Rocky Mountains, Intermountain West region, north into the Canadian Rockies and east into mountain "islands" of north‑central Montana. These are subalpine forests where the dominance of *Pinus contorta* is related to fire history and topo‑edaphic conditions. Following stand‑replacing fires, *Pinus contorta* will rapidly colonize and develop into dense, even‑aged stands. Most forests in this ecological system occur as early‑ to mid‑successional forests which developed following fires. This system includes *Pinus contorta*‑dominated stands that, while typically persistent for >100‑year time frames, may succeed to spruce‑fir; in the southern and central Rocky Mountains it is seral to Rocky Mountain Subalpine Dry‑Mesic Spruce‑Fir Forest and Woodland (CES306.828). More northern occurrences are seral to Rocky Mountain Subalpine Mesic‑Wet Spruce‑Fir Forest and Woodland (CES306.830). Soils supporting these forests are typically well‑drained, gravelly, coarse‑textured, acidic, and rarely formed from calcareous parent materials. These forests are dominated by *Pinus contorta* with shrub, grass, or barren understories. Sometimes there are intermingled mixed conifer/*Populus tremuloides* stands, with the latter occurring with inclusions of deeper, typically fine‑textured soils. The shrub stratum may be conspicuous to absent; common species include *Arctostaphylos uva‑ursi, Ceanothus velutinus, Linnaea borealis, Mahonia repens, Menziesia ferruginea* (in northern occurrences), *Purshia tridentata, Rhododendron albiflorum* (in northern occurrences), *Spiraea betulifolia, Spiraea douglasii, Shepherdia canadensis, Vaccinium cespitosum, Vaccinium scoparium, Vaccinium membranaceum, Symphoricarpos albus*, and *Ribes* spp. In southern interior British Columbia, this system is usually an open lodgepole pine forest found extensively between 500 and 1600 m elevation in the Columbia Range. In the Interior Cedar Hemlock and Interior Douglas‑fir zones, *Tsuga heterophylla* or *Pseudotsuga menziesii* may be present. In Alberta, species composition indicates the transition to more boreal floristics, including such species as *Empetrum nigrum, Ledum groenlandicum, Leymus innovatus*, and more abundant lichens or mosses such as *Cladonia* spp., *Hylocomium splendens*, and *Pleurozium schreberi*.

DISTRIBUTION

**Range:** This system occurs at upper montane to subalpine elevations of the Rocky Mountains, Intermountain West region, north into the Canadian Rockies, and east onto mountain "islands" of north‑central Montana. In Washington, this system occurs mostly on the east side of the Cascade Crest. In Oregon, this system only occurs in the Blue Mountains; all Oregon Cascades lodgepole pine forests are included in other systems.

**Divisions:** 304:C, 306:C

**TNC Ecoregions:** 7:C, 8:C, 9:C, 11:C, 18:C, 20:C, 26:C, 68:C

**Nations:** CA, US

**Subnations:** AB, BC, CO, ID, MT, NV, OR, UT, WA:S3S4, WY

**Map Zones:** 1:C, 8:?, 9:C, 10:C, 16:C, 18:C, 19:C, 20:C, 21:C, 22:C, 23:?, 28:C, 29:C, 33:?

**USFS Ecomap Regions:** 331A:CC, 331G:CC, 331J:CC, 331K:C?, 342A:CC, 342B:C?, 342C:CC, 342D:CC, 342E:CC, 342F:CC, 342G:CC, 342H:CC, 342J:CC, M242B:CC, M242C:CC, M242D:CC, M331A:CC, M331B:CC, M331D:CC, M331E:CC, M331F:CP, M331G:CP, M331H:CC, M331I:CC, M331J:CC, M332A:CC, M332B:CC, M332D:CC, M332E:CC, M332F:CC, M332G:CC, M333A:CC, M333B:CC, M333C:CC, M333D:CC, M341B:CC

CONCEPT

**Environment:** This system occurs in the upper montane to subalpine elevations of the Rocky Mountains, north into the Canadian Rockies and east into mountain "islands" of north‑central Montana. Elevations range from just over 900 m in the northeastern Cascades to well over 3100 m in the Uinta Mountains in Utah and the southern Colorado Rockies. Temperature regimes are extreme throughout this region and frequent growing season frosts occur. Annual precipitation in these montane and subalpine habitats ranges from less than 40 cm to over 150 cm, usually with the majority falling as snow. Late‑melting snowpacks provide the majority of growing‑season moisture.

Soils are variable but are typically well‑drained, gravelly, coarse‑textured, acidic, rarely from calcareous parent materials with occasionally inclusions of deeper, typically fine‑textured soils. Other stands occur on excessively well‑drained pumice deposits, glacial till and alluvium on valley floors where there is cold‑air accumulation, warm and droughty shallow soils over fractured quartzite bedrock, and shallow moisture‑deficient soils with a significant component of volcanic ash.

**Vegetation:** These forests are dominated by *Pinus contorta* with shrub, grass, or barren understories. Sometimes there are intermingled mixed conifer/*Populus tremuloides* stands, with the latter occurring with inclusions of deeper, typically fine‑textured soils. The shrub stratum may be conspicuous to absent; common species include *Arctostaphylos uva‑ursi, Ceanothus velutinus, Linnaea borealis, Mahonia repens, Menziesia ferruginea* (in northern occurrences), *Purshia tridentata, Rhododendron albiflorum* (in northern occurrences), *Spiraea betulifolia, Spiraea douglasii, Shepherdia canadensis, Vaccinium cespitosum, Vaccinium scoparium, Vaccinium membranaceum, Symphoricarpos albus*, and *Ribes* spp. In southern interior British Columbia, this system is usually an open lodgepole pine forest found extensively between 500 and 1600 m elevation in the Columbia Range. In the Interior Cedar Hemlock and Interior Douglas‑fir zones, *Tsuga heterophylla* or *Pseudotsuga menziesii* may be present. In Alberta, species composition indicates the transition to more boreal floristics, including such species as *Empetrum nigrum, Ledum groenlandicum, Leymus innovatus*, and more abundant lichens or mosses such as *Cladonia* spp. (= *Cladina* spp.), *Hylocomium splendens*, and *Pleurozium schreberi*.

**Dynamics:** *Pinus contorta* is an aggressively colonizing, shade‑intolerant conifer which usually occurs in lower subalpine forests in the major ranges of the western United States. Establishment is episodic and linked to stand‑replacing disturbances, primarily fire. The incidence of serotinous cones varies within and between varieties of *Pinus contorta*, being most prevalent in Rocky Mountain populations. Closed, serotinous cones appear to be strongly favored by fire, and allow rapid colonization of fire‑cleared substrates (Burns and Honkala 1990a). Hoffman and Alexander (1980, 1983) report that in stands where *Pinus contorta* exhibits a multi‑aged population structure, with regeneration occurring, there is typically a higher proportion of trees bearing nonserotinous cones.

LANDFIRE developed a state‑and‑transition vegetation dynamics VDDT model for this system which has five classes in total (LANDFIRE 2007a, BpS 2810500). These are summarized as:

A) Early Development 1 All Structures (20% of type in this stage): Tree cover is 0‑80%. Stand initiation: Grasses, forbs, low shrubs and lodgepole seedlings‑saplings. This class does not last long; young lodgepole grows fast. If aspen is present, it grows faster and dominates lodgepole. Cover of trees (seedlings‑saplings) varies widely.

B) Mid Development 1 Closed (20% of type in this stage): Tree cover is 51‑100%. Stem exclusion (RMLANDS: Rocky Mountain Landscape Simulator): Moderate to dense pole‑sized trees, sometimes very dense (dog‑hair); longest time in this class without disturbance. Aspen usually not present.

C) Mid Development 1 Open (tree‑dominated ‑ 20% of type in this stage): Tree cover is 21‑50%. Understory reinitiation: Variety of lodgepole size classes, some mature trees, often somewhat patchy. If aspen is present, lodgepole usually dominates it.

D) Late Development 1 Open (20% of type in this stage): Tree cover is 61‑100%. Many mature lodgepole pine with closed canopy. Trees may vary in age, but consistent in size, diameters and heights.

E) Late Development 1 All Structures (10% of type in this stage): Tree cover is 31‑60%. Many mature lodgepole pine, somewhat patchy, variety of lodgepole size classes, open canopies overall but patches of denser trees. Dead and downed woody materials increasing in volume, young trees infilling openings.

Before fire suppression began in the early 20th century, most fires were low‑intensity, creeping, surface fires, whereas most fires today are high‑intensity crown fires that occur during severe fire weather (dry and windy) (Lotan et al. 1985). The stand‑replacing fire interval in lodgepole pine forests is about 215 years (LANDFIRE 2007a, BpS 2810500).

Nutrient cycling, specifically carbon cycling, is an important ecological process within many ecological systems. Biological decomposition in lodgepole pine forests is more limited than biological production, resulting in accumulation of organic materials, especially in the absence of fire (Harvey 1994).

SOURCES

**References:** Alexander 1986, Alexander et al. 1987, Amman 1977, Anderson 1999a, Anderson 2003b, Arno et al. 1985, Banner et al. 1993, Barrows et al. 1977, Burns and Honkala 1990a, CNHP 2010, Comer et al. 2003*, Dale et al. 2001, DeLong 1996, DeLong et al. 1993, Despain 1973a, Despain 1973b, Ecosystems Working Group 1998, Eyre 1980, Garfin et al. 2014, Harvey 1994, Hess and Alexander 1986, Hess and Wasser 1982, Hoffman and Alexander 1976, Hoffman and Alexander 1980, Hoffman and Alexander 1983, Johnson and Clausnitzer 1992, Johnston 1997, Kingery 1998, LANDFIRE 2007a, Lloyd et al. 1990, Lotan et al. 1985, MacKinnon et al. 1990, Mauk and Henderson 1984, McKenzie et al. 2004, McKenzie et al. 2008, Mehl 1992, Meidinger and Pojar 1991, Moir 1969a, Mote et al. 2014, NCC 2002, Nachlinger et al. 2001, Neely et al. 2001, Pfister et al. 1977, RMLANDS 2018, Romme et al. 1986, Shafer et al. 2014, Steele et al. 1981, Steele et al. 1983, Steen and Coupé 1997, Stevens‑Rumann et al. 2017, WNHP unpubl. data 2018, Westerling et al. 2006, Whipple 1975, Williams and Smith 1990, van der Kamp and Hawksworth 1985

**Version:** 23 May 2018	**Stakeholders:** Canada, Midwest, West

**Concept Author:** M.S. Reid	**LeadResp:** West

CES306.828 Rocky Mountain Subalpine Dry‑Mesic Spruce‑Fir Forest and Woodland

**Primary Division:** Rocky Mountain (306)

**Land Cover Class:** Forest and Woodland

**Spatial Scale & Pattern:** Matrix

**Required Classifiers:** Natural/Semi‑natural; Vegetated (>10% vasc.); Upland

**Diagnostic Classifiers:** Montane [Upper Montane]; Forest and Woodland (Treed); Acidic Soil; Ustic; Very Long Disturbance Interval [Seasonality/Summer Disturbance]; F‑Patch/High Intensity; F‑Landscape/High Intensity; Needle‑Leaved Tree; Abies lasiocarpa ‑ Picea engelmannii; RM Subalpine Mesic Spruce‑Fir; Long (>500 yrs) Persistence

**National Mapping Codes:** EVT 2055; ESLF 4242; ESP 1055

**Concept Summary:** Engelmann spruce and subalpine fir forests comprise a substantial part of the subalpine forests of the Cascades and Rocky Mountains from southern British Columbia east into Alberta, and south into New Mexico and the Intermountain region. They also occur on mountain "islands" of north‑central Montana. They are the matrix forests of the subalpine zone, with elevations ranging from 1275 m in its northern distribution to 3355 m in the south (4100‑11,000 feet). They often represent the highest elevation forests in an area. Sites within this system are cold year‑round, and precipitation is predominantly in the form of snow, which may persist until late summer. Snowpacks are deep and late‑lying, and summers are cool. Frost is possible almost all summer and may be common in restricted topographic basins and benches. Despite their wide distribution, the tree canopy characteristics are remarkably similar, with *Picea engelmannii* and *Abies lasiocarpa* dominating either mixed or alone. *Pseudotsuga menziesii* may persist in occurrences of this system for long periods without regeneration. *Pinus contorta* is common in many occurrences, and patches of pure *Pinus contorta* are not uncommon, as well as mixed conifer/*Populus tremuloides* stands. In some areas, such as Wyoming, *Picea engelmannii*‑dominated forests are on limestone or dolomite, while nearby codominated spruce‑fir forests are on granitic or volcanic rocks. Upper elevation examples may have more woodland physiognomy, and *Pinus albicaulis* can be a seral component. What have been called "ribbon forests" or "tree islands" by some authors are included here; they can be found at upper treeline in many areas of the Rockies, including the central and northern ranges in Colorado and the Medicine Bow and Bighorn ranges of Wyoming. These are more typically islands or ribbons of trees, sometimes with a krummholz form, with open‑meadow areas in a mosaic. These patterns are controlled by snow deposition and wind‑blown ice. Xeric species may include *Juniperus communis, Linnaea borealis, Mahonia repens*, or *Vaccinium scoparium*. In the Bighorn Mountains, *Artemisia tridentata* is a common shrub. More northern occurrences often have taller, more mesic shrub and herbaceous species, such as *Empetrum nigrum, Rhododendron albiflorum*, and *Vaccinium membranaceum*. Disturbance includes occasional blowdown, insect outbreaks and stand‑replacing fire. Mean return interval for stand‑replacing fire is 222 years as estimated in southeastern British Columbia.

**Comments:** It has been proposed to split out the tree island or ribbon forests of high timberline in the drier mountain ranges of north‑central Colorado, southern Wyoming and north‑central Wyoming (the Bighorns) into a new Southern Rocky Mountain Parkland system. With further discussion, this may be implemented, but for now these areas are still included in this existing system.

DISTRIBUTION

**Range:** This system is found in the Cascades and Rocky Mountains from southern interior British Columbia east into Alberta, south into New Mexico and the Intermountain region. This type tends to be very limited in the northern Oregon Cascades.

**Divisions:** 304:C, 306:C

**TNC Ecoregions:** 4:C, 7:C, 8:C, 9:C, 11:C, 20:C, 21:C, 26:C, 68:C

**Nations:** CA, US

**Subnations:** AB, AZ, BC, CO, ID, MT, NM, NV, OR, UT, WA:S3S5, WY

**Map Zones:** 1:C, 6:?, 7:C, 9:C, 10:C, 12:C, 15:C, 16:C, 17:C, 18:C, 19:C, 20:C, 21:C, 22:C, 23:P, 24:P, 25:C, 27:C, 28:C, 29:C

**USFS Ecomap Regions:** 313A:CC, 313B:CC, 315A:PP, 321A:CC, 331J:CC, 341A:CC, 341B:CC, 341D:CC, 341E:CP, 341F:CC, 341G:CC, 342A:CC, 342B:CP, 342C:CC, 342D:CC, 342E:CC, 342F:CC, 342G:CC, 342H:CC, 342I:CP, 342J:CC, M242B:CC, M242C:CC, M242D:CC, M313A:CC, M313B:CC, M331A:CC, M331B:CC, M331D:CC, M331E:CC, M331F:CC, M331G:CC, M331H:CC, M331I:CC, M331J:CC, M332A:CC, M332B:CC, M332D:CC, M332E:CC, M332F:CC, M332G:CC, M333A:CC, M333B:CC, M333C:CC, M333D:CC, M341A:CC, M341B:CC, M341C:CC, M341D:CC

CONCEPT

**Dynamics:** *Picea engelmannii* can be very long‑lived, reaching 500 years of age. *Abies lasiocarpa* decreases in importance relative to *Picea engelmannii* with increasing distance from the region of Montana and Idaho where maritime air masses influence the climate. Fire is an important disturbance factor, but fire regimes have a long return interval and so are often stand‑replacing. *Picea engelmannii* can rapidly recolonize and dominate burned sites, or can succeed other species such as *Pinus contorta* or *Populus tremuloides*. Due to great longevity, *Pseudotsuga menziesii* may persist in occurrences of this system for long periods without regeneration. Old‑growth characteristics in *Picea engelmannii* forests will include treefall and windthrow gaps in the canopy, with large downed logs, rotting woody material, tree seedling establishment on logs or on mineral soils unearthed in root balls, and snags. Landfire VDDT models: #RSPFI.

SOURCES

**References:** Alexander and Ronco 1987, Alexander et al. 1984a, Alexander et al. 1987, Anderson 1999a, Brand et al. 1976, Clagg 1975, Comer et al. 2002, Comer et al. 2003*, Cooper et al. 1987, Daubenmire and Daubenmire 1968, DeVelice et al. 1986, Ecosystems Working Group 1998, Eyre 1980, Fitzgerald et al. 1994, Fitzhugh et al. 1987, Graybosch and Buchanan 1983, Hess and Alexander 1986, Hess and Wasser 1982, Hoffman and Alexander 1976, Hoffman and Alexander 1980, Hoffman and Alexander 1983, Hopkins 1979a, Hopkins 1979b, Johnson and Clausnitzer 1992, Johnson and Simon 1987, Komarkova et al. 1988b, Lillybridge et al. 1995, Major et al. 1981, Mauk and Henderson 1984, Mehl 1992, Meidinger and Pojar 1991, Muldavin et al. 1996, NCC 2002, Nachlinger et al. 2001, Neely et al. 2001, Peet 1978a, Peet 1981, Pfister 1972, Pfister et al. 1977, Romme 1982, Schaupp et al. 1999, Steele and Geier‑Hayes 1995, Steele et al. 1981, Tuhy et al. 2002, Veblen 1986, WNHP unpubl. data 2018, Whipple and Dix 1979, Williams and Lillybridge 1983, Williams et al. 1995, Wong and Iverson 2004, Wong et al. 2003, Youngblood and Mauk 1985

**Version:** 25 Jan 2007	**Stakeholders:** Canada, Midwest, West

**Concept Author:** M.S. Reid	**LeadResp:** West

CES306.830 Rocky Mountain Subalpine Mesic‑Wet Spruce‑Fir Forest and Woodland

**Primary Division:** Rocky Mountain (306)

**Land Cover Class:** Forest and Woodland

**Spatial Scale & Pattern:** Large patch

**Required Classifiers:** Natural/Semi‑natural; Vegetated (>10% vasc.); Upland

**Diagnostic Classifiers:** Montane [Upper Montane]; Forest and Woodland (Treed); Acidic Soil; Udic; Very Long Disturbance Interval [Seasonality/Summer Disturbance]; F‑Patch/High Intensity; F‑Landscape/Medium Intensity; Abies lasiocarpa ‑ Picea engelmannii; RM Subalpine Dry‑Mesic Spruce‑Fir; Long (>500 yrs) Persistence

**National Mapping Codes:** EVT 2056; ESLF 4243; ESP 1056

**Concept Summary:** This is a high‑elevation system of the Rocky Mountains, dry eastern Cascades and eastern Olympic Mountains dominated by *Picea engelmannii* and *Abies lasiocarpa*. It extends westward into the northeastern Olympic Mountains and the northeastern side of Mount Rainier in Washington, and as far east as mountain "islands" of north‑central Montana. It also occurs northward into the Upper Foothills subregion of western Alberta. *Picea engelmannii* is generally more important in southern forests than those in the Pacific Northwest. Occurrences are typically found in locations with cold‑air drainage or ponding, or where snowpacks linger late into the summer, such as north‑facing slopes and high‑elevation ravines. They can extend down in elevation below the subalpine zone in places where cold‑air ponding occurs (as low as 970 m [3180 feet] in the Canadian Rockies); northerly and easterly aspects predominate. These forests are found on gentle to very steep mountain slopes, high‑elevation ridgetops and upper slopes, plateau‑like surfaces, basins, alluvial terraces, well‑drained benches, and inactive stream terraces. In the northern Rocky Mountains of northern Idaho and Montana, *Tsuga mertensiana* occurs as small to large patches within the matrix of this mesic spruce‑fir system and only in the most maritime of environments (the coldest and wettest of the more Continental subalpine fir forests). In the Olympics and northern Cascades, the climate is more maritime than typical for this system, but due to the lower snowfall in these rainshadow areas, summer drought may be more significant than snowpack in limiting tree regeneration in burned areas. *Picea engelmannii* is rare in these areas. Mesic understory shrubs include *Menziesia ferruginea, Vaccinium membranaceum, Rhododendron albiflorum, Amelanchier alnifolia, Rubus parviflorus, Ledum glandulosum, Phyllodoce empetriformis*, and *Salix* spp. Herbaceous species include *Actaea rubra, Maianthemum stellatum, Cornus canadensis, Erigeron eximius, Gymnocarpium dryopteris, Rubus pedatus, Saxifraga bronchialis, Tiarella* spp., *Lupinus arcticus ssp. subalpinus, Valeriana sitchensis*, and graminoids *Luzula glabrata var. hitchcockii* or *Calamagrostis canadensis*. In Alberta, species composition indicates the transition to more boreal floristics, including such species as *Ledum groenlandicum* and *Leymus innovatus*, and more abundant mosses such as *Hylocomium splendens* and *Pleurozium schreberi*. Disturbances include occasional blowdown, insect outbreaks (30‑50 years), mixed‑severity fire, and stand‑replacing fire (every 150‑500 years). The more summer‑dry climatic areas also have occasional high‑severity fires.

**Comments:** This system is similar to Rocky Mountain Subalpine Dry‑Mesic Spruce‑Fir Forest and Woodland (CES306.828) but is distinguished by its occurrence on mesic to wet microsites within the matrix of the drier (and warmer) subalpine spruce‑fir or lodgepole pine forests. The microsites include north‑facing slopes, swales or ravines, toeslopes, cold pockets, and other locations where available soil moisture is higher or lasts longer into the growing season. This system is NOT confined to the northern Rocky Mountains or Pacific Northwest (it is not geographically defined, rather by topographic settings in the subalpine).

While the name of this system suggests a Rocky Mountain distribution, floristic affinities of Engelmann spruce‑subalpine fir forests in western Washington and the Oregon Cascades are such that the spruce‑fir forests of those regions are included in this system. The subalpine fir‑dominated forests of the northeastern Olympic Mountains and the northeastern side of Mount Rainier are included here. They are more similar to subalpine fir forests on the eastern slopes of the Cascades than they are to mountain hemlock forests.

DISTRIBUTION

**Range:** This system is found at high elevations of the Rocky Mountains, extending west into the northeastern Olympic Mountains and the northeastern side of Mount Rainier in Washington, and as far east as mountain "islands" of north‑central Montana. It also occurs north into the Canadian Rockies of Alberta and British Columbia.

**Divisions:** 204:C, 304:C, 306:C

**TNC Ecoregions:** 1:C, 4:C, 7:C, 8:C, 9:C, 11:C, 20:C, 21:C, 26:C, 68:C

**Nations:** CA, US

**Subnations:** AB, AZ, BC, CO, ID, MT, NM, NV, OR, UT, WA:S5, WY

**Map Zones:** 1:C, 6:?, 7:C, 8:?, 9:C, 10:C, 12:C, 15:C, 16:C, 17:C, 18:C, 19:C, 20:C, 21:C, 22:P, 23:P, 24:P, 25:C, 27:C, 28:C, 29:C

**USFS Ecomap Regions:** 242A:CC, 313A:CC, 313B:CC, 315A:??, 331J:CC, 341A:CC, 341B:CC, 341D:CC, 341E:CP, 341F:CC, 341G:CC, 342A:CC, 342B:CC, 342C:CP, 342D:CC, 342E:CC, 342H:CC, 342I:C?, 342J:CC, M242A:CC, M242B:CC, M242C:CC, M242D:CC, M313A:CC, M313B:CC, M331A:CC, M331B:CC, M331D:CC, M331E:CC, M331F:CC, M331G:CC, M331H:CC, M331I:CC, M331J:CC, M332A:CC, M332B:CC, M332D:CC, M332E:CC, M332F:CC, M332G:CC, M333A:CC, M333B:CC, M333C:CC, M333D:CC, M341A:CC, M341B:CC, M341C:CC, M341D:CC

CONCEPT

**Dynamics:** Landfire VDDT models: #RSPFI and #RABLA.

SOURCES

**References:** Alexander and Ronco 1987, Alexander et al. 1984a, Alexander et al. 1987, Anderson 1999a, BCMF 2006, Banner et al. 1993, Brand et al. 1976, Clagg 1975, Comer et al. 2002, Comer et al. 2003*, Cooper et al. 1987, Daubenmire and Daubenmire 1968, DeVelice et al. 1986, Ecosystems Working Group 1998, Eyre 1980, Fitzgerald et al. 1994, Graybosch and Buchanan 1983, Henderson et al. 1989, Hess and Alexander 1986, Hess and Wasser 1982, Hoffman and Alexander 1976, Hoffman and Alexander 1980, Hoffman and Alexander 1983, Johnson and Clausnitzer 1992, Johnson and Simon 1987, Komarkova et al. 1988b, Lillybridge et al. 1995, MacKinnon et al. 1990, Major et al. 1981, Mauk and Henderson 1984, Mehl 1992, Meidinger and Pojar 1991, Muldavin et al. 1996, NCC 2002, Neely et al. 2001, Peet 1978a, Peet 1981, Pfister 1972, Pfister et al. 1977, Romme 1982, Schaupp et al. 1999, Steele and Geier‑Hayes 1995, Steele et al. 1981, Steen and Coupé 1997, Tuhy et al. 2002, Veblen 1986, WNHP unpubl. data 2018, Whipple and Dix 1979, Williams and Lillybridge 1983, Williams et al. 1995, Wong and Iverson 2004, Wong et al. 2003, Youngblood and Mauk 1985

**Version:** 30 Mar 2010	**Stakeholders:** Canada, West

**Concept Author:** M.S. Reid	**LeadResp:** West

CES306.819 Rocky Mountain Subalpine‑Montane Limber‑Bristlecone Pine Woodland

**Primary Division:** Rocky Mountain (306)

**Land Cover Class:** Forest and Woodland

**Spatial Scale & Pattern:** Large patch

**Required Classifiers:** Natural/Semi‑natural; Vegetated (>10% vasc.); Upland

**Diagnostic Classifiers:** Ridge/Summit/Upper Slope; Calcareous; Very Shallow Soil; Mineral: W/ A‑Horizon <10 cm; Aridic; W‑Patch/High Intensity; W‑Landscape/High Intensity; Needle‑Leaved Tree; Pinus flexilis, P. aristata; Upper Treeline

**National Mapping Codes:** EVT 2057; ESLF 4244; ESP 1057

**Concept Summary:** This ecological system occurs throughout the Rocky Mountains, south of Montana, on dry, rocky ridges and slopes near upper treeline above the matrix spruce‑fir forest. It extends down to the lower montane in the northeastern Great Basin mountains where dominated by *Pinus flexilis*. Sites are harsh, exposed to desiccating winds, with rocky substrates and a short growing season that limit plant growth. Higher‑elevation occurrences are found well into the subalpine‑alpine transition on wind‑blasted, mostly west‑facing slopes and exposed ridges. Calcareous substrates are important for *Pinus flexilis*‑dominated communities in the northern Rocky Mountains and possibly elsewhere. The open tree canopy is often patchy and is strongly dominated by *Pinus flexilis* or *Pinus aristata* with the latter restricted to southern Colorado, northern New Mexico and the San Francisco Mountains in Arizona. In the Wyoming Rockies and northern Great Basin, *Pinus albicaulis* is found in some occurrences, but is a minor component. Other trees such as *Juniperus* spp., *Pinus contorta, Pinus ponderosa*, or *Pseudotsuga menziesii* are occasionally present. *Arctostaphylos uva‑ursi, Cercocarpus ledifolius, Juniperus communis, Mahonia repens, Purshia tridentata, Ribes montigenum*, or *Vaccinium* spp. may form an open shrub layer in some stands. The herbaceous layer, if present, is generally sparse and composed of xeric graminoids, such as *Calamagrostis purpurascens, Festuca arizonica, Festuca idahoensis, Festuca thurberi*, or *Pseudoroegneria spicata*, or more alpine plants.

**Comments:** This system is distinguished from lower montane and foothill limber pine stands in Wyoming and Montana. The foothill system Rocky Mountain Foothill Limber Pine‑Juniper Woodland (CES306.955) is found at the lower treeline, below the zone of continuous *Pinus ponderosa* or *Pseudotsuga menziesii* woodlands and forest, and extends out into the eastern portions of these states in the foothill zones of mountain ranges, along rock outcrops, breaks along rivers, and on sheltered sites where soil moisture is slightly higher than surrounding grasslands.

This system needs to be more clearly distinguished from Northern Rocky Mountain Subalpine Woodland and Parkland (CES306.807), which also includes woodlands of *Pinus flexilis* and *Pinus albicaulis* and occurs in similar environmental settings of the northern Rocky Mountains, particularly northwestern Wyoming, Montana, and north into Alberta and British Columbia. There is a proposal to include the dry, subalpine *Pinus albicaulis* woodlands of the Blue Mountains (Oregon) and northern Nevada into this system, Rocky Mountain Subalpine‑Montane Limber‑Bristlecone Pine Woodland (CES306.819). For Landfire, these *Pinus albicaulis* woodlands were included in the subalpine parkland system, but ecologically and floristically they are more similar to Rocky Mountain dry subalpine woodlands.

DISTRIBUTION

**Range:** This system occurs throughout the Rocky Mountains south of Montana on dry, rocky ridges and slopes near upper treeline, including the Uinta and northern Wasatch mountains, and the Jarbridge Mountains in northeastern Nevada. It also occurs farther east, in the Bighorn Range of north‑central Wyoming, although it is not common there.

**Divisions:** 303:C, 304:C, 306:C

**TNC Ecoregions:** 6:C, 7:C, 8:C, 9:C, 20:C, 21:C, 26:C, 68:P

**Nations:** CA, US

**Subnations:** CO, ID?, MT?, NM, NV, OR?, UT, WY

**Map Zones:** 9:C, 12:P, 15:P, 16:C, 18:C, 19:C, 23:P, 24:C, 25:C, 27:C, 28:C, 29:C

**USFS Ecomap Regions:** 313B:CC, 331J:CC, 341G:PP, 342J:??, M242B:CP, M242C:CC, M313A:CC, M313B:CC, M331A:CC, M331B:CC, M331D:CC, M331E:CP, M331F:CC, M331G:CC, M331H:CC, M331I:CC, M332G:CC, M341A:CC, M341B:CC

CONCEPT

**Environment:** This system is found throughout the Rocky Mountains, south of Montana, on dry, rocky ridges and slopes near upper treeline above the matrix spruce‑fir forest. It extends down to the lower montane in the northeastern Great Basin mountains. Sites are harsh, exposed to desiccating winds with rocky substrates and a short growing season that limit plant growth. Higher elevation occurrences are found well into the subalpine ‑ alpine transition on wind‑blasted, mostly south‑ to west‑facing slopes and exposed ridges. *Pinus aristata* forests are typically found on steep, south‑facing slopes from 2700 to 3700 m (8850‑12,140 feet) elevation. *Pinus flexilis* woodlands occupy similar habitats, but may occur at lower elevations than *Pinus aristata*. When found in the same landscape, stands dominated by *Pinus aristata* occur at higher elevation.

**Vegetation:** The open tree canopy is often patchy and is strongly dominated by *Pinus flexilis* or *Pinus aristata* with the latter restricted to southern Colorado, northern New Mexico and the San Francisco Mountains in Arizona. In the Wyoming Rockies and northern Great Basin, *Pinus albicaulis* is found in some occurrences, but is a minor component. Other trees such as *Juniperus* spp., *Pinus contorta, Pinus ponderosa*, or *Pseudotsuga menziesii* are occasionally present. *Arctostaphylos uva‑ursi, Cercocarpus ledifolius, Juniperus communis, Mahonia repens, Purshia tridentata, Ribes montigenum*, or *Vaccinium* spp. may form an open shrub layer in some stands. The herbaceous layer, if present, is generally sparse and composed of xeric graminoids, such as *Calamagrostis purpurascens, Festuca arizonica, Festuca idahoensis, Festuca thurberi*, or *Pseudoroegneria spicata*, or more alpine plants.

**Dynamics:** Both *Pinus flexilis* and *Pinus aristata* are short‑statured, slow‑growing, long‑lived species in which individuals may live for 1000 or more years in fire‑protected areas. They are adapted to cold, drought and extremely windy conditions with flexible branches that likely reduce wind damage. Fire is an important source of disturbance that facilitates stand regeneration in this system. Older woodlands are often broadly even‑aged stands where seedlings are nearly absent, while areas that have recently burned may have abundant seedlings. Bristlecone pine is somewhat more tolerant of fire than is limber pine; however, both species appear to depend on fire for regeneration. Post‑fire regeneration of bristlecone pine tends to be near burn edges and/or under surviving trees (Coop and Schoettle 2011). Regeneration of limber pine on burned areas is largely due to the germination of seeds cached primarily by Clark's nutcrackers (*Nucifraga columbiana*) and jays (i.e., corvid family), but also small mammals such as squirrels (Lanner and Vander Wall 1980, Tomback 2001, Lanner 2007, CNHP 2010b). Dispersal of the smaller winged seeds of bristlecone pine is primarily by wind, but seeds are likely to also be dispersed by birds (Coop and Schoettle 2011).

Fire occurrence in this ecosystem is low frequency and mixed severity. In the absence of wind, fires are likely limited in extent (two acres or less). Understories are often sparse, with little to carry fires across the surface (Landfire 2007a). Stand‑replacement fires are usually wind‑driven, especially in mid‑ and late‑serial classes. Landfire (2007a) review estimated replacement fires occurring between 35‑100+ years and 200+ years (Fire Regime Groups IV and V) with surface fires occurring every 1000 years. However, in northern New Mexico, some open stands transition into subalpine grasslands and have more frequent, less severe fires (Coop and Schoettle 2011).

SOURCES

**References:** Baker 1992, Baumeister and Callaway 2006, Beasley and Klemmedson 1980, Brunstein and Yamaguchi 1992, CNHP 2010, Comer et al. 2003*, Coop and Schoettle 2011, Eyre 1980, Gibson et al. 2008, Keane et al. 2011, Knight 1994, Krebs 1972, LANDFIRE 2007a, LaMarche and Mooney 1972, Lanner 2007, Lanner and Vander Wall 1980, McKinney et al. 2007, NCC 2002, Neely et al. 2001, Ranne 1995, Ranne et al. 1997, Schoettle and Sniezko 2007a, Schoettle et al. 2008, Steele et al. 1983, TNC 2013, Tomback 2001

**Version:** 14 Jan 2014	**Stakeholders:** Canada, West

**Concept Author:** M.S. Reid	**LeadResp:** West

M021. Sierra Madre High Montane Forest

CES305.283 Bosque Subalpino de Oyamel de la Sierra Madre

**Primary Division:** Sierra Madre (305)

**Land Cover Class:** Forest and Woodland

**Spatial Scale & Pattern:** Large patch

**Required Classifiers:** Natural/Semi‑natural; Vegetated (>10% vasc.); Upland

**Diagnostic Classifiers:** Alpine/AltiAndino [Alpine/AltiAndino]; Forest and Woodland (Treed); Ustic

**Concept Summary:** This system occurs on high volcanic and montane slopes from the southern Sierra Madre Occidental, Transvolcanic ranges, and high mountain slopes extending into northern Central America. These forests are dominated by *Pinus hartwegii* (Hartweg's pine), but other pines, alder, and evergreen oaks intermingle with patchy shrublands on most upper‑elevation slopes (3350‑3570 m elevation). The following list of species is diagnostic for this system: *Abies religiosa, Abies concolor, Abies guatemalensis, Pinus* spp., *Cupressus* spp., *Quercus* spp., *Pseudotsuga* spp., *Alnus* spp., *Arbutus* spp., *Salix* spp., *Senecio angulifolius, Roldana barba‑johannis*, and *Senecio toluccanus*.

DISTRIBUTION

**Range:** This system occurs on high volcanic and montane slopes from the southern Sierra Madre Occidental, Transvolcanic ranges, and high mountain slopes extending into northern Central America.

**Divisions:** 305:C

**Nations:** GT, MX

**Subnations:** MXGU, MXJA?, MXMI, MXOA

CONCEPT

**Environment:** This is most common on steep outer volcanic slopes between 3000‑3500 m elevation. These areas typically include deep soils with a thick litter layer.

**Vegetation:** These typically include a dense overstory up to 30 m high, and shrub‑herb layer (0.5‑3 m high), and a dense ground layer of rosaceous herbs and mosses.

**Dynamics:** Natural disturbance regimes in these forests are not well‑documented.

SOURCES

**References:** INEGI 2005, Josse et al. 2003*, Rzedowski 1986, Velazquez et al. 2000

**Version:** 08 Jan 2015	**Stakeholders:** Latin America

**Concept Author:** C. Josse	**LeadResp:** Latin America

CES403.327 Bosque Subalpino de Pino de la Sierra Madre

**Primary Division:** Meso‑American Seasonal Highlands (403)

**Land Cover Class:** Forest and Woodland

**Spatial Scale & Pattern:** Large patch

**Required Classifiers:** Natural/Semi‑natural; Vegetated (>10% vasc.); Upland

**Diagnostic Classifiers:** Alpine/AltiAndino [Alpine/AltiAndino]; Forest and Woodland (Treed); Ustic

**Concept Summary:** This system occurs on high volcanic and montane slopes from the southern Sierra Madre Occidental, Transvolcanic ranges, and high mountain slopes extending into northern Central America. These forests are dominated by *Pinus hartwegii*, but other pines, alder, and evergreen oaks intermingle with patchy shrublands on most upper‑elevation slopes (3350‑3570 m). La siguiente lista de las especies es de diagnóstica para este sistema: *Pinus hartwegii, Pinus montezumae, Alnus firmifolia, Quercus laurina, Lupinus* spp., *Muhlenbergia* spp., *Calamagrostis* spp.

DISTRIBUTION

**Divisions:** 403:C

**Nations:** GT, MX

**Subnations:** MXCP, MXGU, MXJA?, MXMI, MXOA

CONCEPT

**Environment:** This is most common on volcanic slopes below subalpine pine forest. Most often, they are best developed in canyons or below escarpments between lava flows where they are somewhat protected from direct solar exposure and wind. Soils are typically rich in ash and organic matter.

**Vegetation:** These typically include an overstory up to 20 m high, and a dense ground layer of herbs and bunchgrasses.

**Dynamics:** Hartweg's pine is presumed to be late‑seral. Forests of similar composition often invade severly burned fir forests.

SOURCES

**References:** INEGI 2005, Josse et al. 2003*, Rzedowski 1986, Velazquez et al. 2000

**Version:** 08 Jan 2015	**Stakeholders:** Latin America

**Concept Author:** C. Josse	**LeadResp:** Latin America

M022. Southern Rocky Mountain Lower Montane Forest

CES306.823 Southern Rocky Mountain Dry‑Mesic Montane Mixed Conifer Forest and Woodland

**Primary Division:** Rocky Mountain (306)

**Land Cover Class:** Forest and Woodland

**Spatial Scale & Pattern:** Matrix

**Required Classifiers:** Natural/Semi‑natural; Vegetated (>10% vasc.); Upland

**Diagnostic Classifiers:** Montane [Montane, Lower Montane]; Forest and Woodland (Treed); Aridic; Intermediate Disturbance Interval; F‑Patch/Medium Intensity; F‑Landscape/Medium Intensity; Needle‑Leaved Tree; RM Montane Mesic Mixed Conifer; Moderate (100‑500 yrs) Persistence

**National Mapping Codes:** EVT 2051; ESLF 4238; ESP 1051

**Concept Summary:** This is a highly variable ecological system of the montane zone of the Rocky Mountains. It occurs throughout the southern Rockies, north and west into Utah, Nevada, Wyoming and Idaho. These are mixed‑conifer forests occurring on all aspects at elevations ranging from 1200 to 3300 m. Rainfall averages less than 75 cm per year (40‑60 cm), with summer "monsoons" during the growing season contributing substantial moisture. The composition and structure of the overstory are dependent upon the temperature and moisture relationships of the site and the successional status of the occurrence. *Pseudotsuga menziesii* and *Abies concolor* are most frequent, but *Pinus ponderosa* may be present to codominant. *Pinus flexilis* is common in Nevada. *Pseudotsuga menziesii* forests occupy drier sites, and *Pinus ponderosa* is a common codominant. *Abies concolor*‑dominated forests occupy cooler sites, such as upper slopes at higher elevations, canyon sideslopes, ridgetops, and north‑ and east‑facing slopes which burn somewhat infrequently. *Picea pungens* is most often found in cool, moist locations, often occurring as smaller patches within a matrix of other associations. As many as seven conifers can be found growing in the same occurrence, and there are a number of cold‑deciduous shrub and graminoid species common, including *Arctostaphylos uva‑ursi, Mahonia repens, Paxistima myrsinites, Symphoricarpos oreophilus, Jamesia americana, Quercus gambelii*, and *Festuca arizonica*. This system was undoubtedly characterized by a mixed‑severity fire regime in its "natural condition," characterized by a high degree of variability in lethality and return interval.

**Comments:** The transition between this system and Middle Rocky Mountain Montane Douglas‑fir Forest and Woodland (CES306.959) in Wyoming needs to be further clarified, both in terms of floristics and distribution details. For now, it is assumed that this system does not occur in the Bighorn Range or in the Yellowstone region, but its occurrence in isolated ranges of central and western Wyoming is possible.

DISTRIBUTION

**Range:** This system occurs throughout the southern Rockies, north and west into Utah, Nevada, eastern Wyoming (very southern in the Laramie Range and possibly on Sheep Mountain) and Idaho. Although not common, it does occur in southeastern Oregon but does not extend farther west into the Cascades.

**Divisions:** 304:C, 306:C

**TNC Ecoregions:** 6:?, 7:?, 8:?, 9:C, 11:C, 18:C, 19:C, 20:C, 21:C, 26:C

**Nations:** US

**Subnations:** AZ, CO, ID, NM, NV, OR, UT, WY

**Map Zones:** 6:P, 9:?, 12:C, 13:C, 15:C, 16:C, 17:C, 18:C, 21:?, 22:C, 23:C, 24:P, 25:C, 27:C, 28:C, 29:C, 33:?

**USFS Ecomap Regions:** 313A:CC, 313B:CC, 313C:CC, 313D:CP, 315A:C?, 315H:CC, 321A:??, 322A:CC, 331B:CC, 331G:CC, 331H:CC, 331I:CC, 331J:CC, 331N:CP, 341A:CC, 341B:CC, 341C:CP, 341D:CC, 341E:CC, 341F:CC, 341G:CP, 342A:CC, 342B:CC, 342C:CC, 342D:CC, 342E:CC, 342F:CC, 342G:CC, 342H:CC, 342I:CC, 342J:CC, M313A:CC, M313B:CC, M331A:CP, M331B:CC, M331D:CC, M331E:CC, M331F:CC, M331G:CC, M331H:CC, M331I:CC, M331J:CC, M332A:C?, M332E:CP, M332G:CC, M341A:CC, M341B:CC, M341C:CC, M341D:CC

CONCEPT

**Environment:** These are mixed‑conifer forests occurring on all aspects at elevations ranging from 1200 to 3300 m. Landforms are variable and can include canyons, plateaus, draws, benches, hills, mesas, ravines, shoulders, sideslopes and toeslopes. Slopes can be gentle to extremely steep. Rainfall averages less than 75 cm per year (40‑60 cm), with summer "monsoons" during the growing season contributing substantial moisture. Geologic substrates include volcanic andesite, rhyolite, rhyolitic tuffs, colluvium, shale gneiss, granite, sandstone and limestone. Soils are variable from cobbles, clay loam, silt loam, sandy loam, sand, and gravel.

**Vegetation:** This highly variable ecological system comprises mixed‑conifer forests at montane elevations throughout the Intermountain West region. The four main alliances in this system are found on slightly different, but intermingled, biophysical environments: *Abies concolor* dominates at higher, colder locations; *Picea pungens* represents mesic conditions; and *Pseudotsuga menziesii* dominates intermediate zones. As many as seven conifers can be found growing in the same occurrence, with the successful reproduction of the diagnostic species determining the association type. Common conifers include *Pinus ponderosa, Pinus flexilis, Abies lasiocarpa var. lasiocarpa, Abies lasiocarpa var. arizonica, Juniperus scopulorum*, and *Picea engelmannii*. *Populus tremuloides* is often present as intermingled individuals in remnant aspen clones or in adjacent patches. The composition and structure of the overstory are dependent upon the temperature and moisture relationships of the site and the successional status of the occurrence (DeVelice et al. 1986, Muldavin et al. 1996).

Several cold‑deciduous shrub and graminoid species are found in many occurrences (e.g., *Arctostaphylos uva‑ursi, Mahonia repens, Paxistima myrsinites, Symphoricarpos oreophilus, Jamesia americana, Quercus gambelii*, and *Festuca arizonica*). Other important species include *Acer glabrum, Acer grandidentatum, Amelanchier alnifolia, Arctostaphylos patula, Holodiscus dumosus, Jamesia americana, Juniperus communis, Physocarpus monogynus, Quercus arizonica, Quercus rugosa, Quercus x pauciloba, Quercus hypoleucoides, Robinia neomexicana, Rubus parviflorus*, and *Vaccinium myrtillus*. Where soil moisture is favorable, the herbaceous layer may be quite diverse, including graminoids *Bromus ciliatus (= Bromus canadensis), Calamagrostis rubescens, Carex geyeri, Carex rossii, Carex siccata (= Carex foenea), Festuca occidentalis, Koeleria macrantha, Muhlenbergia montana, Muhlenbergia straminea (= Muhlenbergia virescens), Poa fendleriana, Pseudoroegneria spicata*, and forbs *Achillea millefolium, Arnica cordifolia, Erigeron eximius, Fragaria virginiana, Linnaea borealis, Luzula parviflora, Osmorhiza berteroi, Packera cardamine (= Senecio cardamine), Thalictrum occidentale, Thalictrum fendleri, Thermopsis rhombifolia, Viola adunca*, and species of many other genera, including *Lathyrus, Penstemon, Lupinus, Vicia, Arenaria, Galium*, and others.

**Dynamics:** Forests in this ecological system represent the gamut of fire tolerance. Formerly, *Abies concolor* in the Utah High Plateaus were restricted to rather moist or less fire‑prone areas by frequent surface fires. These areas experienced mixed fire severities, with patches of crowning in which all trees are killed, intermingled with patches of underburn in which larger *Abies concolor* survived (www.fs.fed.us/database/feis/). With fire suppression, *Abies concolor* has vigorously colonized many sites formerly occupied by open *Pinus ponderosa* woodlands. These invasions have dramatically changed the fuel load and potential behavior of fire in these forests. In particular, the potential for high‑intensity crown fires on drier sites now codominated by *Pinus ponderosa* and *Abies concolor* has increased. Increased landscape connectivity, in terms of fuel loadings and crown closure, has also increased the potential size of crown fires.

*Pseudotsuga menziesii* forests are the only true "fire‑tolerant" occurrences in this ecological system. *Pseudotsuga menziesii* forests were probably subject to a moderate‑severity fire regime in presettlement times, with fire‑return intervals of 30‑100 years. Many of the important tree species in these forests are fire‑adapted (*Populus tremuloides, Pinus ponderosa, Pinus contorta*) (Pfister et al. 1977), and fire‑induced reproduction of *Pinus ponderosa* can result in its continued codominance in *Pseudotsuga menziesii* forests (Steele et al. 1981). Seeds of the shrub *Ceanothus velutinus* can remain dormant in forest occurrences for 200 years (Steele et al. 1981) and germinate abundantly after fire, competitively suppressing conifer seedlings. Successional relationships in this system are complex. *Pseudotsuga menziesii* is less shade‑tolerant than many northern or montane trees such as *Tsuga heterophylla, Abies concolor, Picea engelmannii*, and seedlings compete poorly in deep shade. At drier locales, seedlings may be favored by moderate shading, such as by a canopy of *Pinus ponderosa*, which helps to minimize drought stress. In some locations, much of these forests have been logged or burned during European settlement, and present‑day occurrences are second‑growth forests dating from fire, logging, or other occurrence‑replacing disturbances (Mauk and Henderson 1984, Chappell et al. 1997).

*Picea pungens* is a slow‑growing, long‑lived tree which regenerates from seed (Burns and Honkala 1990a). Seedlings are shallow‑rooted and require perennially moist soils for establishment and optimal growth. *Picea pungens* is intermediate in shade tolerance, being somewhat more tolerant than *Pinus ponderosa* or *Pseudotsuga menziesii*, and less tolerant than *Abies lasiocarpa* or *Picea engelmannii*. It forms late‑seral occurrences in the subhumid regions of the Utah High Plateaus. It is common for these forests to be heavily disturbed by grazing or fire.

In general, fire suppression has lead to the encroachment of more shade‑tolerant, less fire‑tolerant species (e.g., climax) into occurrences and an attendant increase in landscape homogeneity and connectivity (from a fuels perspective). This has increased the lethality and potential size of fires.

<br />LANDFIRE developed a state‑and‑transition vegetation dynamics VDDT model for this system which has five classes in total (LANDFIRE 2007a, BpS 2810510). These are summarized as:

A) Early Development 1 All Structures (15% of type in this stage): Shrub cover is 0‑80%. Succession after a lethal fire will depend on what vegetation was on site before. In a general conifer‑dominated scenario, some ponderosa pines are likely to survive. Fire will be an opportunity for new ponderosa pine establishment. On site Gambel oak will resprout. White fir will also be regenerating. If aspen cover is 50‑100% prior to disturbance, the stand would regenerate back to aspen.

B) Mid Development 1 Closed (tree‑dominated ‑ 15% of type in this stage): Tree cover is 51‑80%. If aspen is dominant the stand will achieve a mid‑closed stage. Conifers such as white fir and Douglas‑fir could be regenerating with it. Any surviving conifers such as ponderosa pine would be canopy dominants. If aspen canopy cover is 50‑100%.

C) Mid Development 1 Open (tree‑dominated ‑ 10% of type in this stage): Tree cover is 21‑50%. Ponderosa pine is the canopy dominant with an understory dominated by white fir. Douglas‑fir present and some of its regeneration is entering the canopy. If aspen were present, the stand would have undergone some self‑thinning that would have opened up the canopy. The conifers in the stand create a more flammable litter bed with their needles so that patchy surface fire could carry. Any fire would further open the stand by thinning aspen and fir. Eventually the aspen stand would become very open sharing the canopy with ponderosa pine and Douglas‑fir.

D) Late Development 1 Open (conifer‑dominated ‑ 50% of type in this stage): Tree cover is 21‑50%. Ponderosa pine is the canopy dominant. Douglas‑fir can also be a canopy dominant. Recurrent fire maintains white fir as an understory tree, but a rare white fir will join the other two species in the canopy. If aspen is present, its numbers are few. Low levels of suckering may keep it in the stand. Open aspen stands are not common in the warm/dry mixed conifer.

E) Late Development 1 Closed (tree‑dominated ‑ 10% of type in this stage): Tree cover is 51‑80%. Aspen stand is mature to over‑mature with a heavy understory of conifers, mainly white fir and some Douglas‑fir.

This BpS has a fire regime very similar to ponderosa pine. Frequent low‑intensity surface fire is the dominant mode of disturbance. Fire intervals range from 2‑71 years with a mean of 15 years. Lethal fires can occur on a limited scale, but this is not the norm unless aspen is involved. These will be characterized as mixed fires because they most likely occur as a part of a more widespread surface fire. Bark beetles may impact this BpS in isolated areas at small scales (LANDFIRE 2007a, BpS 2810510).

Nutrient cycling, specifically carbon cycling, is an important ecological process within many ecological systems. Biological decomposition in ponderosa pine forests is more limited than biological production, resulting in accumulation of organic materials, especially in the absence of fire (Harvey 1994, Graham and Jain 2005).

SOURCES

**References:** Alexander et al. 1984b, Alexander et al. 1987, Amman 1977, Anderson 2003b, Boyce 1977, Bunin 1975c, Burns and Honkala 1990a, CNHP 2010, Chappell et al. 1997, Comer et al. 2002, Comer et al. 2003*, Dale et al. 2001, DeVelice et al. 1986, Eyre 1980, Fitzhugh et al. 1987, Garfin et al. 2014, Giese 1975, Graham and Jain 2005, Harvey 1994, Heinze et al. 1962, Hess 1981, Hess and Alexander 1986, Hess and Wasser 1982, Hoffman and Alexander 1980, Hoffman and Alexander 1983, Howard 2003b, Howard 2003c, Johnson 2001, Kaufmann et al. 2001, Komarkova et al. 1988b, LANDFIRE 2007a, Mauk and Henderson 1984, McKenzie et al. 2004, McKenzie et al. 2008, Mote et al. 2014, Muldavin et al. 1996, Nachlinger et al. 2001, Neely et al. 2001, Shafer et al. 2014, Steele et al. 1983, Steinberg 2002e, Stevens‑Rumann et al. 2017, Tuhy et al. 2002, Westerling et al. 2006, Youngblood and Mauk 1985, Zouhar 2001a

**Version:** 23 May 2018	**Stakeholders:** West

**Concept Author:** M.S. Reid	**LeadResp:** West

CES306.825 Southern Rocky Mountain Mesic Montane Mixed Conifer Forest and Woodland

**Primary Division:** Rocky Mountain (306)

**Land Cover Class:** Forest and Woodland

**Spatial Scale & Pattern:** Large patch

**Required Classifiers:** Natural/Semi‑natural; Vegetated (>10% vasc.); Upland

**Diagnostic Classifiers:** Forest and Woodland (Treed); Ravine; Stream terrace (undifferentiated); Toeslope; Mesotrophic Soil; Ustic; Long Disturbance Interval; F‑Patch/Low Intensity; F‑Landscape/Low Intensity; Needle‑Leaved Tree; RM Montane Dry‑Mesic Mixed Conifer

**National Mapping Codes:** EVT 2052; ESLF 4239; ESP 1052

**Concept Summary:** These are mixed conifer forests of the Rocky Mountains west into the ranges of the Great Basin, occurring predominantly in cool ravines and on north‑facing slopes. Elevations range from 1200 to 3300 m. Occurrences of this system are found on cooler and more mesic sites than Southern Rocky Mountain Dry‑Mesic Montane Mixed Conifer Forest and Woodland (CES306.823). Such sites include lower and middle slopes of ravines, along stream terraces, moist, concave topographic positions and north‑ and east‑facing slopes which burn somewhat infrequently. *Pseudotsuga menziesii* and *Abies concolor* are most common canopy dominants, but *Picea engelmannii, Picea pungens*, or *Pinus ponderosa* may be present. This system includes mixed conifer ‑ *Populus tremuloides* stands. A number of cold‑deciduous shrub species can occur, including *Acer glabrum, Acer grandidentatum, Alnus incana, Betula occidentalis, Cornus sericea, Jamesia americana, Physocarpus malvaceus, Robinia neomexicana, Vaccinium membranaceum*, and *Vaccinium myrtillus*. Herbaceous species include *Bromus ciliatus, Carex geyeri, Carex rossii, Carex siccata, Muhlenbergia straminea, Pseudoroegneria spicata, Erigeron eximius, Fragaria virginiana, Luzula parviflora, Osmorhiza berteroi, Packera cardamine, Thalictrum occidentale*, and *Thalictrum fendleri*. Naturally occurring fires are of variable return intervals and mostly light, erratic, and infrequent due to the cool, moist conditions.

**Comments:** This system will need to be modeled to separate from similar dry‑mesic system.

DISTRIBUTION

**Range:** This system is found in the southern Rocky Mountains of Arizona and New Mexico north and west into the ranges of the Great Basin, Wyoming and southeastern Idaho, occurring predominantly in cool ravines and on north‑facing slopes.

**Divisions:** 304:C, 306:C

**TNC Ecoregions:** 8:C, 9:C, 11:C, 18:C, 19:C, 20:C, 21:C

**Nations:** US

**Subnations:** AZ, CO, ID, NM, NV, OR?, UT, WY

**Map Zones:** 6:?, 9:?, 12:P, 13:C, 15:C, 16:C, 17:P, 18:C, 21:?, 22:P, 23:C, 24:P, 25:C, 27:C, 28:C, 29:P

**USFS Ecomap Regions:** 313A:CC, 313B:CC, 313C:CC, 313D:CP, 315A:C?, 315H:CC, 321A:??, 322A:CC, 331B:CC, 331G:C?, 331H:CP, 331I:CC, 331J:CC, 341A:CC, 341B:CC, 341C:CP, 341D:CC, 341F:CC, 342A:CP, 342B:CP, 342D:CP, 342E:CC, 342F:CP, 342G:CP, 342H:CC, 342I:C?, 342J:CP, M313A:CC, M313B:CC, M331A:CP, M331B:CC, M331D:CC, M331E:CC, M331F:CC, M331G:CC, M331H:CC, M331I:CC, M331J:CC, M332G:CC, M341A:CC, M341B:CC, M341C:CC, M341D:CC

CONCEPT

**Environment:** This system includes conifer, mixed conifer, and some deciduous montane forests of the southern Rocky Mountains west into the ranges of the Great Basin. Stands occur predominantly in cool ravines and on north‑facing slopes with elevations from 1200 to 3300 m. Occurrences of this system are found on cooler and more mesic sites than those in Southern Rocky Mountain White Fir ‑ Douglas‑fir Dry Forest Group (G226). Such sites include lower and middle slopes of ravines, along stream terraces, moist, concave topographic positions, and north‑ and east‑facing slopes. Naturally occurring fires are of variable return intervals and mostly light, erratic, and infrequent due to the cool, moist conditions.

**Vegetation:** *Pseudotsuga menziesii* and *Abies concolor* are most common canopy dominants, but *Picea engelmannii, Picea pungens*, or *Pinus ponderosa* may be present. This system includes mixed conifer ‑ *Populus tremuloides* stands. Several cold‑deciduous shrub species can occur, including *Acer glabrum, Acer grandidentatum, Alnus incana, Betula occidentalis, Cornus sericea, Jamesia americana, Physocarpus malvaceus, Robinia neomexicana, Vaccinium membranaceum*, and *Vaccinium myrtillus*. Herbaceous species include *Bromus ciliatus, Carex geyeri, Carex rossii, Carex siccata, Muhlenbergia straminea (= Muhlenbergia virescens), Pseudoroegneria spicata, Erigeron eximius, Fragaria virginiana, Luzula parviflora, Osmorhiza berteroi, Packera cardamine, Thalictrum occidentale*, and *Thalictrum fendleri*.

**Dynamics:** Fire is the primary disturbance although insects can also play a major role especially in tree‑gap dynamics. Fire frequencies are variable with a mixed‑severity fire regime in the relatively cool/moist environments where this system occurs. In the absence of stand‑replacing disturbance such as fire, this mesic mixed conifer and aspen forest system will slowly convert to forests dominated by more shade‑tolerant trees such as *Picea pungens* and *Abies concolor*. However, these forests are linked to smaller, gap‑forming disturbances, such as mixed‑severity fire or windthrow facilitated by insect outbreaks and disease. These gaps allow regeneration of *Populus tremuloides* and other less shade‑tolerant species such as *Pinus ponderosa* and *Pseudotsuga menziesii* and limits the abundances of *Abies concolor* (Mueggler and Campbell 1986, Mueggler 1988).

<br />LANDFIRE developed a state‑and‑transition vegetation dynamics VDDT model for this system which has five classes in total (LANDFIRE 2007a, BpS 2810520). These are summarized as:

A) Early Development 1 All Structures (10% of type in this stage): Post‑lethal fire vegetation will depend on what was on site before it burned. Aspen may or may not be present, depending on what was present prior to the fire or other replacement disturbance. The site will start as grass/forb/shrub; aspen may also be present. Fire will maintain or prolong this stage. Conifers may be present. Any surviving conifers will be seed source. This class may look like a pure aspen stand from above.

B) Mid Development 1 Closed (tree‑dominated ‑ 40% of type in this stage): Tree cover is 41‑100%. If present, aspen will be over 10 feet tall and very dense. Seedling‑medium‑sized conifers can be found mixed with aspen, if present. Understory may include mountain snowberry, common juniper, wild rose, and many species of grasses and forbs.

<br />C) Mid Development 1 Open (tree‑dominated ‑ 25% of type in this stage): Tree cover is 11‑40%. If present, aspen will be over 10 feet tall and patchy. Seedling‑medium‑sized conifers can be found mixed with aspen, if present. Understory may include mountain snowberry, common juniper, wild rose, and many species of grasses and forbs. Canopy cover is low.

D) Late Development 1 Open (tree‑dominated ‑ 10% of type in this stage): Tree cover is 11‑40%. Aspen will be rare and mid‑level. Understory will be sparse.<br />

E) Late Development 1 Closed (tree‑dominated ‑ 15% of type in this stage): Tree cover is 41‑100%. Dense conifer stand. Blue spruce and subalpine fir can come in. Aspen present in small amounts. Lots of dead and downed material. Understory possibly depauperate.

Fire is the primary disturbance although insects can also play a major role. Fire frequencies are variable and the cool/moist conditions support a mixed fire regime. Mixed‑severity fires occurred every 6‑60 years. Lethal fires are usually at longer intervals, 100+ years (LANDFIRE 2007a, BpS 2810520).

Nutrient cycling, specifically carbon cycling, is an important ecological process within many ecological systems. Biological decomposition in ponderosa pine forests is more limited than biological production, resulting in accumulation of organic materials, especially in the absence of fire (Harvey 1994, Graham and Jain 2005).

SOURCES

**References:** Agree 1982, Alexander et al. 1984a, Alexander et al. 1984b, Alexander et al. 1987, Amman 1977, Anderson 1999a, Anderson 2003b, Boyce 1977, Bunin 1975c, Burns and Honkala 1990a, CNHP 2010, Comer et al. 2002, Comer et al. 2003*, Cooper et al. 1987, Dale et al. 2001, DeVelice and Ludwig 1983c, DeVelice et al. 1986, Dieterich 1979, Eyre 1980, Fitzhugh et al. 1987, Fowells 1965, Garfin et al. 2014, Giese 1975, Graham and Jain 2005, Harvey 1994, Heinze et al. 1962, Hess 1981, Hess and Alexander 1986, Hess and Wasser 1982, Hoffman and Alexander 1980, Hoffman and Alexander 1983, Hopkins 1982, Howard 2003b, Howard 2003c, Komarkova et al. 1988b, LANDFIRE 2007a, Mauk and Henderson 1984, McKenzie et al. 2004, McKenzie et al. 2008, Moir and Ludwig 1979, Mueggler 1988, Mueggler and Campbell 1986, Nachlinger et al. 2001, Neely et al. 2001, Parson and DeBenedetti 1979, Pavek 1993d, Pfister 1972, Steinberg 2002e, Stevens‑Rumann et al. 2017, Tuhy et al. 2002, Westerling et al. 2006, Youngblood and Mauk 1985, Zouhar 2001a

**Stakeholders:** Midwest, West

**Concept Author:** M.S. Reid	**LeadResp:** West

CES306.649 Southern Rocky Mountain Ponderosa Pine Savanna

**Primary Division:** Rocky Mountain (306)

**Land Cover Class:** Steppe/Savanna

**Spatial Scale & Pattern:** Large patch

**Required Classifiers:** Natural/Semi‑natural; Vegetated (>10% vasc.); Upland

**Diagnostic Classifiers:** Woody‑Herbaceous; Shallow Soil; Aridic; Short Disturbance Interval; F‑Patch/Low Intensity; F‑Landscape/Low Intensity; Needle‑Leaved Tree; Graminoid; Pinus ponderosa with grassy understory

**National Mapping Codes:** EVT 2117; ESLF 5406; ESP 1117

**Concept Summary:** This ecological system is found predominantly in the Colorado Plateau region, west into scattered locations in the Great Basin, and north along the eastern front of the southern Rocky Mountains into southeastern Wyoming. These savannas occur at the lower treeline/ecotone between grassland/or shrubland and more mesic coniferous forests typically in warm, dry, exposed sites. Elevations range from less than 1900 m in central and northern Wyoming to 2800 m in the New Mexico mountains to well over 2700 m on the higher plateaus of the Southwest. It is found on rolling plains, plateaus, or dry slopes usually on more southerly aspects. This system is best described as a savanna that has widely spaced (<25% tree canopy cover) (>150 years old) *Pinus ponderosa* (primarily *var. scopulorum* and *var. brachyptera*) as the predominant conifer. It is maintained by a fire regime of frequent, low‑intensity surface fires. A healthy occurrence often consists of open and park‑like stands dominated by *Pinus ponderosa*. Understory vegetation in the true savanna occurrences is predominantly fire‑resistant grasses and forbs that resprout following surface fires; shrubs, understory trees and downed logs are uncommon. Important and often dominant species include *Festuca arizonica, Koeleria macrantha, Muhlenbergia montana, Muhlenbergia straminea*, and *Pseudoroegneria spicata*. Other important grasses, such as *Andropogon gerardii, Bouteloua gracilis, Elymus elymoides, Festuca idahoensis, Piptatheropsis micrantha*, and *Schizachyrium scoparium*, dominate less frequently. A century of anthropogenic disturbance and fire suppression has resulted in a higher density of *Pinus ponderosa* trees, altering the fire regime and species composition. Presently, many stands contain understories of more shade‑tolerant species, such as *Pseudotsuga menziesii* and/or *Abies* spp., as well as younger cohorts of *Pinus ponderosa*. Northern Rocky Mountain Ponderosa Pine Woodland and Savanna (CES306.030) in the eastern Cascades, Okanogan, and Northern Rockies regions receives winter and spring rains, and thus has a greater spring "green‑up" than the drier woodlands in the Central Rockies.

**Comments:** The Pine Escarpment regions of northwestern and central Nebraska are no longer included within this system; they have been lumped into Northwestern Great Plains‑Black Hills Ponderosa Pine Woodland and Savanna (CES303.650). Because this ecological system has undergone some important changes in its concept, the original system (CES306.826) was archived, and this new system was created to account for the new concept of ponderosa pine savannas in the southern Rocky Mountains.

The FRIS site describes different varieties of *Pinus ponderosa* and associated species. This system is mostly *Pinus ponderosa var. scopulorum* and *Pinus ponderosa var. brachyptera*. Johansen and Latta (2003) have mapped the distribution of two varieties (vars. *scopulorum* and *ponderosa*) using mitochondrial DNA. Hybridization along the Continental Divide in Montana backs up the FRIS information.

DISTRIBUTION

**Range:** This ecological system is found predominantly in the Colorado Plateau region, west into scattered locations of the Great Basin, and north along the eastern front of the Rocky Mountains of Colorado and Wyoming. Pine woodlands and savannas of the Black Hills and central Montana are now included in Northwestern Great Plains‑Black Hills Ponderosa Pine Woodland and Savanna (CES303.650), as are woodlands and savannas in Nebraska and northeastern Colorado.

**Divisions:** 303:C, 304:C, 306:C

**TNC Ecoregions:** 18:C, 19:C, 20:C, 21:C, 26:P

**Nations:** US

**Subnations:** AZ, CO, NM, NV, UT, WY

**Map Zones:** 15:C, 16:?, 22:C, 23:C, 24:C, 25:C, 26:C, 27:C, 28:P, 29:C, 33:P

**USFS Ecomap Regions:** 315A:CC, 315B:CC, 315H:CP, 321A:PP, 331B:CC, 331G:C?, 331H:CC, 331I:CC, 331J:CP, 342F:CC, M313B:PP, M331B:CC, M331F:CC, M331G:CP, M331I:CC

CONCEPT

**Environment:** These savannas occur at the lower elevation ecotone between pinyon conifer woodlands, grassland/or shrubland and upper elevation, more mesic coniferous forests typically in warm, dry, exposed sites. Elevations range from less than 1900 m in central and northern Wyoming to 2800 m in the New Mexico mountains to well over 2700 m on the higher plateaus of the Southwest. It is found on rolling plains, plateaus, or dry slopes usually on more southerly aspects; however, it can occur on all slopes and aspects. Stands occur on soils derived from igneous, metamorphic, and sedimentary material, including basalt, andesite, intrusive granitoids and porphyrites, and tuffs (Youngblood and Mauk 1985). Characteristic soil features include good aeration and drainage, coarse textures, circumneutral to slightly acidic pH, an abundance of mineral material, and periods of drought during the growing season. Surface textures are highly variable in this ecological system ranging from sand to loam and silt loam. Exposed rock and bare soil consistently occur to some degree in all the associations. Annual precipitation is 25‑60 cm (8‑24 inches), mostly through winter storms and some monsoonal summer rains. Typically, a seasonal drought period occurs throughout this system distribution as well.

**Vegetation:** This system is best described as a savanna that has widely spaced (<25% tree canopy cover) (>150 years old) *Pinus ponderosa* (primarily *var. scopulorum* and *var. brachyptera*) as the predominant conifer. It is maintained by a fire regime of frequent, low‑intensity surface fires. A healthy occurrence often consists of open and park‑like stands dominated by *Pinus ponderosa*. Understory vegetation in the true savanna occurrences is predominantly fire‑resistant grasses and forbs that resprout following surface fires; shrubs, understory trees and downed logs are uncommon. Important and often dominant species include *Festuca arizonica, Koeleria macrantha, Muhlenbergia montana, Muhlenbergia straminea (= Muhlenbergia virescens)*, and *Pseudoroegneria spicata*. Other important grasses, such as *Andropogon gerardii, Bouteloua gracilis, Elymus elymoides, Festuca idahoensis, Piptatheropsis micrantha (= Piptatherum micranthum)*, and *Schizachyrium scoparium*, dominate less frequently. A century of anthropogenic disturbance and fire suppression has resulted in a higher density of *Pinus ponderosa* trees, altering the fire regime and species composition.

**Dynamics:** *Pinus ponderosa* is a drought‑resistant, typically open‑grown conifer, which usually occurs at lower treeline in the major ranges of the western United States. Mature trees have thick bark that protects the cambium layer from fire. Historically, fires and drought were influential in maintaining open‑canopy conditions in these woodlands. Low‑intensity surface fire would burn through these stands every 5‑15 year, killing young trees, but not the fire‑resistant mature ponderosa pine trees or grass understory maintaining an open park‑like stand (Harrington and Sackett 1992, Mehl 1992, Swetnam and Baisan 1996). Infrequent stand‑replacement fire on the order of a few hundred years (300‑500 years) is possible (LANDFIRE 2007a). Drought and other weather events (e.g., blowdown), parasites and disease may play a minor role, and have very long rotations (LANDFIRE 2007a). Impacts from insects such as mountain pine beetles (*Dendroctonus ponderosae*) may be significant during outbreaks, but infrequent in occurrence (LANDFIRE 2007a). Beetles attack less vigorously growing trees, e.g., old, crowded, diseased, damaged, or growing on poor sites) especially during droughts (Leatherman et al. 2013). Winter mortality of beetles is a significant factor; however, a severe freeze of at least ‑30 degrees F is necessary for at least five days during midwinter (Leatherman et al. 2013).

LANDFIRE developed a state‑and‑transition vegetation dynamics VDDT model for this system which has five classes in total (LANDFIRE 2007a, BpS 2811170). These are summarized as:

A) Early Development 1 All Structures (Shrub‑dominated ‑ 10% of type in this stage): Bunchgrass‑dominated (0‑49 years). Some ponderosa pine individuals also becoming established.

B) Mid Development 1 Closed (tree‑dominated ‑ 5% of type in this stage): Small and medium‑sized ponderosa pine (50‑149 years), still with high bunchgrass cover. Closed canopy defined as >50%.

C) Mid Development 1 Open (tree‑dominated ‑ 20% of type in this stage): Small and medium‑sized ponderosa pine (50‑149 years), with moderate bunchgrass cover. Open canopy defined as 10‑49%.

D) Late Development 1 Open (conifer‑dominated ‑ 60% of type in this stage): Large and very large old‑growth ponderosa pine, with medium to high cover of bunchgrasses. Old‑growth attributes prominent, including downed wood, snags and diseased trees.

<br />E) Late Development 1 Open (conifer‑dominated ‑ 5% of type in this stage): Large and very large old‑growth ponderosa pine, with medium cover of bunchgrasses. Old‑growth attributes prominent, including downed wood, snags and diseased trees.

Mean composite surface fire intervals have been found to be 5‑15 years (Swetnam and Baisan 1996a). Infrequent stand‑replacement fire on the order of a few hundred years possible (300‑500 years?). Drought and other weather events (e.g., blowdown), parasites and disease may play a minor role, and have very long rotations. Insects may be a significant, but infrequent occurrence (LANDFIRE 2007a, BpS 2811170).

Nutrient cycling, specifically carbon cycling, is an important ecological process within many ecological systems. However, biological decomposition in ponderosa pine forests is more limited than biological production, resulting in accumulation of organic materials, especially in the absence of fire (Harvey 1994, Graham and Jain 2005).

SOURCES

**References:** Burns and Honkala 1990a, CNHP 2010, Comer et al. 2003*, Dale et al. 2001, Eyre 1980, Garfin et al. 2014, Graham and Jain 2005, Harrington and Sackett 1992, Harvey 1994, Howard 2003b, Howard 2003c, Johansen and Latta 2003, LANDFIRE 2007a, Leatherman et al. 2013, McKenzie et al. 2004, McKenzie et al. 2008, Mehl 1992, Reid et al. 1999, Smith 2006, Stevens‑Rumann et al. 2017, Swetnam and Baisan 1996a, TNC 2013, Youngblood and Mauk 1985

**Version:** 23 May 2018	**Stakeholders:** Midwest, West

**Concept Author:** M.S. Reid	**LeadResp:** West

CES306.648 Southern Rocky Mountain Ponderosa Pine Woodland

**Primary Division:** Rocky Mountain (306)

**Land Cover Class:** Forest and Woodland

**Spatial Scale & Pattern:** Matrix

**Required Classifiers:** Natural/Semi‑natural; Vegetated (>10% vasc.); Upland

**Diagnostic Classifiers:** Ridge/Summit/Upper Slope; Very Shallow Soil; Mineral: W/ A‑Horizon <10 cm; Sand Soil Texture; Aridic; Intermediate Disturbance Interval [Periodicity/Polycyclic Disturbance]; F‑Patch/Medium Intensity; Needle‑Leaved Tree; Pinus ponderosa with shrubby understory

**National Mapping Codes:** EVT 2054; ESLF 4241; ESP 1054

**Concept Summary:** This very widespread ecological system is most common throughout the cordillera of the Rocky Mountains, from the Greater Yellowstone region south. It is also found in the Colorado Plateau region, west into scattered locations of the Great Basin. Its easternmost extent in Wyoming is in the Bighorn Mountains. These woodlands occur at the lower treeline/ecotone between grassland or shrubland and more mesic coniferous forests typically in warm, dry, exposed sites. Elevations range from less than 1900 m in northern Wyoming to 2800 m in the New Mexico mountains. Occurrences are found on all slopes and aspects; however, moderately steep to very steep slopes or ridgetops are most common. This ecological system generally occurs on soils derived from igneous, metamorphic, and sedimentary material, with characteristic features of good aeration and drainage, coarse textures, circumneutral to slightly acidic pH, an abundance of mineral material, rockiness, and periods of drought during the growing season. Northern Rocky Mountain Ponderosa Pine Woodland and Savanna (CES306.030) in the eastern Cascades, Okanogan, and Northern Rockies regions receives winter and spring rains, and thus has a greater spring "green‑up" than the drier woodlands in the Central Rockies. *Pinus ponderosa* (primarily *var. scopulorum* and *var. brachyptera*) is the predominant conifer; *Pseudotsuga menziesii, Pinus edulis, Pinus contorta, Populus tremuloides*, and *Juniperus* spp. may be present in the tree canopy. The understory is usually shrubby, with *Artemisia nova, Artemisia tridentata, Arctostaphylos patula, Arctostaphylos uva‑ursi, Cercocarpus montanus, Purshia stansburiana, Purshia tridentata, Quercus gambelii, Symphoricarpos* spp., *Prunus virginiana, Amelanchier alnifolia* (less so in Montana), and *Rosa* spp. common species. *Pseudoroegneria spicata, Pascopyrum smithii*, and species of *Hesperostipa, Achnatherum, Festuca, Muhlenbergia*, and *Bouteloua* are some of the common grasses. Mixed fire regimes and surface fires of variable return intervals maintain these woodlands, depending on climate, degree of soil development, and understory density.

**Comments:** This system intergrades with Southern Rocky Mountain Ponderosa Pine Savanna (CES306.649). They are distinguished by the high‑frequency, surface‑fire regime, less steep or rocky environmental setting, and more open grassy understory structure of the savanna system. Ponderosa pine woodlands, savannas, and "escarpments'" of central and eastern Montana, eastern Wyoming, the Black Hills region, western Dakotas, and Nebraska are now included in Northwestern Great Plains‑Black Hills Ponderosa Pine Woodland and Savanna (CES303.650).

Because this ecological system has undergone some important changes in its concept, the original system (CES306.032) was archived, and this new system was created to account for the new concept of ponderosa pine woodlands in the Southern Rocky Mountains.

DISTRIBUTION

**Range:** This system is found throughout the southern Rocky Mountains and extends into northern Utah and western Wyoming, in the Uinta and Wasatch ranges, and south into New Mexico. It also occurs in northern Arizona on the Mogollon Rim, north on the high plateaus and ranges in the Colorado Plateau region and scattered locations of the Great Basin.

**Divisions:** 304:C, 306:C

**TNC Ecoregions:** 8:C, 9:C, 10:C, 11:C, 18:C, 19:C, 20:C, 21:C, 25:C, 26:C, 33:?

**Nations:** US

**Subnations:** AZ, CO, ID?, NM, NV, UT, WY

**Map Zones:** 12:?, 14:?, 15:C, 16:C, 17:?, 22:C, 23:C, 24:C, 25:C, 26:?, 27:C, 28:C, 29:C, 33:P

**USFS Ecomap Regions:** 313A:CC, 313B:CC, 313C:CC, 313D:CC, 315A:CC, 315B:CC, 315H:CC, 321A:CC, 331B:CC, 331F:CP, 331G:CP, 331H:CC, 331I:CC, 331J:CC, 341A:CP, 341B:CC, 341F:CC, 342F:CC, 342G:CC, M313A:CC, M313B:CC, M331B:CC, M331D:CP, M331E:CC, M331F:CC, M331G:CC, M331H:CC, M331I:CC, M341A:CP, M341B:CC, M341C:CC, M341D:C?

CONCEPT

**Environment:** This ecological system within the region occurs in the southern Rocky Mountains at the lower treeline/ecotone between grassland or shrubland and more mesic coniferous forests. Stands are typically found in warm, dry, exposed sites at elevations ranging from 1980‑2800 m (6500‑9200 feet).

*Climate*: Climate is temperate with cold winter and warm summers. Precipitation generally contributes 25‑60 cm annually to this system, mostly through winter snow and some monsoonal summer rains. Typically, a seasonal drought period occurs throughout this system as well.

*Physiography/Landform*: Stands can occur on all slopes and aspects; however, it commonly occurs on moderately steep to very steep slopes or ridgetops in foothills and lower montane slopes.

*Soil/substrate/hydrology*: Soils are variable. This ecological system generally occurs on soils derived from igneous, metamorphic, and sedimentary material, including basalt, basaltic, andesitic flows, intrusive granitoids and porphyrites, and tuffs (Youngblood and Mauk 1985). Characteristic soil features include good aeration and drainage, coarse textures, circumneutral to slightly acidic pH, an abundance of mineral material, and periods of drought during the growing season. Some occurrences may occur as edaphic climax communities on very skeletal, infertile, and/or excessively drained soils, such as pumice, cinder or lava fields, and scree slopes. Surface textures are highly variable in this ecological system ranging from sand to loam and silt loam. Exposed rock and bare soil consistently occur to some degree in all the associations. *Pinus ponderosa / Arctostaphylos patula* represents the extreme with typically a high percentage of rock and bare soil present.

Fire plays an important role in maintaining the characteristics of these open‑canopy woodlands. However, soil infertility and drought may contribute significantly in some areas as well.

**Vegetation:** *Pinus ponderosa* (primarily *var. scopulorum* and *var. brachyptera*) is the predominant conifer; *Pseudotsuga menziesii, Pinus edulis, Pinus contorta, Populus tremuloides*, and *Juniperus* spp. may be present in the tree canopy. The understory is usually shrubby, with *Artemisia nova, Artemisia tridentata, Arctostaphylos patula, Arctostaphylos uva‑ursi, Cercocarpus montanus, Purshia stansburiana, Purshia tridentata, Quercus gambelii, Symphoricarpos* spp., *Prunus virginiana, Amelanchier alnifolia* (less so in Montana), and *Rosa* spp. common species. *Pseudoroegneria spicata, Pascopyrum smithii*, and species of *Hesperostipa, Achnatherum, Festuca, Muhlenbergia*, and *Bouteloua* are some of the common grasses.

**Dynamics:** *Pinus ponderosa* is a drought‑resistant, shade‑intolerant conifer which usually occurs at lower treeline in the major ranges of the western United States. Historically, surface fires and drought were influential in maintaining open‑canopy conditions in these woodlands. With settlement and subsequent fire suppression, occurrences have become denser. Presently, many occurrences contain understories of more shade‑tolerant species, such as *Pseudotsuga menziesii* and/or *Abies* spp., as well as younger cohorts of *Pinus ponderosa*. These altered structures have affected fuel loads and alter fire regimes. Presettlement fire regimes were primarily frequent (5‑ to 15‑year return intervals), low‑intensity surface fires triggered by lightning strikes or deliberately set fires by Native Americans. With fire suppression and increased fuel loads, fire regimes are now less frequent and often become intense crown fires, which can kill mature *Pinus ponderosa* (Reid et al. 1999).

Establishment is erratic and believed to be linked to periods of adequate soil moisture and good seed crops, as well as fire frequencies, which allow seedlings to reach sapling size. Longer fire‑return intervals have resulted in many occurrences having dense subcanopies of overstocked and unhealthy young *Pinus ponderosa* (Reid et al. 1999). Mehl (1992) states the following: "Where fire has been present, occurrences will be climax and contain groups of large, old trees with little understory vegetation or down woody material and few occurring dead trees. The age difference of the groups of trees would be large. Where fire is less frequent, there will also be smaller size trees in the understory giving the occurrence some structure with various canopy layers. Dead, down material will be present in varying amounts along with some occurring dead trees. In both cases the large old trees will have irregular open, large branched crowns. The bark will be lighter in color, almost yellow, thick and some will like have basal fire scars."

Grace's warbler, pygmy nuthatch, and flammulated owl are indicators of a healthy ponderosa pine woodland. All of these birds prefer mature trees in an open woodland setting (Winn 1998, Jones 1998, Levad 1998 as cited in Rondeau 2001).

LANDFIRE developed a state‑and‑transition vegetation dynamics VDDT model for this system which has five classes in total (LANDFIRE 2007a, BpS 2810540). These are summarized as:

A) Early Development 1 All Structures (pole‑sized tree‑dominated ‑ 10% of type in this stage): Openings with up to 10% cover by overstory dominated by ponderosa pine and sometimes Douglas‑fir. Some openings may persist.

B) Mid Development 1 Closed (tree‑dominated ‑ 10% of type in this stage): Greater than 50% canopy cover in the northern Front Range (above c. 6500 feet) and >30% canopy cover in the southern Front Range.

C) Mid Development 1 Open (tree‑dominated ‑ 25% of type in this stage): Greater than 50% canopy cover in the northern Front Range (above c. 6500 feet) and <30% canopy cover in the southern Front Range

D) Late Development 1 Open (tree‑dominated ‑ 40% of type in this stage): Less than 50% canopy cover in the northern Front Range (above c. 6500 feet) and <30% canopy cover in the southern Front Range.

E) Late Development 1 Closed (tree‑dominated ‑ 15% of type in this stage): Less than 50% canopy cover in the northern Front Range (above c. 6500 feet) and <30% canopy cover in the southern Front Range.

Mixed‑severity fire regime ‑ typically an average fire frequency ranges from 40‑100 years (5‑100 ha) (Kaufmann et al. 2000, Veblen et al. 2000, Ehle and Baker 2003, Sherriff 2004). These fires range from low‑severity to high‑severity fires, and the forest structure was shaped by the pattern of fire at a landscape scale. Drought and other weather events (e.g., blowdown); insects such as mountain pine beetle, Douglas‑fir beetle and western spruce budworm (Swetnam and Lynch 1993, Negron 1998, 2004); and pathogens such as dwarf mistletoe (Hawksworth 1961) also play important roles in this type.

Replacement‑fire rotation uncertain, and this affects the amount of forest in each class. Cheesman Lake ‑ fire rotation (all fires 75 years) and stand‑replacement (460 years) estimation (LANDFIRE 2007a, BpS 2810540).

Nutrient cycling, specifically carbon cycling, is an important ecological process within many ecological systems. However, biological decomposition in ponderosa pine forests is more limited than biological production, resulting in accumulation of organic materials, especially in the absence of fire (Harvey 1994, Graham and Jain 2005).

SOURCES

**References:** Burns and Honkala 1990a, CNHP 2010, Comer et al. 2002, Comer et al. 2003*, Dale et al. 2001, DeVelice et al. 1986, Ehle and Baker 2003, Eyre 1980, Garfin et al. 2014, Graham and Jain 2005, Harvey 1994, Hawksworth 1961, Hess and Alexander 1986, Hoffman and Alexander 1976, Howard 2003b, Howard 2003c, Johansen and Latta 2003, Kaufmann et al. 2001, Komarkova et al. 1988b, LANDFIRE 2007a, Marriott and Faber‑Langendoen 2000, Mauk and Henderson 1984, McKenzie et al. 2004, McKenzie et al. 2008, Mehl 1992, Muldavin et al. 1987, Muldavin et al. 1996, Nachlinger et al. 2001, Neely et al. 2001, Negron 1998, Negron and Popp 2004, Reid et al. 1999, Rondeau 2001, Sherriff 2004, Stevens‑Rumann et al. 2017, Swetnam and Lynch 1993, Tuhy et al. 2002, Veblen et al. 2000, Westerling et al. 2006, Youngblood and Mauk 1985

**Version:** 23 May 2018	**Stakeholders:** West

**Concept Author:** M.S. Reid	**LeadResp:** West

1.B.2.Nc. Western North American Pinyon ‑ Juniper Woodland & Scrub

M026. Intermountain Singleleaf Pinyon ‑ Juniper Woodland

CES304.082 Columbia Plateau Western Juniper Woodland and Savanna

**Primary Division:** Inter‑Mountain Basins (304)

**Land Cover Class:** Forest and Woodland

**Spatial Scale & Pattern:** Large patch

**Required Classifiers:** Natural/Semi‑natural; Vegetated (>10% vasc.); Upland

**Diagnostic Classifiers:** Montane [Lower Montane]; Lowland [Foothill]; Forest and Woodland (Treed); Ridge/Summit/Upper Slope; Aridic; Juniperus occidentalis

**National Mapping Codes:** EVT 2017; ESLF 4204; ESP 1017

**Concept Summary:** This woodland system is found along the northern and western margins of the Great Basin, from southwestern Idaho, along the eastern foothills of the Cascades, south to the Modoc Plateau of northeastern California. Elevations range from under 200 m along the Columbia River in central Washington to over 1500 m. Generally, soils are medium‑textured, with abundant coarse fragments, and derived from volcanic parent materials. In central Oregon, the center of distribution, all aspects and slope positions occur. Where this system grades into relatively mesic forest or grassland habitats, these woodlands become restricted to rock outcrops or escarpments with excessively drained soils. The vegetation is characterized by an open stand of *Juniperus occidentalis* with an understory of open shrub‑steppe (big sage, bitterbrush and/or rabbitbrush) with perennial bunchgrasses representing the dominant vegetation. *Pinus monophylla* is not present in this region, so *Juniperus occidentalis* is typically the only tree species, although *Pinus ponderosa* or *Pinus jeffreyi* may be present in some stands. *Cercocarpus ledifolius* may occasionally codominate. *Artemisia tridentata* is the most common shrub; others are *Purshia tridentata, Ericameria nauseosa, Chrysothamnus viscidiflorus, Ribes cereum*, and *Tetradymia* spp. Graminoids include *Carex filifolia, Festuca idahoensis, Poa secunda*, and *Pseudoroegneria spicata*. These woodlands are generally restricted to rocky areas where fire frequency is low. Throughout much of its range, fire exclusion and removal of fine fuels by grazing livestock have reduced fire frequencies and allowed *Juniperus occidentalis* seedlings to colonize adjacent alluvial soils and expand into the sagebrush shrub‑steppe and grasslands. *Juniperus occidentalis* savanna may occur on the drier edges of the woodland where trees are intermingling with or invading the surrounding grasslands and where local edaphic or climatic conditions favor grasslands over shrublands.

**Comments:** These woodlands are composed of two very different types. There are old‑growth *Juniperus occidentalis* woodlands with trees and stands often over 1000 years old, with fairly well‑spaced trees with rounded crowns. There are also large areas where juniper has expanded into sagebrush steppe and bunchgrass‑dominated areas, with young, pointed‑crowned trees growing closely together. These two types correspond to the *Juniperus occidentalis*‑dominated portion of the *persistent pinyon‑juniper woodland* (open to denser of tree canopy occurring on shallow rocky soils) and *wooded shrubland* (open tree canopy with well‑developed shrub stratum and variable grass‑forb cover) described by Romme et al. (2009). Currently, these two very different types are about equally distributed across the landscape, with *Juniperus occidentalis* continuing to expand, either from the combination of fire exclusion, past grazing or climate change. *Juniperus occidentalis* has also expanded into *Pinus ponderosa* and *Pinus ponderosa ‑ Pinus contorta* stands in central Oregon.

DISTRIBUTION

**Range:** This woodland and savanna system is found along the northern and western margins of the Great Basin, from southwestern Idaho, along the eastern foothills of the Cascades, south to the Modoc Plateau of northeastern California (Tirmenstein 1999h, Sawyer et al. 2009). It also occurs in scattered localities of northern Nevada and south‑central Washington. This system is most abundant in central and south‑central Oregon (Franklin and Dyrness 1973, Tirmenstein 1999h, Sawyer et al. 2009).

**Divisions:** 304:C

**TNC Ecoregions:** 6:C, 7:C, 68:C

**Nations:** US

**Subnations:** CA, ID, NV, OR, WA:S3S4

**Map Zones:** 6:C, 7:C, 9:C, 12:C, 18:C

**USFS Ecomap Regions:** 331A:??, 341G:CC, 342B:CC, 342C:CC, 342D:CP, 342H:CC, 342I:CC, 342J:CC, M242C:CC, M261A:C?, M261D:CC, M261E:CP, M261G:CC, M332G:CC

CONCEPT

**Environment:** This woodland system is found along the northern and western margins of the Great Basin, from southwestern Idaho, along the eastern foothills of the Cascades, south to the Modoc Plateau of northeastern California (Tirmenstein 1999h, Sawyer et al. 2009). Elevations range from under 200 m along the Columbia River in central Washington to over 1500 m. In northwestern California stands range from 700 to 2300 m elevation (Tirmenstein 1999h, Sawyer et al. 2009).

*Climate:* Throughout the range the climate is cool, semi‑arid, continental with 200‑360 mm of precipitation annually, with the majority falling in winter. The temperature regime is cool in summer, with a wide range in diurnal temperatures and night frosts occurring most of the year. Summer lightning storms and associated fire are common and are presumably important in structuring the vegetation. (Franklin and Dyrness 1973).<br />

*Physiography/landform:* In central Oregon, the center of the woodland's range, stands are found on all aspects and slope positions. Where this type grades into relatively mesic forest or grassland habitats, the vegetation becomes restricted to rock outcrops or escarpments with excessively drained soils.

*Soils/substrate/hydrology:* *Juniperus occidentalis* stands occur on a wide variety of soil types. Generally, soils are well‑drained, shallow and stony with rock outcrops common, but soils may be deeper. They are medium‑textured, with abundant coarse fragments, and derived from volcanic parent materials such as basalt, andesite, rhyolite, pumice, volcanic ash, tuff, welded tuff, as well as colluvial, alluvial, or eolian material (Tirmenstein 1999h, LANDFIRE 2007a). Soils derived from pumice ash are the most common edaphic characteristic of this woodland (LANDFIRE 2007a). Origins of the pumice sands are Mount Mazama and Newberry Crater (Miller et al. 1999). In most other areas, it occurs on rimrock, shallow soil scablands and in other isolated pockets.

**Vegetation:** *Pinus monophylla* is not present in this region, so *Juniperus occidentalis* is the only tree species, although *Pinus ponderosa* or *Pinus jeffreyi* may be present in some stands. *Cercocarpus ledifolius* may occasionally codominate. *Artemisia tridentata* is the most common shrub; others are *Purshia tridentata, Ericameria nauseosa, Chrysothamnus viscidiflorus, Ribes cereum*, and *Tetradymia* spp. Graminoids include *Carex filifolia, Festuca idahoensis, Poa secunda*, and *Pseudoroegneria spicata*.

**Dynamics:** *Juniperus occidentalis* is a long‑lived tree that can exceed 3000 years in age in rocky, fire‑protected areas such as along rimrock (Waigchler et al. 2001, Thorne et al. 2007). These fire sensitive trees do not sprout following fire and are typically killed by moderate to severe fires (Tirmenstein 1999h, Sawyer et al. 2009). Young junipers have thin bark and are readily killed by surface fires (Martin et al. 1978), whereas mature trees with thicker bark are described as "moderately resistant" (Fowells 1965). Reproductive age begins at about 20 years, peaks after 50 years and continues for many years (Miller and Rose 1995, Tirmenstein 1999h). Following stand‑replacing fire, recovery time is relatively slow and depends on stand maturity, the size and season of burn, fire severity and juniper mortality, the persistence of the seeds in the seed bank, location of seed source, the presence of animal dispersers such as Clark's nutcrackers, competition from herbaceous species and shrubs, and the amount of post‑fire precipitation (Burkhardt and Tisdale 1976, Tirmenstein 1999h). Large burns and long distances from seed sources slow recovery rates because seed dispersal is dependent on water and animals (Tirmenstein 1999h).

*Juniperus occidentalis* woodlands become "closed" at about 40% canopy cover when lateral tree roots fill interspaces between trees (Young et al. 1982, Thorne et al. 2007). At this stage cover of shrub and herbaceous layers begin to rapidly decline (Thorne et al. 2007).

*Juniperus occidentalis* savanna often occurs on the drier edges of the woodland where trees are intermingling with or invade the surrounding grasslands where local edaphic or climatic conditions favor grasslands over shrublands. Stands occur between the ponderosa zones and the sagebrush moisture zones and are expanding into big sagebrush steppe areas at a fairly rapid rate, creating extensive young stands, increasing the acreage of this type by more than five times (LANDFIRE 2007a, BpS 0910170). Western juniper woodlands and savannas experienced both large‑ and small‑scale natural disturbances (LANDFIRE 2007a). Small‑scale fires (less than 5 acres) and insects and disease kill single trees to small patches of trees throughout the stand on a frequent interval. Large‑scale fires (>1000 acres) are less common, occurring once every 500 years or more (Miller et al. 1999). Drought can cause dieback and death of trees.

Areas where this system occurs contain some of the largest concentrations of ancient trees. Individuals may exceed 2000 years of age. These ancient western juniper woodlands provide important wildlife habitat. Cavities form in older trees and are important for many neotropical migrants. Western juniper cone‑berries provide food for many animals, including elk, deer, coyotes, and small mammals such as mice, chipmunks, rabbits, squirrels, and woodrats; many such as coyotes serve as important dispersing agents of the junipers (Schupp et al. 1997, Tirmenstein 1999h). They are also used by wintering birds such as the American robin and Townsend solitaire (Burkhardt and Tisdale 1969, Eddleman 1984, Tirmenstein 1999h). This juniper is also an important food source for insects with 25 species of bark and wood boring beetles identified (Miller et al. 2005).

LANDFIRE developed a VDDT model for this system which has five classes (LANDFIRE 2007a, BpS 0910170):

A) Early Development (herbaceous‑dominated with 0‑60% cover ‑ 2% of type in this stage): Herbaceous plants dominate this stage immediately following disturbance. Perennial bunchgrasses dominate the plant community. However, in the first few years following disturbance annual plants may dominate while perennial grasses and forbs recover. Succession to class B after 30 years. (Replacement and mixed fires).

B) Mid Development 1 Open (shrub‑dominated with 0‑30% cover ‑ 5% of type in this stage): Shrubs dominate this stage. The composition of the shrub layer will be dependent on soil depth and climatic factors. Rabbitbrush will most likely be the dominant shrub following disturbance. However, big sagebrush, bitterbrush and wax current may also be found. Western juniper seedlings and saplings are present throughout the shrub layer. Western juniper has established below the canopy of the shrub layer. Shrub cover is approaching 20% on more productive sites but is most likely <15%. Herbaceous plants are being suppressed by the increase in woody plants. Succession to class C after 45 years. (Mixed and replacement fires).

C) Mid Development 2 Open (shrub/tree mix, tree cover 0‑20% ‑ 15% of type in this stage): Western juniper forms an even‑aged woodland. Trees are characterized by regular conical shapes. Shrubs are being suppressed by the emerging woodland. Herbaceous vegetation is also being suppressed by the competition from woody plants. Succession to class E (late closed) after 45 years. (Mixed and replacement fires. Certain sites are edaphically constrained and thus transition to class D ‑ late‑open).

D) Late Development 2 Open (shrub/tree mix, tree cover 0‑20% ‑ 35% of type in this stage): Ancient western juniper savanna or open woodland composed of multiple structural layers. Some western juniper trees have dead portions in their canopies. Canopies are irregular in shape. Young trees can be found in open areas where recent small‑scale disturbances occurred. Edaphic factors often maintain wide spacing between junipers. Understory grasses remain dominant and variable. (Maintains in class D. Many disturbances cause transitions to younger or more open conditions).

E) Late Development 1 Open (tree‑dominated 20‑40% cover ‑ 43% of type in this stage): Ancient western juniper woodland composed of multiple structural layers. Some western juniper trees have dead portions in their canopies. Canopies are irregular in shape. Young trees can be found in open areas where recent small‑scale disturbances occurred. Understory grasses are variable, based on slope, aspect and soil depth. (Maintains in class E. Many disturbances cause transitions to younger or more open conditions) (LANDFIRE 2007a).

SOURCES

**References:** Bates et al. 2014, Belnap 2001, Belnap et al. 2001, Burkhardt and Tisdale 1976, Chambers et al. 1999, Comer et al. 2003*, D'Antonio and Vitousek 1992, D'Antonio et al. 2009, Eddleman 1984, Everett 1986, Eyre 1980, Fowells 1965, Franklin and Dyrness 1973, Furniss and Carolin 2002, Kartesz 1988, LANDFIRE 2007a, Mack 1981b, Martin et al. 1978, Miller and Rose 1995, Miller et al. 1999, Miller et al. 2005, Romme et al. 2009, Rosentreter and Belnap 2003, Sawyer et al. 2009, Schupp et al. 1997, Shiflet 1994, Stevens 1999a, Stevens 1999b, Stevens and Monsen 2004, Thorne et al. 2007, Tirmenstein 1999h, Vander Wall 1990, WNHP unpubl. data 2018, Waigchler et al. 2001, Young et al. 1982

**Version:** 04 Nov 2015	**Stakeholders:** West

**Concept Author:** M.S. Reid	**LeadResp:** West

CES304.773 Great Basin Pinyon‑Juniper Woodland

**Primary Division:** Inter‑Mountain Basins (304)

**Land Cover Class:** Forest and Woodland

**Spatial Scale & Pattern:** Matrix

**Required Classifiers:** Natural/Semi‑natural; Vegetated (>10% vasc.); Upland

**Diagnostic Classifiers:** Montane [Lower Montane]; Lowland [Foothill]; Forest and Woodland (Treed); Foothill(s); Piedmont; Plateau; Ridge/Summit/Upper Slope; Aridic; Pinus monophylla, Juniperus osteosperma

**National Mapping Codes:** EVT 2019; ESLF 4206; ESP 1019

**Concept Summary:** This ecological system occurs on dry mountain ranges of the Great Basin region and eastern foothills of the Sierra Nevada extending south in scattered locations throughout southern California. This woodland is typically found at lower elevations ranging from 1600‑2800 m. These woodlands occur on warm, dry sites on mountain slopes, mesas, plateaus and ridges. Woodlands dominated by a mix of *Pinus monophylla* and *Juniperus osteosperma*, pure or nearly pure occurrences of *Pinus monophylla*, or woodlands dominated solely by *Juniperus osteosperma* comprise this system, but in some regions of southern California, *Juniperus osteosperma* is replaced by *Juniperus californica*. *Cercocarpus ledifolius* is a common associate. On the east slope of the Sierras in California, *Pinus jeffreyi* and *Juniperus grandis* may be components of these woodlands. Understory layers are variable. Associated species include shrubs such as *Arctostaphylos patula, Artemisia arbuscula, Artemisia nova, Artemisia tridentata, Cercocarpus ledifolius, Cercocarpus intricatus, Coleogyne ramosissima, Yucca brevifolia, Quercus gambelii, Quercus turbinella, Quercus john‑tuckeri, Juniperus californica, Quercus chrysolepis*, and bunchgrasses *Hesperostipa comata, Festuca idahoensis, Pseudoroegneria spicata, Leymus cinereus*, and *Poa fendleriana*. This system occurs at lower elevations than Colorado Plateau Pinyon‑Juniper Woodland (CES304.767) where sympatric.

**Comments:** Where sympatric with Colorado Plateau Pinyon‑Juniper Woodland (CES304.767) in southwestern Utah at Zion National Park, this system (CES304.773) occurs on warmer, drier lower elevation sites and Colorado Plateau Pinyon‑Juniper Woodland (CES304.767) is restricted to cooler higher plateaus and mesatops. Hybrid trees of both species may occur in transition areas.

DISTRIBUTION

**Range:** This system occurs on dry mountain ranges of the Great Basin region and eastern foothills of the Sierra Nevada, typically at lower elevations ranging from 1600‑2800 m. It extends southwest in California to the northern Transverse Ranges (Ventura County) and San Jacinto Mountains (Riverside County).

**Divisions:** 206:C, 304:C

**TNC Ecoregions:** 6:C, 11:C, 12:C, 18:C, 19:C

**Nations:** US

**Subnations:** AZ, CA, ID, NV, UT

**Map Zones:** 4:C, 6:C, 7:?, 9:C, 12:C, 13:C, 14:C, 15:C, 16:C, 17:C, 18:C, 23:C

**USFS Ecomap Regions:** 313A:CC, 322A:CC, 322B:CC, 341A:CC, 341D:CC, 341E:CC, 341F:CC, 341G:CC, 342B:CC, 342C:CC, 342D:CC, 342J:CC, M242C:??, M261D:C?, M261E:CC, M261G:CC, M331D:CC, M341A:CC, M341C:CC, M341D:CC

CONCEPT

**Environment:** This system occurs on dry mountain ranges of the Great Basin region and eastern foothills of the Sierra Nevada extending south into the Mojave Desert ranges and southwest in to the northern Transverse Ranges and San Jacinto Mountains. Elevations range from 1000 to 2800 m. Upper elevation limits are determined by local climate and/or the presence of competing tree species. Stands generally occur on sites with shallow rocky soils or rock‑dominated sites that are protected from frequent fire (rocky ridges, broken topography and mesatops).

*Climate:* Climate is temperate, continental, and semi‑arid with cold winters. Precipitation ranges from 20 to 45 cm annually, mostly occurring during fall and winter months (Brown 1982a). Summers are typically dry and there is usually extreme variation in annual precipitation. Severe climatic events occurring during the growing season, such as frosts and drought, are thought to limit the distribution of pinyon‑juniper woodlands to relatively narrow altitudinal belts on mountainsides.

*Physiography/landform:* These woodlands occur on warm, dry sites on mountain slopes, mesas, plateaus, ridges, foothills, and upper alluvial fans.

*Soil/substrates/hydrology:* Soils supporting this system vary in texture, ranging from stony, cobbly, gravelly sandy loams to clay loam or clay. Adjacent upland systems include Inter‑Mountain Basins Montane Sagebrush Steppe (CES304.785), Inter‑Mountain Basins Curl‑leaf Mountain‑mahogany Woodland and Shrubland (CES304.772), Inter‑Mountain Basins Aspen‑Mixed Conifer Forest and Woodland (CES304.776) above and at lower elevations, Great Basin Xeric Mixed Sagebrush Shrubland (CES304.774), Inter‑Mountain Basins Big Sagebrush Shrubland (CES304.777), and Mojave Mid‑Elevation Mixed Desert Scrub (CES302.742).

**Vegetation:** These woodlands are characterized by an open to moderately dense tree canopy typically composed of a mix of *Pinus monophylla* and *Juniperus osteosperma*, but either tree species may dominate to the exclusion of the other. In some regions of southern California, *Juniperus osteosperma* is replaced by *Juniperus californica*. *Cercocarpus ledifolius* is a common associate and may occur in tree or shrub form. On the east slope of the Sierra Nevada in California, *Pinus jeffreyi* and *Juniperus grandis (= Juniperus occidentalis var. australis)* may be components of these woodlands. Understory layers are variable, but shrubs such as *Artemisia tridentata* frequently form a moderately dense short‑shrub layer. Other associated shrubs include *Arctostaphylos patula, Artemisia arbuscula, Artemisia nova, Cercocarpus intricatus, Coleogyne ramosissima, Quercus gambelii*, and *Quercus turbinella*. Bunch grasses such as *Poa fendleriana, Hesperostipa comata, Festuca idahoensis, Pseudoroegneria spicata, Leymus cinereus (= Elymus cinereus)*, and *Bouteloua gracilis* are commonly present and may form an herbaceous layer. In the southern extent, *Arctostaphylos patula, Ceanothus greggii, Garrya flavescens, Quercus john‑tuckeri, Juniperus californica, Purshia stansburiana, Quercus chrysolepis, Yucca baccata*, and *Yucca brevifolia* are common.

**Dynamics:** *Pinus monophylla, Juniperus osteosperma*, and *Juniperus scopulorum* are slow‑growing, long‑lived trees (about 650 years for *Juniperus osteosperma*, 300 years for *Juniperus scopulorum*, and 800 years for *Pinus monophylla*, although older individuals are known) (Burns and Honkala 1990a, Zlatnik 1999e, Zouhar 2001b, Scher 2002, Sawyer et al. 2009). These trees are killed by severe fire because of thin bark and lack of self‑pruning; however, mature trees can survive low‑intensity fires (Zouhar 2001b, Sawyer et al. 2009). Although there is variation in fire frequency because of the diversity of site characteristics, stand‑replacing fire was uncommon in this ecological system historically, with an average fire‑return interval (FRI) of 100‑1000 years occurring primarily during extreme fire behavior conditions and during long droughts (Zouhar 2001b) (LF BpS model 1210190). Mixed‑severity fire (average FRI of 100‑500 years) was characterized as a mosaic of replacement and surface fires distributed through stands in patches at a fine scale (<0.1 acre) (LF BpS model 1210190).

Fire rotation in the San Bernardino Mountains was determined to be 480 years (Wangler and Minnich 2006). These woodlands have a truncated long fire‑return interval of 200+ years with surface to passive crown fires of medium size, low complexity, high intensity, and very high severity (Sawyer et al. 2009). After a stand‑replacing fire, the site is usually colonized by herbaceous plants and shrubs. The shrubs act as nurse plants, with *Pinus monophylla* seedlings establishing 20‑30 years post fire after shrub density increases, and then a tree canopy forms after 100‑150 years (Minnich 2007). As tree canopy becomes denser there is a decline in shrub cover (Minnich 2007). Fires are associated with herbaceous fuel buildup following a wet period (Minnich 2007).

Other important ecological processes include drought, insect infestations, pathogens, herbivory, and seed dispersal by birds and mammals. Juniper berry and pinyon nut crops are primarily utilized by birds and small mammals (Johnsen 1962, McCulloch 1969, Short et al. 1977, Salomonson 1978, Balda 1987, Gottfried et al. 1995). Large mammals, such as mule deer (*Odocoileus hemionus*), white‑tailed deer (*Odocoileus virginianus*) and elk (*Cervus elaphus*), eat leaves and seeds of both species and they browse woodland grasses, forbs and shrubs, including *Artemisia tridentata, Cercocarpus montanus, Quercus gambelii*, and *Purshia stansburiana* (Short and McCulloch 1977).

The principal dispersers of juniper and pinyon seeds are birds, although many mammals also feed on them. These animals consume juniper berries and excrete viable scarified juniper seeds over extensive areas, which germinate faster than uneaten seeds (Johnsen 1962, Meeuwig and Bassett 1983). Primary juniper seed dispersers are Bohemian waxwing (*Bombycilla garrulus*), cedar waxwing (*Bombycilla cedrorum*), American robin (*Turdus migratorius*), turkey (*Meleagris gallopavo*), and five species of jays (Scher 2002). Pinyon seeds are a critically important food source for western scrub jay (*Aphelocoma californica*), pinyon jay (*Gymnorhinus cyanocephalus*), Steller's jay (*Cyanocitta stelleri*) and Clark's nutcracker (*Nucifraga columbiana*). These birds are primary dispersers of pinyon seeds and during mast crop years cache hundreds of thousands of pinyon seeds, many of which are never recovered (Balda and Bateman 1971, Vander Wall and Balda 1977, Ligon 1978). Many mammals are also known to eat singleleaf pinyon seeds, including several species of mice (*Peromyscus* spp.), woodrats (*Neotoma* spp.), squirrels (*Sciurus* spp.), chipmunks (*Neotamias* spp.), deer, black bear (*Ursus americanus*), and desert bighorn sheep (*Ovis canadensis nelsoni*) (Christensen and Whitham 1993, Zouhar 2001b). Because singleleaf pinyon seeds are heavy and totally wingless, seed dispersal is dependent on vertebrate dispersers that store seeds in food caches, where unconsumed seeds may germinate. This seed dispersal mechanism is a good example of a co‑evolved, mutualistic, plant‑vertebrate relationship (Vander Wall et al. 1981, Evans 1988, Lanner 1996) and would be at risk with loss of trees or dispersers.

There are many insects, pathogens, and plant parasites that attack pinyon and juniper trees (Gottfried et al. 1995, Rogers 1995, Weber et al. 1999). Juniper mistletoe (*Phoradendron juniperinum*) occurs on junipers and pinyon dwarf mistletoe (*Arceuthobium divaricatum*) occurs on pines. Both mistletoes reduce vigor and cause dieback but rarely cause mortality (Meeuwig and Bassett 1983). For pinyon, there are at least seven insects, and fungi such as blackstain root‑rot (*Leptographium wageneri*), pinyon needle rust (*Coleosporium ribicola*), and pinyon blister rust (*Cronartium occidentale*) (Skelly and Christopherson 2003). The insects are normally present in these woodland stands, and during drought‑induced water stress, outbreaks may cause local to regional mortality (Wilson and Tkacz 1992, Gottfried et al. 1995, Rogers 1995). Most insect‑related pinyon mortality in the West is caused by pinyon Ips bark beetle (*Ips confusus*) (Rogers 1993). The current epidemic of ips beetles in many areas that has killed numerous pinyons has created high fuel loads that further threaten stands (Thorne et al. 2007).

LANDFIRE modelers predict severe weather (usually drought), insects and tree pathogens are coupled disturbances that thin trees to varying degrees and kill small patches every 250‑500 years on average, with greater frequency in more closed stands (LF BpS model 1210190).

LANDFIRE developed a state‑and‑transition vegetation dynamics VDDT model for this system which has five classes in total (LANDFIRE 2007a, BpS 1210190). These are summarized as:

A) Early Development 1 Open (herbaceous‑dominated ‑ 5% of type in this stage): Herbaceous cover is 0‑15%. Shrub cover is 0%. Initial post‑fire community dominated by annual grasses and forbs. Later stages of this class contain greater amounts of perennial grasses and forbs. Evidence of past fires (burnt stumps and charcoal) should be observed. Duration is 10 years with succession to class B, mid‑development closed. Replacement fire occurs every 300 years on average.

<br />B) Mid Development 1 Open (shrub‑dominated ‑ 5% of type in this stage): Shrub cover is 5‑20%. Dominated by shrubs, perennial forbs and grasses. Tree seedlings starting to establish on favorable microsites. Total cover remains low due to shallow unproductive soil. Duration is 20 years with succession to class C unless infrequent replacement fire (FRI of 200 years) returns the vegetation to class A. It is important to note that replacement fire at this stage does not eliminate perennial grasses. Mixed‑severity fire (average FRI of 200 years) thins the woody vegetation but does not change its succession age.

C) Mid Development 2 Open (shrub‑dominated ‑ 20% of type in this stage): Tree cover is 5‑20%. Tree height <5 m. Shrub‑ and tree‑dominated community with young juniper and pinyon seedlings becoming established. Duration is 70 years with succession to class D unless replacement fire (average FRI of 250 years) causes a transition to class A. It is important to note that replacement fire at this stage does not eliminate perennial grasses. Mixed‑severity fire as in class B. Mortality from insects, pathogens, and drought occurs at a rotation of approximately 500 years and causes a transition to class B by killing older trees.

D) Late Development 1 Open (conifer‑dominated ‑ 35% of type in this stage): Tree cover is 5‑40%. Tree height <10 m. Community dominated by young to mature juniper and pine of mixed age structure. Juniper and pinyon becoming competitive on site and beginning to affect understory composition. Duration 200 years with succession to class E unless replacement fire (average FRI of 1000 years) causes a transition to class A. Mixed‑severity fire is less frequent than in previous states (500 years). Surface fire (mean FRI of 500 years) is infrequent and does not change successional dynamics. Tree pathogens and insects such as pinyon Ips become more important for woodland dynamics occurring at a rotation of 250 years, including both patch mortality (500‑year rotation) and thinning of isolated individual trees (500‑year rotation).

E) Late Development 2 Open (conifer‑dominated ‑ 35% of type in this stage): Tree cover is 5‑50%. Tree height 5‑25 m. Some sites dominated by widely spaced old juniper and pinyon, while elsewhere there are dense, old‑growth stands with multiple layers. May have all‑aged, multi‑storied structure. Occasional shrubs with few grasses and forbs and often much rock. Understory depauperate and high amounts of bare ground present. Grasses present on microsites with deeper soils (>50 cm [20 inches]) with restricting clay subsurface horizon. Potential maximum overstory replacement fire and mixed‑severity fires are rare (average FRIs of 1000 and 500 years, respectively). Surface fire occurs when especially dry years follow wet years (500‑year rotation) and will scar ancient trees. Tree pathogens and insects associated with drought conditions kill patches of trees (1000‑year rotation), with succession to class C, and individual trees (1000‑year rotation) with succession to class D. Duration 800+ years.

Most pinyon‑juniper woodlands in the southwest have high soil erosion potential (Baker et al. 1995). Several studies have measured present‑day erosion rates in pinyon‑juniper woodlands, highlighting the importance of herbaceous cover and cryptogamic soil crusts (Baker et al. 1995, Belnap et al. 2001) in minimizing precipitation runoff and soil loss in pinyon‑juniper woodlands.

SOURCES

**References:** Baker et al. 1995, Balda 1987, Balda and Bateman 1971, Belnap 2001, Belnap et al. 2001, Blackburn and Tueller 1970, Breshears et al. 2005, Brooks and Minnich 2006, Brown 1982a, Burns and Honkala 1990a, Chambers 2001, Christensen and Whitham 1993, Comer et al. 2003*, Evans 1988, Evans and Belnap 1999, Eyre 1980, Furniss and Carolin 2002, Gottfried et al. 1995, Hollander and Vander Wall 2004, Johnsen 1962, Keeler‑Wolf and Thomas 2000, LANDFIRE 2007a, Lanner 1996, Ligon 1978, McCulloch 1969, Meeuwig and Bassett 1983, Minnich 2007b, Rogers 1993, Rogers 1995, Rosentreter and Belnap 2003, Salomonson 1978, Sawyer et al. 2009, Scher 2002, Shiflet 1994, Short and McCulloch 1977, Short et al. 1977, Skelly and Christopherson 2003, Swetnam and Baisan 1996a, Tausch and West 1988, Tausch et al. 1981, Thorne et al. 2007, Vander Wall and Balda 1977, Vander Wall et al. 1981, Wangler and Minnich 2006, Weber et al. 1999, Weisberg et al. 2007, Wilson and Tkacz 1992, Zlatnik 1999e, Zouhar 2001b

**Version:** 29 Aug 2015	**Stakeholders:** West

**Concept Author:** K.A. Schulz	**LeadResp:** West

CES304.772 Inter‑Mountain Basins Curl‑leaf Mountain‑mahogany Woodland and Shrubland

**Primary Division:** Inter‑Mountain Basins (304)

**Land Cover Class:** Forest and Woodland

**Spatial Scale & Pattern:** Large patch

**Required Classifiers:** Natural/Semi‑natural; Vegetated (>10% vasc.); Upland

**Diagnostic Classifiers:** Montane [Lower Montane]; Lowland [Foothill]; Aridic; Cercocarpus ledifolius

**National Mapping Codes:** EVT 2062; ESLF 4303; ESP 1062

**Concept Summary:** This ecological system occurs in hills and mountain ranges of the Intermountain West basins from the eastern foothills of the Sierra Nevada northeast to the foothills of the Bighorn Mountains. It typically occurs from 600 m to over 2650 m in elevation on rocky outcrops or escarpments and forms small‑ to large‑patch stands in forested areas. Most stands occur as shrublands on ridges and steep rimrock slopes, but they may be composed of small trees in steppe areas. Scattered junipers or pines may also occur. This system includes both woodlands and shrublands dominated by *Cercocarpus ledifolius*. *Artemisia tridentata ssp. vaseyana, Purshia tridentata*, with species of *Arctostaphylos, Ribes*, or *Symphoricarpos* are often present. Undergrowth is often very sparse and dominated by bunchgrasses, usually *Pseudoroegneria spicata* and *Festuca idahoensis*. *Cercocarpus ledifolius* is a slow‑growing, drought‑tolerant species that generally does not resprout after burning and needs the protection from fire that rocky sites provide.

DISTRIBUTION

**Range:** This system occurs in hills and mountain ranges of the Intermountain West basins from the eastern foothills of the Sierra Nevada northeast to the foothills of the Bighorn Mountains.

**Divisions:** 206:?, 304:C, 306:C

**TNC Ecoregions:** 6:P, 9:C, 10:P, 11:C, 12:C

**Nations:** US

**Subnations:** CA, CO, ID, MT, NV, OR, UT, WA:S1, WY

**Map Zones:** 2:C, 6:P, 7:C, 8:C, 9:C, 10:C, 12:C, 16:C, 17:C, 18:C, 19:C, 21:C, 22:C, 23:?, 29:C, 30:?

**USFS Ecomap Regions:** 313A:CC, 331A:CC, 331G:CC, 341A:CC, 341B:CP, 341D:CC, 341E:CC, 341F:CC, 341G:CC, 342B:CC, 342C:CC, 342D:CC, 342E:CC, 342F:CP, 342G:CC, 342H:CC, 342I:CP, 342J:CC, M242C:CC, M261E:CC, M261G:CC, M331A:C?, M331B:CC, M331D:CC, M331E:CC, M331J:C?, M332A:CC, M332B:C?, M332D:C?, M332E:CC, M332F:CC, M332G:CC, M333D:PP, M341A:CC, M341B:CC, M341C:CC, M341D:CC

CONCEPT

**Environment:** This ecological system is widespread in semi‑arid hills and mountain ranges of the intermountain western U.S. from the eastern foothills of the Sierra Nevada and Cascade Range east into the Rocky Mountains including the foothills of the Bighorn Mountains. It also occurs south into the Mojave Desert and the Grand Canyon in northern Arizona. Stands mostly occur below montane conifer forests and above desert scrub from 1500 to 3200 m in elevation, extending down to 600 m in the north (Gucker 2006c). Higher‑elevation stands typically occur on warmer and drier southerly slopes. Annual precipitation averages 25‑45 cm, with a significant proportion falling as winter snow. Sites typically have shallow to deep, well‑drained, often rocky, nutrient‑poor, sandy loam soils frequently derived primarily from carbonate sediments (limestone or dolomite) or on sandstones rich in calcium carbonate (Reid et al. 1999). Other rock types include quartz, gneiss, and basalt.

**Vegetation:** This system is includes both short and tall shrublands and short woodlands dominated by *Cercocarpus ledifolius*. Some stands occur as scattered shrub communities in steppe or on rocky outcrops or steep escarpments within forests and woodlands, especially on upper slopes and ridges. The woodlands occur mostly in the eastern Sierra Nevada and ranges in the Great Basin. Common shrub associates are *Artemisia tridentata* and *Purshia tridentata*, with species of *Amelanchier, Arctostaphylos, Holodiscus, Prunus, Ribes*, and *Symphoricarpos* commonly present. Scattered trees may also be present, including *Pinus monophylla, Juniperus* spp., *Pinus ponderosa, Pinus flexilis, Pinus jeffreyi, Pseudotsuga menziesii*, or *Abies concolor*. Undergrowth is often very sparse and dominated by bunchgrasses, usually *Achnatherum hymenoides (= Oryzopsis hymenoides), Achnatherum occidentale (= Stipa occidentalis), Hesperostipa* spp., *Poa fendleriana, Poa secunda, Pseudoroegneria spicata*, and *Festuca idahoensis*, and at higher elevations *Calamagrostis rubescens* and *Festuca idahoensis*.

*Cercocarpus ledifolius* woodlands and shrublands are poorly distinguished in the literature, as most authors describe the species as having either a tall‑shrub or small‑tree growth form within a single association. Some associations may have shrub‑dominated stands in one area and also have a woodland physiognomy in another. The woodland physiognomy appears to be more typical, based on available literature. Near the northern edge of its range in Montana and Idaho, *Cercocarpus ledifolius* is described as occurring primarily in the shrub form (Mueggler and Stewart 1980, Tisdale 1986). These northern variants are the only described stands which appear to be clearly distinct from the woodland alliance.

The woodland stands may be dominated by different varieties of *Cercocarpus ledifolius* than shrubland stands. In Wyoming, the Natural Heritage Program is proposing to recognize two *Cercocarpus ledifolius* alliances, based upon varieties of *Cercocarpus ledifolius*. The most widespread proposed alliance (in Wyoming) is dominated by *Cercocarpus ledifolius var. ledifolius*, which grows up to about 1.5 m tall. The other proposed alliance, dominated by *Cercocarpus ledifolius var. intercedens*, is found only along the western border of the state, and the growth form is as small trees 4‑5 m tall. The two taxa are obviously different in Wyoming, in stature and leaf characteristics, and are easily separated (Reid et al. 1998). The shorter variety, *Cercocarpus ledifolius var. ledifolius*, is not reported from Nevada or California (USDA NRCS 2011).

**Dynamics:** *Cercocarpus ledifolius* is a slow‑growing, drought‑tolerant species which can inhabit very poor sites, such as cliffs, stony slopes, and outcrops. Stands are often small and clumped near ridgetops. These sites may also afford the species some protection from fire as the oldest individuals have been observed in these stands (Ross 1999). Succession in these stands is variable depending on site conditions and disturbance as *Cercocarpus ledifolius* is both a primary early‑successional colonizer that rapidly invades bare mineral soils after disturbance and the dominant long‑lived species in mid‑ and late‑seral stands (Duncan 1975, Gruell et al. 1985). Shade tolerance is low so higher‑elevation stands on sites where conifers can grow will eventually be overtopped by taller conifer trees forming woodlands with a *Cercocarpus ledifolius* subcanopy or shrub layer until replaced by more shade‑tolerant shrubs such as *Physocarpus malvaceus* or *Acer glabrum* (Gruell et al. 1985, Steele and Geier‑Hayes 1995).

Mature *Cercocarpus ledifolius* have thick bark and may survive "light" fires (Schultz 1987). However, more often they are killed by fire, and regeneration is by seedling establishment as sprouts following fire are rare and short‑lived (Gruell et al. 1985, Gucker 2006c). Range expansion of this system in the last century has been attributed to decreased fire frequency (Gruell 1982, Gruell et al. 1994). From 1750 to the early 1900s, a mean fire‑return interval was between 13 and 22 years, and stands were likely restricted to rocky sites where fuel levels were low. Since 1900 the fire‑return interval has increased substantially because of fine fuel reductions with heavy livestock grazing, fire exclusion practices, and/or decreased human‑caused fires (Arno and Wilson 1986). However, in the Petersen Mountains of western Nevada, the extent of curl‑leaf mountain‑mahogany has "decreased dramatically" from 1954 to 1997 as a result of increased fire incidence linked to increased cheatgrass dominance (Ross 1999).

*Cercocarpus ledifolius* is highly favored by native ungulates for winter range. Excessive browsing by deer and other wildlife has "high‑lined" individual shrubs and reduced regeneration (West and Young 2000). Seeds are consumed by a variety of small mammals (Plummer et al. 1968). Mortality from bark damage (drilling) by red‑breasted sapsuckers has been reported from Bald Mountain near the California‑Nevada border (Ross 1999).

LANDFIRE developed a state‑and‑transition vegetation dynamics VDDT model for this system which has five classes in total (LANDFIRE 2007a, BpS 1210620). These are summarized as:

A) Early Development 1 All Structures (10% of type in this stage): Curl‑leaf mountain‑mahogany rapidly invades bare mineral soils after fire. Litter and shading by woody plants inhibits establishment. Bunchgrasses and disturbance‑tolerant forbs and resprouting shrubs, such as snowberry, may be present. Rabbitbrush and sagebrush seedlings are present. Vegetation composition will affect fire behavior, especially if chaparral species are present. Replacement fire (average FRI of 500 years), mixed‑severity fire (average FRI of 100 years) and native herbivory of seedlings (2 out every 100) all affect this class. Replacement fire and native herbivory will reset the ecological clock to zero. Mixed‑severity fire does not affect successional age. Succession to class C after 20 years.

B) Mid Development 1 Closed (10% of type in this stage): Young curl‑leaf mountain‑mahogany are common, although shrub diversity is very high. One out of every 1000 mountain‑mahogany are taken by herbivores but this has no effect on model dynamics. Replacement fire (mean FRI of 150 years) causes a transition to class A. Mixed‑severity fire can result in either maintenance (mean FRI of 80 years) in the class or a transition to class D (mean FRI of 200 years). Succession to class E after 90 years.

C) Mid Development 1 Open (15% of type in this stage): Curl‑leaf mountain‑mahogany may codominate with mature sagebrush, bitterbrush, snowberry and rabbitbrush. Few mountain‑mahogany seedlings are present. Replacement fire (mean FRI is 150 years) will cause a transition to class A, whereas mixed‑severity fire (mean FRI of 50 years) will thin this class but not cause a transition to another class. Native herbivory of seedlings and young saplings occurs at a rate of 1/100 seedlings but does not cause an ecological setback or transition. Succession to class B after 40 years.

D) Late Development 1 Open (20% of type in this stage): Moderate cover of mountain‑mahogany. This class represents a combined Mid2‑Open and Late1‑Open cover and structure combination resulting from mixed‑severity fire in class C (note: the combined class results in a slightly inflated representation in the landscape). Further, this class describes one of two late‑successional endpoints for curl‑leaf mountain‑mahogany that is maintained by surface fire (mean FRI of 50 years). Evidence of infrequent fire scars on older trees and presence of open savanna‑like woodlands with herbaceous‑dominated understory are evidence for this condition. Other shrub species may be abundant, but decadent. In the absence of fire for 150 years (2‑3 FRIs for mixed‑severity and surface fires), the stand will become closed (transition to class E) and not support a herbaceous understory. Stand‑replacement fire every 300 years on average will cause a transition to class A. Class D maintains itself with infrequent surface fire and trees reaching very old age.

E) Late Development 1 Closed (45% of type in this stage): High cover of large shrub or tree‑like mountain‑mahogany. Very few other shrubs are present and herb cover is low. Duff may be very deep. Scattered trees may occur in this class. This class describes one of two late‑successional endpoints for curl‑leaf mountain‑mahogany. Replacement fire every 500 years on average is the only disturbance and causes a transition to class A. Class will become old‑growth with trees reported to reach 1000+ years.

Curl‑leaf mountain‑mahogany is easily killed by fire and does not resprout (Marshall 1995b, Gucker 2006c). It is a primary early succession colonizer rapidly invading bare mineral soils after disturbance. Fires are not common in early‑seral stages, when there is little fuel, except in chaparral. Replacement fires (mean FRI of 150‑500 years) become more common in mid‑seral stands, where herbs and smaller shrubs provide ladder fuels. By late succession, two classes and fire regimes are possible depending on the history of mixed‑severity and surface fires. In the presence of surface fire (FRI of 50 years) and past mixed‑severity fires in younger classes, the stand will adopt a savanna‑like woodland structure with a grassy understory, spiny phlox and currant. Trees can become very old and will rarely show fire scars. In late, closed stands, the absence of herbs and small forbs makes replacement fires uncommon (FRI of 500 years), requiring extreme winds and drought. In such cases, thick duff provides fuel for more intense fires. Mixed‑severity fires (mean FRI of 50‑200 years) are present in all classes, except the late‑closed one, and more frequent in the mid‑development classes (LANDFIRE 2007a, BpS 1210620).

Ungulate herbivory: Heavy browsing by native medium‑sized and large mammals reduces mountain‑mahogany productivity and reproduction (Marshall 1995b, Gucker 2006c). This is an important disturbance in early‑ and mid‑seral stages, when mountain‑mahogany seedlings are becoming established. Browsing by small mammals has been documented (Marshall 1995b, Gucker 2006c), but is relatively unimportant and was incorporated as a minor component of native herbivory mortality.

Avian‑caused mortality: In western Nevada, for ranges in close proximity to the Sierra Nevada, sapsucker's drilling of young curl‑leaf mountain‑mahogany has been observed to cause stand‑replacement mortality (C. Ross, NV BLM, pers. comm. 2018). Windthrow and snow creep on steep slopes are also sources of mortality.

SOURCES

**References:** Arno and Wilson 1986, Baker 1983c, Baker and Kennedy 1985, Barbour and Major 1977, Barbour et al. 2007a, Barlow 1977, Barnett and Crawford 1994, Belnap 2001, Belnap et al. 2001, Brown 1982a, Comer et al. 2003*, Cooper et al. 1995, Crawford et al. 2004, Dale et al. 2001, Dealy 1975, Dealy 1978, Drut et al. 1994, Duncan 1975, Ersch 2009, Evans and Belnap 1999, Garfin et al. 2014, Gregg and Crawford 2009, Gruell 1982, Gruell et al. 1985, Gruell et al. 1994, Gucker 2006c, Knight 1994, Knight et al. 1987, LANDFIRE 2007a, Lewis 1975a, Lewis 1975b, Marshall 1995b, Maser et al. 1984, Minnich 2007b, Mote et al. 2014, Mueggler and Stewart 1980, NatureServe Explorer 2011, Phillips et al. 1964, Plummer et al. 1968, Reid et al. 1999, Rich 1980, Rosentreter and Belnap 2003, Ross 1999, Ross pers. comm., Sawyer and Keeler‑Wolf 1995, Sawyer et al. 2009, Schultz 1987, Shafer et al. 2014, Shaw et al. 2004, Shiflet 1994, Steele and Geier‑Hayes 1995, Stevens‑Rumann et al. 2017, Tisdale 1986, USDA NRCS 2011, USFS 1994b, WNHP unpubl. data 2018, Wauer 1977, West and Young 2000

**Version:** 24 May 2018	**Stakeholders:** West

**Concept Author:** K.A. Schulz	**LeadResp:** West

CES304.782 Inter‑Mountain Basins Juniper Savanna

**Primary Division:** Inter‑Mountain Basins (304)

**Land Cover Class:** Steppe/Savanna

**Spatial Scale & Pattern:** Large patch

**Required Classifiers:** Natural/Semi‑natural; Vegetated (>10% vasc.); Upland

**Diagnostic Classifiers:** Temperate [Temperate Continental]; Intermediate Disturbance Interval; F‑Landscape/Medium Intensity; Evergreen Sclerophyllous Tree; Graminoid

**National Mapping Codes:** EVT 2115; ESLF 5404; ESP 1115

**Concept Summary:** This ecological system occupies dry foothills and sandsheets of western Colorado, northwestern New Mexico, northern Arizona, Utah, and west into the Great Basin of Nevada and southern Idaho. It is typically found at lower elevations ranging from 1000‑2300 m. This system is generally found at lower elevations and more xeric sites than Great Basin Pinyon‑Juniper Woodland (CES304.773) or Colorado Plateau Pinyon‑Juniper Woodland (CES304.767). These occurrences are found on lower mountain slopes, hills, plateaus, basins and flats often where juniper is expanding into semi‑desert grasslands and steppe. The vegetation is typically open savanna, although there may be small‑patch inclusions of juniper woodlands. This savanna is typically dominated by an open canopy of *Juniperus osteosperma* trees with high cover of perennial bunchgrasses and forbs, with *Bouteloua gracilis, Hesperostipa comata*, and *Pleuraphis jamesii* being most common. In the southern Colorado Plateau, *Juniperus monosperma* or juniper hybrids may dominate the tree layer. Pinyon trees are typically not present because sites are outside the ecological or geographic range of *Pinus edulis* and *Pinus monophylla*. It has been suggested that all *Juniperus osteosperma* stands in Wyoming be placed in Colorado Plateau Pinyon‑Juniper Woodland (CES304.767). This savanna system does not occur in Wyoming. Extensive *Juniperus osteosperma* woodlands should be included in one of the pinyon‑juniper woodland systems or Rocky Mountain Foothill Limber Pine‑Juniper Woodland (CES306.995).

**Comments:** *Juniperus californica* savannas in the Central Valley of California and around the fringes of the Mojave Desert are not part of this ecological system. In many cases, they are the result of some disturbance removing an oak component from one of the several oak woodland, chaparral, or savanna systems of California. This juniper savanna system does not include sparse juniper stands on rock outcrops or woodlands. It corresponds to the *Juniperus osteosperma*‑dominated portion of the *pinyon‑juniper savanna* type described by Romme et al. (2009) with low to moderate cover of trees, well‑developed graminoid understory, generally a minor shrub component, growing on deeper soils most abundantly in areas with a large proportion of growing‑season precipitation.

DISTRIBUTION

**Range:** This juniper savanna occurs from northwestern New Mexico, northern Arizona, western Colorado, Utah, west into the Great Basin of Nevada and southern Idaho. Where it occurs in California, it is found only in the far eastern edges of the state adjacent to other Great Basin systems.

**Divisions:** 304:C, 306:C

**TNC Ecoregions:** 6:C, 9:C, 10:C, 11:C, 18:C, 19:C, 20:C, 21:C

**Nations:** US

**Subnations:** AZ, CA, CO, ID, NM, NV, OR, UT, WY

**Map Zones:** 7:?, 9:C, 12:P, 13:P, 14:P, 15:C, 16:P, 17:P, 18:C, 19:P, 21:?, 22:?, 23:C, 24:C, 25:C, 28:P

**USFS Ecomap Regions:** 313A:CC, 313B:CC, 313C:CC, 313D:CC, 315H:CC, 321A:CC, 322A:CC, 341A:C?, 341D:C?, 341E:C?, 341F:C?, 341G:CC, 342B:CC, 342C:CC, 342D:CC, 342E:CP, 342G:CC, 342J:CC, M313A:CC, M331D:CC, M331E:C?, M331G:CP, M331H:CC, M331I:CP, M331J:CP, M332E:CC, M341A:CC, M341D:CP

CONCEPT

**Environment:** This widespread ecological system occupies dry foothills and sandsheets of western Colorado, northwestern New Mexico, northern Arizona, Utah, and west into the Great Basin of Nevada and southern Idaho. It is typically found at lower elevations ranging from 1000‑2300m, but may extend up to 2650 m.

*Climate:* Climate is cool, semi‑arid, and continental. Summers are generally hot and dry. Winters are typically cold with occasional snow and there can be extended periods of freezing temperatures. Mean annual precipitation is 25‑35 cm, but the seasonal distribution varies across the range of the system. Generally, winter precipitation in the form of westerly storms is maximal along the northwest edge of the range, and summer moisture increases to the east and south (monsoons). Annual precipitation on the Colorado Plateau has a bimodal distribution with moisture peaking in winter and summer.

*Physiography/landform:* Stands occur on lower to middle elevation mountain slopes and foothills of the many ranges and plateaus of the region.

*Soil/substrate/hydrology:* Substrates are typically moderately deep to deep, coarse‑ to fine‑textured soils that readily support a variety of growth forms, including trees, grasses, and other herbaceous plants (Stuever and Hayden 1997a, Romme et al. 2009).

**Vegetation:** The vegetation is typically open savanna, although there may be small‑patch inclusions of juniper woodlands. This savanna is typically dominated by an open canopy of *Juniperus osteosperma* trees with high cover of perennial bunchgrasses and forbs, with *Bouteloua gracilis, Hesperostipa comata*, and *Pleuraphis jamesii* being most common. In the southern Colorado Plateau, *Juniperus monosperma* or juniper hybrids may dominate the tree layer. Pinyon trees are typically not present because sites are outside the ecological or geographic range of *Pinus edulis* and *Pinus monophylla*.

**Dynamics:** *Juniperus osteosperma* is a relatively short (generally <10 m tall), shade‑intolerant, drought‑tolerant, slow‑growing, long‑lived tree (up to 650 years old) (Meeuwig and Bassett 1983, Zlatnik 1999e). *Juniperus osteosperma* is non‑sprouting and may be killed by fire (Wright et al. 1979). Litter from juniper has an allelopathic effect on some grasses such as *Bouteloua gracilis, Festuca idahoensis*, and *Poa secunda* (Jameson 1970, Zlatnik 1999e).

Within a given region, the density of juniper trees, both historically and currently, is strongly related to topo‑edaphic gradients. Less steep sites, especially those with finer‑textured soils are where savannas, grasslands, and shrub‑steppes have occurred in the past. Stands in this system occurred on these gentler slopes and historically may have been large and savanna‑like with a very open upper canopy and high grass production. Juniper savanna is usually distributed across the landscape in patches that range from 10s to 100s of acres in size (LANDFIRE 2007a). In areas with very broken topography and/or mesa landforms, this type may have occurred in patches of several hundred acres (LANDFIRE 2007a). In Utah and Nevada pinyon and juniper landscape patches tended to be 10‑100s of acres in size (LANDFIRE 2007a).

Key ecological processes are fire, climate fluctuations, grazing/herbivory, and insect/disease outbreaks. The effect of a fire on these stands is largely dependent on the tree height and density, fine‑fuel load on the ground, weather conditions and season (Wright et al. 1979). Large trees generally survive unless the fire gets into the crown due to heavy fuel loads in the understory. In this system fire acts to open stands, kill young trees, increase diversity and productivity in understory species, and create a mosaic of stands of different sizes and ages across the landscape (Bradley et al. 1992).

Uncertainty exists about the fire frequencies of this ecological system, though it is predominantly Fire Regime Group III (fire frequency 30‑100 years) (LANDFIRE 2007a); the fire regime is primarily determined by fire occurrence in the surrounding matrix vegetation (LANDFIRE 2007a). Lightning‑ignited fires were common but typically did not affect more than a few individual trees. Replacement fires were uncommon to rare (average FRI of 100‑500 years) and occurred primarily during extreme fire behavior conditions (LANDFIRE 2007a). Mixed‑severity fire (average FRI of 100‑500 years) was characterized as a mosaic of replacement and surface fires distributed through the patch at a fine scale (<0.1ac) (LANDFIRE 2007a). Surface fires could occur in stands where understory grass cover is high and provides adequate fuel. Surface fires were primarily responsible for producing fire scars on juniper trees and killing juniper seedlings and saplings (average FRI of 100 years).

LANDFIRE developed a state‑and‑transition vegetation dynamics VDDT model for this system which has five classes in total (LANDFIRE 2007a, BpS 2411150). The model was reviewed and references to pinyon were removed, then summarized as:

A) Early Development 1 Open (herbaceous‑dominated ‑ 5% of type in this stage): Initial post‑fire community dominated by annual forbs. Later stages of this class contain greater amounts of perennial grasses and forbs. Duration 10 years with succession to class B, mid‑development closed. Replacement fire occurs every 100 years on average. Infrequent mixed‑severity fire (average FRI of 300 years) thins vegetation.

B) Mid Development 1 Open (herbaceous‑dominated ‑ 5% of type in this stage): Dominated by perennial forbs and grasses. Total cover remains low due to shallow, unproductive soil. Duration 20 years with succession to class C unless infrequent replacement fire (FRI of 100 years) returns the vegetation to A. It is important to note that replacement fire at this stage does not eliminate perennial grasses, thus, succession age in A after this type of fire would be older than zero and <10. Mixed‑severity fire (average FRI of 100 years) thins the woody vegetation.

C) Mid Development 2 Open (15% of type in this stage): Shrub‑dominated community with young juniper seedlings becoming established. Duration 70 years with succession to class D unless replacement fire (average FRI of 200 years) causes a transition to class A. It is important to note that replacement fire at this stage does not eliminate perennial grasses, thus, succession age in class A after this type of fire would be older than zero and <10. Mixed‑severity fire as in class B.

D) Late Development 1 Open (tree‑dominated ‑ 35% of type in this stage): Community dominated by young juniper of mixed age structure. Juniper becoming competitive on site and beginning to affect understory composition. Duration 300 years with succession to class E unless replacement fire (average FRI of 500 years) causes a transition to class A. Mixed‑severity fire is less frequent than in previous states (200 years), whereas surface fire every 100 years on average becomes more important at this age in succession.

E) Late Development 2 Open (tree‑dominated ‑ 40% of type in this stage): Site dominated by widely spaced old juniper trees. Grasses (e.g., *Bouteloua gracilis, Hesperostipa comata*) present on microsites sites with deeper soils (>20 in) with restricting clay subsurface horizon. Replacement fire and mixed‑severity fires are rare (average FRIs of 500 years). Surface fire every 100 years on average will scar ancient trees. Duration 600+ years.

<br />Drought is an important ecological process which limits seedling recruitment and survival and causes mortality of mature trees (Romme et al. 2009). Other important ecological variables include insect infestations, pathogens, herbivory, and seed dispersal by birds and mammals. Juniper berries crops are primarily utilized by birds and small mammals (Johnsen 1962, McCulloch 1969, Short et al. 1977, Salomonson 1978, Balda 1987, Gottfried et al. 1995). The most important dispersers of juniper seeds are birds although mammals also feed on them (Scher 2002). These animals consume juniper berries and excrete viable scarified juniper seeds, which germinate faster than uneaten seeds, over extensive areas (Johnsen 1962, Meeuwig and Bassett 1983). Primary juniper seed dispersers are Bohemian waxwings (*Bombycilla garrulus*), but cedar waxwings (*Bombycilla cedrorum*), American robins (*Turdus migratorius*), turkeys (*Meleagris gallopavo*), and several species of jays are also dispersers (Scher 2002).

There are several insects, plant parasites and pathogens (*Cercospora sequoiae*, a blight, and *Gymnosporangium* spp., stem rusts) that attack juniper trees (Burns and Honkala 1990a, Rogers 1995). Two insects, western cedar borer (*Trachykele blondeli*) and juniper twig pruner (*Styloxus bicolor*), damage mature trees and can cause mortality (Rogers 1995). Juniper mistletoe (*Phoradendron juniperinum*) occurs on junipers where it reduces vigor and causes dieback, but rarely causes mortality (Meeuwig and Bassett 1983).

Biological soils crusts (BSC) are important for soil fertility, soil moisture, and soil stability in many semi‑arid ecosystems and may be important on juniper savanna sites, especially on those with more exposed soil surface and less herbaceous and litter cover, and low disturbance (Belnap et al. 2001, Belnap and Lange 2003). Cyanobacteria (especially *Nostoc*) fix large amounts of soil nitrogen and carbon (Evans and Belnap 1999, Belnap 2001).

SOURCES

**References:** Balda 1987, Bell et al. 2009, Belnap 2001, Belnap and Lange 2003, Belnap et al. 2001, Bradley et al. 1992a, Burns and Honkala 1990a, CNHP 2010, Comer et al. 2003*, Evans and Belnap 1999, Eyre 1980, Gottfried et al. 1995, Jameson 1970, Johnsen 1962, LANDFIRE 2007a, McCulloch 1969, Meeuwig and Bassett 1983, Rogers 1995, Romme et al. 2003, Romme et al. 2009, Rosentreter and Belnap 2003, Salomonson 1978, Scher 2002, Shiflet 1994, Short et al. 1977, Stuever and Hayden 1997b, West 1999b, Wright et al. 1979, Zlatnik 1999e

**Version:** 04 Nov 2015	**Stakeholders:** West

**Concept Author:** K.A. Schulz	**LeadResp:** West

M027. Southern Rocky Mountain‑Colorado Plateau Two‑needle Pinyon ‑ Juniper Woodland

CES304.766 Colorado Plateau Pinyon‑Juniper Shrubland

**Primary Division:** Inter‑Mountain Basins (304)

**Land Cover Class:** Shrubland

**Spatial Scale & Pattern:** Matrix

**Required Classifiers:** Natural/Semi‑natural; Vegetated (>10% vasc.); Upland

**Diagnostic Classifiers:** Lowland [Foothill]; Mesa; Ridge/Summit/Upper Slope; Sedimentary Rock; Temperate [Temperate Xeric]; Aridic; Pinus edulis, Juniperus osteosperma

**National Mapping Codes:** EVT 2102; ESLF 5308; ESP 1102

**Concept Summary:** This ecological system is characteristic of the rocky mesatops and slopes on the Colorado Plateau and western slope of Colorado, but these stunted tree shrublands may extend further upslope along the low‑elevation margins of taller pinyon‑juniper woodlands. Sites are drier than Colorado Plateau Pinyon‑Juniper Woodland (CES304.767). Substrates are shallow/rocky and shaly soils at lower elevations (1200‑2000 m). Sparse examples of the system grade into Colorado Plateau Mixed Bedrock Canyon and Tableland (CES304.765). The vegetation is dominated by dwarfed (usually <3 m tall) *Pinus edulis* and/or *Juniperus osteosperma* trees forming extensive tall shrublands in the region along low‑elevation margins of pinyon‑juniper woodlands. Other shrubs, if present, may include *Artemisia nova, Artemisia tridentata ssp. wyomingensis, Chrysothamnus viscidiflorus*, or *Coleogyne ramosissima*. Herbaceous layers are sparse to moderately dense and typically composed of xeric graminoids.

**Comments:** Species composition is similar to Colorado Plateau Pinyon‑Juniper Woodland (CES304.767); however, stands of Colorado Plateau Pinyon‑Juniper Shrubland (CES304.766) are composed of an open canopy of short/drought‑stunted trees and typically occur at lower elevations on rocky, xeric sites. Lower elevation/more xeric site stands become sparse and transition into Colorado Plateau Mixed Bedrock Canyon and Tableland (CES304.765). Although this system has a shrubland/stunted woodland structure, it corresponds to the *Juniperus osteosperma*‑ and/or *Pinus edulis*‑dominated portion of the *persistent pinyon‑juniper woodland* type from Romme et al. (2009) that occurs on rocky uplands with shallow, coarse‑textured, and often skeletal soils that support relatively sparse herbaceous cover and rarely burn.

DISTRIBUTION

**Range:** This system occurs on rocky mesatops and slopes on the Colorado Plateau.

**Divisions:** 304:C, 306:?

**TNC Ecoregions:** 18:C, 19:C, 20:?

**Nations:** US

**Subnations:** AZ, CO, NM, UT

**Map Zones:** 13:P, 15:P, 16:C, 23:C, 24:C, 25:C, 28:P

**USFS Ecomap Regions:** 313A:CC, 313B:CC, 313D:CP, 322A:??, 341B:CC, 341C:CC, 342G:CC, M331D:CC, M331E:CC, M331G:CC, M331H:CC, M341B:CC, M341C:CC

CONCEPT

**Environment:** This tree‑dominated ecological system is characteristic of the dry, lower elevation sites in the rocky canyons of the Colorado Plateau and Western Slope of Colorado (1200‑1600 m elevation), but these stunted‑tree shrublands may extend further upslope to 2000 m on locally xeric sites (Stuever and Hayden 1997a).

*Climate:* Climate is semi‑arid to arid with hot summers and cold winters. Based on data from Moab, Utah, average annual precipitation is approximately 25 cm. Precipitation mostly occurs as rain during monsoons (late July to October) and spring (March to May). June is the driest month.

<br />*Physiography/landform:* Stands occur on the rocky mesatops, canyon rims, and dry slopes and ridges that are too dry for woodlands.

*Soil/substrate/hydrology:* Substrates are shallow/rocky and shaly soils at lower elevations. Sandstone is the most common parent material.

**Vegetation:** The vegetation is dominated by dwarfed (usually <3 m tall) *Pinus edulis* and/or *Juniperus osteosperma* trees forming extensive tall shrublands in the region along low‑elevation margins of pinyon‑juniper woodlands. Other shrubs, if present, may include *Artemisia nova, Artemisia tridentata ssp. wyomingensis, Chrysothamnus viscidiflorus*, or *Coleogyne ramosissima*. Herbaceous layers are sparse to moderately dense and typically composed of xeric graminoids.

**Dynamics:** *Pinus edulis* is extremely drought‑tolerant and slow‑growing (Little 1987). It is also non‑sprouting and may be killed by fire (Wright et al. 1979, Wright and Bailey 1982a). This shrubland or stunted woodland (<3 m tall) is characteristic of the drier, hotter low‑elevation sites (usually <1600 m), rock outcrops and sites with shallow soils that limit tree growth. The understory is typically sparser than Colorado Plateau Pinyon‑Juniper Woodland (CES304.767) and this system is more affected by drought than fires; however, occurrences of this system will burn under extreme fire conditions. The effect of fire on a stand is largely dependent on tree height and density, fine‑fuel load on the ground, weather conditions, and season (Dwyer and Pieper 1967, Wright et al. 1979, Wright and Bailey 1982a). Trees are more vulnerable in open stands where fires frequently occur in the spring, when the relative humidity is low, wind speeds are over 10‑20 mph, and there are adequate fine fuels to carry fire (Wright et al. 1979, Wright and Bailey 1982a). Under other conditions, burns tend to be spotty with low tree mortality. Large trees are generally not killed unless fine fuels, such as tumbleweeds, have accumulated beneath the trees to provide ladder fuels for the fire to reach the crown (Jameson 1962). Closed‑canopy stands burn infrequently because they typically do not have enough understory or wind to carry fire (Wright et al. 1979).

LANDFIRE developed a state‑and‑transition vegetation dynamics VDDT model for this system which has five classes in total (LANDFIRE 2007a, BpS 2311020). These are summarized as:

A) Early Development 1 Open (herbaceous‑dominated ‑ 5% of type in this stage): Initial post‑fire community dominated by annual forbs. Later stages of this class contain greater amounts of perennial grasses and forbs. Duration 10 years with succession to class B, mid‑development closed. Replacement fire occurs every 100 years on average. Infrequent mixed‑severity fire (average FRI of 300 years) thins vegetation.<br />

B) Mid Development 1 Open (shrub‑dominated ‑ 5% of type in this stage): Dominated by shrubs, perennial forbs and grasses. Total cover remains low due to shallow, unproductive soil. Duration 20 years with succession to class C unless infrequent replacement fire (FRI of 100 years) returns the vegetation to class A. It is important to note that replacement fire at this stage does not eliminate perennial grasses, thus, succession age in class A after this type of fire would be older than zero and <10. Mixed‑severity fire (average FRI of 100 years) thins the woody vegetation but does not cause a transition to another class.

C) Mid Development 2 Open (shrub‑dominated ‑ 10% of type in this stage): Shrub‑dominated community with young juniper and pinyon seedlings becoming established. Duration 70 years with succession to class D unless replacement fire (average FRI of 200 years) causes a transition to class A. It is important to note that replacement fire at this stage does not eliminate perennial grasses, thus, succession age in class A after this type of fire would be older than zero and <10. Mixed‑severity fire as in class B.

D) Late Development 1 Open (conifer‑dominated ‑ 35% of type in this stage): Community dominated by young and stunted juniper and pinyon of mixed age structure. Juniper and pinyon becoming competitive on site and beginning to affect understory composition. Duration 300 years with succession to E unless replacement fire (average FRI of 500 years) causes a transition to A. Mixed‑severity fire is less frequent than in previous states (200 years), whereas surface fire every 100 years on average becomes more important at this age in succession.

E) Late Development 2 Open (conifer‑dominated ‑ 45% of type in this stage): Site dominated by widely spaced old and stunted juniper and pinyon. Understory depauperate and high amounts of bare ground and rock present. Grasses present on microsites with deeper soils (>50 cm [20 inches]) with restricting clay subsurface horizon. Potential maximum overstory coverage is greater in those stands with pinyon as compared to those with only juniper. Replacement fire and mixed‑severity fires are rare (average FRIs of 500 years). Surface fire every 100 years on average will scar ancient stunted trees. Duration 600 years+.

<br />Other important ecological processes include drought, insect infestations, pathogens, herbivory and seed dispersal by birds and mammals. Juniper berries and pinyon nut crops are primarily utilized by birds and small mammals (Johnsen 1962, McCulloch 1969, Short et al. 1977, Salomonson 1978, Balda 1987, Gottfried et al. 1995). The most important dispersers of juniper and pinyon seeds are birds, although many mammals also feed on them. These animals consume juniper berries and excrete viable scarified juniper seeds, which germinate faster than uneaten seeds, over extensive areas (Johnsen 1962, Meeuwig and Bassett 1983). Primary juniper seed dispersers are Bohemian waxwing (*Bombycilla garrulus*), but others include cedar waxwing (*Bombycilla cedrorum*), American robin (*Turdus migratorius*), turkey (*Meleagris gallopavo*), and several species of jays (Scher 2002). Pinyon seeds are a critically important food source for scrub jay (*Aphelocoma californica*), pinyon jay (*Gymnorhinus cyanocephalus*), Steller's jay (*Cyanocitta stelleri*) and Clark's nutcracker (*Nucifraga columbiana*). These birds are the primary dispersers of pinyon pine seeds and, during mast crop years, cache hundreds of thousands of pinyon seeds, many of which are never recovered (Balda and Bateman 1971, Vander Wall and Balda 1977, Ligon 1978). Because pinyon seeds are heavy and totally wingless, seed dispersal is dependent on vertebrate dispersers that store seeds in food caches, where unconsumed seeds may germinate. This dispersal mechanism is a good example of a co‑evolved, mutualistic, plant‑vertebrate relationship (Vander‑Wall et al. 1981, Evans 1988, Lanner 1996) and would be at risk with loss of trees or dispersers. Many mammals are also known to eat pinyon seeds, such as several species of mice (*Peromyscus* spp.), woodrats (*Neotoma* spp.), squirrels (*Sciurus* spp.), chipmunks (*Neotamias* spp.), and desert bighorn sheep (*Ovis canadensis nelsoni*) and, although less effective, they may inadvertently disperse seeds (Anderson 2002).

Although *Pinus edulis* is drought‑tolerant, prolonged droughts will weaken trees and promote mortality by secondary agents. Periodic die‑offs of pinyon pine caused by insects, such as the pinyon ips beetle (*Ips confusus*), or fungal agents, such as blackstain root‑rot (*Leptographium wageneri*), tend to be correlated with droughts (Anhold 2005). These mortality events may be localized or widespread but can result in 50 to 90% mortality of *Pinus edulis* in affected areas (Harrington and Cobb 1988). There are many insects, pathogens, and plant parasites that attack pinyon and juniper trees (Meeuwig and Bassett 1983, Gottfried et al. 1995, Rogers 1995, Weber et al. 1999). Juniper mistletoe (*Phoradendron juniperinum*) occurs on junipers and pinyon dwarf mistletoe (*Arceuthobium divaricatum*) occurs on pines. Both mistletoes reduce vigor and cause dieback but rarely cause mortality (Meeuwig and Bassett 1983). For pinyon and juniper, there are at least seven insects, and fungi such as black stain root‑rot (*Leptographium wageneri*), and pinyon needle rust and pinyon blister rust (Skelly and Christopherson 2003). The insects are normally present in these woodland stands and during drought‑induced water stress, outbreaks may cause local to regional mortality (Wilson and Tkacz 1992, Gottfried et al. 1995, Rogers 1995). Most insect‑related pinyon mortality in the West is caused by pinyon ips bark beetle (*Ips confusus)* (Rogers 1993).

Most pinyon‑juniper woodlands and shrublands in the Southwest have high soil erosion potential (Baker et al. 1995). Several studies have measured present‑day erosion rates in pinyon‑juniper woodlands, highlighting the importance of herbaceous cover and biological soil crusts in minimizing precipitation runoff and soil loss (Baker et al. 1995, Ladyman and Muldavin 1996, Belnap et al. 2001).

SOURCES

**References:** Anderson 2002, Anhold 2005, Baker et al. 1995, Balda 1987, Balda and Bateman 1971, Belnap 2001, Belnap et al. 2001, Betancourt et al. 1993, Breshears et al. 2005, Burns and Honkala 1990a, CNHP 2010, Comer et al. 2003*, Davis and Turner 1986, Dwyer and Pieper 1967, Evans 1988, Furniss and Carolin 2002, Gori and Bate 2007, Gottfried et al. 1995, Harrington and Cobb 1988, Jameson 1962, Johnsen 1962, LANDFIRE 2007a, Ladyman and Muldavin 1996, Lanner 1996, Ligon 1978, Little 1987, McAuliffe and Van Devender 1998, McCulloch 1969, Meeuwig and Bassett 1983, Mehringer and Wigand 1990, Miller and Tausch 2001, Pieper and Wittie 1990, Rogers 1993, Rogers 1995, Romme et al. 2009, Rosentreter and Belnap 2003, Salomonson 1978, Scher 2002, Schwinning et al. 2008, Shiflet 1994, Short et al. 1977, Skelly and Christopherson 2003, Stevens 1999a, Stuever and Hayden 1997b, Swetnam and Baisan 1996a, Tausch 1999, Tausch and Hood 2007, Van Devender 1977, Van Devender 1990, Vander Wall and Balda 1977, Vander Wall et al. 1981, Weber et al. 1999, Wright and Bailey 1982a, Wright et al. 1979

**Version:** 04 Nov 2015	**Stakeholders:** West

**Concept Author:** K.A. Schulz	**LeadResp:** West

CES304.767 Colorado Plateau Pinyon‑Juniper Woodland

**Primary Division:** Inter‑Mountain Basins (304)

**Land Cover Class:** Forest and Woodland

**Spatial Scale & Pattern:** Matrix

**Required Classifiers:** Natural/Semi‑natural; Vegetated (>10% vasc.); Upland

**Diagnostic Classifiers:** Montane [Lower Montane]; Lowland [Foothill]; Mesa; Ridge/Summit/Upper Slope; Sedimentary Rock; Temperate [Temperate Xeric]; Aridic; Pinus edulis, Juniperus osteosperma

**National Mapping Codes:** EVT 2016; ESLF 4203; ESP 1016

**Concept Summary:** This ecological system occurs in dry mountains and foothills of the Colorado Plateau region including the Western Slope of Colorado to the Wasatch Range, south to the Mogollon Rim, and east into the northwestern corner of New Mexico. It is typically found at lower elevations ranging from 1500‑2440 m. These woodlands occur on warm, dry sites on mountain slopes, mesas, plateaus, and ridges. Soils supporting this system vary in texture, ranging from stony, cobbly, gravelly sandy loams to clay loam or clay. *Pinus edulis* and/or *Juniperus osteosperma* dominate the tree canopy. In the southern portion of the Colorado Plateau in northern Arizona and northwestern New Mexico, *Juniperus monosperma* and hybrids of *Juniperus* spp. may dominate or codominate the tree canopy. *Juniperus scopulorum* may codominate or replace *Juniperus osteosperma* at higher elevations. Understory layers are variable and may be dominated by shrubs, graminoids, or be absent. Associated species include *Arctostaphylos patula, Artemisia tridentata, Cercocarpus intricatus, Cercocarpus montanus, Coleogyne ramosissima, Purshia stansburiana, Purshia tridentata, Quercus gambelii, Bouteloua gracilis, Pleuraphis jamesii, Pseudoroegneria spicata, Poa secunda*, or *Poa fendleriana*. This system occurs at higher elevations than Great Basin Pinyon‑Juniper Woodland (CES304.773) and Colorado Plateau shrubland systems where sympatric.

**Comments:** This system is similar to Southern Rocky Mountain Pinyon‑Juniper Woodland (CES306.835) as *Pinus edulis* is a diagnostic species for both. However, this system typically occurs within the range of *Juniperus osteosperma* within the Colorado Plateau ecoregion.

DISTRIBUTION

**Range:** This system occurs on dry mountains and foothills of the Colorado Plateau region from the Western Slope of Colorado to the Wasatch Range, south to the Mogollon Rim, and east into the northwestern corner of New Mexico. It is typically found at lower elevations, ranging from 1500‑2440 m. In Wyoming, it would occur only in the southern portions of mapzone 22.

**Divisions:** 304:C, 306:C

**TNC Ecoregions:** 18:C, 19:C, 20:?

**Nations:** US

**Subnations:** AZ, CO, NM, UT, WY?

**Map Zones:** 13:P, 15:C, 16:C, 17:P, 22:C, 23:C, 24:C, 25:C, 28:C

**USFS Ecomap Regions:** 313A:CC, 313B:CC, 313C:CC, 313D:CC, 315H:CC, 321A:CC, 322A:CC, 341A:CC, 341B:CC, 341C:CC, 341F:CP, 342E:CP, 342G:CC, M313A:CC, M313B:CC, M331D:CC, M331E:CC, M331G:CC, M331H:CC, M331I:CC, M341B:CC, M341C:CC

CONCEPT

**Environment:** This ecological system occurs in dry mountains and foothills of the Colorado Plateau region, including the western slope of Colorado to the Wasatch Range, south to the Mogollon Rim, and east into the northwestern corner of New Mexico. It is typically found at lower elevations ranging from 1500‑2440 m (Hess and Wasser 1982, Stuever and Hayden 1997a).

<br />*Climate:* Climate is semi‑arid. Annual precipitation is usually from 30‑55 cm in the form of rain and snow. Severe climatic events occurring during the growing season, such as frosts and drought, are thought to limit the distribution of pinyon‑juniper woodlands to relatively narrow altitudinal belts on mountainsides.

*Physiography/landform:* These woodlands occur on warm, dry sites on mountain slopes, mesas, plateaus, and ridges. Stands occur on a variety of aspects and slopes. Slope may range from nearly level to steep (up to 80%).

*Soil/substrates/hydrology:* Soils supporting this system vary in depth and texture, ranging from shallow, stony, cobbly, gravelly sandy loams to often deeper clay loam or clay. Parent materials likewise vary widely from granite, basalt, limestone, and sandstone to mixed alluvium (Springfield 1976). Soil depths may range from shallow to deep.

**Vegetation:** *Pinus edulis* and/or *Juniperus osteosperma* dominate the tree canopy. In the southern portion of the Colorado Plateau in northern Arizona and northwestern New Mexico, *Juniperus monosperma* and hybrids of *Juniperus* spp. may dominate or codominate the tree canopy. *Juniperus scopulorum* may codominate or replace *Juniperus osteosperma* at higher elevations. Understory layers are variable and may be dominated by shrubs, graminoids, or be absent. Associated species include *Arctostaphylos patula, Artemisia tridentata, Cercocarpus intricatus, Cercocarpus montanus, Coleogyne ramosissima, Purshia stansburiana, Purshia tridentata, Quercus gambelii, Bouteloua gracilis, Pleuraphis jamesii, Pseudoroegneria spicata, Poa secunda*, or *Poa fendleriana*.

**Dynamics:** Key ecological processes are drought, fire, herbivory, and insect/disease outbreaks. Both *Pinus edulis* and *Juniperus osteosperma* are relatively short (generally <15 m tall), shade‑intolerant, drought‑tolerant, slow‑growing, long‑lived trees (especially *Juniperus osteosperma* can reach 650 years old) (Meeuwig and Bassett 1983, Little 1987, Zlatnik 1999e, Romme et al. 2003). Both tree species are also non‑sprouting and may be killed by fire (Wright et al. 1979). The effect of a fire on these stands is largely dependent on the tree height and density, fine fuel load on the ground, weather conditions and season (Wright et al. 1979). Large trees generally survive unless the fire gets into the crown due to heavy fuel loads in the understory. In this system fire acts to open stands, increase diversity and productivity in understory species, and create a mosaic of stands of different sizes and ages across the landscape while maintaining the boundary between woodlands and adjacent shrublands or grasslands (Bradley et al. 1992).<br />

As modeled by LANDFIRE (2007a), the fire regime is characterized by somewhat frequent mixed‑severity mosaic fires (mean FRI of 150‑200 years) with very infrequent replacement fires (mean FRI of 200‑500 years) (Rondeau 2001). Surface fire occurs only in the earliest succession class every 200 years on average (LANDFIRE 2007a). There is frequent fire spread from adjacent types (LANDFIRE 007a). Severe climatic events occurring during the growing season, such as frosts and drought, are thought to limit the distribution of pinyon‑juniper woodlands to relatively narrow altitudinal belts on mountainsides. Weather‑related stress thins trees every 145 years on average in more closed stands (LANDFIRE 2007a). Insects/disease has a similar effect, but with a greater frequency in closed stands (mean return interval of 100 years) than open ones (mean return interval of 1000 years) (LANDFIRE 2007a). Competition from grasses and older trees in late‑open stands is also included as a disturbance that maintains stand openness (LANDFIRE 2007a).

<br />LANDFIRE developed a state‑and‑transition vegetation dynamics VDDT model for this system which has five classes in total (LANDFIRE 2007a, BpS 2310160). These are summarized as:

A) Early Development 1 All Structures (10% of type in this stage): Grass/forb/shrub/seedling ‑ usually post‑fire. Cover is 0‑30%. Shrub height 0.5 m. Both replacement fire and surface fire occur in this class (mean FRI of 200 years for both). The dominant succession path is to class C (mid, open) after 60 years, although the model allows for an alternate succession pathway to class B (mid, closed) 1/100 times to represent tree invasion.

B) Mid Development 1 Closed (20% of type in this stage): Tree cover is 40‑70%. Tree height <5 m. Mid‑development, dense (>40% cover) pinyon‑juniper woodland; understory is sparse. Replacement fire occurs every 400 years on average. Three disturbances cause a transition to class C (mid, open): mixed‑severity fire (mean FRI of 150 years), insects/disease (mean return interval of 100 years) and weather‑related stress (mean return interval of 150 years). Succession to class E, late‑closed, after 120 years.

C) Mid Development 1 Open (25% of type in this stage): Tree cover is 10‑40%. Tree height <5 m. Mid‑development, open (<40% cover) pinyon‑juniper stand with mixed shrub/herbaceous community in understory. The mean FRI for replacement fire is 500 years. Mixed‑severity fire (mean FRI of 200 years) and insects/disease (mean return interval of 1000 years) maintain stand structure. Primary succession pathway to class D, late‑open, after 100 years, although an alternate succession pathway to class B 2/100 times is included to represent tree invasion;

D) Late Development 1 Open (35% of type in this stage): Tree cover is 10‑40%. Tree height 5‑10 m. Late‑development, open juniper‑pinyon stand with "savanna‑like" appearance; mixed grass/shrub/herbaceous community. Replacement fire is infrequent (mean FRI of 500 years). Mixed‑severity fire (mean FRI of 200 years), insects/disease (mean return interval of 1000 years) and competition (1/100 prob/year) maintain vegetation in class D, which is the primary succession endpoint. Alternate succession to class E, late‑closed, occurs 1/200 times to represent tree invasion;

E) Late Development 2 Open (conifer‑dominated ‑ 35% of type in this stage): Tree cover is 40‑70%. Tree height 5‑10 m. Dense, old‑growth stands with multiple layers. Late‑development, closed pinyon‑juniper forest. May have all‑aged, multi‑storied structure. Moderate mortality within stand. Occasional shrubs with few grasses and forbs and often rock or bare soil. The mean FRI of replacement fire is 500 years. Mixed‑severity fire (mean FRI of 150 years), insects/disease (mean return interval of 100 years) and weather‑related stress (mean return interval of 100 years) thin tree cover, therefore causing a transition to class D. Succession maintains vegetation in class E.

Other important ecological processes include drought, insect infestations, pathogens, herbivory, and seed dispersal by birds and mammals. Juniper berry and pinyon nut crops are primarily utilized by birds and small mammals (Johnsen 1962, McCulloch 1969, Short et al. 1977, Salomonson 1978, Balda 1987, Gottfried et al. 1995). Large mammals, such as mule deer (*Odocoileus hemionus*), white‑tailed deer (*Odocoileus virginianus*) and elk (*Cervus elaphus*), eat leaves and seeds of both species and they browse woodland grasses, forbs and shrubs, including *Artemisia tridentata, Cercocarpus montanus, Quercus gambelii*, and *Purshia stansburiana* (Short and McCulloch 1977).

The most important dispersers of juniper and pinyon seeds are birds, although many mammals also feed on them. These animals consume juniper berries and excrete viable scarified juniper seeds over extensive areas, which germinate faster than uneaten seeds (Johnsen 1962, Meeuwig and Bassett 1983). Primary juniper seed dispersers are Bohemian waxwing (*Bombycilla garrulus*), cedar waxwing (*Bombycilla cedrorum*), American robin (*Turdus migratorius*), turkey (*Meleagris gallopavo*), and several species of jays (Scher 2002). Pinyon seeds are a critically important food source for western scrub jay (*Aphelocoma californica*), pinyon jay (*Gymnorhinus cyanocephalus*), Steller's jay (*Cyanocitta stelleri*) and Clark's nutcracker (*Nucifraga columbiana*). These birds are the primary dispersers of pinyon seeds and during mast crop years cache hundreds of thousands of pinyon seeds, many of which are never recovered (Balda and Bateman 1971, Vander Wall and Balda 1977, Ligon 1978). Many mammals are also known to eat pinyon seeds, such as several species of mice (*Peromyscus* spp.), woodrats (*Neotoma* spp.), squirrels (*Sciurus* spp.), chipmunks (*Neotamias* spp.), deer, black bear (*Ursus americanus*), and desert bighorn sheep (*Ovis canadensis nelsoni*) (Anderson 2002). Because pinyon seeds are heavy and totally wingless, seed dispersal is dependent on vertebrate dispersers that store seeds in food caches, where unconsumed seeds may germinate. This dispersal mechanism is a good example of a co‑evolved, mutualistic, plant‑vertebrate relationship (Vander Wall et al. 1981, Evans 1988, Lanner 1996) and would be at risk with loss of trees or dispersers.<br />

There are many insects, pathogens, and plant parasites that attack pinyon and juniper trees (Meeuwig and Bassett 1983, Gottfried et al. 1995, Rogers 1995, Weber et al. 1999). For pinyon and juniper, there are at least seven insects, plus a fungus (blackstain root‑rot (*Leptographium wageneri*)), juniper mistletoe (*Phoradendron juniperinum*) and pinyon dwarf mistletoe (*Arceuthobium divaricatum*). Both mistletoes reduce vigor and cause occasional dieback but rarely cause mortality (Meeuwig and Bassett 1983). The insects are normally present in these woodland stands, and during drought‑induced water stress periods, outbreaks may cause local to regional mortality (Wilson and Tkacz 1992, Gottfried et al. 1995, Rogers 1995). Most insect‑related pinyon mortality in the West is caused by pinyon ips beetle (*Ips confusus*) (Rogers 1993). Pinyons cannot repel pinyon ips beetles when weakened by drought and many are killed. During the drought of 2002‑2003, populations of ips beetles increased to epidemic levels that killed millions of pinyon trees in the southwestern U.S. (Thorne et al. 2007).

Most pinyon‑juniper woodlands in the southwest have high soil erosion potential (Baker et al. 1995). Several studies have measured present‑day erosion rates in pinyon‑juniper woodlands, highlighting the importance of herbaceous cover and cryptogamic soil crusts (Baker et al. 1995, Belnap et al. 2001) in minimizing precipitation runoff and soil loss in pinyon‑juniper woodlands.

SOURCES

**References:** Anderson 2002, Baker et al. 1995, Balda 1987, Balda and Bateman 1971, Belnap 2001, Belnap et al. 2001, Betancourt et al. 1993, Bradley et al. 1992a, Breshears et al. 2005, Brooks and Minnich 2006, Burns and Honkala 1990a, CNHP 2010, Comer et al. 2003*, Davis and Turner 1986, Evans 1988, Evans and Belnap 1999, Eyre 1980, Furniss and Carolin 2002, Gori and Bate 2007, Gottfried et al. 1995, Hess and Wasser 1982, Hollander and Vander Wall 2004, Johnsen 1962, LANDFIRE 2007a, Lanner 1996, Ligon 1978, Little 1987, McAuliffe and Van Devender 1998, McCulloch 1969, Meeuwig and Bassett 1983, Miller and Tausch 2001, Pieper and Wittie 1990, Rogers 1993, Rogers 1995, Romme et al. 2003, Rondeau 2001, Rosentreter and Belnap 2003, Salomonson 1978, Scher 2002, Shiflet 1994, Short and McCulloch 1977, Short et al. 1977, Springfield 1976, Stevens 1999a, Stuever and Hayden 1997b, Swetnam and Baisan 1996a, Tausch 1999, Tausch and Hood 2007, Thorne et al. 2007, Van Devender 1977, Van Devender 1990, Vander Wall and Balda 1977, Vander Wall et al. 1981, Weber et al. 1999, Wilson and Tkacz 1992, Wright et al. 1979, Zlatnik 1999e

**Version:** 29 Aug 2015	**Stakeholders:** West

**Concept Author:** K.A. Schulz	**LeadResp:** West

CES306.834 Southern Rocky Mountain Juniper Woodland and Savanna

**Primary Division:** Rocky Mountain (306)

**Land Cover Class:** Steppe/Savanna

**Spatial Scale & Pattern:** Large patch

**Required Classifiers:** Natural/Semi‑natural; Vegetated (>10% vasc.); Upland

**Diagnostic Classifiers:** Lowland [Foothill]; Woody‑Herbaceous; Shallow Soil; Mineral: W/ A‑Horizon <10 cm; Aridic; Needle‑Leaved Tree; Graminoid; Juniperus monosperma and grasses

**National Mapping Codes:** EVT 2119; ESLF 5408; ESP 1119

**Concept Summary:** This ecological system occupies the lower and warmest elevations, growing from 1370 to 1830 m in a semi‑arid climate, primarily along the east and south slopes of the Southern Rockies and Arizona‑New Mexico mountains. It is best represented just below the lower elevational range of ponderosa pine and often intermingles with grasslands and shrublands. This system is best described as a savanna that has widely spaced, mature (>150 years old) juniper trees and occasionally *Pinus edulis*. *Juniperus monosperma* and *Juniperus scopulorum* (at higher elevations) are the dominant tall shrubs or short trees. These savannas may have inclusions of denser juniper woodlands and they have expanded into adjacent grasslands during the last century. Graminoid species are similar to those found in Western Great Plains Shortgrass Prairie (CES303.672), with *Bouteloua gracilis* and *Pleuraphis jamesii* being most common. In addition, succulents such as species of *Yucca* and *Opuntia* are typically present.

**Comments:** This system corresponds to the *Juniperus monosperma*‑dominated portion of the *pinyon‑juniper savanna* type described by Romme et al. (2009) with low to moderate cover of trees, well‑developed graminoid understory, generally a minor shrub component, growing on deeper soils most abundantly in areas with a large proportion of growing‑season precipitation. Denser woodland areas are the result of infilling of juniper trees and small‑patch inclusions of the denser of juniper occurring on shallow rocky soils that resemble the *persistent pinyon‑juniper woodland* type from Romme et al. (2009).

DISTRIBUTION

**Range:** This system occupies the lower and warmest elevations, growing from 1370 to 1830 m elevation in a semi‑arid climate, primarily along the east and south slopes of the Southern Rockies and central New Mexico mountains. This includes the Sacramento Mountains, especially the east side; the west side has Madrean elements but is mostly southern Rocky Mountains. This system also occurs in the canyons and tablelands of the southwestern Great Plains extending some distance from the mountains. It may occur along the Cimarron River in the panhandle regions of Oklahoma and Texas, and in the very southwestern corner of Kansas.

**Divisions:** 303:C, 304:C, 306:C

**TNC Ecoregions:** 20:C, 21:C, 27:C

**Nations:** US

**Subnations:** CO, KS?, NM, OK?, TX?

**Map Zones:** 24:P, 25:C, 26:?, 27:C, 28:C, 34:P

**USFS Ecomap Regions:** 315A:CC, 315B:CC, 315H:CC, 321A:PP, 331B:CC, 331C:C?, 331I:CC, 331J:CC, M313B:CC, M331F:CC, M331G:CC

CONCEPT

**Environment:** This ecological system occupies the lower and warmest elevations, growing from 1370 to 1830 m primarily along the east and south slopes of the Southern Rockies and Arizona‑New Mexico mountains. It is best represented just below the lower elevational range of ponderosa pine and often intermingles with grasslands. In the canyons and tablelands of the southern Great Plains, this system forms extensive cover at some distance from the mountain front.

*Climate:* Climate is cool‑temperate, continental, and semi‑arid. Precipitation ranges from approximately 33‑46 cm (13‑18 inches) annually and has a bimodal distribution with moisture peaking in winter and summer. However, most precipitation generally occurs during the summer growing season.

<br />*Physiography/landform:* Stands occur on gentle upland and transitional valley locations, where soil conditions favor grasses (or other grass‑like plants) but can support at least some tree cover. Some savannas apparently have sparse tree cover because of edaphic or climatic limitations on woody plant growth (Romme et al. 2009).

*Soil/substrate/hydrology:* Savannas are found on moderately deep to deep, coarse‑ to fine‑textured soils that readily support a variety of growth forms, including trees, grasses, and other herbaceous plants, and in regions that receive reliable summer rainfall that fosters growth of warm‑season grasses (Romme et al. 2009). This type appears to be especially prevalent in the basins and foothills of northeastern New Mexico, where a large portion of annual precipitation comes in the summer via monsoon rains (Romme et al. 2009).

**Vegetation:** Vegetation structure is typically a savanna with widely spaced, mature (>150 years old) juniper trees and moderately dense perennial grasses in between trees but includes inclusions (patches) of denser juniper woodlands with less herbaceous cover. Vegetation is dominated by an open tree canopy of 2‑ to 10‑m tall *Juniperus monosperma*. *Juniperus scopulorum* may be present or dominant at higher elevations. Occasional *Pinus edulis* trees may be present but have low cover and are typically restricted to mesic microsites. The open to dense herbaceous layer is dominated by perennial grasses that vary with environments. Grass species are similar to those found in adjacent shortgrass prairie and piedmont grasslands. *Bouteloua gracilis, Bouteloua curtipendula*, and *Pleuraphis jamesii* are most common with *Koeleria macrantha, Lycurus phleoides, Muhlenbergia torreyi*, and *Piptatheropsis micrantha* often present. Midgrasses such as *Achnatherum hymenoides, Hesperostipa comata*, or *Hesperostipa neomexicana* are more common in foothills and piedmont stands. *Bouteloua eriopoda* and *Bouteloua hirsuta* are more common grass in the southern extent, while *Andropogon hallii* and *Muhlenbergia pungens* are characteristic of deep sandy sites. Forbs such as *Astragalus* spp., *Cryptantha cinerea var. jamesii (= Cryptantha jamesii), Eriogonum jamesii, Erigeron divergens, Hymenopappus filifolius, Ipomopsis multiflora, Mentzelia* spp., and *Penstemon* spp. are also common. Shrubs are poorly represented or absent except the ruderal subshrub *Gutierrezia sarothrae* and succulents such as *Cylindropuntia imbricata, Opuntia phaeacantha, Opuntia polyacantha, Yucca baccata*, and *Yucca glauca*. Other occasional shrubs may include *Artemisia bigelovii, Rhus trilobata*, or *Cercocarpus montanus*.

**Dynamics:** *Juniperus monosperma* is a long‑lived, slow‑growing, drought‑tolerant small tree (3‑12 m in height) that also occurs as a tall shrub (Johnson 2002). It is more drought tolerant than *Pinus edulis* and often occurs without pinyon on more xeric, lower elevation sites (Johnson 2002). It is also non‑sprouting and may be killed by fire (Wright et al. 1979). Juniper stands at cooler, higher elevation sites typically occur on xeric microsites that are too arid for pinyon or on post‑disturbance sites such as where extended drought or ips beetle (*Ips confusus*) epidemics have eliminated pinyon from mixed pinyon‑juniper stands. In this situation junipers and shrubs may act a nurse plants providing shade for pinyon germination and re‑establishment, converting a juniper woodland to pinyon‑juniper woodland.

Within a given region, the density of trees, both historically and currently, is strongly related to topo‑edaphic gradients. Less steep sites, especially those with finer‑textured soils, are where savannas, grasslands, and shrub‑steppes have occurred in the past. Juniper stands on these gentler slopes may have been larger but more savanna‑like, with very open upper canopy and high grass production. Expansion of juniper into previously non‑wooded areas occurred prior to European settlement on some sites, although this expansion may have been more extensive in the 20th century versus the previous. However, loss of juniper from marginal sites also occurred historically and recently in some areas (Romme et al. 2009). Especially in areas in which trees were historically rare or absent, there have been type conversions such that the historical condition is unidentifiable/replaced today. An important result of expansion into formerly non‑wooded areas in many regions is that formerly heterogeneous mosaics of small patches of woodland, shrubland, and grassland are becoming more homogeneous as trees become established in the shrubland and grassland patches (Romme et al. 2009).

Past fire regimes in southwestern juniper woodlands were mixed, having both surface and crown fires, reflecting variable intensity and frequency depending on site productivity. "Productive sites" could sustain patchy fires at intervals of 10‑50 years and could have attained densities sufficient to carry crown fires at intervals of 200‑300 years. In open stands, where grass cover was continuous, fire intervals might have been 10 years or less, and probably maintained grasslands and savannas (Gottfried et al. 1999). Romme et al. (2009) state that low‑severity fires were probably uncommon except in savannas and in small patches in persistent woodlands.

Soil texture drives the fire regime. Sites with higher potential for graminoid understory will have higher fine‑fuel loading and create the spread component for more frequent and lower intensity fires. Sites with shallow, gravelly soils produce less grass and more shrub components, less fire frequency, more lethal when wind‑driven events occur (LANDFIRE 2007a).

LANDFIRE developed a state‑and‑transition vegetation dynamics VDDT model for this system which has five classes in total (LANDFIRE 2007a, BpS 2711190). The model was reviewed and reference to pinyon were removed then summarized as:

A) Early Development 1 All Structures (10% of type in this stage): Grass/forb/shrub/seedling ‑ usually post‑fire. Cover is 0‑30%. Shrub height is 0‑5 m. This class succeeds to B, a mid‑open stage after approximately 70 years; however, it could be much longer depending on size of burn. Recruitment is even more episodic in response to optimal climate conditions than in ponderosa. An alternate successional pathway could take this class to class C, a mid‑development closed stage, with a probability of 0.015. Replacement fire occurs infrequently, every 400 years. Competition/maintenance can maintain this stage, with a probability of 0.01.

B) Mid Development 1 Open (10% of type in this stage): Tree cover is 11‑40%. Tree height is 5.1‑10 m. Mid‑development, open (<40% cover) juniper stand with mixed shrub/herbaceous community in understory. Review for MZ27 suggested this might even be lower canopy cover to 20%. This class succeeds to class E, a late‑open stage after approximately 170 years. An alternate successional pathway could take this class to class D, a late closed stage, with a low probability of 0.002. Replacement fire occurs infrequently, every 500 years. Surface fire occurs every 25 years. Mixed fire occurs every 300 years. Competition/maintenance can maintain this class in class B, with a probability of 0.007.

C) Mid Development 1 Closed (10% of type in this stage): Tree cover is 41‑70%. Tree height is 5 m. Mid‑development, dense (>40% cover) pinyon‑juniper woodland; understory being lost. Review for Map zone 27 suggested this might even be lower canopy cover to 30%. This class succeeds to D, a late‑closed stage after 100 years. Mixed fire in this stage either causes no transition (every 1000 years) or brings it to an open mid stage (every 200 years). Surface fire occurs infrequently (every 1000 years) and causes no transition. Replacement fire also occurs infrequently (every 500 years).

D) Late Development 1 Closed (5% of type in this stage): Tree cover is 41‑70%. Tree height is 10.1‑25 m. Dense, old‑growth stands with multiple layers. Late‑development, closed pinyon‑juniper forest. May have all‑aged, multi‑storied structure. Moderate mortality within stand. Occasional shrubs with few grasses and forbs and often much rock. Review for MZ27 suggested this might even be lower canopy cover to 11‑35%. This class can persist. Mixed fire can cause this class to move to a late open stage, class E, but very infrequently ‑ every 200 years. Replacement fire occurs very rarely (6‑700 years), and surface fire also occurs very, very rarely. Insect/disease can also open this class and cause a transition to the late‑open stage, class E, every 200 years. This interval may be even longer. Also, drought likely plays a major role, but it was not modeled here.

E) Late Development 1 Open (conifer‑dominated ‑ 65% of type in this stage): Tree cover is 11‑40%. Tree height is 10‑25 m. Late‑development, open juniper‑pinyon stand with "savannah‑like" appearance; mixed grass/shrub/herbaceous community. This class persists. Replacement fire occurs infrequently ‑ every 500 years. Mixed fire also occurs infrequently ‑ every 200 years, and surface fire every 25 years, but neither cause a transition. Insect/disease occurs every 200 years but causes no transition. This interval may be even longer. Also, drought likely plays a major role, but it was not modeled here.

Other important ecological processes include drought, insect infestations, pathogens, herbivory and seed dispersal by birds and mammals. Juniper berries crops are primarily utilized by birds and small mammals (Johnsen 1962, McCulloch 1969, Short et al. 1977, Salomonson 1978). The most important dispersers of juniper seeds are birds, although many mammals also feed on them. These animals consume juniper berries and excrete viable scarified juniper seeds, which germinate faster than uneaten seeds, over extensive areas (Johnsen 1962, Meeuwig and Bassett 1983). Primary juniper seed dispersers are Bohemian waxwing (*Bombycilla garrulus*), but cedar waxwing (*Bombycilla cedrorum*), American robin (*Turdus migratorius*), turkey (*Meleagris gallopavo*), and several species of jays are also dispersers (Johnson 2002, Scher 2002).

There are several insects, pathogens, and plant parasites that attack juniper trees (Meeuwig and Bassett 1983, Gottfried et al. 1995, Rogers 1995, Weber et al. 1999). For juniper, there are several insects, plus the fungus blackstain root‑rot (*Leptographium wageneri*) and juniper mistletoe (*Phoradendron juniperinum*). Mistletoe reduces vigor and causes occasional dieback but rarely causes mortality (Meeuwig and Bassett 1983). The insects are normally present in these woodland stands, and during drought‑induced water‑stress periods, outbreaks may cause local to regional mortality (Gottfried et al. 1995)<br />

Many juniper savannas and woodlands in the Southwest have high soil erosion potential (Baker et al. 1995). Several studies have measured present‑day erosion rates in juniper woodlands, highlighting the importance of herbaceous cover and cryptogamic soil crusts (Baker et al. 1995, Belnap et al. 2001) in minimizing precipitation runoff and soil loss in juniper woodlands.

SOURCES

**References:** AOU 1983, Baker et al. 1995, Belnap 2001, Belnap et al. 2001, Blackburn and Tueller 1970, Breshears et al. 2005, Burns and Honkala 1990a, CNHP 2010, Comer et al. 2003*, Commons et al. 1999, Eager 1999, Eyre 1980, Gottfried et al. 1995, Gottfried et al. 1999, Johnsen 1962, Johnson 2002, LANDFIRE 2007a, McCulloch 1969, Meeuwig and Bassett 1983, Rogers 1995, Romme et al. 2009, Rosentreter and Belnap 2003, Salomonson 1978, Scher 2002, Shiflet 1994, Short et al. 1977, Weber et al. 1999, West 1999b, Wright and Bailey 1982a

**Version:** 04 Nov 2015	**Stakeholders:** Midwest, Southeast, West

**Concept Author:** M.S. Reid	**LeadResp:** West

CES306.835 Southern Rocky Mountain Pinyon‑Juniper Woodland

**Primary Division:** Rocky Mountain (306)

**Land Cover Class:** Forest and Woodland

**Spatial Scale & Pattern:** Matrix

**Required Classifiers:** Natural/Semi‑natural; Vegetated (>10% vasc.); Upland

**Diagnostic Classifiers:** Forest and Woodland (Treed); Very Shallow Soil; Shallow Soil; Mineral: W/ A‑Horizon <10 cm; Aridic; Long Disturbance Interval; Needle‑Leaved Tree; Pinus edulis, Juniperus monosperma

**National Mapping Codes:** EVT 2059; ESLF 4246; ESP 1059

**Concept Summary:** This southern Rocky Mountain ecological system occurs on dry mountains and foothills in southern Colorado east of the Continental Divide, in mountains and plateaus of north‑central New Mexico, and extends out onto limestone breaks in the southeastern Great Plains. These woodlands occur on warm, dry sites on mountain slopes, mesas, plateaus, and ridges. Soils supporting this system vary in texture ranging from stony, cobbly, gravelly sandy loams to clay loam or clay. *Pinus edulis* and/or *Juniperus monosperma* dominate the tree canopy. *Juniperus scopulorum* may codominate or replace *Juniperus monosperma* at higher elevations. Stands with *Juniperus osteosperma* are representative of the Colorado Plateau and are not included in this system. In southern transitional areas between Madrean Pinyon‑Juniper Woodland (CES305.797) and Southern Rocky Mountain Pinyon‑Juniper Woodland (CES306.835) in central New Mexico, *Juniperus deppeana* may be present. Understory layers are variable and may be dominated by shrubs, graminoids, or be absent. Associated species are more typical of southern Rocky Mountains than the Colorado Plateau and include *Artemisia bigelovii, Cercocarpus montanus, Quercus gambelii, Achnatherum scribneri, Bouteloua gracilis, Festuca arizonica*, or *Pleuraphis jamesii*.

**Comments:** This system corresponds to the *Juniperus monosperma*‑ and/or *Pinus edulis*‑dominated portion of the *persistent pinyon‑juniper woodland* type from Romme et al. (2009) that occurs on rocky uplands with shallow, coarse‑textured, and often skeletal soils that support relatively sparse herbaceous cover and rarely burn. This system is similar to Colorado Plateau Pinyon‑Juniper Woodland (CES304.767) as *Pinus edulis* is a diagnostic species for both. However, this system occurs east of the Continental Divide and outside the range of *Juniperus osteosperma*.

DISTRIBUTION

**Range:** This system occurs on dry mountains and foothills east of the Continental Divide in southern Colorado, in mountains and plateaus of northern New Mexico and Arizona, and extends out onto breaks in the Great Plains. It extends south to the Sacramento Mountains, especially the eastern side. The western side of the Sacramento Mountains has Madrean elements (*Quercus grisea*) and may be classified as Madrean woodland.

**Divisions:** 303:C, 304:C, 306:C

**TNC Ecoregions:** 20:C, 21:C, 22:P, 27:C, 28:C

**Nations:** US

**Subnations:** CO, NM, OK, TX

**Map Zones:** 14:?, 15:P, 24:?, 25:C, 27:C, 28:C, 34:P

**USFS Ecomap Regions:** 313B:CC, 315A:CC, 315B:CC, 315H:CC, 321A:CC, 322B:CC, 331B:CC, 331H:CP, 331I:CC, 331J:CC, M313B:CC, M331F:CC, M331G:CC, M331H:CC, M331I:CC

CONCEPT

**Environment:** This southern Rocky Mountain ecological system occurs on dry mountains and foothills in southern Colorado east of the Continental Divide, in mountains and plateaus of north‑central New Mexico, and extends out onto limestone breaks in the southeastern Great Plains. Elevations range from near 1500 to 2900 m with high‑elevation stands restricted to relatively warm, dry ridges and south and west aspects. Lower‑elevation stands are often restricted to cooler north‑ and east‑facing slopes.

<br />*Climate:* Climate is cool‑temperate, continental, and semi‑arid. Precipitation ranges from approximately 33‑46 cm (13‑18 inches) annually. Most of the precipitation occurs during the summer growing season. Severe climatic events occurring during the growing season, such as frosts and drought, are thought to limit the distribution of pinyon‑juniper woodlands to relatively narrow altitudinal belts on mountainsides.

*Physiography/landform:* These woodlands occur on warm, dry sites on mountain slopes, mesas, plateaus, and ridges.

*Soil/substrate/hydrology:* Soils supporting this system vary in texture ranging from stony, cobbly, gravelly sandy loams to clay loam or clay.

**Vegetation:** *Pinus edulis* and/or *Juniperus monosperma* dominate the tree canopy. *Juniperus scopulorum* may codominate or replace *Juniperus monosperma* at higher elevations. Stands with *Juniperus osteosperma* are representative the Colorado Plateau and are not included in this system. In southern transitional areas between Madrean Pinyon‑Juniper Woodland (CES305.797) and Southern Rocky Mountain Pinyon‑Juniper Woodland (CES306.835) in central New Mexico, *Juniperus deppeana* becomes common. Understory layers are variable and may be dominated by shrubs, graminoids, or be absent. Associated species are more typical of southern Rocky Mountains than the Colorado Plateau and include *Artemisia bigelovii, Cercocarpus montanus, Quercus gambelii, Achnatherum scribneri, Bouteloua gracilis, Festuca arizonica*, or *Pleuraphis jamesii*.

**Dynamics:** Both *Pinus edulis* and *Juniperus monosperma* are relatively short (generally <15 m tall), shade‑intolerant, drought‑tolerant, slow‑growing, long‑lived trees (Meeuwig and Bassett 1983, Little 1987, Anderson 2002, Johnson 2002, Romme et al. 2003). Both tree species are also non‑sprouting and may be killed by fire (Wright et al. 1979).

Pinyon‑juniper woodlands are influenced by drought, fires, grazing, and insect‑pathogen outbreaks (West 1999b). Stands vary considerably in appearance and composition, both elevationally and geographically. Juniper tends to be more abundant at the warmer/drier lower elevations, pinyon tends to be more abundant at the higher elevations, and the two species share dominance within a broad middle‑elevation zone (Woodin and Lindsey 1954).

The effect of fire on a stand is largely dependent on the tree height and density, fine‑fuel load on the ground, weather conditions, and season (Dwyer and Pieper 1967, Wright et al. 1979). Some large trees may survive unless the fire gets into the crown due to heavy fuel loads in the understory or extreme fire conditions.

Site conditions affects the successional pathway following a disturbance. Succession on a site is influenced by the severity and size of the disturbance, and by the composition, longevity, and density of any surviving plants and propagules within the disturbed area and the characteristics of plant communities in adjacent undisturbed areas. According to Gottfried et al. (1999) junipers are the first to return in secondary succession but are often followed and replaced by pinyon.

Site conditions influence the stand density. Sites with fewer trees typically have relatively deep soils and support a dense herbaceous level; those with more trees have shallow, rocky soils and often occur on steeper slopes. Stands may range from even‑aged to uneven‑aged stands. Some stands may have closed canopies with little or no understory, but many stands are open with widely scattered trees with a wide variety of understory vegetation (Rondeau 2001).

LANDFIRE developed a state‑and‑transition vegetation dynamics VDDT model for this system which has four classes in total (LANDFIRE 2007a, BpS 2710590). These classes are summarized as:

A) Early Development 1 Open (10% of type in this stage): Total cover is 0‑20% (grass cover <20%, shrub cover <15%, tree cover <10%). Shrub height 0‑0.5 m. There would be very little of this class historically. Initial post‑fire community grass‑ and shrub‑dominated, consisting of mountain‑mahogany with Gambel oak sprouts, perennial grass and various forbs. Pinyon and juniper seedlings and saplings will be in low density. Evidence of past fires may be observed, including charcoal and resprouting woody plants. Duration 50 years with succession to class B, mid‑development stand of small trees. Trees exert very little influence until about 50 years in this system. Replacement fire occurs every few centuries. Drought occurs every 30 years and maintains the class but does not set it back to the beginning.

B) Mid Development 1 Open (20% of type in this stage): Tree cover is 0‑40%. Tree height <3 m. Young juniper saplings are increasing and growing. Grass and shrubs are still dominant. Grass species that would be present are: blue grama, little bluestem, western wheatgrass, and needlegrass. Pinyon seedlings delayed until shade occurs for better growth. Mixed‑severity fire also occurs because sometimes grass density is sufficient to result in pinyon and juniper scorch as well as mortality. Mixed fire occurs every 100‑200 years. Replacement fires every several hundred years. This class probably lasts approximately 100 years, i.e., 50 to 150 years. Might remain in class until 10‑20‑year heavy moisture cycle; this increases seedling production, and juveniles mature. Drought occurs every 30 years but does not cause a transition.

C) Mid Development 1 Closed (45% of type in this stage): Tree cover is 21‑70%. Tree height 5.1‑10 m. Junipers reaching pole‑size, and pinyon pine seedlings and saplings are growing dependent on rainfall patterns and shade. Pinyon having rapid growth in this stage. Gambel oak is also forming stand patches. Thinning effect for mountain‑mahogany due to space/nutrient competition. Very little recruitment of junipers in this stage. This class lasts from approximately 150‑250 years of age, so spending 50‑100 years in this class. For the model, this class will last 75 years. Replacement fire unlikely in this class due to open canopy. Mixed fire also modeled infrequently. Drought occurs every 30 years, also maintaining this class.

D) Late Development 1 Closed (25% of type in this stage): Tree cover is 10‑40%. Tree height 5‑10 m. Mature juniper mixed with maturing pinyon. Understory declining due to canopy closing. Small amount of fine fuels. There is a shift in dominance from juniper to pinyon. This class can persist. Pinyon would be susceptible to drought mortality, disease, and insects. Drought creates conditions for insect disturbance to occur in pinyon pine. Drought itself, however, can impact the understory separate from the insect component. Optional 1 is drought plus insect effect. This takes it back to class C, because pinyon lost but still have mature junipers. Modeled at every 50 years, or 2% of the class each year. Regular drought modeled as every 30 years, as in other classes, not causing a transition. Mistletoe might also be influenced by the drought but not being modeled due to lack of information.

Other important ecological processes include drought, insect infestations, pathogens, herbivory, and seed dispersal by birds and mammals. Juniper berry and pinyon nut crops are primarily utilized by birds and small mammals (Johnsen 1962, McCulloch 1969, Short et al. 1977, Salomonson 1978, Balda 1987, Gottfried et al. 1995). Large mammals, such as mule deer (*Odocoileus hemionus*), white‑tailed deer (*Odocoileus virginianus*) and elk (*Cervus elaphus*), eat leaves and seeds of both species and they browse woodland grasses, forbs and shrubs, including *Artemisia tridentata, Cercocarpus montanus, Quercus gambelii*, and *Purshia stansburiana* (Short and McCulloch 1977).

The most important dispersers of juniper and pinyon seeds are birds, although many mammals also feed on them. These animals consume juniper berries and excrete viable scarified juniper seeds over extensive areas, which germinate faster than uneaten seeds (Johnsen 1962, Meeuwig and Bassett 1983). Primary juniper seed dispersers are Bohemian waxwing (*Bombycilla garrulus*), cedar waxwing (*Bombycilla cedrorum*), American robin (*Turdus migratorius*), turkey (*Meleagris gallopavo*), and several species of jays (Anderson 2002, Johnson 2002, Scher 2002). Pinyon seeds are a critically important food source for western scrub jay (*Aphelocoma californica*), pinyon jay (*Gymnorhinus cyanocephalus*), Steller's jay (*Cyanocitta stelleri*) and Clark's nutcracker (*Nucifraga columbiana*). These birds are the primary dispersers of pinyon seeds and, during mast crop years, cache hundreds of thousands of pinyon seeds, many of which are never recovered (Balda and Bateman 1971, Vander Wall and Balda 1977, Ligon 1978, Evans 1988, Hall and Balda 1988, Ronco 1990). Many mammals are also known to eat pinyon seeds, such as several species of mice (*Peromyscus* spp.), woodrats (*Neotoma* spp.), squirrels (*Sciurus* spp.), chipmunks (*Neotamias* spp.), deer, black bear (*Ursus americanus*), and desert bighorn sheep (*Ovis canadensis nelsoni*) (Anderson 2002). Because pinyon seeds are heavy and totally wingless, seed dispersal is dependent on vertebrate dispersers that store seeds in food caches, where unconsumed seeds may germinate. This dispersal mechanism is a good example of a co‑evolved, mutualistic, plant‑vertebrate relationship (Vander Wall et al. 1981, Evans 1988, Lanner 1996) and would be at risk with loss of trees or dispersers.

There are many insects, pathogens, and plant parasites that attack pinyon and juniper trees (Meeuwig and Bassett 1983, Gottfried et al. 1995, Rogers 1995, Weber et al. 1999). For pinyon and juniper, there are at least seven insects, plus fungus blackstain root‑rot (*Leptographium wageneri*), juniper mistletoe (*Phoradendron juniperinum*) and pinyon dwarf mistletoe (*Arceuthobium divaricatum*). Both mistletoes reduce vigor and cause occasional dieback but rarely cause mortality (Meeuwig and Bassett 1983). The insects are normally present in these woodland stands, and during drought‑induced water‑stress periods, outbreaks may cause local to regional mortality (Wilson and Tkacz 1992, Gottfried et al. 1995, Rogers 1995). Most insect‑related pinyon mortality in the West is caused by pinyon ips beetle (*Ips confusus*) (Rogers 1993). Pinyons cannot repel pinyon ips beetles when weakened by drought and many are killed. During the drought of 2002‑2003, populations of ips beetles increased to epidemic levels that killed millions of pinyon trees in the southwestern U.S.

Most pinyon‑juniper woodlands in the Southwest have high soil erosion potential (Baker et al. 1995). Several studies have measured present‑day erosion rates in pinyon‑juniper woodlands, highlighting the importance of herbaceous cover and biological soil crusts (Baker et al. 1995, Belnap et al. 2001) in minimizing precipitation runoff and soil loss in pinyon‑juniper woodlands.

SOURCES

**References:** Anderson 2002, Baker et al. 1995, Balda 1987, Balda and Bateman 1971, Belnap 2001, Belnap et al. 2001, Betancourt et al. 1993, Breshears et al. 2005, Burns and Honkala 1990a, CNHP 2010, Comer et al. 2003*, Davis and Turner 1986, Evans 1988, Eyre 1980, FNA Editorial Committee 1997, Furniss and Carolin 2002, Gori and Bate 2007, Gottfried et al. 1995, Gottfried et al. 1999, Hall and Balda 1988, Johnsen 1962, Johnson 2002, LANDFIRE 2007a, Lanner 1996, Ligon 1978, Little 1987, McAuliffe and Van Devender 1998, McCulloch 1969, Meeuwig and Bassett 1983, Mehringer and Wigand 1990, Miller and Tausch 2001, Rogers 1993, Rogers 1995, Romme et al. 2009, Ronco 1990, Rondeau 2001, Rosentreter and Belnap 2003, Salomonson 1978, Scher 2002, Shiflet 1994, Short and McCulloch 1977, Short et al. 1977, Stevens 1999a, Swetnam and Baisan 1996a, Tausch 1999, Tausch and Hood 2007, USFWS 1985, Van Devender 1977, Van Devender 1990, Vander Wall and Balda 1977, Vander Wall et al. 1981, Weber et al. 1999, West 1999b, Wilson and Tkacz 1992, Woodin and Lindsey 1954, Wright et al. 1979

**Version:** 04 Nov 2015	**Stakeholders:** Southeast, West

**Concept Author:** M.S. Reid	**LeadResp:** West

1.B.2.Nd. Vancouverian Forest & Woodland

M886. Southern Vancouverian Dry Foothill Forest & Woodland

CES204.085 East Cascades Oak‑Ponderosa Pine Forest and Woodland

**Primary Division:** North American Pacific Maritime (204)

**Land Cover Class:** Forest and Woodland

**Spatial Scale & Pattern:** Matrix

**Required Classifiers:** Natural/Semi‑natural; Vegetated (>10% vasc.); Upland

**Diagnostic Classifiers:** Ridge/Summit/Upper Slope; Very Shallow Soil; Mineral: W/ A‑Horizon <10 cm; Aridic; Intermediate Disturbance Interval [Periodicity/Polycyclic Disturbance]; F‑Patch/Medium Intensity

**National Mapping Codes:** EVT 2060; ESLF 4301; ESP 1060

**Concept Summary:** This narrowly restricted ecological system appears at or near lower treeline in foothills of the eastern Cascades in Washington and Oregon within 65 km (40 miles) of the Columbia River Gorge. It also appears in the adjacent Columbia Plateau ecoregion. Elevations range from 460 to 1920 m. Most occurrences of this system are dominated by a mix of *Quercus garryana* and *Pinus ponderosa* or *Pseudotsuga menziesii*. Isolated, taller *Pinus ponderosa* or *Pseudotsuga menziesii* over *Quercus garryana* trees characterize parts of this system. Clonal *Quercus garryana* can create dense patches across a grassy landscape or can dominate open woodlands or savannas. The understory may include dense stands of shrubs or, more often, be dominated by grasses, sedges or forbs. Shrub‑steppe shrubs may be prominent in some stands and create a distinct tree / shrub / sparse grassland habitat, including *Purshia tridentata, Artemisia tridentata, Artemisia nova*, and *Chrysothamnus viscidiflorus*. Understories are generally dominated by herbaceous species, especially graminoids. Mesic sites have an open to closed sodgrass understory dominated by *Calamagrostis rubescens, Carex geyeri, Carex rossii, Carex inops*, or *Elymus glaucus*. Drier savanna and woodland understories typically contain bunchgrass steppe species such as *Festuca idahoensis* or *Pseudoroegneria spicata*. Common exotic grasses that often appear in high abundance are *Bromus tectorum* and *Poa bulbosa*. These woodlands occur at the lower treeline/ecotone between *Artemisia* spp. or *Purshia tridentata* steppe or shrubland and *Pinus ponderosa* and/or *Pseudotsuga menziesii* forests or woodlands. In the Columbia River Gorge, this system appears as small to large patches in transitional areas in the Little White Salmon and White Salmon river drainages in Washington and Hood River, Rock Creek, Moiser Creek, Mill Creek, Threemile Creek, Fifteen Mile Creek, and White River drainages in Oregon. *Quercus garryana* can create dense patches often associated with grassland or shrubland balds within a closed *Pseudotsuga menziesii* forest landscape. Commonly the understory is shrubby and composed of *Ceanothus integerrimus, Holodiscus discolor, Symphoricarpos albus*, and *Toxicodendron diversilobum*. Fire plays an important role in creating vegetation structure and composition in this habitat. Decades of fire suppression have led to invasion by *Pinus ponderosa* along lower treeline and by *Pseudotsuga menziesii* in the gorge and other oak patches on xeric sites in the east Cascade foothills. In the past, most of the habitat experienced frequent low‑severity fires that maintained woodland or savanna conditions. The mean fire‑return interval is 20 years, although variable. Soil drought plays a role, maintaining an open tree canopy in part of this dry woodland habitat.

**Comments:** Mapping this system presents a typical scale problem. Areas of pure ponderosa pine are found directly adjacent to oak stands. This system is a matrix type with stands of *Pinus ponderosa, Quercus garryana, Pinus ponderosa ‑ (Pseudotsuga menziesii) ‑ Quercus garryana*; still need to get a mapping protocol and concept to distinguish *Pseudotsuga menziesii* with *Quercus garryana* patches in the east gorge White Salmon. The Little White Salmon drainage near Augspurger Mountain is the transition area between North Pacific Oak Woodland (CES204.852) and this system (Dog Mountain is the westernmost in Washington).

DISTRIBUTION

**Range:** This narrowly restricted ecological system appears at or near lower treeline in foothills of the eastern Cascades in Washington and Oregon within 65 km (40 miles) of the Columbia River Gorge. It also appears in the adjacent Columbia Plateau ecoregion. Disjunct occurrences in Klamath and Siskiyou counties, Oregon, have more sagebrush and bitterbrush in the understory, along with other shrubs.

**Divisions:** 204:C, 304:C

**TNC Ecoregions:** 4:C, 6:C

**Nations:** CA, US

**Subnations:** BC, OR, WA:S1S2

**Map Zones:** 1:C, 7:C, 8:C

**USFS Ecomap Regions:** 342H:CC, 342I:CC, M242B:C?, M242C:CC, M242D:CC

CONCEPT

**Environment:** This narrowly restricted ecological system appears at or near lower treeline in foothills of the eastern Cascades in Washington and Oregon within 65 km (40 miles) of the Columbia River Gorge. It also appears in the adjacent Columbia Plateau ecoregion. Elevations range from 460 to 1920 m. In the Columbia River Gorge, this system appears as small to large patches in transitional areas in the Little White Salmon and White Salmon river drainages in Washington and Hood River, Rock Creek, Moiser Creek, Mill Creek, Threemile Creek, Fifteen Mile Creek, and White River drainages in Oregon. *Quercus garryana* can create dense patches often associated with grassland or shrubland balds within a closed *Pseudotsuga menziesii* forest landscape.

**Vegetation:** Most occurrences of this system are dominated by a mix of *Quercus garryana* and *Pinus ponderosa* or *Pseudotsuga menziesii*. Isolated, taller *Pinus ponderosa* or *Pseudotsuga menziesii* over *Quercus garryana* trees characterize parts of this system. Clonal *Quercus garryana* can create dense patches across a grassy landscape or can dominate open woodlands or savannas. The understory may include dense stands of shrubs or, more often, be dominated by grasses, sedges or forbs. Shrub‑steppe shrubs may be prominent in some stands and create a distinct tree / shrub / sparse grassland habitat, including *Purshia tridentata, Artemisia tridentata, Artemisia nova*, and *Chrysothamnus viscidiflorus*. Understories are generally dominated by herbaceous species, especially graminoids. Mesic sites have an open to closed sodgrass understory dominated by *Calamagrostis rubescens, Carex geyeri, Carex rossii, Carex inops*, or *Elymus glaucus*. Drier savanna and woodland understories typically contain bunchgrass steppe species such as *Festuca idahoensis* or *Pseudoroegneria spicata*. Common exotic grasses that often appear in high abundance are *Bromus tectorum* and *Poa bulbosa*.

**Dynamics:** Fire plays an important role in creating vegetation structure and composition in this habitat. Decades of fire suppression have led to invasion by *Pinus ponderosa* along lower treeline and by *Pseudotsuga menziesii* in the gorge and other oak patches on xeric sites in the east Cascade foothills. Most of the habitat experienced frequent low‑severity fires that maintained woodland or savanna conditions. The mean fire‑return interval is 20 years, although variable. LANDFIRE VDDT models: #R OAP1 Oregon White Oak‑Ponderosa Pine model describes general successional pathways treating drier pine succession separate from more mesic Douglas‑fir pathways.

LANDFIRE developed a state‑and‑transition vegetation dynamics VDDT model for this system which has five classes in total (LANDFIRE 2007a, BpS 0710600). These are summarized as:

A) Early Development 1 All Structures (tree‑dominated ‑ 10% of type in this stage): Shrub cover is 0‑40%. The early stage is the initial post‑disturbance community dominated by white oak sprouts from coppice origin. Bunchgrasses and associated forbs dominate understory with bare ground and rock/gravel abundant in interspaces. Native herbivory may maintain oak sprouts in "shrub" form for extended period. Early stage includes oak sprouts or seedling/saplings growth to 4‑6 inches dbh. Occasional sites with ponderosa pine or Douglas‑fir will have diameters up to 8 inches. Succeeds to class C (mid/open) after about 50 years. Herbivory and surface fires maintain the stand in class A. About a tenth of this area is wet enough to succeed to class B.

B) Mid Development 1 Closed (tree‑dominated ‑ 5% of type in this stage): Tree cover is 41‑80%. The mid‑seral, closed stage occurs at the more mesic end of the environmental gradient and supports a dense canopy of oak and ponderosa pine and/or Douglas‑fir. Oak diameter ranges from 6‑12 inches dbh with crown closure approaching 70%. Ponderosa pine and Douglas‑fir may be 8‑20 inches dbh. Sod‑forming grasses and shade‑tolerant shrubs will be prominent on the majority of sites. Species from more arid sites may be remnants of earlier, more open post‑fire communities. Lasts up to 150 years in this class. Replacement fire about every few hundred years; mixed fire opens the stand (to class C).

C) Mid Development 1 Open (tree‑dominated ‑ 10% of type in this stage): Tree cover is 10‑40%. The mid‑seral, open stage occurs on arid slopes and benches and represents that portion of the environmental gradient where fire‑tolerant communities develop as oak woodlands. Usually the dry site conditions limit tree density and canopy closure is relatively low (between 10‑30%). Conifers may occur sporadically at low coverage. Oak diameter ranges from 6‑10 inches dbh. Bunchgrasses and shade‑intolerant shrubs, notably antelope bitterbrush, will be prominent on the majority of sites. Replacement fire is infrequent; surface fire maintains it in class C. Moist sites can fill in to late/closed conditions (class E).

D) Late Development 1 Open (tree‑dominated ‑ 65% of type in this stage): Tree cover is 10‑40%. The late‑seral, open stage is characterized by large, principally multi‑stemmed (now, although historically wider spaced, giant‑trunked trees were more common), white oaks in open stands with bunchgrass, forb, and shrub understories. These woodlands support crown closure between 10‑30%. Diameters range from 10‑18 inches dbh with ages over 350 years for those individuals surviving fires. Mature, large conifers may occur sporadically at low coverage. Bunchgrasses (*Pascopyrum smithii* and *Festuca idahoensis*) and shade‑intolerant shrubs, notably antelope bitterbrush, will be prominent on the majority of sites. Surface fires maintain it in class D. Replacement fire resets to class A.

E) Late Development 1 Closed (tree‑dominated ‑ 10% of type in this stage): Tree cover is 41‑80%. This stage has mature overstory ponderosa pine and/or Douglas‑fir as emergents over a lower canopy layer of white oak. The conifers have survived a few burn cycles and may show fire scars; dbhs are 21+ inches. Oregon white oak may reach its largest diameters in eastside ecosystems in these river and stream terraces attaining a dbh of 18‑20 inches. Canopy closure is high (60‑80%) with a dense understory dominated by sod‑forming grasses and shrubs. Mixed fire opens the stand.

Historical fire frequency is between 5‑30 years in this type. Fire intensities were probably low in open stands but increased in severity as woodland vegetation transitioned to a denser, closed‑canopy type along water courses. Canopy is fire‑tolerant and therefore fire severity is low. The natural fire regime was a type I regime in the upland. In the more mesic river terraces and draws, fire frequency probably decreased with a fire interval of 50‑60 years. With dense vegetation and the occurrence of fuel ladders, fire severity would become mixed. The fire regime may reflect a type III in this more mesic habitat (LANDFIRE 2007a, BpS 0710600).

Insects and disease may impact individual trees (either ponderosa pine or white oak) locally. Armillaria root rot, western pine beetle, western oak looper, western tent caterpillar, and the pine engraver have the greatest potential for damage (LANDFIRE 2007a, BpS 0710600).

Nutrient cycling, specifically carbon cycling, is an important ecological process within many ecological systems. However, biological decomposition in ponderosa pine forests is more limited than biological production, resulting in accumulation of organic materials, especially in the absence of fire (Harvey 1994, Graham and Jain 2005).

SOURCES

**References:** Burns and Honkala 1990a, Comer et al. 2003*, Dale et al. 2001, Eyre 1980, Graham and Jain 2005, Habeck 1992a, Habeck 1992d, Harvey 1994, John and Tart 1986, LANDFIRE 2007a, Lillybridge et al. 1995, McKenzie et al. 2004, McKenzie et al. 2008, Mote et al. 2014, Sawyer et al. 2009, Schmid 1988, Stevens‑Rumann et al. 2017, Topik et al. 1988, WNHP 2011, WNHP unpubl. data 2018, Westerling et al. 2006

**Version:** 24 May 2018	**Stakeholders:** Canada, West

**Concept Author:** R. Crawford	**LeadResp:** West

CES204.845 North Pacific Dry Douglas‑fir‑(Madrone) Forest and Woodland

**Primary Division:** North American Pacific Maritime (204)

**Land Cover Class:** Forest and Woodland

**Spatial Scale & Pattern:** Large patch, Small patch

**Required Classifiers:** Natural/Semi‑natural; Vegetated (>10% vasc.); Upland

**Diagnostic Classifiers:** Forest and Woodland (Treed); F‑Patch/Medium Intensity; Pseudotsuga menziesii; Arbutus menziesii

**National Mapping Codes:** EVT 2035; ESLF 4222; ESP 1035

**Concept Summary:** This ecological system is most common in the Puget Trough ‑ Willamette Valley ecoregion of Oregon and Washington but also occurs in adjacent ecoregions. It occupies small patches associated with dry sites or larger areas in prairie landscapes. This system historically had moderate‑ to low‑severity fires moderately frequently. Historically, these communities were either part of larger forested landscapes or occupied sheltered topographic positions in prairie‑dominated landscapes. They now also occur on some sites that formerly supported prairies or tall shrublands (*Corylus cornuta*) with scattered trees. In the mountains, this type occurs locally on dry sites within dry to mesic (for the coastal areas) climates up to about 1220 m (4000 feet) elevation. This is a forest or woodland primarily dominated by the long‑lived conifer *Pseudotsuga menziesii*. The broadleaf evergreen *Arbutus menziesii*, the short‑lived conifer *Pinus contorta*, the broadleaf deciduous *Acer macrophyllum*, and the shade‑tolerant conifer *Abies grandis* are local dominant or codominant species. These sites are too dry and warm or have been too frequently and extensively burned for anything more than small amounts of *Tsuga heterophylla* or *Thuja plicata* to be present as regeneration. *Arbutus menziesii* dominance is favored by high‑severity fires on sites where it occurs, and *Pseudotsuga menziesii* can be locally eliminated by logging and hot fire or repeated high‑severity fires. *Calocedrus decurrens* is absent. *Abies grandis* can be an important subcanopy or sapling tree, especially in and around the Willamette Valley and in the driest portions of the Georgia Basin (Coastal Douglas‑fir Zone).

**Comments:** Originally named Dry Douglas‑fir and Madrone Forest and Woodland, its name was changed as many areas occur without madrone. However, note that the description states we can have madrone stands with no Douglas‑fir; these are less common than the former.

DISTRIBUTION

**Range:** This system is limited to the foothill transition zone of the Puget Trough ‑ Willamette Valley ‑ Georgia Basin ecoregion.

**Divisions:** 204:C

**TNC Ecoregions:** 1:C, 2:C

**Nations:** CA, US

**Subnations:** BC, OR, WA:S2

**Map Zones:** 1:C, 2:C, 3:?, 7:C

**USFS Ecomap Regions:** 242A:CC, 242B:CC, 263A:PP, M242A:CC, M242B:CC, M242D:CP, M261A:CC

CONCEPT

SOURCES

**References:** Chappell and Christy 2004, Comer et al. 2003*, Eyre 1980, Franklin and Dyrness 1973, Green and Klinka 1994, WNHP unpubl. data 2018

**Version:** 02 Feb 2007	**Stakeholders:** Canada, West

**Concept Author:** C. Chappell	**LeadResp:** West

CES204.852 North Pacific Oak Woodland

**Primary Division:** North American Pacific Maritime (204)

**Land Cover Class:** Forest and Woodland

**Spatial Scale & Pattern:** Large patch, Small patch

**Required Classifiers:** Natural/Semi‑natural; Vegetated (>10% vasc.); Upland

**Diagnostic Classifiers:** Forest and Woodland (Treed); Temperate [Temperate Oceanic]; Quercus garryana

**National Mapping Codes:** EVT 2008; ESLF 4101; ESP 1008

**Concept Summary:** This ecological system is limited to the southern portions of the North Pacific region. It occurs primarily in the Puget Trough and Willamette Valley but trickles down into the Klamath ecoregion and into California. This system is associated with dry, predominantly low‑elevation sites and/or sites that experienced frequent presettlement fires. In the Willamette Valley, soils are mesic yet well‑drained, and the type is clearly large patch in nature. In the Puget Lowland and Georgia Basin, this system is primarily found on dry sites, typically either shallow bedrock soils or deep gravelly glacial outwash soils. It occurs on various soils in the interior valleys of the Klamath Mountains, and on shallow soils of "bald hill" toward the coast. Even where more environmentally limited, the system is strongly associated with a pre‑European settlement, low‑severity fire regime. Succession in the absence of fire tends to favor increased shrub dominance in the understory, increased tree density, and increased importance of conifers, with the end result being conversion to a conifer forest. The vegetation ranges from savanna and woodland to forest dominated by deciduous broadleaf trees, mostly *Quercus garryana*. Codominance by the evergreen conifer *Pseudotsuga menziesii* is common, and *Pinus ponderosa* is important in some stands. In the south, common associates also include *Quercus kelloggii* and *Arbutus menziesii*. This system merges into Mediterranean California Lower Montane Black Oak‑Conifer Forest and Woodland (CES206.923) on sites that support more conifer cover, and into Mediterranean California Mixed Oak Woodland (CES206.909) in the southern portion of its distribution. This system is borderline between small patch and large patch in its dynamics.

**Comments:** East of the Cascade Crest is a different system dominated by Oregon white oak (i.e., East Cascades Oak‑Ponderosa Pine Forest and Woodland (CES204.085)). While *Quercus garryana* does occur in California, it is uncertain that this system (a Garry oak‑dominated woodland) does not occur that far south. Garry oak in California may be mostly shrubby form around the edges of balds or else mixed into woodlands dominated by other species; this needs further review.

DISTRIBUTION

**Range:** This system occurs primarily in the Puget Trough and Willamette Valley and extends southward at low elevations in the Klamath Mountains on both sides of the Oregon/California stateline.

**Divisions:** 204:C

**TNC Ecoregions:** 1:C, 2:C, 5:C, 14:C

**Nations:** CA, US

**Subnations:** BC, CA, OR, WA:S1

**Map Zones:** 1:C, 2:C, 3:C, 6:?, 7:C

**USFS Ecomap Regions:** 242A:CC, 242B:CC, 263A:??, M242A:CC, M242B:CC, M242C:CP, M242D:CP, M261A:CC, M261D:CC

CONCEPT

**Environment:** This type is associated with dry, predominantly low‑elevation sites and/or sites that experienced frequent presettlement fires. In the Willamette Valley, soils are mesic yet well‑drained, and the type is clearly large patch in nature. In the Puget Lowland and Georgia Basin, this system is primarily found on dry sites, typically either on shallow bedrock soils or deep gravelly glacial outwash soils. It occurs on various soils in the interior valleys of the Klamath Mountains, and on shallow soils of "bald hills" toward the coast.

**Dynamics:** Even where more environmentally limited, the system is strongly associated with a pre‑European settlement, low‑severity fire regime. Succession in the absence of fire tends to favor increased shrub dominance in the understory, increased tree density, and increased importance of conifers, with the end result being conversion to a conifer forest. Landfire (2007a) model: Fire Regime I, primarily short‑interval (e.g., <10 years) surface fires. Surface fires every 3‑10 years maintained an open savanna‑like structure. Fires can be mixed‑severity especially when closed‑canopy conditions or additional species such as conifers and shrubs are present. Native American burning was a significant factor in fire frequency of this type, but fire frequency may have decreased significantly with a little distance from native settlements and valley bottoms. Landfire VDDT models: #R OWOA Oregon White Oak applies to southern occurrences. Dissemination of acorns by squirrels and chipmunks is thought to be the most important long‑distance dispersal mechanism for the oaks (WNHP 2011).

SOURCES

**References:** Alverson 2009, Chappell and Christy 2004, Chappell and Kagan 2001, Comer et al. 2003*, Eyre 1980, Franklin and Dyrness 1973, Fuchs 2001, LANDFIRE 2007a, Littell et al. 2009, Pike 1973, Thysell and Carey 2001, WNHP 2011, WNHP unpubl. data 2018

**Version:** 14 Jan 2014	**Stakeholders:** Canada, West

**Concept Author:** C. Chappell	**LeadResp:** West

M023. Southern Vancouverian Montane‑Foothill Forest

CES206.918 California Montane Jeffrey Pine‑(Ponderosa Pine) Woodland

**Primary Division:** Mediterranean California (206)

**Land Cover Class:** Forest and Woodland

**Spatial Scale & Pattern:** Large patch

**Required Classifiers:** Natural/Semi‑natural; Vegetated (>10% vasc.); Upland

**Diagnostic Classifiers:** Forest and Woodland (Treed); Shrubland (Shrub‑dominated); Mediterranean [Mediterranean Xeric‑Oceanic]; F‑Patch/Low Intensity; Needle‑Leaved Tree; Broad‑Leaved Evergreen Shrub; Pinus jeffreyi

**National Mapping Codes:** EVT 2031; ESLF 4218; ESP 1031

**Concept Summary:** These forests are found on relatively xeric sites in mountains and plateaus from southern Oregon (600‑1830 m [1800‑5000 feet] elevation) south into the Sierra Nevada, throughout the Transverse Ranges of California, and into northern Baja California (1200‑2740 m [4000‑8300 feet]), Mexico. While the two dominant pines tend to segregate by soil fertility and temperature regimes, they may co‑occur in certain areas (e.g., Modoc Plateau). These stands are more common on the east side of the Sierra Nevada, although they do occur on the west side. Stands are pure *Pinus jeffreyi, Pinus ponderosa*, or a mix of the two. Ponderosa pine and/or Jeffrey pine on the west slope of the Sierras with other conifer species are part of Mediterranean California Dry‑Mesic Mixed Conifer Forest and Woodland (CES206.916). This system includes sites where *Pinus ponderosa* and/or *Pinus jeffreyi* are the predominant conifers and other tree species do not occur in high abundance, if at all. The exception to this is in southern California on the edges of the Mojave Desert where *Pinus monophylla* or *Juniperus californica* might occur in a subcanopy under *Pinus ponderosa* or *Pinus jeffreyi*. *Pinus jeffreyi* is more tolerant of colder, drier and poorer sites and replaces *Pinus ponderosa* as the dominant at higher elevations. In the north, *Pinus jeffreyi* may be replaced by *Pinus ponderosa var. washoensis* (Carson Range and Warner Mountains). Throughout California, pure stands of ponderosa pine are relatively uncommon. Only on the Modoc Plateau do these pines co‑occur in mixed stands. *Juniperus grandis* [in the south] and *Juniperus occidentalis* can co‑occur in these stands but typically are not dominant. On moister and cooler sites, *Abies lowiana* can be present in some stands. There can be well‑developed shrub understories with strong Great Basin affinities; species can include *Artemisia tridentata, Purshia tridentata, Symphoricarpos rotundifolius var. parishii, Arctostaphylos patula, Ceanothus cordulatus, Ceanothus prostratus, Ceanothus integerrimus, Chrysolepis sempervirens, Eriogonum wrightii, Quercus vacciniifolia*, and *Lupinus elatus*. *Cercocarpus ledifolius* is common on steeper slopes throughout the range. Historically, frequent localized surface fires maintained these systems. Stands of ponderosa pine on the east side of the Cascades transition into East Cascades Oak‑Ponderosa Pine Forest and Woodland (CES204.085), or Northern Rocky Mountain Ponderosa Pine Woodland and Savanna (CES306.030) north of the Warm Springs Reservation of central Oregon.

**Comments:** *Pinus ponderosa* forests with *Calocedrus decurrens* found on the west side of the Sierra Nevada and in the Klamath Mountains are accommodated in Mediterranean California Dry‑Mesic Mixed Conifer Forest and Woodland (CES206.916).

DISTRIBUTION

**Range:** This system occurs in foothills and mountains from southern Oregon south into the Sierra Nevada, throughout the Transverse Ranges of California and into northern Baja California, Mexico.

**Divisions:** 206:C

**TNC Ecoregions:** 5:C, 12:C, 14:C, 15:C, 16:C

**Nations:** MX, US

**Subnations:** CA, MXBC, NV, OR

**Map Zones:** 2:C, 3:P, 4:C, 5:P, 6:C, 7:C, 12:C, 13:?

**USFS Ecomap Regions:** 263A:CC, 341D:CC, 342B:CC, M242A:PP, M242B:PP, M242C:PP, M261A:CC, M261B:CC, M261C:CP, M261D:CC, M261E:CC, M261F:CC, M261G:CC

CONCEPT

**Environment:** This system occupies xeric (mean annual rainfall 200‑430 mm, as winter snow), cool (cold winters; January minimums range from ‑13° to ‑5°C), and nutrient‑poor sites in mountains and plateaus (600‑2740 m elevation), in the rainshadow of the Sierra Nevada. Frequent (8‑10 years) low‑intensity and moderately frequent (44 years) mixed‑intensity fires maintain this system. Greater moisture increases tree diversity (*Abies lowiana* at higher altitudes).

**Vegetation:** This system includes sites where *Pinus ponderosa* and/or *Pinus jeffreyi* are the predominant conifers and other tree species do not occur in high abundance, if at all. The exception to this is in southern California on the edges of the Mojave Desert where *Pinus monophylla* or *Juniperus californica* might occur in a subcanopy under *Pinus ponderosa* or *Pinus jeffreyi*. *Pinus jeffreyi* is more tolerant of colder, drier and poorer sites and replaces *Pinus ponderosa* as the dominant at higher elevations. In the north, *Pinus jeffreyi* may be replaced by *Pinus ponderosa var. washoensis (= Pinus washoensis)* (Carson Range and Warner Mountains). Throughout California, pure stands of ponderosa pine are relatively uncommon. Only on the Modoc Plateau do these pines co‑occur in mixed stands. *Juniperus grandis (= Juniperus occidentalis var. australis)* [in the south] and *Juniperus occidentalis* can co‑occur in these stands but typically are not dominant. On moister and cooler sites, *Abies lowiana (= Abies concolor var. lowiana)* can be present in some stands. There can be well‑developed shrub understories with strong Great Basin affinities; species can include *Artemisia tridentata, Purshia tridentata, Symphoricarpos rotundifolius var. parishii (= Symphoricarpos parishii), Arctostaphylos patula, Ceanothus cordulatus, Ceanothus prostratus, Ceanothus integerrimus, Chrysolepis sempervirens, Eriogonum wrightii, Quercus vacciniifolia*, and *Lupinus elatus*. *Cercocarpus ledifolius* is common on steeper slopes throughout the range.

**Dynamics:** *Pinus jeffreyi* and *Pinus ponderosa* trees are structurally and physiologically fire‑adapted (Habeck 1992a, d, Gucker 2007). Both species have thick, insulating bark, insulating bud scales that protect terminal buds, self‑pruning branches, open crowns, and high moisture content of needles, which make them moderately fire‑resistant as saplings and highly fire‑resistant as mature trees (Habeck 1992a, d, Gucker 2007). Historically, frequent localized surface fires maintained open canopy woodland stands in this system.

LANDFIRE developed a state‑and‑transition vegetation dynamics VDDT model for this system which has five classes in total (LANDFIRE 2007a, BpS 1210310). These are summarized as:

A) Early Development 1 All Structures (shrub‑dominated ‑ 15% of type in this stage): Shrub cover is 0‑100%. Fire‑dependent shrubs such as greenleaf manzanita and mountain whitethorn resprout and germinate from seed vigorously after fire. Scattered Jeffery pine seedlings sprout but may take several years to dominate over the shrub community. Perennial bunchgrasses and some forbs cover small portions of the area.

B) Mid Development 1 Closed (tree‑dominated ‑ 5% of type in this stage): Tree cover is 51‑90%. This class has developed after escaping significant fire and it is modeled as an alternative pathway when three fire cycles have been missed. In the absence of fire, a closed forest with a dense stand of multi‑layered pole and medium‑sized Jeffery pine and white fir trees (5‑16 inches dbh) develops. This multi‑layered forest is often dominated by Jeffery pine in the overstory with white fir dominant in the mid and regeneration layers. The understory vegetation is almost absent due to the lack of sunlight and heavy litter and woody debris accumulations. In some cases, on the east side of the Sierra Nevada, both white fir and Jeffrey pine are pretty equally stocked and have a number of older individuals present suggesting that there is not always a low cover of white fir of small size classes in such settings (e.g., Buckeye Creek and other drainages northeast of Yosemite National Park). The understory vegetation is generally sparse, but not always due to lack of sunlight. *Poa wheeleri* and *Elymus elymoides* can be main understory species.

C) Mid Development 1 Open (tree‑dominated ‑ 20% of type in this stage): Tree cover is 0‑50%. This class has developed with frequent low‑intensity surface fires. Pole to medium‑sized (5‑21 inches dbh) Jeffery pine has become dominant over the shrub layer. Several conifer species could also be present depending on location. Shrubs are prevalent in the understory with scattered forbs and perennial grasses. East of the Sierra crest (e.g., Truckee Basin north of Tahoe), this class can have substantial amounts of white fir, but usually exists where the shrubs are mostly *Purshia tridentata* and other Great Basin species.

D) Late Development 1 Open (conifer‑dominated ‑ 65% of type in this stage): Tree cover is 0‑50%. This class is a continuation of class C which has developed with frequent low‑intensity surface fires. Large to very large (>21 inches dbh) Jeffery pine is dominant with an open canopy. Scattered shrubs are found in the canopy openings, with a diversity of forbs such as lupines and woolly mule's‑ears. Perennial grasses are also present.

E) Late Development 1 Open (conifer‑dominated ‑ 5% of type in this stage): Tree cover is 51‑90%. This class has developed in time from class B or class D after escaping significant fire (>3 years fire‑return intervals). In the absence of fire a closed forest structure continues to develop with a dense stand of multi‑layered medium‑ to large‑sized Jeffery pines and white fir trees (16+ inches dbh). The diameter remains smaller than in the open forest due competition. This overstory canopy is often codominated by Jeffery pine and white fir, with white fir dominating the understory. There is severe competition for sunlight and water. This stress combined with insect and disease infestation create a high level of tree mortality. The understory vegetation is almost absent due to the lack of sunlight and heavy litter and woody debris accumulations. Current conditions where there are large Jeffery pine trees along with multi‑age classes of white fir suggest that historically there were low‑intensity fires that maintained stands without killing white fir, but more recently white fir has become dominant in the understory.

Where stands are relatively dense and sufficient fuels are available, this type is dependent on relatively frequent low‑intensity surface fire intervals of about 30 years (LANDFIRE 2007a, BpS 1210310). The mixed‑intensity fire interval is about 130 years, and the stand‑replacement fire interval is 250 years. The mean fire interval for all fires is 20 years with a range from 8‑28 years. Intervals may be longer for relatively open stands with low understory fuels, as over shallow granitic soils in the Kern Plateau or over serpentine substrate in the Klamath Mountains. The fire regimes in this type are more variable and somewhat longer than the ponderosa pine types, due to slower fuel accumulation rates (LANDFIRE 2007a, BpS 1210310).

SOURCES

**References:** Barbour and Billings 2000, Barbour and Major 1988, Belnap 2001, Belnap et al. 2001, Comer et al. 2003*, Dale et al. 2001, Eyre 1980, Garfin et al. 2014, Gucker 2007, Habeck 1992a, Habeck 1992d, Holland and Keil 1995, Jenkinson 1990, LANDFIRE 2007a, McKenzie et al. 2004, McKenzie et al. 2008, Mote et al. 2014, Rosentreter and Belnap 2003, Sawyer and Keeler‑Wolf 1995, Sawyer et al. 2009, Schmid 1988, Shiflet 1994, Stevens‑Rumann et al. 2017, Tomback 1977, Vander Wall 1992, Vander Wall 1995, Vander Wall 2002, Westerling et al. 2011

**Version:** 24 May 2018	**Stakeholders:** Latin America, West

**Concept Author:** P. Comer and T. Keeler‑Wolf	**LeadResp:** West

CES206.917 Klamath‑Siskiyou Lower Montane Serpentine Mixed Conifer Woodland

**Primary Division:** Mediterranean California (206)

**Land Cover Class:** Forest and Woodland

**Spatial Scale & Pattern:** Large patch

**Required Classifiers:** Natural/Semi‑natural; Vegetated (>10% vasc.); Upland

**Diagnostic Classifiers:** Forest and Woodland (Treed); Serpentine; Mediterranean [Mediterranean Pluviseasonal‑Oceanic]; Ultramafic with low Ca:Mg ratio

**National Mapping Codes:** EVT 2021; ESLF 4208; ESP 1021

**Concept Summary:** This system occurs throughout the Klamath‑Siskiyou region below 1500 m (4550 feet) elevation on thin, rocky, ultramafic (gabbro, peridotite, serpentinite) soils below winter snow accumulations and typically experiences hot and dry summers. Soils are not always rocky; they can be loamy, up to 76 cm (30 inches) in depth, and can be heavy clay. Not all ultramafic outcrops support distinct vegetation; only those with very low Ca:Mg ratios impact biotic composition. These woodlands are highly variable and spotty in distribution. These sites are more productive and can support large‑statured (dbh, height) trees, although they tend to be widely spaced. Common species include *Pseudotsuga menziesii, Pinus sabiniana, Pinus lambertiana, Pinus jeffreyi, Pinus attenuata, Notholithocarpus densiflorus var. echinoides, Calocedrus decurrens, Arctostaphylos* spp., *Quercus vacciniifolia*, and *Xerophyllum tenax*. Perennial grasses such as *Festuca idahoensis* may also be characteristic. *Chamaecyparis lawsoniana* communities can occur within occurrences of this system in mesic and linear riparian zones. Herbaceous‑dominated serpentine fens (and bogs) are treated in Mediterranean California Serpentine Fen (CES206.953).

**Comments:** It has been proposed to merge this system with the similar Klamath‑Siskiyou Upper Montane Serpentine Mixed Conifer Woodland (CES206.914), as they are similar in composition and structure. For now, they are kept as separate systems pending further review and comment from California ecologists.

DISTRIBUTION

**Range:** This system occurs throughout the Klamath‑Siskiyou mountains region below 1500 m (4550 feet) elevation.

**Divisions:** 206:C

**TNC Ecoregions:** 5:C

**Nations:** US

**Subnations:** CA, OR

**Map Zones:** 2:C, 3:C, 7:?

**USFS Ecomap Regions:** 263A:CC, M242A:CP, M242B:CC, M242C:C?, M261A:CC, M261B:CC, M261C:CP, M261D:CC

CONCEPT

**Environment:** This system occurs throughout the Klamath‑Siskiyou region below 1500 m (4550 feet) elevation on thin, rocky, ultramafic (gabbro, peridotite, serpentinite) soils below winter snow accumulations and typically experiences hot and dry summers. Soils are not always rocky; they can be loamy, up to 76 cm (30 inches) in depth, and can be heavy clay. Not all ultramafic outcrops support distinct vegetation; only those with very low Ca:Mg ratios impact biotic composition. Soils on ultramafics are usually shallow and skeletal, with little profile development. Ultramafic soils impose the following stresses on plants: imbalance of calcium and magnesium, magnesium toxicity, low availability of molybdenum, toxic levels of heavy metals, sometime high alkalinity, low concentrations of some essential nutrients, and low soil water storage capacity (Kruckeberg 1984, Sanchez‑Mata 2007). In some cases, the steepness of the slopes and general sparseness of the vegetation result in continual erosion.

**Vegetation:** Common species include *Pseudotsuga menziesii, Pinus sabiniana, Pinus lambertiana, Pinus jeffreyi, Pinus attenuata, Notholithocarpus densiflorus var. echinoides (= Lithocarpus densiflorus var. echinoides), Calocedrus decurrens, Arctostaphylos* spp., *Quercus vacciniifolia*, and *Xerophyllum tenax*. Perennial grasses such as *Festuca idahoensis* may also be characteristic.

**Dynamics:** Sites are productive and can support large‑statured trees, although they will generally be widely spaced. Trees tend to grow very slowly due to the soil chemistry and textural characteristics which limit available nutrients.

Several important trees in this systems are fire‑adapted, but the system as a whole is an edaphically‑controlled type. Fire regimes vary depending on the slope position, elevation, fire history, and successional stage. *Chamaecyparis lawsoniana*‑dominated stands have a low frequency of stand‑replacing fires with an age class distribution showing >50% of stands are more than 300 years old (Jimerson et al. 1995). Other forest types in this system have more frequent stand‑replacing fires. *Pseudotsuga menziesii* woodlands age class distribution shows >80% of stands were older than 175 years. *Pinus jeffreyi* occurs on drier sites and has more frequent fires, age classes are evenly distributed from young to old; while *Pinus lambertiana* has highest age class frequency of stands <175 years. *Pinus lambertiana* stands burn more frequently due to upper slope positions (Jimerson et al. 1995). Native dwarf mistletoe (*Arceuthobium* spp.) infest many trees within this system; generally they do not cause mortality but weaken trees sufficiently for bark or engraver beetles or wood borers to successfully attack and kill the tree.

Parker (1990) suggests that species growing on serpentine sites may suffer greater mortality and poorer recruitment after a fire than the same species on adjacent sandstone soils. Landfire (2007a): This type has a very limited distribution and consequently limited information for fire occurrence history. Adjacent mixed conifer forest types have similar characteristics and are detailed below. Surface and mixed‑severity fires occur at an average of about 10‑15 years (Taylor and Skinner 1998, 2003, Sensenig 2002). Kilgore and Taylor (1979) reported a FRI=19‑39 years (N/NE aspects), which may favor mixed fires. Replacement fires with longer (70‑110 years) return intervals are possible (Frost and Sweeney 2000). With historic fire regimes, insect outbreaks may have been much reduced compared to current conditions. Snow breakage occurs in the mid‑seral closed state about every 5 years. While model is aspatial, most medium‑ and high‑severity fire may actually occur on mid and upper slope positions (Taylor and Skinner 1998, Taylor 2000, Bekker and Taylor 2001).

SOURCES

**References:** Atzet et al. 1996, Barbour and Major 1988, Barbour et al. 2007a, Bekker and Taylor 2001, Comer et al. 2003*, Eyre 1980, Frost and Sweeney 2000, Holland and Keil 1995, Jimerson 1993, Jimerson 1994, Jimerson and Daniel 1999, Jimerson et al. 1995, Kilgore and Taylor 1979, Kruckberg 1984, LANDFIRE 2007a, PRBO Conservation Science 2011, Parker 1990, Sanchez‑Mata 2007, Sawyer and Keeler‑Wolf 1995, Sensenig 2002, Taylor 2000, Taylor and Skinner 1998, Taylor and Skinner 2003

**Version:** 14 Jan 2014	**Stakeholders:** West

**Concept Author:** P. Comer, T. Keeler‑Wolf	**LeadResp:** West

CES206.914 Klamath‑Siskiyou Upper Montane Serpentine Mixed Conifer Woodland

**Primary Division:** Mediterranean California (206)

**Land Cover Class:** Forest and Woodland

**Spatial Scale & Pattern:** Large patch

**Required Classifiers:** Natural/Semi‑natural; Vegetated (>10% vasc.); Upland

**Diagnostic Classifiers:** Montane [Upper Montane]; Forest and Woodland (Treed); Mediterranean [Mediterranean Pluviseasonal‑Oceanic]; Ultramafic with low Ca:Mg ratio; Very Shallow Soil; Ustic

**National Mapping Codes:** EVT 2022; ESLF 4209; ESP 1022

**Concept Summary:** This system occurs throughout the Klamath‑Siskiyou mountains region above 1500 m (4550 feet) elevation on thin, rocky, ultramafic (gabbro, peridotite, serpentinite) soils in dry‑mesic conditions. Not all ultramafic outcrops support distinct vegetation; only those with very low Ca:Mg ratios impact biotic composition. Although ultramafics may be relatively dry and have a moderate to high grass component, they do not burn often where the serpentine syndrome is severe. The problem is not just the calcium:magnesium ratio, but heavy metals and sometimes high clay content limit biomass production. These systems are highly variable and spotty in distribution. Common species include *Pinus monticola, Pinus balfouriana, Quercus vacciniifolia, Pinus jeffreyi, Ceanothus pumilus, Arctostaphylos* spp., *Notholithocarpus densiflorus var. echinoides, Abies magnifica var. shastensis*, and *Callitropsis nootkatensis*. Stands of stunted (up to 12 m [40 feet]) but straight *Pinus contorta* are also possible. *Chamaecyparis lawsoniana* communities can occur in this system in mesic and linear riparian zones. Herbaceous‑dominated serpentine fens (and bogs) are treated in Mediterranean California Serpentine Fen (CES206.953).

**Comments:** It has been proposed to merge this system with the similar Klamath‑Siskiyou Lower Montane Serpentine Mixed Conifer Woodland (CES206.917), as they are similar in composition and structure. For now, they are kept as separate systems pending further review and comment from California ecologists.

DISTRIBUTION

**Range:** This system occurs throughout the Klamath‑Siskiyou mountains region above 1500 m (4550 feet) elevation.

**Divisions:** 206:C

**TNC Ecoregions:** 5:C

**Nations:** US

**Subnations:** CA, OR

**Map Zones:** 2:C, 3:C, 7:?

**USFS Ecomap Regions:** M242A:??, M261A:CC, M261B:CC, M261C:CP, M261D:CC

CONCEPT

**Environment:** This system occurs throughout the Klamath‑Siskiyou mountains region above 1500 m (4550 feet) elevation on thin, rocky, ultramafic (gabbro, peridotite, serpentinite) soils in dry‑mesic conditions. Not all ultramafic outcrops support distinct vegetation; only those with very low Ca:Mg ratios impact biotic composition. Although ultramafics may be relatively dry and have a moderate to high grass component, they do not burn often where the serpentine syndrome [see Kruckeberg (1984)] is severe. The problem is not just the calcium:magnesium ratio, but heavy metals and sometimes high clay content limit biomass production.

**Vegetation:** These systems are highly variable and spotty in distribution. Common species include *Pinus monticola, Pinus balfouriana, Quercus vacciniifolia, Pinus jeffreyi, Ceanothus pumilus, Arctostaphylos* spp., *Notholithocarpus densiflorus var. echinoides (= Lithocarpus densiflorus var. echinoides), Abies magnifica var. shastensis*, and *Callitropsis nootkatensis (= Chamaecyparis nootkatensis)*. Stands of stunted (up to 12 m [40 feet]) but straight *Pinus contorta* are also possible.

SOURCES

**References:** Barbour and Major 1988, Comer et al. 2003*, Eyre 1980, Holland and Keil 1995, Jimerson 1993, Jimerson 1994, Jimerson and Daniel 1999, Jimerson et al. 1995, Kruckeberg 1984, Sawyer and Keeler‑Wolf 1995

**Version:** 25 Apr 2006	**Stakeholders:** West

**Concept Author:** P. Comer and T. Keeler‑Wolf	**LeadResp:** West

CES206.916 Mediterranean California Dry‑Mesic Mixed Conifer Forest and Woodland

**Primary Division:** Mediterranean California (206)

**Land Cover Class:** Forest and Woodland

**Spatial Scale & Pattern:** Matrix

**Required Classifiers:** Natural/Semi‑natural; Vegetated (>10% vasc.); Upland

**Diagnostic Classifiers:** Montane [Lower Montane]; Forest and Woodland (Treed); Mediterranean [Mediterranean Xeric‑Oceanic]; Ustic; Needle‑Leaved Tree

**National Mapping Codes:** EVT 2027; ESLF 4214; ESP 1027

**Concept Summary:** These mixed‑conifer forests, always with at least two conifer species codominating, occur on all aspects in lower montane zones (600‑1800 m elevation in northern California; 1200‑2150 m in southern California). This system occurs in a variety of topo‑edaphic positions, such as upper slopes at higher elevations, canyon sideslopes, ridgetops, and south‑ and west‑facing slopes which burn relatively frequently. Often, several conifer species co‑occur in individual stands. *Pseudotsuga menziesii, Pinus ponderosa*, and *Calocedrus decurrens* are the most common conifers. Other conifers that can occasionally be present include *Pinus jeffreyi, Pinus attenuata*, and *Pinus lambertiana* (not as common in this as in Mediterranean California Mesic Mixed Conifer Forest and Woodland (CES206.915)). Common subcanopy trees include *Quercus chrysolepis* and *Quercus kelloggii*. *Arbutus menziesii* and *Notholithocarpus densiflorus* may be common with the oaks in northern areas. *Pseudotsuga macrocarpa* and *Pinus coulteri* can be present but are not dominant species in this system in the Transverse Ranges of southern California. Codominant *Abies lowiana ‑ Calocedrus decurrens* communities in southern California are also included in this system. In the Transverse Ranges, where Great Basin and Mojavean elements are transitioning into the montane zones, *Juniperus californica* and *Pinus monophylla* can be mixed with the other conifers. Understories are variable, except in the Sierra Nevada, where in some stands there can be dense understory mats of *Chamaebatia foliolosa* (and other low, spreading shrubs) which foster relatively high‑frequency, low‑intensity surface fires. In Oregon, shrubs such as *Holodiscus discolor, Toxicodendron rydbergii, Mahonia nervosa, Mahonia aquifolium*, and *Symphoricarpos mollis* are common in addition to graminoids such as *Festuca californica, Elymus glaucus*, and *Danthonia californica*. In the north, where *Calocedrus decurrens* and *Pinus ponderosa* drop out, this system shifts to North Pacific Dry Douglas‑fir‑ (Madrone) Forest and Woodland (CES204.845).

**Comments:** This forest is more dense, with a greater richness of canopy tree species than Mediterranean California Lower Montane Black Oak‑Conifer Forest and Woodland (CES206.923).

DISTRIBUTION

**Range:** This system occurs in lower montane zones (600‑1800 m elevation in northern California; 1200‑2150 m in southern California), including the eastern Klamath‑Siskiyou, interior Coast Ranges, Transverse Ranges and Sierra Nevada.

**Divisions:** 206:C

**TNC Ecoregions:** 5:C, 12:C, 14:C, 15:C, 16:C

**Nations:** US

**Subnations:** CA, NV, OR

**Map Zones:** 2:C, 3:C, 4:C, 6:C, 7:C, 12:P, 13:?

**USFS Ecomap Regions:** 263A:PP, 322A:PP, 341D:CC, 342B:CC, M242A:CC, M242B:CC, M242C:CC, M261A:CC, M261B:CC, M261C:CC, M261D:CC, M261E:CC, M261F:CC, M261G:CC

CONCEPT

**Environment:** In the lower montane of the Sierra Nevada, 33% of the annual precipitation falls as snow and nearly all of it occurs in the fall, winter and spring months. Throughout California, conifers have to contend with summer drought. Winter precipitation is stored as soil moisture, and available water is virtually depleted by the end of September. The dry‑mixed conifer system occurs where there is plenty of moisture but not prolonged cold. Shade tolerance, drought tolerance and response to fire of dominant tree species seedlings are important factors governing the composition and successional patterns of this forest system. Ponderosa pine seedlings are intolerant of shade compared to Douglas‑fir, white fir, incense‑cedar and sugar pine. In fact, abundant evidence indicates that incense‑cedar and white fir have increased in ponderosa pine forests since the turn of the twentieth century, with more significant changes seen on xeric locations relative to mesic sites (Barbour et al. 2007). Historically, surface fires occurred every 5‑10 years and mixed‑severity fires occur about every 50 years. Fire suppression has led to an increase in forest canopy coverage and tree density, but a decrease in trees with >60 cm dbh. In addition, species composition has shifted due to targeted logging of preferred species.

**Vegetation:** *Pseudotsuga menziesii, Pinus ponderosa*, and *Calocedrus decurrens* are the most common conifers. Other conifers that can occasionally be present include *Pinus jeffreyi, Pinus attenuata*, and *Pinus lambertiana*. Common subcanopy trees include *Quercus chrysolepis* and *Quercus kelloggii*. *Arbutus menziesii* and *Notholithocarpus densiflorus (= Lithocarpus densiflorus)* may be common with the oaks in northern areas. *Pseudotsuga macrocarpa* and *Pinus coulteri* can be present but are not dominant species in this system in the Transverse Ranges of southern California. Codominant *Abies lowiana ‑ Calocedrus decurrens* communities in southern California are also included in this system. In the Transverse Ranges, where Great Basin and Mojavean elements are transitioning into the montane zones, *Juniperus californica* and *Pinus monophylla* can be mixed with the other conifers. Understories are variable, except in the Sierra Nevada, where in some stands there can be dense understory mats of *Chamaebatia foliolosa* (and other low, spreading shrubs) which foster relatively high‑frequency, low‑intensity surface fires. In Oregon, shrubs such as *Holodiscus discolor, Toxicodendron rydbergii, Mahonia nervosa, Mahonia aquifolium*, and *Symphoricarpos mollis* are common in addition to graminoids such as *Festuca californica, Elymus glaucus*, and *Danthonia californica*.

**Dynamics:** Historically, frequent and low‑intensity fires maintained these woodlands. Due to fire suppression, the majority of these forests now have closed canopies, whereas in the past, a moderately high fire frequency (every 20‑30 years) formerly maintained an open forest of many conifers.

SOURCES

**References:** Barbour and Billings 2000, Barbour and Major 1988, Barbour et al. 2007a, Comer et al. 2003*, Eyre 1980, Fites 1993, Holland and Keil 1995, Sawyer and Keeler‑Wolf 1995

**Version:** 12 Jan 2012	**Stakeholders:** West

**Concept Author:** P. Comer and T. Keeler‑Wolf	**LeadResp:** West

CES206.915 Mediterranean California Mesic Mixed Conifer Forest and Woodland

**Primary Division:** Mediterranean California (206)

**Land Cover Class:** Forest and Woodland

**Spatial Scale & Pattern:** Matrix

**Required Classifiers:** Natural/Semi‑natural; Vegetated (>10% vasc.); Upland

**Diagnostic Classifiers:** Montane [Lower Montane]; Toeslope/Valley Bottom; Mediterranean [Mediterranean Xeric‑Oceanic]; Udic

**National Mapping Codes:** EVT 2028; ESLF 4215; ESP 1028

**Concept Summary:** This ecological system occurs in cool ravines and north‑facing slopes (typically with 100‑150 cm annual precipitation; 50% as snow). It is found from 800‑1000 m (2400‑3000 feet) elevation in the Sierra Nevada and 1250‑2200 m (3800‑6700 feet) in the Klamath Mountains. The most characteristically co‑occurring conifers are *Abies lowiana, Calocedrus decurrens*, and *Pinus lambertiana*. *Pinus jeffreyi, Pinus ponderosa*, and *Pseudotsuga menziesii* occur frequently but are not dominant. In limited locations in the central Sierra Nevada, *Sequoiadendron giganteum* dominates, usually with *Abies lowiana*, and at the highest elevations also with *Abies magnifica*. *Acer macrophyllum* is common in lower elevation mesic pockets; *Chrysolepis chrysophylla* also occurs in the western Klamaths. Common understory species include *Corylus cornuta, Cornus nuttallii*, and at higher elevations *Chrysolepis sempervirens*. In areas of recent fire or other disturbance, *Arctostaphylos patula, Ceanothus integerrimus, Ceanothus cordulatus, Ceanothus parvifolius*, and *Ribes* spp. are more common. Fire of highly variable patch size and return interval maintains the structure of these woodlands

**Comments:** The presence of *Abies lowiana* with other conifers is a strong indicator for this system in central California's Coast and Transverse ranges.

DISTRIBUTION

**Range:** This system is found from 800‑1000 m (2400‑3000 feet) elevation in the Sierra Nevada and 1250‑2200 m (3800‑6700 feet) in the Klamath Mountains.

**Divisions:** 206:C

**TNC Ecoregions:** 5:C, 12:C, 14:C

**Nations:** US

**Subnations:** CA, NV, OR

**Map Zones:** 2:C, 3:C, 4:?, 6:C, 7:C, 12:?

**USFS Ecomap Regions:** 263A:CC, 342B:PP, M242A:CC, M242B:CC, M242C:CC, M261A:CC, M261B:CC, M261C:CC, M261D:CC, M261E:CC, M261F:CP, M261G:CC

CONCEPT

**Environment:** This ecological system occurs in cool ravines and north‑facing slopes (typically with 100‑150 cm annual precipitation; 50% as snow). It is found from 800‑1000 m (2400‑3000 feet) elevation in the Sierra Nevada and 1250‑2200 m (3800‑6700 feet) in the Klamath Mountains. Throughout California, conifers have to contend with summer drought. Winter precipitation is stored as soil moisture, and available water is virtually depleted by the end of September. The mesic‑mixed conifer system occurs where there is plenty of moisture but not prolonged cold. Shade tolerance, drought tolerance and response to fire of dominant tree seedlings are important factors governing the composition and successional patterns of this forest system. Ponderosa pine seedlings are intolerant of shade compared to Douglas‑fir, white fir, incense‑cedar and sugar pine. In fact, abundant evidence indicates that incense‑cedar and while fir have increased in ponderosa pine forests since the turn of the twentieth century, with more significant changes seen on xeric locations relative to mesic sites (Barbour et al. 2007). Historically, surface fires occurred every 10‑20 years and mixed‑severity fires occurs about every 19‑39 years. Fire suppression has led to an increase in forest canopy coverage and tree density, but a decrease in trees >60 cm dbh. In addition, species composition has shifted due to targeted logging of preferred species.

**Vegetation:** The most characteristically co‑occurring conifers are *Abies lowiana (= Abies concolor var. lowiana), Calocedrus decurrens*, and *Pinus lambertiana*. *Pinus jeffreyi, Pinus ponderosa*, and *Pseudotsuga menziesii* occur frequently but are not dominant. In limited locations in the central Sierra Nevada, *Sequoiadendron giganteum* dominates, usually with *Abies lowiana*, and at the highest elevations also with *Abies magnifica*. *Acer macrophyllum* is common in lower elevation mesic pockets; *Chrysolepis chrysophylla* also occurs in the western Klamaths. Common understory species include *Corylus cornuta, Cornus nuttallii*, and at higher elevations *Chrysolepis sempervirens*. In areas of recent fire or other disturbance, *Arctostaphylos patula, Ceanothus integerrimus, Ceanothus cordulatus, Ceanothus parvifolius*, and *Ribes* spp. are more common.

**Dynamics:** Fire of highly variable patch size and return interval maintains the structure of these woodlands

SOURCES

**References:** Barbour and Billings 2000, Barbour and Major 1988, Comer et al. 2003*, Eyre 1980, Holland and Keil 1995, Sawyer and Keeler‑Wolf 1995

**Version:** 12 Jan 2012	**Stakeholders:** West

**Concept Author:** P. Comer and T. Keeler‑Wolf	**LeadResp:** West

CES206.928 Mediterranean California Mesic Serpentine Woodland and Chaparral

**Primary Division:** Mediterranean California (206)

**Land Cover Class:** Steppe/Savanna

**Spatial Scale & Pattern:** Large patch

**Required Classifiers:** Natural/Semi‑natural; Vegetated (>10% vasc.); Upland

**Diagnostic Classifiers:** Mediterranean [Mediterranean Xeric‑Oceanic]; Ultramafic with low Ca:Mg ratio; Shallow Soil; Udic; Broad‑Leaved Evergreen Shrub; Cupressus sargentii

**National Mapping Codes:** EVT 2034; ESLF 4221; ESP 1034

**Concept Summary:** This ecological system occurs in Mediterranean California in the north and south Coast Ranges and the northern Sierra Nevada, on cool northerly and concave slopes and toeslopes with thin, rocky, ultramafic (gabbro, peridotite, serpentinite) soils. Not all ultramafic outcrops support distinct vegetation; only those with very low Ca:Mg ratios impact biotic composition. These systems are highly variable and spotty in distribution, and the composition of individual stands can be very diverse, especially the shrubs (often individual species have low cover). *Hesperocyparis sargentii, Pinus sabiniana, Garrya congdonii, Quercus durata, Umbellularia californica*, and *Frangula californica ssp. tomentella* are characteristic. Common associates include *Heteromeles arbutifolia, Adenostoma fasciculatum*, and the California endemics *Arctostaphylos viscida ssp. pulchella* and *Ceanothus jepsonii*. In some settings *Arctostaphylos glauca, Styrax redivivus*, or *Cercocarpus montanus var. glaber* can be common. Occasionally, *Chamaecyparis lawsoniana* may be present. Common grasses and forbs can include *Melica torreyana, Festuca idahoensis, Iris* spp., and locally endemic serpentine forbs (*Senecio* spp. and others). Structurally, this system is sometimes woodland in character, but it can also be an arborescent chaparral, depending on fire history. Herbaceous‑dominated serpentine fens (and bogs) are treated in Mediterranean California Serpentine Fen (CES206.953).

DISTRIBUTION

**Range:** This system occurs throughout Mediterranean California except in the Klamath Mountains and possibly into Oregon.

**Divisions:** 206:C

**TNC Ecoregions:** 5:C, 12:P, 14:C, 15:P

**Nations:** US

**Subnations:** CA, OR?

**Map Zones:** 2:C, 3:C, 4:C, 6:C, 7:P

**USFS Ecomap Regions:** M261A:CC, M261B:CC, M261C:CC, M261D:CC, M261E:CC

CONCEPT

**Environment:** This ecological system occurs in Mediterranean California in the northern and southern Coast Ranges and the northern Sierra Nevada, on cool northerly and concave slopes and toeslopes with thin, rocky, ultramafic (gabbro, peridotite, serpentinite) soils. Not all ultramafic outcrops support distinct vegetation; only those with very low Ca:Mg ratios impact biotic composition. Soils on ultramafics are usually shallow and skeletal, with little profile development (Kruckeberg 1984). Ultramafic soils impose the following stresses on plants: imbalance of calcium and magnesium, magnesium toxicity, low availability of molybdenum, toxic levels of heavy metals, sometime high alkalinity, low concentrations of some essential nutrients, and low soil water storage capacity (Kruckeberg 1984, Sanchez‑Mata 2007). In some cases, the steepness of the slopes and general sparseness of the vegetation result in continual erosion.

**Vegetation:** These systems are highly variable and spotty in distribution, and the composition of individual stands can be very diverse, especially the shrubs (often individual species have low cover). *Hesperocyparis sargentii (= Cupressus sargentii), Pinus sabiniana, Garrya congdonii, Quercus durata, Umbellularia californica*, and *Frangula californica ssp. tomentella (= Rhamnus tomentella ssp. tomentella)* are characteristic. Common associates include *Heteromeles arbutifolia, Adenostoma fasciculatum*, and the California endemics *Arctostaphylos viscida ssp. pulchella* and *Ceanothus jepsonii*. In some settings *Arctostaphylos glauca, Styrax redivivus (= Styrax officinalis)*, or *Cercocarpus montanus var. glaber (= Cercocarpus betuloides)* can be common. Occasionally, *Chamaecyparis lawsoniana* may be present. Common grasses and forbs can include *Melica torreyana, Festuca idahoensis, Iris* spp., and locally endemic serpentine forbs (*Senecio* spp. and others). Structurally, this system is sometimes woodland in character, but it can also be an arborescent chaparral, depending on fire history.

**Dynamics:** Structurally, this system is sometimes woodland in character, but it can also be an arborescent chaparral, depending on fire history. Landfire (2007a): Stand‑replacing fires occur mostly in the shrub‑dominated stages. In the conifer‑dominated late‑seral closed stage, surface fire is also important. Mean FRI is generally greater than that of the surrounding forested landscape (including the lower elevation California Mesic Chaparral (CES206.926) ‑ perhaps double (Nagel and Taylor 2005) ‑ due to the lack of flammability of many young shrub fields without a long history of fuel accumulation.

Within this system, *Hesperocyparis sargentii* dominates some occurrences as woodlands or as dense shrubby thickets (Griffin and Critchfield 1976). This tree begins bearing cones by 3‑7 years of age, and abundant cone crops are produced that require 2 years to mature. The serotinous cones remain closed on the tree until opened by the heat of a fire or from desiccation due to age. Seeds establish best on bare mineral soil. Seedling mortality is high on shaded sites with abundant litter because of damping‑off fungi (Esser 1994b, Barbour 2007). *Hesperocyparis sargentii* has serotinous cones. Burned trees usually release large quantities of seed after fire, and seedlings establish as dense thickets. No information was available on fire‑return intervals. To maintain a stand, fire‑return intervals of greater than 7 years will allow new cone crops to develop (Esser 1994b).

The mesic chaparral stage of this system generally burns in high‑intensity, stand‑replacing crown fires that may burn thousands of acres in a single event (Landfire 2007a). However, there is a considerable range in the flammability of shrub species (e.g., *Adenostoma fasciculatum* is "flashier" than *Arctostaphylos* spp.). Large, stand‑replacement events can interact with seed availability and, hence, influence post‑fire successional pathways differently than for smaller, less severe fires. Mean fire‑return intervals are highly variable across the range of this system depending on species composition and other factors. Sediment cores taken from the Santa Barbara Channel in central California dating from the 16th and 17th centuries indicate that large fires burned the Santa Ynez and Santa Lucia mountains every 40‑60 years. Season of burning plays a large part in species composition. Occasionally, frost affects mortality and increases fuel buildup.

*Quercus durata* is an important shrub in this system. Plants sprout from swollen root crowns and root suckers after damage to their trunks; they sprout rapidly following fire (Sawyer et al. 2009). Small mammals and jays cache acorns, which other wildlife also eat.

SOURCES

**References:** Barbour 2007, Barbour and Major 1988, Barbour et al. 2007a, Batten et al. 2006, Brooks and Minnich 2006, Comer et al. 2003*, Esser 1994b, Eyre 1980, Griffin and Critchfield 1976, Holland and Keil 1995, Kruckberg 1984, LANDFIRE 2007a, Nagel and Taylor 2005, PRBO Conservation Science 2011, Safford and Harrison 2004, Sanchez‑Mata 2007, Sawyer and Keeler‑Wolf 1995, Sawyer et al. 2009

**Version:** 14 Jan 2014	**Stakeholders:** West

**Concept Author:** P. Comer and T. Keeler‑Wolf	**LeadResp:** West

CES204.101 Sierran‑Intermontane Desert Western White Pine‑White Fir Woodland

**Primary Division:** North American Pacific Maritime (204)

**Land Cover Class:** Forest and Woodland

**Spatial Scale & Pattern:** Large patch

**Required Classifiers:** Natural/Semi‑natural; Vegetated (>10% vasc.); Upland

**Diagnostic Classifiers:** Woody‑Herbaceous; Very Shallow Soil; Aridic; Short Disturbance Interval; F‑Patch/Low Intensity; F‑Landscape/Low Intensity; Needle‑Leaved Tree

**National Mapping Codes:** EVT 2172; ESLF 4269; ESP 1172

**Concept Summary:** This interior Pacific Northwest ecological system occurs on the Modoc Plateau and Warner Mountains of California, north into the Fremont National Forest along the east slope of the southern Cascades in Oregon, and may also occur in isolated high‑elevation ranges of northern Nevada. These forests and woodlands range from just above the zone of ponderosa pine in the montane zone, to the upper montane zone. Elevations range from 1370 m to over 2135 m (4500‑7000 feet). Occurrences are found on all slopes and aspects, although more frequently on drier areas, including northwest‑ and southeast‑facing slopes, but also occurs on northerly slopes and ridges. This ecological system generally occurs on basalts, andesite, glacial till, basaltic rubble, colluvium, or volcanic ash‑derived soils, and sometimes on granitics (Carson Range). These soils have characteristic features of good aeration and drainage, coarse textures, circumneutral to slightly acidic pH, an abundance of mineral material, rockiness, and periods of drought during the growing season. Climatically, this system occurs somewhat in the rainshadow of the Sierras and Cascades and has a more continental regime, similar to the northern Great Basin. This system tends to be more woodland than forest in character, and the undergrowth is more open and drier, with little shrub or herbaceous cover. Tree regeneration is less prolific than in other mixed‑montane conifer systems of the Cascades, Sierras and California Coast Ranges. *Pinus monticola* is the dominant conifer in most places, but *Abies lowiana (= Abies concolor var. lowiana)* is usually present, at least in the understory, and occasionally as the dominant in the canopy, replacing *Pinus monticola*, particularly at lower elevations, and *Pinus ponderosa* is also often present. In the Warner Mountains, the *Abies lowiana* stands range from 1675 to 2135 m (5500‑7000 feet) in elevation, and the mixed *Pinus monticola ‑ Abies lowiana* is usually above 2135 m (7000 feet). Mixed stands with *Pinus contorta*, in moister locations, as well as *Pinus jeffreyi* and sometimes *Populus tremuloides* occasionally occur. Southern stands (around Babbitt Peak and in the Carson Range) can sometime have *Abies magnifica* in them, sometimes replacing *Abies lowiana*. These forests and woodlands are marked by the absence of *Pseudotsuga menziesii, Pinus lambertiana*, and *Calocedrus decurrens*, and the generally drier, continental climatic conditions. In addition, the overall floristic affinities are with the Great Basin rather than Pacific Northwest. Understories are typically open, with moderately low shrub cover and diversity, and include *Arctostaphylos patula, Arctostaphylos nevadensis, Chrysolepis sempervirens, Ceanothus* sp., and *Ribes viscosissimum*. Common herbaceous taxa include *Arnica cordifolia, Festuca* sp., *Poa nervosa, Carex inops, Pyrola picta*, and *Hieracium albiflorum*. In openings, *Wyethia mollis* can be abundant.

**Comments:** An alternative name could be Modoc Plateau Western White Pine ‑ White Fir Woodland. This system is very similar to Mediterranean California Dry‑Mesic Mixed Conifer Forest and Woodland (CES206.916), Mediterranean California Mesic Mixed Conifer Forest and Woodland (CES206.915) and Northern Rocky Mountain Dry‑Mesic Montane Mixed Conifer Forest (CES306.805). Justification for splitting this system includes the following: it is *Abies lowiana* (as opposed to being *Abies grandis*, which is found further east and north; hence it's probably not the northern Rocky Mountain system); it lacks Douglas‑fir completely which is an important component of the Californian mixed conifer systems in the Sierras; and the understory composition suggests it is drier (due to: lower elevations? volcanic‑derived ash/tuff soils? rainshadow of the Cascades?) than the Californian systems.

DISTRIBUTION

**Range:** This ecological system is found in the transition zone from the northern Sierra Nevada of California and Oregon, east into the Modoc Plateau and Intermountain region of northwestern Nevada. It is found in the Fremont National Forest east of Lake View in Oregon, and in the Modoc Plateau and Warner Mountains of California. It continues farther south in California to the Diamond Mountains south of Honey Lake (a northeast extension of the Sierras), on Babbitt Peak between Lake Tahoe and Sierra Valley, and also in the Carson Range in Nevada east of Lake Tahoe Scattered stands may occur on Hart Mountain and Steens Mountain in Oregon and possibly a few isolated places in the northern Great Basin and the Jarbridge Mountains of Nevada.

**Divisions:** 204:C, 304:P

**TNC Ecoregions:** 4:C, 6:C

**Nations:** US

**Subnations:** CA, NV, OR

**Map Zones:** 6:C, 7:C, 9:?

**USFS Ecomap Regions:** M242C:CC, M261D:CP, M261E:CC, M261G:CC

CONCEPT

**Dynamics:** The open nature of the stands suggests regeneration and establishment is slow and sporadic. Stand‑replacing events are not frequent; most fire is probably partial stand disturbance. These stands are relatively high elevation, and there are generally widely spaced large and somewhat fire‑resistant individuals. Also the discontinuous understory and only patchy regeneration suggests non‑stand‑replacing fire as the norm., rather patchy burns with isolated trees surviving regularly. Local windthrow, insects, disease (blister rust), and individual lightning strikes probably make up most of the disturbances.

SOURCES

**References:** Comer et al. 2003*, Eyre 1980, Hopkins 1979a, Volland 1985

**Version:** 23 Jan 2006	**Stakeholders:** West

**Concept Author:** M.S. Reid	**LeadResp:** West

M024. Vancouverian Coastal Rainforest

CES206.921 California Coastal Redwood Forest

**Primary Division:** Mediterranean California (206)

**Land Cover Class:** Forest and Woodland

**Spatial Scale & Pattern:** Large patch

**Required Classifiers:** Natural/Semi‑natural; Vegetated (>10% vasc.); Upland

**Diagnostic Classifiers:** Forest and Woodland (Treed); Toeslope/Valley Bottom; Mediterranean [Mediterranean Pluviseasonal‑Oceanic]; Intermediate Disturbance Interval; F‑Patch/Low Intensity; Needle‑Leaved Tree; Sequoia sempervirens; Long (>500 yrs) Persistence

**National Mapping Codes:** EVT 2015; ESLF 4202; ESP 1015

**Concept Summary:** This system occurs from the Klamath Mountains south to Monterey Bay, California. The coastal redwood forest generally can be found in areas of within the fog belt. In the northern portion, it occurs on upland slopes and in riparian zones and on riverine terraces that are flooded approximately every 50‑100 years. In the southern portion of the range, annual precipitation may be as little as 500 mm, and the system is limited to coves and ravines. It is commonly found on moderately well‑drained marine sediments (non‑metamorphosed siltstones, sandstones, etc.). This system forms the tallest forests in North America, with individuals reaching 100 m high (tallest being 106‑110 m [350‑360 feet]). Typically, mature stands of *Sequoia sempervirens* produce a deep shade, so understories can be limited, but coarse woody debris from past disturbance can be quite large. *Pseudotsuga menziesii* is the common associate among the large trees. *Tsuga heterophylla* is found in old‑growth stands in northern sections, and *Notholithocarpus densiflorus* occurs as a subcanopy in almost all stands (possibly as a result of fire suppression). *Sequoia sempervirens* mixes with *Arbutus menziesii, Notholithocarpus densiflorus, Pseudotsuga menziesii* and *Umbellularia californica*. The moist, coastal *Chamaecyparis lawsoniana* stands from southwestern Oregon and northwestern California, often mixed with *Sequoia sempervirens, Pseudotsuga menziesii*, or *Tsuga heterophylla*, are included in this system, as ecologically they function in the same way and have a similar overall floristic composition. Shade‑tolerant understory species include *Rubus parviflorus, Oxalis oregana, Aralia californica, Mahonia nervosa, Gaultheria shallon*, and many ferns, such as *Blechnum spicant, Polystichum* spp., and *Polypodium* spp. Historically, surface fires likely exposed mineral soil for redwood seed germination. Less frequent disturbance can result in increases in *Tsuga heterophylla* in northern occurrences, as it is sensitive to fire and is a decreaser with fire and flood. Fire suppression has tended to result in increasing abundance of *Notholithocarpus densiflorus, Umbellularia californica, Alnus rubra, Arbutus menziesii*, and *Acer macrophyllum*; all respond favorably to fire, flood, wind and slides, becoming more abundant in areas of frequent disturbance.

**Comments:** Stands dominated or codominated with *Chamaecyparis lawsoniana* that are within 25 km (15 miles) of the coast are part of either California Coastal Redwood Forest (CES206.921) (extreme southern Oregon and northern California) or North Pacific Seasonal Sitka Spruce Forest (CES204.841) (central and northern coastal Oregon). Stands in these areas may or may not have redwood or Sitka spruce present. Stands away for the coast and not on serpentine soils are considered part of North Pacific Maritime Mesic‑Wet Douglas‑Fir‑Western Hemlock Forest (CES204.002).

DISTRIBUTION

**Range:** This system occurs from the Klamath Mountains south to Monterey Bay, California.

**Divisions:** 206:C

**TNC Ecoregions:** 14:C, 15:C

**Nations:** US

**Subnations:** CA

**Map Zones:** 2:C, 3:C, 4:C

**USFS Ecomap Regions:** 263A:CC, M242A:PP, M261A:CP, M261B:CC

CONCEPT

**Environment:** Climate is wet, mild maritime. Forests along the immediate coast experience uniformly wet and mild climate, where precipitation averages 2000‑3000 mm/year (500 mm for some of the driest redwood occurrences) with frequent fog and low clouds during warmer months; additional moisture from fog‑drip can be significant. The coastal redwood system generally can be found in areas of lower rainfall than other coastal rainforests in this macrogroup, but still within the fog belt. In the northern portion, it occurs on upland slopes and in riparian zones and on riverine terraces that are flooded approximately every 50‑100 years. In the southern portion of the range, annual precipitation may be as little as 500 mm, and the system is limited to coves and ravines. It is commonly found on moderately well‑drained marine sediments (non‑metamorphosed siltstones, sandstones, etc.). Redwood forests are limited to the north by ultramafic soils of the Klamath Mountains (Sawyer 2007).

**Vegetation:** Typically, mature stands of *Sequoia sempervirens* produce a deep shade, so understories can be limited, but coarse woody debris from past disturbance can be quite large. *Pseudotsuga menziesii* is the common associate among the large trees. *Tsuga heterophylla* is found in old‑growth stands in northern sections, and *Notholithocarpus densiflorus (= Lithocarpus densiflorus)* occurs as a subcanopy in almost all stands (possibly as a result of fire suppression). *Sequoia sempervirens* mixes with *Arbutus menziesii, Notholithocarpus densiflorus, Pseudotsuga menziesii* and *Umbellularia californica*. The moist, coastal *Chamaecyparis lawsoniana* stands from southwestern Oregon and northwestern California, often mixed with *Sequoia sempervirens, Pseudotsuga menziesii*, or *Tsuga heterophylla*, are included in this system, as ecologically they function in the same way and have a similar overall floristic composition. Shade‑tolerant understory species include *Rubus parviflorus, Oxalis oregana, Aralia californica, Mahonia nervosa (= Berberis nervosa), Gaultheria shallon*, and many ferns, such as *Blechnum spicant, Polystichum* spp., and *Polypodium* spp. Historically, surface fires likely exposed mineral soil for redwood seed germination. Less frequent disturbance can result in increases in *Tsuga heterophylla* in northern occurrences, as it is sensitive to fire and is a decreaser with fire and flood. Fire suppression has tended to result in increasing abundance of *Notholithocarpus densiflorus, Umbellularia californica, Alnus rubra, Arbutus menziesii*, and *Acer macrophyllum*; all respond favorably to fire, flood, wind and slides, becoming more abundant in areas of frequent disturbance.

**Dynamics:** Historically, surface fires likely exposed mineral soil for redwood seed germination. Less frequent disturbance can result in increases in *Tsuga heterophylla* in northern occurrences, as it is sensitive to fire and is a decreaser with fire and flood. Landfire (2007a) model: Redwood forests typically burned in the summer and early fall in low‑ to moderate‑intensity surface fires that consumed irregular patches of surface fuel and understory vegetation. The great height of the canopy and separation of surface and crown fuels resulted in a pattern where fire rarely resulted in canopy tree mortality. Fire intervals ranged from less than 10 years in interior and upland locations to 100 years or more along the coast in the fog belt. More recent research funded by Save the Redwoods League suggests that fire has been historically quite variable with much lower frequencies in the extreme north coastal portion of redwood range (as low as 1 every 500 years) and very high in the southern end where ravine redwood stands occur adjacent to California chaparral and grasslands (T. Keeler‑Wolf pers. comm. 2013). Native Americans are thought to have contributed to the ignitions (perhaps as much as every 5‑8 years) since lightning is relatively infrequent in the area, especially in the fog belt. Flooding events that undermine trees may be a significant disturbance, but it's not known for certain this is the case.

SOURCES

**References:** Barbour and Billings 2000, Barbour and Major 1988, CNRA 2009, Comer et al. 2003*, Eyre 1980, Faber‑Langendoen et al. 2008b, Holland and Keil 1995, Keeler‑Wolf pers. comm., LANDFIRE 2007a, PRBO Conservation Science 2011, Save the Redwoods League 2013, Sawyer 2007, Sawyer and Keeler‑Wolf 1995, Sawyer et al. 2009, Sillett and Bailey 2003, Sillett and Van Pelt 2000, WNHP 2011

**Version:** 14 Jan 2014	**Stakeholders:** West

**Concept Author:** P. Comer and T. Keeler‑Wolf	**LeadResp:** West

CES204.846 North Pacific Broadleaf Landslide Forest and Shrubland

**Primary Division:** North American Pacific Maritime (204)

**Land Cover Class:** Forest and Woodland

**Spatial Scale & Pattern:** Large patch, Small patch

**Required Classifiers:** Natural/Semi‑natural; Vegetated (>10% vasc.); Upland

**Diagnostic Classifiers:** Forest and Woodland (Treed); Broad‑Leaved Deciduous Tree

**National Mapping Codes:** EVT 2063; ESLF 4304; ESP 1063

**Concept Summary:** These forests and shrublands occur throughout the northern Pacific mountains and lowlands, becoming less prominent in the northern half of this region. They occur on steep slopes and bluffs that are subject to mass movements on a periodic basis. They are found in patches of differing age associated with different landslide events. The vegetation is deciduous broadleaf forests, woodlands, or shrublands, sometimes with varying components of conifers. *Alnus rubra* and *Acer macrophyllum* are the major tree species. *Rubus spectabilis, Rubus parviflorus, Ribes bracteosum*, and *Oplopanax horridus* are some of the major shrub species. Shrublands tend to be smaller in extent than woodlands or forests. Small patches of sparsely vegetated areas or herbaceous‑dominated vegetation (especially *Petasites frigidus*) also often occur as part of this system. On earthflows, once stable, vegetation may succeed to dominance by conifers.

**Comments:** Early‑successional shrubby patches dominated by *Alnus* or *Acer* not associated with landslide disturbance are removed from this system and are placed within the forest types they are successional to, for example see North Pacific Maritime Dry‑Mesic Douglas‑fir‑Western Hemlock Forest (CES204.001). More stable patches generally belong to North Pacific Montane Shrubland (CES204.087). For other disturbance driven shrublands, see North Pacific Avalanche Chute Shrubland (CES204.854). This system has not been determined to occur in Alaska, so for now that state is removed from its distribution.

DISTRIBUTION

**Range:** This system occurs throughout the northern Pacific mountains and lowlands (latter especially adjacent to coastlines), becoming less prominent in the northern half of this region.

**Divisions:** 204:C

**TNC Ecoregions:** 1:C, 3:C, 69:C, 81:C

**Nations:** CA, US

**Subnations:** BC, OR, WA:S2S3

**Map Zones:** 1:C, 2:C, 3:?, 7:C

**USFS Ecomap Regions:** 242A:CC, 242B:CC, 342I:??, M242A:CC, M242B:CC, M242C:CC, M242D:CC, M261A:CC, M261D:CC

CONCEPT

SOURCES

**References:** Chappell and Christy 2004, Comer et al. 2003*, Eyre 1980, Franklin and Dyrness 1973, WNHP unpubl. data 2018

**Version:** 25 Apr 2006	**Stakeholders:** Canada, West

**Concept Author:** C. Chappell	**LeadResp:** West

CES204.098 North Pacific Dry‑Mesic Silver Fir‑Western Hemlock‑Douglas‑fir Forest

**Primary Division:** North American Pacific Maritime (204)

**Land Cover Class:** Forest and Woodland

**Spatial Scale & Pattern:** Matrix

**Required Classifiers:** Natural/Semi‑natural; Vegetated (>10% vasc.); Upland

**Diagnostic Classifiers:** Forest and Woodland (Treed); Tsuga heterophylla ‑ Abies amabilis

**National Mapping Codes:** EVT 2174; ESLF 4272; ESP 1174

**Concept Summary:** This forested system occurs only in the Pacific Northwest mountains, primarily west of the Cascade Crest. It generally occurs in an elevational band between *Pseudotsuga menziesii ‑ Tsuga heterophylla* forests and *Tsuga mertensiana* forests. It dominates mid‑montane dry to mesic maritime and some submaritime climatic zones from northwestern British Columbia to northwestern Oregon. In British Columbia and in the Olympic Mountains, this system occurs on the leeward side of the mountains only. In the Washington Cascades, it occurs on both windward and leeward sides of the mountains (in other words, it laps over the Cascade Crest to the "eastside"). Stand‑replacement fires are regular with mean return intervals of about 200‑500 years. Fire frequency tends to decrease with increasing elevation and continentality but still remains within this typical range. A somewhat variable winter snowpack that typically lasts for 2‑6 months is characteristic. The climatic zone within which it occurs is sometimes referred to as the "rain‑on‑snow" zone because of the common occurrence of major winter rainfall on an established snowpack. *Tsuga heterophylla* and/or *Abies amabilis* dominate the canopy of late‑seral stands, though *Pseudotsuga menziesii* is usually also common because of its long lifespan, and *Callitropsis nootkatensis (= Chamaecyparis nootkatensis)* can be codominant, especially at higher elevations. *Abies procera* forests (usually mixed with silver fir) are included in this system and occur in the Cascades from central Washington to central Oregon and rarely in the Coast Range of Oregon. *Pseudotsuga menziesii* is a common species (unlike the mesic western hemlock‑silver fir forest system) that regenerates after fires and therefore is frequent as a codominant, except at the highest elevations; the prevalence of this species is an important indicator in relation to the related climatically wetter North Pacific Mesic Western Hemlock‑Silver Fir Forest (CES204.097). *Abies lasiocarpa* sometimes occurs as a codominant on the east side of the Cascades and in submaritime British Columbia. Understory species that tend to be more common or unique in this type compared to the wetter North Pacific Mesic Western Hemlock‑Silver Fir Forest (CES204.097) include *Achlys triphylla, Mahonia nervosa, Xerophyllum tenax, Vaccinium membranaceum, Rhododendron macrophyllum*, and *Rhododendron albiflorum*. *Vaccinium ovalifolium*, while still common, only dominates on more moist sites within this type, unlike in the related type where it is nearly ubiquitous.

**Comments:** Unlike North Pacific Mesic Western Hemlock‑Silver Fir Forest (CES204.097), the dominant natural process here is stand‑replacing fires which occur on average every 200‑500 years. Where old‑growth does exist, it is mostly "young old‑growth" 200‑500 years in age. Natural‑origin stands less than 200 years old are also common. More mixed‑severity fires occur to the south in this system, so structure, patch size and proportions will be different; further north is more stand‑replacing fires. In mapzone 7 this system will get modeled as 2 different BpS because of the differences in regimes. In Oregon there are more mixed‑severity fires.

DISTRIBUTION

**Range:** This system only occurs in the Pacific Northwest mountains, on the leeward side of coastal mountains in both British Columbia and in the Olympic Mountains of Washington. It occurs throughout most of the Washington Cascades on both west and east sides (sporadically on the east) and in the western Cascades of northern to central Oregon. It occurs very sporadically in the Willapa Hills of southwestern Washington and in the northern Oregon Coast Range. This type may also occur on the east side of the Oregon Cascades north of 45°N latitude (Mount Hood National Forest ‑ Hood River and Barlow ranger districts, and possibly the northern edge of Warm Springs Reservation in part of the McQuinn Strip).

**Divisions:** 204:C

**TNC Ecoregions:** 1:C, 3:C, 69:C, 70:C, 81:C

**Nations:** CA, US

**Subnations:** BC, OR, WA:S5

**Map Zones:** 1:C, 2:C, 3:?, 7:C

**USFS Ecomap Regions:** 242A:CC, 342I:PP, M242A:CC, M242B:CC, M242C:CC, M242D:CC

CONCEPT

**Dynamics:** Landfire VDDT models: R#ABAMlo; they use *Pseudotsuga menziesii* as an indicator so some of the eastside *Abies amabilis* are included with *Picea engelmannii* or *Pinus monticola*.

SOURCES

**References:** Comer et al. 2003*, DeMeo et al. 1992, DeVelice et al. 1999, Eyre 1980, Franklin and Dyrness 1973, Martin et al. 1995, Viereck et al. 1992, WNHP unpubl. data 2018

**Version:** 23 Jan 2006	**Stakeholders:** Canada, West

**Concept Author:** C. Chappell	**LeadResp:** West

CES204.842 North Pacific Hypermaritime Western Red‑cedar‑Western Hemlock Forest

**Primary Division:** North American Pacific Maritime (204)

**Land Cover Class:** Forest and Woodland

**Spatial Scale & Pattern:** Matrix

**Required Classifiers:** Natural/Semi‑natural; Vegetated (>10% vasc.); Upland

**Diagnostic Classifiers:** Forest and Woodland (Treed); Temperate [Temperate Hyperoceanic]; Tsuga heterophylla, Thuja plicata

**National Mapping Codes:** EVT 2178; ESLF 4271; ESP 1178

**Concept Summary:** These forests occupy the outer coastal portions of British Columbia, southeastern Alaska, and northwestern Washington. Their center of distribution is the central coast of British Columbia, as *Thuja plicata* approaches its northernmost limit in the southern half of southeastern Alaska. These forests occur mainly on islands but also fringe the mainland. They are generally less than 25 km from saltwater; elevation ranges from 0 to 600 m, and below 245 m in Alaska (above 200 m, *Callitropsis nootkatensis (= Chamaecyparis nootkatensis)* replaces *Thuja plicata*). The climate is hypermaritime, with cool summers, very wet winters, abundant fog, and without a major snowpack. Fire is absent from this system in Alaska and rare throughout the rest of the range. These forests are influenced by gap disturbance processes and intense windstorms and not much by fire. The terrain is mostly gentle to rolling, of low topographic relief, and often rocky. Soils typically have a distinct humus layer overlying mineral horizons or bedrock; where the system is best developed in central British Columbia, the humus layers are very thick (mean 17‑35 cm). Soils are often imperfectly drained, but this is not a wetland system. *Thuja plicata* and *Tsuga heterophylla* are the dominant tree species throughout, and *Callitropsis nootkatensis* joins them from northern Vancouver Island north. Canopy cover of trees is typically over 60%. *Pinus contorta* and *Tsuga mertensiana* can be present in some locations in the central and northern portion of the range. *Abies amabilis* occurs in British Columbia and northern Washington stands but is not typically found in southeastern Alaska. In Washington, nearly pure stands of *Tsuga heterophylla* are common and seem to be associated with microsites most exposed to intense windstorms. A shrub layer of *Gaultheria shallon, Vaccinium ovalifolium*, and *Menziesia ferruginea* is usually well‑developed. The fern *Blechnum spicant* in great abundance is typical of hypermaritime conditions. *Oxalis oregana* (absent in Alaska) is important in the understory of moist sites in Washington. *Polystichum munitum* occurs at the northern end of its range in southeastern Alaska on well‑drained sites. The abundance of *Thuja plicata* in relation to other conifers is one of the diagnostic characters of these forests; the other is the low abundance of *Pseudotsuga menziesii* (absent in Alaska) and *Picea sitchensis*. Where these forests are best developed, they occur in a mosaic with forested wetlands, bogs, and Sitka spruce forests (the latter in riparian areas and on steep, more productive soils).

**Comments:** Yellow‑cedar usually replaces western red‑cedar in southern southeast Alaska at an elevation of about 152 m (500 feet). When yellow‑cedar is mixed with western hemlock, and western red‑cedar has dropped out, the occurrence is classified as North Pacific Mesic Western Hemlock‑Yellow‑cedar Forest (CES204.843). Below 152 m elevation, western red‑cedar predominates; however, yellow‑cedar may be present.

DISTRIBUTION

**Range:** This system is found in the outer coastal portions of British Columbia and southern southeast Alaska, as well as northwestern Washington.

**Divisions:** 204:C

**TNC Ecoregions:** 1:C, 3:?, 69:C

**Nations:** CA, US

**Subnations:** AK, BC, WA:S1S2

**Map Zones:** 1:C, 2:C, 78:C

**USFS Ecomap Regions:** 242A:CC, M242A:CC, M242D:CC

CONCEPT

**Environment:** These forests occur mainly on islands but also fringe the mainland and coastal fjords. They are generally less than 25 km from saltwater; elevation ranges from 0 to 600 m, and below 245 m in Alaska (above 200 m, *Callitropsis nootkatensis* replaces *Thuja plicata*). Climate is characterized by moist mild air from the Pacific. Frequent winter storms produce abundant precipitation as they encounter rising mountain slopes. In summer, large high‑pressure areas off the coast produce prolonged spells of fine weather (Taylor 1997). The climate is classified as hypermaritime, with cool summers, very wet winters, abundant fog, and without a major snowpack (Meidinger and Pojar 1991). Rainfall is relatively high for the region at 254‑380 cm (100‑150 inches) rain annually, rarely as snow (Landfire 2007a). The terrain is mostly gentle to rolling, of low topographic relief, and often rocky. This type generally occurs on relatively old, acidic, humic soils with a distinct humus layer overlying mineral horizons or bedrock; where the system is best developed in central British Columbia, the humus layers are very thick (mean 17‑35 cm) (Banner et al. 1993, Green and Klinka 1994, Steen and Coupe 1997). Soils are often imperfectly drained, but this is not a wetland system. Where these forests are best developed, they occur in a mosaic with forested wetlands, bogs, and Sitka spruce forests (the latter in riparian areas and on steep, more productive soils). This system represents the upper end of the productivity gradient within the Cedar‑Hemlock Ecological Zone and the lower end of the Western Hemlock Ecological Zone (DeMeo et al. 1992).

**Dynamics:** Fire is absent from this system in Alaska and rare throughout the rest of the range, e.g., British Columbia's north coast (Banner et al. 1993, Landfire 2007a). These forests are primarily influenced by gap disturbance processes (gaps created by the death of individual trees, or small patches due to disease, insect damage and treefall following mortality). On the most exposed areas of the coastline, occasional hurricane force winds and severe storms result in major windthrow events. Less severe winds may cause breakage or early blowdown of diseased trees. The ground surface often has pit‑and‑mound microtopography that is formed by windthrow events. Storms are generally from the southwest and sweep across the low country of southwestern Washington, and strike either the front range of the Cascades or the southwest face of the Olympics. Wind damage tends to repeat at certain locations either due to direct exposure or due to the funneling of winds around topographic features. Wind damage tends to be more significant on the coast than further inland. Studies by USFS in southeastern Alaska show lots of broken boles as cause of tree mortality (Hennon 2008).

SOURCES

**References:** Banner et al. 1993, Bigley and Hull 1995, Comer et al. 2003*, DeMeo et al. 1992, DeVelice et al. 1999, Dorner and Wong 2003, Eyre 1980, Green and Klinka 1994, Haughian et al. 2012, Hebda 1997, Hennon 2008, Karl et al. 2009, LANDFIRE 2007a, Littell et al. 2009, Martin et al. 1995, Meidinger and Pojar 1991, Minore 1990, PRBO Conservation Science 2011, Packee 1990, Rodenhuis et al. 2009, Spittlehouse 2008, Steen and Coupé 1997, Taylor 1997, Viereck et al. 1992, WNHP 2011, WNHP unpubl. data 2018, Werner 2011

**Version:** 14 Jan 2014	**Stakeholders:** Canada, West

**Concept Author:** G. Kittel and C. Chappell	**LeadResp:** West

CES204.073 North Pacific Lowland Mixed Hardwood‑Conifer Forest

**Primary Division:** North American Pacific Maritime (204)

**Land Cover Class:** Forest and Woodland

**Spatial Scale & Pattern:** Large patch, Small patch

**Required Classifiers:** Natural/Semi‑natural; Vegetated (>10% vasc.); Upland

**Diagnostic Classifiers:** Forest and Woodland (Treed); Needle‑Leaved Tree; Broad‑Leaved Deciduous Tree

**Concept Summary:** This lowland mixed hardwood ‑ conifer forest system occurs throughout the Pacific Northwest. It occurs on valley terraces, margins, and slopes at low elevations in the mountains of the Pacific Northwest Coast and interior valleys west of the high Cascade Range. These forests are composed of large conifers, including *Pseudotsuga menziesii, Thuja plicata, Abies grandis, Tsuga heterophylla*, and/or *Picea sitchensis*, with deciduous hardwood trees present and usually codominant. Major dominant broadleaf species are *Acer macrophyllum, Quercus garryana, Alnus rubra, Frangula purshiana*, and *Cornus nuttallii*. Conifers tend to increase with succession in the absence of major disturbance although the hardwoods, particularly *Acer macrophyllum*, persist in the overstory. The understory is characterized by deciduous shrubs such as *Acer circinatum, Corylus cornuta, Oemleria cerasiformis, Rubus ursinus, Symphoricarpos albus*, and *Toxicodendron diversilobum*, but evergreen shrubs, including *Gaultheria shallon* and *Mahonia nervosa* and forbs, such as *Polystichum munitum* and *Oxalis oregana*, can be dominant.

DISTRIBUTION

**Range:** This system occurs throughout the Pacific Northwest elevationally below the Silver Fir Zone.

**Divisions:** 204:C

**TNC Ecoregions:** 1:C, 69:C, 81:C

**Nations:** CA, US

**Subnations:** BC, OR, WA

CONCEPT

**Environment:** In some places, hardwoods are truly only found in early‑seral conditions. This is more true the farther north you get, so in Washington, there are a few places where hardwoods persist, outside of the dry Douglas fir ‑ madrone forests around the Willamette Valley, Puget Trough and the western Oregon Interior Valleys. In the Coast Ranges and Cascades, there are hardwoods (mostly alder and bigleaf maple) found in most of the valley toeslopes. They also occur in areas with exposed talus, exposed rocks, and in dry places, and often with Oregon white oak and Oregon ash. This mix of deciduous hardwoods and conifers is a climax forest in many areas, while in others it is successional, with the conifers completely overtaking the hardwoods after 200 years or so without disturbance.

**Vegetation:** These forests are composed of large conifers, including *Pseudotsuga menziesii, Thuja plicata, Abies grandis, Tsuga heterophylla*, and/or *Picea sitchensis*, with deciduous hardwood trees present and usually codominant. Major dominant broadleaf species are *Acer macrophyllum, Quercus garryana, Alnus rubra, Frangula purshiana*, and *Cornus nuttallii*. Conifers tend to increase with succession in the absence of major disturbance although the hardwoods, particularly *Acer macrophyllum*, persist in the overstory. The understory is characterized by deciduous shrubs such as *Acer circinatum, Corylus cornuta, Oemleria cerasiformis, Rubus ursinus, Symphoricarpos albus*, and *Toxicodendron diversilobum*, but evergreen shrubs, including *Gaultheria shallon* and *Mahonia nervosa* and forbs, such as *Polystichum munitum* and *Oxalis oregana*, can be dominant.

SOURCES

**References:** Chappell and Christy 2004, Comer et al. 2003*, Franklin and Dyrness 1973

**Version:** 29 Oct 2007	**Stakeholders:** Canada, West

**Concept Author:** J. Kagan	**LeadResp:** West

CES204.001 North Pacific Maritime Dry‑Mesic Douglas‑fir‑Western Hemlock Forest

**Primary Division:** North American Pacific Maritime (204)

**Land Cover Class:** Forest and Woodland

**Spatial Scale & Pattern:** Matrix

**Required Classifiers:** Natural/Semi‑natural; Vegetated (>10% vasc.); Upland

**Diagnostic Classifiers:** Forest and Woodland (Treed); Temperate [Temperate Oceanic]; Tsuga heterophylla, Pseudotsuga menziesii

**National Mapping Codes:** EVT 2037; ESLF 4224; ESP 1037

**Concept Summary:** This ecological system comprises much of the major lowland forests of western Washington, northwestern Oregon, eastern Vancouver Island, and the southern Coast Ranges in British Columbia. In southwestern Oregon, it becomes local and more small‑patch in nature. It occurs throughout low‑elevation western Washington, except on extremely dry or moist to very wet sites. In Oregon, it occurs on the western slopes of the Cascades, around the margins of the Willamette Valley, and in the Coast Ranges. These forests occur on the drier to intermediate moisture habitats and microhabitats within the Western Hemlock Zone of the Pacific Northwest. Climate is relatively mild and moist to wet. Mean annual precipitation is mostly 90‑254 cm (35‑100 inches) (but as low as 20 inches in the extreme rainshadow) falling predominantly as winter rain. Snowfall ranges from rare to regular, and summers are relatively dry. Elevation ranges from sea level to 610 m (2000 feet) in northern Washington to 1067 m (3500 feet) in Oregon. Topography ranges from relatively flat glacial tillplains to steep mountainous terrain. This is generally the most extensive forest in the lowlands on the west side of the Cascades and forms the matrix within which other systems occur as patches. Throughout its range it occurs in a mosaic with North Pacific Maritime Mesic‑Wet Douglas‑fir‑Western Hemlock Forest (CES204.002); in dry areas it occurs adjacent to or in a mosaic with North Pacific Dry Douglas‑fir‑(Madrone) Forest and Woodland (CES204.845), and at higher elevations it intermingles with either North Pacific Dry‑Mesic Silver Fir‑Western Hemlock‑Douglas‑fir Forest (CES204.098) or North Pacific Mesic Western Hemlock‑Silver Fir Forest (CES204.097).

Overstory canopy is dominated by *Pseudotsuga menziesii*, with *Tsuga heterophylla* generally present in the subcanopy or as a canopy dominant in old‑growth stands. *Abies grandis, Thuja plicata*, and *Acer macrophyllum* codominants are also represented. In the driest climatic areas, *Tsuga heterophylla* may be absent, and *Thuja plicata* takes its place as a late‑seral or subcanopy tree species. *Gaultheria shallon, Mahonia nervosa, Rhododendron macrophyllum, Linnaea borealis, Achlys triphylla*, and *Vaccinium ovatum* typify the poorly to well‑developed shrub layer. *Acer circinatum* is a common codominant with one or more of these other species. The fern *Polystichum munitum* can be codominant with one or more of the evergreen shrubs on sites with intermediate moisture availability (mesic). If *Polystichum munitum* is thoroughly dominant or greater than about 40‑50% cover, then the stand is probably in the more moist North Pacific Maritime Mesic‑Wet Douglas‑fir‑Western Hemlock Forest (CES204.002). Young stands may lack *Tsuga heterophylla* or *Thuja plicata*, especially in the Puget Lowland. *Tsuga heterophylla* is generally the dominant regenerating tree species. Other common associates include *Acer macrophyllum, Abies grandis*, and *Pinus monticola*. In southwestern Oregon, *Pinus lambertiana, Calocedrus decurrens*, and occasionally *Pinus ponderosa* may occur in these forests. Soils are generally well‑drained and are mesic to dry for much of the year. This is in contrast to North Pacific Maritime Mesic‑Wet Douglas‑fir‑Western Hemlock Forest (CES204.002), which occurs on sites where soils remain moist to subirrigated for much of the year and fires were less frequent. Fire is (or was) the major natural disturbance. In the past (pre‑1880), fires were less commonly high‑severity, typically mixed‑severity or moderate‑severity, with natural return intervals of 100 years or less in the driest areas, to a few hundred years in areas with more moderate to wet climates. In the drier climatic areas (central Oregon Cascades, Puget Lowlands, Georgia Basin), this system was typified by a (mixed) moderate‑severity fire regime involving occasional stand‑replacing fires and more frequent moderate‑severity fires. This fire regime would create a complex mosaic of stand structures across the landscape.

DISTRIBUTION

**Range:** This system comprises the major lowland and low montane forests of western Washington, northwestern Oregon, and southwestern British Columbia. In British Columbia and Washington, it is uncommon to absent on the windward side of the coastal mountains where fire is rare. It also occurs locally in far southwestern Oregon (Klamath ecoregion) as small to large patches.

**Divisions:** 204:C

**TNC Ecoregions:** 1:C, 3:C, 5:C, 69:C, 81:C

**Nations:** CA, US

**Subnations:** BC, OR, WA:S2S3

**Map Zones:** 1:C, 2:C, 7:C

**USFS Ecomap Regions:** 242A:CC, 242B:CC, 342I:PP, M242A:CC, M242B:CC, M242C:CC, M242D:CC, M261A:CC

CONCEPT

**Environment:** This system occurs throughout low‑elevation western Washington, except on extremely dry or moist to very wet sites. These forests occur on the drier to intermediate moisture habitats and microhabitats within the Western Hemlock Zone of the Pacific Northwest. Climate is relatively mild and moist to wet. Mean annual precipitation is mostly 90‑254 cm (35‑100 inches) (but as low as 50 cm [20 inches] in the extreme rainshadow) falling predominantly as winter rain. Snowfall ranges from rare to regular, and summers are relatively dry. Elevation ranges from sea level to 610 m (2000 feet) in northern Washington to 1067 m (3500 feet) in Oregon. Topography ranges from relatively flat glacial tillplains to steep mountainous terrain. This is generally the most extensive forest in the lowlands on the west side of the Cascades and forms the matrix within which other systems occur as patches.

**Vegetation:** Overstory canopy is dominated by *Pseudotsuga menziesii*, with *Tsuga heterophylla* generally present in the subcanopy or as a canopy dominant in old‑growth stands. *Abies grandis, Thuja plicata*, and *Acer macrophyllum* codominants are also represented. In the driest climatic areas, *Tsuga heterophylla* may be absent, and *Thuja plicata* takes its place as a late‑seral or subcanopy tree species. *Gaultheria shallon, Mahonia nervosa, Rhododendron macrophyllum, Linnaea borealis, Achlys triphylla*, and *Vaccinium ovatum* typify the poorly to well‑developed shrub layer. *Acer circinatum* is a common codominant with one or more of these other species. The fern *Polystichum munitum* can be codominant with one or more of the evergreen shrubs on sites with intermediate moisture availability (mesic). Young stands may lack *Tsuga heterophylla* or *Thuja plicata*, especially in the Puget Lowland. *Tsuga heterophylla* is generally the dominant regenerating tree species. Other common associates include *Acer macrophyllum, Abies grandis*, and *Pinus monticola*. In southwestern Oregon, *Pinus lambertiana, Calocedrus decurrens*, and occasionally *Pinus ponderosa* may occur in these forests.

**Dynamics:** Fire is (or was) the major natural disturbance process. In the past (pre‑1880), fires were high‑severity or, less commonly, moderate‑severity, with natural return intervals of 100 years or less in the driest areas, to a few hundred years in areas with more moderate to wet climates. In the drier climatic areas (central Oregon Cascades, Puget Lowlands, Georgia Basin), this system was typified by a (mixed) moderate‑severity fire regime involving occasional stand‑replacement fires and more frequent moderate‑severity fires. This fire regime would create a complex mosaic of stand structures across the landscape. Landfire VDDT models: #RDFHEdry Douglas‑fir Hemlock dry mesic describes general successional stage relationship with bias to OR (Landfire 2007a).

SOURCES

**References:** Cadrin pers. comm., Comer et al. 2003*, Eyre 1980, Haughian et al. 2012, Karl et al. 2009, LANDFIRE 2007a, Littell et al. 2009, PRBO Conservation Science 2011, Packee 1990, Spittlehouse 2008, WNHP 2011, WNHP unpubl. data 2018, Werner 2011

**Version:** 14 Jan 2014	**Stakeholders:** Canada, West

**Concept Author:** G. Kittel and C. Chappell	**LeadResp:** West

CES204.002 North Pacific Maritime Mesic‑Wet Douglas‑fir‑Western Hemlock Forest

**Primary Division:** North American Pacific Maritime (204)

**Land Cover Class:** Forest and Woodland

**Spatial Scale & Pattern:** Matrix, Large patch

**Required Classifiers:** Natural/Semi‑natural; Vegetated (>10% vasc.); Upland

**Diagnostic Classifiers:** Forest and Woodland (Treed); Temperate [Temperate Oceanic]; Tsuga heterophylla, Pseudotsuga menziesii

**National Mapping Codes:** EVT 2039; ESLF 4226; ESP 1039

**Concept Summary:** This ecological system is a significant component of the lowland and low montane forests of western Washington, northwestern Oregon, and southwestern British Columbia. It occurs throughout low‑elevation western Washington, except on extremely dry sites and in the hypermaritime zone near the outer coast where it is rare. In Oregon, it occurs on the western slopes of the Cascades, around the margins of the Willamette Valley, and on the west side of the Coast Ranges, and is reduced to locally small patches in southwestern Oregon. In British Columbia, it occurs on the eastern (leeward) side of Vancouver Island, commonly and rarely on the windward side, and in the southern Coast Ranges. These forests occur on moist habitats and microhabitats, mainly lower slopes or valley landforms, within the Western Hemlock Zone of the Pacific Northwest. They differ from North Pacific Maritime Dry‑Mesic Douglas‑fir‑Western Hemlock Forest (CES204.001) primarily in having more hydrophilic undergrowth species, moist to subirrigated soils, high abundance of shade‑ and moisture‑tolerant canopy trees, as well as higher stand productivity, due to higher soil moisture and lower fire frequency. Climate is relatively mild and moist to wet. Mean annual precipitation is mostly 90‑254 cm (35‑100 inches) (but as low as 20 inches in the extreme rainshadow) predominantly as winter rain. Snowfall ranges from rare to regular (but consistent winter snowpacks are absent or minimal), and summers are relatively dry. Elevation ranges from sea level to 610 m (2000 feet) in northern Washington to 1067 m (3500 feet) in Oregon. Topography ranges from relatively flat glacial tillplains to steep mountainous terrain. This is an extensive forest in the lowlands on the west side of the Cascades. In some wetter climatic areas, it forms the matrix within which other systems occur as patches, especially riparian wetlands. In many rather drier climatic areas, it occurs as small to large patches within a matrix of North Pacific Maritime Dry‑Mesic Douglas‑fir‑Western Hemlock Forest (CES204.001); in dry areas, it can occur adjacent to or in a mosaic with North Pacific Dry Douglas‑fir‑(Madrone) Forest and Woodland (CES204.845), and at higher elevations it intermingles with either North Pacific Dry‑Mesic Silver Fir‑Western Hemlock‑Douglas‑fir Forest (CES204.098) or North Pacific Mesic Western Hemlock‑Silver Fir Forest (CES204.097).

Overstory canopy is dominated by *Pseudotsuga menziesii, Tsuga heterophylla*, and/or *Thuja plicata*, as well as *Chamaecyparis lawsoniana* in western Oregon, away from the coast. *Pseudotsuga menziesii* is usually at least present to more typically codominant or dominant. *Acer macrophyllum* and *Alnus rubra* (the latter primarily where there has been historic logging disturbance) are commonly found as canopy or subcanopy codominants, especially at lower elevations. In a natural landscape, small patches can be dominated in the canopy by these broadleaf trees for several decades after a severe fire. *Polystichum munitum, Oxalis oregana, Rubus spectabilis*, and *Oplopanax horridus* typify the poorly to well‑developed herb and shrub layers. *Gaultheria shallon, Mahonia nervosa, Rhododendron macrophyllum*, and *Vaccinium ovatum* are often present but are generally not as abundant as the aforementioned indicators; except where *Chamaecyparis lawsoniana* is a canopy codominant, they may be the dominant understory. *Acer circinatum* is a very common codominant as a tall shrub. Forested stands with abundant *Lysichiton americanus*, an indicator of seasonally flooded or saturated soils, belong in North Pacific Hardwood‑Conifer Swamp (CES204.090). Stands included are best represented on lower mountain slopes of the coastal ranges with high precipitation, long frost‑free periods, and low fire frequencies. Young stands may lack *Tsuga heterophylla* or *Thuja plicata*, especially in the Puget Lowland. *Tsuga heterophylla* is generally the dominant regenerating tree species. Other common associates include *Abies grandis*, which can be a codominant especially in the Willamette Valley ‑ Puget Trough ‑ Georgia Basin ecoregion. Soils are moist to somewhat wet but not saturated for much of the year and are well‑drained to somewhat poorly drained. Typical soils for *Polystichum* sites would be deep, fine‑ to moderately coarse‑textured, and for *Oplopanax* sites, soils typically have an impermeable layer at a moderate depth. Both types of soils are well‑watered from upslope sources, seeps, or hyperheic sources. This is in contrast to North Pacific Maritime Dry‑Mesic Douglas‑fir‑Western Hemlock Forest (CES204.001), which occurs on well‑drained soils, south‑facing slopes, and dry ridges and slopes where soils remain mesic to dry for much of the year. Fire is (or was) the major natural disturbance in all but the wettest climatic areas. In the past (pre‑1880), fires were less commonly high‑severity, typically mixed‑severity or moderate‑severity, with natural return intervals of a few hundred to several hundred years. This system was formerly supported by occasional, stand‑replacing fires. More frequent moderate‑severity fires would generally not burn these moister microsites.

**Comments:** Stands dominated or codominated with *Chamaecyparis lawsoniana* that are within 25 km (15 miles) of the coast are part of either California Coastal Redwood Forest (CES206.921) (extreme southern Oregon and northern California) or North Pacific Seasonal Sitka Spruce Forest (CES204.841) (central and northern coastal Oregon). Stands in these areas may or may not have redwood or Sitka spruce present. Stands away for the coast and not on serpentine soils are considered part of North Pacific Maritime Mesic‑Wet Douglas‑fir‑Western Hemlock Forest (CES204.002).

DISTRIBUTION

**Range:** This system is a significant component of the lowland and low montane forests of western Washington, northwestern Oregon, and southwestern British Columbia. This system may also occur as very small patches in northern California, in the northern Coast Ranges.

**Divisions:** 204:C

**TNC Ecoregions:** 1:C, 3:C, 5:C, 69:C, 81:C

**Nations:** CA, US

**Subnations:** BC, OR, WA:S3S4

**Map Zones:** 1:C, 2:C, 3:?, 7:C, 9:C

**USFS Ecomap Regions:** 242A:CC, 242B:CC, 263A:CC, 342I:??, M242A:CC, M242B:CC, M242C:CC, M242D:CC, M261A:CC

CONCEPT

**Dynamics:** Fire is (or was) the major natural disturbance in all but the wettest climatic areas. In the past (pre‑1880), fires were high‑severity or, less commonly, moderate‑severity, with natural return intervals of a few hundred to several hundred years. This system was formerly supported by occasional, stand‑replacing fires. More frequent moderate‑severity fires would generally not burn these moister microsites. Wind may be equally as important as fire, and in the Bull Run Watershed more important.

SOURCES

**References:** BCMF 2006, Comer et al. 2003*, Eyre 1980, Steen and Coupé 1997, WNHP unpubl. data 2018

**Version:** 23 Jan 2006	**Stakeholders:** Canada, West

**Concept Author:** G. Kittel and C. Chappell	**LeadResp:** West

CES204.097 North Pacific Mesic Western Hemlock‑Silver Fir Forest

**Primary Division:** North American Pacific Maritime (204)

**Land Cover Class:** Forest and Woodland

**Spatial Scale & Pattern:** Matrix

**Required Classifiers:** Natural/Semi‑natural; Vegetated (>10% vasc.); Upland

**Diagnostic Classifiers:** Montane; Forest and Woodland (Treed); Temperate [Temperate Oceanic]; Tsuga heterophylla ‑ Abies amabilis

**National Mapping Codes:** EVT 2042; ESLF 4229; ESP 1042

**Concept Summary:** This forested system occurs only in the Pacific Northwest mountains entirely west of the Cascade Crest from coastal British Columbia to Washington, and probably occurs in southeastern Alaska. It generally occurs in an elevational band between *Pseudotsuga menziesii ‑ Tsuga heterophylla* or hypermaritime zone forests and *Tsuga mertensiana* forests. It dominates mid‑montane maritime climatic zones on the windward side of Vancouver Island, the Olympic Peninsula, and the wettest portions of the North Cascades in Washington (north of Snoqualmie River). A somewhat variable winter snowpack that typically lasts for 2‑6 months is characteristic. The climatic zone within which it occurs is sometimes referred to as the "rain‑on‑snow" zone because of the common occurrence of major winter rainfall on an established snowpack. *Tsuga heterophylla* and/or *Abies amabilis* dominate the canopy of late‑seral stands, and *Callitropsis nootkatensis (= Chamaecyparis nootkatensis)* can be codominant, especially at higher elevations. *Thuja plicata* is also common and sometimes codominates in British Columbia. In Alaska, *Abies amabilis* occurs in nearly pure stands and in mixture with *Picea sitchensis* and *Tsuga heterophylla*. *Pseudotsuga menziesii* is relatively rare to absent in this system, as opposed to the similar but drier North Pacific Dry‑Mesic Silver Fir‑Western Hemlock‑Douglas‑fir Forest (CES204.098). The major understory dominant species is *Vaccinium ovalifolium*. Understory species that help distinguish this system from the drier silver fir system (they are much more common here) include *Oxalis oregana, Blechnum spicant*, and *Rubus pedatus*. Windthrow is a common small‑scale disturbance in this system, and gap creation and succession are important processes.

**Comments:** Jan Henderson suggests using 90 inches mean precipitation at sea level (with modification for topographic moisture) to distinguish wet and dry silver fir systems. Fire regime is significantly different at regional scale between the dry and mesic; this difference appears to be consistent throughout the range of the types. The mesic rarely, if ever, burns; it is dominated by what is sometimes called "old old‑growth" stands that run from 700 to over 1000 years in age. Research in British Columbia indicates these coastal rainforests may burn an average of once every 2000 years. The major processes then are small‑scale gap dynamics, not stand‑replacement fires. This difference is related to climate, not site moisture, with the mesic having a very wet climate that is more coastal, less continental, with cooler summers, and warmer winters on average.

DISTRIBUTION

**Range:** This system occurs only in the Pacific Northwest mountains (Coastal and westside Cascades). It occurs on the windward side of coastal mountains in both British Columbia and in the Olympic Mountains and north Cascade Range of Washington. It may also extend north to about 56°N latitude in southeastern Alaska. *Abies amabilis* has a limited distribution in Alaska, apparently confined to the extreme southern mainland and a few islands south of 56°N latitude.

**Divisions:** 204:C

**TNC Ecoregions:** 1:C, 3:C, 69:P, 81:C

**Nations:** CA, US

**Subnations:** AK?, BC, WA:S5

**Map Zones:** 1:C, 2:C, 3:?, 7:C, 78:C

**USFS Ecomap Regions:** 242A:??, M242A:CC, M242B:CC, M242C:CC, M242D:CC

CONCEPT

**Dynamics:** Stand‑replacing fires are relatively infrequent to absent, with return intervals of several hundred or more years. More mixed‑severity fires occur in the southern parts of this system, so that forest structure, patch size and proportions will be different from northern stands. Further north, stand‑replacing fires are also infrequent but are a more common fire event.

SOURCES

**References:** Banner et al. 1993, Comer et al. 2003*, DeMeo et al. 1992, DeVelice et al. 1999, Eyre 1980, Franklin and Dyrness 1973, Martin et al. 1995, Steen and Coupé 1997, Viereck et al. 1992, WNHP unpubl. data 2018

**Version:** 22 Aug 2008	**Stakeholders:** Canada, West

**Concept Author:** G. Kittel	**LeadResp:** West

CES204.841 North Pacific Seasonal Sitka Spruce Forest

**Primary Division:** North American Pacific Maritime (204)

**Land Cover Class:** Forest and Woodland

**Spatial Scale & Pattern:** Large patch

**Required Classifiers:** Natural/Semi‑natural; Vegetated (>10% vasc.); Upland

**Diagnostic Classifiers:** Forest and Woodland (Treed); Temperate [Temperate Hyperoceanic]; Picea sitchensis

**National Mapping Codes:** EVT 2036; ESLF 4223; ESP 1036

**Concept Summary:** This ecological system is restricted to the hypermaritime climatic areas near the Pacific Coast from Point Arena, California, north to northern Vancouver Island, British Columbia. These forests are typically dominated or codominated by *Picea sitchensis* and often have a mixture of other conifers present, such as *Tsuga heterophylla, Thuja plicata, Pseudotsuga menziesii*, or *Callitropsis nootkatensis*. *Tsuga heterophylla* is very often codominant. In the southern extent (in Oregon, but not in California), *Abies grandis, Acer circinatum, Alnus rubra, Acer macrophyllum, Chamaecyparis lawsoniana*, and *Frangula purshiana* can be associates, while *Callitropsis nootkatensis* is completely absent. Wet coastal environments that support stands of *Chamaecyparis lawsoniana* in the absence of *Picea sitchensis* are also part of this system. The understory is rich with shade‑tolerant shrubs and ferns, including *Gaultheria shallon, Vaccinium ovatum, Polystichum munitum, Dryopteris* spp., and *Blechnum spicant*, as well as a high diversity of mosses and lichens. This ecological system is restricted to the hypermaritime climatic areas near the Pacific Coast from Point Arena, California, north to northern Vancouver Island and Smith Sound on the mainland coast of British Columbia. They are generally limited to areas within 25 km or so of saltwater and are most abundant along the coast of Vancouver Island, southern portions of coastal mainland British Columbia, and the Olympic Peninsula of Washington. This ecosystem is defined as the "Seasonal Rain Forest" by Wolf et al. (1995), as the climate has abundant rainfall in the winter months and very little in the summer months. At the northern boundary this Sitka spruce forest ecosystem merges into Alaskan Pacific Maritime Sitka Spruce Forest (CES204.151) which has more continuous year‑round rainfall and lacks Douglas‑fir. North Pacific Seasonal Sitka Spruce Forest (CES204.841) occurs on outermost coastal fringe where salt spray is prominent, riparian terraces and valley bottoms near the coast where there is major fog accumulation, and on steep, well‑drained productive slopes not directly adjacent to the outer coast but within the hypermaritime zone. Annual precipitation ranges from 65 to 550 cm, with the majority falling as rain. Winter rains can be heavy. When summer drought occurs it is typically short in duration and ameliorated by frequent, dense coastal fog and cloud cover. In fact the fog belt becomes more and more important in the southern half of this ecosystem's distribution. In Washington and Oregon, it is found mostly below 300 m elevation. It also occurs as a very narrow strip or localized patches along the southern Oregon and northern California coasts. The disturbance regime is mostly small‑scale windthrow or other gap mortality processes (though there are occasional widespread intense windstorms) and very few fires, the latter mainly in Oregon.

**Comments:** Stands dominated or codominated with *Chamaecyparis lawsoniana* that are within 25 km (15 miles) of the coast are part of either California Coastal Redwood Forest (CES206.921) (extreme southern Oregon and northern California) or North Pacific Seasonal Sitka Spruce Forest (CES204.841) (central and northern coastal Oregon). Stands in these areas may or may not have redwood or Sitka spruce present. Stands away for the coast and not on serpentine soils are considered part of North Pacific Maritime Mesic‑Wet Douglas‑fir‑Western Hemlock Forest (CES204.002).

DISTRIBUTION

**Range:** This ecological system is restricted to the hypermaritime climatic areas near the Pacific Coast from Point Arena, California, north to northern Vancouver Island and Smith Sound on the mainland coast of British Columbia (S. Saunders pers. comm. 2013), where it merges with its northern counterpart, Alaskan Pacific Maritime Sitka Spruce Forest (CES204.151).

**Divisions:** 204:C

**TNC Ecoregions:** 1:C, 69:C

**Nations:** CA, US

**Subnations:** BC, CA, OR, WA:S1S2

**Map Zones:** 1:C, 2:C, 3:C

**USFS Ecomap Regions:** 242A:CC, M242A:CC, M242D:CC, M261A:??

CONCEPT

**Environment:** From Vancouver Island south, the forest is not confined to fjords, but a marked orographic effect from the Coast and Cascade ranges limits its interior extent. At its southern extent, the zone narrows again, confined to the fog belt not by mountains but by moisture. It is restricted to the hypermaritime climatic areas (Meidinger and Pojar 1991) near the Pacific Coast, along a fog belt from Point Arena, California, north to northern Vancouver Island, British Columbia. These forests are generally restricted to areas within 25 km of saltwater and are most abundant along the coast of Vancouver Island, southern portions of coastal British Columbia, and the Olympic Peninsula of Washington. Sites include the outermost coastal fringe where salt spray is prominent, riparian terraces and valley bottoms near the coast where there is major fog accumulation, and in the northern half of its range starting in central British Columbia, steep, well‑drained productive slopes not directly adjacent to the outer coast but within the hypermaritime zone (Banner et al. 1993, Green and Klinka 1994, Steen and Coupe 1997). Annual precipitation ranges from 65 to 550 cm, with the majority falling as rain. Winter rains can be heavy. The climate has more seasonal rainfall than coastal areas to the north, with a pronounced drought in summer months. Summer drought does occur, but it is typically short in duration and ameliorated by frequent, dense coastal fog and cloud cover. This forest type also dominates lower elevations (to 350 m) on the leeward side of the Queen Charlotte Islands in British Columbia. In Washington and Oregon, it is found mostly below 300 m elevation. It also occurs as a very narrow strip or localized patches along the southern Washington, Oregon and northern California coasts.

**Vegetation:** These forests are typically dominated or codominated by *Picea sitchensis* and often have a mixture of other conifers present, such as *Tsuga heterophylla, Thuja plicata, Pseudotsuga menziesii*, or *Callitropsis nootkatensis (= Chamaecyparis nootkatensis)*. *Tsuga heterophylla* is very often codominant. In the southern extent (in Oregon, but not in California), *Abies grandis, Acer circinatum, Alnus rubra, Acer macrophyllum, Chamaecyparis lawsoniana*, and *Frangula purshiana (= Rhamnus purshiana)* can be associates, while *Callitropsis nootkatensis* is completely absent. Wet coastal environments that support stands of *Chamaecyparis lawsoniana* in the absence of *Picea sitchensis* are also part of this system. The understory is rich with shade‑tolerant shrubs and ferns, including *Gaultheria shallon, Vaccinium ovatum, Polystichum munitum, Dryopteris* spp., and *Blechnum spicant*, as well as a high diversity of mosses and lichens.

**Dynamics:** The disturbance regime is mostly small‑scale windthrow or other gap mortality processes (though there are occasional widespread intense windstorms) and very few fires, the latter mainly in Oregon. Sitka spruce acts as an early colonizer of disturbed sites, such as land slumps, fluvial deposits, recently deglaciated areas. Seeds germinate best on bare mineral soil, a mixture of mineral soil and organic soil, and nurse‑logs (Sawyer et al. 2009). Landfire (2007a) model: The disturbance regime is mostly small‑scale windthrow or other gap mortality processes (though there are occasional widespread intense windstorms) and very few fires, the latter mainly in Oregon. Where fire does occur, it is usually stand‑replacing, with a fire return interval of 300‑1000 years or longer. In most of the range of the type, windthrow is a more significant catastrophic disturbance than wildfire. Windthrow "rotation" is estimated to be between 100‑200 years, (but can be up to 1000 years due to patchiness). The effects of windthrow are strongly correlated with topography and adjacent land use (e.g., clearcuts). Landfire VDDT models: R#SSHE Sitka spruce ‑ hemlock.

SOURCES

**References:** Agee 1993, Banner et al. 1993, Comer et al. 2003*, Dorner and Wong 2003, Eyre 1980, Franklin and Dyrness 1973, Green and Klinka 1994, Harcombe et al. 2004, Harris 1990a, Haughian et al. 2012, Henderson et al. 1989, Holland and Keil 1995, Karl et al. 2009, LANDFIRE 2007a, Littell et al. 2009, McCain and Diaz 2002a, Meidinger and Pojar 1991, PRBO Conservation Science 2011, Packee 1990, Rodenhuis et al. 2009, Saunders pers. comm., Sawyer et al. 2009, Spittlehouse 2008, Steen and Coupé 1997, WNHP 2011, WNHP unpubl. data 2018, Werner 2011, Western Ecology Working Group n.d., Wolf et al. 1995

**Version:** 14 Jan 2014	**Stakeholders:** Canada, West

**Concept Author:** G. Kittel, P. Comer, D. Vanderschaaf	**LeadResp:** West

CES204.883 North Pacific Wooded Volcanic Flowage

**Primary Division:** North American Pacific Maritime (204)

**Land Cover Class:** Forest and Woodland

**Spatial Scale & Pattern:** Large patch

**Required Classifiers:** Natural/Semi‑natural; Vegetated (>10% vasc.); Upland

**Diagnostic Classifiers:** Rock Outcrops/Barrens/Glades; Very Shallow Soil; Lava Flow

**National Mapping Codes:** EVT 2173; ESLF 4329; ESP 1173

**Concept Summary:** This ecological system is found from foothill to subalpine elevations and includes woodland to sparsely vegetated landscapes (generally >10% plant cover) on recent lava flows, excessively well‑drained lahars, debris avalanches and pyroclastic flows. The characteristic feature of this system is the substrate limiting characteristic that creates an environment for a more open vegetation than the surrounding closed matrix forest. Examples are recent lava flows (3500‑8200 years ago) on the north side of Mount Adams (andecite) and the big lava beds (basalt) south of Indian Heaven west of Mount Adams, Washington, and lahars (200‑2000 years old) at Old Maid Flat west of Mount Hood, Oregon. These areas support open to sparse tree cover; characteristic species include *Pseudotsuga menziesii, Pinus contorta, Pinus monticola*, and *Abies lasiocarpa*. Tree cover can range from scattered (5%) up to 70% or occasionally even more. There may be scattered to dense shrubs present, such as *Acer circinatum, Vaccinium membranaceum, Arctostaphylos uva‑ursi* (very characteristic), *Mahonia nervosa, Amelanchier alnifolia*, and *Xerophyllum tenax*. Soil development is limited, and mosses and lichens often cover the soil or rock surface.

**Comments:** This system will include areas that fit the sparsely vegetated system type definition but are included here and delineated by the boundary of lava or other volcanic flowage. Elevation range (>3350 m) for this system is great, but the specialized substrate is the overriding factor defining it. These are mid‑stages of primary succession that differ in degree of forest cover, soil development and productivity. Early primary succession on these substrates are included in North Pacific Active Volcanic Rock and Cinder Land (CES204.092). Later primary succession stages (increased soil development) are included in appropriate matrix forest systems.

DISTRIBUTION

**Range:** This uncommon system is found in the east and west Cascades of Washington and Oregon, and may occur in small patches in northern California in the vicinity of Mount Lassen or Mount Shasta.

**Divisions:** 204:C

**TNC Ecoregions:** 3:P, 4:C, 81:C

**Nations:** US

**Subnations:** CA?, OR, WA:S4

**Map Zones:** 1:C, 2:P, 6:P, 7:C

**USFS Ecomap Regions:** 242A:CC, 342I:PP, M242B:CC, M242C:CC, M242D:CP, M261D:CP, M261G:CC

CONCEPT

SOURCES

**References:** Comer et al. 2003*, Eyre 1980, WNHP unpubl. data 2018

**Version:** 31 Aug 2005	**Stakeholders:** West

**Concept Author:** R. Crawford	**LeadResp:** West

M025. Vancouverian Subalpine‑High Montane Forest

CES206.913 Mediterranean California Red Fir Forest

**Primary Division:** Mediterranean California (206)

**Land Cover Class:** Forest and Woodland

**Spatial Scale & Pattern:** Large patch

**Required Classifiers:** Natural/Semi‑natural; Vegetated (>10% vasc.); Upland

**Diagnostic Classifiers:** Montane [Upper Montane]; Forest and Woodland (Treed); Mediterranean [Mediterranean Pluviseasonal‑Oceanic]; Deep Soil; Ustic; Long Disturbance Interval; Abies magnifica (= var. magnifica)

**National Mapping Codes:** EVT 2032; ESLF 4219; ESP 1032

**Concept Summary:** This ecological system includes high‑elevation (1600‑2700 m [4850‑9000 feet]) forests and woodlands dominated by *Abies magnifica var. magnifica, Abies magnifica var. shastensis*, and/or *Abies procera*. This system is typically found on deep, well‑drained soils throughout this elevation zone from the central Sierra Nevada north and west into southern Oregon. Heavy snowpack is a major source of soil moisture throughout the growing season. The limiting factors can be either cold‑air drainages or ponding, or coarser soils (pumice versus ash, for example). Other conifers that can occur in varying mixtures with *Abies magnifica* include *Pinus contorta var. murrayana, Pinus monticola, Tsuga mertensiana, Pinus jeffreyi*, and *Abies lowiana*. At warmer and lower sites of the North Coast Ranges and Sierra Nevada, *Abies lowiana* can codominate with *Abies magnifica*. *Pinus contorta* in Oregon indicates lower productivity where it intergrades with *Abies magnifica var. shastensis*. This system ranges from dry to moist, and some sites have mesic indicator species, such as *Ligusticum grayi* or *Thalictrum fendleri*. Common understory species include *Quercus vacciniifolia, Ribes viscosissimum, Chrysolepis sempervirens, Ceanothus cordulatus* (in seral stands), *Vaccinium membranaceum, Symphoricarpos mollis*, and *Symphoricarpos rotundifolius*. Characteristic forbs include *Eucephalus breweri, Pedicularis semibarbata*, and *Hieracium albiflorum*. This system commonly occurs above mixed conifer forests with *Abies lowiana* and overlaps in elevation with forests and woodlands of *Pinus contorta var. murrayana*. On volcanic sites of lower productivity, stands may be more open woodland in structure and with poor‑site understory species such as *Wyethia mollis*. Driving ecological processes include occasional blowdown, insect outbreaks and stand‑replacing fire.

DISTRIBUTION

**Range:** This system is typically found on deep, well‑drained soils throughout the high‑elevation zone (1600‑2700 m [4850‑8200 feet]) from the central Sierra Nevada north and west into southern Oregon.

**Divisions:** 206:C

**TNC Ecoregions:** 5:C, 12:C

**Nations:** US

**Subnations:** CA, NV, OR

**Map Zones:** 2:C, 3:C, 6:C, 7:C, 12:?

**USFS Ecomap Regions:** 341D:CC, M242B:CC, M242C:CC, M261A:CC, M261B:CC, M261D:CC, M261E:CC, M261F:CC, M261G:CC

CONCEPT

**Environment:** Red fir forests occur at high elevations (1600‑2700 m [4850‑9000 feet]), and are typically found on deep, well‑drained soils throughout this elevational zone from the central Sierra Nevada north and west into southern Oregon. Heavy snowpack is a major source of soil moisture throughout the growing season. Climate is relative mild for high‑elevation forest with summer temperatures rarely exceeding 29°C (85°F) and winter temperatures rarely fall below ‑29°C (‑20°F). Summers are dry (4‑5 months). Between May (or April) and October summer thunderstorm precipitation is negligible, almost all precipitation occurs from October to March, 80% as snow. Snowpack can exceed 4 m (13 feet). Total ppt per year ranges 750‑1500 mm (30‑60 inches).

**Vegetation:** These forests and woodlands are dominated by *Abies magnifica var. magnifica, Abies magnifica var. shastensis*, and/or *Abies procera*. Other conifers that can occur in varying mixtures with *Abies magnifica* include *Pinus contorta var. murrayana, Pinus monticola, Tsuga mertensiana, Pinus jeffreyi*, and *Abies lowiana (= Abies concolor var. lowiana)*. At warmer and lower sites of the North Coast Ranges and Sierra Nevada, *Abies lowiana* can codominate with *Abies magnifica*. *Pinus contorta* in Oregon indicates lower productivity where it intergrades with *Abies magnifica var. shastensis (= Abies x shastensis)*. This system ranges from dry to moist, and some sites have mesic indicator species, such as *Ligusticum grayi* or *Thalictrum fendleri*. Common understory species include *Quercus vacciniifolia, Ribes viscosissimum, Chrysolepis sempervirens, Ceanothus cordulatus* (in seral stands), *Vaccinium membranaceum, Symphoricarpos mollis*, and *Symphoricarpos rotundifolius*. Characteristic forbs include *Eucephalus breweri, Pedicularis semibarbata*, and *Hieracium albiflorum*. This system commonly occurs above mixed conifer forests with *Abies lowiana* and overlaps in elevation with forests and woodlands of *Pinus contorta var. murrayana*. On volcanic sites of lower productivity, stands may be more open woodland in structure and with poor‑site understory species such as *Wyethia mollis*.

**Dynamics:** Stand‑replacing fire is important but so are moderately frequent (about once every 40 years) low‑ to moderate‑severity fires. The whole system is characterized by a "moderate‑severity fire regime" (Agee 1993), i.e., high variability in severity and moderate frequency of fires. See also Chappell and Agee (1996), Pitcher (1987), and Taylor and Halpern (1991) for documentation of fire regime in these forests. Windthrow causes tree‑sized gaps that release already established individuals in the understory.

TNC model information: At higher elevations and in the southern Sierra Nevada, fuels are relatively more discontinuous than northern locations because the terrain is broken up by natural breaks such as rock outcrops, lava reefs, wet meadows, etc. Fuels may be more continuous at the northern end of the range, where this vegetation type is found at lower elevations. Primarily Fire Regime Group III, but because of slow fuel accumulation rates, it is possible to have 35‑ to 150‑year frequency surface fire in some classes (lower frequency for these settings as a whole). The discontinuous nature of the fuels limits the extent of fires, and while fires may burn less often, they may burn at high severities. Larger and more frequent moderate‑intensity fires occur on average every 60‑70 years. High‑intensity crown fires are rare, occurring every few hundred years; overall mean fire‑return interval is approximately 35‑50 years (Pitcher 1987, Skinner 2000, Taylor 2000, Bekker and Taylor 2001). Replacement fire likely varies with slope position (upper slope > midslope > lower slope), and landscapes with greater topographic variation are likely to experience more stand‑replacement fires. A considerable range of values has been reported in the literature for mixed and surface fires (Taylor and Halpern 1991, Taylor 1993, Bekker and Taylor 2001, Taylor and Solem 2001).

SOURCES

**References:** Agee 1993, Barbour and Billings 2000, Barbour and Major 1988, Bekker and Taylor 2001, Chappell and Agee 1996, Comer et al. 2003*, Eyre 1980, Holland and Keil 1995, Pitcher 1987, Sawyer and Keeler‑Wolf 1995, Skinner 2000, Taylor 1993, Taylor and Halpern 1991, Taylor and Solem 2001

**Version:** 12 Jan 2012	**Stakeholders:** West

**Concept Author:** P. Comer and T. Keeler‑Wolf	**LeadResp:** West

CES206.910 Mediterranean California Subalpine Woodland

**Primary Division:** Mediterranean California (206)

**Land Cover Class:** Forest and Woodland

**Spatial Scale & Pattern:** Large patch

**Required Classifiers:** Natural/Semi‑natural; Vegetated (>10% vasc.); Upland

**Diagnostic Classifiers:** Forest and Woodland (Treed); Temperate [Temperate Oceanic]; Very Shallow Soil; W‑Landscape/High Intensity; Krummholz

**National Mapping Codes:** EVT 2033; ESLF 4220; ESP 1033

**Concept Summary:** This ecological system occurs on ridges and rocky slopes around timberline at 2900 m (9500 feet) elevation in the southern Sierra Nevada and Transverse and Peninsular ranges, up to 3500 m (11,500 feet) in the Sierra Nevada, and 2450 m (8000 feet) in the southern Cascades. Tree species often occur as krummholz growth forms with a wind‑pruned, prostrate, and/or shrublike appearance, but in more protected sites they form true woodland physiognomy. Stands are dominated by *Pinus albicaulis* and/or *Pinus contorta var. murrayana*; other important conifers and locally dominant species include *Pinus balfouriana* (only in the Klamath Mountains and southern Sierra Nevada where it may replace *Pinus albicaulis*), *Pinus flexilis* (but only in small patches on the eastern flank of the Sierra Nevada escarpment when it does occur), *Pinus monticola* (not in Transverse or Peninsular ranges), and *Juniperus grandis* (mostly in the central and southern Sierra Nevada but not in the Klamath Mountains). Important shrubs include *Arctostaphylos nevadensis, Chrysolepis sempervirens*, and *Holodiscus discolor*. Grasses and forbs include *Carex rossii, Carex filifolia, Poa wheeleri, Eriogonum incanum, Penstemon newberryi*, and *Penstemon davidsonii*. Due to landscape position and very thin soils, these are harsh sites exposed to desiccating winds with ice and snow blasts, and rocky substrates. In addition, a short growing season limits plant growth. The highest tree diversity occurs in the Klamath Mountains, with sometimes five or more conifers sharing codominance in one stand.

DISTRIBUTION

**Range:** This system occurs on ridges and rocky slopes around timberline at 2900 m (9500 feet) elevation in the southern Sierra Nevada and Transverse and Peninsular ranges and 2450 m (8000 feet) in the southern Cascades.

**Divisions:** 204:P, 206:C

**TNC Ecoregions:** 4:C, 5:C, 12:C, 16:C

**Nations:** MX, US

**Subnations:** CA, MXBC, NV, OR

**Map Zones:** 2:C, 3:C, 4:C, 6:C, 7:C, 12:C, 13:P

**USFS Ecomap Regions:** 322A:??, 341D:CC, 341F:CC, M242B:CC, M242C:CC, M261A:CC, M261D:CC, M261E:CC, M261G:CC

CONCEPT

**Environment:** Dry, thin soils and exposure to winds are key ecological environmental factors that drive the structure and appearance of this subalpine forest. These forests are at the limit of tree growth in terms of exposure to cold and desiccating winds in the winter (Arno and Huff 1990). Climate is predicted to get warmer in the Sierra Nevada (Fried et al. 2004, as cited in Barbour et al. 2007). This may lead to reduced growth and vigor of trees in this already stressed environment. However, it is the winter cold and desiccating winds that keep trees in a krummholz form. If winter low temperatures increase, these woodlands may increase in growth and vigor, if adequate moisture continues to be available. Soils are thin and poorly developed, usually low in nitrogen‑fixing bacteria which is apparently restricted by low soil temperature and high acidity of many sites. Increased temperatures may increase soil nitrogen availability (Arno and Huff 1990). However, this may result in increased competition from invading native tree species rather than an increase in those typically dominant on these sites.

**Vegetation:** Stands are dominated by *Pinus albicaulis* and/or *Pinus contorta var. murrayana*; other important conifers and locally dominant species include *Pinus balfouriana* (only in the Klamath Mountains and southern Sierra Nevada where it may replace *Pinus albicaulis*), *Pinus flexilis* (but only in small patches on the eastern flank of the Sierra Nevada escarpment when it does occur), *Pinus monticola* (not in Transverse or Peninsular ranges), and *Juniperus grandis (= Juniperus occidentalis var. australis)* (mostly in the central and southern Sierra Nevada but not in the Klamath Mountains). Important shrubs include *Arctostaphylos nevadensis, Chrysolepis sempervirens*, and *Holodiscus discolor (= Holodiscus microphyllus)*. Grasses and forbs include *Carex rossii, Carex filifolia, Poa wheeleri, Eriogonum incanum, Penstemon newberryi*, and *Penstemon davidsonii*.

**Dynamics:** Due to landscape position and very thin soils, these are harsh sites exposed to desiccating winds with ice and snow blasts, and rocky substrates. In addition, a short growing season limits plant growth. The highest tree diversity occurs in the Klamath Mountains, with sometimes five or more conifers sharing codominance in one stand.

SOURCES

**References:** Arno and Hoff 1990, Barbour and Billings 2000, Barbour and Major 1988, Barbour et al. 2007a, Comer et al. 2003*, Eyre 1980, Fried et al. 2004, Holland and Keil 1995, Logan et al. 2010, Sawyer and Keeler‑Wolf 1995, Shiflet 1994

**Version:** 12 Jan 2012	**Stakeholders:** Latin America, West

**Concept Author:** P. Comer and T. Keeler‑Wolf	**LeadResp:** West

CES204.837 North Pacific Maritime Mesic Subalpine Parkland

**Primary Division:** North American Pacific Maritime (204)

**Land Cover Class:** Forest and Woodland

**Spatial Scale & Pattern:** Large patch, Small patch

**Required Classifiers:** Natural/Semi‑natural; Vegetated (>10% vasc.); Upland

**Diagnostic Classifiers:** Montane [Upper Montane]; Tsuga mertensiana; Late‑lying snowpack

**National Mapping Codes:** EVT 2038; ESLF 4225; ESP 1038

**Concept Summary:** This ecological system occurs throughout the mountains of the Pacific Northwest, from the southern Cascades of Oregon to the mountains of southeastern Alaska bordering British Columbia. It occurs at the transition zone of forest to alpine, forming a subalpine forest‑meadow ecotone. Mountain hemlock forests, as they approach treeline, become open patches of mature‑height trees surrounded by mesic and wet meadows rich in dwarf‑shrubs and forbs. Clumps of trees to small patches of forest interspersed with low shrublands and meadows characterize this system. Krummholz often occurs near the upper elevational limit of this system where it grades into alpine vegetation. Associations include woodlands, forested, and subalpine meadow types. It occurs on the west side of the Cascade Range and is a transitional open forest into the true alpine on the interior side of the Coast Mountains of British Columbia where deep, late‑lying snowpack is the primary environmental factor. Major tree species are *Tsuga mertensiana, Abies amabilis, Callitropsis nootkatensis (= Chamaecyparis nootkatensis)*, and *Abies lasiocarpa*. This system includes British Columbia Hypermaritime and Maritime Parkland (*Tsuga mertensiana*). Dominant dwarf‑shrubs include *Phyllodoce empetriformis, Cassiope mertensiana*, and *Vaccinium deliciosum*. Dominant herbaceous species include *Lupinus arcticus ssp. subalpinus, Valeriana sitchensis, Carex spectabilis*, and *Polygonum bistortoides*. There is very little disturbance, either windthrow or fire. The major process controlling vegetation is the very deep long‑lasting snowpacks (deepest in the North Pacific region) limiting tree regeneration. Trees get established only in favorable microsites (mostly adjacent to existing trees) or during drought years with low snowpack. It is distinguished from more interior dry parkland primarily by the presence of *Tsuga mertensiana* or *Abies amabilis* and absence or paucity of *Pinus albicaulis* and *Larix lyallii*.

**Comments:** This system includes what the Alaska Natural Heritage Program called Maritime Subalpine Fir‑Mountain Hemlock Forest. It is very localized in its occurrence in Alaska, occurring in the eastern portion of the panhandle at high elevations.

DISTRIBUTION

**Range:** This system occurs throughout the mountains of the Pacific Northwest, from the central Oregon Cascades (Diamond Peak, 30 miles north of Crater Lake National Park), north to the Coast Mountains of British Columbia, where it can occur on the east side, facing the interior of British Columbia, as well as north to the mountains along the border of Alaska.

**Divisions:** 204:C, 207:C, 306:C

**TNC Ecoregions:** 1:C, 4:C, 7:C, 69:?, 70:C, 81:C, 144:C

**Nations:** CA, US

**Subnations:** AK, BC, OR, WA:S4

**Map Zones:** 1:C, 7:P, 78:C

**USFS Ecomap Regions:** 242A:CC, M242A:CC, M242B:CC, M242C:CP, M242D:CC

CONCEPT

SOURCES

**References:** BCMF 2006, Banner et al. 1993, Comer et al. 2003*, Eyre 1980, Franklin and Dyrness 1973, Green and Klinka 1994, Viereck et al. 1992, WNHP unpubl. data 2018

**Version:** 06 Feb 2009	**Stakeholders:** Canada, West

**Concept Author:** G. Kittel	**LeadResp:** West

CES204.838 North Pacific Mountain Hemlock Forest

**Primary Division:** North American Pacific Maritime (204)

**Land Cover Class:** Forest and Woodland

**Spatial Scale & Pattern:** Matrix

**Required Classifiers:** Natural/Semi‑natural; Vegetated (>10% vasc.); Upland

**Diagnostic Classifiers:** Forest and Woodland (Treed); Temperate [Temperate Oceanic]; Tsuga mertensiana

**National Mapping Codes:** EVT 2041; ESLF 4228; ESP 1041

**Concept Summary:** This forested ecological system occurs throughout the mountains of the North Pacific, from the southern Cascades of Oregon north to southwestern British Columbia. It is the predominant forest of subalpine elevations in the coastal mountains of British Columbia, western Washington and western Oregon. It also occurs on mountain slopes on the outer coastal islands of British Columbia. It lies between the Western Hemlock, Pacific Silver Fir, or Shasta Red Fir zones and the Subalpine Parkland or Alpine Tundra Zone, at elevations ranging from 300 to 2300 m (1000‑7500 feet). The lower and upper elevational limits decrease from south to north and from east to west. The climate is generally characterized by short, cool summers, rainy autumns and long, cool, wet winters with heavy snow cover for 5‑9 months. The heavy snowpack is ubiquitous, but at least in southern Oregon and perhaps the eastern Cascades, summer drought is more significant. Fire is very rare or absent across the majority of the range of the system. *Tsuga mertensiana* is one of the dominant tree species throughout, and *Abies amabilis* becomes an important associated species in the southern portion of the range (British Columbia, Washington, and northwestern Oregon). *Tsuga heterophylla* often occurs at lower elevations in this system but is much less abundant than *Tsuga mertensiana*. *Callitropsis nootkatensis (= Chamaecyparis nootkatensis)* occurs in the more coastal portions, while *Abies lasiocarpa* is found inland and becomes increasingly common near the transition to the Subalpine Fir‑Engelmann Spruce Zone in the Cascades and British Columbia. On the leeward side of the Cascades, this is usually a dense canopy composed of *Abies lasiocarpa* and *Tsuga mertensiana*, with some *Picea engelmannii* or *Abies amabilis*. In the Cascades of central to southern Oregon, *Abies magnifica var. shastensis (= Abies x shastensis)* is typically present and often codominant. *Picea sitchensis* and *Thuja plicata* are occasionally present. Deciduous trees are rare. Common understory species include *Vaccinium ovalifolium, Menziesia ferruginea, Elliottia pyroliflora*, and *Blechnum spicant*. Parklands (open woodlands or sparse trees with dwarf‑shrub or herbaceous vegetation) are not part of this system but of North Pacific Maritime Mesic Subalpine Parkland (CES204.837) or Alaskan Pacific Maritime Subalpine Mountain Hemlock Woodland (CES204.143).

**Comments:** Farther inland, *Tsuga mertensiana* becomes limited to the coldest and wettest pockets of the more continental subalpine fir forests, described from the eastern Cascades and northern Rocky Mountains. In the northern Rocky Mountains of northern Idaho and Montana, *Tsuga mertensiana* occurs as patches within the matrix of Rocky Mountain Subalpine Mesic‑Wet Spruce‑Fir Forest and Woodland (CES306.830) only in the most maritime of environments and is included in the spruce‑fir system. In the northern Rocky Mountains, this forest system is codominated by *Abies lasiocarpa* and/or *Picea engelmannii*. Mountain hemlock forests in Alaska are placed into Alaskan Pacific Maritime Mountain Hemlock Forest (CES204.142) or Alaskan Pacific Maritime Subalpine Mountain Hemlock Woodland (CES204.143).

DISTRIBUTION

**Range:** This system occurs from coastal British Columbia to the southern Cascades of Oregon.

**Divisions:** 204:C, 306:C

**TNC Ecoregions:** 1:C, 3:C, 69:C, 81:C

**Nations:** CA, US

**Subnations:** BC, OR, WA:S4S5

**Map Zones:** 1:C, 2:C, 3:?, 6:C, 7:C

**USFS Ecomap Regions:** 242A:??, M242A:CC, M242B:CC, M242C:CC, M242D:CC, M261A:CC, M261D:C?, M261G:C?

CONCEPT

**Dynamics:** In the more summer‑dry climatic areas (Cascades), occasional high‑severity fires occur, with return intervals of 400‑600 years (J. Kertis pers. comm. 2006, K. Kopper pers. comm. 2006). On drier sites, *Abies lasiocarpa* and *Pinus contorta* can be the first forests to develop after stand‑replacing fire. These early‑seral stages, with lodgepole pine dominant in the upper canopy, could be classified and mapped as Rocky Mountain Lodgepole Pine Forest (CES306.820) but should be considered part of this system if other tree species listed above are present, as it will succeed as a mixed pine type, then mountain hemlock becomes characteristic. Landfire VDDT models: R#ABAMup.

SOURCES

**References:** Banner et al. 1993, Comer et al. 2003*, Ecosystems Working Group 1998, Eyre 1980, Franklin 1988, Kertis pers. comm., Klinka and Chourmouzis 2002, Kopper pers. comm., Steen and Coupé 1997, WNHP unpubl. data 2018

**Version:** 08 Dec 2008	**Stakeholders:** Canada, West

**Concept Author:** G. Kittel and C. Chappell	**LeadResp:** West

CES206.911 Northern California Mesic Subalpine Woodland

**Primary Division:** Mediterranean California (206)

**Land Cover Class:** Forest and Woodland

**Spatial Scale & Pattern:** Large patch

**Required Classifiers:** Natural/Semi‑natural; Vegetated (>10% vasc.); Upland

**Diagnostic Classifiers:** Montane [Upper Montane]; Temperate [Temperate Oceanic]; Udic

**National Mapping Codes:** EVT 2044; ESLF 4231; ESP 1044

**Concept Summary:** This ecological system occurs on ridges and rocky slopes around timberline at 2600 m (7900 feet) elevation in the central Sierra Nevada and 2450 m (8000 feet) in the southern Cascades. These woodlands are found on concave or mesic slopes in areas with long‑lasting snowpack and better soil development than other drier and more exposed subalpine woodlands. The tree canopy is characterized by *Tsuga mertensiana* and may include *Abies magnifica, Abies procera, Pinus albicaulis*, and *Pinus monticola*. Mesic‑site shrubs will include *Cassiope mertensiana, Phyllodoce breweri, Phyllodoce empetriformis, Vaccinium membranaceum*, and others. *Juniperus communis* is found in most stands of the northern Sierra Nevada. *Penstemon davidsonii*, as well as patches of grasses, sedges, and forbs grade into adjacent meadows.

DISTRIBUTION

**Range:** This system occurs on ridges and rocky slopes around timberline at 2600 m (7900 feet) elevation in the central Sierra Nevada and 2450 m (8000 feet) in the southern Cascades.

**Divisions:** 204:C, 206:C

**TNC Ecoregions:** 4:C, 5:P, 12:C, 81:P

**Nations:** US

**Subnations:** CA, NV, OR

**Map Zones:** 6:C, 7:C

**USFS Ecomap Regions:** 341D:CC, M242B:??, M261E:CC

CONCEPT

SOURCES

**References:** Barbour and Billings 2000, Barbour and Major 1988, Comer et al. 2003*, Eyre 1980, Holland and Keil 1995, Means 1990, Peterson and Peterson 2001, Potter 1994, Sawyer and Keeler‑Wolf 1995

**Version:** 07 Oct 2005	**Stakeholders:** West

**Concept Author:** P. Comer and T. Keeler‑Wolf	**LeadResp:** West

CES206.912 Sierra Nevada Subalpine Lodgepole Pine Forest and Woodland

**Primary Division:** Mediterranean California (206)

**Land Cover Class:** Forest and Woodland

**Spatial Scale & Pattern:** Large patch

**Required Classifiers:** Natural/Semi‑natural; Vegetated (>10% vasc.); Upland

**Diagnostic Classifiers:** Montane [Upper Montane]; Mediterranean [Mediterranean Xeric‑Oceanic]; Shallow Soil; Xeric; Short Disturbance Interval [Periodicity/Irregular Disturbance]; Pinus contorta

**National Mapping Codes:** EVT 2058; ESLF 4245; ESP 1058

**Concept Summary:** This ecological system is widespread in glacial basins at upper montane to subalpine elevations of the central and northern Sierra Nevada and Transverse and Peninsular ranges where cold‑dry conditions exist (1800‑2450 m [6000‑8000 feet] in the north and 2450‑3600 m [8000‑12,000 feet] in the south). It also occurs on extensive broad ridges and pumice plateaus of the southern Cascades in Oregon (the broad ridges that form the Cascade crest in southern Oregon tend to be dominated by extensive stands of lodgepole pine). Soils are often shallow and coarse‑textured. These forests and woodlands are dominated by *Pinus contorta var. murrayana* with shrub, grass or barren understories. Avalanche as well as tree mortality from insect outbreak and disease, drought and associated wildfire are drivers of community structure and composition. Understories are open, with scattered shrubs and herbaceous species, which do not carry fire should one get started. Trees can be very large and old and can attain diameters of 1.2 m (4 feet). Associated plant species include *Arctostaphylos nevadensis, Ceanothus cordulatus, Cercocarpus ledifolius* (although not that common, just occasional in drier sites), *Chrysolepis sempervirens, Phyllodoce breweri*, and *Ribes montigenum*. Common graminoids include *Poa wheeleri, Carex filifolia, Carex rossii*, and *Carex exserta*. Fire‑return intervals are many hundreds of years. This system occurs in less severe settings than Mediterranean California Subalpine Woodland (CES206.910) and Northern California Mesic Subalpine Woodland (CES206.911) and is made up of trees that are not usually krummholz. Avalanches are less of a factor except in association with the volcanic peaks. Low‑elevation stands of *Pinus contorta* in the pumice zone of Oregon are included in Rocky Mountain Poor‑Site Lodgepole Pine Forest (CES306.960).

DISTRIBUTION

**Range:** This system occurs in glacial basins at upper montane to subalpine elevations of the central and northern Sierra Nevada and Transverse and Peninsular ranges where cold‑dry conditions exist (1800‑2450 m [6000‑8000 feet] in the north and 2450‑3600 m [8000‑12,000 feet] in the south). It also extends south into Baja California, Mexico, in the San Pedro Martir Mountains.If present in Oregon, the most likely location is the southern Oregon Cascades. The broad ridges that form the Cascade Crest in southern Oregon tend to be dominated by extensive stands of lodgepole pine (south of Crater Lake and north maybe to Mount Bachelor). There are also relatively large areas of lodgepole pine along the broad crest from Mt. Jefferson to a little ways north of Olallie Butte that may also fit this type better than the Rocky Mountain lodgepole pine type, as these stands are more likely dominated by *Pinus contorta var. murrayana* than *Pinus contorta var. latifolia*. Understory species are probably different from those listed, however.

**Divisions:** 206:C

**TNC Ecoregions:** 4:C, 5:C, 12:C

**Nations:** MX, US

**Subnations:** CA, MXBC, NV, OR

**Map Zones:** 4:C, 6:C, 7:C, 12:C

**USFS Ecomap Regions:** 341D:CC, 342B:??, M242B:CC, M242C:CC, M261A:CP, M261D:CC, M261E:CC, M261G:CC

CONCEPT

**Environment:** Upper montane to subalpine elevations of the central and northern Sierra Nevada and Transverse and Peninsular ranges where relatively cold‑dry conditions exist (1800‑2450 m [6000‑8000 feet] in the north and 2450‑3600 m [8000‑12,000 feet] in the south). It is often located on benches but also occurs on moderate slopes, and on extensive broad ridges and pumice plateaus of the southern Cascades in Oregon (the broad ridges that form the Cascade crest in southern Oregon tend to be dominated by extensive stands of lodgepole pine). The climate regime is Mediterranean with wet winters (November‑April), with precipitation occurring as snow, and dry summers, although summer thunderstorms occur sporadically.

**Vegetation:** These forests and woodlands are dominated by *Pinus contorta var. murrayana* with shrub, grass or barren understories. Avalanche as well as tree mortality from insect outbreak and disease, drought and associated wildfire are drivers of community structure and composition. Understories are open, with scattered shrubs and herbaceous species, which do not carry fire should one get started. Trees can be very large and old and can attain diameters of 1.2 m (4 feet). Associated plant species include *Arctostaphylos nevadensis, Ceanothus cordulatus, Cercocarpus ledifolius* (although not that common, just occasional in drier sites), *Chrysolepis sempervirens, Phyllodoce breweri*, and *Ribes montigenum*. Common graminoids include *Poa wheeleri, Carex filifolia, Carex rossii*, and *Carex exserta*.

**Dynamics:** LANDFIRE model information: Disturbance patterns have been poorly studied in Sierran lodgepole pine. Sierran lodgepole has been described as not being a fire type (Barbour and Minnich 2000) or as having long intervals between fires (Keeley 1980, Parker 1986, Potter 1998). Avalanche as well as tree mortality from insect outbreak and disease, drought and associated wildfire are the main drivers of community structure and composition. Somewhat similar wet lodgepole types in the Klamath Mountains and Oregon had a fire‑return interval range of 70‑100 years. Season of fire is generally late summer to early fall. Stand‑replacement fire occurs at long interval, resulting in low stand complexity. Mixed‑severity fire occurs when fuel conditions remain moist and result in mixed‑age stands. Very infrequently, surface fires can occur. Forest understory is typically sparse with few shrubs and low to moderate herbaceous cover. Fuel is considered sparse (Parker 1986, van Wagtendonk 1991). Stands in the southern Sierra Nevada have been described as self‑perpetuating (regeneration from treefall gaps) with long intervals between fires (Keeley 1980, Parker 1986, Potter 1998). Sparse fuels are believed to limit ignition and fire spread (Parker 1986). In contrast, fire history studies from dry subalpine lodgepole pine forest in the southern Sierra Nevada have found moderate fire‑return intervals in some stands (Keifer 1991, Caprio 2008 and unpubl. data). Intervals ranged from 31‑74 years (Chagoopa Plateau, Sequoia National Park and Palisades Canyon, Kings Canyon National Park). Fire severity was mixed and ranged from understory burns on areas up to 100s of ha to high‑severity crown fires in patches up to 10s of ha (FRG of III). Season of fires was late summer or early fall. Seasonal fire scar positions on Chagoopa and Palisades (SEKI) was 40.7% and 15% latewood and 59.3% and 80% dormant, respectively (Caprio unpubl. data). Other important disturbance agents in this system include the lodgepole needle miner, windthrow and stress from extreme climatic events.

SOURCES

**References:** Agee 1993, Barbour and Billings 2000, Barbour and Major 1988, Barbour and Minnich 2000, Caprio 2008, Comer et al. 2003*, Eyre 1980, Holland and Keil 1995, Keeley 1980, Parker 1986b, Potter 1998, Sawyer and Keeler‑Wolf 1995, Sheppard and Lassoie 1998, van Wagtendonk 1991

**Version:** 12 Jan 2012	**Stakeholders:** Latin America, West

**Concept Author:** P. Comer and T. Keeler‑Wolf	**LeadResp:** West

1.B.2.Ne. North American Great Plains Forest & Woodland

M151. Great Plains Forest & Woodland

CES205.688 Eastern Great Plains Tallgrass Aspen Parkland

**Primary Division:** Eastern Great Plains (205)

**Land Cover Class:** Forest and Woodland

**Spatial Scale & Pattern:** Large patch

**Required Classifiers:** Natural/Semi‑natural; Vegetated (>10% vasc.); Upland

**National Mapping Codes:** EVT 2331; ESLF 4137; ESP 1331

**Concept Summary:** This system is found primarily on part of the Glacial Lake Agassiz plain in northwestern Minnesota, ranging into southern Canada. Calcareous glacial drift overlain with lacustrine soils ranging from loamy to gravelly is characteristic of the lakeplain within the range of this system. Historically this system included a mosaic of tallgrass prairie, wet prairie, brush prairie and aspen‑oak woodlands. It is dominated by *Populus tremuloides* with scattered *Quercus macrocarpa* and *Betula papyrifera*. Shrubs such as willow (*Salix* spp.) and hazel (*Corylus* spp.) are also common. The dominant tallgrass species is *Andropogon gerardii* often associated with *Sorghastrum nutans, Calamagrostis* spp., and *Sporobolus heterolepis*. Fire is the most important natural dynamic in this system and helps maintain the open parkland or brush nature of this system. Wind and grazing are also important dynamics. Conversion to agriculture and fire suppression have decreased the range of this system and allowed more shrubs and trees to establish.

DISTRIBUTION

**Range:** This system is found primarily on part of the Glacial Lake Agassiz plain in northwestern Minnesota, ranging into southern Canada.

**Divisions:** 201:P, 205:C

**TNC Ecoregions:** 35:C, 46:?, 47:P, 66:P

**Nations:** CA, US

**Subnations:** MB, MN, ND

**Map Zones:** 39:P, 40:C, 41:P

**USFS Ecomap Regions:** 222N:CC, 251A:PP

CONCEPT

**Environment:** This system occurs largely on the lakeplain of Glacial Lake Agassiz. This landscape is very flat with soils ranging from fine to somewhat coarse. Drainage is moderate to poor at most sites.

**Vegetation:** Vegetation is dominated by *Populus tremuloides* with scattered *Quercus macrocarpa* and *Betula papyrifera*. Shrubs such as willow (*Salix* spp.) and hazel (*Corylus* spp.) are also common. The dominant tallgrass species is *Andropogon gerardii* often associated with *Sorghastrum nutans, Calamagrostis* spp., and *Sporobolus heterolepis*.

**Dynamics:** The interaction of fire and regional climate shaped this system. Aspen parklands occur on the margin of the northern prairies and northern forests. The climate will support tallgrass, tree and shrub species, and aspen parklands are a mix of these lifeforms. Frequent fires favor the spread of tallgrass species and reduce woody cover (Svedarsky et al. 1986). Sites not burned as often, due to a fire‑protected position on the landscape or to a reduction in fire frequency across the entire landscape, tend to become dominated by trees and shrubs. An average fire‑return interval of 10‑15 years was estimated by Landfire modelers (Landfire 2007a), though individual areas would have burned less or more often. This system occurs on a very flat landscape and minor variations in topography can create wet prairie or wet shrub pockets within the parkland.

SOURCES

**References:** Comer et al. 2003*, Eyre 1980, LANDFIRE 2007a, MNNHP 1993, Svedarsky et al. 1986

**Version:** 14 Jan 2014	**Stakeholders:** Canada, Midwest

**Concept Author:** S. Menard	**LeadResp:** Midwest

CES303.680 Great Plains Wooded Draw and Ravine

**Primary Division:** Western Great Plains (303)

**Land Cover Class:** Mixed Upland and Wetland

**Spatial Scale & Pattern:** Linear

**Required Classifiers:** Natural/Semi‑natural; Vegetated (>10% vasc.); Upland; Wetland

**National Mapping Codes:** EVT 2385; ESLF 4328; ESP 1385

**Concept Summary:** This ecological system is typically found associated with permanent or ephemeral streams though it may occur on steep northern slopes or within canyon bottoms that do not experience periodic flooding. Soil moisture and topography allow greater moisture conditions compared to the surrounding areas. Occurrences can be either tree‑dominated or predominantly shrubland. *Fraxinus pennsylvanica* with *Ulmus rubra* or *Ulmus americana* typically dominate this system, although *Juniperus scopulorum* can dominate the canopy in the western Great Plains and *Juniperus virginiana* in the east. *Populus tremuloides, Betula papyrifera*, or *Acer negundo* are commonly present in portions of the northwestern Great Plains, for example in areas of central and eastern Montana. In south‑central and east‑central portions of the Great Plains, *Quercus macrocarpa* can also be present. Wetter areas within this system can have significant amounts of *Populus deltoides*. Component shrubs can include *Cornus sericea, Crataegus douglasii, Crataegus chrysocarpa, Crataegus succulenta, Elaeagnus commutata, Prunus virginiana, Rhus* spp., *Rosa woodsii, Shepherdia argentea, Symphoricarpos occidentalis*, or *Viburnum lentago*. Common grasses can include *Calamagrostis stricta, Carex* spp., *Pascopyrum smithii, Piptatheropsis micrantha, Pseudoroegneria spicata*, or *Schizachyrium scoparium*. This system was often subjected to heavy grazing and trampling by both domestic animals and wildlife and can be heavily degraded in some areas. In addition, exotic species such as *Ulmus pumila* and *Elaeagnus angustifolia* can invade these systems.

**Comments:** More information from the broader division and from the Rocky Mountain division will be needed to determine if those areas dominated by ash and elm should be separated from areas dominated by *Juniperus scopulorum*. Those areas dominated by *Juniperus* are typically found in the Badlands and the western portions of North Dakota and Nebraska, and should probably be described based on data from the Great Plains Steppe or Rocky Mountain division. However, *Juniperus* can occur in stands with elm and ash in Nebraska and North Dakota. Expanded range into the central Great Plains (parts of Provinces 332 and 251). Possibly consider splitting the western Great Plains stands from the central Great Plains stands but there are currently not enough floristic and environmental differences known between stands in the two areas to justify that. In Texas, examples of this system may also include *Populus deltoides* and *Quercus fusiformis* in the canopy with *Fraxinus pennsylvanica, Ulmus americana*, and *Juniperus* spp.

DISTRIBUTION

**Range:** This system is found throughout the Western Great Plains Division and east into the western tallgrass prairie zone of the central United States. In Wyoming, it occurs in the northeastern foothills of the Bighorns and across far‑northeastern Wyoming into the northern fringes of the Black Hills. It has also been identified in the High Plains of Texas.

**Divisions:** 205:P, 303:C

**TNC Ecoregions:** 26:C, 27:C, 28:C, 33:C, 34:C, 36:C, 37:C

**Nations:** US

**Subnations:** IA, KS, MO, MT, ND, NE, OK, SD, TX, WY

**Map Zones:** 20:C, 27:P, 28:P, 29:C, 30:C, 31:C, 33:C, 34:C, 35:?, 38:C, 39:C, 40:C, 43:C

**USFS Ecomap Regions:** 251C:CC, 251F:CC, 251H:CC, 331D:CP, 331E:CP, 331F:CC, 331G:CP, 331H:C?, 331K:CC, 331L:CC, 331M:CP, 331N:C?, 332C:CC, 332E:CC, M331B:??, M331I:??, M334A:PP

CONCEPT

**Environment:** This system is associated with permanent or ephemeral streams. It also can occur on steep northern slopes or within canyon bottoms that do not experience periodic flooding. Soils are primarily wet to mesic, and the more sheltered and lower landscape position allows for greater moisture conditions compared to the surrounding areas.

**Vegetation:** Species composition can vary across the range of this system. *Fraxinus pennsylvanica* and *Ulmus* spp. typically dominate this system. In some western areas of the Great Plains Division, *Juniperus* spp. can dominate, and in the south‑central and east‑central portions of the Great Plains, *Quercus macrocarpa* can also be important. *Tilia americana* and *Ostrya virginiana* are also common associates in the eastern portion of this system's range. Component shrubs can include *Cornus sericea, Crataegus douglasii, Crataegus chrysocarpa, Crataegus succulenta, Elaeagnus commutata, Prunus virginiana, Rhus* spp., *Rosa woodsii, Shepherdia argentea, Symphoricarpos occidentalis*, or *Viburnum lentago*. Common grasses can include *Calamagrostis stricta, Carex* spp., *Pascopyrum smithii, Piptatheropsis micrantha (= Piptatherum micranthum), Pseudoroegneria spicata*, or *Schizachyrium scoparium*. This system was often subjected to heavy grazing and trampling by both domestic animals and wildlife and can be heavily degraded in some areas. Exotic species, such as *Ulmus pumila* and *Elaeagnus angustifolia*, can be present in degraded examples. Wetter areas within this system can have significant amounts of *Populus deltoides*.

**Dynamics:** Fire can influence this system; however, grazing is the most prevalent dynamic process influencing this system. Overgrazing can heavily degrade this system, particularly the understory, and allow for the invasion of exotic species.

SOURCES

**References:** Bell 2005, Comer et al. 2003*, Elliott 2013, Eyre 1980, Rice et al. 2012b, Rolfsmeier and Steinauer 2010

**Version:** 02 Oct 2014	**Stakeholders:** Midwest, Southeast, West

**Concept Author:** S. Menard and K. Kindscher	**LeadResp:** Midwest

CES303.681 Northwestern Great Plains Aspen Forest and Parkland

**Primary Division:** Western Great Plains (303)

**Land Cover Class:** Mixed Upland and Wetland

**Spatial Scale & Pattern:** Matrix

**Required Classifiers:** Natural/Semi‑natural; Vegetated (>10% vasc.); Upland; Wetland

**National Mapping Codes:** EVT 2009; ESLF 4146; ESP 1009

**Concept Summary:** This system ranges from the North Dakota/Manitoba border west to central Alberta and is considered part of the boreal‑mixedgrass prairie grassland transition region. The climate in this region is mostly subhumid low boreal with short, warm summers and cold, long winters. Much of this region is covered with undulating to kettled glacial till. *Populus tremuloides* dominates this system. Common associates are *Betula papyrifera* and *Populus balsamifera* with an understory of mixedgrass species and tall shrubs. More poorly drained sites may contain willow (*Salix* spp.) and sedges (*Carex* spp.). Fire constitutes the most important dynamic in this system and prevents boreal conifer species such as *Picea glauca* and *Abies balsamea* from becoming too established in this system.

**Comments:** This system can grade into Eastern Great Plains Tallgrass Aspen Parkland (CES205.688) to the east, which has a predominance of tallgrass species in the understory compared to the more mixedgrass species in this system. More data from Canada are needed to fully describe this system. In spring 2006, it was determined by Steve Cooper, Marion Reid and Gwen Kittel that this system does not occur in north‑central Montana, mapzone 20. However, it does occur along the lower‑elevation slopes of the Montana Front Range, in mapzone 19.

DISTRIBUTION

**Range:** This system is found in the boreal‑grassland transition region from the North Dakota/Manitoba border west to central Alberta. and south along the eastern slopes of the Front Range of Montana, where it occurs below lower treeline.

**Divisions:** 205:C, 303:C

**TNC Ecoregions:** 34:?, 66:C, 67:C

**Nations:** CA, US

**Subnations:** AB, MB, MT, ND, SK

**Map Zones:** 19:C, 30:?, 40:P

**USFS Ecomap Regions:** 331D:CC, M333C:CC

CONCEPT

**Environment:** Climate in the range of this system is mostly subhumid low boreal with short, warm summers and long, cold winters. Undulating to kettled glacial till predominates this region.

**Vegetation:** *Populus tremuloides* dominates this system. Common associates are *Populus balsamifera* and *Betula papyrifera* along with an understory of mixedgrass and tall‑shrub species.

**Dynamics:** Fire is likely the most important natural dynamic allowing for a more open structure and preventing this system from containing more conifer species.

SOURCES

**References:** Barbour and Billings 1988, Comer et al. 2003*, Eyre 1980, Greenall 1995, Ricketts et al. 1999, Shiflet 1994

**Version:** 20 Apr 2006	**Stakeholders:** Canada, Midwest, West

**Concept Author:** S. Menard	**LeadResp:** Midwest

CES303.667 Western Great Plains Dry Bur Oak Forest and Woodland

**Primary Division:** Western Great Plains (303)

**Land Cover Class:** Forest and Woodland

**Spatial Scale & Pattern:** Large patch

**Required Classifiers:** Natural/Semi‑natural; Vegetated (>10% vasc.); Upland

**National Mapping Codes:** EVT 2013; ESLF 4106; ESP 1013

**Concept Summary:** This system is dominated by *Quercus macrocarpa* and is found in upland areas in the northern part of the Western Great Plains. It often occurs as small to large patches on buttes, escarpments, and in foothill zones, usually on northerly‑facing slopes. Other species, such as *Tilia americana* (not in the Dakotas), *Populus tremuloides, Juniperus virginiana*, and *Fraxinus* spp., may be present. The herbaceous layer can vary from sparsely to moderately vegetated and is composed of prairie grasses or woodland *Carex* spp. Shrub associates can include *Prunus virginiana, Corylus cornuta, Amelanchier alnifolia*, or *Symphoricarpos* spp. Historically, higher cover of grass species occurred as these stands were more open due to more frequent fires. Few good examples of this system likely remain because of past timber harvesting and heavy grazing. Where it occurs at elevations above 915 m (3000 feet), *Pinus ponderosa* woodlands are probably adjacent.

**Comments:** Stands of bur oak can also be included within Central Mixedgrass Prairie (CES303.659); however, that system would only include small patches or single trees protected by fire. Any stands of bur oak or more substantial woodlands should be included within this system.

DISTRIBUTION

**Range:** This system is found throughout the northern part of the Western Great Plains Division. In Wyoming, it occurs in the Bear Lodge Mountains and around Devils Tower National Monument. In North Dakota, it is found in the Killdeer Mountains, and it may occur in the Pine Ridge region of Nebraska.

**Divisions:** 303:C

**TNC Ecoregions:** 25:P, 26:C, 27:C, 33:C, 34:C

**Nations:** US

**Subnations:** MT, ND, NE?, SD, WY

**Map Zones:** 29:C, 30:C, 31:C, 33:C, 38:?, 39:C, 40:C

**USFS Ecomap Regions:** 251B:CC, 251H:CC, 331C:CC, 331E:CC, 331F:CC, 331M:CP, 332B:CC, 332C:CC, 332D:CC, 332E:CC, M334A:CC

CONCEPT

**Environment:** This system is found in upland areas throughout the northern part of the Western Great Plains. Soils are predominately dry to mesic. It usually occurs on protected eastern or northern slopes of buttes or river valleys (Rolfsmeier and Steinauer 2010).

**Vegetation:** This system is typified by the predominance of *Quercus macrocarpa* constituting at least 10% of the vegetation cover in any given example of this system. Other species, such as *Tilia americana, Juniperus virginiana*, and *Fraxinus* spp., may be also present. Understory vegetation can range from sparsely vegetated to more dense and usually exemplifies the surrounding prairie grassland vegetation.

**Dynamics:** This system is primarily driven by fire. This system occurs in a landscape where fire is common but the sites it occupies are somewhat sheltered so fire frequency is less than the surrounding prairie uplands. Fire‑return intervals have been estimated at 15‑25 years (Landfire 2007a). Fire reduces woody species regeneration and shrub cover and allows prairie grasses to grow under the open tree canopy.

SOURCES

**References:** Barbour and Billings 1988, Comer et al. 2003*, Eyre 1980, Girard et al. 1989, LANDFIRE 2007a, Rolfsmeier and Steinauer 2010, Tolstead 1947

**Version:** 14 Jan 2014	**Stakeholders:** Midwest, West

**Concept Author:** S. Menard and K. Kindscher	**LeadResp:** Midwest

1.B.3. Temperate Flooded & Swamp Forest

1.B.3.Na. Eastern North American‑Great Plains Flooded & Swamp Forest

M029. Central Hardwood Floodplain Forest

CES202.608 Central Appalachian River Floodplain

**Primary Division:** Central Interior and Appalachian (202)

**Land Cover Class:** Mixed Upland and Wetland

**Spatial Scale & Pattern:** Large patch

**Required Classifiers:** Natural/Semi‑natural; Vegetated (>10% vasc.); Upland; Wetland

**Diagnostic Classifiers:** Forest and Woodland (Treed); Toeslope/Valley Bottom; Riverine / Alluvial; Broad‑Leaved Deciduous Tree; Intermittent Flooding; Short (<5 yrs) Flooding Interval

**Concept Summary:** This system encompasses floodplains of medium to large rivers in Atlantic drainages from southern New England to Virginia. This system can include a complex of wetland and upland vegetation on deep alluvial deposits and scoured vegetation on depositional bars and on bedrock where rivers cut through resistant geology. This complex includes floodplain forests in which *Acer saccharinum, Populus deltoides*, and *Platanus occidentalis* are characteristic, as well as herbaceous sloughs, shrub wetlands, riverside prairies and woodlands. Microtopography and soil texture determine how long the various habitats are inundated. Depositional and erosional features may both be present depending on the particular floodplain.

**Comments:** This system is distinguished from related floodplain systems; northward, Laurentian‑Acadian Floodplain Forest (CES201.587) is characterized by the lack or unimportance of *Platanus occidentalis* and *Betula nigra*, for example; and westward, North‑Central Interior Floodplain (CES202.694) drains to the midwestern rivers rather than northeastern rivers. Determining the distinctions from South‑Central Interior Large Floodplain (CES202.705), which overlaps the southern and western portions of this system, needs work.

DISTRIBUTION

**Range:** Southern New England west to Lake Erie and south to Virginia. The James River in Virginia marks the southern extent of this system.

**Divisions:** 201:C, 202:C

**TNC Ecoregions:** 49:C, 52:C, 59:C, 60:C, 61:C

**Nations:** US

**Subnations:** CT, MA, MD, NH, NJ?, NY, OH, PA, VA, VT, WV

**Map Zones:** 53:C, 59:C, 60:C, 61:C, 62:C, 63:C, 64:C, 65:C

**USFS Ecomap Regions:** 211F:CC, 211G:CC, 211I:CC, 211J:CC, 221A:CC, 221B:CC, 221D:CC, 232Ac:CCP, M221A:CC, M221B:CC

CONCEPT

**Environment:** This system forms on broad, relatively flat floodplains along medium‑sized to large rivers. Rivershores often exhibit development of one or more terraces formed in relation to hydroperiod and height from river channel. Backswamps may occur in poorly drained depressions behind the main river channel, where substrate is deep muck. Soils range from sandy and silty on point bars to deep muck in backswamps.

**Vegetation:** This complex includes floodplain forests in which *Acer saccharinum, Platanus occidentalis*, and *Populus deltoides* are characteristic, as well as herbaceous sloughs, shrub wetlands, riverside prairies and woodlands. Other trees may include *Acer rubrum, Acer negundo, Betula nigra, Carpinus caroliniana, Fraxinus pennsylvanica, Liriodendron tulipifera, Platanus occidentalis, Populus deltoides*, and *Ulmus americana*. Herbs and shrubs may include *Ageratina altissima, Boehmeria cylindrica, Carex trichocarpa, Carex torta, Cyperus squarrosus, Elymus virginicus, Eragrostis hypnoides, Hypericum prolificum, Ionactis linariifolius, Laportea canadensis, Eubotrys racemosa (= Leucothoe racemosa), Lindera benzoin, Lindernia dubia, Ludwigia palustris, Matteuccia struthiopteris, Onoclea sensibilis, Peltandra virginica, Physocarpus opulifolius, Schizachyrium scoparium*, and *Solidago simplex var. racemosa*.

**Dynamics:** Spring and summer flooding brings large amounts of sediment carried from tributaries, as well as other debris that is deposited on the floodplain as flood waters recede. Floodplain canopy trees often topple as a result of prolonged saturation of sediments; vegetation structure is highly variable and dynamic as a result. Dynamic disturbance regime and high fertility make this system highly susceptible to invasions of non‑native plants.

SOURCES

**References:** Comer et al. 2003*, Edinger et al. 2014a, Eyre 1980, Faber‑Langendoen et al. 2011, Kearsley 1999c, PNHP 2002, Rhoads and Block 1999, Sperduto and Nichols 2004, Swain and Kearsley 2011, Zimmerman 2011m, Zimmerman et al. 2012

**Version:** 02 Jan 2015	**Stakeholders:** East, Midwest, Southeast

**Concept Author:** S.C. Gawler	**LeadResp:** East

CES202.609 Central Appalachian Stream and Riparian

**Primary Division:** Central Interior and Appalachian (202)

**Land Cover Class:** Mixed Upland and Wetland

**Spatial Scale & Pattern:** Linear

**Required Classifiers:** Natural/Semi‑natural; Vegetated (>10% vasc.); Upland; Wetland

**Diagnostic Classifiers:** Lowland; Riverine / Alluvial; Very Short Disturbance Interval; Flood Scouring; Intermittent Flooding

**Concept Summary:** This riparian system ranges from southern New England to Virginia and West Virginia and occurs over a wide range of elevations. It develops on floodplains and shores along river channels that lack a broad flat floodplain due to steeper sideslopes, higher gradient, or both. It may include communities influenced by flooding, erosion, or groundwater seepage. The vegetation is often a mosaic of forest, woodland, shrubland, and herbaceous communities. Common trees include *Betula nigra* and *Platanus occidentalis*. Open, flood‑scoured rivershore prairies feature *Panicum virgatum* and *Andropogon gerardii*, and *Carex torta* is typical of wetter areas near the channel.

**Comments:** This is a high‑gradient system, unlike the low‑gradient system described in Central Appalachian River Floodplain (CES202.608). To the south in the Appalachians and interior, this system is replaced by South‑Central Interior Small Stream and Riparian (CES202.706).

DISTRIBUTION

**Range:** This system ranges from southern New England west to Lake Erie and south to Virginia and West Virginia. The James River in Virginia marks its southern extent.

**Divisions:** 202:C

**TNC Ecoregions:** 49:C, 52:C, 59:C, 60:C, 61:C

**Nations:** US

**Subnations:** CT, DE, MA, MD, NH, NJ?, NY, OH, PA, VA, VT, WV

**Map Zones:** 53:C, 60:C, 61:C, 62:C, 63:P, 64:P, 65:C

CONCEPT

**Environment:** This alluvial system forms on the shores of rivers and streams influenced by flood scour and deposition. It includes vegetation on various substrates ranging from silty sediments low on the channel to rock outcrops, gorge walls, and cobbles.

**Vegetation:** The vegetation is often a mosaic of forest, woodland, shrubland, and herbaceous communities. Common trees include *Betula nigra, Platanus occidentalis*, and *Acer negundo*. Open, flood‑scoured rivershore prairies feature *Panicum virgatum* and *Andropogon gerardii*, and *Carex torta* is typical of wetter areas near the channel.

**Dynamics:** High‑gradient waterflow causes scouring of rivershores, removing soils and depositing them in slower‑moving portions of the river. High amounts of debris cause flood‑battering of trees and shrubs, and removal of woody vegetation during extreme flooding events. Seepage from uplands may emerge from shores, and the often specialized flora of these environments is maintained by repeated removal, or prevention of establishment, of woody vegetation. Flood‑battering of trees prevents succession; scouring by water, and sometimes ice, exposes substrate.

SOURCES

**References:** Comer et al. 2003*, Edinger et al. 2014a, Eyre 1980, Faber‑Langendoen et al. 2011, PNHP 2002, Sperduto and Nichols 2004, Zimmerman 2011j, Zimmerman 2011m, Zimmerman and Podniesinski 2008, Zimmerman et al. 2012

**Version:** 14 Jan 2014	**Stakeholders:** East, Midwest, Southeast

**Concept Author:** S.C. Gawler	**LeadResp:** East

CES202.694 North‑Central Interior Floodplain

**Primary Division:** Central Interior and Appalachian (202)

**Land Cover Class:** Mixed Upland and Wetland

**Spatial Scale & Pattern:** Linear

**Required Classifiers:** Natural/Semi‑natural; Vegetated (>10% vasc.); Upland; Wetland

**Concept Summary:** This system is found along rivers across the glaciated Midwest. It occurs from river's edge across the floodplain or to where it meets a wet meadow system. It can have a variety of soil types found within the floodplain from very well‑drained sandy substrates to very dense clays. It is this variety of substrates and flooding that creates the mix of vegetation that includes *Acer saccharinum, Populus deltoides*, willows, especially *Salix nigra* in the wettest areas, and *Fraxinus pennsylvanica, Ulmus americana*, and *Quercus macrocarpa* in more well‑drained areas. Within this system are oxbows that may support *Nelumbo lutea* and *Typha latifolia*. Understory species are mixed, but include shrubs, such as *Cornus drummondii* and *Asimina triloba* (in Kansas), sedges and grasses, which sometimes help form savanna vegetation. Flooding is the primary dynamic process, but drought, grazing, and fire have all had historical influence on this system. Federal reservoirs have had a serious and negative effect on this system, along with agriculture that has converted much of this system to drained agricultural land.

**Comments:** The distribution limit northward is considered to be the Laurentian region boundary. This system is distinguished from floodplain systems northeastward, Laurentian‑Acadian Floodplain Forest (CES201.587), and eastward, Central Appalachian River Floodplain (CES202.608). *Celtis* and *Populus deltoides* are absent (or essentially so) from the Laurentian‑Acadian type.

DISTRIBUTION

**Range:** This system is found along medium and large river floodplains throughout the glaciated Midwest ranging from eastern Kansas and western Missouri to western Ohio and north along the Red River basin in Minnesota and the eastern Dakotas. This system is essentially restricted to USFS Provinces 251 and 222, though it may go further west in larger rivers in the Great Plains, notably the Missouri and Platte rivers.

**Divisions:** 202:C, 205:C

**TNC Ecoregions:** 35:C, 36:C, 45:C, 46:C, 47:?, 48:?

**Nations:** US

**Subnations:** IA, IL, IN, KS, MI, MN, MO, ND, NE, OH, SD, WI

**Map Zones:** 38:C, 39:C, 40:C, 42:C, 43:C, 44:P, 47:C, 49:C, 50:C, 51:C, 52:C

**USFS Ecomap Regions:** 222H:CC, 222I:CC, 222Ja:CCC, 222Jb:CCC, 222Jc:CCC, 222Je:CCC, 222Jg:CCC, 222Jh:CCC, 222Ji:CCC, 222K:CC, 222L:CC, 222M:CC, 222Ua:CCC, 222Ud:CCC, 222Ue:CCC, 223A:CC, 251B:CC, 251E:CC, 251F:CC, 251H:CC, 255A:CC, 332B:CC, 332C:CC, 332D:CC, 332E:CC

CONCEPT

**Environment:** This ecological system occurs in floodplains of medium to large rivers. It is found on alluvial soils ranging from sandy to very dense clays. Soil texture reflects the upstream substrate through which the river and its tributaries flow and water velocity. Sandy sediments can be carried by faster‑flowing water, while slow‑moving rivers can only carry fine‑textured sediment. Water velocity and volume change greatly during the year as rains and snowmelt deliver pulses of water and seasonal droughts (typically including winter in the northern portion of this system's range when most precipitation is frozen) result in low water. Within a short distance on a river floodplain, different soil textures can be found. Coarser‑textured soils are typically adjacent to the main channel where they are deposited first by rising or falling floodwaters. Finer‑textured soils are further away from the main channel, deposited when floodwaters have spread out and slowed down. Within the space of a few years, floods of differing magnitude can deposit sand over silt or vice versa, resulting in complex soil topology.

**Vegetation:** The variety of soil properties associated with this system can create a mixture of vegetation. *Acer saccharinum* occurs on the wetter soils of floodplains in the eastern portion of this system, with *Populus deltoides* and willows, especially *Salix nigra*, occurring more in the western range of this system. *Fraxinus pennsylvanica, Ulmus americana*, and *Quercus macrocarpa* occur in more well‑drained areas. Understory species can vary across the range of this system but can include shrubs such as *Cornus drummondii* and *Asimina triloba*, and sedge and grass species. Oxbows within this system may have species such as *Nelumbo lutea* and *Typha latifolia*.

**Dynamics:** This system is primarily controlled by moderate to frequent flooding. Flood frequency depends on precipitation patterns within the watershed and proximity to the main channel. Areas adjacent to the main channel or low islands within the channel can be flooded every year or even more than once per year. Those areas further from the channel on terraces or behind natural levees may only be flooded once every several years. Free‑flowing rivers migrate across their floodplain, cutting new channels or eroding the bank on one side while building up the bank on the other, so the flooding regime of any one point in the floodplain will change over time. Flooding redeposits alluvium, eroding some areas and aggrading others, can bury or wash away small plants, and redistributes nutrients, especially in less frequently flooded zones where silt and clay tend to be deposited. These processes open up new areas for colonization. Where trees can grow (i.e., not in permanent or semi‑permanent backwater wetlands), there is a common succession sequence of annual herbaceous species followed by shrub *Salix* spp., followed by *Populus deltoides, Salix nigra*, and *Acer saccharinum*, followed by a number of trees, including *Acer negundo, Carya illinoinensis, Celtis laevigata, Celtis occidentalis, Fraxinus pennsylvanica, Quercus macrocarpa*, and *Ulmus americana*. This sequence can be reset by major floods and erosion/deposition. Frequent minor to moderate flooding holds the system at the intermediate forest stage, and large areas of this floodplain system are dominated by *Populus deltoides, Salix nigra*, and *Acer saccharinum*.

Fire could impact parts of this system. Most of the forests in this system were not fire‑prone due to the lack of litter, frequent flooding, and relatively protected landscape position in the river valley with wetlands often near, but forests on higher, coarser soils or wet‑mesic prairies on the margins of the floodplain could become dry in late summer and burn, if an ignition source was present (Weaver 1960).

SOURCES

**References:** Bragg and Tatschi 1977, Comer et al. 2003*, DeSantis et al. 2012, Eyre 1980, Herms et al. 2010, Johnson 1992, Kost et al. 2007, Nelson 2010, ONHD unpubl. data, Rolfsmeier and Steinauer 2010, WDNR 2015, Weaver 1960, Yin and Nelson 1996

**Version:** 14 Jan 2014	**Stakeholders:** Canada, Midwest, Southeast

**Concept Author:** S. Menard and K. Kindscher	**LeadResp:** Midwest

CES202.705 South‑Central Interior Large Floodplain

**Primary Division:** Central Interior and Appalachian (202)

**Land Cover Class:** Mixed Upland and Wetland

**Spatial Scale & Pattern:** Linear

**Required Classifiers:** Natural/Semi‑natural; Vegetated (>10% vasc.); Upland; Wetland

**Diagnostic Classifiers:** Riverine / Alluvial

**Concept Summary:** This floodplain system is found in the Interior Highlands as far west as eastern Oklahoma, as well as throughout the Interior Low Plateau, Cumberlands, Southern Ridge and Valley, and Western Allegheny Plateau, and lower elevations of the Southern Blue Ridge. Examples occur along large rivers or streams where topography and alluvial processes have resulted in a well‑developed floodplain. A single occurrence may extend from river’s edge across the outermost extent of the floodplain or to where it meets a wet meadow or upland system. Many examples of this system will contain well‑drained levees, terraces and stabilized bars, and some will include herbaceous sloughs and shrub wetlands resulting, in part, from beaver activity. A variety of soil types may be found within the floodplain from very well‑drained sandy substrates to very dense clays. It is this variety of substrates in combination with different flooding regimes that creates the mix of vegetation. Most areas, except for the montane alluvial forests, are inundated at some point each spring; microtopography determines how long the various habitats are inundated. Although vegetation is quite variable in this broadly defined system, examples may include *Acer saccharinum, Platanus occidentalis, Liquidambar styraciflua, Populus deltoides*, and *Quercus* spp. Understory species are mixed, but include shrubs, such as *Cephalanthus occidentalis* and *Arundinaria gigantea*, and sedges (*Carex* spp.). This system likely floods at least once annually and can be altered by occasional severe floods. Impoundments and conversion to agriculture can also impact this system.

**Comments:** Montane alluvial forests may be difficult to place within this system because they share traits with both this system and Southern and Central Appalachian Cove Forest (CES202.373), at least in the Southern Appalachians. This split from Central Appalachian River Floodplain (CES202.608) may appear somewhat arbitrary but is based on the freshwater systems classification, using roughly the Mid‑Continental Divide. This means that Ecoregions 50 and 51 are included in this system, whereas Ecoregions 52 and 59 are considered part of Central Appalachian River Floodplain (CES202.608) (except for a small part of southernmost Ecoregion 59 in West Virginia that drains to the Ohio River).This system grades into Western Great Plains Floodplain (CES303.678) in the Crosstimbers region of east‑central Oklahoma as eastern cottonwood (*Populus deltoides*) and willows (*Salix* spp.) become more dominant. Ozark‑Ouachitas are included in this system.

DISTRIBUTION

**Range:** This system ranges from the Ozarks, Arkansas River Valley, and Interior Low Plateau to the Southern Blue Ridge and north into the Western Allegheny Plateau.

**Divisions:** 202:C, 205:C

**TNC Ecoregions:** 32:P, 37:C, 38:C, 39:C, 44:C, 49:C, 50:C, 51:C, 59:C

**Nations:** US

**Subnations:** AL, AR, GA, IL, IN, KY, MO, NC, OH, OK, PA, SC, TN, VA, WV

**Map Zones:** 32:P, 37:P, 38:?, 43:C, 44:C, 47:C, 48:C, 49:C, 53:C, 57:C, 61:C, 62:C

**USFS Ecomap Regions:** 212H:CC, 212Z:CC, 221E:CC, 221F:CC, 221H:CC, 221J:CC, 223A:CC, 223B:CC, 223D:CC, 223E:CC, 223F:CC, 223G:CC, 231C:CC, 231D:CC, 251E:CC, 251F:CP, 255A:CP, 255B:CP, 255C:CP, 255E:CC, M221A:CC, M221B:CC, M221C:CC, M221D:CC, M223A:CC, M231A:CC

CONCEPT

**Environment:** This system inhabits broad floodplains along large creeks and rivers that are usually inundated for at least part of each year. Flood frequency depends on precipitation patterns within the watershed and proximity to the main channel. Areas adjacent to the main channel or low islands within the channel can be flooded every year or even more than once per year. Those areas further from the channel on terraces or behind natural levees may only be flooded once every several years. Free‑flowing rivers migrate across their floodplain, cutting new channels or eroding the bank on one side while building up the bank on the other, so the flooding regime of any one point in the floodplain will change over time. Flooding redeposits alluvium, eroding some areas and aggrading others, can bury or wash away small plants, and redistributes nutrients, especially in less frequently flooded zones where silt and clay tend to be deposited. These processes open up new areas for colonization.

**Vegetation:** Vegetation varies quite widely, encompassing shrubby and herbaceous communities, as well as forested communities with a wide array of canopy types. Examples may include *Acer saccharinum, Platanus occidentalis, Liquidambar styraciflua*, and *Quercus* spp. Understory species are mixed but include shrubs, such as *Cephalanthus occidentalis* and *Arundinaria gigantea (= ssp. gigantea)*, and sedges (*Carex* spp.).

**Dynamics:** Flooding dynamics are an important factor in the development and maintenance of this system. Flood frequency depends on precipitation patterns within the watershed and proximity to the main channel. Areas adjacent to the main channel or low islands within the channel can be flooded every year or even more than once per year. Those areas further from the channel on terraces or behind natural levees may only be flooded once every several years. Free‑flowing rivers migrate across their floodplain, cutting new channels or eroding the bank on one side while building up the bank on the other, so the flooding regime of any one point in the floodplain will change over time. Flooding redeposits alluvium, eroding some areas and aggrading others, can bury or wash away small plants, and redistributes nutrients, especially in less frequently flooded zones where silt and clay tend to be deposited. These processes open up new areas for colonization.

SOURCES

**References:** Comer et al. 2003*, DeSantis et al. 2012, DuMond 1970, Edwards et al. 2013, Evans et al. 2009, Eyre 1980, Herms et al. 2010, Johnson 1992, Mackie pers. comm., Nelson 2010, ONHD unpubl. data, Schafale 2012, Schafale and Weakley 1990, Simon 2011, Simon 2015, Woods et al. 2002

**Version:** 14 Jan 2014	**Stakeholders:** East, Midwest, Southeast

**Concept Author:** S. Menard, M. Pyne, R. Evans, R. White	**LeadResp:** Midwest

CES202.706 South‑Central Interior Small Stream and Riparian

**Primary Division:** Central Interior and Appalachian (202)

**Land Cover Class:** Mixed Upland and Wetland

**Spatial Scale & Pattern:** Linear

**Required Classifiers:** Natural/Semi‑natural; Vegetated (>10% vasc.); Upland; Wetland

**Diagnostic Classifiers:** Seepage‑Fed Sloping; Riverine / Alluvial

**Concept Summary:** This system is found throughout the Interior Low Plateau, Southern Ridge and Valley and Cumberland Plateau, Western Allegheny Plateau, lower elevations of the Southern Blue Ridge, and parts of the Cumberlands. Examples occur along small streams and floodplains with low to moderately high gradients. There may be little to moderate floodplain development. Flooding and scouring both influence this system, and the nature of the landscape prevents the kind of floodplain development found on larger rivers. This system may contain cobble bars with adjacent wooded vegetation and rarely have any marsh development, except through occasional beaver impoundments. The vegetation is a mosaic of forests, woodlands, shrublands, and herbaceous communities. Canopy cover can vary within examples of this system, but typical tree species may include *Platanus occidentalis, Acer rubrum var. trilobum, Betula nigra, Liquidambar styraciflua*, and *Quercus* spp. Shrubs and herbaceous layers can vary in richness and cover. Some characteristic shrubs may include *Hypericum densiflorum, Salix* spp., and *Alnus* spp. Small seeps dominated by sedges (*Carex* spp.), cinnamon and royal ferns (*Osmunda* spp.), and other herbaceous species can often be found within this system, especially at the headwaters and terraces of streams.

**Comments:** This system is closely related to Central Appalachian Stream and Riparian (CES202.609) but has been distinguished based on the precepts of the Freshwater Systems classification. This system has been divided from Central Appalachian Riparian roughly by the Mid‑Continental Divide. This means that TNC Ecoregions 50 and 51 are included in this system, whereas TNC Ecoregions 52 and 59 are considered part of Central Appalachian Riparian (except for a small part of southernmost TNC Ecoregion 59 in West Virginia that drains to the Ohio River). In contrast to floodplain systems, this system has little to no floodplain development. In comparison with South‑Central Interior Large Floodplain (CES202.705), this system typically has somewhat higher gradients, is sometimes rocky, and may experience flash floods. Stands from somewhat larger rivers have been placed here if the river lacks substantial floodplain development (e.g., the New River of West Virginia and the Ocoee Gorge of Tennessee). This system overlaps with Ozark‑Ouachita Riparian (CES202.703), which has high gradients and flash floods, but this system has low to moderately high gradients. Bruce Hoagland (pers. comm. 2014) would like to eliminate Southeastern Great Plains Floodplain Forest (CES205.710) from Oklahoma, but Texas is using that system north of the West Gulf Coastal Plain.

DISTRIBUTION

**Range:** This system ranges from the Interior Low Plateau to the Southern Blue Ridge and north into the Western Allegheny Plateau and portions of the Cumberlands. There would be limited and peripheral presence in the Upper East Gulf Coastal Plain. It also is present on Crowley's Ridge, an anomalous and distinct upland topographic feature that is embedded within the Mississippi River Alluvial Plain.

**Divisions:** 202:C, 203:C

**TNC Ecoregions:** 42:C, 43:C, 44:C, 49:C, 50:C, 51:C, 59:C

**Nations:** US

**Subnations:** AL, AR, GA, IL, IN, KY, NC, OH, PA, SC, TN, VA, WV

**Map Zones:** 45:C, 46:P, 47:C, 48:C, 49:C, 53:C, 57:C, 61:C, 62:C

**USFS Ecomap Regions:** 221Hc:CCC, 221Hd:CCC, 221J:CC, 231Ag:CCC, 231C:CC, 231D:CC, 234Db:CCC, M221Ab:CCC, M221Cc:CCC, M221Cd:CCC, M221Ce:CCC, M221D:CC

CONCEPT

**Environment:** This system is found along fairly high‑energy streams and rivers with steep banks, this system is subject to frequent flooding and can be subject to scouring depending upon the substrate. Some associations do not flood but instead are saturated zones or patches near the streams.

**Vegetation:** There is wide variation in vegetation depending upon the frequency of the flooding cycle (more frequent flooding creates a better environment for forbs and shrubs, less frequent may create a better environment for the establishment of trees). Typical tree species may include *Platanus occidentalis, Acer rubrum var. trilobum, Betula nigra, Liquidambar styraciflua*, and *Quercus* spp. Shrubs and herbaceous layers can vary in richness and cover. Some characteristic shrubs may include *Hypericum densiflorum, Salix* spp., and *Alnus* spp. Small seeps dominated by sedges (*Carex* spp.), ferns (*Osmunda* spp.), and other herbaceous species can often be found within this system, especially at the headwaters and terraces of streams. These areas are not typically flooded or scoured but saturated.

**Dynamics:** Flooding and seed propagule dispersal caused by flooding events are the two most important processes affecting this system. The two processes vary widely depending upon size of stream, upstream land use and topography, presence or absence of invasive exotics that may displace native community types, etc.

SOURCES

**References:** Comer et al. 2003*, Edwards et al. 2013, Evans et al. 2009, Eyre 1980, Gettman 1974, Nelson 1986, Nelson 2010, ONHD unpubl. data, Schafale 2012, Schafale and Weakley 1990, Simon 2011, Simon 2015, Stevens and Cummins 1999, Tobe et al. 1992

**Version:** 30 Jun 2016	**Stakeholders:** East, Midwest, Southeast

**Concept Author:** S. Menard, M. Pyne, R. Evans, R. White, D.	**LeadResp:** Southeast Faber‑Langendoen

M503. Central Hardwood Swamp Forest

CES202.018 Central Interior Highlands and Appalachian Sinkhole and Depression Pond

**Primary Division:** Central Interior and Appalachian (202)

**Land Cover Class:** Woody Wetland

**Spatial Scale & Pattern:** Small patch

**Required Classifiers:** Natural/Semi‑natural; Vegetated (>10% vasc.); Wetland

**Diagnostic Classifiers:** Lowland [Lowland]; Depressional [Pond, Sinkhole]; Muck; Mineral: W/ A‑Horizon >10 cm

**Concept Summary:** This system of ponds and wetlands is found in the Interior Highlands of the Ozark, Ouachita, and Interior Low Plateau regions, and ranges north from the Southern and Central Appalachians to the northern Piedmont regions. Stands occur in basins of sinkholes or other isolated depressions on uplands. Soils are very poorly drained, and surface water may be present for extended periods of time, rarely becoming dry. Water depth may vary greatly on a seasonal basis and may be a meter deep or more in the winter. Some examples become dry in the summer. Soils may be deep (100 cm or more), consisting of peat or muck, with parent material of peat, muck or alluvium. Ponds vary from open water to herb‑, shrub‑, or tree‑dominated. Tree‑dominated examples typically contain *Quercus* species, *Platanus occidentalis, Fraxinus pennsylvanica, Acer saccharinum*, or *Nyssa* species, or a combination of these. In addition, *Liquidambar styraciflua* may be present in southern examples. *Cephalanthus occidentalis* is a typical shrub component. The herbaceous layer is widely variable depending on geography.

**Comments:** Many of these ponds have their geologic origin as a more‑or‑less complete karst collapse feature. Some of them may display this geologic origin in a more explicit manner, with definite walls and exposed limestone or dolomite at the surface ("sinkholes"). Others are more subtle, and exist as more gentle depressions, with no exposed surface geology ("depression ponds"). This includes the "sagponds" of northwestern Georgia and adjacent Alabama. Rare examples in the Ridge and Valley of Georgia (Coosa Valley) are included here. These occur on limestones or dolomites of the Chickamauga Group. Matt Elliott (pers. comm.): "I would put Ridge and Valley sagponds in with Interior Highlands ponds rather than Piedmont, as they are essentially karst features. R&V sagponds are generally pretty rare but are common in parts of Bartow County, Georgia, and a few other places. The shallower ones are dominated by willow oak, the deeper ones *Nyssa biflora*. On the Cumberland Plateau, the ones I have seen usually have sweetgum and *Nyssa sylvatica*, but I think willow oak and possibly *Nyssa biflora* might occur in some of the deeper ones. A lot of the plateau ponds seem more like swales than deep ponds, but they still may be related to underlying karst features. The Ridge and Valley sagponds may be somewhat different from those on the plateau ‑ often deeper and with even more Coastal Plain elements; it also includes sinkhole ponds of northern New Jersey (K. Strakosch‑Walz pers. comm.) and possibly ponds of the Ridge and Valley in Pennsylvania. These are very similar to Shenandoah sinkhole ponds of Virginia and are in Maryland as well (L. Sneddon pers. comm.). The only documented occurrence in Pennsylvania is the Maple Hills sinkhole in Lycoming County; "there are plenty of other sinkholes in Pennsylvania, but they have not been associated with any specific plant community" (G. Podniesinski pers. comm. 2010).

DISTRIBUTION

**Range:** This system is found from the Ozark and Ouachita mountains east to the Southern and Central Appalachians and the northern Piedmont regions (?), including the unglaciated Interior Low Plateau and Ridge and Valley. It ranges from Missouri, West Virginia, Pennsylvania, and Delaware south to Arkansas, Alabama and Georgia.

**Divisions:** 202:C

**TNC Ecoregions:** 38:C, 39:C, 44:C, 50:C, 51:C, 59:C, 61:C

**Nations:** US

**Subnations:** AL, AR, DE, GA, IL, IN, KY, MD, MO, NJ, OH, PA, TN:S2S3, VA, WV

**Map Zones:** 44:C, 47:C, 48:C, 49:C, 53:C, 57:C, 61:C, 62:P, 64:P

**USFS Ecomap Regions:** 221F:CC, 221H:CC, 221J:CC, 223A:CC, 223D:CC, 223E:CC, 223F:CC, 231C:CC, 231D:CC, M221A:CC, M221D:CC, M223A:CC, M231A:CC

CONCEPT

**Environment:** Examples of this system occur in basins of sinkholes or other isolated depressions on uplands. Soils are very poorly drained, and surface water may be present for extended periods of time, rarely becoming dry. The watershed of these sites is typically small so water depth may vary greatly on a seasonal basis, and may be a meter deep or more in the winter (Homoya and Hedge 1985). Some examples become dry in the summer. The rate of water level rise and fall may also be related to whether these sites have internal drainage within the karst features or are essentially closed depressions (Wolfe 1996). Soils may be deep (100 cm or more), consisting of peat or muck, with parent material of peat, muck or alluvium. Many of these ponds have their geologic origin as a more‑or‑less complete karst collapse feature. Some of them may display this geologic origin in a more explicit manner, with definite walls and exposed limestone or dolomite at the surface ("sinkholes"). Others are more subtle and exist as more gentle depressions, with no exposed surface geology ("depression ponds").

**Vegetation:** Ponds vary from open water to herb‑, shrub‑, or tree‑dominated types. Tree‑dominated examples typically contain *Quercus* species, *Platanus occidentalis, Fraxinus pennsylvanica, Acer saccharinum*, or *Nyssa* species, or a combination of these. In addition, *Liquidambar styraciflua* may be present in southern examples. *Cephalanthus occidentalis* is a typical shrub component. The herbaceous layer is widely variable depending on geography.

**Dynamics:** Water depth may vary greatly on a seasonal basis, and may be a meter deep or more in the winter. Some examples become dry in the summer.

SOURCES

**References:** Comer et al. 2003*, Elliott, M. pers. comm., Evans et al. 2009, Eyre 1980, Faber‑Langendoen et al. 2011, Homoya and Hedge 1985, Nelson 2010, TDNH unpubl. data 2018, Wharton 1978, Wolfe 1996

**Version:** 14 Jan 2014	**Stakeholders:** East, Midwest, Southeast

**Concept Author:** M. Pyne, S. Menard, D. Faber‑Langendoen	**LeadResp:** Midwest

CES202.605 North‑Central Interior and Appalachian Rich Swamp

**Primary Division:** Central Interior and Appalachian (202)

**Land Cover Class:** Woody Wetland

**Spatial Scale & Pattern:** Small patch

**Required Classifiers:** Natural/Semi‑natural; Vegetated (>10% vasc.); Wetland

**Diagnostic Classifiers:** Temperate; Depressional; Broad‑Leaved Deciduous Tree; Mesotrophic Water; Saturated Soil

**Concept Summary:** These forested wetlands are scattered throughout the north‑central Midwest (south of the Laurentian region), the north‑central Appalachians and southern New England at low to mid elevations. They are found in basins where higher pH and/or nutrient levels are associated with a rich flora. Species include *Acer rubrum, Fraxinus nigra*, as well as calciphilic herbs. Conifers include *Larix laricina*, but typically not *Thuja occidentalis*, which is characteristic of more northern wetland systems. There may be shrubby or herbaceous openings within the primarily wooded cover. The substrate is primarily mineral soil, but there may be some peat development.

**Comments:** This system occurs south of the Laurentian‑Acadian region, and these circumneutral or enriched swamps are often rather distinctive and discrete elements of the landscape. They are related to Laurentian‑Acadian Alkaline Conifer‑Hardwood Swamp (CES201.575) but have more temperate elements and generally lack *Thuja occidentalis*. More alkaline shrub/herb fens are treated as part of North‑Central Interior Shrub‑Graminoid Alkaline Fen (CES202.702).

DISTRIBUTION

**Range:** This system is found from central New England to the southern Great Lakes and south‑central Minnesota south to northern Illinois, Indiana, Ohio, and Pennsylvania. It is not known to extend south into the Southern Blue Ridge.

**Divisions:** 202:C

**TNC Ecoregions:** 45:C, 46:C, 48:C, 49:P, 59:C, 60:?, 61:C

**Nations:** CA, US

**Subnations:** CT, DE?, IL, IN, MA, MD, MI, MN, NH, NJ, NY, OH, ON, PA, RI, VT, WI

**Map Zones:** 41:C, 49:C, 50:C, 51:C, 52:C, 53:C, 61:C, 62:C, 63:C, 64:C, 65:C

**USFS Ecomap Regions:** 212Hb:CCP, 222H:CC, 222Ja:CCC, 222Jb:CCC, 222Jc:CCC, 222Je:CCC, 222Jg:CCC, 222Jh:CCC, 222Ji:CCC, 222K:CC, 222L:CC, 222M:CC, 222Ua:CCC, 222Ud:CCC, 222Ue:CCC, M211Cc:CCC

CONCEPT

**Environment:** Water can come from nutrient‑rich groundwater or surface runoff. Sites are basins or low areas in floodplains, usually near the edge of the floodplain in a localized basin or at the base of a bluff where groundwater emerges. Soils are muck or fine‑textured mineral. Small hummocks and depressions, created from tree tip‑ups, sluggish streams, or tree root build up, create drier and wetter microsites within the system. Sites are usually flooded in the spring, and low areas may remain wet for all or most of the growing season, but if stands remain under water for multiple years, the trees die (Kost et al. 2007). The microsite differences allow a mixture of wet‑mesic upland species and wetland species to exist in the herbaceous layer of this system (WDNR 2015).

**Dynamics:** The hydrologic regime is critical to maintenance of this system. Sites must be wet or flooded for part of the growing season but not completely saturated or under water for too long over a large portion of the site. Periodic sustained floods or droughts can kill canopy trees and allow the mostly shade‑intolerant canopy trees (*Fraxinus nigra, Fraxinus pennsylvanica, Larix laricina*) to regenerate. Trees are shallowly rooted in this system so wind can blow canopy trees over relatively easily. This creates gaps in the canopy and allows smaller trees enough light to reach the canopy. Windthrow contributes to hummock‑and‑hollow microtopography, which generates small‑scale gradients in soil moisture and chemistry, contributing to floristic diversity.

SOURCES

**References:** Comer et al. 2003*, DeSantis et al. 2012, Edinger et al. 2014a, Eyre 1980, Faber‑Langendoen et al. 2011, Herms et al. 2010, Kost et al. 2007, ONHD unpubl. data, Sperduto and Nichols 2004, WDNR 2015, Ward et al. 2006, Zimmerman et al. 2012

**Version:** 14 Jan 2014	**Stakeholders:** Canada, East, Midwest, Southeast

**Concept Author:** S.C. Gawler	**LeadResp:** East

CES202.700 North‑Central Interior Wet Flatwoods

**Primary Division:** Central Interior and Appalachian (202)

**Land Cover Class:** Woody Wetland

**Spatial Scale & Pattern:** Small patch

**Required Classifiers:** Natural/Semi‑natural; Vegetated (>10% vasc.); Wetland

**National Mapping Codes:** EVT 2518; ESLF 9186; ESP 1518

**Concept Summary:** This small‑patch system is found throughout the northern glaciated Midwest ranging east into Lower New England and the Champlain Valley. It usually occurs on somewhat poorly drained uplands or in depressions associated with glacial features such as tillplains, lakeplains or outwash plains. Soils often have an impermeable or nearly impermeable clay layer that can create a shallow, perched water table. Saturation can vary, with ponding common during wetter seasons, and drought possible during the summer and autumn months. Microtopography and fluctuating moisture levels can lead to complexes of forest upland and wetland species occurring within this system. *Quercus palustris* and/ or *Quercus bicolor* typically dominate the wetter portions and are often associated with *Acer rubrum*. *Quercus alba, Quercus rubra*, and *Fagus grandifolia* are common in the better‑drained areas. *Carya ovata* is a characteristic tree in the Champlain Valley. *Liquidambar styraciflua, Nyssa sylvatica, Acer saccharinum, Fraxinus americana*, and *Fraxinus pennsylvanica* are also common associates, though their occurrence varies somewhat by region. Understory herbaceous and shrub species present in examples of this system can vary. Stands with more dense tree cover have less shrub and herbaceous cover, while those with moderate tree canopy cover tend to have a dense understory. Some common species in the wetter portions include *Carex* spp., *Osmunda cinnamomea, Cephalanthus occidentalis, Alnus* spp., and *Ilex* spp. Flooding, windthrow, drought, and fire can influence this system.

**Comments:** These are mostly north of the glacial line, but one association is in the Interior Low Plateau and that placement may need to be reviewed. Some examples in Michigan, Indiana, Ohio, Vermont, and southern Ontario are dominated by *Fagus grandifolia*, oak (primarily *Quercus alba* and *Quercus rubra*) and maple species (*Acer* spp.). Vermont's Valley Clayplain Forest is placed here tenuously as it has more of an upland component and occurs at a local matrix scale, not as a small‑patch element.

DISTRIBUTION

**Range:** This system is found in the northern Midwest, southern Ontario, and portions of the northeastern U.S.

**Divisions:** 201:P, 202:C

**TNC Ecoregions:** 36:C, 44:C, 45:C, 47:?, 48:C, 49:P, 59:P, 61:C, 64:C

**Nations:** CA, US

**Subnations:** CT, IA, IL, IN, MA, MI, MO, NH, NY, OH, ON, PA, VT

**Map Zones:** 41:?, 42:C, 43:C, 44:P, 47:C, 49:?, 50:?, 51:C, 52:C, 53:P, 61:C, 62:P, 63:C, 64:P, 65:C

**USFS Ecomap Regions:** 211E:CC, 211F:CP, 221A:CC, 221B:CP, 222I:CP, 222Jh:CCC, 222Ua:CCC, 222Ue:CC?

CONCEPT

**Environment:** This system usually occurs on poorly drained uplands or in depressions associated with glacial features such as tillplains, lakeplains, or outwash plains. Soils often have an impermeable or nearly impermeable clay layer that impedes waterflow. This favors flooding or ponding in the spring or after heavy rains. It also restricts subsurface water movement into the system and slows the growth of roots through it. Both of these factors lead to water deficits for the vegetation in the late summer and fall. These fluctuating moisture levels can lead to complexes of forest upland and wetland species occurring within this system. Overall topographic relief is very flat in this system though small tip‑up mounds and depressions can occur from windthrow and often create small pockets with vegetation more typical of upland or swamp forest, respectively.

**Vegetation:** *Quercus palustris* and/or *Quercus bicolor* typically dominate the wetter portions and are often associated with *Acer rubrum*. *Quercus alba, Quercus rubra, Fagus grandifolia*, and *Acer saccharum* are common in the better‑drained areas, seen in some examples around the southern Great Lakes and Lake Champlain. *Carya ovata* is a characteristic tree in the Champlain Valley. *Liquidambar styraciflua, Nyssa sylvatica, Fraxinus americana*, and *Fraxinus pennsylvanica* are also common associates, though their occurrence varies somewhat by region. Understory herbaceous and shrub species present in examples of this system can vary. Stands with more dense tree cover have less shrub and herbaceous cover, while those with moderate tree canopy cover tend to have a dense understory. Some common species include *Carex* spp., *Osmunda cinnamomea, Cephalanthus occidentalis, Alnus* spp., and *Ilex* spp. In the clayplain forests of Vermont, characteristic herbs include *Waldsteinia fragarioides* and *Moehringia lateriflora (= Arenaria lateriflora)*.

**Dynamics:** The large seasonal change in local available moisture is key to the development and maintenance of this system. Plants must be able to tolerate the excessive available moisture (surface flooding or saturation) and drought conditions that occur in most growing seasons. Fire can occur after the system dries, typically late in the growing season. Fires rarely start in this system but under favorable conditions can spread from nearby fire‑prone systems (typically prairies, oak savannas, or oak woodlands). Under proper hydrologic conditions, this system can be self‑maintaining (Tecic and McCain 2001). With the often shallowly‑rooted trees, strong winds can create canopy openings. Small‑scale windthrow is a characteristic disturbance in flatwoods that influences composition and structure by creating canopy gaps that are suitable for the colonization and growth of light‑dependent tree seedlings and saplings, shrubs, and herbs. Windthrow also tips and uproots trees, creating pit‑and‑mound topography that provides suitable microhabitats for a diversity of plant species (Slaughter et al. 2010).

SOURCES

**References:** Bowles et al. 2003, Braun 1950, Comer et al. 2003*, Eyre 1980, Faber‑Langendoen et al. 2011, Kost et al. 2007, Nelson 2010, ONHD unpubl. data, Slaughter et al. 2010, Sperduto and Nichols 2004, Stroke and Anderson 1992, Tecic and McCain 2001

**Version:** 14 Jan 2014	**Stakeholders:** Canada, East, Midwest, Southeast

**Concept Author:** S. Menard	**LeadResp:** Midwest

CES202.336 Piedmont Upland Depression Swamp

**Primary Division:** Central Interior and Appalachian (202)

**Land Cover Class:** Woody Wetland

**Spatial Scale & Pattern:** Small patch

**Required Classifiers:** Natural/Semi‑natural; Vegetated (>10% vasc.); Wetland

**Diagnostic Classifiers:** Depressional

**Concept Summary:** This system represents isolated wetlands primarily of the Piedmont in small, shallow basins in upland settings where water pools due to limited soil drainage. Most known examples occur over mafic bedrock. The typical hydrology is seasonally flooded. Most examples consist of forests of wetland oaks, but a few are treeless or open‑canopied ponds. Vegetation in open ponds is typically zoned with an outer ring of trees, a more interior ring of shrubs, herbs and vines, and a central area with or without standing water year round depending on precipitation. This system also includes the wet hardwood forests ("Iredell Flatwoods" or "Gabbro Glades") which occur on gently sloping terrain or shallowly depressed upland flats over gabbro‑derived clays in the Piedmont of Georgia and South Carolina. A few examples of this system occur in the adjacent Southern Blue Ridge; these are extremely rare and small‑patch examples.

**Comments:** This system is distinct from all other Piedmont systems in its ponded wetland hydrology in upland settings. The vegetation is generally also distinct from all other Piedmont systems. Though apparently quite different, Piedmont Hardpan Woodland and Forest (CES202.268) is closely related by the importance of an impermeable clay hardpan, and some intermediate gradations occur. A few examples of this system (CES202.336) appear to be closely related to Coastal Plain depressional wetlands, sharing some flora, but most are more distinct. The system has significant variation in vegetation and environment. The forested swamps and open pools represent well‑marked subtypes. There is a more subtle distinction between the basic and acidic soil swamps. There is substantial variation among the pools, related to substrate, basin morphology, and geographic location. This system includes the depression swamps of the Triassic Basins of Virginia and Maryland. A few disjunct examples may occur in the Southern Blue Ridge because of similarity in topographic setting and general structure. They do, however, occur on different substrates (quartzite and sandstone) than any examples in the Piedmont. Their vegetation is different from other examples but not in having more montane flora. Their vegetation is no more different than most other pools are from each other.

DISTRIBUTION

**Range:** This system ranges throughout the Piedmont, from Virginia to Alabama. A few examples attributable to this system are found in the adjacent Southern Blue Ridge.

**Divisions:** 202:C

**TNC Ecoregions:** 51:C, 52:C, 59:?

**Nations:** US

**Subnations:** AL, GA, MD, NC, SC, VA

**Map Zones:** 54:C, 57:C, 59:C, 60:C, 61:C

**USFS Ecomap Regions:** 221D:CC, 231A:CC, 231I:CC

CONCEPT

**Environment:** This system occurs in small, shallow basins or gentle swales on flat to rolling upland sites, and occasionally in depressions on narrow, steeper ridgetops. Soils have a dense clay hardpan or occasionally bedrock which limits internal drainage. Rainwater accumulates in the basins and persists through the wet season, occasionally persisting all year. Only a few kinds of rock are known to form these depressions. Most examples occur on soils derived from mafic rocks such as gabbro or diabase, but a few occur over slates or mafic to felsic tuffs where a dense clay hardpan has formed, and a few occur over unfractured bedrock. Some sites in Georgia have soils with shrink‑swell tendencies which allow for both wetness and extreme dryness and inhibit the survival of trees, promoting an open woodland structure (Edwards et al. 2013). Examples in North Carolina may lack trees in the deepest water parts of basins. A few occur over bedrock of other kinds. Rock chemistry affects soil chemistry and influences variation in vegetation, but hydroperiod is a more important influence.

**Vegetation:** Vegetation consists either of swamps dominated by wetland oaks, or of more open‑canopy pools with sparse trees and with substantial shrub or herbaceous vegetation. Swamps are most often dominated by *Quercus phellos*, with a substantial minority dominated by *Quercus lyrata* and a few having *Quercus bicolor, Quercus michauxii*, or other species. Examples that have been logged or cleared may be dominated by *Acer rubrum* or *Liquidambar styraciflua*. Lower strata are generally sparse in the swamps, often just a few shrubs such as *Vaccinium* spp., patches of *Smilax*, and a few wetland herbs. Open ponds may have the same canopy species on the edges, but a few have *Nyssa sylvatica* or other wetland species. The lower strata are better developed in the open pools, with *Cephalanthus occidentalis, Eubotrys racemosa (= Leucothoe racemosa), Vaccinium* spp., or other wetland species occurring as thickets along the edge or scattered in the interior. Large *Smilax* tangles sometimes occur. Herbs are usually still sparse or patchy, but may include dense beds of various graminoids or ferns, as well as scattered clumps. *Sphagnum* is sometimes extensive in parts of the pools. These isolated seasonal wetlands are often important breeding sites for amphibians.

**Dynamics:** The dynamics of water levels are the most important factor in these systems, differentiating them from the surrounding uplands and differentiating the various communities within the system. These wetlands typically have very small watersheds, and input of water comes largely from rainfall. Variation in rainfall patterns will drive variation in duration of flooding, though most upland depression swamps have an outlet that limits water depth. Fire may be naturally rare in some examples of these systems, such as in North Carolina. Though they could naturally be exposed to fires occurring in the surrounding uplands, standing water and lack of continuous fuel would limit fires to the edges, expect perhaps very rarely in early fall. Other examples in South Carolina and Georgia are naturally prone to drying out and to fire, and have an open woodland structure with a grassy understory, typical of wet flatwoods which naturally burned (Edwards et al. 2013). Presumably important as a dynamic process is the migration of amphibians, which concentrate in these systems for breeding. Ecosystem dynamics may be strongly affected by the suitability of surrounding uplands for amphibian adult habitat.

SOURCES

**References:** Comer et al. 2003*, Edwards et al. 2013, Eyre 1980, Nelson 1986, Peet et al. 2013, Schafale 2012, Schafale and Weakley 1990, Wharton 1978

**Version:** 14 Jan 2014	**Stakeholders:** East, Southeast

**Concept Author:** M. Schafale	**LeadResp:** Southeast

CES203.479 South‑Central Interior / Upper Coastal Plain Flatwoods

**Primary Division:** Gulf and Atlantic Coastal Plain (203)

**Land Cover Class:** Forest and Woodland

**Spatial Scale & Pattern:** Large patch

**Required Classifiers:** Natural/Semi‑natural; Vegetated (>10% vasc.); Upland; Wetland

**Diagnostic Classifiers:** Forest and Woodland (Treed); Pimple mounds; Extensive Wet Flat; Broad‑Leaved Deciduous Tree

**National Mapping Codes:** EVT 2326; ESLF 4132; ESP 1326

**Concept Summary:** This system represents mostly *Quercus stellata*‑dominated "xerohydric flatwoods" of limited flat areas of the most inland portions of the East Gulf Coastal Plain in western Kentucky, as well as in the nearby Shawnee Hills in the western Interior Low Plateau. The core of the area is referred to as the Jackson Purchase or "Jackson Plain." There is some local variability in the expression of this system along a hydrologic/microtopographic gradient. The elevated areas are composed of somewhat coarser‑textured soils and retain less moisture than do the lower areas, although both occur in a tight local mosaic. The soils appear to have well‑developed subsurface hardpans. Thus, soil moisture fluctuates widely throughout the growing season, from saturated to very dry.

**Comments:** The component associations are poorly known and described. More work is needed to clarify which types are present.

DISTRIBUTION

**Range:** This system occurs in limited areas of the most inland portions of the East Gulf Coastal Plain in western Kentucky and adjacent Tennessee (the "Jackson Purchase" or "Jackson Plain" region; 222Cb; 74b in part), as well as in the nearby "Shawnee Hills" of the Interior Low Plateau (222Dh, 222Di; 72c) of Kentucky and adjacent Indiana. The core of the area from which this system was initially described is referred to as the Jackson Purchase or "Jackson Plain," where these areas have long been recognized as a distinctive subdivision within this region (Davis 1923, Bryant and Martin 1988). It is known from the Clarks River National Wildlife Refuge (KSNPC 2009).

**Divisions:** 203:C

**TNC Ecoregions:** 43:C, 44:C

**Nations:** US

**Subnations:** IL?, IN, KY, TN

**Map Zones:** 46:P, 47:C, 49:?

**USFS Ecomap Regions:** 223D:CC, 223E:CC, 231H:CC

CONCEPT

**Environment:** The soils appear to have well‑developed subsurface hardpans, the impermeability of which contributes to shallowly perched water tables during portions of the year when precipitation is greatest and evapotranspiration is lowest (not due to overbank flooding). Thus, soil moisture fluctuates widely throughout the growing season, from saturated to very dry, a condition sometimes referred to as xerohydric (M. Evans pers. comm. 2006). Examples of this system occur along the northeastern flank of the Upper East Gulf Coastal Plain ecoregion where loess deposits thin out and gravelly or sandy soils predominate. Examples occur on relatively high flat areas that are not directly affected by overbank flooding. These environments include ancient Quaternary or Tertiary post‑glacial meltwater lakebeds and high terraces of the Upper Gulf Coastal Plain. The most typical soil is Okaw Silt Loam. The same system is found in the Shawnee Hills of Kentucky (M. Evans pers. comm. 2006). The lakes were originally formed by glacial damming of the Ohio River. It could also occur on upland plains and flat ridgetops (KSNPC 2009).

**Vegetation:** Stands of this system are dominated by *Quercus stellata*, a somewhat fire‑tolerant oak. In addition, *Quercus alba, Carya ovata, Carya glabra*, and *Quercus velutina* may be present. The presence of *Quercus falcata* indicates longer fire‑return times. The presence of *Quercus imbricaria* indicates that the stands were formerly more open. *Pinus* spp. are not prevalent in this area, but could invade from nearby plantations. Herbaceous cover is sparse to moderate; leaf litter is the dominant ground cover. Some shrubs include *Crataegus viridis, Ilex decidua*, and *Ulmus alata*. Characteristic grasses could include *Schizachyrium scoparium, Sorghastrum nutans*, and *Andropogon* spp. Some other typical herbs include *Manfreda virginica, Croton michauxii var. ellipticus (= Croton willdenowii), Danthonia spicata, Gillenia stipulata (= Porteranthus stipulatus)*, and *Pycnanthemum tenuifolium* (Hendricks et al. 1991). Lower areas (drainage ways and depressions) have *Quercus michauxii, Quercus pagoda, Quercus phellos, Liquidambar styraciflua*, or even *Taxodium distichum*. Local herb dominance in depressions is of wetland species such as *Juncus* spp. and *Carex* spp. For this related and possibly juxtaposed wetland vegetation, see South‑Central Interior / Upper Coastal Plain Wet Flatwoods (CES203.480).

**Dynamics:** Fire was an important natural process in this system, and well‑burned examples tend to be relatively open‑canopied with well‑developed herbaceous layers (M. Evans pers. comm. 2006). The natural dynamics of wetness and drought and the patchy variation in soil wetness probably led to patchy fires in this habitat. Due to subsurface hardpans, tree rooting is restricted which makes trees more prone to windthrow. High wind and ice storms contribute to forest openings (Landfire 2007a).

SOURCES

**References:** Bryant and Martin 1988, Comer et al. 2003*, Davis 1923, Evans et al. 2009, Evans, M. pers. comm., Eyre 1980, Hendricks et al. 1991, KSNPC 2009, LANDFIRE 2007a, NatureServe Ecology ‑ Southeastern U.S. unpubl. data, Nelson 2005

**Version:** 14 Jan 2014	**Stakeholders:** Midwest, Southeast

**Concept Author:** R. Evans and M. Evans	**LeadResp:** Southeast

CES203.480 South‑Central Interior / Upper Coastal Plain Wet Flatwoods

**Primary Division:** Gulf and Atlantic Coastal Plain (203)

**Land Cover Class:** Woody Wetland

**Spatial Scale & Pattern:** Large patch

**Required Classifiers:** Natural/Semi‑natural; Vegetated (>10% vasc.); Upland; Wetland

**Diagnostic Classifiers:** Forest and Woodland (Treed); Extensive Wet Flat; Broad‑Leaved Deciduous Tree

**National Mapping Codes:** EVT 2457; ESLF 9126; ESP 1457

**Concept Summary:** This system represents predominantly wet flatwoods of limited areas of the most inland portions of the East Gulf Coastal Plain in western Kentucky, as well as related broad, flat areas of the western Interior Low Plateau. This part of the Coastal Plain is referred to as the Jackson Purchase or "Jackson Plain." They tend to be confined to relatively small areas near the eastern flank of the region where loess deposits thin out. Unlike South‑Central Interior / Upper Coastal Plain Flatwoods (CES203.479) of the same general region (which is typified by complex microtopography), this system occupies broad flats underlain by fragipans. These fragipans impede the downward migration of water, resulting in wet conditions for portions of the year. Fire was important, probably maintaining relatively open‑canopied stands. Stands are dominated by hardwood trees, including *Acer rubrum, Fagus grandifolia, Liquidambar styraciflua, Quercus falcata, Quercus pagoda*, and *Quercus palustris*.

**Comments:** The primary range of this system is limited areas of the "Jackson Purchase" or "Jackson Plain" of Kentucky and possibly related areas in adjacent western Tennessee, as well as related broad, flat areas of the western Interior Low Plateau. According to Bryant and Martin (1988) the "Flatwoods" portion of the Jackson Purchase (which is primarily where the "Wet Flatwoods" are located in that area) occupies less than 2% of the total area, but localized occurrences could have been present in other parts of the region. These apparently related wet flatwoods in the western end of the Moulton Valley of Alabama are found in northeastern Franklin and extreme western Lawrence counties, from 10 to 20 km east of Russellville. More information is needed. In Alabama, this system is apparently found in the Moulton Valley region (A. Schotz pers. comm. 2006), which is technically part of TNC Ecoregion 50 but ambiguously placed there.

DISTRIBUTION

**Range:** The primary range of this system is limited areas of the "Jackson Purchase" or "Jackson Plain" of Kentucky and possibly related areas in adjacent western Tennessee, as well as related broad, flat areas of the western Interior Low Plateau. Examples in the Pennyroyal Plain (of the western Interior Low Plateau) have been known for many years and referred to as "pondywoods" or "crawfishy land" (Chester et al. 1995). They are also known from the Shawnee Hills of Kentucky, on periglacial lakebeds (M. Evans pers. comm. 2006), and related wet flatwoods have been discerned from wetland modeling and confirmed in the Moulton Valley of Alabama (A. Schotz pers. comm. 2006) and are included here. It is assumed to cross the Ohio River into adjacent Indiana.

**Divisions:** 203:C

**TNC Ecoregions:** 43:C, 44:C, 50:C

**Nations:** US

**Subnations:** AL, IL?, IN?, KY, TN

**Map Zones:** 46:P, 47:C, 48:C, 49:?

**USFS Ecomap Regions:** 223D:CC, 223E:CC, 223G:CC, 231B:CC, 231H:CC

CONCEPT

**Environment:** These flatwoods have long been recognized as the primary vegetation type of a distinctive subdivision within the Upper East Gulf Coastal Plain region (Davis 1923, Bryant and Martin 1988), as well as related areas of the western Interior Low Plateau. Within the "Jackson Plain" portion of the Upper East Gulf Coastal Plain, these flatwoods tend to be confined to relatively small areas near the eastern flank of the "Jackson Plain" region where the loess deposits thin out. Like drier *Quercus stellata* flatwoods of these areas (which are typified by microtopographic variation), this system occupies broad flats underlain by fragipans. These fragipans impede the downward migration of water resulting in wet conditions for longer portions of the year. In the Jackson Plain area the soils include Henry silt loam, Routon silt loam (Bryant and Held 2001) and Calloway silt loam (Karathanasis et al. 2003). Fire is probably relatively infrequent in this system (M. Evans pers. comm.). In the Pennyroyal Plain, this system occurs on upland flats and depressions with poor drainage, underlain by limestone; soils include Robertsville silt loam (Chester et al. 1995) and Henry silt loam (M. Evans pers. comm.).

**Vegetation:** Stands are typically dominated by various combinations of oaks and other hardwoods, including *Quercus pagoda, Quercus stellata, Carya ovata, Prunus serotina, Diospyros virginiana, Ulmus alata, Ulmus americana, Quercus palustris* (Bryant 1999), *Quercus michauxii, Liquidambar styraciflua, Carya* spp., *Nyssa sylvatica*, and *Acer rubrum* (Chester et al. 1995). Most stands of this system have been severely altered or destroyed, and the characteristic herbs are poorly known. *Campsis radicans* may be found, along with *Carex* spp., including *Carex leptalea* and *Carex cherokeensis*. Other herbs may include *Leersia* spp. and *Cardamine bulbosa*. *Quercus phellos* and/or *Quercus lyrata* may also be present in stands of this system in Kentucky (M. Evans pers. comm. 2006). Some stands placed here are dominated by *Quercus falcata* (e.g., at Shiloh National Military Park), others (e.g., in the Moulton Valley of Alabama) by a combination of *Quercus phellos* and *Quercus nigra* (A. Schotz pers. comm. 2006).

**Dynamics:** Fire was an important but relatively infrequent natural process in this system, probably maintaining relatively open‑canopied stands (M. Evans pers. comm.). Under such conditions *Andropogon gerardii* and *Chasmanthium* spp. may have dominated the herbaceous ground cover. Flooding and saturation are part of the natural dynamics. Due to the fragipan, deep rooting of trees is limited and the trees are particularly prone to windthrow during storms. This has helped maintain open woodland conditions.

SOURCES

**References:** Bryant 1999, Bryant and Held 2001, Bryant and Martin 1988, Chester et al. 1995, Comer et al. 2003*, Davis 1923, Evans et al. 2009, Evans, M. pers. comm., Eyre 1980, Hendricks et al. 1991, KSNPC 2009, Karathanasis et al. 2003, NRCS 1996, NatureServe Ecology ‑ Southeastern U.S. unpubl. data, Nelson 2010, Schotz pers. comm.

**Version:** 14 Jan 2014	**Stakeholders:** Midwest, Southeast

**Concept Author:** R. Evans and M. Evans	**LeadResp:** Southeast

M028. Great Plains Flooded & Swamp Forest

CES303.676 Northwestern Great Plains Floodplain

**Primary Division:** Western Great Plains (303)

**Land Cover Class:** Woody Wetland

**Spatial Scale & Pattern:** Linear

**Required Classifiers:** Natural/Semi‑natural; Vegetated (>10% vasc.); Wetland

**Concept Summary:** This ecological system is found in the floodplains of medium and large rivers of the northwestern Great Plains, ranging from the Dakotas Mixedgrass Prairie west through the Northern Great Plains Steppe and north into Canada. This system occurs in the upper Missouri River Basin and includes parts of the Niobrara, White, Cheyenne, Little Missouri, Yellowstone, Powder, Bighorn, Milk, and Musselshell rivers. Alluvial soils and periodic, intermediate flooding (every 5‑25 years) typify this system. These are the perennial big rivers of the region with hydrologic dynamics largely driven by snowmelt in the mountains, rather than local precipitation events. Dominant communities within this system range from floodplain forests to wet meadows to gravel/sand flats, however, they are linked by underlying soils and flooding regime. Dominant species are *Populus balsamifera ssp. trichocarpa* or *Populus deltoides* and *Salix* spp. *Fraxinus pennsylvanica, Salix amygdaloides*, and *Ulmus americana* are common in some stands. If present, common shrub species include *Amorpha fruticosa, Cornus drummondii, Cornus sericea, Symphoricarpos occidentalis, Salix exigua, Salix interior*, and *Salix planifolia*. Grass cover underneath the trees is an important part of this system and is a mix of cool‑season graminoid species, including *Carex pellita, Elymus lanceolatus, Pascopyrum smithii*, and *Schoenoplectus* spp., with warm‑season species such as *Panicum virgatum, Schizachyrium scoparium*, and *Spartina pectinata*. This system is often subjected to heavy grazing and/or agriculture and can be heavily degraded. In Montana, most occurrences are now degraded to the point where the cottonwood overstory is the only remaining natural component; undergrowth is dominated by *Bromus inermis*, or a complex of pasture grasses. Another factor is that groundwater depletion and lack of fire have created additional species changes. In most cases, the majority of the wet meadow and prairie communities may be extremely degraded or extirpated from the system.

**Comments:** This system needs to be more clearly delineated from Northwestern Great Plains Riparian (CES303.677). The component plant association list is incomplete. All the riparian/floodplain/alluvial systems of the Great Plains region need to be revisited for naming conventions, along with better definitions of conceptual boundaries. There is much apparent overlap in their concepts and distribution, and the names add to the confusion. In particular, the difference between "riparian" and "floodplain" usage in the names needs revisiting and possible changing. These systems include Northwestern Great Plains Floodplain (CES303.676), Northwestern Great Plains Riparian (CES303.677), Western Great Plains Floodplain (CES303.678), and Western Great Plains Riparian (CES303.956).

DISTRIBUTION

**Range:** This system is found in the northwestern Great Plains, north of the North Platte River through southern Canada. It is found in eastern Montana along the upper Missouri, Yellowstone, Bighorn, Milk, and Musselshell rivers; in northern Nebraska and the Dakotas on the Niobrara, upper Missouri, White, Cheyenne, and Little Missouri rivers; and in Canada on the Saskatchewan River.

**Divisions:** 205:P, 303:C

**TNC Ecoregions:** 26:C, 34:C, 66:P, 67:P

**Nations:** CA, US

**Subnations:** AB, MB, MT, ND, NE, SD, SK, WY?

**Map Zones:** 20:C, 29:C, 30:C, 31:C, 39:C, 40:C

**USFS Ecomap Regions:** 331D:C?, 331E:C?, 331F:CC, 331G:CP, 331K:CC, 331L:CC, 331M:CC

CONCEPT

**Environment:** This ecological system is found in the floodplains of medium and large rivers of the northwestern Great Plains, ranging from the Dakotas Mixedgrass Prairie west through the Northern Great Plains Steppe and north into Canada. Alluvial soils and periodic, intermediate flooding (every 5‑25 years) typify this system. These are the perennial big rivers of the region with hydrologic dynamics largely driven by snowmelt in the mountains, rather than local precipitation events. Dominant communities within this system range from floodplain forests to wet meadows to gravel/sand flats, however, they are linked by underlying soils and flooding regime.

**Vegetation:** Dominant species are *Populus balsamifera ssp. trichocarpa* or *Populus deltoides* and *Salix* spp. *Fraxinus pennsylvanica, Salix amygdaloides*, and *Ulmus americana* are common in some stands. If present, common shrub species include *Amorpha fruticosa, Cornus drummondii, Cornus sericea, Symphoricarpos occidentalis, Salix exigua, Salix interior*, and *Salix planifolia*. Grass cover underneath the trees is an important part of this system and is a mix of cool‑season graminoid species, including *Carex pellita (= Carex lanuginosa), Elymus lanceolatus, Pascopyrum smithii*, and *Schoenoplectus* spp., with warm‑season species such as *Panicum virgatum, Schizachyrium scoparium*, and *Spartina pectinata*.

**Dynamics:** This system is often subjected to heavy grazing and/or agriculture and can be heavily degraded. In Montana, most occurrences are now degraded to the point where the cottonwood overstory is the only remaining natural component; undergrowth is dominated by *Bromus inermis*, or a complex of pasture grasses. Another factor is that groundwater depletion and lack of fire have created additional species changes. In most cases, the majority of the wet meadow and prairie communities may be extremely degraded or extirpated from the system.

SOURCES

**References:** Comer et al. 2003*, Eyre 1980, Rice et al. 2012b, Rolfsmeier and Steinauer 2010, Shiflet 1994

**Version:** 23 Jan 2008	**Stakeholders:** Canada, Midwest, West

**Concept Author:** S. Menard and K. Kindscher	**LeadResp:** Midwest

CES303.677 Northwestern Great Plains Riparian

**Primary Division:** Western Great Plains (303)

**Land Cover Class:** Mixed Upland and Wetland

**Spatial Scale & Pattern:** Linear

**Required Classifiers:** Natural/Semi‑natural; Vegetated (>10% vasc.); Upland; Wetland

**Concept Summary:** This system is found in the riparian areas of medium and small rivers and streams throughout the northwestern Great Plains. It is likely most common in the Northern Great Plains Steppe. This system occurs in the Upper Missouri and tributaries starting at the Niobrara, White, Cheyenne, Belle Fourche, Moreau, Grand, Heart, Little Missouri, Yellowstone, Powder, Tongue, Bighorn, Wind, Milk, Musselshell, Marias, and Teton rivers; and in Canada, the Southern Saskatchewan, Red Deer and Old Man rivers to where they extend into Rocky Mountain Lower Montane‑Foothill Riparian Woodland and Shrubland (CES306.821) or Northern Rocky Mountain Lower Montane Riparian Woodland and Shrubland (CES306.804). These are found on alluvial soils in highly variable landscape settings, from deep cut ravines to wide, braided streambeds. Hydrologically, these tend to be more flashy with less developed floodplain than on larger rivers, and typically dry down completely for some portion of the year. Dominant vegetation shares much with generally drier portions of larger floodplain systems downstream, but overall abundance of vegetation is generally lower. Communities within this system range from riparian forests and shrublands to gravel/sand flats. Dominant species include *Populus deltoides, Populus balsamifera ssp. trichocarpa, Salix* spp., *Artemisia cana ssp. cana*, and *Pascopyrum smithii*. These areas are often subjected to heavy grazing and/or agriculture and can be heavily degraded. Another factor is that groundwater depletion and lack of fire have created additional species changes.

**Comments:** This system needs to be more clearly delineated from Northwestern Great Plains Floodplain (CES303.676). The component plant association list is incomplete. All the riparian/floodplain/alluvial systems of the Great Plains region need to be revisited for naming conventions, along with better definitions of conceptual boundaries. There is much apparent overlap in their concepts and distribution, and the names add to the confusion. In particular, the difference between "riparian" and "floodplain" usage in the names needs revisiting and possible changing. These systems include Northwestern Great Plains Floodplain (CES303.676), Northwestern Great Plains Riparian (CES303.677), Western Great Plains Floodplain (CES303.678), and Western Great Plains Riparian (CES303.956).

DISTRIBUTION

**Range:** This system occurs throughout the northwestern Great Plains, north of the North Platte River basin in eastern Wyoming. It is found in eastern Wyoming and eastern Montana along the upper Missouri, Yellowstone, Powder, Tongue, Bighorn, Wind, Milk, Musselshell, Marias, and Teton rivers; in northern Nebraska and the Dakotas on the Niobrara, upper Missouri, White, Cheyenne, Belle Fourche, Moreau, Grand, Heart, Little Missouri rivers; and in Canada the Southern Saskatchewan, Red Deer and Old Man rivers.

**Divisions:** 205:P, 303:C

**TNC Ecoregions:** 10:C, 26:C, 34:C, 66:P, 67:P

**Nations:** CA, US

**Subnations:** AB, MB, MT, ND, NE, SD, SK, WY

**Map Zones:** 20:C, 22:P, 29:C, 30:C, 31:C, 39:C, 40:C

**USFS Ecomap Regions:** 331D:CC, 331F:CP, 331G:CC, 331K:CC, 331L:CC, 331M:C?, 331N:CC, 342A:CC, 342F:CC, M334A:CC

CONCEPT

**Environment:** This system is found in the riparian areas of medium and small rivers and streams throughout the northwestern Great Plains. It is likely most common in the Northern Great Plains Steppe. Stands are found on alluvial soils in highly variable landscape settings, from deep cut ravines to wide, braided streambeds. Hydrologically, these tend to be more flashy with less developed floodplain than on larger rivers, and typically dry down completely for some portion of the year.

**Vegetation:** Dominant vegetation shares much with generally drier portions of larger floodplain systems downstream, but overall abundance of vegetation is generally lower. Communities within this system range from riparian forests and shrublands to gravel/sand flats. Dominant species include *Populus deltoides, Populus balsamifera ssp. trichocarpa, Salix* spp., *Artemisia cana ssp. cana*, and *Pascopyrum smithii*.

**Dynamics:** These areas are often subjected to heavy grazing and/or agriculture and can be heavily degraded. Another factor is that groundwater depletion and lack of fire have created additional species changes.

SOURCES

**References:** Comer et al. 2003*, Rolfsmeier and Steinauer 2010, Shiflet 1994

**Version:** 01 Oct 2007	**Stakeholders:** Canada, Midwest, West

**Concept Author:** S. Menard	**LeadResp:** West

CES303.678 Western Great Plains Floodplain

**Primary Division:** Western Great Plains (303)

**Land Cover Class:** Woody Wetland

**Spatial Scale & Pattern:** Linear

**Required Classifiers:** Natural/Semi‑natural; Vegetated (>10% vasc.); Wetland

**Concept Summary:** This ecological system is found in the floodplains of medium and large rivers of the western Great Plains. It occurs on the lower reaches of the North and South Platte, Platte, Arkansas, and Canadian rivers, among others. Alluvial soils and periodic, intermediate flooding (every 5‑25 years) typify this system. These are the perennial big rivers of the region with hydrologic dynamics largely driven by snowmelt in the mountains, instead of local precipitation events. Dominant communities within this system range from floodplain forests to wet meadows to gravel/sand flats; however, they are linked by underlying soils and the flooding regime. Dominant species include *Populus deltoides* and *Salix* spp. Grass cover underneath the trees is an important part of this system and is a mix of tallgrass species, including *Panicum virgatum* and *Andropogon gerardii*. Sometimes, *Tamarix* spp. and less desirable or exotic grasses and forbs can invade degraded areas within the floodplains, especially in the western portion of the province. These areas are often subjected to heavy grazing and/or agriculture and can be heavily degraded. Groundwater depletion and lack of fire have created additional alterations in species composition. In most cases, the majority of the wet meadow and prairie communities may be extremely degraded or extirpated from examples of this system.

**Comments:** All the riparian/floodplain/alluvial systems of the Great Plains region need to be revisited for naming conventions, along with better definitions of conceptual boundaries. There is much apparent overlap in their concepts and distribution, and the names add to the confusion. In particular, the difference between "riparian" and "floodplain" usage in the names needs revisiting and possible changing. These systems include Northwestern Great Plains Floodplain (CES303.676), Northwestern Great Plains Riparian (CES303.677), Western Great Plains Floodplain (CES303.678), and Western Great Plains Riparian (CES303.956). Need to review if there needs to be another split of this system into a Central Great Plains floodplain system and a Southern Great Plains floodplain system. Will need to review in conjunction with Northwestern Great Plains Floodplain (CES303.676).

DISTRIBUTION

**Range:** This system is found along major river floodplains in the southern and central portions of the Western Great Plains Division. This system occurs on the middle to lower reaches of the North and South Platte, Platte, Arkansas, and Canadian rivers, among others. Major river floodplains of eastern Wyoming and Montana are included in Northwestern Great Plains Floodplain (CES303.676) and not this system.

**Divisions:** 205:C, 303:C

**TNC Ecoregions:** 27:C, 28:C, 32:C, 33:C, 37:C

**Nations:** US

**Subnations:** CO, KS, NE, OK, TX

**Map Zones:** 22:C, 25:?, 26:C, 27:C, 28:P, 31:C, 32:C, 33:C, 34:C, 35:C, 36:C, 38:C, 43:C

**USFS Ecomap Regions:** 251B:CC, 251F:CP, 251H:CC, 315A:CC, 315B:CC, 315F:CC, 331B:CC, 331C:CC, 331H:CC, 331I:CC, 332B:CC, 332C:CC, 332D:CC, 332E:CC, 332F:CC, M331F:C?, M331I:C?

CONCEPT

**Environment:** This system is found primarily in Quaternary alluvium along floodplains of medium and large rivers. Soils are primarily alluvial and range from sandy to dense clays. This system occurs on valley floors of large rivers and perennial streams where significant alluvial deposition occurs, and tends to occupy broad valley bottoms with deep alluvial deposits. In Texas, this system is found within the Clear Fork of the Middle Brazos watersheds and occurs on Loamy Bottomland, Clayey Bottomland, and Draw ecoclasses. Broad alluvial deposits commonly occur and are generally mapped as bottomland soils (Elliott 2011). Water velocity and volume change greatly during the year as rains and snowmelt deliver pulses of water and seasonal droughts (typically including winter in the northern portion of this system's range when most precipitation is frozen) result in low water. Within a short distance on a river floodplain, different soil textures can be found. Within the space of a few years, floods of differing magnitude can deposit sand over silt or vice versa, resulting in complex soil topology.

**Vegetation:** This system is variable in its expression and can occur as various cover types, including forest, woodland, shrubland, as well as herbaceous vegetation. Herbaceous variants include marshes, which may develop in the floodplain soils, or mesic prairie dominated by *Andropogon gerardii* and *Panicum virgatum* which also may be conspicuous (Elliott 2011). *Populus deltoides, Sapindus saponaria var. drummondii, Prosopis glandulosa, Salix nigra, Ulmus americana*, and/or *Celtis laevigata* may be important components of forests or woodlands of this system. In parts of Texas, *Juniperus ashei, Juniperus pinchotii*, and/or *Quercus fusiformis* may be present to dominant (Elliott 2011). Species such as *Quercus fusiformis* and *Ulmus americana* occur in the system at the western edge of their range, and may not be represented further west within the range of the system. Shrubland examples may also have *Prosopis glandulosa* and *Salix nigra* as important components. Some woodlands and shrublands may be dominated by non‑native *Tamarix* spp., *Ulmus pumila*, or *Elaeagnus angustifolia*. Herbaceous vegetation may include marshes occupying floodplain sites, with species such as *Schoenoplectus* spp. and/or *Typha* spp. Some sites may be dominated by tallgrass species such as *Andropogon gerardii* and *Panicum virgatum*. Sparsely vegetated areas, such as gravel and sand flats, are also included within this system. In Texas, sites lacking significant woody cover may be dominated by *Pleuraphis mutica, Nassella leucotricha*, and *Panicum obtusum*. Non‑native graminoids are also commonly encountered and include *Cynodon dactylon, Sorghum halepense, Bromus arvensis*, and *Bothriochloa ischaemum var. songarica*.

**Dynamics:** Periodic and intermediate flooding (i.e., every 5‑25 years) constitutes the major process influencing this system. Flood frequency depends on precipitation patterns within the watershed and proximity to the main channel. Areas adjacent to the main channel or low islands within the channel are flooded most often, while areas further from the channel or on terraces may only be flooded once every several years. Free‑flowing rivers migrate across their floodplain, cutting new channels or eroding the bank on one side while building up the bank on the other, so the flooding regime of any one point in the floodplain will change over time. Flooding redeposits alluvium, eroding some areas and aggrading others, can bury or wash away small plants, and redistributes nutrients, especially in less frequently flooded zones where silt and clay tend to be deposited. These processes open up new areas for colonization. In the newly exposed or reworked areas, there is a common succession sequence of annual herbaceous species followed by shrub *Salix* spp., followed by *Populus deltoides* and *Salix amygdaloides*, followed by a number of trees, including *Acer negundo, Carya illinoinensis, Celtis laevigata, Celtis occidentalis, Fraxinus pennsylvanica*, and *Ulmus americana* (Bellah and Hulbert 1974). This sequence can be reset by major floods and erosion/deposition.

Fire could impact parts of this system. Most of the forests in this system were not fire‑prone due to the lack of litter, frequent flooding, and relatively protected landscape position in the river valley with wetlands often near, but forests on higher, coarser soils or wet‑mesic prairies on the margins of the floodplain could become dry in late summer and burn, if an ignition source was present.

SOURCES

**References:** Bellah and Hulbert 1974, Comer et al. 2003*, DeSantis et al. 2012, Elliott 2011, Elliott 2013, Eyre 1980, Herms et al. 2010, Johnson 1992, Lauver et al. 1999, Nelson 2010, Rolfsmeier and Steinauer 2010, Shiflet 1994

**Version:** 02 Oct 2014	**Stakeholders:** Midwest, Southeast, West

**Concept Author:** S. Menard and K. Kindscher	**LeadResp:** Midwest

CES303.956 Western Great Plains Riparian

**Primary Division:** Western Great Plains (303)

**Land Cover Class:** Mixed Upland and Wetland

**Spatial Scale & Pattern:** Linear

**Required Classifiers:** Natural/Semi‑natural; Vegetated (>10% vasc.); Upland; Wetland

**Diagnostic Classifiers:** Woody‑Herbaceous; Riverine / Alluvial; Very Short Disturbance Interval; Flood Scouring; Riparian Mosaic

**Concept Summary:** This ecological system is found in the riparian areas of medium and small rivers and streams throughout the western Great Plains. It is most common in the Shortgrass Prairie and extends west as far as the Rio Grande in New Mexico, north into the Wyoming Basins and east into southwestern Nebraska, western Kansas and panhandles of Oklahoma and Texas. It includes primarily small, often narrow feeder streams that originate on the plains. However, it also includes reaches of major rivers, including the North and South Platte, portions of the Arkansas, Cimarron, Canadian and upper Pecos rivers, that have relatively narrow floodplains when compared to the Platte, for example. This system is found on alluvial soils in highly variable landscape settings, from deep cut ravines to wide, braided streambeds. The smaller streams hydrologically tend to be flashy and may dry down completely for some portion of the year. Main‑stem larger rivers have a less well‑developed floodplain than their downstream counterparts (e.g., the Platte and Missouri rivers), that are classified as floodplain systems. Water sources for this riparian system include snowmelt runoff, springs and summer rains. This system includes numerous smaller prairie rivers and streams that are often groundwater‑fed, such as the Arikaree River and the Republican River. Dominant vegetation shares much with generally drier portions of larger floodplain systems downstream, but overall abundance of vegetation is generally lower. Communities within this system range from riparian forests and shrublands to herbaceous vegetation and gravel/sand bars. Dominant species include *Artemisia cana ssp. cana, Forestiera pubescens, Panicum obtusum, Panicum virgatum, Pascopyrum smithii, Populus deltoides, Salix amygdaloides, Salix exigua, Schizachyrium scoparium*, and *Sporobolus cryptandrus*. On the North Platte in southeastern Wyoming, *Fraxinus pennsylvanica* may be present to dominant. These areas are often subjected to heavy grazing and/or agriculture and can be heavily degraded. *Tamarix* spp., *Elaeagnus angustifolia*, and less desirable grasses and forbs can invade degraded examples up through central Colorado. Groundwater depletion and reduction in overbank flooding has resulted in additional species changes. In Texas, several cover types are represented within this system, including forest, woodland, shrubland, and herbaceous vegetation. Forests and woodlands may have species such *Populus deltoides, Salix nigra, Celtis laevigata, Juniperus ashei*, and *Juniperus pinchotii*. *Quercus fusiformis* occurs here at the western edge of its range but may be locally dominant. Shrubland portions are frequently dominated by *Prosopis glandulosa* but may also contain species such as *Salix nigra* and *Sapindus saponaria var. drummondii*. Herbaceous vegetation may be represented by marshes associated with small drainages and dominated by *Schoenoplectus* spp., *Eleocharis* spp., and other sedges.

**Comments:** All the riparian/floodplain/alluvial systems of the Great Plains region need to be revisited for naming conventions, along with better definitions of conceptual boundaries. There is much apparent overlap in their concepts and distribution, and the names add to the confusion. In particular, the difference between "riparian" and "floodplain" usage in the names needs revisiting and possible changing. These systems include Northwestern Great Plains Floodplain (CES303.676), Northwestern Great Plains Riparian (CES303.677), Western Great Plains Floodplain (CES303.678), and Western Great Plains Riparian (CES303.956).

DISTRIBUTION

**Range:** This system is found in riparian areas of medium and small rivers and streams throughout the western Great Plains. It is most common in the Central Shortgrass Prairie and Southern Shortgrass Prairie, but extends west as far as the Rio Grande in New Mexico and into the Wyoming Basins. This system occurs on the North Platte, South Platte, Cache La Poudre, Arkansas, Purgatoire, middle Rio Grande, the upper reaches of the Cimarron, Canadian, and Pecos rivers, and smaller prairie rivers and streams, such as the Arikaree and Republican rivers. Its occurrence is confirmed for Texas (Elliott 2011).

**Divisions:** 303:C, 304:P

**TNC Ecoregions:** 10:P, 26:C, 27:C, 28:C

**Nations:** US

**Subnations:** CO, KS, NE, NM, OK, TX, WY

**Map Zones:** 24:P, 25:C, 26:P, 27:C, 28:C, 32:C, 33:C, 34:C, 35:C

**USFS Ecomap Regions:** 313B:PP, 315A:CC, 315B:CC, 315H:CC, 321A:CC, 331B:CC, 331C:CC, 331E:CP, 331H:CC, 331I:CC, 331J:CC, 342A:PP, 342F:PP, M313A:C?, M313B:CC, M331B:CC, M331F:CC, M331G:CP, M331I:CC

CONCEPT

**Environment:** This riparian system lacks a broad, well‑developed floodplain. It includes primarily small, often narrow feeder streams that originate on the plains. However, it also includes reaches of major prairie rivers, including the North and South Platte, portions of the Arkansas, Cimarron, Canadian and upper Pecos rivers, that have relatively narrow, less well‑developed floodplain when compared to their downstream counterparts (e.g., the Platte and Missouri rivers) that are classified as floodplain systems. Water sources for this riparian system include snowmelt runoff, springs and summer rains. The substrates are highly variable depending on landscape settings that range from deep‑cut ravines to wide, braided streambeds, but tend to occur on relatively young alluvial substrates. In Texas this system occurs along headwater streams and generally occurs over upland soils that have developed in place over a variety of bedrock types, often limestone in parts of Texas (TPWD Phase 1). This system occurs along drainages that may be intermittent and tend to be dominated by erosional processes (as opposed to depositional processes) for example within the drainage of the Clear Fork of the Middle Brazos River of Texas (Elliott 2011). As this system is mapped by TPWD (Elliott 2011), it by definition occurs outside of areas mapped as bottomland soils. Soils are therefore mapped with soils of the surrounding uplands.

**Vegetation:** In Texas, the TPWD Phase 1 mapping represents the southeasternmost part of the range of the system and shares some similarity with Edwards Plateau Riparian (CES303.652) and Southeastern Great Plains Riparian Forest (CES205.709) to which it is adjacent. In the Phase 1 mapping project, this system is restricted to the drainages of the Clear Fork of the Middle Brazos River (Elliott 2011). Several cover types are represented within this system, including forest, woodland, shrubland, and herbaceous vegetation. Forests and woodlands may have species such *Populus deltoides, Salix nigra, Celtis laevigata, Juniperus ashei*, and *Juniperus pinchotii*. *Quercus fusiformis* occurs here at the western edge of its range but may be locally dominant (Elliott 2011). Shrubland portions are frequently dominated by *Prosopis glandulosa* but may also contain species such as *Salix nigra* and *Sapindus saponaria var. drummondii*. Herbaceous vegetation may be represented by marshes associated with small drainages and dominated by *Schoenoplectus* spp., *Eleocharis* spp., and other sedges. Grasslands associated with riparian corridors may also be present and will generally be somewhat more mesic than grasslands of the surrounding landscape; commonly encountered grasses include *Pleuraphis mutica, Nassella leucotricha, Bothriochloa laguroides ssp. torreyana*, and *Schizachyrium scoparium* (Elliott 2011, 2013). The non‑natives *Tamarix* spp., *Elaeagnus angustifolia*, and *Ulmus pumila* may also be commonly encountered in Texas examples of this system.

**Dynamics:** Hydrologically, these sites tend to have a more flashy flood regime hydrology and narrow, less well‑developed floodplains than those found on larger rivers, which are classified as floodplain systems. These streams may dry down completely for some portion of the year. Water sources for this riparian system are largely snowmelt runoff, springs and summer rains. This system includes numerous smaller prairie rivers and streams that are often also groundwater‑fed, such as the Arikaree River and the Republican River.

From CNHP (2010b): Fluvial processes such as channel narrowing, meandering, and flood deposition play a key role in the dynamics of Western Great Plains streams (Friedman et al. 1996, Scott et al. 1996). Various combinations of these three factors may be acting at any particular site, depending on geologic and climate factors, including flow variability, sediment load, and gradient. Channel narrowing results when the stream abandons a portion of the former channel bed or when flow ceases in a channel. Narrowing happens when a period low flow prevents the reworking of the entire channel bed, and allows vegetation to establish. Newly established vegetation reduces erosion and promotes the deposition of fine sediment. On meandering streams, cutbanks on the outside bends gradually erode and the sediments are deposited downstream as point bars on the insides of bends. Vegetation is able to establish on these newly created moist surfaces. Flood deposition can produce bare, moist surfaces for tree establishment that are above the normal channel bed, and protected from normal flow‑related disturbance.

Streamflows are highly variable in Western Great Plains streams. It is not known how much flows have changed since settlement, but a certain amount of intra‑ and inter‑annual variation appears to be normal (Matthews 1988). Nearly all prairie steams are susceptible to lack of water during some years, if not annually. Although most streams receive groundwater inflow, recharge to groundwater is low due to limited precipitation, and water loss to evapotranspiration can be significant. The minimal to moderate groundwater inflow and the large loss of both groundwater and surface water to evapotranspiration resulted in many high plains streams having little to no flow under presettlement conditions, except during spring floods (Covich et al. 1997). Since settlement, variation in water flow is regulated by dams and diversions, groundwater levels have been reduced, agricultural activities have increased siltation rates and introduced both non‑native species and chemical changes, and native grazers have been largely replaced by domestic cattle. Possible effects of altered fire regime in uplands are not known.

Additional factors affecting the dynamics of this system include drought and grazing. Riparian vegetation is affected by climatic drought that reduces soil moisture in the unsaturated zone and decreases streamflows, which reduces recharge and lowers the alluvial water table (Friedman et al. 1997). The elimination of beavers from most of the plains watersheds probably decreased water storage and increased variability in plains streams, although some of these changes were later reversed by dam construction (Friedman et al. 1997). The replacement of native grazers, especially bison, with fenced cattle has changed the regeneration patterns of cottonwood since settlement.

SOURCES

**References:** CNHP 2010, Carsey et al. 2003a, Comer et al. 2003*, Covich et al. 1997, Elliott 2011, Elliott 2013, Eyre 1980, Friedman et al. 1996, Friedman et al. 1997, Kittel et al. 1999b, Manci 1989, Matthews 1988, Muldavin et al. 2000a, Scott et al. 1996, Stromberg and Tellman 2009, TNC 2013

**Version:** 02 Oct 2014	**Stakeholders:** Canada, Midwest, Southeast, West

**Concept Author:** P. Comer, G. Kittel	**LeadResp:** West

M504. Laurentian‑Acadian‑North Atlantic Coastal Flooded & Swamp Forest

CES201.562 Acadian Sub‑boreal Spruce Flat

**Primary Division:** Laurentian‑Acadian (201)

**Land Cover Class:** Mixed Upland and Wetland

**Spatial Scale & Pattern:** Large patch

**Required Classifiers:** Natural/Semi‑natural; Vegetated (>10% vasc.); Upland; Wetland

**Diagnostic Classifiers:** Lowland; Forest and Woodland (Treed); Toeslope/Valley Bottom; Glaciated; Picea (glauca, mariana, rubens) ‑ Abies

**National Mapping Codes:** EVT 2465; ESLF 9134; ESP 1465

**Concept Summary:** These spruce‑fir forests are found in the colder regions of the northern Appalachians‑Acadian region, in areas of imperfectly drained soils where they often form extensive flats along valley bottoms. The nutrient‑poor acidic soils are typically saturated at snowmelt but are moderately well‑drained for much of the growing season and may be reasonably dry at the soil surface. The mostly closed‑canopy forests have *Picea rubens, Picea mariana*, and *Abies balsamea* as the dominant trees; other conifers are often present. Bryophytes are abundant in the ground layer; other layers are typically rather sparse. Many occurrences may be jurisdictional wetlands due to seasonal saturation, but the vegetation is primarily made up of upland or facultative species. The distribution in the Laurentian‑Acadian Division is mostly Canadian.

**Comments:** This might be considered as a component of Acadian Low‑Elevation Spruce‑Fir‑Hardwood Forest (CES201.565) but differs from that type *sensu stricto* in its hydrology (wetland vs. upland) and in that its range is somewhat more boreal. Alternatively, it shares some characteristics with Northern Appalachian‑Acadian Conifer‑Hardwood Acidic Swamp (CES201.574) but is more boreal in nature and appears to be typically not on consistently saturated soils. Information from Quebec and New Brunswick would be helpful in assessing its placement.

DISTRIBUTION

**Range:** This system is found in the northernmost parts of New England, north and east into Canada.

**Divisions:** 103:C, 201:C

**TNC Ecoregions:** 63:C

**Nations:** CA, US

**Subnations:** ME, NB, NH, NY, QC, VT

**Map Zones:** 64:C, 66:C

CONCEPT

SOURCES

**References:** Comer et al. 2003*, Edinger et al. 2014a, Eyre 1980, Gawler and Cutko 2010, Sperduto and Nichols 2004

**Version:** 29 Oct 2008	**Stakeholders:** Canada, East

**Concept Author:** S.C. Gawler	**LeadResp:** East

CES201.726 Great Lakes Wooded Dune and Swale

**Primary Division:** Laurentian‑Acadian (201)

**Land Cover Class:** Mixed Upland and Wetland

**Spatial Scale & Pattern:** Large patch

**Required Classifiers:** Natural/Semi‑natural; Vegetated (>10% vasc.); Upland; Wetland

**Diagnostic Classifiers:** Forest and Woodland (Treed); Dune (Substrate); Glaciated; Sand Soil Texture; 30‑180‑day hydroperiod; Coastal Dune Mosaic

**National Mapping Codes:** EVT 2466; ESLF 9135; ESP 1466

**Concept Summary:** This system is found in nearly 100 occurrences throughout the Great Lakes shorelines of the United States and Canada. It consists of a foredune, followed by a series of low to high dunes (uplands) and swales (wetlands). The system is often best developed where post‑glacial streams entered an embayment and provide a dependable sand source. The combination of along‑shore currents, waves, and winds form foredunes along the shoreline. The foredunes of most dune‑and‑swale complexes are commonly 1‑2 m high, with *Ammophila breviligulata, Calamovilfa longifolia, Salix serissima, Salix cordata*, and *Populus balsamifera* most common. The swale immediately behind the foredune is influenced by short‑term variation in lake levels and can be partially or occasionally completely filled by dune sands following major storm events. Species common to this first swale include *Juncus arcticus ssp. littoralis, Juncus pelocarpus, Juncus nodosus, Eleocharis acicularis*, and *Schoenoplectus americanus*. Occasionally, such swales may contain lake‑influenced, calcareous sands and may contain moderately alkaline indicators.

A low dune field with more advanced plant succession often follows the first open dunes and swales. *Pinus banksiana, Pinus strobus*, and *Pinus resinosa* often form a scattered overstory canopy, while *Juniperus communis, Juniperus horizontalis, Arctostaphylos uva‑ursi*, and *Koeleria macrantha* form a scattered ground layer. Following the dune‑field zone, both dunes and swales are typically forested. Moist swales are often forested, and soil organic material has often begun to accumulate. *Thuja occidentalis, Alnus incana, Salix* spp., and *Acer rubrum* dominate the partial overstory canopy and understory. In contrast to the dry or moist swales, wetter swales (where standing water is present through most of the year) may be dominated by Carices, such as *Carex aquatilis* and *Carex stricta*. Forested beach ridges, with soils of medium to course sand, tend to be dominated by species common to dry‑mesic and mesic northern forest. Complexes located in embayments protected from prevailing winds tend to be formed entirely of low, water‑lain beach ridges. As a result, even the beach ridges within these complexes support wetland vegetation.

Six major subtypes of Great Lakes Dune and Swale were described for Michigan, including the Lake Superior high dune type, the Lake Superior low dune type, the North Lake Michigan high dune type, Northern Lake Huron‑Lake Michigan low dune type, the Southern Lake Huron type, and the Northern Great Lakes low dune type. These subtypes represent patterns of floristic variation resulting from latitude and sand dune/beach ridge characteristics that constrain floristic and structural attributes. High dune types may support predominantly upland vegetation, while low dune types may support predominantly wetland vegetation.

**Comments:** Six major subtypes of Great Lakes Dune and Swale were described for Michigan, including the Lake Superior high dune type, the Lake Superior low dune type, the North Lake Michigan high dune type, Northern Lake Huron‑Lake Michigan low dune type, the Southern Lake Huron type, and the Northern Great Lakes low dune type. These subtypes represent patterns of floristic variation resulting from latitude and sand dune/beach ridge characteristics that constrain floristic and structural attributes.

This system has rather strong variation between northern and southern Great Lakes examples (north and south of Bailey's 210‑220 division line). Those occurring along the southern Lake Michigan shoreline of Indiana and Illinois have been altered significantly, but likely reflect a distinct ecological system type with oak woodland and savanna on beach ridges and wet prairie in swales.

DISTRIBUTION

**Range:** This system occurs throughout the Great Lakes shorelines of the United States and Canada. In Pennsylvania, this is only on Presque Isle.

**Divisions:** 201:C, 202:C

**TNC Ecoregions:** 48:C

**Nations:** CA, US

**Subnations:** IL, IN, MI, MN, NY, OH?, ON, PA, WI

**Map Zones:** 41:C, 49:C, 50:C, 51:C, 52:C, 62:C, 63:C, 64:C

**USFS Ecomap Regions:** 211Ee:PPP, 212Ha:CCC, 212Hf:CCC, 212Hl:CCC, 212J:CC, 212L:CC, 212Ra:CCC, 212Rc:CCC, 212Re:CCC, 212Sb:CCC, 212Sc:CCC, 212Sn:CCC, 212Sq:CCC, 212Te:CCC, 212Ya:CCC, 212Z:CC, 222Ib:CCP, 222Ie:CCC, 222Ud:CCC, 222Ue:CCC

CONCEPT

**Environment:** The system consists of a foredune, followed by a series of low to high dunes (uplands) and swales (wetlands). The system is often best developed where post‑glacial streams entered an embayment and provide a dependable sand source. The combination of along‑shore currents, waves, and winds form foredunes along the shoreline. With gradual long‑term drops in water level, combined with post‑glacial uplifting of the earth's crust, these low dunes gradually rise above the direct influence of the lakes, and new foredunes replace them. Over several thousand years, a series of ridges and swales is created. For most complexes, the flow of surface streams and groundwater maintain the wet conditions in the swales. With time, plant succession has proceeded to the point where the beach ridges are now forested while the wet swales are either forested or open wetlands. Along the Lake Superior shoreline, where post‑glacial uplift is greatest, many of the complexes consist primarily of dry, forested swales. The dunes and swales differs depending on fetch and the amount of sediment available. The influence of Great Lakes water‑level fluctuations is probably limited to the first few swales inland from the shoreline. For most of the complexes, the water occupying the swales comes from streams flowing from the adjacent uplands or from groundwater seepage.

**Vegetation:** The foredunes of most dune‑and‑swale complexes are commonly 1‑2 m high, with *Ammophila breviligulata, Calamovilfa longifolia, Salix serissima, Salix cordata*, and *Populus balsamifera* most common. The swale immediately behind the foredune is influenced by short‑term variation in lake levels and can be partially or occasionally completely filled by dune sands following major storm events. Species common to this first swale include *Juncus arcticus ssp. littoralis (= Juncus balticus), Juncus pelocarpus, Juncus nodosus, Eleocharis acicularis*, and *Schoenoplectus americanus (= Scirpus americanus)*. Occasionally, such swales may contain lake‑influenced, calcareous sands, and the shallow swale may contain moderately alkaline indicators, such as *Cladium mariscoides, Myrica gale, Dasiphora fruticosa ssp. floribunda (= Pentaphylloides floribunda)*, and others.

A low dune field with more advanced plant succession often follows the first open dunes and swales. *Pinus banksiana, Pinus strobus*, and *Pinus resinosa* often form a scattered overstory canopy, while *Juniperus communis, Juniperus horizontalis, Arctostaphylos uva‑ursi*, and *Koeleria macrantha* form a scattered ground layer.

Following the dune‑field zone, both dunes and swales are typically forested. Moist swales are often forested, and soil organic material has often begun to accumulate. *Thuja occidentalis, Alnus incana, Salix* spp., and *Acer rubrum* dominate the partial overstory canopy and understory. In contrast to the dry or moist swales, wetter swales (where standing water is present through most of the year) may be dominated by Carices, such as *Carex aquatilis* and *Carex stricta*.

Forested beach ridges, with soils of medium to course sand, tend to be dominated by species common to dry‑mesic and mesic northern forest. Soil moisture conditions appear to change dramatically with slight elevational changes and are reflected in the development of soil organic material and changing plant species. On higher, drier ridges, soils often have less than 3 cm of organic material. *Pinus resinosa, Pinus strobus*, and *Quercus rubra* are often codominant, while *Betula papyrifera, Populus grandidentata, Abies balsamea*, and *Acer rubrum* are subdominant or understory species. *Pteridium aquilinum, Gaylussacia baccata, Vaccinium myrtilloides, Cornus canadensis*, and *Gaultheria procumbens* occur in the shrub and ground layers.

Complexes located in embayments protected from prevailing winds tend to be formed entirely of low, water‑lain beach ridges. As a result, even the beach ridges within these complexes support wetland vegetation.

**Dynamics:** Foredune and immediate back dune areas are influenced by active dune processes of wind‑caused "blowouts" and subsequent restabilization. Forested beach ridges may support fire regimes characteristic of similar upland forest systems outside of these complexes. Due to lakeshore proximity, heavy winds and resultant windthrow are common in forested ridges. Great Lakes water‑level fluctuations likely influence water levels in swales closest to the shoreline, if at all. The hydrology of interdunal swales is driven largely by lateral flow through the porous beach ridges. Older swales (farthest from current lakeshores) in larger complexes support peat‑forming bogs.

SOURCES

**References:** Comer and Albert 1993, Comer et al. 2003*, Eyre 1980, Kost et al. 2007, Lichter 1998, MNFI 1999, ONHD unpubl. data, WDNR 2015

**Version:** 11 Apr 2007	**Stakeholders:** Canada, East, Midwest

**Concept Author:** P. Comer and D. Albert	**LeadResp:** Midwest

CES202.069 High Allegheny Wetland

**Primary Division:** Central Interior and Appalachian (202)

**Land Cover Class:** Woody Wetland

**Spatial Scale & Pattern:** Large patch

**Required Classifiers:** Natural/Semi‑natural; Vegetated (>10% vasc.); Wetland

**Diagnostic Classifiers:** Montane; Bog; Depression; Fen; Marsh; Swamp; Peat and mud; Temperate; Extensive Wet Flat; Depressional; Palustrine; Unglaciated; >180‑day hydroperiod; Bog and Fen Mosaic

**Concept Summary:** This system occurs along the high plateau of the Allegheny Mountains, immediately west of the Allegheny Front at elevations between 730 and 1430 m. Wetlands in this system are drained by low‑gradient, meandering, intermittent to small headwater streams. Drainage is impounded in high, flat‑lying basins by natural dams or "knickpoints" of resistant sandstone. In addition to poor moisture drainage, cold air drains from the surrounding uplands to pool in the flat basins, which function as frost pockets. Rainfall is plentiful, averaging about 1300 mm/year. Communities in this system may have substrates of shallow to deep peat or, less commonly, mineral soil. Soils are acidic to circumneutral. These high Allegheny wetlands form complex mosaics ranging in size from a few hectares to 6000 hectares. Forested swamps occupy the less disturbed margins or slightly higher "islands." This system has a distinctly northern character in its resemblance to bogs and swamps of New England. However, the striking absence of *Chamaedaphne calyculata* and *Picea mariana*, two abundant and common species of northern bogs and swamps, as well as the presence of species characteristic of the Southern Appalachians, such as *Hypericum densiflorum, Vaccinium erythrocarpum*, and *Rhododendron maximum*, distinguishes this system from its northern counterpart. Ombrotrophic bogs are rare but occur in undisturbed portions of a few of the larger wetlands. The more central, flood‑ or beaver‑influenced portions contain shrub swamps, sedge fens, wet meadows, and open marshes. Forested swamps are dominated by *Picea rubens*, with varying cover by *Acer rubrum, Tsuga canadensis*, and *Betula alleghaniensis var. alleghaniensis*. It is likely that the role of *Pinus strobus* played a greater role in the structure and function of this system historically than it does today (Maryland Geological Survey and Curran 1902). Residual white pines in remote areas of this system in Cranesville Swamp in Maryland also suggest this possibility. Where limestone or calcareous shale influences seepage water, *Abies balsamea* and *Fraxinus nigra* are typical canopy dominants. Common shrub species are *Viburnum nudum var. cassinoides, Rhododendron maximum, Vaccinium myrtilloides, Alnus incana ssp. rugosa, Hypericum densiflorum, Ilex verticillata*, and *Aronia melanocarpa*. Herbaceous species frequently include *Rubus hispidus, Solidago uliginosa, Juncus effusus, Eriophorum virginicum, Osmunda cinnamomea var. cinnamomea, Polygonum sagittatum, Carex folliculata, Carex gynandra, Leersia oryzoides, Galium tinctorium, Solidago rugosa, Symplocarpus foetidus, Lycopus uniflorus var. uniflorus, Scirpus cyperinus, Carex scoparia var. scoparia*, and *Carex trisperma var. trisperma*. *Sphagnum* spp. and *Polytrichum* spp. dominate the bryophyte layer. This system is maintained by a spatially complex mix of seepage, low‑energy flooding, beaver activity, and rainfall. Undisturbed examples exist (e.g., Cranberry Glades), where old‑growth swamp buffers the central peatlands, which have been dated to 10,000 years. In presettlement time, some wetland mosaics in this system had significant forested components (e.g., Canaan Valley, Cranesville Swamp in West Virginia; Finzel Swamp, Hammel Glades in Maryland), while others (e.g., Cranberry Glades, Big Run Bog) were largely open peatlands with forested swamp only on the margins.

DISTRIBUTION

**Range:** The system occurs in a southwest/northeast‑trending band about 40 km wide and 200 km long along the high, flat plateau of the Allegheny Mountains. The eastern boundary is the Allegheny Front, and the western boundary is the heavily dissected, lower elevation Allegheny Plateau. Minimum elevations range from 730 m in the north to 940 m in the south (Droop Mountain, West Virginia). The maximum elevation is 1422 m on Mount Porte Crayon, West Virginia.

**Divisions:** 202:C

**TNC Ecoregions:** 59:C

**Nations:** US

**Subnations:** MD, PA, VA?, WV

**Map Zones:** 61:C

**USFS Ecomap Regions:** M221Ba:CCC, M221Bd:CCC, M221Be:CCC, M221Bf:CCC

CONCEPT

**Environment:** This system occurs along the high plateau of the Allegheny Mountains, immediately west of the Allegheny Front at elevations between 610 and 1430 m. Wetlands in this system are drained by low‑gradient, meandering, intermittent to small streams that form the headwaters of larger (often high‑gradient) mountain rivers. The system is underlain by gently folded sedimentary rocks of Carboniferous and Devonian age. Drainage is impounded in high, flat‑lying basins by natural dams or "knickpoints" of resistant sandstone (Pottsville and Price formations). These sandstone layers come to the surface along the gently dipping axes of breached anticlines or synclines, or occasionally on the gently dipping limb of a fold. Cold air drains from the surrounding uplands to pool in the flat basins, which function as frost pockets. Rainfall is plentiful, averaging about 1300 mm/year. Communities in this system may have substrates of shallow to deep peat (a few centimeters to up to 3 m depth) or, less commonly, mineral soil. Soils are acidic to circumneutral, with pH ranging from 3.1 to 6.5. High values for soil organic matter, total exchange capacity, exchangeable nitrogen, soluble sulphur, and phosphorus are typical. Most soils are low in boron, copper, potassium, and manganese.

**Vegetation:** These High Allegheny wetlands form complex mosaics of small‑patch communities. Forested swamps occupy the less disturbed margins or slightly higher "islands" in the wetland mosaic. Ombrotrophic bogs are rare but occur in undisturbed portions of a few of the larger wetlands. The more central, flood‑ or beaver‑influenced sections contain shrub swamps, sedge fens, wet meadows, and open marshes. A number of species have northern affiliations, including some that are disjunct (e.g., *Abies balsamea, Larix laricina*, and *Andromeda polifolia var. glaucophylla*). The shrub strata include characteristic central Appalachian species (e.g., *Rhododendron maximum*), Appalachian endemic species (e.g., *Ilex collina*), and species with a more southern affiliation (e.g., *Vaccinium erythrocarpum*). Forested swamps are dominated by *Picea rubens*, with varying cover by *Acer rubrum, Tsuga canadensis*, and *Betula alleghaniensis var. alleghaniensis*. Where limestone or calcareous shale influences seepage water, *Abies balsamea* and *Fraxinus nigra* are typical canopy dominants. Common shrub species are *Viburnum nudum var. cassinoides, Rhododendron maximum, Vaccinium myrtilloides, Alnus incana ssp. rugosa, Hypericum densiflorum, Ilex verticillata, Aronia melanocarpa (= Photinia melanocarpa), Viburnum recognitum*, and *Kalmia latifolia*. Herbaceous species frequently include *Rubus hispidus, Solidago uliginosa, Juncus effusus, Eriophorum virginicum, Osmunda cinnamomea var. cinnamomea, Polygonum sagittatum, Carex folliculata, Carex gynandra, Leersia oryzoides, Galium tinctorium, Solidago rugosa, Symplocarpus foetidus, Lycopus uniflorus var. uniflorus, Scirpus cyperinus, Carex scoparia var. scoparia, Carex trisperma var. trisperma, Carex stipata*, and *Calamagrostis canadensis var. canadensis*. *Sphagnum* spp. and *Polytrichum* spp. dominate the bryophyte layer.

**Dynamics:** This system is maintained by a spatially complex mix of seepage, low‑energy flooding, beaver activity, and rainfall. Drainage in the flat headwater basins is partly impounded by resistant sandstone at the basin outlet. Low‑gradient, meandering headwater streams provide regular low‑energy inundation. Seepage from surrounding forests provides nutrients at the margins of the wetland mosaic, and where limestone or calcareous shale is present, circumneutral wetlands are maintained. Beaver activity encourages the cycling of early‑ to mid‑successional types. In the rare ombrotrophic bogs, rainfall is the only source of moisture. Many of the forested swamps in this system were logged during 1880‑1920, and some were subsequently burned and/or heavily grazed. Flat headwater basins function as frost pockets, catchment areas for cold air draining from surrounding uplands. Cool temperatures (mean annual temperature 6.7‑9.4 degrees C) and high rainfall (1220‑1680 mm/year) are characteristic. Floristic diversity is controlled by underlying sedimentary rocks that weather to form high diversity of nutrient, acidity, and drainage conditions (Byers et al. 2007).

SOURCES

**References:** Byers et al. 2007, Comer et al. 2003*, Darlington 1943, Edens 1973, Eyre 1980, Faber‑Langendoen et al. 2011, Furedi 2011e, Grafton and Eye 1982, Maryland Geological Survey and Curran 1902, McPherson 2011a, Robinette 1964, Walbridge 1982, Walbridge and Lang 1982, Wieder et al. 1981, Zimmerman et al. 2012

**Version:** 14 Jan 2014	**Stakeholders:** East, Southeast

**Concept Author:** E.A. Byers and S. Gawler	**LeadResp:** East

CES201.575 Laurentian‑Acadian Alkaline Conifer‑Hardwood Swamp

**Primary Division:** Laurentian‑Acadian (201)

**Land Cover Class:** Woody Wetland

**Spatial Scale & Pattern:** Large patch

**Required Classifiers:** Natural/Semi‑natural; Vegetated (>10% vasc.); Wetland

**Diagnostic Classifiers:** Depressional; Thuja occidentalis ‑ Fraxinus nigra; Mesotrophic Water; Circumneutral Water

**National Mapping Codes:** EVT 2481; ESLF 9345; ESP 1481

**Concept Summary:** These forested wetlands are found across northern New England and the upper Midwest and eastern to south‑central Canada in basins or floodplains where higher pH and/or nutrient levels are associated with a rich flora. The substrate is typically mineral soil, but there may be some peat; often, there is an organic epipedon over mineral soil. *Thuja occidentalis* is a diagnostic canopy species and may dominate the canopy or be mixed with other conifers or with deciduous trees, most commonly *Acer rubrum* or *Fraxinus nigra* but also *Tsuga canadensis, Larix laricina*, and *Betula alleghaniensis*. Some examples can be almost entirely deciduous and dominated by *Fraxinus nigra*. *Cornus sericea* is a common shrub. The herb layer tends to be more diverse than in acidic swamps. Small open fenny areas may occur within the wetland. Seepage may influence parts of the wetland, but the hydrology is dominated by the basin setting.

**Comments:** This system encompasses both wet forests (on saturated mineral soils) and forest rich peatlands. Areas dominated by *Fraxinus nigra* and found throughout the Laurentian area in Minnesota and north of Green Bay in Wisconsin are included in this system.

DISTRIBUTION

**Range:** Scattered locations from New England and adjacent Canada west to the Great Lakes and northern Minnesota.

**Divisions:** 201:C

**TNC Ecoregions:** 47:C, 48:C, 60:C, 61:C, 63:C, 64:C

**Nations:** CA, US

**Subnations:** CT, ME, MI, MN, NH, NY, ON, VT, WI

**Map Zones:** 40:C, 41:C, 50:C, 51:C, 63:C, 64:C, 65:C, 66:C

**USFS Ecomap Regions:** 211Aa:CCC, 211Ab:CCC, 211Ba:CCC, 211Bb:CCC, 211Ca:CCC, 211Cb:CCP, 211Ea:CCC, 211Eb:CCP, 211Ec:CCC, 211Ed:CCC, 211Ee:CCC, 211Fb:CCC, 211Ja:CCP, 211Jb:CCP, 211Jc:CCP, 211Jd:CCC, 212Ha:CCC, 212Hb:CCC, 212Hc:CCC, 212Hd:CCC, 212He:CCC, 212Hf:CCC, 212Hg:CCC, 212Hh:CCC, 212Hi:CCC, 212Hj:CCC, 212Hk:CCC, 212Hl:CCC, 212Hm:CCC, 212Ra:CCC, 212Rb:CCC, 212Rc:CCC, 212Rd:CCC, 212Re:CCC, 222Ib:CCC, 222Ic:CCC, 222Id:CCP, 222Ie:CCC, 222Ja:CCC, 222Ue:CCC, M211Aa:CCP, M211Ab:CCC, M211Ac:CCP, M211Ad:CCP, M211Ae:CCC, M211Af:CCC, M211Ba:CCC, M211Bb:CCP, M211Ca:CCC, M211Cb:CCC, M211Da:CCC, M211Db:CCC, M211Dc:CCC, M211Dd:CCC, M211De:CCC

CONCEPT

**Environment:** This system is typically found in basins or in floodplains with higher pH and/or nutrient levels. Groundwater typically keeps these sites saturated or nearly so through most of the growing season. Surface water, either overland flow or from nearby lakes and streams, often contributes to the hydrologic regime, especially through flooding in the spring or after heavy rains. Some movement of groundwater is important in maintaining the dominant trees in this system (Schwintzer 1981, Johnson and Booker 1983). Soils are mineral or muck (well‑decomposed peat) with sometimes a thin layer of peat over mineral soil. There is often pronounced microtopographic relief between hummock/mounds created by tree boles and roots and rotting fallen logs and small depressions. These provide different microhabitats and contribute to the diversity of the system.

**Dynamics:** Cold, nutrient‑rich and alkaline groundwater is important in maintaining this system. While water chemistry is similar to alkaline fens (Laurentian‑Acadian Alkaline Fen (CES201.585)), this is a treed conifer, conifer‑hardwood, or hardwood swamp versus a shrub‑ or graminoid‑dominated fen, implying other factors beyond just water chemistry are important in creating differing vegetation (Schwintzer and Tomberlin 1982). Other factors are likely hydrologic regime (length and degree of soil saturation), site history, and degree of water movement. Patchy windthrow creates small‑scale canopy gaps. These swamps often occur on structurally weak organic soils where trees root shallowly due to anaerobic conditions and are thus particularly susceptible to windthrow (Slaughter et al. 2007). Fire was very infrequent in this system but could occur in very dry periods. If other factors remain the same, this system could regenerate after fire since *Thuja occidentalis* and many other dominants grow well on exposed mineral soil (Johnson and Booker 1983). Beaver (*Castor canadensis*) flooding can also shape conifer‑hardwood swamp structure, species composition, and direct successional pathways.

SOURCES

**References:** Comer and Albert 1997, Comer et al. 1995a, Comer et al. 1998, Comer et al. 2003*, DeSantis et al. 2012, Edinger et al. 2014a, Eyre 1980, Faber‑Langendoen et al. 2011, Gawler and Cutko 2010, Herms et al. 2010, Johnson and Booker 1983, Kost et al. 2007, Rooney 2001, Rooney et al. 2002, Schwintzer 1981, Schwintzer and Tomberlin 1982, Slaughter et al. 2007, Sperduto and Nichols 2004, WDNR 2015, Ward et al. 2006

**Version:** 14 Jan 2014	**Stakeholders:** Canada, East, Midwest

**Concept Author:** S.C. Gawler	**LeadResp:** East

CES201.587 Laurentian‑Acadian Floodplain Forest

**Primary Division:** Laurentian‑Acadian (201)

**Land Cover Class:** Mixed Upland and Wetland

**Spatial Scale & Pattern:** Large patch

**Required Classifiers:** Natural/Semi‑natural; Vegetated (>10% vasc.); Upland; Wetland

**Diagnostic Classifiers:** Forest and Woodland (Treed); Shrubland (Shrub‑dominated); Woody‑Herbaceous; Herbaceous; Riverine / Alluvial; Flood Scouring; Short (<5 yrs) Flooding Interval [Short interval, Spring Flooding]

**Concept Summary:** This system encompasses north‑temperate floodplains in the northeastern and north‑central U.S. and adjacent Canada at the northern end of the range of silver maple. They occur along medium to large rivers where topography and process have resulted in the development of a complex of upland and wetland temperate alluvial vegetation on generally flat topography. This complex includes floodplain forests, with *Acer saccharinum* characteristic, as well as herbaceous sloughs and shrub wetlands. In areas subject to more scour, sparse non‑wetland vegetation may develop on sandbars or exposed rock. Most areas are underwater each spring; microtopography determines how long the various habitats are inundated. Associated trees include *Acer rubrum* and *Carpinus caroliniana*, the latter frequent but never abundant. On terraces or in more calcareous areas, *Acer saccharum* or *Quercus rubra* may be locally prominent, with *Betula alleghaniensis* and *Fraxinus* spp. *Salix nigra* is characteristic of the levees adjacent to the channel. Common shrubs include *Cornus amomum* and *Viburnum* spp. The herb layer in the forested portions often features abundant spring ephemerals, giving way to a fern‑dominated understory in many areas by mid‑summer. Non‑forested wetlands associated with these systems include shrub‑dominated and graminoid‑herbaceous vegetation.

**Comments:** These floodplains are similar to those to the south in the Central Interior, North‑Central Interior Floodplain (CES202.694) and Appalachian Division, Central Appalachian River Floodplain (CES202.608) in having *Acer saccharinum* as a characteristic species; however, they are generally more depauperate and lack certain tree species that characterize central Appalachian floodplains such as *Platanus occidentalis, Betula nigra*, and *Quercus palustris*. This system can include areas of scour along sandbars or rivershore rock outcrops as well as the more typical floodplain vegetation.

DISTRIBUTION

**Range:** Central and northern New England and adjacent Canada west to the Great Lakes.

**Divisions:** 103:C, 201:C

**TNC Ecoregions:** 47:C, 48:C, 61:C, 63:C, 64:C

**Nations:** CA, US

**Subnations:** MA?, ME, MI, MN, NB, NH, NY, VT, WI

**Map Zones:** 41:C, 50:C, 51:C, 63:C, 64:C, 65:C, 66:C

**USFS Ecomap Regions:** 211A:CC, 211B:CC, 211C:CC, 211D:CC, 211E:CC, 211Ia:C??, 211Jb:CCC, 211Jc:CCC, 212H:CC, 212J:CC, 212K:CC, 212L:CC, 212M:CC, 212N:CC, 212Q:CC, 212Ra:CCC, 212Rb:CCP, 212Rc:CCC, 212Rd:CCC, 212Re:CCC, 212Sb:CCP, 212Sc:CCP, 212Sn:CCC, 212Sq:CCP, 212Tb:CCP, 212Tc:CCC, 212Te:CCC, 212Xb:CCP, 212Xc:CCP, 212Xq:CCC, 212Ya:CCC, 212Z:CC, 222N:CC, 251A:CC, M211A:CC, M211B:CC, M211C:CC, M211D:CC

CONCEPT

SOURCES

**References:** Comer et al. 2003*, Edinger et al. 2014a, Eyre 1980, Gawler and Cutko 2010, Kost et al. 2007, Sperduto and Nichols 2004, WDNR 2015

**Version:** 05 Jun 2008	**Stakeholders:** Canada, East, Midwest, Southeast

**Concept Author:** S.C. Gawler	**LeadResp:** East

CES202.604 North‑Central Appalachian Acidic Swamp

**Primary Division:** Central Interior and Appalachian (202)

**Land Cover Class:** Woody Wetland

**Spatial Scale & Pattern:** Large patch

**Required Classifiers:** Natural/Semi‑natural; Vegetated (>10% vasc.); Wetland

**Diagnostic Classifiers:** Forest and Woodland (Treed); Extensive Wet Flat; Needle‑Leaved Tree; 30‑180‑day hydroperiod

**Concept Summary:** These swamps are distributed from central New England through the Central Appalachians south to Virginia and west to Ohio. They are found at low to mid elevations (generally <700 m) in basins or on gently sloping seepage lowlands. The acidic substrate is mineral soil, often with a component of organic muck; if peat is present, it usually forms an organic epipedon over the mineral soil rather than a true peat substrate (although peat layers up to 1 m deep have been found in some of these swamps). *Tsuga canadensis* is usually present and may be dominant. It is often mixed with deciduous wetland trees such as *Acer rubrum* or *Nyssa sylvatica*. *Sphagnum* is an important component of the bryoid layer. Basin swamps tend to be more nutrient‑poor and less species‑rich than seepage swamps; in some settings, the two occur adjacent to each other with the basin swamp vegetation surrounded by seepage swamp vegetation on its upland periphery.

**Comments:** This system excludes swamps with *Chamaecyparis thyoides*, a tree more characteristic of the Coastal Plain but which sometimes occurs inland. See Northern Atlantic Coastal Plain Basin Peat Swamp (CES203.522). Some examples of this system may appear similar to Southern and Central Appalachian Bog and Fen (CES202.300) or North‑Central Interior and Appalachian Acidic Peatland (CES202.606); those systems are distinguished by their deeper peat substrate and overall partly forested character compared to the shallower organic soil and generally forested nature of the present system. Wetlands on the Allegheny Plateau, at higher elevations, are a distinct system, High Allegheny Wetland (CES202.069). There are many species with this type, but it is distinguished by occurring as a mosaic of open wetlands and smaller forest patches with a distinctive hydrology.

DISTRIBUTION

**Range:** This system occurs from central New England south to western Virginia (the Central Appalachians region) and west to Ohio.

**Divisions:** 202:C

**TNC Ecoregions:** 49:C, 52:C, 59:C, 60:C, 61:C, 63:C

**Nations:** US

**Subnations:** CT, MA, MD, NH, NJ, NY, OH, PA, RI, VA, VT

**Map Zones:** 53:C, 60:C, 61:C, 62:C, 63:C, 64:C, 65:C, 66:P

**USFS Ecomap Regions:** 211E:CP, 211F:CC, 211G:CC, 211I:CC, 211J:CC, 221A:CC, 221B:CC, 221D:CC, 222I:CC, M211A:CP, M211B:CC, M211C:CC, M221A:CC

CONCEPT

**Environment:** These swamps are found at low to mid elevations (generally <700 m) in basins or on gently sloping seepage lowlands. The acidic substrate is mineral soil, often with a component of organic muck; if peat is present, it usually forms an organic epipedon over the mineral soil rather than a true peat substrate (although peat layers up to 1 m deep have been found in some of these swamps).

**Vegetation:** *Tsuga canadensis* is usually present and may be dominant. It is often mixed with deciduous wetland trees such as *Acer rubrum* or *Nyssa sylvatica*. Other trees that may be present include *Abies balsamea, Betula alleghaniensis, Fraxinus americana, Fraxinus pennsylvanica, Picea rubens*, and *Tsuga canadensis*. Shrubs may include *Clethra alnifolia, Ilex verticillata, Kalmia latifolia, Lindera benzoin, Ilex mucronata (= Nemopanthus mucronatus), Rhododendron maximum, Rhododendron viscosum*, and *Vaccinium corymbosum*. Forbs and graminoids may include *Carex lacustris, Carex scabrata, Carex stricta, Eriophorum virginicum, Oclemena acuminata, Onoclea sensibilis, Osmunda cinnamomea, Symplocarpus foetidus*, and *Veratrum viride*. *Sphagnum* is an important component of the bryoid layer. This system excludes swamps with *Chamaecyparis thyoides*, a tree more characteristic of the Coastal Plain but which sometimes occurs inland.

SOURCES

**References:** Comer et al. 2003*, Edinger et al. 2014a, Eyre 1980, ONHD unpubl. data, Sperduto and Nichols 2004, Zimmerman et al. 2012

**Version:** 02 Jan 2015	**Stakeholders:** East, Midwest, Southeast

**Concept Author:** S.C. Gawler	**LeadResp:** East

CES201.574 Northern Appalachian‑Acadian Conifer‑Hardwood Acidic Swamp

**Primary Division:** Laurentian‑Acadian (201)

**Land Cover Class:** Woody Wetland

**Spatial Scale & Pattern:** Large patch

**Required Classifiers:** Natural/Semi‑natural; Vegetated (>10% vasc.); Wetland

**Diagnostic Classifiers:** Extensive Wet Flat; Picea (rubens, mariana) ‑ Acer rubrum; Acidic Water

**Concept Summary:** These forested wetlands are found in temperate northeastern and north‑central U.S., primarily in glaciated regions in the eastern Laurentian‑Acadian region. They occur on mineral soils that are nutrient‑poor; there may be an organic epipedon, but the substrate is generally not deep peat. These basin wetlands remain saturated for all or nearly all of the growing season, and may have standing water seasonally. There may be some seepage influence, especially near the periphery. *Acer rubrum, Fraxinus* spp., *Picea rubens* (rarely *Picea mariana*), and *Abies balsamea* are the most typical trees. The herbaceous and shrub layers tend to be fairly species‑poor. *Ilex mucronata (= Nemopanthus mucronatus)* and *Osmunda* spp. are typical shrub and herb species.

**Comments:** Acadian Sub‑boreal Spruce Flat (CES201.562) is related but is more northern and occurs on imperfectly drained but not persistently saturated soils. *Picea rubens* in the East versus *Picea mariana* in the West and North may be helpful in distinguishing between this type and the more boreal acidic swamp, Boreal‑Laurentian Conifer Acidic Swamp and Treed Poor Fen (CES103.724). This type is distributed in the Acadian and Northern Appalachian region of the U.S. and Canada, whereas Eastern Boreal‑Sub‑boreal Conifer Acidic Swamp and Treed Poor Fen (CES103.724) is found in the Upper Great Lakes region and into Canada. The attribution of CEGL006380 to this system is questionable given that it is a seepage wetland, not a basin swamp association, but is included because portions of these wetlands may have seepage influence.

DISTRIBUTION

**Range:** This system occurs in New England and adjacent Canada west through New York. Occurrences in Massachusetts, Connecticut, and Pennsylvania are at higher elevations and peripheral to the range.

**Divisions:** 201:C

**TNC Ecoregions:** 48:C, 60:C, 61:C, 63:C

**Nations:** CA, US

**Subnations:** CT, MA, ME, NB, NH, NY, ON, PA, VT

**Map Zones:** 63:C, 64:C, 65:C, 66:C

**USFS Ecomap Regions:** 211A:CC, 211B:CC, 211C:CC, 211D:CC, 211I:CC, 211J:CC, 221A:CC, 221B:CC, M211A:CC, M211D:CC

CONCEPT

SOURCES

**References:** Comer et al. 2003*, Edinger et al. 2014a, Eyre 1980, Gawler and Cutko 2010, Golet et al. 1993, Sperduto and Nichols 2004

**Version:** 05 May 2008	**Stakeholders:** Canada, East, Midwest

**Concept Author:** S.C. Gawler and D. Faber‑Langendoen	**LeadResp:** East

CES203.522 Northern Atlantic Coastal Plain Basin Peat Swamp

**Primary Division:** Gulf and Atlantic Coastal Plain (203)

**Land Cover Class:** Woody Wetland

**Spatial Scale & Pattern:** Large patch

**Required Classifiers:** Natural/Semi‑natural; Vegetated (>10% vasc.); Wetland

**Concept Summary:** This system comprises acidic peat swamps formed in basins of various sizes, predominantly Atlantic white‑cedar swamps, occurring on the northern portion of the Atlantic Coastal Plain from Massachusetts south to Virginia. The hydrology is saturated, as evidenced by *Sphagnum*‑dominated hummock‑and‑hollow microtopography. *Chamaecyparis thyoides* is characteristic and often dominant. *Acer rubrum* may also be an important species, especially after logging.

**Comments:** Atlantic white‑cedar swamps do occur inland of the coastal plain and are considered inland disjuncts of this type. Where *Chamaecyparis* is present but not dominant inland, and other coastal plain indicators are absent, North‑Central Appalachian Acidic Swamp (CES202.604) is the more appropriate system.

DISTRIBUTION

**Range:** This system occurs on the northern portion of the Atlantic Coastal Plain from Massachusetts south to Virginia, with sporadic occurrences north to mid‑coast Maine, and occasional disjunct occurrences inland; it is historic in eastern Pennsylvania.

**Divisions:** 201:C, 202:C, 203:C

**TNC Ecoregions:** 58:C, 60:P, 61:C, 62:C, 63:C

**Nations:** US

**Subnations:** CT, DE, MA, MD, ME, NH, NJ, NY, VA

**Map Zones:** 60:C, 65:C, 66:C

**USFS Ecomap Regions:** 211Da:CCC, 221Ad:CCC, 221Ae:CCC, 221Ah:CCC, 221Ai:CCC, 221Ak:CCC, 221Al:CCC, 232A:CC, 232H:CC, M211Bc:CCC

CONCEPT

**Environment:** Topographic depression.

**Dynamics:** Seasonal to saturated hydrology.

SOURCES

**References:** Comer et al. 2003*, Edinger et al. 2014a, Eyre 1980, Gawler and Cutko 2010, Sperduto and Nichols 2004

**Version:** 14 Jan 2014	**Stakeholders:** East, Southeast

**Concept Author:** R. Evans	**LeadResp:** East

CES203.520 Northern Atlantic Coastal Plain Basin Swamp and Wet Hardwood Forest

**Primary Division:** Gulf and Atlantic Coastal Plain (203)

**Land Cover Class:** Woody Wetland

**Spatial Scale & Pattern:** Large patch

**Required Classifiers:** Natural/Semi‑natural; Vegetated (>10% vasc.); Wetland

**Diagnostic Classifiers:** Forest and Woodland (Treed); Seepage‑Fed Sloping; North Atlantic Coastal Plain

**Concept Summary:** This system comprises nonriverine hardwood swamps of seasonally flooded habitats, including relatively shallow groundwater‑influenced depressions and Coastal Plain terraces. It ranges from the southern glaciated Atlantic Plain of Long Island, New York, south along the northern Coastal Plain to Virginia. Although supporting some seepage indicators, it is also affected by overland flow. The substrate is mineral soil overlain by a variable organic but non‑peaty layer. Characteristic tree species include *Acer rubrum, Liquidambar styraciflua, Nyssa sylvatica, Quercus michauxii, Quercus pagoda, Quercus palustris*, and *Quercus phellos*. *Pinus taeda* is not uncommon south of Delaware Bay.

**Comments:** Vegetation along streams is accommodated in Northern Atlantic Coastal Plain Riparian and Floodplain (CES203.070).

DISTRIBUTION

**Range:** It ranges from Long Island, New York, south to Virginia.

**Divisions:** 203:C

**TNC Ecoregions:** 58:C, 62:C

**Nations:** US

**Subnations:** DE, MD, NH, NJ, NY, PA, VA

**Map Zones:** 60:C, 65:C

**USFS Ecomap Regions:** 221An:CCC, 232A:CC, 232H:CC, 232L:CC

CONCEPT

**Environment:** This system occurs in low‑lying areas, such as stream headwaters or depressions, or along water courses.

**Vegetation:** Characteristic tree species include *Acer rubrum, Liquidambar styraciflua, Nyssa sylvatica, Quercus phellos*, and *Fraxinus pennsylvanica*. *Pinus taeda* is not uncommon south of Delaware Bay.

**Dynamics:** This system occurs on extensive, flat terraces and very wide, ancient floodplains that are no longer subject to alluvial processes. Its hydrology is seasonally to nearly permanently saturated, with occasional ponding or groundwater sheetflows, and is maintained by a high water table rather than riverine or estuarine flooding.

SOURCES

**References:** Comer et al. 2003*, Edinger et al. 2014a, Eyre 1980, Faber‑Langendoen et al. 2011, Metzler and Barrett 2006, NYNHP 2013c, Rhoads and Block 2011d, Sperduto and Nichols 2004, Stevens 1992, Zimmerman et al. 2012

**Version:** 14 Jan 2014	**Stakeholders:** East, Southeast

**Concept Author:** R. Evans	**LeadResp:** East

CES203.374 Northern Atlantic Coastal Plain Pitch Pine Lowland

**Primary Division:** Gulf and Atlantic Coastal Plain (203)

**Land Cover Class:** Woody Wetland

**Spatial Scale & Pattern:** Large patch

**Required Classifiers:** Natural/Semi‑natural; Vegetated (>10% vasc.); Wetland

**National Mapping Codes:** EVT 2456; ESLF 9125; ESP 1456

**Concept Summary:** This system comprises wetland pine barrens vegetation and coastal plain peatlands from the New Jersey Pine Barrens south into the Delmarva Peninsula and upper Chesapeake Bay. Although this system can be extensive, components often co‑occur as a mosaic with upland pine barrens vegetation as well. The vegetation is characterized by associations having variable hydroperiods, occurring on a range of substrates from saturated deep peats to seasonally saturated mineral soils. Physiognomy of the component associations is similarly widely variable, ranging from wet grasslands dominated by *Calamovilfa brevipilis*, to boggy shrublands characterized by *Gaylussacia dumosa, Chamaedaphne calyculata, Eubotrys racemosa*, and others, to seasonally saturated pine forests characterized by mesic species such as *Clethra alnifolia*. Fire frequency, as well as hydrology, has a profound influence on the vegetation. Where fire frequency is high, woody vegetation is impeded, favoring the development of large wet grasslands.

**Comments:** Ponded wetlands with standing water (which may drop over the course of the season) and mineral soils are treated as Northern Atlantic Coastal Plain Pond (CES203.518).

DISTRIBUTION

**Range:** This system is best developed in the New Jersey Pine Barrens, but occurrences are present south to the Inner Coastal Plain of Maryland.

**Divisions:** 203:C

**TNC Ecoregions:** 58:C, 62:C

**Nations:** US

**Subnations:** DE?, MD, NJ

**Map Zones:** 60:C

**USFS Ecomap Regions:** 232A:CC, 232Hb:CCC, 232Hc:CCC, 232Hd:CCC

CONCEPT

**Environment:** This system occurs within the larger matrix of pitch pine ‑ scrub oak barrens of the New Jersey Pinelands. Hydrology is primarily groundwater‑controlled; vegetation composition is a reflection of depth to water table.

**Vegetation:** The vegetation is characterized by associations having variable hydroperiods, occurring on a range of substrates from saturated deep peats to seasonally saturated mineral soils. Physiognomy of the component associations is similarly widely variable, ranging from wet grasslands dominated by *Calamovilfa brevipilis*, to boggy shrublands characterized by *Gaylussacia dumosa, Chamaedaphne calyculata, Eubotrys racemosa (= Leucothoe racemosa)*, and others, to seasonally saturated pine forests characterized by mesic species such as *Clethra alnifolia*.

**Dynamics:** This system and the composition and structure of its mosaic of patch types are influenced by depth to water table (Ehrenfeld 1986). Pitch pines are also structured by fires, but fire regime differs from uplands in that in the wet environment, fire frequency is lower, but the high shrub density often leads to crown fires. In high‑intensity fires, pitch pines are killed, and even the organic layer may be consumed during periods of drought. Successional pathways following fire depend on depth of remaining organic layer and proximity of seed source (Little 1979c).

SOURCES

**References:** Comer et al. 2003*, Ehrenfeld 1986, Eyre 1980, Faber‑Langendoen et al. 2011, Little 1979c, Zampella et al. 1992

**Version:** 14 Jan 2014	**Stakeholders:** East, Southeast

**Concept Author:** R. Evans and L. Sneddon	**LeadResp:** East

CES203.070 Northern Atlantic Coastal Plain Riparian and Floodplain

**Primary Division:** Gulf and Atlantic Coastal Plain (203)

**Land Cover Class:** Mixed Upland and Wetland

**Spatial Scale & Pattern:** Linear

**Required Classifiers:** Natural/Semi‑natural; Vegetated (>10% vasc.); Upland; Wetland

**Diagnostic Classifiers:** Riverine / Alluvial

**Concept Summary:** This ecological system is found throughout the northern Atlantic Coastal Plain, ranging from Virginia to New Jersey. Examples occur along low‑gradient streams and rivers. Floodplain development varies from little to moderate according to stream size. This system is influenced by overbank flooding, groundwater seepage and occasional beaver impoundments. The vegetation is a mosaic of forests, woodlands, shrublands, and herbaceous communities. Canopy composition and cover can vary within and among examples of this system, but typical tree species may include *Quercus palustris, Quercus phellos, Chamaecyparis thyoides, Acer rubrum, Fraxinus pennsylvanica, Nyssa sylvatica, Betula nigra, Liquidambar styraciflua*, and *Platanus occidentalis*. Shrubs and herbaceous layers can vary in richness and cover. Some characteristic shrubs may include *Alnus maritima, Carpinus caroliniana, Lindera benzoin*, and *Viburnum nudum*. Seepage forests dominated by *Acer rubrum* and *Magnolia virginiana* can often be found within this system, especially at the headwaters and terraces of streams.

**Comments:** New Jersey's Pine Barrens riverside savannas, not covered in other ecological systems, fit this concept and are explicitly included, pending further review.

DISTRIBUTION

**Range:** This system occurs on the mid‑Atlantic Coastal Plain from Virginia to New Jersey.

**Divisions:** 203:C

**TNC Ecoregions:** 57:C, 58:C, 62:C

**Nations:** US

**Subnations:** DE, MD, NJ, VA

**Map Zones:** 60:C

**USFS Ecomap Regions:** 232A:CC, 232H:CC

CONCEPT

**Environment:** This system occurs on Coastal Plain flood terraces of streams and rivers, and is also influenced by groundwater seepage. In New Jersey, this system occurs in the Pine Barrens matrix.

**Dynamics:** This system is hydrologically influenced primarily by groundwater seepage, but is also subjected to periodic overbank flooding. The system is maintained by a natural disturbance regime of flooding and periodic fires of varied intensity. High‑intensity fires may consume peat and limit re‑establishment of Atlantic white‑cedar.

SOURCES

**References:** Comer et al. 2003*, Eyre 1980, Faber‑Langendoen et al. 2011, Laderman 1989, Smith 2012, Strakosch‑Walz 2004, Wacker 1979, Walz et al. 2006c

**Version:** 14 Jan 2014	**Stakeholders:** East, Southeast

**Concept Author:** NCR Review Team	**LeadResp:** East

CES203.282 Northern Atlantic Coastal Plain Tidal Swamp

**Primary Division:** Gulf and Atlantic Coastal Plain (203)

**Land Cover Class:** Woody Wetland

**Spatial Scale & Pattern:** Small patch

**Required Classifiers:** Natural/Semi‑natural; Vegetated (>10% vasc.); Wetland

**Diagnostic Classifiers:** Forest and Woodland (Treed); Tidal / Estuarine

**Concept Summary:** This system encompasses freshwater to oligohaline tidally‑flooded deciduous forests and shrublands in lower river floodplains and edges of estuaries of the North Atlantic Coastal Plain. This system is restricted to narrow zones along upper tidal reaches of Inner Coastal Plain rivers and tributaries which have sufficient volumes of freshwater and short flooding to support tree canopies. These areas are influenced by lunar tides up to 1 m (3 feet), but diluting freshwater flows from upstream, keeping salinity levels below 0.5 ppt. Deciduous hardwood species predominate, especially *Nyssa biflora* and/or *Fraxinus profunda* or *Fraxinus pennsylvanica*. In Maryland and Virginia, *Taxodium distichum* may be locally dominant.

**Comments:** The range of this system is generally conceived as Chesapeake Bay and northward (e.g., in the Coastal Plain from the James River, Virginia, northward to New Jersey and Massachusetts). Examples of tidal swamp forests south of this region are treated under Southern Atlantic Coastal Plain Tidal Wooded Swamp (CES203.240); the boundaries may overlap somewhere in Virginia.

DISTRIBUTION

**Range:** This system ranges from the James River, Virginia, northward to the New Jersey Coastal Plain. Possible occurrence of this system in Pennsylvania requires additional study. Examples are probably most common in the Chesapeake Bay region.

**Divisions:** 203:C

**TNC Ecoregions:** 58:C, 62:C

**Nations:** US

**Subnations:** DE, MA, MD, NJ, NY, PA?, VA

**Map Zones:** 60:C, 65:C

**USFS Ecomap Regions:** 232Ad:CCC, 232Ha:CCC, 232Hb:CCC, 232Ib:CCC

CONCEPT

**Environment:** This association occurs along fresh reaches of tidal rivers, usually receiving diurnal or irregular tidal flooding. There is distinct hummock‑and‑hollow microtopography with hollows flooded during higher tides. Soil is generally organic‑rich and contains a frequently deep organic horizon over silty alluvial deposits. Pronounced hummock‑and‑hollow microtopography is characteristic. Hollows are inundated by diurnal tides; hummocks may be only irregularly flooded, and the tops of hummocks are only rarely (less than annually) submerged (Rheinhardt and Hershner 1992).

**Vegetation:** Deciduous hardwood species predominate, especially *Nyssa biflora* and/or *Fraxinus profunda* or *Fraxinus pennsylvanica*. In Maryland and Virginia, *Taxodium distichum* may be locally dominant.

**Dynamics:** Development and persistence of this association appears to be limited downstream by halinity and upstream by the availability of sufficient sediment. Hence, tidal hardwood swamps are associated primarily with the upper (higher halinity) end of the freshwater portion of the halinity gradient and typically occur on higher landscape positions adjacent to tidal freshwater marshes (Rheinhardt and Hershner 1992). These swamps are maintained by regular biomass input deposited by regular tidal flow.

SOURCES

**References:** Comer et al. 2003*, Edinger et al. 2014a, Eyre 1980, Faber‑Langendoen et al. 2011, NYNHP 2013d, Rheinhardt and Hershner 1992

**Version:** 14 Jan 2014	**Stakeholders:** East, Southeast

**Concept Author:** R. Evans and P. Coulling	**LeadResp:** East

1.B.3.Nb. Southeastern North American Flooded & Swamp Forest

M161. Pond‑cypress Basin Swamp

CES203.245 Atlantic Coastal Plain Clay‑Based Carolina Bay Wetland

**Primary Division:** Gulf and Atlantic Coastal Plain (203)

**Land Cover Class:** Woody Wetland

**Spatial Scale & Pattern:** Small patch

**Required Classifiers:** Natural/Semi‑natural; Vegetated (>10% vasc.); Wetland

**Diagnostic Classifiers:** Forest and Woodland (Treed); Depressional; Graminoid

**National Mapping Codes:** EVT 2459; ESLF 9128; ESP 1459

**Concept Summary:** This system consists of wetlands associated with ovoid, shallow depressions with nearly flat bottoms in parts of the Atlantic Coastal Plain. Often called Carolina bays, these areas are most numerous and extensive in South Carolina but are also present in adjacent Georgia and the Inner Coastal Plain of North Carolina. These flat‑bottomed depressions have mineral soils with clay hardpans, fragipans, or some other drainage‑impeding mechanism that traps and retains water from a combination of rainfall and exposure of a high regional water table. Some examples are essentially permanently flooded, while others support water levels that vary substantially from year to year and over longer climatic cycles. Vegetation includes a series of primarily herbaceous and woodland associations. The wettest sites have open water and floating‑leaved aquatic vegetation, or marsh vegetation of tall graminoids. Drier sites often have an open canopy of *Taxodium ascendens*, with a dense, often fairly species‑rich herbaceous layer beneath. In a very few cases, *Taxodium ascendens* is replaced by *Taxodium distichum*. A few occurrences are shrubby, but none contain the dense shrub layers of characteristic pocosin species that occur in the bays with organic soils. Vegetational composition often varies substantially from year to year, in response to differences in water levels and drawdown times. Variation in hydroperiod is the most important dynamic, causing rapid major changes in the herbaceous vegetation. Unlike the steeper‑sided solution depressions, where many different hydroperiods are present within a short distance and vegetation zones simply shift, the flat‑bottomed Carolina bays experience drastic yearly changes in hydroperiod over most of their extent. Fire periodically spreads into the bays from adjacent uplands when conditions are dry, helps prevents invasion by less water‑tolerant trees during dry periods, and interacts with flooding to affect vegetational composition. Where fire is removed, *Pinus taeda* often invades the bays. Fire may also be important in preventing buildup of organic matter on the soil surface.

**Comments:** Despite the use of the term "clay‑based" there seem to be other edaphic factors or components that contribute to the extensive and long‑duration ponding of water in some of the Carolina bays. A report by Harrison (1983) contributes to this discussion, and mentions a "fragipan" in the sandy clay loam soil of the McColl soil series, as well as a report of an "iron hardpan" in one case. In addition, a personal communication by Otte (cited by Harrison 1983) refers to a substance called "humate" which is a mixture of organic substances which can act to bind soil particles into a relatively water‑tight layer which can contribute to the perched water table. Harrison also cites a report of soil cores taken in the McColl soil series at Antioch Bay (North Carolina) which had a layer of kaolinitc clay 30‑70 cm (12‑28 inches) deep and about 12‑25 cm (5‑10 inches) below the soil surface, with one core revealing a possible spodic horizon. A possible conclusion to be drawn here is that the use of the term "clay‑based" may be too specific in terms of describing the mechanism whereby some bays develop a perched water table resulting in the extensive and long‑duration ponding of water observed in some bays.

The distinction between the central concepts of this system and Southern Atlantic Coastal Plain Depression Pond (CES203.262) is well marked, with basin morphology, geographic range, and prevailing communities differing. However, there is a common set of plant species, including some rare ones, that occur in both systems. Thus, there may be difficulty in defining the local boundary, and some atypical depressions may have to be placed in one system or the other based on the preponderance of evidence. This system is related to Northern Atlantic Coastal Plain Pond (CES203.518) which occurs farther north in the Coastal Plain, and to some of the flat‑bottomed basin wetlands of Florida which occur outside the range of this system to the south.

DISTRIBUTION

**Range:** This system is found in the Inner to Middle Coastal Plain, from southern North Carolina, through South Carolina, and into adjacent Georgia. It is most numerous and extensive in South Carolina.

**Divisions:** 203:C

**TNC Ecoregions:** 56:C, 57:C

**Nations:** US

**Subnations:** GA, NC, SC

**Map Zones:** 55:C, 58:C

**USFS Ecomap Regions:** 232C:CC, 232I:CC, 232J:CC

CONCEPT

**Environment:** Examples of this system occur in Carolina bays with mineral soils and with seasonal to permanent standing water. Carolina bays are oriented, oval, shallow depressions with nearly flat bottoms, which range from North Carolina through South Carolina, and into adjacent Georgia. The general thought has been that most of the Carolina bays in the Outer Coastal Plain occur in sandy sediments and are filled with peat, while most Carolina bays in the Inner Coastal Plain occur in loamy sediments and have mineral soils with clay hardpans, but the situation may be more complex than this. These depressions hold water, due to a combination of rainfall and exposure of a high regional water table. Some are essentially permanently flooded. Others contain water well into the growing season in most years, but water levels vary substantially from year to year and over longer climatic cycles. Fire is an important natural influence in dry times. The McColl soil series (a fine, kaolinitic, thermic Typic Fragiaquult) is the soil most consistently associated with Carolina bays which are not dominated by "pocosin‑like" vegetation (M. Schafale pers. comm.). Its depth to fragic soil properties is 30‑90 cm (12‑36 inches); the depth to a fragipan is 38‑100 cm (15‑40 inches). Some pedons have few to common concretions of ironstone.

**Vegetation:** Vegetation includes a series of primarily herbaceous and woodland associations. The wettest sites have open water and floating‑leaved aquatic vegetation, or marsh vegetation of tall graminoids. Drier sites often have an open canopy of *Taxodium ascendens*, with a dense, often fairly species‑rich herbaceous layer beneath. In a very few cases, *Taxodium ascendens* is replaced by *Taxodium distichum* (Bennett and Nelson. 1991). A large number of annual species are present. Showy, characteristic plants include species of *Symphyotrichum, Boltonia, Xyris, Ludwigia*, and *Solidago* (Bennett and Nelson 1991). Some sites have similar herbaceous vegetation without trees. A few occurrences are shrubby, but none contain the dense shrub layers of characteristic pocosin species that occur in the bays with organic soils. Vegetational composition often varies substantially from year to year, in response to differences in water levels and drawdown times. Seed banking plays an important role in component communities. The system is also important as amphibian breeding habitat and may support a distinctive aquatic invertebrate community.

**Dynamics:** Variation in hydroperiod is the most important dynamic, causing rapid major changes in the herbaceous vegetation. Unlike the steeper‑sided solution depressions, where many different hydroperiods are present within a short distance and vegetation zones simply shift, the flat‑bottomed Carolina bays experience drastic yearly changes in hydroperiod over most of their extent. Many plants persist in seed banks for periods of years when conditions are not suitable. Fire is also an important process, spreading into the bays from adjacent uplands when conditions are dry. Fire prevents invasion by less water‑tolerant trees during dry periods, and interacts with flooding to affect vegetational composition. Where there is a lack of fire, *Pinus taeda* often invades the bays. Fire may also be important in preventing buildup of organic matter on the soil surface.

SOURCES

**References:** Bennett and Nelson 1991, Comer et al. 2003*, EPA 2004, Elliott, M. pers. comm., Engeman et al. 2007, Eyre 1980, Harrison 1983, Kirkman et al. 2012, Martin et al. 1993a, Nelson 1986, Prouty 1952, Richardson and Gibbons 1993, Schafale 2012, Schafale and Weakley 1990, Schafale pers. comm., Sharitz 2003, Sharitz and Gibbons 1982, Turner 2010, Van De Genachte and Cammack 2002, Wharton 1978

**Version:** 23 Apr 2015	**Stakeholders:** Southeast

**Concept Author:** M. Schafale and R. Evans	**LeadResp:** Southeast

CES411.365 South Florida Cypress Dome

**Primary Division:** Caribbean (411)

**Land Cover Class:** Woody Wetland

**Spatial Scale & Pattern:** Small patch

**Required Classifiers:** Natural/Semi‑natural; Vegetated (>10% vasc.); Wetland

**Diagnostic Classifiers:** Depressional

**National Mapping Codes:** EVT 2447; ESLF 9116; ESP 1447

**Concept Summary:** This ecological system is found primarily in the Everglades and Big Cypress regions of Florida. This system consists of small forested wetlands in poorly drained depressions which are underlain by an impervious layer that impedes drainage and traps precipitation. *Taxodium ascendens* is the dominant tree, with the oldest and largest individuals characteristically occupying the center, and smaller and younger individuals around the margins. Pools of stagnant, highly acidic water may stand in the center of these depressions ranging from 0.3‑1.2 m (1‑4 feet) in depth, but becoming increasingly shallow along the margins. The understory flora is typified by species with tropical affinities. These ponds are important for many wildlife species.

DISTRIBUTION

**Range:** Endemic to south Florida.

**Divisions:** 411:C

**TNC Ecoregions:** 54:C

**Nations:** US

**Subnations:** FL

**Map Zones:** 56:C

**USFS Ecomap Regions:** 411A:CC

CONCEPT

**Environment:** This system occurs in areas of low relief, occupying poorly drained to permanently wet depressions. Pools of stagnant, highly acidic water may stand in the center of these depressions ranging from 0.3‑1.2 m (1‑4 feet) in depth, but becoming increasingly shallow along the margins.

**Vegetation:** In addition to *Taxodium ascendens*, other taxa that may be present include *Annona glabra, Chrysobalanus icaco, Ficus aurea, Persea palustris*, and *Bacopa caroliniana*.

**Dynamics:** Cypress domes get their common name from the unique dome‑shaped appearance in which trees in the center are higher than those around the sides (Monk and Brown 1965). The water draws down more frequently along the edges than in the deeper center. This allows for more frequent recruitment of *Taxodium ascendens* seedlings along the edges, which are also exposed to more frequent wildland fire than the center of the ponds which remain flooded for longer durations. These two factors are reflected in the presence of large trees in the center and smaller trees closer to the edges of the ponds.

SOURCES

**References:** Comer et al. 2003*, Eyre 1980, FNAI 2010a, Monk and Brown 1965

**Version:** 14 Jan 2014	**Stakeholders:** Southeast

**Concept Author:** R. Evans	**LeadResp:** Southeast

CES411.290 South Florida Dwarf Cypress Savanna

**Primary Division:** Caribbean (411)

**Land Cover Class:** Woody Wetland

**Spatial Scale & Pattern:** Large patch

**Required Classifiers:** Natural/Semi‑natural; Vegetated (>10% vasc.); Wetland

**Diagnostic Classifiers:** Woody‑Herbaceous; Extensive Wet Flat

**National Mapping Codes:** EVT 2445; ESLF 9114; ESP 1445

**Concept Summary:** The scrub or dwarf cypress system covers extensive areas of south Florida, especially in the Big Cypress Swamp region of southwest Florida. These stunted stands of *Taxodium ascendens* grow on shallow sands or marl soils above limestone bedrock. Individual trees are usually quite small and widely scattered, with canopy coverage ranging from 30‑45%. The understory shares much overlap with wet prairies of the region and is dominated by the following genera: *Rhynchospora, Cyperus, Muhlenbergia*, and *Cladium*. The open, stunted aspect is maintained in part by stresses imposed by extreme seasonal water level changes and low‑nutrient soils. This type has a hydroperiod of approximately 6 months.

**Comments:** Related vegetation occurs in north Florida on clay soils of Tates Hell Swamp.

DISTRIBUTION

**Range:** This systems is endemic to south Florida and covers extensive areas, especially in the Big Cypress Swamp region of southwest Florida.

**Divisions:** 411:C

**TNC Ecoregions:** 54:C

**Nations:** US

**Subnations:** FL

**Map Zones:** 56:C

**USFS Ecomap Regions:** 411A:CC

CONCEPT

**Environment:** These stunted stands of *Taxodium ascendens* grow on shallow sands or marl soils above limestone bedrock.

**Vegetation:** Individual trees are usually quite small and widely scattered, with canopy coverage ranging from 30‑45% (Flohrschutz 1978). The understory shares much overlap with wet prairies of the region (Drew and Schomer 1984) and is dominated by the following genera: *Rhynchospora, Cyperus, Muhlenbergia*, and *Cladium*.

**Dynamics:** The open, stunted aspect is maintained in part by stresses imposed by extreme seasonal water level changes and low‑nutrient soils (Anonymous 1978). Ewel (1990b) suggests a hydroperiod of approximately 6 months for this type.

SOURCES

**References:** Anonymous 1978, Comer et al. 2003*, Drew and Schomer 1984, Ewel 1990b, Eyre 1980, FNAI 2010a, Flohrschutz 1978, Lodge 1994

**Version:** 14 Dec 2004	**Stakeholders:** Southeast

**Concept Author:** R. Evans	**LeadResp:** Southeast

CES203.251 Southern Coastal Plain Nonriverine Cypress Dome

**Primary Division:** Gulf and Atlantic Coastal Plain (203)

**Land Cover Class:** Woody Wetland

**Spatial Scale & Pattern:** Small patch

**Required Classifiers:** Natural/Semi‑natural; Vegetated (>10% vasc.); Wetland

**Diagnostic Classifiers:** Forest and Woodland (Treed); Depressional; Needle‑Leaved Tree

**National Mapping Codes:** EVT 2460; ESLF 9129; ESP 1460

**Concept Summary:** This system consists of small forested wetlands, typically dominated by *Taxodium ascendens*, often with a dome‑shaped appearance in which trees in the center of the depression are taller than those around the exterior. Examples are known from the Southern Coastal Plain (Omernik Ecoregion 75 and adjacent 65) of Florida and Georgia, extending into Alabama, Mississippi and Louisiana. Examples occupy poorly drained depressions which are most often embedded in a matrix of pine flatwoods or mesic to dry pine woodlands. The oldest and largest individual trees typically occupy the center of these domed wetlands, with smaller and younger individuals around the margins. Pools of stagnant, highly acidic water may stand in the center of these depressions ranging from 30‑120 cm (1‑4 feet) in depth, but becoming increasingly shallow along the margins. These sites are underlain by an impervious clay pan which impedes drainage and perches precipitation. Depending on fire regime and hydroperiod, some examples may have thick (50‑100 cm) organic layers. In addition to *Taxodium ascendens*, other woody species may include *Cephalanthus occidentalis, Clethra alnifolia, Hypericum chapmanii, Hypericum myrtifolium, Ilex myrtifolia, Eubotrys racemosa, Liquidambar styraciflua, Lyonia lucida, Morella cerifera, Nyssa biflora*, and *Styrax americanus*.

**Comments:** The original range of this system was thought to include only the East Gulf Coastal Plain (TNC Ecoregion 53) and was named accordingly. Examples were later confirmed in central Florida (TNC Ecoregion 55) and the South Atlantic Coastal Plain portion of Florida (A. Johnson pers. comm.) (TNC Ecoregion 56), whereupon the name was broadened to Southern Coastal Plain Nonriverine Cypress Dome. Cypress "stringers" are included here as well; these are more‑or‑less linear features that are parts of disconnected drainageways that arise in a pine flatwoods landscape (e.g., CEGL007419). The vegetation of the "stringers" is somewhat analogous to that of the edges of the true "dome swamps."

DISTRIBUTION

**Range:** Examples are known from the Southern Coastal Plain (Omernik Ecoregion 75 and adjacent 65) (EPA 2004) of Florida and Georgia, extending into Alabama, Mississippi and Louisiana.

**Divisions:** 203:C

**TNC Ecoregions:** 53:C, 55:C, 56:C

**Nations:** US

**Subnations:** AL, FL, GA, LA, MS

**Map Zones:** 55:C, 56:C, 99:C

**USFS Ecomap Regions:** 232B:CC, 232C:CC, 232D:CC, 232G:CC, 232J:CC, 232K:CC, 232L:CC, 234A:CC

CONCEPT

**Environment:** This system occurs in areas of low relief, occupying poorly drained to permanently wet depressions in uplands such as pine flatwoods or mesic to dry pine woodlands. Pools of stagnant, highly acidic water may stand in the center of these depressions ranging from 30‑120 cm (1‑4 feet) in depth, but becoming increasingly shallow along the margins (Monk and Brown 1965). Some examples may have thick (50‑100 cm) organic layers (Drew et al. 1998). Some of the depressions are fed by groundwater, while others are dependent on local precipitation.

**Vegetation:** According to Drew et al. (1998), dominant plant taxa include *Taxodium ascendens, Nyssa biflora, Cephalanthus occidentalis, Liquidambar styraciflua, Clethra alnifolia, Lyonia lucida*, and *Styrax americanus*. A few less typical upland depression ponds in Florida dominated by *Nyssa sylvatica* are also accommodated in this system for now (A. Johnson pers. comm.). Other species found in this system can include *Nyssa ursina, Hypericum chapmanii, Hypericum myrtifolium, Ilex myrtifolia, Eubotrys racemosa (= Leucothoe racemosa), Morella cerifera, Lobelia floridana, Polygala cymosa, Carex striata*, and *Carex turgescens*.

**Dynamics:** Cypress domes get their common name from the dome‑shaped appearance in which trees in the center are taller than those around the sides (Monk and Brown 1965). The water draws down more frequently along the shallow margins than in the deeper center. This allows for more frequent recruitment of *Taxodium ascendens* seedlings along the edges, which are also exposed to more frequent wildland fire than the center of the ponds which remain flooded for longer durations. These two factors are reflected in the presence of large trees in the center and smaller trees closer to the edges of the ponds (FNAI 2010a), and greater amounts of herbaceous graminoid plants along the margins of the depression. Where fires are more frequent, open herbaceous vegetation is favored. Without periodic fires *Taxodium ascendens* may become less dominant as hardwood or bay canopy species increase and peat accumulates. *Taxodium ascendens* has fairly thick, fire‑resistant bark and is tolerant of light surface fires; however, the seedlings and small *Taxodium ascendens* trees are vulnerable to fire (FNAI 2010a). When the forest canopy is harvested, the disturbed vegetation can transition to an herbaceous graminoid‑dominated wetland, such as represented by the ecological systems East Gulf Coastal Plain Depression Pond (CES203.558) or Southern Atlantic Coastal Plain Depression Pond (CES203.262). Transitions like this can also occur in response to the natural disturbance dynamics of Coastal Plain depressions, in which the influences of flooding, hurricanes and occasional wildland fire (or lack of fire) can lead to vegetation transition from wooded to herbaceous, or without canopy disturbance, succession from herbaceous to wooded or wetland forest vegetation.

SOURCES

**References:** Comer et al. 2003*, Drew et al. 1998, EPA 2004, Eyre 1980, FNAI 2010a, Johnson, A. pers. comm., Monk and Brown 1965

**Version:** 14 Jan 2014	**Stakeholders:** Southeast

**Concept Author:** R. Evans	**LeadResp:** Southeast

M033. Southern Coastal Plain Basin Swamp & Flatwoods

CES203.557 East Gulf Coastal Plain Southern Loblolly‑Hardwood Flatwoods

**Primary Division:** Gulf and Atlantic Coastal Plain (203)

**Land Cover Class:** Woody Wetland

**Spatial Scale & Pattern:** Large patch

**Required Classifiers:** Natural/Semi‑natural; Vegetated (>10% vasc.); Wetland

**Diagnostic Classifiers:** Forest and Woodland (Treed); Extensive Wet Flat

**National Mapping Codes:** EVT 2455; ESLF 9124; ESP 1455

**Concept Summary:** This forested system occurs on broad upland flats in the East Gulf Coastal Plain of Alabama and Mississippi, as well as western parts of the lower terraces of the East Gulf Coastal Plain ("Florida Parishes"; EPA Ecoregion 74d) of Louisiana, and likely occurs in other parts of the region as well. Its status and extent in this intervening terrain is unknown. Known examples in the Alabama/Mississippi parts of the range include a mosaic of open forests dominated by *Pinus taeda* interspersed with patches of *Quercus phellos* and sometimes other tree species. The ground surface displays an evident microtopography of alternating mounds and swales occurring in a tight local mosaic. These mounds are most likely "gilgai" resulting from vertic or shrink‑swell properties of the Luinn soil series. Known examples display a range of moisture conditions from dry to wet. The wettest examples trap significant moisture from local rainfall events. These areas have ponded water for a minimum of several days at an interval and potentially for long periods of the year, especially when evapotranspiration is lowest. The vegetation of this system supports relatively low vascular plant diversity and thus may appear floristically similar to other pine‑hardwood vegetation of the region. The dry portion of this vegetational mosaic is dominated by grassy ground cover (*Chasmanthium sessiliflorum*) with scattered emergent greenbriars (*Smilax* spp.) underneath a nearly pure *Pinus taeda* overstory. The historical composition of this type is unknown, but it seems likely that *Pinus taeda* was a natural and even dominant component of this system, as it is in related systems in the West Gulf Coastal Plain. Wetter areas are dominated by an overstory of *Quercus phellos* with an abundance of *Sabal minor* in the understory. Although the specific role of fire in this system is unknown, low‑intensity surface fires may have been ecologically important. Such fires could have originated in the surrounding East Gulf Coastal Plain Interior Shortleaf Pine‑Oak Forest (CES203.506).

In the western parts of the lower terraces of the East Gulf Coastal Plain ("Florida Parishes") of Louisiana (EPA Ecoregion 74d and adjacent 75a), the flatwoods vegetation tends to be dominated primarily by hardwoods in the most western portion, and a mixture of *Pinus glabra* and *Pinus taeda* in the intermediate portion to the east of this (Smith 1996b). In this "Louisiana Florida Parishes Spruce Pine Flatwoods Forest" some characteristic species include *Pinus glabra, Quercus laurifolia, Quercus michauxii, Quercus nigra, Quercus pagoda, Quercus virginiana, Pinus taeda*, and *Magnolia grandiflora*. Some important understory trees and shrubs include *Crataegus opaca, Sabal minor* (which may often be very abundant or dominant), and *Arundinaria tecta*.

**Comments:** The description of associations in the NVC for this system is undoubtedly incomplete. Classification work is in progress, but more information is needed.

DISTRIBUTION

**Range:** This forested system occurs on broad upland flats in the East Gulf Coastal Plain of Alabama and Mississippi, as well as western parts of the lower terraces of the East Gulf Coastal Plain ("Florida Parishes") in Louisiana. The complete and detailed range of this system is being developed and is not completely understood. It is not thought to extend into the Mississippi River Alluvial Plain of Louisiana (P. Faulkner pers. comm.).

**Divisions:** 203:C

**TNC Ecoregions:** 43:C, 53:P

**Nations:** US

**Subnations:** AL, GA?, LA, MS

**Map Zones:** 46:C, 99:C

**USFS Ecomap Regions:** 231Bb:CCC, 232La:CCC, 234Ad:CCC

CONCEPT

**Environment:** In the Alabama/Mississippi parts of this system's range, the ground surface displays an evident microtopography of alternating mounds and swales occurring in a tight local mosaic. In Louisiana, the soils are described as Hydric, acidic silt loams (including the Encrow, Gilbert, and Springfield series). The setting is broad, low flats, in small to large depressions, and along small, ill‑defined drainages locally known as "slashes" (Smith 1996b).

**Vegetation:** Known examples of this system in the Alabama/Mississippi parts of its range include a mosaic of open forests dominated by *Pinus taeda* interspersed with patches of *Quercus phellos* and sometimes other tree species. The vegetation of this system supports a relatively low vascular plant diversity and thus may appear floristically similar to other pine‑hardwood vegetation of the region. The dry portion of this vegetational mosaic is dominated by grassy ground cover (e.g., *Chasmanthium sessiliflorum*) with scattered emergent greenbriars (*Smilax* spp.) underneath a nearly pure *Pinus taeda* overstory. The historical composition of this type is unknown, but it seems likely that *Pinus taeda* was a natural and even dominant component of this system, as it is in related systems in the West Gulf Coastal Plain (R. Evans pers. obs., T. Foti pers. comm.). Wetter areas are dominated by an overstory of *Quercus phellos* with an abundance of *Sabal minor* in the understory.

In the western parts of the lower terraces of the East Gulf Coastal Plain ("Florida Parishes") of Louisiana, the flatwoods vegetation tends to be dominated primarily by hardwoods in the most western portion, and a mixture of *Pinus glabra* and *Pinus taeda* in the intermediate portion to the east of this. In this "Louisiana Florida Parishes Spruce Pine Flatwoods Forest" stands contain *Pinus glabra, Quercus laurifolia, Quercus phellos, Quercus michauxii, Quercus nigra, Quercus pagoda, Quercus virginiana, Pinus taeda, Nyssa biflora, Nyssa sylvatica, Magnolia grandiflora, Salix nigra, Liquidambar styraciflua, Carya glabra, Acer rubrum*, and *Fraxinus pennsylvanica*. Understory trees and shrubs include *Crataegus opaca* and *Sabal minor* (which may often be very abundant or dominant), as well as *Arundinaria tecta (= Arundinaria gigantea ssp. tecta), Cephalanthus occidentalis, Diospyros virginiana, Cornus foemina, Crataegus viridis, Ilex opaca var. opaca, Ilex decidua, Itea virginica, Morella cerifera (= Myrica cerifera), Sambucus nigra ssp. canadensis, Styrax americanus*, and *Viburnum dentatum* (Smith 1996b).

SOURCES

**References:** Comer et al. 2003*, Eyre 1980, Smith 1996b

**Version:** 14 Jan 2014	**Stakeholders:** Southeast

**Concept Author:** R. Evans	**LeadResp:** Southeast

CES203.193 Lower Mississippi River Flatwoods

**Primary Division:** Gulf and Atlantic Coastal Plain (203)

**Land Cover Class:** Woody Wetland

**Spatial Scale & Pattern:** Large patch

**Required Classifiers:** Natural/Semi‑natural; Vegetated (>10% vasc.); Wetland

**National Mapping Codes:** EVT 2513; ESLF 9191; ESP 1513

**Concept Summary:** This system encompasses forests, prairies and woodlands on Pleistocene terraces in the Mississippi Alluvial Plain of Arkansas, Missouri and Louisiana. It occurs primarily west of Crowley's Ridge on Pleistocene glacial outwash deposits in Arkansas and Missouri, and on Macon Ridge in Louisiana and adjacent Arkansas. The sites are above modern floodplains, but have poor internal drainage and are flat with poor runoff, leading to very wet conditions in winter and spring. They also often have a claypan that restricts both internal drainage and, later in the year, water availability. Therefore, they are very wet in the winter/spring and very dry in the summer, a moisture regime termed hydroxeric. Because of this moisture regime, the communities are variable, ranging from willow oak flats to post oak flats to prairies. In the 1940s, the Arkansas Game and Fish Commission produced a wildlife habitat map of Arkansas in which these sites were classified as "terrace hardwood forests." These communities have a large variety of upland and lowland tree species, ranging from post oak to overcup oak in a small area. Such species diversity may be explained by regeneration of species with dramatically different moisture tolerances on the same site in dry and wet years on these hydroxeric sites. Because the sites are above current floodplains and susceptible to being drained, they have been cleared at an even greater rate than nearby floodplain forests.

**Comments:** T. Foti (pers. comm. 2007): "I think it does encompass the Louisiana Mesic Hardwood Flatwoods, and the species listed in that description look good for the whole system. Do we want to leave the potential for prairies in this system or include them in the Grand Prairie system? I am inclined to think that small prairie inclusions should remain in this system and larger, individually definable prairies, such as those formerly across the White River from the Grand Prairie proper, could be included in that system. That distinction might be mentioned in the description. The Grand Prairie should be listed as a similar ecological system."

DISTRIBUTION

**Range:** This system is found in the Mississippi Alluvial Plain from the Missouri "bootheel" south to Louisiana. It occurs primarily west of Crowley's Ridge on Pleistocene glacial outwash deposits in Arkansas and Missouri. In southeastern Arkansas and northeastern Louisiana it is found on Macon Ridge (Ecoregion 73j (EPA 2004, LNHP 2009)). It is not reported from Kentucky, Tennessee, or Mississippi.

**Divisions:** 203:C

**TNC Ecoregions:** 42:C

**Nations:** US

**Subnations:** AR, LA, MO

**Map Zones:** 45:C, 98:P

**USFS Ecomap Regions:** 234A:CC, 234D:CC

CONCEPT

**Environment:** The sites where this system is found are above modern floodplains, but have poor internal drainage and are flat with poor runoff, leading to very wet conditions in winter and spring. They also often have a claypan that restricts both internal drainage and, later in the year, water availability. Therefore, they are very wet in the winter/spring and very dry in the summer, a moisture regime termed hydroxeric. In Louisiana, distinct mesic and wet community variants are recognized (LNHP 2004, 2009).

**Vegetation:** The communities of this system are variable, ranging from willow oak flats to post oak flats to prairies. In examples on Macon Ridge (Louisiana), overstory dominants include *Carya tomentosa (= Carya alba), Nyssa sylvatica, Quercus alba, Quercus pagoda, Quercus nigra, Quercus michauxii*, and *Liquidambar styraciflua*. In addition, *Quercus shumardii* and *Quercus falcata* are fairly frequent but not usually abundant. Common midstory trees include *Cornus florida, Ostrya virginiana, Aralia spinosa, Ulmus alata, Sassafras albidum*, and *Acer rubrum*. Important shrubs/small trees are *Vaccinium arboreum, Vaccinium virgatum, Viburnum rufidulum, Crataegus marshallii, Aesculus pavia, Frangula caroliniana, Asimina triloba, Hypericum hypericoides*, and *Euonymus americanus*. Although infrequent, *Hamamelis virginiana* can be locally abundant. Important woody vines include *Toxicodendron radicans, Parthenocissus quinquefolia, Vitis rotundifolia, Vitis aestivalis*, and *Smilax smallii*. *Toxicodendron radicans* and *Parthenocissus quinquefolia* are usually thick on the ground, as well as being represented by high climbing individuals. Common and characteristic herbaceous plants include *Chasmanthium sessiliflorum, Dichanthelium boscii, Podophyllum peltatum, Carex cherokeensis, Elephantopus carolinianus, Elephantopus tomentosus, Scleria oligantha, Aristolochia serpentaria, Botrychium virginianum, Passiflora lutea, Dioscorea villosa, Clitoria mariana, Sanicula canadensis, Geum canadense, Galium circaezans, Agrimonia rostellata, Spigelia marilandica, Clematis virginiana, Phryma leptostachya, Ruellia caroliniensis*, and *Smallanthus uvedalius* (LNHP 2004).

SOURCES

**References:** Comer et al. 2003*, EPA 2004, Foti pers. comm., LNHP 2009, Nelson 2010

**Version:** 14 Jan 2014	**Stakeholders:** Midwest, Southeast

**Concept Author:** T. Foti and M. Pyne	**LeadResp:** Southeast

CES203.304 Southern Atlantic Coastal Plain Nonriverine Swamp and Wet Hardwood Forest

**Primary Division:** Gulf and Atlantic Coastal Plain (203)

**Land Cover Class:** Woody Wetland

**Spatial Scale & Pattern:** Large patch

**Required Classifiers:** Natural/Semi‑natural; Vegetated (>10% vasc.); Wetland

**Diagnostic Classifiers:** Forest and Woodland (Treed); Extensive Wet Flat; Needle‑Leaved Tree; Broad‑Leaved Tree

**National Mapping Codes:** EVT 2501; ESLF 9310; ESP 1501

**Concept Summary:** This system consists of poorly drained, organic or mineral soil flats of the Atlantic Outer Coastal Plain. These areas are saturated by rainfall and seasonal high water tables without influence of river or tidal flooding. Fire is generally infrequent but may be important for some associations. Vegetation consists of hardwood or mixed forests of *Taxodium distichum, Nyssa* spp., bottomland oaks, *Acer rubrum*, or other wetland trees of similar tolerance. The lower strata have affinities with pocosin or baygall systems rather than the river floodplain systems that have affinities with the canopy. The combination of hardwood/deciduous canopy dominants and nonriverine, non‑seepage hydrology distinguishes this system from other Coastal Plain systems. Stands with a high cover of *Chamaecyparis thyoides* formerly occupied much of the acreage of this system. This phase is presently only present in high‑quality examples, and it helps distinguish this system from other Coastal Plain systems. Disturbed and fire‑disrupted examples (those dominated by *Nyssa* spp., bottomland oaks, *Acer rubrum*) may be hard to distinguish from other wetland forests based purely on canopy composition.

**Comments:** This system contains two to three distinctive subgroups within it. A wetter group has communities containing significant amounts of *Taxodium* or *Nyssa*. A drier group has communities with *Quercus* as a significant component. Within this group the calcareous association (CEGL007316) is very distinctive floristically.

The combination of canopy species with nonriverine hydrology distinguishes this system from all others. This system is distinguished from the various floodplain systems, with which the canopy shares affinities, by the distinctive hydrology and the differences in nutrient dynamics and other ecosystem process that follow from it. The overall flora is usually distinct and reflects these differences in nutrient status. The invertebrate fauna is likely very distinct. This system is distinguished from Atlantic Coastal Plain Peatland Pocosin and Canebrake (CES203.267), which can share organic soils and a number of shrub and herb species, by the canopy dominants and the lack of *Pinus serotina* and evergreen hardwoods as major canopy components. Fire frequency is an important difference. It is unclear if fire frequency determines the difference in vegetation or if the different flammability of the vegetation determines the fire regime.

The boundary between this system and Southern Coastal Plain Hydric Hammock (CES203.501) of Florida and Georgia may need clarification. Similarly, the boundary between this and Northern Atlantic Coastal Plain Basin Peat Swamp (CES203.522) has not been precisely delineated. Great Dismal Swamp is included with this system (CES203.304).

DISTRIBUTION

**Range:** This system ranges from Maryland to Georgia. This system is most abundant in the Embayed Region of northeastern North Carolina and southeastern Virginia (south of the James River), where it covers large expanses.

**Divisions:** 203:C

**TNC Ecoregions:** 56:C, 57:C

**Nations:** US

**Subnations:** GA, NC, SC, VA

**Map Zones:** 55:C, 58:C, 60:C

**USFS Ecomap Regions:** 232C:CC, 232H:CC, 232I:CC, 232J:CC

CONCEPT

**Environment:** This system occurs on flat areas of the Atlantic Outer Coastal Plain from Maryland to Georgia, where soils are seasonally to nearly semipermanently saturated because of low relief, poor soil drainage, and seasonal high water table. The largest areas are on broad interfluvial flats, but substantial areas occur on organic deposits in drowned river valleys in the Embayed Region of North Carolina and Virginia, beyond the reach of the influence of wind tides. Hydrology is dominated by rainfall and sheetflow, and overbank flooding, tidal flooding, and seepage are a secondary influence, if at all. Soils may be loamy to clayey, or may be shallow to deep organic. A distinctive small subset has soils with limestone near the surface, influencing soil chemistry. Natural fire is infrequent in this system, and varies from a minor to a significant influence on vegetational composition and structure. Infrequency of fire may be an important factor in differentiating this system from Atlantic Coastal Plain Peatland Pocosin and Canebrake (CES203.267) and the various wet longleaf pine forest systems. In a phase or component of this system on mucky peat soils (Terric or Typic Medisaprists) up to 3 m deep and occasionally on mucky sand or wet mineral soils with an organic epipedon, *Chamaecyparis thyoides* was the most common dominant species; it occurred in a fire‑generated patch mosaic in which the various patch dominants are a variable combination of *Acer rubrum, Chamaecyparis thyoides, Nyssa biflora, Pinus serotina*, and *Taxodium*, most frequently *Taxodium ascendens*. While this is fire‑dominated, it is only found in substantially fire‑sheltered portions of the landscape where scarps or water bodies prevent easy access by fire, resulting in a long fire‑return interval. The original vegetation constituted a true shifting mosaic. The original extent was up to 1 million acres of which at least 400,000 acres were Atlantic white‑cedar in Mapzones 58 and 60. This is a long‑interval, fire‑dependent, forested peatland with its greatest extent found on the Pamlico Terrace of Virginia and North Carolina. The largest sites lie at less than 9 m (30 feet) above sea level (C. Frost pers. comm.).

**Vegetation:** Vegetation is a closed‑canopy forest of wetland trees. The wetter sites are dominated by combinations of *Taxodium distichum, Nyssa biflora*, and occasionally *Nyssa aquatica, Pinus taeda, Chamaecyparis thyoides, Liquidambar styraciflua*, and *Liriodendron tulipifera*. Less wet sites have canopies of wetland oaks such as *Quercus laurifolia, Quercus michauxii*, and *Quercus pagoda*. Most communities have a well‑developed shrub layer that has more floristic affinities with pocosins or baygalls than with river floodplain communities that have similar canopies. The shrub layer is usually dominated by *Clethra alnifolia, Leucothoe axillaris*, or species shared with pocosins. The herb layer is not usually well‑developed but may be dense where shrubs are atypically sparse. Wetland ferns, such as *Osmunda regalis* and *Woodwardia areolata*, and *Carex* spp. usually dominate. In the Atlantic white‑cedar‑related phase of this system, stands that regenerated from crown fire often have nearly pure cover of *Chamaecyparis thyoides*. The most common subcanopy species are *Acer rubrum, Persea palustris*, and *Magnolia virginiana*. Typical shrubs include *Ilex glabra, Ilex coriacea, Eubotrys racemosa (= Leucothoe racemosa), Itea virginica*, and *Lyonia lucida*. Herbs, chiefly ferns and sedges, are typically sparse, but mosses may be common (C. Frost pers. comm.).

**Dynamics:** Fire is an important influence in a subset of this system. Communities dominated by *Chamaecyparis thyoides* depend on fire for regeneration of the canopy trees. The occurrence of fires on the time scale of several decades to a century or more may determine the mosaic of *Chamaecyparis thyoides* forests and other associations. Some areas may once have been canebrakes, with dominance of *Arundinaria* determined by more frequent fire. In the oak‑dominated communities and in wetter *Taxodium* and *Nyssa* communities, fire is probably of little ecological significance because the vegetation is not flammable. Without fire as a major factor, most communities probably occur naturally as old‑growth multi‑aged forests dominated by gap‑phase regeneration. Hurricanes may create larger canopy gaps, and sometimes cause more extensive damage. Examples in drowned river valleys are subject to influence by rising sea level and can be expected to evolve into tidal swamp systems, sometimes fairly quickly.

In specific relation to the *Chamaecyparis thyoides*‑dominated phase of this system, succession pathways depend on water table depth at time of replacement fire. Having the water table at the surface results in regeneration of *Chamaecyparis thyoides* from the seedbank. If the water table is slightly to moderately below the surface, the seedbank is destroyed and succession is dominated by some combination of *Acer rubrum, Nyssa biflora, Pinus taeda*, and related taxa. If the water table is well below the surface, the seedbank is destroyed and a deeper hole is created in the peat. In this case, succession is dominated by *Taxodium distichum* and a deeper water area is created with *Chamaecyparis thyoides* only on the edge.

SOURCES

**References:** Comer et al. 2003*, Eyre 1980, Frost 1987, Frost pers. comm., Nelson 1986, Schafale 2012, Schafale and Weakley 1990, Southeastern Ecology Working Group n.d.

**Version:** 13 Nov 2018	**Stakeholders:** East, Southeast

**Concept Author:** M. Schafale and R. Evans	**LeadResp:** Southeast

CES203.384 Southern Coastal Plain Nonriverine Basin Swamp

**Primary Division:** Gulf and Atlantic Coastal Plain (203)

**Land Cover Class:** Woody Wetland

**Spatial Scale & Pattern:** Large patch

**Required Classifiers:** Natural/Semi‑natural; Vegetated (>10% vasc.); Wetland

**Diagnostic Classifiers:** Forest and Woodland (Treed); Herbaceous; Depressional

**Concept Summary:** This ecological system occupies large, seasonally inundated basins with peaty substrates in the southern and outermost portions of the Coastal Plain of the southeastern United States. These basins are nonriverine and do not receive overbank flooding. The southern limit of this system extends into central Florida, especially along the Atlantic Coast in Volusia and Brevard counties. Examples are generally forested; the vegetation is characterized by *Taxodium distichum, Nyssa biflora*, evergreen "bay" shrubs, and/or mixed hardwoods. Emergent *Pinus elliottii* may also be present. Some characteristic shrubs include *Cliftonia monophylla, Cyrilla racemiflora, Lyonia lucida*, and *Smilax laurifolia*.

**Comments:** Manifestations of this in the Atlantic and Gulf coastal plains are not differentiated at this time. There may be some minor floristic differences, particularly between the northernmost and southernmost examples, but these are not thought to warrant any subdivision of the type. Examples of this system differ from Southern Coastal Plain Hydric Hammock (CES203.501) by the absence of oaks (especially swamp laurel oak and live oak) and other less flood‑tolerant species such as sweetgum (A. Johnson pers. comm.). In addition, this type is found in basins with peaty substrates as opposed to limestone‑influenced substrates.

DISTRIBUTION

**Range:** This system is found in the southern portions of the Atlantic and East Gulf coastal plains, extending down the Florida peninsula. The southern limit of this system extends into central Florida along the Atlantic Coast in Volusia and Brevard counties (A. Johnson pers. comm.).

**Divisions:** 203:C

**TNC Ecoregions:** 53:C, 55:C, 56:C, 57:C

**Nations:** US

**Subnations:** AL, FL, GA, LA?, MS, SC

**Map Zones:** 55:C, 56:C, 58:C, 99:C

**USFS Ecomap Regions:** 232B:CC, 232C:CC, 232D:CC, 232G:CC, 232J:CC, 232K:CC, 232L:CC

CONCEPT

**Environment:** This system occupies large, seasonally inundated basins with peaty substrates. These basins are nonriverine and do not receive overbank flooding. Even though the ecological system tends to occur in large basins, the basin may become full of water, and then there will be some flowout. This is due to high rainfall, and probably is more common in winter, when evapotranspiration is lower than summer. During periods of drought, the amount of water flowing out of a basin swamp may be quite low or none at all, and parts of the basin may become dry. The water tends to be nutrient‑poor and acidic, and often it appears tea‑colored from tannins in the water (called blackwater).

**Vegetation:** Examples are generally forested; the vegetation is characterized by *Taxodium distichum, Nyssa biflora*, evergreen "bay" shrubs, and/or mixed hardwoods (FNAI 2010a). Emergent *Pinus elliottii* may also be present. Some characteristic shrubs include *Cliftonia monophylla, Cyrilla racemiflora, Lyonia lucida*, and *Smilax laurifolia*. Some examples (e.g., Okefenokee Swamp) have extensive open herbaceous areas dominated by various combinations of *Panicum hemitomon, Sagittaria* spp., *Dulichium arundinaceum, Sarracenia* spp., *Carex glaucescens, Carex striata, Orontium aquaticum, Woodwardia virginica, Eriophorum virginicum, Eriocaulon compressum*, and *Peltandra virginica*. In addition, other floating and emergent aquatic plants are present including *Nuphar orbiculata (= Nuphar lutea ssp. orbiculata), Nymphaea odorata ssp. odorata, Nymphoides aquatica, Habenaria repens*, and *Utricularia* spp. (Wharton 1978). These herbaceous zones are called "prairies" or "sphagnum bogs" depending on their composition.

**Dynamics:** The primary source of water in basin swamps is local rainfall, with additional input from runoff and seepage from the surrounding uplands (FNAI 2010a). Flooding is a regular dynamic process. These basins are prone to long periods of inundation with limited waterflow. The deep parts of basin swamps may go without fire for decades or even centuries, while the drier outer edges can be more susceptible to frequent fire. Basin swamps within mesic flatwoods will burn more frequently than basin swamps within a matrix of mesic or hydric hammock. Without fire, bay shrubs and hardwoods increase in density and peat accumulates more rapidly. *Taxodium* and *Pinus* trees are tolerant of light surface fires, but muck fires burning into the peat can kill the trees, lower the ground surface, and transform a swamp into a pond, lake, marsh, or shrub bog (FNAI 2010a).

SOURCES

**References:** Comer et al. 2003*, Eyre 1980, FNAI 2010a, Fowlkes et al. 2003, Nelson 1986

**Version:** 14 Jan 2014	**Stakeholders:** Southeast

**Concept Author:** R. Evans	**LeadResp:** Southeast

CES203.548 West Gulf Coastal Plain Nonriverine Wet Hardwood‑Pine Flatwoods

**Primary Division:** Gulf and Atlantic Coastal Plain (203)

**Land Cover Class:** Woody Wetland

**Spatial Scale & Pattern:** Large patch

**Required Classifiers:** Natural/Semi‑natural; Vegetated (>10% vasc.); Wetland

**Diagnostic Classifiers:** Forest and Woodland (Treed); Hardpan; Depressional; Silt Soil Texture; West Gulf Coastal Plain

**National Mapping Codes:** EVT 2506; ESLF 9350; ESP 1506

**Concept Summary:** This ecological system represents predominantly wet hardwood and hardwood‑pine flatwoods of the West Gulf Coastal Plain of southern Arkansas, eastern Texas, and western Louisiana. Examples may be somewhat more common in the inland portions of the region but are also found in the Outer Coastal Plain as well. These areas are usually found on Pleistocene high terraces (EPA Ecoregion 35c) primarily associated with the Red and Mississippi rivers that are located above the current floodplain. The hydrology is controlled by local rainfall events and not by overbank flooding. Soils are fine‑textured, and hardpans may be present in the subsurface. The limited permeability of these soils contributes to perched water tables during fairly substantial portions of the year (when precipitation is greatest and evapotranspiration is lowest). Saturation occurs not from overbank flooding but typically whenever precipitation events occur. The local landscape is often a complex of ridges and swales, usually occurring in close proximity. There is vegetation variability related to soil texture and moisture and disturbance history. Most examples support hardwood forests or swamps, which are often heavily oak‑dominated. Important species are tolerant of inundation. They include *Liquidambar styraciflua, Quercus laurifolia, Quercus michauxii*, and *Quercus phellos*, with sparse coverage of wetland herbs such as *Carex glaucescens*. Some swales support unusual pockets of *Fraxinus caroliniana* and *Crataegus* spp. Some examples can contain *Pinus taeda*.

**Comments:** This system may grade upslope into West Gulf Coastal Plain Pine‑Hardwood Flatwoods (CES203.278) and down into West Gulf Coastal Plain Flatwoods Pond (CES203.547). Apparently, this system occurs within the historic range of longleaf pine [see USFS ecomap attributions]. Within this range, more information is needed to identify the toposequence between longleaf pine‑dominated flatwoods/savannas/uplands and hardwood/loblolly‑dominated flatwoods. The distribution of this system in the South Central Plains Flatwoods and Southern Tertiary Uplands (EPA 35e and f) needs to be better defined.

DISTRIBUTION

**Range:** This system is found in the West Gulf Coastal Plain, Upper West Gulf Coastal Plain, and Mississippi River Alluvial Plain (P. Faulkner pers. comm.).

**Divisions:** 203:C

**TNC Ecoregions:** 31:?, 40:C, 41:C, 42:C

**Nations:** US

**Subnations:** AR, LA, TX

**Map Zones:** 36:?, 37:C, 44:C, 45:C, 98:C

**USFS Ecomap Regions:** 231E:CC, 232F:CC

CONCEPT

**Environment:** This system is found on the wettest inclusions of Pleistocene terraces in the West Gulf Coastal Plain of southern Arkansas, eastern Texas, and western Louisiana. The geology of this system is similar to that of West Gulf Coastal Plain Pine‑Hardwood Flatwoods (CES203.278), being associated with high Pleistocene terraces of the Lissie and upper Beaumont formations, as well as the Quaternary Fluviatile Terrace Deposits to the north. In terms of landforms, this system represents the lowest topographic position within the level to very gently undulating terraces occupied by flatwoods. Hydrology is controlled by local rainfall, not overbank flooding of nearby streams. Soils are fine‑textured, with an impermeable subsurface horizon, which leads to a perched water table. Because of the lower topographic position of these flatwoods, saturated soil conditions tend to occur over extended periods of the year (Elliott 2011).

**Vegetation:** This system represents the wetter end of the wooded toposequence of the flatwoods and occurs within low positions of swales and other wet circumstances. Stands are closed‑canopy forests, typically dominated by deciduous hardwoods, including *Quercus michauxii*. Important species are tolerant of inundation. The canopy is often dominated by *Liquidambar styraciflua, Quercus laurifolia, Quercus lyrata, Quercus michauxii, Quercus nigra, Quercus phellos*, and *Ulmus alata*. *Pinus taeda* may also be present in the canopy and other strata. *Triadica sebifera* is a commonly encountered non‑native species invading this system. The understory and herbaceous layers of this system are not well‑developed, as the canopy tends to be closed (Elliott 2011). There is sparse coverage of wetland herbs such as *Carex glaucescens*. Some swales support unusual pockets of *Fraxinus caroliniana* and *Crataegus* spp. Some examples can contain *Pinus taeda*.

**Dynamics:** The predominant ecological processes affecting this system are related to soil texture and moisture and disturbance history. These are wetlands that hold standing water for variable periods during the year after rainfall events. The wettest examples were likely not affected to a large degree by fires; however, they are often embedded in pyrogenic landscapes which did burn frequently (R. Evans pers. obs., T. Foti pers. comm.). The difference in the dynamics between this system and the "non‑wet" (dry‑mesic, xero‑hydric) flatwoods of the region (CES203.278) is their different structure: the wetter type occurs as a closed forest, the dry/mesic one as a more open forest or woodland (with an open canopy, a full herbaceous expression, and few shrubs). The fire regime is different as well: the xero‑hydric type is short‑interval, low‑intensity, low‑severity versus medium‑ to long‑interval, low‑intensity, high‑severity for the wet one (D. Zollner pers. comm. 2006).

SOURCES

**References:** Comer et al. 2003*, Elliott 2011, Evans, R. pers. comm., Eyre 1980, Foti pers. comm., Marks and Harcombe 1981, Zollner pers. comm.

**Version:** 20 Aug 2015	**Stakeholders:** Midwest, Southeast

**Concept Author:** R. Evans	**LeadResp:** Southeast

CES203.278 West Gulf Coastal Plain Pine‑Hardwood Flatwoods

**Primary Division:** Gulf and Atlantic Coastal Plain (203)

**Land Cover Class:** Mixed Upland and Wetland

**Spatial Scale & Pattern:** Large patch

**Required Classifiers:** Natural/Semi‑natural; Vegetated (>10% vasc.); Upland; Wetland

**Diagnostic Classifiers:** Forest and Woodland (Treed); Pimple mounds; Extensive Wet Flat; Needle‑Leaved Tree; Broad‑Leaved Deciduous Tree

**National Mapping Codes:** EVT 2458; ESLF 9127; ESP 1458

**Concept Summary:** This ecological system represents predominantly mesic to dry flatwoods of limited areas of inland portions of the West Gulf Coastal Plain. These areas are usually found on Pleistocene high terraces that are located above current floodplains. The hydrology is controlled by local rainfall events and not by overbank flooding. Soils are fine‑textured, and hardpans may be present in the subsurface. The limited permeability of these soils contributes to shallowly perched water tables during portions of the year when precipitation is greatest and evapotranspiration is lowest. Soil moisture fluctuates widely throughout the growing season, from saturated to very dry, a condition sometimes referred to elsewhere as xerohydric. Saturation occurs not from overbank flooding but typically whenever precipitation events occur. Local topography is a complex of ridges and swales, often in close proximity to one another. Ridges tend to be much drier than swales, which may hold water for varying periods of time. Within both ridges and swales, there is vegetation variability relating to soil texture and moisture and disturbance history. The driest ridges support *Pinus taeda* and *Quercus stellata*; more mesic ridges have *Pinus taeda* with *Quercus alba* and species such as *Symplocos tinctoria* and *Viburnum dentatum*. Fire may have been an important natural process in some examples of this system.

**Comments:** Embedded swales tend to support hardwood forests or swamps, often heavily oak‑dominated with species tolerant of some inundation, such as *Quercus phellos* and *Quercus laurifolia*, with sparse coverage of wetland herbs such as *Carex glaucescens*. Some swales support unusual pockets of *Fraxinus caroliniana* and *Crataegus* spp. These latter vegetation types are linked to West Gulf Coastal Plain Nonriverine Wet Hardwood‑Pine Flatwoods (CES203.548). In Arkansas (at least), this system is most closely affiliated with these Soil Associations: "Adaton‑Felker‑Gore" (MUID=AR035); "Wrightsville‑Acadia‑Louin" (MUID=AR036); "Amy‑Pheba‑Savannah" (MUID=038); "Amy‑Pheba‑Guyton" (MUID=AR040); "Smithdale‑Savannah‑Sacul" (MUID= AR041); "Sacul‑Savannah‑Sawyer" (MUID= AR042); "Calloway‑Henry‑Grenada" (MUID=AR044); "Wrightsville‑Kolin‑Gore" (MUID=AR063); "Bussy‑Tillou‑Guyton" (MUID=AR069). Apparently, this system occurs within the historic range of longleaf pine [see USFS ecomap attributions]. Within this range, more information is needed to identify the toposequence between longleaf pine‑dominated flatwoods/savannas/uplands and hardwood/loblolly‑dominated flatwoods. The distribution of this system in the South Central Plains Flatwoods and Southern Tertiary Uplands (EPA 35e and f) needs to be better defined.

DISTRIBUTION

**Range:** This system is found in the inland portions of the West Gulf Coastal Plain, on nonriverine, Pleistocene high terraces.

**Divisions:** 203:C

**TNC Ecoregions:** 32:C, 40:C, 41:C

**Nations:** US

**Subnations:** AR, LA, OK, TX

**Map Zones:** 36:C, 37:C, 44:C

**USFS Ecomap Regions:** 231E:CC, 232Fb:CCC, 234E:??, 255Da:CCC

CONCEPT

**Environment:** Areas occupied by this system are usually found on nonriverine, Pleistocene high terraces. These are mapped in the northern portion of East Texas as Quaternary Fluviatile Terrace (or Tile) deposits. It is found on very gently undulating to flat surfaces, with local topographic relief provided by ridges and swales. Soils tend to be fine‑textured and typically have a somewhat impermeable subsurface horizon, which leads to a perched water table. Saturation results from local rainfall run‑on, and alternates with seasonal drying, leading to a xerohydric hydroperiod. The limited permeability of these soils contributes to shallowly perched water tables during portions of the year when precipitation is greatest and evapotranspiration is lowest. Soil moisture fluctuates widely throughout the growing season, from saturated to very dry, a condition sometimes referred to elsewhere as xerohydric. Saturation occurs not from overbank flooding but typically whenever precipitation events occur. Local topography is a complex of ridges and swales, often in close proximity to one another. Ridges tend to be much drier than swales, which may hold water for varying periods of time.

**Vegetation:** There is vegetation variability between and among ridges and swales, as well as within them, relating to soil texture and moisture and disturbance history. This woodland or forest system is often dominated by more mesic species on interior ridges, including *Pinus taeda, Pinus echinata, Pinus elliottii, Quercus stellata, Quercus alba, Quercus falcata*, and *Carya texana* (Elliott 2011). The driest ridges support *Pinus taeda* and *Quercus stellata*; more mesic ridges have *Pinus taeda* with *Quercus alba* and understory species such as *Symplocos tinctoria* and *Viburnum dentatum*. On the somewhat wetter sites of the swales, species such as *Quercus nigra, Quercus phellos, Quercus laurifolia, Nyssa sylvatica, Liquidambar styraciflua*, and *Fraxinus pennsylvanica* may be dominant. Sites that are even wetter would likely be mapped as West Gulf Coastal Plain Nonriverine Wet Hardwood‑Pine Flatwoods (CES203.548). Midstory species that may be encountered include *Acer rubrum, Ilex opaca, Ulmus alata*, and small members of the overstory. *Morella cerifera, Ilex decidua*, and *Ilex vomitoria* are commonly encountered shrubs. Herbaceous cover is generally sparse, with species such as *Chasmanthium* spp., *Andropogon glomeratus*, and *Gelsemium sempervirens*. Sites dominated by *Pinus taeda* or *Pinus elliottii* may often represent plantations or managed forests (Elliott 2011). *Triadica sebifera* may invade this system. Embedded swales (which are, in effect, smaller interfingered examples of West Gulf Coastal Plain Nonriverine Wet Hardwood‑Pine Flatwoods (CES203.548)) tend to support hardwood forests or swamps, often heavily oak‑dominated with species tolerant of some inundation, such as *Quercus phellos* and *Quercus laurifolia*, with sparse coverage of wetland herbs, such as *Carex glaucescens*. Some swales support unusual pockets of *Fraxinus caroliniana* and *Crataegus* spp.

**Dynamics:** The difference in the dynamics between this system and the "wet" hardwood flatwoods of the region, i.e., West Gulf Coastal Plain Nonriverine Wet Hardwood‑Pine Flatwoods (CES203.548), is the different structure: the wetter type occurs as a closed forest, the dry/mesic (xero‑hydric) one as a more open forest or woodland (with an open canopy, a full herbaceous expression, and few shrubs). The fire regime is different as well: the xero‑hydric type is short‑interval, low‑intensity, low‑severity versus medium‑ to long‑interval, low‑intensity, high‑severity for the wet one (D. Zollner pers. comm. 2006).

SOURCES

**References:** Comer et al. 2003*, EPA 2004, Elliott 2011, Eyre 1980, Foti pers. comm., Hoagland pers. comm., Singhurst pers. comm., Zollner pers. comm.

**Version:** 18 Feb 2011	**Stakeholders:** Midwest, Southeast

**Concept Author:** R. Evans	**LeadResp:** Southeast

M032. Southern Coastal Plain Evergreen Hardwood ‑ Conifer Swamp

CES203.252 Atlantic Coastal Plain Streamhead Seepage Swamp, Pocosin and Baygall

**Primary Division:** Gulf and Atlantic Coastal Plain (203)

**Land Cover Class:** Woody Wetland

**Spatial Scale & Pattern:** Large patch

**Required Classifiers:** Natural/Semi‑natural; Vegetated (>10% vasc.); Wetland

**Diagnostic Classifiers:** Forest and Woodland (Treed); Seepage‑Fed Sloping; Mid and Southern Atlantic Coastal Plain

**National Mapping Codes:** EVT 2468; ESLF 9137; ESP 1468

**Concept Summary:** This ecological system encompasses seepage‑fed wetlands in dissected landscapes of the Atlantic Coastal Plain, from southeastern Virginia south through South Carolina and into the Inner Coastal Plain of Georgia. Examples are usually associated with ravines or along headwater streams. Overbank flooding is a negligible influence. Fire may be an important force in some associations and not in others. Vegetation consists of open to closed forests or woodlands of acid‑tolerant wetland hardwoods or pine. Generally there is a dense shrub layer consisting primarily of species shared with Atlantic Coastal Plain Peatland Pocosin and Canebrake (CES203.267).

**Comments:** This system is very heterogeneous in vegetation and in the role of fire, as well as extensive in geographic range. It might be appropriate to split it into two or even three systems. The streamhead pocosins of the Fall‑line Sandhills region of North Carolina and northern South Carolina (EPA 65c), as well as related areas of Georgia and southern Virginia are distinctive in being strongly fire‑dominated, having pine as a major canopy dominant, and having a flora consisting largely of pocosin species. The closely related white‑cedar‑ and cane‑dominated associations would also fit into this system. A second set of associations ranging from South Carolina through the Gulf Coastal Plain has vegetation that suggests less influence by fire, including hardwood canopies and shrub layers that are primarily pocosin species but share some other wetland species. A third set, from a wider variety of topographic settings throughout the region, has hardwood canopies and shrub and herb layers with less peatland affinities, more closely related to floodplain communities. Their flora suggests a minor role for fire.

This system is distinguished from Atlantic Coastal Plain Sandhill Seep (CES203.253) by the predominance of woody vegetation indicative of less frequent fire. Where the two co‑occur, it occurs in larger and topographically lower patches. This system is distinguished from Atlantic Coastal Plain Peatland Pocosin and Canebrake (CES203.267), which may have fairly similar flora, by having seepage‑dominated hydrology and occurring in dissected landscapes.

DISTRIBUTION

**Range:** This ecological system is found in the Atlantic Coastal Plain, from southeastern Virginia south through South Carolina and into the Inner Coastal Plain of Georgia, primarily in the Fall‑line Sandhills region; rarely in dissected terrain in the Outer Coastal Plain.

**Divisions:** 203:C

**TNC Ecoregions:** 56:C, 57:C

**Nations:** US

**Subnations:** GA, NC, SC, VA

**Map Zones:** 55:C, 58:C, 59:C, 60:C

**USFS Ecomap Regions:** 232C:CC, 232H:CC, 232I:CC, 232Ja:CCC

CONCEPT

**Environment:** This system occurs in dissected Coastal Plain terrain on sites saturated by seepage of shallow groundwater. Seasonal to permanent saturation combined with fire of only moderate to low frequency and woody vegetation are the unifying characteristics of this system. A stream is often present draining the site, but it is small, and overbank flooding is a negligible influence. Most examples are in bottoms of ravines, but some are on sideslopes or flats at the base of slopes. Most examples are in sandy areas where rapid soil drainage in the surrounding landscape supplies the seepage. Soils within the system itself are generally mucky sands or clays, or deeper organic soils. This system occurs in landscapes that had frequent fire under natural conditions.

**Vegetation:** Vegetation is dominated by woody plants. An open to closed tree canopy is usually present and consists of a mixture of acidic‑tolerant wetland trees such as *Nyssa biflora, Acer rubrum, Pinus serotina, Magnolia virginiana, Liriodendron tulipifera*, and *Chamaecyparis thyoides*. There is generally a dense shrub layer that is dominated by species shared with pocosins or baygalls, such as *Cyrilla racemiflora, Leucothoe axillaris, Lyonia lucida, Lyonia ligustrina, Clethra alnifolia, Cliftonia monophylla, Ilex glabra*, and *Arundinaria tecta (= Arundinaria gigantea ssp. tecta)*, but includes some species of other saturated wetlands, such as *Toxicodendron vernix, Morella caroliniensis, Persea palustris*, and *Viburnum nudum*. *Smilax laurifolia* may be abundant. The herb layer, if well‑developed at all, generally consists of large wetland ferns, such as *Osmunda cinnamomea, Osmunda regalis var. spectabilis, Woodwardia virginica*, and *Woodwardia areolata*, with *Carex* spp. Some examples (canebrakes) are dominated by *Arundinaria tecta*.

**Dynamics:** Seepage is the most important ecological factor determining where this system occurs. Seepage provides a steady source of water, so that soils remain saturated but seldom have surface flooding. The importance of fire varies widely in this system. Fire is the most important dynamic process in many examples, but is of minor importance in others, and is probably an important driver of the different vegetation associations. Fire frequency and intensity vary among associations, from moderately frequent intense fires to infrequent low‑intensity fires. This system occurs within larger upland landscapes that had frequent fire in the past, but the wetness of these headwater wetlands often limits fire spread into them. Associations dominated by *Pinus serotina* and evergreen shrubs such as *Ilex, Lyonia, Gaylussacia, Persea, Morella, Arundinaria tecta* and *Cyrilla*, or canebrakes dominated by *Arundinaria tecta* can have intense canopy fires that are the dominant influence on vegetation structure. Those dominated by *Chamaecyparis thyoides* have infrequent fire that may catastrophically kill the canopy trees, while also promoting *Chamaecyparis thyoides* regeneration. Associations with hardwood canopies, such as *Acer rubrum, Liriodendron tulipifera*, or *Nyssa biflora*, especially those with limited shrub abundance, are not very flammable and usually burn with low intensity and limited effect. Wind can be an important natural disturbance. Forests of *Chamaecyparis thyoides* are susceptible to heavy windthrow that can affect a substantial part of the canopy. Wind damage in hardwood and pine forests tends to consist mainly of small to medium‑sized canopy gaps. In ravine bottom sites that have some streamflow, beavers can be an important influence. Beaver ponds convert the forested vegetation to open water. Upon abandonment, beaver pond sites go through a succession that may lead to a long‑lasting mire community, or to regeneration of a swamp canopy and lower strata.

SOURCES

**References:** Comer et al. 2003*, Engeman et al. 2007, Eyre 1980, Fleming et al. 2005, LANDFIRE 2007a, Nelson 1986, Schafale 2012

**Version:** 23 Apr 2015	**Stakeholders:** East, Southeast

**Concept Author:** M. Schafale and R. Evans	**LeadResp:** Southeast

CES203.501 Southern Coastal Plain Hydric Hammock

**Primary Division:** Gulf and Atlantic Coastal Plain (203)

**Land Cover Class:** Woody Wetland

**Spatial Scale & Pattern:** Linear

**Required Classifiers:** Natural/Semi‑natural; Vegetated (>10% vasc.); Wetland

**Concept Summary:** This ecological system occupies flat lowlands along the southern and outermost portions of the Coastal Plain of the southeastern United States, usually over limestone substrates. The vegetation of this system is characterized by mixed hardwood species, often with hydric oak species common. In Florida, examples of this system are often found adjacent to the floodplain of spring‑fed rivers with relatively constant flows. In some areas, such as the Big Bend region of Florida, they occupy large areas of broad, shallow, mucky or seepy wetlands but generally do not receive overbank flooding. In Alabama, this system is apparently confined to floodplains of the Mobile‑Tensaw, where examples are topographically higher than the surrounding floodplains.

**Comments:** The original name of this system was too geographically restrictive and was broadened to Southern Coastal Plain to better reflect the range of this system. Confirmed in South Atlantic Coastal Plain portion of Florida by Ann Johnson (pers. comm.).

DISTRIBUTION

**Range:** As currently documented, this system occurs in Florida, Georgia and rarely in southern Alabama. In Alabama, this system is apparently confined to floodplains of the Mobile‑Tensaw (A. Schotz pers. comm.).

**Divisions:** 203:C

**TNC Ecoregions:** 53:C, 55:C, 56:C

**Nations:** US

**Subnations:** AL, FL, GA, MS?

**Map Zones:** 55:C, 56:C, 99:C

**USFS Ecomap Regions:** 232B:CC, 232C:CC, 232D:CC, 232G:CC, 232J:CC, 232K:CC, 232L:CC

CONCEPT

**Environment:** Examples of this system are associated with limestone‑rich sites. Soils may range from sand to clay to organic (FNAI 2010a). In Florida, examples of this system are often found adjacent to the floodplain of spring‑fed rivers with relatively constant flows. In some areas, such as the Big Bend region of Florida, they occupy large areas of broad, shallow, mucky or seepy wetlands but generally do not receive overbank flooding (A. Johnson pers. comm.). In Alabama, this system is apparently confined to floodplains of the Mobile‑Tensaw, where examples are topographically higher than the surrounding floodplains (A. Schotz pers. comm.).

**Vegetation:** The vegetation of this system is characterized by mixed hardwood species, often with hydric oak species common (FNAI 2010a, A. Johnson pers. comm.). Stands may be dominated by a variety of wetland and upland tree species, including *Chamaecyparis thyoides, Sabal palmetto*, and *Quercus laurifolia*, as well as *Quercus virginiana, Magnolia virginiana*, and *Ulmus americana*. Some shrubs and understory trees include *Ilex cassine* and *Morella cerifera*.

**Dynamics:** Saturation, but usually not inundation, is characteristic of the hydrology of some hydric hammocks; lower areas generally are prone to more flooding. The distributions of trees within hydric hammocks are influenced by the timing and depth of flooding (Vince et al. 1989). These are sites which are only occasionally subject to wildland fire (FNAI 2010a) and are dominated by mixed evergreen and deciduous forest, often with *Sabal palmetto* which is fire‑tolerant.

SOURCES

**References:** Comer et al. 2003*, Eyre 1980, FNAI 2010a, Johnson, A. pers. comm., Schotz pers. comm., Vince et al. 1989

**Version:** 21 May 2014	**Stakeholders:** Southeast

**Concept Author:** R. Evans	**LeadResp:** Southeast

CES203.505 Southern Coastal Plain Seepage Swamp and Baygall

**Primary Division:** Gulf and Atlantic Coastal Plain (203)

**Land Cover Class:** Woody Wetland

**Spatial Scale & Pattern:** Large patch

**Required Classifiers:** Natural/Semi‑natural; Vegetated (>10% vasc.); Wetland

**Diagnostic Classifiers:** Forest and Woodland (Treed); Seepage‑Fed Sloping; Broad‑Leaved Evergreen Tree; East Gulf Coastal Plain

**National Mapping Codes:** EVT 2461; ESLF 9130; ESP 1461

**Concept Summary:** This wetland system consists of forested wetlands in acidic, seepage‑influenced habitats of the East Gulf and Atlantic coastal plains, extending from Mississippi and the Florida Parishes of Louisiana east into southern Georgia and central Florida. These are mostly evergreen forests generally found at the base of slopes or other habitats where seepage flow is concentrated. Resulting moisture conditions are saturated or even inundated. The vegetation is characterized by *Magnolia virginiana* and *Nyssa biflora*. Examples occur in the outer portions of the Coastal Plain within the range of *Persea palustris*, and where *Magnolia virginiana* is an important or even dominant species. To the north this system grades into East Gulf Coastal Plain Northern Seepage Swamp (CES203.554), where evergreen species are largely replaced by deciduous species in the canopy. Due to excessive wetness, these habitats are normally protected from fire except those which occur during extreme droughty periods. These environments are prone to long‑duration standing water, and tend to occur on highly acidic, nutrient‑poor soils.

**Comments:** Some authors have treated *Persea palustris* (of wetlands) and *Persea borbonia* (of uplands) as one taxon under a broadly conceived *Persea borbonia*. We recognize the two distinct taxa, following Godfrey (1988), Kartesz (1999) and Weakley (2005).

DISTRIBUTION

**Range:** This system occurs in the East Gulf and Atlantic coastal plains, extending from Mississippi and the Florida Parishes of Louisiana east into the Outer Coastal Plain of southern Georgia and into central Florida.

**Divisions:** 203:C

**TNC Ecoregions:** 43:C, 53:C, 55:C, 56:C

**Nations:** US

**Subnations:** AL, FL, GA, LA, MS

**Map Zones:** 55:C, 56:C, 99:C

**USFS Ecomap Regions:** 231H:CC, 232B:CC, 232C:CC, 232D:CC, 232G:CC, 232J:CC, 232K:CC, 232L:CC, 234A:CC

CONCEPT

**Environment:** These wetlands may occur in poorly developed upland drainages, narrow ravine bottoms, bases of steepheads, and small headwaters stream bottoms. In most cases, these wetlands are embedded in uplands with deep sandy soils. When this system is associated with streams, they tend to be low‑gradient, with narrow, often braided channels and diffuse drainage patterns. Habitat also includes baygall vegetation in oval depressions (Carolina bays) in southern Georgia (e.g., in Liberty and Long counties, Georgia).

**Vegetation:** The vegetation is characterized by *Magnolia virginiana* and *Nyssa biflora*. Examples occur in the outer portions of the Coastal Plain within the range of *Persea palustris*, and where *Magnolia virginiana* is an important or even dominant species. Dominant trees in some stands may include *Quercus laurifolia, Liquidambar styraciflua*, and *Liriodendron tulipifera*. In addition, some stands may be dominated by *Cyrilla racemiflora* and/or *Cliftonia monophylla*. Other shrubs include *Ilex coriacea, Leucothoe axillaris, Lyonia lucida, Morella caroliniensis, Morella inodora*, and *Viburnum nudum var. nudum*. Herbs include *Carex atlantica ssp. capillacea, Carex glaucescens, Carex lonchocarpa, Chasmanthium ornithorhynchum, Polygala cymosa, Solidago patula var. strictula*, and *Sphagnum* spp.

**Dynamics:** Due to excessive wetness, these habitats are normally protected from fire except those which occur during extreme droughty periods. These environments are prone to long‑duration standing water and tend to occur on highly acidic, nutrient‑poor soils and saturated peat (FNAI 2010a). This system occurs in landscapes that had frequent fire in the past, but the wetness usually limited fire spread, creating an infrequent fire‑return interval. While infrequent, fire intensity varies among associations; those dominated by evergreen shrubs such as *Ilex, Lyonia, Illicium, Cliftonia, Gaylussacia, Persea, Morella, Arundinaria*, and *Cyrilla* and with *Pinus serotina* or *Chamaecyparis thyoides* can produce intense canopy fire when they burn (especially when ladder fuels are present), while others probably experience only low‑intensity surface fires because of low flammability. When severe drought has allowed the peat to dry, wildfire can burn out the peat. If shrubs survive, they will resprout, but if the roots of shrubs are killed, the site may respond to the intense fire and transition to herbaceous marsh or eventually *Taxodium ‑ Nyssa* swamp vegetation (FNAI 2010a).

SOURCES

**References:** Comer et al. 2003*, Engeman et al. 2007, Eyre 1980, FNAI 2010a, Godfrey 1988, Kartesz 1999, LNHP 2009, Weakley 2005

**Version:** 14 Jan 2014	**Stakeholders:** Southeast

**Concept Author:** R. Evans and M. Pyne	**LeadResp:** Southeast

CES203.372 West Gulf Coastal Plain Seepage Swamp and Baygall

**Primary Division:** Gulf and Atlantic Coastal Plain (203)

**Land Cover Class:** Woody Wetland

**Spatial Scale & Pattern:** Large patch

**Required Classifiers:** Natural/Semi‑natural; Vegetated (>10% vasc.); Wetland

**Diagnostic Classifiers:** Seepage‑Fed Sloping; Broad‑Leaved Tree; West Gulf Coastal Plain

**National Mapping Codes:** EVT 2462; ESLF 9131; ESP 1462

**Concept Summary:** This West Gulf Coastal Plain ecological system consists of forested wetlands (often densely wooded) in acidic, seepage influenced wetland habitats. These wetlands may occur in poorly developed upland drainages, toeslopes, and small headwaters stream bottoms. These environments are prone to long duration standing water, and tend to have highly acidic, nutrient‑poor soils. The vegetation is characterized by an overstory of *Magnolia virginiana, Nyssa sylvatica, Nyssa biflora*, and *Acer rubrum*, although there is some variation according to latitude. Understory vegetation throughout the region consistently supports the vines *Smilax laurifolia* and *Smilax walteri*, and a dense abundance of ferns, such as *Osmunda cinnamomea, Osmunda regalis var. spectabilis*, and *Woodwardia areolata*. In most cases, these wetlands are embedded in uplands with deep sandy soils, recharge areas for this wetland system. When these communities are associated with streams, they tend to be low gradient, with narrow, often braided channels and diffuse drainage patterns. Due to excessive wetness, these habitats are normally protected from fire except those which occur during extreme droughty periods. The limited examples in Oklahoma are somewhat depauperate and lack some of the more southern and eastern taxa (e.g., *Magnolia virginiana, Nyssa biflora*).

DISTRIBUTION

**Range:** This system is restricted to eastern Texas, western Louisiana, southern Arkansas, and extreme southeastern Oklahoma.

**Divisions:** 203:C

**TNC Ecoregions:** 40:C, 41:C

**Nations:** US

**Subnations:** AR, LA, OK, TX

**Map Zones:** 37:C, 44:P, 98:C

**USFS Ecomap Regions:** 231E:CC, 232F:CC, 234C:PP, 234E:PP

CONCEPT

**Environment:** This system occurs on saturated soils associated with springs and seepage flow in a variety of landscape positions. In the Outer Coastal Plain, these settings tend to be low landscape positions typically along low‑gradient creeks, headwaters of drainages, or local depressions (Elliott 2011). The low‑gradient creek channels tend to be highly meandering, often with multiple channels and extremely shallow banks. Nixon et al. (1983a) measured stream depths of 0.3‑0.6 m and widths of less than 1 m in a study of this system. Inner Coastal Plain examples tend to be embedded within deep sandy slopes and uplands, and may also occur in association with flatwoods drainages (Martin et al. 1990, Martin and Smith 1991, Smith 1996a, Singhurst pers. comm. 2013). It may occur on a range of geological formations, including intermediate to high Pleistocene terraces, Eocene sands, the Catahoula Formation, and the Wilcox Formation. Soils are typically sandy to loamy soils, often with an impermeable subsurface layer that restricts water percolation. These sites are typically semipermanently saturated. These are typically soils of medium to strong acidity, with low available nutrients and significant organic accumulation (Elliott 2011). The deep, poorly drained, strongly acidic, loamy fine sand soils have high organic matter content (Brooks et al. 1993). Van Kley (1999a) indicates that these habitats, sometimes mapped as the Betis soil series and Guyton soil complex, are notably low in calcium and magnesium. Soils of other examples may be mapped as Lovelady (Arenic Glossudalf), Rentzel (Arenic Plinthaquic Paleudult), Corrigan (Typic Albaqualf), Melhomes (Humaqueptic Psammaquent), and Osier (Typic Psammaquent). This system is known from the Pleistocene Terraces and Tertiary uplands in Louisiana, Texas, Arkansas and to a limited extent in Oklahoma. Geologic formations where this system occurs include: Bentley (Intermediate Pleistocene Terraces), Willis (High Pleistocene Terraces), Fleming (Miocene), Catahoula (Oligocene), Cockfield (Eocene), Sparta (Eocene), Carrizo (Eocene), Wilcox (Eocene), Queen City (Eocene) and possibly the Vicksburg (Oligocene) and other formations.

**Vegetation:** Examples of this system are characterized by overstory species such as *Magnolia virginiana, Nyssa biflora*, and *Acer rubrum*. Other species in the overstory may include *Fraxinus pennsylvanica, Quercus nigra, Liquidambar styraciflua*, and *Quercus laurifolia*. A well‑developed woody understory is often present and includes species such as *Morella caroliniensis, Itea virginica, Persea palustris, Rhododendron prinophyllum, Rhododendron canescens, Ilex decidua, Vaccinium fuscatum, Ilex opaca, Toxicodendron vernix, Viburnum nudum, Morella cerifera, Alnus serrulata, Smilax laurifolia*, and *Vitis rotundifolia*. There is some variation with latitude. Southerly examples generally consist of broad‑leaved evergreen forests, while more northerly examples support more mixed evergreen‑deciduous forests. In addition, evergreen species are especially pronounced in the shrub layer of southern examples. Southern expressions of the type are more likely to have *Ilex coriacea* and/or *Cyrilla racemiflora*. The herbaceous layer is often dominated by ferns such as *Woodwardia areolata, Osmunda regalis, Osmunda cinnamomea*, and *Athyrium filix‑femina*. *Carex* spp., *Rhynchospora* spp., and *Eleocharis* spp. are also frequently encountered. *Sphagnum* occurs in patches throughout, and other bryophytes are common. The rare species *Bartonia texana* may be encountered in this system, along with other interesting forbs such as *Burmannia biflora* and *Apteria aphylla* (Elliott 2011).

**Dynamics:** This system is maintained by groundwater seepage. Soils have high available water capacity and surface runoff is very slow to ponded. This ecological system is embedded within fire‑maintained systems. The role of fire in this system was probably minimal except during droughts or in narrow occurrences where fire may have maintained an example of this system dominated by *Arundinaria gigantea*.

SOURCES

**References:** Ajilvsgi 1979, Brooks et al. 1993, Comer et al. 2003*, Elliott 2011, Eyre 1980, Hoagland 2000, LDWF 2005, Marks and Harcombe 1981, Martin and Smith 1991, Nixon et al. 1983a, Soil Conservation Service 1990, Van Kley 1999a

**Version:** 14 Jan 2014	**Stakeholders:** Midwest, Southeast

**Concept Author:** R. Evans	**LeadResp:** Southeast

M031. Southern Coastal Plain Floodplain Forest

CES203.247 Atlantic Coastal Plain Blackwater Stream Floodplain Forest

**Primary Division:** Gulf and Atlantic Coastal Plain (203)

**Land Cover Class:** Woody Wetland

**Spatial Scale & Pattern:** Linear

**Required Classifiers:** Natural/Semi‑natural; Vegetated (>10% vasc.); Wetland

**Diagnostic Classifiers:** Riverine / Alluvial [Blackwater]

**Concept Summary:** This Atlantic Coastal Plain system, which is most abundant in the Carolinas and Georgia, occurs in floodplains of small streams that carry little mineral sediment (blackwater streams). These streams occur in low areas within sandy portions of the Coastal Plain. The water is usually strongly stained by tannins and other dissolved organics and has little suspended mineral sediment. Depositional landforms may be absent or present in limited variety and of small size. Soils are usually strongly acidic. The duration of flooding is long (semipermanent) in the wettest areas, and shorter in slightly higher gradient small streams. Some small blackwater streams near the Fall‑line Sandhills have most of their flow from sandhill seepage and have limited fluctuation in water levels. But other blackwater stream channels may dry out during the late summer. In these cases, water tables are not far below the channel, and are high enough that the deeper depressions may still hold water. Vegetation varies from north to south, but generally consists almost entirely of forests of wetland trees, but occasional, small shrub‑dominated sloughs may also be present. A variety of tree species may be present; wetter examples (especially toward the northern range limits of this system) are often strongly dominated by *Taxodium distichum* and *Nyssa biflora*. Other examples have mixtures of these species with *Quercus* spp. and other bottomland hardwoods tolerant of blackwater conditions. Species richness ranges from low to moderate, but is lower than in comparable brownwater systems. Flooding is an important ecological factor in this system and may be the most important factor separating it from adjacent systems. However, the high water table supported by inflow from adjacent areas also maintains these areas as wetlands. Flooding excludes non‑flood‑tolerant species. Unlike river systems, flooding tends to be variable and of shorter duration.

**Comments:** The distinction between brownwater and blackwater streams is sometimes problematic. A number of plant species are characteristic of brownwater floodplains and not blackwater. Well‑developed blackwater streams may be confined to areas with primarily sandy soils. The boundary between systems based on river/stream size is necessarily somewhat arbitrary, but is based on significant differences which correspond with river size. Small streams have small watersheds, which tend to lead to more irregular flooding. Depositional landforms are small enough that they do not differentiate communities well, and communities tend to have more of a mixture of species that are segregated on the larger floodplains. The boundary between this system and Atlantic Coastal Plain Streamhead Seepage Swamp, Pocosin and Baygall (CES203.252) may be somewhat gradual. It should be based on the predominance of seepage influence over flooding influence, but vegetational differences may also be partly determined by fire regime. Southern Coastal Plain Spring‑run Stream Aquatic Vegetation (CES203.275) shares many characteristics with this system, but differs in having calcareous water and more steady flows.

DISTRIBUTION

**Range:** This system is potentially found throughout the Atlantic Coastal Plain north to about the James River in Virginia, but it is most abundant in the Carolinas and Georgia.

**Divisions:** 203:C

**TNC Ecoregions:** 55:C, 56:C, 57:C

**Nations:** US

**Subnations:** FL, GA, NC, SC, VA

**Map Zones:** 55:C, 58:C, 60:C

**USFS Ecomap Regions:** 232A:CC, 232C:CC, 232H:CC, 232I:CC, 232J:CC

CONCEPT

**Environment:** Examples of this system occur in floodplains of small streams of the Atlantic Coastal Plain that carry little mineral sediment (blackwater streams). These streams occur in low areas within sandy portions of the Coastal Plain (Smock and Gilinsky 1992). The water is usually strongly stained by tannins but has little suspended clay and is not turbid. Depositional landforms may be absent or may be present in limited variety and of small size. Soils are generally sandy in drier portions of the floodplain, mucky in wetter portions, or may be uniform organic soils. Soils are usually strongly acidic, but spring‑fed rivers or streams may have local components with calcareous water and non‑acidic soils. Flooding ranges from semipermanent in the wettest floodplains to intermittent and short in slightly higher areas and along higher gradient streams. Some small blackwater streams near the Fall‑line Sandhills have most of their flow from sandhill seepage and have limited fluctuation in water levels, but other blackwater stream channels may dry out during the late summer. In these cases, water tables are not far below the channel, and are high enough that the deeper depressions may still hold water (Smock and Gilinsky 1992). Sediment oxygen demand is high in blackwater swamp areas which have long‑duration flooding and high amounts of total organic carbon in the soil and sediments. Evidence suggests that blackwater streams may naturally be low in dissolved oxygen (Todd et al. 2010).

The fluvial features of riverine floodplains occur less frequently along small streams. These features, such as river terraces, oxbows, alluvial flats, point bars, and streamside levees, may occur, but on a smaller scale and sometimes are poorly developed. Fine‑scale alluvial floodplain features may be abundant. In pre‑European settlement forests, community diversity in these streamside systems was much more complex than in the modified landscapes of today. Fire and beaver activity created a mosaic whose elements included canebrakes, beaver ponds and grass‑sedge meadows in abandoned beaver clearings, as well as the streamside zones and mixed hardwood and/or *Pinus* spp. forests that make up more than 95% of the cover that exists today. The most prominent evergreen south of Virginia is the shade‑intolerant *Pinus taeda*, which manages to maintain itself by reproducing in larger (multi‑tree) treefall gaps.

**Vegetation:** Vegetation in the wetter zones consists of forests composed of obligate wetland trees. Composition is more diverse and variable in other areas that are less prone to flooding. Wetter examples are strongly dominated by *Taxodium distichum* and *Nyssa biflora*. Other examples have mixtures of these species with *Quercus* spp. and other bottomland hardwoods tolerant of some flooding and drier conditions in the summer (Burke et al. 2003). Except in the very wet examples, understory, shrub, and herb layers are generally well‑developed, and woody vines are also prominent. Species richness ranges from low to moderate but is lower than in comparable brownwater systems.

**Dynamics:** Flooding is an important ecological factor in this system and may be the most important factor separating it from adjacent systems. Flooding brings nutrients and excludes non‑flood‑tolerant species. Unlike river systems, flooding tends to be variable and of shorter duration. It is unclear how important aquatic fauna are when the system is flooded, but they may be important. The small flows, low gradient, and binding of sediment by vegetation limit channel shifts and sediment movement, but floods may cause local disturbance by scouring. The areas flooded for the longest durations tend to have accumulations of organic sediments which deplete levels of aquatic dissolved oxygen (Todd et al. 2010). Most of these forests would exist naturally as multi‑aged old‑growth forests driven by gap‑phase regeneration. Windthrow is probably the most important cause of canopy gaps.

Fire is probably more important than in larger river systems, because distances to uplands are short and because stream channels and sloughs are smaller and less effective as firebreaks. However, most of the vegetation is not very flammable and usually will not carry fire. Some of these areas apparently were once canebrakes, which presumably were maintained by periodic fire. Fire‑return interval varied highly in this system. Except in canebrakes, most fires were very light surface fires, creeping in hardwood or pine litter with some thin, patchy cover of bottomland grasses such as *Chasmanthium laxum* and *Chasmanthium latifolium*. Flame lengths are typically 15‑30 cm (6‑12 inches) (Landfire 2007a). Even so, fire‑scarred trees can be found in most small stream sites except in the wettest microsites. Stand‑replacement fires are unknown in this type. Except where Native American burning was involved, fires likely occurred primarily during drought conditions and then often only when fire spread into bottomlands from more pyrophytic uplands. Trees may be partially girdled by fire in duff, followed by bark sloughing. While fire rarely killed the tree, this allowed entry of rot, which, in the moist environment, often resulted in hollow trees, providing nesting and denning habitat for many species of birds and animals. Surface fires occurred on a frequency ranging from about 3 to 8 years in streamside canebrake, streamside hardwood/canebrake, or pine, to 25 years or more in hardwood litter. Low areas having a long hydroperiod, islands, and areas protected from fire by backswamps and oxbows were virtually fire‑free. Fire effects were largely limited to top‑kill of shrubs and tree saplings less than 5 cm (2 inches) diameter, and the formation of hollow trees.

The distinctive dynamics of stream flooding and protected topographic position dominate the distinctive vegetation of this system. The small watersheds and sometimes higher gradients on these streams may limit floods to fairly short duration. Flooding is most common in the winter, but may occur in other seasons. The sorting of plants by depositional landforms of different heights suggests that wetness or depth of flood waters has significance. In higher gradient streams, flood waters have significant energy. Scouring and reworking of sediment make up an important factor on the streambanks, and channels may occasionally change course. In addition to disturbance, floods bring nutrient input, deposit sediment and disperse plant seeds. However, because of the limited sediment transport, nutrient input is less in blackwater stream systems than in other floodplains. Stream flooding rarely leads to canopy tree mortality.

The most significant natural disturbance along small streams is wind. Winds create gaps, usually of small to medium size, in which trees regenerate and where smaller vegetation temporarily proliferates. Winds affect streamside forests because of wet sandy or mucky soils, and trees that are shallow‑rooted. Canopy tree mortality was generally limited to tree‑by‑tree or small group replacement. Windthrow formed the primary cause of tree mortality in bottomlands. The frequency of these events equates with major hurricanes occurring at approximately 20‑year intervals. Tornado tracks can be found passing across uplands and bottomlands, leaving narrow swaths of felled trees. The majority of windthrow seems to have been the result of hurricanes and tornadoes spawned by them. However, some of the most abundant tree species of Coastal Plain blackwater stream floodplains, *Taxodium distichum* and *Nyssa biflora*, are notably stable in strong winds. Susceptibility to wind mortality may depend on the species composition of a given community.

Beavers were once an important part of the dynamics of these systems, one which is returning to higher frequency in some areas. Beavers can dam the main channel of many small streams, and create ponds which can cover the entire width of the floodplain for a stretch (M. Schafale pers. comm. 2013). Ponds are often built in series, so that as much as a kilometer or two of the stream may be affected. Most of the crucial parameters of beaver dynamics under natural conditions are unknown or poorly known. Abundance of beavers, duration of a colony in a given place, and whether dam sites were chosen at random or whether specific favorable sites were repeatedly used would have had major effects on the ecology of this system. The existence of a diverse flora of native aquatic plants of ponds, which appear to take long times to colonize a pond (they are found in greater diversity in 100+‑year‑old millponds than in younger impoundments) hints that beaver ponds may have been long‑lasting features. Impoundment drowns the lower strata of plants and displaces non‑aquatic fauna, leading to colonization by aquatic plants and shade‑intolerant marsh herbs and shrubs. However, *Taxodium distichum* and *Nyssa biflora* trees in the swamp forest may survive to provide a partial to complete tree canopy. When beavers abandon a pond, the dam will eventually breach, but sometimes remains and at least partially impounds the area for a long time. With the limited mineral sediment input, long‑standing ponds fill with muck, sometimes developing boggy vegetation that may persist for many years (M. Schafale pers. comm. 2013).

SOURCES

**References:** Burke et al. 2003, Comer et al. 2003*, Devall 1998, Engeman et al. 2007, Eyre 1980, Harris 1989, LANDFIRE 2007a, Nelson 1986, Rosen et al. 2006, Schafale 2012, Schafale pers. comm., Schuster 1974, Sharitz and Mitsch 1993, Smock and Gilinsky 1992, Todd et al. 2010

**Version:** 23 Apr 2015	**Stakeholders:** East, Southeast

**Concept Author:** M. Schafale and R. Evans	**LeadResp:** Southeast

CES203.249 Atlantic Coastal Plain Small Blackwater River Floodplain Forest

**Primary Division:** Gulf and Atlantic Coastal Plain (203)

**Land Cover Class:** Woody Wetland

**Spatial Scale & Pattern:** Linear

**Required Classifiers:** Natural/Semi‑natural; Vegetated (>10% vasc.); Wetland

**Diagnostic Classifiers:** Riverine / Alluvial [Blackwater]

**Concept Summary:** This ecological system encompasses the floodplains of small to medium blackwater rivers in the Atlantic Coastal Plain which are intermediate in size between the smaller streams and the largest rivers. Blackwater rivers originate in the sandy areas of the Coastal Plain, carry little sediment, and have less well‑developed depositional alluvial landforms. The water is usually strongly stained by tannins but has little suspended clay and is not turbid. Soils are sandy or mucky, acidic, and infertile. Vegetation is a mosaic of cypress and gum swamps and bottomland hardwoods dominated by a limited set of oaks and other species. The lowest, wettest areas have some combination of *Taxodium distichum, Taxodium ascendens*, and *Nyssa biflora*. *Nyssa aquatica* is generally scarce or absent. Higher portions of the floodplain have forests with combinations of a small set of wetland oaks and other species, including *Quercus laurifolia, Quercus lyrata, Quercus nigra, Liquidambar styraciflua, Pinus taeda, Magnolia virginiana*, and other species. In general, vegetation is low in species richness.

**Comments:** The distinction between brownwater and blackwater rivers is sometimes problematic. A number of plant species are characteristic of brownwater floodplains and not blackwater. Well‑developed blackwater rivers may be confined to areas with primarily sandy soils. The boundary between systems based on river/stream size is necessarily somewhat arbitrary, but is based on significant differences which correspond with river size. Smaller streams have smaller watersheds, which tend to lead to more variable water levels and irregular flooding. Depositional landforms are small enough that they do not differentiate communities well, and communities tend to have more of a mixture of species that are segregated on the larger floodplains. Large rivers have greater variation in water levels and have flood regimes that integrate the effects of very large watersheds. Depositional landforms are larger, and communities can be more segregated.

DISTRIBUTION

**Range:** This system is potentially found throughout the Atlantic Coastal Plain from Georgia north to about the James River in Virginia, but it is most abundant in North Carolina and South Carolina.

**Divisions:** 203:C

**TNC Ecoregions:** 53:?, 56:C, 57:C, 58:?

**Nations:** US

**Subnations:** FL, GA, NC, SC

**Map Zones:** 55:C, 58:C, 60:P

**USFS Ecomap Regions:** 232A:CC, 232C:CC, 232H:CC, 232I:CC, 232J:CC

CONCEPT

**Environment:** Examples of this system occur in floodplains of medium to small coastal plain rivers that carry little mineral sediment (blackwater rivers). These rivers have their headwaters in sandy portions of the Atlantic Coastal Plain. The water is usually strongly stained by tannins but has little suspended clay and is not turbid. Depositional landforms such as natural levees and backswamps are usually not well‑developed, but point bars, ridge‑and‑swale systems (scrollwork), and sloughs caused by river meandering may be prominent. Soils are generally sandy in drier portions of the floodplain, mucky in wetter portions, and are very acidic (Smock and Gilinsky 1992). Spring‑fed rivers may have calcareous water and non‑acidic soils. Flooding ranges from semipermanent in the wettest areas to intermittent and short on the higher portions of the floodplain. Sediment oxygen demand is high in blackwater swamp areas which have long‑duration flooding and high amounts of total organic carbon in the soil and sediments. Evidence suggests that blackwater streams may naturally be low in dissolved oxygen (Todd et al. 2010). The sandy soils may make some higher areas within the floodplain well‑drained and dry when not flooded. The highest terraces may no longer flood at all and belong to a different system.<br />

Saturation and flooding by acidic water, high in tannins is a key process. These waters carry very little sediment, and are the color of dark tea. This is a linear to large‑patch ecological system; stands may be contiguous over thousands of acres. The largest examples could be called matrix examples of this ecological system. Examples are by nature linear, and tend to be narrow. The Satilla River in Georgia is about 375 km in length, and may be the largest example. The lower floodplain is about 2 km across; an approximate size of 750 km<sup>2</sup> could be used as a working upper bound. There may be limited areas with trees greater than 150 years. Probably there are many stands aged 70‑100 years, and many that are younger than 70 years. Stands that have not had extensive timber removal will probably have more woody debris and constitute better habitat for component animal and plant species.

**Vegetation:** Vegetation consists largely of forests dominated by wetland trees species. Non‑forested vegetation is present only on recently deposited bars and in oxbow lakes. The lowest, wettest areas have some combination of *Taxodium distichum, Taxodium ascendens*, and *Nyssa biflora*. *Nyssa aquatica* is generally scarce or absent. Higher portions of the floodplain have forests with combinations of a small set of wetland oaks and other species, including *Quercus laurifolia, Quercus lyrata, Quercus nigra, Liquidambar styraciflua, Pinus taeda, Magnolia virginiana*, and other species. Overall canopy species richness in a given site and over the system as a whole is lower than in comparable brownwater river systems. The distinctive levee assemblage of trees in brownwater river systems is largely lacking, though *Betula nigra, Salix nigra, Salix caroliniana*, and *Planera aquatica* may dominate banks and bars. The wettest forests are sometimes simple in structure, with an understory but little shrub or herb layer, but the other communities tend to have well‑developed understories, shrub, and herb layers. Woody vines are usually prominent. Areas that have been logged may become dominated by *Pinus taeda, Liquidambar styraciflua*, and *Acer rubrum* with a common shrub being *Morella cerifera*.

**Dynamics:** Flooding is the most important ecological factor in this system. Frequency and duration of flooding determine the occurrences of different associations and separate the system from other kinds of wetlands. Flooding brings nutrients and excludes non‑flood‑tolerant species. When flooded, the system may have a substantial aquatic faunal component, with high densities of invertebrates, and may play an important role in the life cycle of fish in the associated river. Unusually long or deep floods may stress vegetation or act as a disturbance for some species. Larger floods cause local disturbance by scouring and depositing sediment along channels, and occasionally causing channel shifts. However, the low‑gradient and binding of sediment by vegetation generally make these processes much slower and less frequent than in river systems of most other regions. The areas flooded for the longest durations tend to have high amounts of total organic carbon in the soil and sediments which deplete levels of aquatic dissolved oxygen (Todd et al. 2010).

Except for primary successional communities such as bars, most forests exist naturally as multi‑aged old‑growth forests driven by gap‑phase regeneration. Windthrow is probably the most important cause of gaps. In addition to periodic flooding, the formation of windfall gaps is a dominant ecological processes in bottomland hardwood forests. Windfall gaps occur from the local scale (a single mature canopy tree) to the landscape scale (effects of tornadoes and hurricanes). When canopy trees fall, seedlings in the understory are released and compete for a spot in the canopy. This leads to dense areas of herbaceous and woody vegetation in windfall gaps of all sizes. This is a major process in forest regeneration in bottomland hardwood forests.

Flooding is more frequent on the lower terraces but frequently floods higher terraces (Wharton zones IV and V). Catastrophic floods can cause the loss of canopy over large areas. Canopy decline and reproductive failure can create late‑seral open stands. Duration of flooding varies with the placement of a site in the landscape and is a dominant process affecting vegetation on a given site. Flooding can deposit alluvium or scour the ground, depending on the landscape position of a site and the severity of the flood event.

Fire is not believed to be important, due to low flammability of much of the vegetation, wetness, and abundance of natural firebreaks. Fire is infrequent on the lower terraces, but was frequent historically on older terraces outside the floodplain and crept into the floodplains. Putnam (1951, cited in Wharton et al. 1982) states that a serious fire season occurs on an average of about every 5 to 8 years in the bottomland hardwood forests of the Mississippi Alluvial Plain. Some areas of bottomlands apparently were once occupied by canebrakes, which presumably were maintained through deliberate fall burning by Native Americans. Infrequent, mild surface fires would occur in the system; however, they would not alter species composition or structure.

Changes in hydrology due to the activities of beaver are also an important ecological process in bottomland hardwood forests. Beaver impoundments kill trees (sometimes over large areas) and may create open‑water habitat, cypress‑tupelo stands, or cause stand replacement. Meandering streams are dynamic and frequently change course, eroding into the floodplain and depositing new point bars, thus creating new habitat for early‑seral plant communities. Insect outbreaks would occur infrequently in closed‑canopy states.

SOURCES

**References:** Burke et al. 2003, Comer et al. 2003*, Engeman et al. 2007, Eyre 1980, FNAI 2010a, Harris 1989, LANDFIRE 2007a, Nelson 1986, Putnam 1951, Rosen et al. 2006, Schafale 2012, Schuster 1974, Sharitz and Mitsch 1993, Smock and Gilinsky 1992, Todd et al. 2010, Wharton 1978, Wharton et al. 1982

**Version:** 23 Apr 2015	**Stakeholders:** East, Southeast

**Concept Author:** M. Schafale and R. Evans	**LeadResp:** Southeast

CES203.250 Atlantic Coastal Plain Small Brownwater River Floodplain Forest

**Primary Division:** Gulf and Atlantic Coastal Plain (203)

**Land Cover Class:** Woody Wetland

**Spatial Scale & Pattern:** Linear

**Required Classifiers:** Natural/Semi‑natural; Vegetated (>10% vasc.); Wetland

**Diagnostic Classifiers:** Riverine / Alluvial [Brownwater]

**Concept Summary:** This ecological system encompasses the floodplains of small to medium brownwater rivers of the Atlantic Coastal Plain which are intermediate in size between the smaller streams and the largest rivers. Brownwater rivers originate in clayey areas and carry substantial amounts of mineral sediment, creating well‑developed deposition alluvial landforms and fertile soils. These rivers have their headwaters in the Piedmont, Blue Ridge, Interior Plateaus, or in portions of the Coastal Plain where fine‑textured sediment predominates. Vegetation is a mosaic of cypress and gum swamps, oak‑dominated bottomland hardwoods, and mixed levee forests, with only local examples of embedded non‑forested communities. The lowest, wettest areas are dominated by a combination of *Taxodium distichum* and *Nyssa aquatica*. Natural levees and riverfronts have a diverse mixture of trees, including *Platanus occidentalis, Celtis laevigata, Fraxinus pennsylvanica, Acer negundo*, and others. Moderate to high parts of the floodplain away from the levee are usually dominated by bottomland hardwoods, including wetland oaks such as *Quercus laurifolia, Quercus michauxii, Quercus pagoda*, and sometimes a number of other species including *Liquidambar styraciflua*.

**Comments:** The distinction between brownwater and blackwater rivers is sometimes problematic. A number of plant species are characteristic of brownwater floodplains and not blackwater. Well‑developed blackwater rivers may be confined to areas with primarily sandy soils. The boundary between systems based on river/stream size is necessarily somewhat arbitrary, but is based on significant differences which correspond with river size. Smaller streams have smaller watersheds, which tend to lead to more variable water levels and irregular flooding. Depositional landforms are small enough that they don't differentiate communities well, and communities tend to have more of a mixture of species that are segregated on the larger floodplains. Large rivers have greater variation in water levels and have flood regimes that integrate the effects of very large watersheds. Depositional landforms are larger, and communities can be more segregated.

This system as defined covers a large geographic range. There are some significant biogeographic differences across this range, leading to a large number of associations. However, more plant species are shared across the region in this system than in most other systems in the region.

DISTRIBUTION

**Range:** This ranges throughout the Atlantic Coastal Plain from Georgia, north to about the James River in Virginia.

**Divisions:** 203:C

**TNC Ecoregions:** 56:C, 57:C

**Nations:** US

**Subnations:** GA, NC, SC, VA

**Map Zones:** 55:C, 58:C, 60:C

**USFS Ecomap Regions:** 232A:CC, 232C:CC, 232H:CC, 232I:CC, 232J:CC

CONCEPT

**Environment:** Examples of this system occur in floodplains of medium to small Coastal Plain rivers that carry significant mineral sediment (brownwater or redwater rivers). These rivers have their headwaters in the Piedmont, Blue Ridge, Interior Plateaus, or in portions of the Coastal Plain where fine‑textured sediment predominates. The water generally carries substantial amounts of silt, clay, and sometimes sand. Depositional landforms such as point bars, natural levees, backswamps, and ridge‑and‑swale systems (scrollwork) are well‑developed and form patterns of significant variation in flooding duration and nutrient input. Soil texture varies from sandy to clayey. Soils are generally fertile and not strongly acidic. Flooding ranges from semipermanent in the wettest areas to intermittent and short on the higher portions of the floodplain. The highest terraces may no longer flood at all and belong to a different system.

**Vegetation:** Vegetation consists largely of forests dominated by wetland tree species. Non‑forested vegetation is present only on recently deposited bars and in oxbow lakes. Three distinct groups of associations can be recognized. The lowest, wettest areas have some combination of *Taxodium distichum* and *Nyssa aquatica* dominating. Natural levees and riverfronts have a diverse mixture of trees that typically includes *Platanus occidentalis, Celtis laevigata, Fraxinus pennsylvanica, Acer negundo*, and other species that benefit from the high light levels and heavy alluvial deposition of these sites. Moderate to high parts of the floodplain away from the levee are usually dominated by bottomland hardwoods, various mixtures of wetland oaks, including *Quercus laurifolia, Quercus michauxii, Quercus pagoda*, and sometimes a number of other oak species, along with *Liquidambar styraciflua*, but other species are sometimes codominant. The wettest forests are sometimes simple in structure, with an understory but little shrub or herb layer, but the other communities tend to have well‑developed understories, shrub, and herb layers. Woody vines are usually prominent.

**Dynamics:** Flooding is the most important ecological factor in this system. Frequency and duration of flooding determines the occurrences of different associations and separates the system from other kinds of wetlands. Flooding brings nutrients and excludes non‑flood‑tolerant species. When flooded, the system has a substantial aquatic faunal component, with high densities of invertebrates, and may play an important role in the life cycle of fish in the associated river. Unusually long or deep floods may stress vegetation or act as a disturbance for some species. Larger floods cause local disturbance by scouring and depositing sediment along channels, and occasionally causing channel shifts. However, the low gradient and binding of sediment by vegetation generally makes these processes much slower and less frequent than in river systems of most other regions. Except for primary successional communities such as bars, most forests exist naturally as multi‑aged old‑growth forests driven by gap‑phase regeneration. Windthrow is probably the most important cause of gaps. Fire is not believed to be important, due to low flammability of much of the vegetation, wetness, and abundance of natural firebreaks. However, some areas of bottomlands apparently were once canebrakes, which presumably were maintained by periodic fire.

SOURCES

**References:** Comer et al. 2003*, Eyre 1980, Harris 1989, Nelson 1986, Schafale 2012, Schuster 1974, Sharitz and Mitsch 1993, Smock and Gilinsky 1992, Wharton 1978, Wharton et al. 1982

**Version:** 14 Jan 2014	**Stakeholders:** East, Southeast

**Concept Author:** M. Schafale and R. Evans	**LeadResp:** Southeast

CES203.299 East Gulf Coastal Plain Freshwater Tidal Wooded Swamp

**Primary Division:** Gulf and Atlantic Coastal Plain (203)

**Land Cover Class:** Woody Wetland

**Spatial Scale & Pattern:** Large patch

**Required Classifiers:** Natural/Semi‑natural; Vegetated (>10% vasc.); Wetland

**Diagnostic Classifiers:** Forest and Woodland (Treed); Riverine / Alluvial; Tidal / Estuarine

**Concept Summary:** This ecological system encompasses the tidally flooded portions of river floodplains which flow into the northern Gulf of Mexico east of the Mississippi River. Large outflows of freshwater keep salinity levels at a minimum, and flooding is of short enough duration to allow survival of tree canopies. Stands are dominated by a combination of *Nyssa aquatica, Nyssa biflora, Taxodium distichum*, and *Fraxinus pennsylvanica*. Other plants that are typically present include *Magnolia virginiana, Sabal palmetto, Juniperus virginiana var. silicicola, Cyrilla racemiflora, Quercus laurifolia, Sabal minor, Taxodium ascendens, Cliftonia monophylla, Pinus elliottii var. elliottii, Chamaecyparis thyoides, Hypericum nitidum, Cladium mariscus ssp. jamaicense*, and *Persea palustris*. These swamps may be regularly flooded at least twice daily.

DISTRIBUTION

**Range:** This system includes river floodplains which flow into the northern Gulf of Mexico east of the Mississippi River, including the Appalachicola, the Ochlockonee, the St. Marks, the Suwanee, and the Wakulla rivers.

**Divisions:** 203:C

**TNC Ecoregions:** 53:C

**Nations:** US

**Subnations:** AL, FL, LA, MS

**Map Zones:** 55:C, 99:C

**USFS Ecomap Regions:** 232D:CC, 232L:CC

CONCEPT

**Environment:** This system occurs in lower reaches of river floodplains and along estuary shorelines, in places regularly or irregularly flooded by lunar or wind tides. The water has little salt content, due to distance from the ocean and/or strong freshwater input. Soils may be mineral or organic. Soils are generally permanently saturated even when the tide is low. The transition of the hydrology to flood dominance rather than tidal dominance may be very gradual.

**Vegetation:** Stands are dominated by a combination of *Nyssa aquatica, Nyssa biflora, Taxodium distichum*, and *Fraxinus pennsylvanica*. Other plants that are typically present include *Magnolia virginiana, Sabal palmetto, Juniperus virginiana var. silicicola, Cyrilla racemiflora, Quercus laurifolia, Sabal minor, Taxodium ascendens, Cliftonia monophylla, Pinus elliottii var. elliottii, Chamaecyparis thyoides, Hypericum nitidum, Cladium mariscus ssp. jamaicense*, and *Persea palustris*.

**Dynamics:** Regular or irregular tidal flooding with freshwater is the ecological factor that makes this system distinct. These swamps may be regularly flooded at least twice a day for several hours and remain inundated for days during flood or storm events (Wharton et al. 1982, FNAI 1990). River floods may also seasonally affect this system. Wind and flooding are the dominant disturbance agents in this type and this includes wind damage from hurricanes and tornadoes as well as inundation of young stands. Canopy gaps can be created by high winds, such as from nor'easters, tropical storms and hurricanes (Nordman 2013). Infrequent intrusion of saltier water, which is stressful or fatal to many of the plant species, is an important periodic disturbance created by storms. Insect outbreaks would occur infrequently in these closed‑canopy forests (Landfire 2007a). This system generally appears to be in a shifting relationship with tidal freshwater marshes of the same region. Most marshes have standing dead trees in them, suggesting they recently were swamps. But, conversely, some marshes are being invaded with trees and may be turning into swamps. Freshwater tidal marshes generally occur at the shallow edge of tidal rivers and streams, where river and tidal flow is high, and the vegetation is affected by the changing meanders of the tidal channel. Rising sea level is driving shifts in the communities of this system, causing upstream non‑tidal swamps to develop into this system (as they become subject to tides) and causing parts of this system to turn into brackish marshes. In areas not too strongly affected by saltwater intrusion or drowning by rising sea level, these communities can be expected to exist as old‑growth, multi‑aged forests.

SOURCES

**References:** Comer et al. 2003*, Edwards et al. 2013, Eyre 1980, FNAI 2010a, Harris 1989, LANDFIRE 2007a, Nordman 2013, Odum et al. 1984, Schafale pers. comm., Wharton et al. 1982

**Version:** 11 Jun 2014	**Stakeholders:** Southeast

**Concept Author:** R. Evans	**LeadResp:** Southeast

CES203.489 East Gulf Coastal Plain Large River Floodplain Forest

**Primary Division:** Gulf and Atlantic Coastal Plain (203)

**Land Cover Class:** Woody Wetland

**Spatial Scale & Pattern:** Linear

**Required Classifiers:** Natural/Semi‑natural; Vegetated (>10% vasc.); Wetland

**Diagnostic Classifiers:** Forest and Woodland (Treed); Riverine / Alluvial [Brownwater]

**Concept Summary:** This system represents a geographic subset of Southern Floodplain Forest. Examples may be found along large rivers of the East and Upper East Gulf Coastal Plain, especially the Apalachicola, Alabama/Cahaba, Tombigbee, Pascagoula, and Pearl rivers, all of which ultimately drain into the Gulf of Mexico. Several distinct plant communities can be recognized within this system that may be related to the array of different geomorphologic features present within the floodplain. Some of the major geomorphic features associated with different community types include natural levees, point bars, meander scrolls, oxbows, and sloughs. Vegetation generally includes forests dominated by bottomland hardwood species and other trees tolerant of flooding. However, herbaceous and shrub vegetation may be present in certain areas as well.

**Comments:** In the Upper East Gulf Plain of Kentucky, this system is represented in the Ecoregions of Kentucky map (Woods et al. 2002) by the lower part of the Wabash‑Ohio bottomlands (72a). In the lower Gulf Coastal Plain, this includes at least EPA (Omernik) Level IV ecoregions 65p and 75i (EPA 2004).

DISTRIBUTION

**Range:** This system is found in the East and Upper East Gulf coastal plains, and includes the Apalachicola, Alabama, Tombigbee, Pascagoula, and Pearl rivers, all of which ultimately drain into the Gulf of Mexico.

**Divisions:** 203:C

**TNC Ecoregions:** 43:C, 53:C

**Nations:** US

**Subnations:** AL, FL, GA, KY, MS, TN

**Map Zones:** 46:C, 47:C, 55:C, 99:C

**USFS Ecomap Regions:** 231B:CC, 232B:CC, 232J:CC, 232L:CC

CONCEPT

**Environment:** This system represents a geographic subset of Kuchler's (1964) Southern Floodplain Forest. Examples of this system are generally forested with stands of bottomland hardwood species and other trees tolerant of flooding. Local composition varies depending upon actual position within the floodplain, disturbance history, and underlying soils and geology. Although most examples of this system may be thought of as acidic, some examples of this system flow through regions with sufficient calcareous influence to effect vegetation composition. Some of the major geomorphic features associated with different community types include natural levees, point bars, meander scrolls, oxbows, and sloughs (Sharitz and Mitsch 1993).

**Vegetation:** Common canopy trees may include *Betula nigra, Carya illinoinensis, Celtis laevigata, Fraxinus pennsylvanica, Liquidambar styraciflua, Nyssa aquatica, Nyssa biflora, Platanus occidentalis, Populus deltoides, Quercus laurifolia, Quercus lyrata, Quercus nigra, Quercus pagoda, Quercus phellos, Quercus texana, Ulmus americana, Salix caroliniana*, and *Taxodium distichum*. Smaller trees may include *Acer negundo, Carpinus caroliniana, Gleditsia triacanthos, Nyssa ogeche, Planera aquatica*, and *Ulmus crassifolia*. Shrubs may include *Cephalanthus occidentalis, Crataegus viridis, Halesia diptera, Ilex decidua*, and *Lyonia lucida*.

**Dynamics:** In pre‑European settlement forests, community diversity in these bottomland systems was much more complex than in the modified landscapes of today. Fire, beaver activity, and flooding of varied intensity and frequency created a mosaic whose elements included canebrake, grass and young *Betula‑Platanus* beds on reworked gravel or sand bars, beaver ponds, and grass‑sedge meadows in abandoned beaver clearings, as well as the streamside zones and mixed hardwood and/or pine forests that make up more than 95% of the land cover that exists today.

The dominant ecological processes in bottomland hardwood forests are windfall gaps and periodic flooding. Windfall gaps occur on the local scale (the fall of a single mature canopy tree) as well as the landscape scale (storms, hurricanes). When canopy trees fall, seedlings in the understory are released and compete for a spot in the canopy. This leads to dense areas of herbaceous and woody vegetation in windfall gaps of all sizes. This is a major process in forest regeneration in bottomland hardwood forests. Canopy decline and reproductive failure can create late‑seral open stands.

<br />Flooding is more frequent on the lower terraces but frequently impacts higher terraces as well (Wharton et al. (1982) zones IV & V). Catastrophic floods can cause the loss of canopy over large areas, and large coastal areas are also impacted by storm surges from hurricanes and tropical storms as well as by salt deposition in the immediate coastal area. The duration of flooding varies with the placement of a particular site in the landscape and is a dominant process affecting vegetation on a given site. Flooding can deposit alluvium or scour the ground, depending on the landscape position of a site and the severity of the flood event.

Fire is infrequent and of limited importance in lower, wetter areas, but was historically important in the older and higher terraces, especially areas adjacent to upland pine or pine flatwoods, and also crept into the floodplains. Putnam (1951 as cited in Wharton et al. 1982) states that a serious fire season occurs on an average of about every 5 to 8 years in the bottomland hardwood forests of the Mississippi Alluvial Plain. It is conjectured that Native Americans maintained canebrakes by deliberate fall burning. Infrequent, mild surface fires would occur in the system and would cause changes in composition and structure due to low fire tolerance.

Changes in hydrology due to the activities of beaver are also an important ecological process in bottomland hardwood forests. Beaver impoundments kill trees (sometimes over large areas) and may create open water habitat, cypress‑tupelo stands, or cause stand replacement. Meandering streams are dynamic and frequently change course, eroding into the floodplain and depositing new point bars, thus creating new habitat for early‑seral plant communities. In addition, insect outbreaks would occur infrequently in closed‑canopy states, opening up the canopy at least temporarily.

SOURCES

**References:** Comer et al. 2003*, EPA 2004, Evans et al. 2009, Eyre 1980, FNAI 2010a, Harris 1989, Küchler 1964, LANDFIRE 2007a, Putnam 1951, Sharitz and Mitsch 1993, Wharton et al. 1982, Woods et al. 2002

**Version:** 30 Jun 2016	**Stakeholders:** Southeast

**Concept Author:** R. Evans and A. Schotz	**LeadResp:** Southeast

CES203.559 East Gulf Coastal Plain Small Stream and River Floodplain Forest

**Primary Division:** Gulf and Atlantic Coastal Plain (203)

**Land Cover Class:** Woody Wetland

**Spatial Scale & Pattern:** Linear

**Required Classifiers:** Natural/Semi‑natural; Vegetated (>10% vasc.); Wetland

**Diagnostic Classifiers:** Forest and Woodland (Treed); Seepage‑Fed Sloping; Riverine / Alluvial [Brownwater]; Intermittent Flooding

**Concept Summary:** This is a predominantly forested system of the East Gulf Coastal Plain associated with small brownwater rivers and creeks. In contrast to East Gulf Coastal Plain Large River Floodplain Forest (CES203.489), it has fewer major geomorphic floodplain features typically associated with large river floodplains. Those features that are present tend to be smaller and more closely intermixed with one another, resulting in less obvious vegetational zonation. Bottomland hardwood tree species are typically important and diagnostic, although mesic hardwood species are also present in areas with less inundation, such as upper terraces and possibly second bottoms. As a whole, flooding occurs annually, but the water table usually is well below the soil surface throughout most of the growing season. Areas impacted by beaver impoundments are also included in this system.

**Comments:** This is primarily a linear system, with some variability as to the size type of the associations included within it. Most are temporarily flooded, with the possible addition of smaller‑scale seasonally flooded features such as beaver‑created herbaceous wetlands and shrub‑dominated features. It is confined to floodplains or terraces of streams and creeks. This system is dependent on a natural hydrologic regime, especially annual to episodic flooding. These landscapes usually encompass a variety of habitats resulting from natural hydrological spatial patterns (i.e., meander scars, sloughs, old depressions, and/or oxbows are present).

DISTRIBUTION

**Range:** This system is found in the East Gulf Coastal Plain, from the coast northward and inland to the extent of unconsolidated sediments in Kentucky.

**Divisions:** 203:C

**TNC Ecoregions:** 43:C, 53:C

**Nations:** US

**Subnations:** AL, FL, GA, KY, MS, TN

**Map Zones:** 46:C, 47:C, 55:C, 99:C

**USFS Ecomap Regions:** 231B:CC, 231H:CC, 232B:CC, 232D:CC, 232J:CC, 232K:CC, 232L:CC, 234A:CC

CONCEPT

**Environment:** This system is associated with small brownwater rivers and creeks of the East Gulf Coastal Plain. It is confined to floodplains or terraces of streams and creeks. This system is dependent on a natural hydrologic regime, especially annual to episodic flooding. These landscapes usually encompass a variety of habitats resulting from natural hydrological spatial patterns (i.e., meander scars, sloughs, gravel bars, old depressions, and/or oxbows are present). Most component associations are temporarily flooded, with the possible addition of smaller‑scale seasonally flooded features such as beaver‑created herbaceous wetlands and shrub‑dominated features. Some larger examples of this system include the Escambia, the Yellow (Alabama, Florida), the Choctawhatchee, the Chattahoochee, and the Flint rivers.

**Vegetation:** Examples of this system may include a number of different plant communities, each with distinctive floristic compositions. Drew et al. (1998) described vegetation attributable to this systems as including the following species: *Carya glabra, Magnolia grandiflora, Quercus virginiana, Liquidambar styraciflua, Acer floridanum (= Acer barbatum), Fraxinus americana, Fraxinus caroliniana, Celtis laevigata, Sabal minor, Ditrysinia fruticosa (= Sebastiania fruticosa), Serenoa repens*, and *Itea virginica*. Smaller‑scale features may be dominated by shrubs (*Cephalanthus occidentalis, Decodon verticillatus*) and/or perennial and annual herbs.

**Dynamics:** In pre‑European settlement forests, community diversity in these bottomland systems was much more complex than in the modified landscapes of today. Fire, beaver activity, and flooding of varied intensity and frequency created a mosaic whose elements included canebrake, grass and young *Betula‑Platanus* beds on reworked gravel or sand bars, beaver ponds, and grass‑sedge meadows in abandoned beaver clearings, as well as the streamside zones and mixed hardwood and/or pine forests that make up more than 95% of the land cover that exists today.

Flooding is the principal disturbance in this system. When flooded, these systems may have a substantial aquatic faunal component, with high densities of invertebrates, and may play an important role in the life cycle of fish in the associated river. Unusually long or deep floods may stress vegetation or act as a disturbance for some species. Flood waters have significant energy. Larger floods cause local disturbance by scouring and depositing sediment along channels and occasionally causing channel shifts. There are two general types of floods: occasional catastrophic, prolonged floods (due to beaver activity or other severe event); and more frequent repeated minor flooding (i.e., several minor floods within a 10 year period). Flooding is more frequent on the lower terraces but frequently floods higher terraces (Wharton et al. (1982) zones IV and V). Catastrophic floods can cause the loss of canopy over large areas. Canopy decline and reproductive failure can create late‑seral open stands. Duration of flooding varies with the placement of a site in the landscape and is a dominant process affecting vegetation on a given site. Flooding can deposit alluvium or scour the ground, depending on the landscape position of a site and the severity of the flood event. The sorting of plant communities by depositional landforms of different height suggest that wetness or depth of flood waters helps drive this process. Scouring and reworking of sediment make up an important factor in bar and bank communities. In addition to disturbance, floods bring nutrient input, deposit sediment, and disperse plant seeds (Landfire 2007a).

In addition to periodic flooding, the dominant ecological process in bottomland hardwood forests is the formation of windfall gaps, which can occur on the local scale (a single mature canopy tree) as well as the landscape scale (effects of tornadoes or hurricanes). Except for primary successional communities such as bars, most forests exist naturally as multi‑aged old‑growth forests driven by gap‑phase regeneration. Windthrow is probably the most important cause of gaps, and is the primary cause of mortality in bottomlands. Major storms or hurricanes occurring at approximately 20‑year intervals would have impacted whole stands. When canopy trees fall, seedlings in the understory are released and compete for a spot in the canopy. This leads to dense areas of herbaceous and woody vegetation in windfall gaps of all sizes. This is a major process in forest regeneration in bottomland hardwood forests.

Fire is infrequent and of limited importance in lower, wetter areas, but was historically important in the older and higher terraces, and also crept into the floodplains. Putnam (1951 as cited in Wharton et al. 1982) states that a serious fire season occurs on an average of about every 5 to 8 years in the bottomland hardwood forests of the Mississippi Alluvial Plain. It is conjectured that Native Americans maintained canebrakes by deliberate fall burning. Infrequent, mild surface fires would occur in the system; however, they would not alter species composition or structure. Except in canebrake, most fires were very light surface fires, creeping in hardwood or pine litter with some thin, patchy cover of bottomland grasses such as *Chasmanthium laxum* and *Chasmanthium latifolium*. Flame lengths were mostly 15 to 30 cm (6‑12 inches). Fire‑scarred trees can be found in most small stream sites except in the wettest microsites. Stand‑replacement fires are unknown in this type. Except where Native American burning was involved, fires likely occurred primarily during drought conditions and then often only when fire spread into bottomlands from more pyrophytic uplands. Trees may be partially girdled by fire in duff, followed by bark sloughing. While fire rarely killed the tree, this allowed entry of rot, which, in the moist environment, often resulted in hollow trees, providing nesting and denning habitat for many species of birds and animals. Surface fires occurred on a frequency ranging from about 3 to 8 years in streamside canebrake, streamside hardwood/canebrake, or pine, to 25 years or more in hardwood litter. Low areas having a long hydroperiod, islands, and areas protected from fire by backswamps and oxbows were virtually fire‑free. Fire effects were largely limited to top‑kill of shrubs and tree saplings less than 5 cm (2 inches) in diameter, and formation of hollow trees (Landfire 2007a).

Changes in hydrology due to the activities of beaver is also an important ecological process in bottomland hardwood forests. Beaver impoundments kill trees (sometimes over large areas) and may create open water habitat, cypress‑tupelo stands, or cause stand replacement. Meandering streams are dynamic and frequently change course, eroding into the floodplain and depositing new point bars, thus creating new habitat for early‑seral plant communities. In addition, insect outbreaks would occur infrequently in closed canopy states.

The distinctive dynamics of stream flooding and protected topographic position dominate the forming of the distinctive vegetation of this system. Not all of the factors are well known. Gradients of most of these rivers limit floods to fairly short duration. Flooding is most common in the winter, but may occur in other seasons.

SOURCES

**References:** Comer et al. 2003*, Drew et al. 1998, Evans et al. 2009, Eyre 1980, FNAI 2010a, Harris 1989, LANDFIRE 2007a, Putnam 1951, Sharitz and Mitsch 1993, Wharton et al. 1982

**Version:** 14 Jan 2014	**Stakeholders:** Southeast

**Concept Author:** M. Pyne and R. Evans	**LeadResp:** Southeast

CES203.490 Mississippi River Bottomland Depression

**Primary Division:** Gulf and Atlantic Coastal Plain (203)

**Land Cover Class:** Woody Wetland

**Spatial Scale & Pattern:** Large patch

**Required Classifiers:** Natural/Semi‑natural; Vegetated (>10% vasc.); Wetland

**Diagnostic Classifiers:** Forest and Woodland (Treed); Riverine / Alluvial [Brownwater]; Needle‑Leaved Tree; Broad‑Leaved Deciduous Tree

**Concept Summary:** This system represents semipermanently flooded to saturated depressional areas of the lower Mississippi River Alluvial Valley, from southern Illinois south to Mississippi and Louisiana. These areas have a distinctly longer hydroperiod than other parts of the landscape. Typical and characteristic trees in examples of this system include *Acer rubrum var. drummondii, Carya aquatica, Fraxinus profunda, Gleditsia aquatica, Nyssa aquatica, Nyssa biflora, Planera aquatica, Quercus lyrata, Quercus palustris, Salix nigra*, and *Taxodium distichum*. Some characteristic shrubs include *Cephalanthus occidentalis, Cornus foemina, Decodon verticillatus, Forestiera acuminata, Itea virginica*, and *Planera aquatica*. Herbs are uncommon, but *Ludwigia peploides, Sagittaria lancifolia, Ceratophyllum* spp., *Elodea* spp., *Potamogeton* spp., and *Lemna minor* may be found. It includes the "green ash ponds" on Macon Ridge in northeastern Louisiana.

DISTRIBUTION

**Range:** This system is found in the Mississippi Alluvial Plain from southern Illinois south to Mississippi and Louisiana.

**Divisions:** 203:C

**TNC Ecoregions:** 42:C

**Nations:** US

**Subnations:** AR, IL, KY, LA, MO, MS, TN

**Map Zones:** 45:C, 47:?, 98:C

**USFS Ecomap Regions:** 232E:CC, 234A:CC, 234C:CC, 234D:CC, 234E:CC

CONCEPT

**Environment:** Examples of this system are found in depressions and backswamps of the lower Mississippi River Alluvial Valley, from southern Illinois south to Mississippi and Louisiana. These areas have a distinctly longer hydroperiod than other parts of the landscape. Along the Macon Ridge in northeast Louisiana, ponds dominated by *Fraxinus pennsylvanica* occur only in small depressions of generally less than an acre to only a few acres. They are considered isolated wetlands since they do not receive alluvial flooding (LNHP 2009).

**Vegetation:** Typical and characteristic trees in examples of this system include *Acer rubrum var. drummondii, Carya aquatica, Fraxinus profunda, Gleditsia aquatica, Nyssa aquatica, Nyssa biflora, Planera aquatica, Quercus lyrata, Quercus palustris, Salix nigra*, and *Taxodium distichum*. Some characteristic shrubs include *Cephalanthus occidentalis, Cornus foemina, Decodon verticillatus, Forestiera acuminata, Itea virginica*, and *Planera aquatica*. Herbs are uncommon, but *Ludwigia peploides, Sagittaria lancifolia, Ceratophyllum* spp., *Elodea* spp., *Potamogeton* spp., and *Lemna minor* may be found.

**Dynamics:** Flooding is more frequent and of longer duration in these depressions and on the lower terraces than the upper ones. Catastrophic floods of long duration as well as wind events can cause the loss of canopy over large areas, and large coastal areas are also impacted by storm surges from hurricanes. The duration of flooding varies with the placement of a particular site in the landscape and is a dominant process affecting vegetation on a given site. Flooding can deposit alluvium or scour the ground, depending on the landscape position of a site and the severity of the flood event. Fire is infrequent and of limited importance in these lower, wetter areas, but could affect them during periods of prolonged drought. Changes in hydrology due to the activities of beaver are also an important ecological process in bottomland hardwood forests. Beaver activity can add to the dynamics of the system, altering habitat over large areas. Beaver impoundments kill trees (sometimes over large areas) but may also create open water habitat, cypress‑tupelo stands, or cause stand replacement.

SOURCES

**References:** Comer et al. 2003*, Evans et al. 2009, Eyre 1980, Harris 1989, Heineke 1987, LNHP 2009, Nelson 2010, Sharitz and Mitsch 1993

**Version:** 20 Aug 2015	**Stakeholders:** Midwest, Southeast

**Concept Author:** T. Foti and R. Evans	**LeadResp:** Southeast

CES203.195 Mississippi River Low Floodplain (Bottomland) Forest

**Primary Division:** Gulf and Atlantic Coastal Plain (203)

**Land Cover Class:** Woody Wetland

**Spatial Scale & Pattern:** Linear

**Required Classifiers:** Natural/Semi‑natural; Vegetated (>10% vasc.); Wetland

**Diagnostic Classifiers:** Riverine / Alluvial [Brownwater]

**Concept Summary:** These "low bottomlands" are usually seasonally flooded in backswamps, with flooding more frequent than every five years, usually more frequently than every two years, generally by still water that may be impounded behind natural levees, and are classed as Low Gradient Riverine Backwater wetlands in hydrogeomorphic classifications. Low bottomlands occur along the Mississippi River and its tributaries in the Mississippi River Alluvial Plain ecoregion. Prolonged flooding dominates this system, and its duration is greater than in the adjacent Mississippi River Riparian Forest. *Quercus lyrata* is the characteristic dominant species, with *Carya aquatica, Forestiera acuminata*, and other species characteristic of longer hydroperiod environments. Soils are clayey with poor internal drainage.

DISTRIBUTION

**Range:** This system is found in the Mississippi Alluvial Plain from southern Illinois south to Mississippi and Louisiana.

**Divisions:** 203:C

**TNC Ecoregions:** 42:C

**Nations:** US

**Subnations:** AR, IL, KY, LA, MO, MS, TN

**Map Zones:** 45:C, 47:C, 98:C

**USFS Ecomap Regions:** 232E:CC, 234A:CC, 234C:CC, 234D:CC, 234E:CC

CONCEPT

**Environment:** These "low bottomlands" are usually seasonally flooded in backswamps, with flooding more frequent than every five years, usually more frequently than every two years, generally by still water that may be impounded behind natural levees, and are classed as Low Gradient Riverine Backwater wetlands in hydrogeomorphic classifications (Klimas et al. 1981).

**Dynamics:** Changes in soils and vegetation of this system are much slower than in the adjacent Mississippi River riparian forest. Flooding is the principal disturbance in this system. Unusually long or deep floods may stress vegetation or act to regenerate some species. Larger floods cause local disturbance by scouring and depositing sediment along channels and occasionally causing channel shifts. Duration of flooding varies with the placement of a site in the landscape and is a dominant process affecting vegetation on a given site. Flooding can deposit alluvium or scour the ground, depending on the landscape position of a site and the severity of the flood event. Flooding occurs in lower terraces more frequently than in the higher ones. Occasional, long‑duration flooding can cause the loss of canopy over large areas. This canopy decline and reproductive failure can create late‑seral open stands. Duration of flooding varies with the placement of a site in the landscape and is a dominant process affecting vegetation on a given site. Flooding can deposit alluvium or scour the ground, depending on the landscape position of a site and the severity of the flood event.

<br />In addition to periodic flooding, the dominant ecological process in bottomland hardwood forests is the formation of windfall gaps, which can occur on the local scale (a single mature canopy tree) as well as the landscape scale (effects of tornadoes or hurricanes). When canopy trees fall, seedlings in the understory are released and compete for a spot in the canopy. This leads to dense areas of herbaceous and woody vegetation in windfall gaps of all sizes. This is a major process in forest regeneration in bottomland hardwood forests.

The fire history of this type is poorly understood, in part because there has been the widespread assumption that fire was not a factor in its ecological dynamics. However, the presence of extensive cane understories and canebrakes indicates that fire was much more common than is generally believed. These canebrakes exist as a patch community maintained by wind and fire. Fire presumably played a lesser role in this system than in the related "high bottomlands." This system is also bordered by a number of upland communities from which fire would have occasionally burned down into the bottoms, especially in drought years. Beaver activity causes changes in hydrology, and this is an important ecological process in bottomland hardwood forests; the effects are poorly understood at the landscape level, especially in the presettlement context. Beaver impoundments can kill trees (sometimes over large areas) and may create open water habitat or cypress‑tupelo stands, or cause stand replacement. Meandering streams are dynamic and frequently change course, eroding into the floodplain and depositing new point bars, thus creating new habitat for early‑seral plant communities. In addition, insect outbreaks would occur infrequently in closed‑canopy states.

SOURCES

**References:** Comer et al. 2003*, Evans et al. 2009, Eyre 1980, Foti 2001, Harris 1989, Heineke 1987, Klimas et al. 1981, LANDFIRE 2007a, Nelson 2010, Rudis 2001a, Sharitz and Mitsch 1993

**Version:** 14 Jan 2014	**Stakeholders:** Midwest, Southeast

**Concept Author:** T. Foti and M. Pyne	**LeadResp:** Southeast

CES203.190 Mississippi River Riparian Forest

**Primary Division:** Gulf and Atlantic Coastal Plain (203)

**Land Cover Class:** Woody Wetland

**Spatial Scale & Pattern:** Linear

**Required Classifiers:** Natural/Semi‑natural; Vegetated (>10% vasc.); Wetland

**Diagnostic Classifiers:** Riverine / Alluvial [Brownwater]

**Concept Summary:** This ecological system consists of riverfront vegetation, which is generally temporarily (but rarely seasonally) flooded, on point bars and natural levees adjacent to the river that formed them. The period between floods is less than five years, and the flooding is caused by water flowing directly from the channel. Examples occur along the lower Mississippi River and its tributaries in the Mississippi River Alluvial Plain ecoregion. They are classed as Low Gradient Riverine Overbank wetlands in a hydrogeomorphic classification. The flooding is of shorter duration than on adjacent backswamps where water is impounded behind riverfront natural levees, and is of longer duration than on adjacent high bottomlands that are typically temporarily flooded. Soils are typically sandier than those of low bottomlands. *Arundinaria gigantea* is a common understory component in these forests on natural levees and higher point bars, and may become dominant after thinning or removal of the overstory. Willow and cottonwood sandbars may have an open‑canopy (woodland) structure.

DISTRIBUTION

**Range:** This system is found in the Mississippi Alluvial Plain from southern Illinois south to Mississippi and Louisiana.

**Divisions:** 203:C

**TNC Ecoregions:** 42:C

**Nations:** US

**Subnations:** AR, IL, KY, LA, MO, MS, TN

**Map Zones:** 45:C, 47:C, 98:C

**USFS Ecomap Regions:** 232E:CC, 234A:CC, 234C:CC, 234D:CC, 234E:CC

CONCEPT

**Environment:** Stands of this system are generally temporarily (but rarely seasonally) flooded on point bars and natural levees adjacent to the river that formed them, with flooding more frequent than every five years, by flowing water directly from the stream. They are classed as Low Gradient Riverine Overbank wetlands in a hydrogeomorphic classification (Klimas et al. 2004). Flooding is of lower duration than on adjacent backswamps where water is impounded behind riverfront natural levees. Flooding is of longer duration than on adjacent high bottomlands that are typically temporarily flooded. Soils are typically sandier than those of low bottomlands.

**Vegetation:** Some of the most typical and characteristic tree species found in stands of this system include *Acer negundo, Acer saccharinum, Platanus occidentalis, Populus deltoides*, and *Salix nigra*. Other trees may include *Celtis laevigata, Carya illinoinensis, Fraxinus pennsylvanica, Gleditsia triacanthos, Liquidambar styraciflua, Quercus nigra, Quercus pagoda, Quercus texana, Ulmus americana*, and *Ulmus crassifolia*. In addition, *Quercus virginiana* may be present within its range. *Arundinaria gigantea (= ssp. gigantea)* is a common understory component in these forests on natural levees and higher point bars, and may become dominant after thinning or removal of the overstory.

**Dynamics:** Often on sites with rapid soil deposition and, therefore, with rapid development of vegetation from low‑diversity willow‑ and cottonwood‑dominated communities to more diverse communities dominated by sycamore, pecan, sugarberry, green ash or Nuttall oak. Regeneration is through small treefall gaps or large tornado tracks.

SOURCES

**References:** Comer et al. 2003*, Evans et al. 2009, Eyre 1980, Klimas et al. 1981, Nelson 2010

**Version:** 17 Mar 2009	**Stakeholders:** Midwest, Southeast

**Concept Author:** T. Foti, M. Pyne	**LeadResp:** Southeast

CES203.065 Red River Large Floodplain Forest

**Primary Division:** Gulf and Atlantic Coastal Plain (203)

**Land Cover Class:** Woody Wetland

**Spatial Scale & Pattern:** Matrix

**Required Classifiers:** Natural/Semi‑natural; Vegetated (>10% vasc.); Wetland

**Diagnostic Classifiers:** Forest and Woodland (Treed)

**Concept Summary:** This floodplain forest system is specifically restricted to the main stem of the Red River in the West Gulf Coastal Plain and Upper West Gulf Coastal Plain of southwestern Arkansas, adjacent Texas, and Louisiana. Several distinct plant communities can be recognized within this system that may be related to the array of different geomorphic features present within the floodplain. Some of the major geomorphic features associated with different community types within the system include natural levees, point bars, meander scrolls, oxbows, and sloughs. The vegetation generally includes forests dominated by bottomland hardwood species and other trees tolerant of flooding, including bald‑cypress and water tupelo. Herbaceous and shrub vegetation may also be present in certain areas. Some canopy trees that may occur in examples of this system include *Betula nigra, Platanus occidentalis, Fraxinus pennsylvanica, Celtis laevigata, Liquidambar styraciflua, Ulmus americana, Nyssa biflora, Populus deltoides, Salix nigra*, and *Quercus texana*. Components with longer hydroperiods may contain *Quercus lyrata, Gleditsia aquatica, Carya aquatica, Nyssa aquatica*, and *Taxodium distichum*. Smaller trees include *Quercus similis, Quercus sinuata var. sinuata, Ulmus crassifolia*, and *Carpinus caroliniana*. Shrubs include *Alnus serrulata, Forestiera acuminata, Planera aquatica, Cephalanthus occidentalis, Ilex decidua, Crataegus viridis, Sabal minor*, and *Itea virginica*. Herbs are limited due to the length of flooding, but some examples are *Boehmeria cylindrica, Mikania scandens*, and *Lysimachia radicans*. Typical floating aquatic plants include *Nelumbo lutea, Nuphar advena, Nymphaea odorata*, and *Lemna minor*.

**Comments:** This system is generally similar in concept to West Gulf Coastal Plain Large River Floodplain Forest (CES203.488) but is distinct both from it and from the floodplain forests of the Mississippi River Alluvial Plain primarily because of the difference in magnitude between the typical large rivers (such as the Trinity, Neches, and Sabine), on the one hand, and the Mississippi River on the other. In Arkansas (at least), this system is most closely affiliated with the "Billyhaw‑Perry‑Portland" Soil Association (MUID=AR033 in STATSGO). This system is related to the Mississippi River system at its lower end, and in the Texas and Oklahoma border region, it blends into West Gulf Coastal Plain river systems. Based on vegetation it seems very arbitrary to split this system, and Red River floodplain is not that bigger than the Arkansas River (which is in South‑Central Interior Large Floodplain (CES202.705)).

DISTRIBUTION

**Range:** This system is restricted to the main stem of the Red River in the West Gulf Coastal Plain and Upper West Gulf Coastal Plain of southwestern Arkansas, adjacent Texas, and Louisiana. Its range is conceptually coincident with the vast majority of Subsection 234Ai of Keys et al. (1995), excluding the portion of 234Ai within TNC Ecoregion 42 (Mississippi River Alluvial Plain). Its range is also coincident with EPA Ecoregion 35g (Red River Bottomlands) (EPA 2004). The portion of the Red River to the west (231Em of Keys et al. 1995) is treated as part of West Gulf Coastal Plain Large River Floodplain Forest (CES203.488).

**Divisions:** 203:C

**TNC Ecoregions:** 40:C, 41:C

**Nations:** US

**Subnations:** AR, LA, TX

**Map Zones:** 37:C, 98:C

**USFS Ecomap Regions:** 231E:CC, 232F:CC

CONCEPT

**Environment:** Some of the major geomorphic features associated with different community types within the system include natural levees, point bars, meander scrolls, oxbows, and sloughs (Sharitz and Mitsch 1993). The "flatwoods" of the upper terraces within the floodplain are a different system. The geology is Quaternary alluvial deposits. Landforms include the floodplains of the Red River and its major tributaries. Some local topographic variation exists and includes terraces and oxbows. The soils include loams and other bottomland soils (Elliott 2011).

**Vegetation:** The vegetation generally includes forests dominated by bottomland hardwood species and other trees tolerant of flooding, including *Taxodium distichum* and *Nyssa aquatica*. Herbaceous and shrub vegetation may also be present in certain areas. Riverfront sites, and newly exposed or disturbed sites, are occupied by *Platanus occidentalis, Populus deltoides, Salix nigra, Betula nigra, Acer negundo*, and *Fraxinus pennsylvanica*. Some portions of the system are seasonally flooded, and these may contain species such as *Quercus lyrata, Carya aquatica, Taxodium distichum, Nyssa aquatica, Nyssa biflora, Quercus phellos, Gleditsia aquatica*, and *Planera aquatica*. Less frequently flooded areas may be dominated by numerous hardwood species, including *Liquidambar styraciflua, Quercus nigra, Quercus phellos, Quercus shumardii, Quercus macrocarpa, Quercus michauxii, Quercus falcata, Carya illinoinensis, Celtis laevigata, Ulmus alata, Ulmus americana, Ulmus crassifolia, Ulmus rubra, Gleditsia triacanthos, Nyssa sylvatica*, and *Fraxinus pennsylvanica*. In addition, *Juniperus virginiana, Pinus taeda*, and, to a lesser extent, *Pinus echinata* may also be found in the canopy. A midstory component may include young individuals of the overstory, as well as *Quercus similis, Quercus sinuata var. sinuata, Carpinus caroliniana, Ostrya virginiana, Acer rubrum, Sassafras albidum, Maclura pomifera*, and *Morus rubra*. The wetland shrub *Cephalanthus occidentalis* may dominate some open sites within the floodplain. In addition to these species, shrubs such as *Alnus serrulata, Forestiera acuminata, Crataegus viridis, Crataegus marshallii, Callicarpa americana, Ilex decidua, Sabal minor, Itea virginica*, and *Arundinaria gigantea* may be found in the understory of forests, as well as the exotic shrub *Ligustrum sinense*. Numerous woody vines may be encountered, including *Smilax rotundifolia, Brunnichia ovata, Berchemia scandens, Lonicera japonica, Nekemias arborea (= Ampelopsis arborea)*, and *Toxicodendron radicans*. Herbaceous species may be present in the understory of forest, occur as marshy areas, or occupy herbaceous‑dominated sites on areas less frequently flooded. *Saururus cernuus, Nymphaea odorata, Rhynchospora* spp., *Carex* spp., *Dichanthelium* spp., *Chasmanthium* spp., *Juncus* spp., *Leersia* sp., *Geum canadense, Sanicula canadensis, Woodwardia areolata, Boehmeria cylindrica, Mikania scandens*, and *Polygonum* spp. are among the herbaceous species that may be commonly encountered in this system. Typical floating aquatic plants include *Nelumbo lutea, Nuphar advena, Nymphaea odorata*, and *Lemna minor* (Elliott 2011). The forests of the Red River are thought to differ from those of other systems because of the greater presence of "riverfront" species (P. Faulkner pers. comm.). More information is needed, including a review of the affiliations of associations to this system versus West Gulf Coastal Plain Large River Floodplain Forest (CES203.488).

**Dynamics:** This system is maintained by natural large river hydrological processes (e.g., meanders, flooding, backswamps, natural levees). Occasional, long duration flooding can cause the loss of canopy over large areas. This canopy decline and reproductive failure can create late‑seral open stands. Duration of flooding varies with the placement of a site in the landscape and is a dominant process affecting vegetation on a given site. Meandering rivers are dynamic and change course, eroding into the floodplain and depositing new point bars, thus creating new habitat for early‑seral plant communities. Changes in hydrology due to the activities of beaver are also an important ecological process in bottomland hardwood forests. Beaver activity causes changes in hydrology, and this is an important ecological process in bottomland hardwood forests; the effects are poorly understood at the landscape level, especially in the presettlement context. Beaver impoundments kill trees (sometimes over large areas) but may also create open water habitat, cypress‑tupelo stands, or cause stand replacement. In addition to periodic flooding, the dominant ecological process in bottomland hardwood forests is the formation of windfall gaps, which can occur on the local scale (a single mature canopy tree) as well as the landscape scale (tornadoes or hurricanes). When canopy trees fall, seedlings in the understory are released and compete for a spot in the canopy. This leads to dense areas of herbaceous and woody vegetation in windfall gaps of all sizes. This is a major process in forest regeneration in bottomland hardwood forests. This system is also bordered by a number of upland communities from which fire would have occasionally burned down into the bottoms, especially in drought years.

SOURCES

**References:** Comer et al. 2003*, EPA 2004, Elliott 2011, Eyre 1980, Foti pers. comm., Harris 1989, Keys et al. 1995, Küchler 1964, Post 1969, Sharitz and Mitsch 1993

**Version:** 14 Jan 2014	**Stakeholders:** Southeast

**Concept Author:** M. Pyne, R. Evans, T. Foti	**LeadResp:** Southeast

CES203.240 Southern Atlantic Coastal Plain Tidal Wooded Swamp

**Primary Division:** Gulf and Atlantic Coastal Plain (203)

**Land Cover Class:** Woody Wetland

**Spatial Scale & Pattern:** Large patch

**Required Classifiers:** Natural/Semi‑natural; Vegetated (>10% vasc.); Wetland

**Diagnostic Classifiers:** Forest and Woodland (Treed); Tidal / Estuarine

**Concept Summary:** This system encompasses the tidally flooded areas in lower river floodplains and edges of estuaries of the Atlantic Coastal Plain from southeastern Virginia southward to northern Florida that have sufficiently freshwater and short enough flooding to be able to support tree canopies. *Taxodium, Nyssa*, or *Fraxinus* generally dominate. Swamps may be either regularly flooded by lunar tides or irregularly flooded by wind tides.

**Comments:** This system is distinguished from all adjacent systems by the combination of tidal flooding and tree‑dominated vegetation. It is related to East Gulf Coastal Plain Freshwater Tidal Wooded Swamp (CES203.299) but is distinguished because of differences in the tidal flooding regime between the Gulf and Atlantic and because of biogeographic differences.

DISTRIBUTION

**Range:** This system is found along the Atlantic Coast from southeastern Virginia southward to northern Florida.

**Divisions:** 203:C

**TNC Ecoregions:** 56:C, 57:C

**Nations:** US

**Subnations:** FL, GA, NC, SC, VA

**Map Zones:** 55:C, 58:C, 60:C

**USFS Ecomap Regions:** 232C:CC, 232I:CC

CONCEPT

**Environment:** This system occurs in lower reaches of river floodplains and along estuary shorelines, in places regularly or irregularly flooded by lunar or wind tides. The water has little salt content, due to distance from the ocean and/or strong freshwater input. Soils may be mineral or organic. Soils are generally permanently saturated even when the tide is low. The transition of the hydrology to flood dominance rather than tidal dominance may be very gradual.

**Vegetation:** Vegetation is forest or woodland with canopies of the most water‑tolerant tree species, generally *Taxodium distichum, Nyssa* spp., or *Fraxinus* spp. Lower strata generally are denser and more species‑rich than those of river or nonriverine swamps, containing species from those systems as well as a variety of shrubs and herbs shared with freshwater marshes. The tall‑shrub stratum may contain *Juniperus virginiana var. silicicola*. The evergreen shrubs *Morella cerifera* and *Rosa palustris* are often characteristic.

**Dynamics:** Regular or irregular tidal flooding with freshwater is the ecological factor that makes this system distinct. These swamps may be regularly flooded at least twice a day for several hours and remain inundated for days during flood or storm events (Wharton et al. 1982, FNAI 1990). River floods may also seasonally affect this system. Wind and flooding are the dominant disturbance agents in this type and this includes wind damage from hurricanes and tornadoes as well as inundation of young stands. Canopy gaps can be created by high winds, such as from nor'easters, tropical storms and hurricanes (Nordman 2013). Infrequent intrusion of saltier water, which is stressful or fatal to many of the plant species, is an important periodic disturbance created by storms. Insect outbreaks would occur infrequently in these closed‑canopy forests (Landfire 2007a). This system generally appears to be in a shifting relationship with tidal freshwater marshes of the same region. Most marshes have standing dead trees in them, suggesting they recently were swamps. But, conversely, some marshes are being invaded with trees and may be turning into swamps. Freshwater tidal marshes generally occur at the shallow edge of tidal rivers and streams, where river and tidal flow is high, and the vegetation is affected by the changing meanders of the tidal channel. Rising sea level is driving shifts in the communities of this system, causing upstream non‑tidal swamps to develop into this system (as they become subject to tides) and causing parts of this system to turn into brackish marshes. In areas not too strongly affected by saltwater intrusion or drowning by rising sea level, these communities can be expected to exist as old‑growth, multi‑aged forests.

SOURCES

**References:** Comer et al. 2003*, Edwards et al. 2013, Eyre 1980, Harris 1989, LANDFIRE 2007a, Nelson 1986, Nordman 2013, Odum et al. 1984, Schafale 2012, Schafale pers. comm., Southeastern Ecology Working Group n.d., Wharton et al. 1982

**Version:** 23 Apr 2015	**Stakeholders:** East, Southeast

**Concept Author:** M. Schafale and R. Evans	**LeadResp:** Southeast

CES203.493 Southern Coastal Plain Blackwater River Floodplain Forest

**Primary Division:** Gulf and Atlantic Coastal Plain (203)

**Land Cover Class:** Woody Wetland

**Spatial Scale & Pattern:** Linear

**Required Classifiers:** Natural/Semi‑natural; Vegetated (>10% vasc.); Wetland

**Diagnostic Classifiers:** Riverine / Alluvial [Blackwater]

**Concept Summary:** This ecological system occurs along certain river and stream drainages of the southern Coastal Plain of Florida, Alabama, Mississippi, and southwestern Georgia that are characterized by dark waters high in particulate and dissolved organic materials, and that generally lack floodplain development. In most cases these are streams that have their headwaters in sandy portions of the Outer Coastal Plain. Consequently, they carry little mineral sediment or suspended clay particles and are not turbid except after the heaviest rain events. The water is classically dark in color due to concentrations of tannins, particulates, and other materials derived from drainage through swamps or marshes. In comparison with spring‑fed rivers and brownwater rivers of the region, this system tends to be much more acidic in nature and generally lacks extensive and continuous floodplains and levees. Steep banks alternating with floodplain swamps are more characteristic. This system includes mixed rivers, with a mixture of blackwater and spring‑fed tributaries such as the Suwannee River. Canopy trees typical of this system are obligate to facultative wetland species such as *Taxodium distichum, Nyssa aquatica*, and *Chamaecyparis thyoides*.

**Comments:** A new ecological system is needed for Florida spring‑fed streams/rivers, such as the Wakulla River, Ichetucknee River, etc.

DISTRIBUTION

**Range:** This system is found in the East Gulf Coastal Plain of Alabama, Mississippi, southwestern Georgia, Florida, and adjacent portions of central Florida.

**Divisions:** 203:C

**TNC Ecoregions:** 53:C, 55:C

**Nations:** US

**Subnations:** AL, FL, GA, MS

**Map Zones:** 46:C, 55:C, 56:C

**USFS Ecomap Regions:** 232B:CC, 232C:CC, 232D:CC, 232G:CC, 232J:CC, 232K:CC, 232L:CC, 234A:CC

CONCEPT

**Environment:** The rivers in which this system occurs are characterized by dark waters high in particulate and dissolved organic materials, and that generally lack floodplain development. In most cases these are streams that have their headwaters in sandy portions of the Outer Coastal Plain (Smock and Gilinsky 1992). Consequently, they carry little mineral sediment or suspended clay particles and are not turbid except after the heaviest rain events. The water is classically dark in color due to concentrations of tannins, particulates, and other materials derived from drainage through swamps or marshes (FNAI 1990). In comparison with spring‑fed rivers and brownwater rivers of the region, this system tends to be much more acidic in nature and generally lacks extensive and continuous floodplain and levees; steep banks alternating with floodplain swamps are more characteristic (FNAI 1990). This system includes mixed rivers, with a mixture of blackwater and spring‑fed tributaries such as the Suwannee River.

This is a linear to large‑patch ecological system; stands may be contiguous over thousands of acres. The largest examples could be called matrix examples of this ecological system. Examples are by nature linear and tend to be narrow. The Satilla River in Georgia is about 375 km in length and may be the largest example. The lower floodplain is about 2 km across, an approximate size of 750 square km could be used as a working upper bound. There may be limited areas with trees greater than 150 years. Probably there are many stands aged 70‑100 years, and many that are younger than 70 years. Stands that have not had extensive timber removal will probably have more woody debris and constitute better habitat for component animal and plant species. Areas that have been logged may become dominated by *Pinus taeda, Liquidambar styraciflua*, and *Acer rubrum* with a common shrub being *Morella cerifera*.

**Dynamics:** Flooding is the most important ecological factor in this system. Frequency and duration of flooding determine the occurrences of different associations and separate the system from other kinds of wetlands. Flooding brings nutrients and excludes non‑flood‑tolerant species. When flooded, the system may have a substantial aquatic faunal component, with high densities of invertebrates, and may play an important role in the life cycle of fish in the associated river. Unusually long or deep floods may stress vegetation or act as a disturbance for some species. Larger floods cause local disturbance by scouring and depositing sediment along channels, and occasionally causing channel shifts. However, the low gradient and binding of sediment by vegetation generally makes these processes much slower and less frequent than in river systems of most other regions. The areas flooded for the longest durations tend to have high amounts of total organic carbon in the soil and sediments which deplete levels of aquatic dissolved oxygen (Todd et al. 2010).

Except for primary successional communities such as bars, most forests exist naturally as multi‑aged old‑growth forests driven by gap‑phase regeneration. Windthrow is probably the most important cause of gaps. In addition to periodic flooding, the formation of windfall gaps is a dominant ecological processes in bottomland hardwood forests. Windfall gaps occur from the local scale (a single mature canopy tree) to the landscape scale (effects of tornadoes and hurricanes). When canopy trees fall, seedlings in the understory are released and compete for a spot in the canopy. This leads to dense areas of herbaceous and woody vegetation in windfall gaps of all sizes. This is a major process in forest regeneration in bottomland hardwood forests.

Flooding is more frequent on the lower terraces but frequently floods higher terraces (Wharton et al. (1982) zones IV and V). Catastrophic floods can cause the loss of canopy over large areas. Canopy decline and reproductive failure can create late‑seral open stands. Duration of flooding varies with the placement of a site in the landscape and is a dominant process affecting vegetation on a given site. Flooding can deposit alluvium or scour the ground, depending on the landscape position of a site and the severity of the flood event.

<br />Fire is not believed to be important, due to low flammability of much of the vegetation, wetness, and abundance of natural firebreaks. Fire is infrequent on the lower terraces, but was frequent historically on older terraces outside the floodplain and crept into the floodplains. Putnam (1951 as cited in Wharton et al. 1982) states that a serious fire season occurs on an average of about every 5 to 8 years in the bottomland hardwood forests of the Mississippi Alluvial Plain. Some areas of bottomlands apparently were once occupied by canebrakes, which presumably were maintained through deliberate fall burning by Native Americans. Infrequent, mild surface fires would occur in the system; however, they would not alter species composition or structure.

Changes in hydrology due to the activities of beaver are also an important ecological process in bottomland hardwood forests. Beaver impoundments kill trees (sometimes over large areas) and may create open water habitat, cypress‑tupelo stands, or cause stand replacement. Meandering streams are dynamic and frequently change course, eroding into the floodplain and depositing new point bars, thus creating new habitat for early‑seral plant communities. Insect outbreaks would occur infrequently in closed canopy states.

SOURCES

**References:** Comer et al. 2003*, Eyre 1980, FNAI 2010a, Harris 1989, LANDFIRE 2007a, Putnam 1951, Schuster 1974, Sharitz and Mitsch 1993, Smock and Gilinsky 1992, Todd et al. 2010, Wharton et al. 1982

**Version:** 14 Jan 2014	**Stakeholders:** Southeast

**Concept Author:** R. Evans and A. Schotz	**LeadResp:** Southeast

CES202.324 Southern Piedmont Large Floodplain Forest

**Primary Division:** Central Interior and Appalachian (202)

**Land Cover Class:** Woody Wetland

**Spatial Scale & Pattern:** Linear

**Required Classifiers:** Natural/Semi‑natural; Vegetated (>10% vasc.); Wetland

**Diagnostic Classifiers:** Riverine / Alluvial

**Concept Summary:** This ecological system consists of vegetated communities along Piedmont rivers, south of the James River in Virginia, where flooding and flood‑related environmental factors affect vegetation composition and dynamics. Well‑developed examples of this system occur in the Triassic basins. The vegetation includes both non‑forested bar and scour communities and the more extensive forested floodplain communities. Forests are generally differentiated by depositional landforms such as levees, sloughs, ridges, terraces, and abandoned channel segments. The system is affected by flooding through wetness, scouring, deposition of material, and input of nutrients. Piedmont floodplain systems are generally quite distinct from Coastal Plain ones, with steeper river gradients, harder rocks and more limited floodplain development. The near absence of *Taxodium distichum, Nyssa aquatica*, and other species of the Coastal Plain corresponds well to the geologic boundary in most places.

**Comments:** This system is distinguished from Southern Piedmont Small Floodplain and Riparian Forest (CES202.323) by having well‑developed fluvial landforms which differentiate vegetation. The smaller rivers are less differentiated both because the fluvial landforms are smaller and because the flooding regime is more variable. This system is distinguished from upland systems by the significant presence of plants indicative of alluvial or bottomland settings. This suite of species is absent or occurs incidentally in upland sites.

Piedmont floodplain systems are generally quite distinct from those of the Coastal Plain, with more limited development of floodplains and depositional features, because of the steeper river gradients and harder rocks. The near absence of *Taxodium distichum, Nyssa aquatica*, and other species largely confined to the Coastal Plain corresponds well to the geologic boundary in most places. The floodplains on Triassic sediments have some similarity to those in the Coastal Plain because of their more extensive floodplain development. The break with South‑Central Interior Large Floodplain (CES202.705) is less sharp. The presence of Appalachian mesophytic plant species is often the best indicator.

Distinctive subgroups within this system, which could potentially be the basis for further subdivision, include the Triassic Basin floodplains and the distinction between forests and non‑forested communities. The non‑forested communities, maintained by periodic severe disturbance, have very different dynamics as well as vegetation structure, but are always associated with the forests and share the flooding regime. Triassic Basin floodplains have large floodplains with small streams. They likely have differences in flooding regime, including longer duration of flooding. Swamp forests, where periods of standing water are an important environmental influence, occupy larger portions of Triassic Basin floodplains than of other floodplains.

DISTRIBUTION

**Range:** This system is widespread in the Piedmont, from Alabama to southern Virginia. The northern boundary in Virginia is not well‑determined, but it extends approximately to (but does not include) the James River.

**Divisions:** 202:C

**TNC Ecoregions:** 52:C, 57:C

**Nations:** US

**Subnations:** AL, GA, NC, SC, VA

**Map Zones:** 54:C, 59:C, 61:C

**USFS Ecomap Regions:** 231A:CC, 231I:CC

CONCEPT

**Environment:** Examples of this ecological system occur near rivers, on floodplains and terraces affected by river flooding and on emergent bars and banks within channels. The site usually includes distinct depositional landforms, including levees, sloughs, ridges, terraces, and abandoned channel segments. The relative extent of these features varies among the stretches of different rivers depending on factors such as channel morphology (Edwards et al. 2013). The substrate is primarily alluvium. Soils are usually sandy to loamy, but include local clayey and gravelly areas. Soils are generally fertile, among the most nutrient‑rich in the Piedmont region. Emergent and vegetated bars of gravel to cobbles are included here as well, as are scoured bedrock areas. Floods are generally of short duration, and wetness is a major influence only within channels and where water is ponded in local depressions. The geologic substrate may be of any kind, but geological substrates in the Piedmont are primarily acidic. A special case is the soft Triassic sedimentary rocks of the Piedmont, where even small streams develop large floodplains with well‑developed fluvial landforms and therefore fall into this category.

**Vegetation:** Most of the extent of the system is forest vegetation. The forest canopy is usually dominated by a mix of characteristic alluvial bottomland species such as *Platanus occidentalis, Betula nigra, Acer negundo, Celtis laevigata, Fraxinus pennsylvanica, Liquidambar styraciflua, Populus deltoides, Quercus michauxii*, and *Quercus pagoda*. Some more widespread species such as *Liriodendron tulipifera* and *Acer rubrum* are also abundant. Mesophytic species such as *Fagus grandifolia* are a component on the driest areas. Successional areas are often dominated by *Pinus taeda, Pinus virginiana, Liquidambar styraciflua*, or *Liriodendron tulipifera*. Lower strata in the forests are similarly dominated by bottomland species, but may contain more mesophytic species. *Lindera benzoin, Xanthorhiza simplicissima, Elymus hystrix, Elymus canadensis, Chasmanthium latifolium*, and *Boehmeria cylindrica* are among the characteristic species. Non‑forested vegetation is generally limited to small patches or bands along the channel, and is quite variable in structure and composition. Partly submerged bars and rocky shoals may be dominated by *Justicia americana*. Frequently reworked gravel bars may be dominated by young *Salix nigra, Platanus occidentalis*, or *Betula nigra*, or they may have sparse vegetation of a wide variety of annual and perennial herbs of weedy habits. The few extensive bedrock‑scour areas in gorges have distinctive vegetation dominated by perennial herbs rooted in pockets and crevices.

**Dynamics:** The dynamics of river flooding influence the distinctive vegetation of this ecological system. The large rivers have the largest watersheds in the region, but the gradients of most of these rivers limit floods to fairly short duration. Flooding is most common in the winter, but may occur in other seasons. The sorting of plant communities by depositional landforms of different height suggest that duration of wetness or depth of flood waters may be of significance, though it has much less influence than in the Coastal Plain. Flood waters have significant energy, and scouring and reworking of sediment are an important factor in bar and bank communities. However, in the forested floodplains, flood disturbances that kill established woody plants are rare, and canopy population dynamics are dominated by windthrow. In addition to disturbance, floods bring nutrient input, deposit sediment, and disperse plant seeds.<br />

Wind disturbance is at least as important in this system as other Piedmont forests, perhaps more important than in uplands because of frequently wet and less dense soils and more shallowly‑rooted trees. Fire does not appear to be a dominant factor, and most floodplain vegetation is not very flammable. However, historical references to canebrakes dominated by *Arundinaria gigantea* suggest that fire may have once been more possible and more important in at least some portions.

These systems are commonly subject to a variety of indirect modern human influences beyond those that affect most forests. A large fraction of the large Piedmont rivers have been dammed, and power generation and regulation of waterflow create unnatural flood regimes. Extensive erosion of uplands, caused by poor agricultural practices dating back to colonial times, transported large amounts of sediment into floodplains (Edwards et al. 2013). As in uplands, large floodplains often have substantial areas in cultivation. River bottoms were the focus of agriculture among Native Americans, so some of these systems have a long history of human clearing. A number of exotic plant species have invaded floodplains, more than in any other Piedmont ecological system. These include *Ligustrum sinense*, which can form extensive and continuous stands in the understories of floodplain forests (Edwards et al. 2013).

SOURCES

**References:** Comer et al. 2003*, Edwards et al. 2013, Eyre 1980, Harris 1989, Nelson 1986, Peet et al. 2013, Schafale 2012, Schafale and Weakley 1990, Sharitz and Mitsch 1993, Simon and Hayden 2014

**Version:** 02 Oct 2015	**Stakeholders:** East, Southeast

**Concept Author:** M. Schafale and R. Evans	**LeadResp:** Southeast

CES202.323 Southern Piedmont Small Floodplain and Riparian Forest

**Primary Division:** Central Interior and Appalachian (202)

**Land Cover Class:** Woody Wetland

**Spatial Scale & Pattern:** Linear

**Required Classifiers:** Natural/Semi‑natural; Vegetated (>10% vasc.); Wetland

**Diagnostic Classifiers:** Riverine / Alluvial

**Concept Summary:** This ecological system consists of vegetated communities along streams and small rivers in the Piedmont of the southeastern United States where flooding and flood‑related environmental factors affect vegetation composition and dynamics. The vegetation includes both non‑forested bar and scour communities, as well as more extensive forested floodplain communities. The forests of these smaller floodplains and bottomlands are not differentiated by depositional landforms such as levees, sloughs, ridges, terraces, and abandoned channel segments, because these features are small and flooding regimes are variable. The system is affected by flooding through wetness, scouring, deposition of material, and input of nutrients. Piedmont floodplain systems are generally quite distinct from Coastal Plain ones, with steeper river gradients, harder rocks and more limited floodplain development. The near absence of *Taxodium distichum, Nyssa* spp., and other species of the Coastal Plain corresponds well to the geologic boundary in most places.

**Comments:** This system is distinguished from Southern Piedmont Large Floodplain Forest (CES202.324) by lacking well‑developed fluvial landforms which differentiate vegetation. The smaller rivers are less differentiated both because the fluvial landforms are smaller and because the flooding regime is more variable. The large floodplains created by small streams in Triassic sediments are included with Southern Piedmont Large Floodplain Forest (CES202.324). Both of the Piedmont floodplain systems are distinguished from upland systems by the significant presence of plants indicative of alluvial or bottomland settings. This suite of species is absent or occurs incidentally in upland sites.

Piedmont floodplain systems are generally quite distinct from those of the Coastal Plain, with more limited development of floodplains and depositional features, because of the steeper river gradients and harder rocks. The near absence of *Taxodium distichum, Nyssa* spp., and other species largely confined to the Coastal Plain corresponds well to the geologic boundary in most places. The break with South‑Central Interior Large Floodplain (CES202.705) is less sharp. The presence of Appalachian mesophytic species is often the best indicator. The floodplains of the westernmost Piedmont generally belong to South‑Central Interior Large Floodplain (CES202.705).

DISTRIBUTION

**Range:** This system is widespread in the Piedmont, from Alabama to southern Virginia. The northern boundary in Virginia is roughly the watershed of the James River.

**Divisions:** 202:C

**TNC Ecoregions:** 52:C

**Nations:** US

**Subnations:** AL, GA, NC, SC, VA

**Map Zones:** 49:P, 54:C, 59:C, 61:C

CONCEPT

**Environment:** Examples occur on moderately to very high‑gradient streams over a wide range of elevations, near streams and small rivers, on floodplains and terraces affected by river flooding and includes emergent bars and banks within channels. Depositional landforms, including levees, sloughs, ridges, terraces, and abandoned channel segments may be present, but occur less frequently and are smaller than the scale of the communities of the floodplain. Fine‑scale alluvial floodplain features are abundant. The substrate is primarily alluvium. Soils are usually sandy to loamy, but include local clayey and gravelly areas. Soils are generally fertile, among the most nutrient‑rich in the Piedmont region. Alluvial soils may be as important a factor as ongoing flooding in differentiating these systems from adjacent uplands. Emergent and vegetated bars of gravel to cobbles occur occasionally but are generally not extensive or as distinctive as they are on larger rivers. Floods are generally of short duration, and wetness is a major influence only within channels and where water is ponded in local depressions. The geologic substrate may be of any kind, but areas on Triassic sediments tend to have large floodplain systems even on fairly small streams.

**Vegetation:** Almost all of the extent of the system is naturally forested. The forest canopy is usually a mix of mesophytic and widespread species such as *Liriodendron tulipifera, Liquidambar styraciflua*, and *Acer rubrum*, along with characteristic alluvial and bottomland species such as *Platanus occidentalis, Betula nigra, Acer negundo, Celtis laevigata, Fraxinus pennsylvanica, Liquidambar styraciflua, Quercus michauxii*, and *Quercus pagoda*. *Fagus grandifolia* may be present in drier portions, mixed with the other species. Successional areas are often strongly dominated by *Pinus taeda, Pinus virginiana, Liquidambar styraciflua*, or *Liriodendron tulipifera*. Lower strata in the forests may be either primarily of mesophytic species shared with moist uplands systems, or a mix of mesophytic and bottomland species. Non‑forested vegetation is generally limited to very small patches or bands along the channel, and seldom forms distinct communities.

**Dynamics:** The distinctive dynamics of stream flooding are presumably the primary reason for the distinctive vegetation of this system, though not all of the factors are well known. Small rivers and streams with small watersheds have more variable flooding regimes that larger rivers. Floods tend to be of short duration and unpredictably variable as to season and depth. Flood waters may have significant energy in higher gradient systems, but scouring and reworking of sediment rarely affect more than small patches. They are important in maintaining the small non‑forested patches. In the forested floodplains, flood disturbances that kill established woody plants are rare, and canopy population dynamics are dominated by windthrow. In addition to disturbance, floods bring nutrient input, deposit sediment, and disperse plant seeds.

In pre‑European settlement forests, community diversity in these streamside systems was much more complex than in the modified landscapes of today. Fire, beaver activity, and flooding of varied intensity and frequency created a mosaic whose elements included canebrake, grass and young *Betula‑Platanus* beds on reworked gravel or sand bars, beaver ponds, and grass‑sedge meadows in abandoned beaver clearings, as well as the streamside zones and mixed hardwood and/or pine forests that make up more than 95% of the land cover that exists today.

Flooding is the major disturbance process affecting the vegetation, with the substrate more rapidly drained than in flat floodplain areas. The higher gradients of most of these streams and rivers limit floods to fairly short duration. Flooding is most common in the winter, but may occur in other seasons particularly in association with hurricanes, tornados, or microbursts from thunderstorms. Flood waters may have significant energy in higher gradient systems, but scouring and reworking of sediment are important in maintaining the small non‑forested patches of the bar and bank communities. Flooding can act as a replacement disturbance in areas where beavers impounded a channel or in rare years with severe prolonged flood events. There are two general types of floods: occasional catastrophic, prolonged floods (due to beaver activity or other severe event); and more frequent repeated minor flooding (i.e., several minor floods within a 10‑year period).<br />

The wind disturbance associated with flooding is very significant along small streams because of wet and less dense soils and shallow‑rooted trees. Canopy tree mortality from more common windstorms would have resulted in tree‑by‑tree or small group replacement. Windthrow is the primary cause of mortality in bottomlands. Major storms or hurricanes occurring at approximately 20‑year intervals would have impacted whole stands. Tornado tracks can be found passing across uplands and bottomlands [see one such indicated on a map of Umstead State Park, Raleigh, NC], leaving narrow swaths of felled trees (Landfire 2007a). The majority of windthrow in the Piedmont seems to have been the result of hurricanes and tornadoes spawned by them. Even though the Piedmont is removed from the coast by 25 to over 100 miles, extensive windthrow occurred in middle‑aged and old‑growth trees in Piedmont bottomlands following Hurricane Fran in 1996 (Xi et al. 2008). Bottomland *Quercus* species, even though seemingly in more sheltered positions, were much more heavily affected than hardwoods on adjacent uplands. Gaps as large as one hectare were seen intermixed in areas with extensive single‑tree windthrow. Windthrow may also occur because of thunderstorm microbursts or tornados. In addition, ice damage is an infrequent but potentially catastrophic disturbance.

Fire does not appear to be a dominant factor, and most floodplain vegetation is not very flammable. However, historical references to canebrakes dominated by *Arundinaria gigantea* suggest that fire may have once been more possible and more important in at least some portions.

SOURCES

**References:** Comer et al. 2003*, Eyre 1980, Glenn 1911, Ireland et al. 1939, LANDFIRE 2007a, Mulholland and Lenat 1992, Nelson 1986, Peet et al. 2013, Schafale 2012, Schafale and Weakley 1990, Simon and Hayden 2014, Wharton 1978, Xi et al. 2008

**Version:** 02 Oct 2015	**Stakeholders:** East, Southeast

**Concept Author:** M. Schafale and R. Evans	**LeadResp:** Southeast

CES203.488 West Gulf Coastal Plain Large River Floodplain Forest

**Primary Division:** Gulf and Atlantic Coastal Plain (203)

**Land Cover Class:** Woody Wetland

**Spatial Scale & Pattern:** Linear

**Required Classifiers:** Natural/Semi‑natural; Vegetated (>10% vasc.); Wetland

**Diagnostic Classifiers:** Forest and Woodland (Treed); Riverine / Alluvial [Brownwater]; Broad‑Leaved Deciduous Tree; West Gulf Coastal Plain

**Concept Summary:** This system represents a geographic subset of Kuchler's (1964) Southern Floodplain Forest found west of the Mississippi River. Examples may be found along large rivers of the West Gulf Coastal Plain and Upper West Gulf Coastal Plain, especially the Trinity, Neches, Sabine, and others. Several distinct plant communities can be recognized within this system that may be related to the array of different geomorphic features present within the floodplain. Some of the major geomorphic features associated with different community types include natural levees, point bars, meander scrolls, oxbows, and sloughs. Vegetation generally includes forests dominated by bottomland hardwood species and other trees tolerant of flooding, including bald‑cypress and water tupelo. Some other trees which may be associated with examples of this system include *Acer rubrum var. drummondii, Betula nigra, Carya aquatica, Celtis laevigata, Fraxinus pennsylvanica, Liquidambar styraciflua, Platanus occidentalis, Gleditsia aquatica, Nyssa aquatica, Nyssa biflora, Pinus taeda, Populus deltoides, Quercus laurifolia, Quercus lyrata, Quercus michauxii, Quercus nigra, Quercus pagoda, Quercus phellos, Quercus similis, Quercus texana, Salix nigra, Ulmus americana*, and *Ulmus crassifolia*. Smaller areas of herbaceous‑ and shrub‑dominated vegetation may also be present in certain areas. Shrubs and small trees include *Alnus serrulata, Arundinaria gigantea, Carpinus caroliniana, Cephalanthus occidentalis, Clethra alnifolia, Cornus foemina, Crataegus viridis, Forestiera acuminata, Ilex decidua, Itea virginica, Morella cerifera, Planera aquatica, Sabal minor*, and *Ditrysinia fruticosa*. Vines may include *Berchemia scandens* and *Smilax bona‑nox*. Herbaceous species may include *Boehmeria cylindrica, Carex complanata, Carex debilis, Carex intumescens, Carex joorii, Leersia virginica, Lycopus virginicus, Mikania scandens, Saccharum baldwinii*, and *Typha latifolia*. Aquatic and floating herbs include *Lemna minor, Nelumbo lutea, Nuphar advena*, and *Nymphaea odorata*.

**Comments:** It is unclear to what system the Brazos and Colorado rivers belong. A new system is apparently required to accommodate these and other rivers in the Coastal Plain south and west of Galveston Bay. Or would they go into West Gulf Coastal Plain Near‑Coast Large River Swamp (CES203.459)?

DISTRIBUTION

**Range:** This system occurs along large rivers of the West and Upper West Gulf coastal plains, especially the Trinity, Neches, Sabine, and others, as well as the portion of the Red River represented by Keys et al. (1995) (231Em) at the Oklahoma‑Texas border.

**Divisions:** 203:C

**TNC Ecoregions:** 31:C, 40:C, 41:C

**Nations:** US

**Subnations:** AR, LA, OK, TX

**Map Zones:** 37:C, 44:C, 98:C

CONCEPT

**Environment:** Some of the major geomorphic features associated with different community types within this system include natural levees, point bars, meander scrolls, oxbows, and sloughs (Sharitz and Mitsch 1993). This system typically occupies Quaternary Alluvial geology along major rivers including the Trinity (downstream of Cobb Creek), Neches, Angelina, Sabine, Sulphur, and San Jacinto, and a few of their major tributaries. Landforms include broad floodplains with significant development of bottomland soils. These areas include an array of local geomorphic features such as natural levees, point bars, meander scrolls, oxbows, terraces, and sloughs. This system occupies soils of various textures derived from alluvial processes of the associated rivers. The hydrology of these soils is variable, including temporary, seasonal, and semipermanent flooding regimes.

**Vegetation:** This system is typically represented by forests that vary relative to the flooding regime, which is often controlled by local topographic variation and proximity to a river. Swamps are typically represented by forests of *Taxodium distichum*, with other species such as *Nyssa aquatica, Gleditsia aquatica*, and *Carya aquatica* also present. Some semipermanently flooded sites may also be dominated by *Planera aquatica*. Floating aquatics, such as *Lemna minor, Potamogeton* spp., *Ceratophyllum demersum*, and *Nymphaea odorata*, may also be present at those sites. *Quercus lyrata* is characteristic of seasonally flooded bottomlands, but numerous other species are also important components of the canopy, including *Taxodium distichum, Quercus phellos, Fraxinus pennsylvanica, Liquidambar styraciflua, Nyssa biflora, Fraxinus caroliniana*, and *Quercus similis*. Commonly encountered, and sometimes dominant, species of temporarily flooded sites include *Liquidambar styraciflua, Quercus nigra*, and *Fraxinus pennsylvanica*. Numerous other species, such as *Quercus laurifolia, Quercus michauxii, Quercus pagoda, Quercus phellos, Quercus texana, Celtis laevigata, Acer rubrum var. drummondii, Ulmus crassifolia, Ulmus americana*, and *Carya illinoinensis*, may also be important components of the canopy. *Platanus occidentalis, Populus deltoides, Betula nigra*, and *Salix nigra* are more conspicuous as early‑successional species along the riverfront. Understory and shrub cover is variable but is typically relatively low, particularly in more frequently flooded sites and sites with significant overstory canopy. The understory may have small individuals of the overstory, as well as species such as *Alnus serrulata, Arundinaria gigantea, Carpinus caroliniana, Ilex decidua, Ilex opaca, Callicarpa americana, Crataegus viridis, Crataegus marshallii, Crataegus opaca, Styrax americanus, Ditrysinia fruticosa (= Sebastiania fruticosa), Sambucus nigra ssp. canadensis, Clethra alnifolia, Cornus foemina, Forestiera acuminata, Ilex decidua, Itea virginica, Morella cerifera*, and/or *Sabal minor*. Where the overstory canopy is open, *Planera aquatica, Cephalanthus occidentalis*, or *Forestiera acuminata* may form dense stands. Woody vines that may be encountered include *Berchemia scandens, Smilax bona‑nox, Vitis rotundifolia, Toxicodendron radicans*, and *Campsis radicans*. Herbaceous species may include *Boehmeria cylindrica, Saururus cernuus, Saccharum baldwinii, Elymus virginicus, Onoclea sensibilis, Carex cherokeensis, Carex complanata, Carex intumescens, Carex joorii, Carex debilis*, other *Carex* species, *Chasmanthium latifolium, Chasmanthium sessiliflorum, Justicia ovata, Bidens aristosa, Panicum hemitomon, Leersia virginica, Lycopus virginicus, Mikania scandens, Saccharum baldwinii, Typha latifolia*, and numerous others. *Pinus taeda* may be found, particularly on some better‑drained sites and where it has been planted. *Triadica sebifera* sometimes invades this system. Aquatic and floating herbs include *Lemna minor, Nelumbo lutea, Nuphar advena (= Nuphar lutea ssp. advena)*, and *Nymphaea odorata*.

SOURCES

**References:** Comer et al. 2003*, Elliott 2011, Eyre 1980, Keys et al. 1995, Küchler 1964, Marks and Harcombe 1981, Sharitz and Mitsch 1993

**Version:** 17 Feb 2011	**Stakeholders:** Midwest, Southeast

**Concept Author:** R. Evans and T. Foti	**LeadResp:** Southeast

CES203.459 West Gulf Coastal Plain Near‑Coast Large River Swamp

**Primary Division:** Gulf and Atlantic Coastal Plain (203)

**Land Cover Class:** Woody Wetland

**Spatial Scale & Pattern:** Large patch

**Required Classifiers:** Natural/Semi‑natural; Vegetated (>10% vasc.); Wetland

**Diagnostic Classifiers:** Riverine / Alluvial; Tidal / Estuarine; West Gulf Coastal Plain

**Concept Summary:** These swamp forests are found along rivers flowing through the Gulf Coast Prairies and Marshes region of the Outer Coastal Plain of western Louisiana and adjacent Texas. Included are areas where the rivers enter bays and estuaries along the northern Gulf of Mexico that are somewhat tidally influenced. This is restricted to Vermillion Bay in Louisiana west to and including Galveston Bay and Trinity Bay in Texas. Stands of vegetation included in this system are typically dominated by *Taxodium distichum, Nyssa aquatica*, or perhaps a combination of these species. These are forested areas in an area primarily dominated by marshes. Other species are usually more minor components of the canopy, including *Fraxinus pennsylvanica, Acer negundo*, and the exotic tree *Triadica sebifera*. These swamps are typically interspersed with marshes of the coastal region.

DISTRIBUTION

**Range:** This system is found along rivers flowing through the Gulf Coast Prairies and Marshes (TNC Ecoregion 31) of the Outer Coastal Plain of western Louisiana and adjacent Texas. This is restricted to EPA 34g (Texas‑Louisiana Coastal Marshes) from Vermillion Bay in Louisiana west to, and including Galveston Bay and Trinity Bay in Texas (EPA 2004).

**Divisions:** 203:C

**TNC Ecoregions:** 31:C

**Nations:** US

**Subnations:** LA, TX

**Map Zones:** 36:C, 37:C, 98:C

**USFS Ecomap Regions:** 232E:CC, 255D:CC

CONCEPT

**Environment:** The environment of this system consists of rivers flowing through the Gulf Coast Prairies and Marshes ecoregion of the Outer Coastal Plain of western Louisiana and adjacent Texas. This includes somewhat tidally‑influenced areas where the rivers enter bays and estuaries along the northern Gulf of Mexico. The geological substrate consists of Quaternary alluvium deposited within the Beaumont/Deweyville surfaces. Landforms include the large river floodplains of the Sabine, Neches, and Trinity rivers near the coast, often with some tidal influence. Typical soils include bottomland soils of the near‑coast region. Stands are generally distributed downstream of Interstate Highway 10 (a coincidental landmark for the distribution of this system). On the Neches River, this is nearly coincident with the area downstream of the confluence with Pine Island Bayou (Elliott 2011).

**Vegetation:** Stands of vegetation included in this system are typically dominated by *Taxodium distichum, Nyssa aquatica*, or perhaps a combination of these species. These are forested areas in an area primarily dominated by marshes. Other species are usually more minor components of the canopy, including *Fraxinus pennsylvanica, Acer negundo*, and the exotic tree *Triadica sebifera*. These swamps are typically interspersed with marshes of the coastal region (Elliott 2011).

SOURCES

**References:** Comer et al. 2003*, EPA 2004, Elliott 2011, Eyre 1980

**Version:** 17 Feb 2011	**Stakeholders:** Southeast

**Concept Author:** J. Teague and R. Evans	**LeadResp:** Southeast

CES203.487 West Gulf Coastal Plain Small Stream and River Forest

**Primary Division:** Gulf and Atlantic Coastal Plain (203)

**Land Cover Class:** Woody Wetland

**Spatial Scale & Pattern:** Linear

**Required Classifiers:** Natural/Semi‑natural; Vegetated (>10% vasc.); Wetland

**Diagnostic Classifiers:** Forest and Woodland (Treed); Riverine / Alluvial; Intermittent Flooding; West Gulf Coastal Plain

**Concept Summary:** This is a predominantly forested system of the West Gulf Coastal Plain associated with small rivers and creeks. In contrast to West Gulf Coastal Plain Large River Floodplain Forest (CES203.488), examples of this system have fewer major geomorphic floodplain features. Those features that are present tend to be smaller and more closely intermixed with one another, resulting in less obvious vegetational zonation. Bottomland hardwood tree species are typically important and diagnostic, although mesic hardwood species are also present in areas with less inundation, such as upper terraces and possibly second bottoms. As a whole, flooding occurs annually, but the water table usually is well below the soil surface throughout most of the growing season. Areas impacted by beaver impoundments are also included in this system. Stands of this system are typically dominated by hardwood tree species such as *Liquidambar styraciflua, Quercus nigra, Celtis laevigata, Fraxinus pennsylvanica, Betula nigra, Quercus laurifolia, Ulmus americana, Ulmus crassifolia, Ulmus alata, Ulmus rubra, Quercus michauxii, Quercus texana, Quercus pagoda, Quercus falcata, Platanus occidentalis, Diospyros virginiana, Gleditsia triacanthos*, and *Acer rubrum*. Wetter sites tend to be dominated by more flood‑tolerant species such as *Taxodium distichum, Nyssa aquatica, Gleditsia aquatica, Carya aquatica, Quercus lyrata, Quercus similis, Planera aquatica*, and *Quercus phellos*.

DISTRIBUTION

**Range:** West Gulf Coastal Plain.

**Divisions:** 203:C

**TNC Ecoregions:** 31:P, 40:C, 41:C

**Nations:** US

**Subnations:** AR, LA, OK, TX

**Map Zones:** 36:?, 37:C, 44:C, 98:C

CONCEPT

**Environment:** This system is associated with small rivers and creeks in the West Gulf Coastal Plain. It largely occurs on Quaternary alluvium, but may also be found on other mapped geologic surfaces on drainages lacking significant alluvial development. This system occupies small rivers, streams, creeks, and upland drainages. These sites tend to be higher in the watershed where less depositional activity occurs. The local geomorphological variation tends to be less than in West Gulf Coastal Plain Large River Floodplain Forest (CES203.488). Soils are bottomland soils on small streams. A minority of sites are seasonally or semipermanently flooded (Elliott 2011).

**Vegetation:** Stands of this system are typically dominated by hardwood tree species such as *Liquidambar styraciflua, Quercus nigra, Celtis laevigata, Fraxinus pennsylvanica, Betula nigra, Quercus laurifolia, Ulmus americana, Ulmus crassifolia, Ulmus alata, Ulmus rubra, Quercus michauxii, Quercus texana, Quercus pagoda, Quercus falcata, Platanus occidentalis, Diospyros virginiana, Gleditsia triacanthos*, and *Acer rubrum*. Wetter sites tend to be dominated by more flood‑tolerant species such as *Taxodium distichum, Nyssa aquatica, Gleditsia aquatica, Carya aquatica, Quercus lyrata, Quercus similis, Planera aquatica*, and *Quercus phellos*. In addition, *Pinus taeda, Pinus elliottii*, and/or *Juniperus virginiana* may be present in the canopy, or occur as a subcanopy stratum. Rarely, *Fagus grandifolia, Magnolia virginiana, Quercus alba, Quercus muehlenbergii*, and/or *Pinus palustris* may appear with *Chasmanthium sessiliflorum* in mesic, upper terrace examples. Shrubs may form dense patches with species such as *Cephalanthus occidentalis* or *Planera aquatica* being typical. Other shrubs and understory trees may include (depending on length of hydroperiod) *Carpinus caroliniana, Ostrya virginiana, Cornus obliqua, Crataegus marshallii, Ilex opaca, Ilex decidua, Ilex vomitoria, Salix nigra, Morus rubra, Sabal minor, Morella cerifera, Callicarpa americana, Itea virginica, Alnus serrulata, Maclura pomifera*, and *Vaccinium fuscatum*. In addition, *Arundinaria gigantea* may be present. Woody vines may be conspicuous and may include *Berchemia scandens, Brunnichia ovata, Nekemias arborea (= Ampelopsis arborea), Smilax bona‑nox*, and *Toxicodendron radicans*. Some herbs may include *Ambrosia trifida, Bidens aristosa, Boehmeria cylindrica, Carex cherokeensis, Carex debilis, Carex digitalis, Carex joorii, Chasmanthium latifolium, Chasmanthium laxum, Dichanthelium* spp., *Elymus virginicus, Geum canadense, Glyceria striata, Leersia virginica, Panicum virgatum, Paspalum floridanum, Polygonum hydropiperoides, Tripsacum dactyloides*, and *Xanthium strumarium*. Early‑successional woodlands may be mapped as shrublands, due to reduced woody cover. These sites may be dominated by early‑successional species such as *Salix nigra, Gleditsia triacanthos, Platanus occidentalis*, or *Ulmus alata*. Non‑native woody species that may be present include *Triadica sebifera, Lonicera japonica*, and *Ligustrum* spp. Non‑native herbs such as *Cynodon dactylon, Lolium perenne, Paspalum notatum*, and *Sorghum halepense* may be dominant in disturbed examples (Elliott 2011).

SOURCES

**References:** Comer et al. 2003*, Elliott 2011, Eyre 1980, Marks and Harcombe 1981

**Version:** 17 Feb 2011	**Stakeholders:** Midwest, Southeast

**Concept Author:** R. Evans	**LeadResp:** Southeast

M154. Southern Great Plains Floodplain Forest & Woodland

CES203.714 Central Texas Coastal Prairie Riparian

**Primary Division:** Gulf and Atlantic Coastal Plain (203)

**Land Cover Class:** Mixed Upland and Wetland

**Spatial Scale & Pattern:** Linear

**Required Classifiers:** Natural/Semi‑natural; Vegetated (>10% vasc.); Upland; Wetland

**Diagnostic Classifiers:** Forest and Woodland (Treed); Riverine / Alluvial; Broad‑Leaved Deciduous Tree

**Concept Summary:** This system occurs in upland drainages as they coarse through the relatively level landscape of the coastal prairie in Texas. It represents vegetation bordering upland drainages that are mostly incised, erosional features with very little alluvial deposition. Woody vegetation often shares composition with that of floodplains along larger rives and streams of the region. Species may include *Celtis laevigata, Ulmus crassifolia, Carya illinoinensis, Salix nigra, Prosopis glandulosa, Vachellia farnesiana, Quercus nigra, Quercus fusiformis, Prosopis glandulosa, Diospyros texana, Condalia hookeri, Ziziphus obtusifolia*, and *Aloysia gratissima*. The herbaceous layer may contain species such *Elymus virginicus, Chasmanthium latifolium, Calyptocarpus vialis, Verbesina virginica*, and *Chloracantha spinosa*. Though woody vegetation is the predominant vegetation type in this system, it may also include small areas that lack a significant woody component.

**Comments:** The relationship of the vegetation of this system (CES203.714) needs further differentiation from the vegetation of Central Texas Coastal Prairie River Floodplain (CES203.713). They are currently separated more on site conditions and processes rather than vegetation dominants. More information is needed.

DISTRIBUTION

**Range:** This system occurs in upland drainages on the Coastal Prairie surface of the Lissie and Beaumont geologic formations in the Coastal Plain of Texas.

**Divisions:** 203:C

**TNC Ecoregions:** 31:C

**Nations:** US

**Subnations:** TX

**Map Zones:** 34:C

**USFS Ecomap Regions:** 255Cg:CPP, 255Da:CCC, 255Dd:CCC, 315Ed:???

CONCEPT

**Environment:** This system represents vegetation bordering upland drainages occurring in the unconsolidated sediments of the Beaumont or Lissie geologic formations. These areas accumulate flow from the surrounding landscape but rarely accrete significant alluvial deposition. They occupy locally low landscape positions and accumulate moisture from the surrounding landscape but are not bottomland sites.

**Vegetation:** Forested sites typically have a deciduous canopy with species such as *Celtis laevigata, Ulmus crassifolia, Carya illinoinensis, Salix nigra, Prosopis glandulosa, Vachellia farnesiana (= Acacia farnesiana)*, and/or *Quercus nigra*. *Quercus fusiformis* may share, or sometimes dominate, the canopy. The shrub layer may be well‑developed and include species such *Prosopis glandulosa, Vachellia farnesiana, Diospyros texana, Condalia hookeri, Ziziphus obtusifolia*, and/or *Aloysia gratissima*. Some areas may lack a significant overstory and be mapped as shrublands of these species. The herbaceous layer may contain species such *Elymus virginicus, Chasmanthium latifolium, Calyptocarpus vialis, Verbesina virginica*, and *Chloracantha spinosa*.

SOURCES

**References:** Comer et al. 2003*, Elliott 2011

**Version:** 25 Feb 2011	**Stakeholders:** Southeast

**Concept Author:** L. Elliott, D. Diamond, A. Treuer‑kuehn, D. German, J. Teague	**LeadResp:** Southeast

CES203.713 Central Texas Coastal Prairie River Floodplain

**Primary Division:** Gulf and Atlantic Coastal Plain (203)

**Land Cover Class:** Mixed Upland and Wetland

**Spatial Scale & Pattern:** Linear

**Required Classifiers:** Natural/Semi‑natural; Vegetated (>10% vasc.); Upland; Wetland

**Diagnostic Classifiers:** Forest and Woodland (Treed); Riverine / Alluvial; Broad‑Leaved Deciduous Tree

**Concept Summary:** This system occurs in the Gulf Coast Prairies and Marshes region of Texas. It occupies alluvial settings along rivers, streams and larger drainages. This wetland and upland transition area supports a correspondingly similar mixture of wetland/upland vegetation types that are primarily deciduous or mixed evergreen‑deciduous forests. In addition, this system also expresses a transition from northeast to southwest. In the northeastern parts of the range, *Quercus fusiformis* is a common component, as are a few other trees and shrubs with a more eastern affinity such as *Celtis laevigata, Ulmus crassifolia, Sabal minor, Cephalanthus occidentalis, Forestiera acuminata*, and/or *Cornus drummondii*. In the southwestern parts of the range, trees and shrubs with a western and subtropical affinity such as *Fraxinus berlandieriana, Prosopis glandulosa, Vachellia farnesiana*, and *Ehretia anacua* become more prominent.

DISTRIBUTION

**Range:** This system occurs along the Texas coast where it occupies bottomland soils along the coastal portions of the Navidad, Lavaca, Guadalupe, San Antonio, Mission, Aransas, and Nueces rivers (and their tributaries) as they cross the prairie surface of the Lissie and Beaumont geologic formations.

**Divisions:** 203:C

**TNC Ecoregions:** 31:C

**Nations:** US

**Subnations:** TX

**Map Zones:** 34:C

**USFS Ecomap Regions:** 255Cg:CPP, 255Da:CCC, 255Dd:CCC, 315Ed:???

CONCEPT

**Environment:** This system occurs on bottomland soils along rivers, streams and larger drainages. It typically occupies Quaternary alluvium that forms terraces adjacent to the Beaumont or Lissie geologic formations. It is found in bottomland ecological site types with loamy, clayey, and sandy soils.

**Vegetation:** This system is characterized by woodlands and forests dominated by species such as *Celtis laevigata, Celtis laevigata var. reticulata, Ulmus crassifolia, Carya illinoinensis, Ulmus americana, Prosopis glandulosa*, and *Fraxinus berlandieriana* or *Fraxinus pennsylvanica*. The mixed deciduous‑evergreen canopy may include *Quercus fusiformis* and *Ehretia anacua* as significant components of the overstory. Sites dominated by *Quercus fusiformis* typically occur on less‑saturated sites such as slightly elevated situations. Less common species in the canopy may include *Acer negundo, Gleditsia triacanthos, Quercus macrocarpa, Populus deltoides, Platanus occidentalis, Morus rubra*, and *Vachellia farnesiana (= Acacia farnesiana)*. Some wetter sites may be dominated by *Salix nigra* or, less commonly, *Taxodium distichum*. *Acer negundo, Sapindus saponaria var. drummondii, Ungnadia speciosa*, and saplings of the overstory species may form a subcanopy. The shrub layer is often not well‑developed and contains species such as *Sabal minor, Ilex vomitoria, Ilex decidua, Diospyros texana, Sideroxylon lanuginosum, Prosopis glandulosa, Vachellia farnesiana, Condalia hookeri*, and/or *Cornus drummondii*. *Sabal minor* or *Ilex vomitoria* may sometimes form a dense shrub understory. Some sites may represent dense shrublands dominated by *Cephalanthus occidentalis* or *Forestiera acuminata*, with a sparse overstory. Vines are commonly encountered, including species such as *Nekemias arborea (= Ampelopsis arborea), Vitis mustangensis, Smilax bona‑nox, Toxicodendron radicans*, and *Campsis radicans*. The ground layer can be well‑developed and often dominated by graminoids, including *Carex cherokeensis, Carex crus‑corvi, Elymus virginicus, Phanopyrum gymnocarpon, Chasmanthium latifolium, Leersia* spp., *Paspalum langei, Panicum obtusum, Tripsacum dactyloides, Carex bulbostylis, Carex tetrastachya, Oplismenus hirtellus*, and *Dichanthelium sphaerocarpon*. Forbs, such as *Malvaviscus arboreus var. drummondii, Chloracantha spinosa, Verbesina virginica, Calyptocarpus vialis, Commelina erecta, Allium canadense var. canadense, Myosotis macrosperma, Nemophila phacelioides*, and the somewhat rare *Tauschia texana* and *Spigelia texana*, may also be found in these woodlands. *Chloracantha spinosa* may sometimes form large, nearly monotypic in aspect stands. Wetter, herbaceous‑dominated sites occur in these floodplains and may contain species such as *Cyperus* spp., *Eleocharis* spp., *Zizaniopsis miliacea, Paspalum denticulatum*, and *Typha domingensis*. The non‑native *Triadica sebifera* may be present to dominant in the canopy of occurrences of this system. Non‑native grasses such as *Cynodon dactylon, Bothriochloa ischaemum var. songarica, Urochloa maxima*, and *Sorghum halepense* may also be present and sometimes dominate the ground layer to the exclusion of other species.

SOURCES

**References:** Comer et al. 2003*, Elliott 2011

**Version:** 25 Feb 2011	**Stakeholders:** Southeast

**Concept Author:** L. Elliott, D. Diamond, A. Treuer‑kuehn, D. German, J. Teague	**LeadResp:** Southeast

CES203.715 Columbia Bottomlands Forest and Woodland

**Primary Division:** Gulf and Atlantic Coastal Plain (203)

**Land Cover Class:** Mixed Upland and Wetland

**Spatial Scale & Pattern:** Large patch

**Required Classifiers:** Natural/Semi‑natural; Vegetated (>10% vasc.); Upland; Wetland

**Diagnostic Classifiers:** Forest and Woodland (Treed)

**Concept Summary:** This system occupies a generally level landscape encompassing the historic floodplains of the Brazos, Colorado, and San Bernard rivers of the Coastal Prairie region of Texas. The level to gently rolling uplands are punctuated by a series of swales, depressions, terraces, and natural levees. Significant local topographic relief can be associated with these features. Much of the flooding experienced by this system results from seasonal precipitation and tropical storms, not from overbank flooding. A range of communities are expressed along a moisture gradient ranging from the wettest sites along stream margins and depressions, to somewhat drier sites on ridges and natural levees. Soils are frequently clayey or loamy bottomlands.

DISTRIBUTION

**Range:** This system occupies a large area encompassing the historic floodplains of the Brazos, Colorado, and San Bernard rivers in the Coastal Prairie region of the Texas Gulf Coast. Chocolate Bayou represents the eastern extent of this system as the forest grades into systems more closely resembling West Gulf Coastal Plain Small Stream and River Forest (CES203.487) to the northeast. Tres Palacios Creek represents the southwestern limit of this system, as floodplains further south and west share closer affinity to coastal rivers such as the Mission and Aransas.

**Divisions:** 203:C

**TNC Ecoregions:** 31:C

**Nations:** US

**Subnations:** TX

**Map Zones:** 36:C

**USFS Ecomap Regions:** 255Da:CCC, 255Db:CCC, 255Dd:CCC

CONCEPT

**Environment:** This system occurs on Quaternary alluvium and adjacent Pleistocene terraces (Beaumont and Lissie formations) along the Brazos, San Bernard, and Colorado rivers (as they pass through these Pleistocene formations), and adjacent streams such as Oyster Creek, Caney Creek, and Linnville Bayou. It occupies a generally level landscape, punctuated by a series of swales, depressions, and natural levees. Much of the flooding experienced by this system results from seasonal precipitation and tropical storms. Overbank flooding is infrequent, occurring about every 15 to 25 years. Soils are frequently clayey bottomlands (such as Pledger or Brazoria clays) or loamy bottomlands (such as those of the Asa or Norwood series), but also found on blackland and claypan soils within the basin.

**Vegetation:** Herbaceous communities and open water typically characterize the wettest sites in this system, with species such as *Eleocharis quadrangulata, Sagittaria graminea, Sagittaria platyphylla, Ludwigia* spp., *Saururus cernuus, Azolla caroliniana*, and *Lemna obscura*. Such very wet sites may have *Taxodium distichum* and *Salix nigra* in the overstory, or may be shrub swamps dominated by *Cephalanthus occidentalis* and/or *Forestiera acuminata*. Sites inundated somewhat less frequently, such as meander scars, abandoned oxbows, and channels, are often dominated in the overstory by species including *Fraxinus pennsylvanica, Ulmus americana*, and *Carya aquatica*, while the woody understory of these sites are typically open and may be dominated by *Cephalanthus occidentalis* and/or *Forestiera acuminata*. Rarely, *Leitneria floridana* may be a conspicuous component of the shrub layer. Herbaceous cover is often patchy and can include species such as *Phanopyrum gymnocarpon, Echinodorus cordifolius, Carex* spp., *Rhynchospora corniculata, Saururus cernuus, Polygonum punctatum, Hygrophila lacustris, Boehmeria cylindrica, Mikania scandens*, and *Lemna obscura*. Flats and ridges that are only occasionally flooded are often dominated by *Celtis laevigata, Ulmus crassifolia, Quercus nigra*, and *Quercus shumardii*. Shrubs on these sites include *Ilex vomitoria, Sapindus saponaria var. drummondii, Malvaviscus arboreus var. drummondii, Symphoricarpos orbiculatus*, and *Callicarpa americana*. *Sabal minor* and *Carex cherokeensis* are more abundant on these sites, and other species such as *Toxicodendron radicans, Chasmanthium sessiliflorum, Chasmanthium latifolium, Calyptocarpus vialis, Oplismenus hirtellus ssp. setarius*, and *Polygonum virginianum* may be present. Clay backflats in this landscape may be dominated by *Quercus virginiana* and *Carya illinoinensis*, and *Quercus virginiana* may also share dominance with other canopy species on natural levees of these river systems. Blackland soils on the Pleistocene surface (such as those of the Lake Charles series) are often occupied by a forest dominated or codominated by *Quercus nigra, Celtis laevigata, Ulmus crassifolia, Fraxinus pennsylvanica*, and less frequently *Quercus virginiana*. The shrub layer on these sites is often well‑developed and typically dominated by *Ilex vomitoria*, sometimes with *Sabal minor, Cornus drummondii*, and *Prunus caroliniana* also present. Vines are commonly encountered, including species such as *Vitis mustangensis, Toxicodendron radicans, Nekemias arborea (= Ampelopsis arborea)*, and *Berchemia scandens*. *Chasmanthium sessiliflorum, Carex cherokeensis, Carex crus‑corvi, Urochloa platyphylla, Juncus* spp., and numerous other species are commonly found in the herbaceous layer. It is unclear whether these typically prairie‑dominated surfaces are now occupied by woodland and forest due to a disruption in natural fire cycle and disturbance, or whether the unique hydrology or other environmental factors of the Columbia Bottomlands leads to this incongruity. *Tillandsia usneoides* is a frequently encountered epiphyte in these forests. Riverside woodlands, along major rivers, have *Platanus occidentalis* and *Populus deltoides* in the canopy. The non‑native tree *Triadica sebifera* may often be encountered, sometimes as a significant or dominant component of the canopy.

SOURCES

**References:** Comer et al. 2003*, Elliott 2011, Rosen and Miller 2005, Rosen et al. 2008

**Version:** 25 Feb 2011	**Stakeholders:** Southeast

**Concept Author:** L. Elliott, D. Diamond, A. Treuer‑kuehn, D. German, J. Teague	**LeadResp:** Southeast

CES303.651 Edwards Plateau Floodplain Terrace

**Primary Division:** Western Great Plains (303)

**Land Cover Class:** Woody Wetland

**Spatial Scale & Pattern:** Linear

**Required Classifiers:** Natural/Semi‑natural; Vegetated (>10% vasc.); Upland; Wetland

**Diagnostic Classifiers:** Lowland; Forest and Woodland (Treed); Depositional stream terrace; Floodplain

**Concept Summary:** This forest/woodland system occurs on floodplain terraces along perennial rivers and streams in central Texas. Canopy dominants may include *Ulmus crassifolia, Juniperus ashei, Celtis laevigata, Quercus fusiformis, Fraxinus albicans, Platanus occidentalis, Acer negundo, Juglans major, Quercus macrocarpa*, or *Carya illinoinensis*. *Carya illinoinensis* may be more likely to occur in deeper and better‑developed alluvial soils. Occurrences typically have a multi‑layered physiognomy with a woody understory and patchy ground flora. Alluvial sedimentation processes dominate the formation and maintenance of this system. However, overgrazing and/or overbrowsing may influence recruitment of overstory species and composition of the understory and herbaceous layers.

**Comments:** Further field investigation is needed to better develop the association‑level information for this system. It occurs along larger, lower gradient rivers and streams in contrast with Edwards Plateau Riparian (CES303.652) which occurs along smaller, higher gradient streams. Any particular reach of a river would be classified and mapped as one or the other system.

DISTRIBUTION

**Range:** This system occurs along larger permanent rivers and streams throughout the Edwards Plateau of Texas and possibly adjacent ecoregions. It occurs from the Leon watershed in the Limestone Cutplain (EPA 29e) south to the edge of the Bacones Canyonlands (EPA 30c), west through the Edwards Plateau and north to the Pecan Bayou and Concho River watersheds in the lower Limestone Plains (EPA 27j) and lower Crosstimbers (EPA 29c) (EPA 2001).

**Divisions:** 302:C, 303:C

**TNC Ecoregions:** 29:C

**Nations:** US

**Subnations:** TX

**Map Zones:** 32:C, 35:C

**USFS Ecomap Regions:** 255Ec:CCC, 255Ed:CCC, 315Cc:CCC, 315Da:CCC, 315Db:CCC, 315Dc:CCC, 315Ga:CCC, 321B:PP

CONCEPT

**Environment:** This system is found in central Texas and usually occupies Quaternary alluvial deposits often within drainages underlain by Cretaceous limestones, or drainages that receive outwash from landscapes dominated by these limestones (Elliott 2011). Landforms include valley floors of large rivers and perennial streams. This system tends to occupy broad valley bottoms with alluvial deposits on the Edwards Plateau, and rivers and large creeks where outwash from the Edwards Plateau influences the substrate (Elliott 2011). Soils include bottomland soils of various types (loamy, clayey, and sandy).

**Vegetation:** These are forests and woodlands with a canopy dominated or codominated by *Carya illinoinensis, Ulmus crassifolia, Ulmus americana, Celtis laevigata, Celtis laevigata var. reticulata*, and/or *Quercus fusiformis*. *Carya illinoinensis* may be more likely to occur in deeper and better‑developed alluvial soils (Elliott 2011). Apparent dominance of *Carya illinoinensis* may also be an artifact of preferential harvesting of other species, leaving this species in greater abundance. Other species present may include *Fraxinus albicans (= Fraxinus texensis), Fraxinus pennsylvanica, Juglans major, Quercus macrocarpa, Quercus buckleyi, Acer negundo, Sapindus saponaria var. drummondii, Juniperus ashei, Prosopis glandulosa*, and *Platanus occidentalis*. *Quercus stellata* may be dominant on sandy soils within the floodplain at some sites. *Melia azedarach* is a common non‑native tree encountered on floodplains. Woody species in the subcanopy may include *Sideroxylon lanuginosum, Ptelea trifoliata, Cornus drummondii, Morus rubra, Diospyros texana, Parthenocissus quinquefolia, Vitis* spp., *Smilax bona‑nox, Baccharis neglecta, Malvaviscus arboreus var. drummondii, Juniperus ashei*, and *Ilex decidua*. The herbaceous layer may be continuous, though relatively sparse or patchy with species such as *Elymus virginicus, Chasmanthium latifolium, Nassella leucotricha, Verbesina virginica*, and *Carex* spp. Some sites lack, or have very sparse, overstory canopies and represent shrublands or grasslands. Shrublands may be dominated by species in the shrub layer of the surrounding woodlands. Other components or dominants may include species such as *Prosopis glandulosa, Vachellia farnesiana (= Acacia farnesiana), Sapindus saponaria var. drummondii, Juglans microcarpa, Mahonia trifoliolata*, and *Cephalanthus occidentalis*. Native species that may also be present in (and sometimes dominate) these sites include *Panicum virgatum, Andropogon glomeratus, Elymus virginicus, Nassella leucotricha, Hordeum pusillum, Tripsacum dactyloides, Muhlenbergia lindheimeri, Carex* spp., and *Eleocharis* spp. (Elliott 2011). Some grassland sites are frequently dominated by the non‑native species *Cynodon dactylon* and/or *Bothriochloa ischaemum var. songarica*. Floodplain occurrences often include portions that resemble Edwards Plateau Riparian (CES303.652), especially along stream margins, where *Platanus occidentalis, Taxodium distichum, Juglans microcarpa, Brickellia* spp., *Cladium mariscus ssp. jamaicense*, and *Panicum virgatum* are frequently encountered (Elliott 2011).

**Dynamics:** Alluvial sedimentation processes dominate the formation and maintenance of this system. However, overgrazing and/or overbrowsing may influence recruitment of overstory species and composition of the understory and herbaceous layers.

SOURCES

**References:** Comer et al. 2003*, EPA 2004, Elliott 2011, Eyre 1980

**Version:** 28 May 2013	**Stakeholders:** Midwest, Southeast, West

**Concept Author:** L. Elliott and J. Teague	**LeadResp:** Southeast

CES303.652 Edwards Plateau Riparian

**Primary Division:** Western Great Plains (303)

**Land Cover Class:** Woody Wetland

**Spatial Scale & Pattern:** Linear

**Required Classifiers:** Natural/Semi‑natural; Vegetated (>10% vasc.); Wetland

**Diagnostic Classifiers:** Forest and Woodland (Treed); Shrubland (Shrub‑dominated); Woody‑Herbaceous; Herbaceous; Streambed; Flood Scouring

**National Mapping Codes:** EVT 2525; ESLF 9165; ESP 1525

**Concept Summary:** This system occurs in various situations along small and intermittent streams of the Edwards Plateau, with drier representatives occurring in the western plateau and the Stockton Plateau, and moister representatives (such as communities dominated by *Juglans microcarpa* and *Brickellia laciniata*) in the eastern plateau. Representatives of this system typically occur in stream‑scoured situations and vary in the openness of the habitat and physiognomy. Woodland examples may have *Quercus fusiformis, Platanus occidentalis, Taxodium distichum, Fraxinus albicans, Fraxinus pennsylvanica, Ulmus crassifolia, Celtis laevigata* (including *var. reticulata*), *Acer negundo, Prosopis glandulosa, Quercus buckleyi, Juniperus ashei, Salix nigra*, and/or *Sapindus saponaria*. Shrub species that may be encountered in the understory of these woodlands include *Juglans microcarpa, Chilopsis linearis, Baccharis* spp., *Salix nigra, Juniperus ashei, Sapindus saponaria, Cornus drummondii, Sophora secundiflora, Sideroxylon lanuginosum, Diospyros texana, Ungnadia speciosa, Prosopis glandulosa, Cephalanthus occidentalis*, and/or *Aloysia gratissima*. Substantial patches of herbaceous cover may be present and often include species such as *Andropogon glomeratus, Panicum virgatum, Cladium mariscus ssp. jamaicense, Tripsacum dactyloides, Setaria scheelei, Nassella leucotricha, Eleocharis* spp., *Brickellia* spp., *Justicia americana, Hydrocotyle* spp., and/or *Muhlenbergia lindheimeri*.

**Comments:** Further field investigation is needed to better develop the association‑level information for this system. Edwards Plateau Floodplain Terrace (CES303.651) occurs along larger, lower gradient rivers and streams in contrast with Edwards Plateau Riparian (CES303.652) which occurs along smaller, higher gradient streams. Any particular reach of a river would be classified and mapped as one or the other system.

DISTRIBUTION

**Range:** This system is found along minor streams and tributaries throughout the Edwards Plateau.

**Divisions:** 302:C, 303:C

**TNC Ecoregions:** 29:C

**Nations:** US

**Subnations:** TX

**Map Zones:** 32:C, 35:C

**USFS Ecomap Regions:** 255Ba:CCC, 255E:CC, 315C:C?, 315D:CC, 315G:C?, 321B:??

CONCEPT

**Environment:** This system occurs on minor intermittent streams and tributaries throughout the Edwards Plateau of Texas. Its geology is usually Quaternary deposits along headwater streams. These may be alluvial or gravel deposits and are often within drainages dominated by limestone or other calcareous substrates on the Edwards Plateau or where substrate is influenced by outwash from the Edwards Plateau. This riparian system occupies small streams, either intermittent or perennial. These sites tend to be in erosional situations, as opposed to broad alluvial depositional sites. This system was mapped by TPWD in areas upstream of significant development of bottomland soils on soil types of the surrounding uplands. It includes vegetation along very small streams, reaching upstream to spring heads and runs (Elliott 2011).

**Vegetation:** Riparian vegetation may be characterized as woodlands, shrublands, or herbaceous vegetation. These erosional sites may be gravelly, cobbly or rocky and generally occupy the upper reaches of streams. Woodland examples may have *Quercus fusiformis, Platanus occidentalis, Taxodium distichum, Fraxinus albicans (= Fraxinus texensis), Fraxinus pennsylvanica, Ulmus crassifolia, Celtis laevigata* (including *var. reticulata*), *Acer negundo, Prosopis glandulosa, Quercus buckleyi, Juniperus ashei, Salix nigra*, and/or *Sapindus saponaria* (Elliott 2011). Shrub species that may be encountered in the understory of these woodlands include *Juglans microcarpa, Chilopsis linearis, Baccharis* spp., *Salix nigra, Juniperus ashei, Sapindus saponaria, Cornus drummondii, Sophora secundiflora, Sideroxylon lanuginosum, Diospyros texana, Ungnadia speciosa, Prosopis glandulosa, Cephalanthus occidentalis*, and/or *Aloysia gratissima*. In some cases, these species may form shrublands lacking a significant overstory tree canopy (Elliott 2011). Substantial patches of herbaceous cover may be present and often include species such as *Andropogon glomeratus, Panicum virgatum, Cladium mariscus ssp. jamaicense, Tripsacum dactyloides, Setaria scheelei, Nassella leucotricha, Eleocharis* spp., *Brickellia* spp., *Justicia americana, Hydrocotyle* spp., and/or *Muhlenbergia lindheimeri*. Frequently, *Cynodon dactylon* and/or *Bothriochloa ischaemum var. songarica* dominate these grassland sites. *Sorghum halepense* is also a commonly encountered non‑native grass.

SOURCES

**References:** Comer et al. 2003*, Elliott 2011, Eyre 1980

**Version:** 24 Feb 2011	**Stakeholders:** Midwest, Southeast, West

**Concept Author:** L. Elliott and J. Teague	**LeadResp:** Southeast

CES205.710 Southeastern Great Plains Floodplain Forest

**Primary Division:** Eastern Great Plains (205)

**Land Cover Class:** Woody Wetland

**Spatial Scale & Pattern:** Linear

**Required Classifiers:** Natural/Semi‑natural; Vegetated (>10% vasc.); Wetland

**Diagnostic Classifiers:** Forest and Woodland (Treed); Herbaceous; Riverine / Alluvial; Deep (>15 cm) Water; Intermediate (5‑25 yrs) Flooding Interval

**Concept Summary:** This ecological system is found in the floodplains of medium and larger rivers of the East Central Texas Plains, Texas Blackland Prairie Regions, Crosstimbers, and the southeastern edge of the Central Great Plains (Level 3 Ecoregions 33, 32, 29 and 27 respectively). Alluvial soils and sedimentation processes typify this system. Periodic, intermediate flooding and deposition (every 5‑25 years) dominates the formation and maintenance of this system. Dominant communities within this system range from floodplain forests to wet meadows to gravel/sand flats; however, they are linked by underlying soils and the flooding regime. Canopy dominants may include *Carya illinoinensis, Ulmus crassifolia, Ulmus americana, Celtis laevigata, Quercus nigra, Platanus occidentalis, Acer negundo, Quercus macrocarpa, Morus rubra, Fraxinus pennsylvanica, Salix nigra*, and *Sapindus saponaria var. drummondii*. Overgrazing and/or overbrowsing may influence recruitment of overstory species and composition of the understory and herbaceous layers. Shrub species may include *Callicarpa americana, Ilex decidua, Sideroxylon lanuginosum, Diospyros virginiana, Juniperus virginiana, Cornus drummondii*, and *Viburnum rufidulum*, which may occur as dense patches following disturbance, but are otherwise generally fairly sparse. Vines such as *Berchemia scandens, Campsis radicans, Vitis* spp., *Parthenocissus quinquefolia*, and *Nekemias arborea* may be conspicuous. Herbaceous cover includes *Elymus virginicus, Verbesina virginica, Chasmanthium latifolium, Chasmanthium sessiliflorum, Tripsacum dactyloides, Symphyotrichum drummondii var. texanum, Geum canadense, Sanicula canadensis, Panicum virgatum, Galium* spp., and *Carex* sp. Herbaceous cover may be quite high, especially in situations where shrub cover is low. The environment and vegetation of this system become generally and correspondingly drier from east to west with moister representatives (such as communities containing *Quercus phellos, Quercus pagoda, Quercus alba*, and *Quercus lyrata* ) occurring along the eastern and northeastern margins of the range. Representatives of this system may vary in the openness of the habitat and physiognomy.

**Comments:** More data are needed to determine if this system should be split and a new system developed for the southern parts of Ecoregion 32 and 33 (*sensu* EPA; Griffith et al. 2004), south of the Brazos or Colorado rivers. Further field investigation is needed to better develop the association‑level information for this system. This system grades into Edwards Plateau Floodplain Terrace (CES303.651) but can be distinguished by the absence or low cover of western species such as *Juglans major, Juglans microcarpa, Juniperus ashei, Mahonia trifoliolata, Sapindus saponaria var. drummondii*, and the presence or higher cover of more eastern species such as *Maclura pomifera, Ilex decidua, Ilex vomitoria, Quercus nigra, Ulmus americana*, and *Juniperus virginiana*. Species common in both systems include *Ulmus crassifolia, Carya illinoinensis, Platanus occidentalis*, and *Celtis laevigata*. More information is need to better differentiate these systems.

Along the Red River and a few of its tributaries, thin bands of riparian vegetation occurring on sandy floodplain terraces, bluffs and sandbars are significantly different in species composition from riparian communities elsewhere in the region. The floodplains of the Red River have been recognized as a separate system (Red River Large Floodplain Forest (CES203.065)). Occurrences may include *Salix* spp. (especially *Salix exigua*), *Acer saccharinum* (which probably does not occur in any other basin in Texas), *Juniperus virginiana*, and *Populus deltoides*. Adjacent slopes and higher floodplain terraces support woodlands of *Juniperus virginiana, Quercus macrocarpa, Quercus shumardii, Quercus muehlenbergii, Fraxinus albicans, Cornus drummondii*, and *Viburnum rufidulum*.<br />

This system is found in Texas, but not in the West Gulf Coastal Plain and not in the Edwards Plateau. It includes Cross Timbers and the southeastern Tallgrass Prairie region. Interstate 35 goes through the middle of it.

DISTRIBUTION

**Range:** This system is found along major river floodplains in the East Central Texas Plains, Texas Blackland Prairie Regions, Crosstimbers, and the southeastern edge of the Central Great Plains (Level 3 Ecoregions 33, 32, 29 and 27, respectively, *sensu* Griffith et al. (2004)). Rivers such as the Sulphur (and tributaries such as White Oak and Cuthand creeks), Sabine (and Lake Fork), Trinity (and its major tributaries), Navasota, portions of the Lower and Middle Brazos rivers (and major tributaries), portions of the middle and upper Red River, and portions of the Guadalupe, Colorado, and San Antonio rivers downstream of the Edwards Plateau ecoregion may support this system.

**Divisions:** 205:C, 303:C

**TNC Ecoregions:** 32:C

**Nations:** US

**Subnations:** OK, TX

**Map Zones:** 32:C, 35:C, 36:C, 37:C

**USFS Ecomap Regions:** 255Ac:CCP, 255Ad:CCC, 255Af:CCC, 255Ba:CCC, 255Ca:CCC, 255Cc:CCC, 255Cd:CCC, 255Ea:CCC, 255Eb:CCC, 255Ec:CCC, 255Ed:CCC, 255Ee:CCC, 315Cb:CCP, 315Ed:CCC, 315Ga:CCC

CONCEPT

**Environment:** This system occupies relatively broad flats at low topographic positions, along large streams where alluvial deposition dominates. Rivers such as the Sulphur (and tributaries such as White Oak and Cuthand creeks), Sabine (and Lake Fork), Trinity (and its major tributaries), Navasota, and portions of the Lower and Middle Brazos (and its major tributaries), Colorado, Guadalupe, Lavaca, Navidad, and San Antonio rivers may support this system. The geological setting is Quaternary Alluvium (Elliott 2011). It is found in the floodplains of medium and larger rivers of the East Central Texas Plains, Texas Blackland Prairie Regions, Cross Timbers, and the southeastern edge of the Central Great Plains (Level 3 Ecoregions 33, 32, 29 and 27, respectively, *sensu* Griffith et al. (2004)). Bottomland Ecological Sites (including Loamy, Sandy, and Clayey) characterize this system. Soils are primarily alluvial and range from sandy to dense clays.

**Vegetation:** Dominant communities within this system range from floodplain forests to wet meadows to gravel/sand flats; however, they are linked by underlying soils and the flooding regime. Canopy dominants may include *Carya illinoinensis, Fraxinus americana, Quercus nigra, Ulmus crassifolia, Celtis laevigata, Ulmus americana, Quercus fusiformis* or *Quercus virginiana, Platanus occidentalis, Acer negundo, Gleditsia triacanthos, Quercus macrocarpa, Morus rubra, Fraxinus pennsylvanica*, and *Sapindus saponaria var. drummondii*. Especially along river margins, species such as *Platanus occidentalis, Populus deltoides*, and *Salix nigra* may dominate. In this eastern part of the range of the system, *Liquidambar styraciflua, Quercus phellos*, and *Betula nigra* may also be commonly encountered. Seasonally flooded sites, especially within the Trinity River basin, may have *Quercus lyrata* as an overstory component. Overgrazing and/or overbrowsing may influence recruitment of overstory species and composition of the understory and herbaceous layers. Shrub species may include *Callicarpa americana, Cephalanthus occidentalis, Ilex decidua, Ilex vomitoria, Sideroxylon lanuginosum, Diospyros virginiana, Vaccinium arboreum, Juniperus virginiana, Cornus drummondii*, and *Viburnum rufidulum* which may occur as dense patches following disturbance, but are otherwise generally fairly sparse. In the southern expressions of the system, other shrubs such as *Prosopis glandulosa, Vachellia farnesiana (= Acacia farnesiana), Diospyros texana*, and *Condalia hookeri* may be commonly encountered. Vines such as *Berchemia scandens, Campsis radicans, Vitis* spp., *Parthenocissus quinquefolia, Toxicodendron radicans, Smilax bona‑nox*, and *Nekemias arborea (= Ampelopsis arborea)* may be conspicuous. Herbaceous cover includes *Elymus virginicus, Verbesina virginica, Chasmanthium latifolium, Chasmanthium sessiliflorum, Carex cherokeensis, Tripsacum dactyloides, Symphyotrichum drummondii var. texanum, Calyptocarpus vialis, Geum canadense, Sanicula canadensis, Ambrosia trifida, Panicum virgatum, Galium* spp., *Teucrium canadense*, and *Carex* spp. Wetter sites may contain species such as *Zizaniopsis miliacea, Rhynchospora* spp., *Eleocharis* spp., *Nymphaea odorata*, and *Peltandra virginica*. In early‑successional states, growth rates among species are variable with species such as *Acer negundo* having rapid growth rate and species such as *Quercus macrocarpa* growing more slowly. There may be an open canopy resulting from flood events and rare fire events. The environment and vegetation of this system become generally and correspondingly drier from east to west with moister representatives (such as communities containing *Quercus phellos, Quercus pagoda, Quercus alba*, and *Quercus lyrata*) occurring along the eastern and northeastern margins of the range. Non‑native grasses that may dominate these sites include *Cynodon dactylon, Bothriochloa ischaemum var. songarica*, and *Sorghum halepense*. Herbaceous cover may be quite high, especially in situations where shrub cover is low. The non‑native trees *Triadica sebifera* and *Melia azedarach* may be present.

**Dynamics:** Periodic and intermediate flooding is the most significant process controlling this system and is expected every 5 to 25 years. Grazing and conversion to agriculture can significantly impact this system and can lead to the degradation or extirpation of the majority of prairie and wet meadow communities from this system. Fire occurs infrequently relative to surrounding systems. Fuels tend to stay moister due to shady conditions and low topographic position. Other disturbances include ice storm/blowdowns, which are capable of setting back small to large patches; as well as beaver pond flooding, which even though a small‑patch event, is expected to cycle throughout the forest over the long term, perhaps at a scale of hundreds or thousands of years.

SOURCES

**References:** Comer et al. 2003*, Eidson pers. comm., Elliott 2011, Eyre 1980, Griffith et al. 2004

**Version:** 24 Feb 2011	**Stakeholders:** Southeast

**Concept Author:** J. Eidson, M. Pyne, L. Elliott and J. Teague	**LeadResp:** Southeast

1.B.3.Nc. Rocky Mountain‑Great Basin Montane Flooded & Swamp Forest

M034. Rocky Mountain‑Great Basin Montane Riparian & Swamp Forest

CES304.768 Columbia Basin Foothill Riparian Woodland and Shrubland

**Primary Division:** Inter‑Mountain Basins (304)

**Land Cover Class:** Woody Wetland

**Spatial Scale & Pattern:** Linear

**Required Classifiers:** Natural/Semi‑natural; Vegetated (>10% vasc.); Wetland

**Diagnostic Classifiers:** Montane [Lower Montane]; Lowland [Foothill]; Riverine / Alluvial; Short (<5 yrs) Flooding Interval; Short (50‑100 yrs) Persistence

**Concept Summary:** This is a low‑elevation riparian system found on the periphery of the mountains surrounding the Columbia River Basin, along major tributaries and the main stem of the Columbia at relatively low elevations. This is the riparian system associated with all streams at and below lower treeline, including permanent, intermittent and ephemeral streams with woody riparian vegetation. These forests and woodlands require flooding and some gravels for reestablishment. They are found in low‑elevation canyons and draws, on floodplains, or in steep‑sided canyons, or narrow V‑shaped valleys with rocky substrates. Sites are subject to temporary flooding during spring runoff. Underlying gravels may keep the water table just below the ground surface and are favored substrates for cottonwood. Large bottomlands may have large occurrences, but most have been cut over or cleared for agriculture. Rafted ice and logs in freshets may cause considerable damage to tree boles. Beavers crop younger cottonwood and willows and frequently dam side channels occurring in these stands. In steep‑sided canyons, streams typically have perennial flow on mid to high gradients. Important and diagnostic trees include *Populus balsamifera ssp. trichocarpa, Alnus rhombifolia, Populus tremuloides, Celtis laevigata var. reticulata, Betula occidentalis*, or *Pinus ponderosa*. Important shrubs include *Crataegus douglasii, Philadelphus lewisii, Cornus sericea, Salix lucida ssp. lasiandra, Salix eriocephala, Rosa nutkana, Rosa woodsii, Amelanchier alnifolia, Prunus virginiana*, and *Symphoricarpos albus*. Grazing is a major influence in altering structure, composition, and function of the system.

DISTRIBUTION

**Range:** Found on the periphery of the northern Rockies in the Columbia River Basin, along major tributaries and the main stem of the Columbia at relatively low elevations.

**Divisions:** 304:C, 306:C

**TNC Ecoregions:** 6:C, 7:C, 68:C

**Nations:** CA, US

**Subnations:** BC, CA, ID, MT?, NV, OR, UT, WA:S1

**Map Zones:** 1:C, 7:C, 8:C, 9:C, 10:C, 16:?, 17:?, 18:C, 21:?

**USFS Ecomap Regions:** 331A:CC, 342B:CC, 342C:CC, 342D:CC, 342H:CC, 342I:CC, M242C:P?, M242D:PP, M261G:PP, M331A:P?, M331D:PP, M332A:CC, M332E:C?, M332F:CP, M332G:CC, M333A:CC, M333B:CC, M333D:CC

CONCEPT

**Environment:** This is a low‑elevation riparian system found on the periphery of the mountains surrounding the Columbia River Basin, along major tributaries and the main stem of the Columbia at relatively low elevations. This is the riparian system associated with all streams at and below lower treeline, including permanent, intermittent and ephemeral streams with woody riparian vegetation. These forests and woodlands require flooding and some fresh exposed gravel for reestablishment. They are found in low‑elevation canyons and draws, on floodplains, or in steep‑sided canyons, or narrow V‑shaped valleys with rocky substrates. Sites are subject to temporary flooding during spring runoff. Underlying gravels may keep the water table just below the ground surface and are favored substrates for cottonwood. Large bottomlands may have large occurrences, but most have been cut over or cleared for agriculture. Rafted ice and logs in freshets may cause considerable damage to tree boles. Beavers crop younger cottonwood and willows and frequently dam side channels occurring in these stands. In steep‑sided canyons, streams typically have perennial flow on mid to high gradients.

**Vegetation:** Important and diagnostic trees include *Populus balsamifera ssp. trichocarpa, Alnus rhombifolia, Populus tremuloides, Celtis laevigata var. reticulata, Betula occidentalis*, or *Pinus ponderosa*. Important shrubs include *Crataegus douglasii, Philadelphus lewisii, Cornus sericea, Salix lucida ssp. lasiandra, Salix eriocephala, Rosa nutkana, Rosa woodsii, Amelanchier alnifolia, Prunus virginiana*, and *Symphoricarpos albus*.

**Dynamics:** The majority of these forests and woodlands require flooding and freshly deposited gravel/sand for seedling establishment. The natural hydrologic cycle in these reaches includes high spring and early summer flow pulses from snowmelt run off and a natural drawdown into late‑summer and fall months. Spring and early summer months also see a rise of the underlying alluvial groundwater table as well as natural lowering of the groundwater in late summer into fall months. High flows and flooding scour (removal) and deposit sediments that stimulate growth of cottonwoods and willows, replenish nutrients, move seeds and aquatic organisms (Merritt and Wohl 2002). These processes stimulate and revive riparian ecosystems. Some reaches are supported by groundwater discharge where flood disturbances are less vital to long‑term viability.

SOURCES

**References:** Boes and Strauss 1994, Comer et al. 2003*, Ecosystems Working Group 1998, Eyre 1980, Johnson and Simon 1985, Kauffman et al. 2004, Littell et al. 2009, Merritt and Wohl 2002, WNHP 2011, WNHP unpubl. data 2018

**Version:** 14 Jan 2014	**Stakeholders:** Canada, West

**Concept Author:** K.A. Schulz	**LeadResp:** West

CES304.045 Great Basin Foothill and Lower Montane Riparian Woodland and Shrubland

**Primary Division:** Inter‑Mountain Basins (304)

**Land Cover Class:** Woody Wetland

**Spatial Scale & Pattern:** Linear

**Required Classifiers:** Natural/Semi‑natural; Vegetated (>10% vasc.); Wetland

**Diagnostic Classifiers:** Forest and Woodland (Treed); Riverine / Alluvial; Short (<5 yrs) Flooding Interval; Riparian Mosaic

**Concept Summary:** This system occurs in mountain ranges of the Great Basin and along the eastern slope of the Sierra Nevada within a broad elevation range from about 1220 m (4000 feet) to over 2135 m (7000 feet). This system often occurs as a mosaic of multiple communities that are tree‑dominated with a diverse shrub component. The variety of plant associations connected to this system reflects elevation, stream gradient, floodplain width, and flooding events. Dominant trees may include *Abies lowiana, Alnus incana, Betula occidentalis, Populus angustifolia, Populus balsamifera ssp. trichocarpa, Populus fremontii, Salix laevigata, Salix gooddingii*, and *Pseudotsuga menziesii*. Dominant shrubs include *Artemisia cana, Cornus sericea, Salix exigua, Salix lasiolepis, Salix lemmonii*, or *Salix lutea*. Herbaceous layers are often dominated by species of *Carex* and *Juncus*, and perennial grasses and mesic forbs such *Deschampsia cespitosa, Elymus trachycaulus, Glyceria striata, Iris missouriensis, Maianthemum stellatum*, or *Thalictrum fendleri*. Introduced forage species such as *Agrostis stolonifera, Poa pratensis, Phleum pratense*, and the weedy annual *Bromus tectorum* are often present in disturbed stands. These are disturbance‑driven systems that require flooding, scour and deposition for germination and maintenance. Livestock grazing is a major influence in altering structure, composition, and function of the system.

DISTRIBUTION

**Range:** Occurs in mountain ranges of the Great Basin and along the eastern slope of the Sierra Nevada within a broad elevation range from about 1220 m (4000 feet) to over 2135 m (7000 feet).

**Divisions:** 304:C

**TNC Ecoregions:** 6:P, 11:C, 12:C

**Nations:** US

**Subnations:** CA, NV, OR, UT

**Map Zones:** 6:C, 7:C, 9:C, 10:C, 12:C, 13:C, 16:?, 17:C, 18:C, 21:P

**USFS Ecomap Regions:** 322A:CC, 341A:CC, 341D:CC, 341E:CC, 341F:CC, 341G:CC, 342B:CC, 342C:CC, 342D:CC, 342J:CC, M261E:CC, M261G:CP, M331D:??, M332A:??, M341A:CC, M341D:CC

CONCEPT

**Environment:** This system is found in low‑elevation canyons and draws, on floodplains, steep‑sided canyons, or narrow V‑shaped valleys with rocky substrates. This includes both perennial and intermittent streams. Sites are typically subject to temporary flooding during spring or late winter runoff. Overbank flooding and some gravel areas are required for regeneration of these riparian forests and woodlands, especially for cottonwoods.

**Vegetation:** Dominant trees may include *Abies lowiana (= Abies concolor var. lowiana), Alnus incana, Betula occidentalis, Populus angustifolia, Populus balsamifera ssp. trichocarpa, Populus fremontii, Salix laevigata, Salix gooddingii*, and *Pseudotsuga menziesii*. Dominant shrubs include *Artemisia cana, Cornus sericea, Salix exigua, Salix lasiolepis, Salix lemmonii*, or *Salix lutea*. Herbaceous layers are often dominated by species of *Carex* and *Juncus*, and perennial grasses and mesic forbs such *Deschampsia cespitosa, Elymus trachycaulus, Glyceria striata, Iris missouriensis, Maianthemum stellatum*, or *Thalictrum fendleri*. Introduced forage species such as *Agrostis stolonifera, Poa pratensis, Phleum pratense*, and the weedy annual *Bromus tectorum* are often present in disturbed stands.

**Dynamics:** The hydrologic regime is naturally highly variable temporally and spatially among the streams and rivers of this system. Where present, spring discharges from bedrock aquifers provide flows unaffected by rainfall and snowmelt. Otherwise, stream and river flows ‑ where they occur, at what magnitudes, and when and how often ‑ are subject to wide fluctuations as a result of the wide variation in where and when precipitation takes place, what form the precipitation takes (rain versus snow), and where and when snowmelt takes place (e.g., Abell et al. 2000, Levick et al. 2008, Miller et al. 2010a). Intense runoff associated with intense rainfall events is highly erosive, resulting in rapid reconfiguration of aquatic and riparian macrohabitats particularly along reaches with sand and gravel substrates. Fire disturbances occur in riparian zones, but are generally less severe and less often than in neighboring uplands (Reeves et al. 2005).

SOURCES

**References:** Abell et al. 2000, Barbour and Billings 1988, Barbour and Major 1977, Brown and Mote 2009, Cayan et al. 2010, Chambers and Pellant 2008, Chambers and Wisdom 2009, Christensen and Lettenmaier 2007, Comer et al. 2003*, Comer et al. 2013a, Covich 2009, Das et al. 2009, Daubenmire 1952, Dettinger et al. 2009, Eyre 1980, Field et al. 1999, Harper and Peckarsky 2006, Hultine et al. 2007, Jackson et al. 2009, Kittel et al. 1999b, Levick et al. 2008, Manning and Padgett 1989, Martin 2007, McCabe and Wolock 2009, Melack et al. 1997, Millar and Wolfenden 1999, Miller et al. 2010a, Mote 2006, Reeves et al. 2005, Sawyer and Keeler‑Wolf 1995, Seavy et al. 2009, Shiflet 1994, USBOR 2011

**Version:** 14 Jan 2014	**Stakeholders:** West

**Concept Author:** J. Nachlinger and K. Schulz	**LeadResp:** West

CES306.803 Northern Rocky Mountain Conifer Swamp

**Primary Division:** Rocky Mountain (306)

**Land Cover Class:** Woody Wetland

**Spatial Scale & Pattern:** Large patch

**Required Classifiers:** Natural/Semi‑natural; Vegetated (>10% vasc.); Wetland

**Diagnostic Classifiers:** Forest and Woodland (Treed); Seepage‑Fed Sloping [Mineral]; Depressional; Mineral: W/ A‑Horizon <10 cm; Saturated Soil

**National Mapping Codes:** EVT 2161; ESLF 9111; ESP 1161

**Concept Summary:** This ecological system occurs in the northern Rocky Mountains from northwestern Wyoming north into the Canadian Rockies and west into eastern Oregon and Washington. It is dominated by conifers on poorly drained soils that are saturated year‑round or may have seasonal flooding in the spring. These are primarily on flat to gently sloping lowlands, but also occur up to near the lower limits of continuous forest (below the subalpine parkland). It can occur on steeper slopes where soils are shallow over unfractured bedrock. This system is indicative of poorly drained, mucky areas, and areas are often a mosaic of moving water and stagnant water. Soils can be woody peat, muck or mineral but tend toward mineral. Stands generally occupy sites on benches, toeslopes or valley bottoms along mountain streams. Associations present include wetland phases of *Thuja plicata, Tsuga heterophylla*, and *Picea engelmannii* forests. The wetland types are generally distinguishable from other upland forests and woodlands by shallow water tables and mesic or hydric undergrowth vegetation; some of the most typical species include *Athyrium filix‑femina, Dryopteris* spp., *Lysichiton americanus, Equisetum arvense, Senecio triangularis, Mitella breweri, Mitella pentandra, Streptopus amplexifolius, Calamagrostis canadensis*, or *Carex disperma*.

**Comments:** May need to split out calcareous cedar (*Thuja plicata*) swamps from the other conifer swamps‑ needs more review.

DISTRIBUTION

**Range:** This system occurs in the northern Rocky Mountains from northwestern Wyoming and central Montana, north into the Canadian Rockies and west into eastern Oregon and Washington.

**Divisions:** 306:C

**TNC Ecoregions:** 7:C, 8:C, 9:P, 26:C, 68:C

**Nations:** CA, US

**Subnations:** AB, BC, ID, MT, OR, WA:S3, WY

**Map Zones:** 9:C, 10:C, 19:C, 20:C, 21:C, 29:C

**USFS Ecomap Regions:** 331A:PP, M331A:PP, M331D:P?, M332A:CC, M332B:CC, M332D:CP, M332E:CP, M332F:CC, M332G:CP, M333A:CC, M333B:CC, M333C:CC, M333D:CC

CONCEPT

**Environment:** Stands occur on poorly drained soils that are saturated year‑round or may have seasonal flooding in the spring. These are primarily on flat to gently sloping lowlands, but also occur up to near the lower limits of continuous forest (below the subalpine parkland). It can occur on steeper slopes where soils are shallow over unfractured bedrock. This system is indicative of poorly drained, mucky areas, and areas are often a mosaic of moving water and stagnant water. Soils can be woody peat, muck or mineral but tend toward mineral. Stands generally occupy sites on benches, toeslopes or valley bottoms along mountain streams.

**Vegetation:** Associations present include wetland phases of *Thuja plicata, Tsuga heterophylla*, and *Picea engelmannii* forests. The wetland types are generally distinguishable from other upland forests and woodlands by shallow water tables and mesic or hydric undergrowth vegetation; some of the most typical species include *Athyrium filix‑femina, Dryopteris* spp., *Lysichiton americanus, Equisetum arvense, Senecio triangularis, Mitella breweri, Mitella pentandra, Streptopus amplexifolius, Calamagrostis canadensis*, or *Carex disperma*.

SOURCES

**References:** Comer et al. 2003*, Eyre 1980, Meidinger and Pojar 1991, NCC 2002, WNHP unpubl. data 2018

**Version:** 07 Sep 2005	**Stakeholders:** Canada, West

**Concept Author:** M.S. Reid	**LeadResp:** West

CES306.804 Northern Rocky Mountain Lower Montane Riparian Woodland and Shrubland

**Primary Division:** Rocky Mountain (306)

**Land Cover Class:** Woody Wetland

**Spatial Scale & Pattern:** Linear

**Required Classifiers:** Natural/Semi‑natural; Vegetated (>10% vasc.); Wetland

**Diagnostic Classifiers:** Montane [Lower Montane]; Riverine / Alluvial; Short (<5 yrs) Flooding Interval [Short interval, Spring Flooding]

**Concept Summary:** This ecological system of the northern Rocky Mountains and the east slopes of the Cascades consists of deciduous, coniferous, and mixed conifer‑deciduous forests that occur on streambanks and river floodplains of the lower montane and foothill zones. Riparian forest stands are maintained by annual flooding and hydric soils throughout the growing season. Riparian forests are often accompanied by riparian shrublands or open areas dominated by wet meadows. *Populus balsamifera* is the key indicator species. Several other tree species can be mixed in the canopy, including *Populus tremuloides, Betula papyrifera, Betula occidentalis, Picea mariana*, and *Picea glauca*. *Abies grandis, Thuja plicata*, and *Tsuga heterophylla* are commonly dominant canopy species in British Columbia, western Montana and northern Idaho occurrences, in lower montane riparian zones. Shrub understory components include *Cornus sericea, Acer glabrum, Alnus incana, Betula papyrifera, Oplopanax horridus*, and *Symphoricarpos albus*. Ferns and forbs of mesic sites are commonly present in many occurrences, including such species as *Athyrium filix‑femina, Gymnocarpium dryopteris*, and *Senecio triangularis*.

**Comments:** This system is from the Canadian Rockies ecoregion project and represents lower montane riparian in Montana north into Canada. In the Okanagan, this is defined as all the cottonwood‑dominated or ‑codominated riparian systems below subalpine and above the Ponderosa pine zone. This system occurs in fire‑dominated landscapes, which distinguishes it from North Pacific and subalpine/alpine landscapes that have significantly different fire regimes. This system is distinguished from the similar Rocky Mountain Subalpine‑Montane Riparian Woodland (CES306.833) by the floristic component of northern Rocky Mountain species, both in the woody layers and in the herbaceous taxa. This system may occur in northwestern Wyoming where *Populus balsamifera* dominates or codominates some woodlands, but those woodlands may be better placed into Rocky Mountain Lower Montane‑Foothill Riparian Woodland and Shrubland (CES306.821), which lists *Populus balsamifera* as a possible dominant.

DISTRIBUTION

**Range:** This system is found in the northern Rocky Mountains.

**Divisions:** 303:P, 306:C

**TNC Ecoregions:** 7:C, 8:C, 68:C

**Nations:** CA, US

**Subnations:** AB, BC, ID, MT, OR?, WA:S2, WY

**Map Zones:** 8:C, 9:C, 10:C, 18:?, 19:C, 20:C, 21:C, 22:P

**USFS Ecomap Regions:** 331A:P?, 331D:PP, 331N:PP, 342A:??, 342C:??, 342D:??, M242C:PP, M331A:PP, M331B:P?, M332A:CP, M332B:CC, M332D:CC, M332E:CC, M332F:CC, M332G:CP, M333A:CC, M333B:CP, M333C:CC, M333D:CC

CONCEPT

**Environment:** Alluvial soils along perennial and intermittent streams. Valley type is an important variable, as riparian woodlands are mostly found in V‑shaped, steep valleys with many large boulders and coarse soils or U‑shaped gullies formed by glacial processes. These systems can also be found in broad unconfined reaches with deeper soils and more complex geomorphic surfaces. Narrow and steep (i.e., confined) occurrences have minimal to no floodplain development, whereas less steep and wider valley bottoms (i.e., unconfined) occurrences are often associated with substantial floodplain development (Gregory et al. 1991).

**Dynamics:** Natural disturbance regimes are the primary influence on riparian system characteristics. Maintained by the complex interaction of hydrological and geomorphological processes which influence periodic flooding and hydric soils, riparian systems are the most dynamic of all forested, woodland and shrub systems. Hydrogeomorphology determines the form, composition and function of riparian woodland and shrub systems. Typically occurring in watersheds with snow‑dominated hydrological processes, sometimes mixed rain and snow, these riparian systems are further influenced by the variability of inter‑annual and seasonal weather patterns. Typical flow regimes of British Columbia's central interior plateau and mountains are snow‑ (nival) dominated. Precipitation falls as snow and is stored for long periods of time, resulting in low winter flows, and peak flows following snowmelt in May to July (depending on annual temperature variations and snow depth). Glacial snow regimes are similar to nival, except that high flows may continue until August or September (Eaton and Moore 2010). Periods of peak flow have greatest influence on channel morphology and vegetation dynamics. Large woody debris is important for affecting channel morphology.

Beaver can be important hydrogeomorphic driver of montane riparian systems, especially along unconfined reaches. The direct, local presence of beaver creates a heterogeneous complex of wet meadows, marshes and riparian shrublands and increases species richness on the landscape. Naiman et al. (1988) note that beaver‑influenced streams are very different from those not impacted by beaver activity by having numerous zones of open water and vegetation, large accumulations of detritus and nutrients, more wetland areas, having more anaerobic biogeochemical cycles, and in general are more resistance to disturbance.

SOURCES

**References:** Bales et al. 2006, Banner et al. 1993, CNHP 2010, Comer et al. 2003*, DeLong 2003, DeLong et al. 1993, Eaton and Moore 2010, Ecosystems Working Group 1998, Eyre 1980, Gregory et al. 1991, Hansen et al. 1988b, Hansen et al. 1989, Haughian et al. 2012, Karl et al. 2009, Kerns et al. 2009, Knowles et al. 2006, MacKenzie and Moran 2004, MacKinnon et al. 1990, NCC 2002, Naiman et al. 1988, Peterson et al. 2008, Schreiner 1974, Steen and Coupé 1997, Stewart et al. 2004, Stromberg et al. 2010b, WNHP 2011, WNHP unpubl. data 2018, Wiensczyk 2012

**Version:** 14 Jan 2014	**Stakeholders:** Canada, West

**Concept Author:** M.S. Reid	**LeadResp:** West

CES304.060 Northern Rocky Mountain Wooded Vernal Pool

**Primary Division:** Inter‑Mountain Basins (304)

**Land Cover Class:** Woody Wetland

**Spatial Scale & Pattern:** Small patch

**Required Classifiers:** Natural/Semi‑natural; Vegetated (>10% vasc.); Wetland

**Diagnostic Classifiers:** Depressional; Isolated Wetland [Strictly Isolated]

**Concept Summary:** These wooded vernal pools are small shallow circumneutral freshwater wetlands of glacial origin that partially or totally dry up as the growing season progresses. They are documented to occur in northern Idaho and western Montana. These vernal ponds and wetlands usually fill with water over the fall, winter and early spring, but then at least partially dry up towards the end of the growing season. Depending on annual patterns of temperature and precipitation, the drying of the pond may be complete or partial by the fall. These sites are usually shallow and less than 1 m in depth, but can be as much as 2 m deep. The pool substrate is a poorly drained, often clayey layer with shallow organic sediments. The freshwater ponds have pH ranges from 6.2 to 7.8 with most measurements between 6.5 and 7.5, i.e., relatively neutral. The ponds in Montana were thought to be isolated, but it has been shown that in high water years the ponds spill over, and there is an exchange of surface water between ponds. The pools have a ring of trees surrounding the ponds that provide shade and influence their hydrology. A variety of tree species dominant the upper canopy, including *Abies grandis, Abies lasiocarpa, Larix occidentalis, Picea engelmannii, Pinus contorta, Pseudotsuga menziesii*, and the broadleaf trees *Populus balsamifera ssp. trichocarpa, Fraxinus latifolia*, and, to a lesser extent, *Populus tremuloides* and *Betula papyrifera*. Common shrubs include *Alnus incana, Cornus sericea, Rhamnus alnifolia*, and *Salix* spp. *Alopecurus aequalis, Callitriche heterophylla, Carex vesicaria, Eleocharis palustris*, and *Phalaris arundinacea* are common herbaceous plant associates.

DISTRIBUTION

**Range:** These vernal pools are documented to occur in northern Idaho and western Montana.

**Divisions:** 304:C

**TNC Ecoregions:** 7:C, 8:P, 68:C

**Nations:** US

**Subnations:** ID, MT, WA

**Map Zones:** 9:P, 10:C, 19:C, 21:P

**USFS Ecomap Regions:** M332A:??, M332B:??, M332E:??, M332G:??, M333A:PP, M333B:PP, M333C:PP, M333D:PP

CONCEPT

**Environment:** These wooded vernal pools are small shallow circumneutral freshwater wetlands of glacial origin that partially or totally dry up as the growing season progresses. These vernal ponds and wetlands usually fill with water over the fall, winter and early spring, but then at least partially dry up towards the end of the growing season. Depending on annual patterns of temperature and precipitation, the drying of the pond may be complete or partial by the fall. These sites are usually shallow and less than 1 m in depth, but can be as much as 2 m deep. The pool substrate is a poorly drained, often clayey layer with shallow organic sediments. The freshwater ponds have pH ranges from 6.2 to 7.8 with most measurements between 6.5 and 7.5, i.e., relatively neutral. The ponds in Montana were thought to be isolated, but it has been shown that in high water years the ponds spill over, and there is an exchange of surface water between ponds. The pools have a ring of trees surrounding the ponds that provide shade and influence their hydrology.

**Vegetation:** A variety of tree species dominant the upper canopy surrounding the pools, including *Abies grandis, Abies lasiocarpa, Larix occidentalis, Picea engelmannii, Pinus contorta, Pseudotsuga menziesii*, and the broadleaf trees *Populus balsamifera ssp. trichocarpa (= Populus trichocarpa), Fraxinus latifolia*, and, to a lesser extent, *Populus tremuloides* and *Betula papyrifera*. Common shrubs include *Alnus incana, Cornus sericea (= Cornus stolonifera), Rhamnus alnifolia*, and *Salix* spp. *Alopecurus aequalis, Callitriche heterophylla, Carex vesicaria, Eleocharis palustris*, and *Phalaris arundinacea* are common herbaceous plant associates.

SOURCES

**References:** Comer et al. 2003*, Mincemoyer 2005, WNHP unpubl. data 2018

**Version:** 01 Oct 2007	**Stakeholders:** West

**Concept Author:** Western Ecology Group	**LeadResp:** West

CES306.821 Rocky Mountain Lower Montane‑Foothill Riparian Woodland and Shrubland

**Primary Division:** Rocky Mountain (306)

**Land Cover Class:** Woody Wetland

**Spatial Scale & Pattern:** Linear

**Required Classifiers:** Natural/Semi‑natural; Vegetated (>10% vasc.); Wetland

**Diagnostic Classifiers:** Montane [Lower Montane]; Riverine / Alluvial; Mineral: W/ A‑Horizon <10 cm; Unconsolidated; Short (<5 yrs) Flooding Interval; Short (50‑100 yrs) Persistence

**Concept Summary:** This ecological system is found throughout the Rocky Mountain and Colorado Plateau regions within a broad elevational range from approximately 900 to 2800 m. This system often occurs as a mosaic of multiple communities that are tree‑dominated with a diverse shrub component. It is dependent on a natural hydrologic regime, especially annual to episodic flooding. Occurrences are found within the flood zone of rivers, on islands, sand or cobble bars, and immediate streambanks. It can form large, wide occurrences on mid‑channel islands in larger rivers or narrow bands on small, rocky canyon tributaries and well‑drained benches. It is also typically found in backwater channels and other perennially wet but less scoured sites, such as floodplains swales and irrigation ditches. In some locations, occurrences extend into moderately high intermountain basins where the adjacent vegetation is sage steppe. Dominant trees may include *Acer negundo, Populus angustifolia, Populus deltoides, Populus fremontii, Pseudotsuga menziesii, Picea pungens, Salix amygdaloides*, or *Juniperus scopulorum*. Dominant shrubs include *Acer glabrum, Alnus incana, Betula occidentalis, Cornus sericea, Crataegus rivularis, Forestiera pubescens, Prunus virginiana, Rhus trilobata, Salix monticola, Salix drummondiana, Salix exigua, Salix irrorata, Salix lucida, Shepherdia argentea*, or *Symphoricarpos* spp. Exotic trees of *Elaeagnus angustifolia* and *Tamarix* spp. are common in some stands. Generally, the upland vegetation surrounding this riparian system is different and ranges from grasslands to forests. In the Wyoming Basins, the high‑elevation *Populus angustifolia*‑dominated rivers are included here, including along the North Platte, Sweetwater, and Laramie rivers. In these situations, *Populus angustifolia* is extending down into the sage steppe zone of the basins.

**Comments:** This system is physiognomically diverse; because of relatively rapid spatial and temporal shifts in structure and composition, it was too complex to split into different, structurally defined systems (e.g., a shrubland system and a woodland system). This riparian system has been applied to the Green, Yampa, and Colorado rivers (upstream of the Grand Canyon) on the Colorado Plateau. Within and below the Grand Canyon is classified as North American Warm Desert Riparian Woodland and Shrubland (CES302.753). More research is needed to determine if creating a Colorado Plateau riparian woodland and shrubland system is ecologically justified.

DISTRIBUTION

**Range:** This system is found throughout the lower montane Rocky Mountain and Colorado Plateau regions within a broad elevation range from approximately 900 to 2800 m. It is also found in the island mountain ranges of central and eastern Montana.

**Divisions:** 304:C, 306:C

**TNC Ecoregions:** 6:P, 8:C, 9:C, 11:C, 18:C, 19:C, 20:C, 21:C, 25:C, 26:C

**Nations:** US

**Subnations:** AZ, CO, ID, MT, NM, NV, OR, SD, UT, WY

**Map Zones:** 8:?, 9:C, 13:C, 15:C, 16:C, 17:P, 18:C, 20:C, 21:C, 22:C, 23:C, 24:C, 25:C, 26:C, 27:C, 28:C, 29:C, 33:C

**USFS Ecomap Regions:** 313A:CC, 313B:CC, 313D:CC, 315A:CC, 315H:CC, 321A:CC, 331B:CC, 331D:CP, 331F:CC, 331G:CC, 331H:CC, 331I:CC, 331J:CC, 331K:C?, 331N:CP, 341A:CC, 341B:CC, 341C:CC, 341F:CC, 342A:CC, 342D:CC, 342E:CC, 342F:CC, 342G:CC, 342J:CC, M313A:CC, M313B:CC, M331A:CC, M331B:CC, M331D:CC, M331E:CC, M331F:CC, M331G:CC, M331H:CC, M331I:CC, M331J:CC, M332A:CC, M332G:CC, M341B:CC, M341C:CC

CONCEPT

**Environment:** This system is dependent on a natural hydrologic regime, especially annual to episodic flooding. It is found within the flood zone of rivers, on islands, sand or cobble bars, and immediate streambanks. It can form large, wide occurrences on mid‑channel islands in larger rivers or narrow bands on small, rocky canyon tributaries and well‑drained benches. It is also typically found in backwater channels and other perennially wet but less scoured sites, such as floodplains swales and irrigation ditches. It may also occur in upland areas of mesic swales and hillslopes below seeps and springs. The climate of this system is continental with typically cold winters and hot summers. Surface water is generally high for variable periods. Soils are typically alluvial deposits of sand, clays, silts and cobbles that are highly stratified with depth due to flood scour and deposition. Highly stratified profiles consist of alternating layers of clay loam and organic material with coarser sand or thin layers of sandy loam over very coarse alluvium. Soils are fine‑textured with organic material over coarser alluvium. Some soils are more developed due to a slightly more stable environment and greater input of organic matter.

**Vegetation:** Dominant trees may include *Acer negundo, Populus angustifolia, Populus deltoides, Populus fremontii, Pseudotsuga menziesii, Picea pungens, Salix amygdaloides*, or *Juniperus scopulorum*. Dominant shrubs include *Acer glabrum, Alnus incana, Betula occidentalis, Cornus sericea, Crataegus rivularis, Forestiera pubescens, Prunus virginiana, Rhus trilobata, Salix monticola, Salix drummondiana, Salix exigua, Salix irrorata, Salix lucida, Shepherdia argentea*, or *Symphoricarpos* spp. Exotic trees of *Elaeagnus angustifolia* and *Tamarix* spp. are common in some stands. Generally, the upland vegetation surrounding this riparian system is different and ranges from grasslands to forests.

**Dynamics:** This ecological system contains early‑, mid‑ and late‑seral riparian plant associations. It also contains non‑obligate riparian species. Cottonwood communities are early‑, mid‑ or late‑seral, depending on the age class of the trees and the associated species of the occurrence (Kittel et al. 1999b). Cottonwoods, however, do not reach a climax stage as defined by Daubenmire (1952). Mature cottonwood occurrences do not regenerate in place, but regenerate by "moving" up and down a river reach and regeneration is often associated with flooding events. Over time a healthy riparian area supports all stages of cottonwood communities (Kittel et al. 1999b).

SOURCES

**References:** Baker 1988, Baker 1989a, Baker 1989b, Baker 1990, Bales et al. 2006, CNHP 2010, Comer et al. 2002, Comer et al. 2003*, Comer et al. 2013a, Crowe and Clausnitzer 1997, Daubenmire 1952, Elmore and Kauffman 1994, Eyre 1980, Flenniken et al. 2001, Karl et al. 2009, Kittel et al. 1999b, Knopf et al. 1990, Knowles et al. 2006, Kovalchik 1987, Kovalchik 1993, Manning and Padgett 1995, Merritt and Wohl 2002, Muldavin et al. 2000a, Nachlinger et al. 2001, Neely et al. 2001, Padgett et al. 1989, Parsons et al. 2005, Patten 1998, Peterson et al. 2008, Rising 1996, Rondeau pers. comm., Sedgwick and Knopf 1987, Shiflet 1994, Stewart et al. 2004, Stromberg et al. 2010a, Szaro 1989, Tuhy et al. 2002, Tuhy pers. comm., WNHP 2011, Walford 1996, Walford et al. 1997, Walford et al. 2001

**Version:** 14 Jan 2014	**Stakeholders:** Canada, Midwest, West

**Concept Author:** NatureServe Western Ecology Team	**LeadResp:** West

CES306.833 Rocky Mountain Subalpine‑Montane Riparian Woodland

**Primary Division:** Rocky Mountain (306)

**Land Cover Class:** Woody Wetland

**Spatial Scale & Pattern:** Linear

**Required Classifiers:** Natural/Semi‑natural; Vegetated (>10% vasc.); Wetland

**Diagnostic Classifiers:** Montane [Upper Montane, Montane]; Forest and Woodland (Treed); Riverine / Alluvial; Short (<5 yrs) Flooding Interval; RM Subalpine/Montane Riparian Shrubland

**Concept Summary:** This riparian woodland system comprises seasonally flooded forests and woodlands found at montane to subalpine elevations of the Rocky Mountain cordillera, from southern New Mexico north into Montana, and west into the Intermountain West region and the Colorado Plateau. It occurs throughout the interior of British Columbia and the eastern slopes of the Cascade Range. This system contains the conifer and aspen woodlands that line montane streams. These are communities tolerant of periodic flooding and high water tables. Snowmelt moisture in this system may create shallow water tables or seeps for a portion of the growing season. Stands typically occur at elevations between 1500 and 3300 m (4920‑10,830 feet), farther north elevation ranges between 900 and 2000 m. This is confined to specific riparian environments occurring on floodplains or terraces of rivers and streams, in V‑shaped, narrow valleys and canyons (where there is cold‑air drainage). Less frequently, occurrences are found in moderate‑wide valley bottoms on large floodplains along broad, meandering rivers, and on pond or lake margins. Dominant tree species vary across the latitudinal range, although it usually includes *Abies lasiocarpa* and/or *Picea engelmannii*; other important species include *Pseudotsuga menziesii, Picea pungens, Picea engelmannii x glauca, Populus tremuloides*, and *Juniperus scopulorum*. Other trees possibly present but not usually dominant include *Alnus incana, Abies concolor, Abies grandis, Pinus contorta, Populus angustifolia, Populus balsamifera ssp. trichocarpa*, and *Juniperus osteosperma*.

DISTRIBUTION

**Range:** This system is found at montane to subalpine elevations of the Rocky Mountain cordillera, from southern New Mexico north into Montana, Alberta and British Columbia, and west into the Intermountain region and the Colorado Plateau.

**Divisions:** 204:P, 304:C, 306:C

**TNC Ecoregions:** 4:P, 6:P, 7:C, 8:C, 9:C, 11:C, 18:C, 19:C, 20:C, 21:C, 25:C, 68:C

**Nations:** CA, US

**Subnations:** AB, AZ, BC, CO, ID, MT, NM, NV, OR, SD, UT, WA:S4, WY

**Map Zones:** 1:C, 6:P, 7:?, 9:C, 10:C, 12:C, 16:P, 17:P, 18:P, 19:C, 20:C, 21:C, 22:C, 23:C, 24:C, 25:C, 26:P, 27:C, 28:C, 29:C

**USFS Ecomap Regions:** 313B:CC, 331A:C?, 331J:CC, 341A:CP, 341D:CP, 341F:CP, 341G:CC, 342A:CC, 342B:CP, 342C:CC, 342D:CC, 342E:CP, 342F:CC, 342G:CC, 342H:CC, 342I:CC, 342J:CC, M242C:CC, M242D:CC, M261E:CC, M331A:CC, M331B:CC, M331D:CC, M331E:CC, M331F:CC, M331G:CC, M331H:CC, M331I:CC, M331J:CC, M332A:CC, M332B:CC, M332D:CC, M332E:CC, M332F:CC, M332G:CC, M333A:CC, M333B:CC, M333C:CC, M333D:CC, M341A:CC, M341D:CC

CONCEPT

SOURCES

**References:** Baker 1988, Baker 1989a, Baker 1989b, Baker 1990, Comer et al. 2002, Comer et al. 2003*, Crowe and Clausnitzer 1997, Ecosystems Working Group 1998, Eyre 1980, Kittel 1993, Kittel et al. 1994, Kittel et al. 1995, Kittel et al. 1999a, Kittel et al. 1999b, Kovalchik 1987, Kovalchik 1993, Kovalchik 2001, Manning and Padgett 1995, Muldavin et al. 2000a, NCC 2002, Nachlinger et al. 2001, Neely et al. 2001, Padgett 1982, Padgett et al. 1988b, Padgett et al. 1989, Rondeau 2001, Shiflet 1994, Tuhy et al. 2002, WNHP unpubl. data 2018

**Version:** 09 Feb 2005	**Stakeholders:** Canada, Midwest, West

**Concept Author:** M.S. Reid	**LeadResp:** West

1.B.3.Nd. Western North American Interior Flooded Forest

M036. Interior Warm & Cool Desert Riparian Forest

CES206.946 California Central Valley Riparian Woodland and Shrubland

**Primary Division:** Mediterranean California (206)

**Land Cover Class:** Mixed Upland and Wetland

**Spatial Scale & Pattern:** Linear

**Required Classifiers:** Natural/Semi‑natural; Vegetated (>10% vasc.); Upland; Wetland

**Diagnostic Classifiers:** Forest and Woodland (Treed); Mediterranean [Mediterranean Xeric‑Oceanic]; Riverine / Alluvial; Deep Soil; Flood Scouring; Riparian Mosaic

**National Mapping Codes:** EVT 2151; ESLF 9101; ESP 1151

**Concept Summary:** This ecological system occurs in the floodplains of rivers of California's Central Valley. Alluvial soils and late winter/early spring flooding (usually every year) from snowmelt typify this system. Communities are predominantly floodplain woodlands, but also include shrublands, wet meadows and gravel/s and flats. Important trees and shrubs include *Populus fremontii, Platanus racemosa, Quercus lobata, Salix gooddingii, Acer negundo, Cephalanthus occidentalis*, and *Vitis californica*. *Juglans nigra* hybrids and *Ailanthus altissima* are problem invasive trees. *Tamarix* spp. extend as far north as Shasta County. Herbaceous components can include *Carex barbarae, Artemisia douglasiana*, and various marsh species along riverbanks and backwater (*Schoenoplectus californicus, Typha* spp.). *Arundo donax* is another common invasive and introduced forage species that often invades degraded areas within the floodplains. Periodic flooding and associated sediment scour are necessary to maintain growth and reproduction of vegetation. Flooding regimes have been significantly altered in all but a few tributaries that support this system.

DISTRIBUTION

**Range:** Occurs in the floodplains of rivers of California's Central Valley.

**Divisions:** 206:C

**TNC Ecoregions:** 13:C

**Nations:** US

**Subnations:** CA

**Map Zones:** 3:P, 4:C, 5:C, 6:P, 13:P

**USFS Ecomap Regions:** 261B:??, 262A:CC, 263A:??, 322A:??, M261A:C?, M261B:C?, M261C:CC, M261F:CC

CONCEPT

**Environment:** This system is found on alluvial soils adjacent to perennial rivers and streams and their associated floodplains and riverbanks below approximately 550 m (1800 feet).

**Vegetation:** Communities are predominantly floodplain woodlands, but also include shrublands, wet meadows and gravel/s and flats. Important trees and shrubs include *Populus fremontii, Platanus racemosa, Quercus lobata, Salix gooddingii, Acer negundo, Cephalanthus occidentalis*, and *Vitis californica*. *Juglans nigra* hybrids and *Ailanthus altissima* are problem invasive trees. *Tamarix* spp. extend as far north as Shasta County. Herbaceous components can include *Carex barbarae, Artemisia douglasiana*, and various marsh species along riverbanks and backwater (*Schoenoplectus californicus (= Scirpus californicus), Typha* spp.). *Arundo donax* is another common invasive and introduced forage species that often invades degraded areas within the floodplains.

**Dynamics:** Periodic flooding and associated sediment scour are necessary to maintain growth and reproduction of vegetation (Sawyer et al. 2009). Major flood events and consequent flood scour, overbank deposition of water and sediments, and stream meandering are the key fluvial processes that provide new substrates, remove old banks and stimulate renewed growth of cottonwood and willow species (Sawyer et al. 2009). Natural fire‑return interval was long or moderate with low‑intensity surface fires.

SOURCES

**References:** Barbour and Major 1988, Brooks and Minnich 2006, Coffman 2007, Comer et al. 2003*, Holland and Keil 1995, Keeler‑Wolf pers. comm., Ohmart and Anderson 1986, PRBO Conservation Science 2011, Sawyer and Keeler‑Wolf 1995, Sawyer et al. 2009, Shiflet 1994

**Version:** 14 Jan 2014	**Stakeholders:** West

**Concept Author:** P. Comer and T. Keeler‑Wolf	**LeadResp:** West

CES206.944 Mediterranean California Foothill and Lower Montane Riparian Woodland and Shrubland

**Primary Division:** Mediterranean California (206)

**Land Cover Class:** Mixed Upland and Wetland

**Spatial Scale & Pattern:** Linear

**Required Classifiers:** Natural/Semi‑natural; Vegetated (>10% vasc.); Upland; Wetland

**Diagnostic Classifiers:** Forest and Woodland (Treed); Mediterranean [Mediterranean Xeric‑Oceanic]; Riverine / Alluvial; Very Short Disturbance Interval; Flood Scouring; Riparian Mosaic

**Concept Summary:** This system is found throughout Mediterranean California within a broad elevation range from near sea level up to 300 m (900 feet) in the Coast Ranges and inland to 1500 m (4545 feet). This system often occurs as a mosaic of multiple communities that are tree‑dominated with a diverse shrub component and open shrublands. This system includes open channels and bare alluvial bars as well. The variety of plant associations connected to this system reflects elevation, stream gradient, floodplain width, and flooding events. Dominant trees and shrubs may include *Alnus rhombifolia, Acer negundo, Alnus rubra* (in Coast Ranges), *Populus fremontii, Salix laevigata, Salix gooddingii, Pseudotsuga menziesii, Platanus racemosa, Quercus agrifolia*, and *Acer macrophyllum* (in central and south coast). Dominant shrubs include *Salix exigua* and *Salix lasiolepis*. Exotic trees *Ailanthus altissima, Eucalyptus* spp., and herbs such as *Arundo donax* occur. These are disturbance‑driven systems that require flooding, scour and deposition for germination and maintenance.

**Comments:** It is unclear if riparian woodlands and shrublands occur in the upper montane and subalpine regions of the Sierras and possibly the Transverse Ranges, and if they do, if they are significantly different in composition to be distinguished as an ecological system. Some literature indicates that, if they do occur, the woodlands at least are not at all common. For now, there is no "subalpine‑upper montane Sierran riparian" system described. Lower elevation (low montane and foothill) riparian systems on the east side of the Sierras are treated in Great Basin Foothill and Lower Montane Riparian Woodland and Shrubland (CES304.045).

DISTRIBUTION

**Range:** This system is found throughout Mediterranean California within a broad elevation range from near sea level up to 300 m (900 feet) in the Coast Ranges and inland to 1500 m (4545 feet).

**Divisions:** 206:C

**TNC Ecoregions:** 5:C, 12:C, 13:C, 14:C, 16:C

**Nations:** MX, US

**Subnations:** CA, MXBC, OR

**Map Zones:** 2:C, 3:C, 4:C, 5:C, 6:C, 7:C

**USFS Ecomap Regions:** 322A:PP, M261B:CC, M261C:CC, M261D:CC, M261E:CC, M261F:CC, M261G:CC

CONCEPT

**Environment:** This system occurs adjacent to perennial or intermittent streams, streams with at least seasonal channel flow, usually associated with a subsurface groundwater level that is shallower than surrounding uplands. Winter peak and summer discharges can be quite variable. The impact of seasonal high and low flows can be characterized as three regimes: (1) intense disturbances/minimal summer drought (close to channel, or narrow constricted floodplains); (2) moderate disturbances/summer drought (mid distance to channel, or moderate‑sized floodplain); and (3) minimal disturbance/summer drought (greatest distance from channel, or wide floodplains). Type and extent of riparian vegetation are dependent upon the balance between the degree of summer drought as controlled by ground and surface water availability and the intensity of disturbance determined by discharge magnitudes and channel morphology (Ross and Swift 2001). This "distance from channel" can dictate the age and size of the riparian woody species.

**Vegetation:** Dominant trees and shrubs may include *Alnus rhombifolia, Acer negundo, Alnus rubra* (in Coast Ranges), *Populus fremontii, Salix laevigata, Salix gooddingii, Pseudotsuga menziesii, Platanus racemosa, Quercus agrifolia*, and *Acer macrophyllum* (in central and south coast). Dominant shrubs include *Salix exigua* and *Salix lasiolepis*. Exotic trees *Ailanthus altissima, Eucalyptus* spp., and herbs such as *Arundo donax* occur.

**Dynamics:** These are disturbance‑driven systems that require flooding, scour and deposition for germination and maintenance.

SOURCES

**References:** Barbour and Major 1988, Comer et al. 2003*, Holland and Keil 1995, Sawyer and Keeler‑Wolf 1995, Shiflet 1994

**Version:** 13 Jan 2012	**Stakeholders:** Latin America, West

**Concept Author:** P. Comer and T. Keeler‑Wolf	**LeadResp:** West

CES206.945 Mediterranean California Serpentine Foothill and Lower Montane Riparian Woodland and Seep

**Primary Division:** Mediterranean California (206)

**Land Cover Class:** Mixed Upland and Wetland

**Spatial Scale & Pattern:** Linear

**Required Classifiers:** Natural/Semi‑natural; Vegetated (>10% vasc.); Upland; Wetland

**Diagnostic Classifiers:** Serpentine; Mediterranean [Mediterranean Xeric‑Oceanic]; Seepage‑Fed Sloping; Riverine / Alluvial; Cupressus sargentii; Intermittent Flooding; Short (<5 yrs) Flooding Interval

**Concept Summary:** This ecological system is found mostly in the central and inner northern Coast Ranges of California and Sierra Nevada foothills. It includes springs, seeps, and perennial and intermittent streams in serpentine substrates (true serpentinite but also other related substrates). Characteristic species include *Salix breweri, Hesperocyparis sargentii, Frangula californica ssp. tomentella, Umbellularia californica, Cirsium fontinale, Stachys albens, Solidago* spp., *Packera clevelandii, Mimulus glaucescens, Mimulus guttatus, Aquilegia eximia*, and *Carex serratodens*. Riparian portions of this system are disturbance‑driven and require limited flooding, scour and deposition for germination and maintenance.

DISTRIBUTION

**Range:** This system occurs in the central and inner northern Coast Ranges of California and Oregon and Sierra Nevada foothills.

**Divisions:** 206:C

**TNC Ecoregions:** 5:C, 12:P, 14:C, 15:P

**Nations:** US

**Subnations:** CA, OR

**Map Zones:** 2:C, 3:C, 4:C, 6:C

**USFS Ecomap Regions:** M261A:CC, M261B:CP, M261C:CP, M261D:C?, M261F:C?

CONCEPT

**Environment:** This system is found in creek bottoms and stream terraces with serpentine‑derived alluvium. Elevations range from 300‑3000 m. Soils are saturated to moist throughout the growing season (Sawyer et al. 2009).

**Vegetation:** Characteristic species include *Salix breweri, Hesperocyparis sargentii (= Cupressus sargentii), Frangula californica ssp. tomentella (= Rhamnus tomentella), Umbellularia californica, Cirsium fontinale, Stachys albens, Solidago* spp., *Packera clevelandii (= Senecio clevelandii), Mimulus glaucescens, Mimulus guttatus, Aquilegia eximia*, and *Carex serratodens*. Riparian portions of this system are disturbance‑driven and require limited flooding, scour and deposition for germination and maintenance.

**Dynamics:** Steady groundwater flow and fire primarily disturb stands of this ecosystem. Plants resprout after flooding disturbance. Most serpentine riparian areas have moderate rather than large flooding events, and most serpentine riparian has low perennial flows not subject to vacillating events as non‑serpentine areas. Serpentine riparian are less likely to be susceptible to drought and drying since the serpentine geology tends to release water slowly over time (T. Keeler‑Wolf pers. comm. 2013). *Frangula californica* resprouts vigorously after fire (Sawyer et al. 2009). However, it is not known how often fires historically occurred in *Frangula californica*‑dominated systems. Fire is less of a disturbance issue in willow‑dominated systems, but fire does occur, and *Salix* generally resprouts after fires (Stromberg and Rychener 2010). Fires probably occur relatively frequently even though serpentine chaparral surrounding the riparian has lower fuels than typical non‑serpentine chaparral (T. Keeler‑Wolf pers. comm. 2013).

SOURCES

**References:** Barbour and Major 1988, Batten et al. 2006, Brooks and Minnich 2006, CNPS and CDFG 2006, Coffman 2007, Comer et al. 2003*, Eyre 1980, Holland and Keil 1995, Keeler‑Wolf pers. comm., PRBO Conservation Science 2011, Sawyer and Keeler‑Wolf 1995, Sawyer et al. 2009, Shiflet 1994, Stromberg and Rychener 2010

**Version:** 14 Jan 2014	**Stakeholders:** West

**Concept Author:** P. Comer and T. Keeler‑Wolf	**LeadResp:** West

CES302.748 North American Warm Desert Lower Montane Riparian Woodland and Shrubland

**Primary Division:** North American Warm Desert (302)

**Land Cover Class:** Woody Wetland

**Spatial Scale & Pattern:** Linear

**Required Classifiers:** Natural/Semi‑natural; Vegetated (>10% vasc.); Wetland

**Diagnostic Classifiers:** Forest and Woodland (Treed); Shrubland (Shrub‑dominated); Riverine / Alluvial

**Concept Summary:** This ecological system occurs in foothill and mountain canyons and valleys of the warm desert regions of the southwestern U.S. and adjacent Mexico, and consists of mid‑to low‑elevation (1100‑1800 m) riparian corridors along perennial and seasonally intermittent streams. Rivers include upper portions of the Gila, Santa Cruz, Salt, San Pedro, and tributaries of the lower Colorado River (below the Grand Canyon), the lower Rio Grande and Pecos (up to its confluence with Rio Hondo) that occur in the desert portions of their range. The vegetation is a mix of riparian woodlands and shrublands. Dominant trees include *Acer negundo, Populus deltoides ssp. wislizeni, Populus fremontii, Platanus wrightii, Juglans major, Fraxinus velutina*, and *Sapindus saponaria*. Occasionally *Populus angustifolia* may come in from higher elevations. Shrub dominants include *Salix exigua, Shepherdia argentea, Prunus* spp., *Alnus oblongifolia*, and *Baccharis salicifolia*. Vegetation is dependent upon annual or periodic flooding and associated sediment scour and/or annual rise in the water table for growth and reproduction. In Texas, woody species that may be dominant include *Celtis laevigata var. reticulata, Fraxinus velutina, Juglans major, Juglans microcarpa, Populus deltoides ssp. wislizeni, Populus fremontii, Salix gooddingii, Sapindus saponaria var. drummondii*, and *Ungnadia speciosa*. Shrubs commonly encountered include *Acacia constricta, Acacia greggii, Baccharis salicifolia, Brickellia californica, Cephalanthus occidentalis, Fallugia paradoxa, Mimosa aculeaticarpa var. biuncifera, Prosopis glandulosa, Rhus microphylla*, and *Salix gooddingii*. Some sites with sparse woody overstory may be dominated by grasses such as *Aristida* spp., *Bothriochloa laguroides ssp. torreyana, Bouteloua curtipendula, Bouteloua gracilis, Distichlis spicata, Muhlenbergia porteri, Muhlenbergia rigens, Pleuraphis mutica*, and *Sporobolus airoides*.

DISTRIBUTION

**Range:** This system occurs in southern Arizona, New Mexico, and adjacent Mexico, as well as in the desert mountain ranges of southeastern California, at low elevations. It also occurs in southern Nevada and western Texas.

**Divisions:** 302:C

**TNC Ecoregions:** 17:C, 22:C, 23:C, 24:C

**Nations:** MX, US

**Subnations:** AZ, CA, MXBC, MXBS, MXCH, MXSO, NM, NV, TX

**Map Zones:** 12:C, 13:C, 14:C, 15:C, 16:?, 17:P, 24:C, 25:C, 26:C, 27:P

**USFS Ecomap Regions:** 313A:CC, 313C:CC, 315A:CC, 315B:CC, 321A:CC, 322A:CC, 322B:CC, 341F:CC, M261E:CC, M313A:CC, M313B:CC

CONCEPT

**Environment:** This ecological system occurs in foothill and mountain canyons and valleys of the warm desert regions of the southwestern U.S. and adjacent Mexico, and consists of mid‑ to low‑elevation (1100‑1800 m) riparian corridors and their associated perennial and seasonally intermittent streams. Some occurrences originate as, or receive flow from, headwater streams supported by surface runoff and shallow groundwater seepage; others originate at montane springs.

**Vegetation:** The vegetation is a mix of riparian woodlands and shrublands. Dominant trees include *Acer negundo, Populus deltoides ssp. wislizeni, Populus fremontii, Platanus wrightii, Juglans major, Fraxinus velutina*, and *Sapindus saponaria*. Occasionally *Populus angustifolia* may come in from higher elevations. Shrub dominants include *Salix exigua, Shepherdia argentea, Prunus* spp., *Alnus oblongifolia*, and *Baccharis salicifolia*. Vegetation is dependent upon annual or periodic flooding and associated sediment scour and/or annual rise in the water table for growth and reproduction. In Texas, woody species that may be dominant include *Celtis laevigata var. reticulata, Fraxinus velutina, Juglans major, Juglans microcarpa, Populus deltoides ssp. wislizeni, Populus fremontii, Salix gooddingii, Sapindus saponaria var. drummondii*, and *Ungnadia speciosa*. Shrubs commonly encountered include *Acacia constricta, Acacia greggii, Baccharis salicifolia, Brickellia californica, Cephalanthus occidentalis, Fallugia paradoxa, Mimosa aculeaticarpa var. biuncifera, Prosopis glandulosa, Rhus microphylla*, and *Salix gooddingii*. Some sites with sparse woody overstory may be dominated by grasses such as *Aristida* spp., *Bothriochloa laguroides ssp. torreyana, Bouteloua curtipendula, Bouteloua gracilis, Distichlis spicata, Muhlenbergia porteri, Muhlenbergia rigens, Pleuraphis mutica*, and *Sporobolus airoides*.

**Dynamics:** The hydrologic regime is naturally highly variable temporally and spatially among the streams of this ecosystem. Where present, bedrock formations that force alluvial and basin‑fill groundwater to the surface and spring discharges from bedrock aquifers provide flows unaffected by rainfall and snowmelt. Otherwise, stream and river flows are subject to wide fluctuations in where they occur, at what magnitudes, and when and how often as a result of the wide variation in where and when precipitation takes place (cool versus warm season), what form the precipitation takes (rain versus snow), and where and when snowmelt takes place (e.g., Abell et al. 2000, Izbicki and Michel 2004, Levick et al. 2008, Miller et al. 2010a). Intense runoff associated with intense rainfall events are highly erosive, resulting in rapid reconfiguration of aquatic and riparian macrohabitats particularly along reaches with sand and gravel substrates. As a result of this intense regime of fluvial disturbance, occurrences of this ecosystem contain early‑, mid‑ and late‑seral riparian plant associations.

SOURCES

**References:** Abell et al. 2000, Brown 1982a, Brown and Mote 2009, CNHP 2010, Cayan et al. 2010, Chambers and Pellant 2008, Chambers and Wisdom 2009, Christensen and Lettenmaier 2007, Comer et al. 2003*, Comer et al. 2013b, Covich 2009, Das et al. 2009, Daubenmire 1952, Dettinger et al. 2009, Dick‑Peddie 1993, Elliott 2012, Eyre 1980, Field et al. 1999, Harper and Peckarsky 2006, Hultine et al. 2007, Izbicki and Michel 2004, Jackson et al. 2009, Kittel et al. 1999b, Levick et al. 2008, Martin 2007, McCabe and Wolock 2009, Melack et al. 1997, Miller et al. 2010a, Mote 2006, Muldavin et al. 2000a, Reeves et al. 2005, Seavy et al. 2009, Shiflet 1994, Szaro 1989, Thomas et al. 2004, USBOR 2011, WNHP 2011

**Version:** 14 Jan 2014	**Stakeholders:** Latin America, Southeast, West

**Concept Author:** K.A. Schulz	**LeadResp:** West

CES302.753 North American Warm Desert Riparian Woodland and Shrubland

**Primary Division:** North American Warm Desert (302)

**Land Cover Class:** Woody Wetland

**Spatial Scale & Pattern:** Linear

**Required Classifiers:** Natural/Semi‑natural; Vegetated (>10% vasc.); Wetland

**Diagnostic Classifiers:** Lowland [Lowland]; Forest and Woodland (Treed); Shrubland (Shrub‑dominated); Tropical/Subtropical [Tropical Xeric]; Temperate [Temperate Xeric]; Riverine / Alluvial

**Concept Summary:** This ecological system consists of low‑elevation (<1200 m) riparian corridors along medium to large perennial streams throughout canyons and desert valleys of the southwestern United States and adjacent Mexico. Rivers include the lower Colorado (into the Grand Canyon), Gila, Santa Cruz, Salt, lower Rio Grande (below Elephant Butte Reservoir in New Mexico to the Coastal Plain of Texas), and the lower Pecos (up to near its confluence with Rio Hondo in southeastern New Mexico). These are disturbance‑driven plant communities that require flooding, scour and deposition of sands and gravel, and a periodically elevated water table for germination and maintenance. The aquatic communities, in turn, vary with (1) the frequency, intensity, duration and timing of flow, including its often extreme inter‑annual variability; (2) the relative contributions of rainfall, snowmelt, and diffuse groundwater and spring discharges to flow; (3) water temperature and chemistry; (4) channel substrate and form; (5) the extent of the hyporheic zone; and (6) drainage network connectivity. These latter conditions, in turn, vary with elevation, latitude and longitude, channel gradient, floodplain width (a function of topography and geology), and surrounding geology and land cover. The vegetation is a mix of riparian woodlands and shrublands. Species composition varies across the wide range of this system. Dominant trees may include *Celtis laevigata var. reticulata, Fraxinus velutina, Juglans major, Platanus racemosa, Populus fremontii, Populus deltoides ssp. wislizeni, Prosopis glandulosa, Salix amygdaloides, Salix gooddingii, Salix lasiolepis*, and *Sapindus saponaria var. drummondii*. Shrub dominants include *Salix geyeriana* and *Salix exigua*. In Texas, *Baccharis salicifolia, Brickellia laciniata, Celtis ehrenbergiana, Chilopsis linearis, Fallugia paradoxa, Juglans microcarpa*, and *Salix exigua* are present and sometimes patchy. In addition to the woodland and shrubland expression of this system, sparsely vegetated areas also commonly occur. Sparsely vegetated sites may have sparse woody or herbaceous vegetation, including species such as *Brickellia* sp., *Chilopsis linearis, Baccharis* sp., *Prosopis glandulosa*, and *Salvia farinacea*. Vegetation is dependent upon annual or periodic flooding and associated sediment scour and/or annual rise in the water table for growth and reproduction.

DISTRIBUTION

**Range:** This systems occurs throughout canyons and desert valleys of the southwestern United States and adjacent Mexico. Major rivers and tributaries include the lower Colorado (up into the lower portions of the Grand Canyon), Gila, Salt, Rio Grande (from Elephant Butte Reservoir to the Gulf Coastal Plain), and the lower Pecos (near its confluence with Rio Hondo in southeastern New Mexico).

**Divisions:** 302:C

**TNC Ecoregions:** 17:C, 22:C, 23:C, 24:C, 29:P

**Nations:** MX, US

**Subnations:** AZ, CA, MXBC, MXCH, MXSO, NM, NV, TX

**Map Zones:** 4:?, 13:C, 14:C, 15:C, 16:C, 17:?, 23:C, 24:?, 25:C, 26:C, 27:P, 35:P

**USFS Ecomap Regions:** 313A:CC, 313C:CC, 315A:CC, 315B:CC, 321A:CC, 322A:CC, 322B:CC, 322C:CC, 341F:PP, M261E:CC, M313A:CP, M313B:CC

CONCEPT

**Environment:** These are disturbance‑driven plant communities that require flooding, scour and deposition of sands and gravel, and a periodically elevated water table for germination and maintenance. The aquatic communities, in turn, vary with the frequency, intensity, duration and timing of flow, including its often extreme inter‑annual variability; the relative contributions of rainfall, snowmelt, and diffuse groundwater and spring discharges to flow; water temperature and chemistry; channel substrate and form; the extent of the hyporheic zone; and drainage network connectivity. These latter conditions, in turn, vary with elevation, latitude and longitude, channel gradient, floodplain width (a function of topography and geology), and surrounding geology and land cover. In Texas, this system occurs on Loamy Bottomland, Salty Bottomland, and Draw Ecological Sites over Quaternary Alluvium, as well as nearby Cretaceous limestones through which drainages flow.

**Vegetation:** Species composition varies across the wide range of this system. Dominant trees may include *Celtis laevigata var. reticulata, Fraxinus velutina, Juglans major, Platanus racemosa, Populus fremontii, Populus deltoides ssp. wislizeni, Prosopis glandulosa, Salix amygdaloides, Salix gooddingii, Salix lasiolepis*, and *Sapindus saponaria var. drummondii*. Shrub dominants include *Salix geyeriana* and *Salix exigua*. In Texas, *Baccharis salicifolia, Brickellia laciniata, Celtis ehrenbergiana, Chilopsis linearis, Fallugia paradoxa, Juglans microcarpa*, and *Salix exigua* are present and sometimes patchy. In addition to the woodland and shrubland expression of this system, sparsely vegetated areas also commonly occur. Sparsely vegetated sites may have sparse woody or herbaceous vegetation, including species such as *Brickellia* sp., *Chilopsis linearis, Baccharis* sp., *Prosopis glandulosa*, and *Salvia farinacea*. Vegetation is dependent upon annual or periodic flooding and associated sediment scour and/or annual rise in the water table for growth and reproduction.

**Dynamics:** From MBR Ecological Condition Assessment (Comer et al. 2013b): The hydrologic regime is naturally highly variable temporally and spatially among the streams of this ecosystem. Where present, bedrock formations that force alluvial and basin‑fill groundwater to the surface and spring discharges from bedrock aquifers provide flows unaffected by rainfall and snowmelt. Otherwise, stream and river flows are subject to wide fluctuations in where they occur, at what magnitudes, and when and how often as a result of the wide variation in where and when precipitation takes place (cool versus warm season), what form the precipitation takes (rain versus snow), and where and when snowmelt takes place (e.g., Abell et al. 2000, Izbicki and Michel 2004, Levick et al. 2008, Miller et al. 2010a). Intense runoff associated with intense rainfall events are highly erosive, resulting in rapid reconfiguration of aquatic and riparian macrohabitats particularly along reaches with sand and gravel substrates. As a result of this intense regime of fluvial disturbance, occurrences of this ecosystem contain early‑, mid‑ and late‑seral riparian plant associations. Occurrences also contains non‑obligate riparian species. Cottonwood communities are early‑, mid‑ or late‑seral, depending on the age‑class of the trees and the associated species of the occurrence (Kittel et al. 1999b). Cottonwoods, however, do not reach a climax stage as defined by Daubenmire (1952). Mature cottonwood occurrences do not regenerate in place, but regenerate by "moving" up and down a river reach. Over time, a healthy riparian area supports all stages of cottonwood communities (Kittel et al. 1999b). In Texas, the native streamside vegetation along the large drainages is frequently displaced by extensive areas of *Tamarix* sp. and/or *Arundo donax*.

SOURCES

**References:** Abell et al. 2000, Barbour and Major 1988, Brown 1982a, Brown and Mote 2009, CNHP 2010, Cayan et al. 2010, Chambers and Pellant 2008, Chambers and Wisdom 2009, Christensen and Lettenmaier 2007, Comer et al. 2003*, Comer et al. 2013b, Covich 2009, Das et al. 2009, Daubenmire 1952, Dettinger et al. 2009, Dick‑Peddie 1993, Elliott 2012, Eyre 1980, Field et al. 1999, Griffith et al. 2004, Harper and Peckarsky 2006, Holland and Keil 1995, Hultine et al. 2007, Izbicki and Michel 2004, Jackson et al. 2009, Kittel et al. 1999b, Levick et al. 2008, Martin 2007, McCabe and Wolock 2009, Melack et al. 1997, Millar and Wolfenden 1999, Miller et al. 2010a, Mote 2006, Muldavin et al. 2000a, Reeves et al. 2005, Sawyer et al. 2009, Seavy et al. 2009, Szaro 1989, USBOR 2011, WNHP 2011

**Version:** 02 Oct 2014	**Stakeholders:** Latin America, Southeast, West

**Concept Author:** K.A. Schulz	**LeadResp:** West

CES301.716 Rio Grande Delta Thorn Woodland

**Primary Division:** Madrean Semidesert (301)

**Land Cover Class:** Mixed Upland and Wetland

**Spatial Scale & Pattern:** Small patch

**Required Classifiers:** Natural/Semi‑natural; Vegetated (>10% vasc.); Upland; Wetland

**Diagnostic Classifiers:** Lowland [Lowland]; Forest and Woodland (Treed); Tropical/Subtropical; Riverine / Alluvial

**Concept Summary:** This diverse, usually broad‑leaved evergreen woodland is found on resaca banks and old natural levees on the Rio Grande delta in southern Texas. Sites are well‑watered, somewhat elevated relative to the surrounding landscape, and tend to occupy loamy or clayey bottomland soils. This system includes evergreen, mixed and deciduous woodlands and shrublands with a typically open upper canopy of broadleaf evergreen species such as *Ebenopsis ebano* and *Ehretia anacua* and a dense shrub layer of numerous species present. Mature occurrences will often support epiphytes such as *Tillandsia recurvata, Tillandsia usneoides*, and rarely *Tillandsia baileyi*.

**Comments:** Younger occurrences, especially those occupying drier sites, tend to present as shrublands, often dominated by similar, though shorter, canopy species. These occurrences also tend to be less diverse, lack the layered structure, and usually support fewer epiphytes.

DISTRIBUTION

**Range:** This system is currently on known from remnant occurrences in the historic delta of the Rio Grande in southern Texas. It may also occur in Mexico.

**Divisions:** 301:C

**TNC Ecoregions:** 30:C, 31:P

**Nations:** MX?, US

**Subnations:** MXNU?, MXTM?, TX

**USFS Ecomap Regions:** 255Dc:???, 315Ef:CCC, 315Eg:CCC

CONCEPT

**Environment:** This system occurs in the historic floodplain of the Rio Grande delta on Quaternary‑aged alluvium. It is found on slight rises such as old natural levees or resaca banks often on Clayey or Loamy Bottomland Ecological Sites, but occasionally on Clay Loam or Gray Sandy Loam types. Sites are well‑watered, somewhat elevated relative to the surrounding landscape, and tend to occupy loamy or clayey bottomland soils. Occasionally occurrences can be found on clay loams (such as Raymondville or Racombes soils) or gray sandy loams (such as Hidalgo sandy clay loam).

**Vegetation:** The sometimes patchy canopy of these woodlands often contains species such as *Ebenopsis ebano, Ehretia anacua, Celtis laevigata, Ulmus crassifolia*, and *Celtis ehrenbergiana*, and may reach heights of 15 m. Species such as *Phaulothamnus spinescens, Amyris madrensis, Amyris texana, Diospyros texana, Leucaena pulverulenta, Guaiacum angustifolium, Malpighia glabra, Adelia vaseyi, Bernardia myricifolia, Sideroxylon celastrinum, Condalia hookeri, Forestiera angustifolia, Havardia pallens, Iresine palmeri, Trixis inula, Xylosma flexuosa*, and *Randia rhagocarpa* may occur as shrubs or in the subcanopy, and some individuals of a few of these species may reach heights of 4 to 5 m. This shrub or understory layer can be extremely dense, almost impenetrable. Woody cover, including the patchier overstory canopy and the almost continuous shrub/understory layer, often reaches greater than 90%. This system is sometimes referred to as a tall shrubland, since shrubs are often the dominant lifeform, but frequently reach heights resembling the stature of woodland. *Prosopis glandulosa* may occasionally be absent or uncommon in the canopy, and is generally not dominant except in disturbed situations. The herbaceous layer is generally represented by a only a few species and is relatively sparse, with species such as *Rivina humilis, Plumbago scandens, Celosia nitida, Chromolaena odorata, Leersia monandra, Digitaria californica, Setaria* spp., *Salvia coccinea, Petiveria alliacea, Malvastrum americanum, Urtica chamaedryoides, Verbesina microptera, Calyptocarpus vialis*, and *Justicia pilosella* sometimes present. Vines such as *Serjania brachycarpa, Urvillea ulmacea, Cocculus diversifolius, Mikania scandens, Cardiospermum* spp., *Chiococca alba, Cissus trifoliata*, and *Passiflora* spp. may also be commonly encountered. The rather rare epiphyte *Tillandsia baileyi* may be found in these woodlands, along with the more common *Tillandsia recurvata* and *Tillandsia usneoides*. Younger occurrences, especially those occupying drier sites, tend to present as shrublands, often dominated by similar, though shorter, canopy species. These occurrences also tend to be less diverse, lack the layered structure, and usually support fewer epiphytes.

**Dynamics:** The major processes in this system were flooding and drought (Diamond 1998). Freezes can have significant impacts on canopy species such as *Ebenopsis, Sabal* and *Leucaena pulverulenta* in the delta. Hurricanes may not significantly affect dense *Ebenopsis ebano ‑ Ehretia anacua* forest, except through flood effects. Lengthy droughts (lasting 10 years) can influence this system but most species are drought‑tolerant. Infrequent fires may also occur.

This system was modeled as part of the Tamaulipan Riparian Systems group by Landfire (2007a) using three classes: early‑, mid‑ and late‑seral. Fire frequency is likely over‑emphasized in this model for this system, as other ecologists suggest fire was historically infrequent in the Rio Grande delta (Diamond 1998).

Early‑seral class (0‑12 years): Herbaceous species dominant where there are high sunlight conditions. Woody species begin establishing in the understory. Duration of this stage is 12 years. Flooding at an interval of 500 years is modeled for this stage (Landfire 2007a).

Mid‑seral class (13‑33 years): Species composition would contain canopy species of an earlier successional type such as *Acacia farnesiana, Ehretia anacua, Leucaena pulverulenta, Celtis laevigata* and *Celtis ehrenbergiana*. Shrub cover would be increased due to openness of the canopy. Duration of this stage is 20 years. Flooding at an interval of 500 years moves to early‑seral class. Surface fires every 30 years maintain this mid‑seral class (Landfire 2007a).

<br />The late‑seral class (34+ years): Canopy becomes more closed and includes *Ehretia anacua, Ebenopsis ebano, Ulmus crassifolia*, and *Celtis laevigata*. Gap succession does occur as individual tree mortality occurs (maintenance/ every 500 years) and moves class to mid‑seral. Freezes will reduce canopy of ebony and other subtropical species. Freezes (every 50 years) and surface fires (every 30 years) occur but do not cause transitions to other structural or floristic states. Flooding at an interval of 500 years takes class to early‑seral (Landfire 2007a).

SOURCES

**References:** Comer et al. 2003*, Correll and Johnston 1970, Diamond 1987, Diamond 1998, Elliott 2011, Eyre 1980, Jahrsdoerfer and Leslie 1988, LANDFIRE 2007a, Lonard and Judd 2002, McLendon 1991, TNC 2013, Webster 2001

**Version:** 14 Jan 2014	**Stakeholders:** Latin America, Southeast

**Concept Author:** L. Elliott, D. Diamond, A. Treuer‑kuehn, D. German, J. Teague	**LeadResp:** Southeast

CES302.759 Sonoran Fan Palm Oasis

**Primary Division:** North American Warm Desert (302)

**Land Cover Class:** Mixed Upland and Wetland

**Spatial Scale & Pattern:** Small patch

**Required Classifiers:** Natural/Semi‑natural; Vegetated (>10% vasc.); Upland; Wetland

**Diagnostic Classifiers:** Forest and Woodland (Treed); Tropical/Subtropical [Tropical Xeric]; Temperate [Temperate Xeric]; Seepage‑Fed Sloping; Palm or Sabal

**Concept Summary:** This ecological system occurs on highly localized, spring‑fed depressions along canyon waterways and tectonic fault lines below 900 m in elevation in the Sonoran and Mojave deserts. Permanent subsurface water is required to maintain *Washingtonia filifera*, a relict species. Salinity is low in the root zone, but increases near the surface where evaporation leaves salt accumulations. These oases woodlands are distinctively dominated by *Washingtonia filifera* with variable understory conditions. Other trees that may be present include *Platanus racemosa, Quercus chrysolepis, Populus fremontii*, and *Fraxinus velutina*. A subcanopy of *Salix lasiolepis, Salix gooddingii, Salix exigua*, or *Prosopis glandulosa* is often present. Reproduction of *Washingtonia filifera* is limited by water supply, surface salinity, rainfall, and fire. Fan palms are fire‑tolerant, while the understory species are not, and fires open up the understory allowing palm seedlings to establish. Removal of the understory also decreases competition for water. There are currently 80 known occurrences in California and probably 100 throughout its range including Arizona and Nevada.

DISTRIBUTION

**Range:** Below 900 m in elevation in the Sonoran and Mojave deserts.

**Divisions:** 302:C

**TNC Ecoregions:** 17:C, 23:C

**Nations:** MX, US

**Subnations:** AZ, CA, MXBC, MXSO, NV

**Map Zones:** 4:C, 13:C

**USFS Ecomap Regions:** 322A:CC, 322B:CC, 322C:CC

CONCEPT

**Environment:** Desert springs in canyon waterways or along fault lines where underground water is continuously available. Salinity is low in the root zone, but increases near the surface where evaporation leaves salt accumulations.

**Dynamics:** Reproduction of *Washingtonia filifera* is limited by water supply, surface salinity, rainfall, and fire. Fan palms are fire‑tolerant, while the understory species are not, and fires open up the understory allowing palm seedlings to establish. Removal of the understory also decreases competition for water. Fire is essential in the regeneration of *Washingtonia filifera* (Sawyer et al. 2009). Natural fires started by lighting as well as those set by Native Americans prior to 1900s created favorable germination sites and increased the flow of springs. Flash floods probably had similar effect (Sawyer et al. 2009).

SOURCES

**References:** Barbour and Major 1988, Brown 1982a, Comer et al. 2003*, Holland and Keil 1995, MacMahon 1988, PRBO Conservation Science 2011, Sawyer and Keeler‑Wolf 1995, Sawyer et al. 2009, Szaro 1989, Thomas et al. 2004

**Version:** 14 Jan 2014	**Stakeholders:** Latin America, West

**Concept Author:** K.A. Schulz	**LeadResp:** West

CES301.990 Tamaulipan Floodplain

**Primary Division:** Madrean Semidesert (301)

**Land Cover Class:** Woody Wetland

**Spatial Scale & Pattern:** Linear

**Required Classifiers:** Natural/Semi‑natural; Vegetated (>10% vasc.); Wetland

**Diagnostic Classifiers:** Lowland [Lowland]; Forest and Woodland (Treed); Tropical/Subtropical [Tropical Xeric]; Riverine / Alluvial; Intermediate (5‑25 yrs) Flooding Interval

**National Mapping Codes:** EVT 2467; ESLF 9136; ESP 1467

**Concept Summary:** This ecological system is limited to riparian areas of the lower Rio Grande Valley and Rio Corona in southern Texas and northeastern Mexico. Stands occur on riverbanks, floodplains and deltas. Stands are generally deciduous woodlands or forests with tree height reaching to 15 m. Canopy cover is variable, but sometimes reaches near 100%. The canopy may have a conspicuous (sometimes dominant to codominant) evergreen component of species such as *Ebenopsis ebano* and *Ehretia anacua*. Dominant species of the overstory canopy often include one or more of the following: *Celtis laevigata, Ulmus crassifolia, Fraxinus berlandieriana, Prosopis glandulosa, Vachellia farnesiana, Diospyros texana, Leucaena pulverulenta, Celtis ehrenbergiana, Sapindus saponaria var. drummondii, Ebenopsis ebano, Ehretia anacua*, and *Parkinsonia aculeata*. These woodlands are a unique mix of species from southeastern North America and subtropical Central America and are often dominated by *Vachellia farnesiana, Diospyros texana, Ebenopsis ebano, Ehretia anacua, Fraxinus berlandieriana*, or *Ulmus crassifolia*, and many other tree species present to locally dominant. The highly variable understory is dependent on canopy density and may include dense shrub or herbaceous layers.

DISTRIBUTION

**Range:** This system encompasses vegetation of riparian areas of the lower Rio Grande Valley and Rio Corona in southern Texas and northeastern Mexico.

**Divisions:** 301:C

**TNC Ecoregions:** 30:C, 31:P

**Nations:** MX, US

**Subnations:** MXCO, MXNU, MXTM, TX

**Map Zones:** 36:C

**USFS Ecomap Regions:** 255D:PP, 315E:CC

CONCEPT

**Environment:** Stands of this ecological system occur on riverbanks, floodplains, deltas and other riparian areas of the lower Rio Grande Valley and Rio Corona in southern Texas and northeastern Mexico. The geology is Quaternary alluvium. Landforms are floodplains of rivers and large creeks where sediment is deposited. The topography is relatively level with some relief associated with levees and depressions developed from meanders of the waterway, or historical meanders of the Rio Grande (Resaca). It is typically found on alluvial soils of the Bottomland Ecological Sites, including loamy, clayey, and sandy. The Lowland Ecological Site type also supports this system. This ecological system occurs along rivers and major drainages in south Texas from the central portion of the Nueces River south to northeastern Mexico and west to the vicinity of Del Rio, Texas.

**Vegetation:** Stands of this system are generally deciduous woodlands or forests with tree heights reaching to 15 m. Canopy cover is variable but sometimes reaches near 100%. The canopy may have a conspicuous (sometimes dominant to codominant) evergreen component of species such as *Ebenopsis ebano* and *Ehretia anacua*. Dominant species of the overstory canopy often include one or more of the following: *Celtis laevigata, Ulmus crassifolia, Fraxinus berlandieriana, Prosopis glandulosa, Vachellia farnesiana (= Acacia farnesiana), Diospyros texana, Leucaena pulverulenta, Celtis ehrenbergiana, Sapindus saponaria var. drummondii, Ebenopsis ebano, Ehretia anacua*, and *Parkinsonia aculeata* (Elliott 2011). In northern portions of the range of this system, particularly within the Nueces River drainage, *Carya illinoinensis* and *Quercus fusiformis* may be conspicuous components of the overstory. Forests and woodlands may have significant shrub cover, including saplings of the overstory species in addition to species such as *Zanthoxylum fagara, Condalia hookeri, Forestiera angustifolia, Sideroxylon* spp., *Aloysia gratissima, Acacia greggii var. wrightii, Malpighia glabra, Guaiacum angustifolium, Ziziphus obtusifolia*, and *Amyris texana*. Other shrub species, such as *Buddleja sessiliflora, Phaulothamnus spinescens, Lippia alba*, and *Amyris madrensis* may be encountered in southern expressions of the system. *Salix nigra* may dominate sites, especially at river's edge and wet sites. Riverbanks and other sites with a reduced overstory canopy (either from disturbance or prolonged inundation) may also be shrub‑dominated, often with one or few species such as *Baccharis neglecta, Baccharis salicifolia, Arundo donax, Sesbania drummondii*, or *Cephalanthus occidentalis*, and *Salix exigua, Mimosa asperata*, or *Cephalanthus salicifolius* in the lower Rio Grande Valley (Elliott 2011). The herbaceous layer is typically not well‑developed, but may include species such as *Trichloris pluriflora, Setaria scheelei, Panicum virgatum, Paspalum langei, Paspalum denticulatum, Carex crus‑corvi, Cyperus articulatus, Rivina humilis, Calyptocarpus vialis, Chromolaena odorata, Teucrium cubense, Urtica chamaedryoides, Parietaria pensylvanica, Verbesina microptera, Chloracantha spinosa, Parthenium confertum*, and *Malvaviscus arboreus var. drummondii*. Vines such as *Serjania brachycarpa, Cocculus diversifolius, Clematis drummondii*, and *Cissus trifoliata* are frequently encountered, and *Tillandsia usneoides* often drapes the branches of overstory species. Non‑native grasses such as *Cynodon dactylon, Urochloa maxima, Pennisetum ciliare, Bothriochloa ischaemum var. songarica*, and *Bromus catharticus* are often present to dominant, and sometimes to the exclusion of most other herbaceous species (Elliott 2011).

**Dynamics:** Stands occur as linear patches along much of the lower Rio Grande and occupy large patches on the delta (Landfire 2007a). Key ecological processes are succession and disturbance. Disturbance was primarily flooding and, to a lesser extent, fire may have occurred within these woodlands and forests. Occurrence of patches of *Phragmites* spp. may have provided adequate fuel to carry fire to the canopy. Floods may have been annual and were primarily depositional floods rather than scouring floods (Landfire 2007a). Long‑term succession would occur due to deposition and development of this system into more upland characteristics of another system (Landfire 2007a). Extreme floods may have occurred in association with hurricanes. Freezes would have had significant impacts on the largely tropical/subtropical species, though these impacts more directly affect riparian woodlands where tropical species are more common. Drought would also affect this system, and may provide the unusual opportunity for fire to carry in the system (Landfire 2007a).

This system was modeled as part of the Tamaulipan Riparian Systems group by Landfire (2007a) using three classes: early‑, mid‑ and late‑seral. Fire frequency may be over‑emphasized in this model for this system, as other ecologists suggest fire less frequent historically.

The early‑seral class (0‑12 years): Herbaceous cover following 1000‑year scouring flood and replacement fire in mid‑seral class. Herbaceous cover of sedges and rushes develops as sedimentation produces an adequate substrate not continually flooded to allow development of cover. Areas of *Phragmites* spp. may occur in areas where fires would have occurred. Replacement fire‑return interval is approximately 10 years in this class due to the fine fuel (Landfire 2007a).

<br />Mid‑seral class (13‑20 years): Low canopy cover of trees. Shrub layer well‑developed, but composition is similar to the understory of late‑seral class. Scouring floods associated with river channel migration on the delta is modeled as encountering a site every 1000 years, taking the class back to early‑seral. Replacement fire is modeled as occurring at a similar MFRI to surface fires in mid‑seral class (30 years). *Celtis laevigata* is developing as a canopy but still occurs with low cover (Landfire 2007a).

The late‑seral class (21+ years): Dominated by *Celtis laevigata* with 60‑100% canopy, with *Ulmus crassifolia, Celtis ehrenbergiana, Mimosa pellita*, and *Condalia hookeri* in the midstory, and other shrubs in the understory. Scouring floods modeled as occurring every 1000 years take class back to early‑seral. Hurricane of sufficient strength to take out the canopy is modeled as occurring every 50 years, takes class back to mid‑seral. Maintenance surface fires occur every 30 years (Landfire 2007a).

SOURCES

**References:** Comer et al. 2003*, Correll and Johnston 1970, Diamond 1987, Elliott 2011, Eyre 1980, Jahrsdoerfer and Leslie 1988, LANDFIRE 2007a, Lonard and Judd 2002, McLendon 1991, TNC 2013, Webster 2001

**Version:** 14 Jan 2014	**Stakeholders:** Latin America, Southeast

**Concept Author:** K.A. Schulz	**LeadResp:** Southeast

CES301.991 Tamaulipan Palm Grove Riparian Forest

**Primary Division:** Madrean Semidesert (301)

**Land Cover Class:** Woody Wetland

**Spatial Scale & Pattern:** Linear

**Required Classifiers:** Natural/Semi‑natural; Vegetated (>10% vasc.); Wetland

**Diagnostic Classifiers:** Lowland [Lowland]; Forest and Woodland (Treed); Tropical/Subtropical [Tropical Xeric]; Riverine / Alluvial; Palm Tree; Sabal mexicana

**Concept Summary:** This ecological system is limited to riparian areas along the lower Rio Grande and Rio Corona in southern Texas and northeastern Mexico. Stands occur on riverbanks and floodplains. The characteristic species are the neotropical *Sabal mexicana* with *Ebenopsis ebano, Ehretia anacua, Leucaena pulverulenta*, and many other riparian species such as *Acacia farnesiana, Diospyros texana, Fraxinus berlandieriana*, or *Ulmus crassifolia*. The understory is dominated by neotropical species.

DISTRIBUTION

**Range:** This ecological system is limited to riparian areas along the lower Rio Grande and Rio Corona in southern Texas and northeastern Mexico.

**Divisions:** 301:C

**TNC Ecoregions:** 30:C, 31:P

**Nations:** MX, US

**Subnations:** MXTM, TX

**Map Zones:** 36:C

**USFS Ecomap Regions:** 255D:PP, 315E:CC

CONCEPT

**Environment:** Stands of this ecological system occur on riverbanks and floodplains in riparian areas along the lower Rio Grande and Rio Corona in southern Texas and northeastern Mexico. The geologic substrate is Quaternary alluvium. It is currently found on levees and resaca margins and adjacent lower sites near the current Rio Grande channel (Elliott 2011). It was historically more widespread within the Rio Grande delta. Soils are Loamy or Clayey Bottomland Ecological Sites. This system is currently limited to relatively small groves (typically less than 20 hectares) of *Sabal mexicana* (sometimes referred to as *Sabal texana*) located on loamy or clayey bottomland soils, such as those of the Rio Grande, Zalla, and Matamoros series, on the Rio Grande Delta and near the Rio Grande itself in Cameron County, Texas, and similar sites in adjacent Mexico. These often occupy slight elevations along the margins of resacas or old river terraces, but may also occur on level sites. The system may have once occurred along the Rio Grande more than 120 km from its mouth, but is now limited to a few sites near the Gulf of Mexico, with a few small stands identified in extreme southern Hidalgo County, Texas (Elliott 2011).

**Vegetation:** This system is currently limited to relatively small groves (typically less than 20 hectares) of *Sabal mexicana* (sometimes referred to as *Sabal texana*). These forests and woodlands often have a canopy dominated by *Sabal mexicana*, or may share dominance with other floodplain species such as *Ebenopsis ebano, Celtis laevigata, Leucaena pulverulenta, Ulmus crassifolia, Ehretia anacua*, and *Fraxinus berlandieriana*. *Prosopis glandulosa, Sapindus saponaria var. drummondii*, and *Diospyros texana* are often present in the subcanopy (Elliott 2011). The canopy of these forests may reach a height of 15 m, and the subcanopy (to 10 m) may be composed of some of the species mentioned above. The shrub layer can be patchy with some areas extremely dense and containing species such as *Zanthoxylum fagara, Malpighia glabra, Celtis ehrenbergiana, Erythrina herbacea, Ziziphus obtusifolia, Randia rhagocarpa, Parkinsonia aculeata, Havardia pallens, Chiococca alba, Iresine palmeri*, and members of the canopy and subcanopy, and other areas relatively open. In some situations the ground may be covered with a layer of dead palm fronds, restricting the development of an herbaceous layer. In other areas, species including, but not limited to, *Leersia monandra, Salvia coccinea, Petiveria alliacea, Rivina humilis, Plumbago scandens, Tamaulipa azurea, Cocculus diversifolius*, and *Malvaviscus arboreus* may be present in the herbaceous layer (Elliott 2011).

**Dynamics:** Fire may have been an important process in these forests as the sites may become extremely dry and a significant, if patchy, layer of palm thatch may be present. These forests appear to differ from other forests dominated by *Sabal mexicana* further to the south. Martinez‑Ojeda and González‑Medrano (1977) describe a site of limited distribution in the northern part of the Sierra de San José de las Rusias in the Municipio of Soto La Marina in Tamaulipas, Mexico. It occurs at higher altitudes and on Oligocene geologic formations. Their brief description suggests that this is likely different in composition and process from the presently described system. Lopez and Dirzo (2007) describe a site further south in Vera Cruz that also seems to differ relative to composition. Palm groves were once common in the lower Rio Grande Valley 80 miles from the Gulf of Mexico, but have since largely been converted to agriculture.<br />

The major process in this system was flooding. Freezes can have significant impacts on canopy species such as *Ebenopsis, Sabal* and *Leucaena pulverulenta* in the delta. Hurricanes may not significantly affect dense ebony/anacua forest, except through flood effects. Lengthy droughts (lasting 10 years) can influence this system, but most species are drought‑tolerant. Infrequent fires may also occur (Landfire 2007a).

SOURCES

**References:** Comer et al. 2003*, Diamond 1987, Elliott 2011, Gehlbach 1981, Jahrsdoerfer and Leslie 1988, LANDFIRE 2007a, Lopez and Dirzo 2007, Martinez‑Ojeda and González‑Medrano 1977, TNC 2013, Vora 1990, Vora and Messerly 1990, Webster 2001

**Version:** 14 Jan 2014	**Stakeholders:** Latin America, Southeast

**Concept Author:** K.A. Schulz	**LeadResp:** Southeast

M660. Mexican Interior Riparian Forest

CES305.279 Arbustal Ripario Montano Mexicano

**Primary Division:** Sierra Madre (305)

**Land Cover Class:** Forest and Woodland

**Spatial Scale & Pattern:** Linear

**Required Classifiers:** Natural/Semi‑natural; Vegetated (>10% vasc.); Upland

**Diagnostic Classifiers:** Montane [Montane]; Forest and Woodland (Treed); Udic

**Concept Summary:** This riparian system is not well‑documented but is likely composed of seasonally flooded woodlands and shrublands found at montane elevations throughout the Sierra Madres and was historically common throughout the Valley of Mexico. This system includes alder woodlands and shrublands that line montane streams. These are communities tolerant of periodic flooding and high water tables. At high elevations, snowmelt moisture in this system may create shallow water tables or seeps for a portion of the growing season. Stands typically occur at elevations between 2500 and 3500 m and are confined to specific riparian environments occurring on floodplains or terraces of rivers and streams, in V‑shaped, narrow valleys and canyons (where there is cold‑air drainage). Less frequently, occurrences are found in moderate to wide valley bottoms on large floodplains along broad, meandering rivers. The following list of species is diagnostic for this system: *Alnus glabrata, Salix bonplandiana, Fraxinus uhdei, Buddleia cordata, Schinus molle, Populus* spp.

DISTRIBUTION

**Divisions:** 305:C

**Nations:** MX

**Subnations:** MXCH, MXCO, MXCP, MXDU, MXGJ, MXGU, MXHI, MXJA, MXMI, MXNU, MXOA, MXPU, MXSL, MXSO, MXVE, MXZA

CONCEPT

**Environment:** These are found along a wide elevational range along moderate to low gradient streams where river scour occurs and floodplains develop.

**Vegetation:** These are primarily deciduous woodlands and shrublands forming complex canopies as a result of natural riverine disturbance.

**Dynamics:** River scour exposes moist soils and supports regeneration of some riparian species. Periodic flooding and disturbance maintains a dynamic vegetative mosaic.

SOURCES

**References:** Josse et al. 2003*, Rzedowski 1986, Velazquez et al. 2000

**Version:** 17 Jul 2003	**Stakeholders:** Latin America

**Concept Author:** C. Josse	**LeadResp:** Latin America

CES403.316 Bosque y Arbustal Ripario Montano Bajo Mexicano

**Primary Division:** Meso‑American Seasonal Highlands (403)

**Land Cover Class:** Forest and Woodland

**Spatial Scale & Pattern:** Linear

**Required Classifiers:** Natural/Semi‑natural; Vegetated (>10% vasc.); Upland

**Diagnostic Classifiers:** Montane [Lower Montane]; Forest and Woodland (Treed); Udic

**Concept Summary:** This riparian system comprises seasonally flooded woodlands and shrublands found at lower montane elevations to sea level throughout humid and subhumid portions of Mexico. These are communities tolerant of periodic flooding and high water tables. Stands typically are confined to specific riparian environments occurring on floodplains or terraces of rivers and streams in V‑shaped, narrow valleys and canyons (where there is cold‑air drainage). Less frequently, occurrences are found in moderate to wide valley bottoms on large floodplains along broad, meandering rivers. The following list of genera is diagnostic for this system: *Platanus, Populus, Ficus, Astianthus, Bambusa, Inga, Carya, Fraxinus, Pachira, Salix*.

DISTRIBUTION

**Divisions:** 403:C

**Nations:** MX

**Subnations:** MXCP, MXDU, MXGJ, MXGU, MXHI, MXJA, MXMI, MXNU, MXOA, MXPU, MXSL, MXVE, MXZA

CONCEPT

**Environment:** These are found along a wide elevational range along moderate‑ to low‑gradient streams where river scour occurs and floodplains develop.

**Vegetation:** These are primarily deciduous woodlands and shrublands forming complex canopies as a result of natural riverine disturbance.

SOURCES

**References:** Josse et al. 2003*, Rzedowski 1986, Velazquez et al. 2000

**Version:** 17 Jul 2003	**Stakeholders:** Latin America

**Concept Author:** C. Josse	**LeadResp:** Latin America

1.B.3.Ng. Vancouverian Flooded & Swamp Forest

M035. Vancouverian Flooded & Swamp Forest

CES204.090 North Pacific Hardwood‑Conifer Swamp

**Primary Division:** North American Pacific Maritime (204)

**Land Cover Class:** Woody Wetland

**Spatial Scale & Pattern:** Large patch

**Required Classifiers:** Natural/Semi‑natural; Vegetated (>10% vasc.); Wetland

**Diagnostic Classifiers:** Lowland [Lowland]; Forest and Woodland (Treed); Temperate [Temperate Oceanic]; Depressional [Lakeshore]; Needle‑Leaved Tree; Broad‑Leaved Deciduous Tree; Pinus contorta; Sphagnum spp.; Eutrophic Water

**Concept Summary:** This wetland ecological system occurs from southern coastal British Columbia south into coastal Washington and Oregon, west of the coastal mountain summits (not interior). Treed swamps are common in southeastern Alaska (but are placed into different systems than this one), less so farther south. Forested swamps are mostly small‑patch size, occurring sporadically in glacial depressions, in river valleys, around the edges of lakes and marshes, or on slopes with seeps that form subirrigated soils. These are primarily on flat to gently sloping lowlands up to 457 m (1500 feet) elevation but also occur up to near the lower limits of continuous forest (below the subalpine parkland). It can occur on steeper slopes where soils are shallow over unfractured bedrock. This system is indicative of poorly drained, mucky areas, and areas are often a mosaic of moving water and stagnant water. Soils can be woody peat, muck, or mineral. It can be dominated by any one or a number of conifer and hardwood species (*Tsuga heterophylla, Picea sitchensis, Tsuga mertensiana, Callitropsis nootkatensis, Pinus contorta var. contorta, Alnus rubra, Fraxinus latifolia, Betula papyrifera*) that are capable of growing on saturated or seasonally flooded soils. Overstory is often less than 50% cover, but shrub understory can have high cover. In the southern end of the range of this type, e.g., the Willamette Valley, tends to have more hardwood‑dominated stands (especially *Fraxinus latifolia*) and very little in the way of conifer‑dominated stands. While the typical landscape context for the type is extensive upland forests, for the *Fraxinus latifolia* stands, landscapes were very often formerly dominated by prairies and now by agriculture. Many conifer‑dominated stands have been converted to dominance by *Alnus rubra* due to timber harvest.

**Comments:** Shrub swamps are usually not intermixed with the forested swamps and tend to be more wet. Deciduous and conifer forested swamps are often intermixed and more similar to each other in hydrology, and so are combined here in this system.

DISTRIBUTION

**Range:** This system occurs from southern British Columbia south to northwestern Oregon, including the Willamette Valley, west of the Cascade Crest.

**Divisions:** 204:C

**TNC Ecoregions:** 1:C, 2:C, 3:C, 69:C, 81:C

**Nations:** CA, US

**Subnations:** BC, OR, WA:S2

**Map Zones:** 1:C, 2:C, 7:C

**USFS Ecomap Regions:** 242A:CC, 242B:CC, M242A:CC, M242B:CC, M242C:CC, M242D:CC

CONCEPT

**Environment:** This wetland ecological system occurs from southern coastal British Columbia south into coastal Washington and Oregon, west of the coastal mountain summits (not interior). Treed swamps are common in southeastern Alaska (but are placed into different systems than this one), less so farther south. Forested swamps are mostly small‑patch size, occurring sporadically in glacial depressions, in river valleys, around the edges of lakes and marshes, or on slopes with seeps that form subirrigated soils. These are primarily on flat to gently sloping lowlands up to 457 m (1500 feet) elevation but also occur up to near the lower limits of continuous forest (below the subalpine parkland). It can occur on steeper slopes where soils are shallow over unfractured bedrock. This system is indicative of poorly drained, mucky areas, and areas are often a mosaic of moving water and stagnant water. Soils can be woody peat, muck, or mineral.

**Vegetation:** It can be dominated by any one or a number of conifer and hardwood species (*Tsuga heterophylla, Picea sitchensis, Tsuga mertensiana, Callitropsis nootkatensis (= Chamaecyparis nootkatensis), Pinus contorta var. contorta, Alnus rubra, Fraxinus latifolia, Betula papyrifera*) that are capable of growing on saturated or seasonally flooded soils. Overstory is often less than 50% cover, but shrub understory can have high cover. In the southern end of the range of this type, e.g., the Willamette Valley, tends to have more hardwood‑dominated stands (especially *Fraxinus latifolia*) and very little in the way of conifer‑dominated stands. While the typical landscape context for the type is extensive upland forests, for the *Fraxinus latifolia* stands, landscapes were very often formerly dominated by prairies and now by agriculture. Many conifer‑dominated stands have been converted to dominance by *Alnus rubra* due to timber harvest.

SOURCES

**References:** Chappell 1999, Chappell and Christy 2004, Chappell et al. 2001, Comer et al. 2003*, Eyre 1980, Green and Klinka 1994, WNHP unpubl. data 2018

**Version:** 08 Dec 2008	**Stakeholders:** Canada, West

**Concept Author:** K. Boggs, G. Kittel, C. Chappell	**LeadResp:** West

CES204.875 North Pacific Intertidal Freshwater Wetland

**Primary Division:** North American Pacific Maritime (204)

**Land Cover Class:** Herbaceous Wetland

**Spatial Scale & Pattern:** Small patch

**Required Classifiers:** Natural/Semi‑natural; Vegetated (>10% vasc.); Wetland

**Concept Summary:** This ecological system occurs throughout the coastal margin and intertidal zone of the Pacific Northwest Coast of Oregon, Washington and north into British Columbia. It may occur in Alaska, but has not been described from there. Intertidal freshwater wetlands occur as narrow strips to more extensive patches along tidally influenced portions of rivers. There has been little vegetation data collection of this type in this region; a few studies indicate dominant species include *Picea sitchensis, Alnus rubra, Cornus sericea, Myriophyllum hippuroides, Typha angustifolia, Athyrium filix‑femina*, and *Carex lyngbyei*. This system is driven by daily tidal flooding of freshwater and associated soil saturation. Vegetation structure and composition are varied and depend on substrate characteristics and the tidal flooding regime of particular sites. Where small areas of mudflat occur in tidally influenced freshwater areas, they are included in this intertidal freshwater wetland and not in Temperate Pacific Freshwater Mudflat (CES200.878).

**Comments:** It's unclear if this system occurs in Alaska; it was not identified in recent systems classification work.

DISTRIBUTION

**Range:** This system occurs throughout the coastal margin and intertidal zone of the Pacific Northwest coast of Oregon, Washington and north into British Columbia. It may occur in Alaska but has not been described from there.

**Divisions:** 204:C

**TNC Ecoregions:** 1:C, 69:C

**Nations:** CA, US

**Subnations:** BC, OR, WA:S1

**Map Zones:** 1:C, 2:C

**USFS Ecomap Regions:** 242A:CC, M242A:CC

CONCEPT

SOURCES

**References:** Boggs 2000, Boggs 2002, Comer et al. 2003*, Kunze 1994, Viereck et al. 1992, WNHP unpubl. data 2018

**Version:** 08 Dec 2008	**Stakeholders:** Canada, West

**Concept Author:** C. Chappell and G. Kittel	**LeadResp:** West

CES204.869 North Pacific Lowland Riparian Forest and Shrubland

**Primary Division:** North American Pacific Maritime (204)

**Land Cover Class:** Woody Wetland

**Spatial Scale & Pattern:** Linear

**Required Classifiers:** Natural/Semi‑natural; Vegetated (>10% vasc.); Wetland

**Diagnostic Classifiers:** Lowland [Lowland]; Forest and Woodland (Treed); Riverine / Alluvial

**National Mapping Codes:** EVT 2156; ESLF 9106; ESP 1156

**Concept Summary:** Lowland riparian systems occur throughout the Pacific Northwest. They are the low‑elevation, alluvial floodplains that are confined by valleys and inlets and are more abundant in the central and southern portions of the Pacific Northwest Coast. These forests and tall shrublands are linear in character, occurring on floodplains or lower terraces of rivers and streams. Major broadleaf dominant species are *Acer macrophyllum, Alnus rubra, Populus balsamifera ssp. trichocarpa, Salix sitchensis, Salix lucida ssp. lasiandra, Cornus sericea*, and *Fraxinus latifolia*. Conifers tend to increase with succession in the absence of major disturbance. Conifer‑dominated types are relatively uncommon and not well‑described; *Abies grandis, Picea sitchensis*, and *Thuja plicata* are important. Riverine flooding and the succession that occurs after major flooding events are the major natural processes that drive this system. Very early‑successional stages can be sparsely vegetated or dominated by herbaceous vegetation.

**Comments:** This system is driven by snowmelt and rainfall hydrology. It differs from Alaskan Pacific Maritime Floodplain Forest and Shrubland (CES204.154) by the presence of mature black cottonwood gallery forests, and generally narrow linear deciduous riparian forests and shrublands. The Alaskan type includes glacier melt‑driven hydrology, which results in very wide riverine habitats with fewer mature deciduous forests, as well as non‑glacial rivers common on the island archipelago, but also on the mainland, which are narrower and are mostly dominated by Sitka spruce with and without the codominance of black cottonwood.

DISTRIBUTION

**Range:** This system occurs throughout the Pacific Northwest below the Silver Fir Zone in elevation.

**Divisions:** 204:C

**TNC Ecoregions:** 1:C, 81:C

**Nations:** CA, US

**Subnations:** AK, BC, OR, WA:S2

**Map Zones:** 1:C, 2:C, 7:C

**USFS Ecomap Regions:** 242A:CC, 242B:CC, 342I:CC, M242A:CC, M242B:CC, M242C:CC, M242D:CC, M261A:CC, M261D:CP

CONCEPT

**Environment:** Stands occur on low‑elevation, alluvial floodplains on alluvial soils in valleys and inlets, on riverbanks, outer floodplains or low terraces of rivers and streams.

**Dynamics:** Beaver activity is an important driver of hydrological change and subsequent development of a diversity of habitat patches. The contribution of large woody debris (LWD) from riparian or adjacent upland trees is important to maintaining the hydrological and sediment regimes. LWD has a significant impact on the evolution of channel morphology and also contributes to the spatial distribution and diversity of habitat patches within this system (Naiman and Bilby 1998). Major flood events and consequent flood scour, overbank deposition of water and sediments, and stream meandering are the key fluvial processes that provide new substrates, remove old banks and stimulate renewed growth of cottonwood and willow species (Sawyer et al. 2009). Natural fire‑return interval was long or moderate with low‑intensity surface fires.

SOURCES

**References:** Boes and Strauss 1994, Chappell and Christy 2004, Comer et al. 2003*, Eyre 1980, Franklin and Dyrness 1973, Littell et al. 2009, Merritt and Wohl 2002, Naiman and Bilby 1998, Sawyer et al. 2009, WNHP 2011, WNHP unpubl. data 2018

**Version:** 14 Jan 2014	**Stakeholders:** Canada, West

**Concept Author:** G. Kittel and C. Chappell	**LeadResp:** West

CES204.866 North Pacific Montane Riparian Woodland and Shrubland

**Primary Division:** North American Pacific Maritime (204)

**Land Cover Class:** Woody Wetland

**Spatial Scale & Pattern:** Linear

**Required Classifiers:** Natural/Semi‑natural; Vegetated (>10% vasc.); Wetland

**Diagnostic Classifiers:** Forest and Woodland (Treed); Temperate [Temperate Oceanic]; Riverine / Alluvial

**National Mapping Codes:** EVT 2158; ESLF 9108; ESP 1158

**Concept Summary:** This ecological system occurs throughout mountainous areas of the Pacific Northwest coast, both on the mainland and on larger islands. It occurs on steep streams and narrow floodplains above foothills but below the alpine environments, e.g., above 1500 m (4550 feet) elevation in the Klamath Mountains and western Cascades of Oregon, up as high as 3300 m (10,000 feet) in the southern Cascades, and above 610 m (2000 feet) in northern Washington. Surrounding habitats include subalpine parklands and montane forests. In Washington, they are defined as occurring primarily above the *Tsuga heterophylla* zone, i.e., beginning at or near the lower boundary of the *Abies amabilis* zone. Dominant species include *Pinus contorta var. murrayana, Populus balsamifera ssp. trichocarpa, Abies lowiana, Abies magnifica, Populus tremuloides, Alnus incana ssp. tenuifolia, Alnus viridis ssp. crispa, Alnus viridis ssp. sinuata, Alnus rubra, Rubus spectabilis, Ribes bracteosum, Oplopanax horridus, Acer circinatum*, and several *Salix* species. In western Washington, major species are *Alnus viridis ssp. sinuata, Acer circinatum, Salix, Oplopanax horridus, Alnus rubra, Petasites frigidus, Rubus spectabilis*, and *Ribes bracteosum*. This is a disturbance‑driven system that requires flooding, scour and deposition for germination and maintenance. It occurs on streambanks where the vegetation is significantly different than surrounding forests, usually because of its shrubby or deciduous character.

**Comments:** Riparian and floodplain woodlands and shrublands in Alaska have been placed into a different system. Still need to determine where the Alaskan type grades into this one, and whether British Columbian riparian systems should be placed here or in the new Alaskan system.

DISTRIBUTION

**Range:** This system occurs throughout mountainous areas of the Pacific Northwest Coast, both on the mainland and on larger islands, above 1500 m (4550 feet) elevation in the Klamath Mountains and western Cascades, up as high as 3300 m (10,000 feet) in the southern Cascades, and above 610 m (2000 feet) in northern Washington.

**Divisions:** 204:C

**TNC Ecoregions:** 1:C, 3:C, 4:C, 69:?, 81:C

**Nations:** CA, US

**Subnations:** AK, BC, OR, WA:S4

**Map Zones:** 1:C, 2:C, 3:?, 7:C

**USFS Ecomap Regions:** 242A:CC, 242B:C?, M242A:CC, M242B:CC, M242C:CC, M242D:CC, M261A:CC, M261D:CP, M261G:CC

CONCEPT

**Environment:** This ecological system occurs throughout mountainous areas of the Pacific Northwest coast, both on the mainland and on larger islands. It occurs on steep streams and narrow floodplains above foothills but below the alpine environments, e.g., above 1500 m (4550 feet) elevation in the Klamath Mountains and western Cascades of Oregon, up as high as 3300 m (10,000 feet) in the southern Cascades, and above 610 m (2000 feet) in northern Washington. Surrounding habitats include subalpine parklands and montane forests. In Washington, they are defined as occurring primarily above the *Tsuga heterophylla* zone, i.e., beginning at or near the lower boundary of the *Abies amabilis* zone.

**Vegetation:** Dominant species include *Pinus contorta var. murrayana, Populus balsamifera ssp. trichocarpa, Abies lowiana (= Abies concolor var. lowiana), Abies magnifica, Populus tremuloides, Alnus incana ssp. tenuifolia (= Alnus tenuifolia), Alnus viridis ssp. crispa (= Alnus crispa), Alnus viridis ssp. sinuata (= Alnus sinuata), Alnus rubra, Rubus spectabilis, Ribes bracteosum, Oplopanax horridus, Acer circinatum*, and several *Salix* species. In western Washington, major species are *Alnus viridis ssp. sinuata, Acer circinatum, Salix, Oplopanax horridus, Alnus rubra, Petasites frigidus, Rubus spectabilis*, and *Ribes bracteosum*.

**Dynamics:** This is a disturbance‑driven system that requires flooding, scour and deposition for germination and maintenance. It occurs on streambanks where the vegetation is significantly different than surrounding forests, usually because of its shrubby or deciduous character.

SOURCES

**References:** Banner et al. 1993, Comer et al. 2003*, DeLong 2003, DeLong et al. 1993, DeLong et al. 1994, Eyre 1980, Franklin and Dyrness 1973, Holland and Keil 1995, Lloyd et al. 1990, MacKinnon et al. 1990, Meidinger et al. 1988, Steen and Coupé 1997, WNHP unpubl. data 2018

**Version:** 08 Dec 2008	**Stakeholders:** Canada, West

**Concept Author:** G. Kittel	**LeadResp:** West

1.B.4. Boreal Forest & Woodland

1.B.4.Na. North American Boreal Forest & Woodland

M495. Eastern North American Boreal Forest

CES103.022 Boreal Jack Pine-Black Spruce Forest

**Primary Division:** (103)

**Concept Summary:** This conifer forest ecological system is found on nutrient-poor soils in a variety of topographic settings. It ranges from eastern Alberta to eastern Canada, and southward into Minnesota, the Great Lakes region, and very locally into northwestern Maine. Soils are loamy to sandy, varying from thin soil over bedrock to deeper soils, sometimes sandy. Sites are xeric, but less strongly than barrens and sandplains. The dominant fire regime varies from 50-100 years. *Pinus banksiana* and *Picea mariana* are characteristic overstory species. In the Upper Great Lakes region, *Pinus banksiana* may intermix with *Pinus resinosa*. Canopy structure is mostly closed but can be partially open. Conifers typically dominate the canopy, but boreal hardwoods (*Populus tremuloides, Betula papyrifera*) may codominate. As time since fire increases, *Picea mariana* may dominate. The shrub and field layers can be somewhat dense to sparse, and older *Picea mariana* stands may be dominated by feathermosses. Common shrubs include *Alnus viridis, Arctostaphylos uva-ursi, Vaccinium myrtilloides, Vaccinium vitis-idaea, Diervilla lonicera, Rubus* sp., and *Linnaea borealis*. Important bryophytes include *Hylocomium splendens* and *Pleurozium schreberi*.

DISTRIBUTION

**Range:** It ranges from eastern Alberta to eastern Canada, and southward into Minnesota and the Great Lakes region and very locally into northwestern Maine.

**Nations:** CA, US

**Subnations:** AB, MB, ME, MI, MN, ON, QC, SK, WI

CONCEPT

**Environment:**

**Vegetation:**

**Dynamics:**

SOURCES

**References:** Comer et al. 2003*

**Version:** 02 Oct 2014	**Stakeholders:**

**Concept Author:** P. Comer

1.B.5. Boreal Flooded & Swamp Forest

1.B.5.Na. North American Boreal Flooded & Swamp Forest

M299. North American Boreal Conifer Poor Swamp

CES103.724 Eastern Boreal‑Sub‑boreal Conifer Acidic Swamp and Treed Poor Fen

**Primary Division:** Boreal (103)

**Land Cover Class:** Woody Wetland

**Spatial Scale & Pattern:** Large patch

**Required Classifiers:** Natural/Semi‑natural; Vegetated (>10% vasc.); Wetland

**Concept Summary:** This ecological system extends across the boreal regions of central and western Canada, and east and south into northern New England and the Great Lakes region. The system is primarily weakly to moderately minerotrophic (poor fen), though some stands may approach ombrotrophic (bog) conditions. Decomposition is so slow that fibrous or woody peat accumulates, and the water is slightly to very acidic and nutrient‑poor (also called mesotrophic). Acidic (also called poor or transitional) fens have organic soils and are dominated by aquatics, emergents, and dwarf‑shrubs, or raised peat dominated by shrubs and trees. Groundwater, the primary water source, is nutrient‑rich due to its contact with mineral soils, however, acidic fens have less contact with nutrient‑rich waters, as the amount of peat has accumulated to raise the level of the fen, but it remains in contact with groundwater (hence "transitional" on its way to becoming a bog). The water is acidic, with a pH generally between 4.0 and 5.8. This is a forested peatland where the trees form partial to full cover over most or all of the peatland. Stunted to well‑developed *Picea mariana* and *Larix laricina* are the dominant trees. Heaths and sedges are common in the understory, but the dwarf‑shrub layer is less well‑developed than in open acidic peatlands, though it may be prominent in more open parts of the system. *Chamaedaphne calyculata, Kalmia polifolia, Ledum groenlandicum, Vaccinium macrocarpon (= Oxycoccus macrocarpus), Vaccinium vitis‑idaea*, and *Salix* spp. are the dominant dwarf‑shrubs. Other fen indicators also occur, such as *Betula glandulosa* or *Betula pumila*. Other poor fens are graminoid‑dominated with herbaceous indicators such as *Drosera* spp., *Equisetum fluviatile, Maianthemum trifolium, Sarracenia purpurea*, and sedges (*Carex* spp.)

**Comments:** This forested system is most common in poorly drained basins, with some minerotrophic influence. It is sometimes referred to as "muskeg," a flat bog peatland with scattered trees and a fairly dense shrub layer on mounded or hummocky peat, though this system is not, technically, an ombrotrophic bog [see Boreal‑Laurentian Bog (CES103.581)]. Muskeg is probably a complex of bogs and acidic swamps. Black spruce swamps in northeastern Vermont, northern New Hampshire, Adirondack region of New York, and Maine are included here. There appears to be no need for a true Boreal alkaline swamp system, but further review is needed. In Acadia and the Northern Appalachian regions, this system is mostly replaced by the sub‑boreal Northern Appalachian‑Acadian Conifer‑Hardwood Acidic Swamp (CES201.574). Small kettlehole bogs in the northeastern U.S. are attributed to North‑Central Interior and Appalachian Acidic Peatland (CES202.606). In western Canada, this system is uncommon because the substrates are mostly calcareous, resulting in a preponderance of rich, alkaline fens either dominated by *Larix laricina* with some *Picea mariana*, or else shrub‑sedge fens lacking trees.

DISTRIBUTION

**Range:** This system is found in central and eastern Canada, extending into northern New England and the Great Lakes region, particularly in northern Minnesota.

**Divisions:** 103:C, 201:C

**TNC Ecoregions:** 47:C, 48:C, 63:C, 137:?, 140:C, 141:C

**Nations:** CA, US

**Subnations:** AB, MB, ME, MI, MN, NB, NH, NS, NY, ON, PE?, SK, VT, WI

**Map Zones:** 40:C, 41:C, 50:C, 51:C, 64:C, 66:C

**USFS Ecomap Regions:** 211A:CC, 211B:CC, 211C:CC, 211D:CC, 212Ha:CCC, 212Hb:CCC, 212Hc:CCC, 212Hd:CCC, 212He:CCC, 212Hf:CCC, 212Hg:CCC, 212Hh:CCC, 212Hi:CCC, 212Hj:CCC, 212Hk:CCC, 212Hl:CCC, 212Hm:CCC, 212K:CC, 212L:CC, 212M:CC, 212N:CC, 212Ra:CCC, 212Rb:CCC, 212Rc:CCC, 212Rd:CCC, 212Re:CCC, M211A:CC, M211B:CC, M211C:CC, M211D:CC

CONCEPT

SOURCES

**References:** Comer et al. 2003*, Eyre 1980, Glaser and Janssens 1986, Harris et al. 1996, Kost et al. 2007, Smith et al. 2007, Sperduto and Nichols 2004, WDNR 2015

**Version:** 31 Mar 2010	**Stakeholders:** Canada, East, Midwest

**Concept Author:** D. Faber‑Langendoen	**LeadResp:** Midwest

2. Shrub & Herb Vegetation

2.A. Tropical Grassland, Savanna & Shrubland

2.A.1. Tropical Lowland Grassland, Savanna & Shrubland

2.A.1.Ea. Caribbean‑Mesoamerican Lowland Grassland, Savanna & Shrubland

M671. Caribbean Dry Scrub

CES411.422 Caribbean Coastal Thornscrub

**Primary Division:** Caribbean (411)

**Land Cover Class:** Shrubland

**Spatial Scale & Pattern:** Large patch

**Required Classifiers:** Natural/Semi‑natural; Vegetated (>10% vasc.); Upland

**Diagnostic Classifiers:** Latosols

**Concept Summary:** This system occurs either on sandy or rocky substrates, along the Caribbean coasts, or higher in areas of low rainfall. Few species of thorny trees and shrubs form an open canopy with a maximum height of 5 m, and the herb (mainly grasses) layer is conspicuous. Vegetation cover by annual plants varies due to large quantitative and seasonal rain fluctuations. Cacti are codominant; columnar and tree‑shaped cacti are common. Microphyllous shrubs, small succulent trees, plants in rosettes (such as agaves and terrestrial bromeliads) or evergreen and semi‑deciduous shrubs can also be present. In Puerto Rico, the cactus scrub is associated with limestone pavements. In the Bahamas, this type occurs on limestone pavements with sinkholes and "dogtooth" terrain above the water table. In many areas, this vegetation has an open aspect. The following list of species is diagnostic for this system: *Erithalis fruticosa, Plumeria alba, Stenocereus fimbriatus (= Stenocereus hystrix, = Ritterocereus hystrix), Stenocereus griseus (= Ritterocereus griseus, = Ritterocereus deficiens), Opuntia dillenii, Opuntia militaris, Cylindropuntia hystrix, Rhodocactus cubensis, Consolea macracantha, Dendrocereus nudiflorus, Pilosocereus brooksianus, Agave albescens, Agave missionum, Melocactus acunae, Caesalpinia* spp., *Capparis* spp., *Guaiacum officinale, Jacquinia armillaris (= Jacquinia arborea), Gochnatia, Cordia* spp., *Guettarda, Lantana involucrata, Cercidium* sp., and *Bourreria* cumanensis. In Puerto Rico, the Lesser Antilles, and Bahamas, the following species are typical: *Melocactus intortus, Pilosocereus royenii, Stenocereus fimbriatus, Oplonia spinosa, Croton flavens, Eugenia xerophytica, Calliandra purpurea, Comocladia dodonaea, Chrysobalanus icaco, Tabebuia bahamensis, Psidium longipes, Stigmaphyllon sagraeanum, Manilkara jaimiqui ssp. emarginata (= Manilkara bahamensis)*, and *Coccoloba* spp.

DISTRIBUTION

**Range:** This system is found in the Florida Keys, Puerto Rico, Virgin Islands, and most of the islands of the Greater Antilles and the Lesser Antilles.

**Divisions:** 411:C

**Nations:** BS, CU, DO, HT, JM, PR, TT, US, VI, XC, XD

**Subnations:** FL

CONCEPT

**Environment:** This system occurs in areas of rainshadows created by mountains in areas of extreme temperatures. Xeric areas generally have low and highly seasonal precipitation with a range of 800‑1000 mm annual precipitation, with great inter‑annual variation. The rainy season goes from May through November. The driest months are February and March. Common coastal substrates have poor, shallow soils and typically are limestones terraces, dogtooth limestone, or sandy soils. Overall temperature averages at sea level are mostly in the range 25‑27°C. Annual precipitation ranges from 600 to 1500 mm for the distribution range of this macrogroup. The dry season is usually limited to one period that can last for 2‑6 months, or divided into two periods together lasting up to 8 months. The main dry period is usually between January and April; there may be a second dry period in more southerly latitudes in July to September. The limestone substrate has low water‑retention capacity, and rainfall leaches easily after accumulating in cracks and crevices of variable depth. Other substrates where communities of this macrogroup develop also exhibit actual drought during periods of low rainfall and physiological drought due to impeded drainage and waterlogging during periods of high rainfall.

SOURCES

**References:** Areces‑Mallea et al. 1999, Borhidi 1991, Figueroa Colon 1996, Helmer et al. 2002, International Institute of Tropical Forestry n.d., Josse et al. 2003*

**Version:** 30 Oct 2015	**Stakeholders:** Caribbean, Latin America, Southeast, U.S. Territories

**Concept Author:** C. Josse	**LeadResp:** Latin America

CES411.423 Caribbean Dry Karst Shrubland

**Primary Division:** Caribbean (411)

**Land Cover Class:** Shrubland

**Spatial Scale & Pattern:** Large patch

**Required Classifiers:** Natural/Semi‑natural; Vegetated (>10% vasc.); Upland

**Diagnostic Classifiers:** Limestone

**Concept Summary:** This system occurs on bare rock of limestone terraces and on lowland karstic "dogtooth" terrain. Very dense, 2‑ to 3‑m high shrubland with emergent individual trees or groups of trees. Columnar or tree‑shaped cacti may occur but are never dominant. The Cuban scrub on limestone substrate is dominated by the shrubs *Auerodendron cubense, Cordia leucosebestena, Picrodendron macrocarpum, Eugenia cowellii, Polygala guantanamana, Coccothrinax munizii, Jacquinia berteroi , Randia spinifex*, and cacti *Consolea macracantha, Dendrocereus nudiflorus, Pilosocereus brooksianus, Harrisia fernowii, Melocactus acunae*, and *Agave albescens* (Huggins et al. 2007), while this type in Puerto Rico features the endemic *Harrisia portoricensis* and shrubs *Croton discolor, Croton betulinus, Erithalis fruticosa, Plumeria obtusa, Reynosia uncinata* (Rojas‑Sandoval and Meléndez‑Ackerman 2012, Medina et al. 2014). In the Lesser Antilles typical species of the mixed cactus scrub on limestone pavement include *Pilosocereus royenii, Agave karatto, Pisonia subcordata, Pisonia aculeata, Capparis indica, Capparis cynophallophora, Leucaena leucocephala, Pithecellobium unguis‑cati, Haematoxylum campechianum* and *Clerodendrum aculeatum* (Areces‑Mallea et al. 1999). In the Bahamas, when the limestone pavement community occurs right above the water table and there is more moisture available, characteristic species include *Pithecellobium bahamense, Guettarda scabra, Tabebuia bahamensis, Bursera simaruba, Psidium longipes, Coccoloba northropiae, Coccoloba tenuifolia, Sideroxylon americanum (= Bumelia americana), Stigmaphyllon sagraeanum, Manilkara jaimiqui ssp. emarginata (= Manilkara bahamensis), Cephalocereus* sp., *Randia aculeata* and *Cladium mariscus ssp. jamaicense* (Areces‑Mallea et al. 1999).

DISTRIBUTION

**Range:** Coastal areas of Cuba, the Dominican Republic and Puerto Rico.

**Divisions:** 411:C

**Nations:** CU, DO, PR

CONCEPT

**Environment:** [from M671] Overall temperature averages at sea level are mostly in the range 25‑27°C. Annual precipitation ranges from 600 to 1500 mm for the distribution range of this macrogroup. The dry season is usually limited to one period that can last for 2‑6 months, or divided into two periods together lasting up to 8 months. The main dry period is usually between January and April; there may be a second dry period in more southerly latitudes in July to September. The limestone substrate has low water‑retention capacity, and rainfall leaches easily after accumulating in cracks and crevices of variable depth. Other substrates where communities of this macrogroup develop also exhibit actual drought during periods of low rainfall and physiological drought due to impeded drainage and waterlogging during periods of high rainfall.

**Dynamics:** Droughts and hurricanes are the main drivers of the natural dynamics of this system. Low rainfall intensities of 76 mm/d have a recurrence interval of 1 year while high rainfall intensities of >305 mm/d are possible during hurricane conditions or when low‑pressure systems become stationary. These events have a recurrence interval of 100 years (Gómez Gómez 1984). Forests and other natural ecosystems of the limestone region recover quickly from hurricanes and storms (Wadsworth and Englerth 1959, cited in Lugo et al. 2001). Moreover, these events transport vast amounts of freshwater to the island and trigger many ecologically beneficial functions such as the reproduction of karst forest plants and animals, and the maintenance of the hydrologic cycle of the karst area.

SOURCES

**References:** Areces‑Mallea et al. 1999, Borhidi 1991, Franklin and Steadman 2013, Gómez Gómez 1984, Huggins et al. 2007, Josse et al. 2003*, Lugo et al. 2001, Medina et al. 2014, Murphy and Lugo 1995, Rojas‑Sandoval and Meléndez‑Ackerman 2012a

**Version:** 08 Jan 2015	**Stakeholders:** Caribbean, Latin America, U.S. Territories

**Concept Author:** C. Josse	**LeadResp:** Latin America

CES411.464 Caribbean Serpentine Dry Scrub

**Primary Division:** Caribbean (411)

**Land Cover Class:** Shrubland

**Spatial Scale & Pattern:** Small patch

**Required Classifiers:** Natural/Semi‑natural; Vegetated (>10% vasc.); Upland

**Concept Summary:** This system occurs in gently rolling flatlands or hills up to 450 m elevation. Despite the great variation in substrate, climate, and species composition, the physiognomy of the serpentine scrub is very constant throughout its distribution. It occurs on ferrallitic soils, which are derived from serpentine in isolated locations in the case of Cuba's Cajalbana hills and Holguin area. Examples are dominated by stands of dense, thorny 2‑ to 4‑m high shrubland with emergent palms and evergreen microphyllous trees. The proportion of microphylls and spiny elements is very high, with the exception of cacti which are not common in this type of vegetation. Stands of serpentine scrubs that alternate with small grassy clearings also occur, except for the more humid, higher elevation communities which are dominated by microphylls and do not present grassy clearings. These often develop into dwarf‑grass savannas after grazing or human interference. The following list of species is diagnostic for this system: *Neobracea valenzuelana, Phyllanthus orbicularis, Phyllanthus comosus, Annona bullata, Pilosocereus royenii, Thouinia striata var. portoricensis, Rondeletia camarioca, Zanthoxylum dumosum, Passiflora cubensis, Ipomoea cordatotriloba (= Ipomoea carolina), Tabebuia lepidota, Coccothrinax fragrans, Copernicia* spp., *Coccothrinax* spp., *Jacaranda cowellii, Jacquinia shaferi, Myrtus cabanesensis, Hemithrinax savannarum, Hemithrinax rivularis, Acrosynanthus minor, Tabebuia linearis, Antirhea abbreviata, Antirhea orbicularis, Exostema purpureum, Spirotecoma apiculata, Byrsonima bucheri, Sideroxylon cubense (= Dipholis cubensis), Coccoloba* spp., *Paepalanthus brittonii*, and many other very restricted endemics.

DISTRIBUTION

**Range:** This system is found in Cuba and Puerto Rico.

**Divisions:** 411:C

**Nations:** CU, PR

CONCEPT

**Environment:** Ferrallitic soils on isolated sites occurring on the coastal zone and up to lower montane places. Annual precipitation ranges from 1000‑1900 mm, with one or two dry seasons annually. Due to the plant physiology imposed by the limiting factors of soils derived from serpentines, these communities represent a drier degree than a community living in the same climatic conditions but on non‑serpentine rock. Adaptations include xeromorphism or pseudo‑xeromorphism, reduced productivity, reduced structure, and advantage of sclerophyllous evergreen shrubs over deciduous trees/shrubs.

SOURCES

**References:** Areces‑Mallea et al. 1999, Borhidi 1991, Figueroa Colon 1996, Josse et al. 2003*

**Version:** 08 Jan 2015	**Stakeholders:** Caribbean, Latin America, U.S. Territories

**Concept Author:** C. Josse	**LeadResp:** Latin America

M672. Northern Mesoamerican Pine Savanna

CES402.590 Sabana de Pino de Mesoamérica

**Primary Division:** Moist Meso‑America (402)

**Land Cover Class:** Steppe/Savanna

**Spatial Scale & Pattern:** Large patch

**Required Classifiers:** Natural/Semi‑natural; Vegetated (>10% vasc.); Upland

**Diagnostic Classifiers:** Lowland [Lowland]; Grassland, Savanna, Steppe (graminoid‑dominated)

**Concept Summary:** Este sistema representa las sabanas arboladas donde *Pinus caribaea* es la especie distintiva aunque se presenta dispersa y mezclada con otras especies arbustivas y arbóreas. Ocurre en la planicie costera del Caribe de Belice, Honduras y Nicaragua. Esta sabana es del tipo estacional, es decir con deficiencia hídrica durante la estación seca, pero con anegamiento durante la estación lluviosa sobre todo en las partes bajas del terreno. Los suelos son genralmente pobres y ácidos, arcillosos a arcilloso‑arenosos. El sistema está sujeto a quemas y a tala selectiva. La siguiente lista de especies es diagnóstica para este sistema: *Axonopus aureus, Fimbristylis paradoxa, Declieuxia fruticosa, Rhynchospora* spp., *Thrasya trinitensis, Thrasya mosquitensis, Blechnum serrulatum, Acoelorraphe wrightii, Orbignya cohune, Pinus caribaea, Pinus tecunumanii, Quercus oleoides, Byrsonima crassifolia, Calliandra houstoniana, Xilopia frutescens, Myrica cerífera, Miconia* spp., *Ilex guianensis*.

DISTRIBUTION

**Divisions:** 402:C

**Nations:** BZ, HN, NI

CONCEPT

**Environment:** Relieve plano a ligeramente ondulado de la planicie costera o en cerros hasta los 600‑700 msnm (Mountain Pine Forest of western Belize). Suelos de los tipos ultisoles ácidos e inceptisoles, pueden ser higromórficos y generalmente son pobres, en sectores se presentan bancos de grava cuarzosa. La profundidad de la capa laterítica impermeable es variable y sobre ella ocurren suelos arenosos o arcilloso‑arenosos. Clima tropical húmedo. Totalmente de clima estacional con una marcada estación seca, entre febrero y junio. La temperatura varía entre 16°C y 32°C con una precipitación anual de alrededor de 1.500 mm. Suelos de textura gruesa que son típicos de los climas extremadamente estacionales con alguna evidencia de la inundación prolongada durante los períodos húmedos y estaciones secas afectadas por la quema.

**Vegetation:** Sabana arbolada, con mucha diversidad de especies leñosas y también de gramíneas, dependiendo del gradiente de humedad del suelo. Lso pinos generalmene crecen en los sitios menos propensos a la inundación. El estrato leñoso llega a 15 m de alto.

**Dynamics:** Una de las amenazas principales que se ha detectado es los ataques de insectos (*Dendroctonus* spp. e Ips spp.) a los árboles de pino, causando la mortalidad de 60% o mas de los individuos. También son sabanas sujetas a quemas para la producción de pastos, tala y cacería de fauna para subsistencia (Hicks et al. 2011).

SOURCES

**References:** Hicks et al. 2011, Josse et al. 2003*, Meyrat et al. 2001, WWF and IUCN 1997

**Version:** 08 Jan 2015	**Stakeholders:** Latin America

**Concept Author:** C. Josse	**LeadResp:** Latin America

CES402.621 Sabana Inundada de Pino de Mesoamérica

**Primary Division:** Moist Meso‑America (402)

**Required Classifiers:** Natural/Semi‑natural; Vegetated (>10% vasc.)

**Concept Summary:** Este sistema representa una variente de las sabanas de *Pinus caribaea* que ocurre en la planicie costera del Caribe de Belice, Honduras y Nicaragua done hay inundación periódicamente. Esta sabana es del tipo estacional, es decir que todavía hay deficiencia hídrica durante la estación seca, pero con inundación durante la estación lluviosa sobre todo en las partes bajas del terreno. Los suelos son generalmente pobres y ácidos, arcillosos a arcilloso‑arenosos. El sistema está sujeto a quemas y a tala selectiva.

**Nations:** HN, NI

CONCEPT

**Environment:** [de M672] Sarmiento (1983) reconoce tres tipos de sabanas neotropicales: (1) Sabana Semiestacional que se produce en un clima más húmedo, con una o dos temporadas cortas secas e incendios menos frecuentes (algunas de las sabanas amazónicas y de Guayana), (2) Sabana Estacional se caracteriza por una estación seca severa y los frecuentes incendios (por ejemplo, el Cerrado y los Llanos), (3) Sabana Hyperestacional que es el resultado de la sequía y los incendios excesivos durante la temporada seca y grandes inundaciones durante la estación lluviosa. Este tipo sabana es común en las tierras bajas de mal drenaje del Pantanal, Llanos de Moxos, las sabanas de Roraima‑Rupununi, y parte de los Llanos. Rodales de palmeras se encuentran a menudo en las zonas anegadas.<br />

El sistema de la sabana es generalmente heterogéneo, consiste en un mosaico de pastizales puros, parches de árboles o arbustos, bosques secos o semideciduos, bosques de galería y en ocasiones, los humedales. La distribución de las distintas comunidades en un paisaje de sabana a menudo sigue gradientes edáficos (por ejemplo, los tipos de suelo o niveles de la capa freática) (Daly y Mitchell 2000).

Al igual que otras sabanas tropicales, la sabana de pino es un ecosistema estructuralmente simple pero espacialmente irregular. Se caracteriza por una capa de plantas herbáceas ‑principalmente C 4, pastos y juncos, y herbáceas C3 ‑ con diversos grados de arbustos y / o árboles, es decir, un continuo que va desde pastizales sin árboles hasta parches de bosques densos. Las sabanas se dan en climas cálidos con precipitaciones que varían entre (750) 1.000 mm y 2.000 (‑2.500) mm y un período seco de uno a seis meses, ocupan una zona de transición entre el bosque húmedo y la vegetación xerófila. La distribución de las precipitaciones es un factor determinante de los tipos de vegetación de sabana (por ejemplo, pastizales o bosques). La sabana tropical generalmente se desarrolla en suelos deficientes en nutrientes, ácidos con la toxicidad del aluminio y con alteración pronunciada de condiciones húmedas y secas (Fry 1983).

La relación / tallo alto raíz en los ecosistemas de sabana, especialmente en el estrato herbáceo, es una característica que proporciona resistencia al estrés y la perturbación causados por la sequía, el fuego y la herbivoría. Se cree que las sabanas tropicales se han desarrollado bajo factores de perturbación como el fuego, la herbivoría y sequía. La persistencia de las sabanas puede depender de que la perturbación de preservar la estabilización de los componentes y propiedades (Coupland 1992c, Baruch et al. 1996, Silva 1996).

[from M672] Sarmiento (1983) recognizes three types of Neotropical savannas: (1) Semiseasonal savanna which occurs in a rather humid climate with one or two short dry seasons and less frequent fires (some of the Amazonian and Guianan savannas), (2) Seasonal savanna characterized by a severe dry season and frequent fires (e.g., the Cerrado and the Llanos), (3) Hyperseasonal savanna which is the result of excessive drought and fires during dry season and severe flooding during the wet season. This savanna type is common in the poorly drained bottomlands of the Pantanal, Llanos de Moxos, the Roraima‑Rupununi savannas, and part of Llanos. Palm stands are often found in water‑logged areas.

The savanna system is usually heterogeneous, consisting of a mosaic of pure grasslands, patches of trees or shrubs, dry or semi‑deciduous forests, gallery forests, and sometimes wetlands. The distribution of various communities in a savanna landscape often follows edaphic gradients (e.g., soil types or levels of water table) (Daly and Mitchell 2000). Like other tropical savannas, pine savanna is structurally simple but spatially patchy ecosystem. It is characterized by a layer of herbaceous plants‑ mainly C 4 grasses and sedges, and C3 forbs‑ with varying degrees of shrubs and/or trees, that is, a continuum from treeless grassland to dense woodland.

Savannas occur in hot climates with rainfall varying between (750‑) ,000 mm and 2000 (‑2500) mm and a dry period of one to six months, forming a transition zone between moist forest and xerophytic vegetation. Rainfall distribution is a major determinant of the savanna vegetation types (e.g., grassland or woodland). Tropical savanna usually develops on nutrient deficient, acidic soils with aluminum toxicity and pronounced alteration of wet and dry conditions (Fry 1983).

The high root/shoot ratio in savanna ecosystems, especially within the herbaceous layer, is a feature that provides resistance to stress and disturbance from drought, fire, and herbivory. It is believed that tropical savannas have developed under disturbance factors like fire, herbivory and drought. Persistence of savannas may depend on such disturbance to preserve stabilizing components and properties (Coupland 1992, Baruch et al. 1996, Silva 1996).

**Dynamics:** [de M672] Intervalo de incendios: la frecuencia de incendios deben estar dentro de un rango esperado para el tipo (por ejemplo, 5 a 20 años); incendios demasiado frecuentes dan como resultado la pérdida de la diversidad nativa y la supresión de resulta en una densa invasión de especies leñosas (y la pérdida de especies nativas).

La sequía y el régimen de crecidas: Donde están inundadas las sabanas de pino, en general también hay una estación seca de 3‑9 meses. La densidad de arboles y arbustos tiende a ser mayor cuando la la inundación es más profunda. La distribución de clases de edad de los pinos forma una curva de J invertida que refleja una amplia gama de diámetros resultantes de perturbaciones periódicas y regeneración exitosa.

[from M672] Fire Return Interval: fire frequency should fall within an expected range for the type (e.g., 5‑20 years); too frequent fires result in loss of native diversity and fire suppression results in dense woody species encroachment (and loss of native species).

Drought and Flood Regime: Where pine savannas are inundated, there is generally a 3‑9 month dry season. Tree and shrub density tends to be higher with deeper inundation. Age class distribution of pine trees forms an inverted J curve reflecting a full range of diameters resulting from periodic disturbance and successful regeneration.

SOURCES

**References:** Latin American Ecology Working Group n.d.*

**Stakeholders:** Latin America

**Concept Author:** P. Comer	**LeadResp:** Latin America

M673. Northern Mesoamerican Savanna & Shrubland

CES401.310 Sabana de Campeche Veracruz

**Primary Division:** Dry Meso‑America (401)

**Land Cover Class:** Steppe/Savanna

**Spatial Scale & Pattern:** Large patch

**Required Classifiers:** Natural/Semi‑natural; Vegetated (>10% vasc.); Upland

**Diagnostic Classifiers:** Lowland [Foothill]; Grassland, Savanna, Steppe (graminoid‑dominated); Aridic

**Concept Summary:** This system is the most prevalent savanna type of southern Mexico, found on the Caribbean slopes on open plains with dense grasses and scattered trees, and also on rolling hills. Much of their present extension can be of anthropogenic origin. It is likely that natural savannas are the ones occurring on seasonally saturated soils (e.g., surrounding Laguna de Términos in Campeche) or on slopes with shallow soils. The following list of species is diagnostic for this system: Grass species of *Paspalum, Andropogon, Aristida, Imperata, Trichachne, Leptocoryphium, Axonopus, Digitaria*, and woody species: *Curatella americana, Byrsonima crassifolia, Crecentia alata, Crecentia cujete*.

DISTRIBUTION

**Divisions:** 401:C

**Nations:** MX

**Subnations:** MXCA, MXHI, MXOA, MXPU, MXTL, MXVE

CONCEPT

**Environment:** These are typically found on old alluvial plains. Some small portions may be inundated for part of the growing season.

**Vegetation:** Dense grasses dominate, with scattered shrub or short tree species reaching 3‑6m high. Some landscapes support a recurring mosaic of open savanna with "islands" of dense forest composed of the tree species found across the more open savanna.

**Dynamics:** Natural fire regimes are not well‑documented, but likely were within 1‑5 years.

SOURCES

**References:** Josse et al. 2003*, Pennington and Sarukhán 1998, Rzedowski 1986

**Version:** 08 Jan 2015	**Stakeholders:** Latin America

**Concept Author:** C. Josse	**LeadResp:** Latin America

CES401.290 Sabana de Guerrero

**Primary Division:** Dry Meso‑America (401)

**Land Cover Class:** Steppe/Savanna

**Spatial Scale & Pattern:** Large patch

**Required Classifiers:** Natural/Semi‑natural; Vegetated (>10% vasc.); Upland

**Diagnostic Classifiers:** Lowland [Foothill]; Grassland, Savanna, Steppe (graminoid‑dominated); Aridic

**Concept Summary:** This system is a relatively uncommon savanna type of southern Mexico, found on open plains with dense grasses and scattered trees along coastal portions of Guerrero, Oaxaca, Chiapas, and into Guatemala. These savannas extend to the lower slopes of the Sierra Madre Occidental and Sierra Madre del Sur, in isolated occurrences usually growing on shallow soils over quartzitic stone or gravels. The following list of species is diagnostic for this system: Grass species of *Paspalum, Andropogon, Bouteloua, Aristida, Imperata, Trichachne, Leptocoryphium, Axonopus, Digitaria*, and woody species: *Brahea dulcis, Curatella americana, Byrsonima crassifolia, Crescentia alata, Crescentia cujete*.

DISTRIBUTION

**Divisions:** 401:C

**Nations:** GT, MX

**Subnations:** MXCP, MXGU, MXNA, MXOA

CONCEPT

**Environment:** These are typically found on old alluvial plains. Some small portions may be inundated for part of the growing season. The savannas on the lower slopes of the cordillera usually grow on shallow, eroded soils over quarcitic stone or mixed with gravels.

**Vegetation:** Dense grasses dominate, with scattered shrub, short tree, and palm species reaching 3‑6m high. Some landscapes support a recurring mosaic of open savanna with "islands" of dense forest composed of the tree species found across the more open savanna.

**Dynamics:** Natural fire regimes are not well documented, but likely were within 1‑5 years.

SOURCES

**References:** Brown et al. 1998, Josse et al. 2003*, Pennington and Sarukhán 1998, Rzedowski 1986

**Version:** 17 Jul 2003	**Stakeholders:** Latin America

**Concept Author:** C. Josse	**LeadResp:** Latin America

2.A.2. Tropical Montane Grassland & Shrubland

2.A.2.Eb. Caribbean‑Mesoamerican Montane & High Montane Grassland & Shrubland

M691. Mesoamerican Montane Grassland & Shrubland

CES402.610 Herbazal Montano Alto de Talamanca

**Primary Division:** Moist Meso‑America (402)

**Land Cover Class:** Steppe/Savanna

**Spatial Scale & Pattern:** Large patch

**Required Classifiers:** Natural/Semi‑natural; Vegetated (>10% vasc.); Upland

**Diagnostic Classifiers:** Montane [Upper Montane]; Grassland, Savanna, Steppe (graminoid‑dominated); Tropical/Subtropical [Tropical Pluvial]

**Concept Summary:** Este sistema es muy similar al de los páramos andinos, aunque la dominancia de otras especies y en algunos casos, formas de vida diferentes de los pajonales, lo convierten en un sistema distinto. En este caso las herbáceas también son dominantes y entre ellas, una forma bambusoidea del género *Chusquea*. Estos pajonales y herbazales se saturan estacionalmente, especialmente en las partes deprimidas del terreno. La siguiente lista de especies es diagnóstica para este sistema: *Aciachne pulvinata, Lorenzochloa rectifolia, Chusquea subtessellata, Cortaderia apalotricha, Myrrhidendron donnell‑smithii, Rumex costaricensis, Calamagrostis, Carex, Azorella, Alchemilla, Acaena, Castilleja, Centropogon, Coriaria, Vaccinium, Cavendishia, Senecio, Diplostephium, Monnina, Miconia, Relbunium, Arcytophyllum, Pentacalia, Hypericum*.

This system is very similar to the Andean páramos, although the dominance of other species and in some cases, different lifeforms, make it a different system. In this case the forbs are also dominant and among them, the bambu‑like genus *Chusquea*. This steppe and grassland is seasonally saturated, particularly in low areas. The above list of species is diagnostic for this system.

DISTRIBUTION

**Divisions:** 402:C

**TNC Ecoregions:** NT0122:?, NT0167:C

**Nations:** CR, PA

CONCEPT

**Environment:** Ocurren sobre suelos inceptisoles y andosoles, en ocasiones sobre histosoles, estacionalmente pueden saturarse, así como también sufrir déficit hídrico. Las pendientes son moderadas y el drenaje es lento.

Occurs on Inceptisols and Andisols, sometimes on Histosols where seasonally it can be saturated and alternatively, experience drought. Slopes are moderate and drainage is generally slow.

**Vegetation:** Vegetación predominante es herbácea, en forma de pajonal o formando almohadillas. En algunos sitios los individuos de *Chusquea subtessellata* dominan visualmente y a menudo en asociación con el helecho *Blechnum*. También se encuentran arbustos.

**Dynamics:** Frío, la formación de nubes, el viento y la precipitación pesada apoyo developmnet de este ecosistema alpino. El fuego es aparentamente un factor importante en su mantenimiento (League y Horn 2000)

Cold temperature, cloud formation, wind, and heavy precipitation support developmnet of this alpine ecosystem. Fire is apparently an important factor in its maintenance (League and Horn 2000).

SOURCES

**References:** Gómez 1986, Josse et al. 2003*, League and Horn 2000, Meyrat et al. 2001

**Version:** 08 Jan 2015	**Stakeholders:** Latin America

**Concept Author:** C. Josse	**LeadResp:** Latin America

CES305.284 Zacatonal Transvolcánico y de la Sierra Madre

**Primary Division:** Sierra Madre (305)

**Land Cover Class:** Forest and Woodland

**Spatial Scale & Pattern:** Small patch

**Required Classifiers:** Natural/Semi‑natural; Vegetated (>10% vasc.); Upland

**Diagnostic Classifiers:** Montane [Montane]; Forest and Woodland (Treed); Udic

**Concept Summary:** These are high‑elevation meadows found primarily along volcanic slopes around the Valley of Mexico. They are dominated by herbaceous species found on wetter sites with very low‑velocity surface and subsurface flows. They range in elevation from upper montane to alpine (3000‑3300 m). Sites typically are small depressions and flats with deep, poorly drained soils of sandy loam (pH 5.0‑6.2) surrounding volcanic craters. This system often occurs as a mosaic of several plant associations, often dominated by graminoids. Often alpine dwarf‑shrublands are immediately adjacent to these praderas. These systems are typically not subjected to high disturbance events such as flooding. The following list of species is diagnostic for this system: *Potentilla candicans, Stipa ichu, Astragalus micranthus, Reseda luteola, Bidens triplinervia, Hedeoma piperitum, Vulpia myuros, Gnaphalium seemannii, Salvia* spp.

DISTRIBUTION

**Divisions:** 305:C

**Nations:** GT, MX

**Subnations:** MXCH, MXCO, MXCP, MXDU, MXGJ, MXGU, MXHI, MXJA, MXMI, MXNU, MXOA, MXPU, MXSL, MXSO, MXVE, MXZA

CONCEPT

**Environment:** Sites typically are small depressions and flats with deep, poorly drained soils of sandy loam (pH5.0‑6.2) surrounding volcanic craders.

**Vegetation:** These are primarily open meadows with scattered shrubs.

**Dynamics:** River scour exposes moist soils and supports regeneration of some riparian species. Periodic flooding and disturbance maintains a dynamic vegetative mosaic.

SOURCES

**References:** Josse et al. 2003*, Rzedowski 1986, Velazquez et al. 2000

**Version:** 17 Jan 2013	**Stakeholders:** Latin America

**Concept Author:** C. Josse	**LeadResp:** Latin America

2.A.3. Tropical Scrub & Herb Coastal Vegetation

2.A.3.Ee. Caribbean‑Mesoamerican Dune & Coastal Grassland & Shrubland

M700. Caribbean‑Mesoamerican Coastal Dune & Beach

CES402.601 Colinas de Carso del Litoral de Belize

**Primary Division:** Moist Meso‑America (402)

**Land Cover Class:** Barren

**Spatial Scale & Pattern:** Small patch

**Required Classifiers:** Natural/Semi‑natural; Unvegetated (<10% vasc.)

**Diagnostic Classifiers:** Lowland [Lowland]; Shrubland (Shrub‑dominated); Ustic

**Concept Summary:** El sistema representa las comunidades casmófitas que se desarrollan en afloramientos cársticos en la costa Caribe y reciben la brisa marina. La vegetación es dispersa y esclerófila y está compuesta de hierbas y arbustos bajos. La siguiente lista de las especies es de diagnóstica para este sistema: *Coccoloba uvifera, Dactyloctenium aegyptium, Gomphrena* spp., *Jacquinia armillaris (= Jacquinia arborea), Phyla nodiflora (= Lippia nodiflora), Neea psychotrioides, Pancratium littorale, Sesuvium portulacastrum, Stachytarpheta jamaicensis, Tridax procumbens*.

DISTRIBUTION

**Divisions:** 402:C

**Nations:** BZ

CONCEPT

**Environment:** Relieve colinado, sustrato rocoso bien drenado, influencia de brisa marina.

**Vegetation:** Vegetación baja herbácea y arbustiva, escasa, predominan las rocas desnudas.

**Dynamics:** Dinámico

SOURCES

**References:** Josse et al. 2003*, Meyrat et al. 2001

**Version:** 08 Jan 2015	**Stakeholders:** Latin America

**Concept Author:** C. Josse	**LeadResp:** Latin America

CES411.271 South Florida Shell Hash Beach

**Primary Division:** Caribbean (411)

**Land Cover Class:** Barren

**Spatial Scale & Pattern:** Small patch

**Required Classifiers:** Natural/Semi‑natural; Unvegetated (<10% vasc.); Upland

**Diagnostic Classifiers:** Coast; Beach (Substrate); Graminoid

**Concept Summary:** This system represents carbonate sand beaches of the Florida Keys and south Florida mangrove islands (after Johnson and Barbour 1990). The vegetation is poorly known but apparently includes at least one endemic species, *Chamaesyce garberi*. Other diagnostic species may include *Piscidia piscipula* and *Pithecellobium keyense*.

**Comments:** No associations have currently been described in the NVC for this system. More information is needed. See Johnson and Barbour (1990) for more information.

DISTRIBUTION

**Range:** The range of this system includes Cape Sable (the southernmost point of mainland Florida), Ten Thousand Islands (Collier County), Florida Keys, and islands in Biscayne Bay (near Miami).

**Divisions:** 411:C

**TNC Ecoregions:** 54:C

**Nations:** US

**Subnations:** FL

**Map Zones:** 56:C

CONCEPT

SOURCES

**References:** Comer et al. 2003*, FNAI 2010a, Johnson and Barbour 1990

**Version:** 17 Mar 2009	**Stakeholders:** Southeast

**Concept Author:** R. Evans	**LeadResp:** Southeast

CES411.272 Southeast Florida Beach

**Primary Division:** Caribbean (411)

**Land Cover Class:** Barren

**Spatial Scale & Pattern:** Small patch

**Required Classifiers:** Natural/Semi‑natural; Unvegetated (<10% vasc.); Upland

**Diagnostic Classifiers:** Coast; Beach (Substrate); Graminoid

**Concept Summary:** This beach ecological system is the southernmost of its kind along the mainland coast of North America. Its southerly location distinguishes it from other types along the Atlantic Coast, primarily due to the prevalence of the tropical flora it supports. This type is related to Southwest Florida Beach (CES411.276) but is affected directly by much higher wave energy from the Atlantic. This region has some of the highest wave energy along the entire Atlantic Coast.

**Comments:** Apparently few, if any, associations have currently been described in the NVC for this system. More information is needed.

DISTRIBUTION

**Range:** Endemic to south Florida.

**Divisions:** 411:C

**TNC Ecoregions:** 54:C

**Nations:** US

**Subnations:** FL

**Map Zones:** 56:C

CONCEPT

**Environment:** Its southerly location distinguishes this system from others along the Atlantic Coast, primarily due to the prevalence of the tropical flora it supports. This system is affected directly by much higher wave energy from the Atlantic than the beaches on the southwest coast of Florida. The southeast coastal region has some of the highest wave energy along the entire Atlantic Coast (Tanner 1960).

**Dynamics:** This region has some of the highest wave energy along the entire Atlantic Coastal Plain (Tanner 1960). The process of sand movement due to the forces of wind and water are part of the natural dynamics of beach ecosystems. This includes transport of sand along the coast, and movement of sand by wind or water between the dunes, beach and subtidal areas. If not restricted by infrastructure or engineered hard structures, beaches and dunes can migrate as coastlines change over time in response to the action of wind and water. The beaches of southeast Florida are affected by two tides per day.

SOURCES

**References:** Comer et al. 2003*, Defeo et al. 2009, Tanner 1960

**Version:** 14 Jan 2014	**Stakeholders:** Southeast

**Concept Author:** R. Evans	**LeadResp:** Southeast

CES411.276 Southwest Florida Beach

**Primary Division:** Caribbean (411)

**Land Cover Class:** Barren

**Spatial Scale & Pattern:** Small patch

**Required Classifiers:** Natural/Semi‑natural; Unvegetated (<10% vasc.); Upland

**Diagnostic Classifiers:** Coast; Beach (Substrate)

**Concept Summary:** This system ranges from Anclote Key southward to Cape Romano, Florida. Within the northern Gulf of Mexico region, these beaches are distinguished by the highest species richness, greatest cover of succulents, and high cover of *Iva imbricata* and several tropical species. Sands are relatively coarse and, unlike other beach systems of the northern Gulf of Mexico, are extremely rich in calcium from an abundance of calcareous shell fragments.

**Comments:** Apparently, few associations have currently been described in the NVC for this system. More information is needed.

DISTRIBUTION

**Range:** This system ranges from Anclote Key (border of Pasco and Pinellas counties) southward to Cape Romano, Florida (Collier County) (Johnson and Barbour 1990).

**Divisions:** 411:C

**TNC Ecoregions:** 54:C

**Nations:** US

**Subnations:** FL

**Map Zones:** 56:C

CONCEPT

**Environment:** The substrate of these beaches is composed of relatively coarse sands and, unlike other beach systems of the northern Gulf of Mexico, are extremely rich in calcium from an abundance of calcareous shell fragments.

**Vegetation:** Within the northern Gulf of Mexico region, these beaches are distinguished by the highest species richness, greatest cover of succulents, and high cover of *Iva imbricata* and several tropical species (Barbour et al. 1987). These beaches are dominated by *Uniola*, but less so than other beach systems of the northern Gulf of Mexico. Other important species are *Oenothera humifusa, Scaevola plumieri*, and *Sesuvium portulacastrum*.

SOURCES

**References:** Barbour et al. 1987, Comer et al. 2003*, FNAI 2010a, Johnson and Barbour 1990

**Version:** 23 Feb 2010	**Stakeholders:** Southeast

**Concept Author:** R. Evans	**LeadResp:** Southeast

2.A.3.Eg. Tropical Eastern Pacific Dune & Coastal Grassland & Shrubland

M703. Tropical Eastern Pacific Coastal Beach & Dune

CES402.598 Vegetacion de Playas Marinas del Pacifico

**Primary Division:** Moist Meso‑America (402)

**Land Cover Class:** Shrubland

**Spatial Scale & Pattern:** Linear

**Required Classifiers:** Natural/Semi‑natural; Vegetated (>10% vasc.); Upland

**Diagnostic Classifiers:** Lowland [Lowland]; Shrubland (Shrub‑dominated); Tidal / Estuarine [Haline]; Ustic

**Concept Summary:** El sistema incluye tanto a las comunidades que se desarrollan en las playas y sobre dunas, compuestas principalmente por plantas herbáceas esclerófilas, algunas de ellas con hojas suculentas, como los bosques bajos abiertos que se desarrollan sobre los sedimentos costeros muy recientes y moderadamente drenados. Este ambiente está dominado por arbustos y árboles bajos y palmas, tanto nativas como la introducida palma de coco. Las gramíneas están presentes pero no son dominantes en ninguno de los dos ambientes. Generalmente son suelos que no reciben constantemente la influencia de las mareas, salvo aguajes excepcionales, sin embargo por filtración puede haber saturación de capas profundas del suelo. La siguiente lista de especies es diagnóstica para este sistema:*Coccoloba uvifera, Elaeis guianensis, Hibiscus tiliaceus, Caesalpinia bonduc (= Caesalpinia crista), Pithecellobium dulce, Prosopis juliflora, Pithecellobium dulce, Bromelia karatas, Crataeva tapia, Coccoloba floribunda, Pithecellobium oblongum, Hippomane mancinella, Acacia farnesiana, Uniola pittieri, Joubea pilosa, Cenchrus echinatus, Ipomoea pes‑caprae, Heliotropium curassavicum, Calotropis gigantea, Canavalia rosea, (= Canavalia maritima), Vigna peduncularis, Crotalaria* spp., *Opuntia lutea, Croton niveus, Caesalpinia bonduc (= Caesalpinia crista)*.

DISTRIBUTION

**Divisions:** 402:C

**Nations:** CO, CR, EC, NI, PA, SV

CONCEPT

**Environment:** Se encuentra sobre la línea de costa, más arriba que el nivel superior de la marea. En ocasiones cubre dunas y llega a estabilizarlas.

**Dynamics:** Dinámica marina activa e intervención para desarrollo turístico y camaroneras.

SOURCES

**References:** Josse et al. 2003*, Meyrat et al. 2001

**Version:** 08 Jan 2015	**Stakeholders:** Latin America

**Concept Author:** C. Josse	**LeadResp:** Latin America

2.B. Temperate & Boreal Grassland & Shrubland

2.B.1. Mediterranean Scrub & Grassland

2.B.1.Na. Californian Scrub & Grassland

M045. Californian Annual & Perennial Grassland

CES206.942 California Central Valley and Southern Coastal Grassland

**Primary Division:** Mediterranean California (206)

**Land Cover Class:** Herbaceous

**Spatial Scale & Pattern:** Matrix

**Required Classifiers:** Natural/Semi‑natural; Vegetated (>10% vasc.); Upland

**Diagnostic Classifiers:** Herbaceous; Valley; Toeslope/Valley Bottom; Mediterranean [Mediterranean Xeric‑Oceanic]; Eutrophic Soil; Clay Soil Texture; F‑Landscape/Low Intensity; Graminoid; Nassella pulchra

**National Mapping Codes:** EVT 2129; ESLF 7101; ESP 1129

**Concept Summary:** This ecological system is found from 10‑1200 m (30‑3600 feet) elevation, in the Great Central Valley and along the southern coastal regions of California. It receives on average 50 cm (range 25‑100 cm) of precipitation per year, mainly as winter rain. It is found with fine‑textured soils, moist or even waterlogged in winter, but very dry in summer. Historically, these grasslands were common among oak savanna and woodland and probably experienced similar frequent fire regimes. Characteristic plant species include *Nassella pulchra, Aristida* spp., *Achillea millefolium var. borealis, Achyrachaena mollis, Agoseris heterophylla, Bloomeria crocea, Triteleia ixioides, Chlorogalum pomeridianum, Clarkia purpurea, Dodecatheon jeffreyi, Elymus glaucus, Leymus triticoides, Festuca californica, Melica californica, Castilleja attenuata*, and *Poa secunda*.

DISTRIBUTION

**Range:** Found from in California from 10‑1200 m (30‑3600 feet) elevation, in the Great Central Valley and along the southern coastal region.

**Divisions:** 206:C

**TNC Ecoregions:** 13:C, 15:P, 16:P

**Nations:** US

**Subnations:** CA

**Map Zones:** 4:C, 5:C

**USFS Ecomap Regions:** 261B:CC, 262A:CC, M261C:??

CONCEPT

**Environment:** This ecosystem occurs from 10 to 1200 m (30‑3600 feet) in elevation; receiving on average 50 cm (range 25‑100 cm) of precipitation per year, mainly as winter rain. It is found with deep fine‑textured soils, moist or even waterlogged in winter, but very dry in summer (Sawyer et al. 2009).

**Vegetation:** Characteristic plant species include *Nassella pulchra, Aristida* spp., *Achillea millefolium var. borealis (= Achillea borealis), Achyrachaena mollis, Agoseris heterophylla, Bloomeria crocea, Triteleia ixioides (= Brodiaea lutea), Chlorogalum pomeridianum, Clarkia purpurea, Dodecatheon jeffreyi, Elymus glaucus, Leymus triticoides, Festuca californica, Melica californica, Castilleja attenuata (= Orthocarpus attenuatus)*, and *Poa secunda (= Poa scabrella)*.

**Dynamics:** These grasslands have evolved to survive fire and long seasonal droughts (Keeley 2006). Invasion of non‑native annual grasses out‑compete natives through prolific seed production and the ability to re‑seed quickly after fires, which generally means they maintain themselves at the expense of the native grasses and forbs (Sawyer et al. 2009).

SOURCES

**References:** Barbour and Billings 2000, Barbour and Major 1988, Bartolome et al. 2007, Comer et al. 2003*, Holland and Keil 1995, Keeler‑Wolf pers. comm., Keeley 2006a, PRBO Conservation Science 2011, Sawyer and Keeler‑Wolf 1995, Sawyer et al. 2009, Shiflet 1994, Stromberg et al. 2007

**Version:** 14 Jan 2014	**Stakeholders:** West

**Concept Author:** P. Comer and T. Keeler‑Wolf	**LeadResp:** West

CES206.943 California Mesic Serpentine Grassland

**Primary Division:** Mediterranean California (206)

**Land Cover Class:** Herbaceous

**Spatial Scale & Pattern:** Large patch

**Required Classifiers:** Natural/Semi‑natural; Vegetated (>10% vasc.); Upland

**Diagnostic Classifiers:** Herbaceous; Mediterranean [Mediterranean Xeric‑Oceanic]; Ultramafic with low Ca:Mg ratio; Deep Soil; Udic; Calamagrostis ophitidis

**National Mapping Codes:** EVT 2130; ESLF 7102; ESP 1130

**Concept Summary:** These grasslands are of very limited distribution in California within the Coast Ranges, Sierra Nevada, and Transverse Ranges on deep soils with serpentine‑rich parent material. Not all serpentinite outcrops support distinct vegetation; only those with very low Ca:Mg ratios impact biotic composition. In this system, native bunchgrass dominates, though typically in less dense cover than other perennial bunchgrass types. Characteristic species include *Calamagrostis ophitidis, Eschscholzia californica, Vulpia microstachys var. ciliata (= Festuca grayi), Poa secunda (= Poa scabrella), Hemizonia congesta ssp. luzulifolia (= Hemizonia luzulifolia), Nassella cernua*, and *Nassella pulchra*. Historic fire regimes in this system are not well known.

DISTRIBUTION

**Range:** This system is found in the Coast Ranges, Sierra Nevada, and Transverse Ranges of California on deep soils with serpentine‑rich parent material. It may also occur on serpentine in the Klamath Mountains of southern Oregon.

**Divisions:** 206:C

**TNC Ecoregions:** 5:C, 12:C, 14:C, 15:P

**Nations:** US

**Subnations:** CA

**Map Zones:** 1:?, 2:?, 3:C, 4:C, 6:C, 7:P

**USFS Ecomap Regions:** 261B:??, 263A:??, M261A:CP, M261B:CC, M261C:CP, M261E:CC, M261F:C?

CONCEPT

**Environment:** This ecosystem occurs on deep soils with serpentine‑rich parent material. Not all serpentinite outcrops support distinct vegetation; only those with very low Ca:Mg ratios impact biotic composition.

**Dynamics:** Serpentine soils are relatively infertile soils and mycorrhizal relationships are considered important to plant survival (Jimerson et al. 1995). Hopkins 1986 (as cited in Jimerson et al. 1995) found that 98% of the herbaceous plants in the serpentine grassland communities of the Santa Cruz Mountains were mycorrhizal. Ectomycorrhizae are often associated with members of the Ericaceae family, a well‑represented family in the serpentine flora (Jimerson et al. 1995).

SOURCES

**References:** Barbour and Major 1988, CNRA 2009, Comer et al. 2003*, Evens and San 2004, Faber‑Langendoen et al. 2008b, Holland and Keil 1995, Jimerson et al. 1995, Keeler‑Wolf pers. comm., Kruckberg 1984, PRBO Conservation Science 2011, Sawyer and Keeler‑Wolf 1995, Sawyer et al. 2009, Shiflet 1994, WNHP 2011, Weiss 1999

**Version:** 14 Jan 2014	**Stakeholders:** West

**Concept Author:** P. Comer and T. Keeler‑Wolf	**LeadResp:** West

M043. Californian Chaparral

CES206.929 California Maritime Chaparral

**Primary Division:** Mediterranean California (206)

**Land Cover Class:** Shrubland

**Spatial Scale & Pattern:** Large patch

**Required Classifiers:** Natural/Semi‑natural; Vegetated (>10% vasc.); Upland

**Diagnostic Classifiers:** Shrubland (Shrub‑dominated); Mediterranean [Mediterranean Xeric‑Oceanic]; Udic; Evergreen Sclerophyllous Shrub

**National Mapping Codes:** EVT 2096; ESLF 5302; ESP 1096

**Concept Summary:** This ecological system includes chaparral in patches restricted by edaphic conditions (sands, sandstones, other marine sediments, and stabilized sand dunes) within the fog belt throughout the central and northern California coast. This system is characterized by a combination of locally endemic species of *Arctostaphylos* and *Ceanothus*, species that primarily reproduce by seed rather than resprouting. Shrubs vary in height (up to 3 m tall) and occur in variable densities. More open patches support herbaceous vegetation, while occurrences of high shrub density have no understory. Characteristic species include *Arctostaphylos tomentosa, Arctostaphylos nummularia, Arctostaphylos tomentosa ssp. crustacea, Arctostaphylos hookeri, Arctostaphylos pajaroensis, Arctostaphylos montaraensis* (and others), *Ceanothus masonii, Ceanothus griseus*, and *Ceanothus verrucosus*. In occurrences in southern Oregon, *Arctostaphylos hispidula* is the predominant chaparral shrub. Southernmost stands (San Diego County) can include *Cneoridium dumosum* and *Comarostaphylis diversifolia*. Other common widespread woody taxa can include *Adenostoma fasciculatum, Eriogonum fasciculatum, Salvia mellifera, Frangula californica, Rhamnus crocea*, and *Quercus agrifolia*. Controlled burns have resulted in poor survivorship of the *Arctostaphylos* spp., and current theories are that they need long fire‑free intervals to develop a viable seedbank that can reproduce following fire. This system often co‑occurs with California Coastal Closed‑Cone Conifer Forest and Woodland (CES206.922).

DISTRIBUTION

**Range:** This systems occurs within the fog belt from southern California to the Mendocino coast of northern California. It extends north into coastal Oregon in very small patches.

**Divisions:** 206:C

**TNC Ecoregions:** 14:C, 15:C

**Nations:** US

**Subnations:** CA, OR

**Map Zones:** 2:C, 3:C, 4:C, 13:?

**USFS Ecomap Regions:** 261B:CC, 263A:CC, M242A:PP, M261A:PP, M261B:PP

CONCEPT

**Environment:** This system is restricted by edaphic conditions (sands, sandstones, other marine sediments, and stabilized sand dunes) within the summer coastal fog belt throughout the central and northern California coast, usually below 300 m (1000 feet) in elevation (Keeley and Davis 2007, Sawyer et al. 2009). The climate is distinctly Mediterranean, with warm, dry summers and cool, moist winters. Rainfall is rather variable due to the large latitudinal range. Sandy soils with low nutrient levels tend to be the norm, usually within just a few kilometers of the ocean.

**Vegetation:** This system is characterized by a combination of locally endemic species of *Arctostaphylos* and *Ceanothus*, species that primarily reproduce by seed rather than resprouting. Shrubs vary in height (up to 3 m tall) and occur in variable densities. More open patches support herbaceous vegetation, while occurrences of high shrub density have no understory. Characteristic species include *Arctostaphylos tomentosa, Arctostaphylos nummularia (= Arctostaphylos sensitiva), Arctostaphylos tomentosa ssp. crustacea (= Arctostaphylos crustacea), Arctostaphylos hookeri, Arctostaphylos pajaroensis, Arctostaphylos montaraensis* (and others), *Ceanothus masonii, Ceanothus griseus*, and *Ceanothus verrucosus*. In occurrences in southern Oregon, *Arctostaphylos hispidula* is the predominant chaparral shrub. Southernmost stands (San Diego County) can include *Cneoridium dumosum* and *Comarostaphylis diversifolia*. Other common widespread woody taxa can include *Adenostoma fasciculatum, Eriogonum fasciculatum, Salvia mellifera, Frangula californica (= Rhamnus californica), Rhamnus crocea*, and *Quercus agrifolia*.

**Dynamics:** These shrublands are characterized by species that primarily reproduce by seed rather than resprouting, and are fire‑dependent. Infrequent fire results in encroachment of trees and a decline in shrub vigor and seedbank quality. Frequent fire tends to convert the stands to coastal scrub or grassland. Recent studies of many sites that have been fire‑free for decades suggest that at least some of the species of *Ceanothus* may be able to germinate without fire and thus sustain populations for long fire‑free intervals. Controlled burns have resulted in poor survivorship of the *Arctostaphylos* spp., and current theories are that they need long fire‑free intervals to develop a viable seedbank that can reproduce following fire (Keeley and Davis 2007). Most of the dominant shrubs are nitrogen fixers.

Landfire (2007a) model: Chaparral burns in high‑intensity, stand‑replacing crown fires that burn large acreages in a single event. However, there is a considerable range in the flammability of shrub species (e.g., chamise is "flashier" than manzanita). Large, stand‑replacement events can interact with seed availability and, hence, influence post‑fire successional pathways differently than for smaller, less severe fires. Mean fire‑return intervals are variable and longer than intervals of other chaparral types. Fire intervals can exceed 100 years, and the specimens can grow to large size. Season of burning plays a large part in species composition. Occasionally, frost affects mortality and increases fuel buildup.

SOURCES

**References:** Barbour and Billings 2000, Barbour and Major 1988, Barbour et al. 2007a, Comer et al. 2003*, Davis and Borchert 2006, Greelee and Langenheim 1990, Griffin 1978, Holland and Keil 1995, Keeley 2002, Keeley 2006a, Keeley and Davis 2007, Keeley and Fotheringham 2001a, Keeley and Fotheringham 2001b, LANDFIRE 2007a, PRBO Conservation Science 2011, Sawyer et al. 2009, Shiflet 1994, Van Dyke et al. 2001, Wells 1962

**Version:** 14 Jan 2014	**Stakeholders:** West

**Concept Author:** P. Comer and T. Keeler‑Wolf	**LeadResp:** West

CES206.926 California Mesic Chaparral

**Primary Division:** Mediterranean California (206)

**Land Cover Class:** Shrubland

**Spatial Scale & Pattern:** Large patch

**Required Classifiers:** Natural/Semi‑natural; Vegetated (>10% vasc.); Upland

**Diagnostic Classifiers:** Lowland [Foothill]; Shrubland (Shrub‑dominated); Mediterranean [Mediterranean Xeric‑Oceanic]; Udic; Quercus berberidifolia

**National Mapping Codes:** EVT 2097; ESLF 5303; ESP 1097

**Concept Summary:** This ecological system occurs in mesic site conditions, such as north‑facing slopes, concavities, or toeslopes, with well‑drained soils throughout Mediterranean California away from the coastal fog belt. It occurs most commonly on north‑facing slopes up to 1500 m (4550 feet) in elevation and up to 1830 m (6000 feet) in southern California. This system tends to be dominated by a variety of mixed or single‑species, evergreen, sclerophyllous shrubs that resprout from lignotubers following fire. Common species include *Quercus berberidifolia, Quercus wislizeni var. frutescens, Cercocarpus montanus var. glaber, Fraxinus dipetala, Garrya flavescens, Garrya elliptica, Heteromeles arbutifolia, Lonicera* spp., *Prunus ilicifolia, Rhamnus crocea, Rhamnus ilicifolia, Toxicodendron diversilobum, Ribes* spp., and *Sambucus* spp. Weakly re‑sprouting or obligate seeders that also commonly occur in this system include arborescent *Ceanothus* spp., such as *Ceanothus spinosus, Ceanothus oliganthus, Ceanothus tomentosus*, and *Ceanothus leucodermis*. *Umbellularia californica* and *Aesculus californica* can also occur as shrubs and, lacking disturbance, can grow to tree size, as do some of the other chaparral shrubs (some old‑growth stands can reach 10.6 m [35 feet] in height!). Most chaparral species are fire‑adapted, resprouting vigorously after burning or producing fire‑resistant seeds. This is not a system that requires frequent fire for perpetuation.

DISTRIBUTION

**Range:** This system occurs throughout Mediterranean California away from the coastal fog belt. It may occur as very small patches in southwestern Oregon, but it isn't clearly documented from there.

**Divisions:** 206:C

**TNC Ecoregions:** 5:C, 12:C, 13:C, 14:C, 15:C, 16:C

**Nations:** US

**Subnations:** CA

**Map Zones:** 3:C, 4:C, 5:C, 6:C

**USFS Ecomap Regions:** 261B:CC, 262A:??, 263A:CC, M261A:C?, M261B:CC, M261C:CC, M261D:CC, M261E:CC, M261F:CC

CONCEPT

**Environment:** Mesic chaparral is extremely drought‑tolerant and readily reseeds and resprouts after severe fire. It is highly site‑specific; aspect can greatly influence species composition, and the higher elevations of the shrub belt in the Sierra Nevada foothills have greater moisture relative to the chamise‑dominated (dry) chaparral found at lower altitudes.

**Vegetation:** This system tends to be dominated by a variety of mixed or single‑species, evergreen, sclerophyllous shrubs that resprout from lignotubers following fire. Common species include *Quercus berberidifolia, Quercus wislizeni var. frutescens, Cercocarpus montanus var. glaber (= Cercocarpus betuloides), Fraxinus dipetala, Garrya flavescens, Garrya elliptica, Heteromeles arbutifolia, Lonicera* spp., *Prunus ilicifolia, Rhamnus crocea, Rhamnus ilicifolia, Toxicodendron diversilobum, Ribes* spp., and *Sambucus* spp. Weakly re‑sprouting or obligate seeders that also commonly occur in this system include arborescent *Ceanothus* spp., such as *Ceanothus spinosus, Ceanothus oliganthus, Ceanothus tomentosus*, and *Ceanothus leucodermis*. *Umbellularia californica* and *Aesculus californica* can also occur as shrubs and, lacking disturbance, can grow to tree size, as do some of the other chaparral shrubs (some old‑growth stands can reach 10.6 m [35 feet] in height!). Most chaparral species are fire‑adapted, resprouting vigorously after burning or producing fire‑resistant seeds. This is not a system that requires frequent fire for perpetuation.

**Dynamics:** TNC fire model information: Chaparral burns in high‑intensity stand‑replacing crown fires that burn thousands of acres in a single event. However, there is a considerable range in the flammability of shrub species (e.g., chamise is "flashier" than manzanita). Large, stand‑replacement events can interact with seed availability and, hence, influence post‑fire successional pathways differently than for smaller, less severe fires. Mean fire‑return intervals are highly variable across the state depending on species composition and other factors. Sediment cores taken from the Santa Barbara Channel in central California dating from the 16th and 17th centuries indicate that large fires burned the Santa Ynez and Santa Lucia mountains every 40‑60 years. Season of burning plays a large part in species composition. Occasionally, frost affects mortality and increases fuel buildup. In the last century, the high frequency of human ignitions has reduced the mean fire‑return interval to 30‑35 years in southern California.

SOURCES

**References:** Barbour and Billings 2000, Barbour and Major 1988, Comer et al. 2003*, Holland and Keil 1995, Sawyer and Keeler‑Wolf 1995, Shiflet 1994

**Version:** 12 Jan 2012	**Stakeholders:** West

**Concept Author:** P. Comer and T. Keeler‑Wolf	**LeadResp:** West

CES206.927 California Xeric Serpentine Chaparral

**Primary Division:** Mediterranean California (206)

**Land Cover Class:** Shrubland

**Spatial Scale & Pattern:** Large patch

**Required Classifiers:** Natural/Semi‑natural; Vegetated (>10% vasc.); Upland

**Diagnostic Classifiers:** Mediterranean [Mediterranean Xeric‑Oceanic]; Ultramafic with low Ca:Mg ratio; Very Shallow Soil; Xeric; Broad‑Leaved Evergreen Shrub; Cupressus macnabiana

**National Mapping Codes:** EVT 2099; ESLF 5305; ESP 1099

**Concept Summary:** This ecological system occurs throughout Mediterranean California (excluding far southern California) on thin, rocky, ultramafic (gabbro, peridotite, serpentinite) soils and in areas below winter snow accumulations that typically experience hot and dry summers. Not all ultramafic outcrops support distinct vegetation; only those with very low Ca:Mg ratios impact biotic composition. This system is highly variable and spotty in distribution. Characteristic plant species include *Hesperocyparis macnabiana, Quercus durata, Arctostaphylos viscida, Arctostaphylos pungens*, and *Arctostaphylos glauca*. Common associates include *Adenostoma fasciculatum, Ceanothus cuneatus, Fremontodendron californicum, Quercus sadleriana, Quercus vacciniifolia, Garrya* spp., *Umbellularia californica, Ceanothus pumilus, Frangula californica*, and *Arctostaphylos nevadensis*. California endemics such as *Ceanothus jepsonii* also occur. *Pinus sabiniana* can occur at varying cover from trace to more abundant. Many locally endemic and often rare forbs can occur, such as *Streptanthus* spp., *Hesperolinon* spp., *Eriogonum* spp., *Madia* spp., *Mimulus* spp., *Allium* spp., and *Asclepias solanoana*. This chaparral type tends to have fewer trees than mesic chaparral.

**Comments:** Xeric serpentine chaparral shrublands occurring in the Klamath‑Siskiyou region of northwestern California are placed into the similar Klamath‑Siskiyou Xeromorphic Serpentine Savanna and Chaparral (CES206.150). However, the distribution of these two systems, as currently described, overlaps somewhat. Further review and clarification of their differences and differing distributions are desirable.

DISTRIBUTION

**Range:** This system occurs throughout Mediterranean California (excluding far southern California) into Oregon, on thin, rocky, ultramafic soils.

**Divisions:** 206:C

**TNC Ecoregions:** 5:P, 13:P, 14:C, 15:P

**Nations:** US

**Subnations:** CA, OR

**Map Zones:** 2:C, 3:C, 4:C, 5:C, 6:C, 7:?

**USFS Ecomap Regions:** 261B:PP, 262A:PP, 263A:PP, M261A:CC, M261B:CC, M261C:CC, M261D:CC, M261E:CC, M261F:C?

CONCEPT

**Environment:** This system occurs on thin, rocky, ultramafic (gabbro, peridotite, serpentinite) soils and in areas below winter snow accumulations that typically experience hot and dry summers. Not all ultramafic outcrops support distinct vegetation; only those with very low Ca:Mg ratios impact biotic composition. Soils on ultramafics are usually shallow and skeletal, with little profile development. Ultramafic soils impose the following stresses on plants: imbalance of calcium and magnesium, magnesium toxicity, low availability of molybdenum, toxic levels of heavy metals, sometime high alkalinity, low concentrations of some essential nutrients, and low soil water storage capacity (Sanchez‑Mata 2007). In some cases, the steepness of the slopes and general sparseness of the vegetation result in continual erosion.

**Vegetation:** Characteristic plant species include *Hesperocyparis macnabiana (= Cupressus macnabiana), Quercus durata, Arctostaphylos viscida, Arctostaphylos pungens*, and *Arctostaphylos glauca*. Common associates include *Adenostoma fasciculatum, Ceanothus cuneatus, Fremontodendron californicum, Quercus sadleriana, Quercus vacciniifolia, Garrya* spp., *Umbellularia californica, Ceanothus pumilus, Frangula californica (= Rhamnus californica)*, and *Arctostaphylos nevadensis*. California endemics such as *Ceanothus jepsonii* also occur. *Pinus sabiniana* can occur at varying cover from trace to more abundant. Many locally endemic and often rare forbs can occur, such as *Streptanthus* spp., *Hesperolinon* spp., *Eriogonum* spp., *Madia* spp., *Mimulus* spp., *Allium* spp., and *Asclepias solanoana*. This chaparral type tends to have fewer trees than mesic chaparral.

**Dynamics:** Landfire (2007a) model: Due to the poor soil nutrient levels, biomass accumulation tends to be significantly lower in these serpentine systems than in neighboring patches of sandstone chaparral. As a result, fire frequency and fire severity are reduced. A study at the McLaughlin Reserve (Safford and Harrison 2008) found that time since last fire was nearly four times longer than on non‑serpentine sites, and severity was also significantly reduced. The effects of fire on diversity in these systems are less pronounced than in non‑serpentine systems, though they may be longer lasting (Safford and Harrison 2004); these authors found that few species on serpentine depended on fire for germination.

SOURCES

**References:** Barbour and Major 1988, Brooks and Minnich 2006, Comer et al. 2003*, Harrison et al. 2003, Holland and Keil 1995, Kruckberg 1984, LANDFIRE 2007a, PRBO Conservation Science 2011, Safford and Harrison 2004, Safford and Harrison 2008, Sanchez‑Mata 2007, Sawyer and Keeler‑Wolf 1995, Sawyer et al. 2009, Shiflet 1994

**Version:** 14 Jan 2014	**Stakeholders:** West

**Concept Author:** P. Comer and T. Keeler‑Wolf	**LeadResp:** West

CES206.150 Klamath‑Siskiyou Xeromorphic Serpentine Savanna and Chaparral

**Primary Division:** Mediterranean California (206)

**Land Cover Class:** Steppe/Savanna

**Spatial Scale & Pattern:** Large patch

**Required Classifiers:** Natural/Semi‑natural; Vegetated (>10% vasc.); Upland

**Diagnostic Classifiers:** Serpentine; Mediterranean [Mediterranean Pluviseasonal‑Oceanic]; Ultramafic with low Ca:Mg ratio

**National Mapping Codes:** EVT 2170; ESLF 5425; ESP 1170

**Concept Summary:** This ecological system occurs throughout the Klamath‑Siskiyou region below 1500 m (4550 feet) on thin rocky soils below winter snow accumulations and typically experiences hot and dry summers. These savannas and shrublands are almost always found on ultramafic soils (gabbro, peridotite, serpentinite), especially on the Josephine Peridotite Formation in the western Klamaths, with very low Ca:Mg ratio. These systems are highly variable and spotty in distribution. This system represents the most xeromorphic of these environments, generally supporting savannas or shrublands in areas with high rainfall amounts (over 130 cm/year) that would usually support closed‑canopy forests. Landforms can include rocky ridges and ridgetops, south‑facing slopes and river terraces, or gravelly valley bottomlands. These contain mosaics or patches of open‑canopy tree‑savannas with chaparral understories or shrub‑dominated chaparral. Shrubs will often have higher densities than the trees which are more limited due to the rocky/thin soils and are often stunted in growth‑form. These can also be short‑duration chaparrals in previously forested areas that have experienced crownfires. When present, trees tend to have a scattered, open canopy or can be clustered, over a usually continuous, dense shrub layer, but sometimes with a grassy understory. *Pinus jeffreyi* or occasionally *Pinus attenuata* can form a scattered tree layer over bunchgrasses. Dense shrub layers can also be present in some stands, or form their own patches without trees, especially on ridges. *Quercus vacciniifolia, Quercus sadleriana* (coastal and wetter climate but found on xeric sties), *Notholithocarpus densiflorus var. echinoides, Quercus garryana var. fruticosa* (drier, inland), *Ceanothus cuneatus, Ceanothus pumilus, Arctostaphylos viscida, Arctostaphylos x cinerea, Arctostaphylos canescens, Arctostaphylos nevadensis, Frangula californica*, and *Garrya buxifolia* represent some of the many chaparral shrubs that can be found in these habitats. Perennial grasses such as *Festuca idahoensis ssp. roemeri, Achnatherum lemmonii, Melica* sp., and *Danthonia californica* may also be characteristic, although a diverse and often endemic forb component (including rare serpentine endemics) is usually present. This system tends to have lower diversity within stands than in the other serpentine woodland and shrubland systems. Locally occurring, stunted and open stands of *Pinus contorta* and *Pinus monticola* on serpentine at low elevation are included in this system. The grassy understory savannas tend to have understory burns, while shrub‑dense stands will suffer intense, stand‑replacing fires.

**Comments:** While generally occurring on serpentine soils, these also can be found on rocky or shallow, non‑serpentine soils. They are identified by their very dry, open appearance, and hence are distinguished from the similar Klamath‑Siskiyou Lower Montane Serpentine Mixed Conifer Woodland (CES206.917) which occurs on less xeric sties and has a woodland physiognomy.

DISTRIBUTION

**Range:** This system occurs throughout the Klamath‑Siskiyou mountains region below 1500 m (4550 feet), but mostly in the western Klamaths on the Josephine peridotite body.

**Divisions:** 206:C

**TNC Ecoregions:** 5:P

**Nations:** US

**Subnations:** CA, OR

**Map Zones:** 2:C, 3:C, 7:?

**USFS Ecomap Regions:** 263A:??, M242A:??, M242B:??, M261A:CC, M261D:C?

CONCEPT

**Environment:** This ecological system occurs throughout the Klamath‑Siskiyou region below 1500 m (4550 feet) on thin rocky soils below winter snow accumulations and typically experiences hot and dry summers. These savannas and shrublands are almost always found on ultramafic soils (gabbro, peridotite, serpentinite), especially on the Josephine Peridotite Formation in the western Klamaths, with very low Ca:Mg ratio. These systems are highly variable and spotty in distribution. This system represents the most xeromorphic of these environments, generally supporting savannas or shrublands in areas with high rainfall amounts (over 130 cm/year) that would usually support closed‑canopy forests. Landforms can include rocky ridges and ridgetops, south‑facing slopes and river terraces, or gravelly valley bottomlands.

**Vegetation:** Stands contain mosaics or patches of open‑canopy tree‑savannas with chaparral understories or shrub‑dominated chaparral. Shrubs will often have higher densities than the trees which are more limited due to the rocky/thin soils and are often stunted in growth‑form. These can also be short‑duration chaparrals in previously forested areas that have experienced crownfires. When present, trees tend to have a scattered, open canopy or can be clustered, over a usually continuous, dense shrub layer, but sometimes with a grassy understory. *Pinus jeffreyi* or occasionally *Pinus attenuata* can form a scattered tree layer over bunchgrasses. Dense shrub layers can also be present in some stands, or form their own patches without trees, especially on ridges. *Quercus vacciniifolia, Quercus sadleriana* (coastal and wetter climate but found on xeric sties), *Notholithocarpus densiflorus var. echinoides (= Lithocarpus densiflorus var. echinoides), Quercus garryana var. fruticosa (= var. breweri)* (drier, inland), *Ceanothus cuneatus, Ceanothus pumilus, Arctostaphylos viscida, Arctostaphylos x cinerea, Arctostaphylos canescens, Arctostaphylos nevadensis, Frangula californica (= Rhamnus californica)*, and *Garrya buxifolia* represent some of the many chaparral shrubs that can be found in these habitats. Perennial grasses such as *Festuca idahoensis ssp. roemeri (= Festuca roemeri), Achnatherum lemmonii, Melica* sp., and *Danthonia californica* may also be characteristic, although a diverse and often endemic forb component (including rare serpentine endemics) is usually present. This system tends to have lower diversity within stands than in the other serpentine woodland and shrubland systems. Locally occurring, stunted and open stands of *Pinus contorta* and *Pinus monticola* on serpentine at low elevation are included in this system.

**Dynamics:** The grassy understory savannas tend to have understory burns, while shrub‑dense stands will suffer intense, stand‑replacing fires.

SOURCES

**References:** Atzet et al. 1996, Barbour and Major 1988, Comer et al. 2003*, Eyre 1980, Holland and Keil 1995, Jimerson 1993, Jimerson 1994, Jimerson and Daniel 1999, Jimerson et al. 1995, Kagan et al. 2004, Sawyer and Keeler‑Wolf 1995

**Version:** 23 Jan 2006	**Stakeholders:** West

**Concept Author:** J. Kagan and T. Keeler‑Wolf	**LeadResp:** West

CES206.931 Northern and Central California Dry‑Mesic Chaparral

**Primary Division:** Mediterranean California (206)

**Land Cover Class:** Shrubland

**Spatial Scale & Pattern:** Large patch

**Required Classifiers:** Natural/Semi‑natural; Vegetated (>10% vasc.); Upland

**Diagnostic Classifiers:** Mediterranean [Mediterranean Xeric‑Oceanic]; Sand Soil Texture; Ustic; Intermediate Disturbance Interval; F‑Landscape/High Intensity; Ceanothus cuneatus, Adenostoma fasciculatum

**National Mapping Codes:** EVT 2105; ESLF 5311; ESP 1105

**Concept Summary:** This ecological system includes chaparral typically located inland from maritime chaparral up to 1500 m (4550 feet) elevation in central and northern California through the northern end of the Central Valley and north into Oregon. This system includes extensive areas on coarse‑grained soils with annual precipitation up to 75 cm (winter rain but not snow). Adjacent fine‑textured soils support savanna under similar climatic regimes. These areas have supported extensive stand‑replacing wildfires. This system is made up of a mixture of mostly obligate seeders. Characteristic species include *Adenostoma fasciculatum, Ceanothus cuneatus, Arctostaphylos viscida, Arctostaphylos manzanita, Arctostaphylos glauca, Arctostaphylos glandulosa, Arctostaphylos stanfordiana, Fremontodendron californicum, Malacothamnus fasciculatus, Dendromecon rigida*, and *Pickeringia montana*. Common shrubs in Oregon include *Arctostaphylos viscida, Cercocarpus montanus var. glaber*, and *Ceanothus cordulatus*. Fire regimes are intense, stand‑replacing crown fires. Scattered and young trees may occur, such as *Pinus ponderosa, Pinus sabiniana, Pseudotsuga menziesii*, and *Quercus wislizeni*.

DISTRIBUTION

**Range:** This system is located inland from maritime chaparral up to 1500 m (4550 feet) elevation in central and northern California, and southwestern Oregon, through the north end of the California Central Valley.

**Divisions:** 206:C

**TNC Ecoregions:** 5:P, 12:C, 13:C, 14:C, 15:P

**Nations:** US

**Subnations:** CA, OR

**Map Zones:** 2:C, 3:C, 4:C, 5:C, 6:C, 7:C

**USFS Ecomap Regions:** 242B:??, 262A:CC, 263A:CC, M242A:P?, M242B:PP, M261A:CC, M261B:CC, M261C:CC, M261D:CC, M261E:CC, M261F:CC, M261G:CC

CONCEPT

**Environment:** This chaparral occurs inland of coastal chaparral and up to 1500 m (4550 feet) elevation in central and northern California through the northern end of the Central Valley and north into Oregon. This system includes extensive areas on coarse‑grained soils with annual precipitation up to 75 cm (winter rain but not snow).

**Vegetation:** This system is made up of a mixture of mostly obligate seeders. Characteristic species include *Adenostoma fasciculatum, Ceanothus cuneatus, Arctostaphylos viscida, Arctostaphylos manzanita, Arctostaphylos glauca, Arctostaphylos glandulosa, Arctostaphylos stanfordiana, Fremontodendron californicum, Malacothamnus fasciculatus, Dendromecon rigida*, and *Pickeringia montana*. Common shrubs in Oregon include *Arctostaphylos viscida, Cercocarpus montanus var. glaber*, and *Ceanothus cordulatus*. Scattered and young trees may occur, such as *Pinus ponderosa, Pinus sabiniana, Pseudotsuga menziesii*, and *Quercus wislizeni*.

**Dynamics:** Fire regimes are intense, stand‑replacing crown fires.

SOURCES

**References:** Barbour and Billings 2000, Barbour and Major 1988, Comer et al. 2003*, Holland and Keil 1995, Sawyer and Keeler‑Wolf 1995, Sawyer et al. 2009, Shiflet 1994

**Version:** 12 Jan 2012	**Stakeholders:** West

**Concept Author:** P. Comer and T. Keeler‑Wolf	**LeadResp:** West

CES206.930 Southern California Dry‑Mesic Chaparral

**Primary Division:** Mediterranean California (206)

**Land Cover Class:** Shrubland

**Spatial Scale & Pattern:** Large patch

**Required Classifiers:** Natural/Semi‑natural; Vegetated (>10% vasc.); Upland

**Diagnostic Classifiers:** Lowland [Foothill]; Sideslope; Mediterranean [Mediterranean Desertic‑Oceanic]; Ustic; F‑Landscape/High Intensity; Broad‑Leaved Shrub

**National Mapping Codes:** EVT 2110; ESLF 5316; ESP 1110

**Concept Summary:** This ecological system includes chaparral from sea level up to 1500 m (4550 feet) elevation throughout central and southern California and inland portions of Baja Norte, Mexico. It is found in dry‑mesic to mesic site conditions analogous to mesic chaparral. Santa Ana winds drive late‑summer, stand‑replacing fires in these systems. Characteristic species include *Ceanothus megacarpus, Ceanothus crassifolius, Ceanothus leucodermis, Ceanothus greggii, Adenostoma fasciculatum, Adenostoma sparsifolium, Arctostaphylos glauca, Cercocarpus montanus var. glaber (= Cercocarpus betuloides), Cercocarpus montanus var. minutiflorus (= Cercocarpus minutiflorus), Rhus ovata*, and *Xylococcus bicolor*.

DISTRIBUTION

**Range:** This system includes chaparral from sea level up to 1500 m (4550 feet) elevation throughout central and southern California and inland portions of Baja Norte, Mexico.

**Divisions:** 206:C

**TNC Ecoregions:** 13:C, 15:P, 16:C

**Nations:** MX, US

**Subnations:** CA, MXBC

**Map Zones:** 4:C, 5:C

**USFS Ecomap Regions:** 261B:CC, 262A:CC, 322A:PP, 322C:PP

CONCEPT

**Environment:** [from M043] This type occurs on a wide variety of settings. It occurs within the fog belt along the coast of central and northern California on generally nutrient‑poor edaphic conditions (sands, sandstones, other marine sediments, and stabilized sand dunes), the southern California coast and into the western foothills of the Sierra Nevada. It is typically found on arid, south‑facing slopes and ridges, and occasionally on mesic sites, such as north‑facing slopes, concavities, or toeslopes, with well‑drained soils and mafic soils. The more frost‑tolerant species are found at higher, cooler and generally more mesic sites up to approximately 1830 m (6000 feet) elevation. Chaparral is naturally displaced by woodlands on very mesic slopes and by sage scrub on xeric slopes (Keeley and Davis 2007). These shrublands include extensive areas on coarse‑grained soils with annual precipitation up to 75 cm (winter rain, and only intermittent snow).

Californian chaparral is mainly linked to three conditions: climate, soil and dynamics. With regard to climate, Mediterranean climate is the norm, regardless of the total amount of precipitations, because within that macroclimate it can be found under a wide range of rainfall. However, when rainfall is low (roughly below 300 mm/year), chaparral constitutes the late‑seral vegetation, whereas when rainfall is higher, chaparral plays two ecological roles. First, they constitute the edaphic vegetation living on shallow and rocky soils [see Keeley and Davis (2007)], including deep eolian sands and mafic substrates. Second, in areas with higher rainfall (>300 mm), they are successional and linked to fire, forming early‑ and mid‑seral stages of bushlands and pyrophytic chaparral that replace oak woodlands and forests, and mixed‑coniferous forests (M. Peinado pers. comm. 2014).

**Dynamics:** [from M043] The fire regime ranges from root sprouter‑dominated shrubland that survive and regrow after stand‑replacing fires. Other stands are dominated by seed reproducers that need long fire‑free intervals to develop a viable seedbank that can reproduce following fire (Keeley and Davis 2007). Recent studies of many sites that have been fire‑free for decades suggest that at least some of the species of *Ceanothus* may be able to germinate without fire and thus sustain populations during long fire‑free intervals. Other stands are stable and do not need frequent fire to persist. Studies show that frost damage to mature plants and drought stress on seedlings may limit the range and distribution of California chaparral species (Keeley and Davis 2007).

SOURCES

**References:** Barbour and Billings 2000, Barbour and Major 1988, Comer et al. 2003*, Holland and Keil 1995, Sawyer and Keeler‑Wolf 1995, Shiflet 1994

**Version:** 07 Oct 2005	**Stakeholders:** Latin America, West

**Concept Author:** P. Comer and T. Keeler‑Wolf	**LeadResp:** West

M044. Californian Coastal Scrub

CES206.906 Mediterranean California Coastal Bluff

**Primary Division:** Mediterranean California (206)

**Land Cover Class:** Barren

**Spatial Scale & Pattern:** Linear

**Required Classifiers:** Natural/Semi‑natural; Unvegetated (<10% vasc.); Upland

**Diagnostic Classifiers:** Bluff; Headland; Sea cliff; Mediterranean [Mediterranean Xeric‑Oceanic]; Salt Spray; Landslide; W‑Landscape/High Intensity; Succulent Shrub; Dwarf‑Shrub

**Concept Summary:** Areas of sea bluffs and rocky headlands occur just above the tidal zone throughout rugged portions of coastal Oregon, California, Baja Norte, and off‑shore islands (e.g., Channel Islands). Plant communities along these often vertical slopes are typically sparse, with many succulents and prostrate shrubs, and species that readily withstand salt spray and saline soils, as well as seasonal drought. These may include *Baccharis pilularis, Dudleya* spp., *Carpobrotus chilensis, Carpobrotus edulis, Hazardia squarrosa (= Haplopappus squarrosus), Eriogonum parvifolium, Erigeron glaucus, Eriophyllum stoechadifolium*, and *Plantago maritima*. Slope instability and erosion result in severe climate, setting back succession in this system.

DISTRIBUTION

**Range:** Rugged portions of coastal Oregon, California, and off‑shore islands (e.g., Channel Islands), and Baja Norte, Mexico.

**Divisions:** 206:C

**TNC Ecoregions:** 14:C, 15:C, 16:C

**Nations:** MX, US

**Subnations:** CA, MXBC, OR

**Map Zones:** 2:P, 3:C, 4:C

**USFS Ecomap Regions:** 261B:CC, 263A:CC

CONCEPT

SOURCES

**References:** Barbour and Major 1988, Comer et al. 2003*, Holland and Keil 1995, Sawyer and Keeler‑Wolf 1995

**Version:** 17 Mar 2003	**Stakeholders:** Latin America, West

**Concept Author:** P. Comer and T. Keeler‑Wolf	**LeadResp:** West

CES206.932 Northern California Coastal Scrub

**Primary Division:** Mediterranean California (206)

**Land Cover Class:** Shrubland

**Spatial Scale & Pattern:** Large patch

**Required Classifiers:** Natural/Semi‑natural; Vegetated (>10% vasc.); Upland

**Diagnostic Classifiers:** Woody‑Herbaceous; Bluff; Marine Sedimentary; Mediterranean [Mediterranean Xeric‑Oceanic]; Baccharis pilularis

**National Mapping Codes:** EVT 2128; ESLF 5457; ESP 1128

**Concept Summary:** This ecological system includes a variety of mixed and single‑species‑dominated shrublands along a narrow coastal strip with maritime and summer fog influences, on marine sediments, coastal bluffs, terraces, stabilized dunes, and hills below 500 m (1500 feet) elevation from southern Oregon south through central California. It is restricted to coastal plateaus and lower slopes of the Coast Ranges where precipitation ranges from 50‑200 cm annually. These are dominated by evergreen, microphyllous‑leaved or hemi‑sclerophyllous shrub taxa; drought‑deciduous species are unimportant or absent in this system. Dense shrublands typically include a well‑developed woody and herbaceous understory. Characteristic species include *Baccharis pilularis, Lupinus arboreus, Ceanothus thyrsiflorus, Eriophyllum stoechadifolium, Diplacus aurantiacus (= Mimulus aurantiacus), Toxicodendron diversilobum, Rubus ursinus, Rubus parviflorus, Rubus spectabilis, Frangula californica (= Rhamnus californica), Holodiscus discolor, Gaultheria shallon, Heracleum maximum (= Heracleum lanatum)*, and *Polystichum munitum*. These areas have supported extensive stand‑replacing wildfires. This system has direct seral relationships with California Northern Coastal Grassland (CES206.941) as, in the absence of fire and grazing, the grassland will usually succeed to this system. In the absence of fire in this system, conifers (*Abies grandis, Pseudotsuga menziesii*) can invade and become prominent.

**Comments:** Transitions to Southern California Coastal Scrub (CES206.933) begin in the northern San Francisco Bay area where *Artemisia californica* begins to mix with *Baccharis* and others. Further south in the Santa Lucia Range (Big Sur area of Monterey County), *Baccharis pilularis* and *Artemisia californica* tend to codominate, and other southern coastal sage species such as *Salvia leucophylla* and *Eriogonum fasciculatum* become more prominent. South of Monterey County most of the northern California coastal scrub influence is gone, and coastal shrublands shift to Southern California Coastal Scrub (CES206.933). The combination of <30% relative cover of *Artemisia californica* or *Salvia* spp. and high cover *Baccharis* and *Rhamnus*, or having *Polystichum munitum, Gaultheria shallon*, and *Heracleum maximum* makes it Northern California Coastal Scrub (CES206.932).

DISTRIBUTION

**Range:** This system occurs along a narrow coastal strip below 500 m (1500 feet) elevation from southern Oregon south through central California.

**Divisions:** 206:C

**TNC Ecoregions:** 14:C, 15:C

**Nations:** US

**Subnations:** CA, OR

**Map Zones:** 2:P, 3:C, 4:C

**USFS Ecomap Regions:** 263A:CC, M242A:PP

CONCEPT

SOURCES

**References:** Barbour and Billings 2000, Barbour and Major 1988, Comer et al. 2003*, Holland and Keil 1995, Sawyer and Keeler‑Wolf 1995, Shiflet 1994

**Version:** 07 Oct 2005	**Stakeholders:** West

**Concept Author:** P. Comer and T. Keeler‑Wolf	**LeadResp:** West

CES206.933 Southern California Coastal Scrub

**Primary Division:** Mediterranean California (206)

**Land Cover Class:** Shrubland

**Spatial Scale & Pattern:** Large patch

**Required Classifiers:** Natural/Semi‑natural; Vegetated (>10% vasc.); Upland

**National Mapping Codes:** EVT 2092; ESLF 5269; ESP 1092

**Concept Summary:** This ecological system includes mixed coastal shrublands from Monterey, California, south into Baja Norte, Mexico. It is dominated by drought‑deciduous shrubs but at times can have characteristic (constant but not dominant) resprouting, deep‑rooted sclerophyllous shrubs. It occurs below 1000 m (3000 feet) elevation and may extend inland from the maritime zone in hotter, drier conditions than northern (less fog‑drenched) shrublands (e.g., areas with 10‑60 cm of annual precipitation). Soils vary from coarse gravels to clays but typically only support plant‑available moisture with winter and spring rain. Most predominant shrubs include *Artemisia californica, Salvia mellifera, Salvia apiana, Salvia leucophylla, Encelia californica, Eriogonum fasciculatum, Eriogonum cinereum, Opuntia littoralis, Diplacus aurantiacus, Lotus scoparius* (early seral after fire), and *Baccharis pilularis* (in moister, disturbed sites). Characteristic (constant but not dominant) resprouting, deep‑rooted sclerophyllous shrubs include *Malosma laurina, Rhus integrifolia*, and *Rhamnus crocea*. Fire frequency was historically low, but in recent years with adjacency to urban and suburban areas, the fire frequency has increased (a result of arson or cigarette ignition) resulting in type conversion to non‑native and ruderal annual grasslands. *Malosma laurina* and *Rhus integrifolia* are also increasing in abundance because they can continually resprout after repeated fires. In places, *Opuntia littoralis* may proliferate and cover entire slopes in dry rocky areas with repeated fires that have killed the scrub taxa, while *Opuntia littoralis* can resprout and spread to cover large patches.

DISTRIBUTION

**Range:** This system is found from Monterey, California, south into Baja Norte, Mexico. It occurs below 1000 m (3000 feet) elevation and may extend inland from the maritime zone.

**Divisions:** 206:C

**TNC Ecoregions:** 15:C, 16:C

**Nations:** MX, US

**Subnations:** CA, MXBC

**Map Zones:** 4:C, 5:?, 6:?

**USFS Ecomap Regions:** 261B:CC, 262A:CC

CONCEPT

**Environment:** The most important environmental factors are cool‑season precipitation and minimum winter temperature (Rundel 2007, Sawyer et al. 2009). Mean minimum winter temperature is substantially more predictive of southern sage scrub distribution patterns than is mean maximum summer temperature. Southern scrub prefers warm winters and relatively low total precipitation. This means that this system is restricted to low‑elevation areas that receive some marine climatic influence; generally it occurs below 1000 m (3000 feet) elevation and receives about 10‑60 cm of annual precipitation. The coastal region where it occurs has a longer dry season than further north in California. Southern coastal scrub often responds sensitively to aspect on a local scale (for example, in San Diego County sometimes occurring on south‑facing slopes where north‑facing slopes are occupied by chaparral types), but occurs on all aspects when viewed at a regional scale (Sawyer et al. 2009). Species composition of stands varies both with distance from the coast and with latitude. Soils vary from coarse gravels to clays but typically only support plant‑available moisture with winter and spring rain. Stands often form complex mosaics interdigitated with stands of chaparral and grassland types on scales of 10s‑100s of meters.

**Vegetation:** Most predominant shrubs include *Artemisia californica, Salvia mellifera, Salvia apiana, Salvia leucophylla, Encelia californica, Eriogonum fasciculatum, Eriogonum cinereum, Opuntia littoralis, Diplacus aurantiacus (= Mimulus aurantiacus), Lotus scoparius* (early seral after fire), and *Baccharis pilularis* (in moister, disturbed sites). Characteristic (constant but not dominant) resprouting, deep‑rooted sclerophyllous shrubs include *Malosma laurina, Rhus integrifolia*, and *Rhamnus crocea*. Fire frequency was historically low, but in recent years with adjacency to urban and suburban areas, the fire frequency has increased (a result of arson or cigarette ignition) resulting in type conversion to non‑native and ruderal annual grasslands. *Malosma laurina* and *Rhus integrifolia* are also increasing in abundance because they can continually resprout after repeated fires. In places, *Opuntia littoralis* may proliferate and cover entire slopes in dry rocky areas with repeated fires that have killed the scrub taxa, while *Opuntia littoralis* can resprout and spread to cover large patches.

**Dynamics:** This is not an ecosystem type that requires fire for regeneration of the major shrubs (Landfire 2007a, Rundel 2007). Coastal scrub often occupies sites denuded by landslides, slumps, debris flows, and other mass‑wasting events. It sometimes occupies chaparral sites for a number of years after a burn, before the larger, woodier chaparral shrubs reestablish their dominance. The main sage scrub species have seeds that are wind‑dispersed, and recovery of sage scrub communities post‑disturbance may involve dispersal and germination from plants outside the disturbed area (Rundel 2007). Although *Lotus scoparius* can temporarily occupy chaparral sites, *Artemisia californica* could not have the seedbank necessary to be abundant in post‑fire chaparral, except in a case where there were repeated burns over several to many years that opened up the chaparral. Southern coastal scrub can clearly persist on favorable sites for at least a hundred years and probably much longer in the absence of any fire (Rundel 2007).

SOURCES

**References:** Barbour and Billings 2000, Barbour and Major 1988, Brooks and Minnich 2006, Comer et al. 2003*, Holland and Keil 1995, LANDFIRE 2007a, Minnich 1983, Minnich and Dezzani 1998, PRBO Conservation Science 2011, Rundel 2007, Sawyer and Keeler‑Wolf 1995, Sawyer et al. 2009, Shiflet 1994, Taylor 2004

**Version:** 14 Jan 2014	**Stakeholders:** Latin America, West

**Concept Author:** P. Comer and T. Keeler‑Wolf	**LeadResp:** West

2.B.2. Temperate Grassland & Shrubland

2.B.2.Nb. Central North American Grassland & Shrubland

M054. Central Lowlands Tallgrass Prairie

CES205.683 Central Tallgrass Prairie

**Primary Division:** Eastern Great Plains (205)

**Land Cover Class:** Herbaceous

**Spatial Scale & Pattern:** Matrix

**Required Classifiers:** Natural/Semi‑natural; Vegetated (>10% vasc.); Upland

**National Mapping Codes:** EVT 2421; ESLF 7134; ESP 1421

**Concept Summary:** This system is found primarily in the Central Tallgrass Prairie ecoregion ranging from eastern Kansas and Nebraska to northwestern Indiana. This system differs from other prairie systems to the north and south by being the most mesic with primarily deep, rich Mollisol soils. These soils are usually greater than 1 meter deep. This system is dominated by tallgrass species such as *Andropogon gerardii, Sorghastrum nutans*, and *Panicum virgatum*. These species typically grow to 1‑2 m tall in the rich soils found in this system. Other mid‑ and shortgrass species, such as *Bouteloua curtipendula, Hesperostipa spartea*, and *Schizachyrium scoparium*, are usually present and can be common or locally dominant on patches of this system, particularly slopes or other areas with drier habitats. Several forb species are also associated with this system making it one of the most diverse grassland systems. As many as 300 herbaceous plant species could occur on a 10‑ha high‑quality example of this system across its range. Historically, fires limited woody species; however, the current environment and habitat of this system do not prevent invasion by shrubs and trees. High‑quality examples of this system have trees and shrubs widely scattered or clustered in areas that are wetter and/or more sheltered from fire than the surrounding grassland. Fire, drought, and grazing are the primary natural dynamics influencing this system and help prevent woody species from invading. However, conversion to agriculture has been the prime disturbance since European settlement. The rich soils and long growing season make this an ideal location for farming row crops, and as a result very few examples of this system remain.

DISTRIBUTION

**Range:** This system is found primarily in the Central Tallgrass Prairie (TNC Ecoregion 36) ranging from eastern Kansas and Nebraska to north‑central Missouri and northwestern Indiana. In Missouri, it is attributed to EPA 47d, 47f, 72f.

**Divisions:** 205:C

**TNC Ecoregions:** 36:C, 45:C, 46:C

**Nations:** US

**Subnations:** IA, IL, IN, KS, MO, NE, WI

**Map Zones:** 31:?, 38:C, 39:?, 42:C, 43:C, 44:P, 49:C, 50:C, 51:C, 52:C

**USFS Ecomap Regions:** 222Je:CC?, 222Jg:CC?, 222Jh:CCC, 222K:CC, 223A:CC, 251B:CC, 251C:CC, 251F:CC, 251H:CC, 255A:CC, 332C:CC, 332D:CC, 332E:CC, 332F:CC

CONCEPT

**Environment:** This system differs from other prairie systems to the north and south by being the most mesic with primarily deep, rich Mollisol soils. These soils are usually greater than 1 m deep and organic matter is high. Litter can build up if sites are not burned or grazed for several years. This system occurs in a climate that allows the growth of trees and shrubs. These are kept out of the prairies largely by fires and periodic drought, so the prairies tended to be on flat to rolling topography with fewer firebreaks (wetlands, rivers, or steeply dissected topography).

**Vegetation:** This system is dominated by tallgrass species such as *Andropogon gerardii, Sorghastrum nutans*, and *Panicum virgatum*. These species typically grow to 1‑2 m tall in the rich soils found in this system. Other mid‑ and shortgrass species, such as *Bouteloua curtipendula, Hesperostipa spartea*, and *Schizachyrium scoparium*, are usually present and can be common or locally dominant on patches of this system, particularly slopes or other areas with drier habitats. Several forb species are also associated with this system making it one of the most diverse grassland systems. As many as 300 herbaceous plant species could occur in this system across its range. The environment and habitat of this system do not prevent invasion by shrubs and trees. High‑quality examples of this system have trees and shrubs widely scattered or clustered in areas that are wetter and/or more sheltered from fire than the surrounding grassland.

**Dynamics:** Fire, drought, and grazing are the primary natural dynamics influencing this system and help prevent woody species from invading. This system is found in a climate that can support trees and shrubs but woody vegetation is inhibited by frequent fires. Historically, fire‑return intervals were short, estimated at between 2 and 5 years (Stambaugh et al. 2006, Landfire 2007a). The frequent but unpredictable hot fires created a patchwork of habitats across the landscape, with recently burned sites having less litter and forb cover and sites with infrequent fires possibly having more woody species and dense stands of grasses (Kucera and Koelling 1964). This system developed in an area with large numbers of native ungulates, notably bison (*Bos bison*) but including other species (elk and deer), and the grazing of these species affected species composition and the patchwork of habitat. Bison were likely more numerous and thus had more effect in the western portion of this system's range. Bison preferentially favor newly burned areas and graminoids over forbs (Vinton et al. 1993, Coppedge and Shaw 1998). Their grazing, trampling, and wallowing were important in creating habitat diversity across the landscape (Knapp et al. 1999). On unburned sites, grazing removes live and dead vegetation, allowing more light and heat to the soil surface and increasing available moisture thus favoring species, forbs or woody plants, in the case of bison grazing, that were resilient to the effects of grazing or avoided by the grazers (Damoureyeh and Hartnett 1997).

SOURCES

**References:** Barbour and Billings 1988, Comer et al. 2003*, Coppedge and Shaw 1998, Damoureyeh and Hartnett 1997, Heineman and Bragg 1982, Hulbert 1988, Kindscher and Tieszen 1998, Knapp et al. 1999, Kost et al. 2007, Kucera and Koelling 1964, LANDFIRE 2007a, Nelson 2010, ONHD unpubl. data, Rice and Parenti 1978, Ricketts et al. 1999, Rolfsmeier and Steinauer 2010, Samson and Knopf 1994, Stambaugh et al. 2006, Vinton et al. 1993, WDNR 2015

**Version:** 27 May 2016	**Stakeholders:** Midwest, Southeast

**Concept Author:** S. Menard	**LeadResp:** Midwest

CES202.695 North‑Central Interior Sand and Gravel Tallgrass Prairie

**Primary Division:** Central Interior and Appalachian (202)

**Land Cover Class:** Herbaceous

**Spatial Scale & Pattern:** Large patch

**Required Classifiers:** Natural/Semi‑natural; Vegetated (>10% vasc.); Upland

**National Mapping Codes:** EVT 2412; ESLF 7125; ESP 1412

**Concept Summary:** This system is found in the northern Midwest, particularly in Minnesota, Wisconsin, Michigan, and possibly ranging into Ontario. It is often found on glacial features such as kames, eskers, moraines, lakeplains (though excluding the Great Lakes lakeplain) and sandplains, and along eolian dunes. In contrast to the deeper, richer soils supporting other tallgrass systems in the region, the underlying soils in this system tend to be more shallow, sandy, rocky, and/or gravelly outwash soils. Organic content is significantly lower. Grassland species such as *Schizachyrium scoparium, Andropogon gerardii*, and *Bouteloua* spp., varying in cover from sparse to moderately dense, dominate this system. *Hesperostipa spartea* and *Sporobolus heterolepis* are also common components of this system. Woody species more tolerant of droughty conditions may be found in some examples. The most common trees are *Pinus banksiana, Quercus ellipsoidalis, Quercus macrocarpa*, and *Populus tremuloides*. Fire and drought are the major dynamics influencing this system. If fire and periodic drought are not present, woody species begin to invade this system, especially in the eastern parts of its distribution. Wind can also play a role, especially on examples found on sandplains and/or eolian dunes.

DISTRIBUTION

**Range:** This system is found in the northern Midwest possibly ranging into Ontario.

**Divisions:** 202:C, 205:P

**TNC Ecoregions:** 35:C, 36:P, 45:C, 46:C, 47:C, 48:C

**Nations:** CA, US

**Subnations:** IA, IL, IN, MI, MN, MO, ND, ON, SD, WI

**Map Zones:** 39:C, 40:C, 41:P, 42:C, 43:P, 49:P, 50:C, 51:C, 52:C

**USFS Ecomap Regions:** 212Ha:CCC, 212Hb:CCC, 212Hc:CCC, 212Hd:CCC, 212He:CCC, 212Hf:CCC, 212Hg:CCC, 212Hh:CCP, 212Hi:CCC, 212Hk:CCC, 212Hm:CCP, 212K:CP, 212M:CP, 212N:CP, 212Tb:CCC, 222Ja:CCC, 222Jb:CCC, 222Jc:CCC, 222Je:CCC, 222Jf:CCP, 222Jg:CCC, 222Jh:CCC, 222Ji:CCP, 222K:CC, 222L:CC, 222M:CC, 222N:CC, 222R:CP, 222Ua:CCC, 222Ud:CCP, 222Ue:CCP, 251A:CC, 251B:CC

CONCEPT

**Environment:** This system is often found on glacial features such as kames, eskers, moraines, lakeplains (though excluding the Great Lakes lakeplain), and sandplains, and along eolian dunes and river deltas. In contrast to the deeper, richer soils supporting other tallgrass systems in the region, the underlying soils in this system tend to be more shallow, sandy, rocky, and/or gravelly soils. Soil texture is sand or sandy loam. Organic content and soil moisture retention are significantly lower than the more mesic grasslands.

**Vegetation:** Grassland species such as *Schizachyrium scoparium, Andropogon gerardii*, and *Bouteloua* spp., varying in cover from sparse to moderately dense, dominate this system. *Hesperostipa spartea* and *Sporobolus heterolepis* are also common components of this system. Woody species more tolerant of droughty conditions may be found in some examples. The most common trees are *Pinus banksiana, Quercus ellipsoidalis, Quercus macrocarpa*, and *Populus tremuloides*.

**Dynamics:** Fire and drought are the major dynamics influencing this system. If fire and periodic drought are not present, woody species begin to invade this system, especially in the eastern parts of its distribution. Fire‑return intervals were likely 1‑8 years (Landfire 2007a). Drier examples of this system likely could not be maintained in the presence of long‑term short fire‑return intervals due to the lower fertility of the soils. The typical dominant perennial grasses would not have time to recover from repeated burning and shorter‑lived opportunistic species could dominate (Loucks et al. 1985). These sites were maintained as grasslands by the dry soil conditions possibly supplemented by low‑frequency fires, while other areas required fire to eliminate invasion by woody species. Wind can also play a role, especially on examples found on sandplains and/or eolian dunes or during droughts when vegetation cover is low. Blowouts can form, exposing bare sand (Burgess 1965). Productivity is lower on this system than on other tallgrass prairies, so vegetation responds more slowly to disturbance. This system can not persist with the same frequency of reductions in vegetation cover by fire, grazing, drought, or mowing as richer prairies can.

SOURCES

**References:** Burgess 1965, Comer et al. 2003*, Kost et al. 2007, LANDFIRE 2007a, Loucks et al. 1985, MNNHP 1993, Samson and Knopf 1994, Thompson 1940, WDNR 2015

**Version:** 14 Jan 2014	**Stakeholders:** Canada, Midwest, Southeast

**Concept Author:** S. Menard	**LeadResp:** Midwest

CES205.686 Northern Tallgrass Prairie

**Primary Division:** Eastern Great Plains (205)

**Land Cover Class:** Herbaceous

**Spatial Scale & Pattern:** Matrix

**Required Classifiers:** Natural/Semi‑natural; Vegetated (>10% vasc.); Upland

**National Mapping Codes:** EVT 2420; ESLF 7133; ESP 1420

**Concept Summary:** This system is found primarily in the Northern Tallgrass ecoregion ranging along the Red River basin in Minnesota and the Dakotas to Lake Manitoba in Canada. It constitutes the northernmost extension of the "true" prairies. Similar to Central Tallgrass Prairie (CES205.683), this system is dominated by tallgrass species such as *Andropogon gerardii, Sorghastrum nutans*, and *Panicum virgatum*. However, the soils in this region are not as rich nor deep, the growing season length and precipitation are less, and thus this system does not have as much species diversity as grasslands to the south. This system is often found on well‑drained, drier soils and can grade into Eastern Great Plains Tallgrass Aspen Parkland (CES205.688) to the north and east. Grazing and fire influenced this system historically. Much of this system has been converted to agriculture with very few unaltered and highly fragmented examples remaining.

DISTRIBUTION

**Range:** Found primarily in the Northern Tallgrass ecoregion ranging along the Red River basin in Minnesota and the Dakotas to Lake Manitoba in Canada.

**Divisions:** 205:C

**TNC Ecoregions:** 35:C

**Nations:** CA, US

**Subnations:** IA, MB, MN, ND, SD

**Map Zones:** 39:C, 40:C, 41:?, 42:C

**USFS Ecomap Regions:** 222N:CC, 251A:CC, 251B:CC, 251H:C?, 332B:CC, 332D:CC

CONCEPT

**Dynamics:** Fire plays an important role in the maintenance of this prairie system (Curtis 1959, Vogl 1964, Anderson 1990b). Fire promotes seed production and flowering necessary for plant regeneration. Because environmental conditions are suitable for tree growth, without recurrent fire (every 2‑10 years), succession to forest or woodland will occur rapidly (Minnesota DNR 2005b). From Landfire BpS: Frequent fires impacted this prairie system every 1‑3 years, maintaining grass and forb vegetation. Fire could occur throughout the year with larger, less frequent fires occurring during the dormant season and smaller, more frequent fires occurring during the growing season. Native American burning, essential to maintaining the eastern tallgrass prairie, was bimodal in distribution, peaking in April and October with lightning ignition occurring primarily during July and August (Higgins 1986).

Bison grazing as a major disturbance was likely much more limited than prairies further west. Elk probably contributed to the impact of grazing and browsing as well, but it is assumed that the total contributions of these two species was still considerably less than to the west. The elk may have contributed to the reduction of young woody saplings invading prairie adjacent to protected woody areas. Prior to European settlement, episodic grazing by large, native mammals was common and encouraged the persistence of several native grass and forb species (Minnesota DNR 2005b). Insect and small mammal herbivory impacts composition and dominance. From Landfire BpS: Bison, with peripheral help from grasshoppers, elk, antelope and a myriad of smaller animals made herbivory one of the dominating factors of the northern tallgrass prairie (Severson and Sieg 2006). With estimates of 30‑60 million bison in the Northern Great Plains (Isenberg 2000), herbivory by large mammals also was a significant disturbance to the grasslands. Bison herbivory occurred in a mob‑grazing or flash‑grazing method, with extensive herds migrating across the prairie as they graze. Modern rotational grazing systems simulate this by resting areas after intensive grazing. Elk, too, may have played an important role than generally believed, particularly in the eastern portion of the zone. Whether bison or elk, large mammals preferentially grazed recently burned sites.

SOURCES

**References:** Anderson 1990b, Barbour and Billings 1988, Comer et al. 2003*, Curtis 1959, Higgins 1986, Isenberg 2000, Koper et al. 2010, Minnesota DNR 2005b, Ricketts et al. 1999, Severson and Sieg 2006, Vogl 1964

**Version:** 27 May 2016	**Stakeholders:** Canada, Midwest

**Concept Author:** S. Menard	**LeadResp:** Midwest

CES205.685 Southern Tallgrass Prairie

**Primary Division:** Eastern Great Plains (205)

**Land Cover Class:** Herbaceous

**Spatial Scale & Pattern:** Large patch

**Required Classifiers:** Natural/Semi‑natural; Vegetated (>10% vasc.); Upland

**Diagnostic Classifiers:** Herbaceous; Graminoid

**National Mapping Codes:** EVT 2423; ESLF 7136; ESP 1423

**Concept Summary:** This system of tallgrass prairies ranges from Kansas, Missouri, and Oklahoma south into Arkansas and Texas. It includes several subregional units or variants, which are defined biogeographically but with much overlap in floristics and ecological processes. This range includes the Flint Hills (EPA 28a) and portions of the Northern Cross Timbers (EPA 29a) of Kansas and Oklahoma, the Osage Plains (EPA 40b, 40c, 40d) of Kansas, Missouri, and Oklahoma, the southeastern Springfield Plateau (EPA 39a, 39b? of Arkansas and Missouri, the Arkansas Valley Plains (EPA 37d, 37e) of Arkansas and Oklahoma, and the "Grand Prairie" or "Fort Worth Prairie" (EPA 29d) of Texas,; where it is the primary natural system. It is also scattered in the rest of the Cross Timbers (EPA 29b. 29c, 29e, 29f, 29g, 29h), ranging south into the Lampasas Cutplain of Texas (EPA 29e). It is distinguished from Central Tallgrass Prairie (CES205.683) by having more species with southwestern geographic affinities and the presence of a thin soil layer over limestone beds ranging to more acidic substrates, although some areas of deeper soil are found within the region, especially on lower slopes, draws, and terraces. Because of the presence of the rocky substrate close to the surface and the rolling topography, this area is relatively unsuitable for agriculture. The Flint Hills contain one of the largest remaining, relatively intact pieces of Southern Tallgrass Prairie. The vegetation in this system is typified by tallgrass species such as *Andropogon gerardii, Panicum virgatum, Schizachyrium scoparium*, and *Sorghastrum nutans,* which typically form a dense cover. A moderate to high density of forb species also occurs. Species composition varies geographically, with *Oligoneuron rigidum, Liatris punctata, Symphyotrichum ericoides, Lespedeza capitata*, and *Viola pedatifida* commonly occurring in the Flint Hills and Osage Plains. Areas of deeper soil, especially lower slopes along draws, slopes and terraces, can include *Baptisia alba var. macrophylla, Liatris pycnostachya*, and *Vernonia baldwinii*. Shrub and tree species are relatively infrequent and, if present, constitute less than 10% cover. Fire and grazing constitute the major dynamic processes for this region. Although many of the native common plant species still occur, grazing does impact this region. Poor grazing practices can lead to soil erosion and invasion by cool‑season grasses such as *Bromus inermis* within its range.

In the Arkansas Valley Plains (EPA 37d, 37e) the prairies are interspersed with oak or pine‑dominated woodlands. This region is distinctly bounded by the Boston Mountains to the north and the Ouachita Mountains to the south. The valley is characterized by broad, level to gently rolling uplands derived from shales and is much less rugged and more heavily impacted by Arkansas River erosional processes than the adjacent mountainous regions. In addition, the valley receives annual precipitation total of 5‑15 cm (2‑6 inches) less than the surrounding regions due to a rainshadow produced by a combination of prevailing western winds and mountain orographic effects. The shale‑derived soils associated with the prairies are thin and droughty. The combined effect of droughty soils, reduced precipitation, and prevailing level topography create conditions highly conducive to the ignition and spread of fires. Stands are typically dominated by *Andropogon gerardii, Sorghastrum nutans, Panicum virgatum*, and *Schizachyrium scoparium*. Some extant examples of this system remain, but most are small and isolated. They were common on the western edge of the region where precipitation was lower and agriculture conversion was less extensive.

**Comments:** This system includes prairies of the Flint Hills, in addition to prairies in Oklahoma and southwestern Missouri south of the glacial line, as well as those in the "Grand Prairie" or "Fort Worth Prairie" of Texas, ranging south into the Lampasas Cutplain (EPA 29d and 29e), This revised (August 2018) system now includes prairies formerly in Southwestern Ozarks Prairie and Woodland (CES202.326) (also called "Flint Hills Tallgrass Prairie" and originally "Ozark Prairie and Woodland"), which includes grasslands interspersed with woodlands in southwestern Missouri and northwestern Arkansas, and Arkansas Valley Prairie and Woodland (CES202.312), in a similar context in the Arkansas River Valley, which have now been merged in here; Texas Blackland Tallgrass Prairie (CES205.684) remains as a distinctive system.

DISTRIBUTION

**Range:** This system of tallgrass prairies ranges from the Flint Hills (EPA 28a) and portions of the Northern Cross Timbers (EPA 29a) of Kansas and Oklahoma, the Osage Plains (EPA 40b, 40c, 40d) of Kansas, Missouri, and Oklahoma, south through the southeastern Springfield Plateau (EPA 39a, 39b? of Arkansas and Missouri, the Arkansas Valley Plains (EPA 37d, 37e) of Arkansas and Oklahoma, south to the "Grand Prairie" or "Fort Worth Prairie" (EPA 29d; where it is the primary natural system) as well as scattered in the rest of the Cross Timbers (EPA 29b. 29c, 29e, 29f, 29g, 29h), ranging south into the Lampasas Cutplain of Texas (EPA 29e).

**Divisions:** 205:C

**TNC Ecoregions:** 32:C, 36:C, 37:C, 38:C, 39:C

**Nations:** US

**Subnations:** AR, KS, MO, OK, TX

**Map Zones:** 32:P, 35:C, 38:P, 43:C, 44:C

**USFS Ecomap Regions:** 223A:CC, 231G:CC, 251E:CC, 251F:CC, 251H:CC, 255A:CC, 255C:CC, 255E:CC, 332E:CC

CONCEPT

**Environment:** This system is typified by a thin soil layer over limestone beds or acidic substrates such as chert or granite, although areas of deeper soils are possible along lower slopes, draws, and terraces. The topography is rolling and mostly unsuitable for agriculture. In Texas, the typical geology is Lower Cretaceous formations, including various limestones, sands (such as from the Paluxy and Antlers formations), and clays (such as from the Walnut Formation). In contrast to Blackland Prairie, landform surfaces are flat rather than undulating, and valley slopes are angular rather than rounded. The "cuesta" landforms with gentle slopes leading up to relatively abrupt escarpments are characteristic of the Grand Prairie portion of the Southern Tallgrass Prairie in Texas. Much of the region occupied by this prairie in Texas is included in the Blackland Ecological Site, though Clay Loam, Sandy Loam, Shallow, and Claypan Prairie Ecological Sites are also significant. Soils of this area are more frequently characterized as Mollisols, as opposed to the Vertisols more characteristic of the Blackland Prairie. Calcareous clays are commonly encountered (Elliott 2011).

The Arkansas Valley is characterized by broad, level to gently rolling uplands derived from shales and is much less rugged and more heavily impacted by Arkansas River erosional processes than the adjacent mountainous regions. In addition, the valley receives annual precipitation total of 5‑15 cm (2‑6 inches) less than the surrounding regions due to a rainshadow produced by a combination of prevailing western winds and mountain orographic effects (T. Foti pers. comm. 2003). The shale‑derived soils associated with the prairies are thin and droughty. The combined effect of droughty soils, reduced precipitation, and prevailing level topography create conditions highly conducive to the ignition and spread of fires.

**Vegetation:** The vegetation in this system is typified by tallgrass species such as *Andropogon gerardii, Panicum virgatum, Schizachyrium scoparium*, and *Sorghastrum nutans* forming a dense cover. A moderate to high density of forb species also occurs. Species composition varies geographically, with *Oligoneuron rigidum (= Solidago rigida), Liatris punctata, Symphyotrichum ericoides, Lespedeza capitata*, and *Viola pedatifida* commonly occurring in the Flint Hills and Osage Plains. In Texas, *Schizachyrium scoparium* tends to dominate examples of this system, with *Bouteloua curtipendula* as another significant component. Other grasses that are frequently present include *Nassella leucotricha, Bothriochloa laguroides ssp. torreyana, Aristida* spp., *Andropogon gerardii, Andropogon ternarius, Aristida dichotoma, Bouteloua dactyloides (= Buchloe dactyloides), Sporobolus compositus, Bouteloua eriopoda, Bouteloua gracilis, Bouteloua hirsuta, Sorghastrum nutans, Muhlenbergia reverchonii, Chloris verticillata*, and *Erioneuron pilosum*. Forbs species such as *Symphyotrichum ericoides, Ambrosia psilostachya, Tragia ramosa, Amphiachyris dracunculoides, Dyschoriste linearis, Salvia texana, Oenothera* spp., *Hedyotis nigricans var. nigricans (= Stenaria nigricans var. nigricans), Lindheimera texana, Thelesperma* spp., *Dalea* spp., and *Psoralidium* spp. may be encountered (Elliott 2011). In Texas, occurrences often contain, and are sometimes dominated by, the non‑native grass *Bothriochloa ischaemum var. songarica* and/or *Cynodon dactylon*. Other forb species that can occur include *Oligoneuron rigidum, Liatris punctata, Lespedeza capitata, Viola pedatifida, Coreopsis grandiflora, Danthonia spicata, Helianthus grosseserratus, Mentzelia oligosperma, Rudbeckia missouriensis, Silene regia, Croton michauxii var. ellipticus (= Croton willdenowii)*, and *Tradescantia bracteata*. In areas of deeper soils, *Baptisia alba var. macrophylla, Liatris pycnostachya*, and *Vernonia missurica* can also occur within their ranges. Significant areas of this system remain within the Grand Prairie of Texas (Elliot 2011).

In the Arkansas Valley, prairies are typically dominated by *Schizachyrium scoparium, Andropogon gerardii, Sorghastrum nutans*, and *Panicum virgatum*. Other grasses include *Koeleria macrantha, Sporobolus heterolepis, Sphenopholis obtusata, Dichanthelium* spp., *Aristida purpurascens, Panicum brachyanthum*, and *Coelorachis cylindrica*. A rich forb diversity is commonly present and includes *Helianthus mollis, Echinacea pallida, Rudbeckia grandiflora, Silphium laciniatum, Symphyotrichum* spp., *Solidago* spp., *Callirhoe digitata, Asclepias hirtella, Eryngium yuccifolium, Delphinium carolinianum, Castilleja coccinea, Calopogon oklahomensis, Buchnera americana, Dodecatheon meadia, Tephrosia virginiana, Baptisia alba, Baptisia bracteata, Liatris pycnostachya*, and *Liatris squarrosa var. hirsuta*. Wetter areas support a rich diversity of rushes and sedges, including *Carex bicknellii var. opaca (= Carex opaca), Carex oklahomensis, Carex complanata*, and *Eleocharis wolfii* (T. Witsell pers. comm. 2006).

**Dynamics:** Fire and grazing are the prevalent dynamic processes in examples of this system. This system is found in a climate that can support trees and shrubs but woody vegetation is inhibited by frequent fires. Historically, fire‑return intervals were short, estimated at between 2 and 15 years (Abrams 1986, Landfire 2007a). The frequent but unpredictable fires created a patchwork of habitats across the landscape, with recently burned sites having less litter and forb cover, and sites with infrequent fires possibly having more woody species and dense stands of grasses. This system developed in an area occupied by vast numbers of native ungulates, notably bison (*Bos bison*) but including other species, and the grazing of these species affected species composition and the patchwork of habitat. Bison preferentially favor newly burned areas and graminoids over forbs (Vinton et al. 1993, Coppedge and Shaw 1998). Their grazing, trampling, and wallowing were important in creating habitat diversity across the landscape (Knapp et al. 1999). On unburned sites, grazing removes live and dead vegetation, allowing more light and heat to the soil surface and increasing available moisture thus favoring species, forbs or woody plants, in the case of bison grazing, that were resilient to the effects of grazing or avoided by the grazers (Damoureyeh and Hartnett 1997).

SOURCES

**References:** Abrams 1986, Barbour and Billings 1988, Bragg and Hulbert 1976, Briggs and Gibson 1998, Cleland et al. 2007, Comer et al. 2003*, Coppedge and Shaw 1998, Damoureyeh and Hartnett 1997, DeSelm and Murdock 1993, Duffey et al. 1974, EPA 2005, Elliott 2011, Estes et al. 1979, Eyre 1980, Foti pers. comm., Hulbert 1988, Knapp et al. 1999, LANDFIRE 2007a, Lauver et al. 1999, McKinney and Lockwood 1999, Murdock pers. comm., Nelson 2005, Nelson 2010, Nigh and Schroeder 2002, Noss 2013, Rice and Parenti 1978, Ricketts et al. 1999, Samson and Knopf 1994, Taft 1997a, Taft 2009, Taft et al. 1995, Vinton et al. 1993, Wiens and Dyer 1975, Witsell pers. comm.

**Version:** 28 Aug 2018	**Stakeholders:** Midwest, Southeast

**Concept Author:** S. Menard and K. Kindscher	**LeadResp:** Midwest

CES205.684 Texas Blackland Tallgrass Prairie

**Primary Division:** Eastern Great Plains (205)

**Land Cover Class:** Herbaceous

**Spatial Scale & Pattern:** Large patch

**Required Classifiers:** Natural/Semi‑natural; Vegetated (>10% vasc.); Upland

**National Mapping Codes:** EVT 2422; ESLF 7135; ESP 1422

**Concept Summary:** This grassland system is found primarily in the Blackland Prairie region of Texas but may range into southern Oklahoma. It is typified by the presence of dark alkaline Vertisol soils over calcareous parent material, although substantial belts of acidic, sandy clay loam Alfisols and loamy or clay loam Mollisols also occur. These soil types relate directly to the underlying surface geology. Microtopography such as gilgai occurs over Vertisols, and mima mounds occur over Alfisols. These create important microhabitats that increase plant diversity in this system. *Schizachyrium scoparium, Sorghastrum nutans*, and *Andropogon gerardii* are the most common dominants. *Tripsacum dactyloides* and *Panicum virgatum* are common associates on the Vertisol soils, especially on the gilgai microtopography. Fire, drought, and possibly grazing were the major natural dynamics influencing this system.

**Comments:** This system (CES205.684) lies to the east of the floristically related Southern Tallgrass Prairie (CES205.685), and is primarily, if not entirely, found in Texas (and possibly Oklahoma).

DISTRIBUTION

**Range:** This system is restricted to the Blackland Prairie region, part of the Crosstimbers and Southern Tallgrass Prairie Ecoregion, in Texas and possibly adjacent southern Oklahoma.

**Divisions:** 205:C

**TNC Ecoregions:** 32:C

**Nations:** US

**Subnations:** OK?, TX

**Map Zones:** 32:C, 35:C, 36:C, 37:C

**USFS Ecomap Regions:** 255B:CC, 255C:CC, 315E:CC

CONCEPT

**Environment:** This system is typified by the presence of dark alkaline Vertisol soils over calcareous parent material interspersed with patches of acidic, sandy loam Alfisols and Mollisols. The detailed geology includes Cretaceous shales, marls and limestones, such as those of the Pecan Gap Chalk, Marlbrook Marl, Eagle Ford, Gober Chalk, Annona Chalk, and Austin Chalk formations, and Taylor and Navarro groups, as well as portions of the Eocene Midway Group and Wilcox Formation. Also, Miocene formations (Fleming and Oakville Sandstone formations) underlie the southern outlier of Blackland prairie recognized as the Fayette Prairie. Landforms are flat to gently rolling and dissected by drainages, with the most significant ridges associated with harder chalk formations. Microtopography such as gilgai and mima mounds can occur and are important microhabitats that lead to an increase in plant diversity in this system (Diamond and Smeins 1990). Soils are typically Vertisols, but this system may occupy Mollisols or Alfisols with the latter more common. The system generally occurs on calcareous clays, but may also occur on loams, clay loams, or even sandy clay loams or silt loams. Annual rainfall averages 890mm, wettest seasons are spring and fall (Harmel et al. 2003).

**Vegetation:** Currently, only remnants of this system exist, with most of the historical distribution replaced by crop production or improved pasture. *Schizachyrium scoparium* is the most ubiquitous component of occurrences of this system. *Andropogon gerardii* and *Sorghastrum nutans* are also common dominants. Other species commonly encountered include *Bouteloua curtipendula, Carex microdonta, Sporobolus compositus, Nassella leucotricha, Bothriochloa laguroides ssp. torreyana, Eriochloa sericea, Paspalum floridanum*, and *Tridens strictus*. Forbs commonly encountered in this system include *Symphyotrichum ericoides, Stenaria nigricans var. nigricans, Helianthus maximiliani, Rudbeckia hirta, Bifora americana, Acacia angustissima var. hirta, Desmanthus illinoensis*, and many more. Perhaps more commonly encountered species include *Croton monanthogynus, Amphiachyris dracunculoides*, and *Asclepias* spp. Lowland sites and swales are often dominated by *Tripsacum dactyloides* and *Panicum virgatum* (Elliott 2011). Heavy grazing has allowed species such as *Bouteloua dactyloides (= Buchloe dactyloides)* and *Bouteloua rigidiseta* to increase. *Tripsacum dactyloides* and *Panicum virgatum* are common associates on the Vertisols, especially on the gilgai microtopography.

**Dynamics:** Fire, drought and possibly and grazing constitute the major natural dynamics influencing this system. Frequent fires (mean fire‑return interval of 2.5 years) prevent woody species from establishing and favor grassland species adapted to fire for reproduction and vigor (Landfire 2007a) prevent woody species from establishing and favor grassland species adapted to fire for reproduction and vigor. Bison and other ungulates possibly played an important role in the vegetation composition and structure of this system (Eidson and Smeins 1999). Fire suppression and overgrazing have allowed woody species to invade. Heavy grazing has also altered the floristic composition by allowing species such as *Bouteloua dactyloides* and *Bouteloua rigidiseta* to invade. This system is important for a suite of wildlife, many of which are declining, that are dependent on native grasslands (TPWD 2012a).

SOURCES

**References:** Barbour and Billings 1988, Comer et al. 2003*, Diamond and Smeins 1988, Diamond and Smeins 1990, Diggs et al. 1999, Eidson and Smeins 1999, Elliott 2011, Harmel et al. 2003, LANDFIRE 2007a, Ricketts et al. 1999, Riskind and Collins 1975, Smeins and Diamond 1986b, TPDW 2012a

**Version:** 14 Jan 2014	**Stakeholders:** Southeast

**Concept Author:** S. Menard	**LeadResp:** Southeast

CES203.550 Texas‑Louisiana Coastal Prairie

**Primary Division:** Gulf and Atlantic Coastal Plain (203)

**Land Cover Class:** Mixed Upland and Wetland

**Spatial Scale & Pattern:** Matrix

**Required Classifiers:** Natural/Semi‑natural; Vegetated (>10% vasc.); Upland; Wetland

**Diagnostic Classifiers:** Herbaceous; Deep Soil; Graminoid

**National Mapping Codes:** EVT 2434; ESLF 7147; ESP 1434

**Concept Summary:** This system encompasses non‑saline tallgrass prairie vegetation that developed over Pleistocene terraces flanking the Gulf Coast of Louisiana and Texas. It is sometimes characterized by a ridge‑and‑swale or mound‑and‑intermound microtopography and encompasses both upland and wetland plant communities. Upland dominants typically include *Schizachyrium scoparium, Paspalum plicatulum*, and *Sorghastrum nutans*. Wetland dominants in undisturbed occurrences include *Panicum virgatum* and *Tripsacum dactyloides*. Fire is an important ecological process in this system.

**Comments:** The concept of this system includes the Kleberg prairie of Johnston (1963) and the clay prairie concept of McClendon (1991) (in part) that were formerly included in Tamaulipan Clay Grassland (CES301.987).

DISTRIBUTION

**Range:** This system occurs within 50 to 150 miles of the Gulf Coast from southwestern Louisiana to south‑central Texas encompassing approximately 10 million acres.

**Divisions:** 203:C, 301:C

**TNC Ecoregions:** 30:C, 31:C

**Nations:** US

**Subnations:** LA, TX

**Map Zones:** 36:C, 37:C

**USFS Ecomap Regions:** 232E:CC, 255D:CC, 315E:CC

CONCEPT

**Environment:** This mid‑ to tallgrass prairie occupies Pleistocene surfaces of the Texas and Louisiana coast, on non‑saline soils. The occurrence of this system is generally coincident with the distribution of the Pleistocene Beaumont and Lissie formations in Texas (Prairie and Intermediate allogroups in Louisiana). It is usually found on level to gently rolling landscapes, with slopes generally less than 5%. Microtopography plays an important role in local variation in the system, with ridges, swales, mounds, depressions, mima (or pimple) mounds, and gilgai leading to a mosaic of drier and wetter plant communities. Typical soils are non‑saline Vertisols, Alfisols, and (less extensively) Mollisols (Diamond and Smeins 1984, Smeins et al. 1992). Vertisols are often characterized by gilgai, resulting from shrink‑swell attributes of the montmorillonitic clays of which they are composed. Historically, rivers and streams dissected this vegetation type, breaking it into large compartments with species composition shifting across the range. A moisture gradient occurs from northeast (average 120 cm/year) to southwest (average 100 cm/year) across the range of this system (Diamond and Smeins 1984).

**Vegetation:** Stands are dominated by graminoid species, such as *Andropogon gerardii, Andropogon glomeratus, Bouteloua curtipendula, Cyperus entrerianus, Dichanthelium oligosanthes, Fimbristylis puberula, Muhlenbergia capillaris, Panicum virgatum, Paspalum floridanum, Paspalum plicatulum, Paspalum setaceum, Rhynchospora* spp., *Schizachyrium scoparium, Sorghastrum nutans, Sporobolus compositus, Tridens strictus*, and *Tripsacum dactyloides*. *Axonopus* spp., *Andropogon virginicus, Bothriochloa laguroides ssp. torreyana, Nassella leucotricha*, and *Sporobolus indicus* may be particularly noticeable on overgrazed sites; disturbed occurrences may be dominated by *Andropogon glomeratus*. Non‑native graminoids that may be conspicuous to dominant components include *Cynodon dactylon, Bothriochloa ischaemum var. songarica, Dichanthium* spp., *Lolium perenne, Schedonorus arundinaceus (= Schedonorus phoenix), Paspalum notatum*, and *Paspalum dilatatum*. Forbs that may often be encountered include *Liatris* spp., *Sabatia campestris, Ambrosia psilostachya, Euphorbia bicolor, Solidago* spp., *Rudbeckia hirta, Ruellia humilis, Asclepias viridis, Chamaecrista fasciculata, Helianthus angustifolius, Euthamia* spp., *Ratibida columnifera, Symphyotrichum ericoides, Silphium laciniatum, Baptisia* spp., *Iva angustifolia, Eryngium yuccifolium, Boltonia diffusa*, and *Neptunia lutea*. Woody species may invade this typically herbaceous vegetation, including *Rosa bracteata, Vachellia farnesiana (= Acacia farnesiana), Triadica sebifera, Baccharis halimifolia, Celtis laevigata*, and *Prosopis glandulosa* (Elliott 2011).

**Dynamics:** The impacts and interaction of fire, drought, competition, and possibly grazing constitute the major natural dynamics influencing this system (Smeins et al. 1992, USGS 2013). Frequent fires every 2‑5 years of both lightning and anthropogenic origins prevent woody species from establishing and favor grassland species adapted to fire for reproduction and vigor. Microtopographic and moisture variability interacted with fire to produce variable fire effects influencing the distribution of flora and fauna. Grazing by bison and other ungulates also played an important role in maintaining the vegetation composition and structure of this system. This system is important for a suite of wildlife, many of which are declining, that are dependent on native grasslands (TPWD 2012a).

SOURCES

**References:** Bergan 1999, Comer et al. 2003*, Diamond and Smeins 1984, Elliott 2011, Grace et al. 2000, LDWF 2005, Smeins et al. 1992, TPDW 2012a, USFWS and USGS 1999, USGS 2013a

**Version:** 14 Jan 2014	**Stakeholders:** Southeast

**Concept Author:** J. Teague	**LeadResp:** Southeast

CES202.312 Arkansas Valley Prairie and Woodland

**Primary Division:** (202)

**Concept Summary:** This system of prairies and associated woodlands is found in the Arkansas River Valley region of Arkansas and adjacent Oklahoma. This region is distinctly bounded by the Boston Mountains to the north and the Ouachita Mountains to the south, although it has been considered part of the Ouachita Ecoregion. The valley is characterized by broad, level to gently rolling uplands derived from shales and is much less rugged and more heavily impacted by Arkansas River erosional processes than the adjacent mountainous regions. In addition, the valley receives annual precipitation total of 5-15 cm (2-6 inches) less than the surrounding regions due to a rainshadow produced by a combination of prevailing western winds and mountain orographic effects. The shale-derived soils associated with the prairies are thin and droughty. The combined effect of droughty soils, reduced precipitation, and prevailing level topography create conditions highly conducive to the ignition and spread of fires. Stands are typically dominated by *Andropogon gerardii, Sorghastrum nutans, Panicum virgatum*, and *Schizachyrium scoparium*. Some extant examples of this system remain, but most are small and isolated. They were common on the western edge of the region bordering or possibly included in the Crosstimbers and Southern Tallgrass Prairie where precipitation and agriculture conversion were lowest.

DISTRIBUTION

**Range:** This system occurs in the Arkansas River Valley region of Arkansas and adjacent Oklahoma.

**Nations:** US

**Subnations:** AR, OK

CONCEPT

**Environment:** This region is distinctly bounded by the Boston Mountains to the north and the Ouachita Mountains to the south, although it has been considered part of the Ouachita Ecoregion (TNC Ecoregion 39). The valley is characterized by broad, level to gently rolling uplands derived from shales and is much less rugged and more heavily impacted by Arkansas River erosional processes than the adjacent mountainous regions. In addition, the valley receives annual precipitation total of 5-15 cm (2-6 inches) less than the surrounding regions due to a rainshadow produced by a combination of prevailing western winds and mountain orographic effects (T. Foti pers. comm. 2003). The shale-derived soils associated with the prairies are thin and droughty. The combined effect of droughty soils, reduced precipitation, and prevailing level topography create conditions highly conducive to the ignition and spread of fires. Some extant examples of this system remain, but most are small and isolated. They were common on the western edge of the Arkansas Valley region, bordering (or possibly included in) the Crosstimbers (TNC Ecoregion 32) where precipitation and agriculture conversion were lowest (T. Foti pers. comm. 2003). This western portion of the Arkansas Valley region is labeled as part of 231Gc by Cleland et al. (2005) and 37d, 37e by EPA (EPA 2013).

**Vegetation:** These prairies are typically dominated by *Schizachyrium scoparium, Andropogon gerardii, Sorghastrum nutans*, and *Panicum virgatum*. Other grasses include *Koeleria macrantha, Sporobolus heterolepis, Sphenopholis obtusata, Dichanthelium* spp., *Aristida purpurascens, Panicum brachyanthum*, and *Coelorachis cylindrica*. A rich forb diversity is commonly present and includes *Helianthus mollis, Echinacea pallida, Rudbeckia grandiflora, Silphium laciniatum, Symphyotrichum* spp., *Solidago* spp., *Callirhoe digitata, Asclepias hirtella, Eryngium yuccifolium, Delphinium carolinianum, Castilleja coccinea, Calopogon oklahomensis, Buchnera americana, Dodecatheon meadia, Tephrosia virginiana, Baptisia alba, Baptisia bracteata, Liatris pycnostachya*, and *Liatris squarrosa var. hirsuta*. Wetter areas support a rich diversity of rushes and sedges, including *Carex opaca, Carex oklahomensis, Carex complanata*, and *Eleocharis wolfii* (T. Witsell pers. comm. 2006).

**Dynamics:** These prairies and woodlands were historically maintained by frequent fire. Drought cycles and grazing were also likely important ecosystem processes. Fires were frequent, primarily autumnal and of human origin. As *Quercus-Carya* regeneration becomes established, individuals of these species become largely fire-resistant with age. Surface fires within woodland and forest types occurred every 12 to 15 years, reducing duff layers and allowing recruitment of young individuals of *Quercus* and *Carya* species (Landfire 2007a).

SOURCES

**References:** Comer et al. 2003*

**Version:** 02 Oct 2014	**Stakeholders:**

**Concept Author:** P. Comer

M158. Great Plains Comanchian Scrub & Open Vegetation

CES303.041 Edwards Plateau Limestone Shrubland

**Primary Division:** Western Great Plains (303)

**Land Cover Class:** Shrubland

**Spatial Scale & Pattern:** Large patch

**Required Classifiers:** Natural/Semi‑natural; Vegetated (>10% vasc.); Upland

**Diagnostic Classifiers:** Shrubland (Shrub‑dominated); Plain; Plateau; Alkaline Soil; Very Shallow Soil; Xeric

**National Mapping Codes:** EVT 2393; ESLF 5324; ESP 1393

**Concept Summary:** This ecological system occurs as a matrix on relatively thin‑soiled surfaces of plateaus of the massive limestones such as the Edwards limestone. These short to tall shrublands are variable in density depending on the relative amount of, and depth to, bedrock. *Quercus sinuata var. breviloba* is an important component of the system, with some areas dominated by *Quercus fusiformis*. *Juniperus ashei* is often an important component of this system. Important components in western examples may include *Pinus remota, Quercus mohriana, Quercus vaseyana*, and *Juniperus pinchotii*. Herbaceous cover may be patchy and is generally graminoid with species including *Schizachyrium scoparium, Bouteloua curtipendula, Bouteloua rigidiseta, Bouteloua trifida, Hilaria belangeri, Bothriochloa laguroides ssp. torreyana, Nassella leucotricha, Erioneuron pilosum, Aristida* spp., and others. Disturbances such as fire may be important processes maintaining this system. However, it appears to persist on thin‑soiled sites. In the western portions of the Edwards Plateau, more xeric conditions lead to the slow succession of sites to woodlands, resulting in long‑persisting shrublands.

**Comments:** This system represents naturally occurring shrublands that are maintained over long periods (greater than 50 years) as shrublands. It tends to occur on shallow soils over massive hard‑bedded limestone formations and/or in the western and drier portions of the Edwards Plateau of Texas. Early‑successional vegetation of Edwards Plateau Limestone Savanna and Woodland (CES303.660) may exhibit a composition and structure similar to the vegetation described and classified here, but the temporal dynamics are different. This system was modeled for mapzone 26, however, Edwards Plateau Limestone Savanna and Woodland (CES303.660) was not. Both systems occur on the far eastern edge of the mapzone on the Stockton Plateau.

DISTRIBUTION

**Range:** This system is limited in occurrence to the Edwards Plateau of Texas.

**Divisions:** 302:P, 303:C

**TNC Ecoregions:** 24:P, 28:P, 29:C

**Nations:** US

**Subnations:** TX

**Map Zones:** 26:C, 27:?, 34:?, 35:C

**USFS Ecomap Regions:** 255E:CC, 315C:CC, 315D:CC, 315G:CC, 321B:CC

CONCEPT

**Environment:** This system occurs on thin soils over massive limestone such as Edwards or related formations in the Edwards Plateau of Texas. It may occur on plateaus or slopes and may often form a discontinuous band around a plateau edge as it breaks into the adjacent slope. Soils are characterized by Shallow or Very Shallow Ecological Sites, but may also be found on Low Stony Hill Ecological Sites (Elliott 2011).

**Vegetation:** This system may be represented by extensive continuous shrub cover, or occur as a discontinuous shrubland, often with scattered emergent overstory trees. *Quercus sinuata var. breviloba, Quercus fusiformis*, and/or *Juniperus ashei* may be important components of the system. In the interior of the Edwards Plateau ecoregion, *Quercus sinuata var. breviloba* is an important component, with some areas dominated by a shinnery of *Quercus fusiformis*, although monotypic stands of *Quercus fusiformis* occupying the shrub layer are uncommon. In the west, *Pinus remota* may also contribute to a scattered emergent overstory. Shrub cover may be dominated by these species, or may be represented as an assemblage of a rather diverse array of species including *Rhus virens, Rhus lanceolata, Cercis canadensis var. texensis, Forestiera pubescens, Forestiera reticulata, Ungnadia speciosa, Sophora secundiflora, Diospyros texana, Salvia ballotiflora, Mimosa borealis, Condalia hookeri, Rhus trilobata, Opuntia engelmannii var. lindheimeri*, and *Mahonia trifoliolata*. In the northwest corner of the ecoregion, this system may occur as a *Quercus mohriana*‑dominated shrubland (a type more common in the Southern Shortgrass Prairie ecoregion), often sharing dominance with *Juniperus pinchotii*. Towards the southwest, *Quercus vaseyana (= Quercus pungens var. vaseyana)* becomes an important component of the system, and areas dominated by *Sophora secundiflora, Diospyros texana*, and other shrub species become more common. In the southwest corner of the ecoregion, on the Stockton Plateau, this system may be represented by *Acacia berlandieri* shrublands. Where shrub cover is distributed in a patchy mosaic, such sites may be used by black‑capped vireos (*Vireo atricapilla*). Herbaceous cover may be patchy and is generally graminoid‑dominated with species including *Schizachyrium scoparium, Bouteloua curtipendula, Bouteloua rigidiseta, Bouteloua trifida, Hilaria belangeri, Bothriochloa laguroides ssp. torreyana, Nassella leucotricha, Erioneuron pilosum, Aristida* spp., and others (Elliott 2011).

**Dynamics:** This system occurs in a steady state on thin‑soiled xeric sites. Shrub cover can be 100% in patches, but overall cover may be 40‑50%. Patches of dense shrubs may be interspersed with bare rock and grasslands over shallow soil. Farther west this system grades into other shallow‑soiled shrubland systems. Disturbances such as fire may be important processes maintaining this system. However, it appears to persist on thin‑soiled sites. In the western portions of the Edwards Plateau, more xeric conditions lead to the slow succession of sites to woodlands resulting in long‑persisting shrublands.

SOURCES

**References:** Comer et al. 2003*, Elliott 2011, Eyre 1980, TNC 2004b

**Version:** 28 May 2013	**Stakeholders:** Southeast, West

**Concept Author:** L. Elliott and K.A. Schulz	**LeadResp:** Southeast

CES303.725 Llano Estacado Caprock Escarpment and Breaks Shrubland and Steppe

**Primary Division:** Western Great Plains (303)

**Land Cover Class:** Steppe/Savanna

**Spatial Scale & Pattern:** Linear

**Required Classifiers:** Natural/Semi‑natural; Unvegetated (<10% vasc.); Upland

**Diagnostic Classifiers:** Canyon

**Concept Summary:** This ecological system occurs on various surfaces that are sufficiently resistant to erosion to form breaks or escarpments along the eastern edge of the Llano Estacado in Texas. This includes sedimentary deposits such as sandstones, limestones, or shales, or less frequently, igneous formations such as basalt. It is sometimes associated with canyons or drainages, but not always. The system occupies slopes, but may continue over transitions to more level sites upslope and downslope. Soils are variable and this system can occur where there is little soil development. Rough Breaks Ecological Sites are characteristic of this system, but other sites such as Rocky Hill and Gravelly Ecological Sites may also be occupied by this system. The physiognomic character of occurrences ranges from sparsely vegetated to shrubland, to sparse woodland. Bare ground is often conspicuous, and herbaceous cover is usually dominated by mid‑ to short grasses such as *Aristida purpurea, Bouteloua curtipendula, Bouteloua gracilis, Bouteloua hirsuta*, and *Schizachyrium scoparium*. Forbs, including species such as *Artemisia ludoviciana, Calylophus* sp., *Chaetopappa ericoides, Krameria lanceolata*, and *Melampodium leucanthum*, may also be present. Shrub canopy may be dense, with some species reaching tree stature, and on some sites forming sparse woodland. Shrub and tree species include *Juniperus pinchotii, Juniperus ashei, Quercus mohriana, Rhus trilobata, Dalea formosa, Cercocarpus montanus, Prosopis glandulosa*, and *Gutierrezia sarothrae*.

**Comments:** This system is differentiated from Southwestern Great Plains Canyon (CES303.664) by its location along the caprock escarpment along the eastern edge of the Llano Estacado in Texas.

DISTRIBUTION

**Range:** This system occurs along the escarpment breaks on the east side of the Llano Estacado in Texas.

**Divisions:** 303:C

**TNC Ecoregions:** 24:C, 28:C, 29:C

**Nations:** US

**Subnations:** OK?, TX

**Map Zones:** 26:C, 34:C, 35:C

**USFS Ecomap Regions:** 315B:CC, 315C:CC, 315F:CC, 321B:CC

CONCEPT

**Environment:** This system may occur on various surfaces that are sufficiently resistant to erosion to form breaks or escarpments. This includes sedimentary deposits such as sandstones, limestones, or shales, or less frequently, igneous formations such as basalt. Landforms include breaks and escarpments with slopes less than 20% as defined here, sometimes associated with canyons or drainages, but not necessarily. The system occupies slopes but may continue over transitions to more level sites upslope and downslope. The system may occur on various soils, as well as on sites where little soil development has occurred. Rough Breaks Ecological Sites are characteristic of this system, but other sites such as Rocky Hill and Gravelly Ecological Sites may also be occupied (Elliott 2011).

**Vegetation:** The physiognomic character of occurrences ranges from sparsely vegetated to shrubland to sparse woodland. Bare ground is often conspicuous, and herbaceous cover is usually dominated by mid‑ to short grasses such as *Aristida purpurea, Bouteloua curtipendula, Bouteloua gracilis, Bouteloua hirsuta*, and *Schizachyrium scoparium*. Forbs, including species such as *Artemisia ludoviciana, Calylophus* sp., *Chaetopappa ericoides, Krameria lanceolata*, and *Melampodium leucanthum*, may also be present. Shrub canopy may be dense, with some species reaching tree stature and on some sites forming sparse woodland. Shrub and tree species include *Juniperus pinchotii, Juniperus ashei, Quercus mohriana, Rhus trilobata, Dalea formosa, Cercocarpus montanus, Prosopis glandulosa*, and *Gutierrezia sarothrae* (Elliott 2011).

SOURCES

**References:** Comer et al. 2003*, Elliott 2011, Elliott 2012, Eyre 1980

**Version:** 24 Feb 2011	**Stakeholders:** Midwest, Southeast, West

**Concept Author:** L. Elliott	**LeadResp:** Southeast

M051. Great Plains Mixedgrass & Fescue Prairie

CES303.659 Central Mixedgrass Prairie

**Primary Division:** Western Great Plains (303)

**Land Cover Class:** Herbaceous

**Spatial Scale & Pattern:** Matrix

**Required Classifiers:** Natural/Semi‑natural; Vegetated (>10% vasc.); Upland

**National Mapping Codes:** EVT 2132; ESLF 7104; ESP 1132

**Concept Summary:** This mixedgrass prairie system ranges from South Dakota into the Rolling Plains and the western Edwards Plateau of Texas. It is bordered by the shortgrass prairie on its western edge and the tallgrass prairie to the east. The loessal regions in west‑central Kansas and central Nebraska, the Red Hills region of south‑central Kansas and northern Oklahoma are all located within this system. Because of its proximity to other ecoregions, this system contains elements from both shortgrass and tallgrass prairies, which combine to form the mixedgrass prairie ecological system throughout its range. The distribution, species richness and productivity of plant species within the mixedgrass ecological system is controlled primarily by environmental conditions, in particular soil moisture and topography. Grazing and fire are important dynamic processes in this system. The relative dominance of the various grass and forb species within different associations in the system also can strongly depend on the degree of natural or human disturbance. This system can contain grass species such as *Bouteloua curtipendula, Schizachyrium scoparium, Andropogon gerardii, Hesperostipa comata*, and *Bouteloua gracilis*, although the majority of the associations within the region are dominated by *Pascopyrum smithii* or *Schizachyrium scoparium*. Numerous forb and sedge species (*Carex* spp.) can also occur within the mixedgrass system in the Western Great Plains. Although forbs do not always significantly contribute to the canopy, they can be very important. Some dominant forb species include *Ambrosia psilostachya* (grazing increases dominance), *Psoralidium tenuiflorum, Echinacea angustifolia, Helianthus* species, and *Ratibida columnifera*. Oak species such as *Quercus macrocarpa* can occur also in areas protected from fire due to topographic position (usually moister north‑facing slopes). This can cause an almost oak savanna situation in certain areas, although fire suppression may allow for a more closed canopy and expansion of bur oak beyond those sheltered areas. In those situations, further information will be needed to determine if those larger areas with a more closed canopy of bur oak should be considered part of Western Great Plains Dry Bur Oak Forest and Woodland (CES303.667). Likewise, within the mixedgrass system, small seeps may occur, especially during the wettest years. Although these are not considered a separate system, the suppression of fire within the region has enabled the invasion of native woody species such as *Juniperus virginiana, Juniperus pinchotii, Ziziphus obtusifolia, Prosopis glandulosa*, and also allowed for the establishment of *Pinus ponderosa* in some northern areas.

**Comments:** This system is found primarily in the Central Mixed‑grass Prairie (TNC Ecoregion 33); it becomes more restricted to mesic lowlands sites to the west and southwest in the shortgrass prairie region of Texas (S. Menard pers. comm. 2005). This is probably a reference to the Llano Estacado region rather than the Southern Shortgrass Prairie (TNC Ecoregion 28) (J. Teague pers. obs. 2005). The Central Mixed‑grass Prairie (TNC Ecoregion 33) should be extended south to include the Rolling Plains of Texas; being separated from the Southern Shortgrass Prairie (TNC Ecoregion 28) by the Caprock Escarpment (L. Elliott pers. comm. 2005).

DISTRIBUTION

**Range:** This system is found throughout the central and southern areas of the western Great Plains ranging from southern South Dakota into the Rolling Plains and western Edwards Plateau of Texas.

**Divisions:** 205:C, 303:C

**TNC Ecoregions:** 27:P, 28:P, 29:C, 32:C, 33:C, 36:C, 37:P

**Nations:** US

**Subnations:** CO, KS, ND, NE, OK, SD, TX

**Map Zones:** 27:P, 30:C, 31:C, 32:C, 33:C, 34:C, 35:C, 38:C, 39:P, 43:P

**USFS Ecomap Regions:** 223A:??, 251A:CP, 251B:CC, 251E:CP, 251F:CC, 251H:CC, 255A:??, 315F:CC, 331B:CC, 331C:CC, 331E:CC, 331F:CC, 331H:CC, 331I:CC, 331M:CP, 332B:CC, 332C:CC, 332D:CC, 332E:CC, 332F:CC

CONCEPT

**Environment:** Differences in topography and soil characteristics occur across the range of this system. It is often characterized by gently rolling to extremely hilly landscapes with soils developed from loess, shale, limestone or sandstone parent material, including Pennsylvanian formations of the Red Rolling Plains (Elliott 2011). Mollisol soils are most prevalent and range from silt loams and silty clay loams with sandy loams possible on the western edge of the range. The Red Hills region of Kansas and Oklahoma, which contains examples of this system, contains somewhat unique soil characteristics and has developed from a diversity of sources including red shale, red clay, sandy shale, siltstone, or sandstone. These soils have developed a characteristic reddish color from the primary material. These soils can consist of silt, loam, clay loam, or clay and can have textures ranging from a fine sandy loam to a more clayey surface. Ecological Sites include Clay Slopes, Loamy Prairie, Clayey Upland, Claypan Prairie, Sandy Loam, and Clay Loam (Elliott 2011).

**Vegetation:** This system typically contains grass species such as *Bouteloua curtipendula, Schizachyrium scoparium, Andropogon gerardii, Hesperostipa comata, Sporobolus heterolepis*, and *Bouteloua gracilis*, although the majority of the associations within the region are dominated by *Pascopyrum smithii* or *Schizachyrium scoparium*. Isolated patches of *Quercus macrocarpa* also can occur. This system represents the common prairie type in the Rolling Plains of Texas (Elliott 2011). This prairie often has *Schizachyrium scoparium* as a dominant, with *Bouteloua curtipendula, Bouteloua hirsuta, Bouteloua gracilis, Bouteloua dactyloides (= Buchloe dactyloides), Andropogon gerardii, Pascopyrum smithii*, and *Nassella leucotricha* also commonly encountered. Grazing tends to favor shortgrass species such as *Bouteloua dactyloides* and *Bouteloua gracilis*. This system is frequently invaded by juniper (primarily *Juniperus pinchotii*) and *Prosopis glandulosa* (Elliott 2011).

**Dynamics:** Fire, grazing, and drought are the primary processes occurring within the system. The diversity in this mixedgrass system likely reflects both the short‑ and long‑term responses of the vegetation to these often concurrent disturbance regimes (Collins and Barber 1985). Fire is not as common as in more fertile, well‑watered tallgrass prairies further east but is still important. Fire‑return intervals have been estimated at 5‑10 years (K. Kindscher pers. comm.), but fires burn patchily across the landscape, consuming vegetation in some areas and missing others. This combined with the differential responses of species to burning results in greater diversity across the landscape (Wright 1974). Grazing by native ungulates, primarily bison (*Bos bison*) and small mammals, principally prairie dogs (*Cynomys* spp.) added a further degree of patchy disturbance to the mixedgrass prairie (Whicker and Detling 1988, Weltzin et al. 1997). Long‑term precipitation variance affects diversity of the mixedgrass prairie, creating conditions more favorable to shortgrass species during droughts while allowing mixedgrass species to spread during wetter years (Albertson and Tomanek 1965).

SOURCES

**References:** Albertson and Tomanek 1965, Barbour and Billings 1988, Bell 2005, Branson and Weaver 1953, Collins and Barber 1986, Comer et al. 2003*, Elliott 2011, Elliott 2013, Eyre 1980, Kindscher pers. comm., LANDFIRE 2007a, Ricketts et al. 1999, Rolfsmeier and Steinauer 2010, Shiflet 1994, Weaver and Albertson 1956, Weaver and Bruner 1948, Weltzin et al. 1997, Whicker and Detling 1988, Wright 1974

**Version:** 27 May 2016	**Stakeholders:** Midwest, Southeast

**Concept Author:** S. Menard and K. Kindscher	**LeadResp:** Midwest

CES303.451 Northern Great Plains Fescue‑Mixed Grass Prairie

**Primary Division:** Western Great Plains (303)

**Required Classifiers:** Natural/Semi‑natural; Vegetated (>10% vasc.); Upland

**Concept Summary:** This fescue‑mixed grass ecological system is found in the northern Great Plains of Canada and adjacent areas of the United States, with the main area in Saskatchewan, west to Alberta, east to Manitoba and southward to outlier areas in North Dakota and Montana. This midgrass system is typically dominated by *Festuca hallii*. Other common graminoid species include *Hesperostipa comata, Hesperostipa curtiseta, Avenula hookeri, Koeleria macrantha, Pascopyrum smithii*, and upland sedges such as *Carex obtusata, Carex duriuscula, Carex inops ssp. heliophila*, and others. Common herbaceous species tend to be somewhat restricted. *Symphoricarpos occidentalis* and *Rosa arkansana* are common shrub species but may not be readily visible because of the tall growth of the *Festuca hallii*. Other shrubs that may be present include *Rosa woodsii, Artemisia frigida, Amelanchier alnifolia*, and *Rosa acicularis*. Overgrazing can heavily impact species composition and abundance. It usually occurs on nearly level to undulating terrain at elevations between 650 and 1250 m (2130‑4100 feet). Stands tend to be on level sites, hilltops and upper slopes in the southern portion of the range, becoming more restricted to south‑facing sites to the north. They may be on uplands, low‑relief inclines in valleys or in valley settings. Soils may be solonetzic, with an impervious hardpan layer in the subsoil caused by excess sodium (Na+) or may also be clay, silty clay, or loam.

**Comments:** Nearly all of the range of this system occurs in Canada. More information about occurrences in Canada will help refine the definition of this group. The taxonomy of this type has been confused in the past. For example, Sims and Risser (2000) refer to these as *Festuca scabrella* grasslands. Here we follow the recent treatment in Flora of North America. Information provided by Coupland and Brayshaw (1953) and Coupland (1992b) should be consulted to improve the description of this system.

DISTRIBUTION

**Range:** This group is found in the northern Great Plains of Canada and in adjacent areas of the United States, from Manitoba Saskatchewan and Alberta, south to Montana and North Dakota.

**Divisions:** 303:C

**Nations:** CA, US

**Subnations:** AB, MB, MT, ND, SK

CONCEPT

**Environment:** This group occurs on level to undulating topography at elevations between 650 and 1250 m (2130‑4100 feet). Stands tend to be on level sites, hilltops and upper slopes in the southern portion of the range, becoming more restricted to south‑facing sites to the north. They may be on uplands, low‑relief inclines in valleys or in valley settings. Soils may be solonetzic, with an impervious hardpan layer in the subsoil caused by excess sodium (Na+) or may also be clay, silty clay or loam.

**Vegetation:** This midgrass type is typically dominated by *Festuca hallii (= Festuca altaica ssp. hallii)*. The vegetation is characterized by dense to sparse *Festuca hallii*, often with *Hesperostipa curtiseta* or *Hesperostipa comata* codominant. Other species may be rare or there may be interspersed forbs and shrubs between fescue tussocks. Tussocks may be so dense that few other species are present. Some examples may contain considerable leaf litter, bare soil and rock. Other common graminoid species include *Avenula hookeri (= Helictotrichon hookeri), Koeleria macrantha, Pascopyrum smithii*, and upland sedges such as *Carex obtusata, Carex duriuscula, Carex inops ssp. heliophila*, and others. Common herbaceous species tend to be somewhat restricted but may include *Symphyotrichum falcatum, Galium boreale, Achillea millefolium, Artemisia ludoviciana*, and *Cerastium arvense*. *Symphoricarpos occidentalis* and *Rosa arkansana* are common shrub species but may not be readily visible because of the tall growth of the *Festuca hallii*. Other shrubs that may be present include *Rosa woodsii, Artemisia frigida, Amelanchier alnifolia*, and *Rosa acicularis*. Species composition and abundance can shift dramatically with overgrazing.

**Dynamics:** Fire and grazing constitute the primary dynamics affecting this system. Drought can also impact this system. With intensive grazing, species composition and abundance can shift and lead to an increase in dominance of cool‑season exotics such as *Poa pratensis, Bromus inermis*, and *Bromus arvensis*.

SOURCES

**References:** Coupland 1961, Coupland and Brayshaw 1953*, Midwestern Ecology Working Group n.d.

**Version:** 27 May 2016	**Stakeholders:** Canada, Midwest, West

**Concept Author:** R.T. Coupland and T.C. Brayshaw (1953)	**LeadResp:** Midwest

CES303.674 Northwestern Great Plains Mixedgrass Prairie

**Primary Division:** Western Great Plains (303)

**Land Cover Class:** Herbaceous

**Spatial Scale & Pattern:** Matrix

**Required Classifiers:** Natural/Semi‑natural; Vegetated (>10% vasc.); Upland

**National Mapping Codes:** EVT 2141; ESLF 7114; ESP 1141

**Concept Summary:** This system extends from northern Nebraska into southern Canada and westward through the Dakotas to the Rocky Mountain Front in Montana and eastern Wyoming, on both glaciated and non‑glaciated substrates. Soil texture (which ultimately effects water available to plants) is the defining environmental descriptor; soils are primarily fine and medium‑textured and do not include sand, loamy sand, or sandy loam soils. This system occurs on a wide variety of landforms (e.g., rolling uplands stream terraces, ridgetops) and in proximity to a diversity of other systems. Most usually it is found in association with Western Great Plains Sand Prairie (CES303.670) which occupies the coarser‑textured substrates. Northwestern Great Plains Shrubland (CES303.662) is intermixed on the landscape in draws and ravines which receive more precipitation runoff and are somewhat protected from fires. In various locales generally north and east of the Missouri River, the topography where this system occurs is broken by many glacial pothole lakes, and this system may be proximate to Great Plains Prairie Pothole (CES303.661). On the eastern Montana and western Dakota plains, mixedgrass prairie is by far the predominant system. Here it occurred continuously for hundreds of square kilometers, interrupted only by riparian areas or sand prairies, which were associated with gentle rises, eroded ridges, or mesas derived from sandstone. The growing season and rainfall are intermediate to drier units to the southwest and mesic tallgrass regions to the east. Graminoids typically comprising the greatest canopy cover include *Pascopyrum smithii, Nassella viridula*, and *Festuca* spp. In Montana these include *Festuca campestris* and *Festuca idahoensis*. Other commonly dominant species in Montana are *Bouteloua gracilis, Hesperostipa comata*, and *Carex filifolia*, while *Festuca campestris* and *Festuca idahoensis* may be more abundant in the north and foothill/montane grassland transition areas. *Bouteloua curtipendula, Elymus lanceolatus, Muhlenbergia cuspidata*, and *Pseudoroegneria spicata* are common, and sometimes abundant, components of this system. Remnants of *Hesperostipa curtiseta*‑dominated vegetation are found in northernmost Montana and North Dakota associated with the most productive sites (largely plowed to cereal grains); this species, usually in association with *Pascopyrum smithii*, is much more abundant in Canada. Sites with a strong component of *Nassella viridula* indicate a more favorable moisture balance and perhaps a favorable grazing regime as well because this is one of the most palatable of the mid‑grasses. *Hesperostipa comata* is also an important component and becomes increasingly so as improper grazing practices favor it at the expense of (usually) *Pascopyrum smithii*; progressively more destructive grazing can result in the loss of *Pascopyrum smithii* from the system followed by drastic reduction in *Hesperostipa comata* and ultimately the dominance of *Bouteloua gracilis* (or *Poa secunda* and other short graminoids) and/or a lawn of *Selaginella densa*. *Koeleria macrantha*, at least in Montana and southern Canada, is the most pervasive grass; if it has high cover, past intensive grazing is the presumed reason. In the eastern portion of this system's range, tallgrass species, especially *Andropogon gerardii, Panicum virgatum*, and *Sorghastrum nutans*, are often present to common on more mesic sites. Shrub species such as *Symphoricarpos* spp., *Artemisia frigida*, and *Artemisia cana* occur in the western and central portions while *Symphoricarpos* spp. and *Prunus* spp. can be found in the eastern portion. Sites with slightly to moderately saline soils have small to moderate amounts of salt‑tolerant species such as *Distichlis spicata* and *Sporobolus airoides*. Fire, grazing and climate constitute the primary dynamics affecting this system. Drought can also impact this system, in general favoring the shortgrass component at the expense of the mid‑grasses. With intensive grazing, cool‑season exotics such as *Poa pratensis, Bromus inermis*, and *Bromus tectorum* can increase in dominance; both of the rhizomatous grasses have been shown to markedly depress species diversity. Shrub species such as *Juniperus virginiana* can also increase in dominance with fire suppression. This system is one of the most disturbed grassland systems in Nebraska, North and South Dakota, and Canada.

**Comments:** This system is found in the western Great Plains north of the shortgrass prairie and west of the northern tallgrass prairie. Because of its proximity to other prairie types, this system contains elements from both shortgrass and tallgrass prairies, which combine to form the mixedgrass prairie throughout its range. This system was edited to expand the concept for central Montana mixedgrass prairie and to exclude specifically sandy soil grasslands, which are placed into Western Great Plains Sand Prairie (CES303.670). This system is similar to Central Mixedgrass Prairie (CES303.659) and can contain elements of Great Plains tallgrass and shortgrass systems. However, it differs from Central Mixedgrass Prairie (CES303.659) in that the cooler climate in this region allows natural cool‑season grasses to be more important (greater than 50% cover). Cover of native, nongrazing‑induced shrubs typically does not exceed 25% in conjunction with topographic relief (breaks); otherwise the stand would be considered part of Northwestern Great Plains Shrubland (CES303.662). Additional review and commentary by Canadian, Dakotan, and Nebraskan ecologists is needed to flesh out the compositional variation and range of distribution for this important grassland system. In Wyoming, this system transitions into Northern Rocky Mountain Lower Montane, Foothill and Valley Grassland (CES306.040) in the foothills of the northern Wyoming mountains where *Pascopyrum smithii* communities finger up into foothills. If *Festuca idahoensis, Carex rossii, Artemisia nova*, or *Artemisia tripartita ssp. rupicola* occur, then the example is not this system.

DISTRIBUTION

**Range:** This system is found in the western Great Plains north of the shortgrass prairie and west of the northern tallgrass prairie and extends from northern and western Nebraska into southern Canada, and west to central Montana and eastern Wyoming. The U.S. range corresponds to Bailey et al. (1994) sections 331D, 331E, 331F (mostly), 331G, 332A, 332B, 332D, and perhaps minor extensions into 251B, and in Canada to the Moist Mixed Grassland and Fescue Grassland.

**Divisions:** 205:P, 303:C

**TNC Ecoregions:** 26:C, 34:C, 66:P, 67:C

**Nations:** CA, US

**Subnations:** AB, MB, MT, ND, NE, SD, SK, WY

**Map Zones:** 20:C, 22:C, 29:C, 30:C, 31:C, 39:C, 40:C

**USFS Ecomap Regions:** 331D:CC, 331E:CC, 331F:CC, 331G:CC, 331H:CC, 331K:CC, 331L:CC, 331M:CC, 331N:CC, 332B:CC, 332C:CC, 332D:CC, 342A:CP, 342F:CC, 342G:CC, M331A:CP, M331B:CC, M331I:CC, M331J:C?, M334A:CC

CONCEPT

**Environment:** This system extends from northern Nebraska into southern Canada and westward through the Dakotas to the Rocky Mountain Front in Montana and eastern Wyoming, on both glaciated and non‑glaciated substrates. It occurs on a wide variety of landforms (e.g., rolling uplands, mesatops, stream terraces) and in proximity to a diversity of other systems. Elevations range typically from 430‑1220 m, and up to 1980 m in the northwestern extent (LANDFIRE 2007a).

*Climate:* The climate is cool, continental, ranging from hot summers (mean daily temperature in July of 15°C in the northwest to 25°C in the southeast) to cold winters (mean daily temperature of ‑16°C in the northeast to ‑5°C in the southwest). Precipitation increases from west (25 cm) to east (55 cm) with most falling as rain or snow from April through June (LANDFIRE 2007a). Climate and growing season length for the region this system occurs are intermediate to the shortgrass regions to the west and southwest and the tallgrass regions to the east with a shorter growing season and less humid climate compared to the range of Central Mixedgrass Prairie (CES303.659). Moisture conditions are generally semi‑arid.

*Physiography/landform:* Given the system's rather extensive geographic range, it is not surprising to find it occurring on a wide variety of landforms (e.g., rolling uplands, mesatops, stream terraces) and in proximity to a diversity of other systems.

*Soil/substrate/hydrology:* Soils are variable as it occurs on both glaciated and non‑glaciated substrates generally with Entisols in the west and Mollisols in the east (LANDFIRE 2007a). Soil texture (which ultimately effects water available to plants) is the defining environmental descriptor; soils are primarily fine‑ and medium‑textured, ranging from silt and clay loams, silty clay loams, silt loams to gravelly loam and do not include sands, sandy soils, or coarse sandy loams (Rolfsmeier and Steinauer 2010). In unglaciated areas, soils are derived primarily from fine‑textured sedimentary rocks and deposits, primarily Cretaceous Pierre Shales, and to a lesser extent in Tertiary siltstones and chalky shales (Rolfsmeier and Steinauer 2010). Other rock types are included so long as their weathering products are not coarse‑textured, namely not sandy soils. In glaciated areas, this system is found over glacial till and sometimes glacial lakeplains. It is found primarily on planar to gently rolling topography but is found on broken topography hillslopes as well. Some examples may include an impermeable or slowly permeable subsoil claypan layer. Other northern soils may be solonetzic and characterized by a subsoil hardpan layer with an excess of sodium (Adams et al. 2013).

**Vegetation:** This system contains greater than 50% cover of natural, cool‑season grasses such as *Festuca* spp., *Pascopyrum smithii, Elymus lanceolatus, Hesperostipa comata, Hesperostipa curtiseta*, and *Nassella viridula*. *Hesperostipa comata* becomes increasingly important where improper grazing regimes have favored it at the expense of (usually) *Pascopyrum smithii*; progressively more destructive grazing can result in the loss of *Pascopyrum smithii* from the system followed by drastic reduction in *Hesperostipa comata* and ultimately the dominance of *Bouteloua gracilis* (or *Poa secunda* and other short graminoids) and/or a lawn of *Selaginella densa*. *Koeleria macrantha*, at least in Montana and southern Canada, is the most pervasive grass; if it has high cover, past intensive grazing is the presumed reason. Shrub species such as *Symphoricarpos* spp. and *Artemisia frigida* also occur. Cover of native, nongrazing‑induced shrubs typically does not exceed 25% in conjunction with topographic relief (breaks); otherwise the stand would be considered part of Northwestern Great Plains Shrubland (CES303.662). Cool‑season exotics such as *Poa pratensis, Bromus inermis*, and the annual *Bromus tectorum* can increase in dominance with overgrazing; both of the above‑named rhizomatous grasses are sufficiently aggressive to outcompete natives regardless of disturbance regime. Likewise, shrub species such as *Juniperus virginiana* can also increase in dominance with fire suppression.

**Dynamics:** This grassland system evolved with fire, grazing, and drought, which constitute the primary dynamics affecting this system. The diversity in this mixedgrass system likely reflects both the short‑ and long‑term responses of the vegetation to these often concurrent disturbance regimes (Collins and Barber 1985). Drought, rather than fire, is the primary driver maintaining the dry mixed grassland because it occurs more frequently than fire, inhibits expansion of woody shrubs and reduces the abundance of tallgrasses and mesophytic forbs, and prevents an accumulation of fuel that would maintain a frequent fire regime (Sala et al. 1996). Although variable in area, severe drought years in the Great Plains tend to occur in clusters periodically (1890s, 1930s, mid‑1950s, late 1970s, late 1980s to early 1990s, and early 2000s) and have major ecological impacts.

Historic fire‑return intervals have been estimated at 8‑12 years (LANDFIRE 2007a), but fires burn patchily across the landscape, consuming vegetation in some areas and missing others because of natural firebreaks such as badlands, break in topography/ridge, and rivers. Fire‑return intervals were likely longer in the drier, less vegetated central and western portions of this system's range and shorter in the east, near the transition to tallgrass prairie‑dominated landscapes. Grazing and prairie dog towns also reduced fuel loads and fire frequency, size and intensity, with the most substantial impacts in valley bottom shrublands and grasslands, and upland grasslands near water (LANDFIRE 2007a). Historically, the majority of human‑caused ignitions were concentrated in spring and fall seasons, while the more common lightning‑caused fires were concentrated in late summer (Higgins 1984, 1986, LANDFIRE 2007a). This combined with the differential responses of species to burning results in greater diversity across the landscape (Wright and Bailey 1980).

Grazing by native ungulates, primarily bison (*Bos bison*) and small mammals, principally prairie dogs (*Cynomys ludovicianus*) added a further degree of patchy disturbance to the mixedgrass prairie (Whicker and Detling 1988). Available soil moisture drives species composition in this grassland, with a higher percentage of tall grasses on relatively moist, and cooler north‑facing slopes, and mid and short grasses on drier steep and warmer southerly exposures (Rolfsmeier and Steinauer 2010). Long‑term precipitation variance affects diversity of the central mixedgrass prairie, creating conditions more favorable to shortgrass species during droughts while allowing mixedgrass species to spread during wetter years (Sims et al. 1978, Singh et al. 1983). Extended drought in similar mixedgrass prairie in central Kansas caused loss of most forbs and cool‑season grasses, and severe reductions of warm‑season grasses (70‑80%) (Albertson 1937) and likely has the same effects on mixedgrass prairie further north.

The absence of grazing and replacement fire for many years (e.g., 50 years) would lead to an increased shrub component (often *Symphoricarpos* spp. and *Fraxinus pennsylvanica*, but also possibly *Prunus* spp., *Amelanchier alnifolia, Elaeagnus commutata, Dasiphora fruticosa ssp. floribunda*, and *Juniperus horizontalis*) in precipitation zones greater than 35 cm, and a buildup of dead grass (LANDFIRE 2007a). Within the semi‑arid (25‑35 cm) precipitation zones, *Artemisia tridentata ssp. wyomingensis* and *Artemisia cana* may also increase. Productivity of the grasses is decreased, resulting in greater mortality from smoldering fire (LANDFIRE 2007a). Mormon crickets (*Anabrus simplex*), grasshoppers (Orthoptera) and extinct Great Plains locust (*Melanoplus spretus*) probably had more of an impact in this system than currently defined, but the historical impact and frequency are unknown.

<br />LANDFIRE developed a VDDT model for this system which has two classes (LANDFIRE 2007a, BpS 2911410).

A) Early Development 1 All Structures (25% of type in this stage): Herbaceous cover is 0‑40%. Class A is the post‑fire early‑seral stage, combined with the very short‑statured vegetation resulting from prairie dog disturbance or repeated high‑intensity herbivory or trampling (e.g., watering points or buffalo wallows). This class may also be a short‑term response to severe drought, combined with other impacts. This class lasts approximately three years. If in a prairie dog state, then the class would last longer in order to transition out of it; however, this is accounted for by having a prairie dog disturbance in the model, resetting succession and keeping it in this class. The 3‑year interval attempts to capture what would happen post‑fire or post‑drought. Also post‑heavy‑grazing in current conditions would take longer to transition out of this class. Drought can occur every 30 years, not causing a transition. Replacement fire occurs but not as frequently, due to lack of fuel, every 20 years.

B) Mid Development 1 Closed (75% of type in this stage): Herbaceous cover (41‑90%). Class B represents the intact historic plant community functioning under grazing and/or fire, dominated by taller, cool and warm‑season rhizomatous perennial grasses, as well as bunchgrasses. This is the all‑encompassing mid‑to late‑development, functioning final stage. Little below‑ground mortality occurs after replacement fire, and resprouting of perennial grasses and forbs often occurs within days or weeks, depending on season. Grasses show greater vigor; some forb establishment may occur as a result of exposure of mineral soil. Canopy cover recovers quickly after resprouting. Shrub species could be present at 0‑10% cover. Silver sagebrush and winterfat (on deeper soils) are the most common shrub, and would start resprouting. Wyoming big sagebrush can also be a component (on shallower soils) of this BpS, although a small component. Clubmoss might be present in Glaciated Plains at 0‑5% cover, but not on shallow clay sites or dense clay sites, sands, saline upland, saline lowland, subirrigated or wet meadow. Replacement fire occurs every 5‑15 years. Drought occurs every 30 years and maintains this stage. Native grazing by large ungulates could have occurred, including bison grazing. It is likely heavy locally due to increased succulence of young grasses. It might occur with a probability once every five years or 20% of this class each year. Native grazing by prairie dogs could also occur on a small portion of the landscape, bringing this state to A. Insect/disease occurs very infrequently. Grasshoppers and Mormon crickets might have a larger impact historically; however, there is uncertainty of impact and frequency. With a lack of fire, this class might shift to having more shrubs and tree invasion.

In the LANDFIRE BpS 2011410 model (3 Classes), drought was also thought to occur once every 30 years on average (LANDFIRE 2007a). It was also acknowledged that this system occurs within the very same biotope as Inter‑Mountain Basins Big Sagebrush Steppe (CES304.778) or Inter‑Mountain Basins Big Sagebrush Shrubland (CES304.777), the only difference being that fire has not been present where the sagebrush systems occur, a purely stochastic outcome (LANDFIRE 2007a).

SOURCES

**References:** Adams et al. 2013, Albertson 1937, Anderson 1990b, Bailey et al. 1994, Barbour and Billings 1988, Bragg and Steuter 1995, Branson and Weaver 1953, Collins and Barber 1986, Comer et al. 2003*, Davies 2011, Fink and Wilson 2011, Gober 2000, Higgins 1984, Higgins 1986, Hoagland 2006, Kotliar et al. 2006, LANDFIRE 2007a, MTNHP 2002b, Mack et al. 2007, Ogle et al. 2003, Pritekel et al. 2006, Rice et al. 2012b, Ricketts et al. 1999, Rolfsmeier and Steinauer 2010, Sala et al. 1996, Shiflet 1994, Sims et al. 1978, Singh et al. 1983, Weaver 1954, Weaver and Albertson 1956, Whicker and Detling 1988, Wright and Bailey 1980

**Version:** 20 Nov 2015	**Stakeholders:** Canada, Midwest, West

**Concept Author:** S. Menard and K. Kindscher	**LeadResp:** Midwest

CES303.662 Northwestern Great Plains Shrubland

**Primary Division:** Western Great Plains (303)

**Land Cover Class:** Shrubland

**Spatial Scale & Pattern:** Large patch

**Required Classifiers:** Natural/Semi‑natural; Vegetated (>10% vasc.); Upland

**National Mapping Codes:** EVT 2085; ESLF 5262; ESP 1085

**Concept Summary:** This ecological system ranges from South Dakota into southern Canada on moderately shallow to deep, fine to sandy loam soils. These sites are typically more mesic than most of the surrounding area. This system may be located along upper terraces of rivers and streams, gently inclined slopes near breaklands, and upland sandy loam areas throughout its range. This system is dominated by shrub species such as *Amelanchier alnifolia, Rhus trilobata, Symphoricarpos* spp., *Shepherdia argentea, Crataegus douglasii, Elaeagnus commutata, Dasiphora fruticosa ssp. floribunda*, and dwarf‑shrubs such as *Juniperus horizontalis*. Midgrasses such as *Festuca* spp., *Koeleria macrantha*, and *Pseudoroegneria spicata* and species such as *Carex filifolia* can co‑occur. This system differs from Northwestern Great Plains Mixedgrass Prairie (CES303.674) in that it contains greater than 10% cover in conjunction with topographic relief (breaks) of natural shrub species. Fire and grazing constitute the primary dynamics affecting this system; drought can also impact this system. This system may include areas of Northwestern Great Plains Mixedgrass Prairie (CES303.674) where fire suppression has allowed for a greater cover of shrub species. This system is similar to Northern Rocky Mountain Montane‑Foothill Deciduous Shrubland (CES306.994) but occurs in the grassland matrix of the Great Plains, whereas the Rocky Mountain system occurs adjacent to the lower treeline of generally forested mountains and highlands. Floristically their shrub composition is similar, but associated grasses and forbs will differ somewhat given their respective adjacent vegetation types.

**Comments:** This may not be a separate system from the prairie matrix. Those areas that have increased shrub cover due to fire suppression should be considered part of Northwestern Great Plains Mixedgrass Prairie (CES303.674). More information from Canada is probably needed to fully define this system.

DISTRIBUTION

**Range:** This system extends from South Dakota into southern Canada, west into the foothills of north‑central Montana. The U.S. range corresponds to Bailey et al. (1994) sections Northeast Glaciated Plains (332A), Western Glaciated Plains (332B), North Central Glaciated Plains ‑ extreme western part (251B), and in Canada to the Moist Mixed Grassland and Fescue Grassland.

**Divisions:** 303:C

**TNC Ecoregions:** 26:C, 34:C, 66:P, 67:P

**Nations:** CA, US

**Subnations:** AB, MB, MT, ND, SD, SK, WY

**Map Zones:** 20:C, 29:C, 30:C, 31:C, 39:C, 40:C

**USFS Ecomap Regions:** 331D:CC, 331E:CC, 331F:CC, 331G:CC, 331K:CC, 331L:CC, 331M:CP, 331N:CC, 342F:CC, M334A:CC

CONCEPT

**Environment:** Climate and growing season length for the region this system occurs are intermediate to the shortgrass regions to the west and the tallgrass regions to the east with a shorter growing season with semi‑arid moisture conditions. This system occurs on sites more mesic than most of the surrounding area such as upper river terraces, gently inclined slopes, and upland sandy areas. Soils range from shallow to deep and fine to sandy loams.

**Vegetation:** This system is dominated by shrub and dwarf‑shrub species such as *Amelanchier alnifolia, Rhus trilobata, Symphoricarpos* spp., *Dasiphora fruticosa ssp. floribunda*, and *Juniperus horizontalis*. Mid grasses such as *Festuca* spp., *Koeleria macrantha*, and *Pseudoroegneria spicata* can also occur. This system differs from Northwestern Great Plains Mixedgrass Prairie (CES303.674) in that it contains greater than 60% cover of natural shrub species.

**Dynamics:** Fire and grazing constitute the primary dynamics affecting this system. Drought can also impact this system.

<br />LANDFIRE developed a state‑and‑transition vegetation dynamics VDDT model for this system which has three classes in total (LANDFIRE 2007a, BpS 2010850). These are summarized as:

A) Early Development 1 Open (herbaceous‑dominated ‑ 35% of type in this stage): Cover is 0‑50%. Grasses such as little bluestem, western wheatgrass, stipa, bluebunch wheatgrass, sideoats grama and upland sedges dominate this class. This class is a combination of grasses and very short‑statured vegetation resulting also from prairie dog disturbance (maybe only in draws ‑ snowberry). A variety of forb species such as fetid marigold, scarlet globemallow, scarlet gaura, skeleton weed and dotted gayfeather tend to dominate this class. Some sprouting of snowberry, chokecherry and serviceberry. The fuel in this class would be initially too sparse to carry fire, but then fuel increases. This class lasts for 9 years then succeeds to class B, mid‑open state. (Although, if it were a dense stand initially and then re‑sprouted, might take fewer than 9 years to get to class B.) Replacement fire occurs every 30 years, and sets this class back to its beginning stage. Grazing (0.07 probability or 7% of this class each year), the combination of drought and grazing (0.02 probability or 2% of this class each year) and drought modeled as wind/weather/stress (0.05 probability or 5% of this class each year) all occur and maintain this class but don't set it back to its beginning state. Prairie dog impact occurs with a probability of 0.0035 (0.35% of class each year) and returns this class to its beginning. The only shrub that prairie dogs might impact in this BpS would be the snowberry sites and draws/drainageways.

B) Mid Development 1 Open (shrub‑dominated ‑ 25% of type in this stage): Shrub cover is 0‑20%. More open community than late stage. Seedling shrubs. Dominant shrubs coming in include snowberry, chokecherry, skunkbush, creeping juniper and buffaloberry. Western wheatgrass, needlegrasses, little bluestem and upland sedges are common grasses ‑ same as in class A. Bluebunch wheatgrass can be locally common with skunkbush. Common forbs include scurfpea, prairie coneflower, Rocky Mountain beeplant, scarlet globemallow and dotted gayfeather. Herbaceous cover is approximately 30‑70% and approx. 0.5 m in height. This class lasts 9 years and then succeeds to the late‑development stage. Replacement fires occur every 30 years. Grazing (0.02 probability or 2% of this class each year) and the combination of drought and grazing (0.01 probability) occur and cause a transition back to the early stage, class A. Grazing (0.02 probability), the combination of drought and grazing (0.003 probability) and drought modeled as wind/weather stress (0.1 probability) can also occur while maintaining this class in this stage. Prairie dog impact occurs with a probability of 0.0003, taking the class back to class A.

C) Late Development 1 Closed (shrub‑dominated ‑ 40% of type in this stage): Tree cover is 21‑80%. Denser, higher canopy cover. Mature canopy. Vegetation community is similar to previous class. Forbs are present still. Litter layer tends to be relatively continuous. Herbaceous cover 50‑65% and 0.5 m in height. Snowberry average cover could be 65%. Maximum up to 75%, minimum approx. 45%. Skunkbush cover average approximately 25%. Horizontal juniper average 44%, range of 25‑65% cover. Each of the shrub species associated with own habitat type with moisture gradient. Skunkbush is dry end, and snowberry/chokecherry is wet end.

The northern mixed‑grass prairie and shrublands are strongly influenced by wet‑dry cycles. Fire, grazing by large ungulates and small mammals such as prairie dogs and soil disturbances (i.e., buffalo wallows and prairie dog towns) are the major disturbances in this vegetation type. In MZ30, many of these shrubland types occur on moderate to steep slopes (west‑ to northwest‑facing).

From instrumental weather records, droughts are likely to occur about 3 in every 10 years. Historically, there were likely close interactions between fire and grazing since large ungulates tend to be attracted to post‑fire communities. Conversely, fire presumably was less likely in areas recently heavily grazed by herbivory, thus contributing to spatial and temporal variation in fire occurrence.

Average fire intervals are estimated at 8‑25 years, although in areas with very broken topography fire intervals may have been greater than 30 years. The model for MZ20 reflects a 30‑year FRI. This system's FRI should be very similar to 1141 mixedgrass prairie, since this system is just inclusions within 1141. It might be a little less frequent because of moisture; however, it should be similar.

Fires were most common in July and August, but probably occurred from about April to September. Seasonality of fires influences vegetation composition. Early‑season fires (April ‑ May) tend to favor warm‑season species, while late‑season fires (August ‑ September) tend to favor cool‑season species. Replacement fire in our model does remove 75% of the above‑ground cover as assumed in the literature. However, loss of the above‑ground cover by the replacement fire will not necessarily induce a retrogression back to an earlier seral stage from the late stage because the main component of dominant grasses remains unharmed to insure the continuity of the seral stage. The shrub species, however, are sprouters. Fire would remove them, and they would resprout. The exception would be horizontal juniper and skunkbush which would not resprout. It would take longer for them to become re‑established.

Different levels of native ungulate grazing intensities were used in LANDFIRE modelling. Light grazing was assumed to not alter the community enough to change classes but increasing grazing intensity would move the community back to earlier stages. Grazing return interval probably occurred every 7‑10 years but grazing would only result in a class change maybe once every 80‑100 years. Overall, the grazing frequency was modeled at every 20 years ‑ that includes grazing just occurring with no transition resulting, as well as grazing taking the stage back to an earlier class. And, overall, the drought plus grazing impact frequency was modeled as every 70 years ‑ that includes the no‑transition plus transition to early stage (LANDFIRE 2007a, BpS 2010850). In addition to fire, drought, grazing and insect outbreaks (Rocky Mountain locust) would have impacted all classes, historically.

SOURCES

**References:** AOU 1983, Anderson 2001d, Anderson 2004a, Anderson 2004b, Arnold and Higgins 1986, Bailey et al. 1994, Barnett and Crawford 1994, Bent et al. 1968, Blankespoor 1980, Comer et al. 2003*, Crawford et al. 2004, Drut et al. 1994, Ersch 2009, Esser 1994a, Fryer 1997, Gregg and Crawford 2009, Gucker 2006g, Kahl et al. 1985, LANDFIRE 2007a, Shafer et al. 2014, Shiflet 1994, Smith 1963, Vickery 1996, Wiens 1969

**Version:** 24 May 2018	**Stakeholders:** Canada, Midwest, West

**Concept Author:** S. Menard and K. Kindscher	**LeadResp:** Midwest

CES303.817 Western Great Plains Foothill and Piedmont Grassland

**Primary Division:** Western Great Plains (303)

**Land Cover Class:** Herbaceous

**Spatial Scale & Pattern:** Large patch

**Required Classifiers:** Natural/Semi‑natural; Vegetated (>10% vasc.); Upland

**Diagnostic Classifiers:** Lowland [Foothill]; Toeslope/Valley Bottom; Clay Soil Texture; Aridic; Short Disturbance Interval [Periodicity/Irregular Disturbance]; F‑Patch/Low Intensity; Graminoid

**National Mapping Codes:** EVT 2147; ESLF 7120; ESP 1147

**Concept Summary:** This ecological system typically occurs between 1600 and 2200 m in elevation. It is best characterized as a mixedgrass to tallgrass prairie on mostly moderate to gentle slopes, usually at the base of foothill slopes, e.g., the hogbacks of the Rocky Mountain Front Range where it typically occurs as a relatively narrow elevational band between montane woodlands and shrublands and the shortgrass steppe and mixedgrass prairie, but extends east on the Front Range piedmont alongside the Chalk Bluffs near the Colorado‑Wyoming border, out into the Great Plains on the Palmer Divide, and on piedmont slopes below mesas and foothills in northeastern New Mexico. A combination of increased precipitation from orographic rain, temperature, and soils limits this system to the lower elevation zone with approximately 40 cm of precipitation/year. It is maintained by frequent fire and associated with well‑drained clay soils. Usually occurrences of this system have multiple plant associations that may be dominated by *Andropogon gerardii, Schizachyrium scoparium, Nassella viridula, Pascopyrum smithii, Sporobolus cryptandrus, Bouteloua gracilis, Hesperostipa comata*, or *Hesperostipa neomexicana*. In Wyoming, typical grasses found in this system include *Pseudoroegneria spicata, Schizachyrium scoparium, Hesperostipa neomexicana, Hesperostipa comata*, and species of *Poa*. Typical adjacent ecological systems include foothill shrublands, ponderosa pine savannas, juniper savannas, as well as shortgrass prairie.

**Comments:** Need to incorporate northern Rockies information. How does this differ from Northwestern Great Plains Mixedgrass Prairie (CES303.674) which seems pretty similar? In southeastern Wyoming, it is mostly in mapzone 33, along bluffs.

DISTRIBUTION

**Range:** This mixed grassland ecological system occurs in a transitional band between the Rocky Mountains and the Shortgrass Steppe where increased soil moisture from orographic lifting and local topography favor tall and mid‑height grasses. The band is restricted to the Rocky Mountain foothills and piedmont and adjacent plains, extending farther east on the Palmer Divide, north alongside the Chalk Bluffs near the Colorado‑Wyoming border, and south on and below mesas and escarpments in southeastern Colorado, northeastern New Mexico, and the panhandles of Oklahoma and Texas. These grasslands also occur around the edges of the Black Hills uplift, where Schizachyrium scoparium is the dominant grass.

**Divisions:** 303:C, 306:C

**TNC Ecoregions:** 10:C, 20:C, 21:C, 24:C, 25:P, 26:P, 27:C, 28:P

**Nations:** US

**Subnations:** AZ?, CO, NM, OK, SD, TX?, WY

**Map Zones:** 19:?, 21:?, 22:C, 24:?, 25:C, 26:P, 27:C, 28:C, 29:C, 30:P, 31:P, 33:C, 34:?

**USFS Ecomap Regions:** 315A:CC, 315B:CC, 315H:CC, 331B:CC, 331C:CC, 331F:CC, 331G:CC, 331H:CC, 331I:CC, 331J:CC, 342F:CC, M313A:CP, M313B:CC, M331F:CC, M331G:CC, M331I:CC, M341A:CC

CONCEPT

**Environment:** This ecological system occurs between 1600 and 2200 m in elevation. It is best characterized as a mixedgrass to tallgrass grassland on mostly moderate to gentle slopes, usually at the base of foothill slopes, e.g., the hogbacks of the Rocky Mountain Front Range where it typically occurs as a relatively narrow elevational band between montane woodlands and shrublands and the shortgrass steppe and mixedgrass prairie, but extends east on the Front Range piedmont alongside the Chalk Bluffs near the Colorado‑Wyoming border, out into the Great Plains on the Palmer Divide, and on piedmont slopes below mesas and foothills in northeastern New Mexico. This mixed grassland receives more precipitation than shortgrass steppe or occurs on coarser‑textured substrates allowing for increased infiltration and water storage (Noy‑Meir 1973). A combination of increased precipitation from orographic rain, temperature, and soils limits this system to the lower elevation zone with approximately 40 cm of precipitation/year. It is maintained by frequent fire and associated with well‑drained clay soils. Typical adjacent ecological systems include foothill shrublands, ponderosa pine savannas, juniper savannas, as well as shortgrass prairie.

**Vegetation:** Usually occurrences of this system have multiple plant associations that may be dominated by *Andropogon gerardii, Schizachyrium scoparium, Nassella viridula, Pascopyrum smithii, Sporobolus cryptandrus, Bouteloua gracilis, Hesperostipa comata*, or *Hesperostipa neomexicana*. In Wyoming, typical grasses found in this system include *Pseudoroegneria spicata, Schizachyrium scoparium, Hesperostipa neomexicana, Hesperostipa comata*, and species of *Poa*.

**Dynamics:** Relatively frequent surface fire (FRI = 20 years ‑15 years in the southern extent) maintains this ecosystem by reducing seedling survival of shrubs such as *Cercocarpus montanus* and *Rhus trilobata* and trees such as *Pinus ponderosa, Pinus edulis*, and *Juniperus* spp. thus preventing conversion to shrublands and woodlands (Landfire 2007a). There is little information on this natural frequency, size, intensity, or severity of fire in this ecosystem. Ungulate grazing (Landfire 2007a) and herbivory are a key process that includes grazing and browsing by large and small mammals and insects. Soils are naturally disturbed by burrowing mammals such as prairie dogs, rabbits, pocket gophers, ground squirrels, and badgers providing habitat for disturbance‑dependent species. Drought occurs periodically (approximately every 20‑50 years) and can cause shifts in species compositions to more drought‑tolerant species (Landfire 2007a).

SOURCES

**References:** CNHP 2010, Comer et al. 2003*, Hess and Wasser 1982, LANDFIRE 2007a, Lauenroth and Milchunas 1992, Mast et al. 1997, Mast et al. 1998, Neely et al. 2001, Opler and Krizek 1984, Shiflet 1994, TNC 2013, Weaver and Albertson 1956

**Version:** 14 Jan 2014	**Stakeholders:** Midwest, Southeast, West

**Concept Author:** NatureServe Western Ecology Team	**LeadResp:** West

CES303.673 Western Great Plains Tallgrass Prairie

**Primary Division:** Western Great Plains (303)

**Land Cover Class:** Herbaceous

**Spatial Scale & Pattern:** Large patch

**Required Classifiers:** Natural/Semi‑natural; Vegetated (>10% vasc.); Upland

**National Mapping Codes:** EVT 2150; ESLF 7123; ESP 1150

**Concept Summary:** This system can be found throughout the Western Great Plains Division. It is found primarily in areas where soil characteristics allow for mesic conditions more typical of the Eastern Great Plains Division and thus are able to sustain tallgrass species. This system may be small patches interspersed within Northwestern Great Plains Mixedgrass Prairie (CES303.674) or Western Great Plains Shortgrass Prairie (CES303.672) and may also be associated with upland terraces above a floodplain system where these more mesic conditions persist. Soils are primarily loamy Mollisols that are moderately deep and rich. Those areas that contain more sandy soils should be considered part of Western Great Plains Sand Prairie (CES303.670). This system is dominated primarily by *Andropogon gerardii* and may also include *Sorghastrum nutans, Schizachyrium scoparium, Pascopyrum smithii, Hesperostipa spartea*, and *Sporobolus heterolepis*. *Andropogon gerardii* often dominates the lowland regions, although *Pascopyrum smithii* can be prolific if conditions are favorable. Forbs in varying density may also be present. The primary dynamics for this system include fire, climate and grazing. Fire suppression in these areas has allowed for the invasion of woody species such as *Juniperus virginiana* and *Prunus* spp. Grazing also has contributed to these changes and likewise led to a decrease of this system as overgrazing favors shortgrass and mixedgrass systems. Conversion to agriculture likewise has probably decreased the range of this system. Thus, this system likely only occurs in small patches and in scattered locations throughout the division. Large‑patch occurrences are mostly isolated to slopes and swales of rolling uplands where either grazing or cultivation are more problematic.

**Comments:** A granitic woodland association of the Wichita Mountains of Oklahoma (*Quercus fusiformis ‑ Quercus stellata / Schizachyrium scoparium* Granite Woodland (CEGL004937)), formerly included here, now is included in Crosstimbers Oak Forest and Woodland (CES205.682).

DISTRIBUTION

**Range:** This system occurs throughout the Western Great Plains Division, however, grazing and conversion to agriculture have likely decreased its natural range.

**Divisions:** 303:C

**TNC Ecoregions:** 26:C, 27:C, 28:?, 33:C, 34:C

**Nations:** US

**Subnations:** KS, MT, ND, NE, OK, TX, WY

**Map Zones:** 29:C, 30:C, 31:C, 33:C, 34:C, 38:C, 39:C, 40:C

**USFS Ecomap Regions:** 331C:PP, 331H:PP

CONCEPT

**Environment:** This system is found primarily on loam, moderately deep, and rich Mollisols throughout the Western Great Plains Division. These soils tend to be more mesic and deep than the majority of soils within the Western Great Plains and are more typical of the Eastern Great Plains Division. This system requires more moisture than is available from precipitation in the Western Great Plains so it occurs in valleys, on lower slopes, and sometimes on floodplains (Albertson 1937, Heitschmidt et al. 1970). Occurrences are usually medium to small.

**Vegetation:** The mesic, deep soils of this system allow for dominance by *Andropogon gerardii*. Other species, such as *Sorghastrum nutans, Schizachyrium scoparium, Pascopyrum smithii, Hesperostipa spartea*, and *Sporobolus heterolepis*, can also be present. In more lowland areas, *Pascopyrum smithii* can become more prevalent. Fire suppression can lead to the invasion of these areas by woody species such as *Juniperus virginiana* and *Prunus* spp.

**Dynamics:** Fire, climate and grazing constitute the primary dynamic processes impacting this system. Fire may have occurred as often as every 5 years, especially in the wetter eastern portions of this system's range (Landfire 2007a). This system occurred in a landscape dominated by mixedgrass and shortgrass vegetation. These systems do not have the rapid build up of litter that occurs in tallgrass prairies further east and thus do not carry fire as readily so there were fewer fires that could affect this system.

This system developed in an area occupied by vast numbers of native ungulates, notably bison (*Bos bison*) but including other species, and the grazing of these species affected species composition and the patchwork of habitat. Bison preferentially favor newly burned areas and graminoids over forbs (Coppedge and Shaw 1998, Vinton et al. 1993). On unburned sites, grazing removes live and dead vegetation, allowing more light and heat to the soil surface and increasing available moisture thus favoring species, forbs or woody plants, in the case of bison grazing, that were resilient to the effects of grazing or avoided by the grazers (Damoureyeh and Hartnett 1997).

SOURCES

**References:** Albertson 1937, Barbour and Billings 1988, Bell 2005, Branson and Weaver 1953, Comer et al. 2003*, Coppedge and Shaw 1998, Damoureyeh and Hartnett 1997, Heitschmidt et al. 1970, LANDFIRE 2007a, Shiflet 1994, Vinton et al. 1993, Weaver 1954

**Version:** 14 Jan 2014	**Stakeholders:** Midwest, Southeast, West

**Concept Author:** S. Menard and K. Kindscher	**LeadResp:** Midwest

M052. Great Plains Sand Grassland & Shrubland

CES303.670 Western Great Plains Sand Prairie

**Primary Division:** Western Great Plains (303)

**Land Cover Class:** Herbaceous

**Spatial Scale & Pattern:** Large patch

**Required Classifiers:** Natural/Semi‑natural; Vegetated (>10% vasc.); Upland

**National Mapping Codes:** EVT 2148; ESLF 7121; ESP 1148

**Concept Summary:** Sand prairies are often considered part of the tallgrass or mixedgrass regions in the western Great Plains but can contain elements from Western Great Plains Shortgrass Prairie (CES303.672), Central Mixedgrass Prairie (CES303.659), and Northwestern Great Plains Mixedgrass Prairie (CES303.674). The largest expanse of sand prairies (approximately 5 million ha) can be found in the Sandhills of north‑central Nebraska and southwestern South Dakota. These areas are relatively intact. The primary use of this system has been grazing (not cultivation), and areas such as the Nebraska Sandhills can experience less degeneration than other prairie systems. Although greater than 90% of the Sandhills region is privately owned, the known fragility of the soils and the cautions used by ranchers to avoid poor grazing practices have allowed for fewer significant changes in the vegetation of the Sandhills compared to other grassland systems. Nonetheless, the sustained annual grazing within pastures by cattle has altered the mix of vegetation. The unifying and controlling feature for this system is coarse‑textured soils, and the dominant grasses are well‑adapted to this condition. Soils in the sand prairies can be relatively undeveloped and are highly permeable. Soil texture and drainage along with a species' rooting morphology, photosynthetic physiology, and mechanisms to avoid transpiration loss are highly important in determining the composition of the sand prairies. In the northwestern portion of its range, stand size corresponds to the area of exposed caprock sandstone, and small patches predominate, but large patches are also found embedded in the encompassing Northwestern Great Plains Mixedgrass Prairie (CES303.674). Another important feature is their susceptibility to wind erosion. Blowouts and sand draws are some of the unique wind‑driven disturbances in the sand prairies, particularly where there are fine sands, such as in the Nebraska Sandhills (where the rare *Penstemon haydenii* occurs). In most of eastern Montana, substrates supporting this system have weathered in place from sandstone caprock; thus the solum is relatively thin, and the wind‑sculpted features present further east, particularly in Nebraska, do not develop. Graminoid species dominate the sand prairies, although relative dominance can change due to impacts of wind disturbance. *Andropogon hallii* and *Calamovilfa longifolia* are the most common species, but other grass and forb species such as *Hesperostipa comata, Schizachyrium scoparium, Carex inops ssp. heliophila*, and *Panicum virgatum* are often present. Apparently only *Calamovilfa longifolia* functions as a dominant throughout the range of the system. In the western extent, *Hesperostipa comata* becomes more dominant, and *Andropogon hallii* is less abundant but still present. Communities of *Artemisia cana ssp. cana* are included here in central and eastern Montana. Patches of *Quercus havardii* can also occur within this system in the southern Great Plains. Fire and grazing constitute the other major dynamic processes that can influence this system. In the Western Great Plains in Texas, prairies on deep sands and sandhills which currently represent far southern outliers of this system, are dominated by species such as *Andropogon gerardii, Andropogon hallii, Calamovilfa gigantea, Cenchrus spinifex, Hesperostipa comata, Paspalum setaceum, Schizachyrium scoparium, Sporobolus cryptandrus*, and *Sporobolus giganteus*. Some woody species may be present, including *Artemisia filifolia* and *Quercus havardii*. Shrub species such as *Artemisia filifolia, Prunus angustifolia, Rhus trilobata*, and *Quercus havardii* may be present but constitute relatively little cover.

**Comments:** This system was edited to expand the concept to include sandy portions of the mixedgrass prairie of the Montana plains. Although in terms of potentially dominant graminoids there is virtually a complete overlap between the eastern and western extremities of the system, there is a distinct shift from west to east from midgrass species dominance, most notably *Hesperostipa comata*, to tallgrass species dominance, including prominently *Andropogon gerardii* and *Andropogon hallii*. Prevailing patch size also shifts from smaller to larger moving west to east. Current thinking is to include this variation within this system, but with more information and input from other Great Plains ecologists in the U.S. and Canada, this concept is subject to change, including the possibility of creating a new system. In addition, sand prairies in the Western Great Plains in Texas are currently considered far southern outliers of this system, but more information could conclude that a new system is warranted for this vegetation.

DISTRIBUTION

**Range:** This system is found throughout the Western Great Plains Division. The largest and most intact example of this system is found within the Sandhills region of Nebraska and South Dakota. However, it is also common (though occurring in predominantly small patches) farther west into central and eastern Montana. Its western extent in Wyoming is still to be determined, but it does occur in mapzone 29 on weathered‑in‑place sandy soils, where *Calamovilfa longifolia* is found, along with *Artemisia cana*. In addition, outliers have been described from the Western Great Plains in Texas (Monahans Sandhills State Park).

**Divisions:** 303:C

**TNC Ecoregions:** 26:C, 27:C, 28:C, 33:C, 34:C

**Nations:** US

**Subnations:** KS, MT, ND, NE, NM, OK, SD, TX, WY

**Map Zones:** 20:C, 27:P, 29:C, 30:C, 31:C, 32:C, 33:C, 34:C, 38:C, 39:C, 40:C

**USFS Ecomap Regions:** 251F:CC, 251H:CC, 255A:PP, 315A:CC, 315B:CC, 315F:CC, 321A:??, 331B:CC, 331C:CC, 331D:CC, 331E:CC, 331F:CC, 331G:CC, 331H:CC, 331K:CC, 331L:CC, 331M:CP, 331N:C?, 332C:CC, 332D:CC, 332E:CC, 332Fb:CCC

CONCEPT

**Environment:** The distribution, species richness and productivity of plant species within the sand prairie ecological system are controlled primarily by environmental conditions, in particular, the temporal and spatial distribution of soil moisture and topography. Soils in the sand prairies can be relatively undeveloped and are highly permeable. Soil texture and drainage along with a species' rooting morphology, photosynthetic physiology, and mechanisms to avoid transpiration loss are highly important in determining the composition and distribution of communities/associations within the sand prairies. Another important aspect of soils in the sand prairies is their susceptibility to wind erosion. Blowouts and sand draws are some of the unique wind‑driven disturbances in the sand prairies, particularly the Nebraska Sandhills, which can profoundly impact vegetation composition and succession within this system. This tallgrass prairie is found primarily on sandy and sandy loam soils that can be relatively undeveloped and highly permeable as compared to Western Great Plains Tallgrass Prairie (CES303.673), which occurs on deeper loams. This system is usually found in areas with a rolling topography and can occur on ridges, midslopes and/or lowland areas within a region. It often occurs on moving sand dunes, especially within the Sandhills region of Nebraska and South Dakota. In Montana, occurrences are intimately associated with Northwestern Great Plains Mixedgrass Prairie (CES303.674), usually occupying higher positions in local landscapes where sandy members of some geologic formations (that are predominantly marine shales) constitute the highest (and most weathering‑resistant) points in the landscape. In Texas, this system occurs on rolling to level, eolian or alluvial, deep sand deposits classed as Deep Sand or Sandhill Ecological Sites.

**Vegetation:** This system is distinguished by the dominance of graminoids such as *Andropogon hallii* and *Calamovilfa longifolia*. Other graminoids such as *Hesperostipa comata, Carex inops ssp. heliophila*, and *Panicum virgatum* may be present. Characteristic forbs differ by region, but species of *Psoralidium, Pediomelum*, and *Eriogonum* are a common feature, along with sand‑loving annuals such as *Helianthus petiolaris* and *Oenothera rhombipetala*. *Penstemon haydenii* is endemic to the sand prairie system and of special conservation concern because of its probable decline due to grazing and fire suppression. Very diffuse patches of *Rhus trilobata* are found on shallow sandy soils, often associated with breaklands; other shrubs occasionally occurring include *Artemisia cana ssp. cana, Betula occidentalis, Juniperus horizontalis, Prunus pumila var. besseyi (= Prunus besseyi), Prunus angustifolia*, and *Yucca glauca*. Many of the warm‑season graminoids extend at least to the Rocky Mountain Front as dominant components on appropriate sites or as a response to disturbance. All the characteristic species mentioned for Nebraska and South Dakota are also found in Montana stands (and possibly Wyoming and perhaps the rest of the states cited). Some of the communities cited as part of the concept in Nebraska and South Dakota are only marginally present in Montana, but others are found throughout Montana's Great Plains region. In the southern range of this system, patches of *Quercus havardii* can also occur.

**Dynamics:** The distribution, species richness and productivity of plant species within the sand prairie ecological system are controlled primarily by environmental conditions, and in particular, the temporal and spatial distribution of soil moisture and topography. Another important aspect of this system is its susceptibility to wind erosion. Blowouts and sand draws are some of the unique wind‑driven disturbances in the sand prairies, particularly the Nebraska Sandhills, which can profoundly impact vegetation composition and succession within this system.

Fire and grazing constitute the other major disturbances that can influence this system. The most extensive fires are likely to have occurred in years with wet springs followed by hot, dry summers when grazing pressure was low. Wet springs would have resulted in more productive and more continuous plant cover (i.e., fuel) that would have supported and expanded fires ignited under dry conditions occurring later in the season. In addition, litter accumulation over several fire‑free years would also have supported widespread fire, in any conditions. The litter component, a determining factor in fire size and frequency, is correlated with seral stage. Several fire‑free years produce enough litter to carry another fire (LANDFIRE 2007a).

Drought has additional impact in these very sandy soils and the high water table of the sandhills also affects the vegetation and encourages invasive trees (K. Kindscher pers. comm.). Extended periods of severe drought are likely to have affected both species composition and the stability of the sandhill soil, particularly when compounded by temperature, wind and heavy grazing. These conditions may have led to the development of blowouts making it difficult for vegetation to re‑establish quickly. The occurrence of blowout penstemon (*Penstemon haydenii*) suggests long periods when blowouts were common across the landscape although causes resulting in this feature have not been determined (LANDFIRE 2007a).

Overgrazing, fire and trampling that leads to the removal of vegetation within those areas susceptible to blowouts can either trigger a blowout or perpetuate one already occurring. Overgrazing can also lead to significant erosion. The major large grazer, bison (*Bos bison*), occurring in large numbers in this system has largely been replaced by cattle. Both species impact the range by grazing and trampling; however, bison also significantly impacted local areas by wallowing. Unlike elsewhere in the Great Plains mixed and shortgrass prairie dog towns were a minor component of the Sandhills landscape and limited to where soils were finer‑textured and in flat uplands and in valleys and the eastern Sandhills where the water table was not high (LANDFIRE 2007a).

LANDFIRE developed a state‑and‑transition vegetation dynamics VDDT model for this system which has two classes in total (LANDFIRE 2007a, BpS 3111480). These are summarized as:

A) Early Development 1 Open (25% of type in this stage): Herbaceous cover is 0‑20%. Class A represents immediate to three‑year post‑disturbance conditions. Vegetation consists of resprouting and seedling grasses and forbs. Total bare soil is greater than before the disturbance particularly on less productive sites. The vigor of new growth and the specific species affected depend on the season of the disturbance and on pre‑ and post‑disturbance environmental conditions (e.g., available soil moisture). Litter is low initially but increases until, by year three, there is enough to support fire under average burning conditions. Fire was therefore modeled as occurring somewhat less frequently than in class B. In uplands, where soil type is dominated by coarse‑grained sands with low water‑holding capacity, post‑disturbance primary production initially decreases, thus fire may only carry under ideal conditions. Under these conditions, grazing is likely to be light. In lowlands, with finer‑textured soils, primary production is determined largely by moisture availability. *Artemisia cana* can resprout immediately after fire, so it could be present in this stage as well. It could, however, be killed following intensive fires. But since there is not much litter in these sites, possibility of intense fire is reduced. Repeated grazing of these areas will prevent succession to class B. Grazing occurs with a probability of 0.05. Prairie dog grazing was modeled as optional 1, with a very unlikely probability of 0.0007. Both will set succession back to the beginning.

B) Mid Development 1 Closed (80% of type in this stage): Herbaceous cover is 21‑80%. Class B is sandhill grassland, the dominant historical condition. This class has a moderately dense herbaceous layer (20‑80% cover) up to 1 m tall. Fire (every 10 years) would return this class to A, while lack of fire (after 40 years) would move it toward class C. Shrubs may make up to 25% of the cover but is more commonly 0‑10%. Native grazing maintains this class. Severe, multiple‑year drought (every 100 years) moves this to class C by reducing grass cover and fuel loads and giving a competitive advantage to the usually spare shrub cover.

C) Late Development 1 All Structures (shrub‑dominated ‑ 10% of type in this stage): Shrub cover is 21‑100%. Class C is the shrub‑dominated sandhill grassland and differs from the sandhill shrubland (BpS 1094) which is modeled separately based on edaphic differences. Fire returns this to class A (MFRI = 0.10). Dominate shrubs include sand sagebrush, shinnery oak and sand cherry.

SOURCES

**References:** Barbour and Billings 1988, Bell 2005, Comer et al. 2003*, Elliott 2012, Eyre 1980, Hauser 2005, LANDFIRE 2007a, Maser et al. 1984, Rolfsmeier and Steinauer 2010, Rondeau et al. 2016, Rondeau et al. 2018, Shafer et al. 2014, Shiflet 1994, Sims 1988, Tolstead 1942, Weaver 1958b

**Version:** 24 May 2018	**Stakeholders:** Midwest, Southeast, West

**Concept Author:** S. Menard and K. Kindscher	**LeadResp:** Midwest

CES303.671 Western Great Plains Sandhill Steppe

**Primary Division:** Western Great Plains (303)

**Land Cover Class:** Shrubland

**Spatial Scale & Pattern:** Large patch

**Required Classifiers:** Natural/Semi‑natural; Vegetated (>10% vasc.); Upland

**National Mapping Codes:** EVT 2094; ESLF 5271; ESP 1094

**Concept Summary:** This shrubland system is found mostly in south‑central areas of the Western Great Plains Division ranging from southwestern Wyoming and southwestern Nebraska up into the Nebraska Sandhill region, south through eastern Colorado, and New Mexico to central Texas, although some examples may reach as far north as the Badlands of South Dakota. The climate is semi‑arid to arid for much of the region in which this system occurs. This system is found on somewhat excessively to excessively well‑drained, deep sandy soils that are often associated with dune systems and ancient floodplains. In some areas, this system may actually occur as a result of overgrazing in Western Great Plains Tallgrass Prairie (CES303.673) or Western Great Plains Sand Prairie (CES303.670). Typically, this system is characterized by a sparse to moderately dense woody layer dominated or codominated by *Artemisia filifolia*, but other characteristic species may be present, including *Amorpha canescens, Prosopis glandulosa* (southern stands), *Prunus angustifolia, Prunus pumila var. besseyi* (northern stands), *Quercus havardii* (Texas), *Rhus trilobata*, and *Yucca glauca*. Associated herbaceous species can vary with geography, amount and season of precipitation, disturbance, and soil texture. The herbaceous layer typically has a moderate to dense canopy but may include stands with sparse understory. Several mid‑ to tallgrass species characteristic of sand substrates are usually present to dominant, such as *Andropogon hallii, Calamovilfa gigantea, Calamovilfa longifolia, Schizachyrium scoparium, Sporobolus cryptandrus, Sporobolus giganteus*, or *Hesperostipa comata*.

**Comments:** This system is minor in the sandhills region of western Nebraska which is dominated by sand prairie. It may overlap in concept with East‑Central Texas Plains Xeric Sandyland (CES205.897). This system was modeled Monahans and Mescalero Sands of Texas and New Mexico during Landfire workshops, but probably needs significant review because of the complexity of the relationship among tallgrass, shin oak, and sandsage types. This type is probably best represented in mapzone 34.

DISTRIBUTION

**Range:** This system is found primarily within the south‑central areas of the Western Great Plains Division ranging from the Nebraska Sandhills south into central Texas. However, examples of this system can be found as far north as the Badlands in South Dakota.

**Divisions:** 303:C

**TNC Ecoregions:** 26:C, 27:C, 28:C, 33:C

**Nations:** US

**Subnations:** CO, KS, NE, NM, OK, SD?, TX

**Map Zones:** 25:?, 26:C, 27:C, 28:?, 31:C, 33:C, 34:C, 38:C

**USFS Ecomap Regions:** 315A:CC, 315B:CC, 315F:CC, 321A:CC, 331B:CC, 331C:CC, 331H:CC, 331I:CC, 332E:CC, 332F:CC, M313B:PP

CONCEPT

**Environment:** This system is found primarily in semi‑arid to arid areas of the Western Great Plains Division. It occurs on somewhat excessively to excessively well‑drained and deep sandy soils. This system is often found associated with dune systems and/or ancient floodplains but may occur in soils derived from sandstone residuum. In parts of Texas, this system is apparently restricted to thick sandy deposits in the Seymour Formation (a Pleistocene formation formed from ancient channel deposits of the Clear Fork of the Brazos River), and is found on rolling to level uplands. In these areas, it is restricted to Deep Sand, Sand Hills or Sandy Ecological Sites (Elliott 2011).

**Vegetation:** This system is distinguished by a sparse to moderately dense shrub layer (15‑90% canopy cover in Texas) dominated or codominated by *Artemisia filifolia* with *Quercus havardii* and *Prosopis glandulosa* to the south. In addition, *Rhus trilobata, Yucca glauca, Prunus angustifolia*, or *Prunus pumila var. besseyi* (northern stands) may also be conspicuous. Other common shrubs and succulents in Texas include *Atriplex canescens, Penstemon ambiguus, Yucca campestris, Cylindropuntia leptocaulis*, and *Chrysothamnus pulchellus*. Shrub cover may sometimes be sufficient to greatly reduce the cover of herbaceous species in the understory. At some sites, shrub cover may be low, and the herbaceous cover is typically dominated by grass species such as *Schizachyrium scoparium* and *Sporobolus cryptandrus*. Several mid‑ to tallgrass species characteristic of sand substrates are usually present to dominant within areas with low woody cover in this system, including *Andropogon hallii, Calamovilfa gigantea, Calamovilfa longifolia, Cenchrus spinifex, Chloris cucullata, Hesperostipa comata, Panicum havardii, Paspalum setaceum*, and/or *Sporobolus giganteus*. In Texas, forbs may at times constitute an aspect dominant with their prolific show of flowers, including *Cnidoscolus texanus, Dalea lanata, Dimorphocarpa candicans, Eriogonum annuum, Gaillardia pulchella, Helianthus petiolaris, Heliotropium convolvulaceum, Mentzelia nuda*, and *Palafoxia sphacelata*.

**Dynamics:** Fire and grazing constitute the most important processes impacting this system. Burning shrublands reduces cover of *Artemisia filifolia* for several years resulting in grassland patches that form a mosaic pattern with shrublands. Composition of grasslands depends on precipitation and management. Drought stress can also influence this system in some areas. In the southern range of this system, *Quercus havardii* may also be present to dominant and represents one succession pathway that develops over time following a disturbance. *Quercus havardii* is able to resprout following a fire and thus may persist for long periods of time once established, forming extensive clones. Edaphic and climatic factors are the most important dynamic processes for this type, with drought and extreme winds impacting this system significantly in some areas. Because *Quercus havardii* is able to resprout rapidly following fire, fire tends to cause structural changes in the vegetation, and compositional shifts are less significant in most cases. Overgrazing can lead to decreasing dominance of some of the grass species such as *Andropogon hallii, Calamovilfa gigantea*, and *Schizachyrium scoparium*. In the western extent of this system in the shortgrass prairie, more xeric mid‑ and shortgrass species such as *Hesperostipa comata, Sporobolus cryptandrus* and *Bouteloua gracilis* often dominate the herbaceous layer.

SOURCES

**References:** Bell 2005, Comer et al. 2003*, Elliott 2011, Elliott 2012, Eyre 1980, Ramaley 1939b, Shiflet 1994, Sims et al. 1976, Tolstead 1942

**Version:** 02 Oct 2014	**Stakeholders:** Midwest, Southeast, West

**Concept Author:** S. Menard and K. Kindscher	**LeadResp:** Midwest

M053. Western Great Plains Shortgrass Prairie

CES303.668 Western Great Plains Mesquite Scrub Woodland and Shrubland

**Primary Division:** Western Great Plains (303)

**Land Cover Class:** Shrubland

**Spatial Scale & Pattern:** Large patch

**Required Classifiers:** Natural/Semi‑natural; Vegetated (>10% vasc.); Upland

**National Mapping Codes:** EVT 2111; ESLF 5317; ESP 1111

**Concept Summary:** This system is found primarily in the southern portion of the Western Great Plains Division, primarily in Texas, Oklahoma and eastern New Mexico. It is dominated by *Prosopis glandulosa* with shortgrass species in the understory. *Ziziphus obtusifolia* and *Atriplex canescens* can codominate in some examples, as can *Opuntia* species in heavily grazed areas. Shortgrass species *Bouteloua gracilis* or *Bouteloua dactyloides* are typically present. Other grasses may include *Aristida purpurea, Bouteloua curtipendula, Bouteloua eriopoda, Bouteloua hirsuta, Muhlenbergia torreyi, Pleuraphis jamesii, Sporobolus airoides*, and *Sporobolus cryptandrus*. Historically this system probably occurred as a natural component on more fertile soils and along drainages, but it has expanded its range into prairie uplands in recent decades. In Texas, in what are considered the natural alluvial setting of this system, other overstory species may include *Celtis laevigata var. reticulata, Sapindus saponaria var. drummondii, Populus deltoides*, and *Salix nigra*. In these settings, *Prosopis glandulosa* is dominant in the shrub layer, but other shrub species encountered include small representatives of the overstory, and *Ziziphus obtusifolia, Prunus angustifolia*, and *Baccharis* spp. Herbaceous species present in the understory may include *Panicum virgatum, Bothriochloa laguroides ssp. torreyana, Nassella leucotricha*, and *Schizachyrium scoparium*. Non‑native species such as *Cynodon dactylon, Bromus catharticus, Sorghum halepense*, and *Bromus arvensis* are also commonly present and may be dominant.

**Comments:** With fire suppression and grazing, *Prosopis glandulosa* has been able to extend its range and become dense in examples of Western Great Plains Shortgrass Prairie (CES303.672) or Central Mixedgrass Prairie (CES303.659). Those areas should still be considered part of the prairie system. In Landfire mapzone 26 BpS modeling workshops, this was modeled in its limited extent along drainages rather than as the pervasive EVT. Because *Prosopis glandulosa* is the characteristic dominant of this system, and that species can occupy various sites and is thought to have expanded on the landscape as a result of land use, it is difficult to distinguish this system from areas where *Prosopis glandulosa* has invaded.

DISTRIBUTION

**Range:** This system is primarily found in the southern portion of the Western Great Plains division, particularly in Texas, Oklahoma and eastern New Mexico.

**Divisions:** 303:C

**TNC Ecoregions:** 27:?, 28:C, 29:C, 33:C

**Nations:** US

**Subnations:** NM, OK, TX

**Map Zones:** 26:C, 27:C, 34:C, 35:C, 38:P

**USFS Ecomap Regions:** 315A:CC, 315B:CC, 331B:CC, 331I:C?, M313B:??

CONCEPT

**Environment:** This system occurs naturally on deeper or more fertile soils and along drainages.

**Vegetation:** This system is dominated by *Prosopis glandulosa* with *Ziziphus obtusifolia*, and *Atriplex canescens* can codominate. *Opuntia* spp. can be prevalent in areas in heavily grazed examples of this system. Shortgrass species *Bouteloua gracilis* or *Bouteloua dactyloides (= Buchloe dactyloides)* are typically present. In Texas, in what are considered the natural alluvial setting of this system, other overstory species may include *Celtis laevigata var. reticulata, Sapindus saponaria var. drummondii, Populus deltoides*, and *Salix nigra*. In these settings, *Prosopis glandulosa* is dominant in the shrub layer, but other shrub species encountered include small representatives of the overstory, and *Ziziphus obtusifolia, Prunus angustifolia*, and *Baccharis* spp. Herbaceous species present in the understory may include *Panicum virgatum, Bothriochloa laguroides ssp. torreyana, Nassella leucotricha*, and *Schizachyrium scoparium*. Non‑native species such as *Cynodon dactylon, Bromus catharticus, Sorghum halepense*, and *Bromus arvensis* are also commonly present and may be dominant.

**Dynamics:** Historically, fire controlled this system and limited the development of woody cover. Likewise, edaphic conditions and topographic factors limited this system to deep alluvial soils in relatively low topographic positions along broad valley floors.

SOURCES

**References:** Barbour and Billings 1988, Bell 2005, Comer et al. 2003*, Elliott 2013, Eyre 1980, Shiflet 1994

**Version:** 02 Oct 2014	**Stakeholders:** Midwest, Southeast, West

**Concept Author:** S. Menard and K. Kindscher	**LeadResp:** Midwest

CES303.672 Western Great Plains Shortgrass Prairie

**Primary Division:** Western Great Plains (303)

**Land Cover Class:** Herbaceous

**Spatial Scale & Pattern:** Matrix

**Required Classifiers:** Natural/Semi‑natural; Vegetated (>10% vasc.); Upland

**National Mapping Codes:** EVT 2149; ESLF 7122; ESP 1149

**Concept Summary:** This ecological system is found primarily in the western half of the Western Great Plains Division in the rainshadow of the Rocky Mountains and ranges from the Nebraska Panhandle south into Texas and New Mexico, although grazing‑impacted areas appearing as this type may reach as far north as southern Canada where it is a component of Northwestern Great Plains Mixedgrass Prairie (CES303.674). This system occurs primarily on flat to rolling uplands with loamy, ustic soils ranging from sandy to clayey. In much of its range, this system forms the matrix system with *Bouteloua gracilis* dominating. Associated graminoids may include *Aristida purpurea, Bouteloua curtipendula, Bouteloua hirsuta, Bouteloua dactyloides, Carex filifolia, Carex inops ssp. heliophila, Hesperostipa comata, Hesperostipa neomexicana, Koeleria macrantha, Pascopyrum smithii, Pleuraphis jamesii, Sporobolus airoides*, and *Sporobolus cryptandrus*. Although mid‑height grass species may be present, especially on more mesic land positions and soils, they are secondary in importance to the sod‑forming short grasses. Sandy soils have higher cover of *Hesperostipa comata*, and *Sporobolus cryptandrus*. Scattered shrub and dwarf‑shrub species such as *Artemisia filifolia, Artemisia frigida, Artemisia tridentata, Atriplex canescens, Eriogonum effusum, Gutierrezia sarothrae, Lycium pallidum*, and *Yucca glauca* may also be present. Also, because this system spans a wide range, there can be some differences in the relative dominance of some species from north to south and from east to west. Large‑scale processes such as climate, fire and grazing influence this system. High variation in the amount and timing of annual precipitation impacts the relative cover of cool‑ and warm‑season herbaceous species.

In contrast to other prairie systems, fire is less important, especially in the western range of this system, because the often dry and xeric climate conditions can decrease the fuel load and thus the relative fire frequency within the system. However, historically, fires that did occur were often very extensive. Currently, fire suppression and more extensive livestock grazing in the region have likely decreased the fire frequency even more, and it is unlikely that these processes could occur at a natural scale. A large part of the range for this system (especially in the east and near rivers) has been converted to agriculture. Areas of the central and western range have been impacted by the unsuccessful attempts to develop dryland cultivation during the Dust Bowl of the 1930s. The short grasses that dominate this system are extremely drought‑ and grazing‑tolerant. These species evolved with drought and large herbivores and, because of their stature, are relatively resistant to overgrazing. This system in combination with the associated wetland systems represents one of the richest areas for mammals and birds. The endemic bird species of the shortgrass system may constitute one of the fastest declining bird populations in North America.

**Comments:** In Texas, this system occurs on the Llano Estacado and ranges to but does not include the Stockton Plateau. This system occurs on the Pawnee Grasslands LTER site in northeastern Colorado.

DISTRIBUTION

**Range:** This system is found primarily in the western half of the Western Great Plains Division east of the Rocky Mountains and ranges from the Nebraska Panhandle south into the panhandles of Oklahoma and Texas and New Mexico, although some examples may reach as far north as southern Canada where it grades into Northwestern Great Plains Mixedgrass Prairie (CES303.674).

**Divisions:** 303:C

**TNC Ecoregions:** 26:P, 27:C, 28:C, 33:P

**Nations:** US

**Subnations:** CO, KS, NE, NM, OK, TX, WY

**Map Zones:** 22:C, 24:?, 25:C, 26:C, 27:C, 28:C, 29:C, 30:C, 31:P, 33:C, 34:C, 35:P, 38:P

**USFS Ecomap Regions:** 315A:CC, 315B:CC, 315F:CC, 321A:CC, 331B:CC, 331C:CC, 331F:CC, 331H:CC, 331I:CC, 332C:CC, 332E:CC, 332F:CC, M313B:CC, M331F:CC, M331I:CC

CONCEPT

**Environment:** This system forms the matrix grassland in the southwest half of the Great Plains and largely occurs in the rainshadow of the Rocky Mountains. This system occurs on various geologic formations, primarily on flat to rolling uplands. Soils typically are loamy and ustic (bordering on aridic) but can range from sandy to clayey (Scifres 1980, Shiflet 1994).

*Climate:* Climate is temperate, semi‑arid, and continental with mean annual precipitation generally about 300 mm ranging to 500 mm to the east. Annual precipitation has a bimodal distribution occurring mostly before the growing season in winter and early spring and then during summer as monsoon thunderstorms (Sims et al. 1978). In most years, rates of evaporation are greater than precipitation for this system. Most of the annual precipitation occurs during the growing season as thunderstorms. Precipitation events are mostly <10 cm with occasional larger events (Sala and Lauenroth 1982). High variation in amount and timing of annual precipitation impacts the relative cover of cool‑ and warm‑season herbaceous species. This is the driest of the Great Plains grasslands ecosystems. Average daily temperature in July varies from 27°C in the southeast to 21°C in the northwest and along the foothills of the Rocky Mountains. Average daily temperature in January varies from 3°C in the south to ‑6°C in the northwest.

*Physiography/landform:* Stands occur on primarily flat to rolling uplands and to a lesser extent mesatops and plateaus.

*Soil/substrate/hydrology:* Soils are typically well‑drained, shallow to moderately deep, loamy and ustic and range from sandy to clayey (Scifres 1980, Shiflet 1994). In the southeasternmost expression of the system in Texas, it occurs on sites with soils providing relatively dry conditions such as Rough Breaks, Shallow Clay, Very Shallow, Very Shallow Clay, Moderately Alkaline Deep Hardland, and Hardland Ecological Sites (Elliott 2013).

**Vegetation:** This system spans a wide range and thus there can be some differences in the relative dominance of some species from north to south and from east to west. This system is primarily dominated by *Bouteloua gracilis* and *Bouteloua dactyloides (= Buchloe dactyloides)* throughout its range with various associated graminoid species depending on precipitation, soils and management. Associated graminoids may include *Achnatherum hymenoides, Aristida purpurea, Bouteloua curtipendula, Bouteloua hirsuta, Bouteloua dactyloides, Carex filifolia, Hesperostipa comata, Koeleria macrantha (= Koeleria cristata), Muhlenbergia torreyana, Pascopyrum smithii (= Agropyron smithii), Pleuraphis jamesii, Sporobolus airoides*, and *Sporobolus cryptandrus*. In southern examples of this system (Texas), *Bouteloua dactyloides* and *Bouteloua hirsuta* may dominate (especially where soils are rocky) in addition to *Bouteloua gracilis*. In addition, *Bothriochloa laguroides ssp. Torreyana, Bouteloua rigidiseta, Erioneuron pilosum, Hilaria belangeri, Hordeum pusillum, Pleuraphis mutica*, and *Scleropogon brevifolius* may occur in Texas examples (Elliott 2011). Although mid‑height grass species may be present especially on more mesic land positions and soils, they are secondary in importance to the sod‑forming short grasses. Sandy soils have higher cover of *Hesperostipa comata, Sporobolus cryptandrus*, and *Yucca elata*. Scattered shrub and dwarf‑shrub species such as *Artemisia filifolia, Artemisia frigida, Artemisia tridentata, Atriplex canescens, Eriogonum effusum, Gutierrezia sarothrae*, and *Lycium pallidum* may also be present. In Texas examples, shrub cover is generally low but may include species such as *Acacia greggii, Rhus microphylla, Rhus trilobata, Dalea formosa, Mahonia trifoliolata, Juniperus* sp., and *Prosopis glandulosa*. Forbs such as *Calylophus* sp., *Melampodium leucanthum, Krameria lanceolata, Ratibida columnifera, Psoralidium tenuiflorum*, and others are often present. *Gutierrezia sarothrae* may be present with significant cover, especially on sites with intense and continuous grazing (Elliott 2011). High annual variation in amount and timing of precipitation impacts relative cover of herbaceous species. Cover of cool‑season grasses is dependent on winter and early spring precipitation. The vegetation description is based on several other references, including Shaw et al. (1989), Hazlett (1998), and Schiebout et al. (2008).

**Dynamics:** Large‑scale processes such as climate, fire and grazing constitute the primary processes impacting this system. The short grasses that dominate this system are extremely drought‑ and grazing‑tolerant (Lauenroth and Milchunas 1992, Lauenroth et al. 1994a). These species evolved with large herbivores and drought (Milchunas and Lauenroth 2008) and adapted to historical heavy grazing with their low stature making them relatively resistant to overgrazing (Lauenroth et al. 1994a). The return intervals for grazing varied with areas distant from water sources likely grazed less heavily as those near water. However, the shortgrass prairie is probably the system with the highest intensity of grazing than other systems historically (Lauenroth et al. 1994a, Milchunas 2006). This is a drought‑tolerant system. Many shortgrass species are drought‑tolerant and have root systems that extend up near the soil surface where they can utilize low precipitation events (Salas and Lauenroth 1982). If blue grama is eliminated from an area by extended drought (3‑4 years) or disturbance such as plowing, regeneration is slow because of very slow tillering rates (Samuel 1985), low and variable seed production (Coffin and Lauenroth 1992), minimal seed storage in soil (Coffin and Lauenroth 1989) and limited seedling germination and establishment due to particular temperature and extended soil moisture requirements for successful seedling establishment (Hyder et al. 1971, Briske and Wilson 1978, 1980).

In contrast to other prairie systems, fire is less frequent, especially in the western range of this system, because the often dry and xeric climate conditions can decrease the fuel load and reduce lightning events, and thus the relative fire frequency within the system. However, historically, fires that did occur were often very extensive. Wright and Bailey (1982c) suggest that in semiarid areas, big prairie fires usually occurred during drought years that followed one to three years of above average precipitation, because of the abundant and continuous fuel. Consequently, these wildfires could travel far when the winds and air temperatures were high and relative humidity was low. There is debate as to the mean fire‑return interval (MFRI) for this shortgrass system. Because of the lack of long‑lived trees, and trees that do exist are in relatively productive sites, there is absolutely no way to reconstruct a reliable historic fire‑return interval. All estimates of historic fire‑return intervals must be based on those for surrounding vegetation types that do have means for reconstruction, and then extrapolating based on differences in primary production and herbivore removal of fuel loads. Therefore, there is no means to directly obtain the estimate, and the range is varied. It depends on many factors: portions will be drier, and portions will vary in frequency over time and there will be decadal variation. Anderson (2003) reports a broad fire‑return interval (FRI) of <35 years for shortgrass prairie. There is a wide variability of MFRI across this system, based on precipitation, fuel and ignition sources (LANDFIRE 2007a).

LANDFIRE developed a VDDT model for this system which has three classes (LANDFIRE 2007a, BpS 2711490).

A) Mid Development 1 Open (20% of type in this stage): Instead of calling the classes early, mid and late, which do not actually apply in shortgrass prairie and the different stages that we are describing, we are calling all stages "mid‑development." The stages of the grassland are created and/or maintained by disturbances or lack thereof. Class A is the low biomass (0‑1" based on the Robel pole density / visual obstruction method), heavy disturbance‑dependent community. It combines 2 types of communities. One consists of the high cover blue grama‑buffalo grass sod that looks like a golf course (high cover in patches). The other is the low cover bare soil, *Aristida*, and forb stage, which could have taller grasses than the sod, but they are spaced apart due to bare soil between. See biomass in Milchunas and Lauenroth (1989) and Milchunas et al. (1994) and basal cover for sod class by point frame in Milchunas et al. (1989). Please note that this system should be distinguished by on‑the‑ground biomass and not cover, since the cover in class A actually varies from a low, mosaic‑bare‑ground cover to a high sod‑cover, which includes litter too. Due to mapping constraints, we are defining dropdown boxes on cover; however, this stage could go up to 70% cover, including litter, with very low biomass. Basal cover for high cover sod is approximately 45% or higher if including litter. Basal cover for low cover prairie dog areas is approximately 20‑25% cover. On the ground, this class should be distinguished by biomass. There are relatively few cool‑season grasses in this stage. There is always blue grama in this stage, as in the others. Cactus is present (and could even be a dominant in the class A sod depending on soil type). *Aristida* is present, which increases with prairie dog colonies. Annual grasses ‑ sixweeks fescue, red three‑awn, ragweed, annual forbs. [Currently, you would see non‑native annuals in this class such as cheatgrass and kochia ‑ only in the high biomass type. Annuals and exotics are actually less abundant in the sod type than any other class (Milchunas et al. 1989, Milchunas and Lauenroth 1989, Milchunas et al. 1988); the landscape might also have non‑natives of bindweed on prairie dog towns today, but not historically.] On loamier or sandier sites, there is sand dropseed. For the southern, New Mexico version, other indicator species are lemonweed, showy goldeneye, and verbena.<br />

B) Mid Development 2 Closed (60% of type in this stage): Instead of calling the classes early, mid and late, which do not actually apply in shortgrass prairie and the different stages that we are describing, we are calling all stages "mid‑development." The stages of the grassland are created and/or maintained by disturbances or lack thereof. Class B is the mid biomass (2‑4" based on the Robel pole density / visual obstruction method), mid cover stage. See biomass in Milchunas and Lauenroth (1989) and Milchunas et al. (1994). This stage again consists of blue grama. Cactus is often present and could even be the second dominant depending on soil type. There is less needle‑and‑thread and western wheatgrass than in class C. This also includes the "historic climax plant community" with blue grama, buffalograss, and western wheatgrass, galleta grass, green needle grass (not in New Mexico), fringed sage, and New Mexico feather grass in the south. Historically, there would have been more midgrasses (Harvey Sprock et al. pers. comm.). In New Mexico, there would be scatterings of black grama, vine‑mesquite on heavier soils. Fire does occur in this stage. If there is 1‑2 years of no grazing or 4‑10 years of no fire, then 4‑10 years post‑fire, this class would transition to the high biomass class C stage. This was modeled as "alternate succession" occurring as a probability of 0.05, for modeling purposes. Prairie dogs could occur in this stage. If they do, the long‑term prairie dog grazing causes a transition to class A.

C) Mid Development 3 Closed (20% of type in this stage): Instead of calling the classes early, mid and late, which do not actually apply in shortgrass prairie and the different stages that we are describing, we are calling all stages "mid‑development." The stages of the grassland are created and/or maintained by disturbances or lack thereof. Class C is the high biomass (4+" based on the Robel pole density / visual obstruction method), high cover stage. See biomass in Milchunas and Lauenroth (1989) and Milchunas et al. (1994) and basal cover in Milchunas et al. (1989). The same grasses are present as the previous. However, there are also more C3 perennial cool‑season grasses. (However, some have questioned the increase in cool‑season grasses with succession as being speculative. There are definite edaphic differences. Gravelly sites in New Mexico often support *Hesperostipa neomexicana* even under intense grazing regimes.) Blue grama is still present and dominant. Needle‑and‑thread, galleta grass and western wheatgrass are more prominent. Note also that more annuals and exotics occur in the ungrazed than in the heavily grazed sod class (Milchunas et al. 1989, Milchunas et al. 1992). This stage is arrived at through lack of fire and grazing, although while already in this stage, fire would be more likely to occur due to the increased biomass. Fire does occur in this stage. If there is fire and then grazing, this will over time transition to class B, and with long‑term heavy grazing to class A. Fire alone may not cause a transition but can especially on coarser textured soils and also when fire occurs with heavy grazing. Regular grazing can just move the class to class B. Prairie dogs are unlikely to occur in this class, but when they do, they will occur as a patch within the matrix and will cause a transition.

During LANDFIRE modeling workshops, some experts suggest that the MFRI was historically approximately 25‑35 years with small fires at times so fire‑return interval at one spot was longer than expected, i.e., a fire can burn somewhere on the landscape often, but it may not necessarily return to the same spot for 25‑50 years or more (LANDFIRE 2007a). However, other experts thought MFRI was shorter, between 5‑20 years, dependent on the precipitation gradient east to west with shorter FRI (5 years) occurring in the more mesic eastern extent of the shortgrass prairie (LANDFIRE 2007a). A proposed precipitation gradient between drier versus wetter of approximately 350‑375 mm annual precipitation to delineate a change in fuels and fire behavior across the west to east gradient in precipitation / above‑ground primary productivity. The western portion would have a MFRI of 15‑20 years and in the eastern portion, it would be shorter (5‑10 years) (LANDFIRE 2007a). A MFRI of 5 years is similar to mixed and tallgrass prairies (Bragg and Hulbert 1976, Bragg 1986, Umbanhowar 1996, LANDFIRE 2007a).

Black‑tailed prairie dogs are an ecologically important component of the grazing regime in shortgrass prairie and would have occurred extensively (Lauenroth and Milchunas 1992, Milchunas and Lauenroth 2008). There were some very large towns, but there were also areas without any towns. Quantitative historical estimates of black‑tailed prairie dogs abundance are difficult to obtain, but the U.S. Fish and Wildlife Service estimated that about 160 million ha (395 million acres) of potential habitat historically existed in the U.S., and about 20% was occupied at any one time (Gober 2000). Shortgrass has most of the suitable soil types for prairie dogs; in general, they need loamy or clay soil. In historic times, there was frequent and broad‑scale grazing by bison and pronghorn antelope. Through the growing season, bison might have been there for relatively short periods in some years and longer in other years. There were also resident herds of bison in areas of Colorado (LANDFIRE 2007a). Historically, such areas would also have been populated by bison in sufficient numbers to support populations of wolves. Bamforth (1987) suggested that bison herds under relatively undisturbed conditions (prior to 1846) most often ranged in size from several hundred to several thousand. Shaw and Lee (1997) reviewed diaries of European travels in the southern Great Plains from 1806 to 1857. Organized by historical period and biome type, the authors suggest populations of three major large herbivores (bison, elk and pronghorn) changed in the first half of the nineteenth century; bison were most numerous on the shortgrass prairie prior to 1821, pronghorn were most abundant on the shortgrass prairie between 1806 and 1820, again in the 1850s (LANDFIRE 2007a). The dry half of the Great Plains has high interannual rainfall variability, so historically, the population declined faster in dry years (LANDFIRE 2007a). This resulted in a time lag or temporal variability, in which density could be reduced greatly. Bison historically moved nomadically in response to vegetation changes associated with rainfall, fire and prairie dog colonies (LANDFIRE 2007a). The time lag for return movements provided deferment during the regrowth period, which according to both historic and archeological records, may have ranged from 1 to 8 years (Malainey and Sherriff 1996). If there was a series of droughts followed by a wetter year, there would have been little grazing pressure, which would then result in a higher severity or frequency of fire. Drought and grazing were probably most important disturbances historically and greatly influenced fire frequency and extent. Insects such as grasshoppers, range caterpillars, and Mormon crickets were also a natural disturbance agent on the landscape (LANDFIRE 2007a).

Biological soils crusts (BSC) are important for soil fertility, soil moisture, and soil stability in semi‑arid ecosystems such as the drier portions of the shortgrass prairie (Belnap and Lange 2003). Cyanobacteria (especially *Nostoc*) fix large amounts of soil nitrogen and carbon (Evans and Belnap 1999, Belnap and Lange 2003). Generally, BSC are more important on sites with more exposed soil surface and less herbaceous and litter cover; however, cover varies locally with site characteristics, especially disturbance (Belnap et al. 2001, Belnap and Lange 2003).

SOURCES

**References:** Anderson 2003a, Bamforth 1987, Barbour and Billings 1988, Bell 2005, Belnap 2001, Belnap and Lange 2003, Belnap et al. 2001, Bragg 1986, Bragg and Hulbert 1976, Briske and Wilson 1978, Briske and Wilson 1980, CNHP 2010, Coffin and Lauenroth 1989, Coffin and Lauenroth 1992, Comer et al. 2003*, Dick‑Peddie 1993, Elliott 2011, Elliott 2013, Evans and Belnap 1999, Fuhlendorf et al. 2006, Gober 2000, Hazlett 1998, Hoagland 2006, Hyder et al. 1971, Kotliar et al. 2006, LANDFIRE 2007a, Lauenroth and Milchunas 1992, Lauenroth et al. 1994a, Malainey and Sherriff 1996, Milchunas 2006, Milchunas and Lauenroth 1989, Milchunas and Lauenroth 2008, Milchunas et al. 1988, Milchunas et al. 1989, Milchunas et al. 1992, Milchunas et al. 1994, Polley et al. 2013, Ricketts et al. 1999, Rolfsmeier and Steinauer 2010, Rondeau et al. 2018, Rondeau pers. comm., Rosentreter and Belnap 2003, Sala and Lauenroth 1982, Samson and Knopf 1994, Samuel 1985, Schiebout et al. 2008, Scifres 1980, Shaw and Lee 1997, Shaw et al. 1989, Shiflet 1994, Sims et al. 1978, Umbanowar 1996, Wright and Bailey 1982c

**Version:** 27 May 2016	**Stakeholders:** Midwest, Southeast, West

**Concept Author:** S. Menard and K. Kindscher	**LeadResp:** Midwest

2.B.2.Nc. Eastern North American Grassland & Shrubland

M506. Appalachian Rocky Felsic & Mafic Scrub & Grassland

CES202.347 Eastern Serpentine Woodland

**Primary Division:** Central Interior and Appalachian (202)

**Land Cover Class:** Forest and Woodland

**Spatial Scale & Pattern:** Large patch

**Required Classifiers:** Natural/Semi‑natural; Vegetated (>10% vasc.); Upland

**Diagnostic Classifiers:** Forest and Woodland (Treed); Rock Outcrops/Barrens/Glades; Serpentine; Unglaciated; Ultramafic with low Ca:Mg ratio

**National Mapping Codes:** EVT 2375; ESLF 4318; ESP 1375

**Concept Summary:** This system consists of distinct vegetation associated with ultramafic rock substrates in the Piedmont and Blue Ridge of the eastern United States. The bedrock is serpentinite, dunite, or other ultramafic rocks. The soil has unusual and extreme chemical composition that includes strongly skewed calcium‑to‑magnesium ratios and often high levels of heavy metals such as chromium. Most examples are open woodlands with *Pinus rigida, Pinus virginiana*, and/or *Quercus alba, Quercus marilandica*, and *Quercus stellata* in the often stunted canopy. Extreme edaphic conditions lead to locally xerophytic growing conditions that contribute to relatively open canopies and a ground cover dominated by prairie grasses and a variety of forbs. Disjunct species from drier regions and some endemic plant taxa are often present. The unusual and extreme soil chemistry determines the underlying floristics and distinctive flora of the type, but fire frequency, extent, and severity determine the physiognomy of particular examples over time.

**Comments:** While details of flora vary widely among the scattered examples of this system, all associations have in common a composition that is distinct from communities on other substrates and that is more xeric in aspect. Serpentine substrates support distinctive barren vegetation in most places where they occur. This system is distinguished from serpentine barrens in other regions because of the distinctive flora, as well as the climate, lack of glaciation, and other factors distinct to this region. A closely related Piedmont system, Piedmont Hardpan Woodland and Forest (CES202.268), may be only incompletely distinguished from this system. In this Appalachian system *(Quercus stellata) / Schizachyrium scoparium ‑ Packera plattensis ‑ Parthenium auriculatum ‑ Phemeranthus piedmontanus* Wooded Grassland (CEGL006084) occurs in both the Appalachians and in the Piedmont.

<br />Ultramafic rock substrate is apparently not sufficient to create this system. Some Piedmont areas with ultramafic rock outcrops on the most mesic sites support mesic forest vegetation not distinguishable from that on other substrates. It may be that these outcrops have less extreme chemistry, or that sufficient moisture levels or a long period without natural disturbance in the form of fire will override the effects of chemistry. The presence of unusually xerophytic or barren vegetation should be the defining characteristic of this system.

DISTRIBUTION

**Range:** This system is widely scattered throughout the Southern and Central Appalachians and Piedmont, from Pennsylvania to North Carolina.

**Divisions:** 202:C

**TNC Ecoregions:** 51:C, 52:C, 61:C

**Nations:** US

**Subnations:** MD, NC, NY, PA, VA

**Map Zones:** 57:C, 59:C, 60:C, 61:C

**USFS Ecomap Regions:** 221An:CCC, 221Da:CCC, 221Db:CCC, 231Ib:CCC, M221Db:CCC, M221Dc:CCC, M221Dd:CCC

CONCEPT

**Environment:** This system occurs in a variety of topographic settings, perhaps excluding only alluvial sites. The bedrock is serpentinite, dunite, or other ultramafic rocks. The soil has unusual and extreme chemical composition that includes strongly skewed calcium‑to‑magnesium ratios and often high levels of heavy metals such as chromium. Owing to a high level of toxic metals and a deficiency in nutrients, serpentine outcrops are ecologically unique and provide habitat for many plant species that grow nowhere else. The soil may be shallow and rocky, or deep, and is usually very clayey. Seepage may be present locally.

**Vegetation:** Vegetation is generally an open woodland of pines or xerophytic hardwoods. The dominant vegetation is more xerophytic and more open than the topographic setting, soil moisture, and climate would suggest, and contrasts strongly with adjacent vegetation on other kinds of rock. *Pinus rigida* and *Pinus virginiana* are frequent canopy dominants, but *Quercus marilandica, Quercus alba*, and *Quercus stellata* dominate some examples. There is generally not a well‑developed understory. Shrubs may be sparse to dense. The herb layer is usually dense; grasses, including prairie elements such as *Schizachyrium scoparium, Andropogon gerardii*, and/or *Sorghastrum nutans*, usually dominate, but a number of forbs may be present. In the northern portion of this system's range in Pennsylvania and Maryland, *Phlox subulata* and the endemic *Symphyotrichum depauperatum* are characteristic; in the southern Appalachian portion of its range, *Packera plattensis, Hexastylis arifolia var. ruthii*, and *Thalictrum macrostylum* are characteristic. Often, paradoxical mixtures of xerophytic and mesophytic species are present, though the overall plant composition is characteristic of a drier setting. Disjunct species from drier regions and some endemic plant taxa are often present. There is one site where *Pinus palustris* occurs over serpentine (Burks Mountain, Columbus County, Georgia), but this is classed as a "Piedmont Longleaf" site.

**Dynamics:** Although the unique soil chemistry is the crucial determining factor for this system, fire is generally a crucial process influencing species composition and vegetation structure. The unusual and extreme soil chemistry determines the underlying floristics and distinctive flora of the type, but fire frequency, extent, and severity determine the physiognomy of particular examples over time. Without fire, vegetation can sometimes become dense enough to suppress or eliminate the distinctive herbaceous layer, as well as turning a distinctive savanna or woodland structure into dense forest. Southern pine beetle (*Dendroctonus frontalis*) damage is an important factor in examples dominated by *Pinus* species.

SOURCES

**References:** Arabas 2000, Barton and Wallenstein 1997, Brooks 1987, Comer et al. 2003*, Dann 1988, DeSelm and Murdock 1993, Duffey et al. 1974, Estes et al. 1979, Eyre 1980, Harshberger 1903a, Latham 1993, Mansberg and Wentworth 1984, McKinney and Lockwood 1999, Murdock pers. comm., Noss 2013, Pennell 1910, Pennell 1912, Pennell 1929, Radford 1948, Schafale 2012, TNC 1996c, Taft 1997a, Taft 2009, Taft et al. 1995, Wherry 1963, Wiens and Dyer 1975

**Version:** 14 Jan 2014	**Stakeholders:** East, Southeast

**Concept Author:** M. Schafale, R. Evans, S.C. Gawler	**LeadResp:** Southeast

CES202.348 Southern and Central Appalachian Mafic Glade and Barrens

**Primary Division:** Central Interior and Appalachian (202)

**Land Cover Class:** Steppe/Savanna

**Spatial Scale & Pattern:** Small patch

**Required Classifiers:** Natural/Semi‑natural; Vegetated (>10% vasc.); Upland

**Diagnostic Classifiers:** Woody‑Herbaceous; Rock Outcrops/Barrens/Glades; Shallow Soil

**Concept Summary:** This Southern and Central Appalachian system consists of vegetation associated with shallow soils over predominantly mafic bedrock, usually with significant areas of rock outcrop. Bedrock includes a variety of igneous and metamorphic rock types such as greenstone and amphibolite. These areas support a patchy mosaic of open woodland and grassy herbaceous vegetation sometimes with a predominant woody short‑shrub community present.

**Comments:** These glades and barrens are intermediate between other rock outcrop and forest systems, with less dense vegetation than the closed forests supported by the region's climate but with more vegetation than bare rock cover. They may grade very gradually into both kinds of systems. Systems of similar physiognomy and setting but on acidic substrates are generally included in Central Appalachian Pine‑Oak Rocky Woodland (CES202.600).

DISTRIBUTION

**Range:** This system occurs in scattered clusters in the Southern Blue Ridge and adjacent portions of the upper Piedmont and Central Appalachians.

**Divisions:** 202:C

**TNC Ecoregions:** 51:C, 52:C, 59:C

**Nations:** US

**Subnations:** GA?, MD, NC, PA?, SC?, TN, VA

**Map Zones:** 54:C, 57:C, 59:C, 61:C

**USFS Ecomap Regions:** 221D:CC, M221D:CC

CONCEPT

**Environment:** This system occurs on upper to mid slopes, usually on gentle to moderate slopes but occasionally steeper. The ground is mostly shallow soil over bedrock, usually with significant areas of rock outcrop. The rock usually has few fractures but may have a pitted or irregular surface. This rock structure supports more extensive and deeper soil development than in Southern Appalachian Granitic Dome (CES202.297), but has few of the crevices and deeper rooting sites available in Southern Appalachian Rocky Summit (CES202.327). Micro‑scale soil depth and presence of seepage are important factors in determining the vegetation patterns. Shallow soil, unable to support a closed tree canopy, separates this system from forest systems. Bedrock includes a variety of igneous and metamorphic rock types. Some examples are on mafic substrates such as amphibolite, some are on felsic rock such as granitic gneiss but have flora that suggests a basic influence, and a few occur on felsic rocks and are clearly acidic. Rock or soil chemistry appears to be the most important factor affecting different associations on sites that have the physical structure to belong to this system. Elevation may also be an important factor causing variation.

**Vegetation:** Vegetation is a fine mosaic of different physiognomies, with open woodland and grassy herbaceous vegetation or short shrubs predominating. Some instances may have closed canopies of small trees or large shrubs, but no examples have large canopy trees with a closed canopy. Bare rock outcrops are usually present in a minority of the area. The canopy species are species tolerant of dry, shallow soils, most commonly *Quercus montana (= Quercus prinus), Pinus* spp., and *Juniperus virginiana var. virginiana*. Basic examples may also have *Carya glabra, Fraxinus americana*, and other species abundant. Shrubs may be dense, with species determined by soil chemistry. The herb layer is usually fairly dense and dominated by grasses, both in treeless areas and beneath open canopy. An abundant forb component is also usually present, especially in the more basic examples. The forbs include species characteristic of other rock outcrops and grassland species, with a smaller number of forest species present. Other species that may be present include the woody plants *Carya tomentosa (= Carya alba), Diervilla lonicera, Gaylussacia baccata, Kalmia latifolia, Aronia melanocarpa (= Photinia melanocarpa), Physocarpus opulifolius*, and *Quercus stellata*; and herbaceous plants *Aletris farinosa, Allium cernuum, Allium cuthbertii, Carex pensylvanica, Croton michauxii var. ellipticus (= Croton willdenowii), Cheilanthes tomentosa, Deschampsia flexuosa, Helianthus divaricatus, Hylotelephium telephioides, Muhlenbergia sobolifera, Packera paupercula, Phacelia dubia, Pycnanthemum tenuifolium, Saxifraga michauxii, Schizachyrium scoparium, Selaginella rupestris, Sibbaldiopsis tridentata, Solidago simplex var. randii, Solidago ulmifolia, Sorghastrum nutans*, and *Thalictrum revolutum*.

**Dynamics:** The dynamics of this system are complex and poorly understood. These sites, with their shallow soil, would likely be affected by fire, drought, and windstorms. Severe droughts kill tree saplings growing in cracks and potholes, helping to retain the open character of the glades (Quarterman et al. 1993). Fire may be an important influence on the vegetation structure, and may function to keep the vegetation more open in the long run. The patchy distribution of vegetation in examples of this system may limit fire intensity. These glades do not appear to be undergoing the kind of cyclic succession that has been described for granitic domes, but some balance of soil accumulation and destruction may be occurring on a longer term or coarser scale. There may be a zonation or patchiness to glade/barren vegetation, with different zones that may be identified by their characteristic plant species (Quarterman et al. 1993). These zones are apparently relatively stable, with woody plant encroachment evident only in relation to the invasion of shrubs and trees into potholes or crevices where soil accumulates more rapidly. It is possible that the slightly irregular curved surface of some examples represents a late stage in the weathering of old exfoliation surfaces that once supported granitic domes, but most known examples are not spatially associated with existing granitic domes.

SOURCES

**References:** Comer et al. 2003*, DeSelm and Murdock 1993, Duffey et al. 1974, Edwards et al. 2013, Estes et al. 1979, McKinney and Lockwood 1999, Murdock pers. comm., Nelson 1986, Noss 2013, Quarterman et al. 1993, Schafale 2012, Schafale and Weakley 1990, Simon 2011, Simon 2015, South Carolina Endangered Species Program 2010, TNC 1996c, Taft 1997a, Taft 2009, Taft et al. 1995, Wiens and Dyer 1975

**Version:** 29 Apr 2016	**Stakeholders:** East, Southeast

**Concept Author:** M. Schafale, R. Evans, M. Pyne, S.C. Gawler	**LeadResp:** Southeast

CES202.297 Southern Appalachian Granitic Dome

**Primary Division:** Central Interior and Appalachian (202)

**Land Cover Class:** Barren

**Spatial Scale & Pattern:** Large patch

**Required Classifiers:** Natural/Semi‑natural; Unvegetated (<10% vasc.); Upland

**Diagnostic Classifiers:** Rock Outcrops/Barrens/Glades

**Concept Summary:** This ecological system consists of smooth, curved, exfoliated outcrops of massive granite and related rocks in the Southern Blue Ridge and adjacent upper/inner Piedmont. Large areas of smooth rock without crevices distinguish this system. The outcrop surface is largely bare rock but has thin soil mats around the edges and patchily throughout. Mats vary in depth with age and level of development. Granitic domes have a distinctive pattern of cyclical primary succession. The resulting vegetation is a complex of small patches of different species and structure on soil mats of different depths, ranging from mosses and lichens to herbs to shrubs and trees. Deeper soils often have pine‑dominated vegetation with dense shrubs.

**Comments:** Granitic domes are clearly related to other rock outcrop systems in the southern Appalachians. Most similar in the region are Southern and Central Appalachian Mafic Glade and Barrens (CES202.348), which are distinguished by having more continuous vegetation and only a minority of bare rock, resulting from a more irregular rock surface or less steep slope. Glades and barrens occur on a wider range of rock types, but it is possible that granitic domes develop into glades over long periods of time (probably centuries or longer) if exfoliation ceases to occur. Southern Appalachian Montane Cliff and Talus (CES202.330) and Southern Appalachian Rocky Summit (CES202.327) differ in having more fractured rock, with vegetation dominated by plants rooted in fixed microsites related to crevices, ledges, and other small features. Southern Piedmont Granite Flatrock and Outcrop (CES202.329) is most similar to Southern Appalachian Granitic Dome (CES202.297) in occurring on smooth, exfoliated outcrops and having vegetation driven by soil mat dynamics. Some species are shared, but biogeography and climatic differences make for vegetation that is different.

<br />Deeper soils often have pine‑dominated vegetation with dense shrubs, resembling that of Southern Appalachian Montane Pine Forest and Woodland (CES202.331). These communities should be treated as part of this system if they are closely associated with exfoliation outcrops with the more distinctive granitic dome communities. The same is true of closely associated islands and stunted patches of vegetation resembling Southern Appalachian Oak Forest (CES202.886).

While this system as a whole is characterized by sparse vegetation, individual plots may have decidedly non‑sparse vegetation, with as much as 70% total cover, mostly herbaceous; this is a typical problem when scaling up from a plot to a system.

DISTRIBUTION

**Range:** This system is restricted to the Southern Blue Ridge and adjacent upper/inner Piedmont in the Carolinas and Georgia.

**Divisions:** 202:C

**TNC Ecoregions:** 51:C, 52:C

**Nations:** US

**Subnations:** AL, GA?, NC, SC

**Map Zones:** 57:C, 59:C

**USFS Ecomap Regions:** 221D:CC, 231A:CC, 231I:CC

CONCEPT

**Environment:** This system occurs on exfoliated granitic outcrops. In the upper/inner Piedmont, it usually occurs as isolated hills (inselbergs or monadnocks) that stand above the surrounding landscape. In the Blue Ridge, it usually occurs as part of larger mountain ranges but often still as somewhat distinctive knobs. Granite, granitic gneiss, and related rocks without many internal joints tend to fracture in thin sheets parallel to the surface, forming curved outcrops with smooth surfaces largely lacking crevices. Granitic dome outcrops develop on upper to midslopes, and most face south. Most individual outcrops grade from nearly level to very steep. The outcrop surface is largely bare rock but has thin soil mats around the edge and in patches throughout. Mats vary in depth with age and level of development. The smooth rock without crevices is the primary factor in the distinctive ecological character of this system. Distinct microenvironments are created by small irregularities in the rock surface and by areas of seepage at the edge. Elevation is an important factor affecting different associations within the system.

**Vegetation:** Most of the rock surface is bare or has only crustose or foliose lichen cover. Vegetation occurs as a series of small patches in the thin soil mats, with the kind of vegetation closely related to depth of the mat. Bare rock may have moss patches. The thinnest soils usually have a set of fine forbs, many of them annual. Slightly deeper soils often have grasses dominating. Deeper soils support shrubs or small trees. The flora shares some species with other rock outcrops of similar elevations but has some distinctive species and different dominance of species. Some characteristic plants of Southern Appalachian granitic domes include grasses and graminoids *Carex biltmoreana* and *Bulbostylis capillaris*; *Schizachyrium scoparium* is a frequent grass at lower elevations. Forbs include *Pycnanthemum* spp., *Krigia montana, Hypericum gentianoides*, and *Houstonia longifolia*. Some other unusual plants include the lichens *Lasallia papulosa, Lasallia caroliniana (= Umbilicaria caroliniana)*, and the mat‑forming fern allies *Selaginella tortipila* and *Selaginella rupestris*.

**Dynamics:** Granitic domes have a distinctive pattern of cyclical primary succession. Soil mats appear and deepen over time in a process that links vegetational and soil development, but are eventually destroyed by wind throw, drought, other natural disturbances, or simply falling off the rock. The result is a pattern with mats of different levels of development at any given time. Mat dynamics are different in different parts of the rock, with older mats and more permanent patterns near the edges, and sparser and younger mats in the interior. The dynamics are further modified by microtopography and the presence of seepage. The overall vegetation patterns likely respond to climatic cycles and natural disturbance events. The thin soils make these communities sensitive to drought, especially the long‑lived woody species.

SOURCES

**References:** Comer et al. 2003*, Edwards et al. 2013, Eyre 1980, Nelson 1986, Schafale 2012, Schafale and Weakley 1990, Simon 2015

**Version:** 24 Feb 2011	**Stakeholders:** Southeast

**Concept Author:** M. Schafale and R. Evans	**LeadResp:** Southeast

CES202.294 Southern Appalachian Grass and Shrub Bald

**Primary Division:** Central Interior and Appalachian (202)

**Land Cover Class:** Herbaceous

**Spatial Scale & Pattern:** Small patch

**Required Classifiers:** Natural/Semi‑natural; Vegetated (>10% vasc.); Upland

**Diagnostic Classifiers:** Montane; Herbaceous; Graminoid

**National Mapping Codes:** EVT 2414; ESLF 7127; ESP 1414

**Concept Summary:** This ecological system consists of dense herbaceous and shrubland communities in the highest elevational zone of the Southern Appalachians, generally above 1524 m (5000 feet) but occasionally to 1220 m (4000 feet), and at slightly lower elevations at its northern limit in Virginia and West Virginia, and in the Cumberland Mountains along the Virginia‑Kentucky border. Vegetation consists either of dense shrub‑dominated areas (heath balds) or dense herbaceous cover dominated by grasses or sedges (grassy balds). Heath balds are most often dominated by *Rhododendron catawbiense*, but substantial examples are also dominated by *Rhododendron carolinianum, Kalmia latifolia*, or a mixture of shrubs. One large example, dominated by *Alnus viridis ssp. crispa*, has been regarded as related to the heath balds, but is better treated separately due to much greater herbaceous diversity and coverage which is clearly different from typical heath balds. Grassy balds are characteristically dominated by *Danthonia compressa, Deschampsia flexuosa*, or *Carex* spp. Large areas have also become dominated by *Rubus allegheniensis* and/or *Rubus canadensis*, and by mixtures of native grasses with exotic pasture grasses. Most examples of grassy balds have some invading shrubs and trees, often dense enough to threaten the herbaceous vegetation. Heath balds may contain sparse stunted trees barely larger than the shrub canopy. The combination of high‑elevation, non‑wetland sites and dense herbaceous or shrub vegetation without appreciable rock outcrop conceptually distinguishes this system from all others in the Southern Appalachians. However, the widespread areas of degraded spruce‑fir with grass and shrub cover and the invasion of grassy balds by trees blur the distinction somewhat.

**Comments:** Grassy balds and heath balds differ in a number of ways and are often recognized as distinct entities. Whether these need to be split out at the system level, rather than just at the association level, has been questioned (M. Schafale pers. comm.). This system occurs in settings similar to Southern Appalachian Rocky Summit (CES202.327) and might be broadened to encompass that system.

DISTRIBUTION

**Range:** This system ranges from the Balsam Mountains and Great Smoky Mountains of North Carolina and Tennessee northward to Virginia and West Virginia. The system is also of limited extent in the Cumberland Mountains along the Virginia‑Kentucky border. The current status in Georgia is open to question and the ecological system was apparently never extensive in any case. The distribution and classification of grassy balds and high‑elevation pastures has been documented (Gersmehl 1970). Heath balds could be mapped separately from grassy balds as has been done for the Great Smoky Mountains (White et al. 2001). Alder bald can also be mapped separately, but it requires more field verification to map correctly.

**Divisions:** 202:C

**TNC Ecoregions:** 50:C, 51:C, 59:C

**Nations:** US

**Subnations:** GA, KY, NC, TN:S1, VA, WV

**Map Zones:** 57:C, 61:C

**USFS Ecomap Regions:** M221A:CC, M221B:CC

CONCEPT

**Environment:** This system generally occurs at elevations above 1524 m (5000 feet) but may range as low as 1220 m (4000 feet) in the Southern Blue Ridge, with most examples from 1600‑1780 m (5200‑5800 feet) elevation (Mark 1958). It is also of limited extent above 1035 m (3400 feet) in the Cumberland Mountains along the Virginia‑Kentucky border. It occurs on broad ridgetops and narrow spur ridges. Elevation and orographic effects (winds cooling as they rise to create increased condensation) make the climate cool and wet, with heavy moisture input from fog and cloud interception as well as high rainfall and snowfall. Convex slopes and exposure to wind offset the moisture input to some extent. The high peaks of the Southern Appalachians are not above the treeline; balds occur well below the elevation which would be a treeline today. Concentration of air pollutants has been implicated as an important anthropogenic stress in this elevational range in recent years. Soils range from shallow and rocky to fairly deep residual soils. Any kind of bedrock may be present, but most sites have erosion‑resistant felsic igneous or metamorphic rocks, with slate and quartzite particularly frequent. Alder bald tends to occur on areas with thinner and rockier soils than nearby grassy bald (Brown 1941, J. Donaldson pers. comm. 2013), and is distinct from heath bald (Harshberger 1903b, Schafale 2012). The sites that support balds are not obviously different from similar sites that support spruce‑fir forests, so the origin of the balds continues to be fodder for debate. Grazing and/or exposure to the elements may help maintain balds. Grass balds occur on less than one percent of the sites suitable for them (White and Sutter 1999b), and heath balds occur on 4‑9% of the sites suitable for them (White et al. 2001). Forests occur on most of these sites, such as northern hardwood, high‑elevation oak, or spruce‑fir forests.

**Vegetation:** Vegetation consists either of dense shrubs (heath balds or blackberry) or dense herbaceous cover dominated by grasses or sedges (grassy balds). Heath balds are most often dominated by *Rhododendron catawbiense*, but substantial examples are also dominated by *Rhododendron carolinianum, Kalmia latifolia*, or a mixture of other shrubs, including *Prunus pensylvanica, Sorbus americana, Corylus cornuta, Gaylussacia baccata, Pieris floribunda, Vaccinium corymbosum*, and *Leiophyllum buxifolium*. One large example, dominated by *Alnus viridis ssp. crispa*, is generally also regarded as being related to the heath balds. Grassy balds are characteristically dominated by *Danthonia compressa, Carex pensylvanica*, or other *Carex* spp. with forbs including *Minuartia groenlandica, Paronychia argyrocoma, Saxifraga michauxii, Solidago glomerata, Solidago rugosa ssp. aspera, Sibbaldiopsis tridentata*, and others. Large areas have also become dominated by *Rubus allegheniensis*, possibly with other brambles (*Rubus canadensis, Rubus idaeus ssp. strigosus*) and by mixtures of native grasses with exotic pasture grasses (e.g., *Phleum pratense*). Most examples of grassy balds have some invading shrubs and trees, often dense enough to threaten the herbaceous vegetation. Heath balds may contain sparse stunted trees barely larger than the shrub canopy.

**Dynamics:** The dynamics that maintain and that created the communities in this system have been a major topic of debate, so far without resolution. Most grassy bald occurrences show a strong tendency to succeed to shrub or forest vegetation under present conditions, suggesting that some important maintenance process has been lost. Northern hardwood, high‑elevation oak, or spruce‑fir forests may occur adjacent to balds. Grazing by native herbivores (elk and bison) and periodic fire have both been suggested as natural mechanisms to keep out woody vegetation. Others have suggested that all grassy balds are of anthropogenic origin and were never ecologically stable. The most definitive grassy balds have been documented as present at the time of the first European settlement, making documentation of their origin impossible. The presence of shade‑intolerant endemic or disjunct herbaceous plant species in some suggests even greater age. These include *Lilium grayi, Geum radiatum, Packera schweinitziana*, and *Houstonia purpurea var. montana*. Some areas of the spruce‑fir system degraded by a combination of logging, slash fires, and grazing resemble grassy balds, but most do not. The common practice of cattle grazing in grassy balds by early settlers has further obscured their presettlement character and evidence of presettlement disturbance processes.

Heath balds (not including alder balds) are more prone to disturbance by fire (Conkle 2004). However, heavy organic accumulations in the soil suggest great age for some. Most heath balds show limited tendency to succeed to forest, suggesting that the dense heath shrub layer is very competitive with tree seedlings. Spruce‑fir forest stands which burned in historical times have not usually developed vegetation identical to heath balds.

SOURCES

**References:** Billings and Mark 1957, Boggs et al. 2005, Brown 1941, Cain 1930b, Camp 1931, Comer et al. 2003*, Conkle 2004, DeSelm and Murdock 1993, Gates 1941, Gersmehl 1970, Gersmehl 1973, Gilbert 1954, Harshberger 1903b, LANDFIRE 2007a, Lindsay 1976, Lindsay 1977, Lindsay and Bratton 1979a, Lindsay and Bratton 1979b, Mark 1958, Mark 1959, Nodvin et al. 1995, Post 2013, Schafale 2012, Stevens et al. 2004, Sturm et al. 2005, TDNH unpubl. data 2018, Weiss 1999, Wells 1936a, Wells 1936b, Wells 1937, Wells 1961b, White and Sutter 1999b, White et al. 2001

**Version:** 14 Jan 2014	**Stakeholders:** East, Southeast

**Concept Author:** M. Schafale and R. Evans	**LeadResp:** Southeast

CES202.327 Southern Appalachian Rocky Summit

**Primary Division:** Central Interior and Appalachian (202)

**Land Cover Class:** Barren

**Spatial Scale & Pattern:** Large patch

**Required Classifiers:** Natural/Semi‑natural; Unvegetated (<10% vasc.); Upland

**Diagnostic Classifiers:** Rock Outcrops/Barrens/Glades

**Concept Summary:** This system represents treeless rock outcrops of the southern Appalachian Mountains, primarily in western North Carolina and eastern Tennessee. Outcrops may be vertical to horizontal, rugged or fractured rock outcrops of peaks, ridgetops, upper slopes, and other topographically exposed locations. Higher elevation examples occur from 1200 to 2030 m in elevation; other examples may be found at elevations of 305 m (1000 feet) or lower on foothills. These outcrops occur on felsic to mafic rocks and are distinguished from surrounding systems by the prevalence of bare or lichen‑encrusted rocks. The vegetation component of this system is generally characterized by a mixture of low‑growing lifeforms, especially lichens, mosses, and short‑statured forbs. Less commonly, graminoids and low shrubs are encountered. Species common to all outcrop vegetation types include *Carex misera, Saxifraga michauxii*, and *Vaccinium corymbosum*.

**Comments:** The primary variation within this system, which could be the basis for further subdivision, is the distinction between low and high elevation. High‑elevation rocky summits may have a unique biogeographic history of having been adjacent to alpine tundra that existed in the region during the Pleistocene and of now providing a refugium for some of its flora. Their climate is substantially different from the lower elevation examples. However, their structure and the dynamics that results from it are probably similar.

DISTRIBUTION

**Range:** This system is found at a variety of elevations in the southern Appalachian Mountains, primarily in western North Carolina and eastern Tennessee.

**Divisions:** 202:C

**TNC Ecoregions:** 51:C

**Nations:** US

**Subnations:** GA, NC, SC, TN:S1

**Map Zones:** 57:C

CONCEPT

**Environment:** This system occurs on rugged rock outcrops on peaks, ridgetops, upper slopes, and other topographically exposed landforms (Schafale and Weakley 1990). Elevations may range from nearly the highest in the region (1200‑2030 m), down to 305 m (1000 feet) or lower on foothills. The rock outcrops are irregular, with substantial horizontal surfaces, as well as often vertical surfaces, and generally with fractures. This structure allows soil accumulation in local pockets, sometimes to fair depth, even though most of the substrate is bare rock. Bedrock may be a variety of types. Erosion‑resistant rocks such as felsic gneisses and schists or quartzite are most common, but mafic rocks such as amphibolite are also important substrates. Granite and granitic gneiss sometimes form rocky summits, but more often form the smoother outcrops that support Southern Appalachian Granitic Dome (CES202.297) or Southern and Central Appalachian Mafic Glade and Barrens (CES202.348). Moisture conditions are generally quite dry due to lack of soil but may be heterogeneous. Local deep crevices may accumulate water funneled from bare rock. Seepage is occasionally present but is usually minor. Climate varies substantially with elevation and has a strong effect on variation within the system. Higher elevation sites have high rainfall and receive substantial additional moisture from fog and rime ice.

**Vegetation:** Vegetation is sparse or patchy, with substantial expanses of lichen‑covered or bare rock. Vegetation cover may be >25% (i.e., not technically "sparse") in local areas (including some plots), but the overall effect is of sparse vegetation. Species common to all outcrop vegetation types include *Carex misera, Saxifraga michauxii*, and *Vaccinium corymbosum* (Wiser and White 1999). Mosses are usually present but often do not have substantial cover. A suite of typical rock outcrop herbs, including *Saxifraga michauxii, Carex misera, Paronychia argyrocoma, Heuchera villosa, Krigia montana*, and *Hylotelephium telephioides (= Sedum telephioides)*, is usually present, along with more widespread herbs of open areas such as *Danthonia spicata, Danthonia compressa, Schizachyrium scoparium, Potentilla canadensis*, and *Houstonia caerulea*. High‑elevation examples have an additional suite of herbs, which include some northern disjunct species such as *Minuartia groenlandica, Sibbaldiopsis tridentata, Trichophorum cespitosum*, and *Huperzia selago*. A suite of narrow endemic herbs is also characteristic of many high‑elevation examples. Herbs of the adjacent forests may be present in small numbers. Shrubs and stunted trees are usually present in patches, where crevices or deeper soil accumulations are present. A few shrubs, such as *Leiophyllum buxifolium*, are largely limited to this system, but most are widespread species of dry forests and woodlands. Shrubs in the Ericaceae family are particularly prominent. Wiser and White (1999) found that in high‑elevation rocky summits, less than a third of the flora was limited to rock outcrop sites.

**Dynamics:** The dynamics of this system have received little study. Most rocky summit sites are probably stable over long periods of time, but variations in the always stressful environment may disturb and change vegetation. The role of crevices and soil in depressions as the primary rooting site makes for a relatively stable pattern of plant distribution and potentially long‑lived individuals. This is in contrast to the shallow soil mats predominating in granitic domes. Between disturbances, accumulation of soil and succession of vegetation to greater woody abundance may occur. Fire may naturally be uncommon or fairly common. The topographically high location of this system would make it likely that fires would spread into it, though the sparse fuels would allow only patchy burning. Fires have been indicated to be important in preventing dense woody growth from encroaching on open outcrops in at least some instances. Rock falls or other mass movements are rare, but may be important in creating rock outcrops and keeping them open in the long term. Periodic drought is probably a significant disturbance. Animals and freeze‑thaw action may be important disturbances at a local scale. Because of the fragility of soil and vegetation, human disturbance by trampling edges and by climbing may be particularly destructive.

SOURCES

**References:** Comer et al. 2003*, Edwards et al. 2013, Nelson 1986, Schafale 2012, Schafale and Weakley 1990, Simon 2015, TDNH unpubl. data 2018, Wiser and White 1999

**Version:** 18 Apr 2006	**Stakeholders:** Southeast

**Concept Author:** M. Schafale	**LeadResp:** Southeast

CES202.328 Southern Piedmont Glade and Barrens

**Primary Division:** Central Interior and Appalachian (202)

**Land Cover Class:** Steppe/Savanna

**Spatial Scale & Pattern:** Small patch

**Required Classifiers:** Natural/Semi‑natural; Vegetated (>10% vasc.); Upland

**Diagnostic Classifiers:** Woody‑Herbaceous; Rock Outcrops/Barrens/Glades

**Concept Summary:** This glade and barrens system of the southern Piedmont consists of gently to moderately sloping areas with mostly shallow soil over bedrock. Examples usually have significant areas of exposed rock evident. The bedrock potentially includes a variety of igneous and metamorphic rock types, including diabase, mudstone, and shale. Examples support open vegetation of patchy, mixed physiognomy with a significant woody component. Trees may be stunted and/or more widely spaced than in the more typical forests of the region. The shallow soils which impede tree growth help distinguish this system from forest systems of the Piedmont. This system is structurally intermediate between other rock outcrop systems and the more common and typical forest systems. The canopy species are those tolerant of dry, shallow soils, most commonly *Juniperus virginiana* and various oaks and pines, but also including *Fraxinus americana, Ulmus alata*, and *Cercis canadensis* on basic examples. Shrubs may be dense, with species determined by soil chemistry. The herb layer is usually fairly dense and may be dominated by grasses or by a mix of grasses and forbs, both in treeless areas and beneath open canopy. The forbs include species characteristic of other rock outcrops and grassland species, with a smaller number of forest species present. Plant species richness may be fairly high in communities of this system.

**Comments:** The southern Piedmont as defined here consists of TNC Ecoregion 52 (ECOMAP 231A, EPA 45), but within this region, this system is not expected to occur north of about the James River in Virginia. This system is intermediate between other rock outcrops and forest systems, with less dense vegetation than the closed forests supported by the region's climate but with more vegetation than bare rock cover. They may grade very gradually into both kinds of systems. They are analogous to Southern and Central Appalachian Mafic Glade and Barrens (CES202.348), but are distinguished by their climate, flora, and landscape setting. Southern and Central Appalachian Mafic Glade and Barrens (CES202.348) occurs in the hilly upper Piedmont, whereas this system is confined to the eastern and central Piedmont.

This system represents a collection of several different kinds of communities related primarily by structure, and could be further subdivided. The rare diabase glades are flat and have a very distinctive flora. The examples on meta‑mudstone are less well known. Other kinds may occur.

DISTRIBUTION

**Range:** This system is found in scattered clusters in the southern Piedmont, possibly extending north to about the James River in Virginia. However, the overall distribution in this region is not well‑known.

**Divisions:** 202:C

**TNC Ecoregions:** 52:C

**Nations:** US

**Subnations:** AL, GA, NC, SC, VA

**Map Zones:** 54:C, 59:C, 61:P

**USFS Ecomap Regions:** 231A:CC, 231I:CC

CONCEPT

**Environment:** This system occurs on upper to midslopes, usually on moderate slopes but occasionally flat. The ground is mostly shallow soil over bedrock, usually with significant areas of rock outcrop. The rock usually has few fractures but may have a pitted or irregular surface. This rock structure supports more extensive and deeper soil development than in Southern Piedmont Granite Flatrock and Outcrop (CES202.329) or Southern Piedmont Cliff (CES202.386), but has few of the crevices and deeper rooting sites available in Southern Appalachian Rocky Summit (CES202.327). Micro‑scale soil depth and presence of seepage are important factors in determining the vegetation patterns. Shallow soil, unable to support a closed tree canopy, separates this system from forest systems. Bedrock potentially includes a variety of igneous and metamorphic rock types, including diabase, mudstone, and shale. Rock or soil chemistry appears to be the most important factor affecting different associations on sites that have the physical structure to belong to this system.

**Vegetation:** Vegetation is a fine mosaic of different physiognomies, with open woodland and grassy herbaceous vegetation or short shrubs predominating. Bare rock outcrops are usually present in a minority of the area. The canopy species are species tolerant of dry, shallow soils, most commonly *Juniperus virginiana* and various oaks and pines, but also including *Fraxinus americana, Ulmus alata*, and *Cercis canadensis* on basic examples. Shrubs may be dense, with species determined by soil chemistry. The herb layer is usually fairly dense and may be dominated by grasses or by a mix of grasses and forbs, both in treeless areas and beneath open canopy. The forbs include species characteristic of other rock outcrops and grassland species, with a smaller number of forest species present. Plant species richness may be fairly high in communities of this system.

**Dynamics:** The dynamics of this system are not well known. The occurrence of the system appears to be primarily determined by site physical properties, with physical and chemical properties determining vegetational variation. Fire may be an important influence on vegetation, and may in the long run be important for keeping the vegetation structure open, though the patchy distribution of vegetation might limit fire intensity. It is possible that fire would have allowed glade structure and vegetation to extend onto slightly deeper soils and therefore allowed for more extensive glades. Periodic drought and wind storms may also be an important factor limiting canopy density and stature. The shallow soil would make these sites particularly prone to all three. These glades do not appear to be undergoing the kind of cyclic succession that has been described for granitic flatrocks, but some balance of soil accumulation and destruction may be occurring on a longer term or coarser scale.

SOURCES

**References:** Comer et al. 2003*, DeSelm and Murdock 1993, Duffey et al. 1974, Estes et al. 1979, Eyre 1980, LeGrand 1988, McKinney and Lockwood 1999, Murdock pers. comm., Noss 2013, Oakley et al. 1995, Quarterman et al. 1993, Schafale 2012, Schafale and Weakley 1990, Slapcinsky 1994, TNC 1996c, Taft 1997a, Taft 2009, Taft et al. 1995, Wiens and Dyer 1975

**Version:** 14 Jan 2014	**Stakeholders:** East, Southeast

**Concept Author:** M. Schafale and R. Evans	**LeadResp:** Southeast

CES202.329 Southern Piedmont Granite Flatrock and Outcrop

**Primary Division:** Central Interior and Appalachian (202)

**Land Cover Class:** Barren

**Spatial Scale & Pattern:** Small patch

**Required Classifiers:** Natural/Semi‑natural; Unvegetated (<10% vasc.); Upland; Wetland

**Diagnostic Classifiers:** Rock Outcrops/Barrens/Glades

**Concept Summary:** This ecological system consists of smooth, exfoliated outcrops of massive granite and related rocks in the eastern and central Piedmont of the southeastern United States, and rarely in the adjacent Atlantic Coastal Plain, there confined to the fall‑line where erosion has exposed underlying rocks. Examples occur from Virginia south to Alabama but are found most abundant in the upper Piedmont of Georgia. Some noteworthy examples in central Georgia include Stone Mountain, Panola Mountain, and Arabia Mountain in DeKalb, Henry, and Rockdale counties. Depending upon the location, examples may rise above the surrounding landscape by as much as 200 m, or may lie flush with the surrounding land surface. The vegetation is a complex of small‑patch communities of different species and structure occupying different microhabitats which are present on the outcrops, ranging from mosses and lichens, to herbs, to shrubs and trees. In some areas, these microhabitats include solution pits or depressions that retain water and form a distinctive wetland community. This outcrop system supports a relatively large number of endemic plants.

**Comments:** Granitic domes are clearly related to other rock outcrop systems in the Piedmont but are distinct in their flora and vegetation mat succession. The smooth rock surface is crucial to their development. More fractured granitic rocks do not form this distinct system. In contrast, Southern Piedmont Cliff (CES202.386) has vertically oriented rock outcrops that tend to have fractures and ledges. Southern Piedmont Glade and Barrens (CES202.328) is similarly horizontally oriented but has denser vegetation and rather different flora. Southern Appalachian Rocky Summit (CES202.327), which barely overlaps the range of this system, has fractured rock and occurs in topographically high settings in rugged topography.

This system is closely related to Southern Appalachian Granitic Dome (CES202.297), with which it shares the distinctive structure and vegetation mat dynamics of exfoliated outcrops. The flat orientation of the flatrocks makes pools more important in them. Climatic and biogeographic differences lead to floristic differences between the two systems. The massive Piedmont outcrops in the Atlanta vicinity (Wharton 1978) are included here, the distinction between "dome" (CES202.297) and "flatrock" (CES202.329) perhaps being an overly specific use of vague and conceptual terminology.

DISTRIBUTION

**Range:** This system is found scattered in the eastern and central Piedmont, from Alabama to Virginia. Rare examples occur in the upper Piedmont. A few, occurring surrounded by Tertiary sediments in the Fall Zone, may be considered to be in the Coastal Plain.

**Divisions:** 202:C

**TNC Ecoregions:** 52:C, 57:P

**Nations:** US

**Subnations:** AL, GA, NC, SC, VA

**Map Zones:** 54:C, 59:C, 61:C

CONCEPT

**Environment:** This system occurs on exfoliated granitic outcrops; these are Precambrian metamorphic rocks generally found in the Piedmont Plateau (McVaugh 1943). Outcrops are level or gently sloped, occurring as low domes up to 200 m above the surrounding landscape or as flatrocks varying considerably in size (Shure 1999). Smooth rock without crevices is the primary factor in the distinctive ecological character of this system. Granite, granitic gneiss, and related granitoid rocks (Edwards et al. 2013) without many internal joints tend to fracture into thin sheets parallel to the surface, forming outcrops with smooth surfaces largely lacking crevices. The outcrop surface is largely bare rock but has thin soil mats around the edges and in patches throughout. Mats vary in depth with age and level of development. Distinct microenvironments are created by small irregularities in the rock surface and by areas of seepage at the edge. Some examples (e.g., in central Georgia) may have prominent seepage‑related features, where areas of perennial herbaceous vegetation are very wet in the winter and spring. In these cases, the only vegetated areas on the granite outcrop are seepage‑related. One possible substrate is the Lilesville granite.

**Vegetation:** Most of the rock surface is bare or has only crustose or foliose lichen cover. Vegetation occurs as a series of small patches in the thin soil mats, or seasonal pools, with the community type dependent on the nature of the depression and depth of the soil mat (if any). Bare rock may have moss patches or straggling vines including *Gelsemium sempervirens*. The thinnest soils usually have a set of fine forbs, many of them annual. Slightly deeper soils often have grasses dominating. Deeper soils support shrubs or small trees, including *Vaccinium arboreum* and *Pinus virginiana*. A distinctive woodland of pines or pines and oaks occurs on the continuous shallow soils surrounding the outcrop. The flora shares some species with other rock outcrops of the Piedmont, but has some distinctive species and different dominance of species. Some characteristic plant species include the forbs *Minuartia glabra, Minuartia uniflora*, and *Packera tomentosa*; one common grass is *Schizachyrium scoparium*. Other unusual plants include the mat‑forming fern ally *Selaginella rupestris*, the unusual floating aquatic annual *Amphianthus pusillus*, the quillworts *Isoetes melanospora* and *Isoetes tegetiformans*, the diminutive succulents *Diamorpha smallii* and *Phemeranthus teretifolius (= Talinum teretifolium)*, as well as *Cheilanthes lanosa, Croton michauxii var. ellipticus (= Croton willdenowii), Cyperus granitophilus, Danthonia sericea, Danthonia spicata, Diodia teres, Hieracium venosum, Houstonia caerulea, Pleopeltis polypodioides, Saxifraga virginiensis, Vaccinium arboreum*, and *Viola sororia*, and a variety of mosses and lichens.

**Dynamics:** Large numbers of soil island depression may be scattered across the surface of granite outcrops and occasional pools of shallow water may stand in certain depressions which trap rainfall (McVaugh 1943, Shure 1999). Where soil accumulates in depressions formed by exfoliating surface rock, a distinctive and fairly predictable pattern of successional changes occurs [see references in Shure (1999)]. Soil mats appear and deepen over time in a process that links vegetational and soil development, but are eventually destroyed by windthrow, drought, other natural disturbances. The result is a mosaic with mats of different levels of development at any given time. Mat dynamics are different in different parts of the rock, with older mats and more permanent patterns near the edges and sparser and younger mats in the interior. The dynamics are further modified by microtopography and the presence of seepage. The larger vegetation patterns such the relative amount of different stages likely respond to climatic cycles and natural disturbance events. The thin soils make these communities sensitive to drought, especially the long‑lived woody species. Fire is probably rare in the interior, given the sparse fuel, but may be important in determining the size of the open area and may affect the dynamics of the bordering woodlands.

Severe droughts kill tree saplings growing in cracks and potholes, helping to retain the open character of the glades (Quarterman et al. 1993). There is an apparent zonation or patchiness to glade/barren vegetation, with different zones that may be identified by their characteristic plant species (Quarterman et al. 1993). These zones are apparently relatively stable, with woody plant encroachment evident only in relation to the invasion of shrubs and trees into potholes or crevices where soil accumulates more rapidly.

SOURCES

**References:** Baker 1956, Coffey 1964, Comer et al. 2003*, DeSelm and Murdock 1993, Duffey et al. 1974, Edwards et al. 2013, Estes et al. 1979, Eyre 1980, Horn unpubl. data, Mackie pers. comm., McKinney and Lockwood 1999, McVaugh 1943, Murdock pers. comm., Nelson 1986, Noss 2013, Peet et al. 2013, Quarterman et al. 1993, Schafale 2012, Schafale and Weakley 1990, Shure 1999, TNC 1996c, Taft 1997a, Taft 2009, Taft et al. 1995, Wharton 1978, Wiens and Dyer 1975

**Version:** 02 Oct 2015	**Stakeholders:** East, Southeast

**Concept Author:** M. Schafale and R. Evans	**LeadResp:** Southeast

M509. Central Interior Acidic Scrub & Grassland

CES202.692 Central Interior Highlands Dry Acidic Glade and Barrens

**Primary Division:** Central Interior and Appalachian (202)

**Land Cover Class:** Forest and Woodland

**Spatial Scale & Pattern:** Small patch

**Required Classifiers:** Natural/Semi‑natural; Vegetated (>10% vasc.); Upland

**National Mapping Codes:** EVT 2363; ESLF 4305; ESP 1363

**Concept Summary:** This ecological system is primarily found in the Interior Highlands of the Ozark, Ouachita, and Interior Low Plateau regions with small occurrences in northern Missouri. It occurs on flatrock outcrops and along moderate to steep slopes or valley walls of rivers along most aspects. Parent material includes chert, igneous and/or sedimentary (sandstone, shale, siltstone) bedrock with well‑ to excessively well‑drained, shallow soils interspersed with rock and boulders. These soils are typically dry during the summer and autumn, becoming saturated during the spring and winter. Grasses such as *Schizachyrium scoparium* and *Sorghastrum nutans* dominate this system with stunted oak species (*Quercus stellata, Quercus marilandica*) and shrub species such as *Vaccinium* spp. occurring on variable depth soils. *Juniperus virginiana* can be present and often increases in the absence of fire. In Kentucky, this system includes both sandstone glades found in the Shawnee Hills, as well as shale and siltstone glades and barrens found in the Knobs region, both in the Kentucky Interior Low Plateau. It also includes dry *Quercus stellata*‑dominated barrens on Cretaceous‑aged gravel substrates on the northern fringes of the Upper East Gulf Coastal Plain Ecoregion in southern Illinois and western Kentucky. This system is influenced by drought and infrequent to occasional fires. Prescribed fires help manage this system by maintaining an open glade structure.

**Comments:** The occurrence of this system in TNC Ecoregion 43 is apparently confined to southern Illinois and/or Kentucky but does not include any portions of states to the south. Not all examples are acidic. Sometimes a layer of limestone or neutral shale occurs in these and thus are not acidic.

DISTRIBUTION

**Range:** This system is found in the Interior Highlands of the Ozark, Ouachita, and Interior Low Plateau regions, with rare and limited occurrences in the Upper East Gulf Coastal Plain of Kentucky and Illinois. That includes the Shawnee Hills (EPA Ecoregions 71a, 72h of Woods et al. (2002)) and Knobs region (EPA Ecoregions 70d, 71c of Woods et al. (2002)).

**Divisions:** 202:C, 203:C

**TNC Ecoregions:** 36:C, 38:C, 39:C, 43:C, 44:C

**Nations:** US

**Subnations:** AR, IL, IN, KY, MO, OK, TN?

**Map Zones:** 43:P, 44:C, 47:C, 48:C, 49:C, 53:C

**USFS Ecomap Regions:** 221E:CC, 223A:CC, 223B:CC, 223D:CC, 223G:CC, 231H:CC, 251C:CC

CONCEPT

**Environment:** This system occurs on flat rock outcrops and along moderate to steep slopes or valley walls of rivers along most aspects. Parent material includes chert, shale, igneous and/or sedimentary (sandstone, shale, siltstone) bedrock with well‑ to excessively well‑drained, shallow soils interspersed with rock and boulders. These soils are typically dry during the summer and autumn, becoming saturated during the spring and winter.

**Vegetation:** Grasses such as *Schizachyrium scoparium* and *Sorghastrum nutans* dominate this system with stunted oak species (*Quercus stellata, Quercus marilandica*) and shrub species such as *Vaccinium* spp. occurring on variable depth soils. In the Shawnee Hills (EPA Ecoregions 71a, 72h of Woods et al. (2002)) of the Kentucky Interior Low Plateau, *Quercus marilandica, Quercus stellata*, and *Juniperus virginiana* are the dominant trees. *Ulmus alata* may be an understory component. Scattered shrubs, such as *Vaccinium arboreum* and *Chionanthus virginicus*, occur on the margins in patches of deeper soil. *Quercus montana (= Quercus prinus)* may be present in the eastern part of the range. Some other plants that may be associated with these glades include *Andropogon ternarius, Danthonia spicata, Symphyotrichum patens var. patentissimum, Silene rotundifolia, Pityopsis graminifolia var. latifolia, Coreopsis grandiflora, Silene regia, Coreopsis lanceolata, Croton michauxii var. ellipticus (= Croton willdenowii), Sedum nuttallianum, Selaginella rupestris*, and *Portulaca pilosa*.

**Dynamics:** Ericaceous shrubs found here are different from calcareous glades. The thin, dry soil characteristic of this system dries out during the growing season and much of the vegetation dries, as well. This allows fires to spread easily and these fires restrict the abundance of woody species. In high‑quality examples where the natural fire regime operates, small trees and shrubs are limited to the edges of stands or small "islands" of deeper soil that retain more moisture while grasses are the dominant vegetation. Sparsely vegetated areas between the dominant grassy zones contain most of the rare species found in this system (Ware 2002). In the absence of fire, from active suppression or a lack of fuel due to excessive grazing, woody species can increase greatly.

SOURCES

**References:** Baskin et al. 1995, Comer et al. 2003*, Evans et al. 2009, Eyre 1980, Heikens and Robertson 1995, Homoya 1994, Martin and Houf 1993, Nelson 2010, Nelson 2012, ONHD unpubl. data, Ware 2002, Woods et al. 2002

**Version:** 28 Mar 2014	**Stakeholders:** Midwest, Southeast

**Concept Author:** S. Menard and T. Nigh	**LeadResp:** Midwest

CES202.337 Cumberland Sandstone Glade and Barrens

**Primary Division:** Central Interior and Appalachian (202)

**Land Cover Class:** Steppe/Savanna

**Spatial Scale & Pattern:** Small patch

**Required Classifiers:** Natural/Semi‑natural; Vegetated (>10% vasc.); Upland

**Diagnostic Classifiers:** Woody‑Herbaceous; Rock Outcrops/Barrens/Glades; Acidic Soil

**National Mapping Codes:** EVT 2398; ESLF 5414; ESP 1398

**Concept Summary:** This system encompasses a complex of sparsely vegetated rock outcrops, perennial grasslands, and woodlands on shallow soils on the Cumberland Plateau of Kentucky, Tennessee, Alabama, and Georgia. Herbaceous plants, including *Diamorpha smallii* and *Minuartia glabra*, are typical of the outcrops in Tennessee. In Alabama, *Bigelowia nuttallii* and *Schizachyrium scoparium* are important. *Pinus virginiana* and *Acer rubrum* are typical of the current condition of many of the woodlands surrounding these outcrops on the Cumberland Plateau. This dominance pattern may be due to lack of disturbance. *Pinus rigida, Pinus echinata*, and/or *Quercus montana* may also occur. Scattered shrubs, such as *Gaylussacia* spp., *Vaccinium arboreum*, and *Chionanthus virginicus*, occur on the margins in patches of deeper soil. Various mosses and fruticose lichens such as *Cladonia* spp. may be prominent in some examples. To the west, in the Interior Highlands (Ozark, Ouachita, and Interior Low Plateau regions), this system is replaced by Central Interior Highlands Dry Acidic Glade and Barrens (CES202.692) (both are found in Kentucky, with the latter in the Shawnee Hills of the Interior Low Plateau).

DISTRIBUTION

**Range:** This system is found in the Cumberland Plateau of Kentucky, Tennessee, Virginia, Alabama, and Georgia.

**Divisions:** 202:C

**TNC Ecoregions:** 50:C

**Nations:** US

**Subnations:** AL, GA, KY, TN:S2S3, VA

**Map Zones:** 48:C, 53:C

**USFS Ecomap Regions:** 221H:CC

CONCEPT

**Environment:** This suite of glade, barren, and rock outcrop communities are found on flat to gently sloping expanses of sandstone and conglomerate (Edwards et al. 2013) on the surface of the Cumberland Plateau and related formations from Virginia south and west to Alabama. As the cement that holds the sand and conglomerate particles together dissolves and is transported away, sandy particles may collect in crevices and depressions to form sandy soil (Quarterman et al. 1993, Edwards et al. 2013). The sites of this system may be saturated for short times after rainfall, but also experience high temperatures in the summer, creating harsh conditions. Some examples of this system may occur adjacent to sandstone cliff faces.

**Vegetation:** These sandstone glades occur in a matrix of pine‑oak forests. *Pinus virginiana* and *Acer rubrum* are typical of the woodlands surrounding these outcrops on the Cumberland Plateau (Perkins 1981). This dominance pattern may be due to lack of disturbance. Other trees may include *Pinus rigida, Pinus echinata*, and/or *Quercus montana (= Quercus prinus)*. A perennial grass zone is typically present, which contains *Schizachyrium scoparium, Andropogon virginicus, Danthonia sericea*, and *Dichanthelium dichotomum*, in varying proportions. Herbaceous plants which are typical of the outcrops in Tennessee include *Diamorpha smallii* and *Minuartia glabra*. In Alabama, *Bigelowia nuttallii* is important, forming stands with other forbs (Perkins 1981, A. Schotz pers. comm.). Other herbaceous plants which may be found include *Liatris microcephala, Coreopsis pulchra, Eurybia surculosa (= Aster surculosus), Hypericum gentianoides, Phemeranthus mengesii (= Talinum mengesii), Nuttallanthus canadensis (= Linaria canadensis), Opuntia humifusa (= var. humifusa), Sporobolus vaginiflorus*, and *Erigeron strigosus*. Nonvascular plants include *Aulacomnium palustre, Campylopus pilifer, Grimmia* spp., *Polytrichum commune, Polytrichum juniperinum*, and fruticose lichens such as *Cladonia* spp. (= *Cladina* spp.). Scattered shrubs, such as *Gaylussacia* spp., *Vaccinium arboreum*, and *Chionanthus virginicus*, along with oak and hickory regeneration, occur on the margins of more open areas, in patches of deeper soil.

**Dynamics:** Severe droughts kill tree saplings growing in cracks and potholes, helping to retain the open character of the glades (Quarterman et al. 1993). There is an apparent zonation or patchiness to glade/barren vegetation, with different zones that may be identified by their characteristic plant species (Quarterman et al. 1993). These zones are apparently relatively stable, with woody plant encroachment evident only in relation to the invasion of shrubs and trees into potholes or crevices where soil accumulates more rapidly.

SOURCES

**References:** Comer et al. 2003*, DeSelm and Murdock 1993, Duffey et al. 1974, Edwards et al. 2013, Estes et al. 1979, Evans et al. 2009, Eyre 1980, McKinney and Lockwood 1999, Murdock pers. comm., Noss 2013, Perkins 1981, Quarterman et al. 1993, Schotz pers. comm., TDNH unpubl. data 2018, TNC 1996c, Taft 1997a, Taft 2009, Taft et al. 1995, Wiens and Dyer 1975

**Version:** 14 Jan 2014	**Stakeholders:** East, Midwest, Southeast

**Concept Author:** M. Pyne, R. Evans, C. Nordman	**LeadResp:** Southeast

M508. Central Interior Calcareous Scrub & Grassland

CES202.602 Central Appalachian Alkaline Glade and Woodland

**Primary Division:** Central Interior and Appalachian (202)

**Land Cover Class:** Steppe/Savanna

**Spatial Scale & Pattern:** Small patch

**Required Classifiers:** Natural/Semi‑natural; Vegetated (>10% vasc.); Upland

**Diagnostic Classifiers:** Woody‑Herbaceous; Ridge/Summit/Upper Slope; Unglaciated; Alkaline Soil; Shallow Soil

**National Mapping Codes:** EVT 2400; ESLF 5416; ESP 1400

**Concept Summary:** This system occurs at low to moderate elevations from the Central Appalachians (with a few northward incursions into southernmost New York and New England possible) south to the Ridge and Valley and Piedmont. It consists of woodlands and open glades on thin soils over limestone, dolostone or similar calcareous rock. *Juniperus virginiana* is a common tree, often increasing in the absence of fire, and *Quercus muehlenbergii* is indicative of the limestone substrate. *Rhus aromatica, Cercis canadensis*, and *Ostrya virginiana* may occur. Prairie grasses are the dominant herbs (*Andropogon gerardii, Schizachyrium scoparium, Bouteloua* spp.). Forb richness is often high; characteristic forbs include *Asclepias verticillata, Brickellia eupatorioides, Erigeron pulchellus, Monarda fistulosa, Packera obovata, Salvia lyrata*, and *Symphyotrichum oblongifolium*. Fire is sometimes an important natural disturbance factor, but open physiognomies may also be maintained by drought.

DISTRIBUTION

**Range:** This system is known from Pennsylvania and northwestern New Jersey south through the Ridge and Valley to western Virginia, possibly extending to southeasternmost New York and the marble valleys of northwestern Connecticut.

**Divisions:** 202:C

**TNC Ecoregions:** 49:P, 51:C, 59:C, 61:C

**Nations:** US

**Subnations:** CT?, MD, NJ, NY?, OH, PA, VA, WV

**Map Zones:** 57:C, 60:C, 61:C, 64:C, 65:P

**USFS Ecomap Regions:** 221B:CC, 221D:CC, M221A:CC, M221B:CC

CONCEPT

**Environment:** This system occupies mid‑elevation rocky ridges, gentle to steep south‑ and southwest‑facing slopes, and outcrops with thin soils and calcareous bedrock. Large amounts of exposed mineral soils and/or gravel are characteristic. Soils are high in pH and rich in calcium and magnesium. Although these areas are subject to prolonged droughts, local areas of ephemeral vernal seepage occur in microtopographic concavities, and they may have distinctive vegetation (e.g., colonies of Dodecatheon meadia). A series of glades in western Virginia is somewhat distinctive because of the dolostone, which contains a high magnesium content. These glades are located on low dolomite knobs and foothills of Elbrook dolomite that occupy middle to upper slopes and crests of south‑ or southwest‑facing spur ridges at relatively low elevations. In the Allegheny Mountains and along the Allegheny Front of Pennsylvania, the surface geology is primarily sandstone and shale, but the Mauch Chunk formation includes several narrow bands of limestone that outcrop frequently on steep slopes (Berg et al. 1980).

**Vegetation:** In some cases, the woodlands grade into closed‑canopy forests. *Juniperus virginiana* is a common tree, filling in in the absence of fire, and *Quercus muehlenbergii* is indicative of the limestone substrate. *Rhus aromatica, Cercis canadensis*, and *Ostrya virginiana* may occur. Prairie grasses are the dominant herbs (*Andropogon gerardii, Schizachyrium scoparium, Bouteloua* spp.); forb richness is often high. Characteristic forbs include *Asclepias verticillata, Monarda fistulosa, Salvia lyrata, Symphyotrichum oblongifolium*, and *Brickellia eupatorioides* (Braun 1950).

**Dynamics:** Drought stress appears to drive patch dynamics. Fire is likely to have a somewhat lesser impact due to thin soils and sparse vegetation, although fire scars on woody vegetation of barrens in Virginia suggest that fire may also play a role in maintaining the open character of this system (Ludwig 1999), and fire is also thought to contribute to arresting succession by woody species in Pennsylvania (Laughlin 2004, McPherson 2013). Where this system occurs on steep slopes, debris avalanches may cause periodic disturbance, but this process needs further study (Bartgis 1993); anthropogenic disturbance is thought to have played a role in establishment of some occurrences in Pennsylvania; quarrying has been noted to create habitat for the establishment of species characteristic of limestone prairies, but overall this activity poses a threat through outright destruction or habitat degradation (Laughlin 2004, McPherson 2013).

SOURCES

**References:** Bartgis 1993, Berg et al. 1980, Braun 1950, Comer et al. 2003*, Dreese 2010, Edinger et al. 2014a, Eyre 1980, Fike 1999, Laughlin 2004, Ludwig 1999, McPherson 2013

**Version:** 14 Jan 2014	**Stakeholders:** East, Midwest, Southeast

**Concept Author:** S.C. Gawler, G. Fleming, and R. Evans	**LeadResp:** East

CES202.691 Central Interior Highlands Calcareous Glade and Barrens

**Primary Division:** Central Interior and Appalachian (202)

**Land Cover Class:** Steppe/Savanna

**Spatial Scale & Pattern:** Small patch

**Required Classifiers:** Natural/Semi‑natural; Vegetated (>10% vasc.); Upland; Wetland

**Diagnostic Classifiers:** Woody‑Herbaceous; Rock Outcrops/Barrens/Glades; Seepage‑Fed Sloping; Alkaline Soil

**National Mapping Codes:** EVT 2401; ESLF 5417; ESP 1401

**Concept Summary:** This system is found primarily in the Interior Highlands of the Ozark, Ouachita, and Interior Low Plateau regions with scattered occurrences in northern Missouri. It occurs along moderate to steep slopes and steep valleys on primarily southerly to westerly facing slopes. Limestone and/or dolomite bedrock typify this system with shallow, moderately to well‑drained soils interspersed with rocks. These soils often dry out during the summer and autumn, and then become saturated during the winter and spring. *Schizachyrium scoparium* dominates this system and is commonly associated with *Andropogon gerardii, Bouteloua curtipendula*, and calcium‑loving plant species. Stunted woodlands primarily dominated by *Quercus muehlenbergii* interspersed with *Juniperus virginiana* occur on variable‑depth‑to‑bedrock soils. Fire is the primary natural dynamic, and prescribed fires help manage this system by restricting woody growth and maintaining the more open glade structure.

**Comments:** In Tennessee, this system is best developed in the Western Valley of the Tennessee River (a very limited part of EPA 71f) in Decatur and Perry counties. In Alabama, this system is found in the Moulton Valley region, which is technically part of TNC Ecoregion 50, but ambiguously placed there. This region is included in the Interior Plateau (71) of EPA (2004). Also included here, somewhat uncomfortably, is an unusual series of flatrock glades on Silurian dolomite in Bullitt County, Kentucky (71d of Woods et al. (2002)).

DISTRIBUTION

**Range:** This system is found primarily in the Interior Highlands of the Ozark, Ouachita, and the Interior Low Plateau regions ranging east to southern Ohio and including the Knobs region and Cliff section of Kentucky, the Cumberland Plateau escarpment of Tennessee, the Western Valley of the Tennessee River, and the Moulton Valley of northern Alabama.

**Divisions:** 202:C, 203:C

**TNC Ecoregions:** 36:C, 38:C, 39:C, 43:C, 44:C, 50:C

**Nations:** US

**Subnations:** AL, AR, GA, IL, IN, KY, MO, OH, OK, TN

**Map Zones:** 43:P, 44:C, 47:C, 48:C, 49:C, 53:C

**USFS Ecomap Regions:** 221E:CC, 221H:CC, 223A:CC, 223B:CC, 223D:CC, 223E:CC, 223F:CC, M223A:CC

CONCEPT

**Environment:** This system is found primarily along moderate to steep slopes and steep valleys on primarily southerly to westerly facing slopes. Limestone and/or dolomite bedrock typify this system with shallow, moderately to well‑drained soils interspersed with rocks. Soils are affected by the bedrock chemistry and tend to have high levels of calcium and potassium and a relatively high pH. Due to seasonal rainfall patterns and the extremely thin soils, these soils dry out during the summer and autumn and become saturated during the winter and spring. In northern Alabama (Moulton Valley), the stratum on which the system is found is a type of "marl." Seeps may occur where impervious rock strata meet relatively permeable limestone.

**Vegetation:** *Schizachyrium scoparium* dominates this system and is commonly associated with *Andropogon gerardii, Bouteloua curtipendula*, and calcium‑loving plant species. Stunted woodlands primarily dominated by *Quercus muehlenbergii* interspersed with *Juniperus virginiana* occur on variable‑depth‑to‑bedrock soils. The trees typically occur as islands in a wider herbaceous or rocky area. The islands are found in microenvironments where the soil depth and available water are sufficient to support trees (e.g., depressions in the bedrock). Other woody plants associated with this system (within their ranges) include *Quercus shumardii, Cercis canadensis, Ulmus alata, Fraxinus quadrangulata, Juniperus ashei, Acer saccharum*, and *Frangula caroliniana*. Other herbaceous taxa include *Silphium trifoliatum, Silphium terebinthinaceum, Liatris* spp., *Symphyotrichum oblongifolium, Castilleja coccinea, Stenaria nigricans (= Hedyotis nigricans), Phemeranthus (= Talinum)* spp., *Sedum* spp., and *Panicum flexile*. Small‑scale stands of annual *Sporobolus* spp. may be prominent in some examples. In some examples, small‑scale seepage areas may contain *Eleocharis compressa, Nothoscordum bivalve, Isoetes butleri*, and *Hypoxis hirsuta*.

**Dynamics:** The thin, dry soil characteristic of this system dries out during the growing season and much of the vegetation dries, as well. This allows fires to spread easily and these fires restrict the abundance of woody species. In high‑quality examples where the natural fire regime operates, small trees and shrubs are limited to the edges of stands or small "islands" of deeper soil that retain more moisture while grasses are the dominant vegetation. Sparsely vegetated areas between the dominant grassy zones contain most of the rare species found in this system (Ware 2002). In the absence of fire, from active suppression or a lack of fuel due to excessive grazing, woody species can increase greatly.

SOURCES

**References:** Baskin and Baskin 2000, Baskin et al. 1995, Comer et al. 2003*, DeSelm and Murdock 1993, Delcourt and Delcourt 1997, EPA 2004, Erickson et al. 1942, Evans et al. 2009, Eyre 1980, Homoya 1994, Martin and Houf 1993, Nelson 2010, Nelson 2012, Taft 2009, Taft et al. 1995, USFWS 1974, Ware 2002, Webb et al. 1997, Woods et al. 2002

**Version:** 14 Jan 2014	**Stakeholders:** Midwest, Southeast

**Concept Author:** S. Menard, T. Nigh, M. Pyne	**LeadResp:** Midwest

CES202.354 Eastern Highland Rim Prairie and Barrens

**Primary Division:** Central Interior and Appalachian (202)

**Land Cover Class:** Herbaceous

**Spatial Scale & Pattern:** Large patch

**Required Classifiers:** Natural/Semi‑natural; Vegetated (>10% vasc.); Upland; Wetland

**Diagnostic Classifiers:** Herbaceous; Seepage‑Fed Sloping; Extensive Wet Flat; Very Short Disturbance Interval; Graminoid

**National Mapping Codes:** EVT 2417; ESLF 7130; ESP 1417

**Concept Summary:** This system represents "The Barrens" of the Southeast Highland Rim of Tennessee, as well as related areas of Kentucky. This is a distinctive part of Tennessee and the Eastern Highland Rim and includes a series of plant communities with open canopies, ranging from herbaceous‑dominated barrens (some of which are maintained today by mowing instead of fire and grazing) through savanna and woodland types. Open ponds and other wetlands are scattered throughout the landscape. The variety of relatively open habitats which are present here include prairie‑like areas, as well as savanna woodlands and upland depression ponds. Stands may vary in physiognomy from savanna‑grasslands to oak‑dominated woodlands and forests. Many stands are in a forested condition today due to lack of fire. Typical mesic grassland vegetation of the barrens of the southeastern Highland Rim of Tennessee is dominated by *Andropogon gerardii* along with *Schizachyrium scoparium* and *Sorghastrum nutans*. There is also related vegetation in Kentucky (e.g., Hazel Dell Meadow and related sites) which is also included here.

**Comments:** Western Highland Rim Prairie and Barrens (CES202.352), Eastern Highland Rim Prairie and Barrens (CES202.354), Pennyroyal Karst Plain Prairie and Barrens (CES202.355), and Southern Ridge and Valley Patch Prairie (CES202.453) form a series of similar systems in the eastern Interior Highlands and adjacent Ridge and Valley.

DISTRIBUTION

**Range:** This system is restricted to "The Barrens" of the southeastern Highland Rim of Tennessee (today primarily extant in Coffee, Franklin, and Warren counties, Tennessee). This is a small part of Subsection 223Eb (USFS) and EPA Level IV Ecoregion 71g. Also included are related smaller areas in the Eastern Highland Rim of Kentucky.

**Divisions:** 202:C

**TNC Ecoregions:** 44:C

**Nations:** US

**Subnations:** KY, TN:S2

**Map Zones:** 48:C, 53:P

**USFS Ecomap Regions:** 223E:CC

CONCEPT

**Environment:** These various barren communities occur on Fragiudult soils formed in Pleistocene loess over karstic Mississippian Limestone. Their topography is flat to gently sloping. Some proposed factors which have functioned to maintain their openness include the hardpan soils and fire (as well as natural and managed grazing, and modern anthropogenic factors such as mowing for hay, etc.). These barrens include a variety of systems whose primary presettlement environmental factors were specialized soils and extremes of hydrology, as influenced by fire and grazing. The prevalent soils within the polygon labeled "Dickson‑Mountview‑Guthrie" (D32 of Elder and Springer (1978), Springer and Elder (1980)) are generally flatter, wetter, and more likely to have fragipans than adjoining units. Average conditions in the area of The Barrens can be summarized as follows (Wolfe 1996): January is typically the coldest month, with average high and low temperatures of 8.8º C (47.8º F) and 1.9º C (35.4º F), respectively. July is the warmest month, with average high and low temperatures of 31.3º C (88.3º F) and 18.9º C (66.0º F), respectively. Monthly mean temperatures range from 3.5º C (38.3º F) in January to 25.11º C (77.2º F) in July. The mean annual precipitation is 1438 mm (56.6 inches) (Wolfe 1996, Pyne 2000). Precipitation is heaviest from November through May, averaging between 113 and 171 mm (4.4 to 6.7 in) per month. Rainfall is lightest during the months of June through October, with averages ranging from 83 mm (3.3 inches) per month to a minor peak of 122 mm (4.8 inches) in July.

**Vegetation:** Stands may vary in physiognomy from savanna‑grasslands to oak‑dominated woodlands and forests. Many stands are in a forested condition today due to lack of fire. Typical mesic grassland vegetation of the barrens of the southeastern Highland Rim of Tennessee is dominated by *Andropogon gerardii* along with *Schizachyrium scoparium* and *Sorghastrum nutans*. Other graminoid species present include *Andropogon glomeratus, Calamagrostis coarctata, Carex barrattii*, and *Panicum virgatum*. Other dominants may include *Eurybia hemispherica (= Aster paludosus ssp. hemisphericus), Symphyotrichum dumosum (= Aster dumosus), Helianthus angustifolius, Potentilla simplex, Solidago odora, Solidago rugosa, Pteridium aquilinum*, and *Polytrichum commune*; found to a lesser extent are *Aristida purpurascens var. virgata (= Aristida virgata), Chasmanthium laxum, Dichanthelium aciculare (= Dichanthelium angustifolium), Dichanthelium dichotomum, Gymnopogon brevifolius, Panicum anceps, Panicum rigidulum*, and *Panicum verrucosum*. Woody species may include *Quercus alba, Quercus stellata, Quercus falcata, Quercus marilandica, Carya* spp., *Acer rubrum, Rhus copallinum, Rosa setigera, Salix humilis, Diospyros virginiana, Rubus argutus*, and *Smilax glauca*. The Barrens contains a variety of natural, semi‑natural, and managed openings which provide habitat for plants and animals which are unusual in the ecoregion, rare in the state, or globally rare. These include a variety of plants more at home in other ecoregions, most notably the Coastal Plain and the western prairies, including carnivorous plants and other specialized plants of ponds and other wetlands. In addition, globally rare endemic fish and disjunct amphibians and invertebrates call The Barrens their home.

**Dynamics:** Past fire and grazing constitute the major dynamic processes for this system. Fires were frequent (potentially on a five‑year return interval (Guyette et al. 2006), documented over approximately the last 370 years), primarily of human origin, occurring in late summer to early autumn. Forestry activities (including planting of off‑site *Pinus taeda*, which is not truly native to the region) and fire suppression have led to the current forested condition with solar intensity as low as 10%. The current persistence of prairies, shrublands, and grassy‑woodland/savannas is largely dependent on contemporary management regimes. The woodlands, savannas and prairies are often grown up in woody vegetation (e.g., *Acer rubrum, Liquidambar styraciflua*, as well as *Quercus* spp. and *Carya* spp.) due to fire suppression. Woodlands dominated by *Quercus alba, Quercus stellata*, and to a lesser extent *Quercus marilandica* often "fill in" with less fire‑tolerant species (e.g., *Acer rubrum, Liquidambar styraciflua, Nyssa sylvatica, Quercus coccinea, Quercus falcata*, etc.) resulting in a closed‑canopy forest.

SOURCES

**References:** Comer et al. 2003*, DeSelm and Murdock 1993, Duffey et al. 1974, Elder and Springer 1978, Estes et al. 1979, Eyre 1980, Guyette et al. 2006, McKinney and Lockwood 1999, Murdock pers. comm., Noss 2013, Pyne 2000, Springer and Elder 1980, TDNH unpubl. data 2018, TNC 1996c, Taft 1997a, Taft 2009, Taft et al. 1995, Wiens and Dyer 1975, Wolfe 1996

**Version:** 31 Aug 2018	**Stakeholders:** Southeast

**Concept Author:** M. Pyne, R. Evans, C. Nordman	**LeadResp:** Southeast

CES202.334 Nashville Basin Limestone Glade and Woodland

**Primary Division:** Central Interior and Appalachian (202)

**Land Cover Class:** Steppe/Savanna

**Spatial Scale & Pattern:** Small patch

**Required Classifiers:** Natural/Semi‑natural; Vegetated (>10% vasc.); Upland; Wetland

**Diagnostic Classifiers:** Woody‑Herbaceous; Rock Outcrops/Barrens/Glades; Seepage‑Fed Sloping; Alkaline Soil; Graminoid

**National Mapping Codes:** EVT 2397; ESLF 5413; ESP 1397

**Concept Summary:** This system encompasses a range of plant communities associated with thin soils on flat areas of Ordovician limestone in the Nashville Basin of Tennessee (mostly inner basin, also outer basin), with a few disjunct occurrences in Kentucky. The vegetation of this system includes sparsely vegetated rock outcrops, annual *Sporobolus* spp.‑dominated grasslands, *Schizachyrium scoparium*‑dominated perennial grasslands, seasonally wet herbaceous washes and seeps, shrublands, as well as woodlands dominated by *Juniperus virginiana* and oaks. In addition, *Echinacea tennesseensis* and *Astragalus bibullatus* are completely endemic to this system. There are numerous other disjunct and near‑endemic plants.

**Comments:** This system occupies a small portion of the landscape but many associations are only found in this system. The most closely related system is Central Interior Highlands Calcareous Glade and Barrens (CES202.691). Also included here are related disjunct examples in Kentucky on Mississippian limestones (EPA ecoregions 71a, 71e of Woods et al. (2002)).

DISTRIBUTION

**Range:** This system is restricted to flat areas of Ordovician limestone in the Inner Nashville Basin of Tennessee (Ecoregion 71i of Griffith et al. (1998); Subsection 222Ed of Keys et al. (1995)), as well as limited and disjunct examples on flat Mississippian limestones in Kentucky.

**Divisions:** 202:C

**TNC Ecoregions:** 44:C

**Nations:** US

**Subnations:** KY, TN

**Map Zones:** 47:C, 48:C

**USFS Ecomap Regions:** 223Dg:CCC, 223Ec:CCC, 223Ed:CCC, 223Eh:CCC

CONCEPT

**Environment:** This system is associated with thin soils on flat areas of Ordovician limestone in the Inner Nashville Basin of Tennessee (Ecoregion 71i of Griffith et al. 1998 and EPA 2004; Subsection 222Ed of Keys et al. 1995), with a few disjunct occurrences in Kentucky.

**Vegetation:** The vegetation of this system includes sparsely vegetated rock outcrops, annual *Sporobolus* spp.‑dominated grasslands, *Schizachyrium scoparium*‑dominated perennial grasslands, seasonally wet herbaceous washes and seeps, shrublands, as well as woodlands dominated by *Juniperus virginiana* and oaks. Other woody plants associated with this system include *Quercus shumardii, Cercis canadensis, Ulmus alata, Fraxinus quadrangulata*, and *Acer saccharum*. Characteristic shrubs include *Forestiera ligustrina, Rhus aromatica, Hypericum frondosum*, and *Frangula caroliniana*. Other herbaceous taxa include *Andropogon gerardii, Bouteloua curtipendula, Silphium trifoliatum, Silphium terebinthinaceum, Helianthus mollis, Grindelia lanceolata, Liatris* spp., *Stenaria nigricans (= Hedyotis nigricans), Croton capitatus, Heliotropium tenellum, Trichostema brachiatum (= Isanthus brachiatus), Manfreda virginica, Ruellia humilis, Phemeranthus calcaricus (= Talinum calcaricum), Sedum pulchellum*, and *Panicum flexile*. In addition, *Echinacea tennesseensis* and *Astragalus bibullatus* are completely endemic to this system. There are numerous other disjunct and near‑endemic plants, including *Astragalus tennesseensis, Dalea gattingeri*, and *Pediomelum subacaule* (Somers et al. 1986). Small‑scale seepage areas and washes may contain *Dalea foliosa, Eleocharis bifida, Eleocharis compressa, Hypoxis hirsuta, Isoetes butleri, Mecardonia acuminata, Mitreola petiolata, Nothoscordum bivalve*, and *Schoenolirion croceum*, as well as *Juncus filipendulus, Carex crawei, Carex granularis, Leavenworthia torulosa, Leucospora multifida, Lobelia appendiculata var. gattingeri (= Lobelia gattingeri), Muhlenbergia schreberi*, and *Sisyrinchium albidum*.

**Dynamics:** There is an apparent zonation or patchiness to glade/barren vegetation, with different zones that may be identified by their characteristic plant species (Quarterman et al. 1993). These zones are apparently relatively stable, with woody plant encroachment evident only in relation to the invasion of shrubs and trees into potholes or crevices where soil accumulates more rapidly.

<br />Periodic droughts, fire, historic grazing, and ice storms all play a role in the dynamics of the system by restricting woody growth and maintaining the more open glade structure. Historic grazing by wild and domestic ungulate species represented a significant disturbance regime in the past. Regionally significant drought cycles lead to death or decline of *Juniperus virginiana*, as well as affecting the severity of other disturbance regimes. Severe droughts kill tree saplings growing in cracks and potholes, helping to retain the open character of the glades (Quarterman et al. 1993). Fire carries best in zones or areas dominated by perennial grasses, which provide the most abundant and consistent fuel. This zone is also the most vulnerable to succession, with *Juniperus virginiana* and various native (and exotic) shrubs occupying these areas in periods without disturbance (Landfire 2007a).

The ecological processes that maintain these open grasslands and glades within a forested matrix are not completely understood. Clearly periodic drought cycles of varying lengths play a role, along with fire and free‑ranging grazing livestock, at least until the 1940s, when open range laws were changed (DeSelm 1994). Livestock confinement, habitat fragmentation, and the ingrowth of exotic shrubs have caused many examples of these communities to become more densely covered by woody plants, including the native but weedy *Juniperus virginiana var. virginiana*.

Open range laws and the use of fire to clear native grass pastures worked to keep large parts of the rural Nashville Basin in an open, grass‑dominated condition, either as open, prairie‑like areas, or as oak woodlands with a native grass and forb understory. This combination of conditions persisted until about 1945 (DeSelm 1994). In a Missouri study of presettlement fire using composite fire scar chronologies, Guyette and McGinnes (1982 as cited in Frost 1998) reconstructed a presettlement fire frequency of 3.2 years in Missouri cedar glade vegetation.

SOURCES

**References:** Comer et al. 2003*, DeSelm 1994, DeSelm and Murdock 1993, Duffey et al. 1974, EPA 2004, Estes et al. 1979, Evans et al. 2009, Eyre 1980, Frost 1998, Griffith et al. 1998, Guyette and McGinnes 1982, Keys et al. 1995, LANDFIRE 2007a, McKinney and Lockwood 1999, Murdock pers. comm., Noss 2013, Quarterman et al. 1993, Somers et al. 1986, Sutter et al. 2011, TDNH unpubl. data 2018, TNC 1996c, Taft 1997a, Taft 2009, Taft et al. 1995, Wiens and Dyer 1975, Woods et al. 2002

**Version:** 30 Jun 2016	**Stakeholders:** Southeast

**Concept Author:** M. Pyne, R. Evans, C. Nordman	**LeadResp:** Southeast

CES202.024 Southern Ridge and Valley Calcareous Glade and Woodland

**Primary Division:** Central Interior and Appalachian (202)

**Land Cover Class:** Steppe/Savanna

**Spatial Scale & Pattern:** Small patch

**Required Classifiers:** Natural/Semi‑natural; Vegetated (>10% vasc.); Upland

**Diagnostic Classifiers:** Woody‑Herbaceous; Rock Outcrops/Barrens/Glades

**Concept Summary:** This ecological system consists of open glades and surrounding woodlands on shallow, high pH soils of the Ridge and Valley region from southwestern Virginia southward. Examples of related calcareous vegetation from the Cumberland Plateau area of Alabama (231Cd of Ecomap 2007; 68e of EPA) are included here as well. These glades occur in broad valley bottoms, rolling basins, and adjacent slopes where soils are shallow over flat‑lying limestone strata. The flat to rolling terrain and locally xeric soils may have been especially conducive to periodic fires that helped maintain the prairielike openings and savannalike woodlands. Today, much of the system is currently somewhat more closed and brushy, suggesting fire suppression. *Quercus muehlenbergii* and *Quercus stellata* are typical where the canopy is present. Dominant or abundant *Juniperus virginiana var. virginiana* is probably a result of the lack of fire.

**Comments:** This system formerly embodied a narrower concept, being restricted to glades of "valley bottoms," and thereby, at least by implication, not those of slopes. The current concept includes glades in the southern Ridge and Valley on a variety of landforms and slope positions, as they are all sufficiently similar in floristic components and ecological processes to be grouped together. These processes and factors include erosional processes, zonal vegetation patterns, and general ecological dynamics. Examples of related calcareous vegetation from the Cumberland Plateau area of Alabama (231Cd of Ecomap 2007; 68e of EPA) are included here as well based on reports from the Alabama Natural Heritage Program.

DISTRIBUTION

**Range:** This system occurs from southwestern Virginia (roughly Roanoke) south through the southern Ridge and Valley into Georgia (as well as in the Cumberlands of Alabama).

**Divisions:** 202:C

**TNC Ecoregions:** 50:C, 59:?

**Nations:** US

**Subnations:** AL, GA, TN, VA

**Map Zones:** 48:C, 53:C, 57:C, 61:?

**USFS Ecomap Regions:** 221J:CC, 231C:CC, 231D:CC, M221A:CC

CONCEPT

**Environment:** Examples occur on shallow, high pH soils, in broad valley bottoms, rolling basins, and adjacent slopes over limestone strata.

**Vegetation:** The vegetation of typical examples could range from open woodlands of *Quercus muehlenbergii* and *Juniperus virginiana*, with interspersed grasslands dominated by perennial *Schizachyrium scoparium*, to patches dominated by annual grasses such as varieties of *Sporobolus vaginiflorus* (e.g., var. *ozarkanus*, var. *vaginiflorus*). Some other trees that may occur in stands include *Quercus falcata, Quercus shumardii, Quercus stellata*, as well as the understory woody plants *Cercis canadensis, Salix humilis*, and *Viburnum rufidulum*. Some characteristic herbs include *Eryngium yuccifolium, Manfreda virginica*, and *Hypericum dolabriforme*. Dominant or abundant *Juniperus virginiana var. virginiana* is probably a result of the lack of fire.

**Dynamics:** The flat to rolling terrain and locally xeric soils may have been especially conducive to periodic fires that helped maintain the grass‑dominated openings and open woodlands. In addition to occasional fire, periodic drought may also be important in regulating woody plant encroachment into native grasslands. It is believed that these native glade‑grassland systems have evolved under a combined system of grazing, drought, and periodic fire (Duffey et al. 1974, Estes et al. 1979, Noss 2013).

SOURCES

**References:** Cleland et al. 2007, Comer et al. 2003*, DeSelm and Murdock 1993, Duffey et al. 1974, EPA 2013a, Estes et al. 1979, Eyre 1980, Keys et al. 1995, LANDFIRE 2007a, McKinney and Lockwood 1999, Murdock pers. comm., Noss 2013, Quarterman et al. 1993, Sutter et al. 2011, TDNH unpubl. data 2018, TNC 1996c, Taft 1997a, Taft 2009, Taft et al. 1995, Wiens and Dyer 1975

**Version:** 21 Feb 2017	**Stakeholders:** East, Southeast

**Concept Author:** M. Pyne, G. Fleming, R. Evans	**LeadResp:** Southeast

M505. Laurentian‑Acadian Acidic Rocky Scrub & Grassland

CES201.019 Laurentian Acidic Rocky Outcrop

**Primary Division:** Laurentian‑Acadian (201)

**Land Cover Class:** Steppe/Savanna

**Spatial Scale & Pattern:** Small patch

**Required Classifiers:** Natural/Semi‑natural; Vegetated (>10% vasc.); Upland

**Diagnostic Classifiers:** Lowland; Ridge/Summit/Upper Slope; Rock Outcrops/Barrens/Glades; Glaciated; Acidic Soil

**Concept Summary:** This Laurentian and near‑boreal outcrop system is found across central southern Canada and the upper Midwest of the United States. It is found on ridges or summits of resistant acidic bedrock at low to mid elevations. The vegetation is patchy, often a mosaic of woodlands and open glades. The system is typically dominated by various conifers, including *Pinus banksiana* and *Picea mariana*, with occasional *Picea glauca* or *Populus tremuloides*. Hardwoods include *Quercus rubra, Quercus ellipsoidalis*, and *Populus tremuloides*. Structure can vary from treed to low heath shrubs to open lichen woodland. Exposure and occasional fire are the major factors in keeping the vegetation relatively open.

**Comments:** See also Northern Appalachian‑Acadian Rocky Heath Outcrop (CES201.571), which occurs to the east of this system's primary range and is characterized by granitic bedrock.

DISTRIBUTION

**Range:** This system is found in central Canada south to the Great Lakes and northern Minnesota, eastward in Canada to Quebec and a small portion of extreme northeastern New York.

**Divisions:** 103:C, 201:C

**TNC Ecoregions:** 47:C, 48:C, 64:C

**Nations:** CA, US

**Subnations:** MB, MI, MN, NY, ON, QC, WI

**Map Zones:** 41:C, 50:C, 51:C, 64:C

**USFS Ecomap Regions:** 211E:CC, 212Jb:CCC, 212Jc:CCC, 212Jo:CCP, 212K:CC, 212Lb:CCP, 212M:CC, 212Q:CC, 212Sb:CCC, 212Sc:CCP, 212Sn:CCP, 212Sq:CCC, 212Tc:CCC, 212X:CC, 212Ya:CCC

CONCEPT

SOURCES

**References:** Comer et al. 2003*, Eyre 1980, Kost et al. 2007, WDNR 2015

**Version:** 03 Oct 2007	**Stakeholders:** Canada, East, Midwest

**Concept Author:** D. Faber‑Langendoen	**LeadResp:** Midwest

CES201.571 Northern Appalachian‑Acadian Rocky Heath Outcrop

**Primary Division:** Laurentian‑Acadian (201)

**Land Cover Class:** Steppe/Savanna

**Spatial Scale & Pattern:** Small patch

**Required Classifiers:** Natural/Semi‑natural; Vegetated (>10% vasc.); Upland

**Diagnostic Classifiers:** Lowland; Ridge/Summit/Upper Slope; Rock Outcrops/Barrens/Glades; Glaciated; Acidic Soil

**Concept Summary:** This outcrop ecological system ranges across New England and adjacent Canada, and southward at higher elevations to northern Pennsylvania, on ridges or summits of resistant acidic bedrock. Throughout most of its range, it occurs at low to mid elevations (600‑1000 m, lower on the coast of eastern Maine and the Maritimes). The vegetation is patchy, often a mosaic of woodlands and open glades. *Quercus rubra* and various conifers, including *Pinus strobus* and *Picea rubens*, or (especially near the coast) *Picea mariana*, are characteristic trees. Low heath shrubs, including *Kalmia angustifolia, Vaccinium angustifolium, Gaylussacia baccata*, and *Aronia melanocarpa (= Photinia melanocarpa)*, are typically present. Exposure and occasional fire are the major factors in keeping the vegetation relatively open.

**Comments:** This system transitions westward and northward into Laurentian Acidic Rocky Outcrop (CES201.019) and southward into Central Appalachian Pine‑Oak Rocky Woodland (CES202.600). Where their ranges overlap or abut, this system is distinguished from the latter by the presence of more northern elements such as *Picea, Sorbus, Pinus banksiana*, etc., and lack of *Pinus rigida* and *Quercus ilicifolia* which may be found in the Central Appalachian system. This system overlaps with Laurentian Acidic Rocky Outcrop (CES201.019) only in New York state, where the latter occurs in the St. Lawrence ‑ Champlain ecoregion (an extension of its Great Lakes affinities), and the present type occurs primarily in the Northern Appalachian ecoregion. Northward or at higher elevations, this system is replaced by Acadian‑Appalachian Subalpine Woodland and Heath‑Krummholz (CES201.568).<br />

Distribution in Southern New England and the Allegheny region of New York and Pennsylvania needs review. Overlap with rocky woodland systems complicates the application of the system concept, as only one non‑woodland association is attributed to the type.

DISTRIBUTION

**Range:** This system is found in New England and adjacent Canada west to the Adirondacks and south to northern Pennsylvania.

**Divisions:** 201:C

**TNC Ecoregions:** 48:P, 60:C, 61:C, 63:C, 64:P

**Nations:** CA, US

**Subnations:** MA, ME, NB, NH, NS, NY, PA, QC, VT

**Map Zones:** 63:C, 64:C, 65:C, 66:C

**USFS Ecomap Regions:** 211A:CC, 211B:CC, 211C:CC, 211D:CC, 211F:CC, 211I:CC, M211A:CC, M211B:CC, M211C:CC, M211D:CC

CONCEPT

SOURCES

**References:** Comer et al. 2003*, Edinger et al. 2014a, Eyre 1980, Gawler and Cutko 2010, Sperduto and Nichols 2004

**Version:** 05 Oct 2004	**Stakeholders:** Canada, East

**Concept Author:** S.C. Gawler and D. Faber‑Langendoen	**LeadResp:** East

M507. Laurentian‑Acadian Calcareous Scrub & Grassland

CES201.721 Great Lakes Alvar

**Primary Division:** Laurentian‑Acadian (201)

**Land Cover Class:** Steppe/Savanna

**Spatial Scale & Pattern:** Large patch

**Required Classifiers:** Natural/Semi‑natural; Vegetated (>10% vasc.); Upland; Wetland

**National Mapping Codes:** EVT 2409; ESLF 5458; ESP 1409

**Concept Summary:** Alvars are natural systems of humid and subhumid climates, centered around areas of glaciated horizontal limestone/dolomite (dolostone) bedrock pavement with a discontinuous thin soil mantle. These communities are characterized by distinctive flora and fauna with less than 60% tree cover that is maintained by associated geologic, hydrologic, and other landscape processes. In particular, all forms of alvar tend to flood each spring, then experience moderate to severe drought in summer months. They include open pavement, grassland, and shrubland/woodland types. Alvar communities occur in an ecological matrix with similar bedrock and hydrologically influenced communities. Almost all of North America's alvars occur within the Great Lakes basin, primarily in an arc along the Niagaran Escarpment from northern Lake Michigan across northern Lake Huron and eastern Ontario and northwestern New York state.

DISTRIBUTION

**Range:** Alvars occur within the Great Lakes basin.

**Divisions:** 201:C

**TNC Ecoregions:** 48:C, 64:C

**Nations:** CA, US

**Subnations:** MI, NY, OH, ON, WI

**Map Zones:** 41:C, 50:C, 51:C, 52:C, 63:P, 64:C

**USFS Ecomap Regions:** 211Ee:CCC, 212Hl:CCC, 212Rc:CCC, 212Re:CCC, 212Tb:CCC, 212Te:CCC, 222Ie:CCC, 222U:CC

CONCEPT

**Environment:** Alvars are found near Great Lakes shores where flat limestone or dolostone bedrock pavement is exposed. Soils are shallow and discontinuous and tend to accumulate in cracks and shallow depressions in the bedrock. Where present, they are <25 cm deep. In the spring, soils are saturated or even flooded where shallow depressions occur. The thin soils dry quickly and are usually very dry by late summer.

**Dynamics:** The thin soils and large changes in soil moisture during the growing season shape the vegetation of alvars. These conditions favor herbaceous species over woody species. The composition of alvars varies largely with the soil moisture from seasonal herbaceous wetlands to dry grassy areas to sparsely vegetation bedrock. Small shrublands or stunted woodlands can be found where soil accumulates (Reschke et al. 1998). Fires do not carry well on alvars in most years but they did occur with low frequency (Landfire 2007a). Woody species grow slowly on alvars, so even low frequency fires limited their abundance.

SOURCES

**References:** Albert 1990, Comer et al. 2003*, Edinger et al. 2014a, Eyre 1980, Kost et al. 2007, LANDFIRE 2007a, ONHD unpubl. data, Reschke et al. 1998, WDNR 2015

**Version:** 14 Jan 2014	**Stakeholders:** Canada, East, Midwest

**Concept Author:** C. Reschke	**LeadResp:** Midwest

CES201.572 Laurentian‑Acadian Calcareous Rocky Outcrop

**Primary Division:** Laurentian‑Acadian (201)

**Land Cover Class:** Steppe/Savanna

**Spatial Scale & Pattern:** Small patch

**Required Classifiers:** Natural/Semi‑natural; Vegetated (>10% vasc.); Upland

**Diagnostic Classifiers:** Rock Outcrops/Barrens/Glades; Glaciated; Alkaline Soil; Circumneutral Soil

**Concept Summary:** This outcrop system occurs in scattered locations from New England west to the Great Lakes. It occurs on ridges or summits of circumneutral to calcareous bedrock. Sites are often exposed and dry; however, there may be local areas of more moist conditions. The vegetation is often a mosaic of woodlands and open glades. This system may also occur on rocks that are primarily acidic but with a local influence of calcium through weathering.

DISTRIBUTION

**Range:** Scattered locations from New England and adjacent Canada west to the eastern Great Lakes.

**Divisions:** 201:C

**TNC Ecoregions:** 48:C, 63:C

**Nations:** US

**Subnations:** ME, NH, NY, VT

**Map Zones:** 63:C, 64:C, 66:C

CONCEPT

SOURCES

**References:** Comer et al. 2003*, Edinger et al. 2014a, Eyre 1980, Gawler and Cutko 2010, Sperduto and Nichols 2004

**Version:** 09 Jan 2003	**Stakeholders:** East, Midwest

**Concept Author:** S.C. Gawler	**LeadResp:** East

2.B.2.Nf. Western North American Grassland & Shrubland

M048. Central Rocky Mountain Montane‑Foothill Grassland & Shrubland

CES304.792 Columbia Basin Palouse Prairie

**Primary Division:** Inter‑Mountain Basins (304)

**Land Cover Class:** Herbaceous

**Spatial Scale & Pattern:** Large patch

**Required Classifiers:** Natural/Semi‑natural; Vegetated (>10% vasc.); Upland

**Diagnostic Classifiers:** Herbaceous; Loess deposit (undifferentiated); Deep Soil; Mineral: W/ A‑Horizon >10 cm; Graminoid; Cool‑season bunch grasses; Long (>500 yrs) Persistence

**National Mapping Codes:** EVT 2142; ESLF 7115; ESP 1142

**Concept Summary:** This once‑extensive grassland system occurs in eastern Washington and Oregon, and west‑central Idaho, though in very small patches there. In much of its range it is characterized by rolling topography composed of loess hills and plains over basalt plains. The climate of this region has warm‑hot, dry summers and cool, wet winters. Annual precipitation is high, 38‑76 cm (15‑30 inches). The soils are typically deep, well‑developed, and old. The cool‑season bunchgrasses that dominate the vegetation are adapted to this winter precipitation. Characteristic species are *Pseudoroegneria spicata* and *Festuca idahoensis* with *Hesperostipa comata, Achnatherum scribneri, Leymus condensatus, Leymus cinereus, Koeleria macrantha, Pascopyrum smithii*, or *Poa secunda*. Shrubs commonly found include *Amelanchier alnifolia, Rosa* spp., *Eriogonum* spp., *Symphoricarpos albus*, and *Crataegus douglasii*. Excessive grazing, past land use and invasion by introduced annual species have resulted in a massive conversion to agriculture or shrub‑steppe and annual grasslands dominated by *Artemisia* spp. and *Bromus tectorum* or *Poa pratensis*. Remnant grasslands are now typically associated with steep and rocky sites or small and isolated sites within an agricultural landscape.

DISTRIBUTION

**Range:** This system occurs in eastern Washington and Oregon, and west‑central Idaho.

**Divisions:** 304:C, 306:P

**TNC Ecoregions:** 6:C, 8:P

**Nations:** CA?, US

**Subnations:** BC?, ID, OR, WA:S1

**Map Zones:** 8:C, 9:C, 10:P

**USFS Ecomap Regions:** 331A:CC, 342C:C?, 342D:CP, 342H:CC, 342I:CC, M242C:PP, M242D:PP, M332A:CP, M332G:CC, M333A:CC, M333D:CP

CONCEPT

**Environment:** This once‑extensive grassland system occurs in eastern Washington and Oregon, and west‑central Idaho, though in very small patches there. In much of its range it is characterized by rolling topography composed of loess hills and plains over basalt plains. The climate of this region has warm‑hot, dry summers and cool, wet winters. Annual precipitation is high, 38‑76 cm (15‑30 inches). The soils are typically deep, well‑developed, and old. The cool‑season bunch grasses that dominate the vegetation are adapted to this winter precipitation.

**Vegetation:** Characteristic species are *Pseudoroegneria spicata* and *Festuca idahoensis* with *Hesperostipa comata, Achnatherum scribneri, Leymus condensatus, Leymus cinereus, Koeleria macrantha, Pascopyrum smithii*, or *Poa secunda*. Shrubs commonly found include *Amelanchier alnifolia, Rosa* spp., *Eriogonum* spp., *Symphoricarpos albus*, and *Crataegus douglasii*.

**Dynamics:** Fire is the primary disturbance factor. Fires were low intensity due to limited fuel and significant internal spacing between fuel patches. Currently, *Bromus tectorum* and other introduced grasses often invade these habitats after fire, building up a dense fuelbed that creates frequent, high‑intensity fires that are lethal to native perennial grasses (Landfire 2007a). The historic frequency was 50 years to maintain this grassland (Landfire 2007a). Extending fires frequency to >50 years leads to increased shrub cover and shrub regeneration (Landfire 2007a).

SOURCES

**References:** Comer et al. 2003*, Daubenmire 1988, LANDFIRE 2007a, Shiflet 1994, TNC 2013, Tisdale 1982, WNHP unpubl. data 2018

**Version:** 14 Jan 2014	**Stakeholders:** Canada, West

**Concept Author:** K.A. Schulz	**LeadResp:** West

CES204.087 North Pacific Montane Shrubland

**Primary Division:** North American Pacific Maritime (204)

**Land Cover Class:** Shrubland

**Spatial Scale & Pattern:** Large patch

**Required Classifiers:** Natural/Semi‑natural; Vegetated (>10% vasc.); Upland

**Diagnostic Classifiers:** Shrubland (Shrub‑dominated)

**National Mapping Codes:** EVT 2084; ESLF 5261; ESP 1084

**Concept Summary:** This system occurs as small to large patches scattered throughout the North Pacific region, but it is largely absent from the windward sides of the coastal mountains where fires are rare due to very wet climates. It is defined as long‑lived seral shrublands that persist for several decades or more after major wildfires, or smaller patches of shrubland on dry sites that are marginal for tree growth and that have typically also experienced fire. This system occurs on ridgetops and upper to middle mountain slopes and is more common on sunny southern aspects. It occurs from about 152 m (500 feet) elevation up to the lower limits of subalpine parkland. Vegetation is mostly deciduous broadleaf shrubs, sometimes mixed with shrub‑statured trees or sparse evergreen needleleaf trees. It can also be dominated by evergreen shrubs, especially *Xerophyllum tenax* (usually considered a forb). Species composition is highly variable; some of most common species include *Acer circinatum, Arctostaphylos nevadensis, Acer glabrum, Vaccinium membranaceum, Ceanothus velutinus, Holodiscus discolor, Shepherdia canadensis, Sorbus* spp., and *Rubus parviflorus*. On the west side of the Cascades, *Gaultheria shallon* is an important dominant.

DISTRIBUTION

**Range:** This system occurs as small to large patches scattered throughout mountainous regions of the Pacific Northwest, from the southern Cascade and Coast ranges north to southern British Columbia. Its northernmost distribution is not clear, but it does not appear to occur in Alaska.

**Divisions:** 204:C

**TNC Ecoregions:** 1:C, 3:C, 4:C, 81:C

**Nations:** CA, US

**Subnations:** BC, OR, WA:S3S4

**Map Zones:** 1:C, 2:C, 7:C

**USFS Ecomap Regions:** 242A:CC, M242A:CC, M242B:CC, M242C:CC, M242D:CC

CONCEPT

**Environment:** This system occurs as small to large patches scattered throughout the North Pacific region, but it is largely absent from the windward sides of the coastal mountains where fires are rare due to very wet climates. It is defined as long‑lived seral shrublands that persist for several decades or more after major wildfires, or smaller patches of shrubland on dry sites that are marginal for tree growth and that have typically also experienced fire. This system occurs on ridgetops and upper to middle mountain slopes and is more common on sunny southern aspects. It occurs from about 152 m (500 feet) elevation up to the lower limits of subalpine parkland.

**Vegetation:** Vegetation is mostly deciduous broadleaf shrubs, sometimes mixed with shrub‑statured trees or sparse evergreen needleleaf trees. It can also be dominated by evergreen shrubs, especially *Xerophyllum tenax* (usually considered a forb). Species composition is highly variable; some of most common species include *Acer circinatum, Arctostaphylos nevadensis, Acer glabrum, Vaccinium membranaceum, Ceanothus velutinus, Holodiscus discolor, Shepherdia canadensis, Sorbus* spp., and *Rubus parviflorus*. On the west side of the Cascades, *Gaultheria shallon* is an important dominant.

SOURCES

**References:** Chappell and Christy 2004, Comer et al. 2003*, Franklin and Dyrness 1973, Shiflet 1994, WNHP unpubl. data 2018

**Version:** 21 Aug 2008	**Stakeholders:** Canada, West

**Concept Author:** C. Chappell	**LeadResp:** West

CES306.801 Northern Rocky Mountain Avalanche Chute Shrubland

**Primary Division:** Rocky Mountain (306)

**Land Cover Class:** Mixed Upland and Wetland

**Spatial Scale & Pattern:** Small patch

**Required Classifiers:** Natural/Semi‑natural; Vegetated (>10% vasc.); Upland; Wetland

**Diagnostic Classifiers:** Shrubland (Shrub‑dominated); Avalanche chute; Very Short Disturbance Interval [Periodicity/Nonrandom Disturbance]; Avalanche

**National Mapping Codes:** EVT 2168; ESLF 5327; ESP 1168

**Concept Summary:** This ecological system occurs in the mountains throughout the northern Rockies, from Wyoming north and west into British Columbia and Alberta. It is composed of a diverse mix of deciduous shrubs or trees, and conifers found on steep, frequently disturbed slopes in the mountains. Occurrences are found on the lower portions and runout zones of avalanche tracks, and slopes are generally steep, ranging from 15‑60%. Aspects vary, but are more common where unstable or heavy snowpack conditions frequently occur. Sites are often mesic to wet because avalanche paths are often in stream gullies, and snow deposition can be heavy in the run‑out zones. The vegetation consists of moderately dense, woody canopy characterized by dwarfed and damaged conifers and small, deciduous trees/shrubs. Characteristic species include *Abies lasiocarpa, Acer glabrum, Alnus viridis ssp. sinuata* or *Alnus incana, Populus balsamifera ssp. trichocarpa, Populus tremuloides*, or *Cornus sericea*. Other common woody plants include *Paxistima myrsinites, Sorbus scopulina*, and *Sorbus sitchensis*. The ground cover is moderately dense to dense forb‑rich, with *Senecio triangularis, Castilleja* spp., *Athyrium filix‑femina, Thalictrum occidentale, Urtica dioica, Erythronium grandiflorum, Myosotis asiatica, Veratrum viride, Heracleum maximum*, and *Xerophyllum tenax*. Mosses and ferns are often present.

DISTRIBUTION

**Range:** This ecological system occurs in the mountains throughout the northern Rockies, from Wyoming north and west into British Columbia and Alberta. It is likely to occur in the Colorado Rockies, but no association from that area have been classified as "avalanche chute" communities.

**Divisions:** 306:C

**TNC Ecoregions:** 7:C, 8:C, 9:C

**Nations:** CA, US

**Subnations:** AB, BC, CO, MT, OR, WA:S1, WY

**Map Zones:** 9:C, 10:C, 12:?, 19:C, 21:P, 28:P

**USFS Ecomap Regions:** M242D:PP, M331A:CC, M331D:CC, M331E:CP, M331J:CC, M332B:PP, M332E:PP, M332F:PP, M333A:CC, M333B:CC, M333C:CC, M333D:CC

CONCEPT

**Vegetation:** The vegetation consists of moderately dense, woody canopy characterized by dwarfed and damaged conifers and small, deciduous trees/shrubs. Characteristic species include *Abies lasiocarpa, Acer glabrum, Alnus viridis ssp. sinuata* or *Alnus incana, Populus balsamifera ssp. trichocarpa, Populus tremuloides*, or *Cornus sericea*. Other common woody plants include *Paxistima myrsinites, Sorbus scopulina*, and *Sorbus sitchensis*. The ground cover is moderately dense to dense forb‑rich, with *Senecio triangularis, Castilleja* spp., *Athyrium filix‑femina, Thalictrum occidentale, Urtica dioica, Erythronium grandiflorum, Myosotis asiatica (= Myosotis alpestris), Veratrum viride, Heracleum maximum (= Heracleum lanatum)*, and *Xerophyllum tenax*. Mosses and ferns are often present.

SOURCES

**References:** Butler 1979, Butler 1985, Comer et al. 2003*, Malanson and Butler 1984, NCC 2002, WNHP unpubl. data 2018

**Version:** 20 Feb 2003	**Stakeholders:** Canada, West

**Concept Author:** M.S. Reid	**LeadResp:** West

CES306.040 Northern Rocky Mountain Lower Montane, Foothill and Valley Grassland

**Primary Division:** Rocky Mountain (306)

**Land Cover Class:** Herbaceous

**Spatial Scale & Pattern:** Large patch

**Required Classifiers:** Natural/Semi‑natural; Vegetated (>10% vasc.); Upland

**Diagnostic Classifiers:** Lowland [Foothill, Lowland]; Herbaceous; Sideslope; Very Shallow Soil; Loam Soil Texture; Silt Soil Texture; Ustic; Landslide; Graminoid; Cool‑season bunch grasses

**National Mapping Codes:** EVT 2139; ESLF 7112; ESP 1139

**Concept Summary:** This ecological system of the northern Rocky Mountains is found at lower montane to foothill elevations in the mountains and large valleys of northeastern Wyoming and western Montana, west through Idaho into the Blue Mountains of Oregon, and north into the Okanagan and Fraser plateaus of British Columbia and the Canadian Rockies. They also occur to the east in the central Montana mountain "islands," foothills, as well as the Rocky Mountain Front and Big and Little Belt ranges. These grasslands are floristically similar to Inter‑Mountain Basins Big Sagebrush Steppe (CES304.778), Columbia Basin Foothill and Canyon Dry Grassland (CES304.993), and Columbia Basin Palouse Prairie (CES304.792), but are defined by shorter summers, colder winters, and young soils derived from recent glacial and alluvial material. These grasslands reflect a shift in the precipitation regime from summer monsoons and cold snowy winters found in the Southern Rockies to predominantly dry summers and winter precipitation. In the eastern portion of its range in Montana, winter precipitation is replaced by a spring peak in precipitation. They are found at elevations from 300 to 1650 m, ranging from small meadows to large open parks surrounded by conifers in the lower montane, to extensive foothill and valley grasslands below the lower treeline. In the southern extent, some of these valleys may have been primarily sage‑steppe with patches of grassland in the past, but because of land‑use history post‑settlement (herbicide, grazing, fire, pasturing, etc.), they have been converted to grassland‑dominated areas. Soils are relatively deep, fine‑textured, often with coarse fragments, and non‑saline, often with a microphytic crust. The most important species are cool‑season perennial bunchgrasses and forbs (>25% cover), sometimes with a sparse (<10% cover) shrub layer. *Pseudoroegneria spicata, Festuca campestris, Festuca idahoensis*, or *Hesperostipa comata* commonly dominate sites on all aspects of level to moderate slopes and on certain steep slopes with a variety of other grasses, such as *Achnatherum hymenoides, Achnatherum richardsonii, Hesperostipa curtiseta, Koeleria macrantha, Leymus cinereus, Elymus trachycaulus, Bromus inermis var. pumpellianus, Achnatherum occidentale, Pascopyrum smithii*, and other graminoids such as *Carex filifolia* and *Danthonia intermedia*. Other grassland species include *Opuntia fragilis, Artemisia frigida, Carex petasata, Antennaria* spp., and *Selaginella densa*. Important exotic grasses include *Phleum pratense, Bromus inermis*, and *Poa pratensis*. Shrub species may be scattered, including *Amelanchier alnifolia, Rosa* spp., *Symphoricarpos* spp., *Juniperus communis, Artemisia tridentata*, and in Wyoming *Artemisia tripartita ssp. rupicola*. Common associated forbs include *Geum triflorum, Galium boreale, Campanula rotundifolia, Antennaria microphylla, Geranium viscosissimum*, and *Potentilla gracilis*. A soil crust of lichen covers almost all open soil between clumps of grasses; *Cladonia* and *Peltigera* are the most common lichens. Unvegetated mineral soil is commonly found between clumps of grass and the lichen cover. The fire regime of this ecological system maintains a grassland due to rapid fire return that retards shrub invasion or landscape isolation and fragmentation that limits seed dispersal of native shrub species. Fire frequency is variable but is presumed to be generally less than 20 years to reduce shrub cover and maintain grassland. These are extensive grasslands, not grass‑dominated patches within the sagebrush shrub‑steppe ecological system. *Festuca campestris* is easily eliminated by grazing and does not occur in all areas of this system.

**Comments:** This is the same as the Interior Plateau Grassland also called "Northern Plateau Grassland" of the Okanagan Ecoregional Plan. In Wyoming, this is distinguished from Northwestern Great Plains Mixedgrass Prairie (CES303.674) by the presence of *Festuca idahoensis* or *Carex rossii*, the lack of *Bouteloua gracilis* (which is common in CES303.674), or the presence of *Artemisia nova* or *Artemisia tripartita ssp. rupicola*, neither of which occur in CES303.674.

DISTRIBUTION

**Range:** This lower montane, foothill and valley grassland system occurs throughout the southern interior and southern portion of the Fraser Plateau, as well as the valleys around the Fraser River in the Pavilion Ranges, the Nicola River and the Similkameen River in British Columbia. It also occurs in the mountains and large valleys of northwestern Wyoming and western Montana, east to the central Montana Rocky Mountain Front and mountain "island" ranges, west through Idaho into the Blue Mountains of Oregon.

**Divisions:** 207:C, 306:C

**TNC Ecoregions:** 6:P, 7:C, 8:C, 9:P, 26:C, 68:C

**Nations:** CA, US

**Subnations:** AB, BC, ID, MT, OR, WA:S3S4, WY

**Map Zones:** 1:C, 8:C, 9:C, 10:C, 18:C, 19:C, 20:C, 21:C, 22:C, 29:C

**USFS Ecomap Regions:** 331A:CP, 331D:CC, 331N:CC, 341G:CC, 342A:CC, 342B:CC, 342C:CC, 342D:CP, 342H:CC, 342I:CC, 342J:CC, M242C:CC, M242D:CC, M331A:CP, M331B:CC, M331J:CP, M332A:CC, M332B:CC, M332D:CC, M332E:CC, M332F:CC, M332G:CC, M333A:CC, M333B:CP, M333C:CC, M333D:CP, M341A:CC

CONCEPT

**Environment:** This system is found at lower montane to foothill elevations in the mountains and large valleys of northeastern Wyoming and western Montana, west through Idaho into the Blue Mountains of Oregon, and north into the Okanagan and Fraser plateaus of British Columbia and the Canadian Rockies. They also occur to the east in the central Montana mountain "islands" and foothills, as well as the Rocky Mountain Front Range and Big and Little Belt ranges. These grasslands are floristically similar to Inter‑Mountain Basins Big Sagebrush Steppe (CES304.778), Columbia Basin Foothill and Canyon Dry Grassland (CES304.993), and Columbia Basin Palouse Prairie (CES304.792), but are defined by shorter summers, colder winters, and young soils derived from recent glacial and alluvial material. These lower montane and valley grasslands represent a shift in the precipitation regime from summer monsoons and cold snowy winters found in the Southern Rockies to predominantly dry summers and winter precipitation. In the eastern portion of its range in Montana, winter precipitation is replaced by a huge spring peak in precipitation. They are found at elevations from 300 to 1650 m, ranging from small meadows to large open parks surrounded by conifers in the lower montane, to extensive foothill and valley grasslands below the lower treeline. In the southern extent some of these valleys may have been primarily sage‑steppe with patches of grassland in the past, but because of land‑use history post‑settlement (herbicide, grazing, altered fire regime, pasturing, etc.), they have been converted to grassland‑dominated areas. Soils are relatively deep, fine‑textured, often with coarse fragments, and non‑saline, often with a microphytic crust.

**Vegetation:** The most important species are cool‑season perennial bunchgrasses and forbs (>25% cover), sometimes with a sparse (<10% cover) shrub layer. *Pseudoroegneria spicata, Festuca campestris, Festuca idahoensis*, or *Hesperostipa comata* commonly dominate sites on all aspects of level to moderate slopes and on certain steep slopes with a variety of other grasses, such as *Achnatherum hymenoides, Achnatherum richardsonii, Hesperostipa curtiseta, Koeleria macrantha, Leymus cinereus, Elymus trachycaulus, Bromus inermis var. pumpellianus (= Bromus pumpellianus), Achnatherum occidentale (= Stipa occidentalis), Pascopyrum smithii*, and other graminoids such as *Carex filifolia* and *Danthonia intermedia*. Other grassland species include *Opuntia fragilis, Artemisia frigida, Carex petasata, Antennaria* spp., and *Selaginella densa*. Important exotic grasses include *Phleum pratense, Bromus inermis*, and *Poa pratensis*. Shrub species may be scattered, including *Amelanchier alnifolia, Rosa* spp., *Symphoricarpos* spp., *Juniperus communis, Artemisia tridentata*, and in Wyoming *Artemisia tripartita ssp. rupicola*. Common associated forbs include *Geum triflorum, Galium boreale, Campanula rotundifolia, Antennaria microphylla, Geranium viscosissimum*, and *Potentilla gracilis*. A soil crust of lichen covers almost all open soil between clumps of grasses; *Cladonia* and *Peltigera* are the most common lichens. Unvegetated mineral soil is commonly found between clumps of grass and the lichen cover.

**Dynamics:** These are extensive grasslands, not grass‑dominated patches within the sagebrush shrub‑steppe ecological system. *Festuca campestris* is easily eliminated by grazing and does not occur in all areas of this system. The most droughty sites produce little and discontinuous fuel and likely have much longer fire regimes. Isolation of grassland patches by fragmentation may also limit seed dispersal of native shrubs leading to persistence of the grassland. Soil drought and herbivory retard shrub and tree invasion resulting in a patchy distribution of shrubs and trees when present.

The high‑frequency fire regime of this ecological system maintains a grassland due to rapid fire return that retards shrub invasion or landscape isolation and fragmentation that limits seed dispersal of native shrub species. Fire frequency is presumed to be less than 20 years generally. Johnson and Swanson (2005) presumed fire frequency to be less than 35 years in the Blue and Ochoco mountains of Oregon. Wikeem and Wikeem (2004) compiled average fire intervals for interior grasslands in British Columbia which range from 5‑20 years. Klenner et al. (2008) research supports a fire regime of predominantly mixed‑severity fires that maintain grasslands in the dry forest and grasslands ecotone in the southern interior of British Columbia.

Biological soil crust cover is important in these grasslands. It alters the composition of perennial species and increases the establishment of native disturbance‑increasers and annual grasses, particularly *Bromus tectorum* and other exotic annual bromes (WNHP 2011). Crust cover and diversity are greatest where not impacted by trampling, other soil surface disturbance and fragmentation (Belnap et al. 2001, Rosentreter and Eldridge 2002, Tyler 2006).

LANDFIRE developed a state‑and‑transition vegetation dynamics VDDT model for this system which has three classes in total (LANDFIRE 2007a, BpS 1911390). These are summarized as:

A) Early Development 1 All Structures (5% of type in this stage): Graminoid cover is 0‑10%. Post‑fire, early‑seral community dominated by bunchgrasses and forbs. Herbs and forbs will generally have higher cover than pre‑burn and may include milkvetch, balsamroot, lupine, yarrow and prairie junegrass. Cover ranges from 0‑10%. In the absence of fire or heavy animal impact, this condition succeeds to a mid‑development condition (class B). Age ranges from 0‑2 years. Idaho fescue may be present but will recover more slowly than the bluebunch wheatgrass after fire.

B) Mid Development 1 Closed (25% of type in this stage): Graminoid cover is 11‑30%. Mid‑development with moderate canopy closure dominated by bunchgrasses with forb cover generally higher than pre‑burn. Typically lasts 5 years.

C) Late Development 1 Closed (70% of type in this stage): Tree cover is 31‑100%. Late‑development, closed canopy of grasses and forbs. Bunchgrasses dominate with low densities of shrubs (<10%) in some areas, particularly where this BpS transitions to shrub‑ or tree‑dominated communities. Shrub species may include big sagebrush, buckwheat, ceanothus, bitterbrush and snowberry.

This type has frequent replacement fires (fire regime group II). Most species in this type are fire‑adapted and respond favorably to these fire types. Where these systems occur within forested ecosystems, fire frequency will be strongly influenced by the surrounding forest's fire regime (e.g., 10‑20 years). Where these systems occur below lower treeline, fire frequencies may be longer (e.g., 20‑30 years) (LANDFIRE 2007a, BpS 1911390).

SOURCES

**References:** BCCDC unpubl. data 2018, Bell et al. 2009, Belnap et al. 2001, Comer et al. 2003*, Darambazar et al. 2007, Ecosystems Working Group 1998, Johnson and Swanson 2005, Klenner et al. 2008, LANDFIRE 2007a, McKenzie et al. 2004, McKenzie et al. 2008, Mote et al. 2014, Rosentreter and Eldridge 2002, Shafer et al. 2014, Shiflet 1994, Steen and Coupé 1997, Stevens‑Rumann et al. 2017, TNC 2013, Tyler 2006, WNHP 2011, WNHP unpubl. data 2018, Westerling et al. 2006, Wikeem and Wikeem 2004

**Version:** 24 May 2018	**Stakeholders:** Canada, West

**Concept Author:** R. Crawford	**LeadResp:** West

CES306.994 Northern Rocky Mountain Montane‑Foothill Deciduous Shrubland

**Primary Division:** Rocky Mountain (306)

**Land Cover Class:** Shrubland

**Spatial Scale & Pattern:** Large patch

**Required Classifiers:** Natural/Semi‑natural; Vegetated (>10% vasc.); Upland

**Diagnostic Classifiers:** Montane [Lower Montane]; Lowland [Foothill]; Shrubland (Shrub‑dominated); Very Shallow Soil; Broad‑Leaved Deciduous Shrub; Moderate (100‑500 yrs) Persistence

**National Mapping Codes:** EVT 2106; ESLF 5312; ESP 1106

**Concept Summary:** This shrubland ecological system is found in the lower montane and foothill regions around the Columbia Basin, and north and east into the northern Rockies, including Alberta and British Columbia. These shrublands typically occur below treeline, within the matrix of surrounding low‑elevation grasslands and sagebrush shrublands. They also occur in the ponderosa pine and Douglas‑fir zones, but rarely up into the subalpine zone (on dry sites). The shrublands are usually found on steep slopes of canyons and in areas with some soil development, either loess deposits or volcanic clays; they occur on all aspects. Fire, flooding and erosion all impact these shrublands, but they typically will persist on sites for long periods. These communities develop near talus slopes as garlands, at the heads of dry drainages, and toeslopes in the moist shrub‑steppe and steppe zones. *Physocarpus malvaceus, Prunus emarginata, Prunus virginiana, Rosa* spp., *Rhus glabra, Acer glabrum, Amelanchier alnifolia, Symphoricarpos albus, Symphoricarpos oreophilus*, and *Holodiscus discolor* are the most common dominant shrubs, occurring alone or any combination. In the Alberta's Upper and Lower Foothills subregions, common shrubs include *Arctostaphylos uva‑ursi, Juniperus communis, Symphoricarpos* spp., *Amelanchier alnifolia*, and *Rosa* spp. *Rubus parviflorus* and *Ceanothus velutinus* are other important shrubs in this system, being more common in montane occurrences than in subalpine situations. Occurrences in central and eastern Wyoming can include *Artemisia tridentata ssp. vaseyana* and *Cercocarpus montanus*, but neither of these are dominant, and where they occur, the stands are truly mixes of shrubs, often with *Amelanchier alnifolia, Prunus virginiana*, and others being the predominant taxa. In moist areas, *Crataegus douglasii* can be common. *Shepherdia canadensis* and *Spiraea betulifolia* can be abundant in some cases but also occur in Northern Rocky Mountain Subalpine Deciduous Shrubland (CES306.961). *Festuca idahoensis, Festuca campestris, Calamagrostis rubescens, Carex geyeri, Koeleria macrantha, Pseudoroegneria spicata*, and *Poa secunda* are the most important grasses. *Achnatherum thurberianum* and *Leymus cinereus* can be locally important. *Poa pratensis* and *Phleum pratense* are common introduced grasses. *Geum triflorum, Potentilla gracilis, Lomatium triternatum, Balsamorhiza sagittata*, and species of *Eriogonum, Phlox*, and *Erigeron* are important forbs.

**Comments:** Seral shrub fields of comparable composition that typically will develop into a seral stage with trees (within 50 years) are excluded from this shrub system and are included in their appropriate forest system.

DISTRIBUTION

**Range:** This system is found in the lower montane and foothill regions around the Columbia Basin, and north and east into the northern Rockies, including east into central Montana around the "Sky Island" ranges. It also occurs farther south into central and eastern Wyoming, where it forms compositionally diverse shrublands.

**Divisions:** 304:C, 306:C

**TNC Ecoregions:** 6:C, 7:C, 8:C, 26:C, 68:C

**Nations:** CA, US

**Subnations:** AB, BC, ID, MT, OR, WA:S4?, WY

**Map Zones:** 1:C, 7:C, 8:C, 9:C, 10:C, 16:?, 17:?, 18:C, 19:C, 20:C, 21:C, 22:C, 29:C

**USFS Ecomap Regions:** 331A:CC, 331D:CP, 331N:CC, 341G:PP, 342A:CP, 342B:CC, 342C:CC, 342D:CC, 342E:CC, 342H:CC, 342I:CC, 342J:CC, M242C:CC, M242D:CC, M261D:PP, M261G:P?, M331A:CC, M331B:CC, M331D:CC, M331J:CC, M332A:CC, M332B:CC, M332D:CC, M332E:CC, M332F:CC, M332G:CC, M333A:CC, M333B:CC, M333C:CC, M333D:CC, M334A:CC, M341A:PP

CONCEPT

**Environment:** This shrubland ecological system is found in the lower montane and foothill regions around the Columbia Basin, and north and east into the northern Rockies, including Alberta and British Columbia. These shrublands typically occur below treeline, within the matrix of surrounding low‑elevation grasslands and sagebrush shrublands. They also occur in the ponderosa pine and Douglas‑fir zones, but rarely up into the subalpine zone (on dry sites). The shrublands are usually found on steep slopes of canyons and in areas with some soil development, either loess deposits or volcanic clays; they occur on all aspects. Fire, flooding and erosion all impact these shrublands, but they typically will persist on sites for long periods. These communities develop near talus slopes as garlands, at the heads of dry drainages, and toeslopes in the moist shrub‑steppe and steppe zones.

**Vegetation:** *Physocarpus malvaceus, Prunus emarginata, Prunus virginiana, Rosa* spp., *Rhus glabra, Acer glabrum, Amelanchier alnifolia, Symphoricarpos albus, Symphoricarpos oreophilus*, and *Holodiscus discolor* are the most common dominant shrubs, occurring alone or any combination. In the Alberta's Upper and Lower Foothills subregions, common shrubs include *Arctostaphylos uva‑ursi, Juniperus communis, Symphoricarpos* spp., *Amelanchier alnifolia*, and *Rosa* spp. *Rubus parviflorus* and *Ceanothus velutinus* are other important shrubs in this system, being more common in montane occurrences than in subalpine situations. Occurrences in central and eastern Wyoming can include *Artemisia tridentata ssp. vaseyana* and *Cercocarpus montanus*, but neither of these are dominant, and where they occur, the stands are truly mixes of shrubs, often with *Amelanchier alnifolia, Prunus virginiana*, and others being the predominant taxa. In moist areas, *Crataegus douglasii* can be common. *Shepherdia canadensis* and *Spiraea betulifolia* can be abundant in some cases but also occur in Northern Rocky Mountain Subalpine Deciduous Shrubland (CES306.961). *Festuca idahoensis, Festuca campestris, Calamagrostis rubescens, Carex geyeri, Koeleria macrantha, Pseudoroegneria spicata*, and *Poa secunda* are the most important grasses. *Achnatherum thurberianum* and *Leymus cinereus* can be locally important. *Poa pratensis* and *Phleum pratense* are common introduced grasses. *Geum triflorum, Potentilla gracilis, Lomatium triternatum, Balsamorhiza sagittata*, and species of *Eriogonum, Phlox*, and *Erigeron* are important forbs.

SOURCES

**References:** Bell et al. 2009, Comer et al. 2003*, Ecosystems Working Group 1998, Franklin and Dyrness 1973, Hall 1973, Johnson and Clausnitzer 1992, Johnson and Simon 1987, Knight 1994, Lane et al. 2000, Poulton 1955, Shiflet 1994, Tisdale 1986, WNHP unpubl. data 2018, Willoughby 2007

**Version:** 30 Mar 2010	**Stakeholders:** Canada, West

**Concept Author:** M. Reid and J. Kagan	**LeadResp:** West

CES306.961 Northern Rocky Mountain Subalpine Deciduous Shrubland

**Primary Division:** Rocky Mountain (306)

**Land Cover Class:** Shrubland

**Spatial Scale & Pattern:** Large patch, Small patch

**Required Classifiers:** Natural/Semi‑natural; Vegetated (>10% vasc.); Upland

**Diagnostic Classifiers:** Montane [Upper Montane]; Shrubland (Shrub‑dominated); Very Shallow Soil; Broad‑Leaved Deciduous Shrub; Moderate (100‑500 yrs) Persistence

**National Mapping Codes:** EVT 2169; ESLF 5326; ESP 1169

**Concept Summary:** This shrubland ecological system is found within the zone of continuous forest in the upper montane and lower subalpine zones of the northern Rocky Mountains. Soils tend to be moist to wet. Stands are typically initiated by fires and will persist on sites for long periods because of repeated burns and changes in the presence of volatile oils in the soil which impedes tree regeneration. *Menziesia ferruginea, Rhamnus alnifolia, Ribes lacustre, Rubus parviflorus, Alnus viridis, Rhododendron albiflorum, Sorbus scopulina, Sorbus sitchensis, Vaccinium myrtillus, Vaccinium scoparium*, and *Vaccinium membranaceum* are the most common dominant shrubs, occurring alone or in any combination. Other shrubs can include *Shepherdia canadensis* and *Ceanothus velutinus*, but these also commonly occur in Northern Rocky Mountain Montane‑Foothill Deciduous Shrubland (CES306.994). *Rubus parviflorus* and *Ceanothus velutinus* are occasionally present, being more common in montane shrublands than in this subalpine system. Important forbs include *Xerophyllum tenax, Chamerion angustifolium*, and *Pteridium aquilinum*, reflecting the mesic nature of many of these shrublands.

**Comments:** This system is floristically somewhat similar to Northern Rocky Mountain Avalanche Chute Shrubland (CES306.801), but the avalanche chutes originate from very different processes, tend to be more diverse within stands, and are wetter, being driven ecologically by snow‑loading and concomitant snowmelt. Seral shrub fields of comparable composition that typically will develop into a seral stage with trees (within 50 years) are excluded from this shrub system and are included in their appropriate forest system.

DISTRIBUTION

**Range:** This system is found in the subalpine and upper montane zones in the northern Rockies, south and west around the Columbia Basin.

**Divisions:** 304:C, 306:C

**TNC Ecoregions:** 6:C, 7:C, 8:C, 26:C, 68:C

**Nations:** CA, US

**Subnations:** AB, BC, ID, MT, OR, WA:S4, WY

**Map Zones:** 1:C, 8:?, 9:C, 10:C, 18:P, 19:C, 20:C, 21:P, 22:?, 29:C

**USFS Ecomap Regions:** M331A:CP, M331B:CC, M331D:CP, M331E:CP, M331J:C?, M332A:CC, M332B:CC, M332D:CC, M332E:CC, M332F:CC, M332G:C?, M333A:CC, M333B:CC, M333C:CC, M333D:CC

CONCEPT

SOURCES

**References:** Comer et al. 2003*, Ecosystems Working Group 1998, Franklin and Dyrness 1973, Hall 1973, Johnson and Clausnitzer 1992, Johnson and Simon 1987, Poulton 1955, Tisdale 1986, WNHP unpubl. data 2018

**Version:** 26 Jan 2007	**Stakeholders:** Canada, West

**Concept Author:** NatureServe Western Ecology Team	**LeadResp:** West

CES306.806 Northern Rocky Mountain Subalpine‑Upper Montane Grassland

**Primary Division:** Rocky Mountain (306)

**Land Cover Class:** Herbaceous

**Spatial Scale & Pattern:** Large patch

**Required Classifiers:** Natural/Semi‑natural; Vegetated (>10% vasc.); Upland

**Diagnostic Classifiers:** Montane [Upper Montane]; Herbaceous; Deep Soil; Ustic; Intermediate Disturbance Interval; Graminoid; Tussock‑forming grasses

**National Mapping Codes:** EVT 2140; ESLF 7113; ESP 1140

**Concept Summary:** This is an upper montane to subalpine, high‑elevation, lush grassland system dominated by perennial grasses and forbs on dry sites, particularly south‑facing slopes. It is most extensive in the Canadian Rockies portion of the Rocky Mountain cordillera, extending south into western Montana, eastern Oregon, eastern Washington and Idaho. Subalpine dry grasslands are small meadows to large open parks surrounded by conifer trees but lack tree cover within them. In general, soil textures are much finer, and soils are often deeper under grasslands than in the neighboring forests. Grasslands, although composed primarily of tussock‑forming species, do exhibit a dense sod that makes root penetration difficult for tree species. Disturbance such as fire also plays a role in maintaining these open grassy areas. Typical dominant species include *Leymus innovatus, Koeleria macrantha, Festuca campestris, Festuca idahoensis, Festuca viridula, Achnatherum occidentale, Achnatherum richardsonii, Bromus inermis var. pumpellianus, Elymus trachycaulus, Phleum alpinum, Trisetum spicatum*, and a variety of Carices, such as *Carex hoodii, Carex obtusata*, and *Carex scirpoidea*. Important forbs include *Lupinus argenteus var. laxiflorus, Potentilla diversifolia, Potentilla flabellifolia, Fragaria virginiana*, and *Chamerion angustifolium*. This system is similar to Northern Rocky Mountain Lower Montane, Foothill and Valley Grassland (CES306.040) but is found at higher elevations and is more often composed of species of *Festuca, Achnatherum*, and/or *Hesperostipa* with additional floristic components of more subalpine taxa. Occurrences of this system are often more forb‑rich than Southern Rocky Mountain Montane‑Subalpine Grassland (CES306.824).

DISTRIBUTION

**Range:** This system is most extensive in the Canadian Rockies portion of the Rocky Mountain cordillera, extending south into western Montana, central and eastern Oregon, eastern Washington and Idaho. It also occurs in the "island" ranges of central Montana, though it is not common, and is also found in the Bighorn Range of north‑central Wyoming.

**Divisions:** 306:C

**TNC Ecoregions:** 4:P, 7:C, 8:C, 9:P, 26:C, 68:C

**Nations:** CA, US

**Subnations:** AB, BC, ID, MT, OR, WA:S3S4, WY

**Map Zones:** 9:C, 10:C, 18:C, 19:C, 20:C, 21:C, 29:C

**USFS Ecomap Regions:** 331A:??, 341G:CC, 342A:CP, 342C:CC, 342D:CC, 342H:CC, 342I:C?, 342J:CC, M242B:C?, M242C:CP, M242D:CC, M331A:PP, M331B:PP, M332A:CC, M332B:CC, M332D:CC, M332E:CC, M332F:CC, M332G:CP, M333A:CC, M333B:CC, M333C:CC, M333D:CC

CONCEPT

**Environment:** This is an upper montane to subalpine, high‑elevation, lush grassland system dominated by perennial grasses and forbs on dry sites, particularly south‑facing slopes. It is most extensive in the Canadian Rockies portion of the Rocky Mountain cordillera, extending south into western Montana, eastern Oregon, eastern Washington and Idaho. Subalpine dry grasslands are small meadows to large open parks surrounded by conifer trees but lack tree cover within them. In general, soil textures are much finer, and soils are often deeper under grasslands than in the neighboring forests.

**Vegetation:** Grasslands, although composed primarily of tussock‑forming species, do exhibit a dense sod that makes root penetration difficult for tree species. Typical dominant species include *Leymus innovatus (= Elymus innovatus), Koeleria macrantha, Festuca campestris, Festuca idahoensis, Festuca viridula, Achnatherum occidentale (= Stipa occidentalis), Achnatherum richardsonii (= Stipa richardsonii), Bromus inermis var. pumpellianus (= Bromus pumpellianus), Elymus trachycaulus, Phleum alpinum, Trisetum spicatum*, and a variety of Carices, such as *Carex hoodii, Carex obtusata*, and *Carex scirpoidea*. Important forbs include *Lupinus argenteus var. laxiflorus, Potentilla diversifolia, Potentilla flabellifolia, Fragaria virginiana*, and *Chamerion angustifolium (= Epilobium angustifolium)*.

**Dynamics:** Disturbance such as fire also plays a role in maintaining these open grassy areas.

SOURCES

**References:** Comer et al. 2003*, Cooper et al. 1995, Johnson 2004, Lane et al. 2000, NCC 2002, Shiflet 1994, WNHP unpubl. data 2018, Willoughby 2007

**Version:** 07 Sep 2005	**Stakeholders:** Canada, West

**Concept Author:** M.S. Reid	**LeadResp:** West

M168. Rocky Mountain‑Vancouverian Subalpine‑High Montane Mesic Meadow

CES206.940 Mediterranean California Subalpine Meadow

**Primary Division:** Mediterranean California (206)

**Land Cover Class:** Herbaceous

**Spatial Scale & Pattern:** Large patch

**Required Classifiers:** Natural/Semi‑natural; Vegetated (>10% vasc.); Upland

**Diagnostic Classifiers:** Montane [Upper Montane]; Herbaceous; Ustic; W‑Landscape/High Intensity; Late‑lying snowpack

**National Mapping Codes:** EVT 2137; ESLF 7109; ESP 1137

**Concept Summary:** This ecological system occurs at subalpine and montane elevations where finely textured soils, snow deposition, or windswept dry conditions limit tree establishment. It is typically found above 3000 m (9100 feet) elevation in California, western Nevada and Oregon. The soils in these sites can be seasonally moist to saturated in the spring but, if so, will dry out later in the growing season, and overall these are mesic to dry meadows, not wet. Characteristic plant species include *Achillea millefolium var. occidentalis, Artemisia rothrockii, Oreostemma alpigenum, Calamagrostis breweri, Cistanthe umbellata, Carex exserta, Eriogonum incanum, Horkeliella purpurascens*, and *Trisetum spicatum*. Burrowing mammals can increase the forb diversity. Herbs can include *Carex subnigricans, Carex vernacula, Calamagrostis breweri, Antennaria media, Potentilla drummondii, Lewisia pygmaea, Erigeron algidus, Lupinus lepidus, Dodecatheon alpinum*, and *Solidago multiradiata*. Wet meadows of *Carex, Calamagrostis, Camassia, Eleocharis, Juncus, Veratrum*, etc. from montane to subalpine are treated in Temperate Pacific Subalpine‑Montane Wet Meadow (CES200.998).

DISTRIBUTION

**Range:** This system occurs at subalpine elevations where finely textured soils, snow deposition, or windswept dry conditions limit tree establishment, typically above 3000 m (9100 feet) in elevation in California, Nevada and Oregon.

**Divisions:** 206:C

**TNC Ecoregions:** 4:P, 5:P, 12:C

**Nations:** US

**Subnations:** CA, NV, OR

**Map Zones:** 2:C, 3:C, 4:P, 6:C, 7:C

**USFS Ecomap Regions:** M242B:CC, M242C:CC, M261A:CC, M261D:CC, M261E:CC, M261G:CP

CONCEPT

**Environment:** This ecological system occurs at subalpine and montane elevations where finely textured soils, snow deposition, or windswept dry conditions limit tree establishment. It is typically found above 3000 m (9100 feet) elevation in California, western Nevada and Oregon. The soils in these sites can be seasonally moist to saturated in the spring but, if so, will dry out later in the growing season, and overall these are mesic to dry meadows, not wet.

**Vegetation:** Characteristic plant species include *Achillea millefolium var. occidentalis (= Achillea lanulosa), Artemisia rothrockii, Oreostemma alpigenum (= Aster alpigenus), Calamagrostis breweri, Cistanthe umbellata (= Calyptridium umbellatum), Carex exserta, Eriogonum incanum, Horkeliella purpurascens (= Ivesia purpurascens)*, and *Trisetum spicatum*. Burrowing mammals can increase the forb diversity. Herbs can include *Carex subnigricans, Carex vernacula, Calamagrostis breweri, Antennaria media, Potentilla drummondii, Lewisia pygmaea, Erigeron algidus, Lupinus lepidus, Dodecatheon alpinum*, and *Solidago multiradiata*.

SOURCES

**References:** Barbour and Billings 2000, Barbour and Major 1988, Comer et al. 2003*, Holland and Keil 1995, Sawyer and Keeler‑Wolf 1995, Shiflet 1994

**Version:** 16 Jan 2009	**Stakeholders:** West

**Concept Author:** P. Comer and T. Keeler‑Wolf	**LeadResp:** West

CES204.099 North Pacific Alpine and Subalpine Dry Grassland

**Primary Division:** North American Pacific Maritime (204)

**Land Cover Class:** Herbaceous

**Spatial Scale & Pattern:** Large patch

**Required Classifiers:** Natural/Semi‑natural; Vegetated (>10% vasc.); Upland

**Diagnostic Classifiers:** Alpine/AltiAndino [Alpine/AltiAndino]; Montane [Upper Montane]; Herbaceous; Deep Soil; Ustic; Intermediate Disturbance Interval; Graminoid; Tussock‑forming grasses

**National Mapping Codes:** EVT 2171; ESLF 7157; ESP 1171

**Concept Summary:** This high‑elevation, grassland system is dominated by perennial grasses and forbs found on dry sites, particularly south‑facing slopes, typically imbedded in or above subalpine forests and woodlands. Disturbance such as fire also plays a role in maintaining these open grassy areas, although drought and exposed site locations are primary characteristics limiting tree growth. It is most extensive in the eastern Cascades, although it also occurs in the Olympic Mountains. Alpine and subalpine dry grasslands are small openings to large open ridges above or drier than high‑elevation conifer trees. In general, soil textures are much finer, and soils are often deeper under grasslands than in the neighboring forests. These grasslands, although composed primarily of tussock‑forming species, do exhibit a dense sod that makes root penetration difficult for tree species. Typical dominant species include *Festuca idahoensis ssp. idahoensis, Festuca viridula*, and *Festuca idahoensis ssp. roemeri (= Festuca roemeri)* (the latter occurring only in the Olympic Mountains). This system is similar to Northern Rocky Mountain Subalpine‑Upper Montane Grassland (CES306.806), differing in its including dry alpine habitats, more North Pacific floristic elements, greater snowpack, and higher precipitation.

DISTRIBUTION

**Range:** This system occurs only in the Pacific Northwest mountains (Coastal and westside Cascadian).

**Divisions:** 204:C, 306:C

**TNC Ecoregions:** 1:C, 3:C, 4:C, 81:C

**Nations:** CA?, US

**Subnations:** BC?, OR?, WA:S4S5

**Map Zones:** 1:C, 2:?, 7:C

**USFS Ecomap Regions:** 342I:CC, M242B:CC, M242C:CC, M242D:CC

CONCEPT

SOURCES

**References:** Comer et al. 2003*, Ecosystems Working Group 1998, Shiflet 1994, WNHP unpubl. data 2018

**Version:** 31 Mar 2005	**Stakeholders:** Canada, West

**Concept Author:** R. Crawford	**LeadResp:** West

CES204.100 North Pacific Montane Grassland

**Primary Division:** North American Pacific Maritime (204)

**Land Cover Class:** Herbaceous

**Spatial Scale & Pattern:** Large patch

**Required Classifiers:** Natural/Semi‑natural; Vegetated (>10% vasc.); Upland

**Diagnostic Classifiers:** Herbaceous; Temperate [Temperate Oceanic]; Mesotrophic Soil; Shallow Soil; Intermediate Disturbance Interval; F‑Patch/Low Intensity

**National Mapping Codes:** EVT 2138; ESLF 7110; ESP 1138

**Concept Summary:** This ecological system includes open dry meadows and grasslands on the west side of the Cascades Range and northern Sierra Nevada. They occur in montane elevations up to 3500 m (10,600 feet). Soils tend to be deeper and more well‑drained than the surrounding forest soils. Soils can resemble prairie soils in that the A‑horizon is dark brown, relatively high in organic matter, slightly acidic, and usually well‑drained. Dominant species include *Elymus* spp., *Festuca idahoensis*, and *Nassella cernua*. These large‑patch grasslands are intermixed with matrix stands of red fir, lodgepole pine, and dry‑mesic mixed conifer forests and woodlands.

**Comments:** Upon review, Washington Heritage ecologists determined this system does not occur in Washington. Review in November 2008 suggests this ecological system should be lumped with Mediterranean California Subalpine Meadow (CES206.940) and that system be redefined to include the small patches of dry montane grasslands found in the Sierras and southern Cascades. For now, we've retained this as a system pending further review and comment from California ecologists.

DISTRIBUTION

**Range:** This system is found on the west side of the Cascades Range and northern Sierra Nevada, in montane elevations up to 3500 m (10,600 feet).

**Divisions:** 204:C, 206:C

**TNC Ecoregions:** 5:P, 12:C, 81:C

**Nations:** US

**Subnations:** CA, NV, OR

**Map Zones:** 1:C, 2:C, 3:C, 6:C, 7:C, 12:P

**USFS Ecomap Regions:** 242A:CC, 341D:CC, 342B:CP, 342I:CC, M242A:CC, M242B:CC, M242C:CC, M242D:CC, M261D:CP, M261E:CC, M261G:CP, M331D:CC, M332G:CC

CONCEPT

SOURCES

**References:** Barbour and Major 1988, Comer et al. 2003*, Holland and Keil 1995, Sawyer and Keeler‑Wolf 1995, Shiflet 1994

**Version:** 24 Mar 2003	**Stakeholders:** West

**Concept Author:** P. Comer and G. Kittel	**LeadResp:** West

CES306.829 Rocky Mountain Subalpine‑Montane Mesic Meadow

**Primary Division:** Rocky Mountain (306)

**Land Cover Class:** Herbaceous

**Spatial Scale & Pattern:** Large patch

**Required Classifiers:** Natural/Semi‑natural; Vegetated (>10% vasc.); Upland

**Diagnostic Classifiers:** Montane [Upper Montane]; Herbaceous; Silt Soil Texture; Clay Soil Texture; Udic; Forb

**National Mapping Codes:** EVT 2145; ESLF 7118; ESP 1145

**Concept Summary:** This Rocky Mountain ecological system is restricted to sites from lower montane to subalpine where finely textured soils, snow deposition, or windswept dry conditions limit tree establishment. Many occurrences are small patch in spatial character, and are often found in mosaics with woodlands, more dense shrublands, or just below alpine communities. It is typically found above 2000 m in elevation in the southern part of its range and above 600 m in the northern part. These upland communities occur on gentle to moderate‑gradient slopes and relatively moist habitats. The soils are typically seasonally moist to saturated in the spring, but if so will dry out later in the growing season. These sites are not as wet as those found in Rocky Mountain Alpine‑Montane Wet Meadow (CES306.812). Vegetation is typically forb‑rich, with forbs often contributing more to overall herbaceous cover than graminoids. Some stands are composed of dense grasslands, these often being taxa with relatively broad and soft blades, but where the moist habitat promotes a rich forb component. Important taxa include *Erigeron* spp., Asteraceae spp., *Mertensia* spp., *Penstemon* spp., *Campanula* spp., *Lupinus* spp., *Solidago* spp., *Ligusticum* spp., *Thalictrum occidentale, Valeriana sitchensis, Rudbeckia occidentalis, Balsamorhiza sagittata*, and *Wyethia* spp. Important grasses include *Deschampsia cespitosa, Koeleria macrantha*, perennial *Bromus* spp., and a number of *Carex* species. *Dasiphora fruticosa ssp. floribunda* and *Symphoricarpos* spp. are occasional but not abundant. Burrowing mammals can increase the forb diversity.

**Comments:** There are probably quite a number of *Carex*‑ and *Calamagrostis*‑dominated types that could be cited as constituent associations.

DISTRIBUTION

**Range:** This system is very widespread in the Rocky Mountain cordillera from New Mexico north into Canada. It probably occurs in the Black Hills region, as well as the "island ranges" of central Montana.

**Divisions:** 304:C, 306:C

**TNC Ecoregions:** 7:C, 8:C, 9:C, 11:C, 18:C, 19:C, 20:C, 21:C, 26:C, 68:C

**Nations:** CA, US

**Subnations:** AB, AK?, AZ, BC, CO, ID, MT, NM, NV, OR, UT, WA:S3S5, WY

**Map Zones:** 8:?, 9:C, 10:C, 12:C, 13:C, 15:C, 16:C, 17:P, 18:C, 19:C, 20:C, 21:C, 22:P, 23:C, 24:C, 25:C, 27:C, 28:C, 29:C

**USFS Ecomap Regions:** 313A:CC, 313B:CC, 322A:CC, 331A:CC, 331J:CC, 341A:CP, 341B:CC, 341E:CP, 341F:CP, 341G:CC, 342A:CC, 342B:CP, 342C:CC, 342D:CC, 342E:CC, 342H:CC, 342J:CC, M313B:CC, M331A:CC, M331B:CC, M331D:CC, M331E:CC, M331F:CC, M331G:CC, M331H:CC, M331I:CC, M331J:CC, M332A:CC, M332B:CC, M332D:CC, M332E:CC, M332F:CC, M332G:CC, M333A:CC, M333B:CC, M333C:CC, M333D:CC, M334A:??, M341A:CC, M341B:CC, M341C:CC, M341D:CP

CONCEPT

**Environment:** This Rocky Mountain ecological system is restricted to sites from lower montane to subalpine where finely textured soils, snow deposition, or windswept dry conditions limit tree establishment. Many occurrences are small patch in spatial character, and are often found in mosaics with woodlands, more dense shrublands, or just below alpine communities. It is typically found above 2000 m in elevation in the southern part of its range and above 600 m in the northern part. These upland communities occur on gentle to moderate‑gradient slopes and relatively moist habitats. The soils are typically seasonally moist to saturated in the spring, but if so will dry out later in the growing season. These sites are not as wet as those found in Rocky Mountain Alpine‑Montane Wet Meadow (CES306.812).

**Vegetation:** Vegetation is typically forb‑rich, with forbs often contributing more to overall herbaceous cover than graminoids. Some stands are composed of dense grasslands, these often being taxa with relatively broad and soft blades, but where the moist habitat promotes a rich forb component. Important taxa include *Erigeron* spp., Asteraceae spp., *Mertensia* spp., *Penstemon* spp., *Campanula* spp., *Lupinus* spp., *Solidago* spp., *Ligusticum* spp., *Thalictrum occidentale, Valeriana sitchensis, Rudbeckia occidentalis, Balsamorhiza sagittata*, and *Wyethia* spp. Important grasses include *Deschampsia cespitosa, Koeleria macrantha*, perennial *Bromus* spp., and a number of *Carex* species. *Dasiphora fruticosa ssp. floribunda* and *Symphoricarpos* spp. are occasional but not abundant. Burrowing mammals can increase the forb diversity.

SOURCES

**References:** Buckner 1977, Comer et al. 2003*, Ellison 1954, Fritz 1981, Gregory 1983, Hall 1971, Hammerson 1979, Marr 1977a, Meidinger and Pojar 1991, NCC 2002, Nachlinger 1985, Neely et al. 2001, Potkin and Munn 1989, Shiflet 1994, Starr 1974, Steen and Coupé 1997, WNHP unpubl. data 2018

**Version:** 23 Jan 2006	**Stakeholders:** Canada, Midwest, West

**Concept Author:** M.S. Reid	**LeadResp:** West

CES306.824 Southern Rocky Mountain Montane‑Subalpine Grassland

**Primary Division:** Rocky Mountain (306)

**Land Cover Class:** Herbaceous

**Spatial Scale & Pattern:** Large patch

**Required Classifiers:** Natural/Semi‑natural; Vegetated (>10% vasc.); Upland

**Diagnostic Classifiers:** Herbaceous; Acidic Soil; Mineral: W/ A‑Horizon >10 cm; Loam Soil Texture; Silt Soil Texture; Aridic; Short Disturbance Interval; Graminoid; Cool‑season bunch grasses

**National Mapping Codes:** EVT 2146; ESLF 7119; ESP 1146

**Concept Summary:** This Rocky Mountain ecological system typically occurs between 2200 and 3000 m elevation on flat to rolling plains and parks or on lower sideslopes that are dry, but it may extend up to 3350 m on warm aspects. Soils resemble prairie soils in that the A‑horizon is dark brown, relatively high in organic matter, slightly acidic, and usually well‑drained. An occurrence usually consists of a mosaic of two or three plant associations with one of the following dominant bunchgrasses: *Danthonia intermedia, Danthonia parryi, Festuca idahoensis, Festuca arizonica, Festuca thurberi, Muhlenbergia filiculmis*, or *Pseudoroegneria spicata*. The subdominants include *Muhlenbergia montana, Bouteloua gracilis*, and *Poa secunda*. These large‑patch grasslands are intermixed with matrix stands of spruce‑fir, lodgepole pine, ponderosa pine, and aspen forests. In limited circumstances (e.g., South Park in Colorado), they form the "matrix" of high‑elevation plateaus. Small‑patch representations of this system do occur at high elevations of the Trans‑Pecos where they present as occurrences of *Festuca arizonica ‑ Blepharoneuron tricholepis* Grassland (CEGL004508). These occurrences often occupy sites adjacent to Madrean Oriental Chaparral (CES302.031).

**Comments:** Montane grasslands are very similar and intergrade with their subalpine counterparts, but are separated here to represent those species that do not occur at higher altitudes. Southern outliers of this system have been identified in Texas as small patches in high‑elevation sideslopes and local level plains of the Guadalupe, Chisos, and Davis mountains. These occurrences may be dominated by *Festuca arizonica, Bouteloua gracilis*, and *Blepharoneuron tricholepis*. *Allium cernuum, Commelina dianthifolia, Koeleria macrantha, Muhlenbergia montana*, and *Silene laciniata ssp. greggii* may be present. In Texas, occurrences have been identified on loams of high mountains over Tertiary volcanic formations of the Davis Mountains and Permian limestone of the Guadalupe Mountains.

DISTRIBUTION

**Range:** This system occurs between 2200 and 3000 m (7200‑10,000 feet) elevation in the Colorado Rockies. Where it transitions in Wyoming to Northern Rocky Mountain Subalpine‑Upper Montane Grassland (CES306.806) still needs to be clarified. Southern outliers of this system also occur in small patches in high elevations of the mountains of the Trans‑Pecos of Texas.

**Divisions:** 302:C, 304:C, 306:C

**TNC Ecoregions:** 18:C, 19:C, 20:C, 21:C, 24:C

**Nations:** US

**Subnations:** AZ, CO, ID, NM, TX, UT, WY

**Map Zones:** 12:C, 15:C, 16:C, 17:P, 21:P, 22:C, 23:C, 24:C, 25:C, 26:C, 27:C, 28:C, 29:C, 33:P

**USFS Ecomap Regions:** 313A:CC, 313B:CC, 313C:CP, 313D:CP, 315A:CC, 315H:CP, 321A:PP, 322A:??, 331B:CC, 331G:CC, 331H:CC, 331I:CC, 331J:CC, 341A:CC, 341B:CC, 341C:CC, 341F:CP, 342A:CC, 342E:CC, 342F:CC, 342G:CC, 342J:CC, M313A:CC, M313B:CC, M331A:CP, M331D:CC, M331E:CC, M331F:CC, M331G:CC, M331H:CC, M331I:CC, M331J:CC, M341A:CC, M341B:CC, M341C:CC

CONCEPT

**Environment:** This Rocky Mountain ecological system typically occurs between 2200 and 3000 m elevation on flat to rolling plains and parks or on lower sideslopes that are dry, but it may extend up to 3350 m on warm aspects. These are typically grasslands of forest openings and park‑like expanses in the montane and subalpine coniferous forests. Although smaller montane grasslands are scattered throughout the southern Rocky Mountains and high plateaus in the Colorado Plateaus, the largest occurrence by far (over a million acres) is on the valley floor of South Park in central Colorado. Soils resemble prairie soils in that the A‑horizon is dark brown, relatively high in organic matter, slightly acidic, and usually well‑drained.

**Vegetation:** Occurrences of this system are often a mosaic of different bunchgrass associations and may be dominated by *Blepharoneuron tricholepis, Bouteloua gracilis, Danthonia intermedia, Danthonia parryi, Festuca idahoensis, Festuca arizonica, Festuca thurberi, Muhlenbergia filiculmis*, or *Pseudoroegneria spicata*.

**Dynamics:** This system is found in areas that inhibit the establishment of woody species. A variety of factors, including fire, wind, cold‑air drainage, climatic variation, soil properties, fluctuating summer snowbanks (drought sequences), snow avalanches, competition with graminoids, and grazing, have been proposed as mechanisms that maintain open grasslands and parks in forest surroundings. Observations and repeat photography studies in sites throughout the southern Rocky Mountains indicate that trees do invade open areas, but that the mechanisms responsible for this trend may differ from site to site. Anderson and Baker (2005) discounted fire suppression as the cause of tree invasions in Wyoming's Medicine Bow Mountains, concluding that edaphic conditions were the most likely factor limiting tree establishment. In the San Juan Mountains of southeastern Colorado, Zier and Baker (2006) also found that the probability of tree invasion varied with forest type. Climatic variation, fire exclusion, and grazing appear to interact with edaphic factors to facilitate or hinder tree invasion in these grasslands (Zier and Baker 2006). In the Gunnison Basin, Schauer et al. (1998) identified seedling mortality as the primary factor preventing invasions of Engelmann spruce, but did not determine if this was due to competition from established grassland plants, or to edaphic conditions. The work of Coop and Givnish (2007) in the Jemez Mountains of northern New Mexico suggests that both changing disturbance regimes and climatic factors are linked to tree establishment in some montane grasslands. Pocket gophers (*Thomomys* spp.) are a widespread source of disturbance in montane‑subalpine grasslands. The activities of these burrowing mammals result in increased aeration, mixing of soil, and infiltration of water, and are an important component of normal soil formation and erosion (Ellison 1946). In addition, Cantor and Whitham (1989) found that below‑ground herbivory of pocket gophers restricted establishment of aspen to rocky areas in Arizona mountain meadows. The interaction of multiple factors indicates that management for the maintenance of these montane and subalpine grasslands may be complex.

<br />Historically, much of the montane grasslands where this system occurs were heavily grazed by livestock, primarily cattle and sheep (also at subalpine elevation) (Shepherd 1975). Under moderate grazing, the shorter grasses such *Muhlenbergia filiculmis* may have had a competitive advantage over the taller and more palatable *Festuca arizonica* (West 1992). Season of use is also important. In stands with cool‑season *Festuca arizonica* or *Hesperostipa comata* and warm‑season *Muhlenbergia montana*, fall grazing will favor the cool‑season grasses over the later‑blooming, warm‑season *Muhlenbergia montana* (Clary 1978). The reverse is true if grazing is always limited to late summer. Overgrazing will reduce or eliminate *Festuca arizonica, Hesperostipa comata, Muhlenbergia filiculmis, Muhlenbergia montana*, and the other palatable species, leaving the more grazing‑tolerant *Bouteloua gracilis* and less palatable plants such as *Hymenoxys, Artemisia*, and *Chrysothamnus* species to dominate the site (West 1992). Clary (1978) reported that complete natural recovery of montane *Festuca arizonica* may require over 100 years, based on areas where recovery had reached only the "half‑shrub" stage after 10 years. Because of the long time needed for recovery, much of the range may be in a seral state. If the range is properly managed, *Muhlenbergia* and *Festuca arizonica* grasslands could potentially become more common.

Higher‑elevation grasslands are dominated by *Festuca thurberi* and typically have sharp ecotones with adjacent *Picea engelmannii*‑ and *Abies lasiocarpa*‑dominated subalpine forests. There is rarely any invasion by tree seedlings in the adjacent grasslands. These high‑elevation meadows are typically dry with southern or western aspects. The soils are deep and well‑developed, typical of sites with long histories of being grassland. They may need catastrophic disturbance, such as forest‑destroying crown fire, to be created. It is unclear how these grasslands were maintained in the subalpine forest zone; however, it is thought to be by a combination of factors such as herbivory, fire, deep soils, early summer drought and competition from grass species (Moir 1967, Andrews 1983). In addition, south‑ and west‑facing clearcuts are often difficult to reforest because seedlings are damaged by full sun. The ecotones between stands adjacent to *Populus tremuloides*‑dominated subalpine forests are not as sharp because the forest understory consists of the same graminoid and forb species (Andrews 1983).

Where the soil is thinner and rockier in these subalpine parks, *Danthonia parryi* becomes the dominant species with *Festuca thurberi* and *Artemisia* spp. subdominant (Andrews 1983). The spread of the exotic species *Poa pratensis* and *Taraxacum officinale* in subalpine parks is likely from heavy grazing by livestock (Moir 1967, Andrews 1983). These species are more common in heavily grazed bottomlands and near trails in the uplands (Moir 1967).

LANDFIRE developed a state‑and‑transition vegetation dynamics VDDT model for this system which has three classes in total (LANDFIRE 2007a, BpS 2811460). These are summarized as:

A) Early Development 1 All Structures (graminoid‑dominated ‑ 10% of type in this stage): Herb cover is 0‑30%. Low cover and frequency of Thurber fescue, Arizona fescue, sheep fescue, mountain muhly, timber/Parry's oatgrass, Kentucky bluegrass, nodding brome, tufted hairgrass, and various sedges in moist (concave) sites. Pine dropseed is common.

B) Mid Development 1 Closed (graminoid‑dominated ‑ 30% of type in this stage): Herb cover is 31‑70%. Thurber fescue, Arizona fescue, sheep fescue, mountain muhly, timber/Parry's oatgrass, Kentucky bluegrass, nodding brome, tufted hairgrass, and various sedges in moist (concave) sites.

C) Late Development 1 Closed (graminoid‑dominated ‑ 50% of type in this stage): Tree cover is 71‑100%. Thurber fescue, Arizona fescue, sheep fescue, mountain muhly, timber/Parry's oatgrass, Kentucky bluegrass, nodding brome, tufted hairgrass, and various sedges in moist (concave) sites.<br />

Predicted historic stand‑replacement fire regime of approximately 30‑60 years based upon historic photographic analysis (B. Johnston‑R2 pers. comm. 2018) and inference from mean/max and min fire regimes of adjacent forest types (*Pinus ponderosa)* 3‑12 years, *Abies concolor/Pseudotsuga menziesii* 14‑46 years, *Picea engelmannii / Abies lasiocarpa* 60‑180+ years). Anthropogenic (pre‑European cf.) fire use ignitions 5‑15 years, current regime greater than 60 years in montane and 100 years in subalpine systems (LANDFIRE 2007a, BpS 2811460).

SOURCES

**References:** Anderson and Baker 2005, Andrews 1983, Bowns and Bagley 1986, CNHP 2010, Cantor and Whitham 1989, Clary 1978, Comer et al. 2002, Comer et al. 2003*, Coop and Givnish 2007, Ellison 1946, Garfin et al. 2014, Hess 1981, Hess and Wasser 1982, LANDFIRE 2007a, McKenzie et al. 2004, McKenzie et al. 2008, Moir 1967, Neely et al. 2001, Passey et al. 1982, Schauer et al. 1998, Shepherd 1975, Stevens‑Rumann et al. 2017, Stewart 1940, Tuhy et al. 2002, Turner 1975, Turner and Dortignac 1954, West 1992, Westerling et al. 2006, Zier and Baker 2006

**Version:** 24 May 2018	**Stakeholders:** Midwest, Southeast, West

**Concept Author:** M.S. Reid	**LeadResp:** West

M049. Southern Rocky Mountain Montane Shrubland

CES306.818 Rocky Mountain Gambel Oak‑Mixed Montane Shrubland

**Primary Division:** Rocky Mountain (306)

**Land Cover Class:** Shrubland

**Spatial Scale & Pattern:** Large patch

**Required Classifiers:** Natural/Semi‑natural; Vegetated (>10% vasc.); Upland

**Diagnostic Classifiers:** Shrubland (Shrub‑dominated); Shallow Soil; Mineral: W/ A‑Horizon <10 cm; Loam Soil Texture; Sand Soil Texture; Ustic; Unconsolidated; Intermediate Disturbance Interval [Periodicity/Polycyclic Disturbance]; Broad‑Leaved Deciduous Shrub

**National Mapping Codes:** EVT 2107; ESLF 5313; ESP 1107

**Concept Summary:** This ecological system occurs in the mountains, plateaus and foothills of the southern Rocky Mountains and Colorado Plateau, including the Uinta and Wasatch ranges and the Mogollon Rim. These shrublands are most commonly found along dry foothills, lower mountain slopes, and at the edge of the western Great Plains from approximately 2000 to 2900 m in elevation and are often situated above pinyon‑juniper woodlands. Substrates are variable and include soil types ranging from calcareous, heavy, fine‑grained loams to sandy loams, gravelly loams, clay loams, deep alluvial sand, or coarse gravel. The vegetation is typically dominated by *Quercus gambelii* alone or codominant with *Amelanchier alnifolia, Amelanchier utahensis, Artemisia tridentata, Cercocarpus montanus, Prunus virginiana, Purshia stansburiana, Purshia tridentata, Robinia neomexicana, Symphoricarpos oreophilus*, or *Symphoricarpos rotundifolius*. There may be inclusions of other mesic montane shrublands with *Quercus gambelii* absent or as a relatively minor component. This ecological system intergrades with the lower montane‑foothills shrubland system and shares many of the same site characteristics. Density and cover of *Quercus gambelii* and *Amelanchier* spp. often increase after fire. In Texas, this system includes high mountain shrublands dominated by the deciduous oak species *Quercus gambelii*. This species often forms nearly monotypic shrublands, but other species present may include *Cercocarpus montanus, Robinia neomexicana, Symphoricarpos oreophilus*, and *Rhus trilobata*. These shrubland patches represent southern outliers of the extensive and diverse system further north.

**Comments:** LANDFIRE modeled this BpS with Coahuilan Chaparral (Madrean Oriental Chaparral (CES302.031)). *Quercus gambelii* apparently occurs as a significant component of a shrubland of the Trans‑Pecos of Texas. This system was not previously attributed to Texas, as it seems more appropriate to modify the description of CES302.031 to allow for the presence of *Quercus gambelii* as a significant component of some occurrences. All disjunct *Quercus gambelii*‑dominated shrublands found in the Davis Mountains are included in the concept of Madrean Oriental Chaparral (CES302.031). However, information gathered as part of the 2007‑2013 Texas ecological systems mapping project supports a role for *Quercus gambelii* in two additional ecological systems in the Texas Chisos and Guadalupe mountains based on associated species and landscape position: Rocky Mountain Gambel Oak‑Mixed Montane Shrubland (CES306.818) and Madrean Lower Montane Pine‑Oak Forest and Woodland (CES305.796). *Quercus gambelii / Symphoricarpos oreophilus* Shrubland (CEGL001117) is an association found in the Trans‑Pecos and included in Madrean Lower Montane Pine‑Oak Forest and Woodland (CES305.796). Also, there is a need to clarify the relationship with Rocky Mountain Lower Montane‑Foothill Shrubland (CES306.822).

DISTRIBUTION

**Range:** This system occurs in the mountains, plateaus and foothills of the southern Rocky Mountains and Colorado Plateau, including the Uinta and Wasatch ranges and the Mogollon Rim. It also extends into the high mountains of the Trans‑Pecos of Texas.

**Divisions:** 302:C, 304:C, 306:C

**TNC Ecoregions:** 10:P, 18:C, 19:C, 20:C, 21:C, 24:C

**Nations:** US

**Subnations:** AZ, CO, NM, TX, UT, WY

**Map Zones:** 12:?, 15:C, 16:C, 17:C, 22:C, 23:C, 24:C, 25:C, 26:C, 27:C, 28:C, 33:?

**USFS Ecomap Regions:** 313A:CC, 313B:CC, 313C:C?, 313D:CC, 315A:CC, 315B:CC, 315H:CC, 321A:CC, 322A:CC, 331B:CC, 331F:CC, 331G:CC, 331I:CC, 331J:CC, 331M:CC, 341A:CC, 341B:CC, 341C:CC, 341F:CC, 342A:CC, 342E:CC, 342G:CC, 342J:CC, M313A:CC, M313B:CC, M331D:CC, M331E:CC, M331F:CC, M331G:CC, M331H:CC, M331I:CC, M334A:??, M341A:CC, M341B:CC, M341C:CC

CONCEPT

**Environment:** This ecological system typically occupies the lower slope positions of the foothill and lower montane zones. They may occur on level to steep slopes, cliffs, escarpments, rimrock slopes, rocky outcrops, and scree slopes. Climate is semi‑arid and characterized by mostly hot‑dry summers with mild to cold winters and annual precipitation of 25 to 70 cm. Precipitation mostly occurs as winter snows but may also consist of some late‑summer rains. Soils are typically poorly developed, rocky to very rocky, and well‑drained. Parent materials include alluvium, colluvium, and residuum derived from igneous, metamorphic, or sedimentary rocks such as granite, gneiss, limestone, quartz, monzonite, rhyolite, sandstone, schist, and shale. Although this is a shrub‑dominated system, some trees may be present. In older occurrences, or occurrences on mesic sites, some of the shrubs may acquire tree‑like sizes. Adjacent communities often include woodlands or forests of *Abies concolor, Pinus ponderosa, Pseudotsuga menziesii*, or *Populus tremuloides* at higher elevations, and *Pinus edulis* and *Juniperus osteosperma* on the lower and adjacent elevations. Shrublands of *Artemisia tridentata* or grasslands of *Festuca* sp., *Stipa* sp., or *Pseudoroegneria* sp. may also be present at the lower elevations. In Texas, this system primarily occurs on limestone formations on slopes and rolling landforms of the Trans‑Pecos mountains, on Limestone Hill and Mountain and High Montane Conifer Ecological Sites.

**Vegetation:** Vegetation types in this system may occur as sparse to dense shrublands composed of moderate to tall shrubs. Occurrences may be multi‑layered, with some short shrubby species occurring in the understory of the dominant overstory species. In many occurrences of this system, the canopy is dominated by the broad‑leaved deciduous shrub *Quercus gambelii*, which occasionally reaches small tree size. Occurrences can range from dense thickets with little understory to relatively mesic mixed‑shrublands with a rich understory of shrubs, grasses and forbs. These shrubs often have a patchy distribution with grass growing in between. Scattered trees are occasionally present in stands and typically include species of *Pinus* or *Juniperus*. Characteristic shrubs that may co‑occur, or be singularly dominant, include *Amelanchier alnifolia, Amelanchier utahensis, Arctostaphylos patula, Artemisia tridentata, Cercocarpus montanus, Ptelea trifoliata, Prunus virginiana, Purshia stansburiana, Robinia neomexicana, Rosa* spp., *Symphoricarpos oreophilus*, and *Symphoricarpos rotundifolius*. The herbaceous layer is sparse to moderately dense, ranging from 1‑40% cover. Perennial graminoids are the most abundant species, particularly *Bouteloua curtipendula, Bouteloua eriopoda, Bouteloua gracilis, Aristida* spp., *Carex inops, Carex geyeri, Elymus arizonicus, Eragrostis* spp., *Festuca* spp., *Koeleria macrantha, Muhlenbergia* spp., and *Stipa* spp. Many forb and fern species can occur, but none have much cover. Commonly present forbs include *Achillea millefolium, Artemisia* spp., *Geranium* spp., *Maianthemum stellatum, Thalictrum fendleri*, and *Vicia americana*. Ferns include species of *Cheilanthes* and *Woodsia*. Annual grasses and forbs are seasonally present, and weedy annuals are often present, at least seasonally.

**Dynamics:** Fire typically plays an important role in this system, causing die‑back of the dominant shrub species in some areas, promoting stump sprouting of the dominant shrubs in other areas, and controlling the invasion of trees into the shrubland system. Natural fires typically result in a system with a mosaic of dense shrub clusters and openings dominated by herbaceous species. In some instances, these associations may be seral to the adjacent *Pinus ponderosa, Abies concolor*, and *Pseudotsuga menziesii* woodlands and forests. Ream (1964) noted that on many sites in Utah, Gambel oak may be successional and replaced by bigtooth maple (*Acer grandidentatum*).

LANDFIRE developed a state‑and‑transition vegetation dynamics VDDT model for this system which has four classes in total (LANDFIRE 2007a, BpS 2311070). These are summarized as:

A) Early Development 1 All Structures (shrub‑dominated ‑ 5% of type in this stage): Shrub cover is 0‑20%. Post‑replacement sprouts to approximately 2 feet high. Dense resprouting with high number of stems/acre. Abundant grass and forb cover.

B) Mid Development 1 Closed (tree‑dominated ‑ 50% of type in this stage): Tree cover is 21‑70%. Oak 3‑6 feet tall to 3 inches dbh. There will be some stem mortality due to competition and self‑thinning, with slight decrease in understory species due to shading. Grasses and forbs declining.

C) Mid Development 1 Open (tree‑dominated ‑ 15% of type in this stage): Tree cover is 51‑70%. This class has >6 feet tall and >3 inches dbh oak. Small stands <30 m across usually scattered throughout a grassland or shrub type (Brown 1958).

D) Late Development 1 Closed (shrub‑dominated ‑ 30% of type in this stage): Tree cover is 71‑100%. This class has >6 feet tall and 3 inches dbh. Nearly continuous stand two or more hectares in size with only occasional openings (Brown 1958).<br />

Fire regime group IV or III. The primary disturbance mechanism is replacement fire, resulting in >75% top‑kill. Gambel oak responds to fire with vigorous sprouting from the root crown. Larger forms may survive low‑intensity surface fire. Extended drought also contributes to disturbance (LANDFIRE 2007a, BpS 2311070).

SOURCES

**References:** Anderson 1988, Barnett and Crawford 1994, Brown 1958, CNHP 2010, Christensen 1955, Clary and Tiedeman 1986, Comer et al. 2002, Comer et al. 2003*, Crawford et al. 2004, Drut et al. 1994, Elliott 2012, Ersch 2009, Garfin et al. 2014, Gregg and Crawford 2009, Harper et al. 1985, Johnston and Hendzel 1985, Kunzler and Harper 1980, Kunzler et al. 1981, LANDFIRE 2007a, McKell 1950, McKenzie et al. 2004, McKenzie et al. 2008, Neely et al. 2001, Neilson and Wullstein 1986, Price and Brotherson 1987, Rasmussen 1941, Ream 1960, Ream 1964, Rondeau 2001, Shepperd 1990, Shiflet 1994, Simonin 2000d, Stevens‑Rumann et al. 2017, Tirmenstein 1999c, Tuhy et al. 2002, Westerling et al. 2006

**Stakeholders:** Southeast, West

**Concept Author:** M.S. Reid	**LeadResp:** West

CES306.822 Rocky Mountain Lower Montane‑Foothill Shrubland

**Primary Division:** Rocky Mountain (306)

**Land Cover Class:** Shrubland

**Spatial Scale & Pattern:** Large patch

**Required Classifiers:** Natural/Semi‑natural; Vegetated (>10% vasc.); Upland

**Diagnostic Classifiers:** Montane [Lower Montane]; Lowland [Foothill]; Shrubland (Shrub‑dominated); Very Shallow Soil; Aridic; Intermediate Disturbance Interval [Periodicity/Polycyclic Disturbance]

**National Mapping Codes:** EVT 2086; ESLF 5263; ESP 1086

**Concept Summary:** This ecological system is found in the foothills, canyon slopes and lower mountains of the Rocky Mountains and on outcrops and canyon slopes in the western Great Plains. It ranges from southern New Mexico, extending north into Wyoming, and west into the Intermountain West region. These shrublands occur between 1500 and 2900 m elevation and are usually associated with exposed sites, rocky substrates, and dry conditions, which limit tree growth. It is common where *Quercus gambelii* is absent, such as the northern Colorado Front Range and in drier foothills and prairie hills. This system is generally drier than Rocky Mountain Gambel Oak‑Mixed Montane Shrubland (CES306.818) but may include mesic montane shrublands where *Quercus gambelii* does not occur. *Cercocarpus montanus* dominates pure stands in parts of Wyoming and Colorado. Scattered trees or inclusions of grassland patches or steppe may be present, but the vegetation is typically dominated by a variety of shrubs, including *Amelanchier utahensis, Cercocarpus montanus, Purshia tridentata, Rhus trilobata, Ribes cereum, Symphoricarpos oreophilus*, or *Yucca glauca*. Grasses are represented as species of *Muhlenbergia, Bouteloua, Hesperostipa*, and *Pseudoroegneria spicata*. Fires play an important role in this system as the dominant shrubs usually have a severe die‑back, although some plants will stump sprout. *Cercocarpus montanus* requires a disturbance such as fire to reproduce, either by seed sprout or root‑crown sprouting. Fire suppression may have allowed an invasion of trees into some of these shrublands, but in many cases sites are too xeric for tree growth. In Wyoming, stands where *Cercocarpus montanus* is a component of mixed shrublands are placed in Northern Rocky Mountain Montane‑Foothill Deciduous Shrubland (CES306.994).

**Comments:** Some reviewers have requested that this system be renamed in such a way as to more strongly indicate that it is dominated primarily by *Cercocarpus montanus*. However, while *Cercocarpus montanus* is an important shrub in this system, it is not the only dominant, and in many occurrences is not found at all.

DISTRIBUTION

**Range:** This system is found in the foothills, canyon slopes and lower mountains of the Rocky Mountains and on outcrops and canyon slopes in the western Great Plains. It ranges from southern New Mexico, extending north into Wyoming, and west into the Intermountain West region.

**Divisions:** 303:C, 306:C

**TNC Ecoregions:** 10:C, 19:C, 20:C, 21:C, 25:C, 26:C, 27:C

**Nations:** US

**Subnations:** AZ, CO, ID, MT, NE, NM, NV, SD, WY

**Map Zones:** 1:C, 15:C, 16:C, 18:C, 21:P, 22:C, 23:C, 25:C, 26:C, 27:C, 28:C, 29:C, 30:?, 31:P, 33:C, 34:P

**USFS Ecomap Regions:** 313A:CC, 313B:CC, 315A:CC, 315B:CC, 315H:CP, 321A:CC, 331B:CC, 331F:CC, 331G:CC, 331H:CC, 331I:CC, 331J:CC, 341B:CC, 341C:CC, 342E:CC, 342F:CC, 342G:CC, M313B:CC, M331A:CP, M331B:CP, M331E:CC, M331F:CC, M331G:CC, M331H:CC, M331I:CC, M331J:CC, M332G:??, M334A:??, M341B:CC

CONCEPT

**Environment:** This ecological system is found in the foothills, canyon slopes and lower mountains of the Rocky Mountains and on outcrops and canyon slopes in the western Great Plains. It ranges from southern New Mexico, extending north into Wyoming, and west into the Intermountain West region. These shrublands occur between 1500 and 2900 m elevation and are usually associated with exposed sites, rocky substrates, and dry conditions, which limit tree growth. It is common where *Quercus gambelii* is absent, such as the northern Colorado Front Range and in drier foothills and prairie hills.

**Vegetation:** *Cercocarpus montanus* dominates pure stands in parts of Wyoming and Colorado. Scattered trees or inclusions of grassland patches or steppe may be present, but the vegetation is typically dominated by a variety of shrubs, including *Amelanchier utahensis, Cercocarpus montanus, Purshia tridentata, Rhus trilobata, Ribes cereum, Symphoricarpos oreophilus*, or *Yucca glauca*. Grasses are represented as species of *Muhlenbergia, Bouteloua, Hesperostipa*, and *Pseudoroegneria spicata*.

**Dynamics:** Fires play an important role in this system as the dominant shrubs usually have a severe die‑back, although some plants will stump sprout. *Cercocarpus montanus* requires a disturbance such as fire to reproduce, either by seed sprout or root‑crown sprouting. Fire suppression may have allowed an invasion of trees into some of these shrublands, but in many cases sites are too xeric for tree growth.

LANDFIRE developed a state‑and‑transition vegetation dynamics VDDT model for this system which has five classes in total (LANDFIRE 2007a, BpS 2810860). These are summarized as:

A) Early Development 1 All Structures (grass‑dominated ‑ 15% of type in this stage): Grass cover is 0‑10%. Early succession, usually after moderately frequent stand‑replacement fires; grasses and forbs dominant.

B) Mid Development 1 Closed (shrub‑dominated ‑ 15% of type in this stage): Shrub cover is 11‑80%. Greater than 10% shrub cover (i.e., line intercept method) by weakly sprouting and seed‑producing shrubs; grasses/forbs dominant in scattered openings.

C) Mid Development 1 Open (10% of type in this stage): Shrub cover is 0‑10%, with grasses/forbs dominant in extensive openings.

D) Late Development 1 Open (10% of type in this stage): Shrub cover is 0‑10%, with over‑matured shrubs as patchy dominant overstory (e.g., in rock outcrops); grasses/forbs dominant in extensive openings.

E) Late Development 1 Closed (shrub‑dominated ‑ 50% of type in this stage): Shrub cover is 11‑80%. Greater than 10% shrub cover; all age classes present but dominated by over‑matured shrubs (e.g., in rocky draws).

<br />Historically, this type may have been in a Fire Regime IV or II ‑‑ primarily moderate‑interval (e.g., 20‑50 years) stand‑replacement fires in the shrub‑dominated layer. Nearly all the dominant species in this BpS have the capability to resprout after disturbance (LANDFIRE 2007a, BpS 2810860).

SOURCES

**References:** Barnett and Crawford 1994, CNHP 2010, Comer et al. 2003*, Crawford et al. 2004, Dick‑Peddie 1993, Drut et al. 1994, Ersch 2009, Garfin et al. 2014, Gregg and Crawford 2009, Gucker 2006e, Hess 1981, Hess and Wasser 1982, Hoffman and Alexander 1987, Knopf et al. 1990, LANDFIRE 2007a, Marriott and Faber‑Langendoen 2000, McKenzie et al. 2004, McKenzie et al. 2008, Mueggler and Stewart 1980, Muldavin 1994, Muldavin et al. 2000b, Neely et al. 2001, Rising 1996, Roughton 1972, Sedgwick and Knopf 1987, Shiflet 1994, Stevens‑Rumann et al. 2017, Thilenius et al. 1995, Welsh et al. 2008, Westerling et al. 2006

**Version:** 24 May 2018	**Stakeholders:** Midwest, West

**Concept Author:** NatureServe Western Ecology Team	**LeadResp:** West

M050. Southern Vancouverian Lowland Grassland & Shrubland

CES206.941 California Northern Coastal Grassland

**Primary Division:** Mediterranean California (206)

**Land Cover Class:** Herbaceous

**Spatial Scale & Pattern:** Large patch

**Required Classifiers:** Natural/Semi‑natural; Vegetated (>10% vasc.); Upland

**Diagnostic Classifiers:** Lowland [Lowland]; Herbaceous; Terrace; Mediterranean [Mediterranean Xeric‑Oceanic]; Very Short Disturbance Interval [Periodicity/Nonrandom Disturbance]; F‑Patch/Low Intensity; Graminoid

**National Mapping Codes:** EVT 2131; ESLF 7103; ESP 1131

**Concept Summary:** This ecological system is found in discontinuous patches below 300 m (1000 feet) elevation from San Francisco Bay north into Oregon, on coastal terraces and ridgeline balds in the Coast Ranges and Klamath Mountains. Small patches have been documented as far south as Santa Barbara and San Luis Obispo counties. It has a similar distribution to coastal shrublands (Northern California Coastal Scrub (CES206.932)) in areas that receive more rainfall than other California grasslands of the interior or southern coastal California. In recent centuries, these were fire‑dominated systems, and there is a known history of Native American use of fire in these areas. While still present, annual grasses and forbs are not as prevalent in these grasslands as elsewhere in California. With fire suppression, *Baccharis pilularis* and other shrub components of north coastal scrub often invade and can replace these grasslands with scrub‑dominated systems. *Agrostis* spp., *Bromus carinatus, Calamagrostis nutkaensis, Danthonia californica, Festuca rubra, Festuca idahoensis, Deschampsia cespitosa, Koeleria macrantha, Trisetum canescens*, and perennial forbs such as *Iris douglasiana, Sisyrinchium bellum, Grindelia hirsutula*, and *Sanicula arctopoides* are characteristic.

DISTRIBUTION

**Range:** This system is found below 300 m (1000 feet) elevation from San Francisco Bay (and possibly farther south) north into Oregon, on coastal terraces and ridgeline balds in the Coast Ranges and Klamath Mountains.

**Divisions:** 206:C

**TNC Ecoregions:** 5:C, 14:C

**Nations:** US

**Subnations:** CA, OR

**Map Zones:** 2:C, 3:C, 4:C

**USFS Ecomap Regions:** 263A:CC, M261A:CC, M261B:CC

CONCEPT

**Environment:** This ecosystem occurs on coastal terraces and ridgeline balds in the Coast Ranges in small patches in areas that receive more rainfall than the Central Valley grasslands or those of southern coastal California (south of Santa Barbara County), and wherever the cooling influence of the Pacific Ocean moderates summer drought (Ford and Hayes 2007). Soils are rich and moist, on terraces on the coast line and balds on inland ridges and hilltops (Sawyer et al. 2009).

**Vegetation:** *Agrostis* spp., *Bromus carinatus, Calamagrostis nutkaensis, Danthonia californica, Festuca rubra, Festuca idahoensis, Deschampsia cespitosa, Koeleria macrantha, Trisetum canescens*, and perennial forbs such as *Iris douglasiana, Sisyrinchium bellum, Grindelia hirsutula*, and *Sanicula arctopoides* are characteristic.

**Dynamics:** Coastal prairies are maintained by salt spray that limits woody growth, and burning, likely annual ignitions by Native Americans (Stuart and Stephens 2006). Historical frequent fire, salt‑laden wind, and windy ridgetops inhibit forest development in these areas (Franklin and Dyrness 1973, as cited in Sawyer et al. 2009). Fire is a useful management tool for control of non‑native invasive species (Sawyer et al. 2009).

SOURCES

**References:** Barbour and Billings 2000, Barbour and Major 1988, Barbour et al. 2007a, Comer et al. 2003*, Ford and Hayes 2007, Franklin and Dyrness 1973, Holland and Keil 1995, PRBO Conservation Science 2011, Sawyer and Keeler‑Wolf 1995, Sawyer et al. 2009, Shiflet 1994, Stuart and Stephens 2006

**Version:** 14 Jan 2014	**Stakeholders:** West

**Concept Author:** P. Comer and T. Keeler‑Wolf	**LeadResp:** West

CES204.089 North Pacific Herbaceous Bald and Bluff

**Primary Division:** North American Pacific Maritime (204)

**Land Cover Class:** Herbaceous

**Spatial Scale & Pattern:** Small patch

**Required Classifiers:** Natural/Semi‑natural; Vegetated (>10% vasc.); Upland

**Diagnostic Classifiers:** Herbaceous; Bluff; Ridge/Summit/Upper Slope

**Concept Summary:** This system consists of mostly herbaceous‑dominated areas located primarily on shallow soils from eastern Vancouver Island and the Georgia Basin south to at least the southern end of the Willamette Valley and adjacent slopes of the Coast Ranges and western Cascades, excluding areas adjacent to the outer coastline (hypermaritime climate). They are largely, if not completely, absent from the windward side of Vancouver Island, the Olympic Peninsula, and the Coast Ranges of Washington and Oregon. Due to shallow soils, steep slopes, sunny aspect, and/or upper slope position, these sites are dry and marginal for tree establishment and growth except in favorable microsites. Rock outcrops are a typical small‑scale feature within balds and are considered part of this system. Sites with many favorable microsites can have a "savanna" type structure with a sparse tree layer of *Pseudotsuga menziesii* or, less commonly, *Quercus garryana*. The climate is relatively dry to wet (20 to perhaps 100 inches annual precipitation), always with a distinct dry summer season when these sites usually become droughty enough to limit tree growth and establishment. Seeps are a frequent feature in many balds and result in vernally moist to wet areas within the balds that dry out by summer. Vegetation differences are associated with relative differences in soil moisture. Most sites have little snowfall, but sites in the *Abies amabilis* zone (montane *Tsuga heterophylla* in British Columbia) can have significant winter snowpacks. Snowpacks would be expected to melt off sooner on these sunny aspect sites than surrounding areas. Fog and salt spray probably have some influence (but less than in the hypermaritime) on exposed slopes or bluffs adjacent to saltwater shorelines in the Georgia Basin, where soils on steep coastal bluffs sometime deviate from the norm and are deep glacial deposits. Slightly to moderately altered serpentine soils occur rarely. Fires, both lightning‑ignited and those ignited by Native Americans, undoubtedly at least occasionally burn all these sites. Lower elevation sites in the Georgia Basin, Puget Trough, and Willamette Valley probably were burned somewhat more frequently and in some cases intentionally. Because of this fire history, the extent of this system has declined locally through tree invasion and growth, as areas formerly maintained herbaceous by burning have filled in with trees.

Grasslands are the most prevalent vegetation cover, though forblands are also common especially in the mountains. Dwarf‑shrublands occur commonly, especially in mountains or foothills, as very small patches for the most part, usually in a matrix of herbaceous vegetation, most often near edges. Dominant or codominant native grasses include *Festuca idahoensis ssp. roemeri, Danthonia californica, Achnatherum lemmonii, Festuca rubra* (near saltwater), and *Koeleria macrantha*. Forb diversity can be high. Some typical codominant forbs include *Camassia quamash, Camassia leichtlinii, Triteleia hyacinthina, Mimulus guttatus* (seeps), *Plectritis congesta, Lomatium martindalei, Allium cernuum*, and *Phlox diffusa* (can be considered a dwarf‑shrub). Important dwarf‑shrubs are *Arctostaphylos uva‑ursi, Arctostaphylos nevadensis*, and *Juniperus communis*. Small patches and strips dominated by the shrub *Arctostaphylos columbiana* are a common feature nested within herbaceous balds. Significant portions of some balds, especially on rock outcrops, are dominated by bryophytes (mosses) and to a lesser degree lichens.

DISTRIBUTION

**Range:** This system occurs in the Willamette Valley, Puget Trough, Georgia Basin, eastern and northern Olympic Mountains, eastern side of Vancouver Island, western and northwestern Cascades of Washington, probably on the leeward side of the Coast Mountains in British Columbia (submaritime climates)?, Old Cascades of western Oregon, and Oregon Coast Ranges (but not the coast itself).

**Divisions:** 204:C

**TNC Ecoregions:** 1:C, 2:C, 3:P, 81:C

**Nations:** CA, US

**Subnations:** BC, OR, WA:S3

**Map Zones:** 1:C, 2:C, 7:C

**USFS Ecomap Regions:** 242A:CC, 242B:C?, M242A:CC, M242B:CC, M242C:CP, M242D:CC

CONCEPT

**Environment:** This system consists of mostly herbaceous‑dominated areas located primarily on shallow soils from eastern Vancouver Island and the Georgia Basin south to at least the southern end of the Willamette Valley and adjacent slopes of the Coast Ranges and western Cascades, excluding areas adjacent to the outer coastline (hypermaritime climate). They are largely, if not completely, absent from the windward side of Vancouver Island, the Olympic Peninsula, and the Coast Ranges of Washington and Oregon. Due to shallow soils, steep slopes, sunny aspect, and/or upper slope position, these sites are dry and marginal for tree establishment and growth except in favorable microsites. Rock outcrops are a typical small‑scale feature within balds and are considered part of this system. Sites with many favorable microsites can have a "savanna" type structure with a sparse tree layer of *Pseudotsuga menziesii* or, less commonly, *Quercus garryana*. The climate is relatively dry to wet (20 to perhaps 100 inches annual precipitation), always with a distinct dry summer season when these sites usually become droughty enough to limit tree growth and establishment. Seeps are a frequent feature in many balds and result in vernally moist to wet areas within the balds that dry out by summer. Vegetation differences are associated with relative differences in soil moisture. Most sites have little snowfall, but sites in the *Abies amabilis* zone (montane *Tsuga heterophylla* in British Columbia) can have significant winter snowpacks. Snowpacks would be expected to melt off sooner on these sunny aspect sites than surrounding areas. Fog and salt spray probably have some influence (but less than in the hypermaritime) on exposed slopes or bluffs adjacent to saltwater shorelines in the Georgia Basin, where soils on steep coastal bluffs sometime deviate from the norm and are deep glacial deposits. Slightly to moderately altered serpentine soils occur rarely.

**Vegetation:** Grasslands are the most prevalent vegetation cover, though forblands are also common especially in the mountains. Dwarf‑shrublands occur commonly, especially in mountains or foothills, as very small patches for the most part, usually in a matrix of herbaceous vegetation, most often near edges. Dominant or codominant native grasses include *Festuca idahoensis ssp. roemeri (= Festuca roemeri), Danthonia californica, Achnatherum lemmonii, Festuca rubra* (near saltwater), and *Koeleria macrantha*. Forb diversity can be high. Some typical codominant forbs include *Camassia quamash, Camassia leichtlinii, Triteleia hyacinthina, Mimulus guttatus* (seeps), *Plectritis congesta, Lomatium martindalei, Allium cernuum*, and *Phlox diffusa* (can be considered a dwarf‑shrub). Important dwarf‑shrubs are *Arctostaphylos uva‑ursi, Arctostaphylos nevadensis*, and *Juniperus communis*. Small patches and strips dominated by the shrub *Arctostaphylos columbiana* are a common feature nested within herbaceous balds. Significant portions of some balds, especially on rock outcrops, are dominated by bryophytes (mosses) and to a lesser degree lichens.

**Dynamics:** Fires, both lightning‑ignited and those ignited by Native Americans, undoubtedly at least occasionally burn all these sites. Lower elevation sites in the Georgia Basin, Puget Trough, and Willamette Valley probably were burned somewhat more frequently and in some cases intentionally. Because of this fire history, the extent of this system has declined locally through tree invasion and growth, as areas formerly maintained herbaceous by burning have filled in with trees.

SOURCES

**References:** Chappell and Christy 2004, Comer et al. 2003*, Franklin and Dyrness 1973, WNHP unpubl. data 2018

**Version:** 04 Apr 2005	**Stakeholders:** Canada, West

**Concept Author:** C. Chappell	**LeadResp:** West

CES204.088 North Pacific Hypermaritime Shrub and Herbaceous Headland

**Primary Division:** North American Pacific Maritime (204)

**Land Cover Class:** Herbaceous

**Spatial Scale & Pattern:** Small patch

**Required Classifiers:** Natural/Semi‑natural; Vegetated (>10% vasc.); Upland

**Diagnostic Classifiers:** Herbaceous; Bluff; Ridge/Summit/Upper Slope

**Concept Summary:** This system consists of herbaceous‑ and shrub‑dominated areas directly adjacent to the outer Pacific Coast from central Oregon north to Vancouver Island. These are very windy sites where wind and salt spray combine to limit tree growth. The climate is very wet, relatively warm in winter, and cool and foggy. In Oregon, fires apparently set by Native Americans also contributed to the open character of many of these sites. The relative prevalence of grasslands versus shrublands increases to the south. Steep slopes on coastal bluffs, headlands, or small islands are typical, though sometimes this system occurs on relatively level tops of headlands or islands. Soils can be shallow to bedrock or of glacial or marine sediment origin. Vegetation is dominated by perennial bunchgrasses or shrubs. Dominant species include *Vaccinium ovatum, Gaultheria shallon, Rubus spectabilis, Calamagrostis nutkaensis*, and *Festuca rubra*. Scattered stunted trees, especially *Picea sitchensis*, are often present.

**Comments:** California Northern Coastal Grassland (CES206.941) is somewhat similar to the grassland part of this but is more extensive (larger patches) and extends further inland and higher in elevation. In southern Oregon, the climate gets warmer and drier and the grasslands start climbing well up into the hills, picking up some southern elements of vegetation. Probably corresponds with where Northern California Coastal Scrub (CES206.932) starts also, somewhere south of Coos Bay.

DISTRIBUTION

**Range:** This system occurs from the southern Oregon coast north to Vancouver Island.

**Divisions:** 204:C

**TNC Ecoregions:** 1:C

**Nations:** CA, US

**Subnations:** BC, OR, WA:S3S4

**Map Zones:** 1:C, 2:C

**USFS Ecomap Regions:** 242A:CC, M242A:CC, M242D:CP, M261A:??

CONCEPT

SOURCES

**References:** Chappell and Christy 2004, Comer et al. 2003*, Franklin and Dyrness 1973, Shiflet 1994, WNHP unpubl. data 2018

**Version:** 04 Apr 2005	**Stakeholders:** Canada, West

**Concept Author:** C. Chappell and K. Boggs	**LeadResp:** West

CES204.858 Willamette Valley Upland Prairie and Savanna

**Primary Division:** North American Pacific Maritime (204)

**Land Cover Class:** Steppe/Savanna

**Spatial Scale & Pattern:** Small patch, Large patch

**Required Classifiers:** Natural/Semi‑natural; Vegetated (>10% vasc.); Upland

**Diagnostic Classifiers:** Woody‑Herbaceous; Short Disturbance Interval; F‑Landscape/Low Intensity

**National Mapping Codes:** EVT 2120; ESLF 5409; ESP 1120

**Concept Summary:** This grassland system is endemic to the Puget Trough and Willamette Valley. It formed a complex mosaic of varying patch sizes with wet prairies and riparian forests over much of the Willamette Valley during the pre‑European settlement era. In parts of the Puget Trough, it occurred as large patches in more forested landscapes, usually associated with deep, coarse outwash deposits. Historically, it also occurred as large patches on glacially associated soils of variable texture in localized portions of the Georgia Basin in both Washington and British Columbia. It occurs on well‑drained deep soils and was maintained historically by frequent anthropogenic burning. Landforms are usually flat, rolling, or gently sloping, and often part of extensive plains. Dominant vegetation is perennial bunchgrasses, especially *Festuca idahoensis ssp. roemeri* and, to a lesser degree, *Danthonia californica*, with abundant and diverse forbs. Scattered deciduous (*Quercus garryana*) and/or coniferous (*Pseudotsuga menziesii, Pinus ponderosa*) trees are rarely found now, but such savannas historically covered about one‑third of the total acreage. In the absence of disturbance, many of them have succeeded to forest and others continue to do so.

DISTRIBUTION

**Range:** This system is endemic to the Puget Trough and Willamette Valley.

**Divisions:** 204:C

**TNC Ecoregions:** 2:C

**Nations:** US

**Subnations:** OR, WA:S1

**Map Zones:** 1:C, 2:C, 3:?, 7:C

**USFS Ecomap Regions:** 242A:CC, 242B:CC, M242A:??, M242B:??, M261A:CC, M261D:CC

CONCEPT

**Environment:** This ecosystem occurs on well‑drained deep soils and was maintained historically by frequent anthropogenic burning. Landforms are usually flat, rolling, or gently sloping, and often part of extensive plains.

**Dynamics:** Fires are thought to have occurred every few years (Chappell and Kagan 2001, as cited in WNHP 2011). Annual soil drought during the summer made it difficult for woody species (especially trees) to establish in these grasslands. However, occasionally *Quercus garryana* and *Pseudotsuga menziesii* would establish and survive long enough to be resistant to frequent fires thereby creating savanna conditions (Chappell and Kagan 2001, as cited in WNHP 2011). Following European settlement of the region, anthropogenic fire became less frequent resulting in widespread encroachment of the prairies and savannas by woody vegetation, especially conifers (WNHP 2011).

SOURCES

**References:** Chappell and Christy 2004, Chappell and Kagan 2001, Comer et al. 2003*, Littell et al. 2009, PRBO Conservation Science 2011, WNHP 2011, WNHP unpubl. data 2018

**Version:** 14 Jan 2014	**Stakeholders:** West

**Concept Author:** C. Chappell	**LeadResp:** West

2.B.2.Ng. Western North American Interior Chaparral

M094. Cool Interior Chaparral

CES206.925 California Montane Woodland and Chaparral

**Primary Division:** Mediterranean California (206)

**Land Cover Class:** Shrubland

**Spatial Scale & Pattern:** Large patch

**Required Classifiers:** Natural/Semi‑natural; Vegetated (>10% vasc.); Upland

**Diagnostic Classifiers:** Shrubland (Shrub‑dominated); Mediterranean [Mediterranean Xeric‑Oceanic]; Shallow Soil; Short (50‑100 yrs) Persistence

**National Mapping Codes:** EVT 2098; ESLF 5304; ESP 1098

**Concept Summary:** This ecological system includes chaparral or open shrubby woodlands found among montane forests above 1500 m (4550 feet) elevation from the southern Cascades of Oregon to the Peninsular Ranges of California into Baja California, Mexico, where much annual precipitation occurs as snow. These are often locations with steep, exposed slopes with rocky and/or shallow soils, often glaciated. Stands are not found in the foothills but rather occur commonly above 1524 m (5000 feet) in elevation. These are mosaics of woodlands with chaparral understories, shrub‑dominated chaparral, or short‑lived chaparral with conifer species invading, if good seed source is available. Shrubs will often have higher densities than the trees, which are more limited due to the rocky/thin soils. These can also be short‑duration chaparrals in previously forested areas that have experienced crown fires. Trees tend to have a scattered open canopy or can be clustered, over a usually continuous dense shrub layer. Trees can include *Pinus jeffreyi, Abies lowiana (= Abies concolor var. lowiana), Abies magnifica, Pinus monticola, Pinus lambertiana, Pinus coulteri, Pinus attenuata, Hesperocyparis forbesii (= Cupressus forbesii), Hesperocyparis stephensonii (= Cupressus arizonica ssp. stephensonii)*, and *Hesperocyparis nevadensis (= Cupressus nevadensis)*. Typical sclerophyllous chaparral shrubs include *Arctostaphylos nevadensis, Arctostaphylos patula, Arctostaphylos glandulosa, Ceanothus cordulatus, Ceanothus diversifolius, Ceanothus pinetorum, Ceanothus velutinus*, and *Chrysolepis sempervirens (= Castanopsis sempervirens)*. Some stands can be dominated by winter deciduous shrubs, such as *Prunus emarginata, Prunus subcordata* and *Ceanothus sanguineus* (in Oregon), *Prunus virginiana, Ceanothus integerrimus, Holodiscus discolor (= Holodiscus microphyllus)*, and *Quercus garryana var. fruticosa (= var. breweri)*. Most chaparral species are fire‑adapted, resprouting vigorously after burning or producing fire‑resistant seeds. Occurrences of this system likely shift across montane forested landscapes with catastrophic fire events.

**Comments:** Two phases are recognized: first, early‑seral and post‑fire shrub fields with conifers, and second, edaphically controlled sites, with soils that are too dry or shallow‑soiled for trees, hence sites where shrubs stay dominant (such as *Quercus vacciniifolia, Arctostaphylos patula, Chrysolepis sempervirens*). This treatment combines "interior closed‑cone conifer" woodlands (obligate fire‑reproducing species) with montane chaparral and may need to be revisited.

DISTRIBUTION

**Range:** This system occurs above 1500 m (4550 feet) elevation from the southern Cascades of Oregon to the Klamath Mountains and Peninsular Ranges of California into Baja California, Mexico.

**Divisions:** 206:C

**TNC Ecoregions:** 5:C, 12:C, 14:C, 15:C, 16:C

**Nations:** MX, US

**Subnations:** CA, MXBC, OR

**Map Zones:** 2:C, 3:C, 4:C, 6:C, 7:C, 12:P, 13:?

**USFS Ecomap Regions:** 261B:CC, 263A:CC, 322A:??, 341D:CC, 342B:CC, M242B:??, M261A:CC, M261B:CC, M261C:CC, M261D:CC, M261E:CC, M261F:CC, M261G:CC

CONCEPT

**Environment:** [from M094] These are chaparral or open shrublands found at montane elevations throughout much of the western U.S., from the Sierra Nevada and Cascades and into the western Great Basin, Colorado Plateau, and Rocky Mountains. They occur in summer‑dry habitats from 800 to 3000 m elevation. Can occur as low as 50 m in California, but mostly is found above 1500 m. Much of the precipitation comes as winter snow, and summer drought‑stress is characteristic. These shrublands are mostly found on steep, usually south‑facing or exposed slopes, where soils are rocky, shallow and well‑drained, often glaciated. These are typically zonal disclimax or, occasionally, edaphic climax brushfields which occur in association with dry needle‑leaved evergreen forests or woodlands. These shrublands are typically established after stand‑replacing fires or clearcut logging in montane conifer forests or pinyon‑juniper woodlands, and may be seral to forest after several decades. Excessively rocky or droughty, fire‑prone sites in the forest may support relatively persistent stands of this macrogroup. These are in mosaics of woodlands and chaparral and may have conifer species invading if good seed source is available.

**Dynamics:** [from M094] Two phases are recognized: first, early‑seral and post‑fire or post‑logging shrub fields with few conifers; and second, edaphically controlled sites, with soils that are too dry or shallow‑soiled for trees, hence sites where shrubs stay dominant (such as *Quercus vacciniifolia, Chrysolepis sempervirens*). Most chaparral species are fire‑adapted, resprouting vigorously after burning or producing fire‑resistant seeds. Occurrences of this macrogroup likely shift across montane forested landscapes with catastrophic fire events. Clearcut logging can also trigger regeneration of some of the chaparral species.

SOURCES

**References:** Barbour and Billings 2000, Barbour and Major 1988, Comer et al. 2003*, Eyre 1980, Holland and Keil 1995, Sawyer and Keeler‑Wolf 1995, Shiflet 1994

**Version:** 12 Jan 2012	**Stakeholders:** Latin America, West

**Concept Author:** P. Comer and T. Keeler‑Wolf	**LeadResp:** West

CES304.001 Great Basin Semi‑Desert Chaparral

**Primary Division:** Inter‑Mountain Basins (304)

**Land Cover Class:** Shrubland

**Spatial Scale & Pattern:** Large patch

**Required Classifiers:** Natural/Semi‑natural; Vegetated (>10% vasc.); Upland

**Diagnostic Classifiers:** Montane [Lower Montane]; Lowland [Foothill]; Shrubland (Shrub‑dominated); Temperate [Temperate Continental]; Broad‑Leaved Evergreen Shrub

**National Mapping Codes:** EVT 2103; ESLF 5309; ESP 1103

**Concept Summary:** This system includes chaparral on sideslopes transitioning from low‑elevation desert landscapes up into pinyon‑juniper woodlands of the western and central Great Basin. There are limited occurrences extending as far west as the inner Coast Ranges in central California. These are typically fairly open‑canopy shrublands with open spaces either bare or supporting patchy grasses and forbs. Characteristic species may include *Arctostaphylos patula, Arctostaphylos pungens, Ceanothus greggii, Ceanothus velutinus, Cercocarpus montanus var. glaber, Cercocarpus intricatus, Eriogonum fasciculatum, Garrya flavescens, Quercus turbinella, Purshia stansburiana*, and *Rhus trilobata*. *Cercocarpus ledifolius* is generally absent. Typical fire regime in these systems varies with the amount of organic accumulation.

DISTRIBUTION

**Range:** Western and central Great Basin.

**Divisions:** 206:C, 304:C

**TNC Ecoregions:** 11:C, 12:C, 15:P

**Nations:** US

**Subnations:** CA, NV

**Map Zones:** 4:?, 6:C, 7:P, 9:C, 12:C, 13:C, 15:C, 16:?, 17:C, 18:C

**USFS Ecomap Regions:** 313A:??, 341A:CP, 341D:CC, 341E:CC, 341F:CC, 341G:CC, 342B:CC, 342C:CC, 342J:CP, M261E:CC, M341A:CC, M341D:CP

CONCEPT

**Environment:** This chaparral system is found in the western and central Great Basin, and east slopes of the Sierra Nevada and Cascades on slopes between lower‑elevation desert landscapes and higher‑elevation pinyon‑ or juniper‑dominated woodlands. It is also found in limited, small‑patch occurrences in the montane zone of many mountain ranges in the western U.S. and a few small pockets in the inner Coast Ranges of central California. These shrublands occur in summer‑dry habitats from 800 to 3000 m elevation, typically on piedmont slopes, foothills, plateaus and mountains. Much of the precipitation comes as winter snow, and summer drought‑stress is characteristic. These shrublands are mostly found on steep, usually south‑facing slopes, where soils are rocky and well‑drained. These are typically zonal disclimax or, occasionally, edaphic climax brushfields which occur in association with dry needle‑leaved evergreen forests or woodlands. These shrublands are typically established after stand‑replacing fires or clearcut logging in *Pinus ponderosa, Abies concolor*, or *Pseudotsuga menziesii* forests or pinyon‑juniper woodlands, and are seral to forest after several decades. Excessively rocky or droughty, fire‑prone sites in the forest may support relatively persistent stands of this system. In the Rocky Mountains, stands are found in small patches within a matrix of montane conifer forest and woodland. Adjacent systems in alpine include California Montane Jeffrey Pine‑(Ponderosa Pine) Woodland (CES206.918), Southern Rocky Mountain Dry‑Mesic Montane Mixed Conifer Forest and Woodland (CES306.823), Great Basin Pinyon‑Juniper Woodland (CES304.773), and Rocky Mountain Cliff, Canyon and Massive Bedrock (CES306.815) above and Mojave Mid‑Elevation Mixed Desert Scrub (CES302.742) or Great Basin Xeric Mixed Sagebrush Shrubland (CES304.774) below. The environmental description is based on several other references, including Kauffman (1986), Tirmenstein (1989), Pavek (1993), Holland and Keil (1995), Reid et al. (1999), Zouhar (2000), Anderson (2001a, 2004a), League (2005), Barbour et al. (2007), Hauser (2007), and Sawyer et al. (2009).

**Vegetation:** The vegetation in stands of this system are dominated by sclerophyllous shrubs that are adapted to freezing temperatures and cold winters. These are typically open‑canopy shrublands 1‑3 m tall with interspaces of either bare ground or patchy grasses and forbs. Characteristic species include *Arctostaphylos patula, Arctostaphylos pungens, Ceanothus greggii, Ceanothus velutinus, Ceanothus leucodermis, Ceanothus martinii, Ceanothus prostratus, Cercocarpus intricatus, Eriogonum fasciculatum, Purshia stansburiana, Purshia tridentata*, and *Rhus trilobata*. *Cercocarpus ledifolius* is generally absent. Sometimes other shrubs such as *Amelanchier utahensis, Artemisia tridentata ssp. vaseyana, Artemisia tridentata ssp. wyomingensis, Cercocarpus montanus, Ephedra viridis, Fremontodendron californicum, Garrya flavescens*, or *Quercus gambelii* may be present to codominant. The herbaceous layer is variable but typically sparse. The vegetation description is based on several references, including Kauffman (1986), Tirmenstein (1989), Pavek (1993), Holland and Keil (1995), Reid et al. (1999), Zouhar (2000), Anderson (2001a, 2004a), League (2005), Barbour et al. (2007), Hauser (2007), and Sawyer et al. (2009).

**Dynamics:** Disturbance dynamics in this system are variable because of variation in the compositions; however, most dominant shrubs are evergreen species that are adapted to medium‑frequency, medium‑ to large‑sized and medium‑ to high‑intensity fire in late summer or fall (Hauser 2007, Sawyer et al. 2009). Some species, such as *Arctostaphylos patula, Ceanothus velutinus, Ceanothus leucodermis*, and *Fremontodendron californicum*, are generally top‑killed in burns, but then vigorously resprout from rootcrowns or buried lignotubers. Most have seeds stored in soil and duff that need fire scarification to germinate (Pavek 1993, Anderson 2001a, Hauser 2007, Sawyer et al. 2009). Other chaparral shrubs, such as *Arctostaphylos pungens* and *Ceanothus greggii*, are killed or sprout only weakly after fire and regenerate from fire‑scarified seeds in the seedbank (Zouhar 2000, League 2005, Sawyer et al. 2009). The shorter‑lived species such as *Ceanothus leucodermis* are dependent on fire for regeneration and will disappear after 40‑70 years if not burned (Minnich 1976, Tirmenstein 1989). Higher‑severity fires cause greater seedling establishment than lower‑severity fires in chaparral (Kauffman 1986). Some deciduous species such as *Rhus trilobata* are also adapted to fire, vigorously resprout after burning and have fire‑scarified seeds (Anderson 2004a). Fire‑return interval (FRI) for this systems is medium (10‑100 years) on most of the dominant species (Sawyer et al. 2009).

SOURCES

**References:** Anderson 2001a, Anderson 2004a, Barbour and Major 1977, Barbour et al. 2007a, Brown 1982a, Comer et al. 2003*, Hauser 2007a, Holland and Keil 1995, Kauffman 1986, League 2005a, Minnich 1976, NatureServe Explorer 2011, Pavek 1993a, Reid et al. 1999, Sawyer and Keeler‑Wolf 1995, Sawyer et al. 2009, Shiflet 1994, Tirmenstein 1989a, USFS 1937, Zouhar 2000

**Version:** 02 Apr 2014	**Stakeholders:** West

**Concept Author:** K.A. Schulz and P. Comer	**LeadResp:** West

M091. Warm Interior Chaparral

CES302.905 Chaparral Transvolcanico

**Primary Division:** North American Warm Desert (302)

**Required Classifiers:** Natural/Semi‑natural; Vegetated (>10% vasc.)

**Concept Summary:** Asociación generalmente densa, de arbustos resistentes al fuego, que se desarrolla principalmente en laderas de cerros por arriba del nivel de los matorrales de zonas áridas y semiáridas de Pastizales Naturales y en ocasiones mezclada con los Bosques de Pino y Encino. Esta formada por especies arbustivas de *Quercus* spp. (Encinillo, Charrasquillo), *Adenostoma* spp. (Chamizos), *Arctostaphylos* spp. (Manzanita), *Cercocarpus* spp. (Rosa de castilla), etc.

DISTRIBUTION

**Divisions:** 302:C

**Nations:** MX

CONCEPT

**Environment:** [from M091] This type occurs across central Arizona (Mogollon Rim) and southern New Mexico, east in mountains across Trans‑Pecos Texas, and south into the Madrean Occidentale and Madrean Oriental in northern Mexico. Occurrences are also found in desert mountains in the Sonoran and Mojave deserts. Stands are found on foothills, xeric mountain slopes and canyons in hotter and drier habitats. They often dominate along the mid‑elevation (1000‑2500 m) transition zone between desert scrub and montane woodlands (encinal, pine‑oak, and ponderosa pine). Sites are variable but often steep and rocky. Sometimes this macrogroup occurs in thickets along upper canyon watercourses and northerly upland slopes within the pinyon‑juniper woodland zone.

<br />*Climate:* This macrogroup occurs in warm semi‑desert regions in the southwestern U.S. The climate is hot and may have a somewhat bi‑modal precipitation regime with spring rains and warm‑season monsoonal rains as well. Frosts occur in winter, and even sometime snows, which will melt rapidly. *Soil/substrate/hydrology:* Parent materials are varied. This macrogroup is found on igneous intrusives and extrusives, sedimentary, and metamorphic including andesite, basalt, diabase, gneiss, schist, shale, slate, rhyolite, sandstone, tuff, and, more commonly, limestone and coarse‑textured granitic substrates.

**Dynamics:** [from M091] Many of the communities in this macrogroup are dominated by fire‑adapted shrubs. *Quercus cornelius‑mulleri* sprouts vigorously from root crowns after fire. Since *Quercus cornelius‑mulleri* chaparral occurs in areas of lower rainfall and sparser vegetation cover, it typically has less frequent fire and slower recovery rates than typical cismontane chaparral types elsewhere in California. *Quercus turbinella* in Arizona and New Mexico is a fire‑type; it sprouts vigorously from the root crown and rhizomes. Typical fire intervals in Arizona exceed 74 years (Reid et al. 1999, Tirmenstein 1999d). Plants in the New York Mountains of California are treelike, suggesting that fires have been absent for perhaps greater than 100 years. Instead, flooding has initiated stem breakage and sprouting of some canyon bottom stands. *Ceanothus greggii* is an obligate seeder and germinates from seed after fire, and older stands will lose dominance of this shrub to other longer‑lived sprouting shrubs.

<br />Site conditions aside, the dynamics of fire within chaparral are still complex. In southern California, it has been suggested that the even‑aged and large size of modern chaparral patches are a function of 20th century fire suppression feedbacks whereby intensive suppression has led to large fuel buildups over large areas of landscape leading to large stand‑replacement fires of ever increasing size (Minnich 1983, 2001). Others contend that the large patch patterns are within that natural range of variability, and that they are driven more by climate trends, prevailing weather patterns, increased human ignition frequencies with increased population density, changes in land use, and landscape characteristics rather than suppression (Keeley and Fotheringham 2001a, 2001c, Moritz 2003). The pattern of chaparral distribution in southern New Mexico suggests that the latter scenario might be the case here. Because of the rugged country, effective suppression has been minimal. Hence, the large patches of chaparral may be representative of a more or less natural fire regime, but one possibly modified by increased human caused fires and fire suppression on neighboring forested lands. More frequent, intense fires leads to the decline of the grassy woodland savannas on the ridge top summits and a favoring of shrublands (possibly enhanced by increased fine fuels with the cessation of livestock grazing). In this type of fire regime, Keeley and Fotheringham (2001a) and Moritz (2003) contend that prescribed burning may be useless or even harmful and that fire suppression, at least in the short term, may be more appropriate for maintaining an ecosystem near its natural state. Minnich (2001) would likely argue the opposite saying it is fire suppression that generates the large patch pattern and that prescribed fire is needed to restore a small patch mosaic with imbedded natural fuel firebreaks. Detailed fire history studies that focus on chaparral patch age structure in a landscape context would be useful (and perhaps necessary) to help resolve these conflicting viewpoints and generate management options that are tailored to interior chaparral.

At the other end of the elevation spectrum, repeated burning of chaparral, particularly Pinchot juniper, has been suggested as a way to increase grass cover in shrubland communities (Ahlstrand 1982). Most of our understanding of how to manage of Pinchot juniper comes from the high Plains of Texas where it is seen as an invader of fine textured plains grasslands soils, and where management has focused on control and eradication to increase livestock forage. Research from the high plains indicates that the effectiveness of fire in controlling Pinchot juniper is a function of fire intensity, climatic conditions and position of the bud zone above or below the soil (Steuter and Britton 1983). Fire was particularly effective in inducing mortality in young plants with exposed buds on rocky sites, but this dropped off significantly with older plants. In addition, increased grass cover (grama grasses) can inhibit reproduction (Smith et al. 1975). As Ahlstrand (1982) has shown, fires can lead to at least short‑term increases in grass cover, but because Pinchot juniper can recover 50% or more of its original cover within six or seven years of a burn, repeated prescribed fires at 10‑ to 15‑year intervals would be needed to sustain a grassland type.

SOURCES

**References:** Barbour and Billings 2000, Barbour and Major 1988, Comer et al. 2003, INEGI 2005, Latin American Ecology Working Group n.d.*, Shiflet 1994

**Version:** 08 Jan 2015	**Stakeholders:** Latin America

**Concept Author:** P. Comer	**LeadResp:** Latin America

CES302.031 Madrean Oriental Chaparral

**Primary Division:** North American Warm Desert (302)

**Land Cover Class:** Shrubland

**Spatial Scale & Pattern:** Large patch

**Required Classifiers:** Natural/Semi‑natural; Vegetated (>10% vasc.); Upland

**Diagnostic Classifiers:** Montane [Lower Montane]; Shrubland (Shrub‑dominated); Shallow Soil; Xeric; F‑Patch/High Intensity

**National Mapping Codes:** EVT 2101; ESLF 5307; ESP 1101

**Concept Summary:** This ecological system occurs in mountains across southeastern New Mexico (Guadalupe Mountains), Trans‑Pecos Texas (Chisos and Davis mountains) and Madrean Oriental in northern Mexico. It often dominates along the mid‑elevation transition from the Chihuahuan Desert into mountains (1700‑2500 m). It occurs on foothills, mountain slopes and canyons in drier habitats below the encinal and pine woodlands, and is often associated with more xeric and coarse‑textured substrates such as limestone, basalt or alluvium, especially in transition areas with more mesic woodlands. The moderate to dense shrub canopy includes many shrub oak species, such as *Quercus emoryi, Quercus grisea, Quercus intricata, Quercus invaginata, Quercus laceyi, Quercus mohriana, Quercus pringlei, Quercus pungens*, and *Quercus vaseyana*, and several widespread chaparral species, such as *Arctostaphylos pungens, Ceanothus greggii, Cercocarpus montanus, Fallugia paradoxa*, and *Garrya wrightii*; other species characteristic of this system include *Arbutus xalapensis, Fraxinus greggii, Fendlera rigida, Garrya ovata, Purshia mexicana, Rhus virens var. choriophylla, Salvia lycioides, Salvia roemeriana*, and *Salvia regla*. In the Trans‑Pecos of Texas, disjunct *Quercus gambelii* may occur as a significant component of this shrubland. In addition, Texas occurrences may also include *Agave lechuguilla, Aloysia wrightii, Ceanothus greggii, Cercocarpus montanus, Chrysactinia mexicana, Dasylirion leiophyllum, Fallugia paradoxa, Fraxinus greggii, Garrya wrightii, Juniperus pinchotii, Nolina texana, Opuntia engelmannii var. engelmannii, Pinus cembroides* or *Pinus edulis* (in the Guadalupe Mountain region), *Quercus turbinella, Quercus x pauciloba, Rhus virens*, and *Viguiera stenoloba*. Most chaparral species are fire‑adapted, resprouting vigorously after burning or producing fire‑resistant seeds. Stands occurring within montane woodlands are seral and a result of recent fires. Grass cover may be significant. Dominant grasses often include *Bouteloua curtipendula, Bouteloua hirsuta*, and *Muhlenbergia emersleyi*. In Texas, the herbaceous cover is patchy and bare rock is frequently visible. Where present, graminoids dominate the herbaceous layer with species such as *Bouteloua curtipendula, Bouteloua hirsuta, Muhlenbergia emersleyi, Muhlenbergia pauciflora, Muhlenbergia setifolia, Achnatherum lobatum, Muhlenbergia dubia*, and *Heteropogon contortus*.

**Comments:** The similar Mogollon chaparral system has floristics mostly derived from the Sierra Madre Occidental, whereas floristics of this system are derived from the Sierra Madre Oriental. However, this system is not mattoral (thornscrub) as it is typically dominated by shrubby evergreen oaks and chaparral species, not thornscrub species. More survey is needed to determine if *Quercus turbinella*, common in the Mogollon Chaparral system, also occurs in the Madrean Oriental Chaparral. In Texas, distinguishing this shrubland system from Madrean Encinal (CES305.795) is sometimes difficult.

DISTRIBUTION

**Range:** This system is found on mountains across southeastern New Mexico, Trans‑Pecos Texas and northern Mexico. It often dominants along the mid‑elevation transition from the Chihuahuan Desert into mountains (1700‑2500 m elevation).

**Divisions:** 301:P, 302:C, 305:P, 306:C

**TNC Ecoregions:** 21:P, 22:P, 24:P

**Nations:** MX, US

**Subnations:** MXCH, MXCO, NM, TX

**Map Zones:** 25:C, 26:C, 27:P

**USFS Ecomap Regions:** 315A:PP, 321A:CC, M313B:CC

CONCEPT

**Environment:** This system occurs at elevations above desert shrublands on dry rocky habitats of foothills, mountains, and canyons. In Texas, it often occurs at elevations coincident with the occurrence of Madrean Encinal and Madrean coniferous woodlands, but typically occupies more xeric sites, often with steeper slopes and less soil development.

**Vegetation:** The moderate to dense shrub canopy includes many shrub oak species, such as *Quercus emoryi, Quercus grisea, Quercus intricata, Quercus invaginata, Quercus laceyi, Quercus mohriana, Quercus pringlei, Quercus pungens*, and *Quercus vaseyana*, and several widespread chaparral species, such as *Arctostaphylos pungens, Ceanothus greggii, Cercocarpus montanus, Fallugia paradoxa*, and *Garrya wrightii*; other species characteristic of this system include *Arbutus xalapensis (= Arbutus texana), Fraxinus greggii, Fendlera rigida (= Fendlera linearis), Garrya ovata, Purshia mexicana, Rhus virens var. choriophylla (= Rhus choriophylla), Salvia lycioides (= Salvia ramosissima), Salvia roemeriana*, and *Salvia regla*. In the Trans‑Pecos of Texas, disjunct *Quercus gambelii* may occur as a significant component of this shrubland. Most chaparral species are fire‑adapted, resprouting vigorously after burning or producing fire‑resistant seeds. Stands occurring within montane woodlands are seral and a result of recent fires. Grass cover may be significant. Dominant grasses often include *Bouteloua curtipendula, Bouteloua hirsuta*, and *Muhlenbergia emersleyi*.

**Dynamics:** [from M091] Many of the communities in this macrogroup are dominated by fire‑adapted shrubs. *Quercus cornelius‑mulleri* sprouts vigorously from root crowns after fire. Since *Quercus cornelius‑mulleri* chaparral occurs in areas of lower rainfall and sparser vegetation cover, it typically has less frequent fire and slower recovery rates than typical cismontane chaparral types elsewhere in California. *Quercus turbinella* in Arizona and New Mexico is a fire‑type; it sprouts vigorously from the root crown and rhizomes. Typical fire intervals in Arizona exceed 74 years (Reid et al. 1999, Tirmenstein 1999d). Plants in the New York Mountains of California are treelike, suggesting that fires have been absent for perhaps greater than 100 years. Instead, flooding has initiated stem breakage and sprouting of some canyon bottom stands. *Ceanothus greggii* is an obligate seeder and germinates from seed after fire, and older stands will lose dominance of this shrub to other longer‑lived sprouting shrubs.

<br />Site conditions aside, the dynamics of fire within chaparral are still complex. In southern California, it has been suggested that the even‑aged and large size of modern chaparral patches are a function of 20th century fire suppression feedbacks whereby intensive suppression has led to large fuel buildups over large areas of landscape leading to large stand‑replacement fires of ever increasing size (Minnich 1983, 2001). Others contend that the large patch patterns are within that natural range of variability, and that they are driven more by climate trends, prevailing weather patterns, increased human ignition frequencies with increased population density, changes in land use, and landscape characteristics rather than suppression (Keeley and Fotheringham 2001a, 2001c, Moritz 2003). The pattern of chaparral distribution in southern New Mexico suggests that the latter scenario might be the case here. Because of the rugged country, effective suppression has been minimal. Hence, the large patches of chaparral may be representative of a more or less natural fire regime, but one possibly modified by increased human caused fires and fire suppression on neighboring forested lands. More frequent, intense fires leads to the decline of the grassy woodland savannas on the ridge top summits and a favoring of shrublands (possibly enhanced by increased fine fuels with the cessation of livestock grazing). In this type of fire regime, Keeley and Fotheringham (2001a) and Moritz (2003) contend that prescribed burning may be useless or even harmful and that fire suppression, at least in the short term, may be more appropriate for maintaining an ecosystem near its natural state. Minnich (2001) would likely argue the opposite saying it is fire suppression that generates the large patch pattern and that prescribed fire is needed to restore a small patch mosaic with imbedded natural fuel firebreaks. Detailed fire history studies that focus on chaparral patch age structure in a landscape context would be useful (and perhaps necessary) to help resolve these conflicting viewpoints and generate management options that are tailored to interior chaparral.

At the other end of the elevation spectrum, repeated burning of chaparral, particularly Pinchot juniper, has been suggested as a way to increase grass cover in shrubland communities (Ahlstrand 1982). Most of our understanding of how to manage of Pinchot juniper comes from the high Plains of Texas where it is seen as an invader of fine textured plains grasslands soils, and where management has focused on control and eradication to increase livestock forage. Research from the high plains indicates that the effectiveness of fire in controlling Pinchot juniper is a function of fire intensity, climatic conditions and position of the bud zone above or below the soil (Steuter and Britton 1983). Fire was particularly effective in inducing mortality in young plants with exposed buds on rocky sites, but this dropped off significantly with older plants. In addition, increased grass cover (grama grasses) can inhibit reproduction (Smith et al. 1975). As Ahlstrand (1982) has shown, fires can lead to at least short‑term increases in grass cover, but because Pinchot juniper can recover 50% or more of its original cover within six or seven years of a burn, repeated prescribed fires at 10‑ to 15‑year intervals would be needed to sustain a grassland type.

SOURCES

**References:** Brown 1982a, Comer et al. 2003*, Dick‑Peddie 1993, Elliott 2012, Muldavin et al. 1994a, Muldavin et al. 2000b, Muldavin et al. 2003a, Shiflet 1994

**Version:** 02 Oct 2014	**Stakeholders:** Latin America, Southeast, West

**Concept Author:** K.A. Schulz and P. Comer	**LeadResp:** West

CES302.741 Mogollon Chaparral

**Primary Division:** North American Warm Desert (302)

**Land Cover Class:** Shrubland

**Spatial Scale & Pattern:** Matrix

**Required Classifiers:** Natural/Semi‑natural; Vegetated (>10% vasc.); Upland

**Diagnostic Classifiers:** Montane [Lower Montane]; Lowland [Foothill]; Intermediate Disturbance Interval; F‑Patch/High Intensity; Evergreen Sclerophyllous Shrub

**National Mapping Codes:** EVT 2104; ESLF 5310; ESP 1104

**Concept Summary:** This ecological system occurs across central Arizona (Mogollon Rim), western New Mexico, and southern Utah and Nevada. It often dominates along the mid‑elevation transition from the Mojave, Sonoran, and northern Chihuahuan deserts into mountains (1000‑2200 m). It occurs on foothills, mountain slopes and canyons in hotter and drier habitats below the encinal and *Pinus ponderosa* woodlands. Stands are often associated with more xeric and coarse‑textured substrates such as limestone, basalt or alluvium, especially in transition areas with more mesic woodlands. The moderate to dense shrub canopy includes species such as *Quercus turbinella, Quercus toumeyi, Cercocarpus montanus var. paucidentatus, Canotia holacantha, Ceanothus greggii, Garrya wrightii, Purshia stansburiana, Rhus ovata, Rhus trilobata*, and *Arctostaphylos pungens* and *Arctostaphylos pringlei* at higher elevations. Scattered remnant pinyon and juniper trees may be present. Most chaparral species are fire‑adapted, resprouting vigorously after burning or producing fire‑resistant seeds. Stands occurring within montane woodlands are seral and a result of recent fires.

DISTRIBUTION

**Range:** This system occurs across central Arizona (Mogollon Rim), western New Mexico and southern Utah. It often dominates along the mid‑elevation transition from the Mojave, Sonoran, and northern Chihuahuan deserts into mountains (1000‑2200 m elevation). It does not occur as far west as California.

**Divisions:** 302:C, 304:P, 306:P

**TNC Ecoregions:** 17:C, 19:C, 21:C, 22:C, 23:C, 24:C

**Nations:** MX?, US

**Subnations:** AZ, MXSO?, NM, NV, UT

**Map Zones:** 12:?, 13:P, 14:C, 15:C, 16:P, 17:C, 23:C, 24:C, 25:C, 26:C, 27:P, 28:?

**USFS Ecomap Regions:** 313A:CC, 313B:CC, 313C:CC, 313D:CC, 315A:CC, 315H:CC, 321A:CC, 322A:CC, 322B:CC, 341A:CP, 341F:CC, M313A:CC, M313B:CC, M341C:CC

CONCEPT

**Environment:** This chaparral system occurs across central Arizona (Mogollon Rim), western New Mexico, and southern Utah and Nevada. It does not occur as far west as California. It often dominates along the mid‑elevation transition from the eastern Mojave, Sonoran, and northern Chihuahuan deserts into mountains (1000‑2200 m). It occurs on foothills, mountain slopes and canyons in hotter and drier habitats below the encinal and *Pinus ponderosa* woodlands. Stands are often associated with more xeric and coarse‑textured substrates such as limestone, basalt or alluvium, especially in transition areas with more mesic woodlands. Adjacent upland systems include Southern Rocky Mountain Ponderosa Pine Woodland (CES306.648), Madrean Encinal (CES305.795), Madrean Pinyon‑Juniper Woodland (CES305.797) or Great Basin Pinyon‑Juniper Woodland (CES304.773) above and at lower elevations, Sonoran Mid‑Elevation Desert Scrub (CES302.035) and Mojave Mid‑Elevation Mixed Desert Scrub (CES302.742). The environmental description is based on several references, including Cable (1975a), Carmichael et al. (1978), Brown (1982), Dick‑Peddie (1993), Reid et al. (1999), Tirmenstein (1999d), Comer et al. (2003), and NatureServe Explorer (2011).

**Vegetation:** The vegetation is characterized by a moderate to dense, evergreen‑dominated shrub canopy with *Quercus turbinella* or *Quercus toumeyi* often dominant or codominant. Other shrubs that may be present to dominant include *Cercocarpus montanus, Canotia holacantha, Ceanothus greggii, Forestiera pubescens (= Forestiera neomexicana), Garrya flavescens, Garrya wrightii, Purshia stansburiana, Rhus ovata, Rhus trilobata, Arctostaphylos pungens*, and at higher elevations *Arctostaphylos pringlei*. Scattered remnant pinyon and juniper trees may be present, especially *Pinus edulis, Juniperus deppeana, Juniperus monosperma*, and *Juniperus osteosperma* (Carmichael et al. 1978). The herbaceous understory is variable depending on density of the shrub layer, but is usually low and dominated by perennial graminoids such as *Aristida purpurea var. fendleriana (= Aristida fendleriana), Aristida ternipes, Bouteloua curtipendula, Bouteloua hirsuta, Bothriochloa barbinodis, Eragrostis intermedia, Leptochloa dubia, Lycurus phleoides, Poa fendleriana ssp. longiligula (= Poa longiligula)*, and *Digitaria californica (= Trichachne californica)* with scattered forb species of *Boerhavia, Heterotheca*, and *Penstemon* (Brown 1982). The floristic description is based on several references, including Cable (1975a), Carmichael et al. (1978), Brown (1982), Dick‑Peddie (1993), Reid et al. (1999), Tirmenstein (1999d), Comer et al. (2003), and NatureServe Explorer (2011).

**Dynamics:** Most chaparral species are fire‑adapted, resprouting vigorously after burning or producing fire‑resistant seeds. Stands occurring within montane woodlands are seral and a result of recent fires. Disturbance dynamics in this system are variable because of variation in composition of dominant species; however, most dominant shrubs are evergreen species that are adapted to medium frequency, medium to large‑sized and medium‑ to high‑intensity fire, in late summer or fall. Some species such as *Cercocarpus montanus, Garrya wrightii*, and *Quercus turbinella* are generally top‑killed in burns, but then vigorously resprout from root crown or buried lignotubers (Uchytil 1990, Tirmenstein 1999d, Gucker 2006e). Most also have seeds stored in soil and duff that need fire scarification to germinate. Other chaparral shrub such as *Arctostaphylos pungens, Ceanothus greggii*, and *Purshia stansburiana* are killed or sprout only weakly after fire and regenerate from fire‑scarified seeds in the seedbank (Howard 1995, Zouhar 2000, League 2005). Some deciduous species such as *Rhus trilobata* are also adapted to fire, vigorously resprout after burning and have fire‑scarified seeds (Anderson 2004a). Fire‑return interval (FRI) for this systems is medium (5‑70 years) on most of the dominant species (Howard 1995, Tirmenstein 1999d, Zouhar 2000, Anderson 2004a, League 2005, Gucker 2006e). Recovery times after fire for *Quercus turbinella*‑dominated chaparral stands range from 4 to 8 years or more (Tiedemann and Schmutz 1966). Cable (1957) observed that this shrub regained preburn density within 5 years after fire in Arizona.

The foliage of most of these chaparral shrubs is utilized as browse at least to some degree (new growth) by big game species with *Ceanothus greggii, Cercocarpus montanus, Purshia stansburiana, Garrya wrightii*, and *Rhus trilobata* being especially important (Uchytil 1990, Howard 1995, Zouhar 2000, Anderson 2004a, Gucker 2006e). Small mammal and birds use the acorns and fruits of many of the dominant chaparral species (Cable 1975a, Howard 1995, Tirmenstein 1999d, Zouhar 2000, Anderson 2004a, League 2005, Gucker 2006e).

SOURCES

**References:** Anderson 2004a, Brown 1982a, Cable 1957, Cable 1975a, Carmichael et al. 1978, Comer et al. 2003*, Dick‑Peddie 1993, Gucker 2006e, Howard 1995, League 2005a, Muldavin et al. 1994a, Muldavin et al. 2000b, NatureServe Explorer 2011, Reid et al. 1999, Shiflet 1994, Tiedemann and Schmutz 1966, Tirmenstein 1999d, USFS 1937, Uchytil 1990a, Zouhar 2000

**Version:** 02 Apr 2014	**Stakeholders:** Latin America, West

**Concept Author:** K.A. Schulz	**LeadResp:** West

CES302.757 Sonora‑Mojave Semi‑Desert Chaparral

**Primary Division:** North American Warm Desert (302)

**Land Cover Class:** Shrubland

**Spatial Scale & Pattern:** Large patch

**Required Classifiers:** Natural/Semi‑natural; Vegetated (>10% vasc.); Upland

**Diagnostic Classifiers:** Montane [Lower Montane]; Lowland [Foothill]; Shrubland (Shrub‑dominated); Tropical/Subtropical [Tropical Xeric]; Temperate [Temperate Xeric]; Intermediate Disturbance Interval; F‑Patch/High Intensity; Evergreen Sclerophyllous Shrub

**National Mapping Codes:** EVT 2108; ESLF 5314; ESP 1108

**Concept Summary:** This ecological system is composed of evergreen shrublands or dwarf‑woodlands on sideslopes transitioning from low‑elevation desert landscapes up into woodlands of the western Mojave and Sonoran deserts. It extends from northeast Kern County, California, into Baja Norte, Mexico. Associated species include *Quercus john‑tuckeri, Quercus cornelius‑mulleri, Quercus berberidifolia, Arctostaphylos patula, Arctostaphylos pungens, Arctostaphylos glauca, Rhus ovata, Cercocarpus montanus var. glaber, Ceanothus greggii, Garrya flavescens, Juniperus californica*, and *Nolina parryi*. Sometimes *Juniperus californica* forms an open, shrubby tree layer over the evergreen oaks and other shrubs.

DISTRIBUTION

**Range:** This system occurs in the western Mojave and Sonoran deserts, from northeast Kern County, California, into Baja Norte, Mexico.

**Divisions:** 302:C

**TNC Ecoregions:** 17:C, 23:C

**Nations:** MX, US

**Subnations:** AZ, CA, MXBC, MXSO, NV

**Map Zones:** 4:C, 6:P, 12:P, 13:C, 14:C, 17:P

**USFS Ecomap Regions:** 322A:CC, 322B:CC, 322C:CC, 341F:??, M261E:CC

CONCEPT

**Environment:** This ecological system occurs on sideslopes transitioning from low‑elevation desert landscapes up into woodlands of the western Mojave and Sonoran deserts. It extends from northeastern Kern County, California, into Baja Norte, Mexico. This system includes chaparral on sideslopes transitioning from low‑elevation desert landscapes up into pinyon‑juniper and ponderosa pine woodlands of the western Great Basin between 1220 and 2135 m (4000‑7000 feet) elevation. Adjacent upland systems include Madrean Encinal (CES305.795), Madrean Pinyon‑Juniper Woodland (CES305.797) or Great Basin Pinyon‑Juniper Woodland (CES304.773) above and at lower elevations, Sonoran Mid‑Elevation Desert Scrub (CES302.035) and Mojave Mid‑Elevation Mixed Desert Scrub (CES302.742). The environmental description is based on several references, including Cable (1975a), Carmichael et al. (1978), Brown (1982a), Cope (1992b), Howard (1993), Reid et al. (1999), Comer et al. (2003), League (2005), Gucker (2006e), Hauser (2007), Sawyer et al. (2009), and NatureServe Explorer (2011).

**Vegetation:** This ecological system is composed of evergreen shrublands or dwarf‑woodlands. Associated species include *Quercus john‑tuckeri, Quercus cornelius‑mulleri, Quercus berberidifolia, Arctostaphylos patula, Arctostaphylos pungens, Arctostaphylos glauca, Rhus ovata, Cercocarpus montanus var. glaber (= Cercocarpus betuloides), Ceanothus greggii, Garrya flavescens, Juniperus californica*, and *Nolina parryi*. Sometimes *Juniperus californica* forms an open, shrubby tree layer over the evergreen oaks and other shrubs. The floristic description is based on several references including Cable (1975a), Carmichael et al. (1978), Brown (1982a), Cope (1992b), Howard (1993), Reid et al. (1999), Comer et al. (2003), League (2005), Gucker (2006e), Hauser (2007), Sawyer et al. (2009), and NatureServe Explorer (2011).

**Dynamics:** Most chaparral species are fire‑adapted, resprouting vigorously after burning or producing fire‑resistant seeds. Stands occurring within montane woodlands are seral and a result of recent fires. Disturbance dynamics in this system are variable because of variation in composition of dominant species; however, most dominant shrubs are evergreen species that are adapted to medium frequency, medium to large‑sized and medium‑ to high‑intensity fire, in late summer or fall. Some species, such as *Arctostaphylos patula, Cercocarpus montanus, Garrya flavescens, Quercus cornelius‑mulleri, Quercus berberidifolia, Quercus john‑tuckeri*, and *Rhus ovata*, are generally top‑killed in burns, but then vigorously resprout from root crown or buried lignotubers (Gucker 2006e, Hauser 2007, Sawyer et al. 2009). Most also have seeds stored in soil and duff that need fire scarification to germinate. Other chaparral shrub, such as *Arctostaphylos glauca, Arctostaphylos pungens, Ceanothus greggii*, and *Juniperus californica*, are killed or sprout only weakly after fire and regenerate from fire‑scarified seeds in the seedbank (Cope 1992b, Howard 1993, Zouhar 2000, League 2005, Sawyer et al. 2009). Some deciduous species such as *Cercocarpus montanus* are also adapted to fire, vigorously resprout after burning and have fire‑scarified seeds (Gucker 2006e). Fire‑return interval (FRI) for this systems is medium (10‑100 years) on most of the dominant species (Howard 1993, Zouhar 2000, League 2005, Gucker 2006e, Sawyer et al. 2009). Fire‑return intervals for *Juniperus californica*‑dominated stands are between 100 and 200 years (Sawyer et al. 2009).

The foliage of most of these chaparral shrubs is utilized as browse at least to some degree (new growth) by big game species with *Ceanothus greggii, Cercocarpus montanus*, and *Garrya wrightii* being especially important (Howard 1993, Zouhar 2000, Gucker 2006e). Small mammals and birds use the acorns and fruits of many of the dominant chaparral species (Cable 1975a, Howard 1993, Zouhar 2000, League 2005, Gucker 2006e).

SOURCES

**References:** Barbour and Major 1988, Barbour et al. 2007a, Brown 1982a, Cable 1957, Cable 1975a, Carmichael et al. 1978, Comer et al. 2003*, Cope 1992b, Gucker 2006e, Hauser 2007a, Holland and Keil 1995, Howard 1993a, League 2005a, MacMahon 1988, NatureServe Explorer 2011, Reid et al. 1999, Sawyer et al. 2009, Shiflet 1994, Thomas et al. 2004, USFS 1937, Zouhar 2000

**Version:** 02 Apr 2014	**Stakeholders:** Latin America, West

**Concept Author:** K.A. Schulz	**LeadResp:** West

2.B.2.Nh. Southeastern North American Grassland & Shrubland

M162. Florida Peninsula Scrub & Herb

CES203.380 Florida Dry Prairie

**Primary Division:** Gulf and Atlantic Coastal Plain (203)

**Land Cover Class:** Herbaceous

**Spatial Scale & Pattern:** Matrix

**Required Classifiers:** Natural/Semi‑natural; Vegetated (>10% vasc.); Upland

**Diagnostic Classifiers:** Herbaceous; Short Disturbance Interval; Graminoid

**National Mapping Codes:** EVT 2425; ESLF 7138; ESP 1425

**Concept Summary:** This system, which is endemic to subtropical Florida, is characterized by nearly treeless plains with dense cover of grasses and low shrubs, primarily stunted *Serenoa repens* and a wide variety of grasses and forbs. Examples occur on flat, low‑lying terrain over moderately to poorly drained soils with sandy surfaces overlying organic hardpans or clayey subsoil. This type was historically expansive in several regions of Florida. Early surveyors noted large expanses of this system on the plains near the Kissimmee River, north from Lake Okeechobee, and in the area west of Lake Okeechobee (Fisheating Creek). The original extent has been heavily reduced by clearing for agriculture and conversion for forage production. Intact examples have been further altered by fire suppression which changes the proportion of grasses and shrubs and may further alter species composition. Frequent fires were an important natural process in this system, with an estimated frequency of 1‑4 years.

**Comments:** This system grades into mesic pine flatwoods and may have nearly identical composition except for the absent or nearly absent overstory layer (Abrahamson and Hartnett 1990, FNAI 1990, Huffman and Judd 1998).

The Florida Gap program recognizes a single map unit which is apparently analogous to this type.

DISTRIBUTION

**Range:** This system occurs in southern Florida mainly north of the Everglades and Big Cypress area. For instance, it is found on the plains near the Myakka River, Kissimmee River, as well as north of Lake Okeechobee and near Fisheating Creek (west of Lake Okeechobee). This type was historically expansive in several regions of Florida (Harper 1927). For more detail, see map of historic extent in the report of the Florida Dry Prairie Conference (Bridges 2006).

**Divisions:** 203:C

**TNC Ecoregions:** 55:C

**Nations:** US

**Subnations:** FL

**Map Zones:** 56:C

**USFS Ecomap Regions:** 232Gc:CCC, 232Gd:CCC

CONCEPT

**Environment:** The climate where this ecological system occurs is subtropical, characterized by hot, wet summers and mild, dry winters. Average annual rainfall is about 127 cm and occurs mostly in June through September. It occurs on flat, moderately to poorly drained sandy soils with sandy surfaces overlying organic hardpans or clayey subsoil (FNAI 1990). These extensive flat prairies are seldom inundated but may flood with several centimeters of water in the wet summer. Frequent spring fires followed by summer flooding may have limited the survival of *Pinus elliottii var. densa* (Platt et al. 2006a). The normal water table is several centimeters (in summer and fall) to several meters (in winter and spring) below the ground surface (Duever and Brinson 1984a, Abrahamson and Hartnett 1990, Hardin 1990). Soils consist of 0.1‑0.9 m of undifferentiated quartz sand with a spodic horizon or clayey subsoil 30‑107 cm below the surface. These acidic, nutrient‑poor sands have few weatherable minerals and low clay nutrients in the surface soil (Abrahamson and Hartnett 1990). Soils supporting these sparse shrublands are classified as Arenic Haplaquods and include such series as Smyrna; types are Myakka (sandy, siliceous, hyperthermic Aeric Alaquod), Wabasso (sandy, siliceous, hyperthermic Alfic Alaquod), Oldsmar (sandy, siliceous, hyperthermic Alfic Arenic Alaquod), Immokalee (sandy, siliceous, hyperthermic Arenic Alaquod), Leon, Adamsville, and Keri sands (Moore and Swindel 1981, Duever and Brinson 1984a).

**Vegetation:** Intact examples of this system are generally open and essentially treeless areas dominated by *Serenoa repens* (which is often stunted) and low shrubs, including *Asimina reticulata, Bejaria racemosa, Gaylussacia dumosa, Hypericum tenuifolium (= Hypericum reductum), Ilex glabra, Lyonia fruticosa, Lyonia lucida, Morella cerifera (= Myrica cerifera var. pumila), Quercus minima, Vaccinium darrowii*, and *Vaccinium myrsinites* (Carr 2007). A variety of grasses are also present, including *Aristida beyrichiana, Schizachyrium scoparium var. stoloniferum, Sorghastrum secundum, Andropogon ternarius, Aristida spiciformis, Dichanthelium dichotomum var. ensifolium, Dichanthelium strigosum, Paspalum setaceum*, and others (Huffman and Judd 1998), as well as numerous forbs, including *Piloblephis rigida, Pityopsis graminifolia, Polygala* spp., *Rhexia* spp., and *Xyris* spp., (Orzell and Bridges 2006a, Carr 2007). At least 5 fairly discrete phases or "states" of this system can be identified (Huffman and Werner 2000): good conditions are typified by abundant herbaceous cover and relatively low (<40%) cover of shrubs, especially *Serenoa repens*; degraded conditions resulting from long fire‑free intervals result in reduced herbaceous cover and increased shrub coverage, to the eventual exclusion of all herbaceous cover.

**Dynamics:** Like the floristically and ecologically related pine flatwoods, the open structure and species composition of dry prairies is maintained by frequent fire. However, the natural fire frequency is thought to be greater than in the surrounding mesic pine flatwoods (Duever et al. 1982, Abrahamson and Hartnett 1990, Hardin 1990). Dry prairie is readily invaded by woody vegetation in the absence of fire, especially in the absence of fires which occur during the dry portions of early spring. In "good condition" this system has abundant herbaceous cover and relatively low cover (<40%) of *Serenoa repens*; degraded conditions are indicated by reduced herbaceous cover and increased cover of *Serenoa repens* (Huffman and Werner 2000). Outright replacement of dry prairies by oak ‑ palmetto stands has been well documented at Myakka River State Park (Huffman and Blanchard 1990). Some sources suggest that examples of this system may be the result of anthropogenic factors that provided an unnaturally high fire frequency or removed vegetation through logging or grazing (Hardin 1990). Bridges (2006) asserts that the system is a natural one and does not result from logging; it may be maintained by grazing, however.

SOURCES

**References:** Abrahamson and Hartnett 1990, Bridges 2006, Carr 2007, Comer et al. 2003*, Duever and Brinson 1984a, Duever et al. 1982, Eyre 1980, FNAI 2010a, FNAI 2010e, Hardin 1990, Harper 1927, Huffman and Blanchard 1990, Huffman and Judd 1998, Huffman and Werner 2000, Huffman pers. comm., LANDFIRE 2007a, Moore and Swindel 1981, Orzell and Bridges 2006a, Orzell and Bridges 2006b, Penfield 2006, Platt et al. 2006a, Watts et al. 2006

**Version:** 03 Jul 2014	**Stakeholders:** Southeast

**Concept Author:** R. Evans	**LeadResp:** Southeast

CES203.057 Florida Peninsula Inland Scrub

**Primary Division:** Gulf and Atlantic Coastal Plain (203)

**Land Cover Class:** Shrubland

**Spatial Scale & Pattern:** Large patch

**Required Classifiers:** Natural/Semi‑natural; Vegetated (>10% vasc.); Upland

**Diagnostic Classifiers:** Forest and Woodland (Treed); Xeric; F‑Patch/High Intensity; Needle‑Leaved Tree

**National Mapping Codes:** EVT 2387; ESLF 5318; ESP 1387

**Concept Summary:** This system appears in many forms, but generally consists of xeromorphic shrub vegetation (mostly evergreen oak species) with or without an emergent overstory of *Pinus clausa*. The shrubs can be very thick in places, but usually there are open patches. Ground cover is always sparse, and bare soil patches are typically evident. It is found on a sequence of sand ridges and ancient dune fields which are oriented essentially north‑south in the Florida Peninsula. The appearance, floristics, and boundary of Florida scrub may contrast dramatically with the "high pine" or sandhill vegetation which is often adjacent, although lack of fire can blur these boundaries.

DISTRIBUTION

**Range:** This system is endemic to the Florida Peninsula. It is most common in two discrete islands or patches, the Big Scrub of Ocala and the Lake Wales Ridge, which is now highly fragmented and mostly lost to agriculture and development (Weekley et al. 2008).

**Divisions:** 203:C

**TNC Ecoregions:** 55:C

**Nations:** US

**Subnations:** FL

**Map Zones:** 55:C, 56:C

**USFS Ecomap Regions:** 232D:CC, 232G:CC, 232K:CC

CONCEPT

**Environment:** This system is restricted to a sequence of north/south‑trending sand ridges, ancient dune fields, and former shorelines in the Florida peninsula. The largest inland scrub is found in two primary areas, essentially isolated from one another. The so called "Big Scrub" of the Ocala National Forest is the largest expanse of this system, with a somewhat smaller, more southerly area associated with the Lake Wales Ridge. According to Myers (1990), inland scrub occurs on Quartzipsamments which are excessively well‑drained, nearly pure siliceous sands low in nutrients. Although all scrub soils are Entisols, there is considerable variation in soil color. This color variation appears to be related to the amount of leaching which has taken place, and appears to be related to the amount of time a site has been occupied by scrub vegetation. Excessive leaching, due to inferred long occupation by scrub vegetation, is believed to bleach upper soil horizons and develop pure white soils (such as the St. Lucie series), while moderate leaching, due to shorter occupation by scrub, contributes to less bleaching and consequently more yellow‑colored soils (Paola and Orsino series).

**Vegetation:** This system is dominated by xeromorphic, evergreen shrub species with or without an emergent layer of *Pinus clausa*. The shrub layer composition is relatively constant, as is the abundance of individual species. *Quercus myrtifolia, Quercus inopina, Serenoa repens, Quercus geminata, Quercus chapmanii, Lyonia ferruginea*, and *Ceratiola ericoides* are the most important species. Myers (1990) indicates that much of the variability in Florida scrub is due to variation in fire‑return interval, ranging from once every 10 to 100 years. Ground cover is always sparse but typically includes *Licania michauxii, Rhynchospora megalocarpa, Andropogon floridanus*, and a variety of lichens (*Cladonia* species). There are a number of endemic plant species which may occur in inland Florida scrubs, including at least 13 federally listed species; many of the rarest scrub species are found only in the Lake Wales region.

This system has long been noted for its unique and interesting vegetation by authors such as Vignoles (1823), Harper (1914), Mulvania (1931), Kurz (1942), and Laessle (1958, 1968). More recent treatments by Myers (1990) and Menges (1999) have provided the most comprehensive summaries of scrub available. According to Harper (1927), "the nearly pure white sand of the ground surface, when viewed from a short distance, gives the impression of a thin rift of wind‑driven snow. The vegetation is mostly dwarfed, gnarled and crooked, and presents a tangled, scraggly aspect." The appearance, floristics, and boundary of Florida scrub contrast dramatically with the "high pine" or sandhill vegetation which is often adjacent (Laessle 1968), although lack of fire can blur these boundaries. Scrub generally consists of xeromorphic shrub vegetation, such as evergreen *Ceratiola ericoides, Lyonia* spp., *Quercus* spp., *Sabal etonia, Sabal palmetto*, and *Serenoa repens*, with or without an emergent overstory of *Pinus clausa*.

**Dynamics:** Florida scrub is a pyrogenic system with floral and faunal components adapted to fire. Unlike most ecological systems of the Gulf and Atlantic coastal plains, this system is maintained by high‑intensity, infrequent fires. Litter‑fall rates are high, while turnover rates are low, contributing to fuel buildup (Lugo and Zucca 1983, Schmalzer and Hinkle 1996). However, scrub typically lacks fine‑textured fuels necessary to ignite fires; most scrub fires ignite in other adjacent ecological systems. If fire spreads into scrub it is often under severe conditions of high wind, low humidity, and low fuel moisture. When fires occur in scrub they can be stand‑replacing events. *Pinus clausa*, if present, is killed outright but may regenerate from seed released from serotinous cones. In parts of fires that burn completely, the shrub layer is typically killed back to ground layer but rapidly resprouts and returns to prefire levels of cover (Abrahamson 1984, Schmalzer and Hinkle 1992b). Other species such as *Ceratiola ericoides* may regenerate from seeds stored in soil (Johnson 1982). *Eryngium cuneifolium* and *Dicerandra christmanii* are narrowly endemic herb species which exhibit peaks in survival, recruitment, and density after fire (Menges 1999, Menges et al. 1999, Menges and Quintana‑Ascencio 2004). Many scrub fires burn heterogeneously with resulting patches of unburned fuels. This gap dynamics can be significant (Weekley and Menges 2003), especially in the most xeric types like rosemary scrub (Menges 1994). In the sustained absence of fire, smaller shrubs and herbs may be lost as a consequence of increasing dominance of oak stems (Menges et al. 1993).

This system has likely persisted on fossil dunes since the Pleistocene (Laessle 1968), but remaining examples are merely remnants of an ecosystem once expansive in the late Pleistocene (Myers 1990). The stature and appearance of Florida scrub may be due primarily to nutrient‑poor soils, to which many of the scrub species have adapted evergreen habits (Monk 1966). Drought stress is most likely during winter and early spring, but frequent fog during these periods may ameliorate such conditions (Menges 1994). Surprisingly, given the excessively well‑drained soils, drought stress may not be an important ecological factor except to limit seedling establishment (Myers 1987, 1990).

SOURCES

**References:** Abrahamson 1984, Breininger et al. 1996, Comer et al. 2003*, Eyre 1980, FNAI 2010a, Harper 1914, Harper 1927, Hokit et al. 1999, Johnson 1982, Kurz 1942, Laessle 1958, Laessle 1968, Lugo and Zucca 1983, MacAllister and Harper 1998, Menges 1994, Menges 1999, Menges 2007, Menges and Gordon 2010, Menges and Quintana‑Ascencio 2004, Menges et al. 1993, Menges et al. 1999, Monk 1966, Mulvania 1931, Myers 1987, Myers 1990a, Schmalzer and Hinkle 1992b, Schmalzer and Hinkle 1996, Vignoles 1823, Weekley and Menges 2003, Weekley et al. 2008

**Version:** 14 Jan 2014	**Stakeholders:** Southeast

**Concept Author:** R. Evans	**LeadResp:** Southeast

M309. Southeastern Coastal Plain Patch Prairie

CES203.478 Southern Coastal Plain Blackland Prairie and Woodland

**Primary Division:** Gulf and Atlantic Coastal Plain (203)

**Land Cover Class:** Herbaceous

**Spatial Scale & Pattern:** Large patch

**Required Classifiers:** Natural/Semi‑natural; Vegetated (>10% vasc.); Upland

**Diagnostic Classifiers:** Circumneutral Soil; Deep Soil; Clay Soil Texture; Graminoid

**National Mapping Codes:** EVT 2430; ESLF 7143; ESP 1430

**Concept Summary:** This system includes natural grassland vegetation and associated wooded vegetation found primarily in two relatively small natural regions in the southeastern Coastal Plains, primarily in Alabama and Mississippi (with one of these extending barely into southern Tennessee), and a related area of southern Georgia. The larger of these, the so‑called Black Belt, is approximately 480 km (300 miles) long and 40‑50 km (25‑30 miles) wide, and is delineated as the Black Belt Subsection 231Ba and the Blackland Prairie EPA Ecoregion 65a. The smaller and more southerly one of the two is known as the Jackson Prairie region, is found on younger geologic strata and is delineated as the Jackson Hills Subsection (231Bj) and as the Jackson Prairie EPA Ecoregion (65r). The vegetation of this system is comproed of natural grasslands and associated wooded vegetation (woodlands and savannas). The Black Belt region derives its name from the nearly black, rich topsoil that developed over Selma Chalk, and has long been noted as a distinct topographic region in the state of Mississippi. In Alabama, the formations on which this system primarily occurs are Demopolis Chalk and Mooreville Chalk (members of the Selma Group). In Tennessee, only Demopolis Chalk is mapped. Examples occur over relatively deep soils (as opposed to "glades and barrens" on or adjacent to rock outcrops), with circumneutral surface soil pH. Vegetation of this ecological system includes evergreen *Juniperus virginiana*‑dominated forests and deciduous *Quercus*‑dominated woodlands of varying densities, interspersed with native prairielike grasslands. Much of the natural vegetation of the region has been converted to pasture and agricultural uses, but even old‑field vegetation reflects the distinctive composition of the flora and ecological dynamics. In most cases individual prairie openings are small and isolated from one another, but were formerly more extensive prior to European settlement, forming a mosaic of grasslands and woodlands under frequent fire regimes. The flora has much in common with other prairies of the East Gulf Coastal Plains, as well as the classic Midwestern prairies. Within this natural region, there are pockets of acidic soils which produce more typical pine‑oak woodland or forest vegetation. The Jackson Prairie component of the system includes natural grassland vegetation and associated wooded vegetation in the Jackson Hills Subsection (231Bj), also called the Jackson Prairie EPA Ecoregion (65r), a relatively small natural region of Mississippi and adjacent Alabama. This system occurs on montmorillonitic Vertisols, which are deep, slowly permeable soils formed in residuum weathered from marl or chalk. Examples occur in a larger matrix of primarily acidic soils and of generally *Pinus taeda*‑dominated forest vegetation. In most cases individual prairie openings are small and isolated from one another but were formerly more extensive prior to European settlement, forming a mosaic of grassland and woodland under frequent fire regimes. Much of the natural vegetation of the region has been converted to pasture and agricultural uses, with concomitant destruction of most prairie remnants.

**Comments:** "Blackland Prairies" occur primarily in two discrete areas of the East Gulf Coastal Plain: Jackson Prairie and the Black Belt. There is also an area in the Atlantic Coastal Plain of Georgia which is included here. It is a "blackland" but not a "black belt" prairie. Much of the natural vegetation of the region has been converted to pasture and agricultural uses, with concomitant destruction of most prairie remnants (DeSelm and Murdock 1993). Of the approximately 100,000 acres of Blackland Prairies mapped during the general land surveys of the early and mid 1800s in Mississippi, probably less than 500 acres of Jackson Prairie vegetation exists today, even if one considers grazed areas and vacant agricultural lands with a semblance of prairie species (R. Wieland pers. comm.). Almost all of the lands were converted to agriculture. Some of the lands are now reverting back to prairie after being abandoned. More recently, lands are being converted to fescue pasture; other abandoned lands have become stands of eastern red‑cedar. The number of acres in good condition is probably less than 100. The flora has much in common with other prairies of the East Gulf Coastal Plains as well as the classic Midwestern prairies.

DISTRIBUTION

**Range:** This system has several distinct components. The Black Belt Prairie component is primarily restricted to the Black Belt (Subsection 231Ba of Keys et al. 1995) or Blackland Prairie area (EPA Ecoregion 65a) and Flatwoods/Blackland Prairie Margins area (EPA Ecoregion 65b) of Griffith et al. (2001). This region is primarily in Alabama and Mississippi, ranging north in a depauperate form to southern Tennessee (McNairy County) (DeSelm 1989b). The Jackson Prairie component of this system is found in a relatively small natural region of Mississippi, known as the Jackson Hills Subsection 231Bj of Keys et al. (1995) and the Jackson Prairie Ecoregion 65r of EPA (EPA 2004). There is also a recently recognized component found in limited parts of Georgia (e.g., on both sides of the Ocmulgee River on the Fort Valley Plateau of Bleckley, Houston, Peach, and Twiggs counties). There are also outlying occurrences southward in the Chunnenuggee Hills and Red Hills (both of these parts of the Southern Hilly Coastal Plain ‑EPA Ecoregion 65d), and Buhrstone/Lime Hills (EPA Ecoregion 65q) of southern Alabama (in Washington, Wilcox, Monroe, and Clark counties). There are some limited examples in EPA Ecoregion 65i (Fall Line Hills; e.g., Jones Bluff in Alabama).

**Divisions:** 203:C

**TNC Ecoregions:** 43:C, 53:?, 56:C

**Nations:** US

**Subnations:** AL, GA, MS, TN

**Map Zones:** 46:C, 55:C

**USFS Ecomap Regions:** 231B:CC, 232B:CC

CONCEPT

**Environment:** The Black Belt component of this system generally occurs on Cretaceous age chalk, marl and calcareous clay. This includes calcareous soils of the Sumter, Binnsville, and Demopolis series, described as beds of marly clay over Selma Chalk (including the Demopolis and Mooreville formations). The area has an average annual precipitation of 130‑140 cm and a frost‑free period of 200‑250 days. The soils of the Jackson Prairie openings are presently mapped as the Maytag Series, a fine montmorillonitic, thermic Entic Chromudert. This deep slowly permeable soil has formed in residuum weathered from marl of chalk of the Blackland Prairies (Wieland 1995). Examples occur in a larger matrix of primarily acidic soils and of generally Pinus taeda‑dominated forest vegetation (Jones 1971).

**Vegetation:** Vegetation of this ecological system includes evergreen *Juniperus virginiana*‑dominated forests and deciduous *Quercus*‑dominated woodlands of varying densities, interspersed with native prairie‑like grasslands. Much of the natural vegetation of the region has been converted to pasture and agricultural uses, but even old‑field vegetation reflects the distinctive composition of the flora and ecological dynamics. The oak woodlands of the Black Belt component typically contain *Quercus stellata, Quercus muehlenbergii*, and *Quercus marilandica*. Other woody components include *Quercus falcata, Carya tomentosa (= Carya alba), Carya glabra, Fraxinus americana, Celtis laevigata, Cercis canadensis var. canadensis, Crataegus engelmannii, Diospyros virginiana, Ilex decidua, Prunus angustifolia, Frangula caroliniana, Sideroxylon lycioides*, and *Ulmus alata*. Prairie forbs and grasses may persist in small openings and in edge situations in the more heavily forested areas of the Black Belt. The presence of *Juniperus virginiana*‑dominated zones may represent invasion by this species in the absence of sufficiently frequent or intense fire (DeSelm and Murdock 1993). Pines are generally absent, being inhibited by the higher surface soil pH. In the grass‑dominated areas of the Black Belt, *Schizachyrium scoparium* and *Sorghastrum nutans* are the principal herbs. Other herbaceous taxa include *Andropogon glomeratus, Andropogon virginicus, Bouteloua curtipendula, Panicum virgatum*, and *Schizachyrium scoparium*, with lesser amounts of *Paspalum floridanum, Setaria parviflora*, and *Sporobolus indicus* (exotic). Other common species include *Arnoglossum plantagineum, Symphyotrichum dumosum (= Aster dumosus), Symphyotrichum patens (= Aster patens), Crotalaria sagittalis, Dalea candida, Dalea purpurea, Desmanthus illinoensis, Desmodium ciliare, Dracopis amplexicaulis, Liatris aspera, Liatris squarrosa, Liatris squarrulosa, Neptunia lutea, Ratibida pinnata, Ruellia humilis, Silphium terebinthinaceum, Silphium trifoliatum var. latifolium*, and *Solidago nemoralis*. In depressions and drainages, *Andropogon gerardii* and/or *Panicum virgatum* will have greater importance (DeSelm and Murdock 1993). At this more mesic end of the continuum, invasion by woody plants is a more serious threat to the system. Moist, seepy inclusions within this system are often dominated by *Rhynchospora colorata* and *Scleria verticillata*; *Rhynchospora divergens, Lythrum alatum var. lanceolatum, Mitreola petiolata*, and *Mecardonia acuminata* also occur but much less frequently (A. Schotz pers. comm.). The most prominent tall grasses of the Jackson Prairie component are *Andropogon gerardii, Schizachyrium scoparium, Sorghastrum nutans*, and *Panicum virgatum*. Additional tall grasses include *Tripsacum dactyloides, Andropogon glomeratus*, and *Paspalum floridanum*. Along with *Schizachyrium scoparium*, two other species provide over 50% cover in prairie openings: *Carex cherokeensis* and *Helenium autumnale*. Other plants closely affiliated with less disturbed prairie openings include *Dalea purpurea, Dalea candida, Sporobolus compositus var. macer, Muhlenbergia capillaris, Penstemon laxiflorus (= Penstemon australis ssp. laxiflorus), Symphyotrichum novae‑angliae (= Aster novae‑angliae), Echinacea purpurea, Manfreda virginica, Ruellia purshiana, Desmanthus illinoensis*, and *Spiranthes magnicamporum* (Wieland 1995).

**Dynamics:** In the presettlement landscape and throughout the nineteenth century, a combination of fire and grazing (first by native ungulates and then by free‑ranging cattle) kept these sites open and grass‑dominated (DeSelm and Murdock 1993).

<br />Blackland prairie and woodland occurs on eponymous rich, black, circumneutral topsoils formed over clayey, heavy, usually calcareous subsoils with carbonatic or montmorillonitic mineralogy. The system occurs in association with formations of the Tertiary Jackson (Yazoo Clay), Claiborne (Cook Mountain) and Fleming groups, and the Cretaceous Selma group (Selma, Mooreville or Demopolis chalks). The matrix around the blackland prairies is pine‑oak forests growing in acidic, sandier soils with less clay (recent STATSGO soils maps).

Floristic similarity among sites across this geographic range generally appears to be 50% or greater, although a number of different alliances within this type have been recognized according to dominant, codominant, and diagnostic species. Extant prairies occur in single patches as well as mosaics less than one acre to over several hundred acres in response to soil depth, slope and fire. Mosaics may include virtually treeless patches associated with other patches of widely scattered trees, open deciduous woodlands and evergreen thickets (eastern red‑cedar "balds"). This vegetation is a mosaic of *Juniperus virginiana* woodland, *Quercus stellata ‑ Quercus marilandica* woodland, and *Schizachyrium scoparium ‑ Sorghastrum nutans* herbaceous alliances. It is a rare and imperiled vegetation type consisting of scattered remnants. Most of the original cover has been destroyed or altered by conversion to agriculture and the exclusion of fire (Landfire 2007a).

For the last 500‑1000 years, fires were probably annual in most of the system, many if not most set by Native Americans. Fires were probably used to clear prairies for agricultural planting, to eliminate woody growth, and to aid in hunting. The modern landscape shows a tendency toward erosion, creating shallow‑soil areas known as "cedar balds" where soil erosion, presumably from historic agriculture or overgrazing, has reduced topsoil. These areas often show exposures of underlying chalk. Such areas may have resulted (albeit at much lower frequencies) from aboriginal agriculture or overgrazing by native herbivores (Landfire 2007a).

SOURCES

**References:** Comer et al. 2003*, DeSelm 1989b, DeSelm and Murdock 1993, Duffey et al. 1974, EPA 2004, Estes et al. 1979, Eyre 1980, Griffith et al. 2001, Hardeman 1966, Jones 1971, Keys et al. 1995, LANDFIRE 2007a, Lowe 1921, McKinney and Lockwood 1999, Murdock pers. comm., Noss 2013, Quarterman et al. 1993, Schotz and Barbour 2009, TNC 1996c, Taft 1997a, Taft 2009, Taft et al. 1995, Wieland 1995, Wieland pers. comm., Wiens and Dyer 1975

**Version:** 14 Jan 2014	**Stakeholders:** Southeast

**Concept Author:** A. Schotz, R. Evans, M. Pyne, R. Wieland	**LeadResp:** Southeast

CES203.377 West Gulf Coastal Plain Northern Calcareous Prairie

**Primary Division:** Gulf and Atlantic Coastal Plain (203)

**Land Cover Class:** Herbaceous

**Spatial Scale & Pattern:** Large patch

**Required Classifiers:** Natural/Semi‑natural; Vegetated (>10% vasc.); Upland

**Diagnostic Classifiers:** Herbaceous; Circumneutral Soil; Deep Soil; Graminoid; West Gulf Coastal Plain

**National Mapping Codes:** EVT 2428; ESLF 7141; ESP 1428

**Concept Summary:** This is one of two described calcareous prairie ecological systems which occur within the pine‑dominated portions of the Coastal Plain west of the Mississippi River. This type is the more northerly ranging of the two [compare against West Gulf Coastal Plain Southern Calcareous Prairie (CES203.379)]. This system includes natural grassland vegetation and associated wooded vegetation in a relatively small natural region of the Upper West Gulf Coastal Plain of Arkansas and adjacent Oklahoma. Although other calcareous prairies are found west of the Mississippi River, this system represents some of the largest known and highest quality remaining examples. Plant communities in this system occur over relatively deep soils (as well as shallow soils over chalk and limestone) with circumneutral surface soil pH, which is unusual given the predominance of acidic, generally forested soils in the region. In most cases individual prairie openings are small and isolated from one another, but were formerly more extensive prior to European settlement, forming a mosaic of grassland and woodlands under frequent fire regimes. The flora has much in common with other prairie systems of the East Gulf Coastal Plains as well as classic Midwestern prairies.

DISTRIBUTION

**Range:** This system is known only from a relatively small natural region of the Upper West Gulf Coastal Plain of Arkansas and adjacent Oklahoma.

**Divisions:** 203:C

**TNC Ecoregions:** 40:C

**Nations:** US

**Subnations:** AR, OK

**Map Zones:** 37:?, 44:C

**USFS Ecomap Regions:** 232E:CC

CONCEPT

**Environment:** This system is characterized by deep to shallow soils with circumneutral surface soil pH that have developed over Cretaceous‑aged calcareous substrates. Soils vary from well‑drained to poorly drained clays, silty clays, silty clay loams, and fine sandy loams, and are typically excessively dry in summer exhibiting high shrink‑swell potential. Within this general landscape, fine‑scale abiotic characteristics in conjunction with ecological processes, frequent fire in particular, supported a mosaic of grasslands and short‑statured woodlands comprising the ecological system.

**Vegetation:** Dominant plants in stands of this system vary from example to example; there are several subtypes and associations with variability among these. Typical trees include *Quercus stellata, Quercus muehlenbergii, Quercus shumardii, Quercus pagoda, Quercus sinuata, Carya illinoinensis, Carya myristiciformis, Juniperus virginiana var. virginiana*, and *Maclura pomifera*. Some typical shrubs include *Forestiera ligustrina, Symphoricarpos orbiculatus, Ilex decidua*, and *Rhus aromatica*. Herbs may include *Sorghastrum nutans, Bouteloua curtipendula, Andropogon glomeratus, Leersia virginica, Panicum anceps, Panicum flexile, Sporobolus compositus, Fimbristylis puberula var. puberula, Carex cherokeensis, Carex microdonta, Echinacea pallida, Liatris aspera, Marshallia caespitosa, Silphium integrifolium, Silphium laciniatum, Solidago auriculata, Symphyotrichum lanceolatum, Packera tampicana, Thelesperma filifolium, Nemastylis geminiflora, Dalea purpurea, Lythrum alatum, Allium canadense var. mobilense*, and *Zigadenus nuttallii*.

**Dynamics:** The composition and structure of this grassland and open woodland ecological system are primarily maintained by edaphic conditions, fire, and climate. Fires less than every four or so years are necessary to maintain the grassland and open woodland states. Under normal weather conditions, eight to ten years without fire will result in a shrub‑dominated physiognomy. Continued fire suppression under normal climate conditions will result in a closed‑canopy condition. Tight soils provide a barrier to root penetration and limit water availability during dry periods, thereby also inhibiting the establishment and growth of woody plants, but soils alone cannot limit woody growth. Historically, native grazers or browsers also played a role in the maintenance of this system.

SOURCES

**References:** Comer et al. 2003*, Eyre 1980, LANDFIRE 2007a, Zollner pers. comm.

**Version:** 14 Jan 2014	**Stakeholders:** Southeast

**Concept Author:** T. Foti and R. Evans	**LeadResp:** Southeast

CES203.379 West Gulf Coastal Plain Southern Calcareous Prairie

**Primary Division:** Gulf and Atlantic Coastal Plain (203)

**Land Cover Class:** Herbaceous

**Spatial Scale & Pattern:** Small patch

**Required Classifiers:** Natural/Semi‑natural; Vegetated (>10% vasc.); Upland; Wetland

**Diagnostic Classifiers:** Herbaceous; Circumneutral Soil; Deep Soil; Graminoid; West Gulf Coastal Plain

**National Mapping Codes:** EVT 2429; ESLF 7142; ESP 1429

**Concept Summary:** This is one of two described calcareous prairie ecological systems which occur within the pine‑dominated portions of the West Gulf Coastal Plain west of the Mississippi River. This type is the more southerly‑ranging of the two [compare against West Gulf Coastal Plain Northern Calcareous Prairie (CES203.377)]. Examples include natural grassland vegetation and adjacent wooded vegetation in a relatively small natural region of Arkansas, Louisiana and Texas. Although most examples are typically upland, some include small stream bottoms or riparian areas that bisect the prairies. Plant communities in this system occur over relatively deep soils that are unusual in the local landscape because they are much less acidic than the soils of the surrounding forests. Stands are dominated by perennial grasses and graminoids, including *Carex cherokeensis, Carex microdonta, Muhlenbergia expansa, Schizachyrium scoparium, Schizachyrium tenerum, Sorghastrum nutans*, and *Sporobolus silveanus*. Historically, this system is thought to have occupied large patches (up to a couple thousand acres), but currently, most individual prairie openings are small and isolated from one another.

DISTRIBUTION

**Range:** This system is restricted to a relatively small natural region of Arkansas, Louisiana and Texas.

**Divisions:** 203:C

**TNC Ecoregions:** 40:C, 41:C

**Nations:** US

**Subnations:** AR, LA, TX

**Map Zones:** 37:C

**USFS Ecomap Regions:** 231E:CC, 232F:CC

CONCEPT

**Environment:** This system is best documented from the Fleming geologic formation, but is also known from the Cook Mountain Formation in Louisiana. Examples from the Jackson Group (in Louisiana) are also included here, as well as the Morse Clay Calcareous Prairie of northwestern Louisiana and adjacent Arkansas. It occupies deep vertic soils with circumneutral surface pH, a condition uncommon in a region of predominantly acidic, forested soils. It typically occurs on upper slopes and broad uplands in gently undulating landscapes. Soils are circumneutral to moderately alkaline, including vertic soils such as Ferris, Houston Black, or Wiergate clays (Elliott 2011). Occurrences may reflect a relationship to the Blackland Prairie further to the west (including the Fayette Prairie), and some consider these small‑patch prairies to be outliers of the Blackland Tallgrass Prairie. In Arkansas, it also occurs on the Gore silt loam and McKamie silt loam, as well as the Morse clay (Foti 1987). Within this general landscape, fine scale abiotic characteristics in conjunction with ecological processes, frequent fire in particular, supported a mosaic of grasslands and short stature woodlands comprising the ecological system. Prior to European settlement this system is believed to have occupied patches up to a couple thousand acres.

**Vegetation:** The flora has much in common with that of other prairie systems of the East Gulf Coastal Plain as well as that of classic Midwestern prairies, although there is variability among examples. Stands are dominated by perennial grasses and graminoids, including *Schizachyrium scoparium, Schizachyrium tenerum, Muhlenbergia expansa, Sorghastrum nutans, Bothriochloa laguroides ssp. torreyana, Bouteloua curtipendula, Andropogon gerardii, Nassella leucotricha, Paspalum pubiflorum, Panicum virgatum, Carex cherokeensis*, and *Carex microdonta*. Much of the typical flora are species uncommon in the rest of the Pineywoods region, such as *Dalea compacta var. compacta, Rudbeckia hirta, Rudbeckia missouriensis, Acacia angustissima, Croton monanthogynus, Liatris punctata var. mucronata (= Liatris mucronata), Eustoma exaltatum ssp. russellianum (= Eustoma russellianum), Grindelia lanceolata, Agalinis heterophylla, Stenosiphon linifolius, Neptunia lutea, Indigofera miniata (= Indigofera miniata var. leptosepala), Palafoxia reverchonii, Indigofera miniata, Arnoglossum plantagineum, Coreopsis tinctoria, Symphyotrichum* spp., *Onosmodium bejariense var. occidentale (= Onosmodium occidentale)*, and *Euphorbia bicolor*. Some of these species are found either most commonly or exclusively in the more western examples of this system when compared with those in Louisiana. Arkansas examples may contain *Mimosa nuttallii (= Mimosa quadrivalvis var. nuttallii), Dalea purpurea, Neptunia lutea, Baptisia nuttalliana, Arnoglossum plantagineum, Eryngium yuccifolium, Manfreda virginica, Silphium laciniatum, Nemastylis geminiflora*, and other prairie species (Foti 1987). Various woody species from the surrounding landscape, including *Pinus taeda, Ulmus alata, Liquidambar styraciflua, Juniperus virginiana, Crataegus spathulata, Crataegus crus‑galli, Sideroxylon lanuginosum*, and others, may invade these prairies. Non‑native woody species such as *Rosa bracteata* may also invade, this presumably being a result of long‑term fire suppression (Elliott 2011). Non‑native grasses such as *Bothriochloa ischaemum var. songarica, Bromus arvensis, Cynodon dactylon*, and/or *Lolium perenne* may be conspicuous to dominant (Elliott 2011).

**Dynamics:** The composition and structure of this grassland and open woodland ecological system are primarily maintained by edaphic conditions, fire, and climate. Examples historically formed a mosaic of grassland and open woodlands under frequent fire regimes. With fire suppression, trees invade from surrounding pine forests. As a result, some evidence suggests that soil properties are modified, especially the surface pH and nutrient dynamics. Fires every four or so years are necessary to maintain the grassland and open woodland states. Under normal weather conditions, 15 to 20 years without fire will result in a shrub‑dominated physiognomy. Continued fire suppression under normal climate conditions will result in a closed‑canopy condition. Tight soils provide a barrier to root penetration and limit water availability during dry periods, thereby also inhibiting the establishment and growth of woody plants.

SOURCES

**References:** Comer et al. 2003*, Elliott 2011, Eyre 1980, Foti 1987, LANDFIRE 2007a, LDWF 2005, LNHP 2009, Newton 1972, Smith 1993, Smith, L. pers. comm.

**Version:** 14 Jan 2014	**Stakeholders:** Southeast

**Concept Author:** R. Evans and T. Foti	**LeadResp:** Southeast

M308. Southern Barrens & Glade

CES203.364 West Gulf Coastal Plain Catahoula Barrens

**Primary Division:** Gulf and Atlantic Coastal Plain (203)

**Land Cover Class:** Steppe/Savanna

**Spatial Scale & Pattern:** Large patch

**Required Classifiers:** Natural/Semi‑natural; Vegetated (>10% vasc.); Upland

**Diagnostic Classifiers:** Woody‑Herbaceous; Rock Outcrops/Barrens/Glades; Very Shallow Soil

**National Mapping Codes:** EVT 2403; ESLF 5419; ESP 1403

**Concept Summary:** This system is confined to the Catahoula geologic formation of eastern Texas and western Louisiana. It includes a vegetational mosaic ranging from herbaceous‑dominated areas on shallow soil and exposed sandstone to deeper soils with open woodland vegetation. Woodlands include a post oak‑dominated overstory grading into longleaf pine‑dominated areas. Seasonal droughtiness, shallow soils, aluminum toxicity, and periodic fires are important factors that influence the composition and structure of this system. Vegetation associated with thin soils over the tuffaceous sandstone of the Catahoula Formation is primarily herbaceous. But where the soil is deeper, or fire is excluded for long periods, it can display significant woody cover, with usually stunted representatives of species such as *Pinus palustris, Pinus taeda, Pinus echinata, Quercus stellata, Quercus marilandica*, and *Carya texana* dominating the canopy. Shrubs may form a patchy, discontinuous layer. Open sites may have significant herbaceous cover, usually dominated by graminoid species.

**Comments:** The western boundary of this system is unclear. The Catahoula Formation extends into the Crosstimbers region as well as the Pineywoods, but it is not clear whether these areas should be considered the same system.

DISTRIBUTION

**Range:** This system is endemic to areas where sandstones of the Catahoula Formation occur near and at the surface in western Louisiana and eastern Texas. Sandstone glades are estimated to have historically covered less than 2000 acres in Louisiana and today 50‑75% of that historic distribution is thought to remain (Smith 1993).

**Divisions:** 203:C

**TNC Ecoregions:** 40:C, 41:C

**Nations:** US

**Subnations:** LA, TX

**Map Zones:** 37:C

**USFS Ecomap Regions:** 231E:CC, 232F:CC

CONCEPT

**Environment:** The habitat of this system includes shallow soil and exposed sandstone, which tend to an herbaceous‑dominated vegetation expression, as well as zones of deeper soils with open woodland vegetation. Examples of this system are restricted to surface outcrops of the Oligocene Catahoula geologic formation, an often tuffaceous sandstone. Sites are generally level to gently undulating (but sometimes steep), with surface or near‑surface exposure of the underlying sandstone bedrock. Soils are shallow loams, such as Browndell‑Rock outcrop. Soils may contain montmorillonitic clays. These thin soils can be extremely xeric during dry periods, but can also be saturated during wetter months (Elliott 2011).

**Vegetation:** Vegetation associated with thin soils over the tuffaceous sandstone of the Catahoula Formation is primarily herbaceous. But where the soil is deeper, or fire is excluded for long periods, it can display significant woody cover, with usually stunted representatives of species such as *Pinus palustris, Pinus taeda, Pinus echinata, Quercus stellata, Quercus marilandica*, and *Carya texana* dominating the canopy. Shrubs may form a patchy, discontinuous layer with species such as *Ilex vomitoria, Morella cerifera, Vaccinium arboreum, Forestiera ligustrina, Gelsemium sempervirens*, and *Crataegus* spp. commonly encountered. Maintenance of fire in the landscape will reduce woody cover in these sites, with herbaceous‑dominated sites displaying increased species richness. On open sites, there may be exposed patches of bedrock or mineral soils, or areas of patchy cover of foliose and/or fruticose lichens. Open sites may have significant herbaceous cover, usually dominated by graminoid species such as *Schizachyrium scoparium, Sporobolus clandestinus, Sporobolus silveanus, Schizachyrium tenerum, Tridens strictus, Scleria* spp., and/or *Aristida* spp. Forbs, including *Bigelowia nuttallii, Plantago* spp., *Minuartia drummondii, Chaetopappa asteroides, Lechea san‑sabeana, Sabatia campestris, Croton michauxii, Croton monanthogynus, Krameria lanceolata, Selaginella arenicola ssp. riddellii, Phemeranthus parviflorus*, and a variety of other herbaceous species, may also be present. Lack of fire tends to lead to closing of the woody canopy and a reduction in diversity in the herbaceous layer. More wooded sites may have an herbaceous cover that contains species such as *Chasmanthium sessiliflorum, Ranunculus fascicularis*, and *Piptochaetium avenaceum* (Elliott 2011). Undisturbed examples are dominated by *Bigelowia nuttallii, Aristida longespica, Schizachyrium scoparium, Croton michauxii (= Crotonopsis linearis)*, and *Sporobolus silveanus* (Marietta and Nixon 1984). Woodlands include a *Quercus stellata*‑dominated overstory grading into *Pinus palustris*‑dominated areas.

**Dynamics:** Seasonal droughtiness, shallow soils, aluminum toxicity, and periodic fires are important factors that influence the maintenance of this system as one with primarily herbaceous composition and structure. This ecological system is maintained by a combination of edaphic factors and natural disturbances including severe drought and fire. The outcrops themselves are relatively extreme environments for plant growth due to mild alkalinity, exfoliation of rock surfaces, and surface moisture and temperature fluctuations. Severe droughts kill tree saplings growing in cracks and potholes, helping to retain the open character of the glades (Quarterman et al. 1993). There is an apparent zonation or patchiness to glade/barren vegetation, with different zones that may be identified by their characteristic plant species (Quarterman et al. 1993). These zones are apparently relatively stable, with woody plant encroachment evident only in relation to the invasion of shrubs and trees into potholes or crevices where soil accumulates more rapidly.

SOURCES

**References:** Bridges and Orzell 1989a, Comer et al. 2003*, Duffey et al. 1974, Elliott 2011, Estes et al. 1979, Eyre 1980, LDWF 2005, MacRoberts and MacRoberts 1993a, MacRoberts and MacRoberts 1993b, Marietta and Nixon 1984, McKinney and Lockwood 1999, Noss 2013, Quarterman et al. 1993, Smith 1993, Taft 1997a, Taft 2009, Taft et al. 1995

**Version:** 14 Jan 2014	**Stakeholders:** Southeast

**Concept Author:** R. Evans	**LeadResp:** Southeast

CES203.371 West Gulf Coastal Plain Nepheline Syenite Glade

**Primary Division:** Gulf and Atlantic Coastal Plain (203)

**Land Cover Class:** Steppe/Savanna

**Spatial Scale & Pattern:** Small patch

**Required Classifiers:** Natural/Semi‑natural; Vegetated (>10% vasc.); Upland

**Diagnostic Classifiers:** Woody‑Herbaceous; Rock Outcrops/Barrens/Glades; Very Shallow Soil

**National Mapping Codes:** EVT 2405; ESLF 5421; ESP 1405

**Concept Summary:** This glade system is present only in Saline and Pulaski counties, Arkansas, on distinctive, massive outcrops of igneous substrate ("nepheline syenite"). Some typical dominant grasses include *Aristida purpurascens, Piptochaetium avenaceum, Schizachyrium scoparium*, and *Sporobolus clandestinus*. Other herbs may include *Camassia scilloides, Clinopodium arkansanum, Delphinium carolinianum, Sabatia campestris*, and *Phemeranthus calycinus*. Lichens are common on the rocky substrate of some examples. Some examples will have open stands of *Quercus stellata*, but trees may be absent. Zonal vegetation communities are present around the outcrops. Interior herbaceous‑dominated zones can be mesic to wet as springs and small ephemeral streams flow across the rock outcrops and water pools in flat areas. Deeper, more heavily wooded vegetation develops along the flat or slightly sloping outcrop edges.

DISTRIBUTION

**Range:** This system is present only in the Upper West Gulf Coastal Plain of Saline and Pulaski counties, Arkansas. It may have existed historically in Garland and Hot Spring counties (and thereby at least partly in the Ouachita region). Less than 10 occurrences of this ecological system are known to persist.

**Divisions:** 203:C

**TNC Ecoregions:** 39:?, 40:C

**Nations:** US

**Subnations:** AR

**Map Zones:** 37:C, 44:?

**USFS Ecomap Regions:** 231E:CC

CONCEPT

**Environment:** This ecological system is found where the igneous rock nepheline syenite occurs at or near the surface in the Upper West Gulf Coastal Plain of Arkansas. This glade system is characterized by patches of bare rock interspersed with areas of shallow soil imbedded within a matrix of deeper soil supporting forested ecosystems. Slope varies from gentle to flat. Gently sloping areas are often extremely xeric whereas flatter areas can accumulate moisture, creating seasonally wet microhabitats. Exposed bedrock may have abundant lichen and moss cover and limited vascular plants. At the edges of the rock outcrops, areas with shallow soils support grasslands with scattered stunted trees. As soils become deeper, grasslands grade into open woodlands (Witsell 2007).

**Vegetation:** Some examples will have open stands of *Quercus stellata*, but trees may be absent. Some typical dominant grasses include *Aristida purpurascens, Piptochaetium avenaceum, Schizachyrium scoparium*, and *Sporobolus clandestinus*. Other herbs may include *Camassia scilloides, Clinopodium arkansanum, Delphinium carolinianum, Sabatia campestris*, and *Phemeranthus calycinus (= Talinum calycinum)*. Lichens are common on the rocky substrate of some examples.

**Dynamics:** This ecological system is maintained by a combination of edaphic factors and natural disturbances, including severe drought and fire (Witsell 2007). The outcrops themselves are relatively extreme environments for plant growth due to mild alkalinity, exfoliation of rock surfaces, and surface moisture and temperature fluctuations. Severe droughts kill tree saplings growing in cracks and potholes, helping to retain the open character of the glades (Quarterman et al. 1993). There is an apparent zonation or patchiness to glade/barren vegetation, with different zones that may be identified by their characteristic plant species (Quarterman et al. 1993). These zones are apparently relatively stable, with woody plant encroachment evident only in relation to the invasion of shrubs and trees into potholes or crevices where soil accumulates more rapidly.

SOURCES

**References:** Arkansas Geological Commission 2006, Comer et al. 2003*, Duffey et al. 1974, Estes et al. 1979, McKinney and Lockwood 1999, Noss 2013, Quarterman et al. 1993, Taft 1997a, Taft 2009, Taft et al. 1995, Witsell 2007

**Version:** 02 Jan 2015	**Stakeholders:** Southeast

**Concept Author:** R. Evans	**LeadResp:** Southeast

2.B.4. Temperate to Polar Scrub & Herb Coastal Vegetation

2.B.4.Na. Eastern North American Coastal Scrub & Herb Vegetation

M060. Eastern North American Coastal Beach & Rocky Shore

CES203.266 Florida Panhandle Beach Vegetation

**Primary Division:** Gulf and Atlantic Coastal Plain (203)

**Land Cover Class:** Barren

**Spatial Scale & Pattern:** Large patch

**Required Classifiers:** Natural/Semi‑natural; Unvegetated (<10% vasc.); Upland; Wetland

**Diagnostic Classifiers:** Coast; Beach (Substrate); Graminoid; East Gulf Coastal Plain

**Concept Summary:** The panhandle beach system ranges from northwestern Florida (Ochlockonee River) to southeastern Mississippi. It includes the outermost zone of coastal vegetation extending seaward from foredunes. Within the northern Gulf of Mexico, the natural boundaries of this system are fairly distinct; the western boundary is mineralogical and the eastern is defined by a region of sunken, flooded coast line where beaches are absent. In addition, these beaches are distinguished by high cover of *Uniola paniculata* and *Schizachyrium maritimum*, along with local endemic species of *Chrysoma* and *Paronychia*.

DISTRIBUTION

**Range:** Ranges from northwestern Florida (Ochlockonee River) to southeastern Mississippi.

**Divisions:** 203:C

**TNC Ecoregions:** 53:C

**Nations:** US

**Subnations:** AL, FL, MS

**Map Zones:** 55:C, 99:C

**USFS Ecomap Regions:** 232L:CC

CONCEPT

**Environment:** The beach includes the sand intertidal shore and the low‑gradient sand above the daily high tide line, which is between the foredune and the Gulf of Mexico. This area of upper beach is affected by wind and salt spray, seasonal high tides, and storm surge. These beaches are rich in pyroxene, epidote, and garnet (Barbour et al. 1987). Within the northern Gulf of Mexico, the sandy substrate of this system is uniquely rich in medium, nutritionally poor sands. Especially low concentrations of potassium may be of great importance to plant growth and species distributions (Barbour et al. 1987).

**Vegetation:** These beaches are distinguished by high cover of *Uniola paniculata* and *Schizachyrium maritimum*, along with local endemic species of *Chrysoma* and *Paronychia* (Barbour et al. 1987).

**Dynamics:** The natural coastal dynamics include the movement of sand from wind, tides, and storm surge. This includes transport of sand along the coast (primarily from east to west), and movement of sand by wind or water between the dunes, beach and subtidal areas, and the movement of sand from the foredunes to the interior. If not restricted by infrastructure or engineered hard structures, beaches and dunes can migrate as coastlines change over time in response to the action of wind and water. The Gulf of Mexico coast is affected by one tide per day. Wrack and seaweed deposited along the shore is an important source of nutrients for the coastal ecosystem, and helps promote revegetation in newly disturbed areas (Defeo et al. 2009).

SOURCES

**References:** Allen et al. 2001b, Barbour et al. 1987, Comer et al. 2003*, Davis et al. 2001, Defeo et al. 2009, FNAI 2010a

**Version:** 06 Feb 2014	**Stakeholders:** Southeast

**Concept Author:** R. Evans	**LeadResp:** Southeast

CES203.544 Gulf Coast Chenier Plain Beach

**Primary Division:** Gulf and Atlantic Coastal Plain (203)

**Land Cover Class:** Barren

**Spatial Scale & Pattern:** Linear

**Required Classifiers:** Natural/Semi‑natural; Unvegetated (<10% vasc.); Upland; Wetland

**Concept Summary:** This ecological system includes sparsely vegetated ocean beaches along the Gulf of Mexico from the mainland shores of the Chenier Plain of Louisiana and Texas north of Boliver peninsula. These beaches are generally eroding and narrow, and constitute the outermost zone of coastal vegetation in this area. Although these habitats are situated just above the mean high tide limit, they are constantly impacted by waves and flooded by storm surges. Dynamic disturbance regimes largely limit the vegetation to pioneering, salt‑tolerant, succulent annuals or perennial vines (e.g. *Ipomoea* spp.). These beaches are generally unstable and highly impacted by attempts to limit the natural erosional processes. Sediment is carried by westerly‑moving longshore currents from Louisiana to Texas, and these beaches have all been impacted by the reduction of sediment related to the altered deltaic processes of the Mississippi River.

**Comments:** This system is distinguished more by the ecological processes that helped develop and maintain it than by its floristics. Based on floristics, it might warrant merger with other beaches of the northern Gulf of Mexico.

DISTRIBUTION

**Range:** This system ranges from the mainland shores of the Chenier Plain of Louisiana and Texas.

**Divisions:** 203:C

**TNC Ecoregions:** 31:C

**Nations:** US

**Subnations:** LA, TX

**Map Zones:** 37:C

**USFS Ecomap Regions:** 232E:CC

CONCEPT

**Environment:** This ecological system includes the typically sparsely vegetated, sandy, back beach area of the mainland as it transitions into more stabilized communities. Examples are found on recent deposits of sand resulting from ongoing coastal sediment transport, as well as clays remaining on the Gulf margin after longshore transport of sand off of the sites. Sites are gently sloping towards the Gulf, with some development of foreshore dunes. This system occurs in the Chenier Plain region of Louisiana and Texas. Beaches along this part of the coast currently tend to be eroding and narrow, though historically these beaches were part of a system of alternating prograding barrier ridges and eroding tidal flats (Owen 2008). Soils are clays and sands. It is found on the narrow margin of mostly unvegetated sands receiving frequent inundation, erosion, or sediment deposition from eolian processes. The topography is low, and the substrate is dynamic, leading to reduced vegetation development. It is impacted by salt spray, tidal inundation, storm surge, and wind.

**Vegetation:** Dynamic disturbance regimes largely limit the vegetation to pioneering, salt‑tolerant, succulent annuals or perennial vines (e.g., *Ipomoea* spp.). Other species that may be present include *Cakile geniculata, Spartina patens, Distichlis spicata, Panicum amarum*, and *Hydrocotyle bonariensis*. Other species that may be encountered include *Sesuvium* spp., *Ipomoea pes‑caprae*, and *Ipomoea imperati*.

**Dynamics:** These beaches are generally eroding and narrow, and they are constantly impacted by waves and may be flooded by storm surges. They are generally unstable and highly impacted by attempts to limit the natural erosional processes. Sediment is carried by westerly‑moving longshore currents from Louisiana to Texas, and these beaches have all been impacted by the reduction of sediment related to the altered deltaic processes of the Mississippi River. Historically, the Chenier Plain coast was place where headland ridges were either prograding or eroding based the proximity of sediment deposited by the Mississippi River as it meandered across its delta. Today, since new sediments are limited because of the control of the Mississippi River, existing headlands that comprise this ecological system are eroding (Morton et al. 2004).

SOURCES

**References:** Comer et al. 2003*, Defeo et al. 2009, Elliott 2011, Morton et al. 2004, Owen 2008

**Version:** 14 Jan 2014	**Stakeholders:** Southeast

**Concept Author:** J. Teague	**LeadResp:** Southeast

CES201.586 Laurentian‑Acadian Lakeshore Beach

**Primary Division:** Laurentian‑Acadian (201)

**Land Cover Class:** Barren

**Spatial Scale & Pattern:** Small patch

**Required Classifiers:** Natural/Semi‑natural; Unvegetated (<10% vasc.); Upland; Wetland

**Diagnostic Classifiers:** Depressional; Flood Scouring; Broad‑Leaved Shrub; Dwarf‑Shrub; Graminoid; Short (<5 yrs) Flooding Interval [Short interval, Irregular Flooding]

**Concept Summary:** This system encompasses primarily upland vegetation along lakeshores or rivershores in northern New England, southeastern Canada, and the upper Midwest (not including the Great Lakes). Some areas may be briefly inundated during high water periods. The substrate is sandy to gravelly, sometimes consolidated rock; there may be muddy patches. Ice‑scour is not a major influence, although it may be locally important. These shores may be narrow zones of shrubs and/or sparse vegetation on rocks or sandy beaches. Descriptions of these beaches from Maine, New Hampshire, Vermont, and Minnesota suggests a variable structure and composition influenced by exposure, substrate, and how wet the substrate remains. The upper zone often features shrubs; these may include *Myrica gale, Gaylussacia baccata, Salix* spp., and *Aronia melanocarpa*. Creeping shrubs such as *Hudsonia* spp., *Juniperus horizontalis*, and *Prunus susquehanae* may be locally important. The herbaceous flora likewise varies; *Schizachyrium scoparium, Dichanthelium clandestinum, Cyperus* spp., *Dulichium arundinaceum*, and *Spartina pectinata* are representative graminoids; forbs may include *Argentina anserina, Lechea intermedia, Scutellaria lateriflora*, and *Mimulus ringens*, among others.

**Comments:** Very little data on these. May not be defensible as a separate system, keep in for now as a placeholder. If it is combined with the surrounding uplands, the associations tagged to this system may become orphans.

DISTRIBUTION

**Range:** This system ranges across northern New England and northern New York west across the upper Great Lakes to northern Minnesota, and adjacent Canada; and occasional southwards.

**Divisions:** 201:C

**TNC Ecoregions:** 47:C, 48:C, 60:C, 61:C, 63:C, 64:C

**Nations:** US

**Subnations:** MA, ME, MI, MN, NH, NY, RI, VT, WI

**Map Zones:** 41:C, 50:C, 51:C, 63:C, 64:C, 66:C

**USFS Ecomap Regions:** 211A:CP, 211B:CP, 211C:CP, 211D:CP, 211F:CC, 211Jc:CCC, 212Jb:CCC, 212Tb:CCC

CONCEPT

SOURCES

**References:** Comer et al. 2003*, Edinger et al. 2014a, Gawler and Cutko 2010, Kost et al. 2007, Sperduto and Nichols 2004, WDNR 2015

**Version:** 20 Aug 2015	**Stakeholders:** East, Midwest

**Concept Author:** S.C. Gawler	**LeadResp:** East

CES203.469 Louisiana Beach

**Primary Division:** Gulf and Atlantic Coastal Plain (203)

**Land Cover Class:** Barren

**Spatial Scale & Pattern:** Small patch

**Required Classifiers:** Natural/Semi‑natural; Unvegetated (<10% vasc.); Upland; Wetland

**Diagnostic Classifiers:** Coast; Beach (Substrate); Graminoid

**Concept Summary:** Louisiana beaches are predominantly found on remnant barrier islands associated with historic delta lobes of the Mississippi River. Since normal deltaic processes have been altered, the formation of new barrier islands has been halted and Louisiana barrier islands are undergoing deterioration. Within the northern Gulf region, these barrier islands are distinguished by dominance of *Spartina patens* instead of *Uniola paniculata*. Also characteristic are *Cenchrus spinifex* and *Sporobolus virginicus*.

**Comments:** This system is distinguished more by its dependence on the deltaic processes of the Mississippi River than by its floristics. Based on floristics, it might warrant merger with other beaches of the northern Gulf of Mexico.

DISTRIBUTION

**Range:** This system is found on remnant barrier islands associated with historic delta lobes of the Mississippi River. Very few examples remain intact.

**Divisions:** 203:C

**TNC Ecoregions:** 31:C

**Nations:** US

**Subnations:** LA

**Map Zones:** 98:C

CONCEPT

**Environment:** This ecological system includes the usually sparsely vegetated, sandy, back beach area in a microtidal environment (< 0.5m) as it transitions into more stabilized dune or barrier flat communities.

**Vegetation:** These beaches are distinguished by dominance of *Spartina patens*. Also characteristic are *Cenchrus spinifex (= Cenchrus incertus)* and *Sporobolus virginicus* (Barbour et al. 1987).

**Dynamics:** The primary processes controlling this system are the natural deltaic process of the Mississippi River. The process of sand movement due to the forces of wind and water are part of its the natural dynamics. This includes transport of sand along the coast, and movement of sand by wind or water between the dunes, beach and subtidal areas. If the natural supply of sediment is maintained and not restricted by infrastructure or engineered hard structures, beaches and dunes will migrate and cause coastlines to change over time in response to the action of wind and water. Based on the dependence of this system on the natural processes of the Mississippi River, and the current alteration of those processes, this system has been severely degraded; no new beaches are forming and existing beaches are eroding (Morton et al. 2004). The loss of this system will impact the many wildlife that depend on it ‑ terns, shorebirds, wading birds, brown pelican, and sea turtles.

SOURCES

**References:** Barbour et al. 1987, Comer et al. 2003*, Defeo et al. 2009, LDWF 2005, Morton et al. 2004, Smith 1993

**Version:** 14 Jan 2014	**Stakeholders:** Southeast

**Concept Author:** J. Teague	**LeadResp:** Southeast

CES203.301 Northern Atlantic Coastal Plain Sandy Beach

**Primary Division:** Gulf and Atlantic Coastal Plain (203)

**Land Cover Class:** Barren

**Spatial Scale & Pattern:** Linear

**Required Classifiers:** Natural/Semi‑natural; Unvegetated (<10% vasc.); Upland

**Diagnostic Classifiers:** Coast; Beach (Substrate); Graminoid

**Concept Summary:** This ecological system includes sparsely vegetated ocean beaches constituting the outermost zone of coastal vegetation ranging from northern North Carolina (north of Bodie Island) northward to the terminus of extensive sandy coastlines and the beginning of rocky coasts. Examples generally extend seaward from foredunes but may include flats behind breached foredunes. Although these habitats are situated just above the mean high tide limit, they are constantly impacted by waves and may be flooded by high spring tides and storm surges. Constant salt spray and rainwater maintain generally moist conditions. Substrates consist of unconsolidated sand and shell sediments that are constantly shifted by winds and floods. Dynamic disturbance regimes largely limit vegetation to pioneering, salt‑tolerant, succulent annuals. *Cakile edentula ssp. edentula* and *Salsola kali* are usually most numerous and characteristic. Other scattered associates include *Sesuvium maritimum, Polygonum glaucum, Polygonum ramosissimum var. prolificum, Suaeda linearis* and *Suaeda maritima*, and *Atriplex cristata*.

**Comments:** In Virginia, this system is distributed along the ocean side of the Eastern Shore (Accomack and Northampton counties) and on Cape Henry and False Cape (City of Virginia Beach).

DISTRIBUTION

**Range:** This system ranges from northern North Carolina northward to the northern end of extensive sandy coastlines and the beginning of rocky coasts in southern Maine.

**Divisions:** 203:C

**TNC Ecoregions:** 57:C, 58:C, 62:C

**Nations:** US

**Subnations:** CT, DE, MA, MD, ME, NC, NH, NJ, NY, RI, VA

**Map Zones:** 60:C, 65:C, 66:C

**USFS Ecomap Regions:** 211D:CC, 221A:CC, 232A:CC, 232H:CC, 232I:CC

CONCEPT

**Environment:** This system includes sparsely vegetated ocean beaches that constitute the outermost zone of coastal vegetation ranging from northern North Carolina northward to the northern end of extensive sandy coastlines and the beginning of rocky coasts in southern Maine. Examples generally extend seaward from foredunes but may include flats behind breached foredunes. The beach includes the sand intertidal shore and the low‑gradient sand above the daily high tide line, which is between the foredune and the Atlantic Ocean. This area of upper beach is affected by wind and salt spray, seasonal high tides, and storm surge.

**Vegetation:** *Cakile edentula ssp. edentula* and *Salsola kali (= Salsola caroliniana)* are usually most numerous and characteristic. Other scattered associates include *Sesuvium maritimum, Polygonum glaucum, Polygonum ramosissimum var. prolificum, Suaeda linearis* and *Suaeda maritima*, and *Atriplex cristata (= Atriplex pentandra)*.

**Dynamics:** Although these habitats are situated just above the mean high tide limit, they are constantly impacted by waves and may be flooded by high spring tides and storm surges (Fleming et al. 2001). The process of sand movement due to the forces of wind and water are part of the natural dynamics of beach ecosystems. This includes transport of sand along the coast, and movement of sand by wind or water between the dunes, beach and subtidal areas. If not restricted by infrastructure or engineered hard structures, beaches and dunes can migrate as coastlines change over time in response to the action of wind and water. The beaches of the Atlantic coast are affected by two tides per day. Extensive construction of high, artificial dunes along the Atlantic coast has reduced the extent of these habitats by increasing oceanside beach erosion and eliminating the disturbance regime that creates and maintains overwash flats.

SOURCES

**References:** Comer et al. 2003*, Davis et al. 2001, Defeo et al. 2009, Edinger et al. 2014a, Gawler and Cutko 2010, Schafale 2012, Sperduto and Nichols 2004

**Version:** 06 Feb 2014	**Stakeholders:** East, Southeast

**Concept Author:** R. Evans	**LeadResp:** East

CES203.535 Southern Atlantic Coastal Plain Florida Beach

**Primary Division:** Gulf and Atlantic Coastal Plain (203)

**Land Cover Class:** Barren

**Spatial Scale & Pattern:** Linear

**Required Classifiers:** Natural/Semi‑natural; Unvegetated (<10% vasc.); Upland; Wetland

**Concept Summary:** This beach ecological system is found along the Atlantic Coast from the St. Johns River in northeastern Florida south to approximately Cape Canaveral. Unlike Southern Atlantic Coastal Plain Sea Island Beach (CES203.383) north of the St. Johns River, this system is subject to higher wave energy and a greater component of sand. The vegetation of this area is distinct from that farther south along the coast of Florida, lacking the tropical element found south of Cape Canaveral.

**Comments:** Apparently few, if any, associations have currently been described in the NVC for this system. More information is needed.

DISTRIBUTION

**Range:** This system is found along the Atlantic Coast from the St. Johns River in northeastern Florida south to approximately Cape Canaveral.

**Divisions:** 203:C

**TNC Ecoregions:** 55:C, 56:C

**Nations:** US

**Subnations:** FL

**Map Zones:** 55:C, 56:C

**USFS Ecomap Regions:** 232G:CC

CONCEPT

**Environment:** The beach includes the sand intertidal shore and the low‑gradient sand above the daily high tide line, which is between the foredune and the Atlantic Ocean. This area of upper beach is affected by wind and salt spray, seasonal high tides, and storm surge.

**Vegetation:** Characteristic species include mostly annual herbs, such as *Cakile edentula ssp. harperi, Chamaesyce polygonifolia, Chamaesyce bombensis, Sesuvium portulacastrum, Salsola kali ssp. kali (= Salsola caroliniana)*, and *Amaranthus pumilus*. On Cumberland Island National Seashore in southeastern Georgia, perennials such as *Croton punctatus* and *Uniola paniculata* also can be important. The vegetation of this area is distinct from that farther south along the coast of Florida, lacking the tropical element found south of Cape Canaveral (Johnson and Muller 1993a).

**Dynamics:** The process of sand movement due to the forces of wind and water are part of the natural dynamics of beach ecosystems. This includes transport of sand along the coast, and movement of sand by wind or water between the dunes, beach and subtidal areas. If not restricted by infrastructure or engineered hard structures, beaches and dunes can migrate as coastlines change over time in response to the action of wind and water. The beaches of the east coast of Florida are affected by two tides per day.

SOURCES

**References:** Allen et al. 2001b, Comer et al. 2003*, Defeo et al. 2009, FNAI 2010a, Howard and Bodge 2011, Johnson and Muller 1993a, Johnson and Muller 1993b

**Version:** 14 Jan 2014	**Stakeholders:** Southeast

**Concept Author:** R. Evans	**LeadResp:** Southeast

CES203.383 Southern Atlantic Coastal Plain Sea Island Beach

**Primary Division:** Gulf and Atlantic Coastal Plain (203)

**Land Cover Class:** Barren

**Spatial Scale & Pattern:** Linear

**Required Classifiers:** Natural/Semi‑natural; Unvegetated (<10% vasc.); Upland

**Diagnostic Classifiers:** Coastal plain; Beach (Substrate)

**Concept Summary:** This ecological system represents beaches and overwash flats in the Sea Island region of South Carolina and Georgia. The entire region is distinctive, and wave energy is generally lower here than any other point along the Atlantic Coast. Huge quantities of fine‑textured sediments are deposited by the region's alluvial rivers, many of which drain relatively large interior areas of the Piedmont, where clay is an abundant by‑product of weathering and erosion. These beaches are distinguished from others of the Atlantic Coast by the prevalence of fine‑textured sediments. The low wave energy and high tidal range create relatively short barrier islands (as opposed to the long narrow islands of North Carolina and the Gulf of Mexico). In addition, the extensive Continental Shelf coupled with low wave energy contributes to a paucity of shell components of the beach substrates.

DISTRIBUTION

**Range:** This system is found in the Sea Island region of South Carolina and Georgia, extending to the St. Johns River in northern Florida.

**Divisions:** 203:C

**TNC Ecoregions:** 56:C

**Nations:** US

**Subnations:** FL, GA, SC

**Map Zones:** 55:C, 58:C

**USFS Ecomap Regions:** 232C:CC

CONCEPT

**Environment:** Sea island beaches are found on the true barrier islands present in the region. Wave energy is generally lower here than any other point along the Atlantic coast (Tanner 1960). Low wave energy and high tidal range create relatively short barrier islands (as opposed to long narrow islands of North Carolina and the Gulf of Mexico).

**Vegetation:** See descriptions in Hillestad et al. (1975) from Cumberland Island.

**Dynamics:** The process of sand movement due to the forces of wind and water are part of the natural dynamics of beach ecosystems. This includes transport of sand along the coast, and movement of sand by wind or water between the dunes, beach and subtidal areas. If not restricted by infrastructure or engineered hard structures, beaches and dunes can migrate as coastlines change over time in response to the action of wind and water. The beaches of the Atlantic coast are affected by two tides per day.

SOURCES

**References:** Allen et al. 2001b, Comer et al. 2003*, Davis et al. 2001, Defeo et al. 2009, Hillestad et al. 1975, Nelson 1986, Tanner 1960

**Version:** 06 Feb 2014	**Stakeholders:** Southeast

**Concept Author:** R. Evans	**LeadResp:** Southeast

CES203.463 Texas Coast Beach

**Primary Division:** Gulf and Atlantic Coastal Plain (203)

**Land Cover Class:** Barren

**Spatial Scale & Pattern:** Linear

**Required Classifiers:** Natural/Semi‑natural; Vegetated (>10% vasc.); Upland; Wetland

**Concept Summary:** This ecological system includes sparsely vegetated ocean beaches constituting the outermost zone of coastal vegetation ranging from and including Boliver peninsula south to include Padre Island in Texas. These beaches are typically located on barrier islands and peninsulas, and they are generally well‑developed with an established dune system behind them. Examples generally extend seaward from foredunes but may include flats behind breached foredunes. Although these habitats are situated just above the mean high tide limit, they are constantly impacted by wind and salt spray and may be flooded by storm surges. Characteristic dominants are xerophytes and include the perennials *Ipomoea pes‑caprae* and *Ipomoea imperati* and the annual *Cakile geniculata*.

DISTRIBUTION

**Range:** Outermost zone of coastal vegetation ranging from and including Boliver peninsula south to include Padre Island in Texas.

**Divisions:** 203:C, 301:C

**TNC Ecoregions:** 31:C

**Nations:** MX, US

**Subnations:** TX

**Map Zones:** 36:C, 37:C

**USFS Ecomap Regions:** 232E:CC, 255D:CC

CONCEPT

**Environment:** This ecological system includes the typically sparsely vegetated, back beach area of the mainland and barrier islands composed of sand and shell fragments in a microtidal environment (<0.5m) as it transitions into more stabilized coastal communities. These areas generally lie near mean sea level (1 m) and are often found between foredunes and tidal waters. Examples are found on retreating, prograding and aggradating sandy barrier segments. In the case of beaches along bay margins, an active dune system is generally lacking and beaches lie between tidal waters and near‑shore vegetation. Recently deposited sands are transported by gulf currents and distributed and redistributed by onshore winds. Landforms are very gently sloping and restricted to the margins of the Gulf of Mexico as well as interior bays. Soils are recently deposited sands.

**Vegetation:** Species such as *Ipomoea pes‑caprae, Ipomoea imperati, Cakile* spp., and *Tidestromia lanuginosa* provide sparse vegetative cover.

**Dynamics:** This system is dependent on highly dynamic coastal geomorphology. The process of sand movement due to the forces of wind and water are part of the natural dynamics of beach ecosystems. This includes transport of sand along the coast, and movement of sand by wind or water between the dunes, beach and subtidal areas. If not restricted by infrastructure or engineered hard structures, beaches and dunes can migrate as coastlines change over time in response to the action of wind and water. Some beaches in this system are eroding and some are accreting (Morton et al. 2004). Beaches require a supply of sand and in some cases this supply has been altered through control of river outflow into the Gulf of Mexico (e.g., the diversion of the mouth of the Brazos River). Loss of this supply of sand and sediments results in a lack of sand to replenish natural beach erosion and loss of beach systems.

SOURCES

**References:** Comer et al. 2003*, Defeo et al. 2009, Elliott 2011, Morton et al. 2004

**Version:** 10 Jan 2014	**Stakeholders:** Latin America, Southeast

**Concept Author:** J. Teague	**LeadResp:** Southeast

M057. Eastern North American Coastal Dune & Grassland

CES201.573 Acadian‑North Atlantic Rocky Coast

**Primary Division:** Laurentian‑Acadian (201)

**Land Cover Class:** Barren

**Spatial Scale & Pattern:** Linear

**Required Classifiers:** Natural/Semi‑natural; Vegetated (>10% vasc.); Upland

**Diagnostic Classifiers:** Shrubland (Shrub‑dominated); Moss/Lichen (Nonvascular); Rocks and Derived Substrates of the Immediate Coast

**Concept Summary:** This system encompasses non‑forested uplands along the immediate Atlantic Coast, from north of Cape Cod to the Canadian Maritimes. It is often a narrow zone between the high tide line and the upland forest; this zone becomes wider with increasing maritime influence. The substrate is rock, sometimes with a shallow soil layer, and tree growth is prevented by extreme exposure to wind, salt spray, and fog. Slope varies from flat rock to cliffs. Cover is patchy shrubs, dwarf‑shrubs and sparse vascular vegetation, sometimes with a few stunted trees. Many coastal islands have graminoid‑shrub areas that were maintained by sheep grazing and now persist even after grazing has ceased.

DISTRIBUTION

**Range:** Primary range is Maine eastward into the Canadian Maritimes, with peripheral occurrences southward along the New England rocky coast.

**Divisions:** 201:C, 202:C

**TNC Ecoregions:** 62:C, 63:C

**Nations:** CA, US

**Subnations:** CT?, MA, ME, NB, NH

**Map Zones:** 65:C, 66:C

CONCEPT

SOURCES

**References:** Comer et al. 2003*, Gawler and Cutko 2010, Sperduto and Nichols 2004

**Version:** 05 Oct 2004	**Stakeholders:** Canada, East

**Concept Author:** S.C. Gawler	**LeadResp:** East

CES203.500 East Gulf Coastal Plain Dune and Coastal Grassland

**Primary Division:** Gulf and Atlantic Coastal Plain (203)

**Land Cover Class:** Mixed Upland and Wetland

**Spatial Scale & Pattern:** Large patch

**Required Classifiers:** Natural/Semi‑natural; Vegetated (>10% vasc.); Upland; Wetland

**National Mapping Codes:** EVT 2435; ESLF 7148; ESP 1435

**Concept Summary:** This system includes vegetation of coastal dunes along the northern Gulf of Mexico, including the northwestern panhandle of Florida, southern Alabama, and southeastern Mississippi. The vegetation consists largely of herbaceous and embedded shrublands on barrier islands and other near‑coastal areas where salt spray, saltwater overwash, and sand movement are important ecological forces. This vegetation differs from that of other regions of the Gulf, and this region forms a natural unit with similar climate and substrate. There are a number of diagnostic and endemic plant species which characterize this system, including *Ceratiola ericoides, Chrysoma pauciflosculosa, Schizachyrium maritimum, Paronychia erecta*, and *Helianthemum arenicola*.

DISTRIBUTION

**Range:** Coastal dunes along the northern Gulf of Mexico, including the northwestern panhandle of Florida, southern Alabama, and southeastern Mississippi.

**Divisions:** 203:C

**TNC Ecoregions:** 53:C

**Nations:** US

**Subnations:** AL, FL, MS

**Map Zones:** 55:C, 99:C

**USFS Ecomap Regions:** 232L:CC

CONCEPT

**Environment:** The vegetation consists largely of herbaceous vegetation and patches of shrublands on barrier islands and other near‑coastal areas where salt spray, saltwater overwash, and sand movement are important ecological forces. This vegetation differs from that of other regions of the Gulf, and this region forms a natural unit with similar climate and substrate (Johnson 1997).

**Vegetation:** There are a number of diagnostic and endemic plant species which characterize this system, including *Ceratiola ericoides, Chrysoma pauciflosculosa, Schizachyrium maritimum, Paronychia erecta*, and *Helianthemum arenicola* (Johnson and Barbour 1990).

**Dynamics:** The natural coastal dynamics include the movement of sand from wind, tides, and storm surge. This includes transport of sand along the coast (primarily from east to west), and movement of sand by wind or water between the dunes, beach and subtidal areas, and the movement of sand from the foredunes to the interior. If not restricted by infrastructure or engineered hard structures, beaches and dunes can migrate as coastlines change over time in response to the action of wind and water. The Gulf of Mexico coast is affected by one tide per day. Coastal grassland develops as a barrier island builds seaward, developing new dune ridges along the shore which protect the inland ridges from sand burial and salt spray, or as a beach recovers after storm overwash and a new foredune ridge builds up along the shore, protecting the overwashed area behind it from sand burial and salt spray (FNAI 2010a). Wrack and seaweed deposited along the shore is an important source of nutrients for the coastal ecosystem, and helps promote revegetation in newly disturbed areas (Defeo et al. 2009). Fire is rare and local to small areas.

SOURCES

**References:** Allen et al. 2001b, Comer et al. 2003*, Defeo et al. 2009, Eyre 1980, FNAI 2010a, Johnson 1997, Johnson and Barbour 1990, Johnson and Muller 1993a

**Version:** 14 Jan 2014	**Stakeholders:** Southeast

**Concept Author:** R. Evans	**LeadResp:** Southeast

CES201.026 Great Lakes Dune

**Primary Division:** Laurentian‑Acadian (201)

**Land Cover Class:** Barren

**Spatial Scale & Pattern:** Large patch

**Required Classifiers:** Natural/Semi‑natural; Unvegetated (<10% vasc.); Upland

**Concept Summary:** This system occurs along the Great Lakes shores region of the United States and Canada. Component plant communities vary from sparsely vegetated, active dunes to communities dominated by grasses, shrubs, and trees, depending on the degree of sand deposition, sand erosion, and distance from the lake. Many open dunes on Lake Michigan are considered "perched dunes" in that sands were deposited on top of glacial moraine located along the coast. In some instances, dunefields sit several hundred feet above current lake levels. Depositional areas, where Great Lakes beachgrass foredunes are found, are dominated by *Ammophila breviligulata* (or in the eastern part of the range *Ammophila champlainensis*); erosional areas, such as slacks in blowouts and dunefields, by *Calamovilfa longifolia*; and stabilized areas by *Schizachyrium scoparium*. In dunefields and on the most stable dune ridges, especially around northern Lake Michigan and Lake Huron, low evergreen shrubs (*Arctostaphylos uva‑ursi, Juniperus communis, Juniperus horizontalis*) occupy dune crests and also the ground layer in the savanna edge of dunes; elsewhere, deciduous shrubs are dominant, including *Prunus pumila, Salix cordata*, and *Salix myricoides (= Salix glaucophylloides)*. Backdunes tend to succeed to forests and savanna indistinguishable from corresponding types found on sandy substrates further inland.

**Comments:** The system, as described, includes the open grassland, shrubland, and woodland parts of the dune. The lee side of the dunes often contains forests on deep, moist to dry sands that resemble other forested systems. Such forests may include hemlock‑hardwood and red oak forests.

DISTRIBUTION

**Range:** This system occurs along the Great Lakes shores of the United States and Canada on stabilized foredunes, ranging from Wisconsin to Ontario and New York in the Great Lakes, and in isolated occurrences along the shores of Lake Champlain, Vermont.

**Divisions:** 201:C, 202:C

**TNC Ecoregions:** 48:C, 64:C

**Nations:** CA, US

**Subnations:** IL, IN, MI, MN, NY, OH, ON, VT, WI

**Map Zones:** 41:C, 49:C, 50:C, 51:C, 52:C, 62:C, 63:C, 64:C, 65:C, 66:C

**USFS Ecomap Regions:** 211E:CC, 212Ha:CCC, 212Hf:CCC, 212Hl:CCC, 212Ra:CCC, 212Rd:CCC, 212Re:CCC, 212Sb:CCC, 212Sn:CCC, 212Te:CCC, 212Ya:CCC, 222Ja:CCC

CONCEPT

SOURCES

**References:** Albert 1995b, Comer et al. 1995a, Comer et al. 1998, Comer et al. 2003*, Dorr and Eschman 1970, Dorroh 1971, Edinger et al. 2014a, Eyre 1980, Kost et al. 2007, ONHD unpubl. data, WDNR 2015

**Version:** 25 Mar 2003	**Stakeholders:** Canada, East, Midwest

**Concept Author:** D. Faber‑Langendoen	**LeadResp:** Midwest

CES203.264 Northern Atlantic Coastal Plain Dune and Swale

**Primary Division:** Gulf and Atlantic Coastal Plain (203)

**Land Cover Class:** Mixed Upland and Wetland

**Spatial Scale & Pattern:** Large patch, Linear

**Required Classifiers:** Natural/Semi‑natural; Vegetated (>10% vasc.); Upland; Wetland

**Diagnostic Classifiers:** Coast; Beach (Substrate); Graminoid; North Atlantic Coastal Plain

**National Mapping Codes:** EVT 2436; ESLF 7149; ESP 1436

**Concept Summary:** This system consists of vegetation of barrier islands and other coastal areas, ranging from northernmost North Carolina northward to southern Maine (where extensive sandy coastlines are replaced by rocky coasts). A range of plant communities may be present, but natural vegetation is predominately herbaceous, with *Ammophila breviligulata* diagnostic. Shrublands resulting from succession from grasslands may occur in limited areas. Both dune uplands and non‑flooded wetland vegetation of interdunal swales are included in this system. In the northern portion of the range, these swales are often characterized by *Vaccinium macrocarpon*, while south of New Jersey, swales are characterized by a variety of graminoids and forbs, usually including *Schoenoplectus pungens, Fimbristylis castanea, Fimbristylis caroliniana, Juncus* spp. and others. Small patches of natural woodland may also be present in limited areas, especially in the northern range of this system. Dominant ecological processes are those associated with the maritime environment, including frequent salt spray, saltwater overwash, and sand movement.

**Comments:** This system was separated from Southern Atlantic Coastal Plain Dune and Maritime Grassland (CES203.273) to parallel broad‑scale biogeographic and climatic differences believed to be important in this environment. This system occupies the northern part of this broad transition which was labeled by Cowardin et al. (1979) as the Virginian Province, although the demarcated boundary differs somewhat from that used here. A useful vegetation indicator of this transition is the shift in herbaceous dominance on the dunes from *Uniola paniculata* in the south to *Ammophila breviligulata* in the north. Although the location of this shift itself is somewhat imprecise because of widespread planting of both species on artificially enhanced dunes, this boundary appears to be well approximated by Omernik Ecoregion 63g vs. 63d (EPA 2004). There is extensive south‑to‑north turnover of associations in TNC Ecoregion 58 with very little overlap southward. *Quercus virginiana* is only occasional in this system at its extreme southern end (southern Virginia) and should not be thought of as characteristic.

This system is distinguished from Northern Atlantic Coastal Plain Maritime Forest (CES203.302) by the lack of dominant woody vegetation. This distinction becomes blurred where dunes have been artificially enhanced and an unnatural succession to woody vegetation is occurring. The boundary at the northern end is the end of extensive sandy coastlines and the beginning of rocky coasts.

Southeastern Coastal Plain Interdunal Wetland (CES203.258) may occur with this system in northern North Carolina and southern Virginia. Where the ranges overlap, Southeastern Coastal Plain Interdunal Wetland (CES203.258) is distinguished from this system by the presence of standing water for a significant part of the growing season. This corresponds to a break between open‑water and tall‑graminoid marsh vegetation in the ponds and low‑graminoid‑ or forb‑dominated vegetation in the grasslands. North of Virginia, interdunal wetlands are smaller and more integrated into the dune systems and are included in this system.

DISTRIBUTION

**Range:** This system ranges from northernmost North Carolina (EPA ecoregion 63d) and southeastern Virginia to southern Maine. The southern portion is a transition zone from around Kitty Hawk, North Carolina, to the Virginia‑North Carolina border. The northern limit is Merrymeeting Bay, Maine.

**Divisions:** 203:C

**TNC Ecoregions:** 57:C, 58:C, 62:C, 63:C

**Nations:** US

**Subnations:** CT, DE, MA, MD, ME, NC, NH, NJ, NY, RI, VA

**Map Zones:** 60:C, 65:C, 66:C

**USFS Ecomap Regions:** 211Db:CCC, 221Aa:CCC, 221Ab:CCC, 221Ac:CCC, 221Ad:CCC, 221Ak:CCC, 221An:CCC, 232Ab:CCC, 232Ac:CCC, 232Ad:CCC, 232Hb:CCC, 232Hc:CCC, 232I:CC

CONCEPT

**Environment:** This system occurs on coastal strands and barrier islands, on sand dunes and sand flats. Strong salt spray is an important influence on vegetation in many parts. Overwash by sea water during storms is important on sand flats not protected by continuous dunes. On dunes, present or recent sand movement is an important factor. The combination of these factors prevents the dominance of woody vegetation. Sites may be either dry or saturated by freshwater from rainfall and the local water table. Areas connected to tidal influence are placed in other systems. Soils are sandy, with little organic matter and little or no horizon development. Soils may be excessively drained on the higher dunes. Soils are low in nutrient‑holding capacity, but aerosol input of sea salt provides a continuous source of nutrients.

**Vegetation:** Vegetation consists of a set of grassland and herbaceous to shrubby associations. *Ammophila breviligulata* is the characteristic dominant on the youngest dunes and those most exposed to salt spray. Shrublands resulting from succession from grasslands may occur in limited areas, but they are generally not natural components of this system in the southern part of its range (M. Schafale pers. comm.). These communities tend to be low in plant species richness but have a characteristic set of forbs and occasional low shrubs associated with them. Wetter sand flats and dune swales may be dominated by a variety of herbs and sometimes have fairly high species richness.

**Dynamics:** The environment of this system is one of the most dynamic in existence for terrestrial vegetation. Reworking of sand by storms or by slower eolian processes may completely change the local environment in a short time, changing one association to another. Many of these sites are fairly early in the process of primary succession on recent surfaces. Chronic salt spray is an ongoing stress. Overwash and extreme salt spray in storms are frequent disturbances. Vegetation interacts strongly with geologic processes; the presence of grass is an important factor in the development of new dunes. Alteration of dynamic processes, such as artificial enhancement of dunes by planting or sand fencing, can have drastic effects on this system, causing large areas to succeed to woody vegetation. Fire is probably not a major natural factor in this system, but may have been important locally. Most vegetation is too sparse to carry fire well.

SOURCES

**References:** Comer et al. 2003*, Cowardin et al. 1979, Defeo et al. 2009, EPA 2004, Edinger et al. 2014a, Eyre 1980, Gawler and Cutko 2010, Massachusetts Barrier Beach Task Force 1994, NYNHP 2013e, Schafale 2012, Schafale pers. comm., Sperduto and Nichols 2004

**Version:** 21 May 2014	**Stakeholders:** East, Southeast

**Concept Author:** R. Evans	**LeadResp:** East

CES203.895 Northern Atlantic Coastal Plain Heathland and Grassland

**Primary Division:** Gulf and Atlantic Coastal Plain (203)

**Land Cover Class:** Shrubland

**Spatial Scale & Pattern:** Large patch

**Required Classifiers:** Natural/Semi‑natural; Vegetated (>10% vasc.); Upland

**Diagnostic Classifiers:** Shrubland (Shrub‑dominated); Grassland, Savanna, Steppe (graminoid‑dominated); Coastal plain; Glaciated plains; Sandplains/Glacial Outwash or Flats; Temperate; Sand Soil Texture; Very Short Disturbance Interval; North Atlantic Coastal Plain

**National Mapping Codes:** EVT 2522; ESLF 5275; ESP 1522

**Concept Summary:** Sandplain grasslands and heathlands of the southern New England / New York coast are areas of graminoid‑ and shrub‑dominated vegetation maintained by periodic fire or other disturbance, as well as exposure to maritime influences. Developing on acidic, nutrient‑poor, and very well‑drained soils within a few kilometers of the ocean, they may occur as heathlands, grasslands, or support a patchwork of grass and shrub vegetation. Characteristic species include *Gaylussacia baccata, Arctostaphylos uva‑ursi, Corema conradii, Amelanchier nantucketensis, Hudsonia ericoides, Hudsonia tomentosa, Vaccinium angustifolium, Deschampsia flexuosa, Schizachyrium scoparium*, and *Carex pensylvanica*. They provide habitat for several rare or uncommon forbs including *Liatris scariosa var. novae‑angliae* and *Agalinis acuta*. They are important habitat for several bird and other animal species including the short‑eared owl and regal fritillary, and (along with brushy plains and woodlands) provided habitat for the extinct heath hen.

**Comments:** This system includes both the very distinctive Hempstead Plains grasslands of Long Island, New York (which occur a bit further inland than other sites), as well as the maritime heathlands/grasslands of Cape Cod and nearby islands. Grass‑dominated and shrub‑dominated expressions are separated at the association level; they can occur together and intergrade at some sites. This system is related to dune grasslands but occurs on sandplains, not dunes, and lacks significant amounts of *Ammophila breviligulata*. In the absence of disturbance (fire, grazing, mowing), coverage by *Pinus rigida* and *Quercus ilicifolia* can increase, creating vegetation similar to a pitch pine ‑ scrub oak barren (hence the inclusion of CEGL006315 in the associations list); or in some cases, a tall‑shrub community can develop in the absence of fire (CEGL006379). Neither of these associations is core to the concept of this system. Its landscape position and dynamics are sufficiently distinct that it is segregated rather than being treated as a phase or a patch of the coastal pine barrens system.

DISTRIBUTION

**Range:** This system is endemic to a small area ranging from the southern New York coastline north to Cape Cod, Massachusetts.

**Divisions:** 203:C

**TNC Ecoregions:** 62:C

**Nations:** US

**Subnations:** CT?, MA, NY, RI

**Map Zones:** 65:C

**USFS Ecomap Regions:** 221Ab:CCC, 221Ac:CCC, 221Ad:CCP, 221An:CCC

CONCEPT

**Environment:** Sandplain grasslands and heathlands of the southern New England / New York coast are areas of graminoid‑ and shrub‑dominated vegetation maintained by extreme conditions and periodic fire or other disturbance. Developing on acidic, nutrient‑poor, and very well‑drained soils, they may occur as heathlands, grasslands, or support a patchwork of grass and shrub vegetation.

**Vegetation:** Characteristic species include *Gaylussacia baccata, Arctostaphylos uva‑ursi, Corema conradii, Amelanchier nantucketensis, Hudsonia ericoides, Hudsonia tomentosa, Vaccinium angustifolium, Deschampsia flexuosa, Schizachyrium scoparium*, and *Carex pensylvanica*. They provide habitat for several rare or uncommon forbs including *Liatris scariosa var. novae‑angliae* and *Agalinis acuta*.

**Dynamics:** The largely exposed locations experience extreme variations in temperature and moisture, and the sandy, nutrient‑poor soils contribute to prevention of establishment of woody vegetation. Coastal occurrences maintain their open nature with the stress and killing of woody plant tissue caused by high winds, desiccation, and salt spray. Examples that developed in slight depressions are also maintained by frost that persists longer into the growing season (MNHESP 2010a, 2010b). Prior to European settlement, this system is believed to have occurred as small patches in limited areas near the coast (Motzkin and Foster 2002); there may also have been patches in the vicinity of Native American settlements, based on the prevalence of charcoal in some palynological cores (Dunwiddie 1989). Presettlement grasslands appear to have been more likely on portions of Long Island (Hempstead Plains and Montauk) and Martha's Vineyard than on Nantucket, Block Island, or Cape Cod (Motzkin and Foster 2002). This native vegetation is often confused with similar semi‑natural grasslands and heathlands characterized by a mixture of native and exotic species developed as a result of agriculture; some natural occurrences may have resulted as expansions of original native vegetation. They have increased in extent and largely post‑date land clearing following European settlement (Foster et al. 2002). In addition, some heathlands may have developed on severely disturbed soils following the abandonment of agriculture and grazing (Motzkin and Foster 2002). Efforts to reverse the conversion of these heathlands and grasslands to tall shrublands or woodlands have generally used a mixture of prescribed fire and mowing, and less commonly grazing.

SOURCES

**References:** Cain et al. 1937, Chase and Rothley 2007, Dunwiddie 1989, Dunwiddie and Caljouw 1990*, Dunwiddie et al. 1993, Dunwiddie et al. 1996, Dunwiddie et al. 1997, Eastern Ecology Working Group n.d., Edinger et al. 2014a, Eyre 1980, Foster et al. 2002, Harper 1912, Lundgren et al. 2000, MNHESP 2010a, MNHESP 2010b, Motzkin and Foster 2002, NYNHP 2013f, NYNHP 2013j, NYNHP 2013k, Neidich 1980, Swain and Kearsley 2014

**Version:** 14 Jan 2014	**Stakeholders:** East

**Concept Author:** L.A. Sneddon	**LeadResp:** East

CES203.273 Southern Atlantic Coastal Plain Dune and Maritime Grassland

**Primary Division:** Gulf and Atlantic Coastal Plain (203)

**Land Cover Class:** Herbaceous

**Spatial Scale & Pattern:** Large patch

**Required Classifiers:** Natural/Semi‑natural; Vegetated (>10% vasc.); Upland; Wetland

**Diagnostic Classifiers:** Herbaceous; Coast; Graminoid

**National Mapping Codes:** EVT 2426; ESLF 7139; ESP 1426

**Concept Summary:** This ecological system consists primarily of grasslands and related shrublands of Atlantic Coastal Plain barrier islands and related coastal areas from North Carolina south to northern and central Florida. On the Florida coast from south of Cape Canaveral to the sandy portions of the Florida Keys, this system occurs in a more attenuated fashion. This ecological system includes upland dune grasslands and maritime wet grasslands and shrublands, which are not tidal, but may be flooded for short periods of time from storm surge or heavy rain. The environment of this system is highly dynamic. Reworking of sand by storms or by slower eolian processes may completely change the local environment. Vegetation responds to these natural coastal processes through primary succession. The combined effects of chronic and extreme salt spray and periodic ocean overwash by seawater prevent or dramatically inhibit woody plant growth.

**Comments:** This system was separated from Northern Atlantic Coastal Plain Dune and Swale (CES203.264) to parallel broad‑scale biogeographic and climatic differences believed to be important in this environment. The northern part of this broad transition was labeled by Cowardin et al. (1979) as the Virginian Province and the southern region as the Carolinian Province, although the demarcated boundary differs somewhat from that used here. A primary indicator of this transition is the shift in vegetation dominance on the dunes from *Uniola paniculata* in the south to *Ammophila breviligulata* in the north. Although the location of this shift itself is somewhat imprecise because of widespread planting of both species on artificially enhanced dunes, this boundary appears to be well approximated by Omernik Ecoregion 63g vs. 63d (EPA 2004).

DISTRIBUTION

**Range:** This system ranges on the Atlantic Coast from northern North Carolina (Omernik ecoregion 63g, Carolinian Barrier Islands and Coastal Marshes) to central Florida. The northern limit is a transition zone from around Kitty Hawk, North Carolina, to the Virginia‑North Carolina border.

**Divisions:** 203:C

**TNC Ecoregions:** 55:?, 56:C, 57:C

**Nations:** US

**Subnations:** FL, GA, NC, SC

**Map Zones:** 55:C, 56:?, 58:C

**USFS Ecomap Regions:** 232C:CC, 232I:CC

CONCEPT

**Environment:** Occurs on barrier islands and similar coastal strands, on sand dunes and sand flats. Strong salt spray is an important influence on vegetation in many parts. Overwash by sea water during storms is important on sand flats not protected by continuous dunes. On dunes, present or recent sand movement is an important factor. The combination of these factors prevents the dominance of woody vegetation. Sites may be either dry or saturated by freshwater from rainfall and local water table. Areas connected to tidal influence and areas with ponded freshwater are placed in other ecological systems. Soils are sandy, with little organic matter and little or no horizon development. Soils may be excessively drained on the higher dunes. Soils are low in nutrient‑holding capacity, but aerosol input of sea salt provides a continuous source of nutrients. North of the Sea Islands region of coastal Georgia and South Carolina, barrier islands that face south tend to have better developed dune fields, and often have extensive maritime forest systems, and east‑facing barrier islands naturally have less continuous dunes and more overwash flats. On islands that face east, the northern portion tends to experience shoreline and dune erosion and the south end may experience accretion. Many of Georgia's barrier islands (known as Sea Islands) show this pattern.

**Vegetation:** Vegetation consists of a set of grassland and other herbaceous associations. *Uniola paniculata* is the characteristic dominant on the youngest dunes and those most exposed to salt spray and less commonly *Panicum amarum* (Pinson 1973). *Spartina patens* or *Schizachyrium littorale* tend to dominate older dunes and sand flats. Component communities tend to be low in plant species richness, but have a characteristic set of forbs and occasional low shrubs associated with them. Wetter sand flats and dune swales may be dominated by a variety of herbs and sometimes have fairly high species richness. Also included in this system are patches of transition shrub communities or shrub thickets.

**Dynamics:** The environment of this system is one of the most dynamic in existence for terrestrial vegetation. Reworking of sand by storms or by slower eolian processes may completely change the local environment in a short time, changing one association to another or changing this system into a different system. Many of these sites are fairly early in the process of primary succession on recent surfaces. Chronic salt spray is an ongoing stress. Overwash and extreme salt spray in storms is a frequent disturbance. Vegetation interacts strongly with geologic processes; the presence of dune grass is an important factor in the development of new dunes. Artificial enhancement of dunes by sand fencing or planting off‑site species, including *Ammophila breviligulata*, can alter the dynamic processes of the dunes. Fire is probably not a major natural factor in this system, but may have been important locally. Most vegetation is too sparse to carry fire well.

SOURCES

**References:** Allen et al. 2001b, Comer et al. 2003*, Cowardin et al. 1979, Defeo et al. 2009, EPA 2004, Eyre 1980, FNAI 2010a, Johnson and Muller 1993a, Nelson 1986, Pinson 1973, Schafale 2012

**Version:** 14 Jan 2014	**Stakeholders:** Southeast

**Concept Author:** R. Evans	**LeadResp:** Southeast

CES203.539 Southwest Florida Dune and Coastal Grassland

**Primary Division:** Gulf and Atlantic Coastal Plain (203)

**Land Cover Class:** Herbaceous

**Spatial Scale & Pattern:** Large patch

**Required Classifiers:** Natural/Semi‑natural; Vegetated (>10% vasc.); Upland

**Diagnostic Classifiers:** Herbaceous; Coast; Graminoid

**National Mapping Codes:** EVT 2431; ESLF 7144; ESP 1431

**Concept Summary:** This system occurs along the southwest coast of Florida, one of the four distinctive coastal regions of Florida. It includes herbaceous vegetation on dunes and just inland of the dunes, often on recently deposited sands. These are generally upland plant communities and less commonly non‑flooded dune swale wetlands. Although the vegetation is mostly herbaceous, there are typically scattered shrubs of various heights present. The dune vegetation includes *Uniola paniculata, Panicum amarum var. amarulum*, and *Iva imbricata*. *Scaevola plumieri, Chamaesyce mesembrianthemifolia*, and *Coccoloba uvifera* help distinguish this system from similar dune and coastal grasslands found farther north.

**Comments:** The spatial boundary between this system and Florida Panhandle Beach Vegetation (CES203.266) is clearly separated by the Big Bend region (see Tanner 1960, Johnson and Muller 1993a). Within this system, there is a large amount of variation along a north‑to‑south gradient. A finer distinction could be made in the future.

DISTRIBUTION

**Range:** Found along the western coast of Florida south of the Big Bend region to the Florida Keys, one of the four distinctive coastal regions of Florida.

**Divisions:** 203:C, 411:C

**TNC Ecoregions:** 54:C, 55:C

**Nations:** US

**Subnations:** FL

**Map Zones:** 56:C

**USFS Ecomap Regions:** 232D:CC, 411A:CC

CONCEPT

**Environment:** The vegetation consists largely of herbaceous vegetation and patches of shrublands on barrier islands and other coastal areas where salt spray, saltwater overwash, and sand movement are important ecological forces. Soils are sandy, with little organic matter and little or no horizon development. Soils may be excessively drained on the higher dunes. Soils are low in nutrient‑holding capacity, but aerosol input of sea salt provides a continuous source of nutrients. Winter low temperatures are warmer along the southwest coast of Florida, than along the coast further north. Killing frosts are more unusual further south along the coast of the Florida Peninsula.

**Vegetation:** Although the vegetation is mostly herbaceous, there are typically scattered shrubs of various heights present. Although the vegetation may overlap in species composition with other Florida coastal regions, there are important differences based on plant species composition, vegetation structure, and physical site characteristics (Johnson and Muller 1993a). The dune vegetation, like that of other Florida regions, includes *Uniola paniculata, Panicum amarum var. amarulum*, and *Iva imbricata*. *Scaevola plumieri, Chamaesyce mesembrianthemifolia*, and *Coccoloba uvifera* help distinguish this system from those to the north. However, while all other dune communities in Florida have frequently occurring distinctive species which help distinguish them, such species are lacking in this system. However, more inland coastal grassland components of this system sometimes include *Schizachyrium sanguineum (= Schizachyrium semiberbe)* and *Bouteloua hirsuta*, among other species not found in coastal grasslands elsewhere in Florida (Johnson and Muller 1993a).

**Dynamics:** The natural coastal dynamics include the movement of sand from wind, tides, and storm surge along this low‑energy coastline. This includes transport of sand along the coast, and movement of sand by wind or water between the dunes, beach and subtidal areas, and the movement of sand from the foredunes to the interior. If not restricted by infrastructure or engineered hard structures, beaches and dunes can migrate as coastlines change over time in response to the action of wind and water. The Gulf of Mexico coast is affected by one tide per day. Coastal grassland develops as a barrier island builds seaward, developing new dune ridges along the shore which protect the inland ridges from sand burial and salt spray, or as a beach recovers after storm overwash and a new foredune ridge builds up along the shore, protecting the overwashed area behind it from sand burial and salt spray (FNAI 2010a). Wrack and seaweed deposited along the shore is an important source of nutrients for the coastal ecosystem, and helps promote revegetation in newly disturbed areas. Fire is rare and local to small areas.

SOURCES

**References:** Comer et al. 2003*, Defeo et al. 2009, FNAI 2010a, Johnson and Muller 1993a, Tanner 1960

**Version:** 14 Jan 2014	**Stakeholders:** Southeast

**Concept Author:** R. Evans	**LeadResp:** Southeast

CES203.465 Texas Coast Dune and Coastal Grassland

**Primary Division:** Gulf and Atlantic Coastal Plain (203)

**Land Cover Class:** Mixed Upland and Wetland

**Spatial Scale & Pattern:** Matrix

**Required Classifiers:** Natural/Semi‑natural; Vegetated (>10% vasc.); Upland; Wetland

**National Mapping Codes:** EVT 2437; ESLF 7150; ESP 1437

**Concept Summary:** This ecological system consists of wetland and upland herbaceous and shrubland vegetation of barrier islands, near‑coastal areas, and the Coastal Sand Plain along the Texas coast in the northern Gulf of Mexico. Plant communities of primary and secondary dunes, interdunal swales, barrier flats, and adjacent mainland are included. Salt spray, saltwater overwash, and sand movement are important ecological forces. Some examples of this system naturally occurred as an open matrix of midgrass species within native mesquite ‑ acacia shrublands dominated by *Prosopis glandulosa, Acacia farnesiana*, and *Acacia rigidula* but have become shrub‑dominated due to the lack of fire.

DISTRIBUTION

**Range:** This system is found in the northern Gulf of Mexico along the Texas coast.

**Divisions:** 203:C, 301:C

**TNC Ecoregions:** 31:C

**Nations:** US

**Subnations:** TX

**Map Zones:** 36:C, 37:?

**USFS Ecomap Regions:** 232E:CC, 255D:CC, 315E:??

CONCEPT

**Environment:** This system occupies deep eolian sands and Pleistocene barrier island and beach deposits that sit on top of underlying geologic formations, especially the Beaumont Formation. This includes deep sands well inland on the South Texas Sand Sheet, which represents by far the largest continuous patch of this type. It is found on primary and secondary dunes, as well as relatively level areas such as barrier flats, and on the mainland on deep sands of stranded beach ridges. Significant local topography, in the form of swales and pothole wetlands, may be present. Significant surface drainages are generally scarce. Soils are deep or coastal sands (Elliott 2011).

**Vegetation:** This system includes upland, grass‑dominated vegetation on deep sands. Dunes are often dominated by *Uniola paniculata*, with other species such as *Croton punctatus, Panicum amarum, Ipomoea pes‑caprae, Ipomoea imperati, Tidestromia lanuginosa, Cakile* spp., and *Sesuvium portulacastrum* also present. Upland grasslands are often dominated by *Schizachyrium littorale* and *Paspalum monostachyum*. Numerous other species, such as *Sorghastrum nutans, Paspalum plicatulum, Muhlenbergia capillaris, Cenchrus spinifex, Elionurus tripsacoides, Eragrostis secundiflora, Bothriochloa laguroides ssp. torreyana, Heteropogon contortus, Andropogon glomeratus, Spartina patens*, and *Dichanthelium* spp., may also be common. Numerous forbs, including such species as *Heterotheca subaxillaris, Croton* spp., *Chamaecrista fasciculata, Rayjacksonia phyllocephala, Physalis* spp., *Helianthus argophyllus, Gaillardia pulchella, Solidago sempervirens, Baptisia* spp., *Indigofera miniata, Eriogonum multiflorum, Conoclinium betonicifolium*, and *Rudbeckia hirta*, are also commonly encountered. Some woody species are found in the system but typically make up very little cover. Cover of woody species is limited but may include *Baccharis* spp., *Opuntia engelmannii var. lindheimeri, Morella cerifera, Quercus fusiformis, Quercus virginiana*, and stunted *Prosopis glandulosa*. Non‑native woody species such as *Tamarix* spp., *Schinus terebinthifolius*, and *Triadica sebifera* may be present to dominant. Small areas may have sufficient woody cover to be mapped as a shrubland (Elliott 2011).

**Dynamics:** Substrate, hydrology, drought, coastal processes (including tropical storms) and fire play a role in maintaining this ecological system (Lonard et al. 2004, Morton et al. 2004, Britton et al. 2010). Composition and structure vary depending on these processes.

SOURCES

**References:** Bielfelt 2013, Britton et al. 2010, Comer et al. 2003*, Defeo et al. 2009, Elliott 2011, Lonard et al. 2004, Morton et al. 2004

**Version:** 14 Jan 2014	**Stakeholders:** Southeast

**Concept Author:** R. Evans and J. Teague	**LeadResp:** Southeast

CES203.543 Texas Saline Coastal Prairie

**Primary Division:** Gulf and Atlantic Coastal Plain (203)

**Land Cover Class:** Herbaceous Wetland

**Spatial Scale & Pattern:** Matrix

**Required Classifiers:** Natural/Semi‑natural; Vegetated (>10% vasc.); Upland; Wetland

**Diagnostic Classifiers:** Herbaceous; Extensive Wet Flat; Saline Substrate Chemistry; West Gulf Coastal Plain

**National Mapping Codes:** EVT 2486; ESLF 9207; ESP 1486

**Concept Summary:** This system encompasses grassland vegetation occurring along the Gulf Coast of Texas on saline and nonsaline soils on level topography of the Beaumont Formation and in brackish marshes. These areas are often saturated by local rainfall and periodically flooded by saline waters during major storm events. Outliers also occur as scattered patches in salt flats. It is characteristically dominated by *Spartina spartinae*, a tall (1.5 m) warm‑season perennial bunchgrass; other dominants may include *Schizachyrium littorale* and *Muhlenbergia capillaris*. This system also includes depressions often dominated by *Spartina patens*. Saline prairie continues to occupy extensive areas, though quality of the system is often degraded by the invasion of woody shrubs due to the absence of regular fire. Fire is an important ecological process needed to maintain this system, though periodic submersion with saltwater during storm events also helps to control the invasion of woody species.

DISTRIBUTION

**Range:** This system is restricted to the Gulf Coast of Texas.

**Divisions:** 203:C

**TNC Ecoregions:** 31:C

**Nations:** US

**Subnations:** TX

**Map Zones:** 36:C, 37:P

**USFS Ecomap Regions:** 255D:CC, 315E:PP

CONCEPT

**Environment:** This system occurs on saline and nonsaline soils of the Pleistocene Beaumont Formation that are often saturated by local rainfall and periodically flooded by saline waters during major storm events. Landforms are mostly level or very gently undulating, and typically found near the coast. These sites may be inundated by saltwater during storm surges. Pimple mounds may lend some local topographic variation to the otherwise level surface. Soils are very deep, somewhat poorly to poorly drained, often with high salinity and/or sodicity, at least at some depth. These may be loams or clays. These soils may be saturated from local rainfall or occasionally from storm surges (Elliott 2011). This system often forms a band between coastal salt marshes and coastal nonsaline prairie.

**Vegetation:** This system is characteristically dominated by *Spartina spartinae*, which may occur in nearly monotypic stands; other dominants may include *Schizachyrium littorale* and *Muhlenbergia capillaris*. This system includes depressions often dominated by *Spartina patens*. Other graminoids that may be present to abundant include *Schizachyrium scoparium, Andropogon glomeratus, Panicum virgatum*, or *Sporobolus indicus*. On lower, somewhat wetter sites, *Aristida oligantha, Paspalum hartwegianum, Sporobolus virginicus, Paspalum vaginatum*, and *Distichlis spicata* may be common. Forbs are generally uncommon but may include species such as *Borrichia frutescens, Solidago sempervirens, Iva angustifolia, Euthamia* spp., or other species more common to the non‑saline soils nearby or the salt marsh that may also be nearby. Microtopographic highs in the form of pimple mounds often have species more characteristic of less saline adjacent habitats. Shrubby species may invade the prairie, commonly including species such as *Iva frutescens, Prosopis glandulosa, Vachellia farnesiana (= Acacia farnesiana), Lycium carolinianum, Tamarix* sp., and *Baccharis halimifolia* (Elliott 2011).

**Dynamics:** Fire is an important ecological process needed to maintain this system. Periodic submersion with saltwater during storm events also helps to control the invasion of woody species and contributes to higher soil salinity levels.

SOURCES

**References:** Comer et al. 2003*, Elliott 2011, Oefinger and Scifres 1977

**Version:** 14 Jan 2014	**Stakeholders:** Southeast

**Concept Author:** J. Teague	**LeadResp:** Southeast

2.B.4.Nb. Pacific North American Coastal Scrub & Herb Vegetation

M059. Pacific Coastal Beach & Dune

CES302.003 Duna Costera de Sonora y la Península de Baja California

**Primary Division:** North American Warm Desert (302)

**Land Cover Class:** Barren

**Spatial Scale & Pattern:** Linear

**Required Classifiers:** Natural/Semi‑natural; Unvegetated (<10% vasc.); Upland

**Diagnostic Classifiers:** Lowland [Lowland]; Dune (Landform); Dune field; Tropical/Subtropical [Tropical Xeric]; Sand Soil Texture

**Concept Summary:** This system is scattered along the coast of Baja California and Sonoran coast of the Gulf of California. Coastal dunes include beaches, foredunes, sand spits, and active to stabilizing backdunes and sandsheets derived from quartz or gypsum sands. The mosaic of sparse to dense vegetation in dune systems is driven by sand deposition, erosion, and lateral movement. Coastal dunes often front portions of inlets and tidal marshes. They may also occur as extensive dune fields dominating large coastal bays. Dune vegetation typically includes herbaceous, succulent, and low‑shrub species with varying degrees of tolerance for salt spray, wind and sand abrasion, and substrate stability. Dune succession is highly variable, so species composition can vary significantly between occurrences. Common species include *Abronia maritima, Abronia villosa, Astragalus magdalenae, Croton californicus, Dicoria canescens, Euphorbia leucophylla, Helianthus niveus*, and *Jouvea pilosa*.

DISTRIBUTION

**Range:** Scattered along the coast of Baja California and Sonoran coast of the Gulf of California.

**Divisions:** 302:C

**TNC Ecoregions:** 23:C

**Nations:** MX

**Subnations:** MXBC, MXBS, MXSO

CONCEPT

**Environment:** [from M059] This macrogroup occurs on sandy beaches and dunes, with or without salt spray, typically within 2 km of the coast. Soils are usually sandy and well‑drained; some areas may have a cobble layer on top of sand. Forb communities are salt‑tolerant and tend to occur just above mean high tide, while the grasslands tend to occur on cobble beaches and on dunes that become higher and further away from the beach. On the California Channel Islands, communities can be further interior where sand has been moved >2 km inland from high winds. Dwarf‑shrub communities occur on older dunes, usually behind grassland‑dominated dunes.

**Dynamics:** [from M059] Processes that define the macrogroup include sand deposition, salt spray, wind erosion, long‑shore transport, dune formation, and water erosion such as overwash from storm surges. Herbaceous species stabilize the sand deposits (dunes, beaches), and the older deposits support dwarf‑shrubs mixed with herbaceous species.

The beach and open (shrub‑herb) dune vegetation is but one part of the vegetation on dunes, including debris‑line communities, herbaceous rhizomatous vegetation on oligotrophic habitats, consolidated dune scrubs, stabilized dune forests, willow shrubs on dune swales and deflation plains, terophytic sand communities and other specialized groups. The zonal and successional relationships among these communities are complicated and non‑linear.

SOURCES

**References:** Brown 1982a, Comer et al. 2003*, Shreve and Wiggins 1964

**Version:** 24 Mar 2003	**Stakeholders:** Latin America, West

**Concept Author:** NatureServe Western Ecology Team	**LeadResp:** West

CES206.907 Mediterranean California Northern Coastal Dune

**Primary Division:** Mediterranean California (206)

**Land Cover Class:** Barren

**Spatial Scale & Pattern:** Large patch

**Required Classifiers:** Natural/Semi‑natural; Unvegetated (<10% vasc.); Upland

**Diagnostic Classifiers:** Dune field; Dune (Substrate); Mediterranean [Mediterranean Xeric‑Oceanic]; Sand Soil Texture; Very Short Disturbance Interval; W‑Patch/High Intensity; Abronia latifolia; Coastal Dune Mosaic

**Concept Summary:** This coastal system occurs in scattered locations from Point Conception, California, north to Coos Bay, Oregon. Coastal dunes include beaches, foredunes, sand spits, and active to stabilizing backdunes and sandsheets derived from quartz or gypsum sands. The mosaic of sparse to dense vegetation in dune systems is driven by sand deposition, erosion, and lateral movement. Coastal dunes often front portions of inlets and tidal marshes. They may also occur as extensive dune fields dominating large coastal bays. Dune vegetation typically includes herbaceous, succulent, and low‑shrub species with varying degrees of tolerance for salt spray, wind and sand abrasion, and substrate stability. Dune succession is highly variable, so species composition can vary significantly between occurrences. Generally, these dune systems can be dominated by *Leymus mollis, Abronia latifolia, Ambrosia chamissonis, Baccharis pilularis, Calystegia soldanella, Artemisia pycnocephala, Ericameria ericoides, Eriogonum latifolium, Camissonia cheiranthifolia*, and *Carpobrotus chilensis (= Carpobrotus aequilateralus)*. Disturbance processes include dune blowouts caused by wind and occasional wave overwash during storm tidal surges.

DISTRIBUTION

**Range:** Occurs in scattered locations from Point Conception, California, north to Coos Bay, Oregon.

**Divisions:** 204:P, 206:C

**TNC Ecoregions:** 1:P, 14:C, 15:C

**Nations:** US

**Subnations:** CA, OR

**Map Zones:** 2:C, 3:C, 4:C

**USFS Ecomap Regions:** 263A:CC

CONCEPT

**Environment:** Coastal dunes include beaches, foredunes, sand spits, and active to stabilizing backdunes and sandsheets derived from quartz or gypsum sands. The mosaic of sparse to dense vegetation in dune systems is driven by sand deposition, erosion, and lateral movement. Coastal dunes often front portions of inlets and tidal marshes. They may also occur as extensive dune fields dominating large coastal bays. Climate is both Mediterranean and maritime; temperatures are moderate year‑round. Most precipitation occurs in the winter months, followed by summer drought, and mild winter temperatures permit growing season throughout most of the year (Wiedemann 1984, Christy et al. 1998, Pickart and Barbour 2007). Clouds and fog are present throughout much of the year, with fog becoming increasingly common to the south (Wiedemann 1984). The dune localities are generally associated with nearby rivers, estuaries or bays; rivers deposit sediment which is carried by ocean currents and wind and deposited on flat coastline areas with onshore winds (Pickart and Barbour 2007). Dune sands are very poor soils, with no organic matter accumulation (Wiedemann 1984). pH is about neutral and the nutrient status is so low as to be almost unmeasurable. Dune sands have poor moisture‑holding capacity. A salinity gradient appears to be important in California dunes, and germination or emergence stages are more vulnerable to soil salinity or washover of saltwater than established plants. Pickart and Barbour (2007) provide a summary of studies of the physiological ecology of dune plants.

**Dynamics:** Disturbance processes include dune blowouts caused by wind and occasional wave overwash during storm tidal surges. Cyclical dune activity is apparently triggered by cyclical changes in sea level associated with tectonic events (Wiedemann 1984, Christy et al. 1998, Pickart and Barbour 2007). Subsidence or uplift of 1.8 to 2.7 m (6‑9 feet) associated with earthquakes would initiate new successional pathways after destruction of existing dune formations and vegetation (Thilenius 1995). Generally it appears that major earthquakes occur along this coastal region at 300‑ to 700‑year intervals, and sometimes cause tsunamis (Carver et al. 1998, as cited in Pickart and Barbour 2007).

Wind is the other major disturbance process in this system. It drives seasonal movement of large dunes, in turn causing burial of forest vegetation along the eastern edge of the dune sheet and exhumation of previously buried vegetation in interdunal troughs. Storm winds lead to windthrow of many trees in exposed areas, and windfall is commonly seen in senescing stands of *Pinus contorta var. contorta*. Wind‑driven sand and salt stunt and abrade plants, and can kill both buds and leaves of shrubs or conifers. Removal of vegetation exposes the sand to wind erosion, leading to the formation of blowouts or the complete destruction of stabilized dunes. Wind patterns are an important factor; in this system the northerly summer winds are associated with the North Pacific High and bring generally fair weather with occasional high‑velocity land‑sea breezes in the afternoon (Wiedemann 1984). In the winter, the low pressure systems commonly occurring further north are less important in this system. These wind patterns are modified by sheltering headlands and capes in places.

Fire, insects, and pathogens appear to have relatively minor roles in this system, although some *Pinus contorta var. contorta* stands are even‑aged and result from stand‑replacing fires; others result from primary succession (Christy et al. 1998). Pickart and Barbour (2007) provide a summary of recent work on plant‑animal interactions and the roles of nitrogen‑fixing plants in California dune ecosystems; they include topics such as rodent herbivory, the roles of ground‑nesting bees in providing soil nutrients and pollination, cryptogamic soil crusts, obligate or facultative relationships between insects and plants, and others.

SOURCES

**References:** Barbour and Major 1988, Brown 1990, Christy et al. 1998, Comer et al. 2003*, Holland and Keil 1995, PRBO Conservation Science 2011, Pickart 1987, Pickart and Barbour 2007, Pickart and Sawyer 1998, Sawyer and Keeler‑Wolf 1995, Thilenius 1995, WNHP 2011, Wiedemann 1984

**Version:** 14 Jan 2014	**Stakeholders:** West

**Concept Author:** P. Comer and T. Keeler‑Wolf	**LeadResp:** West

CES206.908 Mediterranean California Southern Coastal Dune

**Primary Division:** Mediterranean California (206)

**Land Cover Class:** Barren

**Spatial Scale & Pattern:** Linear

**Required Classifiers:** Natural/Semi‑natural; Unvegetated (<10% vasc.); Upland

**Diagnostic Classifiers:** Blowout; Dune field; Foredune; Dune (Substrate); Mediterranean [Mediterranean Xeric‑Oceanic]; Salt Spray; W‑Patch/High Intensity; Abronia maritima; Coastal Dune Mosaic

**Concept Summary:** This coastal system occurs in scattered locations from Point Conception, California, south to north‑central Baja California. Coastal dunes include beaches, foredunes, sand spits, and active to stabilizing backdunes and sandsheets derived from quartz or gypsum sands. The mosaic of sparse to dense vegetation in dune systems is driven by sand deposition, erosion, and lateral movement. Coastal dunes often front portions of inlets and tidal marshes. They may also occur as extensive dune fields dominating large coastal bays. Dune vegetation typically includes herbaceous, succulent, and low‑shrub species with varying degrees of tolerance for salt spray, wind and sand abrasion, and substrate stability. Dune succession is highly variable, so species composition can vary significantly between occurrences. Generally, this dune system includes fewer perennial grasses and more suffrutescent plants than more northern dune systems. This system can be dominated by *Abronia maritima, Abronia umbellata, Atriplex leucophylla, Isocoma menziesii (= Haplopappus venetus), Distichlis spicata, Croton californicus, Lupinus chamissonis*, and *Carpobrotus chilensis (= Carpobrotus aequilateralus)*. Disturbance processes include dune blowouts caused by wind and occasional wave overwash during storm tidal surges.

DISTRIBUTION

**Range:** Occurs in scattered locations from Point Conception, California, south to north‑central Baja California.

**Divisions:** 206:C

**TNC Ecoregions:** 16:C, NT1301:C

**Nations:** MX, US

**Subnations:** CA, MXBC

**Map Zones:** 4:C

**USFS Ecomap Regions:** 261B:CC

CONCEPT

**Environment:** Coastal dunes include beaches, foredunes, sand spits, and active to stabilizing backdunes and sandsheets derived from quartz or gypsum sands. The mosaic of sparse to dense vegetation in dune systems is driven by sand deposition, erosion, and lateral movement. Coastal dunes often front portions of inlets and tidal marshes. They may also occur as extensive dune fields dominating large coastal bays. The climate is both Mediterranean and maritime; temperatures are moderate year‑round. Most precipitation occurs in the winter months, followed by summer drought, and mild winter temperatures permit growing season throughout most of the year. Clouds and fog are present throughout the year. The dune localities are generally associated with nearby rivers, estuaries or bays; rivers deposit sediment which is carried by ocean currents and wind and deposited on flat coastline areas with onshore winds (Pickart and Barbour 2007). Dune sands are very poor soils, with no organic matter accumulation (Wiedemann 1984), and poor moisture‑holding capacity. pH is about neutral and the nutrient status is so low as to be almost unmeasurable. A salinity gradient appears to be important in California dunes, and germination or emergence stages are more vulnerable to soil salinity or washover of saltwater than established plants. Pickart and Barbour (2007) provide a summary of studies of the physiological ecology of dune plants.

**Dynamics:** Disturbance processes include dune blowouts caused by wind and occasional wave overwash during storm tidal surges. Wind is the major disturbance process in this system. It drives seasonal movement of large dunes, in turn causing burial of forest vegetation along the eastern edge of the dune sheet and exhumation of previously buried vegetation in interdunal troughs. Storm winds lead to windthrow of many trees in exposed areas. Wind‑driven sand and salt stunt and abrade plants, and can kill both buds and leaves of shrubs or conifers. Removal of vegetation exposes the sand to wind erosion, leading to the formation of blowouts or the complete destruction of stabilized dunes. Wind patterns are an important factor; in this system the northerly summer winds are associated with the North Pacific High and bring generally fair weather with occasional high‑velocity land‑sea breezes in the afternoon (Wiedemann 1984). In the winter, the low pressure systems commonly occurring further north are less important in this system. These wind patterns are modified by sheltering headlands and capes in places.

Fire, insects, and pathogens appear to have relatively minor roles in this system. Pickart and Barbour (2007) provide a summary of recent work on plant‑animal interactions and the roles of nitrogen‑fixing plants in California dune ecosystems; they include topics such as rodent herbivory, the roles of ground‑nesting bees in providing soil nutrients and pollination, cryptogamic soil crusts, obligate or facultative relationships between insects and plants, and others.

SOURCES

**References:** Barbour and Major 1988, Brooks and Minnich 2006, Brown 1990, Comer et al. 2003*, Holland and Keil 1995, PRBO Conservation Science 2011, Pickart and Barbour 2007, Sawyer and Keeler‑Wolf 1995, WNHP 2011, Wiedemann 1984

**Version:** 14 Jan 2014	**Stakeholders:** Latin America, West

**Concept Author:** P. Comer and T. Keeler‑Wolf	**LeadResp:** West

CES200.881 North Pacific Maritime Coastal Sand Dune and Strand

**Primary Division:** (200)

**Land Cover Class:** Barren

**Spatial Scale & Pattern:** Large patch

**Required Classifiers:** Natural/Semi‑natural; Unvegetated (<10% vasc.); Upland

**Diagnostic Classifiers:** Herbaceous; Dune (Substrate); Beach (Substrate); Temperate [Temperate Oceanic]; Salt Spray

**Concept Summary:** Coastal sand dunes are found throughout the northern Pacific Coast, from south‑central Alaska to the central Oregon coast (roughly Coos Bay). This system covers large areas of the southern Washington and central Oregon coasts, but coastal dunes in Alaska have been placed into a different system. Coastal dunes include beach strand (not the beach itself but sparsely or densely vegetated areas behind the beach), foredunes, sand spits, and active to stabile backdunes and sandsheets derived from quartz or gypsum sands. The mosaic of sparse to dense vegetation in dune systems is driven by sand deposition, erosion, and lateral movement. Disturbance processes include dune blowouts caused by wind and occasional wave overwash during storm tidal surges. Coastal dunes often front portions of inlets and tidal marshes. Dune vegetation typically includes herbaceous, succulent, shrub, and tree species with varying degrees of tolerance for salt spray, wind and sand abrasion, and substrate stability. Dune succession is highly variable, so species composition can vary significantly among occurrences. These dunes can be dominated by *Leymus arenarius (= Elymus arenarius), Festuca rubra, Leymus mollis*, or various forbs adapted to salty dry conditions. *Gaultheria shallon* and *Vaccinium ovatum* are major shrub species. Forested portions of dunes are included within this system and are characterized (at least in the south) by *Pinus contorta var. contorta* early in succession, *Picea sitchensis* somewhat later in the sere, and in some cases *Tsuga heterophylla* later still. *Pseudotsuga menziesii* sometimes codominates in Oregon. In many cases, occurrences have thin, fragile layers of lichens and mosses covering the sand in between clumps of grasses or shrubs. Disturbance processes include dune blowouts caused by wind and occasional wave overwash during storm tidal surges. Late‑sere forests, dominating stabilized dune systems where active dune processes are nearly absent and that compositionally represent the adjacent matrix system, are excluded from this dune system. Interdunal wetlands occur commonly within the matrix of this system and sometimes are extensive in deflation plains or old dune troughs, but are considered part of various separate wetland ecological systems depending on their hydrology, and are not part of this upland system.

**Comments:** Concept has been revised with input from John Christy. We include forested dunes here and put wetlands in other wetland systems. Forested dunes eventually become very similar to the matrix forest, i.e., *Picea sitchensis* basically late‑successional forest dunes. Mapping issues are expected as forested dunes lose the *Pinus contorta var. contorta* component and become completely dominated by *Picea sitchensis* and/or *Tsuga heterophylla* (old‑growth or late‑successional forest composition). As long as *Pinus contorta var. contorta* is a prominent component, the forested dune continues to be part of the dune system.

DISTRIBUTION

**Range:** This system is found throughout the northern Pacific Coast, including large inlets such as Puget Sound, from south‑central British Columbia to the central Oregon coast (roughly Coos Bay).

**Divisions:** 204:C

**TNC Ecoregions:** 1:C, 2:C, 69:P

**Nations:** CA, US

**Subnations:** BC, OR, WA:S1

**Map Zones:** 1:C, 2:C, 3:C

**USFS Ecomap Regions:** 242A:CC, M242A:CC

CONCEPT

**Environment:** These dunes are found in about 23 localities along the North American Pacific Northwest Coast, from just north of Coos Bay, Oregon, north into Washington near the Copalis River (Wiedemann 1984). Coastal dunes include beach strand (not the beach itself but sparsely or densely vegetated areas behind the beach), foredunes, sand spits, and active to stabile backdunes and sandsheets derived from quartz or gypsum sands. Climate is both Mediterranean and maritime; temperatures are moderate year‑round. Most precipitation occurs in the winter months, followed by summer drought, and mild winter temperatures permit growing season throughout most of the year (Wiedemann 1984, Christy et al. 1998). Clouds and fog are present throughout the year, with fog becoming increasingly common to the south (Wiedemann 1984). The dune localities are generally associated with nearby rivers, estuaries or bays (Wiedemann 1984); rivers deposit sediment which is carried by ocean currents and wind and deposited on flat coastline areas with on‑shore winds. Dune sands are very poor soils, with no organic matter accumulation (Wiedemann 1984). pH is about neutral and the nutrient status is so low as to be almost unmeasurable. In this system, the rainfall is so high that, combined with rapid drainage, salinity is not an important factor even in areas just above the beach (Wiedemann 1984). Dune sands have poor moisture‑holding capacity; however, these dunes are underlain by groundwater aquifers that maintain a high water table (Christy et al. 1998).

**Dynamics:** The north Pacific coastal dunes are dynamic, transgressive, wind‑controlled systems in their natural condition (citations in Zarnetske et al. 2010). These communities are dependent upon longshore drift and wind (WNHP 2011). Most occurrences are spits or berms behind sandy beaches. The mosaic of sparse to dense vegetation in dune systems is driven by sand deposition, erosion, and lateral movement. Disturbance processes include dune blowouts caused by wind and occasional wave overwash during storm tidal surges. Cyclical dune activity is apparently triggered by cyclical changes in sea level associated with glaciation and tectonic events (Wiedemann 1984, Christy et al. 1998). Subsidence or uplift of 1.8 to 2.7 m (6‑9 feet) associated with earthquakes would initiate new successional pathways after destruction of existing dune formations and vegetation (Thilenius 1995). Generally it appears that major earthquakes occur along this coastal region at 300‑ to 700‑year intervals (Christy et al. 1998), and sometimes cause tsunamis.

<br />Wind is the major disturbance process in this system. It drives seasonal movement of large dunes, in turn causing burial of forest vegetation along the eastern edge of the dune sheet and exhumation of previously buried vegetation in interdunal troughs. Storm winds lead to windthrow of many trees in exposed areas, and windfall is commonly seen in senescing stands of *Pinus contorta var. contorta*. Wind‑driven sand and salt stunt and abrade plants, and can kill both buds and leaves of shrubs or conifers. Removal of vegetation exposes the sand to wind erosion, leading to the formation of blowouts or the complete destruction of stabilized dunes. Wind patterns are an important factor; in this system northerly summer winds are associated with the North Pacific High and bring generally fair weather with occasional high‑velocity land‑sea breezes in the afternoon (Wiedemann 1984). In the winter low pressure systems dominate the weather patterns, bringing heavy rains and strong southerly winds. These wind patterns are modified by sheltering headlands and capes in places.

Fire, insects, and pathogens appear to have relatively minor roles in this system, although some *Pinus contorta var. contorta* stands are even‑aged and result from stand‑replacing fires; others result from primary succession (Christy et al. 1998).

SOURCES

**References:** Brown 1990, Chappell and Christy 2004, Christy et al. 1998, Comer et al. 2003*, Eyre 1980, Holland and Keil 1995, Karl et al. 2009, Littell et al. 2009, PRBO Conservation Science 2011, Pickart 1987, Pickart 1997, Pickart and Barbour 2007, Pickart and Sawyer 1998, Thilenius 1995, WNHP 2011, WNHP unpubl. data 2018, Wiedemann 1984, Wiedemann 1990, Zarnetske et al. 2010

**Version:** 14 Jan 2014	**Stakeholders:** Canada, West

**Concept Author:** K. Boggs, C. Chappell, G. Kittel	**LeadResp:** West

M058. Pacific Coastal Cliff & Bluff

CES204.094 North Pacific Coastal Cliff and Bluff

**Primary Division:** North American Pacific Maritime (204)

**Land Cover Class:** Barren

**Spatial Scale & Pattern:** Linear

**Required Classifiers:** Natural/Semi‑natural; Unvegetated (<10% vasc.); Upland

**Diagnostic Classifiers:** Bluff; Headland; Sea cliff; Talus (Substrate); Rock Outcrops/Barrens/Glades; Oligotrophic Soil; Salt Spray; Very Shallow Soil; Landslide; W‑Landscape/High Intensity

**Concept Summary:** This ecological system includes unvegetated or sparsely vegetated rock cliffs and very steep bluffs of glacial deposits along the Pacific Ocean and associated marine and estuarine inlets. It is restricted to degrading slopes from southwestern British Columbia south into central Oregon. It is composed of barren and sparsely vegetated substrates, typically including exposed sediments, bedrock, and scree slopes. Exposure to waves, eroding and desiccating winds, slope failures and sheet erosion create gravelly to rocky substrates that are often unstable. There can be sparse cover of forbs, grasses, lichens and low shrubs.

**Comments:** Small areas of rock outcrop within a mosaic of vegetated systems are best considered part of an adjacent system, e.g., within herbaceous balds and bluffs. In Washington, North Pacific Hypermaritime Shrub and Herbaceous Headland (CES204.088) and this cliff system sometimes occur adjacent or in a mosaic together, but not always. It is quite frequent to get cliffs without the vegetated part. The shrub and herbaceous component is less common. In Mediterranean California these two types of systems are split from each other (coastal grassland from coastal bluff). As far as biodiversity goes, the vegetated versus the nonvegetated are very different. This system is distinguished from Mediterranean California Coastal Bluff (CES206.906) by being further north in areas where summers are typically cooler, and winters may include some snow.

DISTRIBUTION

**Range:** This system is found from central Oregon north along the immediate coast into British Columbia.

**Divisions:** 204:C

**TNC Ecoregions:** 1:C, 69:C

**Nations:** CA, US

**Subnations:** BC, OR, WA:S4

**Map Zones:** 1:C, 2:C

**USFS Ecomap Regions:** 242A:CC, 263A:??, M242A:CC, M261A:??

CONCEPT

SOURCES

**References:** Comer et al. 2003*, Franklin and Dyrness 1973, WNHP unpubl. data 2018

**Version:** 30 Mar 2005	**Stakeholders:** Canada, West

**Concept Author:** R. Crawford and C. Chappell	**LeadResp:** West

2.C. Shrub & Herb Wetland

2.C.2. Temperate to Polar Bog & Fen

2.C.2.Na. North American Bog & Fen

M877. North American Boreal & Subboreal Alkaline Fen

CES201.585 Laurentian‑Acadian Alkaline Fen

**Primary Division:** Laurentian‑Acadian (201)

**Land Cover Class:** Woody Wetland

**Spatial Scale & Pattern:** Small patch

**Required Classifiers:** Natural/Semi‑natural; Vegetated (>10% vasc.); Wetland

**Diagnostic Classifiers:** Organic Peat (>40 cm); Mesotrophic Water; Alkaline Water; Circumneutral Water

**Concept Summary:** These fens, distributed across glaciated eastern and central North America, develop in open basins where bedrock or other substrate influence creates circumneutral to calcareous conditions. They are most abundant in areas of limestone bedrock, and widely scattered in areas where calcareous substrates are scarce. Shore fens, which are peatlands that are occasionally flooded along stream and lakeshores, are also included here because flooding tends to create moderately alkaline conditions. The vegetation may be graminoid‑dominated, shrub‑dominated, or a patchwork of the two; *Dasiphora fruticosa ssp. floribunda* is a common diagnostic shrub. The herbaceous flora is usually species‑rich and includes calciphilic graminoids and forbs. *Sphagnum* dominates the substrate in many sites though in Michigan a patchy to continuous carpet of brown mosses is more typical; *Campylium stellatum* is an indicator bryophyte. The edge of the basin may be shallow to deep peat over a sloping substrate, where seepage waters provide nutrients.

**Comments:** Need to clarify the conceptual boundaries between this and the boreal fens in central and eastern Canada. Alkaline wooded swamps, some of which have fen‑like characteristics, are treated under Laurentian‑Acadian Alkaline Conifer‑Hardwood Swamp (CES201.575).

DISTRIBUTION

**Range:** Scattered locations from New England and adjacent Canada west to the Great Lakes and northern Minnesota.

**Divisions:** 201:C, 202:C

**TNC Ecoregions:** 47:C, 48:C, 60:C, 61:C, 63:C, 64:P

**Nations:** CA, US

**Subnations:** MA, ME, MI, MN, NB, NH, NY, PA?, VT, WI

**Map Zones:** 41:C, 50:C, 51:C, 63:C, 64:C, 65:P, 66:C

**USFS Ecomap Regions:** 211A:CP, 211E:CC, 211F:CP, 211I:CC, 211Jb:CCC, 211Jc:CCC, 212Ha:CCP, 212Hb:CCP, 212Hc:CCP, 212Hd:CCP, 212He:CCC, 212Hf:CCC, 212Hg:CCC, 212Hh:CCP, 212Hi:CCC, 212Hj:CCP, 212Hk:CCC, 212Hl:CCC, 212Hm:CCP, 212J:CC, 212K:CC, 212L:CC, 212M:CC, 212N:CC, 212Q:CP, 212Ra:CCP, 212Rb:CCC, 212Rc:CCC, 212Rd:CCC, 212Re:CCC, 212S:CC, 212Tb:CCC, 212Te:CCP, 212X:CC, 212Y:CC, 212Z:CC, 221A:CC, 221B:CC, 222I:CC, M211A:CP, M211B:CP, M211C:CC

CONCEPT

**Environment:** This system usually occurs where there is flat, highly calcareous bedrock near the surface. Water slowly moves along this bedrock and, where it comes to the surface, fens can form in the cold, mineral‑rich, anoxic water. Soils are organic and saturated most or all of the growing season. Waterflow through this system is slow but greater than in bogs (Schwintzer and Tomberlin 1982). Some fens in this system occur on the shore of lakes or ponds where wave action is low.

**Dynamics:** The presence of cold, mineral‑rich, alkaline groundwater which promotes the formation of peat and marl is key to the formation and maintenance of this system. Where cold, mineral‑rich groundwater emerges as diffuse seeps, decomposition of plant matter is slowed and peat can accumulate. Marl forms under sustained flow of calcium‑ and magnesium‑rich water. Peat can form hummocks which have microenvironments that are drier and more acidic than the bulk of the fen. The hummock‑and‑hollow microtopography, which generates small‑scale gradients in soil moisture and chemistry, contributes to fen floristic diversity. The high pH of the bulk of the fens strongly shapes the floristic composition.

SOURCES

**References:** Comer et al. 2003*, Edinger et al. 2014a, Eyre 1980, Faber‑Langendoen et al. 2011, Gawler and Cutko 2010, Kost et al. 2007, Schwintzer and Tomberlin 1982, Sperduto and Nichols 2004, WDNR 2015

**Version:** 14 Jan 2014	**Stakeholders:** Canada, East, Midwest

**Concept Author:** S.C. Gawler	**LeadResp:** East

CES306.831 Rocky Mountain Subalpine‑Montane Fen

**Primary Division:** Rocky Mountain (306)

**Land Cover Class:** Herbaceous Wetland

**Spatial Scale & Pattern:** Small patch

**Required Classifiers:** Natural/Semi‑natural; Vegetated (>10% vasc.); Wetland

**Diagnostic Classifiers:** Moss/Lichen (Nonvascular); Seepage‑Fed Sloping [Peaty]; Organic Peat (>40 cm); Graminoid; Bryophyte; Extreme (Mineral) Rich and Iron‑Rich; Saturated Soil

**Concept Summary:** This system occurs infrequently throughout the Rocky Mountains from Colorado north into Canada. It is confined to specific environments defined by groundwater discharge, soil chemistry, and peat accumulation of at least 40 cm. This system includes extreme rich fens and iron fens, both being quite rare. Fens form at low points in the landscape or near slopes where groundwater intercepts the soil surface. Groundwater inflows maintain a fairly constant water level year‑round, with water at or near the surface most of the time. Constant high water levels lead to accumulation of organic material. In addition to peat accumulation and perennially saturated soils, the extreme rich and iron fens have distinct soil and water chemistry, with high levels of one or more minerals such as calcium, magnesium, or iron. These fens usually occur as a mosaic of several plant associations dominated by *Carex aquatilis, Carex limosa, Carex lasiocarpa, Betula glandulosa, Kobresia myosuroides, Kobresia simpliciuscula*, and *Trichophorum pumilum*. *Sphagnum* spp. (peatmoss) is indicative of iron fens. The surrounding landscape may be ringed with other wetland systems, e.g., riparian shrublands, or a variety of upland systems from grasslands to forests.

**Comments:** Need to clarify this system in relation to Boreal Fen system. In Montana, small fens are found in scattered locations in the plains and the small isolated mountain ranges of the central part of the state; these are included here.

DISTRIBUTION

**Range:** This system occurs infrequently throughout the Rocky Mountains from Colorado north into Canada. In Montana, small fens included here are found in scattered locations in the plains and the small isolated mountain ranges of the central part of the state. Similarly, recent inventory in Wyoming has revealed the occurrence of small fens throughout the mountain ranges of that state.

**Divisions:** 304:C, 306:C

**TNC Ecoregions:** 7:C, 8:P, 9:P, 11:P, 18:C, 19:P, 20:C, 21:P, 68:P

**Nations:** CA, US

**Subnations:** AB, AZ, BC, CO, ID, MT, NV, OR, UT, WA:S3, WY

**Map Zones:** 9:P, 10:C, 12:?, 16:C, 17:?, 19:C, 20:C, 21:C, 22:?, 23:?, 24:?, 25:?, 27:?, 28:C, 29:C

**USFS Ecomap Regions:** 331J:CC, 331N:C?, M331A:CC, M331B:CC, M331D:CC, M331E:CP, M331F:CC, M331G:CC, M331H:CC, M331I:CC, M332A:CP, M332B:CP, M332D:CC, M332E:CP, M332F:CP, M332G:CP, M333A:PP, M333B:PP, M333C:PP, M333D:PP

CONCEPT

**Environment:** The montane fen ecological system is a small‑patch system composed of mountain wetlands that support a unique ecology of rare plants not found in other types of wetlands. These fens are confined to specific environments defined by groundwater discharge, soil chemistry, and peat accumulation of at least 40 cm. Fens form at low points in the landscape or near slopes where groundwater intercepts the soil surface (CNHP 2010b). Groundwater inflows maintain a fairly constant water level year‑round, with water at or near the surface most of the time. Constant high water levels lead to accumulations of organic material (CNHP 2010b).Within the region this system occurs at montane elevations ranging from 2440‑3500 m (8000‑11,480 feet) and is characterized by mosaics of plant communities. These communities typically occur in seeps and wet sub‑irrigated meadows in narrow to broad valley bottoms. Surface topography is typically smooth to concave with slopes ranging from 0‑10%. The soils within this system are organic Histosols with 40 cm or more of organic material. These Histosols range in texture from clayey‑skeletal to loamy‑skeletal and fine‑loams. They may occur on a variety of parent materials including alluvial and colluvial deposits of granitic and gneiss origins. The pH of wetlands within this system in generally between 4.8 and 6.0‑7.0.

**Vegetation:** hese fens usually occur as a mosaic of several plant associations dominated by *Carex aquatilis, Carex limosa, Carex lasiocarpa, Betula glandulosa, Kobresia myosuroides, Kobresia simpliciuscula*, and *Trichophorum pumilum (= Scirpus pumilus)*. *Sphagnum* spp. (peatmoss) is indicative of iron fens. The surrounding landscape may be ringed with other wetland systems, e.g., riparian shrublands, or a variety of upland systems from grasslands to forests.

**Dynamics:** Groundwater inflows maintain a fairly constant water level year‑round, with water at or near the surface most of the time. Constant high water levels lead to accumulations of organic material. Peatlands in the southern Rocky Mountains are fens that remain saturated primarily as a result of discharging groundwater, seasonal and/or perennial surface water input, or due to their location on the fringes of lakes and ponds (Cooper 1990). Thus, peatlands only occur in confining basins, near persistent groundwater‑discharge sites, or near permanent waterbodies such as lakes, ponds, and streams. Due to the limited amount of precipitation and low humidity in the southern Rocky Mountains, true bogs do not occur in the region (Cooper 1990).

Snowmelt maintains high water tables through June in many wetland types (wet meadows, fens, riparian areas, etc.); however, only those areas with soil saturation or a water table within 30 cm of the soil surface through July and August accumulate peat (Cooper 1990, Chimner and Cooper 2003). Thus, a distinguishing characteristic between wet meadows and fens is the depth of the water table in these months. Even in fens, the water table begins to drop in late‑July and August. However, late‑summer precipitation often replenishes local aquifers thereby raising water tables, suggesting summer precipitation may be important to maintaining high water tables in Southern Rocky Mountain fens (Cooper 1990). In the Northern Rocky Mountains that lack late‑summer rains, continuous groundwater discharge is important (Chadde et al. 1998).

Mountain fens function as natural filters cleaning ground and surface water. Fens also act as sponges by absorbing heavy precipitation, slowly releasing it downstream, minimizing erosion and recharging groundwater systems (Windell et al. 1986). The persistent groundwater and cold temperatures allow organic matter to accumulate (forming peat) which allows classification of wetlands within this system as fens. Fens in the Southern Rockies produce peat that accumulates at the rate of 20 to 28 cm (8‑11 inches) per 1000 years, making peatlands a repository of 10,000 years of post‑glacial history (Windell et al. 1986).

SOURCES

**References:** Bales et al. 2006, Bryce et al. 2012, CNHP 2010, Chadde et al. 1998, Chimner and Cooper 2003, Comer et al. 2003*, Cooper 1986b, Cooper 1990, Cooper 1993b, Cooper and Sanderson 1997, Cooper et al. 1998, Cooper et al. 2005, Garfin et al. 2010, Johnson 1996, Jones 2003, Karl et al. 2009, Knowles et al. 2006, NCC 2002, Neely et al. 2001, Peterson et al. 2008, Rondeau 2001, Steen and Coupé 1997, Stewart et al. 2004, WNHP unpubl. data 2018, Windell et al. 1986, Woods 2001

**Version:** 14 Jan 2014	**Stakeholders:** Canada, West

**Concept Author:** M.S. Reid	**LeadResp:** West

M876. North American Boreal & Subboreal Bog & Acidic Fen

CES201.580 Acadian Maritime Bog

**Primary Division:** Laurentian‑Acadian (201)

**Land Cover Class:** Woody Wetland

**Spatial Scale & Pattern:** Large patch

**Required Classifiers:** Natural/Semi‑natural; Vegetated (>10% vasc.); Wetland

**Diagnostic Classifiers:** Organic Peat (>40 cm); Dwarf‑Shrub; Graminoid; Maritime Climate

**Concept Summary:** These ombrotrophic acidic peatlands occur along the north Atlantic Coast from downeast Maine east into the Canadian Maritimes. When these form in basins, they develop raised plateaus with undulating sedge and dwarf‑shrub vegetation. *Trichophorum cespitosum* may form sedge lawns on the raised plateau. The system may also occur as "blanket bogs" over a sloping rocky substrate in extreme maritime settings; here, dwarf‑shrubs and *Sphagnum* are the dominant cover. Species characteristic of this maritime setting include *Empetrum nigrum* and *Rubus chamaemorus*. Typical bog heaths such as *Kalmia angustifolia, Kalmia polifolia, Gaylussacia baccata, Ledum groenlandicum*, and *Gaylussacia dumosa* are also present. Morphological characteristics and certain coastal species distinguish these from more inland raised bogs. The distribution is primarily Canadian, and these peatlands are rare in the U.S.

DISTRIBUTION

**Range:** This system occurs near the coast from eastern Maine (Mount Desert Island) eastward into the Canadian Maritimes.

**Divisions:** 201:C

**TNC Ecoregions:** 63:C

**Nations:** CA, US

**Subnations:** ME, NB

**Map Zones:** 66:C

**USFS Ecomap Regions:** 211Cb:CCC

CONCEPT

SOURCES

**References:** Comer et al. 2003*, Damman and French 1987, Eyre 1980, Gawler and Cutko 2010, Worley 1980a

**Version:** 09 Jan 2003	**Stakeholders:** Canada, East

**Concept Author:** S.C. Gawler	**LeadResp:** East

CES201.583 Eastern Boreal‑Sub‑boreal Acidic Basin Fen

**Primary Division:** Laurentian‑Acadian (201)

**Land Cover Class:** Woody Wetland

**Spatial Scale & Pattern:** Large patch

**Required Classifiers:** Natural/Semi‑natural; Vegetated (>10% vasc.); Wetland

**Diagnostic Classifiers:** Depressional; Organic Peat (>40 cm); Broad‑Leaved Shrub; Dwarf‑Shrub; Graminoid; Picea mariana ‑ Larix laricina; Acidic Water

**Concept Summary:** This peatland system ranges over a broad geographic area across the glaciated Northeast to the Great Lakes and upper Midwest. The fens have developed in open or closed, relatively shallow basins with nutrient‑poor and acidic conditions. Many occur in association with larger lakes or streams. Some occur as kettlehole fens (usually called kettlehole "bogs") associated with eskers or other glacial deposits. The substrate is *Sphagnum*, and vegetation typically includes areas of graminoid dominance and dwarf‑shrub dominance. *Chamaedaphne calyculata* is usually present and often dominant. Scattered stunted trees may be present. These fens often develop adjacent to open water and may form a floating mat over water.

Particularly distinctive are the ribbed bogs or fens in which a pattern of narrow (2‑ to 3‑m wide), low (less than 1 m deep) ridges are oriented at right angles to the direction of the drainage (National Wetlands Working Group 1988). Wet pools or depressions occur between the ridges. These patterned peatlands may include string bog, Atlantic ribbed fen, or northern ribbed fen (National Wetlands Working Group 1988). They develop almost entirely north of 46°N latitude in east‑central Canada and the adjacent U.S. They are minerotrophic peatlands in which the vegetation has developed into a pattern of strings (raised, usually linear features) and flarks (wet depressions separating the strings). The substrate chemistry is entirely acidic in some peatlands; in others, where bedrock or other substrate influence creates circumneutral to calcareous conditions, peatland chemistry may be entirely calcareous or vary from acidic to calcareous within the same peatland. In acidic portions, typical bog heaths predominate mixed with sedges. *Dasiphora fruticosa ssp. floribunda* is diagnostic of circumneutral to calcareous conditions. These peatlands usually develop in open basins and flat plains, and the patterned portion may occupy only a fraction of the entire peatland. The edge of the basin may be shallow to deep peat over a sloping substrate, where seepage waters provide nutrients.

**Comments:** Need to clarify the conceptual boundaries between this and the boreal fens in central and eastern Canada. This system is also similar to acidic peatlands in the southern edge of the glaciated region, which are treated under North‑central Interior and Appalachian Acidic Peatland (CES202.606); those often tend to be smaller‑patch landscape elements. USFS sections are used to differentiate the ranges.

DISTRIBUTION

**Range:** This system is found in New England and adjacent Canada west to the Great Lakes and Minnesota, north of the glacial boundary.

**Divisions:** 103:C, 201:C, 202:C

**TNC Ecoregions:** 47:C, 48:P, 61:C, 63:C

**Nations:** CA, US

**Subnations:** MA, ME, MI, MN, NB?, NH, NS?, NY, QC, VT, WI

**Map Zones:** 41:C, 50:C, 51:C, 63:P, 64:C, 65:C, 66:C

**USFS Ecomap Regions:** 211A:CC, 211B:CC, 211C:CC, 211D:CC, 211E:CC, 212Ha:CCP, 212Hb:CCP, 212Hc:CCP, 212Hd:CCP, 212He:CCP, 212Hf:CCC, 212Hg:CCC, 212Hh:CCP, 212Hi:CCP, 212Hj:CCP, 212Hk:CCC, 212Hl:CCP, 212Hm:CCP, 212J:CP, 212Lb:CCP, 212Ra:CCC, 212Rb:CCC, 212Rc:CCP, 212Rd:CCP, 212Re:CCC, 212S:CP, 212T:CP, 212X:CP, 212Ya:CCP, M211A:CC, M211B:CC, M211C:CC, M211D:CC

CONCEPT

SOURCES

**References:** Comer et al. 2003*, Damman and French 1987, Edinger et al. 2014a, Eyre 1980, Gawler and Cutko 2010, Kost et al. 2007, Sperduto and Nichols 2004, WDNR 2015

**Version:** 05 Jun 2008	**Stakeholders:** Canada, East, Midwest

**Concept Author:** S.C. Gawler	**LeadResp:** East

CES202.606 North‑Central Interior and Appalachian Acidic Peatland

**Primary Division:** Central Interior and Appalachian (202)

**Land Cover Class:** Woody Wetland

**Spatial Scale & Pattern:** Small patch

**Required Classifiers:** Natural/Semi‑natural; Vegetated (>10% vasc.); Wetland

**Diagnostic Classifiers:** Shrubland (Shrub‑dominated); Organic Peat (>40 cm); Acidic Water; >180‑day hydroperiod

**Concept Summary:** These *Sphagnum* and shrub peatlands occur in basins south of the Laurentian‑Acadian region down to near the glacial boundary in the northeastern and north‑central U.S. Unlike the true raised bogs of boreal regions, the vegetation is not raised above the groundwater level. They are found in colder regions, mostly in areas where glacial stagnation left coarse deposits and glacial depressions (many are "kettleholes"). The basins are generally closed, i.e., without inlets or outlets of surface water, and typically small in area. The nutrient‑poor substrate and the reduced throughflow of water create oligotrophic conditions fostering the development of *Sphagnum* peat and the growth of peatland vegetation. In deeper basins, the vascular vegetation grows on a *Sphagnum* mat over water, with no mineral soil development. Ericaceous shrubs and dwarf‑shrubs (e.g., *Chamaedaphne calyculata*) dominate, with patches of graminoid dominance. Some peatlands may have a sparse tree layer. Although these are often called bogs, in most cases they are technically fens (albeit nutrient‑poor ones), as the vegetation remains in contact with the surface water.

**Comments:** This system occurs south of the Laurentian‑Acadian division in the Midwest, south of the Northern Appalachian‑Boreal ecoregion in the Northeast, and inland from the Coastal Plain, and these acidic peatlands are distinctive and discrete elements of the landscape. They are related to Northern Appalachian‑Acadian Conifer‑Hardwood Acidic Swamp (CES201.574) and Eastern Boreal‑Sub‑boreal Acidic Basin Fen (CES201.583), but occur in a different landscape setting and often have some more temperate floristic elements to distinguish them. They include treed, shrub, and graminoid associations, often occurring in a mosaic. In the Midwest, it may be necessary to split off the shrub/graminoid acid peatland (poor fen) types.

DISTRIBUTION

**Range:** This system is found from central New England to the Great Lakes and south‑central Minnesota southward, generally associated with the glacial terminus or stagnation zones, and interior from the Coastal Plain.

**Divisions:** 202:C

**TNC Ecoregions:** 45:P, 46:P, 48:P, 49:P, 60:C, 61:C, 64:C

**Nations:** CA, US

**Subnations:** CT, IL, IN, MA, ME, MI, MN, NH, NJ, NY, OH, ON, PA, RI, VT, WI

**Map Zones:** 41:?, 49:P, 50:P, 51:P, 52:P, 61:C, 62:C, 63:C, 64:C, 65:C, 66:P

**USFS Ecomap Regions:** 211F:CC, 211I:CP, 211J:CC, 221A:CC, 221B:CC, 221D:CC, 221E:CC, 221Fa:CCC, 222I:CC, 222Ja:CCC, 222Jb:CCC, 222Jc:CCC, 222Je:CCC, 222Jg:CCC, 222Jh:CCC, 222Ji:CCC, 222R:CC, 222Ua:CCP, 222Ud:CCC, 222Ue:CCC

CONCEPT

**Environment:** These peatlands occur in kettle depressions on pitted outwash and moraines and in flat areas and shallow depressions on glacial outwash and glacial lakeplain. Groundwater and surface water feed these temperate peatlands. It is not strongly calcareous and may be acidic in some places but not as much as boreal sites. These peatlands occurred in landscapes dominated by either forest or grassland/savanna. The fire regime is not well known but periodic surface fires likely helped limit the cover by trees. The basins in which these occur tend to be small and, where open water is still present, these peatlands form where wave energy is low (Swinehart 1997). These peatlands are characterized by organic soils composed of saturated peat that contains partially decomposed sphagnum mosses and frequently fragments of sedges and wood. The peat soils are acidic, cool, and characterized by low nutrient availability and oxygen levels. The water‑retaining capacity of sphagnum peat is tremendous and as a result these are saturated, anoxic systems with water tables near the surface (Kost et al. 2007).

**Vegetation:** Trees include *Acer rubrum, Picea mariana*, and *Pinus rigida*. Shrubs may include *Alnus incana, Chamaedaphne calyculata, Decodon verticillatus, Gaylussacia baccata, Gaylussacia dumosa, Ilex verticillata, Larix laricina, Myrica gale, Aronia melanocarpa (= Photinia melanocarpa), Spiraea tomentosa, Vaccinium corymbosum, Vaccinium macrocarpon, Vaccinium myrtilloides, Vaccinium oxycoccos*, and *Viburnum nudum*. Forbs and graminoids may include *Calla palustris, Carex lasiocarpa, Carex oligosperma, Carex pauciflora, Carex utriculata, Dulichium arundinaceum, Eriophorum vaginatum, Eriophorum virginicum, Lysimachia terrestris, Osmunda regalis, Triadenum virginicum, Utricularia* sp., and *Woodwardia virginica*.

**Dynamics:** The cool, nutrient‑poor water which feeds into this system favors peat development. This water can come from surface runoff or groundwater. Basins in which these peatlands occur are small, which limits the amount of nutrients that can be brought in by surface water. Groundwater sources flow through nutrient‑poor, neutral to somewhat acidic substrates. Once peat begins to develop, it tends to create conditions favorable for continued peat development by contributing to the acidic, anoxic character of the water.

SOURCES

**References:** Comer et al. 2003*, Damman and French 1987, Edinger et al. 2014a, Eyre 1980, Faber‑Langendoen et al. 2011, Gawler and Cutko 2010, Kost et al. 2007, ONHD unpubl. data, Sperduto and Nichols 2004, Swinehart 1997, Swinehart and Starks 1994, WDNR 2015

**Version:** 02 Jan 2015	**Stakeholders:** Canada, East, Midwest, Southeast

**Concept Author:** S.C. Gawler	**LeadResp:** East

M063. North Pacific Bog & Fen

CES206.953 Mediterranean California Serpentine Fen

**Primary Division:** Mediterranean California (206)

**Land Cover Class:** Herbaceous Wetland

**Spatial Scale & Pattern:** Small patch

**Required Classifiers:** Natural/Semi‑natural; Vegetated (>10% vasc.); Wetland

**Diagnostic Classifiers:** Moss/Lichen (Nonvascular); Mediterranean [Mediterranean Pluviseasonal‑Oceanic]; Seepage‑Fed Sloping; Organic Peat (>40 cm); Bryophyte; Darlingtonia californica; Ultramafic; Bog and Fen Mosaic

**Concept Summary:** This ecological system is found uncommonly throughout coastal lowlands and high mountains of the Klamath Mountains and surrounding landscapes where serpentine soils are common in cool and moist environments. This system includes unique assemblages of wetlands species restricted to serpentine and ultramafic substrates. These sites remain moist or wet throughout the year and may have substantial *Sphagnum* accumulation. Some may be bogs in the sense of nutrients and moisture primarily coming from rainfall, or more commonly they are seeps or fens maintained by groundwater discharge. Soils are acidic and often derived from ultramafic parent materials. The acidic (6.5‑6.7 pH) and nutrient‑poor substrates produce severe nitrogen deficiency which favors insectivorous plants. Characteristic plant species include *Darlingtonia californica, Drosera rotundifolia, Eleocharis quinqueflora, Calliscirpus criniger, Carex californica*, and *Deschampsia cespitosa*. Around the edges of these fens *Chamaecyparis lawsoniana* can occur and form part of the fen. Burning is essential to maintain healthy stands. *Darlingtonia* fens are important habitat for rare species that respond positively to burning. Burning at least eliminates some of the tree invaders (*Pinus jeffreyi, Pseudotsuga menziesii, Chamaecyparis lawsoniana*) and maintains a high water table, essential for the fen‑dependent plants.

DISTRIBUTION

**Range:** This system is found uncommonly throughout coastal lowlands and high mountains of the Klamath Mountains of California and Oregon and surrounding landscapes where serpentine soils are common in cool and moist environments.

**Divisions:** 206:C

**TNC Ecoregions:** 5:C, 14:P

**Nations:** US

**Subnations:** CA, OR

**Map Zones:** 2:C, 3:C, 7:P

**USFS Ecomap Regions:** M261A:CC, M261B:CP, M261D:CP

CONCEPT

**Environment:** This system comprises wetlands located on serpentine soils, where a layer of sphagnum moss overlays serpentine soils and parent material. Soils are acidic and often derived from ultramafic parent materials. The acidic (6.5‑6.7 pH) and nutrient‑poor substrates produce severe nitrogen deficiency.

**Vegetation:** Characteristic plant species include *Darlingtonia californica, Drosera rotundifolia, Eleocharis quinqueflora, Calliscirpus criniger (= Eriophorum crinigerum), Carex californica*, and *Deschampsia cespitosa*. Around the edges of these fens *Chamaecyparis lawsoniana* can occur and form part of the fen. Burning is essential to maintain healthy stands. *Darlingtonia* fens are important habitat for rare species that respond positively to burning. Burning at least eliminates some of the tree invaders (*Pinus jeffreyi, Pseudotsuga menziesii, Chamaecyparis lawsoniana*) and maintains a high water table, essential for the fen‑dependent plants.

**Dynamics:** Consistently high water table and spring flows to maintain wet soils, water quantity and quality are very important, and stable groundwater, surface water, or precipitation inputs are crucial for continual integrity of these organic soils.

SOURCES

**References:** Barbour and Major 1988, Comer et al. 2003*, Cooper and Wolf 2005, Cooper and Wolf 2006a, Cooper and Wolf 2006b, Cooper and Wolf 2006c, Holland and Keil 1995, Littell et al. 2009, PRBO Conservation Science 2011, Sawyer and Keeler‑Wolf 1995, Sikes et al. 2010, Sikes et al. 2011, Sikes et al. 2012, Sikes et al. 2013, WNHP 2011

**Version:** 14 Jan 2014	**Stakeholders:** West

**Concept Author:** P. Comer and T. Keeler‑Wolf	**LeadResp:** West

CES206.952 Mediterranean California Subalpine‑Montane Fen

**Primary Division:** Mediterranean California (206)

**Land Cover Class:** Herbaceous Wetland

**Spatial Scale & Pattern:** Small patch

**Required Classifiers:** Natural/Semi‑natural; Vegetated (>10% vasc.); Wetland

**Diagnostic Classifiers:** Temperate [Temperate Oceanic]; Seepage‑Fed Sloping; Muck; Circumneutral Water; Extreme (Mineral) Rich and Iron‑Rich; Bog and Fen Mosaic

**Concept Summary:** This system is found in montane to subalpine elevations confined to specific environments defined by groundwater discharge, soil chemistry, and peat accumulation. This system includes extreme rich fens which are quite rare. Fens form at low points in the landscape or near slopes where groundwater intercepts the soil surface. Groundwater inflows maintain a fairly constant water level year‑round, with water at or near the surface most of the time. Constant high water levels lead to accumulation of organic material. In addition to peat accumulation and perennially saturated soils, the extreme rich fens have distinct soil and water chemistry, with high levels of one or more minerals such as calcium and/or magnesium. They usually occur as a mosaic of several plant associations dominated by species of *Carex, Betula, Kobresia*, or *Schoenoplectus*. The surrounding landscape may be ringed with other wetland systems, e.g., riparian shrublands, or a variety of upland systems from grasslands to forests.

DISTRIBUTION

**Range:** These fens are found in montane to subalpine elevations of California mountains, in the Sierra Nevada, northwestern California coastal mountains, and possibly the Klamath‑Siskiyou mountains.

**Divisions:** 206:C

**TNC Ecoregions:** 5:P, 12:P, 14:P

**Nations:** US

**Subnations:** CA, NV, OR

**Map Zones:** 2:P, 3:C, 4:?, 6:C, 7:C, 12:P

**USFS Ecomap Regions:** 341D:PP, M261A:C?, M261B:C?, M261D:C?, M261E:CC

CONCEPT

**Environment:** This system is found in montane to subalpine elevations confined to specific environments defined by groundwater discharge, soil chemistry, and peat accumulation. This system includes extreme rich fens which are quite rare. Fens form at low points in the landscape or near slopes where groundwater intercepts the soil surface. Groundwater inflows maintain a fairly constant water level year‑round, with water at or near the surface most of the time. Constant high water levels lead to accumulation of organic material. In addition to peat accumulation and perennially saturated soils, the extreme rich fens have distinct soil and water chemistry, with high levels of one or more minerals such as calcium and/or magnesium.

**Vegetation:** Vegetation occurs as a mosaic of several plant associations dominated by species of *Carex, Betula, Kobresia*, or *Schoenoplectus*. The surrounding landscape may be ringed with other wetland systems, e.g., riparian shrublands, or a variety of upland systems from grasslands to forests.

SOURCES

**References:** Barbour and Major 1988, Comer et al. 2003*, Hickman 1993, Holland and Keil 1995, Sawyer and Keeler‑Wolf 1995, Shiflet 1994

**Version:** 14 Dec 2004	**Stakeholders:** West

**Concept Author:** P. Comer and T. Keeler‑Wolf	**LeadResp:** West

CES204.063 North Pacific Bog and Fen

**Primary Division:** North American Pacific Maritime (204)

**Land Cover Class:** Woody Wetland

**Spatial Scale & Pattern:** Small patch

**Required Classifiers:** Natural/Semi‑natural; Vegetated (>10% vasc.); Wetland

**Diagnostic Classifiers:** Lowland [Foothill]; Shrubland (Shrub‑dominated); Temperate [Temperate Oceanic]; Depressional; Organic Peat (>40 cm); Sphagnum spp.

**Concept Summary:** This wetland system occurs in peatlands along the Pacific Coast from British Columbia south to northern California, in and west of the coastal mountain summits but including the Puget Sound lowlands. Elevations are mostly under 457 m (1500 feet), and annual precipitation ranges from 890‑3050 mm (35‑120 inches). These wetlands are relatively abundant in British Columbia but diminish rapidly in size and number farther south. They occur in river valleys, around lakes and marshes, or on slopes. The organic soils are characterized by an abundance of sodium cations from oceanic precipitation. Poor fens and bogs are often intermixed except in a few calcareous areas in British Columbia where rich fen vegetation may dominate. *Sphagnum* characterizes poor fens and bogs (pH <5.5), and the two are lumped here, while "brown mosses" and sedges characterize rich fens (pH >5.5). Mire profiles in British Columbia may be flat, raised (domed), or sloping, but most occurrences in Washington and Oregon are flat with only localized hummock development. Vegetation is usually a mix of conifer‑dominated swamp, shrub swamp, and open sphagnum or sedge mire, often with small lakes and ponds interspersed. Vegetation includes many species common to boreal continental bogs and fens, such as *Ledum groenlandicum, Vaccinium uliginosum, Myrica gale, Andromeda polifolia, Vaccinium oxycoccos, Equisetum fluviatile, Comarum palustre*, and *Drosera rotundifolia*. However, it is also distinguished from boreal continental bogs and fens by the presence of Pacific coastal species, including *Callitropsis nootkatensis, Pinus contorta var. contorta, Picea sitchensis, Tsuga heterophylla, Ledum glandulosum, Thuja plicata, Gaultheria shallon, Spiraea douglasii, Carex aquatilis var. dives, Carex lyngbyei, Carex obnupta, Carex pluriflora, Darlingtonia californica, Sphagnum pacificum, Sphagnum henryense*, and *Sphagnum mendocinum*.

**Comments:** This system is distinguished and split from Boreal Depressional Shrub Bog (CES103.871) and Western Canadian Boreal Shrub‑Sedge Rich and Alkaline Fen (CES103.872). The communities comprising this system are not well‑described or classified. Several bog or fen ecological systems have recently (2007‑2008) been classified for southeastern Alaska, hence this system no longer is considered to occur in Alaska. Where the "muskeg" of northern British Columbia should be placed is unclear and needs review from ecologists there. Discussion is that muskeg in the hypermaritime region is extensive, covering large areas of landscape, whereas this system in Oregon and Washington tends to occur in much smaller patches. How distinct is the hypermaritime muskeg of northern British Columbia from bogs and fens from central Vancouver Island south?

DISTRIBUTION

**Range:** This system occurs along the Pacific Coast from British Columbia south to northern California, west of the coastal mountain summits but including the Puget Sound lowlands. Occurrences diminish rapidly in size and number south of British Columbia.

**Divisions:** 204:C, 206:P

**TNC Ecoregions:** 1:C, 2:C, 3:C, 69:P, 81:C

**Nations:** CA, US

**Subnations:** BC, OR, WA:S2

**Map Zones:** 1:C, 2:C, 7:C

**USFS Ecomap Regions:** 242A:CC, 242B:CP, 342C:P?, 342H:PP, M242A:CC, M242B:CC, M242C:CC, M242D:CC, M332G:CC

CONCEPT

**Environment:** Elevations are mostly under 457 m (1500 feet), and annual precipitation ranges from 890‑3050 mm (35‑120 inches). These wetlands are relatively abundant in British Columbia but diminish rapidly in size and number farther south. They occur in river valleys, around lakes and marshes, or on slopes. The organic soils are characterized by an abundance of sodium cations from oceanic precipitation. Poor fens and bogs are often intermixed except in a few calcareous areas in British Columbia where rich fen vegetation may dominate. *Sphagnum* characterizes poor fens and bogs (pH <5.5), and the two are lumped here, while "brown mosses" and sedges characterize rich fens (pH >5.5). Mire profiles in British Columbia may be flat, raised (domed), or sloping, but most occurrences in Washington and Oregon are flat with only localized hummock development.

**Vegetation:** Vegetation is usually a mix of conifer‑dominated swamp, shrub swamp, and open sphagnum or sedge mire, often with small lakes and ponds interspersed. Vegetation includes many species common to boreal continental bogs and fens, such as *Ledum groenlandicum, Vaccinium uliginosum, Myrica gale, Andromeda polifolia, Vaccinium oxycoccos, Equisetum fluviatile, Comarum palustre*, and *Drosera rotundifolia*. However, it is also distinguished from boreal continental bogs and fens by the presence of Pacific coastal species, including *Callitropsis nootkatensis (= Chamaecyparis nootkatensis), Pinus contorta var. contorta, Picea sitchensis, Tsuga heterophylla, Ledum glandulosum, Thuja plicata, Gaultheria shallon, Spiraea douglasii, Carex aquatilis var. dives, Carex lyngbyei, Carex obnupta, Carex pluriflora, Darlingtonia californica, Sphagnum pacificum, Sphagnum henryense*, and *Sphagnum mendocinum*.

**Dynamics:** Successional patterns of wet meadows to fens to bogs in Alaska have been documented as follows, and are likely to be similar in this ecological system. Species that dominate the early stages of succession in newly formed ponded basins include *Equisetum variegatum, Equisetum fluviatile*, and *Comarum palustre*. *Sphagnum* species invade the surface and help in forming peat. Acidic and nutrient‑poor‑tolerant vascular species eventually dominate the sites, such as *Myrica gale, Empetrum nigrum, Vaccinium uliginosum, Andromeda polifolia*, and *Vaccinium oxycoccos*. The late‑successional stage of a peatland supports various community types, depending on the pH, waterflow, and nutrient status of a site such as *Myrica gale / Empetrum nigrum* and *Picea sitchensis / Sphagnum* plant associations. Peat buildup, patterned ground, and changes in water table are recurrent aspects of peatland development rather than unidirectional successional events. It is unlikely that any of the late‑seral peatland communities are stable in the sense of climax vegetation.

SOURCES

**References:** Banner et al. 1993, Comer et al. 2003*, DeLong et al. 1990, DeLong et al. 1994, Eyre 1980, Steen and Coupé 1997, WNHP unpubl. data 2018

**Version:** 08 Dec 2008	**Stakeholders:** Canada, West

**Concept Author:** J.C. Christy	**LeadResp:** West

2.C.2.Nb. Atlantic & Gulf Coastal Plain Pocosin

M065. Southeastern Coastal Bog & Fen

CES203.893 Atlantic Coastal Plain Northern Bog

**Primary Division:** Gulf and Atlantic Coastal Plain (203)

**Land Cover Class:** Woody Wetland

**Spatial Scale & Pattern:** Small patch

**Required Classifiers:** Natural/Semi‑natural; Vegetated (>10% vasc.); Wetland

**Concept Summary:** This system comprises dwarf‑shrub sphagnum bogs dominated by *Chamaedaphne calyculata* occurring on Cape Cod (Massachusetts), Long Island (New York), and the Coastal Plain and near‑coastal areas of northern New Jersey. North of the glacial border, this system typically occurs in isolated glacial kettleholes and in New Jersey in similar isolated basins, generally in regions of deep sands. The system is characterized by acidic, tannic water supporting a floating or grounded *Sphagnum* mat over which *Chamaedaphne calyculata, Gaylussacia dumosa*, and other dwarf‑shrubs have rooted. Taller shrubs such as *Vaccinium corymbosum* may occur at the periphery of the bog, and *Decodon verticillatus* often forms a distinct zone adjacent to open water. Scattered individuals of *Pinus rigida, Pinus strobus*, or less often *Chamaecyparis thyoides* or *Picea mariana* may form a partial and stunted tree layer. Rooted hydromorphic plants such as *Nymphaea odorata* occur in open water.

**Comments:** This system is separated from Northern Atlantic Coastal Plain Pitch Pine Lowland (CES203.374) based on the different landscape setting of the latter (pine barrens) and the implications for a different fire regime.

DISTRIBUTION

**Range:** This system occurs on Cape Cod (Massachusetts), Long Island (New York), and possibly on the Coastal Plain of New Jersey north of the Pine Barrens region.

**Divisions:** 203:C

**TNC Ecoregions:** 62:C

**Nations:** US

**Subnations:** MA, NH, NJ, NY

**Map Zones:** 60:C, 65:C, 66:C

CONCEPT

**Environment:** North of the glacial border, this system typically occurs in isolated glacial kettleholes and in New Jersey in similar isolated basins, generally in regions of deep sands.

**Vegetation:** The system is characterized by acidic, tannic water supporting a floating or grounded *Sphagnum* mat over which *Chamaedaphne calyculata, Gaylussacia dumosa*, and other dwarf‑shrubs have rooted. Taller shrubs such as *Vaccinium corymbosum* may occur at the periphery of the bog, and *Decodon verticillatus* often forms a distinct zone adjacent to open water. Scattered individuals of *Pinus rigida, Pinus strobus*, or less often *Chamaecyparis thyoides* or *Picea mariana* may form a partial and stunted tree layer. Rooted hydromorphic plants such as *Nymphaea odorata* occur in open water.

SOURCES

**References:** Comer et al. 2003*, Edinger et al. 2014a, Eyre 1980, Gawler and Cutko 2010, Sperduto and Nichols 2004

**Version:** 05 May 2008	**Stakeholders:** East

**Concept Author:** L. Sneddon	**LeadResp:** East

CES203.267 Atlantic Coastal Plain Peatland Pocosin and Canebrake

**Primary Division:** Gulf and Atlantic Coastal Plain (203)

**Land Cover Class:** Woody Wetland

**Spatial Scale & Pattern:** Large patch

**Required Classifiers:** Natural/Semi‑natural; Vegetated (>10% vasc.); Wetland

**Diagnostic Classifiers:** Forest and Woodland (Treed); Shrubland (Shrub‑dominated); Extensive Wet Flat

**National Mapping Codes:** EVT 2452; ESLF 9121; ESP 1452

**Concept Summary:** This system includes wetlands of organic soils, occurring on broad flats or gentle basins, primarily on the outer terraces of the Atlantic Coastal Plain of the Carolinas and southeastern Virginia. Under current conditions, the vegetation is predominantly dense shrubland and very shrubby open woodlands. A characteristic suite of primarily evergreen shrubs, *Smilax* species, and *Pinus serotina* dominates. These shrubs include *Cyrilla racemiflora, Ilex coriacea, Ilex glabra, Lyonia lucida*, and *Zenobia pulverulenta*, along with *Smilax laurifolia*. *Pinus serotina* is the characteristic tree, along with *Gordonia lasianthus, Magnolia virginiana*, and *Persea palustris*. Herbs are scarce and largely limited to small open patches. Under pre‑European settlement fire regimes, stands of *Arundinaria tecta* (canebrakes) would have been more common and extensive. Soil saturation, sheet flow, and peat depth create a distinct zonation, with the highest stature woody vegetation on the edges and lowest in the center. Catastrophic fires are important in this system, naturally occurring at moderate frequency. Fires generally kill all above‑ground shrubs in large patches. Mortality of *Pinus serotina* varies, creating a shifting mosaic. Vegetation structure and biomass recover rapidly in most of the burned areas, primarily by sprouting. *Pinus serotina* can regenerate from serotinous cones if killed.

**Comments:** Related vegetation occurs in Atlantic Coastal Plain Streamhead Seepage Swamp, Pocosin and Baygall (CES203.252), which may share many plant species but which has hydrology driven by seepage. This system (CES203.267) has three recognizable landscape patterns within it: domed peatlands, peat‑filled Carolina bays, and small swales. Vegetational and ecological differences between these have not been demonstrated but may warrant further investigation. There are differences in landscape pattern among them. The "small swale" manifestation of this exists in smaller patches.

DISTRIBUTION

**Range:** This system is found primarily in North Carolina, extending into Georgia and southeastern Virginia.

**Divisions:** 203:C

**TNC Ecoregions:** 56:C, 57:C

**Nations:** US

**Subnations:** GA, NC, SC, VA

**Map Zones:** 58:C, 60:C

**USFS Ecomap Regions:** 232C:CC, 232I:CC

CONCEPT

**Environment:** This system occurs on broad interfluvial flats and in small to large, very gentle basins and swales, largely on the outermost terraces of the Outer Coastal Plain. Some occurrences are in large to small peat‑filled Carolina bays (Bennett and Nelson 1991, Nifong 1998). Smaller patches occur in shallow swales associated with relict coastal dune system or other irregular sandy surfaces. Soils range from wet mineral soils with mucky surface layers to peats several meters deep. Most of the largest occurrences are domed peatlands with the deepest peat associated with topographic highs in the center, but deep peats are also associated with buried drainage channels. Hydrology is driven by rainfall and sheet flow. The low hydraulic conductivity of the organic material limits interaction with the groundwater. The raised center of domed peatlands is fed only by rainwater and is therefore a true ombrotrophic bog. More peripheral portions are fed by sheet flow from the center, and so receive only acidic water low in nutrients. Occurrences in Carolina bays and other basins appear to be similarly isolated from surface or groundwater inflow from adjacent areas. Soils are normally saturated throughout the winter and well into the growing season, though the organic material may dry enough to burn during droughts. Standing water is limited to local depressions and disturbed areas. Soil saturation and peat depth, with its corresponding nutrient limitation, are the primary drivers of vegetational zonation as well as the distinction between this system and adjacent ones, but their effect may be modified by drainage patterns.

**Vegetation:** Vegetation is a series of distinctive associations known as pocosins. Under current conditions, the vegetation is predominantly dense shrubland and very shrubby open woodlands, ranging to nearly closed forests. Herbaceous associations are present only as small patches. Vegetation is typically zoned. The lowest stature vegetation occurs in the center of the system, with woodlands on the edges and in the smaller occurrences. The communities have in common a dense shrub layer of wetland shrubs tolerant of the organic soils, low nutrient conditions, and fire. *Ilex glabra, Lyonia lucida, Lyonia mariana, Cyrilla racemiflora, Ilex coriacea*, and *Zenobia pulverulenta* are characteristic and usually dominant in some combination, along with *Smilax laurifolia*. *Pinus serotina* is the characteristic tree, and it along with a set of evergreen hardwoods, including *Gordonia lasianthus, Magnolia virginiana*, and *Persea palustris*, are generally the only trees present. Under pre‑European settlement fire regimes, stands of *Arundinaria tecta (= Arundinaria gigantea ssp. tecta)* (canebrakes) would have been more common and extensive. Component communities tend to be low in plant species richness, and woody species richness exceeds herbaceous in most associations, with herbs being limited to small open patches. These areas would have formerly been more extensive under pre‑European settlement fire regimes. The dominance of pond pine and evergreen shrubs as opposed to a canopy of deciduous hardwoods distinguishes this system from nonriverine swamp forests (CES203.304).

**Dynamics:** Fire is an important factor in these systems, with the pre‑settlement fire regime probably being very different from that observed under current conditions. Natural fire‑return intervals are not well known, but are probably on the order of a decade or two in the wettest areas. Peripheral areas may be subject to fire as often as the surrounding vegetation burns, which may naturally have been an average of 3 years. Fires are typically intense due to density and flammability of the vegetation; all above‑ground vegetation is often killed, though *Pinus serotina* are resilient to fire and may survive. Fires are followed by vigorous root sprouting by shrubs and hardwoods, leading to recovery of standing biomass within a few years. *Pinus serotina* recovers by epicormic sprouting or by regeneration from seeds released from serotinous cones. Fires during droughts may ignite peat, forming holes that take longer to recover. Herb‑dominated openings in pocosins may depend on peat fires for their creation, though this is not well documented. Natural fires occur in large patches, creating a shifting patch structure in the system that interacts with the vegetational zonation created by peat depth. The intensity of fire in these systems makes fire control difficult; prescribed burning is seldom done, and wildfires during drought continue to be a significant influence. The larger peatlands are believed to have been created by paludification following natural blocking of drainage (Otte 1981). Peat buildup raises the water table in the center, creating the domed structure of the largest peatlands and allowing the wetland to spread out as wetness is increased at the edges. Many of the deeper pocosin peats contain fossil logs that indicate dominance by a swamp forest in past millennia. Otte (1981) noted that peat fires likely limit the height to which the peat can accumulate, in proportion to how high it can raise the local water table.

SOURCES

**References:** Bennett and Nelson 1991, Christensen et al. 1981, Comer et al. 2003*, Engeman et al. 2007, Eyre 1980, Nelson 1986, Nifong 1998, Otte 1981, Richardson 2003, Schafale 2012, Sharitz and Gibbons 1982, Weakley and Schafale 1991

**Version:** 23 Apr 2015	**Stakeholders:** East, Southeast

**Concept Author:** M. Schafale and R. Evans	**LeadResp:** Southeast

2.C.3. Tropical Freshwater Marsh, Wet Meadow & Shrubland

2.C.3.Ef. Caribbean‑Mesoamerican Freshwater Marsh, Wet Meadow & Shrubland

M710. Caribbean Freshwater Marsh, Wet Meadow & Shrubland

CES411.467 Caribbean Emergent Herbaceous Estuary

**Primary Division:** Caribbean (411)

**Land Cover Class:** Herbaceous Wetland

**Spatial Scale & Pattern:** Small patch

**Required Classifiers:** Natural/Semi‑natural; Vegetated (>10% vasc.); Wetland

**Diagnostic Classifiers:** Aluvial; Dark gleyized soil; Riverine / Alluvial; Tidal / Estuarine [Oligohaline]

**Concept Summary:** This system is dominated by tall grasses growing along the shores of meandering streams and on (semi‑) permanently flooded plains. *Cladium* is an indicator of alkaline chemistry caused by underlying calcareous rock or brackish tidal influence. The following list of species is diagnostic for this system: *Typha domingensis, Cyperus giganteus, Cladium mariscus ssp. jamaicense, Urochloa mutica (= Brachiaria mutica), Hymenachne amplexicaulis, Sacciolepis striata (= Panicum aquaticum), Paspalidium geminatum (= Panicum geminatum)*, and *Vallisneria americana*.

DISTRIBUTION

**Range:** This system is found in Bahamas, Cuba, the Greater Antilles, Puerto Rico, and Venezuela.

**Divisions:** 411:C

**Nations:** BS, CU, PR, VE, XC

CONCEPT

SOURCES

**References:** Areces‑Mallea et al. 1999, Borhidi 1991, Dansereau 1966, Huber and Alarcón 1988, Josse et al. 2003*

**Version:** 07 Dec 2004	**Stakeholders:** Caribbean, Latin America, U.S. Territories

**Concept Author:** C. Josse	**LeadResp:** Latin America

CES411.286 South Florida Everglades Sawgrass Marsh

**Primary Division:** Caribbean (411)

**Land Cover Class:** Herbaceous Wetland

**Spatial Scale & Pattern:** Matrix

**Required Classifiers:** Natural/Semi‑natural; Vegetated (>10% vasc.); Wetland

**Diagnostic Classifiers:** Extensive Wet Flat; Graminoid

**National Mapping Codes:** EVT 2483; ESLF 9204; ESP 1483

**Concept Summary:** This marsh system was a dominant type throughout much of the Everglades region of southeastern Florida. It consists largely of herbaceous marsh vegetation across a range of soil and hydrologic conditions, i.e., hydroperiod of 225‑275 days per year, maximum wet‑season water level of 40 cm, and occurrence on peat soils. Several individual marsh community associations have been recognized based on species composition, structure, and aspect. Variations are largely due to the interrelated effects of fire, soils, and hydroperiod. Sawgrass beds or "glades" may have been the single most extensive component of this system, and large areas may have the appearance of nearly monotypic stands of *Cladium mariscus ssp. jamaicense*. However, local variation in composition and stature are also often apparent. For example, two broad aspect types of *Cladium* marsh are often recognized based on density and/or height with denser and taller stands typically occurring on higher topographic positions and deeper organic soils, while sparser, shorter stands occur in lower topography on shallower soils. In addition, other marsh types are also interfingered in the sawgrass matrix where wetter depressions are found and/or where fires have burned away peat soils.

**Comments:** The term "wet prairie" has often been used to describe a variety of marsh types which are included in the concept of this system. We follow the definition of Duever et al. (1986) in which prairies occupy mineral soils and marshes occupy peats. The community components of these systems are largely based on Davis (1943) and Hilsenbeck et al. (1979). Open and emergent marshes of the region are generally covered by South Florida Slough, Gator Hole and Willow Head (CES411.485); these are generally small patches included in the sawgrass matrix.

DISTRIBUTION

**Range:** This system is endemic to south Florida.

**Divisions:** 411:C

**TNC Ecoregions:** 54:C

**Nations:** US

**Subnations:** FL

**Map Zones:** 56:C

**USFS Ecomap Regions:** 411A:CC

CONCEPT

**Environment:** A range of conditions are present, but generally falls within conditions outlined by Duever et al. (1986). Soils vary from shallow marl to relatively deep peat. Hydroperiod ranges from 5‑12 months, with maximum wet‑season water level of 40 cm. The effect of fire is influenced by both factors and affects them in turn. For example, peat accumulates in the absence of fire, but under certain conditions, fires may burn away accumulated sawgrass peat resulting in a thin, residual, marly soil and relative increase of effective water depth (resulting in community change).

**Vegetation:** Marsh communities present in this system include tall and short‑statured *Cladium mariscus ssp. jamaicense*, spikerush ‑ beaksedge flats, and maidencane flats. In the absence of fire, portions of stands will become dominated by *Salix caroliniana*. If fire continues to be absent, these areas may succeed to *Acer rubrum* until a replacement fire or mechanical activity restores the marsh.

Several individual marsh community associations have been recognized based on species composition, structure, and aspect. Variations are largely due to the interrelated effects of fire, soils, and hydroperiod. Sawgrass beds or "glades" may have been the single most extensive component of this system (Hilsenbeck et al. 1979), and large areas may have the appearance of nearly monotypic stands of *Cladium mariscus ssp. jamaicense*. However, local variation in composition and stature are also often apparent. For example, two broad aspect types of *Cladium* marsh are often recognized based on density and/or height (Kushlan 1990, Gunderson and Loftus 1993) with denser and taller stands typically occurring on higher topographic positions and deeper organic soils, while sparser, shorter stands occur in lower topography on shallower soils. In addition, other marsh types are also interfingered in the sawgrass matrix where wetter depressions are found and/or where fires have burned away peat soils.

**Dynamics:** In the absence of fire, portions of stands will become dominated by *Salix caroliniana*. If fire continues to be absent, these areas may succeed to *Acer rubrum* until a replacement fire or mechanical activity restores the marsh.

SOURCES

**References:** Comer et al. 2003*, Davis 1943, Duever et al. 1986, FNAI 2010a, Gunderson and Loftus 1993, Hilsenbeck et al. 1979, Kushlan 1990

**Version:** 05 Jul 2006	**Stakeholders:** Southeast

**Concept Author:** R. Evans	**LeadResp:** Southeast

CES411.485 South Florida Slough, Gator Hole and Willow Head

**Primary Division:** Caribbean (411)

**Land Cover Class:** Herbaceous Wetland

**Spatial Scale & Pattern:** Small patch

**Required Classifiers:** Natural/Semi‑natural; Vegetated (>10% vasc.); Wetland

**Diagnostic Classifiers:** Woody‑Herbaceous; Herbaceous; Depressional [Peaty]; >180‑day hydroperiod

**Concept Summary:** This ecological system includes a series of wetlands of southern Florida, ranging in physiognomy from open and herbaceous‑dominated to tree‑dominated patches, including nearly monospecific stands of *Salix caroliniana*. These wetlands hold water for much of the year and have some of the longest hydroperiods (8‑12 months) in a region characterized by wetlands. Most are maintained, at least historically, by American alligators. Alligators were such a dominant disturbance force in many plant communities of southern Florida that their role has been compared with that of bison in the prairies. Through constant movement, they create numerous small pools and ponds (analogous to buffalo wallows), as well as trails to and from these pools through sawgrass marshes. These paths eventually widen and deepen into creeks. Many of these small freshwater creeks have been invaded by mangroves and hardwoods, including *Salix caroliniana*, in the absence of fire and with decreases in alligator populations. Some emergent wetlands included within the concept of this system may also have originated from soil and topographic changes in former sawgrass marshes following severe fires that consume organic substrate and decrease soil elevation. One component of this system ("heads") may originate as circular or oval‑shaped solution holes or basins, being maintained and possibly enhanced by the alligator activity. Without this activity, there would be a tendency for the hole or basin to fill with organic material and succeed to other systems. Soils are mucky peats. In addition, *Salix caroliniana* seeds are readily dispersed by wind and may rapidly colonize wet depressions and disturbed areas. In the absence of fire and disturbance, these areas may remain in a forested condition. Otherwise, they would cycle between different physiognomic states, including sawgrass marsh.

DISTRIBUTION

**Range:** This system is endemic to south Florida.

**Divisions:** 411:C

**TNC Ecoregions:** 54:C

**Nations:** US

**Subnations:** FL

**Map Zones:** 56:C

CONCEPT

**Environment:** Examples of this system may originate as solution holes in sawgrass marsh, with a longer hydroperiod, but expand and contract in size and extent with disturbance, including fire and American alligator activity. Some examples are directly caused by alligator activity and/or the effect of severe fire in sawgrass marshes, South Florida Everglades Sawgrass Marsh (CES411.286) (Craighead 1971, Hilsenbeck et al. 1979). At least some examples attributed to this system occupy "marshes" with long hydroperiods (8‑12 months) and deep organic soils (Hilsenbeck et al. 1979).

**Vegetation:** A number of discrete communities may be recognized as part of this system. Two of the most common types can be considered cattail marshes and flag ‑ pickerelweed communities (Hilsenbeck et al. 1979). Also included are nearly monospecific stands of *Salix caroliniana* (Davis 1943, Loveless 1959, Craighead 1971) called "willow heads." Aquatic and wetland plants that may be present include *Bacopa caroliniana, Ceratophyllum demersum, Najas guadalupensis, Utricularia inflata, Nuphar advena, Nymphaea odorata, Chara* sp., *Pistia stratiotes, Pontederia cordata, Sagittaria lancifolia*, and *Thalia geniculata*. Ferns include *Acrostichum danaeifolium, Nephrolepis exaltata*, and *Blechnum serrulatum*. Grasses and graminoids may include *Schoenoplectus tabernaemontani, Typha domingensis*, and *Zizaniopsis miliacea*. Trees include *Sabal palmetto, Quercus virginiana, Ulmus americana, Ficus aurea*, and *Salix caroliniana*.

**Dynamics:** The American alligators was a dominant force that helped maintain this system, at least historically. Their role has been compared with that of bison in the prairies (Craighead 1971). Through constant movement they create numerous small pools and ponds (analogous to buffalo wallows) as well as trails to and from these pools through sawgrass marshes. These paths eventually widen and deepen into creeks. Many of these small freshwater creeks have been invaded by mangroves and hardwoods in the absence of fire and decrease in alligator populations (Craighead 1971). Some emergent wetlands included within the concept of this system may also have originated from soil and topographic changes in former sawgrass marshes following severe fires that consume organic substrate and decrease soil elevation (Gunderson and Loope 1982b).

SOURCES

**References:** Comer et al. 2003*, Craighead 1971, Davis 1943, Eyre 1980, FNAI 2010a, Gunderson and Loope 1982b, Hilsenbeck et al. 1979, Loveless 1959

**Version:** 17 Mar 2009	**Stakeholders:** Southeast

**Concept Author:** R. Evans and C. Nordman	**LeadResp:** Southeast

CES411.370 South Florida Wet Marl Prairie

**Primary Division:** Caribbean (411)

**Land Cover Class:** Herbaceous Wetland

**Spatial Scale & Pattern:** Large patch

**Required Classifiers:** Natural/Semi‑natural; Vegetated (>10% vasc.); Wetland

**Diagnostic Classifiers:** Graminoid

**National Mapping Codes:** EVT 2484; ESLF 9205; ESP 1484

**Concept Summary:** This system includes marl prairies of the southern Florida Everglades region and related vegetation of the Florida Keys. This system occurs only on shallower soils with bedrock close to the surface. Composition and variability in this system is heavily influenced by hydrology. Especially unusual are small‑patch communities found on elevated areas of oolitic rocks referred to as pinnacle rock or table rock. This system also includes embedded solution holes (depressions formed from limestone collapse).

**Comments:** Plant community components have been variously and often confusingly described. For example, the term wet prairie has often been used to describe a variety of marsh types which are NOT included in the concept of this system [see South Florida Everglades Sawgrass Marsh (CES411.286)]. We follow the definition of Duever et al. (1986) in which prairies occupy mineral soils and marshes occupy peats.

DISTRIBUTION

**Range:** Southern Florida Everglades region and related vegetation of the Florida Keys. Marl prairies with scattered dwarf cypress cover large areas of Big Cypress National Preserve in Collier and Monroe counties. In the Everglades region, marl prairie forms the border between the Miami Rock Ridge and the lower slough and glades marsh and occurs in the narrow finger glades on Long Pine Key (FNAI 2010a).

**Divisions:** 411:C

**TNC Ecoregions:** 54:C

**Nations:** US

**Subnations:** FL

**Map Zones:** 56:C

**USFS Ecomap Regions:** 411A:CC

CONCEPT

**Environment:** This system occurs only on shallower soils with bedrock close to the surface (Gunderson and Loftus 1993). Composition and variability in this system are heavily influenced by hydrology, with the predominant community types occurring on marl substrates which are seasonally inundated (2‑4 months per year). With diminished hydroperiod, species composition changes (Hilsenbeck et al. 1979). Examples of this ecological system can include elevated areas of oolitic rocks referred to as pinnacle rock (Gunderson and Loftus 1993) or table rock (Hilsenbeck et al. 1979), and also include embedded solution holes (depressions formed from limestone collapse).

**Dynamics:** Composition and variability in this system are heavily influenced by hydrology; with shortened hydroperiod, species composition changes (Hilsenbeck et al. 1979). Marl prairie depends on a hydroperiod of two to four months. Longer hydroperiods favor the development of peat and the dominance of *Cladium mariscus ssp. jamaicense*; shorter hydroperiods permit the invasion of woody species (FNAI 2010a). Marl prairie normally dries out during the winter and is subject to fires at the end of the dry season, in late spring. These late‑spring fires promote flowering of the dominant grasses (FNAI 2010a). Biomass recovers to pre‑fire levels after two years. The natural fire frequency may be once every two to six years, or up to ten years for marl prairies with sparse herbaceous vegetation, such as is found on shallow soils (FNAI 2010a).

SOURCES

**References:** Comer et al. 2003*, Duever et al. 1986, FNAI 2010a, Gunderson and Loftus 1993, Hilsenbeck et al. 1979

**Version:** 14 Jan 2014	**Stakeholders:** Southeast

**Concept Author:** R. Evans	**LeadResp:** Southeast

M711. Mesoamerican Freshwater Marsh, Wet Meadow & Shrubland

CES402.589 Vegetacion Palustre Mesoamérica

**Primary Division:** Moist Meso‑America (402)

**Land Cover Class:** Herbaceous Wetland

**Spatial Scale & Pattern:** Small patch

**Required Classifiers:** Natural/Semi‑natural; Vegetated (>10% vasc.); Wetland

**Diagnostic Classifiers:** Lowland [Lowland]; Grassland, Savanna, Steppe (graminoid‑dominated); Riverine / Alluvial [Whitewater]; Aquic

**Concept Summary:** Este sistema es un complejo de comunidades herbáceas acuáticas enraizadas o que rodean cuerpos de agua, sobre todo en orillas de cauces lentos y planicies inundables. Las diferentes comunidades tienden a ser claramente dominadas por una especie. La fisonomía es variable, desde pantanos herbáceos dominados por *Gynerium sagittatum* hasta carrizales pantanosos. En partes de su distribución pueden encontrarse también arbustos o arbolitos creciendo entre las herbáceas. Este sistema ecológico es a menudo adyacente con bosques higrófilos y pantanosos y por eso, algunas de las especies leñosas, salpican su fisonomía herbácea predominante. Los suelos son saturados hidromórficos y puede formarse una capa de turba. La siguiente lista de especies es diagnóstica para este sistema:*Thalia geniculata, Aeschynomene sensitiva, Ipomoea aquatica (= Ipomoea reptans), Heliconia* spp., Marantaceae spp., *Calathea* spp., *Hymenachne amplexicaulis, Agrostis danaefolia, Cyperus ligularis, Cyperus odoratus, Rhynchospora macrostachya, Typha latifolia, Typha domingensis, Polygonum hispidum, Polygonum acuminatum, Myrica mexicana, Cyrilla racemiflora, Camnosperma panamensis, Clusia* sp., *Sabal* sp., *Montrichardia arborescens, Blechnum serrulatum, Thelypteris* sp., *Scleria pterota, Scleria secans, Panicum* sp., and *Acrostichum aureum*.

DISTRIBUTION

**Divisions:** 402:C

**Nations:** BZ, CO, CR, EC, GT, HN, NI, PA

CONCEPT

**Environment:** El sistema es un complejo que cubre una serie de ambientes, todos caracterizados por suelos hidromórficos pesados, con alto contenido orgánico y ácidos. Periodos de inundación muy largos o casi permanentes, casi siempre por aguas dulces aunque puede haber según la situación geográfica, influencia de aguas salobres. Orillas de cauces meándricos, de canales secundarios o ríos grandes de cauce lento y planicies de inundación. Orillas de lagunas o depresiones en terrenos bajos.

**Vegetation:** Vegetación herbácea enraizada y con estructuras flotantes, especies graminoides altas y forbias. Arbustos o árboles bajos dispersos y en ciertos casos, agrupaciones monoespecíficas de palmas rodeando marginalmente a las comunidades herbáceas.

SOURCES

**References:** Ellison 2001, Josse et al. 2003*, Meyrat et al. 2001, Zuluaga 1987

**Version:** 08 Jan 2015	**Stakeholders:** Latin America

**Concept Author:** C. Josse	**LeadResp:** Latin America

2.C.4. Temperate to Polar Freshwater Marsh, Wet Meadow & Shrubland

2.C.4.Nb. Western North American Temperate & Boreal Freshwater Marsh, Wet Meadow & Shrubland

M888. Arid West Interior Freshwater Marsh

CES304.059 Inter‑Mountain Basins Interdunal Swale Wetland

**Primary Division:** Inter‑Mountain Basins (304)

**Land Cover Class:** Herbaceous Wetland

**Spatial Scale & Pattern:** Small patch

**Required Classifiers:** Natural/Semi‑natural; Vegetated (>10% vasc.); Wetland

**Diagnostic Classifiers:** Dune (Landform); Dune field; Dune (Substrate); Temperate [Temperate Xeric]; Depressional; Isolated Wetland [Partially Isolated]; Sand Soil Texture; W‑Landscape/High Intensity; Graminoid

**Concept Summary:** This ecological system occurs within dune fields in the Intermountain western U.S. as small (usually less than 0.1 ha) interdunal wetlands that occur in wind deflation areas, where sands are scoured down to the water table. Small ponds may be associated. The water table may be perched over an impermeable layer of caliche or clay or, in the case of the Great Sand Dunes of Colorado, a geologic dike that creates a closed basin that traps water. These wetland areas are typically dominated by common emergent herbaceous vegetation such as species of *Eleocharis, Juncus*, and *Schoenoplectus*. Dune field ecological processes distinguish these emergent wetlands from similar non‑dune wetlands.

**Comments:** This system was originally included within Inter‑Mountain Basins Active and Stabilized Dune (CES304.775). These small‑scale wetlands were pulled out into their own system because they are isolated wetlands and support completely different biota than the surrounding dry dunes. Many dune fields in the Great Basin are associated with playas and playa lakes such as Washoe Lake, Great Salt Lake, and Mono Lake. At Great Sand Dunes National Monument, Colorado, isolated interdunal swale wetlands occur where winds scour sand to expose wet sand at the water table, largely on the west side (windward) of the main dune field. The same groundwater source also feeds springs that form intermittent creeks that are not part of this interdunal swale system.

DISTRIBUTION

**Range:** The system occurs in some dune fields across the Intermountain western U.S., including the Great Sand Dunes in southern Colorado and the Pink Coral Dunes in Utah. Interdunal wetlands may also occur in dune fields in northeastern Arizona and the Great Basin, as well as in southwestern Wyoming in the Killpecker Dunes and Ferris Dunes, and southern Idaho.

**Divisions:** 304:C, 306:C

**TNC Ecoregions:** 6:?, 10:?, 11:?, 19:?, 20:C

**Nations:** US

**Subnations:** AZ?, CO, ID, NV, UT, WY

**Map Zones:** 7:P, 8:P, 9:P, 12:P, 16:?, 17:P, 18:?, 22:C, 23:?, 24:?, 28:C

**USFS Ecomap Regions:** 313A:PP, 321A:CC, 331J:CC, 342B:PP, 342C:PP, 342D:PP, 342H:P?, M332E:PP

CONCEPT

**Environment:** Occurs in wet interdunal swales.

**Vegetation:** A variety of emergent herbaceous vegetation may occur including, *Juncus arcticus ssp. littoralis (= Juncus balticus), Schoenoplectus pungens, Typha* spp., *Cyperus* spp., *Eleocharis* spp., and *Salix exigua*.

**Dynamics:** The dunes are shaped by the wind and continue to change. The size and exact location of the wet swales may change as the sand dunes shift, due to active dune migration. Dune "blowouts" and subsequent stabilization through succession are characteristic processes of the active dunes which surround the interdunal swales.

SOURCES

**References:** Bowers 1982, Bowers 1984, Bowers 1986, Brand and Sanderson 2002, Cooper and Severn 1992, Hammond 1998*, Pineda 2000, Pineda et al. 1999, Rondeau 2001, Western Ecology Working Group n.d.

**Version:** 12 May 2005	**Stakeholders:** West

**Concept Author:** D.J. Hammond (1998)	**LeadResp:** West

CES300.729 North American Arid West Emergent Marsh

**Primary Division:** (300)

**Land Cover Class:** Herbaceous Wetland

**Spatial Scale & Pattern:** Small patch

**Required Classifiers:** Natural/Semi‑natural; Vegetated (>10% vasc.); Wetland

**Diagnostic Classifiers:** Herbaceous; Depressional [Lakeshore, Pond]; Mineral: W/ A‑Horizon >10 cm; Aquatic Herb; Graminoid; Deep (>15 cm) Water; Saturated Soil

**Concept Summary:** This widespread ecological system occurs throughout much of the arid and semi‑arid regions of western North America, typically surrounded by savanna, shrub‑steppe, steppe, or desert vegetation. Natural marshes may occur in depressions in the landscape (ponds, kettle ponds), as fringes around lakes, and along slow‑flowing streams and rivers (such riparian marshes are also referred to as sloughs). Marshes are frequently or continually inundated, with water depths up to 2 m. Water levels may be stable, or may fluctuate 1 m or more over the course of the growing season. Water chemistry may include some alkaline or semi‑alkaline situations, but the alkalinity is highly variable even within the same complex of wetlands. Marshes have distinctive soils that are typically mineral, but can also accumulate organic material. Soils have characteristics that result from long periods of anaerobic conditions in the soils (e.g., gleyed soils, high organic content, redoximorphic features). The vegetation is characterized by herbaceous plants that are adapted to saturated soil conditions. Common emergent and floating vegetation includes species of *Scirpus* and/or *Schoenoplectus, Typha, Juncus, Potamogeton, Polygonum, Nuphar*, and *Phalaris*. This system may also include areas of relatively deep water with floating‑leaved plants (*Lemna, Potamogeton*, and *Brasenia*) and submerged and floating plants (*Myriophyllum, Ceratophyllum*, and *Elodea*).

**Comments:** This ecological system occurs in the arid and semi‑arid regions of western North America, where semipermanently flooded habitats are found as small patches in the matrix of a relatively dry landscape. Except for stands in the semi‑arid portions of the western Great Plains, emergent marsh found in the Great Plains should be classified into one of the Western Great Plains depressional wetland systems. In the Texas ecological systems map, this system includes margins of lakes and streams frequently or continuously inundated by freshwater, marshes occupying stock tanks and other man‑made depressions, and other moist to wet sites other than marshes dominated by *Ceratophyllum demersum, Chara* spp., *Cladium mariscus ssp. jamaicense, Cynodon dactylon, Echinochloa crus‑galli, Eleocharis montevidensis, Juncus* spp., *Phalaris caroliniana, Phragmites australis, Polygonum* spp., *Polypogon monspeliensis, Potamogeton* spp., *Schoenoplectus acutus, Schoenoplectus pungens var. longispicatus*, and *Typha domingensis*.

DISTRIBUTION

**Range:** This system occurs throughout much of the arid and semi‑arid regions of western North America, extending east peripherally into the semi‑arid portions of the western Great Plains.

**Divisions:** 301:C, 302:C, 303:C, 304:C, 305:C, 306:C

**TNC Ecoregions:** 6:C, 7:C, 8:C, 9:C, 11:C, 17:C, 18:C, 19:C, 20:C, 21:C, 23:C, 24:C, 26:?, 27:C, 28:C, 29:?, 30:C, 68:C

**Nations:** CA, MX, US

**Subnations:** AB, AZ, BC, CA, CO, ID, MT, MXBC, MXCH, MXSO, NM, NV, OR, TX, UT, WA:S2, WY

**Map Zones:** 1:C, 2:C, 5:C, 6:P, 7:C, 8:C, 9:C, 10:C, 12:C, 13:C, 14:C, 15:C, 16:C, 17:C, 18:C, 19:C, 20:P, 21:C, 22:C, 23:P, 24:P, 25:C, 26:C, 27:C, 28:C, 29:P, 31:?, 33:C, 34:C, 35:P, 36:P

**USFS Ecomap Regions:** 313A:CC, 313B:CC, 313C:C?, 313D:CC, 315A:CC, 315B:CC, 315H:CP, 321A:CC, 322A:CC, 322B:CC, 322C:CP, 331A:CP, 331B:CC, 331C:CC, 331D:CC, 331E:CC, 331F:CC, 331G:CC, 331H:CC, 331I:CC, 331J:CC, 331K:CC, 331L:CC, 331M:CC, 331N:CC, 341A:CC, 341B:CC, 341C:CC, 341D:CC, 341E:CC, 341F:CC, 341G:CC, 342A:CC, 342B:CC, 342C:CC, 342D:CC, 342E:CC, 342F:CC, 342G:CC, 342H:CP, 342I:CC, 342J:CC, M261E:CP, M261G:CC, M313A:CC, M313B:CC, M331A:CC, M331B:C?, M331D:CC, M331E:CC, M331F:CP, M331G:CC, M331H:CC, M331I:CC, M331J:C?, M332A:CP, M332B:C?, M332D:CC, M332E:CC, M332F:CC, M332G:C?, M333A:CP, M333C:CC, M341A:CC, M341B:CP, M341D:CC

CONCEPT

**Environment:** Natural marshes may occur in depressions in the landscape (ponds, kettle ponds), as fringes around lakes, and along slow‑flowing streams and rivers (such riparian marshes are also referred to as sloughs). Marshes are frequently or continually inundated, with water depths up to 2 m. Water chemistry may include some alkaline or semi‑alkaline situations, but the alkalinity is highly variable even within the same complex of wetlands. Marshes have distinctive soils that are typically mineral, but can also accumulate organic material. Soils have characteristics that result from long periods of anaerobic conditions in the soils (e.g., gleyed soils, high organic content, redoximorphic features).

**Vegetation:** The vegetation is characterized by herbaceous plants that are adapted to saturated soil conditions. Common emergent and floating vegetation includes species of *Scirpus* and/or *Schoenoplectus, Typha, Juncus, Potamogeton, Polygonum, Nuphar*, and *Phalaris*. This system may also include areas of relatively deep water with floating‑leaved plants (*Lemna, Potamogeton*, and *Brasenia*) and submerged and floating plants (*Myriophyllum, Ceratophyllum*, and *Elodea*).

**Dynamics:** Water levels may be stable, or may fluctuate 1 m or more over the course of the growing season. Some marshes draw down completely on an annual or semi‑annual cycle, or longer 5‑20 year cycle. During the "dry" period, different plant species may become established, encouraging seedlings and discouraging others, in fact, allowing for natural changes in water levels leads to higher diversity of structure and composition of the marsh ecosystems (Mitsch and Gosselink 2000). Fire also has profound effects on marsh vegetation (Kirby et al. 1988). Literature on the specifics of natural fire frequency and effects in western U.S. non‑tidal wetlands is very limited (Kirby et al. 1988, Clark and Wilson 2001).

SOURCES

**References:** Bell 2005, Bell et al. 2009, Brown 1982a, Clark and Wilson 2001, Comer et al. 2003*, Comer et al. 2013a, Cooper 1986b, Dick‑Peddie 1993, Elliott 2012, Faber‑Langendoen et al. 1997, Hansen et al. 1995, Jahrsdoerfer and Leslie 1988, Johnson, J. pers. comm., Kirby et al. 1988, Kittel et al. 1994, Kittel et al. 2012b, Melack et al. 1997, Mitsch and Gosselink 2000, Neely et al. 2001, PRBO Conservation Science 2011, Padgett et al. 1989, Rondeau 2001, Szaro 1989, Ungar 1965, Ungar 1972, WNHP 2011, WNHP unpubl. data 2018

**Version:** 14 Jan 2014	**Stakeholders:** Canada, Latin America, Midwest, Southeast, West

**Concept Author:** M.S. Reid	**LeadResp:** West

CES302.747 North American Warm Desert Cienega

**Primary Division:** North American Warm Desert (302)

**Land Cover Class:** Herbaceous Wetland

**Spatial Scale & Pattern:** Small patch

**Required Classifiers:** Natural/Semi‑natural; Vegetated (>10% vasc.); Wetland

**Diagnostic Classifiers:** Lowland [Lowland]; Seep; Seepage‑Fed Sloping [Mineral]; Alkaline Water

**Concept Summary:** This ecological system occurs at low elevations (<2000 m) across the warm deserts of western North America. "Ciénegas" are freshwater spring‑fed wetlands, characterized by non‑fluctuating shallow surface water; the term ciénega was applied to riparian marshlands by Spanish explorers. Ciénegas are characterized by permanently saturated, highly organic, reducing soils and a relatively simple flora dominated by low‑statured herbaceous hydrophytes (water‑loving plants), with only occasional patches of trees. Evaporation often creates saline conditions especially on the margins as evidenced by salt‑tolerant species such as *Distichlis spicata* and *Sporobolus airoides*. Typically, low‑elevation examples are too warm to accumulate a deep organic layer. The type of vegetation depends on depth of water. In shallow margins, emergent plants typical of riparian vegetation are present including species of *Carex, Juncus*, and *Schoenoplectus*. In adjacent deeper waters, emergent marsh can be characteristic. The hydrology is controlled by permanently saturated hydrosols, with reducing conditions limiting the type of plant life that may grow there. The dense vegetation can slow surface waterflow, reducing the erosive power of flood waters and increase sedimentation within the ciénega. Soils can have many meters of organic deposition. Plant life is limited to low shallow‑rooted semi‑aquatic sedges such as *Eleocharis* spp., *Juncus* spp., *Carex* spp., a few grasses, and more rarely, *Typha* spp. Forbs include *Hydrocotyle verticillata* and *Ludwigia repens*, which can be rooted in patches of gravel below organic root zone in pool bottoms. Few trees and shrubs may be present but may include *Salix gooddingii, Populus fremontii, Fraxinus velutina*, and *Cephalanthus occidentalis*.

**Comments:** In Texas, this predominately herbaceous system occurs on drainages fed by freshwater springs. Evaporative processes may create saline conditions leading to the presence and/or dominance of species such as *Sporobolus airoides, Distichlis spicata, Sesuvium verrucosum, Trianthema portulacastrum*, and *Limonium limbatum*. Other moist‑soil species include *Schoenoplectus pungens var. longispicatus, Juncus* spp., and *Eleocharis* spp. Composition of the occurrence is dependent on the depth and availability of water associated with the originating spring. At some sites, rare species such as *Helianthus paradoxus, Nesaea longipes*, and *Agalinis calycina* may be found. The non‑native grass *Cynodon dactylon* is often encountered. While the cienegas themselves often occur within Quaternary alluvium, the springs that feed the marshes and moist‑soil habitats emanate from contacts often of Cretaceous limestone with less permeable formations. It is often associated with Draw (Desert Grassland) Ecological Sites (Elliott 2012).

DISTRIBUTION

**Range:** Occurs at low elevations (<1000 m) across the warm deserts of western North America, including the Mojave, Sonoran, and Chihuahuan.

**Divisions:** 302:C

**TNC Ecoregions:** 17:C, 22:C, 23:C, 24:C

**Nations:** MX, US

**Subnations:** AZ, CA, MXCH, MXSO, NM, NV, TX

**Map Zones:** 4:?, 13:C, 14:C, 15:C, 17:?, 25:C, 26:C, 27:?

**USFS Ecomap Regions:** 313C:CC, 321A:CC, 322A:CC, 322B:CC, 322C:CC, M313A:CC, M313B:CC

CONCEPT

**Environment:** This spring‑fed marsh ecosystem occurs at mid to low elevations (<2000 m [6562 feet]) across the warm deserts of western North America. "Ciénegas" are freshwater spring‑fed wetlands, characterized by non‑fluctuating shallow surface water (PAG 2001, Stromberg et al. 2009). Ciénegas are characterized by permanently saturated, highly organic, reducing soils (Hendrickson and Minckley 1984, Stromberg et al. 2009, Stevens et al. 2012).

**Vegetation:** Ciénegas are characterized by permanently saturated, highly organic, reducing soils and a relatively simple flora dominated by low‑statured herbaceous hydrophytes (water‑loving plants), with only occasional patches of trees (Hendrickson and Minckley 1984, Stromberg et al. 2009, Stevens et al. 2012). Evaporation often creates saline conditions especially on the margins as evidenced by salt‑tolerant species such as *Distichlis spicata* and *Sporobolus airoides*. Typically, low‑elevation examples are too warm to accumulate a deep organic layer. The type of vegetation depends on depth of water. In shallow margins, emergent plants typical of riparian vegetation are present including species of *Carex, Juncus*, and *Schoenoplectus*. In adjacent deeper waters, emergent marsh can be characteristic. The hydrology is controlled by permanently saturated hydrosols, with reducing conditions limiting the type of plant life that may grow there. The dense vegetation can slow surface waterflow, reducing the erosive power of flood waters and increase sedimentation within the ciénega. Soils can have many meters of organic deposition (Stromberg et al. 1996). Plant life is limited to low shallow‑rooted semi‑aquatic sedges such as *Eleocharis* spp., Juncus spp., *Carex* spp., a few grasses, and more rarely, *Typha* spp. (Stromberg et al. 2009, Stevens et al. 2012). Forbs include *Hydrocotyle verticillata* and *Ludwigia repens (= Ludwigia natans)*, which can be rooted in patches of gravel below organic root zone in pool bottoms (Stromberg et al. 2009, Stevens et al. 2012). Few trees and shrubs may be present but may include *Salix gooddingii, Populus fremontii, Fraxinus velutina*,and *Cephalanthus occidentalis* (Stromberg et al. 2009, Stevens et al. 2012).

**Dynamics:** Ciénegas described here are isolated spring‑fed wetlands found at the outer edge of floodplains and valley floors. Therefore, they have very stable surface hydrologic dynamics. As such they are entirely dependent on groundwater flow to their source spring, and are sensitive to changes in groundwater levels (Hendrickson and Minckley 1984, Stromberg et al. 1996, 1997, 2009, Bagstad et al. 2005, Noonan 2013). Overland surface flow from intense monsoon rains in the summer may deliver sediments into the ciénega, depending on the amount of vegetation and exposed soils on hillslopes above. Winter storms are less intense and are more likely to result in soil moisture absorption, groundwater recharge, and less surface runoff. Groundwater level stability is key to maintaining ciénegas (Hendrickson and Minckley 1984, Stromberg et al. 1996, 1997, 2009, Bagstad et al. 2005, Noonan 2013)

SOURCES

**References:** ACCAG 2006, AFRTF 2010, Abell et al. 2000, Anning et al. 2009, Archer and Predick 2008, Bagstad et al. 2005, Berkman and Rabeni 1987, Brown 1982a, CNRA 2009, Calamusso 2005, Christensen et al. 2007, Comer and Hak 2009, Comer et al. 2003*, Comer et al. 2013b, Debinski and Holt 2000, Dominguez et al. 2009, EPA 2005, Elliott 2012, Faber‑Langendoen et al. 2006b, Faber‑Langendoen et al. 2008b, Heinz Center 2011, Hendrickson and Minckley 1984, Hirschboeck 2009, IPCC 2007c, McKinney and Anning 2009, Mol and Ouboter 2004, Noonan 2013, PAG 2001, Patten 1998, Poff et al. 2010, Price et al. 2005, Rinne 1995, Shafroth et al. 2010, Solomon et al. 2009, Stevens and Meretsky 2008, Stevens et al. 2012, Stromberg 1998, Stromberg and Tellman 2009, Stromberg et al. 1996, Stromberg et al. 1997, Stromberg et al. 2009, Theobald et al. 2010, USCCSP and the Subcommittee on Global Change Research 2009, Unnasch et al. 2009, WNHP 2011, Webb and Leake 2006, Weltzin et al. 2003

**Version:** 14 Jan 2014	**Stakeholders:** Latin America, Southeast, West

**Concept Author:** K.A. Schulz	**LeadResp:** West

M073. Vancouverian Lowland Marsh, Wet Meadow & Shrubland

CES204.854 North Pacific Avalanche Chute Shrubland

**Primary Division:** North American Pacific Maritime (204)

**Land Cover Class:** Shrubland

**Spatial Scale & Pattern:** Large patch

**Required Classifiers:** Natural/Semi‑natural; Vegetated (>10% vasc.); Upland

**Diagnostic Classifiers:** Montane [Montane]; Shrubland (Shrub‑dominated); Avalanche

**National Mapping Codes:** EVT 2083; ESLF 5260; ESP 1083

**Concept Summary:** This tall shrubland system occurs throughout mountainous regions of the Pacific Northwest, from the southern Cascades and Coast Ranges north into the mountains of British Columbia. This system occurs on sideslopes of mountains on glacial till or colluvium. These habitats range from moderately xeric to wet and occur on snow avalanche chutes at montane elevations. In the mountains of Washington, talus sites and snow avalanche chutes very often coincide spatially. On the west side of the Cascades, the major dominant species are *Acer circinatum, Alnus viridis ssp. sinuata, Rubus parviflorus*, and small trees, especially *Callitropsis nootkatensis*. Forbs, grasses, or other shrubs can also be locally dominant. *Prunus virginiana, Amelanchier alnifolia, Vaccinium membranaceum* or *Vaccinium scoparium*, and *Fragaria* spp. are common species on drier avalanche tracks on the east side of the Cascades. The main feature of this system is that it occurs on steep, frequently disturbed (snow avalanches) slopes. Avalanche chutes can be quite long, extending from the subalpine into the montane and foothill toeslopes.

**Comments:** Avalanche slopes in the Cascades and mountains of southern British Columbia are probably drier than those found further north in Alaska, where the precipitation regime does not have a seasonal component to it. Hence, these have been split into two different systems. Exactly where they transition from one to another is yet to be determined.

DISTRIBUTION

**Range:** This system occurs throughout mountainous regions of the Pacific Northwest, from the southern Cascades and Coast Ranges north to the mountains of British Columbia.

**Divisions:** 204:C

**TNC Ecoregions:** 1:C, 3:C, 4:C, 69:C, 81:C

**Nations:** CA, US

**Subnations:** AK, BC, OR, WA:S4

**Map Zones:** 1:C, 2:C, 6:?, 7:C

**USFS Ecomap Regions:** 242A:CC, 242B:CP, 342I:PP, M242A:CC, M242B:CC, M242C:CC, M242D:CC, M332G:CC

CONCEPT

**Environment:** This system occurs on sideslopes of mountains on glacial till or colluvium. These habitats range from moderately xeric to wet and occur on snow avalanche chutes at montane elevations. In the mountains of Washington, talus sites and snow avalanche chutes very often coincide spatially. The main feature of this system is that it occurs on steep, frequently disturbed (snow avalanches) slopes. Avalanche chutes can be quite long, extending from the subalpine into the montane and foothill toeslopes.

**Vegetation:** On the west side of the Cascades, the major dominant species are *Acer circinatum, Alnus viridis ssp. sinuata, Rubus parviflorus*, and small trees, especially *Callitropsis nootkatensis (= Chamaecyparis nootkatensis)*. Forbs, grasses, or other shrubs can also be locally dominant. *Prunus virginiana, Amelanchier alnifolia, Vaccinium membranaceum* or *Vaccinium scoparium*, and *Fragaria* spp. are common species on drier avalanche tracks on the east side of the Cascades.

SOURCES

**References:** Banner et al. 1993, Comer et al. 2003*, Ecosystems Working Group 1998, Franklin and Dyrness 1973, WNHP unpubl. data 2018

**Version:** 08 Dec 2008	**Stakeholders:** Canada, West

**Concept Author:** K. Boggs and G. Kittel	**LeadResp:** West

CES204.865 North Pacific Shrub Swamp

**Primary Division:** North American Pacific Maritime (204)

**Land Cover Class:** Woody Wetland

**Spatial Scale & Pattern:** Large patch

**Required Classifiers:** Natural/Semi‑natural; Vegetated (>10% vasc.); Wetland

**Diagnostic Classifiers:** Forest and Woodland (Treed); Depressional [Lakeshore]; Broad‑Leaved Deciduous Tree; Broad‑Leaved Deciduous Shrub; Eutrophic Water

**National Mapping Codes:** EVT 2663; ESLF 9173; ESP 1663

**Concept Summary:** Swamps vegetated by shrublands occur throughout the Pacific Northwest Coast, from Cook Inlet and Prince William Sound, Alaska, to the southern coast of Oregon. These are deciduous broadleaf tall shrublands that are located in depressions, around lakes or ponds, or river terraces where water tables fluctuate seasonally (mostly seasonally flooded regime), in areas that receive nutrient‑rich waters. These depressions are poorly drained with fine‑textured organic, muck or mineral soils and standing water common throughout the growing season. *Alnus viridis ssp. sinuata* often dominates the shrub layer, but many *Salix* species may also occur. The shrub layer can have many dead stems. However, various species of *Salix, Spiraea douglasii, Malus fusca, Cornus sericea, Alnus incana ssp. tenuifolia (= Alnus tenuifolia), Alnus viridis ssp. crispa (= Alnus crispa)*, and/or *Alnus viridis ssp. sinuata (= Alnus sinuata)* can be the major dominants. They may occur in mosaics with marshes or forested swamps, being on average more wet than forested swamps and more dry than marshes. However, it is also frequent for them to dominate entire wetland systems. Hardwood‑dominated stands (especially *Fraxinus latifolia*) may be considered a shrub swamp when they are not surrounded by conifer forests but do not occur in Alaska. Typical landscape for the *Fraxinus latifolia* stands were very often formerly dominated by prairies and now by agriculture. Wetland species, including *Carex aquatilis var. dives (= Carex sitchensis), Carex utriculata, Equisetum fluviatile*, and *Lysichiton americanus*, dominate the understory. On some sites, *Sphagnum* spp. are common in the understory (Stikine, Yakutat Forelands, Copper River Delta).

**Comments:** Shrub swamps are usually not intermixed with the forested swamps and tend to be more wet. Deciduous and conifer forested swamps are often intermixed and more similar to each other in hydrology, and so are combined into North Pacific Hardwood‑Conifer Swamp (CES204.090). This system includes what is known by the Alaska Natural Heritage Program as Maritime Tall‑Shrub Swamp. Associations found in this system in Alaska need to be identified and added to the list. Deciduous shrub swamps in the Cook Inlet Basin are better placed in Western North American Boreal Deciduous Shrub Swamp (CES105.122).

DISTRIBUTION

**Range:** This system occurs throughout the Pacific Northwest Coast, from Cook Inlet Basin and Prince William Sound, Alaska, to the southern coast of Oregon.

**Divisions:** 204:C

**TNC Ecoregions:** 1:C, 2:C, 3:C, 4:C, 69:C, 70:C, 81:C

**Nations:** CA, US

**Subnations:** AK, BC, OR, WA:S3

**Map Zones:** 1:C, 2:C, 3:P, 7:C, 9:?, 77:C, 78:C

**USFS Ecomap Regions:** 242A:CC, 242B:CC, 342I:??, M242A:CC, M242B:CC, M242C:CC, M242D:CC, M261A:CC

CONCEPT

SOURCES

**References:** Boggs 2002, Chappell and Christy 2004, Comer et al. 2003*, Franklin and Dyrness 1973, Viereck et al. 1992, WNHP unpubl. data 2018

**Version:** 10 Dec 2008	**Stakeholders:** Canada, West

**Concept Author:** G. Kittel, P. Comer, K. Boggs, C. Chappell	**LeadResp:** West

CES200.877 Temperate Pacific Freshwater Emergent Marsh

**Primary Division:** (200)

**Land Cover Class:** Herbaceous Wetland

**Spatial Scale & Pattern:** Small patch

**Required Classifiers:** Natural/Semi‑natural; Vegetated (>10% vasc.); Wetland

**Diagnostic Classifiers:** Herbaceous; Temperate [Temperate Continental]; Depressional [Pond]

**National Mapping Codes:** EVT 2662; ESLF 9260; ESP 1662

**Concept Summary:** Freshwater marshes are found at all elevations below timberline throughout the temperate Pacific Coast and mountains of western North America. In the Pacific Northwest, they are mostly small‑patch, confined to limited areas in suitable floodplain or basin topography. They are mostly semipermanently flooded, but some marshes have seasonal hydrologic flooding. Water is at or above the surface for most of the growing season. Soils are muck or mineral (in Alaska typically muck over a mineral soil), and water is high‑nutrient. Occurrences of this system typically are found in a mosaic with other wetland systems. It is often found along the borders of ponds, lakes or reservoirs that have more open basins and a permanent water source throughout all or most of the year. Some of the specific communities will also be found in floodplain systems where more extensive bottomlands remain. By definition, freshwater marshes are dominated by emergent herbaceous species, mostly graminoids (*Carex, Scirpus* and/or *Schoenoplectus, Eleocharis, Juncus, Typha latifolia*) but also some forbs. Common emergent and floating vegetation includes species of *Scirpus* and/or *Schoenoplectus, Typha, Eleocharis, Sparganium, Sagittaria, Bidens, Cicuta, Rorippa, Mimulus*, and *Phalaris*. Maritime Alaska freshwater marshes are described as having *Carex rostrata, Equisetum fluviatile* (often pure stands), *Carex aquatilis var. dives, Menyanthes trifoliata, Comarum palustre, Eleocharis palustris*, and *Schoenoplectus tabernaemontani*. In relatively deep water, there may be occurrences of the freshwater aquatic bed system, where there are floating‑leaved genera such as *Lemna, Potamogeton, Polygonum, Nuphar, Hydrocotyle*, and *Brasenia*. A consistent source of freshwater is essential to the function of these systems.

**Comments:** In Alaska, freshwater marshes found in floodplain wetland mosaics are not included in this system. Also, freshwater marshes on Kodiak Island, Alaska, are placed into Aleutian Freshwater Marsh (CES105.235). This system encompasses a very large geographic range. We may want to split it into two types, on a north‑south gradient. However, the species composition and environmental settings of freshwater marshes throughout the temperate Pacific region are markedly similar. Where to make a split that would make sense biogeographically is hard to determine. For now, they are maintained as one ecological system.

DISTRIBUTION

**Range:** This system occurs throughout the temperate Pacific Coast and coastal mountains of western North America, from southern coastal California north into coastal areas of British Columbia and Alaska.

**Divisions:** 204:C, 206:C

**TNC Ecoregions:** 1:C, 2:C, 3:C, 4:C, 12:P, 13:C, 14:C, 15:C, 16:C, 69:C, 70:C, 81:C

**Nations:** CA, US

**Subnations:** AK, BC, CA, OR, WA:S2

**Map Zones:** 1:C, 2:C, 3:C, 4:C, 6:P, 7:C, 8:P, 9:P, 77:C, 78:C

**USFS Ecomap Regions:** 242A:CC, 242B:CC, 342B:CC, 342H:CP, 342I:CC, M242A:CC, M242B:CC, M242C:CC, M242D:CC, M261D:CC, M261G:CC, M332G:CC

CONCEPT

**Environment:** In Alaska marshes, standing water is usually persistent throughout the growing season and is generally at least 10 cm above the ground surface.

**Vegetation:** By definition, freshwater marshes are dominated by emergent herbaceous species, mostly graminoids (*Carex, Scirpus* and/or *Schoenoplectus, Eleocharis, Juncus, Typha latifolia*) but also some forbs. Common emergent and floating vegetation includes species of *Scirpus* and/or *Schoenoplectus, Typha, Eleocharis, Sparganium, Sagittaria, Bidens, Cicuta, Rorippa, Mimulus*, and *Phalaris*. Maritime Alaska freshwater marshes are described as having *Carex rostrata, Equisetum fluviatile* (often pure stands), *Carex aquatilis var. dives (= Carex sitchensis), Menyanthes trifoliata, Comarum palustre, Eleocharis palustris*, and *Schoenoplectus tabernaemontani*. In relatively deep water, there may be occurrences of the freshwater aquatic bed system, where there are floating‑leaved genera such as *Lemna, Potamogeton, Polygonum, Nuphar, Hydrocotyle*, and *Brasenia*. A consistent source of freshwater is essential to the function of these systems.

SOURCES

**References:** Banner et al. 1986, Banner et al. 1993, Boggs 2000, Chappell and Christy 2004, Comer et al. 2003*, Holland and Keil 1995, Lloyd et al. 1990, MacKinnon et al. 1990, Shephard 1995, Shiflet 1994, Steen and Coupé 1997, Viereck et al. 1992, WNHP unpubl. data 2018

**Version:** 22 Aug 2008	**Stakeholders:** Canada, West

**Concept Author:** C. Chappell and G. Kittel	**LeadResp:** West

CES200.878 Temperate Pacific Freshwater Mudflat

**Primary Division:** (200)

**Land Cover Class:** Barren

**Spatial Scale & Pattern:** Small patch

**Required Classifiers:** Natural/Semi‑natural; Unvegetated (<10% vasc.); Wetland

**Diagnostic Classifiers:** Herbaceous; Temperate [Temperate Oceanic]; Extensive Wet Flat

**Concept Summary:** Freshwater mudflats are found scattered throughout the temperate regions of the Pacific Coast of North America. In the Pacific Northwest, they occur primarily in seasonally flooded shallow lakebeds on floodplains, especially along the lower Columbia River. During any one year, they may be absent because of year‑to‑year variation in river water levels. Mudflats must be exposed before the vegetation develops from the seedbank. They are dominated mainly by low‑stature annual plants. They range in physiognomy from sparsely vegetated mud to extensive sods of herbaceous vegetation. The predominant species include *Eleocharis obtusa, Lilaeopsis occidentalis, Crassula aquatica, Limosella aquatica, Gnaphalium palustre, Eragrostis hypnoides*, and *Ludwigia palustris*.

**Comments:** Revised to eliminate overlap with North Pacific Intertidal Freshwater Wetland (CES204.875) and to better clarify the type, with input from John Christy.

DISTRIBUTION

**Range:** This system is found throughout the temperate regions of the Pacific Coast of North America.

**Divisions:** 204:C, 206:C

**TNC Ecoregions:** 2:C, 14:C, 15:C, 16:C

**Nations:** US

**Subnations:** AK, CA, OR, WA:S1

**Map Zones:** 1:C, 2:P, 3:C, 4:C, 7:C, 8:C, 9:C

**USFS Ecomap Regions:** 242A:CC, 242B:CC, M242A:CC, M242C:CP, M242D:CP

CONCEPT

SOURCES

**References:** Chappell and Christy 2004, Comer et al. 2003*, Holland and Keil 1995, WNHP unpubl. data 2018

**Version:** 07 Feb 2005	**Stakeholders:** West

**Concept Author:** C. Chappell	**LeadResp:** West

CES204.874 Willamette Valley Wet Prairie

**Primary Division:** North American Pacific Maritime (204)

**Land Cover Class:** Herbaceous Wetland

**Spatial Scale & Pattern:** Small patch

**Required Classifiers:** Natural/Semi‑natural; Vegetated (>10% vasc.); Wetland

**Concept Summary:** This system is largely restricted to the Willamette Valley of Oregon and adjacent Washington. It is nearly extirpated from the Puget Trough of Washington. These are high‑nutrient wetlands that are temporarily and seasonally flooded. They are dominated primarily by graminoids, especially *Deschampsia cespitosa, Camassia quamash, Carex densa*, and *Carex unilateralis*, and to a lesser degree by forbs (e.g., *Isoetes nuttallii*) or shrubs (e.g., *Rosa nutkana*). Wet prairies historically covered large areas of the Willamette Valley where they were maintained by a combination of wetland soil hydrology and frequent burning. They have been reduced to tiny fragments of their former extent.

DISTRIBUTION

**Range:** Restricted to the Willamette Valley of Oregon and adjacent Washington.

**Divisions:** 204:C

**TNC Ecoregions:** 2:C

**Nations:** US

**Subnations:** OR, WA:S1

**Map Zones:** 1:C, 2:C, 7:C

**USFS Ecomap Regions:** 242A:CC, 242B:CC, M242A:??, M261A:CC, M261D:C?

CONCEPT

**Environment:** This ecosystem occurred in areas with seasonally high water tables often perched on clay‑rich soils (e.g., local depressions, swales and low‑gradient riparian areas) within the matrix of a fire‑maintained prairie landscape.

**Dynamics:** Given their location within a fire‑maintained, open grassland landscape, these wet prairies experienced periodic fire, which is what distinguishes them from similar wetland types found elsewhere in western Washington and Oregon. This system was productive and likely dynamic due to frequency of fire.

SOURCES

**References:** Chappell and Christy 2004, Comer et al. 2003*, Easterly et al. 2005, Littell et al. 2009, WNHP 2011, WNHP unpubl. data 2018

**Version:** 14 Jan 2014	**Stakeholders:** West

**Concept Author:** C. Chappell	**LeadResp:** West

M893. Western North American Montane Marsh, Wet Meadow & Shrubland

CES304.084 Columbia Plateau Silver Sagebrush Seasonally Flooded Shrub‑Steppe

**Primary Division:** Inter‑Mountain Basins (304)

**Land Cover Class:** Woody Wetland

**Spatial Scale & Pattern:** Small patch

**Required Classifiers:** Natural/Semi‑natural; Vegetated (>10% vasc.); Upland; Wetland

**Diagnostic Classifiers:** Montane [Upper Montane, Montane, Lower Montane]; Lowland [Lowland]; Playa; Temperate [Temperate Xeric]; Depressional; Impermeable Layer; Intermittent Flooding

**Concept Summary:** This ecological system includes sagebrush communities occurring at lowland and montane elevations in the Columbia Plateau‑northern Great Basin region, east almost to the Great Plains. These are generally depressional wetlands or non‑alkaline playas, occurring as small‑ or occasionally large‑patch communities, in a sagebrush or montane forest matrix. Climate is generally semi‑arid, although it can be cool in montane areas. This system occurs in poorly drained depressional wetlands, the largest characterized as playas, the smaller as vernal pools, or along seasonal stream channels in valley bottoms or mountain meadows. *Artemisia cana ssp. bolanderi* or *Artemisia cana ssp. viscidula* are dominant, with *Artemisia tridentata ssp. tridentata, Artemisia tridentata ssp. wyomingensis*, or *Artemisia tridentata ssp. vaseyana* occasionally codominant; *Dasiphora fruticosa ssp. floribunda* can also be codominant. Understory graminoids and forbs are characteristic, with *Poa secunda, Poa cusickii, Festuca idahoensis, Muhlenbergia filiformis, Muhlenbergia richardsonis*, and *Leymus cinereus* dominant at the drier sites; *Eleocharis palustris, Deschampsia cespitosa*, and *Carex* species dominate at wetter or higher‑elevation sites.

DISTRIBUTION

**Range:** This ecological system includes sagebrush communities occurring at lowland and montane elevations in the Columbia Plateau‑northern Great Basin region, east almost to the Great Plains.

**Divisions:** 304:C, 306:C

**TNC Ecoregions:** 6:C, 7:C, 8:C, 9:C, 12:C, 18:C, 19:C, 20:C

**Nations:** US

**Subnations:** CA, ID, MT, NV, OR, UT?, WA?, WY

**Map Zones:** 7:C, 8:C, 9:C, 12:?, 18:P, 21:?, 22:?

**USFS Ecomap Regions:** 331A:??, 341G:CC, 342B:CC, 342C:CC, 342D:CC, 342H:CC, 342I:CC, 342J:CC, M242C:CC, M261D:CC, M261G:CC, M331A:??, M332A:C?, M332E:C?, M332F:C?, M332G:CC, M333A:PP, M341A:??

CONCEPT

**Environment:** This ecological system includes sagebrush communities occurring at lowland and montane elevations in the Columbia Plateau‑northern Great Basin region, east almost to the Great Plains. These are generally depressional wetlands or non‑alkaline playas, occurring as small‑ or occasionally large‑patch communities, in a sagebrush or montane forest matrix. Climate is generally semi‑arid, although it can be cool in montane areas. This system occurs in poorly drained depressional wetlands, the largest characterized as playas, the smaller as vernal pools, or along seasonal stream channels in valley bottoms or mountain meadows.

**Vegetation:** *Artemisia cana ssp. bolanderi* or *Artemisia cana ssp. viscidula* are dominant, with *Artemisia tridentata ssp. tridentata, Artemisia tridentata ssp. wyomingensis*, or *Artemisia tridentata ssp. vaseyana* occasionally codominant; *Dasiphora fruticosa ssp. floribunda* can also be codominant. Understory graminoids and forbs are characteristic, with *Poa secunda (= Poa nevadensis), Poa cusickii, Festuca idahoensis, Muhlenbergia filiformis, Muhlenbergia richardsonis*, and *Leymus cinereus* dominant at the drier sites; *Eleocharis palustris, Deschampsia cespitosa*, and *Carex* species dominate at wetter or higher‑elevation sites.

SOURCES

**References:** Comer et al. 2003*, Shiflet 1994, WNHP unpubl. data 2018

**Version:** 28 Sep 2007	**Stakeholders:** West

**Concept Author:** J. Kagan	**LeadResp:** West

CES306.812 Rocky Mountain Alpine‑Montane Wet Meadow

**Primary Division:** Rocky Mountain (306)

**Land Cover Class:** Herbaceous Wetland

**Spatial Scale & Pattern:** Small patch

**Required Classifiers:** Natural/Semi‑natural; Vegetated (>10% vasc.); Wetland

**Diagnostic Classifiers:** Alpine/AltiAndino [Alpine/AltiAndino]; Montane [Upper Montane]; Herbaceous; Seepage‑Fed Sloping [Mineral]; Depressional [Lakeshore, Pond]; Graminoid

**Concept Summary:** These are high‑elevation communities found throughout the Rocky Mountains and Intermountain regions, dominated by herbaceous species found on wetter sites with very low‑velocity surface and subsurface flows. They range in elevation from montane to alpine (1000‑3600 m). These types occur as large meadows in montane or subalpine valleys, as narrow strips bordering ponds, lakes, and streams, and along toeslope seeps. They are typically found on flat areas or gentle slopes, but may also occur on sub‑irrigated sites with slopes up to 10%. In alpine regions, sites typically are small depressions located below late‑melting snow patches or on snowbeds. Soils of this system may be mineral or organic. In either case, soils show typical hydric soil characteristics, including high organic content and/or low chroma and redoximorphic features. This system often occurs as a mosaic of several plant associations, often dominated by graminoids, including *Calamagrostis stricta, Caltha leptosepala, Cardamine cordifolia, Carex illota, Carex microptera, Carex nigricans, Carex scopulorum, Carex utriculata, Carex vernacula, Deschampsia cespitosa, Eleocharis quinqueflora, Juncus drummondii, Phippsia algida, Rorippa alpina, Senecio triangularis, Trifolium parryi*, and *Trollius laxus*. Often alpine dwarf‑shrublands, especially those dominated by *Salix*, are immediately adjacent to the wet meadows. Wet meadows are tightly associated with snowmelt and typically not subjected to high disturbance events such as flooding.

**Comments:** Similar systems to this one include Temperate Pacific Subalpine‑Montane Wet Meadow (CES200.998) and Western North American Boreal Wet Meadow (CES105.124). Rocky Mountain Alpine‑Montane Wet Meadow (CES306.812) occurs to the east of the coastal and Sierran mountains, in the semi‑arid interior regions of western North America. Boreal wet meadow systems occur farther north and east in boreal regions where the climatic regime is generally colder than that of the Rockies or Pacific Northwest regions. Floristics of these three systems are somewhat similar, but there are differences related to biogeographic affinities of the species composing the vegetation.

DISTRIBUTION

**Range:** This system is found throughout the Rocky Mountains and Intermountain West regions, ranging in elevation from montane to alpine (1000‑3600 m).

**Divisions:** 304:C, 306:C

**TNC Ecoregions:** 7:C, 8:C, 9:C, 11:C, 18:C, 19:C, 20:C, 21:C, 22:P, 25:C, 68:C

**Nations:** CA, US

**Subnations:** AB, AZ, BC, CO, ID, MT, NM, NV, OR, SD, UT, WA:S3, WY

**Map Zones:** 9:C, 10:C, 12:P, 13:C, 15:?, 16:C, 17:P, 18:P, 19:C, 21:C, 22:P, 23:C, 24:P, 25:C, 27:C, 28:C, 29:P

**USFS Ecomap Regions:** 313A:CP, 313B:CC, 313D:C?, 315A:C?, 315B:C?, 315H:CC, 321A:??, 322A:CC, 331H:CP, 331I:CP, 331J:CC, 341A:CC, 341B:CC, 341C:CC, 341F:CP, 341G:CP, 342B:CC, 342C:CC, 342D:C?, 342E:CC, 342F:CP, 342G:CC, 342H:CC, 342J:CP, M242D:PP, M313A:CC, M313B:CC, M331A:CC, M331B:CC, M331D:CC, M331E:CC, M331F:CC, M331G:CC, M331H:CC, M331I:CC, M331J:CC, M332A:CC, M332B:CC, M332D:CC, M332E:CC, M332F:CC, M332G:CC, M333A:CC, M333B:CC, M333C:CC, M333D:CC, M334A:PP, M341A:CP, M341B:CC, M341C:CC, M341D:CC

CONCEPT

**Environment:** Moisture for these wet meadow community types is acquired from groundwater, stream discharge, overland flow, overbank flow, and on‑site precipitation. Salinity and alkalinity are generally low due to the frequent flushing of moisture through the meadow. Depending on the slope, topography, hydrology, soils and substrate, intermittent, ephemeral, or permanent pools may be present. These areas may support species more representative of purely aquatic environments. Standing water may be present during some or all of the growing season, with water tables typically remaining at or near the soil surface. Fluctuations of the water table throughout the growing season are not uncommon, however. On drier sites supporting the less mesic types, the late‑season water table may be one meter or more below the surface.

Soils typically possess a high proportion of organic matter, but this may vary considerably depending on the frequency and magnitude of alluvial deposition (Kittel et. al. 1999b). Organic composition of the soil may include a thin layer near the soil surface or accumulations of highly sapric material of up to 120 cm thick. Soils may exhibit gleying and/or mottling throughout the profile. Wet meadow ecological systems provide important water filtration, flow attenuation, and wildlife habitat functions.

**Vegetation:** This system often occurs as a mosaic of several plant associations, often dominated by graminoids, including *Calamagrostis stricta, Caltha leptosepala, Cardamine cordifolia, Carex illota, Carex microptera, Carex nigricans, Carex scopulorum, Carex utriculata, Carex vernacula, Deschampsia cespitosa, Eleocharis quinqueflora, Juncus drummondii, Phippsia algida, Rorippa alpina, Senecio triangularis, Trifolium parryi*, and *Trollius laxus*. Often alpine dwarf‑shrublands, especially those dominated by *Salix*, are immediately adjacent to the wet meadows.

**Dynamics:** Wet meadows are tightly associated with snowmelt and typically not subjected to high disturbance events such as flooding. Associations in this ecological system are adapted to soils that may be flooded or saturated throughout the growing season. They may also occur on areas with soils that are only saturated early in the growing season, or intermittently. Typically these associations are tolerant of moderate‑intensity surface fires and late‑season livestock grazing (Kovalchik 1987). Most appear to be relatively stable types, although in some areas these may be impacted by intensive livestock grazing.

SOURCES

**References:** Comer et al. 2002, Comer et al. 2003*, Cooper 1986b, Crowe and Clausnitzer 1997, Kittel et al. 1999b, Komarkova 1976, Komarkova 1986, Kovalchik 1987, Kovalchik 1993, Manning and Padgett 1995, Meidinger and Pojar 1991, NCC 2002, Nachlinger 1985, Nachlinger et al. 2001, Neely et al. 2001, Padgett et al. 1989, Reed 1988, Sanderson and Kettler 1996, Shiflet 1994, Tuhy et al. 2002, WNHP unpubl. data 2018

**Version:** 14 Dec 2004	**Stakeholders:** Canada, Midwest, West

**Concept Author:** M.S. Reid	**LeadResp:** West

CES306.832 Rocky Mountain Subalpine‑Montane Riparian Shrubland

**Primary Division:** Rocky Mountain (306)

**Land Cover Class:** Woody Wetland

**Spatial Scale & Pattern:** Linear

**Required Classifiers:** Natural/Semi‑natural; Vegetated (>10% vasc.); Wetland

**Diagnostic Classifiers:** Montane [Upper Montane, Montane]; Shrubland (Shrub‑dominated); Riverine / Alluvial; Broad‑Leaved Deciduous Shrub; Short (<5 yrs) Flooding Interval; RM Subalpine/Montane Riparian Woodland; Short (50‑100 yrs) Persistence

**Concept Summary:** This system is found throughout the Rocky Mountain cordillera from New Mexico north into Montana and northwestern Alberta, and also occurs in mountainous areas of the Intermountain West region and Colorado Plateau. These are montane to subalpine riparian shrublands occurring as narrow bands of shrubs lining streambanks and alluvial terraces in narrow to wide, low‑gradient valley bottoms and floodplains with sinuous stream channels. Generally, the system is found at higher elevations, but can be found anywhere from 1500‑3475 m, and may occur at even lower elevations in the Canadian Rockies. Occurrences can also be found around seeps, fens, and isolated springs on hillslopes away from valley bottoms. Many of the plant associations found within this system are associated with beaver activity. This system often occurs as a mosaic of multiple communities that are shrub‑ and herb‑dominated and includes above‑treeline, willow‑dominated, snowmelt‑fed basins that feed into streams. The dominant shrubs reflect the large elevational gradient and include *Alnus incana, Betula glandulosa, Betula occidentalis, Cornus sericea, Salix bebbiana, Salix boothii, Salix brachycarpa, Salix drummondiana, Salix eriocephala, Salix geyeriana, Salix monticola, Salix planifolia*, and *Salix wolfii*. Generally the upland vegetation surrounding these riparian systems are of either conifer or aspen forests.

DISTRIBUTION

**Range:** This system is found throughout the Rocky Mountain cordillera from New Mexico north into Montana and the Canadian Rockies of Alberta and British Columbia (including the isolated "island" mountain ranges of central and eastern Montana), and also occurs in mountainous areas of the Intermountain West and Colorado Plateau.

**Divisions:** 304:C, 306:C

**TNC Ecoregions:** 6:P, 7:C, 8:C, 9:C, 11:C, 18:C, 19:C, 20:C, 21:C, 25:C, 26:C, 68:C

**Nations:** CA, US

**Subnations:** AB, AZ, BC, CO, ID, MT, NM, NV, OR, SD, UT, WA:S3, WY

**Map Zones:** 1:C, 6:?, 7:?, 8:?, 9:C, 10:C, 12:C, 15:?, 16:C, 17:P, 18:C, 19:C, 20:C, 21:C, 22:C, 23:C, 24:C, 25:C, 26:P, 27:C, 28:C, 29:C

**USFS Ecomap Regions:** 313A:CC, 313B:CC, 313D:CC, 315A:P?, 315H:PP, 321A:PP, 331A:C?, 331B:C?, 331J:CC, 341A:CP, 341B:CP, 341C:CP, 341D:CP, 341F:CC, 342A:CC, 342B:CP, 342C:CC, 342D:CC, 342E:CC, 342F:CC, 342G:CC, 342H:CC, 342J:CC, M242C:CP, M242D:CC, M261E:CC, M313A:CC, M313B:CC, M331A:CC, M331B:CC, M331D:CC, M331E:CC, M331F:CC, M331G:CC, M331H:CC, M331I:CC, M331J:CC, M332A:CC, M332B:CC, M332D:CC, M332E:CC, M332F:CC, M332G:CC, M333A:CC, M333B:CC, M333C:CC, M333D:CC, M341B:CC, M341C:CC

CONCEPT

SOURCES

**References:** Baker 1988, Baker 1989a, Baker 1989b, Baker 1990, Comer et al. 2002, Comer et al. 2003*, Crowe and Clausnitzer 1997, Kittel 1993, Kittel 1994, Kittel et al. 1996, Kittel et al. 1999a, Kittel et al. 1999b, Kovalchik 1987, Kovalchik 1993, Kovalchik 2001, Manning and Padgett 1995, Muldavin et al. 2000a, NCC 2002, Nachlinger et al. 2001, Neely et al. 2001, Padgett 1982, Padgett et al. 1988b, Padgett et al. 1989, Rondeau 2001, Shiflet 1994, Steen and Coupé 1997, Szaro 1989, Tuhy et al. 2002, WNHP unpubl. data 2018, Walford 1996, Willoughby 2007

**Version:** 31 Mar 2010	**Stakeholders:** Canada, Midwest, West

**Concept Author:** M.S. Reid	**LeadResp:** West

CES200.998 Temperate Pacific Subalpine‑Montane Wet Meadow

**Primary Division:** (200)

**Land Cover Class:** Herbaceous Wetland

**Spatial Scale & Pattern:** Small patch

**Required Classifiers:** Natural/Semi‑natural; Vegetated (>10% vasc.); Wetland

**Diagnostic Classifiers:** Herbaceous; Muck; Graminoid; 30‑180‑day hydroperiod

**Concept Summary:** Montane and subalpine wet meadows occur in open wet depressions, basins and flats among montane and subalpine forests from California's Transverse and Peninsular ranges north to British Columbian coastal forests at varying elevations depending on latitude. Sites are usually seasonally wet, often drying by late summer, and many occur in a tension zone between perennial wetlands and uplands, where water tables fluctuate in response to long‑term climatic cycles. They may have surface water for part of the year, but depths rarely exceed a few centimeters. Soils are mostly mineral and may show typical hydric soil characteristics, and shallow organic soils may occur as inclusions. This system often occurs as a mosaic of several plant associations with varying dominant herbaceous species that may include *Camassia quamash, Carex bolanderi, Carex utriculata, Carex exsiccata, Dodecatheon jeffreyi, Glyceria striata, Carex nigricans, Calamagrostis canadensis, Juncus nevadensis, Caltha leptosepala ssp. howellii, Veratrum californicum*, and *Scirpus* and/or *Schoenoplectus* spp. Trees occur peripherally or on elevated microsites and include *Picea engelmannii, Abies lasiocarpa, Abies amabilis, Tsuga mertensiana*, and *Callitropsis nootkatensis*. Common shrubs may include *Salix* spp., *Vaccinium uliginosum, Betula glandulosa*, and *Vaccinium macrocarpon*. Wet meadows are tightly associated with snowmelt and typically are not subjected to high disturbance events such as flooding.

**Comments:** Rocky Mountain Alpine‑Montane Wet Meadow (CES306.812) occurs to the east of the coastal and Sierran mountains, in the semi‑arid interior regions of western North America. Boreal wet meadow systems occur further north and east in boreal regions where the climatic regime is generally colder than that of the Rockies or Pacific Northwest regions. Floristics of these three systems are somewhat similar, but there are differences related to biogeographic affinities of the species composing the vegetation. Wet meadows in southeastern Alaska have been placed into a new system (2008), Alaskan Pacific Maritime Fen and Wet Meadow (CES204.158).

DISTRIBUTION

**Range:** This system is found from California's Transverse and Peninsular ranges north to British Columbian coastal forests at varying elevations depending on latitude.

**Divisions:** 204:C, 206:C

**TNC Ecoregions:** 3:C, 4:C, 5:C, 12:C, 16:C, 69:?, 81:C

**Nations:** CA, US

**Subnations:** BC, CA, NV, OR, WA:S4

**Map Zones:** 1:C, 2:C, 3:C, 4:C, 6:C, 7:C, 8:?, 9:P, 12:?

**USFS Ecomap Regions:** 262A:PP, 263A:PP, 322A:CC, 331A:PP, 341D:CC, 342B:C?, 342H:CC, 342I:CP, M242A:CC, M242B:CC, M242C:CC, M242D:CC, M261A:CC, M261B:CC, M261D:CC, M261E:CC, M261F:CC, M261G:CC

CONCEPT

**Environment:** Deep mineral soils (often overlain with organic soil that is <40 cm thick) and seasonal high water table are the driving forces of this system. Soil oxygen levels can be low for much of the growing season, and in mature stands, graminoid root density provides extreme competition for seedling establishment. Soils can dry out by the end of the growing season. Prolonged drought will lower the water table and reduce plant vigor.

**Vegetation:** This system often occurs as a mosaic of several plant associations with varying dominant herbaceous species that may include *Camassia quamash, Carex bolanderi, Carex utriculata, Carex exsiccata, Dodecatheon jeffreyi, Glyceria striata (= Glyceria elata), Carex nigricans, Calamagrostis canadensis, Juncus nevadensis, Caltha leptosepala ssp. howellii, Veratrum californicum*, and *Scirpus* and/or *Schoenoplectus* spp. Trees occur peripherally or on elevated microsites and include *Picea engelmannii, Abies lasiocarpa, Abies amabilis, Tsuga mertensiana*, and *Callitropsis nootkatensis (= Chamaecyparis nootkatensis)*. Common shrubs may include *Salix* spp., *Vaccinium uliginosum, Betula glandulosa*, and *Vaccinium macrocarpon*.

**Dynamics:** Wet meadows are tightly associated with snowmelt and typically are not subjected to high disturbance events such as flooding.

SOURCES

**References:** Banner et al. 1993, Barbour and Major 1988, Comer et al. 2003*, DeLong 2003, DeLong et al. 1990, DeLong et al. 1993, Holland and Keil 1995, Lloyd et al. 1990, MacKenzie and Moran 2004, MacKinnon et al. 1990, Meidinger et al. 1988, Sawyer and Keeler‑Wolf 1995, Shiflet 1994, Skinner 1997, Steen and Coupé 1997, WNHP unpubl. data 2018

**Version:** 13 Jan 2012	**Stakeholders:** Canada, West

**Concept Author:** P. Comer	**LeadResp:** West

M074. Western North American Vernal Pool

CES304.057 Columbia Plateau Vernal Pool

**Primary Division:** Inter‑Mountain Basins (304)

**Land Cover Class:** Herbaceous Wetland

**Spatial Scale & Pattern:** Small patch

**Required Classifiers:** Natural/Semi‑natural; Vegetated (>10% vasc.); Wetland

**Diagnostic Classifiers:** Depressional [Vernal Pool]; Impermeable Layer; 1‑29‑day hydroperiod; Vernal Pool Mosaic

**Concept Summary:** This system includes shallow ephemeral waterbodies found in very small (3 m2 to 1 acre) to large depressions (1500 m2 to a square mile, average size of vernal pools is 1600 m2, while average size on non‑alkaline playa lakes is 5‑10 acres) throughout the exposed volcanic scablands of the Columbia Plateau in Washington, Oregon, and northern Nevada. Most of these pools and lakes are located on massive basalt flows exposed by Pleistocene floods; southward they also occur on andesite or rhyodacite caprock. Inundation is highly irregular, sometimes not occurring for several years. Depressions usually (but not always) fill with water during winter and spring. They are generally dry again within 9 months, though in exceptional times they can remain inundated for two years in a row. Water is from rainfall and snowmelt in relatively small closed basins, on average probably no more than 5‑15 times the area of the ponds themselves. Because these pools and playas are perched above the general surrounding landscape, they are not generally subject to runoff from major stream systems. They typically have silty clay soils, sometimes with sandy margins. Pools are often found within a mounded or biscuit‑swale topography with *Artemisia* shrub‑steppe or rarely *Pinus ponderosa* savanna. In the northern Columbia Plateau, characteristic species are predominantly annual and diverse. Floristically akin to California vernal pool flora (one‑third), however, many of the most abundant species are not reported in Californian pools. The Columbia Plateau vernal pools have many floristic similarities to their California counterparts. In one study, it was found that 34% of the native taxa and 65% of the genera also occurred in a comprehensive listing of California vernal pool. Characteristic species of these vernal pools include *Callitriche marginata, Camissonia tanacetifolia, Cuscuta californica var. breviflora, Elatine californica, Elatine chilensis, Elatine rubella, Juncus uncialis, Myosurus minimus, Plagiobothrys* spp., *Polygonum polygaloides ssp. confertiflorum, Polygonum polygaloides ssp. polygaloides, Psilocarphus brevissimus, Psilocarphus elatior, Psilocarphus oregonus*, and *Trifolium cyathiferum*. *Artemisia ludoviciana ssp. ludoviciana* can occur on better developed soils. In northern Nevada, most of the species by biomass are perennials and include *Polygonum, Rumex, Juncus arcticus ssp. littoralis, Eleocharis, Carex douglasii, Muhlenbergia richardsonis*, and *Polyctenium* species, in addition to *Camissonia tanacetifolia* and *Psilocarphus brevissimus*. Endemic plant species *Navarretia leucocephala ssp. diffusa* and *Polyctenium williamsiae* may occur.

**Comments:** This includes Bjork (1997) vernal pool annual‑dominated, vernal pool perennial‑dominated and rain pools.

DISTRIBUTION

**Range:** This system is restricted to the northern Columbia Plateau ecoregion commonly called the Columbia Basin and perhaps the Okanagan Valley in British Columbia, and to the western Great Basin.

**Divisions:** 304:C

**TNC Ecoregions:** 6:C, 68:P

**Nations:** CA?, US

**Subnations:** BC?, NV, OR, WA:S2S3, WY?

**Map Zones:** 7:C, 8:C, 9:P, 18:C

**USFS Ecomap Regions:** 331A:PP, 342B:CC, 342C:CC, 342D:C?, 342H:C?, 342I:CC, M242C:CC, M333A:??

CONCEPT

**Environment:** This system includes shallow ephemeral waterbodies found in very small (3 m2 to 1 acre) to large depressions (1500 m2 to a square mile; average size of vernal pools is 1600 m2, while average size on non‑alkaline playa lakes is 5‑10 acres) throughout the exposed volcanic scablands of the Columbia Plateau in Washington, Oregon, and northern Nevada. Most of these pools and lakes are located on massive basalt flows exposed by Pleistocene floods; southward they also occur on andesite or rhyodacite caprock. Inundation is highly irregular, sometimes not occurring for several years. Depressions usually (but not always) fill with water during winter and spring. They are generally dry again within 9 months, though in exceptional times they can remain inundated for two years in a row. Water is from rainfall and snowmelt in relatively small closed basins, on average probably no more than 5‑15 times the area of the ponds themselves. Because these pools and playas are perched above the general surrounding landscape, they are not generally subject to runoff from major stream systems. They typically have silty clay soils, sometimes with sandy margins. Pools are often found within a mounded or biscuit‑swale topography with *Artemisia* shrub‑steppe or rarely *Pinus ponderosa* savanna. Winters are colder (coldest average median temperature month in the high 20°F) than California vernal pools and are climatically defined by wet winters (November through January, sporadically so southward) and severe summer drought (July‑September), although May or June can be wet. The northernmost vernal pools are adapted to cold spring and long summer days (18 hours).

**Vegetation:** In the northern Columbia Plateau, characteristic species are predominantly annual and diverse. Floristically akin to California vernal pool flora (one‑third), however, many of the most abundant species are not reported in Californian pools. The Columbia Plateau vernal pools have many floristic similarities to their California counterparts. In one study, Crowe et al. (2004) found that 34% of the native taxa and 65% of the genera also occurred in a comprehensive listing of California vernal pool (Keeler‑Wolf et al. 1998). Characteristic species of these vernal pools include *Callitriche marginata, Camissonia tanacetifolia, Cuscuta californica var. breviflora (= Cuscuta occidentalis), Elatine californica, Elatine chilensis, Elatine rubella, Epilobium densiflorum (= Boisduvalia densiflora), Eryngium vaseyi, Juncus uncialis, Myosurus minimus (= Myosurus clavicaulis), Plagiobothrys* spp., *Polygonum polygaloides ssp. confertiflorum, Polygonum polygaloides ssp. polygaloides, Psilocarphus brevissimus, Psilocarphus elatior, Psilocarphus oregonus*, and *Trifolium cyathiferum*. *Artemisia ludoviciana ssp. ludoviciana* can occur on better developed soils. In northern Nevada, most of the species by biomass are perennials and include *Polygonum, Rumex, Juncus arcticus ssp. littoralis (= Juncus balticus), Eleocharis, Carex douglasii, Muhlenbergia richardsonis*, and *Polyctenium* species, in addition to *Camissonia tanacetifolia* and *Psilocarphus brevissimus*. Endemic plant species *Navarretia leucocephala ssp. diffusa* (Bjork 2002) and *Polyctenium williamsiae* may occur.

**Dynamics:** Vernal pools are precipitation‑filled seasonal wetlands inundated during the growing season, allowing for plant growth, followed by a brief water‑logged terrestrial stage and culminating in complete drying of surface and subsurface soils. Inundation during the growing season eliminates establishment of upland species in the pool basins, and the dry period prevents the establishment of many typical wetland taxa (Keeley and Zedler 1998). Pool filling is a combination of direct precipitation and lateral flow among pools within a complex of pools and subsurface flow from uplands which buffers pool volume, keeping them filled into the dry season (Hanes and Stromberg 1998). Soils are relatively shallow (10‑30 cm) underlain by basalt bedrock that prevents drainage (Crowe et al. 1994, Bjork and Dunwiddie 2004). What is unique about vernal pools is the seasonality of the wetting period followed by the desiccation period, which generally is supported by a Mediterranean climate (Crowe et al. 1994, Keeley and Zedler 1998), resulting in the development of diverse and highly endemic vegetation (Barbour et al. 2003, 2005, Solomeshch et al. 2007). Natural fire regimes for vernal pools are generally unknown, but are assumed to be similar to their surrounding upland grassland fire regimes (Wills 2006). Fire can have a positive effect on vernal pool vegetation as it can results in robust response by native grasses and can reduce non‑native invasive woody and non‑native herbaceous species (Pollak and Kan 1998).

SOURCES

**References:** Barbour 1998, Barbour et al. 2003, Barbour et al. 2005, Bjork 1997, Bjork 2002, Bjork and Dunwiddie 2004, Brown 1999, Comer et al. 2003*, Crowe et al. 1994, Dalton et al. 2013, Dlugolecki 2010, Hanes and Stromberg 1998, Keeley and Zedler 1998a, Pollak and Kan 1998, Rocchio pers. comm., Solomeshch et al. 2007, WNHP unpubl. data 2018, Wills 2006

**Version:** 10 Jan 2014	**Stakeholders:** Canada, West

**Concept Author:** R. Crawford	**LeadResp:** West

CES206.948 Northern California Claypan Vernal Pool

**Primary Division:** Mediterranean California (206)

**Land Cover Class:** Herbaceous Wetland

**Spatial Scale & Pattern:** Small patch

**Required Classifiers:** Natural/Semi‑natural; Vegetated (>10% vasc.); Wetland

**Diagnostic Classifiers:** Depressional; Forb; Circumneutral Water; Saline Water Chemistry; Impermeable Layer; Vernal Pool Mosaic

**Concept Summary:** These systems are shallow ephemeral waterbodies found in depressions (up to several hectares in size) among grasslands and open woodlands throughout the northern Central Valley of California. Northern claypan vernal pools include a clay hardpan that retains water inputs throughout some portion of the spring, but typically the depression dries down entirely into early summer months. They tend to be circumneutral to alkaline and slightly saline wetlands with characteristic plant species including *Downingia bella, Downingia insignis, Cressa truxillensis, Plagiobothrys leptocladus (= Allocarya leptoclada), Pogogyne douglasii, Eryngium aristulatum, Veronica peregrina, Lasthenia ferrisiae, Lasthenia glaberrima*, and *Spergularia salina (= Spergularia marina)*. Due to draw‑down characteristics, vernal pools typically form concentric rings of similar forb‑rich vegetation. Given their relative isolation in upland‑dominated landscapes, many endemic and Federally‑listed plant species are common in California vernal pools.

DISTRIBUTION

**Range:** Found in depressions among grasslands and open woodlands throughout the northern Central Valley of California.

**Divisions:** 206:C

**TNC Ecoregions:** 13:C

**Nations:** US

**Subnations:** CA, OR

**Map Zones:** 2:P, 5:C, 7:C

**USFS Ecomap Regions:** 262A:CC, M261C:??, M261F:??

CONCEPT

**Environment:** These systems are shallow ephemeral waterbodies found in depressions (up to several hectares in size) among grasslands and open woodlands throughout the northern Central Valley of California. Northern claypan vernal pools include a clay hardpan that retains water inputs throughout some portion of the spring, but typically the depression dries down entirely into early summer months. They tend to be circumneutral to alkaline and slightly saline wetlands. Pool depth rarely exceeds 50 cm (Solomeshch et al. 2007).

**Dynamics:** Vernal pools are precipitation‑filled seasonal wetlands inundated during the growing season, allowing for plant growth, followed by a brief water‑logged terrestrial stage and culminating in complete drying of surface and subsurface soils. Inundation during the growing season eliminates establishment of upland species in the pool basins and the dry period prevents the establishment of many typical wetland taxa (Keeley and Zedler 1998). Pool filling is a combination of direct precipitation and lateral flow among pools within a complex of pools and subsurface flow from uplands which buffers pool volume, keeping them filled into the dry season (Hanes and Stromberg 1998). Subsurface soils have a hard pan of clay that prevents drainage. What is unique about vernal pools is the seasonality of the wetting period followed by the desiccation period, which generally is supported by a Mediterranean climate (Keeley and Zedler 1998) and resulted in development of diverse and highly endemic vegetation (Barbour et al. 2003, 2005, Solomeshch et al. 2007). Natural fire regimes for vernal pools are generally unknown, but are assumed to be similar to their surrounding upland grassland fire regimes (Wills 2006). Fire can have a positive effect on vernal pool vegetation as it can results in robust response by native grasses and can reduce non‑native invasive woody and non‑native herbaceous species (Pollak and Kan 1998).

SOURCES

**References:** Barbour 1998, Barbour and Major 1988, Barbour et al. 2003, Barbour et al. 2005, Bjork and Dunwiddie 2004, Brown 1999, Calderaro 2011, Comer et al. 2003*, Dlugolecki 2010, Hanes and Stromberg 1998, Holland 1998, Holland 2009, Holland and Keil 1995, Keeley and Zedler 1998a, Marty 2005, PRBO Conservation Science 2011, Pollak and Kan 1998, Robins and Vollmar 2002, Rocchio pers. comm., Sawyer and Keeler‑Wolf 1995, Shiflet 1994, Solomeshch et al. 2007, Wills 2006

**Version:** 14 Jan 2014	**Stakeholders:** West

**Concept Author:** P. Comer and T. Keeler‑Wolf	**LeadResp:** West

2.C.4.Nc. Southwestern North American Warm Desert Freshwater Marsh & Bosque

M076. Warm Desert Lowland Freshwater Marsh, Wet Meadow & Shrubland

CES302.752 North American Warm Desert Riparian Mesquite Bosque

**Primary Division:** North American Warm Desert (302)

**Land Cover Class:** Woody Wetland

**Spatial Scale & Pattern:** Linear

**Required Classifiers:** Natural/Semi‑natural; Vegetated (>10% vasc.); Wetland

**Diagnostic Classifiers:** Lowland [Lowland]; Toeslope/Valley Bottom; Tropical/Subtropical [Tropical Xeric]; Temperate [Temperate Xeric]; Riverine / Alluvial; Prosopis spp.‑dominated

**Concept Summary:** This ecological system consists of low‑elevation (<1100 m) riparian corridors along perennial and intermittent streams in valleys of the warm desert regions of the southwestern U.S. and adjacent Mexico. Rivers include the lower Colorado (within and downstream of the Grand Canyon), Gila, Santa Cruz, Salt, lower Rio Grande, Pecos (up to near its confluence with Rio Hondo), and their tributaries that occur in the desert portions of their range. Dominant trees include *Prosopis glandulosa* and *Prosopis velutina*. Shrub dominants include *Baccharis salicifolia, Pluchea sericea*, and *Salix exigua*. Woody vegetation is relatively dense, especially when compared to drier washes. Vegetation, especially the mesquites, tap groundwater below the streambed when surface flows stop. Vegetation is dependent upon annual rise in the water table for growth and reproduction.

**Comments:** This system is reported to occur, or to have historically occurred, along the Rio Grande and is/was dominated by *Prosopis glandulosa* forming a woodland canopy. Modification of the flood cycle and introduction of *Tamarix* spp. may have influenced the distribution of this system on the Rio Grande. No examples of this system were mapped during the mapping of the ecological systems of Texas (Elliott 2012).

DISTRIBUTION

**Range:** This system is found along perennial and intermittent streams in valleys of southern Arizona, southern Nevada, southeastern California, New Mexico, western Texas, and adjacent Mexico. Major rivers include the lower Colorado (within and downstream of the Grand Canyon), Gila, Santa Cruz, Salt, lower Rio Grande, Pecos (up to near its confluence with Rio Hondo), and their tributaries that occur in the desert portions of their range.

**Divisions:** 302:C

**TNC Ecoregions:** 17:C, 22:C, 23:C, 24:C

**Nations:** MX, US

**Subnations:** AZ, CA, MXBC, MXCH, MXSO, NM, NV, TX

**Map Zones:** 13:C, 14:C, 15:C, 23:?, 25:C, 26:C, 27:?

**USFS Ecomap Regions:** 313C:CC, 321A:CC, 322A:CC, 322B:CC, 322C:CC, M313A:PP, M313B:PP

CONCEPT

**Environment:** This system occurs in low‑elevation (<1100 m) riparian corridors along perennial and intermittent streams in valleys of the warm desert regions of the southwestern U.S. and adjacent Mexico. Rivers include the lower Colorado (within and downstream of the Grand Canyon), Gila, Santa Cruz, Salt, lower Rio Grande, Pecos (up to near its confluence with Rio Hondo), and their tributaries that occur in the desert portions of their range.

**Vegetation:** Dominant trees include *Prosopis glandulosa* and *Prosopis velutina*. Shrub dominants include *Baccharis salicifolia, Pluchea sericea*, and *Salix exigua*. Woody vegetation is relatively dense, especially when compared to drier washes.

**Dynamics:** Vegetation is dependent upon annual rise in the water table for growth and reproduction.

SOURCES

**References:** Barbour and Major 1988, Brown 1982a, Comer et al. 2003*, Dick‑Peddie 1993, Elliott 2012, Muldavin et al. 2000a, Muldavin et al. 2000b, Szaro 1989, Thomas et al. 2004

**Version:** 02 Oct 2014	**Stakeholders:** Latin America, Southeast, West

**Concept Author:** K.A. Schulz	**LeadResp:** West

2.C.4.Nd. Eastern North American Temperate & Boreal Freshwater Marsh, Wet Meadow & Shrubland

M061. Eastern North American Cool Temperate Seep

CES202.300 Southern and Central Appalachian Bog and Fen

**Primary Division:** Central Interior and Appalachian (202)

**Land Cover Class:** Woody Wetland

**Spatial Scale & Pattern:** Small patch

**Required Classifiers:** Natural/Semi‑natural; Vegetated (>10% vasc.); Wetland

**Diagnostic Classifiers:** Shrubland (Shrub‑dominated); Bog; Fen; Seepage‑Fed Sloping; Depressional

**Concept Summary:** This system consists of wetlands associated with flat sites in the Southern Blue Ridge, Central Appalachians, Cumberland Mountains, and possibly upper Piedmont and adjacent Ridge and Valley. These sites occur at elevations below 1220 m (4000 feet) in poorly drained bottomlands on soils which are often saturated and mucky. Wetness results from a combination of groundwater input, seepage from adjacent slopes, rainfall and impeded drainage. The amount of seepage water input is variable among examples, and these wetlands are typically primarily depressional. Vegetation is at least partially open, with herbaceous‑dominated areas as well as shrub thickets and often forested zones. Vegetation is a complex of zones or patches with a mix of physiognomies. The wettest areas have herbaceous vegetation dominated by *Carex* spp., usually with abundant *Sphagnum*. Scattered trees and shrubs may be present in the herbaceous zones. Most examples also have a dense shrub zone around the edges. Some examples have forested zones as well, around the edges or as a matrix in which numerous small herbaceous openings are embedded. Characteristic tree species are *Tsuga canadensis, Acer rubrum, Nyssa sylvatica*, and *Pinus rigida*. Characteristic shrubs include *Rhododendron maximum, Alnus serrulata, Viburnum nudum var. cassinoides*, and *Toxicodendron vernix*.

**Comments:** This system includes communities known locally as either "bogs" or "fens," but it is uncertain as to how their hydrology and soils relate to the definitions of these terms as used in other regions. Bogs are typically defined as rainwater‑fed peatlands, and fens as groundwater‑fed peatlands, ranging from poor to rich, with from weakly minerotrophic to strongly minerotrophic groundwater, respectively. In the Southern Appalachians, there are a few examples with obvious calcareous groundwater input, but more often the vegetation and flora are more characteristic of northern poor fens than either "bogs" or northern "rich fens," although mossy peat layers may be relatively thin. All of these wetland associations are placed in the same system despite the variation in vegetation dominance and uncertainly about the hydrology and soils.

This system is distinguished from Southern Appalachian Seepage Wetland (CES202.317) by patterns of flora and vegetation. Though both systems have heterogeneous and variable vegetation, they share few or no associations. The setting also differs, with Southern and Central Appalachian Bog and Fen (CES202.300) occurring on flat sites such as valley bottoms, where impeded drainage is important, while the seeps occur on sloping sites where water flow is freer and more groundwater flow is needed to create a wetland. High‑elevation wetlands in West Virginia are placed in High Allegheny Wetland (CES202.069).

DISTRIBUTION

**Range:** This system ranges from the southern Appalachians of northern Georgia and South Carolina north to Virginia. It is also found in the Cumberland Mountains of Kentucky.

**Divisions:** 202:C

**TNC Ecoregions:** 50:C, 51:C, 52:?, 59:C

**Nations:** US

**Subnations:** GA, KY, NC, SC, TN:S2, VA

**Map Zones:** 53:C, 54:C, 57:C, 59:C, 61:C

CONCEPT

**Environment:** This system occurs in patches in flat valley bottoms, usually on the outer edges of stream floodplains at elevations below 1220 m (4000 feet). The soil is saturated most or all of the year, at least in the wettest parts, and may be very mucky. Although sites rarely flood, wetness results from a combination of groundwater input, rainfall, seepage from adjacent slopes, and impeded drainage. The groundwater is usually highly acidic and low in dissolved bases, but one or a few examples have somewhat calcareous water input because groundwater flows through mafic rock substrates. Overland flow and stream flooding are presumably only rare events. The geologic substrate is usually alluvium. Often, but not always, there is an adjacent slope with a seep at its base or some visible microtopographic feature, such as a stream levee or ridge, that impedes water drainage out of the area. Some occurrences have substantial microtopography of abandoned stream channels or ridge‑and‑swale systems that pond water in low areas.

**Vegetation:** Vegetation is a complex of zones or patches with a mix of physiognomies. The wettest areas have herbaceous vegetation dominated by *Carex* spp., usually with abundant *Sphagnum*. Scattered trees and shrubs may be present in the herbaceous zones. Most examples also have a dense shrub zone around the edges. Some examples have forest zones as well, around the edges or as a matrix in which numerous small herbaceous openings are embedded. Characteristic tree species are *Tsuga canadensis, Acer rubrum, Nyssa sylvatica*, and *Pinus rigida*. Characteristic shrubs include *Rhododendron maximum, Alnus serrulata, Viburnum nudum var. cassinoides*, and *Toxicodendron vernix*. A number of plant species are shared with northern bogs, including some that are disjunct long distances and occur in the south only in bogs. Other species are narrow endemics, such as *Sarracenia rubra ssp. jonesii*. In the more southern examples, some species are shared with bog communities in the Coastal Plain. The very rare richer fen examples have very distinctive vegetation, sharing a number of species with northern rich fens. Some characteristic bog/fen species include *Carex atlantica, Carex echinata, Carex leptalea, Eriophorum virginicum, Chelone cuthbertii*, and *Solidago patula*. In addition, *Symplocarpus foetidus* occurs in bogs, but it is rare south of Virginia. Other bog plants include *Carex collinsii, Carex folliculata, Drosera rotundifolia, Eriocaulon decangulare, Juncus gymnocarpus, Picea rubens, Rosa palustris, Sarracenia purpurea var. montana, Symphyotrichum puniceum, Toxicodendron vernix, Vaccinium macrocarpon*, and *Sphagnum* spp. When found in wetlands, *Pinus rigida* and *Nyssa sylvatica* seem to be characteristic of bogs and not seeps.

**Dynamics:** The natural dynamics of this system are not well known and are subject to debate. The factors that created and naturally maintain this system are unclear. Most examples show a strong tendency at present for shrubs and trees to increase in density in the open areas, threatening to eliminate the characteristic herb species. This suggests that an important process has been altered or lost. One hypothesis is that bogs are an ephemeral feature developing from abandoned beaver ponds. Another hypothesis is that they result from a narrow combination of moisture and nutrient conditions, which have been widely altered in an obscure way that has changed ecosystem stability. The cattle grazing that was nearly universal in examples of this system in the past appears to have delayed woody succession but may also have altered the natural characteristics. Fire is sometimes considered as a factor, but most examples do not appear flammable enough to burn. Besides woody encroachment, bogs may be altered by changes in adjacent drainage, such as entrenchment by streams.

SOURCES

**References:** Comer et al. 2003*, Edwards et al. 2013, Evans et al. 2009, Evans, M. pers. comm., Nelson 1986, Richardson and Gibbons 1993, Schafale 2012, Schafale and Weakley 1990, Simon 2015, TDNH unpubl. data 2018, Wharton 1978

**Version:** 29 Apr 2016	**Stakeholders:** East, Southeast

**Concept Author:** M. Schafale and R. Evans	**LeadResp:** Southeast

M069. Eastern North American Marsh, Wet Meadow & Shrubland

CES205.687 Eastern Great Plains Wet Meadow, Prairie and Marsh

**Primary Division:** Eastern Great Plains (205)

**Land Cover Class:** Herbaceous Wetland

**Spatial Scale & Pattern:** Small patch

**Required Classifiers:** Natural/Semi‑natural; Vegetated (>10% vasc.); Wetland

**National Mapping Codes:** EVT 2488; ESLF 9213; ESP 1488

**Concept Summary:** This system is found along creeks and streams from Nebraska and Iowa to Illinois, and from Minnesota to Texas. It is also found in depressions and along lake borders, especially in the northern extension of its range into Minnesota. It is often adjacent to a floodplain system but is devoid of trees and riparian vegetation. It is also distinguished from upland prairie systems by having more hydrology, especially associated with silty, dense clay soils that are often hydric, classified as Vertic Haplaquolls. The landform is usually floodplain or poorly drained, relatively level land. The vegetation is dominated by *Spartina pectinata, Tripsacum dactyloides*, numerous large sedges, such as *Carex frankii* and *Carex hyalinolepis*, and in wetter areas, *Eleocharis* spp. Other emergent marsh species such as *Typha* spp. can be associated with this system. Forbs can include *Helianthus grosseserratus, Vernonia fasciculata*, and *Physostegia virginiana*. Some parts of this system may be saline and have species such as *Distichlis spicata* and *Bolboschoenus maritimus (= Schoenoplectus maritimus)*. Fire has been the primary influence in keeping these wet areas free of trees. Other dynamic processes include grazing and flooding (often in late spring). Many areas have been converted to agricultural, but this usually requires some sort of drainage.

DISTRIBUTION

**Range:** This system is found throughout the northeastern Great Plains ranging from eastern Kansas to western Illinois and north into Minnesota.

**Divisions:** 205:C

**TNC Ecoregions:** 35:C, 36:C, 45:P, 46:P

**Nations:** US

**Subnations:** IA, IL, KS, MN, MO, ND, NE, OK, SD, TX?

**Map Zones:** 31:P, 38:C, 39:C, 40:C, 41:P, 42:C, 43:C, 49:C, 50:C, 51:P, 52:P

**USFS Ecomap Regions:** 251A:CC, 251B:CC, 251E:CC, 251F:CC, 251H:CC, 255A:PP, 332B:CP, 332C:CC, 332D:CC, 332E:CC, 332F:C?

CONCEPT

**Environment:** This system is found primarily on silty and/or dense clay, hydric soils, usually classified as Vertic Haplaquolls. It is found within poorly drained, relatively level areas.

**Vegetation:** *Spartina pectinata, Tripsacum dactyloides*, and numerous large sedges, such as *Carex frankii* and *Carex hyalinolepis*, dominate this system. In wetter areas, *Eleocharis* spp. and *Typha* spp. may be significant. Forbs such as *Helianthus grosseserratus, Vernonia fasciculata*, and *Physostegia virginiana* also may be common. Shrub species can be present, especially in the northern range of this system; however, they are usually insignificant compared to the prairie and meadow species.

**Dynamics:** Fire and grazing can affect this system. Fire could spread from adjacent upland prairie, especially in the fall when water levels tended to be low and vegetation was driest. The wet prairie/wet meadow zone burned most frequently, but in the fall, dense, dry tall emergent vegetation in shallow or deep marshes could carry fire, as well. These fires could remove standing dead vegetation, allowing more light to reach the ground and returning nutrients to the soil, but they did not result in a conversion to a different system. In the eastern portion of this system's range, fire was more important in keeping woody species from invading. Native ungulates grazed the margins of potholes and used them as water sources. Muskrats live in larger, wetter potholes and, when populations get high, can have significant effects on the vegetation by eating *Typha* spp. and substantially reducing its cover. Flooding or saturation of sites for part of the growing season is required for the dominant species to survive over time. Grazing during the late summer or other dry periods can result in significant reduction in herbaceous cover but, in general, grazing is of lower importance than fire and flooding in maintaining this system.

SOURCES

**References:** Comer et al. 2003*, Kost et al. 2007, Lauver et al. 1999, Nelson 2010, Rolfsmeier and Steinauer 2010

**Version:** 10 Jan 2014	**Stakeholders:** Canada, Midwest, Southeast

**Concept Author:** S. Menard and K. Kindscher	**LeadResp:** Midwest

CES202.033 Great Lakes Freshwater Estuary and Delta

**Primary Division:** Central Interior and Appalachian (202)

**Land Cover Class:** Herbaceous Wetland

**Spatial Scale & Pattern:** Large patch

**Required Classifiers:** Natural/Semi‑natural; Vegetated (>10% vasc.); Wetland

**Diagnostic Classifiers:** Herbaceous; Riverine / Alluvial; Tidal / Estuarine

**Concept Summary:** This system is found throughout the southern Great Lakes Basin in the United States and Canada. It can include many associated wetlands occurring along portions of tributary rivers and streams that are directly affected by Great Lakes water regimes. It also forms much of the St. Clair River delta. Species distributions and community patterns are determined by multiple abiotic factors, including the type of aquatic system (major river channels, smaller tributary rivers, major deltas, or estuarine), Great Lakes water‑level fluctuations, surficial bedrock, glacial landform, climate, and land use. Although wetland species are generally widely distributed, those of more temperate prairie regions are found in the southern parts of the basin. Vegetation types found across this diverse set of abiotic factors can be placed into a number of zones, though not all are present at a given site. The first four zones are typically inundated directly by lake waters: (a) submergent marsh; (b) emergent marsh; (c) shore fen; and (d) shoreline or strand. The next set of zones are inland from the water's edge and include (e) herbaceous and shrubby wet meadows and (f) shrub or wooded swamps.

This system can be divided into a number of geographical variants, based on the various community types found across the range of the system: (1) Lake Michigan Lacustrine Estuary; (2) Lake Erie‑St. Clair Lakeplain Marsh; (3) Lake Ontario Lagoon Marsh; and (4) St. Lawrence River Estuary.

DISTRIBUTION

**Range:** Throughout the southern Great Lakes Basin in the United States and Canada.

**Divisions:** 201:?, 202:C

**TNC Ecoregions:** 48:C

**Nations:** CA, US

**Subnations:** MI, NY, OH, ON, PA

**Map Zones:** 41:P, 49:C, 50:C, 51:C, 52:C, 62:C, 63:C, 64:C

**USFS Ecomap Regions:** 212Ha:CCC, 212Hf:CCC, 212Hj:CCC, 212Hl:CCC, 212Lb:CCP, 212Ra:CCC, 212Rc:CCC, 212Rd:CCC, 212Re:CCC, 212Sc:CCC, 212Sn:CCC, 212Sq:CCC, 212Te:CCC, 212Y:CC, 222Ja:CCC, 222Ua:CCC, 222Ud:CCC, 222Ue:CCC

CONCEPT

**Environment:** Species distributions and community patterns are determined by multiple abiotic factors. Great Lakes water‑level fluctuations, surficial bedrock, glacial landform, climate, and land use. Great Lakes water level fluctuate over at least three temporal time scales: first, short‑term fluctuations caused by winds or barometric pressures; second, seasonal fluctuations reflecting the annual hydrologic cycle in the basin; and third, interannual fluctuations in lake level as a result of variable precipitation and evaporation within the drainage basin. Interannual fluctuations can be as much as 1.3‑2.5 m, with apparently little or no periodicity. These fluctuations, which also alter turbidity, nutrient availability, ice scour zones, etc., cause locational shifts in vegetation zones, but also in the composition of these zones, as species have individual tolerance limits.

The major bedrock distinction in the Great Lakes Basin is between igneous and metamorphic bedrock of the Precambrian period and younger (Paleozoic) sedimentary bedrock. The igneous and metamorphic bedrock form the rugged north shore of Lake Superior and Georgian Bay, and line much of the St. Lawrence River; they are locally present on the south shore of western Lake Superior. They lack the shallow protected waters and fine‑textured substrates that support broad coastal wetlands. Where such bedrock is at or near the surface, it forms soils that are nutrient‑poor and acidic. The rest of the basin is dominated by softer, sedimentary bedrock, which, with its broad, horizontal depositions, favors broad zones of shallow waters. The sedimentary rocks are typically more alkaline (calcareous), forming soils that are nutrient‑ and moisture‑rich loams and clays. Bedrock patterns are overlaid by glacial landforms that, in combination with recent long‑shore transport processes, create the prevalent physiographic features of the shorelines. In the lakes themselves, sand lakeplains, clay lakeplains, and moraines are shaped by currents, and the long‑shore transportation of sediments has created sand‑spit embayments and swales, dune‑swale complexes, and tombolos. Channels and rivers contain channel‑side wetlands, embayments, and deltas, and estuaries form as either open or barred river mouths. It is this diversity of landforms that has given rise to a diverse set of vegetation types.

<br />Finally, regional patterns of climate affect the basin. The strong latitudinal gradient from southern Lake Erie to northern Lake Superior creates marked differences in length of growing season and solar radiation. Although wetland species are generally widely distributed, those of more temperate and prairie regions are found in the southern parts.

**Vegetation:** Vegetation types found across this diverse set of abiotic factors vary in any number of ways, but they can be placed into a number of zones, though not all are present at a given site. The first four zones are typically inundated directly by lake waters: (a) submergent marsh ‑ containing submergent and/or floating vegetation; (b) emergent marsh ‑ characterized by shallow water or semipermanently flooded soils, and typically dominated by bulrushes, cattails, and other emergent species, but also containing submergent and/or floating vegetation; (c) shore fen ‑ saturated vegetation mats characterized by groundwater influence from shoreline habitats but affected by lake level fluctuations, and dominated by herbaceous or shrubby species; and (d) shoreline or strand ‑ a narrow zone at or just above the water level where seasonal water‑level fluctuations and waves cause erosion, and which is dominated by annual or pioneer herbaceous species. The next set of zones are inland from the water's edge and include (e) herbaceous and shrubby wet meadows ‑ characterized by saturated or seasonally flooded soils, and typically dominated by sedges, grasses, and other herbs, but occasionally dominated by shrubs; and (f) shrub or wooded swamps ‑ characterized by seasonal flooding and dominated by woody species. Species assemblages in these zones change depending on the interaction of factors across the Great Lakes Basin.

SOURCES

**References:** Comer et al. 2003*, Kost et al. 2007, ONHD unpubl. data

**Version:** 26 Mar 2003	**Stakeholders:** Canada, East, Midwest

**Concept Author:** D. Albert and L. Minc	**LeadResp:** Midwest

CES202.027 Great Lakes Wet‑Mesic Lakeplain Prairie

**Primary Division:** Central Interior and Appalachian (202)

**Land Cover Class:** Mixed Upland and Wetland

**Spatial Scale & Pattern:** Large patch

**Required Classifiers:** Natural/Semi‑natural; Vegetated (>10% vasc.); Upland; Wetland

**National Mapping Codes:** EVT 2411; ESLF 7124; ESP 1411

**Concept Summary:** This system is found on the lakeplain near the southern central Great Lakes of the United States and Canada. Stands occur on level, sandy glacial outwash, sandy glacial lakeplains, and deposits of dune sand in silty/clayey glacial lakeplains. The soils are sands and sandy loams, loams with poor to moderate water‑retaining capacity, typically occurring over less permeable silty clays . There is often temporary inundations after heavy rains or in the spring, followed by dry conditions throughout much of the remaining growing season. The vegetation of this system is dominated by graminoid species typically 1‑2 m high. Trees and shrubs are very rare. There is very little bare ground. *Andropogon gerardii, Calamagrostis canadensis, Carex* spp. (*Carex aquatilis, Carex bicknellii, Carex buxbaumii, Carex pellita*), *Panicum virgatum, Spartina pectinata, Schizachyrium scoparium*, and *Sorghastrum nutans* are the most abundant graminoid species. Many of the sites that this system formerly occupied are now urban and/or agricultural. Areas around Chicago and Detroit were likely in this system but are heavily converted now and few sites remain. Remnant sites have been impacted by woody encroachment of native and non‑native species.

DISTRIBUTION

**Range:** This system is found near the southern central Great Lakes of the United States and Canada, from southeastern Wisconsin and northeastern Illinois to southern Michigan and southwestern Ontario. This does not go farther east than northwestern Ohio (glacial Lake Maumee).

**Divisions:** 202:C

**TNC Ecoregions:** 48:C

**Nations:** CA, US

**Subnations:** IL, IN, MI, OH, ON, WI

**Map Zones:** 41:?, 49:C, 50:C, 51:C, 52:C

**USFS Ecomap Regions:** 222Ja:CCC, 222K:CC, 222Ua:CCC, 222Ud:CCC, 222Ue:CCC

CONCEPT

**Environment:** Stands occur on level, sandy glacial outwash, sandy glacial lakeplains, and deposits of dune sand in silty/clayey glacial lakeplains. The soils are sands, sandy loams, and loams with poor to moderate water‑retaining capacity and typically occur over less permeable silty clays. The shallow, less permeable silty clays and the flat landscape combine to favor temporary inundations after heavy rains or in the spring. The coarser surface soils then dry out throughout much of the remaining growing season. These occurred in a patchy landscape of both drier oak woodland/savanna and more mesic beech‑maple forest. Pin oak depressions were common in these prairies.

**Vegetation:** The vegetation of this system is dominated by graminoid species typically 1‑2 m high. Trees and shrubs are very rare. There is very little bare ground. *Andropogon gerardii, Calamagrostis canadensis, Carex* spp. (*Carex aquatilis, Carex bicknellii, Carex buxbaumii, Carex pellita (= Carex lanuginosa)*), *Panicum virgatum, Spartina pectinata, Schizachyrium scoparium*, and *Sorghastrum nutans* are the most abundant graminoid species. Many of the sites that this system formerly occupied are now urban and/or agricultural. Areas around Chicago and Detroit were likely in this system but are heavily converted now and few sites remain. Remnant sites have been impacted by woody encroachment of native and non‑native species.

**Dynamics:** The cycle of soils being temporarily inundated and then drying out during the growing season is important for this system. Great Lakes water levels also affected this system with longer‑term increases and decreases creating wetter and drier baseline conditions, respectively. Graminoids and forbs can thrive under these conditions but woody species are inhibited. The dry conditions and abundance of herbaceous vegetation creates conditions well‑suited for burning and fires further reduced woody vegetation. Drier sites and those in a drier landscape burned more frequently. Fires were most likely in dry years after a productive year(s) when biomass was higher. Fire regime was probably related to the adjacent oak savannas but likely a little less frequent. Water levels were highly variable and boundaries of this system probably shifted across the landscape in response to fire, Great Lakes water levels, and wetter or drier climatic cycles.

SOURCES

**References:** Chapman 1984, Comer et al. 1995b, Comer et al. 2003*, Faber‑Langendoen and Maycock 1987, Faber‑Langendoen and Maycock 1994, Kost et al. 2007, ONHD unpubl. data, WDNR 2015

**Version:** 14 Jan 2014	**Stakeholders:** Canada, Midwest

**Concept Author:** K. Chapman, D. Faber‑Langendoen, P. Comer	**LeadResp:** Midwest

CES201.594 Laurentian‑Acadian Freshwater Marsh

**Primary Division:** Laurentian‑Acadian (201)

**Land Cover Class:** Herbaceous Wetland

**Spatial Scale & Pattern:** Large patch

**Required Classifiers:** Natural/Semi‑natural; Vegetated (>10% vasc.); Wetland

**Diagnostic Classifiers:** Depressional [Lakeshore]; Riverine / Alluvial; Graminoid; Shallow (<15 cm) Water; >180‑day hydroperiod

**Concept Summary:** These freshwater emergent and/or submergent marshes are dominated by herbaceous vegetation. They are common throughout the northeastern United States and adjacent Canadian provinces. Freshwater marshes occur in closed or open basins that are generally flat and shallow. They are associated with lakes, ponds, slow‑moving streams, and/or impoundments or ditches. The herbaceous vegetation does not persist through the winter. Scattered shrubs are often present and usually total less than 25% cover. Trees are generally absent and, if present, are scattered. The substrate is typically muck over mineral soil. Examples of vegetation in the Delaware Estuary freshwater marsh communities include *Typha latifolia, Typha angustifolia, Phragmites australis, Schoenoplectus americanus, Thelypteris palustris, Impatiens capensis, Carex* spp., *Vallisneria americana, Potamogeton perfoliatus, Nuphar advena (= Nuphar lutea ssp. advena)*, and *Nymphaea odorata*.

DISTRIBUTION

**Range:** This system occurs in New England and northern New York west across the upper Great Lakes to Minnesota, and adjacent Canada, mostly north of the glacial boundary.

**Divisions:** 201:C, 202:C

**TNC Ecoregions:** 47:C, 48:C, 49:C, 59:C, 60:C, 61:C, 63:C, 64:C

**Nations:** CA, US

**Subnations:** MA, ME, MI, MN, NB, NH, NY, ON, QC, VT, WI

**Map Zones:** 41:C, 49:?, 50:C, 51:C, 52:?, 60:C, 61:C, 62:P, 63:C, 64:C, 65:C, 66:C

**USFS Ecomap Regions:** 212Ha:CCC, 212Hb:CCC, 212Hc:CCC, 212Hd:CCC, 212He:CCC, 212Hf:CCC, 212Hg:CCC, 212Hh:CCC, 212Hi:CCC, 212Hj:CCC, 212Hk:CCC, 212Hl:CCC, 212Hm:CCC, 212Ra:CCC, 212Rb:CCC, 212Rc:CCC, 212Rd:CCC, 212Re:CCC

CONCEPT

SOURCES

**References:** Comer and Albert 1997, Comer et al. 2003*, Edinger et al. 2014a, Gawler and Cutko 2010, Kost et al. 2007, Sperduto and Nichols 2004, WDNR 2015

**Version:** 20 Aug 2015	**Stakeholders:** Canada, East, Midwest

**Concept Author:** S.C. Gawler and D. Faber‑Langendoen	**LeadResp:** East

CES201.582 Laurentian‑Acadian Wet Meadow‑Shrub Swamp

**Primary Division:** Laurentian‑Acadian (201)

**Land Cover Class:** Herbaceous Wetland

**Spatial Scale & Pattern:** Large patch

**Required Classifiers:** Natural/Semi‑natural; Vegetated (>10% vasc.); Wetland

**Diagnostic Classifiers:** Depressional [Lakeshore]; Riverine / Alluvial; Broad‑Leaved Shrub; Graminoid; Shallow (<15 cm) Water

**Concept Summary:** This system encompasses shrub swamps and wet meadows on mineral soils of the Northeast and upper Midwest. They are often associated with lakes and ponds, but are also found along streams, where the water level does not fluctuate greatly. They are commonly flooded for part of the growing season but often do not have standing water throughout the season. The size of occurrences ranges from small pockets to extensive acreages. The system can have a patchwork of shrub and graminoid dominance; typical species include *Salix* spp., *Cornus amomum, Alnus incana, Spiraea alba, Calamagrostis canadensis*, tall *Carex* spp., and *Juncus effusus*. Trees are generally absent and, if present, are scattered.

**Comments:** Compared to North‑Central Interior Wet Meadow‑Shrub Swamp (CES202.701), this system is more often dominated by *Alnus* spp. rather than the *Cornus* spp. dominance of the latter.

DISTRIBUTION

**Range:** This system is found in New England and northern New York west across the upper Great Lakes to Minnesota, and adjacent Canada, mostly north of the glacial boundary.

**Divisions:** 201:C

**TNC Ecoregions:** 47:C, 48:C, 49:C, 59:C, 60:C, 61:C, 63:C, 64:C

**Nations:** CA, US

**Subnations:** MA, ME, MI, MN, NB, NH, NY, ON, QC, RI, VT, WI

**Map Zones:** 41:C, 49:?, 50:C, 51:C, 52:?, 60:C, 61:C, 62:P, 63:C, 64:C, 65:C, 66:C

**USFS Ecomap Regions:** 211Jb:CCC, 211Jc:CCC, 212Ha:CCC, 212Hb:CCC, 212Hc:CCC, 212Hd:CCC, 212He:CCC, 212Hf:CCC, 212Hg:CCC, 212Hh:CCC, 212Hi:CCC, 212Hj:CCC, 212Hk:CCC, 212Hl:CCC, 212Hm:CCC, 212J:CC, 212K:CC, 212L:CC, 212M:CC, 212N:CC, 212Q:CC, 212Ra:CCC, 212Rb:CCC, 212Rc:CCC, 212Rd:CCC, 212Re:CCC, 212S:CC, 212T:CC, 212X:CC, 212Y:CC, 212Z:CC, 222K:CC, 222M:CC, 222R:CC, 222Ue:CCC

CONCEPT

SOURCES

**References:** Comer et al. 2003*, Edinger et al. 2014a, Gawler and Cutko 2010, Kost et al. 2007, Sperduto and Nichols 2004, WDNR 2015

**Version:** 20 Aug 2015	**Stakeholders:** Canada, East, Midwest

**Concept Author:** S.C. Gawler and D. Faber‑Langendoen	**LeadResp:** East

CES202.899 North‑Central Interior Freshwater Marsh

**Primary Division:** Central Interior and Appalachian (202)

**Land Cover Class:** Herbaceous Wetland

**Spatial Scale & Pattern:** Small patch

**Required Classifiers:** Natural/Semi‑natural; Vegetated (>10% vasc.); Wetland

**Diagnostic Classifiers:** Depressional [Lakeshore]; Graminoid; Shallow (<15 cm) Water; >180‑day hydroperiod

**Concept Summary:** This system is found throughout the northern Midwest ranging into southern Canada. It is typically found on glacial potholes, along small streams, ponds, channels in glacial outwash and on lakeplains. This system contains a deep to shallow area of freshwater marsh dominated by emergent and submergent species. Stands may be open ponds with floating or rooted aquatics, or deep marsh with bulrush or cattails, and range from fairly small to several acres. It contains hydric soils flooded by water ranging from several centimeters to over 1 meter for most of the growing season. Emergent marsh species such as *Typha* spp. and *Schoenoplectus* spp. dominate this system with an occasional scattering of tall *Carex* spp. and forbs that can vary from dense to open cover. Trees are generally absent and, if present, are scattered. Submergent wetlands include a variety of macrophytes.

**Comments:** Some of the specific communities will also be found in the floodplain system and should not be considered a separate system in that case [see North‑Central Interior Floodplain (CES202.694)]. Many of these marshes also may have a border of shrubby wet‑meadow species similar to North‑Central Interior Wet Meadow‑Shrub Swamp (CES202.701), but only those areas with a relatively narrow border (<5‑10 m) should be included with this system.

DISTRIBUTION

**Range:** This system is found in the northern Midwest and southern Canada.

**Divisions:** 201:C, 202:C

**TNC Ecoregions:** 35:C, 36:C, 45:C, 46:C, 47:C, 48:C, 49:?

**Nations:** CA?, US

**Subnations:** IA, IL, IN, MI, MN, MO, ND, OH, ON?, SD, WI

**Map Zones:** 39:C, 40:C, 41:P, 42:C, 43:C, 44:P, 49:C, 50:C, 51:C, 52:C, 62:P

**USFS Ecomap Regions:** 222Ja:CCC, 222Jb:CCC, 222Jc:CCC, 222Je:CCC, 222Jg:CCC, 222Jh:CCC, 222Ji:CCC, 222Ua:CCC, 222Ud:CCC, 222Ue:CCC

CONCEPT

**Environment:** This system is typically found on glacial potholes, along small streams, ponds, channels in glacial outwash, and on lakeplains. This system contains a deep to shallow area of freshwater marsh dominated by emergent and submergent species. It contains hydric soils flooded by water ranging from several centimeters to over 1 meter for most of the growing season.

**Vegetation:** This system contains a deep to shallow area of freshwater marsh dominated by emergent and submergent species. Stands may be open ponds with floating or rooted aquatics, or deep marsh with bulrush or cattails, and range from fairly small to several acres. Emergent marsh species such as *Typha* spp. and *Schoenoplectus* spp. dominate this system with an occasional scattering of tall *Carex* spp. and forbs that can vary from dense to open cover. Trees are generally absent and, if present, are scattered. Submergent wetlands include a variety of macrophytes.

SOURCES

**References:** Comer and Albert 1997, Comer et al. 2003*, Kost et al. 2007, Nelson 2010, ONHD unpubl. data, WDNR 2015

**Version:** 18 Jul 2006	**Stakeholders:** Canada, Midwest, Southeast

**Concept Author:** S. Menard	**LeadResp:** Midwest

CES202.701 North‑Central Interior Wet Meadow‑Shrub Swamp

**Primary Division:** Central Interior and Appalachian (202)

**Land Cover Class:** Woody Wetland

**Spatial Scale & Pattern:** Small patch

**Required Classifiers:** Natural/Semi‑natural; Vegetated (>10% vasc.); Wetland

**Diagnostic Classifiers:** Depressional [Lakeshore]; Broad‑Leaved Shrub; Graminoid

**Concept Summary:** This system is found throughout the northern Midwest ranging into southern Canada. It is typically found on glacial potholes, river valleys, ponds, channels in glacial outwash, and on lakeplains. This system contains a deep to shallow area of freshwater marsh dominated by emergent species surrounded by a zone of wet meadow. The emergent marsh zone within this system contains hydric soils flooded by water ranging from several centimeters to over 1 meter for most of the growing season. Emergent marsh species such as *Typha* spp. and *Schoenoplectus* spp. dominate the core of this system. Wet meadows can surround the emergent marsh core along wet mineral soils or shallow peat with the water table typically just below the surface for most of the growing season. The vegetation in this zone of the system is dominated by sedges (*Carex* spp.) and grasses such as *Calamagrostis canadensis*. This system also can contain a zone of wet prairie species such as *Spartina pectinata*. Shrub swamps can also be associated with the wet meadows within this system. Typical shrub species include *Cornus* spp., *Salix* spp., and/or *Cephalanthus occidentalis*. Trees are generally absent and, if present, are scattered. Fire originating in adjacent uplands, as well as hydrology, can influence this system. In the absence of fire, drought and/or ditching can increase the proportion of shrubs compared to the wet meadow or prairie species.

**Comments:** If examples of these associations are found within a medium to large floodplain, they should be considered part of North‑Central Interior Floodplain (CES202.694). The freshwater marsh component was removed from this system to create a new system, North‑Central Interior Freshwater Marsh (CES202.899).

DISTRIBUTION

**Range:** This system is found in the northern Midwest and southern Canada.

**Divisions:** 201:C, 202:C

**TNC Ecoregions:** 35:C, 36:C, 45:C, 46:C, 47:C, 48:C, 49:?

**Nations:** CA, US

**Subnations:** IA, IL, IN, MI, MN, MO, ND, OH, ON, SD, WI

**Map Zones:** 39:C, 40:C, 41:C, 42:C, 43:C, 44:P, 49:C, 50:C, 51:C, 52:C, 62:P

**USFS Ecomap Regions:** 212Hb:CCP, 222Ja:CCC, 222Jb:CCC, 222Jc:CCC, 222Je:CCC, 222Jg:CCC, 222Jh:CCC, 222Ji:CCC, 222Ua:CCC, 222Ud:CCC, 222Ue:CCC

CONCEPT

**Environment:** This system is typically found on glacial potholes, river valleys, ponds, channels in glacial outwash, and on lakeplains. It contains a deep to shallow area of freshwater marsh dominated by emergent species surrounded by a zone of wet meadow. The emergent marsh zone within this system contains hydric soils flooded by water ranging from several centimeters to over 1 meter for most of the growing season.

**Vegetation:** Emergent marsh species such as *Typha* spp. and *Schoenoplectus* spp. dominate the core of this system. Wet meadows can surround the emergent marsh core along wet mineral soils or shallow peat with the water table typically just below the surface for most of the growing season. The vegetation in this zone of the system is dominated by sedges (*Carex* spp.) and grasses such as *Calamagrostis canadensis*. This system also can contain a zone of wet prairie species such as *Spartina pectinata*. Shrub swamps can also be associated with the wet meadows within this system. Typical shrub species include *Cornus* spp., *Salix* spp., and/or *Cephalanthus occidentalis*. Trees are generally absent and, if present, are scattered.

**Dynamics:** Fire originating in adjacent uplands, as well as hydrology, can influence this system. In the absence of fire, drought and/or ditching can increase the proportion of shrubs compared to the wet meadow or prairie species.

SOURCES

**References:** Comer and Albert 1997, Comer et al. 2003*, Kost et al. 2007, Nelson 2010, ONHD unpubl. data, WDNR 2015

**Version:** 18 Jul 2006	**Stakeholders:** Canada, Midwest, Southeast

**Concept Author:** S. Menard	**LeadResp:** Midwest

CES201.722 Northern Great Lakes Coastal Marsh

**Primary Division:** Laurentian‑Acadian (201)

**Land Cover Class:** Herbaceous Wetland

**Spatial Scale & Pattern:** Large patch

**Required Classifiers:** Natural/Semi‑natural; Vegetated (>10% vasc.); Wetland

**Concept Summary:** This system is found throughout the northern Great Lakes Basin in the United States and Canada. This system, which can include many associated wetlands, occurs along the Great Lakes shoreline directly affected by Great Lakes water regimes. Species distributions and community patterns are determined by multiple abiotic factors, including Great Lakes water‑level fluctuations, surficial bedrock, glacial landform, climate, and land use. Although wetland species are generally widely distributed, those of more boreal and subarctic regions are found in the northern parts of the basin.

Vegetation types found across this diverse set of abiotic factors vary in any number of ways, but they can be placed into a number of zones, though not all are present at a given site. The first four zones are typically inundated directly by lake waters: (a) submergent marsh; (b) emergent marsh; (c) shore fen; and (d) shoreline or strand. The next set of zones are inland from the water's edge and include (e) herbaceous and shrubby wet meadows and (f) shrub or wooded swamps.

This system can be divided into a number of geographical variants, based on the various community types found across the range of the system: (1) Lake Superior Poor Fen; (2) Northern Rich Fen; (3) Northern Great Lakes Marsh; (4) Green Bay Disturbed Marsh; (5) Lake Michigan Lacustrine Estuary; (6) Saginaw Bay Lakeplain Marsh; (7) Lake Erie‑St. Clair Lakeplain Marsh; (8) Lake Ontario Lagoon Marsh; and (9) St. Lawrence River Estuary.

**Comments:** Differs from Great Lakes Freshwater Estuary and Delta (CES202.033) based on its lakeshore setting; the estuary system occurs along rivers, where there is typically more nutrient (e.g., from silts).

DISTRIBUTION

**Range:** This system is found throughout the northern Great Lakes Basin in the United States and Canada.

**Divisions:** 201:C

**TNC Ecoregions:** 48:C

**Nations:** CA, US

**Subnations:** MI, ON, WI

**Map Zones:** 41:C, 49:?, 50:C, 51:C

**USFS Ecomap Regions:** 212Ha:CCC, 212Hf:CCC, 212Hj:CCC, 212Hl:CCC, 212J:CC, 212Lb:CCP, 212Ra:CCC, 212Rc:CCC, 212Rd:CCC, 212Re:CCC, 212Sc:CCC, 212Sn:CCC, 212Sq:CCC, 212Te:CCC, 212Tf:CCC, 212Y:CC, 212Z:CC, 222Ja:CCC, 222Ua:CCC, 222Ud:CCC, 222Ue:CCC

CONCEPT

**Environment:** Species distributions and community patterns are determined by multiple abiotic factors. Great Lakes water‑level fluctuations, surficial bedrock, glacial landform, climate, and land use. Great Lakes water level fluctuate over at least three temporal time scales: first, short‑term fluctuations caused by winds or barometric pressures; second, seasonal fluctuations reflecting the annual hydrologic cycle in the basin; and third, interannual fluctuations in lake level as a result of variable precipitation and evaporation within the drainage basin. Interannual fluctuations can be as much as 1.3‑2.5 m, with apparently little or no periodicity. These fluctuations, which also alter turbidity, nutrient availability, ice scour zones, etc., cause locational shifts in vegetation zones, but also in the composition of these zones, as species have individual tolerance limits.

The major bedrock distinction in the Great Lakes Basin is between igneous and metamorphic bedrock of the Precambrian period and younger (Paleozoic) sedimentary bedrock. The igneous and metamorphic bedrock form the rugged north shore of Lake Superior and Georgian Bay, and line much of the St. Lawrence River; they are locally present on the south shore of western Lake Superior. They lack the shallow protected waters and fine‑textured substrates that support broad coastal wetlands. Where such bedrock is at or near the surface, it forms soils that are nutrient‑poor and acidic. The rest of the basin is dominated by softer, sedimentary bedrock, which, with its broad, horizontal depositions, favors broad zones of shallow waters. The sedimentary rocks are typically more alkaline (calcareous), forming soils that are nutrient‑ and moisture‑rich loams and clays. Bedrock patterns are overlaid by glacial landforms that, in combination with recent long‑shore transport processes, create the prevalent physiographic features of the shorelines. In the lakes themselves, sand lakeplains, clay lakeplains, and moraines are shaped by currents, and the long‑shore transportation of sediments has created sand‑spit embayments and swales, dune‑swale complexes, and tombolos. Channels and rivers contain channel‑side wetlands, embayments, and deltas, and estuaries form as either open or barred river mouths. It is this diversity of landforms that has given rise to a diverse set of vegetation types.

<br />Finally, regional patterns of climate affect the basin. The strong latitudinal gradient from southern Lake Erie to northern Lake Superior creates marked differences in length of growing season and solar radiation. Although wetland species are generally widely distributed, those of more boreal and subarctic regions are found in the northern parts of the basin, whereas those of more temperate and prairie regions are found in the southern parts.

**Vegetation:** Vegetation types found across this diverse set of abiotic factors vary in any number of ways, but they can be placed into a number of zones, though not all are present at a given site. The first four zones are typically inundated directly by lake waters: (a) submergent marsh ‑ containing submergent and/or floating vegetation; (b) emergent marsh ‑ characterized by shallow water or semipermanently flooded soils, and typically dominated by bulrushes, cattails, and other emergent species, but also containing submergent and/or floating vegetation; (c) shore fen ‑ saturated vegetation mats characterized by groundwater influence from shoreline habitats but affected by lake level fluctuations, and dominated by herbaceous or shrubby species; and (d) shoreline or strand ‑ a narrow zone at or just above the water level where seasonal water‑level fluctuations and waves cause erosion, and which is dominated by annual or pioneer herbaceous species. The next set of zones are inland from the water's edge and include (e) herbaceous and shrubby wet meadows ‑ characterized by saturated or seasonally flooded soils, and typically dominated by sedges, grasses, and other herbs, but occasionally dominated by shrubs; and (f) shrub or wooded swamps ‑ characterized by seasonal flooding and dominated by woody species. Species assemblages in these zones change depending on the interaction of factors across the Great Lakes Basin.

SOURCES

**References:** Comer et al. 2003*, Kost et al. 2007, Minc and Albert 1998, WDNR 2015

**Version:** 11 Apr 2007	**Stakeholders:** Canada, East, Midwest

**Concept Author:** D. Albert	**LeadResp:** Midwest

M881. Eastern North American Riverscour Vegetation

CES202.036 Cumberland Riverscour

**Primary Division:** Central Interior and Appalachian (202)

**Land Cover Class:** Woody Wetland

**Spatial Scale & Pattern:** Linear

**Required Classifiers:** Natural/Semi‑natural; Vegetated (>10% vasc.); Wetland

**Diagnostic Classifiers:** Riverine / Alluvial; Graminoid; Short (<5 yrs) Flooding Interval; Riparian Mosaic

**Concept Summary:** Examples of this riverscour‑influenced system may occur on high‑gradient and very high‑gradient streams in the gorges of the Cumberland Plateau, the Cumberland Mountains, and the more rugged parts of the Ridge and Valley in Kentucky, Tennessee, and Alabama, and possibly in Georgia. The succession of woody plants (particularly trees) is retarded by the force of "flashy," high‑velocity water traveling down the stream channels. This system may occur on flood‑scoured acidic or calcareous bedrock, cobble, pebble, or sandbar substrates of sandstone, limestone, dolomite, and possibly other sedimentary and weakly metamorphosed geologies. The most distinctive parts of the system are dominated by shrubs, perennial grasses, and forbs. In some areas, a riparian woodland composed of *Betula nigra* and *Platanus occidentalis* may be a component association. Some common shrubs include *Alnus serrulata, Betula nigra, Cephalanthus occidentalis, Cornus amomum, Fothergilla major, Itea virginica, Salix caroliniana, Rhododendron arborescens, Toxicodendron radicans*, and *Juniperus virginiana var. virginiana*. Some grasses (typical of prairies) include *Andropogon gerardii, Sorghastrum nutans, Schizachyrium scoparium, Chasmanthium latifolium, Tripsacum dactyloides*, and/or *Panicum virgatum*. Forbs are diverse and variable from occurrence to occurrence. This system is affected by flood‑scouring in some areas and deposition in others. There is typically a gradient from dry, nutrient‑poor conditions upslope to moist and relatively enriched conditions downslope. A variety of these conditions may exist at any one site. Some areas are prone to severe drought periods that may stress or kill some (particularly woody) vegetation. Flood‑scouring is a powerful and ecologically important abrasive force along the riverbanks where this system is found.

**Comments:** Examples of the system are sometimes called "scoured riverbank prairies," "riverside prairies," "linear prairies," "rivershore grasslands," or "scoured riverine bluff prairie." River systems where it is found include the Cumberland and its tributaries, the Obed, the Obey, Chickasaw Creek (Tennessee), the Cahaba (Alabama), the Red River Gorge (Kentucky), Rockcastle River (Kentucky), the Big South Fork of the Cumberland (Kentucky/Tennessee) and its tributaries, and others.

DISTRIBUTION

**Range:** This system is found in the Cumberland Plateau, the Cumberland Mountains, and the more rugged parts of the Ridge and Valley, in Kentucky, Tennessee, and Alabama, and possibly in Georgia.

**Divisions:** 202:C

**TNC Ecoregions:** 50:C

**Nations:** US

**Subnations:** AL, GA?, KY, TN, WV

**Map Zones:** 48:C, 53:C

**USFS Ecomap Regions:** 211E:CC, 221H:CC, 231C:CC, M221C:CC

CONCEPT

**Environment:** Examples may occur on high‑gradient and very high‑gradient streams in the gorges of the Cumberland Plateau, the Cumberland Mountains, and rugged parts of the Ridge and Valley, in Kentucky, Tennessee, and Alabama, and possibly in Georgia. The succession of woody plants (particularly trees) is retarded by the force of "flashy," high‑velocity water traveling down the stream channels. This system may occur on flood‑scoured acidic or calcareous bedrock, cobble, pebble, or sandbar substrates of sandstone, limestone, dolomite, and possibly other sedimentary and weakly metamorphosed geologies. It is presumably more extensive and better developed in materials derived from sandstone, where the erodibility creates more material circulating in the stream to create the sandbar/gravelbar areas where the system may occur in extensive patches, and where the extremely well‑drained qualities of the coarse sediments further help to retard woody plant succession.

**Vegetation:** Examples of this system are typically dominated by shrubs, perennial grasses, and forbs. In some areas, a riparian woodland composed of *Betula nigra* and *Platanus occidentalis* may be a component association. Some common shrub component species include *Alnus serrulata, Betula nigra, Cephalanthus occidentalis, Cornus amomum, Fothergilla major, Itea virginica, Salix caroliniana, Rhododendron arborescens, Toxicodendron radicans*, and *Juniperus virginiana var. virginiana*. More southern examples may contain *Hydrangea quercifolia, Hypericum densiflorum*, and *Morella cerifera (= Myrica cerifera var. cerifera)*. Some grasses and forbs include *Andropogon gerardii, Sorghastrum nutans, Schizachyrium scoparium, Chasmanthium latifolium, Tripsacum dactyloides, Panicum virgatum, Baptisia australis, Conoclinium coelestinum (= Eupatorium coelestinum), Coreopsis pubescens, Coreopsis tripteris, Elephantopus carolinianus, Helenium autumnale, Hydrocotyle* sp., *Ludwigia leptocarpa, Lycopus* spp., *Orontium aquaticum, Osmunda regalis var. spectabilis, Oxypolis rigidior, Phlox carolina, Pityopsis graminifolia var. latifolia, Rhynchospora colorata (= Dichromena colorata), Rudbeckia laciniata*, and *Vernonia gigantea*. Patches of *Carex torta* may be present in some examples. Distinctive shoals with *Hymenocallis coronaria* and *Justicia americana* may be present as well. Some of these species are typical of prairies, and thrive in the well‑lit environment.

**Dynamics:** This system is prone to flooding in the upper regions and deposition in the topographically lower areas. There is typically a gradient from dry acidic conditions higher on the bank to moist, fairly enriched conditions lower down may exist at any one site. It is prone to severe drought periods that may stress or kill some vegetation. Flood scouring is a powerful and ecologically important abrasive force along the riverbanks where this system is found. Soils in sandstone areas are rapidly drained Psamments, and may be restricted to the narrow interstices of tightly packed boulders, or to small crevices in bedrock exposures. Within the system the various species are distributed patchily probably due to microsite conditions.

SOURCES

**References:** Bailey and Coe 2001, Comer et al. 2003*, Evans et al. 2009, Eyre 1980, NatureServe Ecology ‑ Southeastern U.S. unpubl. data

**Version:** 14 Jan 2014	**Stakeholders:** East, Midwest, Southeast

**Concept Author:** R. Evans and M. Pyne	**LeadResp:** Southeast

CES202.703 Ozark‑Ouachita Riparian

**Primary Division:** Central Interior and Appalachian (202)

**Land Cover Class:** Mixed Upland and Wetland

**Spatial Scale & Pattern:** Linear

**Required Classifiers:** Natural/Semi‑natural; Vegetated (>10% vasc.); Upland; Wetland

**Diagnostic Classifiers:** Ozark/Ouachita

**Concept Summary:** This system is found along streams and small rivers within the Ozark and Ouachita regions. In contrast to larger floodplain systems, this system has little to no floodplain development and often contains cobble bars and steep banks. It is higher gradient than larger floodplains and experiences periodic, strong flooding. It is often characterized by a cobble bar with forest immediately adjacent with little to no marsh development. Canopy cover can vary within examples of this system, but typical tree species include *Liquidambar styraciflua, Platanus occidentalis, Betula nigra, Acer* spp., and *Quercus* spp. The richness of the herbaceous layer can vary significantly, ranging from species‑rich to species‑poor. Likewise, the shrub layer can vary considerably, but typical species may include *Lindera benzoin, Alnus serrulata*, and *Hamamelis vernalis*. Small seeps and fens can often be found within this system, especially at the headwaters and terraces of streams. These areas are typically dominated by primarily wetland obligate species of sedges (*Carex* spp.), ferns (*Osmunda* spp.), and other herbaceous species such as *Impatiens capensis*. Flooding and scouring strongly influence this system and prevent the floodplain development found on larger rivers.

**Comments:** A separate Ozark‑Ouachita fen/seep system (CES202.052) has also been developed.

DISTRIBUTION

**Range:** This system is found within the Ozarks and the Ouachita Mountains of Missouri, Arkansas and Oklahoma.

**Divisions:** 202:C

**TNC Ecoregions:** 38:C, 39:C

**Nations:** US

**Subnations:** AR, MO, OK

**Map Zones:** 32:C, 44:C

**USFS Ecomap Regions:** M223A:CC, M231A:CC

CONCEPT

**Environment:** This system has little to no floodplain development and often contains cobble bars and steep banks. It is often characterized by a cobble bar with forest immediately adjacent with little to no marsh development. Because these habitats are moister than adjacent uplands, the streamside zones have much higher plant and animal diversity. Orchids and many other species of mesic habitats can be found here. At the larger end of the size continuum, these streams can have gravel and even sand bottoms that support a range of species, including *Salix* spp., *Justicia americana*, and others. Pools provide refugia for invertebrate and vertebrate species that can then rapidly recolonize the stream during high water.

**Vegetation:** Typical tree species in examples of this system include *Liquidambar styraciflua, Platanus occidentalis, Betula nigra*, maples (*Acer* spp.), and oaks (*Quercus* spp.). The richness of the herbaceous layer can vary significantly, ranging from species‑rich to species‑poor. Likewise, the shrub layer can vary considerably, but typical species may include *Lindera benzoin, Alnus serrulata*, and *Hamamelis vernalis*.

**Dynamics:** Flooding and scouring strongly influence this system and prevent the floodplain development found on larger rivers. It is traditionally higher gradient than larger floodplains and experiences periodic strong flooding. The distinctive dynamics of stream flooding are presumably the primary reason for the distinctive vegetation of this system, though not all of the factors are well known. Small rivers and streams, with small watersheds, have more variable flooding regimes that larger rivers. Floods tend to be of short duration and unpredictably variable as to season and depth. In addition to disturbance, floods bring nutrient input, deposit sediment, and disperse plant seeds. Fire does not appear to be a dominant factor, and most floodplain vegetation is not very flammable. Historical references to canebrakes dominated by *Arundinaria gigantea* suggest that fire may have once been more possible and more important in at least some portions.

Flooding is the major process affecting the vegetation, with the substrate more rapidly drained than in flat floodplain areas. The higher gradients of most of these streams and rivers limit floods to fairly short duration. Flooding is most common in the winter, but may occur in other seasons particularly in association with hurricanes, tornados, or microbursts from thunderstorms. Flood waters may have significant energy in higher gradient systems, but scouring and reworking of sediment are important in maintaining the small non‑forested patches of the bar and bank communities. Flooding can act as a replacement disturbance in areas where beavers impounded a channel or in rare years with severe prolonged flood events. There are two general types of floods: occasional catastrophic, prolonged floods (due to beaver activity or other severe event); and more frequent repeated minor flooding (i.e., several minor floods within a 10‑year period).

The wind disturbance associated with flooding is very significant along small streams because of wet and less dense soils and shallow‑rooted trees. Canopy tree mortality from more common windstorms would have resulted in tree‑by‑tree or small group replacement. Windthrow is the primary cause of mortality in bottomlands. Major storms or hurricanes occurring at approximately 20 year intervals would have impacted whole stands.

In this system, the fire‑return interval varies greatly. Except in canebrakes, most fires were very light surface fires, creeping in hardwood or pine litter with some thin, patchy cover of bottomland grasses. Flame lengths were mostly 15‑30 cm (6‑12 inches). Fire‑scarred trees can be found in most small stream sites except in the wettest microsites. Stand‑replacement fires are almost unknown in this type. Except where Native American burning was involved, fires likely occurred primarily during drought conditions and then often only when fire spread into bottomlands from more pyrophytic uplands. Trees may be partially girdled by fire in duff, followed by bark sloughing. While fire rarely killed the tree, this allowed entry of rot, which, in the moist environment, often resulted in hollow trees, providing nesting and denning habitat for many species of birds and animals. Surface fires occurred on a frequency ranging from about 3‑8 years in streamside canebrake, streamside hardwood/canebrake, or pine, to 25 years or more in hardwood litter. Low areas having a long hydroperiod, islands, and areas protected from fire by back swamps and oxbows were virtually fire‑free. Fire effects were largely limited to top‑kill of shrubs and tree saplings less than 5 cm (2 inches) diameter, and formation of hollow trees.

SOURCES

**References:** Arkansas Forestry Commission 2010, Comer et al. 2003*, Eyre 1980, Nelson 2010

**Version:** 14 Jan 2014	**Stakeholders:** Midwest, Southeast

**Concept Author:** S. Menard	**LeadResp:** Midwest

M071. Great Plains Marsh, Wet Meadow, Shrubland & Playa

CES303.654 Edwards Plateau Playa

**Primary Division:** Western Great Plains (303)

**Land Cover Class:** Herbaceous Wetland

**Spatial Scale & Pattern:** Small patch

**Required Classifiers:** Natural/Semi‑natural; Vegetated (>10% vasc.); Upland; Wetland

**Diagnostic Classifiers:** Depression; Depressional

**Concept Summary:** This system includes shallow wetlands formed over limestone on the Edwards Plateau of Texas. Variable in size and duration of inundation, these wetlands are typically found on level uplands. Dominant vegetation includes both graminoids and forbs tolerant of wet periods but not necessarily wetland‑dependent. Dominant species may include *Pleuraphis mutica, Bouteloua dactyloides, Sedum pulchellum, Sedum nuttallianum, Sporobolus vaginiflorus, Chaetopappa bellidifolia, Paronychia* spp., and the alga *Nostoc commune*. Some larger occurrences of this wetland system are found in Crocket, Reagan, Schleicher, Irion and Sterling counties in the northwest Edwards Plateau (the Eldorado Plateau). Formation of these occurrences is apparently from solution of the underlying limestone.

**Comments:** The solution ponds of the Eldorado Plateau are superficially similar to the playa lakes of the Llano Estacado, but the underlying geology of the Edwards Plateau (Cretaceous limestone) occurrences is different from the Llano Estacado (Pliocene and late Tertiary) conglomerates and caliche of the Ogallala Formation and is mantled by a thick layer of wind‑deposited Pleistocene sands and silty sands. Further field investigation is needed to better develop the association‑level information for this system.

DISTRIBUTION

**Range:** This system is found throughout the Edwards Plateau of Texas. Some larger occurrences of this wetland system are found in Crockett, Reagan, Schleicher, Irion and Sterling counties in the northwest Edwards Plateau (the Eldorado Plateau). In TPWD Phase 1, they are found primarily in Runnels, Concho, and Sutton counties (Elliott 2011).

**Divisions:** 303:C

**TNC Ecoregions:** 29:C

**Nations:** US

**Subnations:** TX

**Map Zones:** 35:C

CONCEPT

**Environment:** This system occurs in shallow depressions over massive Cretaceous limestones, such as the Edwards Formation in the Edwards Plateau of Texas. These are internally draining depressions of karstic origin on level plateau surfaces. Soils are loams and clay loams, often mapped as Lakebed ecoclass (Elliott 2011). This system includes shallow wetlands formed over limestone on the Edwards Plateau of Texas. Variable in size and duration of inundation, these wetlands are typically found on level uplands. Formation of these occurrences is apparently from solution of the underlying limestone.

**Vegetation:** Dominant vegetation includes both graminoids and forbs tolerant of wet periods but not necessarily wetland‑dependent. Dominant species may include *Pleuraphis mutica, Bouteloua dactyloides (= Buchloe dactyloides), Tridens albescens, Sedum pulchellum, Sedum nuttallianum, Sporobolus vaginiflorus, Chaetopappa bellidifolia, Ambrosia psilostachya, Paronychia* spp., and the alga *Nostoc commune*. *Panicum obtusum, Bothriochloa barbinodis, Pascopyrum smithii, Bouteloua gracilis, Chenopodium album, Helianthus ciliaris*, and *Solanum elaeagnifolium* may also be present (Elliott 2011).

SOURCES

**References:** Comer et al. 2003*, Elliott 2011

**Version:** 24 Feb 2011	**Stakeholders:** Midwest, Southeast, West

**Concept Author:** J. Teague	**LeadResp:** Southeast

CES303.661 Great Plains Prairie Pothole

**Primary Division:** Western Great Plains (303)

**Land Cover Class:** Mixed Upland and Wetland

**Spatial Scale & Pattern:** Large patch

**Required Classifiers:** Natural/Semi‑natural; Vegetated (>10% vasc.); Wetland

**National Mapping Codes:** EVT 2482; ESLF 9203; ESP 1482

**Concept Summary:** The prairie pothole system is found primarily in the glaciated northern Great Plains of the United States and Canada, and is characterized by depressional wetlands formed by glaciers scraping the landscape during the Pleistocene era. This system is typified by several classes of wetlands distinguished by changes in topography, soils and hydrology. Many of the basins within this system are closed basins and receive irregular inputs of water from their surroundings (groundwater and precipitation), and some export water as groundwater. Hydrology of the potholes is complex. Precipitation and runoff from snowmelt are the principal water sources, with groundwater inflow secondary. Evapotranspiration is the major water loss, with seepage loss secondary. Most of the wetlands and lakes contain water that is alkaline (pH >7.4). The concentration of dissolved solids result in water that ranges from fresh to extremely saline. The flora and vegetation of this system are a function of the topography, water regime, and salinity. In addition, because of periodic droughts and wet periods, many wetlands within this system undergo vegetation cycles. This system includes elements of aquatic vegetation, emergent marshes, and wet meadows that develop into a pattern of concentric rings. This system is responsible for a significant percentage of the annual production of many economically important waterfowl in North America and houses more than 50% of North American's migratory waterfowl, with several species reliant on this system for breeding and feeding. Much of the original extent of this system has been converted to agriculture, and only approximately 40‑50% of the system remains undrained.

**Comments:** More data from Canada is needed to really define this system completely.

DISTRIBUTION

**Range:** This system can be found throughout the northern Great Plains ranging from central Iowa northwest to southern Saskatchewan and Alberta, and extending west into north‑central Montana. It encompasses approximately 870,000 square km with approximately 80% of its range in southern Canada. It is also prevalent in North Dakota, South Dakota, and northern Minnesota.

**Divisions:** 205:C, 303:C

**TNC Ecoregions:** 26:C, 34:C, 35:C, 66:C, 67:C

**Nations:** CA, US

**Subnations:** AB, IA, MB, MN, MT, ND, SD, SK

**Map Zones:** 20:C, 29:C, 38:?, 39:C, 40:C, 41:C, 42:P

**USFS Ecomap Regions:** 251A:CC, 251B:CC, 331D:CC, 331E:CC, 331K:CC, 331L:CC, 331M:CC

CONCEPT

**Environment:** This system is characterized by closed basins, potholes, that receive irregular inputs of water from the surroundings and may export water as groundwater. The climate for the range of this system is characterized by mid‑continental temperature and precipitation extremes. Across the range of this system, precipitation triples from 30‑90 cm (west to east) and average annual temperature increases from 1‑10°C (north to south). Snowmelt and spring rains typically fill many of the potholes in examples of this system. The region in the range of this system is distinguished by a thin mantle of glacial drift overlying stratified sedimentary rocks of the Mesozoic and Cenozoic ages; these form a glacial landscape of end moraines, stagnation moraines, outwash plains and lakeplains. The glacial drift ranges 30‑120 m thick and forms steep to slight local relief with fine‑grained, silty to clayey soils. Soils in outwash plains are coarser. Limestone, sandstone, and shales predominate as bedrock, and highly mineralized water can discharge from these rocks. The hydrology of this system is complex with salinity ranging from fresh to saline, and chemical characteristics varying seasonally and annually. Sites with substantial surface or groundwater outlet are typically fresh while sites with little or no outlet tend to accumulate salts. Rain and snowmelt are the primary water sources with evapotranspiration being the source of major water loss. Some potholes are connected to groundwater sources and can serve as groundwater recharge sources, some receive groundwater outflow, and some have both. Water depth in most potholes is shallow. Many have a maximum depth of <2 m and most are <1 m deep (Sloan 1970). Seasonal water level fluctuations mean that the depth during much of the growing season is less than these maximums.

**Vegetation:** The vegetation within this system is highly influenced by hydrology, salinity and dynamics. Potholes found within this system can vary in depth and duration, which will determine the local gradient of species. Likewise, plant species found within individual potholes of this system will be strongly influenced by periodic drought and wet periods. Deeper potholes with standing water throughout most of the year have a central zone of submersed aquatic vegetation. Potholes that dry during droughty times can have central zones dominated by either tall emergents or mid‑height emergents depending on the depth of the marsh. Wet meadow species such as grasses, forbs and sedges can be found in potholes that are only flooded briefly in the spring. All of these types of potholes can be found within an example of this system. Grazing, draining, and mowing of this system can influence the distribution of these types of potholes and plant species within this system.

**Dynamics:** A cycle of flooding and drying is the primary natural dynamic influencing this system. Snowmelt contributes substantially to the seasonal water input. In addition to runoff from snow melting within the watershed, snow tends to accumulate within the pothole due to the slightly more sheltered landscape position and the typically heavier and taller vegetation cover present in at least parts of the pothole. Spring rains contribute additional water, and potholes consistently have their yearly maximum water depth in late spring. Heavy rains in the summer can fill potholes, but the tendency is for water levels to fall as the growing season progresses. This fluctuation of water level during the year results in very different flooding regimes for different parts of the pothole. At the driest edge, the ground may be flooded or saturated for only a few weeks during the growing season, while the wettest parts of some potholes are flooded year‑round. These different flooding regimes create environments favoring different types of plants and many potholes have strong zonation of vegetation (Johnson et al. 1987). From driest to wettest, these zones are wet meadow, shallow marsh, deep marsh, aquatic, and deep water. Many potholes do not have enough water to support the wetter vegetation zones so individual potholes may have shallow marsh at the center with a ring of wet meadow or deep marsh surrounded by shallow marsh which in turn is surrounded by wet meadow. The changes in water volume in a given pothole are also reflected in the salinity of the water. Prairie potholes are least saline in the spring when snowmelt and spring rains fill the wetland, and possibly flush water out of the basin through seasonal overflow, but salinity increases as evapotranspiration reduces the volume of water in the basin throughout the growing season (Stewart and Kantrud 1972).

In addition to seasonal water level fluctuations, there are longer‑term changes in water levels that affect prairie potholes (Kantrud et al. 1989a). Multi‑year patterns of above or below average precipitation result in shifting vegetation zones within a single site. A multi‑year dry period will cause a pothole to shrink, and the environments suitable for each vegetation zone will move towards the center, possibly eliminating the wettest zones altogether. A multi‑year wet period will fill potholes, moving the environments conducive to each vegetation zone away from the center and possibly creating habitats for new, wetter zones in the middle. Changes in water depth of several feet are possible over a few to several years (Stewart and Kantrud 1972). These multi‑year changes in the location of vegetation zones promote floristic diversity by creating shifting environments at any one place on the landscape. During the wetter seasonal or multi‑year periods, temporary connections may be formed among otherwise discontinuous wetlands, allowing the spread of species and possibly affecting water chemistry through flushing of salts or other dissolved chemicals into or out of basins (Leibowitz and Vining 2003).

Fire and grazing can affect this system. Fire could spread from adjacent upland prairie, especially in the fall when water levels tended to be low and vegetation was driest. The wet prairie/wet meadow zone burned most frequently, but in the fall, dense, dry tall emergent vegetation in shallow or deep marshes could carry fire, as well. These fires could remove standing dead vegetation, allowing more light to reach the ground and returning nutrients to the soil, but they did not result in a conversion to a different system. In the eastern portion of this system's range, fire was more important in keeping woody species from invading. Native ungulates grazed the margins of potholes and used them as water sources. Muskrats live in larger, wetter potholes and, when populations get high, can have significant effects on the vegetation by eating *Typha* spp. and substantially reducing its cover (Kantrud et al. 1989b).

SOURCES

**References:** Adamus and Hairston 1996, Comer et al. 2003*, Johnson et al. 1987, Johnson et al. 2005, Kantrud et al. 1989a, Kantrud et al. 1989b, Leibowitz and Vining 2003, Lesica 1989, Millet et al. 2009, Preston et al. 2013, Sloan 1970, Stewart and Kantrud 1972

**Version:** 14 Jan 2014	**Stakeholders:** Canada, Midwest, West

**Concept Author:** S. Menard	**LeadResp:** Midwest

CES303.666 Western Great Plains Closed Depression Wetland & Playa

**Primary Division:** Western Great Plains (303)

**Land Cover Class:** Herbaceous Wetland

**Spatial Scale & Pattern:** Small patch

**Required Classifiers:** Natural/Semi‑natural; Vegetated (>10% vasc.); Wetland

**Concept Summary:** Communities associated with the playa lakes in the southern areas of this province and the rainwater basins in Nebraska characterize this system. They are primarily upland depressional basins. This hydric system is typified by the presence of an impermeable layer such as a dense clay, hydric soil and is usually recharged by rainwater and nearby runoff. They are rarely linked to outside groundwater sources and do not have an extensive watershed. Ponds and lakes associated with this system can experience periodic drawdowns during drier seasons and years, and are often replenished by spring rains. *Eleocharis* spp., *Hordeum jubatum*, along with common forbs such as *Coreopsis tinctoria, Symphyotrichum subulatum*, and *Polygonum pensylvanicum* are common vegetation in the wetter and deeper depression, while *Pascopyrum smithii* and *Bouteloua dactyloides* are more common in drier playas such as shallow depressions in rangeland. Species richness can vary considerably among individual examples of this system and is especially influenced by adjacent land use, which is often agriculture, and may provide nutrient and herbicide runoff. Dynamic processes that affect these depressions are hydrological changes, grazing, and conversion to agricultural use. Additional species found in Texas examples include *Ambrosia grayi, Chenopodium leptophyllum, Helianthus ciliaris, Heteranthera limosa, Marsilea vestita, Oenothera canescens, Panicum obtusum, Phyla nodiflora, Sagittaria longiloba, Schoenoplectus* spp., and *Typha domingensis*.

**Comments:** Open and emergent marshes may be a separate system from wet meadows and wet prairies. This system needs to be more clearly distinguished from the similar open depressional wetlands of the western Great Plains, as well as from Great Plains Prairie Pothole (CES303.661).

DISTRIBUTION

**Range:** This system can be found throughout the eastern portion of the Western Great Plains Division, however, it is most prevalent in the central states of Nebraska, Kansas, Oklahoma, and Texas. In addition, it does occur farther to the west, in central and eastern Montana and eastern Wyoming.

**Divisions:** 205:P, 303:C

**TNC Ecoregions:** 26:C, 27:C, 28:C, 32:P, 33:C

**Nations:** US

**Subnations:** CO, KS, MT, NE, NM, OK, SD, TX, WY

**Map Zones:** 20:?, 22:C, 25:?, 26:P, 29:P, 30:P, 31:P, 32:P, 33:C, 34:C, 35:?, 36:P, 38:C

**USFS Ecomap Regions:** 251F:CC, 251H:CC, 315F:PP, 331B:CP, 331C:CC, 331D:C?, 331E:CC, 331F:CC, 331G:CP, 331H:CC, 331K:CP, 331L:CP, 331M:CP, 332B:CC, 332C:CC, 332D:CC, 332E:CC, 332F:CC

CONCEPT

**Environment:** This system is typified by circular upland depressional basins with an impermeable layer such as dense clay which slows infiltration and promotes retention of water. Soils are hydric and fine‑grained. Rainwater and runoff recharge this system and it is rarely linked to outside groundwater sources. Water is lost through both evapotranspiration and percolation to aquifers such as the Ogallala Aquifer in the Texas High Plains. It has been estimated that 20‑80% of water in playas infiltrates into the aquifers, principally along the margins of the wetlands where subsurface clay content is less (Osterkamp and Wood 1987). Playas are shallow, generally less than 1 m deep, with very shallow sloping sides. This results in nearly equal water depths throughout the playa, and small changes in water depth have effects across a relatively large surface area. Playas in the Southern High Plains average 6.3 ha in area. These ephemeral wetlands have small watersheds. The average watershed size is 55.5 ha (Guthery and Bryant 1982). Playas are isolated, with no surface outflow except in unusually wet periods. In Texas, playas are typically lined by Vertisols included in Playa, Lakebed, or in some cases Clay Flat ecoclasses.

**Vegetation:** Species richness varies considerably among individual examples of this system. Commonly, *Eleocharis* spp., *Hordeum jubatum*, along with *Coreopsis tinctoria, Symphyotrichum subulatum (= Aster subulatus)*, and *Polygonum pensylvanicum (= Polygonum bicorne)* are found in the wetter and deeper depression. Shallower depressions in rangelands commonly contain *Pascopyrum smithii* and *Bouteloua dactyloides (= Buchloe dactyloides)*.

**Dynamics:** Playas have a large change in hydrologic status over much of their areas. That is, most of the area of an individual playa is wet or flooded at one point in the growing season and also dry during another point in the growing season. Some do have deeper areas that are wet or flooded for nearly the entire growing season. More common is having multiple wet‑dry cycles during one growing season in response to rain and dry periods. This rapid change in available moisture and in exposed soil limits the species that can grow. This often results in strong dominance by a few perennial species able to tolerate these conditions or by annuals that can go through their life cycle before conditions change (Haukos and Smith 1993). However, the unconnected nature of playas combined with the variable environmental conditions throughout the year favors the formation of differing assemblages of vegetation at any one time on playas across the landscape. This contributes to regional diversity of plant and animal habitats throughout the year (Haukos and Smith 1994). Fire can spread into this system from surrounding grasslands but it is uncommon. The surrounding grasslands are typically short and do not have sufficient fuel to carry fire well, and while playas usually have more dense vegetation cover than the adjacent uplands, they may be wet.

SOURCES

**References:** Bolen et al. 1979, Comer et al. 2003*, Elliott 2013, Faber‑Langendoen et al. 2011, Guthery and Bryant 1982, Haukos and Smith 1993, Haukos and Smith 1994, Hoagland 2000, Lauver et al. 1999, Luo et al. 1997, Osterkamp and Wood 1987, Rolfsmeier and Steinauer 2010, Shiflet 1994

**Version:** 02 Oct 2014	**Stakeholders:** Midwest, Southeast, West

**Concept Author:** S. Menard and K. Kindscher	**LeadResp:** Midwest

CES303.675 Western Great Plains Open Freshwater Depression Wetland

**Primary Division:** Western Great Plains (303)

**Land Cover Class:** Herbaceous Wetland

**Spatial Scale & Pattern:** Small patch

**Required Classifiers:** Natural/Semi‑natural; Vegetated (>10% vasc.); Wetland

**Concept Summary:** This Great Plains emergent marsh ecological system is composed of lowland depressions; it also occurs along lake borders that have more open basins and a permanent water source through most of the year, except during exceptional drought years. These areas are distinct from Western Great Plains Closed Depression Wetland & Playa (CES303.666) by having a large watershed and/or significant connection to the groundwater table. A variety of species are part of this system, including emergent species of *Typha, Carex, Eleocharis, Juncus, Spartina*, and *Schoenoplectus*, as well as floating genera such as *Potamogeton, Sagittaria, Stuckenia*, or *Ceratophyllum*. The system includes submergent and emergent marshes and associated wet meadows and wet prairies. These types can also drift into stream margins that are more permanently wet and linked directly to the basin via groundwater flow from/into the pond or lake. Some of the specific communities will also be found in the floodplain system and should not be considered a separate system in that case. These types should also not be considered a separate system if they are occurring in lowland areas of the prairie matrix only because of an exceptional wet year.

**Comments:** This system occurs widely throughout the western Great Plains, but in the arid shortgrass region, it is replaced by North American Arid West Emergent Marsh (CES300.729). Open and emergent marshes may be a separate system from wet meadows and wet prairies. More clarification needs to be made between this system and other depressional wetlands occurring in Wyoming and Montana, such as Inter‑Mountain Basins Alkaline Closed Depression (CES304.998), Great Plains Prairie Pothole (CES303.661), and the other western Great Plains depressional wetland systems. In the Texas Systems Map Project, this system may have also been attributed to anthropogenic ponds and lakes.

DISTRIBUTION

**Range:** This system can occur throughout the Northwestern Great Plains Division but not in the arid shortgrass region.

**Divisions:** 205:P, 303:C

**TNC Ecoregions:** 26:C, 28:C, 29:C, 33:C, 34:C, 37:?, 66:?, 67:?

**Nations:** US

**Subnations:** CO, KS, MT, ND, NE, OK, SD, TX, WY

**Map Zones:** 25:P, 26:C, 27:C, 29:C, 30:C, 31:C, 33:C, 34:C, 38:C

**USFS Ecomap Regions:** 331F:??

CONCEPT

**Environment:** This system is found within lowland depressions and along lakes that have more permanent water sources throughout the year. These areas typically have a large watershed and are connected to the groundwater sources, resulting in a relatively consistent source of water for the semi‑arid climate they occur in. Examples may also drift into stream margins that are more permanently wet and linked to a basin via groundwater flow from/into a pond or lake. Those areas that are found within larger prairie matrix that are only lowland or wet because of an exceptional wet year are not part of this system. This system occurs south of the limit of recent glaciation. Salinity ranges from fresh to brackish. Soils range from clay and silt to sandy loam. Marshes with coarser soils are usually connected more directly to the water table, which prevents rapid draining of the wetland.

**Vegetation:** Many species can be associated with this system with *Typha* spp. and *Schoenoplectus* spp. being common.

**Dynamics:** Hydrology is the primary process influencing this system. Examples of this system have a core area that is saturated or flooded much or all of the growing season. In some sites, water levels exceed 1 m throughout the growing season. Examples of this system receive water from groundwater flow, surface drainage from the watershed, and direct precipitation. In the northern half of the range of this system, snowmelt can cause a relatively large influx of water in the spring. Water levels are typically highest in the spring and generally fall throughout the growing season, with occasional refilling of the basin after very heavy summer rains. Changes in precipitation over a period of years or decades (wetter or drier periods) will increase or decrease the extent of individual examples of this system and can move the range of the entire system slightly.

Fires can occur in this system, often spreading from adjacent upland prairie. Fire is more common in the fall when water levels are lower and the vegetation has dried out. Fire is also more common in the eastern portion of the range of this system where surrounding uplands had more dense upland tallgrass prairie rather than the sparser mixedgrass uplands typical of the western range of this system.

SOURCES

**References:** Comer et al. 2003*, Elliott 2013, Gleason and Euliss 1998, Hoagland 2000, Lauver et al. 1999, Preston et al. 2013, Rolfsmeier and Steinauer 2010

**Version:** 14 Jan 2014	**Stakeholders:** Midwest, Southeast, West

**Concept Author:** S. Menard and K. Kindscher	**LeadResp:** Midwest

2.C.4.Ne. Atlantic & Gulf Coastal Marsh, Wet Meadow & Shrubland

M066. Atlantic & Gulf Coastal Fresh‑Oligohaline Tidal Marsh

CES203.259 Atlantic Coastal Plain Embayed Region Tidal Freshwater Marsh

**Primary Division:** Gulf and Atlantic Coastal Plain (203)

**Land Cover Class:** Herbaceous Wetland

**Spatial Scale & Pattern:** Small patch

**Required Classifiers:** Natural/Semi‑natural; Vegetated (>10% vasc.); Wetland

**Diagnostic Classifiers:** Tidal / Estuarine [Freshwater]; Graminoid

**Concept Summary:** Embayed region tidal freshwater marshes are characterized by fresh to oligohaline waters which are driven by irregular wind tides, with minimal lunar tidal influence. They are the predominant marsh system in the drowned creeks and inland estuary shores of the embayed region of northeastern North Carolina and adjacent Virginia. This system typically occurs as complexes of several associations dominated by large graminoids such as *Spartina cynosuroides, Cladium mariscus ssp. jamaicense, Schoenoplectus pungens, Typha angustifolia, Typha latifolia*, and *Juncus roemerianus*, sometimes with species‑rich associations of shorter graminoids, forbs, and floating or submerged aquatic plants. While some association dominants are tolerant of brackish water, they are associated with plants restricted to oligohaline or freshwater. Irregular flooding and fire are both important forces in this system, and rising sea level is a particularly important driver of long‑term trends.

**Comments:** The distinction between this system and other tidal freshwater marsh systems is based on the distinctive dynamics of the irregular wind tidal flooding.

DISTRIBUTION

**Range:** This system is restricted to the embayed region of North Carolina and Virginia, with the best development in coastal areas along the North Carolina‑Virginia border. The transition to areas with more lunar tidal influence is fairly gradual to the south over a space of 50 miles.

**Divisions:** 203:C

**TNC Ecoregions:** 57:C

**Nations:** US

**Subnations:** NC, VA

**Map Zones:** 58:C, 60:C

**USFS Ecomap Regions:** 232I:CC

CONCEPT

**Environment:** The embayed region of the Mid‑Atlantic Coastal Plain stretches along northeastern North Carolina and adjacent areas of Virginia. Estuaries in drowned river valleys are unusually extensive here. The barrier islands along the coast are unusually continuous and the ocean's tidal range modest. This produces estuaries where irregular wind tides are the dominant hydrological process. The water is oligohaline to fresh over most of the tidal areas, with brackish water near the coast and saltwater only on or near the barrier island inlets. Rainfall may be an important influence in marsh interiors for significant periods of time between high wind tides. Soils appear to be essentially always saturated, with shallow flooding for periods of several days at all times of year. Due to limited sediment transport, marsh soils are primarily organic. Marshes occur in small to large patches or bands along the drowned creeks and rivers. Most give way to tidal swamps inland and upstream, but some occur on islands. Those near the transition to brackish water may grade to wind tide‑influenced brackish marshes downstream.

**Vegetation:** This system consists largely of wetland vegetation dominated by large graminoid herbs that are tolerant of constant saturation but intolerant of too much salt. *Spartina patens, Cladium mariscus ssp. jamaicense, Schoenoplectus pungens, Typha angustifolia*, and *Typha latifolia* dominate large areas. *Juncus roemerianus* is sometimes a dominant, especially in areas that have become fresh in the last 100 years as a result of coastal inlet changes. All of these dominants are accompanied by at least a few other plants intolerant of saltwater. Vegetation dominated by smaller graminoids, wetland forbs, submerged or floating aquatics, shrubs, or open stands of trees may also be present. Individual marshes usually are mosaics or zoned complexes of patches of the component associations.

**Dynamics:** Hydrology is the most important driving process, with the constant saturation determining the potential vegetation, and the variable flooding and variations in salinity in the fresh to brackish range a primary disturbance. Wind tides flood or expose the marshes at irregular intervals and transport nutrients and organic matter. Storm surges and unusually high tides associated with storms may bring saltier water into these systems, disturbing the less salt‑tolerant plants. These disturbances may be an important factor determining the boundary between this system and adjacent tidal swamps, but this is not well documented. Rising sea level is an important driver of longer term vegetation trends, including expansion into adjacent swamp areas. Fire is also an important natural process in all but the smallest and most isolated patches. Frost (pers. comm.) estimates that many marshes burned as often as every three years in presettlement times and were an important source of ignition for adjacent communities. Marshes that have not burned recently have lower species richness, are more strongly dominated by the large graminoids, and are believed to be poorer habitat for waterfowl. Marshes often show evidence of transition to or from tree‑dominated communities, in the form of young invading trees and shrubs or standing dead older trees. Lack of fire appears to be allowing sufficient tree invasion to eventually produce a swamp forest in some upstream examples, but the trend in most places is toward development of marshes in former swamp areas.

SOURCES

**References:** Bertness et al. 2004, Comer et al. 2003*, Schafale 2012

**Version:** 21 May 2014	**Stakeholders:** East, Southeast

**Concept Author:** R. Evans, M. Schafale, G. Fleming	**LeadResp:** Southeast

CES203.507 Florida Big Bend Fresh and Oligohaline Tidal Marsh

**Primary Division:** Gulf and Atlantic Coastal Plain (203)

**Land Cover Class:** Herbaceous Wetland

**Spatial Scale & Pattern:** Large patch

**Required Classifiers:** Natural/Semi‑natural; Vegetated (>10% vasc.); Wetland

**Diagnostic Classifiers:** Tidal / Estuarine [Freshwater]; Graminoid

**Concept Summary:** This ecological system includes tidal freshwater and oligohaline marshes of the northern Gulf of Mexico along the Florida Big Bend area (roughly from Wakulla County to the Pasco/Hernando county line on Florida's west coast). The tidal range in this region is higher than in the western Panhandle, and wave energy is low; lunar, wind and seasonal tides make flooding irregular. In comparison to the matrix‑forming salt and brackish marshes of the same region, this system is confined to small patches that are generally restricted to areas near the mouths of rivers where freshwater is abundant. This system is dominated by herbaceous graminoids tolerant of tidal flooding, but not tolerant of saltwater and with only a limited tolerance of true brackish conditions.

DISTRIBUTION

**Range:** Endemic to Florida from Wakulla County (Apalachicola Bay) to Pasco/Hernando county line, north of Tampa Bay.

**Divisions:** 203:C

**TNC Ecoregions:** 53:C

**Nations:** US

**Subnations:** FL

**Map Zones:** 55:C, 99:C

**USFS Ecomap Regions:** 232D:CC, 232L:CC

CONCEPT

**Environment:** The flooding regime is tidal (irregular) but influenced by the freshwater flows of rivers. This system occurs where there is adequate river flow and discharge to maintain fresh to oligohaline conditions, while still within tidal range. These marshes occur near the mouths and upstream, well inside the mouths of tidal creeks and rivers.

**Vegetation:** This system is dominated by herbaceous graminoids tolerant of tidal flooding, but not tolerant of saltwater and with only a limited tolerance of true brackish conditions. It does not include the abundant salt marshes of *Spartina alterniflora* and *Juncus roemerianus* (brackish).

**Dynamics:** The tidal range in this region is higher than in the western Panhandle, and wave energy is low; lunar, wind and seasonal tides make flooding irregular (Montague and Wiegert 1990). In times of drought and low freshwater flows, brackish water will reach upstream further, into areas which would normally be freshwater. This can be a disturbance which alters community structure, decreasing populations or fecundity of those species intolerant of brackish water.

SOURCES

**References:** Bertness et al. 2004, Comer et al. 2003*, FNAI 2010a, Montague and Wiegert 1990

**Version:** 21 May 2014	**Stakeholders:** Southeast

**Concept Author:** R. Evans and C. Nordman	**LeadResp:** Southeast

CES203.467 Gulf Coast Chenier Plain Fresh and Oligohaline Tidal Marsh

**Primary Division:** Gulf and Atlantic Coastal Plain (203)

**Land Cover Class:** Herbaceous Wetland

**Spatial Scale & Pattern:** Large patch

**Required Classifiers:** Natural/Semi‑natural; Vegetated (>10% vasc.); Wetland

**Diagnostic Classifiers:** Tidal / Estuarine [Freshwater]; Aquatic Herb; Graminoid

**Concept Summary:** This system includes large expanses of tidal marshes, strongly influenced by freshwater, along the Chenier Plain of Louisiana and Texas. Fresh to oligohaline marsh is the most common marsh type of the Chenier Plain because of the unique geomorphology of the area. The Chenier Plain is characterized by a prograding coastline replenished by sediments carried to the Gulf of Mexico by the Atchafalaya and other rivers. It is void of barrier islands, and shoreline sediments are reworked by waves into beach ridges. This process has been continuing since the last glacial retreat, and as the coastline progrades, older beach ridges are left as interior ridges surrounded by marsh. Historically, there were very few natural connections between the marshes and the ocean, resulting in a predominance of fresh to oligohaline salinity. This is a highly threatened system in coastal Louisiana.

**Comments:** This system also includes some flotant marshes formerly recognized as a distinct ecological group.

DISTRIBUTION

**Range:** This system extends from Vermillion Bay, Louisiana, through Jefferson County, Texas. It does not extend into Galveston Bay. Approximately 3000 square km of marshes were present in the Chenier Plain of Louisiana in 1997 and the majority of these were fresh to oligohaline marshes (Visser et al. 2000).

**Divisions:** 203:C

**TNC Ecoregions:** 31:C

**Nations:** US

**Subnations:** LA, TX

**Map Zones:** 37:C, 98:C

**USFS Ecomap Regions:** 232E:CC, 255D:CC

CONCEPT

**Environment:** This system occupies coastal sites with mucky soils and salinities generally less than 4 ppt. Soils are recent alluvial deposits. It occurs along bay margins and outlets of coastal rivers where freshwater inflow is sufficient to drive marsh composition. Sites may be interspersed with areas of open water. Soils are saturated, very deep, mineral soils, often with high organic content, at least at the surface. Ecoclasses (from Ecological Site Descriptions) include various fresh and intermediate marsh types (Elliott 2011). Coastal Louisiana contains about "37% of the estuarine herbaceous marshes in the conterminous U.S." (Glick et al. 2013).

**Vegetation:** Dominant plant species are graminoids, including *Panicum hemitomon, Paspalum vaginatum, Zizaniopsis miliacea, Typha latifolia, Spartina patens, Schoenoplectus* spp., and *Phragmites australis*. Other wetland species such as *Sagittaria* spp., *Ludwigia* spp., and *Vigna luteola* may also be present. Some occurrences may have some woody cover with species such as *Iva frutescens* or *Baccharis halimifolia* (Elliott 2011).

**Dynamics:** Historically, the deltaic processes of the Mississippi River helped to build and maintain this system. Sediments brought to the coast by the Mississippi River and its distributaries were carried west along the coast by longshore currents. The shifting over time of the location of the Mississippi River deltas resulted in variations in sediment availability which, along with other coastal processes, caused an alternating prograding and retreating chenier plain coastline. Sediments were reworked into beach ridges that trapped freshwater flowing coastward off the mainland resulting in a chenier plain of beach ridges (cheniers) alternating with large expanses of fresh to oligohaline marshes. Today, sediments from the Atchafalaya River are again forming mudflats in the chenier plain but not to the extent that was associated with Mississippi River deltaic processes (Gosselink et al. 1998). One of the few areas of coastal accretion in Louisiana is located in the eastern Chenier Plain, a process fed by sediments from the Atchafalaya. Given the predominance of coastal loss and subsidence in Louisiana, the accretion of the eastern Chenier Plain is unusual (Gosselink et al. 1998, Draut et al. 2005). In addition to local rainfall, freshwater entering the chenier plain marshes comes from rivers and streams, and is dependent on functioning hydrological processes in those systems. This marsh system is dependent upon freshwater input, sediment input and organic matter build‑up. Species richness is typically higher in oligohaline marshes than in brackish marshes. In addition to these natural barriers limiting waterflow, many human‑made impoundments exist in the Chenier Plain. These impoundments tend to support fresh marsh because water loss through evapotranspiration is less than local rainfall (Gosselink et al. 1979).

SOURCES

**References:** Bernier 2013, Comer et al. 2003*, Couvillion and Beck 2013, Couvillion et al. 2011, Draut et al. 2005, Elliott 2011, Glick et al. 2013, Gosselink et al. 1979, Gosselink et al. 1998, Howard and Mendelssohn 1999, LDWF 2005, Neubauer 2013, Smith 1993, Visser et al. 2000, Williams 2013, Willis and Hester 2004

**Version:** 14 Jan 2014	**Stakeholders:** Southeast

**Concept Author:** J. Teague and R. Evans	**LeadResp:** Southeast

CES203.470 Mississippi Delta Fresh and Oligohaline Tidal Marsh

**Primary Division:** Gulf and Atlantic Coastal Plain (203)

**Land Cover Class:** Herbaceous Wetland

**Spatial Scale & Pattern:** Large patch

**Required Classifiers:** Natural/Semi‑natural; Vegetated (>10% vasc.); Wetland

**Diagnostic Classifiers:** Tidal / Estuarine [Freshwater]; Graminoid

**Concept Summary:** This system includes tidal marshes strongly influenced by freshwater producing a fresh to oligohaline chemistry. These areas can occupy small to large patches of the Mississippi Delta. A unique type of floating fresh marsh (flotant) is also included in this system. This system has a heterogeneous physiognomy including shrublands, grasslands, and aquatic herbs. Significant fresh marsh loss has occurred in the deltaic plain of the Mississippi River. These losses are related to natural and anthropogenic causes. Subsidence and loss of wetlands are a natural part of the deltaic process, but they have been exacerbated by the reduction in sediment load and freshwater input into coastal areas caused by the impoundment and channelization of streams and rivers. In addition dredged channels in the marsh facilitate saltwater intrusion, and spoil banks prevent marshes from draining. Increases in salinity cause shifts in composition to species more tolerant of salinity, ultimately resulting in loss of species diversity and open saline waters.

DISTRIBUTION

**Range:** This system occurs in the Mississippi River deltaic plain. Marshes in the Mississippi River deltaic plain encompass 22% of the marshes in the conterminous U.S. and about half of these are fresh to oligohaline marshes (Gosselink 1984, Visser et al. 1998).

**Divisions:** 203:C

**TNC Ecoregions:** 31:C

**Nations:** US

**Subnations:** LA

**Map Zones:** 98:C

**USFS Ecomap Regions:** 232E:CC

CONCEPT

**Environment:** This system occurs in the Mississippi River deltaic plain where freshwater inflow is greatest ‑ near the mouths of distributary channels for the Mississippi River and near the mainland where freshwater flow from upland runoff and smaller creeks and rivers enters the marshes.

**Vegetation:** Species found in examples of this system include *Colocasia esculenta, Eleocharis baldwinii, Eleocharis rostellata, Hydrocotyle ranunculoides, Hydrocotyle umbellata, Ludwigia* spp., *Morella cerifera, Panicum hemitomon, Paspalum vaginatum, Phragmites australis, Sagittaria lancifolia, Sagittaria latifolia, Sagittaria platyphylla, Schoenoplectus californicus, Spartina patens, Vigna luteola, Typha domingensis*, and *Zizaniopsis miliacea*.

**Dynamics:** Historically, the deltaic processes of the Mississippi River helped to build and maintain this system. Today, in addition to the large fresh to oligohaline marshes that hug the mainland, two active deltas, the Atchafalaya and the Mississippi Balize deltas, support fresh to oligohaline marshes areas. However, these areas of sediment accumulation and accretion are an anomaly compared to the predominance of coastal loss and subsidence in the Mississippi River deltaic plain (Gosselink et al. 1998, Draut et al. 2005). The natural sediment load and freshwater entering the deltaic marshes are dependent on functioning hydrological processes in the Mississippi River. This marsh system is dependent upon freshwater input, sediment input and organic matter build‑up. Species richness is typically higher in oligohaline marshes than in brackish marshes. Much of this system is characterized by floating mats of vegetation (Visser et al. 1998). Some studies suggest that marshes may convert to floating mats as a result of rapid subsidence.

SOURCES

**References:** Bernier 2013, Comer et al. 2003*, Couvillion and Beck 2013, Couvillion et al. 2011, Deegan et al. 1984, Draut et al. 2005, Glick et al. 2013, Gosselink 1984, Gosselink et al. 1998, Howard and Mendelssohn 1999, LDWF 2005, Neubauer 2013, Smith 1993, Visser et al. 1998, Williams 2013, Willis and Hester 2004

**Version:** 14 Jan 2014	**Stakeholders:** Southeast

**Concept Author:** J. Teague and R. Evans	**LeadResp:** Southeast

CES203.516 Northern Atlantic Coastal Plain Fresh and Oligohaline Tidal Marsh

**Primary Division:** Gulf and Atlantic Coastal Plain (203)

**Land Cover Class:** Herbaceous Wetland

**Spatial Scale & Pattern:** Large patch

**Required Classifiers:** Natural/Semi‑natural; Vegetated (>10% vasc.); Wetland

**Diagnostic Classifiers:** Tidal / Estuarine; Graminoid

**Concept Summary:** This system includes freshwater tidal vegetation occurring on the upper reaches of large rivers influenced by tidal flooding, but beyond the reach of the salt or brackish waters. The system is well‑developed on the Chesapeake and Delaware Bay drainages, including the rivers of southern New Jersey, then extends northeast and includes inland portions of the Hudson, Connecticut, Merrimack, Kennebec, and Penobscot rivers and their tributaries. The vegetation includes tall marshes dominated by tall grasses such as *Zizania aquatica*, marshes of lower stature dominated by forbs such as *Amaranthus cannabinus, Hibiscus moscheutos* and others, and vegetation characterized by short‑statured and rosette‑forming forbs such as *Eriocaulon parkeri* and *Isoetes riparia*. Associations are distributed by proximity to tidal waters and thus duration and force of flooding. Sediments of more protected and isolated vegetation is composed of finer‑grained materials that are poorly drained, or of well‑consolidated peat deposits. Vegetation exposed to greater flooding force and scouring action is supported by mineral substrates such as sand and gravel.

DISTRIBUTION

**Range:** This system is best developed on the Chesapeake and Delaware Bay drainages, including the rivers of southern New Jersey, but extends northeast and includes inland portions of the Hudson, Connecticut, Merrimack, Kennebec, and Penobscot rivers and their tributaries.

**Divisions:** 203:C

**TNC Ecoregions:** 58:C, 62:C, 63:C

**Nations:** US

**Subnations:** CT, DE, MA, MD, ME, NJ, NY, PA, VA

**Map Zones:** 60:C, 65:C, 66:C

**USFS Ecomap Regions:** 211Db:CCC, 211Dc:CCC, 221A:CC, 221B:CC, 232Ad:CCC, 232B:CC, 232Ha:CCC, 232Hb:CCC, 232Ib:CCC

CONCEPT

**Environment:** This system forms on the upper reaches of large rivers regularly inundated by tidal flooding but beyond the effects of saline waters.

**Dynamics:** This system is characterized by a mosaic of patches controlled by a combination of substrate particle size, amount of organic matter deposition, and the degree of stress (persistent natural processes) versus disturbance (episodic and disruptive processes) (Barrett 1994). Tidal amplitude in the Delaware Estuary supporting this system is approximately 2m.

SOURCES

**References:** Barrett 1994, Comer et al. 2003*, Edinger et al. 2014a, Faber‑Langendoen et al. 2011, Gawler and Cutko 2010, NYNHP 2013d, Rhoads and Block 2011c, Zimmerman et al. 2012

**Version:** 14 Jan 2014	**Stakeholders:** East, Southeast

**Concept Author:** R. Evans and L. Sneddon	**LeadResp:** East

CES203.376 Southern Atlantic Coastal Plain Fresh and Oligohaline Tidal Marsh

**Primary Division:** Gulf and Atlantic Coastal Plain (203)

**Land Cover Class:** Herbaceous Wetland

**Spatial Scale & Pattern:** Small patch

**Required Classifiers:** Natural/Semi‑natural; Vegetated (>10% vasc.); Wetland

**Diagnostic Classifiers:** Tidal / Estuarine [Freshwater, Oligohaline]; Graminoid

**Concept Summary:** This ecological system represents tidally‑influenced freshwater herbaceous marshes and tidal shrublands ranging from the vicinity of Morehead City, Carteret County, North Carolina (south of the Embayed Region), south to the vicinity of Marineland or Daytona Beach (Flagler/Volusia counties) in northern Florida. This system occurs where there is adequate riverflow and discharge to maintain fresh to oligohaline conditions, while still within tidal range. These marshes most often occur well inside the mouths of tidal creeks and rivers. Different vegetation types occupy areas of slightly different elevations within the marsh.

**Comments:** Montague and Wiegert (1990) state that "Northeast Florida salt marshes" occur "south to Marineland," which is in northern Flagler County, Florida. These "Northeast Florida salt marshes" are assumed to be the Florida part of Southern Atlantic Coastal Plain Salt and Brackish Tidal Marsh (CES203.270) which ranges north to southern North Carolina. It is further assumed that the range of CES203.270 and Southern Atlantic Coastal Plain Fresh and Oligohaline Tidal Marsh (CES203.376) are basically identical. Some other sources refer to Daytona Beach (Volusia County) as the southern limit. Atlantic Coastal Plain Indian River Lagoon Tidal Marsh (CES203.257) is found to the south.

DISTRIBUTION

**Range:** This system ranges from the vicinity of Morehead City, Carteret County, North Carolina (south of the Embayed Region), south to the vicinity of Marineland or Daytona Beach (Flagler/Volusia counties) in northern Florida (Montague and Wiegert 1990).

**Divisions:** 203:C

**TNC Ecoregions:** 55:C, 56:C, 57:C

**Nations:** US

**Subnations:** FL, GA, NC, SC

**Map Zones:** 55:C, 58:C

**USFS Ecomap Regions:** 232C:CC, 232G:CC, 232I:CC

CONCEPT

**Environment:** This system occurs where there is adequate riverflow and discharge to maintain fresh to oligohaline conditions, while still within tidal range. These marshes most often occur upstream, well inside the mouths of tidal creeks and rivers. Most of the region where this system occurs consists of marshy shores and sea islands.

**Vegetation:** Shrubs may include *Alnus serrulata, Baccharis halimifolia, Iva frutescens, Morella cerifera*, and *Ilex vomitoria*. Dominant grasses and graminoids may include *Carex stricta, Schoenoplectus pungens, Spartina cynosuroides, Spartina patens, Zizania aquatica*, and *Zizaniopsis miliacea*. In addition, *Juncus roemerianus* may be present, but will not occur in extensive patches. Forbs may include *Borrichia frutescens, Peltandra virginica, Sagittaria lancifolia ssp. media, Sagittaria latifolia*, and the fern *Osmunda regalis var. spectabilis*.

**Dynamics:** Tidal flooding with freshwater is the ecological factor that distinguishes this system from others. Tides bring nutrients, making the regularly‑flooded marshes fertile. Rising sea level associated with climate change will affect this system strongly, drowning some marsh areas, promoting shoreline erosion, and causing salt or brackish marshes to spread inland upstream into areas that have been freshwater marsh areas. Some limited natural shifting between this system and tidal swamps may occur, as trees are killed by storm‑driven salt water intrusion and later trees may gradually regenerate. Fire may also have affected this boundary in the past ‑ flammable marsh vegetation and non‑flammable swamp vegetation may both have affected fire regimes in ways that helped maintain them, for instance when dry, herbaceous marsh vegetation may promote the spread of fires, which kill trees. Tidal swamps which have a shaded understory rarely have adequate dry fine fuels to carry fire.

SOURCES

**References:** Bertness et al. 2004, Comer et al. 2003*, FNAI 2010a, Montague and Wiegert 1990, Nelson 1986, Odum et al. 1984, Schafale 2012

**Version:** 21 May 2014	**Stakeholders:** Southeast

**Concept Author:** R. Evans	**LeadResp:** Southeast

CES203.472 Texas Coast Fresh and Oligohaline Tidal Marsh

**Primary Division:** Gulf and Atlantic Coastal Plain (203)

**Land Cover Class:** Herbaceous Wetland

**Spatial Scale & Pattern:** Small patch

**Required Classifiers:** Natural/Semi‑natural; Vegetated (>10% vasc.); Wetland

**Diagnostic Classifiers:** Tidal / Estuarine [Freshwater]; Graminoid

**Concept Summary:** This ecological system includes tidal marshes strongly influenced by freshwater producing a fresh to oligohaline chemistry, where salinity is maintained sufficiently low through freshwater inflows to produce fresh to oligohaline water chemistry. These marshes typically occur as small patches along bay margins and river or bayou mouths of inflowing rivers from Galveston Bay in Chambers County, Texas, south to approximately Corpus Christi Bay. Some characteristic plant species include *Paspalum vaginatum, Spartina patens, Schoenoplectus americanus, Phragmites australis, Sagittaria platyphylla, Vigna luteola*, and *Typha* spp.

DISTRIBUTION

**Range:** This fresh and oligohaline marsh system ranges along the Texas coast south of the Chenier Plain. It is best developed along the central and upper coast of Texas from Galveston Bay in Chambers County, Texas, south to approximately Corpus Christi Bay.

**Divisions:** 203:C

**TNC Ecoregions:** 31:C

**Nations:** US

**Subnations:** TX

**Map Zones:** 36:C

**USFS Ecomap Regions:** 232E:CC, 255D:CC

CONCEPT

**Environment:** The typical geology is young Quaternary alluvium. Characteristic landforms include the mouths of rivers and bayous emptying into bays of the Galveston Bay system. The soils on which this system is found are the typical soils of the Tidal Flats and Salt Marsh Ecological Sites where they occur in areas of sufficient freshwater inflow (Elliott 2011).

**Vegetation:** Some characteristic plant species include *Paspalum vaginatum, Spartina patens, Schoenoplectus americanus, Phragmites australis, Sagittaria platyphylla, Vigna luteola*, and *Typha* spp.

**Dynamics:** This marsh system is dependent upon freshwater input, sediment input and organic matter build‑up. Species richness is higher in oligohaline marshes than in mesohaline and polyhaline marshes.

SOURCES

**References:** Bernier 2013, Comer et al. 2003*, Couvillion and Beck 2013, Elliott 2011, Howard and Mendelssohn 1999, Neubauer 2013, Willis and Hester 2004

**Version:** 14 Jan 2014	**Stakeholders:** Southeast

**Concept Author:** J. Teague	**LeadResp:** Southeast

M067. Atlantic & Gulf Coastal Plain Wet Prairie & Marsh

CES203.890 Central Florida Herbaceous Pondshore

**Primary Division:** Gulf and Atlantic Coastal Plain (203)

**Land Cover Class:** Herbaceous Wetland

**Spatial Scale & Pattern:** Small patch

**Required Classifiers:** Natural/Semi‑natural; Vegetated (>10% vasc.); Wetland

**Diagnostic Classifiers:** Herbaceous; Depressional; Graminoid

**National Mapping Codes:** EVT 2514; ESLF 9275; ESP 1514

**Concept Summary:** This system includes a variety of seasonal depression ponds in central Florida, especially along the Lake Wales Ridge. Examples are rounded or irregularly shaped, shallow depressions from tens to hundreds of meters in diameter. Extensive variation is present based on the variety of soils and hydroperiods. Most examples have vegetation in zones, and nearly all are ringed by *Serenoa repens*. Characteristic or dominant species associated with the interior of the ponds include *Panicum hemitomon, Panicum abscissum, Hypericum edisonianum*, and *Andropogon brachystachyus*.

**Comments:** Compare to East Gulf Coastal Plain Depression Pond (CES203.558), found to the north.

DISTRIBUTION

**Range:** Endemic to central Florida.

**Divisions:** 203:C

**TNC Ecoregions:** 55:C

**Nations:** US

**Subnations:** FL

**Map Zones:** 55:C, 56:C

**USFS Ecomap Regions:** 232K:CC

CONCEPT

**Environment:** Most examples are known from the Lake Wales Ridge area of central Florida. These are shallow depressions from tens to hundreds of meters in diameter, found on a variety of different soils with different hydroperiods (Abrahamson et al. 1984).

**Vegetation:** Most depression ponds accommodated in this system display distinct vegetational zonation. At least four vegetational zones can be readily distinguished (Abrahamson et al. 1984); the community types need to be further reconciled into associations.

**Dynamics:** Variation in the duration and depth of flooding is part of the natural dynamics of the ponds in central Florida. The herbaceous pondshore or rim can burn with fires that burn the surrounding uplands or flatwoods. These fires help maintain the diversity of plants which can occur along the herbaceous pondshore or rim which circles the pond.

SOURCES

**References:** Abrahamson et al. 1984, Comer et al. 2003*

**Version:** 14 Jan 2014	**Stakeholders:** Southeast

**Concept Author:** R. Evans	**LeadResp:** Southeast

CES203.491 Central Florida Wet Prairie and Herbaceous Seep

**Primary Division:** Gulf and Atlantic Coastal Plain (203)

**Land Cover Class:** Herbaceous Wetland

**Spatial Scale & Pattern:** Small patch

**Required Classifiers:** Natural/Semi‑natural; Vegetated (>10% vasc.); Wetland

**Diagnostic Classifiers:** Seepage‑Fed Sloping; Graminoid

**Concept Summary:** This system includes herbaceous seepage wetlands and nearly treeless plains over poorly drained soils in central Florida. Although examples of this system are similar to other wetland ecological systems, these are characterized by the presence of subtropical plant species not occurring in herbaceous‑dominated wetlands farther north, especially *Panicum abscissum*. At least some examples have dense cover of grasses and low shrubs, with fairly high species diversity. Examples may be most common along the southern part of the Lake Wales Ridge area.

**Comments:** East Gulf Coastal Plain Savanna and Wet Prairie (CES203.192) is a closely related system found farther north.

DISTRIBUTION

**Range:** Endemic to central Florida, mainly found in the southern Lake Wales Ridge.

**Divisions:** 203:C

**TNC Ecoregions:** 55:C

**Nations:** US

**Subnations:** FL

**Map Zones:** 55:P, 56:C

**USFS Ecomap Regions:** 232D:CC, 232G:CC, 232K:CC

CONCEPT

**Environment:** Associated with saturated soils caused by seepage or high water tables; some examples may be saturated for 50‑100 days/year. Seepage‑influenced examples tend to occur in areas of greater topographic relief than wet prairies.

**Vegetation:** Usually dominated by *Panicum abscissum*.

**Dynamics:** Frequent fires were an important natural process in this system, with an estimated frequency of 1‑4 years (FNAI 1990, FNAI 2010a).

SOURCES

**References:** Comer et al. 2003*, FNAI 2010a

**Version:** 14 Jan 2014	**Stakeholders:** Southeast

**Concept Author:** R. Evans and C. Nordman	**LeadResp:** Southeast

CES203.558 East Gulf Coastal Plain Depression Pond

**Primary Division:** Gulf and Atlantic Coastal Plain (203)

**Land Cover Class:** Herbaceous Wetland

**Spatial Scale & Pattern:** Small patch

**Required Classifiers:** Natural/Semi‑natural; Vegetated (>10% vasc.); Wetland

**Concept Summary:** This small‑patch herbaceous or shrub dominated wetland ecological system occupies upland depressions (ponds and pondshores) in the East Gulf Coastal Plain. Included here are shallow ponds of various geomorphic origins in a variety of substrates (e.g., limesinks, Grady Ponds, Citronelle Ponds, flatwoods depression marshes) which are not separately distinguished as ecological systems. This ecological system only includes herbaceous or shrub ponds and pondshores in more‑or‑less isolated upland settings, not those in riparian or floodplain environments. They may serve as the origin of a stream system in a general way, releasing water gradually into the stream drainage system during periods of very wet weather. These tend to occupy basins that were formed by subsidence of surface sediments caused by solution in underlying limestone or as swales in eolian sand deposits. In some examples, a distinct zonation of vegetation is present, in others the zones are not distinct or the differing associations are present in a complex mosaic. Most seasonal depression ponds are composed of mosaics of several plant associations. The vegetation includes various zones which become exposed as water levels decline, as well as emergent (rising out of the water) or submergent/floating plants. Some typical species are *Dichanthelium wrightianum, Dichanthelium erectifolium, Eleocharis equisetoides, Eleocharis microcarpa, Juncus effusus, Juncus repens, Leersia* spp., *Ludwigia* spp., *Rhynchospora corniculata, Rhynchospora inundata, Panicum hemitomon, Panicum verrucosum, Proserpinaca* spp., *Pluchea* spp., *Saccharum* spp., *Rhexia* spp., and *Sabatia angularis*. Coastal dune lakes and related wetlands of barrier islands are covered by another system, Southeastern Coastal Plain Interdunal Wetland (CES203.258).

**Comments:** In Mississippi, this system is apparently confined to the "Pamlico Plain" (this is meant to refer to the Outer Coastal Plain) where it is very rare and small scale in occurrence (R. Wieland pers. comm.). It is unknown how distinct these depressions are from so‑called "Grady Ponds" (e.g., Cottonmouth Savanna site). This system is closely related to Southern Atlantic Coastal Plain Depression Pond (CES203.262) of the Atlantic Coastal Plain. This system also has karstic origins in common with Southern Coastal Plain Sinkhole (CES203.495) but occupies comparatively much shallower depressions and lacks exposed limestone. Compare to Central Florida Herbaceous Pondshore (CES203.890) to the south.

DISTRIBUTION

**Range:** This ecological system is found in the East Gulf Coastal Plain, including the Gulf Coast Flatwoods (i.e., EPA Level IV Ecoregion 75a (EPA 2004)), as well as more inland portions (EPA Level III Ecoregion 65). In particular, there are clusters of large ponds in parts of the Southern Pine Plains and Hills (EPA 65f), the Dougherty Plain (EPA 65g), and Tallahassee Hills/Valdosta Limesink (EPA 65o), and the Okefenokee Plain (EPA 75e).

**Divisions:** 203:C

**TNC Ecoregions:** 42:P, 43:C, 53:C

**Nations:** US

**Subnations:** AL, FL, GA, LA?, MS

**Map Zones:** 45:P, 46:C, 55:C, 99:C

CONCEPT

**Environment:** Examples of this ecological system occur in relatively shallow depressions or basins that were formed by subsidence of surface sediments caused by solution in underlying limestone or were formed as swales in eolian sand deposits. However, sinkholes with steep, vertical, exposed limestone walls are accommodated by another ecological system, as are sandhill ponds that develop on extreme sandy sites in the East Gulf Coastal Plain of Florida and adjacent Alabama. Hydroperiod can vary substantially from year to year, and vegetation can similarly vary significantly in aspect and dominants. Highly variable hydroperiods help maintain herbaceous vegetation, and prevent the succession to forest.

**Vegetation:** Most seasonal depression ponds are usually composed of mosaics of several plant associations. The vegetation includes various zones which become exposed as water levels decline. These are occupied sequentially by various graminoids and/or forbs, as well as emergent (rising out of the water) and submergent/floating plants. Some typical dominant species in component associations include *Aristida palustris, Dichanthelium wrightianum, Dichanthelium erectifolium, Eleocharis elongata, Eleocharis equisetoides, Eleocharis microcarpa, Fuirena scirpoidea, Juncus repens, Rhynchospora chapmanii, Rhynchospora corniculata, Rhynchospora harperi, Rhynchospora inundata, Rhynchospora filifolia, Rhynchospora tracyi, Proserpinaca* spp., *Juncus abortivus, Juncus effusus, Panicum hemitomon, Pluchea* spp., *Ludwigia* spp., *Saccharum* spp., *Panicum verrucosum, Rhexia* spp., and *Sabatia angularis*. In addition, associations dominated by *Polygonum* spp., *Leersia* spp., and *Typha* spp. may be present but are not characteristic. Other characteristic species include *Rhexia cubensis, Panicum rigidulum, Panicum verrucosum, Carex striata, Lachnanthes caroliana, Bartonia verna, Lachnocaulon minus*, and *Centella erecta*. Woody plants which may be present (particularly on margins) include *Cephalanthus occidentalis, Hibiscus* spp., *Hypericum chapmanii, Hypericum fasciculatum, Hypericum tenuifolium (= Hypericum reductum), Ilex myrtifolia*, and *Nyssa ursina*. Some stands with trees contain *Fraxinus pennsylvanica, Populus heterophylla, Ulmus americana*, and *Quercus texana*. Vegetation may exhibit distinct zonation in response to variation in duration of flooding. Communities can range from floating aquatic types (in the centers of the deepest basins) to emergent herbaceous zones (in semipermanent water drawdown zones) to sparse, yet diverse, small graminoid and forb herbaceous vegetation to bald‑cypress woodland edges. Some examples may have emergent trees throughout their extent.

**Dynamics:** The seasonal fluctuation in the water levels in these ponds controls both the overall vegetation composition as well as the composition of the zones of the vegetation, which may be quite distinct from one another. Hydroperiod can vary substantially from year to year, and vegetation can similarly vary significantly in aspect and dominants. Highly variable hydroperiods help maintain herbaceous vegetation and prevent the succession to forest. Fire is an important natural disturbance, and the outer, drier portions of the depressions burn most frequently. Fires may sweep through the interior of many examples during dry periods. Today, prescribed fire is important for the management of the pineland landscapes which include these herbaceous or shrub wetland ecological systems.

SOURCES

**References:** Comer et al. 2003*, EPA 2004, Eyre 1980, FNAI 2010a, Kirkman et al. 2012, Peet and Allard 1993, Southeastern Ecology Working Group n.d., Wharton 1978, Wieland pers. comm.

**Version:** 14 Jan 2014	**Stakeholders:** Southeast

**Concept Author:** M. Pyne	**LeadResp:** Southeast

CES203.192 East Gulf Coastal Plain Savanna and Wet Prairie

**Primary Division:** Gulf and Atlantic Coastal Plain (203)

**Land Cover Class:** Herbaceous Wetland

**Spatial Scale & Pattern:** Large patch

**Required Classifiers:** Natural/Semi‑natural; Vegetated (>10% vasc.); Wetland

**Diagnostic Classifiers:** Extensive Wet Flat; Very Short Disturbance Interval; Graminoid

**National Mapping Codes:** EVT 2485; ESLF 9206; ESP 1485

**Concept Summary:** This ecological system of western Florida and adjacent Alabama and Mississippi has been called "lush grassland," "grass‑sedge savannah," wet prairie, or wet savanna. As implied by these names, this system consists of primarily herbaceous vegetation with relatively thick cover of grasses and sedge species. Examples occupy low, flat plains on poorly drained soils, often saturated for 50‑100 days per year. Frequent fires, including growing‑season burns, are essential for maintenance of this system. Some examples have a sparse tree component of *Pinus elliottii* or *Pinus palustris* and scattered shrubs, such as *Morella caroliniensis*.

**Comments:** Related vegetation of central Florida is covered by another ecological system.

DISTRIBUTION

**Range:** Western Florida and adjacent Alabama and Mississippi.

**Divisions:** 203:C

**TNC Ecoregions:** 53:C

**Nations:** US

**Subnations:** AL, FL, MS

**Map Zones:** 55:C, 99:C

**USFS Ecomap Regions:** 232B:CC, 232D:CC, 232K:CC, 232L:CC

CONCEPT

**Environment:** This system occupies low, flat plains on poorly drained Ultisols. Sites are saturated for 50‑100 days per year (FNAI 2010a). Other soil orders may include Ultisols, Spodosols, Inceptisols, and Entisols (Collins et al. 2001); some of these soils have an argillic horizon which impedes drainage and contributes to high water tables. On Eglin Air Force Base, this system is found on the Rutledge series (Kindell et al. 1997).

**Vegetation:** Collins et al. (2001) documented less than 10 trees per acre (*Pinus elliottii* and *Pinus palustris*) in examples of this system on the Apalachicola National Forest. *Magnolia virginiana, Acer rubrum*, and *Morella cerifera* are often present in sometimes locally dense patches, especially when managed with infrequent fires (Collins et al. 2001, FNAI 2010a). *Aristida beyrichiana, Ctenium aromaticum, Rhexia alifanus, Rhynchospora* spp., and *Eriocaulon* spp. are typical species. This ecological system may be considered a "lush grassland" (Kindell et al. 1997), "grass‑sedge savannah" (Clewell 1981), wet prairie (FNAI 2010a), or wet savanna (Collins et al. 2001).

**Dynamics:** Wet prairies are seasonally inundated or saturated for 50 to 100 days a year (FNAI 2010a). Fire‑return intervals have been estimated to be 2‑3 years (FNAI 2010a). Wet prairies can be large areas which would have been naturally prone to frequent fire. Today prescribed fire is needed to maintain high‑quality examples of wet prairies. Without frequent fire, shrubs and trees can dominate the site, and this leads to a decline in the herbaceous plant diversity.

SOURCES

**References:** Clewell 1981, Collins et al. 2001, Comer et al. 2003*, Eyre 1980, FNAI 2010a, Hubricht 1985, Kindell et al. 1997, Norquist 1984

**Version:** 22 May 2014	**Stakeholders:** Southeast

**Concept Author:** R. Evans and C. Nordman	**LeadResp:** Southeast

CES203.077 Floridian Highlands Freshwater Marsh

**Primary Division:** Gulf and Atlantic Coastal Plain (203)

**Land Cover Class:** Herbaceous Wetland

**Spatial Scale & Pattern:** Large patch

**Required Classifiers:** Natural/Semi‑natural; Vegetated (>10% vasc.); Wetland

**Diagnostic Classifiers:** Depressional [Sinkhole]; Graminoid

**National Mapping Codes:** EVT 2489; ESLF 9214; ESP 1489

**Concept Summary:** This system represents non‑tidal marsh vegetation in the peninsula of Florida and in the Tallahassee area. These highland marshes occupy different types of depressions such as former lake basins, shallow peat‑filled valleys, and zones around existing natural lakes. The marshes and the basins they occur within are unstable over time due to subsurface subsidence and drainage pattern changes. In some examples, surface waterflow is generally lacking due to the presence of limestone near the surface, but water levels have fluctuated greatly over time. Soils range from mucky surfaces to sandy loams or sands, but slowly permeable subsoils contribute to the presence of standing water for much of the year. The vegetation mosaic includes a range of mostly herbaceous plant communities that may be referred to as marshes, meadows, and prairies, collectively comprising a relatively diverse number of associations. Permanent water bodies support a range of submerged and floating aquatic species. Areas with approximately a meter of standing water tend to support dense stands of emergent herbaceous perennials, often in monospecific stands; species include *Typha latifolia, Pontederia cordata, Nelumbo lutea*, and others. Where there is less water (usually present only during wet season), more graminoid vegetation is present, with species such as *Panicum hemitomon, Leersia hexandra*, and others. With historic water level fluctuations, the vegetation mosaic has also changed, sometimes quite rapidly.

**Comments:** This system was originally intended to cover Paynes Prairie only, but the concept was greatly expanded to include other non‑tidal marsh vegetation of Florida, including that around natural lakes, as well as the large Kissimmee and St. Johns River marshes. The Kissimmee and St. Johns River marshes also occur within floodplains but are influenced by somewhat different processes than typical highland marshes. These were formerly considered part of Florida River Floodplain Marsh (CES203.055).

DISTRIBUTION

**Range:** This system is found in the Florida Peninsula and in the Tallahassee Hills/Valdosta Limesink area, possibly ranging into adjacent Georgia. See map in Kushlan (1990, p. 327).

**Divisions:** 203:C

**TNC Ecoregions:** 53:C, 55:C

**Nations:** US

**Subnations:** FL, GA?

**Map Zones:** 55:C, 56:C

**USFS Ecomap Regions:** 232B:CC, 232D:CC, 232G:CC, 232J:CC, 232K:CC

CONCEPT

**Environment:** These highland marshes occupy different types of depressions such as former lake basins, shallow peat‑filled valleys, and zones around existing natural lakes (Kushlan 1990). The marshes and the basins they occur within are unstable over time due to subsurface subsidence and drainage pattern changes. Soils range from mucky surfaces to sandy loams or sands, but slowly permeable subsoils contribute to the presence of standing water for much of the year.

**Vegetation:** A relatively diverse assemblage of vegetation is present, ranging from open water communities to emergent and graminoid marshes, and scattered shrublands. Placing all component associations is difficult due to a number of factors; the current list (12‑02) is incomplete. In the absence of fire, portions of stands will become dominated by *Salix caroliniana*. If fire continues to be absent, these areas may succeed to *Acer rubrum* until a replacement fire or mechanical activity restores the marsh.

The concentric zones or bands of vegetation are related to length of the hydroperiod and depth of flooding. The outer, or driest, zone is often occupied by sparse herbaceous vegetation consisting of *Aristida palustris, Rhynchospora microcarpa, Rhynchospora cephalantha, Rhynchospora tracyi, Rhynchospora filifolia*, etc., *Xyris elliottii*, the subshrub *Hypericum myrtifolium*, and patches of *Amphicarpum muehlenbergianum* or *Spartina bakeri*. This sparse zone may be followed downslope by a sparse to dense zone of *Hypericum fasciculatum, Stillingia aquatica*, and scattered herbs, such as *Xyris fimbriata, Eriocaulon compressum, Eriocaulon decangulare, Rhynchospora inundata*, and *Eleocharis baldwinii*. The innermost, deepest zone is occupied by *Panicum hemitomon, Pontederia cordata, Sagittaria lancifolia*, or *Cladium mariscus ssp. jamaicense (= Cladium jamaicense)*. Floating‑leaved plants, such as *Nymphaea odorata*, may be found in open water portions of the marsh. Depending on depth and configuration, depression marshes can have varying combinations of these zones and species within each zone.

**Dynamics:** In some examples, surface waterflow is generally lacking due to the presence of limestone near the surface, but water levels have fluctuated greatly over time (Patton and Judd 1986). In the absence of fire, portions of stands will become dominated by *Salix caroliniana*. If fire continues to be absent, these areas may succeed to *Acer rubrum* until a replacement fire or mechanical activity restores the marsh. Paynes Prairie is a large permanently protected example of highland marsh. Water‑control structures allow the manipulation of water levels in Paynes Prairie to achieve ecosystem management goals (Kushlan 1990).

SOURCES

**References:** Comer et al. 2003*, FNAI 2010a, Kushlan 1990, Patton and Judd 1986

**Version:** 21 Feb 2017	**Stakeholders:** Southeast

**Concept Author:** R. Evans	**LeadResp:** Southeast

CES203.258 Southeastern Coastal Plain Interdunal Wetland

**Primary Division:** Gulf and Atlantic Coastal Plain (203)

**Land Cover Class:** Herbaceous Wetland

**Spatial Scale & Pattern:** Small patch

**Required Classifiers:** Natural/Semi‑natural; Vegetated (>10% vasc.); Wetland

**Diagnostic Classifiers:** Coast; Depressional

**Concept Summary:** This system encompasses the wettest dune swales and basins on barrier islands and coastal areas, supporting pond or marsh‑like vegetation, from the Coastal Plain of Texas to southern Virginia. Most examples are permanently or semipermanently flooded with freshwater but are affected by salt spray or overwash during periodic storm events. It is broadly defined in terms of floristic composition and is wide‑ranging along the Atlantic and Gulf coasts of the United States. These are graminoid‑dominated sites, with species such as *Andropogon glomeratus, Cladium mariscus ssp. jamaicense, Distichlis spicata, Eleocharis* spp., *Fimbristylis castanea, Panicum virgatum, Paspalum monostachyum, Rhynchospora colorata, Rhynchospora* spp., *Schoenoplectus pungens*, and *Typha domingensis*.

**Comments:** This system is currently defined with a much broader geographic range than most other coastal systems in the Southeast. The extreme variability within even a limited geographic range limits the ability to find broader vegetational patterns. Examples may vary regionally with regard to the amount of wind or salt spray and the texture of the sand. The northern end of the range is not clearly defined.

DISTRIBUTION

**Range:** Ranges along the Atlantic and Gulf coasts, from southern Texas to Florida and southeastern Virginia.

**Divisions:** 203:C

**TNC Ecoregions:** 31:C, 53:C, 54:?, 55:C, 56:C, 57:C

**Nations:** US

**Subnations:** AL, FL, GA, LA, MS, NC, SC, TX, VA

**Map Zones:** 36:C, 37:C, 55:C, 56:C, 58:C, 60:C, 99:C

**USFS Ecomap Regions:** 232C:CC, 232D:CC, 232E:CC, 232G:CC, 232H:CP, 232I:CC, 232L:CC, 255D:CC, 315E:CC, 411A:CC

CONCEPT

**Environment:** These wetlands occur on topographic lows, including dune swales or other basins, in nearly level to steeply rolling landscapes on sands and deep sands on barrier islands along the coast and inland on the South Texas Sand Sheet. These ponds have standing water well into the growing season, and most are permanently flooded. The water is from rainfall or the local water table and is fresh, except perhaps during storm events that produce overwash. Soils are sand, sometimes with a thin layer of muck accumulated in the pond. The geology includes coastal eolian sands, extending inland on the South Texas Sand Sheet, as well as Pleistocene barrier island and beach deposits of the Beaumont Formation, such as on the Ingleside Barrier. Examples occupy topographic lows of interdunal swales and potholes. Soils are deep sands and coastal sands (Elliott 2011).

**Vegetation:** Vegetation is characterized by emergent or drawdown wetland plants, often tall graminoids. Vegetation varies substantially from one example to the next. These sites are alternately wet and dry (due to seasonal rainfall events) and generally lack tidal influence, but may contain halophytic species due to the influence of salt spray and repeated inundation and evaporation. They are graminoid‑dominated sites, with species such as *Spartina patens, Andropogon glomeratus, Panicum virgatum, Paspalum monostachyum, Distichlis spicata, Fimbristylis castanea, Rhynchospora colorata, Eleocharis* spp., *Rhynchospora* spp., *Typha* spp., and *Schoenoplectus pungens*. Forbs such as *Hydrocotyle bonariensis, Centella erecta, Phyla nodiflora, Samolus ebracteatus, Bacopa monnieri*, and *Pluchea foetida* may be conspicuous. Woody species such as *Batis maritima, Sesbania* spp., *Prosopis glandulosa*, and *Baccharis* spp. may be present but do not typically constitute significant cover (Elliott 2011).

**Dynamics:** This system occurs in a geologically dynamic environment, where wind and waves may change landforms and hydrology quickly (Feagin et al. 2010). However, ponds usually occur in stable portions of islands, where they may last for decades. Salt spray, salt overwash, and heavy rainfall from storms may affect component communities, sometimes limiting vegetation to species that are somewhat salt‑tolerant. Severe storms may bring about major changes in the landforms and hydrology (Feagin et al. 2010).

SOURCES

**References:** Comer et al. 2003*, Elliott 2011, FNAI 2010a, Feagin et al. 2010, Nelson 1986, Schafale 2012

**Version:** 14 Jan 2014	**Stakeholders:** East, Southeast

**Concept Author:** M. Schafale and R. Evans	**LeadResp:** Southeast

CES203.044 Southeastern Coastal Plain Natural Lakeshore

**Primary Division:** Gulf and Atlantic Coastal Plain (203)

**Land Cover Class:** Woody Wetland

**Spatial Scale & Pattern:** Large patch

**Required Classifiers:** Natural/Semi‑natural; Vegetated (>10% vasc.); Wetland

**Diagnostic Classifiers:** Woody‑Herbaceous; Coastal plain; Depressional [Pond]

**Concept Summary:** This system consists of wetland vegetation along large natural lakeshores in the Outer Coastal Plain of the southeastern United States. Natural lakes are generally rare features throughout most of this region. However, examples range northward to the Atlantic Coastal Plain in southeastern Virginia and North Carolina, but no examples are known from South Carolina and Georgia. However examples are present in Florida, where they are apparently found on smaller lakes than those to the north. Hydroperiod remains relatively constant from year to year, especially when compared to smaller limesink depressions of the region. Vegetation may appear to be zonal in relationship to distance from the lakeshore and may range from open water or floating‑leaved aquatics in the deeper waters of the lakes, to emergent marsh zones along the edges. In some cases there are wet hardwood swamps present.

**Comments:** This system is related to Southern Atlantic Coastal Plain Depression Pond (CES203.262) which is found primarily on limesink depressions that are smaller in scale and have greater hydrologic fluctuation. This system is also related to Atlantic Coastal Plain Clay‑Based Carolina Bay Wetland (CES203.245) in that some of the natural lake basins it occurs on are thought to be Carolina bays (Bennett and Nelson 1991).

DISTRIBUTION

**Range:** This system is found in the Outer Coastal Plain of Virginia (apparently from a single site, Lake Drummond) and North Carolina, apparently absent from South Carolina and Georgia, but examples are present in Florida (i.e., Ocean Pond on Osceola National Forest).

**Divisions:** 203:C

**TNC Ecoregions:** 53:?, 55:C, 57:C

**Nations:** US

**Subnations:** FL, GA?, NC, SC?, VA

**Map Zones:** 55:C, 56:C, 58:C, 60:C, 99:C

**USFS Ecomap Regions:** 232C:CC, 232D:CC, 232G:CC, 232I:CC, 232L:CC

CONCEPT

**Environment:** Occurs along the edges of lakes, where the water is flooded for long durations, but may dry out during dry summer months, or during drought.

**Dynamics:** Long hydroperiod flooding is characteristic of these wetlands which occur along the shore of coastal plain natural lakes. Most of these habitats are naturally nutrient‑poor.

SOURCES

**References:** Bennett and Nelson 1991, Comer et al. 2003*, Eyre 1980, FNAI 2010a, Schafale 2012

**Version:** 06 Feb 2014	**Stakeholders:** East, Southeast

**Concept Author:** M. Schafale and R. Evans	**LeadResp:** Southeast

CES203.262 Southern Atlantic Coastal Plain Depression Pond

**Primary Division:** Gulf and Atlantic Coastal Plain (203)

**Land Cover Class:** Herbaceous Wetland

**Spatial Scale & Pattern:** Small patch

**Required Classifiers:** Natural/Semi‑natural; Vegetated (>10% vasc.); Wetland

**Diagnostic Classifiers:** Woody‑Herbaceous; Coastal plain; Depressional [Pond]

**Concept Summary:** This ecological system consists of wetlands in small basins formed in unconsolidated sediments of the Atlantic Coastal Plain, from southeastern Virginia to Florida. Many basins are formed by subsidence of surface sediments caused by solution in underlying limestone. Others may be formed as swales in mainland eolian sands, natural blockage of small drainages by sediment movement, and more obscure causes. Soils are generally sandy, with mucky surfaces in the wettest areas. Vegetation is often zonal in response to variation in duration of flooding in different parts of the depression pond. Vegetation usually ranges from open water or floating‑leaved aquatic plants in the center of the deepest basins, to emergent marsh zones in semipermanent water, to drawdown zones with diverse small graminoid and forb vegetation, to dense shrub or woodland edges. A smaller number of basins may have emergent trees throughout their extent. Hydroperiod can vary substantially from year to year, and vegetation can similarly vary significantly in aspect and dominants. In addition to flooding, fire is an important natural disturbance to vegetation in the outer, drier portions of the pond.

**Comments:** The boundary of this system with adjacent upland or wetland systems occurs where vegetation begins to reflect the influence of regular flooding and basin hydrology. This system shares much of its character with Atlantic Coastal Plain Clay‑Based Carolina Bay Wetland (CES203.245) but generally accommodates all "limesink depressions" as opposed to mineral soil Carolina bay wetlands. Other basins, especially broad, gently sloped basins on the Outer Coastal Plain and steep‑sided depressions farther inland, will need to be placed based on the preponderance of evidence. The northern and southern range limits of this system are not well known. It is tentatively placed at the mouth of Chesapeake Bay and in south Georgia. Northern Atlantic Coastal Plain Pond (CES203.518) of Virginia and Maryland and the wet prairies of north‑central Florida are closely related systems.

DISTRIBUTION

**Range:** This system is found from southeastern Virginia to Florida, primarily in the Outer Coastal Plain, but occasional depressions in the Inner Coastal Plain and Sandhills could be included.

**Divisions:** 203:C

**TNC Ecoregions:** 56:C, 57:C

**Nations:** US

**Subnations:** FL, GA, NC, SC, VA

**Map Zones:** 55:C, 58:C, 60:C

CONCEPT

**Environment:** This system occurs in small basins, primarily in sandy terrain of the Atlantic Coastal Plain, from southeastern Virginia to Florida. Most basins are formed by subsidence of surface sediments caused by solution in underlying limestone. Others may be formed as dune swales in mainland eolian sands, natural blockage of small drainages by sediment movement, and more obscure causes. Basins often occur in complexes of a few to dozens, which vary in size, depth, and steepness of sides. Most of these basins are considered groundwater windows, with water levels matching the level of the local water table. Rainfall is probably also a substantial contributor. The water is acidic and is apparently not influenced by the underlying limestone or deeper groundwater. Hydroperiods vary substantially, with the deepest ponds having permanent water in the center, and the shallowest normally holding water only in the winter and spring. However, water levels can fluctuate substantially over the course of a year and from year to year in response to rainfall and longer term droughts. Soils have a mucky surface layer in the centers of basins that hold water most or all of the year and are generally sandy in smaller basins and in the outer drawdown zones that are more frequently exposed. Fire is potentially an important, if infrequent, influence in the system, penetrating the portions of the basin that are dry when adjacent communities burn. The northern range limit of this system is generally consistent with the northern limit of *Pinus palustris*, although this species is not a component.

**Vegetation:** This system consists of wetland vegetation that is often strongly zoned within single basins and may vary substantially among basins even in close proximity. Most of the associations are herbaceous, but woody associations may be present. The center of the deepest basins generally is open water or floating‑leaved aquatics. Semipermanently flooded zones may have marsh vegetation of medium to large emergents. Outer, mineral soil drawdown zones often have a species‑rich flora of small to medium graminoids and forbs. These include a number of specialized species that are rare in states, some that are globally rare, and some that are widespread but nowhere common. The aspect of this vegetation may vary substantially from year to year depending on when water level drops. Some basins have a dense shrubby edge zone. Some trees or shrubs tolerant of standing water, especially bald‑cypress (*Taxodium distichum*), pond‑cypress (*Taxodium ascendens*) or swamp blackgum (*Nyssa biflora*), may grow within the basins, either as scattered individuals, as a distinct zone, or forming an open canopy over the whole basin. Because the basins are isolated from larger water bodies and most dry out at least occasionally, their aquatic fauna does not include fish unless fish have been artificially introduced. These systems are well known as important breeding sites for amphibians, and may support important aquatic invertebrate communities as well.

**Dynamics:** Flooding hydrology is the most important dynamic process. Standing water excludes plants not characteristic of the system. Variation in hydroperiod and amount of drawdown drive vegetation changes from year to year. Because ponds are connected to the local water table, hydroperiods respond to seasonal and long‑term cycles in rainfall as much as, perhaps more than, single rainfall events. They may also be affected by regional drainage and groundwater extraction which lowers the water table. Fire is also an important dynamic process in the drier portions of this system. Fire may be important for preventing invasion of trees such as *Pinus taeda* during long‑running droughts, as well as for driving variation in herbaceous species.

SOURCES

**References:** Comer et al. 2003*, Eyre 1980, FNAI 2010a, Kirkman et al. 2012, Schafale 2012, Southeastern Ecology Working Group n.d.

**Version:** 14 Jan 2014	**Stakeholders:** East, Midwest, Southeast

**Concept Author:** M. Schafale and R. Evans	**LeadResp:** Southeast

CES203.078 Southern Coastal Plain Herbaceous Seep and Bog

**Primary Division:** Gulf and Atlantic Coastal Plain (203)

**Land Cover Class:** Herbaceous Wetland

**Spatial Scale & Pattern:** Small patch

**Required Classifiers:** Natural/Semi‑natural; Vegetated (>10% vasc.); Wetland

**Diagnostic Classifiers:** Seepage‑Fed Sloping; Graminoid

**National Mapping Codes:** EVT 2515; ESLF 9404; ESP 1515

**Concept Summary:** This small‑patch ecological system includes wet, fire‑maintained, seepage communities in the outermost portions of the East Gulf Coastal Plain, east of the Mississippi River in Louisiana, Mississippi, Alabama, and extending across northern Florida. These wetlands are generally found on gentle, almost imperceptible slopes maintained by constant seepage zones and/or perched water tables. Examples are typically grass and sedge dominated, and are often species‑rich. *Sarracenia* spp. are notable indicators of many community types in this system. Shrubs frequently encroach in the absence of fire; due to greater topographic isolation, the most interior examples are often naturally shrubbier.

**Comments:** Known from Clay County (Jennings State Forest) and Nassau County (Ralph E. Simmons State Forest), Florida, based on EORs reported from Florida (A. Johnson pers. comm.).

DISTRIBUTION

**Range:** This systems is found in the northern Gulf of Mexico region, east of the Mississippi River in Louisiana, Mississippi, Alabama, and extending across northern Florida.

**Divisions:** 203:C

**TNC Ecoregions:** 53:C, 56:C

**Nations:** US

**Subnations:** AL, FL, LA, MS

**Map Zones:** 55:C, 99:C

**USFS Ecomap Regions:** 232B:CC, 232C:CC, 232D:CC, 232J:CC, 232K:CC, 232L:CC, 234A:CC

CONCEPT

**Environment:** Kindell et al. (1997) document examples in the western Panhandle of Florida on the Leefield, Albany, Pactolus, Pamlico, Rutledge, and Pansey soil series. Albany loamy sand is a common soil type in Florida (FNAI 2010a). Clewell (1981) describes these bogs as commonly occurring between bay swamps and pine flatwoods. These habitats occur on gentle slopes, where seepage water maintains saturated conditions most of the time.

**Vegetation:** Examples are typically grass‑ and sedge dominated, and are often species‑rich. Pitcher plants (*Sarracenia* spp.) are notable indicators of many community types in this system. *Rhynchospora* spp. are dominant or codominant in many examples. Shrubs (e.g., *Lyonia lucida, Ilex glabra, Cyrilla racemiflora*) frequently encroach in the absence of fire. Scattered *Pinus elliottii var. elliottii, Pinus palustris*, and/or *Pinus serotina* may be present.

**Dynamics:** These habitats are kept continuously moist by groundwater seepage (FNAI 2010a). Plants found here tolerate saturated wetland conditions. Frequent fires are necessary to maintain this system. In the absence of fire, shrubs encroach, eventually shading out understory plants. Fires may have occurred every 1‑4 years (FNAI 2010a).

SOURCES

**References:** Clewell 1981, Comer et al. 2003*, Engeman et al. 2007, Eyre 1980, FNAI 2010a, Folkerts 1982, Kindell et al. 1997

**Version:** 22 May 2014	**Stakeholders:** Southeast

**Concept Author:** R. Evans	**LeadResp:** Southeast

CES203.541 Texas‑Louisiana Coastal Prairie Pondshore

**Primary Division:** Gulf and Atlantic Coastal Plain (203)

**Land Cover Class:** Herbaceous Wetland

**Spatial Scale & Pattern:** Large patch

**Required Classifiers:** Natural/Semi‑natural; Vegetated (>10% vasc.); Wetland

**Diagnostic Classifiers:** Hardpan; Depressional; Graminoid; West Gulf Coastal Plain

**National Mapping Codes:** EVT 2487; ESLF 9208; ESP 1487

**Concept Summary:** This ecological system includes small to moderately large ponds and swales in the coastal prairie of southeastern Texas and adjacent Louisiana. These wetlands contain surface water during much of the year, desiccating only in the driest summer months. They are often fed by water runoff but may result from percolation from adjacent sandy areas. Soils in the basins are finer‑textured than surrounding areas and may be underlain by pans that enhance perched water tables in the winter. These wetlands occur within the coastal prairie matrix of southeastern Texas and Louisiana and are wetter than wet prairie dominated by *Tripsacum dactyloides* and *Panicum virgatum*. These wetlands may be dominated by *Eleocharis quadrangulata*. Other species that may be present include *Sagittaria papillosa, Sagittaria longiloba, Steinchisma hians, Panicum virgatum, Cyperus haspan, Cyperus virens, Ludwigia glandulosa, Ludwigia linearis, Fuirena squarrosa, Xyris jupicai, Leersia hexandra, Centella erecta, Symphyotrichum subulatum, Sesbania* spp., and *Rhynchospora* spp. Open areas in the ponds may contain floating and submersed aquatic vegetation, including *Utricularia gibba, Stuckenia pectinata, Ceratophyllum demersum, Brasenia schreberi, Nymphoides aquatica, Nuphar advena*, and *Nelumbo lutea*.

DISTRIBUTION

**Range:** This system is restricted to the coastal prairie of southeastern Texas and Louisiana.

**Divisions:** 203:C

**TNC Ecoregions:** 31:C

**Nations:** US

**Subnations:** LA, TX

**Map Zones:** 36:C, 37:C, 98:C

**USFS Ecomap Regions:** 232E:CC, 255D:CC

CONCEPT

**Environment:** This small‑patch system occurs in shallow depressions (microtopographic lows) on coastal Pleistocene terraces, including the Beaumont and Lissie formations, within the matrix of the generally level landscape of the Texas‑Louisiana Coastal Prairie (CES203.550). Soils tend to be fine‑textured, or are characterized by a relatively impermeable subsurface horizon (Elliott 2011). Examples of this system are often fed by water runoff but may result from groundwater percolation from adjacent sandy areas. Studies have shown that coastal prairie ponds may collectively occupy a large percentage of the land area within the coastal prairie (Enwright et al. 2011).

**Vegetation:** This system occurs as ponds or swales within the coastal prairie matrix. These wetlands are primarily herbaceous, sometimes with sparse woody cover, and are composed of various species, including *Eleocharis quadrangulata* (which may dominate some stands), *Fuirena squarrosa, Cyperus haspan, Cyperus virens, Rhynchospora* spp., *Leersia hexandra, Steinchisma hians, Panicum virgatum, Andropogon glomeratus, Xyris jupicai, Centella erecta, Sagittaria papillosa, Sagittaria longiloba, Ludwigia glandulosa, Ludwigia linearis, Bacopa* spp., *Hydrocotyle* spp., *Symphyotrichum subulatum (= Aster subulatus)*, and *Sesbania* spp. Large areas of some of the occurrences may be relatively homogeneous, dominated by one or a few species. Areas of open water within the ponds may contain floating and submerged aquatic species, including *Stuckenia pectinata, Ceratophyllum demersum, Brasenia schreberi, Nymphoides aquatica*, and *Nelumbo lutea*. Occurrences are wetter than *Tripsacum dactyloides*‑ or *Panicum virgatum*‑dominated prairie sites of Texas‑Louisiana Coastal Prairie (CES203.550).

**Dynamics:** These wetlands are part of the larger hydrologic cycle of the coastal prairie ecosystem. They store surface water and in some cases groundwater during much of the year, desiccating only in the driest summer months. Soils in the basins are finer‑textured than surrounding areas and may be underlain by pans that enhance perched water tables in the winter. They have been shown to play a role in landscape‑level water quality regulation (Forbes et al. 2012). The herbaceous wetlands are maintained by fire.

SOURCES

**References:** Comer et al. 2003*, Elliott 2011, Enwright et al. 2011, Forbes et al. 2012, LDWF 2005, Moulton et al. 1997, TPDW 2012b, USFWS and USGS 1999

**Version:** 14 Jan 2014	**Stakeholders:** Southeast

**Concept Author:** J. Teague	**LeadResp:** Southeast

2.C.5. Salt Marsh

2.C.5.El. Eastern Pacific Coastal Salt Marsh

M737. Mesoamerican‑South American Pacific Coastal Salt Marsh

CES402.591 Herbazal Pantanoso Halófilo Costero de Mesoamérica

**Primary Division:** Moist Meso‑America (402)

**Land Cover Class:** Herbaceous Wetland

**Spatial Scale & Pattern:** Small patch

**Required Classifiers:** Natural/Semi‑natural; Vegetated (>10% vasc.); Wetland

**Diagnostic Classifiers:** Lowland [Lowland]; Grassland, Savanna, Steppe (graminoid‑dominated); Tidal / Estuarine [Haline]; Aquic

**Concept Summary:** Este sistema representa las comunidades herbáceas costeras que soportan inundación durante buena parte del año y que tienen de mediana a alta salinidad. Pueden encontrarse por detrás de los manglares y a menudo son producto de alteración natural o tala de éstos. La siguiente lista de especies es diagnóstica para este sistema: *Eleocharis* sp., *Blechnum serrulatum, Centrosema* sp., *Crinum erubescens, Hyptis* sp., *Ludwigia* spp., *Mimosa pigra, Sagittaria lancifolia, Thalia geniculata*. Variante rica en suculentas: *Batis maritima, Distichlis spicata, Fuirena* sp., *Juncus* spp., *Sarcocornia perennis (= Salicornia perennis), Sesuvium portulacastrum, Spartina cynosuroides*. Variante pobre en suculentas: *Eleocharis acutangula, Cyperus ligularis, Spartina spartinae, Fimbristylis spathacea, Thrinax radiata*, juveniles o individuos enanos de especies de mangle.

DISTRIBUTION

**Divisions:** 402:C

**Nations:** BZ, CR, PA

CONCEPT

**Environment:** Planicie costera de alta salinidad.

**Vegetation:** Pantano herbáceo, que en elevaciones del terreno puede presentar leñosas agrupadas de hasta 6 m de alto.

SOURCES

**References:** Ellison 2001, Josse et al. 2003*, Meyrat et al. 2001

**Version:** 08 Jan 2015	**Stakeholders:** Latin America

**Concept Author:** C. Josse	**LeadResp:** Latin America

CES402.592 Salina Mesoamérica

**Primary Division:** Moist Meso‑America (402)

**Land Cover Class:** Barren

**Spatial Scale & Pattern:** Small patch

**Required Classifiers:** Natural/Semi‑natural; Unvegetated (<10% vasc.)

**Diagnostic Classifiers:** Lowland [Lowland]; Tidal / Estuarine [Haline]; Aquic

**Concept Summary:** Este sistema agrupa las asociaciones vegetales dispersas que se desarrollan en las depresiones que se forman por detrás de las dunas costeras o entre bancos arcillosos que por la acción de la marea varían en posición y forma. El primer caso ocurre en ambientes de litoral marino y el segundo en zonas esturinas. Estas lagunas salobres reciben agua de lluvias, aguas dulces de los ríos o de vertientes y agua salada de filtración o por la marea. Esto hace que la salinidad sea variable y estacional. Los suelos son generalmente arcillosos, a veces con una capa de limo y arena en la superficie, en la época seca se forma una capa de sal cristalina en la superficie. La siguiente lista de las especies es de diagnóstica para este sistema: *Avicennia germinans, Conocarpus erectus, Bromelia pinguin, Tillandsia flexuosa, Chloris barbata (= Chloris inflata), Urochloa fusca (= Brachiaria fasciculata), Cordia curassavica, Marsdenia rotheana, Opuntia eliator, Acanthocereus tetragonus (= Acanthocereus pentagonus), Acacia costaricensis, Parkinsonia aculeata, Pavonia sessiliflora, Waltheria indica, Jacquinia macrocarpa, Sesuvium portulacastrum, Salicornia* spp.

DISTRIBUTION

**Divisions:** 402:C

**Nations:** CO, EC, HN, MX, NI, PA

CONCEPT

**Environment:** Depresiones en la línea de costa o boca de los estuarios. Suelos arcillosos, arenosos y alcalinos. Alternan entre inundados con aguas salobres y secos, con clima estacional hasta xérico.

**Dynamics:** Dinámica marina activa y alteración por construcción de piscinas camaroneras.

SOURCES

**References:** Ellison 2001, Josse et al. 2003*, Meyrat et al. 2001

**Version:** 08 Jan 2015	**Stakeholders:** Latin America

**Concept Author:** C. Josse	**LeadResp:** Latin America

M736. Mexican Pacific Coastal Salt Marsh

CES302.005 Herbazal Pantanoso Costero de Sonora y la Península de Baja California

**Primary Division:** North American Warm Desert (302)

**Land Cover Class:** Herbaceous Wetland

**Spatial Scale & Pattern:** Linear

**Required Classifiers:** Natural/Semi‑natural; Vegetated (>10% vasc.); Wetland

**Diagnostic Classifiers:** Lowland [Lowland]; Marsh; Tidal flat; Tropical/Subtropical [Tropical Xeric]; Saltwater (Polyhaline)

**Concept Summary:** The ecological system includes barren and typically sparsely vegetated tidal flats of Baja California and Sonoran coasts of the Gulf of California and coastal marsh along the Colorado River Delta, and possibly other river deltas such as the Rio Yaqui. Substrates are typically fine‑textured and saline alluvium. Vegetation is generally sparse, but can be relatively dense locally. It is composed of halophytic species such as *Allenrolfea occidentalis, Atriplex* spp., *Batis maritima, Distichlis spicata, Frankenia* spp., *Pluchea* spp., *Limonium californicum, Ruppia maritima, Salicornia* spp., and *Zostera marina*.

DISTRIBUTION

**Range:** Baja California and Sonoran coasts of the Gulf of California and coastal marsh along the Colorado River Delta, and possibly other river deltas such as the Rio Yaqui.

**Divisions:** 302:C

**TNC Ecoregions:** 23:C

**Nations:** MX

**Subnations:** MXBC, MXBS, MXSO

CONCEPT

SOURCES

**References:** Brown 1982a, Comer et al. 2003*, Shreve and Wiggins 1964

**Version:** 24 Mar 2003	**Stakeholders:** Latin America, West

**Concept Author:** NatureServe Western Ecology Team	**LeadResp:** West

2.C.5.Na. North American Great Plains Saline Marsh

M077. Great Plains Saline Wet Meadow & Marsh

CES303.669 Western Great Plains Saline Depression Wetland

**Primary Division:** Western Great Plains (303)

**Land Cover Class:** Herbaceous Wetland

**Spatial Scale & Pattern:** Small patch

**Required Classifiers:** Natural/Semi‑natural; Vegetated (>10% vasc.); Wetland

**Concept Summary:** This ecological system is very similar to Western Great Plains Open Freshwater Depression Wetland (CES303.675) and Western Great Plains Closed Depression Wetland & Playa (CES303.666). However, strongly saline soils cause both the shallow lakes and depressions and the surrounding areas to be more brackish. Salt encrustations can occur on the surface in some examples of this system, and the soils are severely affected and have poor structure. Species that typify this system are salt‑tolerant and halophytic species such as *Distichlis spicata, Sporobolus airoides*, and *Hordeum jubatum*. Other commonly occurring taxa include *Puccinellia nuttalliana, Salicornia rubra, Bolboschoenus maritimus, Schoenoplectus americanus, Suaeda calceoliformis, Spartina* spp., *Triglochin maritima*, and shrubs such as *Sarcobatus vermiculatus* and *Krascheninnikovia lanata*. During exceptionally wet years, an increase in precipitation can dilute the salt concentration in the soils of some examples of this system which may allow for less salt‑tolerant species to occur. Communities found within this system may also occur in floodplains (i.e., more open depressions) but probably should not be considered a separate system unless they transition to areas outside the immediate floodplain. In Texas, these less saline *Sporobolus cryptandrus, Aristida purpurea*, and *Ziziphus obtusifolia* communities found within this system may also occur in floodplains (i.e., more open depressions) but probably should not be considered a separate system unless they transition to areas outside the immediate floodplain.

**Comments:** Open and emergent saline marshes may be a separate system from saline wet meadows and prairies. This system is often intimately associated (in space) with greasewood flats, and there is some overlap in the associations between the two. This system tends to be more of an herbaceous wetlands, whereas Inter‑Mountain Basins Greasewood Flat (CES304.780) is more strongly shrub‑dominated with patches of herb‑dominance.

DISTRIBUTION

**Range:** This system can occur throughout the western Great Plains but is likely more prevalent in the south‑central portions of the division. Its distribution extends as far west as central Montana and eastern Wyoming where it occurs in the matrix of Northwestern Great Plains Mixedgrass Prairie (CES303.674).

**Divisions:** 303:C

**TNC Ecoregions:** 26:C, 27:C, 28:C, 33:C, 34:?

**Nations:** CA, US

**Subnations:** CO, KS, MT, ND, NE, NM, OK, SD, SK, TX, WY

**Map Zones:** 20:C, 25:?, 26:C, 27:C, 29:C, 30:C, 31:C, 33:C, 34:C, 35:?, 38:C, 39:?, 40:?

**USFS Ecomap Regions:** 315A:CC, 315B:CC, 315F:CC, 321A:CC, 331B:CC, 331C:CC, 331D:CP, 331E:CP, 331F:C?, 331G:CP, 331H:C?, 331I:CC, 331K:CC, 331L:CP, 331M:CP, 332E:CC, 332F:C?, M313B:CC

CONCEPT

**Environment:** This system is found in basins and low parts of floodplains where water collects. The soils and water are moderately to strongly saline (>0.5‑1%) (Ungar 1967, 1970). The salts are leached from saline soils in the watershed or, rarely, come from saline groundwater discharge. Salts accumulate as the water in which they were dissolved evaporates. Salt crusts are present on the soil surface of some stands. Soils are fine‑grained, typically with a silt or clay component, and poorly drained. The wettest examples of this system are flooded through most or all of the growing season and can support aquatic species. Other aspects of the system can be flooded or saturated for short periods (Dodd and Coupland 1966, Stewart and Kantrud 1971). In Texas, this system may occur on High Lime, Salty Bottomland, and Wet Saline Ecological Sites, and some of these lakes were thought to form from wind deflation and/or dissolution of subsurface strata.

**Vegetation:** Salt‑tolerant and halophytic species such as *Distichlis spicata, Sporobolus airoides*, and *Hordeum jubatum* typify the system. Additional species that may be found in occurrences of this system in Texas include *Sporobolus pyramidatus, Bolboschoenus maritimus (= Schoenoplectus maritimus), Schoenoplectus americanus, Suaeda suffrutescens, Allenrolfea occidentalis*, and the exotics *Bassia scoparia* and *Salsola tragus*. Shrublands may also develop with species such *Atriplex* spp., *Tamarix* spp., and *Prosopis glandulosa*. During periods of high rainfall, as the salt concentrations are diluted or as one moves further from the salt‑encrusted surfaces or into surrounding habitats, species composition becomes less dominated by halophytes and in Texas, species such as *Bothriochloa laguroides ssp. torreyana, Sporobolus cryptandrus, Aristida purpurea*, and *Ziziphus obtusifolia* may occur.

**Dynamics:** Unusually wet periods or high spring snowmelt may flush some salt away, shifting the boundaries of this system temporarily until more salt accumulates. Salinity varies during the growing season, decreasing in the spring or after heavy rains and increasing during dry periods. The increased salinity due to concentration of the salt as the water evaporates ‑ common in the late summer and early fall ‑ creates a seasonally shifting environment. Species composition is strongly linked to salinity and soil moisture, so there is usually notable zonation within this system with the species tolerant of the wettest and most saline conditions in the center, grading towards midgrass prairie at the edges (Ungar 1967, 1970). Fire may spread into this system from adjacent upland prairies and can burn areas with higher vegetation cover, but the low vegetation cover and wet soils typical of many stands do not carry fire well.

SOURCES

**References:** Comer et al. 2003*, Dodd and Coupland 1966, Elliott 2013, Faber‑Langendoen et al. 2011, Hoagland 2000, Lauver et al. 1999, Rolfsmeier 1993a, Rolfsmeier and Steinauer 2010, Shiflet 1994, Stewart and Kantrud 1971, Ungar 1967, Ungar 1970

**Version:** 02 Oct 2014	**Stakeholders:** Canada, Midwest, Southeast, West

**Concept Author:** S. Menard and K. Kindscher	**LeadResp:** Midwest

2.C.5.Nb. North American Atlantic & Gulf Coastal Salt Marsh

M079. North American Atlantic & Gulf Coastal Salt Marsh

CES201.578 Acadian Coastal Salt Marsh

**Primary Division:** Laurentian‑Acadian (201)

**Land Cover Class:** Herbaceous Wetland

**Spatial Scale & Pattern:** Small patch

**Required Classifiers:** Natural/Semi‑natural; Vegetated (>10% vasc.); Wetland

**Diagnostic Classifiers:** Tidal / Estuarine; Graminoid; Spartina (patens, alterniflora); Saltwater (Polyhaline)

**Concept Summary:** This system ranges from northern Massachusetts on the Gulf of Maine north to Newfoundland, along the immediate ocean shore and near estuary mouths, where salinity regime is polyhaline. Sometimes called "salt meadows," these marshes display strong graminoid dominance, with patchy forbs. *Spartina patens* and *Spartina alterniflora* are the major dominants. Characteristic associates include *Puccinellia maritima, Juncus arcticus ssp. littoralis (= Juncus balticus), Plantago maritima var. juncoides (= Plantago juncoides)*, and *Juncus gerardii*. These marshes may be extensive where the local topography allows their development; they are generally not associated with sand beach and dune systems, being more characteristic of the primarily rocky portions of the Gulf of Maine coast. Where the coastal topography becomes more dissected, they are more commonly seen as a fairly narrow fringe along tidal shorelines. These marshes are typically less extensive and with some different floristic elements than the marshes southward along the Atlantic Coast from Cape Cod to Chesapeake Bay.

DISTRIBUTION

**Range:** This system occurs along the coastline of the Gulf of Maine, from northern Massachusetts north and east to Newfoundland, with the northern border at the Strait of Belle Isle between Labrador and Newfoundland.

**Divisions:** 201:C, 202:C

**TNC Ecoregions:** 62:C, 63:C

**Nations:** CA, US

**Subnations:** LB, MA, ME, NB, NF, NH, NS

**Map Zones:** 65:C, 66:C

**USFS Ecomap Regions:** 211Cb:CCC, 211Db:CCC, 211Dc:CCC, 221Aa:CCC, 221Ak:CCC

CONCEPT

**Environment:** Forms behind barrier beaches or at the outer mouths of tidal rivers where freshwater input is minimal and where vegetation is protected from high‑energy wave action. Substrate is organic peat, which can reach 1‑2 m in depth in low marsh.

**Dynamics:** Tidal flooding is regulated by elevation; flooding is diurnal in low marshes, decreasing to more irregular flooding in high marsh and fringing salt shrublands. Ponded water remains in depressions, causing hypersaline conditions and panne formation. In the northern portion of the range, ice‑rafting of large boulders creates a barrier, behind which salt marshes form. Ice‑scour causes substantial impacts on the structure of salt marshes, causing patches of marsh to be removed. Furrows and ridges are physical features formed by ice movement. Strong onshore wind causes berm development in winter, with a transition to offshore winds in summer that level the berms. Such processes result in a substantial amount of sediment transport. Large amounts of wrack are deposited annually. Geese also impact the marshes (Roberts and Robertson 1986).

SOURCES

**References:** Chapman 1937, Comer et al. 2003*, Faber‑Langendoen et al. 2011, Gawler and Cutko 2010, Jevrejeva et al. 2010, Kennish 2001, Manomet Center for Conservation Sciences and the National Wildlife Federation 2012, Roberts and Robertson 1986, Sperduto and Nichols 2004, Vermeer and Rahmstorg 2009

**Version:** 14 Jan 2014	**Stakeholders:** Canada, East

**Concept Author:** S.C. Gawler	**LeadResp:** East

CES201.579 Acadian Estuary Marsh

**Primary Division:** Laurentian‑Acadian (201)

**Land Cover Class:** Herbaceous Wetland

**Spatial Scale & Pattern:** Small patch

**Required Classifiers:** Natural/Semi‑natural; Vegetated (>10% vasc.); Wetland

**Diagnostic Classifiers:** Tidal / Estuarine; Graminoid; Spartina (patens, alterniflora); Brackish (Mesohaline)

**Concept Summary:** This brackish marsh system is found along mesohaline reaches of estuaries of the Gulf of Maine north to southern Newfoundland. Emergent and submergent vegetation characterizes this system. Dominance ranges from extensive grasslands to sparsely vegetated mudflats, all tidally influenced. Characteristic species include *Carex paleacea, Crassula aquatica, Juncus arcticus, Lilaeopsis chinensis, Limosella australis, Samolus valerandi ssp. parviflorus (= Samolus parviflorus), Bolboschoenus robustus (= Schoenoplectus robustus), Schoenoplectus tabernaemontani, Spartina pectinata*, and *Triglochin maritima*. These marshes grade into the salt marsh system at the mouth of estuaries. They are typically less extensive and more floristically depauperate than the marshes southward along the Atlantic Coast to Chesapeake Bay.

**Comments:** Differences between marshes in Division 202 and Division 201 may be sufficient to distinguish them as separate systems; however, data on estuarine marshes in Division 201 (Laurentian‑Acadian) is very sketchy and should be better documented before such a split is made.

DISTRIBUTION

**Range:** This system is found along the coastline of the Gulf of Maine, from Cape Cod north and east to southern Newfoundland, extending upstream in estuaries to the brackish water limit.

**Divisions:** 201:C, 202:C

**TNC Ecoregions:** 62:C, 63:C

**Nations:** CA, US

**Subnations:** MA, ME, NB, NF, NH, NS

**Map Zones:** 65:C, 66:C

**USFS Ecomap Regions:** 211Cb:CCC, 211Db:CCC, 211Dc:CCC, 221Aa:CCC, 221Ak:CCC

CONCEPT

**Environment:** This vegetation develops on tidal reaches of large rivers where freshwater from inland alluvial inputs mixes with marine saltwater incursion. Salinity levels are variable but generally range from 0.5 to 18 ppt. These marshes most commonly form on freely drained river levees. The substrate is moderately consolidated peat (Barrett 1989).

**Dynamics:** Tidal flooding by mesohaline waters; alluvial deposition of sediments forms levees where this vegetation develops.

SOURCES

**References:** Angradi et al. 2001, Barrett 1989, Comer et al. 2003*, Faber‑Langendoen et al. 2011, Gawler and Cutko 2010, Roberts and Robertson 1986, Sperduto and Nichols 2004

**Version:** 14 Jan 2014	**Stakeholders:** Canada, East

**Concept Author:** S.C. Gawler	**LeadResp:** East

CES203.260 Atlantic Coastal Plain Embayed Region Tidal Salt and Brackish Marsh

**Primary Division:** Gulf and Atlantic Coastal Plain (203)

**Land Cover Class:** Herbaceous Wetland

**Spatial Scale & Pattern:** Large patch

**Required Classifiers:** Natural/Semi‑natural; Vegetated (>10% vasc.); Wetland

**Diagnostic Classifiers:** Tidal / Estuarine [Haline]; Graminoid

**Concept Summary:** This ecological system encompasses the brackish to salt intertidal marshes of the Embayed Region of southeastern Virginia and adjacent North Carolina. It is distinguished by the extensive brackish water and tidal flooding driven by winds which are characteristic of that region. Low in plant diversity, these marshes are found on intertidal flats generally cut off from direct oceanic influence by a series of protective barrier islands. Embedded within the matrix of marshes are smaller hypersaline areas or salt pannes. Vegetation is primarily herbaceous marsh, most of it dominated by *Juncus roemerianus*. Areas near tidal inlets have salt marsh dominated by *Spartina alterniflora*. The marshes are low in plant species richness and are strongly dominated by a single plant species. Also part of the system are more limited communities such as hypersaline flats dominated by *Distichlis spicata* and *Sarcocornia*, as well as salt‑tolerant shrublands and a few tree‑dominated hammocks that occur on small elevated areas closely associated with the marshes.

**Comments:** This system is distinguished from the salt marsh systems to the north and south because of the characteristic hydrology of the embayed region and its implications to ecosystem dynamics. However, the species‑poor vegetation is not notably different. There is some question whether the few salt marshes on near inlets on the barrier islands in this region should be considered part of this system. They have regular lunar tidal flooding and full strength saltwater, both not characteristic of most of the region. However, lunar tidal flooding is muted compared to other regions. Submerged aquatic vegetation (*Ruppia*, etc.) is covered under Atlantic Coastal Plain Embayed Region Seagrass Bed (CES203.243).

DISTRIBUTION

**Range:** Endemic to southeastern Virginia and adjacent North Carolina.

**Divisions:** 203:C

**TNC Ecoregions:** 57:C

**Nations:** US

**Subnations:** NC, VA

**Map Zones:** 58:C, 60:C

**USFS Ecomap Regions:** 232I:CC

CONCEPT

**Environment:** Occurs on intertidal flats that are tidally flooded with salt to brackish water in the Embayed Region of the Mid‑Atlantic Coastal Plain in North Carolina and Virginia. The Embayed Region is characterized by very extensive sounds cut off from the ocean by long barrier islands with few tidal inlets. A low tidal range in the ocean in this region limits tidal exchange at the inlets. Saltwater is present only in limited areas near the inlets. Brackish water prevails in most of the southern part of the region and some of the seaward side of the northern part of the region, grading to oligohaline and freshwater inland and northward, as well as upstream in tidal creeks. Lunar tidal fluctuation is negligible in most of the Embayed Region, and the irregular flooding of wind tides dominates. Soils are generally organic, but mineral soils are present in the more regularly flooded areas.

**Vegetation:** Vegetation is primarily herbaceous marsh, most of it dominated by *Juncus roemerianus*. Areas near tidal inlets have salt marsh dominated by *Spartina alterniflora*. The marshes are low in species richness and are strongly dominated by a single species. Also part of the system are more limited communities such as hypersaline flats dominated by *Distichlis spicata* and *Sarcocornia*, salt‑tolerant shrublands, and a few hammocks that occur on small elevated areas closely associated with the marshes.

**Dynamics:** Tidal flooding is an environmental process which distinguishes this system from others. Because the wind tides are irregular and shifts not as frequent or as strong as in lunar tide‑dominated areas, sediment transport and probably productivity are lower in these marshes. Storms may drive increased amounts of salt into the sounds, stressing or killing plants in the brackish marshes. For marshes on the back of barrier islands, overwash in storms may deposit sand in the marsh. Marshes usually recover from this, but if sufficient sand is deposited, a different system may develop on the site, such as Northern Atlantic Coastal Plain Dune and Swale (CES203.264). Fire is a natural force in the larger and less isolated patches of marsh, removing dead material, stimulating growth, and increasing species richness slightly but not altering overall composition. Rising sea level will affect this system, drowning some marsh areas, promoting shoreline erosion, and causing salt or brackish marshes to spread into freshwater marsh areas. However, elevated atmospheric CO2 increases the productivity of marsh grasses, which can lead to marsh elevation gain (Langley et al. 2009). The marsh snail (*Littoraria irrorata*) is a native and characteristic part of the marsh ecosystem, and is eaten by blue crabs. The disruption of marsh snail predation by blue crabs can lead to a trophic cascade (Silliman and Bertness 2002, Bertness et al. 2004).

SOURCES

**References:** Bertness et al. 2004, Comer et al. 2003*, Eyre 1980, Hackney and Cleary 1987, Langley et al. 2009, Schafale 2012, Silliman and Bertness 2002

**Version:** 22 May 2014	**Stakeholders:** East, Southeast

**Concept Author:** R. Evans, M. Schafale, G. Fleming	**LeadResp:** Southeast

CES203.257 Atlantic Coastal Plain Indian River Lagoon Tidal Marsh

**Primary Division:** Gulf and Atlantic Coastal Plain (203)

**Land Cover Class:** Herbaceous Wetland

**Spatial Scale & Pattern:** Large patch

**Required Classifiers:** Natural/Semi‑natural; Vegetated (>10% vasc.); Wetland

**Diagnostic Classifiers:** Tidal / Estuarine; Graminoid

**Concept Summary:** This tidally influenced marsh system of the Indian River Lagoon along Florida's Atlantic Coast supports approximately 10% of the salt marshes in Florida. It is endemic to the Atlantic Coast of Florida where it ranges from central Volusia County, southward through Brevard, Indian River, St. Lucie, and northern Martin counties, beginning in the vicinity of Daytona Beach and extending south from there. The bulk of these are "high marshes" wholly above mean high water levels. They are protected from direct exposure to the Atlantic Ocean by perched barrier islands, and consequently receive natural inundation only from wind tides and seasonal sea level changes. A berm or levee generally separates these high marshes from lower fringing marshes of *Spartina alterniflora* (to the north) and *Rhizophora mangle* (to the south). Landward of this berm, salt flats or hypersaline zones often develop with *Salicornia, Distichlis spicata, Borrichia frutescens, Batis maritima*, and *Paspalum vaginatum*. In some areas these species occur in monospecific zones, while in others they co‑occur, grading into occasional *Avicennia germinans*. These zones are followed by a typical *Juncus roemerianus* zone, and the most inland fringes may be dominated by *Spartina bakeri*. Marshes of this region have been heavily altered by mosquito control impoundments of the 1950s and 1960s.

DISTRIBUTION

**Range:** This system is endemic to the Atlantic Coast of Florida where it ranges from central Volusia County, southward through Brevard, Indian River, St. Lucie, and northern Martin counties. This area begins in the vicinity of Daytona Beach and extends south from there.

**Divisions:** 203:C

**TNC Ecoregions:** 55:C

**Nations:** US

**Subnations:** FL

**Map Zones:** 55:C, 56:C

**USFS Ecomap Regions:** 232G:CC

CONCEPT

**Environment:** Tidal amplitudes in this region range from 0.6‑1.5 m. Tides have a minute range in the north contributing to a very narrow intertidal zone, which is sometimes occupied by *Spartina alterniflora*. In the south where tidal range is greater, mangroves occupy the intertidal zone, replacing *Spartina alterniflora*.

**Vegetation:** *Spartina alterniflora* zone is dominant, with lesser area of *Juncus roemerianus* and *Spartina patens*.

**Dynamics:** Tidal flooding is the ecological factor that distinguishes this system, but tidal amplitudes along the east coast of Florida are low. Due to evaporation in the Indian River Lagoon, salt flats with *Batis maritima, Salicornia depressa*, and *Salicornia bigelovii* were a common feature (Rey and Connelly 2012). Some of these salt flats were lost to mosquito‑control impoundments (Rey and Connelly 2012). Tides bring nutrients, making the regularly flooded marshes fertile. Storms may push saltwater into brackish areas and higher zones, acting as a disturbance to vegetation. For marshes on the back of barrier islands, storm overwash may deposit sand in the marsh. Marshes usually recover from this, but if sufficient sand is deposited, a different system may develop on the site. Fire may be a natural force in some patches that are connected to the mainland. Prescribed fire has been used to manage tidal marshes for wildlife. Rising sea level will affect this system, drowning some marsh areas, promoting shoreline erosion, and causing salt or brackish marshes to spread inland into freshwater marsh areas. However, elevated atmospheric CO2 increases the productivity of marsh grasses, which can lead to marsh elevation gain (Langley et al. 2009). The marsh snail (*Littoraria irrorata*) is a native and characteristic part of the marsh ecosystem, and is eaten by blue crabs. The disruption of marsh snail predation by blue crabs can lead to a trophic cascade (Silliman and Bertness 2002, Bertness et al. 2004).

SOURCES

**References:** Bertness et al. 2004, Comer et al. 2003*, FNAI 2010a, Langley et al. 2009, Montague and Wiegert 1990, Rey and Connelly 2012, Silliman and Bertness 2002

**Version:** 14 Jan 2014	**Stakeholders:** Southeast

**Concept Author:** R. Evans	**LeadResp:** Southeast

CES203.508 Florida Big Bend Salt and Brackish Tidal Marsh

**Primary Division:** Gulf and Atlantic Coastal Plain (203)

**Land Cover Class:** Herbaceous Wetland

**Spatial Scale & Pattern:** Large patch

**Required Classifiers:** Natural/Semi‑natural; Vegetated (>10% vasc.); Wetland

**Diagnostic Classifiers:** Tidal / Estuarine; Graminoid

**Concept Summary:** This ecological system represents salt and brackish marshes of the northern Gulf of Mexico along the Florida Big Bend (roughly from Wakulla County [Apalachicola Bay] to the Pasco/Hernando county line [more or less to Tampa Bay] on Florida's west coast). The tidal range here is higher than in the western Panhandle, and wave energy is low; lunar, wind and seasonal tides make flooding irregular. The bulk of these marshes are composed of monospecific stands of *Juncus roemerianus* that often exhibit tall‑ and short‑growth zones. Patches of *Spartina alterniflora* are less common, and may be confined to the edges of creeks or in other pockets of low elevation. Small patches of *Distichlis spicata* may also be present near berms or levees.

DISTRIBUTION

**Range:** This system is endemic to Florida from Wakulla County (Apalachicola Bay) to the Pasco/Hernando county line, north of Tampa Bay. (To the west of Apalachicola Bay, where the tides are diurnal instead of semi‑diurnal, Mississippi Sound Salt and Brackish Tidal Marsh (CES203.471) replaces this system.)

**Divisions:** 203:C

**TNC Ecoregions:** 53:C, 55:C

**Nations:** US

**Subnations:** FL

**Map Zones:** 55:C, 99:C

**USFS Ecomap Regions:** 232D:CC, 232L:CC

CONCEPT

**Environment:** Irregularly tidal; wind, lunar, and seasonal influences are important.

**Vegetation:** This system consists of salt marshes characterized by *Spartina alterniflora, Juncus roemerianus*, and *Distichlis spicata* and brackish marshes dominated by *Juncus roemerianus*. The bulk of these marshes are composed of monospecific stands of *Juncus roemerianus* that often exhibit tall‑ and short‑growth zones. Patches of *Spartina alterniflora* are less common, and may be confined to the edges of creeks or in other pockets of low elevation. Small patches of *Distichlis spicata* may also be present near berms or levees (Montague and Wiegert 1990). The brackish marshes are in areas slightly higher than the salt marshes, where flooding is greater.

**Dynamics:** The tidal range here is higher than in the western Panhandle, and wave energy is low; lunar, wind and seasonal tides make flooding irregular (Montague and Wiegert 1990).

SOURCES

**References:** Bertness et al. 2004, Cavanaugh et al. 2013, Comer et al. 2003*, FNAI 2010a, Hackney and Cleary 1987, Montague and Wiegert 1990

**Version:** 06 Feb 2014	**Stakeholders:** Southeast

**Concept Author:** R. Evans and C. Nordman	**LeadResp:** Southeast

CES203.468 Gulf Coast Chenier Plain Salt and Brackish Tidal Marsh

**Primary Division:** Gulf and Atlantic Coastal Plain (203)

**Land Cover Class:** Herbaceous Wetland

**Spatial Scale & Pattern:** Small patch

**Required Classifiers:** Natural/Semi‑natural; Vegetated (>10% vasc.); Wetland

**Diagnostic Classifiers:** Tidal / Estuarine [Haline]; Graminoid; Saltwater (Polyhaline); Brackish (Mesohaline)

**Concept Summary:** This system includes brackish to salt intertidal marshes in the Chenier Plain of Louisiana and Texas. This area was characterized historically by a prograding coastline replenished by sediments carried to the Gulf of Mexico by the Mississippi, Atchafalaya and other rivers. It is void of barrier islands. Shoreline sediments are deposited by longshore currents and reworked by waves into alternating beach ridges and mudflats depending on the amount of sediment input. This process started after the last glacial retreat and, as the coastline prograded over time, older beach ridges were left as interior ridges surrounded by marsh. Historically, natural connections between the marshes and the ocean were limited by these beach ridges, resulting in an abundance of fresh to oligohaline (intermediate) marsh, not as much brackish marsh, and even less salt marsh. In more recent times, with the increase of dredged canals connecting the marsh system to the gulf, an increase in salinity has occurred, to the detriment of plants adapted to freshwater environments. Significant fresh marsh loss has occurred in this area. Increases in salinity levels may be caused by saltwater intrusion and/or freshwater diversion. Both water level and salinity influence species composition. Salt marshes (about 16 ppt) receive regular daily tides and are typically dominated by *Spartina alterniflora*. Brackish marshes (about 8 ppt), under slightly less tidal influence and moderately influenced by freshwater, are typically dominated by *Spartina patens*, and degraded by saltwater intrusion. Brackish occurrences may be found along tidal creeks, smaller ponds and at the upper reaches of daily tides or in areas more influenced by wind tides. Inclusions of *Juncus roemerianus* and other brackish species are found in small to large patches. Through the control of the Mississippi River, historic chenier processes have been lost. Historically a progradational shoreline, today the Chenier Plain shoreline is dominated by erosion. However, coastal processes of progradation are still present in limited areas fed by sediments from the Atchafalaya River.

DISTRIBUTION

**Range:** This system extends from Vermillion Bay, Louisiana, to East Bay, Texas. Salt marsh is limited to areas fringing saltwater shorelines. Brackish marshes are found landward of the salt marshes (typically between fresh to oligohaline marshes and salt marshes) and are more prominent around coastal lakes.

**Divisions:** 203:C

**TNC Ecoregions:** 31:C

**Nations:** US

**Subnations:** LA, TX

**Map Zones:** 37:C

**USFS Ecomap Regions:** 232E:CC, 255D:CC

CONCEPT

**Environment:** Salt marshes on the Gulf Coast receive regular daily microtides. Brackish marshes, under slightly less tidal influence and moderately influenced by freshwater, are degraded by saltwater intrusion. This ecological system is found flanking large bays, along tidal creeks, between saltwater and fresh to oligohaline marshes, and in areas more influenced by wind tides. Examples are found on recent alluvial deposits of coastlines, bay margins, bay inlets, along dredged canals, creeks, and river inlets where tidal influence is adequate to maintain mesohaline to polyhaline conditions. Soils are fine‑textured, sometimes with high organic content at the surface. Ecoclasses (from Ecological Site Descriptions) include brackish and salt marsh types in Texas (Elliott 2011). Though progradation has been reduced from the loss of sediment as a result of the control of the Mississippi River, the Chenier Plain is prograding in some places, most notably west of the mouth of the Atchafalaya River. Historically a progradational shoreline, the Chenier Plain shoreline is now dominated by erosion.

**Vegetation:** This typically herbaceous‑dominated system has a species composition that varies depending on the salinity of the environment and the depth of frequent tidal flooding. Marshes that are frequently flooded by tides (low marshes) tend to be strongly dominated by *Spartina alterniflora*. Occasionally these sites may have significant cover of *Avicennia germinans*, though freezes tend to reduce the cover of mangrove. Some patches of *Juncus roemerianus* may be interspersed. Higher marshes of saline to brackish sites tend to be somewhat more diverse, with *Spartina patens* a common dominant. *Spartina alterniflora* may be present but is typically not strongly dominant. Other species that may be present or sometimes dominant include *Spartina spartinae, Distichlis spicata, Batis maritima, Salicornia* spp., *Bolboschoenus robustus (= Schoenoplectus robustus), Schoenoplectus americanus, Paspalum vaginatum, Sporobolus virginicus*, and *Borrichia frutescens*. *Iva frutescens* and *Baccharis halimifolia* are commonly encountered woody species (Elliott 2011).

**Dynamics:** Historic natural processes of the Chenier Plain were tied to the deltaic processes of the Mississippi River and the natural hydrological processes of other rivers along the western coast of Louisiana and eastern coast of Texas. These natural processes have all been altered, but processes of freshwater and sediment input still persist even though in an altered state. Sediment input is critical to marsh persistence and becomes even more important under accelerated sea‑level rise scenarios. Marsh vegetation plays an equally important role in maintaining marsh elevation (Baustian et al. 2012). The Chenier Plain of Louisiana has unusually high relative sea‑level rise (Draut et al. 2005). This is a microtidal environment. Salt and brackish marshes are important habitats for many animal species.

SOURCES

**References:** Baustian et al. 2012, Comer et al. 2003*, Couvillion et al. 2011, Draut et al. 2005, Elliott 2011, Glick et al. 2013, Gosselink et al. 1979, LDWF 2005, Neubauer 2013, Osland et al. 2013, Smith 1993, USGS 2013b, Visser et al. 2000, Williams 2013

**Version:** 14 Jan 2014	**Stakeholders:** Southeast

**Concept Author:** J. Teague and R. Evans	**LeadResp:** Southeast

CES301.461 Laguna Madre Salt and Brackish Tidal Flat

**Primary Division:** Madrean Semidesert (301)

**Land Cover Class:** Barren

**Spatial Scale & Pattern:** Large patch

**Required Classifiers:** Natural/Semi‑natural; Unvegetated (<10% vasc.); Wetland

**Diagnostic Classifiers:** Tidal / Estuarine [Haline]

**National Mapping Codes:** EVT 2500; ESLF 3113; ESP 1500

**Concept Summary:** This system includes regularly to irregularly flooded hypersaline tidal flats (often exceeding a thousand acres in size). Dominants include a variety of vascular and nonvascular species. The cyanobacteria (blue‑green algae) *Lyngbya* spp. may dominate thousands of acres. Total vegetative cover is quite variable, from near total absence of vascular plants to a dense cover of one of several dominant species including *Batis maritima, Monanthochloe littoralis, Spartina spartinae, Borrichia frutescens*, and *Sarcocornia perennis*. In addition to the dominants, other halophytic plants of this system include *Atriplex matamorensis, Distichlis spicata, Sarcocornia perennis, Sporobolus virginicus, Maytenus phyllanthoides, Prosopis reptans, Borrichia frutescens, Suaeda linearis, Suaeda conferta, Monanthochloe littoralis, Lycium carolinianum var. quadrifidum, Spartina spartinae, Sesuvium portulacastrum, Rayjacksonia phyllocephala*, and *Blutaparon vermiculare*. In addition to dominating non‑vegetated areas, algal mats of blue‑green and sometimes green algae are characteristically present, visible even in densely vegetated pannes. Blue‑green algae may contribute significantly more biomass than vascular species. Widely scattered *Avicennia germinans* (and, less frequently, other mangroves) may occur.

DISTRIBUTION

**Range:** This system ranges south of Corpus Christi Bay along the northern Gulf of Mexico.

**Divisions:** 301:C

**TNC Ecoregions:** 31:C

**Nations:** US

**Subnations:** TX

**Map Zones:** 36:C

**USFS Ecomap Regions:** 255D:CC, 315E:??

CONCEPT

**Environment:** This system occurs in tidal and other hypersaline situations along upper marsh edges and in tidal flats ranging in scale from narrow bands to hundreds of hectares along the Gulf Coast of southern Texas and Mexico. It is regularly to irregularly flooded by shallow brackish waters as a result of lunar, wind and storm tides. As these waters evaporate, high concentrations of salt accumulate, producing hypersaline conditions, forming "salt pannes." It is found on recent wind‑distributed coastal sands along barrier island and mainland shores of hypersaline lagoons and bays where evaporation often exceeds freshwater input. Tidal fluctuations and wind continue to redistribute these sands. Landforms are extensive, very gentle (nearly flat) slopes.

**Vegetation:** Some sites may have sparse vegetation consisting of *Salicornia bigelovii, Salicornia depressa, Batis maritima, Suaeda linearis, Sesuvium portulacastrum, Monanthochloe littoralis*, and/or *Distichlis spicata*, but are typically unvegetated or covered by a layer of *Lyngbya* spp. The development of vast areas dominated by *Lyngbya* spp. occurs with appropriate frequency and duration of inundation. Higher flats may be too dry to support the algae, and at lower elevation, flats may remain inundated for extended periods. Occasionally flats (usually not those supporting extensive blue‑green algae) may develop a substantial herbaceous cover, especially during years of increased rainfall. Development of significant areas of marsh grasses such as *Spartina patens* or *Spartina alterniflora* is generally lacking. Scattered individuals of *Avicennia germinans* occur within these flats.

SOURCES

**References:** Comer et al. 2003*, Elliott 2011, Eyre 1980, Jahrsdoerfer and Leslie 1988

**Version:** 18 Feb 2011	**Stakeholders:** Southeast

**Concept Author:** J. Teague	**LeadResp:** Southeast

CES203.471 Mississippi Delta Salt and Brackish Tidal Marsh

**Primary Division:** Gulf and Atlantic Coastal Plain (203)

**Land Cover Class:** Herbaceous Wetland

**Spatial Scale & Pattern:** Matrix

**Required Classifiers:** Natural/Semi‑natural; Vegetated (>10% vasc.); Wetland

**Diagnostic Classifiers:** Tidal / Estuarine [Haline]; Graminoid; Saltwater (Polyhaline); Brackish (Mesohaline)

**Concept Summary:** This system includes brackish to saline intertidal marshes in the Mississippi Delta area of Louisiana. Both water level and salinity influence species composition. The salt marsh component of this system receives regular daily tides; these areas are typically dominated by large to extensive expanses of *Spartina alterniflora*. Brackish marshes, under slightly less tidal influence and moderate freshwater influence, are typically dominated or codominated by *Spartina patens* and may cover larger expanses than salt marshes in this system. Inclusions of *Juncus roemerianus* and other brackish species are found in small to large patches. Significant brackish marsh loss has occurred in the deltaic plain of the Mississippi River. These losses are related to natural and anthropogenic causes. Subsidence and loss of wetlands are a natural part of the deltaic process, but they have been exacerbated by the reduction in sediment load into coastal areas caused by the impoundment and channelization of the Mississippi River. In addition dredged channels in the marsh facilitate saltwater intrusion, and spoil banks prevent marshes from draining. Increases in salinity cause shifts in composition to species more tolerant of salinity, ultimately resulting in loss of species diversity and potentially open saline waters when marsh accretion is outpaced by a rising sea level.

DISTRIBUTION

**Range:** This system is confined to the deltaic plain of Louisiana. Marshes in the Mississippi River deltaic plain encompass approximately 20% of the marshes in the conterminous U.S. and about half of these are salt and brackish marshes (Gosselink 1984, Field et al. 1991, Visser et al. 1998, Hester et al. 2005).

**Divisions:** 203:C

**TNC Ecoregions:** 31:C

**Nations:** US

**Subnations:** LA

**Map Zones:** 36:C, 98:C

**USFS Ecomap Regions:** 232E:CC

CONCEPT

**Environment:** This system occurs in the Mississippi River deltaic plain. Salt marshes in this system receive regular daily microtides. Brackish marshes, under slightly less tidal influence and moderately influenced by freshwater, are degraded by saltwater intrusion. This ecological system is found flanking large bays, along tidal creeks, between saline waters and fresh to oligohaline marshes, and in areas more influenced by wind tides. Examples are found on recent alluvial deposits of coastlines, bay margins, bay inlets, along dredged canals, creeks, and river inlets where tidal influence is adequate to maintain high salinities. Soils are fine‑textured, sometimes with high organic content at the surface. Historically, these marshes have been protected from the Gulf of Mexico by a series of barrier islands associated with different delta lobes. With the alteration of the Mississippi River deltaic processes, these islands are undergoing increasing deterioration with potential negative effects on the marshes they protect.

**Dynamics:** Historically, the deltaic processes of the Mississippi River helped to build and maintain this system. However, today there is a predominance of coastal loss and subsidence in the Mississippi River deltaic plain (Gosselink et al. 1998, Draut et al. 2005). The natural sediment load and freshwater entering the deltaic marshes are dependent on functioning hydrological processes in the Mississippi River. This marsh system is dependent upon freshwater input, sediment input and organic matter build‑up. Historically, these marshes have been protected from the Gulf of Mexico by a series of barrier islands associated with different delta lobes. With the alteration of the Mississippi River deltaic processes, these islands are undergoing increasing deterioration with potential negative effects on the marshes they protect. Sediment input is critical to marsh persistence and becomes even more important under accelerated sea‑level rise scenarios. Salt and brackish marshes are important habitats for many animal species.

SOURCES

**References:** Comer et al. 2003*, Couvillion et al. 2011, Deegan et al. 1984, Draut et al. 2005, Field et al. 1991, Glick et al. 2013, Gosselink 1984, Gosselink et al. 1998, Hester et al. 2005, LDWF 2005, Neubauer 2013, Osland et al. 2013, Smith 1993, USGS 2013b, Visser et al. 1998, Williams 2013

**Version:** 14 Jan 2014	**Stakeholders:** Southeast

**Concept Author:** J. Teague and R. Evans	**LeadResp:** Southeast

CES203.303 North‑Central Gulf of Mexico Salt and Brackish Tidal Marsh

**Primary Division:** Gulf and Atlantic Coastal Plain (203)

**Land Cover Class:** Herbaceous Wetland

**Spatial Scale & Pattern:** Large patch

**Required Classifiers:** Natural/Semi‑natural; Vegetated (>10% vasc.); Wetland

**Diagnostic Classifiers:** Tidal / Estuarine [Haline]; Graminoid; Saltwater (Polyhaline); Brackish (Mesohaline)

**Concept Summary:** This ecological system includes salt and brackish tidal marshes of the north‑central Gulf of Mexico, ranging from the Pearl River to northwestern Florida. These marshes are typically found bordering protected bays, sounds, lagoons and other low‑energy shorelines. Wind‑dominated tides and low tidal amplitudes (less than 1 m) characterize this region. This system includes predominately brackish marshes and supports what is probably the largest zone of *Juncus roemerianus* in the Atlantic and Gulf coastal plains outside of the North Carolina/Virginia Embayed Region estuarine marshes.

DISTRIBUTION

**Range:** This system is found along the northern Gulf of Mexico in northwestern Florida, southern Alabama, and southern Mississippi. The eastern extent of this system coincides with the range of diurnal tides in the northeastern Gulf of Mexico. (East of Apalachicola Bay, where the tides are semi‑diurnal (Stout 1984), Florida Big Bend Salt and Brackish Tidal Marsh (CES203.508) replaces this system.) To the west, Mississippi Sound Salt and Brackish Tidal Marsh (CES203.471) replaces this system in the Mississippi Delta.

**Divisions:** 203:C

**TNC Ecoregions:** 53:C

**Nations:** US

**Subnations:** AL, FL, MS

**Map Zones:** 46:C, 99:C

**USFS Ecomap Regions:** 232L:CC

CONCEPT

**Environment:** This marsh system occurs along low‑energy shorelines in a region characterized by diurnal tides usually less than 0.5 m in amplitude. Inundation is irregular and depends upon wind speed and direction, and the flow of water from nearby rivers; generally more flooding occurs in the summer than winter (Hackney and de la Cruz 1982). The climate is mixed, with subtropical conditions prevailing during years with mild winters and temperate conditions when strong arctic cold fronts extend to the Gulf of Mexico.

**Vegetation:** Brackish needlerush marshes dominate this system. Communities distinguished by tall and short *Juncus roemerianus* may both be present. A *Spartina* zone occurs in narrow bands only; small‑scale hypersaline tidal flats are frequently present. Other herbaceous species that may be found in stands include *Cladium mariscus ssp. jamaicense, Distichlis spicata, Panicum virgatum, Schoenoplectus americanus, Schoenoplectus pungens, Spartina alterniflora, Spartina patens, Spartina spartinae*, and *Sporobolus virginicus*. Examples of this system may also support inclusions of shrublands containing *Ilex vomitoria* and stunted *Quercus virginiana*.

**Dynamics:** Important processes and interactions in this system include the natural hydrological processes of rivers bringing freshwater and sediments to the coast, diurnal tides, and protection from high‑energy wave actions (Morton et al. 2004). These natural processes have generally all been altered to some degree, but processes of freshwater and sediment input still persist even though in an altered state. Sediment input is critical to marsh persistence and becomes even more important under accelerated sea‑level rise scenarios. Marsh vegetation plays an equally important role in maintaining marsh elevation (Baustian et al. 2012). Salt and brackish marshes are important habitats for many animal species.

SOURCES

**References:** Baustian et al. 2012, Comer et al. 2003*, Eyre 1980, Hackney and de la Cruz 1982, Morton et al. 2004, Stout 1984

**Version:** 14 Jan 2014	**Stakeholders:** Southeast

**Concept Author:** R. Evans	**LeadResp:** Southeast

CES203.519 Northern Atlantic Coastal Plain Tidal Salt Marsh

**Primary Division:** Gulf and Atlantic Coastal Plain (203)

**Land Cover Class:** Herbaceous Wetland

**Spatial Scale & Pattern:** Large patch

**Required Classifiers:** Natural/Semi‑natural; Vegetated (>10% vasc.); Wetland

**Diagnostic Classifiers:** Tidal / Estuarine; Graminoid; North Atlantic Coastal Plain

**Concept Summary:** This system encompasses the mesohaline to saline intertidal marshes of the North Atlantic Coastal Plain, ranging from Chesapeake Bay north to Cape Cod, Massachusetts, and sporadically to the southern Maine coast. It includes a number of different broad vegetation types including salt pannes, salt marshes, and salt shrublands. This system occurs on the bay side of barrier beaches and the outer mouth of tidal rivers where salinity is not much diluted by freshwater input. The typical salt marsh profile, from sea to land, can be summarized as follows: a low regularly flooded marsh strongly dominated by *Spartina alterniflora*; a higher irregularly flooded marsh dominated by *Spartina patens* and *Distichlis spicata*; low hypersaline pannes characterized by *Salicornia* spp.; and a salt scrub ecotone characterized by *Iva frutescens, Baccharis halimifolia*, and *Panicum virgatum*. Salt marsh "islands" of slightly higher elevation also support *Juniperus virginiana*. This system also includes the rare sea‑level fen vegetation, which occurs at the upper reaches of the salt marsh where groundwater seepage creates a freshwater fen that differs from other poor fens in its generally higher species richness, absence of *Chamaedaphne calyculata*, and presence of *Eleocharis rostellata* and *Cladium mariscoides*.

**Comments:** A continuous gradation in salinity presents challenges in separating salt from brackish marsh systems. This system is defined by its landscape position in saltwater bays and outer river mouths as well as actual salinity ranges. Moving up a tidal river, brackish marshes have less cover of *Spartina patens* and increased cover of associated species including tall graminoids such as *Schoenoplectus americanus* and *Typha angustifolia*. Further southward along the East Coast, salt and brackish marshes fall within the same system because the differences in hydrodynamics and landforms in that region produce less distinct habitats.

DISTRIBUTION

**Range:** This system is found from the southern Maine coast south to the Chesapeake Bay.

**Divisions:** 202:C, 203:C

**TNC Ecoregions:** 58:C, 62:C

**Nations:** US

**Subnations:** CT, DE, MA, MD, ME, NH, NJ, NY, RI, VA

**Map Zones:** 60:C, 65:C, 66:C

CONCEPT

**Environment:** Forms behind barrier beaches or at the outer mouths of tidal rivers where freshwater input is minimal and where vegetation is protected from high‑energy wave action. Substrate is organic peat, which can reach 1‑2 m in depth in low marsh.

**Dynamics:** Tidal flooding regulated by elevation; flooding is diurnal in low marshes, decreasing to more irregular flooding in high marsh and fringing salt shrublands. Ponded water remains in depressions, causing hypersaline conditions and panne formation.

SOURCES

**References:** Boon 2012, Comer et al. 2003*, Edinger et al. 2014a, Eyre 1980, Faber‑Langendoen et al. 2011, Gawler and Cutko 2010, Kennish 2001, Manomet Center for Conservation Sciences and the National Wildlife Federation 2012, Sallenger et al. 2012, Sperduto and Nichols 2004

**Version:** 14 Jan 2014	**Stakeholders:** East, Southeast

**Concept Author:** R. Evans	**LeadResp:** East

CES203.270 Southern Atlantic Coastal Plain Salt and Brackish Tidal Marsh

**Primary Division:** Gulf and Atlantic Coastal Plain (203)

**Land Cover Class:** Herbaceous Wetland

**Spatial Scale & Pattern:** Matrix

**Required Classifiers:** Natural/Semi‑natural; Vegetated (>10% vasc.); Wetland

**Diagnostic Classifiers:** Tidal / Estuarine [Haline]; Graminoid

**Concept Summary:** This ecological system encompasses the brackish to saline intertidal marshes of the Atlantic Coast ranging from the vicinity of Morehead City, Carteret County, North Carolina (south of the Embayed Region), south to the vicinity of Marineland or Daytona Beach (Flagler/Volusia counties) in northern Florida. It is dominated by medium to extensive expanses of *Spartina alterniflora*, flooded twice daily by lunar tides. *Juncus roemerianus* and other brackish marshes occur on slightly higher marsh, including upstream along tidal creeks, and a variety of small‑patch associations occur near the inland edges. Examples of this system may also support inclusions of shrublands dominated by either *Baccharis halimifolia* and/or *Borrichia frutescens*, as well as forests or woodlands with *Juniperus virginiana var. silicicola* in the overstory.

**Comments:** This system is distinguished from Atlantic Coastal Plain Embayed Region Tidal Salt and Brackish Marsh (CES203.260) because of the characteristic hydrology of the Embayed Region and what it implies about ecosystem dynamics. This system is dominated by salt marshes regularly flooded by lunar tides, while the Embayed Region is dominated by brackish marshes irregularly flooded by wind tides. This system is distinguished from salt marsh systems of the Gulf Coast because of the differences in tidal dynamics and energy.

The range of this system is somewhat larger than the "Embayed Region" tidal marshes (which range southward only to Cape Lookout). This is due to the fact that submerged aquatic vegetation occurs throughout the region without discernable patterns of change, whereas the tidal marshes do vary across this range.

Montague and Wiegert (1990) state that "Northeast Florida salt marshes" occur "south to Marineland," which is in northern Flagler County, Florida. These "Northeast Florida salt marshes" are assumed to be the Florida part of Southern Atlantic Coastal Plain Salt and Brackish Tidal Marsh (CES203.270) which ranges north to southern North Carolina. It is further assumed that the range of CES203.270 and Southern Atlantic Coastal Plain Fresh and Oligohaline Tidal Marsh (CES203.376) are basically identical. Some other sources refer to Daytona Beach (Volusia County) as the southern limit. Atlantic Coastal Plain Indian River Lagoon Tidal Marsh (CES203.257) is found to the south.

DISTRIBUTION

**Range:** This systems ranges from central North Carolina (Carteret County) south to the vicinity of Flagler and Volusia counties, Florida. The northern boundary is roughly the eastern end of Carteret County, North Carolina.

**Divisions:** 203:C

**TNC Ecoregions:** 55:C, 56:C, 57:C

**Nations:** US

**Subnations:** FL, GA, NC, SC

**Map Zones:** 55:C, 58:C

**USFS Ecomap Regions:** 232C:CC, 232G:CC, 232I:CC

CONCEPT

**Environment:** This system occurs on intertidal flats that are tidally‑flooded with salt to brackish water along the Atlantic Coast south of the Embayed Region of North Carolina, extending to northern Florida (south to the vicinity of Flagler and Volusia counties). Regular tidal flooding occurs over most of the system, with irregular flooding in unusually high tides occurring in the upper zones. Tidal ranges vary but are 60 cm (2 feet) or more. The water is salty over most of the expanse of this system, grading to brackish upstream in tidal rivers and creeks. Upper zones tend to have vegetation suggestive of brackish water as well, but this is apparently the result of a combination of irregular saltwater flooding with freshwater input. Local depressions in upper zones may be hypersaline due to concentration of salt by evaporation. Flooding depth and salinity are the primary determinants of the boundary of this system and of the variation in associations within it. Soils are either sandy or clayey and often are sulfidic and high in organic matter. The input of cations in sea water prevents them from being strongly acidic, but they may rapidly become extremely acidic if drained.

**Vegetation:** Vegetation is primarily marsh. *Spartina alterniflora* is the predominant vegetation. *Juncus roemerianus* may dominate fairly large expanses along brackish portions of tidal creeks and rivers. Upper zones include a few herbaceous and shrubland associations with plants tolerant of occasional to frequent saltwater, and a few herbaceous to sparse vegetation associations in hypersaline depressions. All associations are low in plant species richness. Salt marsh communities are known for their high primary productivity, much of which is exported to estuarine systems with tidal flushing. Examples of this system may also support inclusions of shrublands dominated by either *Baccharis halimifolia* and/or *Borrichia frutescens*, as well as forests or woodlands with *Juniperus virginiana var. silicicola* in the overstory. Some shrub stands containing *Ilex vomitoria* and stunted *Quercus virginiana* may also be included.

**Dynamics:** Tidal flooding is the ecological factor that distinguishes this system from others. Tides bring nutrients, making the regularly flooded marshes fertile. Storms may push saltwater into brackish areas and higher zones, acting as a disturbance to vegetation. In salt marshes, storms locally concentrate debris into piles or bands (wrack) that smother vegetation. For marshes on the back of barrier islands, storm overwash may deposit sand in the marsh. Marshes usually recover from this, but if sufficient sand is deposited, a different ecological system may develop on the site, such as Southern Atlantic Coastal Plain Dune and Maritime Grassland (CES203.273). Fire may be a natural force in some patches that are connected to the mainland, such as *Juncus roemerianus* marsh. *Spartina alterniflora* salt marshes are more often flooded by tides and too wet to burn. Rising sea level will affect this system strongly, drowning some marsh areas, promoting shoreline erosion, and causing salt or brackish marshes to spread inland into freshwater marsh areas. However, elevated atmospheric CO2 increases the productivity of marsh grasses, which can lead to marsh elevation gain (Langley et al. 2009). The marsh snail (*Littoraria irrorata*) is a native and characteristic part of the marsh ecosystem, and is eaten by blue crabs. The disruption of marsh snail predation by blue crabs can lead to a trophic cascade (Silliman and Bertness 2002, Bertness et al. 2004).

SOURCES

**References:** Bertness et al. 2004, Cavanaugh et al. 2013, Comer et al. 2003*, Eyre 1980, FNAI 2010a, Hackney and Cleary 1987, Langley et al. 2009, Montague and Wiegert 1990, Nelson 1986, Schafale 2012, Silliman and Bertness 2002

**Version:** 22 May 2014	**Stakeholders:** Southeast

**Concept Author:** R. Evans	**LeadResp:** Southeast

CES203.473 Texas Coast Salt and Brackish Tidal Marsh

**Primary Division:** Gulf and Atlantic Coastal Plain (203)

**Land Cover Class:** Herbaceous Wetland

**Spatial Scale & Pattern:** Large patch

**Required Classifiers:** Natural/Semi‑natural; Vegetated (>10% vasc.); Wetland

**Diagnostic Classifiers:** Tidal / Estuarine [Haline]; Graminoid

**Concept Summary:** This ecological system encompasses all of the brackish to salt intertidal marshes of the Texas coast south of the Chenier Plain. It ranges from Galveston Bay in Chambers County, Texas, south. These marshes typically occur on the bay side of barrier islands. Representative examples are dominated by *Spartina alterniflora, Juncus roemerianus*, or *Avicennia germinans*. Significant areas of *Avicennia germinans* become more frequent towards the south, while extensive areas of *Spartina alterniflora* become rare south of Corpus Christi Bay. The system also includes extensive irregularly‑flooded tidal flats and salt pannes, some vegetated by succulent herbs such as *Sarcocornia, Salicornia*, and *Batis*; some are nonvegetated.

DISTRIBUTION

**Range:** This salt and brackish marsh system of the Texas coast ranges from Galveston Bay in Chambers County, Texas, south.

**Divisions:** 203:C, 301:C

**TNC Ecoregions:** 31:C

**Nations:** US

**Subnations:** TX

**Map Zones:** 36:C, 37:C

**USFS Ecomap Regions:** 232E:CC, 255D:CC

CONCEPT

**Environment:** These marshes occupy relatively low‑lying, coastal situations on level landforms influenced by microtidal fluctuations. Some sites are only influenced by storm tides or tides resulting from extreme wind events. These marshes typically occur on the bay side of barrier islands. This system also includes extensive irregularly‑flooded tidal flats and salt pannes. The geology consists of recent marine, alluvial and eolian deposits along the coast. Landforms are nearly level to very gentle slopes and flats influenced by tides, including wind tides. Soils are coastal sands, and the system occupies various Salt Marsh Ecological Sites.

**Vegetation:** Representative examples are dominated by *Spartina alterniflora, Juncus roemerianus*, or *Avicennia germinans*. These marshes may be dominated by species such as *Spartina patens, Distichlis spicata, Bolboschoenus robustus (= Schoenoplectus robustus), Schoenoplectus americanus, Sporobolus virginicus, Monanthochloe littoralis*, and *Spartina spartinae*. Shrubs, subshrubs and forbs, such as *Batis maritima, Borrichia frutescens, Sesuvium portulacastrum, Salicornia* spp., *Suaeda linearis, Limonium* spp., and *Lycium carolinianum*, are commonly encountered in these marshes. Some irregularly‑flooded sites may become shrub‑dominated with species such as *Iva frutescens* or *Baccharis halimifolia*. In the south, extensive areas are dominated by *Borrichia frutescens*, and these often occur at very slightly lower elevations and higher salinities than nearby *Spartina spartinae* salty prairie. These *Borrichia* flats may be very infrequently flooded, perhaps only under extreme storm tide conditions. Other species that may be encountered in these situations include *Maytenus phyllanthoides, Prosopis reptans, Monanthochloe littoralis, Distichlis spicata*, and *Batis maritima*. The aspect dominant on these sites is clearly *Borrichia frutescens*. Some examples may have unvegetated patches. Other plants that may be found in examples of this system include *Andropogon hallii, Artemisia filifolia, Iva frutescens ssp. frutescens, Schoenoplectus californicus, Schoenoplectus pungens*, and the exotic shrubs *Tamarix* spp. (Elliott 2011).

**Dynamics:** Important processes and interactions in this system include the natural hydrological processes of rivers bringing freshwater and sediments to the coast, diurnal microtides, and protection from high‑energy wave actions (Morton et al. 2004). The composition of these marshes is primarily influenced by the frequency and duration of tidal inundation. Salinity on some marshes, particularly in the south, is maintained by salt spray from prevailing southeasterly winds. Low marshes are regularly flooded. Areas of decreased frequency and/or duration of tidal inundation are often referred to as high, or irregularly flooded, marsh (Elliott 2011). Freshwater and sediment input are scarce in the southern part of this system's range. Sediment input is critical to marsh persistence and becomes even more important under accelerated sea‑level rise scenarios. Marsh vegetation plays an equally important role in maintaining marsh elevation (Baustian et al. 2012). Salt and brackish marshes are important habitats for many animal species.

SOURCES

**References:** Baustian et al. 2012, Comer et al. 2003*, Elliott 2011, Morton et al. 2004

**Version:** 14 Jan 2014	**Stakeholders:** Southeast

**Concept Author:** J. Teague	**LeadResp:** Southeast

2.C.5.Nc. Temperate & Boreal Pacific Coastal Salt Marsh

M081. North American Pacific Coastal Salt Marsh

CES200.091 Temperate Pacific Tidal Salt and Brackish Marsh

**Primary Division:** (200)

**Land Cover Class:** Herbaceous Wetland

**Spatial Scale & Pattern:** Small patch

**Required Classifiers:** Natural/Semi‑natural; Vegetated (>10% vasc.); Wetland

**Diagnostic Classifiers:** Temperate [Temperate Hyperoceanic, Temperate Oceanic]; Tidal / Estuarine [Haline, Oligohaline]; Saline Water Chemistry; 30‑180‑day hydroperiod

**National Mapping Codes:** EVT 2668; ESLF 9281; ESP 1668

**Concept Summary:** Intertidal salt and brackish marshes are found throughout the Pacific coast, from Kodiak Island and south‑central Alaska to the central California coast. They are primarily associated with estuaries or coastal lagoons. Salt marshes are limited to bays and behind sand spits or other locations protected from wave action. Typically these areas form with a mixture of inputs from freshwater sources into coastal saltwater, so they commonly co‑occur with brackish marshes. This is a small‑patch system, confined to specific environments defined by ranges of salinity, tidal inundation regime, and soil texture. Patches usually occur as zonal mosaics of multiple communities. They vary in location and abundance with daily and seasonal dynamics of freshwater input from inland balanced against evaporation and tidal flooding of saltwater. Summer‑dry periods result in decreased freshwater inputs from inland. Hypersaline environments within salt marshes occur in "salt pans" where tidal water collects and evaporates. Characteristic plant species include *Distichlis spicata, Limonium californicum, Jaumea carnosa, Salicornia* spp., *Suaeda* spp., and *Triglochin* spp. Low marshes are located in areas that flood every day and are dominated by a variety of low‑growing forbs and low to medium‑height graminoids, especially *Salicornia depressa, Distichlis spicata, Bolboschoenus maritimus, Schoenoplectus americanus, Carex lyngbyei*, and *Triglochin maritima*. In Alaska, tidal marshes are often dominated by near‑monotypic stands of *Carex lyngbyei*, while the frequently inundated lower salt marshes are often dominated by *Eleocharis palustris* or *Puccinellia* spp. Other common species in Alaska include *Hippuris tetraphylla, Plantago maritima, Cochlearia groenlandica, Spergularia canadensis, Honckenya peploides*, or *Glaux maritima*. In the Cook Inlet and Alaska Peninsula, *Carex ramenskii* may be an associated species. High marshes are located in areas that flood infrequently and are dominated by medium‑tall graminoids and low forbs, especially *Deschampsia cespitosa, Argentina egedii, Juncus arcticus ssp. littoralis*, and *Symphyotrichum subspicatum*, and in Alaska *Poa eminens, Argentina egedii, Festuca rubra*, and *Deschampsia cespitosa*. Transition zone (slightly brackish) marshes are often dominated by *Typha* spp. or *Schoenoplectus acutus*. *Atriplex prostrata, Juncus mexicanus, Phragmites* spp., *Cordylanthus* spp., and *Lilaeopsis masonii* are important species in California. The invasive weed *Lepidium latifolium* is a problem in many of these marshes. Rare plant species include *Cordylanthus maritimus ssp. maritimus*.

**Comments:** Discussions with John Christy and Todd Keeler‑Wolf led to lumping all West Coast salt and brackish marshes into one system because they co‑occur so intimately and frequently, are not readily distinguished without detailed on‑the‑ground surveys, and are totally intergraded (seemingly continuous variation) in terms of degree of salinity and resulting vegetation. This system encompasses a very large geographic range. We may want to split it into two types, on a north‑south gradient. However, the species composition and environmental settings of tidal marshes throughout the temperate Pacific region are markedly similar. Where to make a split that would make sense biogeographically is hard to determine. For now, they are maintained as one ecological system.

DISTRIBUTION

**Range:** This system is found throughout the Pacific coast, from Kodiak Island and south‑central Alaska to the California coast.

**Divisions:** 204:C

**TNC Ecoregions:** 1:C, 2:C, 3:C, 14:C, 15:C, 16:C, 69:C, 70:C, 71:C

**Nations:** CA, MX, US

**Subnations:** AK, BC, CA, MXBC, OR, WA:S2

**Map Zones:** 1:C, 2:C, 3:C, 4:C, 75:C, 76:C, 77:C, 78:C

**USFS Ecomap Regions:** 242A:CC, 261B:CC, 263A:CC, M242A:CC

CONCEPT

**Environment:** The frequency of tidal flooding and salinity vary widely. Soils are usually fine‑textured and saturated. Tidal marshes have a limited distribution along the Gulf of Alaska and British Columbia coastline due to the topography and geomorphology of the coast, which features steep slopes and deep fjords and offers limited protection from wave action (National Wetlands Working Group 1988).

**Vegetation:** Characteristic plant species include *Distichlis spicata, Limonium californicum, Jaumea carnosa, Salicornia* spp., *Suaeda* spp., and *Triglochin* spp. Low marshes are located in areas that flood every day and are dominated by a variety of low‑growing forbs and low to medium‑height graminoids, especially *Salicornia depressa (= Salicornia virginica), Distichlis spicata, Bolboschoenus maritimus (= Scirpus maritimus), Schoenoplectus americanus (= Scirpus americanus), Carex lyngbyei*, and *Triglochin maritima*. In Alaska, tidal marshes are often dominated by near‑monotypic stands of *Carex lyngbyei*, while the frequently inundated lower salt marshes are often dominated by *Eleocharis palustris* or *Puccinellia* spp. Other common species in Alaska include *Hippuris tetraphylla, Plantago maritima, Cochlearia groenlandica (= Cochlearia officinalis), Spergularia canadensis, Honckenya peploides*, or *Glaux maritima*. In the Cook Inlet and Alaska Peninsula, *Carex ramenskii* may be an associated species. High marshes are located in areas that flood infrequently and are dominated by medium‑tall graminoids and low forbs, especially *Deschampsia cespitosa, Argentina egedii, Juncus arcticus ssp. littoralis (= Juncus balticus)*, and *Symphyotrichum subspicatum (= Aster subspicatus)*, and in Alaska *Poa eminens, Argentina egedii, Festuca rubra*, and *Deschampsia cespitosa*. Transition zone (slightly brackish) marshes are often dominated by *Typha* spp. or *Schoenoplectus acutus*. *Atriplex prostrata (= Atriplex triangularis), Juncus mexicanus, Phragmites* spp., *Cordylanthus* spp., and *Lilaeopsis masonii* are important species in California. The invasive weed *Lepidium latifolium* is a problem in many of these marshes. Rare plant species include *Cordylanthus maritimus ssp. maritimus*.

**Dynamics:** Tidal marsh zonal mosaics of multiple communities vary in location and abundance with daily and seasonal dynamics of freshwater input from inland balanced against evaporation and tidal flooding of saltwater. Summer‑dry periods result in decreased freshwater inputs from inland. Hypersaline environments within salt marshes occur in "salt pans" where tidal water collects and evaporates. High marshes flood infrequently, mid marshes flood usually at higher tides and are usually brackish waters, while low marshes are inundated with saltwater daily.

SOURCES

**References:** Barbour and Major 1988, Boggs 2000, Boggs 2002, Chappell and Christy 2004, Comer et al. 2003*, Holland and Keil 1995, IPCC 2013a, Keeler‑Wolf pers. comm., Littell et al. 2009, National Wetlands Working Group 1988, PRBO Conservation Science 2011, SFBCDC 2011, Sawyer and Keeler‑Wolf 1995, Shiflet 1994, Sparks et al. 1977, Viereck et al. 1992, WNHP 2011, WNHP unpubl. data 2018

**Version:** 14 Jan 2014	**Stakeholders:** Canada, Latin America, West

**Concept Author:** K. Boggs, C. Chappell, G. Kittel	**LeadResp:** West

2.C.5.Nd. North American Western Interior Brackish Marsh, Playa & Shrubland

M082. Warm & Cool Desert Alkali‑Saline Marsh, Playa & Shrubland

CES304.998 Inter‑Mountain Basins Alkaline Closed Depression

**Primary Division:** Inter‑Mountain Basins (304)

**Land Cover Class:** Herbaceous Wetland

**Spatial Scale & Pattern:** Small patch

**Required Classifiers:** Natural/Semi‑natural; Vegetated (>10% vasc.); Wetland

**Concept Summary:** This ecological system occurs on playas that are seasonally to semipermanently flooded, usually retaining water into the growing season and drying completely only in drought years. Many are associated with hot and cold springs, located in basins with internal drainage. Soils are alkaline to saline clays with hardpans. Seasonal drying exposes mudflats colonized by both annual and perennial wetland vegetation. Salt encrustations can occur on the surface in some examples of this system, and the soils are severely affected and have poor structure. Species that typify this system are salt‑tolerant and halophytic species such as *Distichlis spicata, Puccinellia lemmonii, Poa secunda, Muhlenbergia* spp., *Leymus triticoides, Bolboschoenus maritimus, Schoenoplectus americanus, Triglochin maritima*, and *Salicornia* spp. During exceptionally wet years, an increase in precipitation can dilute the salt concentration in the soils of some examples of this system which may allow for less salt‑tolerant species to occur. Communities found within this system may also occur in floodplains (i.e., more open depressions), but probably should not be considered a separate system unless they transition to areas outside the immediate floodplain. Types often occur along the margins of perennial lakes, in alkaline closed basins, with extremely low‑gradient shorelines. This system can occur throughout the Columbia Plateau and the northern Great Basin but is most common in eastern Oregon and northern Nevada. It occurs in the Wyoming basins (central Wyoming) where it is surrounded by sage steppe systems and in Colorado. This system is very similar to Western Great Plains Closed Depression Wetland & Playa (CES303.666).

DISTRIBUTION

**Range:** This system can occur throughout the Columbia Plateau and the northern Great Basin but is most common in eastern Oregon and northern Nevada. It occurs in the Wyoming basins (central Wyoming) where it is surrounded by sage steppe systems and in Colorado.

**Divisions:** 304:C

**TNC Ecoregions:** 6:C

**Nations:** US

**Subnations:** CA, CO, ID, NV, OR, UT, WA?, WY

**Map Zones:** 7:P, 8:P, 9:C, 12:C, 13:?, 16:?, 17:P, 18:C, 22:C, 23:P, 24:?

**USFS Ecomap Regions:** 341A:??, 341D:??, 341E:??, 341G:??, 342B:CC, 342C:CC, 342D:CC, 342H:CC, 342I:CC, 342J:CC, M261G:PP, M341A:??, M341D:??

CONCEPT

**Environment:** This system occurs in cooler context than the playas found further south in the southern Great Basin and Mojave/Sonoran deserts. This ecological system occurs on sites that are seasonally to semipermanently flooded, usually retaining water into the growing season and drying completely only in drought years. Many are associated with hot and cold springs, located in basins with internal drainage. Soils are alkaline to saline clays with hardpans. Seasonal drying exposes mudflats colonized by annual wetland vegetation. The soils are severely affected by salts and have poor structure. This system is distinct from the freshwater depression systems by its brackish nature caused by strongly saline soils. Salt encrustations could occur near the surface in some examples of this system.

**Vegetation:** Species that typify this system are salt‑tolerant and halophytic species such as *Distichlis spicata, Puccinellia lemmonii, Poa secunda, Muhlenbergia* spp., *Leymus triticoides (= Elymus triticoides), Bolboschoenus maritimus (= Schoenoplectus maritimus), Schoenoplectus americanus, Triglochin maritima*, and *Salicornia* spp. It also supports many Federally‑listed plant species.

**Dynamics:** This ecological system is primarily driven by hydrological processes. It occurs on sites that are seasonally to semipermanently flooded, usually retaining water into the growing season, drying completely only in drought years. Increases in precipitation and/or runoff can dilute the salt concentration and allow for less salt‑tolerant species to occur.

SOURCES

**References:** CNHP 2010, Comer et al. 2003*, Cooper and Severn 1992, Dlugolecki 2010, Haukos and Smith 1994, Johnson et al. 2011, Reuter et al. 2013, Shiflet 1994, TNC 2013, WNHP 2011, WNHP unpubl. data 2018

**Version:** 07 Nov 2018	**Stakeholders:** West

**Concept Author:** J. Kagan	**LeadResp:** West

CES304.780 Inter‑Mountain Basins Greasewood Flat

**Primary Division:** Inter‑Mountain Basins (304)

**Land Cover Class:** Mixed Upland and Wetland

**Spatial Scale & Pattern:** Large patch

**Required Classifiers:** Natural/Semi‑natural; Vegetated (>10% vasc.); Upland; Wetland

**Diagnostic Classifiers:** Lowland [Lowland]; Shrubland (Shrub‑dominated); Toeslope/Valley Bottom; Alkaline Soil; Deep Soil; Xeromorphic Shrub

**National Mapping Codes:** EVT 2153; ESLF 9103; ESP 1153

**Concept Summary:** This ecological system occurs throughout much of the western U.S. in intermountain basins and extends onto the western Great Plains and into central Montana. It typically occurs near drainages on stream terraces and flats or may form rings around more sparsely vegetated playas. Sites typically have saline soils, a shallow water table and flood intermittently, but remain dry for most growing seasons. The water table remains high enough to maintain vegetation, despite salt accumulations. This system usually occurs as a mosaic of multiple communities, with open to moderately dense shrublands dominated or codominated by *Sarcobatus vermiculatus*. In high salinity areas, greasewood often grows in nearly pure stands, and on less saline sites, it commonly grows with other shrub species and typically has a grass understory. Other shrubs that may be present to codominant in some occurrences include *Atriplex canescens, Atriplex confertifolia, Atriplex gardneri, Atriplex parryi, Artemisia tridentata ssp. wyomingensis, Artemisia tridentata ssp. tridentata, Artemisia cana ssp. cana*, or *Krascheninnikovia lanata*. Occurrences are often surrounded by mixed salt desert scrub or big sagebrush shrublands. The herbaceous layer, if present, is usually dominated by graminoids. There may be inclusions of *Sporobolus airoides, Pascopyrum smithii, Distichlis spicata* (where water remains ponded the longest), *Calamovilfa longifolia, Eleocharis palustris, Elymus elymoides, Hordeum jubatum, Leymus cinereus, Poa pratensis, Puccinellia nuttalliana*, or herbaceous types. In more saline environments, *Allenrolfea occidentalis, Nitrophila occidentalis*, and *Suaeda moquinii* may be present.

**Comments:** Most *Sarcobatus vermiculatus* stands occur in lowland sites; however, some *Sarcobatus vermiculatus* stands occur in uplands as well as within sandsheet systems. In both situations, stands are usually associated with a seep or shallow water table. According to DeVelice and Lesica (1993) and DeVelice et al. (1995), the *Sarcobatus vermiculatus ‑ Atriplex gardneri* community type is restricted to moderate to steep slopes of "badlands" characterized by acidic shale, bentonite or other highly erodible clayey substrate. These shrublands may be located near seeps and have seasonally saturated soils, but are not intermittently flooded and would more appropriately be classified in Western Great Plains Badlands (CES303.663) (DeVelice and Lesica 1993, Knight 1994, DeVelice et al. 1995).

DISTRIBUTION

**Range:** This system occurs throughout much of the western U.S. in Intermountain basins and extends onto the western Great Plains.

**Divisions:** 303:C, 304:C

**TNC Ecoregions:** 4:C, 6:C, 8:C, 9:C, 10:C, 11:C, 19:C, 20:C, 26:C

**Nations:** US

**Subnations:** AZ, CA, CO, ID, MT, ND, NE, NM, NV, OR, SD, UT, WA:S1, WY

**Map Zones:** 6:P, 7:C, 8:C, 9:C, 10:?, 12:C, 13:C, 15:?, 16:C, 17:C, 18:C, 19:C, 20:C, 21:C, 22:C, 23:C, 24:C, 25:C, 27:C, 28:C, 29:C, 30:P, 33:?

**USFS Ecomap Regions:** 313A:CC, 313B:CC, 313D:CC, 315A:CC, 315H:CC, 321A:??, 322A:CC, 331B:CC, 331C:CP, 331D:CP, 331F:CC, 331G:CC, 331H:CC, 331I:CC, 331J:CC, 331K:CP, 331L:C?, 341A:CC, 341B:CC, 341C:CC, 341D:CC, 341E:CC, 341F:CC, 341G:CC, 342A:CC, 342B:CC, 342C:CC, 342D:CC, 342F:CC, 342G:CC, 342H:CC, 342I:C?, 342J:CC, M242C:??, M261D:CC, M261E:CP, M261G:CC, M313A:CC, M313B:CC, M331A:C?, M331B:CP, M331D:CC, M331E:CC, M331F:CC, M331G:CC, M331H:CC, M331I:CP, M331J:C?, M332A:C?, M332D:CP, M332E:C?, M332G:CC, M341A:CC, M341B:CC, M341C:CC, M341D:CC

CONCEPT

**Environment:** This ecological system occurs throughout much of the intermountain western U.S. from the Mojave Desert and extends onto the western Great Plains and into central Montana. Elevation ranges from 100 to 2400 m. *Sarcobatus vermiculatus* commonly occurs in areas with a seasonally high water table and is often the only green shrub in pluvial desert sites with available groundwater.

*Climate:* This system is tolerant of a wide range of climatic conditions: warm or cool, temperate, semi‑arid and continental, but is most abundant in areas with hot, dry summers. Average annual precipitation ranges from 12.7 to 25.4 cm (5‑10 inches).

*Physiography/landform:* Stands occur on dry, sunny, flat valley bottoms, on lowland floodplains, in ephemeral stream channels, at playa margins, on slopes and in sand dune complexes. Greasewood communities generally occur at lower elevations than moister sagebrush or shadscale zones. In high saline areas, greasewood often grows in nearly pure stands, although on less saline sites, it commonly grows with other shrub species and typically has a grass understory. It typically occurs near drainages on stream terraces and flats or may form rings around more sparsely vegetated playas. Some *Sarcobatus vermiculatus* stands occur on sandsheets when associated with a shallow water table such as near the Great Sand Dunes National Park and Preserve in Colorado.

*Soil/substrate/hydrology:* Sites typically have saline/alkaline soils, with a shallow or perched water table and flood intermittently, seasonally to semipermanently (West 1983b). The water table is usually within 5 m of surface, generally well within the root zone of greasewood and saltbush (Donovan et al. 1996). Sites can become dry for much of the growing season, or remain saturated due to poor drainage; however, the water table generally remains high enough to maintain vegetation, which can thrive despite salt accumulations (West 1983b, Knight 1994). Stands occur on floodplains, along the margins of perennial lakes, and in alkaline closed basins with low‑gradient shorelines. Substrates are fine‑textured saline or alkaline soils, or occasionally coarse‑textured non‑saline soils (USU 2002). Greasewood flats are typically subirrigated and rarely have open water except when associated with playas. As the water evaporates, salinity increases, affecting the biota.

**Vegetation:** This system is characterized by typically open shrublands dominated or codominated by the deciduous, facultative halophytic shrub *Sarcobatus vermiculatus*. Associated species vary with salinity, alkalinity, substrates, and depth to ground water and may create a mosaic of types or form rings surrounding a saline basin, depending on environmental variables. Stands are frequently surrounded by less saline mixed salt desert scrub or big sagebrush shrublands. In higher salinity areas, greasewood often grows in nearly pure stands or with halophytes *Allenrolfea occidentalis, Atriplex gardneri, Nitrophila occidentalis*, or *Suaeda moquinii* present to codominant, often with the salt‑tolerant grass *Distichlis spicata* present in the understory. In less saline, often alkaline sites, it commonly grows mixed with upland shrub species *Atriplex canescens, Atriplex confertifolia, Atriplex parryi, Grayia spinosa, Krascheninnikovia lanata*, or *Picrothamnus desertorum*. In non‑saline sites, *Artemisia tridentata ssp. wyomingensis, Artemisia tridentata ssp. tridentata*, and *Artemisia cana ssp. cana* may be present to codominant, and in highly disturbed areas, such as sand deposits over playas, disturbance‑tolerant species such as *Ericameria nauseosa, Chrysothamnus* spp., or *Gutierrezia sarothrae* are abundant. Herbaceous layers range from absent to a moderately dense canopy of medium‑tall to short bunchgrasses or sod grasses (0‑25% cover). Species include *Bouteloua gracilis, Distichlis spicata, Eleocharis palustris, Elymus elymoides, Hordeum jubatum, Juncus arcticus ssp. littoralis (= Juncus balticus), Leymus cinereus, Pascopyrum smithii, Poa secunda (= Poa juncifolia), Puccinellia nuttalliana*, or *Sporobolus airoides*. Sand deposit sites may have *Achnatherum hymenoides (= Oryzopsis hymenoides)* or other psammophiles. Perennial forbs are typically sparse and often include *Achillea millefolium, Artemisia ludoviciana, Astragalus* spp., *Chenopodium fremontii, Glycyrrhiza lepidota, Grindelia squarrosa, Iva axillaris, Opuntia polyacantha*, and/or *Sphaeralcea coccinea*. Exotic species can be abundant on disturbed weedy sites and include such species as *Bassia scoparia (= Kochia scoparia), Bromus arvensis (= Bromus japonicus), Bromus rubens, Bromus tectorum, Descurainia* spp., *Halogeton glomeratus, Helianthus annuus, Lactuca serriola, Lepidium perfoliatum*, and/or *Poa pratensis*.

**Dynamics:** Greasewood flats are tightly associated with saline soils and groundwater that is near the surface. The primary ecological process that maintains greasewood flats is groundwater recharge, rather than surface water. *Sarcobatus vermiculatus* is a wetland obligate phreatophyte that taps into groundwater generally at less than 5 m, but taproots may reach great depth (>10 m). Hansen et al. (1995) reported that it can tolerate saturated soil conditions for up to 40 days. Like many facultative halophytes, greasewood is tolerant of alkaline and saline soil conditions that allow the species to occur in sites with less interspecific competition (Ungar et al. 1969, Branson et al. 1976).

Floristic variation within *Sarcobatus vermiculatus*‑dominated vegetation varies with depth to water table, salinity and alkalinity, soil texture, and past land use or disturbance. Hanson (1929) described stands in south‑central Colorado and found that pure stands of *Sarcobatus vermiculatus* and *Distichlis spicata* are more common on strongly saline/alkaline sites with fine‑textured soil and shallow water tables, whereas stands with mixed shrubs such as *Chrysothamnus* or *Artemisia* are more common on drier, coarser‑textured, low‑alkaline sites. Understory dominated by *Sporobolus airoides* is found on dry, strongly alkaline sites, while stands dominated by *Pascopyrum smithii* are more common on less alkaline, moist sites in low‑lying areas. The degree of salinity can vary seasonally as well as from year to year. During exceptionally wet years, the salt concentration drops, allowing less salt‑tolerant species to appear, such as cattails (*Typha* spp.) or bulrushes (*Scirpus* and/or *Schoenoplectus* spp.) (Knight 1994). Some areas only flood during wet years, sometimes only once or twice in a decade. Others will have standing water every spring, except in the driest of years. As stands dry out, strong evaporation concentrates salt in the soils.

Fires are uncommon in this system because many stands are open and lack a continuous fuel layer (Sawyer et al. 2009). Severe hot fires can kill *Sarcobatus vermiculatus*, while after low‑ to moderate‑severity fire it commonly sprouts after being top‑killed (Anderson 2004b). Vigorously sprouting following fire can increase growth and stem density, growing up to 0.76 m (2.5 feet) in height within three years, with 90% of the plants surviving one year after burning (Daubenmire 1970, Anderson 2004b, Sawyer et al. 2009). Fire regime for greasewood communities is reported as generally less than a 100‑year return interval (Anderson 2004b) although LANDFIRE (2007a) applied fire regime V (200+ years) and treated fire as a minor ecological driver within this system.

LANDFIRE (2007a) VDDT model for this system (BpS 2311530) has three classes:

A) Early Development 1 All Structures (5% of type in this stage): Shrub cover is 10‑20%. Some grasses, with greasewood sprouts present. Some representation of other sprouting species may be present (creosotebush, rabbitbrush). Grass species vary geographically but include the following for Utah and Nevada: inland saltgrass, bottlebrush squirreltail, Sandberg bluegrass and alkali sacaton. Succession to class B after two years.

B) Mid Development 1 Open (30% of type in this stage): Shrub cover (21‑60%): Greasewood shrubs are maturing, with a good mix of perennial grasses. Other shrub species that may be found with greasewood include creosotebush and rabbitbrush, and in transition zones to Mojave Desert, it may occur with various sagebrush species and salt desert shrub vegetation (shadscale, saltbushes, winterfat, budsage and spiny hopsage). Greasewood communities would stay in this class for 3‑20 years, then succeed to class C. Vegetation will revert to class A with flooding (mean return interval of 75 years) or replacement fire (mean FRI of 200 years).

C) Late Development 1 Closed (65% of type in this stage): Shrubs (41‑70%): Greasewood shrubs have reached maturity and will increase canopy closure. Perennial grasses will still be in the understory. Vegetation will revert to class A with replacement fire (mean FRI of 200 years). Flooding (mean return interval of 75 years) causes two transitions: to class A (50% of the time) or to class B (50% of the time).

There was some question in the model about whether flooding in class C (late‑development) would send the entire system back to class A (early‑development), or Class B (mid‑development). As a compromise, flooding was attributed to take both pathways with equal probability.

SOURCES

**References:** Anderson 2004b, Belnap 2001, Belnap et al. 2001, Branson et al. 1976, Brown and Smith 2000, CNHP 2010, Comer et al. 2003*, Cooper et al. 2006, Daubenmire 1970, DeVelice and Lesica 1993, DeVelice et al. 1995, Donovan et al. 1996, Hansen et al. 1995, Hanson 1929, Knight 1994, LANDFIRE 2007a, Mozingo 1987, Page et al. 1978, Rosentreter and Belnap 2003, Sawyer et al. 2009, Shiflet 1994, USU 2002, Ungar et al. 1969, WNHP 2011, WNHP unpubl. data 2018, West 1983b

**Version:** 26 Jan 2016	**Stakeholders:** Midwest, West

**Concept Author:** K.A. Schulz	**LeadResp:** West

CES304.786 Inter‑Mountain Basins Playa

**Primary Division:** Inter‑Mountain Basins (304)

**Land Cover Class:** Barren

**Spatial Scale & Pattern:** Large patch

**Required Classifiers:** Natural/Semi‑natural; Unvegetated (<10% vasc.); Upland; Wetland

**Diagnostic Classifiers:** Lowland [Lowland]; Playa; Temperate [Temperate Xeric]; Depressional; Alkaline Soil; Saline Substrate Chemistry; Aridic; Alkaline Water; Saline Water Chemistry; Caliche Layer; Impermeable Layer; Intermittent Flooding

**Concept Summary:** This ecological system is composed of barren and sparsely vegetated playas (generally <10% plant cover) found in the intermountain western U.S. Salt crusts are common throughout, with small saltgrass beds in depressions and sparse shrubs around the margins. These systems are intermittently flooded. The water is prevented from percolating through the soil by an impermeable soil subhorizon and is left to evaporate. Soil salinity varies greatly with soil moisture and greatly affects species composition. Characteristic species may include *Allenrolfea occidentalis, Sarcobatus vermiculatus, Grayia spinosa, Puccinellia lemmonii, Leymus cinereus, Distichlis spicata*, and/or *Atriplex* spp.

**Comments:** Bjork (1997) refers to these as vernal lakes in Washington; his one example was ditched and may be artificial. There might have been these in Grand Coulee prior to Columbia Basin irrigation project.

DISTRIBUTION

**Range:** This system occurs throughout the Intermountain western U.S., extending east into the southwestern Great Plains.

**Divisions:** 304:C

**TNC Ecoregions:** 6:C, 10:C, 11:C, 19:C

**Nations:** US

**Subnations:** CA, CO, ID, NM, NV, OR, UT, WA?, WY

**Map Zones:** 6:?, 7:P, 8:P, 9:C, 12:C, 13:P, 15:?, 16:P, 17:C, 18:C, 19:?, 21:?, 22:P, 23:P, 24:C, 25:?, 28:P

**USFS Ecomap Regions:** 313A:CP, 313B:CP, 313D:CC, 322A:??, 331J:CC, 341A:CC, 341B:CC, 341C:CC, 341D:CC, 341E:CC, 341F:CC, 341G:CC, 342B:CC, 342C:CC, 342D:CC, 342E:CP, 342F:CC, 342G:CC, 342H:CC, 342I:C?, 342J:CC, M242C:CC, M261D:P?, M261G:PP, M313A:CC, M331D:??, M331E:??, M332G:CC, M341A:CC, M341B:C?, M341D:CC

CONCEPT

**Dynamics:** Playas are shallow, seasonal wetlands that lie in the lowest point of a closed watershed. Their basins are lined with clay soils, which collect and hold water from rainfall and runoff events. Water evaporates, leaving high salt concentrations in the soils. Some playas will only flood with water during years with high precipitation, sometimes only once or twice in a decade. Others will have standing water every spring, except in the driest of years. During flooded years, some salt‑tolerant marsh plant species may grow, such as cattails (*Typha* spp.) or bulrush (*Scirpus* spp.).

SOURCES

**References:** Bjork 1997, Comer et al. 2003*, Knight 1994, Nachlinger et al. 2001, WNHP unpubl. data 2018

**Version:** 01 Oct 2007	**Stakeholders:** West

**Concept Author:** K.A. Schulz	**LeadResp:** West

CES302.751 North American Warm Desert Playa

**Primary Division:** North American Warm Desert (302)

**Land Cover Class:** Barren

**Spatial Scale & Pattern:** Large patch

**Required Classifiers:** Natural/Semi‑natural; Unvegetated (<10% vasc.); Upland; Wetland

**Diagnostic Classifiers:** Lowland [Lowland]; Playa; Desert Pavement; Tropical/Subtropical [Tropical Xeric]; Temperate [Temperate Xeric]; Depressional; Alkaline Soil; Aridic; Alkaline Water; Saline Water Chemistry; Caliche Layer; Impermeable Layer; Intermittent Flooding

**Concept Summary:** This ecological system is composed of barren and sparsely vegetated playas (generally <10% plant cover) found across the warm deserts of North America, extending into the extreme southern end of the San Joaquin Valley in California. Playas form with intermittent flooding, followed by evaporation, leaving behind a saline residue. Salt crusts are common throughout, with small saltgrass beds in depressions and sparse shrubs around the margins. Subsoils often include an impermeable layer of clay or caliche. Large desert playas tend to be defined by vegetation rings formed in response to salinity. Given their common location in windswept desert basins, dune fields often form downwind of large playas. In turn, playas associated with dunes often have a deeper water supply. Species may include *Allenrolfea occidentalis, Suaeda* spp., *Distichlis spicata, Eleocharis palustris, Oryzopsis* spp., *Sporobolus* spp., *Tiquilia* spp., or *Atriplex* spp. Ephemeral herbaceous species may have high cover periodically. Adjacent vegetation is typically Sonora‑Mojave Mixed Salt Desert Scrub (CES302.749), Chihuahuan Mixed Salt Desert Scrub (CES302.017), Gulf of California Coastal Mixed Salt Desert Scrub (CES302.015), Baja California del Norte Gulf Coast Ocotillo‑Limberbush‑Creosotebush Desert Scrub (CES302.014), or Chihuahuan Creosotebush Desert Scrub (CES302.731).

DISTRIBUTION

**Range:** Found across the warm deserts of North America, extending into the extreme southern end of the San Joaquin Valley in California.

**Divisions:** 302:C

**TNC Ecoregions:** 17:C, 22:C, 23:C, 24:C

**Nations:** MX, US

**Subnations:** AZ, CA, MXBC, MXCH, MXSO, NM, NV, TX

**Map Zones:** 13:C, 14:C, 16:?, 17:?, 25:C, 26:C, 27:P, 28:?

**USFS Ecomap Regions:** 313A:CC, 315A:CC, 315B:CC, 315H:CP, 321A:CC, 322A:CC, 322B:CC, 322C:CP, 341F:CC, M313B:CC

CONCEPT

**Environment:** Playas are internally draining basins often with an impermeable layer of clay or caliche subsoil.

**Vegetation:** Though typically sparsely vegetated, this system can have high cover of ephemeral herbaceous species when conditions are right. Characteristic species include *Allenrolfea occidentalis, Suaeda* spp., *Distichlis spicata, Eleocharis palustris, Oryzopsis* spp., *Sporobolus* spp., *Tiquilia* spp., or *Atriplex* spp. These species include those adapted to droughty and saline environments.

SOURCES

**References:** Barbour and Major 1988, Brown 1982a, Comer et al. 2003*, Dick‑Peddie 1993, Elliott 2012, Holland and Keil 1995, Muldavin et al. 2000b, Shiflet 1994, Thomas et al. 2004

**Version:** 02 Oct 2014	**Stakeholders:** Latin America, Southeast, West

**Concept Author:** K.A. Schulz	**LeadResp:** West

CES301.717 Tamaulipan Saline Lake

**Primary Division:** Madrean Semidesert (301)

**Land Cover Class:** Herbaceous Wetland

**Spatial Scale & Pattern:** Small patch

**Required Classifiers:** Natural/Semi‑natural; Unvegetated (<10% vasc.); Wetland

**Diagnostic Classifiers:** Herbaceous; Isolated Wetland; Saline Substrate Chemistry; Graminoid

**Concept Summary:** This ecological system includes small to medium‑sized, highly saline, interior drainage basins in the Tamaulipan region of south Texas. The edges of these basins may be sparsely to moderately vegetated by halophytic grasses and subshrubs.

DISTRIBUTION

**Range:** This system occurs in the Tamaulipan region of south Texas and possibly Mexico.

**Divisions:** 301:C

**TNC Ecoregions:** 30:C

**Nations:** MX?, US

**Subnations:** MXNU?, MXTM?, TX

**USFS Ecomap Regions:** 315Eb:CCC, 315Ec:CCC, 315Ef:CCC, 315Eg:CCP

CONCEPT

**Environment:** This system occupies broad, gently sloping, interior draining basins receiving runoff from the surrounding landscape. Solution of salts from parent material, deposition from runoff, and subsequent evaporation has lead to a highly saline situation. These areas occur over the Goliad Formation on the edge of the Texas Sandsheet on highly saline sands or sandy loams in south Texas and may be related to the formation of Quaternary‑aged clay dunes that sometimes occur nearby.

**Vegetation:** Sites may be unvegetated or have sparse vegetation. Some areas at very slightly higher landscape positions in the basin are dominated by halophytic grasses such as *Sporobolus airoides, Distichlis spicata, Sporobolus pyramidatus*, and *Monanthochloe littoralis*. Other halophytic forbs and subshrubs may also be present, including *Borrichia frutescens, Batis maritima, Salicornia* spp., *Atriplex matamorensis, Sesuvium portulacastrum*, and *Suaeda linearis*.

SOURCES

**References:** Comer et al. 2003*, Elliott 2011, Johnston 1963

**Version:** 25 Feb 2011	**Stakeholders:** Latin America, Southeast

**Concept Author:** L. Elliott, D. Diamond, A. Treuer‑kuehn, D. German, J. Teague	**LeadResp:** Southeast

2.C.5.Ue. Tropical Atlantic Coastal Salt Marsh

M735. Tropical Western Atlantic‑Caribbean Salt Marsh

CES411.460 Caribbean Salt Flat and Pond

**Primary Division:** Caribbean (411)

**Land Cover Class:** Herbaceous Wetland

**Spatial Scale & Pattern:** Small patch

**Required Classifiers:** Natural/Semi‑natural; Vegetated (>10% vasc.); Wetland

**Diagnostic Classifiers:** Solonetz; Depressional [Pond]

**Concept Summary:** This system is found in semipermanently flooded coastal ponds, or tidally flooded salt flats, as well as sand and mudflats behind barrier beaches and in the surrounding of the mangroves. The following list of species is diagnostic for this system: *Blutaparon vermiculare (= Philoxerus vermicularis), Bacopa monnieri, Sesuvium portulacastrum, Sporobolus virginicus, Heliotropium curassavicum, Amaranthus crassipes, Sesbania sericea, Annona glabra, Atriplex cristata (= Atriplex pentandra), Heterostachys ritteriana*, and *Batis maritima*.

DISTRIBUTION

**Range:** This system is found in the Greater Antilles, Puerto Rico, Virgin Islands, and Venezuela.

**Divisions:** 411:C

**Nations:** PR, US, VE, VI, XC

**Subnations:** FL

CONCEPT

**Environment:** Where hypersaline conditions develop in the upper intertidal zone, extensive salt flats may occur above the level of mangrove (Adams 1990). West (1977) states that extensive salt marshes can occur as (1) a pioneer community on the ocean side of mangroves, (2) as a zone on the inner edge or within a mangrove stand, or (3) as a secondary or disturbance type on disturbed or degraded mangrove stands. These disturbed types may be dominated by *Spartina alterniflora* or the fern *Acrostichum aureum*. Salt marshes and pannes are regularly to irregularly flooded by shallow polyhaline waters as a result of lunar, wind and storm tides. Brackish tidal marshes develop along estuaries where freshwater mixes with ocean saltwater moving up the estuary from the tidal force. They also occur near uplands where freshwater inputs reduce the salinity of the salt marsh. Waters in brackish marshes are generally in the salinity range of 0.5‑18 ppt, and the vegetation is subject to flooding from the twice‑daily tides. Salt marsh soils range from deep mucks with high clay and organic content in the deeper portions to silts and fine sands in higher areas. The organic soils have a high salinity, neutral reaction, and high sulfur content (FNAI 2010a).

**Dynamics:** The main natural factors that are responsible for the vegetation composition and processes in the estuarine and coastal wetland habitats where these marshes develop are freshwater flow, seasonal freshwater pulsing, estuarine salinity, tidal flushing, coastal geomorphology, and depositional area for sediment and nutrient input. Adams (1990) states that there may be a dynamic relationship between mangroves and salt marsh; as the salt marsh advances seaward, so the upper part of the marsh is invaded and replaced by mangrove.

SOURCES

**References:** Adam 1990, Areces‑Mallea et al. 1999, FNAI 2010a, Helmer et al. 2002, Huber and Alarcón 1988, International Institute of Tropical Forestry n.d., Josse et al. 2003*, Ross et al. 1992, TNC 2000, West 1977

**Version:** 30 Oct 2015	**Stakeholders:** Caribbean, Latin America, Southeast, U.S. Territories

**Concept Author:** C. Josse	**LeadResp:** Latin America

3. Desert & Semi‑Desert

3.A. Warm Desert & Semi‑Desert Woodland, Scrub & Grassland

3.A.1. Tropical Thorn Woodland

3.A.1.Ea. Caribbean‑Northern Mesoamerican Xeromorphic Scrub & Woodland

M765. Caribbean‑Northern Mesoamerican Xeromorphic Scrub & Woodland

CES401.291 Matorral Espinoso de Guerrero

**Primary Division:** Dry Meso‑America (401)

**Land Cover Class:** Shrubland

**Spatial Scale & Pattern:** Large patch

**Required Classifiers:** Natural/Semi‑natural; Vegetated (>10% vasc.); Upland

**Diagnostic Classifiers:** Lowland [Lowland]; Shrubland (Shrub‑dominated); Aridic

**Concept Summary:** This tropical thornscrub system is found throughout southern Sonora and western Sinaloa. It is found along low foothills, bajada, and arroyos with sandy alluvial soils. This forms a transition between succulent‑rich Sonoran Desert scrub types and Sinaloan Dry Deciduous Forest (CES401.302). The following list of species is diagnostic for this system: *Prosopis laevigata, Pithecellobium dulce, Acacia cymbispina, Ziziphus amole, Guaiacum coulteri, Cercidium praecox, Haematoxylon brasiletto, Manihot tomatophylla, Backebergia militaris, Opuntia* spp.

DISTRIBUTION

**Divisions:** 401:C

**TNC Ecoregions:** NT0205:C

**Nations:** MX

**Subnations:** MXGJ, MXGU, MXJA, MXMI, MXNU, MXQU, MXSL, MXZA

CONCEPT

**Environment:** These are found along low foothills, bajadas, and flat plains with sandy, alluvial soils. In many instances, soils are deep and rich in organic matter. Precipitation varies considerably, but estimates from Michoacan indicate some 620 mm/year, with 5 dry months (November‑March).

**Vegetation:** Thornscrub includes a complex mixture of low thorn‑bearing trees and shrubs, and some cacti, with tallest individuals typically not exceeding 15 meters in height. Short, microphyllous trees contribute 20‑90% cover.

**Dynamics:** Successional dynamics exist between savanna‑grassland, thornscrub and deciduous forest. Natural fire regimes are not documented.

SOURCES

**References:** Josse et al. 2003*, Rzedowski 1986

**Version:** 08 Jan 2015	**Stakeholders:** Latin America

**Concept Author:** C. Josse	**LeadResp:** Latin America

CES401.308 Matorral Xerofítico del Valle de Motagua

**Primary Division:** Dry Meso‑America (401)

**Land Cover Class:** Steppe/Savanna

**Spatial Scale & Pattern:** Large patch

**Required Classifiers:** Natural/Semi‑natural; Vegetated (>10% vasc.); Upland

**Diagnostic Classifiers:** Lowland [Foothill]; Grassland, Savanna, Steppe (graminoid‑dominated); Xeric

**Concept Summary:** Este sistema corresponde a las comunidades que se encuentran en las laderas áridas y arenosas del Valle de Motagua en Guatemala. La vegetación es la de un matorral o arbustal bajo y abierto con numerosos cactus y especies espinosas. En el fondo del valle, hay comunidades riparias que soportan menor aridez. En la lista de especies diagnósticas de este ecosistema dominan los cactus, algunos de los cuales tienen un papel clave en el mantenimiento de la diversidad ya que sus flores son polinizadas y sirven de alimento a numerosas especies de murciélagos, aves, insectos, e incluso una iguana endémica. La siguientes especies de cactus son son diagnósticas del sistema: *Cephalocereus maxonii, Nyctocereus guatemalensis, Lemairocereus eichlamii, Nopalea guatemalensis, Nopalea lutea, Opuntia decumbens, Pereskiopsis* sp., *Opuntia* spp., *Stenocereus pruinosus, Pilosocereus leucocephalus, Acanthocereus* sp., *Hylocereus, Melocactus, Mamillaria, Mirtillocactus eichlamii*. Entre los arbustos y árboles bajos: *Acacia* spp., *Achatocarpus nigricans, Bursera schlechtendalii, Cordia gerascanthus, Cordia pringlei, Cordia truncatifolia, Guaiacum sanctum, Bucida macrostachya, Cnidoscolus tubulosus, Jacquinia aurantiaca, Jacquinia pungens, Jacquinia macrocarpa, Leucaena diversifolia, Mimosa platycarpa, Mimosa* spp.

DISTRIBUTION

**Divisions:** 401:C

**TNC Ecoregions:** NT1312:C

**Nations:** GT

CONCEPT

**Environment:** Laderas muy inclinadas del valle, con suelos arenosos y muy áridos y fondo del valle. La precipitación promedio anual para esta regiones es de 974 mm, con máxima en ciertos puntos de 1.600 mm, una biotemperatura media anual de entre 19° a 24°C y presentan al menos 5 meses al año con precipitaciones menores a los 100 mm de lluvia (INSIVUMEH 2009). La época lluviosa es entre junio a octubre. Estas zonas son de importancia ecológica tanto por sus singulares sistemas productivos, como por las condiciones de aislamiento geográfico que han desarrollado varios endemismos (Castañeda 2003, Lott y Atkinson 2006).

**Vegetation:** Matorral bajo y espinoso, abierto.

**Dynamics:** La abundancia de cactus que caracteriza al sistema son evidencia de que los incendios no son parte de su dinámica natural, ya que los cactus generalmente no están adaptados para sobrevivir al fuego (Martinez‑Yrízar 1995, Pennington et al. 2004). Sin embargo las condiciones de aridez en que se desarrollan estas comunidades las hacen bastante resilientes a degradaciones severas.

SOURCES

**References:** Ariano et al. 2009, Castañeda 2003, INSIVUMEH 2009, Josse et al. 2003*, Lott and Atkinson 2006, Martinez‑Yrízar 1995, Pennington et al. 2004, WWF and IUCN 1997

**Version:** 08 Jan 2015	**Stakeholders:** Latin America

**Concept Author:** C. Josse	**LeadResp:** Latin America

3.A.2. Warm Desert & Semi‑Desert Scrub & Grassland

3.A.2.Na. North American Warm Desert Scrub & Grassland

M086. Chihuahuan Desert Scrub

CES302.731 Chihuahuan Creosotebush Desert Scrub

**Primary Division:** North American Warm Desert (302)

**Land Cover Class:** Shrubland

**Spatial Scale & Pattern:** Matrix

**Required Classifiers:** Natural/Semi‑natural; Vegetated (>10% vasc.); Upland

**Diagnostic Classifiers:** Shrubland (Shrub‑dominated); Tropical/Subtropical [Tropical Xeric]; Temperate [Temperate Xeric]; Xeromorphic Shrub

**National Mapping Codes:** EVT 2074; ESLF 5251; ESP 1074

**Concept Summary:** This matrix ecological system is the common lower elevation *Larrea tridentata*‑dominated desert scrub that occurs throughout much of the Chihuahuan Desert and has recently expanded into former lower elevation desert grasslands in the northern portion of its range. Stands typically occur in flat to gently sloping desert basins and on alluvial plains, extending up into lower to mid positions of piedmont slopes (bajada). Substrates range from coarse‑textured loams on gravelly plains to finer‑textured silty and clayey soils in basins. Soils are alluvial, typically loamy and non‑saline, and frequently calcareous as they are often derived from limestone, and to a lesser degree igneous rocks. The vegetation is characterized by a moderate to sparse shrub layer (<10% cover on extremely xeric sites) that is typically strongly dominated by *Larrea tridentata* with *Flourensia cernua* often present to codominant. A few additional shrubs or succulents may also be present, such as *Agave lechuguilla, Parthenium incanum, Jatropha dioica, Koeberlinia spinosa, Lycium* spp., and *Yucca* spp. Additionally, *Flourensia cernua* can often be abundant and the sole dominant in silty basins which are included in this ecological system. In general, shrub diversity is low as this ecological system lacks thornscrub and other mixed desert scrub species that are common on the gravelly mid to upper piedmont slopes. However, on deeper soils and along minor drainages, shrub diversity and cover may increase with occasional *Atriplex canescens, Gutierrezia sarothrae*, or *Prosopis glandulosa*. Herbaceous cover is usually low and composed of grasses. Common species may include *Bouteloua eriopoda, Dasyochloa pulchella, Muhlenbergia porteri, Pleuraphis mutica, Scleropogon brevifolius*, and *Sporobolus airoides*. Included in this ecological system are *Larrea tridentata*‑dominated shrublands with a sparse understory that occur on gravelly to silty, upper basin floors and alluvial plains. A pebbly desert pavement may be present on the soil surface.

**Comments:** NRCS Ecological Site Description MLRA 42 SD‑2 Loamy Ecological Site descriptions describe this system on the Jornada Experimental Range with State‑and‑Transition Model showing shifts in species composition with land use. Historic stands are thought to have been *Pleuraphis mutica*‑ and *Bouteloua eriopoda*‑dominated desert grassland with few scrubs present. During LANDFIRE mapzone 25 BpS modeling workshops, experts considered this type to be non‑reference condition, shrub‑invaded Chihuahuan Tobosa Flats and Loamy Plains Desert Grassland (BpS) at the Jornada (LANDFIRE 2007a).

DISTRIBUTION

**Range:** This extensive, lower elevation desert scrub ecological system occurs in the Chihuahuan Desert in broad desert basins and alluvial plains extending up into the lower bajada.

**Divisions:** 302:C

**TNC Ecoregions:** 22:C, 24:C

**Nations:** MX, US

**Subnations:** AZ, MXCH, MXSO?, NM, TX

**Map Zones:** 14:C, 15:P, 24:?, 25:C, 26:C

**USFS Ecomap Regions:** 313C:PP, 315A:CC, 321A:CC, 322B:CC, M313A:CC, M313B:CC

CONCEPT

**Environment:** *Climate:* Climate is semi‑arid to arid with annual precipitation ranging from 200‑250 mm that falls mostly in the summer. Summers are hot and winters can be cold with freezing temperature occurring in the northern extent.

<br />*Physiography/landform:* This ecological system is the common lower elevation desert scrub that occurs throughout much of the Chihuahuan Desert and has recently expanded into former desert grasslands in the northern portion of its range. Elevation ranges from 1000‑2000 m. Stands typically occur in flat to gently sloping, desert basins and on alluvial plains, extending up into the lower to mid positions of piedmont slopes (bajada), sometimes on colluvium.

*Soil/substrate/hydrology:* Substrates range from coarse‑textured loams on gravelly plains to finer‑textured silty and clayey soils in basins. Soils are alluvial, typically loamy and non‑saline, and frequently calcareous as they are often derived from limestone, and to a lesser degree igneous rocks (Brown 1982a, MacMahon and Wagner 1985, Henrickson and Johnston 1986, MacMahon 1988, Dick‑Peddie 1993). In Texas, this system typically occurs on flat and gently rolling landforms, often on gravelly alluvial plains, outwash plains and intermountain basins. A pebbly desert pavement may be present on the soil surface.

**Vegetation:** This desert scrub is common on alluvial plains and is characterized by a moderate to sparse shrub layer (<10% cover on extremely xeric sites) that is typically strongly dominated by *Larrea tridentata* with *Flourensia cernua* often present to codominant (Brown 1982, MacMahon and Wagner 1985, Henrickson and Johnston 1986, MacMahon 1988, Dick‑Peddie 1993). A few scattered shrubs or succulents may also be present such as *Agave lechuguilla, Parthenium incanum, Jatropha dioica, Koeberlinia spinosa, Lycium* spp., and *Yucca torreyi*. Additionally, *Flourensia cernua* will often strongly dominate in silty basins that are included in this ecological system. In general, shrub diversity is low as this ecological system lacks codominant thornscrub and other mixed desert scrub species that are common on the gravelly mid to upper piedmont slopes. However, shrub diversity and cover may increase locally where soils are deeper and along minor drainages with occasional *Atriplex canescens, Gutierrezia sarothrae, Parthenium incanum, Acacia constricta*, or *Prosopis glandulosa*. In Texas, succulents such as *Fouquieria splendens, Agave lechuguilla, Yucca torreyi, Opuntia* spp., and *Echinocereus* spp. may be conspicuous on particularly hot desert sites at low elevations. In the southern Chihuahuan Desert, stands are dominated by *Larrea tridentata* with *Agave parryi (= Agave scabra), Cylindropuntia kleiniae (= Opuntia kleiniae), Cylindropuntia imbricata (= Opuntia imbricata)*, and *Yucca filifera* (Huerta‑Martinez et al. 2004). Herbaceous cover is usually low and composed of grasses. Common species may include *Bouteloua eriopoda, Dasyochloa pulchella (= Erioneuron pulchellum), Muhlenbergia porteri, Pleuraphis mutica, Scleropogon brevifolius*, and *Sporobolus airoides*. Included in this ecological system are *Larrea tridentata*‑dominated shrublands with a sparse understory that occur on gravelly to silty, upper basin floors and alluvial plains. A pebbly desert pavement may be present on the soil surface.

**Dynamics:** This is a stable ecosystem that is well suited to the hot, very dry basins and low hills where it occurs. The dominant and diagnostic species, *Larrea tridentata*, is a very long‑lived species (some clones have been estimated to be over 10,000 years old). It is highly adapted to minimized evapotranspiration both daily and seasonally using stomatal regulation, resinous leaves, and a leaf structure and habit to minimize self‑shading and maximize photosynthesis during favorable growing periods (Hamerlynck et al. 2002, Ogle and Reynolds 2002). *Larrea tridentata* is poorly adapted to fire because of its highly flammable, resinous leaves and limited sprouting ability after burning, although it may survive lower‑intensity fires (Humphrey 1974, Brown and Minnich 1986, Marshall 1995, Paysen et al. 2000). McLaughlin and Bowers (1982) reported that burned individuals surviving a fire regained their former size in five years.

Historic fire regimes for Chihuahuan Creosotebush Desert Scrub are difficult to quantify but fires were rare with a fire‑return interval (FRI) ranging from 300‑1000 years and 500 years on average (from LANDFIRE BpS Model 2510740). The fire characteristics range from low‑ to moderate‑ to high‑intensity, moderate‑severity, stand‑replacing crown fires that occur during spring, summer and fall seasons. Fires tend to be small or medium in size and need unusual conditions (e.g., a drought following an unusually wet year so there are adequate fine fuels that are available to carry a fire) (Brown and Minnich 1986, Paysen et al. 2000).

<br />Weather stress such as drought also affects this community by reducing vegetation cover (especially grasses) every 80 years or so but does cause significant shrub mortality although shrubs may die back some (from LANDFIRE BpS Model 2510740) (Humphrey 1974). Drought is a relatively common occurrence in this desert scrub, generally occurring every 10‑15 years and lasting 2‑3 years with occasional long‑term drought periods (10‑15 years duration). *Larrea tridentata* and other shrubs have extensive root systems that allow them to exploit deep‑soil water that is unavailable to shallower rooted grasses and cacti (Burgess 1995).

Biotic pollination by bees is important for creosotebush (Cane et al. 2000). Seed dispersal is primarily by wind and gravity as fruits are adapted for tumbling (Maddox and Carlquist 1985). However, seed burial by rodents may improve germination and survival of creosotebush (Chew and Chew 1970) so biotic dispersal may enhance regeneration especially in undisturbed, smooth desert pavement areas where seed burial is unlikely. Most seed germination requires between 80‑150 mm (3‑6 inches) of summer precipitation (Marshall 1995).<br />

Herbivory by native herbivores in Chihuahuan Creosotebush Desert Scrub includes small mammals, reptiles and invertebrates. *Larrea* leaves are not edible to most animals; however, seeds are eaten by many small mammals (Paysen et al. 2000).

<br />LANDFIRE developed a VDDT model for this system which has three classes (LANDFIRE 2007a, BpS 2510740):

A) Early Development 1 All Structures (15% of type in this stage): Under natural conditions shrub cover generally represents <10% canopy cover and is likely not affected by disturbance. The grass community may be as low as 10% canopy cover after a combination of drought and fire. Little disturbance was considered in class A, modeled drought every 50 years on average keeping the class in A (option 2). In the historic condition where invasive annual grasses are absent, the fire‑return interval is virtually nonexistent except for areas near the base of mountains experiencing locally higher rainfall and fine fuel buildup. After 100 years, class A transitions to class B. However, if the upper soil horizon and/or microbes are lost, then a longer recovery time is required. Or complete recovery is not possible.

B) Late Development 1 Open (85% of type in this stage): Typically <30% shrub canopy cover. Replacement fire followed by prolonged drought every 500 years (min‑max: 300‑1000 years) on average (Option 1). Wind/weather stress also affected this community on average every 80 years but did not cause a transition to class A. Class B is likely over‑represented on the landscape today.

SOURCES

**References:** Ahlstrand 1979, Belnap 2001, Belnap et al. 2001, Brown 1982a, Brown and Minnich 1986, Buffington and Herbel 1965, Burgess 1995, Cane et al. 2000, Chew and Chew 1970, Comer et al. 2003*, Dick‑Peddie 1993, Donart 1984, Elliott 2013, Evans and Belnap 1999, Finch 2012, Garfin et al. 2013, Gibbens et al. 2005, Hamerlynck et al. 2002, Henrickson and Johnston 1986, Huerta‑Martínez et al. 2004, Humphrey 1974, LANDFIRE 2007a, MacMahon 1988, MacMahon and Wagner 1985, Maddox and Carlquist 1985, Marshall 1995a, McLaughlin and Bowers 1982, Milchunas 2006, Muldavin et al. 1998a, Muldavin et al. 2000b, Muldavin et al. 2002a, NRCS 2006a, Ogle and Reynolds 2002, Paysen et al. 2000, Rosentreter and Belnap 2003, Schlesinger et al. 2006, Stein and Ludwig 1979

**Version:** 27 Jan 2016	**Stakeholders:** Latin America, Southeast, West

**Concept Author:** K.A. Schulz	**LeadResp:** West

CES302.734 Chihuahuan Mixed Desert and Thornscrub

**Primary Division:** North American Warm Desert (302)

**Land Cover Class:** Shrubland

**Spatial Scale & Pattern:** Matrix

**Required Classifiers:** Natural/Semi‑natural; Vegetated (>10% vasc.); Upland

**Diagnostic Classifiers:** Lowland [Foothill, Lowland]; Shrubland (Shrub‑dominated)

**National Mapping Codes:** EVT 2100; ESLF 5306; ESP 1100

**Concept Summary:** This ecological system is the widespread desert scrub that occurs on gravelly mid to upper bajadas, foothills and dissected gravelly alluvial fans in the Chihuahuan Desert and has recently expanded at the expense of desert grasslands in the northern portion of its range. It generally occurs on mid to upper piedmonts above the desert plains Chihuahuan Creosotebush Desert Scrub (CES302.731) and extends up to the chaparral zone. Soils are typically well‑drained, non‑saline, gravelly loams often with a petrocalic layer. Substrates are frequently derived from limestone although igneous rocks are common in some areas. In Texas, this system is best developed over limestone substrates. Vegetation is characterized by the presence of *Larrea tridentata*, typically mixed with thornscrub or other desert scrub such as *Agave lechuguilla, Aloysia wrightii, Baccharis pteronioides, Dasylirion leiophyllum, Flourensia cernua* (not bottomland), *Fouquieria splendens, Koeberlinia spinosa, Krameria erecta, Leucophyllum minus, Mimosa aculeaticarpa var. biuncifera, Mortonia scabrella, Opuntia engelmannii, Parthenium incanum, Prosopis glandulosa*, and *Rhus microphylla* (in drainages). Grasses are common but generally have lower cover than shrubs. Common species may include *Bouteloua curtipendula, Bouteloua eriopoda, Bouteloua gracilis, Bouteloua hirsuta, Bouteloua ramosa, Dasyochloa pulchella*, and *Muhlenbergia porteri*. Stands of *Acacia constricta*‑, *Acacia neovernicosa*‑ or *Acacia greggii*‑dominated thornscrub are included in this system, and limestone substrates appear important for at least these species. If present, *Prosopis glandulosa* has relatively low cover and does not strongly dominate the shrub layer.

This system also includes upper piedmont stands of desert scrub that are strongly dominated by *Larrea tridentata*, as wells as *Larrea tridentata* shrublands with a sparse understory that occur on gravelly piedmont slopes that may extend down gravelly upper basins.

In western Texas, this scrub is best developed over limestone substrates. *Acacia constricta, Agave lechuguilla, Condalia ericoides, Dasylirion leiophyllum, Larrea tridentata, Leucophyllum* spp., *Mimosa aculeaticarpa var. biuncifera, Parthenium incanum, Prosopis glandulosa, Viguiera stenoloba*, and *Yucca torreyi* are often present to dominant, but numerous shrub species may be present. The herbaceous cover is generally low with species such as *Aristida purpurea, Bouteloua curtipendula, Bouteloua eriopoda, Bouteloua ramosa, Bouteloua trifida, Dasyochloa pulchella*, and *Muhlenbergia setifolia*. Historically, much of this desert scrub was thought to be a more open steppe, characterized by perennial desert grasses (typically *Bouteloua eriopoda*) and an open creosotebush ‑ mixed desert shrub layer. Remnant stands of this historic composition of *Larrea tridentata* desert scrub in the Chihuahuan Desert can be seen on remnant early Holocene erosional surfaces that can often have pebbly desert pavement on the soil surface.

**Comments:** A significant expansion of this desert scrub system in the northern extent is thought to be the result of recent invasion of *Larrea tridentata* into former desert grasslands over the last 150 years from the combined effects climate change (increased drought), overgrazing by livestock, and/or decreases in fire frequency over the last 70‑250 years (Buffington and Herbel 1965, Ahlstrand 1979, Donart 1984, Dick‑Peddie 1993, Gibbens et al. 2005). This system now includes large areas of loamy plains that have been converted from *Pleuraphis mutica* and *Bouteloua eriopoda* desert grasslands to *Larrea tridentata* scrub. This system also includes expanding *Flourensia cernua* shrublands that occur in former (now degraded) tobosa (*Pleuraphis mutica*) flats and loamy plains. Presence of *Scleropogon brevifolius* is common on these degraded sites.

<br />LANDFIRE BpS modeling (LANDFIRE 2007a) modeled this broad system using three models. This system (CES302.734) (BpS 2511001) includes mixed desert and thornscrub that is common on more gravelly, rocky, dissected, mid to upper piedmont/bajada, plus the pure creosotebush and creosotebush/tarbush scrub that also occurs there on those landforms.

Chihuahuan Mixed Desert Shrubland (BpS 2511002) was a minor type historically, and thought to occur on gravelly mid to upper bajadas, foothills. It has recently expanded into former desert grasslands in the northern portion of its range as an invasive creosotebush scrub that has invaded loamy plains desert grasslands. Chihuahuan Grama Grass Creosote Steppe (BpS 2511003) is a minor desert scrub steppe that occurs on the bajadas and into foothills in the Chihuahuan Desert and has an open shrub layer characterized by dense perennial grasses (typically black grama). Substrates are generally coarse‑textured, gravelly soils and may have a petrocalic layer.

<br />These three models are in contrast to the related system Chihuahuan Creosotebush Desert Scrub (CES302.731) (BpS 2510740) that represents lower elevation desert scrub in alluvial flats, xeric basins and plains (extending into lower bajada), both on gravelly and loamy (recent invasion/former desert grasslands) that is mostly pure creosotebush and/or tarbush. There are probably small‑patch inclusions of rocky outcrops/breaks and isolated ballenas with mixed desert and thornscrub or succulent desert scrub.

DISTRIBUTION

**Range:** This system occurs in the Chihuahuan Desert (LANDFIRE 2007a).

**Divisions:** 302:C

**TNC Ecoregions:** 22:C, 24:C

**Nations:** MX, US

**Subnations:** AZ, MXCH, MXSO, NM, TX

**Map Zones:** 14:C, 15:C, 24:P, 25:C, 26:C, 27:C

**USFS Ecomap Regions:** 313B:CP, 313C:CC, 315A:CC, 315B:CC, 315H:CC, 321A:CC, 322B:CC, M313A:CC, M313B:CC

CONCEPT

**Environment:** *Climate:* Climate is semi‑arid with annual precipitation ranging from 200‑250 mm that falls mostly in the summer.<br />

*Physiography/landform:* This ecological system is the widespread desert scrub that occurs on gravelly mid to upper bajadas, foothills and dissected gravelly alluvial fans in the Chihuahuan Desert and has recently expanded into former desert grasslands in the northern portion of its range. It generally occurs on mid to upper piedmonts above the xeric basins and plains dominated by Chihuahuan Creosotebush Desert Scrub (CES302.731) and extends up to the chaparral zone.

<br />*Soil/substrate/hydrology:* Soils are typically well‑drained, non‑saline, gravelly loams often with a petrocalic layer. Substrates are frequently derived from limestone, although igneous rocks are common in some areas (Brown 1982a, MacMahon and Wagner 1985, Henrickson and Johnston 1986, MacMahon 1988, Dick‑Peddie 1993).

**Vegetation:** This mid to upper piedmont ecological system is characterized by the presence of *Larrea tridentata* typically mixed with thornscrub or other desertscrub such as *Agave lechuguilla, Aloysia wrightii, Baccharis pteronioides, Dasylirion leiophyllum, Flourensia cernua* (not bottomland), *Fouquieria splendens, Koeberlinia spinosa, Krameria erecta, Leucophyllum minus, Mimosa aculeaticarpa var. biuncifera, Mortonia scabrella (= Mortonia sempervirens ssp. scabrella), Opuntia engelmannii, Parthenium incanum, Prosopis glandulosa*, and *Rhus microphylla* (in drainages). Stands of *Acacia constricta*‑, *Acacia neovernicosa*‑ or *Acacia greggii*‑dominated thornscrub are included in this system, and limestone substrates appear important for at least these species. If present, *Prosopis glandulosa* has lower cover than other shrubs and does not strongly dominate the shrub layer. This system also includes upper piedmont stands of desert scrub that are strongly dominated by *Larrea tridentata*. In Texas, *Acacia constricta, Agave lechuguilla, Condalia ericoides, Dasylirion leiophyllum, Larrea tridentata, Leucophyllum* spp., *Mimosa aculeaticarpa var. biuncifera, Parthenium incanum, Prosopis glandulosa, Viguiera stenoloba*, and *Yucca torreyi* are often present to dominant, but numerous shrub species may be present. Grasses are common but generally have lower cover than shrubs. Common species may include *Bouteloua curtipendula, Bouteloua eriopoda, Bouteloua gracilis, Bouteloua hirsuta, Bouteloua ramosa, Dasyochloa pulchella*, and *Muhlenbergia porteri*. In Texas, herbaceous cover is generally low with species such as *Aristida purpurea, Bouteloua curtipendula, Bouteloua eriopoda, Bouteloua ramosa, Bouteloua trifida, Dasyochloa pulchella*, and *Muhlenbergia setifolia*. Also included in this ecological system are shrublands with a sparse understory of *Larrea tridentata* that occur on gravelly piedmont slopes that may extend down gravelly upper basins. A pebbly desert pavement may be present on the soil surface. This may indicate remnant erosional surfaces from the early Holocene that are thought to be some of the historic distribution of *Larrea tridentata* desert scrub in the Chihuahuan Desert (Muldavin et al. 2000b). Historically, much of this desert scrub was thought to be a steppe characterized by perennial desert grasses such as *Bouteloua eriopoda, Bouteloua ramosa, Muhlenbergia porteri, Bothriochloa barbinodis*, or *Digitaria californica* with an open creosotebush ‑ mixed desert shrub layer.

**Dynamics:** In the U.S., much of this scrub is thought to be a result of recent expansion of *Larrea tridentata* into former desert grasslands and steppe in the last 150 years as a result of drought, overgrazing by livestock, and/or decreases in fire over the last 70‑250 years (Buffington and Herbel 1965, Ahlstrand 1979, Donart 1984, Dick‑Peddie 1993, Gibbens et al. 2005). This expansion has created challenges in determining ecologically historic stands from more recent ones. Dick‑Peddie (1993) suggested that absence of *Flourensia cernua* as codominant and presence of *Dasyochloa pulchella, Acourtia nana*, and *Yucca elata* may be indicators of recent conversion of desert grasslands into desert scrub, but more research is needed. Conversely, sparse understory *Larrea tridentata* shrublands on remnant early Holocene erosional surfaces (often with shallow calcareous soils and a pebbly desert pavement) may indicate historic distributions of *Larrea tridentata* desert scrub in the Chihuahuan Desert (Stein and Ludwig 1979, Muldavin et al. 2000b).

*Larrea tridentata*, a dominant and diagnostic species, is very long‑lived (some clones have been estimated to be over 10,000 years). It is highly adapted to minimized evapotranspiration both daily and seasonally using stomatal regulation, resinous leaves, and a leaf structure and habit to minimize self‑shading and maximize photosynthesis during favorable growing periods (Hamerlynck et al. 2002, Ogle and Reynolds 2002). *Larrea tridentata* is poorly adapted to fire because of its highly flammable, resinous leaves and limited sprouting ability after burning although it may survive lower‑intensity fires (Humphrey 1974, Brown and Minnich 1986, Marshall 1995, Paysen et al. 2000). McLaughlin and Bowers (1982) reported that burned individuals surviving a fire regained their former size in five years. Other dominant shrubs such as *Acacia constricta, Acacia greggii, Acacia neovernicosa, Fouquieria splendens, Flourensia cernua, Mimosa aculeaticarpa var. biuncifera, Mortonia scabrella*, and *Parthenium incanum* are generally top‑killed by low‑ to moderate‑severity fires, while severe fires may kill them. The nitrogen‑fixing ability of *Acacia neovernicosa* and other leguminous shrubs in this system allow it to colonize harsh environments well (Muldavin et al. 1998a).

This system also includes invasive *Flourensia cernua* shrublands that occur in former (degraded) tobosa (*Pleuraphis mutica*) flats and loamy plains (Muldavin et al. 1998a). Presence of *Scleropogon brevifolius* is common in these invasive stands. *Flourensia cernua* is relatively shallow‑rooted and therefore competes strongly with grasses for soil moisture (Muldavin et al. 1998a). Buffington and Herbel (1965) report that *Larrea tridentata* has displaced many stands of *Flourensia cernua* and cite that it may be because *Larrea tridentata* only competes with grasses during the shrub's seedling stage. Muldavin et al. (1998a) state that stands with no graminoid layer are unlikely to develop one; but stands with a graminoid layer are likely to maintain it if not overgrazed. Impermeable caliche and argillic horizons are not uncommon on these sites. These layers restrict deep percolation of soil‑water and may favor the shallower root grasses and shrubs such as *Flourensia cernua* over more deeply rooted shrubs such as *Larrea tridentata* and *Prosopis* spp. (McAuliffe 1995).

Drought is a relatively common occurrence in this desert scrub, generally occurring every 10‑15 years and lasting 2‑3 years with occasional long‑term drought periods (10‑15 years duration). *Larrea tridentata* and other shrubs have extensive root systems that allow them to exploit deep‑soil water that is unavailable to shallower rooted grasses and cacti (Burgess 1995).

LANDFIRE (2007a) developed three VDDT model for this system, all have two classes. (1) Chihuahuan Mixed Desert and Thorn Scrub (BpS 2511001) occurs in basins, plains and into foothills in the Chihuahuan Desert. Substrates are generally fine‑textured, saline soils. Does not do well in poorly aerated soils. Stands of *Acacia constricta*‑, Acacia neovernicosa‑ or *Acacia greggii*‑dominated thornscrub are included in this creosotebush system, and limestone substrates appear important for at least these species.

A) Early Development 1 All Structures (15% of type in this stage): Characterized by low shrub cover (typically 5‑10%). Little disturbance was considered in Class A, except for replacement fire every 300 years on average. In the historic condition where invasive annual grasses are absent, the fire‑return interval is virtually nonexistent except for areas near the base of mountains experiencing locally higher rainfall and fine fuel buildup from native annuals. After 100 years, class A transitions to class B.

B) Late Development 1 Closed (85% of type in this stage): Typically, >10% shrub cover and <10% grass and forb cover; associated with more productive soils. *Larrea tridentata* characteristically dominates shrub layer. *Acacia* species may dominate locally in patches. Few fine fuels are associated with this community, therefore the MFRIs for replacement fire and mixed‑severity fire is 650 years (min‑max: 300‑1000 years). Wind/weather stress also affected this community on average every 80 years, but did not cause a transition to class A.

(2) Chihuahuan Mixed Desert Shrubland (BpS 2511002) a minor desert scrub that occurs on gravelly mid to upper bajadas, foothills and dissected gravelly alluvial fans in the Chihuahuan Desert and has recently expanded into former desert grasslands in the northern portion of its range. It generally occurs on mid to upper piedmonts above the desert plains Chihuahuan Creosotebush Desert Scrub (CES302.731) and extends up to the chaparral zone (LANDFIRE 2007a, BpS 2511002).

A) Early Development 1 Open (25% of type in this stage): Under natural conditions shrub cover represents <20% canopy cover and is likely not affected by disturbance. The grass community may be as low as 10% canopy cover after a combination of drought/fire. Little disturbance was considered in Class A, modeled drought every 50 years on average, resetting the age to zero (Option 2). In the historic condition where invasive annual grasses are absent, the fire‑return interval is virtually nonexistent except for areas near the base of mountains experiencing locally higher rainfall and fine fuel buildup. After 100 years, class A transitions to class B. However, if the upper soil horizon and/or microbes are lost, then a longer recovery time is required or complete recovery is not possible.

B) Late Development 1 Open (75% of type in this stage): Typically, <40% shrub canopy cover and as much as 25% grass and forb canopy cover; associated with more productive soils. Shrubs characteristically dominate the upper layer. Replacement fire followed by prolonged drought every 500 years (min‑max: 300‑1000 years) on average (Option 1). Wind/weather stress also affected this community on average every 80 years, but did not cause a transition to class A.

(3) Chihuahuan Grama Grass‑Steppe (BpS 2511003) a minor desert scrub steppe that occurs on the bajadas and into foothills in the Chihuahuan Desert. Substrates are generally coarse‑textured, gravelly soils and may have a petrocalic layer. This site exhibits a high degree of topographic diversity, including limy uplands (LANDFIRE 2007a, BpS 2511003).

A) Early Development 1 Open (20% of type in this stage): Under natural conditions shrub cover represents <10% canopy cover and is likely not affected by disturbance. The grass community may be as low as 10% canopy cover after a combination of drought/fire. Little disturbance was considered in class A. Modeled drought every 50 years on average, resetting the age to zero (Option 2). In the historic condition where invasive annual grasses are absent, the fire‑return interval is virtually nonexistent except for areas near the base of mountains experiencing locally higher rainfall and fine fuel buildup. After 100 years, class A transitions to class B. However, if the upper soil horizon and/or microbes are lost, then a longer recovery time is required. Or complete recovery is not possible.

B) Late Development 1 Open (80% of type in this stage): Typically, <10% shrub canopy cover and as much as 40% grass and forb canopy cover; associated with more productive soils. Grasses characteristically dominate shrub layer. Replacement fire followed by prolonged drought every 500 years (min‑max: 300‑1000 years) on average (Option 1). Wind/weather stress also affected this community on average every 80 years, but did not cause a transition to class A.

In the northern Chihuahuan Desert, this creosotebush mixed desert and thornscrub shrubland ecological system is thought to occur in presettlement conditions largely as mixed desert shrub‑steppe on upper bajada gravelly soils and dissected gravelly alluvial fans (S. Yanoff pers. comm. 2006). This grama grass steppe with an open canopy of desert scrub species is a mostly historical grama grass steppe BpS that was described during LANDFIRE MZ25 BpS modeling workshops as Chihuahuan Grama Grass Creosote Steppe (LANDFIRE 2007a, BpS 2511003). It is distinct from creosotebush mixed shrublands on similar sites because it has an open shrub layer characterized by dense perennial grasses (typically black grama).

SOURCES

**References:** Ahlstrand 1979, Belnap 2001, Belnap et al. 2001, Brown 1982a, Brown and Minnich 1986, Buffington and Herbel 1965, Burgess 1995, Cane et al. 2000, Comer et al. 2003*, Dick‑Peddie 1993, Donart 1984, Elliott 2012, Evans and Belnap 1999, Finch 2012, Garfin et al. 2013, Gibbens et al. 2005, Hamerlynck et al. 2002, Henrickson and Johnston 1986, Humphrey 1974, LANDFIRE 2007a, MacMahon 1988, MacMahon and Wagner 1985, Marshall 1995a, McAuliffe 1995, McLaughlin and Bowers 1982, Milchunas 2006, Muldavin et al. 1998a, Muldavin et al. 2000b, Muldavin et al. 2002a, NRCS 2006a, Ogle and Reynolds 2002, Paysen et al. 2000, Rosentreter and Belnap 2003, Schlesinger et al. 2006, Shiflet 1994, Stein and Ludwig 1979, Yanoff pers. comm.

**Version:** 27 Jan 2016	**Stakeholders:** Latin America, Southeast, West

**Concept Author:** K.A. Schulz	**LeadResp:** West

CES302.017 Chihuahuan Mixed Salt Desert Scrub

**Primary Division:** North American Warm Desert (302)

**Land Cover Class:** Shrubland

**Spatial Scale & Pattern:** Large patch

**Required Classifiers:** Natural/Semi‑natural; Vegetated (>10% vasc.); Upland

**Diagnostic Classifiers:** Lowland [Lowland]; Shrubland (Shrub‑dominated); Tropical/Subtropical [Tropical Xeric]; Temperate [Temperate Xeric]; Atriplex spp.

**National Mapping Codes:** EVT 2075; ESLF 5252; ESP 1075

**Concept Summary:** This ecological system includes extensive open‑canopied shrublands of typically saline basins in the Chihuahuan Desert. Stands often occur on alluvial flats and around playas, as well as in floodplains along the Rio Grande and Pecos River, possibly also extending into the San Simon of southeastern Arizona. Substrates are generally fine‑textured, saline soils. Vegetation is typically composed of one or more *Atriplex* species, such as *Atriplex canescens, Atriplex obovata*, or *Atriplex polycarpa*, along with species of *Allenrolfea, Flourensia, Salicornia, Suaeda*, or other halophytic plants. Graminoid species may include *Sporobolus airoides, Pleuraphis mutica*, or *Distichlis spicata* at varying densities.

DISTRIBUTION

**Range:** This ecological system occurs in saline basins in the Chihuahuan Desert. Stands often occur around playas and on alluvial flats, as well as in floodplains along the Rio Grande and Pecos River, possibly also extending into the San Simon of southeastern Arizona.

**Divisions:** 302:C

**TNC Ecoregions:** 22:C, 24:C, 28:C, 29:?, 30:P

**Nations:** MX, US

**Subnations:** AZ, MXCH, MXCO, MXDU, MXNU, MXSO, NM, TX

**Map Zones:** 14:C, 15:P, 24:C, 25:C, 26:C, 27:P, 34:?, 35:?, 36:P

**USFS Ecomap Regions:** 313C:CC, 315A:CC, 315B:CC, 315H:CC, 321A:CC, 322B:CC, M313A:CC, M313B:CC

CONCEPT

**Environment:** This system includes extensive open‑canopied shrublands of typically saline basins in the Chihuahuan Desert. Stands often occur on alluvial flats, around playas and floodplains of the Rio Grande and Pecos River, possibly also extending into the San Simon of southeastern Arizona. Sites are flat to gently sloping with slopes up to 3%. Elevation ranges from 1000‑1300 m (3300‑4300 feet). Substrates are generally fine‑textured, saline soils but may include moderately coarse‑textured alluvium in the floodplains. In Texas, this system is associated with Salty desert grassland, Salty Clay Fan, and Salty Bottomland Ecological Sites. Water tables are generally shallow but fluctuate within reach of deep‑rooted plants, and in most places are high enough that salts accumulate on the surface of the soil.

**Vegetation:** Vegetation is typically composed of one or more *Atriplex* species, such as *Atriplex canescens, Atriplex obovata*, or *Atriplex polycarpa*, along with species of *Allenrolfea, Flourensia, Salicornia, Suaeda*, or other halophytic plants. Graminoid species may include *Sporobolus airoides, Sporobolus wrightii, Pleuraphis mutica*, or *Distichlis spicata* at varying densities. Occasional riparian species may be present near watercourses, such as *Prosopis pubescens* or *Populus deltoides ssp. wislizeni*. In Texas, species making up the often relatively sparse vegetative cover include *Allenrolfea occidentalis, Atriplex acanthocarpa, Atriplex canescens, Flourensia cernua, Isocoma pluriflora, Koeberlinia spinosa, Cylindropuntia leptocaulis, Prosopis glandulosa var. torreyana, Sesuvium verrucosum, Suaeda suffrutescens*, and *Ziziphus obtusifolia*. Non‑native halophiles such as *Salsola tragus, Alhagi maurorum, Peganum harmala*, and *Tamarix* spp. are commonly encountered to dominant. Graminoids commonly found, and sometimes constituting significant cover, include *Trichloris crinita, Distichlis spicata, Pappophorum bicolor, Pleuraphis mutica, Scleropogon brevifolius, Sporobolus airoides*, and *Sporobolus wrightii*.

**Dynamics:** [from M086] In the U.S., much of this desert scrubland is thought to be a result of recent expansion of *Larrea tridentata* and *Prosopis glandulosa* into former desert grasslands and steppe in the last 150 years as a result of a combination of drought, overgrazing by livestock, wind and water erosion, and/or decreases in fire over the last 70‑250 years from fire suppression and fine‑fuel removal by livestock, and changes in the seasonal distribution of precipitation (Buffington and Herbel 1965, Herbel et al. 1972, Humphrey 1974, Ahlstrand 1979, McLaughlin and Bowers 1982, Gibbens et al. 1983, Hennessy et al. 1983, Donart 1984, Brown and Archer 1987, Schlesinger et al. 1990, Dick‑Peddie 1993, McPherson 1995, Gibbens et al. 2005). Seed dispersion by livestock is an additional factor in the increase of *Prosopis glandulosa* (Brown and Archer 1987). It is believed that *Prosopis glandulosa* stands formerly occurred in relatively minor amounts and were largely confined to drainages until cattle distributed seed upland from the bosques into desert grasslands (Brown and Archer 1987, 1989). This macrogroup also includes invasive *Flourensia cernua* shrublands that occur in former (degraded) tobosa (*Pleuraphis mutica*) flats and loamy plains. Presence of *Scleropogon brevifolius* is common in these invasive stands. Dick‑Peddie (1993) suggested that absence of *Flourensia cernua* as codominant and presence of *Dasyochloa pulchella, Acourtia nana*, and *Yucca elata* may be indicators of recent conversion of desert grasslands into desertscrub, but more research is needed. Conversely, sparse understory *Larrea tridentata* shrublands on remnant early Holocene erosional surfaces often with shallow calcareous soils and desert pavement may indicate pre‑historic distributions of *Larrea tridentata* desertscrub in the Chihuahuan Desert (Stein and Ludwig 1979, Muldavin et al. 2000b).

Historical natural‑ignition fires were relatively small, probably 10‑15 acres in size. Repeated fire is thought to help maintain a general mosaic pattern between open grassland and shrub‑dominated areas (Johnston 1963). Wright et al. (1976) found that *Prosopis glandulosa* is very fire‑tolerant when only 3 years old. Most plants resprout after being top‑killed by fire. Thus, prior to livestock grazing reducing fire frequency, repeated grassland fires probably maintained lower stature of shrubs and prevented new establishment by killing seedlings.

Drought is a relatively common occurrence in this desertscrub, generally occurring every 10‑15 years and lasting 2‑3 years with occasional long‑term drought periods (10‑15 years duration). *Prosopis* spp. and other shrubs have extensive root systems that allow them to exploit deep‑soil water that is unavailable to shallower rooted grasses and cacti (Burgess 1995). This strategy works well, especially during drought. However, on sites that have well‑developed argillic or calcic soil horizons that limit infiltration and storage of winter moisture in the deeper soil layers, *Prosopis* spp. invasion can be limited to a few, small individuals (McAuliffe 1995). This has implications in plant geography and desert grassland restoration work in the southwestern United States.

On sandsheet and dune sites, *Prosopis glandulosa* is more common on warmer, drier sites on sandsheets with subsoils composed of clays or carbonate substrates, whereas *Artemisia filifolia* is more common on relatively cooler/moisture sites with coarse, deep sand (S. Yanoff pers. comm. 2007). These sites are also more susceptible to grazing pressure.

SOURCES

**References:** Brown 1982a, Comer et al. 2003*, Dick‑Peddie 1993, Elliott 2013, Muldavin et al. 2000b, Muldavin et al. 2002a, Shreve and Wiggins 1964

**Version:** 02 Oct 2014	**Stakeholders:** Latin America, Southeast, West

**Concept Author:** K.A. Schulz	**LeadResp:** West

CES302.737 Chihuahuan Stabilized Coppice Dune and Sand Flat Scrub

**Primary Division:** North American Warm Desert (302)

**Land Cover Class:** Shrubland

**Spatial Scale & Pattern:** Large patch

**Required Classifiers:** Natural/Semi‑natural; Vegetated (>10% vasc.); Upland

**Diagnostic Classifiers:** Lowland [Lowland]; Shrubland (Shrub‑dominated); Plain; Tropical/Subtropical [Tropical Xeric]; Temperate [Temperate Xeric]; Sand Soil Texture; Aridic; Very Short Disturbance Interval; W‑Landscape/High Intensity; Thorn Shrub; Prosopis spp.‑dominated

**National Mapping Codes:** EVT 2076; ESLF 5253; ESP 1076

**Concept Summary:** This ecological system includes the open desert scrub of vegetated coppice dunes and sandsheets associated with eolian sands found in the Chihuahuan Desert. Stands are usually dominated by *Prosopis glandulosa* or *Artemisia filifolia* but also include *Atriplex canescens, Ephedra torreyana, Ephedra trifurca, Poliomintha incana*, and *Rhus microphylla* coppice and sand flat scrub usually with 10‑30% total vegetation cover. *Yucca elata, Gutierrezia sarothrae, Bouteloua eriopoda, Cylindropuntia imbricata*, and *Sporobolus flexuosus* are commonly present. Herbaceous species of the adjacent grasslands may be common. In northern stands, *Artemisia filifolia* dominates and *Prosopis glandulosa* becomes less common or absent. This system includes degraded sandy desert plains grasslands now dominated by *Artemisia filifolia*.

**Comments:** Some occurrences of this system may represent degraded grasslands of North American Warm Desert Active and Stabilized Dune (CES302.744) or Chihuahuan Sandy Plains Semi‑Desert Grassland (CES302.736). Heavy grazing in late 1800s and early 1900s may have caused mesquite to increase. Naturally occurring coppice dunes may have been limited to areas peripheral to active dunes. Coppice dunes in the Tularosa Basin and elsewhere are currently more extensive, resulting from sand movement due to degradation of desert grasslands and steppe. For Landfire mapzone 25 BpS modeling, this system is considered to be historically uncharacteristic of most sites where it occurs. Much of the current extent of this system is thought to have been formerly Chihuahuan Sandy Plains Semi‑Desert Grassland (CES302.736).

DISTRIBUTION

**Range:** This system occurs on dunes and sandsheets found in the Chihuahuan Desert.

**Divisions:** 302:C

**TNC Ecoregions:** 24:C

**Nations:** MX, US

**Subnations:** MXCH, NM, TX

**Map Zones:** 14:P, 15:P, 25:C, 26:C, 27:P

**USFS Ecomap Regions:** 313C:CC, 315A:CC, 315B:CC, 315H:CP, 321A:CC, 322B:??, M313A:CP, M313B:CC

CONCEPT

**Environment:** This system occurs on eolian sandsheets and coppice dunes in the Chihuahuan Desert of North America.

**Vegetation:** Vegetation is typically sparse and dominated by *Prosopis glandulosa* or *Artemisia filifolia*. Other species may include *Atriplex canescens, Ephedra torreyana, Ephedra trifurca, Poliomintha incana, Rhus microphylla, Yucca elata, Gutierrezia sarothrae, Bouteloua eriopoda, Cylindropuntia imbricata*, and *Sporobolus flexuosus*. Herbaceous species of the adjacent grasslands may be common. In northern stands, *Artemisia filifolia* dominates and *Prosopis glandulosa* becomes less common or absent.

**Dynamics:** *Prosopis glandulosa* is more common on warmer, drier sites on sands with clays or carbonate substrates, whereas *Artemisia filifolia* is more common on relatively cooler/moisture sites with coarse, deep sand (S. Yanoff pers. comm. 2007). The composition of this system is similar to the composition of degraded sandy desert plains grasslands now dominated by *Artemisia filifolia*.

SOURCES

**References:** Bowers 1982, Bowers 1984, Comer et al. 2003*, Dick‑Peddie 1993, Elliott 2012, Muldavin et al. 2000b, NRCS 2006a, Shiflet 1994, Yanoff pers. comm.

**Stakeholders:** Latin America, Southeast, West

**Concept Author:** K.A. Schulz	**LeadResp:** West

CES302.738 Chihuahuan Succulent Desert Scrub

**Primary Division:** North American Warm Desert (302)

**Land Cover Class:** Shrubland

**Spatial Scale & Pattern:** Large patch

**Required Classifiers:** Natural/Semi‑natural; Vegetated (>10% vasc.); Upland

**Diagnostic Classifiers:** Temperate [Temperate Xeric]; Succulent Shrub; Cacti‑dominated

**National Mapping Codes:** EVT 2077; ESLF 5254; ESP 1077

**Concept Summary:** This ecological system is found in the Chihuahuan Desert on colluvial slopes, upper bajadas, sideslopes, ridges, canyons, hills and mesas. Sites are hot and dry, typically with southerly aspects. Gravel and rock are often abundant on the ground surface. In Texas, this system is typically associated limestone. The vegetation is characterized by the relatively high cover of succulent species such as *Agave lechuguilla, Dasylirion leiophyllum, Dasylirion texanum, Euphorbia antisyphilitica, Fouquieria splendens, Ferocactus* spp., *Opuntia engelmannii, Cylindropuntia imbricata, Cylindropuntia spinosior, Yucca baccata, Yucca torreyi*, and many others. Perennial grass cover is generally low. The abundance of succulents is diagnostic of this desert scrub system, but desert shrubs are usually present. In Texas, shrub species such as *Larrea tridentata, Parthenium incanum, Viguiera stenoloba*, and *Forestiera angustifolia* may be present. Herbaceous cover is low with grasses such as *Bouteloua eriopoda, Bouteloua ramosa*, and *Bouteloua curtipendula* sometimes present. Fern and fern allies such as *Astrolepis* spp., *Cheilanthes* spp., and *Selaginella lepidophylla* are often common. Stands in rolling topography may form a mosaic with more mesic desert scrub or desert grassland ecological systems that would occur on less xeric northerly slopes. *Agave lechuguilla* is more abundant in stands in the southern part of the mapzone. This system does not include loamy plains desert grasslands or shrub‑steppe with a strong cacti component such as cholla grasslands.

DISTRIBUTION

**Range:** This Chihuahuan Desert ecological system occurs on colluvial slopes, upper bajadas, sideslopes and mesas. It extends east to the Devils River in Texas.

**Divisions:** 302:C

**TNC Ecoregions:** 22:P, 24:C, 29:?, 30:P

**Nations:** MX, US

**Subnations:** AZ, MXCH, NM, TX

**Map Zones:** 14:C, 15:C, 24:P, 25:C, 26:C

**USFS Ecomap Regions:** 313C:CC, 315A:CC, 321A:CC, 322B:CC, M313A:CC, M313B:CC

CONCEPT

**Environment:** Occurrences are found on a variety of hot, dry sites, typically rocky or gravelly slopes with southerly aspects. Gravel and rock are often abundant on the ground surface. In Texas, it is typically associated with limestones, but can also be found on calcareous gravels, igneous and sandstone substrates on rocky or gravelly slopes associated with Igneous Hill and Mountain, Limestone Hill and Mountain, Sandstone Hill, Limestone Hill, Gravelly, and similar ecoclasses.

**Vegetation:** The vegetation is characterized by the relatively high cover of succulent species such as *Agave lechuguilla, Dasylirion leiophyllum, Dasylirion texanum, Euphorbia antisyphilitica, Fouquieria splendens, Ferocactus* spp., *Opuntia engelmannii, Cylindropuntia imbricata (= Opuntia imbricata), Cylindropuntia spinosior (= Opuntia spinosior), Yucca baccata, Yucca torreyi*, and many others. Perennial grass cover is generally low. The abundance of succulents is diagnostic of this desert scrub system, but desert shrubs are usually present. In Texas, shrub species such as *Larrea tridentata, Parthenium incanum, Viguiera stenoloba*, and *Forestiera angustifolia* may be present. Herbaceous cover is low with grasses such as *Bouteloua eriopoda, Bouteloua ramosa*, and *Bouteloua curtipendula* sometimes present. Fern and fern allies such as *Astrolepis* spp., *Cheilanthes* spp., and *Selaginella lepidophylla* are often common. Stands in rolling topography may form a mosaic with more mesic desert scrub or desert grassland ecological systems that would occur on less xeric northerly slopes. *Agave lechuguilla* is more abundant in stands in the southern part of the mapzone.

**Dynamics:** [from M086] In the U.S., much of this desert scrubland is thought to be a result of recent expansion of *Larrea tridentata* and *Prosopis glandulosa* into former desert grasslands and steppe in the last 150 years as a result of a combination of drought, overgrazing by livestock, wind and water erosion, and/or decreases in fire over the last 70‑250 years from fire suppression and fine‑fuel removal by livestock, and changes in the seasonal distribution of precipitation (Buffington and Herbel 1965, Herbel et al. 1972, Humphrey 1974, Ahlstrand 1979, McLaughlin and Bowers 1982, Gibbens et al. 1983, Hennessy et al. 1983, Donart 1984, Brown and Archer 1987, Schlesinger et al. 1990, Dick‑Peddie 1993, McPherson 1995, Gibbens et al. 2005). Seed dispersion by livestock is an additional factor in the increase of *Prosopis glandulosa* (Brown and Archer 1987). It is believed that *Prosopis glandulosa* stands formerly occurred in relatively minor amounts and were largely confined to drainages until cattle distributed seed upland from the bosques into desert grasslands (Brown and Archer 1987, 1989). This macrogroup also includes invasive *Flourensia cernua* shrublands that occur in former (degraded) tobosa (*Pleuraphis mutica*) flats and loamy plains. Presence of *Scleropogon brevifolius* is common in these invasive stands. Dick‑Peddie (1993) suggested that absence of *Flourensia cernua* as codominant and presence of *Dasyochloa pulchella, Acourtia nana*, and *Yucca elata* may be indicators of recent conversion of desert grasslands into desertscrub, but more research is needed. Conversely, sparse understory *Larrea tridentata* shrublands on remnant early Holocene erosional surfaces often with shallow calcareous soils and desert pavement may indicate pre‑historic distributions of *Larrea tridentata* desertscrub in the Chihuahuan Desert (Stein and Ludwig 1979, Muldavin et al. 2000b).

Historical natural‑ignition fires were relatively small, probably 10‑15 acres in size. Repeated fire is thought to help maintain a general mosaic pattern between open grassland and shrub‑dominated areas (Johnston 1963). Wright et al. (1976) found that *Prosopis glandulosa* is very fire‑tolerant when only 3 years old. Most plants resprout after being top‑killed by fire. Thus, prior to livestock grazing reducing fire frequency, repeated grassland fires probably maintained lower stature of shrubs and prevented new establishment by killing seedlings.

Drought is a relatively common occurrence in this desertscrub, generally occurring every 10‑15 years and lasting 2‑3 years with occasional long‑term drought periods (10‑15 years duration). *Prosopis* spp. and other shrubs have extensive root systems that allow them to exploit deep‑soil water that is unavailable to shallower rooted grasses and cacti (Burgess 1995). This strategy works well, especially during drought. However, on sites that have well‑developed argillic or calcic soil horizons that limit infiltration and storage of winter moisture in the deeper soil layers, *Prosopis* spp. invasion can be limited to a few, small individuals (McAuliffe 1995). This has implications in plant geography and desert grassland restoration work in the southwestern United States.

On sandsheet and dune sites, *Prosopis glandulosa* is more common on warmer, drier sites on sandsheets with subsoils composed of clays or carbonate substrates, whereas *Artemisia filifolia* is more common on relatively cooler/moisture sites with coarse, deep sand (S. Yanoff pers. comm. 2007). These sites are also more susceptible to grazing pressure.

SOURCES

**References:** Comer et al. 2003*, Elliott 2012, MacMahon 1988, Muldavin et al. 2000b, Muldavin et al. 2002a, NRCS 2006a, Shiflet 1994

**Version:** 02 Oct 2014	**Stakeholders:** Latin America, Southeast, West

**Concept Author:** K.A. Schulz	**LeadResp:** West

M087. Chihuahuan Semi‑Desert Grassland

CES302.735 Apacherian‑Chihuahuan Semi‑Desert Grassland and Steppe

**Primary Division:** North American Warm Desert (302)

**Land Cover Class:** Steppe/Savanna

**Spatial Scale & Pattern:** Large patch

**Required Classifiers:** Natural/Semi‑natural; Vegetated (>10% vasc.); Upland

**Diagnostic Classifiers:** Lowland [Foothill, Lowland]; Herbaceous; Temperate [Temperate Xeric]; Short Disturbance Interval; F‑Patch/High Intensity [Seasonality/Winter Fire]; Xeromorphic Tree; Thorn Shrub; Graminoid

**National Mapping Codes:** EVT 2121; ESLF 5450; ESP 1121

**Concept Summary:** This ecological system is a broadly defined desert grassland, mixed shrub‑succulent steppe, or xeromorphic oak savanna that is typical of the Borderlands of Arizona, New Mexico and northern Mexico (Apacherian region) but extends west to the Sonoran Desert, north into the Mogollon Rim in Arizona and up the Rio Grande Valley into central New Mexico. It also extends east into the Chihuahuan Desert. It is found on gently sloping alluvial erosional fans and piedmonts (bajadas) that lie along mountain fronts of the isolated basin ranges throughout the Sky Island mountain archipelago and on to foothill slopes up to 1670 m elevation in the Chihuahuan Desert. The vegetation in this mixed semi‑desert grassland ecosystem is variable. It is characterized by the dominance of a typically diverse layer of warm‑season, perennial grasses with scattered stem succulents and shrubs. Frequent species include the grasses *Aristida ternipes, Bouteloua barbata, Bouteloua chondrosioides, Bouteloua curtipendula, Bouteloua eriopoda, Bouteloua gracilis, Bouteloua hirsuta, Bouteloua ramosa, Bouteloua repens, Bouteloua rothrockii, Dasyochloa pulchella, Digitaria californica, Eragrostis intermedia, Heteropogon contortus, Hilaria belangeri, Leptochloa dubia, Muhlenbergia porteri*, with *Muhlenbergia emersleyi, Muhlenbergia setifolia* at upper foothill elevation, rosettophyllous, often succulent species of *Agave, Dasylirion, Nolina, Opuntia*, and *Yucca*, and short‑shrub species of *Calliandra*, and *Parthenium*. Tall‑shrub/short‑tree species of *Acacia, Prosopis, Juniperus, Mimosa*, and various oaks (e.g., *Quercus grisea, Quercus emoryi, Quercus arizonica, Quercus oblongifolia*) may be present with low cover (usually <10%). *Pleuraphis mutica*‑dominated semi‑desert grasslands often with *Bouteloua eriopoda* or *Bouteloua gracilis* occurring on lowlands and loamy plains in the Chihuahuan Desert are classified as Chihuahuan Loamy Plains Desert Grassland (CES302.061). Many of the historical desert grassland and savanna areas have been converted through intensive grazing and other land uses, some to Apacherian‑Chihuahuan Mesquite Upland Scrub (CES302.733) (*Prosopis* spp.‑dominated).

**Comments:** *Dasylirion leiophyllum, Dasylirion wheeleri*, and *Fouquieria splendens* foothill shrublands and oak savannas are included in the concept of this grassland and steppe ecological system. Chihuahuan grassland types that are currently included in this system are: (1) Chino grasslands of mountain slopes on acidic igneous, limestone, or deeper gravelly soils at elevations less than 1070 m (3500 feet). These sites are dominated by *Bouteloua ramosa* with *Euphorbia antisyphilitica, Hechtia texensis, Fouquieria splendens, Jatropha dioica*, and *Agave lechuguilla*. (2) Desert mountain grasslands on mountain slopes between 1070 and 1370 m (3500‑4500 feet) elevation on acidic igneous substrates, but also sometimes on limestone. *Bouteloua eriopoda* and *Bouteloua curtipendula* are constituents of this system. (3) Gravelly piedmont slope grasslands between 1370 and 1670 m (4500‑5500 feet) elevation on Perdiz conglomerate or Tascotal tuff. These grasslands have *Bouteloua eriopoda, Bouteloua gracilis*, and *Dasylirion* as common components. Input from fire ecologist at a Landfire modeling workshop in 2006 suggests a fire‑return interval that is generally long (about 10 years), with pluvial periods providing conditions leading to more rapid fuel development (Landfire 2007a). In Texas, shrub species that may be encountered in these grasslands include *Larrea tridentata, Parthenium incanum, Viguiera stenoloba, Acacia constricta, Mimosa aculeaticarpa var. biuncifera, Condalia ericoides*, and many others. In this region, significant areas dominated by shrubs grade into Chihuahuan Mixed Desert and Thornscrub (CES302.734), Chihuahuan Succulent Desert Scrub (CES302.738), or Chihuahuan Creosotebush Desert Scrub (CES302.731) depending on composition.

DISTRIBUTION

**Range:** This system is found in the Borderlands of Arizona, New Mexico and northern Mexico (Apacherian region), extending to the Sonoran Desert and throughout much of the northern Chihuahuan Desert.

**Divisions:** 302:C

**TNC Ecoregions:** 22:C, 24:C, 28:C

**Nations:** MX, US

**Subnations:** AZ, MXCH, NM, TX

**Map Zones:** 13:C, 14:C, 15:C, 24:C, 25:C, 26:C, 27:C, 28:?, 34:?

**USFS Ecomap Regions:** 313B:CC, 313C:CC, 315A:CC, 315B:CC, 315H:CC, 321A:CC, 322A:CC, 322B:CC, M313A:CC, M313B:CC

CONCEPT

**Environment:** This system is found on gently sloping alluvial erosional fans and piedmonts (bajadas) that lie along mountain fronts of the isolated ranges throughout the Sky Island mountain archipelago and on to foothill slopes from 1000 m to 1670 m and up to 1800 m elevation in the Chihuahuan Desert and up to 2200 m in lower montane grasslands.

*Climate:* Climate is semi‑arid, warm‑temperate with a highly variable, bimodally distributed precipitation. Approximately two‑thirds of the 20‑40 cm mean annual precipitation occurs in the late summer and early fall, usually as localized high‑intensity thunderstorms.

<br />*Physiography/landform:* Sites are typically gently sloping mesas and piedmonts (LANDFIRE 2007a).

<br />*Soil/substrate/hydrology:* Substrates are variable, ranging from fine‑ to coarse‑textured soils depending on site. However, most are typically deep, coarser‑textured, gravelly soils derived from limestone, sandstone, conglomerate or igneous substrates such as tuff.

**Vegetation:** The vegetation in this mixed semi‑desert grassland ecosystem is variable. It is characterized by the dominance of a typically diverse layer of perennial grasses with scattered stem succulents and shrubs. Frequent species include the grasses *Aristida ternipes, Bouteloua curtipendula, Bouteloua eriopoda, Bouteloua gracilis, Bouteloua hirsuta, Bouteloua ramosa, Bouteloua repens, Eragrostis intermedia, Heteropogon contortus, Muhlenbergia porteri*, with *Muhlenbergia emersleyi, Muhlenbergia setifolia* at upper foothill elevation, rosettophyllous, often succulent species of *Agave, Dasylirion, Nolina, Opuntia*, and *Yucca*, and short‑shrub species of *Calliandra*, and *Parthenium*. Tall‑shrub/short‑tree species of *Acacia, Prosopis, Juniperus, Mimosa*, and various oaks (e.g., *Quercus grisea, Quercus emoryi, Quercus arizonica, Quercus oblongifolia*) may be present with low cover.

**Dynamics:** Semi‑desert grasslands are complex with many stands having a shrub or stem succulent component (*Agave* and *Yucca* spp.) under natural conditions (Burgess 1995). This woody component increases in density over time in the absence of disturbance such as fire (Burgess 1995, Gori and Enquist 2003, Schussman 2006a). Under historic natural conditions (also called natural range of variability or NRV), this ecosystem ranges from open perennial grasslands with low cover of shrubs to grasslands with a moderately dense shrub layer and succulent layer (Burgess 1995, Gori and Enquist 2003). An exception is that some stands with deep argillic horizons appear resistant to shrub and tree invasion without disturbance (McAuliffe 1995).

It is well‑documented that frequent stand‑replacing fire (fire‑return interval (FRI) of 2.5 to 10 years) was a key ecological attribute of this semi‑desert grassland ecosystem historically before 1890 (Wright 1980, Bahre 1985, McPherson 1995, Kaib et al. 1996). Other evidence of the importance of fire in maintaining desert grasslands includes the widespread conversion of grasslands to shrublands during the century of fire suppression (McPherson 1995) and the results of prescribed burning on decreasing shrub cover and increasing grass cover (Bock and Bock 1992, Robinett 1994). Additional evidence that frequent fire is a key ecological attribute of this ecosystem is that many common invasive shrubs, subshrubs and cacti are fire‑sensitive and individuals are killed when top‑burned, at least when they are young (<10 years old) (McPherson 1995), while native perennial grasses generally quickly recover from burning (Wright 1980, Martin 1983, Bock and Bock 1992).

Herbivory by native herbivores in the system is varied and ranges from invertebrates and rodents to pronghorn (Parmenter and Van Devender 1995, Whitford et al. 1995, Finch 2004). Soil‑dwelling invertebrates include tiny nematodes and larger termites and ants, are important in nutrient cycling and affect soil properties, such as bulk density (Whitford et al. 1995). Above‑ground invertebrates such as grasshoppers can significantly impact herbaceous cover when populations are high. Herbivory by native mammals also impacts these grasslands. Historically, populations of large mammals such as pronghorn (*Antilocarpa americana*) and mule deer (*Odocoileus hemionus*) were once abundant in this ecosystem (Parmenter and Van Devender 1995). Populations were greatly reduced and, in the case of pronghorn, extirpated during the 1800s and early 1900s, but effective game management has restored many populations, although habitat changes will limit restoration in other areas (Parmenter and Van Devender 1995). The historic impact of large native ungulates on this ecosystem is not known; however, in the case of wintering elk, it may have been significant locally. The current impact is assumed to be relatively small in this ecosystem.

<br />Herbivory from native small mammals such as rodents is significant as they are the dominant mammals in the semi‑desert grassland ecosystem. There is also high diversity of these rodents, especially ground‑dwelling ones such as spotted ground squirrels (*Xerospermophilus spilosoma*), and bannertail and Ord kangaroo rats (*Dipodomys spectabilis* and *Dipodomys ordii*). These burrowing rodents have a substantial effect on vegetation composition, soil structure and nutrient cycling (Parmenter and Van Devender 1995, Finch 2004). Historically, black‑tail prairie dogs (*Cynomys ludovicianus*) had extensive colonies in the Great Plains that extended west to southeastern Arizona but were greatly reduced. Although abundant in southeastern Arizona and southwestern New Mexico in the 1800s, the black‑tailed prairie dog populations were decimated by 1930 and considered extirpated in Arizona by 1960 (Alexander 1932, Hoffman 1986, Parmenter and Van Devender 1995, Van Pelt 1999, Underwood and Van Pelt 2008). Although there have been several reintroductions of black‑tailed prairie dogs, their numbers and impacts are still small in this region. Because of the nature of black‑tail prairie dogs (large towns and major impacts to the local ecosystem), they may have historically functioned as a keystone species in lower elevation stands in the northern extent of Apacherian‑Chihuahuan Semi‑Desert Grassland and Steppe (CES302.735). However, historically black‑tailed prairie dogs were likely more abundant in the deeper soiled Chihuahuan Loamy Plains Desert Grassland (CES302.061) that occurs on lower elevation alluvial flats and plains. More research is needed to determine the role of black‑tailed prairie dogs in these semi‑grassland and steppe systems.

Invertebrate animals are also significant in semi‑desert grassland. They are both abundant and extremely diverse, ranging from single‑celled protozoans, bacterial and soil nematodes and mites to larger arachnids, millipedes, cockroaches, crickets, grasshoppers, ants, beetles, butterflies, moths, flies, bees, wasps, and true bugs (Whitford et al. 1995). Invertebrates are important for nutrient cycling and pollination, and subterranean species of ants and termites can impact soil properties such as bulk density, infiltration permeability and storage (Whitford et al. 1995). Grasshoppers feed on grasses and forbs and can consume significant amounts of forage when their populations are high. Many species of butterflies, flies, bees, and moths are important for pollination. Some species such as Yucca moths (*Tegeticula* spp.) and *Yucca* species have obligate/mutualistic relationships (Whitford et al. 1995, Althoff et al. 2006). In these grasslands, *Yucca* spp. are typically dependent on a single species of *Tegeticula* for pollination, which is usually dependent on a single *Yucca* host plant species for habitat and food for larvae, for example, *Tegeticula baccatella* and *Yucca baccata, Tegeticula carnerosanella* and *Yucca faxoniana, Tegeticula elatella* and *Yucca elata, Tegeticula maderae* and *Yucca x schottii*, and *Tegeticula yuccasella* and *Yucca glauca*. More study and review are needed to fully understand the many functional roles animals have within the semi‑desert grassland ecosystem.

LANDFIRE developed a VDDT model for this system which has three classes (LANDFIRE 2007a, BpS 2711210).

A) Early Development 1 All Structures (20% of type in this stage): Herbaceous cover (0‑20%). Grass and herbs, 0‑5 years (predicated on moisture regime). Early‑succession post‑fire grass and herb community. This class encompasses the time period required to recover sufficient fuel loads to carry fire. Perennial bunchgrasses, annual grass, and herb community. Upper layer of shrubs, canopy cover less than 5%.

B) Mid Development 1 All Structures (35% of type in this stage): Perennial grass species dominate with 35‑50% canopy cover; <0.5 m height. Shrub cover is 5‑10% with shrubs 0‑1 m tall. Grass with some low shrubs, 6‑50 years old. Perennial bunchgrasses regenerated and young shrubs begin growing. Species are perennial bunchgrasses and shrubs. Canopy cover of shrubs is 5‑10%. Maintenance disturbance is drought, occurring approximately every 30 years. Maintenance replacement fire is more frequent with less frequent replacement fire returning to class A. This was modeled to occur every 10 years on average, half the time causing a transition to class A, and half the time maintaining this class.

C) Late Development 1 All Structures (50% of type in this stage): Perennial grass species dominate with 10‑35% canopy cover; 1‑2 m height. Shrubs continue to increase in size and/or number of individuals. Species are perennial bunchgrasses and shrubs. Canopy cover of shrubs is 10‑20%. (Shrub cover will be similar to species composition found in Apacherian‑Chihuahuan Mesquite Upland Scrub (CES302.733)). Shrub species diversity increases. FRI=10 years, half are replacement (to class A) and half take class back to class B. The wind/weather stress in this model is drought, occurring approximately every 30 years. It is thought that this is the class that might result with lack of fire and that more would be present in this class currently versus historically.

In the LANDFIRE BpS 2611210 model, mixed‑severity fire was modeled for MZ26; however, this was removed for MZ27, as it is thought that only patchy replacement fire would occur in this system (LANDFIRE 2007a). It was noted that the amount of moisture following fire has a significant impact on plant response/recovery. Because historical fire data in this system are lacking, there is uncertainty over the role fire plays in maintaining this system. Some modelers think fire has a major impact on control of woody species, whereas others think fire is less important in control of woody species than maintenance of perennial grass cover in this system (LANDFIRE 2007a).

SOURCES

**References:** AGFD 1999, ARPC 2001, Alexander 1932, Althoff et al. 2006, Anable et al. 1992, Bagne and Finch 2013, Bahre 1985, Belnap 2001, Belnap et al. 2001, Bock and Bock 1992, Brown 1982a, Brown and Archer 1987, Brown and Archer 1989, Brown and Archer 1999, Buffington and Herbel 1965, Burgess 1995, Cable 1971, Comer et al. 2003*, Cooke and Reeves 1976, Dick‑Peddie 1993, Elliott 2013, Evans and Belnap 1999, Eyre 1980, Finch 2004, Gibbens et al. 1983, Gori and Enquist 2003, Hennessy et al. 1983, Herbel et al. 1972, Hoffman 1986, Humphrey 1974, Isely 1998, Kaib et al. 1996, LANDFIRE 2007a, Martin 1983, McAuliffe 1995, McLaughlin and Bowers 1982, McPherson 1995, Muldavin et al. 2000b, Muldavin et al. 2002a, NRCS 2006a, Parmenter and Van Devender 1995, Robinett 1994, Rosentreter and Belnap 2003, Schlesinger et al. 1990, Schussman 2006a, Shiflet 1994, TNC 2013, USFWS 1999, Underwood and Van Pelt 2008, Van Pelt 1999, Whitford et al. 1995, Wright 1980, York and Dick‑Peddie 1969

**Version:** 19 Nov 2015	**Stakeholders:** Latin America, Southeast, West

**Concept Author:** K.A. Schulz	**LeadResp:** West

CES302.732 Chihuahuan Gypsophilous Grassland and Steppe

**Primary Division:** North American Warm Desert (302)

**Land Cover Class:** Steppe/Savanna

**Spatial Scale & Pattern:** Large patch

**Required Classifiers:** Natural/Semi‑natural; Vegetated (>10% vasc.); Upland

**Diagnostic Classifiers:** Herbaceous; Tropical/Subtropical [Tropical Xeric]; Temperate [Temperate Xeric]; Alkaline Soil; Gypsiferous; Dwarf‑Shrub; Graminoid

**National Mapping Codes:** EVT 2122; ESLF 5451; ESP 1122

**Concept Summary:** This ecological system is restricted to gypsum outcrops or sandy gypsiferous and often alkaline soils that occur in basins and slopes in the Chihuahuan Desert. Elevation range is from 1100‑2000 m. These typically sparse grasslands, steppes or dwarf‑shrublands are dominated by a variety of gypsophilous plants, many of which are endemic to these habitats. Characteristic species include *Tiquilia hispidissima, Atriplex canescens, Calylophus hartwegii, Ephedra torreyana, Frankenia jamesii, Bouteloua breviseta, Mentzelia perennis, Nama carnosum, Calylophus hartwegii, Selinocarpus lanceolatus, Sporobolus nealleyi, Sporobolus airoides*, and *Sartwellia flaveriae* with gypsophilous species diagnostic of this system. This system does not include the sparsely vegetated gypsum dunes that are included in North American Warm Desert Active and Stabilized Dune (CES302.744). Additional species that may be encountered in this system in Texas include *Anulocaulis* spp., *Atriplex canescens, Calylophus hartwegii, Condalia ericoides, Ephedra torreyana, Gaillardia multiceps, Larrea tridentata, Poliomintha incana, Prosopis glandulosa, Scleropogon brevifolius, Selinocarpus* spp., *Sporobolus airoides, Sporobolus cryptandrus*, and *Yucca torreyi*.

**Comments:** The Texas ecological systems map includes sparsely vegetated gypsum dunes in map units of Chihuahuan Gypsophilous Grassland and Steppe (CES302.732) and not in North American Warm Desert Active and Stabilized Dune (CES302.744). Further investigation is needed to determine the correct classification of these sparsely vegetated gypsum dunes. They range from sparsely vegetated to scattered shrubs with patchy herbaceous cover. In Texas, gypsum dunes include many of the species listed for this system, plus *Andropogon hallii, Artemisia filifolia, Dalea lanata, Dimorphocarpa wislizeni, Krameria lanceolata, Mentzelia* spp., *Poliomintha incana, Psorothamnus scoparius, Sporobolus giganteus, Tidestromia lanuginosa*, and *Yucca elata*. Further investigation is needed to determine the correct classification of the sparsely vegetated gypsum dunes.

DISTRIBUTION

**Range:** This system is found on basins and slopes in the Chihuahuan Desert at elevations ranging from 1100‑2000 m.

**Divisions:** 302:C

**TNC Ecoregions:** 22:P, 24:C

**Nations:** MX, US

**Subnations:** AZ, MXCH, NM, TX

**Map Zones:** 25:C, 26:C, 27:P, 28:?

**USFS Ecomap Regions:** 315A:CC, 315B:CC, 315H:CP, 321A:CC, M313B:CC

CONCEPT

**Environment:** This ecological system is restricted to gypsum outcrops and strongly gypseous soils (Powell and Turner 1974, Henrickson et al. 1985, Meyer 1986, Dick‑Peddie 1993). Sites occur in warm, semi‑desert and desert regions with hot summers, and occasionally cold winters from the Chihuahuan Desert to eastern Mojave Desert and may extend up into the southern Colorado Plateau (Powell and Turner 1974, Meyer 1986, Dick‑Peddie 1993). Elevation range is from 1100‑2000 m. Some occurrences may be windswept gypsum "pavement" where much of the gypsum sand has been removed by wind, but these are not open/moving dunes dominated by eolian processes. Substrates are typically fine‑textured, alkaline clay soils but include some sandy gypsiferous soils that occur in closed basins in the Chihuahuan Desert, but not gypsum dunes at White Sands National Monument (Reid 1980, Dick‑Peddie 1993, Muldavin et al. 2000b). Eolian processes drive the dune system so many of the same common sand scrub plants, e.g., *Atriplex canescens*, may characterize vegetation on both quartz and gypsum active dunes, although some gypsophiles will occur on gypsum dunes (Shields 1956, Reid 1980, 1980, Dick‑Peddie 1993). In Texas, extensive occurrences are associated with the Permian Castile Formation and alluvium within evaporative bolsons; scattered occurrences are associated with exposed gypsite and alluvium of evaporative ponds and swales receiving deposition from eroding gypsiferous formations.

**Vegetation:** These typically sparse grasslands, steppes or dwarf‑shrublands are dominated by a variety of gypsophilous plants, many of which are endemic to these habitats. Characteristic species include *Tiquilia hispidissima, Atriplex canescens, Calylophus hartwegii, Ephedra torreyana, Frankenia jamesii, Bouteloua breviseta, Mentzelia perennis, Nama carnosum, Calylophus hartwegii (= Oenothera hartwegii), Selinocarpus lanceolatus, Sporobolus nealleyi, Sporobolus airoides*, and *Sartwellia flaveriae*.

**Dynamics:** Gypsophile endemism is common in the North American deserts, especially the Chihuahuan Desert where much of the region is underlain by limestone, with occasional gypsum exposures. These gypsum deposits are distributed in a discontinuous, island‑like fashion that facilitates endemism. Gypsum is a difficult substrate for plants to grow on because it typically forms a hard crust when dry, erodes quickly when wet, and is relatively low in available nutrients. However, a large and diverse group of gypsophilous plants only occur on this substrate, several of which are considered rare and at risk.

This is a substrate‑driven ecosystem occurring in extreme environments on chemically harsh substrates. Fire plays little to no role in this ecosystem as vegetation is generally too sparse to carry fire. Normal climate conditions are warm and arid (6‑10 inches annually) with drought not uncommon. Climatic fluctuations (precipitation cycles) have been speculated to affect plant vigor and recruitment (Landfire 2007a), but this is not likely significant considering the hardiness of these plants and the harshness of the environments (E. Muldavin pers. comm.). Variation in abundance of subshrubs and grasses is likely more related to fine‑scale differences in the soil environment then climatic factors (E. Muldavin pers. comm.). Some occurrences may be windswept, but these are not open/moving dunes with eolian processes. Some occurrences may be gypsum "pavement" or outcrop where much of the gypsum sand has been removed by wind.

SOURCES

**References:** Comer et al. 2003*, Dick‑Peddie 1993, Elliott 2012, Henrickson et al. 1985, LANDFIRE 2007a, MacMahon 1988, Meyer 1986, Muldavin et al. 2000b, Muldavin et al. 2002a, Muldavin pers. comm., NRCS 2006a, Powell and Turner 1974, Reid 1980, Shields 1956, TNC 2013

**Version:** 02 Oct 2014	**Stakeholders:** Latin America, Southeast, West

**Concept Author:** K.A. Schulz	**LeadResp:** West

CES302.061 Chihuahuan Loamy Plains Desert Grassland

**Primary Division:** North American Warm Desert (302)

**Land Cover Class:** Herbaceous

**Spatial Scale & Pattern:** Large patch

**Required Classifiers:** Natural/Semi‑natural; Vegetated (>10% vasc.); Upland

**Diagnostic Classifiers:** Lowland; Alluvial flat; Plain; Valley

**National Mapping Codes:** EVT 2503; ESLF 7164; ESP 1503

**Concept Summary:** This ecological system occurs in the northern Chihuahuan Desert and extends into limited areas of the southern Great Plains on alluvial flats, loamy plains, and basins sometimes extending up into lower piedmont slopes. Although there is some localized topography with hills and bluffs, sites are typically flat or gently sloping to moderately steep and may be somewhat mesic if they receive runoff from adjacent areas, but these are not wetlands or mesic, bottomland grassland. Soils are non‑saline, finer textured loams or clay loam. Vegetation is characterized by perennial grasses and is typically dominated by *Pleuraphis mutica* or with *Bouteloua eriopoda* codominant (more historically) or *Bouteloua gracilis*. In degraded stands, *Scleropogon brevifolius, Dasyochloa pulchella*, or *Aristida* spp. may codominate. *Pleuraphis jamesii* may become important in northern stands and *Bouteloua gracilis* in the Great Plains and on degraded stands. If present, mesic graminoids such as *Pascopyrum smithii, Panicum obtusum, Sporobolus airoides*, and *Sporobolus wrightii* typically have low cover and are restricted to drainages and moist depressions (inclusions). Scattered shrubs such as *Ephedra torreyana, Flourensia cernua, Gutierrezia sarothrae, Larrea tridentata, Cylindropuntia imbricata, Prosopis glandulosa*, and *Yucca* spp. may be present, especially on degraded sites.

**Comments:** NRCS Ecological Site Description MLRA 42 SD‑2 Loamy Ecological Site (NRCS 2006) describes this system on the Jornada Experimental Range (1200 m in elevation) with State‑and‑Transition Model showing shifts in species composition with land use. Degraded stands often have scattered desert scrubs such as *Larrea tridentata, Flourensia cernua*, and *Prosopis glandulosa* present.

This upland grassland is similar to the bottomland/depressional wetland system Chihuahuan‑Sonoran Desert Bottomland and Swale Grassland (CES302.746) and grades into Apacherian‑Chihuahuan Semi‑Desert Grassland and Steppe (CES302.735) in the foothills and piedmont desert grasslands. In similar loamy plains land positions in the Great Plains, *Bouteloua gracilis, Bouteloua dactyloides*, or *Pleuraphis jamesii* are dominant grasses in Western Great Plains Shortgrass Prairie (CES303.672).

DISTRIBUTION

**Range:** This grassland system is found from the northern to central Chihuahuan Desert and extends across the Trans‑Pecos and into areas of the southwestern Great Plains. It extends from western Texas across New Mexico and into southeastern Arizona. Stands are described from Jornada del Muerto Basin, Marfa grasslands and Marathon Basin, south to central Chihuahua and Coahuila, Mexico.

**Divisions:** 302:C, 303:C

**TNC Ecoregions:** 22:C, 24:C, 28:C

**Nations:** MX, US

**Subnations:** AZ, MXCH, MXCO, NM, TX

**Map Zones:** 25:C, 26:C

**USFS Ecomap Regions:** 313B:CC, 313C:CC, 315A:CC, 315B:CC, 315H:CC, 321A:CC, 322A:CC, 322B:CC, M313A:CC, M313B:CC

CONCEPT

**Environment:** These upland grasslands occur at approximately 1150‑2320 m (3500‑7610 feet) elevation and are found on various sedimentary and igneous substrates, including alluvial flats, loamy plains, and desert basins sometimes extending up into lower piedmont slopes including mesatops. Sites are typically flat or gently sloping so precipitation does not run off and may be somewhat mesic if they receive runoff from adjacent areas, but these are not wetlands or bottomland grasslands described in Chihuahuan‑Sonoran Desert Bottomland and Swale Grassland (CES302.746). Annual precipitation is usually from 20‑40 cm (7.9‑15.7 inches). Soils are non‑saline, finer‑textured loams or clay loam that are often derived from sedimentary parent materials but are quite variable and may include fine‑textured soils derived from igneous and metamorphic rocks. These grasslands can occur on a variety of aspects and slopes ranging from flat to moderately steep. When they occur near foothill grasslands, they will be at lower elevations (Landfire 2007a). In Texas, this system occurs primarily on Quaternary alluvium but is also found on other formations at higher elevations of mountain foothills. Two somewhat distinct areas are loams of the intermountain basins, and foothill grasslands over shallow soils at the basin edges. The foothill grasslands often occupy Shallow Ecological Sites over Perdiz Conglomerate, but may also occur on gravelly sites.

**Vegetation:** The vegetation in examples of this system is characterized by perennial grasses and is typically dominated by *Pleuraphis mutica* or with *Bouteloua eriopoda* codominant (more historically before heavy grazing reduced this over much of the range) or *Bouteloua gracilis*. In degraded stands, *Scleropogon brevifolius, Dasyochloa pulchella (= Erioneuron pulchellum)*, or *Aristida* spp. may codominate. *Pleuraphis jamesii* may become important in northern stands and *Bouteloua gracilis* in the Great Plains and on degraded stands. If present, mesic graminoids such as *Pascopyrum smithii, Panicum obtusum, Sporobolus airoides*, and *Sporobolus wrightii* typically have low cover and are restricted to drainages and moist depressions (inclusions). Scattered shrubs such as *Ephedra torreyana, Flourensia cernua, Gutierrezia sarothrae, Larrea tridentata, Cylindropuntia imbricata (= Opuntia imbricata), Prosopis glandulosa*, and *Yucca* spp. may be present, especially on degraded sites. In west Texas, this system includes two somewhat distinct grassland types: one on loams of intermountain basins, and the other on foothills and shallow soils at the basin edges. These types are often closely juxtaposed and share graminoid composition but differ in abiotic sites, aspect, and invading shrubs. The loamy grasslands are dominated by species such as *Bothriochloa barbinodis, Bothriochloa laguroides ssp. torreyana, Bouteloua curtipendula, Bouteloua eriopoda, Bouteloua gracilis, Dasyochloa pulchella, Pleuraphis mutica*, and *Scleropogon brevifolius*. These grasslands occur in extensive level plains with deep soils. *Prosopis glandulosa* is the common shrub invader. Other shrubs present to dominant as invaders include *Larrea tridentata, Flourensia cernua*, and *Mimosa aculeaticarpa var. biuncifera*. The foothill grasslands are of similar composition with respect to grasses, but occupy rolling landscapes at slightly higher elevations and are on shallow soils. *Condalia ericoides, Juniperus* spp., and *Acacia constricta* are common invaders.

**Dynamics:** Historic fire frequency in this ecosystem is not known, but is likely less frequent than other denser desert grasslands because of less fuel in this typically open grassland ecosystem (Humphrey 1963). The effects of burning tobosa‑dominated grasslands is variable depending upon soil moisture and plant phenology at the time of the fire, precipitation in the months following the fire, and site characteristics that influence soil moisture availability, and fire intensity based on research in the Great Plains (Innes 2012). However, the dominant grass *Pleuraphis mutica* is likely to survive most fires and can sprout from rhizomes and grow quickly after top‑kill by fire (Britton and Steuter 1983).

These grasslands are prone to flooding during high precipitation events because of slow infiltration. This may result in overland flow and erosion of topsoil and some short‑term loss of vegetative cover. Landfire (2007a) modeled this system and predicted that during a >500‑year flooding event in a swale or stream channel, sites could downcut, thus lowering the water table, and favor woody species in an altered state. Drought cycles likely resulted in a reduction in vegetative cover and production of these sites (Landfire 2007a). Annual growth of woody vegetation depends on annual rainfall; drought negatively affected woody species. Cyclic drought impacts vegetation growth two to three years out of every 10 years, and vegetation‑killing drought has a mean return interval of 100 years (Landfire 2007a).

Some grasslands with deep argillic horizons in the San Rafael valley in Arizona and Animas valley in New Mexico have not shown shrub or tree encroachment and/or conversion in the absence of fire or presence of livestock grazing (McAuliffe 1995, Muldavin et al. 2012c). These deep‑soil systems have maintained open grassland characteristics despite fire suppression, drought, and livestock grazing. However, there are other valley bottom areas that once supported grasslands, such as the San Simon valley, that have been converted to shrublands due to soil erosion. It is unclear exactly what mechanisms are responsible for the resilience seen in some areas and not in others. McAuliffe (1995) highlighted research on the Santa Rita Experimental Range in Arizona that shows sites of the mid‑Pleistocene fan remnants with strongly developed argillic horizons that have not been significantly invaded by deep‑rooted shrubs when compared to nearby younger substrates with weakly developed or absent argillic horizons. McAuliffe (1995) suggested these impermeable argillic layers restrict deep percolation of soil‑water and may favor the shallower‑rooted grasses like tobosa. These soil ‑ water ‑ vegetation relationships may apply to these grasslands in the Chihuahuan Desert.

SOURCES

**References:** Anable et al. 1992, Britton and Steuter 1983, Brown 1982a, Brown and Archer 1999, Cable 1971, Comer et al. 2003*, Cooke and Reeves 1976, Dick‑Peddie 1993, Elliott 2012, Gori and Enquist 2003, Humphrey 1963, Innes 2012, LANDFIRE 2007a, MacMahon and Wagner 1985, McAuliffe 1995, McPherson 1995, Muldavin et al. 1998a, Muldavin et al. 2000b, Muldavin et al. 2012c, NRCS 2006a, Robinett 1994, TNC 2013, Wright 1980

**Version:** 27 May 2016	**Stakeholders:** Latin America, Southeast, West

**Concept Author:** K.A. Schulz, S. Yanoff, and L. Elliott	**LeadResp:** West

CES302.736 Chihuahuan Sandy Plains Semi‑Desert Grassland

**Primary Division:** North American Warm Desert (302)

**Land Cover Class:** Herbaceous

**Spatial Scale & Pattern:** Large patch

**Required Classifiers:** Natural/Semi‑natural; Vegetated (>10% vasc.); Upland

**Diagnostic Classifiers:** Herbaceous; Sand Soil Texture; Graminoid

**National Mapping Codes:** EVT 2133; ESLF 7105; ESP 1133

**Concept Summary:** This ecological system occurs across the Chihuahuan Desert and extends into the southern Great Plains where soils have a high sand content. These dry grasslands or steppe are found on sandy plains and sandy mesatops. The graminoid layer is typically dominated or codominated by *Bouteloua eriopoda* and *Sporobolus flexuosus*. Other common species are *Aristida purpurea, Bouteloua gracilis, Hesperostipa neomexicana* (minor), *Muhlenbergia arenicola, Pleuraphis jamesii, Sporobolus airoides, Sporobolus contractus*, and *Sporobolus cryptandrus*. Typically, there are scattered desert shrubs and stem succulents present, such as *Ephedra torreyana, Ephedra trifurca, Larrea tridentata, Cylindropuntia imbricata, Prosopis glandulosa, Yucca baccata, Yucca elata, Yucca campestris*, and *Yucca torreyi*, that are characteristic of the Chihuahuan Desert. The widespread shrub *Artemisia filifolia* is also frequently present along with *Atriplex canescens*, especially in the northern extent. In Texas, non‑native species *Eragrostis lehmanniana* and *Eragrostis barrelieri* are frequently found in this system.

**Comments:** When degraded, this grassland will convert to open to dense shrublands frequently dominated by *Prosopis glandulosa* or *Artemisia filifolia* (in its northern extent where it is too cold for *Prosopis glandulosa* to be abundant) (S. Yanoff pers. comm. 2006). This degraded type is classified as Chihuahuan Stabilized Coppice Dune and Sand Flat Scrub (CES302.737).

DISTRIBUTION

**Range:** This Chihuahuan Desert ecological system extends into the southern Great Plains where soils have a high sand content.

**Divisions:** 302:C

**TNC Ecoregions:** 22:C, 24:C, 28:C

**Nations:** MX, US

**Subnations:** AZ, MXCH, NM, TX

**Map Zones:** 15:?, 24:C, 25:C, 26:C, 27:C, 34:?

**USFS Ecomap Regions:** 313B:CC, 315A:CC, 315B:CC, 315H:CC, 321A:CC, 322B:??, M313A:CC, M313B:CC

CONCEPT

**Environment:** This grassland or steppe system occurs on sandy, gently sloping, undulating piedmont slopes or plains at elevations ranging from 1065‑1525 m (3500‑5000 feet). Mean annual precipitation ranges from 20‑27 cm (8‑10.5 inches), although rainfall is highly variable ranging from 5‑50 cm (2‑20 inches). Half of the precipitation or more typically falls during summer monsoonal events. Annual frost‑free season exceeds 200 days. Spring southwesterly winds are an important factor for soil/sand distribution (Landfire 2007a). Historically the grassland type was widespread in the northern Chihuahuan Desert occupying sandy sites and dominated by *Bouteloua eriopoda* and other grasses, especially *Sporobolus flexuosus* and *Sporobolus cryptandrus*. Natural spatial variation in the vegetation of this ecological system may be governed by slight variations in soil texture. For example, dropseeds may dominate on loamy sands. Variation in the depth to a restrictive horizon, such as caliche, may also drive variation in grass cover (Landfire 2007a). Frequently, mesquite shrublands have invaded former black grama grassland sites, including the development of coppice dunes (Landfire 2007a). In Texas, this system occurs on eolian sands, sometimes as a thin veneer over surrounding formations, such as caliche, and sandstone. Soils are sandy, loamy sands, and shallow sandy loams.

**Vegetation:** The graminoid layer is typically dominated or codominated by *Bouteloua eriopoda* and *Sporobolus flexuosus*. Other common species are *Aristida purpurea, Bouteloua gracilis, Cenchrus spinifex, Hesperostipa neomexicana* (minor), *Muhlenbergia arenicola, Pleuraphis jamesii, Sporobolus airoides, Sporobolus contractus*, and *Sporobolus cryptandrus*. Typically, there are scattered desert shrubs and stem succulents present, such as *Ephedra torreyana, Ephedra trifurca, Larrea tridentata, Cylindropuntia imbricata (= Opuntia imbricata), Prosopis glandulosa, Yucca baccata, Yucca elata, Yucca campestris*, and *Yucca torreyi*, that are characteristic of the Chihuahuan Desert. The widespread shrub *Artemisia filifolia* is also frequently present along with *Atriplex canescens*, especially in the northern extent. In Texas, non‑native species *Eragrostis lehmanniana* and *Eragrostis barrelieri* are frequently found in this system.

**Dynamics:** Wind is an important disturbance agent in this grassland system. The grassland is highly sensitive to grazing and frequent drought. Fire is relatively infrequent, but can result in a significant change of dominant vegetation (Landfire 2007a). The role of fire in New Mexico's black grama‑dominated grasslands is unclear, as studies of historical records do not document fires in these grasslands (Wright 1960, Buffington and Herbal 1965). However, in contrast to other desert grasslands, fire has been shown to decrease black grama cover (Buffington and Herbel 1965, Drewa and Havstad 2000). Several other New Mexico studies have shown that black grama decreases with other disturbances, such as drought, livestock grazing, and clipping, recovering slowly, if at all, after such events (Buffington and Herbel 1965, Gibbens and Beck 1988, Gosz and Gosz 1996, Whitford et al. 1999, Drewa and Havstad 2000, Gibbens et al. 2005). While drought was a conflicting factor in many of these studies, it is important to note that studies in Arizona were also conducted during times of drought and resulted in longer recovery times, not a lack of recovery in perennial grasses (Schussman 2006a).

*Bouteloua eriopoda* is a key plant due to its dominance under pristine conditions, its high forage value and its consequent sensitivity to grazing. Shifts away from black grama dominance are thought to be due to overgrazing and/or multi‑year periods of summer or spring drought, or due to the introduction of *Prosopis glandulosa* seeds with or without grazing. With continuous heavy grazing, the proportional representation of black grama declines because it is preferred by cattle over species of *Sporobolus, Aristida*, and *Gutierrezia* (Paulsen and Ares 1962). *Sporobolus* spp. are more palatable than *Aristida* spp., so dropseeds may also decline relative to threeawns and *Gutierrezia* spp. Under climatic conditions that are not conducive to black grama reproduction, or due to the loss of components of the soil biota, demographic limitations may lead to persistent absence of black grama, even without shrub invasion. Shrub invasion is, however, very common. Loss of soil stability and/or a reduction in black grama cover may permit either the survival or establishment of mesquite seedlings due to reduced competition or fire frequency. These grasslands have been shown to trend towards shrublands over the last 100 years (Buffington and Herbel 1965, Gibbens et al. 2005). Subsequent grazing by livestock and native herbivores, competition from shrubs, erosion, and concentration of nutrients under adult shrubs eventually lead to persistent reductions of grass cover and mesquite‑dominated coppice dunes with bare or snakeweed‑dominated interdunal areas. A substantial number of studies document states and potential causes of transitions. There are multiple competing and complementary explanations for individual transitions that have not been formally tested. If the operation of these mechanisms is case‑contingent, it may be especially problematic to define the causes of transitions quantitatively (e.g., a threshold cover of black grama). Nonetheless, careful monitoring of black grama health should be a key feature of management. Overall, the high palatability of black grama during times of year when most other species are less palatable, coupled with the limited capacity of this grass to regenerate under current climatic conditions (Neilson 1986), leads to a relatively high probability of transition with poor range management.

As degradation continues, grasses are replaced by shrubs. Current species dominance is sand‑sage and broom dalea in the northern extent and mesquite and broom snakeweed in the southern extent of these grasslands. A significant proportion of the extent of these grasslands have been converted to dune shrubland with mesquite dominance and soil redistribution by wind erosion in the southern portion. There is a lack of research regarding thresholds in response to disturbance and restoration techniques (Landfire 2007a).

SOURCES

**References:** Anable et al. 1992, Buffington and Herbel 1965, Cable 1971, Comer et al. 2003*, Dick‑Peddie 1993, Drewa and Havstad 2000, Elliott 2012, Gibbens and Beck 1988, Gibbens et al. 2005, Gori and Enquist 2003, Gosz and Gosz 1996, LANDFIRE 2007a, Muldavin et al. 2000b, Muldavin et al. 2002a, Muldavin et al. 2012c, Neilson 1986, Paulsen and Ares 1962, Schussman 2006a, Shiflet 1994, TNC 2013, White and Sutter 1999b, Whitford et al. 1999, Wright 1960, Wright 1980, Yanoff pers. comm.

**Version:** 27 May 2016	**Stakeholders:** Latin America, Southeast, West

**Concept Author:** K.A. Schulz	**LeadResp:** West

CES302.746 Chihuahuan‑Sonoran Desert Bottomland and Swale Grassland

**Primary Division:** North American Warm Desert (302)

**Land Cover Class:** Mixed Upland and Wetland

**Spatial Scale & Pattern:** Small patch

**Required Classifiers:** Natural/Semi‑natural; Vegetated (>10% vasc.); Wetland

**Diagnostic Classifiers:** Lowland [Lowland]; Herbaceous; Swale; Toeslope/Valley Bottom; Depressional

**National Mapping Codes:** EVT 2504; ESLF 9411; ESP 1504

**Concept Summary:** This ecological system is named based on the regions (Chihuahuan and Sonoran deserts) where it is best developed and occupies significant areas, however, it does occur well outside these regions, at least as far north and east as the Rolling Plains of Texas. The system occurs in relatively small depressions or swales and along drainages throughout the northern and central Chihuahuan Desert and adjacent Sky Islands and Sonoran Desert, as well as limited areas of the southern Great Plains on broad mesas, plains and valley bottoms that receive runoff from adjacent areas. Occupying low topographic positions, these sites generally have deep, fine‑textured soils that are neutral to slightly or moderately saline/alkaline. During summer rainfall events, ponding is common. Vegetation is typically dominated by *Sporobolus airoides, Sporobolus wrightii, Pleuraphis mutica* (tobosa swales), or other mesic graminoids such as *Pascopyrum smithii* or *Panicum obtusum*. With tobosa swales, sand‑adapted species such as *Yucca elata* may grow at the swale's edge in the deep sandy alluvium that is deposited there from upland slopes. *Sporobolus airoides* and *Sporobolus wrightii* are more common in alkaline soils and along drainages. Other grass species may be present, but these mesic species are diagnostic. Scattered shrubs such as *Atriplex canescens, Prosopis glandulosa, Ericameria nauseosa, Fallugia paradoxa, Krascheninnikovia lanata*, or *Rhus microphylla* may be present.

**Comments:** NRCS Ecological Site Description MLRA 42 SD‑2 Bottomland Ecological Site (NRCS 2006) describes this system on the Jornada Experimental Range with State‑and‑Transition Model showing shifts in species composition with land use.

This bottomland/depressional wetland system can be similar to the upland Chihuahuan Loamy Plains Desert Grassland (CES302.061) but is restricted to moist depressions and intermittently flooded drainage terraces and adjacent flats. Alkali sacaton (*Sporobolus airoides*) is often associated with more alkaline (to gypsic), poorly drained areas and giant sacaton (*Sporobolus wrightii*) with less alkaline better drained areas. *Distichlis spicata, Allenrolfea occidentalis*, and *Suaeda* spp. are characteristic of more saline and alkaline sites.

DISTRIBUTION

**Range:** This system is found in the central and northern Chihuahuan Desert and adjacent Sky Islands and Sonoran Desert, as well as limited areas of the southern Great Plains.

**Divisions:** 302:C, 303:C

**TNC Ecoregions:** 22:C, 23:C, 24:C, 28:C

**Nations:** MX, US

**Subnations:** AZ, MXCH, MXCO, MXSO, NM, TX

**Map Zones:** 14:C, 23:C, 24:C, 25:C, 26:P, 27:C, 34:?, 35:?

**USFS Ecomap Regions:** 313C:PP, 315A:CC, 321A:CC, 322B:CC, M313A:CC, M313B:CC

CONCEPT

**Environment:** This ecological system occurs in relatively small depressions or swales and along drainages on broad mesas, plains and valley bottoms that receive runoff from adjacent areas. These sites occupy low topographic positions and generally have deep, fine‑textured soils that are neutral to slightly or moderately saline/alkaline. The system typically occurs in local topographic lows that may be associated with drainages, or may represent swales or basins, but typically receives run‑off from the surrounding landscape. Soils are generally clayey, and in some cases the shrink‑swell characteristics of the soil may limit the development of woody species. Stands of the system typically occur on Quaternary alluvium, but may be local in nature and mapped within various geological formations. It is generally found on local topographic lows that may be associated with a drainage or may occur as basins or swales. Soils are typically tight ones, and Clay Flat Ecological Sites are typical.

**Vegetation:** The vegetation of this grassland system is typically dominated by *Sporobolus airoides, Sporobolus wrightii, Pleuraphis mutica* (in tobosa swales), or other mesic graminoids such as *Pascopyrum smithii* or *Panicum obtusum*. In tobosa swales, sand‑adapted species such as *Yucca elata* may grow at the swale's edge in the deep sandy alluvium that is deposited there from upland slopes. *Sporobolus airoides* and *Sporobolus wrightii* are more common in alkaline soils and along drainages. Other grass species may be present, but these mesic species are diagnostic. *Prosopis glandulosa* may be present and, in some cases, may develop into a significant canopy. Other scattered shrubs such as *Atriplex canescens, Ericameria nauseosa, Fallugia paradoxa, Krascheninnikovia lanata*, or *Rhus microphylla* may be present. *Sporobolus airoides* is often associated with more alkaline (to gypsic), poorly drained areas and *Sporobolus wrightii* with less alkaline better drained areas. *Distichlis spicata, Allenrolfea occidentalis*, and *Suaeda* spp. are characteristic of more saline and alkaline sites. In Texas, *Pleuraphis mutica* is generally the clear dominant (Elliott 2013).

**Dynamics:** [from M087] During the last century, the area occupied by this desert grassland and steppe decreased through conversion of desert grasslands as a result of drought, overgrazing and *Prosopis glandulosa* seed dispersion by livestock, and/or decreases in fire frequency (Buffington and Herbel 1965, Brown and Archer 1987). It is believed that mesquite formerly occurred in relatively minor amounts and was largely confined to drainages until cattle distributed seed upland into desert grasslands (Brown and Archer 1987, 1989). Shrublands dominated by *Prosopis* spp. have replaced large areas of desert grasslands, especially those formerly dominated by *Bouteloua eriopoda*, in Trans‑Pecos Texas, southern New Mexico and southeastern Arizona (York and Dick‑Peddie 1969, Hennessy et al. 1983). Studies on the Jornada Experimental Range suggest that combinations of drought, overgrazing by livestock, wind and water erosion, seed dispersal by livestock, fire suppression, shifting dunes, and changes in the seasonal distribution of precipitation have caused this recent, dramatic shift in vegetation physiognomy (Buffington and Herbel 1965, Herbel et al. 1972, Humphrey 1974, McLaughlin and Bowers 1982, Gibbens et al. 1983, Hennessy et al. 1983, Schlesinger et al. 1990, McPherson 1995).

Impermeable caliche and argillic horizons are common on these sites. These layers restrict deep percolation of soil water and may favor the shallower rooted grasses over more deeply rooted shrubs such as *Larrea tridentata* and *Prosopis* spp. (McAuliffe 1995). *Pleuraphis mutica* is relatively tolerant of livestock grazing. In west‑central Arizona, livestock have nearly eliminated all native grasses except *Pleuraphis mutica* from semi‑desert grassland (Brown 1982a). Stands codominated by *Scleropogon brevifolius* are characteristic of sites with past heavy grazing by livestock (Whitfield and Anderson 1938).

<br />In gypsophilous grassland *Sporobolus nealleyi* is dominant with *Tiquilia hispidissima* and *Opuntia polyacantha* on crusted gypsum ridges, but not on unstable gypsum dunes (Burgess and Northington 1977). The eolian processes and sand substrate on gypsum dunes may be as important ecologically as the chemical properties of the gypsum parent material as seen by presence of sand‑loving plant species such as *Achnatherum hymenoides, Andropogon hallii, Artemisia filifolia, Muhlenbergia pungens*, and *Psorothamnus scoparius* on gypsum dunes.

SOURCES

**References:** Brown 1982a, Comer et al. 2003*, Dick‑Peddie 1993, Elliott 2011, Elliott 2012, MacMahon and Wagner 1985, Muldavin et al. 1998a, Muldavin et al. 1998d, Muldavin et al. 2000b, NRCS 2006a, Shiflet 1994

**Version:** 24 Feb 2011	**Stakeholders:** Latin America, Southeast, West

**Concept Author:** K.A. Schulz	**LeadResp:** West

M088. Mojave‑Sonoran Semi‑Desert Scrub

CES302.744 North American Warm Desert Active and Stabilized Dune

**Primary Division:** North American Warm Desert (302)

**Land Cover Class:** Barren

**Spatial Scale & Pattern:** Large patch

**Required Classifiers:** Natural/Semi‑natural; Unvegetated (<10% vasc.); Upland

**Diagnostic Classifiers:** Dune (Landform); Dune field; Dune (Substrate); Temperate [Temperate Xeric]; Sand Soil Texture; W‑Landscape/High Intensity

**Concept Summary:** This ecological system occurs across the warm deserts of North America and is composed of unvegetated to sparsely vegetated (generally <10% plant cover) active dunes and sandsheets derived from quartz or gypsum sands. Common vegetation includes *Ambrosia dumosa, Abronia villosa, Artemisia filifolia, Atriplex canescens, Eriogonum deserticola, Larrea tridentata, Pleuraphis rigida, Poliomintha* spp., *Prosopis* spp., *Psorothamnus* spp., *Rhus microphylla*, and *Sporobolus flexuosus*. Dune "blowouts" and subsequent stabilization through succession are characteristic processes. Species composition shifts across the range of this system. Texas examples are characterized by species such as *Amsonia tomentosa var. stenophylla, Aristida purpurea, Artemisia filifolia, Bouteloua eriopoda, Croton dioicus, Dimorphocarpa wislizeni, Eriogonum annuum, Helianthus petiolaris, Heliotropium convolvulaceum, Ipomopsis wrightii, Palafoxia sphacelata, Proboscidea althaeifolia, Prosopis glandulosa, Psorothamnus scoparius, Schizachyrium scoparium, Sporobolus contractus, Sporobolus cryptandrus, Sporobolus flexuosus, Sporobolus giganteus, Tripterocalyx carneus*, and *Yucca elata*.

**Comments:** In the Texas ecological systems map, sparsely vegetated gypsum dunes are included in Chihuahuan Gypsophilous Grassland and Steppe (CES302.732) and not here. Further investigation is needed to determine the correct classification of these sparsely vegetated gypsum dunes.

DISTRIBUTION

**Range:** This system occurs across the warm deserts of North America. In Texas, it is found on deep sands adjacent to the Salt Basin west of the Guadalupe Mountains, and the Hueco Basin along the Rio Grande.

**Divisions:** 302:C

**TNC Ecoregions:** 17:C, 22:C, 23:C, 24:C

**Nations:** MX, US

**Subnations:** AZ, CA, MXBC, MXBS, MXCH, MXSO, NM, NV

**Map Zones:** 13:C, 14:C, 15:?, 24:?, 25:C, 26:C, 27:C

**USFS Ecomap Regions:** 313B:??, 315A:CC, 315B:CC, 315H:CP, 321A:CC, 322A:CC, 322B:CC, 322C:CC, 331B:CC, M313A:CC, M313B:CC

CONCEPT

**Environment:** This ecological system occurs across the warm deserts of North America and is a mosaic of barren active dunes and partially stabilized and stabilized dunes and sandsheets (vegetated). The climate is arid and hot with low annual precipitation ranging from 25 mm in the Gran Desierto to 60‑90 mm at Algodones Dunes, and 205 mm at White Sands National Monument (Felger 1980, Bowers 1982). Summer temperatures usually exceed 40°C. Below freezing temperatures may occur in northern transition zones, but are rare events. The system is defined by the presence of migrating dunes or, where the dunes are entirely anchored or stabilized, evidence that the substrate is eolian and not residual and that the substrate is likely to become actively migrating again with disturbance or increased aridity. There are some smaller, active and partially vegetated dunes along some of the larger washes and on sides of playas and basins (where sand is blown out of a wash or basin and forms dunes) and some larger dunes, but many of the larger dunes were formed during the Pleistocene when sand was blown from large drying lake basins into dunes. Prominent dune systems are the Kelso, Corn Creek and Death Valley dunes in the Mojave Desert; Algodones, Salton Sea, Mohawk, Yuma and the vast Gran Desierto dunes in the Sonoran Desert; and White Sands, Guadalupe Mountains, Samalayuca, Monahans Sandhills and Cuarto Cienegas Dunes in the Chihuahuan Desert. Substrates are usually deep, eolian quartz sand with salinity varying depending on substrate. In Texas, this system occurs on Quaternary eolian sand deposits associated with the Hueco Bolson and the Salt Basin on Sand Hills and Deep Sand Ecological Sites. Several dunefields are composed of pure gypsum in the Chihuahuan Desert. Adjacent systems include various desert scrub systems forming the regional matrix such as Sonora‑Mojave Creosotebush‑White Bursage Desert Scrub (CES302.756), or Chihuahuan Creosotebush Desert Scrub (CES302.731), North American Warm Desert Playa (CES302.751) and rarely North American Warm Desert Cienega (CES302.747) (Cuarto Cienagas wetland). The environmental description is based on several other references, including Powell and Turner (1974), Felger (1980), Reid (1980), Bowers (1982, 1984), MacMahon (1988), Muldavin et al. (1994b), Holland and Keil (1995), Reid et al. (1999), Comer et al. (2003), Thomas et al. (2004), Keeler‑Wolf (2007), Schoenherr and Burk (2007), and Sawyer et al. (2009).

**Vegetation:** This ecological system is characterized by barren and sparsely vegetated active dunes and sandsheets (generally < 10% total plant cover) and small patches of vegetation on stabilized dunes and sandsheets. Most species occupying these environments are adapted to shifting, coarse‑textured substrates and form patchy or open grasslands, shrub‑steppe or scrublands. Within a dunefield some dune species are typical of active sand, such as *Croton wigginsii, Eriogonum deserticola, Palafoxia arida var. gigantea, Panicum urvilleanum*, and *Petalonyx thurberi*, while others are common on stabilized dunes, such as *Pleuraphis rigida, Psorothamnus emoryi, Tiquilia palmeri*, and scattered *Larrea tridentata*. Species composition also varies by region and substrate with the Chihuahuan Desert different from the Sonoran and Mojave deserts and gypsum dunes containing endemic species. Characteristic Chihuahuan Desert species are *Ephedra torreyana, Sporobolus flexuosus*, and *Yucca elata*, and typical species of the Mojave and Sonoran deserts include *Astragalus lentiginosus, Croton wigginsii, Dicoria canescens, Eriogonum deserticola, Palafoxia linearis, Penstemon thurberi, Pleuraphis rigida, Psorothamnus polydenius*, and *Tiquilia plicata*. Characteristic gypsum dune species are *Poliomintha incana, Bouteloua breviseta, Rhus microphylla*, and *Tiquilia hispidissima*. There are also a few wide‑ranging species such as *Atriplex canescens, Artemisia filifolia*, and *Prosopis glandulosa*.

**Dynamics:** The major dynamic process is sand movement. Dune "blowouts" and subsequent stabilization through succession are characteristic processes. Plant species that occur in this system are at risk of burial and excavation by the wind and have evolved adaptions such as rapid growth of stems and rapid elongation of radicals (Bowers 1982). Some plants have extensive lateral roots that anchor the plants and stabilize sand. Symbiotic associations with mycorrhizal fungi or nitrogen‑fixing bacteria are common with many psammophytic plants (Bowers 1982). Salinity and soil moisture are also driving ecological variables that determine species composition. Rapid infiltration of precipitation in dunes reduces evaporation making dunes relatively mesic environments for plants in desert and semi‑desert environments (Bowers 1982).

SOURCES

**References:** Bowers 1982, Bowers 1984, Comer et al. 2003*, Elliott 2012, Elliott 2013, Felger 1980, Holland and Keil 1995, Keeler‑Wolf 2007, MacMahon 1988, Muldavin et al. 1994b, NatureServe Explorer 2011, Powell and Turner 1974, Reid 1980, Reid et al. 1999, Sawyer et al. 2009, Schoenherr and Burk 2007, Thomas et al. 2004

**Version:** 02 Oct 2014	**Stakeholders:** Latin America, Southeast, West

**Concept Author:** K.A. Schulz	**LeadResp:** West

CES302.756 Sonora‑Mojave Creosotebush‑White Bursage Desert Scrub

**Primary Division:** North American Warm Desert (302)

**Land Cover Class:** Shrubland

**Spatial Scale & Pattern:** Matrix

**Required Classifiers:** Natural/Semi‑natural; Vegetated (>10% vasc.); Upland

**Diagnostic Classifiers:** Lowland [Lowland]; Shrubland (Shrub‑dominated); Tropical/Subtropical [Tropical Xeric]; Temperate [Temperate Xeric]; Aridic; Xeromorphic Shrub

**National Mapping Codes:** EVT 2087; ESLF 5264; ESP 1087

**Concept Summary:** This ecological system forms a desert scrub matrix blanketing broad valleys, lower bajadas, plains and low hills in the Mojave and lower Sonoran deserts. This desert scrub is characterized by a sparse to moderately dense layer (2‑50% cover) of xeromorphic microphyllous and broad‑leaved shrubs. *Larrea tridentata* and *Ambrosia dumosa* are typically dominants, but many different shrubs, dwarf‑shrubs, and cacti may codominate or form typically sparse understories. Associated species may include *Atriplex canescens, Atriplex hymenelytra, Encelia farinosa, Ephedra nevadensis, Fouquieria splendens, Lycium andersonii*, and *Opuntia basilaris*. The herbaceous layer is typically sparse but may have abundant seasonal ephemerals. Herbaceous species such as *Chamaesyce* spp., *Eriogonum inflatum, Dasyochloa pulchella, Aristida* spp., *Cryptantha* spp., *Nama* spp., and *Phacelia* spp. are common. This system can often appear as very open sparse vegetation, with the mostly barren ground surface being the predominant feature.

DISTRIBUTION

**Range:** This system occupies broad valleys, lower bajadas, plains and low hills in the Mojave and lower Sonoran deserts.

**Divisions:** 302:C

**TNC Ecoregions:** 17:C, 23:C

**Nations:** MX, US

**Subnations:** AZ, CA, MXBC, MXSO, NV, UT

**Map Zones:** 4:C, 12:C, 13:C, 14:C, 15:C, 16:?, 17:P, 23:C, 24:?, 25:C

**USFS Ecomap Regions:** 313A:CC, 313C:CC, 321A:CC, 322A:CC, 322B:CC, 322C:CC, 341F:CC, M261E:PP, M341A:PP

CONCEPT

**Environment:** *Climate:* Climate is semi‑arid to arid with hot summers and warm to cool winters depending on latitude and elevation.

*Physiography/landform:* This ecological system forms the vegetation matrix in broad valleys, lower bajadas, plains, flats and low hills in the lower Sonoran (Colorado) and Mojave deserts extending into the southeastern Great Basin where it forms the vegetation matrix. Other habitats include minor washes and rills, alluvial fans, and upland slopes. Elevation ranges from ‑75 to 1200 m. Adjacent ecological systems include Mojave Mid‑Elevation Mixed Desert Scrub (CES302.742) above and Inter‑Mountain Basins Playa (CES304.786) below.

*Soil/substrate/hydrology:* Substrates are typically well‑drained, sandy soils derived from colluvium or alluvium, and are often calcareous with a caliche hardpan and/or a pavement surface that is derived from limestone and dolomite (Turner 1982b, Sawyer et al. 2009).

The environmental description is based on several references, including Beatley (1976), Brown (1982a), Turner (1982b), MacMahon (1988), Holland and Keil (1995), Marshall (1995), Reid et al. (1999), Barbour et al. (2007a), Keeler‑Wolf (2007), Schoenherr and Burk (2007), and Sawyer et al. (2009).

**Vegetation:** This desert scrub system occurs as open to intermittent vegetation cover, with the mostly barren ground surface being the predominant feature (Sawyer et al. 2009). It is characterized by a sparse to moderately dense layer (2‑50% cover) of xeromorphic microphyllous and broad‑leaved shrubs that is typically dominated or codominated by *Larrea tridentata* usually with *Ambrosia dumosa*. However, several other shrubs may dominate or codominate this system, including *Atriplex* spp., *Ephedra viridis, Ephedra* spp., *Grayia spinosa*, or *Lycium* spp. Low‑elevation stands typically have low cover and diversity, whereas in higher‑elevation stands, many different shrubs, dwarf‑shrubs, and cacti may be present to codominant or form sparse understories. Associated species may include *Atriplex canescens, Atriplex hymenelytra, Atriplex polycarpa, Croton californicus, Dalea* spp., *Echinocactus polycephalus, Encelia* spp., *Ephedra funerea, Ephedra nevadensis, Lycium andersonii, Opuntia basilaris, Krameria grayi, Krameria erecta, Psorothamnus arborescens, Psorothamnus fremontii, Salazaria mexicana, Senna armata*, and *Viguiera parishii*. Some common disturbance‑related species include *Acamptopappus sphaerocephalus, Bebbia juncea, Cylindropuntia acanthocarpa (= Opuntia acanthocarpa), Ericameria teretifolia, Grayia spinosa*, or *Hymenoclea salsola* (Sawyer et al. 2009). If *Encelia farinosa* or *Yucca schidigera* is present, cover is generally low (< 1‑2% cover). Occasional emergent *Fouquieria splendens* or *Yucca brevifolia* may be present with low cover. The herbaceous layer is typically sparse and intermittent, but may be seasonally abundant with ephemerals. Herbaceous species, such as *Chamaesyce* spp., *Eriogonum inflatum, Dasyochloa pulchella, Aristida* spp., *Cryptantha* spp., *Nama* spp., and *Phacelia* spp., are common. The vegetation description is based on several references, including Beatley (1976), Brown (1982a), Turner (1982), MacMahon (1988), Holland and Keil (1995), Marshall (1995), Reid et al. (1999), Barbour et al. (2007), Keeler‑Wolf (2007), Schoenherr and Burk (2007), and Sawyer et al. (2009).

**Dynamics:** This system covers vast areas of sandy and gravelly alluvial fans and bajadas and rocky slopes in the northwestern Sonoran, Mojave and Colorado deserts (Keeler‑Wolf 2007, Sawyer et al. 2009). The dominant shrub, *Larrea tridentata*, is very long‑lived, with clones living >10,000 years (Keeler‑Wolf 2007) and is very tolerant of drought and high temperatures. It is highly adapted to minimized evapotranspiration both daily and seasonally using stomatal regulation, resinous leaves, and a leaf structure and habit to minimize self‑shading and maximize photosynthesis during favorable growing periods (Hamerlynck et al. 2002, Ogle and Reynolds 2002). It may die back during extreme drought but can sprout from the base (Meinzer et al. 1990). It has low recruitment and is slow to re‑establish from seed (Keeler‑Wolf 2007). *Larrea tridentata* is poorly adapted to fire because of its highly flammable, resinous leaves that burn hot such that fires usually kill the shrub. If the shrub is not killed, it has limited sprouting ability after low‑intensity fires (Humphrey 1974, Brown and Minnich 1986, Marshall 1995, Paysen et al. 2000). McLaughlin and Bowers (1982) reported that burned individuals surviving a fire regained their former size in five years.

The main codominant shrub, *Ambrosia dumosa*, is short‑lived with a relatively shallow root system, and tends to dominate sandy and rocky sites. It can quickly establish after disturbance or drought (Vasek 1980). Post fire, it also has a limited ability to sprout, but can readily re‑establish from seed (Sawyer et al. 2009).

Fire‑return interval is long for this open‑canopied shrub system with typically discontinuous fuels (Sawyer et al. 2009). Fire occurs under extreme conditions often following a wet year when more fine fuels are available. When it burns, fires are usually of high intensity and moderate severity (Sawyer et al. 2009). Fires in historic creosote‑bursage stands were thought to be infrequent except along the margins of the ecological system where it mixed with shrub‑steppe containing greater grass fuel loading. Although bunchgrass species can fill in some of the interspaces between shrubs and provide fine fuels, their distribution is generally patchy and rarely provides fuel continuity sufficient to carry fire (Brooks et al. 2007). Periodic drought is occasionally sufficient to thin grass and shrub cover.

LANDFIRE developed a VDDT model for this system which has two classes (LANDFIRE 2007a, BpS 1310870):

A) Early Development 1 Open (15% of type in this stage): Dominant cover is herbaceous, 5‑10% canopy cover. Creosotebush scrub is characterized by low cover 5‑10%. Little disturbance was considered in class A, except for replacement fire every 300 years on average. Historical condition where invasive annual grasses are absent, the fire‑return interval is virtually nonexistent except for areas near the base of mountains experiencing locally higher rainfall and fine fuel buildup from native annuals. After 100 years, class A transitions to class B.

B) Late Development 1 Closed (shrub‑dominated ‑ 85% of type in this stage): Greater than 15% shrub cover and 20‑40% grass and forb cover; associated with more productive soils. Less fine fuel is associated with this community, therefore the FRIs for replacement fire and mixed‑severity fire is 650 years (min‑max: 300‑1000 years). Wind/weather stress also affected this community on average every 80 years, but did not cause a transition to class A.

<br />LANDFIRE modelers emphasized that pre‑settlement fire conditions in warm desert plant communities are not known. However, it is thought that fires in creosotebush scrub were absent to rare events in pre‑settlement desert habitats, because fine fuels from winter annual plants were probably sparse, only occurring in large amounts during the spring following exceptionally wet winters (LANDFIRE 2007a).

SOURCES

**References:** Barbour and Major 1977, Barbour and Major 1988, Barbour et al. 2007a, Beatley 1976, Belnap 2001, Belnap et al. 2001, Brooks et al. 2007, Brown 1982a, Brown and Minnich 1986, Cane et al. 2000, Comer et al. 2003*, Evans and Belnap 1999, Finch 2012, Garfin et al. 2013, Hamerlynck et al. 2002, Holland and Keil 1995, Humphrey 1974, Keeler‑Wolf 2007, LANDFIRE 2007a, MacMahon 1988, Marshall 1995a, McLaughlin and Bowers 1982, Meinzer et al. 1990, Ogle and Reynolds 2002, Paysen et al. 2000, Reid et al. 1999, Rosentreter and Belnap 2003, Sawyer and Keeler‑Wolf 1995, Sawyer et al. 2009, Schoenherr and Burk 2007, Shiflet 1994, Thomas et al. 2004, Turner 1982b, Vasek 1980

**Version:** 27 Jan 2016	**Stakeholders:** Latin America, West

**Concept Author:** K.A. Schulz	**LeadResp:** West

CES302.760 Sonoran Granite Outcrop Desert Scrub

**Primary Division:** North American Warm Desert (302)

**Land Cover Class:** Shrubland

**Spatial Scale & Pattern:** Large patch

**Required Classifiers:** Natural/Semi‑natural; Vegetated (>10% vasc.); Upland

**Diagnostic Classifiers:** Shrubland (Shrub‑dominated); Granitic Rock; Tropical/Subtropical [Tropical Xeric]; Temperate [Temperate Xeric]; Aridic

**National Mapping Codes:** EVT 2090; ESLF 5267; ESP 1090

**Concept Summary:** This ecological system occurs in foothills and mountains of Sonora, Mexico, and extends north across the border into southern Arizona. It is found on low‑ to mid‑elevation granitic outcrops. Tropical genera of *Jatropha* and *Bursera* become codominants in dense to sparse vegetation transitioning upslope from Sonoran Paloverde‑Mixed Cacti Desert Scrub (CES302.761). Diagnostic species are *Bursera microphylla, Jatropha cuneata, Nolina bigelovii, Parkinsonia microphylla*, or *Rhus kearneyi*.

**Comments:** This ecological system is likely to be a northern expression of a more widely distributed desert scrub system of Sonora, Mexico, where it may not be restricted to rock outcrops or sites, such as in Arizona.

DISTRIBUTION

**Range:** Occurs in foothills and mountains of Sonora, Mexico, and extends north across the border into southern Arizona.

**Divisions:** 302:C

**TNC Ecoregions:** 23:C

**Nations:** MX, US

**Subnations:** AZ, CA, MXBC, MXSO, NV

**Map Zones:** 14:C

**USFS Ecomap Regions:** 322B:CC, 322C:CC

CONCEPT

**Environment:** [from M088] This warm‑temperate to subtropical, semi‑desert type occurs in the southwestern U.S. and adjacent Sonora and Baja California, Mexico. It forms the vegetation matrix in broad valleys, lower bajadas, plains and low hills in the Mojave, western Sonoran and Lower Colorado deserts. Elevation ranges from ‑75 to 1200 m. Sites are gentle to moderately sloping. Substrates are typically well‑drained, sandy soils derived from colluvium or alluvium, and are often calcareous with a caliche hardpan and/or a pavement surface. Precipitation is markedly unimodal with most falling in the winter months associated with winter storm tracks reaching the desert from the Pacific Ocean. Stands extend north into the broad transition with the Great Basin and at higher elevations on desert mountains above *Larrea tridentata ‑ Ambrosia dumosa* desertscrub and below the lower montane woodlands (700‑1800 m elevation) that occurs in the eastern and central Mojave Desert. Stands in the Arizona Sonoran Desert occur on lower slopes of mountains, foothills, hillsides, mesas, upper bajadas, and less commonly in valleys and plains in southern Arizona and extreme southeastern California. Elevations range from 150‑1070 m (Shreve and Wiggins 1964). Climate is semi‑arid. Summers are hot and winters rarely have freezing temperatures. Freezing winter temperatures limit the elevational and northern extent of these stands. Annual precipitation has bimodal distribution with about half of the rain falling during July to September and a third falling from December to March. Farther west, the proportion of summer precipitation decreases until there is not enough summer moisture to sustain *Carnegiea gigantea* (Barbour and Major 1977). Stands in the subtropical central Gulf of California coast and adjacent portions of the lower Colorado River valley region of the Sonoran Desert occur on gentle to steep, rocky sites. It extends north into the extreme southwestern U.S. and northern Sonora.

At Organ Pipe National Monument, stands typically occur on southerly aspects between 550 and 765 m (1800‑2500 feet) elevation. In general, sites have gentle to steep slopes. Sites in northern Baja and southern California occur on isolated maritime coastal bluffs and terraces. Sites in the Vizcaino Region of central Baja California reach several kilometers inland. These areas are frost‑free and receive the least annual precipitation of the California and Baja California coastal shrublands, most of which falls in winter. Climate is extremely arid with mean annual precipitation of less than 100 mm, which occurs mostly in the summer‑early fall season (monsoon). Precipitation is augmented by summer fog drip. Sonoran stands are extremely arid with mean annual precipitation of less than 100 mm, which occurs mostly in the summer‑early fall season (monsoon). Extended drought is common which favors plants with water storage (Turner and Brown 1982). Semi‑desert vegetated and sparsely vegetated sandsheets and dunes that are stabilized or partially stabilized are included in this macrogroup. They occur as small to large patches or as a complex of active and stabilized dunes. These sand deposits often form on the leesides of desert playas and basins that serve as a source for the sand. Substrates are variable, but typically shallow, well‑drained, rocky or gravelly, coarse‑textured soils derived from colluvium or alluvium, except for the sand deposit vegetation included in the macrogroup, which is eolian. Parent material is usually gravelly alluvium and colluvium, derived from basalt and other igneous or metamorphic rocks.

**Dynamics:** [from M088] This type occurs in warm to subtropical semi‑arid regions. Most characteristic species are frost‑sensitive as only vegetation in the Mojave Desert or at high elevation or in the northern extent of the Sonoran Desert experience frost or extended freezing temperatures.

SOURCES

**References:** Barbour and Major 1988, Brown 1982a, Comer et al. 2003*, MacMahon 1988, Thomas et al. 2004

**Version:** 20 Feb 2003	**Stakeholders:** Latin America, West

**Concept Author:** K.A. Schulz	**LeadResp:** West

CES302.035 Sonoran Mid‑Elevation Desert Scrub

**Primary Division:** North American Warm Desert (302)

**Land Cover Class:** Shrubland

**Spatial Scale & Pattern:** Large patch

**Required Classifiers:** Natural/Semi‑natural; Vegetated (>10% vasc.); Upland

**Diagnostic Classifiers:** Montane [Lower Montane]; Shrubland (Shrub‑dominated); Alkaline Soil

**National Mapping Codes:** EVT 2091; ESLF 5268; ESP 1091

**Concept Summary:** This transitional desert scrub system occurs along the northern edge of the Sonoran Desert in an elevational band along the lower slopes of the Mogollon Rim/Central Highlands region between 750 and 1300 m. Stands occur in the Bradshaw, Hualapai, and Superstition mountains, among other desert ranges, and are found above Sonoran Paloverde‑Mixed Cacti Desert Scrub (CES302.761) and below Mogollon Chaparral (CES302.741). Sites range from a narrow strip on steep slopes to very broad areas such as the Verde Valley. Climate is too dry for chaparral species to be abundant, and freezing temperatures during winter are too frequent and prolonged for many of the frost‑sensitive species that are characteristic of Sonoran Paloverde‑Mixed Cacti Desert Scrub (CES302.761), such as *Carnegiea gigantea, Parkinsonia microphylla, Prosopis* spp., *Olneya tesota, Ferocactus* sp., and *Cylindropuntia bigelovii*. Substrates are generally rocky soils derived from parent materials such as limestone, granitic rocks or rhyolite. The vegetation is typically composed of an open shrub layer of *Larrea tridentata, Ericameria linearifolia*, or *Eriogonum fasciculatum* with taller shrub such as *Canotia holacantha* (limestone or granite) or *Simmondsia chinensis* (rhyolite). The herbaceous layer is generally sparse.

**Comments:** Includes Brown's (1982) Jojoba‑Mixed Scrub and Creosotebush‑Crucifixion‑thorn Series.

DISTRIBUTION

**Range:** This system occurs along the northern edge of the Sonoran Desert in an elevational band along the lower slopes of the Mogollon Rim/Central Highlands region between 750 and 1300 m.

**Divisions:** 302:C, 306:P

**TNC Ecoregions:** 22:P, 23:C

**Nations:** MX, US

**Subnations:** AZ, MXSO

**Map Zones:** 13:C, 14:C, 15:C, 25:C

**USFS Ecomap Regions:** 313C:CC, 321A:CC, 322A:CC, 322B:CC, 322C:CP, M313A:CC

CONCEPT

**Environment:** This desert scrub system occurs along the northern edge of the Sonoran Desert and forms an elevational band along the lower slopes of the Mogollon Rim/Central Highlands region between 750 and 1300 m. This system ranges from a narrow strip on steep slopes to very broad areas such as the Verde Valley. Stands also occur in the Bradshaw, Hualapai, and Superstition mountains, among other desert ranges. It is uncommon in the Mojave Desert. This system occurs above Sonoran Paloverde‑Mixed Cacti Desert Scrub (CES302.761) and below Mogollon Chaparral (CES302.741) where climate is too dry for chaparral species to be abundant, and freezing temperatures during winter are too frequent and prolonged for many of the frost‑sensitive species that are characteristic of Sonoran Paloverde‑Mixed Cacti Desert Scrub (CES302.761). Substrates are generally rocky soils derived from parent materials such as limestone, granitic rocks or rhyolite. The environmental description is based on several references, including Brown (1982), Reid et al. (1999), NatureServe Explorer (2011), and Sawyer et al. (2009).

**Vegetation:** The vegetation is typically composed of an open shrub layer of *Larrea tridentata, Ambrosia deltoidea, Ericameria linearifolia*, or *Eriogonum fasciculatum* with taller shrubs such as *Canotia holacantha* (limestone or granite) or *Simmondsia chinensis* (rhyolite). Eastern Sonoran stands may have *Acacia neovernicosa* or *Parthenium incanum* preset that are more typical of Chihuahuan desert scrub. The herbaceous layer is generally sparse. The floristic description is based on several references including Brown (1982), Reid et al. (1999), and Sawyer et al. (2009).

**Dynamics:** Climate is the main driving ecological variable characterizing this system. Sites are too dry for chaparral species to be abundant, and freezing temperatures during winter are too frequent and prolonged for many of the frost‑sensitive species that are characteristic of Sonoran Paloverde‑Mixed Cacti Desert Scrub (CES302.761), such as *Carnegiea gigantea, Parkinsonia microphylla, Prosopis* spp., *Olneya tesota, Ferocactus* sp., and *Cylindropuntia bigelovii*. Fire appears to be infrequent by the presence of the fire‑sensitive dominant shrub *Larrea tridentata*, which is very long‑lived with clones living >10,000 years (Keeler‑Wolf 2007) and very tolerant of drought and high temperatures with small, evergreen, resinous (highly flammable) leaves reducing evapotranspiration (Hamerlynck et al. 2002). It may die‑back during extreme drought, but can sprout from the base (Meinzer et al. 1990). It has low recruitment and is slow to re‑establish from seed (Keeler‑Wolf 2007).

*Simmondsia chinensis* is important forage for wildlife species such as mule deer, jackrabbits, desert bighorn sheep (Gentry 1958, Miller and Gaud 1989), and may provide the best browse available within its range (Matthews 1994).

SOURCES

**References:** Brooks 1978, Brown 1982a, Comer et al. 2003*, Gentry 1958, Hamerlynck et al. 2002, Keeler‑Wolf 2007, Marshall 1995a, Matthews 1994, Meinzer et al. 1990, Miller and Gaud 1989, NatureServe Explorer 2011, Reid et al. 1999, Roundy and Dobrenz 1989, Sawyer et al. 2009

**Version:** 02 Apr 2014	**Stakeholders:** Latin America, West

**Concept Author:** K. Pohs, K. Schulz, P. Comer	**LeadResp:** West

CES302.761 Sonoran Paloverde‑Mixed Cacti Desert Scrub

**Primary Division:** North American Warm Desert (302)

**Land Cover Class:** Shrubland

**Spatial Scale & Pattern:** Matrix

**Required Classifiers:** Natural/Semi‑natural; Vegetated (>10% vasc.); Upland

**Diagnostic Classifiers:** Lowland [Foothill, Lowland]; Shrubland (Shrub‑dominated); Tropical/Subtropical [Tropical Xeric]; Temperate [Temperate Xeric]; Aridic; Xeromorphic Shrub; Succulent Shrub; Cacti‑dominated

**National Mapping Codes:** EVT 2109; ESLF 5315; ESP 1109

**Concept Summary:** This ecological system occurs on hillsides, mesas and upper bajadas in southern Arizona and extreme southeastern California. The vegetation is characterized by a sparse emergent tree layer of *Carnegiea gigantea* (3‑16 m tall) and/or a sparse to moderately dense canopy of xeromorphic deciduous and evergreen tall shrubs codominated by *Parkinsonia microphylla* and *Larrea tridentata*, with *Prosopis* sp., *Olneya tesota*, and *Fouquieria splendens* less prominent. Other common shrubs and dwarf‑shrubs include *Acacia greggii, Ambrosia deltoidea, Ambrosia dumosa* (in drier sites), *Calliandra eriophylla, Jatropha cardiophylla, Krameria erecta, Lycium* spp., *Menodora scabra, Simmondsia chinensis*, and many cacti, including *Ferocactus* spp., *Echinocereus* spp., and *Opuntia* spp. (both cholla and prickly‑pear). The sparse herbaceous layer is composed of perennial grasses and forbs with annuals seasonally present and occasionally abundant. Outliers of this succulent‑dominated ecological system occur as "Cholla Gardens" in transitional areas in the southern and eastern Mojave Desert ecoregion. In this area, the system is characterized by *Cylindropuntia bigelovii, Senna armata*, and other succulents, but it lacks the *Carnegiea gigantea* and *Parkinsonia microphylla* which are typical farther east. *Fouquieria splendens* is present in increasingly diminishing amounts in the system where it occurs further west and north.

DISTRIBUTION

**Range:** This system is found primarily in southwestern Arizona and western Sonora, Mexico, extending east of the Colorado River in southeastern California where locally there is enough summer precipitation (Whipple Mountains).

**Divisions:** 302:C

**TNC Ecoregions:** 23:C

**Nations:** MX, US

**Subnations:** AZ, CA, MXBC, MXSO, NV?

**Map Zones:** 13:C, 14:C, 15:C, 25:C

**USFS Ecomap Regions:** 313C:CC, 321A:CC, 322A:CC, 322B:CC, 322C:CC, M313A:??

CONCEPT

**Environment:** *Climate:* Climate is arid to semi‑arid, continental with mild winters and hot summers (Niering and Lowe 1984). Precipitation has a bimodal distribution with rain in the winter (December‑February) and a summer monsoon (July‑September). Extended periods of drought or episodes of extreme cold limit this type. Specifically, establishment of dominant species is constrained by decadal or longer periods of below‑average precipitation (Turner et al. 1995). Twenty‑four hours of below‑freezing temperature causes nearly total mortality of the dominant plants. At the southern end of the system's range, competition from more mesic species may constrain distribution of this system (Turner et al. 1995).

*Physiography/landform:* This succulent desert scrub ecological system occurs on hillsides, mesas and upper bajadas in southern Arizona and extreme southeastern California. Stands are typically found below 1200 m elevation, with rare occurrences up to 1400 m. Landforms range from steep, rocky slopes of desert mountains to upper and lower bajadas extending out on to alluvial flats. With decreasing elevation, the system typically occurs in xeroriparian habitats (edges of channels and washes) and on rock outcrops.

*Soil/substrate/hydrology:* At higher elevations of bajadas and on steeper surfaces, the system is found on coarse soils that may be associated with poorly developed geomorphic (aka frequently eroded) surfaces; at lower elevations (bottom of bajadas and alluvial fans far from risk of flooding), it is found on very stable geomorphic surfaces. The soils are often underlain by an impervious caliche layer.

**Vegetation:** The vegetation is characterized by a diagnostic sparse, emergent tree layer of *Carnegiea gigantea* (3‑16 m tall) and/or a sparse to moderately dense canopy codominated by xeromorphic deciduous and evergreen tall shrubs *Parkinsonia microphylla* and *Larrea tridentata*, with *Prosopis* sp., *Olneya tesota*, and *Fouquieria splendens* less prominent. Other common shrubs and dwarf‑shrubs include *Acacia greggii, Ambrosia deltoidea, Ambrosia dumosa* (in drier sites), *Calliandra eriophylla, Jatropha cardiophylla, Krameria erecta, Lycium* spp., *Menodora scabra, Simmondsia chinensis*, and many cacti, including *Ferocactus* spp., *Echinocereus* spp., and *Opuntia* spp. (both cholla and prickly‑pear). The sparse herbaceous layer is composed of perennial grasses and forbs with annuals seasonally present and occasionally abundant. On slopes, plants are often distributed in patches around rock outcrops where suitable habitat is present. Outliers of this succulent‑dominated ecological system occur as "Cholla Gardens" in transitional areas in the southern and eastern Mojave Desert ecoregion. In this area, the system is characterized by *Cylindropuntia bigelovii (= Opuntia bigelovii), Senna armata*, and other succulents, but it lacks the *Carnegiea gigantea* and *Parkinsonia microphylla* which are typical farther east. *Fouquieria splendens* is present in increasingly diminishing amounts as the system occurs further west and north.

**Dynamics:** Complex ecological factors determine the occurrence of characteristic species *Carnegiea gigantea*. Major range‑limiting factors are cold winters and dry summers. According to Benson (1982), *Carnegiea gigantea* is killed by extended frosts and does not occur above 1370 m elevation. Its seeds germinate and seedlings and adults grow mostly during the summer monsoon season, so the lack summer moisture further west restricts it from the Mojave Desert. Seedlings require shade from rocks or shrubs called "nurse" plants for seed germination and seedling establishment. The nurse plant protects seedlings from drying out in the intense desert sun, and possibly from frost and predation (Benson 1982, Brown 1982a). As it grows, *Carnegiea gigantea* may inhibit the nurse plant and cause dieback in these shrubs or possibly damage itself significantly (Brown 1982a). In Arizona, north slopes are generally too cold for *Carnegiea gigantea* to germinate; therefore, the best sites are mesic microsites on warm exposures where there is shade and a slight depression to concentrate precipitation. Bats such as lesser long‑nosed bat (*Leptonycteris yerbabuenae*) and Mexican long‑tongued bat (*Choeronycteris mexicana*) pollinate these large night‑blooming cacti. Once the fruit ripens in June, lesser long‑nosed bat, white‑winged dove (*Zenaida asiatica*), Gila woodpecker (*Melanerpes uropygialis*), and other birds or mammals consume the fleshy red pulp and disperse the seeds, which pass through their guts intact (Pavek 1993b). Seed dispersal beneath nurse plant shrub canopies such as *Parkinsonia microphylla* is primarily done by frugivorous birds and is a major factor in saguaro establishment (McAuliffe 1988, 1993). *Carnegiea gigantea* are vulnerable to fire with smaller individuals (<2‑4 m tall) generally killed, especially if large amounts of fuel are present at the plant base, but larger individuals may survive (McLaughlin and Bowers 1982, Pavek 1993b).

This system is not thought to have supported fuel loads to sustain large fires prior to European habitation of the region. Historically, fires in the Sonoran Desert were usually low intensity and uncommon with fire‑return interval greater than 250 years because of limited fuel loads (McLaughlin and Bowers 1982, Thomas 1991). Natural fires are associated with dry lightning coincident with monsoonal storms following years when previous winter precipitation was sufficient to create a thick fine‑fuel bed of annual plants to carry fire. These fires tend to be patchy due to heavier fuel in microsites, or linear when high winds were associated with convection storms (LANDFIRE 2007a). Replacement fires were very rare or absent (average FRI of 100‑1000 years, and perhaps longer) (LANDFIRE 2007a). If they occurred, they did so only during conditions of extreme fire behavior after consecutive years of above‑average winter precipitation when necessary fine fuels accumulate. These rare fires ‑ which may or may not have occurred ‑ had tremendous influence on community structure because the dominant overstory plants are extremely susceptible to fires, even those of low intensity (McLaughlin and Bowers 1982, Esque et al. 2004).

LANDFIRE developed a VDDT model for this system which has three classes (LANDFIRE 2007a, BpS 1411090):

A) Early Development 1 Open (5% of type in this stage): Shrub cover is11‑50%. Initial post‑disturbance community dominated by bursage. Duration 20 years with succession to class B.

B) Mid Development 1 Open (shrub‑dominated ‑ 20% of type in this stage): Dominated by bursage and early‑seral shrubs such as *Encelia farinosa*. Perennial warm‑season grasses are scattered, and dominant succulents and woody plants have established beneath bursage plants. Duration 50‑100 years with succession to class C unless infrequent replacement fire or climatic event (drought, frost) returns vegetation to class A. Lethal freeze and drought are listed as Wind/Weather/Stress in model.

C) Late Development 1 Closed (shrub‑dominated ‑ 75% of type in this stage): Succulent‑ and small tree‑dominated community. Persists until infrequent replacement fire or climatic event (drought, frost) returns vegetation to class A. Lethal freeze and drought are listed as Wind/Weather/Stress in model.

Prolonged weather‑related stress (drought or frost) thinned dominant overstory plants and, in rare cases, led to stand replacement. It is speculated that these events occurred with similar frequency as stand‑replacing fires (LANDFIRE 2007a). Cold stress is more common in stands at the northern extent and at higher elevations on desert mountain ranges. Large (presumably old) saguaro plants are also susceptible to windthrow, particularly after rainstorms saturate the soil (LANDFIRE 2007a). LANDFIRE modelers note there is much uncertainty in model parameters, particularly with respect to the return interval of fire, drought and lethal cold temperatures (LANDFIRE 2007a).

SOURCES

**References:** Alcorn et al. 1961, Belnap 2001, Belnap et al. 2001, Benson 1982, Bowers and McLaughlin 1987, Brown 1982a, Cane et al. 2000, Chambers and Gray 2004, Comer et al. 2003*, Esque et al. 2004, Evans and Belnap 1999, IPCC 2013b, LANDFIRE 2007a, LANDFIRE 2007b, MacMahon 1988, McAuliffe 1988, McAuliffe 1993, McLaughlin and Bowers 1982, Niering and Lowe 1984, Pavek 1993b, Pavek 1994a, Robichaux 1999, Rosentreter and Belnap 2003, Schmidt and Buchman 1986, Shiflet 1994, Shreve and Wiggins 1964, TNC 2013, Thomas 1991, Thomas et al. 2012, Turner et al. 1995, USFS 2002b

**Version:** 27 Jan 2016	**Stakeholders:** Latin America, West

**Concept Author:** K.A. Schulz	**LeadResp:** West

M512. North American Warm Desert Ruderal Scrub & Grassland

CES302.733 Apacherian‑Chihuahuan Mesquite Upland Scrub

**Primary Division:** North American Warm Desert (302)

**Land Cover Class:** Shrubland

**Spatial Scale & Pattern:** Large patch

**Required Classifiers:** Natural/Semi‑natural; Vegetated (>10% vasc.); Upland

**Diagnostic Classifiers:** Lowland [Foothill, Lowland]; Shrubland (Shrub‑dominated); Thorn Shrub; Prosopis spp.‑dominated

**National Mapping Codes:** EVT 2095; ESLF 5301; ESP 1095

**Concept Summary:** This ecological system often occurs as invasive upland shrublands that are concentrated in the extensive desert grassland in foothills and piedmonts of the Chihuahuan Desert, extending into the Sky Island region to the west. Substrates are typically derived from alluvium, often gravelly without a well‑developed argillic or calcic soil horizon that would limit infiltration and storage of winter precipitation in deeper soil layers. *Prosopis* spp. and other deep‑rooted shrubs exploit this deep‑soil moisture that is unavailable to grasses and cacti. Vegetation is typically dominated by *Prosopis glandulosa* or *Prosopis velutina* and succulents. Other desert scrub species that may codominate include *Acacia neovernicosa, Acacia constricta, Juniperus monosperma*, or *Juniperus coahuilensis*. *Larrea tridentata* is typically absent or has low cover. Grass cover is typically low and composed of desert grasses such as *Dasyochloa pulchella, Muhlenbergia porteri, Muhlenbergia setifolia*, and *Pleuraphis mutica*. During the last century, the area occupied by this system has increased through conversion of desert grasslands as a result of drought, overgrazing by livestock, and/or decreases in fire frequency. It is similar to Chihuahuan Mixed Desert and Thornscrub (CES302.734) but is generally found at higher elevations where *Larrea tridentata* and other desert scrub are not codominant. It is also similar to Chihuahuan Stabilized Coppice Dune and Sand Flat Scrub (CES302.737) but does not occur on eolian‑deposited substrates (sandsheets), although some stands may have evidence of wind erosion and deposition.

**Comments:** This system is similar to Chihuahuan Mixed Desert and Thornscrub (CES302.734) but is generally found at higher elevations where *Larrea tridentata* and other desert scrub are not codominant. It is also similar to Chihuahuan Stabilized Coppice Dune and Sand Flat Scrub (CES302.737) but does not occur on eolian‑deposited substrates. This system includes mesquite‑dominated types resulting from conversion of desert grasslands to shrublands. Landfire mapzone 25 modeling workshops limited BpS to naturally occurring mesquite shrublands found on rocky outcrop and foothills. During the last century, the area occupied by the uncharacteristic portion of this system has increased through conversion of desert grasslands as a result of drought, overgrazing and seed dispersion by livestock, and/or decreases in fire frequency. The boundary between Apacherian‑Chihuahuan Mesquite Upland Scrub (CES302.733) and Tamaulipan Mesquite Upland Scrub (CES301.984) needs to be defined.

DISTRIBUTION

**Range:** This system is found on foothills and piedmont in the Chihuahuan Desert, extending into the Sky Island region and into the lower Mogollon Rim to the west.

**Divisions:** 302:C

**TNC Ecoregions:** 22:C, 24:C

**Nations:** MX, US

**Subnations:** AZ, MXCH, MXSO, NM

**Map Zones:** 13:P, 14:C, 15:C, 24:C, 25:C, 26:C, 27:C

**USFS Ecomap Regions:** 313C:CC, 315A:CC, 315B:CC, 315H:CC, 321A:CC, 322A:CC, 322B:CC, 322C:CP, M313A:CC, M313B:CC

CONCEPT

**Environment:** This desert scrub occurs on substrates that are typically derived from alluvium, often gravelly without a well‑developed argillic or calcic soil horizon that would limit infiltration and storage of winter precipitation in deeper soil layers. *Prosopis* spp. and other deep‑rooted shrubs exploit this deep‑soil moisture that is unavailable to grasses and cacti (Burgess 1995).

**Vegetation:** Vegetation is typically dominated by *Prosopis glandulosa* or *Prosopis velutina* and succulents. Other desert scrub species that may codominate include *Acacia neovernicosa, Acacia constricta, Juniperus monosperma*, or *Juniperus coahuilensis*. Grass cover is variable and ranges from sparse to moderately dense. Common species may include *Aristida purpurea, Bothriochloa barbinodis, Bouteloua curtipendula, Dasyochloa pulchella (= Erioneuron pulchellum), Muhlenbergia porteri, Muhlenbergia setifolia, Pleuraphis mutica*, and *Setaria leucopila*. The deeper soils help support good grass cover beneath the shrub canopy. Areas of higher annual rainfall and deeper soils allow for a more diverse plant community.

**Dynamics:** During the last century, the area occupied by this system has increased through conversion of desert grasslands as a result of drought, overgrazing and *Prosopis glandulosa* seed dispersion by livestock, and/or decreases in fire frequency (Buffington and Herbel 1965, Brown and Archer 1987). It is believed that this system formerly occurred in relatively minor amounts and was largely confined to drainages until cattle distributed seed upland from the bosques into desert grasslands (Brown and Archer 1987, 1989). Shrublands dominated by *Prosopis* spp. have replaced large areas of desert grasslands, especially those formerly dominated by *Bouteloua eriopoda*, in Trans Pecos Texas, southern New Mexico and southeastern Arizona (York and Dick‑Peddie 1969, Hennessy et al. 1983). Studies on the Jornada Experimental Range suggest that combinations of drought, overgrazing by livestock, wind and water erosion, seed dispersal by livestock, fire suppression, shifting dunes, and changes in the seasonal distribution of precipitation have caused this recent, dramatic shift in vegetation physiognomy (Buffington and Herbel 1965, Herbel et al. 1972, Humphrey 1974, McLaughlin and Bowers 1982, Gibbens et al. 1983, Hennessy et al. 1983, Schlesinger et al. 1990, McPherson 1995).

Historical natural‑ignition fires were relatively small, probably 10‑15 acres in size. Repeated fire is thought to help maintain a general mosaic pattern between open grassland and shrub‑dominated areas (Johnston 1963). Wright et al. (1976) found that *Prosopis glandulosa* is very fire‑tolerant when only 3 years old. Most plants resprout after being top‑killed by fire. Thus, prior to livestock grazing reducing fire frequency, repeated grassland fires probably maintained lower stature of shrubs and prevented new establishment by killing seedlings.

Drought is a relatively common occurrence in this desert scrub, generally occurring every 10‑15 years and lasting 2‑3 years with occasional long‑term drought periods (10‑15 years duration). *Prosopis* spp. and other shrubs have extensive root systems that allow them to exploit deep‑soil water that is unavailable to shallower rooted grasses and cacti (Burgess 1995). This strategy works well, especially during drought. However, on sites that have well‑developed argillic or calcic soil horizons that limit infiltration and storage of winter moisture in the deeper soil layers, *Prosopis* spp. invasion can be limited to a few, small individuals (McAuliffe 1995). This has implications in plant geography and desert grassland restoration work in the southwestern United States.

SOURCES

**References:** Brown and Archer 1987, Brown and Archer 1989, Buffington and Herbel 1965, Burgess 1995, Comer et al. 2003*, Eyre 1980, Gibbens et al. 1983, Gibbens et al. 2005, Hennessy et al. 1983, Herbel et al. 1972, Humphrey 1974, Johnston 1963, MacMahon 1988, McAuliffe 1995, McLaughlin and Bowers 1982, McPherson 1995, Muldavin et al. 2002a, Schlesinger et al. 1990, Shiflet 1994, Wright et al. 1976, York and Dick‑Peddie 1969

**Version:** 25 Jan 2007	**Stakeholders:** Latin America, Southeast, West

**Concept Author:** K.A. Schulz	**LeadResp:** West

M117. North American Warm Semi‑Desert Cliff, Scree & Rock Vegetation

CES302.743 North American Warm Desert Badland

**Primary Division:** North American Warm Desert (302)

**Land Cover Class:** Barren

**Spatial Scale & Pattern:** Large patch

**Required Classifiers:** Natural/Semi‑natural; Unvegetated (<10% vasc.); Upland

**Diagnostic Classifiers:** Badlands; Badland; Alkaline Soil; Shale and Mudstone; Silt Soil Texture; Clay Soil Texture

**Concept Summary:** This ecological system occurs from Arizona to Texas and adjacent Mexico. It is restricted to barren and sparsely vegetated (generally <10% plant cover) substrates typically derived from marine shale or mudstone (badlands and mudhills). The harsh soil properties, high temperatures and evaporation, low precipitation, and high rate of erosion and deposition are driving environmental variables supporting sparse shrubs and dwarf‑shrubs, e.g., *Atriplex hymenelytra*, and herbaceous vegetation. These conditions often preclude the development of significant vegetative cover.

DISTRIBUTION

**Range:** This ecological system occurs from Arizona to Texas and adjacent Mexico.

**Divisions:** 302:C

**TNC Ecoregions:** 17:C, 22:P, 23:P, 24:C

**Nations:** MX, US

**Subnations:** AZ, MXCH, MXSO, NM, TX

**Map Zones:** 13:C, 14:?, 15:?, 25:P, 26:?

**USFS Ecomap Regions:** 322A:CC, 322B:C?, 322C:C?

CONCEPT

**Environment:** This ecological system is restricted to barren and sparsely vegetated substrates typically derived from marine shales or mudstones (badlands and mudhills). The harsh soil properties, such as high salinity and alkalinity, and high rates of erosion and deposition are driving environmental variables that maintain the barren to sparse vegetation character. Substrates are generally shallow, fine‑textured silty and clayey soils. In Texas, these sites are highly erosional and occupy rolling landscapes frequently cut by drainages. Adjacent systems include Sonora‑Mojave Creosotebush‑White Bursage Desert Scrub (CES302.756), Sonora‑Mojave Mixed Salt Desert Scrub (CES302.749), and North American Warm Desert Playa (CES302.751). The environmental description is based on several references, including Reid et al. (1999), Comer et al. (2003), Thomas et al. (2004), Sawyer et al. (2009), and NatureServe Explorer (2011).

**Vegetation:** This ecological system typically has sparse vegetation (generally < 10% plant cover) characterized by an open to intermittent canopy of shrubs and dwarf‑shrubs that are tolerant of high salinity and alkalinity, e.g., *Atriplex hymenelytra, Cleome isomeris, Ephedra californica*, and *Ericameria linearifolia*. The floristic description is based on several references, including Reid et al. (1999), Comer et al. (2003), Thomas et al. (2004), Sawyer et al. (2009), and NatureServe Explorer (2011).

**Dynamics:** Geomorphic and fluvial processes disturb this system more than fire. Harsh soil properties, such as high salinity and alkalinity, and high rates of erosion and deposition are driving environmental variables supporting the characteristic vegetation pattern (Sawyer et al. 2009). Fire is extremely rare and is only possible after very high winter precipitation produces an abundance of annual vegetation (fine fuels) that can carry a fire. *Atriplex hymenelytra* has low flammability and is fire‑hardy, and *Ephedra californica* is adapted to fire and will vigorously sprout from underground rhizomes after top‑killed from burning (Sawyer et al. 2009).

SOURCES

**References:** Comer et al. 2003*, Elliott 2012, NatureServe Explorer 2011, Reid et al. 1999, Sawyer et al. 2009, Thomas et al. 2004

**Version:** 02 Apr 2014	**Stakeholders:** Latin America, Southeast, West

**Concept Author:** K.A. Schulz	**LeadResp:** West

CES302.745 North American Warm Desert Bedrock Cliff and Outcrop

**Primary Division:** North American Warm Desert (302)

**Land Cover Class:** Barren

**Spatial Scale & Pattern:** Large patch

**Required Classifiers:** Natural/Semi‑natural; Unvegetated (<10% vasc.); Upland

**Diagnostic Classifiers:** Canyon; Cliff (Landform); Rock Outcrops/Barrens/Glades; Temperate [Temperate Xeric]

**Concept Summary:** This ecological system occurs from California to Texas and adjacent Mexico. It is found from subalpine to foothill elevations and includes barren and sparsely vegetated landscapes (generally <10% plant cover) of steep cliff faces, narrow canyons, and smaller rock outcrops of various igneous, sedimentary, and metamorphic bedrock types. Also included are unstable scree and talus slopes that typically occur bellow cliff faces. Species present are diverse and may include *Bursera microphylla, Fouquieria splendens, Nolina bigelovii, Cylindropuntia bigelovii*, and other desert species, especially succulents. Lichens are predominant lifeforms in some areas. May include a variety of desert shrublands less than 2 ha (5 acres) in size from adjacent areas. In the Trans‑Pecos of Texas, this system is well‑developed on rock faces (some of which are 100s of feet tall with slopes greater than 80%) on massive Cretaceous and Permian limestones, but also occupies igneous and sandstone formations. Vegetation is typically restricted to crevices, although crustose lichens may be well‑represented.

DISTRIBUTION

**Range:** This ecological system occurs from California to Texas and adjacent Mexico.

**Divisions:** 302:C

**TNC Ecoregions:** 17:C, 22:C, 23:C, 24:C

**Nations:** MX, US

**Subnations:** AZ, CA, MXBC, MXBS, MXCH, MXSO, NM, NV, TX

**Map Zones:** 12:?, 13:C, 14:C, 15:P, 16:?, 17:P, 23:?, 24:C, 25:C, 26:C, 27:P, 28:?

**USFS Ecomap Regions:** 313A:CC, 313B:CC, 313C:CC, 313D:CC, 315A:CC, 315B:CC, 315H:CC, 321A:CC, 322A:CC, 322B:CC, 322C:CC, 341F:CC, M313A:CC, M313B:CC

CONCEPT

**Environment:** This ecological system occurs across the southwestern U.S. in the Chihuahuan, Sonoran and Mojave deserts. It is restricted to barren and sparsely vegetated sites (generally < 10% plant cover) from smaller rock outcrops in low‑elevation desert hills, on cliff faces in canyons including unstable scree and talus slopes, to higher‑elevation rock outcrops in the foothill and lower montane zones in desert mountain ranges. Substrates are various igneous, sedimentary, and metamorphic bedrock types. Adjacent systems include Chihuahuan Mixed Desert and Thornscrub (CES302.734), Sonoran Paloverde‑Mixed Cacti Desert Scrub (CES302.761), Sonoran Mid‑Elevation Desert Scrub (CES302.035), Mojave Mid‑Elevation Mixed Desert Scrub (CES302.742), and at higher elevation Sonora‑Mojave Semi‑Desert Chaparral (CES302.757) and Great Basin Pinyon‑Juniper Woodland (CES304.773). The environmental description is based on several references, including Shreve and Wiggins (1964), MacMahon and Wagner (1985), Barbour and Major (1988), MacMahon (1988), Dick‑Peddie (1993), Reid et al. (1999), Comer et al. (2003), Thomas et al. (2004), Barbour et al. (2007), Sawyer et al. (2009), and NatureServe Explorer (2011).

**Vegetation:** Vegetation is typically sparse. Species present are diverse and may include *Bursera microphylla, Fouquieria splendens, Jatropha dioica var. graminea, Nolina bigelovii, Cylindropuntia bigelovii (= Opuntia bigelovii), Grusonia schottii (= Opuntia schottii), Petrophytum caespitosum*, and other desert species, especially succulents. Lichens are predominant lifeforms in some areas. It may include a variety of desert shrublands less than 2 ha (5 acres) in size from adjacent areas. The floristic description is based on several references, including Shreve and Wiggins (1964), MacMahon and Wagner (1985), Barbour and Major (1988), MacMahon (1988), Dick‑Peddie (1993), Reid et al. (1999), Comer et al. (2003), Thomas et al. (2004), Barbour et al. (2007), Sawyer et al. (2009), and NatureServe Explorer (2011).

**Dynamics:** In this system growing sites are often limited with plants restricted to cracks in rocks where moisture accumulates.

SOURCES

**References:** Barbour and Major 1988, Barbour et al. 2007a, Comer et al. 2003*, Dick‑Peddie 1993, Elliott 2012, Keeler‑Wolf 2007, MacMahon 1988, MacMahon and Wagner 1985, NatureServe Explorer 2011, Reid et al. 1999, Sawyer et al. 2009, Shreve and Wiggins 1964, Thomas et al. 2004

**Version:** 02 Apr 2014	**Stakeholders:** Latin America, Southeast, West

**Concept Author:** K.A. Schulz	**LeadResp:** West

CES302.750 North American Warm Desert Pavement

**Primary Division:** North American Warm Desert (302)

**Land Cover Class:** Barren

**Spatial Scale & Pattern:** Large patch

**Required Classifiers:** Natural/Semi‑natural; Unvegetated (<10% vasc.); Upland

**Diagnostic Classifiers:** Lowland [Lowland]; Playa; Desert Pavement; Tropical/Subtropical [Tropical Xeric]; Temperate [Temperate Xeric]; W‑Landscape/High Intensity

**Concept Summary:** This ecological system occurs throughout much of the warm deserts of North America and is composed of unvegetated to very sparsely vegetated (<2% plant cover) landscapes, typically flat basins where extreme temperature and wind develop ground surfaces of fine to medium gravel coated with "desert varnish." This sparsely vegetated system may surround playas in valley bottoms or near washes and, less commonly, on dissected, eroding alluvial fans. Very low cover of desert scrub species such as *Larrea tridentata* or *Eriogonum fasciculatum* is usually present. However, ephemeral herbaceous species may have high cover in response to seasonal precipitation, including *Chorizanthe rigida, Eriogonum inflatum*, and *Geraea canescens*.

DISTRIBUTION

**Range:** Occurs throughout much of the warm deserts of North America.

**Divisions:** 302:C

**TNC Ecoregions:** 17:C, 23:C, 24:C

**Nations:** MX, US

**Subnations:** AZ, CA, MXCH, MXSO, NM, NV, TX

**Map Zones:** 13:C, 14:C, 15:?, 24:?, 25:C, 26:C

**USFS Ecomap Regions:** 321A:CC, 322A:CC, 322B:CC, 322C:CC, 341F:PP, M313A:CP, M313B:CC

CONCEPT

**Environment:** This ecological system occurs throughout much of the warm deserts of North America on flat basins and lower bajadas. Elevations range from 1600 m to below sea level. Climate is semi‑arid to arid with hot summers. Potential for freezing winter temperatures depends on latitude and elevation. Desert precipitation varies greatly from year to year with drought not uncommon. In the Mojave Desert, mean annual precipitation is typically <150 mm falling in the winter months (Barbour and Major 1988). In the Sonoran and Chihuahuan deserts, annual precipitation is 230 mm occurring bi‑modally during winter and late‑summer monsoons (Barbour and Major 1988). Substrates are typically gravelly alluvium. In the typically flat intermountain basin sites, extreme temperature and wind develop ground surfaces of fine to medium gravel coated with "desert varnish." This sparsely vegetated system may surround playas or be near washes in valley bottoms, and, less commonly, on dissected, eroding alluvial fans. Adjacent systems include Sonora‑Mojave Creosotebush‑White Bursage Desert Scrub (CES302.756), Sonora‑Mojave Mixed Salt Desert Scrub (CES302.749), and North American Warm Desert Playa (CES302.751). The environmental description is based on several references, including Brown (1982a), MacMahon and Wagner (1985), Barbour and Major (1988), MacMahon (1988), Holland and Keil (1995), Reid et al. (1999), Comer et al. (2003), Thomas et al. (2004), Barbour et al. (2007), Sawyer et al. (2009), and NatureServe Explorer (2011).

**Vegetation:** This sparsely vegetated system is characterized by very low cover (< 2% total cover) of desert scrub. Species such as *Larrea tridentata* or *Eriogonum fasciculatum* are usually present. However, ephemeral herbaceous species may have high cover in response to seasonal precipitation, including *Chorizanthe rigida, Eriogonum inflatum*, and *Geraea canescens*. The floristic description is based on several other references, including Brown (1982a), MacMahon and Wagner (1985), Barbour and Major (1988), MacMahon (1988), Holland and Keil (1995), Reid et al. (1999), Comer et al. (2003), Thomas et al. (2004), Barbour et al. (2007), Sawyer et al. (2009), and NatureServe Explorer (2011).

**Dynamics:** There are several theories about the formation of desert pavement. The more common theory is that pavements are created by the removal of the fine soil particles by the wind and intermittent rain leaving only the larger fragments behind, forming a pebble pavement (McFadden et al. 1987). This pavement acts as a barrier to reduce further erosion. The pavement also reduces water infiltration which reduces soil moisture and increases runoff and concentration of moisture in drainages. The reduced soil moisture likely contributes to sparse cover of *Larrea tridentata* and other deeper‑rooted shrubs (Hamerlynck et al. 2002). Pavement affects the shallower‑rooted *Ambrosia dumosa* and annual plants less (Hamerlynck et al. 2002).

Fire is extremely rare and is only possible after very high winter precipitation produces an abundance of annual vegetation (fine fuels) that can carry a fire. Although very long‑lived, *Larrea tridentata* shrubs are poorly adapted to fire because of highly flammable, resinous foliage and limited ability to sprout after burning (Sawyer et al. 2009).

SOURCES

**References:** Barbour and Major 1988, Barbour et al. 2007a, Brown 1982a, Comer and Hak 2009, Comer et al. 2003*, Elliott 2012, Hamerlynck et al. 2002, Holland and Keil 1995, MacMahon 1988, MacMahon and Wagner 1985, McFadden et al. 1987, McRae 2006, McRae et al. 2008, Musick 1975, NatureServe Explorer 2011, Reid et al. 1999, Sawyer et al. 2009, Thomas et al. 2004

**Version:** 02 Apr 2014	**Stakeholders:** Latin America, Southeast, West

**Concept Author:** K.A. Schulz	**LeadResp:** West

CES302.754 North American Warm Desert Volcanic Rockland

**Primary Division:** North American Warm Desert (302)

**Land Cover Class:** Barren

**Spatial Scale & Pattern:** Large patch

**Required Classifiers:** Natural/Semi‑natural; Unvegetated (<10% vasc.); Upland

**Diagnostic Classifiers:** Lava; Cinder; Basalt; Tropical/Subtropical [Tropical Xeric]; Temperate [Temperate Xeric]

**Concept Summary:** This ecological system occurs across the warm deserts of North America and is restricted to barren and sparsely vegetated (<10% plant cover) volcanic substrates such as basalt lava (malpais) and tuff. Vegetation is variable and includes a variety of species depending on local environmental conditions, e.g., elevation, age and type of substrate. Typically scattered *Larrea tridentata, Atriplex hymenelytra*, or other desert shrubs are present. In Texas, this system occurs on rocky or boulder‑strewn slopes and flats where the rock material is volcanic in origin.

DISTRIBUTION

**Range:** Occurs across the warm deserts of North America.

**Divisions:** 302:C

**TNC Ecoregions:** 17:C, 22:C, 23:C, 24:C

**Nations:** MX, US

**Subnations:** AZ, CA, MXBC, MXCH, MXSO, NM, NV, TX

**Map Zones:** 13:C, 14:C, 15:P, 17:?, 25:C, 26:C

**USFS Ecomap Regions:** 313A:CC, 313C:CP, 321A:CC, 322A:CC, 322B:CC, 322C:C?, 341F:CC, M313A:CP, M313B:CC

CONCEPT

**Environment:** This system occurs on volcanic substrates such as basalt lava (malpais), tuff, and rhyolite. In Texas landforms supporting this system are usually talus slopes, but also relatively level rocky and boulder sites. Soil is generally lacking or reduced to small pockets within the rock matrix.

**Vegetation:** In Texas, scattered individuals of species such as *Larrea tridentata, Fouquieria splendens, Jatropha dioica, Prosopis glandulosa, Yucca torreyi*, and cacti such as *Echinocereus* spp. and *Opuntia rufida* may be present.

**Dynamics:** [from M117] These sparsely vegetated plant communities often represent primary succession on parent materials such as bare rock outcrops or disturbance‑maintained communities such as scree and talus slopes that are frequently disturbed and constantly re‑establishing themselves. Biological soil crusts can improve soil stability and soil fertility, and disturbances such as grazing and non‑native species invasion can negatively impact these crusts (Belnap and Eldridge 2003, Belnap et al. 2006).

SOURCES

**References:** Barbour and Major 1988, Brown 1982a, Comer et al. 2003*, Dick‑Peddie 1993, Elliott 2012, Thomas et al. 2004

**Version:** 02 Oct 2014	**Stakeholders:** Latin America, Southeast, West

**Concept Author:** K.A. Schulz	**LeadResp:** West

M092. North American Warm‑Desert Xeric‑Riparian Scrub

CES302.755 North American Warm Desert Wash

**Primary Division:** North American Warm Desert (302)

**Land Cover Class:** Woody Wetland

**Spatial Scale & Pattern:** Linear

**Required Classifiers:** Natural/Semi‑natural; Vegetated (>10% vasc.); Upland; Wetland

**Diagnostic Classifiers:** Lowland [Lowland]; Shrubland (Shrub‑dominated); Toeslope/Valley Bottom; Tropical/Subtropical [Tropical Xeric]; Temperate [Temperate Xeric]; Riverine / Alluvial; Intermittent Flooding

**Concept Summary:** This ecological system is restricted to intermittently flooded washes or arroyos that dissect bajadas, mesas, plains and basin floors throughout the warm deserts of North America. Although often dry, the intermittent fluvial processes define this system, which are often associated with rapid sheet and gully flow. This system occurs as linear or braided strips within desert scrub‑or desert grassland‑dominated landscapes. The vegetation of desert washes is quite variable, ranging from sparse and patchy to moderately dense, and typically occurs along the banks, but may occur within the channel. The woody layer is typically intermittent to open and may be dominated by shrubs and small trees such as *Acacia greggii, Brickellia laciniata, Baccharis sarothroides, Chilopsis linearis, Fallugia paradoxa, Hymenoclea salsola, Hymenoclea monogyra, Juglans microcarpa, Olneya tesota, Parkinsonia florida, Prosopis* spp., *Psorothamnus spinosus, Prunus fasciculata, Rhus microphylla, Salazaria mexicana*, or *Sarcobatus vermiculatus*. Common upland shrubs such as *Larrea tridentata* and *Ambrosia dumosa* are often present along the edges of these washes. In Texas, woody species found in and adjacent to these washes include *Acacia greggii, Baccharis salicifolia, Brickellia laciniata, Celtis laevigata var. reticulata, Chilopsis linearis, Dasylirion leiophyllum, Fallugia paradoxa, Fraxinus greggii, Juglans microcarpa, Juglans microcarpa, Leucaena retusa, Prosopis glandulosa, Rhus microphylla*, and *Salix gooddingii*. Taller species may form a sparse canopy over the shorter shrubs. In addition, shrubs from the adjacent upland, such as *Larrea tridentata, Viguiera stenoloba, Flourensia cernua*, and *Juniperus pinchotii* may be commonly encountered.

DISTRIBUTION

**Range:** This system is restricted to intermittently flooded washes or arroyos that dissect bajadas, mesas, plains and basin floors throughout the warm deserts of North America.

**Divisions:** 302:C

**TNC Ecoregions:** 17:C, 22:C, 23:C, 24:C

**Nations:** MX, US

**Subnations:** AZ, CA, MXBC, MXCH, MXSO, NM, NV, TX

**Map Zones:** 4:P, 12:P, 13:C, 14:C, 15:P, 16:P, 17:C, 23:?, 24:C, 25:C, 26:C, 27:C

**USFS Ecomap Regions:** 261B:CC, 313A:CC, 313B:CP, 313C:CC, 313D:C?, 315A:CC, 315B:CC, 315H:CC, 321A:CC, 322A:CC, 322B:CC, 322C:CC, 341E:C?, 341F:CC, M261E:PP, M313A:CC, M313B:CC

CONCEPT

**Environment:** This ecological system is restricted to flashy, intermittently flooded, often dry washes and arroyos that dissect bajadas, mesas, plains and basin floors throughout the warm deserts of North America.

**Vegetation:** The vegetation of desert washes is typically characterized by an open layer of shrubs and small trees such as *Acacia greggii, Brickellia laciniata, Baccharis sarothroides, Baccharis salicifolia, Chilopsis linearis, Dasylirion leiophyllum, Fallugia paradoxa, Fraxinus greggii, Hymenoclea salsola, Hymenoclea monogyra, Juglans microcarpa, Leucaena retusa, Olneya tesota, Parkinsonia florida, Prosopis* spp., *Psorothamnus spinosus, Prunus fasciculata, Rhus microphylla, Salazaria mexicana*, or *Sarcobatus vermiculatus*. In addition, shrubs from the adjacent uplands may be commonly encountered. Vegetation cover ranges from sparse and patchy to moderately dense, and typically occurs along the banks, but may occur within the channel. Taller species may form a sparse canopy over the shorter shrubs.

**Dynamics:** Intermittent fluvial processes such as rapid sheet and gully flow define this system.

SOURCES

**References:** Barbour and Major 1988, Brown 1982a, Comer et al. 2003*, Dick‑Peddie 1993, Elliott 2012, MacMahon 1988, Muldavin et al. 2000b, Shiflet 1994, Szaro 1989, Thomas et al. 2004

**Version:** 02 Oct 2014	**Stakeholders:** Latin America, Southeast, West

**Concept Author:** K.A. Schulz	**LeadResp:** West

M130. Tamaulipan Scrub & Grassland

CES301.713 Matorral Crasicaule

**Primary Division:** Madrean Semidesert (301)

**Required Classifiers:** Natural/Semi‑natural; Vegetated (>10% vasc.)

**Concept Summary:** Tipo de vegetación dominada fisonómicamente por cactáceas grandes con tallos aplanados o cilíndricos que se desarrollan principalmente en las zonas áridas y semiáridas del centro y norte del país. Algunas especies comunes son: *Opuntia* spp., *Carnegiea gigantea, Pachycereus pringlei, Stenocereus thurberi*. Se incluyen las asociaciones conocidas como Nopaleras, Chollales, Cardonales, Tetecheras, etc.

El Matorral Crasicaule que se establece en la parte central de Zacatecas y algunas zonas adyacentes de Durango, Aguascalientes, Jalisco, Guanajuato y San Luis Potosí presentan como cubierta vegetal de *Opuntia*, siendo las principales especies dominantes de estas "nopaleras" *Opuntia streptacantha* y *Opuntia leucotricha*.

Esta comunidad se desarrolla preferentemente sobre suelos someros de laderas de cerros de naturaleza volcánica, aunque también desciende a suelos aluviales contiguos. La precipitación media anual varía entre 300 y 600 mm y la temperatura es de 16° a 22°C en promedio anual. En algunas partes de San Luis Potosí y de Guanajuato se le asocia *Myrtillocactus geometrizans* y a veces también *Stenocereus* spp. Por otro lado *Yucca decipiens* puede formar un estrato emergente, mientras que a niveles inferiores conviven muchos arbustos micrófilos, como por ejemplo, especies de *Mimosa, Acacia, Dalea, Prosopis, Rhus, Larrea, Brickelia, Eupatorium, Buddleia, Celtis*, etc.

La altura de este matorral alcanza generalmente de 2 a 4 m, su densidad es variable, pudiendo alcanzar casi 100% de cobertura, y el matorral puede admitir la presencia la numerosa presencia de planta herbáceas.

Generalmente existe ganadería a base de caprinos y bovinos; es igualmente importante la recolección de frutos comestibles, y en el caso de los nopales, de los tallos.

**Nations:** MX

CONCEPT

**Environment:** [from M130] This vegetation is found in semi‑arid and subtropical southern Texas over a variety of soil depths and textures. Rainfall is highly variable both spatially and temporally and can range from 38 to 76 cm (15‑30 inches) annually in a given locality, but all areas are prone to drought and water deficits (Bray 1901, Gilbert 1982, Jahrsdoerfer and Leslie 1988).

SOURCES

**References:** Latin American Ecology Working Group n.d.*

**Version:** 08 Jan 2015	**Stakeholders:** Latin America

**Concept Author:** C. Josse	**LeadResp:** Latin America

CES301.714 Matorral Submontano

**Primary Division:** Madrean Semidesert (301)

**Required Classifiers:** Natural/Semi‑natural; Vegetated (>10% vasc.)

**Concept Summary:** Comunidad arbustiva a veces muy densa, formado por especies inermes o a veces espinosas, caducifolias por un breve período del año, se desarrolla entre los matorrales áridos y los bosques de Encino y la Selva Baja Caducifolia a altitudes de 1500 a 1700 msnm, principalmente en las laderas bajas de ambas vertientes de la Sierra Madre Oriental, desde Querétaro e Hidalgo hacia el norte, penetrando más allá de la frontera política con los Estados Unidos de Norteamérica.

Para el noreste de México se describe la siguiente comunidad de Matorral Submontano: la fisonomía de esta comunidad la proporciona el estrato arbustivo superior, cuya altura varía entre 2.5 a 5 m y alcanza una cobertura hasta de un 70%. Lo caracteriza *Helietta parvifolia* (Barreta), rutácea inerme que le da a la vegetación una estructura relativamente uniforme, pues normalmente es la única dominante, aunque en ocasiones *Acacia berlandieri* (Huajillo) es igual de importante. Las plantas prevalecientes del estrato arbustivo medio (0.5 a 2 m de alto) son *Leucophyllum frutescens* y *Acacia rigidula*. Su cobertura varía de 50 a 80%. El estrato inferior, menor a 0.5 m de altura, es diverso tanto en especies como en cobertura; es notable en los claros de la vegetación, donde *Agave lechuguilla, Euphorbia antisyphilitica* (Candelilla) y las gramíneas amacolladas de los géneros *Bouteloua, Tridens* y *Aristida* son los elementos más importantes. El terreno es pedregoso y es común encontrar plantas creciendo sobre rocas.

Este tipo de vegetación se encuentra en laderas, cañadas y partes altas, sean planas o con pendiente, de las mesetas y lomeríos. Crece sobre suelos someros que a veces presentan una capa superficial de hojarasca y son comunes los afloramientos de la roca madre.

<br />Francisco González Medrano en el trabajo denominado Vegetación al Noreste de Tamaulipas cita al Matorral Submontano como Matorral Alto Subinerme en el cual cita que este tipo de vegetación es muy complejo y variable, ocupando los suelos pedregosos de cerros escarpados y lomeríos con buen drenaje por su inclinación y abundancia de partículas gruesas del suelo. La caracteriza gran número de arbustos a veces subarbóreos, inermes en su gran mayoría, aunque cierto número de especies espinosas se entremezclan con los dominantes. Algunas son caducifolias, pero la mayoría son perennifolias. El elemento característico y dominante de este matorral es *Helietta parvifolia*, asociada con *Gochnatia hypoleuca*. Su límite superior oscila entre 1.800 y 2000 m Crece dentro de una zona con clima BS. La precipitación es inferior a 700 mm anuales, con una temperatura media anual superior a los 23°C.<br />

La dominancia de *Helietta parvifolia* y *Gochnatia hypoleuca* es notoria, forman un matorral denso de 3 a 4 m de altura; aquí se encuentran especies propias del estrato arbustivo del Matorral Espinoso Tamaulipeco con el cual colinda.

<br />Sus principales componentes pueden ser los siguientes: *Helieta parvifolia* (Barreta), *Neopringlea integrifolia* (Corva de gallina), *Cordia boissieri* (Anacahuita), *Havardia pallens (= Pithecellobium pallens)* (Tenaza), *Acacia rigidula* (Gavia), *Gochnatia hypoleuca* (Ocotillo, Olivo), *Karwinskia* spp. (Limoncillo), *Capparis incana* (Vara blanca), *Rhus virens* (Lantrisco), *Flourensia lauriforia, Mimosa leucaeneoides, Mortonia greggii* (Afinador) *Zanthoxylum fagara*, etc.

Su área de distribución ha sido ocupada por la agricultura, ganadería y la explotación forestal.

**Nations:** MX

CONCEPT

**Environment:** [from M130] This vegetation is found in semi‑arid and subtropical southern Texas over a variety of soil depths and textures. Rainfall is highly variable both spatially and temporally and can range from 38 to 76 cm (15‑30 inches) annually in a given locality, but all areas are prone to drought and water deficits (Bray 1901, Gilbert 1982, Jahrsdoerfer and Leslie 1988).

SOURCES

**References:** CONABIO 2003b, INEGI 2005, Latin American Ecology Working Group n.d.*

**Version:** 08 Jan 2015	**Stakeholders:** Latin America

**Concept Author:** C. Josse	**LeadResp:** Latin America

CES301.986 Tamaulipan Calcareous Thornscrub

**Primary Division:** Madrean Semidesert (301)

**Land Cover Class:** Shrubland

**Spatial Scale & Pattern:** Large patch

**Required Classifiers:** Natural/Semi‑natural; Vegetated (>10% vasc.); Upland

**Diagnostic Classifiers:** Lowland [Lowland]; Shrubland (Shrub‑dominated); Ridge; Ridge/Summit/Upper Slope; Tropical/Subtropical [Tropical Xeric]; Calcareous; Very Shallow Soil; Caliche Layer

**National Mapping Codes:** EVT 2392; ESLF 5323; ESP 1392

**Concept Summary:** This xeric thornscrub ecological system is restricted to limestone and calcareous sandstone hills and caliche substrates such as along the Bordas Scarp in southern Texas and northeastern Mexico. Soils are shallow, alkaline, strongly calcareous and underlain by bedrock or a caliche layer. It has a shorter, more open shrub canopy (usually less than 2 m) when compared to more typical thornscrub growing on more favorable sites. However, shrub cover is generally greater than 70% and often greater than 85%. Dominant species include *Leucophyllum frutescens, Acacia berlandieri*, and *Acacia farnesiana* with many other shrub species that may be locally dominant. The sparse to moderately dense herbaceous layer is dominated by perennial graminoids.

DISTRIBUTION

**Range:** Restricted to limestone and calcareous sandstone hills and caliche substrates such as along the Bordas Scarp in southern Texas and northeastern Mexico.

**Divisions:** 301:C, 303:P

**TNC Ecoregions:** 24:C, 29:C, 30:C, 31:P

**Nations:** MX, US

**Subnations:** MXCO, MXNU, MXTM, TX

**Map Zones:** 26:C, 35:C, 36:C

**USFS Ecomap Regions:** 255E:CC, 315A:CC, 315C:C?, 315D:CC, 315G:C?, 321A:CC, 321B:CC

CONCEPT

**Environment:** This system is restricted to xeric, rocky hills, rolling or level plateaus, and ridges composed of limestone and calcareous sandstone, as well as caliche substrates such as of the Goliad Formation or Uvalde gravel along the Bordas Scarp in southern Texas and northeastern Mexico. Soils are thin, alkaline, strongly calcareous and underlain by bedrock or a caliche layer. These are Shallow, Shallow Ridge or Gravelly Ridge Ecological Sites.

**Vegetation:** Sites are most frequently dominated by shrubs between 0.5 and 2 m in height. Shrub canopy can be dense (to about 90% cover), or sparser where rocky exposures reduce substrate for rooting. A sparse overstory, usually <4 m in height, may be present and composed of species such as *Prosopis glandulosa* and, in the south, *Ebenopsis ebano, Cordia boissieri*, and/or *Helietta parvifolia*. *Quercus fusiformis* may form a relatively open canopy in areas in the northeastern part of the South Texas Plains. The shrub layer may be heavily dominated by *Leucophyllum frutescens, Acacia berlandieri*, and/or *Acacia rigidula*. More commonly, a diverse array of shrubs is present, including these three in addition to several of the following species: *Acacia schaffneri, Aloysia macrostachya, Amyris madrensis, Amyris texana, Bernardia myricifolia, Castela erecta ssp. texana, Celtis ehrenbergiana, Condalia spathulata, Croton incanus, Diospyros texana, Ephedra antisyphilitica, Eysenhardtia texana, Forestiera angustifolia, Guaiacum angustifolium, Helietta parvifolia, Jatropha dioica, Karwinskia humboldtiana, Koeberlinia spinosa, Krameria ramosissima, Mahonia trifoliolata, Cylindropuntia leptocaulis, Parkinsonia texana var. macra, Salvia ballotiflora, Sideroxylon celastrinum, Sophora secundiflora, Yucca treculeana*, and others. More southerly occurrences may also contain *Lippia graveolens, Helietta parvifolia, Gochnatia hypoleuca, Croton humilis, Ebenopsis ebano*, and/or *Mortonia greggii*. The herbaceous layer may be somewhat well‑developed, but often bare rock is easily visible through the layer. Many sites are now dominated by non‑native grasses, particularly *Bothriochloa ischaemum var. songarica* and/or *Pennisetum ciliare*. Other grasses are often short grasses, with species such as *Bouteloua rigidiseta, Bouteloua hirsuta, Bouteloua dactyloides, Hilaria belangeri, Aristida purpurea, Bouteloua curtipendula*, and *Setaria leucopila* present. Forbs and subshrubs are conspicuous in the herbaceous layer and include species such as *Tiquilia canescens, Thamnosma texana, Galphimia angustifolia, Polygala alba, Cordia podocephala, Acourtia runcinata, Dalea aurea, Calliandra conferta, Chamaecrista greggii, Heliotropium torreyi, Melampodium cinereum, Hymenopappus scabiosaeus, Desmanthus velutinus, Calylophus hartwegii, Simsia calva, Hermannia texana, Mandevilla macrosiphon (= Macrosiphonia lanuginosa var. macrosiphon), Viguiera stenoloba, Stenaria nigricans, Thymophylla pentachaeta, Wedelia acapulcensis var. hispida (= Wedelia hispida)*, and *Meximalva filipes* (Elliott 2011). Downslope from these sites, soil development increases, soils tend to be tight, a more well‑developed overstory of *Prosopis glandulosa* becomes prominent, and species such as *Castela erecta* and *Ziziphus obtusifolia* increase in cover relative to other species.

**Dynamics:** Erosion occurs on these sites, creating gullies, but not causing a shift in the community. Fire played little to no role in this system, though may have spread into the margins of stands during drought and high wind conditions (Landfire 2007a).

This system was modeled by Landfire (2007a) using a single class. Dense shrubland, generally 40‑90% cover with sparse cover from emergent overstory species. Little natural disturbance affects this shrubland. Low fine fuel loadings make fire spread minimal except under extreme windy and dry conditions when fire may spread into it from surrounding sites. Species are drought‑resistant. However, this system occurs in large patch to matrix scale and marginal fires likely spread little into the interior portions of occurrences.

SOURCES

**References:** CONABIO 2003b, Comer et al. 2003*, Elliott 2011, Jahrsdoerfer and Leslie 1988, LANDFIRE 2007a, McLendon 1991, TNC 2013

**Version:** 14 Jan 2014	**Stakeholders:** Latin America, Southeast

**Concept Author:** K.A. Schulz	**LeadResp:** Southeast

CES301.989 Tamaulipan Caliche Grassland

**Primary Division:** Madrean Semidesert (301)

**Land Cover Class:** Herbaceous

**Spatial Scale & Pattern:** Small patch

**Required Classifiers:** Natural/Semi‑natural; Vegetated (>10% vasc.); Upland; Wetland

**Diagnostic Classifiers:** Lowland [Lowland]; Herbaceous; Plain; Tropical/Subtropical [Tropical Xeric]; Alkaline Soil; Calcareous; Very Shallow Soil; Sand Soil Texture; Caliche Layer

**Concept Summary:** This ecological system is restricted to the Loreto Plain in Tamaulipas, Mexico. It occurs on shallow sandy loam soils with a caliche hardpan subhorizon. These small‑patch grasslands are less than 40 ha in area and are dominated by perennial grasses often with sparse low shrubs within a mosaic of thornscrub. Dominant grasses may include *Aristida purpurea, Bouteloua hirsuta, Bouteloua radicosa, Cenchrus spinifex, Paspalum setaceum*, and *Tridens muticus*. Perennial forbs may be abundant such as *Boerhavia coccinea, Chamaecrista flexuosa, Heliotropium confertifolium*, or *Rhynchosia americana*. Low shrubs are *Calliandra conferta* and *Krameria ramosissima*.

DISTRIBUTION

**Range:** This system is restricted to the Loreto Plain in Tamaulipas, Mexico. It may also be present in Texas on the edge of the sandsheet where it passes over the Goliad Formation in northern Hidalgo and Starr counties (Elliott 2011).

**Divisions:** 301:C

**TNC Ecoregions:** 30:C, 31:C

**Nations:** MX, US

**Subnations:** MXTM, TX

**Map Zones:** 36:C

**USFS Ecomap Regions:** 315E:CC

CONCEPT

**Environment:** This system is described from the vicinity of Loreto in Tamaulipas, Mexico, but the conditions of sand veneer over caliche outcrop may also be present on the edge of the sandsheet where it passes over the Goliad Formation in northern Hidalgo and Starr counties (Elliott 2011). Soils are a reddish sandy loam about 0.3 m in depth or less. These grasslands occur on relatively level sites atop the Goliad Formation. These are areas that have a relatively thin veneer of eolian sand over caliche substrate. Such sites occur on the edge of the South Texas Sand Sheet where it overlies caliche of the Goliad Formation. Soils are shallow sands and sandy loams, sometimes red sandy loams, over caliche substrate. This Tamaulipan ecological system occurs on clay prairies near the Gulf Coast and drier sites further inland. Substrates are fine calcareous clays and clay loam. Occasional fires and root‑pruning from montmorillonitic clay limit shrub invasion, if the grassland is not overgrazed. If overgrazed, the land will convert to stable thornscrub dominated by *Prosopis glandulosa* and *Celtis ehrenbergiana*.

**Vegetation:** Sites may currently be occupied by non‑native grasses such as *Pennisetum ciliare* and *Bothriochloa ischaemum var. songarica*, though invasion by these species is not observed in Mexican occurrences (Chris Best pers. obs.). These grasslands are known to occur within a mosaic of calcareous shrublands. Johnston (1963) describes them as grassland patches (the largest of which are 50 to 100 acres in extent) within a matrix of shrubland. Grasses often dominate sites, including species such as *Schizachyrium littorale, Aristida purpurea, Bouteloua hirsuta, Elionurus tripsacoides, Trachypogon spicatus, Heteropogon contortus, Bouteloua curtipendula, Tridens texanus*, and *Tridens muticus*. *Brachiaria ophryodes* and *Bouteloua radicosa* are also noted from occurrences in Mexico. Shrubs and subshrubs are scattered and sometimes coalesce into larger areas, and include species such as *Calliandra conferta, Krameria ramosissima, Calliandra biflora, Chamaecrista greggii*, and *Mandevilla lanuginosa (= Macrosiphonia lanuginosa)*. Perennial forbs are conspicuous and include species such as *Heliotropium confertifolium, Melampodium cinereum, Simsia calva, Acalypha radians, Cnidoscolus texanus, Galphimia angustifolia, Hermannia texana, Croton capitatus, Rhynchosia americana*, and *Dalea nana*. Scattered shrubs that may be present include *Prosopis glandulosa, Zanthoxylum fagara, Cordia boissieri*, and *Condalia hookeri* (Elliott 2011).

**Dynamics:** Occurrences are naturally small with the larger brush‑free stands only 50‑100 acres in extent. Larger areas occur as mosaics of grassland mixed with brush mottes (Johnston 1963). Fire is a key process that limits invasion by brush (Landfire 2007a). Fire occurs on a frequent 2‑ to 5‑year return interval. The fire regime has frequent replacement fires, both lightning and anthropogenic in origin (Stewart 1951, Lehmann 1965, Drawe 1980, Stewart 2002, Jurney et al. 2004). Fire was dependent on the availability of dry fine fuel sufficient to carry a fire. Both native grazing and wet/dry periods would have dictated whether sufficient dry fine fuels were present to carry a burn and strongly influenced the probable size of burns. Drought may shift composition and cause minor changes in herbaceous cover (Landfire 2007a).

SOURCES

**References:** Best pers. comm., Comer et al. 2003*, Drawe 1980, Elliott 2011, Elliott, L. pers. comm., Johnston 1955, Johnston 1963, Jurney et al. 2004, LANDFIRE 2007a, Lehmann 1965, Stewart 1951, Stewart 2002, TNC 2013

**Version:** 14 Jan 2014	**Stakeholders:** Latin America, Southeast

**Concept Author:** K.A. Schulz	**LeadResp:** Southeast

CES301.987 Tamaulipan Clay Grassland

**Primary Division:** Madrean Semidesert (301)

**Land Cover Class:** Herbaceous

**Spatial Scale & Pattern:** Small patch

**Required Classifiers:** Natural/Semi‑natural; Vegetated (>10% vasc.); Upland

**Diagnostic Classifiers:** Herbaceous; Tropical/Subtropical [Tropical Xeric]; Calcareous; Clay Soil Texture

**National Mapping Codes:** EVT 2440; ESLF 7153; ESP 1440

**Concept Summary:** This Tamaulipan ecological system occurs on clay prairies near the Gulf Coast and drier sites further inland. Substrates are fine calcareous clays and clay loam. Occasional fires and root pruning from montmorillonitic clay limit shrub invasion, if the grassland is not overgrazed. If overgrazed the land will convert to stable thornscrub dominated by *Prosopis glandulosa* and *Celtis ehrenbergiana (= Celtis pallida)*. Vegetation is dominated by perennial mid and short grasses such as *Schizachyrium scoparium, Paspalum* spp., *Trichloris pluriflora (= Chloris pluriflora), Bouteloua dactyloides (= Buchloe dactyloides)*, with other grasses such as *Bothriochloa saccharoides, Bouteloua curtipendula, Chloris andropogonoides, Nassella leucotricha, Schedonnardus paniculatus, Setaria leucopila*, and clumps of *Andropogon gerardii* on less clayey sites. *Prosopis glandulosa* or *Quercus fusiformis* are often present as scattered mottes or are restricted to drainages. *Opuntia engelmannii var. lindheimeri* is often present.

**Comments:** The Texas portion of this system has been subsumed into Texas‑Louisiana Coastal Prairie (CES203.550), leaving it as a placeholder for Mexico.

DISTRIBUTION

**Range:** Thought to occur on clay prairies near the Gulf Coast of Mexico and drier sites further inland.

**Divisions:** 301:?

**TNC Ecoregions:** 30:C

**Nations:** MX

**Subnations:** MXCO?, MXNU?, MXTM?

**Map Zones:** 31:C, 34:C

CONCEPT

**Environment:** [from M130] This vegetation is found in semi‑arid and subtropical southern Texas over a variety of soil depths and textures. Rainfall is highly variable both spatially and temporally and can range from 38 to 76 cm (15‑30 inches) annually in a given locality, but all areas are prone to drought and water deficits (Bray 1901, Gilbert 1982, Jahrsdoerfer and Leslie 1988).

SOURCES

**References:** Comer et al. 2003*, McLendon 1991

**Version:** 26 Nov 2012	**Stakeholders:** Latin America, Southeast

**Concept Author:** NatureServe Western Ecology Team	**LeadResp:** Southeast

CES301.462 Tamaulipan Loma Shrubland and Grassland

**Primary Division:** Madrean Semidesert (301)

**Land Cover Class:** Shrubland

**Spatial Scale & Pattern:** Large patch

**Required Classifiers:** Natural/Semi‑natural; Vegetated (>10% vasc.); Upland

**National Mapping Codes:** EVT 2439; ESLF 7152; ESP 1439

**Concept Summary:** This ecological system occurs on well‑drained portions of clay dunes (lomas) rising above surrounding coastal tidal flats. It is a xeric, subtropical shrubland dominated by thorny evergreen shrubs, generally 2‑4 m tall. Composition of this system is extremely variable, and there is usually no clear dominant, except locally. Local dominants may include *Citharexylum berlandieri, Leucophyllum frutescens, Havardia pallens (= Pithecellobium pallens)*, and *Ebenopsis ebano*. While there is often no clear dominant, *Yucca treculeana* is a constant and conspicuous emergent in many occurrences. Some lomas may be flooded by the sea during severe storm events. Vegetation in this system is sometimes influenced by salt spray, high winds, limited rooting depth, saline water table, and extreme xeric conditions.

DISTRIBUTION

**Range:** This coastal system is known from Aransas County, Texas, south into Mexico.

**Divisions:** 301:C

**TNC Ecoregions:** 31:C

**Nations:** US

**Subnations:** TX

**Map Zones:** 36:C

**USFS Ecomap Regions:** 255D:CC, 315E:??

CONCEPT

**Environment:** This system occupies well‑drained portions of clay dunes (lomas) along the lower Texas coast (and somewhat inland) and adjacent Mexico. These rise above surrounding coastal tidal flats and often develop from deposition of windblown fine sediments, resulting in elevated landforms within a matrix of tidal flats (Elliott 2011). At the time of formation, lomas were located on the leeward side of irregularly flooded lagoons and tidal flats that when dry provided the source for the windblown clayey sediments. The geology consists of Quaternary windblown deposits identified as clay dunes (Qcd). Landforms are round, elliptic, or crescent‑shaped topographic highs, often within a matrix of low flats influenced by wind‑driven tides. Soils include Point Isabel clay loam and Lalinda fine sandy loam, which are often associated with the Coastal Ridge Ecological Site. Lomas are characterized as wind‑formed clay dunes on or near the coast, often surrounded by flats containing halophytic vegetation, coastal grasslands, or unvegetated wind‑tidal flats. They usually occur as topographic highs in the surrounding level landscape, sometimes to 10 m above the surrounding plain and are a small‑patch occurrence.

**Vegetation:** These are typically fairly dense to extremely dense shrublands, often 2‑4 m in height. While there is often no clear dominant, *Yucca treculeana* is a constant and conspicuous emergent in many occurrences (Johnston 1952). Other dominants may include *Acacia rigidula, Castela erecta, Celtis ehrenbergiana, Citharexylum berlandieri, Ebenopsis ebano, Forestiera angustifolia, Guaiacum angustifolium, Jatropha dioica, Karwinskia humboldtiana, Leucophyllum frutescens, Opuntia engelmannii var. lindheimeri, Phaulothamnus spinescens, Prosopis glandulosa, Sideroxylon celastrinum, Zanthoxylum fagara*, and *Ziziphus obtusifolia*. There may be scattered emergent trees of *Ebenopsis ebano* and *Prosopis glandulosa* forming a sparse woodland. Within these shrublands, the herbaceous layer is typically not well‑developed, however, the non‑native *Urochloa maxima* may be conspicuous. A grassland, often dominated by *Sporobolus wrightii* occupies the margins of these clay dunes, as they grade downslope into the surrounding salty flats. These margins may also contain *Sporobolus pyramidatus, Monanthochloe littoralis*, and *Spartina spartinae*. Other somewhat halophytic species, such as *Maytenus phyllanthoides* and *Prosopis reptans*, may also occupy these dunes. The proximity of many of these dunes to active tidal fluctuations and salt spray also influences species composition at these sites.

**Dynamics:** From Landfire (2007a): Hurricanes and tropical storms can affect these sites through tidal surge causing influx of saline waters. Saltwater inundation would be restricted temporally to the period during storm surge and would not likely significantly affect shrub mortality. Also, high‑intensity storms may completely eliminate these sites through erosion. Erosional processes would tend to completely eliminate sites rather than causing changes in the system structure. Fire is not a process important to this system and does not or rarely occurs. Tidal flat islands are important for wildlife such as migratory birds, mollusks and fish (USACE 2013).

<br />This system occurs as small‑patch sites ranging from 10s to a few 100 acres. Disturbances tend to have local effects or completely eliminate a site. This BpS occurs as a stable system.

SOURCES

**References:** Comer et al. 2003*, Crosswhite 1980, Elliott 2011, Jahrsdoerfer and Leslie 1988, Johnston 1952, LANDFIRE 2007a, TNC 2013, USACE 2013

**Version:** 14 Jan 2014	**Stakeholders:** Southeast

**Concept Author:** J. Teague	**LeadResp:** Southeast

CES301.984 Tamaulipan Mesquite Upland Scrub

**Primary Division:** Madrean Semidesert (301)

**Land Cover Class:** Shrubland

**Spatial Scale & Pattern:** Matrix

**Required Classifiers:** Natural/Semi‑natural; Vegetated (>10% vasc.); Upland

**Diagnostic Classifiers:** Lowland [Lowland]; Shrubland (Shrub‑dominated); Tropical/Subtropical [Tropical Xeric]; Prosopis spp. ‑dominated

**National Mapping Codes:** EVT 2391; ESLF 5322; ESP 1391

**Concept Summary:** This ecological system occurs in the Tamaulipan region of northeastern Mexico. Its current concept is dominated by thornscrub that was limited to rocky, broken uplands and drainages that has become widespread in the last 100‑150 years as the result of disturbance to adjacent mesquite savanna grasslands. Severe overgrazing in the mid‑1800s, with subsequent shifts in fire processes and changes in edaphic conditions, has allowed this thornscrub ecological system to be the new steady‑state. The vegetation is characterized by an open to dense tall‑shrub layer dominated by *Prosopis glandulosa* with many other species present to codominant such as *Acacia berlandieri, Vachellia farnesiana, Acacia rigidula, Amyris madrensis, Amyris texana, Celtis ehrenbergiana, Leucophyllum frutescens, Opuntia* spp., *Parkinsonia texana, Yucca* spp., and *Zanthoxylum fagara*. The herbaceous layer is generally sparse, but dense graminoids may dominate the herbaceous layer of stands with open shrub canopies or remnant patches of savanna.

**Comments:** More review is needed to determine the naturalness of this system and its distribution and relationship to other Tamaulipan thornscrub and Chihuahuan Desert systems. It has been removed from the Texas classification per TPWD's comprehensive statewide ecological system mapping project.

DISTRIBUTION

**Range:** This system is a placeholder for relevant vegetation in the Tamaulipan region of northeastern Mexico.

**Divisions:** 301:?

**TNC Ecoregions:** 30:?

**Nations:** MX

**Subnations:** MXCO?, MXNU?, MXTM?

**USFS Ecomap Regions:** 255E:CC, 315C:C?, 315D:CC, 315G:C?

CONCEPT

**Vegetation:** The vegetation is characterized by an open to dense tall‑shrub layer dominated by *Prosopis glandulosa* with many other species present to codominant such as *Acacia berlandieri, Vachellia farnesiana (= Acacia farnesiana), Acacia rigidula, Amyris madrensis, Amyris texana, Celtis ehrenbergiana (= Celtis pallida), Leucophyllum frutescens, Opuntia* spp., *Parkinsonia texana, Yucca* spp., and *Zanthoxylum fagara*. The herbaceous layer is generally sparse, but dense graminoids may dominate the herbaceous layer of stands with open shrub canopies or remnant patches of savanna.

SOURCES

**References:** Brown 1982a, Brown et al. 1998, CONABIO 2003a, Comer et al. 2003*, Eyre 1980, Jahrsdoerfer and Leslie 1988, Webster 2001

**Version:** 26 Nov 2012	**Stakeholders:** Latin America, Southeast

**Concept Author:** NatureServe Western Ecology Team	**LeadResp:** Southeast

CES301.983 Tamaulipan Mixed Deciduous Thornscrub

**Primary Division:** Madrean Semidesert (301)

**Land Cover Class:** Shrubland

**Spatial Scale & Pattern:** Matrix

**Required Classifiers:** Natural/Semi‑natural; Vegetated (>10% vasc.); Upland

**Diagnostic Classifiers:** Lowland [Lowland]; Shrubland (Shrub‑dominated); Tropical/Subtropical [Tropical Xeric]

**National Mapping Codes:** EVT 2390; ESLF 5321; ESP 1390

**Concept Summary:** This thornscrub ecological system occurs throughout much of northeastern Mexico and southern Texas. It occurs on a variety of substrates and landforms. Dominant species include *Acacia roemeriana, Leucophyllum frutescens*, and *Prosopis glandulosa*. Other species present to codominant include *Acacia berlandieri, Vachellia farnesiana, Amyris madrensis, Amyris texana, Celtis ehrenbergiana, Parkinsonia texana*, and cacti such as *Opuntia engelmannii var. lindheimeri*. The herbaceous layer is not well‑developed but *Trichloris pluriflora, Setaria* spp., and *Malpighia glabra* are present. This system generally occurs as a closed shrubland or low woodland, usually lacking a purely open herbaceous component. Soils are clays, clay loams, and clay flats and are often calcareous or alkaline to varying degrees. Some sites are highly saline, and these sites are occupied by Tamaulipan Saline Thornscrub (CES301.711), but transitions between the systems may be subtle.

**Comments:** This shrubland is differentiated from Tamaulipan Savanna Grassland (CES301.985) as it occupies tighter soils, as opposed to the sandier soils of the savanna grassland. The sites are often lower in the landscape compared to nearby savanna grassland or Tamaulipan Calcareous Thornscrub (CES301.986), but would be considered uplands as they are distant from bottomland soils and drainages, and are not well‑developed woodlands typical of the lowest landscape positions. To a large degree, all of these systems share numerous shrub species, but show subtle differences in relative dominance. However, this system generally occurs as a closed shrubland or low woodland, usually lacking a purely open herbaceous component.

DISTRIBUTION

**Range:** Occurs throughout much of northeastern Mexico and southern Texas.

**Divisions:** 301:C

**TNC Ecoregions:** 30:C, 31:C

**Nations:** MX, US

**Subnations:** MXCO, MXNU, MXTM, TX

**Map Zones:** 36:C

**USFS Ecomap Regions:** 315E:CC

CONCEPT

**Environment:** This system is well‑represented on the Eocene Claiborne and Jackson groups and the Pleistocene Beaumont Formation, but is also found on various other formations. Its landforms are gently rolling to nearly level sites, sometimes interdigitated with calcareous ridges and low‑lying drainages and bottomlands. Found on upland sites on tight soils deposited through alluvial processes associated with the Rio Grande, also occurs on uplands away from the delta on deeper soils. Clay, Clay Flat, and Clay Loam Ecological Sites are the typical soils for this system.

**Vegetation:** *Prosopis glandulosa* is very often a conspicuous component of the canopy in stands of this system, sometimes reaching to 6 m in height. This canopy may be dense, but given the open nature of the canopy of individual *Prosopis glandulosa*, significant solar radiation reaches the lower strata. *Vachellia farnesiana (= Acacia farnesiana), Celtis ehrenbergiana, Ebenopsis ebano*, and *Celtis laevigata* may also be components of the canopy, but *Prosopis glandulosa* usually dominates. The overstory canopy may be open with only scattered emergent trees over a dense shrub layer at 1 to 3 m in height. Depending on land‑use history, the shrub understory may be limited to a few species such as *Opuntia engelmannii var. lindheimeri, Ziziphus obtusifolia*, or *Celtis ehrenbergiana (= Celtis pallida)* on relatively recently cleared sites. On more mature sites, a diverse assemblage of species, such as *Acacia rigidula, Castela erecta, Malpighia glabra, Opuntia engelmannii var. lindheimeri, Cylindropuntia leptocaulis, Ziziphus obtusifolia, Celtis ehrenbergiana, Lycium berlandieri, Forestiera angustifolia, Guaiacum angustifolium, Diospyros texana, Amyris texana, Karwinskia humboldtiana, Havardia pallens, Phaulothamnus spinescens, Schaefferia cuneifolia, Condalia hookeri*, and *Zanthoxylum fagara*, may occur. *Leucophyllum frutescens* and *Acacia berlandieri* may be present, but occur as scattered individuals as opposed to dominating the aspect of the community as they sometimes do on some shallow‑soiled calcareous sites. However, like some shallow‑soiled calcareous sites, *Acacia rigidula* is the aspect dominant of the shrub layer. The herbaceous layer is usually fairly sparse. Currently, the herbaceous layer may actually be dense with the non‑native grass *Urochloa maxima*. Other non‑native species, such as *Pennisetum ciliare, Cynodon dactylon, Bothriochloa ischaemum var. songarica*, and *Dichanthium annulatum*, may also be present to dominant. Native grasses, such as *Bothriochloa laguroides ssp. torreyana, Chloris* spp. (= *Trichloris* spp.), and *Pappophorum bicolor*, may be present.

**Dynamics:** Fire plays a role in this system, occurring in situations adjacent to grasslands during dry conditions when fire would jump to the canopy and carry during wind events. Drought would influence fire occurring in the woodland and shrubland classes (Landfire 2007a).

This system was modeled by Landfire (2007a) using three classes: early‑, mid‑ and late‑seral. The early‑seral (0‑5 years) class is dominated by perennial grasses. This class was maintained on higher topographic positions somewhat longer because of slower shrub growth in more xeric situations. Frequent replacement fire (MFRI = 7 years) is the dominant disturbance type in this class (Landfire 2007a). Mid‑seral class is dominated by shrubs (40‑70% cover). In this class, mesquite is a component of the shrub layer along with the other shrubs. Drought is incorporated into the MFRI in that dry conditions would be required for fire to be carried in the canopy. Replacement fire (MFRI = 20 years) is the dominant disturbance type in this class (Landfire 2007a). The late‑seral class has a shrub layer at a height of 2‑4 m and 70‑100% cover. Mesquite canopy is well‑developed in this class. Shrub layer development is extensive forming an almost continuous layer. Replacement fire (MFRI = 30 years) is the dominant disturbance type in this class (Landfire 2007a).

SOURCES

**References:** Brown 1982a, Brown et al. 1998, CONABIO 2003a, Comer et al. 2003*, Crosswhite 1980, Elliott 2011, Jahrsdoerfer and Leslie 1988, LANDFIRE 2007a, TNC 2013, Webster 2001

**Version:** 14 Jan 2014	**Stakeholders:** Latin America, Southeast

**Concept Author:** K.A. Schulz	**LeadResp:** Southeast

CES301.992 Tamaulipan Ramadero

**Primary Division:** Madrean Semidesert (301)

**Land Cover Class:** Woody Wetland

**Spatial Scale & Pattern:** Linear

**Required Classifiers:** Natural/Semi‑natural; Vegetated (>10% vasc.); Wetland

**Diagnostic Classifiers:** Lowland [Lowland]; Shrubland (Shrub‑dominated); Arroyo; Tropical/Subtropical [Tropical Xeric]; Riverine / Alluvial; Intermittent Flooding

**Concept Summary:** This Tamaulipan riparian shrubland system is restricted to drainages in upland areas or ramaderos of southern Texas and adjacent Mexico that are intermittently flooded. Typical stands have a closed canopy (relative to the surrounding landscape) from 5 to 10 m in height. The overstory canopy is typically dominated by species such as *Prosopis glandulosa, Vachellia farnesiana, Celtis ehrenbergiana*, and/or *Parkinsonia aculeata*. In addition, *Celtis laevigata* and/or *Ebenopsis ebano* may also be present in the canopy. Some sites have a relatively open subcanopy, but more commonly the shrub layer is thick, sometimes impenetrable, and varies in height from 1 to 5 m. Species commonly encountered in the shrub layer include *Aloysia gratissima, Phaulothamnus spinescens, Celtis ehrenbergiana, Condalia hookeri, Forestiera angustifolia, Diospyros texana, Ziziphus obtusifolia, Koeberlinia spinosa, Malpighia glabra, Zanthoxylum fagara, Opuntia engelmannii var. lindheimeri, Guaiacum angustifolium, Colubrina texensis*, and *Amyris texana*. Ground cover can be sparse or, in more open stands, may have a fairly continuous grassy cover.

**Comments:** The Ramadero of Jahrsdoerfer and Leslie (1988) is equivalent to this system concept, but could be considered narrower if more information on how it occurs elsewhere in Mexico can be obtained. Not much more information is likely to be found because this system is not sampled very often. Intermittent drainages (arroyos and ramaderos) are not sampled that often because of scale and variability issues. More information is needed to fully characterize this system.

DISTRIBUTION

**Range:** This system occurs in southern Texas and adjacent Mexico.

**Divisions:** 301:C

**TNC Ecoregions:** 30:C

**Nations:** MX, US

**Subnations:** MXCO, MXNU, MXTM, TX

**Map Zones:** 36:C

**USFS Ecomap Regions:** 315E:CC

CONCEPT

**Environment:** This Tamaulipan riparian shrubland system is restricted to drainages in upland areas or ramaderos (isolated strips of dense brush associated with arroyos) that are intermittently flooded. This is a widespread system on various geologic strata. It is typically found in upland drainages in various landscapes. Drainages are extremely flashy from runoff from surrounding landscape. These sites are infrequently flooded during local rainfall events, but because they accumulate runoff, they tend to be slightly more mesic in this otherwise xeric landscape. Soils are various upland soils (but not Bottomland ecological site types). These are sometimes mapped specifically as Ramadero Ecological Site. These woodlands are found along drainages (locally known as ramaderos) that are extremely flashy and are infrequently and briefly flooded during local rain events. The soils are typically clay loams or sandy clay loams

**Vegetation:** Moisture accumulation due to the topographic position of these woodlands promotes the development of a closed canopy (relative to the surrounding landscape) from 5 to 10 m in height. The overstory canopy is typically dominated by species such as *Prosopis glandulosa, Vachellia farnesiana (= Acacia farnesiana), Celtis ehrenbergiana*, and/or *Parkinsonia aculeata*. In addition, *Celtis laevigata* and/or *Ebenopsis ebano* may also be present in the canopy. Some sites have a relatively open subcanopy, but more commonly the shrub layer is thick, sometimes impenetrable, and varies in height from 1 to 5 m (Elliott 2011). Species commonly encountered in the shrub layer include *Aloysia gratissima, Phaulothamnus spinescens, Celtis ehrenbergiana, Condalia hookeri, Forestiera angustifolia, Diospyros texana, Ziziphus obtusifolia, Koeberlinia spinosa, Malpighia glabra, Zanthoxylum fagara, Opuntia engelmannii var. lindheimeri, Guaiacum angustifolium, Colubrina texensis*, and *Amyris texana*. Ground cover can be sparse or, in more open stands, may have a fairly continuous grassy cover. Species encountered in the herbaceous layer include *Clematis drummondii, Parietaria pensylvanica, Salvia coccinea, Calyptocarpus vialis, Rivina humilis, Malvastrum americanum, Ruellia* sp., and *Verbesina microptera*. Grasses include *Trichloris pluriflora, Bothriochloa barbinodis, Bouteloua curtipendula, Setaria scheelei, Setaria macrostachya, Setaria leucopila, Chloris cucullata, Digitaria californica, Pappophorum bicolor, Bouteloua trifida, Bouteloua dactyloides*, and *Hilaria belangeri*. The introduced grasses *Pennisetum ciliare, Urochloa maxima*, and *Cynodon dactylon* often dominate these sites, sometimes to the near exclusion of other herbaceous cover (Elliott 2011). This system may merge downstream with Tamaulipan Floodplain (CES301.990).

**Dynamics:** Intermittent fluvial processes define this system, which are often associated with rapid sheet and gully flow that scours the channel bottoms.

SOURCES

**References:** Comer et al. 2003*, Elliott 2011, Jahrsdoerfer and Leslie 1988

**Version:** 25 Feb 2011	**Stakeholders:** Latin America, Southeast

**Concept Author:** K.A. Schulz	**LeadResp:** Southeast

CES301.711 Tamaulipan Saline Thornscrub

**Primary Division:** Madrean Semidesert (301)

**Land Cover Class:** Shrubland

**Spatial Scale & Pattern:** Small patch

**Required Classifiers:** Natural/Semi‑natural; Vegetated (>10% vasc.); Upland; Wetland

**Diagnostic Classifiers:** Shrubland (Shrub‑dominated); Tropical/Subtropical [Tropical Xeric]; Saline Substrate Chemistry

**Concept Summary:** This system is an open shrubland on gently rolling to level sites where soil salinity is particularly high on saline clays. It occurs in the Tamaulipan region of southern Texas and possibly ranges into Mexico. Scattered *Prosopis glandulosa* usually form an emergent canopy less than 5 m in height, creating an overstory canopy cover of around 10%. A variety of shrubs and subshrubs form the dominant layer with a cover of 20‑70% interspersed in a mosaic with patchy grasses.

DISTRIBUTION

**Range:** This system occurs in the Tamaulipan region of southern Texas and possibly ranges into Mexico.

**Divisions:** 301:C

**TNC Ecoregions:** 30:C

**Nations:** MX?, US

**Subnations:** MXCO?, MXNU?, MXTM?, TX

**Map Zones:** 36:C

**USFS Ecomap Regions:** 315Eb:CCP, 315Ec:CCP, 315Ed:CCP

CONCEPT

**Environment:** This ecological system occurs on gently rolling to low flats, sometimes dissected by minor drainages. It is frequently associated with the Yegua Formation or the Jackson Group and within the Saline Clay and Saline Clay Loam Ecological Sites. Soils are typically saline clays such as Montell, Maverick, and Catarina soils and may have a veneer of gravel over the clay.

**Vegetation:** *Prosopis glandulosa* usually forms a scattered emergent canopy less than 5 m in height, creating an overstory canopy cover of around 10%. Shrubs and subshrubs, such as *Varilla texana, Castela erecta, Acacia rigidula, Atriplex canescens, Isocoma coronopifolia, Condalia spathulata, Jatropha dioica, Suaeda* spp., *Opuntia engelmannii var. lindheimeri, Cylindropuntia leptocaulis, Xylothamia palmeri, Tiquilia canescens*, and *Prosopis reptans*, are conspicuous elements of the relatively open shrubland (20‑70% canopy cover). Patchy grasses typify the herbaceous layer, with such species as *Hilaria belangeri, Sporobolus pyramidatus, Pappophorum bicolor, Bouteloua dactyloides, Bouteloua trifida*, and occasionally *Monanthochloe littoralis*. Forbs such as *Billieturnera helleri, Chamaesyce albomarginata, Heliotropium curassavicum*, and *Thymophylla pentachaeta* may be present and conspicuous. Cacti are sometimes well‑represented in the ground layer, including species such as *Echinocereus reichenbachii ssp. fitchii, Escobaria emskoetteriana, Mammillaria heyderi, Ancistrocactus scheeri (= Sclerocactus scheeri), Echinocactus texensis*, and *Thelocactus setispinus*.

**Dynamics:** Regular fire plays a limited role in this system because of the relatively low cover of fine fuel. During dry conditions it may burn when fire would jump to the shrub layer and canopy and carry during wind events spreading from adjacent grasslands that have more frequent fires. Saline substrates are the driving environmental variable that limits plant growth and species diversity. Substrates are highly erodible saline clay and saline clay loam soils.

SOURCES

**References:** Brown et al. 1998, CONABIO 2003a, Comer et al. 2003*, Elliott 2011, Jahrsdoerfer and Leslie 1988, TNC 2013, Webster 2001

**Version:** 14 Jan 2014	**Stakeholders:** Latin America, Southeast

**Concept Author:** L. Elliott, D. Diamond, A. Treuer‑kuehn, D. German, J. Teague	**LeadResp:** Southeast

CES301.985 Tamaulipan Savanna Grassland

**Primary Division:** Madrean Semidesert (301)

**Land Cover Class:** Herbaceous

**Spatial Scale & Pattern:** Large patch

**Required Classifiers:** Natural/Semi‑natural; Vegetated (>10% vasc.); Upland

**Diagnostic Classifiers:** Lowland [Lowland]; Herbaceous; Tropical/Subtropical [Tropical Xeric]

**National Mapping Codes:** EVT 2438; ESLF 7151; ESP 1438

**Concept Summary:** This Tamaulipan ecological system is dominated by perennial grasses with sparse overstory of mesquite or oak trees and thornscrub. Stands of the system are typically dominated by *Prosopis glandulosa* in the overstory, which may be sparse, giving the aspect of an open grassland with scattered trees and shrubs. Or, more commonly, the system occurs as shrub‑dominated patches within a grassy matrix. There will typically be an emergent canopy ranging to about 6 or more meters in height, composed of *Prosopis glandulosa* sometimes with *Ebenopsis ebano* and/or *Celtis ehrenbergiana*. Sometimes the overstory canopy is well‑developed and would be considered woodland. These patches often coalesce to form significant expanses of shrubland. Dominant grasses are *Cynodon* spp. This system was once a common matrix system, but has largely been converted to desert scrub and exists as remnant patches. Degraded subtropical forests and woodlands may have similar structure but are not included in this system because different ecological processes maintain them.

DISTRIBUTION

**Range:** Examples of the system are found on thinner eolian sands on the western side of the South Texas Sand Sheet in Texas and related areas of Mexico.

**Divisions:** 301:C

**TNC Ecoregions:** 30:C, 31:C

**Nations:** MX, US

**Subnations:** MXCO, MXNU, MXTM, TX

**Map Zones:** 36:C

**USFS Ecomap Regions:** 255D:CC, 315E:CC

CONCEPT

**Environment:** Examples of the system are found on thinner eolian sands on the western side of the South Texas Sand Sheet, as well as other sandy sites such as those of the Eocene sands of the Carrizo, Queen City, and Sparta formations. It may also be found associated with other formations, such as Oakville sandstone and other formations producing sandy residuum. Typical sites are level to gently rolling. This system occurs on sandy soils, including sandy, sandy loam, and loamy sands. Ecological Sites include sandy to sandy loam sites, such as those of the Sandy, Loamy Sand and Sandy Loam Ecological Sites (Elliott 2011).

**Vegetation:** This system is typically dominated by *Prosopis glandulosa* in the overstory, which may be sparse, giving the aspect of an open grassland with scattered trees and shrubs. Or, more commonly, the system occurs as shrub‑dominated patches within a grassy matrix. There will typically be an emergent canopy ranging to about 6 or more meters in height, composed of *Prosopis glandulosa* sometimes with *Ebenopsis ebano* and/or *Celtis ehrenbergiana*. Sometimes the overstory canopy is well‑developed and would be considered woodland. These patches often coalesce to form significant expanses of shrubland. Sites with somewhat tighter soils tend to have a denser shrub stratum, while deep sands and sandy sites tend to be more open, often with sizeable areas lacking significant shrub cover and dominated by a primarily graminoid herbaceous layer. The shrub component of woody patches or shrublands is commonly dominated by species such as *Zanthoxylum fagara, Condalia hookeri, Celtis ehrenbergiana, Opuntia engelmannii var. lindheimeri, Diospyros texana, Colubrina texensis, Cylindropuntia leptocaulis*, and *Vachellia farnesiana (= Acacia farnesiana)* (Elliott 2011). *Prosopis glandulosa* is almost always present, and is often dominant to codominant and occupies the highest canopy position (sometimes sharing that position with few other species), sometimes to 6 m in height. Numerous other species may also occur in the shrub layer, including but not limited to *Schaefferia cuneifolia, Mahonia trifoliolata, Forestiera angustifolia, Lycium berlandieri, Aloysia gratissima, Salvia ballotiflora*, and *Ziziphus obtusifolia*. The diversity of the shrub layer is significantly influenced by land‑use history, with recently cleared areas sometimes being represented by a near monoculture of *Prosopis glandulosa* in the overstory, *Pennisetum ciliare* in the herbaceous layer, and *Opuntia engelmannii var. lindheimeri* as the most conspicuous component of the shrub layer. The herbaceous layer is typically dominated by graminoids and may be quite dense (60‑100% cover). Grasses, such as *Schizachyrium scoparium, Schizachyrium littorale, Chloris cucullata, Paspalum monostachyum, Paspalum plicatulum, Elionurus tripsacoides, Bouteloua rigidiseta, Urochloa ciliatissima, Heteropogon contortus, Eragrostis secundiflora, Bothriochloa laguroides ssp. torreyana, Trichloris pluriflora, Aristida* spp., *Sporobolus cryptandrus*, and/or *Dichanthelium* spp., commonly dominate or codominate the herbaceous layer. Forbs are also common, including species such as *Gaillardia pulchella, Eriogonum multiflorum, Croton* spp., *Cnidoscolus texanus, Aphanostephus skirrhobasis, Rudbeckia hirta, Verbesina encelioides, Clematis drummondii, Cynanchum barbigerum, Thymophylla pentachaeta, Justicia pilosella, Nama jamaicense, Monarda punctata, Palafoxia texana, Florestina tripteris, Zornia bracteata, Croptilon divaricatum, Rhynchosia americana*, and *Wedelia acapulcensis var. hispida (= Wedelia texana)*, though some of these species are restricted to the sandiest sites (Elliott 2011).

**Dynamics:** Fire and drought are key ecological processes in this system. This system was modeled by Landfire (2007a) using three classes: early‑, mid‑ and late‑seral. The early‑seral class (1‑20 years) is dominated by perennial grasses. This class was maintained by frequent replacement fire (MFRI = 5 years) as the dominant disturbance type in this class. Droughts slow progression of this class to mid‑seral class. This class is modeled to last 20 years; this duration is extended due to limited mesquite seed dispersal mechanisms historically (prior to livestock introduction) (Landfire 2007a).

Mid‑seral class (21‑50 years) is the early development of shrub patches, often surrounding a mesquite trees. Tree canopy is sparse, but shrub cover is dense. Herbaceous cover is declining due to increased shrub and overstory canopy. Replacement fire is modeled to occur with a 20‑year return interval. A mixed fire is modeled to occur with a 7‑year return interval. Twenty‑year drought is modeled to slow successional progression to late‑seral class. The mechanism for drought effect may be an enhanced effect of fire. This class is modeled to last 30 years (Landfire 2007a).

The late‑seral class (51+ years) is a closed‑canopy, late‑development stage that represents the continued development of shrub patches as they coalesce into more well‑developed woodlands of *Prosopis glandulosa* (Archer 1989). In these late stages other species begin to colonize into woodlands and shrublands. Species present in mid‑seral class are still present in late‑seral class, but other species begin to colonize, such as *Mahonia trifoliolata, Schaefferia cuneifolia*, and *Lycium berlandieri*. Replacement fire is modeled to occur with a 200‑year return interval. A mixed fire is modeled to occur with a 20‑year return interval. Twenty‑year drought is modeled and may slow increase in patch size but does not cause transition (Landfire 2007a).

SOURCES

**References:** Archer 1989, Brown 1982a, Brown et al. 1998, CONABIO 2003b, Comer et al. 2003*, Elliott 2011, Eyre 1980, LANDFIRE 2007a, TNC 2013, Webster 2001

**Version:** 14 Jan 2014	**Stakeholders:** Latin America, Southeast

**Concept Author:** K.A. Schulz	**LeadResp:** Southeast

CES301.460 South Texas Sand Sheet Grassland

**Primary Division:** Madrean Semidesert (301)

**Concept Summary:** This ecological system includes non-tidal maritime grasslands occurring on barrier islands and mainland areas near the coast of southern Texas, from Matagorda Island and Padre Island south along the northern Gulf of Mexico. This includes grasslands of primary and secondary dunes, interdune swales, barrier flats, and the mainland. Some examples of this system naturally occurred as an open matrix of midgrass species within native mesquite - acacia shrublands dominated by *Prosopis glandulosa, Acacia farnesiana*, and *Acacia rigidula* but have become shrub-dominated due to the lack of fire. In many areas this system has been virtually eliminated due to conversion to tame pasture, cropland, or due to lack of burning.

DISTRIBUTION

**Range:** This system ranges from Matagorda Island south along the northern Gulf of Mexico.

**Divisions:** 301:C

**TNC Ecoregions:** 31:C

**Nations:** US

**Subnations:** TX

**USFS Ecomap Regions:**

CONCEPT

**Environment:**

**Vegetation:**

**Dynamics:**

SOURCES

**References:** Comer et al. 2003*

**Version:** 02 Oct 2014	**Stakeholders:** Southeast

**Concept Author:** P. Comer

M089. Viscaino‑Baja California Desert Scrub

CES302.013 Gulf of California Coast Torchwood‑Cardon Desert Scrub

**Primary Division:** North American Warm Desert (302)

**Land Cover Class:** Shrubland

**Spatial Scale & Pattern:** Matrix

**Required Classifiers:** Natural/Semi‑natural; Vegetated (>10% vasc.); Upland

**Diagnostic Classifiers:** Lowland [Lowland]; Shrubland (Shrub‑dominated); Alluvial fan; Slope; Toeslope/Valley Bottom; Tropical/Subtropical [Tropical Xeric]; Bursera microphylla ‑ Pachycereus pringlei

**Concept Summary:** This desert scrub system is found in disjunct areas along the eastern side of central Baja California south from uplands that divide the peninsula to the Gulf of California, and on the west coast of Sonora inland 20‑40 km from Punta Lobos south to Guaymas, including the major islands. Climate is extremely arid with mean annual precipitation of less than 100 mm in the north increasing to 300 mm in southern Baja. Precipitation occurs mostly in the summer‑early fall season (monsoon). Extended drought is common which favors plants with water storage. This system occurs on coarse substrates such as deep granitic soils and a'a lava flows in broad valley bottoms, alluvial fans and on lower mountain slopes. The vegetation is characterized by an open layer of xeromorphic trees and tall shrubs and tall cacti without a low‑shrub layer common elsewhere in the Sonoran Desert. Species such as *Bursera hindsiana, Bursera microphylla, Parkinsonia microphylla, Fouquieria diguetii, Fouquieria splendens, Pachycereus schottii, Pachycereus pringlei*, and *Stenocereus thurberi* dominate with other scattered shrubs and cacti that include *Ambrosia dumosa, Encelia farinosa, Jatropha cinerea, Jatropha cuneata, Hyptis emoryi, Justicia californica, Cylindropuntia bigelovii*, and *Solanum hindsianum*. Isolated *Fouquieria columnaris*, a species typical of Baja, may occur in this system in Sonora.

DISTRIBUTION

**Range:** Found in disjunct areas along the eastern side of central Baja California south from uplands that divide the peninsula to the Gulf of California, and on the west coast of Sonora inland 20‑40 km from Punta Lobos south to Guaymas, including the major islands.

**Divisions:** 302:C

**TNC Ecoregions:** 23:C

**Nations:** MX

**Subnations:** MXBC, MXBS, MXSO

CONCEPT

**Environment:** This desert scrub system is found in disjunct areas along the eastern side of central Baja California south from uplands that divide the peninsula to the Gulf of California, and on the west coast of Sonora inland 20‑40 km from Punta Lobos south to Guaymas, including the major islands. Climate is extremely arid with mean annual precipitation of less than 100 mm in the north increasing to 300 mm in southern Baja. Precipitation occurs mostly in the summer‑early fall season (monsoon). Extended drought is common which favors plants with water storage. This system occurs on coarse substrates such as deep granitic soils and a'a lava flows in broad valley bottoms, alluvial fans and on lower mountain slopes.

**Vegetation:** The vegetation is characterized by an open layer of xeromorphic trees and tall shrubs and tall cacti without a low‑shrub layer common elsewhere in the Sonoran Desert. Species such as *Bursera hindsiana, Bursera microphylla, Parkinsonia microphylla, Fouquieria diguetii, Fouquieria splendens, Pachycereus schottii (= Lophocereus schottii), Pachycereus pringlei*, and *Stenocereus thurberi* dominate with other scattered shrubs and cacti that include *Ambrosia dumosa, Encelia farinosa, Jatropha cinerea, Jatropha cuneata, Hyptis emoryi, Justicia californica, Cylindropuntia bigelovii (= Opuntia bigelovii)*, and *Solanum hindsianum*. Isolated *Fouquieria columnaris*, a species typical of Baja, may occur in this system in Sonora.

SOURCES

**References:** Brown 1982a, Comer et al. 2003*, Shreve and Wiggins 1964

**Version:** 24 Mar 2003	**Stakeholders:** Latin America, West

**Concept Author:** NatureServe Western Ecology Team	**LeadResp:** West

CES302.740 Magdalena Plain Desert Scrub

**Primary Division:** North American Warm Desert (302)

**Land Cover Class:** Shrubland

**Spatial Scale & Pattern:** Matrix

**Required Classifiers:** Natural/Semi‑natural; Vegetated (>10% vasc.); Upland

**Diagnostic Classifiers:** Shrubland (Shrub‑dominated); Tropical/Subtropical [Tropical Xeric]

**Concept Summary:** This southern Baja California desert scrub is found in the southern part of the Magdalena region along the Pacific Ocean where the narrow coastal strip widens into a broad plain. Climate is arid with less than 200 mm mean annual precipitation (less than 100 mm near the coast) occurring mostly in the summer‑early fall season (monsoon). However, cool coastal sea breezes and fog help ameliorate some of the aridity. Substrates are generally clayey soils with low infiltration that are derived from volcanic rock. Vegetation in this plain is more open than Magdalena Barrancas Desert Scrub (CES302.739) but is composed of similar species with the addition of halophytic species that occur on the many playas (both large and small) in this plain. The lack of *Fouquieria columnaris, Pachycormus discolor*, and low frequency of *Agave* and *Yucca* species separate this vegetation from the Vizcaino region. Common species may include *Jatropha cuneata, Larrea tridentata, Lysiloma candida, Opuntia cholla, Pachycereus pringlei, Parkinsonia microphylla, Prosopis glandulosa var. torreyana, Stenocereus thurberi*, and halophytes such as species of *Lycium* and *Suaeda*.

DISTRIBUTION

**Range:** Southern part of the Magdalena region of Baja California along the Pacific Ocean where the narrow coastal strip widens into a broad plain.

**Divisions:** 302:C

**TNC Ecoregions:** 23:C

**Nations:** MX

**Subnations:** MXBC

CONCEPT

**Environment:** [from M089] This desert scrub type comprises most of the Baja California peninsula and occurs in diverse environments from the coastal and inland plains to alluvial fans, foothills, and mountains. Climate is extremely arid with mean annual precipitation of less than 100 mm in the north increasing to 300 mm in southern Baja. Precipitation occurs mostly in the summer‑early fall season (monsoon). Extended drought is common and favors plants with water storage. Coastal stands west of the mountain divide may benefit from cool sea breezes and fog that help ameliorate some of the aridity. Some vegetation types, such as *Frankenia‑Ocotillo‑Datilillo* desert scrub are limited to the fog belt in the coastal plain of southern Viscaino. Substrates vary from coarse‑textured, deep granitic soils and a'a lava flows in broad valley bottoms, alluvial fans and on lower mountain slopes to finer‑textured, alkaline, sometimes saline, clayey soils with low infiltration that are derived from marine deposit, basalt or other volcanic rock. Some stands occur in narrow valleys and rocky slopes below generally sparsely vegetated volcanic mesas. Soil is present and moisture is available locally in seeps from these mesas. Larger valleys or barrancas are typically drier and have more open vegetation than smaller ones. An exception to the extremely arid environments is the tropical San Lucan thornscrub, which is limited in distribution to central and southern Baja California. These areas receive more precipitation (316‑482 mm) per year, with a dry season from late October through July. Mean monthly temperatures range from 21.5‑23.6°C. Pacific slopes receive greater rainfall and experience generally lower temperatures than the gulf side of the Cape region.

**Dynamics:** [from M089] Periodic severe drought is common in Baja California which favors plants that can store water during the unfavorable times.

SOURCES

**References:** Brown 1982a, Comer et al. 2003*, Shreve and Wiggins 1964

**Version:** 20 Feb 2003	**Stakeholders:** Latin America, West

**Concept Author:** NatureServe Western Ecology Team	**LeadResp:** West

CES302.739 Matorral Desértico de Magdalena Barrancas

**Primary Division:** North American Warm Desert (302)

**Land Cover Class:** Shrubland

**Spatial Scale & Pattern:** Small patch

**Required Classifiers:** Natural/Semi‑natural; Vegetated (>10% vasc.); Upland

**Diagnostic Classifiers:** Lowland [Lowland]; Shrubland (Shrub‑dominated); Toeslope/Valley Bottom; Tropical/Subtropical [Tropical Xeric]

**Concept Summary:** This southern Baja California desert scrub is found the northern part of the Magdalena region from the Pacific Ocean to the crest of the uplands that divide the peninsula. Climate is arid with mean annual precipitation less than 200 mm. Precipitation occurs mostly in the summer‑early fall season (monsoon) . It occurs in narrow valleys and rocky slopes below generally sparsely vegetated volcanic mesas. Soil is present and moisture is available locally in seeps from these mesas. Larger valleys or barrancas are typically drier and have more open vegetation than smaller ones. The lack of *Fouquieria columnaris, Pachycormus discolor*, and low frequency of *Agave* and *Yucca* species separate this vegetation from the Vizcaino region. Common species may include *Bursera fagaroides var. elongata, Fouquieria peninsularis, Jatropha cuneata, Pachycereus pringlei, Parkinsonia microphylla, Prosopis palmeri, Stenocereus thurberi, Opuntia cholla, Acacia brandegeana*, and *Ficus palmeri* and *Lysiloma candida* on rocky slopes.

DISTRIBUTION

**Range:** Magdalena region of Baja California from the Pacific Ocean to the crest of the uplands that divide the peninsula.

**Divisions:** 302:C

**TNC Ecoregions:** 23:C

**Nations:** MX

**Subnations:** MXBC

CONCEPT

**Environment:** [from M089] This desert scrub type comprises most of the Baja California peninsula and occurs in diverse environments from the coastal and inland plains to alluvial fans, foothills, and mountains. Climate is extremely arid with mean annual precipitation of less than 100 mm in the north increasing to 300 mm in southern Baja. Precipitation occurs mostly in the summer‑early fall season (monsoon). Extended drought is common and favors plants with water storage. Coastal stands west of the mountain divide may benefit from cool sea breezes and fog that help ameliorate some of the aridity. Some vegetation types, such as *Frankenia‑Ocotillo‑Datilillo* desert scrub are limited to the fog belt in the coastal plain of southern Viscaino. Substrates vary from coarse‑textured, deep granitic soils and a'a lava flows in broad valley bottoms, alluvial fans and on lower mountain slopes to finer‑textured, alkaline, sometimes saline, clayey soils with low infiltration that are derived from marine deposit, basalt or other volcanic rock. Some stands occur in narrow valleys and rocky slopes below generally sparsely vegetated volcanic mesas. Soil is present and moisture is available locally in seeps from these mesas. Larger valleys or barrancas are typically drier and have more open vegetation than smaller ones. An exception to the extremely arid environments is the tropical San Lucan thornscrub, which is limited in distribution to central and southern Baja California. These areas receive more precipitation (316‑482 mm) per year, with a dry season from late October through July. Mean monthly temperatures range from 21.5‑23.6°C. Pacific slopes receive greater rainfall and experience generally lower temperatures than the gulf side of the Cape region.

**Vegetation:** Common species may include *Bursera fagaroides var. elongata (= Bursera odorata), Fouquieria peninsularis, Jatropha cuneata, Pachycereus pringlei, Parkinsonia microphylla, Prosopis palmeri, Stenocereus thurberi, Opuntia cholla, Acacia brandegeana*, and *Ficus palmeri* and *Lysiloma candida* on rocky slopes.

**Dynamics:** [from M089] Periodic severe drought is common in Baja California which favors plants that can store water during the unfavorable times.

SOURCES

**References:** Brown 1982a, Comer et al. 2003*, Shreve and Wiggins 1964

**Version:** 20 Feb 2003	**Stakeholders:** Latin America, West

**Concept Author:** NatureServe Western Ecology Team	**LeadResp:** West

CES302.006 Matorral Desértico de Maguey y Cirio de la Planicie Costera del Norte de Viscaino

**Primary Division:** North American Warm Desert (302)

**Land Cover Class:** Shrubland

**Spatial Scale & Pattern:** Matrix

**Required Classifiers:** Natural/Semi‑natural; Vegetated (>10% vasc.); Upland

**Diagnostic Classifiers:** Lowland [Lowland]; Shrubland (Shrub‑dominated); Toeslope/Valley Bottom; Igneous Rock; Tropical/Subtropical [Tropical Xeric]; Clay Soil Texture; Agave shawii ‑ Fouquieria columnaris

**Concept Summary:** This desert scrub system is found near the Pacific Coast on the plains and rolling hills of the northern Vizcaino region in central Baja California. Climate is extremely arid with less than 100 mm mean annual precipitation occurring mostly in the fall‑winter. However, cool coastal sea breezes and spring and summer fog help ameliorate the aridity. Substrates are clayey soils with low infiltration that are derived from volcanic rock. Vegetation is an open desert scrub dominated by *Agave shawii* and *Fouquieria columnaris* with abundant low shrubs such as *Ambrosia camphorata, Ambrosia chenopodiifolia, Echinocereus maritimus, Eriogonum fasciculatum*, and *Cylindropuntia prolifera (= Opuntia prolifera)*.

DISTRIBUTION

**Range:** Found near the Pacific Coast on the plains and rolling hills of the northern Vizcaino region in central Baja California.

**Divisions:** 302:C

**TNC Ecoregions:** 23:C

**Nations:** MX

**Subnations:** MXBC

CONCEPT

**Environment:** [from M089] This desert scrub type comprises most of the Baja California peninsula and occurs in diverse environments from the coastal and inland plains to alluvial fans, foothills, and mountains. Climate is extremely arid with mean annual precipitation of less than 100 mm in the north increasing to 300 mm in southern Baja. Precipitation occurs mostly in the summer‑early fall season (monsoon). Extended drought is common and favors plants with water storage. Coastal stands west of the mountain divide may benefit from cool sea breezes and fog that help ameliorate some of the aridity. Some vegetation types, such as *Frankenia‑Ocotillo‑Datilillo* desert scrub are limited to the fog belt in the coastal plain of southern Viscaino. Substrates vary from coarse‑textured, deep granitic soils and a'a lava flows in broad valley bottoms, alluvial fans and on lower mountain slopes to finer‑textured, alkaline, sometimes saline, clayey soils with low infiltration that are derived from marine deposit, basalt or other volcanic rock. Some stands occur in narrow valleys and rocky slopes below generally sparsely vegetated volcanic mesas. Soil is present and moisture is available locally in seeps from these mesas. Larger valleys or barrancas are typically drier and have more open vegetation than smaller ones. An exception to the extremely arid environments is the tropical San Lucan thornscrub, which is limited in distribution to central and southern Baja California. These areas receive more precipitation (316‑482 mm) per year, with a dry season from late October through July. Mean monthly temperatures range from 21.5‑23.6°C. Pacific slopes receive greater rainfall and experience generally lower temperatures than the gulf side of the Cape region.

**Dynamics:** [from M089] Periodic severe drought is common in Baja California which favors plants that can store water during the unfavorable times.

SOURCES

**References:** Brown 1982a, Comer et al. 2003*, Shreve and Wiggins 1964

**Version:** 24 Mar 2003	**Stakeholders:** Latin America, West

**Concept Author:** NatureServe Western Ecology Team	**LeadResp:** West

CES302.007 Northern Viscaino White Bursage‑Agave Inland Low Desert Scrub

**Primary Division:** North American Warm Desert (302)

**Land Cover Class:** Shrubland

**Spatial Scale & Pattern:** Matrix

**Required Classifiers:** Natural/Semi‑natural; Vegetated (>10% vasc.); Upland

**Diagnostic Classifiers:** Lowland [Lowland]; Shrubland (Shrub‑dominated); Toeslope/Valley Bottom; Tropical/Subtropical [Tropical Xeric]; Ambrosia dumosa ‑ Agave cerulata

**Concept Summary:** This desert scrub system is found on rolling hills and plains of the northern Vizcaino region in central Baja California from the Pacific Ocean to the crest of the uplands that divide the peninsula. Climate is extremely arid with less than 100 mm mean annual precipitation occurring mostly in the fall‑winter. However, cool coastal sea breezes help ameliorate some of the aridity, but it occurs too far inland for the fog to influence the vegetation. The vegetation is dominated by low shrubs *Ambrosia dumosa* and *Agave cerulata* with *Encelia californica, Ambrosia camphorata, Eriogonum fasciculatum*, and *Krascheninnikovia lanata*. *Larrea tridentata* is uncommon, but always present. There are only widely scattered taller plants of *Fouquieria columnaris, Fouquieria splendens, Pachycereus pringlei, Pachycormus discolor*, and *Yucca schidigera* because of the aridity. Other common species include *Simmondsia chinensis, Viguiera laciniata, Krameria* spp., and the cacti *Pachycereus schottii (= Lophocereus schottii), Opuntia molesta*, and *Ferocactus* spp.

DISTRIBUTION

**Range:** Found on rolling hills and plains of the northern Vizcaino region in central Baja California from the Pacific Ocean to the crest of the uplands that divide the peninsula.

**Divisions:** 302:C

**TNC Ecoregions:** 23:C

**Nations:** MX

**Subnations:** MXBC

CONCEPT

SOURCES

**References:** Brown 1982a, Comer et al. 2003*, Shreve and Wiggins 1964

**Version:** 24 Mar 2003	**Stakeholders:** Latin America, West

**Concept Author:** NatureServe Western Ecology Team	**LeadResp:** West

CES401.301 San Lucan Thornscrub

**Primary Division:** Dry Meso‑America (401)

**Land Cover Class:** Shrubland

**Spatial Scale & Pattern:** Large patch

**Required Classifiers:** Natural/Semi‑natural; Vegetated (>10% vasc.); Upland

**Diagnostic Classifiers:** Lowland [Lowland]; Shrubland (Shrub‑dominated); Aridic

**Concept Summary:** This tropical thornscrub system is limited in distribution to central and southern Baja California. These are found along low foothills, bajada, and arroyos with sandy alluvial soils derived from granitic rocks. This forms a transition between succulent‑rich Sonoran Desert scrub types and San Lucan Dry Deciduous Forest (CES401.299). The following list of species is diagnostic for this system: *Pachycereus pringlei, Machaerocereus gummosus, Solanum hindsianum, Jatropha cinerea, Bursera microphylla*.

DISTRIBUTION

**Divisions:** 401:C

**Nations:** MX

**Subnations:** MXBS

CONCEPT

**Environment:** These are found along low foothills, bajadas, and arroyos with sandy, alluvial soils derived from granitic rocks. These areas receive 316‑482 mm of precipitation per year, with a dry season from late October through July. Mean monthly temperatures range from 21.5‑23.6°C. Pacific slopes reveive greater rainfall and experience generally lower temperatures than the Gulf side of the Cape region.

**Vegetation:** Thornscrub includes a complex mixture of low thorn‑bearing trees, shrubs, and a high diversity of succulents, including cacti, with tallest individuals typically not exceeding 3 meters in height.

**Dynamics:** Successional dynamics exist between Sonoran desert scrub, thornscrub and dry deciduous forest. Natural fire regimes are not documented.

SOURCES

**References:** Brown et al. 1998, Ffolliott and Ortega‑Rubio 1999, Josse et al. 2003*, Rzedowski 1986

**Version:** 17 Jul 2003	**Stakeholders:** Latin America

**Concept Author:** C. Josse	**LeadResp:** Latin America

3.B. Cool Semi‑Desert Scrub & Grassland

3.B.1. Cool Semi‑Desert Scrub & Grassland

3.B.1.Ne. Western North American Cool Semi‑Desert Scrub & Grassland

M093. Great Basin Saltbush Scrub

CES304.783 Inter‑Mountain Basins Mat Saltbush Shrubland

**Primary Division:** Inter‑Mountain Basins (304)

**Land Cover Class:** Shrubland

**Spatial Scale & Pattern:** Matrix

**Required Classifiers:** Natural/Semi‑natural; Vegetated (>10% vasc.); Upland

**Diagnostic Classifiers:** Lowland [Lowland]; Shrubland (Shrub‑dominated); Alluvial flat; Alluvial plain; Plain; Alkaline Soil; Saline Substrate Chemistry; Calcareous; Silt Soil Texture; Clay Soil Texture; Dwarf‑Shrub; Atriplex spp.

**National Mapping Codes:** EVT 2066; ESLF 5203; ESP 1066

**Concept Summary:** This ecological system occurs on gentle slopes and rolling plains in the northern Colorado Plateau and Uinta Basin on Mancos shale and arid, windswept basins and plains across parts of Wyoming. It is also found in eastern Wyoming in Great Plains areas and may extend north into Montana and Canada. These landscapes typically support dwarf‑shrublands composed of relatively pure stands of *Atriplex* spp., such as *Atriplex corrugata* (in Colorado and Utah), *Atriplex gardneri* (Wyoming and Montana into Canada), or *Atriplex falcata* (Columbia Plateau and northern Great Basin). Other dominant or codominant dwarf‑shrubs may include *Artemisia longifolia, Artemisia pedatifida* (very important in Wyoming, rare in Colorado stands), or *Picrothamnus desertorum*, sometimes with other low shrubs, such as *Krascheninnikovia lanata* or *Tetradymia spinosa*. *Atriplex confertifolia* or *Atriplex canescens* may be present but do not codominate. *Artemisia tridentata ssp. wyomingensis* can occur in local patches within this system. The herbaceous layer is typically sparse. Scattered perennial forbs occur, such as *Oenothera* spp., *Phacelia* spp., *Sphaeralcea grossulariifolia, Stanleya pinnata*, and *Xylorhiza glabriuscula*; perennial grasses *Achnatherum hymenoides, Bouteloua gracilis* (not in Wyoming), *Distichlis spicata, Elymus elymoides, Elymus lanceolatus ssp. lanceolatus, Pascopyrum smithii, Pleuraphis jamesii, Poa secunda*, or *Sporobolus airoides* may comprise the herbaceous layer. In less saline areas, there may be inclusions of grassland patches dominated by *Hesperostipa comata, Leymus salinus, Pascopyrum smithii*, or *Pseudoroegneria spicata*. Substrates are shallow, typically saline, alkaline, fine‑textured soils developed from shale or alluvium and may be associated with shale badlands. Infiltration rate is typically low. In Wyoming and possibly elsewhere, inclusions of non‑saline, gravelly barrens or rock outcrops dominated by cushion plants such as *Arenaria hookeri* and *Phlox hoodii* without dwarf‑shrubs may be present (these are not restricted to this system). Annuals are seasonally present and may include *Eriogonum inflatum, Monolepis nuttalliana, Plantago tweedyi*, and the introduced annual grass *Bromus tectorum*. In Montana, *Atriplex gardneri* also occurs associated with Great Plains badlands, and determining which system it falls into may be difficult.

**Comments:** Reviewers have proposed renaming this system to be more "broad" to include Gardner saltbush (*Atriplex gardneri*) in the name rather than just mat saltbush (*Atriplex corrugata*), but an alternative name has not yet been identified. Recent taxonomic changes need review as to the inclusion of *Atriplex falcata*, formerly a variety or subspecies of *Atriplex nuttallii* and *Atriplex gardneri*. Sparser stands of this system are similar to Inter‑Mountain Basins Shale Badland (CES304.789) and Western Great Plains Badlands (CES303.663), especially when dominated by *Atriplex gardneri*.

DISTRIBUTION

**Range:** This system occurs on gentle slopes and rolling plains in the northern Colorado Plateau and Uinta Basin on Mancos shale and arid, windswept basins and plains across parts of Wyoming, and possibly into Montana and Canada.

**Divisions:** 304:C

**TNC Ecoregions:** 10:C, 19:C

**Nations:** US

**Subnations:** AZ, CO, MT, NM, UT, WY

**Map Zones:** 13:?, 15:?, 16:P, 20:C, 22:C, 23:C, 24:P, 28:P, 29:C

**USFS Ecomap Regions:** 313A:CC, 313B:CC, 341B:CC, 341C:CC, 342E:C?, 342F:C?, 342G:CC, 342J:C?, M331B:CC, M331D:C?, M331E:CC, M331G:CC, M331H:CC, M331J:C?, M341B:CC, M341C:CC

CONCEPT

**Environment:** *Climate:* Climate is temperate and semi‑arid. Summers are generally hot, and freezing temperatures are common in the winter. Mean annual precipitation ranges from 13‑33 cm. In Montana and Wyoming, approximately two‑thirds of the annual precipitation falls in spring and early summer. In Colorado and Utah, over half the precipitation occurs in the late summer monsoons as high‑intensity thunderstorms.

*Physiography/landform:* This ecological system occurs in the intermountain western U.S. on gentle slopes and rolling plains on semi‑arid, windswept plains and basins. Elevation ranges from 1150‑2200 m. Stands occur on shale outcrops and plains and are nearly flat to moderately steep.

*Soils/substrate/hydrology:* Substrates are shallow to moderately deep, typically saline, alkaline, poorly developed, fine‑textured soils but range from sandy loam to clay and may be gravelly. Soil are developed from shale, alluvium, and bentonite and may be associated with shale badlands. Infiltration rate is typically low and erosion rates are high because of poor infiltration and high runoff. In Wyoming and possibly elsewhere, inclusions of non‑saline, gravelly barrens or rock outcrops may be present.

**Vegetation:** This ecological system typically supports dwarf‑shrublands composed of relatively pure stands of *Atriplex* spp., such as *Atriplex corrugata* or *Atriplex gardneri*. Other dominant or codominant dwarf‑shrub may include *Artemisia longifolia, Artemisia pedatifida*, or *Picrothamnus desertorum*, sometimes with a mix of other low shrubs, such as *Krascheninnikovia lanata* or *Tetradymia spinosa*. *Atriplex confertifolia* or *Atriplex canescens* may be present but do not codominate. The herbaceous layer is typically sparse. Scattered perennial forbs occur, such as *Xylorhiza glabriuscula* and *Sphaeralcea grossulariifolia*, and the perennial grasses *Achnatherum hymenoides, Bouteloua gracilis, Elymus elymoides, Elymus lanceolatus ssp. lanceolatus, Pascopyrum smithii*, or *Sporobolus airoides* may dominate the herbaceous layer. In less saline areas, there may be inclusions of grasslands dominated by *Hesperostipa comata, Leymus salinus, Pascopyrum smithii*, or *Pseudoroegneria spicata*. In Wyoming and possibly elsewhere, vegetation dominated by cushion plants such as *Arenaria hookeri* and *Phlox hoodii* without dwarf‑shrubs may be present and occurs on inclusions of non‑saline, gravelly barrens or rock outcrops. Annuals are seasonally present and may include *Eriogonum inflatum, Plantago tweedyi*, and the introduced annual grass *Bromus tectorum*.

**Dynamics:** These are highly saline‑tolerant and drought‑tolerant shrublands. *Atriplex corrugata*‑ and *Atriplex gardneri*‑dominated shrublands are the most saline‑tolerant of the Mancos shale plant communities studied by Branson et al. (1976). Gardner's saltbush has an extensive, highly branched root system, and tolerates poor site conditions (Reed 1993b). Stands are characterized by bare ground and young to mature shrubs that have re‑sprouted or established from nearby seed. Although very slow‑growing, these shrubs can completely dominate these extremely saline sites (Branson et al. 1976). They are true evergreen dwarf‑shrubs retaining leaves for several years. This plant utilizes winter soil moisture, beginning new growth in March when the soils are relatively warm and moist. It flowers in April and by mid‑July fruits are shattered (Branson et al. 1976). If the soils dry out in midsummer, it can go dormant until the late summer monsoon rains begin. Disturbance is characterized by very wet periods that contribute to high shrub mortality every 100 years on average.

Shrub cover may be patchy and discontinuous, but cover is higher than Inter‑Mountain Basin Shale Badland (CES304.789). These shrublands typically occur on flatter slopes with less severe erosion than those occupied by badland communities. This system does not have a fire regime due to discontinuous fuel (LANDFIRE 2007a). Fire can occur in conjunction with wet years possibly once every 100 years on average. Most species of *Atriplex* sprout after fire, recovering fully within 2 to 3 years from root sprouts (Wright 1980).

LANDFIRE developed a VDDT model for this system which has two classes (LANDFIRE 2007a, BpS 2310660):

A) Early Development 1 All Structures (10% of type in this stage): Shrub cover is 0‑5%. Characterized by bare ground and young shrubs that have re‑sprouted or established from nearby seed. May find some ephemeral forbs or grasses at this stage. Disturbance is characterized by very wet periods that contribute to high shrub mortality every 100 years on average. Succession to class B after 12 years.

B) Late Development 1 All Structures (90% of type in this stage): Characterized by mature shrubs (10‑20% cover). Typically lacks understory vegetation. Sites at this stage are very patchy with discontinuous shrubs. Same disturbance as in class A.

SOURCES

**References:** Belnap 2001, Belnap and Lange 2003, Belnap et al. 2001, Blaisdell and Holmgren 1984, Branson et al. 1976, CNHP 2010, Comer et al. 2003*, Knight 1994, LANDFIRE 2007a, Potter et al. 1985, Reed 1993b, Rosentreter and Belnap 2003, Shiflet 1994, Welsh 1957, Wright 1980

**Version:** 26 Jan 2016	**Stakeholders:** West

**Concept Author:** K.A. Schulz	**LeadResp:** West

CES304.784 Inter‑Mountain Basins Mixed Salt Desert Scrub

**Primary Division:** Inter‑Mountain Basins (304)

**Land Cover Class:** Shrubland

**Spatial Scale & Pattern:** Large patch

**Required Classifiers:** Natural/Semi‑natural; Vegetated (>10% vasc.); Upland

**Diagnostic Classifiers:** Lowland [Lowland]; Shrubland (Shrub‑dominated); Alluvial flat; Alluvial plain; Plain; Alkaline Soil; Saline Substrate Chemistry; Calcareous; Silt Soil Texture; Clay Soil Texture; Xeromorphic Shrub; Dwarf‑Shrub; Atriplex spp.

**National Mapping Codes:** EVT 2081; ESLF 5258; ESP 1081

**Concept Summary:** This extensive ecological system includes open‑canopied shrublands of typically saline basins, alluvial slopes and plains across the Intermountain western U.S. This type also extends in limited distribution into the southern Great Plains. Substrates are often saline and calcareous, medium‑ to fine‑textured, alkaline soils, but include some coarser‑textured soils. The vegetation is characterized by a typically open to moderately dense shrubland composed of one or more *Atriplex* species, such as *Atriplex confertifolia, Atriplex canescens, Atriplex obovata, Atriplex polycarpa*, or *Atriplex spinifera*. Other shrubs present to codominant may include *Artemisia tridentata ssp. wyomingensis, Chrysothamnus viscidiflorus, Ericameria nauseosa, Ephedra nevadensis, Grayia spinosa, Krascheninnikovia lanata, Lycium* spp., *Picrothamnus desertorum*, or *Tetradymia* spp. Northern occurrences may lack *Atriplex* species and are typically dominated by *Grayia spinosa, Krascheninnikovia lanata*, and/or *Picrothamnus desertorum*. In Wyoming, occurrences are typically a mix of *Atriplex confertifolia, Grayia spinosa, Artemisia tridentata ssp. wyomingensis, Sarcobatus vermiculatus, Krascheninnikovia lanata*, and various *Ericameria* or *Chrysothamnus* species. Some places are a mix of *Atriplex confertifolia* and *Artemisia tridentata ssp. wyomingensis*. In the Great Basin, *Sarcobatus vermiculatus* is generally absent but, if present, does not codominate. The herbaceous layer varies from sparse to moderately dense and is dominated by perennial graminoids such as *Achnatherum hymenoides, Bouteloua gracilis, Elymus lanceolatus ssp. lanceolatus, Pascopyrum smithii, Pleuraphis jamesii, Pleuraphis rigida, Poa secunda*, or *Sporobolus airoides*. Various forbs are also present.

**Comments:** This system extends south into the southern Colorado Plateau and east into the western Great Plains where the shrub layer often has a herbaceous layer dominated by warm‑season graminoids such as *Bouteloua gracilis, Pleuraphis jamesii, Distichlis spicata, Muhlenbergia asperifolia*, or *Sporobolus airoides*.

DISTRIBUTION

**Range:** This system occurs in the intermountain western U.S., extending in limited distribution into the southern Great Plains. In the Great Basin, this ecological system occupies sites west of the Wasatch Mountains, east of the Sierra Nevada, south of the Idaho batholith and north of the Mojave Desert. In Wyoming, this system occurs in the Great Divide and Bighorn basins.

**Divisions:** 303:C, 304:C, 306:C

**TNC Ecoregions:** 4:?, 6:C, 8:?, 9:C, 10:C, 11:C, 18:C, 19:C, 20:C, 21:C, 26:C, 27:C, 28:C

**Nations:** US

**Subnations:** AZ, CA, CO, ID, MT, NM, NV, OR, UT, WA, WY

**Map Zones:** 6:P, 7:C, 8:P, 9:C, 10:C, 12:C, 13:C, 15:C, 16:C, 17:C, 18:C, 19:C, 21:?, 22:C, 23:C, 24:C, 25:C, 27:P, 28:C, 29:C, 30:?, 33:?, 34:P

**USFS Ecomap Regions:** 313A:CC, 313B:CC, 313D:CC, 315A:CC, 315B:CP, 315H:CC, 321A:CC, 322A:CC, 331A:CP, 331B:CC, 331F:CC, 331G:CC, 331H:CC, 331I:CC, 331J:CC, 341A:CC, 341B:CC, 341C:CC, 341D:CC, 341E:CC, 341F:CC, 341G:CC, 342A:CC, 342B:CC, 342C:CC, 342D:CC, 342E:CC, 342F:CC, 342G:CC, 342H:CC, 342I:CC, 342J:CC, M242C:PP, M261D:CP, M261E:CP, M261G:CC, M313A:CC, M313B:CC, M331A:CC, M331B:CC, M331D:CC, M331E:CC, M331F:CC, M331G:CC, M331H:CC, M331I:CC, M331J:C?, M332A:CP, M332E:CC, M332F:CC, M332G:CP, M341A:CC, M341B:CC, M341C:CC, M341D:CC

CONCEPT

**Environment:** *Climate:* This is a semi‑arid system of extreme climatic conditions, with warm to hot summers and cold winters. Annual precipitation ranges from approximately 13‑33 cm. In much of this shrubland's distribution the season of greatest moisture is mid to late summer, although in the more northern areas a moist period is to be expected in the winter and spring. Precipitation is extremely irregular in the southern part of its distribution, such that long‑term seasonal or monthly averages do not convey the full story (Blaisdell and Holmgren 1984).

*Physiography/landform:* This salt desert shrubland system is a matrix system in the Intermountain West. This system occurs on lowland and upland sites usually at elevations between 1520 and 2200 m (4987‑7218 feet). Sites can be found on all aspects and include valley bottoms, alluvial and alkaline flats, mesas and plateaus, playas, drainage terraces, washes and interdune basins, bluffs, and gentle to moderately steep sandy or rocky slopes. Slopes are typically gentle to moderately steep but are sometimes unstable and prone to surface movement. Many areas within this system are degraded due to erosion and may resemble "badlands." Soil surface is often very barren in occurrences of this system. The interspaces between the characteristic plant clusters are commonly covered by a biological soil crust (West 1982).

<br />*Soils/substrates/hydrology:* Soils are shallow to moderately deep, poorly‑developed, and often alkaline or saline. The soils of much of the area are poorly‑developed Entisols, a product of an arid climate. Vegetation within this system is tolerant of these soil conditions but not restricted to it. Other sites include level pediment remnants where coarse‑textured and well‑developed soil profiles have been derived from sandstone gravel and are alkaline, or on Mancos shale badlands, where soil profiles are typically fine‑textured and non‑alkaline throughout (West and Ibrahim 1968). They can also occur in alluvial basins where parent materials from the other habitats have been deposited over Mancos shale and the soils are heavy‑textured and saline‑alkaline throughout the profile (West and Ibrahim 1968). The environmental description is based on several other references, including Branson et al. (1967, 1976), Beatley (1976), Campbell (1977), Brown (1982), West (1983b), Knight et al. (1987), Knight (1994), Shiflet (1994), Holland and Keil (1995), Reid et al. (1999), Ostler et al. (2000), Barbour et al. (2007), and Sawyer et al. (2009).

**Vegetation:** The vegetation is characterized by a typically open to moderately dense shrubland composed of one or more *Atriplex* species, such as *Atriplex confertifolia, Atriplex canescens, Atriplex polycarpa*, or *Atriplex spinifera*. *Grayia spinosa* tends to occur on coppice dunes that may have a silty component to them. Northern occurrences lack *Atriplex* species and are typically dominated by *Grayia spinosa* and *Krascheninnikovia lanata*. Other shrubs present to codominant may include *Artemisia tridentata ssp. wyomingensis, Chrysothamnus viscidiflorus, Ericameria nauseosa, Ephedra nevadensis, Grayia spinosa, Lycium* spp., *Picrothamnus desertorum*, or *Tetradymia* spp. In Wyoming, occurrences are typically a mix of *Atriplex confertifolia, Grayia spinosa, Artemisia tridentata ssp. wyomingensis, Sarcobatus vermiculatus, Krascheninnikovia lanata*, and various *Ericameria* or *Chrysothamnus* species. In the Great Basin, *Sarcobatus vermiculatus* is generally absent but, if present, does not codominate. The herbaceous layer varies from sparse to moderately dense and is dominated by perennial graminoids such as *Achnatherum hymenoides, Bouteloua gracilis, Elymus lanceolatus ssp. lanceolatus, Pascopyrum smithii, Pleuraphis jamesii, Pleuraphis rigida, Poa secunda*, or *Sporobolus airoides*. The vegetation description is based on several references, including Beatley (1976), Campbell (1977), Brown (1982), West (1979, 1983b), Knight et al. (1987), Knight (1994), Shiflet (1994), Holland and Keil (1995), Reid et al. (1999), Ostler et al. (2000), Barbour et al. (2007), and Sawyer et al. (2009).

**Dynamics:** West (1982) stated that "salt desert shrub vegetation occurs mostly in two kinds of situations that promote soil salinity, alkalinity, or both. These are either at the bottom of drainages in enclosed basins or where marine shales outcrop." However, salt‑desert shrub vegetation may also occur in climatically extremely dry, non‑saline sites, as well as physiologically dry (saline) soils (Blaisdell and Holmgren 1984). Not all salt desert shrub soils are saline, and their hydrologic characteristics may often be responsible for the associated vegetation (Naphan 1966). That is, they are flooded or wetted enough to mobilize but not flush soil salt content, and therefore the ephemeral hydrology precipitates and concentrates salts. Species of the salt desert shrub complex have different degrees of tolerance to salinity and aridity, and they tend to sort themselves out along a moisture/salinity gradient (West 1982). Thus these saltbush shrublands are dependent on a certain amount of ephemeral flooding and warm temperatures causing evaporation. The effects of these physical, chemical, moisture, and topographic gradients on species and communities occur through complex relations that are not well understood and are in need of further study (Blaisdell and Holmgren 1984). In northern, cool desert locations of this system, soil moisture accumulation and storage within this system typically occur in the winter months. There is generally at least one good snowstorm per season that will provide sufficient moisture to the vegetation. The winter moisture accumulation amounts will affect spring plant growth. Plants may grow as little as a few inches to 1 m. Unless more rains come in the spring, the soil moisture will be depleted in a few weeks, growth will slow and ultimately cease, and the perennial plants will assume their various forms of dormancy (Blaisdell and Holmgren 1984). If effective rain comes later in the warm season, some of the species will renew their growth from the stage at which it had stopped. Others, having died back, will start over as if emerging from winter dormancy (Blaisdell and Holmgren 1984). *Atriplex confertifolia* shrubs often develop large leaves in the spring, which increase the rate of photosynthesis. As soil moisture decreases, the leaves are lost, and the plant takes on a dead appearance. During late fall, very small overwintering leaves appear which provide some photosynthetic capability through the remainder of the year (Reid et al. 1999).

The variation of plant communities found within this ecological system is maintained by intra‑ or inter‑annual cycles of flooding followed by extended drought, which favor accumulation of transported salts. The moisture supporting these intermittently flooded communities is usually derived off‑site, and they are dependent upon natural watershed function for persistence (Reid et al. 1999). As a result, these desert communities of perennial plants are dynamic and changing. The composition within this system may change dramatically and may be both cyclic and unidirectional. Superimposed on the compositional change is great variation from year to year in growth of all the vegetation, the sum of varying growth responses of individual species to specific conditions of different years (Blaisdell and Holmgren 1984). Desert plants grow when temperature is satisfactory, but only if soil moisture is available at the same time. Because the amount of moisture is variable from year to year and because different species flourish under different seasons of soil moisture, seldom do all components of the vegetation thrive in the same year (Blaisdell and Holmgren 1984).

Insects are an important component of many shrub steppe and grassland systems. Mormon crickets and grasshoppers are natural components of many rangeland systems (USDA‑APHIS 2003, 2010). There are almost 400 species of grasshoppers that inhabit the western United States with 15‑45 species occurring in a given rangeland system (USDA‑APHIS 2003). Mormon crickets are also present in many western rangelands and, although flightless, are highly mobile and can migrate large distances consuming much of the forage while travelling in wide bands (USDA‑APHIS 2010). Following a high population year for grasshoppers or Mormon crickets and under relatively warm dry spring environmental conditions that favor egg hatching and grasshopper and Mormon cricket survival, there may be large population outbreaks that can utilize 80% or more of the forage in areas as large as 2000 square mile. Conversely, relatively cool and wet spring weather can limit the potential for outbreaks. These outbreaks are naturally occurring cycles and, especially during drought, can denude an area of vegetation leaving it exposed to increased erosion rates from wind and water (USDA‑APHIS 2003).

<br />Disturbance scale was variable during presettlement. Droughts and extended wet periods could be region‑wide, or more local. A series of high water years or drought could affect whole basins. Mormon cricket disturbances could affect hundreds to perhaps thousands of acres for a few years to 1‑2 decades (LANDFIRE 2007a).

LANDFIRE developed a VDDT model for this system which has three classes (LANDFIRE 2007a, BpS 2310810):

A) Early Development 1 All Structures (25% of type in this stage): Shrub cover is 0‑5%. Dominated by continuous grass with widely scattered shrubs and relatively younger shrubs than in classes B and C. Over 10 years, vegetation moves to class B as the primary succession pathway. Replacement fire occurs every 300 years on average, and will set back succession to year zero. Extended wet periods (every 35 years) will also have a stand‑replacing effect. During a drought (mean return interval of 35 years), vegetation will follow an alternative succession pathway to class C.

B) Mid Development 1 Open (45% of type in this stage): Characterized by mature shrubs (5‑20% cover). Discontinuous grass patches and higher shrub canopy cover than in class A. Extended wet periods (every 35 years on average) will cause a stand‑replacing transition to class A. During extended drought periods (every 35 years), vegetation will shift to class C. Replacement fire is rare (mean FRI of 500 years). Class B will be maintained in the absence of disturbance.

C) Mid Development 2 Open (30% of type in this stage): Characterized by mature shrubs (21‑30% cover). Grass is lacking and shrub canopy cover is even higher than class B. During extended wet periods (35 years), vegetation will transition to class A. After 20 years, vegetation moves back to class B through succession. Drought (mean return interval of 35 years) will maintain vegetation in class C. Fire would not carry in this class and is not modeled.<br />

Under reference conditions disturbances were unpredictable, but flooding, drought, insects and fire may all occur in this system. Extended wet periods were modeled as occurring every 35 years, and drought periods every 35 years. Extended wet periods tended to favor perennial grass development, while extended drought tended to favor shrub development. Fire was rare and limited to more mesic sites (and moist periods) with high grass productivity. Mixed‑severity fire was modeled as occurring with a mean FRI of 500‑1000 years (LANDFIRE 2007a).

In summary, desert communities of perennial plants are dynamic and changing. The composition within this system may change dramatically over time and may be both cyclic and unidirectional. Superimposed on the compositional change is great variation from year to year in growth of all the vegetation ‑ the sum of varying growth responses of individual species to specific conditions of different years (Blaisdell and Holmgren 1984). Desert plants grow when temperature is satisfactory, but only if soil moisture is available at the same time. Because amount of moisture is variable from year to year and because different species flourish under different seasons of soil moisture, seldom do all components of the vegetation thrive in the same year (Blaisdell and Holmgren 1984).

SOURCES

**References:** Anderson and Porter 1994, Barbour and Major 1977, Barbour and Major 1988, Barbour et al. 2007a, Beatley 1976, Belnap 2001, Belnap and Lange 2003, Belnap et al. 2001, Benson 1969, Blaisdell and Holmgren 1984, Branson et al. 1967, Branson et al. 1976, Brown 1982a, CNHP 2010, Campbell 1977, Comer et al. 2003*, Cornely et al. 1992, Degenhardt et al. 1996, Francis 1986, Franklin 2005, Grismer 2002, Hammerson 1999, Hoffman et al. 1969, Holland and Keil 1995, INPS 1993, Knight 1994, Knight et al. 1987, LANDFIRE 2007a, Naphan 1966, Nussbaum et al. 1983, Ostler et al. 2000, Reid et al. 1999, Rosentreter and Belnap 2003, Sawyer and Keeler‑Wolf 1995, Sawyer et al. 2009, Shiflet 1994, Stebbins 2003, USDA‑APHIS 2003, USDA‑APHIS 2010, WNHP unpubl. data 2018, Weber 1987, Welsh et al. 1993, Welsh et al. 2003, West 1979, West 1982, West 1983b, West 1983c, West and Ibrahim 1968, Williams 1984

**Version:** 26 Jan 2016	**Stakeholders:** Midwest, West

**Concept Author:** K.A. Schulz	**LeadResp:** West

CES302.749 Sonora‑Mojave Mixed Salt Desert Scrub

**Primary Division:** North American Warm Desert (302)

**Land Cover Class:** Shrubland

**Spatial Scale & Pattern:** Large patch

**Required Classifiers:** Natural/Semi‑natural; Vegetated (>10% vasc.); Upland

**Diagnostic Classifiers:** Lowland [Lowland]; Shrubland (Shrub‑dominated); Basin floor; Toeslope/Valley Bottom; Temperate [Temperate Xeric]; Alkaline Soil; Atriplex spp.

**National Mapping Codes:** EVT 2088; ESLF 5265; ESP 1088

**Concept Summary:** This ecological system includes extensive open‑canopied shrublands of typically saline basins in the Mojave and Sonoran deserts. Stands most often occur around playas and in valley bottoms or basins where evapotranspiration results in saline soils. Substrates are generally fine‑textured, saline soils. Vegetation is typically composed of one or more *Atriplex* species, such as *Atriplex canescens* or *Atriplex polycarpa*, along with other species of *Atriplex*. Species of *Allenrolfea, Salicornia, Suaeda, Krascheninnikovia lanata*, or other halophytic plants are often present to codominant. In some locations, scattered *Yucca brevifolia* may occur, but other Mojavean taxa are typically not present. Graminoid species may include *Sporobolus airoides* or *Distichlis spicata* at varying densities.

DISTRIBUTION

**Range:** This system is found in saline basins of the Mojave and Sonoran deserts.

**Divisions:** 302:C

**TNC Ecoregions:** 17:C, 22:C, 23:C

**Nations:** MX, US

**Subnations:** AZ, CA, MXBC, MXSO, NV, UT

**Map Zones:** 4:C, 5:P, 6:P, 12:C, 13:C, 14:C, 15:?, 17:P, 25:C

**USFS Ecomap Regions:** 261B:??, 262A:CC, 313A:PP, 313C:PP, 321A:PP, 322A:CC, 322B:CC, 322C:CC, 341D:C?, 341F:CC, M261E:CC, M341A:PP

CONCEPT

**Environment:** This ecological system includes extensive open‑canopied shrublands in saline basins, near and around washes, lower bajadas and alluvial fans in the Mojave and Sonoran deserts. Stands most often occur around playas and in valley bottoms or basins where evapotranspiration results in saline soils. Substrates are generally fine‑textured, saline soils. Adjacent systems include Sonora‑Mojave Creosotebush‑White Bursage Desert Scrub (CES302.756) and Sonoran Paloverde‑Mixed Cacti Desert Scrub (CES302.761) above and North American Warm Desert Playa (CES302.751) below. The environmental description is based on several references, including Brown (1982), MacMahon and Wagner (1985), Barbour and Major (1988), MacMahon (1988), Holland and Keil (1995), Reid et al. (1999), Comer et al. (2003), Thomas et al. (2004), Barbour et al. (2007), Keeler‑Wolf (2007), Sawyer et al. (2009), and NatureServe Explorer (2011).

**Vegetation:** Vegetation is typically composed of one or more *Atriplex* species, such as *Atriplex polycarpa, Atriplex lentiformis, Atriplex parryi, Atriplex spinifera, Atriplex canescens* or *Atriplex confertifolia*, which is more common in the Great Basin. Associates include of *Allenrolfea occidentalis, Lycium andersonii, Salicornia* spp., *Suaeda* spp., *Krascheninnikovia lanata*, or other halophytic plants that are often present to codominant. *Ambrosia dumosa, Larrea tridentata*, and other desert scrub may be present on less saline sites and in transitional zones. In some sandy locations, scattered *Yucca brevifolia* may occur, but other Mojavean taxa are typically not present. Graminoid species may include *Sporobolus airoides* or *Distichlis spicata* at varying densities. The floristic description is based on several references, including Brown (1982), MacMahon and Wagner (1985), Barbour and Major (1988), MacMahon (1988), Holland and Keil (1995), Reid et al. (1999), Comer et al. (2003), Thomas et al. (2004), Barbour et al. (2007), Keeler‑Wolf (2007), and Sawyer et al. (2009).

**Dynamics:** West (1982) stated that "salt desert shrub vegetation occurs mostly in two kinds of situations that promote soil salinity, alkalinity, or both. These are either at the bottom of drainages in enclosed basins or where marine shales outcrop." Species and communities are apparently sorted out along physical, chemical, moisture, and topographic gradients with *Atriplex lentiformis* being the most salt‑tolerant, often occurring where the water table is close to the soil surface. It is followed by *Atriplex polycarpa* which has the broadest tolerance (5% salinity to non‑saline soils). *Atriplex canescens* is the least salt‑tolerant and often occurs on well‑drained, sandy soil (Keeler‑Wolf 2007). *Atriplex confertifolia* occurs on both saline bottomland and dry uplands.

SOURCES

**References:** Barbour and Major 1988, Barbour et al. 2007a, Brown 1982a, Comer et al. 2003*, Holland and Keil 1995, Howard 2003a, Keeler‑Wolf 2007, MacMahon 1988, MacMahon and Wagner 1985, Meyer 2005, NatureServe Explorer 2011, Reid et al. 1999, Sawyer et al. 2009, Shiflet 1994, Simonin 2001a, Thomas et al. 2004, West 1982

**Version:** 02 Apr 2014	**Stakeholders:** Latin America, Southeast, West

**Concept Author:** K.A. Schulz	**LeadResp:** West

M171. Great Basin‑Intermountain Dry Shrubland & Grassland

CES304.763 Colorado Plateau Blackbrush‑Mormon‑tea Shrubland

**Primary Division:** Inter‑Mountain Basins (304)

**Land Cover Class:** Shrubland

**Spatial Scale & Pattern:** Large patch

**Required Classifiers:** Natural/Semi‑natural; Vegetated (>10% vasc.); Upland

**Diagnostic Classifiers:** Lowland [Foothill]; Shrubland (Shrub‑dominated); Temperate [Temperate Xeric]; Aridic

**National Mapping Codes:** EVT 2078; ESLF 5255; ESP 1078

**Concept Summary:** This ecological system occurs in the Colorado Plateau on benchlands, colluvial slopes, pediments or bajadas. Elevation ranges from 560‑1650 m. Substrates are shallow, typically calcareous, non‑saline and gravelly or sandy soils over sandstone or limestone bedrock, caliche or limestone alluvium. It also occurs in deeper soils on sandy plains where it may have invaded desert grasslands. This is an evergreen, microphyllous scrub with succulents, half‑shrubs, and scattered deciduous shrubs. The vegetation is characterized by extensive open shrublands dominated by *Coleogyne ramosissima* often with *Ephedra viridis, Ephedra torreyana*, or *Grayia spinosa*. Sandy portions may include *Artemisia filifolia, Eriogonum leptocladon, Poliomintha incana*, or *Quercus havardii var. tuckeri* (relict populations) as codominant. The herbaceous layer is sparse and composed of graminoids such as *Achnatherum hymenoides, Pleuraphis jamesii*, or *Sporobolus cryptandrus*.

**Comments:** This system is similar to stands of Mojave Mid‑Elevation Mixed Desert Scrub (CES302.742) that are dominated by *Coleogyne ramosissima* with *Ephedra* spp. However, this system is restricted to the Colorado Plateau and lacks Mojave indicator species.

DISTRIBUTION

**Range:** Occurs in the Colorado Plateau on benchlands, colluvial slopes, pediments or bajadas. Elevation ranges from 560‑1600 m.

**Divisions:** 304:C

**TNC Ecoregions:** 18:C, 19:C

**Nations:** US

**Subnations:** AZ, NM, UT

**Map Zones:** 13:C, 14:?, 15:C, 16:P, 17:C, 23:C, 24:C, 28:?

**USFS Ecomap Regions:** 313A:CC, 313B:CC, 313D:CC, 322A:CC, 341B:CC, 341C:C?, 341F:CP, M331E:PP, M331H:PP, M341B:CC, M341C:CP

CONCEPT

**Environment:** This shrubland ecological system occurs in the Colorado Plateau at elevations ranging from 580 to 1650 m (1903‑5413 feet) (Bowns and West 1976). *Climate:* This shrubland system occurs in an arid to semi‑arid climate with annual precipitation in the form of summer monsoons and winter storms is generally less than 30 cm, averaging approximately 20 cm.

<br />*Physiography/landform:* Stands occur on gentle to steep, bouldery or rocky colluvial and alluvial slopes of mountains, plateaus, canyons, washes, valley bottoms, and mesas with varying aspects (Anderson 2001a).

<br />*Soils/substrates/hydrology:* Substrates are shallow, well‑drained, typically calcareous, non‑saline and gravelly or sandy soils over sandstone or limestone bedrock, caliche or limestone alluvium, but may include other parent materials such as shale, gneiss, quartzites, and igneous rocks (Anderson 2001a). Effective soil moisture appears to be primarily controlled by regolith depth and position in relation to the water table. This brushland system occupies most sites where regolith is uniformly shallow. In association with blackbrush (*Coleogyne ramosissima*) sites, the soil moisture is concentrated on top of impermeable bedrock at a shallow depth. This perching effect allows for gradual uptake of moisture by the plants roots (Loope and West 1979). This permits growth of plants with more mesic habitat requirements (Warren et al. 1982). On sites with deep soil, blackbrush may occur in almost pure occurrences with only a few associated species (Warren et al. 1982). Dark‑colored biological soil crusts, composed of lichens, mosses, fungi, and algae, are often present in this system in fairly undisturbed areas. Sandy soils may have more biological soil crusts than clayish or silty soil surfaces.

**Vegetation:** This ecological system is dominated by sparse to moderately dense shrubs. Dominant shrubs include *Coleogyne ramosissima, Ephedra nevadensis*, and *Ephedra viridis* (which may codominate with *Grayia spinosa, Salvia dorrii*, and *Lycium andersonii*). There is usually a sparse herbaceous layer with some perennial grasses and forbs. Annual grasses and forbs are present seasonally. Some characteristic species associated with this system include the shrubs *Gutierrezia sarothrae, Chrysothamnus viscidiflorus, Yucca baccata*, and *Krameria grayi*, succulents such as *Ferocactus cylindraceus (= Ferocactus acanthodes), Opuntia* spp., *Echinocereus* spp., *Echinocactus* spp., and *Agave* spp., the graminoid *Pleuraphis rigida*, and perennial forbs such as *Machaeranthera pinnatifida* and *Sphaeralcea ambigua*.

**Dynamics:** Blackbrush is a slow‑growing, long‑lived, drought‑tolerant, evergreen shrub with a diffuse and shallow root system (Bowns 1973, Anderson 2001a). It may lose older leaves during the dry summer season (drought‑deciduous) to reduce water stress and become dormant during dry periods. Unlike many rosaceous species, *Coleogyne ramosissima* is wind‑pollinated and largely self‑incompatible (Pendleton et al. 1995, Pendleton and Pendleton 1998). Blackbrush is a mast species. The resulting fruit crop is a function of available stored energy, producing abundant crops of seeds every few to several years (Pendleton and Meyer 2004).<br />

In general, seed germination and establishment are rare as seedings are uncommon (Anderson 2001a). The germination rate is low, except after a wet spring when soils remain moist for two weeks (Lei 1997). Seeds also require cold stratification (6 weeks) without light to break dormancy (Lei 1997, Meyer and Pendleton 1990). Seeds appear to remain viable for a long time in seed bank. Meyer and Pendleton (2005) observed 80% germination from 15‑year‑old seeds. Abundant seedlings have been observed in clumps from rodent caches (Bowns and West 1976, Lei 1997) or after heavy spring rains, which suggests adaptions to seed caching by small mammals or large runoff events that bury seeds. Kangaroo rats are the main seed dispersers, caching large numbers during mast years (Meyer and Pendleton 2005). Fruits are large and require small mammals or large storm runoff for dispersal (Anderson 2001a).

Blackbrush also provides fair forage for desert bighorn sheep (*Ovis canadensis nelsoni*) and mule deer (*Odocoileus hemionus*) during the winter, and it can tolerate heavy browsing (USFS 1937, Mozingo 1986, Anderson 2001a). Herbaceous forage from understory is generally low.

Fire does not appear to play a role in maintenance of shrublands within this system. Topographic breaks dissect the landscape, and isolated pockets of vegetation are separated by rock walls or steep canyons that protect it from spreading fire. Blackbrush is fire‑intolerant (Loope and West 1979). It does not sprout after fire and is slow to re‑colonized burned sites (Wright 1972). In shallow regolith situations, secondary succession, in the sense of site preparation by seral plants, may not occur at all (Loope and West 1979). In *Coleogyne ramosissima* mixed shrub stands, fire will favor more fire‑tolerant shrubs such as *Artemisia filifolia, Ephedra viridis, Grayia spinosa, Quercus havardii var. tuckeri*, or ruderal species (Tirmenstein 1999j, Anderson 2001a, 2001b, Gucker 2006d).

Biological soil crusts associated with the system are negatively affected by fire, as burning reduced biological soil crusts from 9% cover to less than 1% of total cover, and there was little evidence of recovery postburn after 19 years (Callison et al. 1985). Biological soil crusts are critically important for soil fertility, soil moisture, and soil stability in the many semi‑arid ecosystems in the western U.S. (Belnap and Lange 2003). Biological soil crusts fix large amounts of soil nitrogen (mostly by cyanobacteria) and soil carbon, they protect soils from wind erosion, and rough surface texture slows runoff and allows for more infiltration (Evans and Belnap 1999, Belnap et al. 2001, Belnap and Lange 2003, Johansen 2001). Fires in desert scrub are typically patchy and vary in severity, leaving patches of biological crust organisms to recolonize. Recover rates for biological soil crust organisms vary, e.g., green algae (2 years), cyanobacteria (2‑6 years), mosses (3‑8 years); however, lichens may take decades (Johansen 2001).

Burning blackbrush stands should be minimized because of the unpredictability of successive vegetation, accelerated soil erosion, long‑term or permanent removal of blackbrush, and damage to biological soil crusts (Wright 1980, West 1983d, 1988, Callison et al. 1985).

LANDFIRE (2007a) VDDT model for this system (BpS 2310780) has three classes:

A) Early Development 1 Open (5% of type in this stage): Shrub cover is 0‑5%. Dominated by grasses, shrub seedlings and post‑fire associated forbs. This type typically occurs where fires burn relatively hot in classes B and C. Shrubs (*Coleogyne ramosissima, Ephedra viridis, Ephedra torreyana*, and *Grayia spinosa*) will generally be re‑established after 20‑30 years.

B) Late Development 2 Closed (shrub‑dominated ‑ 30% of type in this stage): Shrub cover (*Coleogyne ramosissima, Ephedra viridis, Ephedra torreyana*, and *Grayia spinosa*) 21‑100%. Greater than 15% shrub cover and 10‑20% herb cover; generally associated with more productive soils. Effects of cumulative drought can cause a shift from this class to class C.

C) Late Development 1 Closed (shrub‑dominated ‑ 65% of type in this stage): Shrubs (*Coleogyne ramosissima, Ephedra viridis, Ephedra torreyana*, and *Grayia spinosa*) are the dominant lifeform with canopy cover of 10‑20%. Less than 15% shrub cover and <10% herb cover generally associated with less productive cobbly and gravelly soils. Effects of cumulative drought can cause a shift from class B to this class.

LANDFIRE modelers emphasized that blackbrush is fire‑intolerant, may be slow to re‑establish following fire such that grasses may dominate immediately following fire. Invasion of non‑native annual grasses following fire is likely under current conditions (LANDFIRE 2007a). LANDFIRE modelers state that generally, the mean fire interval is approximately 75 years with high variability due to annual variation in drying of shrub foliage, shrub mortality and grass and forb production related to drought and moisture cycles (LANDFIRE 2007a). There is also high variation in ignitions and associated fire weather (LANDFIRE 2007a). Fire years are typically correlated with wet years that produce high herbaceous biomass/fine fuel amounts. In areas with high summer moisture from monsoon season rains there are many chances for lightning strikes (LANDFIRE 2007a). Fire‑return intervals would have been much longer in drier geographic areas with return intervals over 200 years (LANDFIRE 2007a). Fire size would have been small because of the discontinuous fuel; frequent topographic breaks that dissect the landscape creating isolated pockets of vegetation are separated by rock walls or steep canyons (LANDFIRE 2007a).

SOURCES

**References:** Anderson 2001a, Anderson 2001b, Belnap 2001, Belnap and Lange 2003, Belnap et al. 2001, Bowns 1973, Bowns and West 1976, Callison et al. 1985, Comer et al. 2003*, Evans and Belnap 1999, Gucker 2006d, Johansen 2001, LANDFIRE 2007a, Lei 1997, Loope and West 1979, McWilliams 2003a, Meyer and Pendleton 1990, Meyer and Pendleton 2005, Mozingo 1987, Pendleton and Meyer 2004, Pendleton and Pendleton 1998, Pendleton et al. 1995, Rondeau 1999, Rosentreter and Belnap 2003, Shiflet 1994, Thatcher 1975, Tirmenstein 1999j, Tuhy and MacMahon 1988, Tuhy et al. 2002, USFS 1937, Warren et al. 1982, West 1983d, West 1988, Wright 1972, Wright 1980

**Version:** 26 Jan 2016	**Stakeholders:** West

**Concept Author:** K.A. Schulz	**LeadResp:** West

CES304.993 Columbia Basin Foothill and Canyon Dry Grassland

**Primary Division:** Inter‑Mountain Basins (304)

**Land Cover Class:** Herbaceous

**Spatial Scale & Pattern:** Large patch

**Required Classifiers:** Natural/Semi‑natural; Vegetated (>10% vasc.); Upland

**Diagnostic Classifiers:** Lowland [Foothill, Lowland]; Sideslope; Very Shallow Soil; Landslide; Graminoid

**National Mapping Codes:** EVT 2134; ESLF 7106; ESP 1134

**Concept Summary:** These grasslands are similar floristically to Columbia Basin Palouse Prairie (CES304.792) but are distinguished by landform, soil, and process characteristics. They occur in the canyons and valleys of the Columbia Basin, particularly along the Snake River canyon, the lower foothill slopes of the Blue Mountains, and along the main stem of the Columbia River in eastern Washington. Occurrences are found on steep open slopes, from 90 to 1525 m (300‑5000 feet) elevation. Annual precipitation is low, ranging from 10 to 25 cm (4‑10 inches). Settings are primarily long, steep slopes of 100 m to well over 400 m, with soils derived from residuum and having patchy, thin, wind‑blown surface deposits. Slope failures are a common process. Fire frequency is presumed to be less than 20 years. The vegetation is dominated by patchy graminoid cover, cacti, and some forbs. *Pseudoroegneria spicata, Festuca idahoensis*, and *Opuntia polyacantha* are common species. Deciduous shrubs *Symphoricarpos* spp., *Physocarpus malvaceus, Holodiscus discolor*, and *Ribes* spp. are infrequent native species that may increase with fire exclusion.

DISTRIBUTION

**Range:** Occurs in the canyons and valleys of the Columbia Basin, particularly along the Snake River canyon, the lower foothill slopes of the Blue Mountains, and along the main stem of the Columbia River in eastern Washington, on steep open slopes, from 90 to 1525 m (300‑5000 feet) elevation.

**Divisions:** 304:C, 306:C

**TNC Ecoregions:** 6:C, 8:C, 68:P

**Nations:** US

**Subnations:** ID, OR, WA:S1S2

**Map Zones:** 1:P, 8:C, 9:C, 10:C, 16:?, 17:?, 18:C

**USFS Ecomap Regions:** 331A:CC, 341G:PP, 342B:CC, 342C:CC, 342D:CC, 342H:CC, 342I:CC, 342J:CC, M242C:CC, M242D:CC, M261D:C?, M261G:CC, M331A:C?, M331D:CP, M332A:CC, M332E:CC, M332F:CC, M332G:CC, M333A:CC, M333B:CP, M333D:CC

CONCEPT

**Environment:** These dry grasslands are distinguished by landform, soil, and process characteristics. Annual precipitation is low, ranging from 12‑25 cm (5‑10 inches) that occurs mostly in the winter, primarily as rain. They occur in the canyons and valleys of the Columbia Basin, particularly along the Snake River canyon, the lower foothill slopes of the Blue Mountains, and along the main stem of the Columbia River in eastern Washington. Occurrences are found on steep open slopes, from 90 to 1525 m (300‑5000 feet) elevation. Landform settings of this grassland are primarily long, steep slopes of 100 m to well over 400 m in length, with colluvial soils derived from residuum and having patchy, thin, wind‑blown surface deposits. Bare ground, gravel and rock between bunches are common features due to frequent soil movement and sun exposure. Biological soil crust cover is usually present but generally decreases with increasing vascular plant cover, elevation, loose surface rock, and coarseness of soil. Elk, deer and bighorn sheep are native large grazers in the canyon who used these grasslands, particularly in winter and spring (Tisdale 1986).

**Vegetation:** The vegetation is dominated by patchy graminoid cover, cacti, and some forbs. *Pseudoroegneria spicata, Festuca idahoensis*, and *Opuntia polyacantha* are common species. Deciduous shrubs *Symphoricarpos* spp., *Physocarpus malvaceus, Holodiscus discolor*, and *Ribes* spp. are infrequent native species that may increase with fire exclusion.

**Dynamics:** This grassland primarily occurs on long, steep slopes. Surface disturbances from slope failure are a common process. Most slips result from saturated soil layers over frozen ground (Tisdale 1986). Fire is the primary disturbance factor. Historically, fire resulted in top‑kill and some mortality, although the overall grassland was not changed. Fires were low intensity due to limited fuel and significant internal spacing between fuel patches. Currently, cheatgrass and other introduced grasses often invade these habitats after fire. The historic frequency was 5‑20 years. Fire frequency is presumed to be less than 20 years; the return interval may have been as low as 5‑10 years (Landfire 2007a).

Biological soil crust cover diminishing or eliminated alters the composition of perennial species and increases the establishment of native disturbance‑increasers and annual grasses, particularly *Bromus tectorum* and other exotic annual bromes (WNHP 2011). Crust cover and diversity are greatest where not impacted by trampling, other soil surface disturbance and fragmentation (Belnap et al. 2001, Rosentreter and Eldridge 2002, Tyler 2006).

SOURCES

**References:** Belnap et al. 2001, Comer et al. 2003*, Darambazar et al. 2007, Davies et al. 2009, Eldridge and Rosentreter 1999, Hall 1973, Johnson and Clausnitzer 1992, Johnson and Simon 1985, LANDFIRE 2007a, Rosentreter and Eldridge 2002, Shiflet 1994, TNC 2013, Tisdale 1986, Tisdale and Bramble‑Brodahl 1983, Tyler 2006, WNHP 2011, WNHP unpubl. data 2018

**Version:** 14 Jan 2014	**Stakeholders:** West

**Concept Author:** R. Crawford, J. Kagan, M. Reid	**LeadResp:** West

CES304.775 Inter‑Mountain Basins Active and Stabilized Dune

**Primary Division:** Inter‑Mountain Basins (304)

**Land Cover Class:** Barren

**Spatial Scale & Pattern:** Large patch

**Required Classifiers:** Natural/Semi‑natural; Unvegetated (<10% vasc.); Upland

**Diagnostic Classifiers:** Dune (Landform); Dune field; Dune (Substrate); Temperate [Temperate Continental]; Sand Soil Texture; Aridic; W‑Landscape/High Intensity

**Concept Summary:** This ecological system occurs in the Intermountain western U.S. on basins, valleys and plains. Often it is composed of a mosaic of migrating, bare dunes; anchored dunes with sparse to moderately dense vegetation (<10‑30% canopy cover); and stabilized dunes. The system is defined by the presence of migrating dunes or, where the dunes are entirely anchored or stabilized, evidence that the substrate is eolian and not residual, that the vegetation is early‑ or mid‑seral, and that the substrate is likely to become actively migrating again with disturbance or increased aridity. In the Colorado Plateau, there are many small active and partially vegetated dunes along some of the larger washes and playas (where sand is blown out of wash and forms dunes) and some larger dunes such as Coral Pink Dunes in southwestern Utah. Substrates are usually eolian sand, but small dunes composed of silt and clay downwind from playas in the Wyoming Basins (which usually support greasewood vegetation) also are included here. Species occupying these environments are often adapted to shifting, coarse‑textured substrates (usually quartz sand) and form patchy or open grasslands, shrublands or steppe, and occasionally woodlands. Vegetation varies and may be composed of *Achnatherum hymenoides, Artemisia filifolia, Artemisia tridentata ssp. tridentata, Atriplex canescens, Ephedra* spp., *Chrysothamnus viscidiflorus, Coleogyne ramosissima, Ericameria nauseosa, Hesperostipa comata, Leymus flavescens, Muhlenbergia pungens, Psoralidium lanceolatum, Purshia tridentata, Redfieldia flexuosa, Sporobolus airoides, Sarcobatus vermiculatus, Tetradymia tetrameres*, or *Tiquilia* spp. Herbaceous species such as *Achnatherum hymenoides, Redfieldia flexuosa*, and *Psoralidium lanceolatum* are characteristic of early‑seral vegetation through much of this system's range. Shrubs are commonly dominant on mid‑ to late‑seral stands, and *Ericameria nauseosa* can be found at any stage.

**Comments:** Rules should be devised for deciding whether shrub or shrub‑steppe vegetation on completely stabilized dunes should be considered part of this active and stabilized dune system, or part of another system. The areas include Inter‑Mountain Basins Mixed Salt Desert Scrub (CES304.784), Inter‑Mountain Basins Big Sagebrush Shrubland (CES304.777), and Inter‑Mountain Basins Big Sagebrush Steppe (CES304.778) on sand, and Inter‑Mountain Basins Greasewood Flat (CES304.780) on clay and silt.

DISTRIBUTION

**Range:** This system occurs in intermountain basins of the western U.S. including southwestern Montana in the Centennial Valley.

**Divisions:** 304:C, 306:C

**TNC Ecoregions:** 6:C, 8:C, 10:C, 11:C, 19:C

**Nations:** US

**Subnations:** AZ, CO, ID, MT, NM, NV, OR, UT, WA:S1, WY

**Map Zones:** 7:P, 8:P, 9:C, 12:P, 13:?, 15:?, 16:C, 17:C, 18:C, 19:C, 20:?, 21:C, 22:C, 23:C, 24:C, 25:C, 27:?, 28:C, 29:?, 33:P

**USFS Ecomap Regions:** 313A:CC, 313B:CC, 313D:CC, 315H:CC, 321A:PP, 322A:??, 331F:CC, 331G:CC, 331J:CC, 341A:CC, 341B:CC, 341D:C?, 341E:CC, 341F:CC, 342B:CC, 342C:CC, 342D:CC, 342F:CC, 342G:CC, 342H:CC, M242C:??, M261G:PP, M313A:CP, M313B:CC, M331A:CC, M331F:CC, M331G:CC, M331H:C?, M331I:CC, M332E:CC, M332G:CC, M341D:??

CONCEPT

**Environment:** This ecological system occurs in the intermountain western U.S. on basins, valleys and plains. Often it is composed of a mosaic of migrating, bare dunes; anchored dunes with sparse to moderately dense vegetation (<10‑30% canopy cover); and stabilized dunes. The system is defined by the presence of migrating dunes or, where the dunes are entirely anchored or stabilized, evidence that the substrate is eolian and not residual, that the vegetation is early‑ or mid‑seral, and that the substrate is likely to become actively migrating again with disturbance or increased aridity. In the Colorado Plateau and Great Basin, there are many small, active and partially vegetated dunes along some of the larger washes and on sides of playas and basins (where sand is blown out of a wash or basin and forms dunes) and some larger dunes, including Coral Pink Dunes in southwestern Utah, Great Sand Dunes in south‑central Colorado, Alkali Lake Dunes in southern Oregon, and many in Nevada, such as Clayton Valley Dunes, Crescent Dunes, Fish Lake Dunes, Sand Mountain, Silver State Dunes, Teel Mountain and Winnemucca Dunes. Substrates are usually eolian sand, but small dunes composed of silt and clay downwind from playas in the Wyoming Basins (which usually support greasewood vegetation) also are included here. Species occupying these environments are often adapted to shifting, coarse‑textured substrates (usually quartz sand) and form patchy or open grasslands, shrublands or steppe, and occasionally woodlands. The environmental description is based on several other references, including Chadwick and Dalke (1965), Bowers (1982), Caicco and Wellner (1983e), Pavlik (1985, 1989), Fryberger et al. (1990), Knight (1994), Pineada et al. (1999), Reid et al. (1999), Marin et al. (2005), Forman et al. (2006), Jones (2006), Hallock et al. (2007), Sawyer et al. (2009).

**Vegetation:** Vegetation cover and species composition are variable and range from absent or sparse on active and recently stabilized dunes and blowouts to 30% canopy cover on stable dunes and sandsheets. Species are often adapted to shifting, coarse‑textured substrates (usually quartz sand) and form patchy or open grasslands, shrublands or steppe, and occasionally woodlands. Characteristic taxa include *Achnatherum hymenoides, Artemisia filifolia, Artemisia tridentata ssp. tridentata, Atriplex canescens, Ephedra* spp., *Chrysothamnus viscidiflorus, Coleogyne ramosissima, Ericameria nauseosa, Hesperostipa comata, Leymus flavescens, Muhlenbergia pungens, Psoralidium lanceolatum, Purshia tridentata, Redfieldia flexuosa, Sporobolus airoides, Sarcobatus vermiculatus, Tetradymia tetrameres*, or *Tiquilia* spp. Herbaceous species such as *Achnatherum hymenoides, Redfieldia flexuosa*, and *Psoralidium lanceolatum* are characteristic of early‑seral vegetation throughout much of this system's range. In the Centennial Valley of southwestern Montana, where the dunes are more stable, *Artemisia tridentata ssp. tridentata* and *Artemisia tripartita ssp. tripartita* contribute a moderate amount of cover and are associated with *Hesperostipa comata* or *Festuca idahoensis* (in more mesic settings). Early‑ and mid‑seral shrub communities in these dunes are dominated by *Ericameria nauseosa, Chrysothamnus viscidiflorus, Purshia tridentata*, and *Hesperostipa comata*. Several rare plant species occur in the Centennial Valley dunes and are associated with early‑successional stages. These dunes are very similar to the St. Anthony Sand Dunes in Idaho. In the Killpecker Dunes in west‑central Wyoming, *Artemisia tridentata ssp. tridentata* dominates late‑seral vegetation and *Ericameria nauseosa* dominates mid‑seral vegetation (Jones 2005). The Great Sand Dunes in southern Colorado consist of an active dunefield surrounded by a stabilized sandsheet dominated by *Ericameria nauseosa, Sarcobatus vermiculatus*, or *Pinus ponderosa* (on a sand ramp). The stabilized areas are periodically disturbed by parabolic dunes tracking across them from blowouts caused by fire or drought (Marin et al. 2005). The vegetation description is based on several other references, including Chadwick and Dalke (1965), Bowers (1982), Caicco and Wellner (1983e), Pavlik (1985, 1989), Fryberger et al. (1990), Knight (1994), Pineada et al. (1999), Reid et al. (1999), Marin et al. (2005), Forman et al. (2006), Jones (2006), Hallock et al. (2007), Sawyer et al. (2009).

**Dynamics:** Periodic drought influences dune migration rates by reducing vegetation cover that anchors dunes (Marin et al. 2005, Forman et al. 2006). Disturbances by fire, heavy grazing, and burrowing are important processes influencing successional dynamics (Lesica and Cooper 1998). A typical primary successional sere on sands appears to be as follows: bare sand or sparse herbaceous vegetation on migrating sand; denser herbaceous vegetation or shrub stands of *Ericameria nauseosa* on anchored or recently stabilized sand; and shrub vegetation of *Artemisia tridentata, Atriplex canescens, Purshia tridentata, Sarcobatus vermiculatus*, and other long‑lived shrub and tree species on longer‑stabilized sands. Vegetation growing on stabilized sandsheets and dunes may be dense enough to carry fire, especially when there is strong wind. Fire reduces vegetation cover and, when stabilized dunes burn followed by wind or drought, local blowouts may occur or stabilized dunes may become active. This creates bare areas capable of supporting early‑successional species.

SOURCES

**References:** Anderson 1999a, Bell et al. 2009, Bowers 1982, Caicco and Wellner 1983a, Caicco and Wellner 1983b, Caicco and Wellner 1983c, Caicco and Wellner 1983e, Chadwick and Dalke 1965, Comer et al. 2003*, Forman et al. 2006, Fryberger et al. 1990, Hallock et al. 2007, Jones 2006, Knight 1994, Lesica and Cooper 1998, Marin et al. 2005, Pavlik 1985, Pavlik 1989, Pineda et al. 1999, Reid et al. 1999, Sawyer et al. 2009, Thomas et al. 2009a, WNHP unpubl. data 2018

**Version:** 02 Apr 2014	**Stakeholders:** West

**Concept Author:** NatureServe Western Ecology Team	**LeadResp:** West

CES304.787 Inter‑Mountain Basins Semi‑Desert Grassland

**Primary Division:** Inter‑Mountain Basins (304)

**Land Cover Class:** Herbaceous

**Spatial Scale & Pattern:** Large patch

**Required Classifiers:** Natural/Semi‑natural; Vegetated (>10% vasc.); Upland

**Diagnostic Classifiers:** Lowland [Foothill, Lowland]; Herbaceous; Temperate [Temperate Xeric]; Alkaline Soil; Aridic; Graminoid

**National Mapping Codes:** EVT 2135; ESLF 7107; ESP 1135

**Concept Summary:** This widespread ecological system includes the driest grasslands throughout the intermountain western U.S. It occurs on xeric sites over an elevation range of approximately 1450 to 2320 m (4750‑7610 feet) on a variety of landforms, including swales, playas, mesas, alluvial flats, and plains. This system may constitute the matrix over large areas of intermountain basins, and also may occur as large patches in mosaics with shrubland systems dominated by *Artemisia tridentata ssp. tridentata, Artemisia tridentata ssp. wyomingensis, Atriplex* spp., *Coleogyne* spp., *Ephedra* spp., *Gutierrezia sarothrae*, or *Krascheninnikovia lanata*. Grasslands in areas of higher precipitation, at higher elevation, typically belong to other systems. Substrates are often well‑drained sandy or loam soils derived from sedimentary parent materials but are quite variable and may include fine‑textured soils derived from igneous and metamorphic rocks. The dominant perennial bunchgrasses and shrubs within this system are all drought‑resistant plants. Dominant or codominant species are *Achnatherum hymenoides, Aristida* spp., *Bouteloua gracilis, Hesperostipa comata, Muhlenbergia* spp., *Pleuraphis jamesii*, or *Sporobolus* spp. Scattered shrubs and dwarf‑shrubs often are present, especially *Artemisia tridentata ssp. tridentata, Artemisia tridentata ssp. wyomingensis, Atriplex* spp., *Coleogyne* spp., *Ephedra* spp., *Ericameria* spp., *Gutierrezia sarothrae*, and *Krascheninnikovia lanata*. This system is typically composed of cool‑season grasses in the western portion of its range where winter precipitation dominates, and a mix of cool‑ and warm‑season grasses where precipitation occurs during both winter and summer seasons (Colorado Plateau). Grasslands in the basins of south‑central and southwestern Wyoming, dominated by *Pseudoroegneria spicata* and *Poa secunda* and containing cushion‑form forbs and other species typical of dry basins, are included in this system.

**Comments:** In the relatively high‑elevation basins of Wyoming and south‑central Montana, grass vegetation dominated or codominated by *Pseudoroegneria spicata* and *Poa secunda* seems to be transitional between more typical Inter‑Mountain Basins Semi‑Desert Grassland (CES304.787) as found farther west and south in the intermountain region and Northern Rocky Mountain Lower Montane, Foothill and Valley Grassland (CES306.040) common on the foothills of the surrounding mountains. That grass vegetation is placed into this semi‑desert grassland system, instead of into the foothill grassland system, for two reasons. The first is composition of the vegetation: *Pseudoroegneria*‑ and *Poa*‑rich vegetation often contains shrubs (*Artemisia tridentata ssp. wyomingensis, Krascheninnikovia lanata*), other grasses (*Achnatherum hymenoides, Hesperostipa comata*), and cushion‑form forbs common in drier vegetation of the same basins, while the species common in the foothills, especially *Festuca idahoensis* and *Leucopoa kingii*, are absent. The second is the setting: patches of the *Pseudoroegneria*‑ and *Poa*‑rich vegetation occur in a mosaic with other basins systems, especially Inter‑Mountain Basins Big Sagebrush Steppe (CES304.778), with which it often merges.

In the Columbia Plateau, this semi‑desert ecological system does not include *Pseudoroegneria spicata*‑dominated or ‑codominated associations such as *Pseudoroegneria spicata ‑ Achnatherum hymenoides* Grassland (CEGL001674) or *Pseudoroegneria spicata ‑ Poa secunda* Grassland (CEGL001677). These associations are included in the similar Columbia Plateau Steppe and Grassland (CES304.083). Additionally, *Poa cusickii* Moist Meadow (CEGL001655) is restricted to relatively mesic sites there and does not occur in this semi‑desert system as it occurs in the Columbia Plateau, but may be found in this system in Wyoming.

DISTRIBUTION

**Range:** This system occurs throughout the intermountain western U.S. on dry plains and mesas, at approximately 1450 to 2320 m (4750‑7610 feet) elevation. In the Bighorn Basin of north‑central Wyoming, there may be some desert grasslands, but this is uncertain.

**Divisions:** 304:C, 306:C

**TNC Ecoregions:** 4:C, 6:C, 8:C, 9:C, 10:C, 11:C, 18:C, 19:C, 20:C, 21:C

**Nations:** US

**Subnations:** AZ, CA, CO, ID, MT?, NM, NV, OR, UT, WA, WY

**Map Zones:** 6:P, 7:C, 8:C, 9:C, 12:C, 13:C, 14:P, 15:C, 16:C, 17:C, 18:C, 22:C, 23:C, 24:C, 25:C, 26:?, 28:C, 29:?

**USFS Ecomap Regions:** 313A:CC, 313B:CC, 313C:CC, 313D:CC, 315A:CC, 315H:CC, 321A:CC, 322A:CC, 331A:CC, 331J:CC, 341A:CC, 341B:CC, 341C:CC, 341D:CC, 341E:CC, 341F:CC, 341G:CC, 342B:CC, 342C:CC, 342D:CC, 342E:CC, 342F:CC, 342G:CC, 342H:CC, 342I:CC, 342J:CC, M242C:CC, M242D:CP, M261E:CC, M261G:CC, M313A:CC, M313B:CC, M331A:CC, M331B:C?, M331D:CC, M331E:CC, M331F:CC, M331G:CC, M331H:CC, M331I:CP, M331J:CP, M332G:CC, M333A:??, M341A:CC, M341B:CC, M341C:CC, M341D:CC

CONCEPT

**Environment:** This widespread semi‑arid ecological system consists of lower‑elevation dry grasslands found on plains, mesas and foothills throughout the intermountain western U.S. Elevation ranges from approximately 1450 to 2320 m (4750‑7610 feet).

<br />*Climate*: Climate usually includes hot summers and cold winters with freezing temperatures and snow common. Annual precipitation is usually from 20‑40 cm (7.9‑15.7 inches). A significant portion of the precipitation falls in July through October during the summer monsoon storms, with the rest falling as snow during the winter and early spring months (bimodal precipitation). However, precipitation in the western portion of this system's range occurs primarily in the winter.

*Physiography/landform:* These grasslands occur on a variety of aspects, slopes and landforms, including swales, playas, mesas, alluvial flats, plains and hillslopes. Stands are found in lowland and upland areas usually on xeric sites. Grasslands in areas of higher precipitation, at higher elevation, typically belong to other systems.

*Soil/substrate/hydrology:* Substrates range from deep to shallow, frequently well‑drained sandy or loam soils derived from sandstone or shale parent materials but are quite variable and may include fine‑textured soils derived from igneous and metamorphic rocks. Some occurrences on sandy soils have a high cover of cryptogams on the soil surface. These cryptogams tend to increase the stability of the highly erodible sandy soils of these grasslands during torrential summer rains and heavy wind storms (Kleiner and Harper 1977). *Muhlenbergia*‑dominated grasslands which flood temporarily, combined with high evaporation rates in this dry system, can have accumulations of soluble salts in the soil. Soil salinity depends on the nature of the parent material and on the amount and timing of precipitation and flooding. Growth‑inhibiting salt concentrations are diluted when the soil is saturated, allowing the growth of less salt‑tolerant species. As the saturated soils dry, the salt concentrates until it precipitates out on the soil surface (Dodd and Coupland 1966, Ungar 1968). The environmental description is based on several other references, including Barbour and Major (1977), Brown (1982), West (1983e), Knight (1994), Reid et al. (1999), West and Young (2000), Tuhy et al. (2002), Barbour et al. (2007), and Sawyer et al. (2009).

**Vegetation:** This grassland system may constitute the matrix over large areas of intermountain basins and may also occur as large patches in mosaics with shrubland systems dominated by *Artemisia tridentata ssp. tridentata, Artemisia tridentata ssp. wyomingensis, Atriplex* spp., *Coleogyne* spp., *Ephedra* spp., *Gutierrezia sarothrae*, or *Krascheninnikovia lanata*. The dominant perennial bunchgrasses and shrubs within this system are all drought‑resistant plants. Dominant or codominant species are *Achnatherum hymenoides, Aristida* spp., *Bouteloua gracilis, Hesperostipa comata, Muhlenbergia* spp., or *Pleuraphis jamesii*. Additional perennial warm‑season grasses found in this system include *Aristida purpurea, Bouteloua curtipendula, Bouteloua eriopoda, Muhlenbergia asperifolia, Muhlenbergia pungens, Muhlenbergia richardsonis, Muhlenbergia torreyi, Pleuraphis rigida, Sporobolus airoides, Sporobolus contractus*, and *Sporobolus cryptandrus*. Scattered shrubs and dwarf‑shrubs often are present, especially *Artemisia tridentata ssp. tridentata, Artemisia tridentata ssp. wyomingensis, Atriplex* spp., *Coleogyne* spp., *Ephedra* spp., *Gutierrezia sarothrae*, and *Krascheninnikovia lanata*. Grasslands in the basins of south‑central and southwestern Wyoming, dominated by *Pseudoroegneria spicata* and *Poa secunda* and containing cushion‑forming forbs and other species typical of dry basins, are included in this system. In the Columbia Plateau, this semi‑desert ecological system does not include *Pseudoroegneria spicata*‑dominated or ‑codominated associations such as *Pseudoroegneria spicata ‑ Achnatherum hymenoides* Grassland (CEGL001674) or *Pseudoroegneria spicata ‑ Poa secunda* Grassland (CEGL001677). Additionally, *Poa cusickii* Marsh (CEGL001655) is restricted to relatively mesic sites there and does not occur in this semi‑desert system as it occurs in the Columbia Plateau, but may be found in this system in Wyoming. The vegetation description is based on several other references, including Barbour and Major (1977), Brown (1982), West (1983e), Knight (1994), Reid et al. (1999), West and Young (2000), Tuhy et al. (2002), Barbour et al. (2007), and Sawyer et al. (2009).

**Dynamics:** Disturbance dynamics in this semi‑arid grassland system are variable because of variation in the composition; however, most are dominated by perennial bunchgrasses that are adapted to low‑ to medium‑frequency (<30 to <100 years) and low‑ to medium‑intensity fires (Howard 1997a, b, Tirmenstein 1999e, Zlatnik 1999a, b, Johnson 2000c, Simonin 2000a, b, c, Anderson 2003a, Sawyer et al. 2009). Most of the species are classified as resistant or tolerant of fire, with the exception of *Bouteloua eriopoda*, which is classified as sensitive, but will recover quickly if there is adequate summer moisture (Simonin 2000a). Season of burn is also important for predicting post‑burn recovery.

The majority of characteristic grass species, such as *Achnatherum hymenoides, Aristida* spp., *Bouteloua eriopoda, Bouteloua gracilis, Hesperostipa comata, Pleuraphis jamesii, Poa secunda, Pseudoroegneria spicata, Sporobolus airoides*, and *Sporobolus cryptandrus*, will be top‑killed after burning, then resprout from rootcrowns unless the fire was very severe (Howard 1997a, b, Tirmenstein 1999e, Zlatnik 1999a, b, Johnson 2000c, Simonin 2000a, b, c, Anderson 2003a, Sawyer et al. 2009). This grassland system is maintained by fires that kill or reduce cover of the more fire‑sensitive shrub species.

The dominant perennial grass species are well‑adapted to the semi‑arid conditions. *Achnatherum hymenoides* is one of the most drought‑tolerant, cool‑season grasses in the western U.S. (USFS 1937, Tirmenstein 1999e). It is also a valuable forage grass in arid and semi‑arid regions. *Hesperostipa comata* is a deep‑rooted, cool‑season grass that uses soil moisture below 0.5 m depth during the dry summers. It is prone to litter accumulations at plant bases, which can increase intensity of fire, making it more susceptible to mortality (Zlatnik 1999a). *Bouteloua gracilis* is a drought‑ and very grazing‑tolerant warm‑season grass that generally forms a short sod. *Pleuraphis jamesii*, also a warm‑season grass, is only moderately palatable to grazers, but decreases when heavily utilized during drought and in the more arid portions of its range where it is the dominant grass (West et al. 1972). This grass reproduces extensively from scaly rhizomes, which make the plant resistant to trampling by large wildlife or livestock and have good soil‑binding properties (Weaver and Albertson 1956, West 1972).

<br />Insects are an important component of many shrub‑steppe and grassland systems. Mormon crickets and grasshoppers are natural components of many rangeland systems (USDA‑APHIS 2003, 2010). There are almost 400 species of grasshoppers that inhabit the western United States with 15‑45 species occurring in a given rangeland system (USDA‑APHIS 2003). Mormon crickets are also present in many western rangelands and, although flightless, are highly mobile and can migrate large distances consuming much of the forage while travelling in wide bands (USDA‑APHIS 2010). Following a high population year for grasshoppers or Mormon crickets and under relatively warm dry spring environmental conditions that favor egg hatching and grasshopper and Mormon cricket survival, there may be large population outbreaks that can utilize 80% or more of the forage in areas as large as 2,000 square miles. Conversely, relatively cool and wet spring weather can limit the potential for outbreaks. These outbreaks are naturally occurring cycles and, especially during drought, can denude an area of vegetation leaving it exposed to increased erosion rates from wind and water (USDA‑APHIS 2003).<br />

LANDFIRE developed this VDDT model for this system for the Great Basin using two classes (LANDFIRE 2007a, BpS 1211350).

A) Early Development 1 Open (grass‑dominated ‑ 20% of type in this stage): Dominated by grasses (*Achnatherum hymenoides, Hesperostipa comata*) and post‑fire‑associated forbs, and remnant *Artemisia tridentata*. Perennial grasses and forbs dominate (generally 25‑40% cover) where woody shrub canopy has been top‑killed/removed by wildfire. Shrub cover is less than 5%. Replacement fire occurs every 120 years on average. Succession to class B after 20 years.

B) Mid Development 1 Open (grass with shrubs ‑ 80% of type in this stage): Dominated by grasses (*Achnatherum hymenoides, Hesperostipa comata*) and *Artemisia tridentata*. Shrubs compose the upper layer lifeform (5‑25% cover) with diverse perennial grass and forb understory dominant. Mean fire‑return interval (FRI) is 75 years with 80% replacement fire (mean FRI of 94 years) and 20% mixed‑severity fire (mean FRI of 375 years). Mixed‑severity fire, insect/disease (return interval of 75 years), and weather‑related stress (return interval of 100 years) maintain vegetation in class B.

SOURCES

**References:** Anderson 2003a, Anderson 2009, Barbour and Major 1977, Barbour et al. 2007a, Bell et al. 2009, Belnap 2001, Belnap et al. 2001, Betts 1990, Brown 1982a, CNHP 2010, Cable 1967, Cable 1969, Cable 1975b, Comer et al. 2003*, Degenhardt et al. 1996, Dodd and Coupland 1966, Greene 1999, Hammerson 1999, Howard 1997a, Howard 1997b, Johnson 2000c, Kleiner and Harper 1977, Knight 1994, LANDFIRE 2007a, Mast et al. 1997, Mast et al. 1998, McClaran and Van Devender 1995, Pellant 1990, Pellant 1996, Quinn 2004, Reid et al. 1999, Rosentreter and Belnap 2003, Saab and Marks 1992, Sawyer and Keeler‑Wolf 1995, Sawyer et al. 2009, Shiflet 1994, Simonin 2000a, Simonin 2000b, Simonin 2000c, Stebbins 2003, TNC 2013, Tennant 1984, Tirmenstein 1999b, Tirmenstein 1999c, Tirmenstein 1999e, Tuhy et al. 2002, USDA‑APHIS 2003, USDA‑APHIS 2010, USFS 1937, USFWS 2004b, Ungar 1968, Vander Haegen et al. 2001, WNHP 2011, WNHP and BLM 2005, WNHP unpubl. data 2018, Weaver and Albertson 1956, West 1983e, West and Young 2000, West et al. 1972, Zlatnik 1999a, Zlatnik 1999b

**Version:** 20 Nov 2015	**Stakeholders:** West

**Concept Author:** NatureServe Western Ecology Team	**LeadResp:** West

CES304.788 Inter‑Mountain Basins Semi‑Desert Shrub‑Steppe

**Primary Division:** Inter‑Mountain Basins (304)

**Land Cover Class:** Steppe/Savanna

**Spatial Scale & Pattern:** Large patch

**Required Classifiers:** Natural/Semi‑natural; Vegetated (>10% vasc.); Upland

**Diagnostic Classifiers:** Lowland [Foothill, Lowland]; Woody‑Herbaceous; Temperate [Temperate Xeric]; Alkaline Soil; Aridic; Very Short Disturbance Interval; G‑Landscape/High Intensity; Graminoid

**National Mapping Codes:** EVT 2127; ESLF 5456; ESP 1127

**Concept Summary:** This ecological system occurs throughout the intermountain western U.S., typically at lower elevations on alluvial fans and flats with moderate to deep soils, and extends into south‑central Montana between the Pryor and Beartooth ranges where a distinct rainshadow effect occurs. This semi‑arid shrub‑steppe is typically dominated by graminoids (>25% cover) with an open shrub to moderately dense woody layer with a typically strong graminoid layer. The most widespread (but not dominant) species is *Pseudoroegneria spicata*, which occurs from the Columbia Basin to the Northern Rockies. Characteristic grasses include *Achnatherum hymenoides, Bouteloua gracilis, Distichlis spicata, Poa secunda, Poa fendleriana, Sporobolus airoides, Hesperostipa comata, Pleuraphis jamesii*, and *Leymus salinus*. The woody layer is often a mixture of shrubs and dwarf‑shrubs, although it may be dominated by a single species. Characteristic species include *Atriplex canescens, Artemisia tridentata, Chrysothamnus greenei, Chrysothamnus viscidiflorus, Ephedra* spp., *Ericameria nauseosa, Gutierrezia sarothrae*, and *Krascheninnikovia lanata*. *Artemisia tridentata* or *Atriplex canescens* may be present but does not dominate. Annual grasses, especially the exotics *Bromus arvensis* and *Bromus tectorum*, may be present to abundant. Forbs are generally of low importance and are highly variable across the range but may be diverse in some occurrences. The general aspect of occurrences may be either open shrubland with patchy grasses or patchy open herbaceous layers. Disturbance may be important in maintaining the woody component. Microphytic crust is very important in some stands.

DISTRIBUTION

**Range:** This system occurs throughout the intermountain western U.S., typically at lower elevations, and extends into Wyoming and Montana across the Great Divide Basin. It barely gets as far north into north‑central Montana (mapzone 20) but is unlikely to be mapped.

**Divisions:** 304:C

**TNC Ecoregions:** 4:C, 6:C, 8:C, 9:C, 10:C, 11:C, 18:C, 19:C, 20:C, 21:C

**Nations:** US

**Subnations:** AZ, CA, CO, ID, MT, NM, NV, OR, UT, WA:S1, WY

**Map Zones:** 6:P, 7:P, 8:C, 9:C, 12:C, 13:C, 14:C, 15:C, 16:C, 17:C, 18:C, 19:?, 22:C, 23:C, 24:C, 25:C, 27:C, 28:C

**USFS Ecomap Regions:** 313A:CC, 313B:CC, 313C:CC, 313D:CC, 315A:CC, 315B:CC, 315H:CC, 321A:CC, 322A:CC, 331B:CC, 331H:CC, 331I:CC, 331J:CC, 341A:CC, 341B:CC, 341C:CC, 341D:CC, 341E:CC, 341F:CC, 341G:CC, 342B:CC, 342C:CC, 342D:CC, 342E:CC, 342F:CC, 342G:CC, 342H:CC, 342I:CP, 342J:CC, M242C:CC, M261E:CC, M261G:CC, M313A:CC, M313B:CC, M331A:CC, M331B:CC, M331D:CC, M331E:CC, M331F:CC, M331G:CC, M331H:CC, M331I:CC, M331J:CC, M332A:CC, M332E:CP, M332G:CC, M333A:??, M341A:CC, M341B:CC, M341C:CC, M341D:CC

CONCEPT

**Environment:** This ecological system occurs throughout the intermountain western U.S., from the western Great Basin to the northern Rocky Mountains and Colorado Plateau and extends into south‑central Montana between the Pryor and Beartooth ranges where a distinct rainshadow effect occurs. Elevation ranges from 300 m up to 2500 m. The climate where this system occurs is generally hot in summers and cold in winters with low annual precipitation, ranging from 18‑40 cm and high inter‑annual variation. Much of the precipitation falls as snow, and growing‑season drought is characteristic. Temperatures are continental with large annual and diurnal variations. Sites are generally alluvial fans and flats with moderate to deep soils. Some sites can be flat, poorly drained and intermittently flooded with a shallow or perched water table often within 1 m depth (West 1983e). Substrates are generally shallow, calcareous, fine‑textured soils (clays to silt loams), derived from alluvium; or deep, fine to medium‑textured alluvial soils with some source of subirrigation during the summer season. Soils may be alkaline and typically moderately saline (West 1983e). Some occurrences are found on deep, sandy loam soils, or soils that are highly calcareous, but not deep sand with active dune fields (Hironaka et al. 1983). The environmental description is based on several references, including Hanson (1929), Branson et al. (1976), Barbour and Major (1977), Brown (1982), Hironaka et al. (1983), West (1983e), Knight (1994), Holland and Keil (1995), Reid et al. (1999), West and Young (2000), Tuhy et al. (2002), Barbour et al. (2007), Sawyer et al. (2009), and NatureServe Explorer (2011).

**Vegetation:** The plant associations in this broadly‑defined system are typically semi‑arid shrub‑steppe dominated by graminoids (>20% cover) with an open shrub to moderately dense woody layer. The typically open woody layer is often a mixture of shrubs and dwarf‑shrubs, although it may be dominated by a single species. This system also includes some open to sparse, mixed‑shrub stands with sparse herbaceous layers that are frequently the result of disturbance. Characteristic woody species include *Artemisia filifolia, Artemisia tridentata, Atriplex canescens, Ephedra cutleri, Ephedra nevadensis, Ephedra torreyana, Ephedra viridis, Ericameria nauseosa, Chrysothamnus viscidiflorus, Gutierrezia sarothrae, Krascheninnikovia lanata*, and *Sarcobatus vermiculatus*. Other shrubs occasionally present include *Purshia tridentata* and *Tetradymia canescens*. Shrubs such as *Artemisia filifolia, Artemisia tridentata, Atriplex canescens*, or *Sarcobatus vermiculatus* may be present but do not dominate as they do in big sagebrush shrublands and steppes, black greasewood or mixed salt desert scrub systems. Trees are very rarely present in this system, but some individuals of *Pinus ponderosa, Juniperus scopulorum, Juniperus occidentalis*, or *Cercocarpus ledifolius* may occur. The typically moderately dense herbaceous layer is dominated by bunchgrasses which occupy patches in the shrub matrix. One of the most widespread species is *Pseudoroegneria spicata*, which occurs from the Columbia Basin to the Northern Rockies. Other locally dominant or important species include *Sporobolus airoides, Leymus cinereus, Festuca idahoensis, Pascopyrum smithii, Bouteloua gracilis, Distichlis spicata, Pleuraphis jamesii, Elymus lanceolatus, Elymus elymoides, Koeleria macrantha, Muhlenbergia richardsonis, Hesperostipa comata*, and *Poa secunda*. Annual grasses, especially the exotics *Bromus arvensis (= Bromus japonicus)* and *Bromus tectorum*, may be present to abundant. Forbs are generally of low importance and are highly variable across the range, but may be diverse in some occurrences. Species that often occur are *Symphyotrichum ascendens (= Aster adscendens), Collinsia parviflora, Penstemon caespitosus, Achillea millefolium, Erigeron compositus, Senecio* spp., and *Taraxacum officinale*. Other important genera include *Astragalus, Oenothera, Eriogonum*, and *Balsamorhiza*. Mosses and lichens may be important ground cover. Forbs are common on disturbed weedy sites. Weedy annual forbs may include the exotics *Descurainia* spp., *Helianthus annuus, Halogeton glomeratus, Lactuca serriola*, and *Lepidium perfoliatum*. The vegetation description is based on several references, including Hanson (1929), Branson et al. (1976), Barbour and Major (1977), Brown (1982), Hironaka et al. (1983), West (1983e), Knight (1994), Holland and Keil (1995), Reid et al. (1999), West and Young (2000), Tuhy et al. (2002), Barbour et al. (2007), and Sawyer et al. (2009).

**Dynamics:** Disturbance dynamics in this system are variable because of variation in the compositions; however, most are dominated by short‑ to long‑lived, deciduous shrubs that are adapted to low‑ to medium‑frequency, medium‑ to large‑sized and low‑ to medium‑intensity fire (Carey 1995, Tirmenstein 1999b, 1999f, 1999g, Anderson 2001b, 2004b, Scher 2001, Sawyer et al. 2009). Some shrubs, such as *Chrysothamnus viscidiflorus, Ephedra torreyana, Ephedra viridis, Ericameria nauseosa, Sarcobatus vermiculatus*, and *Tetradymia canescens*, are generally top‑killed in burns, but then vigorously resprout from rootcrowns unless the fire was very severe (Tirmenstein 1999b, 1999f, 1999g, Anderson 2001b, 2004b, Scher 2001, Sawyer et al. 2009). Other shrubs, such a *Gutierrezia sarothrae* and *Krascheninnikovia lanata*, are more typically killed by fire and only weakly sprout post‑fire, if at all (Carey 1995, Tirmenstein 1999g). However, in most cases, reestablishment generally proceeds rapidly through light wind‑dispersed seeds from adjacent unburned areas, and in some case, these shrub species aggressively invade disturbed open sites, then decline after 15 years to be replaced by longer‑lived species such as *Artemisia tridentata* or *Sarcobatus vermiculatus* (Carey 1995, Tirmenstein 1999b, 1999f, 1999g, Anderson 2001b, 2004b, Scher 2001, Sawyer et al. 2009). Some stands, such as those dominated by *Ericameria parryi*, are too sparse to carry fire (Sawyer et al. 2009). Many stands have a lush herbaceous layer that dries to fine fuels that readily carry fire regardless of shrub density.

Insects are an important component of many shrub‑steppe and grassland systems. Mormon crickets and grasshoppers are natural components of many rangeland systems (USDA‑APHIS 2003, 2010). There are almost 400 species of grasshoppers that inhabit the western United States, with 15‑45 species occurring in a given rangeland system (USDA‑APHIS 2003). Mormon crickets are also present in many western rangelands and, although flightless, are highly mobile and can migrate large distances consuming much of the forage while travelling in wide bands (USDA‑APHIS 2010). Following a high population year for grasshoppers or Mormon crickets and under relatively warm dry spring environmental conditions that favor egg hatching and grasshopper and Mormon cricket survival, there may be large population outbreaks that can utilize 80% or more of the forage in areas as large as 2000 square miles. Conversely, relatively cool and wet spring weather can limit the potential for outbreaks. These outbreaks are naturally occurring cycles and, especially during drought, can denude an area of vegetation leaving it exposed to increased erosion rates from wind and water (USDA 2003).

<br />*Chrysothamnus viscidiflorus* plants are relatively short‑lived (approximately 12‑13 years). Infestation in some densely populated stands by larvae of the beetle *Acamaeodera pulchella* is related to senescence and death. In shrubland stands were *Chrysothamnus viscidiflorus* is scattered, there are lower levels of infestation (Young and Evans 1974).

SOURCES

**References:** Ackerfield 2015, Anderson 2001b, Anderson 2004b, Barbour and Major 1977, Barbour et al. 2007a, Bent et al. 1968, Blankespoor 1980, Branson et al. 1976, Brown 1982a, CNHP 2013, Carey 1995, Comer et al. 2003*, Francis 1986, Hanson 1929, Hironaka et al. 1983, Holland and Keil 1995, Knight 1994, NatureServe Explorer 2011, Reid et al. 1999, Sawyer and Keeler‑Wolf 1995, Sawyer et al. 2009, Scher 2001, Shiflet 1994, Tirmenstein 1999a, Tirmenstein 1999b, Tirmenstein 1999c, Tirmenstein 1999f, Tirmenstein 1999g, Tuhy et al. 2002, USDA‑APHIS 2003, USDA‑APHIS 2010, Vickery 1996, West 1983e, West and Young 2000

**Version:** 02 Apr 2014	**Stakeholders:** West

**Concept Author:** K.A. Schulz	**LeadResp:** West

CES302.742 Mojave Mid‑Elevation Mixed Desert Scrub

**Primary Division:** North American Warm Desert (302)

**Land Cover Class:** Shrubland

**Spatial Scale & Pattern:** Large patch

**Required Classifiers:** Natural/Semi‑natural; Vegetated (>10% vasc.); Upland

**Diagnostic Classifiers:** Lowland [Foothill]; Shrubland (Shrub‑dominated); Evergreen Sclerophyllous Tree

**National Mapping Codes:** EVT 2082; ESLF 5259; ESP 1082

**Concept Summary:** This ecological system is an extensive desert scrub dominated by *Yucca brevifolia* and/or *Coleogyne ramosissima*. It is found in the transition zone between *Larrea tridentata ‑ Ambrosia dumosa* desert scrub and lower montane woodlands (700‑1800 m elevations) that occur in the eastern and central Mojave Desert, and in southern Great Basin. The vegetation in this ecological system is quite variable. Major communities include *Yucca brevifolia* and *Coleogyne ramosissima* scrub. Dominant and diagnostic species include *Coleogyne ramosissima, Ericameria parryi, Ericameria teretifolia, Eriogonum fasciculatum, Ephedra nevadensis, Grayia spinosa, Lycium* spp., *Menodora spinescens, Nolina* spp., *Cylindropuntia acanthocarpa, Salazaria mexicana, Viguiera parishii, Yucca brevifolia*, or *Yucca schidigera*. Less common are stands with scattered Joshua trees and a saltbush short‑shrub layer dominated by *Atriplex canescens, Atriplex confertifolia*, or *Atriplex polycarpa*, or occasionally *Hymenoclea salsola*. In some areas in the western Mojave, *Juniperus californica* is common with the yuccas. Desert grasses, including *Achnatherum hymenoides, Achnatherum speciosum, Muhlenbergia porteri, Pleuraphis jamesii, Pleuraphis rigida*, or *Poa secunda*, may form an herbaceous layer. Scattered *Juniperus osteosperma* or desert scrub species may also be present.

DISTRIBUTION

**Range:** This system is found in the eastern and central Mojave Desert and on lower piedmont slopes in the transition zone into the southern Great Basin.

**Divisions:** 206:P, 302:C, 304:P

**TNC Ecoregions:** 11:C, 12:P, 17:C, 23:P

**Nations:** MX?, US

**Subnations:** AZ, CA, NV, UT

**Map Zones:** 4:C, 6:?, 12:C, 13:C, 14:C, 15:C, 16:?, 17:C, 23:P, 24:?

**USFS Ecomap Regions:** 313A:CC, 322A:CC, 322B:CC, 322C:CC, 341D:CP, 341E:C?, 341F:CC, 342B:PP, M261E:CC, M341A:CC, M341D:C?

CONCEPT

**Environment:** This ecological system is found in the Mojave Desert and in the transition zone into the southern Great Basin. It represents the extensive mid‑elevation desert scrub in the transition zone above the lower elevation creosotebush desert scrub and generally below the foothill and lower montane woodlands (700‑1850 m elevations (Sawyer et al. 2009). Adjacent ecological systems include Great Basin Pinyon‑Juniper Woodland (CES304.773) and Inter‑Mountain Basins Big Sagebrush Shrubland (CES304.777) above and Sonora‑Mojave Creosotebush‑White Bursage Desert Scrub (CES302.756) below.

*Climate:* Climate is semi‑arid with hot summers and cool winters. Annual precipitation is low, averaging between 4 and 25 cm. However, year‑to‑year precipitation variability can be quite large with drought common and rare wet years producing a bloom of desert annuals.

<br />*Physiography/landform:* Stands occur on upper bajada and lower piedmont slopes with smaller patches occurring on rocky ridges and outcrops. Slopes are gentle to moderate. Aspect is variable with higher elevation stands found on warmer south‑ to west‑facing slopes.

*Soil/substrate/hydrology:* Substrates are a mixture of alluvium and colluvium and are variable, ranging from silt to loam to coarse sand, but often shallow, well‑drained, sandy and rocky. Many stands occur on alkaline, calcareous substrates and often have biological crusts and a shallow caliche layer (Sawyer et al. 2009). The environmental description is based on several references, including Beatley (1976), Brown (1982a), Turner (1982b), MacMahon (1988), Holland and Keil (1995), Reid et al. (1999), Ostler et al. (2000), Anderson (2001c), Gucker (2006a, 2006b), Barbour et al. (2007a), Keeler‑Wolf (2007), and Sawyer et al. (2009).

**Vegetation:** The vegetation in this ecological system is quite variable. Major alliances include *Yucca brevifolia* and *Coleogyne ramosissima* scrub. Dominant and diagnostic species include *Coleogyne ramosissima, Ephedra nevadensis, Ericameria parryi, Ericameria teretifolia, Eriogonum fasciculatum, Grayia spinosa, Krameria* spp., *Lycium* spp., *Nolina* spp., *Cylindropuntia acanthocarpa (= Opuntia acanthocarpa), Peucephyllum schottii, Salazaria mexicana, Viguiera parishii, Yucca brevifolia*, or *Yucca schidigera* (Sawyer et al. 2009). Less common are stands with scattered (*Yucca brevifolia* and a saltbush short‑shrub layer dominated by *Atriplex canescens, Atriplex confertifolia, Atriplex polycarpa*, or occasionally *Hymenoclea salsola*. In some areas in the western Mojave, *Juniperus californica* is common with *Yucca brevifolia*. Desert grasses, including *Achnatherum hymenoides, Achnatherum speciosum, Muhlenbergia porteri, Pleuraphis jamesii, Pleuraphis rigida*, or *Poa secunda*, may form an herbaceous layer. Scattered *Juniperus osteosperma* or desert scrub species may also be present. Stands dominated by *Ericameria parryi, Eriogonum fasciculatum, Nolina bigelovii, Nolina parryi, Lycium andersonii, Menodora spinescens*, or *Viguiera parishii* occur on rocky ridges, outcrops, and dry washes and may be too sparse to burn except under extreme conditions (Sawyer et al. 2009). The vegetation description is based on several references, including Beatley (1976), Brown (1982), Turner (1982), MacMahon (1988), Holland and Keil (1995), Reid et al. (1999), Ostler et al. (2000), Anderson (2001c), Gucker (2006a, 2006b), Barbour et al. (2007), Keeler‑Wolf (2007), Sawyer et al. (2009), and NatureServe Explorer (2011).

**Dynamics:** This system occurs on extremely xeric sites and is well‑adapted to prolonged drought and heat stress. Growth slows or stops in winter due to cold and is inhibited at other times by heat. Winter rains are sometimes sufficient to allow ephemeral herbs to flower in the spring. Late summer thunderstorms also contribute moisture.

Disturbance dynamics in this system are variable because of variation in structure and composition, being dominated by open‑ to closed‑canopy scrub to desert grasslands dominated by *Pleuraphis rigida* (<1400 m elevation) and *Pleuraphis jamesii* (>1400 m elevation) sometimes with a *Yucca brevifolia* overstory (Sawyer et al. 2009). Except for the relatively few stands with an herbaceous layer, fire‑return intervals (FRI) also tend to be long because the open stands only burn under extreme conditions. Older *Yucca brevifolia* individuals can tolerate low‑severity fires due to fire‑resistant bark, and both *Yucca brevifolia* and *Yucca schidigera* can sprout if burned (Gucker 2006a, b).

LANDFIRE developed a VDDT model for this system which has two classes (LANDFIRE 2007a, BpS 1410820):

A) Early Development 1 Open (25% of type in this stage): Shrub cover is 0‑50%. Historically, fire was relatively uncommon in this vegetation. The average FRI for replacement fire was 400 years. When burned, the fire‑tolerant/crown‑sprouting shrubs such as spiny menodora, horsebrush and snakeweed will dominate the site. At higher elevations of mesic blackbrush, a big sagebrush‑desert bitterbrush community typically replaces blackbrush for a protracted period. This class can express itself for over a hundred years with varying amounts of blackbrush gradually establishing after decades and eventually succeeding to class B. A few examples of this that have been observed in the field are believed to be over 60+ years. The ground cover varies by elevation and moisture regime with mesic sites being generally 10‑35% with some sites only capable of 10% cover. The thermic sites are generally 10‑15% ground cover with exception going as high as 35%.

B) Late Development 2 Closed (shrub‑dominated ‑ 30% of type in this stage): This community class seems to be stable and occurs after a threshold is crossed. Composition is 50‑70% blackbrush‑dominated. Other species are perennial grasses of desert needlegrass, Indian ricegrass, galleta grass, fluff grass, and threeawn. Lesser shrub composition includes Nevada ephedra, turbinella oak, desert bitterbrush, fourwing saltbush, and Anderson's wolfberry in mesic sites and Nevada ephedra, creosotebush, Mojave buckwheat, snakeweed, prickly pear, white bursage, and spiny menodora in thermic sites. There are other shrubs also. The FRI for replacement fire is 400 years, which causes a rare transition to class A.

Fire‑sensitive shrub species such as the long‑lived *Coleogyne ramosissima, Menodora spinescens, Nolina bigelovii*, or *Nolina parryi* will convert to early‑seral and intermediate shrublands dominated by *Hymenoclea salsola, Grayia spinosa, Gutierrezia sarothrae, Ericameria teretifolia, Ephedra nevadensis, Menodora spinescens, Cylindropuntia acanthocarpa, Salazaria mexicana, Tetradymia* spp., or *Yucca schidigera* which have shorter FRIs (Anderson 2001c, Keeler‑Wolf 2007, Sawyer et al. 2009). LANDFIRE modelers emphasized that blackbrush is fire‑intolerant, may be slow to re‑establish following fire, and grasses may dominate immediately following fire. Invasion of non‑native annual grasses following fire is likely under current conditions (LANDFIRE 2007a).

Some species such as yucca moths (*Tegeticula* spp.) and *Yucca* species have obligate mutualistic relationships (Baker 1986b, Althoff et al. 2006). *Yucca* sp. are typically dependent on one or sometimes two species of *Tegeticula* for pollination, which is usually dependent on one to several *Yucca* host plant species for habitat and food for larvae; for example, *Tegeticula mojavella* and *Tegeticula californica* pollinate *Yucca schidigera*, and *Tegeticula antithetica* and *Tegeticula synthetica* pollinate *Yucca brevifolia*. More study and review are needed to fully understand the many functional roles animals have within this ecosystem.

SOURCES

**References:** Althoff et al. 2006, Anderson 2001c, Baker 1986b, Barbour and Major 1977, Barbour and Major 1988, Barbour et al. 2007a, Beatley 1976, Belnap 2001, Belnap et al. 2001, Brown 1982a, Comer et al. 2003*, Evans and Belnap 1999, Francis 2004, Gucker 2006a, Gucker 2006b, Holland and Keil 1995, Keeler‑Wolf 2007, Keeler‑Wolf and Thomas 2000, Keeler‑Wolf et al. 1998a, LANDFIRE 2007a, MacMahon 1988, Ostler et al. 2000, Pellmyr and Segraves 2003, Reid et al. 1999, Rondeau 1999, Rosentreter and Belnap 2003, Sawyer and Keeler‑Wolf 1995, Sawyer et al. 2009, Shiflet 1994, Thomas et al. 2004, Turner 1982b, West 1983d, West 1988

**Version:** 27 Jan 2016	**Stakeholders:** Latin America, West

**Concept Author:** K.A. Schulz	**LeadResp:** West

CES304.793 Southern Colorado Plateau Sand Shrubland

**Primary Division:** Inter‑Mountain Basins (304)

**Land Cover Class:** Shrubland

**Spatial Scale & Pattern:** Large patch

**Required Classifiers:** Natural/Semi‑natural; Vegetated (>10% vasc.); Upland

**Diagnostic Classifiers:** Lowland [Foothill, Lowland]; Woody‑Herbaceous; Temperate [Temperate Xeric]; Alkaline Soil; Aridic; Very Short Disturbance Interval; G‑Landscape/High Intensity

**National Mapping Codes:** EVT 2093; ESLF 5270; ESP 1093

**Concept Summary:** This large‑patch ecological system is found on the south‑central Colorado Plateau in northeastern Arizona extending into southern and central Utah. It occurs on windswept mesas, broad basins and plains at low to moderate elevations (1300‑1800 m). Substrates are stabilized sandsheets or shallow to moderately deep sandy soils that may form small hummocks or small coppice dunes. This semi‑arid, open shrubland is typically dominated by short shrubs (10‑30 % cover) with a sparse graminoid layer. The woody layer is often a mixture of shrubs and dwarf‑shrubs. Characteristic species include *Ephedra cutleri, Ephedra torreyana, Ephedra viridis*, and *Artemisia filifolia*. *Coleogyne ramosissima* is typically not present. *Poliomintha incana, Parryella filifolia, Quercus havardii var. tuckeri*, or *Ericameria nauseosa* may be present to dominant locally. *Ephedra cutleri* and *Ephedra viridis* often assume a distinctive matty growth form. Characteristic grasses include *Achnatherum hymenoides, Bouteloua gracilis, Hesperostipa comata*, and *Pleuraphis jamesii*. The general aspect of occurrences is an open low shrubland but may include small blowouts and dunes. Occasionally grasses may be moderately abundant locally and form a distinct layer. Disturbance may be important in maintaining the woody component. Eolian processes are evident, such as pediceled plants, occasional blowouts or small dunes, but the generally higher vegetative cover and less prominent geomorphic features distinguish this system from Inter‑Mountain Basins Active and Stabilized Dune (CES304.775).

DISTRIBUTION

**Range:** This system occurs in sandy plains and mesas on the south‑central Colorado Plateau in northeastern Arizona extending into southern and central Utah.

**Divisions:** 304:C

**TNC Ecoregions:** 19:C

**Nations:** US

**Subnations:** AZ, CO?, NM?, UT

**Map Zones:** 15:?, 16:?, 23:C, 24:C, 28:C

**USFS Ecomap Regions:** 313A:CC, 313B:CC, 313D:CC, 341B:CC, M313A:CC, M341B:PP

CONCEPT

SOURCES

**References:** AZGAP unpubl. data 2004, Comer et al. 2003*, UTGAP 2004

**Version:** 08 Sep 2004	**Stakeholders:** West

**Concept Author:** K. Pohs, K. Schulz, J. Kirby	**LeadResp:** West

M170. Great Basin‑Intermountain Dwarf Sagebrush Steppe & Shrubland

CES304.762 Colorado Plateau Mixed Low Sagebrush Shrubland

**Primary Division:** Inter‑Mountain Basins (304)

**Land Cover Class:** Shrubland

**Spatial Scale & Pattern:** Large patch

**Required Classifiers:** Natural/Semi‑natural; Vegetated (>10% vasc.); Upland

**Diagnostic Classifiers:** Montane [Lower Montane]; Lowland [Foothill]; Shrubland (Shrub‑dominated); Ridge/Summit/Upper Slope; Temperate [Temperate Xeric]; Aridic

**National Mapping Codes:** EVT 2064; ESLF 5201; ESP 1064

**Concept Summary:** This ecological system occurs in the Colorado Plateau, Tavaputs Plateau and Uinta Basin in canyons, gravelly draws, hilltops, and dry flats at elevations generally below 1800 m. Soils are often rocky, shallow, and alkaline. This type extends across northern New Mexico into the southern Great Plains on limestone hills. It includes open shrublands and steppe dominated by *Artemisia nova* or *Artemisia bigelovii* sometimes with *Artemisia tridentata ssp. wyomingensis* codominant. Semi‑arid grasses such as *Achnatherum hymenoides, Aristida purpurea, Bouteloua gracilis, Hesperostipa comata, Pleuraphis jamesii*, or *Poa fendleriana* are often present and may form a graminoid layer with over 25% cover.

**Comments:** Diagnostic species for this Wyoming Basins ecological system, *Artemisia nova* and *Artemisia arbuscula*, are shared with three sagebrush dwarf‑shrubland ecological systems from three ecoregions: Wyoming Basins Dwarf Sagebrush Shrubland and Steppe (CES304.794), Colorado Plateau Mixed Low Sagebrush Shrubland (CES304.762), and Great Basin Xeric Mixed Sagebrush Shrubland (CES304.774). Although there is overlap with two dominant species, regional differences in ecosystem processes, total species composition, and environmental variables support distinguishing four dwarf sagebrush ecological systems.

DISTRIBUTION

**Range:** Occurs in the Colorado Plateau, Tavaputs Plateau and Uinta Basin in canyons, gravelly draws, hilltops, and dry flats at elevations generally below 1800 m.

**Divisions:** 303:C, 304:C

**TNC Ecoregions:** 18:C, 19:C, 20:C, 27:C, 28:C

**Nations:** US

**Subnations:** AZ, CO, NM, UT

**Map Zones:** 15:P, 16:C, 17:P, 23:C, 24:C, 25:C, 27:C, 28:C, 34:P

**USFS Ecomap Regions:** 313A:CC, 313B:CC, 313D:CC, 315A:C?, 315B:CC, 315H:CC, 321A:CC, 331B:CC, 341A:CC, 341B:CC, 341C:CC, 342G:??, M313A:CC, M313B:CC, M331D:CC, M331E:CC, M331F:CP, M331G:CC, M331H:CP, M341B:CC, M341C:CC

CONCEPT

**Environment:** This ecological system occurs in the Colorado Plateau, Tavaputs Plateau and Uinta Basin in canyons, gravelly draws, hilltops, mesatops and dry flats at elevations generally below 1800 m. This type extends across northern New Mexico into the southern Great Plains on limestone hills and sandstone breaks. Soils are often rocky, shallow and alkaline. Adjacent upland systems include Colorado Plateau Pinyon‑Juniper Woodland (CES304.767) and Inter‑Mountain Basins Montane Sagebrush Steppe (CES304.785) (deeper soils) at higher elevations and Inter‑Mountain Basins Mixed Salt Desert Scrub (CES304.784) at lower elevations. The environmental description is based on several other references, including Jameson et al. (1962), Brown (1982), West (1983a), Baker and Kennedy (1985), Francis (1986), Dick‑Peddie (1993), West and Young (2000), Howard (2003), Fryer (2009), and NatureServe Explorer (2011).

**Vegetation:** These shrublands are dominated by *Artemisia nova* or *Artemisia bigelovii*, sometimes with *Artemisia tridentata ssp. wyomingensis* codominant. Other shrubs that may be present include *Atriplex canescens, Chrysothamnus viscidiflorus, Ephedra* spp., *Ericameria* spp., *Gutierrezia sarothrae, Lycium* spp., and *Yucca* spp. The herbaceous layer ranges from sparse to moderately dense and is composed of semi‑arid grasses such as *Achnatherum hymenoides, Aristida purpurea, Bouteloua gracilis, Hesperostipa comata, Pleuraphis jamesii*, or *Poa fendleriana* forming a graminoid layer sometimes with over 25% cover. The floristic description is based on several other references, including Jameson et al. (1962), Brown (1982), West (1983a), Baker and Kennedy (1985), Francis (1986), Dick‑Peddie (1993), West and Young (2000), Howard (2003), Fryer (2009), and NatureServe Explorer (2011).

**Dynamics:** The diagnostic species of this system, *Artemisia nova* or *Artemisia bigelovii*, grow in more xeric sites than other *Artemisia* shrublands (Hironaka et al. 1983). This dwarf‑shrubland system is associated with shallow, rocky soils which experience extreme drought in summer. The plants are low and widely spaced, which tends to decrease the risk of fire. Fire is uncommon on drier sites because of discontinuous and low fuel buildup on the generally unproductive sites (Fryer 2009). Fire effects on *Artemisia bigelovii* is not known but assumed to be similar to *Artemisia nova* (Howard 2003), with fire‑return intervals (FRI) ranging from 35 to over 100 years for xeric, low‑productivity sagebrush communities of the Great Basin (Fryer 2009). In general, most sites are thought to have relatively long fire‑return intervals (100‑200 years) according to LANDFIRE models developed by experts (Fryer 2009). Stands in the western Great Plains typically have higher herbaceous cover (Shaw et al. 1989) which may decrease FRI. These shrubs are fire‑sensitive and rarely sprout after burning. They reproduce from light wind‑dispersed seeds from adjacent unburned areas to disturbed areas (Howard 1999, 2003, Fryer 2009). It generally takes around 30 years for a burned *Artemisia nova* stand to recover to pre‑fire density (Hironaka et al. 1983, Fryer 2009). *Artemisia tridentata ssp. wyomingensis* may be present to codominant and shares similar ecological characteristics on these relatively xeric sites (Howard 1999).

Scattered trees may be present in some stands of this system. Fire reduces sagebrush abundance in both sagebrush and pinyon‑juniper systems. Where these systems are adjacent, periodic fire likely prevents establishment of juniper and pinyon trees in sagebrush stands (Wright et al. 1979). In order to maintain dominance of sagebrush, fire‑return interval must be long enough to permit sagebrush stands to mature, but short enough to prevent establishment and growth of trees in these sites. Fire‑return intervals of 150‑250 years for stand‑replacing fire will likely maintain these shrublands. Expansion and contraction of trees into sagebrush shrublands are regulated by a combination of climate, fire, and bark beetle infestations with trees seedlings establishing during wetter periods (Wright et al. 1979, Paysen et al. 2000).

Insects are an important component of many shrub‑steppe and grassland systems. Mormon crickets and grasshoppers are natural components of many rangeland systems (USDA‑APHIS 2003, 2010). There are almost 400 species of grasshoppers that inhabit the western United States with 15‑45 species occurring in a given rangeland system (USDA‑APHIS 2003). Mormon crickets are also present in many western rangelands and, although flightless, are highly mobile and can migrate large distances consuming much of the forage while travelling in wide bands (USDA‑APHIS 2010). Following a high population year for grasshoppers or Mormon crickets and under relatively warm dry spring environmental conditions that favor egg hatching and grasshopper and Mormon cricket survival, there may be large population outbreaks that can utilize 80% or more of the forage in areas as large as 2000 square mile. Conversely, relatively cool and wet spring weather can limit the potential for outbreaks. These outbreaks are naturally occurring cycles and, especially during drought, can denude an area of vegetation leaving it exposed to increased erosion rates from wind and water (USDA‑APHIS 2003).

LANDFIRE developed a state‑and‑transition vegetation dynamics VDDT model for this system which has five classes in total (LANDFIRE 2007a, BpS 2310640). These are summarized as:

A) Early Development 1 All Structures (shrub‑dominated ‑ 10% of type in this stage): Early‑seral community dominated by herbaceous vegetation; less than 6% sagebrush canopy cover; up to 24 years post‑disturbance. Replacement fire occurs every 250 years on average. Succession to class B after 24 years.

B) Late Development 1 Open (shrub‑dominated ‑ 70% of type in this stage): Shrub cover is 0‑10%. Mid‑seral community with a mixture of herbaceous and shrub vegetation; 6‑10% sagebrush canopy cover present; between 20‑59 years post‑disturbance. Replacement fire (FRI of 240 years) causes a transition to class A, whereas mixed‑severity fire (FRI of 100 years) maintains the site in its present condition. In the absence of fire for 120 years, the site will follow an alternative succession path to class C. Otherwise, succession and mixed‑severity fire keeps site in class B.

C) Late Development 1 Open (conifer‑dominated ‑ 20% of type in this stage): Shrub cover is 10‑30%. Late‑seral community with a mixture of herbaceous and shrub vegetation; >10% sagebrush canopy cover present; 75+ years post‑disturbance. Replacement fire is every 200 years on average (transition to class A), whereas mixed‑severity fire happens on average every 140 years due to a diminished herbaceous component compared to class B. Mixed‑severity fire causes a transition to class B. Succession will keep the site in class C without fire.

Black sagebrush generally supports more fire than other dwarf sagebrushes. This type generally burns with mixed severity (average FRI of 100‑140 years) due to relatively low fuel loads and herbaceous cover. Bare ground acts as a micro‑barrier to fire between low‑statured shrubs. Oils and resins present in the foliage and stems of sagebrush allow fire to spread. Stand‑replacing fires (average FRI of 200‑240 years) can occur in this type when successive years of above‑average precipitation are followed by an average or dry year. Stand‑replacement fires dominate in the late‑succession class where the herbaceous component has diminished. Fires may or may not be wind‑driven and only cover small areas. This type fits into Fire Regime Groups IV and III LANDFIRE 2007a, BpS 1210310).

Grazing by wild ungulates occurs in this type due to the high palatability of *Artemisia nova* compared to other browse. Native browsing tends to open up the canopy cover of shrubs but does not often change the succession stage (LANDFIRE 2007a, BpS 1210310).

Prolonged drought may reduce the foliar and basal covers of graminoids but not that of shrubs. Reduced foliar cover of graminoids will affect fire behavior. This effect is assumed minor and not included in the model (LANDFIRE 2007a, BpS 1210310).

SOURCES

**References:** Baker and Kennedy 1985, Belnap 2001, Belnap and Lange 2003, Belnap et al. 2001, Brown 1982a, Comer et al. 2003*, Dick‑Peddie 1993, Francis 1986, Fryer 2009, Garfin et al. 2014, Gucker 2006e, Hironaka et al. 1983, Howard 1999, Howard 2003a, Howard 2003d, Jameson et al. 1962, LANDFIRE 2007a, McKenzie et al. 2004, McKenzie et al. 2008, NatureServe Explorer 2011, Paysen et al. 2000, Rising 1996, Rosentreter and Belnap 2003, Shaw et al. 1989, Shiflet 1994, USDA‑APHIS 2003, USDA‑APHIS 2010, West 1983a, West and Young 2000, Westerling et al. 2006, Wright et al. 1979

**Version:** 24 May 2018	**Stakeholders:** West

**Concept Author:** K.A. Schulz	**LeadResp:** West

CES304.080 Columbia Plateau Low Sagebrush Steppe

**Primary Division:** Inter‑Mountain Basins (304)

**Land Cover Class:** Steppe/Savanna

**Spatial Scale & Pattern:** Large patch

**Required Classifiers:** Natural/Semi‑natural; Vegetated (>10% vasc.); Upland

**Diagnostic Classifiers:** Lowland [Foothill, Lowland]; Shrubland (Shrub‑dominated); Ridge/Summit/Upper Slope; Sideslope; Shallow Soil; Silt Soil Texture; Clay Soil Texture; Aridic; W‑Landscape/High Intensity; Low Artemisia spp.

**National Mapping Codes:** EVT 2124; ESLF 5453; ESP 1124

**Concept Summary:** This ecological system can form the matrix of the landscape and is composed of sagebrush dwarf‑shrub‑steppe that occurs in a variety of shallow‑soil habitats throughout eastern Oregon, northern Nevada and southern Idaho. *Artemisia arbuscula ssp. arbuscula* and close relatives (*Artemisia arbuscula ssp. longiloba* and occasionally *Artemisia nova*) form stands that typically occur on mountain ridges and flanks and broad terraces, ranging from 1000 to 3000 m in elevation. Substrates are shallow, fine‑textured soils, poorly drained clays that occur in thin‑soil areas and are frequently very stony. Other shrubs and dwarf‑shrubs present may include *Purshia tridentata, Eriogonum* spp., and other species of *Artemisia*. Common graminoids include *Festuca idahoensis, Koeleria macrantha, Poa secunda*, and *Pseudoroegneria spicata*. Many forbs also occur and may dominate the herbaceous vegetation, especially at the higher elevations. Isolated individuals of *Juniperus occidentalis* and *Cercocarpus ledifolius* can often be found in this system. This ecological system is closely related to the concept of shallow‑dry sagebrush in the resistance‑resilience framework.

**Comments:** Diagnostic species for this Columbia Plateau ecological system, *Artemisia nova* and *Artemisia arbuscula*, are shared with three other sagebrush dwarf‑shrubland ecological systems from three other ecoregions: Colorado Plateau Mixed Low Sagebrush Shrubland (CES304.762), Great Basin Xeric Mixed Sagebrush Shrubland (CES304.774), and Wyoming Basins Dwarf Sagebrush Shrubland and Steppe (CES304.794). Although there is overlap with two dominant species, regional differences in ecosystem processes, total species composition, and environmental variables support distinguishing four dwarf sagebrush ecological systems.

DISTRIBUTION

**Range:** This system is found throughout the basins of eastern Oregon and southern Idaho, south into northern Nevada and northeastern California.

**Divisions:** 304:C

**TNC Ecoregions:** 6:C, 11:C

**Nations:** US

**Subnations:** CA, ID, MT?, NV, OR, WA:S1S2, WY?

**Map Zones:** 1:?, 7:C, 8:C, 9:C, 10:C, 17:?, 18:C, 19:C, 21:P

**USFS Ecomap Regions:** 331A:CC, 341E:CP, 341G:CC, 342B:CC, 342C:CC, 342D:CC, 342E:CP, 342H:CC, 342I:CC, 342J:CC, M242C:CC, M242D:CC, M261D:CC, M261G:CC, M331D:CC, M332A:CC, M332E:CC, M332F:CC, M332G:CC, M333A:??, M341A:CC

CONCEPT

**Environment:** This system occurs on shallow‑soil habitats, ranging from 1000 to 3000 m in elevation.

*Climate:* Climate is semi‑arid with a large proportion of the 20‑30 cm of annual precipitation falling as winter snow. The temperature regime is continental, with cold winters, warm summers, a large diurnal temperature range, and a short frost‑free season.

<br />*Physiography/landform:* Stands typically occur on mountain ridges and flanks and broad terraces, but may be associated with flats, depressions, and slopes with soils that are either very shallow or quite poorly drained. In the Columbia River Basin, the vegetation in this system occupies the driest habitats of all the *Artemisia*‑dominated stands.

<br />*Soil/substrate/hydrology:* Substrates are generally fine‑textured, usually poorly drained clays that occur in shallow‑soiled areas, which are almost always very stony and characterized by recent rhyolite or basalt. Beetle and Johnson (1982) report that *Artemisia arbuscula ssp. arbuscula* grows in soils with a high volume of gravel (even though soil may be in clay textural class or contain a clay‑rich layer that impedes drainage), and that *Artemisia arbuscula ssp. longiloba* grows in clay soils, often alkaline, that contain no gravels. Soils dominated by *Artemisia nova* are typically alkaline and calcareous.

**Vegetation:** *Artemisia arbuscula ssp. arbuscula* and close relatives (*Artemisia arbuscula ssp. longiloba* and occasionally *Artemisia nova*) form stands. Other shrubs and dwarf‑shrubs present may include *Purshia tridentata, Eriogonum* spp., and other species of *Artemisia*. Common graminoids include *Festuca idahoensis, Koeleria macrantha, Pseudoroegneria spicata*, and *Poa secunda*. Many forbs also occur and may dominate the herbaceous vegetation, especially at the higher elevations. Isolated individuals of *Juniperus occidentalis* (western juniper) and *Cercocarpus ledifolius* (mountain‑mahogany) can often be found in this system.

**Dynamics:** The diagnostic species of this system, *Artemisia arbuscula ssp. arbuscula, Artemisia arbuscula ssp. longiloba*, or *Artemisia nova*, grow in more xeric sites than other *Artemisia* shrubs (Hironaka et al. 1983), and are highly drought‑tolerant. *Artemisia arbuscula* tends to grow where claypan layers exist in the soil profile and soils are often saturated during a portion of the year, while *Artemisia nova* tends to grow where there is a root‑limiting layer in the soil profile (LANDFIRE 2007a). This shrubland system is associated with shallow, rocky soils which experience extreme drought in summer. The plants are low and widely spaced, which tends to decrease the risk of fire (Chappell et al. 1997).

Fire influences the density and distribution of shrubs. In general, fire increases the abundance of herbaceous perennials and decreases the abundance of woody plants (WNHP 2011). The fire interval for this system is 110 years (LANDFIRE 2007a). Anecdotal observations indicate that these patches often are not burned during surrounding forest fires. Fire is uncommon because of discontinuous and low fuel buildup on the generally unproductive sites (Young and Palmquist 1992, Fryer 2009, Sawyer et al. 2009). Most sites are thought to have relatively long fire‑return intervals (100‑200 years) according to LANDFIRE models developed by experts (LANDFIRE 2007a). These shrubs are fire‑sensitive and rarely sprout after burning.

The dominant shrub species can easily colonize burns via wind‑dispersed seeds from adjacent unburned areas into disturbed areas (Howard 1999, Steinberg 2002a, Fryer 2009). It generally takes around 30 years for a burned stand to recover to pre‑fire shrub density (Zamora and Tueller 1973, Hironaka et al. 1983, Howard 1999, Steinberg 2002a, Fryer 2009). However, recovery of this system after fire may take up to 325‑450 years (Baker 2006).

Grazing by wild ungulates occurs in this shrubland system. Native browsing tends to open the canopy cover of shrubs but does not often change the successional stage (LANDFIRE 2007a).

Insects are an important component of many shrub‑steppe and grassland systems. Mormon crickets and grasshoppers are natural components of many rangeland systems (USDA‑APHIS 2003, 2010). There are almost 400 species of grasshoppers that inhabit the western United States with 15‑45 species occurring in a given rangeland system (USDA‑APHIS 2003). Mormon crickets are also present in many western rangelands and, although flightless, are highly mobile and can migrate large distances consuming much of the forage while travelling in wide bands (USDA‑APHIS 2010). Following a high population year for grasshoppers or Mormon crickets and under relatively warm dry spring environmental conditions that favor egg hatching and grasshopper and Mormon cricket survival, there may be large population outbreaks that can utilize 80% or more of the forage in areas as large as 2000 square miles. Conversely, relatively cool and wet spring weather can limit the potential for outbreaks. These outbreaks are naturally occurring cycles and, especially during drought, can denude an area of vegetation leaving it exposed to increased erosion rates from wind and water (USDA‑APHIS 2003).

LANDFIRE developed a VDDT model for this system which has three classes (LANDFIRE 2007a, BpS 0811240 and BpS 0911240). Dominant shrub is *Artemisia arbuscula*. Dominant herbaceous species are *Poa secunda* and *Pseudoroegneria spicata*.

A) Early Development 1 All Structures (10% of type in this stage): Zero to 1% low sagebrush cover. Herbaceous cover of bunchgrasses and forbs would fill to about 20‑30% cover within a few years.

B) Mid Development 1 Open (40% of type in this stage): Dominant lifeform is herb. Minimum cover = 20%, maximum cover = 40%. Minimum height for herbs is 0.6 m. Scattered and usually small low sagebrush is present, but perennial grasses and forbs continue to dominate. The general formation is that of a shrub savanna. Sagebrush cover is usually 1‑5% in this stage.<br />

C) Late Development 1 Open (50% of type in this stage): Sagebrush is codominant with perennial grasses and forbs. Sagebrush and herbaceous cover can be variable depending on site productivity. Bare ground and rock in the interspaces increase on less productive sites. The general formation is that of a shrubland. Expected composition is 50‑60% grass; 5‑10% forbs; 20‑40% shrubs. Windswept ridges with thinner soils may be still more open.

SOURCES

**References:** Baker 2006, Barbour and Major 1988, Beetle and Johnson 1982, Bell et al. 2009, Belnap 2001, Belnap and Lange 2003, Belnap et al. 2001, Blackburn and Tueller 1970, Chappell et al. 1997, Comer et al. 2003*, Fryer 2009, Hironaka et al. 1983, Howard 1999, Johnston 2001, LANDFIRE 2007a, Rosentreter and Belnap 2003, Sawyer et al. 2009, Shiflet 1994, Steinberg 2002a, USDA‑APHIS 2003, USDA‑APHIS 2010, WNHP 2011, WNHP unpubl. data 2018, West 1983a, Young and Palmquist 1992, Zamora and Tueller 1973

**Version:** 26 Jan 2016	**Stakeholders:** West

**Concept Author:** J. Kagan	**LeadResp:** West

CES304.770 Columbia Plateau Scabland Shrubland

**Primary Division:** Inter‑Mountain Basins (304)

**Land Cover Class:** Shrubland

**Spatial Scale & Pattern:** Matrix

**Required Classifiers:** Natural/Semi‑natural; Vegetated (>10% vasc.); Upland

**Diagnostic Classifiers:** Lowland [Lowland]; Shrubland (Shrub‑dominated); Basalt; Shallow Soil

**National Mapping Codes:** EVT 2065; ESLF 5202; ESP 1065

**Concept Summary:** This ecological system is found in the Columbia Plateau region and consists of extensive low shrublands. These xeric shrublands occur under relatively extreme soil‑moisture conditions. Substrates are typically shallow lithic soils with limited water‑holding capacity over fractured basalt. Because of poor drainage through basalt, these soils are often saturated from fall to spring by winter precipitation but typically dry out completely to bedrock by midsummer. Vegetation cover is typically low, generally less than 50% and often much less than that. Vegetation is characterized by an open dwarf‑shrub canopy dominated by *Artemisia rigida* along with other shrub and dwarf‑shrub species, particularly *Eriogonum compositum, Eriogonum douglasii, Eriogonum microthecum, Eriogonum niveum, Eriogonum sphaerocephalum, Eriogonum strictum, Eriogonum thymoides*, and/or *Salvia dorrii*. Other shrubs are uncommon in this system; mixes of *Artemisia rigida* and other *Artemisia* species typically belong to different ecological systems than this. Low cover of perennial bunchgrasses, such as *Danthonia unispicata, Elymus elymoides, Festuca idahoensis*, or primarily *Poa secunda*, as well as scattered forbs, including species of *Allium, Antennaria, Balsamorhiza, Lomatium, Phlox*, and *Sedum*, characterize these sites. Individual sites can be dominated by grasses and semi‑woody forbs, such as *Nestotus stenophyllus*. Annuals may be seasonally abundant, and cover of moss and lichen is often high in undisturbed areas (1‑60% cover).

**Comments:** Sometimes scattered *Artemisia rigida* may occur in non‑scabland grassland system stands. Also, mixes of *Artemisia rigida* and other *Artemisia* species typically belong to different ecological systems than this.

DISTRIBUTION

**Range:** This system occurs in the Columbia Plateau region of southern Idaho, eastern Oregon and eastern Washington, and extreme northern Nevada.

**Divisions:** 304:C

**TNC Ecoregions:** 6:C, 7:C, 68:C

**Nations:** US

**Subnations:** ID, NV, OR, WA:S5

**Map Zones:** 1:C, 7:C, 8:C, 9:C, 12:?, 17:?, 18:P

**USFS Ecomap Regions:** 331A:CC, 341E:C?, 341G:CC, 342B:CC, 342C:CC, 342D:CC, 342H:CC, 342I:CC, 342J:CC, M242C:CC, M242D:CC, M261D:C?, M261G:CC, M332G:CC, M333A:PP, M341A:CC

CONCEPT

**Environment:** This open, low shrubland ecological system is characteristic of the scablands in the Columbia Basin and portions of the Snake River plain. Elevations range from 190‑1830 m.

*Climate:* Climate is semi‑arid and temperate with a winter precipitation peak. Mean annual precipitation ranges from 25‑50 cm and occurs primarily in the winter as snow or rain.

<br />*Physiography/landform:* Stands are found on flat to undulating to rolling plateaus, plains, ridgetops and brows. Sites are nearly level to moderately sloping (to 30%). It occurs on all aspects, but is more common on southern slopes, although given that most sites are flat, aspect is not very significant.

*Soil/substrate/hydrology:* These xeric shrublands occur under relatively extreme soil‑moisture conditions. Substrates are typically shallow lithic soils (7‑30 cm) with a high percentage of rock fragments (10‑70%), limited water‑holding capacity over fractured basalt. This moisture is stored in the soil profile and utilized during the typically dry summers. Because of poor drainage through basalt, these soils are often saturated from fall to spring by winter precipitation but typically dry out completely to bedrock by midsummer. The soils are non‑calcareous, sandy to clay loams, with pH of 6.3‑6.6. Parent material is restricted to colluvium and residuum derived from basalt and acidic lava. Soil surface is mostly rock, erosion pavement (pebble surface), bare ground, and moss. Litter accumulates under the scattered *Artemisia rigida* plants forming moss‑covered mounds up to 20 cm deep. These hummocks persist several years after the death of the dwarf‑shrub (Daubenmire 1970, 1992). Moss and lichen cover a significant amount of the ground surface, often with up to 50% cover.

**Vegetation:** Total vegetation cover is typically low, generally less than 50% and often much less than that. Vegetation is characterized by an open dwarf‑shrub canopy dominated by *Artemisia rigida* along with other shrub and dwarf‑shrub species, particularly *Eriogonum* spp. Other shrubs are uncommon in this system; mixes of *Artemisia rigida* and other *Artemisia* species typically belong to different ecological systems than this. Low cover of perennial bunchgrasses, such as *Danthonia unispicata, Elymus elymoides, Festuca idahoensis*, or primarily *Poa secunda*, as well as scattered forbs, including species of *Allium, Antennaria, Balsamorhiza, Lomatium, Phlox*, and *Sedum*, characterize these sites. Individual sites can be dominated by grasses and semi‑woody forbs, such as *Nestotus stenophyllus (= Stenotus stenophyllus)*. Annuals may be seasonally abundant, and cover of moss and lichen is often high in undisturbed areas (1‑60% cover).

**Dynamics:** This xeric shrubland ecological system is driven by its tolerance of extreme low soil‑moisture conditions and very thin soils that can be easily disturbed or eroded. Stands in this system are generally considered to be late‑seral with species composition controlled by the harsh edaphic conditions of the site (Daubenmire 1970, Johnson and Simon 1987). While these soils are often saturated from fall to spring by the winter precipitation, they typically dry out completely to bedrock by midsummer (Daubenmire 1970, 1992, Johnson and Simon 1987). *Poa secunda*, a typical dominant graminoid, is well‑adapted to these conditions because it starts growing early in the spring and completes its reproductive cycle early while there is still moisture in the soil (Daubenmire 1970, 1992, Johnson and Simon 1987). Also, if there is late summer or early fall precipitation, dormant *Poa secunda* can respond quickly and green up. Daubenmire (1970) and Johnson and Simon (1987) suggest that the basalt bedrock present under these dwarf‑shrub/grassland stands is fractured enough to support deeper‑rooted dwarf‑shrubs. Moss does well in this habitat because of seasonally moist conditions. *Artemisia rigida* is favored winter browse for elk and deer, and moderately palatable to livestock (Johnson and Clausnitzer 1992).

Frost heaving may be severe, causing local soil disturbance in the winter when these thin, saturated soils freeze and push soil and plants up out of the ground. Pedestalled *Artemisia rigida* plants and bunchgrasses are common (Daubenmire 1970, Hironaka et al. 1983).

Fire is thought to be unimportant because it is unlikely that the sparse vegetation in these stands could carry a fire. However, if it does occur the *Artemisia rigida* plants are not tolerant and would be killed (Johnson and Simon 1987, Daubenmire 1992, Johnson and Clausnitzer 1992).

LANDFIRE developed a VDDT model for this system which has three classes (LANDFIRE 2007a, BpS 0810650). This model includes sites where there is potential for pinyon (*Pinus monophylla*) and/or juniper (*Juniperus osteosperma*) establishment in classes C and D.

A) Early Development 1 All Structures (5% of type in this stage): Shrub cover is 0‑10%. This class is dominated by sprouting buckwheats and other hemi‑shrubs, surviving perennial grasses and forbs and annual forbs. Plant cover is typically extremely low. Sagebrush will be absent and patch size is very small in this class. Rock dominates the visual appearance and may dominate satellite imagery. Succession to class B after 10 years.

B) Mid Development 1 Open (5% of type in this stage): Shrub cover is 0‑10%. Young stiff sagebrush appears while the other species reach their more‑or‑less mature sizes. Plant cover remains low but denser patches are now present, composed mostly of the hemi‑shrubs and perennial grasses and forbs. Rock is less dominant visually but may still dominate satellite imagery. Succession to class C after 20 years.

C) Late Development 1 Open (90% of type in this stage): Shrub cover is 0‑10%. Stiff sagebrush is fully mature and visually dominates the scene, particularly after spring leaf out and flowering. Total vegetation cover rarely exceeds 25% and is often <15%. Plant height rarely exceeds 0.5 m.

Replacement fire was modeled as mean fire‑return interval = 250 years in all three classes, with no other disturbances modeled. Severe droughts can temporarily reduce herbaceous vegetation; however, all the species that occupy this BpS are very drought‑tolerant (LANDFIRE 2007a).

SOURCES

**References:** Belnap 2001, Belnap and Lange 2003, Belnap et al. 2001, Bunting et al. 1987, Comer et al. 2003*, Copeland 1980a, Daubenmire 1970, Daubenmire 1992, Ganskopp 1979, Hall 1973, Hironaka et al. 1983, Johnson and Clausnitzer 1992, Johnson and Simon 1985, Johnson and Simon 1987, LANDFIRE 2007a, McWilliams 2003b, Poulton 1955, Rosentreter and Belnap 2003, Shiflet 1994, Tisdale 1986, Tyler 2006, WNHP 2011, WNHP unpubl. data 2018

**Version:** 26 Jan 2016	**Stakeholders:** West

**Concept Author:** J. Kagan	**LeadResp:** West

CES304.794 Wyoming Basins Dwarf Sagebrush Shrubland and Steppe

**Primary Division:** Inter‑Mountain Basins (304)

**Land Cover Class:** Shrubland

**Spatial Scale & Pattern:** Large patch

**Required Classifiers:** Natural/Semi‑natural; Vegetated (>10% vasc.); Upland

**Diagnostic Classifiers:** Lowland [Foothill, Lowland]; Shrubland (Shrub‑dominated); Hill(s); Ridge/Summit/Upper Slope; Sideslope; Shallow Soil; Silt Soil Texture; Clay Soil Texture; Aridic; W‑Landscape/High Intensity; Low Artemisia spp.

**National Mapping Codes:** EVT 2072; ESLF 5209; ESP 1072

**Concept Summary:** This windswept ecological system is composed of dwarf sagebrush shrubland and shrub‑steppe that forms matrix vegetation and large patches on the margins of high‑elevation basins in central and southern Wyoming. Typical sites are gently rolling hills and long, gently sloping pediments and fans. These sites are very windy and have shallow, often rocky soils (*Artemisia nova* and *Artemisia tripartita ssp. rupicola*) or have shallow, poorly drained, fine‑textured soils (*Artemisia arbuscula*). The distinguishing feature of this system is a short‑shrub stratum in which dwarf‑shrubs (<30 cm tall) contribute at least two‑thirds of the woody canopy. Four sagebrush taxa may dominate the shrub stratum: *Artemisia tripartita ssp. rupicola, Artemisia nova, Artemisia arbuscula ssp. longiloba*, and wind‑dwarfed *Artemisia tridentata ssp. wyomingensis*. Two or more of these sagebrushes often codominate, but any of them may occur alone. Where graminoids are common and tall, the vegetation often has the appearance of grassland without shrubs; the presence of shrubs is obvious only when the vegetation is viewed up close. Where graminoids contribute less cover, the vegetation is a compact shrubland. The herbaceous component of the vegetation includes both rhizomatous and bunch‑form graminoids, cushion plants, and other low‑growing forbs. *Bouteloua gracilis*, a common species of Inter‑Mountain Basins Big Sagebrush Steppe (CES304.778) in Wyoming, is absent.

**Comments:** Diagnostic species for this Wyoming Basins ecological system, *Artemisia nova* and *Artemisia arbuscula*, are shared with three sagebrush dwarf‑shrubland ecological systems from three ecoregions: Wyoming Basins Dwarf Sagebrush Shrubland and Steppe (CES304.794), Colorado Plateau Mixed Low Sagebrush Shrubland (CES304.762), and Great Basin Xeric Mixed Sagebrush Shrubland (CES304.774). Although there is overlap with two dominant species, regional differences in ecosystem processes, total species composition, and environmental variables support distinguishing four dwarf sagebrush ecological systems. *Artemisia nova* is restricted to shallow‑soiled, rocky, wind‑exposed ridges in this Wyoming Basins system. Low‑growing, wind‑dwarfed *Artemisia tridentata ssp. wyomingensis* is a common component in this system. Other diagnostic taxa such as *Artemisia tripartita ssp. rupicola* are restricted to this system only.

DISTRIBUTION

**Range:** This system occurs throughout the basins of central and southern Wyoming, extending south into adjacent portions of Colorado. It also occurs on the eastern side of the Continental Divide in Montana, where *Artemisia nova* shrublands are found on calcareous substrates.

**Divisions:** 304:C

**TNC Ecoregions:** 10:C, 26:C

**Nations:** US

**Subnations:** CO, MT, WY

**Map Zones:** 16:?, 21:C, 22:C, 23:C, 29:C

**USFS Ecomap Regions:** 331F:CC, 331G:CC, 331K:CP, 331L:C?, 331N:CP, 341C:??, 342F:CC, 342G:CC, M331A:C?, M331B:CC, M331D:C?, M331E:CC, M331H:CC, M331I:CC, M332D:CC, M341B:PP

CONCEPT

**Environment:** *Climate:* Climate is semi‑arid with 20‑30 (45) cm of annual precipitation. The temperature regime is continental, with cold winters, warm summers, large diurnal ranges, and a short frost‑free season.

*Physiography/landform:* This windswept ecological system of dwarf sagebrush shrubland and shrub‑steppe occurs from 1500 to 3200 m elevation. These sites are very windy, gently rolling hills and long, gently sloping pediments and fans, broad ridgetops, the ridges of low mountains and the margins of high‑elevation basins.

*Soil/substrate/hydrology:* Soils are variable but are often shallow and rocky. *Artemisia nova* generally occupies medium‑ to coarse‑textured soils, often with a large volume of rock fragments and frequently calcareous. *Artemisia arbuscula*‑dominated stands have poorly drained, very heavy, montmorillonite (smectite) clay soils with some coarse fragments, usually effectively very shallow to a hard clay pan, not deep enough to support either big sagebrush or deep‑rooted grasses. Those two sagebrushes do grow together sometimes. *Artemisia tripartita ssp. rupicola*‑dominated stands have coarse‑textured (gravelly), well‑drained shallow soils.

**Vegetation:** This system includes dwarf‑shrublands dominated by one of four species: *Artemisia tripartita ssp. rupicola, Artemisia nova, Artemisia arbuscula ssp. longiloba*, and wind‑dwarfed *Artemisia tridentata ssp. wyomingensis* that characterize different sites. Two or more of these sagebrushes often codominate, but any of them may occur alone. Other often wind‑dwarfed shrubs, such as *Artemisia frigida, Chrysothamnus viscidiflorus, Ericameria nauseosa, Gutierrezia sarothrae*, or *Purshia tridentata*, may be present and occasionally abundant. Where graminoids are common and tall, the vegetation often has the appearance of grassland without shrubs; the presence of shrubs is obvious only when the vegetation is viewed up close. Where graminoids contribute less cover, the vegetation is open to a moderately dense canopy of compact shrubs <30 cm tall. The herbaceous component of the vegetation typically includes cool‑season rhizomatous and bunch‑form graminoids, cushion plants, and other low‑growing forbs. Common graminoids species include *Achnatherum hymenoides (= Oryzopsis hymenoides), Achnatherum speciosum (= Stipa speciosa), Achnatherum thurberianum (= Stipa thurberiana), Elymus elymoides, Elymus lanceolatus, Festuca idahoensis, Hesperostipa comata (= Stipa comata), Koeleria macrantha, Leucopoa kingii, Pascopyrum smithii, Poa fendleriana, Poa secunda*, and *Pseudoroegneria spicata*. Forb cover is typically minor and includes *Antennaria microphylla, Cerastium arvense, Heterotheca villosa, Packera multilobata (= Senecio multilobatus), Phlox hoodii, Senecio integerrimus, Sphaeralcea coccinea*, and *Stenotus armerioides*.

**Dynamics:** The key ecological factors for this system are the harsh, windswept, semi‑arid climate with a short growing season and shallow soils. *Artemisia nova* and *Artemisia tripartita ssp. rupicola* dwarf‑shrublands are associated with shallow, rocky soils which experience extreme drought in summer, whereas *Artemisia arbuscula*‑dominated stands occur on shallow, poorly drained, fine‑textured soils.

Fire is not important in this ecosystem, because it occurs very infrequently. Plants are low and widely spaced so there is little fuel to carry a fire. Replacement fire is predicted to occur every 300 years (LANDFIRE 2007a). Fire effects are variable depending on dominant species. *Artemisia arbuscula ssp. longiloba, Artemisia nova*, and *Artemisia tridentata ssp. wyomingensis* are generally killed by burning and do not resprout, so fire impacts can be severe (Young 1983, Howard 1999, Steinberg 2002a, Fryer 2009). However, *Artemisia tripartita ssp. rupicola* shrubs can sprout from the stump after being top‑killed by fire and will reproduce both by seed and by layering (Tirmenstein 1999k). Hironaka et al. (1983) notes that some populations may have variation in this ability.

LANDFIRE developed a VDDT model for this system which has two classes (LANDFIRE 2007a, BpS 2210720):

A) Early Development 1 All Structures (herbaceous‑dominated‑30% of type in this stage): Grass‑and‑forb‑dominated site for approximately 125 years. Black/low sagebrush seedlings are young and begin to establish towards the end of this seral period. Replacement fire occurs every 300 years

B) Late Development 1 Open (shrub‑dominated‑70% of type in this stage): Black/low sagebrush with mid‑height late‑seral grasses (150 or more years).

Soil erosion caused by native ungulates sometimes can occur in these stands when they trail across them, especially in spring and fall when the sites are wet. The sites are resilient and resistant to trampling in summer and winter, when they are dry or frozen (LANDFIRE 2007a).

SOURCES

**References:** Barbour and Major 1988, Belnap 2001, Belnap and Lange 2003, Belnap et al. 2001, Comer et al. 2003*, Fryer 2009, Hironaka et al. 1983, Howard 1999, Johnston 2001, Jones 1992b, Knight 1994, Knight et al. 1987, LANDFIRE 2007a, Muscha and Hild 2006, Rosentreter and Belnap 2003, Shiflet 1994, Steinberg 2002a, Tirmenstein 1999k, West 1983a, Young 1983

**Version:** 26 Jan 2016	**Stakeholders:** West

**Concept Author:** K.A. Schulz	**LeadResp:** West

M169. Great Basin‑Intermountain Tall Sagebrush Steppe & Shrubland

CES304.083 Columbia Plateau Steppe and Grassland

**Primary Division:** Inter‑Mountain Basins (304)

**Land Cover Class:** Steppe/Savanna

**Spatial Scale & Pattern:** Large patch

**Required Classifiers:** Natural/Semi‑natural; Vegetated (>10% vasc.); Upland

**Diagnostic Classifiers:** Lowland [Foothill, Lowland]; Sideslope; Very Shallow Soil; Landslide; Xeromorphic Shrub; Graminoid

**National Mapping Codes:** EVT 2123; ESLF 5452; ESP 1123

**Concept Summary:** This system occurs throughout much of the Columbia Plateau. It is a bunchgrass‑dominated grassland or steppe that is similar floristically to big sagebrush‑dominated steppe, but is defined by a more frequent fire regime and the absence or low cover of shrubs over large areas. These are large, extensive grasslands, not grass‑dominated patches within the sagebrush shrub‑steppe ecological system. Soils are variable, ranging from relatively deep, fine‑textured often with coarse fragments, and non‑saline often with a microphytic crust, to stony volcanic‑derived clays to alluvial sands. This grassland is dominated by perennial bunchgrasses and forbs (>25% cover), sometimes with a sparse (<10% cover) shrub layer. Associated graminoids include *Achnatherum hymenoides, Elymus elymoides, Elymus lanceolatus ssp. lanceolatus, Hesperostipa comata, Festuca idahoensis, Koeleria macrantha, Poa secunda*, and *Pseudoroegneria spicata*. Common forbs are *Phlox hoodii, Arenaria* spp., and *Astragalus* spp. Shrubs such as *Chrysothamnus viscidiflorus, Ericameria nauseosa, Tetradymia* spp., or *Artemisia* spp. are often present in disturbed stands. Areas with deeper soils are rare because of conversion to other land uses. The rapid fire‑return regime of this ecological system maintains a grassland structure by retarding shrub invasion, and landscape isolation and fragmentation limit seed dispersal of native shrub species. Fire frequency is presumed to be less than 20 years. Through isolation from a seed source, combined with repeated burning, these are "permanently" (more than 50 years) converted to grassland.

**Comments:** These grasslands are similar floristically to Inter‑Mountain Basins Big Sagebrush Steppe (CES304.778) but are defined by a more frequent fire regime and the absence or low cover of shrubs over large areas, occasionally entire landforms. How this differs from Columbia Basin Palouse Prairie (CES304.792) is unclear. This system is more widespread (occurring outside the Palouse region) and is more xeric, occurring on moderate to shallow soils, but shares many of the species.

DISTRIBUTION

**Range:** This system occurs throughout the Columbia Plateau region, from north‑central Idaho, south and west into Washington, Oregon, southern Idaho, and northern Nevada. Whether it also occurs in northeastern California, in the western ranges of Wyoming, or the central Wyoming Basins is unclear.

**Divisions:** 304:C, 306:C

**TNC Ecoregions:** 4:C, 6:C, 8:C, 9:C, 10:P, 11:C

**Nations:** US

**Subnations:** CA?, ID, MT?, NV, OR, UT?, WA:S2, WY?

**Map Zones:** 7:?, 8:C, 9:C, 10:C, 12:P, 17:?, 18:C

**USFS Ecomap Regions:** 331A:CC, 341E:CP, 341G:CC, 342B:CC, 342C:CC, 342D:CC, 342H:CC, 342I:CC, 342J:CC, M242C:CC, M242D:CP, M261G:CC, M331A:??, M332A:CC, M332E:C?, M332F:C?, M332G:CC, M333A:PP

CONCEPT

**Environment:** These are large extensive grassland ecosystems, not grass‑dominated patches within sagebrush shrub‑steppe ecological system. This system occurs throughout much of the Columbia Plateau and is found at slightly higher elevations farther south. Soil depth and soil texture within precipitation zones largely drive the distribution of shrub‑steppe and grassland (WNHP 2011). Geographically (climatically), this steppe system is associated with Inter‑Mountain Basins Big Sagebrush Steppe (CES304.778), rings the driest portion of the basin that supports the big sagebrush shrubland and the semi‑desert shrub‑steppe systems, and is bounded by montane woodlands and the Palouse prairie. It is found in landscapes that favor frequent ignition sources and fuels that spread fire, and few natural firebreaks. Biological soil crust is very important in this ecological system (WNHP 2011).

<br />*Climate:* Climate is semi‑arid, cool temperate with annual precipitation ranging from 18‑40 cm and high inter‑annual variation. Much of the precipitation falls as snow or spring rain; however, growing‑season drought is characteristic. Temperatures are continental with large annual and diurnal variation. Winter precipitation dominates and promotes cool‑season grasses.

<br />*Physiography/landform:* Stands occur on valley floors, alluvial fans, floodplains, stabilized dunes, mesic uplands, swales, and rocky slopes. Slopes are variable from gentle to very steep.

*Soil/substrate/hydrology:* Soils are variable, ranging from relatively deep, fine‑textured often with coarse fragments, and non‑saline often with a biological soil crust, to stony volcanic‑derived clays to alluvial sands. Burrowing animals and their predators likely played important roles in creating small‑scale patch patterns (WNHP 2011).

**Vegetation:** This grassland is dominated by perennial bunchgrasses and forbs (>25% cover), sometimes with a sparse (<10% cover) shrub layer; *Chrysothamnus viscidiflorus, Ericameria nauseosa, Tetradymia* spp., or *Artemisia* spp. may be present in disturbed stands. Associated graminoids include *Achnatherum hymenoides, Elymus elymoides, Elymus lanceolatus ssp. lanceolatus, Hesperostipa comata, Festuca idahoensis, Koeleria macrantha, Poa secunda*, and *Pseudoroegneria spicata*. Common forbs are *Phlox hoodii, Arenaria* spp., and *Astragalus* spp.

**Dynamics:** In the Columbia Plateau this grassland ecosystem occurs in a mosaic with sagebrush steppe vegetation and includes sagebrush steppe habitats where fire has removed the sagebrush; thus, due to change in fire regime, this type has expanded at the expense of sagebrush steppe (LANDFIRE 2007a).

Columbia Plateau ecosystems are more sensitive to grazing than grasslands in the Great Plains as they did not evolve with the same duration, seasonality, and severity of large native ungulate grazing (Mack and Thompson 1982, Burkhart 1996). In general, native ungulate grazing was dispersed and occurred during the winter and spring when forage was available.

These grasslands are defined by a more frequent fire regime and the absence or low cover of shrubs over large areas, occasionally entire landforms. The historic frequency was 30‑100 years (LANDFIRE 2007a). The natural fire regime of this ecological system likely maintains a patchy distribution of shrubs so the general aspect of the vegetation is a grassland. Post‑fire shrub recruitment is limited and rate is estimated to be 25 acres in 50 years under ideal conditions for *Artemisia tridentata* (WNHP 2011). These shrubs produce large quantities of small seeds beginning at age 3‑4 years of which 90% of the seed is dispersed within 9 m (30 feet) of the parent and few seeds are carried more than 30 m (100 feet) (Tirmenstein 1999c). Biological soil crust is very important in this ecological system (LANDFIRE 2007a).

LANDFIRE developed a somewhat different VDDT model for this system which has three classes (LANDFIRE 2007a, BpS 0911230):

A) Early Development 1 All Structures (herbaceous‑dominated ‑ 5% of type in this stage): Herbaceous cover is variable (10‑50%). Grassland having just burned. Young, green vegetation. Lasts one year before natural succession to class B.

B) Mid Development 1 Open (herbaceous‑dominated ‑ 80% of type in this stage): Herbaceous cover 51‑90%. Perennial bunchgrass with solid cryptogam cover, large bluebunch wheatgrasses, lower *Poa secunda* and forb cover, greater forb diversity. Patches are anywhere from 2‑50 years old. Replacement fire is the primary disturbance (MFR=50 years).

C) Late Development 1 Closed (herbaceous‑dominated ‑ 15% of type in this stage): Herbaceous cover 51‑90%. Shrub cover is 0‑30%. Native grassland with shrubs beginning to get a foothold, or small pockets of remnants from the original fire expanding into the grassland. It equals the early‑seral states in Wyoming big sagebrush steppe ecological system. Patches within this matrix die back due to competition/maintenance, but this does not have a profound effect on class condition. Replacement fire occurs every 16‑17 years on average.

Shrubs may increase following heavy grazing and/or with fire suppression, particularly in moist portions in the northern Columbia Plateau where it forms a landscape mosaic pattern with shallow‑soil scabland shrublands.

SOURCES

**References:** Barnett and Crawford 1994, Belnap and Lange 2003, Belnap et al. 2001, Burkhardt 1996, Comer et al. 2003*, Daubenmire 1970, Drut et al. 1994, Ersch 2009, Gregg and Crawford 2009, Howard 1999, Johnson and Swanson 2005, LANDFIRE 2007a, Mack and Thompson 1982, Pellant 1990, Pellant 1996, Quinn 2004, Shiflet 1994, TNC 2013, Tirmenstein 1999c, Vander Haegen et al. 2000, Vander Haegen et al. 2001, WNHP 2011, WNHP unpubl. data 2018

**Version:** 26 Jan 2016	**Stakeholders:** West

**Concept Author:** R. Crawford	**LeadResp:** West

CES304.774 Great Basin Xeric Mixed Sagebrush Shrubland

**Primary Division:** Inter‑Mountain Basins (304)

**Land Cover Class:** Shrubland

**Spatial Scale & Pattern:** Large patch

**Required Classifiers:** Natural/Semi‑natural; Vegetated (>10% vasc.); Upland

**Diagnostic Classifiers:** Lowland [Foothill]; Shrubland (Shrub‑dominated); Ridge/Summit/Upper Slope; Aridic; Low Artemisia spp.

**National Mapping Codes:** EVT 2079; ESLF 5256; ESP 1079

**Concept Summary:** This ecological system occurs in the Great Basin on dry flats and plains, alluvial fans, rolling hills, rocky hillslopes, saddles and ridges at elevations between 1000 and 2600 m. Sites are dry, often exposed to desiccating winds, with typically shallow, rocky, non‑saline soils. Shrublands are dominated by *Artemisia nova* (mid and low elevations), *Artemisia arbuscula ssp. longicaulis*, or *Artemisia arbuscula ssp. longiloba* (higher elevation) and may be codominated by *Artemisia tridentata ssp. wyomingensis* or *Chrysothamnus viscidiflorus*. Other shrubs that may be present include *Atriplex confertifolia, Ephedra* spp., *Ericameria* spp., *Grayia spinosa, Lycium shockleyi, Picrothamnus desertorum*, and *Tetradymia* spp. The herbaceous layer is likely sparse and composed of perennial bunchgrasses, such as *Achnatherum hymenoides, Achnatherum speciosum, Achnatherum thurberianum, Elymus elymoides*, or *Poa secunda*.

**Comments:** Diagnostic species for this Wyoming Basins ecological system, *Artemisia nova* and *Artemisia arbuscula*, are shared with three sagebrush dwarf‑shrubland ecological systems from three ecoregions: Wyoming Basins Dwarf Sagebrush Shrubland and Steppe (CES304.794), Colorado Plateau Mixed Low Sagebrush Shrubland (CES304.762), and Great Basin Xeric Mixed Sagebrush Shrubland (CES304.774). Although there is overlap with two dominant species, regional differences in ecosystem processes, total species composition, and environmental variables support distinguishing four dwarf sagebrush ecological systems.

DISTRIBUTION

**Range:** This system occurs in the Great Basin on dry flats and plains, alluvial fans, rolling hills, rocky hillslopes, saddles and ridges at elevations between 1000 and 2600 m.

**Divisions:** 206:C, 304:C

**TNC Ecoregions:** 6:P, 11:C, 12:C, 18:P

**Nations:** US

**Subnations:** CA, ID?, NV, OR, UT, WY

**Map Zones:** 4:?, 6:P, 7:?, 9:C, 10:C, 12:C, 13:P, 16:C, 17:C, 18:C

**USFS Ecomap Regions:** 322A:CC, 341A:CC, 341D:CC, 341E:CC, 341F:CC, 341G:CC, 342B:CC, 342C:CC, 342D:CC, 342H:CC, 342J:CC, M261E:CC, M261G:C?, M331D:CC, M332G:CC, M341A:CC, M341C:CP, M341D:CC

CONCEPT

**Environment:** *Climate:* Climate is semi‑arid with 20 to 30 cm of annual precipitation and warm summers and cold winters.<br />

*Physiography/landform:* This ecological system is widely distributed in the interior Great Basin of the western United States on dry flats and plains, alluvial fans, rolling hills and foothills, saddles and ridges at elevations between 1000 and 2600 m. Sites are xeric, flat to steep, and often exposed to desiccating winds or with typically shallow, rocky, non‑saline soils. It occupies flat to steeply sloping upland sites, on a wide variety of topographic positions. Sloping sites tend to have southerly aspects.

<br />*Soil/substrate/hydrology:* Sites with low slope tend to have deeper soils, while those with steeper slopes have shallow to moderately deep soils that are well‑drained. Soil texture is loam, sandy loam, or clay loam (Hansen and Hoffman 1988), and there is often a significant amount of coarse fragments in the soil profile. Hironaka et al. (1983) reported that most of the habitat occurred on calcareous soils, often with a cemented duripan. Low sagebrush tends to grow where claypan layers exist in the soil profile and soils are often saturated during a portion of the year; black sagebrush tends to grow where there is a root‑limiting layer in the soil profile, whereas Wyoming sagebrush and basin big sagebrush generally occur on moderately deep to deep soils that are well‑drained (LANDFIRE 2007a). The environmental description is based on several other references, including Blackburn and Tueller (1970), Zamora and Tueller (1973), Hironaka et al. (1983), West (1983a), Barbour and Major (1988), Chappell et al. (1997), Howard (1999), Steinberg (2002a), Barbour et al. (2007a), Fryer (2009), and Sawyer et al. (2009).

**Vegetation:** These shrublands are dominated by *Artemisia nova* (mid and low elevations), *Artemisia arbuscula ssp. longicaulis* (lower elevations), or *Artemisia arbuscula ssp. longiloba* (higher elevations) and may be codominated by *Artemisia tridentata ssp. wyomingensis* or *Chrysothamnus viscidiflorus*. Other shrubs that may be present include *Atriplex confertifolia, Ephedra* spp., *Ericameria* spp., *Grayia spinosa, Lycium shockleyi, Picrothamnus desertorum, Purshia tridentata, Sarcobatus vermiculatus*, and *Tetradymia* spp. The herbaceous layer is likely sparse and composed of perennial bunchgrasses, such as *Achnatherum hymenoides, Achnatherum speciosum, Achnatherum thurberianum, Elymus elymoides, Festuca idahoensis, Hesperostipa comata, Poa fendleriana, Poa secunda*, or *Pseudoroegneria spicata*. The floristic description is based on several other references, including Blackburn and Tueller (1970), Zamora and Tueller (1973), Hironaka et al. (1983), West (1983a), Barbour and Major (1988), Chappell et al. (1997), Howard (1999), Steinberg (2002), Barbour et al. (2007), Fryer (2009), Sawyer et al. (2009), and NatureServe Explorer (2011).

**Dynamics:** The diagnostic species of this system, *Artemisia nova, Artemisia arbuscula ssp. longicaulis*, or *Artemisia arbuscula ssp. longiloba*, grow in more xeric sites than other *Artemisia* shrublands (Hironaka et al. 1983). This shrubland system is associated with shallow, rocky soils which experience extreme drought in summer. The plants are low and widely spaced, which tends to decrease the risk of fire (Chappell et al. 1997). Fire is uncommon because of discontinuous and low fuel buildup on the generally unproductive sites (Young and Palmquist 1992, Fryer 2009, Sawyer et al. 2009). Most sites are thought to have relatively long fire‑return intervals (100‑200 years) according to LANDFIRE models developed by experts (LANDFIRE 2007a). These shrubs are fire‑sensitive and rarely sprout after burning. They reproduce from light wind‑dispersed seeds from adjacent unburned areas into disturbed areas (Howard 1999, Steinberg 2002a, Fryer 2009). It generally takes around 30 years for a burned stand to recover to pre‑fire density (Zamora and Tueller 1973, Hironaka et al. 1983, Howard 1999, Steinberg 2002a, Fryer 2009). *Artemisia tridentata ssp. wyomingensis* may be present to codominant and shares similar ecological characteristics on these relatively xeric sites (Howard 1999).

Scattered trees may be present in some stands of this system. Fire reduces sagebrush abundance in both sagebrush and pinyon‑juniper systems. Where these systems are adjacent, periodic fire likely prevents establishment of juniper and pinyon trees in sagebrush stands (Wright et al. 1979). Blackburn and Tueller (1970) noted rapid invasion of these communities by *Juniperus osteosperma* and *Pinus monophylla* at some sites in Nevada. In order to maintain dominance of sagebrush, fire‑return interval must be long enough to permit sagebrush stands to mature, but short enough to prevent establishment and growth of trees in these sites. Fire‑return intervals of 150‑250 years for stand‑replacing fire will likely maintain these shrublands. Expansion and contraction of trees into sagebrush shrublands are regulated by a combination of climate, fire, and bark beetle infestations with trees seedlings establishing during wetter periods (Wright et al. 1979, Clifford et al. 2008).

The black and low sagebrush type tends to occur adjacent to Inter‑Mountain Basins Big Sagebrush Shrubland (CES304.777). The Wyoming big sagebrush and basin big sagebrush types create a mosaic within the black and low sagebrush types. These big sagebrush types have a different fire regime that acts to carry the fire, with black and low sagebrush serving as firebreaks most of the time (LANDFIRE 2007a).

Black sagebrush (*Artemisia nova*) generally supports more fire than other dwarf sagebrushes (LANDFIRE 2007a). This type generally burns with mixed severity (average FRI of 100‑140 years) due to relatively low fuel loads and herbaceous cover (LANDFIRE 2007a). Bare ground acts as a micro‑barrier to fire between low‑statured shrubs. Stand‑replacing fires (average FRI of 200‑240 years) can occur in this type when successive years of above‑average precipitation are followed by an average or dry year (LANDFIRE 2007a). Stand‑replacement fires dominate in the late‑successional class where the herbaceous component has been diminished or where trees dominate (LANDFIRE 2007a). This type fits best into Fire Regime Group IV (LANDFIRE 2007a).

Grazing by wild ungulates occurs in this shrubland system. Native browsing tends to open up the canopy cover of shrubs but does not often change the successional stage (LANDFIRE 2007a).

Insects are an important component of many shrub‑steppe and grassland systems. Mormon crickets and grasshoppers are natural components of many rangeland systems (USDA‑APHIS 2003, 2010). There are almost 400 species of grasshoppers that inhabit the western United States with 15‑45 species occurring in a given rangeland system (USDA‑APHIS 2003). Mormon crickets are also present in many western rangelands and, although flightless, are highly mobile and can migrate large distances consuming much of the forage while travelling in wide bands (USDA‑APHIS 2010). Following a high population year for grasshoppers or Mormon crickets and under relatively warm dry spring environmental conditions that favor egg hatching and grasshopper and Mormon cricket survival, there may be large population outbreaks that can utilize 80% or more of the forage in areas as large as 2000 square mile. Conversely, relatively cool and wet spring weather can limit the potential for outbreaks. These outbreaks are naturally occurring cycles and, especially during drought, can denude an area of vegetation leaving it exposed to increased erosion rates from wind and water (USDA‑APHIS 2003).

LANDFIRE developed a VDDT model for this system which has three classes (LANDFIRE 2007a, BpS 1210790). This model includes sites where there is potential for pinyon (*Pinus monophylla*) and/or juniper (*Juniperus osteosperma*) establishment in classes C and D.

A) Early Development 1 All Structures (15% of type in this stage): Shrub cover is 0‑5%. Early‑seral community dominated by herbaceous vegetation; less than 6% sagebrush canopy cover; up to 24 years post‑disturbance. Fire‑tolerant shrubs (green/low rabbitbrush) are first sprouters after stand‑replacing, high‑severity fire. Replacement fire (mean FRI of 250 years) maintains vegetation in class A. Prolonged drought every 200 years on average maintains vegetation in class A. Succession to class B after 25 years.

B) Mid Development 1 Open (60% of type in this stage): Mid‑seral community with a mixture of herbaceous and shrub vegetation; 6‑25% sagebrush (sagebrush/brush) canopy cover present; between 20‑59 years post‑disturbance. Drought every 200 years causes two transitions: 50% of times drought thins shrubs while maintaining vegetation in class B, whereas 50% of times drought causes a stand‑replacing event. Replacement fire (FRI of 250 years) causes a transition to class A, whereas mixed‑severity fire (FRI of 100 years) maintains the site in its present condition. In the absence of fire for at least 120 years, the site will follow an alternative successional path to class C. Otherwise, succession and mixed‑severity fire keeps site in class B.

C) Late Development 1 Open (15% of type in this stage): Late‑seral community with a mixture of herbaceous and shrub vegetation; 10‑25% sagebrush canopy cover present; and dispersed conifer seedlings and saplings established at less than 6% cover (*Juniperus osteosperma* and/or *Pinus monophylla*). Insects attack the vegetation in this state every 60 years on average but does not cause a transition to another state. Severe droughts (return interval of 200 years) cause two thinning disturbances: to class B (50% of times) and within class C. Replacement fire is every 200 years on average, whereas mixed‑severity fire is less frequent than in class B (FRI of 130 years). Succession is to class D after 75 years.

D) Late Development 1 Closed (10% of type in this stage): Late‑seral community with a closed canopy of conifer trees (6‑40% cover). The degree of tree canopy closure differs depending on whether it is a low sagebrush (maximum 15%) or black sagebrush (maximum 40%) community. In low sagebrush communities a mixture of herbaceous and shrub vegetation with >10% sagebrush canopy cover would still be present. In black sagebrush communities the herbaceous and shrub component would be greatly reduced (<1%). When Ips beetle outbreaks occur the pinyon pine component is reduced (return interval of 60 years): 75% of times thinning is not intense enough to cause a transition whereas in 25% of cases a transition to class C will occur. The only fire is replacement (FRI of 150 years) and driven by a greater amount of woody fuel than in previous states. Prolonged droughts have the same effect as before.

SOURCES

**References:** Baker and Kennedy 1985, Barbour and Major 1988, Barbour et al. 2007a, Belnap 2001, Belnap and Lange 2003, Belnap et al. 2001, Blackburn and Tueller 1970, Chambers et al. 2013, Chappell et al. 1997, Clifford et al. 2008, Comer et al. 2003*, Fryer 2009, Hansen and Hoffman 1988, Hironaka et al. 1983, Howard 1999, LANDFIRE 2007a, Rosentreter and Belnap 2003, Sawyer et al. 2009, Shiflet 1994, Steinberg 2002a, Tirmenstein 1999c, USDA‑APHIS 2003, USDA‑APHIS 2010, West 1983a, Wright et al. 1979, Young and Palmquist 1992, Zamora and Tueller 1973

**Version:** 26 Jan 2016	**Stakeholders:** West

**Concept Author:** K.A. Schulz	**LeadResp:** West

CES304.777 Inter‑Mountain Basins Big Sagebrush Shrubland

**Primary Division:** Inter‑Mountain Basins (304)

**Land Cover Class:** Shrubland

**Spatial Scale & Pattern:** Matrix

**Required Classifiers:** Natural/Semi‑natural; Vegetated (>10% vasc.); Upland

**Diagnostic Classifiers:** Lowland [Lowland]; Shrubland (Shrub‑dominated); Toeslope/Valley Bottom; Deep Soil; Aridic; Artemisia tridentata ssp. tridentata

**National Mapping Codes:** EVT 2080; ESLF 5257; ESP 1080

**Concept Summary:** This ecological system occurs throughout much of the interior western U.S., typically in broad basins between mountain ranges, plains and foothills between 800 and 2500 m elevation. Soils are typically deep, well‑drained and non‑saline. These shrublands are dominated by *Artemisia tridentata ssp. tridentata* (not as common in Wyoming or Montana but possibly on stabilized part of Killpecker Dunes in Wyoming) and/or *Artemisia tridentata ssp. wyomingensis* (predominant in Wyoming and Montana). Scattered *Juniperus* spp., *Sarcobatus vermiculatus*, and *Atriplex* spp. may be present in some stands. *Ericameria nauseosa, Chrysothamnus viscidiflorus, Purshia tridentata* (not commonly in Montana or Wyoming), or *Symphoricarpos oreophilus* may codominate disturbed stands (e.g., in burned stands, these may become more predominant). Perennial herbaceous components typically contribute less than 25% vegetative cover. Common graminoid species can include *Achnatherum hymenoides, Bouteloua gracilis, Elymus lanceolatus, Festuca idahoensis* (not in Montana or Wyoming), *Hesperostipa comata, Leymus cinereus, Pleuraphis jamesii* (not present in northeastern portions of the range), *Pascopyrum smithii, Poa secunda*, or *Pseudoroegneria spicata* (not in Wyoming). Dunes in the Red Desert have areas of large basin big sage with very dense canopies. In Wyoming, this system is likely to only contain *Artemisia tridentata ssp. tridentata*.

**Comments:** Most *Artemisia tridentata ssp. wyomingensis* communities in Wyoming are placed in Inter‑Mountain Basins Big Sagebrush Steppe (CES304.778); the shrubland system is more restricted in environmental setting than the steppe.

DISTRIBUTION

**Range:** This system occurs throughout much of the interior western U.S., typically in broad basins between mountain ranges, plains and foothills. Its core distribution is in the Great Basin, but it extends north into the Columbia Basin and west into the foothills of the Sierra Nevada and Cascades, and east into the Colorado Plateau, Wyoming Basins and central and eastern Montana, although much of the sagebrush in this region is more steppe in physiognomy.

**Divisions:** 303:C, 304:C, 306:C

**TNC Ecoregions:** 4:C, 6:C, 8:C, 9:C, 10:C, 11:C, 18:C, 19:C, 20:C, 26:C, 27:C

**Nations:** US

**Subnations:** CA, CO, ID, MT, NV, OR, UT, WA, WY

**Map Zones:** 6:P, 7:C, 8:C, 9:C, 10:C, 12:C, 13:C, 15:C, 16:C, 17:C, 18:C, 19:C, 20:C, 21:C, 22:C, 23:C, 24:C, 25:P, 27:?, 28:C, 29:C, 30:P, 31:?, 33:?

**USFS Ecomap Regions:** 313A:CC, 313B:CC, 313D:CC, 315H:CC, 321A:??, 322A:CC, 331A:CC, 331D:CP, 331E:CP, 331F:CC, 331G:CC, 331H:C?, 331J:CC, 331K:CC, 331L:CP, 331M:CP, 341A:CC, 341B:CC, 341C:CC, 341D:CC, 341E:CC, 341F:CC, 341G:CC, 342A:CC, 342B:CC, 342C:CC, 342D:CC, 342E:CC, 342F:CC, 342G:CC, 342H:CC, 342I:CC, 342J:CC, M242C:CC, M242D:CC, M261D:CC, M261E:CC, M261G:CC, M313A:CC, M313B:C?, M331A:CC, M331B:CC, M331D:CC, M331E:CC, M331F:CC, M331G:CC, M331H:CC, M331I:CC, M331J:CC, M332A:CC, M332B:CP, M332D:CC, M332E:CC, M332F:CC, M332G:CC, M333A:CC, M333C:C?, M333D:CC, M341A:CC, M341B:CC, M341C:CC, M341D:CC

CONCEPT

**Environment:** This ecological system occurs throughout much of the interior western U.S., typically in broad basins between mountain ranges, plains and foothills between 1500 and 2500 m elevation.

*Climate:* The climate where this system occurs is semi‑arid with annual precipitation ranging from 18‑40 cm and high inter‑annual variation. Much of the precipitation falls as snow, and growing‑season drought is characteristic. Temperatures are continental with large annual and diurnal variation. In drier regions, these shrublands are usually associated with perennial or ephemeral stream drainages with water tables less than 3 m from the soil surface.<br />

*Physiography/landform:* Sites supporting this system include flat to steeply sloping uplands on alluvial fans and terraces, toeslopes, lower and middle slopes, draws, badlands, and deep, well‑drained alluvial bottomlands foothills and basins and plains (Barker and McKell 1983).

*Soil/substrates/hydrology:* In drier regions, these shrublands are usually associated with perennial or ephemeral stream drainages with water tables less than 3 m from the soil surface. Substrates are typically deep, well‑drained and non‑saline, fine‑ to medium‑textured alluvial soils with some source of subirrigation during the summer season, but moderately deep upland soils with ample moisture storage also support these shrublands. Some stands occur on deep, sandy soils, or soils that are highly calcareous (Hironaka et al. 1983). Although this system may grade into sites with alkaline soils at the edge of internally drained basins, *Artemisia tridentata* is a non‑halophyte and requires low salinity for optimum growth. The importance of perennial bunch grasses, the most typical herbaceous associates, is favored with greater spring and summer rain, which increases northward and eastward.

The environmental description is based on several references, including Brown (1982a), Hironaka et al. (1983), West (1983a), Barbour and Billings (1988), Knight (1994), Shiflet (1994), Holland and Keil (1995), Reid et al. (1999), West and Young (2000), Barbour et al. (2007a), and Sawyer et al. (2009).

**Vegetation:** These shrublands are dominated by *Artemisia tridentata ssp. tridentata* (not as common in Wyoming or Montana but possibly on the stabilized part of Killpecker Dunes in Wyoming) and/or *Artemisia tridentata ssp. wyomingensis* (predominant in Wyoming and Montana). Scattered *Juniperus* spp., *Sarcobatus vermiculatus*, and *Atriplex* spp. may be present in some stands. *Ericameria nauseosa, Chrysothamnus viscidiflorus, Purshia tridentata* (not commonly in Montana or Wyoming), or *Symphoricarpos oreophilus* may codominate disturbed stands (e.g., in burned stands, these may become more predominant). Perennial herbaceous components typically contribute less than 25% vegetative cover. Common graminoid species can include *Achnatherum hymenoides, Bouteloua gracilis, Elymus lanceolatus, Festuca idahoensis* (not in Montana or Wyoming), *Hesperostipa comata, Leymus cinereus, Pleuraphis jamesii* (not present in northeastern portions of the range), *Pascopyrum smithii, Poa secunda*, or *Pseudoroegneria spicata* (not in Wyoming). Some semi‑natural communities are included that often originate on abandoned agricultural land or on other disturbed sites. In these locations, *Bromus tectorum* or other annual bromes and invasive weeds can be abundant. Most *Artemisia tridentata ssp. wyomingensis* communities in Wyoming are placed in Inter‑Mountain Basins Big Sagebrush Steppe (CES304.778); the shrubland system is more restricted in environmental setting than the steppe. Dunes in the Red Desert have areas of large basin big sagebrush with very dense canopies. In Wyoming, this system is likely to only contain *Artemisia tridentata ssp. tridentata*. The vegetation description is based on several references, including Brown (1982a), West (1983a), Barbour and Billings (1988), Knight (1994), Shiflet (1994), Holland and Keil (1995), Reid et al. (1999), West and Young (2000), Barbour et al. (2007a), and Sawyer et al. (2009).

**Dynamics:** Complex ecological interactions of fire regimes and climate patterns result in equally complex patterns of species structure and composition in *Artemisia tridentata* stands. Prolonged drought on the more xeric sites may result in lower shrub cover. Flooding may also cause plant mortality if the soil remains saturated for an extended period of time. The Aroga moth is capable of defoliating large acreages (i.e., >1000 acres, but usually 10‑100 acres). Heavy grazing by wildlife can remove the fine fuels that support mixed‑severity fires and result in woody fuel buildup that leads to severe, stand‑replacement fires (LANDFIRE 2007a, BpS 1210800).

Big sagebrush reproduces from seed only, so stands are inhibited by fire as *Artemisia tridentata* does not sprout after burning (Howard 1999, Tirmenstein 1999c). Increasing fire frequency can eliminate the shrubs from the stands (Daubenmire 1970, Tirmenstein 1999c). With a change in fire frequency, species composition will be altered as well (West 1983a). With a high fire frequency (every 2‑5 years), perennial grasses and shrubs are eliminated and non‑native annual grasses dominate (Whisenant 1990, D'Antonio and Vitousek 1992). At fire‑return intervals of 10‑30 years, short‑lived resprouting shrubs such as *Chrysothamnus* or *Tetradymia* spp. dominate. At fire‑return intervals of 30‑70 years, a mixture of perennial bunch grasses and non‑sprouting shrubs is maintained (Johnson 2000b). Finally, in the complete absence of fire, deep‑rooted shrubs such as *Artemisia tridentata* become dominant. At higher‑elevation sites with absence of fire (>100 years), *Pinus monophylla* and *Juniperus osteosperma* trees may invade and eventually dominate sites (Tirmenstein 1999c).

Insects are an important component of many shrub‑steppe and grassland systems. Mormon crickets and grasshoppers are natural components of many rangeland systems (USDA‑APHIS 2003, 2010). There are almost 400 species of grasshoppers that inhabit the western United States with 15‑45 species occurring in a given rangeland system (USDA‑APHIS 2003). Mormon crickets are also present in many western rangelands and, although flightless, are highly mobile and can migrate large distances consuming much of the forage while travelling in wide bands (USDA‑APHIS 2010). Following a high population year for grasshoppers or Mormon crickets and under relatively warm dry spring environmental conditions that favor egg hatching and grasshopper and Mormon cricket survival, there may be large population outbreaks that can utilize 80% or more of the forage in areas as large as 2000 square mile. Conversely, relatively cool and wet spring weather can limit the potential for outbreaks. These outbreaks are naturally occurring cycles and, especially during drought, can denude an area of vegetation leaving it exposed to increased erosion rates from wind and water (USDA‑APHIS 2003).

<br />LANDFIRE developed a state‑and‑transition vegetation dynamics VDDT model for this system which has five classes in total and two classes (classes D & E) that model conversion to forest systems (LANDFIRE 2007a, BpS 1210800). These are summarized as:

A) Early Development 1 ‑ All Structures (15% of type in this stage): Early development is dominated by grasses and forbs with scattered shrubs representing <10% upper canopy cover. Post‑replacement disturbance; grass‑dominated with scattered shrubs. Fuel loading discontinuous. Surface fire occurs every 200 years on average but has no effect on succession. Succession to class B after 20 years.

B) Mid Development 1 Open (shrub‑dominated ‑ 50% of type in this stage): Shrub cover 11‑50%. Shrubs and herbaceous vegetation can be codominant, fine fuels bridge the woody fuels, but fuel discontinuities are possible. Replacement fire accounts for 80% of fire activity (mean FRI of 125 years), whereas mixed‑severity fire occurs every 500 years on average (20% of fire activity) and maintains vegetation in class B. Succession to class C after 40 years.

C) Mid Development 1 Closed (shrub‑dominated ‑ 25% of type in this stage): Shrubs dominate the landscape; fuel loading is primarily woody vegetation. Shrub density sufficient in old stands to carry the fire without fine fuels. Establishment of pinyon and juniper seedlings and saplings widely scattered. Replacement fire (mean FRI of 100 years) and rare flood events (return interval of 333 years) cause a transition to class A. Prolonged drought (mean return interval of 100 years) and insect/disease (every 75 years on average) cause a transition to class B. Succession to class D after 40 years.

D) Late Development 1 Open (5% of type in this stage): Shrubs may still represent the dominant lifeform with pinyon and juniper saplings common (1‑15% upper canopy cover). Pinyon‑juniper encroachment where disturbance has not occurred for at least 100 years (tree species cover <15%). Saplings and young trees are the dominant lifeform. Sagebrush cover (<25%) and herbaceous cover decreasing compared to class C. Replacement fire occurs every 125 years on average. Insect/disease (every 75 years) and prolonged drought (every 100 years) thin both trees and shrubs, causing a transition to class C. Succession to class E after 50 years.

E) Late Development 1 Closed (5% of type in this stage): Shrubland encroached with mature pinyon and/or juniper (cover 16‑90%) where disturbance does not occur for at least 50 years in class D. Shrub cover <10% and graminoids scattered. Replacement fire occurs every 125 years on average. Prolonged drought thins trees, causing a transition to class B.

SOURCES

**References:** Barbour and Billings 1988, Barbour et al. 2007a, Barker 1983, Barnett and Crawford 1994, Beck et al. 2012, Bell et al. 2009, Belnap 2001, Belnap et al. 2001, Brown 1982a, Bunting et al. 1987, CNHP 2010, Chambers et al. 2007a, Chambers et al. 2013, Comer et al. 2003*, Crawford et al. 2004, D'Antonio and Vitousek 1992, D'Antonio et al. 2009, Daubenmire 1970, Davies et al. 2009, Drut et al. 1994, Ersch 2009, Evans and Belnap 1999, Fertig 1998, Gregg and Crawford 2009, Hironaka et al. 1983, Holland and Keil 1995, Howard 1999, Johnson 2000b, Knight 1994, LANDFIRE 2007a, Mack 1981b, Miller et al. 2014, Pellant 1990, Pellant 1996, Quinn 2004, Reid et al. 1999, Reveal 1989, Rosentreter and Belnap 2003, Rosentreter and Eldridge 2002, Sawyer et al. 2009, Shiflet 1994, Spahr et al. 1991, Tirmenstein 1999b, Tirmenstein 1999c, USDA‑APHIS 2003, USDA‑APHIS 2010, Vander Haegen et al. 2000, Vander Haegen et al. 2001, WNHP 2011, WNHP unpubl. data 2018, Wambolt and Payne 1986, West 1983a, West and Young 2000, Whisenant 1990, Wright et al. 1979, Young and Evans 1971, Young and Evans 1973

**Version:** 29 Aug 2015	**Stakeholders:** Midwest, West

**Concept Author:** NatureServe Western Ecology Team	**LeadResp:** West

CES304.778 Inter‑Mountain Basins Big Sagebrush Steppe

**Primary Division:** Inter‑Mountain Basins (304)

**Land Cover Class:** Steppe/Savanna

**Spatial Scale & Pattern:** Large patch

**Required Classifiers:** Natural/Semi‑natural; Vegetated (>10% vasc.); Upland

**Diagnostic Classifiers:** Lowland [Lowland]; Deep Soil; Aridic; Xeromorphic Shrub; Bunch grasses; Artemisia tridentata ssp. tridentata

**National Mapping Codes:** EVT 2125; ESLF 5454; ESP 1125

**Concept Summary:** This widespread matrix‑forming ecological system occurs throughout much of the Columbia Plateau and northern Great Basin, east into the Wyoming Basins, central Montana, and north and east onto the western fringe of the Great Plains in South Dakota. It is found at slightly higher elevations farther south. Relative to other portions of the distribution, in central Montana this system occurs in areas with more summer rain than winter snow precipitation, more overall annual precipitation, and it occurs on glaciated landscapes. Across the entire distribution of this type, soils are typically deep and non‑saline, often with a microphytic crust. This shrub‑steppe is dominated by perennial grasses and forbs (>25% cover) with *Artemisia tridentata ssp. tridentata* (this is not at all important in Wyoming occurrences), *Artemisia tridentata ssp. xericensis, Artemisia tridentata ssp. wyomingensis, Artemisia tripartita ssp. tripartita* (Snake River valley in Wyoming), *Artemisia cana ssp. cana*, and/or *Purshia tridentata* dominating or codominating the open to moderately dense (10‑40% cover) shrub layer. *Atriplex confertifolia, Chrysothamnus viscidiflorus, Ericameria nauseosa, Sarcobatus vermiculatus, Tetradymia* spp., or *Artemisia frigida* may be common especially in disturbed stands. In Montana and Wyoming, stands are more mesic, with more biomass contributed by grasses, have less shrub diversity than stands farther west, and 50 to 90% of the occurrences are dominated by *Artemisia tridentata ssp. wyomingensis* with *Pascopyrum smithii*. Associated graminoids can include *Achnatherum hymenoides, Calamagrostis montanensis, Elymus lanceolatus ssp. lanceolatus, Koeleria macrantha, Poa secunda, Pascopyrum smithii, Hesperostipa comata, Nassella viridula, Bouteloua gracilis*, and *Pseudoroegneria spicata*. Important rhizomatous species include *Carex filifolia* and *Carex duriuscula*, which are very common and important in the eastern distribution of this system in both Wyoming and Montana. *Festuca idahoensis* is uncommon in this system, although it does occur in areas of higher elevations/precipitation; *Festuca campestris* is also uncommon. In Wyoming, both *Nassella viridula* and *Pseudoroegneria spicata* rarely occur, with the latter typically found in eastern Wyoming on ridgetops and rocky slopes outside of this system. In Montana, there is an absence of *Festuca* spp., except *Vulpia octoflora*. Common forbs are *Phlox hoodii, Arenaria* spp., *Opuntia* spp., *Sphaeralcea coccinea, Dalea purpurea, Liatris punctata*, and *Astragalus* spp. Areas with deeper soils more commonly support *Artemisia tridentata ssp. tridentata* but have largely been converted for other land uses. The natural fire regime of this ecological system likely maintains a patchy distribution of shrubs, so the general aspect of the vegetation is a grassland. Shrubs may increase following heavy grazing and/or with fire suppression, particularly in moist portions of the northern Columbia Plateau where it forms a landscape mosaic pattern with shallow‑soil scabland shrublands. Where fire frequency has allowed for shifts to a native grassland condition, maintained without significant shrub invasion over a 50‑ to 70‑year interval, the area would be considered Columbia Basin Foothill and Canyon Dry Grassland (CES304.993). This ecological system is closely related to the warm‑dry sagebrush in the resistance‑resilience framework.

**Comments:** *Artemisia cana ssp. cana* is listed as a component shrub of this system, but this statement needs a bit of review as to whether it is accurate. In addition, in Wyoming and Montana, *Artemisia tripartita ssp. tripartita* associations are not part of this system but occur at higher elevations as components of Inter‑Mountain Basins Montane Sagebrush Steppe (CES304.785). Farther west, they are included in this system (CES304.778), but perhaps this should be reviewed by ecologists familiar with the Columbia Basin region.

DISTRIBUTION

**Range:** This system occurs throughout much of the Columbia Plateau, the northern Great Basin, central and southeastern Montana, and Wyoming, and is found at slightly higher elevations farther south.

**Divisions:** 304:C, 306:C

**TNC Ecoregions:** 4:C, 6:C, 8:C, 9:C, 10:C, 11:C, 20:C, 26:C

**Nations:** CA, US

**Subnations:** BC, CA, CO, ID, MT, NV, OR, UT, WA:S2, WY

**Map Zones:** 1:C, 4:C, 6:?, 7:C, 8:C, 9:C, 10:C, 12:C, 16:C, 17:C, 18:C, 19:C, 20:C, 21:C, 22:C, 28:P, 29:C, 30:?, 33:P

**USFS Ecomap Regions:** 331A:CC, 331D:CC, 331E:CP, 331F:CC, 331G:CC, 331H:CC, 331K:CC, 331L:CC, 331M:CC, 331N:CP, 341A:CP, 341D:CP, 341E:CC, 341F:CC, 341G:CC, 342A:CC, 342B:CC, 342C:CC, 342D:CC, 342E:CC, 342F:CC, 342G:CC, 342H:CC, 342I:CC, 342J:CC, M242C:CC, M242D:CC, M261A:C?, M261D:CC, M261E:CP, M261G:CC, M331A:CC, M331B:CC, M331D:CC, M331E:CC, M331H:CC, M331I:CC, M331J:CC, M332A:CC, M332B:CP, M332D:CC, M332E:CC, M332F:CC, M332G:CC, M333A:CC, M333C:CP, M333D:CP, M334A:CC, M341A:CC, M341B:CP, M341C:CP, M341D:CC

CONCEPT

**Environment:** This widespread matrix‑forming ecological system occurs throughout much of the Columbia Plateau and northern Great Basin, east into the Wyoming Basins, central Montana, and north and east onto the western fringe of the Great Plains in Montana and South Dakota. It is found at slightly higher elevations farther south.

*Climate:* Climate is semi‑arid and continental with annual precipitation ranging from 18‑40 cm and with high inter‑annual variation. Precipitation amount and time vary depending on location, with stands in the western portion of its range receiving winter/spring precipitation and very little summer precipitation, whereas stands in the eastern portion of its range receive both winter and summer precipitation. Much of the precipitation falls as snow, and growing‑season drought is characteristic. Temperatures are continental with large annual and diurnal variation. In central Montana, this system differs slightly, with more summer rain than winter precipitation, more precipitation annually, and it occurs on glaciated landscapes.

*Physiography/landform:* Stands occur on stream terraces, point bars, valley floors, alluvial fans, floodplains, washes, gullies, stabilized dunes, mesic uplands, swales, and rocky slopes. Slopes are variable from gentle to very steep.

*Soil/substrates/hydrology:* Soils are typically deep and non‑saline, often with a microphytic crust.

<br />The environmental description is based on several references, including Daubenmire (1970), Mueggler and Stewart (1980), Brown (1982a), Hironaka et al. (1983), West (1983c), Barbour and Billings (1988), Knight (1994), Holland and Keil (1995), Howard (1999), Tirmenstein (1999c), West and Young (2000), Barbour et al. (2007a), and Sawyer et al. (2009).

**Vegetation:** This shrub‑steppe is dominated by perennial grasses and forbs (>25% cover) with *Artemisia tridentata ssp. tridentata* (not at all important in Wyoming occurrences), *Artemisia tridentata ssp. xericensis, Artemisia tridentata ssp. wyomingensis, Artemisia tripartita ssp. tripartita* (Snake River valley in Wyoming), *Artemisia cana ssp. cana*, and/or *Purshia tridentata* dominating or codominating the open to moderately dense (10‑40% cover) shrub layer. *Atriplex confertifolia, Chrysothamnus viscidiflorus, Ericameria nauseosa, Sarcobatus vermiculatus, Tetradymia* spp., or *Artemisia frigida* may be common, especially in disturbed stands. In Montana and Wyoming, stands are more mesic, with more biomass of grass, have less shrub diversity than stands farther west, and 50 to 90% of the occurrences are dominated by *Artemisia tridentata ssp. wyomingensis* with *Pascopyrum smithii*. In addition, *Bromus arvensis (= Bromus japonicus)* and *Bromus tectorum* are indicators of disturbance, and *Bromus tectorum* is typically not as abundant as in the Intermountain West, possibly due to a colder climate. Associated graminoids can include *Achnatherum hymenoides, Calamagrostis montanensis, Elymus lanceolatus ssp. lanceolatus, Koeleria macrantha, Poa secunda, Pascopyrum smithii, Hesperostipa comata, Nassella viridula, Bouteloua gracilis*, and *Pseudoroegneria spicata*. Important rhizomatous species include *Carex filifolia* and *Carex duriuscula*, which are very common and important in the eastern distribution of this system in both Wyoming and Montana. *Festuca idahoensis* is uncommon in this system, although it does occur in areas of higher elevations/precipitation; *Festuca campestris* is also uncommon. In Wyoming, both *Nassella viridula* and *Pseudoroegneria spicata* rarely occur, with the latter typically found in eastern Wyoming on ridgetops and rocky slopes outside of this system. In Montana, there is an absence of *Festuca* spp., except *Vulpia octoflora*. Common forbs are *Phlox hoodii, Arenaria* spp., *Opuntia* spp., *Sphaeralcea coccinea, Dalea purpurea, Liatris punctata*, and *Astragalus* spp. Areas with deeper soils more commonly support *Artemisia tridentata ssp. tridentata* but have largely been converted for other land uses. The natural fire regime of this ecological system likely maintains a patchy distribution of shrubs, so the general aspect of the vegetation is a grassland. Shrubs may increase following heavy grazing and/or with fire suppression, particularly in moist portions of the northern Columbia Plateau where it forms a landscape mosaic pattern with shallow‑soil scabland shrublands. Where fire frequency has allowed for shifts to a native grassland condition, maintained without significant shrub invasion over a 50‑ to 70‑year interval, the area would be considered Columbia Basin Foothill and Canyon Dry Grassland (CES304.993). The floristic description is based on several references, including Daubenmire (1970), Mueggler and Stewart (1980), Brown (1982a), Hironaka et al. (1983), West (1983c), Barbour and Billings (1988), Knight (1994), Holland and Keil (1995), Howard (1999), Tirmenstein (1999c), West and Young (2000), Barbour et al. (2007a), and Sawyer et al. (2009).

**Dynamics:** The natural fire regime of this ecological system likely maintains a patchy distribution of shrubs, so the general aspect of the vegetation is a grassland. Shrubs may increase following heavy grazing and/or with fire suppression, particularly in moist portions of the northern Columbia Plateau where it forms a landscape mosaic pattern with shallow‑soil scabland shrublands. Response to grazing can be variable depending on the type of grazer and the season in which grazing occurs. *Hesperostipa comata* can increase in abundance in response to either grazing or fire. In central and eastern Montana (and possibly elsewhere), complexes of prairie dog towns are common in this ecological system. Microphytic crust is very important in this ecological system.

<br />Complex ecological interactions of fire regimes and climate patterns result in equally complex patterns of species structure and composition in *Artemisia tridentata* stands. Prolonged drought on the more xeric sites may reduce shrub cover. Flooding may also cause mortality if the soil remains saturated for an extended period of time. The Aroga moth is capable of defoliating large acreages (i.e., >1000 acres, but usually 10‑100 acres). Heavy grazing by wildlife can remove the fine fuels that support mixed‑severity fires and result in woody fuel buildup that leads to severe, stand‑replacement fires (LANDFIRE 2007a, BpS 1210800).

Big sagebrush stands are inhibited by fire as *Artemisia tridentata* does not sprout after burning (Tirmenstein 1999c). Conversely, increasing fire frequency significantly will eliminate the shrubs from the stands (Daubenmire 1970, Tirmenstein 1999c). With a change in fire frequency, species composition will be altered as well (West 1983c). With a high fire frequency (every 2‑5 years), perennial grasses and shrubs are eliminated and non‑native annual grasses dominate. At fire‑return intervals of 10‑30 years, short‑lived resprouting shrubs such as *Chrysothamnus* or *Tetradymia* spp. dominate. At fire‑return intervals of 30‑70 years, a mixture of perennial bunchgrasses and non‑sprouting shrubs is maintained (Johnson 2000b). Finally, in the complete absence of fire, deep‑rooted shrubs such as *Artemisia tridentata* become dominant. At higher‑elevation sites with absence of fire (>100 years), *Pinus monophylla* and *Juniperus osteosperma* trees may invade and eventually dominate sites (Tirmenstein 1999c).

<br />Insects are an important component of many shrub‑steppe and grassland systems. Mormon crickets and grasshoppers are natural components of many rangeland systems (USDA‑APHIS 2003, 2010). There are almost 400 species of grasshoppers that inhabit the western United States with 15‑45 species occurring in a given rangeland system (USDA‑APHIS 2003). Mormon crickets are also present in many western rangelands and, although flightless, are highly mobile and can migrate large distances consuming much of the forage while travelling in wide bands (USDA‑APHIS 2010). Following a high population year for grasshoppers or Mormon crickets and under relatively warm dry spring environmental conditions that favor egg hatching and grasshopper and Mormon cricket survival, there may be large population outbreaks that can utilize 80% or more of the forage in areas as large as 2000 square miles. Conversely, relatively cool and wet spring weather can limit the potential for outbreaks. These outbreaks are naturally occurring cycles and, especially during drought, can denude an area of vegetation leaving it exposed to increased erosion rates from wind and water (USDA‑APHIS 2003).<br />

Climatic variability may have been as important a disturbance agent as fire in these areas. Prolonged drought may have helped to reduce the density and cover of sagebrush. The size of the area affected by the drought would vary from 100s‑1000s of acres and may be related to soil type (LANDFIRE 2007a).

LANDFIRE developed a state‑and‑transition vegetation dynamics VDDT model for this system which has four classes in total (LANDFIRE 2007a, BpS 0911250). These are summarized as:

A) Early Development 1 All Structures (15% of type in this stage): Herbaceous canopy cover is variable (0‑50%). This class is dominated by forbs with varying presence of grasses. Post‑fire cover and recovery rates vary greatly depending on fire severity and post‑fire precipitation amounts and timing as well as pre‑fire species composition. This stage lasts 9‑15 years, depending on how quickly sagebrush is able to begin reoccupying the area. Replacement fire (MFRI= 100 years) resets.

B) Mid Development 1 Open (30% of type in this stage): Dominant lifeform is herbaceous (20‑40% cover), shrub cover 0‑10%. Scattered and usually small sagebrush are present, but perennial grasses and forbs continue to dominate. The general formation is that of a shrub savanna. Sagebrush cover is usually 1‑5% in this stage. Stands are 15‑35 years old. Succession to class C. Replacement fire (MFRI= 100 years) reset to class A. Surface fires (MFRI=1000 years) maintain in class B.

C) Late Development 1 Open (35% of type in this stage): Shrubs have canopy cover of 11‑20%. Sagebrush is codominant with the perennial grasses and forbs. The general formation is that of a shrub‑steppe. Stands are 35‑70 years old; succession to class D. Replacement fire (MFRI=100 years) reset to class A. Mixed fire (MFRI= 50 years) opens the stand to class B. Surface fire (MFRI=1000 years) keeps in class C.

D) Late Development 1 Closed (20% of type in this stage): Shrubs have canopy cover of 21‑30%. Sagebrush is dominant with relatively low cover of perennial grasses and forbs. Sagebrush cover can be variable, with the lowest productivity sites reaching only about 15% canopy cover with large areas of bare ground and rock in the interspaces. The general formation is that of a shrubland. Stands are greater than about 70 years old. Replacement fire (MFRI=85 years) reset to class A. Mixed fire (MFRI=85 years) opens the stand to class B.

SOURCES

**References:** Barbour and Billings 1988, Barbour et al. 2007a, Barnett and Crawford 1994, Beck et al. 2012, Bell et al. 2009, Belnap 2001, Belnap et al. 2001, Brown 1982a, CNHP 2010, Chambers et al. 2007a, Chambers et al. 2013, Comer et al. 2003*, Condon et al. 2011, Crawford et al. 2004, D'Antonio and Vitousek 1992, D'Antonio et al. 2009, Daubenmire 1970, Davies et al. 2009, Drut et al. 1994, Ecosystems Working Group 1998, Ersch 2009, Evans and Belnap 1999, Gober 2000, Gregg and Crawford 2009, Hironaka et al. 1983, Hoagland 2006, Holland and Keil 1995, Howard 1999, Johnson 2000b, Knick et al. 2003, Knight 1994, Kotliar et al. 2006, LANDFIRE 2007a, Mack 1981b, Miller et al. 2011, Miller et al. 2014, Mueggler and Stewart 1980, Pellant 1990, Pellant 1996, Quinn 2004, Rosentreter and Belnap 2003, Rosentreter and Eldridge 2002, Sawyer et al. 2009, Shiflet 1994, Tirmenstein 1999c, USDA‑APHIS 2003, USDA‑APHIS 2010, Vander Haegen et al. 2000, Vander Haegen et al. 2001, Veblen et al. 2011, WNHP 2011, WNHP unpubl. data 2018, Wambolt and Payne 1986, West 1983c, West and Young 2000, Young and Evans 1971, Young and Evans 1973

**Version:** 29 Aug 2015	**Stakeholders:** Canada, Midwest, West

**Concept Author:** K.A. Schulz	**LeadResp:** West

CES304.785 Inter‑Mountain Basins Montane Sagebrush Steppe

**Primary Division:** Inter‑Mountain Basins (304)

**Land Cover Class:** Steppe/Savanna

**Spatial Scale & Pattern:** Matrix

**Required Classifiers:** Natural/Semi‑natural; Vegetated (>10% vasc.); Upland

**Diagnostic Classifiers:** Montane [Upper Montane, Montane, Lower Montane]; Woody‑Herbaceous

**National Mapping Codes:** EVT 2126; ESLF 5455; ESP 1126

**Concept Summary:** This ecological system includes sagebrush communities occurring at foothills (in Wyoming) to montane and subalpine elevations across the western U.S. from 1000 m in eastern Oregon and Washington to over 3000 m in the Southern Rockies. In Montana, it occurs on isolated mountains in the north‑central portion of the state and possibly along the Boulder River south of Absarokee and at higher elevations. In British Columbia, it occurs between 450 and 1650 m in the southern Fraser Plateau and the Thompson and Okanagan basins. Climate is cool, semi‑arid to subhumid. This system primarily occurs on deep‑soiled to stony flats, ridges, nearly flat ridgetops, and mountain slopes. In general, this system is found on fine‑textured soils, some source of subsurface moisture or more mesic sites, zones of higher precipitation, and areas of snow accumulation. Across its range, this is a compositionally diverse system. It is composed primarily of *Artemisia tridentata ssp. vaseyana, Artemisia cana ssp. viscidula*, and related taxa such as *Artemisia tridentata ssp. spiciformis*. *Purshia tridentata* may codominate or even dominate some stands. *Artemisia arbuscula ssp. arbuscula*‑dominated shrublands commonly occur within this system on rocky or windblown sites. Other common shrubs include *Symphoricarpos* spp., *Amelanchier* spp., *Ericameria nauseosa, Peraphyllum ramosissimum, Ribes cereum*, and *Chrysothamnus viscidiflorus*. *Artemisia tridentata ssp. wyomingensis* may be present to codominant if the stand is clearly montane as indicated by montane indicator species such as *Festuca idahoensis, Leucopoa kingii*, or *Danthonia intermedia*. Most stands have an abundant perennial herbaceous layer (over 25% cover, in many cases over 50% cover), but this system also includes *Artemisia tridentata ssp. vaseyana* shrublands. Common graminoids include *Danthonia intermedia, Festuca arizonica, Festuca idahoensis, Hesperostipa comata, Poa fendleriana, Elymus trachycaulus, Bromus carinatus, Poa secunda, Deschampsia cespitosa, Calamagrostis rubescens*, and *Pseudoroegneria spicata*. Species of *Achnatherum* are common, including *Achnatherum nelsonii ssp. dorei, Achnatherum nelsonii ssp. nelsonii, Achnatherum hymenoides*, and others. In many areas, wildfires can maintain an open herbaceous‑rich steppe condition, although at most sites, shrub cover can be unusually high for a steppe system (>40%), with the moisture providing equally high grass and forb cover. This ecological system is closely related to the cool‑moist sagebrush in the resistance‑resilience framework.

**Comments:** In Wyoming and Montana, *Artemisia tripartita ssp. tripartita* associations are part of this system, occurring at higher elevations than Inter‑Mountain Basins Big Sagebrush Steppe (CES304.778). Farther west, they are included in that system, but perhaps this should be reviewed by ecologists familiar with the Columbia Basin region.

DISTRIBUTION

**Range:** This system is found at montane and subalpine elevations across the western U.S. from 1000 m in eastern Oregon and Washington to over 3000 m in the Southern Rockies. In British Columbia, it occurs in the southern Fraser Plateau and the Thompson and Okanagan basins. This system occurs in mapzone 20 on the Rocky Mountain island ranges and on the western edge with mapzone 19.

**Divisions:** 304:C, 306:C

**TNC Ecoregions:** 6:C, 7:C, 8:C, 9:C, 12:C, 18:C, 19:C, 20:C, 26:C, 68:C

**Nations:** CA, US

**Subnations:** AZ?, BC, CA, CO, ID, MT, NM, NV, OR, UT, WA:S3S4, WY

**Map Zones:** 1:C, 3:?, 4:P, 6:C, 7:C, 8:C, 9:C, 10:C, 12:C, 13:P, 16:C, 17:C, 18:C, 19:C, 20:C, 21:C, 22:C, 23:C, 24:?, 25:C, 27:?, 28:C, 29:C

**USFS Ecomap Regions:** 313A:CC, 313B:CP, 315A:CC, 315H:CC, 321A:??, 322A:CC, 331B:C?, 331F:CC, 331G:CC, 331J:CC, 331M:C?, 331N:CP, 341A:CC, 341B:CC, 341C:CC, 341D:CC, 341E:CC, 341F:CC, 341G:CC, 342A:CC, 342B:CC, 342C:CC, 342D:CC, 342E:CC, 342F:CC, 342G:CC, 342H:CC, 342I:CC, 342J:CC, M242C:CC, M242D:CC, M261A:CC, M261D:CC, M261E:CC, M261F:C?, M261G:CC, M313A:CP, M313B:CC, M331A:CC, M331B:CC, M331D:CC, M331E:CC, M331F:CC, M331G:CC, M331H:CC, M331I:CC, M331J:CC, M332A:CC, M332B:CC, M332D:CC, M332E:CC, M332F:CC, M332G:CC, M341A:CC, M341B:CC, M341C:CC, M341D:CC

CONCEPT

**Environment:** This ecological system includes sagebrush communities occurring at foothills (in Wyoming) to montane and subalpine elevations across the western U.S. from 1000 m elevation in eastern Oregon and Washington to over 3000 m in the Southern Rockies. In Montana, it occurs in isolated mountains in the north‑central portion of the state and possibly along the Boulder River south of Absarokee and at higher elevations. In British Columbia, it occurs between 450 and 1650 m in the southern Fraser Plateau and the Thompson and Okanagan basins.

*Climate:* Climate is cool, semi‑arid to subhumid with yearly precipitation ranging from 25 to 90 cm/year. Much of this precipitation falls as snow. In general, this system occurs on fine‑textured soils, some source of subsurface moisture or more mesic sites, zones of higher precipitation, and areas of snow accumulation.

<br />*Physiography/landform:* This system primarily occurs on deep‑soiled to stony flats, ridges, nearly flat ridgetops, and mountain slopes. Stands occur on all aspects, but the higher‑elevation occurrences may be restricted to south‑ or west‑facing slopes.

*Soil/substrates/hydrology:* Soils generally are moderately deep to deep, well‑drained, and of loam, sandy loam, clay loam, or gravelly loam textural classes; soils often have a substantial volume of coarse fragments and are derived from a variety of parent materials.

The environmental description is based on several other references, including Daubenmire (1970), Young et al. (1977), Mueggler and Stewart (1980), Brown (1982a), Hironaka et al. (1983), West (1983c), Barbour and Billings (1988), Padgett et al. (1989), Knight (1994), Hansen et al. (1995), Holland and Keil (1995), Howard (1999), Johnson (2000b), West and Young (2000), Barbour et al. (2007a), and Sawyer et al. (2009).

**Vegetation:** Across its range of distribution, this is a compositionally diverse system. It is composed primarily of *Artemisia tridentata ssp. vaseyana, Artemisia cana ssp. viscidula*, and related taxa such as *Artemisia tridentata ssp. spiciformis (= Artemisia spiciformis)*. *Purshia tridentata* may codominate or even dominate some stands. *Artemisia arbuscula ssp. arbuscula*‑dominated shrublands commonly occur within this system on rocky or windblown sites. Other common shrubs include *Symphoricarpos* spp., *Amelanchier* spp., *Ericameria nauseosa, Peraphyllum ramosissimum, Ribes cereum*, and *Chrysothamnus viscidiflorus*. *Artemisia tridentata ssp. wyomingensis* may be present to codominant if the stand is clearly montane as indicated by montane indicator species such as *Festuca idahoensis, Leucopoa kingii*, or *Danthonia intermedia*. Most stands have an abundant perennial herbaceous layer (over 25% cover, in many cases over 50% cover), but this system also includes *Artemisia tridentata ssp. vaseyana* shrublands that generally have lower herbaceous cover and may have higher shrub cover. Common graminoids include *Achnatherum nelsonii ssp. dorei, Achnatherum nelsonii ssp. nelsonii, Achnatherum hymenoides, Bromus carinatus, Calamagrostis rubescens, Carex* spp., *Danthonia intermedia, Danthonia parryi, Deschampsia cespitosa, Elymus trachycaulus, Festuca arizonica, Festuca idahoensis, Festuca thurberi, Hesperostipa comata, Koeleria macrantha, Leucopoa kingii, Pascopyrum smithii, Poa fendleriana, Poa secunda*, or *Pseudoroegneria spicata*. Forbs are often numerous and an important indicator of health. Forb species may include species of *Castilleja, Potentilla, Erigeron, Phlox, Astragalus, Geum, Lupinus*, and *Eriogonum*, as well as *Balsamorhiza sagittata, Achillea millefolium, Antennaria rosea, Eriogonum umbellatum, Fragaria virginiana, Artemisia ludoviciana, Hymenoxys hoopesii (= Helenium hoopesii)*, etc. In many areas, wildfires can maintain an open herbaceous‑rich steppe condition, although at most sites, shrub cover can be unusually high for a steppe system (>40%), with the moisture providing equally high grass and forb cover. The vegetation description is based on several other references, including Daubenmire (1970), Young et al. (1977), Mueggler and Stewart (1980), Brown (1982a), Hironaka et al. (1983), West (1983c), Barbour and Billings (1988), Padgett et al. (1989), Knight (1994), Hansen et al. (1995), Holland and Keil (1995), Howard (1999), Johnson (2000b), West and Young (2000), Barbour et al. (2007a), and Sawyer et al. (2009).

**Dynamics:** Complex ecological interactions of fire regimes and climate patterns result in equally complex patterns of species structure and composition in *Artemisia tridentata* stands (Johnson 2000b). Healthy stands often have a very productive herbaceous understory that is high quality forage for livestock.

Like other big sagebrush subspecies, *Artemisia tridentata ssp. vaseyana* stands are inhibited by fire as *Artemisia tridentata* does not sprout after being top‑killed by fire and may take over 10 years to form stands with 20% or more cover (Johnson 2000b, Sawyer et al. 2009). Winward (1991) suggests *Artemisia tridentata ssp. vaseyana* shrublands have a natural fire regime of 10‑30 years. Presettlement fires tended to be patchy, forming a mosaic of different age and density of shrubs because of different fire intensity across the landscape (Winward 1991, Tart 1996). Regeneration of mountain big sagebrush is from on‑site or off‑site seed and, depending on circumstances of the environment and seed source, *Artemisia tridentata ssp. vaseyana* seeds may sprout abundantly or very sparsely the following spring after burning (Johnson 2000b). Establishment after severe fires may proceed more slowly (Bunting et al. 1987, Johnson 2000b). Increasing fire frequency significantly will eliminate the shrubs from the stands (Daubenmire 1970, Johnson 2000b). Stand species composition will be altered with changes in fire frequency (West 1983c). With a high fire frequency (every 2‑5 years), perennial grasses and shrubs are eliminated and non‑native annual grasses dominate. At fire‑return intervals of 10‑30 years, short‑lived resprouting shrubs such as *Chrysothamnus* or *Tetradymia* spp. dominate. At fire‑return intervals of 30‑70 years, a mixture of perennial bunch grasses and non‑sprouting shrubs is maintained (Johnson 2000b). Finally, in the complete absence of fire, deep‑rooted shrubs such as *Artemisia tridentata* become dominant. At higher‑elevation sites with absence of fire (>100 years), trees such as *Pinus monophylla, Juniperus occidentalis*, and *Juniperus osteosperma* may invade and eventually dominant sites (Young et al. 1977, Bunting 1990, Johnson 2000b).

In addition, prolonged drought on the more xeric sites may reduce shrub cover. Flooding may also cause mortality if the soil remains saturated for an extended period of time. The Aroga moth is capable of defoliating large acreages (i.e., >1000 acres), but usually affected areas are relatively small (10‑100 acres). Of the three big sagebrush subspecies and black sagebrush, *Artemisia tridentata ssp. vaseyana* was found to be the most palatable browse for elk (Wambolt 1995, 1996). These big game preference differences may make it more sensitive to effects of browsing. Heavy grazing in these mountain shrub‑steppes may decrease fire frequency due to consumption of herbaceous forage (fine fuels), resulting in increased shrub density (woody fuel buildup) that leads to severe, stand‑replacement fires (LANDFIRE 2007a, BpS 1211260).

LANDFIRE developed a state‑and‑transition vegetation dynamics VDDT model for this system which has five classes in total and two classes (classes D & E) that model conversion to forest systems (LANDFIRE 2007a, BpS 1211260). These are summarized as:

A) Early Development 1 Open (herbaceous‑dominated ‑ 20% of type in this stage): Herbaceous cover is variable but typically >50% (50‑80%). Shrub cover is 0‑5%. Replacement fire (mean fire return interval (FRI) of 80 years) setbacks succession by 12 years. Succession to class B after 12 years.

B) Mid Development 1 Open (shrub‑dominated ‑ 50% of type in this stage): Shrub cover 6‑25%. Mountain big sagebrush cover up to 20%. Herbaceous cover is typically >50%. Initiation of conifer seedling establishment. Replacement fire mean FRI is 40 years. Succession to class C after 38 years.

C) Late Development 1 Closed (shrub‑dominated ‑ 15% of type in this stage): Shrubs are the dominant lifeform with canopy cover of 26‑45+%. Herbaceous cover is typically <50%. Conifer (juniper, pinyon‑juniper, ponderosa pine or white fir) cover <10%. Insects and disease every 75 years on average will thin the stand and cause a transition to class B. Replacement fire occurs every 50 years on average. In the absence of fire for 80 years, vegetation will transition to class D. Otherwise, succession keeps vegetation in class C.

D) Late Development 1 Open (conifer‑dominated ‑ 10% of type in this stage): Conifers are the upper lifeform (juniper, pinyon‑juniper, ponderosa pine, limber pine or white fir). Conifer cover is 11‑25%. Shrub cover generally less than mid‑development classes but remains between 26‑40%. Herbaceous cover <30%. The mean FRI of replacement fire is 50 years. Insects/diseases thin the sagebrush, but not the conifers, every 75 years on average, without causing a transition to other classes. Succession is from class C to class D after 50 years.

<br />E) Late Development 2 Closed (conifer‑dominated ‑ 5% of type in this stage): Conifers are the dominant lifeform (juniper, pinyon‑juniper, ponderosa pine, limber pine or white fir). Conifer cover ranges from 26‑80% (pinyon‑juniper 36‑80% (Miller and Tausch 2001), juniper 26‑40% (Miller and Rose 1999) and white fir 26‑80%). Shrub cover 0‑20%. Herbaceous cover <20%. The mean FRI for replacement fire is longer than in previous states (75 years). Conifers are susceptible to insects/diseases that cause diebacks (transition to class D) every 75 years on average (LANDFIRE 2007a). The woodland systems that this montane sagebrush system would succeed into vary by location.

SOURCES

**References:** Barbour and Billings 1988, Barbour et al. 2007a, Barnett and Crawford 1994, Belnap 2001, Belnap et al. 2001, Brown 1982a, Bunting 1990, Bunting et al. 1987, CNHP 2010, Comer et al. 2003*, Crawford et al. 2004, Daubenmire 1970, Drut et al. 1994, Ecosystems Working Group 1998, Ersch 2009, Evans and Belnap 1999, Gregg and Crawford 2009, Hansen et al. 1995, Hironaka et al. 1983, Holland and Keil 1995, Howard 1999, Johnson 2000b, Knick et al. 2003, Knight 1994, LANDFIRE 2007a, Miller and Rose 1999, Miller and Tausch 2001, Mueggler and Stewart 1980, Padgett et al. 1989, Rosentreter and Belnap 2003, Sawyer et al. 2009, Shiflet 1994, Tart 1996, Tirmenstein 1999c, Veblen et al. 2011, WNHP unpubl. data 2018, Wambolt 1995, Wambolt 1996, West 1983c, West and Young 2000, Winward 1991, Young et al. 1977

**Version:** 29 Aug 2015	**Stakeholders:** Canada, Midwest, West

**Concept Author:** K.A. Schulz	**LeadResp:** West

M095. Great Basin‑Intermountain Xeric‑Riparian Scrub

CES304.781 Inter‑Mountain Basins Wash

**Primary Division:** Inter‑Mountain Basins (304)

**Land Cover Class:** Barren

**Spatial Scale & Pattern:** Linear

**Required Classifiers:** Natural/Semi‑natural; Unvegetated (<10% vasc.); Upland; Wetland

**Diagnostic Classifiers:** Lowland [Lowland]; Shrubland (Shrub‑dominated); Wash; Toeslope/Valley Bottom; Riverine / Alluvial; Alkaline Soil; Xeromorphic Shrub; Sarcobatus vermiculatus

**Concept Summary:** This barren and sparsely vegetated (generally <10% plant cover) ecological system is restricted to intermittently flooded streambeds and banks that are often lined with shrubs such as *Sarcobatus vermiculatus, Ericameria nauseosa, Fallugia paradoxa, Artemisia tridentata ssp. tridentata*, and/or *Artemisia cana ssp. cana* (in more northern and mesic stands) that form relatively dense stringers in open dry uplands. *Grayia spinosa* may dominate in the Great Basin. Shrubs form a continuous or intermittent linear canopy in and along drainages but do not extend out into flats. Typically it includes patches of saltgrass meadow where water remains for the longest periods. In parts of Wyoming, stringers or patches of *Artemisia tridentata ssp. tridentata* are large and distinct enough from surrounding upland vegetation due to the influence of the wash that they can be classified separately. However, small intermittent washes may also be included with adjacent uplands if vegetation is not different enough floristically or structurally from uplands (e.g., just a little denser canopy). Soils are variable but are generally less alkaline than those found in the playa system. Desert scrub species (e.g., *Acacia greggii, Prosopis* spp.) that are common in the Mojave, Sonoran and Chihuahuan desert washes are not present. This type can occur in limited portions of the southwestern Great Plains.

**Comments:** Where the stringers or patches of *Artemisia tridentata ssp. tridentata* are large enough to be mapped separately from both the wash and from the adjacent upland, then they should be mapped as Inter‑Mountain Basins Big Sagebrush Shrubland (CES304.777) or Inter‑Mountain Basins Big Sagebrush Steppe (CES304.778) . Compare this wash system with Inter‑Mountain Basins Greasewood Flat (CES304.780); the wash should be restricted to the periphery of the wash and distinct from adjacent vegetation. If not, consider including with greasewood flat. Invasive, exotic shrubs shrub as *Tamarix* spp. or *Chamaebatiaria millefolium* may be present to dominant in these washes where disturbed.

DISTRIBUTION

**Range:** This system occurs throughout the Intermountain western U.S. extending east into the western Great Plains.

**Divisions:** 303:C, 304:C, 306:C

**TNC Ecoregions:** 4:C, 6:C, 8:C, 9:C, 10:C, 11:C, 19:C, 20:C, 26:C

**Nations:** US

**Subnations:** AZ, CA, CO, ID, MT, NV, OR, UT, WA:S3, WY

**Map Zones:** 8:?, 9:?, 12:C, 16:C, 17:C, 18:?, 22:?, 23:C, 24:C, 25:C, 27:P, 28:?, 29:?, 33:?

**USFS Ecomap Regions:** 313A:CC, 313B:CC, 313D:CC, 315H:PP, 331B:CC, 331H:C?, 331I:CC, 331J:CC, 341A:CC, 341B:CC, 341C:CC, 341D:CC, 341E:CC, 341F:CC, 341G:CC, 342A:CC, 342E:CC, 342F:CC, 342G:CC, M313A:CC, M313B:CP, M331I:??, M341A:CC, M341B:CC, M341C:CC, M341D:CC

CONCEPT

SOURCES

**References:** Comer et al. 2003*, Knight 1994, Shiflet 1994, WNHP unpubl. data 2018, West 1983b

**Version:** 01 Oct 2007	**Stakeholders:** Midwest, West

**Concept Author:** K.A. Schulz	**LeadResp:** West

M118. Intermountain Basins Cliff, Scree & Badland Sparse Vegetation

CES304.765 Colorado Plateau Mixed Bedrock Canyon and Tableland

**Primary Division:** Inter‑Mountain Basins (304)

**Land Cover Class:** Barren

**Spatial Scale & Pattern:** Matrix

**Required Classifiers:** Natural/Semi‑natural; Unvegetated (<10% vasc.); Upland

**Diagnostic Classifiers:** Montane [Lower Montane]; Lowland [Foothill]; Shrubland (Shrub‑dominated); Ridge/Summit/Upper Slope; Sedimentary Rock; Temperate [Temperate Xeric]; Alkaline Soil; Aridic

**Concept Summary:** The distribution of this ecological system is centered on the Colorado Plateau where it is composed of barren and sparsely vegetated landscapes (generally <10% plant cover) of steep cliff faces, narrow canyons, and open tablelands of predominantly sedimentary rocks, such as sandstone, shale, and limestone. Some eroding shale layers similar to Inter‑Mountain Basins Shale Badland (CES304.789) may be interbedded between the harder rocks. The vegetation is characterized by very open tree canopy or scattered trees and shrubs with a sparse herbaceous layer. Common species includes *Pinus edulis, Pinus ponderosa, Juniperus* spp., *Cercocarpus intricatus*, and other short‑shrub and herbaceous species, utilizing moisture from cracks and pockets where soil accumulates.

**Comments:** Geographically restricted and distinct from the related, but broader Inter‑Mountain Basins Cliff and Canyon (CES304.779). Shale areas are not as extensive as in shale badlands.

DISTRIBUTION

**Range:** Colorado Plateau.

**Divisions:** 304:C

**TNC Ecoregions:** 18:C, 19:C, 20:?

**Nations:** US

**Subnations:** AZ, CO, NM, UT, WY

**Map Zones:** 13:C, 14:P, 15:C, 16:C, 17:C, 22:P, 23:C, 24:C, 25:P, 27:?, 28:P

**USFS Ecomap Regions:** 313A:CC, 313B:CC, 313C:CC, 313D:CC, 315H:CC, 321A:CC, 322A:CC, 341A:CC, 341B:CC, 341C:CC, 341F:CP, M313A:CC, M313B:CC, M331D:CC, M331E:CC, M331G:CC, M331H:CC, M341B:CC, M341C:CC

CONCEPT

**Environment:** This system includes limestone escarpments and plateaus occurring in a relatively narrow band of unvegetated or sparsely vegetated badlands formed by the red beds of the Claron (Wasatch) Formation along the eastern edge of the Pausaugunt Plateau (Bryce Canyon) and the western edge of the Markagunt Plateau (Cedar Breaks National Monument) (Graybosch and Buchanan 1983). It includes areas of which often 90% of the exposed surface consists of barren rock. It forms, or includes, areas of fixed bedrock forming the vertical or near‑vertical parts on the plateau faces. The rocks forming such areas are predominantly limestone‑capped plateaus. These areas are highly erodible and form the basic scenic structure of Bryce Canyon and Cedar Breaks national parks. The area is generally too steep to allow any significant soil development. Scattered plants obtain a precarious foothold in the crevices of the rocks. Knolls may form at the base of the cliffs.

This ecological system also includes sandstone and shale escarpments, which form, or include, areas of fixed bedrock forming the vertical or near‑vertical parts of canyon walls and plateau faces. The scenic cliffs of the East Tavaputs area, e.g., the Book Cliffs, are excellent examples of this. The rocks forming such areas are predominantly sandstone and shale with some limestone and marlstone. These areas are unstable and rocks are frequently rolling down onto the talus slopes below (often forming Inter‑Mountain Basins Shale Badland (CES304.789)). The area is generally too steep to allow any significant soil development. Scattered plants obtain a precarious foothold in the crevices of the rocks. Knolls may form at the base of the cliffs. The larger drainages (e.g., East Fork Parachute Creek) plunge several hundred feet at this escarpment, which creates scenic and lush hanging gardens. Many of these escarpments are over 305 m (1000 feet) in height and provide excellent habitat for cliff‑nesting birds such as peregrine falcons and golden eagles.

The Claron limestone, a Tertiary deposit, is divisible into Red Eocene beds and White Oligocene beds, which differ somewhat in presence or absence of pigmentation in the form of iron and manganese oxides, and in amounts of sand and conglomerates in the limestone (Graybosch and Buchanan 1983). The Claron Formation is characterized by a rapid rate of erosion, largely a function of creep resulting from winter freeze‑thaw activity and wash away by summer thunderstorm runoff (Graybosch and Buchanan 1983). Freeze‑thaw cycles are most pronounced on south‑facing slopes. Soil development is limited. Infiltration rates are low and runoff high.

**Vegetation:** For the most part, this system is sparsely vegetated. Small patches of scattered trees and shrubs may occur. These small vegetated patches are usually dominated by conifer trees and may include *Abies concolor, Juniperus scopulorum, Picea pungens, Pinus flexilis, Pinus longaeva, Pinus ponderosa*, and *Pseudotsuga menziesii*. If a shrub layer exists, it may include *Acer glabrum, Amelanchier utahensis, Arctostaphylos patula, Ceanothus martinii, Cercocarpus montanus, Cercocarpus intricatus, Juniperus communis, Mahonia repens, Purshia tridentata, Ribes cereum*, and *Gutierrezia sarothrae*. Grasses and forbs, if present, may include *Astragalus kentrophyta, Cirsium arizonicum, Clematis columbiana, Leymus salinus, Eriogonum panguicense, Achnatherum hymenoides*, and *Linum kingii*.

This ecological system is noted for its high rate of endemic species of forbs, especially in Bryce Canyon. Nine of the eleven endemic species occur in the *Pinus longaeva* community, three are found in the *Pinus ponderosa ‑ Arctostaphylos patula* plant association, and two occur in the mixed conifer type. Species that occur only in the *Pinus longaeva* type have the narrowest geographic distributions, although *Eriogonum panguicense var. panguicense* is an exception (Graybosch and Buchanan 1983). Within Bryce Canyon, most of these endemics are restricted to the Claron Formation (Graybosch and Buchanan 1983). The majority of endemic species found in southern Utah are restricted to substrates derived from a specific geologic formation (Welsh 1979). Welsh notes that most of these taxa are found in areas of exposed parent material. The distribution of endemic species in Utah is not a random one; fine‑textured substrates support more species than coarser ones, and desert and foothill vegetation is richer in endemic species than montane communities (Welsh 1979).

**Dynamics:** This ecological system has a naturally high rate of erosion. Fires are infrequent and not an important ecological process.

SOURCES

**References:** Comer et al. 2003*, Eyre 1980, Graybosch and Buchanan 1983, LaMarche and Mooney 1972, Shiflet 1994, Shute and West 1978, Thorne Ecological Institute 1973a, Welsh 1979, Welsh and Chatterly 1985

**Version:** 20 Feb 2003	**Stakeholders:** West

**Concept Author:** K.A. Schulz	**LeadResp:** West

CES304.081 Columbia Plateau Ash and Tuff Badland

**Primary Division:** Inter‑Mountain Basins (304)

**Land Cover Class:** Barren

**Spatial Scale & Pattern:** Large patch

**Required Classifiers:** Natural/Semi‑natural; Unvegetated (<10% vasc.); Upland

**Diagnostic Classifiers:** Lowland [Lowland]; Badlands; Alkaline Soil; Silt Soil Texture; Clay Soil Texture

**Concept Summary:** This ecological system of the Columbia Plateau region is composed of barren and sparsely vegetated substrates (<10% plant cover) typically derived from highly eroded volcanic ash and tuff. Landforms are typically rounded hills and plains that form a rolling topography. The harsh soil properties and high rate of erosion and deposition are driving environmental variables supporting sparse dwarf‑shrubs and forbs. Characteristic species include *Grayia spinosa, Artemisia tridentata, Salvia dorrii, Achnatherum* sp., *Eriogonum* sp., *Sarcobatus vermiculatus, Purshia tridentata*, and *Atriplex confertifolia*. Characteristic forbs are short‑lived annuals, including *Cleome, Mentzelia, Camissonia*, and *Mimulus* species, although these habitats often support endemic perennial forbs.

**Comments:** Associations assigned to this system are not well‑classified, but as many support G1 and G2 plant taxa, they are well sampled.

DISTRIBUTION

**Range:** This system is found on the Columbia Plateau of southern Idaho west into southern Oregon, northern Nevada, and extreme northeastern California.

**Divisions:** 304:C

**TNC Ecoregions:** 4:P, 6:C

**Nations:** US

**Subnations:** CA, ID, NV, OR, WA?

**Map Zones:** 7:P, 8:C, 9:C, 10:P, 12:P, 18:C

**USFS Ecomap Regions:** 342B:CC, 342C:CC, 342D:CC, M331A:??

CONCEPT

**Environment:** This ecological system of the Columbia Plateau region is composed of barren and sparsely vegetated substrates (<10% plant cover) typically derived from highly eroded volcanic ash and tuff. Landforms are typically rounded hills and plains that form a rolling topography. The harsh soil properties and high rate of erosion and deposition are driving environmental variables supporting sparse dwarf‑shrubs and forbs.

**Vegetation:** Characteristic species include *Grayia spinosa, Artemisia tridentata, Salvia dorrii, Achnatherum* sp., *Eriogonum* sp., *Sarcobatus vermiculatus, Purshia tridentata*, and *Atriplex confertifolia*. Characteristic forbs are short‑lived annuals, including *Cleome, Mentzelia, Camissonia*, and *Mimulus* species, although these habitats often support endemic perennial forbs.

SOURCES

**References:** Comer et al. 2003*, WNHP unpubl. data 2018

**Version:** 08 Sep 2004	**Stakeholders:** West

**Concept Author:** J. Kagan	**LeadResp:** West

CES304.779 Inter‑Mountain Basins Cliff and Canyon

**Primary Division:** Inter‑Mountain Basins (304)

**Land Cover Class:** Barren

**Spatial Scale & Pattern:** Large patch

**Required Classifiers:** Natural/Semi‑natural; Unvegetated (<10% vasc.); Upland

**Diagnostic Classifiers:** Cliff (Landform); Rock Outcrops/Barrens/Glades

**Concept Summary:** This ecological system ranges from Wyoming and Utah west to the Pacific states. It is found from foothill to subalpine elevations and includes barren and sparsely vegetated landscapes (generally <10% plant cover) of steep cliff faces, narrow canyons, and smaller rock outcrops of various igneous, sedimentary, and metamorphic bedrock types. Also included is vegetation of unstable scree and talus slopes that typically occurs below cliff faces. Widely scattered trees and shrubs may include *Abies concolor, Pinus edulis, Pinus flexilis, Pinus monophylla, Juniperus* spp., *Artemisia tridentata, Purshia tridentata, Cercocarpus ledifolius, Ephedra* spp., *Holodiscus discolor*, and other species often common in adjacent plant communities.

DISTRIBUTION

**Range:** This system ranges from Wyoming and Utah west to the Pacific states.

**Divisions:** 304:C

**TNC Ecoregions:** 4:?, 6:C, 11:C, 18:C

**Nations:** US

**Subnations:** CA, ID, NV, OR, UT, WA:S5, WY

**Map Zones:** 1:C, 6:?, 7:P, 8:C, 9:C, 10:P, 12:C, 13:C, 16:C, 17:C, 18:P, 21:P, 22:C, 23:?, 24:?

**USFS Ecomap Regions:** 322A:CC, 331A:CC, 331G:CP, 341A:CC, 341C:C?, 341D:CC, 341E:CC, 341F:CC, 341G:CC, 342A:CC, 342B:CC, 342C:CC, 342D:CC, 342E:CC, 342F:CC, 342G:CC, 342H:CC, 342I:CC, 342J:CC, M242C:??, M331A:CC, M331B:CC, M331D:CC, M331E:CP, M331G:CC, M331H:CC, M331I:CC, M331J:CC, M332A:C?, M332G:CC, M333A:CC, M333D:C?, M341A:CC, M341B:C?, M341C:C?, M341D:CC

CONCEPT

**Environment:** This sparsely vegetated ecological system occurs in the interior western U.S. and ranges up to subalpine elevations. It includes barren and sparsely vegetated sites (generally < 10% plant cover) of steep cliff faces, canyons, and smaller rock outcrops of various igneous, sedimentary, and metamorphic bedrock types. Also included is vegetation of unstable scree and talus slopes that typically occur below cliff faces. Establishment and growth of sparse vegetation occur on these sites because of limited soil moisture in extremely well‑drained sites, lack of suitable sites, or relatively high soil moisture in the case of escarpment woodlands growing on outcrops on lower‑elevation, semi‑arid sites (Knight 1999). Other low‑moisture soils include those with heavy clay and clay loam textures that limit water infiltration. Environmental information is compiled from Hess and Wasser (1982), Holland and Keil (1995), Knight (1999), Reid et al. (1999), Barbour et al. (2007), and Sawyer et al. (2009).

**Vegetation:** The biota reflects what is surrounding it, unless it is an extreme parent material. There is often very high cover of nonvascular lichens and, in wetter places, mosses. There may be small patches of dense vascular vegetation and can include scattered trees and shrubs. Characteristic trees include species from the surrounding landscape, such as *Abies concolor, Abies lasiocarpa, Picea engelmannii, Pinus flexilis, Pinus ponderosa, Populus tremuloides*, and *Pseudotsuga menziesii* at high elevations, or *Pinus edulis, Pinus monophylla*, and *Juniperus* spp. at lower elevations. There may be scattered shrubs present such as *Artemisia tridentata, Cercocarpus intricatus, Cercocarpus ledifolius, Ephedra* spp., *Glossopetalon spinescens, Holodiscus discolor, Physocarpus monogynus, Purshia tridentata*, or *Rhus trilobata*. Herbaceous cover is composed of various grasses such as *Leymus salinus, Poa fendleriana, Pseudoroegneria spicata*, and forbs, especially cushion or mat‑forming species. Characteristic nonvascular species information is not available. Floristic information is compiled from Hess and Wasser (1982), Holland and Keil (1995), Knight (1999), Reid et al. (1999), Barbour et al. (2007), and Sawyer et al. (2009).

**Dynamics:** Plant species are variable with various life history traits, although most can colonize harsh sites and many are fairly long‑lived. Vegetation establishment and growth are limited on these sparsely vegetated sites for different ecological reasons. In relatively mesic climates in the montane zone, there may be a lack of suitable sites for establishment or frequent disturbance may limit plant growth on unstable substrates such as talus and repeatedly disturbed sites such as avalanche chutes. Soil moisture may limit plant growth on sites with excessively well‑drained, shallow soils. On lower‑elevation, semi‑arid sites, deeper‑rooted trees and shrubs may establish in rock cracks and well‑drained coarse‑textured soils because these sites are relatively mesic when compared to surrounding sites. When it rains, runoff collects in superficial cracks in rocky escarpments and infiltrates deeply in coarse‑textured soils where deep‑rooted woody plants can access soil moisture (Knight 1999). This soil moisture/texture relationship is termed "inverse texture effect" (Noy‑Meir 1973, Sala et al. 1988).

Burning is generally not a significant factor on the vegetated sites because fuel amounts are too low and discontinuous to carry fire (Knight 1999). Fire‑return intervals would be very long.

SOURCES

**References:** Barbour et al. 2007a, Comer et al. 2003*, Hess and Wasser 1982, Holland and Keil 1995, Knight 1994, Knight 1999, NatureServe Explorer 2011, Noy‑Meir 1973, Reid et al. 1999, Sala et al. 1988, Sawyer et al. 2009, WNHP unpubl. data 2018

**Version:** 02 Apr 2014	**Stakeholders:** Midwest, West

**Concept Author:** K.A. Schulz	**LeadResp:** West

CES304.789 Inter‑Mountain Basins Shale Badland

**Primary Division:** Inter‑Mountain Basins (304)

**Land Cover Class:** Barren

**Spatial Scale & Pattern:** Large patch

**Required Classifiers:** Natural/Semi‑natural; Unvegetated (<10% vasc.); Upland

**Diagnostic Classifiers:** Lowland [Lowland]; Badlands; Badland; Alkaline Soil; Shale and Mudstone; Silt Soil Texture; Clay Soil Texture

**Concept Summary:** This widespread ecological system of the Intermountain western U.S. is composed of barren and sparsely vegetated substrates (<10% plant cover) typically derived from marine shales but also includes substrates derived from siltstones and mudstones (clay). In southern Wyoming , the shales are not marine in origin, but often have bentonite, derived from volcanic ash deposition that occurred during several eruptions of the Yellowstone volcanic fields. Landforms are typically rounded hills and plains that form a rolling topography. The harsh soil properties and high rate of erosion and deposition are driving environmental variables supporting sparse dwarf‑shrubs, e.g., *Atriplex corrugata, Atriplex gardneri, Artemisia pedatifida*, and herbaceous vegetation.

**Comments:** Exactly where this transitions to Western Great Plains Badlands (CES303.663) in central Wyoming needs to be clarified.

DISTRIBUTION

**Range:** This system is found in the intermountain western U.S., from Arizona and New Mexico north to Idaho and Montana. It is confirmed by Oregon and Washington reviewers to not occur in either of those states.

**Divisions:** 304:C, 306:C

**TNC Ecoregions:** 6:P, 9:C, 10:C, 11:C, 12:?, 18:C, 19:C, 20:C, 21:C

**Nations:** US

**Subnations:** AZ, CA, CO, ID, MT, NM, NV, UT, WY

**Map Zones:** 8:?, 9:C, 12:?, 13:P, 15:?, 16:C, 17:P, 18:P, 21:?, 22:C, 23:C, 24:C, 25:P, 27:?, 28:C

**USFS Ecomap Regions:** 313A:CC, 313B:CC, 313D:CC, 315H:CC, 322A:??, 331F:CC, 331G:CC, 331I:C?, 331J:CP, 341A:CP, 341B:CC, 341C:CC, 342A:CC, 342B:C?, 342C:CP, 342D:CC, 342F:CC, 342G:CC, 342H:CC, 342I:C?, 342J:C?, M261D:??, M313A:PP, M313B:PP, M331B:C?, M331D:CC, M331E:CC, M331F:CC, M331G:CC, M331H:CC, M331I:CC, M331J:CP, M334A:CC, M341B:CC, M341C:CC

CONCEPT

SOURCES

**References:** Comer et al. 2003*, DeVelice and Lesica 1993, Knight 1994, Knight et al. 1987

**Version:** 29 Jan 2007	**Stakeholders:** West

**Concept Author:** NatureServe Western Ecology Team	**LeadResp:** West

CES304.791 Inter‑Mountain Basins Volcanic Rock and Cinder Land

**Primary Division:** Inter‑Mountain Basins (304)

**Land Cover Class:** Barren

**Spatial Scale & Pattern:** Large patch

**Required Classifiers:** Natural/Semi‑natural; Unvegetated (<10% vasc.); Upland

**Diagnostic Classifiers:** Cinder cone; Lava flow (undifferentiated); Lava; Cinder; Basalt; Temperate [Temperate Continental]

**Concept Summary:** This ecological system occurs in the intermountain western U.S. and is limited to barren and sparsely vegetated volcanic substrates (generally <10% plant cover) such as basalt lava (malpais), basalt dikes with associated colluvium, basalt cliff faces and uplifted "backbones," tuff, cinder cones or cinder fields. It may occur as large‑patch, small‑patch and linear (dikes) spatial patterns. Vegetation is variable and includes a variety of species depending on local environmental conditions, e.g., elevation, age and type of substrate. At montane and foothill elevations scattered *Pinus ponderosa, Pinus flexilis*, or *Juniperus* spp. trees may be present. Shrubs such as *Ephedra* spp., *Atriplex canescens, Eriogonum corymbosum, Eriogonum ovalifolium*, and *Fallugia paradoxa* are often present on some lava flows and cinder fields. Species typical of sand dunes such as *Andropogon hallii* and *Artemisia filifolia* may be present on cinder substrates.

DISTRIBUTION

**Range:** This system occurs in the Intermountain western U.S. and is limited to barren and sparsely vegetated volcanic substrates. It occurs in Montana along the Rocky Mountain Front (east of the Continental Divide).

**Divisions:** 304:C

**TNC Ecoregions:** 4:C, 6:C, 8:C, 9:C, 11:C, 18:C, 19:C, 20:C, 21:C

**Nations:** US

**Subnations:** AZ, ID, MT, NM, NV, OR, UT, WY

**Map Zones:** 7:?, 8:?, 9:C, 10:C, 12:P, 13:P, 15:P, 16:C, 17:C, 18:C, 19:C, 20:C, 23:C, 24:C, 25:P, 27:C

**USFS Ecomap Regions:** 313A:CC, 313B:CC, 313D:CC, 315H:CC, 322A:PP, 331B:CC, 331D:CP, 331J:CC, 341A:CC, 341B:CC, 342B:CC, 342C:CC, 342D:CC, 342H:CC, 342J:CP, M242C:PP, M313A:CC, M331A:C?, M331F:CC, M331G:C?, M332D:CC, M332F:CC, M332G:CC, M341C:CC

CONCEPT

**Dynamics:** This ecological system is relatively young (geologically speaking). Lichens are the primary erosion process in this system, and therefore, soil buildup is a slow process. Lichens are susceptible to changes in air quality (Brodo et. al. 2001) and are considered a good indicator of air quality.

SOURCES

**References:** Barbour and Billings 2000, Bell et al. 2009, Brodo et al. 2001, Comer et al. 2003*, Day and Wright 1985, Hansen et al. 2004c, Tisdale et al. 1965

**Version:** 23 Jan 2006	**Stakeholders:** West

**Concept Author:** K.A. Schulz	**LeadResp:** West

4. Polar & High Montane Scrub, Grassland & Barrens

4.B. Temperate to Polar Alpine & Tundra Vegetation

4.B.1. Temperate & Boreal Alpine Tundra

4.B.1.Na. Eastern North American Alpine Tundra

M131. Eastern North American Alpine Tundra

CES201.567 Acadian‑Appalachian Alpine Tundra

**Primary Division:** Laurentian‑Acadian (201)

**Land Cover Class:** Shrubland

**Spatial Scale & Pattern:** Large patch

**Required Classifiers:** Natural/Semi‑natural; Vegetated (>10% vasc.); Upland

**Diagnostic Classifiers:** Alpine/AltiAndino; Shrubland (Shrub‑dominated); Moss/Lichen (Nonvascular); Ridge/Summit/Upper Slope

**National Mapping Codes:** EVT 2386; ESLF 5210; ESP 1386

**Concept Summary:** Restricted to the Northern Appalachians and the Gaspe Peninsula, this system encompasses vegetation above treeline on northeastern mountains. In New Hampshire, climatic treeline occurs at 1495 m (4900 feet) or greater in elevation, following the 10‑12°C July isotherm, but can also occur at lower elevations with high wind exposure, fire history, or shallow soils. Wind, snow, and cloud‑cover fog are prominent environmental factors. Most of the cover is dwarf‑shrubland, lichen, or sparse vegetation; islands of taller shrubs may occur in protected spots. The dominant plants are ericads (*Vaccinium uliginosum* is diagnostic and often dominant, with several other alpine‑restricted ericads such as *Phyllodoce caerulea* and *Loiseleuria procumbens*) and cushion‑plants such as *Diapensia lapponica*. *Carex bigelowii* is a characteristic and, in some places, locally dominant sedge. This system includes wetland depressions, small alpine bogs, within the surrounding upland matrix.

DISTRIBUTION

**Range:** This system is found at higher summits of the northern Appalachian Mountains, from northern New England and the Adirondacks into the Canadian maritimes, including Labrador, Nova Scotia and the Gaspé Peninsula.

**Divisions:** 201:C

**TNC Ecoregions:** 63:C

**Nations:** CA, US

**Subnations:** ME, NH, NY, QC, VT

**Map Zones:** 64:C, 66:C

**USFS Ecomap Regions:** M211A:CC, M211B:CC, M211C:CC, M211D:CC

CONCEPT

**Environment:** This system is restricted to high elevations above climatic treeline, ranging from 1460 m (4900 feet) in New Hampshire to 730 m (2400 feet) at Gros Morne National Park in Labrador.

**Dynamics:** Low temperature, snow accumulation, atmospheric moisture, topography, aspect, and degree of exposure to wind are the primary agents of disturbance to these systems. The degree of wind exposure and snow accumulation is directly related to topographic position. Summits and steep slopes are exposed to high winds, and receive less snow accumulation than more gentle slopes. Ravines collect abundant snowpack, which serves to protect the underlying plants from extreme weather conditions well into the spring (Sperduto and Kimball 2011). The alpine ‑ treeline ecotone is controlled by a variety of climate variables; exposure as a result of topography and mechanical damage caused by ice and wind appear to be largely responsible for the ecotone (Kimball and Weihrauch 2000).

SOURCES

**References:** Bliss 1963, Brouillet et al. 1998, Comer et al. 2003*, Edinger et al. 2014a, Faber‑Langendoen et al. 2011, Forbes 1953, Gawler and Cutko 2010, Kimball and Weihrauch 2000, Lambert and McFarland 2004, Macoun 1883, NYNHP 2013i, Sperduto and Cogbill 1999, Sperduto and Kimball 2011, Sperduto and Nichols 2004, Wipf et al. 2009

**Version:** 14 Jan 2014	**Stakeholders:** Canada, East

**Concept Author:** S.C. Gawler	**LeadResp:** East

4.B.1.Nb. Western North American Alpine Tundra

M099. Rocky Mountain‑Sierran Alpine Tundra

CES206.899 Mediterranean California Alpine Bedrock and Scree

**Primary Division:** Mediterranean California (206)

**Land Cover Class:** Barren

**Spatial Scale & Pattern:** Large patch

**Required Classifiers:** Natural/Semi‑natural; Unvegetated (<10% vasc.); Upland

**Diagnostic Classifiers:** Alpine/AltiAndino [Alpine/AltiAndino]; Ridge/Summit/Upper Slope; Temperate [Temperate Oceanic]; Nonvascular; Alpine Mosaic

**Concept Summary:** This system occurs in limited alpine environments mostly concentrated in the Sierra Nevada, but also on Mount Shasta and as far south as the Peninsular Ranges and White Mountains. Alpine elevations begin around 3500 m (10,600 feet) in the southern mountain ranges and 2700 m (8200 feet) in the southern Cascades. These are barren and sparsely vegetated alpine substrates, typically including both bedrock outcrops and scree slopes, with nonvascular (lichen)‑dominated communities. This also encompasses a limited area of "alpine desert" with unstable sandy substrates and scattered individuals of *Astragalus* spp., *Arabis* spp., *Draba* spp., and *Oxytropis* spp., which mostly fall to the east of the Sierra Nevada crest. Exposure to desiccating winds, rocky and sometimes unstable substrates, and a short growing season limit plant growth.

DISTRIBUTION

**Range:** Concentrated in the Sierra Nevada, but also on Mount Shasta and as far south as the Peninsular Ranges and White Mountains. Alpine elevations begin around 3500 m (10,600 feet) in the southern mountain ranges and 2700 m (8200 feet) in the southern Cascades.

**Divisions:** 206:C

**TNC Ecoregions:** 5:C, 12:C, 16:P

**Nations:** MX, US

**Subnations:** CA, MXBC, NV, OR

**Map Zones:** 2:C, 3:?, 4:C, 6:C, 7:C, 12:P

**USFS Ecomap Regions:** 341D:CC, 342B:??, M261D:CP, M261E:CC

CONCEPT

SOURCES

**References:** Barbour and Major 1988, Comer et al. 2003*, Hickman 1993, Holland and Keil 1995, Sawyer and Keeler‑Wolf 1995

**Version:** 17 Mar 2003	**Stakeholders:** Latin America, West

**Concept Author:** P. Comer and T. Keeler‑Wolf	**LeadResp:** West

CES206.939 Mediterranean California Alpine Dry Tundra

**Primary Division:** Mediterranean California (206)

**Land Cover Class:** Herbaceous

**Spatial Scale & Pattern:** Large patch

**Required Classifiers:** Natural/Semi‑natural; Vegetated (>10% vasc.); Upland

**Diagnostic Classifiers:** Alpine/AltiAndino [Alpine/AltiAndino]; Herbaceous; Temperate [Temperate Oceanic]; Udic; W‑Landscape/High Intensity; Graminoid; Alpine Mosaic

**National Mapping Codes:** EVT 2136; ESLF 7108; ESP 1136

**Concept Summary:** These dry meadows typically occur between 3200 and 4500 m (9700‑13,600 feet) elevation in the northern Sierra Nevada, Klamath Mountains and Cascade Range. They are typically found on gentle to steep slopes, flat ridges and upper basins where the soil is thin and the water supply is constant and strongly regulated by snowpatch patterns. These sites are generally very well‑drained and xeric once the snow melts. The system is commonly composed of a mosaic of small‑patch plant communities that are dominated by sedges, grasses and forbs. Characteristic species include *Phlox diffusa, Phlox covillei, Erigeron pygmaeus, Podistera nevadensis, Carex congdonii, Calamagrostis purpurascens, Eriogonum incanum, Carlquistia muirii (= Raillardella muirii), Castilleja nana, Erigeron compositus, Eriogonum ovalifolium, Eriogonum gracilipes*, etc. There is a rocky mesic version of this system with *Hulsea algida, Saxifraga tolmiei, Carex helleri, Ranunculus eschscholtzii, Polemonium eximium, Salix reticulata* (rarely), *Oxyria digyna, Sibbaldia procumbens*, etc. that could be found near snowmelt patches generally on sheltered, steep, rocky slopes. Alpine dry tundra typically intermingles with alpine bedrock and scree, ice field, fell‑field, alpine dwarf‑shrubland, and alpine/subalpine wet meadows.

DISTRIBUTION

**Range:** This system occurs between 3200 and 4500 m (9700‑13,600 feet) elevation in the northern Sierra Nevada, Klamath Mountains, and Cascade Range of California, Nevada and Oregon.

**Divisions:** 206:C

**TNC Ecoregions:** 4:C, 5:C, 12:C

**Nations:** US

**Subnations:** CA, NV, OR

**Map Zones:** 3:?, 6:C, 7:C

**USFS Ecomap Regions:** M261D:CP, M261E:CC

CONCEPT

SOURCES

**References:** Barbour and Billings 2000, Barbour and Major 1988, Comer et al. 2003*, Holland and Keil 1995, Sawyer and Keeler‑Wolf 1995, Shiflet 1994

**Version:** 07 Oct 2005	**Stakeholders:** West

**Concept Author:** P. Comer and T. Keeler‑Wolf	**LeadResp:** West

CES206.900 Mediterranean California Alpine Fell‑Field

**Primary Division:** Mediterranean California (206)

**Land Cover Class:** Shrubland

**Spatial Scale & Pattern:** Large patch

**Required Classifiers:** Natural/Semi‑natural; Vegetated (>10% vasc.); Upland

**Diagnostic Classifiers:** Alpine/AltiAndino [Alpine/AltiAndino]; Ridge/Summit/Upper Slope; Temperate [Temperate Oceanic]; Very Shallow Soil; Cushion plants; Alpine Mosaic

**National Mapping Codes:** EVT 2067; ESLF 5204; ESP 1067

**Concept Summary:** This ecological system occurs in limited alpine environments mostly concentrated in the Sierra Nevada but also on Mount Shasta and as far south as the Peninsular Ranges and White Mountains. Alpine elevations begin around 3500 m (10,600 feet) in the southern mountain ranges and 2700 m (8200 feet) in the southern Cascades. Wind scours fell‑fields free of snow in the winter, exposing the plants to severe environmental stress. These systems typically have immature soils. Most fell‑field plants are cushioned or matted, frequently succulent, flat to the ground in rosettes, and often densely hairy and thickly cutinized. Common species include *Ribes cereum, Linanthus pungens (= Leptodactylon pungens), Ericameria discoidea, Castilleja nana, Minuartia nuttallii (= Arenaria nuttallii), Phlox condensata, Draba densifolia, Oxyria digyna*, and *Aquilegia pubescens*. Plants cover 15‑50%, while exposed rock makes up the rest. Fell‑fields are usually nested within or adjacent to alpine tundra dry meadows.

DISTRIBUTION

**Range:** This system occurs in limited alpine environments mostly concentrated in the Sierra Nevada but also on Mount Shasta and as far south as the Peninsular Ranges and White Mountains.

**Divisions:** 206:C

**TNC Ecoregions:** 5:C, 12:C, 16:C

**Nations:** MX, US

**Subnations:** CA, MXBC, NV, OR

**Map Zones:** 3:?, 4:P, 6:C, 7:C

**USFS Ecomap Regions:** M261D:CP, M261E:CC

CONCEPT

**Environment:** These are wind‑scoured fell‑fields that are free of snow in the winter, such as ridgetops and exposed saddles, exposing the plants to severe environmental stress. Soils on these windy unproductive sites are shallow, stony, low in organic matter, and poorly developed; wind deflation often results in a gravelly pavement. Fell is Gaelic for stone, and these are stone fields. Sites are stable for 100s to 1000s of years as soils develop. Alpine elevations begin around 3500 m (10,600 feet) in the southern Sierra Nevada and 2700 m (8200 feet) in the southern Cascades.

**Dynamics:** TNC model information: Avalanches on steeper slopes where soil accumulates can cause infrequent soil‑slips, which expose bare ground.

Very small burns of a few square meters (replacement fire) caused by lightning strikes are a rare disturbance, although lighting storms are frequent in those elevations. The calculation of lightning strike frequency was not based on fire‑return intervals but on the number of strikes (in this case, five) per 1000 possible locations per year, thus 0.005.

Alpine rodents (pikas, marmots, etc.) cause common but generally small‑scale disturbances in this system. Native herbivores (Rocky Mountain bighorn sheep, mule deer, and elk) were common in the alpine but probably did not greatly affect vegetation cover because animals move frequently as they reduce vegetation cover.

SOURCES

**References:** Barbour and Billings 2000, Barbour and Major 1988, Comer et al. 2003*, Holland and Keil 1995, Sawyer and Keeler‑Wolf 1995, Shiflet 1994

**Version:** 12 Jan 2012	**Stakeholders:** Latin America, West

**Concept Author:** P. Comer and T. Keeler‑Wolf	**LeadResp:** West

CES306.809 Rocky Mountain Alpine Bedrock and Scree

**Primary Division:** Rocky Mountain (306)

**Land Cover Class:** Barren

**Spatial Scale & Pattern:** Large patch

**Required Classifiers:** Natural/Semi‑natural; Unvegetated (<10% vasc.); Upland

**Diagnostic Classifiers:** Alpine/AltiAndino [Alpine/AltiAndino]; Talus (Substrate); Rock Outcrops/Barrens/Glades; Oligotrophic Soil; Very Shallow Soil; Alpine Slopes

**Concept Summary:** This ecological system is restricted to the highest elevations of the Rocky Mountains, from Alberta and British Columbia south into New Mexico, west into the highest mountain ranges of the Great Basin. It is composed of barren and sparsely vegetated alpine substrates, typically including both bedrock outcrop and scree slopes, with nonvascular‑ (lichen) dominated communities. Exposure to desiccating winds, rocky and sometimes unstable substrates, and a short growing season limit plant growth. There can be sparse cover of forbs, grasses, lichens and low shrubs.

DISTRIBUTION

**Range:** Restricted to the highest elevations of the Rocky Mountains, from Alberta and British Columbia south into New Mexico, west into the highest mountain ranges of the Great Basin.

**Divisions:** 304:C, 306:C

**TNC Ecoregions:** 7:C, 8:C, 9:C, 11:C, 19:C, 20:C, 21:C, 68:C

**Nations:** CA, US

**Subnations:** AB, AK?, AZ, BC, CO, ID, MT, NM, NV, OR, UT, WA:S4?, WY

**Map Zones:** 1:P, 9:P, 10:C, 12:P, 15:P, 16:C, 17:C, 18:?, 19:C, 21:C, 22:P, 23:C, 24:C, 25:C, 28:C, 29:P

**USFS Ecomap Regions:** 331G:PP, 331J:P?, 341A:C?, 341B:CC, 341E:CP, 341F:CP, 341G:CC, 342A:CC, 342B:C?, 342C:C?, 342D:CP, 342H:C?, 342J:CP, M242D:PP, M313A:CC, M331A:CC, M331B:CC, M331D:CC, M331E:CC, M331F:CC, M331G:CC, M331H:CC, M331I:CC, M331J:CC, M332A:CC, M332B:CC, M332D:CC, M332E:CC, M332F:CC, M332G:CP, M333A:CC, M333B:CC, M333C:CC, M333D:CC, M334A:??, M341A:CC, M341B:CC, M341C:CC, M341D:CC

CONCEPT

SOURCES

**References:** Anderson 1999a, Comer et al. 2003*, Cooper et al. 1997, Komarkova 1976, Komarkova 1980, Meidinger and Pojar 1991, NCC 2002, Neely et al. 2001, Nelson 1998, Shiflet 1994, WNHP unpubl. data 2018, Willard 1963

**Version:** 20 Feb 2003	**Stakeholders:** Canada, Midwest, West

**Concept Author:** M.S. Reid	**LeadResp:** West

CES306.810 Rocky Mountain Alpine Dwarf‑Shrubland

**Primary Division:** Rocky Mountain (306)

**Land Cover Class:** Shrubland

**Spatial Scale & Pattern:** Large patch

**Required Classifiers:** Natural/Semi‑natural; Vegetated (>10% vasc.); Upland

**Diagnostic Classifiers:** Alpine/AltiAndino [Alpine/AltiAndino]; Patterned ground (undifferentiated); Glaciated; Acidic Soil; Udic; Very Long Disturbance Interval; Dwarf‑Shrub; Alpine Slopes

**National Mapping Codes:** EVT 2070; ESLF 5207; ESP 1070

**Concept Summary:** This widespread ecological system occurs above upper timberline throughout the Rocky Mountain cordillera, including alpine areas of ranges in Utah and Nevada, and north into Canada. Elevations are above 3360 m in the Colorado Rockies but drop to less than 2100 m in northwestern Montana and in the mountains of Alberta. This system occurs in areas of level or concave glacial topography, with late‑lying snow and subirrigation from surrounding slopes. Soils have become relatively stabilized in these sites, are moist but well‑drained, strongly acidic, and often with substantial peat layers. Vegetation in these areas is controlled by snow retention, wind desiccation, permafrost, and a short growing season. This ecological system is characterized by a semi‑continuous layer of ericaceous dwarf‑shrubs or dwarf willows which form a heath type ground cover less than 0.5 m in height. Dense tuffs of graminoids and scattered forbs occur. *Dryas octopetala* or *Dryas integrifolia* communities are not included here, except for one very moist association, because they occur on more windswept and drier sites than the heath communities. Within these communities, *Cassiope mertensiana, Salix arctica, Salix reticulata, Salix vestita*, or *Phyllodoce empetriformis* can be dominant shrubs. *Vaccinium* spp., *Ledum glandulosum, Phyllodoce glanduliflora*, and *Kalmia microphylla* may also be shrub associates. The herbaceous layer is a mixture of forbs and graminoids, especially sedges, including, *Erigeron* spp., *Luetkea pectinata, Antennaria lanata, Oreostemma alpigenum, Pedicularis* spp., *Castilleja* spp., *Deschampsia cespitosa, Caltha leptosepala, Erythronium* spp., *Juncus parryi, Luzula piperi, Carex spectabilis, Carex nigricans*, and *Polygonum bistortoides*. Fell‑fields often intermingle with the alpine dwarf‑shrubland.

DISTRIBUTION

**Range:** This system occurs above upper timberline throughout the Rocky Mountain cordillera, including alpine areas of ranges in Utah and Nevada, and north into Canada. Elevations are above 3360 m in the Colorado Rockies but drop to less than 2100 m in northwestern Montana.

**Divisions:** 304:C, 306:C

**TNC Ecoregions:** 4:P, 7:C, 8:C, 9:C, 11:C, 19:C, 20:C, 21:C, 68:P

**Nations:** CA, US

**Subnations:** AB, AK?, BC, CO, ID, MT, NM, NV, OR, UT, WA:S4, WY

**Map Zones:** 1:P, 9:P, 10:C, 16:C, 17:C, 18:?, 19:C, 21:C, 22:?, 23:P, 24:P, 25:C, 28:C, 29:?

**USFS Ecomap Regions:** 331J:CC, 341G:PP, 342J:PP, M242B:CC, M242C:C?, M242D:CC, M331A:CC, M331B:CC, M331D:CC, M331E:CC, M331F:CC, M331G:CC, M331H:CC, M331I:CC, M331J:CC, M332A:CC, M332B:CC, M332D:CP, M332E:CC, M332F:CC, M332G:CP, M333A:CC, M333B:CC, M333C:CC, M333D:CC, M341A:PP, M341B:PP, M341C:PP

CONCEPT

**Environment:** This widespread ecological system occurs above upper timberline throughout the Rocky Mountain cordillera, including alpine areas of ranges in Utah and Nevada, and north into Canada. Elevations are above 3360 m in the Colorado Rockies but drop to less than 2100 m in northwestern Montana and in the mountains of Alberta. This system occurs in areas of level or concave glacial topography, with late‑lying snow and subirrigation from surrounding slopes. Soils have become relatively stabilized in these sites, are moist but well‑drained, strongly acidic, and often with substantial peat layers. Vegetation in these areas is controlled by snow retention, wind desiccation, permafrost, and a short growing season.

**Vegetation:** This ecological system is characterized by a semi‑continuous layer of ericaceous dwarf‑shrubs or dwarf willows which form a heath type ground cover less than 0.5 m in height. Dense tuffs of graminoids and scattered forbs occur. *Dryas octopetala* or *Dryas integrifolia* communities are not included here, except for one very moist association, because they occur on more windswept and drier sites than the heath communities. Within these communities, *Cassiope mertensiana, Salix arctica, Salix reticulata, Salix vestita*, or *Phyllodoce empetriformis* can be dominant shrubs. *Vaccinium* spp., *Ledum glandulosum, Phyllodoce glanduliflora*, and *Kalmia microphylla* may also be shrub associates. The herbaceous layer is a mixture of forbs and graminoids, especially sedges, including, *Erigeron* spp., *Luetkea pectinata, Antennaria lanata, Oreostemma alpigenum (= Aster alpigenus), Pedicularis* spp., *Castilleja* spp., *Deschampsia cespitosa, Caltha leptosepala, Erythronium* spp., *Juncus parryi, Luzula piperi, Carex spectabilis, Carex nigricans*, and *Polygonum bistortoides*. Fell‑fields often intermingle with the alpine dwarf‑shrubland.

SOURCES

**References:** Anderson 1999a, Bamberg 1961, Bamberg and Major 1968, Comer et al. 2003*, Cooper et al. 1997, Douglas and Bliss 1977, Ecosystems Working Group 1998, Komarkova 1976, Komarkova 1980, Meidinger and Pojar 1991, NCC 2002, Neely et al. 2001, Schwan and Costello 1951, Shiflet 1994, Thilenius 1975, WNHP unpubl. data 2018, Willard 1963

**Version:** 01 Sep 2005	**Stakeholders:** Canada, West

**Concept Author:** M.S. Reid	**LeadResp:** West

CES306.811 Rocky Mountain Alpine Fell‑Field

**Primary Division:** Rocky Mountain (306)

**Land Cover Class:** Herbaceous

**Spatial Scale & Pattern:** Large patch

**Required Classifiers:** Natural/Semi‑natural; Vegetated (>10% vasc.); Upland

**Diagnostic Classifiers:** Alpine/AltiAndino [Alpine/AltiAndino]; Herbaceous; Ridge/Summit/Upper Slope; Oligotrophic Soil; Very Shallow Soil; Mineral: W/ A‑Horizon <10 cm; Very Short Disturbance Interval; W‑Patch/High Intensity; Cushion plants; Alpine Slopes

**National Mapping Codes:** EVT 2143; ESLF 7116; ESP 1143

**Concept Summary:** This ecological system is found discontinuously at alpine elevations throughout the Rocky Mountains, west into the mountainous areas of the Great Basin, and north into the Canadian Rockies. Small areas are represented in the west side of the Okanagan Ecoregion in the eastern Cascades. These are wind‑scoured fell‑fields that are free of snow in the winter, such as ridgetops and exposed saddles, exposing the plants to severe environmental stress. Soils on these windy unproductive sites are shallow, stony, low in organic matter, and poorly developed; wind deflation often results in a gravelly pavement. Most fell‑field plants are cushioned or matted, frequently succulent, flat to the ground in rosettes and often densely haired and thickly cutinized. Plant cover is 15‑50%, while exposed rocks make up the rest. Fell‑fields are usually within or adjacent to alpine tundra dry meadows. Common species include *Arenaria capillaris, Geum rossii, Kobresia myosuroides, Minuartia obtusiloba, Myosotis asiatica, Paronychia pulvinata, Phlox pulvinata, Sibbaldia procumbens, Silene acaulis, Trifolium dasyphyllum*, and *Trifolium parryi*.

**Comments:** Alpine fell‑fields in the Cascades occur at a very small‑scale spatial pattern not mappable (recognizable) at landscape levels. These small‑scale fell‑fields are conceptually included here.

DISTRIBUTION

**Range:** This system is found discontinuously at alpine elevations throughout the Rocky Mountains, west into the mountainous areas of the Great Basin. Outlier sites occur in the northeastern Cascades and on Mount Rainier in Washington.

**Divisions:** 304:C, 306:C

**TNC Ecoregions:** 7:C, 8:C, 9:C, 11:C, 20:C, 21:C, 68:C

**Nations:** CA, US

**Subnations:** AB, AK?, BC, CO, ID, MT, NM, NV, OR, UT, WA, WY

**Map Zones:** 1:P, 9:P, 10:C, 12:?, 16:C, 17:C, 19:C, 20:?, 21:C, 23:P, 24:P, 25:C, 28:C

**USFS Ecomap Regions:** 331J:CC, 341G:PP, M242B:CP, M242C:CC, M242D:CC, M331A:CC, M331B:CC, M331D:CC, M331E:CC, M331F:CC, M331G:CC, M331H:CC, M331I:CC, M331J:CC, M332A:CC, M332B:CC, M332D:CC, M332E:CC, M332F:CC, M333A:CC, M333B:CC, M333C:CC, M333D:CC, M341A:PP, M341B:PP, M341C:PP

CONCEPT

SOURCES

**References:** Bamberg 1961, Bamberg and Major 1968, Comer et al. 2003*, Cooper et al. 1997, Douglas and Bliss 1977, Hamann 1972, Komarkova 1976, Komarkova 1980, Meidinger and Pojar 1991, NCC 2002, Neely et al. 2001, Shiflet 1994, WNHP unpubl. data 2018, Willard 1963

**Version:** 07 Sep 2005	**Stakeholders:** Canada, West

**Concept Author:** M.S. Reid	**LeadResp:** West

CES306.816 Rocky Mountain Alpine Turf

**Primary Division:** Rocky Mountain (306)

**Land Cover Class:** Herbaceous

**Spatial Scale & Pattern:** Large patch

**Required Classifiers:** Natural/Semi‑natural; Vegetated (>10% vasc.); Upland

**Diagnostic Classifiers:** Alpine/AltiAndino [Alpine/AltiAndino]; Oligotrophic Soil; Very Shallow Soil; Mineral: W/ A‑Horizon <10 cm; Aridic; Very Long Disturbance Interval; Graminoid; Alpine Slopes

**National Mapping Codes:** EVT 2144; ESLF 7117; ESP 1144

**Concept Summary:** This widespread ecological system occurs above upper treeline throughout the Rocky Mountain cordillera, including alpine areas of ranges in Utah and Nevada, and isolated alpine sites in the northeastern Cascades. It is found on gentle to moderate slopes, flat ridges, valleys, and basins, where the soil has become relatively stabilized and the water supply is more or less constant. Vegetation in these areas is controlled by snow retention, wind desiccation, permafrost, and a short growing season. This system is characterized by a dense cover of low‑growing, perennial graminoids and forbs. Rhizomatous, sod‑forming sedges are the dominant graminoids, and prostrate and mat‑forming plants with thick rootstocks or taproots characterize the forbs. Dominant species include *Artemisia arctica, Carex elynoides, Carex siccata, Carex scirpoidea, Carex nardina, Carex rupestris, Festuca brachyphylla, Festuca idahoensis, Geum rossii, Kobresia myosuroides, Phlox pulvinata*, and *Trifolium dasyphyllum*. Many other graminoids, forbs, and prostrate shrubs can also be found, including *Calamagrostis purpurascens, Deschampsia cespitosa, Dryas octopetala, Leucopoa kingii, Poa arctica, Saxifraga* spp., *Selaginella densa, Sibbaldia procumbens, Silene acaulis, Solidago* spp., and *Trifolium parryi*. Although alpine dry tundra is the matrix of the alpine zone, it typically intermingles with alpine bedrock and scree, ice field, fell‑field, alpine dwarf‑shrubland, and alpine/subalpine wet meadow systems.

DISTRIBUTION

**Range:** This system occurs above upper treeline throughout the North American Rocky Mountain cordillera, including alpine areas of ranges in Utah and Nevada, central Wyoming, and isolated alpine sites in the northeastern Cascades.

**Divisions:** 204:P, 306:C

**TNC Ecoregions:** 7:C, 8:C, 9:C, 11:C, 20:C, 21:C, 68:C

**Nations:** CA, US

**Subnations:** AB, AK?, AZ, BC, CO, ID, MT, NM, NV, OR, UT, WA, WY

**Map Zones:** 1:P, 7:P, 9:?, 10:C, 12:P, 16:C, 17:C, 18:?, 19:C, 20:?, 21:C, 22:?, 23:C, 24:C, 25:C, 28:C, 29:C

**USFS Ecomap Regions:** 341E:PP, 341G:PP, 342B:PP, 342J:PP, M242D:PP, M331A:CC, M331B:CC, M331D:CC, M331E:CC, M331F:CC, M331G:CC, M331H:CC, M331I:CC, M331J:CC, M332A:CC, M332B:CP, M332D:CC, M332E:CC, M332F:CC, M332G:CC, M333A:CC, M333B:CC, M333C:CC, M333D:CC, M341A:CC, M341B:CP, M341C:CC, M341D:CC

CONCEPT

**Environment:** This widespread ecological system occurs above upper treeline throughout the Rocky Mountain cordillera, including alpine areas of ranges in Utah and Nevada, and isolated alpine sites in the northeastern Cascades. It is found on gentle to moderate slopes, flat ridges, valleys, and basins, where the soil has become relatively stabilized and the water supply is more‑or‑less constant. Vegetation in these areas is controlled by snow retention, wind desiccation, permafrost, and a short growing season. Stands in Great Basin ranges are often less extensive and sometimes patchy because of more xeric conditions. Adjacent systems include Rocky Mountain Alpine Bedrock and Scree (CES306.809) and Rocky Mountain Alpine Fell‑Field (CES306.811) with Rocky Mountain Subalpine Dry‑Mesic Spruce‑Fir Forest and Woodland (CES306.828) or Rocky Mountain Subalpine‑Montane Limber‑Bristlecone Pine Woodland (CES306.819) near treeline. Inclusions of the small‑patch Rocky Mountain Alpine‑Montane Wet Meadow (CES306.812) occur in wet areas below snow deposition areas and alpine basins. The environmental description is based on several references, including Cox (1933), Schwan and Costello (1951), Bamberg (1961), Willard (1963), Bamberg and Major (1968), Lewis (1970), Thilenius (1975), Komarkova (1976, 1980), Douglas and Bliss (1977), Baker (1980a), Hess (1981), Meidinger and Pojar (1991), Zwinger and Willard (1996), Cooper et al. (1997), Ecosystems Working Group (1998), Reid et al. (1999), Neely et al. (2001), NCC (2002), and NatureServe (2011).

**Vegetation:** The vegetation of this system is characterized by moderate to dense cover of low‑growing, perennial graminoids and forbs. Rhizomatous, sod‑forming sedges are the dominant graminoids, and prostrate and mat‑forming plants such as *Geum rossii* with thick rootstocks or taproots characterize the forbs. Dominant species include *Artemisia arctica, Carex elynoides, Carex siccata, Carex scirpoidea, Carex nardina, Carex rupestris, Festuca brachyphylla, Festuca idahoensis, Geum rossii, Kobresia myosuroides, Phlox pulvinata*, and *Trifolium dasyphyllum*. Many other graminoids, forbs, and prostrate shrubs can also be found, including *Carex scirpoidea, Calamagrostis purpurascens, Deschampsia cespitosa, Dryas octopetala, Leucopoa kingii, Poa arctica, Saxifraga* spp., *Selaginella densa, Sibbaldia procumbens, Silene acaulis, Solidago* spp., and *Trifolium parryi*. Cushion plants are more common on shallow rocky soils near transition zones with alpine fell‑fields. Relatively mesic species such as *Carex haydeniana, Carex siccata, Leucopoa kingii, Potentilla diversifolia, Polygonum bistortoides*, and *Zigadenus elegans* are found in mesic depressions and slopes below snowfields. Some species typical of lower‑elevation grasslands, such as *Festuca idahoensis, Poa fendleriana, Poa lettermanii*, and *Pseudoroegneria spicata*, dominate some stands.

Although alpine dry tundra is the matrix of the alpine zone, it typically intermingles with alpine bedrock and scree, ice field, fell‑field, alpine dwarf‑shrubland, and alpine/subalpine wet meadow systems. The vegetation description is based on several references, including Cox (1933), Schwan and Costello (1951), Bamberg (1961), Willard (1963), Bamberg and Major (1968), Lewis (1970), Thilenius (1975), Komarkova (1976, 1980), Douglas and Bliss (1977), Baker (1980a), Hess (1981), Meidinger and Pojar (1991), Zwinger and Willard (1996), Cooper et al. (1997), Ecosystems Working Group (1998), Reid et al. (1999), Neely et al. (2001), NCC (2002), and NatureServe (2011).

**Dynamics:** Vegetation in these areas is controlled by snow retention, wind desiccation, permafrost and a short growing season. Dry summers associated with major drought years (mean return interval of 100 years) would favor grasses over forbs, whereas wet summers result in a more diverse mixture of forbs and graminoids. Dry turf dominated by graminoids such as *Carex elynoides, Carex rupestris*, or *Kobresia myosuroides* is intolerant of deep snow cover and occurs on wind‑scoured slopes and ridges (Willard 1963). These species are efficient in obtaining water due to the turf‑forming root system. Much of the soil moisture in dry turf is from summer precipitation, whereas mesic alpine slopes and meadows occur on sites with moderate snow accumulation or receive additional moisture from melting snowbeds. Lewis (1970) reports that *Carex rupestris* can send its roots under the edge of boulderfields and rock channels to obtain additional moisture.

*Kobresia myosuroides* is a major late‑seral community in the alpine (Cox 1933, Willard 1963, Hess 1981, Komarkova 1986). Willard (1963) states that the *Kobresia myosuroides* stands on Trail Ridge, Colorado, are very old. Osburn (1958b) estimates that a minimum of 100 years are necessary for the formation of 1 inch of humus soil under present alpine conditions in the Front Range of Colorado. This estimate would suggest that some stands on Trail Ridge are 800 to 1300 years old.

Native large herbivores (Rocky Mountain bighorn sheep, mule deer and elk) are common in the alpine but probably do not greatly affect vegetation cover because animals move frequently as they reduce vegetation cover. Willard (1963) and Komarkova (1976) both remark on the abundance of pocket gopher (*Thomomys talpoides fossor*) activity within stands dominated by *Carex elynoides*. They state that due to the gophers' grazing, small patches of the plant communities are left isolated. Pocket gophers also dig tunnels beneath the soil surface of *Trifolium dasyphyllum* and *Silene acaulis* stands, eating the roots and bulbs of the plants. Pocket gophers kill individual plants in the stands by clipping the roots of the vegetation or smothering the aboveground portion of the plants with soil. The freshly aerated, bare soil is invaded by species from *Carex elynoides ‑ Carex rupestris ‑ Kobresia myosuroides* Rocky Mountain Alpine Turf Alliance (A3155). *Polemonium viscosum* stands are short‑lived, however, possibly due to the loose soil substrate that is subject to erosion by wind and water (Willard 1963, Marr and Willard 1970, Zwinger and Willard 1996). Meadow voles (*Microtus* sp.) live in alpine meadows, feeding on the stems and blades of graminoids. When vole populations are high, however, the small mammals also feed on cushion plants, shredding the centers of *Silene acaulis* and *Trifolium nanum*. Seedlings from erect‑form species, such as *Geum rossii*, become established in the dead parts of the cushion plants. Once established, the erect‑form species shade and outcompete the remaining cushion plants. Willard (1963) suggests that *Geum rossii* stands may be expanding into adjacent cushion plant communities.<br />

Very small burns of a few square meters (replacement fire) caused by frequent lightning strikes may occur as a rare disturbance where there is enough fuel buildup. Lewis (1970) reports that *Carex rupestris* can send its roots under the edge of boulderfields and rock channels to obtain additional moisture.

SOURCES

**References:** Baker 1980a, Bamberg 1961, Bamberg and Major 1968, Comer et al. 2003*, Cooper et al. 1997, Cox 1933, Douglas and Bliss 1977, Ecosystems Working Group 1998, Hess 1981, Komarkova 1976, Komarkova 1980, Komarkova 1986, Lewis 1970, Marr and Willard 1970, Meidinger and Pojar 1991, NCC 2002, NatureServe Explorer 2011, Neely et al. 2001, Osburn 1958b, Reid et al. 1999, Schwan and Costello 1951, Shiflet 1994, Thilenius 1975, WNHP unpubl. data 2018, Willard 1963, Zwinger and Willard 1996

**Version:** 02 Apr 2014	**Stakeholders:** Canada, West

**Concept Author:** M.S. Reid	**LeadResp:** West

M101. Vancouverian Alpine Tundra

CES204.853 North Pacific Alpine and Subalpine Bedrock and Scree

**Primary Division:** North American Pacific Maritime (204)

**Land Cover Class:** Barren

**Spatial Scale & Pattern:** Large patch

**Required Classifiers:** Natural/Semi‑natural; Vegetated (>10% vasc.); Upland

**Diagnostic Classifiers:** Alpine/AltiAndino; Talus (Substrate); Rock Outcrops/Barrens/Glades; Oligotrophic Soil; Very Shallow Soil; Alpine Slopes

**National Mapping Codes:** EVT 2734; ESLF 3118; ESP 1734

**Concept Summary:** This ecological system includes all the exposed rock and rubble above the forest line (subalpine parkland and above) in the North Pacific mountain ranges and is restricted to the highest elevations in the Cascade Range, from southwestern British Columbia south into northern California, and also north into southeastern Alaska. It is composed of barren and sparsely vegetated alpine substrates, typically including both bedrock outcrops and scree slopes, upper mountain slopes, summits and nunataks. Nonvascular‑ (lichen‑) dominated communities are common. Exposure to desiccating winds, rocky and sometimes unstable substrates, and a short growing season limit plant growth. In Alaska, this system usually occurs above alpine dwarf‑shrub, herbaceous meadow, and dwarf‑shrub‑herbaceous systems typically at elevations higher than 915 m (3000 feet) (possibly higher in southeastern Alaska). There can be sparse cover of forbs, grasses, lichens, shrubs and small trees, but the total vascular plant cover is typically less than 25% due to the high cover of exposed rock. Species composition is variable and may include *Artemisia arctica, Astragalus alpinus, Carex microchaeta, Minuartia arctica, Salix rotundifolia, Saxifraga sibirica (= Saxifraga bracteata), Saxifraga bronchialis, Sibbaldia procumbens*, and *Silene acaulis*. Common nonvascular genera include *Racomitrium* and *Stereocaulon*.

**Comments:** This system now includes the type known as Maritime High Alpine Herbaceous by the Alaska Natural Heritage Program.

DISTRIBUTION

**Range:** This ecological system is restricted to the highest elevations in the North Pacific ranges, from southeastern Alaska south into northern California.

**Divisions:** 204:C

**TNC Ecoregions:** 1:C, 2:C, 3:C, 4:P, 69:C, 70:C, 81:C

**Nations:** CA, US

**Subnations:** AK, BC, CA, OR, WA:S4?

**Map Zones:** 1:C, 7:C, 77:C, 78:C

**USFS Ecomap Regions:** 342I:PP, M242A:CC, M242B:CC, M242C:CC, M242D:CC

CONCEPT

SOURCES

**References:** Comer et al. 2003*, Ecosystems Working Group 1998, Meidinger and Pojar 1991, Viereck et al. 1992, WNHP unpubl. data 2018

**Version:** 10 Dec 2008	**Stakeholders:** Canada, West

**Concept Author:** R. Crawford	**LeadResp:** West

CES204.862 North Pacific Dry and Mesic Alpine Dwarf‑Shrubland, Fell‑Field and Meadow

**Primary Division:** North American Pacific Maritime (204)

**Land Cover Class:** Shrubland

**Spatial Scale & Pattern:** Large patch

**Required Classifiers:** Natural/Semi‑natural; Vegetated (>10% vasc.); Upland

**Diagnostic Classifiers:** Alpine/AltiAndino [Alpine/AltiAndino]; Shrubland (Shrub‑dominated)

**National Mapping Codes:** EVT 2068; ESLF 5205; ESP 1068

**Concept Summary:** This ecological system occurs above the environmental limit of trees, at the highest elevations of the mountain regions of the Pacific Northwest coast. It is confined to the coldest, wind‑blown areas above treeline and above the subalpine parkland. This system is found at elevations above 2350 m (7200 feet) in the Klamath Mountains and Cascades north into the Cascade Range and Coast Mountains of British Columbia. It is commonly composed of a mosaic of plant communities with characteristic species including *Cassiope mertensiana, Phyllodoce empetriformis, Phyllodoce glanduliflora, Luetkea pectinata, Saxifraga tolmiei*, and *Carex* spp. It occurs on slopes and in depressions where snow lingers, the soil has become relatively stabilized, and the water supply is more or less constant. Vegetation in these areas is controlled by snow retention, wind desiccation, permafrost, and a short growing season. This system includes all vegetated areas in the alpine zone of the North Pacific. Typically it is a mosaic of dwarf‑shrublands, fell‑fields, tundra (sedge turfs), and sparsely vegetated snowbed communities. Small patches of krummholz (shrub‑form trees) are also part of this system and occur at the lower elevations. Communities are dominated by graminoids, foliose lichens, dwarf‑shrubs, and/or forbs. Vegetation cover ranges from about 5 or 10% (snowbeds) to nearly 100%. The alpine tundra of the northern Cascades has floristic affinities with many mountain regions in western North America. The strongest relationships are with the Arctic and Cordilleran regions to the north and east.

**Comments:** Alpine systems in Alaska are placed into different types than this.

DISTRIBUTION

**Range:** This system occurs above the environmental limit of trees, at the highest elevations of the mountain regions of the Pacific Northwest coast. Alpine systems in Alaska are placed into different types than this.

**Divisions:** 204:C

**TNC Ecoregions:** 1:C, 3:C, 69:?, 81:C

**Nations:** CA, US

**Subnations:** BC, OR, WA:S4

**Map Zones:** 1:C, 2:C, 7:C

**USFS Ecomap Regions:** M242A:CC, M242B:CC, M242C:CC, M242D:CC

CONCEPT

**Dynamics:** Landfire VDDT models: #RALME includes this and Rocky Mountain alpine systems.

SOURCES

**References:** BCMF 2006, Comer et al. 2003*, Ecosystems Working Group 1998, Franklin and Dyrness 1973, Holland and Keil 1995, Shiflet 1994, WNHP unpubl. data 2018

**Version:** 31 Mar 2005	**Stakeholders:** Canada, West

**Concept Author:** K. Boggs, C. Chappell, R. Crawford	**LeadResp:** West

5. Aquatic Vegetation

5.A. Saltwater Aquatic Vegetation

5.A.2. Benthic Macroalgae Saltwater Vegetation

5.A.2.Wb. Temperate Intertidal Shore

M106. Temperate Pacific Seaweed Intertidal Vegetation

CES204.879 Temperate Pacific Intertidal Flat

**Primary Division:** North American Pacific Maritime (204)

**Land Cover Class:** Barren

**Spatial Scale & Pattern:** Linear

**Required Classifiers:** Natural/Semi‑natural; Unvegetated (<10% vasc.); Wetland

**Diagnostic Classifiers:** Temperate [Temperate Hyperoceanic, Temperate Oceanic]; Tidal / Estuarine [Haline, Oligohaline]; Saline Water Chemistry

**National Mapping Codes:** EVT 2669; ESLF 3116; ESP 1669

**Concept Summary:** Coastal flats are found along the north Pacific Coast from Kodiak Island and Cook Inlet, Alaska, south to central California. Tidal flats form a narrow band along oceanic inlets and are more extensive at the mouths of larger rivers. Algae are the dominant vegetation on mud or gravel flats where little vascular vegetation is present due to the daily (in some cases twice daily) tidal flooding of salt or brackish water. Characteristic species include *Vaucheria longicaulis* and *Enteromorpha* spp. Vascular species are sparse, if present, and may include salt‑tolerant species such as *Eleocharis palustris, Salicornia* spp., *Plantago maritima, Glaux maritima*, and other plants common to lower salt marshes; cover is less than 10%. The dominant processes are tectonic uplift or subsidence, isostatic rebound, and sediment deposition.

DISTRIBUTION

**Range:** Along the north Pacific Coast from Kodiak Island and Cook Inlet, Alaska, south to central California.

**Divisions:** 105:P, 204:C

**TNC Ecoregions:** 1:C, 2:C, 69:C, 70:C, 71:C

**Nations:** CA, US

**Subnations:** AK, BC, CA, OR, WA:S3S4

**Map Zones:** 1:C, 2:C, 3:C, 4:?, 75:C, 76:C, 77:C, 78:C

**USFS Ecomap Regions:** 242A:CC, 261B:CC, 263A:CC, M242A:CC

CONCEPT

SOURCES

**References:** Boggs 2002, Comer et al. 2003*, Holland and Keil 1995, Viereck et al. 1992, WNHP unpubl. data 2018

**Version:** 21 Aug 2008	**Stakeholders:** Canada, West

**Concept Author:** K. Boggs and G. Kittel	**LeadResp:** West

5.A.3. Benthic Vascular Saltwater Vegetation

5.A.3.We. Temperate Seagrass Aquatic Vegetation

M184. Temperate Pacific Seagrass Intertidal Vegetation

CES200.882 North Pacific Maritime Eelgrass Bed

**Primary Division:** (200)

**Land Cover Class:** Herbaceous Wetland

**Spatial Scale & Pattern:** Large patch

**Required Classifiers:** Natural/Semi‑natural; Vegetated (>10% vasc.); Wetland

**Diagnostic Classifiers:** Herbaceous; Temperate [Temperate Oceanic]; Aquatic Herb

**National Mapping Codes:** EVT 2670; ESLF 9230; ESP 1670

**Concept Summary:** Eelgrass beds are found throughout the coastal areas of the North Pacific Coast, from southern Oregon (Coos Bay) north into the Gulf of Alaska, Cook Inlet, and Bristol Bay coasts. Intertidal zones are found with clear water in bays, inlets and lagoons, typically dominated by macrophytic algae and marine aquatic angiosperms along the temperate Pacific Coast. Subtidal portions are never exposed while intertidal areas support species that can tolerate exposure to the air. Common substrates include marine silts, but may also include exposed bedrock and cobble, where many algal species become attached with holdfasts. Subtidal/lower intertidal in clear water. Substrate is usually marine silts, but may be cobble. Beds are dominated by *Zostera marina*.

DISTRIBUTION

**Range:** This system is found throughout the coastal areas of the North Pacific Coast, from southern Oregon (Coos Bay) north into the Gulf of Alaska, Cook Inlet, and Bristol Bay coasts.

**Divisions:** 204:C

**TNC Ecoregions:** 1:C, 2:C, 69:C, 70:C, 71:C, 74:C

**Nations:** CA, US

**Subnations:** AK, BC, OR, WA:S3

**Map Zones:** 1:C, 2:C, 75:C, 76:C, 77:C, 78:C

**USFS Ecomap Regions:** 242A:CC, M242A:CC

CONCEPT

SOURCES

**References:** Boggs 2002, Comer et al. 2003*, Holland and Keil 1995, Viereck et al. 1992, WNHP unpubl. data 2018

**Version:** 06 Mar 2003	**Stakeholders:** Canada, West

**Concept Author:** P. Comer, G. Kittel, K. Boggs	**LeadResp:** West

5.B. Freshwater Aquatic Vegetation

5.B.2. Temperate to Polar Freshwater Aquatic Vegetation

5.B.2.Na. North American Freshwater Aquatic Vegetation

M109. Western North American Freshwater Aquatic Vegetation

CES200.876 Temperate Pacific Freshwater Aquatic Bed

**Primary Division:** (200)

**Land Cover Class:** Herbaceous Wetland

**Spatial Scale & Pattern:** Small patch

**Required Classifiers:** Natural/Semi‑natural; Vegetated (>10% vasc.); Wetland

**Diagnostic Classifiers:** Herbaceous; Depressional [Pond]; Aquatic Herb

**National Mapping Codes:** EVT 2664; ESLF 9219; ESP 1664

**Concept Summary:** Freshwater aquatic beds are found throughout the humid temperate regions of the Pacific Coast of North America. They are small patch in size, confined to lakes, ponds, oxbows, and slow‑moving portions of rivers and streams. In large bodies of water, they are usually restricted to the littoral region where penetration of light is the limiting factor for growth. A variety of rooted or floating aquatic herbaceous species may dominate, including *Azolla* spp., *Nuphar polysepala, Polygonum* spp., *Potamogeton* spp., *Ranunculus* spp., and *Wolffia* spp. Submerged vegetation, such as *Myriophyllum* spp., *Ceratophyllum* spp., and *Elodea* spp., is often present. These communities occur in water too deep for emergent vegetation.

**Comments:** The aquatic beds in the Alaska systems classification for the maritime region are included here. However, aquatic beds on Kodiak Island, Alaska, are placed into Aleutian Freshwater Aquatic Bed (CES105.234).

DISTRIBUTION

**Range:** This system is found throughout the humid temperate regions of the Pacific Coast of North America, from the Gulf of Alaska through southeastern Alaska into central California.

**Divisions:** 204:C, 206:C

**TNC Ecoregions:** 1:C, 14:C, 15:C, 69:C, 70:C, 71:C

**Nations:** CA, US

**Subnations:** AK, BC, CA, OR, WA:S3

**Map Zones:** 1:C, 2:P, 3:C, 4:C, 6:P, 7:C, 8:C, 9:C, 10:C, 77:C, 78:C

**USFS Ecomap Regions:** 242A:CC, 242B:CC, 342I:??, M242A:CC, M242B:CC, M242C:CP, M242D:CC, M261G:CC

CONCEPT

SOURCES

**References:** Boggs 2000, Boggs et al. 2008a, Chappell and Christy 2004, Comer et al. 2003*, Holland and Keil 1995, Shephard 1995, Shiflet 1994, Viereck et al. 1992, WNHP unpubl. data 2018

**Version:** 17 Oct 2008	**Stakeholders:** Canada, West

**Concept Author:** G. Kittel, P. Comer, C. Chappell, K. Boggs	**LeadResp:** West

6. Open Rock Vegetation

6.B. Temperate & Boreal Open Rock Vegetation

6.B.1. Temperate & Boreal Cliff, Scree & Other Rock Vegetation

6.B.1.Na. Eastern North American Temperate Cliff, Scree & Rock Vegetation

M111. Eastern North American Cliff & Rock Vegetation

CES202.689 Central Interior Acidic Cliff and Talus

**Primary Division:** Central Interior and Appalachian (202)

**Land Cover Class:** Barren

**Spatial Scale & Pattern:** Small patch

**Required Classifiers:** Natural/Semi‑natural; Unvegetated (<10% vasc.); Upland

**Concept Summary:** This system is found primarily in the Interior Highlands, including the Ozarks, Ouachita, and Interior Low Plateau ecoregions, extending marginally north and west along the Missouri and Mississippi rivers. Sandstone outcrops and talus ranging from moist to dry typify this system. It is typically sparsely vegetated; however, on moister sites with more soil development, several fern species and sedges (*Carex* spp.) can establish. Wind and water erosion are the major dynamic processes influencing this system.

**Comments:** In Kentucky, this system covers the sandstone cliffs of the Shawnee Hills (Interior Low Plateau). In Illinois, one exemplary example is the "Garden of the Gods" in the Shawnee National Forest.

DISTRIBUTION

**Range:** This system is found primarily in the Interior Highlands, including the Ozark, Ouachita, and Interior Low Plateau ecoregions. It extends marginally into the Central Tallgrass Prairie Ecoregion along the Missouri and Mississippi rivers.

**Divisions:** 202:C

**TNC Ecoregions:** 36:C, 38:C, 39:C, 44:C

**Nations:** US

**Subnations:** AR, IA?, IL, IN, KY, MO, TN, WI

**Map Zones:** 43:P, 44:C, 47:C, 48:C, 49:C, 53:C

**USFS Ecomap Regions:** 223A:CC, 223B:CC, 223D:CC, 223E:CC, 223F:CC, 223G:CC, 251C:CC, 251D:CC, M223A:CC, M231A:CC

CONCEPT

**Environment:** Sandstone outcrops and talus ranging from moist to dry typify this system. Examples range from sparsely to moderately well‑vegetated. Soil development is limited to cracks and ledges. Slope aspect and angle are strongly related to the amount of available moisture on a site. Steep, south‑ or west‑facing slopes are drier than less steep east‑ or north‑facing slopes. Some sites have seepage along the cliff face. Shading by adjacent forests can impact cliffs below the height of nearby trees.

**Vegetation:** This system is typically sparsely vegetated; however, on moister sites with more soil development, several fern species and sedges (*Carex* spp.) can establish. Some taxa that could be present include *Ribes cynosbati, Deschampsia flexuosa, Dryopteris marginalis*, and *Dennstaedtia punctilobula*, as well as *Carex interior, Carex lurida, Carex leptalea, Parnassia grandifolia, Rhynchospora capillacea, Osmunda cinnamomea, Rhynchospora capitellata, Heuchera parviflora var. puberula*, and *Xyris jupicai* on wetter sites.

**Dynamics:** Wind and water erosion are the major dynamic processes influencing this system.

SOURCES

**References:** Comer et al. 2003*, Evans et al. 2009, Nelson 2010, WDNR 2015

**Version:** 14 Jan 2014	**Stakeholders:** East, Midwest, Southeast

**Concept Author:** S. Menard, T. Foti, R. Evans	**LeadResp:** Midwest

CES202.690 Central Interior Calcareous Cliff and Talus

**Primary Division:** Central Interior and Appalachian (202)

**Land Cover Class:** Barren

**Spatial Scale & Pattern:** Small patch

**Required Classifiers:** Natural/Semi‑natural; Unvegetated (<10% vasc.); Upland

**Concept Summary:** This system is found primarily in non‑Appalachian portions of the "central interior division" of the United States. It ranges from the Ouachitas east to the Cumberlands and north into the Western Allegheny Plateau and Lake states. Limestone and dolomite outcrops and talus distinguish this system. Examples range from moist to dry and from sparsely to moderately well‑vegetated. Woodland species such as *Thuja occidentalis* can establish along the ridgetops, on ledges, and talus. Understory species can range from grassland species, such as *Andropogon gerardii* on drier slopes, to more mesic species in areas with higher moisture and more soil development. Wind and water erosion along with fire are the primary natural dynamics influencing this system. Some associations included here are rocky openings in forest stands, sometimes with moisture present from groundwater seepage. Also included are wet and dry cliffs. The flora of these wetter examples may include (across the broad range of the system) *Aconitum noveboracense, Adiantum capillus‑veneris, Adoxa moschatellina, Aquilegia canadensis, Asplenium rhizophyllum, Boehmeria cylindrica, Chrysosplenium alternifolium var. sibiricum, Cystopteris bulbifera, Cystopteris bulbifera, Dichanthelium depauperatum, Heuchera americana, Heuchera americana var. hirsuticaulis, Heuchera villosa var. arkansana, Hydrangea arborescens, Impatiens pallida, Lobelia siphilitica, Toxicodendron radicans*, and *Woodsia obtusa*.

**Comments:** Similar examples in the driftless region of Minnesota, Wisconsin, Iowa and Illinois should be considered part of Paleozoic Plateau Bluff and Talus (CES202.704).

DISTRIBUTION

**Range:** This system is found primarily in non‑Appalachian portions of the "central interior division" of the United States, from the Ouachitas east to the Cumberlands and north into the Western Allegheny Plateau and Great Lake states.

**Divisions:** 201:?, 202:C, 205:P

**TNC Ecoregions:** 36:C, 38:C, 39:C, 44:C, 45:C, 46:C, 47:?, 48:C, 49:C

**Nations:** US

**Subnations:** AR, IA, IL, IN, KY?, MI, MN, MO, NY, OH, OK, PA, TN, WI

**Map Zones:** 41:?, 42:P, 43:P, 44:C, 47:C, 48:C, 49:P, 50:C, 51:C, 52:C, 53:C, 61:C, 62:C, 63:C, 64:C

**USFS Ecomap Regions:** 212H:CC, 212Z:CC, 221E:CC, 221F:CC, 222H:CC, 222J:CC, 222K:CC, 222L:CC, 222M:CC, 223A:CC, 223B:CC, 223D:CC, 223E:CC, 223F:CC, 223G:CC, 251C:CC, 251D:CC, M223A:CC, M231A:CC

CONCEPT

**Environment:** Limestone and dolomite outcrops and talus distinguish this system. Examples range from moist to dry and from sparsely to moderately well‑vegetated. Soil development is limited to cracks and ledges. Slope aspect and angle are strongly related to the amount of available moisture on a site. Steep, south‑ or west‑facing slopes are drier than less steep east‑ or north‑facing slopes. Some sites have seepage along the cliff face. Shading by adjacent forests can impact cliffs below the height of nearby trees.

**Vegetation:** Examples range from moist to dry and from sparsely to moderately well‑vegetated. Woodland species such as *Thuja occidentalis* can establish along the ridgetops. Understory species can range from grassland species, such as *Andropogon gerardii* on drier slopes, to more mesic species in areas with higher moisture and more soil development. The flora of some moister examples (e.g., rocky openings in forest stands, with moisture present from groundwater seepage as well as wet cliffs) includes (across the broad range of the system) *Aconitum noveboracense, Adiantum capillus‑veneris, Adoxa moschatellina, Aquilegia canadensis, Asplenium rhizophyllum, Boehmeria cylindrica, Chrysosplenium alternifolium var. sibiricum (= Chrysosplenium iowense), Cystopteris bulbifera, Cystopteris bulbifera, Dichanthelium depauperatum, Heuchera americana, Heuchera americana var. hirsuticaulis, Heuchera villosa var. arkansana, Hydrangea arborescens, Impatiens pallida, Lobelia siphilitica, Toxicodendron radicans*, and *Woodsia obtusa*.

**Dynamics:** Wind and water erosion along with fire are the primary natural dynamics influencing this system. Fires could spread from more vegetated communities adjacent to calcareous cliffs and could burn vegetation on the edges of this community. A study in a similar cliff system in southern Ontario found no relationship between cliff patch size and diversity or richness (Haig et al. 2000).

SOURCES

**References:** Comer et al. 2003*, Evans et al. 2009, Eyre 1980, Haig et al. 2000, Nelson 2010, ONHD unpubl. data, Vanderhorst pers. comm., WDNR 2015

**Version:** 20 Aug 2015	**Stakeholders:** East, Midwest, Southeast

**Concept Author:** S. Menard	**LeadResp:** Midwest

CES202.309 Cumberland Acidic Cliff and Rockhouse

**Primary Division:** Central Interior and Appalachian (202)

**Land Cover Class:** Barren

**Spatial Scale & Pattern:** Small patch

**Required Classifiers:** Natural/Semi‑natural; Unvegetated (<10% vasc.); Upland

**Diagnostic Classifiers:** Cliff (Substrate); Acidic Soil

**Concept Summary:** This sandstone cliff ecological system is found in the Cumberland Plateau and Mountain regions of the southeastern United States. Examples are extremely steep or vertical rock faces exposed along bluffs often associated with rivers. The aspect is variable but best developed on south‑ and west‑facing sites. Plants are infrequent due to the lack of crevices capable of accumulating soil, the highly acidic nature of the bedrock, and the frequent weathering and erosion of the substrate. Lichen cover may be extensive in places, especially on the more exposed portions. These cliffs are also prone to harsh climatic conditions; frequent disturbances include drought stress and wind and storm damage. As a result, examples are characterized by sparse herbaceous cover and few, if any, trees. Vegetation consists of scattered individuals of *Asplenium montanum, Silene rotundifolia*, and other species rooted in crevices and erosion pockets. In some parts of its range, this system is the primary or sole habitat for rare endemic species, such as *Minuartia cumberlandensis* and *Ageratina luciae‑brauniae*. This system includes a mosaic of cavelike features (often called "rockhouses") and associated sandstone box canyons in the western Appalachian foothills regions of Kentucky, Alabama, West Virginia, and possibly southeastern Ohio. Where present, the rockhouses are a prominent and diagnostic feature of the system.

**Comments:** It is unclear whether or not this system should range into the Interior Low Plateau. Also debatable is whether or not wet and dry cliffs should be included as well as the number of different physical settings possible. See also Southern Appalachian Montane Cliff and Talus (CES202.330).

DISTRIBUTION

**Range:** This ecological system occurs in a limited area of the Cumberland Plateau of northern Alabama, northwestern Georgia, eastern Kentucky, eastern Tennessee, West Virginia, and possibly southwestern Virginia. Rockhouses also occur in southeastern Ohio (Rockhouse 349) and in western Pennsylvania (Walck et al. 1996) along Laurel and Chestnut Ridges of the Laurel Highlands in the Central Appalachian Plateau ecoregion (E. Zimmerman pers. comm. 2013).

**Divisions:** 202:C

**TNC Ecoregions:** 50:C

**Nations:** US

**Subnations:** AL, GA, KY, OH, PA, TN, VA?, WV

**Map Zones:** 46:C, 47:C, 48:C, 53:C, 57:C, 62:?

**USFS Ecomap Regions:** 221Ea:CCC, 221Eb:CCC, 221Ec:CCC

CONCEPT

**Environment:** The rockhouses are the most unique and diagnostic feature of the system. These unusual geologic features are created by spray and rock‑cracking from seasonal flowing waterfalls at the heads of canyons amidst thick layers of sandstone from the Mississippian and Pennsylvanian geologic periods. The ceiling of the rockhouse may be 50 m tall, and they can be as much as 50 m deep (Walck et al. 1996, A. Weakley pers. comm. 2006). They require sufficient flowing water and freezing and thawing to weather the thick beds of sandstone. These conditions seem to be restricted to the western margin of the Appalachian Plateau.

**Vegetation:** Examples of this system usually include a vegetational mosaic that includes hemlock bluffs, sandstone cliffs, or overhangs near the base of a cliff (often with a sandy area beneath the overhang which is shaded and protected from direct rainfall, as well as gladelike vegetation at the horizontal portion of the cliffs). The rockhouses in the southern parts of the range are habitats for rare vascular plant species such as *Minuartia cumberlandensis* and *Ageratina luciae‑brauniae* and sometimes support populations of rare nonvascular plants as well.

**Dynamics:** Within rockhouses, there are three distinct habitats: ceiling, backwall, and floor. The dripline defines the outer edge of the ceiling and floor. The ceiling generally slopes back from the dripline to the backwall, which is deeply shaded and generally stays moist. Shading in the rockhouse is greater in the summer when deciduous trees in front of the rockhouse are leafed out. Light levels vary from very low at the backwall (Farrar 1998) to relatively high, especially in winter, when leaves are down (Walck et al. 1996). The combination of shade and the stable and moderate microclimate of rockhouses has maintained a habitat suitable for unusual, disjunct and endemic plants (Walck et al. 1996), including tropical ferns and bryophytes (Farrar 1998) which may have persisted in these habitats since pre‑Pleistocene times when there was a tropical or subtropical climate (Farrar 1998). Temperatures inside rockhouses are higher in the winter and lower in the summer than outside the rockhouses, and while rockhouse habitats are protected from rain, they tend to have higher humidities than the surrounding areas (Walck et al. 1996).

SOURCES

**References:** Comer et al. 2003*, Evans et al. 2009, Eyre 1980, Farrar 1998, Walck et al. 1996, Weakley pers. comm.

**Version:** 14 Jan 2014	**Stakeholders:** East, Midwest, Southeast

**Concept Author:** R. Evans	**LeadResp:** Southeast

CES203.492 East Gulf Coastal Plain Dry Chalk Bluff

**Primary Division:** Gulf and Atlantic Coastal Plain (203)

**Land Cover Class:** Barren

**Spatial Scale & Pattern:** Linear

**Required Classifiers:** Natural/Semi‑natural; Unvegetated (<10% vasc.); Upland

**Diagnostic Classifiers:** Cliff (Substrate)

**Concept Summary:** The ecological system is endemic to the Black Belt region of Alabama and Mississippi. Examples are relatively sheer surfaces of exposed chalk. Some are generally devoid of vegetation. In most cases these bluffs extend directly to the edge of rivers or streams.

DISTRIBUTION

**Range:** This system is endemic to the Black Belt region of Alabama and Mississippi.

**Divisions:** 203:C

**TNC Ecoregions:** 43:C

**Nations:** US

**Subnations:** AL, MS

**Map Zones:** 46:C

**USFS Ecomap Regions:** 231B:CC

CONCEPT

**Vegetation:** More information is needed. See Gunn (1985) and Morris et al. (1993).

SOURCES

**References:** Comer et al. 2003*, Gunn 1985, Morris et al. 1993

**Version:** 17 Mar 2009	**Stakeholders:** Southeast

**Concept Author:** A. Schotz and R. Evans	**LeadResp:** Southeast

CES201.025 Great Lakes Acidic Rocky Shore and Cliff

**Primary Division:** Laurentian‑Acadian (201)

**Land Cover Class:** Barren

**Spatial Scale & Pattern:** Linear

**Required Classifiers:** Natural/Semi‑natural; Unvegetated (<10% vasc.); Upland

**Diagnostic Classifiers:** Coast; Igneous Rock

**Concept Summary:** This system is found in the Great Lakes region of the U.S. and Canada where exposed bedrock dominates the shoreline. The bedrock may consist of acidic igneous, metamorphic, or sedimentary rock. Some bedrock shorelines are solid rock, others more cobbly or fragmented. The bedrock may be relatively horizontal or tilted, rounded or blocky, and sometimes cliff‑like. The leading edge of the shoreline may be heavily impacted by wave action and winter ice movement, decreasing in effect with distance inland. Vegetation varies from sparse nonvascular vegetation to open‑treed or shrubby communities along the same transect.

DISTRIBUTION

**Range:** Found in the Great Lakes region of the U.S. and Canada, where exposed bedrock dominates the shoreline.

**Divisions:** 201:C

**TNC Ecoregions:** 48:C

**Nations:** CA, US

**Subnations:** MI, MN, ON, WI

**Map Zones:** 41:C, 50:C, 51:C

**USFS Ecomap Regions:** 212Lb:CCC, 212Ra:CCP, 212Rd:CCP, 212Sb:CCC, 212Sc:CCC, 212Sn:CCC, 212Sq:CCC, 212Ya:CCC, 222Ud:CCC

CONCEPT

SOURCES

**References:** Albert et al. 1995, Comer et al. 2003*, Kost et al. 2007, WDNR 2015

**Version:** 25 Mar 2003	**Stakeholders:** Canada, East, Midwest

**Concept Author:** D. Albert	**LeadResp:** Midwest

CES201.569 Laurentian‑Acadian Acidic Cliff and Talus

**Primary Division:** Laurentian‑Acadian (201)

**Land Cover Class:** Barren

**Spatial Scale & Pattern:** Small patch

**Required Classifiers:** Natural/Semi‑natural; Vegetated (>10% vasc.); Upland

**Diagnostic Classifiers:** Ridge/Summit/Upper Slope; Cliff (Substrate); Talus (Substrate); Acidic Soil; Landslide

**Concept Summary:** This cliff system occurs at low to mid elevations, well below treeline, from New England west to the Great Lakes. It consists of vertical or near‑vertical cliffs and the talus slopes below, formed on hills of granitic or otherwise acidic bedrock. Most of the substrate is dry and exposed, but small (occasionally large) areas of seepage are often present. Vegetation in seepage areas tends to be more well‑developed and floristically different from the surrounding dry cliffs. The vegetation is patchy and often sparse, punctuated with patches of small trees (e.g., *Betula* and *Picea* spp.). Calciphilic species are absent. In north‑facing or other sheltered settings where cold air accumulates at the bottom of slopes, a shrubland of heaths and reindeer lichens can develop. This system differs from the more southerly North‑Central Appalachian Acidic Cliff and Talus (CES202.601) in the more boreal affinities of its flora, for example *Picea* spp. rather than *Juniperus virginiana*.

DISTRIBUTION

**Range:** This system is found in New England and adjacent Canada west to the Great Lakes.

**Divisions:** 201:C, 202:C

**TNC Ecoregions:** 47:C, 48:C, 61:C, 63:C

**Nations:** CA, US

**Subnations:** MA?, ME, MI, MN, NH, NY, VT, WI

**Map Zones:** 41:C, 50:C, 51:C, 63:C, 64:C, 65:C, 66:C

**USFS Ecomap Regions:** 212Jb:CCC, 212Jc:CCP, 212Jo:CCP, 212K:CC, 212Lb:CPP, 212M:CC, 212Q:CC, 212Ra:CCC, 212Sb:CCC, 212Sc:CCP, 212Sn:CCP, 212Sq:CCC, 212Tb:CCP, 212Tc:CCP, 212X:CP, 212Ya:CCC, 222Jc:CCC

CONCEPT

SOURCES

**References:** Comer et al. 2003*, Edinger et al. 2014a, Eyre 1980, Gawler and Cutko 2010, Kost et al. 2007, Sperduto and Nichols 2004, WDNR 2015

**Version:** 05 Oct 2004	**Stakeholders:** Canada, East, Midwest

**Concept Author:** S.C. Gawler	**LeadResp:** East

CES201.570 Laurentian‑Acadian Calcareous Cliff and Talus

**Primary Division:** Laurentian‑Acadian (201)

**Land Cover Class:** Barren

**Spatial Scale & Pattern:** Small patch

**Required Classifiers:** Natural/Semi‑natural; Unvegetated (<10% vasc.); Upland

**Diagnostic Classifiers:** Ridge/Summit/Upper Slope; Cliff (Substrate); Talus (Substrate); Alkaline Soil; Circumneutral Soil

**Concept Summary:** This cliff system occurs at low to mid elevations, well below treeline, from New England west to the Great Lakes. It consists of vertical or near‑vertical cliffs and the talus slopes below, where weathering and/or bedrock chemistry produce circumneutral to calcareous pH and enriched nutrient availability. The vegetation is often sparse but may include patches of small trees. *Thuja occidentalis* may dominate on some cliffs (and reach very old ages, upwards of 1000 years). *Fraxinus* spp. and *Tilia americana* are woody indicators of the enriched setting.

DISTRIBUTION

**Range:** This system is found in scattered locations from New England and adjacent Canada west to the Great Lakes and northern Minnesota

**Divisions:** 201:C

**TNC Ecoregions:** 47:C, 48:C, 63:C

**Nations:** US

**Subnations:** ME, MI, MN, NH, NY, VT, WI

**Map Zones:** 41:C, 50:C, 51:C, 63:C, 64:C, 66:C

**USFS Ecomap Regions:** 212Hl:CCC, 212Jb:CCC, 212Jc:CCP, 212Jo:CCP, 212Lb:CPP, 212Q:CC, 212Ra:CCC, 212Rc:CCC, 212Re:CCC, 212Sb:CCC, 212Sc:CCC, 212Sn:CCP, 212Sq:CCC, 212Tb:CCC, 212Tc:CCP, 212Tf:CCC, 212X:CP, 212Ya:CCP, 212Z:CC, 222Jc:CCC

CONCEPT

**Vegetation:** *Thuja occidentalis* may dominate on some cliffs (and reach very old ages, upwards of 1000 years). *Fraxinus* spp. and *Tilia americana* are woody indicators of the enriched setting (Kelly and Larson 1997).

SOURCES

**References:** Comer et al. 2003*, Edinger et al. 2014a, Eyre 1980, Gawler and Cutko 2010, Kelly and Larson 1997, Kost et al. 2007, Sperduto and Nichols 2004, WDNR 2015

**Version:** 09 Jan 2003	**Stakeholders:** East, Midwest

**Concept Author:** S.C. Gawler	**LeadResp:** East

CES202.601 North‑Central Appalachian Acidic Cliff and Talus

**Primary Division:** Central Interior and Appalachian (202)

**Land Cover Class:** Barren

**Spatial Scale & Pattern:** Small patch

**Required Classifiers:** Natural/Semi‑natural; Unvegetated (<10% vasc.); Upland

**Diagnostic Classifiers:** Cliff (Substrate); Talus (Substrate); Temperate; Acidic Soil

**Concept Summary:** This system comprises sparsely vegetated to partially wooded cliffs and talus slopes in the Central Appalachians and adjacent ecoregions, occurring on rocks of acidic lithology and lacking any indicators of enriched conditions. This cliff system occurs at low to mid elevations from central New England south to Virginia, and up to 1500 m in West Virginia. It consists of vertical or near‑vertical cliffs and the talus slopes below, formed on hills of granitic, sandstone, or otherwise acidic bedrock. In some cases, especially in periglacial areas, this system may take the form of upper‑slope boulderfields without adjacent cliffs, where talus forms from freeze/thaw action cracking the bedrock. Most of the substrate is dry and exposed, but small (occasionally large) areas of seepage are often present. Vegetation in seepage areas tends to be more well‑developed and floristically different from the surrounding dry cliffs. The vegetation is patchy and often sparse, punctuated with patches of small trees that may form woodlands in places. *Juniperus virginiana* is a characteristic tree species, *Toxicodendron radicans* a characteristic woody vine, and *Polypodium virginianum* a characteristic fern. Within its range, *Pinus virginiana* is often present.

**Comments:** More complete data are needed to clarify the diagnostic differences between this system and similar systems to the north, south, and west: Laurentian‑Acadian Acidic Cliff and Talus (CES201.569), Cumberland Acidic Cliff and Rockhouse (CES202.309), and Central Interior Acidic Cliff and Talus (CES202.689).

DISTRIBUTION

**Range:** This system is found from central New England and New York south to Virginia.

**Divisions:** 202:C

**TNC Ecoregions:** 49:C, 52:?, 59:C, 60:C, 61:C

**Nations:** US

**Subnations:** CT, MA, MD, NH, NJ, NY, OH, PA, VA, WV

**Map Zones:** 60:C, 61:C, 62:C, 63:P, 64:P, 65:C

**USFS Ecomap Regions:** 221E:CC, M221A:CC, M221B:CC, M221D:CC

CONCEPT

**Environment:** This cliff system consists of vertical or near‑vertical cliffs at low to mid elevations and the talus slopes below, formed on hills of granitic, sandstone, or otherwise acidic bedrock. Most of the substrate is dry and exposed, but small (occasionally large) areas of seepage are often present.

**Vegetation:** Vegetation in seepage areas tends to be more well‑developed and floristically different from the surrounding dry cliffs. The vegetation is patchy and often sparse, punctuated with patches of small trees that may form woodlands in places. *Juniperus virginiana* is a characteristic tree species, *Toxicodendron radicans* a characteristic woody vine, and *Polypodium virginianum* a characteristic fern. Additional species that may be present include woody plants *Betula alleghaniensis, Betula lenta, Hydrangea arborescens, Quercus montana (= Quercus prinus)*, and *Quercus rubra*; the vine *Parthenocissus quinquefolia*; herbaceous plants *Asplenium montanum, Corydalis sempervirens, Polypodium virginianum*, and *Sedum ternatum*; and the lichens *Chrysothrix chlorina, Dimelaena oreina, Lasallia papulosa, Lasallia pensylvanica, Melanelia culbersonii, Melanelia stygia, Stereocaulon glaucescens, Umbilicaria mammulata*, and *Umbilicaria muhlenbergii*.

**Dynamics:** Periodic rockslides maintain the open character of this system. Fire is generally not an important factor, since steep slopes and rockslides prevent extensive vegetation development, limiting litter accumulation.

SOURCES

**References:** Comer et al. 2003*, Edinger et al. 2014a, Eyre 1980, ONHD unpubl. data, Sperduto and Nichols 2004

**Version:** 02 Jan 2015	**Stakeholders:** East, Midwest, Southeast

**Concept Author:** S.C. Gawler	**LeadResp:** East

CES202.603 North‑Central Appalachian Circumneutral Cliff and Talus

**Primary Division:** Central Interior and Appalachian (202)

**Land Cover Class:** Barren

**Spatial Scale & Pattern:** Small patch

**Required Classifiers:** Natural/Semi‑natural; Vegetated (>10% vasc.); Upland

**Diagnostic Classifiers:** Cliff (Substrate); Talus (Substrate); Temperate; Alkaline Soil

**Concept Summary:** This cliff system occurs at low to mid elevations from central New England south to Virginia and West Virginia. It consists of vertical or near‑vertical cliffs and steep talus slopes where weathering and/or bedrock lithology produce circumneutral to calcareous pH and enriched nutrient availability. Substrates include limestone, dolomite and other rocks. The vegetation varies from sparse to patches of small trees, in places forming woodland or even forest vegetation. *Fraxinus americana, Tilia americana*, and *Staphylea trifolia* are woody indicators of the enriched setting. *Thuja occidentalis* may occasionally be present but is more characteristic of the related Laurentian‑Acadian system to the north. The herb layer is typically not extensive but includes at least some species that are indicators of enriched conditions, e.g., *Impatiens pallida, Pellaea atropurpurea, Asplenium platyneuron*, or *Woodsia obtusa*.

DISTRIBUTION

**Range:** This system ranges from central New England and New York south to Virginia and West Virginia. The extent of the Virginia range remains to be documented, but it appears to be absent from the Southern Blue Ridge and Southern Ridge and Valley portions of the state.

**Divisions:** 202:C

**TNC Ecoregions:** 52:C, 59:P, 60:?, 61:C

**Nations:** US

**Subnations:** CT, MA, MD, NH, NJ, NY, OH, PA, VA, VT, WV

**Map Zones:** 53:C, 59:P, 61:C, 62:?, 63:P, 64:C, 65:C, 66:P

**USFS Ecomap Regions:** 221Ae:CCP, 221Af:CCP, 221Ag:CCP, 221B:CC, 221D:CC, 221E:CC, M221A:CC, M221B:CC

CONCEPT

**Environment:** This cliff system occurs at low to mid elevations on vertical or near‑vertical cliffs and steep talus slopes where weathering and/or bedrock lithology produce circumneutral to calcareous pH and enriched nutrient availability. Substrates include limestone, dolomite and other rocks.

**Vegetation:** *Fraxinus americana, Tilia americana*, and *Staphylea trifolia* are woody indicators of the enriched setting. *Thuja occidentalis* may occasionally be present but is more characteristic of the related Laurentian‑Acadian system to the north. The herb layer is typically not extensive but includes at least some species that are indicators of enriched conditions, e.g., *Impatiens pallida, Pellaea atropurpurea, Asplenium platyneuron*, or *Woodsia obtusa*.

SOURCES

**References:** Comer et al. 2003*, Edinger et al. 2014a, Eyre 1980, Vanderhorst pers. comm.

**Version:** 02 Jan 2015	**Stakeholders:** East, Midwest, Southeast

**Concept Author:** S.C. Gawler	**LeadResp:** East

CES202.330 Southern Appalachian Montane Cliff and Talus

**Primary Division:** Central Interior and Appalachian (202)

**Land Cover Class:** Barren

**Spatial Scale & Pattern:** Small patch

**Required Classifiers:** Natural/Semi‑natural; Vegetated (>10% vasc.); Upland

**Diagnostic Classifiers:** Moss/Lichen (Nonvascular); Cliff (Substrate)

**Concept Summary:** This system consists of steep to vertical or overhanging rock outcrops (and related steep talus slopes) of the Southern Blue Ridge and adjacent parts of other ecoregions. It occurs on lower slopes, usually in river gorges or bluffs. The sparse vegetation is limited to plants growing on bare rock, small ledges, and crevices. Vegetation is primarily bryophytes, lichens, and herbs, with sparse trees and shrubs rooted in deeper soil pockets and crevices.

**Comments:** This system is distinguished from other rock outcrops by a combination of low topographic position, vertical orientation, large amount of bare rock, and absence of specialized environments such as exfoliated granite, limestone or dolomite, and spray from waterfalls. In contrast, Southern Appalachian Rocky Summit (CES202.327) occurs in high topographic positions; they have more horizontal rock but may have some substantial vertical surfaces. Southern and Central Appalachian Mafic Glade and Barrens (CES202.348) are more horizontally oriented and have much more vegetation cover. Southern Appalachian Granitic Dome (CES202.297) may have steep portions but has smooth, unfractured rock surfaces with soil largely confined to mats adhering to the rock surface. The division of rock outcrop systems may be too fine and warrant combining some; however, each system has distinctive characteristics of structure and some distinctive flora.

The primary variation within this system, which could be the basis for further subdivision, is the distinction between mafic and felsic rock. The distribution north and west needs review. See also Cumberland Acidic Cliff and Rockhouse (CES202.309).

DISTRIBUTION

**Range:** Scattered throughout the Southern Appalachians and incidentally into adjacent ecoregions, from northern Alabama and Georgia through Virginia.

**Divisions:** 202:C

**TNC Ecoregions:** 50:?, 51:C, 52:C

**Nations:** US

**Subnations:** GA, KY, NC, SC, TN, VA

**Map Zones:** 48:?, 53:P, 54:C, 57:C, 59:C, 61:C

**USFS Ecomap Regions:** 231Ag:CCC, 231Ia:CCC, M221Db:CCC, M221Dc:CCC, M221Dd:CCC

CONCEPT

**Environment:** This system occurs on steep rock outcrops on lower slopes and occasionally higher in topographically sheltered sites. River gorges are probably the most common landforms, with bluffs of more open river valleys or meandering rivers also common. The substrate is mostly bare bedrock, which is steep to vertical or overhanging. Most examples are on felsic metamorphic rock such as gneiss or schist, a smaller number on mafic metamorphic rock or felsic or mafic igneous rock. [Examples may occur on any kind of rock except limestone and dolomite, with felsic metamorphic rock the most common in the Southern Blue Ridge and sandstone the most common in the Cumberland Mountains. Mafic metamorphic rocks form a less common but important fraction of examples, along with some more extreme rocks such as quartzite.] The physical structure of cliffs of metamorphic rock is usually irregular, with some ledges and crevices. [Sedimentary rocks often form more vertical cliffs, but with bedding planes and joints forming deep crevices that provide rooting sites.] Moisture levels vary drastically over short distances. Seepage of groundwater from adjacent soils or through rock fractures often creates permanently or seasonally flooded microsites, while lack of soil makes other portions extremely dry. In less sheltered topography, slope aspect affects overall moisture levels to some degree. Rock or soil chemistry appears to be the most important factor affecting different associations on sites that have the physical structure to belong to this system. Elevation may also be an important factor causing variation, though few examples are known at high elevation.

**Vegetation:** Vegetation is sparse. Bryophytes and lichens may cover portions of the open rock. Vascular plants are limited to sparse rooting sites in soil pockets, ledges, and crevices. Some of these microsites may be deep enough to support shrubs or even stunted trees, while most support only herbs. The woody plants are usually species from surrounding forests, and may be mesophytic or xerophytic. The herbs include a suite of rock outcrop specialists such as *Saxifraga michauxii, Hylotelephium telephioides, Asplenium montanum*, and *Polypodium* spp. Mafic rock outcrops have an additional suite of specialist herbs, a number of them rare. Herbs from the surrounding forest are often also present and may make up a significant fraction of the flora.

**Dynamics:** The dynamics of this system have received little study. Most cliff communities are probably stable over long periods of time, with fine‑scale disturbances affecting microsites. Rock falls, slides, and other mass movement are rare, but represent catastrophic disturbance to part or all of a cliff, and may be important in the long term for keeping cliffs open. Animal movements may be locally important. Fire probably has little effect on cliffs, which have too little vegetation to carry fire and which tend to occur in topography that is not conducive to fire spread. Because of the limited natural disturbance and the fragility of soil and vegetation, human disturbance by trampling edges and by climbing may be particularly destructive.

SOURCES

**References:** Comer et al. 2003*, DuMond 1970, Evans et al. 2009, Nelson 1986, Schafale 2012, Schafale and Weakley 1990, Simon 2015

**Version:** 29 Apr 2016	**Stakeholders:** East, Southeast

**Concept Author:** M. Schafale and R. Evans	**LeadResp:** Southeast

CES202.356 Southern Interior Calcareous Cliff

**Primary Division:** Central Interior and Appalachian (202)

**Land Cover Class:** Barren

**Spatial Scale & Pattern:** Small patch

**Required Classifiers:** Natural/Semi‑natural; Unvegetated (<10% vasc.); Upland

**Diagnostic Classifiers:** Cliff (Substrate)

**Concept Summary:** This ecological system encompasses calcareous cliffs of the Southern Ridge and Valley and adjacent areas of the Cumberland Plateau with a few disjunct localities in the Southern Appalachians. This system includes vertical to near‑vertical rock faces of limestone and dolomite. These cliffs are typically dry but may contain relatively small embedded seepage patches. Both wet and, more commonly, dry expressions are included. Due to harsh edaphic conditions, including verticality, these cliffs are nearly unvegetated, however, *Asplenium ruta‑muraria* and *Pellaea atropurpurea* may be characteristic plants. Some cliffs have scattered *Thuja occidentalis* trees which may be very old (>800 years) and more genetically diverse than northern populations. This system also covers a narrow zone of vegetation, often herbaceous, at the horizontal clifftop where growing conditions are harsh and often gladelike.

DISTRIBUTION

**Range:** This system is found in the Southern Ridge and Valley and adjacent areas of the Cumberland Plateau with a few disjunct localities in the Southern Appalachians.

**Divisions:** 202:C

**TNC Ecoregions:** 50:C, 51:C

**Nations:** US

**Subnations:** AL, KY, NC, TN, VA

**Map Zones:** 48:C, 53:C, 57:C

**USFS Ecomap Regions:** 221Hc:CCC, 221Hd:CCC, 221He:CCC, 221J:CC, 231Ag:CCC, 231D:CC, M221Ab:CCC, M221Cc:CCC, M221Cd:CCC, M221Ce:CCC

CONCEPT

**Environment:** This system includes vertical to near‑vertical rock faces of limestone and dolomite. These cliffs are typically dry but may contain relatively small embedded seepage patches. Both wet and, more commonly, dry expressions are included. Disjunct examples in the southern Appalachians attributed to this system include Hot Springs and Linville Caverns area. It presumably includes both the Bull Cave and Calf Cave area in the Smokies.

**Vegetation:** Due to harsh edaphic conditions, including verticality, these cliffs are nearly unvegetated; however, *Asplenium ruta‑muraria* and *Pellaea atropurpurea* may be characteristic plants. Some cliffs have scattered *Thuja occidentalis* trees which may be very old (>800 years) and genetically diverse. This system also covers a narrow zone of vegetation, often herbaceous, at the horizontal clifftop where growing conditions are harsh and often gladelike. Some other plant species that may be found include calciphilic herbs such as *Aquilegia canadensis, Dodecatheon meadia, Symphyotrichum ericoides, Symphyotrichum oblongifolium, Cystopteris bulbifera, Bouteloua curtipendula, Pellaea atropurpurea, Dryopteris marginalis, Allium cernuum, Heuchera americana, Carex oligocarpa, Asplenium trichomanes, Arabis hirsuta, Arabis lyrata, Phlox subulata, Hylotelephium telephioides (= Sedum telephioides)*, and *Saxifraga virginiensis*. Woody species may occur scattered throughout or at the margins; these species include *Juniperus virginiana, Rhus aromatica, Toxicodendron radicans, Hydrangea arborescens, Fraxinus americana, Parthenocissus quinquefolia, Cercis canadensis, Tilia americana, Carya* spp., *Quercus muehlenbergii, Ostrya virginiana*, and *Cornus florida*.

SOURCES

**References:** Comer et al. 2003*, Evans et al. 2009, Eyre 1980, Schafale 2012

**Version:** 23 Feb 2010	**Stakeholders:** East, Southeast

**Concept Author:** R. Evans, C. Nordman, M. Pyne	**LeadResp:** Southeast

CES202.386 Southern Piedmont Cliff

**Primary Division:** Central Interior and Appalachian (202)

**Land Cover Class:** Barren

**Spatial Scale & Pattern:** Small patch

**Required Classifiers:** Natural/Semi‑natural; Unvegetated (<10% vasc.); Upland

**Diagnostic Classifiers:** Cliff (Substrate)

**Concept Summary:** This ecological system consists of steep to vertical or overhanging rock outcrops in the Piedmont. They occur on lower to midslopes, usually in river gorges or bluffs. The sparse vegetation is limited to plants growing on bare rock, small ledges, and crevices. Vegetation is primarily bryophytes, lichens, and herbs, with sparse trees and shrubs rooted in deeper soil pockets and crevices. The types of plants that may grow on cliffs are limited by the harsh conditions to those with adaptations to drought and limited nutrients. Examples of this system occur on steep rock outcrops on lower slopes, occasionally higher in topographically sheltered sites. River bluffs are the primary setting. Cliffs may have any aspect, but north‑facing cliffs seem to be more common. The substrate is mostly bare bedrock, which is steep to vertical or overhanging. Most examples are on felsic metamorphic rock such as gneiss or schist, but a smaller number occur on mafic metamorphic rock, felsic or mafic igneous rock, or sedimentary rock. Vascular plants are limited to sparse rooting sites in soil pockets, ledges, and crevices.

**Comments:** More information is needed on the associations that belong to this system.

This system is distinguished from other rock outcrops by a combination of low topographic position, vertical orientation, large amount of bare rock, and absence of specialized environments such as exfoliated granite, limestone or dolomite, and spray from waterfalls. In contrast, Southern Appalachian Rocky Summit (CES202.327) occurs in high topographic positions; they have more horizontal rock but may have some substantial vertical surfaces. Southern Piedmont Glade and Barrens (CES202.328) is more horizontally oriented and have much more vegetation cover. The division of rock outcrop systems may be too fine and warrant combining some; however, each system has distinctive characteristics of structure and some distinctive flora.

This system is distinguished from Southern Appalachian Montane Cliff and Talus (CES202.330) by floristic differences. Southern Appalachian Montane Cliff and Talus (CES202.330) has a number of species absent or scarce in the Piedmont, differences that may be related to elevation, regional climate, or to biogeography. However, upper Piedmont cliffs that have Appalachian flora are included in that system (CES202.330).

The primary variation within this system, which could be the basis for further subdivision, is the distinction between mafic and felsic rock, with the rare sedimentary rock examples a third category. Sedimentary rock examples in the Piedmont resemble other Piedmont cliffs more than they resemble cliffs of sedimentary rocks in the interior ecoregions.

DISTRIBUTION

**Range:** Scattered throughout the Piedmont and incidentally into the Coastal Plain, from northern Alabama and Georgia north into Virginia.

**Divisions:** 202:C, 203:C

**TNC Ecoregions:** 52:C, 57:C

**Nations:** US

**Subnations:** AL, GA, NC, SC, VA

**Map Zones:** 54:C, 58:C, 59:C, 61:C

**USFS Ecomap Regions:** 232A:CC, 232I:CC

CONCEPT

**Environment:** Examples of this system occur on steep rock outcrops on lower slopes, occasionally higher in topographically sheltered sites. River bluffs are the primary setting. Cliffs may have any aspect, but north‑facing cliffs seem to be more common. The substrate is mostly bare bedrock, which is steep to vertical or overhanging. Most examples are on felsic metamorphic rock such as gneiss or schist, a smaller number on mafic metamorphic rock, felsic or mafic igneous rock, or sedimentary rock. The physical structure of most cliffs in the Piedmont is irregular, with some ledges and crevices, and with steep, vertical, and even overhanging portions intermixed. Moisture levels vary drastically over short distances. Seepage of ground water from adjacent soils or through rock fractures often creates permanently or seasonally flooded microsites, while lack of soil makes other portions extremely dry. In less sheltered topography, slope aspect affects overall moisture levels to some degree. Rock or soil chemistry appears to be the most important factor affecting different associations on sites that have the physical structure to belong to this system.

**Vegetation:** No vascular plant associations have yet been defined for this system. Vegetation is sparse. Bryophytes and lichens may cover portions of the open rock. Vascular plants are limited to sparse rooting sites in soil pockets, ledges, and crevices. Some of these microsites may be deep enough to support shrubs or even trees, while most support only herbs. The woody plants are usually species from surrounding forests, and may be mesophytic or xerophytic. The herbs include a variety of species of open dry habitats, such as *Schizachyrium scoparium, Danthonia spicata, Houstonia purpurea*, and *Coreopsis major*, along with a few rock outcrop specialists such as *Polypodium virginianum, Saxifraga virginiensis*, and *Heuchera* spp. A number of bryophyte species may be present. Mafic rock outcrops have an additional suite of specialist herbs, including *Aquilegia canadensis, Arabis lyrata, Anemone berlandieri*, and *Sedum glaucophyllum*. Herbs from the surrounding forest are often also present and may make up a significant fraction of the flora.

**Dynamics:** The dynamics of this system have received little study. Most cliff communities are probably stable over long periods of time, with fine‑scale disturbances affecting microsites. Rock falls, slides, and other mass movement are rare, but represent catastrophic disturbance to part or all of a cliff, and may be important in the long term for keeping cliffs open. The types of plants that may grow on cliffs are limited by the harsh conditions to those with adaptations to drought and limited nutrients (Edwards et al. 2013). Animal movements may be locally important. Fire probably has little effect on cliffs, which have too little vegetation to carry fire and which tend to occur in topography that is not conducive to fire spread. Because of the limited natural disturbance and the fragility of soil and vegetation, human disturbance by trampling of edges and by climbing may be particularly destructive.

SOURCES

**References:** Comer et al. 2003*, Edwards et al. 2013, McLean 1989, Nelson 1986, Schafale 2012, Schafale and Weakley 1990

**Version:** 06 Feb 2014	**Stakeholders:** East, Southeast

**Concept Author:** M. Schafale	**LeadResp:** Southeast

M115. Great Plains Badlands Vegetation

CES303.663 Western Great Plains Badlands

**Primary Division:** Western Great Plains (303)

**Land Cover Class:** Barren

**Spatial Scale & Pattern:** Large patch

**Required Classifiers:** Natural/Semi‑natural; Unvegetated (<10% vasc.); Upland

**Diagnostic Classifiers:** Badlands; Badland

**Concept Summary:** This ecological system is found within the northern Great Plains region of the United States and Canada with some of the better known and extensive examples in North and South Dakota. In contrast to Western Great Plains Cliff and Outcrop (CES303.665), this system is typified by extremely dry and easily eroded, consolidated clay soils with bands of sandstone or isolated consolidates and little to no cover of vegetation (usually less than 10% but can be as high as 20%). Vegetated patches within the badlands system may have cover higher than 20%. In north‑central Montana, badlands often are a mosaic of bare substrate with small patches of grasses and/or shrubs that may exceed 10% cover. In those areas with vegetation, species can include scattered individuals of many dryland shrubs or herbaceous taxa, including *Grindelia squarrosa, Gutierrezia sarothrae* (especially with overuse and grazing), *Sarcobatus vermiculatus, Atriplex gardneri, Artemisia pedatifida, Eriogonum* spp., *Muhlenbergia cuspidata, Pseudoroegneria spicata*, and *Arenaria hookeri*. Patches of *Artemisia* spp. can also occur. This system can occur where the land lies well above its local base level or below and is created by several factors, including elevation, rainfall, carving action of streams, and parent material.

**Comments:** It has been proposed to change the name of this system to include "shale barrens." As with all predominantly "barren" systems, there will be patches of vegetated areas within the overall system. Small areas of "badlands" or "shale barrens" can also occur without major erosional processes actively taking place. An example location is Bitter Creek Area of Environmental Concern (BLM designation), which is much like a badland but not so eroded. The vegetation is sparse with *Juniperus horizontalis* and much bare ground; there is some grass cover as well. The driving process is erosion. Exactly where this transitions to Inter‑Mountain Basins Shale Badland (CES304.789) in central Wyoming needs to be clarified.

DISTRIBUTION

**Range:** This system ranges throughout the northern Great Plains region of the United States and Canada. Some of the best and well‑known examples occur in North and South Dakota. Its western‑most occurrence in Wyoming needs to be clarified, but it does occur in the eastern portion of that state.

**Divisions:** 303:C

**TNC Ecoregions:** 26:C, 34:P, 66:?, 67:P

**Nations:** CA?, US

**Subnations:** MB?, MT, ND, NE, SD, WY

**Map Zones:** 20:C, 21:?, 22:?, 29:C, 30:C, 31:C, 33:?, 40:P

**USFS Ecomap Regions:** 331E:C?, 331F:CC, 331G:CC, 331H:C?, 331K:CP, 331L:CP, 331M:CC, 342A:CC, 342F:C?, 342G:C?, M331B:CC, M331I:CP, M334A:CC

CONCEPT

**Environment:** A combination of factors such as elevation, rainfall, carving action of streams and parent material can contribute to the development of this system. This system is primarily a type of mature dissection with finely textured drainage pattern and steep slopes. This system contains extremely dry and easily erodible, consolidated clayey soils with bands of sandstone or isolated consolidates. This system is found within an arid to semi‑arid climate with infrequent, but torrential, rains that cause erosion.

**Vegetation:** Vegetation in this system is limited by climate and soils and often is less than 10% cover. Scattered individuals of *Grindelia squarrosa, Gutierrezia sarothrae*, or *Eriogonum* spp. and/or patches of *Artemisia* spp. may occur.

**Dynamics:** This system contains highly erodible soils that can be strongly influenced by infrequent, but often torrential, rains.

SOURCES

**References:** Comer et al. 2003*, Knight et al. 1987, Rice et al. 2012b, Rolfsmeier and Steinauer 2010, Von Loh et al. 1999

**Version:** 29 Jan 2007	**Stakeholders:** Canada, Midwest, West

**Concept Author:** S. Menard and K. Kindscher	**LeadResp:** Midwest

M116. Great Plains Cliff, Scree & Rock Vegetation

CES303.664 Southwestern Great Plains Canyon

**Primary Division:** Western Great Plains (303)

**Land Cover Class:** Barren

**Spatial Scale & Pattern:** Small patch, Large patch

**Required Classifiers:** Natural/Semi‑natural; Unvegetated (<10% vasc.); Upland

**Diagnostic Classifiers:** Canyon

**Concept Summary:** This ecological system occurs in both perennial and intermittent stream canyons of the southwestern Great Plains. Soils can range from deep loams to alluvial to sandy. The mosaic of soil types which have developed from sandstone, limestone, basalt, and shale parent materials creates a complex mosaic of grasslands, shrublands and woodlands within the canyon system (Shaw et al. 1989). Although the system combines many elements from Southern Rocky Mountain Juniper Woodland and Savanna (CES306.834), Rocky Mountain Lower Montane‑Foothill Shrubland (CES306.822), Western Great Plains Shortgrass Prairie (CES303.672), and other shrublands, the varied geology, diverse soil types, and topographic dynamics together form a distinct ecological system characteristic of the canyons and dissected mesas of the southwestern Great Plains.

Vegetation varies both regionally and locally depending on latitude, aspect, slope position and substrate and can range from riparian vegetation to xeric or mesic woodlands and shrublands. Rock outcrops with sparse vegetation are also common. Open to moderately dense pinyon‑juniper woodlands occupy most of the canyonland slopes. Scattered *Pinus edulis* may occur within these community types but are never dominant. Woodlands may be floristically similar to and intergrade with Southern Rocky Mountain Juniper Woodland and Savanna (CES306.834) but are distributed along rocky outcrops, canyon slopes and mesas. *Juniperus monosperma* is the most common tree species and forms extensive woodlands with a grassy understory of *Bouteloua eriopoda, Bouteloua gracilis, Bouteloua hirsuta, Bouteloua curtipendula*, and *Pleuraphis jamesii*, or sometimes with an open shrub layer dominated by *Cercocarpus montanus*. In Kansas, *Juniperus virginiana* can become more dominant and replace *Juniperus monosperma*. Isolated patches of *Pinus ponderosa* or *Populus tremuloides* are found in some locations. Shrublands occur on canyon bottoms, in narrow side canyons, and integrate with woodlands on upper slopes. A mosaic of shrub species is characteristic of canyon walls and slopes and varies with substrate and moisture availability. Common species include *Artemisia bigelovii, Cercocarpus montanus, Rhus trilobata, Ribes* spp., *Ptelea trifoliata, Philadelphus microphyllus*, and *Yucca glauca*. *Frankenia jamesii* and *Glossopetalon spinescens var. meionandrum* form a community restricted to gypsiferous and calciferous soils. Canyon floors often support a degraded shrubby grassland of *Ericameria nauseosa* and *Cylindropuntia imbricata* with a grassy understory.

Because of the varied topography, relatively permanent water along streambeds and southern location, these canyonlands have a rich herpetofauna (Mackessy 1998). This system provides good habitat for a number of snake species that are otherwise uncommon in the Central Shortgrass Prairie ecoregion. Occasional seeps and springs of the canyon walls provide habitat for rare ferns.

**Comments:** Llano Estacado Caprock Escarpment and Breaks Shrubland and Steppe (CES303.725) was created for the escarpment and breaks that follow the eastern edge of the Llano Estacado (Texas Panhandle) and includes the canyons along this escarpment (e.g., Palo Duro Canyon).

DISTRIBUTION

**Range:** This system occurs in dry canyons and mesas in the southwestern portion of the Western Great Plains, ranging from Purgatoire and Apishipa canyons, tributaries of the Arkansas River in Colorado, and east into Kansas, Oklahoma and possibly north Texas.

**Divisions:** 303:C

**TNC Ecoregions:** 27:C, 28:?

**Nations:** US

**Subnations:** CO, KS, OK

**Map Zones:** 26:?, 27:C, 33:C, 34:?, 35:?

**USFS Ecomap Regions:** 315A:CC, 315B:C?, 331B:CC, 331I:CC, M313B:PP

CONCEPT

**Environment:** This ecological system occurs in both perennial and intermittent stream canyons of the southwestern Great Plains. Soils can range from deep loams to alluvial to sandy. The mosaic of soil types which have developed from sandstone, limestone, basalt, and shale parent materials creates a complex mosaic of grasslands, shrublands and woodlands within the canyon system (Shaw et al. 1989).

**Vegetation:** *Juniperus monosperma* is the most common tree species and forms extensive woodlands with a grassy understory of *Bouteloua eriopoda, Bouteloua gracilis, Bouteloua hirsuta, Bouteloua curtipendula*, and *Pleuraphis jamesii*, or sometimes with an open shrub layer dominated by *Cercocarpus montanus*. In Kansas, *Juniperus virginiana* can become more dominant and replace *Juniperus monosperma*. Isolated patches of *Pinus ponderosa* or *Populus tremuloides* are found in some locations. Shrublands occur on canyon bottoms, in narrow side canyons, and integrate with woodlands on upper slopes. A mosaic of shrub species is characteristic of canyon walls and slopes and varies with substrate and moisture availability. Common species include *Artemisia bigelovii, Cercocarpus montanus, Rhus trilobata, Ribes* spp., *Ptelea trifoliata, Philadelphus microphyllus*, and *Yucca glauca*. *Frankenia jamesii* and *Glossopetalon spinescens var. meionandrum* form a community restricted to gypsiferous and calciferous soils. Canyon floors often support a degraded shrubby grassland of *Ericameria nauseosa* and *Cylindropuntia imbricata (= Opuntia imbricata)* with a grassy understory.

SOURCES

**References:** Bell 2005, CNHP 1999, Comer et al. 2003*, Eyre 1980, Mackessy 1998, Shaw et al. 1989

**Version:** 27 May 2004	**Stakeholders:** Midwest, Southeast, West

**Concept Author:** K. Decker, K. Schulz, S. Menard and K. Kindscher	**LeadResp:** Midwest

CES303.665 Western Great Plains Cliff and Outcrop

**Primary Division:** Western Great Plains (303)

**Land Cover Class:** Barren

**Spatial Scale & Pattern:** Small patch

**Required Classifiers:** Natural/Semi‑natural; Unvegetated (<10% vasc.); Upland

**Concept Summary:** This system includes cliffs and outcrops throughout the Western Great Plains Division. Substrate can range from sandstone and limestone, which can often form bands in the examples of this system. Vegetation is restricted to shelves, cracks and crevices in the rock. However, this system differs from Western Great Plains Badlands (CES303.663) in that often the soil is slightly developed and less erodible, and some grass and shrub species can occur at greater than 10%. Common species in this system include short shrubs such as *Rhus trilobata* and *Artemisia longifolia* and mixedgrass species such as *Bouteloua curtipendula* and *Bouteloua gracilis* and *Calamovilfa longifolia*. Drought and wind erosion are the most common natural dynamics affecting this system. Vegetation is typically restricted to shelves, cracks, and crevices where soil can accumulate.

**Comments:** The granite glades and rock outcrops of the Llano Uplift of Texas have been reclassified to Llano Uplift Acidic Forest, Woodland and Glade (CES303.657). The carbonate glades, barrens, and cliffs of the Edwards Plateau of Texas have been reclassified to Edwards Plateau Carbonate Glade and Barrens (CES303.655) and Edwards Plateau Cliff (CES303.653), respectively.

DISTRIBUTION

**Range:** This system ranges throughout the Western Great Plains Division from northern Texas to southern Canada.

**Divisions:** 303:C

**TNC Ecoregions:** 26:C, 27:C, 28:C, 33:C, 37:P, 66:P, 67:P

**Nations:** CA, US

**Subnations:** CO, KS, MB, MT, ND, NE, NM, OK, TX, WY

**Map Zones:** 20:?, 22:?, 25:P, 26:C, 27:C, 28:P, 29:C, 30:C, 31:C, 33:C, 34:C, 35:C, 38:C, 39:?, 43:P

**USFS Ecomap Regions:** 251H:PP, 315A:CC, 315B:CC, 315F:CC, 321A:CC, 331B:CC, 331C:CC, 331F:CC, 331G:CC, 331H:CC, 331I:CC, 331K:CP, 331L:CP, 332B:CP, 332C:CC, 332D:CC, 332E:C?, 342F:PP, M313B:CC, M331B:CC, M331F:CC, M331I:CC

CONCEPT

**Environment:** This system is includes cliff and outcrops with slopes typically greater than 80% throughout the Western Great Plains Division with substrate ranging from sandstone to limestone. These areas are often found along river breaks and escarpments. Areas of shelves, cracks, and crevices accumulate materials and allow soils to develop enough to support more vegetation.

**Vegetation:** Short shrubs and mixedgrass species dominate the vegetation of this system. Common species include *Rhus trilobata, Artemisia longifolia, Bouteloua curtipendula, Bouteloua gracilis*, and *Calamovilfa longifolia*, although species can vary somewhat with substrate and exposure.

**Dynamics:** Drought and wind erosion are the major influences affecting this system.

SOURCES

**References:** Comer et al. 2003*, Elliott 2013, Rolfsmeier and Steinauer 2010

**Version:** 02 Oct 2014	**Stakeholders:** Canada, Midwest, Southeast, West

**Concept Author:** S. Menard and K. Kindscher	**LeadResp:** Midwest

6.B.1.Nb. Western North American Temperate Cliff, Scree & Rock Vegetation

M887. Western North American Cliff, Scree & Rock Vegetation

CES206.903 Central California Coast Ranges Cliff and Canyon

**Primary Division:** Mediterranean California (206)

**Land Cover Class:** Barren

**Spatial Scale & Pattern:** Large patch

**Required Classifiers:** Natural/Semi‑natural; Unvegetated (<10% vasc.); Upland

**Diagnostic Classifiers:** Canyon; Cliff (Substrate); Talus (Substrate); Mediterranean [Mediterranean Xeric‑Oceanic]; Canyon Mosaic

**Concept Summary:** Found from foothill and montane elevations of California's Coast Ranges, these are barren and sparsely vegetated areas (<10% plant cover) of steep cliff faces, narrow canyons, and smaller rock outcrops of various igneous, sedimentary, and metamorphic bedrock. This system also includes unstable scree and talus slopes typically occurring below cliff faces. Scattered vegetation may include *Pseudotsuga menziesii, Pinus contorta var. murrayana, Pinus ponderosa*, and *Pinus jeffreyi*. There may be shrubs including species of *Arctostaphylos* or *Ceanothus*. Soil development is limited as is herbaceous cover.

DISTRIBUTION

**Range:** Found from foothill and montane elevations of California's Coast Ranges.

**Divisions:** 206:C

**TNC Ecoregions:** 14:C, 15:C

**Nations:** US

**Subnations:** CA

**Map Zones:** 3:C, 4:C

**USFS Ecomap Regions:** 262A:PP, M261B:CC, M261C:CC

CONCEPT

SOURCES

**References:** Barbour and Major 1988, Comer et al. 2003*, Holland and Keil 1995, Sawyer and Keeler‑Wolf 1995

**Version:** 17 Mar 2003	**Stakeholders:** West

**Concept Author:** P. Comer and T. Keeler‑Wolf	**LeadResp:** West

CES206.902 Klamath‑Siskiyou Cliff and Outcrop

**Primary Division:** Mediterranean California (206)

**Land Cover Class:** Barren

**Spatial Scale & Pattern:** Large patch

**Required Classifiers:** Natural/Semi‑natural; Unvegetated (<10% vasc.); Upland

**Diagnostic Classifiers:** Woody‑Herbaceous; Moss/Lichen (Nonvascular); Cliff (Substrate); Talus (Substrate); Rock Outcrops/Barrens/Glades; Mediterranean [Mediterranean Xeric‑Oceanic]

**Concept Summary:** Found from foothill to subalpine elevations of the Klamath Range, these are barren and sparsely vegetated landscapes (<10% plant cover) of steep cliff faces, bald ridgetops and shoulder outcrops, narrow canyons, and smaller rock outcrops of various igneous, sedimentary, and metamorphic bedrock. Vegetative cover is dominated by forbs, grasses, mosses, or lichens. This also includes unstable scree and talus slopes typically occurring below cliff faces. Scattered vegetation may include *Pseudotsuga menziesii* and *Acer macrophyllum* along with herbaceous and nonvascular species such as *Achnatherum lemmonii (= Stipa lemmonii), Achnatherum occidentale (= Stipa occidentalis), Elymus elymoides (= Sitanion hystrix), Sedum oregonense*, and *Racomitrium ericoides (= Racomitrium canescens var. ericoides)*. Soil development is limited as is herbaceous cover.

DISTRIBUTION

**Range:** Found from foothill to subalpine elevations of the Klamath Range.

**Divisions:** 206:C

**TNC Ecoregions:** 5:C

**Nations:** US

**Subnations:** CA, OR

**Map Zones:** 2:C, 3:C, 7:C

**USFS Ecomap Regions:** M242A:??, M261A:CC, M261B:CC

CONCEPT

SOURCES

**References:** Barbour and Major 1988, Comer et al. 2003*, Holland and Keil 1995, Sawyer and Keeler‑Wolf 1995

**Version:** 17 Mar 2003	**Stakeholders:** West

**Concept Author:** P. Comer and T. Keeler‑Wolf	**LeadResp:** West

CES206.905 Mediterranean California Serpentine Barrens

**Primary Division:** Mediterranean California (206)

**Land Cover Class:** Barren

**Spatial Scale & Pattern:** Small patch

**Required Classifiers:** Natural/Semi‑natural; Unvegetated (<10% vasc.); Upland

**Concept Summary:** This uncommon system is found in the central and southern Sierra Nevada, Central and Northern Coast Ranges, and Klamath Ranges at elevations between 150 to 1800 m (450‑5500 feet), where serpentine outcrops and related soils are common. Not all serpentinite outcrops support distinct vegetation. Only those with very low Ca:Mg ratio impact biotic composition. This system is usually found on steep slopes with loosely consolidated soils and harsh soil chemical conditions (large rock outcrops and gravelly soil). There is typically a very low cover (<10%) of herbaceous species, including *Streptanthus* spp., *Hesperolinon* spp., *Allium falcifolium, Allium cratericola, Asclepias solanoana, Eriogonum ursinum*, and *Eriogonum nudum*.

**Comments:** This system is very similar to North Pacific Serpentine Barren (CES204.095) of the East and West Cascades (Oregon and Washington) but tends to be more herbaceous in species composition, whereas North Pacific Serpentine Barren has a more developed woody component.

DISTRIBUTION

**Range:** This system is found in the central and southern Sierra Nevada, central and northern Coast Ranges, and Klamath Ranges at elevations between 150 and 1800 m (450‑5500 feet).

**Divisions:** 206:C

**TNC Ecoregions:** 5:C, 12:C, 14:C, 15:C

**Nations:** US

**Subnations:** CA, OR

**Map Zones:** 2:C, 3:C, 6:C, 7:P

**USFS Ecomap Regions:** M261A:CC, M261B:CC, M261C:CC, M261D:CC, M261E:CC

CONCEPT

**Environment:** This system is found in central and southern Sierra Nevada, central and northern Coast Ranges, and Klamath Mountains at elevations between 150 and 1800 m (450‑5500 feet), where serpentine or ultramafic outcrops and related soils are common. Not all serpentinite outcrops support distinct vegetation. Only those with very low Ca:Mg ratio impact biotic composition. This system is usually found on steep slopes where loosely consolidated soils and harsh soil chemical conditions (large rock outcrops and gravelly soil) combine with xeric conditions (low rainfall or south‑ or west‑facing, excessively‑drained). Soils on ultramafics are usually shallow and skeletal, with little profile development. Ultramafic soils impose the following stresses on plants: imbalance of calcium and magnesium, magnesium toxicity, low availability of molybdenum, toxic levels of heavy metals, sometime high alkalinity, low concentrations of some essential nutrients, and low soil water storage capacity (Kruckeberg 1984, Sanchez‑Mata 2007). In some cases, the steepness of the slopes and general sparseness of the vegetation result in continual erosion. Jimerson et al. (1995) found one plant community in this system to occur on convex, ridgetop or spur ridge positions where moisture conditions are extremely dry [often south‑ or west‑facing]; exposed bare ground, gravel and rock have high cover. Occurrences are generally physiognomically distinct from the adjacent predominant forest or shrubland/chaparral vegetation; they can cover 100s of hectares.

**Vegetation:** There is typically a very low cover (<10%) of herbaceous species, including *Streptanthus* spp., *Hesperolinon* spp., *Allium falcifolium, Allium cratericola, Asclepias solanoana, Eriogonum ursinum*, and *Eriogonum nudum*.

**Dynamics:** The low cover of vascular plants combined with exposed sites leads to sheet erosion and soil loss being the major disturbance processes. Fine soil materials may be "captured" by micro‑terraces leading to plant establishment and slow development of an organic soil horizon (Jimerson et al. 1995). Potential for alteration from fire is very low due to the high amount of soil and rock and low vegetation cover. Due the general lack of exotic species which tend to be intolerant of the ultramafic soils, this system is rich in native and endemic flora (Safford et al. 2005, Sanchez‑Mata 2007). The interaction between serpentine soils and soil algae or fungi and small invertebrates is largely unstudied; Pegtel (1980) and Hopkins (1987) found that on barrens such as these the few tolerant plants are likely to be mycorrhizal. Harrison and Shapiro (1988) describe features of population biology of butterflies occurring on serpentines in northern California.

SOURCES

**References:** Barbour and Major 1988, Barbour et al. 2007a, Batten et al. 2006, Brooks and Minnich 2006, Comer et al. 2003*, Harrison and Shapiro 1988, Holland and Keil 1995, Hopkins 1987, Jimerson et al. 1995, Kruckberg 1984, PRBO Conservation Science 2011, Pegtel 1980, Safford et al. 2005, Sanchez‑Mata 2007, Sawyer and Keeler‑Wolf 1995

**Version:** 14 Jan 2014	**Stakeholders:** West

**Concept Author:** P. Comer and T. Keeler‑Wolf	**LeadResp:** West

CES204.092 North Pacific Active Volcanic Rock and Cinder Land

**Primary Division:** North American Pacific Maritime (204)

**Land Cover Class:** Barren

**Spatial Scale & Pattern:** Large patch

**Required Classifiers:** Natural/Semi‑natural; Unvegetated (<10% vasc.); Upland

**Diagnostic Classifiers:** Lava; Cinder; Basalt

**Concept Summary:** This ecological system includes active volcanic landscapes dominated by ash, pyroclastic deposits, lava, landslides and other exposed bare mineral and rock. Periodic eruptions and earthquakes are the primary processes maintaining a primarily barren environment. Decades of inactivity slowly provide opportunity for development of other systems, such as North American Glacier and Ice Field (CES100.728) or North Pacific Wooded Volcanic Flowage (CES204.883), or primary successional stages of surrounding vegetated systems to develop.

**Comments:** Mount St. Helens is the prototype. Barren volcanic landscapes on the Alaska Peninsula and Aleutian Islands have been placed into Aleutian Volcanic Rock and Talus (CES105.308).

DISTRIBUTION

**Range:** This system is found in the Cascade Range from northern California north to Washington and is limited to barren and sparsely vegetated volcanic substrates.

**Divisions:** 204:C

**TNC Ecoregions:** 3:C, 4:C, 5:C, 81:C

**Nations:** US

**Subnations:** CA?, OR, WA:S5

**Map Zones:** 1:C, 2:P, 6:P, 7:C, 8:P, 9:P

**USFS Ecomap Regions:** M242B:C?, M242C:CC, M242D:CP

CONCEPT

SOURCES

**References:** Comer et al. 2003*, WNHP unpubl. data 2018

**Version:** 16 Jan 2009	**Stakeholders:** West

**Concept Author:** R. Crawford	**LeadResp:** West

CES204.093 North Pacific Montane Massive Bedrock, Cliff and Talus

**Primary Division:** North American Pacific Maritime (204)

**Land Cover Class:** Barren

**Spatial Scale & Pattern:** Large patch, Small patch

**Required Classifiers:** Natural/Semi‑natural; Unvegetated (<10% vasc.); Upland

**Diagnostic Classifiers:** Canyon; Cliff (Substrate); Talus (Substrate); Rock Outcrops/Barrens/Glades; Temperate [Temperate Oceanic]

**National Mapping Codes:** EVT 2733; ESLF 3155; ESP 1733

**Concept Summary:** This ecological system is found from foothill to subalpine elevations and includes barren and sparsely vegetated landscapes (generally <10% vascular plant cover) of steep cliff faces, narrow canyons, and larger rock outcrops of various igneous, sedimentary, and metamorphic bedrock types. Also included are unstable scree and talus that typically occur below cliff faces. The dominant process is drought, especially farther south in its distribution, and other extreme growing conditions created by exposed rock or unstable slopes typically associated with steep slopes. Alaskan montane rock and talus probably has a significant component on nonvascular species, and is not drought‑limited. Fractures in the rock surface and less steep or more stable slopes may be occupied by small patches of dense vegetation, typically scattered trees and/or shrubs. Characteristic trees includes *Callitropsis nootkatensis, Tsuga* spp., *Thuja plicata, Pseudotsuga menziesii* (not in Alaska), or *Abies* spp. There may be scattered shrubs present, such as *Acer circinatum, Alnus viridis*, and *Ribes* spp. Soil development is limited as is herbaceous cover. Mosses or lichens may be very dense, well‑developed and display cover well over 10%.

**Comments:** This system was distinguished from montane cliffs and barrens in the Rockies based on a change in floristic division and the apparent abundance of nonvascular cover on rocks compared to drier divisions. It also includes cliffs, barrens and rock outcrops in coastal southeastern Alaska, if they are not covered with snow and ice.

DISTRIBUTION

**Range:** This system occurs from northern California (north of Sierra Nevada Cliff and Canyon (CES206.901)) to southeastern Alaska.

**Divisions:** 204:C

**TNC Ecoregions:** 1:C, 2:C, 3:C, 4:C, 5:P, 69:C, 70:C, 81:C

**Nations:** CA, US

**Subnations:** AK, BC, OR, WA:S4S5

**Map Zones:** 1:C, 2:C, 3:P, 7:C, 77:C, 78:C

**USFS Ecomap Regions:** 242A:CC, 242B:C?, 342D:C?, 342H:CP, 342I:CC, M242A:CC, M242B:CC, M242C:CC, M242D:CC, M261A:CC, M261D:CP

CONCEPT

**Environment:** This ecological system is found from foothill to subalpine elevations and includes barren and sparsely vegetated landscapes (generally <10% vascular plant cover) of steep cliff faces, narrow canyons, and larger rock outcrops of various igneous, sedimentary, and metamorphic bedrock types. Also included are unstable scree and talus that typically occur below cliff faces. The dominant process is drought, especially farther south in its distribution, and other extreme growing conditions created by exposed rock or unstable slopes typically associated with steep slopes.

**Vegetation:** Alaskan montane rock and talus probably has a significant component on nonvascular species, and is not drought‑limited. Fractures in the rock surface and less steep or more stable slopes may be occupied by small patches of dense vegetation, typically scattered trees and/or shrubs. Characteristic trees includes *Callitropsis nootkatensis (= Chamaecyparis nootkatensis), Tsuga* spp., *Thuja plicata, Pseudotsuga menziesii* (not in Alaska), or *Abies* spp. There may be scattered shrubs present, such as *Acer circinatum, Alnus viridis*, and *Ribes* spp. Soil development is limited as is herbaceous cover. Mosses or lichens may be very dense, well‑developed and display cover well over 10%.

SOURCES

**References:** Comer et al. 2003*, WNHP unpubl. data 2018

**Version:** 10 Dec 2008	**Stakeholders:** Canada, West

**Concept Author:** R. Crawford	**LeadResp:** West

CES204.095 North Pacific Serpentine Barren

**Primary Division:** North American Pacific Maritime (204)

**Land Cover Class:** Barren

**Spatial Scale & Pattern:** Small patch

**Required Classifiers:** Natural/Semi‑natural; Unvegetated (<10% vasc.); Upland

**Concept Summary:** This uncommon ecological system is found in the east and west Cascades. It is usually found on steep slopes with loosely consolidated soils and harsh soil chemical conditions (large rock outcrops and gravelly soil), although exposed ridges occur. This system occurs primarily in the Wenatchee Mountains in the east Cascades between 760 and 2100 m elevation (2500‑7000 feet) on thin rocky, ultramafic (peridotite, serpentinite) soils of varying extent up to several square km. Most sites support often stunted conifers, typically stress‑tolerant species. Not all ultramafic outcrops support a distinct vegetation. Only those with very low Ca:Mg ratio impact biotic composition, whereas others reflect increased influence of soil drought on ultramafic material. These systems are highly variable and are described here to include barren slopes to patches of nearly closed forests. Low‑elevation sites support *Pseudotsuga menziesii, Pinus ponderosa*, and *Pinus monticola* trees with a sparse ground cover with *Aspidotis densa, Arctostaphylos nevadensis*, and *Pseudoroegneria spicata*. Higher elevations have *Pinus contorta var. latifolia, Pinus albicaulis, Abies lasiocarpa*, and *Tsuga mertensiana* with *Juniperus communis, Ledum glandulosum, Vaccinium scoparium, Poa curtifolia*, and *Festuca viridula*.

**Comments:** This is very similar to Mediterranean California Serpentine Barrens (CES206.905) of California and southern Oregon but tends to have a more developed woody component, whereas the California serpentine barrens are more herbaceous**.**

DISTRIBUTION

**Range:** This uncommon system is found in the east and west Cascades of Washington.

**Divisions:** 204:C

**TNC Ecoregions:** 3:P, 4:C, 81:C

**Nations:** US

**Subnations:** WA:S4

**Map Zones:** 1:C, 2:C, 7:C

**USFS Ecomap Regions:** M242A:CC, M242B:C?, M242C:CP, M242D:CP

CONCEPT

SOURCES

**References:** Comer et al. 2003*, Kruckeberg 1984, WNHP unpubl. data 2018, del Moral 1982

**Version:** 30 Mar 2005	**Stakeholders:** West

**Concept Author:** R. Crawford	**LeadResp:** West

CES306.815 Rocky Mountain Cliff, Canyon and Massive Bedrock

**Primary Division:** Rocky Mountain (306)

**Land Cover Class:** Barren

**Spatial Scale & Pattern:** Large patch

**Required Classifiers:** Natural/Semi‑natural; Unvegetated (<10% vasc.); Upland

**Diagnostic Classifiers:** Canyon; Cliff (Landform); Ridgetop bedrock outcrop; Talus (Substrate); Rock Outcrops/Barrens/Glades; Oligotrophic Soil; Very Shallow Soil; Landslide

**Concept Summary:** This ecological system of barren and sparsely vegetated landscapes (generally <10% plant cover) is found from foothill to subalpine elevations on steep cliff faces, narrow canyons, and smaller rock outcrops of various igneous (intrusives), sedimentary, and metamorphic bedrock types. It is located throughout the Rocky Mountains and northeastern Cascade Range in North America. Also included are unstable scree and talus slopes that typically occur below cliff faces. In general these are the dry sparsely vegetated places on a landscape. The biota on them reflect what is surrounding them, unless it is an extreme parent material. There may be small patches of dense vegetation, but it typically includes scattered trees and/or shrubs. Characteristic trees include species from the surrounding landscape, such as *Pseudotsuga menziesii, Pinus ponderosa, Pinus flexilis, Populus tremuloides, Abies concolor, Abies lasiocarpa*, or *Pinus edulis* and *Juniperus* spp. at lower elevations. There may be scattered shrubs present, such as species of *Holodiscus, Ribes, Physocarpus, Rosa, Juniperus*, and *Jamesia americana, Mahonia repens, Rhus trilobata*, or *Amelanchier alnifolia*. Soil development is limited, as is herbaceous cover.

**Comments:** This has a very broad elevation range (<3350 m) for a system; consider dividing into foothills/montane and subalpine. And/or by floristic division. This is in the Okanagan and Rockies as the montane sparse. North Pacific Montane Massive Bedrock, Cliff and Talus (CES204.093) includes everything in the Cascades and west, except the northeastern Cascades, where occurrences are this system (CES306.815). Inter‑Mountain Basins Cliff and Canyon (CES304.779) occurs in the dry foothills on the east side of EDC MapZone1.

DISTRIBUTION

**Range:** This system is located throughout the Rocky Mountain, including the isolated island ranges of central Montana, and northeastern Cascade Ranges in North America.

**Divisions:** 306:C

**TNC Ecoregions:** 7:C, 8:C, 9:C, 20:C, 21:C, 25:C, 26:C, 68:C

**Nations:** CA, US

**Subnations:** AB, AK?, AZ, BC, CO, ID, MT, NM, OR, TX?, UT, WA:S4S5, WY

**Map Zones:** 1:C, 8:?, 9:P, 10:C, 12:?, 15:P, 16:C, 17:C, 18:P, 19:C, 20:C, 21:C, 22:C, 23:C, 24:C, 25:C, 26:C, 27:C, 28:C, 29:C, 33:C

**USFS Ecomap Regions:** 313A:CC, 313B:CC, 313D:CC, 315A:CC, 315H:CC, 321A:CC, 331A:C?, 331B:CC, 331D:C?, 331G:CC, 331H:CC, 331I:CP, 331J:CC, 331K:CP, 331N:CP, 341A:CC, 341B:CC, 341C:CC, 341F:CC, 341G:CC, 342A:CP, 342B:CC, 342C:CC, 342D:CP, 342E:CC, 342F:CP, 342G:CP, 342H:CP, 342I:CP, 342J:CC, M242B:CP, M242C:CC, M242D:CC, M313A:CC, M313B:CC, M331A:CC, M331B:CC, M331D:CC, M331E:CC, M331F:CC, M331G:CC, M331H:CC, M331I:CC, M331J:CC, M332A:CC, M332B:CC, M332D:CC, M332E:CC, M332F:CC, M332G:CC, M333A:CC, M333B:CC, M333C:CC, M333D:CC, M334A:CC, M341A:CC, M341B:CC, M341C:CC

CONCEPT

**Environment:** This ecological system of barren and sparsely vegetated landscapes (generally <10% plant cover) is found from foothill to subalpine elevations on steep cliff faces, narrow canyons, and smaller rock outcrops of various igneous (intrusives), sedimentary, and metamorphic bedrock types. Also included are unstable scree and talus slopes that typically occur below cliff faces. In general these are the dry sparsely vegetated places on a landscape. The biota on them reflect what is surrounding them, unless it is an extreme parent material.

**Vegetation:** There may be small patches of dense vegetation, but it typically includes scattered trees and/or shrubs. Characteristic trees include species from the surrounding landscape, such as *Pseudotsuga menziesii, Pinus ponderosa, Pinus flexilis, Populus tremuloides, Abies concolor, Abies lasiocarpa*, or *Pinus edulis* and *Juniperus* spp. at lower elevations. There may be scattered shrubs present, such as species of *Holodiscus, Ribes, Physocarpus, Rosa, Juniperus*, and *Jamesia americana, Mahonia repens, Rhus trilobata*, or *Amelanchier alnifolia*. Soil development is limited, as is herbaceous cover.

SOURCES

**References:** Andrews and Righter 1992, Comer et al. 2003*, Ecosystems Working Group 1998, Hess and Wasser 1982, Larson et al. 2000a, NCC 2002, Neely et al. 2001, Peet 1981, WNHP unpubl. data 2018

**Version:** 04 Apr 2005	**Stakeholders:** Canada, Midwest, Southeast, West

**Concept Author:** M.S. Reid	**LeadResp:** West

CES206.901 Sierra Nevada Cliff and Canyon

**Primary Division:** Mediterranean California (206)

**Land Cover Class:** Barren

**Spatial Scale & Pattern:** Large patch

**Required Classifiers:** Natural/Semi‑natural; Unvegetated (<10% vasc.); Upland

**Diagnostic Classifiers:** Canyon; Cliff (Substrate); Talus (Substrate); Rock Outcrops/Barrens/Glades; Mediterranean [Mediterranean Xeric‑Oceanic]

**Concept Summary:** Found from foothill to subalpine elevations throughout the Sierra Nevada and nearby mountain ranges, these are barren and sparsely vegetated areas (<10% plant cover) of steep cliff faces, narrow canyons, and smaller rock outcrops of various igneous, sedimentary, and metamorphic bedrock. This system also includes unstable scree and talus slopes typically occurring below cliff faces. Scattered vegetation may include *Abies magnifica, Pseudotsuga menziesii, Pinus contorta var. murrayana, Pinus ponderosa, Pinus jeffreyi, Populus tremuloides*, or *Pinus monophylla, Juniperus osteosperma*, and *Cercocarpus ledifolius* at lower elevations. There may be shrubs including species of *Arctostaphylos* or *Ceanothus*. Soil development is limited as is herbaceous cover.

DISTRIBUTION

**Range:** Found from foothill to subalpine elevations throughout the Sierra Nevada and nearby mountain ranges.

**Divisions:** 206:C

**TNC Ecoregions:** 4:C, 5:C, 12:C

**Nations:** US

**Subnations:** CA, NV, OR

**Map Zones:** 4:?, 6:C, 7:C, 12:C

**USFS Ecomap Regions:** 322A:??, 341D:CC, 341E:CC, 341F:CC, 342B:CC, M261D:CC, M261E:CC, M261F:CC

CONCEPT

**Environment:** These are barren and sparsely vegetated areas (<10% plant cover) of steep cliff faces, narrow canyons, and smaller rock outcrops of various igneous, sedimentary, and metamorphic bedrock. This system also includes unstable scree and talus slopes typically occurring below cliff faces. Soil development is limited as is herbaceous cover.

**Vegetation:** Scattered vegetation may include *Abies magnifica, Pseudotsuga menziesii, Pinus contorta var. murrayana, Pinus ponderosa, Pinus jeffreyi, Populus tremuloides*, or *Pinus monophylla, Juniperus osteosperma*, and *Cercocarpus ledifolius* at lower elevations. There may be shrubs including species of *Arctostaphylos* or *Ceanothus*.

SOURCES

**References:** Barbour and Major 1988, Comer et al. 2003*, Holland and Keil 1995, Sawyer and Keeler‑Wolf 1995

**Version:** 17 Mar 2003	**Stakeholders:** West

**Concept Author:** P. Comer and T. Keeler‑Wolf	**LeadResp:** West

CES206.904 Southern California Coast Ranges Cliff and Canyon

**Primary Division:** Mediterranean California (206)

**Land Cover Class:** Barren

**Spatial Scale & Pattern:** Large patch

**Required Classifiers:** Natural/Semi‑natural; Unvegetated (<10% vasc.); Upland

**Diagnostic Classifiers:** Mediterranean [Mediterranean Xeric‑Oceanic]; Xeric; Landslide; Canyon Mosaic

**Concept Summary:** Found from foothill and montane elevations of California's Transverse and Peninsular ranges, these are barren and sparsely vegetated areas (<10% plant cover) of steep cliff faces, narrow canyons, and smaller rock outcrops of various igneous, sedimentary, and metamorphic bedrock type. This system also includes unstable scree and talus slopes typically occurring below cliff faces. Scattered vegetation may include shrub species from surrounding coastal chaparral, such as *Ceanothus megacarpus, Ceanothus leucodermis, Cercocarpus montanus var. minutiflorus (= Cercocarpus minutiflorus), Arctostaphylos glauca*, and *Xylococcus bicolor*. Soil development is limited as is herbaceous cover.

DISTRIBUTION

**Range:** Found from foothill and montane elevations of California's Transverse and Peninsular ranges.

**Divisions:** 206:C

**TNC Ecoregions:** 16:C

**Nations:** MX, US

**Subnations:** CA, MXBC

**Map Zones:** 4:C

**USFS Ecomap Regions:** 261B:PP, 262A:PP, 322C:PP

CONCEPT

SOURCES

**References:** Barbour and Major 1988, Comer et al. 2003*, Holland and Keil 1995, Sawyer and Keeler‑Wolf 1995

**Version:** 17 Mar 2003	**Stakeholders:** Latin America, West

**Concept Author:** P. Comer and T. Keeler‑Wolf	**LeadResp:** West
