# Supplementary material for: Long-term loss in extent and current protection of terrestrial ecosystem diversity in the temperate and tropical Americas
Source: PLoS One. 2020 Jun 30;15(6):e0234960. doi: 10.1371/journal.pone.0234960 (PMC7326196; doi:10.1371/journal.pone.0234960)
Supplement: S3 Appendix — (DOCX) [file pone.0234960.s003.docx]

# S3 Mapping Methods Detail

While vegetation forms one of the most readily observable features of terrestrial ecosystems, approaches to mapping vegetation and other types of land cover vary considerably. Within the remote sensing discipline, mapping approaches are often categorized into two types, including those involving "unsupervised" versus "supervised" techniques (Cihlar 2000). Unsupervised approaches rely on discernable pattern in remotely sensed imagery and other spatial inputs to derive a set of land cover classes, which are subsequently labeled as to their identity (De Caceres and Wiser 2012). Supervised approaches begin with a set of classification concepts, where types on the ground are known and described, and known sites for these types are used in machine learning to “train” the model and generate a map of their distribution (Muchoney et al. 2000). This supervised approach, using *a priori* classification of ecosystem types is well suited to this effort because one primary need was to develop a predictive distribution of described natural ecosystem types prior to intensive human intervention (De Caceres and Wiser 2012).

Given the hemispheric scope of the effort, and substantial differences in existing mapping effort between the United States and all other countries in the hemisphere, two primary mapping approaches were deployed. Within the United States, existing investments in national mapping from U.S. Geological Survey Gap Analysis Program and the interagency LANDFIRE (*Landscape Fire and Resource Management Planning Tools Project)* already completed both potential and current distribution maps of major terrestrial ecological system types (Lowry et al. 2007, Rollins 2009). For this effort, tasks included integration of selected regional map data sets (Tobalske et al. 2002, Lowry et al. 2007, Albert and Comer 2008, Diamond et al. 2009) known to provide more reliable map depictions of either potential/historical or current distributions. In each instance, classifications from other map sources were reconciled to the ecological systems classification. The merged maps were then reviewed by expert ecologists familiar with each area. Occasional manual map editing was completed to spatially reconcile maps where they intersected. After extensive expert review and refinement, these maps were subsequently validated using available georeferenced sample data.

Outside the United States, throughout temperate Canada, Latin America, and the Caribbean, new spatial models of potential distributions were developed and validated, and then current land use classes, derived from globally-available land use maps were combined with potential distribution maps of natural types to estimate current location and extent of all types. Related modeling methods from Lowry et al. (2007), as well as from Sayre et al. (2013) were adapted for this effort. Analytically, RandomForest (Gislason et al. 2006) classification and regression trees (CART) (Breiman et al. 1984) were used to identify predictive pattern for combinations of map surfaces relative to the location of georeferenced samples for each target class from the desired map legend (Hansen et al. 1996, De’ath et al. 2000). A combination of ArcGIS (10.1), ERDAS Imagine, and the data mining tool See 5 (Rulequest Research 2012) was used to develop models representing type distributions.

## Mapping Inputs

Table S3-1 provides a summary of map inputs, including existing map sources for potential distribution modeling. Existing national and regional maps, along with georeferenced field sample data for vegetation types, were all reconciled thematically to the IVC and ecological systems classifications. Both the IVC and ecological systems classifications were used in this effort. IVC macrogroups are substantially more thematically coarse than the ecological systems units. We therefore aimed to use the ecological system concepts where that was feasible (i.e., throughout temperate and tropical North America). Again, given the intent to map potential distribution of “natural” vegetation, only these types were sampled from existing sources. Urban, industrial, and agricultural land use types were not sampled. Given limitations of available field samples, randomized samples were gathered from existing maps in order to provide a robust and spatially balanced representation of each target map class where there was an acceptable level of confidence in the map source. Here we define “acceptable” as being judged sufficiently reliable by project ecologist experienced in the region and familiar with each map source.

Specifically, polygons of a given type with >10km^2^ provided the pool of source areas for sample selection. Selection of the 10km^2^ is again and expert judgment, having evaluated existing maps and concluded that sampling from types depicted in smaller areas risked introducing substantial error. We acknowledge that this risks exclusion of natural rare ecosystem types, but we judged that given the quality of existing map information for this purpose, that was a risk we were willing to assume. This pool of map polygons encompasses 95% of natural landscapes. Stratified random sample selection was weighted by continent-wide area of each type using the log_10_ (area)*100, providing a sample total weighted towards types of lesser area. A total of 683,119 georeferenced samples were generated for the Americas, with an additional 70,380 held aside and used only for map validation.

**Table S3-1.** Map sources of sample points for model development.

| **Mapping Region** | **Map Sources** | **Source MMU** | **Sample Points** |
| --- | --- | --- | --- |
| **Temperate Canada** | Can-VEC, The Nature Conservancy, LANDFIRE | 5ha | 88,168 |
| **United States** | LANDFIRE, USGS GAP, TX, OR, MI | 5ha | * |
| **Caribbean** | Borhidi (1991), The Nature Conservancy | 1ha | 80,539 |
| **Mexico** | INEGI, The Nature Conservancy, ProNatura-Yucatan | 5ha | 41,731 |
| **MesoAmerica** | The Nature Conservancy, ProNatura-Yucatan | 5ha | 56,372 |
| **South America** | The Nature Conservancy, NatureServe, WWF | 1000ha | 416,309 |

**validation only*

Explanatory variables, represented as map surfaces, included a series of biophysical factors; including bioclimate, landform, slope, and aspect, as well as surface flow accumulation (Table S3-2). Bioclimates, as modeled by Metzger et al. (2012), reflect the categorization of temperature and precipitation regime to globally-available remotely sensed data, resulting in a total of 125 unique bioclimates at 1km^2^ spatial resolution. Geophysical map surfaces were developed using 90m x 90m digital elevation data. Slope and aspect were measured in terms of degrees. The methodology for the landform class derivation employed a variable grid cell moving neighborhood analysis window to assess relative relief and followed other regional scale approaches to model macro-landforms (Dikau et al. 1991; True et al. 2000). Landforms as discrete units were derived from Weiss (2001) that used the 90m continental digital elevation, with all pixels assigned into one of the following regional physiographic types: "canyons, deeply incised streams", "midslope drainages, shallow valleys", "upland drainages, headwaters", "u-shaped valleys plains", "open slopes", "upper slopes, mesas", "local ridges, hills in valleys", "midslope ridges, small hills in plains", and "mountain tops, high ridges."

Land surface flow accumulation was derived from existing continental data (Lehner et al. 2006) that is a 90m x 90m hydrologically conditioned digital elevation. This data set specifically aims to use detailed topographic surfaces to indicate stream flow direction and flow accumulation for use in analysis of stream, wetland, and riparian ecosystems. EarthSat NaturalVue is multi-year composite surface of red-green-blue translation of 6 bands from LANDSAT 5-7 at 150m pixel resolution. These data were used as the only modeling input of spectral data. The intent of its use was to assist with differentiating location of natural types tending to occur in close proximity, and therefore, similar biophysical settings.

**Table S3-2.** Inputs to mapping vegetation *types (as needed for modeling, each layer was rescaled to summarize variable per 90m pixel)*.

| Dataset Name | Data Type | Units of Analysis | Spatial Resolution | | Source (citation) |
| --- | --- | --- | --- | --- | --- |
| Climate | raster | 125 | 1km^2^ | Metzger (2012) | |
| Slope | raster | 89 | 90m x 90m | NatureServe, from SRTM digital elevation | |
| Aspect | raster | 1-360^О^ | 90m x 90m | NatureServe, from SRTM digital elevation | |
| Landform | raster | 11 | 90m x 90m | NatureServe, from SRTM digital elevation | |
| Lithology | raster | 9-40 | 450m x 450m | Sayre et al. (2008), INEGI (*Mexico*), USGS (*Caribbean*) | |
| Soils | raster | 259 | 90m x 90m | CanVec (Natural Resources Canada) | |
| Surface Flow Accumulation | raster | 156 | 90m x 90m | HydroSHEDS (Lehner et al. 2006) | |
| EarthSat NatureVue Imagery | raster | 3 (0-255 per band) | 150m x 150m | ESRI | |
| Map Samples | raster | 683,119 | 90m x 90m | LANDFIRE, JOSSE et al. (2009), Borhidi (1991), and others | |
| Hexagon Grid | vector | 320,561 | 96km^2^ | NatureServe, DGGRID, Sahr (2013) | |

## Modeling in Sequence using Vegetation Hierarchy

We used a sequential mapping process where maps derived for multiple broader levels of the IVC classification hierarchy were then used as input to modeling distributions of types defined at lower hierarchical levels. In this application, the first thematic level for inductive modeling was the IVC Division (Main text Table 1). For example, in South America the 80 types may be viewed as continental expressions of vegetation formations; with vegetation responding most directly to global climate pattern. On average, 1,032 (Min=53, Max=5,724) samples per map class were used to generate the South America portion of this map. No satellite imagery was used in the development of the IVC division-level map output. Macrogroups were subsequently modeled using an average of 1,234 (Min=33, Max=3,433) samples per map class. Both the IVC division map output and EarthSat NaturalVue imagery were used as map inputs for the macrogroup model. Terrestrial ecological systems, being most numerous and most finely differentiated among the classification units used in this effort, were modeled using the macrogroup map as an additional model input. Once completed, the modeled terrestrial ecological systems layer is the finest thematic scale achievable using this technique. Since these units could be conceptually nested into IVC macrogroup concepts, the “bottom-up” aggregation of maps depicting these units should provide the most reliable map of macrogroups. This aggregation was then reviewed and edited to finalize the distribution of each IVC macrogroup. As noted previously, in North America, the ecological system-scaled units were edited to finalize those distributions. Numbers of map classes by region and classification level are listed in Table S3-3.

**Table S3-3** Numbers of mapped classification units by region and level of ecological classification *(including types with regionally overlapping distributions).*

| Region | Number of IVC Division | Number of IVC Macrogroup | Number of Terrestrial Ecological System |
| --- | --- | --- | --- |
| Caribbean | 4 | 14 | 66 |
| USA and Canada | 45 | 112 | 531 |
| Mexico | 32 | 55 | 126 |
| Central America | 20 | 33 | 65 |
| South America | 80 | 190 | *NA* |

## **Map Editing and Refinement**

Over-prediction is a common source of error in CART-based inductive modeling of land cover (Lowry et al. 2007). This could be anticipated in this particular application where there is high similarity in predictor variable combinations for vegetation types that naturally occur nearby each other. In these instances, predicted distributions may be skewed in favor of some over other types in some portions of their range of occurrence. Because of this, some form of expert-based review and map refinement is unavoidable. We assumed that over-prediction and skew errors would be concentrated in regional landscapes with extensive land use history and only fragmentary remnants of natural vegetation types. However, as noted above, since the generalized distribution of each terrestrial ecological system type had been previously documented in terms of country and WWF ecoregion (Josse et al. 2003), this knowledge was used in expert type-by-type review and refinement. Draft model outputs were attributed as extent measures per WWF ecoregion. These distributions were compared against known ecoregion distributions to identify likely error. Types found to be in error had their pixel distributions recoded to most-likely correct types for each WWF ecoregion. In turn, a second phase expert review and refinement followed the same procedure as for ecoregions but was applied to each type using a common grid of 100km^2^ hexagons (Sahr 2013). Again, with each type attributed to the hexagon grid, type-by-type review led to final recoding of pixels to most-likely correct types. Final map products were produced at 30m, 90m and 270m pixel resolutions, with 30m resolution limited to current extent and land cover within the USA, and both 90m and 270m data developed for the remainder of the study area.

## Potential Distribution Map Validation

As noted above, during initial sample data collection from map sources, georeferenced samples of each vegetation type were gathered and then set aside for use in model validation. These samples were only gathered for types that had existing polygons in regional/local maps >10 km^2^ in size in South America and 5 hectares for temperate and tropical North America. Of the 315 Macrogroup map classes in North and South America, 284 were quantitatively assessed. Within the USA we relied on field-based georeferenced sample data for all analysis. However, some 39% of terrestrial ecological system types (247 of 623 concentrated in the North America) lacked sufficient numbers of samples for rigorous evaluation.

Once map edits were finalized, these samples were used to score the degree of agreement for each map class at three spatial scales. For each sample, circular buffers were created encompassing 1km^2^ (within 6 pixels of center) and 5 km^2^ (within 28 pixels of center) respectively and point samples with the centroid of each pixel of the 270m version of each map. Overlay of these samples on the final map product generated tabular summaries to determine whether or not the mapped class matched the type labeled to each sample; (i.e., the same types co-occur within the buffered area). While truly independent samples could not be acquired to evaluate a spatial model depicting “potential/historical” extent of these vegetation types, this technique provides one initial measure of map quality, and serves as a primary input to decisions regarding use of the map for type-by-type assessment. Thus, the percentage of agreement between validation samples and maps can indicate the degree of map reliability for use with a practical minimum map unit of 270m vs. 1km^2^ vs. 5km^2^.

Table S3-4 provides a summary of validation statistics for potential distribution maps of macrogroups. A detailed summary for each class is found in Supplemetary Information (**S5 Appendix)**. For the 315 mapped macrogroup types, per class numbers of samples for the 1km^2^ validation sample area varied from a high of 972 to a low of 10. Summary of validation statistics indicate high (>80%) to moderate (>60%) map accuracy overall, and on a per map class basis at 270m vs. 1km^2^ vs. 5km^2^ map resolutions. Using the most demanding “point” (or 270m) validation sample area, 8 types scored at 100% agreement, 11 types scored >90% agreement, 16 types scored >80% agreement, 39 types scored >70% agreement and 52 types scored >60% agreement. A total of 158 types (56% of all assessed map classes) scored below 50% agreement.

Using the 1km^2^ validation sample area in North America only, 44 types scored at 100% agreement, 32 types scored >90% agreement, 14 types scored >80% agreement, 8 types scored >70% agreement and 9 types scored >60% agreement. A total of 12 types (10% of all assessed map classes) scored <50% agreement. For 1km^2^ samples, the total sample agreement was 85% and the median level of map class agreement for the types assessed was 88%.

Using the 5km^2^ validation sample area, 160 types scored at 100% agreement, 50 types scored >90% agreement, 23 types scored >80% agreement, 20 types scored >70% agreement and 15 types scored >60% agreement. A total of 16 types (6% of all assessed map classes) scored below 50% agreement. For 5km^2^ samples, the total sample agreement was 85% and the median level of map class agreement was 92%.

**Table S3-4. Summary validation statistics for 284 (315 total mapped) assessed macrogroups in North and South America.**

| Validation Sample Resolution | No. with 90-100% Agreement | No. with 80-90% Agreement | No. with 70-80% Agreement | No. with 60-70% Agreement | No. with 50-60% Agreement | No. with <50% Agreement |
| --- | --- | --- | --- | --- | --- | --- |
| 270m (point) *n=284* | 8 | 11 | 16 | 39 | 52 | 158 |
| *1 km^2^ *n=131* | 44 | 32 | 14 | 8 | 9 | 12 |
| 5 km^2^ *n=284* | 160 | 50 | 23 | 20 | 15 | 16 |

** North America only*

A total of 623 natural terrestrial ecological system types extend over the temperate North America, MesoAmerica, and the Caribbean. Of these, 443 types had sufficient sample data to report on map validation. Table S3-5 provides a summary of validation statistics. A detailed summary for each class is found in Supplementary Information (**S6 Appendix)**. Using the 270m point validation sample area, 10 types scored at 100% agreement, 16 types scored >90% agreement, 21 types scored >80% agreement, 26 types scored >70% agreement and 46 types scored >60% agreement. A total of 324 types (73% of all assessed map classes) scored <50% agreement. For point validation samples, the total sample agreement was Kappa=43% and the median level of map class agreement for the 443 types assessed was 28%.

Using the 1km^2^ validation sample area, 11 types scored at 100% agreement, 93 types scored >90% agreement, 61 types scored >80% agreement, 29 types scored >70% agreement and 21 types scored >60% agreement. A total of 128 types (29% of all assessed map classes) scored below 50% agreement. For the 1 km^2^ sample area, the total sample agreement was Kappa=75% and the median level of map class agreement was 78%.

Using the 5km^2^ validation sample area, 186 types scored at 100% agreement, 81 types scored >90% agreement, 35 types scored >80% agreement, 19 types scored >70% agreement and 12 types scored >60% agreement. A total of 110 types (25% of all assessed map classes) scored <50% agreement. For 5km^2^ samples, the total sample agreement was Kappa=81% and the median level of map class agreement was 86%.

**Table S3-6. Summary validation statistics for 443 (623 total mapped) assessed ecological systems in temperate and tropical North America and Caribbean.**

| Validation Sample Resolution | No. with 90-100% Agreement | No. with 80-90% Agreement | No. with 70-80% Agreement | No. with 60-70% Agreement | No. with 50-60% Agreement | No. with <50% Agreement |
| --- | --- | --- | --- | --- | --- | --- |
| 270m (point) | 10 | 16 | 21 | 26 | 46 | 324 |
| 1 km^2^ | 111 | 93 | 61 | 29 | 21 | 128 |
| 5 km^2^ | 186 | 81 | 35 | 19 | 12 | 110 |

These results, for both the macrogroup map and the ecological systems map, suggest that map reliability is limited on a per pixel basis (@ 270m pixels), but within relatively small clusters of adjacent pixels, the reliability of the map increases for most map classes.

However, given results of validation and further expert map review, 191 ecological system map classes were removed from subsequent analysis of the Temperate and Tropical North America study area. This left 433 types for subsequent analysis and reporting. While these removed classes amounted to 30% of map classes, they encompassed just 5.2% of the total study area. Most were types with estimated potential extent below 500km^2^. These types, commonly occurring in more localize environmental settings are less well suited for continental-scale modeling then are types that cover more extensive areas. Targeted type-specific modeling and mapping, as well as field inventory, are needed to adequately document trends in these types. Again, we acknowledge that this risks exclusion of naturally rare ecosystem types, but we judged that was a risk we were willing to assume. We have identified these types as needing additional effort in order to adequately evaluate mapped information before bringing them into subsequent conservation-related analyses.

# References

Albert DA, Comer PJ. (Cartography: Helen Enander). Atlas of Early Michigan’s Forests, Grasslands, and Wetlands: anAn Interpretation of the General Land Office Surveys 1816-1856. Michigan State University Press; 2008.

Borhidi AL. Phytogeography and vegetation ecology of Cuba. Budapest: Akademiai Kiado 858p.-illus., col. illus.. ISBN. 1991;1040618365.

Breiman L, Friedman J, Stone CJ, Olshen RA. Classification and regression trees. CRC press; 1984.

Cihlar J. Land cover mapping of large areas from satellites: status and research priorities. International journal of remote sensing; 2000 Jan 1;21(6-7):1093-114.

De Cáceres M, Wiser SK. Towards consistency in vegetation classification. Journal of Vegetation Science. 2012 Apr 1;23(2):387-93.

De'ath G, Fabricius KE. Classification and regression trees: a powerful yet simple technique for ecological data analysis. Ecology; 2000 Nov;81(11):3178-92.

Diamond DD, Elliot LF, Blodgett CD, True D, German D. 2009. Existing Vegetation Type Modeling and Map for Central and East Texas. Interpertive brochure and GIS data layers deliverd to Texas Parks abd Wildlife Department, Austin, TX.

Dikau R, Brabb EE, Mark RM. Landform classification of New Mexico by computer. US Dept. of the Interior, US Geological Survey; 1991.

Gislason PO, Benediktsson JA, Sveinsson JR. Random forests for land cover classification. Pattern Recognition Letters. 2006 Mar 1;27(4):294-300.

Hansen M, Dubayah R, DeFries R. Classification trees: an alternative to traditional land cover classifiers. International journal of remote sensing; 1996 Mar 1;17(5):1075-81.

Josse C, Navarro G, Comer P, Evans R, Faber-Langendoen D, Fellows M, Kittel G, Menard S, Pyne M, Reid M, Schulz K. J Teague (2003) Ecological Systems of Latin America and the Caribbean: A Working Classification of Terrestrial Systems.

Josse C, Cuesta F, Navarro G, Barrena V, Cabrera E, Chacón-Moreno E, Ferreira W, Peralvo M, Saito J, Tovar A. Mapa de ecosistemas de los Andes del norte y centrales. Bolivia, Colombia, Ecuador, Perú y Venezuela. Programa Regional ECOBONA-Intercooperation, CONDESAN-Proyecto páramo Andino, Programa BioAndes, EcoCiencia, NatureServe, IAvH, LTA-UNALM, ICAE-ULA, CDC-UNALM, RUMBOL SRL, Lima; 2009.

Lehner B, Verdin K, Jarvis A. Technical Documentation Version 1.0. HydroSHEDS. World Wildlife Fund and partners. 2006. Available: <http://hydrosheds.cr.usgs.gov/index.php>. *Accessed 26 August 2013*.

Lowry J, Ramsey RD, Thomas K, Schrupp D, Sajwaj T, Kirby J, Waller E, Schrader S, Falzarano S, Langs L, Manis G. Mapping moderate-scale land-cover over very large geographic areas within a collaborative framework: a case study of the Southwest Regional Gap Analysis Project (SWReGAP). Remote Sensing of Environment. 2007 May 15;108(1):59-73.

Metzger MJ, Bunce RG, Jongman RH, Sayre R, Trabucco A, Zomer R. A high‐resolution bioclimate map of the world: a unifying framework for global biodiversity research and monitoring. Global Ecology and Biogeography; 2013 May 1;22(5):630-8.

Muchoney D, Borak J, Chi H, Friedl M, Gopal S, Hodges J, Morrow N, Strahler A. Application of the MODIS global supervised classification model to vegetation and land cover mapping of Central America. International Journal of Remote Sensing. 2000 Jan 1;21(6-7):1115-38.

Rulequest Research Data Mining tools See5 and C5.0; 2012 <http://www.rulequest.com/see5-info.html>

Sahr, K. DGGRID, Version 6.1: User documentation for discrete global grid software. Available: [http://webpages.sou.edu/~sahrk/dgg/dggrid.v6/dggridManual.v61.pdf. *Accessed 30 January 2013*](http://webpages.sou.edu/~sahrk/dgg/dggrid.v6/dggridManual.v61.pdf.%20Accessed%2030%20January%202013)*.*

Tobalske C, Christy J, Alverson E. Historic Vegetation Cover of Oregon. Oregon Natural Heritage Program; 2002.

True D, Gordon T, Diamond D. How the size of a sliding window impacts the generation of landforms. Missouri Resources Assessment Partnership; 2000.

Weiss A. Topographic position and landforms analysis. InPoster presentation, ESRI user conference, San Diego, CA 2001 Jul 9 (Vol. 200).
