# Supplementary material for: Long-term loss in extent and current protection of terrestrial ecosystem diversity in the temperate and tropical Americas
Source: PLoS One. 2020 Jun 30;15(6):e0234960. doi: 10.1371/journal.pone.0234960 (PMC7326196; doi:10.1371/journal.pone.0234960)
Supplement: S4 Appendix — (DOCX) [file pone.0234960.s004.docx]

S6 Appendix - Supplementary Materials

Current Land Use/Land Cover Map

A composite map for current land use *circa* 2010 was developed by combining products from LANDFIRE (30m pixel resolution, circa 2003 [in the USA], Globecover (270m pixel resolution, circa 2009), and GlobeLand30 (30m pixel resolution circa 2000-2010)^[[1]](#footnote-2)^. Investigation of GlobeLand30 indicated inaccuracies in predicting deforestation especially in tropical forest regions. Table 1 indicates how classes from GlobeCover and GlobeLand30 were combined to depict developed, agriculture, and surface water classes. Likewise, substantial areas of ruderal vegetation (vegetation with no natural analog resulting from prior land clearing and abandonment) common in portions of the USA were not reflected in wither GlobeCover or GlobeLand30 data sets. Therefore, a combined map product was developed and summarized at 270m pixel resolution to best approximate urban, industrial, agricultural, and ruderal land cover. This layer was then combined with the potential distribution map of vegetation Macrogroups to indicate current extent of Macrogroups and land use classes *circa* 2010.

Table 1 – Combinations of GlobeCover and GlobeLand30 use to depict current land use classes.

| **GlobeLand30** | **GlobeCover** | **Code** | **Land Cover Name** |
| --- | --- | --- | --- |
| Artificial Surface | Artifical areas | 24 | developed |
| Artificial Surface | Bare areas | 24 | developed |
| Artificial Surface | Closed broadleaved deciduous forest | 24 | developed |
| Artificial Surface | Closed broadleaved forest permanently flooded | 24 | developed |
| Artificial Surface | Closed to open broadleaved evergreen or semi-deciduous | 24 | developed |
| Artificial Surface | Closed to open broadleaved forest regularly flooded | 24 | developed |
| Artificial Surface | Closed to open grassland | 24 | developed |
| Artificial Surface | Closed to open mixed broadleaved and needle leaved | 24 | developed |
| Artificial Surface | Closed to open shrubland | 24 | developed |
| Artificial Surface | Closed to open vegetation regularly flooded | 24 | developed |
| Artificial Surface | Mosaic croplands/Vegetation | 24 | developed |
| Artificial Surface | Mosaic Forest-shrubland-grassland | 24 | developed |
| Artificial Surface | Mosaic grassland-forest-shrubland | 24 | developed |
| Artificial Surface | Mosaic vegetation/croplands | 24 | developed |
| Artificial Surface | Open broadleaved deciduous forest | 24 | developed |
| Artificial Surface | Open Water | 24 | developed |
| Artificial Surface | Permanent snow and ice | 24 | developed |
| Artificial Surface | Rainfed croplands | 24 | developed |
| Artificial Surface | Sparse vegetation | 24 | developed |
| Bareland | Rainfed croplands | 80 | agriculture |
| Cultivated land | Artifical areas | 80 | agriculture |
| Cultivated land | Bare areas | 80 | agriculture |
| Cultivated land | Closed broadleaved deciduous forest | 81 | pasture |
| Cultivated land | Closed broadleaved forest permanently flooded | 81 | pasture |
| Cultivated land | Closed to open broadleaved evergreen or semi-deciduous | 81 | pasture |
| Cultivated land | Closed to open broadleaved forest regularly flooded | 80 | agriculture |
| Cultivated land | Closed to open grassland | 80 | agriculture |
| Cultivated land | Closed to open shrubland | 81 | pasture |
| Cultivated land | Mosaic croplands/Vegetation | 80 | agriculture |
| Cultivated land | Mosaic Forest-shrubland-grassland | 80 | agriculture |
| Cultivated land | Mosaic grassland-forest-shrubland | 81 | pasture |
| Cultivated land | Mosaic vegetation/croplands | 80 | agriculture |
| Cultivated land | Rainfed croplands | 80 | agriculture |
| Cultivated land | Sparse vegetation | 80 | agriculture |
| Forest | Bare areas | 81 | pasture |
| Forest | Mosaic croplands/Vegetation | 81 | pasture |
| Forest | Mosaic vegetation/croplands | 81 | pasture |
| Forest | Rainfed croplands | 80 | agriculture |
| Grassland | Artifical areas | 81 | pasture |
| Grassland | Mosaic croplands/Vegetation | 81 | pasture |
| Grassland | Mosaic vegetation/croplands | 81 | pasture |
| Grassland | Rainfed croplands | 81 | pasture |
| Shrubland | Bare areas | 81 | pasture |
| Shrubland | Mosaic croplands/Vegetation | 81 | pasture |
| Shrubland | Mosaic vegetation/croplands | 81 | pasture |
| Shrubland | Rainfed croplands | 81 | pasture |
| Water Bodies | Artifical areas | 11 | water |
| Water Bodies | Bare areas | 11 | water |
| Water Bodies | Closed broadleaved deciduous forest | 11 | water |
| Water Bodies | Closed broadleaved forest permanently flooded | 11 | water |
| Water Bodies | Closed to open broadleaved evergreen or semi-deciduous | 11 | water |
| Water Bodies | Closed to open broadleaved forest regularly flooded | 11 | water |
| Water Bodies | Closed to open grassland | 11 | water |
| Water Bodies | Closed to open shrubland | 11 | water |
| Water Bodies | Closed to open vegetation regularly flooded | 11 | water |
| Water Bodies | Mosaic croplands/Vegetation | 11 | water |
| Water Bodies | Mosaic Forest-shrubland-grassland | 11 | water |
| Water Bodies | Mosaic grassland-forest-shrubland | 11 | water |
| Water Bodies | Mosaic vegetation/croplands | 11 | water |
| Water Bodies | Open Water | 11 | water |
| Water Bodies | Permanent snow and ice | 11 | water |
| Water Bodies | Rainfed croplands | 11 | water |
| Water Bodies | Sparse vegetation | 11 | water |
| Wetland | Rainfed croplands | 81 | pasture |

1. <http://ionia1.esrin.esa.int/page_landcoverdata.php>; <http://glc30.tianditu.com/> [↑](#footnote-ref-2)
